
JOURNAL INFORMATION
==============================
NLM Title Abbreviation: Insights Imaging
Journal ID: Insights Imaging
NLM Journal ID: 101532453

Insights into Imaging

EISSN: 1869-4101
Publisher: Springer

ARTICLE INFORMATION
==============================
PMCID: PMC9803397
PMID: 36585546
DOI: 10.1186/s13244-022-01337-x
Article ID: 1337
Article version: 2
Subjects: Meeting Abstracts

ECR 2022 Book of Abstracts

Electronic publication date: 2022 Dec 31
Publication date: 2022 Dec
Volume: 13
Issue: Suppl 4
Issue sponsor: Publication of this supplement was funded by the European Congress of Radiology.
Electronic Location ID: 205
Copyright: © The Author(s) 2022
License: Open AccessThis article is licensed under a Creative Commons Attribution 4.0 International License, which permits use, sharing, adaptation, distribution and reproduction in any medium or format, as long as you give appropriate credit to the original author(s) and the source, provide a link to the Creative Commons licence, and indicate if changes were made. The images or other third party material in this article are included in the article's Creative Commons licence, unless indicated otherwise in a credit line to the material. If material is not included in the article's Creative Commons licence and your intended use is not permitted by statutory regulation or exceeds the permitted use, you will need to obtain permission directly from the copyright holder. To view a copy of this licence, visit http://creativecommons.org/licenses/by/4.0/.
License URL: https://creativecommons.org/licenses/by/4.0/

Conference name: European Congress of Radiology 2022
Conference location: Virtual and Vienna, Austria
Conference date: 2-6 March, 13-17 July 2022
issue-copyright-statement: © European Society of Radiology (ESR) 2022

==============================

EUROPEAN CONGRESS OF RADIOLOGY

### ECR 2022

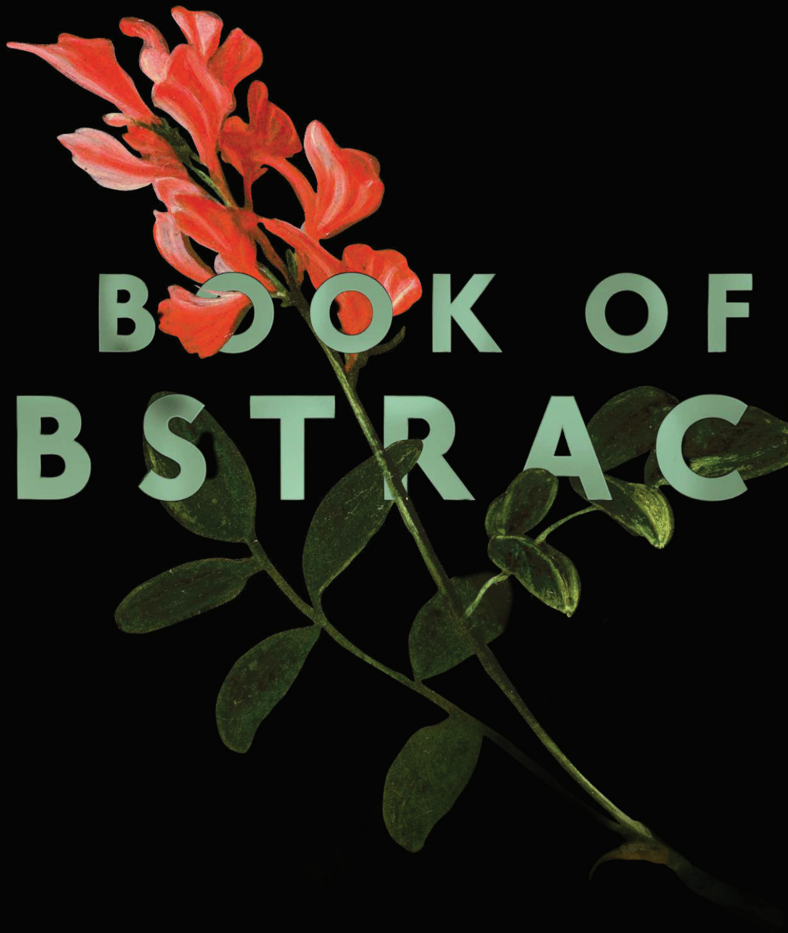

### BOOK OF ABSTRACTS

INSIGHTS INTO IMAGING | VOLUME 13 | SUPPLEMENT 4

### CONTENTS

| | |
|---|---|
| 3 - 70 | ECR 2022 Overture - Abstract-based Programme - March 2-6, 2022 (A) |
| 71 - 454 | ECR 2022 Abstract-based Programme - July 13-17, 2022 (B) |

the annual meeting of
ESR
EUROPEAN SOCIETY OF RADIOLOGY
www.myESR.org

Springer

**Disclaimer**

The ECR 2022 Book of Abstracts is published by the European Society of Radiology (ESR) and summarises the presentations accepted to be held at the European Congress of Radiology 2022 (Vienna, Austria, March 2 - March 6, 2022 and Vienna, Austria, July 13 – July 17, 2022).

Abstracts were submitted by the authors warranting that good scientific practice, copyrights and data privacy regulations have been observed and relevant conflicts of interest declared.
Abstracts reflect the authors' opinions and knowledge. The ESR does not give any warranty about the accuracy or completeness of medical procedures, diagnostic procedures or treatments contained in the material included in this publication. The views and opinions presented in ECR abstracts and presentations, including scientific, educational and professional matters, do not necessarily reflect the views and opinions of the ESR.

In no event will the ESR be liable for any direct or indirect, special, incidental, consequential, punitive or exemplary damages arising from the use of these abstracts.

The Book of Abstracts and all of its component elements are for general educational purposes for health care professionals only and must not take the place of professional medical advice. Those seeking medical advice should always consult their physician or other medical professional.

In preparing this publication, every effort has been made to provide the most current, accurate, and clearly expressed information possible. Nevertheless, inadvertent errors in information can occur. The ESR is not responsible for typographical errors, accuracy, completeness or timeliness of the information contained in this publication.

The ECR 2022 Book of Abstracts is a supplement to Insights into Imaging (1869-4101) and published under Creative Commons Attribution 4.0 International License.

Insights Imaging (2022) 13 (Suppl 4)
https://doi.org/10.1186/s13244-022-01337-x
Published: 31 December 2022

### A

### ECR 2022
### Abstract-based Programme
### Overture - March 2-6, 2022

Research Presentation Sessions (RPS)

**Wednesday, March 2 .................. 4**
**Thursday, March 3.....................17**
**Friday, March 4 .........................26**
**Saturday, March 5 .....................35**
**Sunday, March 6.......................45**

### Wednesday, March 2

08:00-09:00 **Channel 4**

### Research Presentation Session: Chest

### RPS 104

### Lung nodules

**Moderators**
L. Musayeva; Baku/AZ
L. E. Derchi; Genoa/IT

### RPS 104-3

**Deep learning for estimating pulmonary nodule malignancy risk using prior CT examinations in lung cancer screening**

*K. V. Venkadesh*, T. A. Aleef, A. Schreuder, E. Scholten, B. Van Ginneken, M. Prokop, C. Jacobs; Nijmegen/NL
*(kiranvaidhya.venkadesh@radboudumc.nl)*

**Purpose:** Nodule size, morphology, and growth are important factors for accurately estimating nodule malignancy risk in lung cancer screening CT examinations. In this work, we aimed to develop a deep learning (DL) algorithm that uses a current and a prior CT examination to estimate the malignancy risk of pulmonary nodules.
**Methods or Background:** We developed a dual time-point DL algorithm by stacking the nodules from the current and prior CT examinations in the input channels of convolutional neural networks. We used 3,011 nodules (286 malignant) and 994 nodules (73 malignant) as development and hold-out test cohorts from the National Lung Screening Trial, respectively. The reference standard was set by histopathologic confirmation or CT follow-up of more than two years. We compared the performance of the algorithm against PanCan model 2b and a previously published single time-point DL algorithm that only processed a single CT examination. We used the area under the receiver operating characteristic curve (AUC) to measure discrimination performance and a standard permutation test with 10,000 random permutations to compute p-values.
**Results or Findings:** The dual time-point DL algorithm achieved an AUC of 0.94 (95% CI: 0.91 – 0.97) on the hold-out test cohort. The algorithm outperformed the single time-point DL algorithm and the PanCan model, which had AUCs of 0.92 (95% CI: 0.89 – 0.95; $p = 0.055$) and 0.88 (95% CI: 0.85 – 0.91; $p < 0.001$), respectively.
**Conclusion:** Deep learning algorithms using current and prior CT examinations have the potential to accurately estimate the malignancy risk of pulmonary nodules.
**Limitations:** External validation is needed on other screening datasets to generate further evidence.
**Ethics committee approval:** Institutional review board approval was obtained at each of the 33 centers involved in the NLST.
**Funding for this study:** Research grant from MeVis Medical Solutions AG.
**Author Disclosures:**
Mathias Prokop: Speaker: Canon Medical SysteOther: disclosed royalties from Mevis Medical Solutions Research/Grant Support: Canon Medical SysteResearch/Grant Support: Siemens Healthineers Speaker: Siemens Healthineers Other: disclosed travel cost paid to author from Siemens Healthineers Other: disclosed travel cost paid to author from Canon Medical SystePatent Holder: Canon Medical Systems
Kiran Vaidhya Venkadesh: Other: Employee stock option holder at Predible Health
Anton Schreuder: Nothing to disclose
Ernst Scholten: Nothing to disclose
Bram Van Ginneken: Research/Grant Support: Botnar Foundation Shareholder: Thirona Other: disclosed money to author's institution for royalties from Delft Imaging Other: disclosed money to author's institution for royalties from Thirona Other: disclosed money to author's institution for royalties from MeVis Medical Solutions
Tajwar Abrar Aleef: Nothing to disclose
Colin Jacobs: Other: disclosed money to author's institution for royalties from MeVis Medical Solutions

### RPS 104-4

**Radiomics for classifying minimally invasive lung adenocarcinoma and invasive lung adenocarcinoma presenting as ground glass nodules based on contrast-enhanced computed tomography (CECT)**

*D. Tian*, Y. He, J. Zhang, Q. Song, A. Liu, Z. Li; Dalian/CN
*(istiandi@163.com)*

**Purpose:** Radiomics for classifying minimally invasive lung adenocarcinoma and invasive lung adenocarcinoma presenting as ground-glass nodules based on contrast-enhanced computed tomography.
**Methods or Background:** We retrospectively included 98 ground glass nodules (GGNs) that were surgically confirmed as minimally invasive adenocarcinomas (MIAs) or invasive adenocarcinomas (IAs). Each GGO was segmented manually using 3D-slicer, and texture features were extracted from the enhanced venous phase image. The least absolute shrinkage and selection operator (LASSO) method was applied to select optimal radiomics features whose performance was assessed by the area under the receiver operating characteristic curve (AUC-ROC). The radiomics model was compared to the radiographic model and the radiomics-radiographic CT combined model using univariate and multivariate logistic regression analysis.
**Results or Findings:** The radiomics model in CT-enhanced venous phase showed better discriminative performance (training AUC,0.85; test AUC,0.85) than the radiographic CT model (training AUC,0.78; test AUC,0.62). The combined model (training AUC,0.85; test AUC,0.85) did not demonstrated improved performance compared with the radiomics model.
**Conclusion:** A radiomics model based on contrast-enhanced CT imaging have the best diagnostic performance to distinguish MIAs from IAs in GGNs when compare with the radiographic CT model.
**Limitations:** It was a single-center retrospective study, and there is deviation between collection and inclusion.
**Ethics committee approval:** The ethics committee of the First Affiliated Hospital of Dalian Medical University.
**Funding for this study:** No funding support.
**Author Disclosures:**
Jingyu Zhang: Nothing to disclose
Ailian Liu: Nothing to disclose
Zhiyong Li: Nothing to disclose
Qingwei Song: Nothing to disclose
Yifan He: Nothing to disclose
Di Tian: Nothing to disclose

### RPS 104-5

**Lung nodule volumetry: the effect of deep learning versus iterative reconstruction at different dose levels**

*C. Franck*[1], F. Zanca[2], K. Carpentier[1], H. El Addouli[1], M. C. Niekel[1], M. Spinhoven[1], A. Van Hoyweghen[1], A. Snoeckx[1]; [1]Edegem/BE, [2]Heverlee/BE

**Purpose:** Deep learning image reconstruction (DLIR) has been shown to reduce radiation dose compared to iterative reconstruction (IR) for chest CT. It is unknown, however, whether the nodule volume measurements with DLIR are comparable with IR at different dose levels.
**Methods or Background:** An anthropomorphic chest phantom (Lungman, Kyoto Kagaku), containing six spherical, six lobulated, and six spiculated 3D-printed solid nodules (volume range 28-392 $mm^3$), was scanned at six dose levels (0.2, 0.4, 0.8, 1.5, 3, 6 mGy). Images were 1.25 mm reconstructed with ASIR-V 60% and three levels of DLIR (TrueFidelity Low, Medium, High). The volumes of 432 nodules (18 nodules x 6 doses x 4 reconstructions) were measured by five independent observers in a semi-automatic fashion. Mean percentage error in nodule volume measurements was assessed for all reconstructions and dose levels, with respect to the ground truth (high dose scan, 11 mGy). Subsequently, data were stratified per nodule type. A smaller absolute percentage error indicates a higher accuracy.
**Results or Findings:** In general, mean % errors decreased with increasing dose. On average, errors were significantly lower with TrueFidelity (3.6/3.4/3.0% for Low/Medium/High) than with ASIR-V (4.1%), for all dose levels (p=0.001). With increasing DLIR level, errors decreased for the lower dose range (0.2-0.8 mGy), while for higher doses (1.5-6 mGy) values were comparable. When stratifying per morphology, the largest error was found for lobulated nodules (4.8%), followed by spiculated (3.3%) and spherical (2.8%) nodules.
**Conclusion:** In chest CT, volume measurements with TrueFidelity showed a significantly higher accuracy compared to ASIR-V, for all dose levels and all nodule types. Lobulated nodules showed the highest absolute error in volume measurements.
**Limitations:** Only phantom images were used.
**Ethics committee approval:** Not applicable.
**Funding for this study:** Not applicable.
**Author Disclosures:**
Astrid Van Hoyweghen: Nothing to disclose
Caro Franck: Nothing to disclose
Annemie Snoeckx: Nothing to disclose
Maarten Christian Niekel: Nothing to disclose
Maarten Spinhoven: Nothing to disclose
Haroun El Addouli: Nothing to disclose
Ken Carpentier: Nothing to disclose
Federica Zanca: Nothing to disclose

### RPS 104-6

**The value of virtual monoenergetic images and electron density map derived from dual-layer spectral detector CT in differentiating benign from malignant pulmonary ground glass nodules**

*J. Qiu*[1], X. Chen[2], X. Xin[1], B. Zhang[1]; [1]Nanjing/CN, [2]Suzhou/CN
*(jsqiu163@163.com)*

**Purpose:** To investigate the clinical value of virtual monoenergetic images (VMI) and electron density map (EDM) derived from the dual-layer spectral detector CT (DLCT) in the differential diagnosis of benign and malignant pulmonary ground glass nodules (GGN).
**Methods or Background:** 27 benign and 38 malignant GGN were retrospectively studied. The 120kVp polyenergetic image (PI), EDM, and 40-80keV VMI were reconstructed, in which the CT value, electron density (ED), and CT features were analysed between benign and malignant lesions. The CT features included lesion size, location, shape, edge, internal structure, adjacent structure, and nodule type. The statistically significant CT signs and quantitative parameters were analysed by logistic regression analysis to obtain the independent risk factors for GGN malignancy, then all the independent risk factors were united to analyse by ROC curve.
**Results or Findings:** The lesion shape, spiculation, lobulation, location, size, CT value in PI, 40-80 keV VMI, and ED were significant different between two groups (P<0.05), which were enrolled in logistic regression analysis by taking if GGN was malignant as the dependent variable. The EDM had higher efficacy than other VMI with AUC 0.722. Logistic regression analysis results showed that ED (P=0.044), lesion size (P=0.004), and spiculation (P=0.002) were independent risk factors for GGN malignancy. ROC analysis showed that the AUC of ED, size, spiculation, and a combination of the three were 0.722, 0.772, 0.698, and 0.885, respectively. The AUC of combined diagnosis was the largest with sensitivity specificity of 92.1% and 74.1%, respectively.
**Conclusion:** The diagnostic efficiency of DLCT images in differential diagnosis of pulmonary GGN, EDM has a higher efficiency than other VMI, and the diagnostic efficiency is further improved when EDM combines with lesion size and spiculation is analysed comprehensively.
**Limitations:** Not applicable.
**Ethics committee approval:** Not applicable.
**Funding for this study:** Not applicable.
**Author Disclosures:**
Xingbiao Chen: Author: Jiansheng Qiu:Nothing to dislose
Jiansheng Qiu: Speaker: Jiansheng Qiu:Nothing to dislose
Bing Zhang: Author: Jiansheng Qiu:Nothing to dislose
Xiaoyan Xin: Author: Jiansheng Qiu:Nothing to dislose

### RPS 104-7

**CT texture analysis of pulmonary neuroendocrine tumours: associations with tumour grading and proliferation**

*J. Leonhardi*, J. Pappisch, A-K. Höhn, H. Wirtz, T. Denecke, A. Frille, H-J. Meyer; Leipzig/DE
*(jakob.leonhardi@medizin.uni-leipzig.de)*

**Purpose:** Texture analysis derived from computed tomography (CT) might be able to provide clinically relevant imaging biomarkers and might be associated with histopathology features in tumours. The present study sought to elucidate possible associations between texture features derived from CT images with proliferation index Ki-67 and grading in pulmonary neuroendocrine tumours.
**Methods or Background:** 38 patients (n= 22 females, 58%) with a mean age of 60.8 ± 15.2 years were included into this retrospective study. Texture analysis was performed using the free available Mazda software. All tumours were histopathologically confirmed. Discrimination and correlation analyses were performed.
**Results or Findings:** In discrimination analysis, "S(1,1)SumEntrp" was significantly different between typical and atypical carcinoids (mean 1.74 ± 0.11 versus 1.79 ± 0.14, p=0.007). The correlation analysis revealed a moderate positive association between Ki-67 index with the first order parameter kurtosis (r=0.66, p=0.001). Several other texture features were associated with Ki-67 index, the highest correlation coefficient showed S(4,4)InvDfMom" (r=0.59, p=0.004).
**Conclusion:** Several texture features derived from CT were associated with proliferation index Ki-67 and might therefore be a valuable novel biomarker in pulmonary neuroendocrine tumours. "Sumentrp" might be a promising parameter to aid in the discrimination between typical and atypical carcinoids.
**Limitations:** First, it is a retrospective study with possible known inherent bias. Second, the patient sample is rather small based upon pNET low prevalence among lung tumours. Third, only 60% of patients had a Ki-67 index available, which further reduces the size of the patient sample.
**Ethics committee approval:** It received ethical approval from the local ethics committee at the Medical Faculty (IRB00001750, AZ: 259/18-ek) on July 31 2018.
**Funding for this study:** No funding was provided for this study.
**Author Disclosures:**
Timm Denecke: Nothing to disclose
Johanna Pappisch: Nothing to disclose
Hubert Wirtz: Nothing to disclose
Hans-Jonas Meyer: Nothing to disclose
Armin Frille: Nothing to disclose
Anne-Kathrin Höhn: Nothing to disclose
Jakob Leonhardi: Nothing to disclose

### RPS 104-8

**Impact of an automatic lung nodule detection algorithm on the evaluation of routine chest x-ray examinations and referral for chest CT: a retrospective observational study**

*A. Favril*, L. Vael, C. Franck, A. Snoeckx; Edegem/BE
*(favrilalexander@gmail.com)*

**Purpose:** Chest x-ray is frequently performed in daily clinical practice. The detection of lung nodules on radiographs is an indication for chest CT referral. Since lung nodule detection in chest x-ray has a low accuracy, the sensitivity can be improved by computer aided diagnosis (CAD) systems. However, the risk of large false positive rates can lead to redundant referrals for chest CT and contribute to unnecessary radiation exposure. In this retrospective study, we investigated the impact of an automatic lung nodule detection (ALND) algorithm on the evaluation of chest radiographs and referral for chest CT.
**Methods or Background:** Between July and September 2020, 1468 consecutive routine PA chest radiographs of adult, non-pregnant patients were retrospectively collected at a tertiary care center. Images were screened for positive ALND annotations in combination with referral for chest CT. In addition, the correlation of the ALND result with a true positive lung nodule was investigated.
**Results or Findings:** The ALND algorithm detected 591 lung nodules on 524 (36%) chest radiographs. Among these, 12 were indicated as known lesions, 301 were reported as false positive, whereas 220 ALND findings were not reported. On 9% (49/524) of the radiographs, 58 nodules were deemed suspicious and referred for CT. In 55% (27/49) of the cases a CT examination was actually performed of which in 56% (15/27) of the patients a nodular lesion was found.
**Conclusion:** In approximately 1 out of 10 patients, a positive annotation by an ALND algorithm leads to chest CT referral. When a subsequent chest CT is performed, the suspected nodule correlates with a true lung nodule in more than half of the investigated patients.
**Limitations:** Not applicable.
**Ethics committee approval:** Approved.
**Funding for this study:** Not applicable.
**Author Disclosures:**
Caro Franck: Nothing to disclose
Alexander Favril: Nothing to disclose
Annemie Snoeckx: Nothing to disclose
Leo Vael: Nothing to disclose

**09:30-10:30** **Channel 4**

### Research Presentation Session: Hybrid, Molecular and Translational Imaging

### RPS 206

### Advanced prostate cancer imaging

**Moderators**
T. Barrett; Cambridge/UK
L. E. Derchi; Genoa/IT

### RPS 206-3

**A pilot study of 68Ga-PSMA11 and 68Ga-RM2 PET/MRI for evaluation of prostate cancer response to high intensity focused ultrasound (HIFU) therapy**

*H. Duan*, V. Ferri, P. Ghanouni, B. Daniel, G. Davidzon, C. Mari Aparici, G. Sonn, C. Kunder, A. Iagaru; Stanford, CA/US
*(heying@stanford.edu)*

**Purpose:** High intensity focused ultrasound (HIFU) is a non-invasive local treatment procedure that uses thermal energy to ablate prostate cancer (PC) lesions. In this study, we evaluated 68Ga-RM2 and 68Ga-PMSA11 PET/MRI before and after treatment with HIFU to assess the accuracy of localisation and response to treatment.

Wednesday

**Methods or Background:** Fourteen men with newly diagnosed PC were prospectively enrolled. Pre HIFU, patients underwent prostate biopsy, multiparametric MRI (mpMRI), 68Ga-PSMA11 and 68Ga-RM2 PET/MRI. Response to HIFU treatment was assessed with 68Ga-PSMA11 and 68Ga-RM2 PET/MRI. For localisation, the prostate was divided into 12 segments (apex, mid, and base, medial and lateral respectively, left and right) using PET/MRI data and MIM software. Maximum standardised uptake values (SUVmax) of PC lesions and the background of each segment were collected.
**Results or Findings:** Pre HIFU biopsy revealed 23 lesions of which 18 were clinically significant with Gleason score (GS) ≥7 and mpMRI showed 15 lesions with 13 being ≥PIRADS 4. 68Ga-PSMA11 and 68Ga-RM2 PET/MRI each identified 25 positive lesions including all index lesions. Post-HIFU imaging was available in 9 participants. 68Ga-PSMA11 and 68Ga-RM2 PET/MRI were negative for the respective treated area in all patients. SUVmax of target lesions decreased significantly after HIFU for both radiopharmaceuticals (68Ga-PSMA11: from 12.63±10.63 [3.27–36.90] to 3.58±3.37 [1.65–12.23], P=0.03; 68Ga-RM2: from 8.56±4.96 [1.92–17.37] to 2.72±0.95 [1.49–4.12], P=0.00). Pretreatment prostate-specific antigen (PSA) and PSA density were 8.23±3.60ng/mL and 0.19±0.09ng/ml2, respectively, and decreased significantly after HIFU by 55% to 4.00±2.42ng/mL (0.10–7.97ng/mL, P=0.00) and 0.10±0.07ng/mL2 (0.00–0.24ng/mL2, P=0.00).
**Conclusion:** Our preliminary results show that both 68Ga-PSMA11 and 68Ga-RM2 PET/MRI identified the dominant lesion for HIFU ablation pretreatment and were able to accurately verify response to treatment in 100%.
**Limitations:** This study is limited by the small number of participants.
**Ethics committee approval:** The ethics committee approval was obtained.
**Funding for this study:** This study is partially funded by GE Healthcare.
**Author Disclosures:**
Christian Kunder: Nothing to disclose
Geoffrey Sonn: Nothing to disclose
Pejman Ghanouni: Nothing to disclose
Carina Mari Aparici: Nothing to disclose
Valentina Ferri: Nothing to disclose
Guido Davidzon: Nothing to disclose
Bruce Daniel: Nothing to disclose
Andrei Iagaru: Nothing to disclose
Heying Duan: Nothing to disclose

### RPS 206-4

**A pilot study of 68Ga-PSMA11 and 68Ga-RM2 PET/MRI for biopsy guidance in patients with suspected prostate cancer**

*H. Duan*, V. Ferri, P. Ghanouni, B. Daniel, G. Davidzon, C. Mari Aparici, G. Sonn, C. Kunder, A. Iagaru; Stanford, CA/US
*(heying@stanford.edu)*

**Purpose:** Targeting of lesions seen on multiparametric MRI (mpMRI) improves prostate cancer (PC) detection at biopsy. However, 20-65% of highly suspicious lesions on MRI prove to be false positives (FP) at biopsy. We evaluated the potential utility of 68Ga-PSMA11 and 68Ga-RM2 PET/MRI for biopsy guidance in patients with suspected PC and prior negative biopsy or equivocal MRI.
**Methods or Background:** Ten men with suspected PC were prospectively enrolled to undergo 68Ga-PSMA11 and 68Ga-RM2 PET/MRI, including mpMRI. The prostate was divided into 12 segments (apex, mid, and base, lateral and medial, respectively, left and right) using PET/MRI data and MIM software. Maximum standardised uptake values (SUVmax) of suspected PC lesions and background for each segment were collected. Biopsies after PET/MRI included 1 core through each of the 12 segments and targeted sampling of any lesions seen on PET.
**Results or Findings:** PSA and PSA density were 10.77±6.27 ng/mL and 0.19±0.11 ng/mL2, respectively. mpMRI was negative in 5 patients, 4 showed PIRADS 4 and 1 PIRADS 5. 68Ga-PSMA11 identified 25 lesions of which 52% were verified PC and 68Ga-RM2 PET/MRI showed 26 lesions with PC verification in 50%. PET/MRI guided biopsy led to the additional finding of 3 clinically significant tumours and 2 GS 6 cancers. For 68Ga-PSMA11, mean SUVmax for true positives (TP) was slightly higher than FP, however not statistically significant whereas for 68Ga-RM2, SUVmax of TP PC lesions were significantly higher than FP (11.56 ± 9.11 [5.57 – 40.69] vs 7.93 ± 3.74 [3.73–18.21], P=0.007).
**Conclusion:** Our preliminary results show that 68Ga-PSMA11 and 68Ga-RM2 PET/MRI are feasible for biopsy guidance in suspected PC, and most importantly identified additional clinically significant cancers not seen on mpMRI.
**Limitations:** This study is limited by the small number of patients.
**Ethics committee approval:** The ethics committee approval was obtained.
**Funding for this study:** This study is partially funded by GE Healthcare.
**Author Disclosures:**
Christian Kunder: Nothing to disclose
Geoffrey Sonn: Nothing to disclose
Pejman Ghanouni: Nothing to disclose
Carina Mari Aparici: Nothing to disclose
Valentina Ferri: Nothing to disclose
Guido Davidzon: Nothing to disclose
Bruce Daniel: Nothing to disclose
Andrei Iagaru: Nothing to disclose
Heying Duan: Nothing to disclose

### RPS 206-5

**Hyperpolarised [1-13C]lactate production correlates with the percent gleason pattern 4 in human prostate cancer**

*N. Sushentsev*, M. McLean, A. Warren, F. A. Gallagher, T. Barrett; Cambridge/UK
*(ns784@medschl.cam.ac.uk)*

**Purpose:** To evaluate the ability of hyperpolarised [1-13C]pyruvate magnetic resonance imaging (HP 13C-MRI) to visualize biopsy-proven areas of prostate cancer (PCa) and correlate tumour [1-13C]lactate production with standard pathologic and imaging biomarkers of tumour aggressiveness.
**Methods or Background:** Patients with MR-visible biopsy-proven PCa scheduled for radical prostatectomy underwent 3T HP 13C-MRI, with tumour-derived signal-to-noise ratios (SNR) of pyruvate, lactate, and total carbon calculated in addition to [1-13C]pyruvate-to-[1-13C]lactate conversion rate (kPL). Multiparametric MRI (mpMRI) of the prostate was subsequently performed on the same magnet, with apparent diffusion coefficient (ADC) values calculated automatically and extracted from the same tumour regions. Whole-mount surgical sections were stained with haematoxylin and eosin, with tumour grade and per cent Gleason pattern 4 (%GP4) evaluated by an experienced genitourinary pathologist.
**Results or Findings:** The study included 10 patients with 15 lesions, of which 2, 11, and 2 harboured grade 1, 2, and 3 disease, respectively. 2/15 lesions were not reported prospectively on mpMRI and were detected retrospectively on HP 13C-MRI using whole-mount pathology as reference. Spearman's correlation analysis revealed the presence of strong correlations between lactate SNR and %GP4 (rs=0.65, P=0.03), lactate SNR and ADC (rs=-0.69, P=0.02), %GP4 and ADC (rs=-0.62, P=0.03), as well as total carbon SNR and kPL (rs=0.62, P=0.04).
**Conclusion:** HP 13C-MRI was superior to mpMRI for evaluating the true burden of disease in patients with multifocal PCa, with lesion-derived [1-13C]lactate production acting as a metabolic surrogate of tumour aggressiveness. Non-invasive assessment of %GP4 using HP 13C-MRI may be used clinically to improve the selection of suitable candidates for active surveillance, for which %GP4 is critical.
**Limitations:** Not applicable.
**Ethics committee approval:** NREC East of England, 16/EE/0205.
**Funding for this study:** This study was funded by the Prostate Cancer UK, Cancer Research UK.
**Author Disclosures:**
Nikita Sushentsev: Nothing to disclose
Mary McLean: Nothing to disclose
Tristan Barrett: Nothing to disclose
Anne Warren: Nothing to disclose
Ferdia Aidan Gallagher: Research/Grant Support: General Electric

### RPS 206-6

**Differential hyperpolarised [1-13C]Lactate labelling in benign and malignant prostate is driven by a complex interplay between perfusion, cellularity, and cell metabolism**

*N. Sushentsev*, M. McLean, A. Warren, T. Barrett, F. A. Gallagher; Cambridge/UK
*(ns784@medschl.cam.ac.uk)*

**Purpose:** To identify biological mechanisms underpinning differential hyperpolarised [1-13C]lactate labelling in prostate cancer (PCa) and the benign prostate (BP) in patients undergoing radical prostatectomy following hyperpolarised [1-13C]pyruvate magnetic resonance imaging (HP-13C-MRI).
**Methods or Background:** All patients underwent 3T HP-13C-MRI, with a signal-to-noise ratio (SNR) of lactate derived from areas of PCa and contralateral BP as derived from whole-mount histopathology (WMH). Multiparametric MRI of the prostate was subsequently performed in the same sitting, with apparent diffusion coefficient (ADC) and Ktrans values extracted from identical regions-of-interest. Matching WMH sections were used for immunohistochemical analysis of monocarboxylate transporters (MCT) 1 and 4. RNAscope was used to quantify mRNA expression of lactate dehydrogenase (LDH) subunits A and B, alongside pyruvate dehydrogenase E1 subunit alpha 1 (PDHA1).

**Results or Findings:** The study included 8 patients with 10 low-to-intermediate risk lesions. Lactate SNR, Ktrans, and WMP-derived cell density were significantly higher in PCa compared to BP (10.76 vs 1.66, 0.39 min-1 vs 0.11 min-1, and 3227.0 cells/mm2 vs 1944.0 cells/mm2; P<0.0001, 0.002, and 0.005, respectively), and ADC values were significantly lower in PCa compared to BP (922.4 10-6 mm2/s vs 1351.0 10-6 mm2/s; P=0.002). MCT1 and MCT4 did not differ between the two tissue types (P=0.796 and 0.684, respectively). Total LDH density and LDHA/PDHA1 mRNA expression ratio were, however, significantly higher in BP compared to PCa (8483.0 103 copies/mm2 vs 5389.0 103 copies/mm2 and 1.15 vs 0.68; P=0.004 and 0.03, respectively).
**Conclusion:** Lactate SNR was significantly higher in areas of low-to-intermediate risk PCa despite significant overexpression of glycolytic enzymes in BP. This may be explained by the increased perfusion/permeability and cellularity of tumour areas, leading to the increased hyperpolarised [1-13C]pyruvate delivery and [1-13C]lactate labelling.
**Limitations:** Not applicable.
**Ethics committee approval:** NREC East of England, 16/EE/0205.
**Funding for this study:** This study was funded by the Prostate Cancer UK, CRUK.
**Author Disclosures:**
Nikita Sushentsev: Nothing to disclose
Mary McLean: Nothing to disclose
Tristan Barrett: Nothing to disclose
Anne Warren: Nothing to disclose
Ferdia Aidan Gallagher: Research/Grant Support: General Electric

### RPS 206-7
**68Ga PSMA PET/CT initial Egyptian experience in newly diagnosed cancer prostate patients: how we report and stage our patients**
*N. Taha*, M. Shalaby, J. AbdAllah, Y. O. H. Omar; Cairo/EG
*(nohataha85@hotmail.com)*

**Purpose:** To evaluate the role of 68Ga PSMA PET/CT in the staging of patients with pathologically proved prostatic adenocarcinoma.
**Methods or Background:** Prostate cancer is the 2nd most common male malignancy and is the 4th leading cause of death from malignancies. The key issue for optimal patient management is accurate pretreatment staging. 50 patients with pathologically proven prostate cancer, without any treatment or intervention, were included in this study. The PET/CT findings were correlated with the pathology reports, Gleason score and PSA levels. The patients were evaluated for local staging (T stage), including prostatic lesions extra-prostatic extension and seminal vesicles invasion, the regional nodal staging (N stage), and distant metastatic spread (M stage), including distant nodal, osseous and visceral metastases.
**Results or Findings:** A significant statistical relationship was found between PSA level, Gleason score and the findings in PET/CT including the pathological grade of the prostatic lesions with their extensions, the regional nodal deposits and the distant metastatic deposits. 68Ga PSMA shows 94% sensitivity in detecting prostatic lesions and its sensitivity increased with the increase of the PSA level of the patient, also it shows a significant P-value in detecting extra-prostatic spread either regional or distant.
**Conclusion:** 68Ga PSMA PET/CT is a powerful staging tool showing high sensitivity and specificity in the staging of patients with recently diagnosed prostate cancer.
**Limitations:** Nothing significant.
**Ethics committee approval:** This study was ethically approved from the ethical committee at the Faculty of Medicine, Ain Shams University.
**Funding for this study:** No funding was received for this study.
**Author Disclosures:**
Mennatallah Shalaby: Nothing to disclose
Noha Taha: Nothing to disclose
Jilan AbdAllah: Nothing to disclose
Yehia Omar Hussein Omar: Nothing to disclose

### RPS 206-8
**Detection of prostatic cancer lymph nodes metastases using radiomics in 68 Ga-PSMA PET/CT**
*X. A. G. Ballesteros*, A. E. Mercado Sánchez, H. Solis Lara; Monterrey/MX
*(goba.xavier@gmail.com)*

**Purpose:** Prostate cancer is the third leading cause of death in men who die from malignant neoplasia, so new tools are being sought to objectively predict the probability of malignant infiltration of the lymph nodes for the adequate staging of the disease. Radiomics is the process of converting medical images into data allowing the extraction of quantitative characteristics. The aim of the study was the analysis of radiomic features of lymph nodes, using 68Ga-PSMA PET/CT, as a standard reference.
**Methods or Background:** The design was a retrospective, cross-sectional, analytical study, which was developed with a total of 41 patients with prostate cancer diagnosis randomly selected from a database of 253 patients. 3DSlicer software was used to obtain radiomic features. Sixteen nodes were segmented per patient (pelvis, retroperitoneum, mediastinal, axillary, and cervical). The nodes segmented were those with the highest SUVmax value in the positive studies and those with the highest short axis in the negative studies.
**Results or Findings:** Thirty-three variables of shape and textural analysis of radiomic tomographic features were found that allow differentiating between positive and negative lymph nodes for malignancy.
**Conclusion:** Radiomic analysis of lymph nodes in prostate cancer could detect the nodal metastatic disease even in lymph nodes of normal morphology and size.
**Limitations:** The limitations of our study were the small sample size and that it had a retrospective design, but it was performed by a blinded observer. Also, the PET/CT images were evaluated and segmented by a single observer, so the reproducibility of the results between different observers was not evaluated.
**Ethics committee approval:** This study was approved by the ethics in investigation committee with the number RA20-00009.
**Funding for this study:** This study was funded by the radiology department of "Dr. José Eleuterio González" University Hospital.
**Author Disclosures:**
Hugo Solis Lara: Nothing to disclose
Aurora Eloisa Mercado Sánchez: Nothing to disclose
Xavier Alejandro Gonzalez Ballesteros: Nothing to disclose

### RPS 206-9
**Combined use of 68Ga-PSMA-11 PET/CT and multiparametric MR imaging in patients with prostate cancer**
*M. Maksudov*, U. Khaydarov; Tashkent/UZ
*(muzmac@list.ru)*

**Purpose:** To present the results of gallium 68 (68Ga) prostate-specific membrane antigen (PSMA)-11 PET/CT and magnetic resonance (MR) imaging in patients with prostate cancer.
**Methods or Background:** Forty-five men who were scheduled for radical prostatectomy with pelvic lymph node dissection were recruited for this study. Multiparametric MR imaging (including diffusion-weighted imaging, T2-weighted imaging, and dynamic contrast material–enhanced imaging) and PET/CT data were correlated with results of final pathologic examination and pelvic nodal dissection to yield diagnostic accuracy. PET/CT with 68Ga-PSMA-11 was performed according to the whole-body protocol. Interpretation of images was carried out visually and quantitatively with the calculation of SUVmax. A mean dose of 3.8 mCi ± 0.6 (140.6 MBq ± 22.2) of 68Ga-PSMA-11 was administered. Whole-body images were acquired starting 60-90 minutes after injection by using a Philips Ingenuity TF PET/CT scanner. Metabolic parameters were compared by using a paired t-test and were correlated with clinical and histopathologic variables.
**Results or Findings:** High focal (24 cases) or diffuse (21 cases) 68Ga-PSMA-11 uptake was found in the prostate gland in all patients with primary prostate cancer. Whereas multiparametric MR imaging depicted PI-RADS (Prostate Imaging Reporting and Data System) 4 or 5 lesions in 36 patients and PI-RADS 3 lesions in five patients. Pathologic examination confirmed prostate cancer in all patients. In 18 patients, nodal metastases were additionally diagnosed.
**Conclusion:** 68Ga-PSMA-11 PET/CT has a high potential in the work-up of prostate cancer patients, including primary diagnosis and staging, while MR imaging provides detailed anatomic guidance. The combined use of both imaging methods provides valuable diagnostic information and may inform the need for pelvic node dissection.
**Limitations:** Limitations are not required.
**Ethics committee approval:** Approved by Ministry of Health of Uzbekistan
**Funding for this study:** No funding was received for this study.
**Author Disclosures:**
Muzaffar Maksudov: Nothing to disclose
Umar Khaydarov: Nothing to disclose

11:30-12:30 Channel 4

### Research Presentation Session: Abdominal Viscera

### RPS 301a

### Upper abdominal imaging: what's new?

**Moderators**
C. Stoupis; Männedorf/CH
V. Vilgrain; Clichy/FR

### RPS 301a-3

**Quantitative ultrasound assessment of hepatic steatosis with TAI and TSI in NAFLD patients**

*A. D. Rónaszéki*, B. K. Budai, R. Stollmayer, B. Csongrády, K. Hagymási, I. Kalina, G. Györi, P. Maurovich-Horvat, P. N. Kaposi-Novák; Budapest/HU
*(ronaszeki.aladar@gmail.com)*

**Purpose:** To assess the feasibility of tissue attenuation imaging (TAI) and tissue scatter distribution imaging (TSI) for ultrasound-based quantification of liver steatosis in nonalcoholic fatty liver disease (NAFLD).
**Methods or Background:** We prospectively enrolled 101 participants with suspected NAFLD. RS85A ultrasound scanner was used with TAI and TSI applications. Patients were divided into ≤5%, 5%-10%, and ≥10% groups based on magnetic resonance imaging proton density fat-fraction (MRI-PDFF). Spearman's correlation, logistic regression and area under the receiver operating characteristic curve (AUROC) were calculated for both TAI and TSI. Interobserver reliability was assessed with the intraclass correlation coefficient (ICC).
**Results or Findings:** Both TAI (ρ=0.78, P<0.001) and TSI (ρ=0.68, P<0.001) showed a significant correlation with MRI-PDFF. TAI overperformed TSI in the detection of both ≥5% MRI-PDFF (AUROC=0.89 vs. 0.87) and ≥10% (AUROC=0.93 vs. 0.86). In linear regression analysis, MRI-PDFF proved to be an independent predictor of both TAI (β=1.03; P<0.001) and TSI (β=50.9; P<0.001; while liver stiffness (β=-0.86; P<0.001) was negatively associated with TSI. Interobserver analysis showed excellent reproducibility with TAI (ICC=0.95) and moderate reproducibility with TSI (ICC=0.73).
**Conclusion:** TAI and TSI could be used successfully to measure the severity of hepatic steatosis in clinical practice.
**Limitations:** Limitations were single-centre study design, a small number of participants, mixed aetiology of NAFLD, selection bias, lack of histological samples.
**Ethics committee approval:** The present study has been approved by the regional and institutional committee of science and research ethics of our University (SE-RKEB 140/2020).
**Funding for this study:** The authors received no specific funding.
**Author Disclosures:**
Pál N. Kaposi-Novák: Nothing to disclose
Krisztina Hagymási: Nothing to disclose
Barbara Csongrády: Nothing to disclose
Aladár David Rónaszéki: Nothing to disclose
Pál Maurovich-Horvat: Nothing to disclose
Ildikó Kalina: Nothing to disclose
Róbert Stollmayer: Nothing to disclose
Gabriella Györi: Nothing to disclose
Bettina Katalin Budai: Nothing to disclose

### RPS 301a-4

**Spleen volume-based non-invasive criteria can identify compensated cirrhotic patients at high risk of decompensation: a multi-centre study**

*Q. Yu*, Y. Wang, S. Ju; Nanjing/CN
*(yuu.q@foxmail.com)*

**Purpose:** Non-invasive criteria to stratify liver decompensation risk remained an unmet need in patients with compensated cirrhosis. We aimed to develop and validate a non-invasive model based on spleen volume and simple serum markers to predict decompensation in compensated cirrhotic patients.
**Methods or Background:** 239 compensated cirrhotic patients were enrolled from three centres in China from January 2016 to June 2020 in this retrospective study. They were followed up until the occurrence of liver decompensation. Abdominal CT and laboratory workup were collected at baseline. Spleen volume was measured automatically using an in-house AI algorithm with Dice > 0.98 in spleen segmentation. We used these data to develop a spleen volume-based model to determine the risk of decompensation in the first centre comprising 66 patients (Training cohort). We validated it in the other two centres comprising 94 and 79 patients (Test cohorts 1 and 2). And compared with Child-Pugh score, MELD, and FIB-4.
**Results or Findings:** 58 patients (24%) developed liver decompensation over a median follow-up of 25 months. Using a combination of spleen volume, PLT, GGT, and Hb, we developed a Spleen-Alert model (Model score > 2.14) to identify patients with compensated cirrhosis at risk of liver decompensation. HR for decompensation in patients with high risk was 13.4 in training and 6.1 and 12.2 in test, respectively. The Spleen-Alert model has good performance to predict high-risk compensated patients at risk of liver decompensation (C-indexes of 0.82 in training and 0.82, 0.77 in two test cohorts), outperforming traditional non-invasive tests (C- indexes from 0.51 to 0.74).
**Conclusion:** Spleen-Alert model, as a simple and non-invasive criterion, showed considerable performance in stratifying the individual risk of liver decompensation in patients with compensated cirrhosis.
**Limitations:** No limitations were identified.
**Ethics committee approval:** The ethics committee approved this study.
**Funding for this study:** Not applicable.
**Author Disclosures:**
Qian Yu: Nothing to disclose
Shenghong Ju: Nothing to disclose
Yuancheng Wang: Nothing to disclose

### RPS 301a-5

**Differential diagnosis of adrenal adenomas and metastases using spectral parameters in dual-layer detector spectral CT**

*W. Lei-Di*[1], P. Han[2], H. Meng-Ting[2], Y. Hong-Li[2], W. Lin-Xia[2], X. Yue[2], Y. Ming[2], X. Zhang[3]; [1]Zhongshan/CN, [2]Wuhan/CN, [3]Shanghai/CN
*(wuleidi1994@163.com)*

**Purpose:** To assess the diagnostic value of spectral parameters in differentiating adrenal adenomas from metastases based on dual-layer detector spectral CT (DLCT).
**Methods or Background:** One hundred patients with 64 adrenal adenomas and 36 metastases were included eventually. Several spectral parameters of tumours such as the CT values of virtual non-contrast images (CTVNC), slopes of spectral HU curves (s-SHC), and iodine-to-CTVNC ratios were measured on spectral based images in each phase. Receiver operating characteristic (ROC) curves were used to compare the diagnostic values of combined or independent spectral parameters between adenomas and metastases.
**Results or Findings:** In the venous phase, all spectral parameters were significantly different between adenomas and metastases. Combined spectral parameters showed a better diagnostic performance in the venous phase compared with the arterial or delayed phase. In the venous phase, higher AUC was obtained for iodine-to-CTVNC value compared with the other spectral parameters in the differential diagnosis of adenomas and metastases, with diagnostic sensitivity and specificity of 90.5% and 73.8%, respectively. Higher AUC was also obtained for CTVNC value compared with the other spectral parameters in the differential diagnosis of lipid-rich adenomas and metastases, with diagnostic sensitivity and specificity of 90.9% and 81.8%, respectively. In the differential diagnosis of lipid-poor adenomas and metastases, the AUC for s-SHC value was higher than those of other parameters, with diagnostic sensitivity and specificity of 96.6% and 75.0%, respectively.
**Conclusion:** On DLCT images, spectral parameters in the venous phase such as iodine-to-CTVNC, CTVNC or s-SHC values have great clinical value in differential diagnosis of adrenal adenomas and metastases.
**Limitations:** This was a single-centre clinical trial with a relatively small sample and the metastasis group was not pathologically confirmed.
**Ethics committee approval:** This study was approved by IRB.
**Funding for this study:** There was no funding of this study.
**Author Disclosures:**
Wu Lin-Xia: Nothing to disclose
Xiaofei Yue: Nothing to disclose
Huang Meng-Ting: Nothing to disclose
Wu Lei-Di: Nothing to disclose
Yang Ming: Nothing to disclose
Ping Han: Nothing to disclose
Yang Hong-Li: Nothing to disclose
Xiaohui Zhang: Nothing to disclose

### RPS 301a-6

**Adrenal morphology as an indicator for long-term disease control in adults with classic congenital adrenal hyperplasia**

*H. J. Lee*, T. M. Kim, J. H. Kim, H. Chang, M. H. Choi, J. Y. Cho, S. Y. Kim; Seoul/KR
*(hjrosetta@gmail.com)*

**Purpose:** Monitoring adults with classical congenital adrenal hyperplasia (CAH) is challenging due to variability in clinical and laboratory settings. Moreover, guidelines for adrenal imaging in CAH are not yet available. We evaluated the relationship between adrenal morphology and disease control status in classical CAH.
**Methods or Background:** This retrospective, cross-sectional study included 90 adult CAH patients and age and sex-matched healthy controls (n=270). Adrenal volume, width, and tumour presence were assessed using abdominal computed tomography. Correlations of adrenal volume and width with hormonal status were evaluated. The diagnostic performance of adrenal volume and width of patients with CAH for identifying disease control was assessed.
**Results or Findings:** Adrenal morphology of CAH patients showed hypertrophy (45.6%), normal size (42.2%), and hypotrophy (12.2%). Adrenal tumours were detected in 12 patients (13.3%). The adrenal volume and width of CAH patients were significantly larger than that in the control group (18.2±12.2 vs. 7.1±2.0cc, 4.7±1.9 vs. 3.3±0.5mm, P<0.001 for both). 17α-hydroxyprogesterone (17-OHP) and androstenedione were the highest in patients with adrenal hypertrophy, followed by normal adrenal gland and adrenal hypotrophy (P<0.05 for both). The adrenal volume and width correlated positively with adrenocorticotropic hormone, 17-OHP, 11β-hydroxytestosterone, pregnenolone sulfate, and dehydroepiandrosterone sulfate in both male and female patients (r = 0.33–0.95, P<0.05 for all). We obtained the optimal cutoff values of 10.7cc and 4mm for classifying well-controlled patients using adrenal volume and width, respectively (AUC 0.82–0.88, P<0.001 for both).
**Conclusion:** We may use both adrenal volume and width as reliable quantitative parameters for monitoring patients with classical CAH.
**Limitations:** Measuring the adrenal volume is time-consuming and laborious compared with measuring the width. To apply in clinical practice, automatic or semi-automatic volumetric assessment of the adrenal gland is warranted.
**Ethics committee approval:** IRB approval.
**Funding for this study:** Not applicable.
**Author Disclosures:**
Hanna Chang: Nothing to disclose
Jeong Yeon Cho: Nothing to disclose
Hyo Jeong Lee: Nothing to disclose
Sang Youn Kim: Nothing to disclose
Jung Hee Kim: Nothing to disclose
Taek Min Kim: Nothing to disclose
Man Ho Choi: Nothing to disclose

### RPS 301a-7

**Abdominal ischaemia and haemorrhage in COVID-19 patients: uncommon but potentially life-treating complications**

*A. Bonanomi*, P. A. Bonaffini, P. N. Franco, C. Valle, P. Marra, A. Falanga, S. Sironi; Milan/IT
*(a.bonanomi10@campus.unimib.it)*

**Purpose:** To report ischaemic and haemorrhagic abdominal complications in COVID-19 patients. To correlate these complications with lung involvement, laboratory tests, comorbidities and eventual anticoagulant treatment.
**Methods or Background:** Thirty COVID-19 patients underwent an abdomen CECT for not lung-related clinical symptoms (i.e. abdominal pain) between March 10th and April 26th 2020, were retrospectively evaluated. Ischaemic and haemorrhagic complications were recorded. For lung involvement, a parenchymal stage (early, progressive, peak, absorption) was assigned. Blood coagulation values, eventual anticoagulant therapy, comorbidities and eventual presence of PE were assessed.
**Results or Findings:** Ischaemic complications had been demonstrated in 10 patients: 6/10 small bowel ischaemia (thickened, poorly-enhanced intestinal wall and/or pneumatosis intestinalis) with 1 concomitant small bowel obstruction and 1 perforation; 4/10 ischaemic colitis (layered wall and submucosal oedema). Main mesenteric vessels were patent but 1 case with superior mesenteric vein thrombosis. Two ischaemia cases also presented concurrent splenic infarctions. Bleeding complications were found in 20 patients: spontaneous haematomas in soft tissues in 15, retroperitoneal hematomas in 2 and gastro-intestinal bleeding in 3. Ten presented foci of active bleeding. Platelet and lymphocyte counts were in their normal range. D-Dimer levels demonstrated a significant difference between the two groups (p=0.005), being higher in ischaemic cases. Most of the patients had severe lung disease (45% peak, 29% absorption). Only 2 patients had evidence of PE. 22 patients (hospitalised) were under anticoagulant treatment.
**Conclusion:** Ischaemic and haemorrhagic abdominal complications may occur in COVID-19 patients, particularly during extended lung disease. CT plays a key role in the diagnosis of these potentially life-treating conditions.
**Limitations:** Retrospective nature of the study. Small population study.
**Ethics committee approval:** Not applicable.
**Funding for this study:** Not applicable.
**Author Disclosures:**
Anna Falanga: Nothing to disclose
Alice Bonanomi: Nothing to disclose
Clarissa Valle: Nothing to disclose
Paolo Marra: Nothing to disclose
Pietro Andrea Bonaffini: Nothing to disclose
Paolo Niccolò Franco: Nothing to disclose
Sandro Sironi: Nothing to disclose

**13:00-14:00** **Channel 4**

### Research Presentation Session: Breast

### RPS 402

### Updates in the evaluation of response to neoadjuvant therapy

**Moderator**
H. Preibsch; Tübingen/DE
**Author Disclosures:**
Heike Preibsch: Research Grant/Support: restricted research fund, GE healthcare

### RPS 402-2

**Radiomic changes after the first cycle using DISCO DCE-MRI for early predicting tumour response to neoadjuvant chemotherapy for breast cancer**

L. Guo, S. Du, *L. Zhang*; Shenyang/CN

**Purpose:** To investigate the value of radiomics-based tumour heterogeneity changes after one cycle of neoadjuvant chemotherapy (NAC) using a high spatiotemporal resolution DCE-MRI for early prediction of pathological complete response (pCR) in patients with breast cancer.
**Methods or Background:** A total of 140 patients (training: test=7:3) with breast cancer underwent Differential Subsampling with Cartesian Ordering (DISCO) DCE-MRI before (t0) and after one cycle (t1) of NAC. Radiomic features were extracted from postcontrast early (CEe), peak (CEp) and delay (CEd) CE-MRI phases, respectively. Feature changes (tΔ) were computed for each phase. For training, the strongest features that associated with pCR were selected. Logistic regression classifiers based on DCE-MRI at t0, t1, tΔ were constructed for differentiating pCR patients. The performance under different contrast phases was evaluated and compared. Clinicopathological information and the optimal imaging classifier were fused to enhance the predictive performance.
**Results or Findings:** Imaging classifiers using radiomic feature changes (tΔ) achieved superior performance in both training and test cohort for CEe (AUC=0.840/0.825), CEp (AUC=0.772/0.744) and CEd (AUC=0.757/0.728) compared with DCE-MRI at t0 (CEe: AUC=0.725/0.689; CEp: AUC=0.609/0.572; CEd: AUC=0.678/0.611), with a significant difference of NRI and IDI (all p<0.05 in the training cohort). For DCE-MRI at t0 and tΔ, the CEe was the optimal contrast phase for pCR classification. After adding receptor status to CEe at tΔ, the classifier showed the highest AUC of 0.828 in the testing.
**Conclusion:** Using DISCO DCE-MRI, changes in radiomic features after one cycle of NAC, that reflect tumour heterogeneity changes could provide a non-invasive approach for early prediction of breast cancer response regardless of knowledge of the receptor status.
**Limitations:** Single-centre research; no voxel-level analysis; no biological explanation.
**Ethics committee approval:** Approved by the Ethics Committee of First Affiliated Hospital of China Medical University
**Funding for this study:** Funding was received from the National Scientific Foundation of China (81971695).
**Author Disclosures:**
Liangcun Guo: Nothing to disclose
Siyao Du: Nothing to disclose
Lina Zhang: Nothing to disclose

### RPS 402-3

**Quantitative intratumoural habitats analysis of triple-negative breast cancer treated with combination talimogene laherparepvec (TVEC) neoadjuvant immunotherapy and neoadjuvant chemotherapy (NAI/NAC)**

*R. J. Weinfurtner*, N. Raghunand, O. Stringfield, M. Abdalah, B. Niell, M. C. Lee, H. Han, B. Czerniecki, H. Soliman; Tampa, FL/US
*(Tigerphage@yahoo.com)*

**Purpose:** To quantitatively evaluate perfusion-based intratumoural habitats on dynamic contrast-enhanced (DCE) breast MRI to predict triple-negative breast cancer (TNBC) NAI/NAC response.
**Methods or Background:** TVEC is a modified oncolytic herpes simplex 1 virus. Subjects with TNBC in this phase II trial underwent ultrasound-guided intratumoral TVEC injections followed by neoadjuvant chemotherapy prior to surgery. Baseline and post-treatment breast MRIs were evaluated for partial vs complete response (mCR). MRI quantitative analysis was performed on dynamic contrast-enhanced T1-weighted images with voxels assigned 8 habitats based on two criteria: high (H) or low (L) maximum contrast enhancement per Otsu algorithm, and the sequentially numbered dynamic sequence of maximum enhancement (H1-4, L1-4). Then, the % habitat makeup (%HM) of tumour and whole breasts (%HM of habitat X=habitat X

voxels/total voxels in the segmented volume) were evaluated and correlated with pathologic response (PR). Statistical analyses were performed using paired and unpaired t-tests, with p<0.05 considered statistically significant.
**Results or Findings:** Twenty patients were included in the study, and 11 achieved pathologic complete response (pCR). Prediction of pCR with mCR yielded accuracy of 65% (sensitivity 66.7%, specificity of 63.6%, PPV 60% and NPV 70%). Pre-NAI/NAC tumour %HM for each habitat differed significantly from whole breasts (p=0.001 or less). The %HM of habitat H1 (early phase, high enhancement) decreased the most after treatment (-18%, p=0.0004) followed by H2 and H3 (-14% and -4%, respectively). Conversely, %HM H4 (late phase, high enhancement) increased (12%, p=0.031). The H1-3 combination decreased 34%, and this was more pronounced in patients with pCR (-44% vs -22%, p=0.036).
**Conclusion:** In patients undergoing TVEC NAI/NAC treatment for TNBC, a decrease in %HM of early and mid-phase high enhancement habitats correlates with pathologic response.
**Limitations:** Sample size.
**Ethics committee approval:** IRB-approved.
**Funding for this study:** Funding was received from the Moffitt Cancer Center.
**Author Disclosures:**
Robert Jared Weinfurtner: Nothing to disclose
M Catherine Lee: Nothing to disclose
Mahmoud Abdalah: Nothing to disclose
Hatem Soliman: Nothing to disclose
Brian Czerniecki: Nothing to disclose
Hyo Han: Nothing to disclose
Olya Stringfield: Nothing to disclose
Bethany Niell: Nothing to disclose
Natarajan Raghunand: Nothing to disclose

### RPS 402-4

**Can machine-learning models using baseline breast ultrasound radiomics and clinical features aid in predicting response to neoadjuvant chemotherapy?**

*P. Kapetas*, P. Clauser, T. H. Helbich, R-I. Milos, P. A. Baltzer; Vienna/AT
*(panagiotis.kapetas@meduniwien.ac.at)*

**Purpose:** To evaluate whether machine-learning (ML) models using selected clinical and radiomic features from pre-therapeutic, B-mode breast ultrasound (US) can predict response to neoadjuvant chemotherapy (NAC).
**Methods or Background:** 253 patients with invasive breast cancer undergoing NAC were included. One B-mode US image of each tumour from the baseline examination was selected. Tumours were manually segmented (without-ROI1 and with inclusion of surrounding tissue-ROI2) and overall 851 radiomic features were extracted using dedicated software. Two analyses were performed: first, principal component analysis was used for feature reduction and a multilayer perceptron neural network was trained using remaining radiomic and selected clinical features. 1/3 of the cases were used as an external validation set. Second, a decision tree using the exhaustive chi-squared automatic interaction detection method with 10-fold cross-validation was constructed. Postoperative histology was the reference standard. Diagnostic performance was evaluated using the area under the ROC curve (AUC).
**Results or Findings:** 104 patients (41.1%) achieved pathological complete response. In the first model, the combination of radiomic features, age and molecular subtype showed the highest AUC, both for ROI1 (0.715; 95%CI: 0.606-0.809) and for ROI2 (0.709; 95%CI: 0.599-0.803; p>0.05) in the validation set. The model resulted in 66.3% correct classifications considering ROI1 and 55.4% using ROI2 in the validation set. On the other hand, decision trees based on molecular subtype and 5-6 radiomic features showed AUCs of 0.852 (95%CI: 0.802-0.893) for ROI1 and 0.896 (95%CI: 0.852-0.931) for ROI2 (p>0.05). The corresponding correct classifications were 83.4% and 82.6%.
**Conclusion:** ML models based on radiomic features from baseline B-mode breast US and simple clinical features have the potential to predict response to NAC.
**Limitations:** This is a retrospective, monocentric study with a limited patient number.
**Ethics committee approval:** This was an IRB-approved retrospective study.
**Funding for this study:** Not applicable.
**Author Disclosures:**
Pascal A.T. Baltzer: Nothing to disclose
Thomas H. Helbich: Nothing to disclose
Ruxandra-Iulia Milos: Nothing to disclose
Panagiotis Kapetas: Nothing to disclose
Paola Clauser: Nothing to disclose

### RPS 402-5

**Correlation between MRI morphological-response patterns and histopathological tumour regression after neoadjuvant endocrine therapy in locally advanced breast cancer: a randomised phase-II trial**

*J. R. D. Reis*, O. Thomas, M. Lyngra, H. Schandiz, J. Boavida, K-I. Gjesdal, T. Sauer, J. Geisler, J. T. Geitung; Lørenskog/NO

**Purpose:** To correlate MRI morphological-response patterns with histopathological tumour regression grading system based on tumour cellularity in locally advanced breast cancer (LABC) treated neoadjuvant with third-generation aromatase inhibitors.
**Methods or Background:** Fifty postmenopausal patients with ER-positive/HER-2 negative LABC treated with neoadjuvant letrozole and exemestane were given sequentially in an intra-patient cross-over regimen for at least 4 months with MRI response monitoring at baseline as well as after at least 2 and 4 months on treatment. The MRI morphological response pattern was classified into 6 categories: 0/complete imaging response, I/concentric shrinkage, II/fragmentation, III/diffuse, IV/stable and V/progressive. Histopathological tumour regression was assessed based on the recommendations from The Royal College of Pathologists regarding tumour cellularity.
**Results or Findings:** Following 2 and 4 months with therapy, the most common MRI pattern was pattern II (24/50 and 21/50, respectively). After 4 months of therapy, the most common histopathological tumour regression grade was grade 3 (21/50). After 4 months an increasing correlation is observed between MRI patterns and histopathology. The overall correlation, between the largest tumour diameter obtained from MRI and histopathology, was moderate and positive (r=0.50, P-value=2e-04). Among them, the correlation was highest in type IV (r=0.53).
**Conclusion:** The type II MRI pattern “fragmentation” was more frequent in the histopathological responder group; and types I and IV in the non-responder group. Type II pattern showed the best endocrine responsiveness and a relatively moderate correlation between sizes obtained from MRI and histology, whereas type IV pattern indicated endocrine resistance but the strongest correlation between MRI and histology.
**Limitations:** A single-centre study.
**Ethics committee approval:** The NEOLETEXE trial was registered on March 23rd, 2015 and approved by the regional ethical committee of the South-Eastern Health Region in Norway (registration number: REK-SØ-84-2015).
**Funding for this study:** Funding was received from the Bodil and Magne`s Cancer Research Fund.
**Author Disclosures:**
Marianne Lyngra: Nothing to disclose
Joana Roque Dos Reis: Nothing to disclose
Joao Boavida: Nothing to disclose
Kjell-Inge Gjesdal: Nothing to disclose
Jonn Terje Geitung: Nothing to disclose
Hossein Schandiz: Nothing to disclose
Torill Sauer: Nothing to disclose
Owen Thomas: Nothing to disclose
Jürgen Geisler: Nothing to disclose

### RPS 402-6

**Breast cancer surgical treatment prediction after neoadjuvant chemotherapy: the main role of magnetic resonance and digital mammography**

*G. Cimino*, M. Conti, A. Franco, A. De Filippis, C. Esposito, E. Bufi, D. Terribile, P. Belli, R. Manfredi; Rome/IT

**Purpose:** To investigate the influence of radiological features measured on magnetic resonance imaging (MRI), digital mammography (DM), in addition to clinical factors on surgeons' choice between breast conservative surgery (BCS), oncoplastic surgery (OPS) or conservative mastectomy (CM), in patients treated with neoadjuvant chemotherapy (NACT).
**Methods or Background:** Preoperative MRI and DM of 255 women who underwent BCS (118 patients), OPS (50 patients) or CM (87 patients) after NACT in 2016-2021 were retrospectively reviewed. Preoperative radiological features were analysed including the extent of microcalcifications, tumour volume/breast volume ratio (TVBVR) on DM and MRI, multifocality/multicentricity, in addition to histotype, grading and clinical features (menopause, BRCA mutations, ptosis, previous breast surgery). All parameters were correlated with final surgeons’ choice.
**Results or Findings:** On univariate analysis BCS has proved to be the surgeons’ choice in patients with a unifocal tumour, the extent of microcalcifications <54,9 mm, TVBVR pre-NACT <0,42 % on DM and <3,53 % on MRI. OPS showed a significant correlation with multifocal tumour, the extent of microcalcifications 55-79,9 mm, TVBVR ratio pre-NACT 0,4-1,38 % on DM and 3,54-10,27 % on MRI. CM was correlated with multicentric tumours, the extent of microcalcifications >80 mm, TVBVR pre-NACT >1,38 % on DM and >10,27 % on MRI. On multivariate analysis these results were confirmed and were used to create a specific score to define which type of surgery to suggest to patients. ROC curves were produced for each type of surgery (AUC BCS=0,840, OPS=0,751 and MC=0.871).
**Conclusion:** Radiological features measured on DM and MRI play a fundamental role in the surgeons' choice between BCS, OPS or CM in patients treated with NACT.
**Limitations:** No limitations were identified.
**Ethics committee approval:** Not applicable.
**Funding for this study:** Not applicable.
**Author Disclosures:**
Anotnio Franco: Nothing to disclose
Alessandra De Filippis: Nothing to disclose
Carlo Esposito: Nothing to disclose
Marco Conti: Nothing to disclose
Giovanni Cimino: Nothing to disclose
Riccardo Manfredi: Nothing to disclose
Enida Bufi: Nothing to disclose
Daniela Terribile: Nothing to disclose
Paolo Belli: Nothing to disclose

### RPS 402-7

**ADCdiff, ADCmax, ADCmin and ADCmean in breast carcinoma before NACT and in early tumour response assessment on DWI-MRI**

*M. Nadrljanski*, I. Krusac, V. Urban, M. Mihajlovic; Belgrade/RS

**Purpose:** Manually defined ROIs for computation of ADC-values may be technically challenging, prone to sampling errors and operator-dependent. Different ADC parameters (ADCdiff, ADCmax, ADCmin, ADCmean) may provide a more objective approach. ADCdiff corresponding to the level of intratumoral heterogeneity points more objectively to invasive components and may have prognostic significance.
**Methods or Background:** Retrospective analysis of (N=34) consecutive patients undergoing neoadjuvant chemotherapy (NACT) on 1.5T breast DWI-MRI (b50, b850 [s/mm2]), for: a.) Pre-treatment and b.) Early response assessment (after 2nd cycle of NACT) for morpho-dynamic and DWI parameters: ADCdiff, ADCmax, ADCmin, ADCmean, including the analysis of subgroups of patients with pCR (n1=11) and non-pCR (n2=23) following NACT.
**Results or Findings:** ADCdiff and ADCmean are significantly different between n1 and n2 on pre-treatment exam [10-6 x mm2/s]: (403.64+/-122.05 vs. 285.56+/-69.47; p=.008); (808.36+/-60.50 vs. 1015.69+/-78.97; p<.00001) and after the 2nd cycle: (407.36+/-92.00 vs. 261.78+/-60.69; p=.001); (963.27+/-67.51 vs. 1059.65+/-73.63; p=.002). In n1, ADCdiff is not different before and after 2nd cycle of NACT: (403.64+/-122.05 vs. 407.36+/-92.00; p=.096). In n2, ADCdiff is significantly different after 2nd cycle of NACT: (285.56+/-69.47 vs. 261.78+/-60.69; p=.048).
**Conclusion:** There are complex factors determining the response to NACT. There is no standardised ADC cut-off value determining the response. ADCdiff with indirect interpretation of microvascular changes and the rate of growth may also provide information regarding the response to NACT, as higher ADCdiff values in pCR group on pre-treatment MRI and early response assessment exam may contribute to better response and more adequate assessment.
**Limitations:** A relatively small number of patients in a single-centre retrospective study.
**Ethics committee approval:** Referent board approval was obtained for retrospective analysis.
**Funding for this study:** No funding was provided for this study.
**Author Disclosures:**
Vladimir Urban: Nothing to disclose
Iva Krusac: Nothing to disclose
Mirjan Nadrljanski: Nothing to disclose
Marko Mihajlovic: Nothing to disclose

### RPS 402-8

**Mammographic density to predict response to neoadjuvant chemotherapy for breast cancer**

C. Depretto, *C. De Berardinis*, G. Della Pepa, S. Di Cosimo, R. Miceli, G. Pruneri, S. Folli, G. P. Scaperrotta; Milan/IT
*(cla.deberardinis@gmail.com)*

**Purpose:** We aimed to investigate the clinical value of mammographic density (MD) in a large consecutive cohort of breast cancer (BC) patients treated with neoadjuvant chemotherapy (NAC).
**Methods or Background:** We prospectively collected data on all NAC treated BC patients in our Institute from May 2009 to April 2020. We evaluated MD on mammograms as categorised by the Breast Imaging-Reporting and Data System (BI-RADS), according to the following categories: A (almost entirely fat), B (scattered areas of fibroglandular density), C (heterogeneously dense), D (extremely dense). Multivariable logistic regression was used to assess the odds ratios (OR) for pathological complete response (pCR), comparing BI-RADS categories with adjustment for patient age and BMI, and pre-NAC tumour characteristics.
**Results or Findings:** We analysed 442 patients; 120 (27.1%) attained a pCR following NAC. At multivariable analysis, cases classified as BI-RADS C showed an increased likelihood of pCR as compared to A (OR= 2.79), B (OR= 1.70), and D (OR= 1.47) independently of age, BMI (OR underweight vs normal= 3.76), clinical N and T (OR T1/Tx vs T4= 3.87), molecular subtype (HER2 vs luminal= 10.74; triple-negative vs luminal= 8.19). In subgroup analyses, the strongest association of MD with pCR was observed in triple-negative (ORs of B, C, and D versus A: 1.85, 2.49, and 1.55) and HER2 BC cases (ORs 2.70, 3.23, and 1.16). No significant differential effect of MD with respect to pCR was observed in luminal (ORs of B, C, and D versus A: 0.88, 2.01, and 1.58).
**Conclusion:** Patients with dense breasts are more likely to attain a pCR after NAC at net of other current clinical and pathological predictive factors.
**Limitations:** The single centre design study.
**Ethics committee approval:** The study was approved by the Institutional Review Board. Informed consent was obtained.
**Funding for this study:** No funding was received for this study.
**Author Disclosures:**
Gianmarco Della Pepa: Nothing to disclose
Giancarlo Pruneri: Nothing to disclose
Rosalba Miceli: Nothing to disclose
Claudia De Berardinis: Nothing to disclose
Gianfranco Paride Scaperrotta: Nothing to disclose
Catherine Depretto: Nothing to disclose
Serena Di Cosimo: Nothing to disclose
Secondo Folli: Nothing to disclose

**15:00-16:00** **Channel 4**

### Research Presentation Session: Abdominal Viscera

### RPS 501a

### New insights in pancreatic imaging

**Moderators**
C. Matos; Lisbon/PT
V. Vilgrain; Clichy/FR

### RPS 501a-3

**Characterising potential radiological errors of missed pancreatic ductal adenocarcinoma on pre-diagnostic cross-sectional imaging**

*M. M. L. Engels*[1], S. Hoogenboom[2], A. Chuprin[1], J. van Hooft[3], J. Legout[1], M. Wallace[1], C. Bolan[1]; [1]Jacksonville, FL/US, [2]Amsterdam/NL, [3]Leiden/NL

**Purpose:** To analyse the radiological errors associated with pre-diagnostic pancreatic ductal adenocarcinoma (PDAC).
**Methods or Background:** This is a single-centre, retrospective, case-control study of 87 pre-diagnostic cross-sectional imaging studies (CT (N=60) and MRI (N=27)) of patients diagnosed with PDAC <3 years later, and 338 matched control studies (CT (N=235), MRI (N=103)). Two board-certified radiologists, blinded to case/control status, independently reassessed each exam. One radiologist was then unblinded and utilised the 2016 revised RADPEER criteria to grade radiological errors and further classify them according to the classification proposed by Kim and Mansfield (AJR, 2014).
**Results or Findings:** Pancreatic masses were retrospectively suspected in around 57.0% of CT and MRI cases, and 1.6% of controls. Respectively, in comparison to available original CT and MRI reports, the reviewers concurred with the original report in 43.8% and 26.8% (RADPEER 1), with understandable discrepancies in 29.2% and 33.3% (RADPEER 2) and unacceptable discrepancies in 27.0% and 38.1% (RADPEER 3). The 'under-reading' error was by far the most common, occurring in 57.2% of CT and 45.5% of MRI cases. Errors of 'satisfaction of search' occurred in 31.9% of MRI cases and 10.7% of CT cases, and 'faulty reasoning' in 17.9% of CT cases and 4.5% of MRI cases.
**Conclusion:** Unidentified, early findings of PDAC that should not have been overlooked occur up to 27.0% of CT and 38.1% of MRI cases. Given the detrimental effects of a delayed diagnosis in this deadly disease, radiologists must be aware of these findings and be trained to prevent the most commonly occurring errors.
**Limitations:** Retrospective design and selection bias.
**Ethics committee approval:** Mayo Clinic IRB approval #18-002403.
**Funding for this study:** Funding received by Champions for Hope / Funk-Zitiello foundation.
**Author Disclosures:**
Sanne Hoogenboom: Nothing to disclose
Jeanin van Hooft: Speaker: Abbvie Research/Grant Support: Cook Medical Consultant: Boston Scientific, Medtronics and Cook Medical

Anthony Chuprin: Nothing to disclose
Candice Bolan: Nothing to disclose
Jordan Legout: Nothing to disclose
Megan Maria Lynn Engels: Nothing to disclose
Michael Wallace: Shareholder: Virgo Inc. Other: Synergy Pharmaceuticals, Boston Scientific, Cook Medical. Research/Grant Support: Fujifilm, Boston Scientific, Olympus, Medtronic, Cosmo Pharmaceuticals Consultant: Verily Inc., Virgo Inc, Cosmo Pharmaceuticals, Medtronic, GI Supply. On behalf of Mayo Clinic for: GI Supply (2018), Endokey, Endostart, Boston Scientific, Microtek.

### RPS 501a-5

**Analysis of CT findings in patients with different serum pancreatic amylase trends after major pancreatic resections**

*F. Mambrin*, M. Bariani, G. Zamboni, M. C. Ambrosetti, E. Bannone, G. Marchegiani, G. Mansueto; Verona/IT

**Purpose:** The ISGPS has recently defined that a diagnosis of post-pancreatectomy acute pancreatitis (PPAP) requires: sustained postoperative hyperamylasemia, clinically relevant features and radiological alterations consistent with PPAP. Our purpose is to analyse the CT features that correlate with early postoperative serum pancreatic amylase (spAMY) trends after partial pancreatic resections.
**Methods or Background:** Patients who underwent major pancreatic resections between 2016 and 2021, and had a contrast-enhanced postoperative MDCT between POD3 and POD15 were included. Patients were divided into 3 groups based on their spAMY patterns: spAMY1: values always within/below the reference range or with a single increase in spAMY>upper limit of normal at any POD; spAMY2: sustained increase on POD0+1; spAMY3: sustained increase in spAMY including POD1+2. Two readers in consensus, blinded to spAMY trends, analysed the exams and logged arterial enhancement homogeneity, main pancreatic duct (MPD) diameter, peripancreatic oedema, and fluid collections.
**Results or Findings:** 473 patients were included (288 males, 185 females, mean age 63 years). Inhomogeneous arterial enhancement was more common in spAMY3 patients (49%) than in spAMY2 or spAMY1 patients (both 40%, $p<0.001$). MPD was larger in spAMY1 (mean calibre 2.40±1.68mm; $p<0.001$). Peripancreatic fat stranding ($p=0.005$) and fluid collections, especially peripancreatic ($p<0.001$), were significantly more common in spAMY3.
**Conclusion:** A clear definition of the CT features of PPAP is fundamental for the radiological description of this entity, which will lead to its accurate and early diagnosis and thus to better treatment.
**Limitations:** SpAMY evaluation was conducted within a single centre, with homogeneous surgical approaches and postoperative management.
**Ethics committee approval:** Approved by the Ethics Committee of the Provinces of Verona and Rovigo; approval number: 1101CESC.
**Funding for this study:** No financial help was obtained for this study.
**Author Disclosures:**
Elisa Bannone: Nothing to disclose
Giulia Zamboni: Nothing to disclose
Matilde Bariani: Nothing to disclose
Maria Chiara Ambrosetti: Nothing to disclose
Giancarlo Mansueto: Nothing to disclose
Francesca Mambrin: Nothing to disclose
Giovanni Marchegiani: Nothing to disclose

### RPS 501a-7

**Gastroenteropancreatic neuroendocrine neoplasms (GEP-NENs): a radiomic model to predict tumour grade**

*G. Chiti*, F. Flammia, G. Grazzini, P. Tortoli, S. Bettarini, V. Miele; Florence/IT

**Purpose:** This single-centre retrospective study aims to assess whether contrast-enhanced computed tomography (CECT) radiomics analysis is predictive of gastroenteropancreatic neuroendocrine neoplasms (GEP-NENs) grade based on the 2019 World Health Organization (WHO) classification and to establish a tumour grade (G) prediction model.
**Methods or Background:** Preoperative CECT images of 72 patients with GEP-NENs were retrospectively reviewed and divided into two groups (G1-G2 in class 0, G3-NEC in class1). A total of 107 radiomics features were extracted from each neoplasm ROI in CECT arterial phases acquisitions with a 3DSlicer. The Mann-Whitney test and the LASSO regression method were performed in R for feature selection and reduction to build the radiomic-based predictive model. The model was developed for a training cohort (75% of the total) and validated on the independent validation cohort (25%). ROC curves and AUC values were generated on training and validation cohorts.
**Results or Findings:** 40 features were found to be significant in class distinction. From the LASSO regression, 3 features were identified as suitable for group classification and used to construct the tumour grade radiomic-based prediction model: MajorAxisLength, Mean and 90Percentile, also individually statistically significant for the group differentiation (p-values respectively 0.001, <0.001 and <0.001). The prediction model resulted in AUC values of 0.84 (95% CI: 0.72-0.97) and 0.82 (95% CI: 0.62-1) for the training and validation cohorts, respectively.
**Conclusion:** CT-radiomics analysis may aid in differentiating the histological grade for GEP-NENs.
**Limitations:** Due to the low incidence of the disease, the study population is numerically limited to perform 4 groups (G1, G2, G3, NEC separately) classification statistics.
**Ethics committee approval:** Approved by the Ethics Committee of our Institution (register number 13261_oss, approved on 02/02/2021).
**Funding for this study:** No funding was received for this study.
**Author Disclosures:**
Giulia Grazzini: Nothing to disclose
Silvia Bettarini: Nothing to disclose
Federica Flammia: Nothing to disclose
Vittorio Miele: Nothing to disclose
Giuditta Chiti: Nothing to disclose
Paolo Tortoli: Nothing to disclose

### RPS 501a-8

**Intraductal papillary mucinous neoplasm (IPMN): are volumetry and other novel imaging features able to improve malignancy prediction compared to well-established resection criteria?**

*R. M. Pozzi-Mucelli*[1], C. F. Moro[1], M. Del Chiaro[2], R. Valente[3], L. K. Blomqvist[1], N. Papanikolaou[4], J-M. Löhr[1], N. Kartalis[1]; [1]Stockholm/SE, [2]Aurora, CO/US, [3]Umeå/SE, [4]Lisbon/PT

**Purpose:** Current guidelines base the management of IPMN on well-established resection criteria (RC), including cyst size. However, malignancy may occur in small cysts. Since branch-duct (BD) IPMN aren't perfect spheres, the volumetric and morphologic analysis might better correlate with mucin production and grade of dysplasia. Nonetheless, their role in malignancy (high-grade dysplasia/invasive cancer) prediction has been poorly investigated. Previous studies evaluating RC also included patients with IPMN-associated/concomitant pancreatic cancer (PC), which may affect RC's yield. This study aimed to assess the role of volume, morphology, and other well-established RC in malignancy prediction in patients with BD- and mixed-type IPMN after excluding IPMN-associated/concomitant PC.
**Methods or Background:** Retrospective study of 106 patients (2008-2019) with histopathological diagnosis of BD- and mixed-type IPMN (without associated/concomitant PC) and preoperative MRI available. Standard imaging and clinical features were collected and cyst volume and elongation value [EV=1-(width/length)] calculated on T2-w images. Logistic regression analysis was performed and predicted probabilities (PP) were calculated. Statistical significance set at two-tails, $p<0.05$.
**Results or Findings:** Neither volume [Odds ratio (OR)=1.01, 95%CI:0.99-1.02, p=0.12) nor EV (OR=0.38, 95%CI:0.02-5.93, p=0.49) were associated to malignancy. Contrast-enhancing mural nodules (MN), main pancreatic duct (MPD)>=5mm and elevated CA19-9 serum levels (>37$\mu$mol/L) were associated to malignancy [MN OR:4.32, 95%CI:1.18-15.76, p=0.02; MPD>=5mm OR:4.2, 95%CI:1.34-13.1, p=0.01; CA19-9 OR:6.72, 95%CI:1.89-23.89, p=0.003]. The PP for malignancy in a hypothetical male >=70-years-old increased from 0.08 (95%CI:0.02-0.27) with no risk-factors, to 0.92 (95%CI:0.63-0.99) with all three risk-factors.
**Conclusion:** Volume and EV cannot predict malignancy in BD- and/or mixed-type IPMN. MN, MPD>=5mm and elevated CA19-9 are associated with higher malignancy risk even after the exclusion of IPMN-associated/concomitant PC.
**Limitations:** Small sample, only including operated patients with IPMN-diagnosis.
**Ethics committee approval:** Institutional Review Board approval was obtained (EPN 2015/1544–31/4).
**Funding for this study:** This study has received funding from Stockholm Regional Council.
**Author Disclosures:**
Nikolaos Kartalis: Nothing to disclose
Johannes-Matthias Löhr: Nothing to disclose
Raffaella Maria Pozzi-Mucelli: Nothing to disclose
Roberto Valente: Nothing to disclose
Carlos Fernández Moro: Nothing to disclose
Nickolas Papanikolaou: Owner: Nikolaos Papanikolaou is owner of MRIcons LTD company.
Lennart K. Blomqvist: Founder: Lennart Blomqvist is Cofounder and CMO at Collective Minds Radiology.
Marco Del Chiaro: Grant Recipient: Marco del Chiaro has been awarded an industry grant (Haemonetics, Inc) to conduct a multi-center study for the evaluation of the prognostic implications of TEG in pancreas cancer. Further, he is a co-PI of a sponsored Boston Scientific study on the use of intra-operative pancreatoscopy in IPMN's patients.

### RPS 501a-9

**Differentiation between pancreatic cancer and focal pancreatitis using MRI – A retrospective assessment of diagnostic performance**

*A. Messner*, A. Kristic, A. J. Herold, R. Ambros, N. Bastati-Huber, S. Pötter-Lang, M. Schindl, A. Ba-Ssalamah; Vienna/AT

**Purpose:** To evaluate the efficacy of MRI to differentiate pancreatic malignancy from focal pancreatitis.
**Methods or Background:** Ninety-six patients underwent dynamic contrast-enhanced MRI, DWI and secretin-enhanced MRCP on a 3 Tesla unit. Features including lesion morphology, margins, vascular infiltration, signal intensity, enhancement, measurements of lesion and duct and duct penetrating sign and double duct sign, were evaluated by one reader blinded to clincal and histopathological data. Imaging diagnosis was compared to the ground truth, histopathology. Analysis was performed using descriptive statistics as well as the Chi-square test, Student's t-test and logistic regression.
**Results or Findings:** Histology was benign in 43 subjects (45%), malignant in 53 subjects (55%). Benign lesions included chronic pancreatitis (84%), autoimmune pancreatitis (4,7%), abscess (4,7%), pseudocyst (2,3%), lymphocytic infiltrate (2,3%) and cavernous lymphangioma (2,3%). Malign lesions included ductal adenocarcinoma (89,7%), invasive carcinoma (3,8%), neuroendocrine tumor (3,8%) and metastasis (1,9%). MRI evaluated by an expert yielded a sensitivity of 94.3%, specificity of 74.4%, positive predictive value of 82.0%, negative predictive value of 91.4% and an accuracy of 85.4%. Logistic regression found that ductal stenosis (OR=0.243, p=0.015), ill defined lesion margins (OR=0.186, p=0.012) and absence of the duct penetrating sign (OR=46.854, p<0.001) were associated with malignancy.
**Conclusion:** Contrast-enhanced MRI, including dynamic sequences, DWI and secretin-enhanced MRCP can distinguish benign from malignant pancreatic lesions. While sensitivity is high for an expert radiologist, there are many useful imaging characteristics that can aid less experienced radiologists in distinguishing benign from malignant lesions.
**Limitations:** Singe center. Single Reader. Retrospective.
**Ethics committee approval:** IRB-approved (EK Nr. 1627/2017).
**Funding for this study:** None.
**Author Disclosures:**
Antonia Kristic: Nothing to disclose
Sarah Pötter-Lang: Nothing to disclose
Alexander Johannes Herold: Nothing to disclose
Raphael Ambros: Nothing to disclose
Martin Schindl: Nothing to disclose
Ahmed Ba-Ssalamah: Consultant: Bayer Speaker: Bayer
Nina Bastati-Huber: Nothing to disclose
Alina Messner: Nothing to disclose

**16:30-17:30** **Channel 4**

### Research Presentation Session: Oncologic Imaging

### RPS 616

### Imaging for response assessment and prognostication

**Moderators**
C. Caramella; Villejuif/FR
A. G. Rockall; London/UK

### RPS 616-3

**Quantitative washout in patients with hepatocellular carcinoma undergoing TACE: an imaging biomarker for predicting prognosis?**

*L. Müller*[1], F. Hahn[1], F. Jungmann[1], F. Stoehr[1], M. C. Halfmann[1], C. Düber[1], D. Pinto dos Santos[2], R. Kloeckner[1], A. Mähringer-Kunz[1]; [1]Mainz/DE, [2]Cologne/DE
*(dott.andrea.agostini@gmail.com)*

**Purpose:** The delayed percentage attenuation ratio (DPAR) was recently identified as a novel predictor of an early complete response in patients with hepatocellular carcinoma (HCC) undergoing transarterial chemoembolization (TACE). In this study, we aimed to validate the role of DPAR as a predictive biomarker for short-, mid- and long-term outcomes after TACE.
**Methods or Background:** We retrospectively reviewed laboratory and imaging data for 103 treatment-naïve patients undergoing initial TACE treatment at our tertiary care centre between January 2016 and November 2020. DPAR and other wash-in and washout indices were quantified in the triphasic computed tomography performed before the initial TACE. The influence of the DPAR on the 6-, 12-, 18-, and 24-month survival rates and the median overall survival (OS) was compared to other established washout indices and estimates of tumour burden and remnant liver function.
**Results or Findings:** The DPAR was significantly higher in patients who survived the first 6 months after TACE (122 vs 115, p=0.04). In addition, the number of patients with a DPAR > 120 was significantly higher in this group (n=38 vs n=8; p=0.03). However, no significant differences were observed in the 12-, 18-, and 24-month survival rates after the initial TACE. Regarding the median OS, no significant difference was observed for patients with a high DPAR compared to those with a low DPAR (18.7 months vs 12.7 months, p=0.260).
**Conclusion:** Our results confirm DPAR as the most relevant washout index for predicting the short-term outcome of patients with HCC undergoing TACE. However, DPAR and the other washout indices were not predictive of mid- and long-term outcomes.
**Limitations:** Single center study and retrospective design.
**Ethics committee approval:** The ethics committee of the Medical Association of Rhineland Palatinate, Mainz, Germany, approved this study (permit number 2021-16013).
**Funding for this study:** Not applicable.
**Author Disclosures:**
Daniel Pinto dos Santos: Nothing to disclose
Roman Kloeckner: Nothing to disclose
Fabian Stoehr: Nothing to disclose
Christoph Düber: Nothing to disclose
Aline Mähringer-Kunz: Nothing to disclose
Felix Hahn: Nothing to disclose
Moritz Christian Halfmann: Nothing to disclose
Florian Jungmann: Nothing to disclose
Lukas Müller: Nothing to disclose

### RPS 616-4

**Evaluation of treatment response to chemoembolization of hepatocellular carcinoma (HCC) with mRECIST and LI-RADS Treatment Response v2018 (LRTRv2018): correlation with pathology on explanted liver**

S. Capodagli-Colarizi, L. Pierpaoli, *A. Agostini*, A. Borgheresi, L. Ottaviani, D. Nicolini, C. Floridi, M. Vivarelli, A. Giovagnoni; Ancona/IT
*(onofriocatalano@yahoo.it)*

**Purpose:** To evaluate the correlation of treatment response to chemoembolization with drug-eluting beads (debTACE) of hepatocellular carcinoma (HCC) assessed with mRECIST and LI-RADS Treatment Response v2018 (LRTRv2018), with pathological necrosis on explanted livers.
**Methods or Background:** Patients who underwent debTACE for HCC before liver transplantation between June 2018 and December 2019 were retrospectively included. All the patients had a contrast-enhanced CT or MRI examination at baseline and 1 month after treatment; patients lacking radiological data or pathological reports of the explanted liver were excluded. All the lesions were evaluated at baseline according to LI-RADSv2018 criteria, and the treatment response was evaluated according to mRECIST and LRTRv2018 criteria by two expert radiologists (8 and 10 years) in consensus. The pathological necrosis was calculated as a percentage on the nodular section. The statistics were performed with ROC curve analysis.
**Results or Findings:** Thirty-four nodules (median diameter 19mm, range 12-75mm) in 30 patients (26M:4F; median age 57 y.o., range 51-62) were evaluated. At baseline, 4 nodules were classified as LR-M, 19 nodules as LR-5 and 8 nodules as LR-4. 20 nodules (59%) had a complete response according to mRECIST and 18 (53%) according to LRTRv2018. At pathology 13 nodules (38%) had complete necrosis. mRECIST criteria achieved a sensitivity and specificity respectively of 94% and 53% (AUC 0,732, p=0,0005) for pathological necrosis >90% while LRTRv2018 criteria recorded values of 79% and 60% (AUC 0,693, p=0,0159) respectively, without significant differences (p=0,3432).
**Conclusion:** mRECIST and LRTRv2018 criteria demonstrated a comparable correlation with pathological necrosis of treated HCC.
**Limitations:** Retrospective, single centre study.
**Ethics committee approval:** The study was approved by the local IRB.
**Funding for this study:** Not applicable.
**Author Disclosures:**
Daniele Nicolini: Nothing to disclose
Chiara Floridi: Nothing to disclose
Luca Pierpaoli: Nothing to disclose
Andrea Agostini: Speaker: Siemens
Alessandra Borgheresi: Nothing to disclose
Letizia Ottaviani: Nothing to disclose
Andrea Giovagnoni: Nothing to disclose
Marco Vivarelli: Nothing to disclose
Simone Capodagli-Colarizi: Nothing to disclose

### RPS 616-5

**PET/MRI vs the standard of care imaging in the diagnosis of peritoneal carcinomatosis**

*O. A. Catalano*[1], F. S. Furtado[1], M. Wu[1], S. Esfahani[1], M. Anderson[1], A. Mojtahed[1], B. R. Rosen[2], U. Mahmood[1], C. Ferrone[1]; [1]Boston, MA/US, [2]Charlestown, MA/US
*(Julian.Kirchner@med.uni-duesseldorf.de)*

**Purpose:** To compare positron emission tomography (PET)/magnetic resonance imaging (MRI) to the standard of care imaging (SCI) for the diagnosis of peritoneal carcinomatosis (PC) in primary abdominopelvic malignancies.
**Methods or Background:** Adult subjects were prospectively and consecutively enrolled from 4/2019 to 1/2021. Inclusion criteria were: (a) acquisition of whole-body contrast-enhanced (CE) 18F-fluorodeoxyglucose PET/MRI and (b) pathologically confirmed primary abdominopelvic malignancies. Exclusion criteria were: (a) greater than 4 weeks interval between SCI and PET/MRI and (b) unavailable follow-up. SCI consisted of whole-body contrast-enhanced (CE) PET/computed tomography (CT) with diagnostic quality CT, and/or CE-CT of the abdomen and pelvis, and/or CE-MRI of the abdomen ± pelvis. If available, pathology or surgical findings served as the primary reference standard; otherwise, imaging follow-up was used. When SCI and PET/MRI results disagreed, medical records were checked for management changes. Follow-up data were collected until 8/2021.
**Results or Findings:** One hundred sixty-four subjects were included, eighty-five (52%) were female, and the median age was 60 years (IQR 50–69). At a subject level, PET/MRI had higher sensitivity (0.97, 95% CI 0.86–1.00) than SCI (0.54, 95% CI 0.37–0.71), $p<0.001$, without a difference in specificity, of 0.95 (95% CI 0.90–0.98) for PET/MRI and 0.98 (95% CI 0.93–1.00) for SCI, $p=0.25$. Fused PET/MRI was more sensitive than stand-alone MRI, $p=0.023$, and stand-alone PET, $p<0.001$.
**Conclusion:** PET/MRI improves detection of PC compared to SCI, which frequently changes management.
**Limitations:** Study enrollment occurred at the discretion of the subjects' treating oncologist or surgeon, and potential biases in the primary malignancy location and extent of disease are not accounted for in this study.
**Ethics committee approval:** This study was approved by the institutional review board (protocols 2019P000410/2020P001367), and written informed consent was obtained.
**Funding for this study:** This study received no specific funding.
**Author Disclosures:**
Mark Wu: Nothing to disclose
Felipe Sahb Furtado: Nothing to disclose
Shadi Esfahani: Nothing to disclose
Umar Mahmood: Nothing to disclose
Onofrio Antonio Catalano: Nothing to disclose
Amirkasra Mojtahed: Nothing to disclose
Mark Anderson: Nothing to disclose
Cristina Ferrone: Nothing to disclose
Bruce R. Rosen: Nothing to disclose

### RPS 616-6

**18F-FDG PET/MRI and machine learning for axillary staging in newly diagnosed breast cancer patients**

J. Morawitz[1], N-M. Bruckmann[1], P. Baltzer[2], B. Sigl[2], K. Herrmann[3], L. Umutlu[3], C. Rubbert[1], *J. Kirchner*[1], J. Caspers[1]; [1]Düsseldorf/DE, [2]Vienna/AT, [3]Essen/DE
*(sejin711@gmail.com)*

**Purpose:** 1. To investigate the diagnostic accuracy of machine learning algorithms and experienced radiologists in detecting axillary lymph node metastases of primary breast cancer in MRI and PET/MRI. 2. To assess which morphologic and metabolic lymph node features in MRI and PET/MRI are the most informative to determine lymph node dignity. 3. To determine whether an adjusted threshold can increase the sensitivity of PET/MRI to exclude lymph node involvement with sufficient confidence to spare node-negative patients and invasive procedures such as SLNB.
**Methods or Background:** 255 patients from two centres were included for the training sample, and 48 patients from a third centre were included for the validation sample. (PET/)MRI datasets were evaluated regarding axillary lymph nodes. Histopathology of axillary lymph nodes served as the reference standard. For the assessment of the diagnostic performance, sensitivity, specificity, positive and negative predictive value and accuracy were calculated. Diagnostic performances were compared by a McNemar test.
**Results or Findings:** There were no significant differences in the diagnostic performance of radiologists and machine learning algorithms in MRI ($p=0.671$) and PET/MRI ($p=0.683$). The most important lymph node feature was a tracer uptake (ratio of SUVmax of the lymph node/SUVmax of Aorta ascendes, cut-off 1.3-fold of mediastinal blood pool), followed by lymph node size (cut-off 7.5 mm).
**Conclusion:** The diagnostic performance of a random forest classifier in detecting axillary lymph node metastases is comparable to that of an experienced radiologist. A size of 7.5 mm and a tracer uptake of 1.3-times the mediastinal blood pool are the most important features to determine the dignity of a lymph node. By adjusting the threshold, the sensitivity of the random forest classifier can be increased in a way that 54.5% of patients can be spared an invasive procedure such as an SLNB.
**Limitations:** No limitations identified.
**Ethics committee approval:** Institutional Research Committee Votum.
**Funding for this study:** Deutsche Forschungsgemeinschaft.
**Author Disclosures:**
Ken Herrmann: Nothing to disclose
Julian Caspers: Nothing to disclose
Benjamin Sigl: Nothing to disclose
Christian Rubbert: Nothing to disclose
Lale Umutlu: Nothing to disclose
Julian Kirchner: Nothing to disclose
Janna Morawitz: Nothing to disclose
Pascal Baltzer: Nothing to disclose
Nils-Martin Bruckmann: Nothing to disclose

### RPS 616-7

**Accuracy and precision of diffusion-weighted imaging as quantitative imaging across the variable MRI systems and scan protocols**

*S. J. Choi*, K. W. Kim, D. W. Kim, H. Ahn; Seoul/KR
*(stefaniamariarita.rizzo@eoc.ch)*

**Purpose:** Diffusion-weighted imaging that reflects tumour cellularity is emerging as a preferred method for evaluating the response in oncologic imaging. For the clinical utilization of DWI as a quantitative parameter, the repeatability and accuracy of the ADC value is critical. In this study, we aimed to validate variable MRI systems using Quantitative Imaging Biomarkers Alliance (QIBA)/ National Institute of Standards and Technology (NIST) protocols and to confirm the reliability of currently used clinical DWI protocols.
**Methods or Background:** In this prospective study, a NIST/QIBA developed phantom with 13 PVP vials of known ADC value was used. The validation of the MRI systems was performed on four 1.5T and 3.0T MRI systems using the QIBA and the clinically used DWI protocols. Acquired DWI images were analyzed by commercially available web-based software (CaliberMRI, Inc., qCal-MR QC Software), and the parameters for quality control according to the QIBA claim were assessed. The linearity and bias of ADC measurement for each DWI protocol were calculated with linear regression. The difference of ADC values between the two protocols was evaluated with a paired T-test and a Bland-Altman plot.
**Results or Findings:** The regression slope of estimated ADC values on all MRI systems were 1.01 (range, 1.00–1.02) for the QIBA protocol and 1.02 (0.95–1.02) for the clinical protocol within the QIBA claim. The estimated ADC values were not statistically different between the two DWI protocols.
**Conclusion:** ADC estimation from variable MRI systems using QIBA and clinical protocols was accurate, and ADC could be considered as imaging biomarker in the clinical field.
**Limitations:** This study was performed with a small number of MRI examinations using four MRI systems. Further studies with more examinations and MRI systems are needed to robust DWI's usefulness.
**Ethics committee approval:** This prospective study included no human participants and was exempt from the institutional board review.
**Funding for this study:** Nothing to disclosure here.
**Author Disclosures:**
Hyemin Ahn: Nothing to disclose
Dong Wook Kim: Nothing to disclose
Se Jin Choi: Nothing to disclose
Kyung Won Kim: Nothing to disclose

### RPS 616-8

**Computed tomography-based body composition in patients with ovarian cancer: association with chemotoxicity and prognosis**

*S. Rizzo*[1], M. Del Grande[1], G. M. M. Nicolino[2], I. Colombo[1], L. Rossi[1], M. Biroli[2], L. Manganaro[3], F. Del Grande[1]; [1]Lugano/CH, [2]Milan/IT, [3]Rome/IT

**Purpose:** To assess the association between computed tomography (CT) quantitative measures of body composition profiling and chemotherapy-related complications in patients with ovarian cancer. Secondary purposes were to evaluate association between sarcopenia and survival, and differences in body composition profiling at baseline and after neoadjuvant chemotherapy.
**Methods or Background:** The study population was retrospectively selected from patients with newly diagnosed ovarian cancer. Clinical data were recorded, and CT images at the level of the 3rd lumbar vertebra were stored. By using specific software, skeletal muscle area (SMA), subcutaneous adipose tissue (SAT), visceral adipose tissue (VAT), and skeletal muscle density (SMD) were extracted. The skeletal muscle index (SMI) was calculated. Statistical analysis was performed to identify body composition features predictive of dose reduction, premature end of chemotherapy and cycle delays. Kaplan-

Wednesday

Meier analyses were performed to assess overall survival (OS) and progression-free survival (PFS). Wilcoxon test was performed to compare body composition features before and after neoadjuvant chemotherapy (NACT).
**Results or Findings:** Sixty-nine patients were included. A significant association was found between VAT and cycle delays (OR=1.01, z=2.01, 95%CI: 1.00-1.02, $p < 0.05$), between SMA and early discontinuation of chemotherapy (OR=1.03, z=2.10, 95% CI: 1.00-1.05, $p < 0.05$), and between mean SMD and cycle delays (OR=0.92, z=-2.70, 95%CI: 0.87-0.98, $p < 0.01$). No significant difference emerged for OS in sarcopenic and non-sarcopenic patients, nor in CT body composition features before and after NACT.
**Conclusion:** In ovarian cancer patients, CT-derived body composition profiling might predict the risk of chemotoxicity. In particular, VAT and SMD are associated with chemotherapy cycle delays and SMA with early discontinuation of chemotherapy.
**Limitations:** No limitations identified.
**Ethics committee approval:** The ethics committee approval was obtained.
**Funding for this study:** No funding was received for this study.
**Author Disclosures:**
Maria Del Grande: Nothing to disclose
Matteo Biroli: Nothing to disclose
Filippo Del Grande: Nothing to disclose
Ilaria Colombo: Nothing to disclose
Stefania Rizzo: Nothing to disclose
Lorenzo Rossi: Nothing to disclose
Gabriele Maria Maria Nicolino: Nothing to disclose
Lucia Manganaro: Nothing to disclose

### Thursday, March 3

**08:00-09:00** **Channel 4**

### Research Presentation Session: Cardiac

### RPS 703

### Measuring myocardial extracellular volume (ECV) with CT and MR

**Moderators**
C. Lücke; Leipzig/DE
C. Catalano; Rome/IT

### RPS 703-3

**Early prediction of cardiac complications in acute myocarditis by means of extracellular volume quantification with the use of dual-energy computed tomography**

*S. A. Si-Mohamed*, A. Congi, A. Ziegler, D. Tomasevic, S. Boccalini, T. Bochaton, E. Bonnefoy-Cudraz, L. Boussel, P. Douek; Lyon/FR
*(salim.si-mohamed@chu-lyon.fr)*

**Purpose:** To evaluate in acute myocarditis different extracellular volume (ECV) biomarkers of myocardial inflammation burden quantified by dual-energy CT (DECT) in comparison to biological parameters, and to determine the best ECV cut-off for predicting the occurrence of major adverse event (MAE) during the early phase.
**Methods or Background:** Consecutive patients confirmed with acute myocarditis on CMR from May 2018 to September 2020 who underwent a cardiac DECT were included in this retrospective monocentric study. Global/maximal ECVs of the whole heart and global/maximal ECVs per layer were measured on delayed phase iodine maps. MAE included death, heart failure and serious rhythm disturbance. Pearson correlation between investigated biomarkers, univariate and multivariate analyses for prediction of MAE and log-rank test for survival curve (Kaplan Meier) at one-year follow-up were calculated.
**Results or Findings:** Sixty-one patients (78.3% male, 30 (25-42) years) were recruited. Delay between symptoms onset and DECT was 1 (0-3) days. Eight MAEs were recorded during the first week of symptoms onset. No patient was lost to follow-up. ECVs were increased in MAE group (P values<0.05). Highest correlation was observed between global ECV and troponin peak (r=0.79, P<0.0001). A global ECV cut-off ≥39.5% was associated with MAE (log-rank <0.0001) in univariate (P<0.0001) and multivariate (P<0.05) analyses.
**Conclusion:** ECV should be considered as a good DECT biomarker for inflammation burden assessment and prediction of MAE during the early phase of an acute myocarditis.
**Limitations:** While we are reporting a limited number of MAE in our study, we were able to demonstrate an association between ECV and MAE using both univariate and multivariate analyses.
**Ethics committee approval:** Written inform consent was waived by the local IRB (Hospices Civiles de Lyon).
**Funding for this study:** No funding was provided for this study.
**Author Disclosures:**
Danka Tomasevic: Nothing to disclose
Arthur Ziegler: Nothing to disclose
Salim Aymeric Si-Mohamed: Nothing to disclose
Eric Bonnefoy-Cudraz: Nothing to disclose
Loïc Boussel: Nothing to disclose
Philippe Douek: Nothing to disclose
Sara Boccalini: Nothing to disclose
Anaelle Congi: Nothing to disclose
Thomas Bochaton: Nothing to disclose

### RPS 703-5

**Endogenous assessment of myocardial injuries using magnetic resonance T1-rho mapping: comparison to T2 mapping and contrast-enhanced imaging**

A. Bustin[1], *X. Pineau*[1], S. Sridi[1], P. Jais[1], M. Stuber[2], H. Cochet[1]; [1]Pessac/FR, [2]Lausanne/CH

**Purpose:** Magnetic Resonance (MR) myocardial T1-rho mapping has emerged as a promising tool for detecting myocardial injuries without an exogenous contrast agent. Yet, the parameters influencing changes in T1-rho and the applicability of the technique to the broad spectrum of acute and chronic myocardial injury remains unexplored territory. The aims of this study were to identify clinical correlates of myocardial T1-rho and to examine how T1-rho mapping performs against conventional MR sequences under various diseases.
**Methods or Background:** The 113 subjects comprised 69 patients with known ischemic (N=18) and non-ischemic (N=51) heart disease and 44 healthy controls. MR was performed on a 1.5T System (Aera, Siemens Healthcare, Erlangen). Injured and remote areas were defined by an expert on LGE images. Quantitative analysis was achieved by tracing regions of interest on pre-contrast T1-rho and T2 maps, and on post-contrast T1 and ECV maps within remote and injured areas, on short-axis slices.
**Results or Findings:** In healthy controls, the mean myocardial T1-rho was 47±2ms. T1-rho positively related to age (R=0.4, P<0.01) and showed higher values in females than males (48 vs 46ms, P<0,01). T1-rho increased significantly in patients with acute focal myocardial injury (69 vs 48ms in remote, P<0.01, N=9), with focal fibrosis (64 vs 47ms in remote, P<0.01, N=27), and with diffuse myocardial involvement (52 vs 47ms in controls, P<0.01, N=8) with a positive correlation with ECV (R=0.312, P<0.01).
**Conclusion:** Myocardial T1-rho values are gender and age-dependent. The technique appears to be sensitive to acute, chronic, focal, and diffuse myocardial injuries, and may thus be a contrast-free adjunct to LGE for gaining new and quantitative insight into acute and chronic myocardial structural disorders.
**Limitations:** Single center study.
**Ethics committee approval:** CHU Bordeaux.
**Funding for this study:** ANR-11-EQPX-0030, ANR-10-IAHU04-LIRYC, ERC-715093, Lefoulon-Delalande Foundation.
**Author Disclosures:**
Soumaya Sridi: Nothing to disclose
Aurelien Bustin: Nothing to disclose
Hubert Cochet: Nothing to disclose
Pierre Jais: Nothing to disclose
Xavier Pineau: Nothing to disclose
Matthias Stuber: Nothing to disclose

### RPS 703-7

**T1, T2 and ECV mapping improve sensitivity of CMR and offer pathophysiological insights in patients with acute myocarditis with non infarct-like presentation: results from the MIAMI study**

*D. Vignale*[1], A. Palmisano[1], M. Gatti[2], R. Faletti[2], N. Galea[3], F. De Cobelli[1], A. Esposito[1]; [1]Milan/IT, [2]Turin/IT, [3]Rome/IT
*(vignale.davide@hsr.it)*

**Purpose:** To investigate the sensitivity of cardiac magnetic resonance (CMR) with T1, T2 and ECV mapping for the diagnosis of acute myocarditis (AM) and to investigate the characteristics of mapping alterations according to AM clinical presentation.
**Methods or Background:** Prospective multicenter study involving 97 consecutive patients with AM confirmed by a multidisciplinary heart team evaluation based on multimodality imaging (comprising CMR performed according to 2018 LLc), laboratory testing, clinical features, and endomyocardial biopsy (perfomed in 33(34%) patients).
**Results or Findings:** Male:female ratio was 67:30. Median age was 38 years [IQR=27-44]. All patients had increased troponinT (404[81-887]ng/L). 77(79%) patients had infarct-like (IL) presentation with chest pain. The remaining 20(21%) patients had non-IL presentations with heart failure (HF) in 14(14%) and arrhythmia in 6(6%) patients. Ejection fraction was reduced in 20(21%) patients, with significantly lower values in HF group (27% vs 57% and 63% in IL and arrhythmic presentation, p=0.021). 2018 LLc were positive in 97(100%) patients. 2009 LLc were positive in 77(100%) patients with IL presentation, 10(92%) with HF presentation, and 4(67%) with arrhythmic presentation. 2018 LLc significantly improved CMR sensitivity in non-IL presentation (Fisher's exact test p-value=0.001). Patients with non-IL presentations had lower scar burden (3[1-11]% vs 9[5-13]%, p=0.005) and higher global native-T1 (1103[1059-1197]ms vs 1066[1026-1099]ms, p=0.012), percentage of segments with abnormal native-T1 (94[45-1], p=0.008), global ECV (32.5[27.5-36.8]% vs 28.6[26.3-31.8]%, p=0.014), and percentage of segments with abnormal ECV (100[68.8-100]% vs 68.8[32.5-100]%, p=0.014).
**Conclusion:** In patients with AM, CMR performed according to 2018 LLc significantly improves sensitivity in non-IL presentation. In this group, despite the smaller volume of scar, parametric mapping shows more intense and diffuse myocardial alterations.
**Limitations:** Limited number of patients with non-IL presentation.
**Ethics committee approval:** Approved by the Institutional Review Board (27/INT/2016).
**Funding for this study:** Partially funded by the Italian Health Ministry.
**Author Disclosures:**
Davide Vignale: Nothing to disclose
Marco Gatti: Nothing to disclose
Antonio Esposito: Nothing to disclose
Riccardo Faletti: Nothing to disclose
Anna Palmisano: Nothing to disclose
Francesco De Cobelli: Nothing to disclose
Nicola Galea: Nothing to disclose

**10:30-11:30** **Channel 4**

### Research Presentation Session: Head and Neck

### RPS 808

### Temporal bone imaging

**Moderator**
B. De Foer; Antwerp/BE

### RPS 808-3

**Pre-intraoperative evaluation in patients affected by cholesteatoma using CT-MR fusion protocol**

*S. Palizzi*, A. Romano, F. Dellepiane, A. Bozzao; Rome/IT
*(serenapalizzi@gmail.com)*

**Purpose:** Cholesteatoma is a serious pathological condition that requires surgical excision as definitive treatment, with the least invasive technique. CT is the first level examination for the anatomical evaluation of the middle ear; however, it does not allow the differentiation of cholesteatoma margins from surrounding inflammatory tissue. On the contrary, RM-DWI has high specificity, with a reduced spatial resolution. The purpose of the study is to evaluate whether CT-MRI fusion images can increase the accuracy in the diagnosis and localization of cholesteatoma, with the aim of improving pre-operative planning
**Methods or Background:** 83 patients with cholesteatoma were included in our study in the time frame between October 2019 and May 2021; 20 underwent CT-MRI examination for pre-surgical planning. MR and CT images were co-registered using a dedicated console (BrainLab, Feldkirchen, Germany). The images with hypersignal in DWI-SE compatible with cholesteatoma were used to construct a three-dimensional "cholesteatoma", to be displayed on multiplanar and 3D co-registered CT images.
**Results or Findings:** A statistically significant difference was found between the volume of the cholesteatoma tissue and the surrounding inflammatory tissue (mean cholesteatoma volume: 0.03cm3; mean inflammatory tissue volume: 1.8cm3; p <0.05). In 80% of cases, the surgical approach to the patient was modified according to the information obtained from CT-MRI fusion images. No tissue residue was detected in the group of 20 patients surgically treated using the information from CT-MRI fusion. In the control group (63 patients), residual cholesteatoma was present in 6 cases (9.5%).
**Conclusion:** CT and MRI images are complementary to each other. In selected cases, they should be co-registered in the pre-operative evaluation of cholesteatoma in order to optimize surgical time and aim at radical surgical resection.
**Limitations:** No limitations identified.
**Ethics committee approval:** Not applicable.
**Funding for this study:** No funding was provided for this study.
**Author Disclosures:**
Serena Palizzi: Nothing to disclose
Andrea Romano: Nothing to disclose
Alessandro Bozzao: Nothing to disclose
Francesco Dellepiane: Nothing to disclose

### RPS 808-5

**Temporal bone imaging using clinical photon-counting CT: image quality, artefact assessment and radiation dose after cochlear implant surgery**

*A. L. Schönenberger*[1], T. Flohr[2], B. Schmidt[2], S. Winklhofer[1], H. Alkadhi[1];
[1]Zurich/CH, [2]Forchheim/DE

**Purpose:** Assessing the image quality, artefact severity and radiation dose after cochlear implantation of the temporal bone in clinical photon-counting CT (PCCT) depending on the imaging acquisition and postprocessing.
**Methods or Background:** PCCT was performed on a human cadaveric temporal bone after surgical cochlear implantation using the QuantumPlus mode at three different CTDIvol levels (15.3, 9.7, 5.8 mGy). Image reconstructions were performed using four different reconstruction kernels (Hr72, 84, 96, 98) and additional four different Quantum Iterative Reconstruction levels (QIR level 1-4) resulting in a total of 60 different imaging data sets. Quantitative (ROI for noise and metal artefacts) and qualitative image evaluation (5-point Likert scale for anatomical and electrode delineation). Image noise quantification using the standard deviation of attenuation in the surrounding fluid.
**Results or Findings:** The 15.3mGy CTDIvol demonstrated a superior anatomical delineation and reduced quantitative artefacts compared to the two lower radiation levels (mean 3.8 / 2.9 / 1.6; 406 / 471 / 506HU; each p>0.05). Image noise showed no significant differences. Image artefacts decreased with higher iterative levels (no QIR = 512HU, QIR level 4 =379HU, p>0.05). Hr72 showed the best anatomical delineation (median 3.8 / 3.2-1.8) and lowest image noise (68 vs. 127-320HU) compared to Hr84, 96, and 98 (each p>0.05). Hr72 and 84 demonstrated the lowest artefact levels compared to Hr96 and 98 (388 and 357HU / 515 and 582HU, each p>0.05). Electrode delineation showed no significant difference with perfect visibility in all radiation levels.
**Conclusion:** In clinical PCCT of the temporal bone after cochlear implantation, a sufficiently good electrode delineation was seen in all radiation levels. Optimal anatomical delineation and artefact reduction on higher radiation levels and lower kernels (Hr72) and quantitative artefacts reduction by higher iterative levels.
**Limitations:** Single subject.
**Ethics committee approval:** Approved.
**Funding for this study:** Not applicable.
**Author Disclosures:**
Thomas Flohr: Consultant: Siemens Healthineers, Forchheim, Germany
Amadéa Laurence Schönenberger: Nothing to disclose
Bernhard Schmidt: Consultant: Siemens Healthineers, Forchheim, Germany
Sebastian Winklhofer: Nothing to disclose
Hatem Alkadhi: Nothing to disclose

### RPS 808-6

**CT evaluation of labyrinth structures in patients with incomplete partition type II anomaly**

*I. Sel*, Y. Karagöz, D. Ö. Ö. Aksoy, E. Ateş, A. S. Mahmutoglu; Istanbul/TR
*(ipek_sel@yahoo.com)*

**Purpose:** Incomplete partition type II (IP II) is one of the most frequently encountered congenital inner ear malformations in cochlear implant candidates. It is characterized by modiolar apical defects and mild vestibular enlargement. Anomalous child will develop sensorineural hearing loss (SNHL) worsening progressively with ageing supposedly with minor traumas. Radiological diagnosis of malformation could be difficult with equivocal changes in tiny labyrinthine structures, especially with inexperienced viewers.
**Methods or Background:** Our study was a retrospective case-control study including 34 SNHL patients with IP type II (66 ears) and 24 patients (48 ears) with normal audiographic findings who underwent temporal CT imaging. A normal group was examined tomographically for inflammatory conditions or trauma not involving labyrinthine structures. Measurements of defined inner ear structures were performed by two observers separately; observer 1 is a senior neuroradiologist and observer 2 is a novice radiologist with 1 year of experience in neuroradiology. The parameters measured in patient and control groups were compared and interobserver reliability was calculated.
**Results or Findings:** Lateral interscalar notch angle, lateral scalar height, medial interscalar notch depth, medial scalar height, vestibular aqueduct (VA) width at the middle, VA width at operculum level, vestibular height and width were found to be significantly larger while lateral interscalar notch depth and LSSC bone island surface area were significantly smaller in the patient group (p<0.05). These findings especially the lateral cochlear measurements are valuably proving structural changes secondary to apical modiolar deficiency.
**Conclusion:** CT measurements in SNHL could help to diagnose IP type II anomaly especially in patients with equivocal audiological and radiological findings.
**Limitations:** No limitations identified.
**Ethics committee approval:** Yes.
**Funding for this study:** There was no need for any funding.
**Author Disclosures:**
Direnç Özlem Özlem Aksoy: Nothing to disclose
Yeşim Karagöz: Nothing to disclose
Ece Ateş: Nothing to disclose
Abdullah Soydan Mahmutoglu: Nothing to disclose
Ipek Sel: Nothing to disclose

### RPS 808-7

**Is hearing necessary for cochlear nerve myelination?**

*M. B. Eser*, B. Atalay, M. T. Kalcioglu; Istanbul/TR
*(bilgineser@hotmail.com)*

**Purpose:** This study aimed to investigate whether there is a relationship between cochlear nerve myelination and hearing loss in children with congenital non-syndromic sensorineural profound hearing loss.
**Methods or Background:** Patients who underwent cochlear implant surgery in our university hospital were retrospectively evaluated, and 33 patients (19 boys, 14 girls) with congenital non-syndromic sensorineural profound hearing loss (>100db) were included. Current knowledge has shown that the myelinated cochlear nerve can be measured with MRI T2-weighted sequences. So that, the experienced observer measured cochlear nerve circumference (CNC) and surface area (CNSA) from FIESTA-C images taken in the sagittal oblique plane of these patients. Pearson correlation was done to understand the relationship between age and CNC and CNSA, and with t-test was done to compare means of 24 months under-over children of CNC and CNSA.
**Results or Findings:** The mean age of the individuals is 22.52 (SD: 12.37, Range: 5 - 55) months. CNC in male patients is 3.88 (SD: 0.10) mm and in female patients 3.71 (SD: 0.09) mm, and there was no difference. The mean CNC was 3.81 (SD: 0.41) mm. The mean CNSA we found was 1.08 (SD: 0.23)

Thursday mm2. A moderate positive correlation was found between age and CNC (r=0.38, p=0.03), CNSA (r=0.39, p=0.03). Although not significant on the t-test (p=0.06), children under 24 months (1.02, SD: 0.15 mm2) had a smaller CNSA than those over 24 months (1.17, SD: 0.32 mm2).
**Conclusion:** This study provides weak evidence that myelination continues in its normal course in children with a congenital non-syndromic sensorineural profound hearing loss despite the absence of a hearing signal.
**Limitations:** Retrospective design.
**Ethics committee approval:** Exist (DN:2018/0177).
**Funding for this study:** Not applicable.
**Author Disclosures:**
Mehmet Bilgin Eser: Nothing to disclose
M. Tayyar Kalcioglu: Nothing to disclose
Başak Atalay: Nothing to disclose

**12:30-13:30** **Channel 4**

### Research Presentation Session: Interventional Radiology

### RPS 909

### Interventional management of malignant liver lesions

**Moderators**
M. Meijerink; Amsterdam/NL
V. Vilgrain; Clichy/FR
**Author Disclosures:**
Martijn Meijerink: Author: IRE in clinical practice - Springer Verlag; CEO: Director interventional oncology solutions (IOS); Consultant: Angiodynamics, Johnson & Johnson, Medtronic; Grant Recipient: Angiodynamics, Johnson & Johnson, Medtronic; Research Grant/Support: Angiodynamics, Johnson & Johnson, Medtronic, Bristol-Myers-Squibb; Speaker: Angiodynamics, Johnson & Johnson, Medtronic, Guerbet, EXIOH

### RPS 909-3

**Prospective study on the immunological effects of conventional transarterial chemoembolization in patients with hepatocellular carcinoma: an interim analysis**
*R. Schmidt*, B. Gebauer, C. Roderburg, G. Ardila Pardo, E. Can, F. Tacke, B. Hamm, L. Hammerich, L. J. Savic; Berlin/DE
*(robin.schmidt@charite.de)*

**Purpose:** To characterize immune cell profiles in patients with hepatocellular carcinoma (HCC) and alterations induced by conventional transarterial chemoembolization (cTACE).
**Methods or Background:** This interim report of an ongoing prospective clinical trial included 31 patients with HCC, who received 58 cTACE procedures between 09/2020-08/2021. Peripheral blood was sampled before, 24h, and 8 weeks after cTACE for fluorescence-activated cell sorting (FACS) analysis. A 24-color multiplex staining panel was employed to quantify lymphoid cells and checkpoint-molecule expression including CD3, CD4, CD8, CD45 and PD-1 staining. Baseline MRI and post-cTACE non-contrast CT were registered and Hounsfield units (HU) in a segmented tumour mask were quantified. The mean HU of the entire cohort was defined as a threshold for tumoural Lipiodol uptake. Statistics included normality and paired mixed-effects with post-hoc testing.
**Results or Findings:** Compared to baseline (CD4+: 69.7% of CD45+CD3+ T cells (TC); CD8+: 23.3% of TC), CD4+ helper TC decreased (64.6%, p=0.001), whereas CD8+ effector TC increased (26.9%, p=0.01) 24h post-cTACE. A greater increase of CD8+ TC 24h post-cTACE was observed in patients with below threshold tumoural Lipiodol uptake (p=0.001) but values returned to baseline levels at 8 weeks (p<0.001), as compared to patients with above Lipiodol uptake (p=0.043), where levels remained increased 8 weeks post-cTACE (p>0.999). However, in patients with below threshold Lipiodol uptake at 24h, CD8+ TC expressing the exhaustion marker PD1 were higher than in patients with above threshold Lipiodol uptake (p=0.019), but not 8 weeks post-cTACE (p=0.293).
**Conclusion:** These preliminary results demonstrate possibly favorable cTACE-induced antitumoural T cell response, suggesting Lipiodol as an imaging biomarker for the functional TC status. The findings may help exploit cTACE-induced immune-activation to guide personalized treatments using combinations with immuno-oncological therapies.
**Limitations:** Single-site.
**Ethics committee approval:** Obtained.
**Funding for this study:** Guerbet (project-related funding).
**Author Disclosures:**
Bernd Hamm: Nothing to disclose
Linda Hammerich: Nothing to disclose
Frank Tacke: Nothing to disclose
Gracia Ardila Pardo: Nothing to disclose
Elif Can: Nothing to disclose
Christoph Roderburg: Nothing to disclose
Bernhard Gebauer: Nothing to disclose
Robin Schmidt: Nothing to disclose
Lynn Jeanette Savic: Nothing to disclose

### RPS 909-4

**Improved survival after transarterial radioembolisation for hepatocellular carcinoma (HCC) gives the procedure added value**
*A. De Cinque*, C. Mosconi, A. Cucchetti, A. Cappelli, G. Vara, C. Pettinato, L. Strigari, R. Golfieri; Bologna/IT

**Purpose:** Transarterial Radioembolisation (TARE) requires multidisciplinary experience and skill to be effective. The aim of this study was to identify the determinants of survival in patients with hepatocellular carcinoma (HCC), focusing on the learning curve, technical advancements, patient selection and role of subsequent therapies. Thus, TARE results achieved in the initial period (2005-2012) were compared to those obtained in recent years (2012-2020).
**Methods or Background:** From 2005 to 2020, 253 patients were treated with TARE. From 2005 to 2012, delivered activity was calculated using body surface area (BSA) formula while, after 2012, partition model was used and, for the most part, selective treatments were performed.
**Results or Findings:** Of 253 patients, 68 were treated before January 2012 and 185 after 2012. In the second period, patients had an ECOG–PS score of 1 (p=0.025) less frequently, less liver involvement (p=0.006) and a less advanced degree of vascular invasion (p=0.019). The median overall survival (OS) of patients treated before 2012 was 11.2 months and that of patients treated beginning in 2012 was 25.7 months. After reweighting to isolate the effect of the treatment period, the median OS of patients before 2012 increased to 16 months. The median OS of patients with an adsorbed dose <120Gy was 15.7 months and that of patients with an adsorbed dose ≥120Gy was 26.0 months. Of subsequent therapies, only surgery provided a survival advantage.
**Conclusion:** Better patient selection, refinement of technique and adoption of personalized dosimetry improved survival after TARE. Conversely, the addition of sorafenib after TARE did not impact life expectancy.
**Limitations:** Limitations intrinsic to retrospective studies.
**Ethics committee approval:** The study conformed to the ethical guidelines of the Declaration of Helsinki and was approved by the Institutional Review Board of the centre. All patients provided informed written consent.
**Funding for this study:** No funding received.
**Author Disclosures:**
Rita Golfieri: Nothing to disclose
Alessandro Cucchetti: Nothing to disclose
Giulio Vara: Nothing to disclose
Cristina Mosconi: Nothing to disclose
Antonio De Cinque: Nothing to disclose
Alberta Cappelli: Nothing to disclose
Cinzia Pettinato: Nothing to disclose
Lidia Strigari: Nothing to disclose

### RPS 909-5

**A new biodegradable stent to improve the management of biliary strictures in paediatric split liver transplantation**
*L. Dulcetta*, P. Marra, F. S. Carbone, P. A. Bonaffini, R. Muglia, L. D'Antiga, S. Sironi; Bergamo/IT
*(l.dulcetta@campus.unimib.it)*

**Purpose:** Cholestasis due to benign biliary strictures is the most common biliary complication after paediatric split liver transplantation (SLT), decreasing graft survival, but consensus about its management is lacking. Percutaneous transhepatic cholangiography (PTC), bilioplasty and internal-external biliary drainage (IEBD) are standard treatments. The aim of this report is to present the preliminary experience with a new biodegradable biliary stent in the management of post SLT biliary strictures.
**Methods or Background:** In addition to the standard treatment, 6 paediatric patients (4 males; median age 8 years, interquartile range 6.25-9.75) with SLT underwent percutaneous transhepatic implantation of an innovative 10F helical-shaped biodegradable biliary stent, featuring a slow degradation profile. To our knowledge the device is unique and the first to be CE-marked for the use in this indication.
**Results or Findings:** Percutaneous stent implantation was technically successful in all 6 patients. In the first case, early stent dislodgement and migration in the bowel was demonstrated with X-rays after 72 hours, without sequelae. No complications occurred during a 6-month follow-up.

**Conclusion:** Preliminary data suggest that implantation of a new biodegradable biliary stent is feasible and safe, to be considered in the management of post SLT cholestasis in paediatric patients. Some technical considerations must be done during implantation. This device may prolong biliary drainage and may relieve the discomfort of long-term IEBD.
**Limitations:** Small cohort study.
**Ethics committee approval:** The Human Investigation Committee (IRB) of Bergamo approved this study.
**Funding for this study:** Not applicable.
**Author Disclosures:**
Sandro Sironi: Nothing to disclose
Paolo Marra: Nothing to disclose
Pietro Andrea Bonaffini: Nothing to disclose
Francesco Saverio Carbone: Nothing to disclose
Riccardo Muglia: Nothing to disclose
Lorenzo D'Antiga: Nothing to disclose
Ludovico Dulcetta: Nothing to disclose

### RPS 909-6

**The effect of Microwave/Radiofrequency Ablation (MWA/RFA) on liver volume in patients with primary and secondary liver tumours: a single centre retrospective analysis**

*R. Knapen*, R. Korenblik, S. James, G. Dams, S. De Boer, R. Van Dam, C. Van Der Leij; Maastricht/NL
*(robrecht.knapen@mumc.nl)*

**Purpose:** Before liver surgery, hypertrophy-inducing procedures, such as Portal Vein Embolization (PVE), can be performed to stimulate future liver remnant (FLR) hypertrophy. This is to reduce the chance of post-hepatectomy liver failure (PHLF). Thermal ablation can induce liver hypertrophy as well, but the exact influence of Radiofrequency or Microwave Ablation (RFA/MWA) on liver hypertrophy remains unclear. Therefore, liver volume changes after RFA/MWA were retrospectively analyzed.
**Methods or Background:** Consecutive patients, with primary or secondary liver lesions, treated with RFA/MWA between January 2015 and May 2021 were included. Patients with earlier liver treatment or insufficient imaging were excluded. Total Liver Volume (TLV), volume of segment II+III, ablation volume and TLV minus ablation volume (absolute liver volume, ALV) were calculated using OsiriX DICOM viewer and Syngo.via. ALV change after >9 weeks was used as primary outcome variable. Wilcoxon tests were used for analysing volume changes.
**Results or Findings:** 56 patients were analysed, 36 patients with primary and 20 patients with secondary liver lesions. ALV in patients with secondary liver lesions increased with an average of 74.80mL (p=0.052), an average of 104.08% (SD=93.80-114.36%). Segment II+III increased with an average of 10.30mL (p=0.044), percentage average of 104.34% (SD=93.37-115.31%). ALV and segment II+III in patients with primary liver lesions remained stable with an average of 100.98% (SD=92.02-110.94%, p=0.863) and 101.82% (SD+85.22-118.42%, p=0.551), respectively.
**Conclusion:** Patients with secondary lesions ALV and segment II+III increased after MWA/RFA with 4%, while livers with primary liver lesions remained stable. This suggests that performing an ablation in livers with secondary liver lesions could further increase the FLR, and can be additional to FLR-hypertrophy inducing procedures. This might further help decrease PHLF, but needs to be validated in clinical trials.
**Limitations:** No limitations identified.
**Ethics committee approval:** Not applicable.
**Funding for this study:** Not applicable.
**Author Disclosures:**
Sanne De Boer: Nothing to disclose
Sinead James: Nothing to disclose
Remon Korenblik: Nothing to disclose
Glenn Dams: Nothing to disclose
Robrecht Knapen: Nothing to disclose
Ronald Van Dam: Nothing to disclose
Christiaan Van Der Leij: Nothing to disclose

### RPS 909-7

**Interventional oncological treatment of hepatocellular carcinoma (HCC): a monocentric long-term evaluation over 27 years**

*T. J. Vogl*, J. Freichel, T. Gruber-Rouh, W. Bechstein, S. Zeuzem; Frankfurt a. Main/DE
*(t.vogl@em.uni-frankfurt.de)*

**Purpose:** To retrospectively evaluate the development and technical progress in local oncological treatments of hepatocellular carcinoma (HCC) regarding survival rates between 1993 and 2020.
**Methods or Background:** In total, 1.046 HCC patients (824m/222f) were treated in 3.287 sessions with transarterial chemoembolization (TACE) from 1996-2000, laser-induced thermotherapy (LITT) from 1993-2011, microwave ablation (MWA) from 2008-2020, combined LITT+TACE or MWA+TACE. 25 patients (19m/6f, median: 69 years, range: 36-82) received 35 LITTs (1.4 cycles/patient). 67 patients (50m/17f, median: 68 years; range: 42-85) combined LITT+TACE (96 LITTs, 1.4 cycles/patient; 367 TACEs, 5.48 cyles/patient, range: 1-17). 228 patients (179m/49f; median 65 years; range: 32-85) received 385 MWAs (1.7 cyles/patient), 108 patients (90m, 18f; median: 69 years; range: 20-88) MWA+TACE (227 MWAs; 2.1 cycles/patient; 769 TACEs; 7.1 cycles/patient. 618 patients (486m/132f; median: 67 years; range: 15-91) received TACE only (2544 cycles; 4.1 cycles/patient). Results were evaluated regarding overall survival (OS) according to Kaplan-Meier and log-rank test for comparing the groups. A p-value ≤0.05 was considered significant.
**Results or Findings:** Median survival was 593 days for LITT with 1-/3-/5-year survival of 64.0%/24.0%/20.0% and 959 days for combined LITT+TACE with 83.6%/40.3%/14.9%. The total complication rate per LITT treatment was 8.57%; LITT+TACE 6.25%. Complications included pleural effusion, subcapsular haematoma and cutaneous wound infections, but no mortality. Median survival for MWA+TACE was 1,505 days. In the MWA group 1-/3-/5-year survival rates were 91.2%/76.9%/75.0%; for MWA+TACE 83.3%/ 55.7%/ 48.3%. The median survival time for TACE only was 474 days with 1-/3-/5-year survival rates of 58.1%/34.7%/30.2%.
**Conclusion:** Survival rates of MWA and MWA+TACE are significantly higher with less complication rates vs. TACE monotherapy and both LITT groups. However, prospective data remain necessary to further evaluate the superiority of either modality.
**Limitations:** Retrospective study design.
**Ethics committee approval:** Approval of the institutional review board was obtained.
**Funding for this study:** No funding was provided for this study.
**Author Disclosures:**
Stefan Zeuzem: Nothing to disclose
Thomas J. Vogl: Nothing to disclose
Tatjana Gruber-Rouh: Nothing to disclose
Wolf Bechstein: Nothing to disclose
Jason Freichel: Nothing to disclose

### RPS 909-8

**Image-guided microwave ablation of liver lesions using a new 150-W generator: preliminary technical evaluation**

*V. Cignini*, G. Risi, M. Calandri, F. Menchini, F. Coi, C. Maglia, A. N. A. Serafini, C. Gazzera, P. Fonio; Turin/IT

**Purpose:** To assess the safety and efficacy of a new 150-W generator for microwave ablation of liver lesions. Volume, sphericity of the ablation zone as well as adverse events were evaluated. No available data are present in the literature.
**Methods or Background:** Prospective collection of data was performed including all the patients who underwent microwave ablation, treated using the 150-W generator since March 2021. Procedures were performed under conscious sedation in the CT room for immediate control of the ablation zone; the microwave ablation antenna (Emprint Medtronic 14G) was inserted under US guidance. Collected data included among the others: ablation mean time, pre- and post-ablation volume, sphericity index, ablation margins, presence of gas artefacts in the veins, THAD. Adverse events were evaluated according to Clavien-Dindo Classification.
**Results or Findings:** 16 hepatic tumours (32,1 mm +/- 15,1) from 14 patients (M=11; F=3, age 66,4 +/- 5,6) who underwent percutaneous MWA where included in this observational prospective study. Ablation time was 5,8 min. The mean ablation volume was 36,2 +/- 21,3 cm^3, larger than pre-ablation volume (18,6 +/- 26,5 cm^3) and ablation volumes of previously reported series using the 100 W generator with the same antenna. Mean sphericity index was 0,8125 (range 0,7-1,1), similar to the manufacturer chart and other series using the 100 W generator. Vascular abnormalities at the immediate CT control were observed in 5 patients (3 THAD, 4 gas artefacts in the veins). No adverse events were registered.
**Conclusion:** The new 150-W generator provides effective and large ablation volume with predictable sphericity index and high safety profile, in absence of severe adverse events.
**Limitations:** Monocentric study.
**Ethics committee approval:** Not applicable.
**Funding for this study:** Not applicable.
**Author Disclosures:**
Marco Calandri: Nothing to disclose
Valentina Cignini: Nothing to disclose
Carlo Gazzera: Nothing to disclose
Gaetano Risi: Nothing to disclose
Claudio Maglia: Nothing to disclose
Paolo Fonio: Nothing to disclose
Francesca Menchini: Nothing to disclose
Fabrizio Coi: Nothing to disclose
Alessandro Niccolò Annibale Serafini: Nothing to disclose

**14:00-15:00** **Channel 4**

### Research Presentation Session: Physics in Medical Imaging

### RPS 1013

### Computed tomography (CT)

**Moderators**
K. Perisinakis; Iraklion/GR
B. Brkljačić; Zagreb/HR
**Author Disclosures:**
Boris Brkljačić: Advisory Board: Contextflow; Board Member: ISR, IS3R; Research Grant/Support: Croatian Science Foundation

### RPS 1013-3

**Commissioning of a whole-body photon counting spectral CT scanner: physico-technical tests**

*H. Bosmans*, L. Dewulf, J. Binst, H. Verhoeven, D. Petrov, K. Merken, N. Marshall, W. Coudyzer, C. Van Ongeval; Leuven/BE
*(hilde.bosmans@uzleuven.be)*

**Purpose:** To verify the performance of a newly installed, new generation photon-counting CT scanner for potential applications in high-resolution CT scanning and spectral imaging.
**Methods or Background:** A quality control program was developed for commissioning a Siemens-Healthineers NAEOTOM Alpha CT including tests for 2 specific applications: virtual non-contrast imaging (VNC) and ultra-high resolution (UHR). Results were compared to Siemens-Healthineers Force (CT_F) and Edge CT (CT_E). Automatic exposure choices were verified for different phantoms/conditions. The measurements used an in-house phantom with different iodine concentrations, the Catphan phantom, and QRM phantom with liver lesion inserts for model observer (MO) detectability. Results for 120kV spectral imaging (4 dose levels/3 matrix sizes) and 120kV UHR scanning are reported.
**Results or Findings:** Median and range of iodine HUs in the VNC of iodine-filled tubes were respectively -4.7 HU and 9.6 HU for the NAEOTOM, 0.6 HU, and 18.9 for CT_F and -14.7 and 17.7 for CT_E. HU difference showed less variation versus dose for the NAEOTOM. The 50% value of the task transfer function (TTF) for the Catphan air insert was 0.38mm-1 for all three scanners in standard mode, while this value was 1.41 mm-1 for the NAEOTOM UHR. Reconstructed volume size impacted MO results: images with slice 1024x1024 slice voxel matrix had significantly lower threshold diameter of 4.22mm ± 0.08mm, compared to 4.49mm ± 0.07mm for slices reconstructed at 512x512 ($p<0.03$).
**Conclusion:** The results confirmed the added value of the new generation scanner. Added value is expected from the UHR scanning and the improved spectral characteristics. Virtual non-contrast images have a minimal fraction of iodine contamination; many choices for the pre-programmed parameters selections were confirmed.
**Limitations:** More test result will be presented at the conference.
**Ethics committee approval:** Not applicable.
**Funding for this study:** Not applicable.
**Author Disclosures:**
Nicholas Marshall: Nothing to disclose
Walter Coudyzer: Nothing to disclose
Hilde Bosmans: Founder: Co-founder of Qaelum NV, Belgium
Chantal Van Ongeval: Nothing to disclose
Karen Merken: Nothing to disclose
Dimitar Petrov: Nothing to disclose
Hannelore Verhoeven: Nothing to disclose
Lore Dewulf: Nothing to disclose
Joke Binst: Nothing to disclose

### RPS 1013-4

**Task-based study of dose reduction using different kernels and model-based iterative reconstruction levels for low-contrast lesion**

G. Muti, *D. Curto*, S. Riga, F. Rizzetto, M. Felisi, C. De Mattia, P. E. Colombo, A. Vanzulli, A. Torresin; Milan/IT
*(denisecurto@live.it)*

**Purpose:** We aimed to establish the possible dose reduction for Siemens abdomen CT-protocol combining different level of advanced model-based iterative-reconstruction (MBIR, ADMIRE) and three soft-tissue kernels (Br40/Br32/Qr36), keeping the same detectability for low-contrast lesions (HCC, liver metastasis).
**Methods or Background:** We scanned a phantom at three dose levels: standard dose (13mGy), reduced dose (9mGy), and low dose (4mGy). Raw data was reconstructed using three different soft tissue kernels for FBP and MBIR at three levels. The noise power spectrum (NPS), the normalised one (nNPS), and the task-based transfer function (TTF) for one phantom's insert were computed following the method proposed by AAPM-TaskGroup-233. Detectability of simulated HCC lesion (low-contrast task;|ΔHU|=20) was calculated using non-pre-whitening with eye-filter model observer (NPWE).
**Results or Findings:** The nNPS, NPS, and TTF changed differently depending on kernels and MBIR levels. The NPS for images reconstructed using the highest level of MBIR showed a reduction of 50% for the noise magnitude, but also a shift of about 30% in peak frequency. The noise magnitude using Qr36 and Br32 kernels was 25% and 40% less than Br40. These variation, combined with the different TTFs, had an effect on detectability, which increased by about 10% and 5%, respectively. For each dose and kernel, the use of MBIR increased the detectability by more than 40% if used at the highest level. However, NPS's alterations, due to different MBIR levels and kernels, turned out in a different image texture, which was not always well accepted in clinical setting.
**Conclusion:** Starting from the detectability of standard protocol, we could reduce the dose choosing a new kernel-IR combination maintaining the same detectability. Depending on MBIR's level currently used, the dose reduction ranged from 15% up to 55%.
**Limitations:** Not applicable.
**Ethics committee approval:** Not applicable.
**Funding for this study:** Not applicable.
**Author Disclosures:**
Francesco Rizzetto: Nothing to disclose
Gaia Muti: Nothing to disclose
Angelo Vanzulli: Nothing to disclose
Alberto Torresin: Nothing to disclose
Denise Curto: Nothing to disclose
Paola E. Colombo: Nothing to disclose
Cristina De Mattia: Nothing to disclose
Marco Felisi: Nothing to disclose
Stefano Riga: Nothing to disclose

### RPS 1013-5

**Evaluation of the impact of ICRP 135 calculation methodology on LDRL deriving from a large cohort of oncology patients undergoing three phases multiregional CT**

*A. Kuchcinska*, J. Jasieniak, P. Rybarczyk, M. Dedecjus, P. Czuchraniuk, K. Wrzesien, D. Kiprian, A. Cieszanowski; Warsaw/PL
*(akuchcinska@wp.pl)*

**Purpose:** ICRP 135 suggest to calculate typical doses by excluding 5% of lowest and highest values from all dose data distribution. In current dose tracking systems, statistical parameters are calculated based on full distribution of the patients doses. The aim of the study is to check the differences between 75th percentiles of both distributions and evaluate the possible impact for decision whether mean dose exceeds LDRL.
**Methods or Background:** Dose data collected by DTS was exported to excel files and subsequently statistical parameters were recalculated. Analysed data concern, according to ICRP 135 recommendation, total DLP form whole examination that means 1st phase without contrast agent (chest, abdomen, pelvis), 2nd phase after 35s (chest + abdomen), and 3rd phase after 60s (abdomen + pelvis). Mean, median, and 75th percentile were calculated for 2110 patients divided by 6 relevant group of BMI (total 1567 patients) and one group of 543 patients for which BMI data wasn't available.
**Results or Findings:** The impact of the ICRP 135 methodology, result in change of the LDRL by the factor -1,1%±0,8% which is around 10,3%±8% of the interval between median and 75th percentile of dose distribution. For the cohort without BMI data, change in LDRL is 0,7%, which is 1,9% of the 36% dose difference between median (825,9 mGy*cm) and 75th percentile 1302,7 mGy*cm (full distribution) and 1293,4 mGy*cm (distribution modified according to ICRP 135).
**Conclusion:** Calculations provided by DTS, which do not implement the ICRP 135 approach, are connected with small statistical error and therefore should not impact the main purpose of the dose management, which is optimisation and general check if LDRL is exceeded e.g. due to equipment issues or changes in protocols.
**Limitations:** No limitations identified.
**Ethics committee approval:** Not applicable as it is a retrospective analysis of dose data.
**Funding for this study:** Not applicable.

**Author Disclosures:**
Agnieszka Kuchcinska: Nothing to disclose
Jakub Jasieniak: Nothing to disclose
Karolina Wrzesien: Nothing to disclose
Piotr Czuchraniuk: Nothing to disclose
Andrzej Cieszanowski: Nothing to disclose
Dorota Kiprian: Nothing to disclose
Pawel Rybarczyk: Nothing to disclose
Marek Dedecjus: Nothing to disclose

### RPS 1013-6

**Task-based study of detectability for dose reduction using different model-based iterative reconstruction for three computed tomography systems**

*G. Muti*, S. Riga, D. Curto, F. Rizzetto, M. Felisi, C. De Mattia, P. E. Colombo, A. Vanzulli, A. Torresin; Milan/IT
*(gaia.muti@unimi.it)*

**Purpose:** We aimed to evaluate the dose reduction achievable to obtain same detectability across computed tomography scanners with different model-based iterative-reconstruction (MBIR) algorithm developed by three manufacturers.
**Methods or Background:** Three CT system with full (IMR, Philips), partial (Asir-V, GE), and advanced (ADMIRE, Siemens) MBIR were used to scan a phantom at three doses: standard dose (13mGy), reduced dose (9mGy), and low dose (4mGy). The study was conducted using a standard kernel across different vendors and raw data were reconstructed using filtered-back projection (FBP) and three IR level (low/medium/high). The noise power spectrum (NPS), the normalised one (nNPS), and the task-based transfer function (TTF) for one phantom's insert were computed following the method proposed by AAPM-TaskGroup-233. Detectability of small lesion for soft tissue (contrast-task $|\Delta HU|=100$, 5mm diameter) was calculated using non-pre-whitening with eye-filter model observer (NPWE).
**Results or Findings:** NPS, nNPS, and TTF changed differently depending on CT systems. The highest value of detectability was found for advanced-MBIR, followed by full-MBIR, and last partial-MBIR for same dose and iterative strength: detectability of GE and Philips were about 40% and 25% lower than Siemens, respectively. For each system, same detectability of standard dose FBP reconstruction was achieved with lower doses choosing a higher iterative level. For Siemens there was a reduction of 10%, 30%, and 50% using an ADMIRE level 1, 3, and 5; for Philips there was a reduction of 40%, 45%, and 50% using an IMR level 1, 2, and 3; for GE there was a reduction of 15%, 20%, and 30% using an Asir-V level of 30%, 50%, and 70%, respectively.
**Conclusion:** Different MBIR algorithms were compared and the possible dose reduction using higher iterative levels was evaluated by a task-based metric.
**Limitations:** Not applicable.
**Ethics committee approval:** Not applicable.
**Funding for this study:** Not applicable.
**Author Disclosures:**
Francesco Rizzetto: Nothing to disclose
Denise Curto: Nothing to disclose
Gaia Muti: Nothing to disclose
Angelo Vanzulli: Nothing to disclose
Alberto Torresin: Nothing to disclose
Paola E. Colombo: Nothing to disclose
Cristina De Mattia: Nothing to disclose
Marco Felisi: Nothing to disclose
Stefano Riga: Nothing to disclose

### RPS 1013-7

**Empirical scatter correction (ESC): a universal scatter reduction method for cone-beam CT (CBCT) without prior knowledge**

*P. M. Trapp*, J. Maier, M. Susenburger, S. Sawall, M. Kachelrieß; Heidelberg/DE
*(philip.trapp@dkfz.de)*

**Purpose:** To correct for scatter artifacts in cone-beam CT (CBCT) scans without requiring specific knowledge of the scanning system and without the need for Monte Carlo simulations or trained neural networks.
**Methods or Background:** The image quality of CBCT scans often suffers from scattered radiation. Scatter corrections can be done using Monte Carlo simulations or the deep scatter estimation [Maier et al., Med. Phys. 46(1):238-249], for example. A drawback of such methods is that they require detailed knowledge about the CT system to be able to model scatter accurately enough. We propose the empirical scatter correction (ESC), which generates scatter-like basis projections from each projection image. A linear combination of these basis projections is subtracted from the measured projections in intensity domain. The log is taken and an FDK reconstruction is performed. The coefficients needed for the linear combination are determined automatically such that the reconstructed images show almost no scatter artifacts. We demonstrate the potential of ESC by correcting simulated and measured data.
**Results or Findings:** ESC is able to improve the image quality of the simulated data and the CBCT scan significantly. In soft tissue areas with severe scatter artifacts, the CT values closely match those of the ground truth (e.g. for the phantom measurement: difference to slit scan: -13 HU corrected, -289 HU uncorrected).
**Conclusion:** As the simulation results and the comparison to a slit scan show, ESC is able to reduce artifacts caused by scatter solely based on the projection data. Knowledge about the x-ray spectra or the materials involved is not required.
**Limitations:** ESC requires many reconstructions to find the optimum linear combination and thus may be suitable for offline use only.
**Ethics committee approval:** Not applicable.
**Funding for this study:** BMBF (grant number 13N14804, funding program: Photonics Research Germany (KMU Innovative)).
**Author Disclosures:**
Philip Maurice Trapp: Nothing to disclose
Marc Kachelrieß: Nothing to disclose
Stefan Sawall: Nothing to disclose
Markus Susenburger: Nothing to disclose
Joscha Maier: Nothing to disclose

### RPS 1013-8

**Multicentric comparative study of computed tomography dose indicators using an "in vivo" optical fiber detection system**

C. Devic[1], M. Munier[1], F. Pilleul[2], H. Rousseau[3], *C. Popotte*[1]; [1]Entzheim/FR, [2]Lyon/FR, [3]Toulouse/FR
*(christian.popotte@gmail.com)*

**Purpose:** Real-time personalised dosimetry specific of both the equipment and the patient may lead to a paradigm shift in computed tomography dosimetry. The aim of this work is to compare dose index measured by a new real-time in vivo dosimeter under clinical CT conditions and estimated dose index displayed by scanners, and to detect any anomalies during CT procedures. Specific cases are presented.
**Methods or Background:** A multicentric study was conducted in 4 French medical centers and 5 CT scans from 3 different manufacturers. An innovative detector, based on a scintillating optical fiber (IVIscan®, FIBERMETRIX, France) has been used routinely to measure CTDI and DLP. The IVIscan® system was also used to determine the z-axis CTDI mapping and distribution for procedures including those involving several acquisitions.
**Results or Findings:** Dose indexes were generally consistent with those displayed by the scanners independently of the manufacturer, which validates the use of the IVIscan® device under clinical conditions. However, large differences have been observed for some examinations and allowed us to detect scanner modulation failures and poor patient positioning leading to overdoses up to +300%. In addition, CTDI mapping allows a better assessment of the dose actually delivered during a CT procedure compared to the average CTDI usually used especially for procedures with several acquisitions.
**Conclusion:** Thanks to reliable real-time measured dose indexes and an innovative CTDI mapping system, IVIscan® is an independent dosimetric monitoring tool which allows to detect dose heterogeneities and identify possible malfunctions of the CT device or unusual practices.
**Limitations:** Headrest head CT could not be integrated in this study.
**Ethics committee approval:** The data used in this study do not require ethics committee approval.
**Funding for this study:** This study is part of the development of the material used and did not require specific research funding.
**Author Disclosures:**
Frank Pilleul: Nothing to disclose
Hervé Rousseau: Nothing to disclose
Christian Popotte: Employee: Fibermetrix
Clément Devic: Employee: Fibermetrix
Mélodie Munier: Founder: Fibermetrix

16:00-17:00 **Channel 4**

### Research Presentation Session: Emergency Imaging

### RPS 1117

### Detection and interpretation of notable imaging findings in emergency setting

**Moderators**
A. Platon; Geneva/CH
L. E. Derchi; Genoa/IT

### RPS 1117-3

**Contrast medium allergy resulting in suboptimal CTs: the experience at a tertiary hospital**

*M. C. B. Morgan*, E. Okonkwo, F. Yarar, S. Liu, E. C. P. M. Serrao, T. Sadler; Cambridge/UK
*(michael.morgan9@nhs.net)*

**Purpose:** Iodinated contrast (IC) mediums are vital in various imaging modalities. Reported allergies to IC can lead to suboptimal diagnostic workups and the erroneous denial of indicated procedures. We aimed to determine the number of suboptimal CT studies due to IC allergy in our institution and understand whether referral to the immunology allergy service provided any benefit.
**Methods or Background:** The electronic medical records (EMR) of all patients who underwent a CT and were documented to be "allergic to IC", in a two-year period at a single tertiary care hospital were retrospectively reviewed. EMRs were evaluated for documented allergies to 'brand' and 'generic' IC, type of allergy and relevant allergy clinic referral. Allergies were coded as mild, moderate, severe, physiological or unknown.
**Results or Findings:** 141652 CT studies were performed over the period with 1% (545 patients) meeting the study criteria. Suboptimal imaging was acquired in 40.5% of the relevant CTs (221 patients). 75/221 patients had a 'mild' allergy. A total of 26 patients were referred to an allergy clinic, with 54% cleared upon review. Cleared patients had a 'mild' or 'unknown' allergy severity in 79% of the cases.
**Conclusion:** IC medium is essential for providing accurate diagnoses and is relatively safe. An erroneous allergy to IC can deprive patients of crucial diagnostic imaging. We found that allergies occurred in 1% of our CT volume and patients with a 'mild' or 'unclear' allergy gain the most from allergy testing referral, with 79% being cleared of their allergy.
**Limitations:** External allergy testing would not be captured by our EMR leading to a potential underestimate in the number of our patients who have undergone testing.
**Ethics committee approval:** Approval received.
**Funding for this study:** No funding was received for this study.
**Author Disclosures:**
Shizhang Liu: Nothing to disclose
Timothy Sadler: Nothing to disclose
Ekene Okonkwo: Nothing to disclose
Feyza Yarar: Nothing to disclose
Eva Carolina Pereira Mendes Serrao: Nothing to disclose
Michael Christopher Bentley Morgan: Nothing to disclose

### RPS 1117-4

**Expert-raters agreement in the assessment of early ischaemic changes in the territory of the middle cerebral artery (MCA)**

*P. Andropova*, D. G. Cheremisin, P. Gavrilov; St. Petersburg/RU
*(polin.and@icloud.com)*

**Purpose:** The objective of this study was to describe the agreement in the assessment of early ischaemic changes in the middle cerebral artery (MCA) territory between expert raters of various qualifications.
**Methods or Background:** We identified patients presenting with hemiparesis or aphasia at the emergency department who underwent CT. Eight raters of different skill levels and various qualifications reviewed and scored the anonymised CT scans of 100 patients. The list of expert raters included: two neuroradiology fellows, two radiologists (with three years of experience in emergency neuroradiology), two radiologists (with three years of experience not in urgent medicine), and two experienced emergency neuroradiologists (more than eight years experience). The expert raters completed a preliminary interpretation form that included their classification of the interpretations as follows: "presence," "absence," of signs of an MCA stroke. And also, the presence or absence of such signs as (1) hyperdense MCA sign, (2) sulcal effacement, (3) parenchymal hypoattenuation, and (4) focal swelling or mass effect were appreciated. Interobserver agreement was measured with the kappa statistic, sensitivity, and specificity.
**Results or Findings:** The measured interobserver agreement had an interval of 0.2-0.7 for any aforementioned early ischaemic changes. The detection of early signs of ischaemia changes with CT had a mean accuracy of 85%, a sensitivity of 81%, and a mean specificity of 88%.
**Conclusion:** There's a clear necessity in further elaboration of determination of most reliably detected signs and understanding the role of scoring systems in detection improvement.
**Limitations:** The experts were not presented with data on the clinical manifestation indicating the ischaemic hemisphere.
**Ethics committee approval:** Ethical approval for this study was obtained from the Institute of Human Brain of RAS.
**Funding for this study:** This study did not receive any specific grant from funding agencies in the public, commercial, or not-for-profit sectors.
**Author Disclosures:**
Polina Andropova: Author: PA is the first author
Pavel Gavrilov: Author: PG has revised the manuscript
Dmitriy Grigorievich Cheremisin: Author: DC has revised the manuscript

### RPS 1117-5

**CT after emergency surgery in penetrating trauma: a seven-year experience in a level I Nordic trauma center**

K. H. Halldorsson[1], M. T. Nummela[2], S. Þórisdóttir[3], G. Oladottir[3], *S. K. Koskinen*[1]; [1]Stockholm/SE, [2]Helsinki/FI, [3]Reykjavik/IS
*(sekakos@gmail.com)*

**Purpose:** Patients with severe penetrating trauma may require emergency surgery on arrival, and postoperative CT can reveal additional significant injuries. To determine the utility of postoperative CT performed within 48 hours of emergency surgery after penetrating trauma.
**Methods or Background:** Trauma registry data was retrieved retrospectively over a seven-year period (2013-2019) at a single level 1 trauma center. All patients, 17-years and older, admitted with penetrating injury, who underwent urgent surgery and postoperative CT imaging within 48 hours, were included. Preoperative and intra-operative medical records were compared to CT findings.
**Results or Findings:** Patients with gun shot wounds (GSWs) had a longer ICU length of stay and were more severely injured than patients with stab wounds (SWs) (mean ISS, 23.00; range, 1-75; for GSWs, 27.50; for SWs, 20.58, $p = 0.08460$, mean NISS, 31.53; range, 3-75; for GSWs, 38.80; for SWs, 28.19, $p = 0.0422$). 20 out of 38 patients (52.6%) had additional findings at postoperative CT, most of which were minor. Six patients (15.8%) had previously unidentified or underestimated findings at CT that were severe enough to warrant additional surgery or interventional angiography.
**Conclusion:** CT imaging after emergency surgery in penetrating trauma is an important tool in evaluating the injury as a whole and revealing previously undiagnosed and unexpected injuries. Six out of 38 (15.8%) patients had findings at postoperative CT that warranted additional surgical or angiographic intervention.
**Limitations:** Our analysis is retrospective and patients were only selected from a single level 1 trauma center. Small cohort size with limited statistical power. No control group.
**Ethics committee approval:** Approval from the local ethics committee was obtained for this retrospective study (Dnr 2017/1018-31/2, 2020-02164).
**Funding for this study:** Not applicable.
**Author Disclosures:**
Kolbeinn Hans Halldorsson: Nothing to disclose
Mari T. Nummela: Nothing to disclose
Seppo K. Koskinen: Nothing to disclose
Sigurveig Þórisdóttir: Nothing to disclose
Gudrun Oladottir: Nothing to disclose

### RPS 1117-6

**Imaging findings of elder abuse on the trauma service: a retrospective case-control study**

*A. W. P. Wong*, E. Sun, E. Goralnick, A. Salim, B. Khurana; Boston, MA/US

**Purpose:** The WHO estimates that one out of six elders has experienced some form of abuse over the past year, with impact ranging from diminished quality of life to physical injury and death. Screening is a recognised strategy for detection, but less effective in elders with a diminished ability to communicate or psychosocial reasons to fear disclosure. This study aimed to identify imaging findings of elder abuse.
**Methods or Background:** This retrospective case control study identified 12 patients above 60 years of age with keywords "assault" and "abuse" from a multi-institutional trauma registry database from 2015-2020, as well as 12 age and gender matched controls. Demographic information, clinical information, and imaging findings were reviewed.

**Results or Findings:** Our case series demonstrated excellent agreement with known risk factors for elder abuse. Rib fractures, extra-axial head injuries, and upper extremity injuries were most commonly seen, consistent with prior reports of successfully prosecuted cases of elder abuse. In one case, radiologists identified injuries occult on physical examination but extensive on imaging in a patient who was reported to have fallen.
**Conclusion:** This study demonstrated that radiologists can be helpful in the assessment of elder abuse, identified head injuries and upper extremity injuries as warning signs of elder abuse, and highlighted the importance of further research to identify imaging findings of elder abuse.
**Limitations:** This case control study was limited by the small number of patients identified as victims of elder abuse, drawn from the northeastern United States, and limited assessment of patient and abuser risk factors.
**Ethics committee approval:** IRB approved with informed consent waived.
**Funding for this study:** Not applicable.
**Author Disclosures:**
Ali Salim: Nothing to disclose
Bharti Khurana: Nothing to disclose
Eric Goralnick: Nothing to disclose
Andrew Wei Ping Wong: Nothing to disclose
Ellen Sun: Nothing to disclose

### RPS 1117-7

**Abdominal cocoon in tuberculosis: a diagnostic challenge**

*R. Rastogi*; Moradabad/IN
*(rajulrst@yahoo.co.in)*

**Purpose:** Abdominal cocoon is an uncommon condition referring to encapsulation of small bowel by a fibro-collagenous membrane leading ultimately to acute or chronic bowel obstruction. Preoperative recognition of the condition is difficult but imperative to institute early management. We did a pilot study, involving 10 cases of abdominal cocoon, all secondary to abdominal tuberculosis and evaluated the most common radiological findings.
The aim of the study was to evaluate the most common findings of cocoon secondary to Koch's abdomen.
**Methods or Background:** All ten patients underwent radiological evaluation in the form of ultrasonography (USG) followed by cross-sectional imaging in the form of computed tomography (CT scan) or magnetic resonance imaging (MRI). All the radiological findings were recorded and compared with clinico-pathological findings.
**Results or Findings:** In all ten patients, the disease was diagnosed accurately with radiological investigations using clinical and pathological findings as the gold-standard. In eight out of ten cases, follow-up imaging following ATT revealed complete resolution while in rest 2 case; diagnosis was confirmed with biopsy and ATT following laparotomy done for intestinal obstruction.
**Conclusion:** Prior to imaging era, the correct diagnosis of abdominal cocoon was usual only at surgery where the abdominal cocoon appeared as a dense grey white capsule containing a part or whole of the small bowel. The bowel loops within the cocoon can usually be easily freed in spite of adhesions. Late cases, however, may be associated with necrotic changes in the bowel wall requiring bowel resection. Abdominal cocoon is an uncommon feature of tuberculosis. Thick fibrotic peritoneal membrane wrapping the small bowel is pathognomonic. This condition can be diagnosed with confidence by utilising various radiological investigations. Early recognition results in proper management and prevents the need for bowel resection.
**Limitations:** The limited sample size.
**Ethics committee approval:** Not applicable.
**Funding for this study:** Not applicable.
**Author Disclosures:**
Rajul Rastogi: Nothing to disclose

### RPS 1117-8

**CT in acute colitis: is this a valuable tool to reduce overtreatment?**

*G. Piga*, M. Scaglione, A. Contena, A. Canu, A. Achene, M. Conti, P. Crivelli; Sassari/IT
*(g.piga88@virgilio.it)*

**Purpose:** The goal of this study was to establish how CT influenced subsequent therapeutic choices in patients with suspected acute colitis.
**Methods or Background:** Between January and June 2021, we retrospectively reviewed the CT scans of 48 patients admitted to our Emergency Department, with a suspected clinical diagnosis of colitis. CT findings were compared to the clinical, surgical, or endoscopic findings. The severity of the CT findings was assessed on the basis of the wall patterns and/or associated complications. The type of treatment each subject underwent in relation to the CT findings was then evaluated.
**Results or Findings:** All the patients showed transmural wall thickening at CT. Thirty/48 patients (62,5 %) had target and/or halo sign and treated conservatively. Two people (4,1 %), one with pneumatosis and one with reduced wall enhancement, underwent surgical colonic resection. Two/48 patients with an abscess collection (4,1 %) were treated with percutaneous drainage. Three patients (6,2 %), one with perforation secondary to wall ischemia and peritonitis and another two with severe and extensive pneumatosis, rapidly died. Therefore, the CT signs that changed the clinical decision were pneumatosis, ischemic wall changes or abscess. Based on the CT findings, 46/48 (95,8 %) patients were treated conservatively.
**Conclusion:** High performance of CT allows to direct the patient towards NOM (Non-Operative Management), alternatively to surgery or percutaneous drainage, allowing a timely differential diagnosis, reducing the number of unnecessary laparotomies and the number of intra and extra-hospital mortality.
**Limitations:** Retrospective study, with limited sample.
**Ethics committee approval:** Approved by the ethics committee.
**Funding for this study:** No funding was received for this study.
**Author Disclosures:**
Paola Crivelli: Nothing to disclose
Alessio Contena: Nothing to disclose
Antonio Achene: Nothing to disclose
Antonio Canu: Nothing to disclose
Mariano Scaglione: Nothing to disclose
Giorgio Piga: Nothing to disclose
Maurizio Conti: Nothing to disclose

### Friday, March 4

**08:00-09:00** **Channel 4**

### Research Presentation Session: Radiographers

### RPS 1214

### Patient-focused radiography practice

**Moderators**
K. Borg Grima; Msida/MT
M. Vernooij; Rotterdam/NL

### RPS 1214-3

**Radiographers' attitudes and opinions towards management and care of patients with dementia**

C. Devereux, R. Young, *M. F. F. McEntee*; Cork/IE
*(mark.mcentee@ucc.ie)*

**Purpose:** Dementia is an umbrella symptom, encompassing a number of different diseases. It results in progressive cognitive decline. Diagnostic imaging plays a major role in the diagnosis of dementia, however patients with dementia attend from imaging for diagnosis of all diseases and injuries. Caring for them in a short visit can be challenging. The aim of this study is to determine Irish radiographers' opinions towards caring for patients with dementia in the radiology department and to examine the protocols that exist.
**Methods or Background:** This was a qualitative study, which utilised two focus groups to collect study data. A total of eleven radiographers participated in the data collection. The focus groups were transcribed and thematically analysed using NVivo software.
**Results or Findings:** Participants reported being apprehensive in caring for patients with dementia. No guidelines existed for caring for patients with dementia in Irish radiology departments. The themes that emerged were: (1) practice concerns (patient care, distress, quality of imaging fear, physical violence, safety, consent, justification, compassion), (2) change management of patient care (improving practice, willingness to change), (3) infrastructure and staffing (time, scheduling, carers, support) and (4) knowledge and attitudes (experience, expectations, knowledge, understanding, stigma).
**Conclusion:** Radiographers have concerns about caring for patients with dementia. There is a lack of knowledge about dementia care amongst the participants. Improvements in knowledge should be addressed. The development and implementation of best practice guidelines to care for patients with dementia would standardise the care of patients.
**Limitations:** Only one researcher interpreted and analysed the focus group data. Going forward it would be worth assessing radiographers in multiple hospitals, both within the public and private sectors.
**Ethics committee approval:** The ethical approval for this study was obtained from the Social Research Ethics Committee, UCC, Ireland, (CT-SREC-2020-41).
**Funding for this study:** No funding was received for this study.
**Author Disclosures:**
Mark F. F. McEntee: Nothing to disclose
Rena Young: Nothing to disclose
Caitlin Devereux: Nothing to disclose

### RPS 1214-4

**Implementation of a telemedicine, stroke evaluation service**

*E. Kjelle*, A. M. Myklebust; Drammen/NO
*(elin.kjelle@usn.no)*

**Purpose:** To assess how healthcare managers and personnel experience the quality, organisation and value of a rural telemedicine, remote-controlled CT stroke evaluation service.
**Methods or Background:** Semi-structured interviews were conducted and covered ten individual and one focus-group interview including managers, paramedics, radiographers, and junior doctors. The interview guide consisted of the following themes: experience of working with the service, task shifting, quality, management and challenges. Interviews were recorded and transcribed before thematic content analysis was used to develop a narrative of the findings.
**Results or Findings:** Findings were categorised into teamwork, quality, value of the service, organisation of the project, and from project to permanent service. Participants perceived the service as valuable for patients and the local community. The service included task shifting where paramedics positioned the patient in the CT-scanner, while the radiographer ran the scan remotely. This required education, training and changing of routines to facilitate the telemedicine service. The participants experienced the process as both challenging and interesting. The service was considered to improve patient care and health services in the community.
**Conclusion:** The service was perceived as valuable to the local community and of high quality. Communication, training, flexibility, and cooperation within and between the departments locally, as well as with the external hospitals appears to be a key factor for a successful implementation and long-term sustainability of the service.
**Limitations:** This study has a combination of one focus group and several individual interviews. This was due to two factors: the ongoing COVID-19 pandemic and the difficulty in recruitment. This combination may lead to a different depth in data obtained from managers in the focus group compared to managers and personnel in the individual interviews.
**Ethics committee approval:** Yes - NSD 358427.
**Funding for this study:** Not applicable.
**Author Disclosures:**
Elin Kjelle: Nothing to disclose
Aud Mette Myklebust: Nothing to disclose

### RPS 1214-5

**Meeting the imaging service demand of an increasingly ageing population with cancer care needs**

*A. Chukwuani*[1], D. Omiyi[2], A. Ginigeme[3], A. Umunna[4]; [1]Birmingham/UK, [2]Bradford/UK, [3]Washington, DC/US, [4]Manchester/UK
*(anselm.chukwuani@gmail.com)*

**Purpose:** The aim of this paper is to review the current state of imaging service delivery for elderly cancer patients, and examine how to shape the future direction of clinical imaging service delivery to cater for an increasingly ageing population.
**Methods or Background:** Due to ageing, cancer is majorly a disease of the elderly. It is also clearly established that clinical imaging plays a central role in the diagnosis and management of cancer. Clinical imaging is utilised at all stages of the cancer patient pathway: diagnosis, staging, selecting the appropriate therapy, and follow up. In a nutshell, an increasingly ageing population means more cancer cases, resulting in more demand for imaging service needs.
**Results or Findings:** The elderly have special needs, and the imaging study of the elderly poses unique challenges. The current heterogenous models of care are majorly adapted to local priorities, needs and available resources. There is an urgent need to draw up models of care specially tailored to address the needs of the elderly in imaging. With the NHS Long Term Plan mapped out, it is hoped that the care for elderly cancer patients should see a significant boost in resource allocation. Some of these resources could be channelled into drawing up plans on how to ensure the provision of dedicated imaging service for elderly cancer patients.
**Conclusion:** Looking into the future, imaging service will require a lot of investment in imaging equipment, and human resources - skillfully trained to effectively understand and cater for the special needs of an increasingly ageing population.
**Limitations:** This paper is the result of review of available literature, and focused on the UK experience.
**Ethics committee approval:** Ethics Committee approval was obtained for this study.
**Funding for this study:** This study was self-funded by the researchers.
**Author Disclosures:**
Anselm Chukwuani: Nothing to disclose
David Omiyi: Nothing to disclose
Anthony Umunna: Nothing to disclose
Anita Ginigeme: Nothing to disclose

### RPS 1214-6

**Autism-friendly MRI: the patients' perspective**

*N. Stogiannos*[1], J. Harvey-Lloyd[2], A. Brammer[3], C. Papadopoulos[4], B. J. Nugent[1], J. McNulty[5], C. S. d. Reis[6], K. Cleaver[7], T. O'Regan[1], C. M. Simcock[1], S. Parveen[1], K. Marais[1], G. Pavlopoulou[1], D. Bowler[1], S. Gaigg[1], C. Malamateniou[1]; [1]London/UK, [2]Suffolk/UK, [3]Manchester/UK, [4]Luton/UK, [5]Dublin/IE, [6]Lausanne/CH, [7]Greenwich/UK
*(nstogiannos@yahoo.com)*

**Purpose:** To map out the perspectives, needs, and preferences of autistic service users who have experienced an MRI examination in the UK. To gain an insight into the main barriers and facilitators to inclusive and safe MRI examinations when scanning autistic adults or children.
**Methods or Background:** Two online surveys were used, one for autistic individuals over 16 years of age, and the other for parents/carers of autistic individuals, with prior MRI experience. Snowball sampling was employed; the surveys were distributed through the researchers' networks and through the autistic community on Twitter, LinkedIn and Facebook, the National Autistic Society and the London Autism Group. The surveys were open between February 1st and April 30th, 2021. Patient and public involvement was employed during all stages of the project. The SPSS software was used for statistical analyses.

**Results or Findings:** A total of 128 valid responses were received (112 autistic adults and 16 parents/carers of autistic children). The main barrier to a successful MRI scan was poor communication either between healthcare services or between patients and practitioners. Non-disclosure of autism occurred in more than half of the responses (53,6%). Failure to provide customised MRI examinations or autism-friendly MRI environments with reasonable adjustments (82.9%) were major contributing factors to a poor patient experience.
**Conclusion:** Current practice in MRI scanning is not taking into account the autistic service user's needs. Optimal communication throughout and provision of reasonable environment adjustments is vital to ensure inclusive MRI scanning practices.
**Limitations:** Both research design and methodology, and recruitment of participants were impacted by COVID-19 restrictions. Convenience sampling means results should be interpreted with caution.
**Ethics committee approval:** School of Health Sciences, City, University of London Research Ethics Committee [ETH1920-1988].
**Funding for this study:** Society and College of Radiographers CORIPS grant scheme [SCoR 155-50011HY].
**Author Disclosures:**
Claudia Sa dos Reis: Nothing to disclose
Tracy O'Regan: Nothing to disclose
Karen Cleaver: Nothing to disclose
Keith Marais: Nothing to disclose
Jonathan McNulty: Nothing to disclose
Georgia Pavlopoulou: Nothing to disclose
Sophia Parveen: Nothing to disclose
Chris Papadopoulos: Nothing to disclose
Nikolaos Stogiannos: Nothing to disclose
Dermot Bowler: Nothing to disclose
Barbara J. Nugent: Nothing to disclose
Sebastian Gaigg: Nothing to disclose
Clare Marie Simcock: Nothing to disclose
Andrea Brammer: Nothing to disclose
Jane Harvey-Lloyd: Nothing to disclose
Christina Malamateniou: Nothing to disclose

### RPS 1214-7
**Autism-friendly MRI: the radiographers' perspective through a UK-wide survey**
*N. Stogiannos*[1], J. Harvey-Lloyd[2], B. J. Nugent[3], A. Brammer[4], S. Carlier[5], K. Cleaver[6], J. McNulty[7], C. S. d. Reis[5], C. Malamateniou[1]; [1]London/UK, [2]Suffolk/UK, [3]Edinburgh/UK, [4]Manchester/UK, [5]Lausanne/CH, [6]Greenwich/UK, [7]Dublin/IE
*(nstogiannos@yahoo.com)*

**Purpose:** To explore radiographic practices, training/educational needs, as well as the UK radiographers' perspectives when scanning autistic service users with MRI.
**Methods or Background:** An online survey was constructed on Qualtrics and pilot-tested by field experts. All UK-based MRI radiographers were invited to participate. The snowball sampling technique was employed. The survey was distributed by three recruitment agencies between December 2020 and February 2021 on social media. Descriptive and inferential statistics were used to analyse the results using the SPSS software.
**Results or Findings:** This study received 130 valid responses. Effective communication between the patient and the MRI radiographer, adjusted MRI unit environment, and customisation of the MRI examination were found to be beneficial for a successful MRI examination. However, a persistent lack (but also desire) of autism-related training was noted (75.6%). Poor patient-practitioner communication, lack of training (41.5%), lack of Special Educational Needs experts (38.6%), and lack of specific guidelines (37.7%), were the main barriers to a successful MRI examination.
**Conclusion:** Reasonable adjustments are required when scanning autistic individuals, mainly in the context of communication and the MRI unit environment. Formal training is required for MRI radiographers, and guidelines should also be established to assist them in clinical practice.
**Limitations:** The number of responses and the use of convenience sampling mean that the results cannot be seen as representative of the UK-based MRI radiographers, but they still offer some useful insights. Also, the COVID-19 pandemic has negatively impacted the recruitment of radiographers as they were working on the frontline during the second national lockdown.
**Ethics committee approval:** School of Health Sciences, City University of London Research Ethics Committee [ETH1920-1988].
**Funding for this study:** The Society and College of Radiographers CORIPS grant scheme [SCoR 155-50011HY].
**Author Disclosures:**
Sarah Carlier: Nothing to disclose
Claudia Sa dos Reis: Nothing to disclose
Karen Cleaver: Nothing to disclose
Jonathan McNulty: Nothing to disclose
Nikolaos Stogiannos: Nothing to disclose
Barbara J. Nugent: Nothing to disclose
Andrea Brammer: Nothing to disclose
Jane Harvey-Lloyd: Nothing to disclose
Christina Malamateniou: Nothing to disclose

### RPS 1214-8
**Evaluating the effect of music on anxiety during mammography cancer screening**
S. Ellul[1], *F. Zarb*[2], K. B. Borg Grima[2], D. Mizzi[2]; [1]Zurrieq/MT, [2]Msida/MT
*(francis.zarb@um.edu.mt)*

**Purpose:** This study aimed to investigate whether the introduction of music medicine (MM) during mammography examinations, has an effect on the anxiety level experienced by clients undergoing breast cancer screening.
**Methods or Background:** This study followed a quantitative, prospective, and experimental design. Participants were imaged according to the local breast cancer screening protocol, with the experimental group being exposed to MM, selected based on the literature. Anxiety levels were measured before and after each mammogram via the State-Trait Anxiety Inventory for Adults research tool.
**Results or Findings:** Participants in both experimental and control groups experienced a statistically significant increase in anxiety levels before the mammogram, when compared to their normal anxiety levels ($p=<0.001$). Both groups experienced a statistically significant decrease in anxiety levels after the mammogram when compared to their anxiety levels before the mammogram (experimental group: $p=0.005$; control group: $p=0.001$). No significant statistical difference ($p=0.907$) in the anxiety levels after the mammograms was recorded between the experimental and control groups.
**Conclusion:** Anxiety levels indicated that mammography screening induces anxiety and that anxiety levels were reduced in both groups after the mammography examination. A variable contributing to this reduction could be MM. Thus, MM could be used in the clinical setting since it is non-invasive and cheap. Nonetheless, in this study MM had no statistical significant effect in decreasing anxiety levels during mammography screening.
**Limitations:** Due to the cost of the research tool, the study had a small sample size (n=50). Thus, only MM was investigated as a variable and no music choice was given to participants. Further studies with a larger sample size, a choice of music from different genres and investigating other variables which affect anxiety levels are recommended.
**Ethics committee approval:** Approval was obtained from the University of Malta Research Ethics Committee (code:562601062020).
**Funding for this study:** Not applicable.
**Author Disclosures:**
Karen Borg Borg Grima: Author: Not applicable
Deborah Mizzi: Author: Not applicable
Francis Zarb: Nothing to disclose
Sara Ellul: Author: Not applicable

**10:30-11:30** **Channel 4**

### Research Presentation Session: Musculoskeletal

### RPS 1310
### Knee joint

**Moderators**
M. Tzalonikou; Athens/GR
L. E. Derchi; Genoa/IT

### RPS 1310-3
**Sensitivity of DECT in ACL tears: a prospective study with arthroscopy as reference method**
*A-S. Björkman*[1], H. Gauffin[1], A. Persson[1], S. K. Koskinen[2]; [1]Linköping/SE, [2]Stockholm/SE

**Purpose:** To investigate the diagnostic accuracy of dual-energy CT (DECT) for the detection of ACL tears in the acutely and subacutely injured knee with arthroscopy as reference method.
**Methods or Background:** Patients with suspected ACL injury were imaged with DECT (Somatom Force, Siemens Healthcare, Germany) and 3.0 T MRI (Ingenia, Philips Medical Systems, Best, The Netherlands). Clinically blinded images were independently read by two radiologists. ACL was classified as normal or abnormal. Arthroscopy served as reference standard. Sensitivity and positive predictive value (PPV) were calculated. Also, sensitivity between DECT and MRI was assessed.
**Results or Findings:** 48 patients (26 M, 22 F, mean age 23 years, range 15-37 years) were imaged a mean 25 days following trauma. Of these, 21 patients underwent arthroscopy with a mean of 195 days after trauma. Arthroscopy revealed 19 ACL tears and 2 ACLs with no tear. The sensitivity was 76.3% (95% CI 66.8-85.9) and 86.8 (95% CI 71.9-95.6) for DECT and MRI, respectively (p=0.223). The positive predictive value (PPV) was 93.5 (95% CI 84.3-98.2) and 91.7 (95% CI 77.5-98.3) for DECT and MRI, respectively.
**Conclusion:** DECT has lower sensitivity to detect an ACL rupture than MRI but the difference was not statistically significant. The PPV was high in both methods.
**Limitations:** The age of the subjects (15-40 years) limits the generalizability of the results to older populations. The low number of true negatives did not allow to calculate specificity.
**Ethics committee approval:** Ethical approval from the regional ethical review board (2016/44-31 and 2017/221-32) and radiation protection committee was obtained.
**Funding for this study:** This NACOX-cohort study is supported by the Swedish Medical Research Council, the Swedish Research Council for Sport Science, the Medical Research Council of Southeast Sweden and ALF Grants Region Östergötland
**Author Disclosures:**
Anders Persson: Nothing to disclose
Håkan Gauffin: Nothing to disclose
Seppo K. Koskinen: Nothing to disclose
Ann-Sofi Björkman: Nothing to disclose

### RPS 1310-4

### MRI in patients with verified anterior cruciate ligament tears: evaluation of frequency of ramp lesions and anterolateral ligament injuries and correlation with combined injuries of the knee

*N. Stranger*[1], C. Kaulfersch[2], G. P. Mattiassich[2], J. Mandl[1], P. A. Hausbrandt[1], D. H. M. Szolar[1], H. Schöllnast[1]; [1]Graz/AT, [2]Schladming/AT

**Purpose:** To assess the frequency of ramp lesions (RL) and anterolateral ligament (ALL) injuries in MRI of patients with verified anterior cruciate ligament (ACL) tears, and to describe the coexistence of these injuries with further injuries of the knee.
**Methods or Background:** In this retrospective study, 163 patients with surgical repair of ACL tears were included. MRI scans were reviewed for RL, which were defined according to Greif et al., and ALL injuries. In addition, all coexisting meniscal injuries, injuries of the medial and lateral collateral band and bone marrow oedema were recorded. The correlation of RL and ALL-injuries with coexisting injuries was tested for statistical significance using Pearson's chi-square test. After Bonferroni correction for multiple testing a p<0.003 was defined as statistically significant.
**Results or Findings:** RL occurred in 52 patients (31.9%). RL were significantly associated with tears of the posterior horn of the medial meniscus (67.3% versus 19.8% of patients with/without RL, respectively) and with subchondral bone marrow oedema in the postero-medial tibia plateau (63.5% versus 0% of patients with/without RL, respectively). ALL-injuries were detected in 52 patients (31.9%). ALL-injuries were significantly associated with a tear of the posterior horn of the lateral meniscus (51.9% versus 16.2% of patients with/without ALL-injury, respectively), tear of the medial collateral ligament (40.4% versus 16.2% of patients with/without ALL-injury, respectively) and tear of the lateral collateral ligament (19.2% versus 0.9% of patients with/without ALL-injury, respectively).
**Conclusion:** The findings of our study demonstrate that in patients with ACL-tears, the frequency of RL and ALL-injuries is about 32%. Both injuries are frequently associated with further injuries of the knee, which are different between RL and ALL-injuries.
**Limitations:** Lesions not confirmed with arthroscopy.
**Ethics committee approval:** Approval of the Ethics Committee of the Medical University of Graz.
**Funding for this study:** No funding was received for this study.
**Author Disclosures:**
Nikolaus Stranger: Nothing to disclose
Helmut Schöllnast: Nothing to disclose
Peter Anton Hausbrandt: Nothing to disclose
Christian Kaulfersch: Nothing to disclose
Georg Philipp Mattiassich: Nothing to disclose
Dieter H. M. Szolar: Nothing to disclose
Jürgen Mandl: Nothing to disclose

### RPS 1310-5

### Untangling the nature of subchondral bone marrow lesions of the knee with the use of deep learning: a multi-centre cross-sectional study

*M. Klontzas*[1], E. E. Vassalou[1], G. A. Kakkos[1], K. Spanakis[1], A. Zibis[2], K. Marias[1], A. H. Karantanas[1]; [1]Heraklion/GR, [2]Larissa/GR
*(miklontzas@gmail.com)*

**Purpose:** Bone marrow lesions (BMLs) of the knee are commonly found in the context of subchondral insufficiency fractures (SIF) and advanced osteoarthritis (OA). The purpose of our work was to utilize deep learning in the form of convolutional neural networks to differentiate between the two conditions.
**Methods or Background:** The study dataset consisted of MRIs of knees with BMLs in the context of SIF (n=212) and OA (n=102), which were retrospectively collected and augmented to create a final dataset of 1174 images. Transfer learning was applied by utilizing an ImageNet-pretrained InceptionV3 convolutional neural network (CNN) which was fine-tuned with the use of the aforementioned MRI dataset. CNN performance was assessed on a validation cohort of 87 images of each group and was compared to that of two MSK radiologists with the use of receiver operating characteristics (ROC) curves and areas under the curve (AUC). Precision, recall and f1-scores were computed for the CNN and expert readers.
**Results or Findings:** InceptionV3 achieved an AUC of 93.68%, correctly classifying 82/87 OA and 81/87 SIF validation images. The first of the two MSK radiologists performed equally to the CNN, achieving an AUC of 91.95%, whereas the performance of the second expert MSK radiologist was significantly lower compared to both the CNN and the other reader (P<0.001) reaching an AUC of 82.76%.
**Conclusion:** A CNN model was highly accurate in differentiating between SIF and OA, achieving a higher or equal performance to MSK radiologists.
**Limitations:** Not applicable.
**Ethics committee approval:** Approved by the University Hospital of Heraklion (No 360/08/29-04-2020).
**Funding for this study:** Not applicable.
**Author Disclosures:**
Michail Klontzas: Nothing to disclose
George Anthony Kakkos: Nothing to disclose
Kostas Marias: Nothing to disclose
Konstantinos Spanakis: Nothing to disclose
Evangelia E. Vassalou: Nothing to disclose
Apostolos H. Karantanas: Nothing to disclose
Aristeidis Zibis: Nothing to disclose

### RPS 1310-7

### On the interchangeability of standard 2D and accelerated 3D knee MRI: is it time to consider a paradigm shift?

*C. Smekens*[1], E. De Smet[2], E. Roelant[1], T. Vande Vyvere[1], A. Snoeckx[2], P. Van Dyck[2]; [1]Wilrijk/BE, [2]Edegem/BE
*(celine.smekens@uantwerpen.be)*

**Purpose:** To evaluate the interchangeability of multi-contrast 3D controlled aliasing in parallel imaging results in higher acceleration (CAIPIRINHA) sampling perfection with application optimized contrast using different flip angle evolutions (SPACE) TSE and standard 2D TSE knee MRI in a large patient population.
**Methods or Background:** 250 symptomatic subjects prospectively underwent knee MRI on a 3T system with a 15-channel knee coil. The imaging protocol consisted of PD-weighted, T1-weighted and fat-suppressed T2-weighted standard 2D TSE acquisitions (12:14 minutes) and a commercially available multi-contrast 3D CAIPIRINHA SPACE TSE protocol (9:26 minutes). Approximately 13% of the patients (n=33) previously underwent anterior cruciate ligament and/or meniscus surgery. Two experienced musculoskeletal radiologists independently evaluated all datasets for technical image quality and identified pathologies of knee structures using a 4-point Likert scale representing the level of diagnostic confidence. The interchangeability of 2D and 3D protocols was tested under the same-reader scenario using a bootstrap percentile confidence interval. Finally, interreader reliability and intermethod concordance were assessed for all detected pathologies.
**Results or Findings:** Although 2D acquisitions scored better in terms of image quality and diagnostic confidence, standard 2D and accelerated 3D protocols were found interchangeable for diagnosing structural abnormalities, except for patellar (6.8% difference; 95%-CI: 4.0-9.6) and trochlear (3.6% difference; 95%-CI: 0.8-6.6) cartilage defects. Additionally, interreader reliability was high for both 2D and 3D protocols (range κ, 0.785-1 and κ, 0.725-0.964, respectively) and the intermethod concordance was very good for all diagnoses (range κ, 0.817-0.986).
**Conclusion:** This study contributes to the growing evidence that accelerated 3D protocols are a valuable time-saving alternative for standard 2D knee MRI. Yet, the radiologists' preference for 2D images needs to be further challenged to achieve a paradigm shift.
**Limitations:** Not applicable.
**Ethics committee approval:** The ethics committee approval was obtained.
**Funding for this study:** CS: B-Q MINDED (EU H2020 MSCA ETN #764513), UAntwerpen SEP #44883.
**Author Disclosures:**
Céline Smekens: Nothing to disclose
Annemie Snoeckx: Nothing to disclose
Thijs Vande Vyvere: Nothing to disclose
Ella Roelant: Nothing to disclose
Eline De Smet: Nothing to disclose
Pieter Van Dyck: Nothing to disclose

**12:30-13:30** **Channel 4**

### Research Presentation Session: Breast

### RPS 1402

### Predictive and prognostic models in breast imaging

### RPS 1402-1

**Moderators**
I. Biondić Špoljar; Zagreb/HR
M. H. Fuchsjäger; Graz/AT

### RPS 1402-3

**Combining DCE-MRI pharmacokinetic parameters at early time points with prognostic factors improves the prediction of pathologic response to neoadjuvant chemotherapy in breast cancer**

G. C. Baxter, J. C. Carmona-Bozo, R. Manavaki, A. Colarieti, R. Woitek, R. Bedair, J. Abraham, M. J. Graves, *F. J. Gilbert*; Cambridge/UK
*(fjg28@cam.ac.uk)*

**Purpose:** To explore the additional value of pharmacokinetic parameters from DCE-MRI at early time-points in predicting pathologic complete response (pCR) to neo-adjuvant chemotherapy (NACT) in breast cancer.
**Methods or Background:** Women >18 years receiving NACT prior to surgery for breast cancer underwent baseline and post cycle 1 DCE-MRI examinations at 3T. DCE-MRI series were analysed using the extended Tofts' model to derive Ktrans, kep, ve and hotspot Ktrans (hs-Ktrans). pCR was defined as no residual invasive cancer in the breast at surgery but allowing for the presence of in situ carcinoma. The area under the curve (AUC) was calculated to evaluate the predictive performance of logistic regression models including standard prognostic factors (histology, grade, molecular subtype) with and without the addition of DCE-MRI parameters.
**Results or Findings:** Data from 82 patients (86 lesions) were analysed. The majority were invasive ductal carcinomas (ductal: 71/86, 83%; lobular: 3/86, 3%; other: 12/86, 14%), hormone receptor (HR)-positive (57/86, 66%), with 31% HER2-positive. All tumours were either grade 2 or 3. 27/86 (31%) lesions showed pCR. Across all cancers, adding baseline hs-Ktrans increased AUC from 0.77 to 0.80, while the inclusion of Ktrans after 1 treatment cycle yielded the highest increase in AUC (0.72 to 0.76). For the HR+ group, the largest increase in AUC was observed for baseline hs-Ktrans (0.80 to 0.85). The addition of baseline hs-Ktrans and post cycle-1 kep showed the best predictive performance in triple-negative cancers (hs-Ktrans: 0.76 vs 0.59; kep: 0.93 vs 0.70).
**Conclusion:** The addition of DCE-MRI pharmacokinetic parameters at early time-points to standard prognostic factors can improve pCR prediction in HR+ and triple-negative breast cancer.
**Limitations:** Relatively small sample size from single site.
**Ethics committee approval:** NRES Committee South East (13/LO/0411).
**Funding for this study:** NIHR Cambridge Biomedical Research Centre.
**Author Disclosures:**
Anna Colarieti: Nothing to disclose
Jean Abraham: Nothing to disclose
Ramona Woitek: Nothing to disclose
Martin J Graves: Nothing to disclose
Roido Manavaki: Nothing to disclose
Julia Carlota Carmona-Bozo: Nothing to disclose
Fiona J. Gilbert: Nothing to disclose
Reem Bedair: Nothing to disclose
Gabrielle Carmen Baxter: Nothing to disclose

### RPS 1402-7

**The value of diffusion-weighted imaging (DWI) in pathological complete response (pCR) prediction in addition to dynamic contrast-enhanced (DCE) MRI in HER2-positive breast cancer patients**

*A. van der Voort*[1], K. van der Hoogt[1], R. Wessels[2], R-J. Schipper[3], G. Sonke[1], R. M. Mann[4]; [1]Amsterdam/NL, [2]The Hague/NL, [3]Eindhoven/NL, [4]Nijmegen/NL
*(a.vd.voort@nki.nl)*

**Purpose:** To investigate the added value of DWI to identify pCR in stage I-III HER2+ breast cancer patients with radiological complete response (rCR) after neoadjuvant chemotherapy (NAC) on DCE-MRI.
**Methods or Background:** We retrospectively identified patients treated with trastuzumab-containing NAC between January 2015 until September 2019 who had rCR (absence of pathologic enhancement) on post-chemotherapy DCE-MRI-breast in the Netherlands Cancer Institute. Baseline and post-NAC MRI's (Philips 1.5/3.0T) were evaluated by a dedicated breast radiologist blinded for the pathological outcome. We re-evaluated rCR on DCE-MRI and visually evaluated response on high b-value DW-images (b800 and higher). ADC values were measured within the original tumour region. We calculated the negative predictive value (NPV) for pCR (ypT0/is) with a corresponding 95% standard logit confidence interval. Fisher's exact and Mann-Whitney's U test were used for comparison between groups.
**Results or Findings:** DCE showed rCR in 102 patients of whom 76 had a pCR. A pCR was more common in HR+/HER2+ than HR-/HER2+ patients (40 of 46 vs 36 of 56, p=0.01). Residual DWI signal was visible in 7 patients. NPVs for DCE and for DWI among patients with rCR on DCE, were respectively 74.5% and 77.9% (95%CI: 75.4-80.2%) overall, 64.3% and 70.0% (95%CI: 64.3-75.1%) in HR+ and 86.9% and 86.7 (95%CI: 86.1-87.2%) in HR- patients. Within HR+ patients with visual residual DWI signal only 1 of 6 had a pCR (16.7%, 95%CI: 2.5-61.5%). The relative mean ADC-difference in HR+ patients was 80.1% (IQR 41.1-128.6%) and 114.7%, respectively with and without pCR (IQR 25.1-191.7%; p=0.36).
**Conclusion:** Standardised DWI evaluation after NAC could potentially help to identify more HR+/HER2+ patients with residual invasive disease.
**Limitations:** Double reader analysis will be performed before ECR. Multiple DWI-scan protocols were used.
**Ethics committee approval:** Approved by the IRB.
**Funding for this study:** No funding was received for this work.
**Author Disclosures:**
Gabe Sonke: Research/Grant Support: Novartis Research/Grant Support: Merck Research/Grant Support: Roche Research/Grant Support: AstraZeneca
Ronni Wessels: Nothing to disclose
Kay van der Hoogt: Nothing to disclose
Ritse Maarten Mann: Research/Grant Support: Siemens Research/Grant Support: Bayer Healthcare Research/Grant Support: Seno medical Research/Grant Support: BD Research/Grant Support: Medtronic Research/Grant Support: Koning Research/Grant Support: Screenpoint medical
Robert-Jan Schipper: Nothing to disclose
Anna van der Voort: Nothing to disclose

### RPS 1402-8

**MRI morphological criteria and ADC value in predicting axillary lymph node (ALN) response after neoadjuvant chemotherapy (NAC): are we nearly there?**

*M. C. Sciandrello*, M. Durando, G. Bartoli, E. Regini, A. Santonocito, A. Pittaro, I. Castellano, P. Fonio; Turin/IT
*(mariaclotilde.sciandrello@gmail.com)*

**Purpose:** To identify which MRI criteria can predict residual ALN disease in breast cancer patients undergone NAC.
**Methods or Background:** From 2014 to 2021, pre-and post-NAC 1,5 T MRIs of 164 patients with locally advanced breast cancer were retrospectively analysed by two dedicated radiologists in consensus, blinded to histological results. We evaluated both quantitative (number, diameter) and qualitative (irregular margins, absence of fatty hilum, cortical thickness>3mm, perifocal oedema, rim enhancement, asymmetry comparing with contralateral side) criteria and ADC value related to ALNs before and after NAC. ALNs status was compared before NAC with ALN biopsy and with sentinel ALN biopsy or axillary dissection after NAC; nodal pathological response is classified according to Pinder's criteria [complete response (pCR) versus no-complete response(no-pCR)]. Statistical analysis (Chi-square or Fisher's exact tests for categorical variables, non-parametric Mann-Whitney test for continuous variables) was performed.
**Results or Findings:** At pre-therapy MRI, the two parameters that best correlated with positive ALN biopsy were irregular margins and the absence of fatty hilum (p= 0,0003 and p=0,0014 respectively), while, after NAC, relating the different parameters with pCR or no-pCR, the only statistically significant data was the irregularity of margins (p= 0,0003). The other variables, although at the univariate analysis they seemed to demonstrate a statistically significant correlation, did not confirm this data at the multivariate analysis.
**Conclusion:** Based on our results, irregular ALNs margins seem to be the most reliable parameter associated to pre-therapy ALNs disease and no-pCR after NAC.
**Limitations:** Retrospective study.
**Ethics committee approval:** Not required.
**Funding for this study:** No funding was provided for this study.
**Author Disclosures:**
Manuela Durando: Nothing to disclose
Paolo Fonio: Nothing to disclose
Elisa Regini: Nothing to disclose
Alessandra Pittaro: Nothing to disclose
Isabella Castellano: Nothing to disclose
Ambra Santonocito: Nothing to disclose
Maria Clotilde Sciandrello: Nothing to disclose
Germana Bartoli: Nothing to disclose

**14:00-15:00** **Channel 4**

### Research Presentation Session: Imaging Informatics / Artificial Intelligence and Machine Learning

### RPS 1505b

### Technical advances and imaging biomarkers

**Moderators**
W. J. Niessen; Rotterdam/NL
C. Catalano; Rome/IT
**Author Disclosures:**
Wiro J. Niessen: Advisory Board: Scientific Lead Quantib BV; Founder: Quantib BV; Share holder: Quantib BV

### RPS 1505b-3

**Clinical trial: OPERA (orthogonal phase encoding reduction artefact)**
*A. Dell'Orso*; Empoli/IT
*(dellorsoandrea@gmail.com)*

**Purpose:** Artefacts in MRI represent a significant problem leading to loss of diagnostic information and substantial costs. A post-processing algorithm, for orthogonal phase encoding reduction of artefact (OPERA) is proposed and tested in the clinical setting.
**Methods or Background:** The OPERA procedure is based on the key concept that noise-induced signal intensity alterations are randomly distributed, whereas the position of ghosts and aliasing is predictable along with columns or rows of pixels. OPERA combines the intensity values of two images acquired with the same parameters, but with orthogonal phase encoding directions, to correct artefacts. The efficacy of the OPERA procedure on MRI artefacts reduction was tested at a medium-sized general public hospital by using an Espree Siemens 1.5T MR scanner for a total period of 14 months. The procedure was tested on a total of 1003 MR images [55 randomly selected patients (56.4% females; mean age 54.6±16.7 years)]. OPERA corrected images were compared with the corresponding reference-image (Ri) by computing signal-to-noise (SNR) and contrast-noise-ratio (CNR). Images (OPERA vs Ri) were shown in blind at two radiologists with a long-standing MRI expertise by using a better-worse Likert-type scale response, to evaluate artefacts, SNR and CNR.
**Results or Findings:** OPERA application did not significantly affect SNR (+4.3%; IQR:2.61-5.27%) and CNR (+4.30%; IQR: 2.86-6.04%). The two radiologists observed: artefact reduction (responses 4 and 5 of the Likert-scale) between 82.4% and 83.4% (inter-rater agreement, weighed K=0.766); perceived SNR improvement (82.8% to 88.5% K=0.714) and contrast improvement (86.9% to 88.9% K=0.722).
**Conclusion:** The testing of OPERA in the daily MRI practice indicates the efficacy of the algorithm in reducing MRI artefacts and improving perceived image quality.
**Limitations:** Not tested on the heart and superior abdomen. Single-centre study.
**Ethics committee approval:** OSS_15_145, December 14, 2015.
**Funding for this study:** No funding has been received for this study.
**Author Disclosures:**
Andrea Dell'Orso: Nothing to disclose

### RPS 1505b-4

**Multi-organ abnormalities in long-COVID**
A. Dennis, *A. Roca-Fernandez*, J. Mcgonigle, A. Jandor, G. Ralli, V. Carapella, R. Banerjee; Oxford/UK
*(Adriana.Roca@perspectum.com)*

**Purpose:** In a prospective longitudinal observational study in individuals who had recovered from COVID-19, we set out to assess the degree of organ impairment in the heart, lungs and visceral organs using quantitative MRI and explore potential links with ongoing symptoms.
**Methods or Background:** Quantitative MRI data were collected with CoverScanMD across two sites in the UK (Siemens 1.5T and 3T). The 30 min scan assesses: inflammation of the heart, kidneys, liver and pancreas with T1-relaxation; lung function with a dynamic structural T2-weighted scan measuring the difference between max inspiration and expiration; fat in the liver and pancreas using PDFF. Impairment for each organ was considered when the metric was outside of pre-defined reference ranges. Associations between organs and symptoms were explored with logistic regression adjusted by time from first symptoms to MRI.
**Results or Findings:** In N=451 (44yrs, 74% female, 89% white, median 179 days following infection), inflammation was observed in the heart (14%), liver (12%), pancreas (6%) and kidney (4%); fat in liver (22%) and pancreas (30%); estimated lung capacity was reduced in 11%. 21% had evidence of abnormality in 2 or more organs and the number of abnormal metrics negatively correlated with the length of time between initial symptoms and scan (r = -0.23, P<.001) suggesting some recovery with time. 66% reported ongoing severe breathlessness or fatigue which was significantly associated with increased white cell count (WCC) (P=.03) and marginally with myocarditis (P=0.06).
**Conclusion:** Coronavirus is associated with multi-organ dysfunction 6 months after infection. Organ inflammation was associated with symptoms. Multi-organ MRI may provide a diagnostic tool to stratify patients with long COVID and aid clinical management.
**Limitations:** Study population was limited by ethnicity.
**Ethics committee approval:** The study protocol was approved by a UK ethics committee (20/SC/0185).
**Funding for this study:** Funding was received from Perspectum Ltd.
**Author Disclosures:**
Adriana Roca-Fernandez: Author: Perspectum Employee: Perspectum
Rajarshi Banerjee: CEO: Perspectum
Valentina Carapella: Employee: Perspectum
John Mcgonigle: Employee: Perspectum
Arun Jandor: Employee: Perspectum
George Ralli: Employee: Perspectum
Andrea Dennis: Employee: Perspectum

### RPS 1505b-5

**Estimation of bias of deep learning-based chest X-ray classification algorithm**
*D. C. Bastos*[1], E. P. Reis[1], M. Rosa[1], H. M. H. Lee[1], G. Szarf[1], A. Gupta[2], V. K. Venugopal[2], V. Mahajan[2], S. Dey[2], R. Verma[2]; [1]São Paulo/BR, [2]New Delhi/IN

**Purpose:** To evaluate the bias in the diagnostic performance of a deep learning-based chest X-ray classification algorithm on previously unseen external data.
**Methods or Background:** 632 chest X-rays were randomly collected from an academic centre hospital and anonymised selectively, leaving out fields needed for the bias estimation (manufacturer name, age, and gender). They were from six different vendors – AGFA (388), Carestream (45), DIPS (21), GE (31), Philips (127), and Siemens (20). The male and female distribution was 376 and 256. The X-rays were read for consolidation ground truth establishment on CARING analytics platform (CARPL). These X-rays were run on open-sourced chest X-ray classification model. Inferencing results were analysed using Aequitas, an open-source python-based package to detect the presence of bias, fairness of algorithms. Algorithms' performance was evaluated on the three metadata classes – gender, age group, and brand of equipment. False omission rate (FOR) and false-negative rate (FNR) metrics were used for calculating the inter-class scores of bias.
**Results or Findings:** AGFA, 60 to 80 age group, and male were the dominant entities and hence considered as baseline for evaluation of bias towards other classes. Significant false omission rate (FOR) and false negative rate (FNR) disparities were observed for all vendor classes except Siemens as compared to AGFA. No gender disparity was seen. All groups show FNR parity whereas all classes showed disparity with respect to false omission rate for age.
**Conclusion:** We demonstrate that AI algorithms may develop biases, based on the composition of training data. We recommend bias evaluation check to be an integral part of every AI project. Despite this, AI algorithms may still develop certain biases, some of those difficult to evaluate.
**Limitations:** Limited pathological classes were evaluated.
**Ethics committee approval:** IRB approved.
**Funding for this study:** None.
**Author Disclosures:**
David Caetano Bastos: Nothing to disclose
Abhishek Gupta: Employee: Carpl.ai
Sudip Dey: Nothing to disclose
Ritu Verma: Nothing to disclose
Gilberto Szarf: Nothing to disclose
Henrique Min Ho Lee: Nothing to disclose
Marcela Rosa: Nothing to disclose
Eduardo Pontes Reis: Nothing to disclose
Vasantha Kumar Venugopal: Consultant: Carpl.ai
Vidur Mahajan: Other: Mahajan Imaging Pvt Ltd Other: Carpl.ai

**RPS 1505b-6**

**Virtual non-contrast image generation from pre-clinical photon-counting spectral CT - a phantom study to evaluate the algorithm performance**

F. K. Yeung, W. Y. Ip, *V. Vardhanabhuti*; Hong Kong/HK

**Purpose:** Virtual non-contrast (VNC) as a concept has been used to create non-contrast images from contrast studies. In the context of spectral CT, accurate VNC helps with the accurate material decomposition (MD) properties. The aim of the study is to evaluate the performance of VNC images generated from pre-clinical photon-counting CT in a phantom.
**Methods or Background:** Commercially available iodine contrast agent (Iopamiro 370) was diluted into various concentrations of iodine solutions (185, 93, 46, 23, 10, 8, 6, 4, 2, 1, 0.5 mg/ml), placed in a phantom and scanned using a pre-clinical photon-counting spectral CT scanner (MARS Bioimaging Ltd., Christchurch, New Zealand) with pre-calibrated MD protocol of multi-energy range. Iodine was decomposed by the scanner's reconstruction algorithm and the measured iodine concentration was compared with the known diluted concentrations as reference. The images were further processed by a custom-made MATLAB (Mathworks, Natick, MA) program to explore the relationship between CT number (in HU) and iodine concentration with subsequent correlation analysis. After iodine removal by VNC post-processing, the corresponding CT number of iodine vials of VNC and original contrast images were compared with the ground truth water vial respectively.
**Results or Findings:** For iodine images, measured iodine concentration was comparable with calculated ones (mean absolute difference of 9.1%). The HU values and iodine concentration was linearly and highly correlated (adjusted R squared of 0.9974 with p-value of <0.001). At 32-49.9 keV energy range, the mean absolute HU difference between water and VNC image of typical 10 mg/ml iodine solution was 27±13 HU while comparing to contrast image of 605±12 HU.
**Conclusion:** Virtual non-contrast technique is feasible in spectral CT scanner with good accuracy and correlation with known concentrations in phantom study.
**Limitations:** No limitations were identified.
**Ethics committee approval:** Not applicable.
**Funding for this study:** Not applicable.
**Author Disclosures:**
Varut Vardhanabhuti: Nothing to disclose
Wing Yan Ip: Nothing to disclose
Fu Ki Yeung: Nothing to disclose

**RPS 1505b-7**

**An online tool for semi-automatic comprehensible characterisation of dynamic contrast-enhanced magnetic resonance imaging studies**

*S. Ellmann*, K. Hellwig, M. Eckstein, A. Hartmann, R. Fietkau, M. Hecht, M. Uder, T. Bäuerle; Erlangen/DE
*(stephan.ellmann@uk-erlangen.de)*

**Purpose:** Dynamic contrast enhancement (DCE) evaluation in MRI is subject to various difficulties, as the signal intensities reflect contrast media concentration non-linearly. Moreover, individual outliers in the time-intensity curves may compromise the semiquantitative values of the enhancement dynamics. In addition, the plethora of analysis methods are hardly comparable with each other. Thus, the aim of this work was the establishment of a web-based tool for the quantitative and semiquantitative evaluation of DCE-MRI.
**Methods or Background:** An interactive web application was programmed using R to load DCE-MRI studies and semiautomatically fit the raw data to a Brix model using the Levenberg-Marquardt method. Goodness-of-fit was assessed by R2. From the fitted curves, the Brix-equation was extracted, along with (semi-)quantitative parameters: A, kel, kep, time to peak, peak-enhancement, area-under-the-curve. The maximum and minimum of a fitted curve's first derivative were defined as wash-in and wash-out, respectively. In an exemplary case series, 22 neck tumour patients were retrospectively analysed with regard to their response to immunotherapy. For this purpose, pre-therapeutic DCE-MRIs covering the tumour were assessed using the developed online tool. The above parameters were compared between treatment responders and non-responders.
**Results or Findings:** Fitting the Brix model to the raw DCE-MRI data yielded a mean R2 of 0.939 (95%CI 0.922-0.956). Regarding the case series, treatment responders' tumours featured a significantly higher time to peak (p=0.048) and wash-out (p=0.031) compared to non-responders.
**Conclusion:** Raw DCE-MRI data can be fit to pharmacokinetic models semiautomatically with the presented online tool. In a case series, fitting was accurate, and the resulting parameters could be used to identify responders to immunotherapy in neck cancer patients.
**Limitations:** Proof-of-concept-study with a fully functional web application, but only supported by a small case series.
**Ethics committee approval:** Approved by the local ethics committee.
**Funding for this study:** Internal funding.
**Author Disclosures:**
Tobias Bäuerle: Nothing to disclose
Stephan Ellmann: Nothing to disclose
Arndt Hartmann: Nothing to disclose
Markus Hecht: Nothing to disclose
Michael Uder: Nothing to disclose
Konstantin Hellwig: Nothing to disclose
Markus Eckstein: Nothing to disclose
Rainer Fietkau: Nothing to disclose

**RPS 1505b-8**

**Dual-layer spectral CT fat quantification in the liver and the skeletal muscle: experimental development and first in-patient validation**

*I. Molwitz*[1], G. Campbell[1], J. Yamamura[1], T. Knopp[1], R. Fischer[1], J. Wang[2], A. Busch[1], M. Grosser[1], P. Szwargulski[1]; [1]Hamburg/DE, [2]Dallas, TX/US
*(I.Molwitz@gmx.net)*

**Purpose:** To develop a material decomposition algorithm for detector-based dual-layer spectral CT (dlsCT) fat quantification, which so far has only been implemented for source-based dual-energy CT techniques, in phantoms and validate it in first patients.
**Methods or Background:** Phantoms were created with 0, 5, 10, 25, 40, 100% fat and 0, 4.9, 7.0 mg/ml iodine, respectively. Scans were performed with the IQon Spectral CT (Philips, The Netherlands), and 3T MR chemical-shift relaxometry (MRR). Based on maps of the photoelectric effect and Compton scattering, three-material decomposition, including fat and iodine, was done in the image space. After written consent, n=10 patients (mean age 55 years ± 18; six men) in need of a CT staging were prospectively included, received contrast-enhanced abdominal dlsCT scans at 120kV, and MRI scans for MRR. As reference tissue for the liver and the skeletal muscle, retrospectively available non-contrast-enhanced spectral CT data sets were employed. Agreement between dlsCT and MR was evaluated for the phantoms, three hepatic and two muscular regions of interest per patient by intraclass correlation coefficients (ICC) and Bland-Altman analyses.
**Results or Findings:** The ICC was excellent in the phantoms (0.978[95%CI 0.937-0.993]) and the skeletal muscle (0.956[95%CI 0.890-0.982]). The ICC was moderate for log-transformed liver fat values (0.75[95%CI 0.48-0.881]). The Bland-Altman analysis yielded a mean difference of -0.714%[95%CI -4.512-3.085] for the liver and 0.501%[95%CI -4.319-5.321] for the skeletal muscle. Interobserver and intraobserver agreements were excellent (>0.9).
**Conclusion:** DlsCT fat quantification delivers reliable results for the liver and the skeletal muscle, which provide information about steatosis hepatis and muscle quality as parameters of prognostic relevance, in retrospectively available spectral data sets of routine exams.
**Limitations:** Experimental development and proof of concept study with low patient numbers.
**Ethics committee approval:** Available.
**Funding for this study:** Funded by Hamburg Research Center for Medical Technology (04fmthh2020).
**Author Disclosures:**
Alina Busch: Nothing to disclose
Isabel Molwitz: Nothing to disclose
Graeme Campbell: Employee: Philips Healthcare. All data handling and analyzes were performed by the independent authors Molwitz I, Szwargulski P.
Tobias Knopp: Nothing to disclose
Roland Fischer: Nothing to disclose
Patryk Szwargulski: Nothing to disclose
Jin Yamamura: Nothing to disclose
Mirco Grosser: Nothing to disclose
Jerry Wang: Nothing to disclose

16:00-17:00 Channel 4

### Research Presentation Session: Head and Neck

### RPS 1608

### Orbital and paranasal sinus imaging

**Moderator**
P. De Graaf; Amsterdam/NL

### RPS 1608-2

**Intra voxel incoherent motion (IVIM) 3T MRI for orbital lesion characterisation**

*A. Lecler*, L. Duron, M. Zmuda, O. Berges, J. Savatovsky, L. S. Fournier; Paris/FR
*(alecler@for.paris)*

**Purpose:** To determine the diagnostic accuracy of MRI Intravoxel incoherent motion (IVIM) when characterising orbital lesions, which is challenging due to a wide range of locations and histologic types.
**Methods or Background:** This IRB-approved prospective single-centre study enrolled participants presenting with an orbital lesion undergoing a 3 Tesla MRI prior to surgery from December 2015 to July 2019. An IVIM sequence with 15 b-values ranging from 0 to 2000 s/mm² was performed. Two neuroradiologists, blinded to clinical data, individually analysed morphological MRIs. They drew one region of interest inside each orbital lesion, providing Apparent Diffusion Coefficient (ADC), true diffusion coefficient (D), perfusion fraction (f) and pseudo diffusion coefficient (D*) values. T-test, Mann-Whitney U test and receiver operating characteristic curve analyses were performed to discriminate between orbital lesions and to determine the diagnostic accuracy of the IVIM parameters.
**Results or Findings:** 156 participants (84 women and 72 men, mean age 54.4 +/-17.5 years) with 167 orbital lesions (98/167 [59%] benign lesions including 54 orbital inflammations and 69/167 [41%] malignant lesions including 32 lymphomas) were included in the study. ADC and D were significantly lower in malignant than in benign lesions: 0.8x10-3mm2/s [0.45] versus 1.04x10-3mm2/s [0.33], p<0.001 and 0.75x10-3mm2/s [0.40] versus 0.98x10-3mm2/s [0.42], p<0.001, respectively. D* was significantly higher in malignant lesions than in benign ones: 12.8x10-3mm2/s [20.17] versus 7.52x10-3mm2/s [7.57], p=0.005. Area Under Curve were of 0.73, 0.74, 0.72 and 0.81 for ADC, D, D* and a combination of D, f and D*, respectively.
**Conclusion:** Our study showed that IVIM might help better characterise orbital lesions.
**Limitations:** - Only participants who underwent surgery - We used 15 b-values for our IVIM acquisition
**Ethics committee approval:** This study was approved by an IRB (IRB 2015-A00364-45). Signed informed consent was obtained from all subjects.
**Funding for this study:** No funding was provided for this study.
**Author Disclosures:**
Laure S. Fournier: Nothing to disclose
Loïc Duron: Nothing to disclose
Mathieu Zmuda: Nothing to disclose
Olivier Berges: Nothing to disclose
Julien Savatovsky: Nothing to disclose
Augustin Lecler: Nothing to disclose

### RPS 1608-3

**The impact of the CT data analysis on treatment tactics in orbital trauma**

*O. Pavlova*, N. S. Serova, D. Davydov, S. K. Ternovoy; Moscow/RU
*(dr.olgapavlova@gmail.com)*

**Purpose:** To investigate the influence of orbital volume and globe position on the outcome in patients with orbital trauma using CT data postprocessing.
**Methods or Background:** A total of 107 patients with orbital trauma (100%) were admitted to the Sechenov University hospital. The patients were divided into 3 groups: 50 patients (47%) in the acute period (up to 2 weeks after the trauma), period of developing of posttraumatic deformities (n=30; 28%) (up to 3 months), period of posttraumatic deformities (n=27; 25%) (after 3 months). MSCT was performed using Canon Aquilion One 640, CT data processing was performed using workstation Vitrea Core. Along with the classical bone and soft tissue trauma evaluation on CT, the examined parameters of CT analysis included orbital volume measurement and evaluation of globe position.
**Results or Findings:** CT revealed the bone and soft tissue structures trauma in 100% of patients, however, the parameters of CT data analysis allowed to additionally reveal increased traumatic orbital volume in 21 patients (19%) and additional enophthalmos in 9 patients (8%), which helped to change the treatment tactic in 12 patients (11%).
**Conclusion:** The postprocessing of CT data allowed developing new specific criteria such as orbital volume measurement and globe position analysis which increase the efficiency of preoperative planning and help to reduce the possibility of postoperative complications in patients with orbital trauma.
**Limitations:** No limitations were identified.
**Ethics committee approval:** Approved by Sechenov University ethics committee.
**Funding for this study:** No funding was provided for this study.
**Author Disclosures:**
Sergey K Ternovoy: Nothing to disclose
Dmitry Davydov: Nothing to disclose
Natalya S Serova: Nothing to disclose
Olga Pavlova: Nothing to disclose

### RPS 1608-4

**Utility of quantitative magnetic resonance imaging of extraocular muscle in the evaluation of disease activity and severity of thyroid-associated ophthalmopathy**

*C. Shen*; Beijing/CN
*(shencheng4649@bjhmoh.cn)*

**Purpose:** Accurate staging of thyroid-associated ophthalmology (TAO) is crucial for the choice of treatment. Conventional MRI has only limited utility on this purpose. We proposed that quantitative imaging methods are superior in proper staging of TAO.
**Methods or Background:** Twenty patients with TAO were retrospectively enrolled, and clinical activity score (CAS) was used to determine the disease activity and severity. Conventional MRI, fat fraction, T1ρ imaging and T2 mapping were acquired. Volumes of intra-orbital fat were measured in T1FLAIR sequences. Fat fraction, T1ρ and T2 relaxation time in each extraocular muscle were measured. Independent sample t-test, and Spearman correlation analysis were used for statistical analysis.
**Results or Findings:** No significant difference was found in intra-orbital fat volumes between active (CAS ≥ 3) and inactive (CAS < 3) TAO patients (p=0.816), the fat volumes showed no correlation with CAS scores (p=0.2). T2 relaxation time of inferior rectus (IR) was higher in the active group (p=0.03). Fat fraction of medial rectus (MR) decreased with CAS, T1ρ of IR, MR and lateral rectus (LR) increased with CAS, and T2 relaxation time of MR and LR increased with CAS (all p<0.05).
**Conclusion:** Intra-orbital fat volumes might not help to differentiate the activity and severity of TAO.
Quantitative magnetic resonance imaging (MRI) might provide accurate parameters delineating the activity and severity of TAO, thus aiding the appropriate choice of treatment.
**Limitations:** More cases would be involved into the analysis, as well as long-term follow-up in order to assess the role of quantitative MRI in predicting the progression and prognosis of TAO.
**Ethics committee approval:** This research had been approved by our institutional review board.
**Funding for this study:** No funding was provided for this study.
**Author Disclosures:**
Cheng Shen: Nothing to disclose

### RPS 1608-5

**Imaging in unilateral proptosis as a marker of COVID associated mucormycosis (CAM) in post-COVID status**

*K. A. Bhagwat*[1], K. Peethambaram[1], V. K. Hancginal[2]; [1]Davangere/IN, [2]Gadag/IN
*(bhagwatkishan@gmail.com)*

**Purpose:** Unilateral proptosis in post-COVID is one of the clinical signs which needs imaging. The purpose of this study was (1) the early identification of mucormycosis on imaging - that is essential, (2) assessment of the spectrum of ocular involvement of mucormycosis in post-COVID status causing proptosis, and (3) to identify cavernous sinus involvement/thrombosis leading to dilatation of superior ophthalmic vein.
**Methods or Background:** Retrospective data analysis has been done from three district teaching hospitals, where CT/MRI have been performed for post-COVID patients presenting with unilateral proptosis to evaluate for the rhinoocculocerebral mucormycosis. 1.5 T MRI / 16 Slice MDCT scanner have been used for orbital imaging. We have considered all post-COVID patients who underwent imaging for unilateral proptosis for evaluation. Two radiologists, with 16 and 8 years of experience, have read PACS images.
**Results or Findings:** A total of 119 patients have undergone imaging for mucormycosis among whom 65 patients had presented with unilateral proptosis. Involvement of mucormycosis soft tissue with erosive changes in the medial wall (from ethmoid sinus), floor (maxillary sinus), orbital apex (sphenoid sinus) leading to proptosis was found in 61 patients. Dilated superior ophthalmic vein due to cavernous sinus thrombosis was identified in four patients as the cause of proptosis with coexisting sinus involvement. Preseptal oedema, premaxillary oedema, oedema of soft tissue around the orbit and sinuses were found in 30 patients.

Friday

**Conclusion:** Imaging early in unilateral proptosis helps identify mucormycosis. Assessment of involvement of orbital wall and erosive changes helps as a surgical road map.
**Limitations:** Since only unilateral proptosis is considered as a criterion. Other early symptoms/signs of facial swelling, preseptal oedema leading to imaging would identify mucor much earlier.
**Ethics committee approval:** Retrospective study of images on PACS, approval taken.
**Funding for this study:** No funding was received for this study.
**Author Disclosures:**
Kishan Ashok Bhagwat: Nothing to disclose
Krishnarjun Peethambaram: Nothing to disclose
Veeresh Karabasappa Hancginal: Nothing to disclose

### RPS 1608-6

**CT picture of rhino-orbito-cerebral mucormycosis (ROCM), associated in patients with COVID-19**

*L. Yunusova*, S. Valiyev; Tashkent/UZ
*(lolita_yunusova@mail.ru)*

**Purpose:** Report CT manifestations and outcomes of rhino-orbital cerebral mucormycosis (ROCM) in patients with COVID-19.
**Methods or Background:** We investigated 110 patients of invasive ROCM between July 2020 -September 2021, who were treated for the Maxillofacial Surgery of the TMA. The examined patients were from 46 to 52 years old and were men - 59.5%, women - 40.5%.
**Results or Findings:** Clinical features of these patients presents: sixty-eight (61,8%) patients had onset of their disease with toothache with one developing submandibular abscess later, one with otitis externa and perichondritis followed by ophthalmoplegia, and one with catarrh. Twenty-eight (25,4%) patients had altered sensorium at presentation, while two others developed it during their hospital stay, one due to internal carotid artery occlusion, and the other due to hydrocephalus. Ophthalmoplegia (89%) was the most frequent presentation followed by proptosis (83%). Visual loss (80%) was observed in 26 (23,6%) patients at presentation and two patients developed it during their hospital stay. One patient had bilateral proptosis, ophthalmoplegia, and visual loss. On CT imaging, all patients had evidence of paranasal sinuses involvement. The ethmoid (86%) and maxillary sinuses (80%) were most commonly involved, followed by sphenoid and frontal sinus in fifty-six (50,9%) each and pansinusitis in seventy-five (68,1%). Forty-eight (43,6%) patients had a gas shadow over the maxilla suggestive of secondary bacterial infection. Intracranial extension with cerebral lobe involvement presenting as a hypodense area with or without rim enhancement was observed in seven (40%) patients. One of these patients had a massive cerebral infarct with marked perilesional oedema resulting in hydrocephalus, which required ventriculoperitoneal shunt placement, and the other had internal carotid artery occlusion.
**Conclusion:** Computed tomography imaging is a useful modality to assess the extent of the ROCM.
**Limitations:** No limitations were identified.
**Ethics committee approval:** This study was approved by the ethics committee.
**Funding for this study:** No funding was provided for this study.
**Author Disclosures:**
Shakhzod Valiyev: Nothing to disclose
Lolita Yunusova: Author: Author describes results of conducted research. The author of this scientific work is responsible and will also present it

### Saturday, March 5

08:00-09:00 **Channel 4**

### Research Presentation Session: Paediatric

### RPS 1712

### Imaging in children: realistic benefits of artificial intelligence (AI)

**Moderators**
C. E. de Lange; Gothenburg/SE
L. E. Derchi; Genoa/IT

### RPS 1712-3

**AI denoising significantly improves image quality in ultra-low-dose paediatric thorax computed tomography scans and improves radiological workflows**

*A. S. Brendlin*, U. Schmid, S. Afat, I. Tsiflikas; Tübingen/DE
*(andreas.brendlin@gmail.com)*

**Purpose:** To explore the potential impact of an AI-based denoising software solution on image quality and workflows in paediatric thorax ultra-low-dose computed tomography (ULDCT) scans.
**Methods or Background:** From 01.04.2021-01.10.2021, 60 consecutive paediatric patients with ultra-low-dose CT were included. Images were reconstructed using weighted filtered back projection (wFBP), advanced modelled iterative reconstruction (ADMIRE) strength 2, and wFBP with a novel denoising postprocessing algorithm (PixelShine). Three readers with varying experience levels independently rated subjective image quality on a 5-point Likert scale (1=worst - 5=best). An intraclass correlation coefficient was used to measure inter-rater-agreement. Image noise (standard deviation of Hounsfield Units from regions of interest in homogenous paraspinal muscles) was compared for objective image quality. An additional phantom scan was performed to investigate the dose-reduction capabilities of PixelShine. Statistical analysis ensued using a mixed-effects model. Time to diagnosis was measured for all reconstruction methods.
**Results or Findings:** Subjective image quality was significantly higher for PixelShine vs ADMIRE 2 vs wFBP (4 IQR [4-5] vs 3 IQR [2-3] vs 2 IQR [2-3]; each $p<0.001$) with almost perfect agreement (ICC=0.94, $p=0.001$). The noise was significantly lower for PixelShine vs ADMIRE 2 vs wFBP (33.73±0.85 vs 59.96± 1.23 vs 76.89±1.53 HU; each $p=0.001$). The phantom scan revealed 100% radiation dose PixelShine to have equal noise as 325% ADMIRE 2 and 550% wFBP (each $p=0.999$). Overall, time to diagnosis was significantly shorter when using PixelShine vs. ADMIRE 2 vs wFBP (2.28±1.56 vs 2.45±1.9 vs 2.66 ±2.31 min; each $p<0.001$).
**Conclusion:** Compared to conventional reconstruction methods, AI postprocessing denoising may positively impact subjective and objective image quality in pediatric ULDCT thorax scans and improve radiological workflows.
**Limitations:** This is a retrospective study with 60 patients. Dose reduction capabilities were only measurable with a phantom.
**Ethics committee approval:** IRB approved.
**Funding for this study:** No funding was received for this study.
**Author Disclosures:**
Andreas Stefan Brendlin: Nothing to disclose
Saif Afat: Nothing to disclose
Ulrich Schmid: Nothing to disclose
Ilias Tsiflikas: Nothing to disclose

### RPS 1712-4

**A machine learning assessment using laboratory: US and MR findings to predict long-term outcome in patients with biliary atresia after Kasai portoenterostomy**

*M. Caruso*, C. Ricciardi, G. D. Paoli, F. Di Dato, V. Romeo, M. Petretta, R. Iorio, A. Brunetti, S. Maurea; Naples/IT

**Purpose:** Biliary atresia (BA), a rare obliterative cholangiopathy, leads to a progressive biliary cirrhosis and Kasai portoenterostomy (KP) represents its first-line treatment. Our objective was to compare the accuracy of quantitative parameters extracted from the laboratory, ultrasound (US) and magnetic resonance (MR) using machine learning (ML) algorithms in predicting long-term outcome of native liver survivor patients with BA after KP.
**Methods or Background:** Twenty-four patients were evaluated according to clinical and laboratory data at initial evaluation (median follow-up=9.7 years) after KP as with ideal (n=15) or non-ideal (n=9) medical outcome, successively they were re-evaluated after additional 4 years and classified in Group 1 (n=12) as stable and Group 2 (n=12) as non-stable in the disease course. Laboratory and quantitative US and MR imaging parameters were merged to test ML algorithms.
**Results or Findings:** The only statistically significant parameters between Group 1 and 2 were total and direct bilirubin (TB and DB) as laboratory parameters, while US stiffness as an imaging parameter. The values of TB and DB were still in the normal range, but tend towards the upper limit. Naïve Bayes was the best algorithm in terms of accuracy, sensitivity, specificity values and AUCROC to predict long-term outcome, selecting only laboratory parameters (TB and DB), while Random Forest and k-Nearest Neighbors algorithms reached the same results using either laboratory or imaging parameters.
**Conclusion:** The ML evaluation, merging laboratory and quantitative imaging findings, shows the pivotal role of TB and DB values in predicting long-term outcome of BA patients after KP, even though their values may be within normal range. Therefore, clinicians should be alert when the values of these laboratory parameters show subtle changes.
**Limitations:** The small sample size and the retrospective type of the investigation.
**Ethics committee approval:** The ethics approval was obtained.
**Funding for this study:** No funding was received for this study.
**Author Disclosures:**
Fabiola Di Dato: Nothing to disclose
Mario Petretta: Nothing to disclose
Valeria Romeo: Nothing to disclose
Gregorio Delli Paoli: Nothing to disclose
Arturo Brunetti: Nothing to disclose
Raffaele Iorio: Nothing to disclose
Martina Caruso: Nothing to disclose
Carlo Ricciardi: Nothing to disclose
Simone Maurea: Nothing to disclose

### RPS 1712-5

**Assessment of an AI aid in detection of paediatric appendicular skeletal fractures by senior and junior radiologists**

R. Maarek, A-L. Hermann, A. Kamoun, A. Marchi, R. Khelifi, M. Collin, A. Jaillard, *T. Nguyen*, H. Ducou Le Pointe; Paris/FR
*(nguyentoan5989@gmail.com)*

**Purpose:** The number of conventional X-ray examinations in paediatric emergency departments is constantly increasing, leading to avoidable errors in interpretation by the radiologist. The use of artificial intelligence (AI) could improve the interpretation workflow by prioritising pathological radiographs and providing assistance in fracture detection.
**Methods or Background:** A cohort of 300 anonymised radiographs performed for peripheral skeletal fracture detection in patients aged 2 to 21 years was retrospectively collected. The gold standard was established for each examination after an independent review by two radiologists experts in musculoskeletal imaging. In case of disagreement, a consensual review with a third expert radiologist was performed. Out of the 300 examinations, 150 presented at least a fracture. All radiographs were then read by 3 senior radiologists and 5 junior radiologists in training between the 2nd and 4th year of residency without and immediately after with the help of an AI. Poor quality radiographs were excluded from the cohort. Sensitivity and specificity for each group of radiologists were calculated without and with the help of AI.
**Results or Findings:** The standalone sensitivity and specificity of the AI were respectively 91% and 90%. The mean sensitivity for all groups was 73.3% without AI, it increased by almost 10% to 82.8% with the aid of the AI. For the junior radiologists, it increased from 71.9% to 82.2% (+10.3%) and for the seniors from 75.6% to 83.8% (+8.2%). On average, the specificity increased from 89.6% to 90.3% (+0.7%) and from 86.2% to 87.6% (+1.4%) for juniors. For senior radiologists, the average specificity slightly decreased from 95.1% to 94.9% (-0.2%).
**Conclusion:** The aid of the AI increased sensitivity by an average of 10% without decreasing specificity.
**Limitations:** No limitations identified.
**Ethics committee approval:** IRB approval n°20202256.
**Funding for this study:** No funding was received for this study.
**Author Disclosures:**
Mégane Collin: Nothing to disclose
Toan Nguyen: Nothing to disclose
Richard Maarek: Nothing to disclose
Amina Kamoun: Nothing to disclose
Aliénor Jaillard: Nothing to disclose
Redha Khelifi: Nothing to disclose
Hubert Ducou Le Pointe: Nothing to disclose
Antoine Marchi: Nothing to disclose
Anne-Laure Hermann: Nothing to disclose

### RPS 1712-6

**Computational fluid dynamic modelling of airways after laryngotracheal stenosis**

B. Elders[1], H. Sadafi[2], J. Costa[2], J. de Backer[2], H. A. W. M. Tiddens[1], P. Wielopolski[1], B. Pullens[1], *P. Ciet*[1]; [1]Rotterdam/NL, [2]Kontich/BE
*(p.ciet@erasmusmc.nl)*

**Purpose:** The aim of this study was to use Magnetic Resonance Imaging (MRI) based Computational Fluid Dynamic (CFD) modelling of the upper airways after digital surgery to predict the effect of various surgical interventions on airflow patterns and resistance in children post Laryngotracheal Stenosis (LTS) repair.
**Methods or Background:** After open airway surgery for LTS several complex anatomical changes of the airway can remain, leading to altered upper airway airflow patterns and increased airway resistance for which re-operation might be needed. In this study, CFD analyses were performed on free-breathing (FB) and inspiratory (Insp) MRI scans of a healthy volunteer, and of two patients post LTS repair. Digital surgery was executed to predict the effect of 1) widening of the vocal cords, 2) removal of the tracheal deformation (TD), 3) both widening of the vocal cords and TD removal.
**Results or Findings:** Patient 1 had severe vocal cord stenosis and mild TD. Patient 2 had severe vocal cord stenosis and severe TD. Compared to the healthy volunteer during FB and Insp, patient 1 showed an increased total airway resistance of 269% and 180%, and patient 2 showed an increase of 735% and 1548%. In patient 1 the best airway resistance was achieved when just the vocal cords were widened (FB:-20.2 Pa.s/L (45.6%), Insp:-17.9 Pa.s/L (68.8%)). In patient 2 the best results were obtained when both the vocal cords were widened and the TD was removed (FB:-71.1 Pa.s/L (71.0%), Insp:-133.9 Pa.s/L (87.3%)).
**Conclusion:** Our findings suggest that CFD modelling can be used to study the effect of virtual surgical upper airway interventions in patients with complex airway pathology.
**Limitations:** Proof of concept study in a low number of subjects.
**Ethics committee approval:** IRB approved.
**Funding for this study:** Funding was received from Vrienden van het Sophia (SSWO).
**Author Disclosures:**
Bas Pullens: Nothing to disclose
Hosein Sadafi: Nothing to disclose
Pierluigi Ciet: Nothing to disclose
Bernadette Elders: Nothing to disclose
Harm A W M Tiddens: Nothing to disclose
Piotr Wielopolski: Nothing to disclose
Joana Costa: Nothing to disclose
Jan de Backer: Nothing to disclose

### RPS 1712-7

**3D ultrasound kidney volume segmentation in paediatric hydronephrosis: interrater agreement and correlation to hydronephrosis grading**

*M. Esser*[1], I. Tsiflikas[1], J. Jago[2], L. Rouet[3], A. Stebner[4], J. F. Schäfer[1]; [1]Tübingen/DE, [2]Bothell, WA/US, [3]Suresnes/FR, [4]Münsterlingen/CH

**Purpose:** To evaluate the interrater agreement of kidney volume segmentation by three-dimensional ultrasound (3D-US) in children with hydronephrosis and comparison to hydronephrosis grading.
**Methods or Background:** 48 kidney volumes were acquired in 45 patients with hydronephrosis by freehand 3D-US (6-1 MHz volumetric sector array, electronic rotation) in prone position (35 male; median age, 4 years; range, one month to 16 years). Semi-automated kidney segmentation (prototype software) was performed afterwards by two readers providing volumes for total kidney volume, dilated collective system and renal parenchyma (total kidney volume minus dilated collective system), as well as hydronephrosis index (renal parenchyma divided by total kidney volume). Interrater agreement was evaluated with a two-way intraclass correlation coefficient (ICC). The maximum anteroposterior diameter of renal pelvis was measured on transverse 2D B-mode images, and hydronephroses were classified grade 1-4.
**Results or Findings:** Most hydronephroses were grade 2 (n=29), followed by grade 3 (n=15) and grade 1 (n=4). The most frequent patient history included pelvic ureteric junction stenosis (n=21). Interrater agreement for total kidney volume, collective system, hydronephrosis index and renal parenchyma was good to excellent with ICC of 0.94, 0.87, 0.83 and 0.91 respectively (p<0.001 each). There was a positive correlation between hydronephrosis grading and ultrasonic hydronephrosis index and between 2D renal pelvis diameter and volume of the collective system (p<0.001 both).
**Conclusion:** Novel 3D-US volumetric analysis has a high degree of interrater agreement providing parenchyma volume in hydronephrotic kidneys. Volumes of the collective system and hydronephrosis index correlate with the extent of hydronephrosis.
**Limitations:** No interrater agreement was assessed.
**Ethics committee approval:** This prospective study was approved by the local ethics committee.
**Funding for this study:** The study was supported by a research grant in the framework of a collaboration contract with Philips Ultrasound, Inc.
**Author Disclosures:**
Michael Esser: Nothing to disclose
James Jago: Research/Grant Support: Philips Healthcare, Ultrasound General Imaging
Jürgen F. Schäfer: Grant Recipient: Collaboration contract with Philips Ultrasound, Inc
Alexander Stebner: Nothing to disclose
Ilias Tsiflikas: Nothing to disclose
Laurence Rouet: Research/Grant Support: Philips Research France

**10:30-11:30** **Channel 4**

### Research Presentation Session: Imaging Informatics / Artificial Intelligence and Machine Learning

### RPS 1805a

### Artificial intelligence (AI) in abdominal imaging

**Moderators**
M. Zins; Paris/FR
A. G. Rockall; London/UK

### RPS 1805a-3

**Artificial intelligence-based VS standard acquisition in upper abdomen MRI: quantitative and qualitative image analysis**

*B. Masci*, M. Zerunian, F. Pucciarelli, M. Polici, G. Piccinni, D. Polverari, E. Iannicelli, D. Caruso, A. Laghi; Rome/IT
*(benedetta.masci93@gmail.com)*

**Purpose:** To compare T2 and diffusion-weighted images (DWI) in upper abdomen magnetic resonance imaging (MRI) with AIR Recon Deep Learning (ARDL) algorithm with standard acquisition (non-ARDL), in terms of quantitative and qualitative image analysis and scanning time.
**Methods or Background:** Thirty consecutive healthy volunteers (23 female, mean age 55±22 years) were included and underwent unenhanced upper abdomen MRI (1.5 Tesla) from May 2021 to October 2021. Exclusion criteria were: contraindications to MRI and severe artefacts on MRI sequences. Examinations included T2 and DWI axial sequences with both standard protocol and ARDL. A radiologist evaluated objective image quality by drawing fixed regions of interest (ROIs) in the liver parenchyma, gallbladder and background to calculate signal-to-noise-ratio(SNR) and contrast to-noise-ratio(CNR). Then, subjective image quality was assessed by two radiologists independently with a five-point Likert scale including parameters as parenchyma edge sharpness, contrast, truncation and motion artefacts and overall image quality. Acquisition timing was also recorded and analysed.
**Results or Findings:** The objective analysis showed no significant differences between SNR and CNR in ARDL-T2 sequences vs non-ARDL-T2 (SNR=282.23 vs 249.6 and CNR=1122.18vs877, P= 0.5038 and 0.57 respectively) and in DWI sequences with ARDL vs non-ARDL (SNR=677.91 vs 509.35 and CNR=408.75 vs 421.30, P=0.6884 and 0.6435 respectively). For subjective analysis, ARDL sequences showed significantly better overall image quality with lower motion and truncation artefacts and higher sharpness and contrast (all p<0,0001) with the good inter-rater agreement (k=0,83925). Acquisition timing was significantly lower in both ARDL sequences compared to non-ARDL ones (T2=19.07s vs 24s and DWI=197.93s vs 498.52s, all p<0.0001).
**Conclusion:** ARDL sequences showed significantly higher overall image quality with reduced acquisition timing, in particular for DWI.
**Limitations:** Reduced number of sequences tested and small population sample.
**Ethics committee approval:** This study was approved by our local institutional review board; written informed consent was obtained from all study participants.

**Funding for this study:** No funding was received for this study.
**Author Disclosures:**
Damiano Caruso: Nothing to disclose
Benedetta Masci: Nothing to disclose
Francesco Pucciarelli: Nothing to disclose
Marta Zerunian: Nothing to disclose
Daniele Polverari: Nothing to disclose
Giulia Piccinni: Nothing to disclose
Andrea Laghi: Nothing to disclose
Michela Polici: Nothing to disclose
Elsa Iannicelli: Nothing to disclose

### RPS 1805a-4

### Deep learning training with prior and current CECT scans improves liver lesions detection and segmentation

L. Joskowicz, A. Szeskin, S. Rochman, R. Lederman, *J. Sosna*; Jerusalem/IL
*(jacobs@hadassah.org.il)*

**Purpose:** In clinical practice, radiologists compare the prior current study with improved detection of lesions. We aimed to verify if liver lesions detection and segmentation in a CECT is more accurate with deep learning classification trained on pairs of current and prior scans.
**Methods or Background:** We developed two deep learning 3D R2U-Net classifiers. The singles classifier is trained with individual CECT liver lesion annotations; the pairs classifier is trained with pairs of registered prior and current CECT liver lesion annotations of the same patient. While the singles classifier discriminates lesion voxels based on absolute appearance, the pairs classifier discriminates based on relative lesion/healthy tissue appearance differences. We evaluated the performance of the singles and pairs classifiers with 174 retrospective clinical CECTs of 59 patients from two centres with metastatic disease with a total of 2,897 liver metastases manually delineated by an expert radiologist. The singles and pairs classifiers were trained/validated/tested with 126/15/33 single CECTs and 350/35/101 pairs of CECTs created from the same 174 CECTs. Their performance was quantified by comparing the test sets results of each to the manual expert annotation with the liver metastasis precision/recall detection and the segmentation Dice coefficient and average symmetric surface distance (ASSD).
**Results or Findings:** The pairs classifier yields significantly better Dice, ASSD and precision and similar recall results than the singles classifier for liver metastases whose diameter is > 5mm: Dice 0.82±0.16 vs 0.79±0.17 (4% improvement, p-value=0.0015), ASSD 1.08±1.36mm vs 1.80±1.81mm (36% improvement, p-value=0.0031, precision 0.64±0.28 vs 0.37±0.28 (73% improvement, p-value=0.00001) and recall 0.87±0.15 vs 0.87±0.16 (same, p-value=0.985).
**Conclusion:** Deep learning classification of liver lesions trained on pairs of registered current and prior scans increases lesion detection and segmentation accuracy.
**Limitations:** Single observer annotations, two centres.
**Ethics committee approval:** IRB waiver informed consent.
**Funding for this study:** No funding was received for this study.
**Author Disclosures:**
Richard Lederman: Nothing to disclose
Leo Joskowicz: Nothing to disclose
Adi Szeskin: Nothing to disclose
Shalom Rochman: Nothing to disclose
Jacob Sosna: Nothing to disclose

### RPS 1805a-5

### Limited impact of scanner variability on CT radiomic features in predicting outcome for pancreatic cancer patients

E. Scalco[1], *M. M. Mori*[2], D. Palumbo[2], M. Falconi[2], M. Reni[2], F. De Cobelli[2], G. Rizzo[1], C. Fiorino[2]; [1]Segrate/IT, [2]Milan/IT
*(mori.martina@hsr.it)*

**Purpose:** To assess the impact of features harmonisation accounting for inter-scanner variability in stratifying patients with pancreatic carcinoma according to the risk of early distant relapse after surgery.
**Methods or Background:** Pre-surgical CT images of 114 patients who underwent upfront pancreaticoduodenectomy were acquired in a single institute using 3 different scanners. Tumour volumes were delineated by expert radiologists and 181 IBSI-consistent radiomic features were computed. The ComBat harmonisation method was adopted to correct for batch effect related to the acquisition scanner; significant differences among scanners groups were assessed through ANOVA test before and after harmonisation. A previously validated and published 3-variable model including clinical and radiomic features was applied to the original and harmonised dataset. During the original model's training, radiomic features were reduced by removing those with low inter-observer reproducibility and retaining those (3 morphological, 4 texture, 1 statistical) most significantly associated with the endpoint. The prediction performances of the features retained in the first selection were also compared considering original and harmonised values.
**Results or Findings:** The ComBat method was able to remove differences among the 3 scanners groups (28/181 features with p_value<0.05 before harmonisation; none after harmonisation). Despite this improvement, the model's performances were almost equal before/after harmonisation: the hazard ratios resulting from applying the published score to stratify low-vs-high-risk patients in multivariable logistic regression analysis was 4.33 (95%CI 2.31-8.11) against 4.07 (2.16-7.64) before/after harmonisation. Univariate logistic regression performed on the 8 most relevant radiomic features confirmed that very similar performances (regression coefficients, p-value, odds ratio and 95% CI) were obtained before/after harmonisation.
**Conclusion:** The performances of predictive radiomic features and of a multi-variate model built on pancreatic CT images were poorly affected by inter-scanner variability.
**Limitations:** Homogeneous acquisition protocols across the scanners.
**Ethics committee approval:** Ethics committee approval was obtained. Reference: 28/INT/2015.
**Funding for this study:** This study was funded - AIRC-grant-IG23015.
**Author Disclosures:**
Claudio Fiorino: Nothing to disclose
Giovanna Rizzo: Nothing to disclose
Diego Palumbo: Nothing to disclose
Massimo Falconi: Nothing to disclose
Francesco De Cobelli: Nothing to disclose
Martina Mori Mori: Nothing to disclose
Elisa Scalco: Nothing to disclose
Michele Reni: Nothing to disclose

### RPS 1805a-6

### Pre-treatment computed tomography radiomics for predicting the response to neoadjuvant chemoradiation in locally advanced rectal cancer

*X. Yi*[1], Y. Fu[1], B. T. Chen[2]; [1]Changsha/CN, [2]Duarte, CA/US
*(yixiaoping@csu.edu.cn)*

**Purpose:** Pretreatment computerised tomography (CT) scans are commonly acquired prior to neoadjuvant chemoradiotherapy (nCRT). This study assessed the usefulness of pretreatment CT-based radiomics for predicting pathological complete response (pCR) of locally advanced rectal cancer (LARC) to nCRT, and to develop a predictive model.
**Methods or Background:** Patients with LARC who underwent nCRT followed by total mesorectal excision surgery from July 2010 to December 2018 were retrospectively enrolled. A total of 340 radiomics features were extracted from pretreatment contrast-enhanced CT images. The most relevant features to pCR were selected using the least absolute shrinkage and selection operator (LASSO) method and a radiomics signature was generated. Predictive models were built with radiomic features and clinicopathological variables. Model performance was evaluated using discrimination, calibration, and decision curve analysis, and validated in an independent cohort.
**Results or Findings:** pCR was achieved by 44 of the 216 consecutive patients (20.4%) in this study. The model with the best performance used both radiomics and clinical variables, including radiomic signatures, distance to the anal verge, lymphocyte-to-monocyte ratio, and carcinoembryonic antigen. This combined model discriminated between patients with and without pCR with an area under the curve of 0.926 and 0.872 in the training and the validation cohorts, respectively. The combined model also showed better performance than models built with radiomic or clinical variables alone.
**Conclusion:** Our combined predictive model was robust in differentiating patients with and without response to nCRT.
**Limitations:** This was a retrospective study, making case selection bias possible and affecting the performance of our predictive model. Future prospective, multicentre studies are needed to validate our results.
**Ethics committee approval:** This retrospective study was reviewed and approved by our hospital (IRB No.201910070), and written informed consent was waived.
**Funding for this study:** Not applicable.
**Author Disclosures:**
Yan Fu: Nothing to disclose
Bihong T Chen: Nothing to disclose
Xiaoping Yi: Nothing to disclose

### RPS 1805a-7

### Deep-learning-based differentiation of benign and premalignant colorectal polyps in CT colonography

*S. Grosu*, P. Wesp, A. Graser, S. Maurus, B. Schachtner, C. C. Cyran, J. Ricke, P. M. Kazmierczak, M. Ingrisch; Munich/DE

**Purpose:** To evaluate the potential of deep-learning algorithms to differentiate premalignant and benign colorectal polyps detected by CT colonography (CTC).
**Methods or Background:** In this retrospective analysis polyps of all size categories and morphologies were manually segmented on supine and prone CTC images and classified as benign (hyperplastic polyp or regular mucosa) or

Saturday premalignant (adenoma) according to histopathology. Two models based on convolutional neural networks were trained to predict the polyp class (benign vs premalignant). Model SEG was trained on 3D CTC image subvolumes covering individual polyps and additionally polyp segmentation masks. Model noSEG was solely trained on the CTC subvolumes. The diagnostic performance of both models for the determination of premalignant from benign was then validated in an external multicenter test sample. The operating point for both models was selected individually to maximize the respective Youden-Index.
**Results or Findings:** The training set consisted of 107 colorectal polyps in 63 patients (mean age: 63±8 years, 40 men) comprising 169 polyp segmentations. The external test set included 77 polyps in 59 patients comprising 118 polyp segmentations. Model SEG achieved a ROC-AUC of 0.83, the sensitivity of 66%, and specificity of 92% on the external test set for determination of premalignant from benign polyps. Model noSEG yielded a ROC-AUC of 0.75, the sensitivity of 65%, and specificity of 79% on the external test set.
**Conclusion:** Deep-learning-based image analysis in CTC enabled the differentiation of premalignant and benign colorectal polyps, with the potential to facilitate the identification of high-risk colorectal polyps as an automated second reader.
**Limitations:** The sample size was small. Results of this study are only applicable to polyps clearly detectable in CT colonography and a selection bias cannot be fully ruled out.
**Ethics committee approval:** Approved by institutional review board.
**Funding for this study:** This study was funded by FöFoLe, Faculty of Medicine, Ludwig-Maximilians-Unversity, Munich.
**Author Disclosures:**
Anno Graser: Nothing to disclose
Philipp M Kazmierczak: Nothing to disclose
Balthasar Schachtner: Nothing to disclose
Clemens C. Cyran: Nothing to disclose
Sergio Grosu: Nothing to disclose
Stefan Maurus: Nothing to disclose
Michael Ingrisch: Nothing to disclose
Philipp Wesp: Nothing to disclose
Jens Ricke: Nothing to disclose

### RPS 1805a-8

**Automatic detection and measurement of renal cysts for abdominal ultrasound examination: deep learning approach**

*Y. Kanauchi*[1], M. Hashimoto[2], N. Toda[2], S. Okamoto[2], H. Haque[3], M. Jinzaki[2], Y. Sakakibara[1]; [1]Yokohama/JP, [2]Tokyo/JP, [3]Hino/JP
*(yurie@dna.bio.keio.ac.jp)*

**Purpose:** The aim of this study was to develop a deep learning model (DLM) that can automatically detect renal cysts in ultrasound images and predict the appropriate position of a pair of measurement markers (MM) to measure their size.
**Methods or Background:** In total, 2664 ultrasonographic B-mode images with MM of renal cysts obtained from January 2019 to May 2020 were split into 70/30% for training and testing. The DLM adopted a fine-tuned YOLOv5 for detection of renal cysts and a pretrained UNet++ for prediction of heat maps representing the position of MMs. Preprocessed ultrasound images were input to YOLOv5, and images cropped inside the bounding box detected from the input image by YOLOv5 were input to UNet++. Three sonographers manually placed MM on 100 unseen test data. The MM positions annotated by a board-certified radiologist were used as the ground truth. We have evaluated and compared the accuracy of sonographers and the DLM. Their performances were evaluated using precision-recall metrics and measurement errors.
**Results or Findings:** The evaluation result (precision, recall, position-error, and length-error) for DLM was 0.87, 0.90, 3.73±3.68 mm, and 1.26±1.06 mm respectively. For the first sonographer, the respective metrics were 0.86, 0.87, 2.56±2.76 mm and 1.21±1.36 mm; for the second sonographer, they were 0.83, 0.84, 2.34±2.63 mm, and 0.96±1.07 mm.
**Conclusion:** Our DLM was able to detect renal cysts with the comparable level of accuracy as sonographers, and the measurement prediction was slightly less accurate. Hence, it might help to automate the process of measurement in ultrasound analysis.
**Limitations:** No external validation.
**Ethics committee approval:** Ethics committee approval was obtained; #20170018.
**Funding for this study:** This study was funded by GEHealthcare.
**Author Disclosures:**
Masahiro Jinzaki: Research/Grant Support: Radiologist
Saori Okamoto: Nothing to disclose
Hasnine Haque: Employee: GE Healthcare
Masahiro Hashimoto: Research/Grant Support: Radiologist
Yurie Kanauchi: Nothing to disclose
Naoki Toda: Research/Grant Support: Ph.D student

**12:30-13:30 Channel 4**

### Research Presentation Session: Physics in Medical Imaging

### RPS 1913

### Magnetic resonance imaging (MRI)

**Moderators**
G. Hagberg; Tübingen/DE
M. H. Fuchsjäger; Graz/AT

### RPS 1913-3

**Varying MRI-contrast in solid 3D printed structures without adding contrast agents**

*A. Valladares*, G. Oberoi, T. Beyer, A. Berg, E. Unger, I. Rausch; Vienna/AT
*(alejandra.valladares@meduniwien.ac.at)*

**Purpose:** The development of MRI phantoms presents several challenges in terms of materials and is mainly limited to water-filled compartments and gel-based components. Here, we present a novel design to additively manufacture solid objects with adjustable MRI contrast based on the integration of MRI-visible supporting material.
**Methods or Background:** Eleven homogeneous cubic probes and one spherical object mimicking a necrotic tumour were designed and printed using additive manufacturing. Each probe comprises a solid internal structure and a specific percentage of commonly-used support material. The models were scanned using standard spin- and gradient-echo pulse sequences on a 3T PET/MRI system. We evaluated MRI visibility and T1 and T2 relaxation rates over time.
**Results or Findings:** For most probes, measurable signal intensities were obtained. T2 values ranged from 22 ms to 40 ms, comparable to reported values for some body tissues at 3T. T1 values were relatively short compared to similar reports with relaxation times of 109 ms to 131 ms. T1 and T2 relaxation times were stable over three months.
**Conclusion:** The proposed design allowed the adjustment of imaging contrast in MRI, based mainly on the spin density contribution of the MRI-visible supporting material within a voxel. Furthermore, we demonstrated the possibility of creating a one-compartment object to mimic necrosis in tumours, which is advantageous to create complex models for tumour heterogeneity in MRI and PET/MRI.
**Limitations:** The current design does not allow mimicking the varying contrast with different T1 and T2- weighing MRI sequences.
**Ethics committee approval:** Not applicable.
**Funding for this study:** This work has received funding from the Horizon 2020 programme under the Marie Skłodowska-Curie grant agreement No.764458. AM infrastructure was supported by the Austrian FFG within the M3dRES project (Grant-No.858060).
**Author Disclosures:**
Gunpreet Oberoi: Nothing to disclose
Alejandra Valladares: Nothing to disclose
Andreas Berg: Nothing to disclose
Thomas Beyer: Nothing to disclose
Ivo Rausch: Nothing to disclose
Ewald Unger: Nothing to disclose

### RPS 1913-4

**Comparisons between simulated and experimental data in quantitative susceptibility maps in a 3.0T MRI-scanner**

*E. G. Cuña*[1], E. Tuzzi[2], L. Biagi[3], P. Bosco[3], M. Garcia[1], J. Mattos[1], K. Scheffler[2], M. Tosetti[3], G. E. Hagberg[2]; [1]Montevideo/UY, [2]Tübingen/DE, [3]Pisa/IT
*(enrique.cuna@cudim.org)*

**Purpose:** To compare quantitative susceptibility quantification between simulated and experimental phantom containing iron. This would help to adjust processing pipeline parameters to obtain quantitative susceptibility mapping (QSM).
**Methods or Background:** A phantom was constructed by inserting small vials into a cylindrical container. Vials contained FeCl2 at four different concentrations (range 0.22-1.79mM) in two forms: clustered and free. The phantom was scanned at 3.0T (Siemens Germany). Multi-echo-GRE-images (TE=6:6:30ms; TR=53ms; nominal FA=18°; voxel=600x600x600µm; GRAPPA=2). For simulations, the same geometry as the real phantom was used. Susceptibility values were assigned to three different regions: background (1ppm), body (0ppm) and iron vials. A molar susceptibility of 1ppm/mM was assigned. For clustered iron vials, random noise (with a uniform probability distribution, from 0 to 1) was generated and multiplied by the corresponding iron concentration. Phase images at TE=6ms were obtained by convolution of the phantom geometry with the macroscopic unit-dipole function. Morphology enabled dipole inversion (MEDI)2 was applied to the earliest echo-time phase images (6ms) to obtain QSM for experimental and simulated data using lambda1=1000 and lambda2=10 for background regularisation.
**Results or Findings:** For measured QSM images, clustered iron led to highly localised field effects, also captured by simulations. For experimental data, best curve fittings were obtained after applying a Laplacian-based unwrapping pre-processing, with determination coefficients >0.88. In the simulations, QSM-values in both clustered and free iron showed a linear increase with iron (determination coefficient >0.99). In both cases, estimated molar susceptibility was lower with clustered iron.
**Conclusion:** Our simulation method captures the effect of iron clustering in QSM calculations as seen in experimental phantom acquisitions.
**Limitations:** QSM processing pipelines need to be refined to achieve higher accuracy for local field effects, as also seen in Alzheimer's beta-amyloid plaques.
**Ethics committee approval:** Not applicable.
**Funding for this study:** Validation EU-LACH #16/T01-0118.
**Author Disclosures:**
Michela Tosetti: Nothing to disclose
Paolo Bosco: Nothing to disclose
Elisa Tuzzi: Nothing to disclose
Javier Mattos: Nothing to disclose
Margarita Garcia: Nothing to disclose
Gisela E Hagberg: Nothing to disclose
Klaus Scheffler: Nothing to disclose
Laura Biagi: Nothing to disclose
Enrique Gustavo Cuña: Nothing to disclose

### RPS 1913-5

**Validation of a novel segmentation tool (segfatMR) for semiautomatic volumetry of adipose tissue in MR images**
*A. Linder*, T. Eggebrecht, N. P. Linder, T. Denecke, H. Busse; Leipzig/DE
*(anna.linder@medizin.uni-leipzig.de)*

**Purpose:** Subcutaneous (SAT) and visceral adipose tissue (VAT) are considered as biomarkers for a variety of clinical disciplines and questions. Segmentation and volumetry are generally time-consuming, creating a need for rapid but accurate analysis tools. This work aims to present a new segmentation tool (developed under a custom framework, DicomFlex [RS]) that combines the speed of automatic pre-segmentation with the reliability of a brief interactive revision. Results were validated against a widely used commercial tool (SliceOmatic).
**Methods or Background:** Validation was performed on MRI datasets (1.5 T Achieva XR, Philips Healthcare) from 20 patients (10 women/men), 25.1-63.1 (mean 48.5) years old, with BMIs between 28.3 and 58.8 (mean 36.8) kg/m2. Two independent readers analysed the abdominopelvic datasets (30-40 slices, mean 35.8 ) with both tools (segfatMR, SliceOmatic). Coefficients of determination (R2) as well as bias and limits of agreement (Bland Altman) were determined.
**Results or Findings:** Segmentation performance (R2 between methods) was excellent for both readers for SAT (> 0.99) and very high for VAT (around 0.90). The novel method was almost twice as fast as the reference – 19 (R2) and 25 (R1) s/slice vs 34 and 40 s/slice. On a subjective level, readers appreciated the intuitive workflow resulting from the standardised interface.
**Conclusion:** The open-source semiautomatic PC segmentation tool enables a fast and accurate quantification of whole abdominopelvic adipose tissue volume. Development under a standardised software framework (Dicomflex) allows for further extensions or adaptations.
**Limitations:** Limitations include single-centre-design, limited number of readers (n=2) and specific patient selection based on BMI(>25kg/m²).
**Ethics committee approval:** MRI study was under IRB approval.
**Funding for this study:** There was no specific funding for this study.
**Author Disclosures:**
Timm Denecke: Nothing to disclose
Harald Busse: Nothing to disclose
Anna Linder: Nothing to disclose
Nicolas Peter Linder: Nothing to disclose
Tobias Eggebrecht: Nothing to disclose

### RPS 1913-6

**Skin temperature changes in pregnant women during foetal MRI**
*L. Wachholz*, H-J. Mentzel, A. Heinrich, H. Proquitte, E. Schleussner, P. Schlattmann, U. K. M. Teichgräber, C-H. Cho-Nöth; Jena/DE

**Purpose:** Foetal MRI will be performed as an adjunct to sonography in foetal pathologies. Generally, MRI is related with heat, caused by radiofrequency waves produced during MRI scans. The purpose of this prospective study was to confirm that there is no heating in pregnant women during MRI.
**Methods or Background:** 20 pregnant women (aged between 19 and 45 years) in a range of 20 to 35 pregnancy week underwent foetal MRI for various indications. Imaging was performed on a 1.5 T MRI (AERA, SIEMENS, GERMANY) without sedation or contrast media. Imaging time was between 30 and 45 minutes. Two probes Tesla M3 (Mammendorfer Institut für Physik und Medizin, GERMANY) were used for measuring the skin temperature: one on the leg outside the field of view (FOV) (TL) and the second one next to the belly button within the FOV (TFOV). Before and after each MRI investigation the body core temperature was estimated by evaluation of the ear temperature (Tc).
**Results or Findings:** Both probes demonstrated in all 20 pregnant women an increase of the skin temperature of 0.1°C per imaging minute. TFOV increased 3.6°C (mean; min 1.8, max 5.6°C), TL 1.7°C (mean; min 0.1°C, max 2.6°C), Tc 0.3°C (mean; min -0.3°C, max 1.2°C).
**Conclusion:** We observed a significant increase in temperature in pregnant women during MRI. We don't know if there is any side effect on the foetus. Therefore, possible health consequences, also for the fetus, should be checked.
**Limitations:** The limitations are the missing online rectal and intrauterine temperature measurements.
**Ethics committee approval:** The ethics committee approval was obtained.
**Funding for this study:** Not applicable, institutional study.
**Author Disclosures:**
Chie-Hee Cho-Nöth: Nothing to disclose
Hans-Joachim Mentzel: Nothing to disclose
Peter Schlattmann: Nothing to disclose
Andreas Heinrich: Nothing to disclose
Laura Wachholz: Nothing to disclose
Hans Proquitte: Nothing to disclose
Ekkehard Schleussner: Nothing to disclose
Ulf Karl Martin Teichgräber: Nothing to disclose

### RPS 1913-8

**Analysis of slice profile effects for super resolution thin-slice multi-slice MRI**
*S. J. Riederer*, E. Borisch, R. Grimm; Rochester, MN/US

**Purpose:** Application of super-resolution methods to the slice select direction attempts to recover through-plane resolution finer than the slice thickness. Critical in this process is the correction for the slice profile. However, conventional "rect" profiles have zeroes in the kZ passband, causing undesirable attenuation of high spatial frequencies in the slice direction. The purpose of this work is to investigate alternative slice profiles which allow improved fidelity.
**Methods or Background:** First, several alternative RF profiles were identified with no zeroes in the kZ passband. These are a Gaussian and a (1+cos) (raised cosine, or "rCos"). Second, experimental images using these were compared with a standard "rect" profile using a phantom with resolution patterns varying from 0.15 to 0.50 lp/mm. Third, sagittal images were acquired of the prostate in vivo from several subjects. Axial sampling using 3mm thick slices with the various slice profiles was simulated using several levels of additive noise, super-resolution reconstruction performed, and results compared with the original sagittal image. Finally, results were compared with sagittal reformats made from the actual axial acquisition.
**Results or Findings:** For 3mm thick slices, both Gaussian and rCos RF pulses allow full restoration of spatial frequencies up to 0.50 1/mm using SR reconstruction, equivalent to 1mm slice thickness. rCos excitation consistently provides improved sharpness vs Gaussian over all noise levels. Sagittal reformats of SR-reconstructed axially acquired data well resemble direct sagittal acquisition.
**Conclusion:** Alternative (non-rect) RF slice profiles such as Gaussian and rCos allow improved fidelity of high spatial frequencies in the slice direction with resolution finer than that in the acquired slices.
**Limitations:** The number of subjects studied is limited. Finer (<1mm) slice resolution is desirable.
**Ethics committee approval:** Approved by Institutional Review Board (IRB).
**Funding for this study:** Funding for this study was institutional.
**Author Disclosures:**
Stephen J. Riederer: Nothing to disclose
Eric Borisch: Nothing to disclose
Roger Grimm: Nothing to disclose

**14:00-15:00** **Channel 4**

### Research Presentation Session: Genitourinary

### RPS 2007

### Ovarian imaging today: what's new?

**Moderators**
T. M. Cunha; Lisbon/PT
A. G. Rockall; London/UK
*(wmd328@126.com)*

### RPS 2007-3

**CT radiomics in differentiating histological subtypes of epithelial ovarian carcinoma**

*M. Wang*[1], J. A. U. Perucho[1], L. Han[2], E. M. F. Wong[1], G. Ho[1], P. Ip[1], E. Y. P. Lee[1]; [1]Hong Kong/CN, [2]Guangzhou/CN

**Purpose:** To evaluate the ability of contrast-enhanced CT (ceCT)-based radiomic features in differentiating high-grade serous carcinoma (HGSC) and non-HGSC of epithelial ovarian carcinoma (EOC).
**Methods or Background:** A total of 341 patients with EOC were enrolled in this multicentre retrospective study. Centre A with 282 patients was designated as the internal cohort and was divided into an internal training set (n = 225) and an internal test set (n = 57). Centre B with 59 patients, was designated as the external cohort and was used for external testing. To address the class imbalance between the distribution of HGSC and non-HGSC, synthetic balanced samples were generated using Random Over-Sampling Examples (ROSE). Tumours were manually delineated on each slice of ceCT images to encompass the entire tumour. Radiomic features with Laplacian of Gaussian (LoG) and wavelet transforms were extracted. Feature selection was performed using Mann-Whitney U tests and least absolute shrinkage and selection operator (LASSO) regression. Selected features were used to build the logistic regression (LR) models for differentiating HGSC and non-HGSC. The performances of the models were assessed by receiver operating characteristic curves (ROC) and the area under the curve (AUC).
**Results or Findings:** Thirty-four radiomic features were selected for modelling. The AUCs of LR models for differentiating HGSC and non-HGSC were 0.944 for the internal training set, 0.945 for the internal test set and 0.805 for the external test set.
**Conclusion:** Radiomic features extracted from ceCT were helpful in histological subtyping of EOC. The LR models offered outstanding diagnostic efficiency in the internal cohorts, and excellent in the external cohort.
**Limitations:** Centre B has a limited sample size. The distribution of HGSC and non-HGSC was different between Centre A and B.
**Ethics committee approval:** Institutional review board (No. UW 20-251).
**Funding for this study:** Health and Medical Research Fund (no. 08192106).
**Author Disclosures:**
Grace Ho: Nothing to disclose
Jose Angelo Udal Perucho: Nothing to disclose
Elaine Yuen Phin Lee: Nothing to disclose
Philip Ip: Nothing to disclose
Mandi Wang: Nothing to disclose
Lujun Han: Nothing to disclose
Esther Man Fung Wong: Nothing to disclose

### RPS 2007-4

**Comparative performance of ORADS MRI score vis-a-vis ADNEX MR score for characterisation of ovarian-adnexal masses, was the transition justified? A comparative evaluation in an Indian cohort**

*S. Patra*[1], S. B. Grover[2], H. Grover[3], P. Mittal[1], G. Khanna[1]; [1]New Delhi/IN, [2]Greater Noida/IN, [3]New York, NY/US
*(patrasayantan92@gmail.com)*

**Purpose:** To compare the diagnostic performance of ORADS-MRI score vis-a-vis ADNEX-MR score for characterisation of ovarian-adnexal tumours as benign or malignant using histopathology as a reference standard. The secondary objective was to justify the transition from ADNEX-MR to ORADS-MRI.
**Methods or Background:** The ORADS-MRI algorithm was recently proposed for characterising ovarian tumours, directing the management of malignant tumours towards specialised oncology teams and benign tumours towards restricted intervention. This study was designed as a cross-sectional, observational study, with the retrospective application of ORADS-MRI and ADNEX-MR scores to a prospectively acquired data set during an IRB approved study on ovarian-adnexal masses conducted during the last 2 years. We recruited patients with ovarian-adnexal tumours, who had undergone 3T MRI with morphological, functional and dynamic contrast-enhanced sequences and who had final histopathology diagnosed. Major exclusion criteria at recruitment were age below 18 years, deranged renal functions, and metallic implants. Two trained radiologists initially blinded to histopathology diagnosis assessed ORADS-MRI and ADNEX-MR scores in each tumour. The final score and the diagnosis were assigned by consensus, ORADS 1-3 and ADNEX 1-3 as benign, ORADS 4/5 and ADNEX 4/5 as malignant, and subsequently correlated with histopathology.
**Results or Findings:** The cohort comprised 45 patients with 63 tumours, histopathology diagnosis was 36 benign and 27 malignant tumours. ORADS-MRI vis-a-vis ADNEX-MR had sensitivity 96.3% vis-a-vis 96.3%, specificity 72.2% vis-a-vis 63.9%, PPV 72.2% vis-a-vis 66.7%, NPV 96.3% vis-a-vis 95.8% and diagnostic accuracy 82.5% vis-a-vis 77.78%.
**Conclusion:** Performance of ORADS-MRI was superior in specificity, PPV and diagnostic accuracy and further, since ORADS-MRI is more definitive for risk assignment in tumours with score 5 (ORADS >90%, ADNEX >50%) the transition from ADNEX-MR score towards ORADS-MRI algorithms is adequately justified.
**Limitations:** Small sample size, single centre study.
**Ethics committee approval:** Ethics approval was obtained.
**Funding for this study:** No funding was received for this study.
**Author Disclosures:**
Sayantan Patra: Author: Nothing to disclose
Shabnam Bhandari Grover: Author: Nothing to disclose
Geetika Khanna: Nothing to disclose
Hemal Grover: Nothing to disclose
Pratima Mittal: Nothing to disclose

### RPS 2007-5

**Monitoring treatment response in epithelial ovarian carcinoma by PET/CT following neoadjuvant chemotherapy**

*E. Y. P. Lee*, J. A. U. Perucho, G. S. Kwok, K. Y. Tse, P. Ip; Hong Kong/CN
*(eyplee77@hku.hk)*

**Purpose:** To evaluate the accuracy of PET/CT in monitoring treatment response in epithelial ovarian carcinoma (EOC) after neoadjuvant chemotherapy (NACT).
**Methods or Background:** Patients with advanced EOC (FIGO III-IV) were prospectively recruited to undergo PET/CT after 3-6 cycles of NACT before interval debulking surgery. PET/CT was evaluated using a 5-point Likert scale. Nineteen regions in the abdominopelvic cavity were evaluated on PET/CT (subdiaphragmatic spaces, perihepatic, liver serosa and hilum, gastric serosa, pancreas and lesser sac, splenic serosa, splenic hilum, paracolic gutters, omentum, bowel mesentery, paraaortic and pelvic lymph nodes, and central pelvis) and correlated with surgical evaluation and histopathological assessment. Histopathological specimens were taken as the gold standard. In the event where resection was not performed and biopsy could not be safely taken (e.g. miliary serosal disease), the intra-operative surgical findings were taken as the standard of reference. A region-based analysis was performed. The diagnostic characteristics of PET/CT were described by accuracy, sensitivity, specificity, positive (PPV) and negative predictive values (NPV).
**Results or Findings:** The average accuracy of region-based analysis was 0.88 (sensitivity 0.38, specificity 0.96, PPV 0.56 and NPV 0.91) based on 285 regions evaluated in 15 patients with EOC. Sensitivity in disease detection was low in all regions evaluated, but PET/CT showed high specificity and NPV, except for disease in the omentum (specificity 0.71, NPV 0.56) and central pelvis (specificity 0.71, NPV 0.63).
**Conclusion:** PET/CT was highly specific with a high NPV in evaluating the abdominopelvic cavity of advanced EOC following NACT. These metrics could be useful in surgical planning in stratifying patients for less aggressive and extensive debulking surgery when the disease was deemed to be absent on PET/CT.
**Limitations:** The study was limited by small a sample size.
**Ethics committee approval:** Institutional review board (No. UW 18-604).
**Funding for this study:** Health and Medical Research Fund (No. 06171706).
**Author Disclosures:**
Ka Yu Tse: Nothing to disclose
Jose Angelo Udal Perucho: Nothing to disclose
Elaine Yuen Phin Lee: Nothing to disclose
Philip Ip: Nothing to disclose
Gladys S.T. Kwok: Nothing to disclose

### RPS 2007-6

**T2*-weighted imaging performance in detection of deep endometriosis: a comparison with standard MRI sequences**

*P. N. Franco*[1], S. Annibali[2], C. Cazzella[1], S. Viganò[1], A. Bonanomi[1], P. A. Bonaffini[1], S. Sironi[1]; [1]Milan/IT, [2]Bergamo/IT
*(p.franco@campus.unimib.it)*

**Purpose:** To evaluate the diagnostic performance of T2*-weighted sequence compared to conventional MRI protocol, used as a reference standard, in the assessment of deep endometriosis.
**Methods or Background:** Patients who underwent a pelvic MRI on a 3T scan for clinical and/or ultrasound suspicion of deep endometriosis were prospectively enrolled (December 2020 - August 2021). Two radiologists (with 10 and 2 years of experience, respectively) qualitatively evaluated in consensus standard MRI sequences to detect endometriotic lesions. The most experienced radiologist assessed the presence of signal voids on T2*-weighted sequences: in case of discrepancy between standard and T2* sequences, MRI images were re-evaluated in consensus. Equivocal lesions were assessed through second-look ultrasound and clinical evaluation.
**Results or Findings:** Forty patients (mean age 35.5 years) with a total of 78 endometriosis foci were included in the study. 24,4% (19/78) of endometriotic lesions detected through morphologic standard sequences showed a signal loss in T2*-sequences. At radiologic re-evaluation, 13/19 lesions associated with signal voids (68.4%) have been related to artefacts (including air or previous surgery) or hemorrhagic foci not linked to endometriosis (i.e., hemorrhagic corpus luteum). Among lesions whose nature on T2* sequences was still unclear but not artefact-related (31.6%; 6/19), only 4 resulted consistent with endometriosis at the ultrasound and clinical follow-up and none was an isolated lesion.
**Conclusion:** T2*-weighted sequences, even if can allow the detection of hemosiderin deposits in endometriotic foci, do not seem to provide an added value in the assessment of deep lesions. Furthermore, artefacts caused by undesirable sources of magnetic signal voids may lead to diagnostic overestimation.
**Limitations:** The limited sample size. Lack of surgical correlation.
**Ethics committee approval:** Not applicable.
**Funding for this study:** No funding was provided for this study.
**Author Disclosures:**
Simona Annibali: Nothing to disclose
Alice Bonanomi: Nothing to disclose
Pietro Andrea Bonaffini: Nothing to disclose
Paolo Niccolò Franco: Nothing to disclose
Caterina Cazzella: Nothing to disclose
Sandro Sironi: Nothing to disclose
Sara Viganò: Nothing to disclose

### RPS 2007-7

**Attention to diaphragmatic endometriosis diagnostics: simple extension of pelvic endometriosis MRI protocol**

*K. H. Härmä*, S. Imboden, F. Siegenthaler, M. Mueller, J. T. Heverhagen; Bern/CH
*(kirsihannele.haermae@insel.ch)*

**Purpose:** Diaphragmatic endometriosis on MRI.
**Methods or Background:** In this retrospective single-centre study from 2017 to 2021, nineteen patients with detected diaphragmatic endometriosis (DE) on MRI were consecutively included. Among them (1) patients without DE symptoms referred for pelvic endometriosis MRI (peMRI), including upper abdomen chemical shift sequence (uaCSS) and (2) symptomatic patients suspicious for DE referred for diaphragm endometriosis MRI (diMRI). The image analysis was followed-up by a gynecologic radiologist. Location, number, size, laterality and accuracy of DE findings, as clinical symptoms and correlation with pelvic/bowel deep infiltrating endometriosis (DIE), were investigated. The MR-graphic findings were intra-operative and histopathologically proven. Descriptive statistics, means, medians and ranges were calculated for continuous variables; frequencies for categorical variables. Confusion matrix/PPV/NPV (Excel 2016).
**Results or Findings:** MRI detected 49 DE suspicious lesions in 19 patients. 47% (9/19) of the patients were referred for diMRI, being suspicious for DE. 53% (10/19) patients were referred for peMRI with uaCSS without any suspicion on DE but pelvic endometriosis. 57% of DE lesions were located posteriorly, 33% laterally, and 10% anteriorly. The mean size of all DE lesions was 13.9 mm (range 3mm-38mm). Until now, nine patients (9/19) underwent a laparoscopic operation resulting in a PPV of 75% for DE detection on MRI. 5/9 operated patients were referred to pelvic MRI. DE was proven in 6/9 patients intra-operatively and histopathologically. False-positive findings in two patients were identified as a diaphragmatic post-operative scar. Both DE and pelvic or bowel DIE was diagnosed in 58%/53% of the women.
**Conclusion:** The current study demonstrated the importance of incorporating diaphragmatic imaging to pelvic endometriosis MRI. It allows the DE diagnosis in an early stage, offers optimal patient counselling, management of the surgery and anticipating its complexity.
**Limitations:** Small patient population.
**Ethics committee approval:** The ethics committee approvals was obtained.
**Funding for this study:** No funding was received for this study.
**Author Disclosures:**
Johannes T. Heverhagen: Nothing to disclose
Michel Mueller: Nothing to disclose
Sara Imboden: Nothing to disclose
Kirsi Hannele Härmä: Nothing to disclose
Franziska Siegenthaler: Nothing to disclose

### RPS 2007-8

**Multiparametric MRI in the differential diagnosis of epithelial ovarian tumours: association between quantitative DCE parameters, ADC and immunohistochemical markers**

*A. Solopova*, Y. Nosova, G. Khabas; Moscow/RU
*(dr.solopova@mail.ru)*

**Purpose:** To explore the correlation between quantitative parameters from DCE-MRI and ADC with Ki-67 proliferation status, p53 and p16 expression in patients with ovarian tumours.
**Methods or Background:** This prospective study, approved by the local ethical committee, enrolled 102 patients (26 benign (BOT), 24 borderline (BEOT) and 52 malignant epithelial ovarian tumours (MEOT)) from 2018-2020. Preoperative MRI was performed in all patients, DCE-MRI quantitative parameters (Ktrans, Kep, Ve) and ADC (mean) were measured, compared, and correlated with Ki-67, p53, p16 expression between the groups of BOT, BEOT, MEOT (according to the histologic verification). Statistical analyses were performed using the Mann–Whitney U-test, ROC-curves, and Spearman's correlation by drawing ROIs.
**Results or Findings:** The Ktrans, Kep and Ve was significantly higher in MEOTs than in BOTs (0,39 (0,36-0,50), 0,25 (0,230-0,270) и 0,22 (0,210-0,240) vs 0,2 (0,16-0,22), 0,016 (0,016-0,051) and 0,1 (0,060-0,130), respectively). ADC values were significantly higher in BOTs than in MEOTs (1,277 (1,2-1,4) x10-3 vs 0,826 (0,7-0,9) x10-3) (p<0,0001). The Ktrans and ADC (mean) value were the most informative parameters for the differentiation between BOTs and MEOTs, providing the sensitivity, specificity, and accuracy of 88,9%, 94,7% and 90,6% for Ktrans and 85,1%, 87,5%, and 85,7% for ADC (mean), respectively; the combination of Ktrans and ADC mean value provides 91,1%, 94,7% and 92,2%, respectively. Ktrans and Kep were positively correlated with Ki-67 expression (r=0,6 and 0,671, respectively; p<0,01), ADC values were correlated with Ki-67 and p16 expression (r=0,624 and 0,6, respectively; p<0,01).
**Conclusion:** Preliminary findings demonstrate that DCE MRI and ADC are an effective additional tool for differentiating BOTs from MEOTs, and are correlated with Ki-67 and p16 expression.
**Limitations:** This is a single-centre study.
**Ethics committee approval:** All studies were approved by the local ethics committee.
**Funding for this study:** Grant of the President of Russian Federation (MD-130.2019.7).
**Author Disclosures:**
Grigory Khabas: Nothing to disclose
Alina Solopova: Nothing to disclose
Yulia Nosova: Nothing to disclose

**16:00-17:00** **Channel 4**

### Research Presentation Session: Vascular

### RPS 2115

### Advances in thoracoabdominal and peripheral vascular MRI

**Moderators**
A. Redheuil; Paris/FR
C. Catalano; Rome/IT

### RPS 2115-4

**4D flow MRI in Marfan patients: association of Z-score and altered aortic haemodynamics**

*A. Lenz*, C. Riedel, F. Wright, I. Ristow, S. Zhang, B. Schönnagel, G. Adam, Y. von Kodolitsch, P. Bannas; Hamburg/DE
*(a.lenz@uke.de)*

**Purpose:** To determine the association of normalised aortic root diameters (Z-score) and 4D flow MRI-derived aortic haemodynamics in Marfan patients.
**Methods or Background:** We prospectively performed a 4D flow MRI at 3T of the thoracic aorta in 100 Marfan patients (36±14 years). Patients were divided into two groups according to Z-score of the aortic root: i) Z-score <2 (n=43), or ii) Z-score >2 (n=57). The degree of helical and vortical flow in the ascending aorta was evaluated according to a 3-point scale (0: no, 1: rotation <360º, 2: rotation >360º). In addition, we assessed wall shear stress (WSS) and flow eccentricity at the level of the sinotubular junction (STJ), mid-ascending aorta (midAAo), and proximal arch (proxAA). Results were statistically compared using unpaired t-tests or Mann-Whitney U tests.
**Results or Findings:** Flow eccentricity at the level of STJ was significantly higher in Marfan patients with Z-score >2 (0.15±0.07 vs 0.12±0.07; p=0.02) when compared to Marfan patients with Z-score <2. WSS at the level of STJ was significantly lower in Z-score >2 patients compared to Z-score <2 patients (0.39±0.11 N/m2 vs 0.46±0.12 N/m2; p=0.002). Aberrant vortical and helical flow patterns in the ascending aorta were more pronounced in Z-score >2 patients; however, those differences in vortical (0.5±0.7 vs 0.4±0.6; p=0.3) and helical flow (0.8±0.7 vs 0.7±0.7; p=0.4) were not statistically significant.
**Conclusion:** Marfan patients with pathologically increased Z-scores reveal increased flow eccentricity and reduced WSS compared to Marfan patients with physiological Z-scores.
**Limitations:** Visual assessment of helical and vortical flow as well as manual positioning of 2D analyses planes for assessment of WSS and flow eccentricity may introduce a subjective bias and affect both qualitative and quantitative results.
**Ethics committee approval:** The study was approved by the local research ethics committee.
**Funding for this study:** The German Heart Research Foundation funded this study.
**Author Disclosures:**
Christoph Riedel: Nothing to disclose
Felicia Wright: Nothing to disclose
Alexander Lenz: Nothing to disclose
Peter Bannas: Nothing to disclose
Björn Schönnagel: Nothing to disclose
Shuo Zhang: Employee: Philips GmbH
Gerhard Adam: Nothing to disclose
Inka Ristow: Nothing to disclose
Yskert von Kodolitsch: Nothing to disclose

### RPS 2115-5

**Validation of 4D flow MRI in TIPS stent-grafts using a 3D-printed flow phantom**

*C. Riedel*, A. Lenz, I. Ristow, F. Wright, B. Schönnagel, G. Adam, P. Bannas; Hamburg/DE
*(ch.riedel@uke.de)*

**Purpose:** To validate 4D flow MRI-derived flow measurements in transjugular intrahepatic portosystemic shunt (TIPS) stent-grafts using a 3D-printed flow phantom.
**Methods or Background:** A flow phantom mimicking the portal venous vasculature was 3D-printed using Clear Resin (Formlabs). The model consisted of the superior mesenteric vein (SMV) and the splenic vein (SV) draining into the extrahepatic portal vein (PV), the intrahepatic TIPS-tract, and the liver vein. A Viatorr® stent-graft was positioned within the TIPS tract. SMV and SV served as inlets for blood-mimicking fluid and were connected over a flow regulator and a flow sensor to a pump. TIPS flow rates ranging from 0.8-2.8 L/min were preset and 4D flow MRI acquisitions were performed at 3T using a velocity encoding (venc) of both 100cm/s and 200cm/s. 4D flow MRI-derived datasets were evaluated at predefined levels including SMV, SV, PV, the uncovered part of the stent-graft as well as the covered stent-graft (distal, central, proximal). 4D flow MRI-derived flow rates were compared to preset flow rates as a standard of reference.
**Results or Findings:** At a venc of 200cm/s, 4D flow MRI-derived flow rates were significantly correlated with the preset flow rates at all vascular levels and within the stent-graft (all r>0.98, p<0.001). At a venc of 100cm/s, aliasing artefacts were present within the stent-graft at flow rates ≥2.0L/min. Lower preset flow rates were also significantly correlated with 4D flow MRI-derived flow rates (all r>0.99, p<0.05). In the uncovered stent-graft, 4D flow MRI underestimated the flow rate at a venc of 100cm/s by 1-6%.
**Conclusion:** 4D flow MRI enables valid flow evaluation within TIPS stent-grafts and warrants further in vivo validation studies to determine its clinical usefulness for monitoring TIPS function and patency.
**Limitations:** Not applicable.
**Ethics committee approval:** Not applicable.
**Funding for this study:** Not applicable.
**Author Disclosures:**
Christoph Riedel: Nothing to disclose
Felicia Wright: Nothing to disclose
Alexander Lenz: Nothing to disclose
Peter Bannas: Nothing to disclose
Björn Schönnagel: Nothing to disclose
Gerhard Adam: Nothing to disclose
Inka Ristow: Nothing to disclose

### RPS 2115-6

**Comparison of image quality of non-contrast MR pulmonary angiography with different voxel sizes**

*J. Liu*, J. Qiu, W. Li, K. Zhao, Z. Bi; Beijing/CN
*(liujia8877@126.com)*

**Purpose:** To compare the image quality of non-contrast MR pulmonary angiography (MRPA) with two different voxel sizes.
**Methods or Background:** This study consisted of 29 healthy volunteers (13 males, mean age: 44.1±8.0 years) who were given written informed consent. All non-contrast MRPA data were collected on a 1.5T MR scanner (MAGNETOM Aera, Siemens Healthcare, Erlangen, Germany) with an 18-channel body coil and an integrated 32-channel spine matrix coil. A free-breathing 3D turbo spin-echo (TSE) with variable-flip-angle sequence (SPACE) was used to acquire non-contrast MRPA data with the following two sequences of different parameters: 1) voxel size=1.2×1.2×4 mm3. 2) voxel size=2×2×2 mm3. The subjective image quality assessment of normal pulmonary arteries was evaluated by one experienced radiologist on primary coronary images and constructed axial images separately. The image quality of the two groups was compared by the paired t-test.
**Results or Findings:** The mean image quality scores of the main, left, and right pulmonary arterial trunks on primary coronary images were 3.0±0.6, 3.0±0.6, 3.0±0.6 (group 1) and 2.4±0.6, 2.5±0.6, 2.5±0.8 (group 2). The mean image quality scores of the main, left, and right pulmonary arterial trunks on constructed axial images were 2.2±0.5, 1.4±0.5, 1.6±0.5 (group 1) and 2.0±0.8, 2.1±0.7, 2.1±0.7 (group 2). The branch image quality of group 1 was better than group 2 on primary coronal images (p<0.05). However, the image quality of the left and right pulmonary artery trunk in group 2 was better than group 1 on constructed axial images (p<0.05).
**Conclusion:** Non-contrast MRPA images with higher resolution within a coronal plane can provide acceptable image quality but images with isotropy can acquire better constructed axial image quality.
**Limitations:** No limitations.
**Ethics committee approval:** The ethics approval was obtained.
**Funding for this study:** No funding was received for this study.
**Author Disclosures:**
Wei Li: Nothing to disclose
Zhongxu Bi: Nothing to disclose
Kai Zhao: Nothing to disclose
Jianxing Qiu: Nothing to disclose
Jia Liu: Nothing to disclose

### RPS 2115-7

**Quiescent-interval single-shot magnetic resonance angiography outperforms carbon dioxide angiography as a nephroprotective imaging method of chronic lower extremity arterial disease**

*J. Csőre*, F. I. Suhai, M. Gyánó, A. A. Pataki, G. Juhász, M. Vecsey-Nagy, D. Pál, D. M. Fontanini, C. Csobay-Novák; Budapest/HU
*(csore.judit@gmail.com)*

**Purpose:** Patients with lower extremity arterial disease (LEAD) often present with chronic kidney disease (CKD), in whom the use of nephroprotective imaging is of utmost importance. We compared the diagnostic performance of two such modalities: the non-contrast quiescent-interval single-shot (QISS) magnetic resonance angiography (MRA) and carbon dioxide digital subtraction angiography (CO2-DSA).
**Methods or Background:** CO2-DSA and QISS-MRA images of patients with chronic LEAD scheduled for elective diagnostic imaging were compared. A 19-segment lower extremity arterial model was used to assess the degree of stenosis (none, <50%, 50-70%, 70%<) and the image quality (5-point Likert scale: 1-non-diagnostic, 5-excellent) per-segment. Four radiologists evaluated the images. Intra-class correlation coefficient (ICC) was calculated for both inter-rater and intra-rater reliability. Three regions (aorto-iliac, femoro-popliteal, tibio-peroneal) were created by the segments for the final analysis. Diagnostic accuracy and interpretability were also evaluated.
**Results or Findings:** 523 segments were evaluated in 28 patients (mean age: 71±9 years). Subjective image quality of QISS-MRA was significantly better compared to CO2-DSA for all regions [aorto-iliac: 4(4-5) vs 3(3-4); femoro-popliteal: 4(4-5) vs 4(3-4); tibio-peroneal: 4(3-5) vs 3(2-3), all regions: 4(4-5) vs 3(3-4), p<0.001]. QISS-MRA out-performed CO2-DSA regarding interpretability (98.3% vs 86.2%, p<0.001). Diagnostic accuracy parameters of QISS-MRA for >70% luminal stenosis as compared to CO2-DSA: sensitivity 77.8%, specificity 95.2%, positive predictive value 83.2%, negative predictive value 93.3%. ICC regarding the degree of stenosis: QISS-MRA: 0.97; CO2-DSA: 0.82. Intraobserver variability for each investigator: CO2-DSA: 0.88, 0.93; QISS-MRA: 0.86, 0.91.
**Conclusion:** QISS-MRA had a better diagnostic value than CO2-DSA in subjective assessment of image quality in all regions studied, proved to be an excellent reproducible method for the assessment of LEAD.
**Limitations:** Single-centre study with a relatively small number of patients.

**Ethics committee approval:** Approved by the National Institute of Pharmacy and Nutrition (OGYEI/7984/2020).
**Funding for this study:** No funding was received for this study.
**Author Disclosures:**
Milán Vecsey-Nagy: Nothing to disclose
Georgina Juhász: Nothing to disclose
Ferenc Imre Suhai: Nothing to disclose
Daniele Mariastefano Fontanini: Nothing to disclose
Csaba Csobay-Novák: Nothing to disclose
Judit Csőre: Nothing to disclose
Akos Andras Pataki: Nothing to disclose
Marcell Gyánó: Nothing to disclose
Dániel Pál: Nothing to disclose

### RPS 2115-8

**Diagnostic value of standardised MR angiography protocol in the evaluation of thoracic outlet syndrome**

*M. Ragusi*[1], C. Talei Franzesi[1], C. Maino[1], D. G. Gandola[1], T. P. Giandola[1], D. Ippolito[1], S. Sironi[2]; [1]Monza/IT, [2]Bergamo/IT
*(maria.ragusi@gmail.com)*

**Purpose:** To evaluate the diagnostic performance of a standardised MR angiography (MRA) protocol in the study of thoracic outlet syndrome (TOS).
**Methods or Background:** A total of 20 patients who underwent MRA study to investigate vascular TOS were retrospectively enrolled. Protocol sequences include Balanced Fast Field Echo M2D, Turbo Spin Echo T1 weighted, high-resolution TSE T1 weighted, HR TSE DIXON T2 weighted, contrast-enhanced MRA (CE- MRA), pre- and post-contrast T1-weighted high-resolution isotropic volume examination. Baseline sequences were performed with the arms in adduction, CE-MRA sequences were performed both with arms in adduction and abduction, with an injection of contrast media repeated for each arms' position.
**Results or Findings:** Sixteen patients showed unilateral TOS (n=16, 80%), with the left side more frequently involved (n=10, 64.5%) than the right one (n=6, 45.5%). Thirteen patients showed venous compression (vTOS) (65%), 3 patients arterial TOS (aTOS) (15%), only in one case an overlap between vTOS-aTOS (5%) was reported. Eight patients showed compression with the arm in abduction (50%), 8 with the arm both in adduction and abduction (50%). In 5 cases TOS was caused by an osseus abnormalities both post-traumatic or post-surgical. In 6 patients (30%) vTOS was associated with thrombosis. Twenty per cent of TOS were caused by muscle hypertrophy or wrong insertion. Five out of sixteen cases involved the scalene triangle (31%), 8/16 the costoclavicular space (50%) and 3/16 patients the subacromial-pectoralis space (19%). In 4/20 patients vascular TOS was not identified (20%).
**Conclusion:** A standardised MRI protocol with CE-MRA sequence with arms in adduction and abduction allows identifying the presence of vascular TOS, along with the identification of the intrinsic and extrinsic abnormalities causing pathology.
**Limitations:** Not applicable.
**Ethics committee approval:** Not applicable.
**Funding for this study:** Not applicable.
**Author Disclosures:**
Sandro Sironi: Nothing to disclose
Davide Giacomo Gandola: Nothing to disclose
Cesare Maino: Nothing to disclose
Maria Ragusi: Nothing to disclose
Cammillo Talei Franzesi: Nothing to disclose
Teresa Paola Giandola: Nothing to disclose
Davide Ippolito: Nothing to disclose

### Sunday, March 6

**08:00-09:00** **Channel 2**

### Research Presentation Session: Musculoskeletal

### RPS 2210

### Arthritis, inflammation, and sarcopenia

**Moderator**
L. E. Derchi; Genoa/IT

### RPS 2210-2

**Value of contrast administration in patients with rheumatoid arthritis receiving a 3T MRI scan of the finger joints**

*M. Frenken*, D. B. B. Abrar, A. Mewes, L. Wollschläger; Düsseldorf/DE

**Purpose:** Rheumatoid arthritis (RA) is the most common inflammatory joint disease worldwide and leads to the destruction of bone and cartilage. This prospective study helps to evaluate the value of gadolinium-containing contrast agent application in magnetic resonance imaging (MRI) of the finger joints in patients with RA using the rheumatoid arthritis MRI score (RAMRIS) and its predictive value on treatment response and remission.
**Methods or Background:** 31 therapy-naïve patients with RA underwent pre-therapeutic MRI (t0) of finger joints and follow-up MRI at 3 (t1) and 6 months (t2) after therapy initiation. CRP was measured to determine treatment response and remission. All MRI examinations were evaluated according to RAMRIS for metacarpophalangeal joints. Synovitis as part of the RAMRIS was evaluated after contrast application as standard and additionally with a STIR sequence instead. The extent to which RAMRIS of finger joints is a predictor of treatment response or remission was investigated for both MRI protocols, with and without contrast administration. Differences were validated by Mann-Whitney-U test.
**Results or Findings:** As expected, standard RAMRIS with contrast administration was a good predictor for treatment response and remission at t0 and t1 (response/non-response: t0: p=0.0014, t1: p=0.016, remission/non-remission: t0: p=0.092, t1: p=0.0037). The pre-therapeutic and the 3 months after therapy STIR-based RAMRIS also showed differences between responders/non-responders and remission and non-remission (response: t0: p=0.001, t1: p=0.01, remission: t0: p=0.087, t1: p=0.0023). Neither score showed significant differences between responders/non-responders or remission/non-remission at t2.
**Conclusion:** In terms of predictive value for treatment response and remission, RAMRIS of finger joints with and without contrast administration showed similar diagnostic accuracy. The results indicate that native STIR sequences may replace contrast administration in RA imaging.
**Limitations:** Other RA-predilection sites besides the hand could be examined.
**Ethics committee approval:** The ethics committee approval was obtained by HHU-Düsseldorf.
**Funding for this study:** Not applicable.
**Author Disclosures:**
Alexander Mewes: Nothing to disclose
Lena Wollschläger: Nothing to disclose
Miriam Frenken: Nothing to disclose
Daniel B. Benjamin Abrar: Nothing to disclose

### RPS 2210-3

**50 shades of backfill: new bone formation in axial spondyloarthritis**

*T. Diekhoff*, C. Niedermeyer, D. Poddubnyy, K-G. A. Hermann; Berlin/DE

**Purpose:** Several MRI findings of the sacroiliac joint (SIJ) space in axial spondyloarthritis (axSpA) were previously described such as inflammation or fatty metaplasia inside an erosion, i.e. “backfill”. This study aims to link the aforementioned changes to CT and to understand, which findings represent new bone formation.
**Methods or Background:** Out of 178 patients from two prospective studies that included CT and MRI of the SIJs, all patients with the axSpA were selected. MRI was screened by two senior musculoskeletal radiologists in consensus for joint-space related findings and in three categories, Type A: hyperintense in STIR and hypointense in T1 (inflammation inside erosion), Type B: hyperintense in both sequences and Type C: hypointense in STIR and hyperintense in T1 (backfill). By using image fusion techniques and one-by-one comparison, the Hounsfield Units (HU) of those lesions, normal cartilage and spongeous and cortical bone were measured in CT.
**Results or Findings:** Ninety-nine axSpA were identified, and 48 Type A, 88 Type B and 84 Type C lesions were included. The HU values of cartilage were 73.6±15.0, spongiosa 188.0±69.9, cartilage 1086.0±100.3, Type A 341.2±96.7, Type B 359.3±153.5 and Type C 446.8±123.0, respectively. The lesion values were significantly higher than cartilage and spongiosa but lower than cortical bone (p<0.001). Type A and B showed similar HU (p=0.93), whereas Type C lesions were less dense (p<0.001).
**Conclusion:** All joint space lesions (Type A to C) showed a calcified matrix and, thus, resemble new bone formation with gradually higher values in Type C lesions, i.e. typical backfill. Therefore, the nomenclature of those lesions should be critically re-assessed.
**Limitations:** Image fusion between MRI and CT scans was done manually by landmarks. Up to four lesions per patient were measured.
**Ethics committee approval:** This study was approved by the ethics committee (EA1/086/16, EA1/073/10), and written informed consent was obtained from patients.
**Funding for this study:** Not applicable.
**Author Disclosures:**
Denis Poddubnyy: Nothing to disclose
Torsten Diekhoff: Speaker: Canon MS
Christoph Niedermeyer: Nothing to disclose
Kay-Geert A. Hermann: Nothing to disclose

### RPS 2210-4

**The use of dual-energy CT to quantitatively assess osteomyelitis in patients with diabetic foot ulcers**

*M. Mens*, A. de Geus, R. Wellenberg, G. Streekstra, S. Bus, T. Busch-Westbroek, M. Nieuwdorp, M. Maas; Amsterdam/NL

**Purpose:** The purpose of this study was to evaluate if dual-energy CT (DECT) can be used to quantitatively assess osteomyelitis in patients with diabetic foot ulcers.
**Methods or Background:** All patients with a diabetic foot ulcer and suspected osteomyelitis that underwent a DECT-scan (dual-source 80kVp and Sn150kVp) between January 2018 and January 2021 were retrospectively included. Two observers independently measured CT-values in Hounsfield Units (HU) of the bone adjacent to the ulcer and of a reference bone, either the same location on the contralateral foot or, if unavailable, the talus in virtual non-calcium images. Subjects were divided into two groups, “osteomyelitis” or “no-osteomyelitis”, based on the conventional CT report. CT-values were compared between groups and between affected and reference bone within both groups. Observer agreement was tested using an intraclass correlation coefficient (ICC).
**Results or Findings:** Fifty-seven foot ulcers were identified of which twenty-four were suspected for osteomyelitis based on the radiology report. The mean CT-value at the suspected location in the osteomyelitis group was -16.55 HU (SD 34.35) and -67.71 HU (SD 51.62) in the no-osteomyelitis group. This difference was statistically significant (p<0.001). In the osteomyelitis group, the difference between affected bone and reference bone was statistically significant (p<0.001); this was not the case in the no-osteomyelitis group (p=0.51). The observer agreement was good for affected bone measurements (ICC=0.840) and moderate for reference bone measurements (ICC=0.584).
**Conclusion:** Dual-energy CT seems a promising diagnostic tool for the quantitative assessment of osteomyelitis in patients with diabetic foot ulcers.
**Limitations:** The main limitation is the absence of comparison with MRI since this is considered the gold standard.
**Ethics committee approval:** Ethical approval was waived by the medical ethical committee of our institute (W21_401#21.446).
**Funding for this study:** No funding was received for this study.
**Author Disclosures:**
Sicco Bus: Nothing to disclose
Tessa Busch-Westbroek: Nothing to disclose
Ruud Wellenberg: Nothing to disclose
Geert Streekstra: Nothing to disclose
Marieke Mens: Nothing to disclose
Mario Maas: Nothing to disclose
Max Nieuwdorp: Nothing to disclose
Anna de Geus: Nothing to disclose

### RPS 2210-5

**Evaluation of bone marrow eodema using spectral photon-counting CT**

*K. M. Chapagain*, M. Rajeswari Amma, J. Clarke, C. Lowe, S. Dahal, T. E. Kirkbride, S. Gieseg, P. Butler, A. Butler; Christchurch/NZ
*(Krish22chapagain@gmail.com)*

**Purpose:** The purpose of this study was to evaluate water and lipid component measurement from spectral photon-counting CT for the detection of bone marrow oedema in acute bone injury.
**Methods or Background:** Patients with acute bone injury were imaged using high-resolution spectral photon-counting CT in the early phase of injury. Physical phantoms were developed to mimic bone marrow and validate water and lipid measurements. The phantoms contained a two-material mixture (water gel, peanut oil) and a three material mixture (water gel, oil and hydroxyapatite nanopowder). Lipid and water maps were generated by harnessing the spectral information contained in the photon-counting CT images. For both phantoms and human images, regions of interest (ROIs)

Sunday were drawn in the target areas and reference areas to quantitatively measure the water and lipid concentrations. The estimated values from the photon-counting CT were compared with reference values using linearity plots, and the agreement between reference and estimated values were analysed with Bland-Altman plots.
**Results or Findings:** Estimated water and lipid mass density values had a linear correlation with reference values (linearity=0.98, 0.99) The measurements were not significantly different from reference values (p=0.63, 0.91) with average quantification errors (Bias) (-1.9% and -0.4%), upper limit of agreement (11.5%, 8.7%), and lower limit of agreement (-14.7%, -7.9%) for water and lipid component estimation respectively. Similar to phantom results, the targeted regions in human images showed an increase in water mass density.
**Conclusion:** Lipid and water components measured from the system are validated using phantom measurements to demonstrate the bone marrow oedema in patients with an acute injury.
**Limitations:** Comparisons with MRI is not done at this stage, which will be performed in the next phase.
**Ethics committee approval:** The ethics committee approval was received (18/STH/221).
**Funding for this study:** This study is funded by MBIE, New Zealand.
**Author Disclosures:**
Chiara Lowe: Employee: MARS Bioimaging LTD.
Steven Gieseg: Investigator: MARS Bioimaging LTD.
Shishir Dahal: Investigator: MARS Bioimaging
Jennifer Clarke: Employee: MARS Bioimaging LTD.
Philip Butler: CEO: MARS Bioimaging LTD.
Maya Rajeswari Amma: Employee: MARS Bioimaging LTD.
Anthony Butler: Board Member: MARS Bioimaging LTD.
Tracy Elizabeth Kirkbride: Investigator: MARS Bioimaging LTD.
Krishna Mani Chapagain: Investigator: MARS Bioimaging LTD.

### RPS 2210-8

**The diagnostic accuracy of AI for ruling out C-spine fractures: are we there yet?**

*G. van den Wittenboer*[1], A. de Wit[1], E. Langius-Wiffen[1], B. van der Kolk[1], I. M. Nijholt[1], R. van Dijk[1], M. Podlogar[1], M. Maas[2], M. F. Boomsma[1]; [1]Zwolle/NL, [2]Amsterdam/NL

**Purpose:** To assess the diagnostic accuracy of a cervical spine (C-spine) artificial intelligence (AI) application (Aidoc Medical, Tel Aviv, Israel) for identifying C-spine fractures on CT scans.
**Methods or Background:** A retrospective diagnostic accuracy study was performed in a level one trauma centre. Consecutive trauma patients (age ≥18 years; 2007-2014) were screened with CT for C-spine fractures. To set the reference standard, one radiologist and three neurosurgeons verified scans considered positive by the radiologist on-call and two radiologists verified negative scans that were flagged positively by the AI application. The index test was defined as detection of ≥1 fracture(s) per scan by the FDA approved and CE marked AI application. The proportion of patients with missed fractures that received stabilising therapy was determined to highlight therapeutic consequences of missed fractures.
**Results or Findings:** The AI application analysed 2331 patients. After verification, the on-call radiologist's report was adjusted for 25 patients initially considered negative and flagged positive by the AI (1.2% of all negative scans), increasing the total number of fractures by 13%. The AI application detected 159/211 patients with fractures, resulting in a sensitivity of 75% (95% confidence interval (CI) 69-81%). 16/52 (31%) patients with fractures missed by the AI had received stabilising therapy. Specificity of the AI application was 99% (95% CI 98-99%), overall diagnostic accuracy 97% (95% CI 96-97%), positive predictive value 86% (95% CI 81-90%) and negative predictive value 98% (95% CI 97-98%).
**Conclusion:** The moderate sensitivity of the AI and the high-miss rate of injuries that received stabilising therapy makes a stand-alone application for screening purposes less expedient, however, as a concurrent reader, AI could aid the radiologist by detecting previously unnoticed fractures, thus increasing the diagnostic yield.
**Limitations:** Not applicable.
**Ethics committee approval:** Not applicable.
**Funding for this study:** Not applicable.
**Author Disclosures:**
Martin Podlogar: Nothing to disclose
Rogier van Dijk: Nothing to disclose
Britt van der Kolk: Nothing to disclose
Eline Langius-Wiffen: Nothing to disclose
Martijn Franklin Boomsma: Nothing to disclose
Ingrid M. Nijholt: Nothing to disclose
Aranka de Wit: Nothing to disclose
Mario Maas: Nothing to disclose
Gaby van den Wittenboer: Nothing to disclose

**08:00-09:00** **Channel 3**

### Research Presentation Session: Neuro

### RPS 2211

### Neuroimaging in various diseases

**Moderator**
J. Frühwald-Pallamar; St. Pölten/AT

### RPS 2211-2

**Evidence of nerve hypertrophy in patients with inclusion body myositis on lower limb MRI**

*M. Elmansy*[1], J. M. Morrow[1], S. Shah[1], S. Wastling[1], S. Saleh El-Essawy[2], E. Helmy[2], M. G Hanna[1], J. S. Thornton[1], T. A. Yousry[1]; [1]London/UK, [2]Mansoura/EG
*(sasamansy90@gmail.com)*

**Purpose:** To quantify the cross-sectional area (CSA) of the sciatic and tibial nerves in patients with IBM compared with Charcot-Marie-Tooth disease type IA (CMTIA) and healthy controls using MRI and correlate these nerve measurements with the clinical data and disease scores.
**Methods or Background:** MRI of the sciatic and tibial nerves was performed at 3T using magnetisation-prepared rapid gradient-echo sequence (MPRAGE) and 2D Dixon. The nerve CSA was measured at the mid-thigh and upper-calf regions by an observer blinded to the diagnosis. The measurements were correlated with clinical parameters.
**Results or Findings:** 20 patients with IBM, 20 CMT1A patients and 29 healthy controls (age and sex-matched) were studied. Sciatic nerve CSA was significantly enlarged in patients with IBM and CMT1A compared to controls (sciatic nerve mean CSA 62.3±22.9mm (IBM) vs 35.5±9.9mm (controls), p=0.001; and 97.6±35.5 (CMT1A) vs 35.5±9.9mm (controls); p=0.001. Similarly, tibial nerve CSA was also enlarged in IBM and CMT1 patients compared to controls. There was no significant correlation between CSA nerve measurements in patients with IBM and clinical disease scores. However, in CMT1A patients, sciatic nerve CSA correlated positively with age r=0.53-0.65 (p≤0.02) as well as the duration of disease r=0.56-0.61 (p≤0.01).
**Conclusion:** MRI reveals significant hypertrophy of the sciatic and tibial nerves in patients with IBM and CMT1A compared to controls. This study is the first study to demonstrate and quantify nerve hypertrophy in IBM patients using MRI. Further studies are needed to validate this feature, correlate with neurophysiological evaluation, and assess its diagnostic value.
**Limitations:** No gross limitations.
**Ethics committee approval:** Not applicable.
**Funding for this study:** Not applicable.
**Author Disclosures:**
Jasper M. Morrow: Nothing to disclose
Stephen Wastling: Nothing to disclose
Tarek A. Yousry: Nothing to disclose
John S. Thornton: Nothing to disclose
Saleh Saleh El-Essawy: Nothing to disclose
Sachit Shah: Nothing to disclose
Eman Helmy: Nothing to disclose
Michael G Hanna: Nothing to disclose
Mostafa Elmansy: Nothing to disclose

### RPS 2211-3

**Detection of regional white-matter cingulum alterations in breast cancer patients**

*A. S. C. Verde*, J. Ruivo, B. Sousa, A. Oliveira-Maia, F. Cardoso, N. Papanikolaou; Lisbon/PT

**Purpose:** Volumetry studies have shown that breast cancer patients undergoing treatment present reduced volumes in the bilateral thalamus, putamen, frontal and temporal lobes, compared with healthy controls (HC). Since cingulum fibers are interconnecting some of these structures, our aim was to identify local fractional anisotropy (FA) changes in patients which may explain the cancer-related cognitive impairment.
**Methods or Background:** Brain MRI examinations of 32 non-metastatic breast cancer patients - 17 endocrine-treated (ET) and 15 chemotherapy-treated (CT) - were prospectively acquired on a 3T scanner at two timepoints, namely, before (t0) and 6 months after treatment (t1). Additionally, 19 age-matched female HC, from two OpenNeuro datasets, were included in the analysis. A b-value of 1000 s/mm2 and 64 or 128 diffusion encoding directions were used as acquisition parameters for the DTI sequence. For regional-tract analysis, local differences in FA for the bilateral cingulum were quantified between groups by adapting an available MATLAB code.

**Results or Findings:** Visual analysis of fiber tracking results has depicted local changes in FA, which motivated the cingulum regional analysis. Overall, it was found a bilateral cingulum anisotropy reduction in patients compared with HC, both at t0 and t1. Particularly, there was a point-group interaction, statistically significant after multiple comparisons correction, with patients showing lower FA in the middle of the cingulum compared with HC. No significant differences were found between ET and CT patients.
**Conclusion:** White-matter integrity loss for the bilateral cingulum in breast cancer patients was shown. This tract belongs to a limbic circuit involved in memory and emotions, which may be associated with symptoms reported by patients.
**Limitations:** Use of external HC datasets.
**Ethics committee approval:** MRI acquisitions approved by the local ethics committee.
**Funding for this study:** BOUNCE project funded by the EU Horizon 2020 research and innovation programme.
**Author Disclosures:**
Albino Oliveira-Maia: Nothing to disclose
Fátima Cardoso: Nothing to disclose
Berta Sousa: Nothing to disclose
Nickolas Papanikolaou: Advisory Board: Advantis Medical SysteFounder: MRIcons
Joana Ruivo: Nothing to disclose
Ana Sofia Castro Verde: Nothing to disclose

### RPS 2211-5

**A quantitative imaging study of amide proton transfer weighted in diabetes-associated cognitive dysfunction in type 2 diabetes mellitus rat**
*W. J. Shao*, S. Xiang, J. Fang, W. Su, Y. Yang, Y. Xiong, J. Li; Kunming/CN
*(wave.forever@yeah.net)*

**Purpose:** To evaluate the feasibility of amide proton transfer weighted (APTw) in reflecting the pathological changes of brain tissue, cognitive impairment in T2DM rat.
**Methods or Background:** 48 Sprague-Dawley male rats were divided into control and T2DM groups. Cognitive function was assessed using the Morris water maze experiment. The APTw signal intensity (SI)(%) was measured by APTw . Tau expression was determined using immunofluorescence and Immunohistochemistry. Pearson and Spearman correlation analysis were used to study the relationship between hippocampal APTw SI (%), tau protein expression and cognitive function.
**Results or Findings:** The escape latency time significantly reduced in the T2DM group. The APTw SI(%) in bilateral hippocampus in T2DM group was significantly higher than that in NC group ($P < 0.05$). Compared with control group, the expression of t-Tau and p-Tau ser199 increased in T2DM group ($P < 0.05$). The expression of t-Tau protein was positively correlated with escape latency time (rho = 0.425, P = 0.0486). There was a positive correlation between APTw SI(%) and t-Tau protein expression. APTw SI (%) is negatively correlated with platform crossings times.
**Conclusion:** T2DM may result in increase the expression of t-Tau and p-Tauser199 protein in hippocampus. The increase of t-Tau protein may be the cause of DACD. MRI APTw technology can be used as an imaging biomarker for the pathological changes of brain parenchyma and cognitive function in T2DM.
**Limitations:** This study only focuses on amide proton concentration and does not make an in-depth study on pH
**Ethics committee approval:** All animal experiments conformed to the internationally accepted principles for the care and use of laboratory animals (Kunming Medical University Institutional Review Board, Approval No. kmmu 2020410).
**Funding for this study:** The Endocrine Clinical Medical Center of Yunnan Province, No. ZX20190202
**Author Disclosures:**
Yuxin Xiong: Nothing to disclose
Shutian Xiang: Nothing to disclose
Jianbo Li: Nothing to disclose
Jing Fang: Nothing to disclose
Wei Su: Nothing to disclose
Wei Ju Shao: Nothing to disclose
Ying Yang: Nothing to disclose

### RPS 2211-6

**A novel application of neurite orientation dispersion and density imaging (NODDI) to differentiate cognitively recovered vs non-recovered in mild traumatic brain injury (mTBI)**
*P. Swaminathan*, N. Hamzah, N. Mohd Ramli, V. Narayanan, T. Li Kuo, K. Rahmat; Kuala Lumpur/MY
*(prasathswaminathan.rad@gmail.com)*

**Purpose:** DTI can detect changes of microstructural brain damage in mTBI, however subtle changes in recovery process remains a challenge. NODDI measures orientation dispersion index (ODI), neurite density index (NDI) and isotropic volume fraction (ISOVF) which may elucidate the process in mTBI recovery.
**Methods or Background:** 56 mTBI and 19 healthy controls (HC) were recruited. Neuropsychological assessment battery screening module (S-NAB) performance were assessed 2 weeks post-trauma and at 3 months. The mTBI group was then divided into recovered (REC; S-NAB ≥85) and non-recovered (NREC; S-NAB <85), whereby domains affected were mainly attention and language. DTI and NODDI were done at 3 months. Using tract-based spatial statistics (TBSS), DTI and NODDI parameters were obtained for 50 white matter tracts (WMTs). Data was analysed using SPSS.
**Results or Findings:** NODDI demonstrated significant changes ($p<0.050$) in multiple WMTs. Significantly lower NDI was demonstrated in REC (0.4260, 0.4034) vs NREC (0.4540, 0.4389) in both cingulate gyri suggestive of ongoing reparative process in the still-recovering NREC WMTs. Significantly higher ISOVF was seen in REC (0.0716, 0.1349) than NREC (0.0526, 0.0983) in the right external capsule and left fornix/stria terminalis, which may represent increased CSF surrounding healed WMTs. No significant difference between REC and NREC was found in DTI.
**Conclusion:** NODDI detected more microstructural WMT changes than DTI at 3 months. We postulate that at three months post-mTBI, there is concurrent axonal degeneration and astrogliosis following trauma, which was more abundant in the NREC group. The significantly affected WMTs are comparable to tracts seen in previous studies on mTBI.
**Limitations:** Significant number of participants whom defaulted.
**Ethics committee approval:** Medical Research Ethics Committee of the University of Malaya Medical Centre (MREC No. 2018315-6133).
**Funding for this study:** Malaysian Ministry of Science, Technology and Innovation (MOSTI) Flagship Program Project No. FP0911F001
**Author Disclosures:**
Norlisah Mohd Ramli: Nothing to disclose
Vairavan Narayanan: Nothing to disclose
Prasath Swaminathan: Nothing to disclose
Tan Li Kuo: Nothing to disclose
Kartini Rahmat: Nothing to disclose
Norhamizan Hamzah: Nothing to disclose

### RPS 2211-7

**Role of hippocampal volumetry and T2 relaxometry in mesial temporal lobe epilepsy**
*N. Sharma*; Gwalior/IN
*(niharika.sharmaepsilon@gmail.com)*

**Purpose:** Aim of our study was to evaluate the role of hippocampal volumetry and T2 Relaxometry in MTLE and compare the relative value of visual assessment, hippocampal volumetry, and T2 relaxometry individually and in combinations.
**Methods or Background:** This was one-year tertiary care teaching hospital-based case-control study. MRI analysis was done in 40 non-epileptic controls and 40 patients with intractable epilepsy on 1.5T scanner. Visual assessment and hippocampal volumetry were done on oblique coronal IR/T2W and oblique coronal FLAIR images, respectively. T2 relaxation times were measured using 16-echo Carr-Purcell-Meiboom-Gill sequence. All cases were correlated with EEG findings for lateralisation of the epileptic focus.
**Results or Findings:** The study showed that the highest percentage of MTLE cases were seen in patients with seizures onset in a group of 11-20 years of age and no sex predilection was noted (M:F= 1.5:1). Mean right and left hippocampal volume of 2.19 cm3 and 2.10 cm3 (P-value of <0.001) were found in the cases of study, which was decreased compared to control. T2 relaxation time was increased in MTLE cases (P-value of <0.001). Two other parameters hippocampal volume ratio (HVR) and hippocampal volume difference (HVD) were included in study to detect unilateral cases of mesial temporal sclerosis.
**Conclusion:** There was an increase in the sensitivity of detection of mesial temporal sclerosis in epilepsy patients on including quantitative methods like T2 relaxometry and hippocampal volumetry with conventional MRI. Use of quantitative methods leads to early diagnosis and helps treatment.

**Limitations:** Manual error
**Ethics committee approval:** I have taken approval from Member Secretary, Institutional ethics committee, Assam Medical College, Dibrugarh dated 21/09/2019. (I am unable to attach the same approval as there is no such option available for attachment)
**Funding for this study:** No funding is given for our study.
**Author Disclosures:**
Niharika Sharma: Nothing to disclose

### RPS 2211-8
**Anatomic study of the medial calcaneal nerve using ultrasonography**
*C. Deniel*, D. Guenoun, T. Le Corroller, P. Champsaur; Marseille/FR
*(cecile.deniel@hotmail.fr)*

**Purpose:** To evaluate the possibility and accuracy of the medial calcaneal nerve assessment using ultra sound examination. Secondary objective was to define anatomical landmarks to facilitate the medial calcaneal nerve study for non musculoskeletal specialised radiologists.
**Methods or Background:** This study was first undertaken in eight cadaveric specimens then followed by high resolution ultrasonographic examination of 20 healthy volunteers (40 legs) by a fellow musculoskeletal radiologist. The location and course of the medial calcaneal nerve was depicted, as well as its relationship to the adjacent anatomic structures.
**Results or Findings:** High resolution ultrasonography permitted effective study of the medial calcaneal nerve throughout its course. The medial calcaneal nerve mainly branches from the tibial nerve. The level at which it branches is highly variable with a mean distance from the medial malleolus tip of 14.1mm (range -7 – 75). More distally, at the level of the medial calcaneal side, it ends close to the abductor hallucis muscle with a mean distance of 1.28mm (range 0 – 2.8).
**Conclusion:** The medial calcaneal nerve study is achievable using high resolution ultrasonography. The definition of easy to use anatomical landmarks allows physicians to routinely explore the MCN as a differential diagnosis for heel pain.
**Limitations:** Only a short number of cadavers were included. We did not analyse intra- or inter-observer differences.
**Ethics committee approval:** All healthy volunteers gave their written consent.
**Funding for this study:** No funds were received for this study
**Author Disclosures:**
Pierre Champsaur: Nothing to disclose
Daphné Guenoun: Nothing to disclose
Cécile Deniel: Nothing to disclose
Thomas Le Corroller: Nothing to disclose

**08:00-09:00** **Channel 4**

### Research Presentation Session: Chest

### RPS 2204

### New techniques for pulmonary imaging

**Moderator**
M. Eberhard; Zurich/CH

### RPS 2204-2
**X-ray dark-field computed tomography allows for the detection of radiation-induced lung damage in early stages**
*F. T. Gassert*, R. Burkhardt, T. Gora, D. Pfeiffer, A. Fingerle, A. Sauter, M. Makowski, J. Wilkens, F. Pfeiffer; Munich/DE
*(florian.gassert@t-online.de)*

**Purpose:** The aim of this study was to show the benefit of dark-field CT imaging for the detection of radiation-induced lung damage in early stages.
**Methods or Background:** We compared attenuation based CT imaging to dark-field (DF) CT imaging in a murine model of radiation-induced lung damage in the right lung (n=6) and a control group (n=6). Animals were scanned before irradiation and 12 weeks, 16 weeks, 20 weeks and 24 weeks thereafter. Three radiologists assessed the images twice for the presence of lung damage and rated their confidence on a scale from 1 to 5. The inter-rater- and intra-rater-reliability was determined and rated with Cohen's $\kappa$ respectively Fleiss' $\kappa$. For the quantitative analysis the ratio of the mean pixel value of the right and left lung ($\langle R \rangle$=mright/mleft) was calculated. Results of the irradiated group were compared with the respective control group by using a t-test.
**Results or Findings:** The sensitivity of DF CT for radiation-induced lung damage in the reader study was significantly higher at 12 weeks (Att: 36.7%, DF: 53.3%, p=0.023) and at 16 weeks (Att: 50.0%, DF: 91.7%, p<0.001). The overall confidence of the readers was significantly higher when reading DF images (Att: 3.48, DF: 4.77, p<0.001). Both the average intra-rater-reliability (Att: $\kappa$=0.82 , DF: $\kappa$=0.91) and the inter-rater-reliability (Att: $\kappa$=0.66, DF: $\kappa$=0.75) were higher for DF imaging. For attenuation based imaging the difference of the ratio $\langle R \rangle$ between the control group and the irradiated group became significant after 20 weeks (p=0.011), while for DF imaging it was already highly significant after 16 weeks (p=0.003).
**Conclusion:** This small animal study demonstrates that dark-field CT imaging allows for the detection of radiation-induced lung damage in early stages and, in that respect, is superior to conventional CT.
**Limitations:** This study is limited by its small cohort only.
**Ethics committee approval:** The ethics committee approval was obtained.
**Funding for this study:** Not applicable.
**Author Disclosures:**
Andreas Sauter: Nothing to disclose
Florian Tilman Gassert: Nothing to disclose
Rico Burkhardt: Nothing to disclose
Jan Wilkens: Nothing to disclose
Alexander Fingerle: Nothing to disclose
Franz Pfeiffer: Nothing to disclose
Marcus Makowski: Nothing to disclose
Daniela Pfeiffer: Nothing to disclose
Thomas Gora: Nothing to disclose

### RPS 2204-4
**Feasibility of human lung imaging with a large field-of-view spectral photon-counting CT system**
*S. A. Si-Mohamed*[1], S. Boccalini[1], P-A. Rodesch[1], R. A. K. Dessouky[2], P. Coulon[3], E. Lahoud[4], M. Villien[1], L. Boussel[1], P. Douek[1]; [1]Lyon/FR, [2]Zagazig/EG, [3]Dourdan/FR, [4]Haifa/IL
*(salim.si-mohamed@chu-lyon.fr)*

**Purpose:** To characterise the technical capabilities of a clinical spectral photon-counting CT (SPCCT) and to evaluate its feasibility on a first human volunteer for high-resolution lung imaging.
**Methods or Background:** Measurement of a modulation transfer function (MTF) and acquisition of a line pairs phantom were performed. An anthropomorphic lung nodules phantom was scanned under standard, low, and ultra-low radiation doses. A human volunteer underwent scans, at 120 kVp standard (62 mAs) and low (11 mAs) dose. High-resolution (HR) images were reconstructed with 1024 matrix, 300mm FOV and 0.25mm slice thickness. Lung structures conspicuity and sharpness, image noise and overall image quality were independently analysed by three radiologists and compared to a previous scan (120kVp, 10mAs).
**Results or Findings:** 10 % MTF was measured at 22.3lp/cm with a cut-off at 31lp/cm. Up to 28lp/cm were depicted. While mixed and solid nodules were depicted on standard and low dose images with FBP, ultra-low dose imaging necessitated the use of iDose and 1mm slice thickness to allow visualisation of the ground-glass component. In a human volunteer, standard dose SPCCT images were of greater overall image quality and lung structures conspicuity and sharpness than conventional CT images and comparable image noise. Low dose SPCCT images were of greater or similar conspicuity and sharpness of lung structures, of equivalent overall image quality, of lower but acceptable image noise despite a flux reduction of 89%.
**Conclusion:** An SPCCT prototype demonstrated high-resolution technical capabilities and high image quality on a human volunteer for lung imaging.
**Limitations:** Exhaustive evaluation of image quality was out of the scope of the present study opening the way for furthermore investigations.
**Ethics committee approval:** IRB approved the study.
**Funding for this study:** This study was funded by the European Union Horizon 2020 grant No 668142.
**Author Disclosures:**
Marjorie Villien: Employee: Philips
Salim Aymeric Si-Mohamed: Nothing to disclose
Pierre-Antoine Rodesch: Nothing to disclose
Loïc Boussel: Nothing to disclose
Philippe Douek: Nothing to disclose
Sara Boccalini: Nothing to disclose
Elias Lahoud: Employee: Philips
Riham Amir Kamal Dessouky: Nothing to disclose
Philippe Coulon: Employee: Philips

### RPS 2204-5

**Virtual non-contrast images in clinical photon-counting detector CT for emphysema quantification: proof of concept**

*L. Jungblut*, T. D. J. Sartoretti, D. Kronenberg, V. Mergen, A. Euler, H. Alkadhi, T. Frauenfelder, K. Martini; Zurich/CH

**Purpose:** The purpose of this study was to evaluate the accuracy of emphysema quantification as performed on post-processed virtual non-contrast images derived from photon-counting detector computed tomography (PCD-CT).
**Methods or Background:** Sixty-five patients who underwent a three-phase chest CT on a first-generation, clinical dual-source PCD-CT were retrospectively included. Scans were performed in the multi-energy (QuantumPlus) mode at 120kV with a weight-adjusted intravenous contrast agent. Virtual non-contrast images (VNC) were post-processed from the venous as well as from the arterial phase. Images were assessed quantitatively (global noise index (GNI)) and qualitatively by independent readers (overall image quality, emphysema assessment, delineation of small structures). Emphysema quantification (with a threshold of -950 HU) was performed for non-contrast images, contrast-enhanced images (arterial and venous phase) and the post-processed virtual non-contrast images (generated from both; the atrial and the venous phase) by commercially available software. Non-contrast images served as a reference standard for emphysema quantification.
**Results or Findings:** Virtual non-contrast images post-processed from the arterial phase (p=0,409) as well as from the venous phase (p=0.093) showed no significant difference in emphysema quantification relative to true non-contrast images while there was a highly significant difference compared for the contrast-enhanced scans (arterial and venous; p<0.001). GNI showed no significant difference between the virtual non-contrast image from the arterial and venous phase and the true non-contrast image. The score of subjective assessment was highest for the true non-contrast image (p<0.001) while there was no significant difference between both virtual non-contrast reconstructions.
**Conclusion:** Computer-aided emphysema quantification with PCD-CT is feasible for virtual non-contrast-enhanced images post-processed from the venous as well as the arterial phase.
**Limitations:** This study has been performed as a single-centre study.
**Ethics committee approval:** The ethics committee approved this study.
**Funding for this study:** No funding was received for this study.
**Author Disclosures:**
Daniel Kronenberg: Nothing to disclose
Thomas Frauenfelder: Nothing to disclose
Victor Mergen: Nothing to disclose
Katharina Martini: Nothing to disclose
Thomas Daniel Jean Sartoretti: Nothing to disclose
Lisa Jungblut: Nothing to disclose
Andre Euler: Nothing to disclose
Hatem Alkadhi: Nothing to disclose

### RPS 2204-6

**Spectral CT quantification of airway contrast enhancement with virtual monoenergetic reconstructions**

*A. Bodenberger*, P. Konietzke, O. Weinheimer, H-U. Kauczor, W. Stiller, T. F. Weber, T. D. Do, M. O. Wielpütz; Heidelberg/DE

**Purpose:** Chronic-obstructive airway diseases show progressive remodelling of airway dimensions. Inflammatory activity may be visualised by contrast enhancement in computed tomography (CT) examinations, which has not been systematically studied to date. Spectral CT offers the possibility to study contrast enhancement without the need for multiphase acquisitions.
**Methods or Background:** 234 lung healthy patients underwent dual-layer spectral CT (Philips iQon) with four retrospective groups: non-enhanced (NE), pulmonary venous (PV), pulmonary arterial (PA) and systemic arterial (SA) standardised contrast phase. Ten virtual monoenergetic series were reconstructed at 40-160 keV. Fully automatic segmentations were carried out using validated in-house software. Attenuation of the airway wall was assessed in Hounsfield Units (HU) for airway generations G2, G3, G4 and combined G5-10. The slope of the spectral attenuation was calculated by λ=(HU40keV–HU100keV)/60.
**Results or Findings:** Slopes were significantly different for all airway generations between NE (λNE,G5-10=0.3HU/keV) vs. contrast-enhanced acquisitions PV (λPV,G5-10=0.67HU/keV), PA (λPA,G5-10=1.83HU/keV) and SA (λSA,G5-10=1.97HU/keV) (λNE,G5-10-λPV,G5-10 p=0.006; λNE,G5-10-λPA,G5-10 p<0.001; λNE,G5-10-λSA,G5-10 p<0.001). Additionally, the slope differs between PV and PA phase (pG5-10<0.001) but does not vary between PA and SA phase (pG5-10>0.999). Wall thickness (WT) for generation G5-10 did not change significantly between groups at any keV level (40 keV: WTNE=1.14 mm, WTPV=1.22 mm, WTPA=1.33 mm, WTSA=1.22 mm, p=0.054).
**Conclusion:** Spectral CT may quantify airway wall attenuation with a single acquisition by determining the slope of spectral enhancement, and may separate arterial and venous enhancement. Further studies are warranted to analyse spectral CT for inflammatory airway diseases.
**Limitations:** The spectral slope is only assessed in airway-healthy patients.
**Ethics committee approval:** This retrospective study was approved by the institutional ethics committee (S-924/2019).
**Funding for this study:** This study was supported by grants from the Bundesministerium für Bildung und Forschung (BMBF) to the German Center for Lung Research (DZL) (82DZL004A, 82DZL004A2).
**Author Disclosures:**
Mark O. Wielpütz: Nothing to disclose
Thuy Duong Do: Nothing to disclose
Oliver Weinheimer: Other: Parts of the lobe segmentation algorithm that are used for labeling of the airways have been licensed to the company Imbio, LCC.
Wolfram Stiller: Board Member: W. Stiller is a member of the CT Advisory Board of Philips Medical Systems.
Hans-Ulrich Kauczor: Nothing to disclose
Tim Frederik Weber: Nothing to disclose
Arndt Bodenberger: Nothing to disclose
Philip Konietzke: Nothing to disclose

### RPS 2204-7

**Deep learning reconstruction vs hybrid-type and model-based iterative reconstructions: radiation dose reduction of lung density evaluation on ultra-high resolution and area-detector CTs as QIBA study**

*Y. Ohno*[1], N. Akino[2], Y. Ito[2], H. KImata[2], K. Fujii[2], Y. Fujisawa[2], Y. Kataoka[1], K. Murayama[1], H. Toyama[1]; [1]Toyoake/JP, [2]Otawara/JP
*(yohno@fujita-hu.ac.jp)*

**Purpose:** To compare radiation dose reduction capability of ultra-high resolution CT (UHR-CT) and area-detector CT (ADCT) for lung density evaluation among hybrid-type and model-based iterative reconstruction (IR) and developed deep learning reconstruction (DLR) at Quantitative Imaging Biomarkers Alliance (QIBA) recommended phantom study.
**Methods or Background:** QIBA recommended phantom was scanned by UHR-CT with normal resolution (NR: 0.5mm×80 rows/896 channels), high resolution (HR: 0.5mm×80 rows/1792 channels) and super-high resolution (SHR: 0.25mm×160 rows/1792 channels) and ADCT (0.5mm×80 rows/896 channels) at 400mA, 230mA, 100mA, 50mA, 20mA and 6mA in five times. Then, all CT data were reconstructed as 0.5mm and 1mm section thicknesses by each reconstruction. Then, CT values of all density forms and CT value within lung density form were determined by ROI measurements five times. Pierson's correlation was analysed between measured CT density and each form density on all CT protocols. To compare the capability for radiation dose reduction on each CT data with ADCT obtained by 400mA and reconstructed with hybrid-type IR (i.e. standard protocol), ΔCT of each protocol was compared with that of the standard protocol by paired t-test.
**Results or Findings:** There was a significant and excellent correlation with standard reference on each protocol (0.99<r<1, p<0.0001). At each section thickness, there were no significant differences of ΔCT between all CT protocols except 6mA and standard protocol (p>0.05).
**Conclusion:** For lung density assessment, hybrid-type and model-based IR, as well as DLR on UHR-CT and ADCT, can reduce 95% radiation dose with keeping accuracy as compared with standard CT protocol and QIBA profile.
**Limitations:** Not applicable.
**Ethics committee approval:** This study was a phantom study and no need for IRB approval.
**Funding for this study:** This study was financially and technically supported by Canon Medical Systems Corporation.
**Author Disclosures:**
Kazuhiro Murayama: Research/Grant Support: Canon Medical SysteCorporation
Kenji Fujii: Employee: Canon Medical SysteCorporation
Yasuko Fujisawa: Employee: Canon Medical SysteCorporation
Hirona KImata: Employee: Canon Medical SysteCorporation
Hiroshi Toyama: Research/Grant Support: Canon Medical SysteCorporation
Yuya Ito: Employee: Canon Medical SysteCorporation
Yoshiharu Ohno: Research/Grant Support: Canon Medical SysteCorporation
Yumi Kataoka: Nothing to disclose
Naruomi Akino: Employee: Canon Medical SysteCorporation

### RPS 2204-8

**Comparison of conventional and dark-field chest radiography for the diagnosis of pulmonary emphysema**

*T. Urban*[1], A. Sauter[2], M. Frank[1], K. Willer[1], T. Koehler[3], F. T. Gassert[2], M. Makowski[2], D. Pfeiffer[2], F. Pfeiffer[1]; [1]Garching/DE, [2]Munich/DE, [3]Hamburg/DE
*(theresa.urban@tum.de)*

**Purpose:** Dark-field radiography can provide information on the condition of the lungs' alveolar structure. It has recently been translated from the lab to the clinical stage. Here, we evaluate its performance for the detection of emphysema, and compare its diagnostic value with conventional radiography.
**Methods or Background:** We included 91 patients after a medically indicated CT scan, either without any lung impairment or with varying stages of emphysema. As a reference standard, visual scores based on the Fleischner scale for emphysema severity (absent, trace, mild, moderate, confluent, advanced destructive emphysema) were assigned to all CTs by three radiologists. For dark-field chest radiography, we employed a clinical prototype, which is capable of acquiring dark-field and attenuation-based radiographs simultaneously at a dose in the range of 0.035mSv. Both modalities, displayed individually and simultaneously, were rated for presence and severity of emphysema (no, mild, moderate, severe) by three radiologists. Statistical analysis included receiver-operator-characteristics and comparison of adjacent groups using two-sided Mann-Whitney-U-tests with a significance level of 0.05.
**Results or Findings:** The dark-field images showed a decrease in signal strength with emphysema severity. Compared to conventional images (AUC=0.73), readers were better able to identify mild emphysema when reading dark-field images (AUC=0.86). While the differentiation between trace and mild emphysema was not possible reading conventional radiographs, readers could differentiate between these stages based on dark-field radiographs.
**Conclusion:** Dark-field radiography increases the diagnostic value of attenuation-based radiography for the identification and staging of emphysema, especially in the early stages.
**Limitations:** There is only a limited number of participants. Emphysema was the only lung pathology under investigation.
**Ethics committee approval:** Approval of the Institutional Review Board was obtained prior to this study (IRB reference number 166/20S). All participants gave written informed consent.
**Funding for this study:** This study was funded by the European Research Council, Philips Medical Systems DMC GmbH, Karlsruhe Nano Micro Facility.
**Author Disclosures:**
Andreas Sauter: Nothing to disclose
Florian Tilman Gassert: Nothing to disclose
Thomas Koehler: Employee: Philips GmbH Innovative Technologies
Manuela Frank: Nothing to disclose
Konstantin Willer: Nothing to disclose
Theresa Urban: Nothing to disclose
Franz Pfeiffer: Nothing to disclose
Marcus Makowski: Nothing to disclose
Daniela Pfeiffer: Nothing to disclose

**09:30-10:30** **Channel 2**

### Research Presentation Session: Imaging Informatics / Artificial Intelligence and Machine Learning

### RPS 2305a

### Artificial intelligence (AI) in liver imaging

**Moderator**
M. Wagner; Paris/FR

### RPS 2305a-2

**Doing more with less: combining manually annotated and automated liver segmentations to train deep neural network segmentation algorithms**

*M. Gross*[1], M. Spektor[2], A. Jaffe[2], A. S. Kücükkaya[1], S. Iseke[3], S. Haider[4], M. Strazzabosco[2], J. Chapiro[2], J. A. Onofrey[2]; [1]Berlin/DE, [2]New Haven, CT/US, [3]Rostock/DE, [4]Munich/DE

**Purpose:** Manual liver segmentation is time-consuming and expensive. In this study, a DCNN was trained using a novel framework that incorporates a small set of manually annotated imaging with a large set of automated segmentations to supplement segmentation performance.
**Methods or Background:** This study included 617 arterial-phase T1-weighted MR images of which 219 had corresponding manual liver segmentations. The 219 annotated images were split into 50/15/35% (n=109/33/77) training/validation/testing subsets, respectively. First, ten proportions (10-100%, increments of 10%) of manual liver segmentations from the training pool were used to train ten baseline (BL) DCNN models with identical 3D U-net architectures. The BL models were used to generate automated liver segmentations on the unlabeled dataset (n=398) and on the remaining portion of the training pool that was not in their respective training set. Second, these automated segmentations were combined with the manually annotated images into new training sets and used to train ten enhanced-training (ET) DCNNs. The dice similarity coefficient (DSC) was used to quantify segmentation performance and a Wilcoxon signed-rank test was used for comparisons.
**Results or Findings:** BL models trained with more manual segmentations substantially outperformed BL models trained with fewer segmentation data. ET models significantly outperformed their respective BL models (for all training sets including 10-50% of available manual segmentations: $p<0.05$). Some ET models significantly outperformed BL models trained with more manual segmentations (ET 30% vs BL 40%, ET 70% vs BL 90%, $p<0.05$).
**Conclusion:** This new training approach reduced the overfitting of models trained on smaller training sets and achieved satisfactory liver segmentation performance even with fewer expert annotations, outperforming models that were trained with more manual segmentations.
**Limitations:** One network architecture was used and a single contrast phase.
**Ethics committee approval:** This study was approved by the local IRB.
**Funding for this study:** This study was funded, NIH grant P30 KD034989.
**Author Disclosures:**
Julius Chapiro: Nothing to disclose
Stefan Haider: Nothing to disclose
Ariel Jaffe: Nothing to disclose
Mario Strazzabosco: Nothing to disclose
Michael Spektor: Nothing to disclose
Ahmet Said Kücükkaya: Nothing to disclose
Simon Iseke: Nothing to disclose
John Aaron Onofrey: Nothing to disclose
Moritz Gross: Nothing to disclose

### RPS 2305a-3

**Deep-learning based hepatic tumour load analysis of neuroendocrine liver metastases in Gd-EOB MRI**

*U. Fehrenbach*[1], S. Xin[1], T. A. Auer[1], H. Jann[1], H. Amthauer[1], D. Geisel[1], T. Denecke[2], B. Wiedenmann[1], T. Penzkofer[1]; [1]Berlin/DE, [2]Leipzig/DE
*(uli.fehrenbach@charite.de)*

**Purpose:** Fast and exact quantification of hepatic metastasis is an unmet medical need in patients with secondary liver malignancies. We therefore present a deep-learning 3D-quantification model of neuroendocrine liver metastases (NELM) using gadoxetic-acid (Gd-EOB)-enhanced MRI.
**Methods or Background:** In 149 patients, manual segmentations of NELM and livers were used to train a neural network (278 Gd-EOB MRI scans). Clinical utility was evaluated in another 33 patients which were discussed in our multidisciplinary cancer conference (MCC) and received a Gd-EOB MRI both at baseline and as follow-up examination (n = 66). The model's measurements (NELM volume; hepatic tumour load (HTL)) with corresponding absolute (ΔabsNELM; ΔabsHTL) and relative changes (ΔrelNELM; ΔrelHTL) between baseline and follow-up were compared to MCC decisions of therapy response.
**Results or Findings:** Internal and external validation of the model's accuracy showed a high overlap for NELM and livers (Matthew's correlation coefficient (phi): 0.76/0.95 (internal), 0.86/0.96 (external)) with higher phi in larger NELM volume (phi= 0.80 vs. 0.71; $p = 0.003$). MCC decisions were significantly differentiated by all response variables (ΔabsNELM; ΔabsHTL; ΔrelNELM; ΔrelHTL) ($p < 0.001$). ΔrelNELM and ΔrelHTL showed optimal discrimination between therapy success or failure (AUC:1.000; $p < 0.001$).
**Conclusion:** The deep-learning based model shows high accuracy in 3D-quantification of NELM and HTL in Gd-EOB-MRI. The model's measurements correlated well with the evaluation of therapeutic response of an expert MCC.
**Limitations:** The 3D assessment approach needs to be further evaluated in direct comparison to 2D measurements and its impact on clinical endpoints in larger cohorts. The ground truth of accuracy is based on manual segmentation of liver metastasis. Due to the sometimes pronounced, even small foci of liver metastases, manual segmentation is not perfect.
**Ethics committee approval:** Approved by the Institutional Review Board of Charité Berlin.
**Funding for this study:** No funding was received for this study.
**Author Disclosures:**
Siyi Xin: Nothing to disclose
Tobias Penzkofer: Nothing to disclose
Timm Denecke: Nothing to disclose
Holger Amthauer: Nothing to disclose

Uli Fehrenbach: Speaker: GE, Bayer, IPSEN, Siemens
Dominik Geisel: Nothing to disclose
Henning Jann: Nothing to disclose
Timo Alexander Auer: Nothing to disclose
Bertram Wiedenmann: Nothing to disclose

### RPS 2305a-4

**Accuracy and efficiency of right-lobe graft volume estimation with deep learning-based CT volumetry in a large cohort of living right liver donors**

*R. Park*, S. S. Lee, Y. S. Sung, J. S. Yoon, H-I. Suk, H. J. Kim, S. H. Choi; Seoul/KR

**Purpose:** To devise construct graft volume-to-weight conversion formula and to evaluate efficiency and accuracy of DLA-assisted CT volumetry in right lobe (RL) graft in a large cohort of LDLT.
**Methods or Background:** We retrospectively enrolled 581 RL donors and divided them into development and validation groups. The CT was analysed using DLA-assisted software. The graft volume-to-weight conversion formula was derived from the development group by linear regression. The agreement between estimated and measured graft weights and inter-reader agreement were assessed using CCC and 95% Bland-Altman LOA in the validation group. To assess factors influencing estimation error, multivariable linear regression was performed in the validation group.
**Results or Findings:** Segmentation correction was required in 28.6% cases with short correction time (mean, 12.8±33.6 seconds) and small change in volume (95% LOA, -3.0% to 3.0%). The total process time ranged from 1.3 to 8.0 minutes (mean, 1.8±0.6 minutes). The conversion formula was as follows: estimated graft weight (g)=206.3+0.653 x CT-measured graft volume (ml) (r=0.878, p<.001). Between estimated and measured graft weights, CCC was 0.834 and 95% LOA was -1.7%±17.1% (P=.002). Of the inter-reader agreement of RL volume, CCC was 0.998 and 95% LOA was 0.2%±1.8% (P=.069). The sex (coefficient;-3.47) and BMI (coefficient;-0.58) have a significant independent association with estimation error, while age, hepatosteatosis, CT interval and graft type did not.
**Conclusion:** We proposed a graft volume-to-weight conversion formula that would be useful in preoperative graft weight estimation. The DLA-assisted CT volumetry is a highly efficient method for preoperative graft weight estimation. The error margin of RL graft weight is within approximately 17% of graft weight.
**Limitations:** First, it was retrospective. Second, we evaluated only RL graft donors. Third, development and validation groups were enrolled in the same institution.
**Ethics committee approval:** IRB waived the requirement for informed consent.
**Funding for this study:** This research was supported by a National Research Foundation of Korea (NRF) grant, funded by the Korean government (MSIT) (2020R1F1A1048826).
**Author Disclosures:**
Seung Soo Lee: Nothing to disclose
B.S. Jee Seok Yoon: Nothing to disclose
Hyoung Jung Kim: Nothing to disclose
Sang Hyun Choi: Nothing to disclose
Heung-Il Suk: Nothing to disclose
Yu Sub Sung: Nothing to disclose
Rohee Park: Nothing to disclose

### RPS 2305a-5

**Fully automatic calculation hepato-renal index in ultrasound images using deep learning**

*M. Ghelichoghli*[1], S. M. Bagheri[2], A. Akhavan[1], V. Ashkani Chenarlogh[1], N. Sirjani[1], I. Shiri[3], A. Shabanzadeh[1]; [1]Karaj/IR, [2]Tehran/IR, [3]Geneva/CH
*(m.g31_mesu@yahoo.com)*

**Purpose:** In this study, we have proposed and validated a fully automatic approach for the quantification of fatty liver disease using ultrasound images based on hepato-renal-index (HRI) calculation. The procedure includes segmentation of kidney and liver, detection of an ROI in the renal parenchyma region and liver at the same depth, and HRI calculation.
**Methods or Background:** We proposed a highly accurate and fast convolutional neural network, named Fast-Unet, for the segmentation of kidneys and liver. The main superiority of Fast-Unet model is low response time, which is appropriate in ultrasound image analysis that needs on-site measurement by radiologists. We used a superpixel algorithm to find the lowest variance region in parenchyma as the renal ROI. At the next step, all pixels with the same depth as renal ROI centrum were found using the intersection of two borders of the convex probe sector. This step is conducted because if the renal and liver ROIs were not in the same depth resulting HRI is not accurate due to the ultrasound depth attenuation effect. Finally, an ROI in the liver with the same depth was found and HRI was calculated.
**Results or Findings:** The train-test dataset contained 752 ultrasound images. The Dice and Jaccard coefficients were used to evaluate the segmentation step, and 94% and 89% for the kidney and 97% and 91% for the liver were achieved respectively. The predicted HRI values were also validated with a radiologist's report using the root-mean-square-error (RMSE) metric and 0.04 was achieved.
**Conclusion:** Automation of HRI calculation speeds up the fatty liver diagnosis and helps novice radiologists to interpret ultrasound images more accurately.
**Limitations:** There is no limitation in this study.
**Ethics committee approval:** Med Fanavaran Plus co. ethics committee approved this study.
**Funding for this study:** Med Fanavaran Plus Co. funded this study.
**Author Disclosures:**
Nasim Sirjani: Author: image processing and deep learning part
Vahid Ashkani Chenarlogh: Author: image processing and deep learning part
Mostafa Ghelichoghli: Author: image processing and deep learning part
Ardavan Akhavan: Author: data preparing
Isaac Shiri: Author: image processing and deep learning part
Ali Shabanzadeh: Author: data preparing
Seyed Morteza Bagheri: Author: data preparing

### RPS 2305a-6

**Deep learning-based automated assessment of hepatic fibrosis on magnetic resonance images and non-image data**

*W. Li*[1], Y. Zhu[1], G. Zhao[1], X. Chen[1], X. Zhao[2], Q. Xie[1], F. Yan[1]; [1]Shanghai/CN, [2]Guangdong/CN
*(lwxyjck@126.com)*

**Purpose:** To evaluate the performance of fully automated deep learning (DL) algorithm for staging hepatic fibrosis and distinguishing fibrosis from normal people based on MR images with or without non-image information.
**Methods or Background:** 500 patients were retrospectively enrolled from two hospitals. Model DL were built using delay phase MR images to assess fibrosis stages. In addition, different models of model DL combined with non-image information including biomarkers (APRI and FIB-4), virus status (hepatitis B and C virus tests), and MR information (manufactures and static magnetic field).The AUROCs were compared between different models using Delong test, the sensitivity and specificity of both model DL and model Full (model DL combined with all non-image information) were compared with experienced radiologists and biomarkers using McNemar's test.
**Results or Findings:** In the test set, the AUROC (with 95% confidence intervals) values of model Full for diagnosing fibrosis stages F0-4, F1-4, F2-4, F3-4 and F4 were 0.99 (0.94-1.00), 0.98 (0.93-0.99), 0.90 (0.83-0.95), 0.81 (0.73-0.88) and 0.84 (0.76-0.90), respectively, which outperformed model DL on diagnosing F0-4 and F1-4.Compared with the radiologists, model Full showed better specificity for fibrosis stage F0-4, better sensitivity for the other four classification tasks. While compared with biomarkers, both model DL and model Full showed significantly higher specificity in staging F3-4 and F4.
**Conclusion:** DL using MR images with or without non-image data provides a promising non-invasive assessment tool for the staging liver fibrosis, and for distinguishing liver fibrosis patients from normal people, with a performance superior to experienced radiologists and biomarkers.
**Limitations:** The sample among fibrosis stage was unbalanced.
**Ethics committee approval:** Ruijin Hospital affiliated to Shanghai JiaoTong University School of Medicine
**Funding for this study:** National Natural Science Foundation of China (grant numbers 81401406) and Innovative research team of high-level local universities in Shanghai.
**Author Disclosures:**
Qing Xie: Nothing to disclose
Yaying Zhu: Nothing to disclose
Weixia Li: Nothing to disclose
Xiangtian Zhao: Nothing to disclose
Fuhua Yan: Nothing to disclose
Xiaoyan Chen: Nothing to disclose
Gangde Zhao: Nothing to disclose

### RPS 2305a-7

**CNN-based tumour progression prediction after thermal ablation with CT imaging**

M. Taghavi, F. C. Staal, M. Maas, R. G. H. Beets-Tan, *S. H. Benson*; Amsterdam/NL

**Purpose:** For solitary small (< 3 cm) tumors, ablation of liver metastases is now part of international guidelines. However, a significant number of patients experience regrowth due to insufficient treatment or inadequate ablation of tumour margins. Local tumour progression (LTP): defined as regrowth /recurrent disease after ablation is a threat to overall survival. The aim of this study was to create a deep learning model using CT images obtained at baseline and directly after treatment in order to predict which patients would later experience regrowth.
**Methods or Background:** For this study, we retrospectively included 79 patients (120 lesions) with colorectal liver metastasis (CRLM) who were treated by thermal ablation consisting of either radiofrequency ablation (RFA), or microwave ablation (MWA) for liver metastases (LM). Exclusion criteria were based on the ESMO guidelines. The pre- and post-treatment scans were used as input to a multi-channel Convolutional Neural Network (CNN). The manual lesion delineation was used to identify a 3D region of interest (RoI) around each lesion. We employed transfer learning in order to train a deep learning model for the dataset in question. A 19-layer CNN from the Visual Geometry Group (VGG-19) was found to perform best.
**Results or Findings:** The area under the receiver operating characteristic curve (AUC) was found to be 0.72, 95% confidence interval: (0.64, 0.79).
**Conclusion:** We have demonstrated that it is possible to use transfer learning together with CNN models in order to predict tumour progression and also demonstrated that it is possible to employ state-of-the-art methods to avoid overfitting.
**Limitations:** Small cohort size of 79 patients, therefore impacting the size of the AUC confidence interval.
**Ethics committee approval:** The informed consent requirement was waived by the Institutional Review Board due to the retrospective nature of the study.
**Funding for this study:** Not applicable.
**Author Disclosures:**
Femke C.R. Staal: Nothing to disclose
Monique Maas: Nothing to disclose
Sean Harry Benson: Nothing to disclose
Marjaneh Taghavi: Nothing to disclose
Regina G. H. Beets-Tan: Nothing to disclose

### RPS 2305a-8

**Hepatic CT-based radiomics phenotypes associate with response to anti-angiogenics in neuroendocrine tumours**
*M. Ligero*, E. Delgado, J. Hernando, A. Garcia-Alvarez, X. Merino Casabiel, M. Escobar, J. Capdevila, R. Perez Lopez; Barcelona/ES
*(mligero@vhio.net)*

**Purpose:** To define and validate CT-based radiomics phenotypes associating with response to anti-angiogenic treatment in patients with gastroenteropancretic neuroendocrine tumors(GEP-NET). To investigate if multi-phase radiomics model or the combination of radiomics with clinical data improves response prediction.
**Methods or Background:** A predictive CT-based radiomics signature was developed in 57 patients included in the TALENT phase II prospective trial of lenvatinib in advanced GEP-NET from October 2015 to August 2020. Radiomics features were extracted from all liver lesions at pre-treatment CT. Features were selected using minimum redundancy maximum relevance (mRMR) and combined in a logistic regression for predicting clinical benefit (progression free survival[PFS]>15 months). A multiphase model including arterial and portal acquisitions was developed. Models were validated internally and tested in an external cohort of 26 patients treated with the VEGFR1-3 inhibitor sunitinib. A regression model was used to combine radiomics and clinical variables. Model interpretability plots were also developed.
**Results or Findings:** In the training and validation set, the model associated with response (area under the curve [AUC] 0.76 and 0.69, respectively). In the test set the model associated with response (AUC = 0.68). The multi-phase didn't improve the prediction capacity (AUC = 0.59). The model combining radiomics and clinical variables slightly improved the capacity for predicting response in the three cohorts (AUC = 0.78, 0.75 and 0.67, respectively). Interpretability plots showed that patients with high radiomics-score presented more spherical and hypervascularised lesions.
**Conclusion:** Single-phase radiomics associates with response to anti-angiogenics based on the quantification of hypervascularisation and tumour shape.
**Limitations:** Further testing populations are needed to validate the prediction capacity of the model.
**Ethics committee approval:** The institutional review board approved this retrospective study. Need for informed consent for the computational analysis of the images was waived.
**Funding for this study:** The TALENT clinical trial was funded by Eisai.
**Author Disclosures:**
Manel Escobar: Nothing to disclose
Jaume Capdevila: Investigator: Eisai
Jorge Hernando: Investigator: Roche Investigator: Pfizer Investigator: LEO Pharma Investigator: AAA Investigator: Ipsen Investigator: Novartis Investigator: Angelini Investigator: Eisai
Raquel Perez Lopez: Nothing to disclose
Xavier Merino Casabiel: Nothing to disclose
Eric Delgado: Nothing to disclose
Marta Ligero: Nothing to disclose
Alejandro Garcia-Alvarez: Investigator: Sanofi Investigator: Pierre Fabre Investigator: Eisai Investigator: Angelini Investigator: Ipsen Investigator: Kyowa Kirin

**09:30-10:30** **Channel 3**

### Research Presentation Session: Cardiac

### RPS 2303

### Advanced techniques in cardiac MR

**Moderator**
C. Treutlein; Erlangen/DE

### RPS 2303-2

**Reducing artefacts in cardiac magnetic resonance imaging in patients with cardiac implantable electronic devices**
*A-M. Vuorinen*, L. Lehmonen, J. Karvonen, M. Holmström, S. Kivistö, T. Kaasalainen; Helsinki/FI

**Purpose:** Cardiac implantable electronic devices (CIED) induce artefacts on MRI and may significantly reduce the diagnostic value of cardiac magnetic resonance imaging (CMR). The study aimed to assess the effect of CIED generator location and a raised-arm imaging position on the CIED-induced artefacts on CMR.
**Methods or Background:** All clinically indicated CMRs performed in our institution for CIED patients with normal cardiac anatomy and permanent CIED with endocardial pacing leads between November 2011 and October 2019 were included (n=171). Cine and late gadolinium enhancement (LGE) images were analysed according to the American Heart Association 17-segment model for artefacts.
**Results or Findings:** Right-sided generator implantation and raised-arm imaging were associated with a significantly increased number of artefact-free segments. In patients with a right-sided pacemaker, the median percentage of artefact-free segments in short-axis balanced steady-state free precession LGE was 93.8% (IQR 9.4%, n=53) compared to 78.1% (IQR 20.3%, n=58) with a left-sided pacemaker (p<0.001). In patients with a left-sided implantable cardioverter defibrillator, the median percentage of artefact-free segments was 87.5% (IQR 6.3%, n=9) with raised-arm imaging, compared to 62.5% (IQR 34.4%, n=9) with arm down in spoiled gradient-echo short-axis cine (p=0.02).
**Conclusion:** Raised-arm imaging is a simple, costless method for reducing CMR artefacts in patients with left-sided CIED, and can be used with other image quality improvement methods. Right-sided generator implantation could be considered in patients who are known to require subsequent CMRs to ensure adequate image quality.
**Limitations:** Assessing artefacts included subjective judgement. Artefacts were not evaluated by specific CIED models.
**Ethics committee approval:** The study was approved by the Helsinki University Hospital Medical Imaging Center review board.
**Funding for this study:** This study was supported by HUS Medical Imaging Center research grant and Ida Montin Foundation research grant.
**Author Disclosures:**
Jarkko Karvonen: Other: Abbott, Medtronic
Touko Kaasalainen: Nothing to disclose
Sari Kivistö: Nothing to disclose
Lauri Lehmonen: Nothing to disclose
Miia Holmström: Nothing to disclose
Aino-Maija Vuorinen: Nothing to disclose

### RPS 2303-3

**Free-breathing cine with motion correction: a new generation of real-time compressed sensing cine**
*B. Longere*[1], N. Abassebay[1], S. Toupin[2], C. V. Gkizas[1], A. Simeone[1], J. Hennicaux[1], M. Schmidt[3], J. Pang[4], F. Pontana[1]; [1]Lille/FR, [2]Saint-Denis/FR, [3]Erlangen/DE, [4]Chicago, IL/US
*(benjamin.longere@chru-lille.fr)*

**Purpose:** The image quality of cine imaging is highly affected by arrhythmia and shortness of breath. This study aimed to evaluate whether a new compressed sensing free-breathing cine sequence (CS-FB) with motion correction (MOCO) can address such limitations.
**Methods or Background:** Forty-eight patients referred for cardiac MRI underwent both conventional bSSFP multi-shot multi-breath-hold cine (BH-SSFP, GRAPPA=2) and prototype free-breathing single-shot bSSFP cine (CS-FB, CS acceleration=9.0-24.0, depending on heart rate) with fully automated MOCO. Short-axis stacks were acquired for each patient with matched parameters. Acquisition and reconstruction times, image quality (Likert scale from 1 to 4), left ventricular (LV) and right ventricular (RV) volumes, LV, and RV ejection fractions (EF) and LV mass were assessed for each sequence.
**Results or Findings:** CS-FB cine's scan time was shorter (2.2±0.6 min vs 4.8±1.2 min for BH-SSFP, p<0.0001), although reconstruction times were longer for CS-FB (4.7±1.5 min vs 0 min for BH-SSFP, p<0.0001). CS-FB achieved higher image quality (3.9±0.3) than BH-SSFP (3.6±0.6, respectively, p=0.0001), especially for patients with arrhythmia or difficulties holding breath

(n=19; 3.9±0.2 vs 3.3±0.7, p=0.002). Regardless of cardiac rhythm, LVEF, LV stroke volumes and mass were similar between both sequences. There was difference in RVEF (CS-FB: 49.9±11.2%; BH-SSFP: 51.5±11.4%; p=0.0007) and RV stroke volume (CS-FB: 73.9±24.4mL;BH-SSFP: 76.8±26.3mL; p=0.005) but not for the RV end-systolic and end-diastolic volumes.
**Conclusion:** Free-breathing CS acquisition with MOCO can be reliably performed in a clinical setting to evaluate LV parameters, with better image quality and shorter scan time than conventional cine, especially for patients with arrhythmia or shortness of breath.
**Limitations:** CS-FB sequence required longer reconstruction times than the reference BH-SSFP cine. However, the robustness of the CS-FB sequence against arrhytmia and respiratory motion avoid the repetition of cine acquisitions.
**Ethics committee approval:** The ethics committee approval was obtained, IRB number: CRM-2103-163.
**Funding for this study:** No funding was received for this study.
**Author Disclosures:**
Jianing Pang: Employee: Siemens Healthcare employee
Benjamin Longere: Nothing to disclose
Christos Vasileiou Gkizas: Nothing to disclose
Arianna Simeone: Nothing to disclose
Justin Hennicaux: Nothing to disclose
Michaela Schmidt: Employee: Siemens Healthcare employee
Solenn Toupin: Employee: Siemens Healthcare employee
François Pontana: Nothing to disclose
Neelem Abassebay: Nothing to disclose

### RPS 2303-5

**Acute impact of an endurance race on biventricular and biatrial myocardial strain in competitive male and female triathletes evaluated by feature-tracking CMR**

*H. Chen*, A. Kisters, M. K. Świderska, E. Cavus, K. Müllerleile, D. Säring, G. K. Lund, G. Adam, E. Tahir; Hamburg/DE
*(hang.chen@stud.uke.uni-hamburg.de)*

**Purpose:** Cardiac adaptation in endurance athletes is a well-known phenomenon, but the acute impact of strenuous exercise is fairly unknown. The purpose of this study was to analyse the alterations in biventricular and biatrial function in triathletes after an endurance race using novel feature-tracking cardiac magnetic resonance (FT-CMR).
**Methods or Background:** Fifty consecutive triathletes (45±10 years; 80% men) and twenty-eight controls were prospectively recruited. All underwent 1.5T CMR examination. The time interval between race completion and CMR was 2.3±1.1 hours (range 1-5 hours). Biventricular and biatrial volumes, left ventricular ejection fraction (LVEF), FT-CMR analysis and late gadolinium enhancement (LGE) imaging were performed. Global systolic longitudinal strain (GLS), circumferential strain (GCS) and radial strain (GRS) were assessed. CMR was performed at baseline and following an endurance race. High-sensitive Troponin T and NT-proBNP were determined.
**Results or Findings:** Post-race Troponin T (P<0.0001) and NT-proBNP (P<0.0001) were elevated. LVEF remained constant (62±6 vs 63±7%, P=0.607). Post-race LV GLS decreased by tendency (-18±2 vs -17±2%, P=0.054), whereas GCS (-16±4 vs -18±4%, P<0.05) and GRS increased (39±11 vs 44±11%, P<0.01). Post-race right ventricular GLS (-19±3 vs -19±3%, P=0.668) remained constant and GCS increased (-7±2 vs -8±3%, P<0.001). Post-race left atrial GLS (30±8 vs 24±6%, P<0.0001) decreased while right atrial GLS remained constant (25±6 vs 24±6%, P=0.519).
**Conclusion:** The different alterations of post-race biventricular and biatrial strain might constitute an intrinsic compensatory mechanism following an acute bout of endurance exercise. The combined use of strain parameters may allow a better characterisation and understanding of ventricular and atrial function in endurance athletes.
**Limitations:** This study had a relatively small sample size and ruled out triathletes with pre-existing cardiovascular disease and systemic diseases, limiting its generalisability.
**Ethics committee approval:** The ethics committee approved the study (PV4764).
**Funding for this study:** No funding was received for this study.
**Author Disclosures:**
Ersin Cavus: Nothing to disclose
Kai Müllerleile: Nothing to disclose
Gunnar K Lund: Nothing to disclose
Gerhard Adam: Nothing to disclose
Anna Kisters: Nothing to disclose
Hang Chen: Nothing to disclose
Dennis Säring: Nothing to disclose
Enver Tahir: Nothing to disclose
Monika Katarzyna Świderska: Nothing to disclose

### RPS 2303-6

**Cardiac remodeling and subclinical left ventricular dysfunction in uncomplicated obese adults: a cardiovascular magnetic resonance study with tissue tracking**

*J. Liu*[1], J. Li[1], H. Pu[1], W. He[1], X. Zhou[2], N. Tong[1], L. Peng[1]; [1]Chengdu/CN, [2]Shanghai/CN
*(dr_liujing@126.com)*

**Purpose:** Obesity increases the risk of heart failure and cardiovascular mortality. However, the specific effects of obesity on cardiac structure and function have not been clarified. This study aimed to evaluate the left ventricular (LV) geometric and functional changes using cardiovascular magnetic resonance (CMR) in uncomplicated obese adults.
**Methods or Background:** A total of 48 obese participants and 25 healthy controls were prospectively enrolled. The LV geometry, global systolic functions, and strains were assessed using CMR. Body composition was measured using dual X-ray absorptiometry.
**Results or Findings:** Compared with healthy controls, the obese patients had greater LV size, mass, myocardial thickness, and impaired myocardial contractility with lower LV global radial, circumferential, and longitudinal peak strains (PS) and LV global circumferential and longitudinal peak diastolic strain rates (PDSR) (all p<0.05). Multivariable linear regression showed that the body mass index (BMI) was independently associated with LV average myocardial thickness (LVAMT), LV maximum myocardial thickness (LVMMT), and concentricity in the obese group (LVAMT: β=0.098, p=0.02; LVMMT: β=0.16, p=0.044; concentricity: β=0.013, p=0.037). In addition, the visceral adipose tissue (VAT) was associated with LV global longitudinal PS and LV longitudinal and circumferential PDSR (longitudinal PS: β =-2.784, p<0.001; longitudinal PDSR: β=-0.202, p=0.001; circumferential PDSR: β=-0.193, p=0.005).
**Conclusion:** LV geometric remodelling and subclinical dysfunction are observed in obese adults with preserved LV ejection fraction. Instead of BMI, VAT is found to be a sensitive predictor for subclinical LV dysfunction.
**Limitations:** A cross-sectional study.
**Ethics committee approval:** The study was approved by the Institutional Review Board of West China Hospital.
**Funding for this study:** This work was supported by the National Natural Science Foundation of China [Grant number.81601462].
**Author Disclosures:**
Wenzhang He: Nothing to disclose
Xiaoyue Zhou: Nothing to disclose
Liqing Peng: Nothing to disclose
Jing Liu: Nothing to disclose
Nanwei Tong: Nothing to disclose
Jing Li: Nothing to disclose
Huaxia Pu: Nothing to disclose

### RPS 2303-7

**Spin-echo diffusion weighted imaging of in-vivo human heart at 3T**

*X-M. Wu*, X-Y. Wu, H. Tan, L-F. Ma, H-P. Dong, Q. Jiang; Shanghai/CN

**Purpose:** The objective of this study was to investigate the possibility of cardiac DWI using conventional spin-echo (SE) EPI.
**Methods or Background:** Seven female and five healthy male subjects (mean age, 39.2 ± 13.0 years; heart rate, 69.8 ± 7.8 bpm) without any contraindication to MR scanning underwent cardiac DWI, which was performed on a 3 Tesla MR scanner (Elition X, Philips Healthcare, Best, the Netherlands, Max G 45 mT/m, Max SR 200 T/m/s) with a 16-channel anterior coil and a 12-channel posterior coil. Diffusion-weighted SE-EPI with respiratory navigation, volume shim, and fat suppression (both SPIR and gradient reversal) was then performed with the following parameters: voxel size = 2.5 × 2.5 × 8 mm3, FOV 320 × 320 mm2, slice number 1, half scan factor 0.6, SENSE factor 2.0, NSA 10, TR 1 heartbeat, 5 b values (200, 300, 500, 800, 1000 s/mm2) were acquired separately with b0, corresponding TEs are 35, 38, 42, 47, 50 ms. Scan time ~1min6s for each b value scan considering navigation efficiency of 60%.
**Results or Findings:** Image quality degraded, and distortion became more severe, with higher b values. When b value > 500 s/mm2, the overall mean image quality score was lower than 2.5.A significant difference was also found between segments (SNR: F=4.59, P=0.001; CNR: F=4.60, P=0.001). The signal was lower in the interventricular septum, especially in the infero-septal part, compared with other segments.
**Conclusion:** Using a clinical 3T scanner, SE-EPI DWI could be successfully performed with careful choice of navigator placement and cardiac trigger delay. Medium b values are recommended to obtain good image quality.
**Limitations:** Only healthy volunteers were involved in this study. Patients should be included to investigate the diagnostic value of cardiac DWI in future studies.
**Ethics committee approval:** Not applicable.
**Funding for this study:** Not applicable.

**Author Disclosures:**
Hai-Peng Dong: Nothing to disclose
Houmin Tan: Nothing to disclose
Li-Fei Ma: Nothing to disclose
Qian Jiang: Nothing to disclose
Xin-Yang Wu: Nothing to disclose
Xiao-Meng Wu: Nothing to disclose

### RPS 2303-8

**Evaluation of novel rapid post-processing vs conventional strain parameters in highly accelerated cardiac magnetic resonance**

*M. C. Halfmann*[1], T. S. Emrich[2], K-F. Kreitner[1]; [1]Mainz/DE, [2]Charleston, SC/US

**Purpose:** Development of compressed sensing (CS) cardiac magnetic resonance (CMR) sequences rapidly accelerated cardiac imaging while showing good reproducibility for volumetric analyses. However, for imaging biomarkers such as strain, CS-derived measurements are significantly altered. Novel prototype rapid strain parameters (junction strain (JS) and long axis strain (RS)) are evaluated for their potential to overcome that bias.
**Methods or Background:** 15 prospectively enrolled healthy volunteers (HV) underwent CMR including a stack of short-axis slices and two orthogonal left ventricular long-axis slices. Acquisitions were based on balanced steady-state free precession Cine (bSSFP) and CS-sequences. Exclusion criteria were any history of cardiac disease and abnormal volumes as characterised by CMR. Dedicated post-processing software (cvi42 Circle) was used to compute JS, RS and global longitudinal strain (GLS).
**Results or Findings:** GLS correlated moderately between bSSFP and CS (r=0.58). JS and RS correlated strongly between acquisitions (r= 0.70/0.69, respectively). Correlation between novel parameters and GLS was moderate in bSSFP (JS/GLS r=0.56; RS/GLS r= 0.53) and weak in CS (JS/GLS r=0.34; RS/GLS r= 0.31). Bland-Altman analysis showed a significant bias for CS-derived GLS with a mean difference of -3.9% and limits of agreement (LoA) from -6.9 to -1.0%. There was no significant bias for both JS and RS (mean differences -1.1/-1.0%; LoA -3.8 to 1.7/-3.8 to 1.8%).
**Conclusion:** Novel rapid strain parameters correlate strongly between bSSFP and CS acquisitions and with established GLS. However, they differ from GLS as there is no significant systematic bias between acquisition types.
**Limitations:** Rapid post-processing strains are still under evaluation, larger studies including patient populations are needed to evaluate the clinical significance of these preliminary results.
**Ethics committee approval:** The study protocol was approved by the local ethics committee with a waiver for informed consent.
**Funding for this study:** No outside funding was acquired for this study.
**Author Disclosures:**
Tilman Stephan Emrich: Other: Siemens Healthineers Speaker: Siemens Healthineers
Moritz Christian Halfmann: Nothing to disclose
Karl-Friedrich Kreitner: Nothing to disclose

**09:30-10:30** **Channel 4**

### Research Presentation Session: Radiographers

### RPS 2314

### Optimising patient referral checks and pre-examination communication

**Moderators**
L. A. Rainford; Dublin/IE
B. M. Verbist; Leiden/NL

### RPS 2314-3

**Referral justification and the role of the radiographer**

L. Bowden, *L. Barreira*, S. Hoare; Dublin/IE

**Purpose:** Recently in Ireland, the role of the radiographer has been revised to include a ‘practitioner status’, as amended into statutory legislation S.I. 256. In effect, this means that radiographers have the qualification and professional judgment to accept, reject or amend a referral according to the clinical information, prior imaging and presentation status of a patient. This ensures that no exam is performed without being justified in advance by the radiographer and documented accordingly. A justification audit was devised in a Tertiary Paediatric Hospital to evaluate at what rate radiographers cancel or modify referrals received, the results of which are presented here.
**Methods or Background:** Any referrals that had been cancelled or modified by radiographers at the time of examination were collected. The reasons for cancellation/modification were coded according to 13 different categories. The origin of the referrals was also recorded allowing the data to establish trends with relation to the clinics/departments where most modified/cancelled referrals originate from.
**Results or Findings:** The majority of referrals that were modified or cancelled had originated from the emergency department (ED) (42%) and from the out-patient department (35%). The audits further demonstrated that the majority of the reasons for cancelling/changing an exam are due to, ‘incorrect exam selected according to clinical indication’, ‘unnecessary additional views requested’, ‘wrong body part’, ‘wrong side requested’, ‘exam not justified by the referrer’.
**Conclusion:** This audit shows that the role of the radiographer as a practitioner is both established and of fundamental importance to positive patient outcomes. Radiographers are not only professionally trained to act as practitioners but also are effective gate-keepers to potential errors in the referral process. Outcomes and learnings from the audit are disseminated among referrers where possible.
**Limitations:** No limitations were identified.
**Ethics committee approval:** Not applicable.
**Funding for this study:** Not applicable.
**Author Disclosures:**
Liliana Barreira: Nothing to disclose
Louise Bowden: Nothing to disclose
Siobhan Hoare: Nothing to disclose

### RPS 2314-4

**Inadequate filling of radiology request form and its impact on patient radiation exposure and waiting time: a preliminary study**

*B. S. Hussain*, I. Garba; Kano/NG
*(shussainreal@gmail.com)*

**Purpose:** The purpose of this study was to evaluate how the failure or inadequacy in filling of radiology request forms leads to an increase in patient radiation exposure resulting from repeat investigations.
**Methods or Background:** A total of 158 patient request cards for conventional X-ray examination were randomly evaluated. Scoring criteria: filled; inadequately filled, and unfilled were used to score each item based on the following: patient demographic information; patient referral details and referring physician details. Repeat examinations due to inadequate filling or lack of filling of the request card were evaluated. Data was analysed based on descriptive statistics using SPSS statistical software.
**Results or Findings:** Patient names were adequately filled on all the request cards (100%). Demographic information related to gender and hospital number was provided in 94.9% and 93.7% of the request forms, respectively. Information related to patient referral details such as previous X-ray examination, blood pressure (BP), and last menstrual period (LMP) were inadequately filled with 4.4%, 2.5%, and 19.7% completion, respectively. Of the 158 request forms assessed, 33 (20.9%) led to repeat due to inadequate information provided. Inadequate information related to clinical history and requested examination had the highest and accounted for 45.5% and 24.2% of the causes of repeat, respectively.
**Conclusion:** Study findings have shown that information related to patient referral details is the most inadequately provided information. Besides, clinical history and the requested examination are the commonest causes of a repeat which is associated with an increase in patient exposure and also in patients' waiting time all of which has medico-legal implications and impact on the overall quality of service rendered.
**Limitations:** The sample size is small considering the large number of requests received on the daily basis.
**Ethics committee approval:** Ethics approval was obtained from the institutional research and ethics board.
**Funding for this study:** No funding was received for this study.
**Author Disclosures:**
Idris Garba: Nothing to disclose
Bashir Shafiu Hussain: Nothing to disclose

### RPS 2314-5

**Assessment of clinical information in examination requests in a radiology department**

S. R. Gonçalves, A. F. C. L. Abrantes, L. P. V. Ribeiro, S. I. Rodrigues, *O. Lesyuk*, J. Pinheiro, A. d. M. Ribeiro, R. P. P. Almeida; Faro/PT

**Purpose:** Clinical information is an important tool to perform correctly imaging studies. Immobilisation devices may difficult the identification of the region of study, therefore, proper communication before an examination to adjust the study to a patient's condition is important. The present study aims to evaluate the existence or absence of clinical information in the examination requests forms in a radiology department.

**Methods or Background:** A retrospective study was carried out with a total of 600 examination requests from two hospitals (300 from the emergency department and 300 from routine procedures), which were analysed in order to assess the presence or absence of clinical information. The instruments used were a checklist of existence or absence of clinical information in the examination request, and software for examination and visualisation of the exam request forms called RADIO from Global Intelligent Technologies (Glintt). In the second phase, a questionnaire “Relevance of prescription/clinical information in the Orto-traumatology services" by Vasconcelos was applied to a total of 34 radiographers.
**Results or Findings:** In this study, we verified that only 44.6% of the examination requests in the context of emergency trauma presented clinical information and 55.3% of them do not present any relevant information. 100% of the radiographers affirm that the requests for examination do not contain enough clinical information or correct anatomic region identification to perform the examinations and correct diagnosis. So, questioning the patient or the physicians is necessary to perform the study, decreasing the workflow in radiology departments.
**Conclusion:** Better communication to ensure a multidisciplinary approach is necessary in order to provide the patient with the best possible care and avoid an inadequate exposure.
**Limitations:** No limitations.
**Ethics committee approval:** No ethics conflicts were identified in this study.
**Funding for this study:** No funding was received for this study.
**Author Disclosures:**
Joao Pinheiro: Nothing to disclose
António Fernando Caldeira Lagem Abrantes: Nothing to disclose
Anabela de Magalhães Ribeiro: Nothing to disclose
Rui Pedro Pereira Almeida: Nothing to disclose
Susana Ribeiro Gonçalves: Nothing to disclose
Sónia Isabel Rodrigues: Nothing to disclose
Oksana Lesyuk: Nothing to disclose
Luís Pedro Vieira Ribeiro: Nothing to disclose

### RPS 2314-6

**The benefits of quality referral information and assessment: the radiographers' perspective**

*C. Chilanga*, H. M. Olerud, K. B. Lysdahl; Drammen/NO

**Purpose:** The purpose of this study was to determine the benefits of quality referral information and involve radiographers in assessing referrals for appropriate imaging, as perceived by radiographers.
**Methods or Background:** An online survey was distributed to radiographers in clinical and non-clinical/academic settings via the International Society of Radiographers and Radiological Technologists (ISRRT) networks. The questionnaire consisted of 5-point Likert scale questions on radiographers' usefulness of referral information (12 reasons listed) and benefits of radiographers assessing referrals (8 benefits suggested).
**Results or Findings:** A total 279 responses were received. Clinical radiographers reported making use of the referral information frequently for a number of reasons, with the highest rank for ‘patient identification’, ‘ensuring imaging of the correct body region’ and ‘correct patient positioning’ (83%, 79%, 66% very frequently responses). Non-clinical/academic radiographers ranked the same reasons high for usefulness (74%, 63%, 52% strongly agreed as useful responses). Benefits of radiographers’ involvement in referral assessment ranked high (strongly agree responses) were the items ‘promotes radiographers' professional responsibility’ (72%) ‘improves radiographers' collaboration with radiologists and referring clinicians’ (67%) and ‘enables efficient use of radiology services’ (57%).
**Conclusion:** Radiographers perceive referral information as useful for many purposes in their clinical practice, all vital for ensuring patient safety and quality radiology services. Radiology departments can benefit from involving radiographers in assessing referrals, through professional development, inter-professional collaboration, and efficiency of services.
**Limitations:** The number of responses is low. Larger sample size could also have been of benefit to capture views of a wider range of radiographers. The study is subject to selection bias as only participants who had information from ISRRT organisation networks were able to view and respond to the survey.
**Ethics committee approval:** The Norwegian Centre for Research Data (NSD) approved this study (reference number 472337).
**Funding for this study:** No funding was provided for this study.
**Author Disclosures:**
Kristin Bakke Lysdahl: Nothing to disclose
Catherine Chilanga: Nothing to disclose
Hilde Merete Olerud: Nothing to disclose

### RPS 2314-7

**Evaluating clinical criteria in patients being referred for a preoperative chest X-ray in a state general hospital in Malta**

F. Vella[1], *K. B. Borg Grima*[2], D. Mizzi[2]; [1]Sliema/MT, [2]Msida/MT
*(karen.borg-grima@um.edu.mt)*

**Purpose:** Preoperative chest X-rays (CXRs) are frequently performed before surgeries. The aim of this study was to investigate adult clinical referral criteria for a preoperative CXR and to determine if referrals adhered to local protocols, international and European guidelines. The association between clinical indications and the result of the preoperative CXR was investigated.
**Methods or Background:** The research design was a prospective, cross-sectional study performed in a state general hospital in Malta. 271 adult participants undergoing elective surgery were recruited, using convenience sampling. The research tools comprised of a close-ended questionnaire in which clinical and medical information was obtained. The questionnaire was complimented by a data sheet completed by intermediaries.
**Results or Findings:** Out of 271 participants, 72 (26.6%) participants were healthy asymptomatic patients, having an American Society of Anaesthesiology (ASA) score I. ASA II was the most common score obtained (45.7%). 44 (16.2%) participants had an abnormal CXR result, with cardiomegaly being the most common abnormality detected. Abnormalities were significantly low in all ASA scores but were seen to increase with an increasing ASA score. Only 11.4% (n=5) of abnormalities were unexpected. A statistically significant association (p=0.007) was found between increasing age and abnormal CXR results. No statistically significant association was found between cardiac or respiratory conditions and CXR abnormalities.
**Conclusion:** 64.2% of CXRs were not requested in accordance with protocols implemented locally on preoperative testing. Whereas, if the international and European guidelines were applied, 70.1% of CXRs were not requested according to guidelines.
**Limitations:** Referring physicians may have had cogent reasons for requesting the preoperative CXR but due to time constraints were unable to provide them in detail.
**Ethics committee approval:** Ethical approval was sought and obtained from the University Research Ethics Committee of the University of Malta.
**Funding for this study:** No funding was received for this study.
**Author Disclosures:**
Karen Borg Borg Grima: Nothing to disclose
Deborah Mizzi: Nothing to disclose
Faith Vella: Nothing to disclose

### RPS 2314-8

**What makes compassion difficult: a narrative review of the evidence for compassion fatigue in diagnostic radiographers**

*S. Robertson*[1], H. McNair[1], E. Olanloye[1], S. Cruickshank[1], A. England[2]; [1]London/UK, [2]Keele/UK

**Purpose:** The objective of this study was to investigate the evidence of occupational stressors in diagnostic radiographers, and whether they are at risk of developing compassion fatigue.
**Methods or Background:** Compassion is an essential concept in healthcare, however, repeated exposure to challenging or traumatic situations can lead to compassion fatigue (CF). A review protocol was developed and registered on PROSPERO. Database and grey literature searches were carried out. No meta-analysis was possible therefore data has been presented as a narrative.
**Results or Findings:** Fifteen studies were selected for review published between 1982 and 2020. Evidence demonstrates that radiographers suffer from high levels of occupational stress. Stress is perceived rather than defined. Common causes of occupational stress were identified as poor patient interactions, and a lack of time to spend with patients. There is a lack of evidence to show how this stress affects radiographers' health or their ability to provide compassionate care.
**Conclusion:** Radiographers are prone to suffering from symptoms that can be attributed to CF. This has been present for an extended period of time, and the main changes have been a decrease in job satisfaction and accomplishment. Patient interaction was identified as a cause, but it is unclear if this affects staff ability to be compassionate. Further work is required to find ways to mitigate these effects and prevent the issue from getting worse.
**Limitations:** It was difficult to find consistency in the range of studies identified in this review, and direct comparisons of the data have not been possible. The wide range of definitions used to cover this topic means that some studies may not have been identified.
**Ethics committee approval:** Not applicable.
**Funding for this study:** SR is supported by a pre-doctoral research fellowship grant from the Royal Marsden Hospital Charity.
**Author Disclosures:**
Elizabeth Olanloye: Nothing to disclose
Scott Robertson: Nothing to disclose
Susanne Cruickshank: Nothing to disclose
Helen McNair: Nothing to disclose
Andrew England: Nothing to disclose

11:30-12:30 Channel 2

### Research Presentation Session: Neuro

### RPS 2411

### Brain tumour: other than glioma

**Moderator**
A. Mazumder; London/UK

### RPS 2411-4

**MRI based radiomics and spatial distribution joint model in the differentiation of autoimmune encephalitis from low-grade diffuse astrocytoma**

*S. Piao*, X. Luo, Y. Bao, B. Hu, Y. Zhu, L. Yang, D. Geng, Y. Li; Shanghai/CN
*(piaosirong@fudan.edu.cn)*

**Purpose:** Preoperative differentiation of autoimmune encephalitis from low grade diffuse astrocytoma is important to guide clinical decision-making. We aim to develop quantitative models integrating both radiomics and spatial distribution features of brain lesions from non-contrast MRI for discrimination of autoimmune encephalitis from low grade diffuse astrocytoma.
**Methods or Background:** The study included patients with autoimmune encephalitis (AE, n=59) and WHO grade II diffuse astrocytoma (AS, n=89) into training and test sets with a ratio of 3:1. Another 22 AE patients and 28 AS patients were allocated as the external set. Hyperintensity T2-FLAIR lesions were segmented manually. 42 radiomics and 11 spatial distribution features were extracted via LASSO, and joint models were constructed using logistic regression algorithms for two diseases differentiations. The discrimination performance of the joint model was compared with neuroradiologists.
**Results or Findings:** The radiomics and spatial distribution feature joint model achieved AUC=0.998/0.974 (training/test set) and prominently outperformed the radiomics model (AUC=0.993/0.971) and the spatial distribution model (AUC=0.989/0.965). The senior and junior neuroradiologists achieved the AUC of 0.951/0.870 and 0.689/0.641, respectively. The diagnostic ability of the joint model suppressed the junior neuroradiologist.
**Conclusion:** The radiomics and spatial distribution joint model could effectively differentiate AE from AS and achieved the diagnostic performance as senior neuroradiologist in the discrimination between the two diseases, with a clear path that is easy to follow in further practice.
**Limitations:** AE mediated by different auto-antibodies was not strictly distinct due to the retrospective nature of the study. Additionally, the results need to be further validated via a multi-centre study with more participants.
**Ethics committee approval:** This study was approved by the Institutional Research Review Board of the Huashan Hospital.
**Funding for this study:** This work was supported by the Science and Technology Commission of the Shanghai Municipality (19ZR1407900, 20S31904300) and Shanghai Hospital Development Center (SHDC2020CR3020A).
**Author Disclosures:**
Yifang Bao: Nothing to disclose
Xiao Luo: Nothing to disclose
Daoying Geng: Nothing to disclose
Yuxin Li: Nothing to disclose
Liqin Yang: Nothing to disclose
Sirong Piao: Nothing to disclose
Bin Hu: Nothing to disclose
Yuqi Zhu: Nothing to disclose

### RPS 2411-6

**Myeline changes caused by neurotoxicity after the first chemotherapy course: a pilot study**

*N. Kriventsova*, P. Menshchikov, N. Kosirnikova, D. Kupriyanov, G. Tereshchenko; Moscow/RU
*(nataliastrumila@gmail.com)*

**Purpose:** Neurotoxicity of the central and peripheral nervous system is a frequent complication of chemotherapy and is characterized by different neurological symptoms. Some previous DTI/DWI studies indirectly indicate the demyelination processes caused by neurotoxicity. Thus, the study's main aim was to directly measure myeline content after the first chemotherapy course.
**Methods or Background:** 6 paediatric patients (mean age–13.7±2.4) with leukaemia and osteosarcoma were enrolled in the study. MRI examination included 2 scanning sessions: 1 week before and after chemotherapy. Each scanning includes the acquisition of the following pulse sequences: (1) macromolecular proton fraction (MPF) – gradient echo (GE) with FA=3° and 20° as well as additional MT-weighted GE with Gaussian saturated prepulse, and (2) water myeline fraction (WMF) – Turbo Spin Echo with TE from 10 to 210ms. MPF, WMF and T2 values were measured in white matter (WM) of parietal, temporal, occipital, frontal lobes, cerebellum and thalamus.
**Results or Findings:** Both MPF and T2 simultaneously increase in WM occipital lobes and thalamus. WMF shows increased values for the cerebellum as well as decreased values in WM parietal lobes.
**Conclusion:** Previously, both MPF and WMF have been shown to reflect myeline concentration. Moreover, some previous studies revealed the influence of region oedema associated with the T2 increase on the MPF. In our study, we have the same effect. Therefore, MPF seems to be ineffective in this case. Reduced WMF indicates a demyelination process in white matter of the parietal lobe. The increase in WMF in the cerebellum may indicate the accumulation of "myelin refuse". Myelin residues are located at the site of degeneration until the complete cleansing by macrophages.
**Limitations:** The main limitation of the study is the small patient cohort.
**Ethics committee approval:** Not applicable.
**Funding for this study:** No funding was received for this study.
**Author Disclosures:**
Petr Menshchikov: Employee: Philips
Galina Tereshchenko: Nothing to disclose
Nadezhda Kosirnikova: Nothing to disclose
Dmitry Kupriyanov: Employee: Philips
Nataliia Kriventsova: Nothing to disclose

### RPS 2411-7

**Clinical outcomes post stereotactic radiosurgery (SRS) treatment for metastases: can pseudocontinuous arterial spin labelling (pCASL) contribute?**

*M. Mashar*, M. Sokolska, J. Markus, M. Kosmin, H. Hyare; London/UK
*(meghavi@hotmail.co.uk)*

**Purpose:** We investigated the utility of pseudocontinuous arterial spin labelling (pCASL), a perfusion-weighted MR technique, in clinically stratifying patients post stereotactic radiosurgery (SRS) for cerebral metastases.
**Methods or Background:** 45 patients (25 female, mean age: 60.2 years) with metastases treated with first SRS treatment (03/17 to 10/19) and pCASL sequence at the first follow-up (mean: 6.35 weeks). pCASL perfusion weighted image (PWI) was visually classified: positive (n=16) or negative (n=29) (ASL+/-). Signal intensity (SI) from the lesion and perilesional area were manually defined on PACS. Ratios of SI were calculated (mean ± standard error); radiological progression as per RANO criteria and 6-month mortality were documented.
**Results or Findings:** The ratio of mean SI in the lesion to perilesional area (contrast to noise ratio) was higher in ASL+ (1.94 ± 0.15) vs ASL- (0.77 ± 0.05) ($P<0.05$) and similarly in ratios of maximum SI [ASL+ (1.81 ± 0.22), ASL- (0.95 ± 0.04)]. Treatment response at first follow-up was similar ASL+, non-progression n=15, 94%; ASL, non-progression n=27, 93%, and 6-month mortality: ASL+, n=2, 13%; ASL-, n=4, 13%. Whilst there was limited follow-up available at 6 months (n=29), a higher proportion in the ASL- group had progressive disease at 6 months: n=10, (50%) vs ASL+, n=2 (22%).
**Conclusion:** Our results indicate that post-SRS for metastases, pCASL can stratify patients at 6 months. Visual assessment of pCASL PWI allows accurate classification of perfusion in metastases indicating the feasibility of use clinically. The association between ASL signal post-treatment and disease non-progression may be due to treatment-related increased vascularity, or that vascular metastases are more radio-sensitive.
**Limitations:** As a preliminary retrospective, small sample study, findings are limited. Assessment of interobserver variability would add robustness to our conclusions and better evaluate utility in clinical practice.
**Ethics committee approval:** Not applicable.
**Funding for this study:** Not applicable.
**Author Disclosures:**
Magdalena Sokolska: Nothing to disclose
Michael Kosmin: Nothing to disclose
Meghavi Mashar: Nothing to disclose
Harpreet Hyare: Nothing to disclose
Julia Markus: Nothing to disclose

11:30-12:30 Channel 3

### Research Presentation Session: GI Tract

### RPS 2401b

### Diagnosis and preoperative planning of colon pathology: advances in knowledge

**Moderator**
E. Neri; Pisa/IT
**Author Disclosures:**
Emanuele Neri: Advisory Board: QUIBIM; Synlab; DeepMammo; Board Member: ESOI; SIRM; Speaker: GE/HEALTHCARE; EBIT/ESAOTE

### RPS 2401b-3

**Low volume reduced bowel preparation regimen for CT colonography: a randomised non-inferiority trial**

*S. Vicini*[1], D. M. Bellini[1], N. Panvini[1], M. Rengo[2], I. Carbone[2]; [1]Latina/IT, [2]Rome/IT
*(simone.vicini@gmail.com)*

**Purpose:** To determine whether the quality of low-volume reduced bowel preparation (LV-RBP) for CT colonography (CTC) is non-inferior to full-volume reduced bowel preparation (FV-RBP) regimen.
**Methods or Background:** In this randomised controlled trial, consecutive participants referred for CTC were randomly assigned to receive LV-RBP (52.5 g of PMF104 in 500 mL of water) or FV-RBP (105 g of PMF104 in 1000 mL of water). Images were independently reviewed by five readers who rated the quality of bowel preparation from 0 (best score) to 3 (worst score). The primary outcome was the non-inferiority of LV-RBP to FV-RBP in the proportion of colonic segments scored 0 for cleansing quality, with a non-inferiority margin of 10%. The volume of residual fluids, colonic distension, lesions and polyps detection rates and patient tolerability were secondary outcomes.
**Results or Findings:** 110 participants (mean age 65 years±14 SD) were allocated to LV-RBP (n=55) or FV-RBP (n=55) arms. There was 92% segment scored 0 in colon cleansing quality in LV-RBP and 94% in FV-RBP for prone scans, and 94% vs 92% for supine scans. The risk difference was -2.1 (95% CI –5.9 to 1.7) and 1.5 (95%CI –2.4 to 5.4) for prone and supine positions, respectively. Residual fuids and colonic distension were also non-inferior in LV-RBP. LV-RBP was associated with a lower number of evacuations during preparation (7±5 vs 10±6, p=0.002).
**Conclusion:** The LV-RBP demonstrated the non-inferior quality of colon cleansing with improved gastrointestinal tolerability compared to the FV-RBP regimen.
**Limitations:** The number of participants enrolled was relatively small. Second, we were not able to evaluate CTC diagnostic accuracy for polyps detection. Third, the quality of colon cleansing was assessed exclusively by subjective analysis.
**Ethics committee approval:** Approval was obtained from the ethics committee of the Sapienza University of Rome.
**Funding for this study:** No funding was received for this study.
**Author Disclosures:**
Nicola Panvini: Nothing to disclose
Iacopo Carbone: Nothing to disclose
Simone Vicini: Nothing to disclose
Marco Rengo: Nothing to disclose
Davide Maria Bellini: Nothing to disclose

### RPS 2401b-5

**Enterocele, a problem for the patient, the surgeon, the radiologist: ten years and more of experience of oral contrast agent in defecography**

*F. Testa*, G. Di Guardia, M. Lo Bello, P. Lasciarrea, V. Verna; Verduno/IT
*(ftesta@aslcn2.it)*

**Purpose:** The oral contrast medium does not seem mandatory in defecography procedures; however, to demonstrate an enterocele (critical information for the surgeon), it is necessary to opacify the small bowel. A decade of case studies to illustrate the opportunity of oral contrast in all patients who undergo the procedure.
**Methods or Background:** Our series of 353 defecographies conducted on patients with various degrees of evacuation disorders were reviewed in detail. For each procedure, we considered the enterocele and its extension. In all of our procedures, contrast medium (barium sulphate or iodate) was administered orally between 40 and 60 minutes before the examination.
**Results or Findings:** 353 defecographs in 333 females and 20 males (mean age 60.3, median age 62) showed a total of 132 cases of enterocele (37.4%), classified in 75 cases of mild enterocele (56.8%, non-interfering with the evacuation dynamics) and 57 of severe enterocele (43.2%), interfering with the evacuation dynamics. No significant differences were recognised in the use of barium sulphate or iodate oral contrast medium.
**Conclusion:** The prevalence of enterocele suggests the use of oral contrast medium in all patients undergoing defecography, with an advantageous cost-benefit balance. The choice between barium and iodate contrast can be made on the basis of the constipation tendency, which tends to be aggravated by barium-based compounds.
**Limitations:** No limitations were identified.
**Ethics committee approval:** No ethics committee approval was needed.
**Funding for this study:** No funding was received for this study.
**Author Disclosures:**
Francesco Testa: Nothing to disclose
Giuseppe Di Guardia: Nothing to disclose
Paolo Lasciarrea: Nothing to disclose
Valter Verna: Nothing to disclose
Michele Lo Bello: Nothing to disclose

### RPS 2401b-6

**Magnetic resonance imaging (MRI) and colonoscopy in evaluation of colorectal diseases**

*R. Rastogi*, V. Khare, A. Mishra; Moradabad/IN
*(rajulrst@yahoo.co.in)*

**Purpose:** Colorectal disease, especially carcinoma, are an important cause of morbidity and mortality in the modern era. With the rising incidence of colorectal diseases and due to limitations of conventional flexible fiberoptic colonoscopy (gold standard tool), imaging plays a significant role in the evaluation of these patients. Recent developments in magnetic resonance imaging (MRI) coupled with its advantages of noninvasive and radiation-free nature, it has recently become a screening tool in colorectal diseases.
**Methods or Background:** Forty-four patients with signs and symptoms of the colorectal disease were evaluated by 1.5T MRI followed by conventional, flexible, fiberoptic colonoscopy on the same day. Bowel preparation was done using polyethene glycol. Data from MRI and colonoscopy were recorded and compared with the final diagnosis.
**Results or Findings:** The majority of patients in the study were in the 21-40yrs age group with male predominance. Altered bowel habits followed by bleeding per rectum were the commonest presentations. Both MRI and colonoscopy overdiagnosed the lesions as malignant with higher errors by MRI. MRI was very effective in the detection of growth, strictures, diverticulosis, mucosal thickening/oedema and extracolonic manifestation but failed in detecting small polyps and ulcers. MRI had high sensitivity and negative predictive value of 100% with an accuracy of more than 70%.
**Conclusion:** MRI with its noninvasive and radiation-free nature along with its high sensitivity and negative predictive value for malignant lesions should be considered over colonoscopy as well as computed tomography in the evaluation of colorectal diseases.
**Limitations:** The study was performed on 1.5T MR scanner.
**Ethics committee approval:** The ethics committee approval was obtained.
**Funding for this study:** No funding was provided for this study.
**Author Disclosures:**
Rajul Rastogi: Nothing to disclose
Vaibhav Khare: Nothing to disclose
Amit Mishra: Nothing to disclose

### RPS 2401b-7

**Magnetic resonance imaging in the local staging of colon cancer: a prospective, blinded study**

*S. R. Rafaelsen*, C. Dam, C. Vagn-Hansen, J. Møller, H. Rahr, M. Sjöström, J. Lindebjerg, T. F. Hansen, M. R. V. Pedersen; Vejle/DK
*(soeren.rafael.rafaelsen@rsyd.dk)*

**Purpose:** The purpose of this study was to investigate the sensitivity and specificity of magnetic resonance imaging (MRI) compared to CT scan.
**Methods or Background:** Patients underwent a standard CT scan. For the MRI scan, a 3 Tesla unit was used, including diffusion-weighted imaging (DWI), with an average scan time of 25 minutes. No intravenous, oral or rectal contrast medium was administered. Prior to the study both the CT and MRI radiologist had received feedback from the pathologist at a weekly colorectal MDT meeting five years prior to inclusion of the first patient. McNemar's test was used to compare the two modalities.
**Results or Findings:** From 2018 to 2021, 134 patients had CT and MRI scans. CT failed to detect 16/134 (12%) colon tumours, half of which were pT3 tumours. whereas MRI detected all tumours. For discriminating between stage T3ab and T3cd the sensitivity of CT was 51.1% and of MRI 80.0% (p = 0.02). CT and MRI had a sensitivity of 21.4% and 46.4% in detecting pT4 tumours and a specificity of 79.0% and 85.0%, respectively. For the evaluation of extravascular involvement, the sensitivity and specificity of CT was 35.0% and 82.0%, whereas MRI had higher values of 50.0% and 81.8%, respectively.

**Conclusion:** MRI detected colonic tumours more often than CT. Compared to CT the sensitivity of MRI was statistically significantly higher in detecting advanced T3cd and T4 tumours. MRI has the potential to be used in the treatment planning of colon cancer.
**Limitations:** Inter-observer variation was not evaluated.
**Ethics committee approval:** The study was approved by the local Science Ethical Committee, and all patients signed the informed consent.
**Funding for this study:** This study was funded by the Danish Cancer Society, MRP grant nr. R231-A14077, and the Region of Southern Denmark, E-fond 152.
**Author Disclosures:**
Chris Vagn-Hansen: Nothing to disclose
Hans Rahr: Nothing to disclose
Mikkel Sjöström: Nothing to disclose
Malene Roland Vils Pedersen: Nothing to disclose
Torben F Hansen: Nothing to disclose
Claus Dam: Nothing to disclose
Jakob Møller: Nothing to disclose
Jan Lindebjerg: Nothing to disclose
Sören R. Rafaelsen: Nothing to disclose

### RPS 2401b-8

**Dose optimisation of iodinated contrast media in the preoperative evaluation of the venous system before CME for right colon cancer**
*L. Asmundo*, M. Marongiu, F. Rizzetto, L. A. Carbonaro, A. Vanzulli; Milan/IT
*(luigi.asm@gmail.com)*

**Purpose:** The objective of this study was to optimise the dose of iodinated contrast media for CT imaging of venous colic vessels for preoperative evaluation before a complete mesocolic excision (CME) technique to treat right colon cancer.
**Methods or Background:** 150 patients with an abdominal venous phase CT scan performed February-April 2021 were retrospectively selected: 50 with i.v. administration of Iobitridol (350mg/ml), 50 with Iohexol (370mg/ml), 50 with administration of Iohexol (350mg/ml). Enhancement values of the superior mesenteric vein, of the right colic vein, and of the ileocolic vein were evaluated with ROIs.
**Results or Findings:** We did not find any significant difference between the groups, in terms of weight of the patients (p=0.504), administered contrast dose (p=0.504), dose of iodine per kilogram (p=0.228), a dose of contrast medium per kilogram (p=0.786). The upper quartile enhancement thresholds (>75%) for each vein were: superior mesenteric vein (170 HU), ileocolic vein (134 HU), right colic vein (102 HU). The average dose (iodium/kg) for upper quartile enhancement thresholds (>75%) of the three veins (564, 557 and 549 mg/kg for superior mesenteric, ileocolic and right colic veins, respectively) proved to be higher than the average dose for the lower percentile (<25%) enhancement thresholds (p<0.001).
**Conclusion:** In order to get the highest enhancement values, a contrast media dose of 1.48 cc/kg of Ioheol 370, and 1.57 cc/kg of Iobitridol 350 and Iohexol 350, should be administered to enable us to outline a protocol of CT image acquisition in order to improve the visualisation of the right colic vein vascular map, before CME.
**Limitations:** This is a retrospective study and no other technical parameters such as the injection flow, nor patients' parameters such as body mass index were evaluated.
**Ethics committee approval:** This study was approved by the local ethical commitee.
**Funding for this study:** No funding was received for this study.
**Author Disclosures:**
Francesco Rizzetto: Nothing to disclose
Luca Alessandro Carbonaro: Nothing to disclose
Angelo Vanzulli: Nothing to disclose
Marta Marongiu: Nothing to disclose
Luigi Asmundo: Nothing to disclose

**11:30-12:30** **Channel 4**

### Research Presentation Session: Imaging Informatics / Artificial Intelligence and Machine Learning

### RPS 2405a

### Artificial intelligence (AI) in cardiovascular imaging

**Moderators**
M. F. Russe; Freiburg/DE
C. Catalano; Rome/IT

### RPS 2405a-3

**Deep convolutional neural networks improve the long term prediction of major cardiovascular events after coronary computed tomography angiography**
*A. Chami*, C. v. Schack, R. Adolf, N. Nano, E. Hendrich, A. Will, S. Martinoff, M. Hadamitzky; Munich/DE
*(alessa.chami@gmx.de)*

**Purpose:** Coronary Computed Tomography Angiography (CCTA) is an established modality for assessing coronary artery disease (CAD). Its role for prognosis assessment is still limited. Deep convolutional neural networks (CNNs) might improve this process by using plaque characteristics that are currently not used.
**Methods or Background:** The Consecutive CCTAs from patients with suspected CAD examined between October 2004 and January 2018 were analyzed. The primary endpoint was a composite of all-cause mortality, myocardial infarction and late revascularization. The training endpoint additionally included early revascularization. The clinical risk was assessed by Morise score; for conventional CCTA assessment, extent of CAD (eoCAD) and segment involvement score (SIS) were used. Semiautomatic post-processing was performed for vessel delineation and annotation of calcified and non-calcified plaque areas. Two-step training of a densenet-121 CNN was done: The full network was trained using the training endpoint, then the feature layer was trained using the primary endpoint. Five times cross-validation was performed to ensure that each CNN was evaluated on an unseen set of data.
**Results or Findings:** The study population comprised 5468 patients. During the follow-up of 7.2 years, 334 patients reached the primary endpoint; in addition, 405 early revascularizations occurred. The outcome correlation of CNN showed an AUC of 0.720±0.010 and 0.631±0.015 for training endpoint and primary endpoint resp. Combining CNN with conventional CT parameters showed an improvement of AUC from 0.791 to 0.821 (p<0.0001) and from 0.766 to 0.773 (p<0.0001) for eoCAD and SIS resp. In a stepwise model including clinical risk, conventional CT parameters and CNN, the latter improved the prediction from 0.813 to 0.819 (p=0.0022) and from 0.772 to 0.775 (p<0.0013) for eoCAD and SIS resp.
**Conclusion:** CNNs are a promising tool to further improve the prediction of major cardiovascular events after CCTA.
**Limitations:** No limitations identified.
**Ethics committee approval:** Ethics committe approval was received.
**Funding for this study:** No funding was received for this study.
**Author Disclosures:**
Alessa Chami: Nothing to disclose
Nejva Nano: Nothing to disclose
Martin Hadamitzky: Nothing to disclose
Claudio v. Schack: Nothing to disclose
Rafael Adolf: Nothing to disclose
Stefan Martinoff: Research/Grant Support: Martinoff reports receiving unrestricted research grants from Siemens Healthineers.
Albrecht Will: Nothing to disclose
Eva Hendrich: Nothing to disclose

### RPS 2405a-4

**Improving the degree of enhancement in coronary computed tomography angiography with a patient-specific trigger delay bolus tracking in a third-generation dual-source scanner**
*Y. Wang*; Zhengzhou/CN
*(13298363780@163.com)*

**Purpose:** To compare coronary CT angiography (CCTA) contrast opacification between a fixed trigger delay and patient-specific trigger delay bolus tracking in a third-generation dual-source scanner.
**Methods or Background:** 100 consecutive patients were randomly divided into two groups to perform CCTA scans in the bolus tracking method; group A

with a fixed trigger delay time of 5 seconds and group B with an automatic patient-specific trigger delay time estimated from monitored CT values. All CT scanning and contrast media injection protocol parameters were kept identical. CT value of aorta root (AO), coronary segments and superior vena cava (SVC) were measured for the objective image quality evaluation. Subjective evaluation of the image quality was performed by two independent blinded reviewers using a 5-point scale (5 = excellent, 1 = poor). Independent sample t-test and the Wilcox-Mann-Whitney test were used to compare quantitative and qualitative data, respectively.
**Results or Findings:** The trigger delay time in group B ranged from 4-8 seconds (mean, 6.6±1.4 seconds). Group B had higher mean enhancement in AO and coronary (407±61Hu vs 360±48Hu for pRCA, 423±58Hu vs 367±57Hu for pLAD, and 408±55Hu vs 351±56Hu for pLCX, all p＜0.05) than group A. The opacification of the SVC was significantly lower in group B than in group A (147Hu vs 261Hu; p<0.05). Subjective image quality was higher in group B than in group A (4.5 vs 4.1; p<0.05).
**Conclusion:** Compared with a fixed delay time, a patient-specific trigger delay bolus tracking estimation provided significantly higher attenuation and improved the image quality for coronary CT angiography.
**Limitations:** The diagnostic accuracy of our study needs to be further validated against invasive angiography.
**Ethics committee approval:** This study protocol was approved by the local ethics committee.
**Funding for this study:** No funding was received for this study.
**Author Disclosures:**
Yiran Wang: Nothing to disclose

### RPS 2405a-5

**The use of a graph convolutional neural network model based on fundus photograph derived vascular biomarkers to predict coronary artery disease based on the CT CAD-RADS scores**

F. Huang, J. Lian, K. S. NG, *V. Vardhanabhuti*; Hong Kong/HK

**Purpose:** The purpose of this study is to utilize a graph convolutional neural network (GCN) to predict the coronary artery disease reporting and data system (CAD-RADS) based on coronary CT angiography (CCTA) using the quantitative vascular biomarkers derived from fundus images of the same subjects.
**Methods or Background:** This prospective single-centre study included 145 subjects who had received both CCTA and fundoscopy examinations on the same day in our local imaging centre in 2019. The CCTA scans were stratified by CAD-RADS scores by expert readers, which were then binarized into two classes (i.e. 0 (normal), 1 (minimal) and 2 (mild) were in class 0, and 3 (moderate), 4 (severe) and 5 (occluded) were in class 1). The vascular biomarkers were extracted from their eye images using a retinal health information and notification system. A graph was constructed, where each graph nodes represented a fundus image, and the node features were the vascular biomarkers relevant to blood vessel width and curvature. The graph edges were determined by the similarity of age and gender of paired subjects. A GCN model was employed on the graph to predict the binarized CAD-RADS score for each node. The image data of 115 subjects (80%) were used for training and 30 subjects (20%) for testing. We also trained multiple traditional machine learning models for comparison.
**Results or Findings:** The GCN model showed a sensitivity, specificity, accuracy and area under the curve of 75%, 81.03%, 79.27% and 0.864, respectively. The performance outperforms the same evaluation metrics obtained from the traditional machine learning models (p<0.01).
**Conclusion:** The changes in fundus vasculature had potential predictive value for CAD-RADS scores and significant coronary artery diseases.
**Limitations:** Small sample size. Proof of concept study.
**Ethics committee approval:** Approved by the local institution.
**Funding for this study:** No funding was received for this study.
**Author Disclosures:**
Varut Vardhanabhuti: Nothing to disclose
Jie Lian: Nothing to disclose
Kei Shing NG: Nothing to disclose
Fan Huang: Nothing to disclose

### RPS 2405a-6

**Image preprocessing and filtering effect on the estimate of myocardial radiomic features from T1 and T2 mapping in hypertrophic cardiomyopathy**

*D. Marfisi*[1], C. Tessa[2], C. Marzi[3], J. Del Meglio[4], S. Linsalata[1], C. Vignali[4], G. Casolo[4], A. C. Traino[1], M. Giannelli[1]; [1]Pisa/IT, [2]Massa/IT, [3]Sesto Fiorentino/IT, [4]Lido di Camaiore/IT

**Purpose:** Radiomics, often combined with artificial intelligence techniques, is emerging as a promising and useful tool for unveiling myocardial tissue characteristics in cardiac magnetic resonance (CMR) imaging. Nonetheless, its proper application deserves some caution and a preliminary assessment of possible radiomic feature dependence on various factors, since each step of the radiomic workflow could influence feature estimation. Accordingly, the purpose of this study was to investigate, for the first time, the effect of image preprocessing and filtering on radiomic feature estimation from quantitative CMR T1 and T2 mapping.
**Methods or Background:** Specifically, T1/T2 maps of 26 patients with hypertrophic cardiomyopathy (HCM) were used to estimate 98 myocardial radiomic features for 7 different resampling voxel sizes (at fixed bin width), 9 different bin widths (at fixed resampling voxel size), and 7 different spatial filters (at fixed resampling voxel size/bin width).
**Results or Findings:** While we found a remarkable dependence of myocardial radiomic features from T1/T2 mapping on image filters, many radiomic features showed a limited sensitivity to resampling voxel size/bin width in terms of intraclass correlation coefficient (>0.75) and coefficient of variation (<30%). The estimate of several textural radiomic features showed a linear significant (p<0.05) correlation with resampling voxel size/bin width.
**Conclusion:** Overall, radiomic features from T2 maps have proven to be less sensitive to image preprocessing than those from T1 maps, especially when varying bin width. Our results might corroborate the potential of radiomics from T1/T2 mapping in HCM and hopefully in other myocardial diseases.
**Limitations:** This was a single-centre study of a relatively small cohort of HCM patients.
**Ethics committee approval:** The study was approved by the local ethics committee of the Azienda USL Toscana Nord Ovest (Pisa, Italy).
**Funding for this study:** This study received no external funding.
**Author Disclosures:**
Stefania Linsalata: Nothing to disclose
Carlo Tessa: Nothing to disclose
Chiara Marzi: Nothing to disclose
Giancarlo Casolo: Nothing to disclose
Antonio Claudio Traino: Nothing to disclose
Jacopo Del Meglio: Nothing to disclose
Marco Giannelli: Nothing to disclose
Daniela Marfisi: Nothing to disclose
Claudio Vignali: Nothing to disclose

### RPS 2405a-7

**Fully automated left ventricular late gadolinium enhancement detection by a convolutional neuronal network in chronic myocardial infarction**

*M. J. Pamminger*, D. Obmann, C. Kremser, P. Poskaite, F. Troger, S. Reinstadler, B. Metzler, M. Haltmeier, A. Mayr; Innsbruck/AT
*(Mathias.Pamminger@tirol-kliniken.at)*

**Purpose:** To compare fully automated segmentation of left ventricular late gadolinium enhancement (LGE) as evaluated by a convolutional neuronal network (CNN) with manual segmentation in chronic myocardial infarction.
**Methods or Background:** Cardiac magnetic resonance imaging, including two-dimensional LGE imaging, was performed in 191 patients on a 1.5 T clinical scanner 12 months after ST-elevation myocardial infarction. LGE images were presented to a trained CNN for automated determination of left ventricular myocardium and consequently LGE volume. Manual LGE segmentation according to the +5-SD method was used as the reference standard. Image quality was assessed according to a 3-point Likert scale (2 = perfect image quality, 1 = some artefacts without impaired LGE delineation, 0 = strong artefacts with impaired LGE delineation). Regression and Bland-Altman analyses were performed.
**Results or Findings:** In 191 included patients (182 male, mean age 57 years), the LGE volume was 9.7 [IQR 3.6 to 16.2] cm3 according to manual segmentation and 8.3 [3.2 to 17.6] cm3 according to CNN segmentation. The Bland-Altman analysis showed little average difference (-0.5 cm3, p=0.257), however, the limits of agreement ranged from -18.4 cm3 to 17.5 cm3. The linear correlation was fair (0.57, p<0.001). The subgroup analysis according to the image quality showed comparable performance of CNN segmentation in all three groups.
**Conclusion:** Our fully automated LGE segmentation based on a CNN in two-dimensional data sets provides measurements with little average difference compared to very time-consuming manual segmentations. However, dispersion is substantially and limits the current application of this approach on a per-patient basis. Image quality does not affect CNN performance.
**Limitations:** Manual segmentation according to the +5-SD method is dependent on investigator experience and is limited in circumferential myocardial LGE.
**Ethics committee approval:** Local ethics committee approval was provided.
**Funding for this study:** No funding was received for this study.
**Author Disclosures:**
Markus Haltmeier: Nothing to disclose
Paulina Poskaite: Nothing to disclose
Mathias Josef Pamminger: Nothing to disclose
Felix Troger: Nothing to disclose
Daniel Obmann: Nothing to disclose
Sebastian Reinstadler: Nothing to disclose
Agnes Mayr: Nothing to disclose
Christian Kremser: Nothing to disclose
Bernhard Metzler: Nothing to disclose

### RPS 2405a-8

**Prediction of low-keV monoenergetic images from dual-energy spectral CT to improve the automatic detection of pulmonary embolism in single-energy CT scans**

*M. A. Fink*[1], C. Seibold[2], H-U. Kauczor[1], R. Stiefelhagen[2], J. Kleesiek[3]; [1]Heidelberg/DE, [2]Karlsruhe/DE, [3]Essen/DE
*(matthias.fink@med.uni-heidelberg.de)*

**Purpose:** We aimed to develop a deep learning (DL) model based on detector-based spectral dual-energy angiography CT (DE-CTPA) data, yielding predictions of low-keV acquisitions to improve automatic pulmonary embolism (PE) detection in conventional single-energy CT scans.
**Methods or Background:** We used two data sets: our institutional DE-CTPA data set D1 comprising standard arterial series and the corresponding virtual monoenergetic images (VMI) at low-energy levels (40 keV) with 7,892 image pairs, and a 10% subset of the RSNA Pulmonary Embolism Detection Challenge (2020) data set D2, which consists of 161,253 polyenergetic images with dichotomous slice-wise annotations (PE/no PE). We trained a fully convolutional 9-block ResNet encoder-decoder network to generate VMI predictions from D1, which are then fed into a ResNet50 network for the PE classification task on single-energy CT scans from D2. We evaluated our VMI reconstruction results in terms of Peak-Signal-to-Noise-Ratio (PSNR) and Structural Similarity Index Measure (SSIM). For PE identification, we performed a binary classification on slice level and reported the area under the curve (AUC).
**Results or Findings:** The quantitative results on the reconstruction ability of the DL model revealed high-quality visual VMI predictions with reconstruction results of 0.984 ± 0.002 (SSIM) and 41.706 ± 0.547 (PSNR). The PE classification yielded an AUC of 0.84 for our framework, which improves PE classification compared to other naïve PE classification approaches with AUCs up to 0.81.
**Conclusion:** Our results demonstrate that the prediction of synthetic VMI from polyenergetic CT scans can improve the automated detection of PE. This could help rescue CTPA studies with suboptimal opacification of the pulmonary arteries from single-energy CT scanners.
**Limitations:** Class imbalance per subset could bias the results.
**Ethics committee approval:** The study was approved by our IRB (S-236/2020).
**Funding for this study:** No funding was received for this study.
**Author Disclosures:**
Jens Kleesiek: Nothing to disclose
Matthias Alexander Fink: Nothing to disclose
Hans-Ulrich Kauczor: Nothing to disclose
Rainer Stiefelhagen: Nothing to disclose
Constantin Seibold: Nothing to disclose

### RPS 2405a-9

**Vessel segmentation on non-contrast liver MRI**

*D. Sobotka*, A. J. Herold, M. Perkonigg, L. Beer, N. Bastati-Huber, A. Sablatnig, A. Ba-Ssalamah, G. Langs; Vienna/AT

**Purpose:** Liver vessel segmentation in MR imaging is crucial for the computational analysis of vascular remodelling. Existing techniques rely on contrast-enhanced MR (MRce), which are not uniformly acquired. Non-contrast images are acquired more frequently, but vessels are hard to distinguish from other structures due to lack of opacification. Here, we propose a convolutional neural network to segment liver vessels on non-contrast images with the help of auxiliary contrast-enhanced data available only during training. The approach improves segmentation accuracy on non-contrast images and reduces the need for annotated examples.
**Methods or Background:** A multi-task learning approach trains a Y-net style convolutional neural network for liver vessel segmentation on MR imaging data. During training, MR with and without contrast together with vessel annotations on a sub-set of the data are available. The auxiliary MRce provides variability to the encoder training of the model. This improves the vessel segmentation accuracy, even if no MRce is available during application. We investigate overall- and vessel thickness specific segmentation accuracy.
**Results or Findings:** Using auxiliary contrast-enhanced sequences improves the Dice score for vessel segmentation on non-contrast MR by 0.10 from 0.45 to 0.55. For small vessels (0-10 mm), the score increases by 0.03, for bigger vessels (>10mm) by 0.13.
**Conclusion:** Highly-informative contrast-enhanced sequences improve vessel segmentation models for non-contrast imaging data. It allows for a reduction of the number of annotated examples necessary for vessel segmentation model training.
**Limitations:** Number of vessel annotations used in evaluating the proposed framework.
**Ethics committee approval:** The local ethics committee approved this study protocol (EK 2027/2017), which was performed in accordance with the Helsinki Declaration.
**Funding for this study:** This study was partially funded by Austrian Science Fund (FWF): P 35189, Vienna Science and Technology Fund (WWTF): LS20-065, Novartis Pharmaceuticals Corporation.
**Author Disclosures:**
Alexander Johannes Herold: Nothing to disclose
Georg Langs: Nothing to disclose
Daniel Sobotka: Nothing to disclose
Lucian Beer: Nothing to disclose
Ahmed Ba-Ssalamah: Nothing to disclose
Matthias Perkonigg: Nothing to disclose
Nina Bastati-Huber: Nothing to disclose
Alina Sablatnig: Nothing to disclose

**13:00-14:00** **Channel 2**

### Research Presentation Session: Interventional Radiology

### RPS 2509

### Interventional neuroradiological management

**Moderator**
I. Gil; Lisbon/PT

### RPS 2509-4

**Clinical consequence of vessel perforations during endovascular treatment for acute ischaemic stroke**

*M. van der Sluijs*[1], R. Su[1], J. Hofmeijer[2], T. van Walsum[1], G. Lycklama[3], A. van Es[4], S. Cornelissen[1], A. Van Der Lugt[1]; [1]Rotterdam/NL, [2]Arnhem/NL, [3]The Hague/NL, [4]Leiden/NL

**Purpose:** Endovascular treatment of acute ischaemic stroke can be complicated by vessel perforation. In this work we study the incidence of this specific complication in clinical practice and its effects on functional outcome, including the relation with the location of a vessel perforation.
**Methods or Background:** All patients in the MR CLEAN Registry who underwent EVT were analysed for the presence of vessel perforation. DSA imaging of cases mentioned by interventionalist or corelab were studied. Additionally, DSAs of SAH cases were reassessed for potential vessel perforations. In cases where an interventionalist mentioned a perforation, but corelab did not find any, perforation was assumed. Functional outcome was measured using the modified Rankin Scale (mRS) at 90 days. The association between vessel perforation and outcome was analysed with ordinal logistic regression models adjusted for confounding parameters, such as NIHSS at baseline, reperfusion and collaterals. Results were described as unadjusted common (cOR) and adjusted common odds ratio (acOR).
**Results or Findings:** Vessel perforation occurred in 74 (2,7%) of 2794 patients who underwent EVT. The proportion of vessel perforations in females was higher compared to non-perforation cases. (63.5% vs 47.5% p=0.009). Anatomical location of perforations was located respectively in ICA-M1 (35%), M2-M3 (45%), posterior (6.3%) and missing in 14.9% of cases. Functional outcome (mRS) was worse in patients with vessel perforations (cOR 0.31, 95%CI 0.20-0.49, acOR 0.50, 95%CI 0.29-0.85) compared to patients without a vessel perforation. No association was observed with anatomical location proximal vs distal (cOR 2.36, 95%CI 0.83-6,73, acOR 1.10, 95%CI 0.29-4.17).
**Conclusion:** Incidence of vessel perforation during EVT is low, but has severe clinical consequences, regardless of the anatomical location of the vessel perforation.
**Limitations:** Potential bias by reviewing SAH patients, therefore, a higher chance of poor outcome.
**Ethics committee approval:** Not applicable.
**Funding for this study:** Not applicable.
**Author Disclosures:**
Theo van Walsum: Nothing to disclose
Ruisheng Su: Nothing to disclose
Aad Van Der Lugt: Nothing to disclose
J. Hofmeijer: Nothing to disclose
Matthijs van der Sluijs: Nothing to disclose
Adriaan van Es: Nothing to disclose
Geert Lycklama: Nothing to disclose
Sandra Cornelissen: Nothing to disclose

### RPS 2509-5

**Analysis of the clinical results of the endovascular treatment of indirect carotid-cavernous fistulae**

*J. Rodríguez Castro*, L. Martínez Camblor, M. Martínez-Cachero García, S. Budiño Torres, E. Murias Quintana, J. M. Jiménez Pérez, J. Chaviano Grajera, F. García Arias, P. Vega Valdés; Oviedo/ES
*(jorocas03@gmail.com)*

**Purpose:** The purpose of this study was to (1) demonstrate that endovascular treatment of indirect carotid-cavernous fistulae is effective and stable in the long term, (2) describe the techniques and materials most used in our centre, (3) evidence that this treatment reduces patient's symptomatology, and (4) display the safety of this treatment.
**Methods or Background:** A retrospective observational study was carried out. A database was made with 39 interventions in 32 patients with indirect carotid-cavernous fistulae treated in our centre from 2006 to 2020. A collection of epidemiological, clinical, intervention-related, and postoperative variables was made for subsequent statistical analysis.
**Results or Findings:** In 28 (72%) of the interventions, complete closure of the fistula was achieved, with 26 patients (81.3%) being achieved in the initial intervention. The fistula was closed with stable treatment in 92% of the cases at 6 months. The access route was also analysed, the most frequent being the venous route through the inferior petrosal sinus (71.9%). Coils were the preferably used material (84.6%). Regarding the improvement of symptoms at 6 months after the intervention, 29 patients (74.3%) had a complete remission of symptoms. Complications were associated barely to 7.7% of the interventions.
**Conclusion:** Endovascular treatment of indirect carotid-cavernous fistulae is effective and stable in the long term. The most used and effective access route in our centre is the venous one through the inferior petrosal sinus. The most widely used and effective material for closing indirect carotid-cavernous fistulae are coils. Most of the patients had symptomatic improvement immediately after the intervention and nearly all had no symptoms 6 months after the procedure. Endovascular treatment is a technique that has few perioperative complications and does not usually require reoperation.
**Limitations:** This study has a small sample size.
**Ethics committee approval:** The ethics committee approval was obtained.
**Funding for this study:** No funding was needed.
**Author Disclosures:**
Luis Martínez Camblor: Nothing to disclose
Pedro Vega Valdés: Nothing to disclose
José María Jiménez Pérez: Nothing to disclose
Juan Chaviano Grajera: Nothing to disclose
Sara Budiño Torres: Nothing to disclose
Miguel Martínez-Cachero García: Nothing to disclose
José Rodríguez Castro: Nothing to disclose
Eduardo Murias Quintana: Nothing to disclose
Faustino García Arias: Nothing to disclose

### RPS 2509-6

**Patients' perception and satisfaction of Vim MRgFUS thalamotomy: comparative evaluation of the influence of interactive video-assisted vs standard informed consent**

*L. Pertici*, F. Sgalambro, V. Pagliei, F. Bruno, A. Gagliardi, C. Fagotti, A. Barile, A. Splendiani, C. Masciocchi; L'Aquila/IT
*(leonardo.pertici@gmail.com)*

**Purpose:** MRgFUS thalamotomy for the treatment of tremor in ET and PD is usually perceived as a simple procedure by patients, who fail to consider it as an ablative procedure with some risks, that, therefore, requires strong compliance from patients. We evaluated the influence of interactive video-assisted vs standard informed consent on patients' treatment perception, understanding and satisfaction.
**Methods or Background:** We prospectively evaluated 58 patients eligible for MRgFUS thalamotomy. Before treatment, patients were randomly assigned to two groups: group A (28 patients, 15 males, mean age 65 y/o, ET/PD 18/10) received the standard written informed consent and group B (30 patients, 16 males, mean age 64 y/o, ET/PD 19/11) the video-assisted consent. Two questionnaires were then given to all study participants: the first one at the end of the consent process (5 items, score 0-4, total score 20), assessing patients' understanding of the procedure, the second one at the end of the treatment (2 items, score 0-4, total score 8), assessing patients' perception and satisfaction based on the expectations they had after the consent information received.
**Results or Findings:** In ET patients, mean total understanding and satisfaction scores were 25.2 and 27.6 in groups B and A respectively (p=.234). In PD patients, scores were 23 and 28 in groups B and A. In younger patients (28-75 y/o) scores were 21 (A) and 28 (B), while in the older patients' group (>75yo) 25 and 22.
**Conclusion:** Video-assisted integrated informed consent increases understanding of the procedure and its risks, as well as satisfaction regarding the treatment, especially in younger patients. In older patients and individuals with mild cognitive impairment, the computer interaction may represent a limitation compared to direct communication.
**Limitations:** Not applicable.
**Ethics committee approval:** Not applicable.
**Funding for this study:** Not applicable.
**Author Disclosures:**
Alessio Gagliardi: Nothing to disclose
Ferruccio Sgalambro: Nothing to disclose
Alessandra Splendiani: Nothing to disclose
Carlo Masciocchi: Nothing to disclose
Leonardo Pertici: Nothing to disclose
Cristina Fagotti: Nothing to disclose
Antonio Barile: Nothing to disclose
Federico Bruno: Nothing to disclose
Valeria Pagliei: Nothing to disclose

### RPS 2509-7

**A novel thrombectomy device: an in vitro evaluation of a prototype catheter**

*Y. Tanyildizi*, S. Krost-Reuhl, A. Heimann, O. Kempski, F. Hahn, R. Kloeckner, M. A. Brockmann; Mainz/DE

**Purpose:** This prototype catheter is a newly-developed distal access catheter featuring a self-expanding, flexible, funnel-shaped tip. The purpose of its design is to reduce the risk of thrombus fragmentation during mechanical thrombectomy and improve first pass recanalisation (TICI 3). In this experimental setup, we preclinically evaluated the effectiveness and navigability of the new catheter.
**Methods or Background:** A vessel model was filled with a blood-like-viscous medium, and the image was projected with the corresponding vessel area by camera transmission to corresponding to the conditions in an angiography. Thrombi from porcine blood were placed into the arteria carotis interna of the vascular model and subsequently mechanically thrombectomised with a stent retriever. In the first part, the prototype was compared to a standard distal-access-catheter without using an external catheter. (N=20 for each catheter). In the second part, the prototype was inserted through a guiding catheter (n=11) to determine the navigability performance.
**Results or Findings:** In the first experimental series, mechanical thrombectomy was successful 19 out of 20 times (95% success rate) for the prototype catheter versus 15 out of 20 times (75% success rate) for the standard distal-access catheter. In the second experimental series, the prototype catheter achieved first-pass recanalisation 10 out of 11 times (91% success rate) and 1 out of 11 times at second pass (9%).
**Conclusion:** This series of experiments demonstrated higher first-pass recanalisation rates for the newly-developed funnel-shaped prototype featuring a self-expanding tip in comparison to a cylindrical standard distal-access-catheter.
**Limitations:** This study is limited by (1) no in vivo testing and (2) the limited number of thrombectomies.
**Ethics committee approval:** The ethics committee approved this study (AZ G 14-1-093 and AZ 23177 – 07 A16 -1-001 AFW).
**Funding for this study:** This study was funded by the WIPANO (Wissens- und Technologietransfer durch Patente und Normen) and Bundesministerium für Wirtschaft und Energie.
**Author Disclosures:**
Yasemin Tanyildizi: Nothing to disclose
Axel Heimann: Nothing to disclose
Samantha Krost-Reuhl: Nothing to disclose
Roman Kloeckner: Nothing to disclose
Marc A Brockmann: Nothing to disclose
Felix Hahn: Nothing to disclose
Oliver Kempski: Nothing to disclose

**13:00-14:00** **Channel 3**

### Research Presentation Session: Neuro

### RPS 2511

### Artificial intelligence (AI) meets the brain

**Moderator**
T. Nunes; Loures/PT

### RPS 2511-2

**AI-enhanced multi-shot multi-contrast EPI protocol: a preliminary clinical experience**

*S. Pistocchi*[1], T. Hilbert[1], D. Rodriguez[1], B. Clifford[2], T. Feiweier[3], Z. Hosseini[4], S. Cauley[2], V. Dunet[1], T. Kober[1]; [1]Lausanne/CH, [2]Boston, MA/US, [3]Erlangen/DE, [4]Atlanta, GA/US
*(silvia.pistocchi@chuv.ch)*

**Purpose:** The duration of MRI acquisitions is a major limitation of this imaging modality, but especially for time-sensitive applications such as stroke or when imaging non-compliant or very young patients. Here we aim to evaluate the image quality of a new fast AI-enhanced protocol utilising a prototype multi-shot, multi-contrast EPI sequence and compare it to the standard imaging protocol at our institution.
**Methods or Background:** Between the 1st and 31st of June 2021, the AI-enhanced multi-shot multi-contrast EPI prototype sequence was added to our standard protocol in 30 brain MRI examinations with mixed clinical indications. The prototype sequence provided five contrasts (2D sagittal T1, axial FLAIR, T2GE, DWI) in a total of two minutes of scan time. Images were prospectively reviewed and independently compared to the standard 7:30 min: sec protocol by two experienced neuroradiologists. Six items (overall image quality, grey-white matter interface, basal ganglia delineation, sulci, motion, and susceptibility artefacts) were assessed on each generated contrast using a 4-point Likert scale. Inter-observer concordance was assessed using the Gwet AC1 coefficient.
**Results or Findings:** The AI-enhanced multi-shot multi-contrast EPI protocol allowed a 73% reduction of acquisition time and showed good to excellent overall image quality (mean score ≥3). Inter-observer concordance was good to excellent (Gwet AC1: 0.52 to 1.0). Motion and susceptibility artefacts were mostly rated as absent or minor with no adverse effect on diagnostic use, but with more heterogeneous inter-observer concordance (Gwet AC1: 0.27 to 0.83).
**Conclusion:** The AI-enhanced multi-shot multi-contrast EPI protocol demonstrated good image quality with a 73% reduction in acquisition time. Further studies evaluating diagnostic performance in time-sensitive clinical applications should be planned.
**Limitations:** This study is monocentric and has a small sample size.
**Ethics committee approval:** Not applicable.
**Funding for this study:** No funding has been used for this study.
**Author Disclosures:**
Stephen Cauley: Nothing to disclose
David Rodriguez: Nothing to disclose
Bryan Clifford: Employee: BC is employee of Siemens Healthcare
Tobias Kober: Employee: TK is employee of Siemens Healthcare
Vincent Dunet: Nothing to disclose
Tom Hilbert: Employee: TH is employee of Siemens Healthcare
Zahra Hosseini: Employee: ZH is employee of Siemens Healthcare
Silvia Pistocchi: Nothing to disclose
Thorsten Feiweier: Employee: TF is employee of Siemens Healthcare

### RPS 2511-3

**Comparison of image quality improvements among deep learning reconstruction, hybrid-type and model-based iterative reconstruction on brain contrast-enhanced CT angiography for ultra-high-resolution CT**

*K. Murayama*[1], Y. Ohno[1], H. Ikeda[1], H. KImata[2], N. Akino[2], K. Fujii[2], Y. Kataoka[1], A. Katagata[1], H. Toyama[1]; [1]Toyoake/JP, [2]Otawara/JP
*(kmura@fujita-hu.ac.jp)*

**Purpose:** To directly compare the capability for image quality improvements on brain contrast-enhanced CT angiography (CE-CTA) for ultra-high-resolution CT (UHR-CT) in intracranial aneurysms patients among deep learning reconstruction (DLR) and hybrid-type iterative reconstruction (IR) and model-based IR.
**Methods or Background:** 21 intracranial aneurysm patients underwent brain CE-CTA and reconstructed by DLR, hybrid-type IR and model-based IR using a UHR-CT system with super-high resolution mode (SHR: 0.25mm×160 rows/1792 channels). CT values at MCA were assessed by ROI measurements. Image J software was used to generate the profile curves. To assess the capability for improvement of spatial resolution with UHR-CT and DLR, full width at half maximum (FWHM), the width of the edge rise distance (ERD) and the edge rise slope (ERS) were measured at each vessel. For qualitative assessment, overall image quality, artefact, aneurysm, and vascular depiction levels were assessed by 5-point scales by two board-certified radiologists. CT values, ERS and all qualitative indexes were compared by Tukey's HSD test. Inter-observer agreements of each method were evaluated by kappa statistics with $\chi 2$ test.
**Results or Findings:** CT values and ERS of model-based IR and DLR were significantly higher than those of hybrid-type IR at MCA ($p<0.05$). Inter-observer agreement of each index by all methods was determined as moderate, substantial or excellent ($0.51 \leq \kappa \leq 0.92$, $p<0.001$). In addition, overall image quality and artefact of DLR were significantly improved as compared with others ($p<0.05$). Aneurysm and vascular depiction levels had no significant difference among all methods ($p>0.05$).
**Conclusion:** DLR has a potential for image quality improvements than hybrid-type and model-based IR on brain CE-CTA for UHR-CT.
**Limitations:** Not applicable.
**Ethics committee approval:** This retrospective study was approved by the Institutional Review Board of Fujita Health University.
**Funding for this study:** This study was financially supported by Canon Medical Systems Corporation.
**Author Disclosures:**
Akio Katagata: Nothing to disclose
Kazuhiro Murayama: Grant Recipient: Canon Medical SysteCorporation
Kenji Fujii: Employee: Canon Medical SysteCorporation
Hirotaka Ikeda: Nothing to disclose
Hirona KImata: Employee: Canon Medical SysteCorporation
Hiroshi Toyama: Grant Recipient: Canon Medical SysteCorporation
Yoshiharu Ohno: Grant Recipient: Canon Medical SysteCorporation
Yumi Kataoka: Nothing to disclose
Naruomi Akino: Employee: Canon Medical SysteCorporation

### RPS 2511-5

**Development and validation of a deep learning-based automatic brain volumetry for parkinsonian syndromes using 3D T1-weighted images**

*S. Kim*, C. Suh, H. Oh, E. P. Hong, S. Park, J. K. Sung, W. H. Shim, S. J. Kim; Seoul/KR
*(closea@naver.com)*

**Purpose:** To develop and validate a deep learning-based automatic brain volumetry (DLABV) for the differentiation of parkinsonian syndromes using 3D T1-weighted brain MR images.
**Methods or Background:** A DLABV was trained using a dataset of 3D T1-weighted brain MR images. 2D U-Net model was used for model architecture. The training dataset which contains 300 cognitively normal subjects (CN, 129 men) was labelled with FreeSurfer 6.0 brainstem substructure module. The test dataset consists of 207 CN, 52 progressive supranuclear palsy (PSP) patients, 65 multiple system atrophy (MSA) patients, and 189 Parkinson disease (PD) patients. The volume of the midbrain, pons, medulla, SCP, the midbrain-pons area ratio (MP) and the midbrain-pons volume ratio (MP_vol) were measured for differentiation of parkinsonian syndromes. Normalised volume using intracranial volume (ICV) was also used. To distinguish between each group, the receiver operating characteristic curve and area under the curve (AUC) was calculated and classification accuracy was measured by support vector machine (SVM).
**Results or Findings:** Compared with simple volumetry, volumetry using ICV normalisation showed more accurate performance in the differentiation of parkinsonian syndromes. The AUC in PSP vs PD using normalised midbrain volume was 0.89. In addition, the AUC in MSA vs PD using normalised pons volume was 0.97. MP_vol in MSA patients were significantly larger than in PSP patients and AUC was 0.98. Using normalised volume and MP showed highest classification accuracy.
**Conclusion:** The DLABV using ICV normalisation allowed an accurate differentiation of parkinsonian syndromes using 3D T1-weighted brain MR images.
**Limitations:** It is unclear whether the early parkinsonian syndromes can be differentiated using brain volumetry since our study did not target early parkinsonian syndrome patients.
**Ethics committee approval:** Our institutional review board approved this study.
**Funding for this study:** This study has received funding by the National Research Foundation of Korea.
**Author Disclosures:**
Jin Kyeong Sung: Author: 6th author
Eun Pyeong Hong: Author: 4th author
HyunWoo Oh: Author: 3rd author
Seongken Kim: Author: Presenter
Sang Joon Kim: Author: 8th author
Woo Hyun Shim: Author: 7th author
Sejin Park: Author: 5th author
ChongHyun Suh: Author: Corresponding author

### RPS 2511-7

**Patient-specific vs normative brain connectivity: a symptom-specific artificial intelligence-based comparison**

*Q. D. Strotzer*, J. Schlaier, A. Beer; Regensburg/DE

**Purpose:** Structural connectivity based on diffusion-weighted magnetic resonance imaging (DWI) is gaining importance in research and clinical use in fields like deep brain stimulation. Individual DWI is often unavailable. Therefore, normative connectomes based on averaged whole-brain tractography are a practical alternative. Comparisons of these concepts are sparse. Here, we compared patient-specific and normative approaches by their ability to predict the effects of deep brain stimulation using a symptom-specific, machine learning-based approach.
**Methods or Background:** Twenty-one patients who received bilateral subthalamic deep brain stimulation for Parkinson's disease were included. For every electrode contact (168 in total), we computed tractography patterns based on individual DWI and two normative connectomes (32 healthy individuals, 90 Parkinson's patients). Connectivity strength to 36 brain structures was calculated for every electrode contact, resulting in a dataset of 168 observations (electrode contacts) with 36 attributes (connectivity strength) for each connectome. Stimulation-associated symptom mitigation and side effects were assessed for every contact. We tested the prediction of stimulation outcomes based on connectivity strength using several supervised learning algorithms.
**Results or Findings:** Support vector machines yielded overall the best results. Averaged across all clinical classes (symptoms, side effects), the individual connectome achieved the highest area under the receiver operating characteristic curve (AUC-ROC; .81) compared to the normative healthy (.76) and disease-matched connectomes (.74). By clinical class, there were significant differences for paresthesia and autonomous side effects in favour of the individual connectome. Results differed considerably between clinical classes, from a mean AUC-ROC of 0.68 for paraesthesia to 0.91 for hyperkinesia.
**Conclusion:** Clinical effects may be mediated by different networks, as revealed by tractography methods based on DWI. Individual connectomes may be superior in predicting stimulation effectiveness.
**Limitations:** This study is done with single-centre data and has a limited sample size.
**Ethics committee approval:** Approval by the local ethics committee.
**Funding for this study:** No funding was received for this study.
**Author Disclosures:**
Juergen Schlaier: Nothing to disclose
Quirin D. Strotzer: Nothing to disclose
Anton Beer: Nothing to disclose

### RPS 2511-8

**Real-world evaluation of artificial intelligence software for cerebral large vessel occlusion detection in CT angiography**

*K. G. van Leeuwen*[1], R. Becks[1], S. Schalekamp[1], B. Van Ginneken[1], M. J. Rutten[2], M. De Rooij[1], F. J. A. Meijer[1]; [1]Nijmegen/NL, [2]'S-Hertogenbosch/NL
*(kicky.vanleeuwen@radboudumc.nl)*

**Purpose:** The commercially available AI tool (StrokeViewer v2, Nicolab) supports the diagnostic process of stroke by detecting large vessel occlusions (LVO) on CTA. We prospectively evaluated this tool in our department to monitor safety and impact.
**Methods or Background:** We implemented the software with the goal to improve the diagnosis of LVO and elevate the diagnostic confidence of the radiologist (resident). We used quantitative measures (data from clinical systems, vendor log files) and qualitative measures (user survey) to analyse diagnostic performance, number of users, login attempts, radiologists' diagnostic confidence, and user experience.
**Results or Findings:** In total, 226 CTAs with a clinical indication of stroke between January-June 2021 were prospectively evaluated. Thirteen cases of posterior circulation and distal vessel occlusions were excluded as they were outside the intended use of the AI tool. The AI tool missed 12 of the 36 occlusions in the middle cerebral or intracranial internal carotid artery (M1=1, M2=10, ICA=1) resulting in an accuracy of 86.4%. Irrespective of location, the sensitivity was 77.8% and specificity 90.4%. The number of monthly unique users varied between 8 and 24 radiologists/residents. Log in attempts dropped after the initial month (which included training) to a monthly average of 44 attempts. The diagnostic confidence did not increase during the use of the tool. The likelihood that users would recommend StrokeViewer to colleagues was rated 4.5/10.
**Conclusion:** Over six months, the use of StrokeViewer dropped and users did not sense improvement of diagnostic confidence. Measures have been taken to stimulate adoption for the latter six months of the trial period.
**Limitations:** Because of the prospective character, no comparison could be made between radiologists supported by AI vs radiologists without AI.
**Ethics committee approval:** Not applicable.
**Funding for this study:** Not applicable.
**Author Disclosures:**
Ruud Becks: Nothing to disclose
Frederick Jan Anton Meijer: Nothing to disclose
Bram Van Ginneken: Nothing to disclose
Kicky Gerhilde van Leeuwen: Nothing to disclose
Matthieu J.C.M. Rutten: Nothing to disclose
Steven Schalekamp: Nothing to disclose
Maarten De Rooij: Nothing to disclose

**13:00-14:00 Channel 4**

### Research Presentation Session: Chest

### RPS 2504

### Pleural disorders

**Moderator**
I. Vollmer; Barcelona/ES

### RPS 2504-2

**Ultrasound-guided percutaneous needle biopsy of pleural and peripheral lung lesion: comparison with computed tomography guided biopsy**

*B. K. Choudhury*; Guwahati/IN
*(choudhury60@gmail.com)*

**Purpose:** Ultrasound (US) guided needle biopsy is a very useful, easily available, less expensive and safer diagnostic technique with real-time monitoring. The purpose of this study was to determine the efficacy, safety and advantages of US-guided biopsy of pleural and peripheral lung lesions abutting pleura and to compare it with CT guidance.
**Methods or Background:** Among 1960 image-guided thoracic biopsies obtained at our hospital between January 2001 and December 2020, 363 were US-guided biopsies for pleural and peripheral lung lesions (278-male, 85-female). Out of these, 343 were performed using US only; the other 20 had initial CT localization. There were 27 pleural lesions and 336 pulmonary lesions with pleural contact. After reviewing the patient, CT scan and coagulation profile, a biopsy was performed using the freehand US technique under real-time visualization. Lesion size, pleural lesion contact, biopsy type, number of passes, procedure time, sample adequacy and complications were recorded.
**Results or Findings:** Among 1960 biopsies, 363 (18.5% ) were US-guided and 1597 (81.5%) were CT-guided procedures. The procedure time was significantly less in the US-guided than under the CT-guided procedures. The post-procedure pneumothorax was significantly less in the US-guided group as observed on 2 out of 363 US-guided procedures (0.55%) and 43 out of 1597 CT-guided procedures (2.7%). Intraparenchymal haemorrhage occurred in 2 out of 363 US-guided biopsies ( 0.55%) and 17 out of 1597 under CT guidance (1.1%).
**Conclusion:** US guidance allows significantly less procedure time and post-procedural complications without the use of ionizing radiation. US guidance can be used as an alternative to CT guidance for the biopsy of thoracic lesions abutting pleura.
**Limitations:** Single-centre retrospective study. The patients were not randomized between the US and CT guidance which may result in selection bias.
**Ethics committee approval:** The ethics committee approval was obtained.
**Funding for this study:** Not applicable.
**Author Disclosures:**
Binoy Kumar Choudhury: Nothing to disclose

### RPS 2504-3

**Artificial intelligence-driven pleural plaque segmentation and volume correlation to lung function**

*K. Groot Lipman*, T. N. Boellaard, N. Bogveradze, E. K. Hong, F. Castagnoli, F. Landolfi, R. G. H. Beets-Tan, S. Burgers, S. Trebeschi; Amsterdam/NL
*(k.groot.lipman@nki.nl)*

**Purpose:** Pleural plaques (PP) are morphological manifestations of long-term asbestos exposure. The relationship between PP and lung function is not well-understood. The time-consuming nature of PP delineation to obtain volume impedes research. To automate this, we aimed to develop automatic Artificial Intelligence (AI)-driven segmentation of PP. Moreover, we want to explore the relationship between the pleural plaque volume in CT scans and pulmonary function tests (PFT).
**Methods or Background:** Radiologists manually delineated pleural plaques in n=422 thoracic CT scans (n=61 363 images) of patients with occupational exposure to asbestos, which were used to develop the AI model. The Pearson correlation coefficient (r) was used for the correlation between PP volume and PFT metrics. When recorded, these were VC, FVC, DLCO, and KCO.

Sunday

**Results or Findings:** We trained the AI system on subjects in the training set (n=322). On the independent test set (n=100), the correlation between the predicted volume and the ground truth was r=0.89, the median overlap was 0.70 Dice Similarity Coefficient. We found weak to no correlations for VC (n=138, r=-0.41), FVC (n=152, r=-0.44), DLCO (n=137, r=-0.16), and KCO (n=119, r=0.15). For DLCO and KCO, no significant differences were found (p>0.05). Significant differences were found for VC (p=0.02) and FVC (p=0.006).
**Conclusion:** We successfully developed an AI algorithm to automatically segment PP in CT images to enable fast volume extraction. The PP volume is associated with loss in VC and FVC. We envision that the AI model could be used to non-invasively gain insight into lung morphology and lung function, which could make PP volume investigation more accessible to other researchers.
**Limitations:** We could not correct for confounders in the correlation between the lung function parameters and the volume, like smoking.
**Ethics committee approval:** Approved by the institutional board.
**Funding for this study:** No funding was received for this study.
**Author Disclosures:**
Sjaak Burgers: Nothing to disclose
Eun Kyoung Hong: Nothing to disclose
Stefano Trebeschi: Nothing to disclose
Thierry N. Boellaard: Nothing to disclose
Kevin Groot Lipman: Nothing to disclose
Regina G. H. Beets-Tan: Nothing to disclose
Nino Bogveradze: Nothing to disclose
Francesca Castagnoli: Nothing to disclose
Federica Landolfi: Nothing to disclose

### RPS 2504-4

**Development and validation of CT-based radiomics nomogram for prognostic prediction in patients with malignant pleural mesothelioma**
*X. Xiaojie*[1], D. Han[1], J. Chen[1], H. Luo[2], J. Jie[1], W. Zhao[1], S. Shen[1], X. Zhang[2], W. Jin[1]; [1]Kunming/CN, [2]Chuxiong/CN
*(1061892797@qq.com)*

**Purpose:** To develop and validate a prognostic model combining the clinical factors and unenhanced CT-based radiomic signature for malignant pleural mesothelioma (MPM).
**Methods or Background:** A total of 164 patients with MPM were enrolled in this multi-centre study from 2007 to 2020 and divided into training (n = 82) and test (n = 82) sets according to the admission time. A clinical model was constructed based on the clinicopathological and CT morphological features. The correlation of the overall survival (OS) with radiomic features, which were extracted from the region of interest (ROI) manually segmented on the three-dimensional CT images were analyzed. The Cox proportional risk model was conducted by combining the radiomic signature with clinical factors to establish a nomogram for prognostic prediction and risk stratification. Harrell's Concordance Index (C-Index) and calibration curve were used to evaluate the model's discrimination and consistency, respectively.
**Results or Findings:** Based on the clinical factors, Cox multivariate analysis showed that albumin reduction (hazard ratio[HR]=1.584), clinical stage IV (HR=3.075) and chemotherapy (HR=0.477) were independent factors for OS prediction. The predictive performance of the clinic-radiomic combined nomogram was superior to the clinical model alone in training (C-index: 0.709 & 0.662) and test (C-index: 0.664 & 0.613) sets. The patients in the training (P<0.001) and test (P=0.007) sets can be successfully stratified into low and high-risk groups with the combined nomogram.
**Conclusion:** The nomogram combined with clinical and CT-based radiomic signature is a new and favourable tool to predict the prognosis for MPM patients, which may provide valuable information for clinical decision making.
**Limitations:** The multi-centre study cannot guarantee the uniformity of clinical and imaging data.
**Ethics committee approval:** Ethical approval No. 2020-L-27.
**Funding for this study:** Joint Program of Yunnan Science and Technology and KMMU (202001AY070001-201).
**Author Disclosures:**
Wenfeng Jin: Nothing to disclose
Shasha Shen: Nothing to disclose
Dan Han: Nothing to disclose
Jianyou Chen: Nothing to disclose
Jiang Jie: Nothing to disclose
Heng Luo: Nothing to disclose
Xie Xiaojie: Author: speaker
Xingwen Zhang: Nothing to disclose
Wen Zhao: Nothing to disclose

### RPS 2504-5

**Training and validation of DL algorithms for the detection of pneumothorax based on data from a competition in diagnostic imaging**
*M. J. Henkel*, B. Stieltjes, A. W. Sauter; Basel/CH

**Purpose:** Competitions are a potential approach in data science to solve real-world problems. It offers organizers the opportunity to get external expertise and participants a great learning opportunity. In this study, we show how this approach can be used to create labelled data for the training of deep learning (DL) algorithms.
**Methods or Background:** A platform to create competitions in diagnostic imaging has been developed. Ten radiology residents competed on the platform in detecting pneumothorax on 1161 chest x-rays. Using this data, multiple DL algorithms for detecting pneumothorax were developed. Classification and localization performance of the models were tested on an internal and NIH ChestX-ray14 dataset.
**Results or Findings:** The AI models F1 scores on the internal and the NIH dataset were 0.87 and 0.44, respectively. Sensitivity was 0.85 and 0.80 for classification and specificity 0.96 and 0.48 for classification. F1 scores were 0.72 and 0.66, sensitivity 0.72 and 0.72.
**Conclusion:** Our results demonstrated that competition derived annotations are a valuable data source for DL algorithm development. Further work is needed to include additional parameters such as user performance, consensus of diagnosis, and quality control in the development pipeline.
**Limitations:** The training data is based on data from a single institution. The "ground truth" in this study is based on the consensus opinion of the residents performing the annotation of the images; no expert review other than the approved report has taken place to confirm the diagnoses. Finally, this is a retrospective study; the model's performance has not yet been prospectively evaluated in a clinical environment.
**Ethics committee approval:** All patient data used in this study were completely anonymized and therefore it does not require ethics committee approval.
**Funding for this study:** This study was funded by Innosuisse - Schweizerische Agentur für Innovationsförderung, Einsteinstrasse 2, 3003 Bern.
**Author Disclosures:**
Bram Stieltjes: Nothing to disclose
Maurice Johannes Henkel: Nothing to disclose
Alexander Walter Sauter: Nothing to disclose

### RPS 2504-7

**Imaging IRAE-pericardial effusion on chest CT: clinical and radiologic manifestations and implications for management**
*K. M. Capaccione*[1], S. Huang[1], Z. Toor[1], A. Deng[2], B. May[1], M. M. Salvatore[1]; [1]New York/US, [2]Charolette, NC/US
*(capacckm@gmail.com)*

**Purpose:** IRAE pericardial effusion has been reported but is not well characterized clinically or radiographically. Here, we described the largest cohort of patients to date who developed IRAE pericardial effusion and analyzed their clinical course and radiologic correlates.
**Methods or Background:** We identified patients treated with checkpoint inhibitor immunotherapy and who developed pericardial effusion during the same time period. We analyzed chronicity to assess if pericardial effusions developed after initiation of therapy; cardiac function on echocardiogram was also assessed to rule out cardiac dysfunction as a cause of pericardial effusion. We analyzed clinical information and serial CT exams for concomitant findings. We performed statistical analysis to evaluate which features were characteristic of or associated with IRAE pericardial effusion.
**Results or Findings:** The majority of IRAE-pericardial effusions were small and not clinically significant, and most commonly followed anti-PD-1 inhibitor therapy; a significant number were seen in patients previously treated with carboplatin. Approximately half were treated with steroids or resolved spontaneously. Approximately half of the patients with IRAE- pericardial effusion had mediastinal adenopathy and/or pleural effusions, however these were not significantly different in the control patients.
**Conclusion:** Contrary to prior case reports and series, our data suggest that IRAE-pericardial effusions are rarely clinically significant and analysis of the risk/benefit ratio does not warrant discontinuation of checkpoint inhibitor therapy.
**Limitations:** Limitations of this study were the relatively small sample size and the retrospective design of the study given that patients may have gone on to have significant developments in their clinical course after the end of the study period.
**Ethics committee approval:** Columbia University IRB AAAS7350 approved 10/29/2020.

**Funding for this study:** No funding was received for this study.
**Author Disclosures:**
Kathleen Mary Capaccione: Nothing to disclose
Benjamin May: Nothing to disclose
Sophia Huang: Nothing to disclose
Aileen Deng: Nothing to disclose
Mary Margaret Salvatore: Grant Recipient: Genentech, Boehringer Ingelheim
Speaker: Genentech, Boehringer Ingelheim
Zeeshan Toor: Nothing to disclose

**15:00-16:00** **Channel 2**

### Research Presentation Session: Neuro

### RPS 2611

### Vascular: other than acute stroke

**Moderator**
A. Majos; Lodz/PL

### RPS 2611-3

**Preliminary study on subclinical brain alterations in patients with asymptomatic carotid vulnerable plaques using DTI**

*S. Yang*; Changsha/CN
*(332170193@qq.com)*

**Purpose:** To assess the alterations in the topological properties of the white matter brain network in carotid vulnerable plaque group and carotid hard plaque group based on magnetic resonance diffusion tensor imaging(DTI).
**Methods or Background:** One hundred and nineteen volunteers were included and performed DTI examination, among who, 58 volunteers had carotid vulnerable plaques, 23 volunteers had carotid hard plaques. The differences in the topological properties among the three groups were explored at both the global and local levels using one-way ANOVA and Bonferroni t-test ($p<0.05$). Then network-based statistic (NBS) method was employed to assess the alterations of the interregional connections among three groups (NBS corrected, $p<0.001$ at voxel level, $p<0.05$ at cluster level, permutated for 5000 times).
**Results or Findings:** Compared with the control group and vulnerable plaque group, the hard plaque group demonstrated significantly increased betweenness centrality in the left supramarginal gyrus region. Compared with the control group and hard plaque group, the vulnerable plaque group demonstrated significantly decreased nodal clustering coefficiency in the left putamen region. The vulnerable-plaque group presented a significantly decreased subnetwork component and two significantly increased subnetwork components in the NBS analysis results.
**Conclusion:** The topological organisation of white matter networks in carotid hard plaque group is different from vulnerable plaque group, which tends to increase the local efficiency of network communication to compensate. Furthermore, the carotid vulnerable plaque group showed more disorder of topological properties.
**Limitations:** First, we were limited by the cross-sectional design and small sample size of this study. Second, we only analysed the anatomical connectivity of white matter. The combination of structural and functional network analysis might provide a more comprehensive perspective for the disorder of topological properties in patients with carotid vulnerable plaques.
**Ethics committee approval:** Approved by the institutional review boards of Xiangya Hospital.
**Funding for this study:** No funding was received for this study.
**Author Disclosures:**
Shuai Yang: Author: The authors declare no conflict of interest.

### RPS 2611-5

**The impact of acceleration factors of compressed sensing on the image quality of 3D-TOF-MRA for cervical vessels**

*Q. Song*; Dalian/CN

**Purpose:** Explore the impact of acceleration factors of CS on the image quality of 3D-TOF-MRA for cervical vessels.
**Methods or Background:** 22 healthy volunteers were recruited and underwent the 3D-TOF MRA scan of neck vessels on a 3.0 T MR scanner. Four groups with different acceleration schemes were set up in our study, group A with a routine clinical setup of SENSE acceleration factor 3, and groups B, C, and D with CS factors of 4, 6, and 8. Regions of interest were placed manually at both sides of the carotid artery and nearby sternocleidomastoid muscle by experienced radiologists for the measurement of SNR and CNR. The two observers used a four-point scoring method to evaluate the quality of the four groups of images. The Kappa statistics were calculated for determining the interobserver agreement. The assessment of intermethod agreement was based on the evaluation of the senior physicians. Kruskal-Wallis test was employed to assess the difference of SNR, CNR and image scores between the 4 groups. Mann-Whitney U test was used to make a pairwise comparison.
**Results or Findings:** There were no statistically significant differences in SNR, CNR between the four groups. However, if CS acceleration factor of 8 was used, the subjective scores decreased obviously ($p < 0.05$, Table. 3). And no significant differences in image quality were detected between conventional SENSE acceleration with a factor of 3 and CS acceleration with factors of 4 and 6.
**Conclusion:** CS acceleration factor of 6 is recommended for clinical 3D-TOF carotid MRA to achieve an optimal balance between imaging time and image quality.
**Limitations:** This study was limited by the few amount of volunteers.
**Ethics committee approval:** This study has been approved by the local IRB.
**Funding for this study:** Not applicable.
**Author Disclosures:**
Qingwei Song: Nothing to disclose

### RPS 2611-6

**Effect of MRI acquisition parameters on accuracy and precision of phase-contrast measurements in a small lumen vessel phantom**

*M. Correia de Verdier*, J. Wikström; Uppsala/SE
*(maria.correiadeverdier@radiol.uu.se)*

**Purpose:** To assess the effects of spatial resolution, number of excitations (NEX) and velocity encoding (VENC) on accuracy and precision of phase-contrast (PC) MRI measurements in a vessel phantom with a small lumen diameter.
**Methods or Background:** A 3 T scanner and a 32-channel head coil were used for all the PC-MRI measurements. An in vitro flow model consisting of a plastic tube (2.3 mm inner diameter) passing through an agar gel was constructed to provide a continuous flow. The flow rate was controlled using a reservoir with a scale and timer and used as the standard reference. A PC-MRI sequence was performed with varying voxel size (0.6 x 0.8 x 5 mm, 1 x 1 x 5 mm, 1.2 x 1.2 x 5 mm), NEX (1, 2, 3) and VENC (200, 300, 400 cm/s). Measurements were repeated 9 times for each setting. Mean flow and peak velocity were calculated for each combination of settings and the least detectable difference (LDD) was computed.
**Results or Findings:** All PC-MRI mean flow measurements were higher than our standard reference (mean values ranging from 7.3 to 9.5 ml/s compared with 6.5 ml/s). Decreasing voxel size improved the accuracy of mean flow measurements, with measured values changing from 9.5 to 7.3 ml/s. LDD for mean flow decreased with increasing voxel size and NEX ($p<0.05$). LDD for peak velocity decreased with increasing voxel size ($p<0.05$). No change in LDD was observed with different VENC settings.
**Conclusion:** Accuracy in PC-MRI flow measurements in a small vessel phantom is low, with higher measured values than control. Improved accuracy is obtained with increased spatial resolution. Improved precision is obtained with decreased spatial resolution and increased NEX.
**Limitations:** Not applicable.
**Ethics committee approval:** Not applicable.
**Funding for this study:** Not applicable.
**Author Disclosures:**
Johan Wikström: Nothing to disclose
Maria Correia de Verdier: Nothing to disclose

### RPS 2611-7

**Collateral status at single-delay arterial spin labelling MRI can non-invasively predict cerebral hyperperfusion after carotid endarterectomy**

*X. Fan*, T. Lin, Z. Lai, J. Qu, H. You, F. Feng; Beijing/CN
*(18801122923@163.com)*

**Purpose:** To explore and compare the predictive ability of collateral score systems assessed with single-delay ASL and conventional CT/MRI protocols for cerebral hyperperfusion after carotid endarterectomy (CEA).
**Methods or Background:** Eighty-five patients who underwent CEA between May 2015 and July 2021 were included (mean age 65.3±7.1 years, 76.5% male). Cerebral hyperperfusion was defined as an increase in cerebral blood flow >100% compared with preoperative values. Preoperative ASL images were scored based on the presence of arterial transit artefacts (ATAs) in 10 regions of interest corresponding to ASPECTS methodology as follows: 0, no or minimal ASL signal; 1, low/moderate ASL signal with ATA; 2, high ASL signal with ATA; and 3, normal perfusion without ATA. The degree of stenosis, primary and secondary collaterals were evaluated on conventional CTA, MRA and T2 FLAIR images.
**Results or Findings:** Cerebral hyperperfusion was presented in 16 (18.8%) patients. Preoperative ASL score was an independent predictor of cerebral hyperperfusion (OR=0.47, 95% CI [0.32-0.71], $p<0.001$). ROC curve analysis revealed that the predictive ability for cerebral hyperperfusion was statistically higher for ASL score (AUC=0.98, 95% CI [0.923-0.998]) than for degree of stenosis (AUC=0.786, 95% CI [0.684-0.868], $p=0.002$), type of circle of Willis

(AUC=0.771, 95% CI [0.667-0.855], p=0.002) or leptomeningeal collaterals (AUC=0.798, 95% CI [0.697-0.877],p=0.004). The ASL score performed as well as the combination of degree of stenosis, type of circle of Willis and leptomeningeal collaterals (AUC=0.947, 95% CI [0.876, 0.984],p=0.258).
**Conclusion:** Single-delay ASL can non-invasively predict cerebral hyperperfusion after CEA in patients with carotid stenosis.
**Limitations:** The sample size was relatively small.
**Ethics committee approval:** This study was approved by the Medical Ethics Committee of the Peking Union Medical College Hospital.
**Funding for this study:** This work was supported by the Beijing Natural Science Foundation grant (L182067) and National Nature Science Foundation of China grant (82071899).
**Author Disclosures:**
Feng Feng: Nothing to disclose
Zhichao Lai: Nothing to disclose
Hui You: Nothing to disclose
Xiaoyuan Fan: Nothing to disclose
Jianxun Qu: Nothing to disclose
Tianye Lin: Nothing to disclose

### RPS 2611-8

**Microbleeds in cerebral fat embolism**
*O. Giyab*, B. L. Balogh, P. P. Bogner, G. Orsi, A. Tóth; Pécs/HU
*(ogiyab@gmail.com)*

**Purpose:** Our aim was to prove our hypothesis according to which cerebral fat embolism commonly presents with a characteristic microbleed pattern on MRI.
**Methods or Background:** We searched the literature and the database of our home institution for cases of cerebral fat embolism (CFE). The hypothesized CFE characteristic microbleed pattern (diffuse presence of round microbleeds of monotonous size in the subcortical white matter involving but not limited to the U-fibers, internal capsule and the corpus callosum, mostly sparing the corona radiata and the non-subcortical centrum semiovale on T2* GRE or SWI images), the starfield pattern as described by Parizel et al (scattered bright spots on a dark background in DWI with diffusion restriction), and confluent diffusion restriction in the corpus callosum were statistically compared. Temporal characteristics of the imaging features were also analysed.
**Results or Findings:** 141 patients with cerebral fat embolism were included. The characteristic "walnut kernel microbleed pattern" was found in 89.74%. Diffusion abnormality in general was seen in 97.64%. A definitive starfield pattern was ascertained in 68.5%. Confluent restricted diffusion was seen in the corpus callosum in 77.27%. The walnut kernel microbleed pattern had a more consistent presence among time periods compared to the starfield pattern.
**Conclusion:** Microbleeds in CFE are very common and mainly occur in a characteristic pattern in SWI or T2*, which along with the starfield pattern and corpus callosum diffusion restriction in DWI/ADC appear to be the most important imaging markers of CFE and may aid the differential diagnosis in clinically equivocal cases.
**Limitations:** More articles investigated diffusion abnormalities than microbleeds.
**Ethics committee approval:** The Institutional Review Board approved the institutional medical database search that was performed related to this study.
**Funding for this study:** Funding was received from the Bolyai Scholarship Hungarian Academy of Science.
**Author Disclosures:**
Bendeguz Lazar Balogh: Nothing to disclose
Gergely Orsi: Nothing to disclose
Omar Giyab: Grant Recipient: Bolyai Scholarship of the Hungarian Academy of Science
Arnold Tóth: Nothing to disclose
Péter Peter Bogner: Nothing to disclose

**15:00-16:00** **Channel 3**

### Research Presentation Session: Oncologic Imaging

### RPS 2616

### Primary and secondary liver tumours

**Moderator**
G. Brancatelli; Palermo/IT
**Author Disclosures:**
Giuseppe Brancatelli: Speaker: Guerbet, GE Healthcare, Bayer

### RPS 2616-2

**Flow-compensated diffusion encoding in MRI for improved focal liver lesion detection**
F. B. Laun[1], T. Führes[1], H. Seuss[2], A. Müller[1], S. Bickelhaupt[1], A. Stemmer[1], T. Benkert[1], M. Uder[1], *M. Saake*[1]; [1]Erlangen/DE, [2]Forchheim/DE

**Purpose:** To intra-individually compare diffusion-weighted imaging (DWI) of the liver acquired with conventional monopolar (MP) and flow-compensated (FloCo) diffusion encoding for as-sessing focal liver lesions (FLLs) in non-cirrhotic patients.
**Methods or Background:** Patients with known or suspected multiple FLLs were included, and DWI with MP and FloCo diffusion encoding was performed (b-values, 50 and 800 s/mm², respectively). DWI images were analysed independently by two board-certified radiologists. They issued Likert-scale ratings (1 = worst, 5 = best) for pulsation artefact severity and counted the difference of lesions visible at b = 800 s/mm² separately for small and large FLLs (i.e., < 1 cm or > 1 cm) and separately for left and right liver lobe. Differences between the two diffusion encodings were assessed with the Wilcoxon signed-rank test.
**Results or Findings:** Forty participants were included in the study. Both readers found a reduction in pulsation artefact in the liver with FloCo encoding (left liver lobe: $p < 0.001$; ratings ≥4: reader 1, 9 of 40 cases for monopolar vs 35 of 40 for FloCo; reader 2, 11 of 40 for monopolar vs 37 of 40 for FloCo ). More small lesions were detected with FloCo diffusion encoding in both liver lobes (left lobe: reader 1, six additional lesions; reader 2, seven additional lesions; $p = 0.063$ for both readers). Both readers found one additional large lesion in the left liver lobe.
**Conclusion:** FloCo is more effective than MP diffusion encoding for the detection of FLLs.
**Limitations:** The study was performed using one scanner from a single vendor.
**Ethics committee approval:** This retrospective study was approved by the IRB.
**Funding for this study:** Financial support by the DFG is gratefully acknowledged (DFG LA 2804/12-1, DFG LA 2804/15-1, DFG SA 4141/1).
**Author Disclosures:**
Tobit Führes: Grant Recipient: Position funded by a DFG grant
Hannes Seuss: Nothing to disclose
Marc Saake: Research/Grant Support: Work was funded by the DFG
Alto Stemmer: Employee: Employed by Siemens Healthcare GmbH
Frederik Bernd Laun: Research/Grant Support: Work was funded by the DFG
Michael Uder: Nothing to disclose
Sebastian Bickelhaupt: Nothing to disclose
Thomas Benkert: Employee: Employed by Siemens Healthcare GmbH
Astrid Müller: Nothing to disclose

### RPS 2616-3

**Feasibility of quantitative dynamic contrast-enhanced MRI for prediction of microvascular invasion in small solitary hepatocellular carcinoma based on a dual-input two-compartment model**
*Y. Zhu*, C. Wei, X. Ma, X. Zhao; Beijing/CN
*(zhuyj04@126.com)*

**Purpose:** Microvascular invasion (MVI) serves as an important prognostic factor for hepatocellular carcinoma (HCC) after an operation. However, predicting MVI in patients with HCC is a clinical challenge as MVI is a histopathological diagnosis. The aim of this study is to investigate the feasibility of quantitative dynamic contrast-enhanced MRI (DCE-MRI) for predicting MVI in small solitary HCC (ssHCC).
**Methods or Background:** A total of 63 patients with pathologically confirmed ssHCC (≤ 3cm) underwent quantitative DCE-MRI studies and received hepatic resection. A dual-input two-compartment exchange model (2CXM) was used to calculate the values of quantitative permeability and perfusion parameters. The differences in parameters between different MVI status groups were analysed. Multivariate logistic regression was used to build the combined prediction model for MVI prediction with the statistically significant parameters. The predictive performance was evaluated using ROC analysis.
**Results or Findings:** Among the 63 patients with ssHCC, 22 (34.9%) exhibited MVI positive. The MVI positive group had higher volume transfer constant (Ktrans), reverse reflux rate constant (kep), portal vein blood flow (BFpv), and lower extracellular extravascular volume fraction (ve), hepatic arterial perfusion index (HPI) values than negative group (1.532 min-1 vs. 0.853 min-1, 0.547 min-1 vs. 0.362 min-1, 84.63 mL/min/100g vs. 34.95 mL/min/100g, 0.316 vs. 0.582, 65.32 vs. 84.59, respectively) (P<0.05). Quantitative parameters Ktrans, kep and BFpv values independently associated with MVI with OR values of 4.36, 2.53 and 3.74 (P<0.05) through multivariate logistic regression. ROC analysis showed that the AUC, sensitivity, specificity in predicting MVI by combined Ktrans, kep and BFv values were 0.903, 87.6%, 95.4%, respectively.
**Conclusion:** Quantitative DCE-MRI derived parameters showed potential value in the prediction MVI in ssHCC.
**Limitations:** The sample size was relatively small.
**Ethics committee approval:** Approved by the Independent Ethics Committee of the Cancer Hospital, CAMS (no. 20/412-2608).
**Funding for this study:** No funding was received for this study.
**Author Disclosures:**
Yongjian Zhu: Nothing to disclose
Xiaohong Ma: Nothing to disclose
Xinming Zhao: Nothing to disclose
Cai Wei: Nothing to disclose

### RPS 2616-7

**Hepatocellular carcinoma: radiomics analysis of contrast-enhanced computed tomography images in prediction tumour grade**

*M. Shantarevich*, E. V. Kondratyev, G. G. Karmazanovsky; Moscow/RU
*(shantarevichm@list.ru)*

**Purpose:** Poor tumour differentiation of hepatocellular carcinoma (HCC) correlates with lower overall and disease-free survival. Therefore, accurate non-invasive preoperative prediction of the tumour histologic grade is crucial for patient prognosis. The purpose of this study was to investigate the value of radiomics analysis of contrast-enhanced computed tomography images (CECT) in estimating the histologic tumour grade before surgery in patients with HCC.
**Methods or Background:** The 36 patients with HCC and preoperative liver CECT who had undergone surgical resection were retrospectively enrolled in the study (25 patients with the tumour Grade 1+Grade2, and 11 patients - with Grade 3). The LIFEx application software (version v7.1.0, www.lifexsoft.org) was used to obtain texture features. 3D ROI that covered the whole tumour was delineated in the images for each patient. Radiomic features were extracted from four phases (native, arterial, portal, and delay). A total of 497 radiomic features were extracted from each CECT phase.
**Results or Findings:** Tree radiomic features: CONVENTIONAL_HUKurtosis, DISCRETIZED_HUExcessKurtosis and GLZLM_SZE derived from native and arterial phases showed significant positive associations with the histologic grade (p<0,05) and were selected after multiple linear regression analysis. The sensitivity and specificity of radiomics-based model in detecting poor-differentiated HCC from well- and moderate- differentiated HCC were 87,5% and 94,7%, respectively (AUC 0,901±0,078 CI: 0,749-1,0).
**Conclusion:** The use of the CECT radiomics-based model reflects a better evaluating performance in the prediction of HCC grade, which may contribute to personalised treatment.
**Limitations:** The limitation of our research is the small number of included patients.
**Ethics committee approval:** Not applicable.
**Funding for this study:** Not applicable.
**Author Disclosures:**
Evgeny V Kondratyev: Nothing to disclose
Grigory G. Karmazanovsky: Nothing to disclose
Mariia Shantarevich: Nothing to disclose

### RPS 2616-8

**Assessment of radiogenomic venous invasion to predict the outcome after loco-regional therapies in patients with hepatocellular carcinoma**

*R. Schmidt*, C. Hamm, H. Xu, V. H. Broukal, L. A. Gottwald, B. Gebauer, L. J. Savic; Berlin/DE
*(robin.schmidt@charite.de)*

**Purpose:** Radiogenomic venous invasion (RVI) is a set of imaging biomarkers indicative of the presence of microvascular invasion. This study aims to investigate the predictive value of RVI regarding response and survival of patients with HCC receiving LRT.
**Methods or Background:** This retrospective study included 95 patients with unresectable HCC, who received ablation using CT-guided high dose-rate brachytherapy alone (n=48) or in combination with transarterial chemoembolisation (TACE, n=47) between 01/2016-12/2017. Patients were stratified according to positive or negative RVI assessed on baseline contrast-enhanced MRI using two decision-tree-models: RVI (art) and RVI (ven) based on the presence of internal vessels in the arterial or portal-venous phase, respectively, and the absence of both a hypointense halo and a sharp tumour-liver-transition in native T1-weighted images. Primary endpoints were overall (OS), progression-free survival (PFS), and time to progression (TTP). Statistics included Fisher's exact test and Kaplan-Meier analysis.
**Results or Findings:** Regarding brachytherapy alone, stratification according to RVI (art) achieved significant separation of OS (p=<0.001) and PFS (p=0.029) but not TTP (p=0.142), revealing poorer outcomes for RVI positive patients. RVI (ven) was predictive of TTP (p=0.032) and PFS (p=0.004) but not OS (p=0.078). On the contrary, both RVI types achieved no significant stratification for any endpoint following TACE/brachytherapy. In patients receiving brachytherapy alone, median OS, PFS, and TTP were shorter for RVI (ven) positive compared to negative patients (12.4 vs 40.4, 5.9 vs 13.2, 6.4 vs 11.8 months). In contrast, no difference could be observed for patients receiving TACE/brachytherapy.
**Conclusion:** While decisions for LRT are currently based on visual assessments of tumour enhancement on baseline MRI, the findings underscore the potential of RVI to identify HCC patients who would benefit from TACE before brachytherapy.
**Limitations:** This study was done retrospectively at a single site.
**Ethics committee approval:** Ethics committee approval was obtained.
**Funding for this study:** No funding was received for this study.
**Author Disclosures:**
Charlie Hamm: Nothing to disclose
Bernhard Gebauer: Nothing to disclose
Han Xu: Nothing to disclose
Luzie Alexandra Gottwald: Nothing to disclose
Veronika Hella Broukal: Nothing to disclose
Robin Schmidt: Nothing to disclose
Lynn Jeanette Savic: Nothing to disclose

**15:00-16:00** **Channel 4**

### Research Presentation Session: Genitourinary

### RPS 2607

### Prostate cancer staging and management: where are we today?

**Moderators**
A. Baur; Berlin/DE
L. E. Derchi; Genoa/IT
**Author Disclosures:**
Alexander Baur: Speaker: b.e.imaging GmbH

### RPS 2607-3

**MR perfusion imaging of the prostate without contrast media using arterial spin labelling**

*M. Boschheidgen*, L. Schimmöller, T. Ullrich, C. Arsov, G. Antoch, H. Wittsack; Düsseldorf/DE
*(matthias.boschheidgen@med.uni-duesseldorf.de)*

**Purpose:** To determine the capability of gadolinium-free arterial spin labelling (ASL) sequence as contrast-free, non-invasive alternative perfusion imaging method to differentiate prostate cancer (PCA) from benign prostate tissue compared to conventional DCE MRI.
**Methods or Background:** Thirty men with histologically confirmed PCA were included in this prospectively enrolled single-centre cohort study. All patients received multiparametric MRI (T2, DWI, DCE) at 3T with additional ASL of the PCA lesion. The primary endpoint was the differentiability of PCA versus normal prostate tissue in ASL in comparison to DCE. Secondary objectives were differences in signal intensities (SI), contrast ratios (CR), and differences in the attenuation pattern of peripheral (PZ) and transition zone (TZ) PCA.
**Results or Findings:** In both ASL and DCE, the average SI of PCA areas differed significantly from SI in reference areas in the TZ and PZ (p<0,01, respectively). ASL had significantly higher CR discerning PCA and benign tissue in PZ and TZ (PZ=5.2; TZ=6.5) compared to DCE (PZ=1.6; TZ=1.4) (p<0.01, respectively). In subjective evaluation, ASL could visualise PCA in 28 patients, compared to 29 in DCE.
**Conclusion:** ASL had significantly higher contrast-ratios discerning PCA and benign tissue in PZ and TZ compared to DCE and visual discrimination of PCA does not differ significantly between the two sequences. As perfusion gadolinium-based contrast media is seen more critical in the last few years, ASL seems to be a promising alternative to DCE in PCA detection.
**Limitations:** Single-centre design. Small sample size. Single slice sequence. Long acquisition time.

**Ethics committee approval:** The study was approved by the local ethics committee.
**Funding for this study:** No funding was received for this study.
**Author Disclosures:**
Tim Ullrich: Nothing to disclose
Christian Arsov: Nothing to disclose
Matthias Boschheidgen: Nothing to disclose
Lars Schimmöller: Nothing to disclose
Gerald Antoch: Nothing to disclose
Hansjoerg Wittsack: Nothing to disclose

### RPS 2607-4

**Diagnostic performance of MRI-derived capsular enhancement sign for the detection of prostate cancer extracapsular extension**

*N. Sushentsev*[1], I. Caglič[1], A. Colarieti[2], A. Warren[1], B. Lamb[1], N. Shah[1], T. Barrett[1]; [1]Cambridge/UK, [2]Milan/IT
*(ns784@medschl.cam.ac.uk)*

**Purpose:** To retrospectively determine the prevalence and diagnostic performance of the capsular enhancement sign (CES) on dynamic contrast-enhanced (DCE) magnetic resonance imaging (MRI) for the detection of prostate cancer (PCa) extracapsular extension (ECE).
**Methods or Background:** This retrospective study included patients who underwent DCE-MRI prior to radical prostatectomy. CES was defined as an area of asymmetrical early hyperenhancement on DCE-MRI that was adjacent to a peripheral zone tumour, matched or exceeded the tumour circumferential diameter, and persisted beyond the washout of contrast within the adjacent tumour. Two expert uro-radiologists evaluated the presence of CES on DCE-MRI, independently and then in consensus, with the interobserver agreement calculated using a bias-adjusted and prevalence-adjusted kappa (PABAK). CES diagnostic performance for prediction of ECE was assessed using sensitivity, specificity, positive predictive value (PPV), and negative predictive value (NPV).
**Results or Findings:** The study included 146 patients, of whom 91/146 (62%) harboured ECE on final surgical pathology. Following the initial review of the images, Reader 1 called 12/146 (8%) CES-positive cases, while Reader 2 reported 14/146 (10%) CES-positive cases, and a total of 15/146 (10%) lesions were subsequently identified as CES-positive following a consensus read. PABAK for CES between the two readers was high at 0.90. All consensus determined CES-positive lesions represented pathological ≥T3a disease, with the overall prevalence of CES among tumours with confirmed ECE being 15/91 (17%). Hence, whilst showing 100% specificity and PPV for ECE detection, CES had sensitivity, NPV, and accuracy of 16.5%, 41.29%, and 47.38%, respectively.
**Conclusion:** The presence of CES on DCE-MRI is highly predictive for the presence of ECE and may improve local staging in the small but significant percentage of patients in whom it is demonstrated.
**Limitations:** Not applicable.
**Ethics committee approval:** This study was funded by the NREC East of England.
**Funding for this study:** Not applicable.
**Author Disclosures:**
Anna Colarieti: Nothing to disclose
Iztok Caglič: Nothing to disclose
Nikita Sushentsev: Nothing to disclose
Benjamin Lamb: Nothing to disclose
Nimish Shah: Nothing to disclose
Tristan Barrett: Nothing to disclose
Anne Warren: Nothing to disclose

### RPS 2607-5

**Utility of computed diffusion-weighted imaging b2000 for detection of prostate cancer**

*Y. Kim*, S. H. Kim, T. Baek, H. Park; Busan/KR
*(kkyeonjung@gmail.com)*

**Purpose:** To compare the diagnostic performance in tumour detection and inter-observer agreement between acquired diffusion-weighted imaging (aDWI) b2000 and computed DWI (cDWI) b2000 for patients with prostate cancer (PCa).
**Methods or Background:** A total of 88 patients (mean age: 68.6 years, range: 47-82 years) who had been diagnosed with PCa by radical prostatectomy and undergone pre-operative 3.0-Tesla magnetic resonance imaging (3T-MRI) including DWI (b values, 0, 100, 1000, 2000 s/mm2) were included in this study. cDWI b2000 was made from aDWI b0, 100 and 1000 under a mono-exponential decay model. Two independent reviewers performed a 4-week-interval review of aDWI b2000 images and then cDWI b2000 in random order for each session. T2-weighted images were presented for both sessions. A region of interest was drawn for an index tumour on each dataset, and a PIRADS score based on PIRADS v2.1 was recorded. Topographic maps served as the reference standard. The McNemar test was performed to compare the sensitivities for tumour detection, and kappa statistics were used to evaluate the inter-observer agreement on the PIRADS score on each dataset.
**Results or Findings:** The study population consisted of Gleason score (GS) 6 (n=16), GS 7 (n=53), GS 8 (n=9) and GS 9 (n=10) patients. For both reviewers, the sensitivities of cDWI b2000 and aDWI b2000 for detection of PCa showed no significant difference (for reviewer 1, both 94% (83/88); for reviewer 2, both 90% (79/88), P = 1.000, respectively). The kappa values of cDWI b2000 and aDWI b2000 for the PIRADS scores were 0.422 (95% CI, 0.240-0.603) and 0.495 (95% CI, 0.308-0.683), respectively.
**Conclusion:** cDWI b2000 showed comparable diagnostic performance and sustained moderate inter-observer agreement with aDWI b2000 for detection of PCa.
**Limitations:** No limitations were identified.
**Ethics committee approval:** This study was approved by the Pertinent institutional review board.
**Funding for this study:** No funding was received for this study.
**Author Disclosures:**
Taewook Baek: Nothing to disclose
Yeonjung Kim: Nothing to disclose
Hyungin Park: Nothing to disclose
Seung Ho Kim: Nothing to disclose

### RPS 2607-6

**Prediction of PET-positive lymph nodes with multiparametric MRI and clinical information in primary staging of prostate cancer**

*A. M. Hötker*[1], U. J. Mühlematter[1], S. Skawran[1], S. Stocker[1], I. A. Burger[2], M. Huellner[1], O. F. Donati[1]; [1]Zurich/CH, [2]Baden/CH

**Purpose:** To predict the presence of PET-positive pelvic lymph nodes in prostate cancer using quantitative parameters of multiparametric MRI (mpMRI) and clinical information.
**Methods or Background:** This study included 35 patients with high suspicion for prostate cancer undergoing multiparametric prostate MRI and PSMA-PET/CT prior to MRI-guided biopsy. All MRI examinations were assessed by a radiologist, and the Apparent Diffusion Coefficient (ADC, mean and volume), capsular contact length, volume and maximal diameter on T2-weighted sequences and parameters of dynamic contrast-enhanced MRI (iAUC, kep, Ktrans, ve) were calculated for the index lesion. Clinical data was extracted from the hospital information system to calculate the Briganti 2018 nomogram scores. PET examinations were evaluated by two board-certified nuclear medicine physicians and served as the standard of reference.
**Results or Findings:** Quantitative imaging parameters of mpMRI mostly demonstrated mediocre to good performance in prediction of PET-positive nodes (AUCs, ADCmean: 0.74, ADCvol: 0.55, iAUC: 0.42, kep: 0.71, Ktrans: 0.64, ve: 0.37, T2capsular: 0.59, T2diameter: 0.58, T2vol: 0.55), while the Briganti 2018 nomogram (including maximum diameter of the index lesion) reached an AUC of 0.78 (95%-CI: 0.61-0.95). Quantitative MR parameter did not provide added value to the Briganti 2018 model alone.
**Conclusion:** The Briganti 2018 model, which includes clinical/pathological data and the maximal tumour length of the index lesion on prostate MRI, performed well in predicting PET-positive lymph nodes and may serve as a tool to stratify patients for primary staging using PSMA-PET.
**Limitations:** The relatively low number of patients. Retrospective study design.
**Ethics committee approval:** Approved by local IRB.
**Funding for this study:** Not applicable.
**Author Disclosures:**
Irene A. Burger: Nothing to disclose
Stephan Skawran: Nothing to disclose
Martin Huellner: Nothing to disclose
Soleen Stocker: Nothing to disclose
Olivio Fabrizio Donati: Nothing to disclose
Urs Jakob Mühlematter: Nothing to disclose
Andreas M. Hötker: Nothing to disclose

### RPS 2607-7

**Independent evaluation of the PI-QUAL score for prostate MRI: does it provide value?**

*N. Pötsch*[1], E. Rainer[1], P. Clauser[1], G. Vatteroni[2], T. H. Helbich[1], P. A. Baltzer[1]; [1]Vienna/AT, [2]Milan/IT

**Purpose:** To test the inter-reader agreement of the Prostate Imaging Quality (PI-QUAL) score for multiparametric prostate MRI and its impact on diagnostic performance.
**Methods or Background:** Prebioptic multiparametric (T2-weighted, DWI and DCE) prostate MRIs (mpMRI) of 50 patients undergoing transrectal ultrasound-guided MRI-fusion and systematic prostate biopsy were included. Two radiologists independently assigned a PI-QUAL score to each patient. PI-RADS categories were assigned in a lesion-based approach, dividing the prostate into six regions (left and right: base/midglandular/apex). Additionally, the diagnostic quality of each sequence was evaluated independently. Inter-reader agreement was calculated by using Cohen's kappa and diagnostic performance was compared by the area under the ROC curve (AUC).

**Results or Findings:** In a total of 274 diagnostic areas, the malignancy rate was 62.2% (22.7% clinically significant prostate cancer ISUP ≥ 2). Inter-reader agreement for the diagnostic quality was only slight for T2w (kappa 0.19) and fair for DWI and DCE (kappa 0.23 and 0.29). For PI-QUAL as such, the inter-reader agreement was moderate (kappa 0.51). For PI-RADS category assignments, the inter-reader agreement was almost perfect (kappa 0.86). Overall diagnostic performance was significantly better in studies with a PI-QUAL score > 3 compared to a score ≤ 3 (P=0.03; AUC 0.805 and 0.839).
**Conclusion:** In conclusion, the diagnostic performance of mpMRI for the detection of prostate cancer does depend on MRI image quality. Though there is room for improvement regarding inter-reader reproducibility, PI-QUAL is a tool that provides value by predicting the accuracy of diagnostic mpMRI results.
**Limitations:** As all patients underwent MRI-US fusion biopsy due to suspicious mpMRI findings, the rate of malignancy is higher compared to routine clinical practice which may bias the outcomes in non-selected patients.
**Ethics committee approval:** IRB waived the need for informed consent.
**Funding for this study:** No funding was received for this study.
**Author Disclosures:**
Pascal A.T. Baltzer: Nothing to disclose
Nina Pötsch: Nothing to disclose
Eva Rainer: Nothing to disclose
Thomas H. Helbich: Nothing to disclose
Giulia Vatteroni: Nothing to disclose
Paola Clauser: Nothing to disclose

### RPS 2607-8

### Quantitative evaluation of diffusion-weighted imaging may help to avoid biopsies for low PI-RADSv2.1 categories in transition zone lesions

*H. Engel*, B. Oerther, A. Sigle, M. Reisert, E. Kellner, F. Bamberg, M. Benndorf; Freiburg im Breisgau/DE

**Purpose:** To analyse whether low PI-RADS categories of transition zone (TZ) lesions can be downgraded based on mean apparent diffusion coefficients (mADC) without risking false-negative results.
**Methods or Background:** This retrospective cohort study consists of consecutive patients with TZ lesions in multiparametric prostate MRI and subsequent MRI-ultrasound-fusion-biopsy between 05/2017-04/2020. Patients with known prostate cancer (PCa) are excluded. All lesions are scored by two blinded radiologists according to PI-RADSv2.1 guidelines. To determine mADC, lesions are manually segmented. Regression and ROC analyses are performed to determine the diagnostic performance of PI-RADSv2.1 categories and mADC.
**Results or Findings:** Among 85 patients with 98 TZ lesions, 33 (33.7%) are PCa and 65 (66.3%) are benign. 24 (72.7%) of the 33 PCa lesions are clinically significant (csPCa, ISUP-grade>1). mADC for PCa are significantly lower than for benign lesions (894 vs 1.091µm2/s, p<0.001). AUC values from ROC analysis with csPCa as outcome variable are 0.916 for PI-RADSv2.1 and 0.806 for mADC. Compared to PI-RADSv2.1 alone, a combination with an mADC cut-off of 950µm2/s for TZ lesions ≤ PI-RADS 3 improves the negative predictive value (0.95 vs 1.00). Among 58 TZ lesions ≤ PI-RADS 3, only 8 (13.8%) have mADC below 950µm2/s, 3 (37.5%) PCa and 1 (12.5%) csPCa.
**Conclusion:** The key question after a prostate MRI is whether a biopsy is indicated: cancer detection rates for PI-RADS 1+2 are very low while being too high for PI-RADS 3 to abandon biopsies completely. Thus, further parameters which allow avoiding unnecessary biopsies are desirable. Our data indicate that by applying an mADC cut-off for TZ lesions ≤ PI-RADS 3 most biopsies could be avoided without overlooking prostate cancer.
**Limitations:** mADC can differ between vendors/algorithms. External validation of our findings is warranted before clinical use.
**Ethics committee approval:** The ethics committee approval was obtained.
**Funding for this study:** No funding was received for this study.
**Author Disclosures:**
Benedict Oerther: Nothing to disclose
August Sigle: Nothing to disclose
Marco Reisert: Nothing to disclose
Hannes Engel: Nothing to disclose
Matthias Benndorf: Nothing to disclose
Fabian Bamberg: Nothing to disclose
Elias Kellner: Nothing to disclose

### RPS 2607-9

### Improving workflow in prostate MRI: AI-based decision-making on biparametric or multiparametric MRI

*A. M. Hötker*, R. Da Mutten, A. Tiessen, E. Konukoglu, O. F. Donati; Zurich/CH

**Purpose:** To develop and validate an artificial intelligence algorithm to decide on the necessity of dynamic contrast-enhanced sequences (DCE) in prostate MRI.
**Methods or Background:** A convolutional neural network (CNN) was developed on 300 prostate MRI examinations. The consensus of two expert readers on the necessity of DCE acted as the reference standard. The CNN was validated in a separate cohort of 100 prostate MRI examinations from the same vendor and 31 examinations from a different vendor. Sensitivity/specificity were calculated using ROC curve analysis and results were compared to decisions made by a radiology technician.
**Results or Findings:** The CNN reached a sensitivity of 94.4% and specificity of 68.8% (AUC: 0.88) for the necessity of DCE, correctly assigning 44%/34% of patients to a biparametric/multiparametric protocol. In 2% of all patients, the CNN incorrectly decided on omitting DCE. With a technician reaching a sensitivity of 63.9% and specificity of 89.1%, the use of the CNN would allow for an increase in sensitivity of 30.5%. The CNN achieved an AUC of 0.73 in a set of examinations from a different vendor.
**Conclusion:** The CNN would have correctly assigned 78% of patients to a biparametric or multiparametric protocol, with only 2% of all patients requiring re-examination to add DCE sequences. Integrating this CNN in clinical routine could render the requirement for on-table monitoring obsolete by performing contrast-enhanced MRI only when needed.
**Limitations:** The decision rendered by the neural network was dichotomous. The performance of the AI could be improved by defining a range of probability values in which it's unsure and prompt the technician to call a radiologist for this particular examination.
**Ethics committee approval:** This study was approved by the institutional review board and the requirement for study-specific informed consent was waived.
**Funding for this study:** Not applicable.
**Author Disclosures:**
Anja Tiessen: Nothing to disclose
Ender Konukoglu: Nothing to disclose
Olivio Fabrizio Donati: Nothing to disclose
Raffaele Da Mutten: Nothing to disclose
Andreas M. Hötker: Nothing to disclose

### B

### ECR 2022
### Abstract-based Programme
### July 13-17, 2022

Poster Presentation Sessions (PP)
Research Presentation Sessions (RPS)

**Wednesday, July 13 .................... 72**
**Thursday, July 14 .......................169**
**Friday, July 15 ...........................226**
**Saturday, July 16.......................300**
**Sunday, July 17 .........................375**

### Wednesday, July 13

**08:00-09:00** **Open Forum #1 (Radiographers)**

### Research Presentation Session: Radiographers

### RPS 114

### Improving radiation protection outcomes through radiographic practice enhancement

**Moderators**
N. Mekis; Ljubljana/SI
M. Raissaki; Iraklion/GR

### RPS 114-3

**Exposure factors prediction model for large size patients in projection radiography**

*S. J. M. Alqahtani*, K. Knapp, J. Meakin, R. Palfrey; Exeter/UK
*(salbeshri@hotmail.com)*

**Purpose:** High and varied radiation doses when imaging obese patients are reported in the literature. This is of concern, especially with the high prevalence of obesity. This study reports new exposure factor prediction models that can be implemented in digital radiography.
**Methods or Background:** Dose optimisation experiments were conducted using a multi-factorial design method. 5 bespoke phantoms with different sizes were used along with a Multix Fusion Max Siemens unit. Image quality was assessed physically and visually, and the DAP used as the dose measure. A mAs prediction model was produced based on the exposure factors (kVp, SID, and filtration) of images with the highest FOM. The data was analysed using Minitab software.
**Results or Findings:** Fixed kVp (75) with 125 cm of SID and 0.3 mm filtration of Cu were observed to produce the best image quality with the lowest dose across all phantoms. The mAs best prediction model was created based on fixed kVp, SID, and filtration with r2=98.
**Conclusion:** The results of the current study prove a promising future for image quality improvement in obese patients. A mAs prediction model can be used as a preliminary guide to optimising image quality and minimising radiation dose in obese patients. More studies need to be conducted using other CR and DR systems.
**Limitations:** Identified limitations of this study were that a single digital radiography system was used, and that this was a phantom study only.
**Ethics committee approval:** No ethical approval is needed as the study is an experimental study.
**Funding for this study:** Funded by the Ministry of Education, Saudi Arabia, Najran University as part of a PhD fund for the first author, Dr Alqahtani.
**Author Disclosures:**
Saeed Jaber M Alqahtani: Nothing to disclose
Karen Knapp: Nothing to disclose
Judith Meakin: Nothing to disclose
Rachel Palfrey: Nothing to disclose

### RPS 114-4

**Cumulative radiation dose from medical imaging in children with congenital heart disease**

E. Shelly, R. Young, *M. F. F. McEntee*; Cork/IE
*(mark.mcentee@ucc.ie)*

**Purpose:** Children with congenital heart disease are exposed to repeated medical imaging throughout their lifetime. Although the imaging contributes to their care and treatment, exposure to ionising radiation is known to increase one's lifetime attributable risk of cancer. Quantifying and tracking the radiation dose that these patients receive is important in order to minimise the lifetime dose.
**Methods or Background:** A systematic search was performed of Science Direct, PubMed, Scopus and Embase with relevant filters applied. Inclusion and exclusion criteria were applied to all relevant papers and 7 papers were deemed to be acceptable for quality assessment and risk of bias assessment. Data extraction was performed on all 7 papers and data analysis was carried out using descriptive analysis.
**Results or Findings:** It was found that cumulative effective dose (CED) varied widely across the patient cohorts, ranging from 0.96mSv to 53.5mSv. It was however evident across many of the included studies that a significant number of patients were exposed to a CED of >20mSv, which is the current occupational exposure limit. Many factors affected the dose which patients received including age and clinical demographics. The imaging modality which contributed the most radiation dose to patients was cardiology interventional procedures.
**Conclusion:** Paediatric patients with congenital heart disease are at an increased risk of receiving a cumulative radiation dose across their lifetime, with a proportion of patients in this review receiving up to 53.5mSv from medical imaging procedures. Further research should focus on identifying risk factors for receiving higher radiation doses, keeping track of dose and minimising the use of medical imaging where possible.
**Limitations:** Meta-analysis could not be carried out due to the nature of the data that was extracted.
**Ethics committee approval:** None needed for a systematic literature review.
**Funding for this study:** No funding was received for this study.
**Author Disclosures:**
Mark F. F. McEntee: Nothing to disclose
Emer Shelly: Nothing to disclose
Rena Young: Nothing to disclose

### RPS 114-5

**Suitable measures for reducing low-value radiological services in Norway: preliminary results from a qualitative multi-professional study**

*E. R. Andersen*, B. M. Hofmann, E. Kjelle; Gjøvik/NO
*(eivind.r.andersen@ntnu.no)*

**Purpose:** To examine which measures service providers consider appropriate for reducing the use of low-value radiology.
**Methods or Background:** Semi-structured interviews were conducted among radiographers, radiologists, general practitioners, in-hospital referrers, radiological department leaders, and health government representatives. The interview guide covered four broad areas: experience with low-value services, facilitators for reducing low-value services, barriers for reducing low-value radiology and measures deemed to be appropriate for reducing low-value radiology. Data were analysed in line with qualitative framework analysis.
**Results or Findings:** 27 participants were included in the final analysis. The use of electronic decision support was considered to be the most prominent measure to reduce low-value services. Altering the financial system was suggested as an effective measure but assessed as difficult to implement in the Norwegian setting. Other measures included guideline revision, better communication between professionals, altering patient expectations, and making clinicians prioritize their patients within limited time slots. In general, appropriate measures should be well-founded at several levels, easy to use, and should not influence the daily practice in terms of delay or increased workload.
**Conclusion:** All participants acknowledged the use of low-value radiology as a problem. Few had specific suggestions on how to reduce this use in their own practice, or how the health care system could be changed to face this challenge without unintended consequences. A multiperspective approach is necessary to achieve effective and long-lasting changes in the use of radiology. While many measures for reducing low-value radiology are available, it is essential that they are well adapted to the context.
**Limitations:** No limitations were identified.
**Ethics committee approval:** This study was approved by the Norwegian Centre for Research Data (NSD), approval number 475812.
**Funding for this study:** Funding was received from the Norwegian Research Council (number 302503).
**Author Disclosures:**
Bjørn Morten Hofmann: Nothing to disclose
Elin Kjelle: Nothing to disclose
Eivind R. Andersen: Nothing to disclose

### RPS 114-6

**Using beam hardening techniques as a dose optimisation tool in digital planar radiography**

K. Mifsud, J. L. Portelli, F. Zarb, *J. G. Couto*; Msida/MT
*(jose.g.couto@um.edu.mt)*

**Purpose:** To evaluate the use of higher kVp and additional copper (Cu) filtration to optimise four digital planar radiographic projections: AP abdomen, AP knee, AP and lateral lumbar spine.
**Methods or Background:** In phase 1, an anthropomorphic phantom was exposed using different combinations of kVp and Cu filtration thicknesses. Apart from exposing the phantom using the standard local protocol, additional exposures were performed using +10kVp and +20kVp as well as 0.1mm or 0.2mm Cu. The dose-area product (DAP), milliampere-second (mAs) and exposure time (s) were recorded after each exposure was terminated by automatic exposure control (AEC). During phase 2, image quality analysis was performed. Objective image quality was measured using contrast-noise ratio (CNR) calculations, while subjective image quality was determined using a sample of radiographers and radiologists who performed visual grading analysis (VGA).

**Results or Findings:** Optimised exposure parameters were established as follows: AP abdomen, 100kVp and 0.2mm Cu; AP knee, 85kVp and 0.1mm Cu; AP lumbar spine, 110kVp and 0.2mm Cu; and lateral lumbar spine 110kVp and 0.2mm Cu. These optimised protocols resulted in a 71.98%, 62.50%, 64.51% and 71.85% reduction in DAP and areas under the curve (AUC) of 0.883, 0.664, 0.708 and 0.516 respectively. The optimised AP abdomen protocol had a significantly better image quality than the standard protocol ($p<0.05$). CNR values decreased linearly as both kVp and Cu filtration thickness increased for all the projections.
**Conclusion:** The results support using beam hardening techniques when imaging the AP abdomen, AP knee, AP and lateral lumbar spine. A significant reduction in DAP was recorded whilst an acceptable or improved level of image quality was maintained.
**Limitations:** Further research is merited to confirm these results clinically and evaluate its application in other radiographic projections.
**Ethics committee approval:** This study was approved by the University of Malta research ethics committee: 5963_3062020.
**Funding for this study:** No funding was received for this study.
**Author Disclosures:**
Jonathan Loui Portelli: Nothing to disclose
Francis Zarb: Nothing to disclose
Katrina Mifsud: Nothing to disclose
Jose Guilherme Couto: Nothing to disclose

### RPS 114-7

**Barriers and enablers to implementation of diagnostic reference levels**

M. O'Driscoll, N. Moore, *M. F. F. McEntee*; Cork/IE
*(mark.mcentee@ucc.ie)*

**Purpose:** Optimisation is imperative in radiography given the potential risk associated with exposure to ionising radiation. In radiography, diagnostic reference levels (DRLs) are utilised to optimise the medical radiation exposure of patients. It is a legislative and regulatory requirement to establish and audit DRLs in hospital sites in the Republic of Ireland. This work aimed to explore radiographers' experiences in establishment and implementation of DRLs.
**Methods or Background:** This study employed a semi-structured interview as the method of data collection. The work included the radiological protection officer and radiological safety officer of clinical sites from four public hospitals. Thus, purposive non-probability sampling was employed. Written, informed consent was obtained. The interviews were recorded transcribed verbatim by the researcher. The transcribed data was then uploaded to data analysis software NVivo and codes were assigned to the appropriate data.
**Results or Findings:** Resources was the prominent theme that emerged from the data. Availability of resources such as time, manpower, dose tracking software, equipment and raw data were all described as important facilitators in establishing and auditing of DRLs. The concept of image quality and its relationship with DRLs was also identified. Participants discussed the relationship between DRLs and low dose radiation, quality assurance and radiologist input. The following themes were identified: resources, relevant national authority guidance, image quality and education.
**Conclusion:** Implementation of DRLs requires resources that are often lacking. The factors that assist implementation include guidelines and education. Staff knowledge of DRLs is a barrier in the implementation and auditing of DRLs in a department.
**Limitations:** This work focussed on government-run hospitals, drivers may be different in a privately run institution
**Ethics committee approval:** This study was approved by the ethics committee of the UCC School of clinical therapies. Ethics CT-SREC–2020-42
**Funding for this study:** No funding was received for this study.
**Author Disclosures:**
Mark F. F. McEntee: Nothing to disclose
Niamh Moore: Nothing to disclose
Maebh O'Driscoll: Nothing to disclose

### RPS 114-8

**Radiation protection measures used in Portuguese interventional radiology departments: a national survey**

*A. S. Pimenta*[1], J. Santos[2], L. Azevedo[1], I. M. Ramos[1]; [1]Porto/PT, [2]Coimbra/PT
*(aspimenta@gmail.com)*

**Purpose:** The aim of this study is to characterise radiation protection (RP) measures used on Portuguese interventional radiology (IR) departments taking into account the European and national recommendations.
**Methods or Background:** A national survey was created and piloted online in order to characterise the fluoroscopy technology, analyse the frequency of fluoroscopy guided intervention procedures (FGIP), the staff education/training and the RP measures used daily.
**Results or Findings:** The majority of the fluoroscopy equipment in FGIP in Portugal used a single flat panel detector. The most frequent FGIP performed in Portugal are percutaneous biliary drainage, percutaneous arterial and venous thrombolysis/thrombectomy, arteriovenous malformations embolisation and percutaneous transluminal AVF balloon angioplasty. Only a few members of the staff reported having postgraduate education in RP. The majority of nurses participating in this study reported no RP training. A lack of harmonisation was found for some RP measures. More than 50% of IR departments do not define patient follow-up based on examination dose values.
**Conclusion:** This study characterises the technological evolution in IR, however it reveals a lack RP education and training in staff members. Some centres need to update RP measures according to the recommendations.
**Limitations:** The response rate from the private institutions was an identified limitation of this study.
**Ethics committee approval:** Ethical approval was obtained from the University and Hospital Committees.
**Funding for this study:** No funding was obtained for this study.
**Author Disclosures:**
Joana Santos: Nothing to disclose
Andrea Susana Pimenta: Nothing to disclose
Luís Azevedo: Nothing to disclose
Isabel M. Ramos: Nothing to disclose

**08:00-09:00** **Open Forum #3 (ESR)**

### Poster Presentation Session

### PP 1

### Bridging from neuroanatomy to anatomy of head and neck and vascular imaging

**Moderator**
R.-I. Milos; Vienna/AT

### PP 1-2

**Viscosity assessment of parotid and submandibular glands in healthy subjects with the novel Viscosity Plane-wave UltraSound (ViPLUS) technique**

*D. D. Muntean*, L. M. Lenghel, S. M. Dudea; Cluj-Napoca/RO

**Purpose:** Viscosity is an important mechanical property directly linked to the shear wave dispersion within tissues. The purpose of this study was to establish the normal viscosity value of the parotid gland (PG) and submandibular gland (SMG) in a group of healthy subjects, using the novel Viscosity Plane-wave UltraSound (ViPLUS) technique, and to assess its potential dependence on gender and age.
**Methods or Background:** The study group included a total of 49 healthy volunteers (median age 31, 65% females) prospectively examined. The viscosity of both PG and SMG was measured using the new Aixplorer MACH 30 ultrasound system (SuperSonic Imagine, Aix-en-Provence, France) equipped with a curvilinear C6-1X transducer. The mean value of three valid measurements was considered (quantified in Pa.s).
**Results or Findings:** The mean viscosity value for the PG was 2.13±0.23 Pa.s, significantly lower than the mean viscosity value of the SMG 2.44±0.35 Pa.s ($p < 0.0001$). A negative low correlation between SMG viscosity and age was found (rho = -0.38, p = 0.006). Viscosity values of the SMG were significantly lower in the age group between 35-77 years (2.12±0.35 Pa.s) than in the age groups 25-34 years (2.52±0.36 Pa.s), and 20-24 years (2.53±0.24 Pa.s), respectively ($p < 0.05$). Viscosity values of both salivary glands did not differ significantly between gender groups.
**Conclusion:** SuperSonic ViPLUS represents an innovative and useful non-invasive method to assess the viscosity of the parotid and submandibular glands. Age is a potential confounding factor in the evaluation of normal SMG viscosity.
**Limitations:** (1) The small size sample. (2) The curvilinear transducer was used because the ViPLUS module was only available on this transducer. However, the focus of this preliminary study was to obtain quantitative data regarding viscosity, and not to assess parenchymal structural changes, where higher frequency transducers are mandatory.
**Ethics committee approval:** This study was approved by an ethics committee.
**Funding for this study:** No funding was received.
**Author Disclosures:**
Sorin Marian Dudea: Nothing to disclose
Delia Doris Muntean: Nothing to disclose
Lavinia Manuela Lenghel: Nothing to disclose

### PP 1-3

**Craniometry and imaging markers of the craniovertebral junction**

DOI: 10.26044/ecr2022/11424

*J. Sá Silva*, V. R. L. S. Abreu, J. Tarrio, E. M. M. Pinto, J. P. Filipe; Porto/PT

**Purpose:** To understand the anatomy of the craniovertebral junction and to learn objective methods of evaluation of this anatomical segment in different imaging techniques.
**Methods or Background:** The craniovertebral junction (CVJ) is an important anatomic landmark and a region where disease can present in multiple ways, be it congenital or acquired. Knowing its normal anatomical appearance and how to assess it should be part of a radiologist's faculties.
**Results or Findings:** Evaluation of the CVJ requires knowledge of some anatomical landmarks, normal measurements and topographic relationships that enable us to perform CVJ craniometry. The basion, opisthion, posterior margin of the hard palate, anterior and posterior arches of atlas, lateral atlas masses, odontoid process, body of axis, jugular tubercles and occipital condyles are the craniometric points used as osseous anatomical landmarks. With these landmarks we can apply radiographic lines and angles to evaluate normal morphology relationships: the Wackenheim's clivus canal line, the Chamberlain's line, the McRae line, the bimastoid and digastric lines, the clivus-canal angle, the basal angle, the anterior atlanto-dens interval and the atlanto-occipital joint axis angle. Anomalies of the normal relationship characteristics may raise the suspicion of disease, and certain patterns of anomalies may indicate certain diagnosis. Most of these evaluations are made in a sagittal plane. Computed tomography (CT) and magnetic resonance (MR) are best for their evaluation, since conventional radiography may have superimposition artifacts precluding correct measuring. Also, assessment of bone anatomy is better accomplished by CT, whereas the evaluation of spinal soft tissues, ligaments and neural structures is better with MR.
**Conclusion:** By routinely using these craniometric measures in the CVJ, we can objectively evaluate this region and report its normality or, if present, the abnormalities that may indict certain diagnosis.
**Limitations:** Not applicable.
**Ethics committee approval:** Not applicable.
**Funding for this study:** No funding was received for this study.
**Author Disclosures:**
João Tarrio: Nothing to disclose
João Pedro Filipe: Nothing to disclose
José Sá Silva: Nothing to disclose
Eduarda Maria Marinho Pinto: Nothing to disclose
Vasco Rafael Lima Sousa Abreu: Nothing to disclose

### PP 1-4

**Brainstem anatomy: from histological staining to in-vivo 7T-MRI**

*G. Donatelli*[1], A. Emmi[2], P. Cecchi[1], V. Macchi[2], A. Porzionato[2], L. Biagi[1], M. Tosetti[1], R. De Caro[2], M. Cosottini[1]; [1]Pisa/IT, [2]Padua/IT

**Purpose:** The brainstem is a complex anatomical structure containing several nuclei and connecting fibre bundles not visible in-vivo at high magnetic field strengths. In ultra-high field, instead, the higher resolution and increased tissue contrast can improve their visibility. Here, we explored the performance of high-resolution 7T-MRI in representing the normal in-vivo anatomy of the brainstem.
**Methods or Background:** One adult volunteer was imaged at 7T to obtain T2*-weighted images, phase images and quantitative susceptibility maps of the brainstem (0.33x0.33x1.2mm3). An ex-vivo formalin-fixed brainstem was scanned with the same MR system, obtaining 3D T2*-weighted and 2D PD-weighted images in midbrain (0.3x0.3x0.3mm3 and 0.33x0.33x1.2mm3, respectively), pons and medulla oblongata (0.2x0.2x0.2mm3 and 0.16x0.16x1mm3, respectively). The sample was then paraffin-embedded, sectioned and stained for myelin (Weigert-Pal) and iron (Perls). Using histological sections as reference, in-vivo and ex-vivo MR images were assessed to identify brainstem nuclei and white matter tracts.
**Results or Findings:** The in-vivo imaging depicted the detailed anatomy of many brainstem structures such as the red nucleus, with the distinction between magnocellular and parvocellular portion, and the substantia nigra, with the identification of the nigrosome 1. Other nuclei and many white matter tracts were also detectable in-vivo, such as the medial and lateral geniculate body, the abducens nucleus, the fibres of the facial nerve, the medial and lateral lemniscus, the spinothalamic tract, the central tegmental tracts, the transverse pontocerebellar fibres, the medial longitudinal fasciculus, the inferior olivary nucleus, the nuclei gracilis and cuneatus and the spinal trigeminal nucleus.
**Conclusion:** 7T-MRI allows an impressive representation of the normal radiological anatomy of many brainstem structures that are not visible in detail at conventional field strength.
**Limitations:** The limitation is the evaluation of only one volunteer and one ex-vivo sample.
**Ethics committee approval:** The local ethics committees approved this study.
**Funding for this study:** No funding was received for this study.
**Author Disclosures:**
Michela Tosetti: Nothing to disclose
Aron Emmi: Nothing to disclose
Andrea Porzionato: Nothing to disclose
Raffaele De Caro: Nothing to disclose
Graziella  Donatelli: Nothing to disclose
Veronica Macchi: Nothing to disclose
Paolo Cecchi: Nothing to disclose
Laura Biagi: Nothing to disclose
Mirco Cosottini: Nothing to disclose

### PP 1-5

**Changes in intra- and extracranial carotid plaque calcification: a 2 years follow-up study**

*T. Zadi*; Rotterdam/NL

**Purpose:** Carotid atherosclerotic calcification has gained rapid interest given its potential plaque-stabilising effects, yet information on temporal changes in calcification remains scarce. Hence, we evaluated changes in carotid plaque calcification over two years in a sample of symptomatic patients with carotid artery disease.
**Methods or Background:** From the PARISK-study, a prospective cohort study with TIA/minor stroke patients with ipsilateral mild-to-moderate carotid artery stenosis(<70%), we included 79 patients (25% women, mean age 66 years) who underwent CT imaging with a two-year interval. We assessed extra- and intracranial carotid artery calcification (ECAC, ICAC). And calculated the difference in ECAC and ICAC volume, using logistic regression analyses to investigate associations of cardiovascular determinants.
**Results or Findings:** We found an increase in ECAC volume in 46% of patients (median: 23.0 mm3[IQR=3.9;40.1]) and in 45% in ICAC volume (median:12.9 mm3[IQR=7.2;27.5]). Larger ECAC volume at baseline was associated with less increase in ECAC over two years (odds ratio (OR):0.72 [95%CI=0.58;0.90]). No statistically significant associations were found for increase in ICAC. Additionally, we observed decreases in ECAC and ICAC in 34% and 25% of the arteries. More ECAC at baseline was related to a decrease in ECAC (OR:2.24 [95%CI=1.60;3.13]. More ICAC at baseline was associated with a decrease in ICAC (OR:2.17 [95%CI=1.48;3.16]).
**Conclusion:** We provide important novel insights into the dynamics of carotid plaque calcification in TIA/minor stroke patients.
**Limitations:** The limitations are a threshold of 600 HU and a cut-off value of 10%, to define both increase and decrease of the calcification volume.
**Ethics committee approval:** This study was approved by the institutional review board and all patients gave written informed consent.
**Funding for this study:** This research was supported by the Dutch Heart Foundation and performed within the framework of the Centre for Translational Molecular Medicine (www.ctmm.nl), project PARISK (Plaque At RISK; grant number 01C-202).
**Author Disclosures:**
Taihra Zadi: Nothing to disclose

### PP 1-6

**Intracranial vascular malformations: what radiologists need to know**

DOI: 10.26044/ecr2022/16796

*A. Manzella*, M. Silva, R. Gonçalves dos Santos, I. d. S. C. Oliveira, A. Alcoforado, N. Seabra, J. Brito, G. Andrade, B. Mota; Recife/BR

**Purpose:** First, to discuss and illustrate the most common intracranial vascular malformations. Second, to identify the imaging features that help differentiate brain arteriovenous malformations (AVMs) from other vascular brain lesions. Third, to discuss the imaging findings that should be included in radiology reports in patients with brain AVMs.
**Methods or Background:** Cerebrovascular malformations are the most common symptomatic vascular malformations. They are predominantly solitary and supratentorial. Their origin remains uncertain, although they are considered multifactorial and often related to congenital factors. They can be classified according to the presence of arteriovenous shunt (AVMs, proliferative cerebral angiopathy, dural arteriovenous fistula and pial arteriovenous fistula, carotid-cavernous fistula and aneurysmal malformation of the vein of Galen) or absence of shunt (venous development anomaly, sinus pericranii, cerebral cavernous malformation and capillary telangiectasia).
**Results or Findings:** The authors will review the anatomy of the superficial and deep venous system, as well as the eloquent areas of the brain parenchyma, which are essential to categorise these malformations according to the Spetzler-Martin classification. The imaging findings will be illustrated using a multimodality approach. Each malformation will be discussed separately.

**Conclusion:** Radiologists must be able to detect the presence of a cerebrovascular malformation. Mimics of brain AVMs need to be identified as such to help guide diagnosis and therapy. In addition, when a brain AVM is found, specific details about the natural risk posed by a brain AVM and the risks related to treatment need to be reported.
**Limitations:** This is a pictorial review with cases from the authors' institution archives.
**Ethics committee approval:** Not applicable.
**Funding for this study:** Not applicable.
**Author Disclosures:**
Isabela de Souza Carvalho Oliveira: Nothing to disclose
Bruno Mota: Nothing to disclose
Josemar Brito: Nothing to disclose
Natalia Seabra: Nothing to disclose
Rennah Gonçalves dos Santos: Nothing to disclose
Maria Silva: Nothing to disclose
Gustavo Andrade: Nothing to disclose
Adonis Manzella: Nothing to disclose
Ayrton Alcoforado: Nothing to disclose

### PP 1-7

**A pictorial essay of cranial neurovascular conflicts: where to look and when to report**
*G. Rotariu*, I. Sava, D. Negru; Iasi/RO

**Purpose:** This educational exhibit aims to bring to attention the cranial neurovascular compression syndromes by illustrating a comprehensive MRI gallery of neurovascular conflicts involving cranial nerves III, V, VI, VII, VIII, IX, XII in order for the radiologist to: (1) review the anatomy of the cranial nerves in the cisternal segment and the physiopathology of this syndrome; (2) differentiate between neurovascular contact and conflict and therefore to know when to report such a finding.
**Methods or Background:** Neurovascular conflicts represent abnormal contacts between the cisternal segment of a cranial nerve and a vessel (usually a redundant artery), causing nerve irritation. Clinically, this translates to oculomotor nerve palsy, trigeminal neuralgia, abducens nerve palsy, hemifacial spasm, tinnitus/vertigo, glossopharyngeal or hypoglossal neuralgia. A neurovascular contact may be an incidental finding on MRI, but there are some useful signs to determine whether it will become clinically relevant and therefore if it should be reported in asymptomatic patients. Attention should be paid to the arteries, which are more expected to produce a significant conflict than veins, and to the effects produced on the nerve (deviation, indentation, atrophy).
**Results or Findings:** A combination of three MRI sequences is considered the standard to visualise the complex neurovascular anatomy in the cisternal space: 3D T2-WI, TOF, and 3D gadolinium-enhanced T1-WI. These sequences allow optimal visualisation of the relation between the cranial nerves and vasculature around them.
**Conclusion:** Familiarity with the anatomy of cranial nerves and vessels within the cisternal space and awareness of the elements that differentiate a normal neurovascular contact from a conflict are key to make specific and confident diagnoses regarding neurovascular compression syndromes.
**Limitations:** No limitations were identified.
**Ethics committee approval:** Not applicable.
**Funding for this study:** Not applicable.
**Author Disclosures:**
Gabriela Rotariu: Nothing to disclose
Dragos Negru: Nothing to disclose
Ionut Sava: Nothing to disclose

### PP 1-8

**NOWinBRAIN: a large, systematic, comprehensive and freely available neuroimage repository bridging neuroradiology, neuroanatomy, neuroscience, neuroeducation and art**
*W. L. Nowinski*; Lomianki/PL

**Purpose:** Neuroanatomy, neuroscience, and/or neurology are reported to be the most difficult in medical education. To facilitate coping with this problem I have created NOWinBRAIN, a large, systematic, comprehensive, beautiful and freely available repository of three-dimensional images of the human brain extended to head and neck bridging neuroradiology, neuroanatomy, neuroscience, neuroeducation (from students to patients) and art.
**Methods or Background:** NOWinBRAIN is derived from a 3D electronic brain atlas (Nowinski WL et al, "The Human Brain, Head and Neck in 2953 Pieces", Thieme, New York, 2015) constructed from multiple 3/7 Tesla MR and high-resolution CT scans of a living brain. This interactive repository contains 8 galleries (denoted G1-G8). Galleries G1-G6 were created earlier; G1-G5 comprise surface anatomy fully parcellated by colour and labelled, and organised into image (appearance and context) sequences, and G6 contains correlated surface and planar anatomy placed in a stereotactic coordinate system. For ECR2022 two new galleries are created, dissection gallery (G7) and functional gallery (G8). G7 provides relationships between the dissected brain and its inner structures, white matter tracts, cranial nerve nuclei, and intracranial vasculature. G8 contains surface anatomy with its functional description, also suitable for patients.
**Results or Findings:** The NOWinBRAIN repository is implemented and contains about 7,500 3D images. It is web-based running on low-cost computers and mobile devices, freely available at www.nowinbrain.org and requires no password/registration. Because of its accessibility and ease of use, the majority of the current visitors are from Asia, Africa, and Latin America.
**Conclusion:** This bridge-building repository is useful for a spectrum of users: clinicians, educators, medical students, and patients. It also serves users from less privileged countries.
**Limitations:** No 3D models, which are suitable e.g. for 3D printing, are available.
**Ethics committee approval:** Not applicable.
**Funding for this study:** None. This is my labour of love.
**Author Disclosures:**
Wieslaw Lucjan Nowinski: Nothing to disclose

### PP 1-9

**Does the extent of FLAIR vessel hyperintensities predict futile recanalisation after mechanical thrombectomy?**
*A. Bani Sadr*; Lyon/FR

**Purpose:** FLAIR vessel hyperintensities (FVH) are thought to be a surrogate of cerebral collateral status. We aimed to assess the relationship between FVH and futile recanalisation in acute ischaemic (AIS) patients with large vessel occlusion treated by mechanical thrombectomy (MT).
**Methods or Background:** HIBISCUS-STROKE cohort includes AIS patients treated by MT after baseline MRI. FVH were assessed using a previously published four-scale classification and were considered poor when grade < 3 (no FVH in frontoparietal sulci). We defined futile recanalisation as a 3-month mRS > 2 despite successful reperfusion (TICI 2B – 3). We performed single and multiple variables logistic regressions to assess predictive factors of futile recanalisation
**Results or Findings:** 128 patients were included. Of them 108 (84.38%) experienced successful recanalisation. Recanalisation was futile in 39 subjects (36.11%). Bivariate analyses indicated that poorly extended FVH (FVH grade < 3) was associated with futile recanalisation (odds ratio (OR) 3.10, 95% confidence interval (CI): [1.38; 7.31], p=0.008) as well as age, hypertension and diabetes, baseline NIHSS score, pre-stroke mRS, lack of intravenous thrombolysis, cerebral microangiopathy and microbleeds. Multivariable analysis confirmed that poor FVH status was independently associated with futile recanalisation (OR 5.32; CI 95% [1.91; 16.79]) in combination with hypertension (OR 5.79, 95% CI [2.13; 17.66]), baseline NIHSS score (OR 1.09, 95% CI [1.01; 1.18]), intravenous thrombolysis (OR 0.21, 95% CI [0.07; 0.56]) and, microbleeds (OR 4.26; 95% CI [1.25; 16.16]).
**Conclusion:** Poorly extended FVH (Grade <3) at baseline MRI is an independent predictor of futile recanalisation.
**Limitations:** We analysed both TICI 2b-3 patients.
**Ethics committee approval:** The local ethics committee approved the study and all subjects or their relatives signed an informed consent form (IRB number: 00009118).
**Funding for this study:** Funding was received for this study by RHU-MARVELOUS (ANR-16-RHUS-0009) of Université de Lyon, within the program “Investissements d'Avenir” (French National Research Agency).
**Author Disclosures:**
Alexandre Bani Sadr: Nothing to disclose

### PP 1-10

**Impact of directions removal on DTI markers used in clinical routine**
J-B. Martini[1], D. Cassereau[1], I. Trimeche[1], *D. Galanaud*[1], L. Velly[2], L. Puybasset[1], V. Perlbarg[1]; [1]Paris/FR, [2]Marseille/FR

**Purpose:** The brainQuant software (BrainTale, France) provides calibrated diffusion tensor imaging (DTI) markers clinically validated and activable in clinical situations. Since signal-dropout is a common artifact in DTI, limiting the use of these markers, the objective of this work is to evaluate the impact of the removal of artifacted directions on provided markers.
**Methods or Background:** 18 patients from the MRI-coma trial (NCT00577954), surviving after cardiac arrest (CA), were examined with 3T MRI scanner including 3DT1 and DTI (50 directions) sequences. For each patient, we have generated eight altered DTI scans by randomly removing up to 40 directions (step of 5). These altered scans have been analysed with the brainQuant software to extract the resulting whole-brain white matter fractional anisotropy (WWM-FA) used for prediction of neurological outcome in CA survivors (Velly et al., Lancet, 2018). Percent root mean square errors (%RMSE) have been calculated between modified and reference scans.
**Results or Findings:** Up to 15 removed directions, our simulations show that %RMSE remains less than 1% between WWM-FA in reference and altered data. Up to 35 removed directions, the %RMSE grows to 5%. For 40 removed directions (among the 50 directions initially present in the reference scans), the %RMSE increases to 10.5%.

**Conclusion:** In case of signal dropout, these results suggest that we can accept a loss of up to 15 directions without significant effects on the robustness of the DTI markers evaluation. This opens the door to a larger use of DTI-derived markers, even in the presence of dropout artifacts.
**Limitations:** The study has been realised on a small number of patients and only one DTI protocol was used.
**Ethics committee approval:** Not applicable.
**Funding for this study:** Funding was received for this study by the French Ministry of Health.
**Author Disclosures:**
Iyed Trimeche: Nothing to disclose
Damien Galanaud: Nothing to disclose
Louis Puybasset: Nothing to disclose
Lionel Velly: Nothing to disclose
Didier Cassereau: Nothing to disclose
Vincent Perlbarg: Nothing to disclose
Jean-Baptiste Martini: Nothing to disclose

### PP 1-11

**APT-weighted MRI, a familiar stranger: uses and applications of APTw MRI technique in CNS tumours**
*J. M. Brenes Castro*, G. A. Lozano, S. Castells, B. Tintaya, B. Matellini Mosca, J. E. Mendez, M. Tecame, B. Gomez-Anson; Barcelona/ES

**Purpose:** Explaining APT MRI basic technical principles. Reviewing the most-used applications of this MRI technique, focusing on the diagnosis and monitoring of CNS tumours. Illustrating those main uses throughout images of patients in our institution.
**Methods or Background:** Amide proton transfer-weighted (APTw) imaging is a relatively recent MRI technique that has consolidated until being part of our hospital's protocols, especially in the monitoring of CNS tumoural lesions. Nevertheless, we can easily feel uncertainty when facing the interpretation of this sequence, not knowing what it could provide us, or even whether it does. This poster is focused on reviewing those potential applications and helping us familiarise ourselves with this new acquaintance that has come to stay.
**Results or Findings:** APTw imaging is a molecular MRI technique that generates images based on the amide groups in mobile cellular proteins and peptides and does not require exogenous contrast agent injection. Currently, the most-used application of APTw MRI is for the study of brain tumours, where sometimes standard MRI sequences (such as T2w, FLAIR and Gd-T1w) are not sufficiently tissue-specific and suffer from several limitations. Some of the principal uses are: in the initial diagnosis of tumours, helping us in the differential diagnosis with other intracranial non-tumoural pathologies (Fig. 1); providing us information about the grading of tumoural lesions (Fig. 2, 3, 4); as a tool to distinguish the cases of recurrent tumours from treatment effects (Fig. 5); as an essential instrument in the evaluation of patients with impaired kidney function.
**Conclusion:** APTw imaging is a valuable tool we can use as an additional technique in the evaluation of CNS tumoural pathology, both in the initial diagnosis and in the monitoring.
**Limitations:** Not applicable.
**Ethics committee approval:** Not applicable.
**Funding for this study:** Not applicable.
**Author Disclosures:**
Jose Manuel Brenes Castro: Nothing to disclose
Briano Matellini Mosca: Nothing to disclose
Benjamin Tintaya: Nothing to disclose
Beatriz Gomez-Anson: Nothing to disclose
Jorge Eliecer Mendez: Nothing to disclose
Sara Castells: Nothing to disclose
Mario Tecame: Nothing to disclose
Gloria Andrea Lozano: Nothing to disclose

**08:00-09:00 Room B**

### Research Presentation Session: Musculoskeletal

### RPS 110

### Musculoskeletal oncology

**Moderator**
V. Vasilevska-Nikodinovska; Skopje/MK

### RPS 110-2

**Intravoxel incoherent motions imaging combined with diffusion kurtosis imaging to assess the response to radiotherapy in rabbit VX2 malignant bone tumor model**
*J. Guo*, W. Xu; Qingdao/CN
*(jnzx_gj@qq.com)*

**Purpose:** To probe the potential of several non-invasive parameters extracted from intravoxel incoherent motion (IVIM) and diffusion kurtosis imaging (DKI) in evaluating the early response to radiotherapy for VX2 malignant bone tumors in rabbits.
**Methods or Background:** 47 models which underwent IVIM and DKI before and after radiotherapy were prospectively enrolled. They were grouped into pre-treatment, good responders (GRs) and poor responders (PRs). IVIM-based parameters (tissue diffusion [Dt], pseudo-diffusion [Dp], perfusion fraction [fp]) and DKI-based parameters (mean diffusion coefficient [MD] and mean kurtosis [MK]) were calculated for each subject. Corresponding changes of MRI parameters before and after radiotherapy in each group were studied with one-way analysis of variance. The most sensitive MRI parameters were screened by using binary logistic regression and using receiver operating characteristic curve (ROC) analysis for evaluating the diagnostic performance.
**Results or Findings:** Dt, fp and MD increased significantly and MK decreased significantly after radiotherapy ($p<0.05$). The differences in Dt, fp and MK between GRs and PRs groups were statistically significant ($p<0.05$). The combination of Dt, fp and MK had the best diagnostic performance in identifying GRs from PRs (AUC=0.972, $p<0.001$).
**Conclusion:** The combination of IVIM- and DKI-based parameters hold great potential for assessing the early response to radiotherapy in rabbit VX2 malignant bone tumors.
**Limitations:** Only treatment changes after 3 days of radiotherapy were studied, and future studies will monitor the long-term dynamic changes. Furthermore, ROI was outlined in single layer, and we will outline the whole tumor in the future to explore the impact on the assessment results.
**Ethics committee approval:** This study was approved by the hospital ethics committee.
**Funding for this study:** Funding was received from the National Natural Science Foundation of China (Grant No. 8167070553).
**Author Disclosures:**
Wenjian Xu: Nothing to disclose
Jia Guo: Nothing to disclose

### RPS 110-3

**MRI radiomics-based machine learning to predict neoadjuvant chemotherapy response in Ewing sarcoma**
*S. Gitto*, V. Corino, M. Bologna, L. Marzorati, E. Milazzo Machado, D. Albano, C. Messina, L. Mainardi, L. M. Sconfienza; Milan/IT
*(sal.gitto@gmail.com)*

**Purpose:** To evaluate 2D and 3D magnetic resonance imaging (MRI) radiomics-based machine learning prediction of neoadjuvant chemotherapy response in Ewing sarcoma.
**Methods or Background:** Thirty patients were retrospectively included at two tertiary bone sarcoma centres. Inclusion criteria were: (i) biopsy-proven Ewing sarcoma treated with neoadjuvant chemotherapy before surgery; (ii) preoperative MRI available; (iii) therapy response evaluated after surgery based on pathological findings. Seven patients were poor responders and 23 were good responders. On T1-weighted and T2-weighted images, manual segmentations were performed by drawing both 2D regions of interest (ROIs) along tumour borders on the slice showing the largest diameter and 3D ROIs including the whole volume. A total of 1702 3D and 958 2D features were extracted. Feature stability was assessed through small geometrical transformations of the ROIs mimicking multiple manual delineations, and intraclass correlation coefficient >0.75 defined feature stability. Feature selection included collinearity and significance analysis. Three machine learning classifiers were considered, such as k-nearest neighbors (k-NN), logistic regression (LR) and random forest (RF). To evaluate the unbiased performance of the classifiers, a cross-validation approach was used with a hold-out partition of 80-20 (80% for training and 20% for test, repeated 100 times). Class balancing was performed to oversample the minority (poor responders) class in the training cohort.
**Results or Findings:** A total of 1303 3D and 620 2D radiomic features were stable to geometrical transformation of the ROI. Four 2D and four 3D features were selected during dimensionality reduction. LR built upon 3D features achieved the best performance with 85% sensitivity, 87% specificity and 85% accuracy (AUC=0.9) in predicting response to chemotherapy.
**Conclusion:** Machine learning showed very good performance in predicting Ewing sarcoma response to neoadjuvant chemotherapy using MRI radiomic features.
**Limitations:** Small sample size.
**Ethics committee approval:** This study was approved by an ethics committee.
**Funding for this study:** No funding was received for this study.
**Author Disclosures:**
Valentina Corino: Nothing to disclose
Luca Mainardi: Nothing to disclose
Carmelo Messina: Nothing to disclose
Salvatore Gitto: Nothing to disclose
Luca Maria Sconfienza: Nothing to disclose
Lorenzo Marzorati: Nothing to disclose
Estevao Milazzo Machado: Nothing to disclose
Marco Bologna: Nothing to disclose
Domenico Albano: Nothing to disclose

### RPS 110-5
**Multi-parametric magnetic resonance imaging for radiotherapy response monitoring in soft-tissue sarcomas: a concept study for a histology and MRI co-registration algorithm**
*M. Jung*, A. Runkel, T. D. Diallo, J. Kiefer, P. Bronsert, P. M. Jungmann, M. Eisenblaetter, F. Bamberg, M. Benndorf; Freiburg/DE
*(matthias.jung@uniklinik-freiburg.de)*

**Purpose:** To establish a spatially exact co-registration procedure between in-vivo multi-parametric MRI (mpMRI) and (immuno-) histopathology of soft-tissue sarcomas (STS) to identify imaging parameters reflecting radiotherapy response of STS.
**Methods or Background:** In selected patients with STS, mpMRI is performed before, during, and after completion of neoadjuvant radiotherapy. mpMRI includes diffusion-weighted (DWI), intravoxel-incoherent-motion (IVIM), and dynamic-contrast-enhanced (DCE) imaging. The resection specimen is embedded in 6.5% agarose after initial fixation in formalin. To ensure an identical alignment of histopathological sectioning and in-vivo imaging, an ex-vivo MRI is fused to the last in-vivo mpMRI. The deviating angulation of the specimen to the in-vivo location of the tumour is determined. The agarose-block is trimmed accordingly. A second ex-vivo MRI in a dedicated localiser is performed, which is matched to a custom-built sectioning machine. Sections are stained with hematoxylin. Immunohistochemical staining is performed with ALDH1A1 as a radioresistance and MIB1 as a proliferation marker. Fusion of the digitised (immuno-) histopathological sections to the in-vivo mpMRI is accomplished via intermediate registration of the histopathological sections to the ex-vivo MRI. Slice-by-slice correlation of mpMRI-parameters with (immuno-) histopathology is performed.
**Results or Findings:** Initial analyses show a strong morphological correlation of the sectioning of the (immuno-) histopathological specimen with in-vivo mpMRI after neoadjuvant radiotherapy. Preliminary analyses in post-therapeutic specimens revealed increased values in the Ktrans-map in vital tumour parts, characterised by MIB1- and ALDH1A1-positive cells, compared with necrotic tumour parts.
**Conclusion:** Our initial results show a promising approach to obtain accurate co-registration between histopathology and MRI for STS. In a larger cohort of patients, the method established here will enable the prospective identification and validation of in-vivo imaging-biomarkers for radiotherapy response monitoring of STS via precise molecular and cellular correlation.
**Limitations:** Preliminary proof-of-concept study with exemplary results.
**Ethics committee approval:** This study was approved by an ethics committee.
**Funding for this study:** No funding was received for this study.
**Author Disclosures:**
Peter Bronsert: Nothing to disclose
Matthias Jung: Nothing to disclose
Matthias Benndorf: Nothing to disclose
Fabian Bamberg: Nothing to disclose
Pia M Jungmann: Nothing to disclose
Michel Eisenblaetter: Nothing to disclose
Alexander Runkel: Nothing to disclose
Jurij Kiefer: Nothing to disclose
Thierno D. Diallo: Nothing to disclose

### RPS 110-6
**CT-guided fine-needle aspiration and core biopsy, prognostic factors and diagnostic accuracy as individual and combined procedures in bone tumours**
*N. A. Ochoa Sambrizzi*, M. Pérez Torán, A. Gimeno, M. De Albert De Delas-Vigo, M. Veintemillas, L. Casas, C. Torrents, R. Dominguez; Barcelona/ES
*(n.ochoa.sambrizzi@gmail.com)*

**Purpose:** We present a retrospective study in which all CT-guided bone biopsies performed at our centre in a span of 3 years were reviewed in order to determine the success rate of fine-needle aspiration (FNA) and core biopsy techniques as individual procedures in addition to their combined diagnostic accuracy.
**Methods or Background:** A total of 172 biopsies were performed from March 2016 to March 2019, with only 121 of the procedures meeting inclusion criteria. Clinical history, imaging studies as well as pathological reports were reviewed in order to determine associated factors affecting the success rate of such techniques. The main outcome of this study was determined as a statistically significant difference between diagnostic rates of both individual procedures and their combined accuracy. As secondary outcomes, we compared diagnostic rates considering the most likely diagnosis reported on the last imaging study, as well as the composition of the lesion (lytic, mixt, or osteoblastic).
**Results or Findings:** FNA extracted enough samples for cytological study in 96 out of the 120 procedures (success rate of 80% [0.73-0.87]) and core biopsy in 112 (success rate of 93% [0.88-0.97]). However, when combined, enough material was successfully obtained in 100% of patients for diagnosis either by cytology or histology. The sensitivity of FNA was 0.95 [0.91-0.99] and the specificity 0.83 [0.75-0.9]. As for biopsy, the sensitivity was 0.98 [0.95-1] and the specificity was 1.
**Conclusion:** Associated performance of FNA and core biopsy, when approaching the diagnosis of a bone tumour, not only notoriously increases the success rate of the procedure and lowers the risk of repetitive interventions, but also improves sample viability and technique sensitivity.
**Limitations:** Incomplete patients records and pathology reports.
**Ethics committee approval:** This study was approved by the ethics committee.
**Funding for this study:** No funding was recieved.
**Author Disclosures:**
Lourdes Casas: Nothing to disclose
Matias De Albert De Delas-Vigo: Nothing to disclose
Alfredo Gimeno: Nothing to disclose
Maite Veintemillas: Nothing to disclose
Carme Torrents: Nothing to disclose
Rosa Dominguez: Nothing to disclose
Manuel Pérez Torán: Nothing to disclose
Nicolás Andrés Ochoa Sambrizzi: Nothing to disclose

**08:00-09:00** **Room E1**

### Research Presentation Session: Neuro

### RPS 111
### Functional MRI in various diseases

**Moderator**
A. Borges; Lisbon/PT

### RPS 111-5
**What changes occur in the brain of veterans? An MRI and 1HMRS study**
*A. Urbanik*[1], I. Kucybała[1], P. Guła[2], M. Brożyna[2], W. Guz[2]; [1]Kraków/PL, [2]Rzeszów/PL

**Purpose:** The aim of the study was to assess changes in magnetic resonance imaging (MRI) of the head of soldiers and to compare the level of 1HMRS (proton magnetic resonance spectroscopy) metabolites in frontal and occipital lobes between soldiers and a healthy age-matched control group.
**Methods or Background:** Overall, 54 professional soldiers who served at least ten years and participated in international missions were included in the study group. Mean age of the study group was 41.4±4.0 years and all soldiers were male. All examinations were performed using a 1.5 Tesla MRI system. Acquired MR sequences were T1-weighted, T2-weighted and PRESS. 1HMRS volumes of interest were located symmetrically in the right and left frontal and occipital lobes. Relative values of lipids (Lip), lactates (Lac), N-acetylaspartate (NAA), choline (Cho) and myoinositol (mI) concentrations were calculated in relation to the creatine (Cr) concentration. The mean relative concentrations of metabolites were compared between the study and the control group, separately for frontal and occipital lobes.

**Results or Findings:** The most frequent findings in the head MRI of the soldiers were: sinusitis (38.9%), paranasal sinus retention cysts (14.8%) and asymmetric lateral cerebral ventricles (11.1%). Brain ischaemic lesions were observed in 5.6% of soldiers. Frontal lobe mI/Cr ratio was significantly higher (p=0.005), while NAA/Cr ratio was markedly lower (p=0.001) in soldiers, in comparison to the control group. In occipital lobes, NAA/Cr ratio was statistically significantly lower (p=0.005) in military personnel than in the control group. Statistically significant differences were not detected for other metabolites.
**Conclusion:** Brain MR spectroscopy of soldiers revealed neuronal loss (reduced NAA/Cr ratio in comparison to controls) and glial proliferation (increased mI/Cr ratio compared to controls). The frequency of brain ischaemic lesions was moderate.
**Limitations:** An identified limitation was the small group size.
**Ethics committee approval:** Jagiellonian University Bioethics Committee (Approval No 1072.6120.196.2019).
**Funding for this study:** No funding was received for this study.
**Author Disclosures:**
Wieslaw Guz: Nothing to disclose
Maciej Brożyna: Nothing to disclose
Przemysław Guła: Nothing to disclose
Iwona Kucybała: Nothing to disclose
Andrzej Urbanik: Nothing to disclose

### RPS 111-6

**Multimodal advanced MRI imaging of nerve roots in lumbar radiculopathy using DWI, DTI and T2 mapping sequences: clinical and neurophysiological correlations**
*F. Bruno*, F. Sgalambro, V. Pagliei, L. Pertici, A. Gagliardi, F. Borea, F. Arrigoni, A. Splendiani, C. Masciocchi; L'Aquila/IT
*(federico.bruno.1988@gmail.com)*

**Purpose:** To assess lumbar nerve root DWI, DTI and T2 mapping MRI metrics in patients with lumbar disc herniation sciatica, and evaluate their correlations with clinical and neurophysiological findings.
**Methods or Background:** We prospectively evaluated 45 patients suffering from unilateral lumbar radiculopathy due to L5-S1 discoradicular conflict.
All patients were submitted to MRI examinations using a standard spine protocol and additional DWI, DTI, and T2 mapping sequences. Relative metrics of ADC, FA, and T2 relaxation times were recorded placing ROIs at the preforaminal, intraforaminal, and post-foraminal level, either at the affected side and the contralateral side, used as control. All patients were also submitted to EMG testing, recording the spontaneous activity, voluntary activity, F-wave amplitude and latency, motor evoked potentials (MEP) amplitude and latency, either at the level of the tibialis anterior and the gastrocnemius muscles. Clinical features (disease duration, pain, sensitivity, strength, osteotendinous reflexes) were also recorded.
**Results or Findings:** Among clinical features, we found positive correlation of pain intensity with ADC values of the lumbar nerve roots. The presence of spontaneous activity correlated with lower ADC values of the affected lumbar nerve root. F-wave and MEP latency correlated with decreased FA values at the foraminal level and increased values at the post-foraminal level. The same neurophysiological measures correlated positively with pre-foraminal T2 mapping values and negatively with post-foraminal T2 mapping values. Increased T2 mapping values at the foraminal level correlated with disease duration.
**Conclusion:** Evaluation of lumbar nerve roots using advanced MRI sequences may provide useful clinical information in patients with lumbar radiculopathy, potentially indicating active inflammation/myelinic damage (DTI, T2 mapping) and axonal damage/cronicity (DWI).
**Limitations:** An identified limitation was the limited study sample.
**Ethics committee approval:** This study was approved by Institutional IRB.
**Funding for this study:** This study was approved by an ethics committee.
**Author Disclosures:**
Alessio Gagliardi: Nothing to disclose
Ferruccio Sgalambro: Nothing to disclose
Alessandra Splendiani: Nothing to disclose
Francesco Arrigoni: Nothing to disclose
Francesco Borea: Nothing to disclose
Carlo Masciocchi: Nothing to disclose
Leonardo Pertici: Nothing to disclose
Federico Bruno: Nothing to disclose
Valeria Pagliei: Nothing to disclose

### RPS 111-8

**Restoration of brain networking following carotid endarterectomy**
*M. Porcu*[1], L. Cocco[1], R. Cau[1], M. Wintermark[2], R. Sanfilippo[1], J. S. Suri[3], L. Saba[1]; [1]Cagliari/IT, [2]Stanford, CA/US, [3]Roseville, CA/US
*(micheleporcu87@gmail.com)*

**Purpose:** The purpose of this rs-fcMRI study is to compare the connectivity state of the population study of patients with extracranial carotid artery stenosis (ECAS) eligible for carotid endarterectomy (CEA) before and 12 months after CEA with the connectivity state of a control population of non-demented healthy subjects (HS).
**Methods or Background:** In this exploratory observational study, 20 patients with extracranial carotid artery stenosis eligible for CEA, and 20 voluntary HS matched for age and sex were prospectively included. Within one week before the CEA procedure and 12 months after the CEA procedure, all patients underwent an MRI examination for rs-fcMRI analysis. A single MRI examination for the same purpose was performed on the control population study. A region-of-interest (ROI) based functional connectivity approach was used for analysing the differences in connectivity between patients and healthy controls, before and following CEA respectively. Parametric multivariate statistics (cluster level inferences) were adopted for analysing the differences of the global connectivity by exploiting a cluster-level p-value corrected for false discovery rate <0.05 as cluster threshold, and p-uncorrected value <0.05 as connection threshold.
**Results or Findings:** The ROI based functional analysis revealed that patients with ECAS before CEA showed two clusters of reduced interhemispheric connectivity between temporal, frontal, and parietal cerebral areas when compared to HS. No statistical significant differences in connectivity were found in the comparison between the study population following CEA procedure and the HS.
**Conclusion:** CEA is associated with restoration of brain networking, particularly interhemispheric connectivity.
**Limitations:** The exploratory nature of the study and the intrinsic huge variability in rs-fcMRI analytical approaches.
**Ethics committee approval:** The institutional review board approved this study.
**Funding for this study:** This research did not receive any specific grant from funding agencies in the public, commercial, or non-profit sectors.
**Author Disclosures:**
Max Wintermark: Nothing to disclose
Riccardo Cau: Nothing to disclose
Roberto Sanfilippo: Nothing to disclose
Luigi Cocco: Nothing to disclose
Jasjit S Suri: Nothing to disclose
Michele Porcu: Nothing to disclose
Luca Saba: Nothing to disclose

**08:00-09:00** **Room F1**

### Research Presentation Session: Genitourinary

### RPS 107

### Solid or cystic, benign or malignant renal lesions: how to make the right diagnosis?

**Moderator**
P. Asbach; Berlin/DE

### RPS 107-2

**CT imaging findings of renal epithelioid lipid-poor angiomyolipoma**
*X. Yi*[1], D. Wang[1], Y. Fu[1], B. T. Chen[2]; [1]Changsha/CN, [2]Duarte, CA/US
*(yixiaoping@csu.edu.cn)*

**Purpose:** To identify the specific imaging and clinicopathological features of a rare potentially malignant epithelioid variant of renal lpAML (E-lpAML) through comparison with other lpAML tumours.
**Methods or Background:** A total of 20 patients with E-lpAML (Group 1) and 43 patients with lpAML (Group 2) were retrospectively included in this study. Computed tomography (CT) imaging features and clinicopathological findings were recorded. Independent predictors for E-lpAML were identified using multivariate logistic regression and were used to construct a diagnostic score for differentiation of E-lpAML from other lpAML.

**Results or Findings:** The E-lpAML group consisted of 6 men and 14 women (median ±SD: 39.45±15.70 years of age, range: 16.0-68.0 years). E-lpAML usually appeared as hyperdense mass lesions located at renal sinus (n=8, 40%) or at renal cortex (n=12, 60%), a "fast-in and slow-out" enhancement pattern (n=20, 100%), cystic degeneration (n=18, 90%), "eyeball" sign (n=11, 55%) and tumour neo-vasculature (n=15, 75%) on CT. Multivariate logistic regression analysis showed that the independent predictors for diagnosing E-lpAML were cystic degeneration on CT imaging and CT Hounsfield value in corticomedullary phase of enhancement. A predictive model was built with the two predictors, achieving an AUC of 93.5% (95% confidence interval [95%CI]: 84.3-98.2%) with a sensitivity of 95.0% (95%CI: 75.1-99.9%) and a specificity of 83.72% (95%CI: 69.3-93.2%).
**Conclusion:** We identified some specific CT imaging features that could contribute to the correct diagnosis of E-lpAML, which may require nephron-sparing surgery from other lpAML tumours which are benign requiring no intervention.
**Limitations:** Firstly, this was a retrospective study and selective bias was unavoidable. Secondly, the underlying pathological basis was not clear. Lastly, other imaging modality such as MRI should be considered.
**Ethics committee approval:** Institutional Review Board approval was obtained.
**Funding for this study:** Not applicable.
**Author Disclosures:**
Bihong T. Chen: Nothing to disclose
Yan Fu: Nothing to disclose
Di Wang: Nothing to disclose
Xiaoping Yi: Nothing to disclose

### RPS 107-3
**Comparison of the diagnostic performance of the Bosniak classification with its 2019 update and the Renal cyst index**
*E. Rainer*, N. Garstka, N. Pötsch, P. Clauser, T. H. Helbich, P. A. Baltzer; Vienna/AT

**Purpose:** To compare the diagnostic performance and kappa agreement of the Bosniak classification with its 2019 update and the Renal cyst index (RCI) proposed by Yaohui Li in 2018 for the diagnosis of renal cystic lesions.
**Methods or Background:** This IRB-approved retrospective study included 73 patients with cystic renal masses (cystic component >70%) who were undergoing CT of the abdomen 6 months prior to surgery (nephrectomy, partial nephrectomy or marsupialisation). Two different readers (R1, R2) scored the CT studies according to the Bosniak classification and its 2019 update and the RCI. Inter-reader agreement was assessed by kappa statistics. Diagnostic measures (sensitivity, specificity, and area under the curve, AUC) were compared for all cases.
**Results or Findings:** Inter-reader agreement for the investigated classification systems did not differ significantly (P>0.05). The AUC´s were as follows: 0.855 (Bosniak 2019, 95%-CI 0.748-0.929), 0.869 (old Bosniak, 95%-CI 0.764-0.939) and 0.891 (RCI, 95%-CI 0.791-0.954).
**Conclusion:** The 2019 Bosniak update and the RCI present with high diagnostic accuracy and inter-reader agreement. They are thus equally applicable in clinical practice.
**Limitations:** Retrospective study design; Patients with Bosniak III and IV cystic lesions underwent surgery more often than patients with Bosniak I and II lesions which may bias the outcome. However, patients with Bosniak I and II lesions which did not undergo surgery were included if they had no progression in follow-up CT.
**Ethics committee approval:** The study is IRB approved (ethics committee number: 2057/2017).
**Funding for this study:** No funding was received for this study.
**Author Disclosures:**
Pascal A.T. Baltzer: Nothing to disclose
Nina Pötsch: Nothing to disclose
Eva Rainer: Nothing to disclose
Thomas H. Helbich: Nothing to disclose
Nathalie Garstka: Nothing to disclose
Paola Clauser: Nothing to disclose

### RPS 107-4
**Contrast-Enhanced Ultrasound (CEUS) in the evaluation of renal masses: results from a prospective single-centre study**
*M. Martino*[1], E. Polito[1], F. M. Drudi[1], F. Angelini[1], D. Fresilli[1], M. Bertolotto[2], V. Cantisani[1], C. Catalano[1]; [1]Rome/IT, [2]Trieste/IT

**Purpose:** To evaluate the diagnostic accuracy of Contrast Enhanced Ultrasound (CEUS) in characterising between malignant and benign renal lesions confirmed by the histological examination.
**Methods or Background:** 110 patients, for a total of 118 renal masses previously identified at CT and MRI underwent CEUS. An expert radiologist evaluated morphological, qualitative and quantitative parameters.
**Results or Findings:** Histological results of 118 renal masses showed 88 (75%) malignant lesions and 30 (25%) benign lesions. Among morphological features, echogenicity was the best predictor of malignancy depicting a sensitivity, specificity, positive predictive value (PPV), negative predictive value (NPV) of 76%, 76%, 88%, and 57%, respectively. Among qualitative parameters, the most reliable parameter was the pseudo-capsule. Here, sensitivity, specificity, positive PPV, NPV were 85%, 86%, 94% and 71%, respectively. Among quantitative parameters, the most reliable parameters were peak intensity (PI) and the area under the ROC curve (AUC) with sensitivity, specificity, PPV, NPV values of 94%, 92%, 96%, 87%, and 99%, 92%, 97%, 97%, respectively. The best combination obtained to distinguish between benign and malignant lesions was restricted to CEUS parameters (PI and AUC). Here, sensitivity, specificity, PPV, NPV and accuracy rate were 93%, 100%, 100%, 83% and 93%, respectively.
**Conclusion:** CEUS increases the US accuracy to discriminate between benign and malignant renal lesions.
**Limitations:** The small sample size, and small percentage of benign tumours: only 25% of our sample. We enrolled patients with an indication for surgery, excluding patients undergoing active surveillance. All cases were evaluated by a single reader with long experience in ultrasound. So our results may overestimate the diagnostic accuracy of this technique.
**Ethics committee approval:** All procedures performed were in accordance with the ethical standards of the institutional and/or national research committee.
**Funding for this study:** Not applicable.
**Author Disclosures:**
Daniele Fresilli: Nothing to disclose
Michele Bertolotto: Nothing to disclose
Francesco Maria Drudi: Nothing to disclose
Milvia Martino: Nothing to disclose
Eleonora Polito: Nothing to disclose
Flavia Angelini: Nothing to disclose
Vito Cantisani: Nothing to disclose
Carlo Catalano: Nothing to disclose

### RPS 107-5
**Diagnostic value of combined use of CT and MRI for the Bosniak classification 2019: impact of reader specialty**
*Y. Arita*[1], H. Akita[1], H. Edo[2], R. Kufukihara[1], K. Shigeta[1], S. Yoshida[1], S. Okuda[1], Y. Fujii[1], M. Jinzaki[1]; [1]Tokyo/JP, [2]Saitama/JP
*(yarita@rad.med.keio.ac.jp)*

**Purpose:** To investigate the utility of combined use of computed tomography and magnetic resonance imaging (CT+MRI) for evaluating cystic renal masses using the Bosniak classification 2019 (BC2019).
**Methods or Background:** We retrospectively included 103 cystic renal masses from 83 consecutive patients assessed with both CT and MRI. Six readers in two groups (three radiologists and three urologists) reviewed the CT, MRI, and CT+MRI images using the BC2019. The interobserver agreement was assessed using Fleiss' kappa values. The overall Bosniak category was determined by consensus in each group of readers, with Bosniak categories III/IV considered malignant. The utility of each or combination of modalities in detecting malignancy was evaluated using weighted generalised score statistics. Radical or partial nephrectomy was used as a reference standard.
**Results or Findings:** The interobserver agreement for CT+MRI was substantial for both reader groups (radiologists: 0.78; urologists: 0.61), whereas that for CT alone was substantial for radiologists, but moderate for urologists (0.77 and 0.58, respectively). The sensitivity for detecting malignancy was significantly higher for CT+MRI than for CT alone among urologists (76.9% vs. 57.6%, p=0.03), while it did not significantly differ between CT+MRI and CT alone among radiologists (84.0% vs. 80.0%, p=0.49).
**Conclusion:** CT+MRI may be useful for diagnosing malignant cystic renal masses using the BC2019 for non-expert readers, whereas radiologists may be able to equally accurately determine the Bosniak category using CT alone.
**Limitations:** Firstly, according to the standard work-up of cystic renal masses in our hospital, both CT and MRI were performed for baseline evaluation. Secondly, the study sample size was relatively small.
**Ethics committee approval:** This study was approved by the Institutional Review Board. The requirement for written informed consent was waived due to the retrospective nature of the study.
**Funding for this study:** No funding was received for this study.
**Author Disclosures:**
Masahiro Jinzaki: Nothing to disclose
Ryohei Kufukihara: Nothing to disclose
Soichiro Yoshida: Nothing to disclose
Keisuke Shigeta: Nothing to disclose
Shigeo Okuda: Nothing to disclose
Yasuhisa Fujii: Nothing to disclose
Yuki Arita: Nothing to disclose
Hirotaka Akita: Nothing to disclose
Hiromi Edo: Nothing to disclose

### RPS 107-6
**Multi-parametric MRI to detect early treatment response of renal cell carcinoma to sunitinib**

*S. Ursprung*[1], A. N. Priest[2], F. Zaccagna[2], G. Stewart[2], A. Warren[2], S. J. Welsh[2], T. Eisen[2], F. A. Gallagher[2], T. Barrett[2]; [1]Tübingen/DE, [2]Cambridge/UK

**Purpose:** The spectrum of therapeutics available for metastatic clear cell renal cell carcinoma (mRCC) is expanding rapidly. Besides immune checkpoint inhibitors, tyrosine kinase inhibitors (TKI) continue to play a major role. Early treatment response assessment may guide treatment decisions and improve patient outcomes. The prospective, phase II NeoSun trial (EudraCtNo: 2005-004602-82) aimed to detect early response to the TKI sunitinib in mRCC.
**Methods or Background:** Patients with treatment-naïve mRCC eligible for surgery underwent multi-parametric MRI before and after 12 days of preoperative sunitinib. The MRI protocol included diffusion-weighted, blood-oxygen level-dependent and dynamic contrast-enhanced imaging. Following nephrectomy, patients recommenced sunitinib until disease progression determined by contrast-enhanced CT. Changes in imaging parameters were assessed with the Wilcoxon signed-rank test and effects on survival with the log-rank test.
**Results or Findings:** 22 patients were screened and 12 fulfilled the inclusion criteria. Following pre-surgical sunitinib, the volume reduced more in less necrotic tumours (-28% in solid and -17% in necrotic tumour areas, p=0.02). However, this was not correlated with progression-free (PFS). The reduction in diffusivity of the solid tumour (median from 1.30x10-3 to 1.20x10-3mm2/s, p=0.03) was significantly associated with PFS and a better RECIST response in the post-surgical follow-up (p=0,031 and 0,018 respectively). Furthermore, an increase in R2* (19 to 28s-1, p=0.001), a decrease in blood vessel permeability (Ktrans 0.415 to 0.305min-1, p=0.01) and microperfusion (perfusion fraction 0.34 to 0.19, p<0.001) were measured following 12 days of anti-angiogenic treatment with sunitinib.
**Conclusion:** Physiological imaging confirmed the efficacy of the anti-angiogenic agent 12 days after initiating therapy and correlated with long-term response and PFS.
**Limitations:** Larger studies are required to confirm the findings of this phase II clinical trial.
**Ethics committee approval:** This study was approved by UK national research ethics committee ref: 2005-004502-82.
**Funding for this study:** Pfizer provided the study drug sunitinib.
**Author Disclosures:**
Tim Eisen: Research/Grant Support: Pfizer and AstraZeneca Shareholder: AstraZeneca and Roche Employee: AstraZeneca and Roche
Sarah J Welsh: Research/Grant Support: Pfizer Other: IPSEN
Fulvio Zaccagna: Nothing to disclose
Tristan Barrett: Nothing to disclose
Andrew N Priest: Other: GE Healthcare
Anne Warren: Advisory Board: Roche
Stephan Ursprung: Nothing to disclose
Grant Stewart: Other: Pfizer Consultant: Merck, Pfizer, EUSA Pharma and CSurgical Grant Recipient: Pfizer, Astrazeneca and Intuitive Surgical
Ferdia Aidan Gallagher: Nothing to disclose

**08:00-09:00** **Room G**

### Research Presentation Session: Abdominal Viscera

### RPS 101

### Abdominal imaging pearls based on solid research

**Moderator**
J. M. Ni Mhuircheartaigh; Limerick/IE

### RPS 101-2
**Simple diagnosis of relevant strictures in primary sclerosing cholangitis patients using gadoxetic acid-enhanced MRI**

*S. Pötter-Lang*, N. Bastati-Huber, A. Messner, R. Ambros, A. Kristic, A. Korajac, G. O. Dovjak, M. Zalaudek, A. Ba-Ssalamah; Vienna/AT

**Purpose:** To compare T1-HBP-MRC to T2-MRCP in distinguishing non-RS from RS and hepatocellular dysfunction (HD), and to correlate these with outcomes in primary sclerosing cholangitis (PSC) patients.
**Methods or Background:** Relevant stricture (RS) in PSC patients has significant clinical and prognostic impact. However, RS definition and diagnosis remain controversial, relying upon morphology on T2-MRCP, which has a high inter-observer variability. We introduce a function-based RS definition on T1-weighted hepatobiliary phase (HBP) images of gadoxetic acid (GA)-enhanced MR cholangiography (T1-HBP-MRC). Six independent readers blinded to patient data reviewed 130 MRIs, diagnosing RS on T2-MRCP using several guidelines. On T1-HBP-MRC images, RS was diagnosed if no GA excretion was seen in the common bile duct or the distal right or left hepatic duct. If liver cirrhosis was additionally present, HD was diagnosed. The three diagnostic groups from T1-HBP-MRC (non-RS=86; RS=32; HD=12) were correlated with clinical scores, labs, splenic volume, and outcomes. Statistical analyses included Kaplan-Meier curves, decision-tree, and Cox regression.
**Results or Findings:** Inter-observer agreement was excellent for non-RS/RS/HD diagnosis on T1-HBP-MRC (κ=0.82), but poor to fair for T2-MRCP (κ=0.13 - 0.31). Laboratory tests and clinical scores were significantly worse in RS and HD versus non-RS patients (p<0.001). There were more clinical events in RS or HD than in non-RS patients (p<0.05). Non-RS/RS/HD diagnoses on T1-HBP-MRC correlated (p<0.001) with outcomes, whereas measurement-based T2-MRCP diagnoses did not (p>0.05). Multivariate analysis found T1-HBP-MRC-derived diagnosis was an independent risk factor for morbidity and mortality.
**Conclusion:** T1-HBP-MRC stratifies PSC patients into non-RS/RS/HD, which correlates with outcomes. Conventional T2-MRCP measurement-based criteria cannot.
**Limitations:** Retrospectiv; single centre.
**Ethics committee approval: Ethics committee approval:** IRB-approved, (EK 2249/2016).
**Funding for this study:** No funding was received for this study.
**Author Disclosures:**
Antonia Kristic: Nothing to disclose
Sarah Pötter-Lang: Nothing to disclose
Raphael Ambros: Nothing to disclose
Aida Korajac: Nothing to disclose
Ahmed Ba-Ssalamah: Speaker: Bayer Consultant: Bayer
DGregor Oliver Dovjak: Nothing to disclose
Nina Bastati-Huber: Nothing to disclose
Alina Messner: Nothing to disclose
Martin Zalaudek: Nothing to disclose

### RPS 101-3
**The adrenal-to-spleen ratio as a short-term predictor of mortality in intensive care patients: a feasibility study**

*D. Fedders*, R-T. Hoffmann, R. Winzer; Dresden/DE
*(dieter.fedders@gmail.com)*

**Purpose:** To validate the adrenal-to-spleen ratio determined in a pilot study in portal venous CT for short-term mortality prediction in ICU patients.
**Methods or Background:** Three hundred and seventy-one portal-venous CT scans of 203 ICU patients (127 men, age: 68.1 ± 14.4 years) were included in the retrospective analysis. ROI-based Hounsfield units of adrenal glands and spleen and their density ratio were evaluated. Matthews correlation coefficient (MCC) and receiver operating characteristic (ROC) analysis were performed to establish a threshold for the adrenal-to-spleen ratio in relation to 72-hour mortality to classify patients as survivors or deceased. In addition, the cohort was classified using the threshold determined in a pilot study (>1.41) and the results were compared. The precision-recall curve (PRC) was used to test the impact of adding patients at low vital risk on ROC. Statistical measures for binary classification tests as well as relative chance and relative risk were determined.
**Results or Findings:** The discriminatory power of the adrenal-to-spleen ratio threshold (>1.37), determined using the current cohort yields a high degree of correct predictions regarding short-term mortality (MCC: 0.87; sensitivity: 83.67%; specificity: 99.07%; PPV: 93.2%; NPV: 97.6%). The results using the threshold determined in the pilot study deviate only slightly.
**Conclusion:** The adrenal-to-spleen ratio on portal-venous CT has high predictive power as a reproducible image-based prognostic marker for short-term ICU mortality. Despite different cohorts, the adrenal-to-spleen ratio varies only slightly. Therefore, it is suitable as a stable indicator for an extraordinarily high risk of dying within three days after imaging.
**Limitations:** Retrospective study design. Measurement inaccuracies, adjacent adipose tissue and partial volume effects may have affected the results.
**Ethics committee approval:** This study was approved by an ethics committee.
**Funding for this study:** No funding was received for this study.
**Author Disclosures:**
Ralf-Thorsten Hoffmann: Nothing to disclose
Robert Winzer: Nothing to disclose
Dieter Fedders: Nothing to disclose

### RPS 101-5

**How to distinguish ITPN with associated invasive carcinoma from conventional ductal adenocarcinomas of the pancreas**

*E. Khristenko*[1], T. Hank[2], M. Gaida[3], H-U. Kauczor[1], T. Hackert[1], M. Klauß[1], P. Mayer[1]; [1]Heidelberg/DE, [2]Vienna/AT, [3]Mainz/DE

**Purpose:** Intraductal tubulopapillary neoplasms (ITPN) are rare pancreatic tumors and are considered to have better prognosis than pancreatic ductal adenocarcinoma (PDAC). The present study aimed to evaluate imaging features of ITPN in computed tomography (CT) and magnetic resonance (MR).
**Methods or Background:** We performed monocentric retrospective analysis of 14 patients with histopathologically verified ITPN. Images were analysed independently by two radiologists, blinded to reports. Imaging features were compared to a matched control group consisting of 43 patients with PDAC (sex and age).
**Results or Findings:** Histopathologic analysis showed invasive carcinoma component in all ITPN patients. CT-attenuation values of ITPN were significantly higher in arterial and venous phases (62.3±14.6 HU and 68±15.6 HU) than in unenhanced phase (39.2±7.9 HU), compatible with solid lesion enhancement. Compared to PDAC, ITPN lesions had significantly higher HU-values in both arterial and venous phases (arterial and venous phases, p<0.001). ITPN were significantly larger than PDAC (4.1±2.0cm versus 2.6±0.84cm, p=0.004). ITPN lesions were significantly better circumscribed (p<0.001). Employing a multiple logistic regression analysis with forward stepwise method, HU density in the venous phase was found to be significant predictor of the histological entity of the lesions (p<0.02).
**Conclusion:** Our study identified key imaging features for differentiation of ITPN and PDAC. Isodensity or moderate hypodensity and well-circumscribed margins favor the diagnosis of ITPN over PDAC. Being familiar with CT-features of these rare pancreatic tumors is essential for radiologists to accelerate the diagnosis and narrow the differentials.
**Limitations:** The major limitation is lack of standardisation of CT/MRI protocols due to the retrospective study design. Another limitation is the relatively small sample size of ITPN lesions, which is comparable to previous studies on ITPN and can be explained by their rarity.
**Ethics committee approval:** The study protocol was approved by the local ethics committee of our institution (S-011/2015).
**Funding for this study:** No funding was received for this study.
**Author Disclosures:**
Matthias Gaida: Nothing to disclose
Miriam Klauß: Nothing to disclose
Hans-Ulrich Kauczor: Nothing to disclose
Thomas Hank: Nothing to disclose
Philipp Mayer: Nothing to disclose
Thilo Hackert: Nothing to disclose
Ekaterina Khristenko: Nothing to disclose

### RPS 101-7

**Post-mortem CT in fatal gunshot wounds: every bit as good as autopsy**

*M. Palermo*, A. G. g. Musumeci, S. Palmucci, C. Pomara, A. Basile; Catania/IT
*(monica.palermo91@gmail.com)*

**Purpose:** The aim of this study is to assess the role of Post-Mortem Computed Tomography (PMCT) and different imaging post-processing techniques in fatal gunshot wounds.
**Methods or Background:** Among the PMCT scans that we performed between December 2018 and September 2021 in our institution, we selected the cases of fatal gunshot wounds (n=15). Fourteen of them were found dead, while in one case the patient died on the operating table immediately after admission in the surgical emergency department. For each case, after the PMCT, we performed multiplanar reformations (MPR) and 3D reconstructions; we then detected entrance and exit wounds, position of bullet or bullet fragments within the body, and depicted the bullet track(s). These data were then compared to forensic autoptic findings.
**Results or Findings:** In all cases, the radiology findings were confirmed on the autoptic study. Entrance and exit wounds were correctly recognised, and the bullet tracks were accurately depicted as well. Detailed images of PMCT, MPR, 3D reconstructions and autopsy are provided.
**Conclusion:** PMCT imaging correctly established entrance/exit wounds, positions of retained bullets and bullets trajectory prior to the forensic autopsy and provided a guide for the physical autopsy. Knowing the bullet's location before physical autopsy reduced the procedure time. Moreover, ballistic trajectory detection was of supreme significance when reconstructing the crime scene.
**Limitations:** In all cases, the corpses were refrigerated to delay decomposition, so that the bodies' conditions were optimal for the tomography study. In case of important post-mortem changes, it could be challenging to recognise the correct bullet track and to determine the nature of superficial wounds.
**Ethics committee approval:** Not applicable.
**Funding for this study:** Not applicable.
**Author Disclosures:**
Andrea Giovanni giovanni Musumeci: Nothing to disclose
Monica Palermo: Nothing to disclose
Cristoforo Pomara: Nothing to disclose
Antonio Basile: Nothing to disclose
Stefano Palmucci: Nothing to disclose

### RPS 101-8

**Increasing the specificity of computed tomography angiography for the diagnosis of hepatic artery occlusion after liver transplantation in suspected patients with Doppler US abnormalities**

*J. S. Kim*, K. W. Kim, D. W. Kim; Seoul/KR
*(truth0508@nate.com)*

**Purpose:** To investigate whether the diagnostic performance of CTA could be improved by modifying the conventional criterion to diagnose hepatic artery occlusion (HAO) after liver transplantation (LT) in suspected patients with Doppler ultrasound (US) abnormalities.
**Methods or Background:** One hundred thirty-four adult LT recipients with suspected HAO on Doppler US (40 HAO and 94 non-HAO according to the reference standards) were included. We evaluated abnormalities in the HA anastomosis, categorized as a cut-off of 50% stenosis at the anastomotic site and abnormalities in the distal run-off including invisibility or irregular, faint, and discontinuous enhancement. The sensitivity, specificity, positive predictive value (PPV), negative predictive value (NPV), and accuracy of the conventional (considering anastomosis site abnormalities alone) and modified CTA criteria (requiring abnormalities in both the anastomosis site and distal run-off) for the diagnosis of HAO were calculated and compared using the McNemar test.
**Results or Findings:** By using the conventional criterion to diagnose HAO, the sensitivity, specificity, PPV, NPV, and accuracy were 100% (40/40), 74.5% (70/94), 62.5% (40/64), 100% (70/70), and 82.1% (110/134), respectively. The modified criterion for diagnosing HAO showed significantly increased specificity (93.6%, 88/94) and accuracy (93.3%, 125/134) compared with the conventional criterion (p=0.001/0.002, respectively), although the sensitivity (92.5%, 37/40) decreased slightly without statistical significance (p=0.250).
**Conclusion:** The modified criterion considering abnormalities in both the anastomosis site and distal run-off improved the diagnostic performance of CTA for HAO in suspected patients with Doppler US abnormalities, particularly by increasing the specificity.
**Limitations:** Firstly, this study is a retrospective design. Secondly, we included only patients who showed an abnormality on Doppler US.
**Ethics committee approval:** This study was approved by the Institutional Review Board.
**Funding for this study:** No funding was received for this study.
**Author Disclosures:**
Jin Sil Kim: Nothing to disclose
Kyoung Won Kim: Nothing to disclose
Dong Wook Kim: Nothing to disclose

**08:00-09:00** **Room K**

### Research Presentation Session: Chest

### RPS 104

### Pulmonary infections

**Moderator**
M. Pirnat; Maribor/SI
**Author Disclosure:**
M. Pirnat: Advisory Board: Bayer

### RPS 104-2

**Automated lung vessel segmentation reveals blood vessel volume redistribution in viral pneumonia**

*J. Poletti*[1], M. Bach[1], S. Yang[1], R. Sexauer[1], B. Stieltjes[1], D. C. Rotzinger[2], J. Bremerich[1], A. W. Sauter[1], T. Weikert[1]; [1]Basle/CH, [2]Lausanne/CH

**Purpose:** It is known from histology studies that lung vessels are affected in viral pneumonia. However, their diagnostic potential as a chest CT imaging parameter has only rarely been exploited. The purpose of this study is to develop a robust method for automated lung vessel segmentation and morphology analysis and apply it to a large chest CT dataset.
**Methods or Background:** In total, 509 non-enhanced chest CTs (NECTs) and 563 CT pulmonary angiograms (CTPAs) were included. Sub-groups were patients with healthy lungs (group_NORM, n=634) and those RT-PCR-positive for Influenza A/B (group_INF, n=159) and SARS-CoV-2 (group_COV, n=279).

A lung vessel segmentation algorithm (LVSA) based on traditional image processing was developed, validated with a point-of-interest approach, and applied to a large clinical dataset. Total blood vessel volume in lung (TBV) and the blood vessel volume percentage (BV%) of three blood vessel size types were calculated and compared between groups: small (BV5%, cross-sectional area <5mm2), medium (BV5-10%, 5-10mm2) and large (BV10%, >10mm2).
**Results or Findings:** Sensitivity of the LVSA was 84.6% (95%CI: 73.9-95.3) for NECTs and 92.8% (95%CI: 90.8-94.7) for CTPAs. In viral pneumonia, besides an increased TBV, the main finding was a significantly decreased BV5% in group_COV (n=14%) and group_INF (n=15%) compared to group_NORM (n=18%) [p<0.001]. At the same time, BV10% was increased (group_COV n=15% and group_INF n=14% vs. group_NORM n=11%; p<0.001).
**Conclusion:** In COVID-19 and Influenza, the blood vessel volume is redistributed from small to large vessels in the lung. Automated LSVA allows researchers and clinicians to derive imaging parameters for large amounts of CTs. This can enhance the understanding of vascular changes, particularly in infectious lung diseases.
**Limitations:** The LVSA does not distinguish between arteries and veins and its performance in the central zone was not as good as in the middle and peripheral zone.
**Ethics committee approval:** This study was approved by EKNZ; IRB -IDs Req-2020-00595 and 2020-00566.
**Funding for this study:** No funding was received for this study.
**Author Disclosures:**
Thomas Weikert: Nothing to disclose
Bram Stieltjes: Nothing to disclose
Julien Poletti: Nothing to disclose
Michael Bach: Nothing to disclose
David Christian Rotzinger: Nothing to disclose
Raphael Sexauer: Nothing to disclose
Alexander Walter Sauter: Nothing to disclose
Jens Bremerich: Nothing to disclose
Shan Yang: Nothing to disclose

### RPS 104-3

### Radiographic findings of non-tuberculous mycobacterial lung disease using a modified CT scoring system

*S. S. Martin*, C. Booz, I. Yel, T. Vogl, T. Biciusca; Frankfurt/DE
*(simartin@outlook.com)*

**Purpose:** The aim of this study was to evaluate CT signs and patterns of patients during the course of non-tuberculous mycobacterial lung disease (non-MAC-NTM) using a modified CT scoring system.
**Methods or Background:** A total of 40 patients (28 men and 12 women; mean age 60 years) with non-MAC-NTM were retrospectively included in this study. We used a modified CT scoring system for pulmonary non-MAC-NTM infections to assess the disease extent and severity during the course of disease. Additionally, the radiographic findings of immunocompromised and immunocompetent patients were compared.
**Results or Findings:** Twelve patients (30%) received anti-NTM therapy according to ATS criteria. CT scores in patients with therapy showed significantly higher values (22.8 vs. 15.5; p=0.043). Additionally, CT scores were significantly higher in the immunocompromised patients compared to the immunocompetent ones (22.2 vs. 15.1; p=0.045). In the subgroup of patients with NTM-therapy, CT-scores showed no significant changes before and after treatment (p=0.715).
**Conclusion:** The modified CT scoring system for pulmonary non-MAC-NTM disease allows to reliably assess the disease severity and extent in immunocompromised and immunocompetent patients.
**Limitations:** Small patient population. MAC-NTM images were not investigated.
**Ethics committee approval:** The IRB of our university hospital approved this study with a waiver for informed consent.
**Funding for this study:** No funding was received for this study.
**Author Disclosures:**
Christian Booz: Nothing to disclose
Ibrahim Yel: Nothing to disclose
Thomas Vogl: Nothing to disclose
Teodora Biciusca: Nothing to disclose
Simon S. Martin: Speaker: Siemens Healthineers

### RPS 104-5

### Investigation of childhood pneumonia with chest ultrasound: a comparison between X-ray and ultrasound

*A. Buz*, M. Yağbasan Tarhan, B. Hoşgören Atalay, A. Kabaalioğlu, S. Girit Kanmis; Istanbul/TR
*(aysenurbuz@gmail.com)*

**Purpose:** To avoid radiation exposure in the pediatric age group with suspected pneumonia, we preferred to evaluate lung parenchyma using ultrasound (US) images with the help of a larger acoustic window than adults.
**Methods or Background:** 60 subjects were assigned to study between March 2019 and July 2021, ten of them were removed consequently, after confirming upper respiratory tract infection or bronchiolitis. The inclusion criteria were defined as being younger than 18 years old, clinically diagnosed with pneumonia, having a chest X-ray within 24 hours, signing an informed consent form. Unstable patients who require mechanical ventilation were excluded. Spearman's correlation tests were used for non-parametric categorical variables. ROC curves are performed to compare the diagnostic accuracy of transthoracic US and chest X-ray.
**Results or Findings:** A total number of 50 children have participated in our study, there was a slight female predominance with a female/male ratio of 1.17. Participants' ages ranged from 2 months to 17 years and 8 months with the median age of 70.5 months (Q1: 28 & Q3: 96.75 months). 37 (74%) of 50 patients had consolidations on US scan and mean consolidation depth was 2.41±1.21 cm (min: 0.3 cm & max: 6 cm). Air bronchogram was present in 30 of 50 cases. There was a great correlation between consolidation depth and air bronchogram sign (CC: 0.730, p: 0.000). The area under the curve was calculated 0.928 for X-ray and 0.951 for the US. Mean shear-wave elastography values in pneumonia cases was 4.32 ± 3.06 kPa.
**Conclusion:** Chest ultrasound can be used as a beneficial tool to diagnose and follow up of the pediatric pneumonia.
**Limitations:** The major limitation of the study is the small sample size.
**Ethics committee approval:** This single-centre, prospective study was approved by our institutional review board (March 2019, project decision number: 2019/0137).
**Funding for this study:** No funding was received for this study.
**Author Disclosures:**
Merve Yağbasan Tarhan: Nothing to disclose
Başak Hoşgören Atalay: Nothing to disclose
Ayşenur Buz: Nothing to disclose
Adnan Kabaalioğlu: Nothing to disclose
Saniye Girit Kanmis: Nothing to disclose

### RPS 104-7

### Reader variability in COVID-19 chest X-ray severity assessment: the need for multi-reader datasets for artificial intelligence development

*G. Tessarin*, M. Zandehshahvar, M. van Assen, E. Muscogiuri, V. Keerthipati, Y. Kiarashi, A. Adibi, C. N. De Cecco; Atlanta, GA/US

**Purpose:** To evaluate the inter- and intra-reader variability among experienced and in-training radiologists for the severity classification of COVID-19 pneumonia on chest X-rays (CXR).
**Methods or Background:** CXRs from patients with positive COVID-19 PCR tests were retrospectively collected from 1 January 2020 to 1 May 2020. Four degrees of severity (normal, mild, moderate, and severe) were defined based on consolidation and extent of lung involvement. The entire dataset was labeled by 6 radiologists and 2 residents, blinded to patients' clinical information. Inter-reader variability was evaluated comparing the labels of the readers among themselves and with the median of radiologists. Intra-reader variability was also evaluated. Kendal rank correlation coefficient (KRCC) was used and confusion matrices over different test-sets were calculated.
**Results or Findings:** A total of 1208 CXR from 396 patients were included. The KRCC among readers varied between 0.66 and 0.78, with residents showing less agreement. Comparing each radiologist to the median of the 5 other radiologists, the KRCC varies between 0.73 and 0.79 for radiologists outside the median and 0.79 to 0.89 for those within the median. Among the assessment by all 6 radiologists, only 18% (220) of the CXRs were classified concordantly, and there was a maximum 1, 2 and 3 class-difference for 59% (707), 21% (259), and 2% (22) CXRs, respectively. For the 2 residents, 62% (750) CXRs were labeled concordantly. For intra-reader variability, the KRCC between the two readings was 0.77; 883 (69%) CXRs were labeled with no class difference.
**Conclusion:** Our study proved the existence of high variability among experts and in-training radiologists in assessing severity of COVID-19 based on CXRs, highlighting the importance of multi-reader datasets for the development of AI algorithms for medical imaging.
**Limitations:** Retrospective collection of data.
**Ethics committee approval:** Study was approved by an ethics committee.
**Funding for this study:** No funding was received.
**Author Disclosures:**
Marly van Assen: Nothing to disclose
Ali Adibi: Nothing to disclose
Carlo Nicola De Cecco: Research/Grant Support: Siemens Healthineers
Giovanni Tessarin: Nothing to disclose
Mohammadreza Zandehshahvar: Nothing to disclose
Emanuele Muscogiuri: Nothing to disclose
Vikrant Keerthipati: Nothing to disclose
Yashar Kiarashi: Nothing to disclose

**08:00-09:00** **Room M 1**

### Research Presentation Session: Cardiac

### RPS 103

### MRI and CT in acquired and genetic cardiac disease

**Moderator**
L. Jamjoom; Jeddah/SA

### RPS 103-2

**Nano-scale 3D morphological characterisation of cardiac micro-thrombi from COVID-19 positive patients via synchrotron CT**

*G. E. Barbone*[1], D. Pellegrini[2], S. Grosu[1], L. Faggi[2], A. Bravin[3], S. Gerevini[2], A. Gianatti[2], G. Guagliumi[2], P. Coan[1]; [1]Munich/DE, [2]Bergamo/IT, [3]Milan/IT
*(giacomo.e.barbone@gmail.com)*

**Purpose:** The scientific objective of this study is to gain novel insight into the specific pathological mechanism observed in the vascular system supplying the heart of COVID-19 patients as an adverse consequence of a SARS-CoV-2 infection, i.e. the development of micro-thrombi within myocardial microvascular capillary beds. A precise mechanistic understanding of the process by which this viral infection induces endothelial dysfunction, leading to inflammation, clotting and remodeling of blood vessels is still needed. To this end, we propose to use synchrotron X-ray phase-contrast CT (X-PCI-CT) to assess the micro- to nanometer scale 3D morphology of heart tissue pathology in COVID-19 patients.
**Methods or Background:** X-PCI-CT is an emerging technique for non-destructive 3D multiscale virtual histology, which is density-based, label-free and multiscale. 10 human autoptic tissue specimens (1 cm^3) from heart (myocardial tissue) of COVID-19 patients, fixed in formalin & embedded in paraffin, were imaged using several synchrotron X-PCI-CT imaging setups to collect 3 to 0.05-micron voxel-size 3D CT datasets.
**Results or Findings:** This approach allowed the 3D visualisation of COVID-19-driven tissue damage to myocardium (ischaemia) and the 3D localisation and characterisation of micro-thrombi within local micro-vasculature. At the highest resolutions, the individual cellular components of the interface between endothelium and micro-thrombus (endothelial cells, thrombotic material, erythrocytes, immune cells) were captured volumetrically, characterised morphologically and quantified.
**Conclusion:** The acquired data represent unprecedented 3D characterisations of the cellular architecture around COVID-19-driven micro-thrombi within cardiac micro-vasculature.
**Limitations:** The obtained ultra-high resolution CTs are post-mortem acquisitions of dissected specimens. Myocardial tissue negative controls were not included in the study protocol.
**Ethics committee approval:** All human samples were collected following ethical committee approval and all institutional and national regulations.
**Funding for this study:** This study was supported by the Klinikum der Universität München.
**Author Disclosures:**
Alberto Bravin: Nothing to disclose
Lara Faggi: Nothing to disclose
Giacomo Edoardo Barbone: Nothing to disclose
Andrea Gianatti: Nothing to disclose
Sergio Grosu: Nothing to disclose
Dario Pellegrini: Nothing to disclose
Giulio Guagliumi: Nothing to disclose
Paola Coan: Nothing to disclose
Simonetta Gerevini: Nothing to disclose

### RPS 103-3

**Prevalence and association of cardiac magnetic resonance detected myocardial injury with cardiac symptoms in patients hospitalised due to COVID-19**

*A. Yar*, S. Vaara, S. Syväranta, V. Uusitalo, T. Heliö, R. Paakkanen, S. Kivistö, M. Holmström, J. Hästbacka; Helsinki/FI

**Purpose:** We investigated the significance of myocardial injury among previously hospitalised coronavirus (COVID-19) patients by examining findings of cardiac magnetic resonance imaging (CMR) and surveying prolonged cardiac symptoms.
**Methods or Background:** We studied a cohort of critically ill and non-critically ill patients hospitalised with COVID-19 between March and December 2020 in Helsinki University Hospital. Eligible patients were recruited for follow-up after discharge. A control group with no history of COVID-19 was recruited for comparison. Participants were subject to symptoms questionnaire, clinical tests and CMR evaluation. Data are presented as mean ± standard deviation or median (interquartile range). Informed consent was obtained from all participants.
**Results or Findings:** Altogether 95 COVID-19 patients and 43 control subjects were included (males 48% vs. 56%, p=0.42). Dyspnea was a significant self-reported symptom (65% among hospitalised vs. 7% in control group, p<0.01). Median time from hospitalisation to CMR acquisition was 9 months (7-10). Myocarditis-pattern scarring was equally frequent in both critical and non-critical patient groups (29% vs. 41%, p=0.23, compared to 12% in control group, p=0.01). When comparing patients with or without scarring, we found no difference in occurrence of decreased left ventricular ejection fraction (LVEF,<50%), (6% vs 5%, p=0.77) or dyspnea (73 % vs 60%, p=0.21).
**Conclusion:** COVID-19 infection was frequently associated with myocardial scarring. LV dysfunction was, however, rare and scarring was unrelated to symptom burden.
**Limitations:** Myocardial injury may precede COVID-19 infection and influence the results of CMR screening.
**Ethics committee approval:** Approved by the ethical board of Helsinki University Hospital (decision HUS-1949-2020).
**Funding for this study:** Funding was received for this study by 2021 Grant from Finnish Society for Study of Infectious Diseases (ITY), 2021-2022 Grant from Helsinki and Uusimaa Hospital District (HUS) Diagnostic Center, 2021-2022 Government grant for University Level Research (TYH 2021310), 2020-2021 Nordforsk, 2021-2023 Tor och Kirsti Johanssons Hjärt- och Cancersstiftelse.
**Author Disclosures:**
Satu Vaara: Nothing to disclose
Aria Yar: Nothing to disclose
Sari Kivistö: Nothing to disclose
Johanna Hästbacka: Nothing to disclose
Riitta Paakkanen: Nothing to disclose
Suvi Syväranta: Nothing to disclose
Miia Holmström: Nothing to disclose
Tiina Heliö: Nothing to disclose
Valtteri Uusitalo: Nothing to disclose

### RPS 103-4

**Clinical course of acute myocarditis and pericarditis after Sars-Cov-2 vaccine with cardiac magnetic resonance**

*N. Galea*, G. Cundari, F. Catapano, L. Conia, G. Mancuso, C. Chimenti, C. Catalano; Rome/IT
*(nicogale2000@yahoo.it)*

**Purpose:** To evaluate outcome in patients with acute myocarditis and pericarditis after Sars-Cov-2 vaccine with cardiac magnetic resonance (CMR).
**Methods or Background:** Eleven consecutive patients (mean age 45.9+/-17.1; 2/11 F) with clinical or bioptic diagnosis of acute myocarditis or pericarditis were prospectively enrolled, within 15 days from Sars-Cov-2 vaccination associated to serum Troponine-T-hs increase and acute chest pain. All patients underwent CMR within 14 days from the onset of symptoms and clinical follow-up for > 3 months; 7/11 patients were also studied with CMR from 4 to 6 months after clinical onset. CMR protocol included short tau inversion recovery (STIR), late gadolinium enhancement (LGE), cine steady state free precession, modified look locker inversion recovery (MOLLI) and T2-3pt-GRE sequences.
**Results or Findings:** Baseline CMR revealed 4/11 patients with acute pericarditis, 5/11 with acute myocarditis, 1/11 with peri-myocarditis according to revised Lake Louise criteria. One patient was negative at CMR. Six/11 presented with subepicardial or mesocardial LGE, among them 4/11 also showed myocardial edema at STIR. Eight/11 demonstrated native T1 mapping and 5/11 T2 mapping increase. Left ventricular function was preserved in 9/11 patients (ejection fraction 57.3%+/-7.67%). At clinical follow-up all patients (11/11) did not show any major cardiovascular event and normal Troponine-T levels. At follow-up CMR 7/7 patients have preserved ejection fraction (60.9%+/-5.9%) and resolution of myocardial edema or pericarditis signs. LGE was present in 6/7 patients and T1 mapping alterations in 5/7.
**Conclusion:** CMR has an important role in the identification of myocarditis and pericarditis after Sars-CoV2 vaccination, which appeared to have a benign clinical course in short-term follow-up.
**Limitations:** The limitations of this study are the small sample size but very rare condition, histological confirmation in only 2 patients (ethical issues), vaccine-induced causal mechanism cannot be demonstrated but only inferred from temporal proximity.
**Ethics committee approval:** Ethics committee approval has been obtained.
**Funding for this study:** No funding was received for this study.
**Author Disclosures:**
Giulia Cundari: Nothing to disclose
Cristina Chimenti: Nothing to disclose
Giuseppe Mancuso: Nothing to disclose
Luca Conia: Nothing to disclose
Federica Catapano: Nothing to disclose
Nicola Galea: Nothing to disclose
Carlo Catalano: Nothing to disclose

### RPS 103-6

**Influence of PCSK-9 inhibitor therapy on CT-FFRML in a patient cohort with familial hypercholesterolemia or contraindications to statin therapy and/ or therapy refractory to statin therapy**

*D. P. Overhoff*[1], S. Baumann[2], S. Ksenija[1], N. Rathmann[1], S. Waldeck[3], D. Lossnitzer[2], D. Daniel Duerschmied[2], S. O. Schönberg[1], M. Kuru[1]; [1]Mannheim/DE, [2]Heidelberg/DE, [3]Koblenz/DE

**Purpose:** The study aimed to evaluate the changes in computed tomography fractional flow reserve machine learning (CT-FFRML) under PCSK-9 inhibitor therapy in a patient cohort with familial hypercholesterolemia and/ or therapy refractory to statin therapy or contraindications to statin therapy.
**Methods or Background:** In our prospective study, we enrolled 35 patients with a high cardiovascular risk profile/ familial hypercholesterolemia. A baseline coronary computed tomography angiography (CCTA) with CT-FFRML was performed at the time of introduction of PCSK-9 inhibitor therapy. After one year a follow-up CCTA with CT-FFRML was performed and the changes in coronary hemodynamics in terms of CT-FFRML were analysed. All CCTA were performed with a 3rd generation dual source CT (Siemens, SOMATOM Force, Siemens Healthineers, Forchheim, Germany).
**Results or Findings:** In the baseline CCTA no hemodynamic significant stenosis (CT-FFRML<0.8) was detected. During the one-year course of PCSK-9 therapy with adequate decrease in serum LDL levels, no significant changes in CT-FFRML of the coronary vessels were observed. This finding could be demonstrated for proximal vessel segments (LAD; LCX; RCA) (LCX: 0.99 vs 0.99 p=0.207), medial vessels (LAD: 0.86 vs 0.87 p=0.664) as well as for distal vessel segments (RCA: 0.83 vs 0.81 p=0.557).
**Conclusion:** PCSK-9 therapy does not seem to influence CT-FFRML in a patient cohort with familial hypercholesterolemia and/ or therapy-refractory to statin therapy or contraindications to statin therapy.
**Limitations:** The limitation of the study is the rather small study population.
**Ethics committee approval:** Local ethics comitte approved the study.
**Funding for this study:** No funding was received for this study.
**Author Disclosures:**
Dirk Lossnitzer: Nothing to disclose
Stefan Oswald Schönberg: Nothing to disclose
Stefan Baumann: Nothing to disclose
Stach Ksenija: Nothing to disclose
Mustafa Kuru: Nothing to disclose
Nils Rathmann: Nothing to disclose
Daniel Pasqual Overhoff: Nothing to disclose
Stephan Waldeck: Nothing to disclose
Daniel Daniel Duerschmied: Nothing to disclose

### RPS 103-7

**Cardiovascular magnetic resonance imaging in Noonan syndrome: case series of young children**

*W. Yang*, Y. Wang, M. Lu; Beijing/CN
*(wenjingyang96@163.com)*

**Purpose:** Noonan syndrome (NS) is an autosomal dominant disorder characterised by distinctive facial dysmorphology, short stature and a wide spectrum of cardiac abnormalities. Our study aimed to evaluate detailed cardiac features of NS by magnetic resonance imaging (MRI).
**Methods or Background:** From September 2020 to August 2021, ten small children with cardiac MRI and genetically confirmed with NS were enrolled. All patients were comprehensively evaluated by MRI including late gadolinium enhancement (LGE), T1mapping and myocardial strain.
**Results or Findings:** Of the 10 children, 70% were females and the median age is 2.9 years ranging from 0.3-6years. PTPN11 mutation was confirmed in seven patients followed by RAF1 and LZTR1. Pulmonary stenosis presented in 5 patients, and affected individuals in our cohort had a high prevalence of biventricular hypertrophy and obstruction to the outflow of both ventricles with LV outflow tract obstruction identified in 80% of patients and right ventricular outflow tract obstruction in 70% of patients. Characteristic LGE pattern, subendocardial LGE in the anterior and anterolateral wall, and elevated native T1 and extracellular volume (1287.8ms and 31.1%, respectively) were detected by cardiac MRI. The global peak systolic radial, circumferential, longitudinal strain (mean, 24.39%; -14.43%; -7.80%, respectively) and peak early systolic and diastolic strain rates of LV were all impaired in NS.
**Conclusion:** Comprehensive evaluation of NS associated cardiac abnormalities defined a vital role of MRI in NS. Characteristic LGE pattern, together with functional and morphological imaging showing PS and biventricular hypertrophy, are helpful to diagnose NS. Interstitial fibrosis confirmed by evaluated ECV and impaired myocardial deformation were also identified in NS, which may contribute to risk stratification and prognosis in NS.
**Limitations:** Not applicable.
**Ethics committee approval:** The study protocol was approved by the institution's Ethics Review Board.
**Funding for this study:** No funding was received for this study.
**Author Disclosures:**
Minjie Lu: Nothing to disclose
Yining Wang: Nothing to disclose
Wenjing Yang: Nothing to disclose

### RPS 103-8

**Added value of the T1 and T2 mapping sequences in the diagnosis of acute myocarditis**

W. Frikha, S. Boukriba, D. M. Dhifallah, A. Eya, S. Gaied Chortane, *H. Mizouni*; Tunis/TN
*(habiba.mizouni@fmt.utm.tn)*

**Purpose:** CMR is the primary tool for non-invasive diagnosis of acute myocarditis. Mapping techniques have become essential criteria for the detection of myocardial injury through quantitative measurement of myocardial relaxation values. Our study aimed to evaluate the value of mapping techniques in the diagnosis of acute myocarditis.
**Methods or Background:** Our study included 30 patients who presented clinical and biological arguments of acute myocarditis. The diagnosis of acute myocarditis was made according to the 2018 Lake Louise criteria. MRI protocol included dynamic sequences SSFP, T2 and T1 mapping sequences realised by a sequence "MOLLI" with synchronisation ECG in three short axis slices (basal, midventricular and apical), as well as late enhancement sequences and T1 post gadolinium sequences. Images were acquired on an artist general electric 1.5 Tesla MRI machine. The post-processing software used was "Circle 42 cvi 42".
**Results or Findings:** Mean age was 32±17 years (range 10-58), male (73 %). All the patients showed high values of T1 and T2 above the reference values established in our centre (1110 ms for T1 and 60 ms for T2). In our study, the diagnosis of acute myocarditis was based on mapping techniques in 13% of cases (4 patients), in the absence of late enhancement. The use of parametric maps increased the MRI sensitivity in the diagnosis of acute myocarditis.
**Conclusion:** Mapping techniques increased the sensitivity of MRI in the detection of acute myocarditis.
**Limitations:** The confirmation of our findings requires larger, longitudinal studies.
**Ethics committee approval:** Our study had the approval of the ethics committee.
**Funding for this study:** Not applicable.
**Author Disclosures:**
Dhifallah Moez Dhifallah: Nothing to disclose
Seifeddine Boukriba: Nothing to disclose
Azouz Eya: Nothing to disclose
Sofiane Gaied Chortane: Nothing to disclose
Habiba Mizouni: Nothing to disclose
Wassim Frikha: Nothing to disclose

**08:00-09:00** **Room M 2**

### Research Presentation Session: Head and Neck

### RPS 108

### Temporal bone imaging

**Moderator**
Z. Rumboldt; Rovinj-Rovigno/HR

### RPS 108-2

**The visibility of extra cranial facial nerve and his anatomical variants in 3D spin echo T2 sequence on a 1.5 Tesla MRI**

S. Boukriba, W. Frikha, B. El Khouni, A. Eya, S. Siala, R. Zainine, R. Bechraoui, S. Gaied Chortane, *H. Mizouni*; Tunis/TN
*(habiba.mizouni@fmt.utm.tn)*

**Purpose:** Our study aimed to determine the visibility of extracranial facial nerve and its anatomical variants in 3D spin echo T2 sequence on a 1.5 Tesla MRI.
**Methods or Background:** It was a descriptive cross-sectional monocentric study between October 2020 and September 2021, that included 60 patients. All patients were imaged with a «3D T2 Cube» sequence. Two radiologists independently segmented the 100 facial nerves using the 3D slicer programme and their segmentations were compared.
**Results or Findings:** The main trunk was visible in all the patients, while the temporo facial branch was visible in 88% and the cervicofacial branch in 92% of the patients with a reasonable inter-observer variability. The mean length of the segmentations was respectively 19.7mm for the main trunk, 11mm for the temporo facial branch and 13.9mm for the cervicofacial branch. In patients presenting a parotid tumour the facial branches inferring with the tumour and the facial nerve proximal to it were visualised in all cases. The study of anatomical variants showed a trifurcation in 5% of cases, a bifurcation in 83% of the cases and a single main trunk for all the patients.
**Conclusion:** Our results suggest that the extracranial facial nerve and its temporofacial and cervicofacial trunks can be routinely identified with a 3D T2 cube imaging protocol even in the presence of a parotid tumour. Extracranial facial nerve imaging with 3D T2 sequences may be of use in the pre-operative evaluation for exofacial parotidectomy to reduce the risk of facial nerve injury.
**Limitations:** Confirmation of our findings requires larger, longitudinal studies.
**Ethics committee approval:** Our study had the approval of the ethics committee.
**Funding for this study:** Not applicable.
**Author Disclosures:**
Rim Bechraoui: Nothing to disclose
Rim Zainine: Nothing to disclose
Balkis El Khouni: Nothing to disclose
Seifeddine Boukriba: Nothing to disclose
Azouz Eya: Nothing to disclose
Selima Siala: Nothing to disclose
Sofiane Gaied Chortane: Nothing to disclose
Habiba Mizouni: Nothing to disclose
Wassim Frikha: Nothing to disclose

### RPS 108-3

**Radiological evaluation of tympanic segment of normal chorda tympani nerve: implications for transcanal middle ear surgery**
*R. Tang*; Beijing/CN

**Purpose:** To visualise course of the tympanic segment of the chorda tympani nerve (CTN) using ultra-high-resolution CT and discuss the surgical implications.
**Methods or Background:** A hundred and four ears with no evident otologic pathologies were recruited. The tympanic CTN was divided into four portions: periannular, posteromalleal, malleal, and anteromalleal portions. Four points of interest (the beginning and end of the posteromalleal and anteromalleal portions) were selected to perform distance measurements relative to the tip of the malleus manubrium. The differences in the lengths and distances were compared in terms of ear laterality and sex.
**Results or Findings:** The length of the periannular portion was 2.16 mm. The beginning of the posteromalleal portion on the right side was located more laterally than on the left side (4.09 mm vs. 3.92 mm, $P = 0.015$), and similar for the beginning of the anteromalleal portion (2.59 mm vs. 2.45 mm, $P = 0.030$). The end of the posteromalleal portion and the beginning of the anteromalleal portion were both more posteriorly located in men than in women (0.04 mm vs. 0.35 mm, $P = 0.005$; 2.04 mm vs. 2.32 mm, $P = 0.024$).
**Conclusion:** The course of the tympanic CTN was comprehensively visualised by ultra-high-resolution CT. Right-sided ears and male sex are more vulnerable to iatrogenic injury during middle ear surgery.
**Limitations:** This was a pilot study of normal ears, and no anatomical correlation was acquired as a reference. And identification of the tympanic segment in normal ears does not necessarily guarantee the exact same visualisation in patients with middle ear diseases.
**Ethics committee approval:** Approved by the local institutional ethics committee (IRB: 2020-P2-061-02).
**Funding for this study:** Funding was received for this study by National Natural Science Foundation of China [grant numbers 61931013 and 61527807]; Beijing Natural Science Foundation [grant number 7212199]; Beijing Scholar [2015]160.
**Author Disclosures:**
Ruowei Tang: Nothing to disclose

### RPS 108-4

**Photon-counting detector CT virtual monoengergetic images for cochlear implant visualisation: a head to head comparison to energy-integrating detector CT**
*S. Waldeck*, L. S. Alizadeh, M. Müller, B. V. Becker, K. Nestler, S. Schmidt, D. P. Overhoff; Koblenz/DE
*(stephanwaldeck@bundeswehr.org)*

**Purpose:** Cochlear implants (CIs) are the primary treatment method in patients with profound sensorineural hearing loss. Interpretation of postoperative imaging with conventional energy-integrating detector computed tomography (EID-CT) following CI surgery remains challenging due to metal artifacts, but photon-counting detector (PCD-CT) is a new emerging technology with the potential to eliminate these problems.
**Methods or Background:** This study evaluated the performance of Virtual Monoenergetic (VME) EID-CT images versus PCD-CT in CI imaging. In this cadaveric study, two temporal bone specimens with implanted CIs were scanned with EID-CT and PCD-CT. The images were assessed according to visibility of interelectrode wire, size of electrode contact and diameter of halo artifacts.
**Results or Findings:** The visibility of interelectrode wire sections was significantly higher when reviewing PCD-CT images ($p < 0.001$). The difference in diameter measurements for electrode contacts between the two CT scanner modalities showed that the PCD-CT technology lead to significantly larger diameter readings in general ($p < 0.001$). The larger measurements were closer to the manufacturer's specifications for the CI electrode. The size of halo artifacts surrounding the electrode contacts did not differ significantly between the two imaging modalities ($p = 0.931$).
**Conclusion:** PCD-CT imaging is a promising technology for CI imaging with improved spatial resolution and better visibility of small structures than conventional EID-CT.
**Limitations:** The limitation is that the study is an ex- vivo study.
**Ethics committee approval:** Not applicable.
**Funding for this study:** No funding was received for this study.
**Author Disclosures:**
Kai Nestler: Nothing to disclose
Benjamin Valentin Becker: Nothing to disclose
Daniel Pasqual Overhoff: Nothing to disclose
Stephan Waldeck: Nothing to disclose
Sandra Schmidt: Nothing to disclose
Mathias Müller: Nothing to disclose
Leona Soraja Alizadeh: Nothing to disclose

### RPS 108-5

**MRI features of spontaneous lateral temporal bone cephaloceles**
*R. Srinivasan*, R. Obholzer, S. Connor; London/UK

**Purpose:** To determine the clinical presentation, location and MRI features of spontaneous lateral temporal bone cephaloceles.
**Methods or Background:** Spontaneous cephaloceles of the lateral temporal bone are an important and emerging clinical phenomenon. Diagnosis is often challenging due to the non-specific symptoms including hearing loss and middle ear fullness. Prompt and accurate diagnosis is essential as patients are at risk of meningitis. Imaging can have a crucial role in its diagnosis.
A retrospective cohort study analysed all adult patients with imaging or surgically confirmed spontaneous temporal bone cephaloceles from 01/01/2006 to 09/02/2022. Non-spontaneous cephaloceles and petrous apex cephaloceles were excluded. All patients with 3D T2W MR imaging of the temporal bones were included for final analysis. Data collection included demographic information, presenting signs and symptoms, surgical history. Two head and neck radiologists analysed MRI predefined features.
**Results or Findings:** Twenty-nine patients were included (M=14, F=15; mean age 57.9, age range 31-88 years). Five patients (17.2%) had bilateral defects and the cohort included a total of 34 lateral temporal bone cephaloceles. Twenty patients (58.9%) underwent surgical repair. Hearing loss (65.6%) was the most common symptom and 4 patients (13.7%) had meningitis. Nine patients (37.5%) had MRI imaging features suggestive of intracranial hypertension. High T2W mastoid signal was found in 30 (88%) cases. A high T2W CSF cleft, either traversing or pointing towards the defect, was identified in 31 (91.1%) cases.
**Conclusion:** The principle clinical and MRI features of spontaneous lateral temporal bone cephaloceles are described with a view to aiding their identification. A high T2W CSF cleft related to the defect is a potentially useful diagnostic feature.
**Limitations:** Data recording was not entirely incomplete due to the retrospective nature of the study.
**Ethics committee approval:** This study was approved by the local institution.
**Funding for this study:** No funding was received for this study.
**Author Disclosures:**
Rupert Obholzer: Nothing to disclose
Steve Connor: Nothing to disclose
Rohit Srinivasan: Nothing to disclose

### RPS 108-6

**Intralabyrinthine hemorrhage as a cause of sudden deafness**
*S. Palizzi*, A. Romano, G. Moltoni, E. Covelli, M. Barbara, A. Bozzao; Rome/IT
*(serenapalizzi@gmail.com)*

**Purpose:** The aim of the study is to evaluate the presence of intralabyrinthine hemorrhage in patients with sudden idiopathic hearing loss by means of an MRI examination performed within fifteen days from symptom onset. Furthermore, the goal of the study is to understand whether intralabyrinthine hemorrhage can be a cause of sudden hearing loss, what type of hearing loss it causes and whether its presence could be a predictor of hearing outcomes and response to therapy.

**Methods or Background:** Twenty patients with sudden hearing loss were included in the study. Patients were divided according to the audiometric curve into four groups: rising, falling, pantonal and anacusis pattern. All patients underwent MRI of the ear within fifteen days from the onset of symptoms and were treated with intratympanic corticosteroid therapy. They underwent tonal audiometry in headphones before and after treatment. Audiological results were evaluated according to the Sigel and Furuhashi criteria. The presence or absence of intralabyrinthine hemorrhage on MRI was correlated with the type of curve and with the response to intratympanic corticosteroid treatment.
**Results or Findings:** In 25% of cases intralabyrinthine hemorrhage was present at MRI, with all of the patients showing anacusis at the audiometric exam. Patients with intralabyrinthine hemorrhage have shown no audiological benefit from intratympanic corticosteroid therapy. In 80% of cases patients without hemorrhage had a complete recovery of the audiological condition.
**Conclusion:** Intralabyrinthine hemorrhage can be considered a cause of sudden deafness, especially in severe forms presenting with anacusis and vertigo. This condition appears to be a negative prognostic factor for auditory recovery after intratympanic corticosteroid therapy.
**Limitations:** Not applicable.
**Ethics committee approval:** Not applicable.
**Funding for this study:** Not applicable.
**Author Disclosures:**
Serena Palizzi: Nothing to disclose
Andrea Romano: Nothing to disclose
Maurizio Barbara: Nothing to disclose
Alessandro Bozzao: Nothing to disclose
Edoardo Covelli: Nothing to disclose
Giulia Moltoni: Nothing to disclose

### RPS 108-7

**Cortical sulcus depth alterations in patients with tinnitus before and after sound therapy: a surface-based morphometry study**

*X. Wei*; Beijing/CN
*(weixuan315@163.com)*

**Purpose:** This study aimed to explore alterations in brain surface-based morphometry sulcal depth in patients with idiopathic tinnitus before and after 24 weeks of sound therapy.
**Methods or Background:** 33 tinnitus patients underwent magnetic resonance imaging (MRI) scans at baseline and after 24 weeks of sound therapy. 26 age- and sex-matched healthy control (HC) individuals also underwent two scans over a 24-week interval. For all participants, 3.0-T MRI and high-resolution three-dimensional (3D) structural images were acquired. Structural image data preprocessing was performed using the DPABISurf toolbox. Tinnitus handicap inventory (THI) scores were used to assess the severity of tinnitus before and after treatment. Two-way mixed-model analysis of variance (ANOVA) and Pearson's correlation analysis were used in the statistical analysis. Student-Newman-Keuls (SNK) tests were used in the post hoc analysis.
**Results or Findings:** Compared to HCs, patients in the tinnitus group at baseline had significantly lower sulcal depth in the left medial temporal cortex (MTC) and right somatosensory and motor cortex (SMC). After 24 weeks of sound therapy, the tinnitus patients demonstrated significantly increased sulcal depth in the left MTC and right SMC. There were no significant differences in sulcal depth between the tinnitus patients after treatment and the HCs.
**Conclusion:** The remodeling of sulcal depth after sound therapy is an indicator of effective sound therapy. These brain regions may provide meaningful neuroimaging targets for effective treatment of tinnitus.
**Limitations:** This study only explores the change of sulcal depth after sound therapy of tinnitus patients. We will continue to explore several other indicators such as cortical area, curvature in the follow-up study.
**Ethics committee approval:** This study was approved by the ethics committees of our Beijing Friendship Hospital.
**Funding for this study:** This work was supported by No. 61801311 from the National Natural Science Foundation of China.
**Author Disclosures:**
Xuan Wei: Nothing to disclose

**08:00-09:00** **Room M 3**

### Research Presentation Session: Imaging Informatics / Artificial Intelligence and Machine Learning

### RPS 105

### Artificial intelligence in prostate and retroperitoneal imaging

**Moderator**
H.-P. Schlemmer; Heidelberg/DE
**Author Disclosure:**
H.-P. Schlemmer: Advisory Board: Siemens Healthineers, Bayer Vital/Healthcare; : Siemens Healthineers, Bayer Vital/Healthcare, Bracco

### RPS 105-2

**Deep learning-based segmentation for prostate cancer risk assessment in magnetic resonance imaging**

*E. H. P. Pooch*, R. G. H. Beets-Tan, S. H. Benson; Amsterdam/NL
*(e.pais.pooch@nki.nl)*

**Purpose:** Delineating prostates in magnetic resonance imaging (MRI) scans and identifying regions of interest (ROIs) is a recurring task when planning the treatment of prostate cancer patients. ROIs are especially useful for biopsy planning to properly stratify patients. However, manual delineation is a very time-consuming task. We evaluate the performance of two deep learning-based segmentation methods, their generalisation to out-of-domain (OOD) data, and their performance delineating ROIs.
**Methods or Background:** The training dataset contains 772 MRI scans of 650 patients. We also use 112 scans from five different centres for OOD validation. We compare two state-of-the-art models, nnUnet, based on convolutional architectures, and nnFormer, based on the transformer architecture.
We use high-scoring ROIs (UCLA score > 2) to train high-risk region segmentation models and assess their overlap with high-grading biopsy results.
**Results or Findings:** The nnUnet and nnFormer models achieve a Dice similarity coefficient of 91.93% and 91.90% respectively for prostate delineation and, on OOD datasets, 85.32% and 86.06%. The Dice for the ROI segmentation was 27.76% and 28.23%, respectively. When analysing the overlap between the abnormal grading biopsy cores (Gleason >= 3+3) and the ROIs, the percentage of positive cores included in the ROIs was 84.85% for human-delineated, 78.86% for nnUnet, and 79.82% for nnFormer regions.
**Conclusion:** Both models perform well for prostate segmentation, with the transformer architecture showing a slight advantage in generalisation and ROI segmentation. The Dice scores for the ROI segmentation were low, but considering the average sensitivity for high-scoring ROIs to abnormal biopsies, the model-predicted ROIs are close to the human delineation.
**Limitations:** The publicly-released dataset only includes T2-weighted MRI sequences, which might not provide enough information for ROI delineation.
**Ethics committee approval:** Not applicable.
**Funding for this study:** Not applicable.
**Author Disclosures:**
Sean Harry Benson: Nothing to disclose
Eduardo H. P. Pooch: Nothing to disclose
Regina G. H. Beets-Tan: Nothing to disclose

### RPS 105-3

**Automatic quality control of diffusion magnetic resonance images for prostate cancer in clinical routine**

*A. Routier*, N. Debs, L. Wood, F. Nicolas, M-M. Rohé; Villepinte/FR

**Purpose:** MRI suffers from several artifacts which often yield images that are of non-diagnostic quality. It can then fool automatic diagnostic algorithms to produce wrong results. This study aims to evaluate a deep neural network designed to automatically detect and reject these images thereby preventing potential mistakes.
**Methods or Background:** Studies from clinical routine were collected across different centres and continents. We used apparent diffusion coefficient (ADC) map as a proxy measure of diffusion MRI quality. Slices were individually annotated and classified as either 'GOOD' or 'BAD' whether it was of diagnostic quality or not. 1510 studies were included in the training dataset and 54 studies were included in an independent test set. In the training (resp. testing) 1177 (resp. 46) had all slices of 'GOOD' quality, 255 (resp. 5) all slices of 'BAD' quality, and 78 (resp. 3) had both slices with 'GOOD' and 'BAD' quality. The network is trained on 2D slices and outputs a probability map for each slice. A threshold is applied to keep ('GOOD') or discard ('BAD') the slice

Wednesday and ADC map is rejected if there is a certain number of consecutively low-quality slices. We evaluate the model using false positive (FP) and false negative (FN) rates on both the slice and the study level.
**Results or Findings:** On the testing dataset, it demonstrated at the slice level a balanced accuracy of 91.3% (FP rate=0.3%, FN rate=17.0%) with an AUC of 0.92. At the study level, it demonstrated a balanced accuracy of 98.9% (FP rate=2.2%, FN rate=0.0%).
**Conclusion:** This method demonstrated high-performance detection on the unseen test dataset and could be used to automatically reject inadequate ADC maps to prevent mistakes of automatic models on non-diagnostic quality studies.
**Limitations:** Not applicable.
**Ethics committee approval:** Not applicable.
**Funding for this study:** Not applicable.
**Author Disclosures:**
Noelie Debs: Employee: Guerbet
Marc-Michel Rohé: Employee: Guerbet
Alexandre Routier: Employee: Guerbet
Laura Wood: Employee: Guerbet
Francois Nicolas: Employee: Guerbet

### RPS 105-4

**Who is best? Comparing radiologists, radiomics and deep learning for the assessment of renal masses on computed tomography**

*S. Bachanek*, H. Timucin, M. Nietert, L. Trojan, J. Lotz, A. Uhlig, J. Uhlig; Göttingen/DE

**Purpose:** To assess the diagnostic performance of radiologists, radiomics and deep learning approaches for discrimination of benign and malignant renal masses on computed tomography (CT).
**Methods or Background:** Patients with renal masses imaged with nephrogenic phase CT at more than 20 radiological institutes were included with histopathological assessment as reference standard. Malignancy assessment of renal masses was performed with 1) radiologists: two blinded independent radiologists rated malignancy probability on a 10-point scale; 2) radiomics: 159 radiomics were derived from manually segmented renal masses and used for prediction of malignancy implementing machine learning algorithms with 10-fold cross-validation (CV); 3) deep learning: a convolutional neural network (CNN Keras library with Tensorflow backend) was trained on CT-slices to predict malignancy with 10-fold CV. Diagnostic performance was calculated on out-of-bag CV samples using AUC and compared using the Delong method.
**Results or Findings:** A total of n=292 patients were included (median age 56.2yrs; 36.6% female) with n=234 malignant renal masses (80.1%; median diameter 3.5cm). The diagnostic performance for discrimination of malignant and benign renal masses was significantly higher for radiomics (AUC=0.72) and deep learning (AUC=0.79) when compared to the radiologist (AUC=0.60; $p<0.001$, each). The diagnostic performance of the deep learning approach was also significantly higher when compared to the radiomics approach (AUC=0.79 vs AUC=0.72, $p<0.001$).
**Conclusion:** A deep learning approach is suitable for assessment of renal masses on CT studies acquired in a clinical routine setting, outperforming experienced radiologists and radiomics approaches. This method, therefore, has the potential to reduce overtreatment of benign renal masses identified by CT.
**Limitations:** Limitations include the small number of benign renal masses and missing external validation dataset.
**Ethics committee approval:** This study received prior approval from the local ethics committee.
**Funding for this study:** Funding was received for this study by the research programme, University Medical Center, University of Göttingen.
**Author Disclosures:**
Annemarie Uhlig: Nothing to disclose
Lutz Trojan: Nothing to disclose
Sophie Bachanek: Nothing to disclose
Manuel Nietert: Nothing to disclose
Johannes Uhlig: Nothing to disclose
Hazal Timucin: Nothing to disclose
Joachim Lotz: Nothing to disclose

### RPS 105-5

**Deep learning for bone lesion detection in CT TAP**

*P. Mlynarski*, A. Marcoux, L. Wood, F. Nicolas, M-M. Rohé, A. Feydy; Paris/FR
*(pawel.mlynarski@guerbet.com)*

**Purpose:** Bone metastases are frequent for several types of cancers (prostate, breast, lung, kidney, thyroid) and can often be observed in CT scans of the thorax, abdomen and pelvis (TAP). Accurate detection of suspicious bone lesions by radiologists is challenging due to the large size of CT TAP, read in a limited amount of time. We evaluate a deep learning model for automatic detection of several types of bone lesions. The problem is particularly challenging because of the variability of lesion appearances and locations.
**Methods or Background:** A multi-centre database of 949 CT TAP from oncology departments was annotated by trained radiologists, localising all types of bone lesions except common degenerative findings. On average, 11 lesions were found per scan. The database was used to train a deep learning model based on convolutional neural networks. The model was evaluated on a set of 100 scans annotated independently by 3 radiologists. We measured the model performance on three sets of ground truth lesions: those found by all three radiologists (GT3, 185 lesions), by at least two (GT2, 341 lesions) and finally by at least one radiologist (GT1, 713 lesions).
**Results or Findings:** The average sensitivity of the model compared to GT3, GT2 and GT1 was respectively 87.6%, 80.3% and 65.3%. The model produced 5 false positives per scan on average. The model was able to detect various types of lesions (metastasis, bone island, hemangioma) located in all bones visible in CT TAP.
**Conclusion:** The model reaches a high sensitivity, especially for lesions for which there is a high inter-reader agreement (GT3). These results suggest the interest in using deep learning to help radiologists in detecting bone lesions in CT TAP in clinical routine.
**Limitations:** The limitation is that no histopathology data is available.
**Ethics committee approval:** Not applicable.
**Funding for this study:** Funding was received for this study by Guerbet.
**Author Disclosures:**
Pawel Mlynarski: Employee: Guerbet
Antoine Feydy: Consultant: Guerbet
Marc-Michel Rohé: Employee: Guerbet
Laura Wood: Employee: Guerbet
Arnaud Marcoux: Employee: Guerbet
Francois Nicolas: Employee: Guerbet

### RPS 105-6

**Multi-stage AI analysis system to support prostate cancer diagnostic imaging across multiple centres and scanners**

*M. Hinton*[1], A. Shah[2], R. Hindley[2], S. le Conte[1], N. Moreira da Silva[1], N. Celik[1], F. Geraci[1], A. Rix[1], E. Sala[1]; [1]Cambridge/UK, [2]Winchester/UK

**Purpose:** To evaluate performance of fully-automated AI software to support analysis of prostate MRI to identify clinically significant prostate cancer (csPCa), using public datasets and real-world retrospective study data from a UK NHS hospital focusing on cross-manufacturer, multi-field strength and multi-centre application.
**Methods or Background:** AI-based software (Lucida Medical, PI v2) was developed, using open datasets (PROSTATEx, 204 patients) and retrospective data from a research study site (PAIR-1/site 1, 87 patients), a total of 291 patients to increase generalisability of the model. Data included 3 scanner models, different acquisition protocols and 1.5 Tesla and 3.0 Tesla field strengths. The data was split into 177 patients for training, 55 patients for development-validation, 59 patients for held-out-test.
**Results or Findings:** For biopsy assessment, the system identified patients with Gleason≥3+4 csPCa with sensitivity 93% (95% CI 84-100%), specificity 66% (56-76%), NPV 94% (87-100%), and AUC 0.86 (0.78-0.92) using multiparametric MRI (mpMRI) data from combined PROSTATEx and PAIR-1 development-validation and held-out-test sets (114 patients). Performance on the held-out-test set (59 patients) was similar. For biopsy targeting, the system identified lesions containing csPCa in the combined PROSTATEx and PAIR-1 validation and test set (176 lesions) with sensitivity 92% (83-98%), specificity 49% (40-57%), NPV 94% (87-99%), and AUC 0.83 (0.76-0.89) using mpMRI data. The biparametric MRI (bpMRI) model performed similarly on the same data compared to mpMRI model. Performance compares well with radiology studies and AI literature.
**Conclusion:** The proposed AI model shows promising preliminary results with open source and real-world retrospective data, at both 1.5 Tesla and 3 Tesla, suggesting it could help improve prostate cancer detection.
**Limitations:** Initial results are based upon limited data. Future work will add more sites, scanner manufacturers, models and protocols.
**Ethics committee approval:** This study was approved by Health Research Approval IRAS number 278640.
**Funding for this study:** Funding was received for this study by Lucida Medical.
**Author Disclosures:**
Aarti Shah: Nothing to disclose
Steffi le Conte: Employee: Lucida Medical Limited
Richard Hindley: Nothing to disclose
Antony Rix: CEO: Lucida Medical Limited
Mark Hinton: Employee: Lucida Medical Limited
Evis Sala: Board Member: Lucida Medical Limited
Fabio Geraci: Employee: Lucida Medical Limited
Nadia Moreira da Silva: Employee: Lucida Medical Limited
Numan Celik: Employee: Lucida Medical Limited

### RPS 105-7

**Does deep-learning software improve the consistency of PI-RADS scoring amongst radiologists with various levels of expertise?**

*D. Alis*, A. Arslan, S. Erdemli, G. Zeybel, M. E. Seker, E. Karaarslan; Istanbul/TR
*(drdenizalis@gmail.com)*

**Purpose:** To investigate whether a commercially available deep learning (DL) software improves the Prostate Imaging-Reporting and Data System (PI-RADS) scoring of less-experienced radiologists using the experienced radiologist as the ground truth.
**Methods or Background:** We retrospectively enrolled consecutive men who underwent multiparametric prostate MRI at 3T scanner due to suspicion of prostate cancer (PCa). Three radiologists, a radiology resident and radiologists with >3 and >20 years of experience, evaluated the bi-parametric MRI scans with and without the DL. The inter-rater agreement amongst the observers was investigated and compared using the kappa statistics. The inter-rater agreement between the standalone DL software and the experienced radiologist was also explored.
**Results or Findings:** In all, 151 men with a mean age of 63.59 years (range, 53-80) were enrolled in the study. There was a fair agreement between the experienced radiologist and the DL software, with a kappa of 0.33. The kappa score amongst the radiologists significantly increased to 0.54 from 0.48 with the DL software (P=0.006). The pair-wise analysis revealed no improvement between the less-experienced and experienced radiologists, while the PI-RADS scoring of the radiology resident with the DL software became significantly more consistent with the others.
**Conclusion:** The commercially available DL software significantly improves the PI-RADS scoring agreement amongst radiologists with varying levels of expertise, primarily improving the consistency of the radiology resident compared with the less experienced and experienced radiologist.
**Limitations:** The limitations were that the sample size was relatively small, covering prostate MRI scans obtained with the 3T scanner from a single tertiary center, and that we used bi-parametric MRI since the DL software used in this study does not use dynamic contrast-enhanced images.
**Ethics committee approval:** The local ethics committee approved this study and waived the need for informed consent.
**Funding for this study:** No funding was received for this study.
**Author Disclosures:**
Servet Erdemli: Nothing to disclose
Deniz Alis: Nothing to disclose
Aydan Arslan: Nothing to disclose
Ercan Karaarslan: Nothing to disclose
Mustafa Ege Seker: Nothing to disclose
Gokberk Zeybel: Nothing to disclose

### RPS 105-8

**Deep learning image reconstruction algorithm improves image quality of abdominal computed tomography: extensive comparison with hybrid iterative reconstruction**

*A. Del Gaudio*, G. Guido, N. Ubaldi, D. Valanzuolo, D. Pugliese, G. Bona, D. De Santis, D. Caruso, A. Laghi; Rome/IT
*(antonella.delgaudio@uniroma1.it)*

**Purpose:** To perform a comprehensive interindividual objective and subjective image quality evaluation of abdominal computed tomography (CT) images reconstructed with deep learning image reconstruction (DLIR) and hybrid iterative reconstruction (ASiR-V).
**Methods or Background:** Consecutive patients undergoing abdominal contrast-enhanced CT were prospectively enrolled from August to September 2021. Exclusion criteria were: contraindication to CT and severe motion artifacts. Thirteen datasets were reconstructed for each patient: DLIR at three strength levels (DLIR_L, DLIR_M, and DLIR_H, respectively) and ASiR-V from 10% to 100% in 10%-increments. Signal-to-noise ratio (SNR) and contrast-to-noise ratio (CNR) were calculated to assess object image quality. Subjective image quality was assessed with a 5-point Likert scale by two independent readers. ANOVA and Kruskal-Wallis H tests were used for statistical comparison, inter-reader agreement was calculated by means of k-statistics. Post-hoc pairwise comparisons were adjusted for multiple comparisons by the Bonferroni correction.
**Results or Findings:** Fifty patients were enrolled (39 male, mean age 67±13 years). DLIR algorithm did not impact vascular attenuation compared with ASiR-V (P > 0.05). DLIR_H showed the lowest noise, comparable with ASiR-V 100% (P = 1) and significantly lower than every other reconstruction (P < 0.05). DLIR_H achieved the highest objective image quality, with SNR and CNR comparable with ASiR-V 100% (P < 0.05). DLIR_H also achieved the highest median overall subjective image quality (score 5; IQR: 4-5) with excellent inter-reader agreement (k = 0.81), comparable with DLIR_M and significantly higher than every ASIR-V dataset.
**Conclusion:** DLIR significantly improves abdominal CT image quality compared to ASiR-V, potentially improving image reconstructions in routine clinical practice.
**Limitations:** The limitation is the small population sample.
**Ethics committee approval:** Local institutional review board approved the study; all study participants gave written informed consent.
**Funding for this study:** No funding was received for this study.
**Author Disclosures:**
Damiano Caruso: Nothing to disclose
Daniela Valanzuolo: Nothing to disclose
Nicolò Ubaldi: Nothing to disclose
Domenico De Santis: Nothing to disclose
Dominga Pugliese: Nothing to disclose
Andrea Laghi: Nothing to disclose
Antonella Del Gaudio: Nothing to disclose
Gisella Guido: Nothing to disclose
Giovanna Bona: Nothing to disclose

**08:00-09:00** **Room M 4**

### Research Presentation Session: Oncologic Imaging

### RPS 116

### Genitourinary oncology

**Moderator**
D. Prezzi; London/UK

### RPS 116-2

**Survival analysis of patients with endometrial cancer using an integrated radiomics model**

X. Li, D. Marcus, *A. Sheeka*, J. Russell, L. Ellis, E. Aboagye, N. Bharwani, S. Ghaem-Maghami, A. G. Rockall; London/UK

**Purpose:** To identify imaging signatures that predict survival in endometrial cancer using baseline T2 weighted MR images and to develop a clinically useful nomogram to provide a more personalised and accurate estimation of survival time.
**Methods or Background:** 413 internal and 82 independent external retrospective patients were included. All patients underwent preoperative MRI and had final histopathology. The internal dataset had at least 3-year survival data. Imaging features were extracted and delineated to predict survival time. A group least absolute shrinkage and selection operator logistic regression (LASSO) was used to select radiomics features. Stratified 10-fold cross-validation was used to calculate accuracy and determine the optimal model. A Cox proportional hazards (CPH) model was used to study the relationship between predictor variables and survival time. Internal and external validation were conducted to evaluate the integrated model for survival time prediction.
**Results or Findings:** In total 958 radiomic and 3 clinical features were extracted from T2 sequences. After applying a bi-level LASSO method, 3 radiomic features and two clinical variables were selected as predictors in the CPH model. Using the internal dataset, the concordance index was 0.797 for the model with age and clinical cancer grade and 0.818 for the integrated model which includes age, cancer grade and 3 radiomic features. Likelihood ratio test showed significant difference (P<0.05) between the two models. Based on external data, these models were still significantly different using likelihood ratio test; the concordance index was 0.792 for the model with age and clinical cancer grade, and 0.882 for the integrated model.
**Conclusion:** The developed radiomic signature is a powerful predictor of survival time, outperforming current clinical cancer grade models. The integrated radiomics nomogram facilitates individualised prediction of survival.
**Limitations:** Not applicable.
**Ethics committee approval:** Not applicable.
**Funding for this study:** Not applicable.
**Author Disclosures:**
Alexander Sheeka: Nothing to disclose
Xingfeng Li: Nothing to disclose
James Russell: Nothing to disclose
Sadaf Ghaem-Maghami: Nothing to disclose
Laura Ellis: Nothing to disclose
Diana Marcus: Nothing to disclose
Eric Aboagye: Nothing to disclose
Andrea Grace Rockall: Board Member: European Society of Radiology
Nishat Bharwani: Nothing to disclose

### RPS 116-3

**De novo radiomics with image augmentation and features from T1 mapping to predict Gleason scores in prostate cancer**

M. Makowski[1], *K. K. Bressem*[2], S. M. Niehues[2], S. Keller[2], D. Rueckert[1], L. C. Adams[2]; [1]Munich/DE, [2]Berlin/DE

**Purpose:** The aims of this study were to discriminate among prostate cancers (PCas) with Gleason scores 6, 7, and ≥8 on biparametric magnetic resonance imaging (bpMRI) of the prostate using radiomics and to evaluate the added value of image augmentation and quantitative T1 mapping.
**Methods or Background:** 85 patients with subsequently histologically proven PCa underwent bpMRI at 3T. The PCa lesions as well as the peripheral and transition zones were segmented pixel by pixel in multiple slices of the 3D MRI data sets (T2w, ADC, T1 maps). To increase the size of the data set, images were augmented for contrast, brightness, noise, and perspective multiple times, effectively increasing the sample size 10-fold. Four different machine learning algorithms, including a random forest (RF), stochastic gradient boosting (SGB), support vector machine (SVM), and k-nearest neighbour, were trained with and without features from T1 maps to differentiate among 3 different Gleason groups.
**Results or Findings:** SVM showed the highest accuracy of 0.92 (95% confidence interval [CI], 0.62-1.00) for classifying the different Gleason scores, followed by RF (0.83; 95%-CI, 0.52-0.98), SGB (0.75; 95%-CI, 0.43-0.95), and k-nearest neighbour (0.50; 95%-CI, 0.21-0.79). Image augmentation resulted in an average increase in accuracy between 0.08 (SGB) and 0.48 (SVM). Removing T1 mapping features led to a decline in accuracy for RF (-0.16) and SGB (-0.25) and a higher generalisation error.
**Conclusion:** When data are limited, image augmentations and features from quantitative T1 mapping sequences might help to achieve higher accuracy and lower generalisation error for classification among different Gleason groups in bpMRI by using radiomics.
**Limitations:** There are several more limitations to the present work, the foremost being the limited number of evaluated PCa patients and lesions (n = 85) and the retrospective study design.
**Ethics committee approval:** This study was approved by an ethics committee.
**Funding for this study:** No funding was received for this study.
**Author Disclosures:**
Keno K. Bressem: Nothing to disclose
Lisa C. Adams: Nothing to disclose
Daniel Rueckert: Nothing to disclose
Sarah Keller: Nothing to disclose
Marcus Makowski: Nothing to disclose
Stefan Markus Niehues: Nothing to disclose

### RPS 116-4

**High-risk imaging features in prostate cancer**

M. Fedchenko[1], B. Giesteira[2], P. Maganinho[2], N. J. Lamas[2], *J. Amorim*[2]; [1]Braga/PT, [2]Porto/PT

**Purpose:** To identify mpMRI imaging features capable of predicting radical prostatectomy pathological features that provide a worse prognosis to prostate cancer patients, such as an unfavourable grade group (UGG) (Gleason grade ≥ 3).
**Methods or Background:** We retrospectively identified 65 prostate cancer patients that performed radical prostatectomy in our centre during 2019. Clinical, laboratory, imaging and pathology data was collected, with subsequent descriptive and interferential analysis, followed by the creation of a binary logistic regression model for the prediction of UGG. To evaluate the discriminatory capacity of the model, we created a ROC curve and identified the best cut-off point.
**Results or Findings:** Of the included patients, 53.8% had un UGG on prostatectomy. Among the patients with favourable (FGG) and unfavourable grade group, we found statistically significant differences in the imaging parameters PI-RADS category (p = .006), diameter of the highest index lesion (p = .002), restricted diffusion (p = .042), ADC values (p = .002), contrast enhancement (p = .008), capsular invasion (p = .036) and benign prostate hyperplasia (BPH) (p = .007). On regression analysis the variables diameter of the highest index lesion, ADC value and BPH were selected as the best predictors of UGG. Our constructed model had a good discriminatory capacity (AUC = 0.86, p < 0.001), with a 90.9% sensitivity, 73.1% specificity and 82.0% accuracy for a cut-off point of 0.45.
**Conclusion:** The combination of the highest index lesion diameter, the ADC value and the presence/absence of BPH could differentiate UGG from FGG with high sensitivity and accuracy.
**Limitations:** The limitations are the population size and retrospective data collection.
**Ethics committee approval:** Ethical approval for this study was obtained.
**Funding for this study:** No funding was received for this study.
**Author Disclosures:**
Nuno Jorge Lamas: Nothing to disclose
Pedro Maganinho: Nothing to disclose
Bruno Giesteira: Nothing to disclose
Maria Fedchenko: Nothing to disclose
João Amorim: Nothing to disclose

### RPS 116-5

**Comparison of diagnostic performance between diffusion kurtosis imaging parameters and mono-exponential ADC for determination of clinically significant cancer in patients with prostate cancer**

*S. H. Kim*, H. Park; Busan/KR
*(radiresi@gmail.com)*

**Purpose:** To compare the diagnostic performance between diffusion kurtosis imaging (DKI) parameters and mono-exponential apparent diffusion coefficient (ADC) for determination of clinically significant cancer (CSC, Gleason score (GS) ≥7) in patients with histologically proven prostate cancer (PCa).
**Methods or Background:** 92 patients (mean age: 71.5 years, range: 47-89 years) who had been diagnosed as PCa and undergone 3T-MRI including DWI (b values, 0, 100, 1000, 2000 s/mm2) were included in this study. The DKI parameters, namely apparent diffusion for non-Gaussian distribution (Dapp) and apparent kurtosis coefficient (Kapp), were calculated by dedicated software using mono-exponential and diffusion kurtosis models for quantitation. The measurement was performed for a whole tumour after segmentation, and pathologic topographic maps or systemic biopsy results served as the reference standard for segmentation. To compare the diagnostic performance of each parameter for determination of CSC, pair-wise comparison of receiver operating characteristic (ROC) curves was performed.
**Results or Findings:** The study population consisted of GS 6 (n=18), GS 7 (n=31), GS 8 (n=25), GS 9 (n=15) and GS 10 (n=3) patients. The AUC of Kapp (0.707, 95% CI, 0.603 – 0.798) for discriminating CSC from non-CSC was not significantly different from those of mono-exponential ADC (0.725, 0.622 – 0.813, P=0.2175) or Dapp (0.726, 0.623 – 0.814, P=0.9628). Diagnostic predictive values of Kapp were estimated to a maximum accuracy of 78%, a sensitivity of 86%, and a specificity of 47%, while those of mono-exponential ADC were 75, 81, and 53%, respectively.
**Conclusion:** The DKI parameters showed a diagnostic performance comparable to mono-exponential ADC for determination of CSC in patients with PCa.
**Limitations:** Half of the references for tumour segmentation were based on systemic biopsy.
**Ethics committee approval:** This study was approved by the Institutional Review Board, and informed consent was waived.
**Funding for this study:** No funding was received for this study.
**Author Disclosures:**
Hyungin Park: Nothing to disclose
Seung Ho Kim: Nothing to disclose

### RPS 116-7

**Comparison of 68 GA PSMA and whole-body diffusion-weighted MR imaging in staging of high-risk prostate cancer**

*B. Raghavan*, R. H. Arafath, D. A. Sundaram, J. Amalachandran, M. K. Logudoss; Chennai/IN
*(drbagyam@gmail.com)*

**Purpose:** The purpose of the study is to compare the sensitivity and specificity of 68 GA PSMA and whole-body diffusion-weighted MR imaging in staging of high-risk prostate cancer.
**Methods or Background:** 63 patients who have been recently diagnosed with high-risk prostate cancer were included in the study. Inclusion criteria included biopsy-proven prostate cancer with PSA of more than 20ng/ml and Gleason score of more than seven. After obtaining informed consent all the patients underwent Ga PSMA or FDG PRT and whole-body diffusion-weighted imaging within 1 week duration.
**Results or Findings:** Regional nodal metastases were detected by 68 GA PSMA in 48 patients and by DWI in 45 patients. Non-regional nodal metastases were detected in 18 patients by 68GA PSMA and in 17 patients in DWI. Skeletal metastases were detected in 24 patients by both 68GA PSMA and whole-body DWI. Both 68GA PSMA and whole-body DWI detected hepatic metastasis in one patient. Additionally, 68GA PSMA detected pulmonary metastasis in one patient, which was missed by DWI.
**Conclusion:** This study suggests that whole-body diffusion-weighted imaging has sensitivity and specificity almost equal to 68GA PSMA in the detection of nodal and distant metastases in high-risk prostate cancer and can be used with multiparametric imaging of the prostate for complete TNM staging of prostate cancers.

Wednesday

**Limitations:** The limitations are the small number and large volume disease.
**Ethics committee approval:** Retrospective study - approved by an ethics committee.
**Funding for this study:** No funding was received for this study.
**Author Disclosures:**
Murali Krishna Logudoss: Nothing to disclose
Rasheed H Arafath: Nothing to disclose
Dayala A Sundaram: Nothing to disclose
Bagyam Raghavan: Nothing to disclose
Jaykanth Amalachandran: Nothing to disclose

### RPS 116-8

**Prediction of axillary lymph node metastasis of early-stage breast cancer by ultrasound and pathology: to avoid unnecessary axillary lymph node dissection**
*L. Yang*, Y. Yin; Nantong/CN

**Purpose:** To develop a clinical model for predicting the number of axillary lymph node metastases in early breast cancer by using ultrasonography (US) and pathology to avoid unnecessary axillary lymph node dissection.
**Methods or Background:** From December 2011 to December 2016, 233 patients who underwent breast-wide local excision and axillary node dissection were included. According to the number of SLN metastasis based on pathology, the patients were divided into two groups: limited nodal burden (0-2 metastatic LNs) and heavy nodal burden (3 metastatic LNs). Univariate logistic regression analysis and multivariate logistic regression analysis of the US results and clinical-pathologic variables were conducted to find the most valuable variables for predicting the number of axillary lymph node metastases. A nomogram was created based on these variables.
**Results or Findings:** We found that cortical thickness (>3 mm), suspicious ALNs and hilum status are indicators of HNB. Coefficient of determination (R2) and the area under the ROC curve (AUC) were shown good calibration and discrimination of the model.
**Conclusion:** The results of this study suggest that the model according to the variables of US and pathology can help select patients with limited nodal burden, for whom axillary lymph node dissection is unnecessary.
**Limitations:** First, it was a single-centre retrospective study. Second, the model was only confirmed in a small sample cohort (n= 233). It needs to be further studied in more groups to evaluate universality and predictive accuracy. In addition, the results of US like cortical thickness measured by different doctors had some differences.
**Ethics committee approval:** This study has been approved by the Ethics Committee of Affiliated Hospital of Nantong University.
**Funding for this study:** No funding was received for this study.
**Author Disclosures:**
Lei Yang: Author: Author
Yifei Yin: Author: corresponding author

**08:00-09:00** **Room N**

### Research Presentation Session: Paediatric

### RPS 112

### Radiological diagnosis and follow-up of congenital disorders in children

**Moderator**
M. Galluzzo; Rome/IT

### RPS 112-2

**Congenital lung abnormalities on magnetic resonance imaging: the CLAM study**
B. Elders, C. Kersten, S. Hermelijn, P. Wielopolski, H. A. W. M. Tiddens, M. Schnater, *P. Ciet*; Rotterdam/NL
*(p.ciet@erasmusmc.nl)*

**Purpose:** The aim of this study was to develop a non-contrast chest Magnetic Resonance Imaging (MRI) protocol to image lung structure and function in paediatric Congenital Lung Abnormalities (CLA). A further aim was to compare MRI at school-age in a cohort of CLA patients to post-natal Computed Tomography (CT).
**Methods or Background:** CT is currently the standard modality for radiological diagnosis and follow up of CLA. This technique is limited by the exposure to ionising-radiation and need for intravenous contrast to visualise vessels. MRI could be a safe alternative with the potential to image lung structure and function in a single examination without the contrast enhancement. Therefore, in this study twenty-one patients with a history of CLA and a mean age of 12.8 (range 9.4-15.9) years underwent spirometry and chest MRI. MRI findings were compared to post-natal CT using in-house developed software that expresses lung abnormalities in percentage of total lung volume. A structured report for CLA was compared between MRI and CT.
**Results or Findings:** MRI successfully visualised all CLA related lung abnormalities, including vascularisation, without contrast enhancement. On school age MRI, compared to post-natal CT, size of the CLA decreased in 41% (n=7), remained stable in 47% (n=8) and increased in 12% (n=2). Associated lung abnormalities, such as atelectasis and hypo-attenuation, disappeared in 41% (n=7), decreased in 29% (n=5), remained stable in 24% (n=4) and increased in 6%(n=1). In 7 patients (41%), diagnoses of the CLA changed between post-natal CT and school-age MRI. The best lesion visualisation was achieved on ZTE scan, while PROPELLER and 3D MR angiography resulted in the best vessel visualisation.
**Conclusion:** Non-contrast enhanced MRI is a feasible and safe imaging method for the long-term follow up of paediatric CLA patients.
**Limitations:** A limitation of the study is that there was no CT comparison at school-age.
**Ethics committee approval:** This study was IRB approved.
**Funding for this study:** Funding was received from Vrienden van het Sophia (SSWO).
**Author Disclosures:**
Marco Schnater: Nothing to disclose
Sergei Hermelijn: Nothing to disclose
Casper Kersten: Nothing to disclose
Pierluigi Ciet: Nothing to disclose
Bernadette Elders: Nothing to disclose
Harm A W M Tiddens: Nothing to disclose
Piotr Wielopolski: Nothing to disclose

### RPS 112-3

**Structure and function at school age in preterm born children with and without bronchopulmonary dysplasia on lung-MRI**
B. Elders, H. A. W. M. Tiddens, M. Pijnenburg, I. Reiss, P. Wielopolski, *P. Ciet*; Rotterdam/NL
*(p.ciet@erasmusmc.nl)*

**Purpose:** Our aim was to develop and test a fast MRI protocol to image lung structure and function of preterm born children with and without bronchopulmonary dysplasia (BPD) at school-age, and compare MRI findings to spirometry.
**Methods or Background:** The most common respiratory complication of extreme prematurity is BPD, but also patients born extremely preterm without BPD may show structural lung changes and impaired respiratory outcomes, which can lead to life-long morbidity. To assess severity of disease and future risks, there is need for a safe imaging modality for preterm born children (with) BPD. In this study nine healthy volunteers (age 11.6, range 8.8-12.8 years), eleven children with BPD (11.0, range 7.2-15.6) and nine preterm born children without BPD (11.1, range 10.7-12.6) underwent MRI. In-house developed software was used to score images on hypo- and hyperintense abnormalities and bronchopathy expressed as percentage total lung volume and architectural distortion, and correlated to spirometry. Fourier Decomposition (FD) sequences were analysed for ventilation and perfusion defects.
**Results or Findings:** Children with BPD showed significantly lower spirometry outcomes compared to premature children without BPD and healthy volunteers (all p≤0.01). On MRI, children with BPD had a higher percentage diseased lung (9.1, IQR 5.9-11.6%) compared to premature children without BPD (3.4, IQR 2.5-5.4%, p<0.001) and healthy volunteers (0.4, IQR 0.1-0.8%, p<0.001). Percentage of diseased lung correlated positively with percentage predicted FEV1 (r=-0.40, p=0.04), FEV1/FVC (r=-0.49, p=0.009) and FEF75 (r=-0.63, p<0.001). FD identified regions of ventilation and perfusion defects corresponding to hypointense regions on expiratory MRI.
**Conclusion:** MRI is a feasible method to identify structural and functional lung damage at school-age in preterm born children with and without BPD, showing good correlation with spirometry. Therefore, MRI could play a role for safe long-term follow up of preterm and BPD children.
**Limitations:** The following limitations were identified: single centre study, no CT comparison.
**Ethics committee approval:** This study was IRB approved.
**Funding for this study:** This study was funded by Vrienden van het Sophia.
**Author Disclosures:**
Pierluigi Ciet: Nothing to disclose
Irwin Reiss: Nothing to disclose
Marielle Pijnenburg: Nothing to disclose
Bernadette Elders: Nothing to disclose
Harm A W M Tiddens: Nothing to disclose
Piotr Wielopolski: Nothing to disclose

### RPS 112-6

**Three-dimensional visualisation of large vessel anomalies in foetuses using multivein super-resolution MR technique**

*F. Prayer*, G. O. Dovjak, P. Brugger, G. Gruber, D. Prayer, G. Kasprian; Vienna/AT

**Purpose:** To assess the feasibility of three-dimensional visualization of foetal large vessels based on multivein MR technique in combination with super-resolution post-processing in normal foetuses, and foetuses with large vessel anomalies.
**Methods or Background:** Foetal MRI data of fifteen normal cases (mean 29+5 gestation weeks), and fifteen cases with anomalies of the large vessels (mean 28+1 GW) were included. Foetal MRI scans were performed on one 1.5 Tesla scanner (Philips Ingenia). Anomalies of the large vessels included tetralogy of Fallot (n=4), aortic coarctation (n=3), right descending aorta (n=2), common arterial trunk (n=2), hypoplastic left heart (n=1), persistent left superior vena cava (n=1), pulmonary agenesis (n=1), and heterotaxy syndrome (n=1). T2-weighted sequences were acquired in three orthogonal planes using radial k-space sampling multivein technique. Super-resolution post-processing was performed to obtain iso-voxel data sets. Manual segmentation of large vessel structures including aorta, pulmonary arteries, large veins, ductus venosus, and ductus arteriosus was performed to generate three-dimensional models. Multivein images were reviewed alone and in combination with three-dimensional models for large vessel anomalies by one foetal imaging expert.
**Results or Findings:** Super-resolution aided three-dimensional visualisation of foetal large vessels based on foetal MRI scans was feasible in all thirty cases. Review of orthogonal T2-weighted multivein sequences alone correctly identified 13 of 15, and, if reviewed alongside three-dimensional models, 15 of 15 vessel anomalies. No vessel anomaly was found in the normal group.
**Conclusion:** Foetal MRI-based three-dimensional visualisation of large vessels including large vessel anomalies using multivein and super-resolution post-processing technique is feasible.
**Limitations:** Foetal motion artefacts may limit the applicability of super-resolution post-processing.
**Ethics committee approval:** This study was approved by the IRB.
**Funding for this study:** No funding was received for this study.
**Author Disclosures:**
Florian Prayer: Nothing to disclose
DGregor Oliver Dovjak: Nothing to disclose
Peter Brugger: Nothing to disclose
Gerlinde Gruber: Nothing to disclose
Daniela Prayer: Nothing to disclose
Gregor Kasprian: Nothing to disclose

### RPS 112-7

**Image quality and radiation dose evaluation of paediatric ECG-triggered cardiovascular computed tomography in congenital heart diseases**

*M. Gulizia*, A. Viry, L. Alamo Mastre, T. Cherpillod, C. Chevallier, E. V. Tenisch; Lausanne/CH
*(marianna.gulizia@chuv.ch)*

**Purpose:** The aim of this study is to provide an inventory of radiation doses delivered by cardiac CTs in children with congenital heart disease and to subjectively and objectively assess the image quality.
**Methods or Background:** 56 children between 0 and 6 months having undergone an ECG-triggered cardiac-CT over a 5-year period were included. Images were acquired at 80 kVp, 100-545 mAs using automatic tube current modulation, 0.28 sec tube rotation after injection of iodinated contrast medium with bolus tracking. Images were reconstructed using ASiR-V 50% (0.625mm slice thickness). Sedation was performed in children older than 3 months. Radiation doses were analysed and compared to previous studies. Quantitative analyses were done calculating CNR and SNR in the ascending aorta and pulmonary trunk. Subjective analyses were done assessing the image quality of various vascular structures. Detectability analyses were done on a phantom with iterative and deep learning reconstructions.
**Results or Findings:** CTDIvol and effective dose were 2.35±0.54 mGy and 0.85±0.22 mSv (range 0.39-1.66) respectively. CNR for pulmonary trunk and ascending aorta were 36.96±2.18 and 32.50±2.02 respectively. SNR ratios were 32.37±2.25 and 37.81±2.04 respectively. Image quality was subjectively very good even for small structures. Detectability analysis provided a 30% optimisation potential thanks to deep learning image reconstructions (AUC=0.995).
**Conclusion:** Our analysis shows a slightly higher radiation doses compared to the literature. Our current practice provides high quality images with acceptable radiation doses. There is a potential to optimise our protocol by reducing radiation doses and contrast media while maintaining a diagnostic image quality.
**Limitations:** It is a unicentric study and our patients had suboptimal arms positioning.
**Ethics committee approval:** This retrospective study has been approved by the Vaud Ethics Committee (Ref CER-VD 2021-00827).
**Funding for this study:** No funding was received for this study.
**Author Disclosures:**
Tyna Cherpillod: Author: mm
Anaïs Viry: Author: mm
Marianna Gulizia: Investigator: mm
Leonor Alamo Mastre: Author: mm
Christine Chevallier: Author: mm
Estelle Valérie Tenisch: Investigator: mm

**09:30-10:30** **Open Forum #1 (Radiographers)**

### Research Presentation Session: Radiographers

### RPS 214

### Optimising oncology imaging, treatment, and the patient experience

**Moderators**
A. Sarchosoglou; Athens/GR
D. Caruso; Rome/IT

### RPS 214-3

**Analysis of dose and acute radio-toxicity in breast cancer patients**

*A. M. Carmo*[1], M. Ramos[2], F. Serra[2], A. F. C. L. Abrantes[2], S. I. Rodrigues[2], L. P. V. Ribeiro[2], B. Vicente[2], R. P. P. Almeida[2]; [1]Olhão/PT, [2]Faro/PT

**Purpose:** Over the last 20 years, there have been significant technological and technical advances in radiotherapy for breast cancer. However acute radiation-induced skin toxicity remains a side effect impacting the quality of life in breast cancer patients. The aim of this study was to verify if there is a relationship between the dose-surface that reaches the skin and the location of acute radio toxicity.
**Methods or Background:** In this study we evaluated 49 patients scheduled to undergo conventional three-dimensional conformal RT after surgery (including breast conserving surgery and mastectomy with breast reconstruction) at a private radiotherapy department. The data collection for this study consisted of two parts: a patient survey and data from the dosimetric planning system. First, the patients were surveyed to describe the toxicity, its symptoms, its location and the week of treatment. Then, the values of the doses of the mapped skin from each patient who was selected in the first part were collected. Subsequently, the location was related to the doses collected from the skin in the dosimetric planning system.
**Results or Findings:** It was found that the location of side effects in the breast quadrants was not correlated with the mean and maximum dose values, and with the values of V30 and V40. There was a weak correlation of 0.200 ($p<0.05$) with V50. Side effects located in the fold zones were not correlated with dose and V30, V40 and V50 values.
**Conclusion:** In this study, a relationship between the dose received by the risk organ, the skin, and the development of acute toxicity was not observed.
**Limitations:** Identified limitation were the sample size and the fact that only one setting was studied.
**Ethics committee approval:** An ethics committee approved the study and written informed consent was obtained from the participants.
**Funding for this study:** No funding was received for this study.
**Author Disclosures:**
Magda Ramos: Nothing to disclose
António Fernando Caldeira Lagem Abrantes: Nothing to disclose
Rui Pedro Pereira Almeida: Nothing to disclose
Bianca Vicente: Nothing to disclose
Sónia Isabel Rodrigues: Nothing to disclose
Ana Maria Carmo: Nothing to disclose
Fábio Serra: Nothing to disclose
Luís Pedro Vieira Ribeiro: Nothing to disclose

### RPS 214-4

**Application of advanced MRI techniques in white matter characterisation of patients affected by meningioma treated with proton therapy**

*L. Anemoni*, I. E. Mascayano, E. Orlandi, L. Preda, M. S. Cadeo, M. E. Piazzolla, S. Imparato, A. Mancin, S. Tampellini; Pavia/IT

**Purpose:** The study aimed to detect, through the processing of data obtained from imaging techniques of diffusion weighted imaging (DWI-MRI) and intravoxel incoherent motion technique (IVIM-MRI), early changes in diffusion, pseudo-diffusion and perfusion properties of normal tissue in patients affected by meningioma treated with proton therapy.

**Methods or Background:** Datasets were analysed before the start of proton therapy and at follow-up at 3, 6 and 9 months after the end of treatment. Multiparametric maps (ADC, D, D* and f) were created, and for each patient the total number of white matter voxels, the total volume included in the contours (mm3) and the average intensity of voxels with the relative standard deviation (SD, σ) was calculated.
**Results or Findings:** The results obtained concerning the mean intensity values (mm2/s) of ADC, D, D* and f show a homogeneous trend with small discrepancies between: ADC=0.79x10-3 mm2/s (±150.3628 mm2/s); D=0.70×10−3 mm2/s (±159.0189 mm2/s) D*=1.3×10^2 mm2/s (±0.0335 mm2/s); f=1.1x10 mm2/s (±0.1052 mm2/s). D has a value similar to ADC, but is larger as it takes into account as a loss contribution to the signal not only perfusion but also the effect of diffusion; D* as a result of micro-perfusion in capillaries is shown to be greater than D.D* as well as f, due to the inability to acquire images at low b-values (0-50 s/mm2), where micro-perfusion makes a greater contribution to signal loss than DWI.
**Conclusion:** In conclusion, it was observed that the ADC value obtained from DWI sequences is (0.79 x 10-3 mm2/s) in line with the expected values in the literature of neuro-oncological studies (0.319-1.05×10-3 mm2/s). DWI-MRI and IVIM-MRI techniques will contribute diagnostic improvement, both in the field of research and clinics.
**Limitations:** Not applicable.
**Ethics committee approval:** Not applicable.
**Funding for this study:** Not applicable.
**Author Disclosures:**
Alice Mancin: Nothing to disclose
Margherita Sofia Cadeo: Nothing to disclose
Sara Imparato: Nothing to disclose
Sara Tampellini: Nothing to disclose
Lorenzo Preda: Nothing to disclose
Luca Anemoni: Nothing to disclose
Ester Orlandi: Nothing to disclose
Ivonne Elenoire Mascayano: Nothing to disclose
Maria Elena Piazzolla: Nothing to disclose

### RPS 214-5

### Women's positive and less positive experiences of mammography and radiotherapy services during their breast cancer care pathway

*E. M. Metsälä*[1], S. Kivistik[2], L. Marmy[3], J. A. Jorge[3], K. Straume[4], B. Strom[4]; [1]Helsinki/FI, [2]Tartu/EE, [3]Lausanne/CH, [4]Bergen/NO
*(eija.metsala@metropolia.fi)*

**Purpose:** The purpose is to describe women's positive and less positive experiences of mammography and radiotherapy services during their breast cancer care pathway.
**Methods or Background:** Data was collected by using open-ended online questionnaires via the websites and social media of national breast cancer patient organisations in four countries. The announcement was targeted at patients having finished their breast cancer treatments a maximum of six months before responding. Data comprising 14 women's responses was analysed by deductive thematic analysis.
**Results or Findings:** Reported positive and less positive aspects of mammography services were associated with how painful or unpleasant women perceived the examination, patient-staff relationships and in addition as a positive aspect, having scheduled the appointment quickly. In relation to radiotherapy, both positive and negative experiences focused on the way treatment was organised and delivered and how staff treated and encountered women. In addition, as a positive aspect easy-going treatment was mentioned, and as a less positive aspect, side-effects of radiotherapy were brought up.
**Conclusion:** Though many of the positive and less positive experiences women had in mammography and radiotherapy were the same, there were also differences. Targeted interventions at each patient contact point in the process should be planned, to improve the quality of breast cancer care.
**Limitations:** Limitations associated with patient-reported data apply to this study. Patient reported experience data should be interpreted with caution, as reported positive experiences might neither reflect high quality care nor satisfied patients.
**Ethics committee approval:** Not applicable.
**Funding for this study:** The study was supported by the European Commission Erasmus+ strategic partnership programme grant number 2020-1-EE01-KA203-077941. For the Swiss associate partner this work was supported by the Swiss national agency MOVETIA.
**Author Disclosures:**
Jose A. Jorge: Nothing to disclose
Eija Metsälä Metsälä: Nothing to disclose
Kjersti Straume: Nothing to disclose
Siret Kivistik: Nothing to disclose
Bergliot Strom: Nothing to disclose
Laurent Marmy: Nothing to disclose

### RPS 214-6

### Patients' perceptions of the skills and competencies of therapeutic radiographers across Europe

*S. L. McFadden*, C. Hughes, T. Flood, A. O Neill, P. McClure; Belfast/UK
*(s.mcfadden@ulster.ac.uk)*

**Purpose:** Variation in training and education of therapeutic radiographers (TRs) across Europe leads to differences in roles and staff autonomy in clinical practice. The aim of this study was to gain insight into patients' perceptions of the skills and competencies of TRs to help inform undergraduate curricula across Europe.
**Methods or Background:** Ethical permission was sought and obtained from Ulster University, Belfast, UK. An electronic survey was performed using Qualtrics® and a hard copy questionnaire was distribution to radiotherapy patients across the UK, Portugal, Malta, and Poland. Patients >18 years currently receiving, or who had received radiotherapy within the last 24 months, were included. Data analysis was performed with the aid of SPSS version 27.
**Results or Findings:** Data collection was ongoing until December 2021. Preliminary results from the UK and Portugal show that 331 survey responses have been collected from patients who have received radiotherapy both pre and post the COVID-19 pandemic. The vast majority of patients felt TRs had the required competencies to listen, understand and communicate compassionately. Conflicting opinions arose over whether patients wanted staff to ask them about their life and be more aware of them 'as a person'.
**Conclusion:** Patients perceive the TR more in terms of their personal attributes rather than their professional competencies. While they may not always remember specific details of what the TRs do with/to them, they tend to remember and focus on how TRs made them feel.
**Limitations:** Not all European countries were involved in this study, hence, the findings may not represent the breadth of patient experiences throughout Europe. Further research could be conducted internationally.
**Ethics committee approval:** Integrated Research Applications System: 277006 REC reference: 20/YH/038; Institute of Nursing Health Research Ethics Committee UU, Ref No: FCNUR-20-035
**Funding for this study:** ERASMUS+ funded this project. There were no conflicts of interest.
**Author Disclosures:**
Ciara Hughes: Nothing to disclose
Patricia McClure: Nothing to disclose
Angela O Neill: Nothing to disclose
Sonyia Lorraine McFadden: Nothing to disclose
Terri Flood: Nothing to disclose

### RPS 214-7

### Image quality assessment of 2D mammograms for missed, discordant and concordant screen-detected cancer, and interval cancers in population-based screening with independent double reading

*R. Gullien*, A. E. Haakull, A. S. Bakken, P. Skaane, J-G. Andersen; Oslo/NO

**Purpose:** To investigate whether reduced image quality (PGMI) or compression force (CF) might have contributed to false negative interpretations of screening-detected (SDC) and interval (IC) cancers.
**Methods or Background:** Oslo Tomosynthesis Screening Trial (OTST) included four independent arms comparing double-reading 2D versus DBT. Exams with prospectively true positive (TP) score in all four arms were excluded (n=100). 130 women with screen-detected cancers (SDC) had at least one false-negative score. 51 women were diagnosed with interval cancer (IC). Four radiologists retrospectively classified the 2D screening mammograms of these 181 women as true negative (TN, cancer not visible), non-specific minimal sign (ns-m.s.), significant minimal sign (s-m.s.), and false negative (FN). 2D mammograms with negative score at double reading, but retrospectively classified as s-m.s. and FN were included for analysis. In a retrospective consensus-meeting three radiographers PGMI assessed image quality of CC+MLO images separately using criteria from the quality-assurance-manual (QAM) from BreastScreen Norway: perfect-good (P+G) >75%, moderately-good (M) <22%, inadequate (I) <3%. QAM recommend compression-force (CF) between 10-18N, usually between 12-15N. CF was registered for all images.
**Results or Findings:** Final material included 2D mammography for 108 women of which 23 had concordant TP, 52 had discordant scores, and 33 FN after excluding TN and ns-m.s. for DBT-only detected cancers (n=28) and IC's (n=45). PGMI result for concordant TP P+G 73%, M 24%, I 3% with CF 12,8N; discordant P+G 72%, M 21%, I 7% with mean CF 12.4N; FN: P+G 73%, M 20%, I 7% with mean CF 12.5N.
**Conclusion:** Our study did not show any significant difference in PGMI scores between concordant or discordant positives and false negatives. The overall lower PGMI scores did not obviously influence cancer detection. Compression-force for all examinations was within recommendations.
**Limitations:** No limitations were identified.

**Ethics committee approval:** ClinicalTrial.gov NCT01248546.
**Funding for this study:** No funding was received for this study.
**Author Disclosures:**
Randi Gullien: Nothing to disclose
Anne Synnøve Bakken: Nothing to disclose
Per Skaane: Speaker: Speaker fees and travel grants from Hologic Inc., GE Healthcare and Pfizer.
Jack-Gunnar Andersen: Nothing to disclose
Anne Emilie Haakull: Nothing to disclose

**09:30-10:30** **Open Forum #3 (ESR)**

### Research Presentation Session: Vascular

### RPS 215

### Artificial intelligence and more for vascular imaging

**Moderator**
S. Duvnjak; Odense/DK

### RPS 215-3

**Artificial intelligence in the evaluation of peripheral arterial disease in lower limbs**
*S. S. Tonpe*[1], H. Warbhe[2]; [1]Secunderabad/IN, [2]Pune/IN
*(sudhanshutonpe@gmail.com)*

**Purpose:** This study evaluates the potential of deep learning algorithm for evaluation of peripheral arterial disease (PAD) in lower limbs with convolutional neural network (CNN).
**Methods or Background:** Colour doppler with spectral tracing scans of lower-limbs were conducted at a tertiary hospital between May 2017 and December 2019 with equivocal findings excluded, resulting in 190 studies. A test set of 45 images was randomly selected from the initial sample (27 abnormal,18 normal) and was not used in the training process. A further 37 abnormal scans were randomly excluded resulting in a training set of 54 normal and 54 abnormal studies. Equal numbers were required in each group for optimal network training and these were converted to JPEG-image format. The initial sample was amplified 26-fold using a combination of horizontal flip, size alteration and rotation. Pre-trained Inception-v3 network was then retrained using amplified images. Training data was randomly split with 80% for training, 10% for validation & 10% for final testing. The model was trained over 2000 iterations with a learning rate of 0.01. The area under receiver-operator-curve (ROC) was calculated using a web-based analysis tool.
**Results or Findings:** Area under ROC for this CNN was 0.87, demonstrating high levels of diagnostic test accuracy. Output from CNN produces a continuous score of between 0 (abnormal) and 1 (normal). Setting the output score threshold to 0.395 results in a test sensitivity of 96.3%, specificity of 66.7%, positive predictive-value of 81.3% and negative predictive-value of 92.3%.
**Conclusion:** This proof of concept study demonstrates that high diagnostic test accuracy can be achieved in an automated analysis of triplex images. Results obtained from this study may help vascular surgeons and radiologists to objectively evaluate patients with PAD and can be adopted across many different forms of imaging.
**Limitations:** In this study, CNN could never outperform the radiologist since the radiologist's opinion was ground truth. Future studies could attempt to use superior ground truth by using a consensus of many experts and clinicopathological data.
**Ethics committee approval:** Not applicable.
**Funding for this study:** Not applicable.
**Author Disclosures:**
Himandri Warbhe: Nothing to disclose
Sudhanshu Sunil Tonpe: Nothing to disclose

### RPS 215-4

**Iterative reconstruction vs. deep learning image reconstruction: comparison of image quality and diagnostic accuracy of arterial stenosis in low-dose lower extremity CT angiography**
*T. T. Qu*; Xi'an/CN

**Purpose:** To compare image quality and diagnostic accuracy of arterial stenosis in low-dose lower extremity CT angiography (CTA) between adaptive statistical iterative reconstruction-V (ASIR-V) and deep learning image reconstruction (DLIR) algorithms.
**Methods or Background:** Forty-six patients undergoing lower extremity artery CTA with low dose scheme were collected. Images were reconstructed using ASIR-V with blending factors of 0% (AV-0), 50% (AV-50) and 100% (AV-100) and DLIR with medium (DL-M), and high (DL-H) settings. CT number, standard deviation (SD) of the abdominal aorta and left popliteal artery were measured. The degrees of quantum mottle and blurring and overall image quality were assessed using a 5-grade method. The stenosis rates were measured on the 5 reconstruction types, and their mean square errors (MSE) against that of DSA were calculated.
**Results or Findings:** The higher the blending factor in ASIR-V or the strength in DLIR, the lower the noise, and the higher the SNR and CNR. The SNR and CNR values of the aorta in DLIR images were significantly higher than those of AV-0 and AV-50 images but lower than those of AV-100 images (all $p<0.001$). Qualitative evaluation revealed that DLIR had much better overall image quality than that of ASIR-V ($p<0.001$). For the stenosis measurement, the results of DLIR algorithms were closer to that of DSA ($p<0.001$), with DL-H having the lowest MSE compared to DSA.
**Conclusion:** Compared with AV-50, DLIR algorithms significantly reduce image noise in low-dose lower extremity CTA, and DL-H provides the best overall image quality and highest accuracy in diagnosing arterial stenosis.
**Limitations:** All our evaluation results were based on low-radiation dose scanning, and the relevant comparison in conventional dose scanning conditions requires further investigation.
**Ethics committee approval:** This study was approved by the ethics committee of the First Affiliated Hospital of Xi'an Jiaotong University.
**Funding for this study:** No funding was received for this study.
**Author Disclosures:**
Ting Ting Qu: Nothing to disclose

### RPS 215-6

**Image fusion guidance for in-situ fenestration during thoracic endovascular repair**
*L. Zhao*, J. Liu, X. Cai, J. Wang; Shanghai/CN
*(76384620@qq.com)*

**Purpose:** To evaluate the clinical benefit of utilising image fusion for in-situ fenestrated TEVAR.
**Methods or Background:** TEVAR with in-situ fenestration has shown potential for LSA revascularisation in recent years. Accurate localisation of perforation is the key to the procedure. 3D fusion image guidance could provide fusion of pre-procedural CTA and fluoroscopy for real-time visualisation of target vessel ostia and facilitate more accurate vessel localisation for non-complex TEVAR procedures. Thirty-four patients with complex thoracic aortic aneurysms or dissections who underwent in-situ fenestrated TEVAR were divided into the fusion group (n=16) and control group (n=18) for this comparative study. 3D fusion of CTA and fluoroscopic image for real-time 3D guidance was used in the fusion group, while regular fluoroscopic images for guidance were used in the control group. Contrast medium volume, overall procedure time, radiation dose and fluoroscopy time were compared between the fusion group and case controls.
**Results or Findings:** 3D fusion imaging guidance was successfully implemented in all patients in the fusion group. Hand-injected contrast medium volume and overall procedure time were significantly lower in the fusion group than in the control group (p=.028 and p=.011). In addition, the fusion group showed significant reduction in time and radiation dose (DAP) for fluoroscopy compared to the control group (p=.010 and p=.004). No significant differences in total radiation dose (DAP) and total contrast medium volume were observed (p=.079 and p=.443).
**Conclusion:** The utilisation of 3D image fusion for in-situ fenestrated TEVAR was associated with a significant reduction in contrast medium, time and radiation exposure for fluoroscopy and overall procedure time.
**Limitations:** The small cohort was an identified limitation of this study.
**Ethics committee approval:** This study was approved by the research ethics committee of the hospital.
**Funding for this study:** Funding was received from Shanghai Jiaotong University clinical scientific research innovation cultivation fund (PYII20-06).
**Author Disclosures:**
Xiaoshu Cai: Nothing to disclose
Ji Wang: Author: Corresponding author
Jidong Liu: Author: Co-first author
Liang Zhao: Author: First Author

09:30-11:00 Room F1

### Research Presentation Session: Imaging Informatics / Artificial Intelligence and Machine Learning

### RPS 205

### Artificial intelligence (AI) in the management of COVID-19

**Moderator**
L. Topff; Amsterdam/NL

### RPS 205-2

**U-Net based denoising of sparsely sampled chest CT scans can be used to reduce radiation dose for detection of COVID-19 pneumonia**

*F. C. D. Hofmann*[1], J. Binder[1], A. Benne[1], M. Schultheiss[1], F. Schaff[1], T. Lasser[2], M. Makowski[1], F. Pfeiffer[1], D. Pfeiffer[1]; [1]Munich/DE, [2]Garching/DE
*(felix.hofmann@tum.de)*

**Purpose:** To demonstrate the accuracy of combined sparsely sampled computed tomography (CT) and U-net-based deep learning approach (U-DL) for ultra-low-dose CT for the diagnosis of COVID-19 pneumonia.
**Methods or Background:** 49 CT chest scans (31 patients suffering from COVID-19 pneumonia; 18 controls) were employed to calculate sparse sampling simulations with 25%, 12.5%, 6.3%, 3.1% and 1.6% of the regular dose. Streak artifacts were then removed with a U-DL using separate training (7471 slices from n = 24 patients), validation (1213 from n = 4) and test (5712 from n = 21) datasets. To include only one slice per subject in the reader study a comparatively large test set was chosen. These slices were evaluated by four blinded, experienced radiologists regarding COVID-19 imaging features. Image quality and diagnostic confidence was ranked using 5-point scales (5, excellent; 1, poor).
**Results or Findings:** Readers were able to diagnose COVID-19 imaging features with high accuracy and high confidence (scores > 4) in sparsely sampled images with U-DL correction down to 12.5% dose level. Image quality was consistently rated higher in U-DL images compared to sparsely sampled images without U-DL processing.
**Conclusion:** The radiation dose of CT scans for detection and severity assessment of COVID-19 pneumonia in clinical routine can be reduced using sparse sampling CT and subsequent artifact reduction using U-DL. U-DL can be used to reliably reduce streak artifacts in chest CT scans.
**Limitations:** In the present study, sparsely sampled image acquisition was simulated. Furthermore, our data set was comparatively small for the U-DL training.
**Ethics committee approval:** Institutional Review Board approval was obtained prior to this study.
**Funding for this study:** Funded by the Federal Ministry of Education and Research, the Free State of Bavaria under the Excellence Strategy of the Federal Government and the States and the TUM Institute for Advanced Study.
**Author Disclosures:**
Manuel Schultheiss: Nothing to disclose
Janko Binder: Nothing to disclose
Franz Pfeiffer: Nothing to disclose
Marcus Makowski: Nothing to disclose
Florian Schaff: Nothing to disclose
Daniela Pfeiffer: Nothing to disclose
Felix Carl Daniel Hofmann: Nothing to disclose
Ashley Benne: Nothing to disclose
Tobias Lasser: Nothing to disclose

### RPS 205-3

**Comparison of clinical, radiological and combined machine learning models in predicting COVID-19 patients who will require intensive care unit**

*M. Gulbay*, B. Yüce Öztürk, E. Özkan; Ankara/TR
*(drgulbay@gmail.com)*

**Purpose:** The aim of this study was to generate and compare models that predict the emergence of ICU (intensive care unit) need in hospitalised COVID-19 patients with severe illness, using clinical features and radiological data.
**Methods or Background:** Symptoms and findings, past medical history, laboratory, and computed tomography of 268 RT-PCR-positive COVID-19 patients hospitalised consecutively in inpatient floor between March 2020 and March 2021 were evaluated. Parenchymal lesions were segmented by using a U-Net model, a convolutional neural network architecture depending on the ResNet-34 backbone. Inflamed pulmonary volume, 18 first-order, 74 second-order radiomics features were calculated from obtained segmentations by following parameters: (1) resampled voxel size of 1x1x1 mm3 by using bicubic interpolation, (2) fixed bin-width of 25, (3) voxel array shift of 1024, (4) distance of 1 voxel and 13-isotropic displacement vectors at angles of 0, 45, 90 and 135 degrees for second order matrices. Clinical (symptoms, history, laboratory), radiologic (volumetric, radiomics) and combined (all parameters) logit fit models were built by Bayesian Information Criterion.
**Results or Findings:** The final study population of 191 patients was randomly divided into the Training and Cross-validation set (n = 152, 79.6%) and the Test set (n = 39, 20.4%). Using Training and Cross-validation set, models were calibrated with 10-fold cross validation and evaluated for overfitting. The Test set was evaluated with the final clinical, radiological and combined models and AUC was 0.736, 0.708, 0.794, specificity was 79.17%, 79.17%, 87.50%, sensitivity was 66.67%, 60%, 73.33%, and F1 was 0.67, 0.62 and 0.76, respectively.
**Conclusion:** COVID-19 patients who would need ICU were best predicted with a model consisting of a combination of clinical features with radiologic parameters based on automated segmentation.
**Limitations:** The results were belonged to a single centre and the number of cases were limited.
**Ethics committee approval:** This study was approved by an ethics committee. SaglikBakanligi: 2021-10-06T21_28_45.
**Funding for this study:** Not applicable.
**Author Disclosures:**
Erdem Özkan: Nothing to disclose
Mutlu Gulbay: Nothing to disclose
Büşra Yüce Öztürk: Nothing to disclose

### RPS 205-5

**CNN-based automatic analysis of chest radiographs for the detection of COVID-19 pneumonia: a prioritising tool in the emergency department, phase I study and preliminary "real world" results**

D. Tricarico[1], M. Calandri[2], *M. Barba*[2], C. Piatti[2], C. Geninatti[2], D. Basile[2], M. Gatti[1], M. Melis[1], A. Veltri[2]; [1]Turin/IT, [2]Orbassano/IT

**Purpose:** Develop an automatic method for the prioritisation of diagnostic workflow for COVID-19 analysing emergency department chest x-rays (CXR).
**Methods or Background:** Proposed Convolutional Neural Network (CNN) method, namely AIppo, has been tested retrospectively on a single-centre set of 542 CXRs, collected and evaluated by radiologists for COVID-19. Positive dataset (n = 234) has been collected between March and April 2020 and is defined by RT-PCR test in the 24h, negative dataset (n = 308) has been collected before the pandemic, in 2019. For each image, the proposed method computes COVID-19 risk indicator used for the prioritisation of the cases and the identification of urgent ones. The resulting prioritisation and identification have been evaluated in terms of Mean Average Precision (MAP) and sensitivity, estimated by cross-validation, and compared with random prioritisation and radiologist's diagnosis performance. After installing the software into hospital RIS, preliminary comparison between local daily COVID-19 cases and daily average of risk indicator on 2918 CXRs in the same period was performed.
**Results or Findings:** Significant improvements obtained for both prioritisation and identification using the proposed method. MAP increased (Mann-Whitney test p-value<;1.21e-21) from 43.79% (40%-48%) with random sorting to 71.75% (63%-81%) with our method. Sensitivity of 78.23% (65%-92%) was obtained, higher than 61.1% (55%-67%) from radiologists; specificity of 64.20% (63%-66%) was observed. In the real-world setting, COVID-19 incidence curve and risk indicator decreased concurrently during the evaluation period, with a significant correlation of 0.873.
**Conclusion:** Proposed CNN-based system effectively prioritises CXRs according to COVID-19 risk in an experimental setting; preliminary "real-world" results revealed high concordance with local pandemic incidence.
**Limitations:** The number of available CXR is limited (only 542 samples) and the data collection involved only one centre.
**Ethics committee approval:** The study was approved by the IRB.
**Funding for this study:** No funding was received for this study.
**Author Disclosures:**
Davide Tricarico: Employee: AITEM Artificial Intelligence Technologies Multipurpose s.r.l.
Domenico Basile: Nothing to disclose
Marco Calandri: Nothing to disclose
Massimiliano Melis: Patent Holder: AITEM Artificial Intelligence Technologies Multipurpose s.r.l.
Marco Gatti: Nothing to disclose
Andrea Veltri: Nothing to disclose
Carlotta Geninatti: Nothing to disclose
Clara Piatti: Nothing to disclose
Matteo Barba: Nothing to disclose

### RPS 205-6

**Artificial intelligence and COVID-19: CT-based radiomics can identify false-negative swabs**

*N. Cardobi*, G. Benetti, G. Cardano, C. Arena, C. Micheletto, C. Cavedon, S. A. Montemezzi; Verona/IT
*(nicolo.cardobi@gmail.com)*

**Purpose:** To identify COVID-19 positive cases among different interstitial pneumonias using lung CT radiomic features.
**Methods or Background:** CT data of 115 patients with respiratory symptoms suspected for COVID-19 disease were retrospectively analysed. Based on the results of nasopharyngeal swab, patients with interstitial pneumonias were divided in three groups: COVID-19 positive (C+), COVID-19 negative (C-) and COVID-19-like (CL). CL patients were negative to the swab test but turned out to be COVID-19 positive in a second time, hence due to a false-negative result. A fully automatic procedure, involving automatic lung contouring (convolutional neural network), radiomic features extraction (pyradiomics) and machine learning (LASSO regression) was used. A 2000-times repeated random test-train splitting and an internal cross-validation guaranteed model stability and performance reliability.
**Results or Findings:** Using a classifier trained on C+ vs C-, the model performed with an AUC of 0.83 (ROC analysis). With this model, the median predicted probability of being COVID-positive for CL cases (0.80) was more similar to C+ (0.92) compared to C- (0.17), indicating that the CL radiomic footprint is compatible with C+. A new model, trained with the inclusion of CL into C+, showed a good performance (AUC = 0.81) on the test set.
**Conclusion:** Whole lung CT radiomics can identify COVID-19 positive patients when based on radiological data only. The model demonstrated its capability of identifying COVID-19 positive patients in case of uncertain results from a swab test.
**Limitations:** Although the study was performed using a 2000-times repeated random test-train splitting, the data were collected from a single institution.
**Ethics committee approval:** Approved by the local IRB for studies on COVID-19 not involving drugs.
**Funding for this study:** No funding was received.
**Author Disclosures:**
Giulio Benetti: Nothing to disclose
Nicolò Cardobi: Nothing to disclose
Carlo Cavedon: Nothing to disclose
Cinzia Arena: Nothing to disclose
Claudio Micheletto: Nothing to disclose
Stefania Anna Montemezzi: Nothing to disclose
Giuseppe Cardano: Nothing to disclose

### RPS 205-7

**Artificial intelligence based severity assessment of COVID-19 pneumonia using a multireader chest x-ray dataset**

*G. Tessarin*, M. Zandehshahvar, M. van Assen, E. Muscogiuri, L. Cotter, Y. Kiarashi, C. N. De Cecco, A. Adibi; Atlanta, GA/US

**Purpose:** To develop a two-stage transfer learning convolutional neural network (CNN) for severity assessment of COVID-19 pneumonia based on chest x-rays (CXRs).
**Methods or Background:** The dataset consists of 1208 CXRs from 396 PCR-confirmed COVID-19 patients. All images were labeled in 4 severity classes (normal, mild, moderate and severe) by 6 radiologists and 2 residents, based on extension of lung consolidations and opacifications. A two-stage transfer-learning method was used to train a VGG-16CNN. First, the CNN was trained on the RNSA pneumonia dataset (25,684 CXRs). Then, fully connected layers were added, trained on our dataset. The median of 5 radiologists was used as the ground truth. Bootstrap sampling was used to create 20 iterations of training and testing, comparing with the 6th radiologist and the 2 residents. In each trial, 800 CXRs were used for training, 100 for validation, and 308 for testing. A pruning approach was used to visualise the most important features extracted by the AI algorithm.
**Results or Findings:** The CNN outperformed the readers in all classes, showing less variation in predictions over different test sets, compared to residents. The sensitivities of the model were 0.77, 0.75, 0.60, and 0.87, for the normal, mild, moderate, and severe classes, respectively. The miss-classification over non-adjacent classes was 0. The 6th radiologist had a sensitivity of 0.97, 0.64, 0.49, 0.61 for the 4 classes, respectively. Only in the normal class, the 6th radiologist reached a higher sensitivity, but with a higher false positive rate than the algorithm (0.33 versus 0.09).
**Conclusion:** The study proved that a two-stage transfer-learning CNN can outperform human readers in classifying severity of COVID-19 in different patients based on CXRs.
**Limitations:** Retrospective collection of data.
**Ethics committee approval:** Study was approved by ethics committee.
**Funding for this study:** No funding received.
**Author Disclosures:**
Marly van Assen: Nothing to disclose
Ali Adibi: Nothing to disclose
Carlo Nicola De Cecco: Research/Grant Support: Siemens Healthineers
Luca Cotter: Nothing to disclose
Giovanni Tessarin: Nothing to disclose
Mohammadreza Zandehshahvar: Nothing to disclose
Emanuele Muscogiuri: Nothing to disclose
Yashar Kiarashi: Nothing to disclose

### RPS 205-8

**Radiomics and machine learning in predicting pathophysiologic changes in patients with COVID-19-associated pneumonia**

Y. Statsenko[1], T. Habuza[1], *S. Meribout*[2], K. Neidl-Van Gorkom[1], T. M. Almansoori[1], F. Al Zahmi[3], L. Uzianbaeva[4], J. Al Koteesh[1], M. Ljubisavljevic[1]; [1]Al Ain/AE, [2]Constantine/DZ, [3]Dubai/AE, [4]Detroit, MI/US
*(s-meribout@hotmail.com)*

**Purpose:** To evaluate the effectiveness of radiomics and machine learning in predicting the pathophysiologic changes in patients with COVID-19 associated pneumonia.
**Methods or Background:** Background: Machine learning allows for prognostication of the clinical severity of patients with COVID-19-associated pneumonia by combining personal risk factors, laboratory findings and medical imaging data. Methods: We enrolled a total number of 605 cases admitted to a governmental hospital from 24 February to 1 July 2020. The patients fulfilled the following inclusion criteria: age ≥18 years; inpatient admission; PCR positive for SARS-CoV-2; lung CT available at PACS. We categorised cases into 4 classes: mild ≤25% of pulmonary parenchymal involvement, moderate 25-50%, severe 50-75%, and critical over 75%. We used three distinct kernels (B30f, B60f, B80f) for the reconstruction of the images from which we retrieved the radiomical data. After removing redundant features, we built regression models predicting the oxygenation level, respiratory and cardiovascular functioning (SpO2, HCO3, breath and heart rate, systolic and diastolic blood pressure, anion gap, serum potassium).
**Results or Findings:** The average accuracy (MAE/range, %) of the models based on radiomics is: 7.069±4.17, on the clinical features (e.g. demographics, laboratory findings, etc.) - 6.593±3.654, and in their combination - 6.454±3.715. The information gain of radiomics is considerably higher in the model predicting SpO2. The least accurate prediction is observed once the images are acquired with B60f kernel, the most accurate - with B30f kernel. However, the accuracy varies insignificantly among disparate reconstruction kernels.
**Conclusion:** Radiomics is reliable in reflecting the pathophysiological changes, depicted by the oxygenation level, and respiratory and cardiovascular functioning, in COVID-19 patients. The settings of the CT reconstruction kernels influence the predictive accuracy of the machine learning algorithms insignificantly.
**Limitations:** Not applicable.
**Ethics committee approval:** This study was approved by an ethics committee: Abu Dhabi DOH/CVDC/2020/889.
**Funding for this study:** Funding was received from Aare19-060.
**Author Disclosures:**
Liaisan Uzianbaeva: Nothing to disclose
Klaus Neidl-Van Gorkom: Nothing to disclose
Taleb M. Almansoori: Nothing to disclose
Milos Ljubisavljevic: Nothing to disclose
Sarah Meribout: Nothing to disclose
Tetiana Habuza: Nothing to disclose
Yauhen Statsenko: Nothing to disclose
Fatmah Al Zahmi: Nothing to disclose
Jamal Al Koteesh: Nothing to disclose

### RPS 205-9

**Artificial intelligence algorithm for automated detection of compression fractures and low vertebral body mineral density using chest CT images during the COVID-19 pandemic**

*A. Petraikin*, A. Zakharov, M. Belyaev, N. Kudryavtsev, Z. Artyukova, A. E. Andreychenko, L. Nizovtsova, A. Vladzymyrskyy, S. Morozov; Moscow/RU
*(alexeypetraikin@gmail.com)*

**Purpose:** To present initial results related to the opportunistic screening for osteoporosis based on chest CT images performed by an artificial intelligence algorithm (AI).
**Methods or Background:** The Genant-IRA, a convolutional neural network-based AI algorithm, successfully passed a qualification testing and integrated into the “Experiment on the use of innovative technologies in the field of computer vision in the healthcare system of Moscow”. Test dataset consisted of anonymised chest CT images of 100 patients, where 50% of patients had osteoporosis vertebral fractures (OVF).

Wednesday

**Results or Findings:** Based on the test dataset, the accuracy of the AI model was ROC AUC = 0.98 at the patient level for OVF detection. The HU-based assignment of a patient to categories with or without fracture showed ROC AUC = 0.877 for expert and 0.870 for the AI algorithm. HU values were converted to bone mineral density (BMD) by asynchronous calibrating with a K2HPO4 solution phantom. We analysed a set of 2.600 chest CT studies as opportunistic AI screening. AI-derived results demonstrate: 30.49% female and 19.66% male patients over 50 years old had osteoporosis, i.e. reduced BMD of less than 80 mg/ml (ACR criteria); 7.6% (male+female) had deformities of vertebrae bodies of more than 25% and BMD less 80 mg/ml.
**Conclusion:** The study presents a rationale for the use of AI algorithms in automated opportunistic screening for osteoporosis using chest CT images obtained during the COVID-19 pandemic. The findings can be used to determine the treatment and prevent possible pathological fractures.
**Limitations:** Some limitations for separating different type of compression deformities by the AI algorithm.
**Ethics committee approval:** Approved of the independent ethics committee of Moscow regional office of the RSRR MRO. d/d 18 February 2021.
**Funding for this study:** Funding was received from: No. EGISU: AAAA-A20-120071090045-7 under the Program of the Moscow Healthcare Department for 2020-2022.
**Author Disclosures:**
Alexey Zakharov: Nothing to disclose
Sergey Morozov: Nothing to disclose
Anna Evgenevna Andreychenko: Nothing to disclose
Lyudmila Nizovtsova: Nothing to disclose
Zlata Artyukova: Nothing to disclose
Mikhail Belyaev: Nothing to disclose
Nikita Kudryavtsev: Nothing to disclose
Alexey Petraikin: Nothing to disclose
Anton Vladzymyrskyy: Nothing to disclose

### RPS 205-10

### Development and validation of a prognosis and intervention prediction model for COVID-19 patients using clinical findings and artificial intelligence interpreted chest radiographs

*J. S. Ahn*[1], J. Lee[1], J. E. Lee[2], E. Y. Kim[3]; [1]Seoul/KR, [2]Gwangju/KR, [3]Incheon/KR
*(johnahn92@lunit.io)*

**Purpose:** To develop and validate a COVID-19 prognosis and intervention prediction model using a multimodal approach, in a 9 multicentre cohort.
**Methods or Background:** 1409 COVID-19 patients' initial clinical findings and CXRs were retrospectively collected from 9 centres in South Korea, between July 2020 and January 2021. The prognosis outcomes collected were ICU admission and mortality. The intervention outcomes collected were the use of oxygen supplementation, mechanical ventilation and Extracorporeal Membrane Oxygenation. A deep-learning algorithm detecting 10 common CXR abnormalities (DLAD-10) were used to inference the CXR. Using the DLAD-10 output and clinical findings, we trained and created random forest models to predict the binary outcome of prognosis and interventions, using data from 799 patients from 5 centres. The models were externally validated by 610 patients from 4 other centres.
**Results or Findings:** For the prediction of prognostic and interventional events in the external validation set, the AUROC of the model using clinical findings and using DLAD-10 output were 0.812 (95% C.I. 0.695-0.902) and 0.751 (0.704-0.904), respectively. The AUROC of the combined model, using both clinical findings and DLAD-10 output, was significantly higher at 0.863 (0.824-0.897), than both models ($p < 0.001$, DeLong's test). The AUROCs for predicting O2 supplementation, mechanical ventilation, ECMO, ICU admission and mortality of the combined model were 0.825, 0.851, 0.775, 0.832 and 0.870, respectively. The most predictive DLAD-10 feature was consolidation and the order of important variables for prediction was age, dyspnoea, consolidation and fever.
**Conclusion:** The use of both the clinical findings and AI CXR output improves the performance of a prediction model for COVID-19 patients.
**Limitations:** We utilised data solely from Korea and similar diagnostic performance with other populations is not guaranteed.
**Ethics committee approval:** This study was approved by an ethics committee. IRB number: GCIRB2021-312.
**Funding for this study:** Funding was received from the Ministry of Science and ICT, Republic of Korea.
**Author Disclosures:**
Jeonghoon Lee: Employee: Lunit
Jong Eun Lee: Nothing to disclose
Jong Seok Ahn: Employee: Lunit
Eun Young Kim: Nothing to disclose

### RPS 205-11

### AI-assisted COVID-19 detection on unsegmented chest CT scans

*L. M. Florescu*, M. S. Șerbănescu, C. T. Streba, D. N. Florescu, R. V. Teica, R. E. Nica, I. A. Gheonea; Craiova/RO

**Purpose:** This paper aims to combine the data from two distinct databases (a private one and a public one) in order to detect COVID-19 on unsegmented chest CT scans using deep learning and two convolutional neural networks (AlexNet and GoogLeNet).
**Methods or Background:** Both private and public databases consisted of axial chest CT images (lung window) that illustrated COVID-19, non-COVID-19 lung infections, lung cancer, and normal lung aspect. The public dataset included 243 images from free online databases like Radiopaedia and Radiology Assistant while the private dataset included 389 images selected from the internal PACS of the Medical Imaging Department of the University of Medicine and Pharmacy of Craiova.
**Results or Findings:** After training and testing each network on the same database that they were trained on, the mean accuracy and standard deviation (SD) when using AlexNet was 95.87± 0.39 on the public dataset and 94.35±0.81 on the private dataset. When using GoogLeNet, the values were somewhat similar (96.12±0.72 on the public database and 91.57±1.64 on the private database). When using cross validation, the highest mean±SD accuracy was obtained when training AlexNet on the private dataset and testing it on the public one (79.81±1.47).
**Conclusion:** Our approach is represented by a completely independent software that is able to greatly decrease the infection rate among medical personnel and boost the daily testing capacity by rapidly providing a test result.
**Limitations:** The size of both private and public databases is the main limiting factor of our study.
**Ethics committee approval:** Approved by the Committee of Ethics and Academic and Scientific Deontology of the University of Medicine and Pharmacy of Craiova, Romania.
**Funding for this study:** Funding was received from the University of Medicine and Pharmacy of Craiova, grant no. 26/10C/13.07.2021.
**Author Disclosures:**
Rossy Vladut Teica: Nothing to disclose
Raluca Elena Nica: Nothing to disclose
Mircea Sebastian Șerbănescu: Nothing to disclose
Dan Nicolae Florescu: Nothing to disclose
Lucian Mihai Florescu: Nothing to disclose
Ioana Andreea Gheonea: Nothing to disclose
Costin Teodor Streba: Nothing to disclose

### RPS 205-12

### An integrated method to detect silent failures in pretrained nnU-Net models for COVID-19 lung lesion segmentation: towards automated failure detection of DNNs

*C. Gonzalez*[1], A. M. Bucher[2], R. Fischbach[2], K. Gotkowski[1], I. Kaltenborn[2], T. Vogl[2], B. Hamm[3], T. Penzkofer[3], A. Mukhopadhyay[1]; [1]Darmstadt/DE, [2]Frankfurt a. Main/DE, [3]Berlin/DE
*(camila.gonzalez@gris.informatik.tu-darmstadt.de)*

**Purpose:** Automatic lung lesion segmentation can quantify COVID-19 lung affection. Yet predictive deep learning models fail silently in out-of-distribution data (OOD), limiting the robustness for large scale use. We propose a novel method for OOD-detection that exploits the Mahalanobis-distance in the feature space.
**Methods or Background:** We trained a patch-based nnU-Net for segmentation of COVID-19 lung lesions on a subset of the challenge-dataset (n = 199; training: 160; validation: 4; test-cases: 35) and evaluated on an in-house dataset (n = 50) and two further public datasets (Mosmed, Radiopedia; n = 70). All cases had known COVID-19 lung affection. For OOD-detection we (a) estimate the Gaussian distribution from training features (b) extract uncertainty masks for test images and (c) calculate subject-level uncertainty scores. We compared our approach to 6 state-of-the-art techniques which assess uncertainty by performing inference on a trained model (SOTA). Expected Segmentation Calibration Error (ESCE) was calculated as an uncertainty metric.
**Results or Findings:** Unlike SOTA, our method did not fail silently by assigning low uncertainties to low-Dice samples. Within the uncertainty boundary defined as 95% true positive rate on the in-distribution validation set, our method displays the lowest false-positive rate (our-method: 0.050; SOTA: 0.183-1.000) and lowest detection error (our-method: 0.082; SOTA: 0.082-0.598). Our method distinguished all OOD-samples below the uncertainty boundary with Dice-scores less than 0.6. ESCE indicates the prediction quality without requiring manual annotations (our-method: 0.125; SOTA: 0.408-0.215).

Wednesday

**Conclusion:** Calculating the Mahalanobis-distance to features in a low-dimensional subspace is a lightweight and flexible way to signal when a model prediction should not be trusted. Our OOD-detection approach can be seamlessly integrated into state-of-the-art segmentation pipelines without changes in model architecture or training procedure.
**Limitations:** Future work should explore better identification of high-quality predictions.
**Ethics committee approval:** IRB approval was obtained.
**Funding for this study:** RACOON is funded by the network-university-medicine (BMBF-grant-number:01KX202).
**Author Disclosures:**
Bernd Hamm: Nothing to disclose
Thomas Vogl: Nothing to disclose
Tobias Penzkofer: Nothing to disclose
Karol Gotkowski: Nothing to disclose
Camila Gonzalez: Nothing to disclose
Ricarda Fischbach: Nothing to disclose
Isabel Kaltenborn: Nothing to disclose
Andreas Michael Bucher: Nothing to disclose
Anirban Mukhopadhyay: Nothing to disclose

**09:30-11:00** **Room G**

### Research Presentation Session: GI Tract

### RPS 201

### Imaging advances in chronic liver disease assessment and treatment

**Moderator**
B. E. Van Beers; Clichy/FR

### RPS 201-2

**Imaging features in patients with porto-sinusoidal vascular disease (PSVD) - radiological evaluation guiding diagnosis**

*K. Lampichler*, G. Semmler, B. Simbrunner, M. Mandorfer, M. Trauner, A. Ba-Ssalamah, T. Reiberger, M. Scharitzer, B. Scheiner; Vienna/AT
*(katharina.lampichler@meduniwien.ac.at)*

**Purpose:** Porto-sinusoidal vascular disease (PSVD) is a recently defined vascular liver disease resulting in portal hypertension (PH). Diagnosis is challenging and PSVD patients are often misdiagnosed as having cirrhosis. We evaluated whether radiological features can be used to distinguish between PSVD and cirrhosis.
**Methods or Background:** Demographic, clinical and laboratory parameters of 54 patients with PSVD and 115 patients with cirrhosis with available CT/MRI scans were retrospectively evaluated. The following imaging features were analyzed: Portal tract abnormalities, splanchnic vein thrombosis, spleen size, collaterals, ascites, FNH-like lesions, changes in liver morphology and liver surface nodularity.
**Results or Findings:** Clinical signs of PH were comparable between PSVD and cirrhosis patients, while PSVD patients were younger, had lower HVPG and liver stiffness, as well as lower MELD. Intrahepatic portal tract abnormalities (52% vs. 17%; p<0.001), splanchnic vein thrombosis (24% vs. 10%; p=0.012) and FNH-like lesions (33% vs. 0%; p<0.001) were significantly more common in PSVD patients. Hypertrophy of segment I (43% vs. 68%; p=0.002), atrophy of segment IV (24% vs. 44%; p=0.01) and nodular liver surface (19% vs. 85%; p<0.001) were more commonly observed in patients with cirrhosis. In patients with available gadoxetic acid-enhanced MRI (PSVD: n=25, cirrhosis: n=75), we identified a new imaging feature in the hepatobiliary phase (HBP) that was very specific for patients with PSVD (termed periportal hyperintensity: 52% in patients with PSVD vs. 1,3% in patients with cirrhosis; p<0.001).
**Conclusion:** Diagnosis of PSVD should be considered in younger patients presenting with clinical features of portal hypertension and portal tract abnormalities, splanchnic vein thrombosis and FNH-like lesions on CT/MRI. Periportal hyperintensity on HBP seems to be a very specific radiological feature of PSVD patients.
**Limitations:** Retrospective analysis.
**Ethics committee approval:** Approved by the ethics committee of the MUV (EK1928/2017, EK1889/2019).
**Funding for this study:** None.
**Author Disclosures:**
Bernhard Scheiner: Other: Travel support
Mattias Mandorfer: Advisory Board: and/or Consultant and/or Speaker: AbbVie, Bristol-Myers Squibb, Gilead, Collective Acumen, and W. L. Gore & Associates
Benedikt Simbrunner: Other: Travel support
Ahmed Ba-Ssalamah: Speaker: Novartis, Siemens and Bayer Consultant: Bayer
Michael Trauner: Advisory Board: and/or Consultant and/or Speaker: Albireo, Boehringer Ingelheim, Bristol-Myers Squibb, Falk, Genfit, Gilead, Intercept, MSD, Novartis, Phenex, Regulus, and Shire Grant Recipient: Albireo, Cymabay, Falk, Gilead, Intercept, MSD, and Takeda
Katharina Lampichler: Nothing to disclose
Georg Semmler: Other: Travel support
Martina Scharitzer: Nothing to disclose
Thomas Reiberger: Advisory Board: and/or Consultant and/or Speaker for AbbVie, Bayer, Boehringer Ingelheim, Gilead, Intercept, MSD, Roche, Siemens, and W. L. Gore & Associates Grant Recipient: AbbVie, Boehringer Ingelheim, Gilead, MSD, Philips, and W. L. Gore & Associates

### RPS 201-3

**Hepatic elastic modulus and haemodynamic liver index: novel non-invasive parameters for prediction of presence and grade of oesophageal varices in cirrhotic patients**

*A. Rana*, A. Malik, V. Krishnan, M. Thakur; Delhi/IN
*(rana07abhilasha@gmail.com)*

**Purpose:** Currently oesophagoscopy is the gold standard for assessment of oesophageal varices. However, it is invasive and no varices are detected in the majority of cirrhotic patients undergoing oesophagoscopy. Hepatic elastic modulus and Doppler Haemodynamic liver index (HDLI), which indirectly measure hepatic fibrosis and portal hypertension, may non-invasively predict presence and grade of varices.
**Methods or Background:** Our cross-sectional study consisted of 60 consecutive consenting cirrhotic patients (diagnosed by clinical, laboratory and ultrasonographic features), who underwent shear wave elastography and portal Doppler for measurement of hepatic elastic modulus and Doppler HDLI (portal vein diameter / mean velocity) respectively. Within three days of ultrasound, all subjects underwent oesophagoscopy for assessment of presence and grade (I-III) of varices. The data was statistically analysed.
**Results or Findings:** Mean elastic modulus of subjects with varices was significantly higher (28.9 vs 12.6kPa, p<0.0001) and progressively increased with variceal grade, being highest in grade-III (51.1kPa). Likewise, mean HDLI was higher in cases with varices (0.72 vs 0.59, p<0.0001) and progressively increased with grade (grade-III 0.77). For predicting the presence of varices, maximum accuracy of shear modulus was at cut-off of 14.5kPa (sensitivity 83.0% and specificity 84.6%) and HDLI at 0.66 (66.0% and 92.3%). These parameters combined in parallel gave maximum accuracy (94.3% and 78.1%). Good interrater agreement was present (0.66).
**Conclusion:** Combination of hepatic elastic modulus and Doppler HDLI is an excellent non-invasive screening modality for predicting presence and grade of oesophageal varices and may obviate the need for routine endoscopic screening in cirrhotic patients.
**Limitations:** Limited sample box depth (8cm) and fixed small dimensions (1.5x0.5cm) led to exclusion of subjects with gross ascites, obesity and small liver size. Subjects unable to hold breath were excluded.
**Ethics committee approval:** This study was approved by the institutional ethics committee.
**Funding for this study:** No funding was received for this study.
**Author Disclosures:**
Amita Malik: Nothing to disclose
Venkatram Krishnan: Nothing to disclose
Manisha Thakur: Nothing to disclose
Abhilasha Rana: Nothing to disclose

### RPS 201-4

**To evaluate the effectiveness and safety of endovascular intervention in the management of nonportal hypertension related gastrointestinal bleeding at a Vietnamese hospital**

*T. H. N. Nguyen*, K. V. Le, T-B. Nguyen, T. Pham, H. Duong, L. Van Phuoc; Ho Chi Minh/VN
*(drtuaninr@gmail.com)*

**Purpose:** The purpose of this study was to evaluate the effectiveness and safety of endovascular intervention in the treatment of nonportal hypertension related gastrointestinal bleeding at a Vietnamese institution.
**Methods or Background:** Case series study of patients diagnosed with nonportal hypertension related gastrointestinal bleeding and treated by endovascular intervention at a Vietnamese hospital from January 2017 to May 2020.

**Results or Findings:** Twenty-four patients were enrolled. The mean age was 54.5 years old, the male/female ratio was 5/1. The rate of upper GI bleeding was more common than lower GI bleeding, in which up to 50% of cases the cause of GI bleeding cannot be found. Abdominal CT scan with contrast media identified the bleeding site, accounting for 73.7%. On digital subtraction angiography (DSA), pseudoaneurysms accounted for the majority with the rate of 66.7%, the most common lesion site was the gastroduodenal artery. The endovascular intervention method for haemostasis achieved good results: complete occlusion of the lesion site accounted for 91.7%. There was one (4.2%) case of partial occlusion and only one case of failure (4.2%) due to vasospasm of tortuous and tiny artery. Embolic agents were commonly coils and Histoacryl glue (NBCA). Clinical symptoms of patients improved well in most cases, accounting for 91.7%; there were only 2 (8.3%) cases of severe clinical progression due to underlying comorbidities.
**Conclusion:** Endovascular intervention appeared to be a safe and effective method for haemostasis of patients with nonportal hypertension related gastrointestinal bleeding who had failed endoscopic therapy or who had contraindications to endoscopy.
**Limitations:** This is a case series study in an institution with a small sample size. Only nonportal hypertension related gastrointestinal bleeding cases were studied.
**Ethics committee approval:** This study was reviewed and approved by our Institutional Ethics Committee (IEC).
**Funding for this study:** No funding was received for this study.
**Author Disclosures:**
Khoa Van Le: Nothing to disclose
Tu Pham: Nothing to disclose
Hoan Duong: Nothing to disclose
Tien-Bao Nguyen: Nothing to disclose
Le Van Phuoc: Nothing to disclose
Tuan Huynh Nhat Nguyen: Nothing to disclose

### RPS 201-5

**TRANS-TACE: prognostic role of the transient hypertransaminasaemia after conventional chemoembolisation for hepatocellular carcinoma**

*N. Brandi*[1], A. Granito[1], A. Facciorusso[2], L. Bartalena[1], C. Mosconi[1], F. Tovoli[1], F. Piscaglia[1], R. Golfieri[1], M. Renzulli[1]; [1]Bologna/IT, [2]Foggia/IT
*(nicolo.brandi@studio.unibo.it)*

**Purpose:** The aim of the present study was to correlate laboratory data and post-procedural parameters after conventional transarterial chemoembolisation (cTACE) for hepatocellular carcinoma (HCC) with the radiological response.
**Methods or Background:** The study consisted of a retrospective analysis of prospectively collected data from 70 consecutive patients who underwent cTACE. Laboratory parameters were assessed daily after cTACE and compared to pre-treatment values. Post-treatment radiological response was assessed using mRECIST at one month from cTACE and factors associated with treatment response (complete and objective response) were assessed by logistic regression analysis.
**Results or Findings:** The optimal cut-off points in predicting the complete response of target lesions were a 52% ALT and a 46% AST increase after cTACE compared to the pre-treatment values. By multivariate analyses, >46% AST and >52% ALT increase respectively to the pre-treatment value, significantly correlated with the objective response (p=0.03 and p=0.04, respectively) and the complete response (p=0.02 and p=0.02, respectively). No patients experienced liver function deterioration after cTACE, and no specific treatment was required.
**Conclusion:** This study shows that post-treatment transient hypertransaminasaemia is an accurate predictor of complete response to superselective cTACE in clinical practice, representing a simple tool to guide the treatment strategy of HCC patients in a tailored approach.
**Limitations:** This is a small series of patients treated with cTACE and the overall survival was not assessed.
**Ethics committee approval:** The study was conducted according to the guidelines of the Declaration of Helsinki, and approved by the Institutional Review Board of IRCCS Azienda Ospedaliero-Universitaria di Bologna (protocol code 098/2014/U/Oss).
**Funding for this study:** No funding was received for this study.
**Author Disclosures:**
Francesco Tovoli: Nothing to disclose
Rita Golfieri: Nothing to disclose
Alessandro Granito: Nothing to disclose
Fabio Piscaglia: Nothing to disclose
Nicolò Brandi: Nothing to disclose
Matteo Renzulli: Nothing to disclose
Cristina Mosconi: Nothing to disclose
Laura Bartalena: Nothing to disclose
Antonio Facciorusso: Nothing to disclose

### RPS 201-6

**Splenic volume as prognostic factor for patients with hepatocellular carcinoma undergoing TACE: fully automated volume assessment using a 3D segmentation convolutional neural network**

L. Müller, F. Stöhr, A. Mähringer-Kunz, C. Düber, A. Weinmann, R. Kloeckner, *F. Hahn*; Mainz/DE

**Purpose:** Initial results indicate splenic volume as a relevant prognostic factor for patients with HCC undergoing TACE. However, manual splenic volume assessment is time-consuming and labour-intensive and therefore not feasible in clinical routine. This study implemented fully automated splenic volume assessment in CT scans using deep learning to investigate splenic volume as a risk factor for HCC patients undergoing TACE.
**Methods or Background:** This retrospective study included 327 treatment-naïve patients that received initial TACE treatment at our tertiary care centre between January 2010 and November 2020. A 3D U-Net (convolutional neural network for segmentation) was trained and validated on the first 100 consecutive cases. Subsequently, the spleen was automatically segmented for the remaining 227 patients, the quality of the segmentation visually assessed by two radiologists and splenic volume correlated with various clinical and image-derived risk factors as well as with overall survival.
**Results or Findings:** The Sørensen-Dice-Score yielded excellent values of 0.96 for training and 0.96 for validation in preliminary results. In the remaining 227 patients, the segmentation was visually approved in 221 out of 227 patients; in 6 patients (3%) the segmentation failed and had to be manually corrected. Regarding survival, compared to axial and craniocaudal splenic size, only a significant cut-off for splenic volume could be derived: patients with a splenic volume >384ml had significantly impaired survival (10.8 months vs 18.8 months, p=0.023).
**Conclusion:** Automated splenic volume assessment is feasible in HCC patients undergoing TACE. Compared to two-dimensional estimates of splenic size, actual splenic volume is superior in survival prediction. Thus, it could function as an additional risk factor available without extra work with every diagnostic CT during initial and follow-up imaging.
**Limitations:** The nature of this work as a singlecentre study and its retrospective design were identified as limiting factors.
**Ethics committee approval:** This study was approved by the ethics committee of the medical association of Rhineland Palatinate, Mainz, Germany (number 2021-15984).
**Funding for this study:** Not applicable
**Author Disclosures:**
Aline Mähringer-Kunz: Nothing to disclose
Roman Kloeckner: Nothing to disclose
Fabian Stöhr: Nothing to disclose
Christoph Düber: Nothing to disclose
Felix Hahn: Nothing to disclose
Lukas Müller: Nothing to disclose
Arndt Weinmann: Nothing to disclose

### RPS 201-7

**Correlation between hepatic and splenic volumes and portal venous pressure in cirrhotic patients candidate for transjugular intrahepatic portosystemic shunt (TIPS)**

*L. M. G. Bianchi*, P. Gemma, S. De Nicola, L. A. Carbonaro, A. Airoldi, A. Vanzulli; Milan/IT
*(lorenzomaria.bianchi@unimi.it)*

**Purpose:** To evaluate the correlation between hepatic and splenic volumes and portal venous pressure in cirrhotic patients, and the use of these volumes to predict portal venous pressure increase.
**Methods or Background:** In this retrospective study, cirrhotic patients who underwent TIPS (2014-2019) were included. Hepatic and splenic volumes were calculated by CT images. Weight, height, presence of ascites, and direct portal venous pressure (dPVP), registered via catheterisation during TIPS, were assessed. Theoretical hepatic volume was determined ((1267,28*weight*height/3600) - 794,41); the formula 17,37 - 4,91*ln (hepatic volume/splenic volume), +3.8 [Iranmanesh et al.] was used to estimate of the hepatic venous pressure gradient (eHVPG). dPVP was compared to hepatic volume, splenic volume, real/theoretical liver volume rate, and the eHVPG. Statistical significance was assessed by Student's t-test and mixed two-way ICC for absolute agreement.
**Results or Findings:** 81 patients were included. Mean dPVP ± standard deviation was 18.2 ± 5.2 mmHg, mean liver volume 1374 ± 446 cc, mean splenic volume 923 ± 501 cc, and dPVP was 26,6 ± 3.2 mmHg; the mean real/theoretical liver volume rate was 88 ± 25%. No statistically significant linear correlation was found between dPVP and hepatic volume (p=0.570); dPVP showed a positive linear correlation with splenic volume (p=0.05), real/theoretical liver volume rate (p=0.027), and eHVPG (p=0.009), although with extreme variability not explained by the model ($R2$=0.061, $R2$=0.096, and $R2$=0.078, respectively), the latter with a poor reliability by absolute agreement (ICC=0.382, p=0.09).

**Conclusion:** Although portal venous pressure showed a direct correlation with splenic volume, real/theoretical liver volume rate and estimated HVPG calculated from hepatosplenic volumes, these measures appeared poorly reliable as a non-invasive surrogate index of portal pressure in cirrhotic patients candidate to TIPS.
**Limitations:** Likelihood of publication bias was identified as a limiting factor.
**Ethics committee approval:** This retrospective study was approved by the ethics committee.
**Funding for this study:** No funding was received for this study.
**Author Disclosures:**
Lorenzo Maria Giuseppe Bianchi: Nothing to disclose
Luca Alessandro Carbonaro: Nothing to disclose
Pietro Gemma: Nothing to disclose
Angelo Vanzulli: Nothing to disclose
Aldo Airoldi: Nothing to disclose
Stella De Nicola: Nothing to disclose

### RPS 201-8

**HCC-MRI score: a simplified criteria for the diagnosis of hepatocellular carcinoma in high-risk patients using contrast-enhanced MRI of liver**
*W. Teerasamit*; Bangkok/TH
*(Mojiamp@gmail.com)*

**Purpose:** To create simplified criteria for the diagnosis of hepatocellular carcinoma (HCC) in high-risk liver using contrast-enhanced MRI liver.
**Methods or Background:** The retrospective study was performed between January 2008 and December 2020. A total of 182 hepatic lesions (HCCs or non-HCCs) were included, which had pathological confirmation (114 lesions) or stable size of benign lesions for 2 years (68 lesions). Nine MRI parameters (including arterial phase hyperenhancement [APHE], washout, enhancing capsule, transitional phase hypointensity, hepatobiliary phase hypointensity, restricted diffusion, mild-moderate T2W hyperintensity, non-targetoid appearance and fat in lesion) were reviewed by two abdominal radiologists. All parameters were analysed by logistic regression for predictors of HCC. The statistically significant parameters were summarised and backward stepwise likelihood ratio analysis was applied for creating the simplified HCC-MRI score. The cut-point score was calculated. ROC curve analyses were used for assessment of diagnostic performance of this score for diagnosis of HCC. The interreader agreement was assessed.
**Results or Findings:** Of the 182 hepatic lesions, 92 lesions (50.5%) were HCC and 90 lesions (49.5%) were non-HCC. The four parameters including APHE, enhancing capsule, transitional phase hypointensity and mild-moderate T2W hyperintensity, were significant predictors of HCC on multivariate analyses. The final simplified HCC-MRI score was described as 1.5 points for APHE, 1 point for enhancing capsule, 2 points for transitional phase hypointensity and 1.5 points for T2W hyperintensity. The cut-point score was 4 (suggested HCC, if more than 4). Overall sensitivity of this MRI-HCC score was 84.71%, specificity 91.0%, PPV 90% and NPV 86.2%. The interreader agreement was excellent (0.909).
**Conclusion:** A HCC-MRI score including four parameters with weighting factor and cut-point score offers a simplified method, with good diagnostic performance, for the diagnosis of HCC in high-risk liver.
**Limitations:** The small sample size was an identified limitation.
**Ethics committee approval:** An ethics committee approved this study.
**Funding for this study:** No funding was received for this study.
**Author Disclosures:**
Wanwarang Teerasamit: Nothing to disclose

### RPS 201-9

**Quantitative MRI in the evaluation of patients with non-alcoholic steatohepatitis (NASH)**
*F. Pucciarelli*, M. Watanabe, M. Zerunian, M. Polici, B. Masci, D. Polverari, L. Gnessi, D. Caruso, A. Laghi; Rome/IT

**Purpose:** To evaluate the reliability of quantitative magnetic resonance imaging (MRI) in the diagnosis and follow-up of patients with NASH.
**Methods or Background:** From March to September 2021 twenty patients who met diagnostic criteria for NASH and twenty healthy volunteers were enrolled and underwent quantitative 1.5T MRI examination of the liver. Acquisition protocol comprised magnetic resonance elastography (MRE) and proton density fat fraction (PDFF). Image post-processing was performed with a dedicated workstation. Quantitative image analysis was performed by a radiologist with 15 years of experience in abdominal MRI; liver stiffness (kPa) and grade of steatosis (%) were collected. Statistical analysis was performed using a dedicated software and a p value <0.05 was considered significant.
**Results or Findings:** Liver stiffness was higher in patients with NASH than in healthy volunteers (10.8±6.42 kPa vs 4.75±1.44 kPa; p=0.0002). PDFF was higher in patients with NASH than in healthy volunteers (2.52±0.56% vs 1.87±0.26%; p=0.0020). Steatosis was present in 100% of patients in the NASH group (15% in healthy volunteers); NASH group showed elevated liver stiffness in 80% of patients (0% in healthy volunteers). Quantitative MRI performance for liver stiffness and PDFF detection showed an area under the curve (AUC) of 0.915 (sensitivity 100%; specificity 75%) and 0.843 (sensitivity 60%; specificity 90%), respectively. The whole time examination was 72 seconds (55 seconds for MRE and 17 seconds for PDFF).
**Conclusion:** Quantitative MRI techniques are reliable in the study of NASH. Furthermore, being very fast and risk-free protocols, these technique could also be used as a screening method in populations at risk of developing diffuse liver disease.
**Limitations:** The small population sample and lack of multivariate clinical-radiological analysis were identified limitations.
**Ethics committee approval:** This study was approved by our local institutional review board; written informed consent was obtained from all study participants.
**Funding for this study:** No funding was received for this study.
**Author Disclosures:**
Damiano Caruso: Nothing to disclose
Benedetta Masci: Nothing to disclose
Francesco Pucciarelli: Nothing to disclose
Lucio Gnessi: Nothing to disclose
Marta Zerunian: Nothing to disclose
Mikiko Watanabe: Nothing to disclose
Daniele Polverari: Nothing to disclose
Andrea Laghi: Nothing to disclose
Michela Polici: Nothing to disclose

### RPS 201-10

**The "stiff rim" sign of hepatocellular carcinoma on shear wave elastography: correlation with pathological features and potential prognostic value**
*X. Zhong*; Guangzhou/CN
*(zhongx35@mail3.sysu.edu.cn)*

**Purpose:** To explore pathologic basis and the influencing factors of the stiff rim sign in hepatocellular carcinoma (HCC), and to explore the potential prognostic value.
**Methods or Background:** HCC patients who underwent tumour two-dimensional shear wave elastography (2D-SWE) examination before resection from February 2019 to January 2020 were enrolled in this prospective study. The presence of stiff rim sign and quantitative stiffness measurements were reviewed. Interobserver and intraobserver variability of stiff rim sign was assessed. The correlation between the stiff rim sign and pathological characteristics was analysed. Multivariate analysis was performed to examine clinical and radiological factors influencing the appearance of stiff rim sign. The Kaplan-Meier method was used to analyse the relationship between progression-free survival (PFS) and quantitative/qualitative characteristics of the stiff rim sign.
**Results or Findings:** The stiff rim sign on 2D-SWE was present in 44.7% of HCC lesions with Emax of the stiff rim of 66.5 (53.8-84.8) kPa. Interobserver agreement and intraobserver agreement for the stiff rim sign were substantial (κ=0.772) and almost perfect (κ=0.895), respectively. Pathologically, the stiff rim sign was associated with capsule status, capsule integrity, capsule thickness, proportion of peritumoral fibrous tissue and peritumoral fibrous arrangement. Multivariate analysis showed that tumour size was an independent clinical predictor for the appearance of stiff rim sign (OR 1.213, p=0.012). Kaplan-Meier analysis showed PFS was significantly poorer in the high stiff rim Emax group than in the low stiff rim Emax group (p=0.025).
**Conclusion:** The stiff rim sign in HCC was mainly correlated with peritumoral fibrous tissue status. The Emax of the stiff rim is a prognostic indicator for HCC.
**Limitations:** Multi-centre validation is needed.
**Ethics committee approval:** This study was approved by the ethics committee of First Affiliated Hospital of Sun Yat-Sen University.
**Funding for this study:** This study was funded by the National Natural Science Foundation of China (No. 92059201).
**Author Disclosures:**
Xian Zhong: Nothing to disclose

### RPS 201-11

**CT-derived quantitative parameters of oesophageal varices for evaluation of severe varices and the risk of bleeding: a pilot study**
*S. Wan*, X. Zhang, B. Song; Chengdu/CN

**Purpose:** We aimed to assess whether quantitative computed tomography (CT)-derived parameters can noninvasively predict the severity of oesophageal varices (EV) and the risk of oesophageal variceal bleeding (EVB).
**Methods or Background:** A total of 136 endoscopically confirmed EV patients were included in this retrospective study and were divided into a conspicuous (mild-to-moderate EV, n=30) and a non-conspicuous EV group (severe EV, n=106), a bleeding (n=89) and a non-bleeding group (n=47). EV grade (EVG), EV diameter (EVD), cross-sectional surface area (CSA), EV volume (EVV), spleen volume (SV), splenic vein (SNV), portal vein (PV), diameter of left gastric vein (DLGV), and the opening type of LGV were measured independently using 3D-slicer. Univariate and multivariate logistic analyses were used to determine the independent factors and the receiver operating characteristic (ROC) curves were produced to evaluate the diagnostic performance.
**Results or Findings:** The difference in EVG, EVD, CSA, EVV, DLGV, SNV between the conspicuous and non-conspicuous EV groups was statistically significant ($p<0.05$), corresponding area under the curves (AUCs) for predicting severe EV were 0.72, 0.772, 0.704, 0.768, 0.707, 0.65 respectively, with corresponding sensitivities of 70.3%, 63.5%, 50%, 74.3%, 52.7%, and 48.6% and specificities of 71.4%, 85.7%, 100%, 71.4%, 81%, and 81% respectively. EVG, CSA (odds ratio: 3.258, 95%CI 1.597-6.647; 1.029, 95%CI 1.008-1.050) were found to be independent predictive factors. However, there was no significant difference of the included indices between the bleeding and non-bleeding group ($p>0.05$).
**Conclusion:** CT can be used as a noninvasive method to predict the severity of EV, which may reduce the invasive screening of endoscopy.
**Limitations:** First, some interobserver disagreements may exist due to the subjective nature of endoscopic grading systems. Additionally, given the retrospective nature of the study, some interval progression of the disease cannot be managed.
**Ethics committee approval:** This study was approved by the Institutional Review Board.
**Funding for this study:** Not applicable.
**Author Disclosures:**
Xin Zhang: Nothing to disclose
Shang Wan: Nothing to disclose
Bin Song: Nothing to disclose

**09:30-11:00** **Room M 1**

### Research Presentation Session: Genitourinary

### RPS 207

### Advances in prostate imaging

**Moderator**
T. Barrett; Cambridge/UK
**Author Disclosure:**
T. Barrett: Speaker: Guerbet

### RPS 207-2

**MRI T1 mapping for the assessment of the prostate: a new diagnostic tool**
O. Al-Bourini, A. Uhlig, J. Balz, L. Heitz, L. Trojan, J. Lotz, J. Frahm, A. Seif Amir Hosseini, *J. Uhlig*; Göttingen/DE
*(johannes.uhlig@med.uni-goettingen.de)*

**Purpose:** To assess the role of MRI T1 mapping of the prostate to distinguish normal prostatic tissues and prostatic cancer (PCA).
**Methods or Background:** Patients with suspected PCA were prospectively enrolled between 10/2021–02/2022. Patients received standardised mpMRI on a 3T Magnetom VIDA scanner and T1 mapping using single-shot inversion recovery fast low-angle shot MRI with radial undersampling and iterative reconstruction. Segmentations were performed on T1 maps for prostate lesions and representative areas of the prostatic peripheral zone (PZ), transitional zone (TZ) and benign prostate hyperplasia nodules (BPN).
**Results or Findings:** A total of n=71 patients were included (median age 67.5y). T1 mapping was successfully performed without artefacts at an average time of 2 min in all patients. PIRADS 3+ lesions were identified in n=44 patients, of which n=28 received subsequent ultrasound-MRI prostate biopsy at time of this preliminary analysis (n=14 PCA histology). Among histologically assessed patients, the median T1 relaxation time was 1586ms(BPH), 1730ms(PZ), 1487ms(TZ) and 1393ms (PCA, an overall difference of $p<0.001$). T1 relaxation time correlated with the Gleason score of the histologically assessed prostatic lesion, with shorter median relaxation time in Gleason 7b (1250ms) and Gleason 7a PCA (1401ms) versus Gleason 6 PCA (1467ms) and PIRADS 3+ lesions with benign histology (1470ms).
**Conclusion:** The presented MRI T1 mapping technique robustly performs at a short examination time, demonstrating variable T1 relaxation times depending on prostatic zones. T1 relaxation was shortened in radiologically suspicious lesions and correlated with PCA Gleason score. T1 mapping could therefore aid in the discrimination of clinically significant vs. non-significant PCA.
**Limitations:** The small patient cohort and short follow-up time limit the findings of this preliminary analysis.
**Ethics committee approval:** This prospective study received prior approval by the local ethics committee. All patients consented to study participation.
**Funding for this study:** No funding was received for this study.
**Author Disclosures:**
Jens Frahm: Nothing to disclose
Annemarie Uhlig: Nothing to disclose
Lutz Trojan: Nothing to disclose
Julia Balz: Nothing to disclose
Johannes Uhlig: Nothing to disclose
Luisa Heitz: Nothing to disclose
Joachim Lotz: Nothing to disclose
Ali Seif Amir Hosseini: Nothing to disclose
Omar Al-Bourini: Nothing to disclose

### RPS 207-3

**Promoting the use of the PRECISE score for prostate MRI during active surveillance: results from the ESOR Nicholas Gourtsoyiannis teaching fellowship**
*F. Giganti*[1], E. Barret[2], A. Ambrosi[3], V. Kasivisvanathan[1], M. Emberton[1], C. Allen[1], A. Kirkham[1], C. M. Moore[1], R. Renard Penna[2]; [1]London/UK, [2]Paris/FR, [3]Milan/IT
*(giganti.fra@gmail.com)*

**Purpose:** The Nicholas Gourtsoyiannis Teaching Fellowship, established by the ESOR, is aimed at radiologists with clinical/academic experience who wish to enhance their teaching and training skills by delivering lectures and undertaking interactive workshops in a foreign environment. The PRECISE criteria for serial MRI of the prostate during active surveillance recommend the use of a dedicated scoring system (PRECISE score) to assess the likelihood of significant radiological change. We report the effect of an interactive teaching course on prostate MRI during active surveillance in assessing radiological change on serial imaging.
**Methods or Background:** Eleven radiology fellows and registrars with different experience in prostate MRI reading participated in a dedicated teaching course where they initially evaluated radiological change (based on their previous training in prostate MRI reading) independently in fifteen patients on active surveillance (baseline and follow-up scan), and then attended a lecture on the PRECISE score. The initial scans were reviewed for teaching purposes and afterwards the participants re-assessed the degree of radiological change on a new set of images (from fifteen different patients) applying the PRECISE score. ROC analysis was performed. Confirmatory biopsies and PRECISE scores given in consensus by two radiologists (involved in the original draft of the PRECISE score) were the reference standard.
**Results or Findings:** There was a significant improvement in the average area under the curve (AUC) for the assessment of radiological change from baseline (AUC: 0.60 [confidence intervals: 0.51 - 0.69]) to post-teaching (AUC: 0.77 [0.70 - 0.84]). This was an improvement of 0.17 ($p = 0.004$).
**Conclusion:** A dedicated teaching course on the use of the PRECISE score improves the accuracy in the assessment of radiological change on serial prostate MRI.
**Limitations:** Small population.
**Ethics committee approval:** This study was approved by an ethics committee.
**Funding for this study:** ESOR 2021 Nicholas Gourtsoyiannis Teaching Fellowship.
**Author Disclosures:**
Raphaele Renard Penna: Nothing to disclose
Eric Barret: Nothing to disclose
Caroline M Moore: Nothing to disclose
Mark Emberton: Nothing to disclose
Alessandro Ambrosi: Nothing to disclose
Clare Allen: Nothing to disclose
Veeru Kasivisvanathan: Nothing to disclose
Alex Kirkham: Nothing to disclose
Francesco Giganti: Research/Grant Support: 2021 ESOR Nicholas Gourtsoyiannis Teaching Fellowship recipient

### RPS 207-4

**Time-series radiomics outperforms conventional radiomics approaches for the prediction of prostate cancer histopathological progression on active surveillance**
*N. Sushentsev*[1], L. A. Abrego Rangel[2], Z. Li[2], L. Rundo[3], A. Zaikin[2], E. Sala[1], O. Blyuss[2], T. Barrett[1]; [1]Cambridge/UK, [2]London/UK, [3]Salerno/IT
*(ns784@medschl.cam.ac.uk)*

**Purpose:** To introduce a novel concept of time-series radiomics (TSR) for the analysis of longitudinal MRI-derived radiological changes in prostate cancer (PCa) patients with and without histopathological progression on active surveillance (AS) and compare its diagnostic performance against conventional baseline (BR) and delta-radiomics (DR) models.

**Methods or Background:** This study included AS patients with biopsy-proven PCa with a minimum follow-up of 2 years and at least one repeat targeted biopsy. Histopathological progression was defined as grade-group progression from diagnostic biopsy. The control group included patients with both radiologically and histopathologically stable disease. T2WI- and ADC-derived radiomic features were computed using all available MRI scans performed on the same 3T system using the same protocol. Predictive modelling was performed using the k-nearest neighbours for BR, parenclitic networks for DR, and recurrent neural networks for TSR. Areas under the ROC curve (AUCs) for differentiating between progressors and non-progressors were calculated and compared using DeLong's test.
**Results or Findings:** The study included 76 patients (29 progressors and 47 non-progressors) with a median follow-up of 42 months. 31, 25, 16, and 4 patients had three, four, five, and six consecutive MRI scans available for TSR analysis, respectively. The AUC for TSR (0.86) was significantly higher than AUCs of 0.62 and 0.75 for BR and DR, respectively ($p < 0.001$ and 0.046, respectively).
**Conclusion:** TSR presents a novel approach to analysing longitudinal MRI-derived radiomics data, showing superior performance to conventional radiomic techniques for predicting PCa progression on AS. These preliminary results will be supplemented by time-series analysis of PSA density that may further improve TSR performance.
**Limitations:** Small sample size; preliminary findings.
**Ethics committee approval:** NREC East of England. The need for informed consent for data analysis was waived.
**Funding for this study:** No funding was received for this study.
**Author Disclosures:**
Luis Alberto Abrego Rangel: Nothing to disclose
Nikita Sushentsev: Nothing to disclose
Leonardo Rundo: Nothing to disclose
Oleg Blyuss: Nothing to disclose
Zonglun Li: Nothing to disclose
Evis Sala: Nothing to disclose
Tristan Barrett: Nothing to disclose
Alexey Zaikin: Nothing to disclose

### RPS 207-5

**Strategy of target biopsy and number of target cores for a PI-RADS 3-5 index lesion to reduce Gleason score underestimation: a propensity score-matching analysis**

*B. K. Park*; Seoul/KR
*(1436park@gmail.com)*

**Purpose:** The aim of our research was to determine the number of target cores and a targeting strategy to reduce GS underestimation.
**Methods or Background:** Between May 2017 and April 2020, a total of 385 patients undergoing target cognitive or image-fusion biopsy of PI-RADS 3-5 index lesions and radical prostatectomies (RP) were 2:1 matched with a propensity score using multiple variables and divided into 1-4 core (n=242) and 5-6 core (n=143) groups, which were obtained with multiple logistic regression with restricted cubic spline curve. Target cores of 1-3 and 4-6 were sampled from central and peripheral areas, respectively. Pathologic outcomes and target cores were retrospectively assessed to analyse the GS difference or changes between biopsy and RP with Wilcoxon signed-rank test.
**Results or Findings:** The median of target cores was 3 and 6 in the 1-4 core and 5-6 core groups, respectively ($p<0.001$). Restricted cubic spline curve showed that GS upgrade was significantly reduced from the 5th core and that there was no difference between 5th and 6th cores. Among the matched patients, 35.4% (136/385; 95% confidence interval, 0.305-0.403) had a GS upgrade after RP. The GS upgrades in the 1-4 core and 5-6 core groups were observed in 40.6% (98/242, 0.343-0.470) and 26.6% (38/143, 0.195-0.346), respectively ($p=0.023$). Although there was no statistical difference between the matched groups in terms of RP GS ($p=0.092$), the 5-6 core group had significantly higher biopsy GS ($p=0.006$) and lower GS change from biopsy to RP ($p=0.027$).
**Conclusion:** Five or more target cores sampled from both the periphery and centre of an index tumour contribute to reducing GS upgrade.
**Limitations:** Retrospective design.
**Ethics committee approval:** Our institutional review boards approved the present study (2020-08-137) and waived the need for informed consent.
**Funding for this study:** No funding was received for this study.
**Author Disclosures:**
Byung Kwan Park: Nothing to disclose

### RPS 207-6

**Vector prostate biopsy: a novel electro-magnetic biopsy technique for mpMRI/US fusion transperineal prostate biopsies under local anaesthesia**

*P. Fletcher*[1], M. De Santis[2], T. Barrett[1], C. Kastner[1]; [1]Cambridge/UK, [2]Rome/IT

**Purpose:** Prostate MRI performed early in the assessment for suspected prostate cancer is well accepted, and MRI-based targeting has revolutionised prostate biopsies. MRI/US fusion transperineal prostate biopsy (TPB) with template grid needle guidance using the BiopSee® system achieves high detection rates but requires general anaesthesia. This study introduces a novel technique, utilising EM needle tracking to replace the template grid to perform MRI/US fusion TPB under local anaesthesia (LA). It maintains optimal image fusion by minimal prostate deformation and alignment of the trajectory of the biopsy needle with the target through two perineal access points.
**Methods or Background:** Using BiopSee® fusion software (Medcom, Germany), electro-magnetic (EM) tracking technology (Vtrax, Civco) and transrectal US (Fujifilm, Japan), 39 patients with MRI lesions and various indications for prostate biopsy underwent the procedure. The stepper-mounted rectal US probe obtained prostate images which were fused with the MRI. Local anaesthetic was applied into two defined perineal tracks, and a needle sheath (BARD) with EM sensor was inserted. Through the sheath, the biopsy needle is directed precisely to the pre-contoured lesion. Targeted and systematic biopsies were taken. Cancer detection (all; grade group GG=>2), side-effects and patient experience measures were recorded.
**Results or Findings:** Of 26 returned questionnaires, 21 reported no or minimal discomfort and no episodes of retention or significant infection have been reported. Detection in PI-RADS 3 lesions is 66.7% (≥GG2 33.3%), and in PI-RADS 4+5 lesions it is 91.2% (≥GG2 67.6%).
**Conclusion:** The novel vector technique optimises the use of MRI/US fusion to achieve high targeting accuracy. It provides a feasible and tolerable procedure for fusion TPB under LA and has demonstrated negligible retention and infection rates.
**Limitations:** Single-centre analysis with a limited patient number.
**Ethics committee approval:** Service evaluation registered with GenesisCare UK Medical director.
**Funding for this study:** No funding was received for this study.
**Author Disclosures:**
Peter Fletcher: Nothing to disclose
Christof Kastner: Nothing to disclose
Tristan Barrett: Nothing to disclose
Marta De Santis: Nothing to disclose

### RPS 207-8

**Analysis of dose distribution at organs at risk in patients with early stage prostate cancer treated with the robotic stereotactic body radiation therapy (SBRT) and pencil-beam scanning proton therapy**

*N. Kataev*, N. Vorobyov, A. Mikhaylov, N. Berezina, M. Cherkashin, K. Suprun, A. Kubasov; Saint Petersburg/RU

**Purpose:** To evaluate differences in dose distribution at organs at risk in patients with early-stage prostate cancer treated with the photon robotic stereotactic body radiation therapy (SBRT) and pencil-beam scanning proton therapy.
**Methods or Background:** This retrospective study describes a comparative analysis of treatment plans of 40 patients with early-stage prostate cancer treated with ionising radiation. 22 patients were treated with pencil-beam scanning proton therapy and 18 patients were treated with robotic SBRT. In each treatment plan, the following organs at risk were assessed: the urinary bladder wall, penile bulb, rectum and femoral heads. All patients were treated with 35 – 40 Gy in 5 fractions. All patients who received proton therapy had their target volume immobilised with endorectal balloon.
**Results or Findings:** The greatest differences were in D66%, which for the rectum was 1.22 Gy and 12.9 Gy ($p<0.01$) for proton and photon irradiation, respectively, and 0.24 Gy and 7.77 Gy ($p<0.01$) for the bladder. Rectal V20Gy volume was significantly higher for proton therapy, 48.24 ±20.42 cc vs 25.81 ±10.69 cc ($p<0.01$), due to the proximity of the rectal intestinal wall to the prostate because of the rectal balloon. The maximum doses for the rectum, penile bulb and bladder wall did not differ significantly ($p>0.05$).
**Conclusion:** Although there is no significant difference in maximum doses in organs at risk, we found that using proton therapy reduces the volume of healthy tissue covered by low and intermediate doses, which may be important in terms of the favorable prognosis for this category of patients.
**Limitations:** Not applicable.
**Ethics committee approval:** Not applicable.

Wednesday

**Funding for this study:** This research received no specific grant from any funding agency in the public, commercial, or not-for-profit sectors.
**Author Disclosures:**
Nikita Kataev: Nothing to disclose
Kirill Suprun: Nothing to disclose
Mikhail Cherkashin: Nothing to disclose
Natalia Berezina: Nothing to disclose
Alexey Mikhaylov: Nothing to disclose
Nikolay Vorobyov: Nothing to disclose
Anton Kubasov: Nothing to disclose

### RPS 207-9

**Effects of dynamic contrast enhancement on transition-zone prostate cancer in PI-RADS version 2.1**

*J. Zhang*, L. Xu; Beijing/CN
*(2469824941@qq.com)*

**Purpose:** To explore the effects of dynamic contrast enhanced (DCE)-MRI on transitional-zone prostate cancer (tzPCa) and clinically significant prostate cancer (csPCa) in PI-RADS version 2.1.
**Methods or Background:** The diagnostic efficiencies of T2WI+DWI, T2WI+DCE and T2WI+DWI+DCE in tzPCa and csPCa were compared using a PI-RADS score of ≥4 as the positive threshold and prostate biopsy as the reference standards.
**Results or Findings:** A total of 425 PCa cases were included in the study, including 203 cases in the tzPCa group and 222 in the non-tzPCa group, 146 in the csPCa group and 279 in the non-csPCa group. The AUCs of T2WI+DWI in diagnosing tzPCa and csPCa were significantly greater than those of T2WI+DCE and T2WI+DWI+DCE (all $P < 0.05$), T2WI+DCE and T2WI+DWI+DCE had similar AUCs in tzPCa and csPCa (all $P > 0.05$). The AUCs were 0.829, 0.764 and 0.771 for tzPCa, 0.789, 0.737 and 0.746 for csPCa in T2WI+DWI, T2WI+DCE and T2WI+DWI+DCE respectively. The sensitivity of T2WI+DCE and T2WI+DWI+DCE in diagnosing tzPCa and csPCa were significantly greater than that of T2WI+DWI, while the converse were true of its specificity (all $P < 0.05$). The sensitivity and specificity of T2WI+DCE and T2WI+DWI+DCE in diagnosing tzPCa and csPCa had no significant differences (all $P > 0.05$).
**Conclusion:** T2WI+DWI is the preferred scoring sequence of PI-RADS v2.1 for prostate transitional-zone lesion. The added information gained by DCE may have a meaningful impact on tzPCa and csPCa detection and surveillance.
**Limitations:** It was a single-centre clinical retrospective analysis, which may have selection bias.
**Ethics committee approval:** The Institutional Review Board (IRB) of our hospital approved this retrospective study (IRB number JS-2114), and the requirement for informed consent was waived.
**Funding for this study:** This study has received funding from the National Natural Science Foundation of China (grant No. 91859119).
**Author Disclosures:**
Jiahui Zhang: Speaker: no
Lili Xu: Nothing to disclose

### RPS 207-10

**Utility of 2D shear-wave elastography for the detection of prostate cancer: a preliminary study**

*C. K. Kim*, M. Y. Kang, B. C. Jeong, J. S. Jeon; Seoul/KR
*(chankyokim@skku.edu)*

**Purpose:** To investigate the utility of two-dimensional shear-wave elastography (2D-SWE) in detecting prostate cancer (PCa).
**Methods or Background:** In this prospective study, all 38 patients with suspected PCa underwent 2D-SWE, followed by a standard systemic 12-core biopsy with and without a targeted biopsy. Tissue stiffness (kPa) on SWE was measured in the targeted lesion and 12 regions of systemic 12-core biopsy. For predicting PCa, the receiver operating characteristics (ROC) curve analysis was performed. The associations between tumour stiffness and clinical parameters were evaluated. For measuring tissue stiffness, interobserver reliability was evaluated using an intraclass correlation coefficient (ICC).
**Results or Findings:** PCa was found in 77 regions of 17 patients. In region-based and patient-based analyses, the mean tissue stiffness of tumours were 47.39 kPa and 68.97 kPa, respectively and were significantly higher than those of benign prostate tissue (29.71 kPa and 30.95 kPa) ($P< 0.001$). For predicting PCa, the area under the ROC curve (AUC) of tissue stiffness was 0.703 (95% confidence interval [CI]: 0.652, 0.749) in the region-based analysis; in the patient-based analysis, the AUC of tissue stiffness was 0.840, followed by PSA density (AUC = 0.717). Tumour tissue stiffness demonstrated significantly weak positive correlation with the Gleason score (correlation coefficient = 0.297, $P = 0.009$). ICC in the benign prostate tissue was 0.928 (95% CI: 0.832, 0.970).
**Conclusion:** As a reproducible tool, 2D-SWE appears to be useful for the prediction of PCa. A larger further study awaits the validation.
**Limitations:** (1) Small numbers of study population; and (2), as a standard reference, no whole mount surgical specimens were used.
**Ethics committee approval:** Our institutional review board approved this prospective study, and written informed consent was obtained from each participant.
**Funding for this study:** This study was supported by a Samsung Medison grant.
**Author Disclosures:**
Jeong Soo Jeon: Nothing to disclose
Chan Kyo Kim: Nothing to disclose
Byong Chang Jeong: Nothing to disclose
Min Youg Kang: Nothing to disclose

### RPS 207-11

**Promoting the use of the PI-QUAL score for prostate MRI quality: results from the ESOR Nicholas Gourtsoyiannis teaching fellowship**

*F. Giganti*[1], A. P. Cole[2], A. Ambrosi[3], M. Emberton[1], V. Kasivisvanathan[1], C. M. Moore[1], C. M. Allen[1], C. M. Tempany-Afdhal[2]; [1]London/UK, [2]Boston, MA/US, [3]Milan/IT
*(giganti.fra@gmail.com)*

**Purpose:** The ESOR Nicholas Gourtsoyiannis teaching fellowship is aimed at radiologists with clinical and academic experience who wish to enhance their teaching and training skills. Selected fellows have the opportunity to deliver lectures and conduct interactive workshops at universities of their choice. The prostate imaging quality (PI-QUAL) score is a new metric to evaluate the diagnostic quality of multiparametric magnetic resonance imaging (MRI) of the prostate. We report the results of a prostate MRI quality training course (under the umbrella of the Nicholas Gourtsoyiannis Teaching Fellowship) on the participant's ability to apply PI-QUAL.
**Methods or Background:** Sixteen participants (radiologists, urologists, physicists, and computer scientists) of varying experience in reviewing diagnostic prostate MRI, all assessed the image quality of ten examinations from different vendors and machines. Then, they attended a dedicated lecture followed by a hands-on workshop on MRI quality assessment using the PI-QUAL score. Five scans assessed by the participants were evaluated in the workshop using the PI-QUAL score for teaching purposes. After the course, the same participants evaluated the image quality of a new set of ten scans applying the PI-QUAL score. Results were assessed using receiver operating characteristic analysis. The reference standard was the PI-QUAL score assessed by one of the developers of PI-QUAL.
**Results or Findings:** There was a significant improvement in average area under the curve for the evaluation of image quality from baseline (0.59 [confidence intervals: 0.50 - 0.66]) to post-teaching (0.96 [confidence intervals: 0.92 - 0.98]), an improvement of 0.36 ($p < 0.001$).
**Conclusion:** A teaching course (dedicated lecture + hands-on workshop) on PI-QUAL significantly improved the application of this scoring system to assess the quality of prostate MRI examinations.
**Limitations:** The limitation is the small number of participants and MR examinations.
**Ethics committee approval:** An approval by an ethics committee is not required.
**Funding for this study:** Funding was received for this study by the European School Radiology.
**Author Disclosures:**
Alexander P Cole: Nothing to disclose
Clare Mary Allen: Nothing to disclose
Caroline M Moore: Nothing to disclose
Mark Emberton: Nothing to disclose
Alessandro Ambrosi: Nothing to disclose
Clare M. Tempany-Afdhal: Nothing to disclose
Veeru Kasivisvanathan: Nothing to disclose
Francesco Giganti: Grant Recipient: ESOR Teaching Fellowship

**09:30-11:00** **Room M 2**

### Research Presentation Session: Physics in Medical Imaging

### RPS 213

### Advances in CT and radiation protection

**Moderator**
F. Zanca; Leuven/BE

### RPS 213-2

**Suppressing beam-hardening artefact using a novel edge-on silicon photon-counting CT**

*D. Crotty*[1], Z. Yin[2], R. Bujila[3], B. Da Silva[3], J. Fan[3], B. Thomsen[3]; [1]Cork/IE, [2]Niskayuna, NY/US, [3]Waukesha, WI/US
*(dominic.crotty@ge.com)*

**Purpose:** A novel edge-on silicon-based photon-counting computed tomography (Si-PCCT) device is being developed to simultaneously obtain high spatial and spectral resolution by exploiting the low charge-sharing and K-fluorescence properties of silicon. We demonstrate that this energy-resolving capability helps to accomplish not just spectral clinical imaging tasks but also helps to reduce beam-hardening artefacts for general imaging.
**Methods or Background:** We imaged a challenging beam-hardening phantom consisting of aluminium rods embedded in a water-equivalent background that mimics a human head. The phantom was additionally imaged using a commercial CT system with an energy-integrating detector (EID) in both single-kV (120kVp) and dual-energy (DECT) modes. Scan settings ensured dose-equivalent acquisitions for all modes. Monoenergetic images were reconstructed from 70-140keV. A derived artefact index (AIdx) metric quantified beam-hardening suppression, defined as the absolute difference in Hounsfield unit (HU) measured in regions of interest (ROIs) located inside the artefact-affected regions and three ROIs located in adjacent artefact-free background material. An AIdx approaching zero indicates improved beam-hardening suppression via increased similarity in CT number between artefact-affected and artefact-free regions.
**Results or Findings:** Beam-hardening in Si-PCCT reconstructed images at 70keV is 80% lower than that of equivalent EID-DECT images (AIdx of 1.7HU vs. 8.0HU), while the AIdx of EID-120kVp images is 24.2HU, indicating a 5- and 16-fold improvement in artefact suppression for Si-PCCT relative to EID-DECT and EID-120kVp, respectively. Across all monoenergetic images, AIdx for Si-PCCT is between 55-80% of the equivalent AIdx for today's state-of-the-art EID-DECT, particularly at lower energies. Reconstructed images visually attest to comparatively improved beam-hardening suppression.
**Conclusion:** By leveraging its energy resolution capabilities, a prototype edge-on Si-PCCT system natively enhances suppression of beam-hardening artefacts, potentially illuminating previously occluded pathologies and structures and leading to improved diagnostic confidence and tissue quantification.
**Limitations:** Prototype.
**Ethics committee approval:** Not applicable.
**Funding for this study:** Commercial.
**Author Disclosures:**
Jiahua Fan: Employee: GE Healthcare
Robert Bujila: Employee: GE Healthcare
Brody Da Silva: Employee: GE Healthcare
Dominic Crotty: Employee: GE Healthcare
Zhye Yin: Employee: GE Healthcare
Brian Thomsen: Employee: GE Healthcare

### RPS 213-3

**Low-dose lung cancer screening with a novel CZT photon-counting CT prototype: a phantom study**

*T. W. Holmes*[1], P. Gleason[1], X. Zhan[2], R. Zhang[2], S. Wu[2], R. Thompson[2], Z. Yu[2], A. Pourmorteza[1]; [1]Atlanta, GA/US, [2]Vernon Hills, IL/US
*(twholme@emory.edu)*

**Purpose:** In this study we investigated the initial image quality of a prototype whole-body photon counting detector (PCD) CT scanner based on cadmium-zinc-telluride technology. PCDs offer improved tissue contrast and lower noise, which make them the prime candidate for low-dose applications such as low-dose lung cancer screening. We assessed non-spectral image quality by assessing PCD-CT images created from all detected photons with energies above 25 keV.
**Methods or Background:** We scanned a lung phantom (COPDGENE2) with seven calibrated inserts to simulate healthy lung tissue, ground-glass opacifications and emphysema. The PCD-CT scan parameters were: 120kVP tube voltage, with three tube current-time values at 50, 100, and 200 mAs; 32x0.6 collimation; and 1-second rotation time. Images were reconstructed with filtered-back projection algorithm with body and sharp kernels. We scanned the same phantom on a commercial energy-integrating detector (EID) CT scanner for comparison. The scan and reconstruction parameters of the EID-CT were closely matched to those of the PCD-CT. We compared CT number stability, contrast and contrast-to-noise ratio (CNR) of the two CT systems.
**Results or Findings:** The PCD-CT images showed more robust CT numbers for all simulated types of lung tissue, with maximum deviation of 0.8 vs 2.5 HU for PCD and EID images, respectively. Contrast and CNR for detection of ground-glass opacifications and emphysema was higher for PCD images compared to the clinical EID measurements.
**Conclusion:** Our initial assessment of the new PCD-CT prototype showed improved performance in CT number stability and CNR, specifically in low-dose lung CT scans. Further studies are warranted to assess the clinical performance of the prototype scanner.
**Limitations:** This was a limited pilot study with 3 phantoms. More experiments with direct comparison to EID are warranted.
**Ethics committee approval:** Not applicable.
**Funding for this study:** Sponsored research agreement with Canon Medical Research USA.
**Author Disclosures:**
Zhou Yu: Employee: Canon Medical Research USA
Amir Pourmorteza: Grant Recipient: sponsored research agreement with Canon Medical Research USA
Thomas Wesley Holmes: Nothing to disclose
Patrick Gleason: Nothing to disclose
Richard Thompson: Employee: Canon Medical Research USA
Ruoqiao Zhang: Employee: Canon Medical Research USA
Xiaohui Zhan: Employee: Canon Medical Research USA
Shuoxing Wu: Employee: Canon Medical Research USA

### RPS 213-4

**Reliability of computed tomographic dose data: comparing the tin filter technique with conventional scanning protocols using thermoluminescence dosimeter measurements**

*S. Schüle*[1], M. Abend[1], M. J. Beer[2], P. Ostheim[1], M. Port[1], C. Hackenbroch[2]; [1]Munich/DE, [2]Ulm/DE

**Purpose:** The use of tin filtering in high-contrast computed tomography (CT) examinations is increasing due to lower radiation exposure while maintaining high diagnostic image quality. Our aim was to compare the applied dose ((TLD measurements (thermoluminescence dosimeter)) of CT protocols with and without tin filtration (Sn) at the same CTDIvol (computed tomography dose index).
**Methods or Background:** TLD measurements were performed in triplicates, centered in a water phantom (diameter: 32cm) on a 3rd-generation dual-source CT scanner. Five different scan protocols (Sn 150 kV, 150 kV, 120 kV, 100 kV and Sn 100 kV) with six (CTDIvol: 29 mGy, 23.3 mGy, 17.4 mGy, 8.5 mGy, 6.3 mGy and 2.8 mGy) dose settings were used. TLD measurement results and the slope of the linear regression of the different protocols were compared. Statistical analysis was performed using ANOVA or Kruskal-Wallis test.
**Results or Findings:** Sn protocols resulted in higher applied doses at all dose settings according to TLD measurements (dose difference: 16% +/- 4%) compared to non-tin filter scan protocols ($p \leq 0.01$). Exceptions were the comparison between the Sn 150 kV and 150 kV protocols and between the Sn 150 kV/Sn 100 kV and 120 kV protocols at a CTDIvol of 29 mGy and 6.3 mGy, respectively ($p \geq 0.055$). An increased slope of the linear regression was shown for the Sn 150 kV protocol compared to all remaining protocols ($p<0.05$).
**Conclusion:** Compared to conventional protocols, the applied dose of Sn protocols is underestimated based on CTDIvol data from the scanner. With Sn protocols, patients are thus exposed to a higher dose compared to conventional CT protocols at the same CTDIvol. For radiation protection reasons, this should be considered when using the Sn technique.
**Limitations:** The water phantom used is comparable but not identical to a patient's body.
**Ethics committee approval:** Not applicable.
**Funding for this study:** Not applicable.
**Author Disclosures:**
Patrick Ostheim: Nothing to disclose
Matthias Port: Nothing to disclose
Simone Schüle: Nothing to disclose
Meinrad Johannes Beer: Nothing to disclose
Carsten Hackenbroch: Nothing to disclose
Michael Abend: Nothing to disclose

### RPS 213-5

**Impact of iterative reconstruction algorithms on the applicability of the Fourier-based detectability index for X-ray CT imaging**

*E. Pouget*, V. Dedieu; Clermont-Ferrand/FR

**Purpose:** The current paradigm for evaluating imaging system performance relies on Fourier methods, which presuppose a linear, wide-sense stationary system. Long-range correlations introduced by iterative reconstruction algorithms may narrow the applicability of Fourier techniques. Differences in the implementation of reconstruction algorithms between manufacturers add further complexity. The present work set out to quantify the errors entailed by the use of Fourier methods, which can lead to design decisions that do not correlate with detectability.
**Methods or Background:** The noise properties and the detectability index of the ideal linear observer were evaluated using the spatial approach and the Fourier-based approach. For this purpose, a homogeneous phantom was imaged on two scanners: the Revolution CT (GE Healthcare) and the Somatom Definition AS+ (Siemens Healthcare) at different exposure levels. Images were reconstructed using different strength levels of IR algorithms available on the systems considered: ASIR-V and SAFIRE.
**Results or Findings:** Our findings highlight that the spatial domain estimate of the detectability index is higher than the Fourier domain estimate. This trend is found to be dependent on the specific regularisation used by IR algorithms as well as the signal to be detected. The eigenanalysis of the noise covariance matrix and of its circulant approximation yields explanation about the evoked trends.
**Conclusion:** The applicability of Fourier techniques is dependent on the specific regularisation used by IR algorithms. These results argue for verifying the assumptions made when using Fourier methods since the Fourier task-based detectability index does not always correlate with signal detectability. Impact of iterative reconstruction algorithms on the applicability of Fourier-based detectability index for X-ray CT imaging. POUGET E., DEDIEU V., Med Phys. 2021 Aug; 48(8): 4229-4241.
**Limitations:** Not applicable.
**Ethics committee approval:** Not applicable.
**Funding for this study:** Not applicable.
**Author Disclosures:**
Véronique Dedieu: Nothing to disclose
Eléonore Pouget: Nothing to disclose

### RPS 213-6

**Image quality and dose reduction potential of an artificial intelligence deep-learning reconstruction algorithm for abdomen CT examinations: a study on phantoms**

*J. Greffier*; Nimes/FR
*(joel.greffier@chu-nimes.fr)*

**Purpose:** To assess the impact of a new artificial intelligence deep-learning reconstruction (Precise Image; AI-DLR) algorithm on image quality against a hybrid iterative reconstruction (IR) algorithm in abdominal CT for different clinical indications.
**Methods or Background:** Acquisitions on phantoms were performed at 5 dose levels (CTDIvol: 13/11/9/6/1.8mGy). Raw data were reconstructed using Level 4 of iDose4 (i4) and 3 levels of AI-DLR (Smoother/Smooth/Standard). Noise power spectrum (NPS), taskbased transfer function (TTF) and detectability index (d') were computed: d' modelled detection of a liver metastasis (LM) and hepatocellular carcinoma at portal (HCCp) and arterial (HCCa) phases. Image quality was subjectively assessed on an anthropomorphic phantom by 2 radiologists
**Results or Findings:** From standard to smoother levels, noise magnitude and average NPS spatial frequency decreased and the detectability (d') of all simulated lesions increased. For both inserts, TTF values were similar for all three AI-DLR levels from 13 to 6 mGy but decreased from standard to smoother levels at 1.8 mGy. Compared to the i4 used in clinical practice, d' values were higher using smoother and smooth levels and close for the standard level. For all dose levels, except for 1.8 mGy, radiologists considered images satisfactory for clinical use for the 3 levels of AI-DLR but rated images too smooth using the smoother level.
**Conclusion:** Using the smooth and smoother levels of AI-DLR reduces image noise, improves detectability of lesions and spatial resolution for standard and low-dose levels. Using the smooth level is apparently the best compromise between the lowest dose level and adequate image quality.
**Limitations:** It was conducted on phantoms that did not precisely mimic the patient's anatomical structures and body morphology.
**Ethics committee approval:** Not applicable. Phantom study.
**Funding for this study:** No funding was received for this study.
**Author Disclosures:**
Joel Greffier: Nothing to disclose

### RPS 213-7

**Radiation dose and image quality in middle and inner ear CT imaging: comparison between multidetector CT and 3D flat-panel rotational acquisitions on two different phantoms**

*L. Bellesi*[1], F. Magoga[2], G. Cancellato[2], E. Ventura[2], A. Cianfoni[2], S. Presilla[1]; [1]Bellinzona/CH, [2]Lugano/CH
*(luca.bellesi@eoc.ch)*

**Purpose:** Middle and inner ear imaging requires high spatial resolution images to assess fine anatomical details. The aim of this study was to compare a 256 multi-detector computed tomography (MDCT) and 3D flat-panel rotational acquisitions (3DRA) in terms of radiation dose and image quality, using two different phantoms.
**Methods or Background:** To assess image quality, MDCT and 3D RA were performed on a CT Catphan phantom and a head anthropomorphic phantom. MDCT standard acquisition was acquired with 120 kV, 60 mAs, pitch 0.3 and slice thickness 0.6 mm. 3DRA standard acquisition was acquired with 80 kV, 258 mAs and 30 frame per second. Image quality was assessed evaluating high contrast spatial resolution on Catphan phantom and by a subjective evaluation of a neuroradiologist on the anthropomorphic phantom, evaluating anatomical details detectability, noise and contour sharpness. To perform dosimetric evaluations CTDI, DLP, DAP were collected while effective doses were calculated, using two different Monte Carlo systems.
**Results or Findings:** 3D RA images showed a better high-contrast spatial resolution in comparison with MDCT, with values of 15 lp/cm and 12 lp/cm respectively. The subjective visual assessment performed by a neuroradiologist on anthropomorphic phantom images resulted in higher image quality for 3D RA. Effective doses calculated with Monte Carlo systems on the head-CTDI phantom standard acquisition were 0.63 mSv for 3D RA and 0.5 mSv for MDCT. The dose measured with pencil-type CT chamber were 367 mGycm for 3D RA, and 292.7 mGycm for MDCT.
**Conclusion:** 3D RA provides higher image quality compared to MDCT in terms of high-contrast resolution and the neuroradiologist's subjective visual assessment in middle and inner hear imaging on phantom models with a comparable radiation dose.
**Limitations:** No limitations were identified.
**Ethics committee approval:** Not applicable.
**Funding for this study:** No funding received.
**Author Disclosures:**
Stefano Presilla: Nothing to disclose
Gaetano Cancellato: Nothing to disclose
Alessandro Cianfoni: Nothing to disclose
Luca Bellesi: Nothing to disclose
Francesco Magoga: Nothing to disclose
Elisa Ventura: Nothing to disclose

### RPS 213-8

**A new performance evaluation method for high-resolution CT using changes in CT values (improved spatial resolution and increased CT value for small objects)**

*K. Tsujioka*[1], K. Yamada[2], M. Niwa[2]; [1]Toyota/JP, [2]Yokkaichi/JP
*(tsujioka@fujita-hu.ac.jp)*

**Purpose:** The modulation transfer function (MTF) is often used as a method for evaluating the spatial resolution of CT devices. However, it is difficult for doctors who make a diagnosis in actual clinical practice to understand the effect on clinical CT images from MTF results. In actual clinical practice, it is known that the improvement of spatial resolution improves the CT value of small blood vessels, but MTF cannot express this phenomenon. We have devised a new evaluation method for spatial resolution, focusing on the improvement of spatial resolution and the increase in CT value of small objects.
**Methods or Background:** We performed a scan using a phantom with a metal wire spirally wound around a cylindrical acrylic pipe. The CT value profile of the wire and the peak CT value of the wire were measured.
**Results or Findings:** The MTF results and the CT value profile and CT value of the spiral wire were highly correlated. When the MTF was high, the CT value profile of the spiral wire was sharp and the peak CT value was high.
**Conclusion:** Improving the spatial resolution of the CT device not only sharpens the image but also increases the CT value of the small blood vessels. This is a phenomenon of partial volume effect in CT. MTF is a physically excellent evaluation method for CT equipment. However, there are some CT phenomena that cannot be evaluated by MTF alone. The method we propose is a clinically-based CT image evaluation method.
**Limitations:** This report is based on the phantom experiment. We are planning to conduct research using human body data.
**Ethics committee approval:** Our experiments have been approved by the ethics committee.

**Funding for this study:** We were not financially funded by any organisation for this report.
**Author Disclosures:**
Katsumi Tsujioka: Nothing to disclose
Masayoshi Niwa: Nothing to disclose
Kyohei Yamada: Nothing to disclose

### RPS 213-9

**Comparison of patient exposure and routine protocols for multidetector computed tomography angiography examinations of patients with peripheral artery disease**

*D. Kostova-Lefterova*, S. Stanev, D. P. Ivanova, B. Chesmedzhieva, S. E. Dineva, E. Georgiev, G. Rashev; Sofia/BG

**Purpose:** The conventional aorto-arteriography (CA) is accepted as "gold standard" imaging modality for diagnosing of intravascular pathologies. The advantages of multidetector computed tomography angiography (MDCTA) as a fast non-invasive method has increased its application for imaging of anatomical and structural details of the vascular system. It allows 3D-reconstruction and post-processing methods for precise treatment planning. The purpose of this survey was to compare patient exposure and routine MDCTA protocols used for patients with peripheral artery disease in different medical imaging departments in Bulgaria.
**Methods or Background:** Nine MDCT units (named A-I) were included in the study. Optimisation of routine CT protocols was performed for 5 of them in the period 2015-2022. The image quality was assessed by radiologists according to the visibility of the contrast in the pedal vessels.
**Results or Findings:** Patients were scanned from celiac artery (mid diaphragm) to toes with median scan length in the range (1048 mm-1371 mm). 12 times difference was observed between the CTDIvol values (1.74 mGy(E)-20.3 mGy(D)) and 13 times-between the DLP values (199.51 mGy.cm - 2640.04 mGy.cm) between the protocols. Optimised protocols reduced patient exposure by a factor of 1.6 (A), 1.4 (D), 2.2 (E) and 5.4 (G), with lowest values when iterative reconstruction methods (B, C, E, G) were used, except for D. Compared to the standard 120 kV acquisition, the vascular enhancement of the same volume of iodinated contrast materia was increased by 25% (100kV) and by 70%(80kV), with 36%(A), 54%(E) and 82%(G) dose reduction.
**Conclusion:** The non-invasive MDCTA has replaced most diagnostic applications of CA. MDCTA can provide anatomic and functional assessment of most intravascular pathologies, which is comparable to CA applications as diagnostic modality.
**Limitations:** Not all of the departments have an optimisation program in process.
**Ethics committee approval:** No ethical approval required for the survey.
**Funding for this study:** No funding was received for this study.
**Author Disclosures:**
Stefan Stanev: Nothing to disclose
Bogomila Chesmedzhieva: Nothing to disclose
Svetla Emilova Dineva: Nothing to disclose
Desislava Kostova-Lefterova: Nothing to disclose
Georgy Rashev: Nothing to disclose
Emil Georgiev: Nothing to disclose
Desislava Petkova Ivanova: Nothing to disclose

### RPS 213-10

**Molecular biological response in peripheral blood cells after tin filtration in computed tomography**

*S. Schüle*[1], C. Hackenbroch[2], M. J. Beer[2], C. Hermann[1], P. Ostheim[1], M. Port[1], H. Scherthan[1], M. Abend[1]; [1]Munich/DE, [2]Ulm/DE

**Purpose:** Tin filtration is an emerging technique in computed tomography (CT) for dose reduction while maintaining high diagnostic image quality. Our goal was to compare the molecular biological response in peripheral blood cells after X-irradiation with and without tin filtration (Sn).
**Methods or Background:** Human peripheral blood was irradiated in vitro (six donors, aged 28-48 years) on a 3rd-generation dual source CT-scanner. Sn 150 kV, 150 kV and 120 kV scan protocols with 20 mGy (full dose, FD) and a Sn 150 kV scan protocol with 16 mGy (low dose, LD) were used. The Sn 150 kV LD and the 120 kV FD protocol showed comparable image noise. Radiation-induced molecular changes were examined by gene expression (GE) measurements of FDXR, DDB2, BAX, CDKN1A, AEN, EDA2R, APOBEC3H and quantification of γH2AX DSB foci (DNA double-strand breaks) relative to unexposed samples. Statistical analysis was performed using the t-test or Mann–Whitney U test, where applicable.
**Results or Findings:** All investigated genes (except APOBEC3H) showed a protocol-independent radiation-induced upregulation of GE and an increased number of double-strand breaks (1.5 to 7-fold, $p<0.05$). There was no significant difference between the 150 kV FD protocol ± Sn ($p>0.19$) nor between the 120 kV FD and Sn 150 kV LD protocol ($p>0.14$). The highest differential gene expression was observed in GE measurements of EDA2R.
**Conclusion:** X-irradiation with tin filtration resulted in similar molecular biological responses compared to conventional CT protocols. Tin filtering allows a dose reduction while maintaining high diagnostic image quality, directly reduces radiation exposure and is therefore preferable to other protocols. Furthermore, EDA2R is particularly suitable for detecting low radiation doses, while APOBEC3H is not.
**Limitations:** In-vitro experiments generally need to be validated in further studies.
**Ethics committee approval:** Approval was obtained by the local ethics committee.
**Funding for this study:** Not applicable.
**Author Disclosures:**
Patrick Ostheim: Nothing to disclose
Matthias Port: Nothing to disclose
Simone Schüle: Nothing to disclose
Cornelius Hermann: Nothing to disclose
Meinrad Johannes Beer: Nothing to disclose
Carsten Hackenbroch: Nothing to disclose
Michael Abend: Nothing to disclose
Harry Scherthan: Nothing to disclose

### RPS 213-11

**An audit on the awareness of radiation risks, radiation legislation and referral guidelines (iRefer) amongst junior doctors**

*S. Subramanian Parameswaran*, D. Platten, A. Malla, A. Aslam; Lincoln/UK

**Purpose:** To assess junior doctors' knowledge of radiation legislation, referral guidelines (iRefer), radiation dose and associated risks in commonly requested (ionising) imaging investigations at United Lincolnshire Hospital Trust.
**Methods or Background:** Literature shows that junior doctors' knowledge of ionising radiation and its effects are limited. Radiation awareness is inadequately covered by the UK undergraduate and postgraduate curricula. Referrers must be aware of iRefer guidelines and their legal responsibilities to ensure appropriate use of NHS resources. Using iRefer guidelines improves imaging appropriateness and reduces unnecessary investigations by approximately 20%. This is particularly important as there is a shortage of UK radiologists. Though risks associated with diagnostic imaging radiation doses are acceptable, stochastic effects can be minimised by increasing awareness.
**Results or Findings:** Data was collected from junior doctors before (n=45) and after intervention (n=30), using a 15-point questionnaire. The initial audit showed poor radiation awareness amongst junior doctors: 75% had no formal teaching on radiation doses or associated risks and 64% were not formally taught how to make an imaging referral. A teaching session was delivered on IRMER 2017 and a recording emailed to junior doctors. The accuracy of the contents was verified by radiation protection staff. A re-audit was completed to evaluate teaching effectiveness. This showed increased knowledge in all areas: radiation doses (42% to 87%); lifetime risk of radiation induced cancer (23% to 93%); heritable defects from radiation exposure to pregnant patients (20% to 80%); concept of ALARA (20% to 93%); Cochrane's Law (14% to 100%); iRefer guidelines (17% to 100%); and referrer legal responsibilities (15.9% to 100%).
**Conclusion:** Radiation awareness amongst junior doctors can be improved using focused teaching based on IRMER 2017. This will ensure appropriate use of NHS resources and promote radiation safety.
**Limitations:** Represents data from one trust in UK.
**Ethics committee approval:** Not applicable.
**Funding for this study:** Not applicable.
**Author Disclosures:**
David Platten: Nothing to disclose
Sathvikha Subramanian Parameswaran: Nothing to disclose
Aqsa Aslam: Consultant: Lincoln County Hospital
Abinash Malla: Nothing to disclose

### RPS 213-12

**ZrO2-reinforced PVA nanofibers produced for radiation ray-shielding studies as a protective vest in the radiology department**

*A. F. F. Tekin*, Y. C. Kartal, A. H. Yardımcı; Istanbul/TR
*(aftrad333@gmail.com)*

**Purpose:** Researchers have always been interested in finding new and effective materials for protection against radiation. This experimental study aimed to design and fabricate new types of nanomaterial- and micromaterial -based shields against the ionising effect of computed tomography (CBCT), diagnostic radiography and digital substraction angiography X-rays.
**Methods or Background:** Our study aims to produce PVA nanofibers containing $ZrO_2$ ceramic nanoparticles and measure their X-ray shielding abilities. First, polyvinyl alcohol is mixed with 20 ml of distilled water at 70°C for 2 hours in a magnetic stirrer. The mixing process continues at room temperature until PVA forms a viscous and homogeneous solution in pure water. Then 0.45 g $ZrO_2$ nanoparticles are added to the PVA solution and mixing is continued for 1 hour. The final solution was stabilized at room temperature for 12 hours to increase homogeneity. The mixed solution was loaded into a 10 ml syringe with a 0.5 mm inner diameter stainless steel needle. Electrospinning under 20 kV potential voltage was carried out by covering the collector rotating at 500 rpm with aluminum foil. $ZrO_2$ reinforced PVA nanofibers produced with this method were collected and used to produce X- and gamma-ray shielding vests.
**Results or Findings:** To measure the half-value layers (HVL) and the linear attenuation coefficient (μ), we sent the fabricating prototype to the Turkish Energy, Nuclear and Mineral Research Agency.
**Conclusion:** Preliminary results have shown us the $ZrO_2$-reinforced PVA nanofiber vest radiation attenuation is better than traditional lead vests.
**Limitations:** No limitations were identified.
**Ethics committee approval:** Not applicable.
**Funding for this study:** Not applicable.
**Author Disclosures:**
Aytül Hande Yardımcı: Author: İnvestigater
Ali Fuat Fuat Tekin: Speaker: İnvestigater
Yiğit Can Kartal: Author: İnvestigater

**09:30-11:00** **Room O**

### Research Presentation Session: Chest

### RPS 204

### COVID: chest radiology beyond the lungs

**Moderator**
G. Aviram; Tel Aviv/IL

### RPS 204-2

**AI dual-energy CT lung analysis can help distinguish COVID-19 infiltrates from visually similar non-infectious pneumonitis and can optimise radiological workflows**

*A. S. Brendlin*, M. Mader, S. Gassenmaier, K. Nikolaou, S. Afat; Tübingen/DE

**Purpose:** To explore the potential impact of an AI dual-energy CT (DECT) prototype on decision-making and workflows by investigating its capabilities to differentiate COVID-19 from similar pneumonitis.
**Methods or Background:** From 03.04.2020 to 12.02.2021, DECT from biometrically matching patients with COVID-19, pneumonitis, and inconspicuous findings were selected from our clinical routine. Three blinded readers independently scored each pulmonary lobe analogous to CO-RADS. Interrater agreement was determined with an intraclass-correlation-coefficient (ICC). Averaged perfusion metrics per lobe (iodine uptake in mg, volume without vessels in ml, iodine concentration in mg/ml) were extracted using manual segmentation and an AI DECT prototype. A generalised linear mixed model was used to investigate metric validity and potential distinctions at equal CO-RADS scores. Multinomial regression measured the contribution of "Reader", "CO-RADS score", and "perfusion metrics" to diagnosis. Time to diagnosis was measured for manual vs AI segmentation.
**Results or Findings:** We included 105 patients (62 ± 13 years, mean BMI 27 ± 2). There were no significant differences between manually and AI-extracted perfusion metrics (p=0.999). Regardless of CO-RADS score, iodine uptake and concentration per lobe were significantly higher in COVID-19 than in pneumonitis (p<0.001). In regression, iodine uptake had a greater contribution to diagnosis than CO-RADS scoring (Odds Ratio [OR]=1.82 [95%CI 1.10-2.99] vs OR=0.20 [95%CI 0.14-0.29]). The AI prototype extracted the relevant perfusion metrics significantly faster than radiologists (10±1 vs 15±2 minutes, p<0.001).
**Conclusion:** The investigated AI prototype positively impacts decision-making and workflows by extracting perfusion metrics that differentiate COVID-19 from visually similar pneumonitis significantly faster than radiologists.
**Limitations:** This study was retrospective with 35 matched patients per group. Iodine metrics extracted from a portal venous phase may be phase-specific. We used a high-end 3rd generation dual-source scanner, so generalisability may be limited.
**Ethics committee approval:** This study was approved by the IRB.
**Funding for this study:** No funding was received.
**Author Disclosures:**
Konstantin Nikolaou: Nothing to disclose
Andreas Stefan Brendlin: Nothing to disclose
Saif Afat: Nothing to disclose
Markus Mader: Nothing to disclose
Sebastian Gassenmaier: Nothing to disclose

### RPS 204-3

**Lung vessel volume evaluated with CALIPER software is an independent predictor of mortality in COVID-19 patients: a multicentric retrospective analysis**

*C. Romei*[1], Z. Falaschi[2], P. Danna[2], M. Tonerini[1], S. C. Fanni[1], R. Arioli[2], A. De Liperi[1], B. Bartholmai[3], A. Carriero[2]; [1]Pisa/IT, [2]Novara/IT, [3]Rochester, MN/US
*(chiara.romei@gmail.com)*

**Purpose:** Computer-Aided Lung Informatics for Pathology Evaluation and Ratings (CALIPER) software has already been widely used in the evaluation of Interstitial lung diseases (ILD) but has not yet been tested in patients affected by COVID-19. Our aim was to use it to describe the relationship between Coronavirus Disease 2019 (COVID-19) outcome and the CALIPER-detected pulmonary vascular-related structures (PVRS).
**Methods or Background:** We performed a multicentric retrospective study enrolling 570 COVID-19 patients who undergone a chest CT in the emergency settings in two different institutions. Fifty-three age and sex matched healthy controls were also identified. Chest CTs were analysed with CALIPER identifying the percentage of PVRS over the total lung parenchyma. Patients were followed for up to 72 days recording mortality and required intensity of care.
**Results or Findings:** There was a statistically significant difference in PVRS between COVID-19 positive patients and the controls (median [iqr] 4.05 [3.74] and 1.57 [0.40] respectively, P = 0.0001). PVRS showed an increasing trend with the severity of care, P < 0.0001. The univariate Cox regression model showed that PVRS increase is a risk factor for mortality (HR 1.17, P < 0.0001). The multivariate analysis demonstrated that PVRS is an explanatory factor of mortality along with age (HR 1.13, P < 0.0001).
**Conclusion:** Our study suggests that PVRS increases with the required intensity of care, and it is an explanatory factor for mortality.
**Limitations:** Restrospective study. Software unable to recognise areas of lung consolidation. Laboratory values unavailable.
**Ethics committee approval:** The internal review boards of both institutions approved the research, protocol numbers 17368 (Pisa) and CE 130/20 (Novara).
**Funding for this study:** No funding has been released for this research.
**Author Disclosures:**
Michele Tonerini: Nothing to disclose
Chiara Romei: Nothing to disclose
Roberto Arioli: Nothing to disclose
Brian Bartholmai: Nothing to disclose
Salvatore Claudio Fanni: Nothing to disclose
Pietro Danna: Nothing to disclose
Alessandro Carriero: Nothing to disclose
Zeno Falaschi: Nothing to disclose
Annalisa De Liperi: Nothing to disclose

### RPS 204-5

**Characteristics of chest CT-scan and outcome in pregnant patients with COVID-19: a cross-sectional study**

M. Hosein Yazdi, *S. Esmaeilian*, A. Teimouri, M. Ardeshiri, R. Jahankhah, F. Rafiei, F. Ghazi Sherbaf; Shiraz/IR
*(kmssd87@gmail.com)*

**Purpose:** Since the beginning of COVID-19 pandemic, researchers have been trying to find risk factors for severe infection and vulnerable health conditions. Pregnancy is one of these conditions with lots of controversies. Also, there is no adequate information regarding features of lung CT scan imaging in these patients.
**Methods or Background:** In this cross-sectional study, we retrospectively assessed pregnant patients with positive results of PCR for COVID-19, between April and July 2020. Data on CT scan characteristics, demographic features and laboratory factors were collected in a pre-designed checklist.
**Results or Findings:** We analysed 23 pregnant women with a mean age of 32.34±8.06 years. Mean $O_2$ saturation was 93.52±4.59% and patients were hospitalised for a mean duration of 6.13±4.07 days. Only one patient was expired. Dyspnoea was the most (65.2%) and generalised pain was the least (8.7%) common symptom. Consolidation was the most common (39.1%) lung CT scan finding followed by ground glass opacity (34.7%). There was no significant difference between presence or location of GGO (p = 0.348) or consolidation (p = 0.391) and patients' outcome. Following the same manner, there was also no significant difference between presence of Halo sign (p = 0.053), nodule (p = 0.087), fibrotic band (p = 0.870), sub-pleural spare (p = 0.13) or atoll sign (p = 0.087) and the final outcome.
**Conclusion:** There is no significant association between characteristics of lung CT scan imaging and clinical manifestations or final outcome in pregnant patients with COVID-19.

**Limitations:** The retrospective nature of study resulted in loss of valuable data and low sample size. This the first study evaluating lung CT scan characteristics of Iranian pregnant women with COVID-19.
**Ethics committee approval:** The study was carried out according to the Declaration of Helsinki and was approved by the Ethics Committee of Shiraz University of Medical Sciences (IR.SUMS.REC.1081).
**Funding for this study:** No funding was received for this study.
**Author Disclosures:**
Meisam Hosein Yazdi: Author: analysis and review
Faranak Rafiei: Consultant: review
Arash Teimouri: Author: data collector
Saeid Esmaeilian: Owner: Idea and data collection and final review
Dear Reza Jahankhah: Author: review
Farzaneh Ghazi Sherbaf: Other: final review
Masoumeh Ardeshiri: Other: data collector

### RPS 204-6

**Correlating COVID-19 disease severity and pulmonary vascular manifestations on CT pulmonary angiography**
W. Y. Chan[1], N. Gowdh[1], K. Rahmat[1], N. Mohd Ramli[2], W. L. Ng[1], M. M. Mohamed[1], *F. B. Fadzli*[1]; [1]Kuala Lumpur/MY, [2]Petaling Jaya/MY
*(farhana.fadzli@yahoo.com)*

**Purpose:** Pulmonary microangiopathy has been deemed a culprit for silent clinical deterioration, "silent hypoxia" [2, 3]. The aim of this study is to identify factors associated with pulmonary embolism (PE) and the features of pulmonary vascular angiopathy in COVID-19 patients.
**Methods or Background:** A cross sectional cohort study of 100 hospitalised consecutive COVID positive patients who underwent pulmonary angiography from January 2021 to April 2021. CTPA features were evaluated for presence, localisation of PE, signs of vascular angiopathy (ie. pulmonary vascular enlargement, vascular tree-in-bud sign, target sign, dandelion sign) and CT severity score. Clinical, demographic and laboratory parameters at time of imaging were collected
**Results or Findings:** 18 patients were positive for PE of which 94.4% (16/18) were at segmental pulmonary artery level and 66.7% (12/18) at lower lobe. All patients had vascular tree in bud pattern. A statistically significant correlation was found between presence of PE and those with premorbid diabetes mellitus ($p = 0.025$), intubated at time of imaging ($p = 0.023$) and pulmonary vascular tree-in-bud sign ($p=0.002$). A significant difference in CTSS score ($p = 0.003$) was noted between the two groups, those with PE and without PE. A highly significant correlation was found between patients with vascular tree-in-bud sign and rising CRP, WBC, serum ferritin and CTSS ($p = 0.010, 0.025, 0.048$ and 0.000 respectively).
**Conclusion:** In our study population pulmonary embolism predominantly affected segmental arteries at lower lobes and was more prevalent in those intubated. Suspicion of PE is raised in those with higher CTSS and vascular tree-in-bud pattern. Therefore, it is a potential indirect indicator of vascular angiopathy in COVID-19 patients.
**Limitations:** Small sample size.
**Ethics committee approval:** This study was approved by an ethics committee.
**Funding for this study:** Funding was received for this study.
**Author Disclosures:**
Norlisah Mohd Ramli: Nothing to disclose
Farhana Binti Fadzli: Nothing to disclose
Kartini Rahmat: Nothing to disclose
Nadia Gowdh: Nothing to disclose
Mariyam Muzna Mohamed: Nothing to disclose
Wei Lin Ng: Nothing to disclose
Wai Yee Chan: Nothing to disclose

### RPS 204-7

**Quantitative evaluation of pulmonary blood flow in patients three months after COVID-19 infection with extensive pulmonary parenchyma involvement**
*A. Zakharova*, D. Kupriyanov, A. Pozdniakov; Saint Petersburg/RU

**Purpose:** To investigate pulmonary perfusion in patients 3 months after recovery from COVID-19 infection with extensive pulmonary involvement based on dynamiccontrast-enhanced MR imaging.
**Methods or Background:** In this IRB-approved cross-sectional study eleven healthy volunteers and nine patients 3 months (± 7 days) after recovery from COVID-19 infection with pneumonia involving >50% of lung parenchyma were included. All patients underwent a lung MR protocol including the post-contrast time-resolved 3D gradient-echo time-resolved pulse sequence in the coronal plane (TFE, TE/TR: 1.6/3.5 ms, flip angle 40°, time resolution 0.6s, 22 series) at 1.5T MRI scanner. A quantitative perfusion analysis was performed based on indicator dilution theory at dedicated software. To evaluate perfusion parameters chest radiologist generated ROIs on every slice of the calculated PBF, PBV, MTT maps. The ROIs measured 10mm in diameter and located in the peripheral regions in the upper, middle, and lower areas of the lung. The mean values of PBF, PBV, MTT, and standard deviations were calculated for upper, middle, and lower areas, compared between groups by one-way ANOVA.
**Results or Findings:** The one-way ANOVA revealed the difference between levels of mean PBF in healthy individuals and post-COVID-19 pneumonia patients in upper, middle, and lower pulmonary areas ($p < 0.05$). The differences in the mean PBV and MTT values were present in lower pulmonary areas ($p < 0.05$), but were not confirmed in upper and middle areas ($p > 0.05$).
**Conclusion:** This study shows that in patients recovered from COVID-19 infection with extensive pulmonary involvement the perfusion pattern 3 months after the disease is characterised by lower PBF values compared to the group of healthy individuals.
**Limitations:** The main limitations in our study are the unavoidable selection bias and small sample size.
**Ethics committee approval:** Approved by Ethics Committee of Saint-Petersburg State Pediatric Medical University.
**Funding for this study:** No external funding was received.
**Author Disclosures:**
Dmitry Kupriyanov: Nothing to disclose
Anna Zakharova: Nothing to disclose
Aleksandr Pozdniakov: Nothing to disclose

### RPS 204-8

**Changing of pulmonary artery diameter in accordance with severity of COVID-19 (assessment based on non-contrast computed tomography)**
A. Aliev, *N. Kudryavtsev*, A. Petraikin, Z. Artyukova, A. Shkoda, S. Morozov; Moscow/RU

**Purpose:** To reveal the correlation between pulmonary artery (PA) diameter and the severity of COVID-19 course in patients of different ages.
**Methods or Background:** This work is a retrospective cohort study performed on a group of patients (n = 511, 267 men, median age 59 years, IQR 49.0-65.0, range 31 to 84 years) treated at the COVID-19 temporary field hospital. Non-contrast CT scans of the chest organs were performed on a mobile CT scanner. Anonymised CT images were independently evaluated by two radiologists with 3 and 9 years of experience. An "empirical" visual scale was used to assess the volume of lung damage (CT0 - 0% pulmonary parenchyma involvement, CT1 - <25%, CT2 25-50%, CT3 - 50-75%, CT4 - >75%). Diameters of the main pulmonary artery (PA), aorta (Ao), their ratio (LA/Ao) were measured.
**Results or Findings:** During the study the following statistically significant regularities were obtained: pulmonary artery (PA) dilation and LA/Ao ratio were associated with increasing degree of pulmonary lesions in COVID-19; aortic dilation (Ao) was significantly associated with increasing age of patients. A significantly higher number of patients with signs of pulmonary hypertension (LA>29 mm) was shown for all age groups.
**Conclusion:** Pulmonary artery dilation and increased LA/Ao ratio are associated with increased severity of lung damage in COVID-19 in all patients age groups.
**Limitations:** This study does not answer the question whether pulmonary hypertension, marked as pulmonary artery dilation, is an initial condition or develops against the background of the course of COVID-19. In this study, there is no control group of patients with diagnosed COVID-19 without lung damage.
**Ethics committee approval:** Study was approved by the Independent Ethics Committee of the Russian Society of Radiographers and Radiologists.
**Funding for this study:** No funding was received for this work.
**Author Disclosures:**
Sergey Morozov: Nothing to disclose
Andrey Shkoda: Nothing to disclose
Zlata Artyukova: Nothing to disclose
Nikita Kudryavtsev: Nothing to disclose
Alexander Aliev: Nothing to disclose
Alexey Petraikin: Nothing to disclose

### RPS 204-9

**The effect of chest CT derived body composition and laboratory parameters on clinical outcomes in COVID-19 patients**
Ö. Özdemir, *R. E. Büyüktoka*, Z. H. Adibelli, A. M. Koc, H. Özkan Özdemir, B. Zengel, Y. Küçükzeybek; Izmir/TR
*(rasiterenbuyuktoka@hotmail.com)*

**Purpose:** We evaluated the effect of body composition and laboratory parameters on clinical outcomes in hospital presentation of COVID-19 patients. We investigated whether the fat/muscle area ratio (FMR), Skeletal-Muscle-Index (SMI), and muscle HU/splenic HU ratio could predict severe progression of COVID-19 during the follow-up period.

Wednesday

**Methods or Background:** This single-centre retrospective study was conducted in patients with possible symptoms and positive RT-PCR for SARS-CoV-2 who admitted to our hospital between March-July 2020 and underwent chest CT scan. Radiological measurements (Paraspinous Muscle Area [PMA], Muscle HU, Subcutaneous Fat Tissue Area, Spleen Density) were made by the radiologist using Horos-Software-v3.3.5 (www.horosproject.org). CT-severity index was measured by two radiologists and the clinical outcome was evaluated by applying the proposed method by Kottlors et al. The standard scale from 1 to 5 for each patient with higher numbers corresponding more severe clinical outcomes or complications.
**Results or Findings:** A total of 231 patients were included in the study, of which 121 were female and 110 were male. The median age of the patients was 47.12 (18-96). There was a significant correlation between muscle HU, muscle HU to spleen HU, paraspinous muscle area with exitus ($p < 0.05$). Mechanical ventilation was required in 20 of the patients. There was a significant correlation between FMR, muscle HU, PMA with mechanical ventilation ($p < 0.05$). A significant correlation was found between patient's age and CT-severity score with length of hospitalisation.
**Conclusion:** The prognostic importance of obesity and body composition is known as a prognostic factor for patients infected with SARS-CoV-2. As a potential surrogate for body composition and obesity, FMR represents prognostic information in COVID-19 patients. Clinical outcome and prognosis of patients may be an additional correlation factor in FMR prediction.
**Limitations:** Retrospective design of the study.
**Ethics committee approval:** This study was approved by the Bozyaka Education and Research Hospital Ethics Committee.
**Funding for this study:** Not applicable.
**Author Disclosures:**
Yüksel Küçükzeybek: Nothing to disclose
Hülya Özkan Özdemir: Nothing to disclose
Ali Murat Koc: Nothing to disclose
Raşit Eren Büyüktoka: Nothing to disclose
Baha Zengel: Nothing to disclose
Özlem Özdemir: Nothing to disclose
Zehra Hilal Adibelli: Nothing to disclose

### RPS 204-11

**Changes in chest CT body composition parameters at 3 and 6 months after severe COVID-19 pneumonia**

*F. Monelli*[1], G. Besutti[1], E. Bonelli[1], M. Ottone[1], M. Pellegrini[2], G. Ligabue[2], G. Guaraldi[2], P. Giorgi Rossi[1], P. Pattacini[1]; [1]Reggio Emilia/IT, [2]Modena/IT
*(mofilippo@hotmail.it)*

**Purpose:** The aims were to describe changes in body composition parameters in severe COVID-19 survivors on CT scans collected at diagnosis, 2/3- and 6/7-months follow-up, and to evaluate the impact of COVID-19 inflammatory burden on these changes.
**Methods or Background:** Baseline (t0), 2/3- (t1) and 6/7-months (t2) follow-up CT scan of severe COVID-19 pneumonia survivors were retrospectively reviewed to measure pectoralis muscle area (PMA) and density (PMD), liver-to-spleen ratio (LSR), and total, visceral, and intermuscular adipose tissue areas (TAT, VAT and IMAT) at T7-T8 vertebrae. COVID-19 inflammatory burden was estimated through the daily C-reactive protein (CRP) measures during hospitalisation summarised as integral of the curve. Its impact on body composition changes was evaluated in multivariable linear regression models adjusting for age, sex, and baseline TAT (index of general adiposity).
**Results or Findings:** At follow-up a decrease in mean PMA and in all mean body fat areas was registered, faster from t0 to t1, and slower from t1 to t2, with the exception of PMD, which increased (i.e. intramuscular fat decreased) only from t1 to t2. Mean VAT decrease was more conspicuous than mean TAT decrease. In adjusted models, increasing CRP integral was significantly associated with larger PMA reduction and smaller PMD increase, larger LSR increase (i.e. stronger steatosis decrease), and larger VAT decrease, but not with TAT decrease. These associations were stronger in patients with baseline higher VAT and lower LSR.
**Conclusion:** Muscle and fat loss in sever COVID-19 survivors is faster in the first months, but slowly continues till 6-7 months. Fat loss is more apparent in visceral compartments. Inflammatory burden is associated with the degree of muscle and visceral/liver fat loss.
**Limitations:** Selection of severe cases, body composition assessed at thoracic level.
**Ethics committee approval:** This study was approved by the AVEN Ethics Committee (855/2020/OSS/AUSLRE).
**Funding for this study:** No funding was received for this study.
**Author Disclosures:**
Pierpaolo Pattacini: Nothing to disclose
Massimo Pellegrini: Nothing to disclose
Filippo Monelli: Nothing to disclose
Guido Ligabue: Nothing to disclose
Efrem Bonelli: Nothing to disclose
Paolo Giorgi Rossi: Nothing to disclose
Giulia Besutti: Nothing to disclose
Marta Ottone: Nothing to disclose
Giovanni Guaraldi: Nothing to disclose

### RPS 204-12

**CT-derived pulmonary enhancement in short-term follow-up of COVID-19 patients: a case-control study**

*M. Mashar*, E. Allan, S. Patel, M. Taylor, A. Ahmed, A. Procter, J. Jacob, M. Duncan, A. Nair; London/UK
*(meghavi@hotmail.co.uk)*

**Purpose:** To assess if relative lung enhancement on subtraction CT pulmonary angiography (sCTPA) is more heterogeneous in COVID-19 patients versus non-COVID-19 patients, as a potential biomarker of microvascular alterations.
**Methods or Background:** Consecutive COVID-19 patients undergoing sCTPA due to persistent dyspnoea were compared with contemporaneous non-COVID-19 patients (patients with acute/chronic thromboembolism, malignancy, any parenchymal abnormalities were excluded). Lung enhancement (Lung_Enh) was measured on the subtraction map dataset of the sCTPA, using 30 regions of interest at five levels. Main (mPA) and lobar (lobPA) pulmonary arterial enhancement were also measured. We calculated the mPA:Lung_Enh, and the lobPA:Lung_Enh (markers of pulmonary vascular resistance) and compared their average values and variance, expressed as median (interquartile range, IQR).
**Results or Findings:** 80 patients (age 52 ± 17 years, 57 [71%] female) were analysed. Between the COVID-19 ($n = 29$) and non-COVID-19 ($n = 51$) cohorts, there was no statistically significant difference in the average mPA:Lung_Enh (9.7 [4.8, 18.0] HU versus 11.7 [8.2, 22.8] HU, $p = 0.115$), or average lobPA:Lung_Enh (8.0 [4.4, 17.8] HU versus 10.3 [6.5, 20.8] HU, $p = 0.185$). Variance of mPA:Lung_Enh (median 23.4 [4.9, 3747.9] HU and 183.6 [13.7, 2717.8] HU respectively, $p = 0.506$) and lobPA:Lung_Enh (median 22.2 [4.3, 3179.6] HU and 195.6 [9.4, 2630.5] HU respectively, $p = 0.519$) was wide for both cohorts, but not statistically different.
**Conclusion:** Manually-measured lung enhancement heterogeneity on subtraction CTPA is not sufficiently different between symptomatic recovering COVID-19 patients and non-COVID-19 patients, indicating that there are either no persistent perfusion alterations, or that this technique is not sufficiently sensitive.
**Limitations:** Manual measurements, only five levels assessed, corroboration with other imaging unavailable as performed during the first wave of COVID-19 in the UK.
**Ethics committee approval:** This study was approved by IRAS Project ID 282063.
**Funding for this study:** No funding was received for this study.
**Author Disclosures:**
Arjun Nair: Nothing to disclose
Asia Ahmed: Nothing to disclose
Magali Taylor: Nothing to disclose
Joseph Jacob: Nothing to disclose
Shivani Patel: Nothing to disclose
Meghavi Mashar: Nothing to disclose
Alex Procter: Nothing to disclose
Mark Duncan: Nothing to disclose
Emma Allan: Nothing to disclose

**09:30-11:00** **Room X**

### Research Presentation Session: Hybrid, Molecular and Translational Imaging

### RPS 206

### PET/MRI, MRS and radiomics

**Moderator**
P. Brader; Graz/AT

### RPS 206-2

**The more the better? Multiparametric simultaneous 18F-FDG PET/MRI vs DCE-MRI for breast cancer diagnosis**

*V. Romeo*[1], P. Clauser[2], P. Kapetas[2], P. A. Baltzer[2], S. Rasul[2], Z. Bago-Horvath[2], K. Pinker-Domenig[3], T. H. Helbich[2]; [1]Naples/IT, [2]Vienna/AT, [3]New York, NY/US
*(valeria.romeo@unina.it)*

**Purpose:** To assess whether simultaneous 18F-FDG PET/MRI may improve the accuracy of DCE-MRI for breast cancer (BC) diagnosis.
**Methods or Background:** 133 patients with 169 breast lesions (35 benign, 134 malignant) were prospectively enrolled and underwent simultaneous breast 18F-FDG PET/MRI. The accuracy of six BC diagnostic methods was explored, the first based on individual PET/MRI images: (1) DCE-MRI trough BI-RADS assessment; (2) quantitative DWI using an ADC cut-off value of 1.25 $\times 10^{-3}$ mm2/s; (3) qualitative PET assessment of lesion uptake. Combinations of 2 and 3 parameters were also explored as follows: (4) DCE-MRI + DWI evaluation and (5) DCE-MRI + PET, where DWI and PET findings were used to up/downgrade BI-RADS categories; (6) DCE-MRI + DWI+ PET, considered as positive if at least two resulted suggestive for malignancy. AUC, sensitivity, and specificity of each method were calculated.
**Results or Findings:** DCE-MRI alone showed AUC, sensitivity and specificity for BC diagnosis of 0.886, 100% and 77.1%, respectively. The combination of DCE-MRI + DWI used to up/downgrade BI-RADS categories resulted as the most accurate with AUC, sensitivity, specificity of 0.953, 99.3%, 91.4%, respectively. Methods including PET evaluation were all affected by a low specificity, the lowest using PET images alone (65.7%), and the highest when PET images were used to up/downgrade BI-RADS 4 categories (85.7%).
**Conclusion:** The combination of DCE-MRI + DWI is the most accurate for BC diagnosis, while the addition of 18F-FDG PET is of limited value. Further research with advanced targeted tracers is necessary to elucidate the full potentials of PET/MRI of the breast.
**Limitations:** Single-institution; limited sample size.
**Ethics committee approval:** This study is IRB-approved.
**Funding for this study:** Fund was received from: H2020 Research and Innovation Framework Programme.
**Author Disclosures:**
Pascal A.T. Baltzer: Speaker: Siemens Healthineers
Katja Pinker-Domenig: Speaker: Siemens Healthineers
Valeria Romeo: Grant Recipient: BRACCO Fellowship
Thomas H. Helbich: Speaker: Guerbet Speaker: Novomed Speaker: siemens Healthineers
Sazan Rasul: Nothing to disclose
Panagiotis Kapetas: Nothing to disclose
Zsuzsanna Bago-Horvath: Nothing to disclose
Paola Clauser: Speaker: Siemens Healthineers

### RPS 206-3

**Multiparametric dual timepoint 18F-FDG PET/MRI imaging for lymph node staging in Figo I/II cervical carcinoma**

*M. Weissinger*, J. Jacoby, S. Kommoss, F. F. Seith, S. Gatidis, S. Brucker, K. Nikolaou, C. la Fougère, H. Ditttmann; Tübingen/DE

**Purpose:** Previous studies have demonstrated limited sensitivity of 18F-FDG-PET/MRI for preoperative detection of lymph node metastases (LNM) in early stage cervical carcinoma. Therefore, the aim of this prospective study was to optimise LNM detection by multiparametric evaluation of PET/MRI data.
**Methods or Background:** 63 consecutive patients underwent whole-body dual-timepoint FDG-PET/MRI ≈60+90min p.i. (Biograph mMR®, Siemens Healthineers). Results were validated by surgical staging or SLN-guided biopsy. Multiparametric analysis was performed using a logistic regression-based malignity score (MS).
**Results or Findings:** In total, 245 LN were evaluated prospectively as consensus of nuclear medicine and radiology experts, quantified multiparametric and correlated to histology one by one. Prevalence of LNM was 17.1% (G1:0/19, G2:18/106, G3:24/120). The parameters “SUVavg, diameter, ADC, histologic grade, dual-timepoint SUV kinetic” showed a synergy and were implemented in the MS. Grading of the primary tumour (G2/G3) revealed a significant impact on visual sensitivity (sens: 8.3%/31%) as well as on the uniparametric (AUC SUVavg: 0.673/0.901) and multiparametric (AUC: 0.769/0.877) detection rate. MS lowered combined false positive and false negative rate (FPR+FNR) from 65.5% to 44.5% in G2 tumours and reduced confidence intervals significantly. Compared to the expert consensus, application of MS tremendously increased the overall sensitivity from 31.0% to 79.3% (Youden optimum) with an acceptable decrease in specificity from 98.3% to 75.6%.
**Conclusion:** Histologic grade of cervical carcinoma has a crucial impact on LNM detection rate in FDG-PET/MRI, which shows high accuracy in G3 tumours. Multiparametric evaluation of PET/MRI seems to be necessary to reduce both false-positive and false-negative findings in G2 tumours. Thus, a solely visual evaluation of FDG-PET/MRI in Figo I/II G2 cervical carcinoma fails to use its full potential.
**Limitations:** Medium sample size.
**Ethics committee approval:** Approved by the institutional review board (registry No.173/2015BO01).
**Funding for this study:** Funding was received from the Deutsche Forschungsgemeinschaft.
**Author Disclosures:**
Konstantin Nikolaou: Nothing to disclose
Stefan Kommoss: Nothing to disclose
Ferdinand Frederic Seith: Nothing to disclose
Sergios Gatidis: Nothing to disclose
Helmut Ditttmann: Nothing to disclose
Christian la Fougère: Nothing to disclose
Matthias Weissinger: Nothing to disclose
Johann Jacoby: Nothing to disclose
Sara Brucker: Nothing to disclose

### RPS 206-4

**Improving the diagnostic performance of 2HG MR spectroscopy utilising creatine as an internal reference**

*D. Juskanic*[1], J. Polakova Mistinova[2], S. Hollý[1], M. Chmelik[3], Z. Berecova[2], T. Hrubá[4], L. Patrovic[1]; [1]Nitra/SK, [2]Bratislava/SK, [3]Presov/SK, [4]Banska Bystrica/SK

**Purpose:** Water peak is used in MR spectroscopy (MRS) for scaling and directly impacts metabolites' concentration estimates. Variable water proportion in different glioma grades is non-negligible and may introduce a systematic bias. In low-concentration metabolites, including 2-hydroxyglutarate (2HG), the impact could be more protuberant. Thus, referencing a 2HG concentration estimate with a more stable internal metabolite appears plausible to partially overcome this issue. Our study compared 2HG concentration estimates and 2HG/Cr ratios regarding their diagnostic performance in the same patient cohort.
**Methods or Background:** Eighteen patients with suspect glioma underwent 3T MR 2HG spectroscopy with Mescher-Garwood point-resolved spectroscopy (MEGA-PRESS) sequence. Spectral postprocessing and evaluation were performed with jMRUI and LCModel. Creatine concentration was estimated from an off-spectrum. 2HG concentration estimates and 2HG/Cr ratios were compared using diagnostic performance parameters and areas under the ROC curves. Immunohistochemistry and genomic analysis were used as a ground truth.
**Results or Findings:** 2HG concentration estimates with 1mM threshold for test positivity resulted in sensitivity 75% (95% CI 0.34-0.97), specificity 90% (95% CI 0.55-0.99). Overall diagnostic accuracy was 83.33% (95% CI 0.58-0.96). The 2HG/Cr ratio with the cutoff value 0.085 improved sensitivity and overall diagnostic accuracy (87.5%, 95% CI 0.47-1.00 and 88.89%, 95% CI 0.65-0.98], respectively).
**Conclusion:** Using creatine as a stable internal reference in central glioma MRS has superior diagnostic performance than water-referenced 2HG concentration estimates.
**Limitations:** Single centre study with a small patient cohort, focusing on presurgical diagnostic workup.
**Ethics committee approval:** This study was approved by the Ethical Committee of Faculty Hospital in Nitra.
**Funding for this study:** Not applicable.
**Author Disclosures:**
Dominik Juskanic: Nothing to disclose
Terézia Hrubá: Nothing to disclose
Lukas Patrovic: Nothing to disclose
Jana Polakova Mistinova: Nothing to disclose
Marek Chmelik: Nothing to disclose
Zuzana Berecova: Nothing to disclose
Samuel Hollý: Nothing to disclose

### RPS 206-6

**Prognostic significance of 68 gallium fibroblast activation protein inhibitor versus 18 fluorodeoxyglucose positron emission tomography/computed tomography in gastric neoplasm subtypes**

*N. G. A. Niyarah*, S. S. S. Shivalingappa, K. Kallur, G. R. Prashanth, M. Ashok Kumar, A. A. R. Kesari, I. Desai, S. Sampangi; Bangalore/IN
*(niyarahalemao99@ymail.com)*

**Purpose:** (1) To evaluate the performance between the two tracers by identifying the SUV max associated with stomach cancer subtypes on each scan. (2) To demonstrate the prognostic significance of FAPI and FDG-PET in stomach cancer subtypes by calculating total lesion glycolysis (TLG), metabolic tumour volume (MTV) and maximum standardised uptake value (SUV max).
**Methods or Background:** A retrospective study with a convenient sample of 30 patients at HCG Cancer Hospital who had undergone Ga 68 FAPI and 18 F FDG PET study and further underwent a histopathological analysis to determine the subtype of gastric neoplasm. These included carcinomas, lymphomas, gastrointestinal stromal tumours, neuroendocrine tumours and metastasis. The SUV max, TLG and MTV was obtained on both the scans. Tumour size, lymph nodal and distant metastasis for each histological subtype and location of primary tumour was noted. Independent t test was used for statistical analysis.
**Results or Findings:** A sample size of 30 was selected for data collection. Tumour SUVmax, MTV and TLG was more for the adenocarcinoma group on FAPI PET scan in comparison to FDG PET scan ($p < 0.005$). On FDG PET scan, lymphoma had higher SUV max. Neuroendocrine tumours showed less SUV max on both scans.Also, higher SUV values on both scans represented poorer prognosis with higher T stage of the primary tumour, lymph nodal and solid organ metastasis.
**Conclusion:** In the present study, it was found that baseline high SUVmax was associated with significantly worse prognosis of patients. Adenocarcinomas, lymphomas and neuroendocrine tumours showed significant differences in SUVmax, MTV and TLG parameters on each scan.
**Limitations:** (1) Case selection bias. (2) Other PET parameters were not considered.
**Ethics committee approval:** Not applicable.
**Funding for this study:** Not applicable.
**Author Disclosures:**
Kumar Kallur: Nothing to disclose
G R Prashanth: Nothing to disclose
Sudhakar Sampangi: Nothing to disclose
Avinash Arjun Rao Kesari: Nothing to disclose
Shivakumar Swamy S Shivalingappa: Nothing to disclose
Indresh Desai: Nothing to disclose
Nadezhda Gloria Alemao Niyarah: Nothing to disclose
Mahesh Ashok Kumar: Nothing to disclose

### RPS 206-7

**Assessing radio-chemotherapy response in glioblastoma tumour models using 2H-labeled fumarate and deuterium magnetic resonance spectroscopic imaging**

*F. Hesse*, A. Wright, V. Somai, F. Bulat, F. Kreis, K. Brindle; Cambridge/UK
*(friederike.hesse@cruk.cam.ac.uk)*

**Purpose:** To investigate whether deuterium metabolic imaging (DMI), a technique that has recently been demonstrated in the clinic, can be used to assess early brain tumour treatment response using a new imaging biomarker of necrotic cell death, [2,3-2H2] fumarate.
**Methods or Background:** Fumarate is hydrated in a reaction catalysed by the enzyme fumarase to produce malate. Loss of plasma membrane integrity during cell necrosis results in fumarate rapidly gaining access to the enzyme and an increased rate of malate production. Previously this has been assessed in vivo by using 13C-MRSI.
**Results or Findings:** Surface-coil localised 2H MR spectroscopy and spectroscopic imaging were used to assess conversion of [2,3-2H2] fumarate to [2,3-2H2] malate in orthotopically implanted tumours following an injection of labelled fumarate (1g/kg) into tumour-bearing mice. Within 1 week of chemo-radiation (20 Gy in total with 4 Gy/fraction, temozolomide (100 mg/kg) the malate/fumarate ratio increased, from $0.051 \pm 0.03$ to $0.22 \pm 0.03$ ($p = 0.04$, $n = 3$), a ~4.3x increase, in patient-derived tumours (A11) and from $0.071 \pm 0.017$ to $0.42 \pm 0.05$ ($p = 0.006$, $n = 3$), a ~6x increase in a cell line model (U87).
**Conclusion:** Tumour malate production increased significantly within seven days of targeted radio-chemotherapy, demonstrating the potential of 2H-labeled fumarate for assessing GB tumour cell death and the early response to treatment.
**Limitations:** The main limitation is the narrow frequency range of 2H MR, which requires the use of relatively high magnetic field strengths, at least 7T.
**Ethics committee approval:** All animal experiments were carried out in compliance with project and personal licenses issued by the Home Office, UK and approved by the Cancer Research UK, Cambridge Institute Animal Welfare and Ethical Review Body.
**Funding for this study:** Funding was received from Cancer Research UK (C197/ A17242, C197/A16465, C9685/A25177). FH is in receipt of a Cambridge European Scholarship from the Cambridge Trust.
**Author Disclosures:**
Kevin Brindle: Nothing to disclose
Alan Wright: Nothing to disclose
Friederike Hesse: Nothing to disclose
Flaviu Bulat: Nothing to disclose
Vencel Somai: Nothing to disclose
Felix Kreis: Nothing to disclose

### RPS 206-9

**ComBat harmonisation of multi-centric 18F-FDG-PET/CT and PET/MRI radiomic data: impact on tissue classification**

*D. Leithner*[1], H. Schoder[1], A. Haug[2], H. A. Vargas[1], P. Gibbs[1], I. Häggström[1], I. Rausch[2], M. Weber[2], M. Mayerhoefer[1]; [1]New York, NY/US, [2]Vienna/AT

**Purpose:** To determine whether ComBat harmonisation improves radiomics-based tissue discrimination in pooled 18F-FDG-PET data from PET/CT and PET/MR scanners and to determine the effects of ComBat on different radiomic feature classes.
**Methods or Background:** 18F-FDG-PET data of 200 patients who had undergone PET/CT (two scanners/vendors; 50 patients each) or PET/MR (2 scanners/vendors; 50 patients each) for clinical purposes were retrospectively included. Fixed-size 2.5-cm³ spherical volumes of interest were placed in the disease-free liver, spleen, and bone marrow, and radiomic features of the following classes were calculated: grey-level histogram (GLH), co-occurrence matrix (GLCM), run-length matrix (GLRLM), size-zone matrix (GLSZM), and neighbourhood grey-tone difference matrix (NGTDM). For each feature class independently, and for a multi-class radiomic signature, tissue classification was performed on both ComBat harmonised and unharmonised pooled data, using a multi-layer perceptron neural network.
**Results or Findings:** ComBat-harmonised PET radiomic features were superior to unharmonised features in terms of separation of liver, spleen and bone marrow. Median accuracies in training/validation sets were: GLH, 71.0/64.4% (harmonised) vs. 64.3/57.8% (unharmonised); GLCM, 83.3/72.8% vs. 58.1/54.4%; GLRLM, 83.1/76.1% vs. 60.7/58.3%; GLSZM, 79.8/75.6% vs. 54.8/55.0%; NGTDM, 66.0/66.1% vs. 52.6/49.4%, and radiomic signature, 83.3/78.3% vs. 68.3/61.7%.
**Conclusion:** ComBat harmonisation may improve 18F-FDG-PET radiomics-based tissue classification for all radiomic feature classes, even when PET data from PET/CT and PET/MR data are pooled. ComBat harmonisation may be useful for multi-centric clinical 18F-FDG-PET radiomics studies that utilise different PET/CT and PET/MR devices.
**Limitations:** Not applicable.
**Ethics committee approval:** This HIPAA-compliant study was approved by the local Institutional Review Boards; informed consent was waived.
**Funding for this study:** This research was funded in part through the NIH/NCI Cancer Center Support Grant P30 CA008748.
**Author Disclosures:**
Heiko Schoder: Consultant: Aileron Therapeutics
Alexander Haug: Nothing to disclose
Marius Mayerhoefer: Speaker: Siemens Speaker: GE Speaker: Bristol-Myers Squibb
Ivo Rausch: Nothing to disclose
H. Alberto Vargas: Nothing to disclose
Ida Häggström: Nothing to disclose
Doris Leithner: Nothing to disclose
Peter Gibbs: Nothing to disclose
Michael Weber: Nothing to disclose

### RPS 206-10

**Fully automated staging via PET-CT of primary Hodgkin Lymphoma using a deep learning approach**

*K. Ekert*[1], A. J. Beer[1], K. Nikolaou[2], S. Gatidis[2]; [1]Ulm/DE, [2]Tübingen/DE

**Purpose:** To apply and evaluate a deep learning framework for automated analysis of FDG positive lymphoma lesions in their corresponding region to result in a fully automated Ann Arbor staging.
**Methods or Background:** 100 primary Hodgkin patients (mean age, 40.16 ±16.91 years; range, 11.0 to 85.5 years; 51 females and 49 males) between 02/2013-02/2020 were retrospectively evaluated. Only patients with histologically newly diagnosed Hodgkin lymphoma prior to therapy and complete whole-body CT as well as corrected PET scan were included. Whole body correct PET-scan in addition to contrasting enhanced whole-body CT were supplied to the algorithm resulting in automatic segmentation of FDG positive lesions in their corresponding body compartment and classified as physiological or pathological. Automatically, a primary Ann Arbor stage was classified. A secondary Ann Arbor stage was calculated with obvious misclassified extra nodal involvement removed (i.e. fat and muscle).

**Results or Findings:** Automatically calculated Ann Arbor stage algorithm without any modification resulted in 35 correctly classified stages, 31 with one degree deviation, 28 with two degrees of deviation and 6 with four degrees of deviation. Sensitivity, PPV was 59.3/40.7%. Secondary calculated Ann Arbor stage, obvious misclassified extranodal involvement removed, resulted 36 correctly classified stages, 35 with one degree deviation, 25 with two degrees of deviation and 4 with four degrees of deviation. Sensitivity, PPV was 52.7/53.3%.
**Conclusion:** Automated staging of primary Hodgkin lymphoma using a deep learning approach is feasible, however more fine tuning is needed to result in a fully automated Ann Arbor staging.
**Limitations:** Only retrospective data was analysed. The deep learning prototype is still under development.
**Ethics committee approval:** The ethics committee approved this study.
**Funding for this study:** Not applicable.
**Author Disclosures:**
Konstantin Nikolaou: Nothing to disclose
Sergios Gatidis: Nothing to disclose
Ambros J. Beer: Nothing to disclose
Kaspar Ekert: Nothing to disclose

### RPS 206-11

**Radiomic features as biomarkers of soft tissue paediatric sarcomas: results of a PET/MR study**
*G. Fichera*, C. Giraudo, R. Stramare, G. Bisogno, R. Motta, L. Evangelista, D. Cecchin, P. Zucchetta; Padua/IT

**Purpose:** To assess the prognostic value of radiomic features extracted from T2w MR images of the primary tumour of paediatric patients affected by soft tissue sarcomas (pSTS) and to evaluate if such features are biomarkers of tumour grade and histotype.
**Methods or Background:** pSTS who underwent PET/MR for staging in our tertiary centre were retrospectively examined. One radiologist with four years of experience in oncological imaging segmented each primary tumour and extracted 33 radiomic features using an open source software (3D Slicer, www.slicer.org). Factor analysis was applied to select highly correlating features; their prognostic value was investigated by logistic regression analysis. The Student's t-test was used to evaluate if differences in radiomic, metabolic, and laboratory variables occurred according to STS grade (subdivided as low and high grade) and histotype (rhabdomyosarcomas vs non-rhabdomyosarcomas); if a significant difference occurred then the diagnostic value of the variable was computed. The applied level of significance was 0.05 for all analyses.
**Results or Findings:** Eighteen patients (11 female; mean age 7.8±4.6 years old; 12 with rhabdomyosarcoma; nine with high grade STS; four deceased) were examined. The following features were selected using factor analysis: minimum, lmc1, cluster shade, long run length emphasis, and variance. None of the radiomic features predicted the outcome ($p > 0.05$, each). Lmc1 was significantly higher in low grade STS (-0.17±0.05 vs -0.13±0.02, $p = 0.045$) and showed 70.4% accuracy in classifying high grade STS (the value -0.14 showed 77.8% Se and 66.7% Sp). Variance was significantly lower in rhabdomyosarcomas (23588±23325 vs 68144±39009, $p = 0.008$) and showed 83.3% accuracy in classifying rhabdomyosarcomas (the value 52314 showed 83% Se and 99% Sp).
**Conclusion:** The radiomic features lmc1 and variance are biomarkers of STS grade and histotype, respectively.
**Limitations:** Not applicable.
**Ethics committee approval:** This study was approved by an ethics committee.
**Funding for this study:** No funding was received for this work.
**Author Disclosures:**
Giulia Fichera: Nothing to disclose
Diego Cecchin: Nothing to disclose
Laura Evangelista: Nothing to disclose
Pietro Zucchetta: Nothing to disclose
Raffaella Motta: Nothing to disclose
Roberto Stramare: Nothing to disclose
Chiara Giraudo: Nothing to disclose
Gianni Bisogno: Nothing to disclose

### RPS 206-12

**The value of 18F-FDG PET/MRI in prediction of microvascular invasion in hepatocellular carcinoma**
A. Görmez[1], *F. Celebi*[1], N. C. Balci[2]; [1]Istanbul/TR, [2]Abu Dhabi/AE

**Purpose:** Investigating the association between standardised uptake value (SUV) and peritumoral/ intratumoral apparent diffusion coefficient (ADC) values and whether these parameters are useful in predicting the preoperative microvascular invasion (MVI) of hepatocellular carcinoma (HCC).
**Methods or Background:** Forty-four patients involving 8 women and 36 men (mean age, 60 ± 10.01 years; range, 21-76 years) with single HCCs (≥2 cm) who underwent preoperative PET/MRI were retrospectively evaluated. The peritumoral and intratumoral ADC values were evaluated on diffusion-weighted images, and the intratumoral SUV values were measured on fusion PET/MRI images. Both the peritumoral and whole-tumour ADC values were measured using ImageJ, an opensource software supported by the National Institutes of Health. Univariate and multivariate logistic regression analyses were performed to determine the most effective factor predicting MVI.
**Results or Findings:** Univariate analysis showed that the histologic grade, tumour size, maximum peritumoral ADC (PTband ADCmax), mean peritumoral ADC (PTband ADCmean), mean intratumoral ADC (IT ADCmean), and maximum SUV (SUVmax) correlated with MVI ($p < 0.05$). On multivariate analysis, the SUVmax was the only independent risk factor for the MVI of HCC. Combining the IT ADCmean and SUVmax further improved differentiation between MVI-positive and MVI-negative HCCs, and the area under the curve was 0.935 (95% confidence interval (CI) = 0.864-1.00), yielding a sensitivity of 86% and specificity of 81%. The ICC values were 0.95-0.99, a nearly perfect level of agreement.
**Conclusion:** 18F-FDG PET/MRI is a useful noninvasive imaging tool for the prediction of the MVI of HCC.
**Limitations:** This a retrospective single-centre study; it is necessary to validate the results from other centres.
**Ethics committee approval:** This study was approved by an ethics committee.
**Funding for this study:** This research did not receive any specific grant from funding agencies in the public, commercial, or not-for-profit sectors.
**Author Disclosures:**
Filiz Celebi: Nothing to disclose
Ayşegül Görmez: Nothing to disclose
Numan Cem Balci: Nothing to disclose

**09:30-11:00** **Room Z**

### Research Presentation Session: Emergency Imaging

### RPS 217

### Emergency patients management and COVID-19 complications: open questions

**Moderator**
J. B. Dormagen; Oslo/NO

### RPS 217-3

**Who gets what imaged: exploring patient age and number of body regions imaged by CT in emergency room patients**
*J. Vosshenrich*, H-C. Breit, D. Boll, E. M. Merkle, T. Heye; Basel/CH
*(jan.vosshenrich@usb.ch)*

**Purpose:** To investigate the relationship between patient age and body regions imaged with CT in emergency room patients.
**Methods or Background:** 27,962 CT examinations ordered from 01/2019-02/2021 by the emergency department in our tertiary care hospital for patients aged 20-99 years were retrospectively analysed. Exams were divided into three groups based on the number of body regions imaged: 1-region CTs (e.g. CT head); 2-region CTs (e.g. CT chest-abdomen/pelvis); and 3-region CTs (e.g. CT neck-chest-abdomen/pelvis). Data was separately aggregated both per year of patient age, and in 5-year or 10-year age groups (e.g. 50-59 years) and visualised using scatter plots and trending line graphs. Relationships between patient age and average number of body regions imaged were analysed using linear correlation and Welch-ANOVA.
**Results or Findings:** There was a clear trend towards more body regions being imaged with increasing patient age (r=0.96 [95% CI: 0.94, 0.98]; p<.001). The average number of body regions imaged with CT increased from 1.2 (age: 20 years) to 1.6 (age: 99 years). Similar results were observed with patients aggregated in 5-year (r=0.99 [95% CI: 0.96,1.00]; p<.001) or 10-year age groups (r=0.99 [95% CI: 0.93,1.00]; p<.001). Welch-ANOVA revealed significant differences between most age groups. Except with directly adjacent age groups (e.g. 30-39 years vs. 40-49 years [mean: 1.25 vs. 1.29; p=.17]), the average number of body regions imaged was different to all other age groups, both when aggregated in 5-year and 10-year age groups (all p<.01).
**Conclusion:** There is a positive linear correlation between patient age and number of body regions imaged with CT in emergency room patients. Further investigation of the causes (e.g. more critically ill patients vs. emergency department workflow optimisation), and potential implications for radiologists' workload and radiation exposure is needed.

**Limitations:** Not applicable.
**Ethics committee approval:** Not applicable.
**Funding for this study:** Not applicable.
**Author Disclosures:**
Tobias Heye: Nothing to disclose
Hanns-Christian Breit: Nothing to disclose
Daniel Boll: Nothing to disclose
Elmar M. Merkle: Consultant: Siemens Healthineers
Jan Vosshenrich: Nothing to disclose

### RPS 217-4

**Whole-body CT scan in polytrauma patients: estimation of radiation dose and cancer risk**

*M. M. Abuzaid*, W. Elshami, S. H. Hamid; Sharjah/AE
*(mabdelfatah@sharjah.ac.ae)*

**Purpose:** This study aims to estimate effective and organ doses and cancer risk associated with whole-body computed tomography (CT) scanning for polytrauma patients.
**Methods or Background:** CT investigations were obtained from a 64-multidetector unit. CT doses were measured in CT dose index (CTDI) and dose length product (DLP). Effective doses were calculated based on figures from publication 103 of the International Commission on Radiological Protection. According to the National Academies' Biological Effects of Ionizing Radiation VII Report, the lifetime attributable risk (LAR) of cancer incidence was estimated and organ dose estimated using NCICT dosimetry software.
**Results or Findings:** Among 76 eligible patients, 84.2% were male and 15.7% were female (mean age=35±12.5 years; height=177±8.5 cm; weight 81.5±12.7 kg). All patients had multiple traumas, with 88% due to road traffic accidents and the rest due to a fall from height. The proposed dose for CTDIvol (mGy) and DLP (mGy cm) is 62 and 2873.4, respectively. The mean effective dose was 31.4 mSv, and the organ doses ranged from 10.2-63.7 mGy.
**Conclusion:** Although WBCT results in fast and accurate diagnosis, it led to the increment of radiation dose >20 mSv, increasing cancer risk regardless of patient age, injury severity and evaluation result. Radiation dose justification and optimisation can positively influence dose reduction. Staff engagement in training and education will improve the practice and increase the awareness of radiation protection.
**Limitations:** The number of patients was an identified limitation.
**Ethics committee approval:** This study was approved by the research ethics committee at the University of Sharjah.
**Funding for this study:** No funding was received for this study.
**Author Disclosures:**
Wiam Elshami: Nothing to disclose
Simaa Hamid Hamid: Nothing to disclose
Mohamed M. Abuzaid: Nothing to disclose

### RPS 217-6

**CT imaging post resuscitation: do we really need CT pulmonary angiography after return of spontaneous circulation?**

*K. Mueller-Peltzer*, F. Bamberg, D. Staudacher; Freiburg/DE
*(katharina.mueller-peltzer@uniklinik-freiburg.de)*

**Purpose:** To evaluate if contrast enhanced computed tomography (CT) following resuscitation needs to include CT pulmonary angiography (CTPA) in patients after return of spontaneous circulation (ROSC).
**Methods or Background:** Over a period of 12 months all consecutive patients treated in the cardiac arrest centre of a maximum care hospital after resuscitation with ROSC were retrospectively included in this study. CT imaging was retrospectively analysed by a board-certified radiologist specialised in emergency imaging. Clinical data was retrospectively retrieved from the clinical information system.
**Results or Findings:** 78 patients (74.4% men, 65.02±15.23 years) with contrast enhanced CT imaging of the thorax performed the day of resuscitation were included in this study. Thoracic CT imaging was performed as dual-rule-out CT angiography (CTA) (42.31%), CTA of the aorta (17.95%), CTPA (16.67%), biphasic arterial and venous phase CT (7.69%) or venous phase CT (15.38%). Pulmonary embolism (PE) was detected in 5 patients (6.4%), level of vessel involvement was categorised as central in 2, lobar in 1 and segmental in 2 patients. PE was detected in dual-rule-out CTA in 3 cases, in CTPA and venous phase CT in 1 case, respectively. PE exclusion was limited to central and lobar pulmonary arteries in only 4 (5.13%) and 11 cases (14.10%), respectively. Segmental and subsegmental pulmonary arteries were evaluable in the vast majority of cases (80.77%).
**Conclusion:** PE is a serious but rare resuscitation cause in patients with ROSC. PE exclusion seems not to be limited to CTPA but to be feasible up to segmental level in the majority of cases with contrast enhanced thoracic imaging after resuscitation.
**Limitations:** The results from this single centre study need to be confirmed in a larger, preferably multi centre, study cohort.
**Ethics committee approval:** This study was approved by the local ethics committee.
**Funding for this study:** No funding was received for this study.
**Author Disclosures:**
Fabian Bamberg: Nothing to disclose
Katharina Mueller-Peltzer: Nothing to disclose
Dawid Staudacher: Nothing to disclose

### RPS 217-7

**Spontaneous muscle haematomas in patients hospitalised for COVID-19 infection in the largest Serbian COVID hospital**

*K. Mijović*, D. Vasin, S. Milenkovic, D. Mašulović; Belgrade/RS
*(mijovicksenija@gmail.com)*

**Purpose:** The aim of this study is to draw attention to the frequency of spontaneous muscle haematomas in patients who are hospitalised for COVID-19 viral infection and to describe their radiological characteristics. These patients are at an increased risk due to protocolary anticoagulant medication administration, given the well-established risk for developing a hypercoagulable state and subsequent thrombotic and thromboembolic complications in COVID-19.
**Methods or Background:** We searched the reports of abdominal and pelvic CT examinations in Covid hospital "Batajnica" with the keyword "haematoma" for a period of 9 months (December 11, 2020 - September 28, 2021), additionally evaluating the characteristics of the haematomas: contrast agent extravasation, localisation and propagation into extramuscular adipose tissue, and the largest diameter of the extramuscular component. Additionally, we searched for eventual surgical treatment data.
**Results or Findings:** Out of a total 394 CT examinations of the abdomen and pelvis in 346 patients aged 23-98, 66 (19%), with an average age of 75, were diagnosed with 78 spontaneous muscular haematomas of different regions, with 11 patients (17%) having haematomas in more than one region. The most common locations were abdominis rectus and iliopsoas muscles. CT signs of active contrast agent extravasation were seen in 34 patients (51%), 13 of which required surgical treatment (19% of patients).
**Conclusion:** Spontaneous muscle haematomas are a common complication in patients hospitalised for COVID-19 viral infection, with at least a fifth of the cases requiring surgical treatment. Hereby we underline the necessity for careful surveillance of possible active haemorrhage in these patients, especially in certain muscle compartments. CT examination can reliably determine the distribution and propagation of haematomas and detect active bleeding.
**Limitations:** Not applicable.
**Ethics committee approval:** The study was approved by the institutional ethic committee.
**Funding for this study:** Not applicable.
**Author Disclosures:**
Sara Milenkovic: Nothing to disclose
Ksenija Mijović: Nothing to disclose
Dragan Vasin: Nothing to disclose
Dragan Mašulović: Nothing to disclose

### RPS 217-8

**COVID-19 gastrointestinal complication: imaging and pathological correlation.**

R. Tintori, *I. Ambrosini*, C. D'Amelio, M. Tonerini, E. Neri; Pisa/IT

**Purpose:** To relate CT findings of intestinal complication in SARS-Cov 2 infected patients to pathological findings.
**Methods or Background:** SARS-Cov 2 infection mainly affects the lungs but many extrapulmonary manifestations have been commonly reported, which may present alone or with respiratory symptoms. Gastrointestinal tract (GI) is the most common extrapulmonary site of involvement; coronavirus has proven to directly infect enterocytes of both the small and large intestine due to their ACE-2 receptor surface expression. GI involvement in SARS-Cov 2 infection has a broad spectrum of clinical severity, it is associated with higher serum lactate levels, higher risk of invasive ventilation and a worse prognosis.
**Results or Findings:** We retrospectively selected six SARS-Cov 2 infected patients who presented to our emergency department with acute abdominal symptoms. In all cases abdominal CT was performed and findings consistent with ischaemic bowel disease where reported and confirmed at surgical/pathological examination. The most common imaging finding was pneumatosis intestinalis; other findings included bowel wall thickening, bowel dilatation, and pneumoperitoneum. At histopathological evaluation pneumatosis (n=5) was confirmed in all cases but one along with ischaemic necrosis; all patients had inflammatory infiltration with a submucosal/perivascular pattern or with full thickness distribution. Fibrin thrombi in the small vessels and ischaemic changes were present in all cases.

**Conclusion:** Pathogenesis of intestinal damage in SARS-Cov 2 infection is not fully understood yet, although a combination of factors is hypothesised. On the one hand there would be a direct viral cytolytic effect on enterocytes and on the other, there would be a local inflammatory activation leading to inflammatory infiltration, increased vascular permeability, microvascular damage and thrombosis. Increased intraluminal pressure may also contribute. Radiologists should be familiar with CT findings associated with bowel ischaemia to warrant an appropriate diagnosis and prompt treatment.
**Limitations:** The small number of patients was an identified limitation.
**Ethics committee approval:** Ethics committee approval was not required for this study.
**Funding for this study:** Not applicable.
**Author Disclosures:**
Claudio D'Amelio: Nothing to disclose
Rachele Tintori: Nothing to disclose
Emanuele Neri: Nothing to disclose
Michele Tonerini: Nothing to disclose
Ilaria Ambrosini: Nothing to disclose

### RPS 217-9

**Correlation between periappendicular fat density value and clinical and radiological CT scores on CT in acute appendicitis cases**
*E. Torun*, Y. Yüksel, T. Ergun; Antalya/TR

**Purpose:** Introduction: the clinical and radiological appendicitis severity index (CRASI) is used to predict complicated appendicitis in adults. However, this evaluation includes many parameters. Periappendicular fat density (PFD) is a numerical value that can be easily measured in CT examination. To our knowledge, there is no study examining the relationship between PFD value and CRASI score. Aim: to investigate the relationship between the CRASI and the PFD value.
**Methods or Background:** CT images of 47 adult patients with surgically confirmed acute appendicitis and 40 adult patients in the control group without clinical and radiological appendicitis were analysed retrospectively. For each patient, CRASI was calculated. Periappendicular, mesenteric, and subcutaneous fat density were measured in CT. The ratio of PFD to other locations (mesenteric region and umbilical region subcutaneous fat) was calculated.
**Results or Findings:** PFD value was significantly higher in patients with acute appendicitis than in the control group (p=0.045). Significant differences were observed between the two groups in terms of PFD/mesenteric fat density and PFD/umbilical region subcutaneous fat density ratio ($p < 0.001$, p=0.034, respectively). A close correlation was found between CRASI and PFD/mesenteric fat density and PFD/umbilical region subcutaneous fat density ratio and PFD value (p=0.002, r=0, -444; p=0.010, r=-0.372, p=0.004, r=0.411, respectively).
**Conclusion:** There is a strong close relationship between the increase in the CRASI, which is an indicator of the tendency to complications, and the density of periappendicular fat in appendicitis cases. PFD value/mesentery fat density ratio is more decisive in this relationship.
**Limitations:** The main limitation is the small number of patients. Significant differences can be detected in different parameters in higher sample groups.
**Ethics committee approval:** This study was approved by Alanya Alaaddin Keykubat University medical ethics committee, (10354421 - 2021/15-04) Turkey.
**Funding for this study:** No funding was received for this study.
**Author Disclosures:**
Ebru Torun: Author: Author
Tarkan Ergun: Author: Author
Yavuz Yüksel: Author: Author

### RPS 217-10

**Observation and repetitive ultrasound: a successful tool to reduce unnecessary CT scans in diagnostics of acute appendicitis**
*R. Luksaite-Lukste*, A. Samuilis, T. Zvirblis, K. Strupas, T. Poskus; Vilnius/LT
*(raminta.luksaite@santa.lt)*

**Purpose:** Acute appendicitis is routinely diagnosed using conditional computed tomography (CT) strategy, which may result in up to 50% unnecessary CT scans for patients revealing no acute pathology. Our purpose is to present our prospective trial results analysing the value of observation and repetitive abdominal ultrasound in the diagnosis of acute appendicitis for low-risk patients.
**Methods or Background:** Single-centre prospective study included patients with suspected acute appendicitis who, after initial clinical evaluation, laboratory tests and ultrasound results, did not achieve a final conclusive diagnosis. All patients went through an 8-12 hour period of observation, later clinical examination, laboratory tests, and abdominal ultrasound were repeated.
**Results or Findings:** The study included 97 patients: 37 male and 60 female. The average age was 31.4 years (SD 13.55), the average duration of symptoms was 13.5 hours (SD 11.24), Alvarado and appendicitis inflammation response score averages were 4.3 (SD 1.87) and 3.8 (SD 1.60) respectively. After repetitive abdominal ultrasound primary, inconclusive diagnosis (probable appendicitis) changed to conclusive results for 70% of cases, primary nonvisualised appendix result changed to visualised or other confirmed acute pathology for 42.4% patients (p<0.005). Finally, a CT scan was avoided for patients in 75% of cases. A negative appendectomy rate was 1.0%, and 2.0% of complicated cases were revealed.
**Conclusion:** Observation and repetitive abdominal ultrasound strategy is a safe tool to increase the rate of final conclusive diagnosis without ionising radiation for low-risk patients and does not increase rates of negative appendectomies and complicated cases.
**Limitations:** Not applicable.
**Ethics committee approval:** Study protocol UADO-1, version 2.0 was approved by the local bioethical committee, approval No: 2019/3-1107-610, date: 2018-06-05. Registered in Clinicaltrials.gov (ID: NCT04117061), October 2019.
**Funding for this study:** Not applicable.
**Author Disclosures:**
Tadas Zvirblis: Nothing to disclose
Artūras Samuilis: Nothing to disclose
Kestutis Strupas: Nothing to disclose
Raminta Luksaite-Lukste: Nothing to disclose
Tomas Poskus: Nothing to disclose

### RPS 217-11

**Acute pelvic pain in girls: how valuable is MR imaging as an adjunct to ultrasound?**
*G. Masselli*, D. Cozzi, E. Guiducci, S. Cardaccio, M. L. De Cicco, C. Andreoli, S. Lanciotti, M. Colaiacomo, P. Ricci; Rome/IT
*(gabriele.masselli@uniroma1.it)*

**Purpose:** To retrospectively evaluate the utility of pelvic MRI following inconclusive pelvic ultrasound in girls with acute pelvic pain.
**Methods or Background:** All MRI examinations of patients who were referred for examination because of acute pelvic pain, after inconclusive ultrasound between June 2018 and September 2021 at one institution, were included our study (n=49). Multi-planar multi-sequence MR images of the pelvis were obtained in each patient. MR images were reviewed at the time of acquisition by an experienced radiologist to determine whether a diagnosis could be made without the administration of IV gadolinium. In 35 of 49 examinations gadolinium was not administered. The prospective clinical MRI interpretations were retrospectively compared with follow-up medical, surgical, and obstetric records to determine the correctness of the interpretation.
**Results or Findings:** Correlation of prospective clinical MRI interpretations with follow-up medical records showed correct identification of disease entities in all 49 patients. The following disease processes were correctly identified using MRI: appendicitis (n=5), ulcerative colitis (n=1), Crohn's disease with acute inflammation (n=3), ovarian torsion (n=4), fallopian tube torsion (n=2), adnexal/ovarian masses (n=4), ovarian rupture with active bleeding (n=2), haematometrocolpos (n=1), Meckel diverticulum (n=2), PID (n=3), and pelvic osteomyelitis (n=1). 21 of the 49 patients had normal findings on MRI examinations and also had unremarkable follow-up.
**Conclusion:** The intrinsic safety of MRI and its ability to accurately establish the cause of acute pelvic pain in girls, make it highly useful in the evaluation of the subset of young female patients without a definitive initial diagnosis on sonography, and in directing medical and surgical treatment.
**Limitations:** Identified limitation were as follows: the relatively limited cohort of patients; longer term follow-up data was not available.
**Ethics committee approval:** The local ethical committee approved this retrospective study.
**Funding for this study:** Not applicable.
**Author Disclosures:**
Sara Cardaccio: Nothing to disclose
Paolo Ricci: Nothing to disclose
Mariachiara Colaiacomo: Nothing to disclose
Elisa Guiducci: Nothing to disclose
Maria Luisa De Cicco: Nothing to disclose
Gabriele Masselli: Nothing to disclose
Silvia Lanciotti: Nothing to disclose
Denis Cozzi: Nothing to disclose
Chiara Andreoli: Nothing to disclose

### RPS 217-12

**Diagnostic errors in imaging and their impact when requesting patients to revisit the emergency room**

*A. Son*, G-S. Hong; Seoul/KR
*(ayeon1230@gmail.com)*

**Purpose:** Investigate diagnostic errors in imaging and their impact when requesting patients to revisit the emergency room
**Methods or Background:** In our hospital, if the resident or clinician's diagnosis in imaging is made incorrectly in the emergency room (ER) patient, if it is a critical finding we use an automatic text message transmission system or call the clinician to notify the diagnostic error. We extracted data from text messages or phone call histories related to diagnostic errors on CT or MR in ER from January 2013 to January 2021, and conducted a descriptive cross-sectional study based on the extracted data.
**Results or Findings:** There were 686 confirmed cases. The main types of diagnostic errors were missed findings (70.4%) and faulty reasoning (19%). The majority of imaging was abdominal imaging (47.8%), followed by neuroimaging (31.3%). Errors were most common during night shifts when a resident worked alone as a radiologist (54.8% on 10pm~8am). Critical findings (related to patient symptoms or a change of treatment options) were 95% and incidental findings were 5%. Of the 686 patients, 149 (21.7%) were hospitalised for errors related to findings or pre-existing underlying diseases. Of these, a total of 6 patients died within 30 days: 5 died from progression of the pre-existing disease, and 1 died from pneumonia after surgery.
**Conclusion:** There were no cases of patient death due to diagnostic errors, but most cases were critical findings, which may affect treatment and prognosis and cause unnecessary further visits to the emergency room. There is a need for a way to reduce diagnostic errors.
**Limitations:** An identified limitation was that patients with a loss of follow-up could not be evaluated.
**Ethics committee approval:** Institutional review boards approved the research.
**Funding for this study:** No funding was received for this study.
**Author Disclosures:**
Ayeon Son: Speaker: speaker for oral presentation Author: data analysis, writing
Gil-Sun Hong: Author: corresponding author

**11:30-12:30** **Open Forum #3 (ESR)**

### Research Presentation Session: Imaging Informatics / Artificial Intelligence and Machine Learning

### RPS 305

### Artificial intelligence (AI) meeting clinical needs in radiology: achievements and challenges

### RPS 305-1

**Moderator**

E. R. Ranschaert; Tilburg/NL

**Author Disclosure:**
E. R. Ranschaert: Advisory Board: Osimis, Diagnose.me; Consultant: Quibim, Contextflow, Oxipit, Unilabs; Investigator: AICT.ai; Other: Visiting professorship Ghent University

### RPS 305-2

**The rise of artificial intelligence solutions in radiology departments in the Netherlands**

*K. G. van Leeuwen*[1], M. De Rooij[1], S. Schalekamp[1], B. Van Ginneken[1], M. J. Rutten[2]; [1]Nijmegen/NL, [2]'S-Hertogenbosch/NL
*(kicky.vanleeuwen@radboudumc.nl)*

**Purpose:** There are over 180 CE-marked artificial intelligence (AI) products for radiology commercially available in Europe, but little is known about the current clinical use. We investigated the clinical use of commercial AI software in radiology departments in the Netherlands over a two-year period.
**Methods or Background:** We consulted the radiology department of all hospital organisations in the Netherlands (n = 69) in February-March 2020 (44 respondents) and February-March 2021 (37 respondents). A representative of the department was asked to fill in a questionnaire about the (planned) clinical use of CE marked AI products for radiology, the available funding for AI, and biggest obstacles for implementation.
**Results or Findings:** From 2020 to 2021 the percentage of respondents that desired the adoption of AI tools in radiology increased from 63% to 86%. In 2020, 14 responding organisations used AI in clinical practice, which increased to 23 (33% of all organisations) in 2021. The total number of AI implementations in clinical practice expanded by 157%, from 19 to 49 implementations. Also, the diversity increased from 8 to 32 unique products. In 2021, 35% of respondents had budgets allocated for AI implementations either on the departmental level or on the institutional level, which was 26% in 2020. The major obstacles for AI adoption shifted from difficulties with the technical integration (2020) to the lack of budgets and an unclear business case (2021). Technical integration remained the second most often listed obstacle.
**Conclusion:** AI adoption is gradually increasing in clinical radiology in the Netherlands. The number of radiology departments using AI has increased to at least a third of all organisations. Also, the number and diversity of AI applications per department grew substantially.
**Limitations:** Results may be influenced by a nonresponse bias.
**Ethics committee approval:** Not applicable.
**Funding for this study:** Not applicable.
**Author Disclosures:**
Bram Van Ginneken: Nothing to disclose
Kicky Gerhilde van Leeuwen: Nothing to disclose
Matthieu J.C.M. Rutten: Nothing to disclose
Steven Schalekamp: Nothing to disclose
Maarten De Rooij: Nothing to disclose

### RPS 305-3

**Adversarial attacks in radiology**

*V. Sorin*[1], S. Soffer[2], B. Glicksberg[3], Y. Barash[1], E. Konen[1], E. Klang[1]; [1]Ramat Gan/IL, [2]Ashdod/IL, [3]New York, NY/US
*(verasrn@gmail.com)*

**Purpose:** Deep learning is increasingly being applied in radiology. Along with the developments in deep learning, questions arise concerning the security of this technology. In particular, adversarial attacks are gaining interest. We aimed to review the literature on adversarial attacks in radiology.
**Methods or Background:** We searched for studies on adversarial attacks in radiology published up-to September 2021. MEDLINE and Google Scholar were used as search databases.
**Results or Findings:** Fourteen studies were published from 2018 to 2021. Attacks were applied to image classification as well as natural language processing (NLP) algorithms. The efficacy of attacks was high. Some attacks reduced the accuracy of formerly well-performing deep learning algorithms to 0% with 100% confidence. Several protective strategies were examined. The most common is training algorithms with adversarial examples.
**Conclusion:** Machine learning models are at risk of adversarial attacks, particularly in radiology. There are numerous potential incentives for cyber-attacks on healthcare frameworks. Thus, the occurrence of these attacks in practice is of concern. Radiologists and policymakers should be aware of adversarial attacks. It is important to prepare for such attacks and influence the formulation of ethical and legal guidelines to ensure the safe use of deep learning technology.
**Limitations:** Not applicable.
**Ethics committee approval:** Not applicable.
**Funding for this study:** Not applicable.
**Author Disclosures:**
Vera Sorin: Nothing to disclose
Eli Konen: Nothing to disclose
Benjamin Glicksberg: Nothing to disclose
Shelly Soffer: Nothing to disclose
Yiftach Barash: Nothing to disclose
Eyal Klang: Nothing to disclose

### RPS 305-4

**Synthesis of medical images and corresponding ROI by paired-GAN**

*Y. Wu*, J. Dong, F. Fangfang, Y. Bai, Y. Lin, M. Wang; Zhengzhou/CN

**Purpose:** The small data size is the major challenge of artificial intelligence for medical imaging. This study aims to use adversarial networks to generate structurally consistent medical images and corresponding regions of interest.
**Methods or Background:** Based on cycleGAN's two-stage cycle consistency, the priori characteristics of the region of interest were added to construct the artificial intelligence model. This method improves the tissue contrast of ROI and achieves the paired generation of high-quality magnetic resonance imaging (MRI) and its corresponding ROI. During the model training, the MRI image with ROI was taken as the input, and the corresponding mask image was taken as the target. The circular consistency function of the network was used to ensure the structural consistency between the synthesis image and the input image, and the quality of the synthesis image was improved through GAN network.

**Results or Findings:** The validation experiment in BRATS 2017 shows that the proposed method can effectively generate pairwise training data for the training of machine learning models. The DICE coefficient of the generated image is 0.9810.11 and the Hausdorff distance is 4.212.84. Compared with cycleGAN model, the image synthesised by this method improved the classification accuracy of the model by 4%.
**Conclusion:** Using GAN to synthesise medical images can generate image data consistent with the distribution of real image data. By combining the quality of paired images, it can effectively increase the training data and improve the performance of machine learning model.
**Limitations:** N/A
**Ethics committee approval:** The institutional ethics committee approved this study and all participants gave informed written consent.
**Funding for this study:** This work was partially supported by National Key R&D Program of China (2017YFE0103600), National Science Foundation of China (81720108021), Zhongyuan Thousand Talents Plan Project (ZYQ201810117), and Zhengzhou Collaborative Innovation Major Project (20XTZX05015).
**Author Disclosures:**
Yan Bai: Nothing to disclose
Fu Fangfang: Nothing to disclose
Jiale Dong: Nothing to disclose
Yusong Lin: Nothing to disclose
Meiyun Wang: Nothing to disclose
Yaping Wu: Nothing to disclose

### RPS 305-5

**Deep learning-based detector row upsampling to reduce windmill artifacts in diagnostic spiral CT**

J. Magonov[1], E. Fournié[1], K. Stierstorfer[1], *M. Kachelrieß*[2]; [1]Forchheim/DE, [2]Heidelberg/DE
*(marc.kachelriess@dkfz.de)*

**Purpose:** To develop a software-based method to reduce windmill artifacts in spiral CT.
**Methods or Background:** Due to longitudinal undersampling spiral CT scans may suffer from windmill artifacts in the reconstructed images. To fulfill the sampling condition and achieve double sampling in z-direction, some CT scanners use the z-flying focal spot (zFFS) technique, a hardware-based solution that effectively doubles the number of detector rows. To obtain a software-based solution we developed a convolutional neural network that is trained in a supervised manner by defining projections containing all (upsampled) rows as the desired output, while every other row of the corresponding projections is used for network input. Patient CT raw data containing various anatomical regions are used for training. Evaluation was performed in image domain by comparing to reconstructions of projections both with zFFS enabled and zFFS disabled.
**Results or Findings:** Interpolation of projection data with the neural network leads overall to slightly better reconstruction results in the image domain compared to disabling the zFFS (up to 10% improvement in RMSE). In areas where little structural change occurs, i.e. homogeneous structures, the network achieved less favorable results. Qualitatively, it was observed that windmill artifacts were less pronounced when projection data were interpolated by the trained network than when zFFS was avoided.
**Conclusion:** The application of a neural network can partially meet the requirements of increasing the sampling of spiral CT projection data and may have a positive effect on avoiding windmill artifacts in the corresponding reconstructions. It cannot, however, outperform the zFFS.
**Limitations:** The zFFS-generated rows were manually removed from projection data. It remains to be investigated how the results of the interpolation look like with real projections acquired without zFFS.
**Ethics committee approval:** Not applicable.
**Funding for this study:** This study is funded by Siemens Healthcare GmbH.
**Author Disclosures:**
Eric Fournié: Employee: Siemens Healthcare GmbH
Jan Magonov: Employee: Siemens Healthcare GmbH
Karl Stierstorfer: Employee: Siemens Healthcare GmbH
Marc Kachelrieß: Nothing to disclose

### RPS 305-6

**Semi-supervised 3D universal lesion segmentation in CT thorax-abdomen scans**

*M. J. J. De Grauw*, B. Van Ginneken; Nijmegen/NL
*(max.degrauw@radboudumc.nl)*

**Purpose:** Extracting volumetric information using automated lesion segmentation could allow for more accurate quantification of disease response in heterogeneous lesions. Using a single model for Universal Lesion Segmentation has the potential for faster inference times compared to multimodel approaches and allows for internal representation of lesion type features.
**Methods or Background:** We compiled eight public datasets with segmentation masks for various lesion types in CT thorax-abdomen scans. Scans were resampled to 1mm isotropic voxel spacing and regions of interest were cropped centered on each lesion. A nnUnet was trained with 3213 lesions from 1481 studies and used to predict 3D segmentation masks for the circa 32,000 partially annotated lesions from the DeepLesion dataset. Masks were further refined by applying the GrabCut algorithm in three orthogonal directions based on the provided long and short-axis diameter measurements. We fine-tuned the nnUnet using the resulting masks and evaluated on a test set with full annotations. We experimented with epoch numbers and learning rate decay. All models were trained using 5-fold cross validation.
**Results or Findings:** Fine-tuning the model using the DeepLesion masks improved segmentation performance from 0.71 to 0.73 Dice compared to the baseline nnUnet. Segmentation performance ranged from 0.53, 0.61, 0.66, 0.77, 0.79 to 0.9 Dice for colon, pancreas, lymph node, lung, liver and kidney lesions.
**Conclusion:** 3D universal lesion segmentation using large, aggregated datasets shows promise as an alternative to lesion specific models. By incorporating partially annotated data in a semi-supervised manner we can further increase data volume and model performance with minimal annotation effort.
**Limitations:** This study used a small number of scans in the test set and did not evaluate model performance on out-of-distribution lesion types.
**Ethics committee approval:** Not applicable.
**Funding for this study:** This research was supported by the Eurostars PIANO project E113829.
**Author Disclosures:**
Max Jacobus Johannes De Grauw: Nothing to disclose
Bram Van Ginneken: Nothing to disclose

### RPS 305-7

**Radiomics and transvaginal ultrasound in adnexal masses: is the next future of diagnostics here?**

*V. Chiappa*, M. Interlenghi, C. Salvatore, R. Fruscio, F. Bertolina, G. Vittori Antisari, C. Paniga, F. Raspagliesi, I. Castiglioni; Milan/IT
*(valentina.chiappa@gmail.com)*

**Purpose:** Multicentre prospective clinical validation of the radiomic machine learning model (TRACE4OC) applied to transvaginal ultrasound (US) in predicting the risk of malignancy of adnexal masses.
**Methods or Background:** From a multicentre prospective consecutive series of women scheduled for surgery of adnexal masses, we collected and evaluated, fully blinded, 102 US images of adnexal masses with the TRACE4OC radiomic model previously developed according to the International Biomarker Standardisation Initiative guidelines, trained and externally validated on a retrospective study of 274 US images of adnexal masses using histopathology as reference standard.
**Results or Findings:** TRACE4OC model showed 82.4% accuracy (95% CI 73.6%-82.2%), 97.7% sensitivity (95% CI 87.7–99.9%)., 71.2% specificity (95% CI 57.9%-82.2%) when tested on the prospective multicentric external datasets of 102 masses (resulting into 43 malignant and 59 benign lesions at histology postsurgery), achieving 71.2% positive predictive value (PPV) (95% CI 57.9–82.2%) versus a 42.2% radiologists' PPV (95% CI 32.4%-52.3%) ($p<0.005$).
**Conclusion:** The radiomic machine learning model can support clinicians in the diagnostic process of benignancy versus malignancy for adnexal masses, providing a strong reduction of the definite surgery rate for benign lesions still warranting very high sensitivity.
**Limitations:** Prevalently Caucasian population.
**Ethics committee approval:** The study was approved by the local ethics committee with the protocol number INT 157/20.
**Funding for this study:** The study did not require external funding.
**Author Disclosures:**
Cristiana Paniga: Nothing to disclose
Isabella Castiglioni: Nothing to disclose
Valentina Chiappa: Nothing to disclose
Francesca Bertolina: Nothing to disclose
Robert Fruscio: Nothing to disclose
Christian Salvatore: Nothing to disclose
Francesco Raspagliesi: Nothing to disclose
Matteo Interlenghi: Nothing to disclose
Giulia Vittori Antisari: Nothing to disclose

11:30-12:30 Room G

### Research Presentation Session: Oncologic Imaging

### RPS 316

### Neuro-oncology

**Moderator**
M. d. F. Vasco Aragão; Recife, PE/BR

### RPS 316-3

**[18F]FET PET/MRI: a novel and improved technique for detection of pituitary microadenoma**

*I. J. Pruis*, S. Neggers, F. A. Verburg, S. E. Veldhuijzen van Zanten; Rotterdam/NL
*(i.pruis@erasmusmc.nl)*

**Purpose:** Pituitary adenoma can cause severely disabling symptoms resulting from hormonal dysregulation. In 40% of patients, diagnostic MRI is inconclusive as microadenoma by definition are <10mm and not always sufficiently contrasting. We here introduce a novel method for detection of microadenoma by O-(2-[18F]-fluoroethyl)-L-tyrosine ([18F]FET) PET/MRI.
**Methods or Background:** Patients with suspected microadenoma underwent PET/MR-imaging at 20 minutes post-injection of 200 (median, range 50-207) MBq [18F]FET. A positive scan was defined as focal uptake exceeding local background (i.e., normal pituitary tissue). Outcomes were compared with results of selective inferior petrosus sinus sampling (IPSS), postoperative pathology reports, and clinical follow-up.
**Results or Findings:** Nineteen patients, 73.7% female, median age 56 (11-68), with Cushing's disease (n=12; 63.2%) or acromegaly (36.8%) but with a negative/inconclusive MRI were included. Fifteen patients (78.9%; 10 Cushing, 5 acromegaly) showed positive focal [18F]FET PET/MRI uptake. Four patients underwent surgery upon which pathology confirmed presence of an adenoma. Nine out of eleven Cushing patients also had positive IPSS, however, in five IPSS could not differentiate between left/right, and in two the designated side by IPSS did not correlate with imaging. In the patients with a negative [18F]FET PET/MRI, one proved true-negative after surgery, one was diagnosed with a Rathke's cleft/non-metabolic cystic adenoma instead, one appeared to have minimal symptoms that not required therapy, and one had a later confirmed microadenoma that was possibly not detected by [18F]FET PET/MRI because of ACTH normalising medication at the time of the scan.
**Conclusion:** [18F]FET PET/MR-imaging shows high accuracy for localising microadenoma in patients with hormonal dysregulation. The diagnostic yield of this hybrid imaging technique showed to exceed that of MRI alone and IPSS. This novel approach herewith provides an important addition for planning of selective transsphenoidal adenomectomy.
**Limitations:** Not applicable.
**Ethics committee approval:** Not applicable.
**Funding for this study:** Not applicable.
**Author Disclosures:**
Sebastian Neggers: Nothing to disclose
Sophie E.M. Veldhuijzen van Zanten: Nothing to disclose
Ilanah Johanna Pruis: Nothing to disclose
Frederik Anton Verburg: Nothing to disclose

### RPS 316-4

**Clinical value of MRI screening for brain metastases in resected stage III melanoma**

*S. Derks*, L. S. Ho, K. de Joode, A. Joosse, M. de Jonge, M. Smits, M. van den Bent, A. van der Veldt; Rotterdam/NL

**Purpose:** Melanoma has a high propensity to metastasize to the brain, especially in patients with extracranial metastases (stage IV). We assessed the number of patients with asymptomatic brain metastases (BMs) at screening in stage III melanoma, after resection of loco-regional disease (resected stage III). With this, we aimed to determine the clinical value of MRI screening in these patients.
**Methods or Background:** In this retrospective cohort analysis, we included all patients with resected stage III melanoma who underwent screening MRI of the brain between 1st August, 2018, and 1st January, 2021. From our hospital's electronic health record system, we collected reported American Joint Committee of Cancer (AJCC) stage at initial referral, diagnosis of extracranial disease on screening CT or PET/CT; diagnosis of BMs on screening MRI (neuro-radiologists' reports) and treatment plan prior to and after MRI.
**Results or Findings:** We identified 208 patients: 27 were initially referred with stage IIIA, 74 with stage IIIB, 99 with stage IIIC, and 8 with stage III of unknown primary (MUP). Brain metastases were discovered at MRI screening in 5 patients: 4 in stage IIIC and 1 in MUP. The number of BMs ranged from 1 to 10 and all lesions were small (diameter range 3.5-18.0mm). Two out of these 5 patients had simultaneous extracranial disease discovered at screening, which was the main factor that resulted in a changed treatment plan. Therefore, in only 3 out of 208 (1.4%) patients with resected stage III melanoma, screening MRI of the brain changed the treatment plan (dual immune checkpoint inhibition, ipilimumab/nivolumab, instead of monotherapy).
**Conclusion:** The clinical value of MRI screening for BMs in patients with resected stage III melanoma is low.
**Limitations:** The retrospective nature of the study.
**Ethics committee approval:** Not applicable.
**Funding for this study:** No funding was received for this study.
**Author Disclosures:**
Maja de Jonge: Nothing to disclose
Astrid van der Veldt: Other: Consultancy fees (paid to the institute): BMS, MSD, Merck, Sanofi, Roche, Novartis, Pierre Fabre, Ipsen, Eisai, Pfizer
Sophie Derks: Nothing to disclose
Karlijn de Joode: Nothing to disclose
Martin van den Bent: Nothing to disclose
Marion Smits: Nothing to disclose
Arjen Joosse: Nothing to disclose
Li Shen Ho: Nothing to disclose

### RPS 316-5

**Analysis of dynamic susceptibility contrast MR perfusion parameters in differentiating radiation necrosis from glioblastoma multiforme recurrence**

*M. K. Radom*, A. Awramienko-Włoczek, A. Hebda, B. Bobek-Billewicz; Gliwice/PL

**Purpose:** Dynamic susceptibility contrast (DSC) MR perfusion in patients with brain tumours is invaluable thus improving methods for analysing this sequence is crucial. In this study, two parameters characterising MR perfusion imaging were investigated: the peak height (PH) and the percentage of signal recovery (PSR) to determine whether they could be a tool in differentiating radiation necrosis from recurrence of glioblastoma multiforme (GBM). Identifying such characteristics in MR imaging can help diagnose patients more accurately without the need for an unnecessary neurosurgical procedure.
**Methods or Background:** 34 patients were included in this study and analysed retrospectively. The key condition for all participants was to undergo teleradiotherapy followed by MR examination with DSC imaging in which a contrast-enhancing lesion was present. For patients with recurrent GBM, confirmation of the diagnosis by histopathology was necessary while for patients with radiation necrosis, certain radiological features of the lesion in subsequent MR examinations were crucial to establish a definitive diagnosis. To obtain signal intensivity-time curves, regions of interest (ROIs) were drawn manually around the contrast-enhancing parts of the lesions. Based on those curves both PH and PSR were calculated and normalised to the contralateral normal appearing white matter.
**Results or Findings:** Mean, maximum, and minimum relative PH (rPH) were significantly higher ($p < 0.05$) in GBM recidivans group than in radiation necrosis group, whereas maximum relative PSR (rPSR) was significantly higher ($p < 0.05$) in radiation necrosis group than in GBM recidivans group. The following cut-off points were distinguished for: mean rPH 1.44 (sensitivity: 100%, specificity: 68,4%), max rPH 2.55 (sensitivity: 92,9%, specificity: 66,7%), min rPH 1.04 (sensitivity: 86,7%, specificity: 83,3%) and max rPSR 118.10%(sensitivity: 80%, specificity: 83,3%).
**Conclusion:** Selected DSC parameters could be helpful in differentiating radiation necrosis from GBM recurrence.
**Limitations:** Limited group of patients. PWI layer thickness and lesion size.
**Ethics committee approval:** Not needed.
**Funding for this study:** No extra funding.
**Author Disclosures:**
Mateusz Krzysztof Radom: Nothing to disclose
Anna Hebda: Nothing to disclose
Barbara Bobek-Billewicz: Nothing to disclose
Aleksandra Awramienko-Włoczek: Nothing to disclose

**RPS 316-6**

**MRI posttreatment surveillance for head and neck squamous cell carcinoma: proposed MR NI-RADS criteria**

*M. M. M. Ashour*, E. Darwish, R. Faheim, T. Taha; Cairo/EG
*(manar.ashour@gmail.com)*

**Purpose:** The goal of this work was to develop MRI NI-RADS criteria by combining diffusion characteristics and T2 signal intensity with the present ACR NI-RADS template.
**Methods or Background:** 69 patients with head and neck squamous cell carcinoma underwent post treatment imaging surveillance with CE-MRI neck were included in this retrospective study. Scans were interpreted by two neuroradiologists, assessment and scoring of the primary tumour site was done using the current ACR NI-RADS lexicon, and rescoring was then performed based on our proposed criteria incorporating T2 signal and diffusion features with the ACR NI-RADS system. The gold standard was a set of criteria including clinical and imaging follow-up, and pathologic assessment.
**Results or Findings:** In post-treatment surveillance for HNSCC the usage of T2 SI and diffusion features as modifying rules to the current ACR NI-RADS showed higher specificity, sensitivity, positive predictive value, negative predictive value, and accuracy (92.3%, 90.7%, 85.7%, 95.1%, and 91.3%, respectively) compared to the ACR NI-RADS lexicon on its own (84.6%, 81.4%, 73.3%, 89.8%, and 82.6%, respectively).
**Conclusion:** Incorporating diffusion features and T2 signal within the American College of Radiology NI-RADS criteria for the primary tumour site as modifying rules enhanced the diagnostic performance of the ACR NI-RADS system.
**Limitations:** Being a retrospective study with a fine variations in follow-up scans timing.
**Ethics committee approval:** Approval was obtained and informed consent was waived.
**Funding for this study:** No funding was received.
**Author Disclosures:**
Reham Faheim: Nothing to disclose
Eman Darwish: Nothing to disclose
Tougan Taha: Nothing to disclose
Manar Maamoun Mohamed Ashour: Nothing to disclose

**11:30-12:30 Room Z**

### Research Presentation Session: Emergency Imaging

### RPS 317

### New technologies and advances in emergency radiology

**Moderator**
F. Macri; Geneva/CH

**RPS 317-2**

**Beneficial usage of dual-energy CT brain imaging to detect intracranial haemorrhage in NCCT scan**

*P. Chiewvit*, C. Ngamsombat, S. Piyapittayanan, K. Ratanakarn; Bangkok/TH
*(pipat8999@gmail.com)*

**Purpose:** To compare diagnostic accuracy, sensitivity and specificity of material decomposition non-contrast DECT brain with stimulated conventional 120 kVp-single energy CT brain (sSECT) in diagnosis ICHs.
**Methods or Background:** Single centre, the post-processing images of 111 patients with 215 lesions were reviewed retrospectively, separately and blindly, generated from DECT acquisition as sSECT, non-overlay blood-subtracted-calcium images [blood (calcium)], overlay blood-subtracted-calcium images in rainbow and greyscale color [blood (calcium) overlay], and combined sSECT and blood (calcium) images, by three neuroradiologists. Reference standards were established by consensus diagnosis correlated with relevant prior and/or following CT or MRI, and clinical information. Accuracy, sensitivity, and specificity were accessed and compared.
**Results or Findings:** Blood (calcium) overlay images increased accuracy (99.6%), sensitivity (99.5%) and specificity (100%) in diagnosis of ICH on non-contrast brain CT and increased observer confident (94.06% certain diagnosis) as compared to sSECT images (p-value < 0.05). Combination of non-overlay blood (calcium) and sSECT images also got higher accuracy (92.8%), sensitivity (90.9%) and specificity (100%) than sSECT images alone (p-value < 0.05). They were very good interrater reliability, while sSECT images were poor.
**Conclusion:** Material decomposition DECT as blood (calcium) overlay images or combination of non-overlay blood (calcium) and conventional images had high diagnostic ability of ICH on non-contrast brain CT scan and benefit especially in emergency setting.
**Limitations:** This study is a retrospective study.
**Ethics committee approval:** This study was approved by the institutional ethics committee, Faculty of Medicine Siriraj hospital, Mahidol university.
**Funding for this study:** No funding was received.
**Author Disclosures:**
Kanyaphak Ratanakarn: Nothing to disclose
Pipat Chiewvit: Nothing to disclose
Siriwan Piyapittayanan: Nothing to disclose
Chanon Ngamsombat: Nothing to disclose

**RPS 317-3**

**Dual Energy CT for differentiation of intraperitoneal haematoma and bowel structures**

*M. T. Winkelmann*, M. Bongers; Tübingen/DE

**Purpose:** The aim of this study was to evaluate the accuracy of dual energy CT (DECT) virtual-unenhanced imaging (VNC) and iodine maps (CM) for differentiation between intraperitoneal haematomas and bowel structures.
**Methods or Background:** 30 patients with intraperitoneal haematomas having received clinically indicated DECT. Quantitative parameters such as VNC (iodine removed), iodine maps (IM) and CT-mixed (equivalent to single energy-CT) values were collected in intraperitoneal haematomas and adjacent bowel structures. Follow-up imaging and operative reports were used as a reference. Mean values of VNC, IM and CT-mixed in DECT images were compared using non-parametric tests. Diagnostic accuracy was assessed by calculating receiver operating characteristics (ROC). Results are given as median with interquartile ranges.
**Results or Findings:** CM values in DECT showed significant differences between intraperitoneal haematomas (3.6 HU [-2.4, 10]) and physiological bowel structures (36.2 HU [22.6, 52.5])($p \leq 0.0001$). Analysis of VNC revealed significant differences between haematomas (53.8 HU [32.1, 69.1]) and bowel structures (26.5 HU [-1, 47.9])($p \leq 0.0001$). No significant differences between haematomas (60 HU [34.9, 73.6]) and bowel structures (64.5 HU [26.3, 77.4]) were detected for CT-mixed. ROC analysis revealed highest AUC values and sensitivity for IM (AUC=100%/sensitivity=100%; threshold ≤ 19.1) and VNC (92%/96.7%; ≥ 34.1) ($p<0.001$) compared to CT-mixed (74%/73%).
**Conclusion:** DECT is suitable for initial differentiation between intraperitoneal haematomas and physiological bowel structures with computation of iodine maps and VNC images.
**Limitations:** Retrospective study design.
**Ethics committee approval:** The study was approved by the local institutional review boad (Project number: 247/2021BO2).
**Funding for this study:** No funding was received.
**Author Disclosures:**
Moritz T. Winkelmann: Nothing to disclose
Malte Bongers: Nothing to disclose

**RPS 317-4**

**CT MBIR applied to emergency radiology: image quality and radiation dose reduction**

*D. Ippolito*[1], M. Ragusi[1], T. P. Giandola[1], C. Talei Franzesi[1], C. Maino[1], D. G. Gandola[1], S. Sironi[2]; [1]Monza/IT, [2]Bergamo/IT
*(davide.atena@tiscalinet.it)*

**Purpose:** To assess if low dose computed tomography (CT) examination using model-based reconstruction algorithms (MBIR) can help reducing radiation dose exposure and improving image quality when compared to standard hybrid iterative algorithms (HIR).
**Methods or Background:** 7000 CT scans in several anatomical districts (brain, thorax, abdomen, and whole body) in patients referred to our department for medical emergencies were retrospectively evaluated. Two radiologists evaluated all CTs with a Likert scale (1 to 4), while signal-to-noise-ratio (SNR) and contrast-to-noise-ratio (CNR) were used to analyse images quantitatively. CT-dose-index (CTDI) and the dose-length-product (DLP) were analysed for each exam and compared on the basis of the acquisition and reconstruction data.
**Results or Findings:** Mean CTDI values were lower with MBIR compared with HIR in different districts: brain (-29%), thorax (-36%), abdomen (-64%) and whole-body (-26%). Totals DLP were lower in MBIR studies when compared to standard CT: brain (-17%), thorax (-25%), abdomen (-60%) and whole-body (-20%). SNR was higher in MBIR in comparison with FBP: brain 16.2 vs 8.6, thorax 61.6 vs 18.73, abdomen 10.72 vs 6.58, whole-body 13.43 vs 6.58. CNR was higher in MBIR when compared with HIR: brain 3.9 vs 2.2, thorax 58.9 vs 9.19, abdomen 4.64 vs 3.25, whole-body 4.84 vs 3.06. Images quality demonstrated a better quality of MBIR scans in comparison to HIR: brain 3 vs 2, thorax 3 vs 2, abdomen 3 vs 2, and whole-body 3 vs 1 (all $p < 0.05$).

**Conclusion:** CT MBIR represent a feasible approach for the evaluation of patients in the emergency setting, achieving high diagnostic image quality and significant radiation dose reduction.
**Limitations:** Not applicable.
**Ethics committee approval:** Not applicable.
**Funding for this study:** Not applicable.
**Author Disclosures:**
Sandro Sironi: Nothing to disclose
Davide Giacomo Gandola: Nothing to disclose
Cesare Maino: Nothing to disclose
Maria Ragusi: Nothing to disclose
Cammillo Talei Franzesi: Nothing to disclose
Teresa Paola Giandola: Nothing to disclose
Davide Ippolito: Nothing to disclose

### RPS 317-5

**Ultra-low-dose CT (ULDCT) of the chest vs. chest x-ray (CXR) in non-traumatic emergency patients**

*C. Wassipaul*, D. Tamandl, H. Prosch, M. Scharitzer, H. Domanovits, K. Janata-Schwatczek, M. Weber, C. J. J. Herold, H. Ringl; Vienna/AT

**Purpose:** Detection rate and positive-predictive-value of CXR and ULDCT in non-traumatic emergency patients.
**Methods or Background:** 294 consecutive non-traumatic emergency patients with a clinically indicated CXR were included in this study. Inclusion criteria were age >18years, informed consent and negative ß-HCG testing for women <55years. All participants received both examinations, CXR and ULDCT, and were randomised into two arms with inverse reporting order. ULDCT examinations were performed at 50 ref.mAs, 100 kV, tin-filtration and activated tube-current-modulation on a 3rd-generation 256-row MD-DSCT scanner. Findings from radiological reports and emergency physicians' diagnoses were reviewed by an expert radiologist and expert emergency physician to build a compound reference standard.
**Results or Findings:** Mean total DLP of ULDCT scans was 12.7 mGy*cm corresponding to a mean effective dose (ED) of 0.21 mSv and relating to twice the mean ED of a CXR in two views of 0.1 mSv in the USA and the EU. ULDCT detected significantly more findings and diagnoses than CXR as first imaging modality with 574 vs. 257 (p < 0.001) at a positive-predictive-value of 95.1% vs. 74.7%. As second imaging modality ULDCT added 365 findings to a prior CXR vs. 32 added by CXR to a prior ULDCT report (p < 0.001) at a PPV of 97.0% vs. 53.1%.
**Conclusion:** ULDCT detected significantly more findings and diagnoses than CXR in both study arms at only twice the mean radiation dose of a CXR in two views in the USA and the EU and might therefore become an alternative primary imaging modality in non-traumatic emergency patients.
**Limitations:** Higher ED of ULDCT than CXR. Subjective image quality wasn't evaluated. Limited number of patients per disease due to elaborate study design.
**Ethics committee approval:** EC at Medical University of Vienna (EK-Nr.2254/2018).
**Funding for this study:** Institution has research support/grants pending from Siemens Healthineers.
**Author Disclosures:**
Christian J. Johannes Herold: Research/Grant Support: Institution has research support/grants pending from Siemens Healthineers.
Helmut Ringl: Research/Grant Support: Former Institution (Medical University of Vienna) has/had research support/grants pending from Siemens Healthineers.
Helmut Prosch: Research/Grant Support: Institution has research support/grants pending from Siemens Healthineers.
Hans Domanovits: Nothing to disclose
Christian Wassipaul: Research/Grant Support: Institution (Medical University of Vienna) has/had grants / grants pending from Siemens Healthineers.
Karin Janata-Schwatczek: Nothing to disclose
Dietmar Tamandl: Research/Grant Support: Institution has research support/grants pending from Siemens Healthineers.
Martina Scharitzer: Nothing to disclose
Michael Weber: Nothing to disclose

### RPS 317-6

**Role of the "fast" whole-body MRI in the diagnosis of mild/moderately injured patients**

*S. F. U. Blum*, K. Reichel, D. Seppelt, G. Jones, A. Röhnert, O. Al-Sadi, K. Kamin, R-T. Hoffmann, J-P. Kühn; Dresden/DE
*(sophta@me.com)*

**Purpose:** Despite the high radiation exposure, the whole-body CT is firmly established to exclude injuries of polytraumatised patients. Nevertheless, the indication for polytrauma CT in mild or moderately injured patients should be critical. The purpose was to investigate the accuracy of a "fast" whole-body MRI protocol in mildly or moderately injured patients as an alternative to CT with the aim to avoid radiation exposure.
**Methods or Background:** In a prospective approach, 35 polytrauma-patients (74% men, 26% women, 47±19,2 years) with an AIS score <3 and a trauma CT according to the guidelines, were included on a voluntary basis. Within a maximum of 5 days after the trauma, a whole-body MRI was performed with a fast protocol (scan time: 25 minutes). Two radiologists independently evaluated injuries using MRI in 7 body regions (cerebral, thoracic, abdominal, skeleton, extremities, vascular, lower extremity/soft tissue mantle). Both radiologists were unaware of the CT findings. The results of the MRI were compared with CT as standard of reference.
**Results or Findings:** On whole-body MRI reading, both readers had substantial agreement (Cohen's k: 0.68, 95% CI: 56.4-80.4). In MRI, 162 true negative, 31 true positive, 10 false negative, and 7 false positive findings were documented. Thus, MRI achieved a sensitivity of 92% and a specificity of 96%, and an accuracy of 92% (95% CI: 87.4-95.2).
**Conclusion:** Compared to the polytrauma CT, the whole-body MRI with a "fast" protocol is an alternative approach, especially for young patients with mild or moderate injuries. A further evaluation is required in order to determine for which injury patterns the sensitivity of the rapid MRI is limited.
**Limitations:** Small number of patients.
**Ethics committee approval:** The local ethics committee approved this study (EK 160052018).
**Funding for this study:** No funding was received for this work.
**Author Disclosures:**
Ralf-Thorsten Hoffmann: Nothing to disclose
Konrad Kamin: Nothing to disclose
Jens-Peter Kühn: Nothing to disclose
Sophia Freya Ulrike Blum: Nothing to disclose
Glenda Jones: Nothing to disclose
Onays Al-Sadi: Nothing to disclose
Anne Röhnert: Nothing to disclose
Danilo Seppelt: Nothing to disclose
Katrin Reichel: Nothing to disclose

**13:00-14:30** **Room F1**

### Research Presentation Session: Breast

### RPS 402

### How to improve lesion characterisation in breast imaging

**Moderator**
L. Graña Lopez; Lugo/ES
**Author Disclosure:**
L. Graña Lopez: Speaker: Mammotome

### RPS 402-2

**Structured reporting and clinical decision rules improve breast MRI assessment even in challenging clinical situations**

*M. Dietzel*[1], P. Clauser[2], H. Bickel[2], R. Schulz-Wendtland[1], E. Wenkel[1], M. Uder[1], T. H. Helbich[2], P. A. Baltzer[2]; [1]Erlangen/DE, [2]Vienna/AT

**Purpose:** In challenging clinical-situations (CCs), such as assessment of non-mass or subcentimetre lesions, breast-MRI diagnosis may be difficult. Clinical-Decision-Rules (CDR) provide evidence-based decision-support and could be of value in CCs. We investigated the diagnostic-performance of two CDR in typical CCs.
**Methods or Background:** Consecutive patients receiving MRI for non-screening indications at our institutions were retrospectively included (506 patients, mean-age: 51.8 years). MRI was re-evaluated by an experienced blinded breast-radiologist. He assigned the empirical Göttingen-score (GS) and the machine-learning derived Kaiser-score to every lesion (KS). Histological verification of suspicious lesions (n=527) served as reference-standard (malignancy-rate: 131/527, 24.9%; confidence interval: 21.3%-28.8%).
Six subgroups of CCs were investigated: subcentimetre-lesions, non-mass-lesions, significant background enhancement, premenopausal status, DCIS, and invasive-lobular cancer.
Performance of GS and KS was compared within each CC (area under the receiver-operating characteristics-curve: AUC, Delong-test). Likewise, potential to avoid unnecessary biopsies of benign lesions based on established high-sensitivity thresholds was compared (KS>4/GS >3; McNemar-test).
**Results or Findings:** The KS (AUC: median= 0.91, standard-deviation SD=0.03) was superior to the GS in all CC (AUC: median=0.83; standard-deviation = 0.04; pre-menopausal: P=0.06, all other CCs P≤ 0.01). False negative-rate for KS (missed: invasive= 3, DCIS = 4) and GS (missed: invasive= 5, DCIS = 1) was similar within CCs (P: 0.13 to 1.0). However, specificity of the KS (median= 78.5%, range 74.7%-84.5%) was superior to the GS in all CCs, (median=40.4%, range 17.8%-56.7; all CCs: P<0.0001).

**Conclusion:** In CCs, CDR provide accurate decision support to distinguish benign from malignant breast-MRI lesions. Most of all, CDR showed promising potential to substantially reduce the number of unnecessary biopsies in CCs.
**Limitations:** Clinical trials are necessary prospectively evaluating the impact of the CDRs on clinical decision making in real live.
**Ethics committee approval:** This study was approved by IRB.
**Funding for this study:** Not applicable.
**Author Disclosures:**
Pascal A.T. Baltzer: Nothing to disclose
Hubert Bickel: Nothing to disclose
Evelyn Wenkel: Nothing to disclose
Thomas H. Helbich: Nothing to disclose
Rüdiger Schulz-Wendtland: Nothing to disclose
Matthias Dietzel: Nothing to disclose
Michael Uder: Nothing to disclose
Paola Clauser: Nothing to disclose

### RPS 402-3

**New suspicious findings on breast MRI during NACT follow-up: radiological-pathological correlation**
*M. Nadrljanski*, V. Urban, I. Krusac, M. Mihajlovic; Belgrade/RS
*(dr.m.nadrljanski@gmail.com)*

**Purpose:** To determine the nature of the new suspicious lesions (masses, non-mass enhancement and foci) detected in breast during neoadjuvant chemotherapy (NACT).
**Methods or Background:** Retrospective analysis of 282 breast MRI exams performed in 166 female patients (from January 2011 to June 2021), examined with FDP (full diagnostic protocol) on 1.5T unit at least twice: before the 1st cycle of NACT (pretreatment MRI) and for the early response assessment (after the 2nd cycle of NACT) and/or in the middle of the treatment (after the 4th cycle of NACT) and/or following the completion of NACT. The nature of the lesions was confirmed either by biopsy (US-CNB), following surgery or after the 2-yr-follow-up period.
**Results or Findings:** There were 21 (7.45%) new lesions categorised as MRI-BI-RADS 4: 4 masses (19.05%; size: 1.02+/-0.15 cm), 12 non-mass enhancement lesions (57.14%; size: 1.61+/-0.42 cm) and 5 foci (23.81%). The majority of the lesions occurred in pre-menopausal patients (15/21, 71.43%). US-CNB was performed in 10 patients (47.62%), surgical removal of the lesion(s) with index tumours (mastectomy) was performed in 8 patients (38.09%) and the lesion regression was detected during the 2-yr-follow-up in 3 patients (14.28%). Radiological-pathological correlation was available for 18 lesions (85.71%). All of the lesions belong to B2 category: fibrocystic change (9/18, 50.00%); sclerosing adenosis (3/18, 16.67%); PASH (2/18, 11.11%) benign intraductal papilloma (2/18, 11.11%) and periductal inflammation (2/18, 11.11%).
**Conclusion:** The new detected lesions during the assessment of tumour response to NACT occur in 7.45%, predominantly as NME lesions, pathologically confirmed as benign (B2). The new lesions developing during NACT are likely to be benign.
**Limitations:** Relatively small number of patients in a single-centre retrospective study.
**Ethics committee approval:** Referent board approval obtained for retrospective analysis.
**Funding for this study:** No funding was provided for this study.
**Author Disclosures:**
Vladimir Urban: Nothing to disclose
Iva Krusac: Nothing to disclose
Mirjan Nadrljanski: Nothing to disclose
Marko Mihajlovic: Nothing to disclose

### RPS 402-4

**Quantification of enhancement intensity and pattern of benign and malignant lesions in contrast enhanced spectral mammography and its correlation with magnetic resonance imaging enhancement patterns**
*I. Allajbeu*, N. Payne, V. Papalouka, N. Healy, P. L. Moyle, K. Morris, F. J. Gilbert; Cambridge/UK

**Purpose:** To quantify the enhancement intensity and pattern of different breast lesions with contrast enhanced spectral mammography (CESM) and to investigate the correlation with enhancement types in breast magnetic resonance imaging (MRI).
**Methods or Background:** Using the CESM studies from the CONTEND and BRAID trials, three experienced breast radiologists calculated contrast-to-noise ratio (CNR) for each lesion identified in the temporally earlier (CC view, CNR1) and later (MLO view, CNR2) where CNR=Sa-Sb/Sb (Sa=maximum pixel value in breast lesion, Sb=mean pixel value of background ). Relative signal difference (RSD) was calculated as (CNR2-CNR1)/(CNR1)x100% and the enhancement patterns were classified as no enhancement, progressive (RSD>10%), plateau (-10%≤RSD≤10%), or wash-out (RSD<-10%). Similarly, MRI enhancement patterns were recorded for 38 lesions that had a clinical breast MRI at the same episode.
**Results or Findings:** From 75 research CESM studies, 70 lesions (3-110 mm) were included in the study, from which 49 were cancers (42 invasive, 7 non-invasive) and 21 normal/benign (7 B1, 12 B2 and 2 B3). The CNR1 and RSD values were significantly lower for benign lesions than for invasive cancers (mean CNR1: 0.046 vs 0.066, $p < 0.0095$; mean RSD: 29.89 vs -8.48, $p < 0.0001$) but not significantly different for DCIS (mean CNR1:0.046 vs 0.058, $p < 0.07$). Full agreement between CESM and MRI enhancement patterns was found in 29 of 38 cases (76.3%) with a Pearson chi-square value $\chi 2=54.5$ and a Cramér's V $\varphi c=0.69$ showing good correlation between the techniques.
**Conclusion:** Quantification of enhancement intensity in CESM may be helpful to differentiate between benign and invasive breast lesions. Furthermore, there seems to be good correlation between MRI and CESM enhancement patterns.
**Limitations:** Limited pathologies. Single centre.
**Ethics committee approval:** This study was approved by an ethics committee. CONTEND: REC ref: 15/EE/0279; BRAID: REC ref: 19/LO/0350.
**Funding for this study:** This study was funded by BRAID: C543/A26884; CONTEND: RQAG/113.
**Author Disclosures:**
Kirsten Morris: Nothing to disclose
Penelope Laura Moyle: Nothing to disclose
Fiona J. Gilbert: Research/Grant Support: Research collaborations with Volpara, Screenpoint, Lunit, Merantix, Curemetrix, Hologic, GE Healthcare, Bayer, GSK, RhinoHealth. Consultant: Consultancy with Google, Kheiron
Iris Allajbeu: Nothing to disclose
Vasiliki Papalouka: Nothing to disclose
Nicholas Payne: Nothing to disclose
Nuala Healy: Nothing to disclose

### RPS 402-5

**Diagnostic efficiency of ultrafast DCE breast MRI in differentiation of malignant and benign breast lesions**
*Y. V. Wong*, K. Rahmat, N. Ab Mumin, M. T. Ramli Hamid, W. Y. Chan, F. I. Rozalli; Kuala Lumpur/MY
*(yeevoon@ummc.edu.my)*

**Purpose:** Ultrafast dynamic contrast-enhanced (DCE) MRI has demonstrated comparable diagnostic accuracy in characterisation of breast lesions. Three-dimensional sequence time-resolved angiography with interleaved stochastic trajectories (TWIST) allows rapid sequential imaging within the first 2 minutes after contrast material injection at high temporal resolution. In this study, we aimed for evaluating the diagnostic performance of ultrafast DCE-MRI-derived kinetic parameters in detection of malignant breast lesions.
**Methods or Background:** Fifty-five patients with suspicious BI-RADS 4 and 5 breast lesions underwent standard breast MRI and ultrafast protocol (TWIST) between July 2020 and May 2021. Images were read by three radiologists in consensus. Ultrafast kinetic parameters were automatically generated, maximum slope (MS), time to enhance (TTE), arteriovenous index (AVI), and maximal relative enhancement (MRE). These parameters were compared using receiver operating characteristics (ROC), with p values <0.05 considered significant.
**Results or Findings:** A total of 83 histopathologically proven lesions in 55 patients were analysed (34 benign, 49 malignant). 69 lesions were visualised on ultrafast MRI, comprising 48 malignant and 21 benign lesions. Of the malignant lesions, invasive carcinoma was most common n=34 (68.4%), followed by ductal carcinoma in situ (DCIS) 22.4% (n=11) with majority high grade (88%, n=8). The AUC for MS and MRE were 0.836, 0.728, with sensitivity of 82.2%, 77.8% and specificity of 64.3% and 57.1% respectively. MS and MRE demonstrated a significant difference between benign and malignant lesions, with p-value < 0.05. No significant difference was observed in TTE and AVI.
**Conclusion:** Ultrafast DCE MRI-derived kinetic parameters, specifically MS and MRE may be reliable in evaluating kinetics and differentiation of breast lesions, with advantages of reduced scanning time and cost-effectiveness.
**Limitations:** No limitations were identified.
**Ethics committee approval:** This study was approved by an ethics committee.
**Funding for this study:** Funding was received for this study by University of Malaya Grants GPF009C-2019 and BK006-2018.
**Author Disclosures:**
Marlina Tanty Ramli Hamid: Nothing to disclose
Kartini Rahmat: Nothing to disclose
Faizatul Izza Rozalli: Nothing to disclose
Yee Voon Wong: Nothing to disclose
Wai Yee Chan: Nothing to disclose
Nazimah Ab Mumin: Nothing to disclose

### RPS 402-6

**A multicentric comparison of the Göttingen-score and the Kaiser-score in the assessment of breast MRI lesions**

*M. Dietzel*[1], P. Clauser[2], R. Schulz-Wendtland[1], H. Bickel[2], M. Uder[1], T. H. Helbich[2], P. A. Baltzer[2]; [1]Erlangen/DE, [2]Vienna/AT

**Purpose:** Clinical-Decision-Rules provide evidence-based decision support for breast-MRI assessment. The Göttingen-score (GS) and the Kaiser-score (KS) represent widely used Clinical-Decision-Rules for breast-MRI. We compared the diagnostic-performance of the GS with the KS to distinguish benign from malignant breast lesions and to compare their potential to avoid unnecessary biopsies.
**Methods or Background:** In this multicentric study consecutive patients received standardised multiparametric breast-MRI for standard indications. One radiologists with 15 years breast-MRI experience retrospectively interpreted the examinations. He assigned the KS and the GS to every histologically verified lesion. Diagnostic-accuracy was evaluated by the area under the receiver-operating characteristics-curve (ROC) and compared between KS and GS (DeLong-test). The potential of boths Clinical-Decision-Rules to help avoid unnecessary biopsies was compared based on established high-sensitivity thresholds (McNemar-test).
**Results or Findings:** A total of 527 lesions in 506 patients (mean-age: 51.8 years, IQR: 43.0–61.0 years) were included, with 131 lesions being malignant (24.9%; CI: 21.3% to 28.8%). The performance of the KS (ROC: 0.921; CI: 0.894 to 0.942) was significantly better than that of the GS (ROC: 0.821; CI: 0.894 to 0.942; P < 0.001). When applying high-sensitivity thresholds for avoiding unnecessary biopsies, the KS (94.7%; CI: 88.5%–97.4%) and the GS (125/131, 95.4%; CI: 89.3%–97.8%) achieved similar levels of sensitivity (P = 1.00). However, the rate of potentially avoidable biopsies was 93.2% higher when using the KS (specificity: 78.5%; CI: 74.2%–82.5%) compared to the GS (specificity: 40.7%; CI: 35.8%–45.7%; P < 0.001).
**Conclusion:** Both the KS and the GS accurately distinguish benign from malignant breast-lesions. However, KS proved superior in this task. Most of all the KS could substantially reduce the rate of unnecessary biopsies.
**Limitations:** Prospective studies are required to evaluate the impact of the GS and the KS on clinical decision making.
**Ethics committee approval:** IRB-approval obtained.
**Funding for this study:** Not applicable.
**Author Disclosures:**
Pascal A.T. Baltzer: Nothing to disclose
Hubert Bickel: Nothing to disclose
Thomas H. Helbich: Nothing to disclose
Rüdiger Schulz-Wendtland: Nothing to disclose
Matthias Dietzel: Nothing to disclose
Michael Uder: Nothing to disclose
Paola Clauser: Nothing to disclose

### RPS 402-8

**Effects of BI-RADS combined with breast contrast-enhanced ultrasound (CEUS) prediction model on biopsy rate and assessing risk of malignancy of breast lesions**

*H. Wu*[1], C. Qin[1], J. Luo[1], L. Tang[2], L. Yang[3], Z. Lv[4], Y. Cheng[1], Y. Lijun[5], B. Bai[6]; [1]Chengdu/CN, [2]Fuzhou/CN, [3]Kunming/CN, [4]Huangshi/CN, [5]Xi'an/CN, [6]Yan'an/CN
*(fkjerrynick@163.com)*

**Purpose:** To evaluate whether the combination of Breast Imaging Report and Data System (BI-RADS) and CEUS prediction models can optimise BI-RADS 4 and 5 lesions by reducing unnecessary biopsy.
**Methods or Background:** 1197 breast lesions provided by 8 centres were examined in the midterm of the study. The enrolled BI-RADS 4 and 5 lesions were evaluated and categorised by two independent physicians groups which included the examination group and the reading group in each centre using 6 prediction models. The malignant lesions and precancerous lesions were defined into biopsy lesions, while benign lesions were defined as follow-up lesions according to histopathological results. The diagnostic efficiency of the category given by each group was compared. The BI-RADS 4A lesions were combined with the prediction model alone to observe its diagnostic value for biopsy lesions.
**Results or Findings:** The category given by examination group achieved the highest diagnostic efficiency and its area under Within all 4A lesions, some were redefined into follow-up category when they were consistent with benign models, while others were redefined into biopsy category when they were consistent with malignant models. Then 80.17% of specificity and 94.50% of NPV in predicting biopsy lesions were achieved and the unnecessary biopsy rate was reduced (from 81.09% to 51.52%) on a base of lower risk of malignancy (from 18.91% to 5.5%).
**Conclusion:** The midterm results of our multicentre study had confirmed CEUS prediction model could assist BI-RADS to improve its diagnostic value and reduce the unnecessary biopsy rate.
**Limitations:** Due to the inconsistent familiarity of the physicians in the study centre with ACR BI-RADS®, the BI-RADS classification of the in-situ lesions is not standardised, especially for the 4A lesions.
**Ethics committee approval:** All hospitals in our multicentre study have received the ethics committee approval.
**Funding for this study:** No funding was received.
**Author Disclosures:**
Yuan Lijun: Nothing to disclose
Jun Luo: Nothing to disclose
Huangshi Central Hospital Zhihong Lv: Nothing to disclose
Lichun Yang: Nothing to disclose
Baoyan Bai: Nothing to disclose
Hao Wu: Nothing to disclose
Lina Tang: Nothing to disclose
Yinrong Cheng: Nothing to disclose
Chen Qin: Nothing to disclose

### RPS 402-10

**Application of delayed-contrast MRI for improved sensitivity in breast cancer diagnosis**

*D. Daniels*[1], D. Last[1], N. Fridman[1], N. Lahat[1], R. Faermann Weidenfeld[1], O. Halshtok[1], D. Samoocha[1], Y. Mardor[2], M. Sklair-Levy[2]; [1]Tel-Hashomer/IL, [2]Ramat-Gan/IL
*(dianne.daniels@sheba.health.gov.il)*

**Purpose:** Contrast-enhanced MRI of the breast provides high sensitivity but variable specificity in detecting breast cancer, and may lead to excessive benign biopsies. Treatment response assessment maps (TRAMs) are calculated from delayed-contrast MRI and reflect delayed-contrast clearance/accumulation. In brain tumour patients, TRAMs provide high sensitivity/specificity (>90%) for differentiating tumour (contrast clearance, blue in TRAMs)/non-tumour (accumulation, red) tissues, currently in routine clinical use. Here, we studied their application for reducing benign biopsies in breast lesions.
**Methods or Background:** 92 women with 133 breast lesions suspected as tumours were scanned by standard (including DCE) and delayed-contrast MRI. Lesions were determined by biopsy (119)/follow-up (18) as malignant/benign. The performance of benign/malignant classifiers calculated from the TRAMs were studied using a supervised machine-learning (ML) algorithm and leave-one-out cross-validation (LOOCV).
**Results or Findings:** 100% of malignant lesions (78) appeared blue in the TRAMs while benign (55) lesions appeared mixed red/blue. The lesion features found to be most predictive were: blue portion, blue intensity and largest blue cluster. The TRAMs-based classifier combining these features resulted in: sensitivity=99%/specificity=60%/PPV=78%/NPV=97%/accuracy=83%, while standard DCE resulted in: sensitivity=100%/ specificity=33%/ PPV=68%/ NPV=100%/ accuracy=72%. Comparison of TRAMs to histology revealed different vascular patterns for blue (vessels with closed/compressed lumens) and red (open lumens) regions.
**Conclusion:** The TRAMs-based classifier may provide improved diagnosis of breast cancer as it resulted in nearly no false negatives and 60% of correctly identified benign tumours, thus allowing significant reduction of benign biopsies. Expanding to more advanced ML methods and a larger cohort is ongoing.
**Limitations:** Our cohort is naturally biased towards malignant lesions. Still, this does not affect the reliability determined by the negligible percentage of false negatives. False positive cases were mainly due to hemangiomas/fat necrosis/fibroadenomas with myxoid components.
**Ethics committee approval:** IRB approved.
**Funding for this study:** Funding was received from the Israel Science Foundation.
**Author Disclosures:**
Yael Mardor: Nothing to disclose
Miri Sklair-Levy: Nothing to disclose
Renata Faermann Weidenfeld: Nothing to disclose
David Samoocha: Nothing to disclose
David Last: Nothing to disclose
Nora Lahat: Nothing to disclose
Dianne Daniels: Nothing to disclose
Osnat Halshtok: Nothing to disclose
Naomi Fridman: Nothing to disclose

**RPS 402-11**
**Incidental lesions detected on magnetic resonance imaging are more common among patients with luminal A-like breast cancers**
*A. Sassi*, M. Tervo, A. Salminen, N. Mäenpää, M. Moisander, L. Tiainen, I. Rinta-Kiikka, O. Arponen; Tampere/FI

**Purpose:** The amount of incidental enhancing lesions in different biological subtypes of breast cancer remains unknown. As incidental enhancing lesions pose a frequent diagnostic and surgical problem, more research is needed to improve their management. We assessed the prevalence and prognostic factors of incidentally detected breast lesions.
**Methods or Background:** Although conventional mammography and ultrasound are the primary imaging modalities in breast cancer diagnostics, breast MRI has gained a growing role in preoperative staging. Breast MRI has superior sensitivity, but limited specificity in detecting malignant breast lesions. The percentual share of patients with incidental only-MRI-detected enhancing lesions ranges between 11-29%. We retrospectively collected all preoperative breast MRI examinations in Tampere University Hospital (TaUH) between the years 2014-2019. Altogether 948 patients with invasive primary breast tumours underwent breast MRI examination, of which 167 had at least one incidental enhancing breast lesion requiring core needle biopsy. We collected clinical and histopathological data from the medical records.
**Results or Findings:** Patients with luminal A invasive breast carcinoma of no special type (NST) had a higher prevalence of all incidental enhancing lesions (p=0.038) and malignant incidental lesions (p=0.011) in breast MRI. Incidental enhancing lesions in the contralateral breast had a lower malignancy rate (27.3% vs. 59.1%, p<0.001).
**Conclusion:** In conclusion, the most substantial finding of this study is the higher incidence of incidental MRI-detected and malignant MRI-detected breast lesions in patients with invasive NST-breast cancer of luminal A biological subtype. Therefore, histological type and biological subtypes of breast cancer should be considered when planning to subject patient to preoperative breast MRI.
**Limitations:** The retrospective study was one-centred.
**Ethics committee approval:** Not applicable due to the retrospective nature of the study.
**Funding for this study:** Funding was received for this study by Emil Aaltonen-foundation and State Research Funding.
**Author Disclosures:**
Annukka Salminen: Nothing to disclose
Otso Arponen: Nothing to disclose
Irina Rinta-Kiikka: Nothing to disclose
Niina Mäenpää: Nothing to disclose
Mikko Moisander: Nothing to disclose
Antti Sassi: Nothing to disclose
Leena Tiainen: Nothing to disclose
Maija Tervo: Nothing to disclose

**RPS 402-12**
**Radiological features of B3 breast lesions in mutation carrier patients: our retrospective experience**
*L. Messina*, C. L. L. Piccolo, R. Stefanucci, M. Tommasiello, M. Sammarra, B. Beomonte Zobel; Rome/IT

**Purpose:** The aim of this study is to evaluate the radiological features of B3 breast lesions in patients with genetic mutations to establish an anatomo-radiological correlation.
**Methods or Background:** We retrospectively enrolled 227 women with histological diagnosis of B3 breast lesion at the Breast Unit of our Institute from 2010 to 2021. Among this group, we identified 21 patients with genetic test positivity for high, intermediate and low penetrance mutated genes associated with breast cancer (BRCA1, BRCA2 and ATM). Mammographic, sonographic and MRI studies were evaluated according to the BI-RADS lexicon (5th edition).
**Results or Findings:** BRCA was the most frequent mutation (n=15) followed by ATM mutation (n=6). Histological findings showed 9 atypical ductal hyperplasia (ADH), 6 lobular neoplasia (LIN) including lobular carcinoma in situ (LCIS), 3 flat epithelial atypia (FEA) and 3 radial scar (RS). Microcalcifications were the most frequent findings in mammography (71%) with a prevalence of 75% in ADH, 50% in LIN and 100% in RS and FEA. Only 6 patients had a negative mammography, but they presented pathological features at MRI. MRI was performed in 12 patients and it demonstrated a “non-mass like” enhancement in all patients, with the final diagnosis of ADH (n=6), FEA (n=3) and LIS (n=3). We did not find sonographic findings in patients with BRCA mutation. All patients with ATM mutation had a hypoechoic nodule with irregular margins, interpreted with a high risk of malignancy (BI-RADS US 5). Definitive histological diagnosis showed RS (n=3) and LIN (n=3).
**Conclusion:** According to our experience, B3 lesions in mutation carrier patients show high suspicious radiological features. Our results are promising but more studies are necessary in the future.
**Limitations:** The study included a small sample of patients.
**Ethics committee approval:** Approval for this study by an ethics committee was not necessary.
**Funding for this study:** No funding was received for this study.
**Author Disclosures:**
Matteo Sammarra: Nothing to disclose
Laura Messina: Nothing to disclose
Bruno Beomonte Zobel: Nothing to disclose
Claudia Lucia Lucia Piccolo: Nothing to disclose
Rita Stefanucci: Nothing to disclose
Manuela Tommasiello: Nothing to disclose

**13:00-14:30** **Room M 1**

### Research Presentation Session: Oncologic Imaging

### RPS 416

### Miscellaneous oncology

**RPS 416-1**
**Moderator**
B. Van Ginneken; Nijmegen/NL
**Author Disclosure:**
B. Van Ginneken: Founder: Thirona; Share holder: Thirona

**RPS 416-2**
**Association of pre-radiotherapy tumour burden and overall survival in newly diagnosed glioblastoma corrected for MGMT methylation status: retrospective analysis of the EORTC CENTRIC-CORE trials**
*A. Alafandi*[1], K. A. van Garderen[1], S. Klein[1], S. van der Voort[1], M. Weller[2], T. Gorlia[3], J. C. Tonn[4], M. Smits[1]; [1]Rotterdam/NL, [2]Zurich/CH, [3]Brussels/BE, [4]Munich/DE
*(a.alafandi@erasmusmc.nl)*

**Purpose:** We retrospectively evaluated the association between pre-radiotherapy tumour burden and overall survival (OS) adjusted for the prognostic value of MGMT promoter methylation in newly diagnosed glioblastoma receiving radio-/chemotherapy with temozolomide.
**Methods or Background:** Patients were included from the EORTC CENTRIC (EORTC-26071) and CORE (EORTC-22072) trials if post-operative MRI scans were available within a timeframe of 3 weeks after surgery and up to 4 weeks before radiotherapy, including both pre- and post-contrast T1w images and at least one T2w sequence (T2w or FLAIR). Automated segmentation was performed on post-operative (residual) pre-radiotherapy tumour to obtain volumetric measurement of (i) contrast-enhancing (CET) and (ii) non-enhancing T2w hyperintensity (NET) tumour components. Cox proportional hazard models and Kaplan Meier estimates were used to assess the prognostic power of CET / NET tumour volume for OS compared to known prognostic factors (age and performance status).
**Results or Findings:** 408 tumour segmentations (MGMT methylated, n=270) were included in the main analysis. Median OS for MGMT unmethylated tumours was 61 weeks versus 116 weeks for MGMT methylated tumors (p<0.001) without significant correlation between MGMT methylation status and CET volumes. When stratified for MGMT methylation status higher CET volume (HR 1.02, 95%CI; 1.013-1.027, p<0.001) and older age (HR 1.66, 95%CI: 1.212-2.278, P=0.002) were significantly associated with shorter OS while NET volume and performance status were not.
**Conclusion:** Pre-radiotherapy contrast enhanced tumour volume was strongly associated with overall survival in patients receiving radio/chemotherapy adjusted for the MGMT promoter methylation status.
**Limitations:** Heterogeneity of image acquisition due to the multi-centric nature of the EORTC trials.
**Ethics committee approval:** Obtained as part of EORTC-trials.
**Funding for this study:** Medical Delta, KWF-EMCR-2017-11026, Hestia.
**Author Disclosures:**
Stefan Klein: Nothing to disclose
Michael Weller: Nothing to disclose
Karin Alida van Garderen: Nothing to disclose
Thierry Gorlia: Nothing to disclose
Sebastian van der Voort: Nothing to disclose
Ahmad Alafandi: Nothing to disclose
Marion Smits: Nothing to disclose
Jörg Christian Tonn: Nothing to disclose

### RPS 416-3

**Development of a standard of quality in medical imaging along the patient pathway**

*E. Schouman-Claeys*[1], J-C. Leclerc[2], L. Boyer[3], J-F. Meder[4];
[1]Neuilly-sur-Seine/FR, [2]Saint-Dizier/FR, [3]Clermont-Ferrand/FR, [4]Paris/FR
*(eschouman@gmail.com)*

**Purpose:** To develop, initially at a national level, a standard on imaging quality, covering the services we offer in radiology structures and patient care before, during and after the examination. The standard should be applicable to any radiological structure, public or private; be as close as possible related to our professional practice; and apprehend the whole patient management and pathway, whatever the imaging technique used.
**Methods or Background:** The initiative is the result of the practical experience we acquired by conducting voluntary quality audits in France since 2004, under the coordination of the profession. It showed the need to broaden our experience and of validated references in a context where most of the current standards are focused on equipment and radiation protection. The work implied the participation of various professionals and stakeholders, including patients, government agencies and regulatory bodies representatives.
**Results or Findings:** The work led to the publication in July 2021 by AFNOR, the French standardisation body, of the standard NF S99-300 "Quality approach in medical imaging".
**Conclusion:** Based on this dynamic, the future step is to try to develop a European dynamic leading to the construction of a common standard. This is the rationale for the proposal of the establishment of a new Technical Committee at the CEN (European Committee for Standardization).
**Limitations:** The extent of the scope and content of the future project is open to discussion.
**Ethics committee approval:** Not applicable.
**Funding for this study:** SFR (French Society of Radiology).
**Author Disclosures:**
Elisabeth Schouman-Claeys: Nothing to disclose
Jean-Charles Leclerc: Nothing to disclose
Louis Boyer: Nothing to disclose
Jean-François Meder: Nothing to disclose

### RPS 416-4

**Use of T2 mDixon water image in oncological examination of soft-tissue MR**

*M. J. de Koning*, A. te Boekhorst, B. C. Heeres; Amsterdam/NL
*(m.d.koning@nki.nl)*

**Purpose:** With T2 SPAIR fat suppression, a relatively low SNR is achieved, and this reduces image quality. The aim of this study was to evaluate the value of T2 TSE mDixon instead of other fat-suppression techniques.
**Methods or Background:** In a tertiary referral centre, 22 patients with soft tissue tumours, such as leiomysarcoma, fibrosarcoma and liposarcoma, were included. They all underwent MRI with standard T2 SPAIR fat suppression on 3T. A T2TSE mDixon sequence was added to the existing scan protocol; the same field of view (FOV) and scan region as for the T2 multivane SPAIR were used. Images were visually analysed by a dedicated soft-tissue radiologist on image quality and artefacts. After every 5 scans, the mDixon sequence was improved by changing various parameters, such as NSA, K-space and slice thickness, to gain an optimal SNR without artefacts and acceptable scan time. All mDixon sequences and the SPAIR were repeated on a phantom which consisted of an apple and bacon to measure signal and noise.
**Results or Findings:** Absolute SNR has improved from 1,88 to 2,278: comparing SPAIR to mDixon. Susceptibility artefacts were eliminated.
Total scan time has been reduced by 34 seconds.
**Conclusion:** T2 TSE mDixon is a good replacement for the T2 SPAIR mv for the soft-tissue protocol on MRI.
This is a great advantage, especially when used in a large FOV.
**Limitations:** Only a small amount of scans were made in a single-centre study. To increase the reliability of the study, a multicentre study can still be considered.
**Ethics committee approval:** No ethics committee approval was needed.
**Funding for this study:** No funding was received for this study.
**Author Disclosures:**
Birthe Christina Heeres: Nothing to disclose
Maria Johanna de Koning: Nothing to disclose
Arjan te Boekhorst: Nothing to disclose

### RPS 416-5

**Image-guided superficial radiation therapy versus non-image guided superficial radiation therapy for treatment of non-melanoma skin cancer (NMSC): a comparison of studies**

*L. Yu*[1], M. Moloney[2]; [1]Smithtown, NY/US, [2]Old Westbury, NY/US
*(lyu@skincureoncology.com)*

**Purpose:** To compare the effectiveness of image-guided superficial radiotherapy to non-image-guided superficial radiotherapy in the treatment of early-stage epithelial cancer.
**Methods or Background:** An image-guided form of superficial radiation therapy (IGSRT) was developed in 2013 where the tumour configuration and depth can be visualised prior to, during and subsequent to treatments, using a 22 megahertz (MHz) dermal ultrasound (U.S.) with a doppler component. We previously published the results using this technology to treat 2917 early-stage epithelial cancers showing a high local control (LC) rate of 99.3%. We compared these results with similar American studies from a comprehensive literature search used in an article/guideline published by the American Society of Radiation Oncology (ASTRO) on curative radiation treatment of basal cell carcinoma (BCC), squamous cell carcinoma (SCC) and squamous cell carcinoma in-situ (SCCIS) lesions from 1988 to 2018. Only U.S. based studies with greater than 100 cases with similar patient/lesion characteristics and stages treated by external beam, electron or superficial/orthovoltage radiation therapy were included in the criteria for selection. The resultant 4 studies had appropriate comparable cases identified and the data analysed/calculated with regard to local control. Logistic regression analysis was performed comparing each study to IGSRT individually and collectively with stratification by histology (BCC, SCC, and SCCIS).
**Results or Findings:** IGSRT LC was found to be statistically superior to each of the 4 non-image-guided radiation therapy studies individually and collectively (as well as stratified by histology subtype) with p-values ranging from $p< 0.0001$ to $p= 0.046$.
**Conclusion:** Results of IGSRT in local control were statistically significantly superior across the board versus non-image-guided radiation modalities in treatment of epithelial NMSC and should be considered a new gold standard for treatment of early-stage cutaneous BCC, SCC, and SCCIS.
**Limitations:** Not applicable.
**Ethics committee approval:** Not applicable.
**Funding for this study:** No funding was received for this study.
**Author Disclosures:**
Mairead Moloney: Nothing to disclose
Lio Yu: Other: Yu has received research, speaking and/or consulting support from companies including Bayer and Skin Cure Oncology.

### RPS 416-6

**Skeletal muscle cut-offs to determine 3-month survival in frail older cancer patients**

*A. Tolonen*, K. Lehtomäki, H. Kerminen, M. Bärlund, P. Österlund, O. Arponen; Tampere/FI

**Purpose:** To examine if optimal skeletal muscle index (SMI) and psoas muscle index (PMI) cut-offs could predict 3-month overall survival (OS) and thus treatability in frail older cancer patients.
**Methods or Background:** Computed tomography (CT) is the gold standard for assessing SMI and PMI. Low SMI and PMI, i.e. sarcopenia, are associated with poor survival in many cancers, but older frail patients are underrepresented in this research. The optimal oncologic treatment decisions for over-75-year-old frail cancer patients are challenging and optimised at Tampere University Hospital by comprehensive geriatric assessment (CGA) and experimental SMI- and PMI-assessment for patients with G8-screen ≤14/17. SMI and PMI were retrospectively assessed from CT scans of 43 males and 37 females; median age 80 (range 75-91) years. Optimal cut-offs were determined using the Younden method to predict 3-month OS-rates. Kaplan-Meier estimates and Cox regression were used. Median reverse Kaplan-Meier follow-up was 31 months.
**Results or Findings:** Optimal cut-offs for SMI were 42.5 cm2/m2 for men and 33.1 cm2/m2 for women, and for PMI 4.39 cm2/m2 and 4.06 cm2/m2, respectively. Three-month OS-rates were 75% vs. 96% for SMI sarcopenic vs. non-sarcopenic; and 71% vs. 95% for PMI, with median OS of 11.5 vs. 14.4 months (HR 1.84, 95% CI 1.1-3.1) for SMI and 11.5 vs. 12.9 months (1.31; 0.7-2.4) for PMI. A Cox model for 3-month OS cut-off showed HR of 3.9 (95% CI 0.7-41) for SMI, 2.5 (0.4-16.8) for ECOG PS 0-2 vs. 3-4, and 1.4 (0.2-9.5) for clinical frailty scale 1-4 vs. 5+ points.
**Conclusion:** SMI and PMI can predict 3-month survival and thus guide in oncological treatment selection for older frail cancer patients.
**Limitations:** Heterogenous and small patient sample.
**Ethics committee approval:** No ethics approval needed. Hospital permissions R19628S/R20503S.

**Funding for this study:** Tampere University Hospital research funds.
**Author Disclosures:**
Maarit Bärlund: Nothing to disclose
Otso Arponen: Nothing to disclose
Pia Österlund: Nothing to disclose
Hanna Kerminen: Nothing to disclose
Kaisa Lehtomäki: Nothing to disclose
Antti Tolonen: Nothing to disclose

### RPS 416-7

**Volumetric virtual non-calcium data of the spine's bone marrow compartment in healthy individuals: an AI-assisted reference cohort of 500 dual-energy scans**

P. Fervers[1], F. Fervers[2], D. Zopfs[1], R. P. Reimer[1], G. Pahn[3], J. Kottlors[1], D. Maintz[1], T. Persigehl[1], *N. Grosse Hokamp*[1]; [1]Cologne/DE, [2]Karlsruhe/DE, [3]Hamburg/DE

**Purpose:** To identify reference values of virtual non-calcium (VNCa) bone marrow images of the spine in a large-scale cohort of healthy individuals.
**Methods or Background:** Methods: The assessment of contrast-enhanced, dual-energy CT was fully automated and deep-learning supported and did not require specific user interaction. The thoracolumbar spine was segmented by a challenge winning, pre-trained convolutional neuronal network. Attenuation histograms of volumetric VNCa data were created for each exam and suppression setting. The percentage overlap of histograms in consecutive scans assessed the intra-individual consistency of our method.
Background: VNCa images have shown high potential to diagnose bone marrow malignancy of the spine, which is frequently disguised by dense trabecular bone on conventional CT. Yet, there is a lack of reference values defining the physiological appearance of the spine on VNCa data.
**Results or Findings:** 500 exams of 168 individuals were included (88 female, patient age 61.0 ± 15.9). In total, 8298 vertebrae were segmented (median 17 [interquartile range (IQR) 16–17] vertebrae per exam). Median overlap of histograms in two consecutive exams was 0.93 [IQR 0.88–0.96]. Bone marrow attenuation presented distinct age- and sex-specific profiles, which are reported in detail as part of the full manuscript.
**Conclusion:** Automated, volumetric assessment is an intra-individually robust method to evaluate the spine's bone marrow using VNCa data. We provide the bone marrow attenuation profiles of a large-scale cohort of healthy individuals as a reference for future studies.
**Limitations:** Normal values are restricted to the specific scanner we used. Yet the methodology is transferable to other imaging protocols.
**Ethics committee approval:** IRB approved study. Informed consent waived due to retrospective design.
**Funding for this study:** No specific funding.
**Author Disclosures:**
Florian Florian Fervers: Nothing to disclose
Gregor Pahn: Employee: Currently working for Philips Healthcare.
David Maintz: Speaker: Speaker for Philips Healthcare.
David Zopfs: Nothing to disclose
Philipp Fervers: Nothing to disclose
Nils Grosse Hokamp: Speaker: Speaker for Philips Healthcare.
Jonathan Kottlors: Nothing to disclose
Thorsten Persigehl: Nothing to disclose
Robert Peter Reimer: Nothing to disclose

### RPS 416-8

**Adherence of diagnostic accuracy studies published in Radiology to the STARD statement: a meta-analysis**

*A-C. Stahl*, A-S. Tietz, B. Kendziora, M. Dewey; Berlin/DE
*(ann-christine.stahl@charite.de)*

**Purpose:** To investigate whether the quality of reporting of diagnostic accuracy studies improved after the use of the STARD guidelines became mandatory.
**Methods or Background:** In 2016 Radiology made the use of the STARD guidelines mandatory for its authors. Our MEDLINE search identified 66 diagnostic accuracy studies published in Radiology in 2015 and 2019. The quality of reporting was assessed by two independent reviewers using the revised STARD statement. Item 11 was excluded because a meaningful decision about adherence was not possible. Studen's t-test for independent samples was used to analyse differences in the mean number of reported STARD items related to publication date, data collection, study design and citation rate.
**Results or Findings:** Oncological imaging was the main topic of most of the analysed studies (65.2%). The mean total number of reported STARD items of all included 66 diagnostic accuracy studies was 18.6 ± 2.4 of 29 items (64.1%). Adherence of diagnostic accuracy studies to the STARD statement significantly improved from 17.7 ± 2.4 in 2015 to 19.9 ± 1.9 in 2019 (p<0.001). No evidence of a difference was found for data collection (p=0.67), study design (p=0.86) and citation rate (p=0.34).
**Conclusion:** Overall adherence of diagnostic accuracy studies to the STARD statement in Radiology is moderate to good with a relevantly better adherence after the use of the STARD guidelines became mandatory.
**Limitations:** We changed the original STARD checklist by excluding item 11 and only focused on studies published in Radiology. Due to these two points, the generalisability of our study may be limited.
**Ethics committee approval:** Not applicable.
**Funding for this study:** Not applicable.
**Author Disclosures:**
Marc Dewey: Other: Dewey is the editor of Cardiac CT, published by Springer Nature, and offers hands-on courses on CT imaging (www.ct-kurs.de). Other: Institutional master research agreements exist with Siemens, General Electric, Philips, and Canon. The terof these arrangements are managed by the legal department of Charité – Universitätsmedizin Berlin. Patent Holder: Professor Dewey holds a joint patent with Florian Michallek on dynamic perfusion analysis using fractal analysis (PCT/EP2016/071551 and USPTO 2021 10,991,109). Other: Dewey is European Society of Radiology (ESR) Research Chair (2019–2022) and the opinions expressed in this article are the author's own and do not represent the view of ESR. Per the guiding principles of ESR, the work as Research Chair is on a voluntary basis and only remuneration of travel expenses occurs. Research/Grant Support: EU (603266-2) DFG (DE 1361/14-1, DE 1361/18-1, BIOQIC GRK 2260/1, Radiomics DE 1361/19-1 [428222922] and 20-1 [428223139] in SPP 2177/1), Berlin University Alliance (GC_SC_PC 27), Berlin Institute of Health (Digital Health Accelerator).
Ann-Christine Stahl: Nothing to disclose
Anne-Sophie Tietz: Nothing to disclose
Benjamin Kendziora: Nothing to disclose

### RPS 416-10

**Do different liver backgrounds affect the diagnosis of focal liver lesions by Sonazoid-contrast enhanced ultrasound? A multicentre, prospective study**

*Y. Li*[1], P. Liang[1], X. Yu[1], X. Jing[2], H. Yang[3], K. Li[4], W. Wu[1], S. Lu[1], H. Ding[5]; [1]Beijing/CN, [2]Tianjin/CN, [3]Nanning/CN, [4]Wuhan/CN, [5]Shanghai/CN

**Purpose:** We aimed to systematically investigate the value of Sonazoid-CEUS for diagnosing FLLs in different liver backgrounds compared to CEMRI/CECT.
**Methods or Background:** This multicentre, prospective study included 915 patients with FLLs from 23 centres in China. SCEUS and CEMRI/CECT were performed between August 2020 and March 2021, and the results were divided into four groups: (I) non-hepatitis and non-fatty liver background, (II) hepatitis and non-fatty liver background, (III) non-hepatitis and fatty liver background, and (IV) hepatitis and fatty liver background. The diagnostic performance between SCEUS and CEMRI/CECT was compared. Diagnostic performance was analysed using the McNemar test and chi-square test.
**Results or Findings:** 915 participants (56.5 years ± 12.2) were enrolled. We found that, regardless of liver backgrounds, SCEUS has high sensitivity and accuracy. Although the specificity in SCEUS, CEMRI/CECT is low in patients with hepatitis, SCEUS could significantly improve specificity (group II: SCEUS vs. CEMRI/CECT, P<0.05; SCEUS vs. CEMRI, P<0.05, respectively). Although the specificity and accuracy in group III between SCEUS and CEMRI/CECT did not show significant difference (P>0.05), it is still lower than that in group I.
**Conclusion:** SCEUS has good performance in the differentiation between malignant and benign FLLs in different liver backgrounds and is a more reliable tool than CEMRI/CECT, especially in patients with hepatitis. Accurate diagnosis using imaging is essential to determine the correct treatment strategy. (Acknowledgements: we thank Prof. Guangjian Liu, Wen Cheng, Guangzhi He, Kai Li, Yan Luo, Liping Liu, Tianan Jiang, Guiming Zhou, Xiaoyan Xie, Zhigang Song and Pharmaceutical Diagnostics GE Healthcare Ltd for their support.)
**Limitations:** Long-time scanning will lead to microbubble rupture and affect the image quality.
**Ethics committee approval:** Chinese PLA General Hospital.
**Funding for this study:** Grant 82172027 from the National Scientific Foundation Committee of China.
Grant 2018ZX10723204-008 from the State Key Project on Infectious Diseases of China.
**Author Disclosures:**
Ping Liang: Nothing to disclose
Xiang Jing: Nothing to disclose
Hong Yang: Nothing to disclose
Hong Ding: Nothing to disclose
Xiaoling Yu: Nothing to disclose
Yunlin Li: Nothing to disclose
ShiChun Lu: Nothing to disclose
Kaiyan Li: Nothing to disclose
Wei Wu: Nothing to disclose

13:00-14:30 Room M 3

### Research Presentation Session: Imaging Informatics / Artificial Intelligence and Machine Learning

### RPS 405

### Artificial intelligence (AI) in chest imaging: part 1

**Moderator**
D. Wormanns; Berlin/DE

### RPS 405-2

**Malignancy risk stratification for pulmonary nodules: comparing a deep-learning approach to multiparametric statistical methods**

*L. Piskorski*, M. Debić, K. Schlamp, L. Welzel, O. v. Stackelberg, H-U. Kauczor, C. P. Heussel, J. Kroschke; Heidelberg/DE
*(lars.piskorski@med.uni-heidelberg.de)*

**Purpose:** Artificial intelligence is a promising tool helping clinicians estimate the risk of malignancy in pulmonary nodules. Previous studies have been performed on highly preselected patient data. We used a clinically derived dataset, to compare the AI-based Optellum lung cancer prediction score (LCP score) to the Brock model and Lung-RADS®. Additionally, we investigated whether underlying pulmonary structural changes due to emphysema or fibrosis affect LCP scoring.
**Methods or Background:** We conducted a retrospective study analysing CT images of incidental pulmonary nodules measuring 5-30 mm. Ground-truth diagnosis was defined by histology (mandatory for malignant nodules) or follow-up stability. Final analysis was performed on 297 patients with 422 eligible nodules, of which 106 nodules in 95 patients were malignant.
**Results or Findings:** Overall AUC score was 91,5% (95% CI 88,8 to 94,3) for LCP; 88,0% (95% CI 84,4 to 91,7) for Brock model; and for 82,9% (95% CI 79,0 to 86,8) Lung-RADS®. LCP score performed better than Brock model (p<0,05) and Lung-RADS® (p<0,005). AUC for assessing nodules in patients with emphysema was slightly lower, with 90,6% (95% CI 86,2 to 95,0), compared to 92,5% (95% CI 89,0 to 96,1), but not significantly (p=0,503). Similarly for patients with fibrosis, an AUC of 88,2% (95% CI 78,6 to 97,8) was achieved, compared to 89,5% (95% CI 79,4 to 99,5) in the control group (p=0,86).
**Conclusion:** We could replicate previous studies showing LCP score outperforming Brock model, even when used in a clinically derived dataset. For the first time, we could show LCP score outperforming Lung-RADS® as well. Additionally, LCP scoring is not significantly affected when analysing nodules in patients with pulmonary emphysema or fibrosis.
**Limitations:** Larger patient numbers needed for confirming the applicability of LCP score in emphysema and fibrosis patients.
**Ethics committee approval:** This study was approved by an ethics committee.
**Funding for this study:** No funding was received for this study.
**Author Disclosures:**
Linn Welzel: Nothing to disclose
Manuel Debić: Nothing to disclose
Claus Peter Heussel: Nothing to disclose
Hans-Ulrich Kauczor: Nothing to disclose
Oyunbileg von Stackelberg: Nothing to disclose
Kai Schlamp: Nothing to disclose
Lars Piskorski: Nothing to disclose
Jonas Kroschke: Nothing to disclose

### RPS 405-3

**Detection of unreported clinically significant pulmonary nodules using a combination of computer vision (CV) algorithm and report processing**

*D. Stav*[1], J. Balcombe[2], G. Aviram[1], D. Mercer[1], G. Levy[1]; [1]Tel Aviv-Yafo/IL, [2]Ramat HaHayal/IL
*(danastav50@gmail.com)*

**Purpose:** Missed pulmonary nodules may progress to lung cancer. AI-aided detection of nodules is helpful but the large volume of unneeded alerts may cause radiologist fatigue. Filtration of AI findings via radiology report analysis will notify radiologists only of unreported nodules, reducing unnecessary alerts.
**Methods or Background:** Retrospective analysis of 1,727 consecutive chest CT scans and corresponding reports. Scans processed by computer vision (CV) nodule detection algorithm (IMedis Ltd.). Associated radiology reports tagged by radiology resident to indicate mention/absence of nodules ≥6 mm. Combined outputs classified by 3 senior radiologists.
**Results or Findings:** Nodules ≥6mm were detected by AI in 588 cases, of which 169 were unreported as per report analysis. Consensus radiologist review confirmed 23 cases with true unreported nodules, 111 cases with findings that were not nodules and 35 cases with nodules that were determined to be insignificant. Radiologist review revealed most non-nodule findings to be multifocal, infectious or inflammatory process. Language for such processes was not considered as positive nodule mention at report processing stage.
**Conclusion:** Dual algorithm combining CV analysis of chest CTs and human report tagging outputs a relatively small number (9.8%) of scans for re-review, with high probability (13.6%) of clinically significant missed nodules. In comparison, a CV only algorithm would produce 3.5 times as many alerts. Overall, in 1.1% (23/1727) of cases an actionable missed nodule was detected. The high yield, small volume output of the dual algorithm may justify radiologist review of these flagged cases, improving detection of actionable pulmonary nodules with minimal increased workload.
**Limitations:** NLP would be the optimal method of report tagging but was not available in the report language (Hebrew).
**Ethics committee approval:** IRB approval was obtained and informed consent was waived for retrospective study.
**Funding for this study:** Not applicable
**Author Disclosures:**
Galit Aviram: Nothing to disclose
Diego Mercer: Nothing to disclose
Dana Stav: Nothing to disclose
Jonathan Balcombe: Employee: IMedis Ltd.
Gad Levy: Nothing to disclose

### RPS 405-4

**Earlier discharge of patients from follow-up for lung cancer screening using artificial intelligence for detection of pulmonary noduli on computed tomography**

*I. Gimbel*, M. Bergsma, M. v. d. Weijer, N. Barlo, P. Algra; Alkmaar/NL
*(ia.gimbel@nwz.nl)*

**Purpose:** Lung cancer is the leading cause of cancer death. Effective screening and early detection are instrumental in reducing mortality. Artificial intelligence (AI) and computer-aided detection (CAD) methods have been proved useful in the diagnosis of pulmonary nodules and early diagnosis of lung cancer. Meanwhile, according to the new BTS guidelines, the use of volumetrics of noduli could result in earlier discharge. Using AI in clinical practice is expensive. Determining the cost-effectiveness of AI is important for implementing these methods in routine clinical practice.
To determine whether using the use of CAD of pulmonary nodules on computed tomography (CT) scan results in earlier discharge from follow-up for lung noduli.
**Methods or Background:** For this retrospective cohort study, consecutive patients screened with thoracic CT for pulmonary nodules follow-up were included. The primary outcome is the percentage of patients that could have been discharged earlier from follow-up based on the current BTS guidelines when using CAD for volumetrics. Secondary outcomes include nodule measurements, time saved when examining CT-scans with the aid of AI, and accuracy of AI in the detection of pulmonary noduli.
**Results or Findings:** A total of 300 patients were included. Preliminary results of 50 patients show that based on current BTS guidelines 10 (20%) patients could have been discharged earlier from follow-up using computer-aided detection volumetrics. Additionally, with the use of AI, thoracic CT scans were examined 13% faster than without. Preliminary results show no missed noduli in the AI analysis of thoracic CT scans.
**Conclusion:** Based on current BTS guidelines, using AI in detecting and measuring pulmonary nodules for lung cancer screening leads to a shorter follow-up period and therefore a reduction of unnecessary CT scans.
**Limitations:** Single-centre, retrospective cohort.
**Ethics committee approval:** Not applicable.
**Funding for this study:** Financial support from Northwest Clinics, Alkmaar.
**Author Disclosures:**
Minke Bergsma: Nothing to disclose
Nicole Barlo: Nothing to disclose
Inge Gimbel: Nothing to disclose
Maarten van de Weijer: Nothing to disclose
Paul Algra: Nothing to disclose

### RPS 405-5

**AI post-COVID study. Predicting post-COVID pulmonary sequelae applying AI to imaging and clinical data: preliminary results of an Italian multicentre study**

N. C. D'Amico[1], M. Alì[1], M. Cellina[2], C. Bortolotto[3], D. Cozzi[4], S. Panella[1], S. Ibba[1], *S. Gonella*[1], S. Papa[1]; [1]Milan/IT, [2]Sesto San Giovanni/IT, [3]Pavia/IT, [4]Florence/IT

**Purpose:** As reported by the WHO, about 33% of COVID-19 patients have reported pulmonary fibrosis after the disease. However, currently it isn't possible to predict which patients will have lung sequelae. Thus, we designed a retrospective, multicentre study with the aim to develop an AI-based algorithm able to predict which patients will have pulmonary sequelae within one year after the disease.
**Methods or Background:** Comorbidities, laboratory, ventilatory and imaging data acquired during the COVID hospitalisation were collected and used to train the algorithms. Moreover, CT-exams performed within 12 months of the disease were evaluated by an expert radiologist to assess the presence of post-COVID pulmonary sequelae. We developed a radiomic model which uses four classifiers: extra decision tree, gradient boosting, random forest, and support vector machine (SVM). After that, an ensemble model, which combines conceptually different machine-learning classifiers and uses a majority vote to predict the class labels, was used.
**Results or Findings:** A total of 118 patients (45 females) with complete clinical, laboratory and imaging data were analysed. Out 118 patients, 44 subjects (37%) with pulmonary sequelae were reported at CT scan. The developed models showed a balanced accuracy of 72% for the extra decision tree, 71% for the gradient boosting, 69% for the random forest and 67% for the SVM. The ensemble model, using a majority voting, obtained a balanced accuracy of 71%, a sensibility of 75%, a specificity of 67% and a f1-score of 77%.
**Conclusion:** The ensemble model showed robust results in predicting pulmonary sequelae in post-COVID patients.
**Limitations:** The main limitation is the dimension of the dataset, but since the data collection is still ongoing further analysis will be made.
**Ethics committee approval:** Ethical committee approval was obtained on September 29, 2021 (Protocol ID 965_2021bis).
**Funding for this study:** No funding was received for this study.
**Author Disclosures:**
Sergio Papa: Nothing to disclose
Marco Alì: Consultant: Bracco Imaging
Simona Ibba: Nothing to disclose
Silvia Panella: Nothing to disclose
Michaela Cellina: Nothing to disclose
Natascha Claudia D'Amico: Nothing to disclose
Silvia Gonella: Nothing to disclose
Diletta Cozzi: Nothing to disclose
Chandra Bortolotto: Nothing to disclose

### RPS 405-7

**Transfer learning with convolutional neural network for pneumonia detection on chest radiographs**

*M. Tsuchiya*[1], S. Ichikawa[1], S. Funayama[2], H. Watanabe[2], K. Onohara[2], H. Imada[1], K. Kubota[1], S. Goshima[1]; [1]Hamamatsu/JP, [2]Yamanashi/JP
*(vd347x@bma.biglobe.ne.jp)*

**Purpose:** To assess the performance of a convolutional neural networks (CNNs) model constructed and verified based on transfer learning for the diagnosis of pneumonia using chest radiographs.
**Methods or Background:** We developed a CNNs model to detect the presence or absence of pneumonia on frontal chest radiographs. The convolution base of pre-trained CNNs model on ImageNet dataset was trained and validated on 26684 chest radiograph images (6012 positive for pneumonia) based on the database from the RSNA Pneumonia Detection Challenge. The performance of the CNNs model was compared with interpretations from four radiologists on 427 random test images using McNemar test for sensitivity and specificity.
**Results or Findings:** For the entire test set, the accuracy of the CNNs model was 76.5%, with the area under the receiver operating characteristic curve of 0.91. Our CNNs model had a significantly higher sensitivity (69.5%) than three radiologists (54.7%, p< .0.01; 62.1%, p< .0.01; 43.0%, p< .0.01) and a significantly higher specificity (81.0%) than one radiologist (79.3%, p< .0.01).
**Conclusion:** Our transfer learning-based CNNs model has a good classification performance for the detection of pneumonia.
**Limitations:** The RSNA Pneumonia Detection dataset is an imbalanced dataset.
**Ethics committee approval:** Required Ethics Committee approvals were obtained for this study.
**Funding for this study:** No funding was received for this study.
**Author Disclosures:**
Kojiro Onohara: Nothing to disclose
Hiroki Imada: Nothing to disclose
Shintaro Ichikawa: Nothing to disclose
Satoshi Funayama: Nothing to disclose
Mitsuteru Tsuchiya: Nothing to disclose
Satoshi Goshima: Nothing to disclose
Hiroaki Watanabe: Nothing to disclose
Kou Kubota: Nothing to disclose

### RPS 405-8

**Chest CT-based deep-learning model for predicting risk of transition to severe disease in patients with initial diagnosis of mild COVID-19**

*X. Yi*; Changsha/CN
*(yixiaoping@csu.edu.cn)*

**Purpose:** It is extremely urgent to identify those patients at higher transition risk to severe and critical disease in patients with no-severe COVID-19 at the first visit. The present study aimed to evaluate whether a chest CT-based deep-learning prediction system (DLPS) could be used to predict the risk of transition to severe illness in patients with initial diagnosis of mild COVID-19 infection.
**Methods or Background:** Patients with laboratory-confirmed COVID-19 with mild common-subtype illness were retrospectively recruited between January 21, 2020 and March 19, 2020. Using a semantic segmentation module to segment muscle areas in chest CT images and a multi-window extraction technique by employing three sub-networks to select three different window widths and window levels of muscle region CT image, the feature-level and decision-level information of the three sub-networks are integrated to obtain the prediction results.
**Results or Findings:** A total of 540 patients were included. Forty-eight of the enrolled patients transitioned to severe illness. We found that the DLPS achieved high performance in identifying the critically ill patient of COVID-19 by using early CT images, e.g. AUC was 0.864 (95% confidence interval (CI): 0.794, 0.918); accuracy was 86.4%; sensitivity was 93.3% and specificity was 78.2%. Compared with other well-known CNN methods, our DLPS has improved 10.9%, 6.5%, 8.9%, and 12.7% on AUC, accuracy, sensitivity and specificity, respectively.
**Conclusion:** We found chest CT-derived automated measurements of thoracic muscle to be associated with higher risk of transition to severe illness in patients affected by COVID-19 who presented initially as having the mild common-subtype infection.
**Limitations:** First, it was a single-centred, retrospective study. Second, the number of cases was relatively small.
**Ethics committee approval:** This single-centred, retrospective study protocol was approved by the Medical Ethics Committee of the Xiangya Hospital.
**Funding for this study:** No funding was received for this study.
**Author Disclosures:**
Xiaoping Yi: Nothing to disclose

### RPS 405-9

**Evaluation and determination of radiological findings of COVID-19 lung lesions in Latvia: study of artificial intelligence performance**

*L. Keisa*, M. Radzina, A. Micena, E. Birkenfelds, A. Agera, Z. Krastina, I. Priedite, L. Zvaigzne; Riga/LV
*(laura_keisha@inbox.lv)*

**Purpose:** After two years of the pandemic, more than 250 million people have been diagnosed with COVID-19. Artificial intelligence(AI) has been recognised as an important tool for diagnosis of lung lesions caused by COVID-19 infection. The aim of this study was to assess the accuracy of AI by evaluating each lung segment and to determine the limitations and benefits of interpretation in a clinical context.
**Methods or Background:** Clinical and imaging data of two groups with a mean age of 65 years(26-99 years): patients with PCR confirmed COVID-19 infection (n=250) and a healthy control group (n=50) of COVID-19 negative patients were retrospectively analysed. Non-enhanced CT of the thorax evaluation was performed by a cloud-based AI software which provides an objective quantification of the degree of lung involvement in the different lung regions and per lesion type as well as by an experienced radiologist.
**Results or Findings:** The highest accuracy (Acc) of AI performance in detection of COVID-19 lesions was in the right lower lobe despite lowest Sp-38% (95% CI 0.23-0.56), with highest Se-97% (95% CI 0.94-0.98), PPV-91%, NPV-66%, Acc-89.96%, p=0.01. Right upper lobe showed highest Sp-92% (95% CI 0.83-0.97), while Se 77% (95% CI 0.70-0.82), PPV-97%, NPV-54%; p=0.01 and Acc-80.8%. Right middle lobe showed Sp-81% (95%CI 0.72-0.88), Se of 82% (95% CI 0.75-0.87), PPV-88%, NPV-72%;p=0.01 and Acc of 82%. The left lower lobe showed Sp 82% (95% CI 0.67-0.91) and Se-83% (95% CI 0.77-0.0.87), PPV-96%, NPV-47%;p=0.01 and Acc-83,2%. The left upper lobe showed Sp-39%(95% CI 0.27-0.53) and Se-91% (95% CI 0.86-0.94), PPV-86%, NPV-52%;p=0.01 and Acc-81,6%. The main reasons for inaccuracy were due to mild or soft ground-glass opacity or markedly intense infiltrations-dense or extensive alveolar consolidations.
**Conclusion:** AI has proven to be an effective tool in assessing the COVID-19 lung damage by providing useful data about the prognostic extent of the disease, although AI results should be supervised by an experienced radiologist due to suboptimal specificity in the right lower lobe and left upper lobe as a result of minor or extensive interstitial involvement affecting calculation precision.
**Limitations:** Not applicable.
**Ethics committee approval:** Approved by the Pauls Stradiņš Clinical University Hospital ethics committee.
**Funding for this study:** Not applicable.
**Author Disclosures:**
Zanda Krastina: Nothing to disclose
Laura Keisa: Nothing to disclose
Aija Agera: Nothing to disclose
Maija Radzina: Nothing to disclose
Ligita Zvaigzne: Nothing to disclose
Edgars Birkenfelds: Nothing to disclose
Arta Micena: Nothing to disclose
Ilze Priedite: Nothing to disclose

### RPS 405-10

**The value of AI based CT severity scoring system in the triage of patients with Covid-19 pneumonia as regards oxygen requirement and place of admission**

*A. KOHLI*, T. Jha, A. B. Pazhayattil; Mumbai/IN
*(dranirudhkohli@gmail.com)*

**Purpose:** An important component of a pandemic like Covid 19 is to triage patients in order to prevent medical establishments from getting overwhelmed. The objective of our study is to find whether an initial HRCT chest can help triage patient by determining their oxygen requirement, the place of treatment, and the risk of mortality and to compare 3 CT scoring systems to find if one is a better predictor of prognosis than the other.
**Methods or Background:** This was a prospective observational study. Data collected included demographics, days from swab positivity to CT scan, comorbidities, place of treatment, laboratory parameters, oxygen requirement and mortality. We divided the patients into mild, moderate and severe based on 3 criteria - 20 point CT score (OS1), 25 point CT score (OS2) and opacity percentage (OP). CT scans were analyzed using CT pneumonia analysis prototype software.
**Results or Findings:** 1721 patients were included in our study. All the 3 scoring systems showed a significant positive correlation with oxygen requirement, place of admission and death. Based on ROC analysis a score of 4 for OS1, 8 for OS2 and 12.65% for OP was determined as the cut off for oxygen requirement.
**Conclusion:** An early CT scan in patients affected by Covid-19 is predictive of the oxygen requirement of the patient. The severity scoring system can be based on lobar involvement or based on percentage of lung involved, as they are all predictive of oxygen requirement. CT severity scoring using an automated deep learning software programme is a boon for triage. As the score increases, the chances of requirement of higher oxygen increases.
**Limitations:** This was a singlecentre study and hence representative of data in a particular community.
**Ethics committee approval:** Ethics committee approval was waived.
**Funding for this study:** No funding was received for this study.
**Author Disclosures:**
Tanya Jha: Nothing to disclose
ANIRUDH KOHLI: Nothing to disclose
Amal Babu Pazhayattil: Nothing to disclose

### RPS 405-11

**Automated detection of emphysema using artificial intelligence on CT scans: accuracy and prognostic value**

*C. Mota*, F. L. C. M. Santos, I. Matos, F. M. Antunes, C. Senra, P. F. G. T. Sousa, I. Marques; Vila Nova de Gaia/PT
*(cristina.sousa.mota@gmail.com)*

**Purpose:** The purpose of this study was to evaluate an AI-based prototype algorithm for fully automated quantification of emphysema on chest CT compared with the performance of the radiologists.
**Methods or Background:** Emphysema is a complex and heterogeneous disease that can benefit from novel approaches to understanding its evolution. Traditional evaluation of emphysema on CT is based on visual assessments, which can be subjective and prone to variation. Artificial Intelligence (AI) algorithms can be useful for the quantification of emphysema and the discrimination between the different types of emphysema. In this work, we conducted a retrospective analysis, and a total of 348 who underwent chest CT acquisition in January of 2021 were randomly included in this study. AI algorithm determines the emphysema index as the volumetric percentage of the areas of low CT attenuation (-950 HU). The visual classification of the emphysema subtype by the radiologists was made accordingly to the Fleischner Society visual emphysema CT patterns.
**Results or Findings:** The results obtained show that AI-based emphysema quantification showed a very strong correlation with the visual classification of the different degrees of emphysema severity by the radiologists and that the AI algorithm mainly failed to report low grades of emphysema. We then performed a receiver operating characteristic curve analysis to determine the cutoff point for each subtype of emphysema.
**Conclusion:** Taken together, our work shows that AI-based fully automated emphysema quantification could be a useful complementary tool to an image-based diagnosis and quantification of emphysema severity.
**Limitations:** Careful replication studies and reevaluation of the AI algorithm are needed.
**Ethics committee approval:** This study was approved by an ethics committee.
**Funding for this study:** No funding was received for this study.
**Author Disclosures:**
Carlos Senra: Nothing to disclose
Pedro Filipe Goncalves Teixeira Sousa: Nothing to disclose
Filipa Lima Carneiro Marques Santos: Nothing to disclose
Cristina Mota: Nothing to disclose
Francisco Miranda Antunes: Nothing to disclose
Inês Matos: Nothing to disclose
Inês Marques: Nothing to disclose

### RPS 405-12

**Measuring the impact of Artificial Intelligence-enhanced x-ray image analysis on the reporting performance of frontline clinicians: the GE critical care suite pneumothorax reader study**

*A. Novak*, A. Gill, P. Aylward, J. Oke, S. Ather, F. Gleeson; Oxford/UK
*(alex.novak@ouh.nhs.uk)*

**Purpose:** To evaluate the impact of artificial intelligence-enhanced image analysis on the performance of acute care clinicians when identifying pneumothoraces on plain chest radiography.
**Methods or Background:** Clinical readers were recruited from 4 different UK hospital sites, representing varying levels of seniority across 6 different clinical specialties (Emergency Medicine, Acute General Medicine, Intensive Care Medicine, Cardiothoracic Surgery, Respiratory Medicine and Radiography). Using an online platform, readers interpreted 400 chest x-ray images with respect to the presence or absence of pneumothorax, then repeated the interpretation after a 3-week washout phase, this time with the addition of an imaging heatmap and confidence score for the presence/absence of a pneumothorax as detected by the GE Critical Care Suite, an AI-enhanced image analysis algorithm. Reporting accuracy was compared in all readers with and without the AI overlay, along with time taken to report the images.
**Results or Findings:** 18 clinical readers participated in the study, with a resultant total of 144,000 image interpretations. Average sensitivity (95% CI) to detect pneumothoraces improved from 65.4% (54.5 to 76.2) to 76.0 (67.0 to 84.9)%, p = 0.003. Average specificity (95% CI) improved from 93.2 (90.2 to 96.3)% to 95.0 (92.5 to 97.6)% but this was not statistically significant (p = 0.25). Average (95% CI) time to complete scans decreased by 11% (9 to 13%) from 33 seconds to 29 seconds.
**Conclusion:** AI-enhanced image analysis can be used to significantly improve frontline clinicians' accuracy in correctly identifying pneumothoraces on plain chest radiography.
**Limitations:** This study used an enriched dataset with an artificially high prevalence of pneumothoraces (50%).
**Ethics committee approval:** This study was deemed not to require ethical approval by the UK Health Research Authority.
**Funding for this study:** This study was sponsored by GE via the National Consortium of Intelligent Medical Imaging (NCIMI).
**Author Disclosures:**
Pete Aylward: Employee: RAIQC Ltd
Avneet Gill: Nothing to disclose
Sarim Ather: CEO: RAIQC Ltd
Fergus Gleeson: Nothing to disclose
Alex Novak: Consultant: GE
Jason Oke: Nothing to disclose

**13:00-14:30** **Room X**

### Research Presentation Session: Cardiac

### RPS 403

### Cardiac MRI of cardiomyopathy: part 1

**Moderator**
T. Acar; Izmir/TR

### RPS 403-2

**Different MRI presentation of arrhytmogenic cardiomyopathy: a challenge**

*E. Listo*[1], C. De Gori[2], R. Licordari[3], G. Trocchio[4], G. Todiere[5], G. Aquaro[5]; [1]Florence/IT, [2]Capannoli/IT, [3]Messina/IT, [4]Genoa/IT, [5]Pisa/IT
*(elisa.listo@gmail.com)*

**Purpose:** This pictorial illustrates four principal MRI patterns of ventricular involvement in arrhythmogenic cardiomyopathy (AC). Different conditions such as dilated cardiomyopathy or acute myocardial injury, particularly acute myocarditis, may mimic a left dominant AC.
**Methods or Background:** The most frequent genetic mutations associated with AC are plakophilin2 and desmoplakin. The most common form of this cardiomyopathy usually affects the right ventricle (RV) but left dominant (LD) or biventricular forms are also described. Fibrofatty replacement of the myocardium and ventricular arrhythmias with preserved systolic function is hallmark of this condition, but in advanced stages eventually, ventricular dysfunction may occur. This cardiomyopathy is more frequent in young adults and clinical manifestations range from no symptoms to life-threatening arrhythmias and sudden cardiac death.
**Results or Findings:** Compared to the International task force criteria, recent “Padua Criteria” include late gadolinium enhancement (LGE) as a major criterion. Acute myocardial damage in the setting of LDAC, mostly associated with desmoplakin mutation, may be indistinguishable from myocarditis, making a diagnosis challenging. The combination of SSFP intramyocardial “India ink”, T2-STIR edema and LGE may help to make this differential diagnosis.
We propose a new classification of AC, depending on the a) ventricular presentation at MRI, b) genetic mutation and c) presence of acute myocardial damage at MRI: desmoplakin chronic (no edema) or active LDAC (with edema), biventricular (both mutations), plakophilin2 lone RVAC, genotype negative/phenotype positive (all presentations) AC.
**Conclusion:** Different MRI patterns of ventricular involvement in AC are associated with different prognoses: desmoplakin LDACs are more arrhythmogenic whereas plakophilin2 AC is more prone to heart failure. MRI is a key tool to identify AC excluding myocarditis, to suggest the potential genetic mutation and to provide prognostic stratification.
**Limitations:** Studies are needed to validate this classification.
**Ethics committee approval:** Not applicable.
**Funding for this study:** No funding was received for this study.
**Author Disclosures:**
Gianluca Trocchio: Nothing to disclose
Giancarlo Todiere: Nothing to disclose
Carmelo De Gori: Nothing to disclose
Giovanni Aquaro: Nothing to disclose
Roberto Licordari: Nothing to disclose
Elisa Listo: Nothing to disclose

### RPS 403-3

**Diastolic dysfunction assessment by CMR strain rate imaging reveals differences between hypertrophic phenotypes of fabry’s disease and hypertensive heart disease**

*M. C. Halfmann*[1], E. Wolf[1], C. Kampmann[1], S. Altmann[1], K-F. Kreitner[1], T. S. Emrich[2]; [1]Mainz/DE, [2]Charleston, SC/US

**Purpose:** Fabry’s disease (FD) and hypertensive heart disease (HHD) lead to myocardial hypertrophy in advanced disease despite significantly different underlying pathomechanisms: FD is characterised by the accumulation of sphingolipids, HHD is caused by remodelling due to chronic pressure overload. In this study, differences in diastolic function between diseases were assessed using feature tracking strain-rate imaging.
**Methods or Background:** 20 FD patients with severe myocardial involvement and 44 patients with HHD were retrospectively identified. 62 prospectively recruited individuals served as healthy controls (HC).
In addition to clinical standard (volumetry, T1-mapping, LGE-assessment), feature-tracking strain analysis was performed and strain rates were utilised to assess diastolic function by passive and active ventricular filling (E-/A-waves) and calculation of E/A strain-rate ratios.
**Results or Findings:** FD and HHD had similar indexed LV myocardial mass (92.5±31.5 g/m2 vs. 87.5±21.1 g/m2), which was significantly higher compared to HC (58.7±8.9 g/m2). FD patients showed similar E/A strain-rate ratios compared to HC, while HHD showed significantly reduced E/A strain-rate ratios compared to both other groups (HHDvsHCvsHD: 1.4±1.3 vs. 4.2±3.6 vs. 2.4±1.6). This was driven by a significant reduction of the A-wave in FD compared to HC/HHD (FDvsHCvsHHD: 0.4±0.2 vs. 0.6±0.2 vs. 0.5±0.3) and a reduction of the E-wave in HHD compared to FD/HC (FDvsHCvsHHD: 0.9±0.4 vs. 1.1±0.3 vs. 0.6±0.3).
**Conclusion:** FD showed a pseudo-normalisation of E/A strain-rate ratios, caused by a lower A-wave, suggesting a co-existing impairment of atrial function. HHD showed a reduction in passive ventricular filling as a sign of predominantly disturbed myocardial relaxation with preserved active filling. Therefore, E/A-ratios should be used carefully to assess diastolic dysfunction in FD.
**Limitations:** This study is a single-centre retrospective study.
**Ethics committee approval:** The study protocol was approved by the local ethics committee with a waiver for informed consent.
**Funding for this study:** No outside funding was acquired for this study.
**Author Disclosures:**
Christoph Kampmann: Nothing to disclose
Tilman Stephan Emrich: Other: Siemens Healthineers Speaker: Siemens Healthineers
Moritz Christian Halfmann: Nothing to disclose
Elias Wolf: Nothing to disclose
Karl-Friedrich Kreitner: Nothing to disclose
Sebastian Altmann: Nothing to disclose

### RPS 403-6

**Modern iron chelation management of thalassemia major patients guided by MRI techniques: real-world data from a long-term cohort study**

S. Bayraktaroğlu, N. Karadaş, *A. Çinkooğlu*, Ş. Önen, D. Yılmaz Karapınar, Y. Aydınok; Izmir/TR
*(acinko@gmail.com)*

**Purpose:** The aim of this study was to share our experience of over a decade with the utilisation of magnetic resonance imaging (MRI) techniques to tailor iron chelation therapy (ICT) management in patients with thalassemia major (TM) and to evaluate longitudinal correlations between various to iron overload (IOL) indices.
**Methods or Background:** We assessed the impact of using MRI techniques to guide ICT on IOL outcomes in a cohort of 99 patients with TM (mean age at baselines 20.7±6.9 years) followed from 2006 to 2019. We also assessed the ability of serum ferritin (SF) trends to predict changes in consecutive liver iron concentration (LIC) and cardiac T2* (cT2*) measurements.
**Results or Findings:** Patients with safe LIC values (<7 mg/g dw) increased from 57% to 77%, and safe cT2* values (>20 ms) increased from 72% to 86%. We obtained the most significant improvement in patients with severe and moderate liver (p=0.006 and p<0.001) and cardiac (p<0.0013 and p<0.0001) IOL at baseline. SF trends were in the same direction in 64% of changes in LIC, but only 42% of changes were proportional. Most of the changes in SF (64%) and LIC (61%) could not predict changes in cT2*. Moreover, downward trends in SF and LIC were associated with worsening cardiac iron in 29% and 23.5% of consecutive cT2* measurements.
**Conclusion:** Liver and cardiac MRI-driven oral iron chelation improved the iron status of subjects with TM and demonstrated the importance of using validated MRI techniques in critical clinical decisions.
**Limitations:** First, it includes retrospective and observational data. Second, MRI study intervals varied among individual patients. Third, in this single-centre experience, the study population is relatively small.
**Ethics committee approval:** The study was approved by Ege University’s Institutional Review Board.
**Funding for this study:** This study did not receive any funding to declare.
**Author Disclosures:**
Selen Bayraktaroğlu: Nothing to disclose
Nihal Karadaş: Nothing to disclose
Deniz Yılmaz Karapınar: Nothing to disclose
Yeşim Aydınok: Author: Conflict of Interest: Bristol Myers Squibb: Research Funding, Membership on an entity’s Board of Directors or advisory committees, Celgene: Research Funding, CRISPR Therapeutics: Consultancy, Imara: Research Funding, Ionis Pharmaceuticals: Research Funding, LaJolla: Research Funding, Novartis: Research Funding, Speakers Bureau, Protagonist: Research Funding, Resonance Health: Research Funding, SLN Therapeutics: Consultancy
Şebnem Önen: Nothing to disclose
Akın Çinkooğlu: Nothing to disclose

### RPS 403-8

**Impacts of T2DM on right ventricular systolic function and interactions between ventricles in patients with essential hypertension: evaluation by CMR tissue tracking**

*X-M. Li*, Z-G. Yang, L. Yang, R. Shi, J. Li; Chengdu/CN
*(393549991@163.com)*

**Purpose:** To evaluate the impact of T2DM on right ventricular (RV) systolic dysfunction and interventricular interaction using cardiac magnetic resonance feature tracking (CMR-FT) in patients with essential hypertension.
**Methods or Background:** Seventy-five hypertensive patients without T2DM [HTN(T2DM−)], forty-eight patients with T2DM [HTN(T2DM+)] and fifty-four normal controls were included in this study. The bi-ventricular global radial, circumferential and longitudinal peak strain (GRS, GCS, GLS) and regional strain of RV including basal-cavity, mid-cavity, apical-cavity strain were calculated and compared among groups. Backwards stepwise multivariable linear regression analyses were used to determine the effect of T2DM and LV strains on RV strains.
**Results or Findings:** Biventricular GLS and apical longitudinal strain of RV deteriorated significantly from controls, through HTN(T2DM-), to HTN(T2DM+) group; middle longitudinal strains of RV in both patient groups were significantly reduced, and LV GRS and GCS and basal longitudinal strain of RV were decreased in HTN(T2DM+) but preserved in HTN(T2DM-) group. In patients with hypertension, multivariable regression analyses adjusted for covariates demonstrated that T2DM was independently associated with LV strains (LV GRS: p=0.011, model R2=0.315; GCS: p=0.019, model R2=0.429; GLS: p=0.01, model R2=0.438, respectively) and RV GLS (p=0.018, model R2=0.136) but not with RV GCS and GRS. When T2DM and LV GLS were included in the multiple regression analysis, LV GLS (β=0.394, p<0.001, model R2=0.194) but not T2DM was independently associated with RV GLS.
**Conclusion:** These findings indicate that T2DM may exacerbate RV systolic dysfunction in patients with hypertension, which may be associated with superimposed LV dysfunction by coexisting T2DM, suggesting adverse interventricular interaction.
**Limitations:** Cross-sectional single-centre study with relatively small sample size and follow-up observation was not conducted.
**Ethics committee approval:** This study was approved by Biomedical Research Ethics Committee of our hospital.
**Funding for this study:** Funding was received for this study by 1·3·5 project for disciplines of excellence, West China Hospital, Sichuan University (ZYGD18013).
**Author Disclosures:**
Ling Yang: Nothing to disclose
Jiang Li: Nothing to disclose
Rui Shi: Nothing to disclose
Xue-Ming Li: Nothing to disclose
Zhi-Gang Yang: Nothing to disclose

### RPS 403-9

**Long-term effects of transjugular intrahepatic portosystemic shunt on cardiac structure and function in cirrhotic patients: a cardiac magnetic resonance imaging study**

*A. Isaak*, M. Praktiknjo, D. Kütting, C. Meyer, U. I. Attenberger, C. Jansen, J. A. Luetkens; Bonn/DE

**Purpose:** To evaluate long-term effects of TIPS implantation on structural and functional myocardial parameters in cirrhotic patients.
**Methods or Background:** In this prospective study, 30 patients with cirrhosis and portal hypertension who had clinical indication for TIPS insertion underwent cardiac magnetic resonance (CMR) imaging. 13 patients (Child-Pugh class A: 5/13, 38%; class B: 6/13, 46%; class C: 2/13, 19%) received CMR follow-up after TIPS procedure (mean time to follow-up: 11±3 months). The CMR protocol incorporated the assessment of left and right ventricular ejection fraction (LVEF, RVEF), end-diastolic volume index (LVEDVI, RVEDVI), left ventricular mass index (LVMI), and LV global longitudinal and circumferential strain (GLS, GCS), myocardial edema, late gadolinium enhancement (LGE), T1 and T2 relaxation times (T1, T2), and extracellular volume fraction (ECV).
**Results or Findings:** Biventricular volumes and LVMI increased at follow-up (LVEDVI: 75±16ml/m² vs. 92±19ml/m², P=0.001; RVEDVI: 71±15ml/m² vs. 92±22ml/m², P<0.001; LVMI: 45±13g/m² vs. 54±13g/m², P=0.005). LVEF and RVEF showed no significant difference between baseline and follow-up studies (63±6% vs. 63±7%, P=0.90; 53±9% vs. 56±10%, P=0.43, respectively). LV strain parameters showed improved values following TIPS procedure (GLS: -20.2±4.3% vs. -25.0±3.4%, P=0.008, GCS: -24.4±5.5% vs. 30.2±5.4%, P=0.003). LGE parameter did not significantly differ between both examinations (visible LGE in 5/13 patients, respectively; LGE%: 5.5±7.2% vs. 5.8±6.6%, P=0.46). Quantitative myocardial tissue parameters decreased at follow-up (T1:999±33ms vs. 975±25ms, P=0.008; ECV:32.3±5.9% vs. 29.1±4.0%, P=0.010; T2:58±3ms vs. 55±3ms, P=0.015).
**Conclusion:** Cardiac volumes, left ventricular mass and myocardial strain increased and signs of diffuse myocardial tissue alterations decreased at follow-up CMR after TIPS. TIPS implantation promotes long-term cardiac remodelling and might reduce effects of cirrhotic cardiomyopathy.
**Limitations:** This study included no histopathological reference and only a small cohort.
**Ethics committee approval:** This study was approved by an ethics committee.
**Funding for this study:** A.I. was supported by an intramural grant from the BONFOR research program (2021-1A-05).
**Author Disclosures:**
Julian Alexander Luetkens: Speaker: speaker fees from Philips Healthcare
Alexander Isaak: Research/Grant Support: supported by a grant from the BONFOR research program (2021-1A-05).
Christian Jansen: Nothing to disclose
Ulrike I. Attenberger: Speaker: speaker fees received from Siemens Healthineers in 2018
Daniel Kütting: Nothing to disclose
Carsten Meyer: Nothing to disclose
Michael Praktiknjo: Nothing to disclose

### RPS 403-10

**Diffuse myocardial inflammation and fibrosis in patients with systemic lupus erythematosus insights from cardiac magnetic resonance T1 mapping**

*H. Pu*, B. Cui, J. Liu, W. He, H. Lin, L. Peng; Chengdu/CN

**Purpose:** Systemic lupus erythematosus (SLE) is an autoimmune multi-organ disorder with frequent cardiovascular involvement. We aimed to evaluate myocardial involvement in SLE patients by cardiac magnetic resonance (CMR) T1 mapping.
**Methods or Background:** 38 SLE patients and 26 healthy age- and gender-matched controls were enrolled and underwent CMR examination. The association of T1 mapping parameters [including native T1 and extracellular volume (ECV)] with native T2, LGE, and clinical variables was analysed.
**Results or Findings:** The native T1 and ECV were significantly higher than that of the control group (1308.26±58.49ms vs. 1227.04±40.61ms, 34.18±4.85% vs. 28.34±3.00%, respectively; all p<0.001). The native T1 was moderately correlated with ECV (r=0.636, p<0.001). Global native T1 and ECV had moderate correlation with native T2 (r = 0.517 and 0.583, respectively; all p <0.001), while they had no correlation with LGE (p>0.05). Both native T1 and ECV had similar degree of correlation with pericardial effusion, pulmonary arterial hypertension (PAH), C-reactive protein (CRP), myoglobin, and troponin T (r = 0.322-0.518, all p <0.05). Besides, native T1 was also associated with creatine kinase-MB, brain-natriuretic peptide (BNP), and valvular regurgitation (r = 0.366-0.496, all p <0.05).
**Conclusion:** CMR T1 mapping enables early detection of diffuse myocardial fibrosis in SLE. The correlation of ECV with elevated T2 indicates that myocardial edema might play a nonignorable role in the extracellular volume expansion in SLE patients. These correlation findings of T1 mapping with CMR-based and clinical parameters might provide additive value for disease monitoring and therapeutic intervention for SLE patients.
**Limitations:** This study included only a small sample.
**Ethics committee approval:** This study was approved by the Biomedical Research Ethics Committee of West China Hospital.
**Funding for this study:** Funding was received for this study by the 1·3·5 Project for Disciplines of Excellence, West China Hospital, Sichuan University [ZYGD1801].
**Author Disclosures:**
Wenzhang He: Nothing to disclose
Liqing Peng: Nothing to disclose
Jing Liu: Nothing to disclose
Beibei Cui: Nothing to disclose
Hui Lin: Nothing to disclose
Huaxia Pu: Nothing to disclose

**15:00-16:00** **Room C**

### Research Presentation Session: Musculoskeletal

### RPS 510

### Musculoskeletal imaging of the lower limbs

**Moderator**
I. S. Örgüc; Manisa/TR

### RPS 510-2

**Basic and advanced metal-artifact reduction techniques at ultra-high field 7 tesla MRI: phantom study investigating feasibility and efficacy**

*C. Germann*, A. Falkowski, C. von Deuster, D. Nanz, R. Sutter; Zurich/CH

**Purpose:** To demonstrate the feasibility and efficacy of basic (increased receive bandwidth) and advanced techniques (view-angle tilting, VAT; slice encoding for metal artifact correction, SEMAC) for metal-artifact-reduction in ultra-high field 7-T MRI.
**Methods or Background:** In this experimental study, we performed 7-T MRI of titanium-alloy phantom models composed of a spinal pedicle screw (phantom 1) and an intervertebral cage (phantom 2) centered in a rectangular LEGO frame, embedded in deionised-water-gadolinium (0.1 mmol/l) solution. The following turbo spin-echo sequences were acquired: 1) non-optimised standard sequence (STA); 2) optimised, i.e., increased receive bandwidth sequence (oBW); 3) VAT; 4) combination of oBW and VAT (oBW-VAT); and 5) SEMAC. Two fellowship-trained radiologists independently evaluated images regarding peri-implant signal void and geometric distortion (a. angle measurement and b. presence of circular shape loss). Statistics included Friedman test and Cochrane Q test with Bonferroni correction for multiple comparisons. P-values <0.05 were considered to represent statistical significance.
**Results or Findings:** All metal-artifact-reduction techniques reduced peri-implant signal voids and diminished geometric distortions with oBW-VAT and SEMAC being most efficient. Compared to non-optimised sequences, oBW-VAT and SEMAC produced significantly smaller peri-implant signal voids (all $p \leq 0.008$) and significantly smaller distortion angles ($p \leq 0.001$). Only SEMAC could significantly reduce distortions of circular shapes in the peri-implant frame ($p \leq 0.006$). Notably, increasing the number of slice-encoding-steps (SES) in SEMAC sequences did not lead to a significantly better metal-artifact reduction (all $p>0.26$).
**Conclusion:** The use of basic and advanced methods for metal-artifact reduction at 7-T MRI is feasible and effective. Both a combination of increased receive bandwidth and VAT as well as SEMAC significantly reduce the peri-implant signal void and geometric distortion around metal implants.
**Limitations:** No limitations were identified.
**Ethics committee approval:** Not applicable.
**Funding for this study:** No funding was received for this study.
**Author Disclosures:**
Anna Falkowski: Nothing to disclose
Daniel Nanz: Nothing to disclose
Constantin von Deuster: Nothing to disclose
Christoph Germann: Nothing to disclose
Reto Sutter: Nothing to disclose

### RPS 510-3

**Three-dimensional analysis for quantification of knee joint space width with weight-bearing CT: comparison with non-weight-bearing CT and weight-bearing radiography**

*B. Fritz*[1], J. Fritz[2], S. Fucentese[1], C. Pfirrmann[1], R. Sutter[1]; [1]Zurich/CH, [2]New York, NY/US
*(benjamin.fritz@balgrist.ch)*

**Purpose:** To compare computer-based 3D-analysis for quantification of the femorotibial joint space width (JSW) using weight-bearing cone beam CT(WB-CT), non-weight-bearing multi-detector CT (NWB-CT), and weight-bearing conventional radiographs (WB-XR).
**Methods or Background:** Twenty-six participants prospectively underwent NWB-CT, WB-CT, and WB-XR of the knee. For WB-CT and NWB-CT, the average and minimal JSW were quantified by 3D-analysis of the minimal distance between any point of the subchondral tibial bone surface and the femur. Associations with mechanical leg axes and osteoarthritis were evaluated. Minimal JSW of WB-CT was further compared to WB-XR. Two-tailed p-values of <0.05 were considered significant.
**Results or Findings:** Significant differences existed of the average medial and lateral JSW between WB-CT and NWB-CT (medial: 4.7 vs. 5.1 mm [p=0.028], lateral: 6.3 vs. 6.8mm [p=0.008]). The minimal JSW on WB-XR (medial: 3.1 mm, lateral: 5.8 mm) was significantly wider compared to WB-CT and NWB-CT (both medial: 1.8 mm, lateral: 2.9 mm, all $p<0.001$), but not significantly different between WB-CT and NWB-CT (all $p \geq 0.869$). Significant differences between WB-CT and NWB-CT existed in participants with varus knee alignment for the average and the minimal medial JSW (p=0.004 and p=0.011) and for participants with valgus alignment for the average lateral JSW (p=0.013). On WB-CT, 25% of the femorotibial compartments showed bone-on-bone apposition, which was significantly higher when compared to NWB-CT (10%, p=0.008) and WB-XR (8%, p=0.012).
**Conclusion:** Combining WB-CT with 3D-based assessment allows detailed quantification of the femorotibial joint space and the effect of knee alignment on JSW. WB-CT demonstrates significantly more bone-on-bone appositions, which are underestimated or even undetectable on NWB-CT and WB-XR.
**Limitations:** Full-length leg radiographs were only available for 22/26 participants. Examinations (CT and XR) were performed in full-leg extension, which may differ from (semi-)flexed examinations regarding JSW.
**Ethics committee approval:** This study was approved by an ethics committee.
**Funding for this study:** No funding was received for this study.
**Author Disclosures:**
Christian Pfirrmann: Nothing to disclose
Jan Fritz: Other: Siemens AG, GE, QED, BTG, IBL, Synthetic MR, Boston Scientific Mirata Pharma Research/Grant Support: Siemens AG, BTG International, Microsoft Corporation, Zimmer Biomed, Synthetic MR, DePuy Synthes, QED; Patent Holder: Johns Hopkins and Siemens AG
Sandro Fucentese: Consultant: Medacta International AG
Benjamin Fritz: Nothing to disclose
Reto Sutter: Nothing to disclose

### RPS 510-4

**Femoral version measurement in slipped capital femoral epiphysis patients using MRI with T1 VIBE DIXON to detect femoral retroversion**

*T. D. Lerch*[1], F. Schmaranzer[1], M. Hanke[1], S. Steppacher[1], K. Siebenrock[1], M. Tannast[2], J. D. Busch[1], K. Ziebarth[1]; [1]Bern/CH, [2]Fribourg/CH

**Purpose:** Slipped capital femoral epiphyses (SCFE) and Perthes disease are paediatric hip diseases. It is unknown if SCFE patients have lower femoral version (FV) or femoral retroversion. Therefore, we report (1) mean FV, (2) prevalence of femoral retroversion (3) side-to-side difference for SCFE patients.
**Methods or Background:** A retrospective MRI analysis involving 52 hips (26 patients, 01/2017-10/2021) was performed. Inclusion criteria were paediatric hip disease (16 SCFE patients and 10 Perthes patients, 10-16 years of age). We evaluated FV using the Murphy method on rapid bilateral T1 VIBE Dixon MRI sequence (AT 32-40 seconds for 3 Tesla and for 1.5 Tesla) of the pelvis and knee (was added to the routine MRI protocol). FV was compared to the contralateral side. Five Perthes patients were treated surgically. Twelve SCFE patients presented with moderate or severe slips and were treated surgically. All SCFE patients underwent contralateral prophylactic pinning.
**Results or Findings:** The results were (1) mean FV of SCFE patients (1±2°) was significantly ($p<0.001$) lower compared to contralateral side (16±14°) and compared to Perthes patients (18±16°). (2) ten SCFE patients (63%) had FV>0°. Six SCFE patients (37%) had femoral retroversion (FV<0°). Of the contralateral side, two patients (12%) had femoral retroversion. Two Perthes patients (20%) had femoral retroversion. (3) Side-to-side difference of FV was higher for SCFE patients (17±16°) compared to Perthes patients (13±9°).
**Conclusion:** One third of SCFE patients, undergoing surgical treatment showed, had femoral retroversion and they had lower FV compared to Perthes patients. SCFE patients are at risk for femoral retroversion that can cause anterior extraarticular hip impingement. Therefore routine radiographic follow-up is recommended for these patients. These findings could be important for radiologists and paediatric surgeons treating SCFE patients.
**Limitations:** The limitations are the low sample size and the retrospective study design.
**Ethics committee approval:** Ethics committee approval was obtained.
**Funding for this study:** Funding was received for this study by the Swiss National Science Foundation.
**Author Disclosures:**
Kai Ziebarth: Nothing to disclose
Till Dominic Lerch: Nothing to disclose
Florian Schmaranzer: Nothing to disclose
Simon Steppacher: Nothing to disclose
Jasmin D. Busch: Nothing to disclose
Markus Hanke: Nothing to disclose
Moritz Tannast: Nothing to disclose
Klaus Siebenrock: Nothing to disclose

### RPS 510-6

**Impact of iterative metal artifact reduction on artifact burden and diagnostic image quality in the setting of external fixation for complex lower extremity fractures**

*H. Almansour*, A. S. Brendlin, F. Springer; Tübingen/DE

**Purpose:** External fixation (EF) of complex lower extremity fractures is a common tool in trauma surgery. Evaluation of perioperative CT scans can be impaired by metal artifacts. Iterative metal artifact reduction (iMAR) algorithms are frequently used tools with different capabilities. However, there is no dedicated preset for EFs. The aim is to qualitatively and quantitatively identify the best preset for improving image quality and reducing artifact burden in EFs with varying geometries.
**Methods or Background:** Seventy-two CTs with three types of EFs of the lower extremity (regular, hybrid and monotube) were included. CT-reconstruction ensued without iMAR (reference standard), and with three iMAR presets (spine, hip, extremity) that might fit the fixator geometry. Subjective image quality and diagnostic confidence (including potentially new artifacts produced by the iMAR algorithm) were independently analysed by two blinded readers on a 4-point-Likert-scale. Spectral artifact quantification was performed. Statistical analysis was performed using repeated measures ANOVA with Bonferroni-corrected post-hoc tests. Inter/intra-reader agreement was determined using an intraclass-correlation-coefficient(ICC). Contributors to image quality were identified via a post-hoc multinomial-regression-model.
**Results or Findings:** A total of 288 CT reconstructions were compared. Inter/intra-reader agreement was good in all image quality assessments ($ICC \geq 0.80, p<0.001$). The presets iMARhip and iMARextremity enhanced image quality significantly more than the other presets across all devices ($p< 0.001$). Although iMAR introduced new artifacts in all cases, diagnostic confidence was not impaired. Regression analysis showed the preset iMAR-extremity to have the greatest contribution to image quality (Odds Ratio≥7, $p<0.001$). Spectral artifact quantification showed iMAR-hip and iMAR-extremity to produce the lowest artifact burden ($p<0.001$).
**Conclusion:** Regardless of fixator geometry, iMAR-extremity showed the highest performance in image quality enhancement and quantitative metal artifact reduction.
**Limitations:** The limitation is the retrospective design and small sample size(288 CTs). This study included MAR algorithms and a CT scanner from a single vendor.
**Ethics committee approval:** This study was approved by an ethics committee.
**Funding for this study:** Not applicable.
**Author Disclosures:**
Andreas Stefan Brendlin: Nothing to disclose
Haidara Almansour: Nothing to disclose
Fabian Springer: Nothing to disclose

### RPS 510-7

**Magnetic resonance imaging of the lower limbs in suspected Idiopathic Inflammatory Myopathy (IIM) patients**

*L. L. Gramegna*, R. Rinaldi, G. Cenacchi, R. D'Angelo, V. Papa, R. Costa, C. Tonon, R. Lodi; Bologna/IT
*(lauraludovica.gramegna@unibo.it)*

**Purpose:** To propose a quantitative Magnetic Resonance Imaging (MRI) score of muscle edema for distinguishing IIM (Idiopathic Inflammatory Myopathies) from other mimics.
**Methods or Background:** MRI can detect muscle edema, which reflects active muscle inflammation, assessing the extent of muscle involvement in IIM. We retrospectively evaluated 85 consecutive patients (mean age 57.4±13.9 years; 48 F) with suspected IIM (i.e. muscle weakness and/or persistent hyper-CK-emia with/without myalgia) who underwent MRI of lower limbs using T2-weighted Fast Recovery-Fast Spin Echo (FR-FSE) images. Muscle inflammation was evaluated in 11 muscles on the upper and 8 muscles on the lower portions of both legs. The edema of each muscle was graded according to a 4-point likert-type scale (0=no edema, 1= slight edema defined as involving 1/3 of muscle area and/or being slightly hyperintense, 2=moderate defined as involving 2/3 of muscle area and/or being moderately hyperintense, or 3=severe edema defined as involving total muscle area and/or being severely hyperintense), totaling up to 114= ((11+8)*3*2). Diagnostic accuracy of the MRI edema total score was explored by computing the area under the ROC curve and measuring sensitivity and specificity.
**Results or Findings:** According to an Expert Consensus Panel (a multidisciplinary team applying the Bohan and Peter diagnostic criteria), 34 (40%) patients were diagnosed with definite IIM (IIM group) whilst 51 (60%) received an alternative diagnosis (non-IIM group). Our results showed that a potential cut-off score ≥18 was able to correctly classify patients with AUC=0.85, specificity=96% and sensitivity=52.9%.
**Conclusion:** Our study shows that assessing the extent of muscle edema in the lower limbs with MRI may be capable of distinguishing IIM from other mimics.
**Limitations:** Different cut-off scores with different specificity and sensitivity values can be proposed.
**Ethics committee approval:** This study was approved by the local ethics committee.
**Funding for this study:** No funding was received for this study.
**Author Disclosures:**
Roberto D'Angelo: Nothing to disclose
Rita Rinaldi: Nothing to disclose
Valentina Papa: Nothing to disclose
Laura Ludovica Gramegna: Nothing to disclose
Glovanna Cenacchi: Nothing to disclose
Raffaele Lodi: Nothing to disclose
Roberta Costa: Nothing to disclose
Caterina Tonon: Nothing to disclose

### RPS 510-8

**Muscle imaging beyond morphology: chemical exchange saturation transfer imaging of lactate**

*D. B. B. Abrar*, K. L. Radke, M. Frenken, G. Antoch, S. Nebelung, A. Müller-Lutz; Düsseldorf/DE

**Purpose:** To establish and optimise a protocol for imaging chemical exchange saturation transfer (CEST) of lactate (LATEST) at 3 Tesla through in-vitro, in-situ, and in-vivo studies.
**Methods or Background:** Using Bloch-McConnell simulations, we optimised LATEST sequences for the optimal detection of lactate at a clinical 3T MRI scanner. The optimised sequences were then evaluated under longitudinal variation of lactate concentrations, analysed using the nonparametric Friedman test and Kendall-Tau b-rank correlation.
**Results or Findings:** The LATEST effect size of 0.4% expected by Bloch-McConnell simulations could be confirmed in in-vitro and in-situ studies. Significant differences ($p < 0.001$) and a strong correlation ($\tau = 0.67$) of the LATEST effect size as a function of intramuscular lactate concentrations were observed in 9 human in-situ lower legs. In a healthy volunteer, an increase in lactate concentration was demonstrated after exercise and recovery in the following 20 minutes using the LATEST technique.
**Conclusion:** In this feasibility study, changes in lactate concentrations were detected using an optimised LATEST imaging protocol in-vitro, in-situ, and in-vivo at a clinical 3T MRI that enables the quantification of altered lactate concentration using non-invasive MR imaging.
**Limitations:** First, the technique is vulnerable to magnetic field inhomogeneities. Second, our sample size was small.
**Ethics committee approval:** This study was approved by an ethics committee.
**Funding for this study:** Funding was received for this study by the Research Committee of the Medical Faculty and the "Deutsche Forschungsgemeinschaft".
**Author Disclosures:**
Sven Nebelung: Nothing to disclose
Karl Ludger Radke: Nothing to disclose
Gerald Antoch: Nothing to disclose
Anja Müller-Lutz: Nothing to disclose
Miriam Frenken: Nothing to disclose
Daniel B. Benjamin Abrar: Nothing to disclose

**15:00-16:00** **Room E1**

### Research Presentation Session: Neuro

### RPS 511

### The developing brain

**Moderator**
Z. Rumboldt; Rovinj-Rovigno/HR

### RPS 511-2

**Quantitative in utero fMRI assessment of functional asymmetries in the developing human brain**

*A. Taymourtash*, E. Schwartz, K-H. Nenning, R. Licandro, G. Kasprian, D. Prayer, G. Langs; Vienna/AT
*(athena.taymourtash@meduniwien.ac.at)*

**Purpose:** Cross-sectional studies of the language system in the early infancy and newborns suggest that the basic neural mechanisms are in place even before birth and the brain is primed for language while in utero. However, it remained unclear whether these functional asymmetries arise in utero, or whether they are shaped principally by postnatal experiences. This work presents, using in utero resting-state fMRI of typically developing fetuses, the assessment of functional asymmetry across different cortical regions.

**Methods or Background:** rs-fMRI was acquired from 72 singleton fetuses between 19 and 39 weeks of gestation. Customised image processing pipelines for fetal population including irregular fetal movement correction, and age-specific segmentation were used. Individualised functional connectivity (FC) matrices were then obtained by correlating regional brain activity over time. 24 cases were automatically excluded due to computational benchmarking of the resulting matrices. We calculated the lateralisation index for each individual subject as the normalised difference in the degree of FC between two hemispheres after thresholding negative and weak connections.
**Results or Findings:** According to the Kostovic timeline of brain development, we divided the observed period of gestation to three intervals of pre-expansion (19-26), expansion (26-32), and post-expansion (32-40). Number of significant functional connections increased significantly from first to second (average %21.5 to %25.7, p-val=0.032), and first to third (average %21.5 to %30.7, p-val=0.0004) period. We found significant leftward laterality in Temporal-Superior (p=0.043, tstat: -2.071, CI=[-0.0567,-0.00082], sd=0.096), Temporal-Medial (p=0.047,tstat: -2.030, CI=[-0.0691,-0.00032], sd=0.118), and Temporal-Inferior (p=0.048, tstat: 2.031, CI=[0.0008,0.1818],sd=0.311) regions after correcting for multiple comparisons. We observed a significant increase in functional laterality with gestation age (slope: 0.025±0.0087,p=0.005) for Temporal-Inferior region with the adjusted R-squared of 0.156.
**Conclusion:** Our results support the hypothesis that functional asymmetries are present during prenatal brain development.
**Limitations:** Only control cases were included in this study.
**Ethics committee approval:** This study was approved by an ethics committee.
**Funding for this study:** Funding was received for this study.
**Author Disclosures:**
Mister Karl-Heinz Nenning: Nothing to disclose
Georg Langs: Nothing to disclose
Athena Taymourtash: Nothing to disclose
Ernst Schwartz: Nothing to disclose
Daniela Prayer: Nothing to disclose
Gregor Kasprian: Nothing to disclose
Roxane Licandro: Nothing to disclose

### RPS 511-3

### Pilot study on brain ASL perfusion in newborns with HIE after hypothermia: a comparison between two different anesthesiologic drugs

L. Monti, *D. Del Roscio*, T. Casseri, F. Tarantino, R. Tinturini, S. Negro, B. Tomasini, P. Galluzzi, A. Rossi; Siena/IT

**Purpose:** The aim of this pilot study, by using arterial spin labeling (ASL) MRI, was to evaluate cerebral blood flow (CBF) changes between different anesthesiologic drugs Dexmedetomidine (DEX) versus other opioids and benzodiazepines (no-DEX) in neonates with hypoxic-ischaemic encephalopathy (HIE) treated with hypothermia.
**Methods or Background:** Twenty neonates (eleven females and nine males) with HIE, studied between December 2019 and December 2020, were included in this retrospective study. These neonates were admitted to the neonatal intensive care unit of our hospital for therapeutic hypothermia. MR examination in sedation was performed within 4-10 days of life. Perfusion imaging data adapted to neonates was developed to perform automated ROI analysis bilaterally on thalamus, basal ganglia, frontal, temporal and occipital grey matter. Twelve neonates were sedated with intranasal DEX (1-2mcg/kg), an alpha-2-adrenergic receptor agonist (DEX-group) and eight neonates with Fentanyl 0,5 mg/Kg/h or Midazolam 0,03-0,05 mg/kg/h in intravenous infusion (no-DEX group).
**Results or Findings:** CBF values in the basal ganglia and thalami were significantly higher in no-DEX than DEX sedation, respectively p-value=0,0088 and p-value=0,0002. No differences between cortical CBF values were observed.
**Conclusion:** This pilot study shows lower basal ganglia and thalamus perfusion in DEX group with respect to no-DEX group. This result is opposite to what has been reported in literature in HIE newborns without hypothermia. Hypothermia, therefore, appears to be the causal factor responsible for these results.
**Limitations:** The limitation of this pilot study is the small cohort of selected patients.
**Ethics committee approval:** Not applicable.
**Funding for this study:** No funding was received for this study.
**Author Disclosures:**
Davide Del Roscio: Nothing to disclose
Lucia Monti: Nothing to disclose
Rebecca Tinturini: Nothing to disclose
Tommaso Casseri: Nothing to disclose
Paolo Galluzzi: Nothing to disclose
Alessandro Rossi: Nothing to disclose
Barbara Tomasini: Nothing to disclose
Francesca Tarantino: Nothing to disclose
Simona Negro: Nothing to disclose

### RPS 511-4

### The transient zones of human fetal telencephalon wall: 3T MR intensities and thicknesses

*I. Pogledic*[1], E. Schwartz[1], M. Bobić-Rasonja[2], C. Mitter[1], P. Baltzer[1], M. Milković-Periša[2], G. Kasprian[1], D. Prayer[1], N. N. Jovanov Milošević[2]; [1]Vienna/AT, [2]Zagreb/HR
*(ivana.pogledic@meduniwien.ac.at)*

**Purpose:** To compare the signal intensity (SI) profiles and the average thicknesses of the developmental transient zones obtained from postmortem MRI (pMRI) and corresponding histological slices of temporal lobe (TL) and frontal lobe (FL), aiming to assess these parameters for monitoring pace of corticogenesis in different brain lobes.
**Methods or Background:** Nine postmortem human fetal brains (19-24 gestational weeks), without any pathology, were scanned on T2-weighted 3TpMRI scans, compared with same slices after hematoxylin-eosin staining and high-resolution scanning by Hamamatsu NanoZoomer 2.0-HT and analysed using ImageJ software, MATLAB, and Statistics Toolbox Release 2013a.
**Results or Findings:** The SI profiles of the transient zones of the TL spatially and temporally correlate to the SI profiles of the FL. The intermediate and subventricular zone of the TL during midgestation are about the size of the subplate zone (SP). The superficial SP shows highest signal intensity and SI profiles that indicate a concurrent course of histogenetic events in the two observed brain lobes during this developmental period.
**Conclusion:** Comparative evaluation of sizes of the transient developmental zones and the SI profiles of different cortical regions using 3TpMRI enables us to observe synchrony or differences in corticogenesis in different brain lobes. Thus, developing MRI methodologies could enable us to observe normal vs. abnormal transient lamination patterns using SI profiles and thicknesses. The MRI advancements in this direction are needed for prenatal detection of malformation of cortical development especially mild ones, as well as white matter injuries of TL due to cytomegalovirus infection.
**Limitations:** A rigorous selection of cases with the highest quality pMRI resulted in the modest number of cases in this study, but it is comparable to or even larger than the number of analyzed cases in other similar studies.
**Ethics committee approval:** This study was approved by an ethics committee (Ek1825/2015, Mfsz380-59-1016-19-111/210).
**Funding for this study:** Funding was received for this study (Cost-Ca16118, Cif-ip-2019-04-3182).
**Author Disclosures:**
Christian Mitter: Nothing to disclose
Mihaela Bobić-Rasonja: Nothing to disclose
Nataša Natasa Jovanov Milošević: Nothing to disclose
Ernst Schwartz: Nothing to disclose
Daniela Prayer: Nothing to disclose
Ivana Pogledic: Nothing to disclose
Marija Milković-Periša: Nothing to disclose
Gregor Kasprian: Nothing to disclose
Pascal Baltzer: Nothing to disclose

### RPS 511-5

### Evaluation of sulcal developmental asymmetry in fetal cerebral isolated ventricolomegalia by fetal magnetic resonance imaging and post-delivery outcomes

S. Sefidbakht[1], M. Hosein Yazdi[1], *S. Esmaeilian*[1], P. Iranpour[1], P. Keshavarz[1], B. Bijan[2]; [1]Shiraz/IR, [2]Sacramento, CA/US
*(kmssd87@gmail.com)*

**Purpose:** To evaluate sulcal asymmetry in fetuses with “isolated” ventriculomegaly using fetal MRI. To compare degree of asymmetry with post-delivery outcome.
**Methods or Background:** This is a retrospective study. Out of 658 Fetal MRI’s performed in our centre, 67 fetuses with isolated ventriculomegaly and 77 fetuses with normal brains based on prenatal ultrasound and MRI were included in the study. Fetal MRI images were retrospectively and blindly reviewed by two radiologists. Sulcal development was scored based on Kriakopoulou Asymmetry. The score is defined as sum of right side cortical development scores minus left side CDS divided by number of visible sulci post-delivery developmental outcomes were assessed using ASQ Questionnaires obtained at least one year post-delivery.
**Results or Findings:** The mean gestational ages were 28.6±6.3 weeks and 28.4±6.1 respectively in the fetuses with and without ventriculomegaly (p= 0.9). The mean sulcal development score was 3.6±1.1 and 4.5±1.3 respectively for fetuses with and without ventriculomegaly (p= 0.03 ). The mean asymmetry scores were 1.4±1.0 and 0.2±1.0 for fetuses with/without ventriculomegaly (0.001). The mean ventricular size was 14.4 ±11.2 mm in fetuses with ventriculomegaly. The ASQ score was 11.3 ± 9.8 months and 22.6 ± 12.8 months in fetuses with and without VMG. At year 2 of age, only 39% of the children with prenatal isolated ventriculomegaly seen in fetal MRI had normal neurodevelopment. Also, 8% of fetuses with ventriculomegaly underwent neonatal death.

**Conclusion:** The fetuses with VMG had a significant delay in sulcation and development compared to the control fetuses. Sulcal asymmetry was more common in fetuses with ventriculomegaly who had delayed development, compared to those with ventriculomegaly but normal developmental outcome.
**Limitations:** The limitation is data missing of imaging in the pacs and evaluation by telephone.
**Ethics committee approval:** This study is approved by the Ethical Committee of Shiraz University IR.SUMS.REC.1732.
**Funding for this study:** No funding was received for this study.
**Author Disclosures:**
Bijan Bijan: Advisory Board: review
Meisam Hosein Yazdi: Author: review
Sepideh Sefidbakht: Owner: Idea and data collection and final review
Saeid Esmaeilian: Author: Idea and data collection and final review
Poya Iranpour: Advisory Board: review
Pedram Keshavarz: Other: other

### RPS 511-6

**GABA-concentration in posterior cingulate cortex increases after acute paediatric concussion**

*A. Manzhurtsev*, P. A. Bulanov, M. Ublinskiy, P. Menshchikov, T. Akhadov, I. Melnikov, O. Bozhko, N. Semenova; Moscow/RU
*(andrey.man.93@gmail.com)*

**Purpose:** Concussion (mild traumatic brain injury (mTBI)) is one of the most widespread types of trauma. The literature data on the [GABA] change after acute mTBI is based on the measurement of GABA+ levels, where GABA signal is contaminated with macromolecular compounds. The aim of this study is to measure GABA in the posterior cingulate cortex (PCC) region in the acute phase of pediatric mTBI.
**Methods or Background:** Nineteen patients with acute mTBI (12-70 hours since the injury, 16.2±1.4 y.o.) and twenty-one healthy control (18.5±2.3 y.o.) participated in the study. Philips Achieva 3.0T was used, standard MRI protocol for TBI patients revealed no pathological lesions in brain tissue of any subject. MRS voxel (50x25x25 mm) was located in PCC. MEGA-PRESS pulse sequence without macromolecular contamination was used: TR = 2000 ms, TE = 80 ms, 180-editing pulses applied on 1.9 ppm and 1.5 ppm, NSA = 288 (acq. time ~10 min). Spectra were processed in Gannet 3.1, [GABA] and [Glx] values were calculated.
**Results or Findings:** [GABA] was statistically significantly higher in mTBI group than in the normal group, while [Glx] was unchanged. No correlations between age and metabolite concentrations were revealed.
**Conclusion:** Previously we have demonstrated that GABA-/Cr increases in anterior cingulate cortex of children with mTBI. To our knowledge, the increase in GABA in PCC in the current study is reported for the first time. The lack of correlations between age and metabolite levels agrees with the literature data and eliminates possible bias in the results caused by group age differences. This study provides insight into metabolic alterations caused by mTBI and may facilitate better understanding of the long-term mTBI consequences.
**Limitations:** There is a small between-group age difference.
**Ethics committee approval:** All studies were approved by the local ethical committee.
**Funding for this study:** No funding was received for this study.
**Author Disclosures:**
Petr Menshchikov: Nothing to disclose
Maxim Ublinskiy: Nothing to disclose
Petr Алексеевич Bulanov: Nothing to disclose
Natalia Semenova: Nothing to disclose
Olga Bozhko: Nothing to disclose
Andrei Manzhurtsev: Nothing to disclose
Tolibjohn Akhadov: Nothing to disclose
Ilya Melnikov: Nothing to disclose

### RPS 511-7

**Synthetic MRI-based fast gray matter acquisition T1 inversion recovery (FGATIR) contrasts identify neonatal brainstem pathways in vivo**

*V. Schmidbauer*, M. S. Yildirim, M. Stümpflen, G. O. Dovjak, K. Goeral, D. Prayer, A. Berger, G. Kasprian; Vienna/AT
*(victor.schmidbauer@meduniwien.ac.at)*

**Purpose:** SyMRI® allows to retrospectively reconstruct different MR imaging contrasts using a single acquisition of a multi-dynamic multi-echo (MDME) sequence. This study aimed to investigate the feasibility of synthetic MRI-based fast gray matter acquisition T1 inversion recovery (FGATIR) contrasts for the qualitative identification of early myelinating neonatal brainstem pathways in vivo.
**Methods or Background:** Thirty-one cases of neonatal MRI (median gestational age at birth: 27+0; range, 23+4–41+6) were collected, which included MDME sequences and conventional T1-weighted/T2-weighted sequence acquisitions as a standard-of-reference. MDME-based FGATIR contrasts (TR/TE/TI: 3000/5/410 ms) were generated using the MR data post-processing software SyMRI®. The identification of seven brainstem pathways/regions of interest was assessed on synthetic FGATIR contrasts and conventionally acquired T1-weighted/T2-weighted imaging data: decussation of superior cerebellar peduncle (DSCP); left/right medial lemniscus (ML); left/right central tegmental tract (CTT); and left/right longitudinal medial fascicle (LMF).
**Results or Findings:** SyMRI® provided FGATIR contrasts of diagnostic quality in 31/31 cases (100%). Based on MDME-based FGATIR contrasts, the DSCP [31/31 (100%)]; left/right ML [31/31 (100%)]; left/right CTT [20/31 (65%)]; and left/right LMF [31/31 (100%)] were reliably identified. Based on conventional T1-weighted contrasts, the DSCP [14/31 (45%)]; left/right ML [25/31 (81%)/23/31 (74%)]; left/right CTT [3/31 (10%)/7/31 (23%)]; and left/right LMF [15/31 (48%)] were reliably identified. Based on conventional T2-weighted contrasts, the DSCP [30/31 (97%)]; left/right ML [30/31 (97%)/29/31 (94%)]; left/right CTT [26/31 (84%)/25/31 (81%)]; and left/right LMF [30/31 (97%)] were reliably identified.
**Conclusion:** Synthetic generation of specific FGATIR contrasts enables reliable radiological identification of neonatal brainstem pathway anatomy in vivo. The investigated MR approach depicts early myelinating tracts more reliable than standard-of-reference contrasts.
**Limitations:** The limitation is the study's small sample size.
**Ethics committee approval:** The local Ethics Commission approved the protocol of this study.
**Funding for this study:** No funding was received for this study.
**Author Disclosures:**
Victor Schmidbauer: Nothing to disclose
Mehmet Salih Yildirim: Nothing to disclose
Gregor Oliver Dovjak: Nothing to disclose
Daniela Prayer: Nothing to disclose
Angelika Berger: Nothing to disclose
Katharina Goeral: Nothing to disclose
Gregor Kasprian: Nothing to disclose
Marlene Stümpflen: Nothing to disclose

### RPS 511-8

**Motion correction and volumetric reconstruction for fetal fMRI**

*D. Sobotka*[1], M. Ebner[2], E. Schwartz[1], T. Vercauteren[2], S. Ourselin[2], G. Kasprian[1], D. Prayer[1], G. Langs[1], R. Licandro[1]; [1]Vienna/AT, [2]London/UK

**Purpose:** Motion correction is an essential preprocessing step in resting-state functional Magnetic Resonance Imaging (rs-fMRI) of the fetal brain, to remove motion artifacts caused by fetal movement and maternal breathing and to suppress erroneous signal correlations. Here, we propose a novel framework including a motion correction and volumetric reconstruction methodology for fetal rs-fMRI. Further, we demonstrate the ability of the proposed framework to improve functional connectivity estimates, reproducibility and signal interpretability.
**Methods or Background:** The motion correction and volumetric reconstruction algorithm consists of three parts. First, a high-resolution 3D reference volume is estimated. Second, a slice-to-volume motion correction procedure is performed, where each slice of a fMRI time point is registered rigidly to the reference volume. Third, motion corrupted time points are reconstructed using a Huber loss-based regularisation scheme.
**Results or Findings:** For the evaluation of the reconstruction performance, the outlier ratio and specific functional connectivity-based metrics were developed to assess reproducibility of the measured BOLD. Our approach was compared against state-of-the-art reconstruction and motion correction schemes, showing decreased outlier ratio (from 12.71% to 8.62% ) and correlation differences (from 0.193 to 0.191).
**Conclusion:** The proposed framework allows the use of motion corrupted fMRI acquisitions for further analysis which would traditionally be discarded. This could mitigate the need for repeated rs-fMRI acquisitions therefore leading to an overall reduction of scan time of fetal imaging.
**Limitations:** The limitation is the number of rs-fMRI acquisitions: 15 subjects with 96 time points are used for evaluation.
**Ethics committee approval:** The local ethics committee approved the protocol of this study performed in accordance with the Declaration of Helsinki.
**Funding for this study:** Funding was received for this study by the Austrian Science Fund FWF (I2714-B31), EU H2020 Marie Sklodowska-Curie No 765148, the Wellcome Trust [WT101957; 203148/Z/16/Z] and the Engineering and Physical Sciences Research Council [NS/A000027/1; NS/A000049/1].
**Author Disclosures:**
Tom Vercauteren: Nothing to disclose
Georg Langs: Nothing to disclose
Daniel Sobotka: Nothing to disclose
Ernst Schwartz: Nothing to disclose
Daniela Prayer: Nothing to disclose
Sebastien Ourselin: Nothing to disclose
Gregor Kasprian: Nothing to disclose
Roxane Licandro: Nothing to disclose
Michael Ebner: Nothing to disclose

**15:00-16:00 Room G**

### Research Presentation Session: Chest

### RPS 504

### Cardiovascular disease in chest imaging

**Moderator**
M. Arzanauskaite; Liverpool/UK

### RPS 504-2

**Ultra-high pitched tin-filtered CT pulmonary angiography in radiation dose reduction for pulmonary embolism investigations in young females**

*S. Rehan*, P. Kutschera, K. K-P. Lau; Clayton/AU
*(saad.rehan@outlook.com)*

**Purpose:** Computed tomography pulmonary angiography (CTPA) investigations, the gold standard to investigate pulmonary embolism (PE), are undergoing a recent surge secondary to COVID-induced hypercoagulable state. This technique carries significant radiation risk in young females because of radiosensitive breast and thymus tissues. An ultra-high pitched CT technique offers significant radiation dose reduction (RDR) through interpolation and minimises breathing artefact. The addition of tube tin-filtration may offer further RDR. This retrospective study's aim was to assess RDR and image quality (IQ) of ultra-high pitch tin-filtered (UHPTF) CTPA against conventional-CTPA.
**Methods or Background:** Consecutive adult females age <50 years undergoing UHPTF-CTPA and conventional-CTPA in the past 12 months were included. CTs in both groups were compared for radiation dose, adequate pulmonary arteries contrast density (Hounsfield units (HU)) and movement artefact with Fisher's exact test, and p<0.05 considered significant. Diagnostic quality was also recorded.
**Results or Findings:** 10 female patients (mean age 33) in UHPTF-CTPA group and 10 female patients (mean age 36) in conventional-CTPA group were included. There was no significant contrast density difference between the two groups in the main, left or right pulmonary arteries (322.72HU, 311.85HU and 319.41HU in UHPTF-CTPA group vs. 418.60HU, 405.10HU and 415.96HU in conventional-CTPA group respectively, p=1.00). 8/10 UHPTF-CTPA group and 10/10 in the control group were >250 HU in all PA; the remaining 2 UHPTF-CTPAs were >210HU. The UHPTF-CTPA group achieved 87% RDR (25.15DLP vs 337.1DLP, p<0.01). All CT scans in both groups were of diagnostic quality and none exhibited movement artifact.
**Conclusion:** This study was the first to demonstrate significant RDR with the UHPTF-CTPA technique whilst maintaining IQ. This technique is particularly beneficial in young females with suspected PE.
**Limitations:** Future work of this UHPTF-CTPA technique includes interobserver-reliability assessment.
**Ethics committee approval:** Ethics committee approved.
**Funding for this study:** No funding required.
**Author Disclosures:**
Kenneth Kwok-Pan Lau: Nothing to disclose
Peter Kutschera: Nothing to disclose
Saad Rehan: Nothing to disclose

### RPS 504-3

**Clinical importance of CT lung parenchymal appearances in chronic thromboembolic pulmonary hypertension: a retrospective cohort study**

*L. F. Abdulaal*, K. Dwivedi, M. Sharkey, S. Alabed, M. Mamalakis, D. D. F. Alkhanfar, R. Condliffe, D. Kiely, A. J. Swift; Sheffield/UK

**Purpose:** To evaluate the association of mosaic perfusion and lung scarring with prognostic indicators in CTEPH.
**Methods or Background:** CT pulmonary angiography (CTPA) is recommended in suspected chronic thromboembolic pulmonary hypertension (CTEPH). Mosaic perfusion and lung scarring are common features in CTEPH, but their clinical relevance is less understood. 291 patients with CTEPH who underwent CTPA were identified. Radiological assessment of mosaic perfusion (nil/minor/mild/moderate-severe), lung scarring (nil/minor/mild/moderate-severe), emboli (proximal/segmental/distal) and CT cardiac measurements were scored blinded to clinical parameters. Right heart catheterisation data, pulmonary vascular resistance (PVR) and mixed venous oxygen saturation (SvO2)(n=118) were collected alongside the transfer factor for carbon monoxide (TLCO)(n=112). The correlation was assessed using Spearman's correlation coefficient. Group comparison was made on dichotomised mosaicism/scarring data (nil/minor vs mild/moderate-severe) using an independent t-test.
**Results or Findings:** Significant correlation was found between both mosaicism and scarring, and PVR (p=0.049, p=0.004) and SVO2 (p=0.026, p=0.001), whereas no correlation was identified with clot location (p=0.534, p=0.370). RV:LV ratio correlated with scarring (p=0.045) but not mosaicism (p=0.354). Patients with greater mosaicism had higher PVR and lower SvO2 (all p<0.05) (mean difference 367 dyns, 95% confidence interval [CI] 91.4 to 643), (-6.56%, -12.04 to -1.08), respectively. Patients with greater than minor scarring had lower SvO2 (p=0.005) (-7.93%, CI -14.12 to -1.75) and lower TLCO (p=0.038) (-1.78 mmol/min/kPa, CI -3.51 to -0.06).
**Conclusion:** CT lung features in CTEPH relate to prognostic indicators and are important to assess disease severity.
**Limitations:** Retrospective single-centre study.
**Ethics committee approval:** Research ethical approval was obtained from Sheffield Research Ethics Committee, database study (ref c06/Q2308/8).
**Funding for this study:** Wellcome Trust (grant numbers: 222930/Z/21/Z; 205188/Z/16/Z)
**Author Disclosures:**
Andrew J Swift: Nothing to disclose
Lojain Faisla Abdulaal: Nothing to disclose
Michael Sharkey: Nothing to disclose
Samer Alabed: Nothing to disclose
David Kiely: Nothing to disclose
Dheyaa Dhahir Farhan Alkhanfar: Nothing to disclose
Michail Mamalakis: Nothing to disclose
Robin Condliffe: Nothing to disclose
Krit Dwivedi: Nothing to disclose

### RPS 504-4

**CT assessment of bronchial arteries in predicting the development of chronic thromboembolic disease (CTED) after acute pulmonary embolism**

*A. Uchevatkin*, A. L. Yudin, E. Yumatova, A. Kondakov, Y. Abovich, N. Afanasieva, N. Ledikhova; Moscow/RU
*(uchevatkin@mail.ru)*

**Purpose:** To compare bronchial arteries after acute pulmonary embolism in patients who developed chronic thromboembolic disease (CTED) in the future and in patients who did not develop CTED in the future.
**Methods or Background:** 44 patients who did not develop chronic thromboembolic disease after acute pulmonary embolism and 43 patients who developed CTED after acute pulmonary embolism were retrospectively selected from 600 patients with suspected pulmonary embolism who underwent computed tomography (forty-row CT scanner, Siemens). CT-angiography scans were evaluated for the diameter, number and type of origin of the bronchial arteries and their role in the predicting the CTED development in the future.
**Results or Findings:** The diameter of the bronchial arteries in the group of patients who developed CTED after acute pulmonary embolism was significantly larger than diameters of the bronchial arteries in the group of patients who did not develop CTED after acute pulmonary embolism (p = 0.0001). There were no differences in the number and type of origin of the bronchial arteries in the group of patients who developed CTED after acute pulmonary embolism and in the group of patients who did not develop CTED after acute pulmonary embolism.
**Conclusion:** A significant difference was found between the diameter of the bronchial arteries in patients who developed CTED after acute pulmonary embolism compared with patients who did not develop CTED after acute pulmonary embolism (p = 0.0001). ROC analysis showed a threshold value of the diameter of the bronchial arteries of 1.5 mm with a sensitivity of the method of 90.24% and a specificity of 95.12%. The presence of bronchial arteries with a diameter of ≥ 1.5 mm in patients after acute pulmonary embolism can be used as a prediction of CTED.
**Limitations:** Retrospective study.
**Ethics committee approval:** Not applicable.
**Funding for this study:** Not applicable.
**Author Disclosures:**
Natalia Ledikhova: Nothing to disclose
Yulia Abovich: Nothing to disclose
Elena Yumatova: Nothing to disclose
Andrey Leonidovich Yudin: Nothing to disclose
Andrey Uchevatkin: Nothing to disclose
Anton Kondakov: Nothing to disclose
Natalia Afanasieva: Nothing to disclose

### RPS 504-5

**Imaging in the detection of pulmonary emboli: are we minimising radiation dose?**

*P. Singhal*, J. Spillane, R. Kulshrestha; Bristol/UK
*(priyankasinghal@hotmail.co.uk)*

**Purpose:** When investigating for pulmonary emboli, CT pulmonary-angiogram (CTPA) delivers a higher radiation dose to breast tissue than perfusion (Q) scans. Q scans are favoured over CTPAs in those who have a normal chest radiograph in pregnant and young patients (Royal College of Obstetrics and Gynaecology, Royal College of Radiologists). We have performed a re-audit with a target standard of 85%. Following on from our previous audit in 2019, we discussed our findings at multiprofessional meetings and created a standardised operating procedure at our tertiary centre, highlighting a pathway where Q scans should be the first line investigation for pulmonary emboli if relevant criteria are met.
**Methods or Background:** We retrospectively collected data on all patients aged 40 years and younger who underwent Q scans and CTPAs between 01/01/2021 and 31/12/2021. Pregnancy/post-partum status was noted. We excluded weekend data as Q scans were unavailable. Data was collected from CRIS and analysed on Microsoft Excel.
**Results or Findings:** Out of a total of 288 patients, 212 patients were excluded due to having an abnormal chest radiograph or were severely unwell e.g. post cardiac arrest. This left a total of 76 patients who could have a Q scan, of which 70% underwent a Q scan in 2021, an improvement from 40% in 2017. The proportion of pregnant patients who underwent Q scans improved from 68% in 2019 to 98% in 2021. The proportion of Q scans in post-partum patients increased from 45% to 77%.
**Conclusion:** We have significantly improved the proportion of patients who underwent a Q scan for the diagnosis of pulmonary emboli, consequently reducing radiation exposure and cost.
**Limitations:** Not applicable.
**Ethics committee approval:** Not applicable.
**Funding for this study:** Not applicable.
**Author Disclosures:**
Priyanka Singhal: Nothing to disclose
Randeep Kulshrestha: Nothing to disclose
John Spillane: Nothing to disclose

### RPS 504-6

**High rate of identification of ruptured mitral valve prolapse on non-ECG-gated chest CT**

S. M. M. Yoo, *M. Son*; Bundang/KR

**Purpose:** Primary imaging modality for the diagnosis of mitral valve prolapse (MVP) is echocardiography supplemented by ECG-gated cardiac CT angiography. However, the authors have recently encountered multiple patients with ruptured MVP, initially identified on non-ECG-gated chest CT. Notably, there has been no study regarding identification of ruptured MVP on non-ECG-gated chest CT. Thus, the purpose of the paper is to evaluate diagnostic accuracy of ruptured MVP on non-ECG gated chest CT.
**Methods or Background:** We retrospectively assessed 50 patients with ruptured MVP and 15 patients without ruptured MVP as control who underwent non-gated chest CT. We analysed CT findings of the MVP including presence or absence of MVP (asymmetrical double line-sign due to overlapping of ruptured and non-ruptured mitral leaflet) and culprit location of MVP (A1, 2, 3, P1, 2, 3). A retrospective evaluation was performed by two blind radiologists in a consensus manner for CT findings.
**Results or Findings:** Double-line sign to predict ruptured MVP on non-ECG-gated chest CT resulted in sensitivity, specificity, negative predictive value and positive predictive valve of 68%, 73%, 41%, and 89%, respectively. In contrast, only 24.6% (n=16/65) was correctly localised as the culprit lesion on non-ECG-gated chest CT.
**Conclusion:** Asymmetrical double line sign may be a suggestive CT finding of ruptured MVP on non-ECG-gated chest CT. Familiarity of this CT finding may lead to prompt diagnosis and surgery of ruptured MVP.
**Limitations:** This study is limited by its small retrospectively selected cohort, which may have led to selection bias.
**Ethics committee approval:** IRB approved the retrospective study.
**Funding for this study:** No funding was received for this study.
**Author Disclosures:**
Seung Min Min Yoo: Nothing to disclose
Minji Son: Nothing to disclose

### RPS 504-7

**Atrial size ratio in computed tomography pulmonary angiography as a prognostic factor in patients with acute pulmonary thromboembolism**

*S. M. Revanna*[1], V. K N[2]; [1]Bangalore, Karnataka/IN, [2]Bangalore/IN
*(sinumr25@gmail.com)*

**Purpose:** Pulmonary thromboembolism (PTE) is a significant cause of mortality and morbidity, with the need for risk stratification. This study aims to correlate atrial size ratio (ASR) with 30-day mortality in patients with acute PTE and compare its accuracy with the pulmonary artery obstruction index (PAOI).
**Methods or Background:** A prospective study was performed on 40 consecutive patients diagnosed with acute PTE on CTPA at Victoria Hospital, BMCRI, Bengaluru. ASR was calculated by the ratio of axial longitudinal diameters of the atria. PAOI was ascertained by scoring the obstruction of the pulmonary arterial tree as given by Qanadli et al. ASR and PAOI were compared with 30-day mortality using area under receiver operator curve.
**Results or Findings:** 11/ 40 patients (27.5%) patients with acute PTE had 30-day mortality. Middle aged and older patients were predominantly affected and majority of the patients in our study were men (male: female= 1.6:1). The mean ASR and PAOI were higher in the non-survivor group and correlated with 30-day mortality. AUC of ASR and PAOI was 0.906 and 0898 respectively. ASR criterion of 1.47 showed the highest accuracy with sensitivity and specificity of 100% and 75.9%, respectively. PAOI of 13 showed the highest accuracy. The sensitivity of PAOI at 13 was lower than ASR (90.9%) but showed higher specificity (86.2%).
**Conclusion:** ASR, a relatively easy technique, is comparable, if not better than, PAOI in predicting 30-day mortality in patients with acute PTE. It furthers the role of CTPA in the management of acute PTE.
**Limitations:** Non-gated images were degraded by cardiac motion and all-cause mortality was assessed irrespective of treatment received by the patient.
**Ethics committee approval:** Institutional ethics committee approval and informed consent were obtained.
**Funding for this study:** No funding was received.
**Author Disclosures:**
Srinivas Maskal Revanna: Nothing to disclose
Venkateswar K N: Nothing to disclose

### RPS 504-8

**Non-invasive detection of severe PH in lung disease using magnetic resonance imaging**

*D. D. F. Alkhanfar*; Sheffield/UK

**Purpose:** Severe pulmonary hypertension (PH) in chronic lung disease (PH-CLD) is associated with high mortality and morbidity. There is increased interest in the identification of patients with severe PH in CLD, as there is new evidence of potential vasodilator therapy response. Echocardiography is the recommended screening test but can be challenging in patients with advanced CLD. The aim of this study was to evaluate the diagnostic role of MRI to diagnose severe PH in CLD.
**Methods or Background:** 167 patients with CLD who underwent baseline cardiac MRI and right heart catheterisation were identified from the ASPIRE registry. In a derivation cohort (n=67), the optimal diagnostic threshold of a previously published multiparameter model [Whitfield model (arbitrary units) = −27.7 + 5.75 loge (interventricular septal angle) + 1.899 loge (right ventricular mass/left ventricular mass) + 0.004 (diastolic pulmonary artery area, mm2)] was identified using the Youden index. In the test cohort (n=100), the diagnostic accuracy was tested using receiver operating characteristic (ROC) analysis, and, to assess the prognostic value, Kaplan-Meier curves were constructed.
**Results or Findings:** The identified Whitfield model threshold ≥1.6 had high accuracy in the test cohort, area under the ROC curve (0.95) (p<0.0001); sensitivity, 80.8%; specificity, 87.2%; positive predictive value (PPV), 87.5%; and negative predictive value (NPV), 80.4%. Patients with Whitfield model ≥1.6 had worse survival; log-rank chi square 44.47, p<0.0001. On Cox regression, adjusting for age, sex and body surface area, the Whitfield model also remained significant (hazard ratio=1.28; 95% CI: 1.17, 1.41, p<0.0001).
**Conclusion:** The Whitfeld MRI model with threshold ≥1.6 applied has high accuracy to detect severe PH in CLD and has strong prognostic value.
**Limitations:** Retrospective, single-centre study.
**Ethics committee approval:** Database study (ref c06/Q2308/8).
**Funding for this study:** Wellcome Trust (grant numbers: 222930/Z/21/Z; 205188/Z/16/Z).
**Author Disclosures:**
Dheyaa Dhahir Farhan Alkhanfar: Nothing to disclose

15:00-16:00 Room K

### Research Presentation Session: Abdominal Viscera

### RPS 501

### The role of CT imaging in primary liver malignancies

**Moderator**
G. Brancatelli; Palermo/IT
**Author Disclosure:**
G. Brancatelli: Consultant: Bayer Healthcare; Speaker: Guerbet, GE Healthcare, Bracco

### RPS 501-4

**Imaging features of histological subtypes of hepatocellular carcinoma: implication for the LI-RADS**
*R. Cannella*[1], M. Dioguardi Burgio[2], A. Beaufrère[2], V. Paradis[2], F. Cauchy[2], M. Bouattour[2], V. Vilgrain[2], R. Sartoris[2], R. Maxime[2]; [1]Palermo/IT, [2]Clichy/FR

**Purpose:** To report LI-RADS-defined imaging features of different HCC subtypes in a cohort of resected tumours. To assess the influence of HCC subtypes on CT/MRI LI-RADS categorisation in the subgroup of high-risk patients.
**Methods or Background:** This retrospective IRB-approved study included patients with resected HCC and available histopathological classification. Three radiologists independently reviewed preoperative CT and MRI exams. The readers evaluated the presence of imaging features according to LI-RADS v2018 definitions and provided an LI-RADS category in patients at high risk of HCC. Differences in LI-RADS features and categorisations were assessed for non-otherwise specified (NOS-HCC), steatohepatitic (SH-HCC), and macrotrabecular-massive (MTM-HCC) HCC.
**Results or Findings:** Two hundred and seventy-seven patients (median 64.0 years old, 215 [77.6%] men) were analysed with 295 HCCs. There were 197 (66.7%) NOS-HCCs, 62 (21.0%) SH-HCCs, 23 (7.8%) MTM-HCCs, and 13 (4.5%) other rare subtypes. The following features were more frequent in MTM-HCC: elevated α-fetoprotein serum levels ($P<0.001$), tumor-in-vein ($P<0.001$ on CT, $P\leq0.052$ on MRI), presence of at least one LR-M feature ($P\leq0.010$ on CT), infiltrative appearance ($P\leq0.032$ on CT), necrosis or severe ischaemia ($P\leq0.038$ on CT) and larger size ($P\leq0.006$ on CT, $P\leq0.011$ on MRI). SH-HCC was associated with fat in mass ($P<0.001$ on CT, $P\leq0.002$ on MRI). The distribution of the LI-RADS major features and categories in high-risk patients did not significantly differ among the three main HCC subtypes.
**Conclusion:** The distribution of LI-RADS major features and categories is not different among the HCC subtypes. Nevertheless, careful analysis of tumour-in-vein, LR-M, and ancillary features as well as clinico-biological data can provide information for the noninvasive diagnosis of HCC subtypes.
**Limitations:** The limitation is the single-centre and retrospective design. Only resected HCC was included in the study.
**Ethics committee approval:** This study was approved by IRB.
**Funding for this study:** No funding was received for this study.
**Author Disclosures:**
Mohamed Bouattour: Speaker: Bayer, IPSEN Consultant: Bayer, IPSEN, ROCHE, SIRTEX Medical, ASTRA-Zeneca, EISAI, BMS
Marco Dioguardi Burgio: Nothing to disclose
Francois Cauchy: Nothing to disclose
Aurélie Beaufrère: Nothing to disclose
Valérie Paradis: Nothing to disclose
Roberto Cannella: Nothing to disclose
Ronot Maxime: Nothing to disclose
Riccardo Sartoris: Nothing to disclose
Valérie Vilgrain: Speaker: Canon Medical, GE Heallthcare, Roche, Sirtex Consultant: Guerbet Other: Bayer, Guerbet, Sirtex, Quantum Surgical

### RPS 501-6

**Predicting symptomatic post-hepatectomy liver failure in patients with hepatocellular carcinoma: development and validation of a preoperative nomogram**
*H. Long*, M. Lin, X. Xie; Guangzhou/CN
*(longhy9@mail.sysu.edu.cn)*

**Purpose:** To develop and validate a nomogram model for predicting symptomatic post-hepatectomy liver failure (PHLF) in patients with hepatocellular carcinoma (HCC).
**Methods or Background:** A total of 190 patients diagnosed as HCC were enrolled prospectively from three tertiary referral hospitals. All patients underwent preoperative laboratory examination to obtain parameters pertaining to liver function, two-dimensional shear wave elastography examination to obtain liver stiffness (LS), and three-dimensional virtual resection of the liver to obtain volume-related parameters. A nomogram was developed by using logistic regression and determined by receiver operating characteristic curve analysis and calibration curve analysis.
**Results or Findings:** A nomogram was constructed with the following variables: liver remnant volume ratio, LS>9.5kPa, child-pugh grade, and the presence of clinically significant portal hypertension (CSPH). This nomogram enabled differentiation of symptomatic PHLF in the derivation cohort (Area under curve [AUC], 0.915), internal 5-fold cross-validation (mean AUC, 0.918), and the validation cohort (AUC, 0.845). The nomogram also showed good calibration in both the derivation and the validation cohorts (Hosmer-Lemeshow goodness-of-fit test, $p=0.641$, and $p=0.127$, respectively). With the best cutoff point of 81, the safe limit of the remnant ratio could be stratified using the nomogram.
**Conclusion:** A simple nomogram model combining both preoperative clinical and imaging features could be useful in predicting postoperative outcomes in HCC.
**Limitations:** First, the sample size was relatively small. Second, further studies should be designed for predicting postoperative mortality or long-term survival.
**Ethics committee approval:** The protocol of this prospective study was approved by the Institutional Review Board of the First Affiliated Hospital of Sun Yat-sen University.
**Funding for this study:** Funding was received for this study by Youth Program of the National Natural Science Foundation of China (Grant No. 81901768); Natural Science Foundation of Guangdong Province (Grant No. 2021A1515012367); Major Program of National Natural Science Foundation of China (Grant No. 92059201).
**Author Disclosures:**
Haiyi Long: Nothing to disclose
Xiaoyan Xie: Nothing to disclose
Manxia Lin: Nothing to disclose

### RPS 501-7

**Preoperative CT-body composition in patients with perihilar cholangiocarcinoma: specific body composition types in correlation to perioperative morbidity and outcome**
*L. K. Segger*, N. L. Beetz, T. A. Auer, D. Geisel, M. Schmelzle, U. Fehrenbach; Berlin/DE

**Purpose:** As sarcopenic cancer patients appear to have a poor outcome during cancer treatments the aim of this study was to evaluate specific body composition types (sarcopenia, sarcopenic obesity and myosteatosis) in patients with perihilar cholangiocarcinoma (pCC) to evaluate the influence on peri- and postoperative morbidity and outcome.
**Methods or Background:** In this retrospective study, preoperative CT datasets of 197 patients with pCC, who underwent extended hemihepatectomy were evaluated. Single slice segmentations (lumbar vertebra 3 as landmark) of the psoas muscle, total abdominal muscle mass (TAMA), visceral and subcutaneous adipose tissue were performed. Lumbar skeletal muscle index (LSMI) was calculated by normalising the TAMA by patients' height and was used to define sarcopenia and sarcopenic obesity. Myosteatosis was based on the density (HU) of the segmented muscles. These body composition parameters were correlated to peri- and postoperative complications and outcomes.
**Results or Findings:** 61,4% (121/197) of the patients were sarcopenic, 3,6% (7/197) had sarcopenic obesity and 20,8% (41/197) had myosteatosis. Neither sarcopenia nor sarcopenic obesity was independent risk factor for the occurrence of peri- or postoperative complications. However, in patients with sarcopenic obesity, the 90-day-survival was significantly decreased ($p=0.009$). Myosteatosis was associated with a prolonged ICU stay ($p=0.014$), poorer disease-free survival ($p=0.003$) and overall survival ($p<0.001$).
**Conclusion:** Sarcopenia does not increase perioperative morbidity in patients with pCC undergoing extended hemihepatectomy, but sarcopenic obesity is associated with a poorer 90-day-survival. Myosteatosis is linked to prolonged ICU stays and decreased disease-free survival and overall survival.
Our study shows the value of identifying certain body composition types to optimise individual risk and outcome assessment, and should encourage their implementation into routine clinical practice.
**Limitations:** The limitation is the single-centre and retrospective design, which indicates that these results may not be generalisable.
**Ethics committee approval:** This study was approved by an ethics committee.
**Funding for this study:** No funding was received for this study.
**Author Disclosures:**
Laura Katharina Segger: Nothing to disclose
Nick Lasse Beetz: Nothing to disclose
Uli Fehrenbach: Nothing to disclose
Dominik Geisel: Nothing to disclose
Timo Alexander Auer: Nothing to disclose
Moritz Schmelzle: Nothing to disclose

### RPS 501-8
**CT features of mixed primary liver cancers with a special focus on the two different histological subtypes**
*A. Pecorelli*, F. Adduci, F. Vasuri, C. Mosconi, A. D'Errico, R. Golfieri; Bologna/IT
*(pecorelli.anna@gmail.com)*

**Purpose:** Mixed primary liver cancer (mPLC) is characterised of both hepatocytic and cholangiocytic differentiation. Despite the increasingly rising incidence, its radiological diagnosis is still a challenge. This study's aim is to provide CT imaging features of mPLC, focusing on the differences between the two histological subtypes recently defined, combined hepatocellular-cholangiocellular carcinoma(cHCC-CC)and undifferentiated carcinoma.
**Methods or Background:** The CT images of patients with a biopsy-proven mPLC diagnosed between 2007-2019 at a single institution, were retrospectively reviewed by two radiologists with 7- and 1-year experience in liver imaging. Dimension, number of nodules, morphology, margins(regular/irregular), enhancement (arterial, intensity and pattern), presence of capsule and internal necrosis were recorded. A comparative analysis between the two histological subtypes was made by using Fisher-exact test for categorical variables and t-Student test for continuous one. The inter-reader agreement was assessed with k-Cohen test.
**Results or Findings:** Twenty-nine patients (18=cHCC-CC;11= undifferentiated) were enrolled. At the diagnosis, the majority of mPLC appeared as a single large nodule (mean diameter 64±39mm), with a lobulated morphology (76%) and smooth margin (86%). The majority of lesions (59%) showed a mild peripheral ring enhancement in arterial phase with progressive enhancement in portal and delayed phase. Necrosis and capsule were present in 45% and 17% of nodules, respectively. The undifferentiated subtype most often affected women (72%vs28%; p=0.027). Despite these lesions not differing in the enhancement pattern, they showed a weaker arterial enhancement (45%vs11%; p=0.050) and a capsule appearance on the portal phase (54%vs0%; p= 0.004). A good agreement was found between the two radiologists (k=0.769).
**Conclusion:** mPLC should enter in the differential diagnosis of large liver lesions with mild, peripheral ring arterial enhancement, progressively increasing in portal-delayed phase. In women, the presence of a capsule on the portal phase should raise the suspicion of undifferentiated subtype.
**Limitations:** The limitation is the retrospective design and the small sample.
**Ethics committee approval:** Not applicable.
**Funding for this study:** Not applicable.
**Author Disclosures:**
Rita Golfieri: Nothing to disclose
Anna Pecorelli: Nothing to disclose
Francesco Vasuri: Nothing to disclose
Cristina Mosconi: Nothing to disclose
Francesco Adduci: Nothing to disclose
Antonia D'Errico: Nothing to disclose

**15:00-16:00** **Room M 1**

### Research Presentation Session: Vascular

### RPS 515

### Vascular imaging: miscellaneous

**Moderator**
T. Rodt; Lüneburg/DE

### RPS 515-2
**Comparison of true-non-contrast and virtual-non-contrast phases derived from arterial and delayed phases of fast-kVp switching dual-energy CT in patients after EVAR**
*W. Kazimierczak*, N. Kazimierczak, Z. Serafin, A. Jawień, E. Nowak, A. Lemanowicz; Bydgoszcz/PL

**Purpose:** To analyse differences in CT numbers, SNR of vascular structures and endoleaks in TNC and VNC images derived from arterial (VNCa) and delayed (VNCd) phase of dual-energy fast-kVp switching CT in patients after EVAR procedure.
**Methods or Background:** CT scans of 97 patients who had prior EVAR procedure performed were enrolled in this study. All CT scans were obtained using a dual-energy fast-kVp switching scanner (Discovery 750 HD, GE Healthcare). TNC phase was obtained using single energy, both VNC phases were obtained using the fast-kVp switching technique. An intravenous administration of 80 ml iodine contrast agent was applied. The delayed phase was performed 60 s after the arterial phase. Simultaneous TNC, VNCa, and VNCd measurements were performed for (among others) aorta, endoleaks. The differences in the mean CT numbers in TNC, VNCa and VNCd were analysed statistically.
**Results or Findings:** The largest differences in the VNCa and VNCd densities were demonstrated in vascular structures. The mean density in the aorta - TNC 49.82 HU, VNCa 55.32 HU, VNCd 42.24 HU; in endoleaks 46.19 HU, 51.24 HU, 39.82 HU, respectively. These differences were statistically significant (p<0.05). The mean SNR measured in the aorta and endoleaks were: 2.11 and 2.04 in VNCa, 1.09 and 1.08 in TNC, 1.5 and 1.44 in VNCd.
**Conclusion:** The VNC images have higher SNR compared to TNC images. There are significant differences in CT numbers of the arteries and endoleaks in TNC, VNCa, VNCd images, with significantly lower densities in VNCd.
The study shows a high diagnostic value of VNC images and suggests use of VNCd images in the assessment of endoleaks.
**Limitations:** The study is limited to one method of dual-energy data acquisition.
**Ethics committee approval:** Our University ethics committee approved this study.
**Funding for this study:** Not applicable.
**Author Disclosures:**
Wojciech Kazimierczak: Nothing to disclose
Adam Lemanowicz: Nothing to disclose
Natalia Kazimierczak: Nothing to disclose
Zbigniew Serafin: Nothing to disclose
Arkadiusz Jawień: Nothing to disclose
Ewa Nowak: Nothing to disclose

### RPS 515-4
**A potential role of venous congestion in COVID-19-related pulmonary embolism distribution**
*D. C. Rotzinger*[1], F. Nevesny[2], A. W. Sauter[3], L. I. Loebelenz[4], L. Schmuelling[3], H. Alkadhi[5], L. Ebner[4], A. Christe[4], S. Qanadli[1]; [1]Lausanne/CH, [2]Dijon/FR, [3]Basel/CH, [4]Bern/CH, [5]Zurich/CH
*(david.rotzinger@chuv.ch)*

**Purpose:** Pulmonary embolism (PE) and non-thrombotic vascular disease, including venous congestion (VC), have been recognised as frequent COVID-19-related abnormalities and proposed as severity markers. However, the pathophysiological mechanisms involved remain a matter of debate. We aimed to describe the relationship between VC, PE and alveolar opacities (AO).
**Methods or Background:** This multicentre observational registry includes 268 patients diagnosed with SARS-CoV-2 pneumonia and subjected to contrast-enhanced CT between March and June 2020. We diagnosed acute PE in 61 (22.8%) patients, 17 females (27.9%), mean age 61.7±14.2 years. We retrieved demographic, laboratory, and outcome data. CT image analysis at the segmental level included VC (qualitatively and quantitatively [diameter]), AO (semi-quantitatively as absent, <50%, or >50% involvement), clot location, and distribution related to VC and AO. Segments with vs. without PE were compared.
**Results or Findings:** Of 411 emboli, 82 (20%) were lobar or more proximal, and 329 (80%) were segmental or sub-segmental. Venous diameters were significantly higher in segments with AO (p=0.031), unlike arteries (p=0.138). At the segmental level, 77% of emboli were associated with VC. Overall, PE occurred in 28.2% of segments with AO vs. 21.8% without (p=0.047). In the absence of VC, however, AO did not affect PE rates (p=0.94).
**Conclusion:** Vascular congestion predominantly affected veins and most pulmonary emboli were located in segments with VC. In the absence of VC, AOs did not influence the PE rate. VC might result from increased flow supported by the hypothesis of pulmonary arteriovenous anastomosis dysregulation as a relevant contributing factor.
**Limitations:** Only patients with contrast-enhanced CT were included potentially missing patients with thromboembolic disease who had no CT.
**Ethics committee approval:** Obtained, written consent was waived.
**Funding for this study:** Currently, no external funding is available.
**Author Disclosures:**
David Christian Rotzinger: Nothing to disclose
Lena Schmuelling: Nothing to disclose
Alexander Walter Sauter: Nothing to disclose
Franck Nevesny: Nothing to disclose
Lukas Ebner: Nothing to disclose
Andreas Christe: Nothing to disclose
Laura Isabel Loebelenz: Nothing to disclose
Salah Qanadli: Nothing to disclose
Hatem Alkadhi: Nothing to disclose

### RPS 515-5

**Simulating the impact of section thickness on automated measurements of pulmonary vascular volume on thin-slice, volumetric CT**

*B. R. Lavon*, R. Godon, I. Kendall, J. De Backer, M. Lanclus, S. Bonte; Kontich/BE
*(benjamin.lavon@fluidda.com)*

**Purpose:** Algorithms to measure the volume of the pulmonary vasculature on volumetric, non-contrast, chest CT have shown promise as imaging biomarkers for pulmonary vascular disease research and clinical decision-making. To estimate the impact of scan section thickness on these measurements, we resliced scans from healthy volunteers into various thicknesses and compared volumes of small pulmonary vessels detected by one such algorithm.
**Methods or Background:** Chest CT scans from 107 COPDGene healthy volunteers, reconstructed with volumetric thin slices (.7 mm) according to a standardised protocol, were resliced using the Mimics (Materialise, Belgium) reslicing tool to approximate reconstructed section thicknesses of 1, 1.5, 2, and 3 mm, and analysed using Fluidda's (Fluidda NV, Belgium) Functional Respiratory Imaging platform to quantify the volume of vessels between 1.25 and 140 mm2 in cross-sectional area. BV5, the volume of vessels smaller than 5 mm2 expressed in milliliters and as % total pulmonary blood volume, was compared between the different resliced cohorts.
**Results or Findings:** 107 subjects' scans were analysed (73 female, 34 male); average age was 62.3 years (+/- 9.1). Mean (SD) BV5 was 137.03 (24.94) ml, 151.4 (27.65) ml, 124.66 (22.01) ml, 88.15 (15.01) ml, and 29.02 (5.7) ml for the .7, 1, 1.5, 2-, and 3- mm resliced cohorts, respectively. BV5 vessels represented a mean of 54.72 (3.39)%, 53.88 (3.56)%, 46.46 (3.47)%, 37.78 (3.07)%, and 16.86 (2.4)% of total vascular volume. In a paired t-test, each resliced cohort differed significantly from every other resliced cohort ($p<.001$).
**Conclusion:** Section thickness likely has a significant impact on the volume of small vessels detected and the proportion of total pulmonary vascular volume they represent. Research to clinically validate these endpoints should consider this impact when studying historical datasets.
**Limitations:** Not applicable.
**Ethics committee approval:** Not applicable.
**Funding for this study:** Not applicable.
**Author Disclosures:**
Rik Godon: Employee: Fluidda
Maarten Lanclus: Employee: Fluidda
Benjamin R. Lavon: Employee: Fluidda
Jan De Backer: CEO: Fluidda
Irvin Kendall: Other: Fluidda
Stijn Bonte: Employee: Fluidda

### RPS 515-6

**Evaluation of MR biomarkers for advanced classification and therapy monitoring of patients with venous malformation**

*F. Haupt*, A. Huber, C. Calastra, A. Tuleja, J. Rössler, I. Baumgartner, H. Von Tengg-Kobligk; Bern/CH
*(fabian.haupt@insel.ch)*

**Purpose:** The purpose of this study is to evaluate morphological and functional MR sequences for the correct classification of venous malformations and for identification of imaging features, which contribute to therapy monitoring.
**Methods or Background:** Background: Congenital vascular malformations (CVM) are defects of the vascular system, which develop during angiogenesis in utero. CVMs are divided into high flow and low flow malformations and are classified by the ISSVA according to the predominant diseased vessel type. Magnetic resonance imaging (MRI) is the best method for the morphological characterisation of CVMs, regarding extent, involved compartments and lesion composition (pure venous vs combined malformation). The utilisation of morphological T1/2w sequences allows tissue characterisation of suspected vascular malformations, i.e. to differentiate between vascular and lymphatic components. MR angiography (MRA) with Gadolinium-based contrast agents allows the visualisation and assessment of the perfused vascular portion of malformations. The continuous acquisition using time-resolved MRA allows semiquantitative analysis of the hemodynamics in vascular malformations. Methods: MRI with morphological and angiographic sequences of 15 patients with simple venous malformations was performed before and after treatment by percutaneous ethanol embolisation. Size of the malformation was measured in T2w, size of vascularisation was measured in MRA. Number of draining veins was counted in MRA.
**Results or Findings:** While T2w was only slightly reduced (n.s.), the vascular volume was reduced impressively. The vascular portion (MRA/T2w) was significantly lower after therapy (81±27% vs 41±28%,$p<0.001$). The number of draining veins was significantly reduced after therapy (1.7±0.3 vs 0.3±0.6%,$p<0.05$).
**Conclusion:** MRI is feasible for therapy monitoring of venous malformations. Vascular volume in MRA is a better endpoint than morphological T2w-based morphometry. Draining veins are another feasible endpoint.
**Limitations:** The limitation is the preliminary data in a small cohort.
**Ethics committee approval:** This study is IRB approved.
**Funding for this study:** Funding was received for this study by the Swiss National Science Foundation (SNF).
**Author Disclosures:**
Fabian Haupt: Nothing to disclose
Jochen Rössler: Nothing to disclose
Camilla Calastra: Nothing to disclose
Aleksandra Tuleja: Nothing to disclose
Adrian Huber: Nothing to disclose
Iris Baumgartner: Nothing to disclose
Hendrik Von Tengg-Kobligk: Nothing to disclose

### RPS 515-7

**Clinical application of high-frame rate vector flow imaging technology in evaluating hemodynamic changes of moderate and severe carotid stenosis**

*W. Wang*, Y. Qiu, D-H. Yang, Q. Zhang, Y. Dong; Shanghai/CN
*(puguang61@126.com)*

**Purpose:** To investigate the value of high-frame-rate vector flow imaging (V flow) technology in evaluating the hemodynamic changes of moderate and severe carotid stenosis caused by atherosclerotic plaque.
**Methods or Background:** In this prospective study, patients with moderate and severe carotid atherosclerotic stenosis were enrolled. Mindray Resona7s ultrasound machine and L9-3 linear array probe were used for V Flow and conventional ultrasound examination before surgery. Changes in vector arrows and streamlines in the carotid stenosis were observed and analysed by V flow imaging. Carotid wall shear stress (WSS) values at the proximal, narrowest and distal of stenosis were measured. Taking the carotid digital subtraction angiography results as gold standard, the diagnostic efficacy of WSS value measured by V flow was analysed.
**Results or Findings:** Finally, 54 patients with carotid stenosis rate ≥ 50% were prospectively included. V flow showed yellow or red vector arrows with a faster velocity at the segmentation of carotid stenosis. Taking WSS value > 1.05 Pa in carotid stenosis segments as the cut-off value, area under the curve (AUC), accuracy, sensitivity and specificity of WSS value in diagnosing severe carotid stenosis was 79.3%, 72.2%, 80.6% and 68.2%.
**Conclusion:** V flow dynamic imaging could reflect more comprehensively the hemodynamic changes caused by carotid atherosclerotic stenosis.
**Limitations:** This is a single-centre study with a limited number of patients.
**Ethics committee approval:** The study protocol of this multicentre study was reviewed and approved by the institutional review boards (No. B2021-145).
**Funding for this study:** Supported by Clinical Research Plan of SHDC (Grant No. SHDC2020CR1031B); Supported by Shanghai Municipal Key Clinical Specialty (Grant No. shslczdzk03501); Supported by Natural Science Foundation Project of Shanghai 'Science and Technology Innovation Action Plan' (Grant No. 20ZR1452800).
**Author Disclosures:**
Qi Zhang: Nothing to disclose
Wenping Wang: Nothing to disclose
Yi Dong: Nothing to disclose
Yijie Qiu: Nothing to disclose
Dao-Hui Yang: Nothing to disclose

### RPS 515-8

**Reliability of Tomographic 3D Ultrasound in measuring carotid plaque volume**

*A. A. Alzahrani*, S. R. Sultan, M. Aslam; London/UK
*(aa114@ic.ac.uk)*

**Purpose:** This study aims to assess the reliability of tomographic 3D ultrasound (t3DUS) for measuring carotid plaque volume (CPV) in patients undergoing carotid endarterectomy.
**Methods or Background:** t3DUS is a promising imaging technique for quantifying carotid plaque by measuring the degree of stenosis and plaque volume. CPV could add benefit in predicting plaque vulnerability.
t3DUS was used to obtain carotid plaque volume from 25 patients before surgery. CPV from the endarterectomy specimen was then measured using a validated water displacement method as a reference test. Intraclass correlation coefficient (ICC) and Bland-Altman plot were used to establish bias and limit of agreement between carotid plaque volume measurements.
**Results or Findings:** There was an excellent agreement between t3DUS and gold standard in measuring carotid plaque volume with an ICC value of 0.98 (95% CI 0.93 – 0.99, $p <0.001$). Bias in measurements was 0.07±0.08 cm3 (95% limit of agreement (LoA) -0.08 – 0.23). Intra-operator agreement of t3DUS CPV measurements was excellent with an ICC value of 0.95 (95% confidence interval (CI) 0.91 – 0.97, $p <0.001$). Bias in measurements was 0.004±0.07 cm3 (95% limit of agreement (LoA) -0.14 – 0.15).
**Conclusion:** t3DUS is a reproducible and accurate imaging method for measuring CPV. Further studies investigating the reliability of t3DUS in assessing plaque morphological characteristics are required.
**Limitations:** Calcified plaques.

**Ethics committee approval:** This study was approved by an ethics committee.
**Funding for this study:** Funding was received for this study by the Saudi Arabia KAMC.
**Author Disclosures:**
Mohammed Aslam: Nothing to disclose
Adel Ahmad Alzahrani: Nothing to disclose
Salahaden R Sultan: Nothing to disclose

**15:00-16:00** **Room M 2**

### Research Presentation Session: Hybrid, Molecular and Translational Imaging

### RPS 506

### Hybrid imaging

**Moderator**
J. Ferda; Plzen/CZ
**Author Disclosure:**
J. Ferda: Advisory Board: Siemens Photon-counting CT project; Board Member: EJR

### RPS 506-2

**Initial nodal staging in newly diagnosed breast cancer: comparability between whole body MRI and [18F]-FDG PET/MRI**

M. M. A. Rezk[1], *B. L. Alsaleh*[2], W. Ibrahim[2], H. Hasan[2], M. A. Abu el Dahab[1]; [1]Cairo/EG, [2]Manama/BH
*(Balsaleh94@gmail.com)*

**Purpose:** To compare the diagnostic potential of whole body MRI and 18F-FDG PET/MRI in nodal staging of newly diagnosed, histopathologically proven breast cancer.
**Methods or Background:** A single centered 100 patients was included in this retrospective study. Patients underwent and departmental whole body magnet resonance imaging MRI protocol and [18F]-FDG PET/MRI in same the scanner. Locoregional and distant nodal lesions were evaluated. Histopathology results were added. Statistical analyses were done for the two imaging modalities.
**Results or Findings:** PET/MRI displayed high specificity indices in locoregional and distant nodal lesions, while whole MRI showed better sensitive indices. No statistically significant difference was seen between whole body MRI and PET MRI.
**Conclusion:** FDG PET/MRI depicted admirable diagnostic performance in nodal staging of newly diagnosed, proven breast cancer patients.
**Limitations:** Heterogenous study populations.
**Ethics committee approval:** Institutional ethics approval.
**Funding for this study:** No funding for this study.
**Author Disclosures:**
Bano Loai Alsaleh: Nothing to disclose
Hossam Hasan: Nothing to disclose
Mohamed Abdu Abu el Dahab: Nothing to disclose
Wael Ibrahim: Nothing to disclose
Mahmoud M. A. Rezk: Nothing to disclose

### RPS 506-3

**Performance of whole-body [18F]-FDG-PET/MRI in lymphoma patients: a single-centre study**

*B. L. Alsaleh*[1], M. M. A. Rezk[2], W. Ibrahim[1]; [1]Manama/BH, [2]Cairo/EG
*(Balsaleh94@gmail.com)*

**Purpose:** To investigate the performance of whole-body [18F]-FDG-PET/MRI in lymphoma patients.
**Methods or Background:** Single-centered, retrospective study for 30 histologically proven lymphoma patients underwent whole-body [18F]-FDG-PET/MRI for staging with routine local institutional protocol . [18F]-FDG-PET and MR scans were evaluated and interpreted by hybrid radiology and nuclear medicine physicians. [18F]-FDG-PET/MRI findings were correlated with available routine imaging studies and clinical investigations. Statistical analysis was done.
**Results or Findings:** 30 patients with 330 nodal and extra-nodal regions were assessed. PET/MRI had high diagnostic indices in terms of sensitivity or specificity parameters. The detection of pleural and bone and liver infiltrations were significantly higher ($P < 0.05$).
**Conclusion:** 18F-FDG PET/MRI displayed higher diagnostic performance correct classification of the disease extent with potential impact on patient and therapy management.
**Limitations:** Number of the study populations with heterogenicity.
**Ethics committee approval:** Institutional committee approval is taken.
**Funding for this study:** No funding for this study.
**Author Disclosures:**
Bano Loai Alsaleh: Nothing to disclose
Wael Ibrahim: Nothing to disclose
Mahmoud M. A. Rezk: Nothing to disclose

### RPS 506-4

**Convolutional neural network-based analysis of PSMA PET/CT for predicting PSA-based response to 177Lu-PSMA radioligand therapy in mCRPC patients**

*T. Geyer*; Munich/DE

**Purpose:** This retrospective study aims to train CNN models which predict response to 177Lu-PSMA radioligand therapy in mCRPC patients on pretherapeutic PSMA PET/CT and to assess early therapy response to 177Lu-PSMA radioligand therapy in mCRPC patients on post-therapeutic PSMA PET/CT.
**Methods or Background:** A CNN model was trained predicting response to 177Lu-PSMA radioligand therapy in mCRPC patients on pretherapeutic PSMA PET/CT with serum PSA levels as the standard of reference. Four clinical relevant experiments have been carried out: CNN trained with whole 3D PET with attenuation corrected CT volumes (PET/CT); with whole 3D PET/CT volumes and stochastic weight averaging (PET/CT+SWA); with whole 3D PET/CT volumes and the corresponding CT volume as second channel (PET/CT+CT); and with rotated and then MIP 2D images (Rotation+MIP). AUROC was calculated for all experiments separately.
**Results or Findings:** The median AUROC for each experiment is was about 0.75, except for Rotation+MIP (median <0.7).
**Conclusion:** In conclusion, it is hard to train a verifiable model, as only using different samples for training and validation show a high volatility in model performance. However, it seems like that the problem of predicting therapy response is solvable by CNNs under the requirement of more samples used for training and validating. Using more information (PET/CT+CT), artificially increasing the dataset size (rotation+MIP) using state-of-the-art optimisation techniques (PET/CT+SWA) increase the model's generalisation and should be taken into account for further research.
**Limitations:** The major limitation of this study is the sample size. Further studies with convolutional neural network-based analyses of larger patient cohorts are needed in order to train the CNN and validate the findings from this study.
**Ethics committee approval:** This study was approved by the ethics committee of the Medical department Ludwig-Maximilians-University Munich (21-0239).
**Funding for this study:** No funding was received for this study.
**Author Disclosures:**
Thomas Geyer: Nothing to disclose

### RPS 506-6

**Quantification of coronary artery atherosclerotic burden and muscle mass: comparison of two software programs**

C. Nappi, *A. Ponsiglione*, R. Megna, F. Volpe, C. G. Mainolfi, M. Klain, M. Imbriaco, M. Petretta, A. Cuocolo; Naples/IT

**Purpose:** We compared the performance of two semiautomatic freeware software, Horos and LIFEx, for the evaluation of coronary artery calcium (CAC) score and muscle mass in 40 patients undergoing whole-body PET/CT.
**Methods or Background:** Sarcopenia and coronary calcification may have a relevant prognostic impact in oncological and non-oncological patients. The use of freeware software is promising for quantitative evaluation of these variables after whole-body positron emission tomography (PET)/computed tomography (CT) and might be useful for one-stop-shop risk stratification without additional charges to health care costs.
**Results or Findings:** The muscle areas obtained by the two software were comparable, showing high correlation by Lin's concordance coefficient (0.9997; 95% confidence intervals, CI: 0.9995-0.9999) and a very good agreement by the Bland-Altman analysis (mean difference = 0.41 cm2, lower limit = -1.06 cm2, upper limit = 1.89). For CAC score, the Lin's concordance correlation coefficient was 0.9976 (95% CI: 0.9965-0.9984) and at Bland-Altman analysis there was a mean difference of 8 (lower limit = -32, upper limit = 48), with a slight overestimation of Horos CAC score as compared to LIFEx. We investigated the possible causes of this discrepancy at the level of region of interest (ROI). The overestimation was due to a different calculation method of the CAC score, being the ROI equal for the two software.
**Conclusion:** Our results demonstrated that off-line analysis performed with freeware software may allow a comprehensive evaluation of the oncological patient, making available the evaluation of parameters, such as muscle mass and calcium score, that may be relevant for the staging and prognostic stratification of these patients, beside standard data obtained by PET/CT imaging. For this purpose, the Horos and LIFEx software seem to be comparable.

**Limitations:** Single-centre.
**Ethics committee approval:** This study was approved by an ethics committee.
**Funding for this study:** No funding was received for this study.
**Author Disclosures:**
Fabio Volpe: Nothing to disclose
Carmela Nappi: Nothing to disclose
Michele Klain: Nothing to disclose
Mario Petretta: Nothing to disclose
Rosario Megna: Nothing to disclose
Alberto Cuocolo: Nothing to disclose
Andrea Ponsiglione: Nothing to disclose
Massimo Imbriaco: Nothing to disclose
Ciro Gabriele Mainolfi: Nothing to disclose

### RPS 506-7

**The diagnosis of b-thalassemia in an early medieval archaeological context: the contribution of radiology**

*A. Lupi*[1], S. Viva[2], G. Vincenti[3], C. Morbiato[1], E. Quaia[1], D. Caramella[4], P. F. Fabbri[2], A. Pepe[1]; [1]Padua/IT, [2]Lecce/IT, [3]Florence/IT, [4]Pisa/IT
*(amalia.lupi@phd.unipd.it)*

**Purpose:** The application of radiology as an aid to paleo-demographic analyses is increasingly widespread. The aim of this study is to examine the radiological aspects of hereditary hemoglobinopathy, such as b-thalassemia, on skeletal samples from an ancient community, using CT.
**Methods or Background:** Within the ERC Advanced project "nEU-Med: Origins of a new Economic Union (7th to 12th centuries): resources, landscapes, and political strategies in a Mediterranean region", a sample of 15 ribs belonging to 9 individuals from early childhood to adult was examined. The samples come from the early medieval archaeological site (X-XI century) of Vetricella (Scarlino, GR), Italy. To complete the macroscopic paleopathological analysis, the bone samples underwent CT investigation, by using 128-layer GE equipment and a layer thickness of less than 1 mm. The measure of the density of the ribs and the alterations of the trabecular component were also evaluated.
**Results or Findings:** The CT analysis of the 15 rib samples examined allowed the identification of findings compatible with the presence of b-thalassemia, such as signs of hyperostosis and the characteristic "rib-within-a-rib appearance" of the anterior and median portions.
**Conclusion:** This preliminary study demonstrates that skeletal changes in advanced stages of b-thalassemia can be assessed by CT on bone samples from ancient communities. Clinical findings of these conditions are rare in industrialised countries but are sometimes still present in geographic areas with little or no access to transfusion and chelation therapy. Therefore, the above analyses may be of interest also to improve our knowledge on the natural history of this pathology that represents the most common genetic disorder worldwide.
**Limitations:** Sample size.
**Ethics committee approval:** Not appropriate.
**Funding for this study:** Not appropriate.
**Author Disclosures:**
Amalia Lupi: Nothing to disclose
Alessia Pepe: Nothing to disclose
Davide Caramella: Nothing to disclose
Serena Viva: Nothing to disclose
Claudia Morbiato: Nothing to disclose
Giorgia Vincenti: Nothing to disclose
Pier Francesco Fabbri: Nothing to disclose
Emilio Quaia: Nothing to disclose

**15:00-16:00** **Room M 3**

### Research Presentation Session: Cardiac

### RPS 503

### MR imaging of myocardial scar

**Moderator**
K.-F. Kreitner; Mainz/DE

### RPS 503-2

**Histopathological validation of semi-automated myocardial scar quantification techniques for dark-blood LGE MRI**

*H. M. J. M. Nies*[1], S. Gommers[1], L. I. B. Heckman[1], F. W. Prinzen[1], C. M. Van De Heyning[2], A. Chiribiri[3], J. E. Wildberger[1], C. Mihl[1], R. J. Holtackers[1]; [1]Maastricht/NL, [2]Antwerp/BE, [3]London/UK

**Purpose:** To evaluate the performance of various semi-automated techniques for quantification of myocardial infarct (MI) size on both conventional bright-blood and novel dark-blood LGE images using histopathology as the reference standard.
**Methods or Background:** In thirteen Yorkshire pigs, reperfused MI was experimentally induced. At 7 weeks post-MI, both bright-blood and dark-blood LGE imaging was performed on a 1.5T MRI scanner. Following MRI, the animals were sacrificed and histopathology was obtained. For both LGE methods, the percentage of infarcted myocardium was assessed per slice using various semi-automated scar quantification techniques, including the signal threshold versus reference mean (STRM, using 3 to 8 standard deviations (SDs) as threshold) and full-width at half-maximum (FWHM) methods, as well as manual contouring. Infarct size obtained by histopathology was used as the reference standard.
**Results or Findings:** In total, 24 paired LGE MRI slices and histopathology samples were available for analysis. For both bright-blood and dark-blood LGE, the STRM method with a threshold of 5 SDs led to the best agreement to histopathology without significant bias (-0.23%, 95% CI [-2.99,2.52%], p=0.862 and -0.20%, 95% CI [-2.12,1.72%], p=0.831, respectively). Manual contouring significantly underestimated infarct size on bright-blood LGE (-1.57%, 95% CI [-2.96,-0.18%], p=0.029), while manual contouring on dark-blood LGE outperformed semi-automated quantification and demonstrated the most accurate quantification in this study (-0.03%, 95% CI [-0.22,0.16%], p=0.760).
**Conclusion:** The signal threshold versus reference mean method with a threshold of 5 standard deviations demonstrated the most accurate semi-automated quantification of infarcted myocardium, without significant bias compared to histopathology, for both conventional bright-blood and novel dark-blood LGE.
**Limitations:** MI induction to achieve the maximum amount of scar tissue led to significant dropouts due to arrhythmia-related deaths.
**Ethics committee approval:** This study was approved by the Experimental Animal Committee (DEC2016-002).
**Funding for this study:** No funding was received for this study.
**Author Disclosures:**
Casper Mihl: Nothing to disclose
Joachim E. Wildberger: Nothing to disclose
Suzanne Gommers: Nothing to disclose
Luuk I. B. Heckman: Nothing to disclose
Caroline M. Van De Heyning: Nothing to disclose
Robert J. Holtackers: Nothing to disclose
Amedeo Chiribiri: Nothing to disclose
Frits W. Prinzen: Nothing to disclose
Hedwig M. J. M. Nies: Nothing to disclose

### RPS 503-3

**Novel 3D dark-blood late gadolinium enhancement MRI for visualisation of atrial scar to guide atrial fibrillation ablation**

*G. P. Bijvoet*, B. Hermans, D. Linz, K. Vernooy, J. E. Wildberger, C. Mihl, S-M. Chaldoupi, R. J. Holtackers; Maastricht/NL

**Purpose:** To evaluate the feasibility and first experience with novel dark-blood LGE CMR to visualise the atrial myocardium.
**Methods or Background:** Background: Late gadolinium enhancement (LGE) MRI is the method of choice for the assessment of myocardial scar. However, novel dark-blood LGE methods proved superior for localisation of subendocardial scar by enhancing the often poor scar-to-blood contrast of conventional bright-blood LGE. Although extensively evaluated in the ventricles, dark-blood LGE has not been applied for the atria yet.
Methods: 45 patients scheduled for atrial fibrillation (AF) ablation with pre-procedural LGE MRI were enrolled in this study. All patients received free-breathing 3D dark-blood PSIR LGE with high spatial resolution (0.63 x 0.63 x 1.00mm reconstructed) and a dynamic inversion time mechanism. LGE

Wednesday datasets were analysed using ADAS-AF software (Figure 1). Signal intensities were expressed as ratio to the mean signal intensity of the blood pool (IIR).
**Results or Findings:** 3D dark-blood LGE was successfully performed in 41/45 participants (91%). Four scans were terminated prematurely due to arrhythmia. Image segmentation and analysis were successful in 39/41 patients (95%). In two patients, the atrial wall was non-interpretable due to artefacts (1 mechanical valve, 1 ICD). Patients without prior ablation (19/39 patients) showed lower IIR as compared to re-do patients. In addition, the IIR distribution suggests that the conventional thresholds for bright-blood LGE (healthy <1.20, scar >1.32) do not differentiate between scar and non-scar in dark-blood LGE (Figure 2).
**Conclusion:** Novel 3D dark-blood LGE of the atria is feasible and could be successfully performed and post-processed in a pre-ablation AF population. Future studies, however, should determine scar thresholds for 3D dark-blood LGE as conventional thresholds do not apply.
**Limitations:** Atrial arrhythmias influenced image quality.
**Ethics committee approval:** Study approved by the local ethics committee (METC2019-1136).
**Funding for this study:** No funding was received for this study.
**Author Disclosures:**
BJM Hermans: Nothing to disclose
Sevasti-Maria Chaldoupi: Nothing to disclose
Casper Mihl: Nothing to disclose
Joachim E. Wildberger: Nothing to disclose
D Linz: Nothing to disclose
K Vernooy: Nothing to disclose
Robert J. Holtackers: Nothing to disclose
Geertruida Petronella Bijvoet: Nothing to disclose

### RPS 503-4

**Role of early cardiac magnetic resonance in patients with acute myocardial infarction**

*O. V. Mochula*, D. Vorobyeva, V. Ryabov, W. Y. Ussov; Tomsk/RU
*(mochula.olga@gmail.com)*

**Purpose:** Purpose was to study the type of left ventricular myocardial damage in patients with AMI according to early CMR.
**Methods or Background:** The study included 66 patients (age 62±11 years; 62% male patients) that were admitted to the emergency department with AMI. CMR was performed in a 1.5 T MR-scanner at a mean of 6.9±3.7 days after admission. For determination of oedema, short-axis slices using a T2-WI were obtained. Presence and type of scar were assessed in LGE.
**Results or Findings:** The admitted patients were diagnosed with AMI with ST-elevation in 68.18% of cases. According to the data of ICA, the patients were divided into two groups: patients with significant stenosis of the coronary arteries (CA) (MICAD) (48.5% n=32), with primarily single-vessel CA (56%); and patients with non-obstructive CA (MINOCA) (n=34, 51.5%). Myocardial damage (MD) detected of LGE was divided into three main types: ischaemic (IT), nonischaemic (NIT) and mixed (MT). According to LGE, patients with MICAD in 84.4% of cases visualised ischaemic type of LV myocardial damage with varying patterns of LGE transmurality. In turn, patients of the MINOCA were characterised predominantly (65%) by non-ischaemic type of MD according to LGE-CMR. Nevertheless, in patients with MINOCA, in one-third of cases there was an IT MD. The combination of ischaemic and non-ischaemic MR patterns of myocardial damage was found to a lesser extent and in general amounted to 11% among all patients.
**Conclusion:** CMR has important diagnostic value for determining the underlying cause of myocardial damage in patients with AMI. This is extremely important because various non-ischaemic diseases have a clinical picture similar to AMI.
**Limitations:** No limitations were identified.
**Ethics committee approval:** Approved by an ethics committee.
**Funding for this study:** The study was supported by a grant from the Russian Science Foundation (project N 21-75-00051, https://rscf.ru/project/21-75-00051/).
**Author Disclosures:**
Daria Vorobyeva: Nothing to disclose
Olga Vital'evna Mochula: Nothing to disclose
Wladimir Yurievich Ussov: Nothing to disclose
Vyacheslav Ryabov: Nothing to disclose

### RPS 503-5

**Myocardial necrosis and inflammation markers of myocardial ischaemia-reperfusion injury in patients with ST-elevation myocardial infarction**

*T. Rusak*, L. Gelis, H. Medvedeva, N. Shibeko, T. Horbat; Minsk/BY
*(tanyarusack@yandex.ru)*

**Purpose:** To evaluate the informative value of biomarkers (cardiac troponin I, C-reactive protein, N-terminal pro-brain natriuretic peptide, stimulating growth factor) as laboratory markers of myocardial ischaemia-reperfusion injury in patients with STEMI endovascular revascularisation.
**Methods or Background:** The study included 115 patients with STEMI who underwent endovascular revascularisation. To identify the informative value of biomarkers, the levels of cTnI, NTproBNP, hsCRP, sST2 were analysed at baseline, as well as after reperfusion therapy on the 2nd (cTnI) and 5th (ST2, hsCRP, NTproBNP) days. Cardiac magnetic resonance imaging was performed 5 days after revascularisation.
**Results or Findings:** Microvascular obstruction was disclosed in 54 patients (47%), of which 24 (44%) patients had a combination of microvascular obstruction and myocardial hemorrhage. In 61 cases (53%), no microvascular damage was registered. It was found that, with an increase in the threshold initial NTproBNP levels >590 pg/ml, the odds ratio (OR) of myocardial reperfusion injury was 12,2 (95% confidence interval (CI), 4,81-30,92, p<0,001). In addition, we analysed biomarker levels in the early period after myocardial infarction, at which threshold values of reperfusion injury parameters were established as follows: for cTnI >8,1 ng/ml, OR=7,17 (95% CI, 3,11-16,53, p=0,001); for hsCRP >14 mg/L, OR=12,71 (95% CI, 5,03-32,08, p=0,001); for NTproBNP >334 pg/ml, OR=11,8 (95% CI, 4,88-28,59, p=0,001); for sST2 >41 ng/ml, OR=7,17 (95% CI, 3,11-16,53, p=0,001). According to multivariate analysis, predictors of microvascular injury were the initial NTproBNP values, as well as the cTnI, hsCRP, and sST2 values in the early postinfarction period (sensitivity, 89,5%; specificity, 83,3%).
**Conclusion:** Thus, the initial NTproBNP, as well as cTnI, hsCRP, sST2 values after endovascular intervention are more informative for assessing the risk of microvascular damage in patients with ST-elevation myocardial infarction.
**Limitations:** Not applicable.
**Ethics committee approval:** Not applicable.
**Funding for this study:** Not applicable.
**Author Disclosures:**
Tatsiana Horbat: Author: collection of material, writing text
Helen Medvedeva: Author: collection of material, processing, writing text
Tatsiana Rusak: Author: collection of material, processing, writing text
Natalia Shibeko: Author: collection of material, processing, writing text
Ludmila Gelis: Author: study concept and design

### RPS 503-7

**Native T1 mapping to detect the extent of acute ST-segment elevation myocardial infarction and viable myocardium compared to delayed gadolinium-enhanced MRI**

*Y. O. Shalaginova*, E. Butorova, O. Stukalova, R. Shakhnovich, I. Staroverov, S. K. Ternovoy; Moscow/RU
*(shalaginovayuliya@gmail.com)*

**Purpose:** To compare native T1 mapping in assessing the extent of myocardial injury in acute STEMI and myocardial viability with delayed gadolinium-enhanced MR-imaging.
**Methods or Background:** 40 patients with revascularised acute STEMI underwent CMR imaging and T1-mapping 3-7 after onset of symptoms using a Siemens Magnetom Aera, 1.5 T MRI scanner. T1 maps were obtained using the modified MOLLI 5(3)3 sequence (Modified Look-Locker Inversion Recovery). Data were analysed on a Siemens workstation by a semiquantitative method. The severity of myocardial damage was obtained by calculating the fibrosis index, which reflects the depth and extent of the lesion.
**Results or Findings:** MR images of 40 patients (680 segments in total) were analysed. 4 patients were excluded due to the unsatisfactory quality of T1 maps. All patients with gadolinium (GD) accumulation zones showed a change in relaxation time (T1). The sensitivity and specificity of T1 mapping in detecting acute MI compared with LGE was 86.5% (95% CI 80.4-91.2) and 90.3% (95% CI 87.1-92.9), respectively. Median T1 for regions with GD accumulation zones was 1211.5 ms [1186.3;1249.6], and for unaffected myocardium it was 1073.9 ms [1049.5.3;1094.6]. T1 for subendocardial and transmural lesions did not differ significantly. Correlation analysis of the severity of myocardial injury on LGE MR imaging and native T1 maps revealed a statistically significant direct moderate correlation (R=0.67, p<0.001).
**Conclusion:** Native T1-mapping may detect ischaemic myocardial injury and assess its extent without using contrast. It's important for patients with chronic kidney disease, allergic reactions to gadolinium-containing contrast agents, and patients with serious conditions because it reduces the study time. However, the assessment of transmurality on native T1 maps requires further research.
**Limitations:** The small cohort. Using a semiquantitative method for detecting myocardial injury.
**Ethics committee approval:** Ethics committee approval obtained.
**Funding for this study:** No funding was received for this work
**Author Disclosures:**
Ekaterina Butorova: Nothing to disclose
Sergey K Ternovoy: Nothing to disclose
Olga Stukalova: Nothing to disclose
Roman Shakhnovich: Nothing to disclose
Yuliya Olegovna Shalaginova: Nothing to disclose
Igor Staroverov: Nothing to disclose

### RPS 503-8
**T1 Mapping in cardiac magnetic resonance in patients with premature ventricular complexes**
*I. Notarangelo*, M. Gravina, M. G. R. Manco, G. Casavecchia, L. Macarini, G. Guglielmi; Foggia/IT
*(ilenia_notarangelo.549379@unifg.it)*

**Purpose:** To analyse the correlation between the gold standard late gadolinium enhancement (LGE) sequences vs. mapping sequences and to evaluate the performance of both techniques in diagnosis and management of patients with premature ventricular complexes.
**Methods or Background:** A total of 39 patients with a diagnosis of premature ventricular complexes were studied by cardiac 1.5 T MRI. The study was performed with cardiac and respiratory gating to compare the PSIR-TFE LGE sequences and the native T1 mapping (MOLLI).
**Results or Findings:** The analysis of LGE values detected variations of signal characteristic of myocardial alteration in 50 cardiac segments in 14 (36%) patients. The native T1 mapping values recognised variations of signal in 278 cardiac segments in 33 (85%) patients.
**Conclusion:** Our results demonstrated that without contrast enhancement and with a reduction in acquisition time, T1 mapping sequences are more sensitive compared to the traditional PSIR-TFE LGE in detecting premature ventricular complexes and in discovering signal alteration in more extensive myocardial areas.
**Limitations:** The limitations included the small number of patients examined, the proper diagnosis of premature ventricular complexes and the analysis of the T1-Mapping results.
**Ethics committee approval:** This study was approved by an ethics committee.
**Funding for this study:** There was no funding for this study.
**Author Disclosures:**
Luca Macarini: Nothing to disclose
Giuseppe Guglielmi: Nothing to disclose
Maria Grazia Rita Manco: Nothing to disclose
Ilenia Notarangelo: Nothing to disclose
Grazia Casavecchia: Nothing to disclose
Matteo Gravina: Nothing to disclose

**15:00-16:00** **Room M 4**

### Research Presentation Session: Genitourinary

### RPS 507

### Advances in Gynaecologic Imaging

**Moderator**
N. Pötsch; Vienna/AT

### RPS 507-3
**Repeatability and reproducibility of quantitative cervical strain elastography (E-Cervix) in pregnancy**
*J. Mlodawski*, M. Mlodawska, G. Swiercz; Kielce/PL
*(kuba.mlodawski@gmail.com)*

**Purpose:** The aim of this study was to assess the repeatability and reproducibility of quantitative cervical strain
elastography (E-Cervix) of the uterine cervix in pregnancy and to assess the correlation of the obtained parameters with selected clinical features of patients in the third trimester of pregnancy.
**Methods or Background:** E-Cervix is a software programme that uses intrinsic compression to excite tissue and allows the evaluation of quantitative parameters on the basis of pixel distribution in an elastogram. In total 222 patients participated in the study. We assessed 5 ultrasound parameters: elasticity index (ECI), hardness ratio (HR), internal os strain (IOS), external os strain (EOS), and IOS/EOS ratio. Each study was performed according to a predetermined standardised protocol.
**Results or Findings:** For all assessed elastographic parameters, we obtained good intra- and interobserver reproducibility. The interclass correlation coefficient (ICC) ranged from 0.77 to 0.838 for intraobserver variability and from 0.771 to 0.826 for interobserver variability. We demonstrated a significant correlation of some obtained elastographic parameters with the basic clinical features of patients, such as age, the number of previous caesarean sections, pregnancy weight, and BMI. In each case, the correlation was very low.
**Conclusion:** Quantitative elastographic assessment with the use of E-Cervix is characterised by good repeatability. Some clinical features may affect the value of the parameters obtained. The clinical relevance of this interference requires further investigation.
**Limitations:** Analysis of correlation in our sample group may be subject to limitations due to the selection of the sample,
which included patients hospitalised at the pregnancy pathology unit rather than a population of patients in
physiological pregnancy.
**Ethics committee approval:** We received approval to examine the patients for research purposes from the bioethics commission at Jan Kochanowski University in Kielce (approval number—55/2019).
**Funding for this study:** No external funding was received for this study.
**Author Disclosures:**
Grzegorz Swiercz: Nothing to disclose
Jakub Mlodawski: Nothing to disclose
Marta Mlodawska: Nothing to disclose

### RPS 507-5
**Diagnostic value of MR for placenta accreta spectrum disorders**
*R. P. Radeva*, G. I. Kirova-Nedyalkova, E. Georgiev, E. R. Panova, E. Naseva; Sofia/BG

**Purpose:** The aim of our study is to evaluate the overall sensitivity and specificity of MR for diagnosing disorders of the placenta accreta spectrum and the pathologic features that have the highest predictive value.
**Methods or Background:** Between March 2018 and October 2021, 66 pregnant women with risk factors of placenta accreta underwent MR examination after inconclusive ultrasound or to determine the size and depth of invasion for already diagnosed placenta accreta. Mean maternal age was 35.2, mean gestational age was 29.4. All MR examinations were reviewed by two radiologists with experience in genitourinary radiology. The standard of reference were intraoperative and pathologic findings. ROC curve analysis was used to test the discriminative ability of MRI features for abnormally adherent and invasive placenta.
**Results or Findings:** MR exhibited high overall sensitivity of 95.7%, specificity of 88.4 %, PPV of 94.4% and NPV of 82% for diagnosing abnormalities of the spectrum of placenta accreta. The features which exhibited the greatest sensitivity and specificity were presence of dark bands, placental-myometrial bulge, vascular lacunes, myometrial thinning, myometrial disruption, serosal vessel sign, bladder vessel sign, bladder tenting. The loss of the dark line between placenta and myometrium and the change of the pear shape of the uterus that are cited to have high sensitivity and specificity in other studies did not show to have high diagnostic value in our study.
**Conclusion:** MR is a helpful adjunct tool to ultrasound in diagnosing placenta accreta spectrum disorders with very high sensitivity and specificity and thus aiding the preoperative planning and patient management.
**Limitations:** The study includes a small number of patients. Intraoperative data might be biased due to adhesions from previous C-section, therefore there might be some intraoperative overdiagnosing of superficial bladder invasion.
**Ethics committee approval:** This study was approved by an ethics committee.
**Funding for this study:** No funding was received for this study.
**Author Disclosures:**
Galina Ivanova Kirova-Nedyalkova: Nothing to disclose
Radina Pavlinova Radeva: Nothing to disclose
Elizabet Rossenova Panova: Nothing to disclose
Emil Georgiev: Nothing to disclose
Emilia Naseva: Nothing to disclose

### RPS 507-6
**Characteristics of unicornuate uterus subtype on MRI and assessment of patient outcomes: an 11-year retrospective study**
*G. Kakar*, S. Murugesu, M. Al-Memar, N. Bharwani; London/UK

**Purpose:** Review imaging features of unicornuate uterus subtypes with associated clinical presentation and patient outcomes in a large London teaching hospital. Assess the possibility of developing a radiological subcategorisation of rudimentary horn attachment to aid surgical planning.
**Methods or Background:** Review of imaging and management of all patients with unicornuate uterus diagnoses between 2010-2021 with a diagnostic MRI via a PACS and electronic records system search (n=33). Data on the following was collated: age at and reason for presentation, unicornuate uterus subtype (AFS classification), anatomical features on MRI (emphasis on attachment of rudimentary horns) and patient outcomes.
**Results or Findings:** 33 female patients, age range at presentation 19-59 years, with mean age at presentation 32.5 years. 18/33 patients had a rudimentary horn, 6 functioning (33%) and 12 nonfunctioning (67%). Those without horns or with nonfunctioning horns presented later with unrelated symptoms, and therefore no intervention was required. Of the 6 functioning horns, 2 communicating horns were detected incidentally, but the 4 noncommunicating horns presented with significant associated symptoms and were offered resection. Myometrial attachments ranged from 7-35 mm, majority being 6-10 mm (n=7). The 2 cases that underwent open resection had myometrial attachments measuring 10 mm and 35 mm, information which is beneficial for planning the surgical approach.
**Conclusion:** This retrospective study demonstrates the anatomical spectrum seen with the unicornuate uterus anomaly. We illustrate the link between presentation and uterine subtype, highlighting the importance of careful imaging review to determine extent of myometrial attachment of rudimentary horns to plan surgical management. With a larger sample size, development of a radiological subcategorisation of myometrial attachment may be possible to aid surgical planning.
**Limitations:** Relatively small case number limits the ability to draw inferences and create myometrial attachment subcategories.
**Ethics committee approval:** Nil. Retrospective review only.
**Funding for this study:** No funding required.
**Author Disclosures:**
Geetanjali Kakar: Nothing to disclose
Maya Al-Memar: Nothing to disclose
Sughashini Murugesu: Nothing to disclose
Nishat Bharwani: Nothing to disclose

**15:00-16:00** **Room X**

### Research Presentation Session: Physics in Medical Imaging

### RPS 513

### Planar radiology (radiography, mammography, fluoroscopy, and interventional)

**Moderator**
O. Rampado; Turin/IT

### RPS 513-2

**Detection and diagnosis of pulmonary emphysema in COPD-patients using x-ray dark-field chest radiography**
K. Willer[1], W. Noichl[1], M. Frank[1], *T. Urban*[1], T. Koehler[2], F. T. Gassert[3], A. Sauter[3], D. Pfeiffer[3], F. Pfeiffer[1]; [1]Garching/DE, [2]Hamburg/DE, [3]Munich/DE
*(theresa.urban@tum.de)*

**Purpose:** X-ray dark-field imaging is a novel imaging technique, which has shown to be very sensitive to emphysema-induced structural lung changes in small-animal disease models and was recently transferred to a first application in humans at our institution. Here, we present a clinical study investigating the diagnostic capabilities of this technique with respect to the detection and staging of emphysema.
**Methods or Background:** A collective of 77 subjects was included. X-ray dark-field and computed tomography (CT) images of the chest were acquired and visually assessed by 5 radiologists with respect to emphysema severity and dark-field signal strength. In addition, pulmonary function and clinical symptoms were recorded. The individual data sets were evaluated in a statistical work-up using the findings from visual CT assessment (Fleischner scheme) as reference standard. Key specifications of our prototype system: simultaneous acquisition of dark-field and attenuation images, field of view: 37x37 cm2, acquisition time: 7 seconds, radiation dose: 0.035 mSv.
**Results or Findings:** The dark-field signal exhibits a good correlation to the lung's diffusion capacity ($\rho$=0.62, p<0.0001). Emphysema assessment based on dark-field radiographs and CT-images yield consistent diagnostic findings. Markers extracted from the dark-field images show improved diagnostic performance in comparison to conventional clinical tests characterising emphysema. Pair-wise comparison of corresponding test parameters between adjacent visual emphysema severity groups yielded higher effect sizes. (Average over group comparisons: COPD assessment test: 0.21; FEV1/FVC: 0.25; CT emphysema-index: 0.35; dark-field based emphysema severity: 0.42).
**Conclusion:** X-ray dark-field chest imaging provides diagnostically relevant information on emphysematous lung impairment in humans.
**Limitations:** The limitation is the limited number of participants.
**Ethics committee approval:** All study participants gave written informed consent. Approval of Institutional Review Board was obtained prior to the study (IRB reference: 166/20S).
**Funding for this study:** Funding was received for this study by the European Research Council, Philips Medical Systems DMC GmbH, Karlsruhe Nano Micro Facility.
**Author Disclosures:**
Andreas Sauter: Nothing to disclose
Florian Tilman Gassert: Nothing to disclose
Thomas Koehler: Employee: Royal Philips
Manuela Frank: Nothing to disclose
Konstantin Willer: Nothing to disclose
Theresa Urban: Nothing to disclose
Franz Pfeiffer: Nothing to disclose
Daniela Pfeiffer: Nothing to disclose
Wolfgang Noichl: Nothing to disclose

### RPS 513-3

**The potential win of including the patient size in large scale outlier analysis for chest radiography**
*A. S. L. Dedulle*, N. Fitousi, H. Bosmans; Leuven/BE
*(andedulle@gmail.com)*

**Purpose:** One of the features of dose management systems is outlier analysis by alerting for doses above threshold levels. Including the patient size in outlier analysis could reduce the number of false positives due to large-sized patients. The purpose of this study was to prove this hypothesis in a large dataset of chest posterior-anterior radiography examinations.
**Methods or Background:** A dataset of 8777 chest posterior-anterior examinations performed on three devices was extracted from a dose management software (DOSE, Qaelum). The patient size in terms of the water equivalent diameter was estimated based on data in the radiographic DICOM header (Dedulle et al. 2021). First, an examination was marked as outlier when the dose area product (DAP) exceeded the target value of 1dGy.cm2. Second, the DAP of the examination was correlated with the patient size and robust regression and outlier removal was used to obtain the outliers of the correlation curve (Graphpad Prism).
**Results or Findings:** The target value was exceeded in 5% of the examinations (397). Of these cases, 60% were also an outlier in the curve analysis. The remaining 40% were larger-sized patients, with a water equivalent diameter above 29cm and for these patients, a higher dose is justified. The curve analysis also gave 114 additional outliers, with main causes distributed as follows: high doses for smaller-sized patients (12%), wrongly indicated view (lateral images, 16%) and a large part of the abdomen included in the examination (35%).
**Conclusion:** The study showed the win of an integrated two-parameter analysis (size and dose area product) in quality assurance of chest radiography; this analysis pointed also to other cases than just size and dose outliers. Some issues would have gone undetected if only doses had been evaluated.
**Limitations:** Not applicable.
**Ethics committee approval:** Not applicable.
**Funding for this study:** Not applicable.
**Author Disclosures:**
An Saskia Luc Dedulle: Employee: Qaelum NV
Hilde Bosmans: Board Member: Qaelum NV
Niki Fitousi: Employee: Qaelum NV

### RPS 513-4

**Dynamic phantom in fluoroscopy for high and low contrast assessment**
N. Paruccini, *R. Villa*, A. Tamiru, E. De Ponti; Monza/IT
*(raffaele.vill@gmail.com)*

**Purpose:** Fluoroscopy imaging represents a real-time guide of moving objects during interventional procedures. The presentation of high and low contrast moving objects is affected by different characteristics of the fluoroscopy device i.e. the typical lag of the detector, the number of frames per second acquired and the recursive filters applied.
**Methods or Background:** A simple phantom has been assembled to simulate the motion of high and low contrast objects and quantify the effect of high and low contrast dynamic objects. Fluoroscopy images from 2 equipment (Siemens Artis Q, Germany and Philips Juno, the Netherlands) have been analysed to quantify lag and blur. A 30 rpm motor allows the rotation of an aluminium plate; high contrast lead disks and a linear steel insert with 0.2 mm of diameter, are assembled on the rotating plate. Lag, defined as the signal persistence of high contrast disks has been evaluated; furthermore, blur or the contrast changes of low contrast detail has been measured.
**Results or Findings:** The lag effect on the closest frame is about 1.3% without recursive filtration while it is about 74% for recursive filters with k=4. The contrast decreases to about 1/10 applying a recursive filter with k=4. Finally, the contrast of the linear steel insert decreases with the k factor as 1/k.
**Conclusion:** This phantom allows a deeper characterisation of the effects of acquisition parameters on dynamic images; it gives both a quantitative and qualitative estimation of blur, lag and distortion, especially regarding their temporal behaviour.

**Limitations:** The limitation is the fixed motor rpm.
**Ethics committee approval:** Not applicable.
**Funding for this study:** Not applicable.
**Author Disclosures:**
Raffaele Villa: Nothing to disclose
Nicoletta Paruccini: Nothing to disclose
Abiy Tamiru: Nothing to disclose
Elena De Ponti: Nothing to disclose

### RPS 513-5

**Helping to know personal occupational doses to improve the interventional practices**

*R. M. Sanchez*, D. Fernandez, E. Vaño, J. M. Fernandez; Madrid/ES
*(robertomariano.sanchez@salud.madrid.org)*

**Purpose:** This study presents a smartphone application prototype for occupational dosimetry in interventional practices where the professionals involved who wear an electronic personal dosimeter (with wireless transmission capacity), can follow their occupational doses received at any time and can compare them with dose limits and with the other working colleagues.
**Methods or Background:** Wearing electronic dosimeters on the chest over the apron, Hp(10) is recorded every second and sent to hubs located at interventional labs. Occupational cumulative dose and dose rate are archived at operator and procedure level, and it is shown to the users, in a comprehensive way, on their smartphones or PCs. A colour code (green, amber, red) indicates if the cumulative dose is well below the dose limits or on the contrary, requires optimisation. A dosimeter located at the C-arm is used as a reference. Results extracted from the first months of use are presented.
**Results or Findings:** Eight interventional laboratories and 29 interventional professionals (interventionalists, radiographers and nurses) are being monitored with the DOSIM prototype. During Jan-Sept 2021 one interventionalist received 25 mSv over the apron, while the average dose for his professional profile is 4.3 mSv. The ratio between the personal cumulative dose and the dose at a reference C-arm dosimeter ranged from 0.2% to 6.6% (a factor of 33) for different interventionalists. These differences suggest different protection habits among operators, as well as a target for optimisation.
**Conclusion:** With this system, professionals have easy access to their occupational dosimetry records using their smartphones, to thereby actively engage in the optimisation process.
**Limitations:** The limitation is the potential lack of use of the personal dosimeters.
**Ethics committee approval:** An approval by an ethics committee is not required.
**Funding for this study:** The authors would like to thank the Spanish National Safety Council under the project EDOCI.
**Author Disclosures:**
Eliseo Vaño: Nothing to disclose
Roberto M Sanchez: Nothing to disclose
Daniel Fernandez: Nothing to disclose
Jose Miguel Fernandez: Nothing to disclose

### RPS 513-6

**Typical diagnostic reference levels assessment for cardiologic, interventional radiology and neuroradiology procedures using a radiation dose tracking system**

*P. E. Colombo*, G. Muti, S. Riga, M. Felisi, A. Macera, E. Piccaluga, F. Barbosa, M. Baroni, A. Torresin; Milan/IT
*(paolae.colombo@ospedaleniguarda.it)*

**Purpose:** This work aims to determine using the radiation dose index monitoring (RDIM) software typical diagnostic reference levels (DLRs) for cardiologic, electrophysiology, interventional radiology and neuroradiology procedures, as recommended in the European Directive 2013/59/EURATOM.
**Methods or Background:** 1-y retrospective study for the thirteen most frequent fluoroscopically-guided procedures is performed. Demographic information and dosimetric data, such as fluoroscopy time (FT), air-kerma at the interventional radiological point (Ka,r) and kerma-area-product (KAP) were collected using the RDIM software NEXO[DOSE]® (Bracco Injeneering SA, Lausanne). The 3800 studies analysed were carried on by four clinical teams and using seven angiographic rooms equipped with two biplane Philips Azurion, two Philips Integris Allura, two GE Innova2100IQ and a Siemens Artis Zeego. Median values of KAP, FT and Ka,r were evaluated and compared to international DRLs. Three neuroradiology procedures (cerebral aneurysm repair, mechanical thrombectomy for acute ischemic stroke MT and cerebral angiography), two electrophysiological procedures (pacemaker and implantable cardioverter-defibrillator implant ICD and transcatheter ablation TCA), three cardiological procedures (coronary angiography CA, percutaneous transluminal coronary angioplasty PTCA, transcatheter aortic valve implantation TAVI) and four interventional radiology procedures (prostatic artery embolisation PAE, transjugular intrahepatic portosystemic shunt TIPS, transarterial chemoembolization TACE, varicocele) were considered.
**Results or Findings:** The typical DRLs established were reported, respectively, for MT (n=213, FT:29min, KAP:137Gy·cm2, Ka,r:1128mGy), cerebral angiography (n=409, FT:13min, KAP:97Gy·cm2, Ka,r:891mGy), cerebral aneurysm repair in election (n=50, FT:44min, KAP:163Gy·cm2, Ka,r:2531mGy) and in emergency (n=38, FT:58min, KAP:221Gy·cm2, Ka,r:2779mGy), ICD implants (n=251, FT:6min, KAP:2Gy•cm2), TCA (n=154, FT:16min, KAP:4Gy·cm2), CA (n=1258, FT:4min, KAP:20Gy·cm2), PTCA (n=760, FT:17min, KAP:62Gy·cm2), TAVI (n=92, FT:22min, KAP:120Gy·cm2), PAE (n=111, FT:47min, KAP:366Gy·cm2, Ka,r:2251mGy), TIPS (n=55,FT:19min, KAP:129Gy·cm2, Ka,r:514mGy), TACE (n=108, FT:21min, KAP:157Gy·cm2, Ka,r:807mGy) and varicocele (n=48, FT:12min, KAP:25Gy·cm2, Ka,r:60mGy).
**Conclusion:** Typical DRL values for diagnostic and therapeutical procedures were evaluated and are lower than European and national DRLs, except for cerebral aneurysm repair in emergency. This work suggests that NEXO[DOSE]® software can be used for effective evaluation of large amount of data.
**Limitations:** Not applicable.
**Ethics committee approval:** Not applicable.
**Funding for this study:** Not applicable.
**Author Disclosures:**
Matteo Baroni: Nothing to disclose
Antonio Macera: Nothing to disclose
Emanuela Piccaluga: Nothing to disclose
Fabiane Barbosa: Nothing to disclose
Gaia Muti: Nothing to disclose
Alberto Torresin: Nothing to disclose
Paola E. Colombo: Nothing to disclose
Marco Felisi: Nothing to disclose
Stefano Riga: Nothing to disclose

### RPS 513-7

**Diagnostic performance and organ dose evaluation in radiological diagnosis and follow-up of Ollier enchondromatosis paediatric patients: a phantom study**

*A. Piai*[1], A. Loria[1], D. Albano[1], A. Mazzilli[2], S. Magnino[1], L. M. Sconfienza[1], A. del Vecchio[1]; [1]Milan/IT, [2]Parma/IT

**Purpose:** The applicability of a low-dose imaging technique based on slot-scanning technology (EOS system; EOS imaging, Paris, France) for diagnosis and follow-up of paediatric Ollier disease is evaluated.
**Methods or Background:** European Directive 2013/59/Euratom included paediatric exposure in special practices that require appropriate optimisation. Ollier disease is characterised by enchondromas, cartilage tumours that begin as benign but may become malignant, and then need long follow-up. Diagnostic and dosimetric performances of EOS are compared to those of digital radiography (DR) and computed tomography (CT). Homemade bone structures are stuck on a 5-year-old anthropomorphic phantom to mimic enchondromas, and imaged with DR, CT, and EOS at three x-ray tube speeds. Experienced radiologists measure the dimensions of the inserts and compare them with those derived from CT and DR. For each technique, Monte Carlo simulations and in-phantom measurements with thermoluminescent dosimeters are performed. Effective dose and Lifetime-Attributable-Risk (LAR) are estimated. Three scanning speeds and two DR acquisition protocols (diagnostic and follow-up) are investigated.
**Results or Findings:** The EOS system performs similarly to DR and CT in detection and measurement of enchondromas-like inserts. The fast protocol provides a reduction of effective and organ doses with respect to DR diagnosis protocol, but not to follow-up protocol. However, the latter is equivalent to the fast protocol in terms of LAR. Doses and LAR of CT are always considerably higher than of the other techniques.
**Conclusion:** EOS has same diagnostic capability as DR and CT. Although no dose reduction is observed with respect to DR follow-up protocol, the fast protocol still provides low doses, and the two techniques are equivalent concerning LAR. Thus, it may be considered a solution for diagnosis and follow-up of Ollier enchondromatosis and other bone pathologies, allowing easier and faster examinations.
**Limitations:** Not applicable.
**Ethics committee approval:** Not applicable.
**Funding for this study:** Not applicable.
**Author Disclosures:**
Aldo Mazzilli: Nothing to disclose
Antonella del Vecchio: Nothing to disclose
Luca Maria Sconfienza: Nothing to disclose
Alessandro Loria: Nothing to disclose
Simone Magnino: Nothing to disclose
Anna Piai: Nothing to disclose
Domenico Albano: Nothing to disclose

### RPS 513-8

**Synthetic mammography detection performance evaluation for different vendors using an anthropomorphic model observer and 3D structured phantom**

*L. Vancoillie*, L. Cockmartin, N. Marshall, H. Bosmans; Leuven/BE
*(liesbeth.vancoillie@uzleuven.be)*

**Purpose:** Task-based evaluation of synthetic mammography (SM) of different vendors using a 3D structured phantom and anthropomorphic model observer (MO).
**Methods or Background:** SM images of a phantom containing 3D models of microcalcifications and masses were acquired at AEC, ½AEC and 2xAEC dose for 5 vendors: S-View (Fujifilm), V-Preview (GEHC), intelligent 2D (Hologic), clarity S2D (Planmed), insight 2D (Siemens). A 4-AFC study was performed for 5 microcalcification and mass diameters. Percentage correct (PC) was calculated using results from 5 readers and used for MO training. A two-layer non-biased Channelized Hotelling Observer (CHO) was tuned separately for each system from a MO developed for DBT: a Laguerre-Gauss localisation CHO followed by a Gabor classification CHO. MO uncertainty was estimated using bootstrapping. Agreement between human and CHO results was evaluated using three criteria: difference between human and CHO scores (mean error (ME)), linear regression slope (a) and Pearson correlation coefficient (r).
**Results or Findings:** Microcalcification results for all systems were in the range -7.8<ME<11.9, a>0.90 and 0.86<r<1.08 suggesting good modelling of human performance. CHO predictions differed for the clarity S2D at ½AEC (ME=-18.2 and a=0.72), probably due to low microcalcification visibility in the human reader results. For the masses, results were -8.4<ME<10.4, a>0.90 and 0.86<r<1.09. The CHO underestimated reader performance for mass detection on V-Preview and Intelligent 2D images, at MEmax=-16.1 and MEmax=-16.5, respectively. However, the trend of increasing PC for increasing diameter was maintained (V-Preview: a>0.80 and 0.89<r<1.07 and intelligent 2D: a>0.91 and 0.86<r<1.08).
**Conclusion:** A two-layer CHO predicted human observer results for microcalcification and mass detection in phantom SM images of 5 vendors. The CHO in combination with the phantom is a promising candidate for periodic, standardised SM performance evaluation.
**Limitations:** Not applicable.
**Ethics committee approval:** Not applicable.
**Funding for this study:** Not applicable.
**Author Disclosures:**
Nicholas Marshall: Nothing to disclose
Liesbeth Vancoillie: Nothing to disclose
Hilde Bosmans: Research/Grant Support: The medical physics team has a research agreement with Siemens-Healthineers and GE HealthCare.
Lesley Cockmartin: Nothing to disclose

**15:00-16:00** **Room Z**

### Research Presentation Session: Interventional Radiology

### RPS 509

### Therapeutic embolotherapy

**Moderator**
G. Maleux; Leuven/BE
**Author Disclosure:**
G. Maleux: Advisory Board: NIPRO; Speaker: Sirtex, W.L. Gore, NIPRO; Other: proctor Sirtex & Terumo

### RPS 509-2

**Splenic artery steal syndrome in patients with orthotopic liver transplant: where to embolise the splenic artery?**

*F. N. N. Fleckenstein*, W. Lüdemann, T. A. Auer, B. Gebauer, B. Hamm, G. Wieners; Berlin/DE
*(florian.fleckenstein@charite.de)*

**Purpose:** This study compared proximal and distal embolisation of the splenic artery (SA) in patients with splenic artery steal syndrome (SAS) after orthotopic liver transplantation (OLT) regarding post-interventional changes in liver function to identify an ideal location of embolisation.
**Methods or Background:** 85 patients with SAS after OLT treated with embolisation were retrospectively reviewed. DSA was used to assess treatment success and to stratify patients according to the site of embolisation. Liver function was assessed using: bilirubin, albumin, gamma-glutamyl transferase, glutamat-pyruvat-transaminase (GPT), glutamic-oxaloacetic transaminase (GOT), alkaline phosphatase (ALP), aPTT, prothrombin-time and thrombocyte count. Descriptive statistics were used to summarise the data. Median laboratory values of pre, 1- and 3-days, as well as 1-week and 1-month post-embolisation were compared using linear mixed model regression analysis.
**Results or Findings:** All procedures were technically successful and showed an improved blood flow in the hepatic artery post-embolisation. Ten patients were excluded due to re-intervention or inconsistent image documentation. Pairwise-comparison using linear-mixed-model-regression analysis showed a significant difference between proximal and distal embolisation for GPT (57.0 (IQR 107.5) vs. 118.0 (IQR 254.0) U/l, p=0.002) and GOT (48.0 (IQR 48.0) vs. 81.0 (IQR 115.0) U/l, p=0.008) 3-days post-embolisation as well as median thrombocyte counts 7-days after embolisation (122 (IQR 108) vs. 83 (IQR 74) in thousands, p=0.014). For all other laboratory values, no statistically significant difference could be shown with respect to the embolisation site.
**Conclusion:** We conclude that a successful embolisation of the SA is independent of the site of embolisation. Choice of technique should therefore be informed by anatomical conditions, safety considerations and preferences of the interventionalist.
**Limitations:** Single-institutional retrospective analysis with a medium-sized cohort with fairly heterogeneous underlying diseases.
**Ethics committee approval:** The study was approved by the local ethics committee.
**Funding for this study:** No funding was received for this study.
**Author Disclosures:**
Bernd Hamm: Nothing to disclose
Gero Wieners: Nothing to disclose
Bernhard Gebauer: Nothing to disclose
Timo Alexander Auer: Nothing to disclose
Willie Lüdemann: Nothing to disclose
Florian Nima Nima Fleckenstein: Nothing to disclose

### RPS 509-3

**Aneurysmal bone cysts treatment: curettage versus transarterial embolisation in the treatment of 320 patients**

*C. Martella*, M. Miceli, G. Facchini, E. Costantino, G. Peta; Bologna/IT
*(c.martella92@gmail.com)*

**Purpose:** To evaluate the outcome of the curettage treatment and trans-arterial embolisation of aneurysmal bone cysts in a cohort of 320 patients, comparing their effectiveness and safety.
**Methods or Background:** We retrospectively studied the data obtained from patients diagnosed with aneurysmal cysts, treated at a single referral centre from 1994 to 2020: 266 received curettage, 54 embolisation. The site, lesion activity status (latent, active, and aggressive) and clinical response were evaluated with a follow-up of approximately 40 months in the curettage group and 121 months in the embolisation one.
**Results or Findings:** The efficacy in the curettage group was 77% (n=205). Cases of recurrence underwent a second treatment with embolisation (n=5), curettage (n=46), infiltration (n=6), with efficacy of 100%, 74% and 83% respectively. 4 patients who experienced a recurrence were no longer treated. From the group of embolised patients, 59% (n=32) were considered healed at first procedure. The remaining patients underwent embolisation (n=12), curettage (n=8) or infiltration (n=2), with a clinical efficacy of the second treatment of 38%, 71,4% and 50%, respectively. The recorded data demonstrated a lower rate of complications (5% vs 9.6%) and days of hospitalisation (1 vs 3) in embolised patients compared to those receiving the other treatments.
**Conclusion:** Selective arterial embolisation represents an attractive option for the management of these lesions. This minimal invasive treatment is correlated with good results and a lower complication rate than the curettage. Furthermore, it is associated with a shorter hospitalisation period.
**Limitations:** The limitation is the retrospective design.
**Ethics committee approval:** The study protocol was approved by the Medical Ethics Committee of our hospital.
**Funding for this study:** No funding was received for this study.
**Author Disclosures:**
Claudia Martella: Author: nothing to disclose
Marco Miceli: Author: nothing to disclose
Errani Costantino: Author: nothing to disclose
Giancarlo Facchini: Author: Nothing to disclose
Giuliano Peta: Author: nothing to disclose

### RPS 509-4

### Management of pseudo-aneurysms in splenic trauma

*S. Batting*[1], C. Barnetson[1], E. Platt[1], J. Ekpe[1], N. Patel[2], E. Kashef[1], D. Frith[1]; [1]London/UK, [2]Toronto, AB/CA

**Purpose:** Splenectomy was the mainstay of management of splenic injury, however, there has been a shift to non-operative management (NOM). Formation of pseudoaneurysms (PSA), delayed bleeding and rupture can lead to failure of NOM. Interventional Radiology (IR) embolisation is now an integrated part of NOM. The aim is to look at incidence and management of PSAs at our institution and the association with failure of NOM.
**Methods or Background:** A single centre retrospective study from a Major Trauma Centre. All adult trauma patients who sustained a splenic injury between January 2014 and September 2019. Protocol in our centre is for imaging at 48 hours and 6 weeks post-injury.
**Results or Findings:** 226 patients with splenic injury were identified. 69 were managed non-operatively, with 41 receiving IR embolisation. NOM was successful in 98.8% of cases. There were 19 cases of PSA (8.4%) in our cohort, with a median time of diagnosis of 2.4 +/- 0.87 days from injury. Higher average grade of injury was seen in PSA patients (3.16 +/- 0.481) compared to the whole cohort (2.5 +/- 0.211). 42.1% of PSA were detected via the imaging protocol. 3 patients with PSA underwent splenectomy. 11 patients with PSA underwent IR embolisation. Persistent PSA, defined as PSA seen on imaging post IR, was seen in 6 patients. One of these patients required further IR intervention, 3 had resolution of PSA on follow-up and 2 did not have further imaging but remained clinically well. 5 patients with PSA were managed conservatively, with half having PSA resolution confirmed on imaging.
**Conclusion:** NOM was successful in 98.8% of cases (compared to 80% in the literature). Aggressive pursuit of PSA in our institution is associated with high success of NOM.
**Limitations:** The limitation is the single-centre design.
**Ethics committee approval:** Not applicable.
**Funding for this study:** Not applicable.
**Author Disclosures:**
Calum Barnetson: Nothing to disclose
Sarah Batting: Nothing to disclose
Jadesola Ekpe: Nothing to disclose
Neeral Patel: Nothing to disclose
Dan Frith: Nothing to disclose
Elika Kashef: Nothing to disclose
Esther Platt: Nothing to disclose

### RPS 509-5

### Arterioportal fistulas in pediatric patients: single-centre experience with interventional radiological versus conservative management and clinical outcomes

*F. S. Carbone*[1], P. Marra[1], L. Dulcetta[1], R. Agazzi[1], R. Muglia[1], P. A. Bonaffini[1], M. Venturini[2], S. Sironi[1]; [1]Bergamo/IT, [2]Varese/IT
*(f.carbone15@campus.unimib.it)*

**Purpose:** To report the 10-year experience of a tertiary referral center for pediatric orthotopic liver transplantation (OLT) in the interventional radiological (IR) and conservative management of acquired arterioportal fistulas (APFs).
**Methods or Background:** A retrospective search was performed to retrieve pediatric patients (<18 years old) with APF diagnosis at color-Doppler ultrasound (CDUS) or computed tomography angiography (CTA) from 2010 to 2020. Criteria for IR treatment were hemodynamic alterations at CDUS (arterial resistive index <0.5; portal flow reversal) or clinical manifestations (bleeding; portal hypertension). Conservatively managed patients served as a control population. Clinical and imaging follow-ups were analysed.
**Results or Findings:** Twenty-three pediatric patients (median age 4 years; interquartile range=11 years; 15 males) with 24 APFs were retrieved. Twenty patients were OLT recipients with acquired APFs (16 iatrogenic). Twelve out of twenty-three patients were managed conservatively. The remaining 11 underwent angiography with confirmation of a shunt in 10, who underwent a total of 16 embolisation procedures (14 endovascular; 2 transhepatic). Technical success was reached in 12/16 (75%) procedures. Clinical success was achieved in 8/11 (73%) patients; three clinical failures resulted in one death and two OLTs. After a median follow-up time of 42 months (range 1-107), successfully treated patients showed an improvement in hemodynamic parameters at CDUS. Conservatively managed patients showed a stable persistence of the shunts in 6 cases, spontaneous resolution in 4, reduction in 1 and mild shunt increase in 1.
**Conclusion:** In pediatric patients undergoing liver interventions, APFs should be investigated. Although asymptomatic in most cases, IR treatment of APFs should be considered whenever hemodynamic changes are found at CDUS.
**Limitations:** The limitation is the retrospective design, the wide time range, and small number of very selected patients.
**Ethics committee approval:** The Ethical Committee of Bergamo authorized this study.
**Funding for this study:** No funding was received for this study.
**Author Disclosures:**
Sandro Sironi: Nothing to disclose
Paolo Marra: Nothing to disclose
Pietro Andrea Bonaffini: Nothing to disclose
Francesco Saverio Carbone: Nothing to disclose
Massimo Venturini: Nothing to disclose
Riccardo Muglia: Nothing to disclose
Roberto Agazzi: Nothing to disclose
Ludovico Dulcetta: Nothing to disclose

### RPS 509-6

### Pulmonary artery embolisation in the management of hemoptysis related to lung tumours

*A. Claudinot*[1], A. David[2], A. Bouvier[3], A. LE GUEN[4], J-F. Heautot[5], F. Thouveny-Paisant[3], F. Douane[2], A. Fohlen[1], J-P. Pelage[1]; [1]Caen/FR, [2]Nantes/FR, [3]Angers/FR, [4]Vannes/FR, [5]Rennes/FR
*(a_claudinot@orange.fr)*

**Purpose:** To evaluate safety and efficacy of pulmonary artery embolisation in patients presenting with hemoptysis related to lung tumours.
**Methods or Background:** All consecutive patients with cancer and at least one episode of hemoptysis that required pulmonary artery embolisation from December 2008 to December 2020 at participating centres were included in this observational retrospective review. The endpoints were technical success, clinical success, recurrence of hemoptysis and complications. Univariate and multivariate analyses were performed to identify variables associated with recurrence or death.
**Results or Findings:** A total of 92 patients were treated with pulmonary artery embolisation with 12 patients presenting recurrence after bronchial artery embolisation and 38 treated with simultaneous pulmonary and bronchial embolisation. Pulmonary artery embolisation was technically successful in 82 (89%) patients and clinically successful in 77 (84%) patients. Recurrence occurred in 49% of patients. Infectious complications occurred in 15 patients (16%). The 30-days mortality rate was 31%. At 3 years, survival rate was 3.6%. Tumour size (p=0.004), tumour cavitation (p=0.004) and necrosis (p=0.04) were associated with recurrence in univariate analysis. Tumour cavitation (p=0.01) and pulmonary artery pseudo-aneurysm (p=0.009) were associated with recurrence using a multivariate analysis. Tumour size (p=0.023), tumour cavitation (p=0.003) and necrosis (p=0.02) were associated with higher mortality. Using a multivariate analysis, tumour cavitation (p=0.001), pulmonary artery pseudo-aneurysm (p=0.007) and treatment with covered stents (p<0.001) were associated with shorter survival.
**Conclusion:** Pulmonary artery embolisation is an effective treatment to initially control hemoptysis in patients with lung carcinoma. Recurrence rate remains high and overall survival remains poor with less than 5% of patients still alive at 3 years.
**Limitations:** This is a retrospective, one-sample cohort with no control group.
**Ethics committee approval:** This study was approved by an ethics committee (registration number 2317).
**Funding for this study:** The study was promoted by the Interbreizh Research Foundation.
**Author Disclosures:**
Frederic Douane: Nothing to disclose
Audrey Fohlen: Nothing to disclose
Jean-François Heautot: Nothing to disclose
Jean-Pierre Pelage: Nothing to disclose
Francine Thouveny-Paisant: Nothing to disclose
Amandine Claudinot: Nothing to disclose
Antoine Bouvier: Nothing to disclose
Arnaud LE GUEN: Nothing to disclose
Arthur David: Nothing to disclose

### RPS 509-7

### Retrospective analysis of the outcome of angiographic embolisation of peptic ulcers

*C. Vanhoenacker*, I. Demedts, E. Hufkens, G. Maleux; Leuven/BE
*(charlottevanhoenacker@live.be)*

**Purpose:** To evaluate the outcome of patients who underwent a transcatheter arterial embolisation (TAE) for severe, gastroduodenal hemorrhage associated with peptic ulcer and refractory to medical and endoscopic therapy; to compare the outcome of different embolic agents and to evaluate factors associated with early recurrent bleeding and 30-day mortality.
**Methods or Background:** A monocentre, retrospective study of 76 consecutive patients who underwent TAE for bleeding gastroduodenal peptic ulcers from 2005-2020. Patient demographics, endoscopy findings, co-morbidities and interventional procedure findings were recorded. The outcome measures were technical and clinical success, procedure-related complications, recurrent bleeding, length of hospital stay, 30-day mortality and overall survival.

**Results or Findings:** The technical success rate was 90.8% and the clinical success was 65,8%. The rebleeding and 30-day mortality were 30,7% and 22,4% respectively. A higher international normalised ratio (INR) was a statistically significant risk factor for 30-day mortality (OR, 7.15; 95% CI, 1.67-30.70; p=0.008). A lower Charlson Comorbidity Index (CCI) and a lower Rockall score were significantly associated with a longer overall survival (HR, 1.24; 95% CI, 1.14-1.35; p =0.0001) and (HR, 1.32; 95% CI, 1.10-1.59; p=0.003) respectively. The occurrence of early rebleeding is significantly associated with a lower overall survival (HR, 2.72; 95% CI, 1.57-4.71; p=0.0004). There were no statistically significant differences in outcome or overall survival using different embolic agents.
**Conclusion:** Type of embolic agent did not influence overall survival. A higher INR was a significant risk factor with a higher 30-day mortality. A lower CCI, a lower Rockall score and the absence of early rebleeding were significantly associated with a longer overall survival.
**Limitations:** The limitation is the retrospective study analysis over a long time period.
**Ethics committee approval:** This retrospective study is approved by the UZ Leuven Ethics Committee.
**Funding for this study:** No funding was received for this study.
**Author Disclosures:**
Ingrid Demedts: Nothing to disclose
Charlotte Vanhoenacker: Nothing to disclose
Eva Hufkens: Nothing to disclose
Geert Maleux: Nothing to disclose

### RPS 509-8

**Pseudoaneurysm development: incidence and predisposing factors in a high-volume cardiovascular centre**
*H. S. Sarkadi*, J. Csőre, D. Veres Sandor, N. Szegedi, L. Molnár, V. Bérczi, E. Dosa; Budapest/HU

**Purpose:** Evaluate factors associated with pseudoaneurysm formation in a relatively large patient population.
**Methods or Background:** Between January 2016 and May 2020, 30196 patients had interventional radiological or cardiac endovascular procedures that required arterial (appr 26362) or venous (appr 15308) puncture; all patients developing PSA were identified. A matched (age, gender, and type of procedure) control group of 134 patients was created to assess factors associated with PSA formation. For statistical analysis, ANOVA and likelihood-ratio tests were calculated; effect sizes were expressed as odds ratios (ORs; with PSA versus without PSA).
**Results or Findings:** 134 PSAs (symptomatic: N = 112; 83.6%) were identified in 134 patients (72 women; mean age: 69.5 ± 15.2 years); IR: 53/6555; 0.8%, coronary: 31/18038; 0.2%, non-coronary cardiac: 25/5603; 0.4%, 25 for unintended arterial puncture. 25.4% of PSAs were localised to the upper limb and 74.6% to the lower limb (vascular closure device used in 37 of these patients). Treatments for upper and lower limb PSAs, respectively: bandage replacement in 25 cases, transducer compression in 5 cases, thrombin injection in 86 cases, and surgical reconstruction in 18 cases. Compared to the matched control group (N = 134), anemia (OR: 0.33; 95% CI: 0.21–0.51; P <0.001), high INR (OR: 12.97; 95% CI: 2.80–72.06; P <0.001) and the use of vascular closure device (OR: 3.5; 95% CI: 2.3–5.2; P = 0.016) was a predictor of PSA formation.
**Conclusion:** The risk of PSA formation is higher in the case of preoperative anemia, high INR, and if vascular closure devices were used.
**Limitations:** Limitations of this study include those inherent to a single centre retrospective analysis.
**Ethics committee approval:** Institutional review board approval was granted (Approval No: 176/2020).
**Funding for this study:** There was no financial support during the study.
**Author Disclosures:**
Nándor Szegedi: Nothing to disclose
Hunor Sandor Sarkadi: Nothing to disclose
Viktor Bérczi: Nothing to disclose
Levente Molnár: Nothing to disclose
Judit Csőre: Nothing to disclose
Edit Dosa: Nothing to disclose
Daniel Veres Sandor: Nothing to disclose

**16:30-17:30 Open Forum #1 (Radiographers)**

### Research Presentation Session: Radiographers

### RPS 614

### Radiography education updates and training considerations

**Moderators**
C. Buissink; Groningen/NL
L. Oleaga Zufiría; Barcelona/ES

### RPS 614-3

**The impact of 3D virtual reality simulation upon radiation protection training for radiography and medical students**
*A. Tcacenco*, J. Potočnik, C. Brophy, A. Lunney, D. Duggan, J. M. Grehan, S. J. Foley, J. McNulty, L. A. Rainford; Dublin/IE
*(Antoninatcacenco@yahoo.com)*

**Purpose:** Virtual Reality (VR) in the context of healthcare education is an immersive and interactive simulated environment. Educators can recreate a clinical environment or experience without exposing the patient to any associated risks and is ideal for students to gain radiation safety knowledge.
**Methods or Background:** A cohort of final year radiography students (n=100) and medical students (n=35) were introduced to 3 Dimensional Virtual Reality (3DVR) software designed to improve the learners' understanding of radiation safety in a radiology interventional suite. The radiography students underwent formal training as part of an academic module and combined this experience and that gained on clinical placement when being assessed in the 3D virtual space. Volunteer medical students attended similar 3DVR activities without assessment. A feedback survey gained insight into how the 3DVR radiation experience had supported their knowledge of radiation safety.
**Results or Findings:** In total 76 survey responses were received from 49 radiography and 27 medical students. Participants reported an increase in confidence in their knowledge of radiation safety and valued the simulation experience. 87% of respondents stated they would recommend the 3DVR as a learning tool to other students. The majority of participants (n = 84%) also agreed that the 3DVR sessions had helped them understand radiation safety matters better and enhanced their critical thinking. 80% of participants enjoyed using 3D VR as part of their learning and 74% stated they would prefer more time using the 3D VR.
**Conclusion:** The students identified the 3DVR radiation safety software as very beneficial and were encouraging of its integration into the curriculum in conjunction to traditional lecture and lab-based teaching in line with universal design learning principles.
**Limitations:** COVID-19 created challenges, medical student participation.
**Ethics committee approval:** This study was approved by an ethics committee: LS-E-21-126-Tcacenco-Rainford.
**Funding for this study:** Funding was received for this study by the Irish National Forum TL19UCD1460/02.
**Author Disclosures:**
Aine Lunney: Nothing to disclose
Louise A. Rainford: Nothing to disclose
Jennifer Mary Grehan: Nothing to disclose
Carmel Brophy: Nothing to disclose
Jaka Potočnik: Nothing to disclose
Jonathan McNulty: Nothing to disclose
Shane J Foley: Nothing to disclose
Diana Duggan: Nothing to disclose
Antonina Tcacenco: Nothing to disclose

### RPS 614-4

**An evaluation of the skills and competencies required by therapeutic radiographers from a patient's perspective**
*S. L. McFadden*, C. Hughes, T. Flood, A. O Neill, P. McClure; Belfast/UK
*(s.mcfadden@ulster.ac.uk)*

**Purpose:** Variation in training and education of therapy radiographers (TRs) across Europe leads to variations in clinical practice. The aim of this study was to gain an insight into patients' perceptions of the required skills and competencies of TRs to inform the development of undergraduate curricula across Europe.
**Methods or Background:** Ethical permission was obtained from Ulster University, Belfast, UK. Online and face to face interviews were conducted with radiotherapy patients across UK, Portugal, and Malta. Patients >18years currently receiving, or had received radiotherapy within the last 24 months, were included in the study. Thematic analysis was performed with the aid of NVivo.
**Results or Findings:** In total, 38 interviews (18 in the UK; 11 in Malta; 19 in Portugal) have been conducted to date. Patients felt that TRs had the required competencies to listen, understand and communicate compassionately with them during their treatment. The importance of psychosocial support and building a relationship with the TRs varied considerably. Patients who developed stronger relationships with their TRs felt safer compared to those patients with less established personal connections to their TRs. Patients expressed a desire for further information from their TRs at the end of their radiotherapy treatment and through the post-treatment phase of their care.
**Conclusion:** Patients need more holistic support at the end of treatment and in post-treatment care. Role extension/specialist roles are needed to support patients.
**Limitations:** As not all European countries were involved in this study, the findings may not represent the breadth of patient experiences throughout Europe.
**Ethics committee approval:** This study was approved by an ethics committee: Integrated Research Applications System reference number: 277006. REC reference: 20/YH/038, Institute of Nursing and Health Research Ethics Committee UU Ref No: FCNUR-20-035.
**Funding for this study:** This study forms part of the ERASMUS+ funded SAFEEUROPE project. There were no conflicts of interest.
**Author Disclosures:**
Ciara Hughes: Nothing to disclose
Patricia McClure: Nothing to disclose
Angela O Neill: Nothing to disclose
Sonyia Lorraine McFadden: Nothing to disclose
Terri Flood: Nothing to disclose

### RPS 614-5

### Development of a framework for radiographer online clinical education (FORCE)

*K. Matthews*[1], J. Atutornu[2], P. Bezzina[3], P. Costa[4], N. Dalen[5], J. T. Huhtanen[6], M. Jaronen[7], C. Kamp[8], L. Rainford[1]; [1]Dublin/IE, [2]Ipswich/UK, [3]Msida/MT, [4]Porto/PT, [5]Bergen/NO, [6]Turku/FI, [7]Tampere/FI, [8]Vienna/AT
*(kate.matthews@ucd.ie)*

**Purpose:** The overall aim of the FORCE project is to develop virtual web-based learning resources where radiography undergraduates can engage in interactive, problem-based development of radiographic knowledge, ability and professional awareness.
**Methods or Background:** It is a pre-requisite of radiographer education that students learn during clinical placements. This is essential for the development of competence and confidence in the emerging graduate. The number of undergraduate radiographers in training poses challenges to clinical placement capacity in many countries, which in turn impacts the quality of the clinical education experience. Solutions are needed to augment the scale and quality of clinical learning. The FORCE project is leveraging a variety of web-based tools to develop a simulation system to augment clinical education, and within this will promote the recognition and validation of the knowledge, skills, and competences thus gained.
**Results or Findings:** Within a web-based virtual learning environment (VLE), simulated patient cases (20 diagnostic, 10 RNI and 10 therapy) that encourage learner interaction are being developed. Each patient case includes learning resources pertaining to patient care; performance of diagnostic radiography or radionuclide imaging or therapy planning; patient safety; image interpretation and radiographer roles and responsibilities.
**Conclusion:** The presentation will summarise some achievements and challenges from the development process.
**Limitations:** The FORCE project is a work in progress and will not complete until May 2023.
**Ethics committee approval:** All image data have been and will be harvested and used in compliance with GDPR and under ethical approval.
**Funding for this study:** €300,000 grant over two years awarded by the European Union for an Erasmus + strategic collaboration between: FH Campus Wien, Instituto Politecnico do Porto, Tampere University of Applied Sciences, Turku University of Applied Sciences, Universita ta Malta, University College Dublin, University of Suffolk, University of Western Norway.
**Author Disclosures:**
Pedro Costa: Grant Recipient: Erasmus+ Strategic Collaboration Grant
Jerome Atutornu: Grant Recipient: Erasmus+ Strategic Collaboration Grant
Kate Matthews: Grant Recipient: Erasmus+ Strategic Collaboration Grant
Christoph Kamp: Grant Recipient: Erasmus+ Strategic Collaboration Grant
Louise Rainford: Grant Recipient: Erasmus+ Strategic Collaboration Grant
Jarno Tapani Huhtanen: Grant Recipient: Erasmus+ Strategic Collaboration Grant
Marja Jaronen: Grant Recipient: Erasmus+ Strategic Collaboration Grant
Nina Dalen: Grant Recipient: Erasmus+ Strategic Collaboration Grant
Paul Bezzina: Grant Recipient: Erasmus+ Strategic Collaboration Grant

### RPS 614-6

### EURAMED rocc-n-roll (EuRnR) project findings: radiation protection education and training across Europe

*L. A. Rainford*[1], J. Santos[2], C. Hoeschen[3], J. Damilakis[4], G. Frija[5], J. S. Andersson[6], J. McNulty[1], S. J. Foley[1], K. Bacher[7], F. Alves[2], J. P. Figueiredo[2], M. Hierath[8], G. Paulo[2]; [1]Dublin/IE, [2]Coimbra/PT, [3]Magdeburg/DE, [4]Iraklion/GR, [5]Paris/FR, [6]Umeå/SE, [7]Gent/BE, [8]Vienna/AT
*(louise.rainford@ucd.ie)*

**Purpose:** EURAMED rocc-n-roll (EuRnR) involves broad stakeholder input and consultation to develop a coordinated European approach for research and innovation in medical applications of ionising radiation and related radiation protection (RP). SWOT/TOWS analysis was performed to understand the status quo of education and training (E&T) in radiation protection in Europe.
**Methods or Background:** The project work package team (n=14) included representatives from six European professional societies, namely: CIRSE; EANM; EFOMP; EFRS; ESR; ESTRO; and from HERCA, the WHO and IAEA; and five clinical experts representing: medical physics; nuclear medicine physicians; radiologists; radiation oncologists and radiographers. Four subgroups performed SWOT/TOWS analysis related to E&T in RP developed under previous EU programmes and on the guidelines on radiation protection education and training of medical professionals in the European Union.
**Results or Findings:** Consensus agreement identified four main themes for strengths and opportunities, namely: (1) existing structures and training recommendations; (2) RP training needs assessment and E&T model(s) development; (3) E&T dissemination, harmonisation, and accreditation; (4) financial supports. Weaknesses and threats analysis resulted in two themes: (1) awareness and prioritisation at a national/global level and (2) awareness and prioritisation by healthcare professional groups and researchers.
**Conclusion:** EuRnR strategic planning needs to consider processes at European, national and local levels and incorporate the multiple factors identified. Clear, efficient governance structures and expert leadership are required, as are finances to facilitate a pan-European radiation protection training network which is sustainable and accredited across multiple national domains.
**Limitations:** Future consideration does need to incorporate the expertise of E&T scientists as the EuRnR framework documentation is progressed.
**Ethics committee approval:** Institutional confirmation of exemption from full ethical approval across all participating partners.
**Funding for this study:** Funding was received for this study: EURAMED rocc-n-roll project is funded by the European Commission, grant nº 899995, under the call NFRP-2019-2020.
**Author Disclosures:**
Graciano Paulo: Nothing to disclose
Klaus Bacher: Nothing to disclose
Guy Frija: Nothing to disclose
Christoph Hoeschen: Nothing to disclose
Joana Santos: Nothing to disclose
Louise A. Rainford: Nothing to disclose
Monika Hierath: Nothing to disclose
Jonathan McNulty: Nothing to disclose
João Paulo Figueiredo: Nothing to disclose
Shane J Foley: Nothing to disclose
Jonas Seth Andersson: Nothing to disclose
John Damilakis: Nothing to disclose
Francisco Alves: Nothing to disclose

### RPS 614-7

### Advanced practice roles amongst therapeutic radiographers: a European survey

*C. M. Oliveira*[1], B. Barbosa[2], J. G. Couto[3], I. Bravo[2], C. Hughes[4], S. L. McFadden[4], R. Khine[5], H. McNair[5]; [1]Vigo/ES, [2]Porto/PT, [3]Msida/MT, [4]Belfast/UK, [5]Utrecht/NL
*(celeste.oliveira1983@gmail.com)*

**Purpose:** This study aimed to assess advanced practice (AP) roles amongst therapeutic radiographers (TRs) and to identify educational gaps for this level across Europe.
**Methods or Background:** A self-designed mix-method cross-sectional online survey (English) was validated by 5 external experts and by 5 TRs from different countries. It was distributed to TRs practicing AP roles across Europe irrespective of recognition as "advanced practitioners" (between December 2021 and March 2022). Practitioners were asked about current AP roles and opinions regarding current and future education needs to underpin these roles. Convenience sampling was used to recruit the qualified TRs working in AP using multiple strategies to disseminate the survey including snowballing. Quantitative data (using SPSS) and qualitative data (thematic analysis using NVivo12) were analysed separately, triangulated and interpreted.
**Results or Findings:** 271 participants responded with 189 valid participants from 21 European countries. Inconsistency was found in role titles, scope of practice, educational backgrounds, and AP roles implementation across countries. These practitioners have a trend to work more in clinical practice domain with a low percentage of time allocated to research. Education needs regarding knowledge about image-guided and adaptive Radiotherapy (RT), multimodal imaging and technologies, and advanced treatment planning were found. Training needs on leadership and management skills, and clinical site-specific expertise were identified.
**Conclusion:** There is an urgent need to standardise AP in RT to uniformise educational and training at the national and European levels.
**Limitations:** The language bias of the survey may have excluded some participants from non-English speaking countries.
**Ethics committee approval:** This research was approved by the Institute of Nursing and Health Research Ethics Committee at Ulster University, Belfast (Project Number: FCNUR-21-080).
**Funding for this study:** This work was co-funded by the SAFE EUROPE project under the Erasmus Sector Skill Alliances programme [grant agreement 2018-2993/001-001].
**Author Disclosures:**
Ricardo Khine: Nothing to disclose
Isabel Bravo: Nothing to disclose
Ciara Hughes: Nothing to disclose
Sonyia Lorraine McFadden: Grant Recipient: This work was co-funded by the SAFE EUROPE project under the Erasmus Sector Skill Alliances programme [grant agreement 2018-2993/001-001].
Helen McNair: Research/Grant Support: Helen McNair is funded by a National Institute for Health Research and Health Education England (HEE/NIHR), Senior Clinical Lectureship and supported by the NIHR Biomedical Research Centre at The Royal Marsden NHS Foundation Trust and the Institute of Cancer Research, London.
Bárbara Barbosa: Nothing to disclose
Celeste Marques Oliveira: Research/Grant Support: The European Commission support for the production of this publication does not constitute an endorsement of the contents which reflects the views only of the authors, and the Commission cannot be held responsible for any use which may be made of the information contained therein. This work was co-funded by the SAFE EUROPE project under the Erasmus Sector Skill Alliances programme [grant agreement 2018-2993/001-001].
Jose Guilherme Couto: Nothing to disclose

### RPS 614-8

**Current benefit-risk communication in Irish medical imaging. Is practice adhering to legislation?**

*C. A. Murphy*, S. J. Foley; Dublin/IE
*(cliodhnamurphy06@gmail.com)*

**Purpose:** To identify whether practitioners and referrers in Irish hospitals have sufficient knowledge about ionising radiation to adequately inform patients on the risks and benefits associated with imaging examinations.
**Methods or Background:** The EU Directive 2013/59/Euratom requires referrers and practitioners explain the benefits and risks associated with medical imaging examinations to their patient, prior to radiation exposure. This has been transposed into national legislation amongst member states. To comply with this legislation, these professionals must have sufficient knowledge about radiation, although published research suggests this is not the case. An online survey was sent to three Irish radiology departments of varying sizes for distribution to radiographers, referring nurses and doctors in each hospital. This asked about participant demographics, ionising radiation knowledge and benefit-risk conversations. Quantitative responses were analysed manually, and SPSS software was used for statistical analysis. Thematic analysis was conducted for qualitative results.
**Results or Findings:** 34 responses were received. Results demonstrated that all three professional groups have a low level of radiation knowledge. Radiographers scored highest on the knowledge-based questions (radiographers mean score: 0.47, medical doctors: 0.32, nurses: 0.28). Legislation compliance was low among participants, with only 5 (17%) indicating they regularly engage in benefit-risk conversations, even though 17 (57%) participants stated they had confidence in doing so. Opinions differed on who should deliver this information to patients and when.
**Conclusion:** Further education and training is required for referrers and radiographers to ensure consistent and accurate benefit risk communication to patients and ensure compliance with key EU radiation legislation.
**Limitations:** Not applicable.
**Ethics committee approval:** Exemption from ethical approval was obtained from University College Dublin.
**Funding for this study:** Not applicable.
**Author Disclosures:**
Cliodhna Alice Murphy: Nothing to disclose
Shane J Foley: Nothing to disclose

### RPS 614-9

**A method to categorise an image bank before use in image interpretation research or education**

K. Matthews, *C. Heckmann*; Dublin/IE
*(caoimhe.heckmann@ucdconnect.ie)*

**Purpose:** When using a bank of images to teach or measure image interpretation, it is important to include images with varying levels of difficulty. Previous authors propose rigorous but resource-intensive methods for establishing these degrees of difficulty. The current study proposes a simple approach in an image bank suitable for an observer study.
**Methods or Background:** Extremity examinations with radiological reports were harvested. Of 94 images, 51 were reported as abnormal, 43 as normal. The report was taken as the true opinion. Insight of these reports, the researchers assigned the level of normality or abnormality as obvious or subtle. The 94 images were then reviewed independently by four radiographers, blind of the reports. Each radiographer assigned an opinion from obvious abnormality, subtle abnormality, not sure, probably normal and definitely normal. Each radiographer's opinions were scored relative to the report opinion on a scale from 0 to 3. Observer scores for each image were summed, and ranked by median values in two groups: radiology normal and radiology abnormal. The number of radiographers agreeing with radiology opinion of normal/abnormal was calculated as a percentage.
**Results or Findings:** Combined consideration of median values and % agreement with radiology opinion allowed categorisation of ease of image interpretation as very easy, easy, moderately difficult, very difficult, too difficult. A random selection of the categorised images was trialed in an image observer study investigating student radiographer image interpretation ability. Student performance correlated moderately with the degree of difficulty assigned, thus validating the method.
**Conclusion:** The method permits reasonable categorisation of image interpretation difficulty for observer studies.
**Limitations:** A larger radiographer observer study would further validate the approach.
**Ethics committee approval:** Ethical approval was granted by UCD School of Medicine Undergraduate Research Ethics Committee.
**Funding for this study:** No funding was received for this study.
**Author Disclosures:**
Caoimhe Heckmann: Nothing to disclose
Kate Matthews: Nothing to disclose

**16:30-17:30** **Open Forum #3 (ESR)**

### Research Presentation Session: Imaging Informatics / Artificial Intelligence and Machine Learning

### RPS 605a

### Artificial intelligence (AI) in oncology

**Moderator**
M. D'Anastasi; Msida/MT
**Author Disclosure:**
M. D'Anastasi: Advisory Board: Keosys Medical Imaging; Consultant: Keosys Medical Imaging; Speaker: Siemens Healthineers

### RPS 605a-2

**CT radiomics for malignancy prediction of renal cysts**

*Z. Salahuddin*[1], H. C. Woodruff[1], R. L. Miclea[1], S. Behr[2], O. Morin[2], P. Lambin[1]; [1]Maastricht/NL, [2]San Francisco, CA/US

**Purpose:** A fully automated radiomics-based classification system has been proposed to predict malignancy of renal cysts using CT scans.
**Methods or Background:** Cystic renal cell carcinoma consists of fluid-filled malignant masses that are diagnosed based on the Bosniak classification system. This CT-based classification system often results in over-diagnosis and consequently the unwanted resection of benign masses and is affected by interreader variability. To overcome these shortcomings, machine learning models were trained using 5 quantitative imaging features selected for their robustness and predictive power. An ensemble of linear SVMs was used to perform classification. The imaging data were collected at two medical centres (Maastricht University Medical Center (MUMC+) and the University of California - San Francisco (UCSF) medical center). Leave One-Out-Cross-Validation was performed on the UCSF dataset. MUMC dataset was used as an external validation dataset. To make them interpretable and facilitate adoption, the models were made explainable via Shapley analysis.

**Results or Findings:** For the UCSF dataset, a precision of 0.97± 0.07 and recall of 0.85 ± 0.15 was obtained for benign cysts (precision 0.60 ± 0.34 and recall 0.82 ± 0.38 for malignant cysts). External validation yielded a precision of 0.84 and recall of 0.88 for benign cysts (precision of 0.79 and recall of 0.73 for malignant cysts). These results are competitive with clinical performance.
**Conclusion:** An interpretable machine learning pipeline based on radiomics features can aid clinicians in reducing the number of unnecessary surgeries.
**Limitations:** The reproducibility of radiomics features should be investigated using phantoms and test-retest patient data.
**Ethics committee approval:** The study was approved by the ethics committees.
**Funding for this study:** Funding was received through ERC-ADG-2015 n° 694812 - Hypoximmuno, EU Horizon 2020 grant agreement: MSCA-ITN-PREDICT n° 766276, CHAIMELEON n° 952172, EuCanImage n° 952103, IMI-OPTIMA n° 10103434, and KWF Kankerbestrijding n ° 12085/2018–2.
**Author Disclosures:**
Olivier Morin: Nothing to disclose
Razvan Lucian Miclea: Nothing to disclose
Zohaib Salahuddin: Author: Student at Maastricht University.
Henry Christian Woodruff: Shareholder: Minority shares in radiomics SA
Spencer Behr: Nothing to disclose
Philippe Lambin: Shareholder: Minority shares in the companies Radiomics SA, Convert pharmaceuticals, Comunicare, and LivingMed Biotech Patent Holder: Co-inventor of two issued patents with royalties on radiomics (PCT/NL2014/050248, PCT/NL2014/050728) licensed to Radiomics SA and one issue patent on mtDNA (PCT/EP2014/059089) licensed to ptTheragnostic/DNAmito, three non-patented invention (softwares) licensed to ptTheragnostic/DNAmito, Radiomics SA and Health Innovation Ventures and three non-issues, non-licensed patents on Deep LearningRadiomics and LSRT (N2024482, N2024889, N2024889).

### RPS 605a-3

**Prospective post-marketing surveillance of AI for breast cancer screening in clinical practice**

*D. Byng*[1], S. Bunk[1], D. Schüler[1], M. Brehmer[1], T. Töllner[2], L. Umutlu[3], K. Pinker-Domenig[4], C. Leibig[1]; [1]Berlin/DE, [2]Stade/DE, [3]Essen/DE, [4]New York, NY/US
*(danalyn.byng@vara.ai)*

**Purpose:** Prospectively-collected post-market surveillance (PMS) data can provide a multifaceted picture of the robustness of an AI device in clinical practice. We introduce an integrated AI system with live monitoring to support PMS in breast cancer screening.
**Methods or Background:** Routinely-documented data on screen-detected cancers and recalls from 6 German screening units using a CE-marked AI system was collected over a 9 month period in 2020/21. Age- and density-stratified crude unadjusted cancer detection and recall rates were calculated and compared between studies read with, and without AI support using Pearson's chi-squared test. Mixed-effects logistic regression was used to investigate the relationship between screening with AI and likelihood of cancer detection or recall. Models were weighted by inverse propensity scores, adjusted for important risk factors (age, breast density, incident or prevalent screening) and clustered by reader ID.
**Results or Findings:** Data on N=23,453 studies read with AI, and N=37,019 studies not read with AI were documented. Unadjusted cancer detection rates and recall rates across subgroups were higher for studies read with AI. When controlling for important risk factors, the likelihood of cancer detection was higher, and the likelihood of recall was lower for studies screened with AI vs studies not screened with AI (odds ratio (OR) for cancer detection 1.11, 95% confidence interval (CI) 0.94–1.30; OR for recall 0.96, 95% CI 0.90–1.03).
**Conclusion:** There is a trend towards a higher cancer detection rate and lower recall rate among studies read with AI. Continuous live monitoring in breast screening units, analogous to PMS, can provide invaluable information to safely roll-out AI systems.
**Limitations:** Not applicable
**Ethics committee approval:** This is a post-market surveillance study of routinely-collected clinical data of a CE-marked medical device. Ethics approval and patient consent was not necessitated.
**Funding for this study:** Funding was received from Vara (Berlin, Germany).
**Author Disclosures:**
Stefan Bunk: Employee: Vara
Katja Pinker-Domenig: Advisory Board: Vara Grant Recipient: NIH/NCI Cancer Center Support Grant Grant Recipient: Breast Cancer Research Foundation
Danalyn Byng: Employee: Vara
Moritz Brehmer: Employee: Vara
Lale Umutlu: Grant Recipient: Siemens Healthcare & Research Advisory Board: Bayer Healthcare Advisory Board: Vara
Christian Leibig: Employee: Vara
Dominik Schüler: Employee: Vara
Thilo Töllner: Nothing to disclose

### RPS 605a-4

**A practical deep learning model in differentiating pneumonia-type lung carcinoma from pneumonia on CT Images: ResNet added with attention mechanism**

*X. Luo*, W. Du, J. Ding, M. Chen; Beijing/CN

**Purpose:** We aimed to introduce a deep learning model with ResNet added with attention mechanism to differentiate pneumonia-type lung carcinoma from pneumonia based on chest CT images.
**Methods or Background:** We retrospectively enrolled 131 patients with pneumonia-type lung carcinoma and 171 patients with pneumonia from October 2019 to February 2021. There were slightly more men than women (162 vs 140), with the average age of 68±15 years old. A Unet model was applied to extract lesion features from chest CT images. The extracted lesions were classified by a designed spatial attention mechanism network. The model AUC and diagnostic accuracy were analysed and compared with radiologists' (junior and senior radiologists) performance.
**Results or Findings:** This model detected and classified each lesion within 6.31 seconds. The average AUC could reach 0.82 through five-fold cross-validation (p<0.01). However, according to lesion texture diversity and boundary ambiguity, it could produce 13.5% of false-positive samples. Its accuracy, sensitivity and specificity (74.2%, 60.4%, and 89.4%) were higher than either junior (61.0%, 48.1%, and 75.0%) or senior (65.0%, 51.9%, and 79.2%) radiologists. With the assistance of this model, both junior (76.0%, 62.8%, and 89.8%) and senior (78.0%, 64.7%, and 91.8%) radiologists' performance were improved.
**Conclusion:** ResNet added with attention mechanism could be a practical approach to detecting and differentiating pneumonia-type lung carcinoma from pneumonia.
**Limitations:** There is an overlap between pneumonia-type lung carcinoma and other pulmonary injuries.
**Ethics committee approval:** This study was approved by an ethics committee.
**Funding for this study:** No funding was received for this study.
**Author Disclosures:**
Wang Du Wang Du: Nothing to disclose
Min Chen: Nothing to disclose
Jia Ding: Nothing to disclose
Xiaojie Luo: Nothing to disclose

### RPS 605a-5

**Artificial intelligence may lead to unexpected changes in the balance of benefits and harms in lung cancer screening**

*V. Gombolevskiy*, R. V. Reshetnikov, I. Blokhin, V. Chernina, A. Nikolaev, A. E. Andreychenko, M. Belyaev, S. Morozov; Moscow/RU
*(g_victor@mail.ru)*

**Purpose:** To develop an understanding of the limitations of applying artificial intelligence (AI) to lung cancer screening (LCS).
**Methods or Background:** LCS has proven to be an effective methodology that results in fewer lung cancer (LC) deaths. However, AI in LCS could change the balance: reducing false negatives can increase unnecessary invasive procedures and complications. AI-based detection of pulmonary nodules, coronary calcium, emphysema may seem beneficial but will inevitably increase the burden on health care.
**Results or Findings:** LCS is an additional burden; scaling up without AI would be impractical or even unfeasible. That being said, only a few LCS studies require immediate action. With the introduction of AI, the benefit-to-harm ratio of LCS will change. The potential harms will be difficult to assess during the initial introduction of AI in LCS. A difficult-to-predict redistribution of patient groups with different management tactics is likely, primarily due to a decrease in patients with normal findings. Proving the benefit of AI in LCS will take years. The algorithms are updating so rapidly that classic lengthy randomised trials on AI in LCS are impractical. To address this race against the machine, we need to develop and use new progressive research designs.
**Conclusion:** First, full-scale lung cancer screening will not be possible without AI. Second, AI could imperceptibly change the balance of benefit and harm in LCS. Third, AI is evolving so rapidly that future multi-year studies evaluating AI in screening are impractical and require new approaches to study design.
**Limitations:** No limitations were identified.
**Ethics committee approval:** Not applicable
**Funding for this study:** No funding was received for this study.
**Author Disclosures:**
Sergey Morozov: Nothing to disclose
Anna Evgenevna Andreychenko: Nothing to disclose
Mikhail Belyaev: Nothing to disclose
Valeria Chernina: Nothing to disclose
Roman V. Reshetnikov: Nothing to disclose
Alexander Nikolaev: Nothing to disclose
Ivan Blokhin: Nothing to disclose
Victor Gombolevskiy: Nothing to disclose

### RPS 605a-6

**Transferability and added benefit between three different diffusion sequences in prostate MRI deep learning for detection of significant prostate cancer**

*N. B. Netzer*, A. Schrader, M. Görtz, C. Schwab, R. Gnirs, M. Hohenfellner, H-P. Schlemmer, D. Bonekamp; Heidelberg/DE

**Purpose:** Deep learning based segmentation of significant prostate cancer (sPC) has been shown to perform similar to PI-RADS assessment. However, questions remain on the transferability of models trained on homogeneous training data to different MRI protocols. Here, we investigate the ability of training using only one diffusion sequence to generalise to two different diffusion sequences, and the benefit of training with all three diffusion sequences.
**Methods or Background:** Bi-parametric (T2w, ADC and high b-value) MRI sequences from 1022 exams were used for training and 246 were used for testing. 3D U-Net based CNN ensembles (5-fold cross-validation) to segment sPC were trained: A) using T2w and same-protocol echo-planar imaging DWI (epiDWI); and B) using T2w, epiDWI, readout-segmented diffusion images (rsDWI) and zoomed-FOV imaging (zDWI) (1613 unique combinations). Testing was performed on three different test sets, T2w paired with a) 246 epiDWI, b) 246 rsDWI, c) 246 zDWI images. Performance was assessed by patient-based prediction of sPC determined by extended systematic and targeted MRI/TRUS fusion biopsy.
**Results or Findings:** On the epiDWI test set, models A and B reached area under the receiver-operating-characteristics (ROC-AUC) of 0.86. On rsDWI test set ROC AUC was 0.84/0.85 for models A/B respectively, and 0.85/0.86 for zDWI. All differences were statistically insignificant in DeLong's test.
**Conclusion:** Epi-only CNN training led to only minor reduction of performance on rsDWI and zDWI test sets, demonstrating that a large fraction of domain-specific knowledge can be transferred from epi-only training. However, small gains in performance and robustness from zDWI and rsDWI enriched training motivate inclusion of multi-sequence data into training the future, especially as no detrimental effect on epi-only test data was found.
**Limitations:** Data was acquired on scanners from the same vendor and institution.
**Ethics committee approval:** Informed consent was waived.
**Funding for this study:** Not applicable
**Author Disclosures:**
Magdalena Görtz: Nothing to disclose
David Bonekamp: Consultant: Bayer Vital Speaker: Bayer Vital
Markus Hohenfellner: Nothing to disclose
Adrian Schrader: Nothing to disclose
Nils Bastian Netzer: Nothing to disclose
Constantin Schwab: Nothing to disclose
Heinz-Peter Schlemmer: Consultant: Curagita, Bayer Grant Recipient: BMBF, Deutsche Krebshilfe, Dietmar-Hopp-Stiftung Board Member: Curagita Speaker: Siemens, Curagita, Profound, Bayer Other: Siemens, Curagita, Profound, Bayer
Regula Gnirs: Nothing to disclose

### RPS 605a-7

**Minimally interactive AI-based segmentation of lymph nodes and soft tissue lesions in follow-up CT**

*A. Hering*[1], F. Peisen[2], A. Othman[2], J. Moltz[3]; [1]Lübeck/DE, [2]Tübingen/DE, [3]Bremen/DE

**Purpose:** Semi-automated and automated lesion segmentation is an ever-growing task in daily routine and scientific radiology, due to the extensive use of imaging techniques, especially among oncologic patients. Several metrics such as RECIST or volume can be derived from a lesion segmentation.
The goal of the study was to develop and evaluate an AI-based algorithm for segmenting lymph nodes and soft tissue lesions in follow-up CT of cancer patients with minimal user interaction.
**Methods or Background:** In this approved retrospective study, we include follow-up CT examinations from 206 stage IV melanoma patients from the University Hospital. Lesions are first identified by a radiologist with one click in the baseline CT scan and then automatically segmented by a neural network in less than a second. In follow-up, the segmentation is created fully automatically by propagating the click point using a co-registration and applying the same neural network. The neural network was trained on 163 patients with overall 3462 lesions and validated on 43 patients with overall 222 lesions. The accuracy of our algorithm was measured in terms of diameter and volume overlap compared to a manual segmentation performed by an experienced radiologist.
**Results or Findings:** The algorithm achieved a median Dice coefficient of 0.81 on baseline and 0.81 on follow-up soft tissue lesions and of 0.79 on baseline and 0.75 on follow-up lymph nodes. The median relative diameter error over all lesions is 0.13 and the median relative volume error is 0.23.
**Conclusion:** Soft tissue lesions and lymph nodes can be successfully segmented by an AI-based algorithm. This can potentially increase reading efficiency in both clinical routine and research.
**Limitations:** The study was performed within a single institution and among patients with a single cancer entity.
**Ethics committee approval:** This study was approved by the ethics committee.
**Funding for this study:** Funding was received from the DFG, German Research Foundation 428216905 SPP2177.
**Author Disclosures:**
Felix Peisen: Nothing to disclose
Alessa Hering: Nothing to disclose
Jan Moltz: Nothing to disclose
Ahmed Othman: Nothing to disclose

### RPS 605a-8

**Liver lesions follow-up changes analysis in CECT by deep learning classification: a phantom study**

L. Joskowicz, A. Szeskin, S. Rochman, R. Lederman, *J. Sosna*; Jerusalem/IL
*(jacobs@hadassah.org.il)*

**Purpose:** Deep learning classification may be affected by scanner type. We aimed to evaluate the accuracy of a novel method that simultaneously identifies and quantifies the changes of liver metastasis in contrast enhanced abdominal CT (CECT) between a current and a prior scan by deep learning classification on a custom phantom scanned with different vendor CT scanners.
**Methods or Background:** The liver phantom (QRM) consisted of a semi-anthropomorphic frame with two custom-designed inserts in which 28 spheres (14 in each insert) of 12 diameters (3-50 mm) and densities were embedded to simulate lesions and lesion changes. It was scanned 3 times on 4 scanners (Philips iCT, Siemens Somatom, Canon Aquilon, GE Optima 660). Pairs of scans corresponding to clinical current and prior scans were processed with a novel method that automatically detects and segments the liver metastases with a simultaneous 3D U-Net classifier. The scan resolutions were 512×512×350-449, 0.75-0.85×0.75-0.85×1.5 mm3 with 0.75 mm slice overlap at 120 kV. The sphere detection, matching changes, individual volume and matching spheres volume difference were computed and compared to the theoretical ground truth values defined by the liver inserts design.
**Results or Findings:** The method correctly detected all 484 (28 spheres × 16 scan pairs) and correctly classified all 32 "new", 32 lesions that "disappeared" and 384 "existing" sphere changes. The mean individual lesion volume error was -18.1% [-100.0,+41.5], 0.19cc [-6.0,+1.4]; the mean pairwise lesion volume difference was -16.6% [-99.6,+42.5], 0.22cc [-0.6,+2.93]. All sphere volumes and sphere volume changes measurements were within ±1/2 voxel spheres diameter imaging resolution (±0.75-0.85 mm).
**Conclusion:** Simultaneous deep learning based detection and quantitative volumetric analysis of liver lesions follow-up changes analysis in CECT provides accurate detection and measurements on a custom phantom among different scanners.
**Limitations:** This was a phantom study.
**Ethics committee approval:** Not applicable
**Funding for this study:** Funding was received from HighRAD Ltd.
**Author Disclosures:**
Richard Lederman: Nothing to disclose
Leo Joskowicz: Nothing to disclose
Adi Szeskin: Nothing to disclose
Shalom Rochman: Nothing to disclose
Jacob Sosna: Consultant: Philips Founder: High-Rad

16:30-17:30 Open Forum #4 (ESR)

### Poster Presentation Session

### PP 6

### Syndromes and joint injuries: cardiac, vascular, chest and musculoskeletal imaging

**Moderator**
S. Harden; Southampton/UK

### PP 6-2

**Monitoring Marfan syndrome and related disorders: accuracy of echocardiography and computed tomography in initial assessment and two-year follow-up**

S. Shnayien, C. Maier, T. D. Trippel, K. Philipp, P. Gehle, *N. L. Beetz*; Berlin/DE

**Purpose:** Patients with Marfan syndrome or related disorders are at risk for aortic dissection and aortic rupture, and therefore require appropriate monitoring. Computed tomography (CT) and transthoracic echocardiography (TTE) are routinely used for initial evaluation and follow-up. The purpose of this study is to compare the accuracy of TTE and CT in initial assessment and two-year follow-up.
**Methods or Background:** This retrospective study included 95 patients aged ≤55 years diagnosed with Marfan syndrome or related disorders. All patients underwent initial assessment including aortic diameter measurement using both TTE and ECG-triggered CT. 42 of the patients did not undergo aortic repair within two years after initial assessment and were monitored by follow-up imaging. Differences between the two methods were compared using Bland-Altman plots. The acceptable clinical limit of agreement was predefined as <+/-2millimeters.
**Results or Findings:** Measurement differences showed wide limits of agreement for initial assessment, with the aortic sinus ranging from +6.3mm to 5.9mm and the ascending aorta from +5.6mm to -8.9mm, and for follow-up, with the aortic sinus from +6.7mm to -5.8mm and the ascending aorta from +8.1mm to -10.1mm. Regarding the ascending aorta, TTE systematically overestimated aortic diameters with a difference bias of -1.6mm for initial assessment and -1.1mm for follow-up. Progressive aortic enlargement was detected in 57% using CT and 40% using TTE. The frequency of measurement differences outside the acceptable clinical agreement limit of difference was highest for the ascending aorta for initial assessment (40%).
**Conclusion:** Monitoring of patients with Marfan syndrome and related disorders should include ECG-triggered CT of the aorta for initial assessment, follow-up, and determination of diameter changes over time to correctly identify aortic enlargement and progressive disease.
**Limitations:** Retrospective dataset, possible selection bias toward patients with less severe disease.
**Ethics committee approval:** This study was approved by an ethics committee.
**Funding for this study:** No funding was received for this study.
**Author Disclosures:**
Tobias Daniel Trippel: Nothing to disclose
Christoph Maier: Nothing to disclose
Petra Gehle: Nothing to disclose
Seyd Shnayien: Nothing to disclose
Karla Philipp: Nothing to disclose
Nick Lasse Beetz: Nothing to disclose

### PP 6-4

**Mind the heart: ECG-gated cardiac CT in the acute phase of ischaemic stroke**

L. A. Rinkel, V. Guglielmi, S. M. Boekholdt, L. F. Beenen, C. B. L. M. Majoie, Y. Roos, *A. Van Randen*, J. Coutinho, N. R. Planken; Amsterdam/NL

**Purpose:** To assess the feasibility and diagnostic yield of cardiac CT in the acute phase of ischaemic stroke.
**Methods or Background:** We performed a single-centre, prospective cohort study in consecutive adult patients with acute ischaemic stroke. Patients underwent prospective ECG-gated cardiac CT, immediately after CT-angiography of the aortic arch, cervical and intracranial arteries. Patients also underwent routine stroke work-up, including transthoracic echocardiography (TTE). Primary endpoint was the proportion of patients with a predefined high-risk cardio-aortic source of embolism on CT versus TTE in patients undergoing both investigations.
**Results or Findings:** 452 patients (59.2% male, median age 72) were included and underwent cardio-aortic CT. The median additional scan time of cardiac CT was 6 (interquartile range 5-7) minutes with good or excellent image quality in 73.2%. In total, 350/452 (77.4%) patients underwent TTE. A high-risk cardio-aortic source of embolism was detected in 40/350 (11.4%) patients on CT, compared to 17/350 (4.9%) patients on TTE (odds ratio 5.60, 95% confidence interval: 2.28-16.33). Cardiac thrombus was the most frequent CT finding (25 patients [7.1%]). The diagnostic yield of cardiac CT in the full study population (n = 452) was 12.2%. Among 175 patients with cryptogenic stroke after routine diagnostic workup, cardiac CT identified a potential cause of stroke in 11 (6.3%).
**Conclusion:** Cardiac CT acquired in the acute phase of ischaemic stroke is technically feasible and has a higher diagnostic yield than TTE to detect high-risk sources of embolism. Cardiac CT may be considered as an alternative to TTE to screen for cardioembolism.
**Limitations:** Compared CT with TTE and not transoesophageal echocardiography (TEE), time difference (median of one day) between CT and TTE.
**Ethics committee approval:** The medical ethics committee of Amsterdam UMC, location AMC approved the study (2018_017#C2018275).
**Funding for this study:** Not applicable.
**Author Disclosures:**
Nils Robrecht Planken: Nothing to disclose
Charles Bernardus Lucia Maria Majoie: Shareholder: Nico.lab
S. Matthijs Boekholdt: Nothing to disclose
Leon A. Rinkel: Nothing to disclose
Valeria Guglielmi: Nothing to disclose
Yvo Roos: Shareholder: Nico.lab
Adrienne Van Randen: Nothing to disclose
Jonathan Coutinho: Nothing to disclose
Ludo F.M. Beenen: Nothing to disclose

### PP 6-7

**Acute aortic syndrome**

*R. Barquet Mur*, N. Cañete, V. Pineda, F. Jaldo-Reyes, S. Pedraza Gutierrez; Girona/ES

**Purpose:** (1) Understand the pathophysiology of the acute aortic syndrome (AAS) for a better understanding of the radiological findings. (2) Identify the most characteristic radiological findings of this pathology and be able to make a correct differential diagnosis, including the most frequent diagnostic errors. (3) Diagnose the most common complications. (4) Understand the pathophysiology of the acute aortic syndrome (AAS) for a better understanding of the radiological findings. (5) Identify the most characteristic radiological findings of this pathology and be able to make a correct differential diagnosis, including the most frequent diagnostic errors. (6) Diagnose the most common complications.
**Methods or Background:** Not applicable.
**Results or Findings:** Acute aortic syndrome (AAS) is made up of three different entities: aortic dissection, aortic intramural haematoma, and penetrating atherosclerotic ulcer; each with characteristic radiological findings. Despite the low incidence of AAS (7.7 cases per 100,000 people/year), it is a pathology with high mortality, reaching up to 57% at 10 years in the case of aortic dissection. An accurate and early diagnosis is of vital importance in the context of this urgency; this is where the role of the radiologist is valuable. The radiological study of the AAS should include a simple thoracic computed tomography (CT) study and subsequently a CT angiography. The radiological study should allow us to identify and interpret the most characteristic radiological findings of the different entities of the SAA, taking into consideration their differential diagnosis and possible image interpretation errors that may occur.
**Conclusion:** SAA is a vital clinical situation, where its prognosis depends on an accurate and quick diagnosis, for an early start of its treatment. The role of the radiologist is of utmost importance in the management of the patient with suspected AAS.
**Limitations:** Not applicable.
**Ethics committee approval:** Not applicable.
**Funding for this study:** Not applicable.
**Author Disclosures:**
Francisco Jaldo-Reyes: Nothing to disclose
Noemí Cañete: Nothing to disclose
Salvador Pedraza Gutierrez: Nothing to disclose
Ramon Barquet Mur: Nothing to disclose
Víctor Pineda: Nothing to disclose

### PP 6-9

**Multimodality imaging of midtarsal joint injuries. A biomechanic-oriented didactic approach**

DOI: 10.26044/ecr2022/18897

*U. Viglino*, A. Muda, D. Orlandi; Genoa/IT

**Purpose:** The purpose of our educational exhibit is to: First, to illustrate the normal anatomy of the ligaments stabilising the midtarsal joint; second, to describe the appearance of these ligaments in different imaging modalities; third, to produce anatomical schemes with didactic purpose and show correlations with sonographic and MR imaging; fourth, to elucidate US-MRI findings of traumatic injuries of midtarsal ligaments.
**Methods or Background:** The midtarsal joint is composed by several bones and ligaments. The evaluation of these structures in post-traumatic injuries could be challenging in many cases. The Bifurcate ligament and the Spring ligament complex have a key role in the stability of the midfoot and the plantar arch of the foot respectively. Bifurcate ligament is a Y-shaped ligament composed by two components: the calcaneo-navicular component extends from the anterior process of the calcaneus to the dorso-lateral navicular and the lateral calcaneo-cuboid component extends from the anterior process of the calcaneus to the dorsomedial cuboid. The plantar Calcaneonavicular Ligament (CNL), also known as the spring ligament complex, extends from the calcaneus to the tarsal navicular and has three components: the SuperoMedial CalcaneoNavicular Ligament (smCNL), the MedioPlantarOblique CalcaneoNavicular Ligament (mpoCNL) and the InferoPlantarLongitudinal CalcaneoNavicular Ligament (iplCNL). Other important stabilising ligaments are: dorsal calcaneo-cuboid ligament, dorsal talo-navicular ligament, dorsal and plantar cuboid-navicular ligaments, and plantar ligaments. These complex structures can be damaged in sprain injuries of the ankle and the midfoot.
**Results or Findings:** We will provide dedicated US-MR images compared with detailed anatomic schemes and dynamic animations showing the most common injury patterns.
**Conclusion:** Dynamic HRUS allows a quick, real-time evaluation of midtarsal ligaments and combined with MRI allows a complete and thorough evaluation of ligaments anatomy and biomechanics of traumatic injuries.
**Limitations:** No limitations were identified in this study.
**Ethics committee approval:** Ethics committee approval was obtained.
**Funding for this study:** No funding was required for this study.
**Author Disclosures:**
Alessandro Muda: Nothing to disclose
Davide Orlandi: Nothing to disclose
Umberto Viglino: Nothing to disclose

### PP 6-10

**The utility of Shear Wave elastography to differentiate physiological joint fluid from inflammatory synovitis of the hand: a preliminary study**

DOI: 10.26044/ecr2022/10346

*S. Marsico*, M. I. Carrión Barberà, A. Agustí Claramunt, J. M. Maiques Llácer, J. Monfort Faure, T. C. Salman Monte, A. Solano López; Barcelona/ES

**Purpose:** To perform a preliminary study and validate Shear Wave elastography as a new imaging tool to identify synovitis, comparing the stiffness of synovial tissue in patients with clinical suspicion of synovitis versus a healthy control group.
**Methods or Background:** In the period between April 2020 and June 2021 an observational retrospective study was carried out including a group of patients with clinical suspicion of synovitis under therapy (n = 50), and a control group of volunteers without clinical suspicion of synovitis (n = 50). All the subjects underwent complete reumathologic examinations and laboratory tests. In all the participants, the biomechanical evaluations were carried out using 2D Shear Wave elastosonographic module of wrist and hand using a multiplanar scanning technique according to EULAR guidelines. A synovial tissue elasticity score was also inserted, which was dependent on the synovial tissue stiffness measured by Shear Wave elastography. Grade 1 0-30 kPa, Grade 2 30-60 kPa, Grade 3 60-90 kPa and Grade 4 90-120 kPa.
**Results or Findings:** In the 50 patients with clinical suspicion of synovitis, detectable joint effusion was found in 46 (92%) of them. The mean synovial tissue stiffness value measured by Shear Wave elastography in this group was 72.18 kPa. In 50 healthy volunteers, joint effusion was found in 25 (50%) of them. The mean synovial tissue stiffness value measured by Shear Wave elastography in this control group was 6.91 kPa.
**Conclusion:** Shear Wave elastosonography is a promising, simple, accurate, reproducible and non-invasive imaging technique that allows differentiating between physiological joint fluid and synovitis.
**Limitations:** First study on the subject with few patients. Necessary evaluation of the different rigidity in specific rheumatological diseases to confirm the usefulness of the technique.
**Ethics committee approval:** The study was approved by an ethics committee.
**Funding for this study:** No funding was received.
**Author Disclosures:**
Anna Agustí Claramunt: Nothing to disclose
José María Maiques Llácer: Nothing to disclose
Salvatore Marsico: Nothing to disclose
Albert Solano López: Nothing to disclose
Tarek Carlos Salman Monte: Nothing to disclose
Jordi Monfort Faure: Nothing to disclose
Maria Irene Carrión Barberà: Nothing to disclose

**16:30-17:30** **Room E1**

### Research Presentation Session: Vascular

### RPS 615

### Aortic imaging: morphology and beyond

**Moderator**
H. von Tengg-Kobligk; Berne/CH

### RPS 615-3

**Clinical evaluation of non-contrast-enhanced magnetic resonance angiography in comparison to computed tomography angiography for evaluating endoleaks after abdominal endovascular aneurysm repair**

*K. Mostafa*, J. A. Pfarr, P. Langguth, P. J. Schäfer, J. Trentmann, M. Both, O. Jansen, F. Bueno Neves, M. Salehi Ravesh; Kiel/DE
*(mostafa.karim86@gmail.com)*

**Purpose:** Clinical evaluation of non-contrast-enhanced radial quiescent-interval slice-selective magnetic resonance angiography (QISS-MRA) in its ability to diagnose and monitor endoleaks and aneurysm size after abdominal endovascular aneurysm repair (EVAR) in direct comparison to contrast-enhanced computed tomography angiography (CE-CTA).
**Methods or Background:** Our study included 20 patients (17 male, median age 79.8 years) who underwent radial QISS-MRA and CE-CTA after non-fenestrated infrarenal EVAR at their first follow-up examination. Two interventional radiologists evaluated datasets from both techniques for each patient concerning presence of endoleaks, types of endoleaks, aneurysm diameters and image quality.
**Results or Findings:** Compared to CE-CTA, all endoleaks after abdominal EVAR were detected and classified correctly on QISS-MRA. The interobserver agreement between CE-CTA and QISS-MRA was almost perfect, except for type 2 endoleaks, where agreement was substantial. Intermodal aneurysm diameter measurements correlate “very strongly” for both observers. All results were statistically significant. Hyperdense imaging artefacts in CE-CTA cause aneurysm diameter measurements to be inaccurate by up to 1 cm. These artifacts were not present in QISS-MRA. Also, QISS-MRA seems to be more sensitive in detecting type II endoleaks. Some type II endoleaks could only be detected in QISS-MRA but not in CE-CTA, which was most likely due to low-flow endoleak characteristics.
**Conclusion:** Radial QISS-MRA is a contrast agent-free technique for diagnosing and monitoring all types of endoleaks and aneurysms in patients after abdominal EVAR. It provides information about specific clinical questions concerning aneurysm size as well as presence and types of endoleaks without exposure to radiation and contrast agents.
**Limitations:** There was only a relatively small number of patients included in the study.
**Ethics committee approval:** The local institutional review board, the "Ethikkommission der Medizinischen Fakultät der Christian-Albrechts-Universität zu Kiel" approved this prospective single-centre study (File No. D576/18).
**Funding for this study:** No funding was received for this study.
**Author Disclosures:**
Patrick Langguth: Nothing to disclose
Marcus Both: Nothing to disclose
Mona Salehi Ravesh: Nothing to disclose
Julian Andreas Pfarr: Nothing to disclose
Philipp Jost Schäfer: Nothing to disclose
Jens Trentmann: Nothing to disclose
Karim Mostafa: Nothing to disclose
Fernando Bueno Neves: Nothing to disclose
Olav Jansen: Nothing to disclose

### RPS 615-4

**Sex-specific hemodynamic normal values of the aorta: a 4D MR flow study**

*M. Ramaekers*[1], J. Westenberg[2], M. Venner[1], J. F. Juffermans[2], S. Schalla[1], J. E. Wildberger[1], H. J. Lamb[2]; [1]Maastricht/NL, [2]Leiden/NL

**Purpose:** Abnormal blood flow may be related to aneurysm growth. However, sex-specific normal values for aortic flow and potential sex differences have not been established yet. This study assesses quantitative flow parameters including velocity, wall shear stress, vorticity, helicity, and flow displacement in healthy males and females.
**Methods or Background:** Forty young healthy volunteers (28.9 ± 5.0 years, 50% male) were prospectively recruited and underwent 4D flow MRI of the total aorta at 3T. Quantitative flow parameters were calculated for all systolic cardiac phases in the following segments: root (AoR), ascending aorta (AAo), arch (AoA), descending aorta (DAo), suprarenal- (SRA) and infrarenal aorta (IRA). Data was compared using a student T-test, p<0.05 was considered statistically significant.
**Results or Findings:** Females show smaller aortic diameters, which are significant for AoR (33.8±2.6 vs. 30.0±2.1 mm, p<0.01), AAo (28.2±2.2 vs. 25.6±2.0 mm, p<0.01), SRA (19.2±1.6 vs. 17.3±1.3 mm, p<0.01), and IRA (16.5±1.4 vs. 14.8±1.0, p<0.01 mm). Maximum velocities are significantly lower in females for the AoA (56.37±9.6 vs. 49.7±5.7 cm/s, p=0.011). Wall shear stress is significantly lower in females for the AAo (p=0.032). Vorticity is higher in females for the AoR, SRA, and IRA (all p<0.05). There are no significant differences in helicity and flow displacement.
**Conclusion:** This study establishes sex-specific hemodynamic normal values for the entire aorta. Significant differences are present between males and females. This stresses the importance of a patient-specific approach for hemodynamic evaluations. Further studies are necessary to evaluate sex-specific cut-off values in aneurysm prognosis.
**Limitations:** The spatial and temporal resolution of 4D flow MRI is limited. However, both groups underwent identical scanning protocol, thus highly comparable.
**Ethics committee approval:** Approved by the local medical ethical committee (NL69521.068.19).
**Funding for this study:** Funding was received for this study by the Dutch Heart Foundation (CVON2017-08-RADAR).
**Author Disclosures:**
Joachim E. Wildberger: Other: Institutional (CTCM / Maastricht UMC+): Agfa, Bard, Bayer, Cook, GE, Philips, Radiomics, Siemens Other: Speaker’s Bureau (Maastricht UMC+ / JW): Bayer
Jos Westenberg: Nothing to disclose
Mitch Ramaekers: Nothing to disclose
Max Venner: Nothing to disclose
Joe Faustino Juffermans: Nothing to disclose
Hildo J. Lamb: Consultant: AI consultant Royal Philips
Simon Schalla: Nothing to disclose

### RPS 615-6

**A tracking method for the reconstruction of the aorta wall from 4DMRI data**

*M. A. Agbalessi*[1], J-J. Christophe[1], A. Lalande[2], D. Lombardi[3], M. A. Fernandez[3]; [1]Quétigny/FR, [2]Dijon/FR, [3]Paris/FR
*(magbalessi@casis.fr)*

**Purpose:** Accurate evaluation of predictive parameters for aortic aneurysms often relies on patient-specific blood flow simulations. The purpose of this study is to present a tracking approach to estimate the local motion of the aortic wall from 4D MRI data, in view of fluid-structure interaction simulation calibration.
**Methods or Background:** At every time step a simplified elastic model-based prediction is corrected with a dynamical state observation derived from the whole 4D MRI data, both as geometrical and kinematic data, with a Kalman filter. Systematic error estimation is provided, assuming that the near-wall velocity error is gaussian. Parameter sensitivity analysis is conducted on 2D academic cases. An elastic vesicle immersed in the cavity flow is simulated to generate time-resolved image sequences with a velocity field. The tracking is then tested for one patient exam. Further validation can be expected on a cohort of 5 patients with aortic aneurysm.
**Results or Findings:** The accuracy of the tracking is evaluated in terms of surface reconstruction (Dice coefficient ) and mesh-point trajectories, in academic test cases. Satisfying surface reconstruction is obtained on slightly blurred images (Dice > 0.92), for different levels of data noise. Similar results are obtained with one patient exam (D = 0.92).
**Conclusion:** The study shows promising preliminary results of a tracking method for the surface reconstruction of the aortic wall, with reasonable parameter sensitivity. It also provides an estimation of the local wall displacement and velocity.
**Limitations:** The method could be improved with further validation and the computation time needs to be optimised for only segmentation purpose.
**Ethics committee approval:** Patient data has been collected as part of a research protocol NCT03817008 on clinicalTrials.gov approved by the local ethics committee. Data has been anonymised to complain about RGPD regulation.
**Funding for this study:** Funding was received for this study by ANRT grant.
**Author Disclosures:**
Mocia A. Agbalessi: Author: Author of the abstract
Advisor Miguel A. Fernandez: Advisory Board: Academic Advisor
Radiologist Alain Lalande: Nothing to disclose
advisor Damiano Lombardi: Advisory Board: Academic Advisor
Jean-Joseph Christophe: CEO: Initiated the project, commercial use expected

### RPS 615-7

**Medical 3D-printing of aortic templates and its appropriateness for clinical use in the interventional treatment of thoraco-abdominal aortic diseases: one centre experience**

P. Rynio, *M. Wojtuń*, Ł. Wójcik, M. P. Kawa, A. Falkowski, P. Gutowski, A. Kazimierczak; Szczecin/PL
*(wojtunmaciek@gmail.com)*

**Purpose:** Applications of 3D-printing for physician-modified endovascular stent grafting (PMEG) in patients with aortic aneurysm are rapidly developing. 3D-printed models guide interventional radiologists to fenestrate stent grafts for fenestrated endovascular aortic aneurysm repair (FEVAR). 3D-printing technology may improve urgent construction of patient-specific PMEG for treatment of complex aortic pathologies and significantly improve quality of modified stent grafts for FEVAR. Preoperatively, 3D-printed models of aortic aneurysms are made according to angio-CT images. However, the accuracy and reliability of personalised 3D-printed models are not established. Dedicated imaging studies are necessary to standardise the measurement methods and to demonstrate safety and reliability of 3D-printed models use for FEVAR.
**Methods or Background:** 16 3D-printed models of aortic arch and 13 3D-printed models of aorta and their corresponding angio-CTs were generated for patients with stable aortic aneurysms that were used in this accuracy study. First, 3D-models were scanned in CT and evaluated by three radiologists experienced in vascular CT. Next, CT-scanned 3D-models were segmented, aligned with patient data, and evaluated for the Hausdorff matrix. Finally, Bland-Altman plots determined the degree of agreement.
**Results or Findings:** Intraclass Correlation Coefficients values were more significant than 0.9 for measurements of aorta diameters in all landmark locations. Therefore, reliability of aortic 3D-models was outstanding. The Bland-Altman plots analysis indicated measurement biases of 0.05 to 0.47 for aortic arch templates and 0.06 to 0.38 for abdominal aortic templates. Arithmetic mean of Hausdorff's mean distances for 3D-arch-templates was 0.47±0.06 mm and for 3D-abdominal-templates was 0.24±0.03 mm.
**Conclusion:** 3D-printed models based on angio-CT images of aortic aneurysms appeared to be reliable. Thus, they can be widely used in interventional radiology and endovascular surgery departments worldwide to accurately guide the PMEGs fenestration for advanced treatment of thoracoabdominal aortic diseases.
**Limitations:** No limitations were identified.
**Ethics committee approval:** This study was approved by an ethics committee.
**Funding for this study:** No funding was received for this study.
**Author Disclosures:**
Maciej Wojtuń: Nothing to disclose
Arkadiusz Kazimierczak: Nothing to disclose
Milosz P. Kawa: Nothing to disclose
Łukasz Wójcik: Nothing to disclose
Piotr Gutowski: Nothing to disclose
Aleksander Falkowski: Nothing to disclose
Paweł Rynio: Nothing to disclose

16:30-17:30 Room E2

### Research Presentation Session: Paediatric

### RPS 612

### Paediatric MSK imaging: rethinking the skeletal survey - repurposing MRI

**Moderator**
S. B. Grover; Greater Noida, NCR-Delhi/IN

### RPS 612-2

**Lateral radiographs of joints in suspected physical abuse: do they add to the diagnosis?**

*R. Meshaka*, D. Garbera, O. Arthurs, S. C. Shelmerdine; London/UK
*(riwa.meshaka@nhs.net)*

**Purpose:** A skeletal survey to screen for fractures is indicated when there are concerns over inflicted injury in a young child. Royal College of Radiologists 2017 guidance on imaging in suspected physical abuse (SPA) includes the addition of lateral radiographs of the joints. Our objective was to determine the additional diagnostic value of lateral radiographs in SPA.
**Methods or Background:** A retrospective review of all SPA skeletal surveys in children aged under 30 months over a 3-year period was carried out. Incomplete and follow-up surveys were excluded. Blinded reviews by two consultant paediatric radiologists were undertaken: firstly, with only frontal views available, secondly with frontal and lateral views. Fractures and confidence scores (1-5) were recorded.
**Results or Findings:** 138/173 surveys met the inclusion criteria, mean age of 8 months, 17/138 (54%) male, 29/128 (21%) live cases. 9/138 (7%) patients had a total of 16 appendicular fractures. 14/15 (93%) and 13/14 (93%) detected fractures were visible on the frontal view alone by Radiologist 1 and Radiologist 2, respectively. A distal radius fracture only seen on lateral view by Radiologist 1 was suboptimally imaged on the frontal view (whole arm view only). There was diagnostic uncertainty over a distal tibial fracture only seen on the lateral view only by Radiologist 2. A significant increase in confidence was demonstrated in normal cases, but not fracture cases.
**Conclusion:** Lateral views led to apparent additional fracture detection rate of 13% (2/16 in our study). The majority of corner metaphyseal fractures were visible on frontal view alone. Lateral views increased reporter confidence in correctly identifying the absence of a fracture in normal cases.
**Limitations:** A single centre study limited by a small number of fractures. Larger, multi-centre studies are required.
**Ethics committee approval:** Approval by an ethics committee was not required.
**Funding for this study:** No funding was received for this study.
**Author Disclosures:**
David Garbera: Nothing to disclose
DSusan Cheng Shelmerdine: Other: Funded by a NIHR Advanced Fellowship Award (NIHR 301322) Other: Funded by a NIHR Advanced Fellowship Award (NIHR 301322) Other: Funded by a NIHR Advanced Fellowship Award (NIHR 301322) Other: Funded by a NIHR Advanced Fellowship Award (NIHR 301322) Other: Funded by a NIHR Advanced Fellowship Award (NIHR 301322)
Owen Arthurs: Other: Funded by a National Institute for Health Research (NIHR) Career Development Fellowship (NIHR-CDF-2017-10-037)
Riwa Meshaka: Nothing to disclose

### RPS 612-3

**Whole body postmortem computed tomography versus the skeletal survey: can computed tomography replace the skeletal survey?**

S. de Vries[1], M. Nagtegaal[1], V. Konijn[2], E. van de Mortel[2], S. G. F. Robben[3], *R. R. Van Rijn*[2]; [1]The Hague/NL, [2]Amsterdam/NL, [3]Maastricht/NL

**Purpose:** Can total body CT scan replace the skeletal survey in a post mortem setting?
**Methods or Background:** In a retrospective observational cohort study we included deceased children aged 0-4 years who underwent both a post mortem skeletal survey and a whole-body post mortem CT (PMCT) as part of a legal autopsy at the Netherlands Forensic Institute between November 2008 and December 2020. The studies were reviewed on a PACS System by one of two experienced paediatric radiologists.
**Results or Findings:** Sixty-three cases were included. The median age was 4.0 months (range 0 – 58 months). The majority of children were younger than 2 years old (91.4%). Most of the children were male (60.5%). Fractures were observed in 36 (44.4%). A total of 202 unique fractures were identified with PMCT and/or the skeletal survey. PMCT (n=182) detected significantly more fractures compared to the skeletal survey (n=75, p=0.011).
**Conclusion:** PMCT is superior for the detection of skull and rib fractures, but missed more metaphyseal corner fractures and hand and foot fractures. The two modalities should be seen as complementary but based on these findings a limited skeletal survey could be performed if a total body CT is obtained.
**Limitations:** The main limitation is that the pathology reports were not structured and that despite the fact that all cases underwent autopsy a pathology correlation was not possible.
**Ethics committee approval:** The Medical Ethics Review Committee of the Academic Medical Centre Amsterdam approved this study (reference number W19_159m date: 25th of April 2019) and, with a waiver of informed consent requirements, confirmed that the Medical Research Involving Human Subjects Act (WMO) does not apply.
**Funding for this study:** No funding was received for this study.
**Author Disclosures:**
Simon G. F. Robben: Nothing to disclose
Selena de Vries: Nothing to disclose
Rick R. Van Rijn: Author: I receive royalties from Springer for Forensic Aspects of Pediatric Fractures. Differentiating Accidental Trauma from Child Abuse.
Veronique Konijn: Nothing to disclose
Michelle Nagtegaal: Nothing to disclose
Esther van de Mortel: Nothing to disclose

### RPS 612-4

**MRI of the proximal tibia epiphysis for the estimation of majority: validation study from Thuringia**

N. Chitavishvili[1], D. Wittschieber[1], I. Papageorgiou[2], A. Malich[2], *H-J. Mentzel*[1]; [1]Jena/DE, [2]Nordhausen/DE
*(hans-joachim.mentzel@med.uni-jena.de)*

**Purpose:** Skeletal age is an important marker of somatic maturity. MRI shows that the evaluation of knee joint epiphyses can allow age estimation, especially with regard to the legally relevant age limit of 18 years. We checked the published age estimation criteria on their practicability in routine MRI data from German patients aged 12-25 years.
**Methods or Background:** MRI of the proximal tibial epiphyses of 413 patients (214 m and 199 f) who had a knee MRI (1.5T and 3.0T MRI) were analysed retrospectively. Quality assessment (Likert scale 1-3) and staging (stages 2-6 according to Vieth et al. 2018) were based on coronary sequences (T1 TSE, FS; T2 TIRM/STIR; slice thickness 3 mm).
**Results or Findings:** 29 of initial 442 cases (6%) could not be classified regarding their stages due to bone marrow edema. Intra-observer agreement was “very good” ($\kappa$=0.931), and inter-observer agreement was “good” ($\kappa$=0.798). MR image quality (assessed on a Likert scale from 1 to 3) did not show a significant influence on the process of stage determination. The first results of the analysed MRIs show significant acceleration in male gender (female sex stage 2 = 12.11yrs, 3 = 12.03yrs, 4 = 12.57yrs, 5 = 14.61yrs, 6 = 18.55yrs; male sex stage 2 = 12.08yrs, 3 = 12.43yrs, 4 = 15.23yrs, 5 = 16.52yrs, 6 = 20.27yrs). Detailed statistics including age minima and maxima of the individual stages are presented with exemplary pictures and discussed.
**Conclusion:** The staging according to Vieth et al. is possible using routine MRI data sets of the knee joint. However, an evaluation and weighting of the individual sequences in the age estimation is necessary.
**Limitations:** The limitation is the small number of volunteers.
**Ethics committee approval:** The local ethics committee approved the study.
**Funding for this study:** No funding was received for this study.
**Author Disclosures:**
Ismini Papageorgiou: Nothing to disclose
Hans-Joachim Mentzel: Nothing to disclose
Daniel Wittschieber: Nothing to disclose
Natia Chitavishvili: Nothing to disclose
Ansgar Malich: Nothing to disclose

### RPS 612-5

**Prevalence of cam deformities and osteoarthritis at longterm radiographic followup of patients with slipped capital femoral epiphysis**

*T. D. Lerch*, M. Hanke, F. Schmaranzer, S. Steppacher, J. D. Busch, K. Ziebarth; Bern/CH

**Purpose:** Patients with slipped-capital-femoral-epiphyses(SCFE) are at risk for development of osseous deformities causing hip pain and osteoarthritis. Therefore, we report on (1) prevalence of cam-deformities, (2) prevalence of reduced femoral head-neck offset and (3) osteoarthritis at long-term follow-up in patients with SCFE.
**Methods or Background:** We performed a retrospective radiographic analysis involving 32 hips (32 patients, 1998-2010). Inclusion criteria were SCFE patients that underwent modified Dunn procedure. We evaluated pelvic radiographs at mean follow-up time of 9±3 years to calculate alpha angle on AP and lateral radiograph, head-neck offset, signs for osteoarthritis, Articulo-trochanteric distance(ATD) and minimal joint space width. Most patients had acute-on-chronic slip. The mean age of SCFE patients was 13±2 years at time of surgery and all patients presented with moderate or severe slips. Four

Wednesday patients underwent subsequent hip preservation surgery for correction of cam-deformities.
**Results or Findings:** First, in total nine patients (28%) had cam deformities. Seven patients (22%) had cam-deformites (alpha-angle>60°) on ap radiographs. Two additional patients had cam-deformites (alpha-angle>55°) on lateral radiographs. Mean alpha-angle of SCFE patients was 51±2° on ap radiograph and 40±2° on lateral radiograph. Second, seven SCFE patients(22%) had reduced femoral head-neck offset<10mm. Mean ATD was 40mm, two patients(6%) had ATD <20 mm. Third, osteoarthritic changes were present in two SCFE patients (OA Grade 2 according to Tönnis). Minimal joint space width was 3.5mm±0.5. Five patients developed periarticular ossifications, one patient had bilateral osseous cysts of the proximal femur.
**Conclusion:** One out of four SCFE patients developed cam-deformities associated with femoroacetabular impingement (FAI). Although we found a low prevalence of osteoarthritis, these patients are at risk for developing hip osteoarthritis. We recommend routine radiographic follow-up for these patients. This could be important for doctors treating SCFE patients.
**Limitations:** The limitation is the retrospective analysis.
**Ethics committee approval:** Ethical approval was obtained.
**Funding for this study:** Funding was received by the Swiss-National-Science-Foundation.
**Author Disclosures:**
Kai Ziebarth: Nothing to disclose
Till Dominic Lerch: Nothing to disclose
Florian Schmaranzer: Nothing to disclose
Simon Steppacher: Nothing to disclose
Jasmin D. Busch: Nothing to disclose
Markus Hanke: Nothing to disclose

### RPS 612-6

**The clinical, biochemical and radiological features of kingella kingae osteoarticular infection in a metropolitan paediatric community**
*N. R. Watson*[1], R. Warne[2], M. D. Mason[2]; [1]Birytina/AU, [2]Perth/AU

**Purpose:** The aim of this study was to evaluate the relevance of radiological findings of kingella kingae (KK) osteoarticular infection in a paediatric population. KK is one of the most frequent causative pathogens of osteoarticular infections in children under the age of 4 years. Infant osteoarticular infection is often insidious in nature and infections with KK usually have unremarkable inflammatory biochemical markers, adding further to the difficult diagnostic dilemma.
**Methods or Background:** This retrospective study reports on 33 paediatric patients in an Australian metropolitan paediatric population with PCR proven KK osteoarticular infection between September 2015 and September 2018. Each individual patient had clinical and biochemical parameters measured; additionally, radiological investigations were undertaken for 32 of the patients.
**Results or Findings:** Often, radiograph imaging alone is not enough to diagnose a KK infection. Radiographs if viewed in insolation were only 50% sensitive to support a KK infection. However, from this study, it was shown that ultrasound, if viewed in insolation, was 96.15% sensitive in supporting a KK infection. Of these ultrasounds, all reported an effusion as one of the findings. 87% of the patients had a white cell count within normal limits and only 97% had a negative blood culture.
**Conclusion:** KK infection is easy to misdiagnose because it affects a paediatric population that can be difficult to assess clinically, often appears relatively well and biochemical markers often within normal limits. It is therefore imperative to consider KK in the correct patient, so appropriate imaging can be performed. Our study concludes that ultrasound is highly sensitive to positive findings and should be performed first if KK infection is suspected. Ultrasound is optimal for the demographic having no radiation side effects and being non-invasive.
**Limitations:** The limitation is the study's sample size.
**Ethics committee approval:** This study was approved by an ethics committee.
**Funding for this study:** Funding was received for this study by Nil.
**Author Disclosures:**
Michael David Mason: Author: Perth Children's Hospital
Nicholas Robert Watson: Author: Sunshine Coast University Hospital
Richard Warne: Author: Perth Childrens Hospital

### RPS 612-7

**Hyperostosis in chronic recurrent multifocal osteomyelitis whole body MRI: a longitudinal case review**
*S. G. Kandemirli*, M. Sebaaly, A. Lenert, P. Ferguson2, S. Sato;
Iowa City, IA/US
*(sedat-kandemirli@uiowa.edu)*

**Purpose:** Chronic Recurrent Multifocal Osteomyelitis (CRMO) is an idiopathic rheumatologic condition characterised by multiple sterile inflammatory bone lesions with relapsing and remitting course. In early stages, lesions may be osteolytic in nature with progressive sclerosis over time resulting in hyperostosis.
**Methods or Background:** We retrospectively evaluated 106 patients with a diagnosis of CRMO that underwent at least 1 whole-body MRI between April 2014 to April 2020. Whole-body MRI and radiography images were assessed for hyperostosis.
**Results or Findings:** 31 sites of hyperostosis in 24 patients were identified. Mean age at symptom onset was 8 years (range 2-14 years), with a F:M ratio of 14/10. Sites of hyperostosis included clavicle in 12, femur in 5, pelvis in 5, humerus in 4, radius in 2, mandible in 2 and scapula in 1. At baseline MRI, 29 lesions had cortical thickening, with accompanying bone marrow edema in 28 and soft-tissue edema in 26 lesions. At baseline whole-body MRI, additional regions of bone marrow edema were identified at 99 sites in 21 patients. X-ray was available in 28 lesions. Baseline x-ray revealed expansion and sclerosis in 25 lesions, accompanied by periosteal reaction in 6 and lytic changes in 2. 3 lesions had only periosteal reaction at baseline. Follow-up MRIs were available for 28 lesions with a mean follow-up of 34 months (5-83 months). At final follow-up, 3 patients suffered from leg discrepancy and 1 patient had restricted jaw opening.
**Conclusion:** Chronic recurrent multifocal osteomyelitis is a difficult clinical and imaging diagnosis. Hyperostosis and periosteal reaction have a predilection for clavicle. Whole-body MRI is an essential tool in diagnosing and monitoring CRMO.
**Limitations:** The limitation is the retrospective nature.
**Ethics committee approval:** The institutional review board approved this HIPAA compliant study; informed consent was waived.
**Funding for this study:** No funding was received for this study.
**Author Disclosures:**
Shawn Sato: Nothing to disclose
Mikhael Sebaaly: Nothing to disclose
Polly Ferguson2: Nothing to disclose
Sedat Giray Kandemirli: Nothing to disclose
Aleksander Lenert: Nothing to disclose

### RPS 612-8

**Value of magnetic resonance imaging for skeletal bone age assessment in healthy male adolescent soccer players**
*L. Basten*[1], D. Leyhr[2], D. Murr[2], D. Lüdin[3], M. Romann[3], O. Höner[2], S. Fischer[1], T. Gruber-Rouh[1], K. Eichler[1]; [1]Frankfurt a. Main/DE, [2]Tübingen/DE, [3]Magglingen/CH

**Purpose:** To comprehensively evaluate value of MRI for skeletal bone age (SBA) assessment in healthy male adolescents.
**Methods or Background:** In this cross-sectional study n=63 male, elite soccer athletes between 10-13 years (mean: 12.35±1.1 years) were examined on two weekends in November 2019. No chronic disease was known. All participants underwent 3.0 Tesla MRI acquiring three unenhanced sequences: coronal T1-TSE, coronal PD-TSE and T1-3D-VIBE. MRIs were evaluated by three independent blinded radiologists: specific pediatric, senior, and resident. SBA was assessed using the common Greulich-Pyle (GP) atlas and Tanner-Whitehouse (TW2) method. Results were statistically processed by computing descriptive statistics, inter-rater reliabilities, T-Test, Wilcoxon-Mann-Whitney-Test and Bland-Altman-plots.
**Results or Findings:** Mean total acquisition time was 5:04±0:47 min. Image quality was sufficient in 63/63 (100%) cases. The T1-3D-VIBE sequence was determined as the preferred diagnosing sequence in 63/63 (100%). MRI appraisal was significantly faster (p<0.001) using GP with mean 1:22±0:08 min (range: 1:04-1:47 min) compared to TW with mean 7:39±0:28 min (range: 6:51-9:42 min). SBA assessment by GP resulted in mean age of 12.8±1.2 years (range: 10-16 years), by TW in 13.0±1.4 years (range: 10.6-16.6 years). Inter-rater reliabilities of all raters were excellent for both GP (ICC=0.912 (95%-CI = [0.868; 0.944]) and TW (ICC=0.988 (95%-CI = [0.980; 0.992]). Inter-rater ICCs were statistically significant (p<0.001).
**Conclusion:** MRI is a clinically feasible, radiation-free and rapidly evaluable method to assess SBA of healthy male children. Using the most common Greulich-Pyle (GP) atlas or the Tanner-Whitehouse (TW2) method reliable results are obtained independent of the radiologist's experience level.
**Limitations:** Due to the study design and the German law, we could not compare to the most common and investigated method conventional x-ray.
**Ethics committee approval:** The study was approved by local ethical committee and the scientific board of the DFB.
**Funding for this study:** No funding was received for this study.
**Author Disclosures:**
Lajos Basten: Nothing to disclose
Dennis Lüdin: Nothing to disclose
Dennis Murr: Nothing to disclose
Daniel Leyhr: Nothing to disclose
Sebastian Fischer: Nothing to disclose
Tatjana Gruber-Rouh: Nothing to disclose
Oliver Höner: Nothing to disclose
Michael Romann: Nothing to disclose
Katrin Eichler: Nothing to disclose

16:30-17:30 Room F1

### Research Presentation Session: Breast

### RPS 602

### Learning from mistakes: breast imaging in the pandemic

**Moderator**
F. Kilburn-Toppin; Cambridge/UK

### RPS 602-2

**Timeline for resolution of incidentally detected sonographic lymphadenopathy reactive to mRNA COVID-19 vaccination**

*Y. Adler- Levy*, G. Zeltzer, A. I. Zaben, S. Marks, N. Lioubashevsky, E. Chernovsky, T. Sella; Jerusalem/IL
*(ayal@hadassah.org.il)*

**Purpose:** COVID-19 vaccination with the Pfizer-BioNTech mRNA is associated with post-vaccination lymphadenopathy, but the timeline for its resolution remains unclear. This study describes the resolution pattern of incidentally detected post-vaccination lymphadenopathy in women referred for routine breast ultrasound examinations.
**Methods or Background:** Between January and March 2021, women referred for breast ultrasound, if demonstrating axillary lymphadenopathy of ≥3 mm cortical thickness, were questioned whether they underwent a recent COVID-19 vaccination. If vaccinated in the ipsilateral arm within 6 weeks, serial follow-up axillary ultrasound was performed every 3 weeks until lymphadenopathy resolved. Data was collected and analysed retrospectively.
**Results or Findings:** Forty-one women were included in the study. Time from the second COVID-19 vaccine injection ranged from 1 day to 6 weeks (mean 2.2; median 2). Cortical thickness at presentation ranged between 3 and 9 mm (mean 4.4). At 3-5 weeks of follow-up (n=38) lymphadenopathy resolved in 17/38 (45%), and persisted in 21/38 (55%) with cortical thickness between 3 and 7 mm. At 6-8 weeks (n=13), lymphadenopathy resolved in 7/13 (54%) and persisted in 6/13 (46%) with cortical thickness ranging between 3 and 5 mm. By 9 weeks (n=5) lymphadenopathy persisted in 3/5 (60%) women, consequently biopsied at 12 weeks, with benign pathology and documented resolution by 21 weeks. Overall, lymphadenopathy persisted ≥6 weeks in 32% and ≥9 weeks in 12%.
**Conclusion:** Post COVID-19 vaccination related sonographically detected axillary lymphadenopathy, persisted in one of three women for ≥6 weeks, and in 12% for ≥9 weeks. This timeline should be considered in guiding management.
**Limitations:** This study involved a relatively small series, with some women lost to follow-up during serial imaging.
**Ethics committee approval:** Due to the retrospective nature of study, informed consent was waived.
**Funding for this study:** This study received academic funding.
**Author Disclosures:**
Natali Lioubashevsky: Nothing to disclose
Galina Zeltzer: Nothing to disclose
Elena Chernovsky: Nothing to disclose
Yael Adler- Levy: Nothing to disclose
Ashwaq Issa Zaben: Nothing to disclose
Shelly Marks: Nothing to disclose
Tamar Sella: Nothing to disclose

### RPS 602-3

**Delays in breast care due to the COVID-19 pandemic**

*K. M. Capaccione*[1], S. Huang[1], A. Deng[2], E. West[1], M. M. Salvatore[1], E. Desperito[1]; [1]New York/US, [2]Charolette, NC/US
*(capacckm@gmail.com)*

**Purpose:** Studies have predicted excess breast cancer mortality as an indirect result of the COVID-19 pandemic. Here, we sought to identity which stage or stages of breast cancer diagnosis were delayed by the pandemic in order to identify vulnerable populations to target for comprehensive follow-up.
**Methods or Background:** Using the radiology search engine MModal Catalyst, we identified patients with BIRADS 0, 4, and 5 mammograms in 2020 and 2019 for comparison. Using the medical record system Epic, we identified dates of key stages of the mammographic abnormality workup to define time intervals. Additional demographic and clinical information were also collected. We analysed these data to assess if there were significant differences in time intervals or other characteristics in 2020 during the pandemic compared to the prior year.
**Results or Findings:** In 2019 the average follow-up interval of a BIRADS 0 mammogram was 31 days (SD= 59.9 days) compared to 140 days (SD= 131.3 days) in 2020, a statistically significant difference (p=0.000003). For patients with BIRADS 4 and 5 mammograms in 2019, the average time intervals from screening mammogram to diagnostic mammogram, biopsy, first surgical consult, and surgery were 12, 15, 33, and 66 days, respectively. In 2020, the same intervals were 24, 14, 36, and 51, respectively. None of these intervals were significantly different between 2019 and 2020.
**Conclusion:** Patients with BIRADs 0 mammograms in 2020 had significantly increased follow-up intervals. Targeting this population for close follow-up may reduce predicted morbidity and mortality stemming from the COVID-19 pandemic.
**Limitations:** Limitations of this study were that patients were drawn from a single health care system, and there was a relatively small sample size.
**Ethics committee approval:** Columbia University Human Research Protection Office and Internal Review Board Protocol AAAT5158 approved 12/18/2020.
**Funding for this study:** No funding was received for this study.
**Author Disclosures:**
Elizabeth West: Nothing to disclose
Kathleen Mary Capaccione: Nothing to disclose
Sophia Huang: Nothing to disclose
Aileen Deng: Nothing to disclose
Mary Margaret Salvatore: Speaker: Genentech, Boehringer Ingelheim Grant Recipient: Genentech, Boehringer Ingelheim
Elise Desperito: Nothing to disclose

### RPS 602-4

**Prevalence and time regression hypothesis of axillary lymphadenopathy (AL) in women undergoing a breast imaging examination at different time intervals after COVID-19 vaccination**

*C. Catanese*[1], A. L. Scarano[2], M. Marcon[1], S. Rizzo[1], M. Manganiello[1], F. Del Grande[1]; [1]Lugano/CH, [2]Bellinzona/CH
*(carolamialaura.catanese@eoc.ch)*

**Purpose:** To evaluate the prevalence of AL in patients for breast imaging examination at different time intervals after COVID-19 vaccination.
**Methods or Background:** In this IRB approved study women undergoing breast imaging from July to Semptember 2021 for any indication were enrolled prospectively. Exclusion criteria was a known metastatic lymphadenopathy. COVID-19 vaccination status, timing and side of vaccination were recorded. AL was considered positive with one or more of the following features in at least one lymphnode ipsilateral to the vaccinated arm: diffuse cortical thickening >3 mm; eccentric cortical thickening; rounded hypoechoic node; complete/partial effacement of fatty hilum; short axis >1 cm; long axis >2 cm. Participants were divided in subgroups based on the following time intervals after COVID-19 vaccination: (a)<6 weeks; (b) 7-8 weeks; (c) 9-10 weeks; (d) 11-12 weeks and (e) >12 weeks. Descriptive statistics and chi-square test with post-hoc comparison were performed to compare proportions. A p-value <0.05 indicated statistical significance.
**Results or Findings:** 162 consecutive women (median age, 56 years; range 23-84 years) were included with the following number of cases at different time interval after vaccination (a) 34 (21%); (b) 25 (15.4%); (c) 31 (19.1%); (d) 24 (14.9%) (e) 48 (29.6%). 44 women in total (27.2%) presented AL with: (a) 19/44 (43.2%); (b) 10/44 (22.7%); (c) 9/44 (20.4%); (d) 4/44 (9%); (e) 2/44 (4.5%). The differences were only significant for the time interval a and e (p-value (p<0.0001). The most common observed pathologic features were a diffuse or eccentric cortical thickening (41/44; 93.1%).
**Conclusion:** After COVID-19 vaccination, AL has the highest prevalence in the first 6 weeks whereas after 12 weeks it is only rarely observed. As literature suggested, 6 weeks after vaccination, AL should be regressed, so we advise to perform breast imaging in asymptomatic patients at least 6 weeks after the possible next vaccination.
**Limitations:** Not applicable
**Ethics committee approval:** This study was approved by an ethics committee.
**Funding for this study:** No funding was received for this study.
**Author Disclosures:**
Carola Catanese: Nothing to disclose
Filippo Del Grande: Nothing to disclose
Magda Marcon: Nothing to disclose
Stefania Rizzo: Nothing to disclose
Angela Lia Scarano: Nothing to disclose
Mario Manganiello: Nothing to disclose

### RPS 602-5

**Impact of the COVID-19 lockdown in France on the diagnosis and staging of breast cancers in a tertiary cancer centre**

*P-A. Linck*, C. Garnier, M-P. Depetiteville, S. Mathoulin-Pelissier, N. Quenel-Tueux, H. Charitansky, M. Boisserie-Lacroix, G. Mac Grogan, F. Chamming'S; Bordeaux/FR
*(pierre.linck@hotmail.fr)*

**Purpose:** To evaluate the impact of the lockdown in France on the diagnosis and staging of breast cancers diagnosed in a tertiary cancer centre.
**Methods or Background:** Our database was searched for all consecutive invasive breast cancers diagnosed in our institution during the lockdown period (36 working days), during equivalent periods of 36 working days immediately before and after the lockdown and during a reference period in 2019 (equivalent to these three periods in term of date range and number of working days). Number and staging of breast cancers diagnosed during and after the lockdown were compared to the pre-lockdown and the reference periods.
**Results or Findings:** In comparison with the reference period, the number of breast cancers diagnosed decreased by 20% during the lockdown. After the lockdown, breast cancers where more often palpable (p=0.001) and demonstrated bigger tumour size (p=0.0008). In comparison to the reference period, the rate of small tumours (T1) was reduced by 38% (p=0.01), the rate of locally advanced cancers (T3, T4) increased by 80% (p=0.04) and the rate of lymph node invasion increased by 64% (p=0.006).
**Conclusion:** The lockdown due to the covid-19 pandemic was associated with a 20% decrease in the number of diagnosed breast cancers. Because of delayed diagnosis, breast cancers detected after the lockdown had poorer prognosis with bigger tumour size and a higher rate of node invasion.
**Limitations:** We only evaluated the impact of the lockdown on the diagnosis of breast cancer in one institution. The study was conducted too early after the lockdown to be able to evaluate its impact on recurrence and mortality rates.
**Ethics committee approval:** Institutional Ethics Review Board approval was obtained for this retrospective study and informed consent was waived.
**Funding for this study:** This research received no specific grant from any funding agency.
**Author Disclosures:**
Pierre-Antoine Linck: Nothing to disclose
Gaëtan Mac Grogan: Nothing to disclose
Simone Mathoulin-Pelissier: Nothing to disclose
Foucauld Chamming'S: Nothing to disclose
Hélène Charitansky: Nothing to disclose
Cassandre Garnier: Nothing to disclose
Martine Boisserie-Lacroix: Nothing to disclose
Nathalie Quenel-Tueux: Nothing to disclose
Marie-Pierre Depetiteville: Nothing to disclose

### RPS 602-6

**"Which research project should I go for?" A strategic management tool for selecting your optimal research project**

*M. Dietzel*, R. Schulz-Wendtland, M. Uder, S. Ellmann; Erlangen/DE

**Purpose:** Successful research is essential for a career in academic radiology. "Selecting" the optimal research-project is therefore key for future success. This "selection" process may benefit from a structured, comprehensive, and objective methodology. Such methodologies are well established in the field of strategic management. Here, they support, for example, product development and marketing campaigns. However, strategic management tools (SMTs) specifically designed for academic radiology are lacking. We developed a SMT for the personalised evaluation of breast-imaging research projects.
**Methods or Background:** The SMT was developed based on principles of strategic management in four steps: First (1), the DEFINITION of essential requirements (brainstorming) (1). Second (2), the IDENTIFICATION of pre-existing tools (systematic search of literature) (2). Third (3), the DESIGN of the SMT as an open-access web-application based on results of steps (1) & (2). Fourth (4), TESTING of the SMT in a timely and controversial topic (breast MRI vs contrast enhanced spectral mammography/CESM)
**Results or Findings:** In step (1) Four perspectives summarising 18 attributes were identified: structure (cross-linked, semi-structured, hierarchical), assessment (extra/financial performance indicators, weighting), and criteria of the SMT were defined (stakeholders, timing, resources). In step (2), key elements of activity-based costing, balanced scorecard, diffusion theory (Rogers) and scoring-models where identified to support the SMT. In step (3), the SMT was designed based on the previous two steps and published open-access (http://35.178.93.39/shiny/rstudio/CESM/). In step (4), the SMT was successfully tested. In our personalised use-case the SMT substantiated strategic advantages of research projects related to CESM over breast MRI.
**Conclusion:** An open-access SMT for the personalised assessment of research-projects in breast-imaging was developed. Methodologies well-established in strategic-management were applied. Accordingly, the SMT provides structured decision support to academic radiologists in selecting optimal research projects.
**Limitations:** Validation studies evaluating the SMT within multiple intuitions, researchers, and research topics are pending.
**Ethics committee approval:** Not applicable
**Funding for this study:** Not applicable
**Author Disclosures:**
Stephan Ellmann: Nothing to disclose
Rüdiger Schulz-Wendtland: Nothing to disclose
Matthias Dietzel: Nothing to disclose
Michael Uder: Nothing to disclose

### RPS 602-7

**Axillary lymph node response after anti-COVID-19 vaccination: mRNA vs viral vector vaccines**

*I. Soriano Aguadero*[1], A. C. Igual Rouilleault[1], L. J. Pina Insausti[1], A. Elizalde[1], P. Quan Lopez[1], C. Sobrido[2]; [1]Pamplona/ES, [2]Madrid/ES
*(nachosoriagua@gmail.com)*

**Purpose:** The objective of this study was to compare the axillary lymph node reaction to anti-COVID-19 vaccination between mRNA and viral vector vaccines.
**Methods or Background:** Between February and July 2021, 512 employees from our centre were invited to participate in this prospective study after receiving complete vaccination with either Pfizer, Moderna or AstraZeneca. Patients were classified into two groups: mRNA (Pfizer and Moderna) and viral vector (AstraZeneca) vaccines. Volunteers were scanned with axillary US within the week after receiving the second vaccine injection. Multiparametric US assessment of lymph nodes included: total number of visible nodes, maximum measurements of the long-axis size and cortical thickness (mm), morphological Bedi's classification (grades 1 to 6) and colour Doppler evaluation (grades 0 to 3). Due to absence of normal distribution of variables, Mann-Whitney U test was used for statistical analysis.
**Results or Findings:** Out of 223 volunteers who accepted to participate in this study, 146 (65.5%) received mRNA vaccines (91 Pfizer and 55 Moderna); while the other 77 participants (35.5%) underwent AstraZeneca vaccination. Statistically significant differences ($p \leq 0.001$) were found in all the US findings evaluated, with higher values in the mRNA vaccine group: the total number of visible nodes (mean 6.38 vs 4.26), maximum diameter (mean 23.46 mm vs 20.58 mm), cortical thickness (mean 4.81 mm vs 2.92 mm), Bedi's classification (grades 4+5+6: 41.8% vs 11.7%) and Doppler (grades 2+3: 73.3% vs 62.3%).
**Conclusion:** Patients who received mRNA vaccines (Pfizer and Moderna) showed significantly higher values in all the axillary lymph node US findings evaluated, compared with viral vector vaccines (AstraZeneca).
**Limitations:** Other lymph node localisations (subclavian, submandibular…) were not assessed.
**Ethics committee approval:** Regional ethics committee approval and the written informed consent of all participants were obtained.
**Funding for this study:** Not applicable
**Author Disclosures:**
Paola Quan Lopez: Nothing to disclose
Ignacio Soriano Aguadero: Nothing to disclose
Arlette Elizalde: Nothing to disclose
Alba Cristina Igual Rouilleault: Nothing to disclose
Carolina Sobrido: Nothing to disclose
Luis Javier Pina Insausti: Nothing to disclose

### RPS 602-8

**A novel score to guide diagnostic and therapeutic path in breast cancer used during COVID-19 emergency: a valuable model applicable in the future**

*A. Abate*, C. Di Bella, M. Gisabella, C. Iacuzzo, C. Chifu, C. Pellitteri, R. Giovanazzi; Monza/IT
*(abate.anna75@gmail.com)*

**Purpose:** San Gerardo Hospital in Monza is one of the centres most affected by the COVID-19 pandemic in Italy. Many non-emergency services were discontinued, elective surgeries were postponed. Limited resources during the pandemic compelled rigorous discipline. Breast patients were classified into groups according to cancer risk, delivering accurate treatment using priority criteria, preventing overtreatment and preserving time and supplies.
**Methods or Background:** A score from 1 to 5 called Correlation Categories (CC) was assigned to patients matching biopsy results and radiological findings. A management score, called Action Categories (AC) ranging from standard follow-up to surgical/oncological treatment was assigned to patients on the basis of CC score during a multidisciplinary meeting discussion.
**Results or Findings:** Using this scoring system we decided the diagnostic and therapeutic path of 237 patients during the COVID-19 pandemic. The majority of the findings showed a concordance. In 2020, surgical activity has decreased in absolute numbers, but not in terms of rate. The 2020 cohort accounted for a higher number of CC1 and CC2 (49.4% and 26.6%) than the 2019 cohort (52.7% and 9.5%), a lower number of CC3 than in 2019 (10.1% versus 26%).

AC distribution did not show substantial differences, but AC4 action (larger volume biopsy) increased (8.9% from 2.3%).
**Conclusion:** This score was successfully applied during the COVID-19 pandemic in our breast unit, reducing procedure repetition, avoiding non-urgent interventions and planning the most suitable follow-up. We believe it could be useful in non-emergency times, especially in low resource environments, with limited treatment facilities and hospitals with a lack of equipment.
**Limitations:** Not applicable
**Ethics committee approval:** Not applicable
**Funding for this study:** Not applicable
**Author Disclosures:**
Cristina Pellitteri: Nothing to disclose
Camillo Di Bella: Nothing to disclose
Mara Gisabella: Nothing to disclose
Camelia Chifu: Nothing to disclose
Anna Abate: Nothing to disclose
Riccardo Giovanazzi: Nothing to disclose
Cristiana Iacuzzo: Nothing to disclose

**16:30-17:30** **Room K**

### Research Presentation Session: Cardiac

### RPS 603

### Advances in cardiac MR infarct imaging

**Moderator**
I. Carbone; Rome/IT

### RPS 603-2

**Association between cine CMR-based radiomics signature and microvascular obstruction in patients with ST-segment elevation myocardial infarction**

J. Gong, *Y. Peng*; Shenzhen/CN

**Purpose:** To investigate the association between cine cardiac magnetic resonance (CMR)-based radiomics signature and microcirculatory obstruction (MVO) in patients with ST-segment elevation myocardial infarction (STEMI).
**Methods or Background:** In this retrospective study, 116 consecutive patients with STEMI, who underwent CMR within 6 days after PCI, were retrospectively enrolled. According to the late gadolinium enhancement (LGE) of CMR, the myocardial infarction (MI) was dichotomised into with and without MVO. Radiomic features were extracted from cine CMR images and a least absolute shrinkage and selection operator (LASSO) algorithm was used for features selection and radiomic signatures construction. Binary logistic regression was used to assess between radiomic signatures and MVO with adjusted for baseline clinical characteristics.
**Results or Findings:** Of 116 patients with STIMI, MI with MVO was found in 50 patients and MI without MVO was found in 66 patients. LASSO regression selected five radiomics features for radiomics signature construction. Logistic regression revealed that radiomics score, high sensitivity C-reactive protein (hs-CRP) and creatine phosphokinases (CPK) were independent risk factors for MVO with odds ratio (OR) of 4.41 (95%CI: 2.26-9.93), 1.02 (95% CI: 1.00-1.01) and 1.03 (95% CI 1.000-1.001), respectively. Area under curve (AUC) of receiver operating characteristic (ROC) of radiomics score to predict MVO was 0.75 (95% CI: 0.68-0.85).
**Conclusion:** Cine CMR-based radiomics signature was an independent predictive factor of MVO in patients with STEMI, which showed the potential of this contrast free radiomics signature to be an imaging biomarker for MVO.
**Limitations:** Although cine CMR-based radiomics signature had the highest odds ratio (4.41 [95% CI 2.26-9.93]) for MVO in this study, only medium predictive preformance (a ROC-AUC of 0.75 [95% CI: 0.68-0.85]) was obtained.
**Ethics committee approval:** This retrospective study was approved by the Institutional Review Board.
**Funding for this study:** Funding was received from the Shenzhen Science and Technology Project (No. GJHZ20180928172002087).
**Author Disclosures:**
Jingshan Gong: Nothing to disclose
Yongjia Peng: Nothing to disclose

### RPS 603-3

**Cine MR-based radiomics to predict myocardial segments with infarction**

*J. Gong*, Y. Peng; Shenzhen/CN
*(jshgong@sina.com)*

**Purpose:** To harness cine CMR-based radiomics to predict myocardial segments with infarction.
**Methods or Background:** Forty-eight patients with acute myocardial infarction (AMI) confirmed by late gadolinium enhancement (LGE) on CMR were included. Radiomic features of the myocardial segments were extracted from the cine CMR images, and the myocardial segments were randomly divided into training and validation sets at a ratio of 0.7:0.3. A least absolute shrinkage and selection operator (LASSO) algorithm was used for feature selection in the training set. Radiomic signatures were constructed in both the training and validation sets, and their predictive performance was assessed using the area under the receiver operating characteristic curve (AUC-ROC).
**Results or Findings:** Of 768 myocardial segments in the 48 patients, there were 291 (38%) segments with MI and 477 (62%) segments without myocardial infarction (MI). After univariate analysis, there were 22 radiomics features related to MI with statistical significance. LASSO regression selected 18 radiomics features for inclusion in the radiomics signature. The AUC-ROC of the radiomic signature for predicting segments with MI was 0.74 (95% CI: 0.69-0.78) and 0.68 (95% CI: 0.60-0.75) in the training and validation sets, respectively. The difference was not statistically significant (p=0.14).
**Conclusion:** Cine MR-based radiomics signatures can achieve good prediction performance for MI and show potential as promising imaging biomarkers for MI with no need for contrast agents.
**Limitations:** Although cine CMR-based radiomics signature could be a contrast-free biomarker to predict myocardial segment with MI, segmentation was time-consuming.
**Ethics committee approval:** This retrospective study was approved by the Ethical Committee of Shenzhen People's Hospital.
**Funding for this study:** Funding was received from the Shenzhen Science and Technology Project (No. GJHZ20180928172002087).
**Author Disclosures:**
Jingshan Gong: Nothing to disclose
Yongjia Peng: Nothing to disclose

### RPS 603-4

**Assessment of the area-at-risk and infarcted-area by T1 mapping sequences in patients with acute myocardial infarction**

*G. C. Pambianchi*[1], N. Galea[1], G. Cundari[1], L. Conia[1], L. Vero[1], M. Francone[2], C. Catalano[1]; [1]Rome/IT, [2]Milan/IT
*(giacomo.pambianchi@gmail.com)*

**Purpose:** Validate the use of myocardial nT1 values for the differentiation and quantitative measurement of the area-at-risk (AAR) and infarcted-area (IA) in patients with AMI (STEMI and NSTEMI).
**Methods or Background:** 29 patients diagnosed with AMI underwent CMR within 10 days of the myocardial infarction. The acquisition protocol included T1 MOLLI, STIR-T2 weighted and PSIR sequences acquired after administration of contrast agent (LGE). The AAR and the IA were identified and measured as areas of increased signal in the STIR-T2 and LGE images. nT1 values were measured in the RA, in the IA in the remote myocardium (RM) and analysed using ANOVA and independent samples t-test. Therefore, the analysis of the ROC curves was carried out to evaluate the capability of nT1 values to distinguish between AAR, IA and RM.
**Results or Findings:** Myocardial T1 values measured in the AAR (1145.6±71.4 ms), the IA (1335.2±76.4 ms), the RM (1025.4±44.8 ms) and in the ventricular cavity (1617.9±102.6 ms) were significantly different from each other ($p<0.001$ for all comparisons). Analysis of ROC curves demonstrated excellent performance of myocardial nT1 to distinguish between the AAR and the RM (AUC: 0.972, $p<0.001$) and between the IA and the AAR (AUC: 0.983, $p<0.001$), with thresholds of 1086 ms (Se: 96.7%, Sp: 91.4%) and 1200ms (Se: 94.8%, Sp: 96.7%) respectively. Applying these threshold values to nT1 maps, the volumes of AAR and IA showed a high correlation with those respectively obtained from the STIR-T2 and LGE images (Pearson's r: 0.927 for the AAR and 0.859 for the IA, $p<0.001$ for both).
**Conclusion:** The T1 mapping sequences allow identification and precise measurement of the extent of myocardial AAR and IA in patients with AMI.
**Limitations:** The small population and manual semi-automatic segmentation of the areas were identified as limitations.
**Ethics committee approval:** This study was approved by an ethics committee.

**Funding for this study:** No funding was received for this study.
**Author Disclosures:**
Giacomo Carlo Pambianchi: Nothing to disclose
Giulia Cundari: Nothing to disclose
Luca Conia: Nothing to disclose
Marco Francone: Nothing to disclose
Laura Vero: Nothing to disclose
Nicola Galea: Nothing to disclose
Carlo Catalano: Nothing to disclose

### RPS 603-5

**Evolution of myocardial tissue injury over a decade after ST-elevation myocardial infarction: a CMR study**

*A. Mayr*, G. Klug, M. Pamminger, P. Poskaite, F. Troger, M. Reindl, S. Reinstadler, B. Metzler; Innsbruck/AT

**Purpose:** This study examined myocardial tissue injury dynamics over a decade after ST-elevation myocardial infarction (STEMI).
**Methods or Background:** Sequential CMR examinations (within the first week after STEMI and at 4, 12 months and 9 years thereafter) were conducted in 74 patients with STEMI treated with primary percutaneous coronary intervention. Left ventricular (LV) function, infarct size (IS) and microvascular obstruction (MVO) were assessed at all time-points. T2* and T2 mapping (n=59) were added at 9 year scan to evaluate the presence of iron and oedema within the infarct core, respectively.
**Results or Findings:** IS decreased progressively and significantly between all CMR time-points (all p<0.001), with an average reduction rate of 5.8% per year [IQR 3.5-8.8] and a relative reduction of 49% [IQR 39-76] over a decade. MVO was present in 61% of patients at baseline, but in none of the follow-up examinations. At 9 year CMR, 17/59 patients (29%) showed iron deposition within the infarct core, whereof 82% revealed persistence of oedema. Persistent iron and oedema, respectively, was associated with greater infarct size on any occasion (all p<0.001) as well as presence of MVO (p<0.001). Patients with persistent iron and oedema showed a lower relative regression of IS (p=0.005 and p=0.032, respectively) and greater end-systolic volumes over a decade (all p<0.012 and p>0.023, respectively).
**Conclusion:** The evolution of IS is a dynamic process that extends well beyond the first few months after STEMI. Persistence of iron and oedema within the infarct core occurs up to a decade after STEMI and is associated with initial infarct severity and poor infarct healing.
**Limitations:** No limitations were identified.
**Ethics committee approval:** The study received approval by the local research ethics committee.
**Funding for this study:** The study was supported by grants from the 'Austrian Society of Cardiology'.
**Author Disclosures:**
Gert Klug: Nothing to disclose
Mathias Pamminger: Nothing to disclose
Martin Reindl: Nothing to disclose
Felix Troger: Nothing to disclose
Sebastian Reinstadler: Nothing to disclose
Paulina Poskaite: Nothing to disclose
Agnes Mayr: Nothing to disclose
Bernhard Metzler: Nothing to disclose

### RPS 603-6

**Accordance of infarct localisation between 12-lead electrocardiography and cardiac magnetic resonance imaging in ST-elevation acute myocardial infarction**

J. E. Park, *J-H. Choi*; Seoul/KR

**Purpose:** This study aimed to establish the assignment of 12-lead electrocardiography (ECG) for localisation of myocardial infarction validated by cardiac magnetic resonance imaging (CMR).
**Methods or Background:** We enrolled a total of 349 patients with ST-elevation myocardial infarction (STEMI) who underwent CMR after primary percutaneous coronary intervention. ST-elevation in each of 12 leads was compared with the presence of late gadolinium enhancement (LGE) using the left ventricular myocardial 17-segment model.
**Results or Findings:** LGE was found in 6±3 segments per patient, and in 2,109 (36%) out of a total of 5,933 myocardial segments. Overall, 1 out of every 5 myocardial segments with LGE did not match with the empirically assigned ECG lead (N=423, 20%). In per-lead analysis, no myocardial segments matched 100% with a specific ECG lead. Leads I, aVR, and aVL corresponded not only to LGE in lateral myocardial segments but also to LGE anterior myocardial segments. We developed a new modified assignment of 12-lead ECG to myocardial 17-segment model. It could additionally match a total of 212 myocardial segments (10%) to ECG leads, and increased the rate of matching between ST-elevation of 12-lead ECG and myocardial 17-segment model from 80 to 90%.
**Conclusion:** ST elevation in leads I, aVR, and aVL corresponded to both anterior and lateral wall myocardial infarction. A newly proposed ECG assignment could correctly reclassify the location of myocardial infarction in 1 out of 10 myocardial segments. Our result suggests that the current standard assignment of 12-lead ECG for infarction localisation may require reappraisal.
**Limitations:** This study was performed at a single centre with a single CMR scanner.
**Ethics committee approval:** The study was approved by the Institutional Review Board of Samsung Medical Center.
**Funding for this study:** This research received no external funding.
**Author Disclosures:**
Jong Eun Park: Nothing to disclose
Jin-Ho Choi: Nothing to disclose

### RPS 603-7

**Simplified image acquisition and detection of ischemic and non-ischemic myocardial fibrosis with fixed short inversion time magnetic resonance late gadolinium enhancement**

*M. Polacin*, M. Karolyi, M. Eberhard, C. Blüthgen, S. Kozerke, H. Alkadhi, R. Manka; Zurich/CH

**Purpose:** Magnetic resonance late gadolinium enhancement with fixed short inversion time (LGE_short) provides excellent tissue contrast with dark scar, grey blood pool, very bright remote myocardium and does not need prior myocardial nulling. We hypothesise better visibility of ischaemic scars and equal visibility of non-ischaemic LGE in LGE_short compared to clinically established LGE (LGE_standard).
**Methods or Background:** LGE_short (fixed inversion time at 110 ms at 1.5 T) and LGE_standard (individually adjusted inversion time) were retrospectively evaluated in 179 patients (3043 segments) with suspected or known coronary artery disease by four blinded readers (reader A: most experienced - D: least experienced). Amount of ischaemic and non-ischaemic LGE as well as visibility (1: very good – 4: poor) of ischaemic LGE was visually assessed.
**Results or Findings:** All readers detected more infarcted segments in LGE_short compared to LGE_standard (378 segments reported as infarcted; A: p= 0.5, B: p= 0.8, C, D: p= 0.03). Scar visibility was scored higher in LGE_short by all readers (A, B: p= 0.03; C, D: p= 0.02), especially visibility of subendocardial infarcts (A, B: p= 0.04, C, D: p= 0.02). Less experienced readers detected significantly more infarcted papillary muscles (C: p= 0.02, D: p= 0.03) and had reduced dataset reading time in LGE_short (C: p=0.04, D: p=0.02). Non-ischaemic LGE was equally visible in both sequences (A: p=0.9, B: p=0.8, C, D: p= 0.6).
**Conclusion:** LGE_short with its maximal operational simplicity detects more ischaemic LGE with improved scar visibility compared to LGE_standard, independent of experience level. The visibility of non-ischaemic LGE is equivalent to LGE_standard. Less experienced readers can diagnose ischaemic and non-ischaemic LGE faster in LGE_short. LGE_short can be used for visualisation of all types of fibrosis – ischaemic and non-ischaemic - instead of LGE_standard, independent of experience level.
**Limitations:** The retrospective nature of the study was identified as a limitation.
**Ethics committee approval:** This retrospective study was approved by local authorities.
**Funding for this study:** No funding was received for this study.
**Author Disclosures:**
Mihaly Karolyi: Nothing to disclose
Sebastian Kozerke: Nothing to disclose
Matthias Eberhard: Nothing to disclose
Christian Blüthgen: Nothing to disclose
Robert Manka: Nothing to disclose
Malgorzata Polacin: Nothing to disclose
Hatem Alkadhi: Nothing to disclose

### RPS 603-8

**Myocardial scar detection in free-breathing Dixon-based fat- and water-separated 3D inversion recovery late-gadolinium enhancement whole heart MRI**

*A. A. Peters*[1], B. Wagner[1], G. Spano[1], F. Haupt[1], L. Ebner[1], M. Schmidt[2], K. Kunze[2], C. Gräni[1], A. Huber[1]; [1]Bern/CH, [2]Erlangen/DE

**Purpose:** Cardiac magnetic resonance imaging is the gold standard for non-invasive detection and quantification of myocardial fibrosis and scars. The aim of this study was to investigate the diagnostic accuracy and reader confidence for late-gadolineum enhancement (LGE) detection of a novel free breathing, respiration-gated 3D LGE sequence with fat-water separation, in comparison to a free breathing motion-corrected 2D LGE sequence in patients with cardiomyopathy.
**Methods or Background:** Patients undergoing cardiac MRI containing both sequences were enrolled. Two independent blinded readers analysed both sequences and compared them regarding image quality, LGE detection/quantification, SNR/CNR. Results were compared by Wilcoxon test for paired samples. For the LGE detection and confidence rating, a JAFROC analysis with fixed readers and random cases was performed, JAFROC figure of merit (FOM) calculated as the area under the AFROC curve.

**Results or Findings:** 47 patients were included (technical failure rate 5/47). The mean sequence times of the 2D sequence were significantly shorter compared to the 3D sequence (p<0.001). 3D-LGE sequences were significantly superior for evaluation of fine anatomical structures such as the atria or pericardium. JAFROC analysis revealed a significantly higher FOM for the 3D LGE-sequence (FOM=0.84) than the 2D LGE-sequence (FOM = 0.77; p=0.003). There were no differences in overall image quality or all the other analysed parameters.
**Conclusion:** The 3D LGE sequence allows the accurate LGE detection with higher confidence and better delineation of fine anatomical structures. Scan acquisition time for the 3D sequence is just slightly longer than the 2D sequence. 3D LGE is ready for implementation in clinical routine protocols.
**Limitations:** The small sample size, the retrospective nature of the analysis as well as the heterogeneity of the underlying etiology (ischemic and non-ischemic cardiomyopathies) were identified as limiting factors.
**Ethics committee approval:** This study was approved by the local institutional review board.
**Funding for this study:** No funding was received for this study.
**Author Disclosures:**
Fabian Haupt: Nothing to disclose
Alan Arthur Peters: Nothing to disclose
Giancarlo Spano: Nothing to disclose
Christoph Gräni: Nothing to disclose
Karl Kunze: Nothing to disclose
Adrian Huber: Nothing to disclose
Michaela Schmidt: Nothing to disclose
Ben Wagner: Nothing to disclose
Lukas Ebner: Nothing to disclose

**16:30-17:30** **Room M 1**

### Research Presentation Session: Interventional Radiology

### RPS 609

### Chemoembolisation and radioembolisation of the liver: new developments

**Moderator**
I. E. Keussen; Stockholm/SE

### RPS 609-2

**Does the use of angio-CT vs cone-beam CT influence the radiation exposure during transarterial chemoembolisation for liver cancer?**
*M. Nowak*, A. Viry, R. Holderbaum do Amaral, G. Heymann, A. F. Ponti, S. Saltiel, N. Villard, G. Tsoumakidou, R. Duran; Lausanne/CH
*(marie.nowak@chuv.ch)*

**Purpose:** The use of cone-beam CT (CBCT) has improved the accuracy of image-guided procedures, in particular intra-arterial therapies such as transarterial chemoembolisation (TACE). More recently, a CT-scanner coupled with a C-arm system has been used in this setting to perform angio-CTs. Although these technological evolutions allow for a better image quality and tumour targeting, they also impact patient radiation exposure. The aim of our study was to compare CBCT and CT modalities on patient exposure during TACE for liver cancer.
**Methods or Background:** A retrospective analysis was performed of patients who underwent TACE with either CBCT (Philips Allura Xper FD20) or Angio-CT (Canon Alphenix 4D-CT) systems at Lausanne University Hospital (2015-2021). Clinical (e.g. BMI) and dosimetric data (e.g. dose area product or dose length product) were collected, and tumour response (modified RECIST) was assessed and compared between the imaging modalities. Effective doses were calculated by multiplying the dosimetric indicators specific to each modality by the associated conversion factor.
**Results or Findings:** Ongoing analysis of 132 TACEs performed (CBCT, n=55; angio-CT, n=77) in 53 patients. Total patient radiation exposure (2D+3D imaging) was 1.4 times lower with Angio-CT when compared to CBCT (median 27.1 vs 37.7mSv) with a clear trend (p=0.076). Angio-CT delivered 17% more dose than CBCT on 3D-imaging (median 19.2 vs 16.4mSv) although this was not statistically significant (median 19.2 vs 16.4mSv; p=0.169). Dose gain was mainly due to fluoroscopy and digital subtraction angiography which was 2.2 times less irradiating with Angio-CT (p<0.001). Treatment targeting (p=0.376) and tumor response (p=0.764) were similar between the groups.
**Conclusion:** A clear trend showed that Angio-CT achieved less patient radiation (2D+3D imaging) than CBCT with similar efficacy. More patients are currently being included.
**Limitations:** Retrospective, single-centre study.
**Ethics committee approval:** IRB-approved study (CER-VD-2021-02213).
**Funding for this study:** No funding was received for this study.
**Author Disclosures:**
Sarah Saltiel: Nothing to disclose
Ricardo Holderbaum do Amaral: Nothing to disclose
Georgia Tsoumakidou: Nothing to disclose
Marie Nowak: Nothing to disclose
Anaïs Viry: Nothing to disclose
Rafael Duran: Nothing to disclose
Dre Greicy Heymann: Nothing to disclose
Nicolas Villard: Nothing to disclose
Alexandre Flavien Ponti: Nothing to disclose

### RPS 609-3

**Hepatic changes following selective internal radiation therapy for liver metastases: a quantitative volumetric assessment at the segment level**
*K. Oung*, Y. Aleman, A. Digklia, N. Halkic, N. Villard, G. Tsoumakidou, S. Boughdad, N. Schaefer, R. Duran; Lausanne/CH

**Purpose:** To quantitatively assess volumetric liver changes in patients with liver metastases treated with Yttrium90-selective internal radiation therapy (SIRT).
**Methods or Background:** Single-centre, institutional review board approved, retrospective study of a prospectively collected database (2010-2020). Patients with liver metastases treated with SIRT and who underwent CT-scan before and 3, 6, 9, 12 months after treatment were included. A 3D quantification of individual liver segments, whole liver, tumour burden and spleen volume was performed for each time-point using a semi-automatic software. Liver segment volumes were precisely obtained based on portal/hepatic vein anatomy. Clinical, laboratory, imaging and SIRT data were analysed.
**Results or Findings:** 142 volumetric assessments were performed on 47 patients. SIRT was administered to the whole liver in 23 patients, right/left liver in 12/6 patients, and other (sectorial/sub/segmental) in 6 patients. Treated liver volume decreased significantly at 3 months from 932+/-367 mL to 865+/-324 mL (p<0.05) whereas untreated liver region volumes increased. When receiving whole liver treatment, liver volume did not change significantly over time (all time-points, p>0.05). Complex segment changes were observed. Tumour volume did not change significantly over time (all time-points p>0.05). Spleen volume increased significantly at all time-points, from baseline 238+/-35 mL to 288+/-63 mL at 12 months (all p<0.05). The evolution of liver, tumour and spleen volumes was not significantly correlated with the administered Y-90 activity at the different time-points (p>0.05).
**Conclusion:** Dynamic and complex changes were observed in liver and spleen volumes of patients with liver metastases treated with SIRT.
**Limitations:** The limitation is the study's retrospective, monocentric design.
**Ethics committee approval:** This study was approved by an ethics committee.
**Funding for this study:** No funding was received for this study.
**Author Disclosures:**
Georgia Tsoumakidou: Nothing to disclose
Yasser Aleman: Nothing to disclose
Sarah Boughdad: Nothing to disclose
Niklaus Schaefer: Nothing to disclose
Nermin Halkic: Nothing to disclose
Antonia Digklia: Nothing to disclose
Rafael Duran: Nothing to disclose
Karine Oung: Nothing to disclose
Nicolas Villard: Nothing to disclose

### RPS 609-4

**Transradial versus transfemoral access for transarterial chemoembolisation in patients with hepatocellular carcinoma: a randomised trial**
*Y. Luo*, X. Zhang, J. Tsauo, X. Li; Beijing/CN

**Purpose:** To compare patient satisfaction, procedural variables, and safety with transradial access (TRA) and transradial access (TRA) in patients undergoing transarterial chemoembolisation (TACE) for hepatocellular carcinoma (HCC).
**Methods or Background:** From February 2019 to August 2021, 130 patients undergoing TACE for HCC were randomly allocated to the TRA (n = 65) or TFA (n = 65) group. All patients completed the post-catheterisation questionnaire and 8-item short-form health survey 1 day after TACE.
**Results or Findings:** Technical success rate, crossover rate, contrast agent dose, fluoroscopy time, procedure time, air kerma, dose-area product, length of hospital stay, and total cost were similar between the two groups (all P > .05). The incidence and severity of adverse events were also similar between the two groups (all P > .05). However, overall discomfort, difficulty going to the bathroom, difficulty feeding or self-caring, difficulty walking, general health, physical function, role physical function, social function, mental health, and role emotional function was better in the TRA group than in the TFA group (all P <

Wednesday

.001). Consequently, more patients preferred the current access for their next procedure in the TRA group than in the TFA group (90.8% vs. 24.6%; P < .001).
**Conclusion:** In patients undergoing TACE for HCC, using TRA instead of TFA can improve patient satisfaction without compromising procedural variables and safety.
**Limitations:** First, most patients had HCC caused by hepatitis B virus. Second, the efficacy of TRA-TACE and TFA-TACE were not compared. Last, the patients and the investigators could not be blinded to allocation.
**Ethics committee approval:** This study was approved by the institutional review board.
**Funding for this study:** This study was supported by a grant from the Beijing Hope Run Special Fund of Cancer Foundation of China (grant No. LC2017B09).
**Author Disclosures:**
Xiao Li: Nothing to disclose
Yingen Luo: Nothing to disclose
Xiaowu Zhang: Nothing to disclose
Jiaywei Tsauo: Nothing to disclose

### RPS 609-5

**A review of MRI appearances of lipiodol in conventional TACE (cTACE) treated hepatocellular carcinomas**
Z. A. Khan, A. Rana, *M. Rauf*; Islamabad/PK
*(mari23392@gmail.com)*

**Purpose:** Lipiodol TACE has become standard treatment for unresectable HCC without vascular invasion. Lipiodol is used as imaging biomarker for computed tomography due to its radiopaque nature. However, data regarding lipiodol TACE imaging via MRI is limited and results are not familiar to radiologists for regular assessment.
**Methods or Background:** We assessed patterns of retention of lipiodol upon different MRI sequences. After taking approval from IRB-EC, all patients who underwent lipiodol TACE, having both 4-6-week post-treatment CT and MRI were included. 25 patient fulfilled criterion. Following confirmation of presence of lipiodol on CT scan, pattern of signal intensities of lipiodol was noted on all MRI sequences.
**Results or Findings:** On T1-weighted MRI, 15 lipiodol retention areas were hyperintense, 7 appeared hypointense while 3 were isointense. On T2-weighted images, 13 appeared hypointense, 8 hyperintense, 1 isointense and 3 showed mixed intensity signals. Restricted diffusion was observed in 2 lesions, facilitated diffusion in 4 lesions, 7 lesions were low on both DWI, ADC while 12 exhibited no specific pattern. Fat suppressed images showed 19 being hypointense, 3 hyperintense and 3 remained isointense.
**Conclusion:** Fat-suppressed T1 were most sensitive in detecting lipiodol retention. On T1-weighted images, lipiodol retention areas appeared hyperintense in 60% of the lesions and on T2 weighted images only 52% of the lesions were hypointense. Diffusion-weighted imaging sequences give a much more variable appearance. Results indicate that while MRI remains most useful and reliable imaging modality for assessing HCC's following cTACE however requires cautious interpretation with knowledge of the variable signal appearance seen on different imaging sequences.
**Limitations:** The limitation is the small sample size.
**Ethics committee approval:** This study was approved by an ethics committee.
**Funding for this study:** No funding required.
**Author Disclosures:**
Atif Rana: Nothing to disclose
Zahid A Khan: Nothing to disclose
Maria Rauf: Nothing to disclose

### RPS 609-6

**Comparison between C-arm cone beam computed tomography and interventional angiography in trans-arterial chemo-embolisation of hepatocellular carcinoma**
*I. A. Diab*; Shebin Elkom/EG
*(dr.diab2@gmail.com)*

**Purpose:** Utomography and interventional angiography in transarterial chemoembolisation of hepatocellular carcinoma.
**Methods or Background:** By using conventional angiography in TACE, we can detect and identify the vascular anatomy of the liver by obtaining 2D images. Recently C-arm cone-beam computed tomography (CBCT) is introduced for obtaining cross-sectional and three-dimensional (3D) images for better visualisation. A prospective study was done on patients diag-nosed with HCC by imaging and laboratory criteria, re-ferred to do TACE as the recommended treat-ment. Inclusion criteria were HCC patients with well-preserved liver function who refused the surgery or who were not suitable for resection, liver transplantation, or percutaneous ablation.
**Results or Findings:** The number of detected focal lesions by angiography was 51 compared to 87 focal lesion detected by CBCT; of those, 45 and 77 were active lesions by both procedures respectively. For lesions, less than 1 cm CBCT detected 23 lesions while angiography detected only one lesion. Angiography detected 87 feeding arterial branches while cone-beam CT-HA detected 130 branches to the same number of target lesions. Feeder tractability and confidence were better by CBCT.
**Conclusion:** CBCT is superior to angiography in tumor detectability, detection of lesions less than 1 cm, feeder detection, and feeder traction; however, conventional angiography and DSA are irreplaceable. Thus, combination of CBCT with angiography during TACE produces better results and less complication.
**Limitations:** The limitation is the small sample size.
**Ethics committee approval:** This study was approved by the Research Ethics Committee of the Faculty of Medicine at Menoufia University in Egypt on 10 October 2017; reference number of approval: 19719RADIO. All patients included in this study gave written informed consent to participate in this research.
**Funding for this study:** This study had no funding from any resource.
**Author Disclosures:**
Ibrahim Ahmed Diab: Author: This study

### RPS 609-7

**Radiochemical feasibility of mixing of Y90 and iohexol contrast for radioembolisation therapy**
*H. H. Liang*, C. Yang, P. I. Ngam, A. Gogna, D. C. E. Ng; Singapore/SG

**Purpose:** To confirm radiochemical feasibility of mixing Y90 microspheres with iohexol to allow real-time visualisation of radioembolisation under fluoroscopy.
**Methods or Background:** Yttrium-90 (Y90) microspheres used to treat liver dominant malignant tumours are infused via catheter into the hepatic artery branches under fluoroscopic guidance. Currently, the microspheres are suspended in radiolucent dextrose 5% (D5) solution which precludes real-time monitoring of their implantation. We investigated whether mixing Y90 microspheres in different concentrations of iohexol diluted with D5 or normal saline is radiochemically acceptable in terms of stability of the Y90 microspheres, leaching of Y90 from the microspheres, and creation of potential by-products. Y90 microspheres (~5 mCi) were suspended in iohexol (350 mg/ml) with D5 or saline solution at various concentrations. Respective mixtures were left to stand for 4 hours and the radiochemical purities determined by instant thin layer chromatography (iTLC) as the stationary phase and saline as the mobile phase. The mixtures were passed through a 0.22 micrometer filter and the filtrate measured for radioactivity compared to the residue. As the microspheres sizes range from 20 to 60 micrometers, the filtrate would only contain leached Y90 activity.
**Results or Findings:** Radiochemical purities on iTLC ranged from 90.92% when iohexol was diluted to 75% with normal saline, to 98.11% when iohexol was diluted to 50% with D5. No significant photopeaks to suggest by-product radioactive species were detected. The proportion of Y90 activity in the residue after filtration was 99%, 96%, 98%, 95% and 93%, in 100%/0% iohexol/D5, 75%/25% iohexol/D5, 50%/50% iohexol/D5, 25%/75% iohexol/saline and 50%/50% iohexol/saline, respectively. Thus, leaching of Y90 from microspheres was less than 5% with D5.
**Conclusion:** Suspension of Y90 in iohexol contrast results in acceptable radiochemical purity, particularly with D5.
**Limitations:** The limitation is that the study is preclinical.
**Ethics committee approval:** Not required.
**Funding for this study:** No funding was received for this study.
**Author Disclosures:**
Apoorva Gogna: Nothing to disclose
David Chee Eng Ng: Other: Sirtex Medical - received honoraria
Changtong Yang: Nothing to disclose
Huang Hian Liang: Nothing to disclose
Pei Ing Ngam: Nothing to disclose

### RPS 609-8

**Efficacy and safety of transarterial chemoembolisation with DC Beads LUMI in the treatment of HCC: experience from a tertiary centre**
*C. Lanza*, S. Carriero, P. Biondetti, A. M. Ierardi, G. Carrafiello; Milan/IT

**Purpose:** The aim of this study was to describe safety and efficacy profiles of TACE using DC Beads LUMI.
**Methods or Background:** Were retrospectively analysed 90 patients with HCC who underwent TACE with DC Bead LUMI. Were evaluated number of treated lesions, dose of DC Beads LUMITM, dose of Epirubicin and DC Beads LUMITM target tumour coverage. The efficacy assessment of TACE was based on reviewing the follow-up imaging for evidence of response in target lesion(s), according to modified response criteria in solid tumours (mRECIST) with following outcomes: complete response (CR), partial response (PR), stable disease (SD) and progressive disease (PD). Safety assessment was classified according to CIRSE classification.

Wednesday

**Results or Findings:** 72 patients were enrolled, 95 procedures were carried out. Target tumour response rate at 1 month has CR, PR, SD, PD of respectively 67,3%, 10,3%, 13,1% and 4,7%. Overall tumour(s) (whole liver) response at 1 month has CR, PR, SD, PD of respectively 58,9%, 12,6%, 10,5% and 17,9%. We found a significant association ($p<0.01$) between tumour CR or CR + PR and number of target lesion(s). CIRSE classification grade I and II complications were recorded in 11.6% and 6.3% procedures. No grade III-IV-V complications occurred.
**Conclusion:** TACE using DC Beads LUMI is a safe and effective treatment option for patients with HCC.
**Limitations:** The limitations of this study are its retrospective nature, limited simple size, and the absence of the control group, which means the impossibility of demonstrating the relative performance of the LUMI with other beads.
**Ethics committee approval:** All procedures performed were in accordance with the 1964 Helsinki Declaration and its later amendments or comparable ethical standards. Institutional review board approval was obtained for this retrospective review.
**Funding for this study:** The authors received no financial support for this study.
**Author Disclosures:**
Anna Maria Ierardi: Nothing to disclose
Pierpaolo Biondetti: Nothing to disclose
Serena Carriero: Nothing to disclose
Carolina Lanza: Nothing to disclose
Gianpaolo Carrafiello: Nothing to disclose

**16:30-17:30** **Room M 2**

### Research Presentation Session: Musculoskeletal

### RPS 610

### Advances in imaging upper extremity

**Moderator**
I. Engele; Riga/LV

### RPS 610-2

**Our experience of cone-beam CT in the management of acute radiocarpal injury**

*M. A. Barry*, G. Orpen, E. Kenny, P. J. Macmahon; Dublin/IE
*(meallabarry@mater.ie)*

**Purpose:** To share our experiences in incorporating cone-beam CT (CBCT), into the evaluation of patients with acute radiocarpal injury. The use of CBCT in patients with suspected radiographically occult fractures optimises visualisation of the carpal bones with a minimal increase in radiation dose. We review the fracture detection rates, cost-effectiveness and radiation dose implications.
**Methods or Background:** Meta-analysis of articles published by the Department of Radiology, Mater Misericordiae University Hospital Dublin.
**Results or Findings:** Incorporating CBCT in the management pathway for acute radiocarpal injury yielded a 50% fracture detection rate in patients with negative wrist radiographs but ongoing concern for radiocarpal fracture. CBCT had a sensitivity of 98.3% and specificity of 100%. Fractures of the trapezium, which are rarely diagnosed on plain film radiographs, were found to be the most common radiographically occult fracture. Cost-effective studies have demonstrated that advanced multiplanar imaging techniques performed shortly after presentation reduce cost and morbidity compared to empirical wrist immobilisation in the setting of scaphoid fracture. CBCT may be the most cost-effective imaging option for initial evaluation. Effective dose at the wrist from standard CBCT has been calculated as 0.7-2.4 times greater than wrist radiographs; however MDCT of the wrist was calculated as 8.6 times greater than plain radiographs.
**Conclusion:** Cross-sectional imaging should be considered in all cases of post-traumatic wrist pain with negative radiographs. CBCT is a cost-effective modality that demonstrates a sensitivity of 98.3% and specificity of 100%, at a lower radiation dose than conventional MDCT. CBCT should be regarded as the new standard of care in the management of patients following acute radiocarpal injury.
**Limitations:** Patient movement, the lack of soft-tissue detail and degraded images when in-cast imaging is performed were identified as limitations of the modality.
**Ethics committee approval:** Not required.
**Funding for this study:** None required.
**Author Disclosures:**
Peter Joseph Macmahon: Nothing to disclose
Mealla Anne Barry: Nothing to disclose
Gerald Orpen: Nothing to disclose
Emer Kenny: Nothing to disclose

### RPS 610-3

**Patient-specific homogenous spectral fat suppression for distal extremity MRI**

*I. Khodarahmi*[1], I. Brinkmann[2], M. Bruno[1], V. Chebrolu[2], J. Fritz[1];
[1]New York, NY/US, [2]Malvern, PA/US

**Purpose:** To develop and apply patient-specific optimised radiofrequency pulses for more homogeneous fat suppression in distal extremity MRI.
**Methods or Background:** Six ankles, six feet, and five hands (in both superman and next-to-torso positions) were imaged at a 3T MRI system with standard and patient-specific FS techniques. For each subject, water and fat resonance frequencies obtained routinely during scanner calibration were used as real-time inputs to apply an individualised radiofrequency pulse with a high time-bandwidth product for homogenous fat suppression. Corresponding MR images obtained with routine and patient-specific FS pulses were compared and ranked by a musculoskeletal radiologist in a blinded fashion. The non-parametric signed test was used to assess the difference between the two techniques.
**Results or Findings:** At the ankle, the images obtained with the patient-specific FS were ranked superior to the routine protocol (46% superior, 51% equal, $p<0.001$). Similarly, the patient-specific FS was preferred at the feet (39% superior, 60% equal, $p<0.001$). For hands in the superman position, the patient-specific FS was ranked superior to the standard FS (27% superior, 57% equal, $p < 0.001$), whereas no statistically significant difference was seen between the two techniques when hands were imaged next to the torso (30% superior, 40% equal, $p>0.05$).
**Conclusion:** Patient-specific optimised FS technique holds promise to improve the homogeneity of fat suppression in distal extremity MRI significantly. Our initial results are most promising in the forefoot, which is more susceptible to inhomogeneous fat suppression due to complex anatomy and off-centre positioning resulting in higher B0 and B1 inhomogeneity.
**Limitations:** The study's limitations are a lack of comparison with other techniques (SPAIR and DIXON) and performance evaluation only at 3T.
**Ethics committee approval:** The study was performed after Institutional Review Board (IRB) approval.
**Funding for this study:** Not applicable.
**Author Disclosures:**
Jan Fritz: Nothing to disclose
Mary Bruno: Nothing to disclose
Venkata Chebrolu: Employee: Siemens Medical Solutions, USA Inc.
Iman Khodarahmi: Nothing to disclose
Dear Inge Brinkmann: Employee: Siemens Medical Solutions, USA Inc.

### RPS 610-4

**Assessment of visibility of bone structures in the wrist using photon counting computed tomography**

R. Booij[1], N. F. Kämmerling[2], E. Oei[1], A. Persson[3], *E. Tesselaar*[3];
[1]Rotterdam/NL, [2]Motala/SE, [3]Linköping/SE
*(erik.tesselaar@liu.se)*

**Purpose:** To quantitatively and subjectively measure the visibility of bone structures in the wrist on photon-counting computed tomography (PCCT) images and to compare it to state-of-the-art energy-integrating CT (EID-CT).
**Methods or Background:** Four human cadaveric wrist specimens were scanned with EID-CT (Siemens SOMATOM Edge Plus) and PCCT (Siemens NAEOTOM Alpha) at comparable CTDIvol of 12.2 mGy, as well as 6.1 mGy (half dose). Axial images were reconstructed using the thinnest possible slice thickness, i.e. 0.4 mm on EID-CT and 0.2 mm on PCCT (UHR-mode) at 50% overlap, with the largest image matrix size possible (512x512 on EID-CT and 1024x1024 on PCCT) using reconstruction kernels optimised for bone (EID-CT: Ur80, PCCT: Br92). The visibility of cortical and trabecular bone structures was measured using CNR, sharpness of the cortical bone-air boundary, by histogram analysis in the trabecular structure. Also, visibility of bone structures and nutrient canals was assessed by five radiologists at two hospitals using visual grading characteristics (VGC) analysis.
**Results or Findings:** At equal dose, images obtained with PCCT had 39±6% lower CNR ($p=0.001$), 42±8% sharper cortical edges ($p=0.002$) and 53±10% less trabecular unsharpness in the radius ($p=0.02$) than those obtained with EID-CT. This was confirmed by VGC analysis showing superior visibility of cortical bone, trabeculae and nutrient canals (AUC>0.89). At half dose, PCCT also yielded superior image quality, both in quantitative measures and based on VGC analysis.
**Conclusion:** Despite a lower CNR, PCCT offers superior visibility of bone structures in the wrist at half dose relative to state-of-the-art EID-CT, due to smaller detector elements, reduced slice thickness, sharper kernels and a larger image matrix.

**Limitations:** Since only cadaveric specimens were scanned, the effect of movement artifacts was not considered.
**Ethics committee approval:** Not applicable.
**Funding for this study:** Not applicable.
**Author Disclosures:**
Anders Persson: Other: CMIV has research agreements with Siemens, Philips, Bayer, SECTRA
Edwin Oei: Other: Erasmus MC receives research support from Siemens Healthineers
Nina Fredäng Kämmerling: Nothing to disclose
Erik Tesselaar: Nothing to disclose
Ronald Booij: Other: Erasmus MC receives research support from Siemens Healthineers

### RPS 610-5

**Genetic variants for hereditary haemochromatosis and risk of musculoskeletal outcomes in UK biobank**

*L. Banfield*, K. Knapp, D. Melzer, J. Atkins; Exeter/UK
*(l.r.banfield@exeter.ac.uk)*

**Purpose:** Hereditary haemochromatosis (HH) caused by the HFE p.C282Y homozygous variant results in iron overload, especially in males with increased risks of joint pain and osteoarthritis. We estimated HH-genotype associations with musculoskeletal outcomes and joint arthroplasties in a large community genotyped cohort; UK biobank.
**Methods or Background:** 451,143 European ancestry participants (mean 56.8yrs) were followed from baseline (40-70yrs) until 2020 in hospital records (mean 11.5yrs). Cox proportional hazards regression models assessed associations between genotypes (HFE p.C282Y/p.H63D) and incidence of musculoskeletal outcomes, adjusting for age, assessment centre, genotyping array, and population genetics substructure. Analyses were stratified by sex.
**Results or Findings:** Participants included 2,890(0.6%) p.C282Y homozygotes. Male homozygotes have increased risk of osteoarthritis (hazard ratio(HR): 2.12[95%CI:1.61-2.80]; P<0.001), hip replacements (HR: 1.84[95%CI:1.49-2.27]; P<0.001), knee replacements (HR: 1.54 [95%CI:1.20-1.98]; P=0.001), ankle replacements (HR: 20.08[95%CI:10.96-36.78] P<0.001) and shoulder replacements (HR: 7.55[95%CI:1.79-31.86] P=0.006), compared to wild-type males. Male p.C282Y homozygotes had increased risk of femoral fractures (HR: 1.72[95%CI:1.03-2.87]; P=0.04) and osteoporosis (HR: 1.71[95%CI:1.11-2.64, P=0.02). The osteoporosis association disappeared when excluding those with a liver fibrosis/cirrhosis diagnosis. Female p.C282Y homozygotes had an increased risk of osteoarthritis only (HR: 1.46[95%CI:1.12-1.89]; P=0.01). In lifetable estimates based on observed 5-year age-band incidence rates from 40-75 years, 15.5% (95%CI:12.6-19.1) of the male p.C282Y homozygotes are projected to undergo hip replacement surgery, compared to 8.7% (95%CI:8.5-9.0) with no variants.
**Conclusion:** p.C282Y homozygotes have significantly increased risk of OA, with male p.C282Y homozygotes also at increased risk of joint replacement surgeries, osteoporosis and femoral fracture, compared to those without mutations. Osteoporosis was associated with a diagnosis of fibrosis/cirrhosis. Results support pursuing earlier diagnosis of HH by testing at-risk individuals for iron levels and HFE genotypes at orthopaedic and fracture clinics.
**Limitations:** The study used a volunteer population, so may be biased towards healthier individuals.
**Ethics committee approval:** This study is an UK Biobank study.
**Funding for this study:** Not applicable.
**Author Disclosures:**
Lucy Banfield: Nothing to disclose
Karen Knapp: Nothing to disclose
David Melzer: Nothing to disclose
Janice Atkins: Nothing to disclose

### RPS 610-6

**Metal artifact reduction in computed tomography: tin filter and dual-energy technique compared to standard protocols in a cadaver study**

S. Schüle[1], M. J. Beer[2], *C. Hackenbroch*[2]; [1]Munich/DE, [2]Ulm/DE

**Purpose:** Our goal was to a) improve image quality of metal implants in CT and b) reduce radiation exposure without compromising diagnostic image quality. New methods of metal artifact reduction (MAR), such as the tin filter (Sn) and dual-energy technique (DECT), were compared with conventional CT examinations for this purpose.
**Methods or Background:** Four human pelvises from body donors with orthopedic implants were tested with 9 different protocols on a 3rd generation DECT scanner - including FD (full dose, CTDIVol 10 mGy) and LD (low dose, CTDIVol 3.3 mGy) protocols. Sn, DE, virtual monoenergetic (VM), and conventional CT images were compared. FD- and LD protocols were evaluated by ten radiology and eight surgical residents. CT images with the highest diagnostic image quality received the maximum score and vice versa. The scores obtained were summed over the four pelvic specimens. Statistical testing was performed using a rANOVA or the Friedman test.
**Results or Findings:** In FD- and LD-protocols, Sn 150 kV CT-images were superior to DE and VM CT-images (p<0.05). There was no difference between the 150 kV protocol with and without tin filteration (p>0.18). Surgeons and radiologists rate protocols equally (p>0.82). Except for the vertebral cement CT-images, 95% of the raters found the LD protocol sufficient for clinical questions (e.g. detection of periprosthetic fracture or prosthetic loosening).
**Conclusion:** Sn-protocols are an excellent method for MAR with optimum image quality of the adjacent structures. DE and VM protocols are inferior to tin filter protocols. A dose reduction of 66% is possible in pelvic imaging with tin filtering. Sn-protocols have their limitations when imaging the cancellous bone of the spine.
**Limitations:** The small number of pelvic cadavers examined (n=4) is a limitation of our study.
**Ethics committee approval:** Approval was obtained by the local ethics committee.
**Funding for this study:** Not applicable.
**Author Disclosures:**
Simone Schüle: Nothing to disclose
Meinrad Johannes Beer: Nothing to disclose
Carsten Hackenbroch: Nothing to disclose

### RPS 610-7

**Comparison of three different positions for bilateral hands non-contrast MR angiography and perfusion**

*A. Yamamoto*[1], W. Bae[2], V. Malis[2], C. Chung[2], M. Miyazaki[2]; [1]Tokyo/JP, [2]San Diego, CA/US

**Purpose:** To investigate the subject comfort and imaging quality of non-contrast MR angiography (NC-MRA) and -perfusion (NC-MRP) for the bilateral hand MR study in prone over-head (PO), supine over-head (SO), and semi-sitting (SS) positions.
**Methods or Background:** Fresh blood imaging (FBI) NC-MRA and time-spatial labeling inversion pulse (Time-SLIP) NC-MRP images were acquired in three different positions on 6 volunteers (3 males, 3 females, 23-48 yo) at 3T scanner. FBI was performed using the DelayTracker (auto-determined trigger delays) and ECG-prep scan (manual). Parameters were TR/TEeff = 3 RR intervals/60 ms, STIR = 200 ms, resolution = 0.5x0.5 mm, and 320x320 matrix for FBI. Time-SLIP parameters were: TI 1000/1500/2000 ms, SPAIR fat suppression, and resolution=0.4x0.4 mm. Contrast-to-noise ratio (CNR), qualitative visual score (VS) of segmented arteries, and the degree of artifact in NC-MRA, qualitatively VS, the degree of artifacts, and quantitatively the arterial lengths from the tag position (AL) in NC-MRP were evaluated with reconstructed MIP images by one experienced radiologist. All the data were statistically analysed.
**Results or Findings:** Pain scores showed a lower trend in SS position (p=0.12). In NC-MRA, the images with the DelayTracker showed significantly high VS in the SS position (p<0.05). In NC-MRP, significantly smaller VS (p<0.05) and shorter AL (p<0.05) at TI = 1500 were observed in SO position. Artifact at TI = 2000 was significantly less in the SS position (p<0.05).
**Conclusion:** The SS position demonstrated significantly higher VS with ECG-prep in NC-MRA and less artifacts in NC-MRP, with the lower pain scale. The SO position presented inferior VS and AL and increased artifacts in NC-MRP.
**Limitations:** The small sample size is the limitation of this study.
**Ethics committee approval:** The ethical committee of the University of California, San Diego approved this study.
**Funding for this study:** Grant from Canon Medical Systems.
**Author Disclosures:**
Vadim Malis: Nothing to disclose
Mitsue Miyazaki: Grant Recipient: Grant from Canon Medical Systewithout remuneration.
Christine Chung: Nothing to disclose
Asako Yamamoto: Nothing to disclose
Won Bae: Nothing to disclose

### RPS 610-8

**Looking beyond the plexus: relevance of extra-plexal findings in patients with traumatic brachial plexopathy**

*V. Upadhyaya*, D. N. Upadhyaya; Lucknow/IN
*(vshali77@yahoo.co.in)*

**Purpose:** The purpose of the study was to correlate the presence of extra-plexal findings with the severity of injury in patients with traumatic brachial plexopathy who underwent an MR neurography of the brachial plexus. This association, if proved, would help the referring clinician predict the course and outcome of the injury, and prognosticate the patient better.
**Methods or Background:** This was a retrospective observational study of imaging data of thirty subsequent patients who underwent MR neurography of the brachial plexus following trauma. The study was conducted over a period of 6 months, from July to December 2021. Patient consent for the use of images for academic and research purposes was obtained. All data was entered into a Microsoft Excel spreadsheet and analysed. No statistical tools were used.

**Results or Findings:** The extra-plexal findings in these patients included fractures of the clavicle, scapula, humerus and vertebrae, shoulder joint effusion and axillary artery thrombosis. Clavicular fractures were most common and were seen in 23.3% of patients followed by scapular fractures in 16.7% of patients and humeral fractures in 6.7% of patients. Vertebral fractures were the least common and were seen in 3.3% of patients. Both shoulder joint effusion and axillary artery thrombosis were seen in 3.3% of cases each.
**Conclusion:** Presence of extra-plexal injuries points to a greater severity and extent of injury to the brachial plexus, and results in poor outcome in patients with traumatic plexopathy.
**Limitations:** The study has a small sample size and more data is required to make any definitive recommendations.
**Ethics committee approval:** Institutional ethical clearance was waived off as no patient identifying factors were present in the current dataset.
**Funding for this study:** Not applicable.
**Author Disclosures:**
Vaishali Upadhyaya: Nothing to disclose
Divya Narain Upadhyaya: Nothing to disclose

**16:30-17:30** **Room M 3**

### Research Presentation Session: Imaging Informatics / Artificial Intelligence and Machine Learning

### RPS 605b

### Artificial intelligence for improvement of patient's care and radiologist's workflow

**Moderator**
E. R. Ranschaert; Tilburg/NL
**Author Disclosure:**
E. R. Ranschaert: Advisory Board: Osimis, Diagnose.me; Consultant: Quibim, Contextflow, Oxipit, Unilabs; Other: Visiting Professorship Ghent University

### RPS 605b-2

**Quantifying operator and hardware variability in diagnostic ultrasound using principle component analysis and k-means clustering**
*C. Zhu*, T. Doyle, M. Noseworthy; Hamilton, ON/CA
*(zhuc@mcmaster.ca)*

**Purpose:** Diagnostic ultrasound (US) is one of the most widely used imaging modalities due to low cost, ability to capture real-time images, portability, and use of non-ionising radiation. The greatest disadvantage, however, is the operator dependence on image acquisition. Another source of variance could be the ultrasound system. The goal of this work was to use an unsupervised learning algorithm to learn 'clusters' of operators and hardware and act as a quantification tool for operator skill and hardware variance from ultrasound images.
**Methods or Background:** A BLUE phantom from CAE Healthcare (Sarasota, FL) with various mock lesions was scanned by three operators of varying skill levels using three different US systems (Siemens S3000, Clarius L15, and Ultrasonix SonixTouch) producing 39013 images. Dimension reduction and feature extraction was done using principal component analysis (PCA). The extracted features were given to a K-means clustering algorithm to determine clusters of operators or clusters of hardware. Silhouette scores and misclassification rates were used to judge model performance. Euclidean distance from cluster centres was used to compare skill levels and hardware types.
**Results or Findings:** The hardware model had a misclassification rate of 0.597%. The operator model had a misclassification rate of 71.4%. The silhouette scores and average distance between cluster centres were higher in the hardware model, implying that the hardware data is more separable.
**Conclusion:** K-means clustering was able to learn clusters of hardware well but the ability to learn operator clusters was limited, suggesting hardware variance is higher than operator variance.
**Limitations:** More operators with varying levels could have strengthened the results. Also the US systems varied widely in age and type.
**Ethics committee approval:** Not applicable.
**Funding for this study:** Funding was received for this study by the Canadian Department of National Defence (DnD): Innovation for Defence Excellence and Security (IDEaS) grant #CFPMN2-17).
**Author Disclosures:**
Calvin Zhu: Nothing to disclose
Thomas Doyle: Nothing to disclose
Michael Noseworthy: Nothing to disclose

### RPS 605b-3

**Detection of unreported clinically significant ascending aortic dilation by combination of computer vision (CV) algorithm and report processing**
*D. Stav*[1], J. Balcombe[2], G. Aviram[1], D. Mercer[1], G. Levy[1]; [1]Tel Aviv-Yafo/IL, [2]Ramat HaHayal/IL
*(danastav50@gmail.com)*

**Purpose:** Missed aortic dilation may progress to aortic aneurysm and rupture. AI-aided detection of aortic dilation may cause a large volume of mostly unneeded alerts, as the radiologist will detect most cases of aortic dilation without AI. Filtration of AI findings via radiologist's report analysis, (as a surrogate to NLP) will notify radiologist only of unreported dilated aortas while reducing unnecessary alerts.
**Methods or Background:** Retrospective analysis of 1,727 consecutive chest CT scans (contrast and non-contrast protocols) and their corresponding reports. Scans were processed by CV aortic measurement algorithm (developed by IMedis Ltd.). Corresponding radiology reports tagged by radiology resident to indicate mention/absence of ascending aortic dilation (≥42mm). Combined outputs were classified blindly by 3 senior radiologists, with consensus round for disagreed cases.
**Results or Findings:** Dilated ascending aorta was detected in 35 cases (2%), of which 19 (54.3%) were unreported. Consensus radiologist review confirmed 16 (84.2%) true positives and 3 (15.8%) false positives.
**Conclusion:** A dual algorithm combining CV analysis of chest CTs and human report tagging alerts for missed ascending aortic dilation with a high true positive rate and minimal false positives or unneeded alerts. The algorithm doubled the number of detected cases of aortic dilation (16 reported cases, 16 unreported cases) while alerting only 1.1% of cases (19/1,727 cases). The high true positive rate of the dual algorithm and the 100% increase in detection justifies radiologist review of these flagged cases, to improve detection of actionable ascending aortic dilation with minimal increased workload.
**Limitations:** NLP would be the optimal method of report tagging but was not employed as it was unavailable in the report language (Hebrew).
**Ethics committee approval:** IRB approval obtained, informed consent waived for retrospective study.
**Funding for this study:** Not applicable.
**Author Disclosures:**
Galit Aviram: Nothing to disclose
Diego Mercer: Nothing to disclose
Dana Stav: Nothing to disclose
Jonathan Balcombe: Employee: IMedis Ltd.
Gad Levy: Nothing to disclose

### RPS 605b-4

**Amplifying the contrast of MR images using a DL method trained on synthetic images**
*S. Colombo Serra*[1], A. Fringuello Mingo[1], D. Bella[1], M. Alì[2], S. Papa[2], G. Valbusa[1], F. Tedoldi[2]; [1]Colleretto Giacosa/IT, [2]Milan/IT
*(sonia.colombo@bracco.com)*

**Purpose:** A deep learning (DL) method to amplify contrast in contrast-enhanced MR (CE-MR) images was evaluated. The proposed training strategy is based on synthetic data not requiring reduced contrast images, i.e. images obtained after the injection of a non-standard dose of Gadolinium Based Contrast Agent (GBCA).
**Methods or Background:** A convolutional neural network (CNN) was used to boost contrast enhancement. Loss function, hyperparameters, and CNN architecture were modified and tuned. CE-MR images for training were artificially generated reducing the contrast in standard CE-MR images of a fraction k. The CNN was then trained to learn the task of amplifying contrast by a factor k using standard CE-MR images as target. 1990 CNS clinical cases were used to train, test and validate the proposed approach. Contrast amplification from standard CE-MR to predicted images amplified using different k factors was characterised qualitatively and quantitatively. The method was also tested in a preclinical setting with a model of human glioma (120 rats).
**Results or Findings:** Increased contrast-to-noise ratio, lesion-to-brain ratio and contrast enhancement is observed in amplified images. Notably, lesions count and diameter remain unvaried between standard-dose and amplified images. Preclinical results show similar performances between CNN trained on synthetic and CNN trained on real data.
**Conclusion:** The proposed DL approach trained on simulated images demonstrates promising performances in amplifying the effects of GBCA. The proposed data simulation strategy can be used to avoid the collection of reduced-dose CE-MR images not included in the standard of care.
**Limitations:** The limitation is that it is a monocentric study at CDI.
**Ethics committee approval:** LOW-DOSE, number 181-2020bis, approved by Comitato Etico Milano area 21.
**Funding for this study:** No profit study.

**Author Disclosures:**
Fabio Tedoldi: Employee: Bracco Imaging SpA
Sergio Papa: Employee: Centro Diagnostico Italiano
Marco Alì: Consultant: Centro Diagnostico Italiano
Giovanni Valbusa: Employee: Bracco Imaging SpA
Sonia Colombo Serra: Employee: Bracco Imaging SpA
Alberto Fringuello Mingo: Employee: Bracco Imaging SpA
Davide Bella: Consultant: Bracco Imaging SpA

### RPS 605b-5

**Designing for appropriate trust in artificial intelligence: making machine learning usable for radiologists**

*R. Verhoeven*, P. M. A. Van Ooijen, F. Cnossen; Groningen/NL
*(r.verhoeven@umcg.nl)*

**Purpose:** Artificial intelligence-based computer-aided detection (AI-CAD) systems are supposed to help radiologists with the detection of pulmonary nodules in medical images. However, inappropriate trust in these systems often leads to an incorrect use, manifested in both under- and over-reliance. Literature suggests that a lack of transparency in the system and insufficient knowledge of the underlying algorithm from the user are the main causes for this concern. The current study tested proposed solutions for the design of CAD systems to counter these causes.
**Methods or Background:** Eleven radiologists took part in an online experiment, detecting pulmonary nodules in thoracic CT slices using AI-CAD. Participants were randomly assigned to one of six conditions, varying in the degree of transparency of the system and whether AI was used as first or second reader. Transparency was reflected in the display of the system's performance, confidence ratings for each mark, and extensiveness of the explanation of the system. The outcomes measured were the subjective evaluations of the radiologists as well as specificity, sensitivity, and additional false positives marked.
**Results or Findings:** Specificity was lower in the least transparent conditions, without affecting sensitivity. Specificity was also lower with CAD as second reader. The evaluation showed that participants did not think the explanation of the system affected their trust. They did indicate that display of the performance and confidence ratings helped them with scanning, pattern recognition, and decision-making processes, leading to more confidence and higher satisfaction.
**Conclusion:** Displaying performance levels and confidence ratings could lead to increased appropriate trust in CAD, as does using CAD as first reader.
**Limitations:** Due to the small sample size, only data exploration was performed. In addition, the experiment was performed without supervision and in a lab setting.
**Ethics committee approval:** This study was approved by an ethics committee.
**Funding for this study:** No funding was received for this study.
**Author Disclosures:**
Fokie Cnossen: Nothing to disclose
Peter M. A. Van Ooijen: Nothing to disclose
Rosa Verhoeven: Nothing to disclose

### RPS 605b-6

**Natural language processing for automatic evaluation of free text answers: a pilot study based on the EDIR examination**

*F. Stöhr*[1], B. Kämpgen[2], V. Junquero[3], C. Merino[3], P. Mildenberger[1], L. Oleaga[3], R. Kloeckner[1]; [1]Mainz/DE, [2]Kaiserlsautern/DE, [3]Barcelona/ES

**Purpose:** Most written medical examinations consist of multiple-choice questions and/or free text answers. The latter requires to be evaluated and rated manually by the examiner, which is time-consuming and potentially error-prone. We tested whether natural language processing (NLP) can be used to semi-automatically analyse and score free text answers in order to support the examiner.
**Methods or Background:** The European Board of Radiology (EBR) provided a representative dataset comprising of questions and their corresponding answer keys, together with original answers and markings from all participants. Based on the official answer key, RadLex terms were used to code free-text answers.
**Results or Findings:** Answers from 96 participants extracted from one sample question were analysed and ranked via a rule-based classification (0-3 points, as in the original answer key). Afterwards, rankings from the NLP engine were compared to the original rankings provided by the EBR. For 0-1 points, the probability for correct classification by NLP yielded 71%.
**Conclusion:** Our feasibility study showed that NLP can be used successfully to automatically analyse and score free-text answers. However, the more complex the question/answer is, the more challenging it is to 1. find "right" terms for coding free-text answers and 2. train the NLP engine. A well-trained NLP engine might not only help improve the review process at first but could also help develop future exams, which can be assessed fully automated.
**Limitations:** As data only contain 96 cases, the NLP engine should be improved with more training and more input data before implementation in the "real" world is feasible.
**Ethics committee approval:** Not applicable.
**Funding for this study:** FS is supported by the Clinician Scientist Fellowship "Else Kröner Research College: 2018_Kolleg.05".
**Author Disclosures:**
Roman Kloeckner: Nothing to disclose
Cristina Merino: Nothing to disclose
Benedikt Kämpgen: Nothing to disclose
Vanesa Junquero: Nothing to disclose
Fabian Stöhr: Nothing to disclose
Peter Mildenberger: Nothing to disclose
Laura Oleaga: Nothing to disclose

### RPS 605b-7

**Additional dose reduction potential of vendor-agnostic deep learning models combined with conventional iterative reconstruction methods: a phantom study**

*J. Kim*[1], W. Chang[1], H. J. Lee[2], H. Y. Kim[1], H. Choi[1], J. Cho[1], Y. J. Lee[1], Y. H. Kim[1]; [1]Seongnam-si/KR, [2]Seoul/KR
*(k.jisu819@gmail.com)*

**Purpose:** To evaluate the additional dose reduction potential (DRP) of a vendor-agnostic deep learning model (DLM, ClariCT.AI) applied to iterative reconstruction (IR) methods of different CT vendors.
**Methods or Background:** Computed tomography (CT) images of a multi-sized image quality phantom (Mercury v4.0) were acquired with two CT machines from different vendors under six radiation dose levels (0.7/1.5/3.0/6.1/12.2/24.4 mGy). Those images were reconstructed using various IRs (advanced modeled iterative reconstruction (ADMIRE), iDose4, iterative model-based reconstruction (IMR)) with two different strength levels (low or medium, high) for each IRs, and were then denoised with DLM. For all IR and DLM images, the detectability index (d') (a task-based detection performance metric) was obtained using a phantom with a diameter of 31 cm, under various combinations of three target sizes (10/5/1 mm), five inlets (CT value difference with the background; -895/50/90/335/1000 HU), and six radiation dose levels. DRP measures the dose reduction made by using DLM while yielding d' equivalent to that of IR at a reference dose level (12.2 mGy).
**Results or Findings:** The mean DRPs were 93% (83–98%) for ADMIRE, 90% (83–93%) for the iDose4 and 88% (81–94%) for the IMR. By inlet type, the mean DRPs were 91% (83–98%) for the highest contrast material (1000HU) and 87% (81–92%) for the lowest contrast materials (50HU). The DRP of the high contrast materials increased with smaller target size, while that of the low contrast materials decreased with smaller target size.
**Conclusion:** Additional dose reduction was possible using the vendor-agnostic DLM applied to images reconstructed with IR methods of different vendors.
**Limitations:** DRPs were evaluated using a phantom. Further research using clinical CT images is needed.
**Ethics committee approval:** Institutional Review Board approval was waived.
**Funding for this study:** No funding was received for this study.
**Author Disclosures:**
Jisu Kim: Nothing to disclose
Hae Young Kim: Nothing to disclose
Jungheum Cho: Nothing to disclose
Yoon Jin Lee: Nothing to disclose
Won Chang: Nothing to disclose
Young Hoon Kim: Nothing to disclose
Hee Jin Lee: Nothing to disclose
Hyunsu Choi: Nothing to disclose

### RPS 605b-8

**NAVIGATOR: an imaging biobank to precisely prevent and predict cancer, and facilitate the participation of oncologic patients to diagnosis and treatment**

*L. Tumminello*[1], A. Barucci[2], R. Carpi[2], S. Colantonio[1], V. Colcelli[2], P. Liò[3], M. A. Mazzei[4], V. Miele[2], E. Neri[1]; [1]Pisa/IT, [2]Florence/IT, [3]Cambridge/UK, [4]Siena/IT
*(lorenzo.tumminello@med.unipi.it)*

**Purpose:** NAVIGATOR is an Italian regional project that aims to boost 4P precision medicine in oncology making it more predictive, preventive, personalised and participatory by advancing translational research based on quantitative imaging and multi-omics analyses. The project's aim is to develop an open imaging biobank for the collection and preservation of a large amount of standardised imaging datasets, including CT, MRI and PET multimodal data, together with the corresponding patient-related and omics-related relevant information extracted from regional healthcare services using an adapted privacy-preserving model.

**Methods or Background:** The project is constructed centring on the open-source imaging Biobank and an open-science-oriented Virtual Research Environment (VRE). A large number of multi-omics and multi-imaging data of three use cases, which incorporate three major abdominal neoplasms (prostate cancer, rectal cancer and gastric cancer), will be collected.
**Results or Findings:** All data confined in Navigator (i.e., imaging biomarkers, non-imaging data, health agency data) will be integrated with prior medical knowledge and will be used to create a digital patient model to support the reliable prediction of the disease phenotype and risk-stratification. The VRE will further provide a multiset infrastructure for processing the multi-omics data, extracting specific radiomics-signatures and for identification and testing of novel imaging biomarkers through Big Data Analytics and Artificial Intelligence.
**Conclusion:** NAVIGATOR Biobank will deliver an infrastructure to collect and preserve a large number of multi-omics and multi-imaging data. It will create a digital patient model that will ensure accurate cancer phenotyping and more comprehensive risk stratification in oncology and will offer advanced VRE for extracting novel radiomics-based biomarkers and defining computation algorithms for big data analysis.
**Limitations:** Not applicable.
**Ethics committee approval:** This study was approved by an ethics committee: TUSCANY region, AREA VASTA NORD OVEST (Prot. n. 18253).
**Funding for this study:** Funding was received for this study by Bando Ricerca Salute Regione Toscana 2018 (DD 15397/2018).
**Author Disclosures:**
Maria Antonietta Mazzei: Nothing to disclose
Emanuele Neri: Nothing to disclose
Vittorio Miele: Nothing to disclose
Valentina Colcelli: Nothing to disclose
Roberto Carpi: Nothing to disclose
Pietro Liò: Nothing to disclose
Lorenzo Tumminello: Nothing to disclose
Andrea Barucci: Nothing to disclose
Sara Colantonio: Nothing to disclose

**16:30-17:30** **Room Z**

### Research Presentation Session: Head and Neck

### RPS 608

### Parotid gland imaging

**Moderator**
E. Loney; Halifax/UK

### RPS 608-2

**T2WI maximum tumour level histogram for differentiating parotid pleomorphic adenoma and malignant tumour**
*M. Zhang*, Y. Zhang, J. Cheng; Zhengzhou/CN

**Purpose:** To assess the value of T2WI maximum tumour level histogram in differentiating pleomorphic adenoma from malignant tumours of parotid gland.
**Methods or Background:** MRI of 64 patients with parotid tumours, including 41 cases of pleomorphic adenomas and 23 cases of malignant tumours confirmed by pathology were analysed retrospectively. Mazda software was used to select ROIs in the maximum tumour level image on axial T2WI. Gray histogram analysis was carried out to obtain 9 characteristic parameters, including mean, variance, kurtosis, skewness, first percentile (perc 1%), tenth percentile (perc 10%), fiftieth percentile (perc 50%), ninetieth percentile (perc 90%) and ninety-ninth percentile (perc 99%). Statistical analysis was performed to compare the characteristic parameters of histogram between pleomorphic adenoma and malignant tumours. ROC curve was drawn to evaluate the effectiveness of the characteristic parameters of histogram in tumours differentiating.
**Results or Findings:** Among 9 characteristic parameters of histogram, perc 1% and perc 10% had significant differences between pleomorphic adenoma and malignant tumours (both $P<0.05$). Both of perc 1% and perc 10% of pleomorphic adenoma were significantly higher than those of malignant tumours. ROC curve analysis showed that perc 10% was the most effective parameter for differential diagnosis. The AUC was 0.07(P=0.01), and the optimal critical value was 76.00. The sensitivity and specificity were 66.70% and 60.00% respectively. Besides, the AUC, optimal critical value, sensitivity and specificity of perc 1% were 0.67(P=0.04), 46.50, 63.90% and 60.00% respectively.
**Conclusion:** T2WI maximum tumour level histogram can be used as an important method to differentiate pleomorphic adenoma from malignant tumours of parotid gland before operation, which can provide valuable references for clinic.
**Limitations:** The limitation is that the sample size is small.
**Ethics committee approval:** This study was approved by the Medical Ethics Committee of First Affiliated Hospital of Zhengzhou University.
**Funding for this study:** No funding was received for this study.
**Author Disclosures:**
Jingliang Cheng: Nothing to disclose
Mengzhe Zhang: Nothing to disclose
Yong Zhang: Nothing to disclose

### RPS 608-3

**Automated machine learning based on intratumoural and peritumoural radiomics features may identify capsular status of parotid pleomorphic adenoma**
*S. Li*, X. Su, Q. Yue; Chengdu/CN

**Purpose:** The capsular status of parotid pleomorphic adenoma (PA) has various forms, including incomplete, capsular penetration, pseudopodia, and satellites nodules, which is considered as a recurrence factor. We aimed to investigate whether employing tumoural and peritumoural radiomics features from computed tomography (CT) images can identify capsular status of parotid PA by applying an automated machine learning (AutoML) approach.
**Methods or Background:** A total of 143 patients with parotid PA (76 patients had complete capsule and 67 patients had other capsular status) were randomly divided into a training set (n=115) and a testing set (n=28). The volumetric region of interest was identified for the tumour and peritumoural parotid parenchyma (2 mm around the tumour) on contract-enhanced CT images. Radiomics features were extracted from the original, Laplacian of Gaussian (LoG)-filtered and wavelet-filtered images. We applied a tree-based pipeline optimisation tool (TPOT) as the AutoML method to the extracted feature sets to obtain the optimal pre-processing steps, classification algorithm, and corresponding hyper-parameters.
**Results or Findings:** Radiomics of peritumour outperformed intratumoural and combined radiomics (intra- and peritumoral). An extra trees classifier using textual features from original and LoG-filtered CT images achieved an area under the receiver operating characteristic curve of 0.934 on the test set.
**Conclusion:** Radiomic features from peritumour regions may identify capsular status of parotid PA by applying an AutoML approach. It could be a promising tool which may assist in the treatment planning for patients with parotid PA.
**Limitations:** The following limitations were identified: a relatively small sample size was used (n=143); there was a lack of further external validation; the fact that MRI images may contain more valuable information.
**Ethics committee approval:** This study was approved by an ethics committee.
**Funding for this study:** This work was supported by the Sichuan Provincial Foundation of Science and Technology (Grant No. 2019YFS0428).
**Author Disclosures:**
Xiaorui Su: Nothing to disclose
Qiang Yue: Nothing to disclose
Shuang Li: Nothing to disclose

### RPS 608-4

**Ultrasound assessment of focal lesions within the major salivary glands in Sjögren syndrome patients with increased lymphoproliferative risk: what is the sensitivity and specificity for lymphoma?**
*M. Lorenzon*, F. Tulipano Di Franco, E. Spina, R. Girometti, C. Zuiani; Udine/IT

**Purpose:** To assess sensitivity and specificity of ultrasound (US) for the diagnosis of lymphoma in primary Sjögren syndrome (pSS) patients at increased lymphoproliferative risk with focal lesions of the major Salivary Glands (SGs).
**Methods or Background:** pSS-patients have an increased risk of lymphoma of SGs (SGs-L), which can appear with a diffuse or focal pattern. In this clinical setting, recent evidence suggests that focal SGs-L are frequently associated with some US features, specifically: OMERACT grade 3; appearance very hypoechoic, homogeneous, with oval shape, well-defined margins, presence of septa, colour-doppler vascularisation and posterior acoustic enhancement. As the number of these features, simultaneously present in a lesion, increases, then also the likelihood of SGs-L increases. We prospectively included all pSS-patients at high risk for SGs-L with focal lesions of SGs that were referred to our Department for US assessment and US-guided core-needle biopsy (CNB) between September 2019 and March 2021. We assessed US sensitivity and specificity for SGs-L when 5/8, 6/8, 7/8 or 8/8 of the features reported above were simultaneously present in each focal lesion.
**Results or Findings:** 16/27 pSS-patients who underwent US-guided CNB had a focal lesion. 9/16 (56%) of them were affected by SGs-L, in 7/16 (44%) the diagnosis was different. Considering increasing simultaneous features, the sensitivity/specificity were respectively: for 5/8 features 100%/42.9%, 6/8 features 88.9%/71.4%, 7/8 features 77.8%/85.7%, 8/8 features 33.3%/100%.

**Conclusion:** In pSS-patients with high risk of SGs-L with focal lesion, US provides useful information to guide CNB. The threshold of 6/8 features suspicious for SGs-L simultaneously present in a lesion represents the best trade-off in terms of sensitivity and specificity.
**Limitations:** The limitation is the single-centre design.
**Ethics committee approval:** This study was approved by an ethics committee.
**Funding for this study:** No funding was received for this study.
**Author Disclosures:**
Erica Spina: Nothing to disclose
Francesco Tulipano Di Franco: Nothing to disclose
Chiara Zuiani: Nothing to disclose
Michele Lorenzon: Nothing to disclose
Rossano Girometti: Nothing to disclose

### RPS 608-5

**Maximum signal intensity ratio on T1-weighted magnetic resonance images for the differential diagnosis of benign and malignant parotid tumours: a control study in two medical centres**

P. Wei, *Z. Ding*, Z. Han; Hangzhou/CN
*(hangzhoudzx73@126.com)*

**Purpose:** To investigate the value of the maximum signal intensity of tumour on T1-weighted magnetic resonance (MR) images for the differential diagnosis of benign and malignant parotid tumours in two medical centres.
**Methods or Background:** MR images of 87 pleomorphic adenomas (PAs), 58 Warthin tumours (WTs), and 38 malignant tumours (MTs) in centre A were retrospectively analysed and compared with 45 PAs, 45 WTs, and 36 MTs in centre B. The maximum signal intensity of tumour and the mean signal intensity of buccal subcutaneous fat were measured on T1-weighted images, then the tumour to fat signal intensity ratio (T1-Max-SIR) was calculated. The distribution differences in T1-Max-SIRs among the three groups of tumours within and between the two medical centres were analysed statistically. The diagnostic performance of T1-Max-SIR to identify parotid tumours was analysed using the receiver operating characteristic curve.
**Results or Findings:** In the two centres, T1-max-SIRs were higher in WTs than in PAs ($P<0.001$) and MTs ($P<0.001$), while there was no statistically significant difference between PAs and MTs (center A: $P=0.257$, center B: $P=0.543$). In centre A and centre B, the AUC, sensitivity, and specificity for differentiating WTs from PAs were 0.862 vs. 0.833, 0.810 vs. 0.844, and 0.793 vs. 0.844, respectively, and the AUC, sensitivity, and specificity for differentiating WTs from MTs were 0.791 vs. 0.788, 0.810 vs. 0.844, and 0.711 vs. 0.778, respectively.
**Conclusion:** T1-max-SIR was helpful in differentiating WTs from PAs and MTs with highly consistent diagnostic efficiency between the two medical centres.
**Limitations:** First, this study did not classify MTs according to pathological type. Second, no combination with other MR imaging parameters.
**Ethics committee approval:** This study was approved by the Ethics Committees.
**Funding for this study:** Funding was received for this study by the National Natural Science Foundation of China (81871337).
**Author Disclosures:**
Zhijiang Han: Nothing to disclose
Peiying Wei: Nothing to disclose
Zhongxiang Ding: Nothing to disclose

### RPS 608-6

**The role of multiparametric ultrasound and FNAC in the preoperative evaluation of parotid gland tumours: a single-centre prospective study**

*O. Guiban*, D. Fresilli, P. Pacini, G. Del Gaudio, V. Dolcetti, M. Martino, V. Cantisani, C. Catalano; Rome/IT
*(olga.guiban@gmail.com)*

**Purpose:** To evaluate the presurgical diagnostic value of Multiparametric Ultrasound (MPUS) and Fine Needle Aspiration Cytology (FNAC) in differentiating parotid gland tumours, comparing the results with histology.
**Methods or Background:** This prospective study included 126 consecutive patients with parotid gland lesions surgically treated in a single tertiary centre. After institutional review board approval, MPUS examination was performed prior to FNAC or surgery using B-mode Ultrasound (B-mode US), Colour-Doppler (CDUS), Ultrasound Elastography (USE) and Contrast-Enhanced Ultrasound (CEUS). We studied the diagnostic performance of the different techniques taken alone and in combination. Histological examination was considered the gold standard.
**Results or Findings:** Histology identified 93 benign tumours and 33 malignancies. In the differential diagnosis between malignant and benign lesions, B-mode US, CDUS, CEUS, Ultrasound Elastography and FNAC showed the following values of sensitivity: 82%, 81%, 86%, 77%, 73% respectively; specificity: 97%, 61%, 95%, 71%, 97% respectively; PPV: 90%, 43%, 86%, 50%, 89% respectively; NPV: 93%, 90%, 95%, 88%, 91% respectively; and accuracy: 89%, 71%, 90%, 78%, 84% respectively.
**Conclusion:** The combination of B-mode US and CEUS greatly improved the sensitivity of the CEUS performed individually and presented remarkable accuracy. USE did not improve the diagnostic performance of the B-mode US, alone or in association with CEUS; however, it revealed the highest diagnostic accuracy in the differentiation between benign lesions. FNAC demonstrated lower values in comparison with CEUS and with USE. Therefore, according to our study, MPUS could be proposed as a valid alternative to FNAC.
**Limitations:** The sample size of the study was limited, Shear-wave elastography was not performed and a comparison between MPUS and MRI was not carried out.
**Ethics committee approval:** All procedures performed were in accordance with the ethical standards of the institutional and/or national research committee.
**Funding for this study:** No external funding was received for this study.
**Author Disclosures:**
Daniele Fresilli: Nothing to disclose
Giovanni Del Gaudio: Nothing to disclose
Patrizia Pacini: Nothing to disclose
Olga Guiban: Nothing to disclose
Milvia Martino: Nothing to disclose
Vincenzo Dolcetti: Nothing to disclose
Vito Cantisani: Speaker: Bracco, Canon
Carlo Catalano: Nothing to disclose

### RPS 608-7

**The role of conventional, diffusion-weighted and dynamic contrast-enhanced MR imaging in the evaluation of parotid tumours and its value in predicting histopathological type**

*N. Janssen*, A. Bernaerts, J. van Dinther, F. Deckers, B. De Foer; Antwerp/BE
*(nick.janssen2@student.uantwerpen.be)*

**Purpose:** The aim of this retrospective study is to provide a summary of the role of conventional MRI, diffusion weighted imaging (DWI) and dynamic contrast enhanced MRI (DCE-MRI) in the diagnosis and histopathological characterisation of parotid tumours.
**Methods or Background:** 38 parotid tumours evaluated with conventional MRI, DWI and DCE-MRI in the period from 2019 to 2021 and confirmed by histopathology, were included in this study. MRI studies were read by two experienced head and neck radiologists. Different morphological features, apparent diffusion coefficient (ADC) and time-intensity curves (TIC) were considered. Washout ratios of the TICs were determined at 300 seconds after gadolinium-contrast administration. Multiple comparisons were made to determine if any differences between the various histopathological types existed.
**Results or Findings:** Some malignant tumours showed benign morphological features, making conventional MRI alone less reliable. Warthin tumours and malignancies had great overlap producing low ADC values. Pleomorphic adenomas and other benign tumours produced higher ADC values and therefore showed little overlap with Warthin tumours and malignancies. TIC patterns were often very characteristic for a specific histopathological type. All pleomorphic adenomas demonstrated a progressive enhancement and no washout. All Warthin tumours showed a fast peak with washout ratios of 50% or more. The malignant tumours also showed a fast peak but variable washout ratios, ranging from a plateau up to nearly 40% washout. Carcinoma ex pleomorphic adenomas were larger lesions, showing higher ADC values compared to other malignancies and had mixed TIC patterns.
**Conclusion:** The combination of conventional MRI, DWI and DCE-MRI has the highest diagnostic accuracy in determining the histopathological type of parotid tumours and discriminating malignancies. To safely differentiate Warthin tumours, cut-off values of at least 50% should be used for the washout ratios.
**Limitations:** Not applicable
**Ethics committee approval:** This study was approved by an ethics committee.
**Funding for this study:** No funding was received for this study.
**Author Disclosures:**
Nick Janssen: Nothing to disclose
Bert De Foer: Nothing to disclose
Anja Bernaerts: Nothing to disclose
Filip Deckers: Nothing to disclose
Joost van Dinther: Nothing to disclose

### Thursday, July 14

08:00-09:00 Room D

### Research Presentation Session: Chest

### RPS 704

### The use of contrast for pulmonary vascular disorders

**Moderator**
H. Prosch; Vienna/AT
**Author Disclosure:**
H. Prosch: Advisory Board: Boehringer Ingelheim, Sanofi, Roche; Speaker: Boehringer Ingelheim, Sanofi, Roche, MSD, Astra Zeneca, Novartis

### RPS 704-2

**The difference in pulmonary veins enhancement as a sign of pulmonary embolism on contrast-enhanced CT**
L. Aker[1], *D. Sibira*[1], L. Abandeh[2], F. Al-Khafaji[1], A. Al-Rashid[1], A. E. Mahfouz[1]; [1]Doha/QA, [2]Seattle, WA/US
*(dsibira@hamad.qa)*

**Purpose:** On contrast-enhanced CT, the lung tissue affected by pulmonary embolism is hypothesised to have slower venous return of the contrast agent to the left atrium. The purpose of the study is to assess the difference in the enhancement of pulmonary veins as a sign of pulmonary embolism on contrast-enhanced chest CT.
**Methods or Background:** One hundred three patients with pulmonary embolism and 104 controls were examined by an identical protocol of contrast-enhanced chest CT. Attenuation values of the four pulmonary veins were measured by a manually placed region of interest on each vein. The ratio between veins of the lowest and highest enhancement (Vmin/Vmax) was calculated, indicative of the difference in attenuation value of the pulmonary veins. Pre-test predictive values and area under the Receiver-Operator-Characteristic Curve (AUC-ROC) were calculated. Also, the means of Vmin/Vmax in patients with pulmonary embolism were compared with the control group.
**Results or Findings:** Vmin/Vmax was statistically lower in patients with pulmonary embolism (mean 0.76, SD 0.12) compared to control group (mean 0.88, SD 0.6), p value < 0.001. AUC-ROC was found to be 0.82. At a cut-off value of 0.8, the sensitivity, specificity, and accuracy of this parameter for diagnosis of pulmonary embolism were 60%, 88%, and 74%, respectively.
**Conclusion:** The difference between enhancement of the individual pulmonary veins with a Vmin/Vmax ratio of 0.8 is a useful diagnostic sign of pulmonary embolism.
**Limitations:** Limitations included: the retrospective nature of the study and the relatively heterogeneous small population.
**Ethics committee approval:** This study was approved by an ethics committee.
**Funding for this study:** No funding was received for this study.
**Author Disclosures:**
Ahmed Emad Mahfouz: Nothing to disclose
Dalal Sibira: Nothing to disclose
Laith Abandeh: Nothing to disclose
Fatima Al-Khafaji: Nothing to disclose
Loai Aker: Nothing to disclose
Amal Al-Rashid: Nothing to disclose

### RPS 704-3

**Contrast medium administration for chest CT: a European protocol survey**
*M. K. Henning*, T. M. Aaløkken, A. C. T. Martinsen, S. Johansen; Oslo/NO
*(meid@ous-hf.no)*

**Purpose:** To investigate different contrast medium (CM) administration regimes for chest CT used by members of the European Society of Thoracic Imaging (ESTI).
**Methods or Background:** An online questionnaire was e-mailed to 650 ESTI members. The survey focused on CM protocols. In particular, questions referred to CM concentration, the use of fixed CM volume or weight/body composition tailored CM administration, injection rate, kV, and fixed delay or use of bolus tracking for chest CT.
**Results or Findings:** The overall response rate was 15.2%. Approximately 44% of the respondents used 350 mgI/ml and 54.5% used fixed CM volume regime. The injection rate varied between 1.5 to 5 ml/sec. Around 42% of respondents used fixed kV varying between 80 to 140 kVp and the majority used kV-modulation. Nearly 52% of the respondents used bolus tracking with a delay of 20-90 seconds.
**Conclusion:** Our survey showed a large variation in CM protocols used across Europe. There is a clear preference for using fixed CM administration regime for thoracic CT. More attention to the optimisation and establishment of a standard CM administration protocol in chest CT is needed.
**Limitations:** A limitation is the low response rate. However, this is in line with response rates in other survey studies.
**Ethics committee approval:** The study was reviewed and approved by the Local Ethics Committee.
**Funding for this study:** The author(s) received no financial support for the research.
**Author Disclosures:**
Mette Karen Henning: Nothing to disclose
Safora Johansen: Nothing to disclose
Trond Mogens Aaløkken: Nothing to disclose
Anne Catrine Traegde Martinsen: Nothing to disclose

### RPS 704-4

**Possibilities of quantitative assessment of regional pulmonary perfusion using three-dimensional ultrafast dynamic contrast-enhanced magnetic resonance imaging: preliminary experience in 10 subjects**
*A. Zakharova*, D. Kupriyanov, A. Pozdniakov; Saint Petersburg/RU
*(ellin-ave@yandex.ru)*

**Purpose:** To assess regional differences in quantitative pulmonary perfusion parameters, i.e., pulmonary blood flow (PBF), mean transit time (MTT), and pulmonary blood volume (PBV) in the entire lung on a voxel-by-voxel basis in 10 volunteers with no signs of interstitial lung damage according to computer tomography data and clinical and laboratory data.
**Methods or Background:** Three-dimensional ultrafast dynamic contrast-enhanced MR imaging using 3D T1-weighted images was performed on 10 healthy volunteers with no signs of interstitial lung damage. Postprocessing methods based on the indicator dilution theory and the central volume limit theorem were applied to the dynamic image series, which enabled evaluating PBF, PBV, and MTT for the targeted regions of interest. Arterial input function (AIF) was used, as well as the time-intensity curves.
**Results or Findings:** Regional PBF, MTT, and PBV showed reliable differences between central and peripheral sections of lung lobes. An applied calculation model can be used to evaluate the entire volume of lung tissue.
**Conclusion:** A three-dimensional ultrafast MRI sequence is feasible for the assessment of regional quantitative pulmonary perfusion parameters in the lung tissue, regardless of physiological features of blood supply mechanisms of different lung regions.
**Limitations:** The main limitations of our study were the unavoidable selection bias and the small sample size.
**Ethics committee approval:** This study is approved by the Ethics Committee of Saint-Petersburg State Pediatric Medical University.
**Funding for this study:** No external funding was received for this study.
**Author Disclosures:**
Dipl.Ing. Dmitry Kupriyanov: Nothing to disclose
Anna Zakharova: Nothing to disclose
Aleksandr Pozdniakov: Nothing to disclose

### RPS 704-6

**Comparability of methods for assessing coronary risks by chest ultra-low dose computed tomography (ultra-LDCT) from lung cancer screening (LCS) and coronary computed tomography angiography (CCTA)**
*A. Nikolaev*[1], V. Gombolevskiy[1], V. Chernina[1], I. Blokhin[1], O. Korkunova[1], M. Suchilova[1], N. Nikolaeva[2], S. Morozov[1]; [1]Moscow/RU, [2]Unterföhring/DE
*(a.e.nikolaev@yandex.ru)*

**Purpose:** Coronary calcification is important incidental finding in ultra-LDCT performed for LCS. In our study we decide to assess the comparability of coronary calcium and coronary risk values measured on LCS ultra-LDCT (>1 mSv) without ECG-synchronization and contrast-enhancement versus a) non-Contrast Cardio Computed Tomography with ECG-synchronization (ECG-Gated Cardiac CT), b) Coronary Computed Tomography Angiography (CCTA).
**Methods or Background:** The study comprised 283 studies: a) 68 patients who underwent ultra-LDCT in LCS and standard ECG-Gated Cardiac CT. Comparison methods: Agatstone index for both CT, visual and quantitative CAC-DRS scales for both CT, and b) 49 patients with LCS ultra-LDCT and standard CCTA, also carried out in one visit, meeting all inclusion/exclusion criteria of the study and in LCS. Comparison methods: visual and quantitative (V&A) CAC-DRS scales for ultra-LDCT, CAD-RADS scale for CCTA.
**Results or Findings:** We have established correlation of Agatston score determined with ultra-LDCT in LCS and ECG-gated Cardiac CT with Spearman Correlation Coefficient (SCC) in 0.930 (p-value (p)< .001) and Kendall's Tau-b (KT-b) score in 0.859 (p< .001). Also, we have established correlation between data of A/V CAC-DRS on ultra-LDCT and A/A CAC-DRS on ECG-gated cardiac CT with SCC in 0.906/0.973 (p)< .001 and KT-b score in 0.887/0.965 (p< .001). Risks identified by the ultra-LDCT fixed by CAC-DRS are comparable to risks identified by CCTA with CAD-RADS with SCC in 0.855 (p)< .001) and KT-b score in 0.821 (p< .001).

**Conclusion:** Methods of coronary calcium assessment with chest ultra-LDCT and CT with ECG synchronisation are comparable. Therefore it is possible to assess coronary calcium in lung cancer screening by ultra-LDCT at a reliable-high level using both quantitative and visual CAC-DRS scales.
**Limitations:** No limitations were identified.
**Ethics committee approval:** The local ethical committee approved this study.
**Funding for this study:** No funding was received for this work.
**Author Disclosures:**
Olga Korkunova: Nothing to disclose
Sergey Morozov: Author: ideological inspirer
Maria Suchilova: Nothing to disclose
Valeria Chernina: Author: Second author
Alexander Nikolaev: Author: Main Author
Nataliia Nikolaeva: Author: Third author
Ivan Blokhin: Nothing to disclose
Victor Gombolevskiy: Nothing to disclose

### RPS 704-7

**Impact of contrast-enhancement and virtual monoenergetic image energy levels on emphysema quantification: experience with photon-counting detector CT**

*L. Jungblut*[1], D. Kronenberg[1], V. Mergen[1], K. Higashigaito[1], B. Schmidt[2], A. Euler[1], H. Alkadhi[1], T. Frauenfelder[1], K. Martini[1]; [1]Zurich/CH, [2]Forchheim/DE

**Purpose:** To evaluate the impact of contrast enhancement and different virtual monoenergetic image energies on automatised emphysema quantification with photon-counting detector computed tomography (PCD-CT).
**Methods or Background:** Sixty patients who underwent contrast-enhanced chest CT on a first-generation, clinical dual-source PCD-CT were retrospectively included. Scans were performed in the multienergy (QuantumPlus) mode at 120kV with weight-adjusted intravenous contrast agent. Virtual non-contrast images (VNC), as well as virtual monoenergetic images (VMI) from 40-80keV obtained in 10keV intervals, were reconstructed. CT attenuation was measured in the aorta; noise was measured in subcutaneous fat and defined as the standard deviation of attenuation. Contrast-to-noise with ROI in the ascending aorta and signal-to-noise ratio in the subcutaneous fat were calculated. Subjective image quality was rated by two blinded radiologists. Emphysema quantification (threshold at -950 HU) was performed by a commercially available software. VNC images served as reference standard for emphysema quantification.
**Results or Findings:** Noise and CNR showed a strong negative correlation ($r=-0.98$; $p<0.01$) to VMI-energies. The score of subjective assessment was highest at 70keV for lung parenchyma and 50keV for pulmonary vessel evaluation ($p<0.001$). The best trade-off for the assessment of emphysema while maintaining reasonable contrast for pulmonary vessel evaluation was determined between 60-70keV. Overall, contrast-enhanced imaging led to significant and systematic underestimation of emphysema as compared to VNC ($p<0.001$) and decreased with increasing VMI-energy ($r=0.98$; $p=0.003$). The least difference in emphysema quantification between contrast-enhanced scans and VNC was found at 80keV.
**Conclusion:** Emphysema quantification was significantly affected by intravenous contrast administration and VMI-energy level. VMI at 80keV yielded most comparable results to VNC. The best trade-off in qualitative as well as in quantitative image quality evaluation was determined at 60/70keV.
**Limitations:** The limitation is the single-centred design.
**Ethics committee approval:** This study was approved by an ethics committee.
**Funding for this study:** No funding was received for this study.
**Author Disclosures:**
Daniel Kronenberg: Nothing to disclose
Thomas Frauenfelder: Nothing to disclose
Victor Mergen: Nothing to disclose
Kai Higashigaito: Nothing to disclose
Katharina Martini: Nothing to disclose
Lisa Jungblut: Nothing to disclose
Bernhard Schmidt: Employee: Siemens
Andre Euler: Nothing to disclose
Hatem Alkadhi: Nothing to disclose

**08:00-09:00** **Room E1**

### Research Presentation Session: Cardiac

### RPS 703

### Cross-sectional imaging in congenital heart disease

**Moderator**
M. Krupiński; Krakow/PL

### RPS 703-2

**Accuracy of fully automated ventricular morpho-functional assessment in patients with tetralogy of fallot**

*D. Capra*, C. B. Monti, M. D. M. Galimberti Ortiz, G. V. D. Amato, F. Sardanelli, F. Secchi; Milan/IT

**Purpose:** Our aim was to assess the accuracy of a fully automated AI-based segmentation of ventricular volumes in patients with Tetralogy of Fallot (ToF) compared to manual measurements.
**Methods or Background:** Patients with ToF who had undergone magnetic resonance at our institution were retrospectively reviewed. Fully automated cardiac segmentation was performed by an AI-based software, to obtain right ventricular (RV) end-diastolic (EDVi) and end-systolic (ESVi) volume indexes, and ejection fraction (EF). A human reader then performed manual segmentation to obtain the same parameters.
**Results or Findings:** 55 patients were included in the study, 23 of whom were females (42%), with a median age of 27 years (interquartile range [IQR] 18-40 years). Median values were: RV EDVi 112 mL/m2 (IQR 93-130 mL/m2), RV ESVi 57 mL/m2 (IQR 46-71 mL/m2), RV EF 50% (IQR 41-56%), for automatic assessment, and RV EDVi 100 mL/m2 (IQR 79-125 mL/m2), RV ESVi 44 mL/m2 (IQR 33-60 mL/m2), RV EF 57% (IQR 49-61%) for manual assessment. Comparisons showed a bias of -9 mL/m2 and a CoR of 65 mL/m2 for RV EDVi, a bias of -12 mL/m2 and a CoR of 45 mL/m2 for RV ESVi, a bias of 7% and a CoR of 32% for RV EF.
**Conclusion:** While the biases for automatic segmentation are within acceptable clinical range, reproducibility is not fully satisfactory due to wide variation ranges.
**Limitations:** The limitation is the retrospective and monocentric design.
**Ethics committee approval:** The local Ethics Committee approved this retrospective study.
**Funding for this study:** No funding was received for this study.
**Author Disclosures:**
Francesco Secchi: Nothing to disclose
Francesco Sardanelli: Speaker: General Electric, Bayer, Bracco Advisory Board: General Electric, Bayer, Bracco Research/Grant Support: General Electric, Bayer, Bracco
Caterina Beatrice Monti: Nothing to disclose
Maria Del Mar Galimberti Ortiz: Nothing to disclose
Gaetano Valerio Davide Amato: Nothing to disclose
Davide Capra: Nothing to disclose

### RPS 703-3

**Four-dimensional phase contrast MRI with accelerated dual velocity encoding in patients with complex congenital heart disease**

*L. Kalifa*, G. Chatelier, A. Fels, E. Gouverneur, C. Pasquet, Y-W. Kim, F. Bouhajja, C. Roux, A. A. Azarine; Paris/FR
*(kalifalaurette@hotmail.fr)*

**Purpose:** To test the feasibility of a dual-velocity encoding (Dual-Venc) 4D Flow MR prototype sequence to assess various vascular flows in complex congenital heart disease patients.
**Methods or Background:** Routine cardiac MRI was performed on 17 young adults mostly followed for complex congenital heart disease on a 3T Magnet MRI (Discovery MR 750, GEMS). The usual 4D flow sequence has been replaced by a Kt-ARC accelerated prototype sequence, using two different velocity encoding (Venc) set to 300/100 cm/s; temporal/spatial resolution = 40-45msec / 2 × 2 × 2.2 mm3, after a triphasic gadolinium-based contrast agent injection (Gadovist, Bayer, Germany). MRI data was anonymised and sent to a cloud-based software (Arterys). After automatic phase offsets and background correction, we assessed velocity-to-noise ratio (VNR), peak velocity and forward flow for different vessels, simultaneously and comparatively between high and low Venc datasets. All patients were informed and signed a consent to test the prototype sequence.

Thursday

**Results or Findings:** All MRIs were successfully acquired in a mean scan time of 15 ± 4 minutes, reducing total scan time by 33% compared to two separate acquisitions. Cloud-based post-processing enabled easy analysis of both low and high Venc heavy datasets. Aliasing artifacts occurred more often for arterial measurements performed at low Venc. VNR at low Venc was always significantly better. The variability of instantaneous net flow measurements was significantly higher at high Venc than at low Venc (p < 0,05), especially for low velocity and medium caliber veins.
**Conclusion:** Kt-ARC accelerated Dual-Venc 4D Flow MR sequence is feasible and particularly well suited for patients with complex congenital heart disease.
**Limitations:** The limitations are a small number of patients and no control subjects.
**Ethics committee approval:** This study was approved by IRB (number 00012157).
**Funding for this study:** No funding was received for this study.
**Author Disclosures:**
Young-Wouk Kim: Nothing to disclose
Caroline Pasquet: Nothing to disclose
Charles Roux: Nothing to disclose
Elodie Gouverneur: Nothing to disclose
Gilles Chatelier: Nothing to disclose
Audrey Fels: Nothing to disclose
Arshid A. Azarine: Nothing to disclose
Laurette Kalifa: Nothing to disclose
Fraj Bouhajja: Nothing to disclose

### RPS 703-4
**Multimodal approach in diagnostics of complex anatomical variants of atrioventricular septal defect**
*Y. Y. Tsasiuk*, L. Shapoval, R. Tammo, T. Yalynska; Kiev/UA
*(tsasyuk1881@gmail.com)*

**Purpose:** Background: diagnostic efficiency of echocardiography in assessing the morpho-functional features of AVSD as an isolated heart pathology reaches 100%. In cases of a combination of AVSD with cardiac and noncardiac anomalies, the indicators of diagnostic efficiency change. Purpose: to evaluate the effectiveness of Echo, MDCT and MRI in the diagnosis of complex anatomical variants of the defect.
**Methods or Background:** 279 patients with different anatomical variants of AVSD.
**Results or Findings:** According to Echo results, 142 (50.1%) patients were diagnosed with additional CHD, 49 (34.5%) more than two and 12 (8.5%) complex CHD, including AVSD, common atrium, TAPVC, TGA, PA, DORV. This complex anatomy has been observed in patients with heterotaxic syndrome. In visualisation of intracardiac anatomy, 48 cases required clarification: in 29 cases, an additional MDCT was performed and 19 cases MRI examination. The overall accuracy of Echo in the diagnosis of pathology did not exceed 89.2% (sensitivity 83.4%, specificity 38.9%; predictability of a positive result 84.7%). False-negative results were obtained in 9 out of 48 cases, false-positive results in 7 patients. Overall accuracy MDCT was 98.9% (S-96.2%, Sp-99.8%; PPR-98.7%). FNR was obtained in 2 of 29 cases. At the same time, concomitant pathological broncho-pulmonary changes were detected in 35% of children. Overall accuracy MRI was 99% (S - 98.9%, Sp - 98%; PPR - 99.7%). FNR was obtained in 1 out of 19 cases.
**Conclusion:** In the diagnosis and identification of pathology concomitant with AVSD, MRI and MDCT have significant advantages over Echo due to the high sensitivity and specificity in visualisation of extracardiac structures. The possibility of diagnosis of concomitant pathological broncho-pulmonary changes in 35% of children is also a significant advantage over Echo.
**Limitations:** The limitation is the retrospective analysis.
**Ethics committee approval:** This study was approved by an ethics committee.
**Funding for this study:** Daily-work.
**Author Disclosures:**
Tetyana Yalynska: Nothing to disclose
Yevhen Yevhenovich Tsasiuk: Nothing to disclose
Raad Tammo: Nothing to disclose
Liudmila Shapoval: Nothing to disclose

### RPS 703-5
**Free-breathing high resolution modified Dixon steady-state angiography with compressed sensing for the assessment of the thoracic vasculature in pediatric patients with congenital heart disease**
*N. Mesropyan*[1], A. Isaak[1], D. Dabir[1], C. Hart[1], D. Kravchenko[1], C. Katemann[2], D. Kütting[1], U. I. Attenberger[1], J. A. Luetkens[1]; [1]Bonn/DE, [2]Hamburg/DE

**Purpose:** This study was aimed to evaluate the diagnostic utility of a respiratory- and electrocardiogram-gated steady-state MR-angiography (SS-MRA) with modified Dixon (mDixon) fat suppression technique and compressed sensing in comparison to standard first-pass (FP) MRA in pediatric patients with congenital heart disease (CHD) at 3.0T.
**Methods or Background:** Pediatric CHD patients, who underwent cardiac MRI with FP-MRA followed by mDixon SS-MRA at 3T were retrospectively analysed. Image quality was assessed using a Likert scale from 5 (excellent) to 1 (non-diagnostic). Blood-to-tissue ratio, fat suppression quality, and quantitative measurements of the thoracic vasculature were assessed. Measurements were performed separately and/or in consensus by two readers. Paired Student t-test, Wilcoxon test, and ICCs were used for statistical analysis.
**Results or Findings:** 32 patients with CHD (mean age: 3.3±1.7 years, 13 female) were included. Overall image quality of mDixon SS-MRA was higher compared to FP-MRA (4.5±0.5 vs. 3.3±0.5; P<0.001). Blood-to-tissue contrast ratio of mDixon SS-MRA was comparable to FP-MRA (7.85±4.75 vs. 6.35±2.23; P=0.133). Fat suppression of mDixon SS-MRA was perfect in 30/32 (94 %) cases. Vessel diameters were greater in FP-MRA compared to mDixon SS-MRA with the greatest differences at the level of pulmonary arteries and veins (e.g., right pulmonary artery for reader 1: 10.4±2.4 vs. 9.9±2.3 mm, P<0.001). Interobserver agreement was higher for mDixon SS-MRA for all measurements compared to FP-MRA (ICCs >0.92). In 9/32 (28%) patients, 10 additional findings were identified on mDixon SS-MRA (e.g., partial anomalous venous return, abnormalities of coronary arteries, subclavian artery stenosis), which were not depicted using FP-MRA.
**Conclusion:** mDixon SS-MRA offers a robust fat suppression, a high image quality, and diagnostic utility for the assessment of the thoracic vasculature in pediatric CHD patients.
**Limitations:** The limitation is the small sample size.
**Ethics committee approval:** Ethics committee approval was received.
**Funding for this study:** No funding was received for this study.
**Author Disclosures:**
Darius Dabir: Nothing to disclose
Julian Alexander Luetkens: Nothing to disclose
Narine Mesropyan: Nothing to disclose
Alexander Isaak: Nothing to disclose
Christopher Hart: Nothing to disclose
Dmitrij Kravchenko: Nothing to disclose
Ulrike I. Attenberger: Nothing to disclose
Daniel Kütting: Nothing to disclose
Christoph Katemann: Nothing to disclose

**08:00-09:00** **Room G**

### Research Presentation Session: Neuro

### RPS 711

### Spine imaging and intervention

**Moderator**
D. Prayer; Vienna/AT

### RPS 711-2
**Comparison of radiation exposure of AIRO intraoperative CT with C-arm fluoroscopy during posterior lumbar interbody fusion**
*B. Van Berkel*[1], S. Van Cauter[2], G. Smets[3], D. Peuskens[2], T. Daenekindt[2], E. Buelens[2], F. Weyns[2]; [1]Leuven/BE, [2]Genk/BE, [3]Hasselt/BE
*(brecht_van_berkel@hotmail.com)*

**Purpose:** Navigation systems used during minimally invasive spine procedures have evolved from uniplanar, two-dimensional C-arm fluoroscopy to multiplanar, 3D intraoperative computed tomography (iCT). IUsing iCT, radiation exposure can significantly be reduced.
**Methods or Background:** The radiation exposure to the patient and operating room staff in posterior intervertebral lumbar fusion procedures is compared between iCT and C-arm fluoroscopy. The effective dose of the surgeon, operating nurse and anaesthesiologist were measured during surgery with personal dosimeters. The effective dose of the patient was calculated and the lateral and abdominal peak skin dose was measured with GafchromicTM films. Time-efficiency of the procedure was evaluated by recording the duration of pedicle screw fixation and the duration of the total surgery time.
**Results or Findings:** A total of 75 patients participated in the study, 30 patients had surgery guided by iCT and 45 by C-arm fluoroscopy. The radiation dose of the surgeon, the operating nurse and the anaesthesiologist was significantly lower with surgeries assisted by iCT, compared to C-arm fluoroscopy. In contrast, the effective dose of the patient significantly increased with iCT.
**Conclusion:** Using iCT, radiation exposure of the operating room staff can be reduced significantly. iCT increases the effective dose of the patient and prolongs the operative time.
**Limitations:** First, there was a significant difference between the iCT and the C-arm fluoroscopy group in terms of the surgeon who performed the MI-PLIF procedures. The AIRO iCT and spinal navigation themselves feature limitations. Bugs in the software sometimes caused the need to restart the AIRO iCT device during surgery.
**Ethics committee approval:** The study was conducted according to the guidelines of the Declaration of Helsinki, and approved by the Institutional Ethics Committee of Ziekenhuis Oost-Limburg Genk, Belgium (eudract/B-nr 66 B371201630400 – approved 17.01.2017).
**Funding for this study:** Funding was not required.
**Author Disclosures:**
Gwendolien Smets: Nothing to disclose
Thomas Daenekindt: Nothing to disclose
Sofie Van Cauter: Nothing to disclose
Dieter Peuskens: Nothing to disclose
Frank Weyns: Nothing to disclose
Eveleen Buelens: Nothing to disclose
Brecht Van Berkel: Nothing to disclose

### RPS 711-3

**Prognostic utility of anterior atlantodens interval widening on cervical spine CT for transverse atlantal ligament integrity**

*P. Fiester*, D. Rao, E. Soule, G. Rahmathulla, P. Orallo; Jacksonville/US

**Purpose:** Post-traumatic atlanto-axial instability is classically associated with tears of the transverse atlantal ligament at C1-C2 and is indirectly evaluated on plain film and cervical spine CT. The purpose of our study was to determine which method is most sensitive in predicting transverse atlantal ligament injury.
**Methods or Background:** Adult and paediatric trauma patients who suffered a transverse atlantal ligament tear on cervical MRI were identified retrospectively using Nuance mPower software. The cervical CT and MRI exams for these patients were reviewed by two neuroradiologists for the following: anterior and lateral atlanto-dens interval widening, lateral C1 mass offset, C1-C2 rotatory subluxation, and transverse atlantal ligament injuries on cervical MRI.
**Results or Findings:** Twenty-seven trauma patients were identified with a tear of the transverse atlantal ligament on cervical MRI. Eleven percent of these patients demonstrated an anterior dens interval measuring greater than 2 mm, 26% of patients demonstrated lateral mass offset of C1 on C2, 18% of patients demonstrated an asymmetry greater than 1 mm between the left and right lateral atlantodens interval, and one patient demonstrated atlanto-axial rotation measuring greater than 20%.
**Conclusion:** An anterior atlantodens interval measuring greater than 2 mm is an unreliable methodology to screen trauma patients for transverse altantal ligament injuries and atlanto-axial instability. Moreover, C1 lateral mass offset, lateral atlantodens asymmetry, and atlanto-axial rotation were all poor predictors of transverse atlantal ligament tears. Our findings underscore the importance of cervical MRI in the diagnostic workup and management of patients with acute, high velocity cervical spine trauma and suggest that anterior atlantodens interval widening on cervical CT is an unreliable screening method for atlanto-axial instability.
**Limitations:** This was a retrospective study featuring a small sample size.
**Ethics committee approval:** Informed consent was waived for this low risk, retrospective study.
**Funding for this study:** Not applicable
**Author Disclosures:**
Peaches Orallo: Nothing to disclose
Dinesh Rao: Nothing to disclose
Gazanfar Rahmathulla: Nothing to disclose
Erik Soule: Nothing to disclose
Peter Fiester: Nothing to disclose

### RPS 711-4

**Comprehensive evaluation of spinal structure changes contributing to foraminal stenosis: a weight-bearing MRI study**

*L. Pertici*, V. Pagliei, F. Bruno, F. Sgalambro, E. Tommasino, F. Borea, A. Barile, C. Masciocchi, A. Splendiani; L'Aquila/IT
*(leonardo.pertici@gmail.com)*

**Purpose:** The purpose of this study is to identify which structure contributes most to determining the worsening of lumbar foraminal stenosis through the study of dynamic magnetic resonance in the supine and orthostatic position.
**Methods or Background:** We retrospectively evaluated 200 patients who underwent standard supine and standing weight-bearing MRI scans of the lumbar spine (on a 0.25T MR scan). The inclusion criteria were grade 1-2-3 foraminal stenosis and complete good quality examinations Foraminal stenosis parameters were manually measured by two radiologists and included: depth of the lateral recess; angle of the lateral recess; area of the lateral canal in sagittal and axial. As explanatory variables, the same researchers manually measured: capsular and midpoint ligamental flava thickness; spinous processes distance; facet joint cross-sectional area and thickness; intervertebral disc area and height; posterior intervertebral disc protrusion; spondylolisthesis. A matched group Hotelling's T2 test was used to compare supine vs orthostatic measurements. The Log-likelihood ratio statistics was used to assess the overall model fitting between foraminal stenosis parameters and explanatory variables.
**Results or Findings:** We found significant differences between supine and orthostatic position for all foraminal parameters and explanatory variables. Foraminal stenosis gets worse by reduction of the depth and angle of the lateral recess and the lateral canal sagittal and axial area (mean decrease 15-20%). The linear mixed model showed significant regression coefficients between foraminal stenosis parameters and ligamental flava midpoint thickness (z -1.96, $p<0.050$), ligamental flava capsular thickness (z 1.85, $p<0.05$), facet joint thickness (z 2.87, p 0.004), spinal processes distance (z 2.87, p 0.033), spondylolisthesis (z 2.40, p 0.016).
**Conclusion:** According to our weight-bearing study, changes of posterior elements induced by load may be the prevalent determinant of foraminal stenosis.
**Limitations:** Not applicable
**Ethics committee approval:** Not applicable
**Funding for this study:** Not applicable
**Author Disclosures:**
Emanuele Tommasino: Nothing to disclose
Ferruccio Sgalambro: Nothing to disclose
Alessandra Splendiani: Nothing to disclose
Francesco Borea: Nothing to disclose
Carlo Masciocchi: Nothing to disclose
Leonardo Pertici: Nothing to disclose
Antonio Barile: Nothing to disclose
Federico Bruno: Nothing to disclose
Valeria Pagliei: Nothing to disclose

### RPS 711-5

**Craniocervical prevertebral effusion in the acute trauma setting: a common sign for anterior atlanto-occipital membrane complex injury**

*P. Fiester*, G. Rahmathulla, M. Jenson, J. Patel, E. Soule; Jacksonville/US

**Purpose:** The purpose of this study was to identify and classify the different types of anterior atlanto-occipital membrane complex injuries on MRI and evaluate for the presence and location of a prevertebral effusion.
**Methods or Background:** Patients who suffered an anterior atlanto-occipital membrane complex injury were identified retrospectively using Nuance mPower software. The cervical CT and MRI exams for these patients were reviewed for the presence and location of a prevertebral effusion. Age-matched positive and negative control groups were obtained.
**Results or Findings:** Fifty patients were identified with an acute anterior atlanto-occipital membrane complex injury. Three distinct patterns of anterior atlanto-occipital membrane complex injury were observed: 1) increased STIR signal and disruption of the anterior atlanto-occipital membrane (19 patients); 2) increased STIR signal and disruption of the anterior atlantoaxial membrane (10 patients); 3) increased STIR signal and disruption of both the anterior atlanto-occipital membrane and anterior atlantoaxial membrane (21 patients). Ninety-four percent of patients with an anterior atlanto-occipital membrane complex injury presented with a prevertebral effusion at the craniocervical junction with an average anteroposterior dimension of 8 mm. Statistical analysis revealed a statistically significant association between anterior atlanto-occipital membrane complex injury and a craniocervical prevertebral effusion.
**Conclusion:** A craniocervical prevertebral effusion was observed in 94% of patients with an anterior atlanto-occipital membrane complex tear. Based on our findings, the presence of a craniocervical prevertebral effusion is 94% sensitive and 94% specific for an anterior atlanto-occipital membrane complex injury in high velocity trauma victims.
**Limitations:** This was a retrospective study with a small sample size.
**Ethics committee approval:** Informed consent was waived.
**Funding for this study:** No funding was received for this study.
**Author Disclosures:**
Jeet Patel: Nothing to disclose
Matthew Jenson: Nothing to disclose
Gazanfar Rahmathulla: Nothing to disclose
Erik Soule: Nothing to disclose
Peter Fiester: Nothing to disclose

### RPS 711-6

**Strong contrast stagnation of unilateral vertebral artery on black blood enhanced MR imaging predicts acute medulla infarction**

*S. M. Cho*, S. Y. Park, H-S. Kwak; Jeonju/KR
*(tytyuuii96@naver.com)*

**Purpose:** The purpose of this study was to evaluate angiographic and contrast enhancement patterns on three-dimensional (3D) black blood (BB) contrast-enhanced magnetic resonance (MR) imaging in patients with acute medulla infarction.
**Methods or Background:** From January 2020 to January 2021, we retrospectively analysed stroke 3D BB contrast-enhanced MR imaging and angiography findings of patients visiting the emergency room for symptom evaluation of acute medulla infarction. In total, 28 patients with acute medulla infarction were enrolled in this study. We divided the four groups according to whether angiography findings suggested occlusion, hypoplasia, or normal and whether vertebral artery (VA) contrast enhancement was shown or not.
**Results or Findings:** Of 28 patients with acute medulla infarction, seven (25.0%) showed delayed positive findings after 24 hours on diffusion-weighted imaging (DWI). Of these patients, 19 (67.9%) showed contrast enhancement of unilateral VA. Of 19 patients with contrast enhancement of VA, 18 showed no visualisation of enhanced VA on MR angiography and one showed hypoplastic VA. Of seven patients with delayed positive findings on DWI, five showed contrast enhancement of unilateral VA and no visualisation of enhanced VA on MR angiography.
**Conclusion:** Unilateral contrast enhancement on 3D BB contrast-enhanced MR imaging and no visualisation of VA on MR angiography are related to recent occlusion of distal VA. These findings suggest that recent occlusion of distal VA is related to acute medulla infarction including delayed visualisation on DWI.
**Limitations:** This is a retrospective study featuring a small case series.
**Ethics committee approval:** This study was approved by the institutional review board (IRB) of our institute, and informed consent was waived.
**Funding for this study:** No funding was received for this study.
**Author Disclosures:**
Seong Min Cho: Nothing to disclose
Hyo-Sung Kwak: Nothing to disclose
Suh Yeon Park: Nothing to disclose

### RPS 711-7
**Interrelationship between craniocervical disassociation spectrum injuries and atlantoaxial instability on trauma cervical MRI exams**
*P. Fiester*, M. Jenson, D. Rao, E. Soule, G. Rahmathulla; Jacksonville/US

**Purpose:** The purpose of our study is to identify adult trauma patients with an acute, traumatic tear of the transverse atlantal ligament on cervical MRI and evaluate for concomitant craniocervical junction ligamentous injuries.
**Methods or Background:** Adult trauma patients who suffered a transverse atlantal ligament injury on cervical MRI were identified retrospectively using Nuance mPower software. The cervical CT and MRI exams for these patients were reviewed by two neuroradiologists for the following: bony fractures of the craniocervical junction, transverse atlantal ligament integrity, major craniocervcial ligamentous integrity (alar ligaments, tectorial membrane, atlanto-occipital capsular ligaments), minor craniocervcial ligamentous integrity (anterior and posterior atlanto-occipital membranes), cervical cord trauma, and intracranial trauma.
**Results or Findings:** Thirty trauma patients presented to the ED with a history of high velocity trauma and an acute, mid-substance transverse atlantal ligament tear on cervical MRI. Twenty seven percent of patients demonstrated a tear in at least one major craniocervical ligament (atlanto-occipital capsular ligaments, alar ligaments, and tectorial membrane) with 17% demonstrating a tear in two major craniocervical ligaments, and no patients demonstrating a tear in all three major craniocervcial ligaments. Minor craniocervical ligament injuries (anterior and posterior atlanto-occipital membrane complex) were common and observed in 73% of patients. Fifty-seven percent of patients had a C1 and/or C2 fracture. Two patients underwent occipital cervical fusion and five patient underwent C1-C2 posterior fusion for their injuries with the remaining patients treated with prolonged external immobilisation.
**Conclusion:** Major and minor craniocervical junction ligamentous injuries are relatively common in the setting of transverse atlantal ligament injury.
**Limitations:** This was a retrospective study featuring a small sample size.
**Ethics committee approval:** Informed consent waived.
**Funding for this study:** No funding was received for this study.
**Author Disclosures:**
Dinesh Rao: Nothing to disclose
Matthew Jenson: Nothing to disclose
Gazanfar Rahmathulla: Nothing to disclose
Erik Soule: Nothing to disclose
Peter Fiester: Nothing to disclose

### RPS 711-8
**Can machine learning predict gender and age by evaluation of the subject's lumbar vertebrae?**
*R. Levi*, F. Garoli, M. Battaglia, G. Savini, G. Mazziotti, M. Grimaldi, L. S. Politi; Pieve Emanuele/IT
*(riccardo.levi@st.hunimed.eu)*

**Purpose:** To assess the accuracy of machine learning approach in predicting gender and age through analysis of radiomics features from CT images of the lumbar vertebrae, and define generalisability across different CT scanners.
**Methods or Background:** Age and gender are known factors that modify bone structure. Radiomics may depict modification of CT trabecular bone that could predict subjects' gender and age, and that could identify bone frailty and vertebral fracture risk. Further, little is known about reproducibility of CT radiomics. We annotated spherical regions-of-interest (ROIs) (9 mm diameter) in the centre of vertebral body of lumbar vertebrae (L1-L5) in 233 subjects undergoing lumbar CT for back pain on 3 different CT scanners, and we evaluated features from each ROIs. Subjects with a history of bone methabolism disorders, cancer and vertebral fractures were excluded. We performed machine learning classification and regression models to evaluate gender and age respectively on each lumbar vertebrae and we provided a model which combined predictions on each vertebrae.
**Results or Findings:** The machine learning model was trained on 173 patients and validated on a testing dataset of 60 patients. Radiomics models were able to predict patients' gender within the same CT scanner (ROC AUC: up to 0.9714), with lower performance on the combined dataset of the 3 scanners (ROC AUC 0.5545). Subjects' age was found to obtain good consistency among scanners (R2 0.568 all scanner) and MAD of 7.232 years, with highest results on a single CT scanner (R2 0.667, MAD 3.296 years).
**Conclusion:** Radiomics features are able to extract subjects' information from lumbar vertebral trabecular bone, and predict subjects' gender and age with great accuracy. However, acquisition from different CT scanners might reduce the accuracy of the analysis.
**Limitations:** The cohort of patients was small.
**Ethics committee approval:** This study was approved by an ethics committee.
**Funding for this study:** No funding was received for this study.
**Author Disclosures:**
Giovanni Savini: Nothing to disclose
Gherardo Mazziotti: Nothing to disclose
Federico Garoli: Nothing to disclose
Marco Grimaldi: Nothing to disclose
Massimiliano Battaglia: Nothing to disclose
Riccardo Levi: Nothing to disclose
Letterio S. Politi: Nothing to disclose

**08:00-09:00** **Room K**

### Research Presentation Session: Breast

### RPS 702

### New technologies in breast imaging

**Moderator**
M. Dietzel; Erlangen/DE
**Author Disclosure:**
M. Dietzel: CEO: radiologie-weiterbildung.de; school-of-radiology.com; Owner: radiologie-weiterbildung.de; school-of-radiology.com

### RPS 702-3
**Towards a disruptive innovation in breast imaging? Initial experience with a next-generation low-field MRI scanner**
*M. Dietzel*, F. B. Laun, E. Wenkel, R. Heiß, M. Wetzl, L. Siegler, S. Bickelhaupt, M. Uder, S. Ohlmeyer; Erlangen/DE

**Purpose:** Breast-MRI is essential for state-of-the-art breast imaging. However, broader clinical use is limited due to high procedural costs, technical complexity, limited availability and substantial requirements on local infrastructure. Low-field breast-MRI may compensate for these disadvantages. We report on our first experiences with a next-generation low-field scanner operating at 0.55 Tesla.
**Methods or Background:** In this prospective IRB-approved study we examined patients based on standard MRI-indications (0.55T MAGNETOM Free.Max, Siemens Healthineers, Erlangen/Germany; multichannel breast-coil, Noras, Höchberg/Germany). Low-field scanner design enables operation beyond traditional MRI departments (height <2 metre, weight <3.2 tons, no quench pipe necessary etc.). Likewise, total cost of ownership is approximately 40% reduced compared to 1.5T systems. We optimised a dedicated multiparametric breast-MRI protocol (T1, T2, DWI, ADC, dynamic contrast-enhanced; +/- fat-sat). A blinded expert reader (>15 years breast-MRI experience) assessed the examinations based on established procedures (BI-RADS, ADC-measurement by Wielema et al., artifact-grading by Fischer et al.).
**Results or Findings:** We examined ten patients (mean: 54.6 years, range: 36-72 years) with typical breast-MRI indications (recurrence vs. scar, therapy-response, preoperative-staging, CUP-syndrome, assessment etc.). In all patients, image quality was compelling and entirely diagnostic. No significant artifacts were noted. Scanning a full, non-abbreviated multiparametric protocol was possible in less than <20 minutes. We will provide clinical cases, technical-, and protocol-specifications at the meeting.
**Conclusion:** We demonstrate feasibility of low-field breast-MRI with compelling image quality. Low-field scanners promise to drive affordability and accessibility of breast-MRI. If upcoming clinical studies confirm our initial observations, the method may emerge as an alternative to high-field breast-MRI. We expect the greatest benefits of low-field breast-MRI for personalised screening.

**Limitations:** A more detailed analysis of low-field breast-MRI (qualitative, quantitative, statistical, multireader, etc.) is required and will be part of future clinical trials.
**Ethics committee approval:** This study was approved by IRB.
**Funding for this study:** Not applicable.
**Author Disclosures:**
Evelyn Wenkel: Nothing to disclose
Lisa Siegler: Nothing to disclose
Frederik Bernd Laun: Nothing to disclose
Matthias Dietzel: Nothing to disclose
Matthias Wetzl: Nothing to disclose
Michael Uder: Nothing to disclose
Sabine Ohlmeyer: Nothing to disclose
Sebastian Bickelhaupt: Nothing to disclose
Rafael Heiß: Nothing to disclose

### RPS 702-4

**Quantification of contrast enhancement of breast cancer in direct converting, photon-counting spiral breast CT: immunohistochemical subtypes and grading**

*S. Ohlmeyer*, M. Wetzl, R. Erber, R. Schulz-Wendtland, M. Uder, E. Wenkel; Erlangen/DE
*(sabine.ohlmeyer@uk-erlangen.de)*

**Purpose:** To quantify contrast media enhancement of breast lesions scanned with a newly introduced direct converting, photon-counting Spiral Breast CT (SBCT) and to evaluate whether tumour contrast enhancement could aid discriminating breast cancer subtypes.
**Methods or Background:** This study included 48 patients with malignant breast lesions. SBCT was performed in all patients before and two minutes after intravenous contrast application. Lesion Hounsfield units (HU) were recorded in the unenhanced scan and in the contrast-enhanced scan of SBCT using a circle tool. Lesion contrast enhancement was calculated as the difference measured in HU. All histopathology results were approved via image-guided biopsy or surgery. Immunohistochemical staining was conducted for expression of estrogen receptor (ER), progesterone receptor (PR), human epidermal growth factor receptor-2 (HER2) and Ki-67 index. Contrast enhancement of breast lesions was correlated with immunohistochemical breast cancer subtypes (Luminal A/B, HER2 positive, triple negative), Ki-67 expression and grading (G1-G3).
**Results or Findings:** Lesions with high Ki-67 (n=35) index showed higher contrast enhancement than those with low Ki-67 index (n=13): 106.8 vs. 94.5 HU (P = .151). Highest contrast enhancement was seen for Luminal B lesions (114.2 HU, n=19) and HER2 positive lesions (102.9 HU, n=9) compared to Luminal A lesions (94.4 HU, n=16) and triple negative lesions (75.0 HU, n=4). G1 lesions (n=11) showed an enhancement of 119.2 HU, G2 (n=20) and G3 tumours (n=17) enhancement was lower with 94.1 HU and 100.7 HU, respectively.
**Conclusion:** Contrast media uptake of malignant breast lesions in SBCT seems to be different depending on immunohistochemical subtype and Ki-67 expression. This could be a valuable benefit in the pre-operative work-up of breast cancer patients.
**Limitations:** Not applicable.
**Ethics committee approval:** This study was approved by IRB.
**Funding for this study:** No funding was received for this study.
**Author Disclosures:**
Evelyn Wenkel: Nothing to disclose
Rüdiger Schulz-Wendtland: Nothing to disclose
Matthias Wetzl: Nothing to disclose
Michael Uder: Nothing to disclose
Sabine Ohlmeyer: Nothing to disclose
Ramona Erber: Nothing to disclose

### RPS 702-6

**SOLUS - smart optical and ultrasound diagnostics of breast cancer: US / DOT hybrid system for the characterisation of breast lesions**

*E. Venturini*, G. Maffeis, P. Taroni, P. Panizza, SOLUS Consortium; Milan/IT
*(eleventurini86@gmail.com)*

**Purpose:** Presentation of technology and first clinical results of a project funded by a Grant Horizon 2020 for the construction of a hybrid ultrasound and optical tomography device, created by SOLUS consortium that unites 9 European partners' experts in optical imaging, ultrasound and images processing.
**Methods or Background:** US/optical hybrid prototype. The device consists of SSI Aixplorer Mach 30 ultrasound system combined with a diffuse optical tomography (DOT) system. SOLUS includes, in a single exam, B mode ultrasound, colour doppler, share wave elastography and DOT, through a probe that has a normal US transducer in the centre, flanked, laterally, by 8 optodes that emit and receive light in the red and infrared spectrum. The optical study allows one to measure the concentrations of water, lipids, collagen, oxygenated hemoglobin (HbO2) and deoxygenated (Hb), the total hemoglobin content (tHb = Hb+HbO2) and saturation (SO2 = HbO2 / tHb), as well as to provide information on cell membranes and subcellular organelles. The clinical study plans to compare, in vivo, the parameters obtained from the evaluation of 20 fibroadenomas and 20 malignant lesions to verify the possibility of differentiating them with this multimodal device.
**Results or Findings:** To date, the cases of multimodal in vivo analysis, performed in the preliminary phase of the study, have confirmed the possibility of acquiring useful information for the characterisation of breast lesions, confirming the expectations.
**Conclusion:** Very initial data, based mainly on DOT findings guided by ultrasound, suggest that a multiparametric analysis may have a role in the characterisation of breast lesions.
**Limitations:** The limitation is very initial data.
**Ethics committee approval:** The Ethical committee of IRCCS San Raffaele approved the study.
**Funding for this study:** The project has received funding from the European Union's Horizon 2020 research and innovation programme under the grant agreement n. 731877.
**Author Disclosures:**
Paola Taroni: Nothing to disclose
Elena Venturini: Speaker: Bayer basic and advanced breast MRI course
SOLUS Consortium: Nothing to disclose
Giulia Maffeis: Nothing to disclose
Pietro Panizza: Speaker: Bayer basic and advanced breast MRI course

### RPS 702-7

**Preliminary evaluation of an optical mammography system for monitoring response to neoadjuvant chemotherapy in breast cancer patients**

*C. Santangelo*, G. Maffeis, S. Calamarà, R. Cubeddu, P. Taroni, P. Panizza; Milan/IT
*(carola.santangelo.md@gmail.com)*

**Purpose:** Preliminary evaluation of the performance of an optical mammography prototype in predicting and monitoring the response to neoadjuvant chemotherapy in patients with breast cancer.
**Methods or Background:** An optical mammography prototype was developed by the Department of Physics of Politecnico of Milano to study the compressed breast in two projections, such as X-ray mammography. It emits pulsed light at seven wavelengths that passes through the breast and is measured by detectors placed on the opposite surface. This device allows non-invasive detection of the concentration values of some parameters such as: oxyhemoglobin, deoxyhemoglobin, water, lipids, collagen. To date, 6 patients undergoing neoadjuvant chemotherapy for breast cancer were evaluated with optical mammography, monitored with mammography, ultrasound and MRI; optical parameters were measured before, during and after treatment.
**Results or Findings:** In the responders, the first data show clear correlation between the response to neoadjuvant chemotherapy and the variation of some of the optical parameters examined, more marked immediately after the first administration of the drug: the percentage of hemoglobin, water and collagen decreases rapidly while that of the lipids increases.
**Conclusion:** The preliminary data confirm the potential of this optical mammography prototype, in the prediction and monitoring of the response to neoadjuvant chemotherapy, in a non-invasive way. These data are such to justify a study on large numbers to optimise the protocol and verify their usefulness in daily clinical practice.
**Limitations:** The limitation are the preliminary results.
**Ethics committee approval:** This study was approved by an ethics committee.
**Funding for this study:** Grant was received for this study from Fondazione Umberto Veronesi.
**Author Disclosures:**
Giulia Maffeis: Nothing to disclose
Rinaldo Cubeddu: Nothing to disclose
Paola Taroni: Nothing to disclose
Sabrina Calamarà: Nothing to disclose
Pietro Panizza: Nothing to disclose
Carolina Santangelo: Nothing to disclose

**RPS 702-8**

**Correlation of quantitative CT-data derived from multiparametric dual-layer spectral CT-maps with molecular biomarkers in patients with invasive breast carcinomas**

*K. B. Krug*[1], B. Schömig-Markiefka[1], W. Malter[1], G. Campbell[2], M. Puesken[1], M. Schlamann[1], D. Maintz[1], M. Hellmich[1]; [1]Cologne/DE, [2]Hamburg/DE
*(Barbara.krug@uk-koeln.de)*

**Purpose:** To examine the correlation of quantitative data derived from dual-layer CT material maps with molecular biomarkers of invasive breast carcinomas.
**Methods or Background:** All patients at the University Breast Cancer Centre who underwent a clinically indicated dual-layer CT-scan (IQon, Philips) for pretherapeutical staging of invasive breast cancer from 03/2017 to 07/2020 were prospectively included. Virtual monoenergetic (VM) images (40/100 keV), iodine concentration maps, and zeffective maps were reconstructed from the CT-image datasets. Spectral attenuation curves were calculated additionally. ROI-based evaluations of the index tumours and the reference "aorta" were performed semi-automatically in identical anatomical positions using dedicated evaluation software. Descriptive statistical analysis was based on Spearman's rank correlations.
**Results or Findings:** Bivariate analysis showed a significant correlation between conventional Houndsfield scores and Her2neu receptor status (p=0.004) molecular subtyping (p=0.041) for all carcinomas, which were both confirmed by the evaluations of iodine content, VM slopes, and zeffective values (p=0.002-0.021). Additionally, iodine content and zeffective values correlated with estrogen (p=0.039,0.028) and progesterone receptor status (p=0.005,0.006). Normalisation of the measured values in the tumours with those in the aorta confirmed significant correlations of iodine content, VM slopes and zeffective-values compared to estrogen, progesterone and Her2neu receptor status (p=0.002-0.024). The results were consistent in 168 carcinomas of the non-significant type (NST).
**Conclusion:** Our preliminary results indicate that quantitative evaluations of iodine content, VM slopes, and zeffective-values on dual-layer CT-examinations allow conclusions on the hormone and Her2neu receptor status of invasive breast carcinomas. These initial results will be further elaborated with the clinical introduction of photon CT-imaging and may be translated into clinical care in the long term.
**Limitations:** The limitation is a low number of cases prohibiting further subgroup analyses.
**Ethics committee approval:** The implementation of the study was approved by the local Institutional Ethical Review Board (file number 17-167).
**Funding for this study:** Not applicable.
**Author Disclosures:**
Birgid Schömig-Markiefka: Nothing to disclose
Graeme Campbell: Nothing to disclose
David Maintz: Nothing to disclose
Michael Puesken: Nothing to disclose
Wolfram Malter: Nothing to disclose
Martin Hellmich: Nothing to disclose
Kathrin Barbara Krug: Nothing to disclose
Marc Schlamann: Nothing to disclose

**08:00-09:00 Room M 1**

### Research Presentation Session: Paediatric

### RPS 712

### Imaging children from head to toe

**Moderator**
M. I. Argyropoulou; Ioannina/GR

**RPS 712-2**

**Imaging cytoarchitectural changes within focal cortical dysplasia using diffusion tensor imaging**

*A. G. Gennari*, D. Cserpan, I. Yakoub, R. Kottke, R. Tuura O'Gorman, G. Ramantani; Zurich/CH
*(gennari_antonio@libero.it)*

**Purpose:** To investigate if diffusion tensor imaging (DTI) indices differ between focal cortical dysplasia (FCD) and contralateral brain parenchyma (CBP) and if alterations in specific clinical features affect DTI indices.
**Methods or Background:** This single-centre retrospective, cross-sectional study, considered children and adolescents with focal structural epilepsy associated with FCD who underwent brain magnetic resonance imaging (MRI), including DTI sequence. FCDs were manually segmented on high-resolution 3D T1-weighted MRI. After normalising the DTI to the anatomical images, fractional anisotropy (FA), mean diffusivity (MD), axial diffusivity (AD), and radial diffusivity (RD) were calculated in both FCD and CBP. DTI indices facilitating an optimal differentiation between FCD and CBP were further analysed to determine if clinical features affected DTI indices.
**Results or Findings:** 32 patients (20 male; mean age at MRI 6.0±4.7 years) were enrolled in this study: FCD was histologically confirmed in 15/32 cases. FA values were lower in FCD compared to CBP (p = 0.028), whereas MD values were higher in FCD than in CBP (p = 0.044). In histologically confirmed FCD, the difference in FA values between FCD and CBP was higher for FCD type IIb than for FCD type I (p = 0.033), and for patients with a positive vs. negative history of status epilepticus (p = 0.015). None of the clinical features influenced the difference in MD values.
**Conclusion:** FA values discriminated FCD from CBP and distinguished between FCD subtypes.
**Limitations: Limitations:** (1) it is a retrospective single-centre study; (2) two different DTI protocols were used; (3) FCDs were manually segmented.
**Ethics committee approval:** Patients and caretakers gave written informed consent to participate in the study (local ethics committee reference: KEK-ZH PB-2019-01854).
**Funding for this study:** Funding was received from: Anna Mueller Grocholski Foundation (G.R.); ESKAS Scholarship, Premio Nino and Hansi Cominotti (A.G.G.). The funders had no role in the design/analysis of the study.
**Author Disclosures:**
Ruth Tuura O'Gorman: Nothing to disclose
Antonio Giulio Gennari: Nothing to disclose
Dorottya Cserpan: Nothing to disclose
Georgia Ramantani: Nothing to disclose
Ilona Yakoub: Nothing to disclose
Raimund Kottke: Nothing to disclose

**RPS 712-4**

**Mild central canal dilatation after spinal concussion in children: a new finding in SCIWORA spectrum**

*c. bodlak*, I. Delgado, Á. Sánchez-Montañez, J. M. Escudero, É. Vazquez; Barcelona/ES
*(carmenbodlak@hotmail.com)*

**Purpose:** To describe and review the literature about central canal dilatation as an isolated radiologic finding in children presenting clinically with a medullary concussion.
**Methods or Background:** We review three paediatric patients presenting with medullary concussion after minor trauma in which isolated central canal dilatation was seen in the MR. We analyse the CT and MR of 3 children who after spinal trauma were presenting with incomplete traumatic cord syndrome (mostly altered sensation). Patients underwent CT for depicting traumatic injuries with no abnormalities found. In MR images only central cord dilatation was found, as an isolated imaging finding. Patients were treated conservatively with complete neurological function restoration. Furthermore, we do a non-systematic review of the available literature. To the best of our knowledge, we are the first ones to describe this entity and its clinical course.
**Results or Findings:** Isolated central canal dilatation was not previously described as an imaging finding after spinal trauma. A condition called presyrinx was described as being the predecessor of the development of syringomyelia. This state was characterised by the presence of extensive spinal cord T2 hyperintensity, a feature that is not seen in our cases. The recently described glymphatic system and the importance of aquaporin 4 in reabsorbing the cerebrospinal fluid and the altered expression of this protein after trauma can be a physiopathological explanation. Other classically described physiopathological mechanisms for the development of syringomyelia are also reviewed and commented on.
**Conclusion:** The isolated central canal dilatation can be an underdiagnosed entity in patients presenting with medullary acute syndromes after trauma categorised as medullary concussions or SCIWORA.
**Limitations:** The limited amount of cases that we compiled and the fact that they are in the paediatric age are current limitations of this report.
**Ethics committee approval:** Not applicable.
**Funding for this study:** No funding was received.
**Author Disclosures:**
carmen bodlak: Nothing to disclose
Jose Miguel Escudero: Nothing to disclose
Ángel Sánchez-Montañez: Nothing to disclose
Élida Vazquez: Nothing to disclose
Ignacio Delgado: Nothing to disclose

### RPS 712-5

**Sonographic image of major salivary glands in children with juvenile Sjögren's syndrome: beyond "black spots"**

*D. Świętoń*, A. Pomorska, N. Irga; Gdańsk/PL
*(dominik.swieton@gumed.edu.pl)*

**Purpose:** Recurrent salivary glands (mostly parotid) enlargement is a common symptom of juvenile Sjögren's syndrome (JSS). In paediatric population JSS symptoms are commonly not obvious, especially in localised symptoms. The diagnostic criteria are still unclear for paediatric population. The previously leading imaging methods, like x-ray sialography or scintigraphy, are avoided nowadays due to radiation and invasiveness. Nowadays ultrasound (US) starts to play an important role, however not included in the diagnostic criteria. In our study we present characteristics of ultrasound findings in major salivary glands in children with JSS.
**Methods or Background:** 13 children with proven JSS and positive salivary gland biopsy (LSGB) were included for the study (5 males and 8 females, the mean age 9 years, range: 4-14 years). The clinical data was collected, including the time of duration and frequency of the symptoms, clinical symptoms xerostomia, ocular dryness, and laboratory exponents of JSS. The ultrasound examination was focused on the major salivary glands, parotids and submandibular, done with high frequency linear probe. The results were compared with clinical disease activity.
**Results or Findings:** All children presented sonographic changes in salivary glands, only in one of the cases the findings were not specific for JSS. In 10/13 patients parotids and submandibular glands were involved. In 2/13 children we observed isolated changes in submandibular and sublingual salivary glands, with parotid glands spared. The changes limited to parotid gland were found in only one child. No correlation of clinical presentation and imaging findings was found.
**Conclusion:** Ultrasound is a very sensitive and specific tool for diagnosis JSS. However, low correlation of clinical symptms and imaging findings needs further prospective studies.
**Limitations:** Low number of patients.
**Ethics committee approval:** The study was approved by the Independent Ethics Committee.
**Funding for this study:** No funding was received.
**Author Disclosures:**
Ninela Irga: Nothing to disclose
Anna Pomorska: Nothing to disclose
Dominik Świętoń: Nothing to disclose

### RPS 712-6

**An update on imaging of musculoskeletal paediatric infections**

*V. T. Paula*, P. V. P. Helito, R. V. Leão, D. G. Leão Edelmuth, L. Suzuki; São Paulo/BR
*(vitortav@gmail.com)*

**Purpose:** The purpose of this presentation is to highlight the current and rational imaging evaluation of musculoskeletal infections in children population.
**Methods or Background:** Pictorial essay containing clinical imaging cases from our paediatric and musculoskeletal radiology departments with correlation to clinical and laboratorial data and also to anatomopathological and culture results from biopsies and drainage interventions.
**Results or Findings:** We reviewed the accepted current guidelines and scientific evidences about imaging evaluation of musculoskeletal infections in children and illustrated that rational concepts with the cases from our radiology department. Common and uncommon presentations of spondylodiscitis, osteomyelitis, pyomyositis and septic arthritis were highlighted and correlated to the diagnostic approach and clinical outcomes. Conventional radiography, computed tomography, ultra-sonography, magnetic resonance and nuclear medicine methods were the most frequently used modalities to assess paediatric musculoskeletal infections and the correspondent results were demonstrated. Inflammatory markers, blood culture and cultures of osseous and soft tissue collections samples were also emphasised.
**Conclusion:** A pictorial essay applying the review of evidence-based concepts about imaging role on musculoskeletal paediatric infections constitutes a valuable tool in order to enhance and renew knowledge of radiologists, especially paediatric and musculoskeletal ones. The conjugation of the theoretical concepts with corresponding imaging findings contributes to didatic learning about rational use of imaging modalities and correct interpretation and reporting on daily practice with paediatric population.
**Limitations:** Limitations of our study include its retrospective aspect and the consequent difficulty to obtaine complete informations about all of the patients.
**Ethics committee approval:** Ethics commitee approval was obtained.
**Funding for this study:** No funding was received.
**Author Disclosures:**
Paulo Victor Partezani Helito: Nothing to disclose
Vitor Tavares Paula: Nothing to disclose
Diogo Guilherme Leão Edelmuth: Nothing to disclose
Lisa Suzuki: Nothing to disclose
Renata Vidal Leão: Nothing to disclose

### RPS 712-7

**Role of MRI in differentiating osteomyelitis from osteonecrosis in children with sickle cell disease: a cross-sectional study from a tertiary care hospital in Riyadh, Saudi Arabia**

*L. A. Alfahad*, A. A. Alboukai, K. Attia, M. Al Boukai; Riyadh/SA

**Purpose:** To evaluate the role of multi-sequential MRI in differentiating medullary osteonecrosis from osteomyelitis in children with sickle cell disease (SCD).
**Methods or Background:** All MRI scans done for children with SCD (ages 0-13 years) admitted for acute bone crisis between 2015-2021 were collected. MRI parameters were noted by 2 radiologists with experience in paediatric and musculoskeletal imaging, providing radiological diagnoses of osteomyelitis, osteonecrosis, or indeterminate. Electronic medical records were reviewed to obtain clinical diagnoses (censuses of haematology, infectious diseases, and orthopaedic surgery). Kappa test was used for clinical/radiological agreement. Fisher's exact test was used to evaluate statistically significant MRI parameters in relation to the radiological and clinical diagnoses of osteomyelitis or osteonecrosis (statistical significance defined at $P < 0.05$).
**Results or Findings:** A total of 50 radiological observations were gathered, and 8 were excluded due to lack of imaging findings or diagnosis of epiphyseal AVN. Agreement between clinical and radiological diagnoses was fair (Kappa = 0.52). MRI parameters that show statistical significance related to both clinical and radiological diagnoses included: presence of cortical defect ($P = 0.001$ and 0.001, respectively), presence of soft tissue inflammation ($P = 0.001$ and 0.007, respectively), soft tissue inflammation relative to bony changes ($P = 0.001$ and 0.001, respectively), the presence of subperiosteal collections ($P = 0.002$ and 0.029, respectively), non-fat saturated T1 signal intensity of the bone marrow ($P = 0.021$ and 0.014, respectively), and bone marrow fluid collection ($P = 0.011$ and 0.004, respectively).
**Conclusion:** MRI can reliably differentiate osteomyelitis from medullary osteonecrosis in children with SCD. Findings that support osteomyelitis include the presence of cortical defects, extensive soft tissue inflammation relative to bony changes, and bone marrow fluid collection. Osteonecrosis is highly suggested with high bone marrow signal intensity on non-fat saturated T1WI, relatively less extensive soft tissue inflammation, and absence of subperiosteal fluid collection.
**Limitations:** Confounding factors, misclassification bias, and missing information related to the retrospective nature of the study. There are also uncertain diagnoses, lack of follow-up and small sample size.
**Ethics committee approval:** Local IRB requirements were fulfilled.
**Funding for this study:** None to report.
**Author Disclosures:**
Kamal Attia: Nothing to disclose
Mohammad Al Boukai: Nothing to disclose
Latifah Ahmad Alfahad: Nothing to disclose
Ahmad Abdulmuttaleb Alboukai: Nothing to disclose

### RPS 712-8

**Imaging signs for granuloma annulare by epifascial mound sign, and subdermal inverse mound sign: first descriptions**

*B. P. Beqo*[1], S. Tschauner[2], E. Q. Haxhija[2]; [1]Boston, MA/US, [2]Graz/AT
*(besiana.beqo@medunigraz.at)*

**Purpose:** Granuloma annulare (GA) is a self-limiting granulomatous disease of skin and subcutaneous tissue of unknown etiology. While localised GA (LGA) affects all ages, subcutaneous GA (SGA) occurs exclusively during childhood. So far, accurate diagnosis of SGA was mainly established by surgical biopsy. This study evaluated potential imaging signs in children with GA.
**Methods or Background:** A retrospective analysis of available imaging in children diagnosed for GA at our institution between 01.01.2001-31.12.2021 was performed.
**Results or Findings:** 56 patients were diagnosed with GA. Only 4/28 patients with LGA received ultrasound imaging, all others were diagnosed clinically. All 28 SGA patients were examined by ultrasound, 16 patients received an x-ray, and 12 patients were examined by MRI. All imaging was inconclusive, and subcutaneous vascular anomalies were the main differential diagnoses. Surgery was done in 18 patients, including all patients examined by MRI. During retrospective analysis of MR images we discovered a specific shape of SGA lesions, resembling an epifascial mound with broad circular fascial base extending centrally into the subdermal/dermal tissue. This characteristic shape of the lesions was present in all MRIs of SGA patients. The same shape was found in the analysis of ultrasound images. Interestingly, in analysis of ultrasound images of LGA patients we found the subdermal inverse mound sign.
**Conclusion:** We describe for the first time, pathognomonic imaging signs for SGA and LGA in children. To the best of our knowledge, there is no other known subcutaneous lesion presenting in the shape of epifascial mound or subdermal inverse mound as we just described. We believe that recognition and awareness of epifascial mound sign will significantly reduce the need for surgery in children with SGA.
**Limitations:** Low number of LGA patients examined by ultrasound.

**Ethics committee approval:** This study was approved by an ethics committee, EK-No.33-126ex20/21.
**Funding for this study:** Funding was received from the Medical University Graz.
**Author Disclosures:**
Besiana P Beqo: Nothing to disclose
Sebastian Tschauner: Nothing to disclose
Emir Q. Haxhija: Nothing to disclose

**08:00-09:00** **Room M 3**

### Research Presentation Session: Imaging Informatics / Artificial Intelligence and Machine Learning

### RPS 705

### Artificial intelligence performance evaluation and new AI methods

**Moderator**
J. J. Visser; Rotterdam/NL
**Author Disclosure:**
J. J. Visser: Advisory Board: Contextflow, Quibim; Consultant: Noaber Foundation, NLC Ventures

### RPS 705-2

**The evolution of uncertainty "hedging" expressions following the introduction of structured reporting**

T. Heye, E. M. Merkle, *J. Vosshenrich*; Basel/CH
*(jan.vosshenrich@usb.ch)*

**Purpose:** To investigate the use of uncertainty "hedging" expressions in radiology reports and how the introduction of structured reporting and a reporting culture with focus on certainty affects hedging expression occurrence.
**Methods or Background:** 1,073,324 reports written from 2013 to 2021 in our department's body (n = 134,817), cardiothoracic (n = 253,924), musculoskeletal (n = 373,090), neuroradiology (n = 207,730) and nuclear medicine section (n = 103,763) were retrieved from the radiological information system (RIS). Nine expressions such as "prominent", "accentuated" and "cannot exclude" considered as hedging were identified by a group of board certified radiologists. Using a text search algorithm the occurrence of hedging expressions including their grammatical variations were searched and counted. The algorithm does only register the occurrence of the expression in a report, not the number of occurrences within a report. 100% structured reporting and a culture using factual standardised reporting language was introduced in the body and cardiothoracic sections in 2015/16.
**Results or Findings:** There was a 60.5% overall decrease in the use of hedging expressions from 2013 (n = 11,859) to 2021 (n = 4,681). In body imaging overall hedging decreased by 73.6%, in cardiothoracic by 72.8%. The occurrence of the expression "prominent" decreased by 97.8% from 2013 to 2021 in body (n = 274 vs. n = 6) and in cardiothoracic imaging by 89.3% (n = 597 vs. n = 64). In musculoskeletal imaging hedging decreased by 74.5%, "prominent" by 98.2% and in neuroradiology by 29.3%, "prominent" by 69%. In nuclear medicine overall hedging increased by 5.8% and "prominent" decreased by 46.3%.
**Conclusion:** The introduction of structured reporting and awareness for certainty using a factual standardised reporting language appears to decrease the use of uncertainty "hedging" expressions.
**Limitations:** Not applicable.
**Ethics committee approval:** Not applicable.
**Funding for this study:** Not applicable.
**Author Disclosures:**
Tobias Heye: Nothing to disclose
Elmar M. Merkle: Nothing to disclose
Jan Vosshenrich: Nothing to disclose

### RPS 705-3

**Diagnostic imaging availability for non-COVID population during COVID-19 pandemic**

*V. Opancina*, S. Jankovic; Kragujevac/RS

**Purpose:** The aim of our study was to assess the healthcare delivery, in regard to diagnostic imaging, to non-COVID-19 population in our conditions.
**Methods or Background:** The pandemic has urged countries to reorganise their healthcare. It has caused cancellation/deferment of elective surgeries, face-to-face consultations, medical inventory shortages, diagnostic modalities delay and limited health maintenance globally. This had major effect on non-COVID-19 conditions, especially on chronic and undiagnosed patients with clinical manifestations. This study was designed as cross-sectional online survey of a sample of adults about their access to the non-COVID19 diagnostic imaging services. It was conducted from July-November, 2021. The survey was posted on Facebook in four public groups with approximately 83.000 members. The study population consisted of adults who fulfilled the following inclusion criteria: older than 18 years and member of one of the target Facebook public groups.
**Results or Findings:** There were in total 364 participants of both sex (m/f:30.5%69.5%), with mean age of 37.94±13.35. Among the participants who responded to the question about chronic condition, one-third had at least one 113/244 (31.6%68.4%). Among the participants who responded to the question about undiagnosed condition, which required diagnostic imaging, one fifth had at least one 79/285 (21.7%78.3%). Waiting period (in days) for imaging modalities was: radiography (11.55±40.82), ultrasound (42.97±88.69), CT (44.34±112.24), MRI (46.66±100.60). Nagelkerke R2 was0.30, and predictors with significant influence on missing an imaging appointment were: female gender and chronic disease (AdjustedOR= 2.070, 1.967, respectively).
**Conclusion:** There is a need for creation of "chronic care model" during crises such as pandemic, due to great morbidity, mortality and economic burden of chronic conditions and the fact that undiagnosed conditions need to undergo imaging in order to start treatment.
**Limitations:** Not applicable.
**Ethics committee approval:** Approved by institutional ethics committee.
**Funding for this study:** Not applicable.
**Author Disclosures:**
Valentina Opancina: Nothing to disclose
Slobodan Jankovic: Nothing to disclose

### RPS 705-4

**Radiology through immersive virtual reality: diagnostic accuracy for 3D CT renderings of basilar skull fractures**

*J. C. Rodríguez Domínguez*, C. A. Calderon Diaz, J. P. Montemayor lozano, S. Tristan; Monterrey/MX
*(cruz.rd@outlook.com)*

**Purpose:** Determine the diagnostic accuracy of radiologists assessing 3D rendered CT studies of basilar skull fractures on an immersive virtual reality headset (IVR) and its comparison with workstation visualisation (WS).
**Methods or Background:** In this retrospective study, two groups of observers conformed each by two radiologists and two residents in their 2nd and 3rd year, assessed a set of 10 non-contrasted head CT of patients diagnosed with linear or comminute fractures alongside control cases in the facilities of IVR or WS. This set was randomised and reviewed again after a week. Only clinical data provided was "traumatic head injury". Fractures were classified as "anterior, middle or posterior fossa fractures" through formularies for statistical analysis applying logistic regression models and ROC curves for posterior comparison.
**Results or Findings:** IVR sensibility was 100%, 46%, 56% and specificity 95%, 97%, 90% for anterior, middle and posterior fossa respectively, on 95% CI. WS sensibility was 100% 78% and 43% and specificity 87% 83% 93% for anterior, middle and posterior fossa respectively, on 95% CI. ROC curves for IVR had values of AUC= 0,979, 0,724 and 0,734 with a p=0,001 for anterior, middle and posterior fossa. The ROC curves for WS had values of AUC=0,938, 0,807 and 0,680 with p= 0,001 for anterior, middle and posterior fossa respectively.
**Conclusion:** AUC on both groups did not have major significant variations, in general, they both had variable sensibility and greater specificity values, this is in accordance with patterns reported by international literature (sensibility 18%-85%, specificity 100%).
**Limitations:** Only assessed linear or comminuted fractures in adults, confirmation by autopsy was not possible.
**Ethics committee approval:** Approved by Dr. Edgar Rosenberg Ortiz Gutierrez, president of ethics in research committee of ISSSTE Regional Hospital Monterrey.
**Funding for this study:** None, no funding to declare.
**Author Disclosures:**
Juan Pablo Montemayor lozano: Nothing to disclose
José Cruz Rodríguez Domínguez: Nothing to disclose
Carlos Alberto Calderon Diaz: Nothing to disclose
Samuel Tristan: Nothing to disclose

### RPS 705-5

**Novel tool for asynchronous communication between radiologist and radiologic technologist: a viable replacement of interruptive phone calls?**

*K. Hergaarden*[1], O. Hertgers[1], S. Vosbergen[2], J. Knoester[2], T. Anten[2], P. Haima[2], M. Sevenster[1], H. J. Lamb[1]; [1]Leiden/NL, [2]Eindhoven/NL
*(K.F.M.Hergaarden@LUMC.nl)*

**Purpose:** To assess the perceived value of an asynchronous communication tool for technologists and radiologists for raising and answering image acquisition questions.
**Methods or Background:** To drive first-time-right acquisition services, it is paramount that radiologists are accessible to technologists. However, in many hospitals radiologists are consulted over phone or in person, leading to frequent interruptions of the diagnostic workflow. This has been associated with increased stress, errors and turn-around-times. We developed an asynchronous communication tool (RadConnect) that enables radiologic technologists to send a "ticket" to a radiology section role account. A ticket comprises a question category, urgency (i.e. desired response time) and imaging status. The tickets appear on a prioritised worklist from which the radiologist can open it to engage with the technologist in a chat channel. Four technologists and five radiologists from an academic hospital in the Netherlands participated in a two-hour usability study. They performed simulated tasks in RadConnect. A member of the research team acted as sending/receiving counterpart. A structured survey was conducted at the end of each session to gauge perceived value on a 5-point Likert scale (1=Strongly disagree, 5=Strongly agree).
**Results or Findings:** Study participants expected to use RadConnect frequently (4.3) and that it will reduce phone communication by >80% (4.3). Radiologists preferred RadConnect over phone for protocolling (4.9) and image review (4.3) questions, and were not concerned about incoming tickets interrupting their workflow (1.7).
**Conclusion:** RadConnect is expected to have impact on reducing workflow interruptions by asynchronous communication between radiologist and radiologic technologist, potentially contributing to a more pleasant work environment and sustainable radiological workforce.
**Limitations:** The study was conducted at a clinical site in a lab setting.
**Ethics committee approval:** This study was approved by the Philips Internal Committee for Biomedical Experiments.
**Funding for this study:** Research support was received from Koninklijke Philips N.V.
**Author Disclosures:**
Omar Hertgers: Research/Grant Support: Koninklijke Philips N.V.
Merlijn Sevenster: Employee: Koninklijke Philips N.V.
K.F.M. Hergaarden: Research/Grant Support: Koninklijke Philips N.V.
Paul Haima: Employee: Koninklijke Philips N.V.
Thomas Anten: Employee: Koninlijke Philips N.V.
Jaap Knoester: Employee: Koninklijke Philips N.V.
Hildo J. Lamb: Research/Grant Support: Koninklijke Philips N.V.
Sandra Vosbergen: Employee: Koninklijke Philips N.V.

### RPS 705-6

**The role of teleradiology during COVID-19 outbreak: Saudi perspectives**

*S. Al Dahery*[1], W. Mofti[1], S. Nawawi[1], F. Alamri[1], W. Alsharif[2], A. A. Qurashi[2]; [1]Jeddah/SA, [2]Madinah/SA
*(shrooq_talal@hotmail.com)*

**Purpose:** This is the first study to evaluate teleradiology at hospitals from Saudi radiologists' perspectives, to evaluate the impact of teleradiology in radiology departments at Saudi hospitals during the COVID-19 pandemic, and to suggest reorganising the workflow at Saudi hospitals.
**Methods or Background:** Teleradiology is an important role during the COVID-19 pandemic to maintain social distancing and reduce cross-infection, activities in radiology departments can be adapted to remote workstations which is also useful in providing consistent quality health service to patients. It is based on digital transmission of medical images from one site to another to provide services remotely, which can result in minimising the expansion of COVID-19.
**Results or Findings:** A total of 102 radiologists participated in this study (56% males and 44% females). The majority (85%) were either residents or consultants. The results showed that qualification is significantly associated with the overall responses. However, no statistical difference was noted between hospital type, years of experience, and responses. Only 41% of participants believed that teleradiology might increase the quality of radiological reports during COVID-19.
**Conclusion:** The results demonstrated that teleradiology can accelerate the interpretation process and improve patients' accessibility to the healthcare services during the pandemic, and could have a future in developing new projects for Saudi hospitals. However, some participants were dissatisfied with the level of communication between healthcare professionals.
**Limitations:** A Saudi radiologist's perspectives only of teleradiology. The results may need to be interpreted with caution due to the small sample size, which may not be generalised to the whole radiologist in Saudi Arabia.
**Ethics committee approval:** Approval was obtained from the Bioethics Committee of Scientific and Medical Research at University of Jeddah: HAP-02-J-094.
**Funding for this study:** No funding was received for this study.
**Author Disclosures:**
Abdulaziz Abdulsalam Qurashi: Nothing to disclose
Shrooq Al Dahery: Nothing to disclose
Fatima Alamri: Nothing to disclose
Wed Mofti: Nothing to disclose
Shahad Nawawi: Nothing to disclose
Walaa Alsharif: Nothing to disclose

### RPS 705-7

**Evaluation of image performance in deep learning reconstruction (increase in spatial resolution and image contrast)**

*K. Tsujioka*[1], K. Yamada[2], M. Niwa[2]; [1]Toyota/JP, [2]Yokkaichi/JP
*(tsujioka@fujita-hu.ac.jp)*

**Purpose:** Deep learning reconstruction (DLR) is a new imaging technology for CT. Among them, DLR for improving spatial resolution for conventional CT targeting ultra-high resolution CT (UHR-CT) was developed. In this report, we examined the image performance of conventional CT, UHR-CT and DLR-CT.
**Methods or Background:** We evaluated changes in spatial resolution and CT values. The wire method was used to measure the spatial resolution. For changes in CT values, CT value profiles and peak CT values were measured in a spiral wire phantom.
**Results or Findings:** The spatial resolution of DLR images was superior to conventional CT, but slightly inferior to UHR-CT. The CT value profile was similar. No characteristic edge undershoot appeared in any of the images. The peak CT value of the wire was higher than that of the conventional CT, but slightly inferior to that of UHR-CT.
**Conclusion:** DLR-CT image was able to obtain spatial resolution close to that of UHR-CT with conventional CT. DLR-CT not only improves the spatial resolution but also has the effect of improving the CT value of small blood vessels. DLR-CT is effective in diagnosing small blood vessels.
**Limitations:** This report is based on the phantom experiment. We are planning to conduct research using human body data.
**Ethics committee approval:** Our experiments have been approved by the ethics committee.
**Funding for this study:** We are not financially funded by any organisation for this report.
**Author Disclosures:**
Katsumi Tsujioka: Nothing to disclose
Masayoshi Niwa: Nothing to disclose
Kyohei Yamada: Nothing to disclose

### RPS 705-8

**Deep learning based co-registration QC of MRI**

*Y. Li*, B. Duffy, K. Datta; Menlo Park, CA/US
*(yiheng@subtlemedical.com)*

**Purpose:** Medical image registration is the process of aligning several medical images based on anatomical structures. It is a widely used preprocessing step for many medical imaging-related clinical applications like image reconstruction, and it strongly affects the performance of algorithms that rely on image alignments, such as multimodal segmentation. Thus, the quality control (QC) of medical image registration is essential for controlling the failure rates of many downstream clinical applications.
**Methods or Background:** This study provided a deep learning-based registration QC model that can correctly classify misaligned image pairs on multiple datasets and can be generalised to multiple modalities/anatomies using the strategy of combined training.
**Results or Findings:** Our approach can perform well on multiple datasets with different contrasts and with randomly synthesised movements. On 3 validation datasets of spine MR image with affine transformations, SVF transformations, and brain MR image with affine transformations, the model can flag misaligned image pairs with 0.98, 0.93, and 0.99 F1 scores.
**Conclusion:** We proposed a feasible method based on deep learning CNN and combined training with synthesised movements, that can achieve automatic, accurate, and fast medical image registration quality control by predicting misaligned image pairs. This method can be applied to multiple contrasts and anatomies.
**Limitations:** The limitations are that data collection-wise, we need to have the corresponding image pairs in the training set for the model to have nice generalisability of that kind of image data.

**Ethics committee approval:** This study utilises public datasets. There is no ethical problem.
**Funding for this study:** This study is a research project supported by Subtle Medical Inc.
**Author Disclosures:**
Keshav Datta: Other: Manager
Yiheng Li: Speaker: Speaker
Ben Duffy: Author: Co-author

**08:00-09:00** **Room M 4**

### Research Presentation Session: Oncologic Imaging

### RPS 716

### Advanced imaging in lung oncology

**Moderator**
M. Rémy-Jardin; Lille/FR
**Author Disclosure:**
M. Rémy-Jardin: Advisory Board: Siemens Healthineers: Research Grant/Support: Siemens Healthineers: Speaker: MSD, Actelion, Boehringer-Engelheim

### RPS 716-2

**Dynamic changes of CT-radiomic and systemic immune-inflammatory features predict the response to immune checkpoint inhibitors in advanced NSCLC patients**
*G. Milanese*, G. Mazzaschi, L. Moron Dalla Tor, L. Leo, M. Balbi, R. E. Ledda, M. Silva, M. Tiseo, N. Sverzellati; Parma/IT

**Purpose:** Clinical biomarkers foreseeing response to immune checkpoint inhibitors (ICIs) can be obtained by decoding tumour heterogeneity and its evolution during treatment. We tested whether longitudinal assessment of radiomic features (RFs) and blood descriptors of systemic inflammation (SI) may predict ICI efficacy in advanced non-small cell lung cancer (NSCLC).
**Methods or Background:** CT-derived RFs and peripheral blood SI parameters, including derived Neutrophil-to-Lymphocyte ratio (dNLR) and lactate dehydrogenase (LDH), were acquired at baseline (T0) and at first disease assessment (T1) for 92 stage IV NSCLC patients undergoing ICIs. Primary endpoint was ICI response per RECIST. T1-T0 variations of 852 RFs and 6 SI indices were challenged into machine learning-based predictive models. RFs pre-processing included redundant features elimination and Z-score standardisation; L2-penalised logistic regression with Monte-Carlo cross-validation was implemented for wrapper-based feature selection and model training/test. Resulting delta-radiomic (ΔR), immune/inflammatory (ΔI) and integrated (ΔInt) models were compared based on performance metrics.
**Results or Findings:** Median overall survival (OS) and progression-free survival (PFS) were 8.1 (95%CI, 4.1-12.2) and 2.6 months (95%CI, 1.1-4.4), respectively; 34 (37%) patients were responders and 58 (63%) non-responders. A model validation calibrated at up to 10 parameters, 5 delta-RFs and delta-LDH was built according to ROC-AUC scores and adopted for ΔR, ΔI and ΔInt models. ROC-AUC and accuracy (±SD) were 0.86 (±0.08) and 0.78 (±0.08) for ΔR model, while 0.78 (±0.09) and 0.67 (±0.09) for ΔI model. ΔInt model yielded highest ROC-AUC and accuracy: 0.88 (±0.07) and 0.82 (±0.08) ($p<0.001$), respectively.
**Conclusion:** A dynamic blood-radiomic predictor of ICI efficacy in advanced NSCLC suggests that current predictive models might be implemented by non-invasive interception of systemic and local events critically implicated in cancer evolution.
**Limitations:** This was a monocentric retrospective study.
**Ethics committee approval:** This study was approved by an ethics committee.
**Funding for this study:** Funding was received from the Fondazione AIRC per la Ricerca sul Cancro (Italian Foundation for Cancer Research).
**Author Disclosures:**
Giulia Mazzaschi: Nothing to disclose
Roberta Eufrasia Ledda: Nothing to disclose
Ludovica Leo: Nothing to disclose
Nicola Sverzellati: Nothing to disclose
Marcello Tiseo: Nothing to disclose
Maurizio Balbi: Nothing to disclose
Gianluca Milanese: Nothing to disclose
Lucas Moron Dalla Tor: Nothing to disclose
Mario Silva: Nothing to disclose

### RPS 716-3

**Evaluation of differential response in patients with metastatic lung cancer treated with immune checkpoint inhibitors: a pilot study**
*A. Antolín Redondo*, M. Ligero, E. Garralda, E. Felip, M. Escobar, R. Perez Lopez; Barcelona/ES
*(antolin.andreu@gmail.com)*

**Purpose:** To assess the rate of differential response between lesions in patients with metastatic lung cancer treated with immune checkpoint inhibitors (ICIs) through a radiomics analysis of CT images at best response and progressive disease timepoints.
**Methods or Background:** In this retrospective study, 102 lesions corresponding to 42 patients with metastatic lung cancer treated with ICIs were included. Lesions were semi-automatically segmented using 3DSlicer at baseline and follow-up contrast-enhanced-CT corresponding to best-response and disease-progression. Volume change was calculated per lesion from baseline CT to both timepoints. Differential response was defined as co-existence of lesions with volume increase or decrease (+30%/-20%) at same timepoint or volume decrease of ≥1 lesions with appearance of new lesions. Statistical analysis was performed using Mann-Whitney U-test and Pearson's chi-squared test for quantitative and categorical variables respectively, with significance at $p<0.05$.
**Results or Findings:** Differential response occurred in 31% of the patients (13/42) at the time of progressive disease. However, there was already a differential response trend in 4 patients at best response timepoint while considered stable-disease (2/4) and partial-response (2/4) by RECIST 1.1. Higher number of lesions, total volume and number of organs affected associated with differential response ($p<0.05$).
**Conclusion:** This study proves the existence of differential response in patients with metastatic lung cancer treated with ICIs, which may limit the clinical efficacy of this therapy due to heterogeneous response. Therefore, the extraction of imaging biomarkers in these patients should not consider general response as an indicator; instead, there should be a move towards personalised analysis per lesion or organ cluster.
**Limitations:** The small number of patients with multiple lesions (n<30) reduces statistical power.
**Ethics committee approval:** The institutional review board approved this retrospective study. Need for informed consent for computational analysis was waived.
**Funding for this study:** M.L. is funded by the Generalitat de Catalunya.
**Author Disclosures:**
Manel Escobar: Nothing to disclose
Elena Garralda: Nothing to disclose
Raquel Perez Lopez: Research/Grant Support: Research Funding: Prostate Cancer Foundation, CRIS Foundation, FERO Foundation, LaMarató Foundation, La Caixa Foundation, Carlos III Instituto de Investigación.Grant support: AstraZeneca, Roche
Andreu Antolín Redondo: Nothing to disclose
Marta Ligero: Nothing to disclose
Enriqueta Felip: Nothing to disclose

### RPS 716-4

**Comparison of performance by whole-body MRI, PET/MRI and PET/CT and conventional staging methods for TNM and VALSG staging of small cell carcinoma patients**
*Y. Ohno*[1], T. Yoshikawa[2], D. Takenaka[2], K. Aoyagi[3], M. Yui[3], T. Ueda[1], H. Ikeda[1], K. Murayama[1], H. Toyama[1]; [1]Toyoake/JP, [2]Akashi/JP, [3]Otawara/JP
*(yohno@fujita-hu.ac.jp)*

**Purpose:** To compare the capabilities for assessment of tumour, node, and metastasis (TNM) and Veterans Administration Lung Cancer Study Group (VALSG) staging of small cell carcinoma (SCLC) patients by whole-body MRI, PET/MRI, PET/CT and conventional staging methods.
**Methods or Background:** A cohort of 98 pathologically diagnosed SCLC patients underwent whole-body MRI, FDG-PET/CT and conventional staging methods. TNM and VALSG stages were determined by a tumour board. All PET/MRIs were generated by means of our proprietary software. Each factor, TNM and VALSG stage were then evaluated by means of consensus reading. Kappa statistics were determined for evaluations of agreements for each clinical stage between each method and final diagnosis. Finally, diagnostic accuracy of TNM and VALSG stage evaluations were statistically compared among all methods by using McNemar's test.
**Results or Findings:** Agreements for each factor, TNM stage and VALSG stage were determined as substantial or almost perfect ($0.67 \leq \kappa \leq 0.94$, $p<0.0001$). Accuracies for TNM stage on whole-body MRI (88.8%) and PET/MRI (86.7%) were significantly higher than that of PET/CT (77.6%: vs MRI, p=0.001; vs PET/MRI, p=0.004) and conventional staging methods (72.4%: vs MRI, p<0.0001; vs PET/MRI, p=0.0001). In addition, accuracies for VALSG stage on whole-body MRI (95.9%), PET/MRI (95.9 %) and PET/CT (98.0%) were significantly higher than that of conventional staging methods (82.7%: vs MRI and vs PET/MRI, p=0.0005; vs PET/CT, p<0.0001).

**Conclusion:** Capabilities of whole-body MRI and PET/MRI for each factor assessment and TNM and VALSG stage evaluations are as high as or higher than those of PET/CT or conventional staging methods.
**Limitations:** Not applicable
**Ethics committee approval:** This retrospective study was approved by Institutional Review Boards of Kobe University Graduate School of Medicine and Fujita Health University School of Medicine in Japan.
**Funding for this study:** This study was financially supported by the Canon Medical Systems Corporation.
**Author Disclosures:**
Kazuhiro Murayama: Grant Recipient: Canon Medical SysteCorporation
Hirotaka Ikeda: Nothing to disclose
Hiroshi Toyama: Research/Grant Support: Canon Medical SysteCorporation
Kota Aoyagi: Employee: Canon Medical SysteCorporation
Takahiro Ueda: Nothing to disclose
Daisuke Takenaka: Nothing to disclose
Masao Yui: Employee: Canon Medical SysteCorporation
Takeshi Yoshikawa: Nothing to disclose
Yoshiharu Ohno: Research/Grant Support: Canon Medical SysteCorporation

### RPS 716-6

**To differentiate pulmonary sclerosing pneumocytoma from atypical lung cancer based-on contrast-enhanced CT: a radiomic analysis**
*L. Wang*, T. Wan, X. Peng; Nanchang/CN
*(836282726@qq.com)*

**Purpose:** To explore the feasibility of radiomic analysis based on computed tomography (CT) plain scan and biphasic contrast-enhanced image in the differentiation of pulmonary sclerosing pneumocytoma (PSP) from atypical lung cancer.
**Methods or Background:** The chest CT image data (plain scan, arterial phase and venous phase) of 67 patients with PSP and 58 patients with atypical lung cancer were collected. The tumour lesions in CT images were manually delineated, and obtained the regions of interest. The two sample t-test and least absolute shrinkage and selection operator regression were applied to the most discriminative features selected. Using the hold-out method, the original set was randomly divided into training dataset (70%) and testing dataset (30%) (the proportion of two group patients in the training and testing datasets was similar). The feature selection and model training were performed based on the training dataset and three machine learning classification models were constructed, including random forest (RF), logistic regression (LR) and support vector machine (SVM). The diagnostic performance of the models in testing dataset were validated by the area under receiver operate curve (AUC).
**Results or Findings:** We got the best LR classification model with 0.89 accuracy (AUC=0.85) by 9 discriminative features selected based on arterial phase image. Combining the plain and contrast-enhanced features, the LR model got the best performance with 0.73 accuracy (AUC=0.79).
**Conclusion:** This study indicated that the radiomic analysis method has a high application value in differentiating PSP from atypical lung cancer.
**Limitations:** A bigger sample size and external validation is needed in the future.
**Ethics committee approval:** This study was approved by the Medical Research Ethics Committee and the Institutional Review Board of the First Affiliated Hospital of Nanchang University.
**Funding for this study:** Not applicable
**Author Disclosures:**
Xinji Peng: Nothing to disclose
Lei Wang: Nothing to disclose
Ty Wan: Nothing to disclose

### RPS 716-7

**Differentiation of benign lung nodules and lung metastases: a radiomics approach in a large cancer patient cohort**
*S. Lennartz*, M. Schöneck, T. Struck, N. Große Hokamp, L. Caldeira, D. Zopfs, T. Persigehl; Cologne/DE
*(simon.lennartz@uk-koeln.de)*

**Purpose:** To investigate differentiation of metastatic and benign lung nodules in cancer patients using radiomics in a large cancer patient cohort.
**Methods or Background:** 537 cancer patients who received contrast-enhanced computed tomography (CT) of the chest were retrospectively included in this singlecentre study. The dataset included thin-slice reconstructions in the lung window derived from multiple CT scanner types. 1135 lung metastases confirmed by either CT follow-up imaging, histopathology or FDG-PET/CT and 426 benign lung nodules confirmed by either histopathology or follow-up CT were included. Semi-automated segmentations of lung nodules were performed. After image resampling and pre-processing, 118 radiomic features were extracted using PyRadiomics. Lesions were split into training (70%) and test (30%) datasets. Powerful features for lung nodule differentiation were selected by means of ANOVA f-test and mutual information and redundant features were excluded by correlation analysis. After feature normalisation, model training was performed with 5-fold cross validation; the best performing model was evaluated on the test dataset. Model performance was evaluated using the balanced accuracy score (BAS), F1 score and sensitivity.
**Results or Findings:** A random forest classifier using features selected based on mutual information yielded the best performance in differentiating metastatic from benign lung nodules (BAS: 0.75, F1 score: 0.78, sensitivity: 0.78). When applied to the test set, the model attained a BAS of 0.71, an F1 score of 0.75, and a sensitivity of 0.75.
**Conclusion:** Radiomics-based differentiation between benign and metastatic lung nodules in contrast-enhanced CT is feasible. We found satisfactory model performance in a large retrospective, multi-scanner cohort. Future scientific efforts should aim to further improve discriminative performance enabling clinical application.
**Limitations:** This was a singlecentre study.
**Ethics committee approval:** Consent was waived for this retrospective study.
**Funding for this study:** Not applicable
**Author Disclosures:**
Simon Lennartz: Research/Grant Support: Philips
Mirjam Schöneck: Nothing to disclose
Liliana Caldeira: Research/Grant Support: Philips
David Zopfs: Nothing to disclose
Nils Große Hokamp: Other: Philips Research/Grant Support: Philips
Thore Struck: Nothing to disclose
Thorsten Persigehl: Nothing to disclose

**08:00-09:00** **Room M 5**

### Research Presentation Session: Emergency Imaging

### RPS 717

### Advances, appropriateness and training in emergency radiology

**Moderator**
K. Katulska; Poznan/PL

### RPS 717-2

**Deep-learning based reconstruction for multiphase CT angiography: a rapid method for diagnosis and prognosis of acute ischaemic stroke**
*Y. Lin*, S. Lv, J. Wang; Xiamen/CN
*(420867402@qq.com)*

**Purpose:** Deep-learning based colour-coded reconstruction for multiphase computed tomography angiography (mCTA) can provide sensitive time-variant blood flow information of culprit vessel and collateral circulation for patients with acute ischaemic stroke (AIS). We aim to compare the usefulness of colour-coded and conventional mCTA for stroke detection and prognosis evaluation of patients with AIS.
**Methods or Background:** Consecutive patients with anterior circulation AIS were reviewed at our stroke centre. Baseline collateral scores of colour-coded mCTA and conventional mCTA were evaluated by a 6-point scale. The imaging to reporting time and detection of “delayed vessel sign” regarding the two mCTA methods were recorded. Receiver operating characteristic (ROC) curves were applied to evaluate the predictive value of colour-coded and conventional mCTA for a favourable outcome of AIS.
**Results or Findings:** A total of 127 patients were included in our study. Patients with favourable prognoses were correlated with abundant collateral circulations on both colour-coded and conventional mCTA (both $P < 0.05$). Compared with that of the conventional method, the imaging to reporting time of colour-coded mCTA was shorter ($186 \pm 23$ secs vs $1287 \pm 192$ secs). While colour-coded mCTA might contribute to the precise detection of "delayed vessel sign" in 17 cases with almost "normal" manifestations on conventional mCTA, ROC curves revealed that colour-coded and conventional mCTA achieved similar performance ($P = 0.427$) in prognosis evaluation.
**Conclusion:** Radiologists can rapidly detect “delayed vessel sign” and reach high diagnostic accuracy when interpreting colour-coded mCTA images. Colour-coded mCTA provides equivalent prognostic information with less time consumption when comparing with that of conventional mCTA in stroke imaging.
**Limitations:** The heterogeneity of baseline stroke severity and time windows in our retrospective study might lead to selection bias.

**Ethics committee approval:** Approved by the Ethical Committee of Xiamen University.
**Funding for this study:** Not applicable.
**Author Disclosures:**
JinAn Wang: Nothing to disclose
Yu Lin: Nothing to disclose
Shaomao Lv: Nothing to disclose

### RPS 717-3

**Diagnostic accuracy and analysis of an artificial intelligence algorithm for the detection of intracranial haemorrhage**

*J. Kiefer*, M. S. May; Erlangen/DE
*(jonas.kiefer@gmx.de)*

**Purpose:** Intracranial haemorrhage requires an immediate diagnosis to optimise patient outcomes and CT is the modality of choice in the emergency setting. Our aim was to evaluate the performance of the first scanner integrated artificial intelligence (AI) algorithm to detect brain haemorrhages in a clinical routine setting.
**Methods or Background:** This retrospective study includes 435 consecutive non-contrast head CT scans. Automatic brain haemorrhage detection was calculated inline as a separate reconstruction job in all cases. The radiological report, always created by a radiology resident and finalised by a senior radiologist, served as reference.
**Results or Findings:** Brain haemorrhage detection was executed successfully in 432 out of 435 patient images. The AI algorithm and reference standard were consistent in 392 (90.7%) of cases. The diagnostic performance was calculated as sensitivity 98.1% (95% CI, 94.3%-100%), specificity 89,7% (95% CI, 86.7%-92.8%), positive predictive value 56.7% (95% CI, 46.4%-66.9%), and negative predictive value (NPV) 99.7% (95% CI, 99.1%-100%). There was a single false negative case with a very small lesion of 0.4x0.2 mm.
**Conclusion:** Execution of scanner integrated AI detection of brain haemorrhage is fast and stable. The diagnostic accuracy has a high specificity and a very high negative predictive value and sensitivity. However, many false positive findings resulted in rather moderate positive predictive value. The limited image quality in the emergency setting by positioning and compliance problems may be the reason for this relative low outcome.
**Limitations:** This study could be limited by the number of only 2 radiologists reviewing the images as the reference standard.
**Ethics committee approval:** This monocentric retrospective study was approved by the local ethics committee. All CT examinations included in the study were clinically indicated.
**Funding for this study:** This study was not funded.
**Author Disclosures:**
Jonas Kiefer: Nothing to disclose
Matthias Stefan May: Nothing to disclose

### RPS 717-4

**Daily prediction of polytrauma-CTs using weather data**

*M. Segeroth*, J. Vosshenrich, C. Breit, D. Winkel, J. Wasserthal, T. Heye; Basel/CH

**Purpose:** To investigate if fluctuations in polytrauma-CT scan occurrence are related to weather changes and thus predictable.
**Methods or Background:** Due to the unpredictability of emergency examinations, any approach for estimation would improve resource planning in a hospital. All polytrauma-CTs between 1.1.2011 and 31.12.2020 (n = 4613) were retrieved from the radiological information system. Daily local weather data was downloaded from meteoblue.com. To account for the increase of the maximum number of polytrauma-CTs per day (2011: 4 to 2020: 11) and meteorological weather changes, all data were normalised (subtraction of mean and division by the difference between maximum and mean of the corresponding year). Data was smoothened using the moving average of the preceding 15 days. Above normal number of daily pCT was defined as above median. Logistic regression analysis was used as a prediction model.
**Results or Findings:** A significant seasonal change in polytrauma-CTs in the winter compared to summer months was observed (normalised median 0.003; IQR -0.16-0.14 vs. normalised median 0.03; IQR -0.11-0.20; p< 0.0001). There were significantly fewer polytrauma-CTs in the lower temperature quartile compared to the upper temperature quartile (normalised median -0.03; IQR -0.16-0.10 vs. normalised median 0.05; IQR -0.11-0.2; p< 0.0001). Temperature (r = 0.39), sunshine duration (r = 0.34) and ultra-violet amount (r = 0.35) correlated positively, wind velocity (r = -0.39) and clouds (r = -0.22) negatively with polytrauma-CT occurrence (correlations were significant p < 0.001). The diagnostic performance of the prediction model for identification of days with above normal number of polytrauma-CTs achieved an area under the curve of 0.74 (p < 0.0001) with a specificity of 71% and a sensitivity of 68%.
**Conclusion:** Using weather data, it is possible to partially predict normal or above normal daily number of polytrauma-CTs and thus improve resource planning.
**Limitations:** Retrospective study.
**Ethics committee approval:** The local IRB approved the study.
**Funding for this study:** No funding.
**Author Disclosures:**
Tobias Heye: Nothing to disclose
David Winkel: Nothing to disclose
Christian Breit: Nothing to disclose
Jacob Wasserthal: Nothing to disclose
Martin Segeroth: Nothing to disclose
Jan Vosshenrich: Nothing to disclose

### RPS 717-5

**Current abdominal x-rays practice in accident and emergency: an audit**

*W. Tam*; Cardiff/UK
*(telex@wstam.uk)*

**Purpose:** A literature review was presented at ECR 2020, which revealed that abdominal x-rays (AXR) performed for the accident and emergency department (A+E), had low sensitivity and high rates of further imaging and non-alignment to the Royal College of Radiologists (RCR) justification guidelines. A single-site audit was performed to investigate the current practice.
**Methods or Background:** An audit was performed at the largest A+E in Wales, in accordance with the RCR audit guidelines. All the AXR requests from A+E, regardless of the patient's age, within the 28 days period commencing 17th November 2021, were retrospectively assessed. Non-A+E patients and abandoned examinations due to uncooperative patients were excluded.
**Results or Findings:** The total number of AXR requests received by the A+E imaging department was 169, with 28/169 falling into the exclusion criteria. Of the 141 included requests, five unjustified requests were correctly rejected; the remaining 136 requests were accepted and subsequently performed. However, only 115/136 had justified indications. The most common justified and unjustified indications were obstruction and renal stones, respectively. Additionally, 45/136 patients had further imaging, with CT being the most popular modality. Only 4% of reported AXR had non-foreign body abnormalities.
**Conclusion:** The small proportion of significant findings agreed with previous studies, suggesting a significant AXR overuse. Over 80% of non-compliant requests were performed, and awareness of the justification guidelines can be increased by clinical governance, posters, or an algorithm previously presented. However, the 32.4 % further imaging rate, as opposed to the 73.7% reported previously, merits attention. Stopping the overuse of AXR can minimise the radiation dose received and relieve the mounting pressure in imaging and reporting, which can serve other patients who would benefit from the services otherwise.
**Limitations:** Single-centre study limits the validity of the result. Going forward, a multi-centre study and a re-audit after implementing the interventions mentioned are warranted.
**Ethics committee approval:** Not applicable.
**Funding for this study:** Not applicable.
**Author Disclosures:**
Winnie Tam: Nothing to disclose

### RPS 717-6

**Diagnostic accuracy of a fast MRI T2-sequence for the diagnosis of internal herniation after Roux-and-Y gastric bypass during pregnancy: a retrospective single centre study**

*B. Van Berkel*, P. Gillardin, V. Sneyers, L. Meylaerts, H. Mertens, H. Vandermaesen, A. Thaens, W. Bouckaert, G. Verswijvel; Genk/BE
*(brecht_van_berkel@hotmail.com)*

**Purpose:** The objective of this retrospective study was to evaluate the diagnostic accuracy of a fast sequence Magnetic Resonance Imaging (MRI) sequence, T2- half-Fourier acquisition single-shot turbo spin echo (HASTE) for the diagnosis of internal herniation (IH) in pregnant patients with a history of Roux-en-Y gastric bypass (RYGB).
**Methods or Background:** Abdominal MRI studies, performed for the clinical suspicion of IH of pregnant patients with a history of RYGB, were analysed by two abdominal radiologists. Both readers independently evaluated the T2 HASTE sequences to determine the presence or absence of IH. Diagnostic performance of T2 HASTE MRI was evaluated compared to the clinical diagnosis of IH as the gold standard.
**Results or Findings:** The results in this study, with 16 patients with IH and 15 patients without IH, portray a high accuracy for the detection of IH, ranging from 88.0% to 90.3%. A high NPV (86.7%-100.0%), sensitivity (88.9%-100.0%) and specificity (80.0%-86.7%). This study also portrayed a substantial interobserver agreement (0.741) for the evaluation of IH.
**Conclusion:** T2 HASTE MRI shows an excellent sensitivity and NPV for the detection of IH in pregnant patients. Fast acquisition time makes this sequence useful in the emergency setting, and it can be used for the diagnosis or exclusion of IH in pregnant patients.

**Limitations:** First, our sample size was rather small. Second, reader bias cannot be avoided as our readers had a high suspicion for internal herniation in this sample. Third, little is known about the MRI findings in asymptomatic patients with a (recent) history of RYGB. Finally, we should address the retrospective nature of our study of patients that did not undergo surgical exploration, indicating possible verification bias.
**Ethics committee approval:** Approval was obtained by the internal ethics committee (Z-2021040).
**Funding for this study:** Not required.
**Author Disclosures:**
Wim Bouckaert: Nothing to disclose
Vincent Sneyers: Nothing to disclose
Geert Verswijvel: Nothing to disclose
Hendrik Mertens: Nothing to disclose
Anke Thaens: Nothing to disclose
Liesbeth Meylaerts: Nothing to disclose
Hendrik Vandermaesen: Nothing to disclose
Brecht Van Berkel: Nothing to disclose
Patrick Gillardin: Nothing to disclose

### RPS 717-7

**Pulmonary embolism in patients with COVID-19 on CT: relationship with the extent of pulmonary parenchymal disease**
*M. L. Urbano*, C. Mendonça, B. Silva, J. J. B. Leitão, J. R. Inacio; Lisbon/PT
*(mluisa.urbano@gmail.com)*

**Purpose:** To evaluate if there is a correlation between the extent of pulmonary parenchymal disease and the presence of pulmonary embolism in COVID-19.
**Methods or Background:** This retrospective study included all COVID-19 patients admitted to the Emergency Department (ED) from June 2020 to January 2021, who underwent computed tomography pulmonary angiography due to pulmonary embolism (PE) suspicion. In total, 224 patients with COVID-19 and suspected PE were classified into two groups based on the presence or absence of pulmonary embolism. All patients were categorised in absence or presence of pulmonary parenchymal disease extent in 25% intervals.
**Results or Findings:** The study cohort consisted of 211 patients positive for SARS-CoV-2, 118 male and 89 female, with mean age 68.7±16.5. The extent of pulmonary disease was 0% in 13 (6%), 1-25% in 84 (39%), 26-50% in 65 (30%), 51-75% in 36 (17%) and >75% in 13 (6%) patients. Pulmonary embolism (PE) was confirmed in 40/211 (19%) patients, with 18/40 (45%) patients showing small vessel PE. The mean age in PE group was 73.4±12 versus 67.6±17,2 in the non PE group (p = 0,015). There was no difference in the extent of pulmonary disease between PE and non PE groups (p = 0.49).
**Conclusion:** In COVID-19 patients, the extent of the pulmonary disease was not correlated with the presence of PE.
**Limitations:** Our study has some limitations. It is a retrospective single-centre and chart review study. We only included patients admitted in the ED, and cannot infer whether these results can be applied to the whole COVID population with PE suspicion. Previous CT exams were not evaluated to differentiate the parenchymal abnormalities from pre-existent parenchymal changes.
**Ethics committee approval:** Due to the retrospective analysis of data, the need for informed consent was waived.
**Funding for this study:** No funding was received.
**Author Disclosures:**
João José Baeta Leitão: Nothing to disclose
Joao Rodrigues Inacio: Nothing to disclose
Carlos Mendonça: Nothing to disclose
Maria Luisa Urbano: Nothing to disclose
Beatriz Silva: Nothing to disclose

### RPS 717-8

**Evaluation of a fully interactive radiology workshop using an online DICOM viewer**
*G. Bailey*[1], M. Naik[2], C. McLeavy[3], E. Dick[2], T. Raupach[4]; [1]Surrey/UK, [2]London/UK, [3]Liverpool/UK, [4]Göttingen/DE
*(georgina@pjcg.co.uk)*

**Purpose:** This study aims to evaluate the effectiveness of a new interactive virtual teaching method comprising live review of radiological cross-sectional trauma imaging cases using an online Digital Imaging and Communications in Medicine (DICOM) viewer.
**Methods or Background:** The European Society of Emergency Radiology ran an online workshop on the topic of blunt trauma CT via a video conferencing service. Thirty delegates spanning 18 countries were given access to online links to cases via the DICOM viewer, Pacsbin, which they could scroll through in real time during the workshop. Interactive discussion was encouraged throughout. The teaching method was evaluated using online quiz before and after the workshop took place, comprising of 10 of the same knowledge-based questions aligned to the learning objectives. They were also asked to rate their level of confidence in reporting blunt trauma CT using a five-point scale. 21 delegates completed the quiz before the workshop and 27 after.
**Results or Findings:** Following the workshop, 74% (20/27) that took the quiz stated that they felt either “very confident' or “extremely confident”, compared to 35% (7/20) describing these levels of confidence before the workshop (39% increase, p = 0.03). The average quiz score from the knowledge-based questions improved from 77% of the pre-workshop quiz to 92% of the post workshop quiz (15% increase, p = 0.15).
**Conclusion:** This evaluation demonstrates the potential of using software with screen-sharing capabilities and an online DICOM viewer to deliver interactive teaching to an international audience. This is likely to have an increasingly integral role in medical, and notably radiological, education.
**Limitations:** Small sample. No paired comparison between delegates before and after the workshop due to the method of data collection. Repeated testing may have influenced quiz results.
**Ethics committee approval:** Not required.
**Funding for this study:** Not required.
**Author Disclosures:**
Mitesh Naik: Nothing to disclose
Elizabeth Dick: Nothing to disclose
Chris McLeavy: Nothing to disclose
Tobias Raupach: Nothing to disclose
Georgina Bailey: Nothing to disclose

**08:00-09:00** **Room N**

### Research Presentation Session: Genitourinary

### RPS 707

### Prostate cancer: will we ever be replaced by machines and urologists?

**Moderator**
J. J. Fütterer; Nijmegen/NL

### RPS 707-2

**The first 5 targeted trans-perineal prostate biopsies diagnose the majority of PROMIS criteria cancer in patients with a Likert 4 or 5 score on mpMRI**
*W. M. Stevens*[1], E. Adiotomre[2], O. Hulson[2], A. Khan[2], R. Lapham[2], P. Melling[2], A. Morrell[2], S. Pierre[2], J. Smith[2]; [1]Bradford/UK, [2]Leeds/UK

**Purpose:** To see if the first 5 trans-perineal (TP) biopsies give a diagnosis of PROMIS criteria cancer in patients with a likely prostate cancer on mpMRI.
**Methods or Background:** NICE guidelines suggest multiple prostate biopsies for histological diagnosis of cancer in those patients with a Likert 4 or 5 score on their mpMRI scan. Depending on the treatment planned, some patients need systematic TP biopsy, but in frail patients, those with limited treatment options or extensive disease, a cancer diagnosis may be all that is required. 375 patients had an mpMRI for suspected prostate cancer between January and June of 2021 in a large volume quaternary centre. 367 were given a Likert score of which 108 were scored Likert 4 or 5. Of these, 94 patients were sent for biopsy. 70 of the biopsied patients were ultimately diagnosed with PROMIS criteria cancer. A separate pot was sent containing the first 2-5 targeted biopsies in 69 of the 70.
**Results or Findings:** The median number of biopsies in all patients sent for biopsy was 12 (range 3-19). The first 2-5 targeted biopsies showed PROMIS criteria cancer in 65 of the 69 (94.2%) Likert 4 and 5 patients diagnosed with cancer. 62/69 (89.9%) showed the maximum length or grade of cancer in the first 2-5 targeted biopsies. All 4 of the missed cancers were ISUP 2 or less and located in the apex of the gland.
**Conclusion:** A cancer diagnosis is usually obtained in the first 2-5 targeted biopsies in patients with a Likert score of 4 or 5. Further biopsies may be required for treatment planning or for lesions in the apex of the gland where cancers can be missed.
**Limitations:** This was a single-centre observational study.
**Ethics committee approval:** Not applicable
**Funding for this study:** Not applicable
**Author Disclosures:**
Ese Adiotomre: Nothing to disclose
Roger Lapham: Nothing to disclose
Oliver Hulson: Nothing to disclose
Sacha Pierre: Nothing to disclose
William Mark Stevens: Nothing to disclose
Philip Melling: Nothing to disclose
Atif Khan: Nothing to disclose
Jonathan Smith: Nothing to disclose
Adam Morrell: Nothing to disclose

### RPS 707-3

**Is Likert scoring of mpMRI scans for suspected prostate cancer different from PI-RADSv2.1 scoring? 5 reporters compare overall scores, number of equivocal cases and predicted biopsy cancer yields**

*W. M. Stevens*[1], C. Parchment-Smith[2], E. Adiotomre[2], O. Hulson[2], A. Khan[2], P. Melling[2], S. Pierre[2], J. Smith[2]; [1]Bradford/UK, [2]Leeds/UK

**Purpose:** To assess if Likert scores are different from PI-RADSv2.1 scores with respect to the number of patients given each score, the predicted number of patients sent for biopsy and cancer yield assuming a standardised biopsy protocol.
**Methods or Background:** 5 experienced reporters in a quaternary referral unit used a standardised reporting template to prospectively collect PI-RADS and Likert scores for all mpMRI querying prostate cancer between January and June 2021. The histology results were recorded for those patients who proceeded to a transperineal biopsy.
**Results or Findings:** 367 of the total 375 patients were allocated a PI-RADS and a Likert score. The number of PI-RADS scores that were changed to a different Likert score varied between reporters (3.3%, 21.7%, 23.5%, 26.9%, 47.3%). The median PI-RADS score was 3 and the median Likert score was 2. The number of equivocal "3" scores given was halved in Likert (13.9%) compared with PI-RADS (26.2%). Assuming a protocol in which all patients scoring 3 or above were advised to proceed to biopsy, fewer patients scored with Likert (43.3%) than with PI-RADS (59.4%) would have been so advised (59 fewer patients). Using the same assumption, the predicted biopsy yield improved when Likert scores were used (48.4%) instead of PI-RADS (35.3%). The number of clinically significant cancers (ISUP 3-5) who were scored 1 or 2 (very unlikely or unlikely to have cancer) was the same (0.3%) whether PI-RADS or Likert was used.
**Conclusion:** PI-RADS and Likert were not the same for any reporter. The Likert score had several outcome advantages compared with PI-RADS.
**Limitations:** This was a single unit study. Cases not randomised. The numbers of scans reported by each reporter varied. Cancer outcomes were only verified in biopsied patients.
**Ethics committee approval:** Not applicable
**Funding for this study:** Not applicable
**Author Disclosures:**
Ese Adiotomre: Nothing to disclose
Oliver Hulson: Nothing to disclose
Sacha Pierre: Nothing to disclose
William Mark Stevens: Nothing to disclose
Philip Melling: Nothing to disclose
Atif Khan: Nothing to disclose
Jonathan Smith: Nothing to disclose
Catherine Parchment-Smith: Nothing to disclose

### RPS 707-4

**In-bore robot assisted MRI guided target biopsy of the prostate: our experience**

*R. Van Stiphout*[1], H. Raat[1], A. van Gorp[1], J. O. Barentsz[2]; [1]Roermond/NL, [2]Nijmegen/NL
*(R.vanstiphout@lzr.nl)*

**Purpose:** In succession to our award-winning presentation on the ECR 2020 we present our expierence and results of the in-bore robot assisted MRI guided target biopsy (MRGB) of the prostate in 500 cases.
**Methods or Background:** Men with elevated PSA levels (>/= 3 ng/ml) are at risk for prostate cancer and will undergo a mpMRI of the prostate. Suspicious lesions are graded according to PIRADS v2.0 classification. In patients with a PIRADS >2 lesion biopsy is advised (EAU). Systematic TRUS biopsy has been the standard for years with a higher risk of sepsis (4%). How accurate and feasible is target biopsy using the Robot controlled manipulator from Soteria Medical to detect high grade prostate cancer?
**Results or Findings:** This method is as feasible and accurate as manually MRGB, though much faster. Our results are equal to the results found in recent literature. By the beginning of 2022, we are able to analyse the data of 500 cases. We will discuss the role of target biopsy compared to TRUS biopsy and the literature about the combination of both methods. The robot-assisted method can be considered in small lesions (<10 mm), lesions in hard to reach locations for TRUS biopsy, such as anteriorly or laterally located lesions and in patients with very high suspicion on high grade prostate cancer and repeatedly negative TRUS biopsies.
**Conclusion:** Robot-assisted MRGB is as feasible and accurate as manual MRGB. It is of great help in hard to reach lesions and small lesions and in patients with repeatedly negative TRUS biopsies. It has much lower risk of sepsis or bleeding.
**Limitations:** Patients should be MRI-compatible and be able to lie down in a prone position.
**Ethics committee approval:** Not applicable
**Funding for this study:** No funding was received for this study.
**Author Disclosures:**
Rogier Van Stiphout: Nothing to disclose
Alexander van Gorp: Nothing to disclose
Jelle O. Barentsz: Nothing to disclose
Henricus Raat: Nothing to disclose

### RPS 707-5

**Machine learning-based multimodal data analysis for optimised treatment planning and surgical outcome prediction of prostate cancer patients**

F. Tollens[1], N. Westhoff[1], J. Moltz[2], F. Derigs[1], *M. Naeimi*[1], P. Kohlmann[2], S. Gatidis[3], S. O. Schönberg[1], D. Nörenberg[1]; [1]Mannheim/DE, [2]Bremen/DE, [3]Tübingen/DE

**Purpose:** To examine the potential of a multimodal feature signature for optimised therapy planning of primary prostate cancer (PCa) and to determine the risk of a positive margin (R0 vs R1-status) after radical prostatectomy (rPx) by applying a machine learning approach.
**Methods or Background:** 175 retrospectively and 30 prospectively identified and histopathologically proven PCa patients underwent pretherapeutic multiparametric MRI, structured reporting and rPx. Data collection, image annotation and machine learning-based analysis were conducted on the cloud-based "International Radiomics Platform" (IRP) as an initiative of the German and Austrian radiological societies. Conventional imaging biomarkers (PI-RADS, mean ADC), selected MR-based radiomics features and clinical (digital rectal examination, PSA density (PSAD)) as well as pathological data (ISUP grade, T-status) were used to predict therapy decision parameters (nerve-sparing surgery) and R-status. Repeated 5-fold cross validation was performed to test robustness of the results.
**Results or Findings:** In total, 112 of the 205 included patients received nerve-sparing surgery and 34 of 205 showed positive resection margins (R1). A signature of 16 multimodal features predicted R1-status after rPx with an AUC of 0.61 (p=0.12) and decision for nerve-sparing surgery with an AUC of 0.69 (p<0.01), compared to a combination of mean ADC, PI-RADS and PSAD with an AUC of 0.55 and 0.53, respectively.
**Conclusion:** In the present study, the feasibility of an integrated, machine-learning based analysis of multimodal medical data on the IRP could be demonstrated. Broadly available multimodal data combined with quantitative image analysis and structured reporting can be used to support risk stratification and surgical planning of primary PCa patients.
**Limitations:** The small number of patients with R1-status and the lack of an external validation cohort were identified as limitations.
**Ethics committee approval:** The study has been approved by local ethics committees.
**Funding for this study:** This study was funded by the Ministry of Labour, Economy and Tourism Baden-Wuerttemberg (Germany).
**Author Disclosures:**
Stefan Oswald Schönberg: Nothing to disclose
Sergios Gatidis: Nothing to disclose
Niklas Westhoff: Nothing to disclose
Dominik Nörenberg: Nothing to disclose
Fabian Tollens: Nothing to disclose
Peter Kohlmann: Nothing to disclose
Jan Moltz: Nothing to disclose
Fabian Derigs: Nothing to disclose
Mahnoosh Naeimi: Nothing to disclose

### RPS 707-6

**Vulnerability of radiomics machine learning models for the detection of clinically significant prostate cancer in heterogeneous MRI datasets**

E. K. Gresser[1], *B. Schachtner*[1], O. Solyanik[1], A. Schreier[1], M. Giuseppe[1], A. Kretschmer[1], J. Ricke[1], M. Ingrisch[1], D. Nörenberg[2]; [1]Munich/DE, [2]Mannheim/DE
*(Balthasar.Schachtner@med.uni-muenchen.de)*

**Purpose:** To investigate the diagnostic accuracy and robustness of advanced machine learning radiomics approaches in heterogenous datasets to characterise suspicious prostate lesions (PI-RADS score ≥ 3) in biparametric MRI for the detection of significant prostate cancer (PCa) compared to conventional imaging biomarkers.
**Methods or Background:** 142 patients with clinical suspicion of PCa underwent 1.5T or 3T biparametric prostate MRI with various scanner types (n=7) in different institutions (n=14) and exhibited suspicious lesions ≥ PI-RADS 3 in peripheral or transitional zones. Whole-gland prostate and index lesion segmentations were performed semi-automatically on T2w- and diffusion-weighted images. 1482 quantitative morphologic, shape, texture, and intensity-based imaging features were calculated to assess the impact of a radiomics signature for non-invasive prediction of PCa aggressiveness. Several bias mitigation techniques were applied and robustness of results was evaluated. Performance was compared with mean ADC (mADC), PI-RADS scores and PSA density (PSAD).

Thursday

**Results or Findings:** Discriminative potential of radiomics machine learning approaches (mean AUCs from 0.77 to 0.84) to differentiate clinically significant PCa from clinically insignificant PCa or benign lesions was comparable to clinical assessment using PI-RADS scoring (AUC=0.78), mADC (AUC=0.72) and PSAD (AUC=0.62). Fivefold cross validation of datasets demonstrated high performance variability even when employing advanced mitigation techniques for the high-dimensional feature space and inherent class imbalance.
**Conclusion:** The clinical applicability of radiomics models on suspicious prostate lesions in a heterogeneous dataset is limited because of low robustness and high variations of results. Radiomics did not reliably improve clinical decision-making compared to conventional imaging biomarkers. Feature variability, robustness and reproducibility of radiomics-based measures should be addressed more transparently in future research to enable broad clinical application.
**Limitations:** Relatively small sample size (n=142), no external validation cohort and semi-automated segmentations. Radical prostatectomy and biopsy included.
**Ethics committee approval:** Ethics committee approval is available.
**Funding for this study:** No funding was received for this study.
**Author Disclosures:**
Andrea Schreier: Nothing to disclose
Balthasar Schachtner: Nothing to disclose
Olga Solyanik: Nothing to disclose
Dominik Nörenberg: Nothing to disclose
Michael Ingrisch: Nothing to disclose
Alexander Kretschmer: Nothing to disclose
Magistro Giuseppe: Nothing to disclose
Eva Kristina Gresser: Nothing to disclose
Jens Ricke: Nothing to disclose

### RPS 707-7

**Convolutional neural networks for automated classification of prostate multiparametric magnetic resonance imaging based on image quality**
*E. Messina*, S. Cipollari, M. Pecoraro, M. Bicchetti, C. Catalano, V. Panebianco; Rome/IT
*(emanuele.messina@uniroma1.it)*

**Purpose:** To develop a convolutional neural networks (CNNs)-based analysis pipeline for the classification of prostate MRI image quality.
**Methods or Background:** Prostate multiparametric magnetic resonance imaging (mpMRI) is technically demanding, requiring high image quality. An automated method to identify diagnostically inadequate images could help optimise image quality. 316 mpMRI scans and 312 men were retrospectively enrolled. MRI scans were reviewed by three experienced genitourinary radiologists. Sequences were labelled as high quality (Q1) or low quality (Q0) and used as the reference standard for all analyses. Sequences were split into training, validation, and testing sets (869, 250, and 120 sequences, respectively). Following preprocessing and data augmentation, 28 CNNs were trained. Model performance was assessed on both a per-slice and a per-sequence basis. A pairwise t-test was performed to compare performances of the classifiers.
**Results or Findings:** The number of sequences labelled as Q0 or Q1 was 38 vs 278 for T2WI, 43 vs 273 for DWI, 41 vs 275 for ADC, and 38 vs 253 for DCE. Interreader agreement was almost perfect for T2WI and DCE and substantial for DWI and ADC. On the per-slice analysis, accuracy was 89.95% 0.02% for T2WI, 79.83% 0.04% for DWI, 76.64% 0.04% for ADC, 96.62% 0.01% for DCE. On the per-sequence analysis, accuracy was 100% 0.00% for T2WI, DWI, and DCE, and 92.31% 0.00% for ADC. The three best algorithms performed significantly better than the remaining ones on every sequence (p-value < 0.05).
**Conclusion:** CNNs achieved high accuracy in classifying prostate MRI image quality on an individual-slice basis and almost perfect accuracy when classifying the entire sequences.
**Limitations:** A relatively small sample size was used for training.
The CNNs were trained on images acquired on a single MR scanner and protocol.
**Ethics committee approval:** This study was approved by an ethics committee.
**Funding for this study:** No funding was received for this study.
**Author Disclosures:**
Marco Bicchetti: Nothing to disclose
Valeria Panebianco: Nothing to disclose
Stefano Cipollari: Nothing to disclose
Emanuele Messina: Nothing to disclose
Martina Pecoraro: Nothing to disclose
Carlo Catalano: Nothing to disclose

### RPS 707-8

**A deep learning masked segmentation alternative to manual segmentation in biparametric MRI prostate cancer radiomics**
*J. Bleker*[1], T. Kwee[1], D. Rouw[1], C. Roest[1], J. Borstlap[2], I. J. de Jong[1], R. Dierckx[1], H. Huisman[3], D. Yakar[1]; [1]Groningen/NL, [2]Hoogeveen/NL, [3]Nijmegen/NL
*(j.bleker@umcg.nl)*

**Purpose:** To determine the value of a deep learning masked (DLM) auto-fixed volume of interest (VOI) segmentation method as an alternative to manual segmentation for radiomics-based diagnosis of clinically significant (CS) prostate cancer (PCa) on biparametric magnetic resonance imaging (bpMRI).
**Methods or Background:** This study included a retrospective multicentre dataset of 524 PCa lesions (of which 204 CS PCa) on bpMRI. All lesions were both semi-automatically segmented with a DLM auto-fixed VOI method (averaging <10 seconds per lesion) and manually segmented by an expert uroradiologist (average 5 minutes per lesion). The DLM auto-fixed VOI method uses a spherical VOI (with its centre at the location of the lowest apparent diffusion coefficient of the prostate lesion as indicated with a single mouse-click) from which non-prostate voxels are removed using a deep learning based prostate segmentation algorithm. Thirteen different DLM auto-fixed VOI diameters (ranging from 6 to 30 mm) were explored. Extracted radiomics data were split into training and test sets (4:1 ratio). Performance was assessed with receiver operating characteristic (ROC) analysis.
**Results or Findings:** In the test set, the area under the ROC curve (AUCs) of the DLM auto-fixed VOI method with a VOI diameter of 18 mm (0.76 [95% CI: 0.66 – 0.85]) was significantly higher (p=0.0198) than that of the manual segmentation method (0.62 [95% CI: 0.52-0.73]).
**Conclusion:** A DLM auto-fixed VOI segmentation can provide a potentially more accurate radiomics diagnosis of CS PCa than expert manual segmentation while also reducing expert time investment by more than 97%.
**Limitations:** Not applicable
**Ethics committee approval:** Not applicable
**Funding for this study:** Not applicable
**Author Disclosures:**
Henkjan Huisman: Nothing to disclose
Jeroen Bleker: Nothing to disclose
Igle Jan de Jong: Nothing to disclose
Dennis Rouw: Nothing to disclose
Thomas Kwee: Nothing to disclose
Jaap Borstlap: Nothing to disclose
Derya Yakar: Nothing to disclose
Rudi Dierckx: Nothing to disclose
Christian Roest: Nothing to disclose

**08:00-09:00** **Room X**

### Research Presentation Session: Head and Neck

### RPS 708

### Head and neck: miscellaneous

**Moderator**
E. Loney; Halifax/UK

### RPS 708-2

**Clinical and radiological picture of ROCM in post-COVID-19 patients**
*L. Yunusova*; Tashkent/UZ
*(lolita_yunusova@mail.ru)*

**Purpose:** To report the outcome of ROCM in patients with diabetes mellitus.
**Methods or Background:** Retrospective analysis of the medical records of 121 patients with ROCM with diabetes.
**Results or Findings:** Gender: M-96/F-25 with a mean (SD). Age of 47.3 (14.4) years was studied. 5 patients had type 1 diabetes mellitus, 30 type 2 diabetes mellitus. 86 patients had ROCM as the first clinical manifestation of diabetes. The mean (SD) blood glucose at presentation was 20.6 (8.3) mmol/l and 17 patients had ketosis/ketoacidosis. Ophthalmic symptoms and signs were pronounced: external ophthalmoplegia (89%), proptosis (83%), visual loss (80%), chemosis (74%), and eye lid gangrene (14%). Non-ophthalmic manifestations included sinusitis (100%), nasal discharge/ulceration (74%), infranuclear VI nerve palsy (46%), palatal necrosis (29%), cerebral lobe involvement (20%), and hemiparesis (17%). Computed tomography/magnetic resonance imaging showed involvement of paranasal sinuses in all patients with ethmoid (86%) and maxillary (80%) sinuses being most frequently involved. Orbital involvement was observed in 80% of patients with cavernous sinus thrombosis in 11%, and internal carotid occlusion and hydrocephalus in

3% each. All were treated with amphotericin B (3-3.5 g) and 26 (31.5%) patients underwent appropriate surgery. Twenty one patients (68%) survived with a mean (SD) follow up of 39.6 (34.1) months. Factors related to poor survival included delay in diagnosis and treatment ($p = 0.05$), facial and/or eye lid gangrene ($p = 0.05$), hemiplegia ($p = 0.05$), cerebral invasion by mucorales ($p = 0.05$), and treatment with amphotericin B alone ($p = 0.05$).
**Conclusion:** In patients with diabetes and ROCM, the ROCM was the main manifestation in one fourth of patients. The clinical picture was dominated by ophthalmic and extensive brain lesions, radiological imaging confirming it.
**Limitations:** No limitations were identified.
**Ethics committee approval:** This study was approved by the ethics committee.
**Funding for this study:** No funding was provided for this study.
**Author Disclosures:**
Lolita Yunusova: Nothing to disclose

### RPS 708-3

**Comparing mucormycosis severity in COVID-19 patients and COVID-19 free patients: an imaging-based diagnosis**

S. Sefidbakht[1], S. B. Hashemi[1], *A. Motealleh*[1], Z. Hemmati[1], H. Hosseinpour[1], B. Bijan[2]; [1]Shiraz/IR, [2]Sacramento, CA/US
*(ali.motealleh3976@gmail.com)*

**Purpose:** Mucormycosis is an opportunistic disease. Literature on the association of rhino-orbital mucormycosis with COVID-19 is limited to a few case reports citing DM as a predisposing risk factor. We describe imaging findings and disease extent in patients with rhino-orbital-cerebral mucormycosis in COVID-positive and COVID-negative groups.
**Methods or Background:** A retrospective study was conducted on the picture archiving and communication system and hospital information system of an ear-nose-throat major tertiary referral centre for COVID-associated and non-COVID-associated mucormycosis from June 2020 till May 2021. COVID diagnosis was conducted through a chest CT scan or PCR test.
**Results or Findings:** Among 60 patients with mucormycosis, imaging from 52 patients was available. Among 20 COVID-positive patients, paranasal sinuses and nasal cavity, orbit, infratemporal area, intracranial, facial soft tissue, and pterygopalatine fossa were involved 100%, 80%, 40%, 20%,10%, and 0%, respectively. Among 32 COVID-negative patients, paranasal sinuses and nasal cavity, orbit, intracranial, infratemporal area and facial soft tissue, and pterygopalatine fossa were involved in 100%, 87%, 37%, 25%, and 18%, respectively. Among 32 COVID-negative patients, erosions of maxillary sinus wall, ethmoid septa, nasal septum, concha in 87%, lamina papiracea in 81%, the floor of orbit in 75%, palate in 50%, cribriform plate in 37%, and roof of orbit in 6% of the patients were seen. Among 20 COVID-positive patients, erosions of maxillary sinus wall, ethmoid septa, nasal septum, concha, and lamina papiracea in 100%, orbit floor and palate in 70%, cribriform plate in 50%, and roof of orbit in 20% of patients were seen.
**Conclusion:** No significant difference was seen in disease extent; however, bone erosion was more extensive in the COVID-positive group, which indicates a more severe disorder.
**Limitations:** No limitations were identified.
**Ethics committee approval:** The institutional ethics committee approved this study.
**Funding for this study:** Funding was received from the Shiraz University of Medical Sciences (SUMS).
**Author Disclosures:**
Bijan Bijan: Nothing to disclose
Sepideh Sefidbakht: Nothing to disclose
Seyed Basir Hashemi: Nothing to disclose
Hamidreza Hosseinpour: Nothing to disclose
Ali Motealleh: Nothing to disclose
Zahra Hemmati: Nothing to disclose

### RPS 708-4

**The incidence and imaging findings of intracranial extension of sino-nasal mucormycosis**

*S. Sefidbakht*[1], S. B. Hashemi[1], A. Motealleh[1], Z. Hemmati[1], B. Bijan[2]; [1]Shiraz/IR, [2]Sacramento, CA/US
*(SepidehSefidbakht@yahoo.com)*

**Purpose:** Many countries have experienced a recent surge of rhinocerebral mucor mycosis following the COVID-19 pandemic. The purpose of this study is to describe the imaging findings and frequency of intracranial extension of sino-nasal mucormycosis.
**Methods or Background:** A retrospective study was done using a tertiary ENT referral hospital using the HIS search system. Over a 24-month period from January 2020 to December 2021, 50 patients were admitted and treated with rhinocerebral mucor mycosis. For all patients, multiple PNS CT scans were available during their hospital course and all had at least one brain MRI. Demographic data, COVID-19 status and underlying disease were recorded. Imaging findings indicating intracranial spread were recorded.
**Results or Findings:** In 12 (24%) patients evidence of intracranial extension was present. This included 5 patients with positive COVID-19 PCR test and 7 with negative PCR test. Imaging findings include cerebritis presenting as T2 hyperintensity of frontal lobe cortex and subcortical white matter in 5 patients, extension to cavernous sinus and cavernous sinus thrombosis in 2 patient, ICA occlusion in 2 patients, frontal lobe meningeal enhancement in 2 patients, and parenchymal haemorrhage in one.
**Conclusion:** A relatively high percentage (24%) of rhinocerebral mucor mycosis can extend intracranially despite timely treatment. Despite the recent surge of mucor mycosis, intracranial extension is not significantly different in patients with positive PCR.
**Limitations:** Number of intracranial extension was low for generalisations. Later a meta-analysis can be helpful. Also there are too many confounding factors to control for between the two groups.
**Ethics committee approval:** The institutional ethics committee approved this retrospective study.
**Funding for this study:** No funding was received for this study.
**Author Disclosures:**
Bijan Bijan: Nothing to disclose
Sepideh Sefidbakht: Nothing to disclose
Seyed Basir Hashemi: Nothing to disclose
Ali Motealleh: Nothing to disclose
Zahra Hemmati: Nothing to disclose

### RPS 708-8

**The potential role of shear wave elastography in the assessment of nodular thyroid disease**

*E. Makai*, F. Kakuja, Z. Fejes, P. Palásti, B. Bozsik, B. Nagy, Z. T. Kincses; Szeged/HU
*(makaieszter8@gmail.com)*

**Purpose:** There is an increasing evidence that tissue rigidity as measured by elastography may aid the differentiation of thyroid tumorous and benign nodules. The aim of this study was to evaluate the role of shear wave elastography (SWE) in the diagnostic algorithm of nodular thyroid disease.
**Methods or Background:** Ultrasonography was used to determine the size of the nodules, the distance from the surface and the EU-TIRADS classification. Standard approach was used for SWE measurements cytological samples from FNAB were evaluated according to the 2017 Bethesda criteria. Principal component analysis (PCA) was used to generate factors for pathological abnormalities and radiological data, their relationship was analysed by Pearson correlation.
**Results or Findings:** 63 patients with thyroid nodules were included (mean age 60.4 year). 62% of the nodules were EU-TIRADS category III. The mean elasticity of the nodules was 31.6 kPa (±10.7). Colloid content explained most of the variability in the pathological factor generated by PCA and the mean elasticity in the radiological factor. There was also significant correlation between pathological and radiological factors ($p = 0.023$).
**Conclusion:** Here we showed that a set of radiological measures can reliably predict the pathological picture of thyroid nodules, the most important of which is the elasticity. Rigid structure of the malignant nodules may indicate cell-rich areas, helping to localise the site of FNAB, and may reduce the number of non-diagnostic and unnecessary FNABs.
**Limitations:** Increasing the size of the patient population could further improve the statistical power.
**Ethics committee approval:** The local ethics committee of the University of Szeged approved the study.
**Funding for this study:** No funding was received for this study.
**Author Disclosures:**
Bence Nagy: Nothing to disclose
Eszter Makai: Nothing to disclose
Bence Bozsik: Nothing to disclose
Flora Kakuja: Nothing to disclose
Zsigmond Tamás Kincses: Nothing to disclose
Zsuzsanna Fejes: Nothing to disclose
Péter Palásti: Nothing to disclose

**10:30-12:00** **Room D**

### Research Presentation Session: Neuro

### RPS 811

### New techniques in neuro imaging

**Moderator**
S. Kristjánsson; Rejkjavik/IS

### RPS 811-2

**A quantitative imaging study of amide proton transfer-weighted imaging in diabetes-associated cognitive dysfunction of type 2 diabetes mellitus**
*W. J. Shao*, S. Xiang, J. Fang, W. Su, Y. Yang, Y. Xiong, J. Li; Kunming/CN
*(wave.forever@yeah.net)*

**Purpose:** To evaluate the feasibility of identifying the occurrence and development of diabetes-associated cognitive dysfunction (DACD) of type 2 diabetes mellitus using Amide proton transfer-weighted (APTw).
**Methods or Background:** 28 patients with mild cognitive impairment (MCI +) in T2DM, 8 patients without mild cognitive impairment (MCI -) in T2DM and 11 normal control (NC) subjects were included. APTw was performed in patients with T2DM and evaluated by the multiple field cognitive function scale. APTw signal intensity(SI) (%) of hippocampus in three groups and the APTw SI (%) of the hippocampus were analysed. The correlation of APTw SI and multiple field cognitive function scale was analysed by Pearson analysis correlation in MCI+ group.
**Results or Findings:** The APTw SI (%) of left hippocampal head, left hippocampal body and right hippocampal tail were statistically different among three groups (P < 0.05). SVF score was positively correlated with APTw SI (%) in left hippocampal head of MCI+ group (rho = 0.414, P = 0.044) , TMT-A score was negatively correlated with APTw SI (%) in left hippocampal tail of MCI+ group (rho = -0.333, P = 0.021), AVLT-H (delaye memory) scores were negatively correlated with APTw SI (%) in left hippocampal tail of MCI+ group (rho = -0.376, P = 0.000）.
**Conclusion:** Findings suggestive of the progression of MCI were found from the APTw SI in different regions of hippocampus. APTw technique may be a noninvasive and potential imaging biomarker for DACD of T2DM at molecular imaging level.
**Limitations:** This study only measured the APTw SI in hippocampus, but not in other brain regions.
**Ethics committee approval:** All procedures performed were in accordance with the Institutional Review Board of the Affiliated Hospital of Yunnan University.
**Funding for this study:** Funding was received for this study by the Endocrine Clinical Medical Center of Yunnan Province, No. ZX20190202.
**Author Disclosures:**
Yuxin Xiong: Nothing to disclose
Shutian Xiang: Nothing to disclose
Jianbo Li: Nothing to disclose
Jing Fang: Nothing to disclose
Wei Su: Nothing to disclose
Wei Ju Shao: Nothing to disclose
Ying Yang: Nothing to disclose

### RPS 811-3

**Early diffusion-weighted MRI at 3Tesla detects ischaemic changes of the optic nerve in anterior ischaemic optic neuropathy**
S. Mournet, T. Sene, F. Charbonneau, G. Poillon, C. Vignal, G. Clavel, J. Guillaume, J. Savatovsky, *A. Lecler*; Paris/FR
*(alecler@for.paris)*

**Purpose:** To assess the impact of timing from visual symptoms' onset to diffusion-weighted (DW) 3T MRI completion to detect ischaemic changes of the optic disc and optic nerve in AION patients.
**Methods or Background:** This IRB-approved retrospective single-centre study included 3T MRI data from 126 patients with AION and 111 controls with optic neuritis treated between January 2015 and May 2020. Two radiologists blinded to all data, individually analysed imaging. A senior neuroradiologist resolved any discrepancies by consensus. The primary judgment criterion was the restricted diffusion of the optic disc and/or the optic nerve assessed subjectively on the ADC maps. ADC values were also measured. Spearman rank correlations were used to examine the relationships between timing from visual symptoms' onset to MRI completion and both the restricted diffusion and the ADC values.
**Results or Findings:** 126 patients (47/126 [37.3%] women and 79/126 [62.7%] men, mean age 69.1 +/- 13.7 years) with AION were included. Restricted diffusion of the optic disc in AION eyes was more frequent in the early MRI group than in the late MRI group: 35/49 (71.4%) eyes versus 3/83 (3.6%) eyes, p<0.001. ADC values of the pathological optic discs and optic nerves were lower in the early MRI group than in the late MRI group: 0.61 [0.52-0.94] x 10-3 mm2/s versus 1.28 [1.01-1.44] x 10-3 mm2/s, p<0.001 and 0.74 [0.61-0.88] x 10-3 mm2/s versus 0.89 [0.72-1.10] x 10-3 mm2/s, p<0.001, respectively.
**Conclusion:** DWI MRI showed good diagnostic performance to detect AION when performed early after the onset of visual symptoms.
**Limitations:** The limitation was the monocentric study design in a tertiary centre specialised in ophthalmological diseases. 3T MRI is not available everywhere. No use of high-resolution DWI.
**Ethics committee approval:** This study is IRB approved and adhered to the tenants of the Declaration of Helsinki.
**Funding for this study:** No funding was received for this study.
**Author Disclosures:**
Frédérique Charbonneau: Nothing to disclose
Jessica Guillaume: Nothing to disclose
Thomas Sene: Nothing to disclose
Sandy Mournet: Nothing to disclose
Gaëlle Clavel: Nothing to disclose
Catherine Vignal: Nothing to disclose
Julien Savatovsky: Nothing to disclose
Augustin Lecler: Nothing to disclose
Guillaume Poillon: Nothing to disclose

### RPS 811-4

**Gadolinium presence in the skin: assessment of intraepidermal nerve fiber density in rat foot skin samples after multiple administrations of Gadolinium-based contrast agents**
J. Boyken, J. Lohrke, G. Jost, T. Frenzel, *H. Pietsch*; Berlin/DE

**Purpose:** Gadolinium (Gd) presence in the body after multiple exposures to Gd-based contrast agents (GBCAs) has been part of intensive research in recent years. While most studies investigated Gd presence in brain, Gd was similarly observed in skin and other organs. The current animal study investigated all marketed extracellular GBCAs and evaluated the skin of rat foot pads for Gd presence and neuro-histological changes after repeated administration of GBCAs.
**Methods or Background:** Rats received 8 intravenous injections of either gadodiamide, gadobenate, gadopentetate, gadobutrol, gadoterate, gadoteridol at a dose of 0.6 mmol Gd/kg body weight or saline. Animals were sacrificed 5 weeks p.i., the amount of Gd was measured by inductively coupled plasma mass spectrometry. The quantification of the intraepidermal nerve fiber density in the footpad was determined by PGP9.5 immunofluorescent staining and automated image analysis which was performed in blinded fashion by an external provider.
**Results or Findings:** The concentrations of Gd [nmol/g] in the skin were highest for linear gadodiamide [10.2±1.8], followed by gadopentetate [3.5±0.7] and gadobenate [1.4±0.3]. Gd concentrations after macrocyclic GBCAs were an order of magnitude lower: gadoteridol [0.12±0.02], gadobutrol [0.080±0.01] and gadoterate [0.076±0.01]. Skin of the control group contained 0.024±0.01 nmol Gd/g tissue. Quantitative analysis detected approximately 15 nerve fibers/mm in the lower epidermis of the footpads of all groups with no significant differences between them.
**Conclusion:** In the current animal study using a multiple dosing regimen and an examination 5 weeks after last GBCA administration, there were no signs of small fiber neuropathy in the skin of the footpads.
**Limitations:** The animal study was performed in healthy rats.
**Ethics committee approval:** The study was performed with the approval of the animal welfare committee in Germany.
**Funding for this study:** Authors are employees of Bayer AG.
**Author Disclosures:**
Gregor Jost: Employee: Bayer AG
Thomas Frenzel: Employee: Bayer AG
Jessica Lohrke: Employee: Bayer AG
Janina Boyken: Employee: Bayer AG
Hubertus Pietsch: Employee: Bayer AG

### RPS 811-5

**Image quality comparison between a novel mobile head computed tomography scanner and current generation stationary computed tomography scanners**
*H. Andersson*, A. Tamaddon, M. Malekian, R. Siemund, J. Wassélius; Lund/SE

**Purpose:** Point-of-care imaging with mobile CT scanners (mobCT) offers advantages, provided the image quality is sufficient. Our aim was to compare image quality of a novel mobCT to stationary scanners for patients at a Neurosurgical Intensive Care Unit (NICU).
**Methods or Background:** From November 2020 to April 2021 all patients examined by brain CT on a mobCT (Somatom On.site, Siemens Healthineers) at a tertiary referral center for neurosurgery were included if they had at least one stationary CT examination during the same hospitalisation.

Quantitative image quality parameters included attenuation (HU) and noise (1SD) in four predefined regions of interest (ROIs), as well as contrast-to-noise ratio (CNR) between grey and whiter matter. Subjective image quality was rated by two neuroradiology fellows and two senior neuroradiologists blinded to scanner parameters.
**Results or Findings:** MobCT images had significantly higher noise in all ROIs and lower CNR compared to images from stationary CT scanners. The mobCT images had lower subjective quality rating (mean 2.1) than stationary CT images (mean 2.4). Two of the raters favoured the stationary CT images, whereas one rater was neutral and one rater slightly favoured the mobCT images. The prevalence of grade 1 (poor image quality) when rating overall image quality was 14% for mobCT images compared to 8% for the stationary CT images.
**Conclusion:** Point-of-care imaging by mobCT was successfully performed with few examinations rated as poor image quality. The quantitative and qualitative image quality parameters favoured stationary CT, but the differences were generally small, and the advantages of point-of-care imaging may potentially outweigh the small reduction in image quality.
**Limitations:** Only one mobC was studied in this single centre study.
All patients had severe intracranial pathology, limiting generalisation to other groups.
**Ethics committee approval:** This study was approved by the Swedish Ethical Review Authority (#2021-01722).
**Funding for this study:** No funding was received for this study.
**Author Disclosures:**
Ashkan Tamaddon: Nothing to disclose
Johan Wassélius: Nothing to disclose
Henrik Andersson: Nothing to disclose
Mazdak Malekian: Nothing to disclose
Roger Siemund: Nothing to disclose

### RPS 811-6

**Oxygen-challenge magnetic resonance study on the dynamic evolution of ischaemic penumbra in MCAO rats**
*Z. Yang*, Q. Lv, Y. Zhang; Zhengzhou/CN

**Purpose:** Imaging in the identification of ischaemic penumbra by oxygen challenge MRI of acute middle cerebral artery occlusion (MCAO) model in rats.
**Methods or Background:** 58 SD rats were selected for MCAO treatment. Ten rats were selected for MRI before and after oxygen challenge at 1h, 3h, 6h and 12h, and the T1 values and T1 rate in different brain regions were measured. Twenty-four MCAO rats were selected for oxygen challenge treatment at the above time points, and another 24 MCAO rats were treated with no oxygen challenge. HE staining and immunohistochemistry were performed before and after oxygen challenge. The expression of RACK1 protein was statistically analysed, and the correlation between protein expression and relaxation value was analysed.
**Results or Findings:** The expression of RACK1 protein in the cerebral ischaemic area at different time points before and after oxygen challenge showed a downward trend, and the expression of RACK1 was higher than that before oxygen inhalation. The difference was statistically significant, $P < 0.05$. There is a positive correlation between the expression of RACK1 protein and T1 value.
**Conclusion:** Oxygen challengeMRI can be used to determine the oxygen metabolism in the brain tissue of rats after infarction. The oxygen challenge MRI T1 value and T1 change rate can help to identify the ischaemic penumbra of MCAO rats. Oxygen stimulation may delay the development of cerebral ischaemia; the expression of RACK1 plays a protective role in acute cerebral ischaemia and is related to the development of hypoxia in brain tissue.
**Limitations:** The sample size is small in this study.
**Ethics committee approval:** This study was approved by the Local Medical Ethics Committee of the First Affiliated Hospital of Zhengzhou University.
**Funding for this study:** No funding was received for this study.
**Author Disclosures:**
Zhengui Yang: Nothing to disclose
Yong Zhang: Nothing to disclose
Qinqqing Lv: Nothing to disclose

### RPS 811-7

**Anatomical and functional alterations following endoscopic third ventriculostomy: the value of phase contrast and b-FFE sequences**
*D. M. El-Mossly*, A. Abdel Latif, H. Abdelkader Ahmed, S. R. R. Hanna, S. A. Mohammad; Cairo/EG
*(dollyelmossly@yahoo.com)*

**Purpose:** To demonstrate functional and anatomical alterations following endoscopic third ventriculostomy (ETV) by using, PC-MRI and bFFE sequences in relation to clinical outcome.
**Methods or Background:** This is a prospective study including twenty patients with hydrocephalus who recently underwent ETV. Patients underwent MRI-study within first postoperative week; and they were followed clinically after procedure (mean=8.6 months). In addition to conventional MR sequences, mid-sagittal 3D-bFFE sequence (at plane of third ventriculostomy); and axial/sagittal PC-MRI were acquired with CSF flow quantitative analysis by using “2D Q-flow” PC-MR angiography software on Philips MR-workstation. Three-month follow-up MRI was available in 5 patients.
**Results or Findings:** The overall ETV success and failure rates were 70% and 30%, respectively. There was statistically significant association between flow identification in PC-MRI sequence with the patients' late clinical outcome ($P<0.001$). Functional patency by PC-MRI was more sensitive in the radiographic prediction of early ETV clinical outcome than the anatomical patency by 3D-bFFE sequence (83.3% and33.3% sensitivity respectively). All CSF quantitative flow data (peak systolic velocity, peak diatolic velocity, absolute stroke volume, forward flow and backward flow) had a statistically significant association ($P<0.001$) with late clinical outcome except for regurgitation fraction and ventriculostomy AP-diameter. Patients with successful clinical outcome who underwent follow-up MRI (4 patients) showed increased most of CSF parameters in their follow-up MRI compared to the only failed ETV.
**Conclusion:** Whenever there is agreement between functional and anatomical patency, clinical success was the outcome. While in case of functional-anatomical mismatch, functional patency had a stronger contribution to clinical outcome, however correlation between them is of ultimate importance. CSF flow quantification in early post-operative study can predict early ETV failure, also the follow-up parameters can do.
**Limitations:** The limitations are the small sample size and short follow up period due to COVID-19.
**Ethics committee approval:** This study was approved by an ethics committee.
**Funding for this study:** No funding was received for this study.
**Author Disclosures:**
Shaimaa Abdelsattar Mohammad: Nothing to disclose
Dalia Mohamed El-Mossly: Nothing to disclose
Samar Ramzy Ragheb Hanna: Nothing to disclose
Assem Abdel Latif: Nothing to disclose
Hanaa Abdelkader Ahmed: Nothing to disclose

### RPS 811-8

**Intraoperative MRI: our experience in one single centre**
*G. Moreno Zamarro*, A. Bolívar, A. T. Vizarreta, C. Ordoñez, J. Amorim; Madrid/ES

**Purpose:** The purpose of this study is (1) to describe the different types of tumours and characteristics in which intraoperative MRI (IOMR) was performed in our centre, (2) to evaluate the frequency of tumour remnants detection, (3) to evaluate the impact of tumour remnant detection on the surgical approach, (4) to describe the type and rate of complications detected by IOMRI.
**Methods or Background:** Settings and participants: Radiological, anatomopathological and surgical reports of intracranial tumours operated with the assistance of IOMR were reviewed from February 2019 to January 2021. Statistical methods: Descriptive analysis of the following variables was performed: type of tumour, extension of the surgery according to the evidence of tumour remnant and complications evidenced in the IOMRI. The association between the presence of tumour remnant evidenced in the IOMRI and the extension of the surgery in the same surgical time was evaluated by a bivariate descriptive study.
**Results or Findings:** 42 patients were included in the study. In 32 (76.2%) cases tumour remnant was detected on IOMRI of which in 30 (71.4%), surgery was extended to optimise the surgical result. Surgical complications were identified in 8 (19%) patients, 5 of which were ischaemic and 3 hemorrhagic. None of them had significant clinical repercussions.
**Conclusion:** Intraoperative brain MRI is a novel technique whose main role is the detection of tumour remnant, allowing an optimised resection at the same surgical act while monitoring for potential complications and providing an updated neuronavigator. In our experience, intraoperative MRI has an impact on surgical management by detecting operable tumor remnants during surgery.
**Limitations:** Further multicentre and prospective studies are needed to assess its implications on the patient's surgical outcomes and survival rates undergoing this technique.
**Ethics committee approval:** Not applicable.
**Funding for this study:** Not applicable.
**Author Disclosures:**
Aurora Bolívar: Nothing to disclose
Cristina Ordoñez: Nothing to disclose
Gonzalo Moreno Zamarro: Nothing to disclose
Anthony Tito Vizarreta: Nothing to disclose
Joaquim Amorim: Nothing to disclose

### RPS 811-9

**The value of spectral quantitative parameters on dual-layer detector CT in ischaemic stroke**

*J. Chen*[1], J. Huang[1], J. Yan[1], X. Wang[1], X. Zhang[2], W. Fang[1], Z. Sheng[3]; [1]Suzhou/CN, [2]Shanghai/CN, [3]Nanjing/CN
*(cjh193026@163.com)*

**Purpose:** To investigate the value of spectral quantitative parameters derived from dual-layer detector CT in differentiating ischaemic area and normal tissue in ischaemic stroke patients considering CTP as reference.
**Methods or Background:** Nine ischaemic stroke patients who underwent one-stop scans including unenhanced scan, CTP, and CTA with dual-layer detector spectral CT were retrospectively analysed. With a commercial perfusion analysis software F-Stroke, ischaemic areas (infarct core and penumbra) and normal tissue were determined in each patient. The spectral quantitative parameters (Z-effective value (Z-eff) and iodine concentration (IC)) were measured and compared between ischaemic areas and normal tissue using Mann-Whitney test. Received operating characteristic (ROC) curves were plotted to determine the optimum threshold and diagnostic efficiency of Z-eff and IC.
**Results or Findings:** Both Z-eff and IC showed significant differences between areas of ischaemic area and normal tissue ($P<0.002$ and $P<0.001$, respectively). The AUC of Z-eff could reach 0.914 (cutoff 7.566, sensitivity 77.8%, specificity 88.9%), and the AUC of IC was even higher with 0.926 (cutoff 0.261, sensitivity 77.8%, specificity 100%).
**Conclusion:** Spectral quantitative parameters of Z-eff and IC on dual-layer detector CT showed good diagnostic performance in determining ischaemic area of ischaemic stroke patients, which could develop to be potential alternative to CTP.
**Limitations:** Not applicable.
**Ethics committee approval:** Not applicable.
**Funding for this study:** Not applicable.
**Author Disclosures:**
Wenxing Fang: Nothing to disclose
Zhihong Sheng: Nothing to disclose
Xiaohui Zhang: Nothing to disclose
Ximing Wang: Nothing to disclose
Jian Huang: Nothing to disclose
Jiulong Yan: Nothing to disclose
Jinghua Chen: Nothing to disclose

### RPS 811-10

**From MRI to microscopy: advances in anatomo-radiological correlations at 7T**

*A. Emmi*[1], G. Donatelli[2], M. Costagli[3], P. Cecchi[2], V. Macchi[1], M. Tosetti[2], R. De Caro[1], M. Cosottini[2], A. Porzionato[1]; [1]Padua/IT, [2]Pisa/IT, [3]Genoa/IT
*(aronemmi@hotmail.it)*

**Purpose:** Light microscopy represents the current gold standard for investigating both morphology and pathology in the ex-vivo human central nervous system. However, recent advancements in ultra-high field (7T) imaging allow for the evaluation of CNS morphology without processing, sectioning and staining. The main challenge, however, consists in the proper correlation of MRI findings with microscopy, especially when considering small structures (i.e. brainstem nuclei) and lesions. Here we present a working pipeline for the anatomo-radiological correlation of 7T ex-vivo MRI findings with anatomical and staining techniques and its application in both morphology and neuropathology.
**Methods or Background:** Human brain specimens deriving from the Body Donation Programme of the University of Padova were employed for the study. The specimens were formalin-fixed, stored in a perfluoropolyether-filled cylinder, and underwent imaging on a 7TMR system equipped with a 2 ch-tx/32ch-rx head coil for whole-brain imaging and with a birdcage custom-built coil for brainstem imaging. We acquired high-resolution sequences for morphologic purposes, as well as images tailored to design sample-specific cutting boxes. These are custom-built 3D printed devices acting as a negative plaster cast for the specimen and provided with guides for sectioning. The resulting sections were paraffin-embedded and stained to evaluate both morphology and histopathology.
**Results or Findings:** Cutting boxes allowed for micron-precise sectioning along with MRI acquisition. Histochemical and immunohistochemical staining was not influenced by immersion in perfluoropolyether. The profile of brainstem nuclei and fiber tracts was preserved and perfectly comparable. Histopathological alterations were revealed in a neuropathology referral case and their MRI correlates were analysed.
**Conclusion:** The presented working pipeline facilitates the correlation between MRI findings and microscopy, revealing both the normal architecture of the human brainstem, as well as fine neuropathological alterations.
**Limitations:** No limitations were identified.
**Ethics committee approval:** This study was approved by Padova: BodyDonationProgram Regulations; CEAVNO:17664.
**Funding for this study:** No funding was received for this study.
**Author Disclosures:**
Mauro Costagli: Nothing to disclose
Michela Tosetti: Nothing to disclose
Aron Emmi: Nothing to disclose
Andrea Porzionato: Nothing to disclose
Raffaele De Caro: Nothing to disclose
Graziella Donatelli: Nothing to disclose
Veronica Macchi: Nothing to disclose
Paolo Cecchi: Nothing to disclose
Mirco Cosottini: Nothing to disclose

### RPS 811-12

**Different approaches for aspartate approximation**

P. A. Bulanov, P. Menshchikov, *A. Manzhurtsev*, A. Yakovlev, M. Ublinskiy, T. Akhadov, N. Semenova; Moscow/RU
*(andrey.man.93@gmail.com)*

**Purpose:** Aspartate (Asp) is an amino acid that plays important role in the functioning of central nervous system and metabolism. Asp signal (in MRS spectrum $\delta \approx 2.72$ ppm) approximation seems to be challenging. Therefore, the main goal of the current study is optimisation of Asp assessment using different approximation approaches.
**Methods or Background:** MRI examination: 75 Asp-edited MEGA-PRESS spectra acquired in healthy volunteers (mean age – 21.2±3.3 years) were retrospectively collected, using Philips Achieva dStream 3.0T MRI scanner. The spectra localisation was: - 33 from anterior cingulate gyrus (ACC) (voxel size: 50x25x25 mm); 23 from dorsolateral pre-frontal area (DLPFA) (voxel size: 50x19x27 mm); 19 from visual cortex (VC) (voxel size: 20x40x30 mm) Processing: one, two, three and four gauss models, simulated spectrum and phantom spectrum were used for Asp approximation. MEGA-PRESS spectra were pre-processed using gannet 3.1 script. We estimated Asp/Cr signal intensity, fitting error values, and group variations.
**Results or Findings:** In case of ACC spectra, one and two gauss models demonstrated the lowest approximation quality (fitting errors: 15.6±2.4% and 13.3±2.4%). Other approaches had satisfactory fitting error values: three gauss - 9.9±2.4%, four gauss - 8.8±2.3%, phantom spectrum -11.2±2.7%, simulated spectrum - 11.1±3.0%.
**Conclusion:** Four gauss models demonstrate the best fitting errors in each case. In addition, we have correlation between fitting errors and variation of Asp/Cr values, therefore four gauss model provides the most accurate Asp concentration determination. Thus, this work presents unique results in the optimisation of the Asp approximation and suggests the most effective approaches.
**Limitations:** In VC and DLPFA low SNR leads to the fact, that all approaches, except four gauss, had no significant differences in fitting errors (average value for DLPFA and VC: 18.3±3.2% and 23.2±2.5%).
**Ethics committee approval:** Not applicable.
**Funding for this study:** Not applicable.
**Author Disclosures:**
Petr Menshchikov: Nothing to disclose
Alexey Yakovlev: Nothing to disclose
Maxim Ublinskiy: Nothing to disclose
Petr Алексеевич Bulanov: Nothing to disclose
Natalia Semenova: Nothing to disclose
Andrei Manzhurtsev: Nothing to disclose
Tolibjohn Akhadov: Nothing to disclose

10:30-12:00 Room E1

### Research Presentation Session: Musculoskeletal

### RPS 810

### Lower extremity

**Moderator**
M. Klontzas; Iraklion/GR

### RPS 810-2

**Deep learning based fully automated 3D models of hip labrum based on MR arthrography are feasible and allow detection of differences in labrum volume among different hip deformities**

*F. Schmaranzer*[1], M. Meier[1], G. Zeng[1], T. D. Lerch[1], G. Nicolas[1], M. Tannast[2], K. Siebenrock[1], S. Steppacher[1]; [1]Bern/CH, [2]Fribourg/CH
*(florian.schmaranzer@insel.ch)*

**Purpose:** To (1) develop and validate a deep learning approach for fully automatic labrum segmentation based on MR arthrography of the hip against manual segmentation; (2) compare labrum volume among different hip deformities.
**Methods or Background:** Sixty patients (mean age 31 ± 7 years, 67% female) with femoroacetabular impingement (FAI) and hip dysplasia (DDH) were included. All patients underwent direct 3T MR arthrography including high-resolution 3D T1-w MP2RAGE (0.5 x 0.5 x 1 mm). Patients were assigned to three subgroups based on acetabular coverage: DDH (n=20), cam deformity with normal acetabular coverage (n=18) and pincer deformity (n=22). Manual segmentations of labrum served as training data for the neural network (3D U-Net) to obtain an automated 3D labrum model. A 5-fold cross-validation was performed and dice-coefficient as measure of overlap, Pearson correlation coefficient and mean bias were calculated. For morphological analysis of the labrum, ANOVA test was performed to compare labrum volume among groups.
**Results or Findings:** Mean dice coefficient was 75 ± 6 %, corresponding to a mean non-significant difference of 24mm³ (95%CI: -107 – 154 mm³, p = 0.716) and a high correlation (rp =0.88, p <0.0001) in labrum volume. Mean labrum volume differed (p = 0.040) among the hip deformities with 2840 ±1332mm³ for DDH, 2064 ±744mm³ for hips with normal coverage and 2223 ± 755mm³ for pincer hips.
**Conclusion:** Automatic segmentation of the hip labrum based on MR arthrograms using deep learning is feasible and accurate. Our results suggest that labrum volume adapts to acetabular coverage. Integrating these 3D models into the daily clinical workflow has the potential to improve surgical decision-making.
**Limitations:** No validation of the segmentation approach in an external dataset was performed.
**Ethics committee approval:** IRB approved this retrospective study.
**Funding for this study:** No funding was received for this study.
**Author Disclosures:**
Malin Meier: Nothing to disclose
Till Dominic Lerch: Nothing to disclose
Florian Schmaranzer: Nothing to disclose
Simon Steppacher: Nothing to disclose
Guodong Zeng: Nothing to disclose
Moritz Tannast: Nothing to disclose
Klaus Siebenrock: Nothing to disclose
Gerber Nicolas: Nothing to disclose

### RPS 810-3

**Visual and quantitative assessment of hip implant-related metal artifacts at low field MRI: a comparative phantom study between 0.55T, 1.5T and 3T**

*H-C. Breit*, J. Vosshenrich, T. Weikert, M. Clauss, M. Bach, D. Harder; Basel/CH

**Purpose:** To visually and quantitatively investigate hip implant-related metal artifacts on a 0.55T scanner system in comparison to 1.5T and 3T imaging with metal artifact reduction sequences from clinical routine.
**Methods or Background:** Femoral components from total hip arthroplasty made of three different alloys were evaluated in a water phantom at 0.55T, 1.5T and 3T. An optimised imaging protocol from clinical routine was used for imaging at 1.5T and 3T and adapted to 0.55T. Artifacts were visually assessed by two musculoskeletal radiologists and one orthopedic surgeon using a 7-point Likert scale. Quantitative assessment was performed by calculating the coefficient of variation and the fraction of voxels within the water phantom lying above or below a standardised threshold. Inter- and intrareader agreement was evaluated using interclass correlation.
**Results or Findings:** Interreader agreement was strong to moderate (0.74-0.82). For titanium implants, artifacts were rated less intense at all field strengths (Likert-score 0.55T/1.5T/3T: 2.44/2.9/2.7) compared to Fe-Cr (Likert-score: 4.1/3.9/5.1; p<.001) and Co-Cr (Likert-score: 4.1/4.1/5.2; p<.001). Artifacts for all implants were lower at 0.55T and 1.5T than at 3T (p<.05). Coefficient of variation showed strong correlation between visual and quantitative assessment (r=0.81; p<.001) and was lowest for titanium implants at all field strengths. The fraction of voxels within a threshold of a control ROI showed strong (25%/50% threshold: r=-0.84; p<.001) or moderate (10%: r=-0.68; p<.001) negative correlation with the visual assessment.
**Conclusion:** Artifact intensity was lowest for titanium implants imaged at 0.55T. For all other alloys, artifact intensities at 0.55T were comparable to MARS imaging at 1.5T but superior to 3T. Therefore, low-field MRI at 0.55T is a promising technique and may further improve image quality in patients with total hip arthroplasty.
**Limitations:** The limitation is that it is a phantom study.
**Ethics committee approval:** This study was approved by Swiss-Ethics: 2021-00166.
**Funding for this study:** No funding was received for this study.
**Author Disclosures:**
Thomas Weikert: Nothing to disclose
Michael Bach: Nothing to disclose
Hanns-Christian Breit: Nothing to disclose
Martin Clauss: Nothing to disclose
Dorothee Harder: Nothing to disclose
Jan Vosshenrich: Nothing to disclose

### RPS 810-4

**Ultrasound-guided periprosthetic biopsy in failed total hip arthroplasty: a novel approach to test infection in patients with dry joints**

*D. Albano*, C. Messina, S. Gitto, L. Zagra, L. M. Sconfienza; Milan/IT
*(albanodomenico@me.com)*

**Purpose:** Our aim was to diagnose periprosthetic joint infection (PJI) preoperatively, ultrasound-guided joint aspiration (US-JA) may not be performed when effusion is minimal or absent. We aimed to report and investigate the diagnostic performance of ultrasound-guided periprosthetic biopsy (US-PB) of synovial tissue to obtain joint samples in patients without fluid around the implants.
**Methods or Background:** One-hundred nine patients (55 men; mean age: 68 ± 13 years) with failed total hip arthroplasty (THA) who underwent revision surgery performed preoperative US-JA or US-PB to rule out PJI.
**Results or Findings:** Sixty-nine of 109 patients had joint effusion and underwent US-JA, while the remaining 40 with dry joints required US-PB. Thirty-five of 109 patients (32.1%) had PJI, while 74/109 (67.9%) had aseptic THA failure. No immediate complications were observed in both groups. Technical success of US-PB was 100%, as the procedure was carried on as planned in all cases. Sensitivity, specificity, positive predictive value, negative predictive value, and accuracy of US-JA were 52.2%, 97.8%, 92.3%, 80.3%, and 82.6%, while for US-PB, they were 41.7%, 100%, 100%, 80%, and 82.5%, respectively, with no significant difference (P = .779). Using the final diagnosis as reference standard, we observed a moderate agreement with both US-JA (k = 0.56) and US-PB (k = 0.50).
**Conclusion:** We present a novel US-guided technique to biopsy periprosthetic synovial tissue of failed THA to rule out PJI. We found similar diagnostic performance as compared with traditional US-JA. This supports future larger studies on this procedure that might be applied in patients without joint effusion.
**Limitations:** The limitations are (1) the retrospective study's design on relatively small sample size, particularly concerning the biopsy group of patients, and (2) more than one biopsy sample might have increased the sensitivity of periprosthetic biopsy.
**Ethics committee approval:** This study was approved by an ethics committee.
**Funding for this study:** No funding was received for this study.
**Author Disclosures:**
Carmelo Messina: Nothing to disclose
Salvatore Gitto: Nothing to disclose
Luca Maria Sconfienza: Nothing to disclose
Luigi Zagra: Nothing to disclose
Domenico Albano: Nothing to disclose

### RPS 810-5

**Feasibility study of the detectability of fiber rotation in muscle injuries using diffusion tensor imaging of skeletal muscle using a standardised defect model**

*M. Frenken*, D. B. B. Abrar, L. Wollschläger; Düsseldorf/DE

**Purpose:** Muscle injuries are common and difficult to quantify diagnostically because muscle edema often masks actual muscle damage. DTI can provide insight into muscle structure and fiber routing. This study used a standardised ex-vivo defect model to investigate the ability of DTI to quantify muscle injury.

**Methods or Background:** Ex vivo, 9 human lower legs (4 right, 5 left, 80y±8, 8f/1m) were subjected to a defect model. A cuboid standardised muscle defect was cut into the tibialis muscle. After rotation by 90°, it was reinserted and closed using subcutaneous fat and skin. 3T-MRI examination was performed before and after defect insertion and included morphologic sequences (T1 TSE cor., STIR cor., T2 TSE trans.) and DTI. Analysis was based on ROIs. Standard DTI parameters (axial diffusivity (AD), mean diffusivity (MD), radial diffusivity (RD), fractional anisotropy (FA), streamline mean) and visual representation of fiber tractography were evaluated.
**Results or Findings:** After 90° rotation of a muscle cube, change of the main fiber direction from longitudinal to transverse is visible in the rotated area. DTI parameters AD, MD and RD showed a significant increase (AD: p=0.003, MD: p=0.0001, RD: p=0.0001). FA and streamline mean showed significant decrease (FA: p=0.003, stream.: p=0.0001).
**Conclusion:** Rotational defects can be imaged both visually and quantitatively with DTI fiber tractography. Significant changes in DTI parameters can be attributed to structural injury. DTI is a promising tool for identification and quantification of muscle defects and may potentially serve for recovery prognosis of muscular injuries in the future.
**Limitations:** The study was performed ex-vivo to obtain basic knowledge about the assessment of DTI parameters in muscular injuries using a standardised defect model. In the following, these findings may be valuable for patients.
**Ethics committee approval:** This study was approved by an ethics committee.
**Funding for this study:** Not applicable.
**Author Disclosures:**
Lena Wollschläger: Nothing to disclose
Miriam Frenken: Nothing to disclose
Daniel B. Benjamin Abrar: Nothing to disclose

### RPS 810-7

### Mass-diagnosing knee osteoarthritis with AI in an entire production year

*A. Lenskjold*, M. W. Brejnebol, J. D. D. Nybing, L. E. Egnell, M. Boesen; Copenhagen/DK

**Purpose:** To investigate knee osteoarthritis (KOA) in an entire production year from 2018 at Bispebjerg and Frederiksberg Hospital, Copenhagen, using an Artificial Intelligence (AI) approach.
**Methods or Background:** We included patients aged 35-79 with weight-bearing radiographs, and RBknee™ AI-software analysed the radiographs to provide Kellgren-Lawrence (KL) grades for the tibiofemoral compartments and presences of distal/proximal patella osteophytes.
**Results or Findings:** In total, 1,737 patients were included and their 1,701 left and 1,698 right posterior-anterior/anterior-posterior and paired lateral (1,718) non-repeated radiographs. Sixteen patients with orthopedic hardware or unknown assignments were excluded. The average age was 58.9 years (SD 11.7), and female patients were overrepresented (1,018). The distribution of KL-grades were 0: 18.9%; 1: 7.5%; 2: 37.7%; 3: 22.2%; 4: 13.7%. Ordinal logistic regression showed that each year of age increased the odds of a higher KL-grade by 1.08 CI95% [1.07-1.09] (p<0.001), and female sex increased the odds by 1.34 CI95% [1.12-1.60] (p<0.01). A tendency for slower advancements was seen in younger males (<60 years). Median KL-grades increased with age from 0 to 2, and higher mean KL-grades were seen among female patients (1.8 vs 1.4). There were no differences between paired lateralities (p>0.05), and 55.5% of patients with no KOA (KL<2) had patella osteophytes.
**Conclusion:** We found that 73.6% of patients had radiographic KOA (KL 2-4), and high age is the predominant risk factor in our consecutive cohort. Furthermore, females had higher KL-grades and showed advancement trends earlier on. Over half of the patients without radiographic KOA had patella osteophytes which could explain their symptoms/referral.
**Limitations:** We did not test RBknee's accuracy, but a previous publication shows performance at a senior MSK radiologist consultant level.
**Ethics committee approval:** The study was approved by the Danish National Committee on Health Research Ethics #2101001.
**Funding for this study:** Funding was received for this study by the Danish government.
**Author Disclosures:**
Mikael Boesen: Research/Grant Support: Subcontractor to EU grant given to Radiobotics Other: Minor provisions have been received by Radiobotics Advisory Board: Advisory board member of Radiobotics
Liv Elisabet Egnell: Employee: Radiobotics
Mathias Willadsen Brejnebol: Research/Grant Support: Subcontractor to EU grant given to Radiobotics Other: Minor provisions have been received by Radiobotics
Anders Lenskjold: Research/Grant Support: Subcontractor to EU grant given to Radiobotics Other: Minor provisions have been received by Radiobotics
Janus Damm Damm Nybing: Research/Grant Support: Subcontractor to EU grant given to Radiobotics Other: Minor provisions have been received by Radiobotics

### RPS 810-9

### True versus pseudo avulsion tear of superficial deltoid ligament of ankle on 3T MRI

*H. Rajendran*[1], H. Duddukuru[2], S. Abubacker[1]; [1]Ahmadi/KW, [2]Pondicherry/IN
*(harrykrish29@rediffmail.com)*

**Purpose:** To determine the accuracy of 3T MRI in detecting true superficial deltoid ligament avulsion tear of the ankle using MRI criteria in correlation with arthroscopy.
**Methods or Background:** A retrospective observation cross-sectional study was conducted between March 2018 to April 2020 in which 50 patients (male n=30, female n=20) of mean age 35 years with medial ankle injury were consented to be imaged on 3T GE MRI scanner using standard three plane PDFS sequence who also underwent diagnostic and/or therapeutic arthroscopy. Multiple established MRI criteria namely, presence or absence of joint recess, depth of the joint recess, width of the joint recess, shape of joint recess, presence of double black line sign & medial malleolus marrow oedema, were used to differentiate true from false-positive avulsion tear of superficial deltoid ligament of the ankle.
**Results or Findings:** Of 50 patients who underwent arthroscopy, 40 patients showed superficial deltoid ligament tear & 10 patients had normal ligament. MRI using established criteria yielded a sensitivity of 99% (39/40) and specificity of 98% (8/10) for superficial deltoid ligament tears of the ankle.
**Conclusion:** MRI using established criteria in 3T scanner showed very high accuracy in diagnosing and differentiating true from pseudo avulsion tear superficial deltoid ligament of the ankle.
**Limitations:** No limitations were identified.
**Ethics committee approval:** This study was approved by an ethics committee.
**Funding for this study:** No funding was received for this study.
**Author Disclosures:**
Shafeek Abubacker: Nothing to disclose
Haritha Duddukuru: Nothing to disclose
Harikrishna Rajendran: Nothing to disclose

### RPS 810-10

### T1rho relaxation of talar cartilage with and without axial loading in situ: an indicator of early degenerative changes in individuals with chronic ankle instability?

*M. Jung*, P. Giesler, P. M. Jungmann, T. Lange, M. Wenning, F. Bamberg, H. Schmal, L. Sturm; Freiburg/DE
*(matthias.jung@uniklinik-freiburg.de)*

**Purpose:** To evaluate T1rho relaxation times of talar cartilage in individuals with chronic mechanical ankle instability (CAI) and healthy controls (HC) with and without axial-loading during MRI acquisition.
**Methods or Background:** Individuals with CAI (n=10) and HC (n=17) underwent 3T-MRI of the ankle with and without in-situ axial-loading of 500N applied by a custom-built ankle arthrometer. T1rho relaxation time measurements of talar cartilage were performed using a 3D fast low-angle shot (FLASH) sequence augmented with variable spin-lock preparation intervals (0/10/20/30/40ms) and mono-exponential pixel-by-pixel T1rho-fitting. Manual segmentation of six talar cartilage regions (ROI) was performed. Median T1rho values, and changes between no-load and load, were compared using Mann-Whitney-U-test.
**Results or Findings:** Age was 22.5±3.1 years (mean±SD; n=20 female). No cartilage defects are depicted on morphological MRI. Without axial-loading, median T1rho values of talar cartilage were significantly higher in all medial ROIs in the CAI-group compared to the HC-group (p<.05). Axial-loading led to a significant decrease of T1rho values of the talar cartilage in the lateral (p=.039), posterolateral (p=.006), medial (p=.023), and posteromedial (p=.006) segment. With axial-loading, significantly higher T1rho relaxation times were observed in the posterolateral ROI of the CAI-group (p=.047). Comparing the CAI- and HC-group, no significant differences were observed between the changes of T1rho values (Δno-load/load).
**Conclusion:** In chronic ankle instability, significantly higher T1rho values were found in medial segments of talar cartilage, indicating early cartilage matrix degeneration. Axial-loading particularly affects the middle and posterior areas of the talar surface. The lack of differences of T1rho values between groups with axial-loading may indicate maintained cartilage shock-absorbing function at this early time-point, where no morphological changes were detected. Following, preventive measures may be effective to halt progression of ankle joint degeneration.
**Limitations:** No limitations were identified.
**Ethics committee approval:** Ethics committee approval was obtained.
**Funding for this study:** Funding was received for this study by Bauerfeind (in part).

Author Disclosures:
Lukas Sturm: Nothing to disclose
Matthias Jung: Nothing to disclose
Hagen Schmal: Nothing to disclose
Fabian Bamberg: Nothing to disclose
Pia M Jungmann: Nothing to disclose
Thomas Lange: Nothing to disclose
Markus Wenning: Nothing to disclose
Paula Giesler: Nothing to disclose

**10:30-12:00** **Room M 1**

### Research Presentation Session: Breast

### RPS 802

### Breast cancer screening and mammography

**Moderator**
M. G. Wallis; Cambridge/UK

### RPS 802-2

**Artificial Intelligence-aided mammograms and detection of malignancy in dense breasts**

*S. A. Mansour*, S. Soliman, A. Kansakar, M. M. H. Hanafy; Cairo/EG
*(sahar_mnsr@yahoo.com)*

**Purpose:** Assess the impact of adding artificial intelligence to the mammogram of the dense breasts on the diagnosis of malignancy.
**Methods or Background:** High breast density is a risk factor for breast cancer and overlapping of glandular tissue can mask lesions thus lowering mammographic sensitivity. Also, dense breasts are more vulnerable to increase recall rate and false positive results. Adding ultrasound to mammography in dense breasts improves the sensitivity and specificity of mammography. New generations of artificial intelligence (AI) have been introduced to the realm of mammography. The study included 6600 mammograms of dense patterns "c" and "d". All the patients were subjected to digital mammography and breast ultrasound, and mammographic images were scanned by AI software. The AI provided color hue for abnormality overlay and a abnormality scoring percentage according to the probability of malignancy as follows: 0 definite non-cancer; 1-25 probably non-cancer; 26–50 possibly non-cancer; 51-75 possibly cancer; 76–99 probably cancer; and 100 definite cancer.
**Results or Findings:** This study presented 4061 breast abnormalities. Diagnostic indices of the sono-mammogram were a sensitivity of 98.71%, a specificity of 88.04%, a positive predictive value of 80.16%, a negative predictive value of 99.29% and an accuracy of 91.5%. AI-aided mammograms presented sensitivity of 88.29%, a specificity of 96.34%, a positive predictive value of 92.2%, a negative predictive value of 94.4% and an accuracy of 94.5% in its ability to read dense mammograms
**Conclusion:** The artificial intelligence algorithm applied on mammogram of the dense breasts showed a notable reduction of sono-mammographic misdiagnosis, so, it can be used as a decision-support tool for breast with heavy glandular tissue.
**Limitations:** Clinical data.
**Ethics committee approval:** This study was approved by an ethics committee.
**Funding for this study:** No funding was received for this study.
**Author Disclosures:**
Sahar Abdelkhalek Mansour: Author: Cairo University Research/Grant Support: Cairo University
Abisha Kansakar: Author: Cairo University
Somia Soliman: Author: Cairo University
Mennatallah Mohamed Hassan Hanafy: Author: Cairo University

### RPS 802-4

**Worldwide mammographic breast density characterisation**

*W. Sanderink*, I. Sechopoulos; Nijmegen/NL

**Purpose:** To determine if, for equal compressed breast thickness (CBT), there is an equal distribution of volumetric breast density (VBD) across screening populations worldwide.
**Methods or Background:** Ten digital mammography screening datasets consisting of the VBD estimates (Volpara Health), CBT, view, side, and anonymised exam identifier were collected. In total, 732,376 screened women ≥50 years old from 10 datasets from 9 different countries were included. The distribution of VBD within each of the 10 different CBT bins (16-25 mm, 26-35 mm, etc.) were analysed to estimate different percentile densities, and 95% CI, for each CBT bin. Differences among the corresponding VBD for the same percentiles across datasets were compared.
**Results or Findings:** The absolute difference in VBDs for the same percentile density and CBT bin across sets had a range of CC: -7.0%–+6.3% and MLO: -7.8%–+10.7% from the worldwide means of the medians, with 942/1190=79.2% being statistically significant differences. For example, the 50th percentile VBD of a 56-65 mm thick CC-view breast varies, across the ten datasets, between 4.2% and 8.5%, with nine of the ten datasets having significant differences from the mean of this median VBDs of 5.7%.
**Conclusion:** Descriptive statistics on breast characteristics are required for dosimetry or cancer risk estimations, but international variations in breast density suggest that a single model may not be widely representative. However, this quantitative data analysis shows that breasts of comparable thickness have similar VBD distributions, with the differences being statistically significant. For dosimetry estimations, this is probably not clinically significant, while, for cancer risk estimations, more accurate predictions may be needed. Therefore, without patient-specific VBD estimates, a single population-based model of VBD as a function of breast thickness may be suitable for many applications.
**Limitations:** Not applicable.
**Ethics committee approval:** Not applicable.
**Funding for this study:** Not applicable.
**Author Disclosures:**
Ioannis Sechopoulos: Research/Grant Support: Volpara Health Ltd.
Wendelien Sanderink: Nothing to disclose

### RPS 802-5

**Missed cases in breast imaging, lesion type, modality and areas to improve**

S. Sefidbakht[1], *L. Ataei Rooyani*[1], V. Zangoori[1], M. Y. Karami[1], S. Tahmasebi[1], P. Iranpour[1], R. Ravanfar Haghighi[1], B. Bijan[2]; [1]Shiraz/IR, [2]Sacramento, CA/US
*(ataee.lobat@gmail.com)*

**Purpose:** To analyse missed breast cancers referred to a sub-specialty breast clinic for lesion type and modality.
**Methods or Background:** Images and biopsies performed over 6 months in a breast-imaging clinic were reviewed for missed cases in a multistep process. (1) Two physicians reviewed the studies and excluded those without priors. (2) Two radiologists reviewed the reports compared with priors and excluded the studies with stable/benign findings. The remaining studies were reviewed for missed findings. The missed cases were categorised by modality, modality-specific lesion type and delay caused by misdiagnosis.
**Results or Findings:** Out of 8974 studies performed over 6 months, 1570 studies had new findings. 151 lesions were missed in previous studies. These included 49 mammographically detected lesions (11 tissue distortions including 5 visible in one view); 18 focal/developing asymmetries; 13 grouped/segmental microcalcifications (6 overlooked, and 7 for which follow-up was recommended); and 7 masses (4 missed in dense background, 3 partly seen due to extreme posterior position and/or quality issues in prior mammograms). Out of 97 missed cases in ultrasound, 34 were additional cancer (multifocal/centric; 18 missed LN (including 14 lower level 1 and 7 level 2)); 7 tissue distortions; 31 non-mass lesions; and 7 masses erroneously considered probably benign for follow-up (including 3 mucinous cancers, 3 mixed-solidcystic masses proven to be papillary carcinoma and one round luminal B cancer). There were 4 cancers detected in re-biopsy of previously biopsied lesions with benign/inconclusive results. 3 of the non-mass lesions missed on ultrasound in women >40 would have been detected if mammogram had been performed first, as per protocol.
**Conclusion:** Most common errors in breast imaging are detection of focal asymmetries and microcalcifications in mammogram; detection of non-mass lesions in ultrasound; characterisation of cystic lesions in ultrasound; tissue-distortions in both; and failure to detect multifocality/multicentricity in breast cancer and lower level-1 axillary LNs.
**Limitations:** Calling a case missed/underdiagnosed is somehow subjective.
**Ethics committee approval:** An institutional ethics committee approved this retrospective study.

**Funding for this study:** No funding was received for this study.
**Author Disclosures:**
Bijan Bijan: Nothing to disclose
Lobat Ataei Rooyani: Nothing to disclose
Sepideh Sefidbakht: Nothing to disclose
Sedigheh Tahmasebi: Nothing to disclose
Poya Iranpour: Nothing to disclose
Rezvan Ravanfar Haghighi: Nothing to disclose
Vahid Zangoori: Nothing to disclose
Mohammad Yasin Karami: Nothing to disclose

### RPS 802-6

**Self-compression mammography in clinical practice: final results of a randomised trial compared to standard compression for patient-centred care**

*V. Iotti*, P. Giorgi Rossi, M. Ottone, V. Marchesi, A. Nitrosi, L. Canovi, M. Guberti, R. Vacondio, P. Pattacini; Reggio Emilia/IT
*(valentina.iotti@ausl.re.it)*

**Purpose:** To test the efficacy of self- compared to radiographer-led compression to reduce the average glandular dose without affecting image quality and compliance to follow-up mammography.
**Methods or Background:** Women presenting for mammography in clinical practice (cancer follow-up, symptoms, opportunistic screening or familial risk) were asked to participate and, if willing, randomised to self-compression or radiographer-led compression. Image quality was assessed blindly by two independent radiologists and two radiographers. Pain and discomfort were measured immediately after mammography and their recall was asked when the women participated in the follow-up mammogram one or two years later.
**Results or Findings:** 495 women (mean age 57 years +/-14) were enrolled, 245 in the self-compression and 250 radiographer-compression arms. Image quality (radiologists' judgement p = 0.62; radiographers' judgement P=.34, slightly better in self-compression for both), dose (0.94 vs. 0.96 mGy, P=.38) and pain (3.8 vs. 4.0, P=.50) were similar in the two arms. Compression was stronger in the self than in the radiographer arm (11.4 vs. 10.2 daN, P<.001), with a small impact on thickness (46.4 vs. 48.1 mm, P=.13). Moderate/severe discomfort was reported by 7.9% vs 9.9% (P=.46). Time was slightly longer with self-compression (11.5 vs. 10.2 minutes, P=.06). Compliance to the subsequent mammography was 95.1% and 97.1% in the self and radiographer arm, respectively (P=.28).
**Conclusion:** Self-compression achieved stronger compression of the breast, with comparable image quality, but did not substantially reduce glandular dose. The proportion of women who attended follow-up mammography was similar in the two groups.
**Limitations:** The study had insufficient power to detect differences in participation in subsequent mammography due to a much higher participation than what was anticipated in the sample-size evaluation, but data do not suggest any increase.
**Ethics committee approval:** Ethics committee approval No. 2017/0103951.
**Funding for this study:** Internal funding.
**Author Disclosures:**
Valentina Iotti: Speaker: GEHS
Monica Guberti: Nothing to disclose
Pierpaolo Pattacini: Nothing to disclose
Andrea Nitrosi: Nothing to disclose
Vanessa Marchesi: Nothing to disclose
Laura Canovi: Nothing to disclose
Rita Vacondio: Nothing to disclose
Paolo Giorgi Rossi: Nothing to disclose
Marta Ottone: Nothing to disclose

### RPS 802-7

**Impact of obtaining a digital breast tomosynthesis (DBT) spot compression view on assessment of equivocal DBT findings**

*F. Deleau*[1], P-A. Linck[1], V. Brouste[1], I. Thomassin-Naggara[2], M-P. Depetiteville[1], M. Boisserie-Lacroix[1], F. Chamming'S[1]; [1]Bordeaux/FR, [2]Paris/FR
*(florian.deleaubdx@gmail.com)*

**Purpose:** A recently introduced DBT device allows obtaining DBT spot compression views using a small paddle during DBT acquisition.
The objective is to evaluate the impact on diagnostic performance of obtaining a DBT spot compression view for assessment of equivocal DBT findings.
**Methods or Background:** This retrospective study included 102 women in whom a DBT spot compression view was obtained to characterise an equivocal finding on DBT, performed between December 2018 and December 2019. Two fellowship-trained radiologists and one breast-imaging fellow independently reviewed examinations. Readers first assigned a BI-RADS category using standard DBT views followed immediately by a category using the DBT spot compression view. Histology and at least 1 year of imaging follow-up served as reference standard. Diagnostic performance was compared between DBT with and without DBT spot compression views using McNemar tests.
**Results or Findings:** Intrareader agreement increased from 0.43 to 0.72, and interreader agreement increased from 0.21 to 0.45, based on kappa coefficients for DBT with and without spot compression views. Compared with standard DBT views, DBT spot compression views yielded significantly increased accuracy for three readers (74% vs 90%, 73% vs 94%, 71% vs. 94%); significantly increased specificity for three readers (69% vs 90%, 75% vs 94%; 68% vs. 93%); and significantly increased sensitivity for one reader (67% vs 94%) without significant change in sensitivity for the two other readers. Radiation dose was 1.97 mGy for the DBT spot compression view, versus 1.78-1.81 mGy for standard DBT view.
**Conclusion:** The DBT spot compression view increased intrareader and interreader agreement and diagnostic accuracy; the view's supplemental dose was slightly higher than that of a standard DBT view.
**Limitations:** It's a retrospective, single-centre study. Population was relatively small.
**Ethics committee approval:** Institutional review board approval was obtained.
**Funding for this study:** Not applicable.
**Author Disclosures:**
Pierre-Antoine Linck: Nothing to disclose
Florian Deleau: Nothing to disclose
Foucauld Chamming'S: Nothing to disclose
Veronique Brouste: Nothing to disclose
Martine Boisserie-Lacroix: Nothing to disclose
Marie-Pierre Depetiteville: Nothing to disclose
Isabelle Thomassin-Naggara: Nothing to disclose

### RPS 802-8

**Optimising the set of pairs of radiologists that double read screening mammograms**

*J. Gommers*[1], C. Abbey[2], F. Strand[3], S. Taylor-Phillips[4], M. Larsen[5], S. Hofvind[5], M. Broeders[1], I. Sechopoulos[1]; [1]Nijmegen/NL, [2]Santa Barbara, CA/US, [3]Stockholm/SE, [4]Coventry/UK, [5]Oslo/NO
*(jessie.gommers@radboudumc.nl)*

**Purpose:** To investigate how radiologist performance characteristics can be leveraged to determine the optimal set of pairs of radiologists for the double reading of screening mammograms.
**Methods or Background:** We analysed three datasets of women who underwent screening mammography in Sweden, the UK and Norway. Any examination that was flagged by either radiologist was classified as abnormal. Cancer detection rates (CDR) and abnormal interpretation rates (AIR) were evaluated. Individual radiologists were divided into four categories: high CDR and low AIR (HL), high CDR and AIR (HH), low CDR and AIR (LL), or low CDR and high AIR (LH). Random pair performance, for which any pair was equally likely, was compared to specific pair performance.
**Results or Findings:** The CDRs for the random and all specific pairings were not significantly different. The Swedish and UK datasets did show a similar significant pattern for AIR: compared to random pairings, pairing strategies involving opposite AIR radiologists resulted in a significant AIR reduction of 3.4% and 2.9%, respectively. For the Swedish dataset, the pairing strategy with fully opposite performance radiologists also resulted in a significant 10.3% AIR reduction when compared to random pairing. The Norwegian dataset showed a different pattern, with no significant differences between the AIRs of specific pairings and the random pairing.
**Conclusion:** Pairing radiologists based on their performance characteristics, as opposed to randomly, may improve grouped screening performance. However, our data showed contradicting patterns for the different pairing strategies. Further analyses with more datasets from different screening settings have to be done to confirm our results.
**Limitations:** Each examination was read by only two radiologists, so only existing pairings were possible to be analysed.
**Ethics committee approval:** Previously acquired ethics-approved data included permission for anonymised follow-up research.
**Funding for this study:** aiREAD project financed by NWO-KWF-HH.
**Author Disclosures:**
Mireille Broeders: Speaker: Siemens, Hologic
Fredrik Strand: Speaker: Lunit
Marthe Larsen: Nothing to disclose
Jessie Gommers: Nothing to disclose
Craig Abbey: Consultant: Canon Medical Research USA, Chicago Illinois, USA
Advisory Board: Izotropic Corp., Vancouver British Columbia, Canada
Sian Taylor-Phillips: Nothing to disclose
Solveig Hofvind: Nothing to disclose
Ioannis Sechopoulos: Research/Grant Support: Siemens Healthcare, Canon Medical, ScreenPoint Medical, Sectra Benelux, Volpara Solutions, Lunit
Speaker: Siemens Healthcare

### RPS 802-9

**Evaluation of dual spectrum digital breast tomosynthesis with iterative reconstruction for dose reduction**

*L. B. van den Oever*, K. Michielsen, M. C. Pinto, I. Sechopoulos; Nijmegen/NL
*(daan.vandenoever@radboudumc.nl)*

**Purpose:** To develop and evaluate a maximum likelihood iterative reconstruction algorithm with material decomposition (ML-MADE) for low-dose digital breast tomosynthesis (DBT) for single-DBT acquisitions involving multiple X-ray spectra.
**Methods or Background:** ML-MADE considers the spectral nature of the beams used to acquire the DBT projections, resulting in two reconstructed basis material images that are combined into a virtual monochromatic DBT volume. DBT projections of a breast phantom containing objects representing different lesion types were acquired for evaluation. Two acquisitions were made: one with all projections using a W/Rh spectrum at 28 kVp, 8.75 mAs/projection; and one at 35 kVp, 3.1 mAs/projection. Three DBT volumes were reconstructed to test our algorithm: one reference volume from only the 28 kVp projections; the second using half the 28 kVp and 35 kVp projections interleaved; and the third with the first half of the projections at 28 kVp and the second half at 35 kVp. Contrast-to-noise (CNR) and signal-to-noise (SNR) ratios were calculated to quantify the image quality of the lesions representing masses. Calcification lesion representation was evaluated qualitatively. The average glandular doses (AGD) were estimated using entry-dose and published-dose conversion factors.
**Results or Findings:** The AGD was reduced by 19% by acquiring dual-spectrum projection sets. The CNR and SNR of the masses in the reference reconstruction were 1.27 and 18.1, and 1.09 and 15.0 for both dual-spectrum acquisitions, respectively. The calcifications from the three reconstructions were not discernibly different.
**Conclusion:** Dual spectrum acquisition is feasible with our ML-MADE algorithm at the 19% lower dose levels by interleaving spectra or switching spectra halfway during acquisition, therefore, requiring no second regular scan. No information appears to be lost as a monochromatic reconstruction is still possible.
**Limitations:** Not applicable.
**Ethics committee approval:** Not applicable.
**Funding for this study:** Not applicable.
**Author Disclosures:**
Marta C. Pinto: Nothing to disclose
Koen Michielsen: Nothing to disclose
Ioannis Sechopoulos: Research/Grant Support: Siemens Healthcare, Canon Medical, ScreenPoint Medical, Sectra Benelux, Volpara Solutions, Lunit Speaker: Siemens Healthcare
Leonardus Bernardus van den Oever: Nothing to disclose

### RPS 802-10

**Breast cancer surveillance with MRI in patients with a history of chest irradiation**

*S. L. van Winkel*, R. M. Mann; Nijmegen/NL
*(Suzanne.vanWinkel@radboudumc.nl)*

**Purpose:** To assess the yield and diagnostic accuracy of breast MR surveillance in women with a history of chest irradiation under 40 years old (RT<40), usually due to childhood cancer.
**Methods or Background:** This retrospective study analysed the performance MR breast cancer screening in RT<40 patients. Women with RT<40 and at least one screening MR in our hospital between 2012 and 2020 were included. Information on indication, number of screenings and age was extracted from the medical record, recall-, biopsy- and cancer detection rate were calculated as the number of positives per 1000 screens. Pathologic outcomes were obtained for biopsied lesions.
**Results or Findings:** 59/1755 women (3,4%) received breast MR because of RT<40 (min.1; max.5; median 2; total 123), maximum follow-up: 61 months, median age at first MR: 38,7 (min.24; max.69). 37 women underwent concurrent mammography screening (min.1 max.5 median: 2; total: 71). Recall rates were 187/1000 for MR- and 211/1000 for mammographic screens. Biopsy rates were 130/1000 for MR and 210/1000 for mammographic exams. In total, 17 biopsies in 16 woman were performed, of which resp. 2 and 1 due to MR and mammography findings only. Cancer was detected in 6 women (2 DCIS, 4 invasive): 1 underwent mammography screening only before her diagnosis; 5 underwent screening by both modalities. All positive cases (5/5) were visible at MR (cancer detection rate 41/1000), 5/6 on mammography. No interval cancers were observed (sensitivity: mammography 83%, MR 100%; specificity: mammography 84%, MR 85%).
**Conclusion:** High breast cancer yield (41/1000 MRI) within women after mediastinal RT justifies intensive screening. Adherence to a structural surveillance program appears to be low.
**Limitations:** More research and data aggregation is necessary to determine the best surveillance approach for these women.
**Ethics committee approval:** Not required.
**Funding for this study:** European Regional Development Fund.
**Author Disclosures:**
Suzanne L. van Winkel: Nothing to disclose
Ritse Maarten Mann: Nothing to disclose

**10:30-12:00** **Room M 3**

### Research Presentation Session: Abdominal Viscera

### RPS 801

### New developments in MR imaging of primary liver tumours

**Moderator**
A. Palkó; Szeged/HU

### RPS 801-3

**Preoperatively, clinical classification of patients with intrahepatic cholangiocarcinoma may guide personalised management and improve prognosis**

*Q. Li*, Y. Wei, B. Song; Chengdu/CN

**Purpose:** Intrahepatic cholangiocarcinoma (ICC) is featured with high heterogeneity and dismal prognosis. We aimed to divide ICC patients into different clinical phenotypes based on preoperative data, and identify ICC patients who benefit most and least from liver resection.
**Methods or Background:** Between January 2009 and December 2017, 308 consecutive patients with pathologically confirmed ICC who underwent preoperative CECT were retrospectively enrolled. Clinical data, imaging findings and radiomics scores associated with prognostic outcome were collected. Exploratory factor analysis was performed to variable selection and identify relative variables. Hierarchical cluster analysis was used to identify preoperative phenotype. A decision tree algorithm was applied to predict the phenotype.
**Results or Findings:** In the hierarchical cluster analysis, four phenotypes of ICC patients were identified. Phenotype 1 (n=78) was associated with biliary dilation and low AFP value, phenotype 2 (n=41) was related to large tumour size and tumour in vein, phenotype 3 (n=80) was featured by satellite nodule and multifocality, while phenotype 4 (n=109) was characterised by small tumour size and a low radiomics score. Phenotype 2 was associated with the poorest prognosis (median survival time: 12.1 months, while phenotype 4 was associated with the longest median survival time of 27.0 months. Biliary dilation, satellite nodule, multifocality, and tumour in vein were used to construct a decision tree with five nodes, and 90.58% of the subjects were assigned to the correct phenotypes.
**Conclusion:** Four clinical phenotypes based on clinical and imaging features were identified with different pathological findings and prognostic outcomes. Moreover, the phenotypes may be useful for preoperative patient selection and personalised management.
**Limitations:** The retrospective design may have resulted in selection bias; also, the study was conducted in a single centre without external validation.
**Ethics committee approval:** This study was approved by the Institutional Review Board of West China Hospital, Sichuan University.
**Funding for this study:** No funding was received for this study.
**Author Disclosures:**
Yi Wei: Nothing to disclose
Qian Li: Nothing to disclose
Bin Song: Nothing to disclose

### RPS 801-4

**Biomechanical phenotype of hepatocellular carcinoma based on multifrequency MR elastography: association with aggressiveness**

*J. Zhou*, R. Li, F. Yan; Shanghai/CN

**Purpose:** To explore correlation of viscoelastic properties of HCC and background liver with aggressive biological behavior.
**Methods or Background:** From June 2020 to August 2021, 81 patients with 85 histopathologically confirmed HCC lesions were included in this prospective study. They were classified as cirrhotic and non-cirrhotic groups. All patients underwent preoperative multifrequency MRE combined with tomoelastography post-processing. Viscoelasticity was quantified as shear wave speed (c, m/s) representing tissue stiffness and loss angle of the complex shear modulus (φ, rad) relating to tissue fluidity. Tumour aggressive signature (differentiation grade, microvascular invasion, Ki-67 and CK19 expression), and fibrosis stages and inflammation activity of background liver were evaluated.

Thursday

Viscoelastic parameters were compared between cirrhotic and non-cirrhotic group. Further subgroup analysis was performed between HCC with different aggressive signature.
**Results or Findings:** Cirrhotic HCC showed higher φ value than non-cirrhotic HCC (1.20 ± 0.24 rad vs 1.08 ± 0.19 rad, p=0.022). For subgroup analysis of cirrhotic HCC, high-grade and high Ki-67 expression group showed increased φ value than those with low-grade and low Ki-67 expression (1.25 ± 0.27 vs 1.12 ± 0.14 rad, p=0.04; and 1.25± 0.24 vs 1.08 ± 0.17, p=0.018; respectively), and CK19 positive HCC showed increased c value than negative HCC (3.16 ± 0.89 vs 2.60 ± 0.58 s/s, p=0.046). For background liver, cirrhotic HCC group showed increased c and φ value compared with non-cirrhotic HCC group ((2.30 ± 0.31 m/sec vs 1.77 ± 0.26 m/sec, p＜0.001; 0.84 ± 0.12 rad vs 0.67 ± 0.11 rad, p＜0.001; respectively).
**Conclusion:** HCC in noncirrhotic liver has a distinct biomechanical phenotype associated with less aggressiveness.
**Limitations:** This was a singlecentre exploratory study.
**Ethics committee approval:** This study was approved by the institutional review board and local ethics committee.
**Funding for this study:** This project was supported by the National Natural Science Foundation of China.
**Author Disclosures:**
Ruokun Li: Nothing to disclose
Fuhua Yan: Nothing to disclose
Jiahao Zhou: Nothing to disclose

### RPS 801-5

### Risk score models for predicting aggressiveness in early-stage hepatocellular carcinoma: the role of gadoxetic acid-enhanced magnetic resonance imaging

*H. Wei*, H. Jiang, B. Song; Chengdu/CN
*(weih_cat@163.com)*

**Purpose:** To investigate the effectiveness of preoperative gadoxetic acid-enhanced magnetic resonance imaging (EOB-MRI) in predicting aggressiveness in early-stage hepatocellular carcinoma (eHCC).
**Methods or Background:** This retrospective study enrolled consecutive treatment-naïve adult patients who underwent EOB-MRI within 3 months before curative resection for eHCC within the Milan criteria between July 2015 and November 2020. All MR images were independently reviewed by two radiologists with respect to the Liver Imaging Reporting and Data System (LI-RADS) version 2018 imaging features, LI-RADS category and several non-LI-RADS imaging features. Univariable and multivariable logistic regression analyses were performed to identify predictors for microvascular invasion (MVI) and poor histologic grade of eHCC. Model performance was estimated by using the area under the receiver operating characteristic (AUC) curve analysis.
**Results or Findings:** One hundred and six patients with pathologically confirmed HCC were included; 28 of 106 (26.4%) patients had MVI, whereas 29 of 106 (27.4%) patients had poor histologic grade. Variables significantly predictive of MVI included fat in mass, more than adjacent liver (odds ratio [OR] = 0.202), marked diffusion restriction (OR = 3.152), and non-smooth tumour margin (OR = 7.187). Variables significantly predictive of poor histologic grade were AFP level > 400 ng/mL (OR = 4.820) and marked diffusion restriction (OR = 2.428). Incorporating the aforementioned predictors, the risk score model achieved an AUC of 0.829 (95% confidence interval [CI]: 0.744, 0.895) for MVI and of 0.716 (95%CI: 0.621, 0.800) for poor histologic grade, respectively.
**Conclusion:** EOB-MRI-based models effectively predict tumour aggressiveness in eHCC and may contribute to a rational therapeutic choice.
**Limitations:** The sample size of this study is small.
**Ethics committee approval:** Institutional Review Board approval was obtained.
**Funding for this study:** This work was supported by the National Natural Science Foundation of China (No. 81971571).
**Author Disclosures:**
Hanyu Jiang: Nothing to disclose
Hong Wei: Nothing to disclose
Bin Song: Nothing to disclose

### RPS 801-6

### Gadoxetic acid-enhanced MR imaging combining with computed tomography-vascular features may improve the diagnosis of hepatocellular carcinoma

*S. Yao*, Y. Wei, H. Tang, B. Song; Chengdu/CN

**Purpose:** To determine whether gadoxetic acid-enhanced MRI combined with vascular features of dynamic enhanced CT can improve the diagnostic performance of hepatocellular carcinoma (HCC) based on various diagnostic criteria.
**Methods or Background:** 142 patients with 169 surgically confirmed nodules (137 HCCs, 32 non-HCCs) were retrospectively enrolled. Included patients underwent gadoxetic acid-enhanced MRI and dynamic enhanced CT preoperatively. All the MR and CT images were anonymised and combined as two protocols for reviewing:1) full gadoxetic acid-enhanced MRI sequences; 2) dynamic CT combined with gadoxetic acid-enhanced MRI but excluded the vascular features-related phase. Three independent reviewers characterised the nodules according to the LI-RADS v2018 and EASL. The clinical impact of CTAP and MRAP images for detecting the arterial phase hyperenhancement (APHE), the sensitivity and specificity of the CTAP-combined and MRAP images were analysed and compared.
**Results or Findings:** For the whole hepatic nodules, CTAP-combined images had a significantly higher detection rate for APHE than MRAP images (87.57% [148/169] vs.75.15% [127/169; p<0.001). For LI-RADS, CTAP-combined images significantly increased the sensitivity to 75.91% (104/137) from 70.80% (97/137) on MRAP images (p=0.016), with the minimal decrease of the specificity to 71.88% (23/32) from 75.00% (24/32) (p=1.000). For EASL, MRAP images showed a sensitivity of 78.10% (107/137) and a specificity of 71.88% (23/32) for HCC diagnosis. However, when determining APHE on CTAP, the sensitivity showed numerical increase to 81.02% (111/137) (p=0.152), whereas the specificity decreased to 75.00% (24/32) which was not statistically significant either (p=1.000).
**Conclusion:** The combined use of dynamic enhanced CT vascular features for HCC diagnosis enables high sensitivity and comparable specificity when compared with the gadoxetic acid-enhanced MRI.
**Limitations:** The limitation is no stratified analysis based on different sizes of lesions.
**Ethics committee approval:** This study was approved by the institutional review board.
**Funding for this study:** Funding was received for this study (2021YFS0144, 2021YFS0021, and NSFC 81901700).
**Author Disclosures:**
Yi Wei: Nothing to disclose
Shan Yao: Nothing to disclose
Hehan Tang: Nothing to disclose
Bin Song: Nothing to disclose

### RPS 801-11

### Intraindividual comparison of LI-RADS category and imaging features on contrast-enhanced CT and MRI

F. Agnello, *R. Cannella*, F. Vernuccio, F. Midiri, G. Brancatelli, M. Galia; Palermo/IT

**Purpose:** To perform an intraindividual comparison of LI-RADS categories and imaging features in patients at high risk for hepatocellular carcinoma (HCC) on contrast-enhanced CT and MRI.
**Methods or Background:** This retrospective study included adult patients meeting the following criteria: 1) diagnosis of cirrhosis; 2) contrast-enhanced CT and MRI performed with a maximum interval of one month; 3) lack of interval treatment for target observations; 4) confirmation of benignity or malignancy by pathology or long-term follow-up. Two blinded radiologists evaluated the observations according to the LI-RADSv2018 diagnostic algorithm. Intraindividual differences were assessed using the McNemar. Intermodality agreement was calculated by using the Cohen's kappa (k) test.
**Results or Findings:** A total of 73 observations (mean size 34.0 ± 32.4 mm) in 53 patients were included. There were no significant differences in major and imaging features between CT and gadoxetate disodium MRI (p≥0.063). Overall intermodality agreement for LI-RADS categories was moderate (k value: 0.56, 95% CI 0.39-0.73). Intermodality agreement between CT and Gd-EOB.DTPA MRI was moderate (k value of 0.52, 95% CI 0.32, 0.72) for all observations, while agreement was poor for the subset of observations ≤20 mm (k value -0.20, 95% CI -0.30, -0.09). Intermodality agreement between CT and MRI with extracellular contrast was moderate (k value: 0.50, 95% CI 0.12-0.87). Agreement between Gd-EOB-DTPA and MRI with extracellular contrast was fair (k value: 0.26, 95% CI 0.06-0.47).
**Conclusion:** The intermodality agreement for LI-RADS categorisation is moderate between CT and MRI, but the agreement is low for observations ≤20 mm.
**Limitations:** The retrospective study design, the composite reference standard and the small number of observations were identified as limitations.
**Ethics committee approval:** This study was approved by the IRB.
**Funding for this study:** No funding was received for this study.
**Author Disclosures:**
Federica Vernuccio: Nothing to disclose
Massimo Galia: Nothing to disclose
Roberto Cannella: Nothing to disclose
Federico Midiri: Nothing to disclose
Francesco Agnello: Nothing to disclose
Giuseppe Brancatelli: Nothing to disclose

10:30-12:00 Room Z

### Research Presentation Session: Interventional Radiology

### RPS 809

### Visceral interventions

**Moderator**
S. C. Spiliopoulos; Athens/GR

### RPS 809-2

**Methotrexate infusion followed by uterine artery embolisation for the management of placental adhesive disorders**

*M. Khaleghi*, M. R. Babaei, I. Mohseni, M. Farasatinasab, P. Arman; Tehran/IR
*(mkh712003@yahoo.com)*

**Purpose:** To evaluate the efficacy and safety of the uterine artery embolisation (UAE) in combination with methotrexate (MTX) for conservative management of placental adhesive disorders.
**Methods or Background:** Patients with placental adhesive disorders (including accreta, increta, and percreta lesions) that were treated with uterine artery embolisation in combination with methotrexate (MTX) were identified and were followed prospectively for outcomes including uterine preservation and complications.
**Results or Findings:** Twenty-six patients were identified who had the diagnosis of abnormal placenta implantation during this study. Fourteen patients were excluded because they were treated by a caesarean hysterectomy. Among remaining 12 patients, successful uterine preservation was observed in seven (58%) cases. Menstruation cycles returned in all successfully treated patients, although they did not have a desire to get pregnant. Five (42%) patients underwent primary or delayed hysterectomy due to severe post-partum haemorrhage in three cases, and intestinal adhesion/peritonitis and secondary post-partum haemorrhage/sepsis in two patients, respectively.
**Conclusion:** Although this interventional method is relatively successful in uterine preservation, the possibility of treatment failure cannot be ignored. Given that there are too little data regarding its efficacy and safety, this method should be reserved for patients who have a strong desire to maintain the uterus and their fertility, or if it is technically difficult to perform hysterectomy due to the extent of the invasion.
**Limitations:** The limitations of this study are the retrospective collection of patients, single study arm, and intervention in an open-label fashion.
**Ethics committee approval:** The study protocol was approved by the local ethics committee (Iran University of Medical Sciences).
**Funding for this study:** Not applicable.
**Author Disclosures:**
Maryam Farasatinasab: Nothing to disclose
Mohammadreza Khaleghi: Nothing to disclose
Pariya Arman: Nothing to disclose
Mohammad Reza Babaei: Nothing to disclose
Iman Mohseni: Nothing to disclose

### RPS 809-3

**A prospective study for assessment of changes in liver stiffness, before and after endovascular interventions in patients with budd-chiari syndrome, using shear wave elastography**

*R. V. Mall*, R. R. Yadav, A. Gupta, S. Singh, A. Israr; Lucknow/IN
*(ranavishwadeep@gmail.com)*

**Purpose:** Increased liver stiffness (LS) is observed in patients with Budd-Chiari Syndrome (BCS). The purpose of this prospective study was to evaluate the serial changes in LS after endovascular interventions in chronic BCS using Shear Wave Elastography (SWE) and to determine its effectiveness as a non-invasive monitoring tool in follow-up.
**Methods or Background:** This prospective study included 32 patients diagnosed with chronic BCS who underwent endovascular therapy. Serial evaluation of LS was performed pre-procedure, post-procedure, at 1 month and at 3 months post-revascularisation. These changes in LS were correlated with Liver Biopsy fibrosis assessment using METAVIR score and Hepatic Venous (HV) Pressure/ Hepatic Venous Pressure Gradient (HVPG) measurements taken during the recanalisation.
**Results or Findings:** Technical success was achieved in 31 patients (96%). A significant reduction (p=0.001) of liver stiffness was observed in patients with chronic BCS after endovascular venous decompression, suggesting that liver congestion could be the primary determinant of liver stiffness, particularly in the cohort of patients with lower grades of fibrosis (METAVIR ≤2). Beyond the 3 to 6 months post-intervention period, the liver stiffness could be suggested by levels of residual fibrosis. Patients with chronic BCS did not show resolution of liver stiffness to non-cirrhotic levels, even at 6-months post-revascularisation.
**Conclusion:** In conclusion, SWE could be used as an effective monitoring tool in follow-up after endovascular therapy for chronic BCS. It could also be used to gauge effective venous decompression relative to the degree of fibrosis.
**Limitations:** Repeat liver biopsies were not performed at follow-up for assessment of residual fibrosis and for correlation with liver stiffness.
**Ethics committee approval:** This single institute prospective study was performed after obtaining approval from Institute Ethics Committee.
**Funding for this study:** No external funding was necessary as it was part of standard protocol at the institute.
**Author Disclosures:**
Somesh Singh: Nothing to disclose
Amrin Israr: Nothing to disclose
Rajanikant R Yadav: Nothing to disclose
Archna Gupta: Nothing to disclose
Rana Vishwadeep Mall: Nothing to disclose

### RPS 809-5

**Potential of pre-interventional magnetic resonance angiography for optimisation of workflow and clinical outcome of prostatic arterial embolisation**

*L. S. Alizadeh*, I. Yel, T. J. Vogl, L. D. Grünewald, V. Koch, C. Booz; Frankfurt a. Main/DE

**Purpose:** Impact of pre-interventional magnetic resonance angiography (MRA) on prostatic artery embolisation (PAE) regarding workflow, radiation dose, and clinical outcome.
**Methods or Background:** Retrospective evaluation of 259 patients (mean age 68 ± 9, range 41-92) with benign prostatic hypertrophy (BPH) undergoing PAE. MRA was performed in 137 cases. In 122 patients, no pre-interventional MRA was performed. Origin of the PA, volumetry of the prostatic gland and ADC values were evaluated. International Prostate Symptom Score (IPSS), Quality of Life (QoL) and International Index of Erectile Function (IIEF) were evaluated before and after PAE.
**Results or Findings:** Origin of the PA was identified in all cases. Significant differences regarding volume reduction (-20±13 ml with MRA vs -17±9 ml without MRA) and ADC value reduction were found (-78±111 10-6mm2/s with MRA vs -45±99 10-6mm2/s without MRA). PAE workflow was modified in 16 patients due to MRA findings. Radiation dose (5518.54±6677.97 µGym2 with MRA vs 23963.50±19792.25 µGym2 without MRA) and fluoroscopy times (19.35±9.01 min. with MRA vs 27.45±12.54 min. without MRA) significantly differed. IPSS reduction improved (-11±8 points with MRA vs -7±9 points without MRA, p<0.001), while QOL (-2±1 points with MRA and -2 ± 2 points without MRA) and IIEF (+2±10 points with MRA and +1±11 points without MRA) showed no significant differences (p>0.05).
**Conclusion:** Pre-interventional MRA facilitates improved workflow, patient safety and clinical outcome of PAE while reducing radiation dose.
**Limitations:** The limitation is the retrospective single-centre design and a limited follow-up time for clinical outcome.
**Ethics committee approval:** Ethics committee approved with a waiver for informed consent.
**Funding for this study:** No funding was received for this study.
**Author Disclosures:**
Christian Booz: Nothing to disclose
Ibrahim Yel: Nothing to disclose
Vitali Koch: Nothing to disclose
Thomas J. Vogl: Nothing to disclose
Leona Soraja Alizadeh: Nothing to disclose
Leon David Grünewald: Nothing to disclose

### RPS 809-6

**Prostatic artery embolisation in benign prostate syndrome (BPS): potential of pre-interventional MR angiography for optimising workflow and clinical outcome**

*T. J. Vogl*, S. El Nemr, L. S. Alizadeh, N-E. A. N-E. Mohammed; Frankfurt a. Main/DE

**Purpose:** To evaluate the impact of pre-interventional MR angiography (MRA) on prostatic artery embolisation (PAE) regarding workflow, radiation dose, and clinical outcome.
**Methods or Background:** 259 patients (mean 68.28±8.69; range 41-92) with severe benign prostatic syndrome (BPS) who had undergone PAE between 01/17 and 12/20, were retrospectively evaluated. Pre-interventional MRA was performed in 137 patients vs. 122 patients without pre-interventional MRA. MR images were evaluated regarding PA origin, volumetry of prostatic gland and ADC values. Impact of MRA on PAE workflow and radiation dose was examined. International-Prostate-Symptom-Score (IPSS), Quality-of-Life (QoL) and International-Index-of-Erectile-Function (IIEF) were evaluated before and after PAE to examine clinical improvement.

**Results or Findings:** PA origin was identified in 100% of cases (31% A. vesicalis inf., 27% A. pudenda int., 7% A. iliaca int., 8% A. obturatoria, 2% A vesicalis sup., 25% other origin). Significant differences regarding volume reduction (-20±13 ml with MRA vs. -17±9 ml without) and ADC value reduction (-78±111 10-6mm2/s with MRA vs. -45±99 10-6 mm2/s without) were detected. Workflow during PAE was changed because of pathological findings in MRA in 16 patients. Radiation dose (5518.54±6677.97µGym2 with MRA vs. 23963.50±19792.25µGym2 without) and fluoroscopy times (19.35±9.01 min with MRA vs 27.45±12.54 min without) were significantly lower with MRA. The MRA group showed significantly higher improvement in IPSS reduction (-11±8 points with MRA vs. -7±9 points without) while QoL (-2±1 points with MRA vs. -2±2 points without) and IEF (2±10 points with MRA vs. 1±11 points without) showed no significant differences.
**Conclusion:** Pre-interventional MRA significantly improved workflow and clinical outcome of PAE while reducing radiation dose.
**Limitations:** The limitation is the retrospective study design.
**Ethics committee approval:** Approval of institutional review board was obtained.
**Funding for this study:** No funding was received for this study.
**Author Disclosures:**
Shirin El Nemr: Nothing to disclose
Thomas J. Vogl: Nothing to disclose
Nour-Eldin Abdelrehim Nour-Eldin Mohammed: Nothing to disclose
Leona Soraja Alizadeh: Nothing to disclose

### RPS 809-7

**Percutaneous transluminal angioplasty for symptomatic hepatic vein-type budd-chiari syndrome: feasibility and long-term outcomes**

*A. Elkilany*[1], M. Al-Warraky[2], T. Denecke[3], D. Geisel[1]; [1]Berlin/DE, [2]Shebin Elkom/EG, [3]Leipzig/DE
*(aboelyazid.elkilany@gmail.com)*

**Purpose:** To determine the feasibility, clinical effectiveness, and long-term outcomes of percutaneous transluminal angioplasty for endovascular treatment of hepatic vein (HV)-type Budd-Chiari syndrome (BCS).
**Methods or Background:** We retrospectively identified 51 patients (31 female, mean age 27.2±9.1 years) with symptomatic short segment (≤30 mm) HV-type BCS who underwent percutaneous transluminal balloon angioplasty (PTBA) with/without stenting between October 2014 and January 2019. Baseline characteristics, technical success, clinical success, complications, and long-term outcomes were analysed.
**Results or Findings:** The intervention was technically successful in 94.1% of cases (48/51) - 32 patients underwent PTBA and 16 patients underwent HV stenting. Procedure-related complications occurred in 14 patients (29.1%). The clinical success rate at 4 weeks was 91.7% (44/48). Nine patients underwent reintervention, six patients due to restenosis/occlusion and 3 patients with clinical failure. Among the 9 patients, 5 underwent HV stent insertion and 4 underwent TIPS insertion. The mean primary patency duration was 64.6±19.9 months (CI, 58.5–70.8; range, 1.2–81.7 months). The cumulative 1-, 2-, and 5-year primary patency rates were 85.4, 74.5, and 58.3%, respectively. The cumulative 1-, 2-, and 5-year secondary patency rates were 93.8, 87.2, and 75%, respectively. The cumulative 1-, 2-, and 5-year survival rates were 97.9, 91.5, and 50%, respectively.
**Conclusion:** Percutaneous transluminal angioplasty with and without stenting is effective and achieves excellent long-term patency and survival rates in patients with symptomatic HV-type BCS. With its lower incidence of re-occlusion and higher clinical success rate, HV angioplasty combined with stenting should be the preferred option, especially in patients with segmental HV-type BCS.
**Limitations:** The limitations are the retrospective study design and the small sample size, especially the subset of patients who underwent HV stenting.
**Ethics committee approval:** Ethics committee approval was obtained.
**Funding for this study:** Not applicable.
**Author Disclosures:**
Timm Denecke: Nothing to disclose
Mohamed Al-Warraky: Nothing to disclose
Aboelyazid Elkilany: Nothing to disclose
Dominik Geisel: Nothing to disclose

### RPS 809-8

**Applications of digital variance angiography and colour-coded digital variance angiography in prostatic artery embolisation**

*L. S. Alizadeh*[1], C. Booz[1], M. Gyánó[2], I. Góg[2], J. P. P. Kiss[2], K. Szigeti[2], S. Osváth[2], T. J. Vogl[1]; [1]Frankfurt a. Main/DE, [2]Budapest/HU

**Purpose:** In previous clinical studies Digital Variance Angiography (DVA) provided higher contrast-to-noise ratio (CNR) and better image quality in lower extremity angiography than Digital Subtraction Angiography (DSA). Our aim was to investigate whether DVA has similar quality advantage in Prostatic Artery Embolisation (PAE). The secondary aim was to explore whether the newly developed colour-coded DVA (ccDVA) technology might be useful in PAE.
**Methods or Background:** We evaluated angiographic acquisitions of 30 patients (mean age 67.47, SD 9.76, range 42-82) undergoing PAE. The CNR of DSA and DVA images was calculated and compared. Visual evaluation of DVA and DSA image quality was performed by four experienced interventional radiologists in a randomised blinded manner. The diagnostic value of DSA and ccDVA images was analysed by the experts using clinically relevant criteria in a paired comparison survey. Data were analysed by the Wilcoxon signed rank test, and the Kendall W analysis or Fleiss Kappa analysis was used to determine interrater agreement.
**Results or Findings:** DVA images provided 4.12 times higher CNR than DSA (median, Q1-Q3 3.29-5.01). The visual evaluation score of DVA images (4.42 ± 0.05) was significantly higher than that of DSA (3.38 ± 0.07, p<0.01). The Kendall W analysis showed moderate but significant agreement in both groups. The overall diagnostic value of ccDVA images was judged superior to DSA in 82% of comparisons with an 85% interrater agreement. The Fleiss Kappa was 0.41 (p<0.01).
**Conclusion:** Our data show that DVA provides higher CNR and better image quality also in PAE. This quality reserve might be used for dose management (reduction of radiation dose and contrast agent volume), and the ccDVA technology has a high potential to assist PAE interventions in the future.
**Limitations:** The limitation is the single centre retrospective evaluation.
**Ethics committee approval:** Waiver for informed consent has been obtained.
**Funding for this study:** No funding was received for this study.
**Author Disclosures:**
Janos Pal P. Kiss: Nothing to disclose
Christian Booz: Nothing to disclose
Krisztián Szigeti: Nothing to disclose
István Góg: Nothing to disclose
Szabolcs Osváth: Nothing to disclose
Thomas J. Vogl: Nothing to disclose
Leona Soraja Alizadeh: Nothing to disclose
Marcell Gyánó: Nothing to disclose

### RPS 809-9

**Centre experience and other determinants of patient radiation exposure during prostatic artery embolisation: a retrospective study in three Scandinavian centres**

*P. Svarc*[1], T. Hagen[2], M. Bläckberg[3], E. Baco[2], M. Taudorf[1], M. A. Røder[1], H. Lindgren[3], N-E. Kløw[2], L. B. Lönn[1]; [1]Copenhagen/DK, [2]Oslo/NO, [3]Helsingborg/SE
*(petrasvarc@yahoo.com)*

**Purpose:** To evaluate the effects of center experience and a variety of patient- and procedure-related factors on patient radiation exposure during prostatic artery embolisation (PAE) in three Scandinavian centres with different PAE protocols and levels of experience. Understanding factors that influence radiation exposure is crucial in effective patient selection and procedural planning.
**Methods or Background:** Data were collected retrospectively for 352 consecutive PAE procedures from January 2015 to June 2020 at the three centres. Dose area product [DAP (Gy·cm2)] was selected as the primary outcome measure of radiation exposure. Multiple patient- and procedure-related explanatory variables were collected and correlated with the outcome variable. A multiple linear regression model was built to determine significant predictors of increased or decreased radiation exposure as reflected by DAP.
**Results or Findings:** There was considerable variation in DAP between the centres. Intended unilateral PAE (p = 0.03) and each 10 additional patients treated (p = 0.02) were significant predictors of decreased DAP. Conversely, increased patient body mass index (BMI, p < 0.001), fluoroscopy time (p < 0.001) and number of digital subtraction angiography (DSA) acquisitions (p < 0.001) were significant predictors of increased DAP.

**Conclusion:** To minimise patient radiation exposure during PAE radiologists may, in collaboration with clinicians, consider unilateral embolisation, pre-interventional CTA for procedure planning, using predominantly anteroposterior (AP) projections and limiting the use of cone beam CT (CBCT) and fluoroscopy.
**Limitations:** The results of the statistical analysis might have been affected by the retrospective study design. Further, radiation dose parameters were obtained from the imaging system and not directly from dosimeters, and therefore should be considered as estimates of the true dose received by the patients.
**Ethics committee approval:** Ethics committee approvals (31-1521-421 in Denmark, 2020-04211 in Sweden and 20/14473 in Norway) were obtained.
**Funding for this study:** No funding was received for this study.
**Author Disclosures:**
Mikkel Taudorf: Nothing to disclose
Nils-Einar Kløw: Nothing to disclose
Petra Svarc: Nothing to disclose
Lars Birger Lönn: Nothing to disclose
Thijs Hagen: Nothing to disclose
Hans Lindgren: Nothing to disclose
Eduard Baco: Nothing to disclose
Martin Andreas Røder: Nothing to disclose
Mats Bläckberg: Nothing to disclose

### RPS 809-11

**Computed tomography-guided transthoracic cutting needle biopsy of small pleural lesions (≤ 10 mm)**
*E. Mršić*, M. Kukuljan; Rijeka/HR
*(enamrsic94@gmail.com)*

**Purpose:** Transthoracic CT-guided cutting needle biopsy is a minimally invasive diagnostic procedure and a preferred radiological method for the diagnosis of pleural lesions. The purpose of this study was to assess the diagnostic accuracy of the aforementioned intervention and also to determine the incidence of complications.
**Methods or Background:** This retrospective study included a total of 56 patients with small pleural lesions, with a thickness of ≤ 10 mm, who underwent the transthoracic needle biopsy at the Department of Radiology, Clinical Hospital Centre Rijeka, from January 2015 to July 2021. One of the inclusion criteria for this study was a pleural effusion.
**Results or Findings:** Pleural effusion was considered a protective factor for complications since no pneumothorax was noted. The sensitivity of CT-guided transthoracic biopsy (TTB) in our study was 84.6 %, specificity 100 %, PPV 100 %, and NPV 73.9 %, while diagnostic accuracy was 89.3 %. The overall diagnostic contribution of TTB in our study is comparable with the results of the other reports.
**Conclusion:** Our study shows a major diagnostic contribution and a zero complication rate in the presence of pleural effusion. These results indicate that CT-guided TTB can be considered the optimal method for the diagnosis of small suspected malignant pleural lesions.
**Limitations:** No limitations were identified.
**Ethics committee approval:** The Ethics Committee has approved the conduct of this research.
**Funding for this study:** No funding was received for this study.
**Author Disclosures:**
Melita Kukuljan: Nothing to disclose
Ena Mršić: Nothing to disclose

### RPS 809-12

**In-office needle arthroscopy (IONA): may a traditionally orthopedic procedure fall in the field of interventional radiology? A pilot study to evaluate knee IONA performed by interventional radiologist**
*F. Arrigoni*, M. G. Mazzoleni, F. Calafiore, A. Barile, L. Zugaro, V. Calvisi, C. Masciocchi; L'Aquila/IT

**Purpose:** Trained musculoskeletal radiologists ensure high level of diagnostic accuracy in MRI of the knee. However, orthopedists still complain of reduced sensibility and specificity in some pathologies involving cartilage, synovium and meniscus. For this reason, since the '90s orthopedists have introduced an arthroscope with reduced caliber, disposable and portable kits to turn diagnostic arthroscopy into an in-office procedure (IONA), routinely performed by orthopedists. Interventional radiologist could be a qualified operator to perform this procedure: knowledge of the MRI anatomy and manual skills allow them to have a short learning curve and to focus their attention on the MRI's dubious findings. Moreover, such procedure can be coupled with biopsies if these are needed to confirm the diagnosis (for example, synovial biopsy in case of clinically unclear arthritis).
**Methods or Background:** Seven procedures of knee IONA were carried out by an interventional radiologist as first operator assisted by a trained orthopedic surgeon. All patients have been previously undergone to high-field MRI with uncertain results, not consistent with painful clinical conditions. All procedures were performed using local anesthesia with the patient awake. One arthroscopy was combined with synovial biopsy to confirm diagnosis prior to total synovectomy.
**Results or Findings:** No complications were recorded. The procedures required one-day hospitalisation; no limitation in function were recorded after the procedure. In four cases, pathological findings not visible on MRI were detected (three meniscal tears and one mucoid degeneration of the ACL). Biopsy was successfully performed.
**Conclusion:** The interventional radiology setting seems adequate to carry out IONA of the knee. The radiologist, in fact, provides the patients with a definitive diagnosis quickly obtained by submitting them to minimally invasive diagnostic procedures shortly after a not diriment MRI.
**Limitations:** Not applicable.
**Ethics committee approval:** Not applicable.
**Funding for this study:** Not applicable.
**Author Disclosures:**
Francesco Calafiore: Nothing to disclose
Francesco Arrigoni: Nothing to disclose
Carlo Masciocchi: Nothing to disclose
Vittorio Calvisi: Nothing to disclose
Manuel Giovanni Mazzoleni: Nothing to disclose
Antonio Barile: Nothing to disclose
Luigi Zugaro: Nothing to disclose

**14:00-15:00** **Open Forum #4 (ESR)**

### Research Presentation Session: GI Tract

### RPS 1001a

### Staging and response assessment in colorectal cancer

**Moderator**
M. Maas; Amsterdam/NL

### RPS 1001a-2

**Is MRI better than MSCT in the diagnosis of preoperative T and N restaging of rectal cancer after neoadjuvant chemoradiotherapy?**
*W. Liu*, Z. Wang; Beijing/CN
*(wenjuanliu@mail.ccmu.edu.cn)*

**Purpose:** The purpose was to evaluate the value of MRI and MSCT in the clinical scene of restaging of rectal cancer after neoadjuvant chemoradiotherapy (NCRT).
**Methods or Background:** Clinical data of 62 patients with rectal cancer were retrospectively analysed. Before surgery, all patients received NCRT, and underwent MSCT as well as MRI successively. Diagnostic accuracy and agreements of the two methods in T and N restaging of rectal cancer after NCRT were assessed with the postoperative pathological staging as golden standard.
**Results or Findings:** The total diagnostic accuracy of MSCT and MRI for T restaging were 51.6% and 41.9%, respectively, and no statistical difference was found between them (P>0.05). The two methods failed to accurately diagnose ypT0~1 staging, and the wrong staging was mainly over staging of ypT0~2. The diagnostic accuracy of CT and MRI for N retaging were 56.5% and 53.2%, respectively, and no statistical difference was found between them (P>0.05). There were fair agreements between MSCT and MRI in T restaging (Kappa=0.583, P<.001) and N restaging (Kappa=0.644, P<.001).
**Conclusion:** MRI is not better than MSCT in the diagnosis of preoperative T and N restaging of rectal cancer after NCRT. Therefore, in order to save medical resources, MRI may be unnecessary sometimes.
**Limitations:** Firstly, our series included a small number of ypT0 and ypT4 lesions. Secondly, while this study is a retrospective case analysis, we only studied the conventional sequence of MRI, and did not study the new sequence as well as functional imaging.
**Ethics committee approval:** This study was approved by the Medical Ethics Review Board of Beijing Friendship Hospital Affiliated to Capital Medical University (reference number 2021-P2-144-01).
**Funding for this study:** This study was supported by No. (2015) 160 from Beijing Scholars Program and No. yyqdktbh2020-9 from Beijing Friendship Hospital, Capital Medical University.
**Author Disclosures:**
Zhenchang Wang: Nothing to disclose
Wenjuan Liu: Nothing to disclose

### RPS 1001a-4

**Predicting response to chemoradiotherapy in rectal cancer via visual assessment on baseline staging MRI: a multicentre and multireader study**

*N. El Khababi*[1], M. Lahaye[1], M. Maas[1], S. Nougaret[2], L. Curvo-Semedo[3], R. G. H. Beets-Tan[1], D. M. J. Lambregts[1]; [1]Amsterdam/NL, [2]Montpellier/FR, [3]Coimbra/PT

**Purpose:** In 2020 van Griethuysen et al. proposed a 5-point confidence level scoring system to predict response to chemoradiotherapy (CRT) on baseline staging MRI based on visual morphologic assessment/staging. The aim was to test this scoring system in a multicentre setting among multiple readers with different experience levels, and compare it to a more simplified and specified 4-point risk score, designed for the purpose of this study.
**Methods or Background:** 22 international radiologists (5 rectal MR-experts, 17 general/abdominal radiologists) retrospectively reviewed the pre-CRT MRIs of n=90 patients (from 10 centres) to estimate the chance of achieving a complete response (CR) post-CRT. Readers first applied the 5-point confidence score by van Griethuysen (0=highly unlikely; 1=unlikely; 2=equivocal; 3=likely; 4=highly likely to achieve CR). They then assigned a risk score from 0-4 with 1-point for the presence of each of the following adverse features: bulky (cT3cd-4) tumour, obvious mesorectal fascia invasion, obvious nodal involvement, obvious extramural vascular invasion. Diagnostic performance to predict CR was assessed using ROC-curves, interobserver agreement (IOA) was calculated using Kendall's coefficient (W). Readers were asked to indicate their preferred scoring method.
**Results or Findings:** Mean (+ranges) area under the ROC-curve (AUC) for the 5-point confidence score was 0.67 (0.60-0.76) for the MR-experts and 0.61 (0.52-0.73) for the abdominal/general radiologists; for the 4-point risk score AUCs were 0.68 (0.62-0.80) and 0.62 (0.52-0.70), respectively. IOA was similar for both methods (W0.65 for the confidence score; W0.66 for the 4-point risk score), but higher for the MR-experts (W0.76 and W0.78, respectively). Most readers (55%) favoured the 4-point risk score.
**Conclusion:** Diagnostic performance to predict a complete response was moderate with similar results for the 5-point confidence score and 4-point risk-score. Diagnostic performance and IOA were better for the more experienced readers.
**Limitations:** The limitation is that the study was retrospective.
**Ethics committee approval:** The study was approved by an ethics committee.
**Funding for this study:** No funding was received for this study.
**Author Disclosures:**
Max Lahaye: Nothing to disclose
Monique Maas: Nothing to disclose
Najim El Khababi: Nothing to disclose
Doenja Marina Johanna Lambregts: Nothing to disclose
Luis Curvo-Semedo: Nothing to disclose
Regina G. H. Beets-Tan: Nothing to disclose
Stephanie Nougaret: Nothing to disclose

### RPS 1001a-5

**Impact of MR image quality on diagnostic performance to assess response after chemoradiotherapy in rectal cancer**

*N. El Khababi*[1], M. Maas[1], M. Lahaye[1], S. Nougaret[2], L. Curvo-Semedo[3], R. Tissier[1], R. G. H. Beets-Tan[1], D. M. J. Lambregts[1]; [1]Amsterdam/NL, [2]Montpellier/FR, [3]Coimbra/PT

**Purpose:** To assess the impact of MR image quality on diagnostic performance to assess rectal tumour response after chemoradiotherapy (CRT) on restaging T2-weighted and diffusion-weighted MRI.
**Methods or Background:** As part of a previous trial, 22 radiologists assessed response to CRT in a multicenter patient group of n=90 (from 10 centers with varying acquisition protocols) on post-CRT T2W-MRI (using the mr tumor regression grade (mrTRG)) and a combination of T2W-MRI+DWI (using a modified-mrTRG incorporating DWI-findings), respectively. Impact of image quality on average sensitivity/specificity/NPV/PPV/accuracy to diagnose a complete response versus residual tumour was calculated using mixed model linear regression. Image quality was graded for each MRI with a 0-6-point score, which was designed for the purpose of this study (based on current guidelines), according to the following criteria: for T2W-MRI (1) slice-thickness (≤3mm), (2) in-plane-resolution (≤0.6x0.6mm), (3) angulation-perpendicular-to-tumor-axis; for DWI (4) high b-value ≥b800, (5) sufficient signal to noise ratio, (6) absence of significant artefacts. A total score of ≤3 was classified as below-standard quality.
**Results or Findings:** Quality of 38/90 (42%) cases was classified as below-standard, which had a significant negative impact on the readers' average specificity (effect size -0.096, P<0.001) and overall accuracy (effect size -0.045, P <0.001), with similar effects when calculated for the mrTRG (T2W-MRI) and modified-mrTRG (T2W-MRI+DWI) scorings. Average overall accuracy was 72% (mrTRG / T2W-MRI) and 77% (modified-mrTRG / T2W-MRI+DWI) for the good-quality scans versus 67% (mrTRG / T2W-MRI) and 72% (modified-mrTRG / T2W-MRI+DWI) for the below-standard quality scans.
**Conclusion:** MR image quality has a significant effect on radiologists' diagnostic performance to assess response of rectal tumours to chemoradiotherapy on restaging MRI. Optimising image acquisition is therefore crucial to ensure optimal diagnostic performance.
**Limitations:** Retrospective; non-validated quality scoring system.
**Ethics committee approval:** Yes.
**Funding for this study:** No funding was received for this study.
**Author Disclosures:**
Max Lahaye: Nothing to disclose
Monique Maas: Nothing to disclose
Renaud Tissier: Nothing to disclose
Najim El Khababi: Nothing to disclose
Doenja Marina Johanna Lambregts: Nothing to disclose
Luis Curvo-Semedo: Nothing to disclose
Regina G. H. Beets-Tan: Nothing to disclose
Stephanie Nougaret: Nothing to disclose

### RPS 1001a-7

**Study on biological characteristics of rectal cancer based on double-layer detector spectral CT quantitative multi-parameter**

*L. Wang*, W. X. Zheng; Lanzhou/CN
*(10206269632@qq.com)*

**Purpose:** To explore the relationship between double-layer detector spectral CT 120Kvp mixed energy images, VNC, 40 keV VMI, anhydrous iodine density, standardised iodine density effective atomic number and vascular nerve invasion of rectal cancer. To evaluate the best DLCT parameters and diagnostic phase for the diagnosis of vascular nerve invasion, in order to expect DLCT to predict the biological behaviour of rectal cancer before operation.
**Methods or Background:** A total of 70 patients with rectal cancer were included. DLCT data of arterial phase and venous phase of rectal cancer were obtained. Vascular nerve invasion, degree of differentiation and lymph node metastasis were obtained. In the results there were significant differences in arterial phase 40 keV VMI.
**Results or Findings:** DLCT data of arterial phase and venous phase of rectal cancer were obtained. Vascular nerve invasion, degree of differentiation and lymph node metastasis were obtained by histopathological histochemical evaluation. Receiver operating characteristic curves were used for statistical analysis.
**Conclusion:** NIC ,40 keV, anhydrous iodine density and Eff-Z of rectal cancer in arteriovenous phase have more clinical value than conventional 120 Kvp PI and VNC in the diagnosis of vascular invasion. It is concluded that the diagnostic efficacy of portal phase spectral CT parameters in the evaluation of vascular invasion is higher than that in arterial phase. Eff-Z in venous phase can reflect the neurological invasion, differentiation and lymph node metastasis of rectal cancer. DLCT multi-parameter quantitative analysis provides a new non-invasive imaging method for preoperative biological behavior evaluation of rectal cancer.
**Limitations:** This study was single-center with a small sample. Patients with rectal cancer in this study had inadequate intestinal preparation.
**Ethics committee approval:** The study was approved by the ethics committee of Gansu Provincial People's Hospital.
**Funding for this study:** Funding was received from Nature Foundation of Gansu Province.
**Author Disclosures:**
Wen Xia Zheng: Nothing to disclose
Lili Wang: Nothing to disclose

### RPS 1001a-8

**Prediction of the response to neoadjuvant chemotherapy in colon cancer by CT tumour regression grading: a preliminary study**

*H. Je*, S. H. Cho, H. S. Oh, A. N. Seo, S. H. Kim, G. Choi; Daegu/KR
*(sepia1411@naver.com)*

**Purpose:** To investigate the potential usefulness of MDCT based tumour regression grading that evaluates response to neoadjuvant chemotherapy in patients with colon cancer.
**Methods or Background:** In a retrospective cohort study, 53 patients who had colon cancer treated by neoadjuvant chemotherapy were analysed for CT tumour regression grade (TRG) and pathologic TRG. Tumour volume change was also assessed by a 3D slicer. We assessed the association between pathologic TRG and CT-based TRG using Spearman's rank correlation coefficient. Independent t-test and chi-square test were used to determine associations between pathologic response and characteristics of the patients. ROC curve analysis was used to compare the prediction accuracy between CT-based TRG and the volume change model.

**Results or Findings:** The CT-based TRG (ctTRG) was significantly correlated with pathologic TRG (pTRG) (Spearman's coefficient rank correlation, -0.54; P<0.01). AUC value to predict pathologic good responder was 0.749 in ctTRG (P<0.01, criterion≤3) and 0.794 in volume change model (P<0.01, criterion≤ -27.1). The difference between the two methods was not significant (P=0.53). AUC value to predict pathologic complete remission was 0.908 in ctTRG and 0.964 in the volume change model. The difference between the two methods was also not significant (P=0.45).
**Conclusion:** There is a significant correlation between ctTRG and pathologic TRG. There are no significant differences in performance between ctTRG and volume change model for predicting pTRG and pathologic complete remission. This study suggested a novel tumor regression grading system by using MDCT.
**Limitations:** The number of the patients was too small to study. This study compared only CT imaging and pathologic result, without direct comparison with survival.
**Ethics committee approval:** This retrospective study received ethics committee approval, and the requirement for written informed consent was waived.
**Funding for this study:** No funding was received for this study.
**Author Disclosures:**
Hyun Seok Oh: Nothing to disclose
See Hyung Kim: Nothing to disclose
Gyu-Seog Choi: Nothing to disclose
Seung Hyun Cho: Nothing to disclose
An Na Seo: Nothing to disclose
Hwanju Je: Nothing to disclose

**14:00-15:00** **Room M 2**

### Research Presentation Session: Breast

### RPS 1002

### New artificial intelligence applications in breast imaging

**Moderator**
V. Romeo; Naples/IT

### RPS 1002-2

**Systematic analysis of changes in radiomics features during dynamic breast-MRI: creating specific biomarkers**
*A. Landsmann*[1], C. M. Ruppert[1], S. Nowakowska[1], P. Hejduk[1], A. Ciritsis[1], K. Borkowski[1], M. Wurnig[2], C. Rossi[1], A. Boss[1]; [1]Zurich/CH, [2]Lachen/CH

**Purpose:** To investigate the changes of radiomics features during dynamic breast magnetic resonance imaging (MRI) for healthy tissue compared to benign and malignant lesions.
**Methods or Background:** Sixty patients with 30 fibroadenomas and 30 breast cancers underwent breast-MRI using a dynamic 3D gradient-echo sequence after injection of 0.2 ml/kg bodyweight gadoteric acid with an injection rate of 1.5 ml/s. Changes of 34 texture features (TF) in benign and malignant lesions were calculated for 5 dynamic datasets and corresponding 4 subtraction datasets. Statistical analysis was performed with ANOVA, systematic changes in features were described by linear and polynomial regression models.
**Results or Findings:** ANOVA revealed significant differences in 13 TF between normal tissue and lesions, compared to 9 TF between benign and malignant lesions. Most TF showed significant differences (p <0.05) and in early dynamic and subtraction image datasets, which seem therefore particularly suitable for TA. TF associated with tissue homogeneity were suitable to discriminate healthy parenchyma and lesions, whereas run-length features were more suitable to discriminate benign and malignant lesions. Run-length nonuniformity (RLN) was the only feature able to distinguish between all three classes with an area under the curve of 88.3 %. Depending on the individual TF, characteristic changes were observed with systematic increase or decrease for most TF with mostly polynomial behaviour. Mean values for the coefficient of determination were higher during subtraction sequences compared to dynamic sequences (benign: 0,98 vs 0, 72; malignant: 0,94 vs 0,74).
**Conclusion:** TF of breast lesions show characteristic changes during dynamic breast-MRI creating new biomarkers. Early dynamic and subtraction datasets are particularly suitable for texture analysis of lesions.
**Limitations:** This was a single-centre retrospective study. We only analysed the influence of 34 TF.
**Ethics committee approval:** In this study, patients signed informed consent.
**Funding for this study:** Funding was received for this study by Swiss National Foundation.
**Author Disclosures:**
Karol Borkowski: Nothing to disclose
Andreas Boss: Nothing to disclose
Moritz Wurnig: Nothing to disclose
Sylwia Nowakowska: Nothing to disclose
Patryk Hejduk: Nothing to disclose
Anna Landsmann: Nothing to disclose
Cristina Rossi: Nothing to disclose
Carlotta Maria Ruppert: Nothing to disclose
Alexander Ciritsis: Nothing to disclose

### RPS 1002-3

**Evaluation of the performance of artificial intelligence (AI) after one year of use in breast cancer screening practice: is the promise being delivered?**
*E. Elías Cabot*[1], S. Romero Martin[1], J. L. Raya Povedano[1], A. Gubern[2], M. A. B. Álvarez Benítez[1]; [1]Cordoba/ES, [2]Nijmegen/NL

**Purpose:** To evaluate the impact of using an AI system as support for human double reading in a breast cancer screening program and its ability to correctly stratify these exams according to probability of cancer.
**Methods or Background:** We reviewed all digital mammography (DM) or digital breast tomosynthesis (DBT) screening examinations between March 2021 and March 2022 that were double read (without consensus) by radiologists concurrently with AI support at our hospital. The AI system used in this study categorises each exam into three categories (low, intermediate, elevated) representing the probability of cancer and highlights suspicious areas (1-100 score). We computed the number of examinations, cancers, recalls and positive predictive value (PPV) of the recalled studies globally and in each AI category, as well as the overall cancer detection rate (CDR) and recall rate (RR) during the study period. We compared these data with the same 12 months period one year earlier (CDR 5.5/1000, RR 6.1%, PPV 9%), prior to the implementation of AI, using the Chi2 test.
**Results or Findings:** 11998 screening examinations were included and classified as low: 7917 (65.9%), intermediate: 3730 (31%) and elevated: 351 (2.9%). 108 cancers were detected, which were categorised as low: 1 (0.9%), intermediate: 32 (29.6%), elevated: 75 (69.4%). AI correctly marked 101 cancers. CDR, RR and PPV were 9/1000 (+3.5/1000, p<0.001); 6.1 % (0 %, p=0.9) and 14.6% (+5.6%, p<0.001), respectively.
**Conclusion:** AI used concurrently in clinical practice is able to stratify the examinations according to probability of cancer. AI increases cancer detection rate and PPV of the recalled women.
**Limitations:** A longer time frame, including interval cancers, is needed to support these findings.
**Ethics committee approval:** This study was approved by an ethics committee.
**Funding for this study:** No funding was received for this study.
**Author Disclosures:**
Marina Alvarez Benito Álvarez Benítez: Nothing to disclose
Albert Gubern: Employee: ScreenPoint Employee
Sara Romero Martin: Nothing to disclose
Esperanza Elías Cabot: Nothing to disclose
Jose Luis Raya Povedano: Nothing to disclose

### RPS 1002-4

**Training of artificial intelligence (AI) networks for identification and classification of microcalcifications in digital mammography (DM): preliminary data**
*G. Signorelli*, C. Trentin, F. Pesapane, F. Ferrari, M. Montesano, S. D'Acquisto, R. Virgoli, L. Nicosia, E. Cassano; Milan/IT
*(giulia.signorelli@unimi.it)*

**Purpose:** Performance of AI networks to 1. classify and 2. detect microcalcifications in DM to develop a tool for improving reading efficiency of DM.
**Methods or Background:** A trained AI system may have clinical value for early detection of cancer, microcalcifications classification, lowering of recall rates and for reducing the number of missed cancers and unnecessary biopsies. From 484 patients undergoing DM (2019-2021) and stereotactic-guided biopsies, we extracted 1036 images (486 microcalcifications clusters) to train AI networks. Data collection includes a. preprocessing and segmentation of microcalcifications, b. regions of interest (ROI) specification, c. feature extraction and classification. ROIs were considered on "microcalcification" type patches (MP) regardless of histological outcome. The same images were used for obtaining patches without microcalcifications (nMP).

**Results or Findings:** Dataset: 11142 MP (5170 benign; 5972 malignant), 11142 nMP. Patches were partitioned into 70% training set, 20% validation set, 10% test set. Results were obtained by evaluating the performance of the networks on a testing set composed for aim 1 by 1114 patches (517 benign, 597 malignant) and for aim 2 by 2288 patches equally distributed. Three networks performed differently; the best network showed 81% of accuracy, 89% of AUC for aim 1; and 95% of accuracy, 99% of AUC for aim 2. The increased complexity of the network used doesn't follow an increase in performance in terms of accuracy or AUC.
**Conclusion:** AI networks can be appropriately trained to classify and detect microcalcifications in mammography. However, AI development is further required, and the result should be tested in clinical practice.
**Limitations:** Not applicable.
**Ethics committee approval:** This study was approved by an ethics committee (IRB approbation (ID trial:3052)).
**Funding for this study:** This study was partially supported by Reply®.
**Author Disclosures:**
Chiara Trentin: Nothing to disclose
Luca Nicosia: Nothing to disclose
Roberto Virgoli: Nothing to disclose
Giulia Signorelli: Nothing to disclose
Filippo Pesapane: Nothing to disclose
Enrico Cassano: Nothing to disclose
Federica Ferrari: Nothing to disclose
Marta Montesano: Nothing to disclose
Silvia D'Acquisto: Nothing to disclose

### RPS 1002-5

**Pretreatment prediction of pathologic complete tumour response to neoadjuvant chemotherapy in triple-negative breast cancer patients using dynamic-contrast-enhanced MRI-based radiomics models**

A. Lenaers, H. C. Woodruff, M. Beuque, S. A. Keek, R. Granzier, S. Vanwetswinkel, S. Engelen, M. Smidt, *T. van Nijnatten*; Maastricht/NL
*(Thiemo.nijnatten@mumc.nl)*

**Purpose:** To explore the potential of radiomics models based on different post-contrast phases of pretreatment dynamic contrast-enhanced (DCE) MRI for prediction of pathologic complete response (pCR) to neoadjuvant chemotherapy in triple-negative breast cancer patients.
**Methods or Background:** A dual-centre cohort consisting of triple-negative patients treated with neoadjuvant chemotherapy that underwent pretreatment DCE-MRI between 2011-2020 was retrospectively collected. After manual tumour segmentation, MR images were preprocessed to homogenise the data. A total of 100 quantitative features were extracted from each phase using pyradiomics. Random forest prediction models were developed for each post-contrast phase separately. Because of our limited sample size, a nested cross-validation strategy was used for model development. The performance regarding pCR prediction was quantified by the area under the receiver operating characteristic curve (AUC) values.
**Results or Findings:** A total of 66 patients with 69 tumours were included from two centres. For each model, three potentially predictive features were selected. Radiomics models based on pretreatment MR images of the first, second and third post-contrast phase yielded AUC values of 0.56 (95% CI: 0.55 - 0.57), 0.55 (95% CI: 0.54 – 0.56) and 0.61 (95% CI: 0.60 – 0.62) respectively.
**Conclusion:** Radiomics features extracted from pretreatment DCE-MRI may be associated with pCR in triple-negative breast cancer. This association appears to be higher for our model based on delayed post-contrast phase pretreatment DCE-MRI compared to the models based on early post-contrast DCE-MRI. Future radiomics studies should include mid- and post-treatment DCE-MRI from larger cohorts for further improvement of pCR prediction.
**Limitations:** Additional research on a larger multi-centric dataset is needed to further establish the predictive performance of our models.
**Ethics committee approval:** Medical ethical committee waived requirement for informed consent.
**Funding for this study:** Not applicable.
**Author Disclosures:**
Marjolein Smidt: Nothing to disclose
Sigrid Vanwetswinkel: Nothing to disclose
Simon Andreas Keek: Nothing to disclose
Henry Christian Woodruff: Nothing to disclose
Anouk Lenaers: Nothing to disclose
Thiemo van Nijnatten: Nothing to disclose
Renée Granzier: Nothing to disclose
Manon Beuque: Nothing to disclose
Sanne Engelen: Nothing to disclose

### RPS 1002-6

**Evaluate performance of two different versions of an artificial intelligence (AI) system for predicting the risk of developing interval cancer (IC) within 6 to 24 months after negative screening exam**

L. Çelik[1], *D. C. Guner*[1], A-K. Brehl[2], N. Janssen[2], M. E. Aribal[1]; [1]Istanbul/TR, [2]Nijmegen/NL
*(davutcanguner@gmail.com)*

**Purpose:** Evaluate performance of two different versions of an artificial intelligence (AI) system for predicting risk of developing interval cancer (IC) within 6 to 24 months after negative screening exam.
**Methods or Background:** This retrospective study was performed with a data cohort between 2016-2019 derived from women between 40-69 who attended a national screening programme. Negative screening exams of 323 women who developed IC before the next screening round were collected. The pathological outcome was retrieved from the national cancer registry. All mammograms were processed by an AI cancer detection system (Transpara, ScreenPoint Medical v1.6and v1.7.1), assigning a score between 1-10 to the exam. The performance of the AI system for detection of IC on negative screening exams was estimated in terms of the area under the receiver operating characteristic curve (AUC), and sensitivity at 90% and 95.0% specificity, with 95% confidence intervals (CI).
**Results or Findings:** More than half of all IC (56.6% and 65.9% respectively for versions 1.6 and 1.7.1) were flagged by the highest AI score 10. The AUCs of AI to detect signs of IC on negative screening exams were 0.86 and 0.81 and the sensitivity was 65.9% and 56.6% at a specificity of 90.0% for versions respectively ($p<0.01$). Highest performance of AI was found for cases that were diagnosed within six months after screening (AUC: 0.82), compared to cases diagnosed 6 to 24 months after screening (AUC: 0.57).
**Conclusion:** AI has the potential to reduce the stable high rate of interval cancer in case AI is applied as a second or third independent reader within a national breast cancer screening programme. Moreover, further developments of AI promise increasing performance towards prediction of interval cancer.
**Limitations:** The limitation is the study's retrospective design.
**Ethics committee approval:** Institutional ethics committee approved this study.
**Funding for this study:** No funding was received for this study.
**Author Disclosures:**
Mustafa Erkin Aribal: Nothing to disclose
Natasja Janssen: Employee: Working at screenpoint medical
Davut Can Guner: Nothing to disclose
Levent Çelik: Nothing to disclose
Anne-Kathrin Brehl: Employee: Working at screenpoint medical

### RPS 1002-7

**Radiomics in spiral breast CT: potential of texture analysis in breast density classification**

*A. Landsmann*[1], C. M. Ruppert[1], J. Wieler[1], P. Hejduk[1], A. Ciritsis[1], K. Borkowski[1], M. Wurnig[2], C. Rossi[1], A. Boss[1]; [1]Zurich/CH, [2]Lachen/CH

**Purpose:** To investigate whether features derived from texture analysis (TA) can distinguish breast density (BD) in spiral breast-computed-tomography (BCT).
**Methods or Background:** 10000 images of 400 BCT-examinations were categorised using a four-level density-scale (A-D). After definition of regions of interest (ROIs), 19 texture features (TF) were calculated to analyse the voxel grey-level distribution in the included image area. Data was statistically evaluated using ANOVA and cluster analysis. Multinomial logistic regression was performed to evaluate the potential of TA for BD assessment. A human readout then was performed on a subset of 60 images to evaluate reliability of the proposed feature set.
**Results or Findings:** Of the 19 features, four first-order features and seven second-order features showed significant correlation with BD and were therefore selected for further analysis. Multinomial logistic regression revealed an overall accuracy of 80% for BD assessment. The majority of features systematically increased or decreased with denser breast tissue. Skewness (-0.81), as a first-order feature and grey-level nonuniformity (GLN, - 0.59), as a second-order feature, showed the strongest correlation with BD and appeared to be independent of other TF. Mean values of skewness, and GLN decreased linearly from density-level A to D. Run-length nonuniformity (RLN), as a second-order feature, showed moderate correlation with BD, but was considered redundant because of its correlation with GLN. All other TF evaluated showed only weak correlation with BD (range - 0.49 to 0.49, p-values <0.001) and were therefore neglected.
**Conclusion:** TA in BCT examinations might be a useful approach in the assessment of BD and may serve as an observer-independent, objective tool.
**Limitations:** This was a retrospective single-centre study. We only examined the influence of 19 TF.
**Ethics committee approval:** In this study, patients signed informed consent.

**Funding for this study:** Funding was received for this study by Swiss National Foundation.
**Author Disclosures:**
Karol Borkowski: Nothing to disclose
Andreas Boss: Nothing to disclose
Moritz Wurnig: Nothing to disclose
Jann Wieler: Nothing to disclose
Patryk Hejduk: Nothing to disclose
Anna Landsmann: Nothing to disclose
Cristina Rossi: Nothing to disclose
Carlotta Maria Ruppert: Nothing to disclose
Alexander Ciritsis: Nothing to disclose

### RPS 1002-8

**The potential of radiomics-based features on screening mammograms in distinguishing molecular subtypes of invasive breast cancer**

*J. Peters*[1], M. Caballo[1], J. van Dijck[1], S. Elias[2], E. Lips[3], J. Wesseling[3], R. Mann[1], M. Broeders[1]; [1]Nijmegen/NL, [2]Utrecht/NL, [3]Amsterdam/NL
*(Jim.Peters@radboudumc.nl)*

**Purpose:** For optimal breast cancer screening, it would be a major gain if we could already distinguish aggressive invasive breast cancer (IBC) from indolent lesions. It is crucial to detect aggressive IBC timely, while overdiagnosis of indolent lesions could be reduced. As breast cancer molecular subtypes are widely used in prognostication, we investigated if these subtypes could be predicted by radiomic features using the screening mammograms of women diagnosed with IBC.
**Methods or Background:** In this consecutive cohort study, screening mammograms of 743 women diagnosed with screen-detected IBC were used. Using information from pathology reports, tumours were considered indolent if they were hormone receptor-positive, Her2-negative and well differentiated (“luminal A-like”), whereas other subtypes (“luminal B-like”, “Her2-enriched-like”,“basal-like”) were considered aggressive. Tumours were manually annotated in both mammographic views. Using pyradiomics, 2,748 radiomics-features were extracted from intra- and peritumoral regions. After feature reduction, an extreme gradient boosting classifier was trained to predict whether a tumour was indolent or aggressive. The model performance was validated using the area under the receiver operating characteristics curve (AUC), using 10 fold, 100x repeated nested cross-validation.
**Results or Findings:** The mean cross-validated AUC was 0.67 (SD±0.04) to distinguish indolent (N=302) from aggressive IBC (N=430). For each cross-validation fold, the model was trained based on 30 features to prevent overfitting. 19 features were selected during more than 50% of repeats. They were related to pixel intensity, shape and texture of both the intra- and peritumoral regions.
**Conclusion:** Radiomic features on screening mammograms show a modest performance in distinguishing indolent from aggressive IBCs. To assess precisely their prognostic value, external validation and investigating other endpoints (e.g. breast cancer-specific survival) are required.
**Limitations:** The limitation of this study is that mammograms prior to interval cancers were not yet incorporated.
**Ethics committee approval:** Not applicable.
**Funding for this study:** Funding was received for this study by Dutch Cancer Society (KWF11835).
**Author Disclosures:**
Esther Lips: Nothing to disclose
Jim Peters: Nothing to disclose
Mireille Broeders: Nothing to disclose
Marco Caballo: Nothing to disclose
Sjoerd Elias: Nothing to disclose
Ritse Mann: Nothing to disclose
Jos van Dijck: Nothing to disclose
Jelle Wesseling: Nothing to disclose

**14:00-15:30** **Room E1**

### Research Presentation Session: Imaging Informatics / Artificial Intelligence and Machine Learning

### RPS 1005

### Artificial intelligence (AI) in musculoskeletal imaging

**Moderator**
M. Klontzas; Iraklion/GR
**Author Disclosure:**
M. Klontzas: Board Member: EuSoMII

### RPS 1005-2

**Added value of artificial intelligence in traumatic radiographic findings detection in emergency settings**

*A. Parpaleix*[1], C. Parsy[1], M. Codari[2], M. Mejdoubi[1]; [1]Valenciennes/FR, [2]San Francisco, CA/US

**Purpose:** To assess the added value of using a commercially available artificial intelligence (AI) tool in the detection of traumatic radiological findings in emergency settings. In particular, we investigated the role of this tool in case of discrepancies between the emergency physician and radiologist reports.
**Methods or Background:** We retrospectively analysed 2580 consecutive musculoskeletal radiological studies (not including the spine or skull). All patients were admitted to the emergency department (ED) at Centre Hospitalier de Valenciennes between 07/2020 and 10/2020. All images were processed anonymously using the Chest|MSK AI tool (Arterys Inc.) to classify each patient according to the presence/absence of any of the following imaging findings: fracture, dislocation, and elbow fat pad. ED clinical reports were analysed to independently classify each patient according to presence/absence of such findings, while the radiologist clinical report was used as a reference standard. We compared the AI tool, ED, and radiologist classifications to investigate the added value of using this AI tool to mitigate disagreements among physicians.
**Results or Findings:** In 1716/2580 (67%) patients, both ED and radiological reports were available. In 1519/1716 (89%) cases the physicians agreed on the patient classification. In 1351/1519 (89%) patients this result was confirmed by the AI tool. The ED classification did not match the radiologist one in 197/1716 (11%) patients. Notably, 178/197 (90%) of disagreements would have been resolved by the AI model, which agreed with the radiologist.
**Conclusion:** The AI model showed promising potential in mitigating image misinterpretation in ED settings. Future studies will focus on confirming these results in multiple centres and prospective patient cohorts.
**Limitations:** Binary classification; single centre.
**Ethics committee approval:** Non-interventional study on data, patient consent was obtained.
**Funding for this study:** No funding was received for this study.
**Author Disclosures:**
Marina Codari: Employee: Arterys
Clémence Parsy: Nothing to disclose
Alexandre Parpaleix: Founder: Milvue
Mehdi Mejdoubi: Nothing to disclose

### RPS 1005-3

**Automated bone age assessment on a multi-site US cohort: agreement between AI and expert readers**

*M. DiFranco*[1], T. S. Chung[1], J. Wood[2], K. Caudill[2], R. Bowling[2], M. Hileman[2], W. Cho[2], M. Kelsey[2], A. Mintz[2]; [1]Vienna/AT, [2]St. Louis, MO/US

**Purpose:** We evaluate the agreement between AI and expert readers for bone age (BA) assessment according to the Greulich & Pyle method on a cohort of children from the clinical workflow.
**Methods or Background:** The radiological determination of bone age (BA) from a left-hand x-ray continues to be the reference standard for skeletal maturity assessment related to short- or long stature, premature or delayed puberty, and underlying conditions. Artificial intelligence (AI) algorithms are becoming more prevalent due to the subjectivity and time-consuming nature of BA assessment. Radiographs of 342 patients were analyzed retrospectively (178 males years, 165 females aged 2 to 16 years). Sampling was performed across multiple sites in the USA associated with Washington University in St. Louis (WUSTL) School of Medicine. Three US board certified pediatric radiologists who were blinded to chronological age made reads of BA using the Greulich & Pyle (GP) method independent of each other and an AI-software.

Performance of AI to readers was assessed using of Bland-Altman limits of agreement (LOA), orthogonal linear regression and interchangeability.
**Results or Findings:** Bland-Altman assessment revealed a mean difference between readers and AI of -0.72 (95% CI -1.46; 0.02) months. Orthogonal linear regression revealed no significant proportional bias across ages (slope = 1.02 [95% CI 1.00; 1.03]). AI was found to be interchangeable with readers based on an equivalence index of -5.8 months.
**Conclusion:** A fully automated AI software shows agreement with expert readers in BA assessment on a multi-site cohort of US children and adolescents, suggesting AI integration into the radiology workflow is possible and could lead to more efficient bone age reading.
**Limitations:** No limitations were identified.
**Ethics committee approval:** This study was approved by Washington University of St. Louis IRB [ID # 202102127].
**Funding for this study:** Imaging clinical trial sponsored by IB Lab GmbH.
**Author Disclosures:**
Michael Hileman: Nothing to disclose
Tek Sin Chung: Employee: IB Lab GmbH
Aaron Mintz: Investigator: Washington University in St. Louis
Woonchan Cho: Nothing to disclose
Matthew Kelsey: Nothing to disclose
Karen Caudill: Nothing to disclose
Rebecca Bowling: Nothing to disclose
Jonathan Wood: Nothing to disclose
Matthew DiFranco: Employee: IB Lab GmbH

### RPS 1005-5

### Automated assessment of lower limb alignment based on artificial intelligence: An independent validation study on pre- and postoperative full leg X-rays

*F. I. Erne*[1], P. Grover[2], M. Dreischarf[2], A. Nüssler[1], M. Reumann[1], F. Springer[1], C. Scholl[2]; [1]Tübingen/DE, [2]Leipzig/DE

**Purpose:** The accurate assessment of lower limb alignment from X-rays is essential for therapy indication and postoperative quality control, but suffers from time-consuming manual measurements. Therefore, this study aims to develop and validate a fully automated, artificial intelligence (AI) based algorithm for measuring lower limb alignment.
**Methods or Background:** In total, 50 preoperative, weight-bearing, anterior-posterior full-leg radiographs were manually measured by two expert human raters (R1 and R2) and twice by one of these raters (R1a, R1b). Additionally, 47 postoperative images (total knee arthroplasty) were measured twice by R1. All radiographs were analysed by the AI-algorithm (R-AI) consisting of five convolutional neural networks, which were trained to determine the following angles: mechanical-femoral-tibial-angle (mFAmTA), mechanical-lateral-distal-femoral-angle, mechanical-medial-proximal-tibial-angle, mechanical-lateral-distal-tibial-angle (mLDTA), and the angle between the anatomical femur axis and mechanical tibia axis. Agreement between human raters and AI was analysed by mean errors (95% confidence interval [CI]) and single-measure intraclass correlation coefficients (ICC) for absolute agreement (ICC>0.75 considered as excellent).
**Results or Findings:** ICC values for intra- (range 0.97-0.99) and inter-rater reliability (0.88-0.98) between human raters reflect excellent agreement. The AI algorithm can determine all parameters in 96% of pre- and 94% of postoperative images with excellent ICC values (PreOP-range: 0.82-0.99; PostOP: 0.86-0.99). Exemplary for R1a vs R-AI, mean errors were smallest for mFAmTA (PreOP: 0.2° [CI: 0-0.3°]); PostOP: (0.1° [0-0.3°]) and largest for mLDTA (PreOP: 0.5 [0.1-0.9°]; PostOP: 0.4° [0-0.8°]).
**Conclusion:** The novel, fully automated AI algorithm determines lower limb alignment pre- and postoperatively with excellent reliability and accuracy. The tool may facilitate routine clinical tasks and independently analyse large-scale datasets.
**Limitations:** The results should be verified on a larger patient cohort from multiple sites, with varying surgical interventions.
**Ethics committee approval:** This study was approved by the ethics committee Tübingen (197/2021BO2).
**Funding for this study:** This study was funded by BMWi (DLR-project; grant-ID 01MK20003A).
**Author Disclosures:**
Marie Reumann: Nothing to disclose
Carolin Scholl: Employee: Raylytic GmbH
Felix Institut Erne: Nothing to disclose
Marcel Dreischarf: Employee: Raylytic GmbH
Priyanka Grover: Employee: Raylytic GmbH
Fabian Springer: Nothing to disclose
Andreas Nüssler: Nothing to disclose

### RPS 1005-6

### Automated analysis of implant alignment following total knee arthroplasty in long leg radiographs

G. M. Schwarz, S. Simon, B. Frank, A. Aichmair, M. Dominkus, J. Hofstaetter, *M. DiFranco*; Vienna/AT

**Purpose:** Evaluation of long leg radiographs (LLR) after total knee arthroplasty (TKA) is highly dependent on expertise. Artificial intelligence (AI) technology may help automate and standardise this process and allows analysis of large datasets. The aim of this study was to develop an AI-software which automatically evaluates limb alignment following TKA.
**Methods or Background:** The AI-system was trained on over 15,000 LLRs. In the evaluation cohort 200 calibrated LLRs of eight different common unconstrained and constrained knee systems were analysed. Accuracy and reproducibility of the software were compared to expert readers regarding the hip-knee-ankle angle (HKA) and femoral and tibial component angles. To assess the software's ability of handling big data 1460 LLRs were evaluated in our testing cohort and the distribution of HKA angle values were investigated.
**Results or Findings:** The AI-software was reproducible on 96 % of LLR and showed excellent reliability in all measured angles (ICC > 0.90). Excellent results were found for primary unconstrained TKAs. In constrained TKAs landmark setting on the femoral and tibial component failed nine times (12.5%). The mean HKA angle in our testing cohort was 0.3° ± 3.1° (n=1460).
**Conclusion:** The AI-software allows for accurate automatic measurement of implant alignment following primary and revision TKAs and is capable of handling large datasets. Visual inspection of results detected that poor AI performance on constrained TKAs was due to the hinge pin, suggesting that the AI requires further training with more representative images.
**Limitations:** Although the AI-software was trained on LLRs from different sites, radiographs in the evaluation and testing cohort originated from only one institution.
**Ethics committee approval:** This study was approved by an ethics committee.
**Funding for this study:** The Michael Ogon Laboratory received financial funding from Image Biopsy Lab.
**Author Disclosures:**
Jochen Hofstaetter: Research/Grant Support: Image Biopsy Lab
Alexander Aichmair: Nothing to disclose
Bernhard Frank: Nothing to disclose
Gilbert Manuel Schwarz: Nothing to disclose
Matthew DiFranco: Nothing to disclose
Martin Dominkus: Nothing to disclose
Sebastian Simon: Nothing to disclose

### RPS 1005-7

### Faster MR imaging with artificial intelligence: a deep learning-based approach for image reconstruction with compressed sensing for imaging of the knee

*A-I. Iuga*[1], P. Rauen[1], F. Siedek[1], N. Grosse Hokamp[1], K. Sonnabend[2], D. Maintz[1], G. Bratke[1]; [1]Cologne/DE, [2]Hamburg/DE
*(iza.iuga@yahoo.com)*

**Purpose:** The main objective of this study was to evaluate clinical feasibility for combining compressed SENSE (CS) with a newly developed deep learning-based algorithm (CS-AI) using Convolutional Neural Networks to accelerate 2D imaging of the knee.
**Methods or Background:** In this prospective study, 20 healthy volunteers were scanned with a 3 Tesla MRI scanner (Ingenia, Philips, Best, The Netherlands). All subjects received a standard fat saturated sagittal 2D proton density (PD) sequence without any acceleration (CS1) and four additional acceleration levels: CS2, CS3, CS4 and CS6. Images were reconstructed with the conventional CS and the new CS-AI algorithm for all acceleration factors. Two independent, blinded readers rated all images using a 5-point-Likert scale. Additionally, signal-to-noise ratio and contrast-to-noise ratio were calculated. The Friedman and Dunn´s multiple comparisons test was used for ordinal data while ANOVA and the Tukey Kramer test was applied for continuous data. Cohen´s kappa was calculated for interrater reliability.
**Results or Findings:** The duration of the sequences was: CS1:317s; CS2:165s; CS3:114s; CS4:89s; CS6:63s. The AI-based reconstruction allows a 64% reduction in scan time compared to the fully unaccelerated sequence (CS-AI3). Except for artifacts, the subjective rating was significantly higher for CS-AI than conventional CS for at least one acceleration factor. Sequences reconstructed using AI were rated better than the time-equivalent conventional CS for almost all acceleration factors. Signal-to-noise and contrast-to-noise were significantly better for all CS-AI reconstructions (all p<0.05).
**Conclusion:** The AI-based reconstruction outperformed the conventional CS in all criteria for all tested acceleration factors resulting in faster imaging with unchanged image quality (-64%).
**Limitations:** The study sample size was small and included only healthy volunteers. This work didn't analyse the feasibility of CS-AI to accelerate 3D imaging of the knee.

**Ethics committee approval:** This study was approved by Drks00024156.
**Funding for this study:** No funding was received for this study.
**Author Disclosures:**
Florian Siedek: Nothing to disclose
David Maintz: Speaker: Philips
Philip Rauen: Nothing to disclose
Nils Grosse Hokamp: Grant Recipient: Philips
Andra-Iza Iuga: Nothing to disclose
Kristina Sonnabend: Employee: Philips GmbH Market DACH
Grischa Bratke: Nothing to disclose

### RPS 1005-8

**Lumbar spine MRI: comparison of novel deep learning algorithm and conventional sequences on 1.5T**

*F. Pucciarelli*, M. Zerunian, M. Polici, B. Masci, D. Polverari, B. Bracci, A. Del Gaudio, D. Caruso, A. Laghi; Rome/IT

**Purpose:** To prospectively compare quantitative and subjective image quality and scanning time between a new deep learning-based reconstruction (DLR) algorithm and standard MRI protocol of the lumbar spine.
**Methods or Background:** Thirty-one healthy volunteers underwent 1.5T MRI examination of lumbar spine from April to September 2021. Protocol acquisition comprised sagittal T1- and T2-weighted and short-tau inversion recovery (STIR) images and axial T2-weighted images. All sequences were acquired with both DLR and standard protocols. The quantitative image analysis with signal-to-noise ratio (SNR) and contrast-to-noise ratio (CNR) was performed by a radiologist drawing regions of interest (ROIs) on the fourth lumbar vertebral body and in the intervertebral disc (L4/5); the qualitative image analysis between the two protocols was performed by two radiologists in blind. Scanning times were also recorded and compared.
**Results or Findings:** The SNR of DLR algorithm was higher in all sequences in the vertebrae and discs compared to standard images (all $p<0.001$). The CNR of the DLR algorithm was superior to conventional T2-weighted images ($p<0.001$), whereas no significant differences were reported for T1-weighted ($p=0.67$) and STIR images ($p=0.40$). Qualitative analysis showed that DLR had greater overall quality in all sequences (all $p<0.001$), with an inter-rater agreement of 0.69 (0.63-0.76). Total protocol scanning time was lower in DLR compared to standard protocol (average acquisition time 6.33 vs 13.06 minutes, $p<0.001$), resulting in acquisition time reduction of 49%.
**Conclusion:** DLR algorithm applied to 1.5T MRI is a feasible method for lumbar spine imaging providing morphologic sequences with a higher SNR, CNR and image quality, compared with standard protocol with a significant scanning time reduction.
**Limitations:** Small population sample and only one district analysed.
**Ethics committee approval:** This study was approved by our local institutional review board; written informed consent was obtained from all study participants.
**Funding for this study:** No funding was received for this study.
**Author Disclosures:**
Damiano Caruso: Nothing to disclose
Benedetta Masci: Nothing to disclose
Francesco Pucciarelli: Nothing to disclose
Marta Zerunian: Nothing to disclose
Daniele Polverari: Nothing to disclose
Benedetta Bracci: Nothing to disclose
Andrea Laghi: Nothing to disclose
Antonella Del Gaudio: Nothing to disclose
Michela Polici: Nothing to disclose

### RPS 1005-9

**Two-fold and three-fold acceleration of compressed sense MR imaging of the ankle using artificial intelligence**

*S. C. Foreman*, J. Neumann, J. Han, N. Harrasser, K. Weiss, D. C. Karampinos, M. Makowski, A. S. Gersing, K. Woertler; Munich/DE

**Purpose:** To evaluate a Compressed-Sensing Artificial Intelligence Framework (CSAI) to accelerate MRI acquisition of the ankle.
**Methods or Background:** Ankles of thirty patients were scanned (15 female, age 19-84 years) at 3T. Axial T2-w, coronal T1-w, and coronal/sagittal intermediate-w sequences with fat-saturation were acquired using C-SENSE only (12:44 min), CSAI with an acceleration factor of 4.6-5.3 (6:45 min, CSAI2x), and CSAI with an acceleration factor of 6.9-7.7 (4:46 min, CSAI3x). Moreover, a high-resolution axial T2-w sequence was obtained using CSAI with the same scan duration of the C-SENSE sequence. Readings were performed independently by two radiologists grading the depiction and presence of abnormalities. Signal-to-noise and contrast-to-noise were calculated. The Wilcoxon-signed-rank test and Cohen's Kappa were used to compare CSAI with conventional C-SENSE sequences.
**Results or Findings:** The correlation between the two sequences was perfect for C-SENSE and CSAI2x ($\kappa= 1.0$) and excellent for C-SENSE and CSAI3x ($\kappa=0.86$-$1.0$). No significant differences were found for the depiction of structures between C-SENSE and CSAI2x and the same abnormalities were detected in both sequences. For CSAI3x the depiction was graded significantly lower ($p<0.05$) compared to C-SENSE, though most abnormalities were also detected. For CSAI2x contrast-to-noise fluid/muscle was higher compared to C-SENSE ($p<0.05$), while no differences were found for other investigated tissues. Signal-to-noise and contrast-to-noise were significantly higher for CSAI3x compared to C-SENSE ($p<0.05$). The high-resolution axial T2-w sequence specifically improved the depiction of tendons, bone and the tibial nerve ($p<0.05$).
**Conclusion:** Acquisition times can be reduced by ~50% using CSAI without decreasing diagnostic image quality. Reducing acquisition times by ~66% is feasible, but should be reserved for specific patients. The depiction of specific structures is improved using a high-resolution axial T2-w CSAI sequence.
**Limitations:** Correlation to an external standard of reference was not available.
**Ethics committee approval:** This study was approved by Nr:42/21S.
**Funding for this study:** No funding was received for this study.
**Author Disclosures:**
Jessie Han: Nothing to disclose
Alexandra Sophia Gersing: Nothing to disclose
Jan Neumann: Nothing to disclose
Kilian Weiss: Nothing to disclose
Marcus Makowski: Nothing to disclose
Norbert Harrasser: Nothing to disclose
Dimitrios C. Karampinos: Nothing to disclose
Sarah C. Foreman: Nothing to disclose
Klaus Woertler: Nothing to disclose

### RPS 1005-10

**Automated Bilsky grading of epidural spinal cord compression on thoracic spine MRI using deep learning: initial results.**

*D. Lim*, J. T. P. D. Hallinan, A. Makmur; Singapore/SG
*(desmond_lim@nuhs.edu.sg)*

**Purpose:** To develop a Deep Learning (DL) model for automated Bilsky grading of metastatic epidural spinal cord compression (ESCC) using thoracic spine MRI. Dichotomous classification of low-grade (no epidural disease/Bilsky 1a/1b) versus high-grade (Bilsky 1c/2/3) disease is useful to identify potential surgical candidates (high-grade).
**Methods or Background:** MRI spines with vertebral metastases (215 studies) were split into training (82%: 177/215) and test sets (18%: 38/215). Axial T2w images of the thoracic spine were evaluated and graded by subspeciality radiologists (training set) using dichotomous classification (low vs high-grade). A two-step DL model was developed with the training set. Firstly, Faster R-CNN with Resnet101 detects a region of interest (ROI). Secondly, a convolutional network with Resnet50 projects ROIs into a high-dimensional embedding space (ES). Class prototype of Bilsky scores are assigned in the ES and the model learns with distance-based cross-entropy loss. The test set was labelled by the DL model, a radiation oncologist (RO), spine surgeon (SS) and a neuroradiologist (NR). Gradings were then compared against a musculoskeletal (MSK) radiologist with 10 years experience (reference standard).
**Results or Findings:** The DL model had 95.5% accuracy in identifying high-grade Bilsky ESCC, with sensitivity of 88.9% and specificity of 96.4%. The specialists had accuracies of 98.1% (RO), 99.0% (SS), 99.1% (NR), with sensitivities/specificities of 100%/97.9% (RO), 95.7%/99.5% (SS), and 96.7%/99.5% (NR) respectively.
**Conclusion:** A DL model for Bilsky grading was successfully built with accuracy comparable to specialists.
**Limitations:** Cervical and lumbar regions have degenerative changes, contributing to stenosis in addition to metastatic disease.
**Ethics committee approval: Ethics committee approval:** Utilization of Machine Learning and Patient Electronic Medical Records (EMR) for image interpretation, diagnostics and clinical prognostication in patients with spinal metastases; DSRB ref: 2020/00835-SRF0001.
**Funding for this study:** Funding was received: Deep learning pathway for the management of spine metastases, Proposal ID: CNIG20nov-0011 (NMRC).
**Author Disclosures:**
Desmond Lim: Nothing to disclose
Andrew Makmur: Nothing to disclose
James Thomas Patrick Decourcy Hallinan: Nothing to disclose

### RPS 1005-11

**Predicting muscular dystrophy mutations using muscle fat infiltration patterns and supervised machine learning models**

*B. Pizarro*[1], D. Gómez-Andrés[2], J. Diaz[1], L. Suazo Rojas[1], V. A. Salinas Angel[1], C. Correa[1], M. F. Eyssautier Susarte[1], J. A. Bevilacqua[1]; [1]Santiago/CL, [2]Barcelona/ES
*(benjaminpizarro@ug.uchile.cl)*

**Purpose:** Muscular dystrophies (MD) are a heterogeneous group of disorders caused by mutations in genes involved in muscular structure and function. They are clinically characterised by muscle weakness and dystrophic changes in muscle histology. MRI has been used to support the diagnosis of MD because of its sensitivity to detect muscle oedema and fat infiltration. Random Forest (RF) has been used to characterise fat infiltration patterns in MD, however, no studies have compared different supervised machine learning (ML) models for MD prediction.
**Methods or Background:** In this retrospective study, we compared different supervised ML models trained on a published European database, and then tested them on a small convenience series of genetically confirmed patients with calpainopathy. Nine hundred and seventy-two European patients corresponding to ten different muscular dystrophies, and six Latin American patients with calpainopathy were included. Linear Discriminant Analysis, Logistic Regressions, Random Forests, Support Vector Machines (SVM), Multi-Layer Perceptron, AdaBoost, Naïve Bayes (NB), and KNeighbors (KN) were compared.
**Results or Findings:** Random Forests outperformed the other models in the European database (96% accuracy), however, it failed to predict the diagnosis in Latin American patients. Even if KN and NB didn't reach the same accuracy as RF in the European database, they outperformed the other models in Latin American patients (83% accuracy).
**Conclusion:** ML models are suitable tools to aid in the diagnosis of MD, however, real-world use requires more robust and generalisable models.
**Limitations:** Our preliminary data show that KN and NB seem to be more robust methods compared to RF, at least in this small series of patients with calpainopathy.
**Ethics committee approval:** The local HCUCH Ethics Committee and the Chilean National Commission of Scientific Research and Technology approved the study protocol.
**Funding for this study:** We thank the FONDECYT 1151383 grant.
**Author Disclosures:**
Jorge Diaz: Nothing to disclose
Benjamín Pizarro: Nothing to disclose
Lionel Suazo Rojas: Nothing to disclose
Claudio Correa: Nothing to disclose
Vicente Alejandro Salinas Angel: Nothing to disclose
Jorge A Bevilacqua: Nothing to disclose
María Fernanda Eyssautier Susarte: Nothing to disclose
David Gómez-Andrés: Nothing to disclose

### RPS 1005-12

**Evaluation of visual explanation for knee MRI pathology classifiers**

*G. D'Assignies*, M. Charachon, T. Vesoul, P. Guetat, E. Pluot, R. Ardon; Paris/FR

**Purpose:** To evaluate an automatic and generic technique to visually explain the decisions of deep learning classifiers trained to identify healthy from pathological cases on knee MRI problems.
**Methods or Background:** Binary classifiers were respectively trained on 23538 and 4458 knee MRI studies to detect meniscal tear and identify pathological cartilages. On test data sets of 5090 and 764 studies, these classifiers achieved 0.84 and 0.85 AUCs on meniscal and cartilage pathologies respectively. On the same training sets, we trained visual explainers to highlight relevant regions for the classifiers on a given input image. For evaluation, we first assessed if the visual explainer can localise relevant regions matching clinician expectations. One expert clinician annotated 71 (respectively 38) test images with boxes tightly bounding pathological regions for meniscal (respectively cartilage) pathologies. We measured the distance between centres of the ground truth and a derived box from visual explanation respectively. We also reported the localisation accuracy defined as the success rate of this explanation to contain the ground truth centre. To solely evaluate visual explanation fidelity to classifiers, we assessed if pixels with the highest values in explanation maps had also the greatest impact on the classifiers when their value was changed.
**Results or Findings:** We reported respective localization accuracies of 0.845 and 0.705 and distance between centres of 5.61+/-4.56mm and 8.21+/-5.9mm. Regarding visual explanation fidelity to classifiers, our method outperformed state-of-the-art techniques in 99% of our tests.
**Conclusion:** Our visual explanation method accurately highlights crucially important regions for classifiers. It thus contributes to classifiers evaluation by non experts and create confidence in their clinical use.
**Limitations:** No limitations were identified.
**Ethics committee approval:** This study was approved by IRB number: CRM-2006-099.
**Funding for this study:** No funding was received for this study.
**Author Disclosures:**
Martin Charachon: Employee: Incepto Medical
Pierre Guetat: Nothing to disclose
Gaspard D'Assignies: Employee: Incepto Medical
Etienne Pluot: Nothing to disclose
Tom Vesoul: Employee: Incepto Medical
Roberto Ardon: Employee: Incepto Medical

**14:00-15:30** **Room M 1**

### Research Presentation Session: GI Tract

### RPS 1001b

### Novelties in hepatobiliary imaging

**Moderator**
F. Vernuccio; Padova/IT

### RPS 1001b-2

**Is there any role for multiparametric MRI of the liver in follow-up of liver-transplant patients?**

*B. Henninger*, E. Josip, B. Schäfer, M. Plaikner, P. Schullian, H. Zoller, C. Kremser; Innsbruck/AT

**Purpose:** Follow-up of liver-transplant patients is a complex process in which many purposes should be fulfilled. Imaging mainly plays a role in detecting malignancy with ultrasound being the method of choice. There are surprisingly little data on the most effective method of follow-up. Our study aims to evaluate multi-parametric MRI (mpMRI) of the liver as a tool in follow-up of transplant recipients.
**Methods or Background:** We retrospectively included 237 liver-transplant recipients (169 male, 68 female; 54.4 years mean age). 91/237 patients had multiple mpMRI scans resulting in a total of 336 MRI investigations being evaluated in this study. All patients received mpMRI for follow-up consisting of Gd-EOB-DTPA contrast-enhanced imaging, MR-elastography, quantification of liver iron (R2*), fat (PDFF) and liver function with relative liver enhancement (RLE). These parameters were calculated and documented. Further, all examinations were re-evaluated with regard to pathological findings with a special focus on malignancies.
**Results or Findings:** 65/336 showed steatosis of the transplant (PDFF>5.6%). In only two patients mild iron overload (R2*>160, i.e. >75umol/g iron) was detected. Fibrosis stage F1 or higher was found in 58/336 with 27/58 showing relevant fibrosis stage F2 or higher. In only 1/27 F2 fibrosis patients concomitant steatosis was found. RLE had a very weak correlation with MRE (r=0.16) and PDFF (r=0.04). In only three cases malignancy was detected by MRI: HCC, metastasis of a neuroendocrine tumour and cholangiocellular carcinoma. These three were also detected during routine sonography.
**Conclusion:** mpMRI is an over-the-top method for liver-transplant patients concerning detection of malignancy. Nevertheless, the quantification and diagnosis of fibrosis or steatosis of the transplant are relevant features that could influence management in this patient cohort.
**Limitations:** The limitation is the study's retrospective character.
**Ethics committee approval:** This study was approved by the local ethics committee.
**Funding for this study:** No funding was received for this study.
**Author Disclosures:**
Peter Schullian: Nothing to disclose
Benedikt Schäfer: Nothing to disclose
Michaela Plaikner: Nothing to disclose
Ena Josip: Nothing to disclose
Christian Kremser: Nothing to disclose
Benjamin Henninger: Nothing to disclose
Heinz Zoller: Nothing to disclose

### RPS 1001b-3

**Golden-angle radial sparse parallel dynamic contrast enhanced MRI: automatic detection and characterisation of liver lesions using contrast agent behaviour**

*M. T. Meyer*[1], H-C. Breit[1], S. Yang[1], K. T. Block[2], E. M. Merkle[1], T. Heye[1]; [1]Basel/CH, [2]New York, NY/US

**Purpose:** Golden-angle Radial Sparse Parallel (GRASP) dynamic contrast-enhanced (DCE) magnetic resonance imaging (MRI) technique combines golden-angle radial k-space sampling, compressed sensing and parallel imaging reconstruction allowing higher spatial and temporal resolution compared with standard DCE-MRI techniques. Reading DCE-MRI data is time-consuming and error-prone since images at different time points need to be evaluated. The aim of this study is to condense contrast agent behaviour of liver lesions in a simple, quick-to-grasp way.
**Methods or Background:** Experiments were conducted on GRASP-MRI scans after the application of gadoxetate disodium contrast agent (CA). The mean temporal resolution was 6.5 – 10.5 s with larger intervals towards the end. 7 patients with previously untreated, hypervascularised hepatocellular carcinoma (HCC; LI-RADS 4 – 5) and 5 patients with benign, late-enhancing lesions were analysed. Using custom-made software, the contrast signal of each voxel was normalised using the signal of surrounding liver tissue to find voxels with aberrant signal behaviour. Within HCC lesions, the mean normalised signal in the early arterial and portal-venous phases was extracted to derive a template signal for early enhancement. To find voxels with similar contrast behaviour, a heatmap was generated by correlating each voxel with the early enhancement template signal. For lesions with late enhancement, an analogous procedure was employed.
**Results or Findings:** Preliminary results of our experiments show that hypervascularised, early-enhancing lesions and late-enhancing, CA-retaining lesions demarcate well on their respective heatmaps. By thresholding the heatmaps, accurate masks of homogenous lesions may be generated. Early experiments with other types of lesions (e.g. cysts, lesions with CA-washout) showed promising results.
**Conclusion:** Using simple methods, distinct and potentially diagnostic patterns of contrast behaviour as well as areas with aberrant spatiotemporal signal behaviour may be visualised in GRASP-DCE-MRI.
**Limitations:** The limitations are the experimental design and preliminary results.
**Ethics committee approval:** Not applicable.
**Funding for this study:** Not applicable.
**Author Disclosures:**
Kai Tobias Block: Nothing to disclose
Tobias Heye: Nothing to disclose
Hanns-Christian Breit: Nothing to disclose
Elmar M. Merkle: Nothing to disclose
Manfred Tobias Meyer: Nothing to disclose
Shan Yang: Nothing to disclose

### RPS 1001b-4

**Quantification of hepatic steatosis with a novel attenuation imaging (ATI) ultrasound technique (QAI): preliminary findings on feasibility, reproducibility and diagnostic accuracy**

*M. Garcovich*, L. Riccardi, M. A. Zocco, M. E. Ainora, F. Pizzolante, N. De Matthaeis, A. Gasbarrini, M. Pompili; Rome/IT
*(matteogarcovich@yahoo.it)*

**Purpose:** In recent years various ultrasound (US) techniques, such as attenuation imaging (ATI), have been developed to quantitively assess the hepatic fat content. The aim of the study was to assess technical feasibility and reproducibility (both intra- and inter-observer) of ATI with QAI (Esaote) in healthy volunteers and in patients with suspected steatosis. The secondary aim was to evaluate the correlation of QAI with hepatic steatosis assessed by standard US and to assess diagnostic accuracy for steatosis quantification.
**Methods or Background:** This prospective study included two different study groups, composed of healthy volunteers (group 1,n=25) and patients with suspected hepatic steatosis (group 2,n=61); among them 28 patients also underwent liver biopsy. In group 1 two operators performed both US and two sessions of ATI respectively. Intra-class correlation coefficients (ICCs) were used to assess the intra-observer and inter-observer reproducibility in group 1. In group 2, QAI values were correlated with the degree of hepatic steatosis using Spearman rank correlation analysis. Temptative cut-off values for steatosis were calculated with ROC analysis as compared to liver histology.
**Results or Findings:** For the intra-observer reproducibility of ATI, the ICC was 0.932 (95%CI,0.854-0.969); inter-observer reproducibility showed an ICC of 0.902 (95%CI,0.793-0.955). QAI measurements showed a significant correlation with the visual grade of hepatic steatosis (rho 0.860;$p<0.001$). ATI enabled the identification of mild steatosis (greater than 0) with an AUC of 0.97 with an optimal cut-off of 0.60 dB/cm/MHz.
**Conclusion:** ATI imaging with QAI showed high intra- and inter-observer reproducibility in healthy volunteers. Correlation between QAI and hepatic steatosis assessed by standard US is very good. Our study identifies for the first time normal values of QAI in healthy volunteers and preliminary cut-off thresholds for steatosis staging in NALFD patients.
**Limitations:** No limitations were identified.
**Ethics committee approval:** This study was approved by an ethics committee.
**Funding for this study:** No funding was received for this study.
**Author Disclosures:**
Fabrizio Pizzolante: Nothing to disclose
Nicoletta De Matthaeis: Nothing to disclose
Maria Elena Ainora: Nothing to disclose
Maurizio Pompili: Nothing to disclose
Matteo Garcovich: Nothing to disclose
Laura Riccardi: Nothing to disclose
Antonio Gasbarrini: Nothing to disclose
Maria Assunta Zocco: Nothing to disclose

### RPS 1001b-5

**Virtual portal vein pressure gradient based on CFD model: a noninvasive procedure with CT angiography for the diagnosis of portal hypertension**

*S. Wan*[1], L. Ren[2], Y. Wei[1], X. He[2], B. Song[1]; [1]Chengdu/CN, [2]Beijing/CN

**Purpose:** We aimed to develop a virtual model for estimating portal vein pressure gradient (PVPG) noninvasively on computed tomography (CT) images using computational fluid dynamics (CFD) analysis, which we termed virtual PVPG (vPVPG), to allow the noninvasive diagnosis of portal hypertension (PH) in patients with cirrhosis.
**Methods or Background:** Sixty-one patients were included in this retrospective study from August 2021 to December 2021, patients were divided into a PH group (n=37) and a non-PH group (n=24) according to the history of portal hypertension. Patients in the PH group underwent the transjugular intrahepatic portosystemic shunt (TIPS) procedure for the transjugular PVPG measurement, which was used as the reference standard for the diagnosis of PH and the CFD simulation. All participants in the two groups underwent CT angiography. The model of the portal venous system was reconstructed using 3D-slicer software, afterwards, vPVPG was computed with the reconstructed three-dimensional model and the CFD simulation.
**Results or Findings:** The ratio of vPVPG was identified statistically significant for discriminating the PH group from the non-PH group ($p<0.05$). The area under the receiver operating characteristic curve (AUC) of vPVPG in the diagnosis of PH was 0.857 (95% confidence interval[CI]: 0.728, 0.93), with a sensitivity of 81.08%, and a specificity of 83.33% when using a cut-off value of 0.659. The inter-observer's agreement was 0.88, suggesting the good reproducibility of vPVPG measurements.
**Conclusion:** The computational model of virtual portal vein pressure gradient (vPVPG) may have the potential for the noninvasive diagnosis of portal hypertension, which may serve as a surrogate of the invasive transjugular PVPG measurement.
**Limitations:** The data processing of this study is relatively time-consuming, the average time for the vPVPG calculation was 1 hour.
**Ethics committee approval:** This study was approved by the West China Hospital Ethics Committee.
**Funding for this study:** Not applicable.
**Author Disclosures:**
Yi Wei: Nothing to disclose
Xiaowei He: Nothing to disclose
Shang Wan: Nothing to disclose
Bin Song: Nothing to disclose
Lixin Ren: Nothing to disclose

### RPS 1001b-6

**Prediction of hepatic decompensation in patients with primary sclerosing cholangitis using body composition model and machine learning-based combined topological and radiomics features**

*Y. Singh*, J. Sobek, P. Korfiatis, J. Eaton, B. J. Erickson; Rochester, AL/US
*(yashbir143@gmail.com)*

**Purpose:** This study aims to develop a tool of a machine learning-based combination of topological and radiomics feature to predict hepatic decompensation status using the Body Composition model.
**Methods or Background:** A total of 24 patients were included: 8 had hepatic decompensation, 8 were allocated to the no hepatic decompensation group during 5-fold cross-validation, and 8 were left out for the external validation set. A body composition model was used to segment subcutaneous adipose tissue, skeletal muscle, visceral adipose tissue, and intermuscular adipose tissue. Using the pyradiomics library, and using persistence homology, we extracted both features, and we trained a logistic regression model using these derived features as input.

**Results or Findings:** The AUC was calculated for both the training data set and the validation data set. The logistic regression classifier obtained an average AUC of 0.8 from 5-fold cross-validation. Then the best performance of AUC =0.8 was calculated for hepatic decompensation in the external validation data set.
**Conclusion:** Using PSC as an example, we demonstrated the potential utility of combining radiomic features and topological features for predicting disease progression and have shown for our case that an approach involving both radiomics and topological features gives improved performance compared to using either type of feature alone.
**Limitations:** We used a much smaller number of patients with hepatic decompensation. The study's inclusion criteria are a diagnosis of PSC, and the availability of an abdomen CT acquired during the portal venous phase.
**Ethics committee approval:** The Institutional Review Board approved this study and the informed consent procedure.
**Funding for this study:** This research work has been supported by Halloran PSC Benefactors Fund.
**Author Disclosures:**
Bradley James Erickson: Nothing to disclose
Panagiotis Korfiatis: Nothing to disclose
Joseph Sobek: Nothing to disclose
John Eaton: Nothing to disclose
Yashbir Singh: Nothing to disclose

### RPS 1001b-7

**Assessment of clinical outcome in patients with portal hypertension: quantitative computed tomographic feature may serve as an effective biomarker**
*S. Wan*, Y. Wei, X. Zhang, B. Song; Chengdu/CN

**Purpose:** The level of hepatic venous pressure gradient (HVPG) is related to patient's outcome, which is invasive and considered the gold standard for evaluating portal hypertension. We aimed to evaluate whether texture features from computed tomography (CT) images could be used as a predictive biomarker of outcome in patients with portal hypertension noninvasively.
**Methods or Background:** A total of 65 patients with portal hypertension were included in this retrospective study and divided into progression-free survival (PFS)(n=14) or non-PFS group (n=51) based on patients with rebleeding or death after transjugular intrahepatic portosystemic shunt procedure. All patients underwent contrast-enhanced CT and the laboratory data were recorded, texture features of liver or spleen were extracted in the portal venous phase. Cox proportional-hazards model and Kaplan-Meier analysis were used to determine PFS-related features and C-index was used to determine the diagnostic capabilities.
**Results or Findings:** We found that only the RobustMeanAbsoluteDeviation feature from liver was the significant prognostic predictor obtaining a C-index of 0.72 (95% CI 0.57-0.89), the KM curve was plotted with high- and low-risk groups based on a cut-off value of 4.15 (p<0.05,log-rank test). The median survival time was 20.5 (interquartile range IQR 10.25-39.75) months and 37 (IQR 32.5-41.5) months for high- and low-risk groups, respectively, the 1-, 2-, 3-year survival probabilities were 66.7%, 50%, 33.3% and 93.2%, 91.5%, 84.4% for high- and low-risk groups, respectively. Clinical variable of international normalised ratio (INR) also indicated a moderate prognostic ability for the high-risk group, with a C-index of 0.68 (95% CI 0.54-0.82) when using a cut-off value of 1.36.
**Conclusion:** CT texture feature from liver and INR may be used as effective biomarker for predicting clinical outcome in patients with portal hypertension.
**Limitations:** The sample size of patients for follow-up was limited.
**Ethics committee approval:** This study was approved by our hospital.
**Funding for this study:** Not applicable.
**Author Disclosures:**
Yi Wei: Nothing to disclose
Xin Zhang: Nothing to disclose
Shang Wan: Nothing to disclose
Bin Song: Nothing to disclose

### RPS 1001b-8

**Development and assessment of prognostic value and agreement of a novel MRCP-score for patients with primary sclerosing cholangitis**
*A. Grigoriadis*[1], K. I. Ringe[2], J. Bengtsson[3], E. Fridh-Baubeta[3], C. Forsman[4], N. Korsavidou-Hult[4], N. Kartalis[1], A. Bergquist[1]; [1]Stockholm/SE, [2]Hannover/DE, [3]Lund/SE, [4]Uppsala/SE
*(aristeidis.grigoriadis@ki.se)*

**Purpose:** To develop, evaluate the reproducibility and prognostic value of a novel MRCP-score for patients with primary sclerosing cholangitis (PSC).
**Methods or Background:** The MRCP-score was developed based on the extent and severity of cholangiographic changes of intrahepatic and extrahepatic bile ducts (range 0-8) in coronal 3D-MRCP-sequences. In this ethics review-board-approved retrospective, multicentre study, three pairs of radiologists (each pair from a national tertiary center) applied the score independently on MRCP-examinations of a total of 220 consecutive patients (103, 52, and 65, respectively) with large-duct PSC from a prospectively collected cohort, with a median follow-up of 7.4 years. Interreader and intrareader agreement was assessed with intraclass correlation coefficient (ICC). After consensus reading, the prognostic value of the score was assessed with Cox-regression analysis and outcome-free survival rates with Kaplan-Meier estimates. Area under the curve (AUC) was calculated and a 10-fold cross-validation was performed.
**Results or Findings:** 41 patients developed outcomes (i.e., liver transplantation and liver-related death). Interreader agreement for radiologists from two centers was good (ICC=0.82, 95%CI: 0.74-0-87 and ICC=0.81, 95%CI: 0.70-0.87, respectively). Intrareader agreement was from good to excellent (ICC=0.93, 95%CI: 0.90-0.95, and ICC=0.87, 95%CI: 0.80-0.92, respectively). Patients with MRCP-score 5-8 had 7.5 times higher risk (HR=7.5; 95%CI:3.6-15.8) for developing outcomes, and significantly lower survival rates (p<0.000), compared to those with MRCP-score 0-4. AUC for the MRCP-score was 0.82. After cross-validation, the results did not change.
**Conclusion:** The novel MRCP-score is reproducible and strongly associated to outcomes which indicates its value for the prognosis of PSC patients.
**Limitations:** The limitation is that there is no external validation.
**Ethics committee approval:** This study was approved by an ethic committee (Dnr: 2011/824-31/2, 2018/1494-31/3, 2018/1497-31/3, 2016/1261-32, 2018/1111-32).
**Funding for this study:** This study has received funding by Stockholm County Council and Cancer Research Funds of Radiumhemmet.
**Author Disclosures:**
Nikolaos Kartalis: Nothing to disclose
Cecilia Forsman: Nothing to disclose
Aristeidis Grigoriadis: Nothing to disclose
Kristina Imeen Ringe: Nothing to disclose
Annika Bergquist: Nothing to disclose
Erik Fridh-Baubeta: Nothing to disclose
Nafsika Korsavidou-Hult: Nothing to disclose
Johan Bengtsson: Nothing to disclose

### RPS 1001b-9

**Early tumour shrinkage as a novel imaging biomarker for patients with hepatocellular carcinoma undergoing immunotherapy**
L. Müller, S. J. Gairing, R. Kloeckner, F. Förster, A. Weinmann, C. Düber, *F. Hahn*; Mainz/DE

**Purpose:** Early Tumour Shrinkage (ETS) has been identified as a promising imaging biomarker for patients undergoing immunotherapy in several cancer entities. This study aimed to validate the potential of ETS as imaging biomarker for patients with hepatocellular carcinoma (HCC) undergoing immunotherapy.
**Methods or Background:** All patients with HCC treated with immunotherapy in first or subsequent lines at our tertiary care centre between 2016 and 2021 were screened for eligibility. ETS was calculated as the size reduction of the target lesions between initial imaging and first FU. ETS was compared to the radiologic response according to mRECIST. Furthermore, the influence of ETS on overall survival (OS), progression-free survival (PFS) and alpha-fetoprotein (AFP) response was calculated.
**Results or Findings:** A total of 39 patients with available cross-sectional imaging at baseline and within 8-14 weeks after the baseline of immunotherapy treatment were included in the final analysis. Median ETS was 5.4% in our study. ETS significantly correlated with the response according to mRECIST and with the AFP response. Patients with an ETS ≥10% had a significantly longer survival after the first FU (15.1 months vs 4.0 months, p=0.008). Additionally, patients with disease control according to mRECIST and an ETS ≥10% had a longer PFS after the initial FU as well (23.6 months vs 2.4 months, p<0.001).
**Conclusion:** ETS was strongly associated with survival outcomes in patients with HCC undergoing immunotherapy. Thus, ETS as an easily assessable imaging biomarker has the potential to timely identify HCC patients benefitting from immunotherapy.
**Limitations:** The limitation was the single-centre and retrospective design.
**Ethics committee approval:** The Ethics Committee of the Medical Association of Rhineland Palatinate, Mainz, Germany approved this study (permit number 837.199.10).
**Funding for this study:** Not applicable.
**Author Disclosures:**
Roman Kloeckner: Nothing to disclose
Christoph Düber: Nothing to disclose
Felix Hahn: Nothing to disclose
Lukas Müller: Nothing to disclose
Friedrich Förster: Nothing to disclose
Simon Johannes Gairing: Nothing to disclose
Arndt Weinmann: Nothing to disclose

### RPS 1001b-10

**Evaluation of various methods of liver measurement in comparison to volumetric segmentation based on computed tomography**

*M. M. Cebula*, A. A. Biernacka, O. Bożek, B. Kokoszka, M. Kulig-Kulesza, S. Modlińska, M. Winder, J. Pilch-Kowalczyk, K. Gruszczyńska; Katowice/PL
*(maciejmichalcebula@gmail.com)*

**Purpose:** Many measurement methods and formulas based on biometric data are used to calculate the liver volume. To date, it has not been established which of these methods is the best, and the search for new assessment methods continues. The main objective of this work is to compare the various methods of liver measurement with semi-automatic volumetric liver segmentation based on computed tomography.
**Methods or Background:** Eight investigators independently performed a series of liver measurements on one hundred and five computed tomography examinations of the abdominal cavity. Another researcher performed liver segmentation with the liver analysis model of syngo.via software, and the result was taken as the gold standard in the study. The patient's sex, age, weight, and height were noted, then BMI and BSA were calculated. Based on the data obtained, the liver volume was calculated using twelve established methods, then compared with each other and the segmentation result.
**Results or Findings:** A significant difference was observed between all but one method and liver segmentation results. No single measurement made it possible to estimate liver volume reliably. Using stepwise regression, we proposed our model to estimate the liver volume based on the collected biomedical data.
**Conclusion:** Reliable determination of liver volume remains a challenge. Despite the time-consuming nature of volumetric segmentation, it seems to be the optimal solution.
**Limitations:** A retrospective character, number of evaluated CT studies, and no re-evaluation to assess repeatability limits the study.
**Ethics committee approval:** The full ethical review and approval, as well as patient consent, was waived for this study by the Ethical Committee of Medical University of Silesia in Katowice, Poland.
**Funding for this study:** No funding to declare.
**Author Disclosures:**
Monika Kulig-Kulesza: Nothing to disclose
Maciej Michał Cebula: Nothing to disclose
Bartosz Kokoszka: Nothing to disclose
Mateusz Winder: Nothing to disclose
Joanna Pilch-Kowalczyk: Nothing to disclose
Angelika Anna Biernacka: Nothing to disclose
Oskar Bożek: Nothing to disclose
Sandra Modlińska: Nothing to disclose
Katarzyna Gruszczyńska: Nothing to disclose

### RPS 1001b-11

**The role of splenic volume as imaging biomarker in patients with hepatocellular carcinoma under immunotherapy**

L. Müller, S. J. Gairing, R. Kloeckner, F. Förster, C. Düber, A. Weinmann, *F. Hahn*; Mainz/DE

**Purpose:** An association of immunotherapy and splenic volume (SV) increase has been described for patients with various cancer entities. Furthermore, SV and SV increase have been proposed as prognostic factors towards patients' survival. This study aimed to evaluate SV, its changes, and its prognostic influence on survival for patients with hepatocellular carcinoma (HCC) under immunotherapy.
**Methods or Background:** All patients with HCC treated with an immunotherapy agent in first or subsequent lines at our tertiary care centre between 2016 and 2021 were included. SV was assessed at baseline and follow-up using a fully automated AI-based tool for spleen segmentation and normalised to body-surface-area. SV was correlated with overall survival (OS) and clinical/laboratory parameters.
**Results or Findings:** In the 50 patients included in this analysis, the median SV prior to treatment was 532ml (IQR 270–784ml). The median OS of patients with high SVs (SV above the median of the patient population) after treatment initiation was 5.1 months, while patients with low SVs had a median OS of 18.1 months (p=0.01). Moreover, patients with a high SV had significantly lower albumin levels, significantly higher bilirubin levels, and significantly lower number of thrombocytes. Increase in SV between treatment start and first follow-up was observed in 28 out of 37 patients (76%) with available follow-up imaging. Change/Increase in SV was no additional prognostic factor for median OS.
**Conclusion:** SV at baseline prior to immunotherapy was a significant prognostic factor and correlated with parameters of liver function. Although a large proportion of HCC patients showed a further increase in SV after immunotherapy, this immuno-modulated SV change did not significantly influence OS in our cohort.
**Limitations:** The limitation is the study's single-centre and retrospective design.
**Ethics committee approval:** The study was approved by the Ethics Committee of the Medical Association of Rhineland Palatinate, Mainz, Germany (number 2021-15984).
**Funding for this study:** Not applicable.
**Author Disclosures:**
Roman Kloeckner: Nothing to disclose
Christoph Düber: Nothing to disclose
Felix Hahn: Nothing to disclose
Lukas Müller: Nothing to disclose
Friedrich Förster: Nothing to disclose
Simon Johannes Gairing: Nothing to disclose
Arndt Weinmann: Nothing to disclose

### RPS 1001b-12

**Myosteatosis can predict unfavourable outcomes in advanced hepatocellular carcinoma patients treated with hepatic artery infusion chemotherapy and anti-PD-1 immunotherapy**

*X. Yi*, H. Lin; Changsha/CN
*(yixiaoping@csu.edu.cn)*

**Purpose:** To evaluate the feasibility of computed tomography (CT) - derived measurements of body composition parameters to predict the risk factor of non-objective response (non-OR) in patients with hepatocellular carcinoma (HCC) undergoing anti-PD-1 immunotherapy and hepatic artery infusion chemotherapy (immune-HAIC).
**Methods or Background:** Patients with histologically confirmed HCC and treated with the immune-HAIC were retrospectively recruited between June 30, 2019, and July 31, 2021. CT-based estimations of body composition parameters were acquired from the baseline unenhanced abdominal CT images at the level of the third lumbar vertebra (L3) and were applied to evaluate its role in predicting the probability of OR. Associations among predictors and gene mutations were also examined by correlation matrix analysis.
**Results or Findings:** Fifty-two patients were recruited to this study cohort, with 30 patients having a OR status after immune-HAIC treatment. Estimations of myosteatosis parameters, like SM-RA (skeletal muscle radiation attenuation), were significantly associated with the probability of predicting OR (P=0.007). The SM-RA combined nomogram model, including serum red blood cells, hemoglobin, creatinine, and the mean CT value of visceral fat (VFmean) improved the prediction probability for OR disease with an AUC of 0.713 (95% CI, 0.75 to 0.95).
**Conclusion:** The CT-based estimations of myosteatosis could be used as an indicator to predict a higher risk of transition to the non-OR disease state in HCC patients treated with immune-HAIC therapy. This study demonstrated the therapeutic relevance of skeletal muscle composition assessments in the overall prediction of treatment response and prognosis in HCC patients.
**Limitations:** First, it was a single-centred retrospective study that might involve the case selection bias. Second, the number of cases with non-OR was relatively small.
**Ethics committee approval:** This study was approved by the Medical Ethics Committee of the Xiangya Hospital (20220111109).
**Funding for this study:** No funding was received for this study.
**Author Disclosures:**
Huashan Lin: Nothing to disclose
Xiaoping Yi: Nothing to disclose

**14:00-15:30** **Room X**

### Research Presentation Session: Physics in Medical Imaging

### RPS 1013

### Photon counting and dual-energy CT

**Moderator**
C. H. McCollough; Rochester, MN/US
**Author Disclosure:**
C. H. McCollough: Board Member: International Society of Computed Tomography; Employee: Mayo Clinic; Equipment Support Recipients: Siemens Healthcare; Research Grant/SupportSiemens Healthcare

### RPS 1013-2

**Phantom imaging evaluations of a prototype full-size photon counting CT system at clinical dose levels**

*X. Zhan*, R. Zhang, X. Niu, B. Budden, I. Hein, S. Wu, Y. Qiang, Z. Yu, R. Thompson; Vernon Hills, IL/US
*(xzhan@mru.medical.canon)*

**Purpose:** Recent studies have demonstrated that semiconductor-based photon counting CT (PCCT) has the potential to provide better contrast and noise performance compared to conventional scintillator-based systems. With multi-energy threshold detection, it can provide additional spectral information and enable material decomposition to better differentiate different materials. With much smaller detector pixel size, the system can be configured at different pixel summing modes, and generate images with different spatial resolutions.
**Methods or Background:** To explore PCCT system benefits and limits, an engineering prototype is designed and built based on a Canon Aqilion ONE ViSION™ system. The CdZnTe-based photon counting detector array populates the full 50cm FOV in the fan angle. A 3x3 macro-pixel grid size is used for standard resolution image reconstruction, and generate equivalent resolution images as the conventional system. Each micro-pixel can output up to 6 energy bins of counting measurements starting from 20keV. Scans were acquired using axial protocol with 120kVp, 1s per rotation, and tube current between 50 – 400mA. Different sizes of phantoms were scanned and reconstructed at different dose levels. The counting images were generated based on the events with photon energy >30keV, and the spectral images were generated using 5 energy bins with threshold setting of 30/45/55/65/80keV respectively.
**Results or Findings:** In this work, comprehensive IQ evaluations including noise, resolution, HU uniformity are presented for both counting and spectral images for various phantoms. Some preliminary results on the high-resolution imaging studies are also presented and compared with the standard mode images.
**Conclusion:** We have designed and built a full-size photon counting CT prototype system, and performed counting and spectral imaging evaluations with various phantoms. The generated images prove its capability to scan at clinical dose levels with excellent quantitative accuracy.
**Limitations:** Not applicable.
**Ethics committee approval:** Not applicable.
**Funding for this study:** No funding was received for this study.
**Author Disclosures:**
Ilmar Hein: Employee: Canon Medical Research, USA
Zhou Yu: Employee: Canon Medical Research, USA
Xiaofeng Niu: Employee: Canon Medical Research, USA
Richard Thompson: Employee: Canon Medical Research, USA
Yi Qiang: Employee: Canon Medical Research, USA
Ruoqiao Zhang: Employee: Canon Medical Research, USA
Xiaohui Zhan: Employee: Canon Medical Research, USA
Brent Budden: Employee: Canon Medical Research, USA
Shuoxing Wu: Employee: Canon Medical Research, USA

### RPS 1013-3

**Quantum iterative reconstruction for low-contrast liver lesion detection in clinical photon-counting detector CT: Impact on noise texture and diagnostic performance**

*D. Racine*[1], A. Viry[1], T. Sartoretti[2], F. Becce[1], V. Mergen[2], D. C. Rotzinger[1], H. Alkadhi[2], A. Euler[2]; [1]Lausanne/CH, [2]Zurich/CH
*(damien.racine@chuv.ch)*

**Purpose:** To assess the impact of a novel iterative reconstruction algorithm (Quantum Iterative Reconstruction, QIR) for clinical dual-source photon-counting detector CT (PCD-CT) on noise texture and diagnostic performance for the detection of low-contrast liver lesions.
**Methods or Background:** An intermediate-sized anthropomorphic abdomen phantom (QRM, Germany) with a liver insert containing two different 5 x 7.5mm cylindrical iodinated liver lesions (contrast of +30 HU and -30 HU) was imaged on a clinical dual-source PCD-CT (NAEOTOM Alpha, Siemens Healthcare GmbH) using a routine abdominal CT protocol at three dose levels (CTDIvol of 5, 2.5 and 1.25mGy). Virtual monoenergetic images (VMI) were reconstructed at two energy levels of 50 and 60keV. Each of those VMIs were reconstructed without QIR (QIR-off) and with QIR at four strength levels and noise texture was characterised by the Noise Power Spectrum (NPS). Lesion detectability was assessed using a channelised Hotelling (CHO) model observer with ddog channels. The area under the ROC curve (AUC) was used as figure of merit.
**Results or Findings:** At each dose level, increasing QIR-levels substantially decreased noise magnitude (from 19% to 64%) while preserving a comparable noise texture to QIR-off. Lesion detectability increased when increasing QIR-level and radiation dose. At 2.5 mGy, detectability was equal to 0.89, 0.90, 0.91, 0.93, 0.94 for QIR-off to QIR-4, respectively, at 50keV and 0.97, 0.98, 0.98, 0.99, 0.99 for QIR-off to QIR-4, respectively, at 60keV. On average, VMI at 60keV provided 2.5%, 7.1% and 14.1% higher detectability as compared to 50keV at 5, 2.5 and 1.25mGy.
**Conclusion:** In abdominal PCD-CT, high levels of QIR in combination with VMI at 60keV decreased image noise and improved lesion detectability without compromising image texture. This holds the potential for radiation dose reduction while preserving diagnostic performance.
**Limitations:** Not applicable.
**Ethics committee approval:** Not applicable.
**Funding for this study:** Not applicable.
**Author Disclosures:**
Victor Mergen: Nothing to disclose
Anaïs Viry: Nothing to disclose
Damien Racine: Nothing to disclose
David Christian Rotzinger: Nothing to disclose
Thomas Sartoretti: Nothing to disclose
Fabio Becce: Other: Research agreement with MARS Bioimaging Other: Research agreement for dual-energy CT with Siemens Healthineers
Andre Euler: Nothing to disclose
Hatem Alkadhi: Nothing to disclose

### RPS 1013-4

**Quantitative image quality comparison between a prototype full-size photon counting CT system and conventional CT systems with energy integrating detectors**

*K. Nomura*[1], K. Fujii[2], Y. Muramatsu[1], T. Kobayashi[1], R. Zhang[3], X. Zhan[3], X. Niu[3], Z. Yu[3], A. Nishijima[4]; [1]Kashiwa/JP, [2]Nagoya/JP, [3]Vernon Hills, IL/US, [4]Otawara/JP
*(kenomura@east.ncc.go.jp)*

**Purpose:** Photon counting CT (PCCT) has been shown to offer improved contrast and noise performance than traditional CT systems with scintillator-based energy integrating detectors (EID-CT), in addition to its capability of spectral separation and material differentiation due to multi-energy detection and acquisition. In this study, we compare a full-size CdZnTe-based engineering prototype PCCT system with conventional EID-CT systems at similar scan conditions and evaluate image quality in multiple aspects.
**Methods or Background:** The prototype PCCT system is built based on a Canon AquilionONE ViSION™ system, which provides coverage of 50cm in fan angle. Summation of 3x3 micro-pixels is used for normal resolution reconstruction, which produces image resolution equivalent to that of conventional EID-CT, while high resolution image is reconstructed using 1x1 micro-pixels. In this study, scans were acquired in axial mode at 120kVp with 1sec rotation and tube current from 50 to 400mA. Phantoms of various sizes, contrast levels, and material concentrations were scanned. CT number consistency, noise, spatial resolution, and material quantification accuracy were evaluated and compared.
**Results or Findings:** PCCT images show consistent CT number across different mA levels at multiple phantoms. At comparable spatial resolution, PCCT reduces noise significantly as compared to EID-CT, especially at low dose. Improved spatial resolution is achieved by PCCT in high resolution mode. PCCT and EID-CT have comparable material quantification accuracy for contrast materials.
**Conclusion:** Quantitative image comparison between a prototype full-size PCCT and conventional EID-CT shows, PCCT reduces image noise considerably as compared to EID-CT at similar spatial resolution, while PCCT also allows for higher image resolution.
**Limitations:** The beam width is different in scanning conditions between CT systems.
**Ethics committee approval:** Not applicable. This presentation is a phantom study.
**Funding for this study:** This is a collaborate research with Canon Medical Systems Corporation.

**Author Disclosures:**
Akira Nishijima: Employee: Canon Medical SysteCorporation
Yoshihisa Muramatsu: Nothing to disclose
Keiichi Nomura: Nothing to disclose
Zhou Yu: Employee: Canon Medical Research USA, Inc.
Xiaofeng Niu: Employee: Canon Medical Research USA, Inc.
Tatsushi Kobayashi: Nothing to disclose
Keisuke Fujii: Nothing to disclose
Ruoqiao Zhang: Employee: Canon Medical Research USA, Inc.
Xiaohui Zhan: Employee: Canon Medical Research USA, Inc.

### RPS 1013-5

**Improved stent imaging & visualisation with photon counting CT: a phantom study**

*J. O Doherty*[1], J. A. Decker[2], T. Flohr[3], B. Schmidt[3], T. Allmendinger[3], U. J. Schoepf[4], A. Varga-Szemes[4], F. Risch[2], T. S. Emrich[4]; [1]Malvern, PA/US, [2]Augsburg/DE, [3]Forchheim/DE, [4]Charleston, SC/US
*(james.odoherty@siemens-healthineers.com)*

**Purpose:** To demonstrate imaging benefits of photon counting CT (PCCT) in the assessment of stent visibility using an in vitro phantom with and without the presence of iodine contrast.
**Methods or Background:** Scans were performed on a Siemens NAEOTOM Alpha PCCT. A phantom was created with stent sizes from 2.5mm-9mm diameter, placed within silicone tubings to simulate vessels. The tubings were placed in a water tank and filled with increasing concentrations of iodine (I_conc) to provide 0, 200, 400 and 600 HU attenuation. Data were reconstructed using 2 kernels as virtual monoenergetic images (VMI) at 70 keV (pixel size=0.4 mm), and a polyenergetic image in ultrahigh resolution (UHR) of 0.2mm. Conventional CT scans were also reconstructed at 0.4mm and 0.6mm. Spectral data was post-processed to examine removal of contrast using a virtual non-iodine (VNI) algorithm. Stent lumen visibility was calculated for all images.
**Results or Findings:** Bv60 reconstruction demonstrated 3-10% higher stent visibility for all values of Iconc compared to a Bv40 kernel. Stent visibility was 5-10% higher for a conventional CT scan at 0.4mm than 0.6mm over all I_conc. VNI images demonstrated successful removal of iodine for all I_conc, with HU equivalent to that of the water only tubings (-6.8±13.9 HU). Stent struts were better distinguished on the UHR scan, including in one case a damaged 2.5 mm stent.
**Conclusion:** PCCT demonstrated excellent imaging characteristics for in vitro imaging of stents in a phantom setup. The best stent lumen visibility was achieved in UHR mode (0.2 mm). Spectral data allowed added processing steps unavailable with conventional CT systems.
**Limitations:** Simulated setup only. Influences such as patient size/thickness, stent direction, presence of calcium, exposure parameters and motion were not evaluated.
**Ethics committee approval:** Not applicable.
**Funding for this study:** This project received support from Siemens Healthineers.
**Author Disclosures:**
Thomas Flohr: Employee: Siemens Healthineers
Franka Risch: Nothing to disclose
Thomas Allmendinger: Employee: Siemens Healthineers
Uwe Joseph Schoepf: Grant Recipient: Medical University of South Carolina
Tilman Stephan Emrich: Research/Grant Support: Medical University of South Carolina
Jim O Doherty: Employee: Siemens Medical Solutions
Bernhard Schmidt: Employee: Siemens Healthineers
Josua A. Decker: Nothing to disclose
Akos Varga-Szemes: Nothing to disclose

### RPS 1013-6

**Performance evaluation of (ultra) high resolution photon-counting CT of the lungs: an anthropomorphic lung vessel phantom study**

*I. Hernandez-Giron*[1], R. Booij[2], A. Odink[2], R. P. J. Budde[2], Z. Zhai[3], W. Veldkamp[1], A. Van Der Lugt[2], B. Stoel[1], M. van Straten[2]; [1]Leiden/NL, [2]Rotterdam/NL, [3]Amsterdam/NL
*(irene.debroglie@gmail.com)*

**Purpose:** To objectively assess and compare the performance of imaging lung vasculature by photon-counting detector (PCD) and energy-integrating detector (EID) CT-scanners in both high and ultra-high resolution (HR, UHR) imaging, combining an anthropomorphic phantom and an automated lung vessel detection algorithm.
**Methods or Background:** A 3D printed, lung-vessel phantom (Visijet_EX-200 material, vessel radii 0.13-4.0mm range), inside a PMMA thorax-shaped holder, was scanned on a PCD-based (Siemens NAEOTOM Alpha) and an EID-based CT (Siemens SOMATOM Force). Two protocols were selected: routine-thorax HR (CTDIvol=2.4mGy) and UHR (CTDIvol=2.0mGy), 5 repetitions, image reconstruction: matching spacing, slice thicknesses, kernels and all iterative strength levels (PCD-QIR: [Q1-Q2-Q3-Q4]; EID-ADMIRE: [20%-40%-60%-80%-100%]). Vessels were automatically segmentated and classified (0.25mm radius intervals) by an in-house developed graph-cuts method with vesselness filters, and median pixel values (MPV, impact on attenuation) and inter-quartile range (IQR, noise) calculated.
**Results or Findings:** The vessel-air contrast-to-noise ratio was higher for PCD- than for EID-based-CT, even at the thinnest slices setup (0.2mm vs 0.6mm). In both systems and protocols, noise (IQR) decreased with increasing IR strength. Vessels attenuation remained virtually constant for radii>1.5mm (UHR). For radii<1.5mm, MPVs dropped due to partial volume effect and false positives in air (noise). For HR-scans (1.0mm), vessels-IQR (noise) did not significantly differ between PCD and EID scanners. For UHR and equivalent IR strenghts, image noise was 30% lower for 0.2mm PCD-images (Q2-IR 50%) than for 0.6mm EID-images (ADMIRE-60%).
**Conclusion:** Thanks to PCD-technology maturity, UHR-chest-CT allows for improved image quality and lower image noise when compared to EID-based-technology in an anthropomorphic phantom study, with the potential of dose reduction in clinical protocols.
**Limitations:** Phantom vessels´ contrast is higher than patients´ due to parenchyma absence. Future phantoms will mimic more realistically a wider range of patients´ morphometries and overall attenuations.
**Ethics committee approval:** Not applicable.
**Funding for this study:** Funding was received from Phantom:CLUES-project-(NWO funding_nr:13592).
**Author Disclosures:**
Ricardo P. J. Budde: Nothing to disclose
Aad Van Der Lugt: Nothing to disclose
Arlette Odink: Nothing to disclose
Marcel van Straten: Nothing to disclose
Zhiwei Zhai: Nothing to disclose
Wouter Veldkamp: Nothing to disclose
Ronald Booij: Nothing to disclose
Irene Hernandez-Giron: Research/Grant Support: Irene Hernandez received a NWO-Talent Programma personnal Veni grant (project 17378-Through the eyes of AI)
Berend Stoel: Nothing to disclose

### RPS 1013-7

**Dose reduction in coronary artery calcium scoring using mono-energetic images from reduced tube voltage dual-source photon-counting CT data: a dynamic phantom study**

M. v. Gent[1], N. R. van der Werf[2], R. Booij[2], D. Bos[2], A. Van Der Lugt[2], R. P. J. Budde[2], M. Greuter[1], *M. van Straten*[2]; [1]Groningen/NL, [2]Rotterdam/NL

**Purpose:** To assess the reproducibility of coronary artery calcium (CAC) quantification for photon-counting computed tomography (PCCT) at reduced tube voltages.
**Methods or Background:** An anthropomorphic thorax phantom with low, medium and high density CAC inserts was scanned on a dual-source PCCT (Siemens NAEOTOM Alpha), using five different imaging protocols: 1) 120 kVp standard dose (reference); 2) 90kVp at standard; 3) 75%; and 4) 45% dose; and 5) Sn100 kVp standard dose. For each protocol, a robotic arm simulated coronary artery movement of heart rates of 0 and 60-75 beats per minute (bpm). Each scan was repeated five times. Images were reconstructed according to the clinical CAC protocol, using mono-energetic (monoE) reconstructions at 70keV (equivalent to 120kVp). For each heart rate Agatston scores were compared with the reference. Deviations in Agatston score of >10% were deemed clinically relevant.
**Results or Findings:** Volumetric CT dose index was 4.06mGy for the reference protocol. Protocols 2, 3, 4, and 5 resulted in 27%, 44%, 67%, and 46% dose reduction, respectively. For the low density CAC at 0 bpm, clinically relevant lower median Agatston scores were found for all 90kVp acquisitions. At 60-75bpm, 90kVp at standard dose overestimated the Agatston score by 15%. For the medium and high density CAC, the used 90 kVp protocols and heart rates revealed no clinically relevant differences in Agatston score based on the 95% confidence intervals.
**Conclusion:** PCCT allows for reproducible Agatston scores at a reduced tube voltage of 90kVp with radiation dose reductions up to 67% for medium and high density CAC.
**Limitations:** Not applicable.
**Ethics committee approval:** Not applicable.
**Funding for this study:** Not applicable.
**Author Disclosures:**
Niels R. van der Werf: Nothing to disclose
Ricardo P. J. Budde: Nothing to disclose
Aad Van Der Lugt: Nothing to disclose
Marcel van Straten: Other: Research collaboration, Siemens Healthineers
Ronald Booij: Other: Ongoing research collaboration on CT with Siemens Healthineers
Daniel Bos: Nothing to disclose
Margo van Gent: Nothing to disclose
Marcel Greuter: Nothing to disclose

**RPS 1013-8**

**Comparison of image quality between spectral photon-counting CT and dual-layer CT for the evaluation of lung nodules: a phantom study**

*S. A. Si-Mohamed*[1], J. Greffier[2], J. Miailhes[1], S. Boccalini[1], P-A. Rodesch[1], N. Van Der Werf[3], L. Boussel[1], J-P. Beregi[2], P. Douek[1]; [1]Lyon/FR, [2]Nimes/FR, [3]Utrecht/NL
*(salim.si-mohamed@chu-lyon.fr)*

**Purpose:** To evaluate the image quality (IQ) of a spectral photon-counting CT (SPCCT) using filtered back-projection (FBP) and hybrid iterative reconstruction (IR) algorithms (iDose4), in comparison with a dual-layer CT (DLCT) system, and to choose the best image quality according to the IR level for SPCCT.
**Methods or Background:** Two phantoms were scanned using a standard lung protocol (120 kVp, 40 mAs) with SPCCT and DLCT systems. Raw data were reconstructed using FBP and 9 iDose4 levels (i1/i2/i3/i4/i5/i6/i7/i9/i11) for SPCCT and 7 for DLCT (i1/i2/i3/i4/i5/i6/i7). Noise-power-spectrum and task-based transfer function (TTF) were computed. Detectability-index (d') was computed for detection of 4 mm ground-glass nodule (GGN) and solid nodule. Two chest radiologists performed an IQ evaluation (noise/nodule sharpness/nodule conspicuity/overall IQ) in consensus, and chose the best image for SPCCT.
**Results or Findings:** Noise magnitude was -47%±2% lower on average with SPCCT than with DLCT for iDose4 range from i1 to i6. Average NPS spatial frequencies increased for SPCCT in comparison with DLCT. TTF also increased, except for the air insert with FBP, and i1/i2/i3. Higher detectability was found for SPCCT for both GGN and solid nodules. IQ for both types of nodule was rated consistently higher with SPCCT than with DLCT for the same iDose4 level. For SPCCT and both nodules, the scores for noise and conspicuity improved with increasing iDose4 level. iDose4 level 6 provided the best subjective IQ for both types of nodule.
**Conclusion:** Higher IQ for GGN and solid nodules was demonstrated with SPCCT compared with DLCT with better detectability using iDose4.
**Limitations:** We did not evaluate the impact of iDose4 depending on the radiation dose: investigations under low and ultra-low dose conditions need to be performed.
**Ethics committee approval:** Not applicable.
**Funding for this study:** Funding was received from European Union Horizon 2020 grant No 668142.
**Author Disclosures:**
Jean-Paul Beregi: Nothing to disclose
Niels Van Der Werf: Nothing to disclose
Salim Aymeric Si-Mohamed: Nothing to disclose
Pierre-Antoine Rodesch: Nothing to disclose
Jade Miailhes: Nothing to disclose
Loïc Boussel: Nothing to disclose
Philippe Douek: Nothing to disclose
Sara Boccalini: Nothing to disclose
Joel Greffier: Nothing to disclose

**RPS 1013-9**

**Iron quantification in dual-source dual-energy photon-counting CT with up to four energy bins**

*S. Sawall*, L. Klein, C. Amato, J. Maier, L. T. Rotkopf, S. Heinze, C. H. Ziener, H-P. Schlemmer, M. Kachelrieß; Heidelberg/DE
*(stefan.sawall@dkfz.de)*

**Purpose:** To evaluate the potential of dual-source dual-energy (DSDE) photon-counting (PC) CT for iron quantification in comparison to today's conventional energy-integrating (EI) DSDE systems.
**Methods or Background:** Images of an abdomen phantom with different sizes (S, M, L) equipped with vials containing iron solutions with varying concentrations were acquired at the SOMATOM CounT CT (Siemens Healthineers, Germany), an experimental photon-counting system housing an EI and PC detector. Acquisitions were performed with 80kV/140kV+Sn and 100kV/140kV+Sn at a dose of 15mGy (CTDI32cm) by subsequent measurements with the PC detector. Besides conventional acquisitions, each source detector thread is capable of acquiring two energy bins simultaneously resulting in a total of 4 bins: [25 keV,Tlow] and [Tlow,eULow] for the low-energy thread and [25keV,Thigh] and [Thigh,140keV] for the high-energy thread with Tx being the used thresholds. Iron maps were computed in a statistically optimal manner exploiting multiple energy bins if available. Reference acquisitions were performed using an energy-integrating DSDE CT (SOMATOM Definition Flash) using the same prefilters, tube voltage combinations and dose levels. The dose- and concentration-normalized contrast-to-noise ratio (CNRDc) was evaluated as figure of merit to compare both technologies.
**Results or Findings:** In acquisitions with only one energy bin per source-detector-thread, DSDE PCCT provides a CNRD increase by a factor of up to 3.3 (S:1.2, M:2.5, L:3.3) compared to EICT. In case of four energy bins and a statistically optimal material decomposition, CNRD is increased by a factor of up to 4.3 (S:2.0, M:3.3, L:4.3) if thresholds are chosen in an optimal manner.
**Conclusion:** Dual-source dual-energy photon-counting CT allows for a highly significant increase in iron detectability compared to today's energy integrating CT.
**Limitations:** No patient data are available since the described experiments would require multiple acquisitions.
**Ethics committee approval:** Not required.
**Funding for this study:** No funding was received for this study.
**Author Disclosures:**
Laura Klein: Nothing to disclose
Christian Herbert Ziener: Nothing to disclose
Lukas Thomas Rotkopf: Nothing to disclose
Sarah Heinze: Nothing to disclose
Carlo Amato: Nothing to disclose
Heinz-Peter Schlemmer: Nothing to disclose
Marc Kachelrieß: Nothing to disclose
Stefan Sawall: Nothing to disclose
Joscha Maier: Nothing to disclose

**RPS 1013-10**

**Comparison of the virtual monoenergetic image quality obtained with two versions of deep learning image reconstruction algorithm for rapid kV-Switching Dual-Energy CT: a phantom study**

*D. Dabli*, J. Frandon, A. Belaouni, P. Akessoul, B. Guiu, J-P. Beregi, J. Greffier; Nîmes/FR
*(Djamel.Dabli@chu-nimes.fr)*

**Purpose:** To compare the image quality of two versions of deep-learning image reconstruction algorithm on virtual monoenergetic images at low-energy levels with rapid kV switching Dual-Energy CT.
**Methods or Background:** Two CT phantoms were scanned with rapid kV-switching Dual-Energy CT (Canon). Three repeated acquisitions on each phantom were performed using classical parameters of abdomen-pelvic examinations and CTDIvol close to 13 mGy. Raw data were reconstructed with two versions of deep-learning image reconstruction algorithm (AiCE V8 and AiCE V10) using three levels (Mild, Standard and Strong). Noise power spectrum (NPS) and task-based transfer function (TTF) were assessed on virtual monoenergetic images (VMI) at 40/50/60/70/80 keV. A detectability index (d') was computed as function of keV to model the detection of iodine concentrations of 0.5, 1 and 2 mg/mL.
**Results or Findings:** The noise magnitude decreased for AiCE V10 compared to V8 at all levels particularly between 40 and 60 keV (-34%±1%) and fav shifted towards the high frequencies. The TTF at 50% increased with all levels of AiCE V10 compared to V8 and all energy levels (58%±9% for Mild, 17%±8% for Standard and 31%±11% for Strong). The d' increased strongly for all AiCE V10 and all iodine concentrations between 40 to 60 keV (133%±39% for 2mg/mL, 86%±26% for 1mg/mL and 50%±24% for 0.5mg/mL). A lower increase was found for 70 and 80 keV (25%±14%, 15%±12%, and 22%±16%, respectively). For all AiCE levels and iodine concentrations, the highest d' was obtained at 60 keV with AiCE V10 and at 70 keV with AiCE V8.
**Conclusion:** Noise magnitude, noise texture, spatial resolution and the detectability of low iodine concentrations were improved in VMI for all levels of AiCE V10 compared to AiCE V8.
**Limitations:** No patient image assessment.
**Ethics committee approval:** Not applicable.
**Funding for this study:** Not applicable.
**Author Disclosures:**
Jean-Paul Beregi: Nothing to disclose
Boris Guiu: Nothing to disclose
Julien Frandon: Nothing to disclose
Joel Greffier: Nothing to disclose
Philippe Akessoul: Nothing to disclose
Asmaa Belaouni: Nothing to disclose
Djamel Dabli: Nothing to disclose

**RPS 1013-11**

**Comparison of virtual monoenergetic imaging between a rapid kilovoltage switching dual-Energy CT with deep-learning and four dual-Energy CTs with iterative reconstruction**

*J. Greffier*[1], S. A. Si-Mohamed[2], B. Guiu[3], J. Frandon[1], P. Douek[2], J-P. Beregi[1], D. Dabli[1]; [1]Nimes/FR, [2]Lyon/FR, [3]Montpellier/FR
*(joel.greffier@chu-nimes.fr)*

**Purpose:** To assess the spectral performance of rapid kV switching Dual-Energy CT (KVSCT-Canon) equipped with a Deep-Learning Spectral Reconstruction (DLSR) algorithm on virtual monoenergetic images (VMIs) at low-energy levels and to compare its performances with four other Dual-Energy CT (DECT) platforms equipped with iterative reconstruction algorithms (IR).
**Methods or Background:** Two CT phantoms were scanned on five DECT platforms: KVSCT-Canon, a fast kV-switching CT (KVSCT-GE), split-filter CT (SFCT), dual-source CT (DSCT), and dual-layer CT (DLCT). Acquisitions were performed on each phantom with a CTDIvol close to 10mGy. Noise power

Thursday spectrum (NPS) and task-based transfer function (TTF) were evaluated from 40 to 80keV of VMIs. A detectability index (d') was computed to model the detection task of two contrast-enhanced lesions as function of keV.
**Results or Findings:** For KVSCT-Canon, the noise magnitude and average NPS spatial frequency (fav) decreased from 40 to 70keV and increased thereafter. Similar noise magnitude outcomes were found for KVSCT-GE but the opposite for fav. For the other DECT platforms, the noise magnitude decreased as the keV increased. For SFCT, DSCT and DLCT, the fav values increased from 40 to 80keV. For all DECT platforms, TTF at 50% (f50) decreased as the keV increased, decreasing spatial resolution. For KVSCT-Canon, d' values peaked at 60 and 70keV for both simulated lesions and from 50 to 70keV for KVSCT-GE. d' decreased between 40 and 70keV for DSCT, DLCT and SFCT. The highest d' values were found for DLCT at 40 and 50keV and for KVSCT-Canon for other keV.
**Conclusion:** For KVSCT-Canon, the highest detectability of contrast-enhanced lesions was found at 60keV. The highest d' values were found for DLCT at 40 and 50 keV and for KVSCT-Canon for other keV.
**Limitations:** Phantom study.
**Ethics committee approval:** Not applicable.
**Funding for this study:** No funding was received for this study.
**Author Disclosures:**
Jean-Paul Beregi: Nothing to disclose
Salim Aymeric Si-Mohamed: Nothing to disclose
Boris Guiu: Nothing to disclose
Julien Frandon: Nothing to disclose
Philippe Douek: Nothing to disclose
Joel Greffier: Nothing to disclose
Djamel Dabli: Nothing to disclose

### RPS 1013-12
**Comparison between three generations of rapid kV-switching dual-energy CT systems on virtual monoenergetic images at low keV: a task-based image quality assessment on phantom data**
*J. Greffier*[1], A. Viry[2], Y. Barbotteau[3], J. Frandon[1], J-P. Beregi[1], D. Dabli[1]; [1]Nimes/FR, [2]Lausanne/CH, [3]Marseille/FR
*(joel.greffier@chu-nimes.fr)*

**Purpose:** To compare the spectral performance of three rapid kV-switching Dual-Energy CT (DECT) systems on virtual monoenergetic images (VMIs) at low-energy levels.
**Methods or Background:** A multi-energy phantom was scanned on three DECT platforms equipped with three different Gemstone Spectral Imaging™ (GSI) platforms: GSI, GSI Pro and GSI Xtream. Acquisitions were performed with a CTDIvol close to 11mGy. For all platforms, raw data were reconstructed using filtered-back projection (FBP) and a hybrid iterative reconstruction algorithm (ASIR-V at 50%; AV50). A deep learning image reconstruction (DLR) algorithm (TrueFidelity™) was used only for the GSI Xtream. The noise power spectrum (NPS) and task-based transfer function (TTF) were evaluated from 40 to 80keV of VMIs. A detectability index (d') was computed to model the detection task of two contrast-enhanced lesions according to the keV level used.
**Results or Findings:** For all GSI platforms, the noise magnitude decreased from 40 to 70keV, and using AV50 compared to FBP. The average NPS spatial frequency (fav) and spatial resolution (TTF50%) were similar from 40 to 70 keV and decreased with AV50 compared to FBP. Compared to AV50, using DLR reduced noise magnitude (-27%±3%) and improved noise texture (10%±0%) without altering spatial resolution (2%±5%). For the two lesions, d' values peaked at 70keV for GSI and GSI-Pro platforms and at 40/50keV for GSI Xtream, for all reconstruction algorithms. The highest d' values were found for the GSI-Xtream with DLR and the lowest for the GSI platform.
**Conclusion:** Differences in image quality were found between the GSI platforms on low keV VMIs. New DLR algorithm on the GSI-Xtream platform reduced noise and improved spatial resolution and detectability without changing the noise texture for VMIs at low keV.
**Limitations:** Phantom study.
**Ethics committee approval:** Not applicable.
**Funding for this study:** No funding was received for this study.
**Author Disclosures:**
Jean-Paul Beregi: Nothing to disclose
Yves Barbotteau: Nothing to disclose
Anaïs Viry: Nothing to disclose
Julien Frandon: Nothing to disclose
Joel Greffier: Nothing to disclose
Djamel Dabli: Nothing to disclose

**16:00-17:00 Room M 2**

### Research Presentation Session: Oncologic Imaging

### RPS 1116
### Pulmonary and skeletal oncology

**Moderator**
G. Milanese; Parma/IT
**Author Disclosure:**
G. Milanese: Speaker: Siemens Healthineers, Bayer Vital/Healthcare, Bracco

### RPS 1116-2
**Association between contrast-enhanced CT radiomic features and gene alterations in advanced non-small cell lung cancer patients**
*L. Rinaldi*, A. Ranghiero, G. Lo Presti, G. Cammarata, I. Attili, F. Botta, G. Funicelli, G. Spitaleri, E. Guerini Rocco; Milan/IT
*(lisa.rinaldi@ieo.it)*

**Purpose:** Targeted therapies have improved the management of advanced non-small cell lung cancer (NSCLC). Non-invasive methods to assess mutational status are warranted to improve therapy personalisation. This study investigated the association between contrast-enhanced CT radiomic features and tumour mutational status assessed from tissue biopsy.
**Methods or Background:** 261 patients affected by advanced NSCLC, tested for EGFR, ALK and KRAS alterations and imaged with CT within +/- 2 months from biopsy and before starting therapy were retrospectively enrolled. 52 patients were prospectively enrolled as validation set. 1118 radiomic features were calculated from the CT images of lung lesions (Pyradiomics). Clinical data were collected including: age, sex, smoke habit, stage at diagnosis, treatments before CT, lesion lobe, lymph node status, presence of distant metastasis, treatment after CT. Using retrospective data as training set, with mutations as outcomes, radiomic features were included in LASSO-logistic regression models. A radiomic score (RS) was created using the coefficients of the LASSO model. Radiomic, clinical and clinical-radiomic multivariable models were tested on prospective data (validation set) using the area under the ROC curve (AUC), with 95% confidence intervals calculated with bootstrap resampling.
**Results or Findings:** The RS was significantly associated with the presence of any driver gene alteration versus no alteration, both at univariate and multivariable analysis including clinical variables. The validation AUC increased from 0.71 to 0.84 when adding the RS to the clinical variables significantly associated with the outcome at univariate analysis (stage and smoke habit).
**Conclusion:** This study suggests a possible significant role of CT radiomics for the non-invasive identification of patients with advanced NSCLC harbouring actionable gene alterations.
**Limitations:** The limitations are a possible difference between training and validation populations and the monocentric study design.
**Ethics committee approval:** This study was approved by the institutional Ethical Committee (R784/18-IEO836).
**Funding for this study:** Funding was received for this study by the Italian Health Ministry (GR-2016-02362050).
**Author Disclosures:**
Gianluigi Funicelli: Nothing to disclose
Francesca Botta: Nothing to disclose
Elena Guerini Rocco: Nothing to disclose
Giuliana Lo Presti: Nothing to disclose
Giulio Cammarata: Nothing to disclose
Alberto Ranghiero: Nothing to disclose
Lisa Rinaldi: Nothing to disclose
Gianluca Spitaleri: Nothing to disclose
Ilaria Attili: Nothing to disclose

### RPS 1116-3
**Association between contrast-enhanced CT radiomic features and prognosis in advanced non-small cell lung cancer patients**
*L. Rinaldi*[1], I. Attili[1], G. Cammarata[1], S. Raimondi[1], S. Mora[1], S. Rizzo[2], F. Botta[1], E. Guerini Rocco[1], G. Spitaleri[1]; [1]Milan/IT, [2]Lugano/CH
*(lisa.rinaldi@ieo.it)*

**Purpose:** Target therapies are emerging for the treatment of advanced non-small cell lung cancer. The identification of novel prognostic biomarkers can improve treatment choice and optimisation. This study investigates the association between CT radiomic features of lung lesions acquired before starting therapy, and overall survival (OS).

**Methods or Background:** 287 patients affected by advanced non-small cell lung cancer were retrospectively enrolled. Clinical data were collected including: age, sex, smoke habit, stage at diagnosis, previous treatments, lesion lobe, lymph node status, presence of distant metastasis, mutational status (tested alterations: EGFR, KRAS, ALK). After CT, 126 patients underwent chemotherapy, 20 immunotherapy, 9 chemo+immunotherapy, 27 local therapy (surgery and/or radiation therapy), 55 Tyrosine-Kinase-Inhibitors. Data were not available for 50 patients. 261 patients (160 stage IV, 101 stage II-III) with adequate CT were included in the analysis. 1118 radiomic features were calculated from CT images with Pyradiomics package. Median follow-up of patients was 57.1 [31.4-150.4] months. A Radiomic Score (RS) for OS prediction was calculated with LASSO-Cox regression model. The accuracy of OS prediction was estimated with the C-index for radiomic, clinical and clinical-radiomic models, after internal cross-validation. Subgroup analysis by stage and mutational status was performed.
**Results or Findings:** Among clinical variables, stage and post-CT therapy were independent predictors of OS (multivariable model C-index 0.64). The RS was significantly associated to OS, both taken alone ($p<0.001$, C-index 0.78) and in the multivariable model in association to clinical variables (C-index 0.80).
**Conclusion:** The significant and independent prognostic role of RS was confirmed both when stratifying patients according to stage (stage IV and stage II-III sub-groups) and according to gene alteration (EGFR versus no-EGFR, KRAS versus no-KRAS sub-groups).
**Limitations:** The limitation is the internal validation.
**Ethics committee approval:** Approved by the institutional Ethical Committee (R784/18-IEO836).
**Funding for this study:** Funding was received for this study by the Italian Health Ministry (GR-2016-02362050).
**Author Disclosures:**
Francesca Botta: Nothing to disclose
Serena Mora: Nothing to disclose
Sara Raimondi: Nothing to disclose
Elena Guerini Rocco: Nothing to disclose
Giulio Cammarata: Nothing to disclose
Stefania Rizzo: Nothing to disclose
Lisa Rinaldi: Nothing to disclose
Gianluca Spitaleri: Nothing to disclose
Ilaria Attili: Nothing to disclose

### RPS 1116-4

**Performance of whole body MRI with background suppression in detecting metastatic lesions in adenocarcinoma of the lung as compared to 18F-FDG PET/CT**

*J. Khoda*[1], L. Niranjanamurthy[1], A. Jajodia[2], J. Goyal[1], R. Jyani [1], S. K. Puri[1], A. K. Chaturvedi[1]; [1]New Delhi/IN, [2]Hamilton, ON/CA
*(jeeviteshkhoda@gmail.com)*

**Purpose:** To see how whole body MRI with background suppression (WB MRI) performs in detecting metastatic lesions in cases of adenocarcinoma of the lung as compared to 18F-FDG PET/CT.
**Methods or Background:** 70 patients of biopsy-proven adenocarcinoma of the lung were enrolled consecutively in this prospective study. The patients who had undergone FDG PET/CT examination underwent WB MRI with DWIBS after the PET/CT. In addition to acquiring the WB MRI diffusion sequence additional T2 haste axial whole body, T1 Dixon axial whole body, T1 Dixon and STIR sagittal sequences of the spine were acquired as well. The lesions were considered true positive only if they were biopsied or showed response or progression on imaging in 6 months follow-up while on chemotherapy. The total number of lesions detected on WB MRI and PET/CT were documented and divided into osseous lesions and non-osseous soft tissue lesions.
**Results or Findings:** Total lesions detected on WB MRI were 279, total lesions on PET/CT 260 and total lesions on standalone WB diffusion only 286. The sensitivity, specificity and accuracy of WB DWI was 95%, 91.77% and 93.2% respectively. The sensitivity, specificity and accuracy of PET/CT was 90.8%, 92.6% and 91.5% respectively. The sensitivity, specificity and accuracy of standalone WB diffusion only was 94.4%, 84.5% and 90.8% respectively. WB MRI detected more lesions accurately than PET/CT when both diffusion and additional sequences were used together for lesion characterisation. Using WB diffusion only led to poorer performance compared to PET/CT due to higher false positives.
**Conclusion:** WB MRI with additional sequences is more accurate than PET/CT in detecting metastatic lesions in adenocarcinoma lung.
**Limitations:** The limitations are the small sample size and limited histopathology (practically cannot biopsy all lesions).
**Ethics committee approval:** Approved with consent of the patients.
**Funding for this study:** Funding was received for this study by Nil.
**Author Disclosures:**
Likhith Niranjanamurthy: Nothing to disclose
Jitin Goyal: Nothing to disclose
Ruchir Jyani : Nothing to disclose
Sunil Kumar Puri: Nothing to disclose
Arvind K Chaturvedi: Nothing to disclose
Ankush Jajodia: Nothing to disclose
Jeevitesh Khoda: Nothing to disclose

### RPS 1116-5

**Imaging findings and value of CT and (DCE-)MRI in monitoring denosumab therapy of giant cell tumours of bone**

T. Van Den Berghe, M. Lejoly, L. Lapeire, W. Huysse, D. Creytens, *K. Verstraete*; Ghent/BE
*(koenraad.verstraete@ugent.be)*

**Purpose:** To determine the value of CT and (DCE-)MRI for monitoring denosumab therapy of giant cell tumours of bone (GCTB).
**Methods or Background:** Twelve patients (8 males, 4 females) with GCTB (4 spine, 8 limbs) were monitored six-monthly alternately with CT and (DCE-)MRI imaging studies and (semi-)quantitative measurements to assess response, relapse and complications.
**Results or Findings:** On CT, good responders showed progressive re-ossification of osteolysis, cortical remodelling and tumour and soft tissue volume reduction. On MRI, first a reduction in contrast enhancement, surrounding bone marrow oedema and volume were observed. Next, focal necrosis, fatty infiltration and progressive re-ossification of the osteolysis appeared. On DCE-MRI, the time-intensity curve evolved from a type IV curve with high first pass, high amplitude and steep wash-out rate over an intermediate type III or V to a slow type I or II curve. A reduction in wash-in slope, maximum signal intensity, area under the curve and amplitude of wash-in was observed, along with an increase in arrival time and time to peak. A decrease in Ktrans, initial area under curve and amplitude A was observed, along with increased Ve and Kep (extended Tofts/Brix models). Patients with poor response or relapse showed the inverse findings of good responders. Three patients showed relapse after initial good response when denosumab was stopped for pregnancy or dental surgery. Regarding evolution in time, one patient with initial good response showed a new cortical breakthrough and extensive bone marrow oedema. Biopsy revealed a highly cellular high-grade conventional osteosarcoma with small interstitial space.
**Conclusion:** Denosumab is effective in inoperable GCTB and in operable cases with large morbidity. CT and (DCE-)MRI allow adequate evaluation of early/late response and detection of therapy failure and malignant transformation.
**Limitations:** No limitations were identified.
**Ethics committee approval:** The study was approved, ICF obtained.
**Funding for this study:** No funding was received for this study.
**Author Disclosures:**
Lore Lapeire: Nothing to disclose
Koenraad Verstraete: Nothing to disclose
Maryse Lejoly: Nothing to disclose
Wouter Huysse: Nothing to disclose
David Creytens: Nothing to disclose
Thomas Van Den Berghe: Nothing to disclose

### RPS 1116-6

**Assessment of a targeted imaging pathway for investigation of pain in patients with known, biochemically stable multiple myeloma**

*A. D. Dragan*, K. Boyd, M. Kaiser, E. Scurr, D-M. Koh, A. Riddell, C. Messiou; London/UK

**Purpose:** Whole-body MRI (WBMRI) is an option for restaging multiple myeloma (MM) patients. New pain does not always indicate progression, particularly in the absence of biochemical progression, in patients under active surveillance and/or ongoing therapy. This study aims to assess whether targeted MRI scans (as opposed to WBMRI) are an appropriate method of investigating new pain in patients with known MM, without biochemical progression.
**Methods or Background:** Retrospective analysis of sequential targeted MRI scans performed over 2 years to investigate new pain in MM patients without biochemical progression at a tertiary referral centre. Patients with nonsecretory/oligosecretory disease or other metastatic cancers were excluded.
**Results or Findings:** 137 encounters/scans were included, from 109 patients. 55 (40%) of scans were performed during active surveillance. 115 (84%) covered a single area (e.g. spine, pelvis etc), while 22 (16%) covered 2 anatomical areas. 100 (73%) of scans showed no active lesion, half (50 scans) showed degenerative changes, 39% vertebral fractures etc. Only 3 of these patients progressed to the next treatment line in the subsequent 3 months. 37 (27%) of all scans reported presumed active disease. Of these, 21 demonstrated new lesions; 6 however also developed biochemical progression

Thursday around the time of the scan. Of the 21 scans with new lesions, a WBMRI was requested in only 11 cases (8% of total), 10 confirming progressing disease. In 61 cases, patients had no recurrence to date (average follow-up of 24 months, min 2, max 41).
**Conclusion:** In myeloma patients presenting with new pain but no biochemical recurrence, targeted scans are an appropriate method of investigation, rather than full restaging WBMRI. This reduces capacity pressures on MRI and the time that patients in pain spend in MRI scanners.
**Limitations:** The limitation is the single-centre retrospective study design.
**Ethics committee approval:** The study was approved by the local institutional review board.
**Funding for this study:** Not applicable.
**Author Disclosures:**
Christina Messiou: Nothing to disclose
Dow-Mu Koh: Nothing to disclose
Erica Scurr: Nothing to disclose
Martin Kaiser: Nothing to disclose
Alina Denisa Dragan: Nothing to disclose
Angela Riddell: Nothing to disclose
Kevin Boyd: Nothing to disclose

### RPS 1116-7

**Prognostic value of myosteatosis assessed by computed tomography in patients with multiple myeloma**

*T. D. Diallo*, F. Bamberg, J. Neubauer; Freiburg/DE

**Purpose:** Fatty infiltration of skeletal muscle, also known as myosteatosis is associated with increased frailty, decreased muscle and mobility function, which seems fairly prevalent in multiple myeloma (MM) patients. This study aimed to determine the prognostic value of myosteatosis assessed by computed tomography (CT) for progression-free survival (PFS) and overall survival (OS).
**Methods or Background:** This IRB-approved cohort study included patients with newly diagnosed MM who were treated at a single university hospital and received CT at baseline. Geriatric assessment was performed via international myeloma working group (IMWG) frailty score and revised myeloma comorbidity index (R-MCI). Myosteatosis was determined through CT measurement of paravertebral skeletal muscle radiodensity. The myeloma outcome was defined via PFS and OS assessment. Statistical analyses included uni- and multivariable Cox proportional hazard models and Kaplan-Meier-method.
**Results or Findings:** A total of 226 newly diagnosed MM patients (median age: 65 years [range: 29-89], 63% males, mean BMI: 25 [14-42]) were analysed. The prevalence of myosteatosis was 60%. Muscle radiodensity was significantly decreased in individuals with international staging system stage III vs. I (p<0.001), indicating higher amounts of intermuscular adipose tissue in patients with advanced disease. Median PFS and OS of the whole population were 3.4 years and 7.6 years, respectively. In univariable analysis, myosteatosis was a statistically valuable predictor of impaired PFS and OS (HR: 1.70; 95%-CI: 1.16-2.50 and HR: 2.44; 95%-CI: 1.45-4.11). Myosteatosis remained an independent predictor of OS in multivariable analyses.
**Conclusion:** Myosteatosis seems highly prevalent in patients with newly diagnosed MM and associated with impaired overall survival.
**Limitations:** The main limitation of the present study is its retrospective design. Prospective clinical trials are required to confirm our findings.
**Ethics committee approval:** The present study was approved by our local Independent Ethics Committee.
**Funding for this study:** No funding was received for this study.
**Author Disclosures:**
Jakob Neubauer: Nothing to disclose
Fabian Bamberg: Nothing to disclose
Thierno D. Diallo: Nothing to disclose

### RPS 1116-8

**Novel free-breathing day-optimising-throughput sequences in comparison with conventional breath-hold examinations during whole-body MRI**

*V. Koch*, S. Zangos, K. Eichler, L. D. Grünewald, C. Booz, I. Yel, T. Vogl, M. C. Langenbach, T. Gruber-Rouh; Frankfurt/DE
*(vitali-koch@gmx.de)*

**Purpose:** To assess and compare novel free-breathing DOT sequences (day optimising throughput) with conventional breath-hold examinations in whole-body magnetic resonance imaging (MRI).
**Methods or Background:** This prospective study included 20 healthy study participants and 6 patients with multiple benign pathologies who had undergone whole body 1.5-Tesla MRI. Image quality, diagnostic confidence, and image noise were evaluated by two experienced radiologists. Diagnostic performance for the overall detection of pathologies in both sequences was determined using the area under the receiver operating characteristics curve (AUC). Additionally, study participants were asked to rate their experience in a satisfaction survey using a 5-point Likert scale.
**Results or Findings:** MRI free-breathing scans were rated as at least equivalent to conventional MRI scans in more than 90% of cases, showing high overall diagnostic accuracy (95%) and performance (AUC 0.968, 95% CI 0.939 to 0.986; P < 0.0001) for the assessment of pathologies. Interrater agreement was excellent for both, free-breathing ($\varkappa$ = 0.96 [95% CI, 0.88–1.00]) and conventional scans ($\varkappa$ = 0.93 [95% CI, 0.84–1.00]). Ratings for image quality, image noise, and diagnostic confidence differed not significantly between the two types of MRI acquisition (all P > 0.05). The mean examination time of whole-body MRI was 56 ± 4 minutes (range, 52–70 minutes) using DOT free-breathing and 73 ± 6 minutes (range, 64–84 minutes) using conventional MRI sequences with breath-hold commands (P < 0.0001).
**Conclusion:** MRI images generated with free-breathing sequences yielded similar diagnostic performance at equivalent image quality and noise levels compared to conventional breath-holding algorithms.
**Limitations:** The limitations are the single-centre study design, the impact of contrast agent, and the patient number.
**Ethics committee approval:** The institutional ethical review board approved this prospective study, which complies with the Declaration of Helsinki. Written informed consent was obtained from each participant.
**Funding for this study:** No funding was received for this study.
**Author Disclosures:**
Stephan Zangos: Nothing to disclose
Christian Booz: Speaker: received speaking fees from Siemens Healthineers.
Ibrahim Yel: Speaker: received a speaking fee from Siemens Healthineers
Thomas Vogl: Nothing to disclose
Vitali Koch: Nothing to disclose
Kathrin Eichler: Nothing to disclose
Tatjana Gruber-Rouh: Nothing to disclose
Marcel Christian Langenbach: Nothing to disclose
Leon David Grünewald: Nothing to disclose

**16:00-17:30** **Room E1**

### Research Presentation Session: Genitourinary

### RPS 1107

### What is best to detect prostate cancer?

**Moderator**
O. F. Donati; Zurich/CH

### RPS 1107-2

**The negative predictive value of PI-RADS V2 for clinically significant prostate cancer on 3T multiparametric MRI**

*Q. Miao*[1], S. Afshari Mirak[2], M. Hosseiny[2], D. Lu[2], K. Sung[2], S. Raman[2]; [1]Shenyang/CN, [2]Los Angeles, CA/US
*(meganmiaoqi@126.com)*

**Purpose:** To assess negative predictive value (NPV) of 3TmpMRI in patients with a suspicion of prostate cancer (PCa) and to identify clinical factors that predict clinically significant PCa (csPCa) in patients with negative mpMRI.
**Methods or Background:** In this IRB approved, HIPAA compliant study, the cohort of 609 patients underwent 3TmpMRI between 2016 to 2020 with all lesions scored as PI-RADS ≤2 followed by 12-14 core systematic biopsy (SBx) within six months. Mann-Whitney test and chi-square test were used to analyse significance of differences in results. Univariate and multivariate logistic regression analyses were performed to identify factors significantly associated with csPCa.
**Results or Findings:** Of the 609 patients, 59 (9.7%) and 111 (18.2%) patients had csPCa and clinically insignificant PCa, respectively. The NPV of 3TmpMRI for csPCa and PCa was 90.3% and 72.1%, respectively. Patients with biopsy proven csPCa were older (p=0.016), had higher prostate specific antigen density (PSAD, p=0.003), lower prostate volume (PV, p<0.001) and greater family history of PCa (p=0.028) compared to Benign + csPCa patients. Univariate analysis showed PV (p=0.002), PSAD (p=0.010), age (p=0.031) and family history of PCa (p=0.031) to be significantly associated with csPCa. Multivariate analysis revealed PV (p<0.001) and age (p=0.001) to be independent predictors of csPCa.
**Conclusion:** In patients suspected of having PCa, 3TmpMRI can be used to reliably predict the absence of csPCa and is a reliable tool to rule out csPCa before biopsy, possibly eliminating the need for biopsy. Prostate volume and age were found to be independent predictors of csPCa in men with negative mpMRI.
**Limitations:** A retrospective analysis, unintended selection bias could have existed. The NPV was calculated to a systematic biopsy standard by necessity since no higher standard such as WMHP was available.

Ethics committee approval: This study was approved by an ethics committee, IRB#10-001863.
**Funding for this study:** No funding was received for this study.
**Author Disclosures:**
Sohrab Afshari Mirak: Nothing to disclose
Kyunghyun Sung: Nothing to disclose
David Lu: Nothing to disclose
Steven Raman: Nothing to disclose
Qi Miao: Nothing to disclose
Melina Hosseiny: Nothing to disclose

### RPS 1107-3

**Differentiation of benign prostate hyperplasia from prostate cancer based on the combination of radiomics signatures of the transition zone in mpMRI and PSA-level elevations**

*B. Oerther*, M. Benndorf; Freiburg/DE

**Purpose:** To determine the prognostic value of radiomics phenotypes of the transition zone in multiparametric MRI in combination with elevated PSA levels to differentiate hyperplastic glandular or stromal changes from prostate cancer.
**Methods or Background:** One hundred and forty consecutive patients scanned for clinically significant prostate cancer (3T) with systematic transperineal biopsy as reference standard are analysed retrospectively in a single-centre study. After volumetric segmentation of the transition zone and peripheral zone in T2w normalisation of signal intensities is achieved through comparison with standardised representative volumes of muscle and fat tissue. Field bias is reduced by N4ITK-correction. Radiomic signatures are then derived from the volumes and fit in a overall regression model analysing the correlation with PSA-level elavations and biopsy results.
**Results or Findings:** A preliminary analysis of a subcohort with non-normalised T2w signal intensities (only N4ITK-corrected) consisting of 36 patients with benign prostate hyperplasia showed significant correlation of a subset of radiomic signatures with PSA-levels (r: 0,53-0,69, p < 0,05) suggesting that larger areas of high signal intensities correlate with elevation of PSA-levels. Application of regression models to a control group of 11 patients with csPCa suggested a trend for positive correlation of radiomics phenotypes with malignancy.
**Conclusion:** Initial results suggest that radiomics signatures of the transition zone can explain elevated PSA levels in patients without PCa in a subcohort and may predict prostate cancer in the transition zone. Application of the model with normalised signal intensities to the whole collective consisting of patients with BPH and TZ-PCa may additionally enable differentiation of glandular and stromal hyperplasia from malignant genesis based on image features and PSA-levels. Confirmation of these preliminary findings may enhance diagnostic accuracy of MRI examinations in both malignant an non-malignant settings.
**Limitations:** Preliminary results of subcohort.
**Ethics committee approval:** This study was approved by an ethics committee.
**Funding for this study:** No funding was received for this study.
**Author Disclosures:**
Benedict Oerther: Nothing to disclose
Matthias Benndorf: Nothing to disclose

### RPS 1107-4

**A randomised controlled trial on the role of MRI for prostate cancer screening: prostate cancer secondary screening in Sapienza, PROSA preliminary results**

*E. Messina*, M. Pecoraro, M. Bicchetti, M. L. Pisciotti, C. Catalano, V. Panebianco; Rome/IT
*(emanuele.messina@uniroma1.it)*

**Purpose:** To investigate the role of MRI with a bi-parametric approach (without injection of paramagnetic contrast medium), as a secondary prevention test for the early diagnosis of prostate cancer, comparing it with the serum PSA test. Comparison of the different combination of screening tests in terms of carcinoma detection rate, non-clinically significant and clinically significant cancer detection rate.
**Methods or Background:** Monocentric, prospective, interventional randomised controlled trial. 221 men have been enrolled and blindly randomised in two different arms: A)113 patients performed MRI with a bi-parametric approach (without contrast medium) regardless their PSA value; B) 108 patients performed MRI with a bi-parametric approach (without contrast medium) due to elevated PSA (>4 ng/ml or 2.5 ng/ml if positive family history). Furthermore, 110 patients were enrolled as control group. Patients with positive MRI defined as bPI-RADS ≥3, have undergone targeted prostate biopsy with the TRUS-MRI image fusion technique. Statistical analysis was performed using both bPI-RADS ≥ 3 and 4 as cut-off for clinically significant prostate cancer.
**Results or Findings:** We found that about 15% of patients from arm A had a bPI-RADS ≥ 3 and were directed to prostate biopsy, among those 71% had clinically significant prostate cancer. Among patients in arm B about 40% were directed to biopsy. The difference in accuracy between arm A and arm B was statistically different (p<0.05). Specificity, sensitivity, PPV and NPV were calculated for both arms and MR-directed biopsy was considered as the gold standard. Inter-reader agreement was excellent (k=0.863).
**Conclusion:** Prostate MRI without contrast media injection showed promising results compared to PSA as screening tool in men between 49-69 years of age.
**Limitations:** Small sample size due to ongoing analysis of the trial.
**Ethics committee approval:** This study was approved by an ethics committee.
**Funding for this study:** No funding was received for this study.
**Author Disclosures:**
Marco Bicchetti: Nothing to disclose
Valeria Panebianco: Nothing to disclose
Martina Lucia Pisciotti: Nothing to disclose
Emanuele Messina: Nothing to disclose
Martina Pecoraro: Nothing to disclose
Carlo Catalano: Nothing to disclose

### RPS 1107-5

**Biparametric versus multiparametric MR imaging of prostate imaging reporting and data system version 2.1 in detection of prostate cancer**

*A. Mohamed Ali Khalil Elhendy*, H. M. Farg, M. A. El-Adalany, T. El-Diasty; Mansoura/EG

**Purpose:** The aim of this work was to assess the diagnostic capability of biparametric MRI (bpMRI) and multiparametric MRI (mpMRI) of PI-RADS V2.1 in detection of prostate cancer (PCa).
**Methods or Background:** Prostate cancer (PCa) is considered to be the commonest cancer among males. Early and precise diagnosis of PCa is essential for adequate treatment. Multiparametric MR imaging (mpMRI) is actually the most precise imaging technique used for early diagnosis of PCa. This prospective study was carried out on 60 male patients with high PSA. BpMRI and mpMRI were performed for all patients using a 3-T MRI scanner. The diagnostic performance of bpMRI of PI-RADS V2.1 was compared to that of mpMRI of PI-RADS V 2.1. The diagnosis of Pca was confirmed by transrectal ultrasound-guided biopsy and the results of open prostatectomy specimens.
**Results or Findings:** When considering PI-RADS categories 1, 2, and 3 as benign and categories 4 and 5 as malignant, mpMRI had higher sensitivity and diagnostic accuracy when compared with bpMRI (sensitivity was 88.6% for mpMRI versus 60% for bpMRI and diagnostic accuracy was 91.7% for mpMRI versus 75% for bpMRI). When considering PI-RADS categories 1 and 2 as benign and PI-RADS categories 3, 4 and 5 as malignant, the sensitivity and diagnostic accuracy of bpMRI and mpMRI were comparable (sensitivity was 94.3% for both bpMRI and mpMRI and diagnostic accuracy was 86.7% for both bpMRI and mpMRI).
**Conclusion:** Considering PI-RADS scores 4 and 5 as malignant, mpMRI had higher sensitivity and diagnostic accuracy when compared with bpMRI; however, when considering PI-RADS scores 3, 4, and 5 as malignant, both bpMRI and mpMRI had similar diagnostic accuracy.
**Limitations:** No limitations were identified.
**Ethics committee approval:** This study was appproved by the IRB of the Mansoura University, Egypt.
**Funding for this study:** No funding was received for this study.
**Author Disclosures:**
Tarek El-Diasty: Consultant: Observation
Ahmed Mohamed Ali Khalil Elhendy: Author: Working on data collection and analysis
Mohamed Ali El-Adalany: Consultant: Observation
Hashim Mohamed Farg: Consultant: Statistical analysis and study design

### RPS 1107-6

**Towards the definition of radiomics and clinical indices to enhance malignant tumour diagnosis in PI-RADS 4 and 5 lesions**

*P. N. Franco*[1], P. A. Bonaffini[1], E. De Bernardi[1], A. Corsi[1], D. Nicoletta[2], R. Simonini[1], A. Bonanomi[1], G. Perugini[3], S. Sironi[1]; [1]Milan/IT, [2]Merate/IT, [3]Bergamo/IT
*(p.franco@campus.unimib.it)*

**Purpose:** To identify promising radiomics features that, combined with routine clinical parameters, may support the detection of clinically significant tumours in PI-RADS 4/5 lesions in prostate MRI studies.
**Methods or Background:** Patients undergoing a 3T MR study for clinical suspicion of prostate cancer or active surveillance were retrospectively enrolled. Inclusion criteria were: presence of at least one PI-RADS 4 and/or 5 lesion (PI-RADS v2.1 criteria), adequate diagnostic imaging, available histopathological data. Pathological results utilising MRI-targeted biopsies or prostatectomy specimens were used as reference. Conventional clinical (age, PSA, PSA density, prostate volume) and MRI parameters (mean ADC in circular 2D ROI) were collected. Lesions were manually contoured with ITK-SNAP on axial T2-weighted images and on ADC maps. Radiomics features were extracted with Pyradiomics. Clinical and radiomics features best

Thursday correlating with histopathological results were selected and assessed with a balanced cross-validation approach over 200 repetitions using Youden thresholds. Sensitivity and specificity were assessed on validation samples.
**Results or Findings:** 103 patients were included. At pathology, 70 PI-RADS 4/5 lesions were identified as clinically significant cancers (Gleason score [GS] ≥7). Features best correlating with histopathology were: PSA-density selected in 78% of trials with a sensitivity of 35% and a specificity of 78% at validation; T2-glszm_SmallAreaLowGrayLevelEmphasis selected in 45% of trials with a sensitivity of 57% and a specificity of 57% at validation; T2-shape_Sphericity selected in 41% of trials with a sensitivity of 77% and a specificity of 45% at validation.
**Conclusion:** PSA-density appears to be the feature with the best specificity while T2-shape_Sphericity the one with the best sensitivity in correlating with histopathology. Multivariate analysis and test on wider samples are needed.
**Limitations:** Study's retrospective design, limited sample size, intra- and inter-observer variance in manual segmentation.
**Ethics committee approval:** This study was approved by Comitato Etico di Bergamo.
**Funding for this study:** No funding was received for this study.
**Author Disclosures:**
Alice Bonanomi: Nothing to disclose
Dario Nicoletta: Nothing to disclose
Pietro Andrea Bonaffini: Nothing to disclose
Giovanna Perugini: Nothing to disclose
Elisabetta De Bernardi: Nothing to disclose
Roberto Simonini: Nothing to disclose
Andrea Corsi: Nothing to disclose
Paolo Niccolò Franco: Nothing to disclose
Sandro Sironi: Nothing to disclose

### RPS 1107-7

### Artificial intelligence and multiparametric (mp)MRI in the assessment of prostate cancer (PCa) aggressiveness

*L. Mercatelli*[1], E. Bertelli[1], C. Marzi[1], E. Pachetti[2], M. A. Pascali[2], S. Colantonio[2], A. Barucci[1], S. Agostini[1], V. Miele[1]; [1]Florence/IT, [2]Pisa/IT

**Purpose:** To investigate the potential role of different AI approaches in predicting PCa aggressiveness from mpMRI.
**Methods or Background:** 85 patients underwent prostate mpMRI (PIRADS: 3-5) and free hand transperineal MRI/US fusion guided targeted biopsy (103 peripheral zone PCa lesions: 76 with ISUP GG<=2 and 27 with ISUP GG>=3). The lesions were segmented on T2w images and ADC maps. We used several ML algorithms and DL architectures, properly trained, validated and tested: 90% of the PCa patients were considered as the development set, and the remaining 10% as the test set.
**Results or Findings:** The results were computed at the lesion level. Employing only the T2w images, the best-performing ML model provided an AUROC of 75%, while the best DL architecture achieved an AUROC of 75.8%. Otherwise, for the ADC maps the AUROC values were 52.3% and 66.7% for the selected ML algorithm and the optimal DL model, respectively. Eventually, for the combination of T2w and ADC the best-performing ML classifier achieved an AUROC of 69.0%, whereas the most successful multimodal network yielded an AUROC of 72.7%. Although PI-RADS is attributed to PZ lesions by simultaneously looking at T2w images and ADC maps, the best performance was obtained using only the information extracted from T2w images, probably due to the fact that ADC maps have a much lower spatial resolution and the quantitative information extracted from them is less relevant and perhaps confounding, especially for ML algorithms.
**Conclusion:** Applying AI in the clinical practice is still a big challenge. However, our results are encouraging, especially those on T2 sequences. ML and DL models could be a valid aid in the diagnostic pathway of PCa.
**Limitations:** Small number of cases.
**Ethics committee approval:** The study was approved by our Institutional Review Board.
**Funding for this study:** Not applicable.
**Author Disclosures:**
Eva Pachetti: Nothing to disclose
Chiara Marzi: Nothing to disclose
Laura Mercatelli: Nothing to disclose
Maria Antonietta Pascali: Nothing to disclose
Vittorio Miele: Nothing to disclose
Elena Bertelli: Nothing to disclose
Simone Agostini: Nothing to disclose
Andrea Barucci: Nothing to disclose
Sara Colantonio: Nothing to disclose

### RPS 1107-8

### An impact of prior treatment with 5-alpha inhibitor on apparent diffusion coefficient values in prostate cancer differentiation

*Y. Mytsyk*, I. Dutka, I. Komnatska, O. Borzhievsky, I. Datz, V. Dmytrienko, A. Borzhievsky; Lviv/UA

**Purpose:** To analyse an impact of prior lower urinary tract symptoms (LUTS) treatment with finasteride on apparent diffusion coefficient (ADC) values in prostate cancer (PCa) differentiational diagnostics.
**Methods or Background:** The retrospective study enrolled 204 patients with histologically verified PCa or benign prostatic hyperplasia (BPH). In 102 cases of PCa and 70 cases of BPH there was no prior treatment with finasteride; in 20 cases of PCa and 12 of BPH treatment with finasteride due to the LUTS before histological diagnosis was conducted. In all cases multiparametric MRI of prostate was performed using 1.5T machine and b-values of 0 and 1000 s/mm2.
**Results or Findings:** The was no difference in mean ADC values between subgroups with PCa previously treated and untreated with finasteride due to LUTS: 0.69±0.15 vs 0.74±0.11 ×10−3 mm2/sec accordingly, p=0,633. Significant difference in mean ADC values in subgroups with BPH treated and untreated with finasteride was observed: 0.84±0.12 vs 1.16±0.16 ×10−3 mm2/sec respectively, p<0.001. There was no difference in mean ADC values between subgroup with BPH treated with finasteride and subgroups with clinically significant and clinically insignificant PCa without finasteride prescription history: 0.84±0.12 vs 0.70±0.05 vs 0.88±0.13×10−3 mm2/sec correspondingly, p>0.05. No observed difference between mean ADC values in subgroups with BPH and insignificant PCa where finasteride was previously prescribed (p>0.05). There was strong inverse correlation between the duration of finasteride use and the ADC value in subgroups of patients with PCa and BPH (r=0.657 and r=0.721 accordingly; p<0.05).
**Conclusion:** Treatment of LUTS with finasteride caused an overlap of mean ADC values in subgroups with BPH and PCa, the result of which is hindering of ADC application in differential diagnostics of PCa.
**Limitations:** The retrospective investigation.
**Ethics committee approval:** Research was approved by local ethics committee.
**Funding for this study:** No funding was received for this study.
**Author Disclosures:**
Olexander Borzhievsky: Nothing to disclose
Yulian Mytsyk: Nothing to disclose
Volodymyr Dmytrienko: Nothing to disclose
Iryna Komnatska: Nothing to disclose
Ihor Datz: Nothing to disclose
Andriy Borzhievsky: Nothing to disclose
Ihor Dutka: Nothing to disclose

### RPS 1107-10

### EPE-score: a comprehensive grading system for predicting pathologic extraprostatic extension of prostate cancer at multi-parametric magnetic resonance imaging

*G. A. Strazzarino*, M. Gatti, R. Faletti, F. Gentile, E. Soncin, A. N. A. Serafini, A. Fornari, S. Cirillo, P. Fonio; Turin/IT

**Purpose:** To investigate the diagnostic accuracy of the PIRADS v2.1 multi-parametric magnetic resonance imaging (mpMRI) features in predicting pathologic extraprostatic extension (EPE) of prostate cancer (PCa), as well as to develop and validate a comprehensive score incorporating all of them.
**Methods or Background:** We retrospectively reviewed all consecutive patients admitted to two institutions for radical prostatectomy for PCa with available records of mpMRI performed between January 2015 and December 2020. Data from one institution was used for investigating diagnostic performance of each mpMRI EPE features, using radical prostatectomy specimensas benchmark. The results were implemented in a comprehensive grading system, labelled EPE-score, based on the scores: capsular abutment: 1; irregular or spiculated margin: 3; bulging prostatic contour, or invasion of the neurovascular bundles, or tumour-capsule interface>1.0 cm: 4; ≥2 of the previous three parameters or measurable EPE: 5. The performance of mpMRI features was evaluated using the five diagnostic parameters and ROC curve analysis.
**Results or Findings:** A total of 200 patients were enrolled at site 1 and 76 at site 2. MpMRI features had poor sensitivities ranging from 0.08 (0.00-0.15) to 0.71 (0.59-0.83), whereas specificity ranged from 0.68 (0.58-0.79) to 1.00. EPE-score showed excellent discriminating ability (AUC>0.8) and sensitivity=0.82, specificity=0.77 with a threshold of 3. EPE-score had AUC comparable to ESUR-score (p=0.59), higher than EPE-GRADE (p=0.04) and early-and-late-EPE (p<0.0001). There were no significant differences between readers of different expertise with EPE-score (p=0.32) or EPE-GRADE (p=0.45), but there were significant differences for ESUR-score (p=0.02) and early-versus-late-EPE (p=0.03).

**Conclusion:** The individual mpMRI features have low sensitivity and high specificity. The use of EPE-score allows for consistent and reliable assessment for EPE.
**Limitations:** 1) Retrospective study; 2) mpMRIs were evaluated by radiologists of the local staff, so we cannot exclude performance disparities; 3) The scores were tested only on examinations done without endorectal coil.
**Ethics committee approval:** Approval from IRB was obtained.
**Funding for this study:** No funding was received for this study.
**Author Disclosures:**
Francesco Gentile: Nothing to disclose
Giulio Antonino Strazzarino: Nothing to disclose
Marco Gatti: Nothing to disclose
Enrico Soncin: Nothing to disclose
Riccardo Faletti: Nothing to disclose
Stefano Cirillo: Nothing to disclose
Alberto Fornari: Nothing to disclose
Alessandro Niccolò Annibale Serafini: Nothing to disclose
Paolo Fonio: Nothing to disclose

### RPS 1107-11

**The role of ADC values in the distinction between clinically significant PCa, insignificant PCa and HPIN lesions by 3T MRI**

*G. Agrotis*[1], M. Fanariotis[2], I. Tsougos[1], M. Michaliou[1], V. Tzortzis[1], M. Vlychou[1], K. Vassiou[1]; [1]Larisa/GR, [2]Skien/NO
*(g.agrotis@hotmail.com)*

**Purpose:** The aim of this study was to investigate the apparent diffusion coefficient (ADC) and apparent diffusion coefficient ratio (ADCratio) relationship between different histological grades of prostate cancer (PCa) and High-Grade Prostatic Intraepithelial Neoplasia (HPIN) lesions.
**Methods or Background:** 3T mpMRI was performed for 46 patients and at least one suspicious lesion was identified and histopathologically confirmed with targeted fusion guided mpMRI-TRUS biopsy. Axial diffusion-weighted sequence (DWI) was performed with b-values of 0, 500, 800, 1000 and 1600 s/mm2. Patients were categorised into Group A: Gleason score ≥ 7; Group B: Gleason score 6; Group C: HPIN lesions. ADC and ADCratio were correlated with Gleason score and HPIN. A threshold value for ADC and ADCratio were obtained to differentiate between Group A, B and C.
**Results or Findings:** For distinction between Group A and Group B, an ADC threshold value of 1.2*10-3 mm2/s and ADCratio of 0.75 were calculated. The mean value of ADC and ADCratio were statistically significant between Group A and B (p=0.03 and p=0.008 respectively). For distinction between Group B and Group C, an ADC threshold value of 1.33*10-3 mm2/s and ADCratio of 0.82 were obtained. The mean value of ADC and ADCratio were not statistically significant. The mean ADC value for Gleason score 6, 7, 8 and 9 was calculated with a weak inverse correlation, not statistically significant.
**Conclusion:** ADC and ADCratio are promising variables to differentiate histological grades in patients at risk for PCa, especially between Group A and B.
**Limitations:** More patients need to be added for statistically significant results between Group B and C.
**Ethics committee approval:** Ethics committee approval was obtained: No. 4861.
**Funding for this study:** Co-financed by the European Union and Greek national funds under the call RESEARCH – CREATE – INNOVATE (project code:T1EDK-03079).
**Author Disclosures:**
Georgios Agrotis: Nothing to disclose
Marianna Vlychou: Nothing to disclose
Vassilios Tzortzis: Nothing to disclose
Michael Fanariotis: Nothing to disclose
Maria Michaliou: Nothing to disclose
Katerina Vassiou: Nothing to disclose
Ioannis Tsougos: Nothing to disclose

**16:00-17:30** **Room M 1**

### Research Presentation Session: Neuro

### RPS 1111

### Neuroimaging in neurodegenerative diseases

**Moderator**
J. Boban; Novi Sad/RS

### RPS 1111-2

**Does macronutrient deficiency affect white matter integrity positively in elderly? Results of a DTI study**

*A. Kaya*[1], P. Soysal[2]; [1]Tekirdag/TR, [2]Istanbul/TR
*(ahmetkaya.md@gmail.com)*

**Purpose:** To investigate whether the white matter integrity in the brain is impaired in macronutrient deficiency in geriatric population with DTI technique.
**Methods or Background:** Geriatric patients who had a mini-nutritional assessment (MNA) test and had brain MRI including DTI sequence were included in the study. The patients were divided into three groups according to the MNA test result: those without malnutrition (group 1), those with malnutrition risk (group 2), and those with malnutrition (group 3). DTI indices (FA, ADC, AD, RD) of white matter tracts were measured by ROI-based method using Syngo workstation (Siemens, Erlangen, Germany).
**Results or Findings:** There were 112 geriatric patients who met the inclusion criteria (group 1= 12, group 2= 50, group 3= 50). In group 3, there was significant increase in FA, and a significant decrease in RD in the right superior longitudinal fasciculus and in the right cingulum compared to group 2. In the left superior longitudinal fasciculus lower ADC and RD values were noted in group 3 compared to group 2. Left inferior fronto-occipital fasciculus showed significantly increased FA, and decreased ADC and RD values in group 3 compared to group 2. In the right anterior limb of the internal capsule FA was significantly higher, and RD was significantly lower in groups 2 and 3 compared to group 1. Moreover, ADC was significantly decreased in the same tract in group 3 compared to group 1. ADC values in the left medial lemniscus were increased significantly in group 2 compared to group 1.
**Conclusion:** The findings revealed a better white matter integrity in the setting of macronutrient deficiency, and might indicate a correlation between calorie restriction and improved microstructural integrity in brain.
**Limitations:** The limitation is the retrospective cross-sectional study design.
**Ethics committee approval:** Institutional ethical committee approval was obtained.
**Funding for this study:** No funding was received for this study.
**Author Disclosures:**
Ahmet Kaya: Nothing to disclose
Pinar Soysal: Nothing to disclose

### RPS 1111-4

**Comparison of the accuracy of quantitative automatic and radiological imaging markers in distinguishing parkinson disease and progressive supranuclear palsy**

*F. Mambrin*[1], E. Cavedo[2], N. Guizard[2], G. Mansueto[1], M. Tinazzi[1], F. B. B. Pizzini[1]; [1]Verona/IT, [2]Paris/FR

**Purpose:** To compare the accuracy of imaging markers measured by QyScore®, an FDA and CE marked medical device, and radiological assessment in distinguishing parkinson disease (PD) from progressive supranuclear palsy (PSP) patients.
**Methods or Background:** 3D T1 MRI images of 18 PD, 9 with PSP and 25 controls (HC) were: (i) radiologically assessed by two radiologists in consensus, who provided the score of 13 indices used in clinical routine; and (ii) automatically segmented using QyScore®, providing volumes and population-normed z-scores for 17 brain structures. Radiological indices and QyScore® markers were compared across groups using Kruskal-Wallis tests, and p-values adjusted for multiple comparisons (Benjamini-Hochberg and Bonferroni methods). The area under the curve (AUC), sensitivity, specificity and the Youden index were calculated and compared for all markers that were significant in distinguishing PD from PSD in group comparisons.
**Results or Findings:** PD patients were younger than HC and PSP (PD 65 yrs, HC 71 yrs, PSP 72 yrs) but the sex proportions were not significantly different. The markers that significantly distinguished PD from PSP were, using QyScore®, the brainstem (BS p<0.001), globus pallidus (GP p<0.001) and thalamus (TH p<0.001); and, using radiological indices, the midbrain surface (p=0.004) and the MRPI 2.0 (p=0.003). Accuracy results of these markers/indexes plus a composite region including BS+GP+TH showed that the composite volume marker had the highest AUC (0.97), followed by GP (0.96), TH (0.95), BS (0.94), midbrain surface (0.89) and MRPI2.0 (0.89).

However, the AUC values for the best QyScore® marker and best visual index were not significantly different (p=0.308).
**Conclusion:** There is no statistically significant difference between radiological evaluation and automated markers quantified using QyScore® in distinguishing PD and PSP patients.
**Limitations:** The limitation is the sample limit.
**Ethics committee approval:** Retrospective study approved by the IRB of our Institution.
**Funding for this study:** No funding was received for this study.
**Author Disclosures:**
Giancarlo Mansueto: Nothing to disclose
Enrica Cavedo: Employee: Qynapse SAS
Nicolas Guizard: Employee: Qynapse SAS
Michele Tinazzi: Nothing to disclose
Francesca Mambrin: Nothing to disclose
Francesca Benedetta Benedetta Pizzini: Nothing to disclose

### RPS 1111-5

**Right cingulum cingulate correlates with gait and cognitive impairments in Parkinson's disease**
*X. Wei*; Beijing/CN
*(weixuan315@163.com)*

**Purpose:** This study aimed to evaluate the alterations of cingulate fiber tracts by using high angular resolution diffusion imaging (HARDI) in patients with Parkinson's disease (PD), and to explore the correlation relationship between mean fractional anisotropy (FA) value with cognitive behavioural and gait measures.
**Methods or Background:** Twenty-four PD patients and twenty-nine healthy controls (HCs) were included. For each participant, two-shell HARDI and high-resolution 3D structural images were acquired with a MPRAGE pulse sequence on a 3T MRI. Diffusion-weighted data preprocessing was performed using Mrtrix3 software, using orientation distribution function to trace the main nerve fibre tracts in PD patients. Automated fibre quantification analysis was performed. Clinical characteristics between PD and HC group were compared, and correlation between the FA value and behavioral data were analysed. Quantitative gait and clinical tests were recorded in PD subjects' medication ON and OFF states.
**Results or Findings:** The FA values of 34-58 equidistant nodes in the right cingulum cingulate (rCC) were positively correlated with mini-mental state examination (MMSE), berg balance scale (BBS)-OFF, BBS-ON scores; and negatively correlated with the MDS-UPDRS-III ON score. In the gait analysis, the FA value significantly correlated with velocity, cadence and stride time of the pace and rhythm domains in both 'ON' and 'OFF' state (P<0.05).
**Conclusion:** We use HARDI data to obtain detailed and accurate whole-brain fibres tracts in segments, and provide important neuroimaging information in association with clinical behavioral measures of PD. HARDI combined with neurocognitive and gait metrics may be a valuable biomarker for identifying gait and cognitive impairments in PD.
**Limitations:** This was a single-centre study with a relatively small sample size.
**Ethics committee approval:** It was approved by the ethics committees of Beijing Friendship Hospital (2019-P2-283-02).
**Funding for this study:** Funding was received for this study by No. 81871322 from National Natural Science Foundation of China.
**Author Disclosures:**
Xuan Wei: Nothing to disclose

### RPS 1111-6

**A critical evaluation of hemodynamic quality by single-time-point ASL in dementia**
F. B. B. Pizzini[1], I. Boscolo Galazzo[1], *V. Natale*[1], M. Scheffler[2], K-O. Loevblad[2], G. Frisoni[2], M. Günther[3]; [1]Verona/IT, [2]Geneva/CH, [3]Bremen/DE
*(valerio.natale01@gmail.com)*

**Purpose:** To validate a new biomarker for cerebrovascular integrity.
**Methods or Background:** Single-timepoint arterial spin labelling (ASL) MRI is recommended for quantifying non-invasively cerebral blood flow (CBF) in patients with dementia. However, this protocol provides complex hemodynamic information, e.g., arrival time, that may affect the end signal. This is a cross-sectional study on 144 memory clinic patients (72±7 years; 53% women) with mild cognitive impairment (n=88), dementia (n=20) and cognitively unimpaired controls (n=36) using single-timepoint pulsed ASL. A visual rating scale was applied to CBF maps, assessing delayed perfusion levels. Statistical parameters describing whole-brain perfusion included a new measure of hemodynamic quality evaluating the proportion of gray matter perfused voxels over their total number (HQIndex). One-way ANOVA analysis was used to compare mean, median, standard deviation(std), maximum and normalised mean and median difference of the calculated whole-brain CBF across delayed perfusion levels. Spearman correlation was performed to study the association of HQIndex with clinical and imaging parameters usually correlated with perfusion.
**Results or Findings:** Four degrees of delayed perfusion were identified, namely no delay (or normal,22), mild (29), moderate (53), and severe delay (40). HQIndex revealed a significant reduction (p<0.05) in the number of perfused voxels across the four visual classes: 90±3% in normal, 85±4% in mild, 79±5% in moderate and 67±8% in severely delayed groups. It was negatively associated with several variables including age(p<0.001), mean(p<0.0001), median(p<0.0001) and std(p<0.0001) of whole-brain CBF. HQIndex was not associated with age-related white matter changes or dementia severity.
**Conclusion:** Arrival time severely affects perfusion maps derived from single-timepoint ASL. The degree of this delay can be assessed by a visual rating scale and the HQIndex, possibly improving clinical interpretation of ASL maps.
**Limitations:** Not applicable.
**Ethics committee approval:** This study was approved by CCER n.2020-00403 on 21/10/2020.
**Funding for this study:** This study was funded by FNS n.320030_182772.
**Author Disclosures:**
Karl-Olof Loevblad: Nothing to disclose
Ilaria Boscolo Galazzo: Nothing to disclose
Valerio Natale: Nothing to disclose
Matthias Günther: Nothing to disclose
Francesca Benedetta Benedetta Pizzini: Nothing to disclose
Max Scheffler: Nothing to disclose
Giovanni Frisoni: Nothing to disclose

### RPS 1111-7

**Impact of the inversion time on regional brain perfusion estimation with clinical arterial spin labelling protocols**
*F. Sanvito*[1], F. Palesi[1], E. Rognone[1], L. Barzaghi[1], L. Pasca[1], V. De Giorgis[1], R. Borgatti[1], C. A. Gandini Wheeler-Kingshott[2], A. Pichiecchio[1]; [1]Pavia/IT, [2]London/UK
*(francesco.sanvito.1992@gmail.com)*

**Purpose:** Evaluating the impact of the inversion time (TI) on regional perfusion estimation in a paediatric cohort using arterial spin labelling (ASL).
**Methods or Background:** Pulsed ASL (PASL) was acquired at 3 T both at TI 1500 ms and 2020 ms from twelve MRI-negative patients (age range 9–17 years). A volume of interest (VOIs) and a voxel-wise approach were employed to evaluate subject-specific TI-dependent cerebral blood flow (CBF) differences, and grey matter CBF Z-score differences. A visual evaluation was also performed.
**Results or Findings:** CBF was higher for TI 1500 ms in the proximal territories of the arteries (PTAs) (e.g. insular cortex and basal ganglia — P < 0.01 and P < 0.05 from the VOI analysis, respectively), and for TI 2020 ms in the distal territories of the arteries (DTAs), including the watershed areas (e.g. posterior parietal and occipital cortex — P < 0.001 and P < 0.01 from the VOI analysis, respectively). Similar differences were also evident when analysing patient-specific CBF Z-scores and at a visual inspection.
**Conclusion:** TI influences ASL perfusion estimates with a region-dependent effect. The presence of intraluminal arterial signal in PTAs and the longer arterial transit time in the DTAs (including watershed areas) may account for the TI-dependent differences. Watershed areas exhibiting a lower perfusion signal at short TIs (~ 1500 ms) should not be misinterpreted as focal hypoperfused areas.
**Limitations:** The limitations are a reduced number of enrolled subjects, and a lack of validation through other perfusion modalities.
**Ethics committee approval:** Institutional Review Board approval was obtained.
**Funding for this study:** Funding was received for this study by the Italian Ministry of Health (RC 2017–2019).
**Author Disclosures:**
Ludovica Pasca: Nothing to disclose
Elisa Rognone: Nothing to disclose
Valentina De Giorgis: Nothing to disclose
Renato Borgatti: Nothing to disclose
Fulvia Palesi: Nothing to disclose
Leonardo Barzaghi: Nothing to disclose
Claudia A.M. Gandini Wheeler-Kingshott: Nothing to disclose
Anna Pichiecchio: Nothing to disclose
Francesco Sanvito: Nothing to disclose

### RPS 1111-8

**Decreased cerebral blood flow and delayed arterial transit are independently associated with white matter hyperintensity**
*R. Zhang*, P. Huang, M. Zhang; Hangzhou/CN
*(zhangruiting@zju.edu.cn)*

**Purpose:** White matter hyperintensities (WMH) and lacunes were important features of cerebral small vessel disease (CSVD), which contributes to 25% of ischaemic strokes and 45% of dementias. We aimed to investigate the role of cerebral blood flow (CBF) and arterial transit in WMH and lacune development.

**Methods or Background:** Ninety-nine CSVD patients were included. We used arterial spin labelling (ASL) with post-labelling delay time (PLD) of 1525ms and 2025ms to measure CBF respectively, and the difference between CBFPLD1.5 and CBF PLD2.0 was recorded as δCBF. We performed regression analysis to understand the contribution of CBF, δCBF to CSVD imaging markers.
**Results or Findings:** We found that CBF derived from both PLDs was associated with WMH volume and the presence of lacune. CBFPLD1.5 was significantly lower than CBFPLD2.0 in CSVD patients, and δCBF was correlated with WMH volume but not the presence of lacune. Furthermore, CBFPLD2.0 and δCBF were both associated with WMH in multiple regression analyses, suggesting an independent effect of delayed arterial transit. δCBF correlated with venous disruption but not large artery stenosis.
**Conclusion:** Both CBF and arterial transit were associated with WMH. ASL with multiple PLDs could provide additional hemodynamic information to CSVD related studies.
**Limitations:** First, we included patients at a single institution who had symptoms, and it might not represent the full spectrum of CSVD patients. Second, we only tested the CBFs derived from two PLDs. Verifying our results using ASL with multiple PLDs is needed.
**Ethics committee approval:** The protocols had been approved by the medical ethics committee of the Second Affiliated Hospital, Zhejiang University School of Medicine.
**Funding for this study:** This study was supported by the National Natural Science Foundation of China (Grant no. 82101987) and the China Postdoctoral Science Foundation (Grant no. 2019M662083).
**Author Disclosures:**
Minming Zhang: Nothing to disclose
Peiyu Huang: Nothing to disclose
Ruiting Zhang: Nothing to disclose

### RPS 1111-9

**Characterisation of normal-appearing white matter over 1-2 years in small vessel disease using quantitative susceptibility mapping and free-water mapping**

*Y. Sun*; Shanghai/CN
*(cjs1119@hotmail.com)*

**Purpose:** The aim of this study was to investigate alterations in the normal-appearing white matter (NAWM) with small vessel disease (SVD) over 1-2 years using quantitative susceptibility (QS) mapping and free-water (FW) mapping.
**Methods or Background:** Fifty-one SVD patients were included. QS, FA, MD, FW, FW-corrected FA (FAT), and FW-corrected MD (MDT) maps within white matter lesions (WMLs) and NAWM were generated for comparison. We also used the ICBM-DTI-81 label atlas as an anatomic guide and calculated the NAWM part in each of the WM tracts.
**Results or Findings:** Over 1-2 years, no significant difference was found between baseline and follow-up in the WMLs. In contrast, the QS values (index of demyelination) increased significantly in the NAWM at follow-up. Among WM tracts, we found the QS values in the NAWM part of the left superior frontal blade (SF), left occipital blade, right uncinate fasciculus, and right corticospinal tract (CST) was higher at follow-up. Results of FW (index of neuroinflammation/edema) analysis revealed that SVD patients at follow-up had increased FW in the NAWM part of the right CST and decreased FW in the NAWM part of the right inferior frontal blade (IF). The right SF and the right IF demonstrated significantly decreased FAT (indices of axonal loss) at follow-up. The degree of FAT changes in the NAWM part of the right IF was positively correlated with MoCA scores changes.
**Conclusion:** The results supported that SVD is a dynamic chronic disease and the NAWM in SVD is still in the progressive injury process. Distinguishing among demyelination, neuronal degeneration and neuroinflammation in the NAWM in vivo may provide a better understanding.
**Limitations:** The limitations are no healthy controls.
**Ethics committee approval:** Approved by the Research Ethics Committee of the Renji Hospital.
**Funding for this study:** Funding was received for this study by the National Natural Science Foundation of China (81901693).
**Author Disclosures:**
Yawen Sun: Nothing to disclose

### RPS 1111-10

**Alterations of brain networks in mild cognitive impairment: a resting-state fMRI study**

A. Kiryanova, *A. Manzhurtsev*, O. Bozhko, N. Kudryavtsev, I. Kolykhalov, N. Cherkasov, N. Semenova, Y. Fedorova; Moscow/RU
*(andrey.man.93@gmail.com)*

**Purpose:** This study is dedicated to the search for the alterations in resting-state networks in mild cognitive impairment (MCI). Previously, we have revealed the metabolic changes in the posterior cingulate cortex (PCC) of patients with MCI. In this study, using functional MRI we investigated the differences in the correlated activity of the PCC with other networks between normal subjects and patients with MCI.
**Methods or Background:** The measurements were carried out on a Philips Ingenia 3.0 T scanner, 29 subjects took part in the study. The subjects were divided into the following groups: 1) 15 patients with diagnosed MCI – 13 women and 2 men (mean age = 68.2± 6.2) and 2) 14 age-matched controls – 12 women and 2 men (mean age = 66.1± 7.5). 3D T1w images were acquired. Functional MRI protocol: EPI pulse sequence, TR = 1.5s, TE = 35 ms, 120 dynamic scans. Preprocessing and statistical analysis of the anatomic and functional MRI data were performed with FMRIB's software library tools. To study the resting-state networks, seed-based analysis was used with the PCC serving as the initial region. Statistical maps were calculated with Z-threshold of 3.1.
**Results or Findings:** The change in the functional connectivity in the splenium of corpus callosum vs PCC was found in patients with MCI compared to the control group.
**Conclusion:** The corpus callosum is an important structure of the brain, and a violation of the activity of this area in MCI can lead to a change in the interhemispheric transmission. Also, this can be critical to the analysis of the incoming information, on which the normal functioning of the human brain depends. The revealed changes are promising.
**Limitations:** No limitations were identified.
**Ethics committee approval:** This study was not approved by an ethics committee.
**Funding for this study:** No funding was received for this study.
**Author Disclosures:**
Igor Kolykhalov: Nothing to disclose
Anna Kiryanova: Nothing to disclose
Natalia Semenova: Nothing to disclose
Olga Bozhko: Nothing to disclose
Andrei Manzhurtsev: Nothing to disclose
Nikita Kudryavtsev: Nothing to disclose
Yana Fedorova: Nothing to disclose
Nikita Cherkasov: Nothing to disclose

### RPS 1111-11

**Using syMRI to explore the biological markers of brain aging**

*S. Bao*; Kunming/CN

**Purpose:** The characteristics of brain tissue change dynamically during aging. The purpose of this study was to evaluate the changing trend of relaxation values in different brain regions during brain aging by synthetic MRI.
**Methods or Background:** We conducted quantitative MRI tests on 1000 healthy people (aged from 20 to 85 years old) from September 2020 to October 2021. T1, T2 and Pd were simultaneously measured in 17 regions of interest (cerebellar cortex, pons, amygdala, hippocampal head, hippocampal tail, temporal lobe, occipital lobe, frontal lobe, caudate nucleus, lentiform nucleus, dorsal thalamus, semiovale center, parietal lobe, precentral gyrus, postcentral gyrus, substantia nigra, red nucleus).
**Results or Findings:** In different brain regions, we found that the T1 value of male was slightly higher than that of female, and the correlation was higher: the T1 value of frontal lobe and semiovale centre increased with age (positive correlation); hippocampal tail and lenticular nucleus decreased with age (negative correlation); frontal lobe and semiovale centre increased with age (positive correlation).
**Conclusion:** We found that the relaxation values of brain regions with differences between different genders in the same age group were slightly higher in males than in females, which may be of reference value for the incidence of some neurodegenerative diseases higher than that in females. Finally, with the increase of age, we found that the relaxation values of different brain regions can be positively correlated, and some of them are negatively correlated, which can provide a basis for us to early judge brain aging and diagnose neurodegenerative diseases.
**Limitations:** Clinical application of the study may need to be verified.
**Ethics committee approval:** The study has passed the ethical review.
**Funding for this study:** Funding was received for this study by the National Nature Fund.
**Author Disclosures:**
Shasha Bao: Author: No conflict of interest

**16:00-17:30** **Room N**

### Research Presentation Session: Chest

### RPS 1104

### Thoracic malignancies

**Moderator**
J. Coolen; Leuven/BE

### RPS 1104-2

**Volume doubling times of pulmonary metastases of bone and soft tissue sarcomas: correlation with subsequent new metastases and survival after metastasectomy**

*Y. Ahn*, S. M. Lee; Seoul/KR

**Purpose:** To investigate the correlation of volume doubling time (VDT) of pulmonary metastases with the subsequent appearance of new metastases and survival after metastasectomy in patients with bone or soft tissue sarcoma.
**Methods or Background:** Patients with bone or soft tissue sarcoma who underwent their first complete pulmonary metastasectomy between July 2010 and December 2020 were included. Volumetric segmentation for pulmonary metastases was performed on two CT scans, and VDTs were calculated. VDTs were compared in patients with and without subsequent new metastases. Cox proportional hazards regression analyses were performed to determine risk factors for recurrence-free survival (RFS) after metastasectomy and for post-metastasectomy survival (PS).
**Results or Findings:** The study cohort consisted of 40 patients (21 women; mean age, 51.1±14.3 years). Of these patients, 23 (57.5%) developed new metastatic nodules and 10 (25%) died during the follow-up period. The median VDT was shorter in patients with rather than without new metastases after metastasectomy (56 vs. 140 days, p=0.002). The VDT of metastasis was associated with the occurrence of subsequent metastases. Multivariable analysis showed that VDT <140 days (hazard ratio [HR], 4.22; p=0.01), female sex (HR, 2.80; p=0.03), and older age (HR, 1.06; p=0.004) were significantly related to a worse RFS. Moreover, age (HR, 1.17; p=0.005) and VDT <50 days (HR, 8.60; p=0.02) were independently correlated with a worse PS. None of the patients with VDT ≥140 days died during the study.
**Conclusion:** The VDT of pulmonary metastasis was associated with subsequent new metastases and survival after pulmonary metastasectomy. VDT may be considered when planning pulmonary metastasectomy.
**Limitations:** Identified limitations of this study were: the small population, retrospective nature, and heterogeneity of treatment after the post-metastasectomy occurrence of new metastases.
**Ethics committee approval:** This retrospective study was approved by the institutional review board.
**Funding for this study:** No funding was received for this study.
**Author Disclosures:**
Yura Ahn: Nothing to disclose
Sang Min Lee: Nothing to disclose

### RPS 1104-3

**Pulmonary MRI with UTE: potential for improving lymph node metastasis prediction capability with and without DWI as compared with thin-section CT and PET/CT in NSCLC patients**

*Y. Ohno*[1], M. Yui[2], K. Yamamoto[2], M. Ikedo[2], Y. Kassai[2], D. Takenaka[3], T. Yoshikawa[3], K. Murayama[1], H. Toyama[1]; [1]Toyoake/JP, [2]Otawara/JP, [3]Akashi/JP
*(yohno@fujita-hu.ac.jp)*

**Purpose:** To compare capability for lymph node metastasis prediction among pulmonary MRI with UTE (UTE-MRI) with single and dual-echo techniques (UTE-MRI Single and UTE-MRI Dual) with and without DWI, DWI only, thin-section CT and FDG-PET/CT in non-small cell lung cancer (NSCLC) patients.
**Methods or Background:** 56 NSCLC patients underwent CT, UTE-MRIs with both techniques, DWI, PET/CT, surgical resection and pathological and follow-up examinations. Then, the solid component within each tumour as a ratio between consolidation and total tumour (C/T ratio) by each method, ADC and SUVmax at all primary lesions were assessed. All indexes were compared between N0 and equal to or more than N1 (N1≤) cases by Student's t test. Multiple regression analysis was performed to determine predictors for differentiating two groups among all MR indexes. Then, ROC analysis and McNemar's test were also performed to compare diagnostic performances among all indexes and combined MR predictors.
**Results or Findings:** There was significant difference of each index between N0 and N1≤ cases (p<0.05). C/T ratio from UTE-MRI Dual and ADC were determined to be significant predictors (p<0.05). AUC of ADC (AUC=0.84), SUVmax (AUC=0.82) or combined MR predictors (AUC=0.90) were significantly larger than that of each C/T ratio (0.68<AUC<0.74, p<0.05). Specificity or accuracy of combined MR predictors were significantly higher than those of others (p<0.05).
**Conclusion:** UTE-MRI Dual is considered as having the capability to be a significant predictor for lymph node metastasis similar to DWI, and combined MR predictors shows significantly higher capability than CT and FDG-PET/CT in this setting.
**Limitations:** Not applicable.
**Ethics committee approval:** This retrospective study was approved by the Institutional Review Board of Fujita Health University School of Medicine in Japan.
**Funding for this study:** This study was financially supported by Canon Medical Systems Corporation.
**Author Disclosures:**
Masato Ikedo: Employee: Canon Medical SysteCorporation
Kazuhiro Murayama: Research/Grant Support: Canon Medical SysteCorporation
Kaori Yamamoto: Employee: Canon Medical SysteCorporation
Hiroshi Toyama: Research/Grant Support: Canon Medical SysteCorporation
Daisuke Takenaka: Nothing to disclose
Masao Yui: Employee: Canon Medical SysteCorporation
Yoshimori Kassai: Employee: Canon Medical SysteCorporation
Takeshi Yoshikawa: Nothing to disclose
Yoshiharu Ohno: Research/Grant Support: Canon Medical SysteCorporation

### RPS 1104-5

**Appropriate b value for computed DWI results in superior capability for N-stage assessment of NSCLC patients than that of actual DWI, STIR imaging and FDG-PET/CT**

*Y. Ohno*[1], M. Yui[2], D. Takenaka[3], T. Yoshikawa[3], Y. Kassai[2], K. Yamamoto[2], H. Ikeda[1], K. Murayama[1], H. Toyama[1]; [1]Toyoake/JP, [2]Otawara/JP, [3]Akashi/JP
*(yohno@fujita-hu.ac.jp)*

**Purpose:** To prospectively determine the appropriate b value for computed DWI (cDWI) to attain a better diagnostic capability for N-staging of non-small cell lung cancer (NSCLC) patients than actual DWI (aDWI), STIR imaging or FDG-PET/CT.
**Methods or Background:** aDWI with b values of 0 and 1000 (aDWI1000) s/mm2, STIR imaging and FDG-PET/CT were administered to 245 consecutive and pathologically diagnosed NSCLC patients. Then, each DWI data generated cDWIs using 400 (cDWI400), 600 (cDWI600), 800 (cDWI800) and 2000 (cDWI2000) s/mm2. Then, 114 metastatic nodes and 114 non-metastatic nodes were selected for evaluation of contrast ratio (CR) for each cDWI and aDWI, ADC, lymph node to muscle ratio (LMR) and SUVmax, as well as probability of lymph node metastasis of each method. ROC analysis was performed on a per node basis. Accuracy of each method for N-stage classification was also compared by using McNemar's test.
**Results or Findings:** Area under the curve (AUC) of CR600 was significantly higher than that of others (p<0.0001). AUCs of qualitatively assessed cDWI600, cDWI800, ADC map, aDWI1000 and STIR imaging were significantly higher than those of cDWI400, cDWI2000 and PET/CT (p<0.05). Comparison of N-staging accuracies (AC) showed CR600 and CR800 were significantly higher than those of others (p<0.05). Moreover, ACs of qualitatively assessed cDWI600 and cDWI800 were significantly higher than those of cDWI400, cDWI2000 and PET/CT (p<0.05).
**Conclusion:** cDWI using an appropriate b value has a potential to improve N-staging accuracy as compared with aDWI and PET/CT and is considered at least as effective as STIR imaging.
**Limitations:** Not applicable.
**Ethics committee approval:** This retrospective study was approved by the Institutional Review Boards of Kobe University Graduate School of Medicine and Fujita Health University School of Medicine in Japan.
**Funding for this study:** This study was financially supported by Canon Medical Systems Corporation.
**Author Disclosures:**
Kazuhiro Murayama: Research/Grant Support: Canon Medical SysteCorporation
Kaori Yamamoto: Employee: Canon Medical SysteCorporation
Hirotaka Ikeda: Nothing to disclose
Hiroshi Toyama: Research/Grant Support: Canon Medical SysteCorporation
Daisuke Takenaka: Nothing to disclose
Masao Yui: Employee: Canon Medical SysteCorporation
Yoshimori Kassai: Employee: Canon Medical SysteCorporation
Takeshi Yoshikawa: Nothing to disclose
Yoshiharu Ohno: Research/Grant Support: Canon Medical SysteCorporation

### RPS 1104-6

**Automatic segmentation and T-staging of oesophageal cancer using deep learning on CT images**

*Y. Wang*, J-z. Wang, Y. Huang, Y-h. Sun, L. Wang, Q-y. Zhao; Jinan/CN
*(kongbeixiaobao@163.com)*

**Purpose:** To investigate the automatic segmentation and T-staging based on CT images in patients with oesophageal squamous cell carcinoma (ESCC).
**Methods or Background:** CT datasets from 600 patients (mean age, 63±8 years; 477 male, 123 female) with pathologically confirmed ESCC between July 2014 and February 2021 were retrospectively collected, and the ratio of the T1, T2, T3 and T4 ESCC patients was 1:1:1:1. The imaging datasets were randomly grouped (4:1 ratio). Dataset 1 (480 cases) was used to construct the automatic segmentation models based on 2D-UNet and 3D-UNet and the T-staging model based on radiomics. Manual segmentation of the primary tumours was delineated as ground truth. Then, the segmentation task and the T-classification task were integrated to construct the artificial intelligence (AI) T-staging tool. Dataset 2 (120 cases) was used for testing, which was unprocessed original DICOM data not exposed in the model training process. Performance of segmentation was evaluated by Dice coefficient and mean Intersection-over-Union (mIoU). Performance of the AI T-staging tool was assessed by the receiver operating characteristic (ROC) curve and compared with the junior and senior radiologists.
**Results or Findings:** Experiments showed that the 3D-UNet outperforms the 2D-UNet based segmentation model (Dice coefficient, 0.875±0.012 vs 0.846±0.007; mIoU, 0.907±0.014 vs 0.885±0.005). The AI T-staging tool reached the areas under the curve of 0.94, 0.76, 0.74, and 0.88 for T1, T2, T3, and T4 ESCC, respectively. The accuracy for T-staging prediction by AI tool, junior radiologist and senior radiologist were 63.33%, 53.33%, and 79.16%, respectively.
**Conclusion:** The 3D-UNet based automatic segmentation model was accurate enough to delineate the primary tumors on CT images. Radiomics features extracted from automatic segmented targets showed good T-staging prediction of ESCC.
**Limitations:** No limitations were identified.
**Ethics committee approval:** This study was approved by an ethics committee.
**Funding for this study:** No funding was received for this study.
**Author Disclosures:**
Ling Wang: Nothing to disclose
Yu-hong Sun: Nothing to disclose
Jin-zhi Wang: Nothing to disclose
Yue Wang: Nothing to disclose
Qi-yu Zhao: Nothing to disclose
Yong Huang: Nothing to disclose

### RPS 1104-8

**Non-cancerous features on chest computed tomography predict overall survival in stage I non-small cell lung cancer (NSCLC) treated with stereotactic body radiotherapy (SBRT)**

I. Tahir, P. Marquardt, N. Mercaldo, G. Sharp, M. M. Wrobel, M. Khandekar, H. Willers, F. Keane, *F. J. Fintelmann*; Boston, MA/US
*(florianfintelmann@gmail.com)*

**Purpose:** To explore the association and predictive value of non-cancerous features on chest CT with overall survival (OS) in patients with non-small cell lung cancer (NSCLC) receiving stereotactic body radiotherapy (SBRT).
**Methods or Background:** This retrospective study included consecutive patients with stage I NSCLC treated with SBRT from 2009 to 2017. We quantified coronary artery calcium (CAC) score, pulmonary artery to aorta (PA:Ao) ratio, emphysema, and skeletal muscle at the level of vertebral bodies T5, T8, and T10. We constructed multivariable Cox models adjusting for age, sex, Eastern Cooperative Oncology Group (ECOG) status, smoking status, body mass index, gross tumour volume, and imaging features. We used LASSO to derive prediction coefficients and evaluated the discriminative ability of models using time-dependent area under the curve (AUC).
**Results or Findings:** Of 282 consecutively included patients, 168 (59.6%) were females (median age 75 years [interquartile range [IQR] 68-81 years]). 148 patients (52.5%) died after a median survival time of 60.8 months (95% confidence interval [CI] 55.8-68.0; median follow up 43.8 months [IQR 29.3-60.2]). PA:Ao ratio (hazard ratio [HR] 1.32 [CI 1.15-1.52], P<0.001, per 0.1 unit increase), CAC score 11-399 (HR 1.81 [CI 1.13-2.89], P=0.01) and ≥400 (HR 1.63 [CI 1.01-2.63], P=0.04) and sum of thoracic muscle index (HR 0.88 [CI 0.78-0.99], P=0.03, per 10 cm²/m² increase) were associated with OS. At 5-years, models incorporating clinical and imaging features outperformed models with clinical features alone (AUC 0.75 [CI 0.68-0.83] vs 0.61 [CI 0.53-0.70]).
**Conclusion:** CAC score, PA:Ao ratio, and thoracic muscle predict OS in stage I NSCLC treated with SBRT.
**Limitations:** Radiotherapy planning CT scans are not optimized for CAC scoring or emphysema densitometry. External data is needed to validate our prediction models.
**Ethics committee approval:** Our IRB approved this HIPAA compliant retrospective study.
**Funding for this study:** No funding was received for this study.
**Author Disclosures:**
Melin Khandekar: Nothing to disclose
Henning Willers: Nothing to disclose
Ismail Tahir: Nothing to disclose
Florian J. Fintelmann: Nothing to disclose
Maria Marta Wrobel: Nothing to disclose
Peter Marquardt: Nothing to disclose
Gregory Sharp: Nothing to disclose
Nathaniel Mercaldo: Nothing to disclose
Florence Keane: Nothing to disclose

### RPS 1104-9

**Bronchiolar adenoma/ciliated muconodular papillary tumour mimicking peripheral lung cancer: CT features of 48 cases**

*Y. Wang*[1], Y. Huang[1], F. Wang[1], H. Guo[2], L. Hu[1], Y-h. Sun[1], J-z. Wang[1], C-l. Zhao[1]; [1]Jinan/CN, [2]Yantai/CN
*(kongbeixiaobao@163.com)*

**Purpose:** To assess the clinical and computed tomography (CT) features of bronchiolar adenoma/ciliated muconodular papillary tumour (BA/CMPT) in 48 patients.
**Methods or Background:** A retrospective study of 48 patients with surgically resected BA/CMPT between January 2018 and September 2021 was performed. Clinical and CT data were reviewed. Two thoracic radiologists evaluated CT features. BA/CMPT is a peripheral pulmonary tumour newly defined by the World Health Organization in 2021. However, the clinical and radiological characteristics have not been well described.
**Results or Findings:** There were 48 patients (30 women and 18 men; mean age, 59±9 years; range, 32-75 years), including 30 (62.5%) patients with BA/CMPT and 18 (37.5%) patients with BA/CMPT coexisting with lung cancer. For the patients with only BA/CMPT, 28 of 30 (93.3%) patients had no obvious symptoms, and the level of lung tumour markers were within the normal range in 21 of 30 (70.0%) patients. All the tumours were located in the subpleural area. 25 of 48 (52.1%) tumours presented as subsolid nodules (mainly dense or heterogeneous GGNs), 13 (27.1%) as solid nodules, 8 (16.7%) as cavitary nodules, and 2 (4.2%) as consolidations. CT showed small size (mean diameter, 10±7 mm; range 3–36 mm), irregular shape (85.4%), lower lobe predominace (66.7%), vacuole (45.8%), vascular sign (37.5%) and air bronchogram (20.8%). However, other CT signs were rare, such as pleural traction (8.3%), speculation (6.3%), lobulation (4.2%) and calcification (2.1%). The BA/CMPT presenting as solid lesions showed delayed significant enhancement, accompanied by angiogram sign in the arterial phase. Preoperative follow-up was available in 14 patients, the tumours remained unchanged or grew slowly.
**Conclusion:** BA/CMPT is characterised by small, irregular, subpleural and slow-growing nodules with morphological diversity.
**Limitations:** Small sample size.
**Ethics committee approval:** This study was approved by the institutional review board.
**Funding for this study:** No funding was received for this study.
**Author Disclosures:**
Fang Wang: Nothing to disclose
Yu-hong Sun: Nothing to disclose
Doctor Hao Guo: Nothing to disclose
Jin-zhi Wang: Nothing to disclose
Yue Wang: Nothing to disclose
Yong Huang: Nothing to disclose
Cheng-long Zhao: Nothing to disclose
Li Hu: Nothing to disclose

### RPS 1104-10

**Value of dual-layer spectral detector CT for delineation of central type lung cancer in patients with atelectasis**

*Y. Qi*[1], Q. Zhang[1], S. Dong[2]; [1]Jinan/CN, [2]Beijing/CN
*(qiyuangang2004@126.com)*

**Purpose:** To investigate the value of dual-layer spectral detector CT (DLSCT) derived virtual monoenergetic images (VMI), iodine density images (IDI) and VMI-IDI overlays (VM-ID) for delineating central type lung cancer in patients accompanied by atelectasis.
**Methods or Background:** Fifty-one patients (37 males, 14 females) with a proven diagnosis of central type lung cancer accompanied by atelectasis in Shandong Cancer Hospital from January to December 2020 were retrospectively enrolled. Chest images were acquired using DLSCT in three staging examinations (unenhanced, arterial-phase and venous-phase). Two radiologists determined presence/absence of tumours independently. Tumour visualization was compared between conventional images (CI), 40keV VMI, IDI and VM-ID in different staging examinations. Quantitative image analysis was

Thursday performed by assessing the maximum diameter of tumours in the four-types of images.
**Results or Findings:** Among three staging examinations, venous-phase images showed significantly higher probability of visualization compared to arterial-phase (x2=52.40, P<0.001) with 4, 5, 5, 6 and 17, 35, 39, 38 tumours detected in CI, VMI40keV, IDI and VM-ID respectively, while all unenhanced images showed uncertain diagnoses because of insufficient contrast. In venous-phase examination, spectral images (40keV VMI, IDI and VM-ID) showed significantly higher diagnostic performances than those of CI (x2=-0.35, -0.43, -0.41 respectively, all P<0.001) but no significant difference among intra-group comparisons (all P>0.05) was observed. Seventeen tumours were visualized in all four-types of images of the venous phase, while the quantitative image analysis was insignificant among all the types of images (x2=3.61, P=0.31).
**Conclusion:** The venous phase spectral images of DLSCT could improve tumor visualization of central type lung cancer accompanied by atelectasis but contribute little to quantitative delineation.
**Limitations:** No limitations were identified.
**Ethics committee approval:** This study was not approved by an ethics committee.
**Funding for this study:** Project of Shandong medical and healthcare technology development plan (2019WS200).
**Author Disclosures:**
Shushan Dong: Author: polished the abstract with no remuneration
Yuangang Qi: Nothing to disclose
Qing Zhang: Nothing to disclose

### RPS 1104-11
**A novel algorithm for accurate lymph node metastasis prediction in synchronous multiple primary lung cancer: a multicentre study**
*W. Zhang*, C. Xie, Q. Li; Guangzhou/CN
*(zhangwb@sysucc.org.cn)*

**Purpose:** This study aimed to develop a multiparametric decision tree algorithm based on CT images and prior clinical knowledge for accurate lymph node (LN) metastasis prediction in synchronous multiple primary lung cancer (sMPLC).
**Methods or Background:** We retrospectively included 139 patients with surgically resected sMPLCs between December 2011 and March 2020 from our hospital. CT-imaging characteristics were analysed using multinomial univariable and multivariable logistic regression analysis to identify discriminating factors for LN metastasis. These factors were used to develop a novel CT-based multi-parameter decision tree algorithm model (CT-DTA). This model was then combined with other clinicopathological characteristics to further evaluate the performance of the CT-DTA model by multivariable logistic regression analysis and receiver operating characteristic (ROC) analysis. Finally, performance of the CT-DTA model was validated with a cohort of 96 sMPLC cases from two other hospitals.
**Results or Findings:** Of the CT-derived characteristics of sMPLC, five radiological parameters including lesions with spiculation, pure solid nodules, long-axis diameters of the solid portions, long-axis diameters of the lesions and consolidation tumor ratio (CTR) were finally included in the decision tree model. AUCs of the training cohort, the validation cohort, and the entire cohort were 0.905, 0.812 and 0.861, respectively. Multivariable analysis showed that the CT-DTA model is an independent factor for predicting lymph node metastasis in a training cohort (OR = 2.09, P = 0.003) and validation cohort (OR = 1.53, P = 0.041).
**Conclusion:** CT-based multi-parameter decision tree algorithm model is potentially a non-invasive and easy-to-use method for LN metastasis status prediction in sMPLC, which can help with clinical decision making.
**Limitations:** This is a retrospective study and is subject to inherent limitations associated with retrospective analyses.
**Ethics committee approval:** This retrospective study was approved by the local ethics committees of each clinical centre.
**Funding for this study:** No funding was received for this study.
**Author Disclosures:**
Chuanmiao Xie: Nothing to disclose
Wenbiao Zhang: Nothing to disclose
Qiong Li: Nothing to disclose

### RPS 1104-12
**Radiomics features of neuroendocrine tumours (NET) of the lung: comparative study using different CT scans**
*E. Bicci*, D. Cozzi, E. Cavigli, G. Danti, S. Bettarini, P. Tortoli, L. N. Mazzoni, S. Busoni, V. Miele; Florence/IT

**Purpose:** The aim of our study is to find a correlation using Radiomics, on images detected with different Computed Tomography (CT) scanners, between texture features of primary lesion of neuroendocrine (NET) lung cancer subtypes (typical and atypical carcinoids, large and small-cell neuroendocrine carcinoma), Ki-67 index and the presence of lymph-nodal mediastinal metastases.
**Methods or Background:** Sixty patients with histological diagnosis of pulmonary NET with known Ki-67 status and metastases who have performed pre-treatment CT in our department were included. After segmentation of primary lesions, quantitative texture parameters of first and higher orders were extracted. Statistical non-parametric tests were conducted.
**Results or Findings:** Regarding the relationship between tumour subtypes and Ki-67 values, statistically significant (p-value<0,05) differences were seen in post-contrast enhanced CT in various first-order and in one second-order extracted features (correlation). For direct acquisition, many second-order features were significant. Concerning the correlation with metastases in post-contrast acquisitions, some first-order features were statistically significant (median, root-mean-squared and 90th percentile). Many second-order features were significant in direct acquisitions, including Maximal Correlation Coefficient (MCC), cluster prominence and strength, also significant in correlating direct images with tumour subtypes and, therefore, the aggressiveness.
**Conclusion:** Some first-order radiomics CT features can be used as a valid and reproducible tool for predicting the subtype of lung NET. CT texture analysis in direct examination is useful in the evaluation of both tumour class and aggressiveness.
**Limitations:** Examinations were performed on different CTs, leading to an inhomogeneous sample which, although a limitation, can be useful in creating reproducible results. Another limitation is the number of patients with this rare neoplasm, accurately selected, to obtain a homogeneous sample in twelve years of data collection.
**Ethics committee approval:** Approved by the ethics committee of our institution (study protocol n:14776_oss).
**Funding for this study:** No funding was received for this study.
**Author Disclosures:**
Lorenzo Nicola Mazzoni: Nothing to disclose
Silvia Bettarini: Nothing to disclose
Ginevra Danti: Nothing to disclose
Vittorio Miele: Nothing to disclose
Eleonora Bicci: Nothing to disclose
Edoardo Cavigli: Nothing to disclose
Simone Busoni: Nothing to disclose
Paolo Tortoli: Nothing to disclose
Diletta Cozzi: Nothing to disclose

**16:00-17:30** **Room Z**

### Research Presentation Session: Imaging Informatics / Artificial Intelligence and Machine Learning

### RPS 1105

### Radiology workflow evolution and new developments

**Moderator**
C. A. Minoiu; Bucharest/RO

### RPS 1105-2
**Exploring automated MR logfile analysis and clinical context to quantify exam changeover time as basis for future workflow optimisation**
*X. Wang*[1], S. C. Chaduvula[2], R. Tellis[2], J. Schmidt[1], J. Borgert[1], T. Amthor[1], A. Frydrychowicz[3], J. Barkhausen[3]; [1]Hamburg/DE, [2]Cambridge, MA/US, [3]Lübeck/DE
*(xinyu.wang@philips.com)*

**Purpose:** Optimising radiology workflow is a challenging topic, due to many factors influencing performance. In this study, we explore how MR logfiles and selected information on clinical context can be used to quantify and even predict workflow elements. Here we focus on the exam changeover time, which typically adds no value to the workflow, and investigate its influencing factors to assist workflow orchestration.
**Methods or Background:** Data consists of pre-processed logfiles of 19680 exams from three MR scanners across two radiology departments and additional context information of 735 exams derived from questionnaires filled out by technologists. All data is de-identified and stored in an in-house database for statistical analysis.
**Results or Findings:** From the logfiles, we quantify the changeover time for various combinations of examined body parts in pairs of sequential exams, revealing their correlations. As an example of the influence of patient characteristics, our analysis identifies that children (<10y) have the longest changeover time, 18.1 min, significantly longer (P<0.01) than other age groups. From the additional clinical context, we conclude that (1) exams of patients with limited mobility have significantly longer changeover time than other exams, 18.9 min vs. 16.1 min; (2) when communication with patients is restricted, a 7-min delay is observed; and (3) setting up pulse oximetry, ECG, or intubation extends changeover time by 8.4 min, 7.9 min and 20.1 min, respectively.
**Conclusion:** Accurate quantification of changeover time reveals different influencing factors that need to be considered in workflow planning and orchestration. Our multidimensional dataset is well suited for future AI-based predictions of changeover time or other workflow parameters. The feasibility of such predictions will be discussed in the presentation.
**Limitations:** Clinical context dataset is limited in size and should be extended in future research.
**Ethics committee approval:** Approval has been obtained.
**Funding for this study:** No additional funding.
**Author Disclosures:**
Siva Chaitanya Chaduvula: Employee: Philips North America
Joachim Schmidt: Employee: Philips GmbH
Ranjith Tellis: Employee: Philips North America
Thomas Amthor: Employee: Philips GmbH
Jörn Borgert: Employee: Philips GmbH
Alex Frydrychowicz: Nothing to disclose
Jörg Barkhausen: Nothing to disclose
Xinyu Wang: Employee: Philips GmbH

### RPS 1105-3

**Sustanaibility in radiology: a model of optimisation for the development of a green, ecofriendly and smart radiology**
*S. Palmucci*, G. Distefano, A. Basile; Catania/IT
*(spalmucci@sirm.org)*

**Purpose:** To propose a model of optimisation for daily routine and management of radiology requests, acquisition of written informed consensus, delivery of reports and Dicom image. To analyse the impact of a "green radiology" in terms of a cost-efficacy analysis, emphasizing the outcome of this model based on ecofriendly resources.
**Methods or Background:** Four main steps of a radiological work-flow need to be focused: 1) the formulation of a request; 2) the acquisition of written informed consensus by patients; 3) the writing of radiological reports; 4) the delivery dicom images for patients. These four steps would be virtually assessed by an “ecofriendly smart radiology”.
**Results or Findings:** In some hospitals, there are still models based on radiology requests edited by general practioners and printed on papersheets: these requests are booked using phone-call services. Eco-friendly models could be based on web-service systems for radiological reservations, emphasising use of dematerialised requests. A great possibility could be achieved using online applications, installed on smartphones and tablets, to book examinations easily and quickly. Detailed information about procedures and examinations could be prudently sent to patients through emails and/or web based interviews, informative materials will no longer be printed, avoiding paper waste. Written informed consensus could be obtained in a dematerialised form, based on a digital signature: this will ensure a more consistent and safe storage. Finally, radiology reports could be accurately sent by email using PDF/A files, avoiding unnecessary trips to-and-from the hospitals. Images could be easily consulted through online links, without increasing costs of digital versatile discs or other paper-based iconograhic supports.
**Conclusion:** An optimised, eco-friendly and green model of radiology should be adopted, increasing the diffusion of economically sustainable and effective models.
**Limitations:** Not applicable.
**Ethics committee approval:** Not applicable.
**Funding for this study:** Not applicable.
**Author Disclosures:**
Giulio Distefano: Nothing to disclose
Antonio Basile: Nothing to disclose
Stefano Palmucci: Nothing to disclose

### RPS 1105-4

**Importance of business analytics for the re-organization of an academic hospital radiology unit during the COVID-19 pandemic**
*M. Polici*, V. Tamburi, F. Pucciarelli, B. Bracci, T. Polidori, G. Guido, M. Zerunian, D. Caruso, A. Laghi; Rome/IT
*(policimichela28@gmail.com)*

**Purpose:** To explore the rapid response of the radiology unit of an academic hospital in Rome during the COVID-19 pandemic. To analyse the ward’s governance decisions, almost all made empirically in the first wave and supported by a dedicated business analytics software with real-time data on imaging workflows in the second wave, then manage both acute emergencies and chronic oncologic/fragile patients.
**Methods or Background:** A retrospective review of the imaging volumes and workflows for 2019 and 2020 was performed. Information was collected from the hospital's data warehouse and evaluated using a business analytics software operating since March 2019. Data was aggregated both per week and per quarter, stratified by patient service location (emergency department, inpatients, outpatients) and imaging modality (x-ray, mammography, densitometry, ultrasound, CT, MRI, and interventional radiology). For the emergency radiology sub-unit, we reviewed average number of examinations per hour, CT examination allocation between the primary and secondary/backup machine, and CT median turnaround time (TAT), especially for weeks 11-15 and 45-48 of both years.
**Results or Findings:** During the first pandemic peak (11-15w 2019 vs 11-15w 2020) significant drops (P<0.001) in emergency (-73.2%), inpatient (-35.4%), and outpatient (-66.4%) examinations were registered. In the second pandemic peak (45-48w 2019 vs 45-48w 2020) significant (P<0.001) drops only in emergency (-41.7%) and inpatient (-10.1%) examinations were registered. For the emergency department, the average number of examinations per hour and CT median TAT during the first and the second pandemic peaks (2019 vs 2020) were -24.7%, +37.3%, +3.4%, and +32.8%, respectively.
**Conclusion:** Business analytics are extremely powerful in providing precise insight of real-time changing scenarios. When applied to a radiology unit it facilitated evidenced-based timely organisational decisions during a crisis, controlling infections, minimizing productivity loss, and maximising efficiency.
**Limitations:** An identified limitation is the retrospective nature of this study.
**Ethics committee approval:** Institutional ethical committee approved the study (ref. nr CE 5773_2020).
**Funding for this study:** No funding was received for this study.
**Author Disclosures:**
Damiano Caruso: Nothing to disclose
Francesco Pucciarelli: Nothing to disclose
Marta Zerunian: Nothing to disclose
Virginia Tamburi: Nothing to disclose
Benedetta Bracci: Nothing to disclose
Tiziano Polidori: Nothing to disclose
Andrea Laghi: Nothing to disclose
Michela Polici: Nothing to disclose
Gisella Guido: Nothing to disclose

### RPS 1105-5

**Structured reporting yields linguistic standardisation and semantic distinguishability of radiology reports**
*J. Vosshenrich*, I. Nesic, H-C. Breit, D. Boll, E. M. Merkle, T. Heye; Basle/CH
*(jan.vosshenrich@usb.ch)*

**Purpose:** To investigate if structured reporting affects linguistic standardisation and allows identification of reported imaging studies based on report content alone.
**Methods or Background:** 767,256 radiology reports dictated from 01/2013-12/2019 were included into analysis. During this time period structured reporting templates were introduced. Plain report texts were extracted from the institutional RIS and converted into a 20-dimension number vector using the doc2vec approach. Vectors closer together in vector-space represent higher report similarity, larger vector distance translates into lower report similarity. To allow for two-dimensional data plotting and assessment, a t-SNE approach was applied. Finally, data was enriched with RIS attributes (e.g. modality and body region) to enable in-depth analysis.
**Results or Findings:** With two-dimensional data visualisation, free-text report vectors were spread along the plot’s x- and y-axis, overlapping between distinct imaging studies and thus indistinguishable from each other. With structured reporting, document vectors were clearly organised into distinguishable clusters, each representing a distinct imaging study (e.g. abdominal ultrasound or prostate MRI). Higher linguistic similarity of specific report types with structured reporting was expressed by lower mean x- and y-coordinate distance of individual reports from the cluster’s centroid (i.e. elements closer together within clusters; e.g. abdominal ultrasound [x-coordinate spread: 5.81 vs 10.23, y-coordinate spread: 7.75 vs 12.49;p<.001] or MR-angiography lower extremities [x-coordinates: 0.92 vs 11.48, y-coordinates: 1.36 vs. 16.39;p<.001]). Higher semantic distinguishability between report types was expressed by larger distance between the centroids’ coordinates (i.e. clusters further apart from each other; e.g. CT abdomen-pelvis vs abdominal ultrasound [x-coordinate distance: 65.33 vs 27.35, y-coordinate distance: 16.22 vs 1.83]).
**Conclusion:** Structured reporting increases linguistic standardisation when reporting specific types of imaging studies. This may allow natural language processing algorithms to identify the reported imaging study based on report content alone.
**Limitations:** Not applicable.
**Ethics committee approval:** Not applicable.

Funding for this study: Not applicable.
**Author Disclosures:**
Tobias Heye: Nothing to disclose
Hanns-Christian Breit: Nothing to disclose
Ivan Nesic: Nothing to disclose
Daniel Boll: Nothing to disclose
Elmar M. Merkle: Consultant: Siemens Healthineers
Jan Vosshenrich: Nothing to disclose

### RPS 1105-6

**A novel approach to a national patient dose registry**

M. Gracia-Ochoa, *J. V. Catret Mascarell*, J. Vilar-Palop, L. Oliver-Cañamás, C. Candela-Juan; Valencia/ES
*(jvcatret@ingesa.sanidad.gob.es)*

**Purpose:** This work is part of the INGESA's (Spanish National Institute of Health Management) project for the creation of a national patient dose registry. The objective is the creation of a working method to establish dynamic diagnostic reference levels (DRLs) based on the doses registered in diagnostic imaging procedures within the Spanish territory.
**Methods or Background:** DRLs are a useful tool in patient dose optimisation. These are established on the basis of the data generated in different Spanish hospitals. As the DRLs are computed from real patient data, the source of information for the project are the different radiation dose management systems, being the anonymisation of these data a key factor for their sharing. The collection of these data has to be carried out through the integration and application of established standards such as DICOM or IHE. The management of the information from a national platform would allow the application of advanced data analysis techniques and, therefore, facilitate the establishment of dynamic Spanish reference levels.
**Results or Findings:** A proof of concept based on synthetic data generated by virtual dose management systems has been developed, to show the main functionalities of the national dose registry such as anonymisation, normalisation, visualisation of results and especially the analysis through interactive control panels.
**Conclusion:** A platform has been created with synthetic data allowing the generation of diagnostic reference levels, which are an effective tool to establish comparisons between different modalities, equipment and territories and to optimise patient doses.
**Limitations:** Not applicable.
**Ethics committee approval:** Not applicable.
**Funding for this study:** Not applicable.
**Author Disclosures:**
Maria Gracia-Ochoa: Nothing to disclose
Laura Oliver-Cañamás: Nothing to disclose
Jorge Vilar-Palop: Nothing to disclose
Juan Vicente Catret Mascarell: Nothing to disclose
Cristian Candela-Juan: Nothing to disclose

### RPS 1105-8

**Automated analysis of CT repeated scans in a large European hospital**

*N. Fitousi*[1], J. Binst[1], T. Szczykutowicz[2], S. Rose[3], W. Coudyzer[1], H. Bosmans[1], C. Van Ongeval[1], J. Jacobs[1]; [1]Leuven/BE, [2]Madison, WI/US, [3]Houston, TX/US
*(niki.fitousi@qaelum.com)*

**Purpose:** Recent work has highlighted the repeat rates in CT as a significant source of excess radiation and time loss in radiology. Therefore, as part of quality optimisation activities, the number of repeated scans was evaluated using a commercially available software equipped with an automated protocol analyser.
**Methods or Background:** For a period of one year, information on examinations from six CT scanners in a large European university hospital was retrieved with a dose management system (DOSE, Qaelum, Belgium). The data were then analysed by a commercial software (FOQAL-CT repeat, Qaelum), which uses a fully automatic algorithm, developed by the University of Wisconsin. The algorithm identifies typical protocols and then tracks outliers and performs a further quantification. Repeat rates (RR) were calculated per acquisition type (spiral, axial, localiser) and per protocol.
**Results or Findings:** Out of 58623 analysed examinations (137658 series), 7.0% were ignored due to low occurrence. In 211 identified protocols, 2619 exams contained a repeat, resulting in an overall RR of 4.8%. In the initial pass of the algorithm through the data, clinically acceptable repeats were identified reflecting acute stroke (29.7%), high resolution chest (inspiration/expiration) (12.3%) and kidney scans with late excretion phase (6.5%). The FOQAL-CT repeat module allowed to label these protocols with clinically acceptable deviations from the standard, resulting in a "real" spiral RR of 1.9%. High localiser RR was found for trauma protocols (overall 4.4% on trauma protocols).
**Conclusion:** The RR analysis correctly detected that for 3 specific protocols radiologists actively adjust the exam to the patient situation. This is considered good practice. The automated tool identified also real outliers, meaning protocols that require adjustments or extra training. The new software is a sophisticated option for true optimisation in imaging.
**Limitations:** Not applicable.
**Ethics committee approval:** Not applicable.
**Funding for this study:** Not applicable.
**Author Disclosures:**
Walter Coudyzer: Nothing to disclose
Jurgen Jacobs: Founder: Qaelum NV Shareholder: Qaelum NV CEO: Qaelum NV
Hilde Bosmans: Board Member: Qaelum NV Founder: Qaelum NV Shareholder: Qaelum NV
Chantal Van Ongeval: Nothing to disclose
Timothy Szczykutowicz: Consultant: AstoCT, GE, Imalogix, RadpiAI, AIDoc, ALARA Medical, FlowHow.ai Research/Grant Support: Canon USA, GE Advisory Board: GE, Imalogix Patent Holder: Qaelum, FlowHow.ai
Sean Rose: Research/Grant Support: Imalogix
Niki Fitousi: Employee: Qaelum NV
Joke Binst: Nothing to disclose

### RPS 1105-9

**Digital evolution of informed patient consent: added value for CT examinations**

*M. S. May*, M. Wetzl, E. Balbach, S. J. Daniel, N. I. El Amrani, M. Uder, M. Kopp; Erlangen/DE
*(matthias.may@uk-erlangen.de)*

**Purpose:** To evaluate the feasibility of digitised informed patient consent (D-IPC) for computed tomography (CT) and to compare digitised information with the conventional, paper-based IPC process (C-IPC).
**Methods or Background:** Consecutively 2459 patients were scheduled for a routine CT examination. Several questions were asked using a mobile tablet device considering general patient history (e.g. prior CT examinations, malignant diseases, cardiovascular disease) and possible contraindication (Red Flags) for a CT examination (e.g. thyroid hyperfunction, kidney malfunction). First, we compared the age between the patients, who were able and unable ($\leq$ 4 answered questions) to sufficiently answer the questionnaire. Second, we compared the prevalence of Red Flags between D-IPC and C-IPC. Third, we analysed the prevalence of the most relevant diseases and complications.
**Results or Findings:** A total of 335 (13.6%) patients answered the questionnaire insufficiently. These patients were older (64 $\pm$ 13.6 years; $p<0.001$) compared to the 2124 included patients (58.6 $\pm$ 14.2 years). In total 395 Red Flags were detected digitally, but only 47 of these were documented in the C-IPC. The congruency for thyroid hyperfunction, kidney malfunction, hemodialysis and claustrophobia was less than 10%. Most prevalent Red Flags were kidney diseases (11.1%), claustrophobia (10%) and allergic reactions against contrast-agents (4.6%). Severe allergic reactions were rare (0.3%).
**Conclusion:** Patients who are unable to complete D-IPC are significantly older. D-IPC can ensure accurate patient history recordings, which is mandatory to perform CT examinations safely. Paper-based archiving of critical data is error-prone.
**Limitations:** D-IPC requires a technical setup and investment costs. Not all patients in a clincal setting can manage D-IPC.
**Ethics committee approval:** IRB approved study.
**Funding for this study:** This study was funded by the Bavarian Government (MED-1810-0020).
**Author Disclosures:**
Matthias Stefan May: Speaker: Siemens Healthcare GmbH
Markus Kopp: Speaker: Siemens Healthcare GmbH
Nouhayla Imad El Amrani: Nothing to disclose
Matthias Wetzl: Nothing to disclose
Michael Uder: Nothing to disclose
Eva Balbach: Speaker: Siemens Healthcare GmbH
Sascha Jakob Daniel: Speaker: Siemens Healthcare GmbH

### RPS 1105-10

**A study on RECIST variability: a mathematical simulation**

*T. Bucho*[1], R. Tissier[1], M. van der Heijden[1], C. Blank[1], J. Haanen[1], T. D. L. Nguyen-Kim[2], R. G. H. Beets-Tan[1], S. Trebeschi[1]; [1]Amsterdam/NL, [2]Zurich/CH

**Purpose:** Tumour response to therapy in clinical trials is typically evaluated with RECIST. These criteria presuppose that the target lesions can be objectively identified and measured on CT scans. This study aims to investigate the extent of target lesion selection variability in RECIST by means of computer simulation models of clinical trials.
**Methods or Background:** We implemented a model for the simulation of clinical trials, by controlling the effects tumour growth, number of lesions and affected organs for multiple virtual patient cohorts. Each patient was assessed by multiple readers, who randomly selected valid target lesions according to RECIST criteria. The particular conditions under which RECIST is inconsistent,

Thursday despite being correctly employed, were then analysed. We further validated the results of the simulation model on a retrospective dataset of 96 cancer patients receiving immunotherapy. Lesion measurements were performed automatically from expert's segmentation.
**Results or Findings:** Variability increases when the number of measurable lesions is large and when lesion growth borders the thresholds for progressive disease and partial response. For 15.6% of the immunotherapy patients the discordance in the selection of target lesions was sufficiently large to result in distinct response classifications (interreader agreement: $\kappa = 0.30$).
**Conclusion:** RECIST evaluations are inconsistent even in the absence of measurement variability. The choice of lesions at baseline alone has a significant impact on RECIST outcomes. Five target lesions alone might not be sufficient to adequately portray overall tumour burden and determine response to treatment.
**Limitations:** Only two timepoints (baseline and first follow-up) were simulated and analysed, which can potentially lead to an imbalance of RECIST categories.
**Ethics committee approval:** Approved by the ethical committee (IRBd19-083).
**Funding for this study:** No funding was received for this study.
**Author Disclosures:**
Teresa Bucho: Nothing to disclose
Stefano Trebeschi: Nothing to disclose
John Haanen: Nothing to disclose
Christian Blank: Nothing to disclose
Thi Dan Linh Nguyen-Kim: Nothing to disclose
Renaud Tissier: Nothing to disclose
Regina G. H. Beets-Tan: Nothing to disclose
Michiel van der Heijden: Nothing to disclose

### RPS 1105-11

**AI co-pilot: content-based image retrieval for the reading of rare diseases in chest CT**

*M. Meetschen*[1], K. Zeng[2], S. Farhand[2], C. Speier[3], S. Stalke[4], H. Steinberg[1], D. Bos[1], S. Zensen[1], J. Haubold[1]; [1]Essen/DE, [2]Malvern, PA/US, [3]Forchheim/DE, [4]Stuttgart/DE
*(meetschen@gmx.de)*

**Purpose:** This study aims to evaluate the impact of the newly developed Similar Patient Search (SPS) web service, which supports reading complex lung diseases on computed tomography, on residents' diagnostic accuracy.
**Methods or Background:** SPS is an image-based search engine that provides rapid access to images with similar pattern from a large database of pre-diagnosed cases matched with corresponding clinical reference content (https://eref.thieme.de). The reference database was created using 13,658 annotated ROIs from chest CT scans of 621 patients, comprising 69 diseases of the lung. For validation, five radiology residents without SPS and three months later with SPS evaluated 50 CT scans. Residents were allowed to submit a maximum of three diagnoses per case.
**Results or Findings:** Without SPS, the residents achieved an average score of 17.6±5.0 points. By using SPS, residents increased their score by 81.8% to 32.0±9.5 points. The score improvement per case was highly significant (0.35 vs 0.64 $p=0.0001$). Residents took an average of 205.9±350.6 seconds per case (21.9% more) when SPS was used. In the second half of cases, after residents were more familiar with SPS, this time increase was reduced to 7%.
**Conclusion:** Residents' reading accuracy on complex chest CT scans improved by over 80% when AI-driven SPS with integrated clinical reference content was used.
**Limitations:** No books or Internet sources were available to the residents as sources of information. Therefore, further studies should investigate how SPS compare with other sources of information.
**Ethics committee approval:** The study was performed in approval of the ethics committee of the investigating hospital.
**Funding for this study:** Siemens Healthcare partially supported this study by providing the laptop and the software with which the study was conducted.
**Author Disclosures:**
Sepehr Farhand: Employee: Siemens Healthineers AG
Denise Bos: Nothing to disclose
Mathias Meetschen: Nothing to disclose
Sarah Stalke: Employee: Georg Thieme Verlag KG
Ke Zeng: Employee: Siemens Healthineers AG
Hannah Steinberg: Nothing to disclose
Johannes Haubold: Research/Grant Support: Research/Grant Support: Research grant within the University Medicine Essen Academy (UMEA) program, funded by the German Research Foundation (DFG; grant FU356/12-1) and the Faculty of Medicine, University of Duisburg-Essen
Sebastian Zensen: Research/Grant Support: Research/Grant Support: Research grant within the University Medicine Essen Academy (UMEA) program, funded by the German Research Foundation (DFG; grant FU356/12-1) and the Faculty of Medicine, University of Duisburg-Essen
Christoph Speier: Employee: Siemens Healthineers AG

### RPS 1105-12

**Contrast media reduction in CT with deep learning utilising a generative adversarial network in an experimental animal study**

*J. Haubold*[1], G. Jost[2], J. Theysohn[1], Y. Li [1], J. Kleesiek[1], B. M. Schaarschmidt[1], H. Pietsch[2], M. Forsting[1], R. Hosch[1]; [1]Essen/DE, [2]Berlin/DE
*(johannes.haubold@uk-essen.de)*

**Purpose:** To reduce the dose of iodine-containing contrast medium (CM) in abdominal CT in a large animal model by virtual contrast enhancement using generative adversarial networks (GAN).
**Methods or Background:** 20 healthy Goettingen minipigs underwent multiphase abdominal low-kV CT (90kV) three times with low (lowCM; 100 mgI/kg) and standard (standardCM; 350 mgI/kg) CM doses (120 examinations) containing an early arterial, late arterial, portal-venous, and venous contrast phase. One animal had to be excluded because of incomplete examinations. Of 19 animals, three were randomly selected and withheld for validation (18 examinations). With the remaining 16 animals (96 examinations), the GAN was trained for an image-to-image conversion from lowCM to standardCM. Subsequently, ROI-measurements were performed in the abdominal aorta, inferior vena cava, portal vein, liver parenchyma, and autochthonous back muscles, and the CNR was calculated. Furthermore, the standardCM and the virtual standard dose data (virtualCM) were demonstrated in a visual-Turing-test (VTT) to 3 radiology consultants, who had to decide whether they would have reported images from both examinations identical and which images originates from the standardCM exam.
**Results or Findings:** Mean CNR significantly ($P<0.0001$) increased by GAN in all contrast phases and was not significantly different from the standardCM examination (1.4±1.0 lowCM, 5.0±4.8 virtualCM, 5.1±5.7 standardCM). In the VTT, on average, the testers reported that the standardCM and virtualCM images were identical in 93% of the examinations. Testers could identify the standardCM data as such in 80% of the cases.
**Conclusion:** This feasibility study on healthy minipigs demonstrates that the amount of CM for abdominal CT might be reduced by about 71.4% using GAN-based contrast enhancement while maintaining comparable image quality.
**Limitations:** The study was performed under highly standardised laboratory conditions and the collective consisted only of healthy animals.
**Ethics committee approval:** The animal experiment has been approved by the local state authority.
**Funding for this study:** The study was performed in cooperation with Bayer AG.
**Author Disclosures:**
Michael Forsting: Nothing to disclose
Jens Theysohn: Nothing to disclose
Jens Kleesiek: Nothing to disclose
Yan Li: Nothing to disclose
Gregor Jost: Employee: Employee at Bayer AG
Benedikt Michael Schaarschmidt: Nothing to disclose
Johannes Haubold: Grant Recipient: Johannes Haubold received financial support by the DFG (German Research Foundation)-funded Clinician Scientist Program of the University Medicine Essen Clinician Scientist Academy (UMEA) (FU 356/12-1).
Hubertus Pietsch: Employee: Employee at Bayer AG
René Hosch: Nothing to disclose

### Friday, July 15

08:00-09:00 Open Forum #1 (Radiographers)

### Research Presentation Session: Radiographers

### RPS 1214

### Optimising CT practice: enhancing examination outcomes

**Moderators**
F. Zarb; Msida/MT
L. Preda; Milan/IT

### RPS 1214-3

**Assessment of image quality and radiation dose in the study of carotid arteries by computed tomography in low-dose protocols**

*A. J. Bylinka*, B. M. Zwierko, R. Pawlik, W. Skura, J. Budzyński; Bydgoszcz/PL

**Purpose:** To compare radiation doses and image quality in low dose and standard dose protocols for CTA of carotid arteries.
**Methods or Background:** The study included a group of 151 patients qualified for medical reasons for CTA of carotid arteries. By randomisation, 50 (33.11%) patients underwent examination using the low-dose protocols using the back projection (group I), and 50 (33.11%) with the iterative reconstruction ASIR 40% (group II). The control group consisted of 51 (33.77%) patients examined with the standard protocol (group III). The diagnostic quality of the obtained images was assessed by 3 medical specialists on the forms prepared for the study.
**Results or Findings:** The radiation dose (DLP) during CTA in group I was 218.4 [mGy cm], while in group II 207.2 [mGy cm]. These doses were statistically significantly lower than in the control group III, which was 317.9 [mGy cm], p <0.001. The mean evaluations of the 4 parameters performed by radiologists in each of the 7 segments of the carotid arteries did not differ statistically in the standard and low dose protocol. However, in the iterative protocol (ASIR 40%), the mean scores in individual arterial sections (initial CCA, intracranial ICA, V1 VA, V4 VA) were statistically significantly higher than in the group I and III.
**Conclusion:** The radiation doses were reduced by about 31% in group I and by 35% in group II. The low-dose protocol using the iterative reconstruction had the highest mean image quality ratings with the lowest radiation dose. Therefore, this method is recomended if its available.
**Limitations:** Protocol details can depend on various computer tomographs.
**Ethics committee approval:** Consent of the local Bioethics Committee No. 764/2019 was obtained.
**Funding for this study:** A low-dose carotid CTA protocol with iterative reconstruction (ASIR 40%) has the best image quality while enabling a dose reduction of 35%.
**Author Disclosures:**
Wojciech Skura: Consultant: assessing the diagnostic quality of the examination, Radiologist
Beata Małgorzata Zwierko: Consultant: substantive support, radiological protection inspector
Rafał Pawlik: Consultant: assessing the diagnostic quality of the examination, Radiologist
Jacek Budzyński: Consultant: assessing the diagnostic quality of the examination, Angiologist, research tutor, supervisor
Agnieszka Julia Bylinka: Author: creator and performer of the study

### RPS 1214-4

**Contrast monitoring techniques in thoracic-abdominal CT examinations: an important and underappreciated contributor to patient dose**

M. Fiebich[1], A. H. Mahnken[2], A. König[2], L. Abou Assali[1], *J. Verbe Zoum*[2]; [1]Gießen/DE, [2]Marburg/DE
*(julius.verbezoum@uni-marburg.de)*

**Purpose:** The purpose of our study was to evaluate the contribution of contrast monitoring techniques to overall dose in a CT exam and to optimise patient dose.
**Methods or Background:** CT retrospective study of 500 patients from a Siemens device (Somatom Definition) were analysed. High dose contrast-monitoring protocols were selected. 170 thoracic-abdominal CT exams presented high monitoring-dose in comparison to other studies. Exposure parameters were retrieved from the radiation dose structure report (RDSR). Two dose optimisation strategies based on parameter settings were implemented and the third is still to be tested in routine exams. Dose and diagnostic quality after each optimisation was assessed. Initial parameter of 120kV and 80mAs was reduced to100kV and 20mAs. Signal-to-noise ratios before and after optimisation were determined. Finally, the contrast media density curve was also evaluated as a measure to delay the scan start after injection. Results before and after optimisation were compared and discussed.
**Results or Findings:** Average DLP dose of monitoring in thoracic-abdominal CT studies was 35mGy*cm using a standard 120kV and 80mAs protocol. After optimisation to 100kV and 20mAs, average DLP dose was reduced to 5mGy*cm for contrast-monitoring scans. Subsequently, minimum Signal-to-noise ratio from initial setting was 4.2±3.3 versus 3.3±1.8 after optimisation. Initial scan start from 12s increased to 15s after injection can be achieved.
**Conclusion:** Despite a short scan length and relatively small DLP, contrast-monitoring techniques set at 120kV can account for 14% of the overall dose from a thoracic-abdominal CT study. By decreasing kilovoltage, tube current and delaying start scan time of contrast-monitoring component, a significant reduction in overall dose can be achieved without affecting diagnostic quality or timing. Results presented should serve as incentive to include monitoring dose, that is both CTDIvol and DLP, when setting diagnostic reference levels for CT examinations.
**Limitations:** Flow rate and haemodynamics were absent.
**Ethics committee approval:** Ethical approval was not sought.
**Funding for this study:** No funding was received for this study.
**Author Disclosures:**
Lilian Abou Assali: Investigator: Student
Alexander König: Employee: Occupational Worker
Martin Fiebich: Consultant: Occupational Worker
Julius Verbe Zoum: Investigator: Occupational Worker
Andreas H. Mahnken: Research/Grant Support: Occupational Worker

### RPS 1214-5

**Cardiac CT Hi-res scan mode in stent patients**

*S. D. Moerup*[1], H. Precht[1], J. Lambrechtsen[2], S. J. Foley[3]; [1]Odense/DK, [2]Svendborg/DK, [3]Dublin/IE
*(sdmo@ucl.dk)*

**Purpose:** To investigate the influence of high-resolution scan mode (Hi-res) on image quality and radiation dose for cardiac computed tomography angiography scanning in patients with stents.
**Methods or Background:** Eighteen patients with coronary stents presenting for cardiac CT were randomly scanned on the Revolution Apex CT scanner (GE Healthcare, USA). Three scan protocols were used according to patient diameters (AP+Lat): 1) Hi-res with 80kVp (39-56.99cm), 2) Hi-res with 100kVp (57-63.5cm), 3) standard protocol with kV assist (<63.6cm). All images were reconstructed with ASiR-V 50% and TrueFidelity in three levels. Image quality was assessed objectively (HU, noise). A cardiologist subjectively evaluated spatial resolution of each stent on all image stacks, which were randomly displayed and scored from 1 (lowest)-4 (highest quality). Effective dose was calculated.
**Results or Findings:** The mean effective dose for the three protocols were 1.35, 1.6 and 3.0mSv, respectively. Hi-res with 80kVp (n=1) showed a decrease in image noise from ASiR-V 50% to TrueFidelity low (42%), medium (61%) and high (67%). Hi-res with 100kVp (n=11) showed a decrease in noise by 39% from ASiR-V 50% to TrueFidelity low, medium (54%) and high (61%). Standard protocol with kV assist (n=6), noise was reduced by 20% for TrueFidelity low, medium (34%) and high (52%). The difference in noise between the three protocols (89.7HU, 64.5HU, 35.5HU) showed that the patients scanned without Hi-res had up to 153% less image noise. The stent evaluation showed that the best visualisation of the stent was obtained at TrueFidelity High, for all but one patient with stent calcifications (TrueFidelity medium).
**Conclusion:** Hi-res scan mode substantially increases image noise which to some extend can be reduced by applying TrueFidelity. Hi-res did not affect subjective spatial resolution around the stent.
**Limitations:** Limited amount of patients.
**Ethics committee approval:** This study was approved by an ethics committee.
**Funding for this study:** No funding was received.
**Author Disclosures:**
Helle Precht: Nothing to disclose
Svea Deppe Moerup: Nothing to disclose
Shane J Foley: Nothing to disclose
Jess Lambrechtsen: Nothing to disclose

### RPS 1214-6

**Advanced diagnostic pathway using CT coronary angiography (CTCA) for the management of a chest pain pathway in the emergency department before and during the covid pandemic: a single centre experience**

*D. W. Reidy*, W. A. Moynihan, P. Sheehy, J. M. O'Brien, S. Atyani; Limerick/IE

**Purpose:** University Hospital Limerick always strives to improve patients' experience. This novel streamlined integrated ED pathway is based on validated, peer-reviewed scientific data representing best possible clinical practice. Patients are cared for by an interdepartmental team in a safe, patient-

Friday focused and cost effective manner. Our aim was to introduce CTCA into ED to facilitate patients who met the criteria of the chest pain pathway in order to reduce admissions and augmenting same day discharge.
**Methods or Background:** This service was first introduced pre-covid pandemic January 2019. We have successfully increased our volume of patients scanned year on year. A 40% increase of CTCA eligible patients were imaged after the covid pandemic began than prior. Patients presenting to ED with a strong clinical suspicion of coronary artery disease and met strict inclusion criteria were eligible to undergo CTCA. All scans were performed using a 128 slice scanner.
**Results or Findings:** 366 patients were scanned in total since the beginning of first implementation. 351 of these patients had same day discharge representing a 96% success rate. 68% of all CTCA scans were performed during the covid pandemic. Radiation reduction dose was found to be 40% when compared to conventional coronary angiography in the cardiac catheterisation laboratory. Average cost effectiveness was found to be 600 euro for CTCA in ED versus 4000 euro for hospital admission and conventional angiography.
**Conclusion:** CTCA has been successfully integrated into the ED clinical decision unit chest pain pathway replacing exercise ECG. CTCA is more accurate, allowing definite identification of coronary artery disease. This initiative is a patient-centred approach that is less invasive, resulting in shorter hospital stays for patients with significant radiation dose reduction while also proving to be cost-effective.
**Limitations:** No limitations were identified.
**Ethics committee approval:** This study was approved by an ethics committee.
**Funding for this study:** Funding was received in the form of education and radiographer specific CTCA course.
**Author Disclosures:**
Said Atyani: Nothing to disclose
Paul Sheehy: Nothing to disclose
Julie Marie O'Brien: Nothing to disclose
David William Reidy: Nothing to disclose
William Anthony Moynihan: Nothing to disclose

### RPS 1214-7

### Detection of incidental adrenal nodules on CT by radiographers

*S. Camilleri*, F. Zarb, V. Micallef, K. B. Borg Grima; Msida/MT
*(sarah.s.camilleri.17@um.edu.mt)*

**Purpose:** The purpose of this research was to investigate whether radiographers' socio-demographic characteristics were affecting the detection rate of incidental adrenal nodules, also known as adrenal incidentalomas (AIs) in Malta. Additionally, local statistics of AI findings were evaluated.
**Methods or Background:** This research consisted of two phases and employed a non-experimental, cross-sectional quantitative approach. Phase 1 comprised a self-designed data collection sheet to retrospectively determine the occurrence of recalled computed tomography (CT) examinations as a result of AI findings. In phase 2, a self-designed structured questionnaire with anonymised CT scan images (n=30) displayed on ViewDex was prospectively completed by CT radiographers (n=23). Moreover, the image quality of each CT examination was evaluated to ensure that the image quality was not affecting the radiographers' detection rate.
**Results or Findings:** In phase 1, AIs were present in 1.4% of contrast-enhanced CT (CECT) examinations (n=12 139), out of which, 79.82% were not acknowledged by the radiographers and had to be recalled for a dedicated adrenal CT examination. In phase 2, a statistically significant relation ($p<0.05$) between the radiographers' educational level, working hours and years of experience, to the detection rate of AIs was determined. Furthermore, no statistical correlation was determined between the image quality and the radiographers' detection rate.
**Conclusion:** Findings suggest that certain socio-demographic characteristics of radiographers affected their ability to recognise AIs. This could have potentially contributed to one of the reasons for recalling patients, which in turn results in an added burden to both the patient and the medical imaging department (MID).
**Limitations:** The filtering process used to acquire the data set was limited to specific keywords, commonly used in the radiologists' reports.
**Ethics committee approval:** This study was performed following ethical permission from the University of Malta Research Ethics Committee.
**Funding for this study:** No funding was received for this study.
**Author Disclosures:**
Karen Borg Borg Grima: Nothing to disclose
Victor Micallef: Nothing to disclose
Francis Zarb: Nothing to disclose
Sarah Camilleri: Nothing to disclose

### RPS 1214-8

### Simulated motion artefacts and reduction of temporal resolution in cardiac CT using only image data

*M. W. Kusk*; Esbjerg/DK
*(martin.weber.kusk@rsyd.dk)*

**Purpose:** To develop a method for introducing motion artefacts and simulate reduced temporal resolution in cardiac CT images. Using original, artefact-free images as reference standard, such a method could be used to quantify the influence of scanner type, heartrate and respiration on for example cardiac volume measurements.
**Methods or Background:** High-quality functional cardiac CT series were used as input. Image processing was done in MATLAB. Respiration was simulated by spatial shifting of portions of the image stack. Pulsation artefacts were produced by conversion to sinograms, and mixing of data from different phases at discrete positions. Temporal resolution reduction was simulated by mixing sinograms from overlapping temporal phases over the entire scan range. Artificial noise was introduced to compensate for increased data volume. Images were reconstructed using inverse Radon transform. 20 real and simulated artefact image pairs were evaluated in blinded fashion by two cardiac CT radiographers.
**Results or Findings:** Simulated artefacts were qualitatively similar to real artfacts and could be analysed at clinical workstation. Artefact severity could be controlled by parameter adjustment. Radiographers were not able to distinguish real from simulated artefacts.
**Conclusion:** Motion artefact simulation is a viable alternative to expensive dynamic phantoms, for evaluation on clinical images, allowing for inter-patient variation.
**Limitations:** Phase sampling interval, heartrate and temporal resolution of input images limits the possibility of artefact introduction in some cases. Some loss of spatial information resulted from the use of the Radon/inverse Radon transform, limiting applicability in high-resolution imaging.
**Ethics committee approval:** Danish National Research Ethics Comittee approved the retrospective use of image data without patient consent (approval no. 2015994).
**Funding for this study:** The study was supported by research grants from the Danish Radiographers' Association ("Radiograf Rådet") and Karola Jørgensen Research fund at the hospital of Soutwest Jutland.
**Author Disclosures:**
Martin Weber Kusk: Nothing to disclose

### RPS 1214-9

### A study to investigate how confident CT trained radiographers are at performing paediatric CT trauma scans

*H. J. Greenberg*, M. D. Davis; Dublin/IE
*(heather-35@hotmail.co.uk)*

**Purpose:** This research aimed to determine how confident qualified CT radiographers are at performing paediatric CT trauma scans at two district general hospitals in Wales, and whether teaching sessions on this topic have assisted in improving the radiographers' perceived confidence.
**Methods or Background:** In 2020, trauma was one of the leading causes of paediatric mortality in England and Wales. Use of CT can provide time efficient and accurate diagnosis, increasing chances of survival, and this has subsequently lead to an increase in the use of CT over the last 25 years. However, whilst the use of CT in assisting in the evaluation of paediatric trauma has been invaluable, it carries a significant radiation risk, largely because paediatric patients have a greater sensitivity with regard to radiation when compared to adults. Although national paediatric trauma workload within the UK is proportionately low, the majority of paediatric patients are conveyed to hospitals which predominately scan adults. Individual questionnaires containing qualitative and quantitative aspects were used to ascertain the radiographers' initial perceived confidence levels and reasons for this figure. Following this, a teaching intervention was formulated by the researchers which was delivered to CT radiographers across the two sites. A post-teaching intervention questionnaire was used to determine whether the radiographers' confidence levels had improved due to the teaching intervention.
**Results or Findings:** The results will provide useful data on confidence levels regarding CT radiographers performing CT paediatric trauma scans.
**Conclusion:** The results will provide useful data on confidence levels regarding CT radiographers performing CT paediatric trauma scans.
**Limitations:** Small sample size, reporters' bias (as it is radiographers' perceived confidence), information bias (might have been introduced during the collecting and measuring stages as the researcher knows some participants).
**Ethics committee approval:** Ethical exemption received.
**Funding for this study:** Not applicable.
**Author Disclosures:**
Michaela Dawn Davis: Nothing to disclose
Heather Jane Greenberg: Nothing to disclose

**08:00-09:00** **Room E1**

### Research Presentation Session: Neuro

### RPS 1211

### Neurodegenerative diseases: dementia

**Moderator**
D. Chourmouzi; Thessaloniki/GR

### RPS 1211-2

**Assessment of the superficial white matter alterations using diffusion tensor imaging and CSF biomarkers in neurodegenerative dementia**

*S. Criscuolo*, V. E. Contarino, S. Siggillino, A. P. Savoldi, S. Casale, L. Caschera, A. Arighi, F. M. Triulzi, G. Conte; Milan/IT
*(stefania.criscuolo@unimi.it)*

**Purpose:** To explore superficial white matter alterations (SWM) using diffusion tensor imaging (DTI) and its relationship to cerebrospinal fluid (CSF) biomarkers in patients with neurodegenerative dementia (ND).
**Methods or Background:** We retrospectively enrolled 97 ND patients who had undergone assessment of CSF levels of amyloid-β 42 (Aβ42), total-tau (T-TAU), and phosphorylated tau (P-TAU), and brain MRI. They were divided into 55 NDs with abnormal Aβ42 (Aβ+) and 38 NDs with normal Aβ42 (Aβ-). Ten healthy controls (HC) were recruited for imaging and Mini-Mental State Examination (MMSE). The following DTI metrics of the SWM were calculated for each lobe: fractional anisotropy (FA), mean diffusivity (MD), axial diffusivity (L1), and radial diffusivity (RD).
**Results or Findings:** Both Aβ+ and Aβ- showed lower MMSE compared to HC ($p < 0.005$). Aβ+ showed lower FA and higher MD, L1, and RD across all lobes compared to HC. Aβ- showed higher FA and lower MD and RD in the frontal, parietal and temporal lobes, and lower L1 in the right frontal lobe compared to HC. Aβ42 showed a positive correlation with FA in all lobes, and a negative correlation with L1, MD, and RD in the parietal lobe bilaterally, with MD in left temporal and right occipital lobes, with RD in left temporal and occipital lobes. T-TAU showed significant positive correlations with L1, MD, and RD bilaterally in the frontal, parietal, and right temporal lobes and with L1 in the right occipital lobe. P-TAU showed positive correlations with L1, MD, and RD in the right frontal, parietal, and temporal lobes.
**Conclusion:** DTI detects and locates SWM alterations, it may be a promising non-invasive biomarker in the preclinical phase of ND.
**Limitations:** Post-mortem identification would be necessary for diagnostic confirmation.
**Ethics committee approval:** The study was approved by the Institutional Review Board.
**Funding for this study:** No funding was received.
**Author Disclosures:**
Andrea Arighi: Nothing to disclose
Silvia Siggillino: Nothing to disclose
Fabio Maria Triulzi: Nothing to disclose
Stefania Criscuolo: Nothing to disclose
Luca Caschera: Nothing to disclose
Anna Paola Savoldi: Nothing to disclose
Giorgio Conte: Nothing to disclose
Valeria E. Contarino: Nothing to disclose
Silvia Casale: Nothing to disclose

### RPS 1211-3

**Anatomically standardised detection of MRI atrophy patterns in early-stage Alzheimer's disease**

*L. Lenhart*[1], S. Seiler[2], G. Göbel[1], M. Wagner[1], P. Dal-Bianco[3], G. Ransmayr[4], R. Schmidt[2], T. Benke[1], C. Scherfler[1]; [1]Innsbruck/AT, [2]Graz/AT, [3]Vienna/AT, [4]Linz/AT

**Purpose:** MRI studies consistently identified atrophy patterns in Alzheimer's disease (AD) by whole-brain voxel-based analysis but efforts to investigate morphometric profiles using anatomically standardised and automated whole-brain ROI analysis performed at the individual subject space are still lacking. In this study we aimed i) to apply atlas-derived measurements of cortical thickness and subcortical volumes including hippocampal subfields to identify atrophy patterns in early-stage AD, and ii) to compare cognitive profiles at baseline and one-year follow-up of those previously identified morphometric AD subtypes to predict disease progression.
**Methods or Background:** From a prospectively recruited multicentre study conducted at four Austrian sites, 120 patients were included with probable AD, a disease onset beyond 60 years and a clinical dementia rating of ≤1. Morphometric measures of T1-weighted images were obtained using FreeSurfer.
**Results or Findings:** Principal component and subsequent cluster analysis identified four morphometric subtypes including i) hippocampal predominant (30.8%), ii) hippocampal-temporo-parietal (29.2%), iii) parieto-temporal (hippocampal sparing, 20.8%) and iv) hippocampal-temporal (19.2%) atrophy patterns that were associated with phenotypes differing predominately in presentation and progression of verbal memory and visuospatial impairments.
**Conclusion:** These morphologically distinct subtypes are based on standardised brain regions, which are anatomically defined and freely accessible to validate its diagnostic accuracy and enhance the prediction of disease progression.
**Limitations:** The inclusion of data from different MRI sites introduces potential confounds due to different magnetic field strengths and scanner specific parameter setups.
**Ethics committee approval:** The study was conducted according to the guidelines of the Declaration of Helsinki and was approved by the corresponding local Ethics Committee of the individual sites at the Medical Universities of Graz, Vienna, Linz and Innsbruck.
**Funding for this study:** This research received no external funding.
**Author Disclosures:**
Christoph Scherfler: Nothing to disclose
Georg Göbel: Nothing to disclose
Lukas Lenhart: Nothing to disclose
Gerhard Ransmayr: Nothing to disclose
Michaela Wagner: Nothing to disclose
Peter Dal-Bianco: Nothing to disclose
Reinhold Schmidt: Nothing to disclose
Thomas Benke: Nothing to disclose
Stephan Seiler: Nothing to disclose

### RPS 1211-4

**Behavioural and psychological symptoms of dementia are associated with structural brain changes in the limbic system**

*L. Melazzini*, L. M. Farina, G. Perini, F. P. Lombardo, E. Ballante, S. Bernini, M. Cotta Ramusino, A. Costa, S. Bastianello; Pavia/IT

**Purpose:** Behavioural and psychological symptoms of dementia (BPSD) are a group of signs and symptoms frequently occurring in patients with dementia. Given the poor evidence available on the neuroimaging correlates of BPSD in the literature, the aim of this study was to evaluate the associations between brain cortical and subcortical volumes and BPSD in mild cognitive impairment (MCI) and several types of dementia.
**Methods or Background:** Eighty-five cognitively impaired patients were prospectively recruited at our Institute of Neurology between June 2018 and February 2021. Patients underwent a thorough neurological assessment, a 3D T1-weighted 3-T brain MRI scan, a neuropsychological evaluation using the Neuropsychiatric inventory (NPI) and a lumbar puncture. Mean cortical thickness and brain subcortical volumes were automatically extracted using FreeSurfer v.7.1.0.
**Results or Findings:** Delusion, hallucination and apathy appeared to be inversely correlated with cortical thickness and volumes in frontal and limbic system areas ($p<0.05$) in all types of dementia. With regard to dementia sub-types, Lewy-body dementia (LBD) patients showed higher hallucination scores compared to MCI and Alzheimer's disease patients ($p<0.001$ and $p<0.05$, respectively). Psychosis cluster also displayed higher NPI scores in LBD with respect to MCI patients ($p<0.05$). A negative significant association among NPI total score and t-tau level (p-value<0.01) was found in a ß-regression model after correction for clinical confounding variables.
**Conclusion:** Our study showed that changes in brain volumes in several brain areas are associated with varying BPSD, with specific associations according to the type of cognitive impairment. Many of the involved areas are part of the fronto-limbic and para-limbic circuits, thus confirming the importance of these networks in the processes underlying the behavioural correlates of dementia.
**Limitations:** Cross-sectional study.
**Ethics committee approval:** This study was approved by an ethics committee.
**Funding for this study:** Funding was received from Ricerca Corrente 2018, Italian Min. of Health, IRCCS Mondino, Pavia.
**Author Disclosures:**
Matteo Cotta Ramusino: Nothing to disclose
Stefano Bastianello: Nothing to disclose
Sara Bernini: Nothing to disclose
Francesca Paola Lombardo: Nothing to disclose
Alfredo Costa: Nothing to disclose
Lisa Maria Farina: Nothing to disclose
Luca Melazzini: Nothing to disclose
Giulia Perini: Nothing to disclose
Elena Ballante: Nothing to disclose

### RPS 1211-5

**European inter-societal Delphi consensus for the biomarker-based aetiological diagnosis of neurocognitive disorders**

*F. B. B. Pizzini*[1], R. Vanninen[2], C. Festari[3], M. Cotta Ramusino[4], F. Massa[5], F. Nobili[5], G. Frisoni[6]; [1]Verona/IT, [2]Kuopio/FI, [3]Brescia/IT, [4]Pavia/IT, [5]Genoa/IT, [6]Geneva/CH
*(francesca.pizzini@aovr.veneto.it)*

**Purpose:** CSF and imaging biomarkers are needed for the aetiological diagnosis of neurocognitive disorders, but evidence is incomplete on their efficient use in the clinic. A European task force is defining a diagnostic algorithm where incomplete evidence on biomarker prioritisation is filled by expert opinion. This abstract reports the preliminary results as of October 2021.
**Methods or Background:** The project started in November 2020. A Delphi panel method was used to create consensus. Eleven pertinent European scientific societies delegated two panellists each to take part at the Delphi rounds and votes. Four rounds have been completed so far.
**Results or Findings:** Panellists agreed on the clinical workspace of the algorithm (specialist outpatient service), the stage of application (prodromal and mild dementia), the patient age window (biomarker use strongly encouraged below 70 years and of limited usefulness over age 85), and 11 clinical profiles driving the choice of first-line biomarkers. FDG PET was first line for the clinical profiles leading to suspect frontotemporal lobar degeneration and motor tauopathies; dopamine SPECT/PET for those leading to suspect Lewy body spectrum; and CSF biomarkers for those leading to suspect Alzheimer's disease and in cases with inconsistent neuropsychological and MRI findings. None of these biomarkers is indicated when clinical profiles suggest vascular cognitive impairment or other neurological disorders.
**Conclusion:** The task force is currently defining second-line biomarkers. The project it set to deliver the final algorithm by October 2022.
**Limitations:** The limitations are linked to the Delphi procedure (e.g. kick-off questions may be driven by the moderator, a subtle phrasing of the questions may drive answers, and 70% threshold for convergence is conventional).
**Ethics committee approval:** Not applicable.
**Funding for this study:** Unrestricted grant from F. Hoffmann-La Roche Ltd., Biogen International GmbH, Eisai Europe Limited, and Life Molecular Imaging GmbH.
**Author Disclosures:**
Cristina Festari: Nothing to disclose
Federico Massa: Nothing to disclose
Francesca Benedetta Benedetta Pizzini: Nothing to disclose
Flavio Nobili: Nothing to disclose
Matteo Cotta Ramusino: Nothing to disclose
Ritva Vanninen: Nothing to disclose
Giovanni Frisoni: Nothing to disclose

### RPS 1211-6

**Qualitative and quantitative comparison of hippocampal volumetric software applications: do all roads lead to Rome?**

*S. Mangesius*[1], L. Haider[2], L. Lenhart[1], R. Steiger[1], F. Prados[3], C. Scherfler[1], E. R. Gizewski[1]; [1]Innsbruck/AT, [2]Vienna/AT, [3]London/UK
*(stephanie.mangesius@tirol-kliniken.at)*

**Purpose:** Brain volumetric software is increasingly suggested for clinical routine. The present study quantifies the agreement across different software applications.
**Methods or Background:** Ten individuals with hippocampal volume z-scores <-1.96 as measured by FreeSurfer (FS) based on an in-house gender and age-adjusted healthy control group, and 10 age-matched healthy controls were chosen, median age: 74 years (25-75% range: 66-77). Hippocampal volumes were computed using 3-Tesla T1-MPRAGE-sequences with FS, Statistical-Parametric-Mapping (SPM; Neuromorphometrics and Hammers atlases), Geodesic-Information-Flows (GIF), Similarity-and-Truth-Estimation-for-Propagated-Segmentations (STEPS), and a commercially available software application. MTA scores were manually rated. Volumetric measures of each individual were compared against the mean of all applications with intraclass-correlation-coefficients (ICC) and Bland-Altman-statistics.
**Results or Findings:** Comparing against the mean of all methods, moderate to low agreement was present considering categorisation of hippocampal volumes into quartiles. ICCs ranged noticeably between software applications (left hippocampus [LH]: from 0.42 [STEPS] to 0.88 [FS]; right hippocampus [RH]: from 0.36 [commercial software application] to 0.86 [FS]). Mean differences between individual methods and the mean of all methods (mm3) was considerable (LH: FS -209, SPM-Neuromorphometrics -820; SPM-Hammers -1474; commercial software application -680; GIF 891; STEPS 2218; RH: FS -232, SPM-Neuromorphometrics -745; SPM-Hammers -1547; commercial software application -723; GIF 982; STEPS 2188).
**Conclusion:** Considerable variability in volumetric measurements between different applications was revealed, even in a cohort with large spread in data (normal aging vs severe atrophy) and absence of structural lesions. Interchangeable use of different volumetric applications is not recommended.
**Limitations:** This pilot study included a limited case number and RRMS cases with minor atrophy. However, the goal was to investigate the agreement of planimetric and volumetric measures in early disease stages. Therefore, high agreement in a small cohort is reassuring.
**Ethics committee approval:** The study was approved by the local Ethics Committee.
**Funding for this study:** No funding was received for this study.
**Author Disclosures:**
Christoph Scherfler: Nothing to disclose
Ruth Steiger: Nothing to disclose
Lukas Lenhart: Nothing to disclose
Mister Ferran Prados: Nothing to disclose
Elke Ruth Gizewski: Nothing to disclose
Lukas Haider: Nothing to disclose
Stephanie Mangesius: Nothing to disclose

### RPS 1211-8

**Building a comprehensive model of normal brain ageing with brain MRI, cognitive tests and machine learning**

Y. Statsenko[1], T. Habuza[1], *S. Meribout*[2], D. Smetanina[1], G. Simiyu[1], K. Neidl-Van Gorkom[1], I. Charykova[3], M. Szolich[1], M. Belghali[1]; [1]Al Ain/AE, [2]Constantine/DZ, [3]Minsk/BY
*(s-meribout@hotmail.com)*

**Purpose:** To get an insight into brain morphometry and psychophysiological performance across the lifespan.
**Methods or Background:** Background: Because of non-specific brain structural and functional changes both in physiological brain ageing and brain disorders, the MRI diagnostics of the latter can be challenging. Establishing a comprehensive definition of normal brain ageing will enhance diagnostic accuracy. Methods: We used the publicly available POBA dataset of 231 subjects (4-84 yo). The exclusion criteria were: organic brain pathology, mental disorders, injury to the head, the MRI signs of neurodegeneration. By using voxel-based morphometry and lesion segmentation along with linear statistics and machine learning, we analysed structural changes of the major brain compartments and modeled dynamics of neurofunctional performance across life. We correlated volumetric changes of major brain compartments with dynamics of psychophysiological performance in age groups.
**Results or Findings:** The percentage of the CSF (CSF%) increases placidly throughout life and its accumulation reflects the atrophy of brain parenchyma. The percentage of the cortical gray matter (cGM%) to total intracranial volume reduces with advancing age. However, the percentage of the total WM (WM%) rises across the lifespan. Decision-making time is associated positively with the CSF volume (for CSF% r=0.16). There is no association between the percentage of WM lesions (WMH%) and psychophysiological performance as well as no correlation is seen between WMH% and age.
**Conclusion:** The strongest correlation between brain structural data and functional outcomes is observed between total CSF% and reaction time in the IRT test which is most cognitively demanding in our battery (r=0.36). As the variables CSF% and age have the highest coefficient of correlation in this study (r=0.8), CSF% can be considered as the most sensitive marker of age-related brain atrophy.
**Limitations:** Not applicable.
**Ethics committee approval:** This study was approved by UAEU Human Ethics.
**Funding for this study:** Funding was received from Aare19-060; Uaeu31M442; G00003264.
**Author Disclosures:**
Inna Charykova: Nothing to disclose
Klaus Neidl-Van Gorkom: Nothing to disclose
Darya Smetanina: Nothing to disclose
Sarah Meribout: Nothing to disclose
Gillian Simiyu: Nothing to disclose
Tetiana Habuza: Nothing to disclose
Yauhen Statsenko: Nothing to disclose
Miklos Szolich: Nothing to disclose
Maroua Belghali: Nothing to disclose

08:00-09:00 Room G

### Research Presentation Session: Cardiac

### RPS 1203

### New developments in cardiac CT and calcium scoring

**Moderator**
E. Zimmermann; Berlin/DE

### RPS 1203-2

**Coronary CT angiography with photon-counting computed tomography: first-in-human results**

*S. A. Si-Mohamed*, S. Boccalini, H. Lacombe, A. Diaw, M. Varasteh, P-A. Rodesch, L. Boussel, J. Greffier, P. Douek; Lyon/FR
*(salim.si-mohamed@chu-lyon.fr)*

**Purpose:** To compare the quality of a coronary CT angiography (CCTA) between a clinical prototype photon-counting CT (PCCT) and an energy-integrating detector dual-layer CT (EID-DLCT).
**Methods or Background:** In this prospective board-approved study with informed consent, participants with coronary artery disease underwent retrospective ECG-gated CCTA on both systems, following injection of 65-75 mL of 400 mg/mL iodinated contrast agent at 5 mL/s. A prior task-based quality assessment of coronary lesions' detectability index was performed in a phantom study. 1024 matrix and 0.25 mm slice thickness were used for PCCT while 512 matrix, 0.67 mm slice thickness for EID-DLCT. Three cardiac radiologists independently performed a blinded analysis using a 5-point quality score (1: insufficient; 5: excellent) for overall quality, diagnostic confidence and quality of calcifications, stents, and non-calcified plaques. Logistic regression model, adjusted on radiologists, was used to evaluate the proportion of improvement in scores with the best method.
**Results or Findings:** Fourteen consecutive participants (12 male, 61±17-years) were enrolled. Scores of overall quality and diagnostic confidence were higher with PCCT images with a median (interquartile range) of 5 (2) and 5 (1) versus 4 (1) and 4 (3), using 255 mAs versus 349±111 mAs for EID-DLCT images ($p<0.01$). Proportions of improvement with PCCT images for quality of calcification, stent and non-calcified plaque were of 100%, 92% and 45%, respectively. In phantom study, detectability indices were 2.3-fold higher for lumen and 2.9-fold higher for non-calcified plaques with PCCT images.
**Conclusion:** Coronary CT angiography imaging with a PCCT system demonstrated in human an improved image quality and diagnostic confidence compared to an EID-DLCT.
**Limitations:** Number of patients was limited.
**Ethics committee approval:** This prospective study has been approved by the institutional review board with informed consent.
**Funding for this study:** Funding was received from the European Union Horizon 2020 grant No 668142.
**Author Disclosures:**
Salim Aymeric Si-Mohamed: Nothing to disclose
Adja Diaw: Nothing to disclose
Pierre-Antoine Rodesch: Nothing to disclose
Loïc Boussel: Nothing to disclose
Philippe Douek: Nothing to disclose
Hugo Lacombe: Nothing to disclose
Sara Boccalini: Nothing to disclose
Joel Greffier: Nothing to disclose
Mohammad Varasteh: Nothing to disclose

### RPS 1203-3

**Coronary stent image quality with a spectral photon counting CT: first results in humans**

*S. Boccalini*[1], S. A. Si-Mohamed[1], H. Lacombe[1], M. Villien[1], A. Diaw[1], K. Erhard[2], Y. Yagil[3], L. Boussel[1], P. Douek[1]; [1]Lyon/FR, [2]Hamburg/DE, [3]Haifa/IL

**Purpose:** The aim of this study was to compare the image quality (IQ) of in-vivo coronary stents between an energy-integrating detectors dual-layer CT (EID-DLCT) and a clinical prototype of spectral photon counting CT (SPCCT).
**Methods or Background:** Between January and June 2021 consecutive patients with coronary stents were prospectively enrolled to undergo a coronary CT with an EID-DLCT and a SPCCT. A retrospectively ECG-gated acquisition was performed with optimised matching parameters and identical injection protocol on the two scanners. Images were reconstructed with slice thickness of 0.67mm, 512 matrix, XCD kernel, iDose 3 for EID-DLCT and 0.25mm slice thickness, 1024 matrix, sharp kernel and iDose 6 for SPCCT. The difference (ΔS-C) between ROIs drawn inside the stent and the adjacent coronary artery was calculated. Measurements of the outer and inner diameters of the stents were used to quantify blooming artefacts. For subjective IQ, three experienced observers graded different parameters with a 4-point scale: coronary wall before stents, stent lumen, stent structure, calcifications surrounding stents, and beam hardening artefacts.
**Results or Findings:** Eight patients (age: 68 [IQ=8]; all males; BMI: 26.2 [IQ=4.2]) with 16 stents were scanned. Five stents were not able to be evaluated due to motion artefacts on the SPCCT. Radiation dose was lower for SPCCT (fixed CTDIvol=25.7mGy vs. CTDIvol=35.7mGy [IQ=13.6]; $p=0.02$). With SPCCT, external diameters were smaller, internal diameters were larger and, consequently, blooming artefacts were reduced ($p<0.05$). The ΔS-C was lower for SPCCT as compared to EID-DLCT-XCD ($p<0.05$). SPCCT received higher IQ subjective scores than EID-DLCT for all parameters (all $p\leq0.05$).
**Conclusion:** SPCCT demonstrated improved objective and subjective image quality as compared to EID-DLCT for the evaluation of coronary stents.
**Limitations:** An identified limitation of this study was that few patients were assessed.
**Ethics committee approval:** This study was approved by the local ethics committee and patients signed an informed consent.
**Funding for this study:** Funding was received from: EUH2020 No. 668142.
**Author Disclosures:**
Marjorie Villien: Employee: Philips
Salim Aymeric Si-Mohamed: Nothing to disclose
Mme Adja Diaw: Nothing to disclose
Yoad Yagil: Employee: Philips
Loïc Boussel: Nothing to disclose
Philippe Douek: Nothing to disclose
Hugo Lacombe: Nothing to disclose
Sara Boccalini: Nothing to disclose
Klaus Erhard: Employee: Philips

### RPS 1203-4

**Benefit of virtual monoenergetic imaging reconstructions for coronary CT angiography on a novel dual-source photon counting detector CT: comparison with energy-integrating detector CT**

*J. A. Decker*, F. Risch, P. Woźnicki, F. Braun, S. Bette, C. Wollny, C. Scheurig-Muenkler, T. J. Kroencke, F. Schwarz; Augsburg/DE
*(Josua.Decker@uk-augsburg.de)*

**Purpose:** To identify the benefit of virtual monoenergetic imaging (VMI) reconstructions for coronary CT angiography (CCTA) on a novel dual-source photon-counting detector CT (PCD-CT) in comparison with an energy-integrating detector CT (EID-CT) and to assess the impact of VMI on calcium blooming and coronary artery stenosis.
**Methods or Background:** Twenty-five consecutive patients with clinically indicated CCTA on a PCD-CT (Naeotom alpha, Siemens Healthineers, Erlangen, Germany) were retrospectively identified and matched for tube voltage to a cohort scanned on an EID-CT (Somatom Flash, Siemens Healthineers) using comparable ECG-synchronisation and contrast protocols. Circular regions of interest were placed in the aorta and 6 proximal coronary artery segments. Contrast-to-noise (CNR) and signal-to-noise ratio (SNR) were calculated for EID-CT and 13 PCD-CT VMI-reconstructions (range: 40-120keV). In segments with calcified plaques, the degree of stenosis and area of calcification was measured for different VMI-reconstructions using profile plots and histograms for CT-values (ImageJ).
**Results or Findings:** VMI-reconstructions at lower keV-levels (40-70) have significantly higher SNR (16.1±3.6 vs 12.3±1.9; $p<0.001$) and CNR (12.1±3.0 vs 10.4±1.5; $p=0.002$) at the PCD-CT compared to EID-CT with a maximum at 40keV. The degree of coronary artery stenosis was lowest at 100-120keV showing an increase of up to ~20% between 60 and 80keV. Similar, calcium blooming was smallest at 100-120keV increasing between 60 and 80keV with a plateau between 40 and 60keV.
**Conclusion:** VMI-reconstructions at low keV-levels (40-70keV) from PCD-CT significantly increase SNR and CNR in CCTA compared to EID-CT but might be more susceptible to blooming artifacts than VMI-reconstructions at higher keV levels. We suggest that the optimal keV level has to be selected based on the extent of coronary artery disease.
**Limitations:** Retrospective single-centre study, small sample size.
**Ethics committee approval:** This study was approved by an ethics committee. Necessity to obtain informed-consent was waived.
**Funding for this study:** No funding was received for this study.
**Author Disclosures:**
Franka Risch: Nothing to disclose
Stefanie Bette: Nothing to disclose
Christian Scheurig-Muenkler: Nothing to disclose
Franziska Braun: Nothing to disclose
Claudia Wollny: Nothing to disclose
Thomas J. Kroencke: Nothing to disclose
Florian Schwarz: Nothing to disclose
Piotr Woźnicki: Nothing to disclose
Josua A. Decker: Nothing to disclose

### RPS 1203-5

**Novel virtual non-contrast reconstruction for coronary calcium scoring derived from coronary CT angiography using dual-layer dual-energy CT**

*I. L. Langenbach*[1], K. Klein[1], C. P. Naehle[1], H. Wienemann[1], A. Bunck[1], J. Holz[1], D. Maintz[1], E. Langzam[2], M. C. Langenbach[1]; [1]Cologne/DE, [2]Haifa/IL
*(breidert.isabel@gmail.com)*

**Purpose:** To evaluate the clinical applicability of a new prototype virtual non-contrast (VNC) reconstruction algorism based on contrast-enhanced coronary CT angiography (cCTA) to assess calcified coronary plaques by calcium scoring (CaSc).
**Methods or Background:** We retrospectively included 80 consecutive patients suspected of coronary artery disease. All patients underwent a cardiac CT at the same dual-layer spectral detector CT system (IQon; Philips Healthcare, The Netherlands) using a standardised acquisition protocol including unenhanced CaSc and contrast-enhanced cCTA. VNC-reconstructions were calculated from the cCTA images using a novel post-processing algorism discriminating calcified plaques from contrast media and soft tissue in the coronary arteries. Reconstructions were made in 2.5 mm, 2.5 of 0.9 mm and 0.9 mm with the same imaging parameters as the unenhanced CaSc sequence. We compared the Agatston score and classifications according to CAC-DRS of all VNC-reconstructions with the TNC-dataset as the gold standard.
**Results or Findings:** In all patients, reconstructions of the VNC-images were performed successfully. We found no significant differences in the Agatston score comparing all VNC-reconstructions with the TNC-dataset (p=0.379). Correlation analysis of the datasets showed a significant excellent correlation of the TNC-results with the different VNC-reconstructions with r=0.871-0.891. By classification according to CAC-DRS, the Kruskal-Wallis-Test revealed no significant difference in classification between all groups (p=0.284). Evaluation of the intra-class correlation coefficient revealed a strong to almost perfect agreement between the two different readers for Agatston scoring of the coronary arteries for all reconstructions.
**Conclusion:** The investigated novel VNC reconstructions algorism of contrast-enhanced routine cCTA allows reliable detection and evaluation of calcified coronary plaques without the requirement for an additional acquisition of an unenhanced CT scan for calcium scoring.
**Limitations:** Retrospective character, small study population.
**Ethics committee approval:** Approval was obtained.
**Funding for this study:** No funding was received for this study.
**Author Disclosures:**
Jasmin Holz: Nothing to disclose
Isabel Luisa Langenbach: Nothing to disclose
David Maintz: Nothing to disclose
Konstantin Klein: Nothing to disclose
Claas Philip Naehle: Nothing to disclose
Eran Langzam: Employee: Philips Healthcare
Marcel Christian Langenbach: Nothing to disclose
Alexander Bunck: Nothing to disclose
Hendrik Wienemann: Nothing to disclose

### RPS 1203-6

**Coronary CTA-based calcium scoring: in-vitro and in-vivo validation of a novel virtual non-iodine reconstruction algorithm on a clinical, first-generation photon counting-detector system**

*T. S. Emrich*[1], G. Aquino[1], U. J. Schoepf[1], J. O'Doherty[2], V. Brandt[1], T. Flohr[3], B. Schmidt[3], T. Allmendinger[3], A. Varga-Szemes[1]; [1]Charleston, SC/US, [2]Malvern, PA/US, [3]Forchheim/DE
*(emrich@musc.edu)*

**Purpose:** To evaluate coronary CTA (CCTA)-based in-vitro and in-vivo coronary artery calcium scoring (CACS) using a novel virtual non-iodine (VNI) reconstruction on a clinical, first-generation photon counting detector (PCD)-CT system compared to virtual non-contrast (VNC) and true non-contrast (TNC) acquisitions.
**Methods or Background:** While CACS and CCTA are well established techniques for the assessment of coronary artery disease, they are complementary acquisitions, translating into increased scan time and radiation dose. Hence, accurate CACS derived from a single CCTA acquisition would be highly desirable. In this study, CACS based on VNI, VNC and TNC reconstructions were evaluated in a CACS phantom (QRM, Moehrendorf, Germany) and in 32 prospectively recruited patients (59.4±14.1 years; 59.3% male) undergoing CCTA with a first generation PCD-CT system (NAEOTOM Alpha, Siemens Healthineers, Germany). CACS were quantified for the three reconstructions and compared using Wilcoxon's test. Agreement was evaluated by Spearmen's correlation and Bland-Altmann analysis. Classification of CACS categories (CACS 0, 1-100, 101-400 and >400) were compared using Cohen's kappa.
**Results or Findings:** Phantom studies demonstrated strong agreement between VNI and TNC (60.7±90.6 vs 67.3±88.3,p=0.01, r=0.98, mean bias: 6.6), while VNC showed a significant underestimation of CACS (42.4±75.3 vs 67.3±88.3, p<0.001, r=0.94, mean bias: 24.9). In-vivo comparison confirmed the high correlation, but revealed a slight underestimation of CACS based on VNI (median [IQR]: 21.7 [0/472.5] vs 9.0 [0/364.6], p<0.001; r=0.99; mean bias: -113.5). In comparison, VNC showed weaker correlation and a larger underestimation (21.7 [0/472.5] vs 0.8 [0/86.6], p<0.001, r=0.93; mean bias: -372.4). VNI showed superior agreement of CACS classification (kappa=0.93), compared to that of VNC (kappa=0.69).
**Conclusion:** Accuracy of CACS quantification and classification based on VNI reconstruction of CCTA outperforms CACS derived from VNC.
**Limitations:** Single-centre study, missing ground-truth for in-vivo scans.
**Ethics committee approval:** This study was approved by an ethics committee. IRB approval: Pro00108359.
**Funding for this study:** Funding was received from Siemens Healthcare.
**Author Disclosures:**
Verena Brandt: Nothing to disclose
Thomas Flohr: Employee: Siemens Healthineers
Jim O'Doherty: Employee: Siemens Healthcare
Thomas Allmendinger: Employee: Siemens Healthineers
Uwe Joseph Schoepf: Research/Grant Support: Bayer, Guerbet, Elucid, Siemens Healthcare, Bracco, HeartFlow
Tilman Stephan Emrich: Speaker: Siemens Healthcare
Bernhard Schmidt: Employee: Siemens Healthineers
Akos Varga-Szemes: Research/Grant Support: Elucid, Siemens Healthcare
Gilberto Aquino: Nothing to disclose

### RPS 1203-7

**Dual-source photon-counting CT in coronary artery calcium imaging: early experience and comparison to current clinical standard**

*F. R. Schwartz*, J. C. Ramirez-Giraldo, M. Daubert, T. D. Tailor, D. Marin; Durham, NC/US
*(fidesschwartz@me.com)*

**Purpose:** The purpose of this study is to assess the performance of a photon-counting CT (PCCT) system for coronary calcium scoring compared to current clinical standard.
**Methods or Background:** Patients received clinical non-contrast CT on a dual-source CT (FORCE) and were consented to undergo a PCCT (NAEOTOM Alpha, both Siemens Healthineers) with similar acquisition parameters (120 kVp, 3 mm slice thickness, filtered back projection) on the same day. Calcium scoring was calculated and radiation dose measurements (CTDIvol and DLP) were documented. Contrast to noise ratio (CNR) and signal to noise ratio (SNR) were calculated. Agatston scores, clinical risk category and radiation dose measures were compared using paired t-tests.
**Results or Findings:** Six male patients were included (age: 68.5 ± 6 years, BMI of 25.7 ± 3.7 kg/m2). The average Agatston score was similar for standard CAC CT: 779 ± 1612 and PCCT: 861 ± 1803 (P=0.35). Coronary artery disease risk stratification the same (1 patient=no risk, 2=mild risk, 1=moderate risk, 2 =severe risk for sex and age). CNR (clinical CT: 8.1 vs PCCT: 7.8) and SNR (clinical: 2.5 vs PCCT 2.9) were not statistically significantly different. All PCCT scans had lower radiation doses than the clinical scans (CTDIvol clinical: 3.6 ± 1.1 vs PCCT: 2.1 ± 0.9 mGy; DLP clinical: 60.7 ± 27.9 vs PCCT: 35.8 ± 10.2 mGy×cm) with an average decrease of 37% in CTDIvol (P=0.039) and a 34% decrease in DLP (P=0.037).
**Conclusion:** This pilot investigation suggests that PCCT affords similar calcium scoring and cardiovascular risk stratification compared to standard CT at a significantly lower radiation dose.
**Limitations:** The cohort included in this study is small and will be expanded in the coming months.
**Ethics committee approval:** This study was approved by the IRB.
**Funding for this study:** Departmental funding was partially provided by Siemens Healthineers.
**Author Disclosures:**
Juan Carlos Ramirez-Giraldo: Employee: Siemens Healthineers
Melissa Daubert: Nothing to disclose
Tina D. Tailor: Nothing to disclose
Daniele Marin: Nothing to disclose
Fides Regina Schwartz: Nothing to disclose

### RPS 1203-8

**Influence of mono-energetic image reconstruction levels for coronary artery calcium scoring on a dual-source photon-counting CT: a dynamic phantom study**

N. R. van der Werf[1], R. Booij[1], D. Bos[1], A. Van Der Lugt[1], R. P. J. Budde[1], M. Greuter[2], *M. van Straten*[1]; [1]Rotterdam/NL, [2]Groningen/NL

**Purpose:** To assess the influence of virtual mono-energetic (monoE) reconstruction levels on coronary artery calcium (CAC) scores for a dual-source photon-counting CT (PCCT).
**Methods or Background:** A dynamic anthropomorphic phantom was scanned using routine clinical CAC protocols on conventional CT and PCCT. Three calcification (equal size [196.3 mm3], but different densities) were translated in a horizontal plane at heart rates of 0 and 60-75 bpm. In addition to the clinical protocol standard of 70 keV, PCCT images were reconstructed at monoE

Friday levels of 72, 74 and 76 keV. CAC was quantified using Agatston and volume scores. PCCT Agatston scores were compared to the CT scores and deviations (95% confidence interval) <10% were deemed to be not clinically relevant. Volume scores were compared with the physical volume.
**Results or Findings:** At 70 keV, static PCCT Agatston scores resulted in clinically relevant deviations for all CAC densities. Increased monoE levels resulted in reduced CAC scores. Low density CAC Agatston scores showed high variability at both increased monoE levels and increased heart rate. For medium density CAC deviations were not relevant at 74 and 72 keV, for 0 and 60-75 bpm, respectively. For static high density CAC, deviations were not relevant at 76 keV, while at 60-75 bpm deviations were not relevant at 72-76 keV. For static CAC, the smallest average deviation from physical volume was -37% at 70 keV, 0% at 74 keV, and 65% at 76 keV for low, medium, and high density, respectively. Deviations from physical volume increased with increased heart rate.
**Conclusion:** At 70 keV clinically relevant differences were found between CT and PCCT. At 72 keV, relevant differences were absent for dynamic medium and high density CAC.
**Limitations:** Not applicable.
**Ethics committee approval:** Not applicable.
**Funding for this study:** Not applicable.
**Author Disclosures:**
Niels R. van der Werf: Nothing to disclose
Ricardo P. J. Budde: Nothing to disclose
Aad Van Der Lugt: Nothing to disclose
Marcel van Straten: Other: The Radiology & Nuclear Medicine department of the Erasmus University Medical Centre has a research agreement with Siemens Healthineers.
Ronald Booij: Nothing to disclose
Daniel Bos: Nothing to disclose
Marcel Greuter: Nothing to disclose

**08:00-09:00** **Room M 1**

### Research Presentation Session: Genitourinary

### RPS 1207

### Advances in urological imaging

**Moderator**
V. Panebianco; Rome/IT

### RPS 1207-2

**Radiomics for the assessment of renal masses: a multicentre CT study with independent validation**
*J. Uhlig*[1], A. Uhlig[1], S. Bachanek[1], J. Lotz[1], L. Trojan[1], A. Leha[1], P. Zeuschner[2], A. Massmann[2]; [1]Göttingen/DE, [2]Homburg/DE
*(johannes.uhlig@med.uni-goettingen.de)*

**Purpose:** To evaluate the diagnostic performance of radiomic analyses (radiomics) for assessment of renal masses on computed tomography (CT) in a multicentre setting.
**Methods or Background:** Patients with histopathologically analysed solid renal masses were retrospectively enrolled from two tertiary referral centres in Germany if imaged with CT in arterial and portalvenous contrast media (CM) phase. All renal masses were manually segmented and a total of n=129 radiomics features were calculated on each CM phase. Machine learning models with class-imbalance handling, feature selection and cross-validation (CV) techniques were used to predict histological subtypes of renal masses. Diagnostic performance was quantified with multiclass area-under-the ROC curve (AUC) for renal mass subtype prediction. Model training was performed on data from one centre. An independent dataset from the other centre was used from model validation.
**Results or Findings:** The training dataset comprised n=173 patients (median age 66y; 41% female; 65% ccRCC; 6% chRCC; 13% pRCC; 8% AML; 9% oncocytoma). The validation dataset contained n=137 patients (median age 65y; 31% female; 58% ccRCC; 4% chRCC; 16% pRCC; 3% AML; 20%). For assessment of renal mass subtype, the machine learning algorithms reached a multiclass AUC=0.77 in the out-of-bag CV samples of the training dataset. The identification of AML vs. other subtypes showed higher diagnostic accuracy (AUC=0.84) compared to oncocytoma vs. other subtypes (AUC=0.74).
In the independent validation dataset, the machine learning algorithm demonstrated a multiclass AUC=0.74.
**Conclusion:** Radiomics and machine learning demonstrate good diagnostic performance for assessment of renal masses in CT studies, and robustly perform on independent multicentre data.
**Limitations:** Study limitations include a low number of benign renal masses and a heterogeneous study cohort.
**Ethics committee approval:** This study received prior approval by the ethics committees of both participating centres.
**Funding for this study:** No funding was received for this study.
**Author Disclosures:**
Annemarie Uhlig: Nothing to disclose
Lutz Trojan: Nothing to disclose
Andreas Leha: Nothing to disclose
Sophie Bachanek: Nothing to disclose
Johannes Uhlig: Nothing to disclose
Joachim Lotz: Nothing to disclose
Alexander Massmann: Nothing to disclose
Philip Zeuschner: Nothing to disclose

### RPS 1207-4

**Can shear wave elastography be a reliable non-invasive tool in diagnosis and follow-up of lupus nephritis?**
R. Mostafa, A. El-Badrawy, A. M. Tawfik, *M. E. Abou El-Ghar*; Mansoura/EG
*(maboelghar@yahoo.com)*

**Purpose:** To assess the role of shear wave elastography (SWE) ultrasound as a non-invasive tool in classification of lupus nephritis (LN) using histopathologic findings as a gold standard.
**Methods or Background:** Thirty-five systemic lupus erythematosus (SLE) patients with renal affection were subjected to SWE ultrasound as well as twenty healthy subjects as a control group.
**Results or Findings:** There was a significant difference in SWE values between the case and the control groups; the mean in the case group was 37.66 ± 11.692 kpa while the mean in the control group was 17.36 ± 3.913 kpa ( p-value < 0.001, AUC= 0.973, cut-off = 27.17 kPa, sensitivity 90.9% and specificity 100%). There was a slight increase in SWE values in classes III and IV compared to other classes, but not statistically significant. Also, there was no correlation between SWE values and the histopathological grade of lupus nephritis (LN).
**Conclusion:** SWE can be used as a reliable non-invasive tool in LN. Increased SWE values at follow-up of LN patients may indicate disease progression and this can provoke an early biopsy before deterioration of the renal function in SLE patients.
**Limitations:** The limitations included; the small number in each group, SWE was not widely evaluated in all LN classes and also the follow-up of the renal changes before and after treatment wasn't carried out. We recommend a follow-up protocol for the patients for better assessment of the role of SWE as a non-invasive parameter for following up on LN progress.
**Ethics committee approval:** This study was approved by the Research Ethics Committee of the Faculty of Medicine at Mansoura University in Egypt on 16 /07 /2019; reference number of approval: MS.19.07.733.
**Funding for this study:** This study had no funding from any resource.
**Author Disclosures:**
Ahmed M Tawfik: Nothing to disclose
Mohamed Ebrahem Abou El-Ghar: Nothing to disclose
Raghda Mostafa: Nothing to disclose
Adel El-Badrawy: Nothing to disclose

### RPS 1207-5

**Spectral attenuation curve analysis for evaluation of incidental small renal lesions using rapid kilovoltage-switching dual-energy computed tomography**
A. M., A. Maneegarn, R. Kaewlai, *S. Thiravit*; Bangkok/TH

**Purpose:** To retrospectively evaluate whether spectral attenuation (SA) analysis on rapid kilovoltage-switching dual-energy CT (KVS-DECT) scan can distinguish enhancing from non-enhancing incidental small renal lesions, compared with conventional attenuation changes (ΔHU).
**Methods or Background:** Patients who underwent contrast-enhanced KVS-DECT for an indication other than assessment of urinary tract disease and had incidental renal lesion with a size of 1-4 cm were included. DE mode was performed during portovenous phase. The slope between virtual monochromatic images (VMI) dataset at different energy-pair and SA curve for pattern assessment (upward, mildly upward and flat) of each renal lesion was created. Final diagnosis was determined by pathology or imaging criteria. Diagnostic accuracies were calculated in cross-validated Mann-Whitney U Test.
**Results or Findings:** 78 (24 enhancing and 54 non-enhancing) renal lesions were included. All the quantitative and qualitative SA parameters significantly differentiated enhancing from non-enhancing lesions (p= <0.001). Sensitivity and specificity of using the optimal slope threshold at all VMI datasets (3.0, 1.8 and 1.2 for dataset between 40-70 keV, 40-100 keV and 40-140 keV, respectively) were 100% and 94%, which were slightly lower than using ΔHU with a threshold of 20HU (sensitivity and specificity of 100%). Visual analysis of the curve pattern yielded 100% sensitivity but 91 % specificity.

**Conclusion:** SA curve on KVS-DECT gives excellent diagnostic performance in differentiating incidental enhancing versus non-enhancing renal lesions. This benefit of DECT will be most helpful when true unenhanced phase is not performed.
**Limitations:** The limitations of the study are a retrospective study performed at single institution, our study did not aim to distinguish between benign or malignant masses, and the ROI measurement is an operator-dependent method.
**Ethics committee approval:** This study was approved by the institutional board.
**Funding for this study:** No funding was received for this study.
**Author Disclosures:**
Rathachai Kaewlai: Nothing to disclose
Arjin Maneegarn: Nothing to disclose
Adisa M.: Nothing to disclose
Shanigarn Thiravit: Board Member: Medical Ultrasonic Society of Thailand (MUST)

### RPS 1207-6

**Influence of ureteral splints on the diagnosis of urinary stones and their composition in dual-energy CT: a phantom study**

*D. P. Overhoff*[1], K. Spornitz[1], R. Stoll[1], A. Hesse[2], K. Nestler[1], H. U. Schmelz[1], T. Nestler[1], S. Waldeck[1]; [1]Koblenz/DE, [2]Bonn/DE

**Purpose:** CT imaging is used for treatment planning or follow-up in cases of urolithiasis. Foreign bodies can worsen the diagnostic quality of the imaging due to artefacts. The aim of this study was to systematically investigate the influence of ureteral splints on urinary stone dual-energy CT(DECT) imaging.
**Methods or Background:** N=45 mixed urinary stones were examined ex-vivo using 3rd generation DECT(Siemens, SOMATOM Force, Siemens Healthineers, Forchheim, Germany). Ureteral splints were inserted into the phantom and scanned (DECT-DJ) with the urinary stones adjected and then examined without the foreign body (DECT-NAT). As standard of reference the urinary stone composition was determined by IR spectroscopy and manually measured by water displacement method and caliper. The DECT measurements were each compared with the standard of reference and the Pearson's correlation coefficient or sensitivity/specificity calculated.
**Results or Findings:** All urinary stones could be detected with DECT images. Comparing the maximum urinary stone diameter the standard of reference showed a median of 6.1mm (range 3.1-11.0mm), in the DECT-DJ 5.7mm (range 2.7-10.9mm; Pearson 0.79,p<0.001) and in the DECT-NAT 6.2mm (range 1.5-10.3mm; Pearson 0.61,p<0.001). For volumetry, the standard of reference yielded 80mm3 (range 10-345mm3) in median, 61mm3 (range 4-363mm3; Pearson 0.89,p<0.001) in DECT-DJ and 68mm3 (range 2-364mm3; Pearson 0.65,p<0.001) in DECT-NAT. Urinary stone composition revealed uric acid in 8 stones and 37 non-uric acid stones in the standard of reference. In DECT-DJ one uric acid stone was not detected (Sens 87.5%,Spec 100%), in DECT-NAT all stones were correctly distinguished(Sens/Spec each 100%).
**Conclusion:** DE-CT can reliably measure maximum urinary stone length and volume in comparison to the standard of reference even with adjected urethral splint. Our results suggest that stone composition may be more difficult to determine with the ureteral splint in place.
**Limitations:** The limitation is that the study is ex-vivo.
**Ethics committee approval:** No approval required.
**Funding for this study:** No funding was received for this study.
**Author Disclosures:**
Katja Spornitz: Nothing to disclose
Kai Nestler: Nothing to disclose
Rico Stoll: Nothing to disclose
Albrecht Hesse: Nothing to disclose
Tim Nestler: Nothing to disclose
Daniel Pasqual Overhoff: Nothing to disclose
Stephan Waldeck: Nothing to disclose
Hans U. Schmelz: Nothing to disclose

### RPS 1207-7

**Diffusion tensor imaging and tractography on non-obstructive azoospermia: a 3T observational study**

*O. Pappa*, C. Bougia, A. C. Tsili, V. Maliakas, A. Kaltsas, N. Sofikitis, M. Argyropoulou; Ioannina/GR
*(raniapappa160@gmail.com)*

**Purpose:** To assess the value of 3T diffusion tensor imaging (DTI) and testicular tractography in the evaluation of patients with non-obstructive azoospermia (NOA).
**Methods or Background:** This retrospective study included 54 NOA patients and a control group of 47 men. A 3T scrotal MRI examination was conducted, including coronal DTI, followed by tractography three-dimensional reconstructions. Nonparametric statistics compared apparent diffusion coefficient (ADC) and fractional anisotropy (FA) between the following groups: a) NOA testes and normal population, b) different NOA histologic subtypes (group 1: hypospermatogenesis; group 2: maturation arrest; and group 3: Sertoli-cell only syndrome) and c) NOA patients with positive and negative sperm retrieval after therapeutic testicular biopsy (TTB).
**Results or Findings:** Higher ADC and FA values were observed in NOA group compared to normal testes (P <0.001). Significant differences in FA were found between groups 1 and 3 (P =0.003) and groups 2 and 3 (P <0.001). Increase in FA was observed in NOA testes with positive sperm retrieval compared to those with negative results after TTB (P <0.029). Tractography demonstrated spermatic tubules' disorientation in NOA testes.
**Conclusion:** 3T testicular DTI provides valuable information regarding the damage of spermatogenesis in NOA testes and more importantly, the presence or absence of viable spermatozoa prior to TTB. Tractography may represent a promising non-invasive tool in visual representation of derangement of the seminiferous tubules.
**Limitations:** The limitation is the small sample size.
**Ethics committee approval:** Institutional review board approval with waiver of informed consent was obtained for this study.
**Funding for this study:** No funding was received for this study.
**Author Disclosures:**
Christina Bougia: Nothing to disclose
Vasileios Maliakas: Nothing to disclose
Ourania Pappa: Nothing to disclose
Maria Argyropoulou: Nothing to disclose
Nikolaos Sofikitis: Nothing to disclose
Aris Kaltsas: Nothing to disclose
Athina C Tsili: Nothing to disclose

### RPS 1207-8

**Clinical low dose photon counting CT for the detection of urolithiasis: evaluation of image quality and radiation dose**

*J. H. Niehoff*, A. Carmichael, M. M. Woeltjen, J. Boriesosdick, I. Lopez Schmidt, A. Michael, H. Piechota, J. Borggrefe, J. R. Kroeger; Minden/DE

**Purpose:** Purpose of the present study was the evaluation of the image quality and the radiation dose parameters of the novel photon-counting CT (PCCT, Naeotom Alpha, Siemens Healthineers) using a low dose scan protocol for the detection of urolithiasis. Standard CT scans were used as reference (S40, Somatom Sensation 40, Siemens Healthineers).
**Methods or Background:** In total, 63 patients were retrospectively enrolled in this study (PCCT n=31, S40 n=32). Radiation dose parameters, as well as quantitative and qualitative image parameters, were evaluated. Presence of urolithiasis, diagnostic certainty and perceived image quality were rated on a 5-point Likert scale by 3 blinded readers.
**Results or Findings:** The PCCT utilised significantly less radiation dose compared to the S40 (2.4±1.0 mSv vs. 3.4±1.0 mSv; p<0.001). The SNR was significantly better on images acquired with the PCCT (13.3±3.3 vs. 8.2±1.9; p<0.001). Likewise, the perceived image quality offered by the PCCT was rated significantly better (4.3±0.7 vs. 2.8±0.6; p<0.001). The diagnostic accuracy for the detection of urolithiasis was excellent (PCCT 97.8%, S40 99%).
**Conclusion:** In high contrast imaging, the PCCT allows a significant reduction of radiation dose compared to a standard CT, while maintaining excellent image quality. Further adjustments of the scan protocol towards ultra-low-dose CT scans appear feasible.
**Limitations:** The size of the calculi as well as the influence of the image quality on the measurement of the calculi was not addressed. The scan parameters of both scanners differed slightly.
**Ethics committee approval:** The ethics committee of the Faculty of Medicine of the Ruhr-University Bochum approved this study.
**Funding for this study:** Not applicable.
**Author Disclosures:**
Jan Boriesosdick: Nothing to disclose
Julius Henning Niehoff: Nothing to disclose
Matthias Michael Woeltjen: Nothing to disclose
Alexandra Carmichael: Nothing to disclose
Jan Robert Kroeger: Research/Grant Support: Philips Advisory Board: Siemens Healthineers Other: Veryan Speaker: GE Healthcare
Ingo Lopez Schmidt: Nothing to disclose
Jan Borggrefe: Speaker: Philips and Siemens
Hansjuergen Piechota: Nothing to disclose
Arwed Michael: Nothing to disclose

**08:00-09:00** **Room M 2**

### Research Presentation Session: Oncologic Imaging

### RPS 1216

### Gastrointestinal oncology

**Moderator**
I. G. Lupescu; Bucharest/RO

### RPS 1216-2

**CT radiomic features of hepatic metastasis for predicting immunohistochemistry molecular classification of primary breast cancer**

*H. Bae*, S-Y. Youn, J-I. Choi; Seoul/KR
*(qogkssk416@naver.com)*

**Purpose:** Breast cancer is classified into four different subtypes based on immunohistochemistry molecular classification, i.e., luminal A/B, human epidermal growth factor receptor 2-positive (HER2+) and triple negative (TN). The purpose of this study is to investigate whether CT radiomic analysis derived from hepatic metastasis can predict immunohistochemistry molecular classification of breast cancer.
**Methods or Background:** 106 patients who underwent abdomen CT with initial or newly diagnosed hepatic metastasis from breast cancer and had immunohistochemistry result within 3 months interval were enrolled. Patients were randomly assigned a training set (n=71) and internal validation set (n=35). Each hepatic mass was manually segmented by volume of interest using prototype RADIOMICS (Siemens Healthineers) and radiomic features were extracted by same software. Best subset of significant features were selected by multivariate analysis. Radiomic classification models for each subtype were built using random forest classifier.
**Results or Findings:** Total 1226 radiomic features were extracted from each training object. Performance of training vs. validation yielded AUROC values of 0.71 vs. 0.75, and 0.62 vs. 0.67 in the tasks of distinguishing between luminal vs. non-luminal, and HER2+ vs. non-HER2+, respectively. Due to small TN population size limitation, performance of discriminating TN type in validation was poor (AUC value = 0.35).
**Conclusion:** Radiomic features extracted from hepatic metastasis are potential predictive tool for molecular subtypes- luminal vs. non-luminal and HER2+ vs. non-HER2+ in breast cancer.
**Limitations:** The limitation is that the study has an internal validation only. We plan to perform the external validation.
**Ethics committee approval:** Approved by the institutional review board of the Catholic Medical Center of Korea.
**Funding for this study:** This work was supported by the National Research Foundation of Korea (NRF) grant funded by the Korea government (MSIT) (No. 2019R1F1A1060566).
**Author Disclosures:**
Seo-Yeon Youn: Nothing to disclose
Joon-Il Choi: Nothing to disclose
Hanna Bae: Nothing to disclose

### RPS 1216-3

**Evaluation and timing optimisation of CT perfusion first pass analysis in comparison to maximum slope model in pancreatic carcinoma**

*N. Vats*, P. Mayer, H-U. Kauczor, W. Stiller, S. Skornitzke; Heidelberg/DE
*(neha.vats@med.uni-heidelberg.de)*

**Purpose:** Performance evaluation and timing optimisation of CT perfusion first pass analysis (FPA) in comparison to maximum slope model (MSM) in pancreatic carcinoma.
**Methods or Background:** 34 dynamic CT perfusion acquisitions were performed at 80kVp in 17 patients with pancreatic carcinoma. Perfusion maps of blood flow (BF (ml/100ml/min)) were calculated in regions of interest in both healthy tissue and carcinoma for all patients with both FPA and MSM. FPA requires only two volume scans at baseline and peak of arterial input function (AIF). The first volume scan was retrospectively selected when AIF CT-numbers exceeded 120 HU. The second volume scan representing the temporal center of the contrast bolus was selected with a delay of half of the injection time plus a dispersion delay, which was incremented from 0 to 6s in 1.5s steps. FPA-BF values were compared with MSM-BF values using Pearson's correlation and difference between healthy tissue and carcinoma was evaluated using Student's t-test. Coefficient of variation (COV) was calculated to measure sensitivity of FPA-BF values to acquisition timing.
**Results or Findings:** Mean FPA-BF values were 69.17ml/100ml/min, 69.17ml/100ml/min, 68.95ml/100ml/min, 53.69ml/100ml/min, 64.81ml/100ml/min for carcinoma and 184.04ml/100ml/min, 180.93ml/100ml/min, 184.53ml/100ml/min, 153.10ml/100ml/min, 158.67ml/100ml/min for healthy tissue, compared to 42.15ml/100ml/min and 107.02ml/100ml/min for MSM, respectively. Pearson's correlation was 0.82, 0.83, 0.70, 0.85, 0.83 between FPA and MSM, indicating highest agreement for 4.5s dispersion delay. Differences between healthy tissue and carcinoma were significant ($p<0.05$) for all 5 time-points. COV between FPA-BF values were 10.25% for carcinoma and 8.79% for healthy tissue, respectively.
**Conclusion:** FPA has the potential to differentiate pancreatic carcinoma from healthy tissue with good correlation to MSM. FPA could allow a decrease in radiation dose required in CT perfusion for diagnosing pancreatic diseases.
**Limitations:** Prospective validation is still necessary.
**Ethics committee approval:** This study was approved by an ethics committee.
**Funding for this study:** Funding was received for this study by BMBF-grant 031L0163.
**Author Disclosures:**
Neha Vats: Nothing to disclose
Wolfram Stiller: Nothing to disclose
Hans-Ulrich Kauczor: Nothing to disclose
Philipp Mayer: Nothing to disclose
Stephan Skornitzke: Shareholder: Investment funds containing stock of healthcare companies

### RPS 1216-4

**Is CT radiomics superior to morphological evaluation for pN0 characterisation? A pilot study in colon cancer**

*I. Nacci*, M. Zerunian, M. Polici, D. De Santis, D. Caruso, E. Iannicelli, A. Laghi; Rome/IT

**Purpose:** Lymph node (LN) involvement is among the most important prognostic factors for patients with colon cancer (CC). However, CT morphological analysis is not a reliable method to assess nodal status. Thus, we compared morphological and radiomic features extracted from contrast-enhanced computed tomography (CECT) images, in assessing regional LNs in histopathologically confirmed patients with stage I to stage IIC CC.
**Methods or Background:** This retrospective study included 100 patients with CC who underwent preoperative portal-venous phase CT examination and diagnosed as pN0 after surgery. Patients with motion artifacts or lack of pathology report were excluded. Regional LNs were scored with a qualitative Likert-scale based on morphological imaging (Node-score with range 0-7) and divided into two groups: low likelihood (0-2 points) and high likelihood (3-7 points) of malignancy. Then, 107 radiomic features were extracted from CECT for each LN. T-test and Mann-Whitney were performed to compare radiomic features between the two groups. Correction for multiple comparisons was performed with Holm-Bonferroni method. $P<0.05$ was considered significant.
**Results or Findings:** A total of 337 negative LNs were analysed and divided into 167 with low likelihood and 170 with high likelihood of malignancy based on the node-score. Radiomic analysis showed 92 features (3/13 shape, 14/19 first order and 75/75 second order features) with no significant difference between the two groups, according to histopathology (all $P>0.05$). On the other hand, 15 features (10/13 shape and 5/19 first order features) were significantly different (all $P<0.04$).
**Conclusion:** Our preliminary experience showed that CT Radiomics characterises nodal status better than CECT morphological evaluation and could be used as a non-invasive preoperative tool in patients with CC.
**Limitations:** Retrospective nature of the study. Small population sample.
**Ethics committee approval:** This study was approved by our local institutional review board; written informed consent was obtained from all study participants.
**Funding for this study:** No funding was received for this study.
**Author Disclosures:**
Damiano Caruso: Nothing to disclose
Marta Zerunian: Nothing to disclose
Domenico De Santis: Nothing to disclose
Ilaria Nacci: Nothing to disclose
Andrea Laghi: Nothing to disclose
Michela Polici: Nothing to disclose
Elsa Iannicelli: Nothing to disclose

### RPS 1216-6

**SSR-PET/CT compared to contrast-enhanced liver MRI in detection of liver metastases in neuroendocrine tumours (NET): a 15-year retrospective European single-centre analysis**

*F. Grawe*, M. Ingenerf, C. Schmid-Tannwald, C. C. Cyran, J. Ricke, J. Rübenthaler, M. P. Fabritius; Munich/DE

**Purpose:** Liver metastases in patients with neuroendocrine tumours (NET) are an indicator of poor prognosis and markedly reduce survival. The aim of this study was to compare the diagnostic accuracy of somatostatin receptor (SSR)-PET/CT to liver MRI as reference standard in the evaluation of hepatic involvement in neuroendocrine tumours.
**Methods or Background:** In this retrospective study, an institutional database was screened for “SSR” imaging studies between 2006 and 2021. 1000 NET Patients (grade 1/2) with 2383 SSR-PET/CT studies and matching liver MRI in an interval of ± 3 months were identified). Medical reports of SSR-PET/CT and MRI were retrospectively evaluated regarding hepatic involvement and either confirmed by both or observed in MRI but not in SSR-PET/CT (false-negative) or in SSR-PET but not in MRI (false-positive).
**Results or Findings:** Hepatic involvement was reported in 1650 (69%) of the total 2383 SSR-PET/CT studies, whereas MRI detected hepatic involvement in 1685 (71%) cases. There were 51 (2%) false-negative and 16 (1%) false-positive cases. SSR-PET/CT demonstrated a sensitivity of 97.0% (95%CI: 96.0%, 97.7%), a specificity of 97.7% (95%CI: 96.3%, 98.7%), a PPV of 99.0% (95%CI: 98.4%, 99.4%) and NPV of 93.0% (95%CI: 91.0, 94.8%) in identifying hepatic involvement. The most frequent reason for false-negative results was the small size of lesions (<1.2 cm).
**Conclusion:** This study confirms the high diagnostic accuracy of SSR-PET/CT in the detection of hepatic involvement in NET patients with a high sensitivity and specificity using liver MRI imaging as reference standard. However, one should be aware of possible pitfalls. Thus, SSR-PET/CT can reduce unnecessary biopsies and optimise clinical patient management.
**Limitations:** This was a retrospectively conducted study and histopathological confirmation was not given in all patients. Expert reading was not blinded since medical reports were generated in clinical routine.
**Ethics committee approval:** This study was approved by an ethics committee.
**Funding for this study:** No funding was received for this study.
**Author Disclosures:**
Matthias Philipp Fabritius: Nothing to disclose
Freba Grawe: Nothing to disclose
Clemens C. Cyran: Nothing to disclose
Maria Ingenerf: Nothing to disclose
Johannes Rübenthaler: Nothing to disclose
Christine Schmid-Tannwald: Nothing to disclose
Jens Ricke: Nothing to disclose

### RPS 1216-7

**Texture analysis for nonlinear characterisation of 18F-FDG PET/CT images in patients with rectal cancer: comparison between tumour and peritumoural tissues**

*V. B. Orel*, A. Ashykhmin, V. E. Orel, O. Rykhalskyi, T. Golovko; Kyiv/UA
*(orel.valeriy@gmail.com)*

**Purpose:** Examination of texture parameters from 18F-Fluorodeoxyglucose (FDG) positron emission tomography/computed tomography (PET/CT) images in the nonlinear characterisation of the tumour and the peritumoural tissue in patients with rectal cancer.
**Methods or Background:** Tumour and peritumoural tissues were compared using texture parameters (heterogeneity, entropy, energy, skewness, kurtosis, standard deviation, average brightness) on PET/CT images obtained from 22 patients with T3aN1M1 rectal cancer (mean age 61 ± 2 years). PET/CT scans were carried out on a Biograph 64 TruePoint scanner (Siemens Medical Solutions). Texture parameters were extracted using MATLAB R2016a (MathWorks, Inc) software. Correlation analysis described the relationship between heterogeneity, tumour size and extramural venous invasion (EMVI) scores. Statistical analyses were performed with Statistica 12.5 (StatSoft, Inc).
**Results or Findings:** Texture analysis of FDG PET/CT images revealed a 23% difference in the heterogeneity parameter between the tumour and the peritumoural tissue ($P < 0.05$). Heterogeneity in the peritumoural tissue showed significant ($P < 0.05$) correlation with EMVI score ($r = 0.64$) and tumour size ($r = -0.48$). Men and women had significantly different correlation coefficients ($P < 0.05$) due to the anatomical position of the rectum, vascular architecture and tumour metabolism.
**Conclusion:** The heterogeneity parameter provided additional quantitative information for the nonlinear characterisation of differences between the rectal tumour and the peritumoural tissue. Quantification of the heterogeneity phenomenon in malignant tumours and peritumoural tissues could be used as a factor involved in metastasising for patient prognosis and reduce the variability for EMVI assessment.
**Limitations:** Further studies focused on the application of image heterogeneity parameters should use deep learning techniques such as neural networks.
**Ethics committee approval:** Regional committee on medical research ethics at the National Cancer Institute of Ukraine.
**Funding for this study:** Funded by the Ministry of Health of Ukraine.
**Author Disclosures:**
Valerii B Orel: Nothing to disclose
Tetiana Golovko: Nothing to disclose
Andrii Ashykhmin: Nothing to disclose
Valerii E. Orel: Nothing to disclose
Oleksandr Rykhalskyi: Nothing to disclose

**08:00-09:00** **Room X**

### Research Presentation Session: Musculoskeletal

### RPS 1210

### Shoulder and upper limb

**Moderator**
J.-L. Drapé; Paris/FR

### RPS 1210-2

**Occupation-ratio underestimates muscle volume in full-thickness supraspinatus tears with medial tendon retraction**

*S. S. Goller*, B. Erber, N. Fink, J. Ricke, A. Heuck; Munich/DE
*(sophia.goller@gmx.de)*

**Purpose:** Supraspinatus (SSP) muscle atrophy is usually estimated from oblique-sagittal MRI on the Y-view by calculating the occupation-ratio (OR) between the cross-section surface of the muscle belly and that of the supraspinatus fossa. Full-thickness tears may cause medial retraction of the supraspinatus tendon leading to underestimation of muscle volumes derived from the OR. The purpose was to investigate the effect of medial retraction in full-thickness SSP tears on the OR.
**Methods or Background:** SSP volumetry was performed using the software mint LesionTM. To verify its accuracy, ten muscle specimens were volumetrised ex-vivo using the physical water displacement method in comparison to the software. The patient cohort included 149 individuals (mean age 55.0 ± 16.0 years, 66 females) with intact SSP tendons, partial and full-thickness tears who obtained MRI scans. OR values were determined by three radiologists independently. An alpha .05 was set as the limit of statistical significance.
**Results or Findings:** Excellent correlation for the variables volumetry using mint LesionTM and physical volumetry was demonstrated, allowing software-based volumetry to serve as the standard of reference. Interreader reliability was between .89 and .92 for OR estimations. Muscle volumes in patients with intact SSP tendons were significantly higher than in partial or full-thickness tears with all degrees of tendon retraction ($p<.001$). Correlation between OR values and muscle volumes was best for intact tendons (.84) and significantly decreased for full-thickness tears with all degrees of tendon retraction (.56-.70).
**Conclusion:** The OR method is appropriate to estimate muscle atrophy in patients without SSP tendon retraction, however, in medial tendon retraction it is not reliable.
**Limitations:** Retrospective study design.
**Ethics committee approval:** This study was approved by the ethics committee of the Medical Faculty, University of Munich (project number 20-0814).
**Funding for this study:** No funding was received for this study.
**Author Disclosures:**
Andreas Heuck: Nothing to disclose
Bernd Erber: Nothing to disclose
Sophia Samira Goller: Nothing to disclose
Nicola Fink: Nothing to disclose
Jens Ricke: Nothing to disclose

### RPS 1210-3

**Accelerated TSE imaging of the shoulder using deep learning image reconstruction**

*J. Herrmann*, S. Gassenmaier, H. Almansour, A. Othman, S. Afat; Tübingen/DE
*(judith.herrmann@med.uni-tuebingen.de)*

**Purpose:** To evaluate the feasibility and diagnostic performance of an accelerated image protocol using turbo spin echo (TSE) imaging with deep learning (DL) reconstruction of the shoulder.
**Methods or Background:** Thirty patients who underwent shoulder MRI at 1.5 and 3 T including standard TSE (TSE_S) and TSE with DL reconstruction (TSE_DL) were prospectively enrolled in this institutional review board-approved study after obtaining written informed consent between October 2020 and June 2021. Two radiologists independently assessed image quality and visualisation of anatomical structures using a Likert scale ranging from 1-5 (5 best) as well as the diagnostic performance concerning pathologic shoulder lesions.
**Results or Findings:** Overall image quality was evaluated to be superior in TSE_DL versus TSE_S, with less noise and improved sharpness in TSE_DL versus TSE_S. No difference was found concerning qualitative diagnostic confidence and quantitative diagnostic performance for pathologic shoulder lesions when comparing the two sequences. The acquisition time of TSE using DL was reduced (1:10 min vs 2.53 min).
**Conclusion:** TSE_DL of the shoulder is feasible, without limitations concerning the diagnostic performance and allows a reduction of scan time of more than 50% compared to TSE_S.
**Limitations:** Single centre study. Small number of included patients.
**Ethics committee approval:** This study was approved by the local ethics committee.
**Funding for this study:** No funding was received for this study.
**Author Disclosures:**
Judith Herrmann: Nothing to disclose
Saif Afat: Nothing to disclose
Haidara Almansour: Nothing to disclose
Ahmed Othman: Nothing to disclose
Sebastian Gassenmaier: Nothing to disclose

### RPS 1210-4

**Optimisation of the Grashey view radiography for the critical shoulder angle measurement: a reliability assessment with zero echo-time MRI**

A. E. Yildiz, Y. Yaraşir, G. Huri, *Ü. Aydingöz*; Ankara/TR
*(ustunaydingoz@yahoo.com)*

**Purpose:** Suboptimal positioning in Grashey view radiography (XR) might limit prognosticating potential of critical shoulder angle (CSA). We aimed to investigate whether XR, when optimised according to current criteria, is reliable for measuring CSA in comparison to MRI featuring zero echo-time imaging (ZTE) that accentuates cortical bone-to-surrounding-soft-tissue contrast with high fidelity.
**Methods or Background:** Patients (age range 25-49 years) with shoulder pain and/or frozen shoulder were prospectively and consecutively enrolled. All underwent XR and shoulder MRI at 3T, which included isotropic 3D ZTE sequence. Acceptability of XRs was established by ratio of transverse-to-longitudinal diameter of lateral glenoid outline (RTL). Unacceptable XRs (RTL ≥0.25) were repeated. Two observers independently measured CSA on both XR and coronal oblique reformatted ZTE images, the latter including verification of measurement points by cross-referencing on images from other planes.
**Results or Findings:** Study comprised 65 patients (35 females, 30 males; mean age 40.2 years). Appropriateness of radiographs according to RTL (range, 0–0.20; mean, 0.09) was attained after a mean number of 1.6 exposures (range, 1/4). Interobserver agreement was excellent for radiography (ICC=0.91; 95% CI: 0.84, 0.94) and good for ZTE MRI (ICC=0.85, 95% CI: 0.71, 0.92). Intermodality agreement for XR and ZTE MRI was moderate (ICC=0.66; 95% CI: 0.48, 0.73). CSA measurements were significantly different between optimal XR and ZTE MRI (P=.005). Subgroup analysis revealed no significant difference between CSA measurements on ZTE and Grashey views with a RTL <0.1 (P=.08).
**Conclusion:** CSA measurement on ZTE MRI with anatomical point cross-referencing is significantly different than on XR (even with optimal positioning performed according to latest research), which might necessitate more than one exposures. An RTL <0.1 ensures reliability of XRs, when other standards of sufficient X-ray exposure are met.
**Limitations:** Small sample size.
**Ethics committee approval:** This study was approved by an ethics committee.
**Funding for this study:** No funding was received for this study.
**Author Disclosures:**
Gazi Huri: Nothing to disclose
Adalet Elcin Yildiz: Nothing to disclose
Yasin Yaraşir: Nothing to disclose
Üstün Aydingöz: Nothing to disclose

### RPS 1210-5

**Performances of a deep learning algorithm for the detection of fracture, dislocation, elbow joint effusion, focal bone lesions on trauma x-rays**

*N-E. Regnard*[1], B. Lanseur[2], L. Lassalle[1], A. Lambert[3], B. Dallaudiere[4], A. Feydy[5]; [1]Lieusaint/FR, [2]Bobigny/FR, [3]Dijon/FR, [4]Bordeaux/FR, [5]Paris/FR

**Purpose:** To appraise the performances of an AI trained to detect and localise skeletal lesions and compare them to the routine radiological interpretation.
**Methods or Background:** We retrospectively collected all radiographic examinations with the associated radiologists' reports performed after a traumatic injury during 3 consecutive months (January to March 2017) in a private imaging group of 14 centres. Each examination was analysed by an AI (BoneView, Gleamer) and its results were compared to those of the radiologists' reports. In case of discrepancy, the examination was reviewed by a senior skeletal radiologist to settle on the presence of fractures, dislocations, elbow effusions, and focal bone lesions (FBL). Lesion-wise sensitivity, specificity, and NPV of the AI and of the radiologists' report were calculated for each lesion type.
**Results or Findings:** A total of 4774 exams were included in the study. Lesion-wise sensitivity was 73.7% for the radiologists' reports vs. 98.1% for the AI (+24.4 points) for fracture detection, 63.3% vs. 89.9% (+26.6 points) for dislocation detection, 84.7% vs. 91.5% (+6.8 points) for elbow effusion detection, and 16.1% vs. 98.1% (+82 points) for FBL detection. The specificity of the radiologists' reports was always 100% whereas AI specificity was 88%, 99.1%, 99.8%, 95.6% for fractures, dislocations, elbow effusions, and FBL respectively. The NPV was measured at 99.5% for fractures, 99.8% for dislocations, and 99.9% for elbow effusions and FBL.
**Conclusion:** AI has the potential to prevent diagnosis errors by detecting lesions that were initially missed in the radiologists' reports.
**Limitations:** Performance of the AI was calculated stand-alone. The concordant examinations between the AI and the radiologists' reports were not reviewed by the Ground Truth.
**Ethics committee approval:** The study received IRB approval n°CRM-2106-177.
**Funding for this study:** Gleamer funded this study.
**Author Disclosures:**
Antoine Feydy: Advisory Board: Gleamer
Nor-Eddine Regnard: Shareholder: Gleamer Founder: Gleamer
Aurélien Lambert: Shareholder: Gleamer
Benjamin Dallaudiere: Shareholder: Gleamer
Boubekeur Lanseur: Nothing to disclose
Louis Lassalle: Shareholder: gleamer Consultant: Gleamer

### RPS 1210-6

**The deep motor branch of the ulnar nerve: high resolution ultrasound beyond the Guyon tunnel**

*L. Tovt*[1], M. Pansecchi[1], S. Sanguinetti[1], F. Pistoia[1], R. Picasso[1], F. Zaottini[1], M. Miguel Perez[2], C. Martinoli[1]; [1]Genoa/IT, [2]Barcelona/ES

**Purpose:** The aim of the present study was to illustrate high-resolution ultrasound (HRUS) findings in patients with ulnar nerve motor branch (UNMB) entrapment beyond the Guyon tunnel.
**Methods or Background:** Firstly, n=3 cadaveric hands were dissected to demonstrate the anatomic course of the UNMB distal to the pisiform. Then, the nerve was examined in a group of n=10 healthy subjects by means of linear 17-5MHz and 18-5MHz transducers at the level of the hamate hook, the pisohamate ligament and within the palm. Finally, a series of n=11 consecutive patients with motor symptoms in the territory of innervation of the UNMB was evaluated with HRUS. These patients had neurophysiologic, 1.5T/3T MRI and surgical correlations.
**Results or Findings:** HRUS was reliable in depicting the UNMB from its origin to the midpalm in volunteers. In patients, it provided essential preoperative information on the injury level. In n=6 patients the UNMB was squeezed against the outside slope of the hamate hook by ganglion cysts. In n=4 patients the UNMB was entrapped at a more distal level in the palm by fibrous bands (n=1) and ganglion cysts (n=3). N=1 patient had multiple neurofibromas affecting the nerve and its divisional branches. In cases of distal entrapment at the level of the midpalm, there was selective denervation of the III/IV dorsal interosseous muscles.
**Conclusion:** HRUS may provide accurate information about the level and the nature of UNMB neuropathy, helping the diagnostic workup and surgical decisions. In patients with suspected zone 2 syndrome, HRUS should include UNMB assessment at the level of the hamate hook and in the midpalm.
**Limitations:** No limitations were identified.
**Ethics committee approval:** Not applicable.
**Funding for this study:** No funding was received for this study.

Friday

**Author Disclosures:**
Federico Zaottini: Nothing to disclose
Carlo Martinoli: Nothing to disclose
Luca Tovt: Nothing to disclose
Michelle Pansecchi: Nothing to disclose
Sara Sanguinetti: Nothing to disclose
Riccardo Picasso: Nothing to disclose
Federico Pistoia: Nothing to disclose
Maribel Miguel Perez: Nothing to disclose

### RPS 1210-7

**Extensor carpi ulnaris tendon pathology and related bone marrow oedema as diagnostic markers of peripheral triangular fibrocartilage tears on wrist MRI**

*M. T. Nevalainen*[1], W. Morrison[2], A. C. Zoga[2], J. B. Roedl[2]; [1]Oulu/FI, [2]Philadelphia, PA/US
*(mika.nevalainen@oulu.fi)*

**Purpose:** To evaluate pathology of extensor carpi ulnaris (ECU) tendon and related subtendinous bone marrow oedema (BMO) as diagnostic markers for peripheral TFC tears.
**Methods or Background:** 133 patients (age range 21-75; 68 females) with wrist MRI and arthroscopy were included in retrospective case-control study. The presence of TFC tears (no tear, central perforation, or peripheral tear), ECU pathology (tenosynovitis, tendinosis, tears and subluxation) and BMO at ulnar styloid process were determined on MRI and correlated with arthroscopy as gold standard. 1.5 T MRI scanners were used, and images interpreted by two experienced fellowship-trained musculoskeletal radiologists. Cross tabulation with chi square test, binary logistic regression and sensitivity, specificity, positive predictive value, negative predictive value and accuracy were used to describe diagnostic efficacy.
**Results or Findings:** On arthroscopy, 46 cases with no tear, 34 cases with central perforation and 53 cases with peripheral TFC tear were identified. Using arthroscopy as the gold standard, BMO was seen in 21.7% (10/46) with no tears, in 23.5% (8/34) with central perforations, and in 88.7% (47/53) with peripheral TFC tears ($p<0.001$); the respective numbers for ECU pathology were 19.6% (9/46), 11.8% (4/34), and 84.9% (45/53) ($p<0.001$). Direct inspection of TFC on MRI provided 90.6% sensitivity and 92.5% specificity for detecting peripheral TFC tears. Out of the 53 arthroscopically proven peripheral TFC tears, 52 had either BMO or ECU pathology (or both), yielding a sensitivity of 98.1%. Furthermore, on MRI 41 cases had both BMO and ECU pathology; 40 of these had peripheral TFCC tear in arthroscopy yielding a specificity of 98.8%.
**Conclusion:** ECU pathology and related BMO are highly associated with peripheral TFC tears, and can be used as secondary signs to confirm the presence of clinically significant TFC tear.
**Limitations:** Not applicable.
**Ethics committee approval:** This study was approved by an ethics committee.
**Funding for this study:** No funding was received for this study.
**Author Disclosures:**
William Morrison: Nothing to disclose
Mika Tapani Nevalainen: Nothing to disclose
Adam C Zoga: Nothing to disclose
Johannes B Roedl: Nothing to disclose

### RPS 1210-8

**High-resolution ultrasound of post-procedural complications in carpal tunnel syndrome surgical release**

*M. Pansecchi*[1], L. Tovt[1], S. Sanguinetti[1], F. Pistoia[1], R. Picasso[1], F. Zaottini[1], M. Miguel-Pérez[2], C. Martinoli[1]; [1]Genoa/IT, [2]Barcelona/ES

**Purpose:** Flexor retinaculum surgical release is a routine and safe procedure to treat the median nerve compression in the carpal tunnel. However, in a not negligible subset of patients undergoing surgery, the carpal tunnel syndrome may persist or recur and in some of them disabling symptoms, not related to median nerve compression, may manifest. We propose an essay of common and uncommon high frequency ultrasound (HFUS) findings in patients with postoperative neuropathic symptoms in hand and wrist.
**Methods or Background:** High resolution ultrasound has gained increasing interest as complementary diagnostic test for carpal tunnel median nerve compression neuropathy. However, in the literature there is no comprehensive report of the sonographic findings of carpal tunnel release complications, despite the valuable information the HFUS can provide. We enrolled n=25 consecutive patients complaining of relapsing carpal tunnel syndrome, pain in the volar aspect of the hand and thenar eminence weakness at 3 months following carpal tunnel surgery. The wrist of each patient was scanned using linear array 24-5 MHz matrix probe. The median nerve cross sectional area was obtained at the point of maximum enlargement, the release of flexor retinaculum was verified and the palmar cutaneous branch and motor recurrent branch of median nerve were systematically evaluated.
**Results or Findings:** We found n=10 incomplete section of flexor retinaculum, n=2 persistent compression of median nerve, n=3 damage of palmar cutaneous branch of median nerve, n=4 hypertrophic scar, n=1 palmar haematoma, n=1 recurrent motor branch lesion with amputation neuroma. In n=4 patients no pathological findings were disclosed.
**Conclusion:** HFUS is accurate in the diagnosis of postprocedural complications of carpal tunnel release and can provide additional information compared to electrophysiology.
**Limitations:** No limitations were identified.
**Ethics committee approval:** The study was conducted in accordance with the declaration of Helsinki.
**Funding for this study:** Not applicable.
**Author Disclosures:**
Federico Zaottini: Nothing to disclose
Carlo Martinoli: Nothing to disclose
Luca Tovt: Nothing to disclose
Maribel Miguel-Pérez: Nothing to disclose
Michelle Pansecchi: Nothing to disclose
Sara Sanguinetti: Nothing to disclose
Riccardo Picasso: Nothing to disclose
Federico Pistoia: Nothing to disclose

**08:00-09:00** **Room Z**

### Research Presentation Session: Physics in Medical Imaging

### RPS 1213

### Advances in CT dosimetry and radiobiology

**Moderator**
M. Kachelrieß; Heidelberg/DE

### RPS 1213-2

**Risk-minimising tube current modulation (riskTCM) for CT: potential dose reduction across different tube voltages**

*L. Klein*[1], C. Liu[2], J. Steidel[1], L. Enzmann[1], S. Sawall[1], J. Maier[1], A. Maier[2], M. Lell[3], M. Kachelrieß[1]; [1]Heidelberg/DE, [2]Erlangen/DE, [3]Nuremberg/DE
*(laura.klein@dkfz.de)*

**Purpose:** To estimate the potential CT dose reduction of a patient radiation risk-minimising tube current modulation (riskTCM) and to compare it with today's standard of minimising the total mAs-value (mAsTCM).
**Methods or Background:** State-of-the-art TCM methods in CT aim at reducing dose by minimising the total mAs-product at constant image quality. This minimises the mAs-value but not necessarily the radiation risk since mAsTCM does not account for varying dose sensitivities of different organs. We propose riskTCM, an algorithm minimising a patient-specific risk measure, for example the effective dose Deff, at constant image quality. It computes the organ dose distributions in real-time using the deep dose estimation (DDE), a convolutional neural network trained on Monte-Carlo simulations, and thereby sets the tube current such that the risk measure is minimised. A total of 20 patient acquisitions are retrospectively evaluated using simulated riskTCM at 70, 100, 120, and 150 kV. The potential Deff reduction is evaluated as figure of merit and compared to mAsTCM in the abdomen and head with arms over it.
**Results or Findings:** For the abdomen, Deff with riskTCM is lower than mAsTCM by 33%, 29%, 29% and 28% for 70 kV, 100 kV, 120 kV, and 150 kV on average. For the head, Deff is lower by about 16%, 14%, 13%, and 13%, respectively.
**Conclusion:** In excentric body regions mAsTCM does a good job in reducing the patient dose besides the mAs-value. In regions with less attenuation differences, for example the abdomen, riskTCM offers enormous benefit over mAsTCM. The proposed riskTCM method can be easily adapted to risk measures other than Deff.
**Limitations:** The proposed method has only been applied retrospectively on measured, forward-projected, noise-injected patient data since riskTCM is not implemented on commercial CT systems yet.
**Ethics committee approval:** Not applicable.

**Funding for this study:** Funding was received from DFG-Grant KA1678/24.
**Author Disclosures:**
Lucia Enzmann: Nothing to disclose
Chang Liu: Nothing to disclose
Laura Klein: Nothing to disclose
Andreas Maier: Nothing to disclose
Jörg Steidel: Nothing to disclose
Michael Lell: Nothing to disclose
Marc Kachelrieß: Nothing to disclose
Stefan Sawall: Nothing to disclose
Joscha Maier: Nothing to disclose

### RPS 1213-3

**A software program for the fully automated contouring of patient outlines for the determination of size specific dose estimate in abdominal CT: results of initial testing on 17 systems**

*E. Pace*[1], C. J. Caruana[1], H. Bosmans[2], K. Cortis[1], M. D'Anastasi[1], G. Valentino[1]; [1]Msida/MT, [2]Leuven/BE
*(ericpace+esr@protonmail.com)*

**Purpose:** The AAPM220 recommendation that water equivalent diameter (WED) is evaluated at every CT slice is too time intensive for manual contouring, prohibiting real-time analysis. Thus an automated approach is desired. Existing studies proposing automated delineation do not explain either the image processing pipeline, or how complex patient anatomy would be handled. This work sought to develop and test an automated open-source tool to contour patient areas across the abdominal region that correctly excludes the patient couch for a variety of CT systems.
**Methods or Background:** The tool performs a sequence of Python based morphological operations using multiple CPUs and was tested on abdominal datasets that together covered 17 CT systems. Results were reviewed by three medical physicists. Computational time was investigated for the feasibility of real-time applications.
**Results or Findings:** The tool provided very accurate contours over all systems including complex clinical scenarios and variety of patient habitus. Imperfect contours occurred in very few isolated slices. On six cores the tool contoured a 300 slice study in 8 seconds.
**Conclusion:** A fast, robust, automated tool for determination of patient abdominal outline is presented. The tool is sufficiently fast to achieve the AAPM220 goal in real-time. The use of morphological operators permits easy possibility to tune performance to other CT systems.
**Limitations:** The tool does not omit slices where there is a truncation artefact and does not consider patient anatomy effects in the z-direction.
**Ethics committee approval:** All necessary ethics approvals were sought and granted.
**Funding for this study:** No funding was received.
**Author Disclosures:**
Kelvin Cortis: Nothing to disclose
Carmel J. Caruana: Nothing to disclose
Hilde Bosmans: Nothing to disclose
Gianluca Valentino: Nothing to disclose
Eric Pace: Nothing to disclose
Melvin D'Anastasi: Nothing to disclose

### RPS 1213-4

**Effects of simvastatin/metformin therapy on double-strand breaks in human lymphocytes in vitro after diagnostic range ionising radiation**

*D. Komljenovic*, C. Engert, M. Uder, T. Bäuerle; Erlangen/DE

**Purpose:** The aim of the present study is to test the effects of lipid-lowering drug simvastatin and oral hypoglycemic agent metformin in lymphocytes in vitro exposed to the diagnostic-range radiation. The amount of radiation-induced gamma-H2AX-positive foci in cell nuclei is a reliable parameter of the rate of the most important DNA lesions induced by the ionising radiation, the double-strand breaks (DSBs). These findings may elucidate the radiation profile of imaging modalities relying on ionising radiation by employing commonly used therapeutic agents with well-studied pharmacokinetics.
**Methods or Background:** Human peripheral blood lymphocytes treated in vitro with simvastatin and metformin as a monotherapy and in combination were subjected to ionising radiation of 2.5, 5, 10 and 20 mGy. Resulting DSBs were analysed 15 minutes after the onset of radiation by means of indirect immunofluorescence using the phosphorylated form of the histone variant H2AX (gamma-H2AX) as a marker and compared to untreated irradiated cells.
**Results or Findings:** The rate of DSBs in simvastatin-treated lymphocytes 15 minutes after the 20 mGy radiation exposure was significantly decreased compared to untreated irradiated lymphocytes ($p<0.01$). This effect was confirmed after the simvastatin/metformin combination therapy ($p<0.01$). Metformin monotherapy indicated the similar trend yet did not reach statistical significance ($p>0.05$). No conclusive effects of treatment of lymphocytes on the DSB rate after the 2.5, 5 and 10 mGy radiation exposure were observed.
**Conclusion:** Present study revealed radioprotective effects of simvastatin as a monotherapy and in combination with metformin in cultured human peripheral blood lymphocytes exposed to ionising radiation at 20 mGy. These results may increase the understanding of biological response of lymphocytes to diagnostic-range ionising radiation and improve the profile of radiation-induced chromosomal alterations in patients undergoing diagnostic X-ray-based imaging procedures.
**Limitations:** In vitro study.
**Ethics committee approval:** Not applicable.
**Funding for this study:** Not applicable.
**Author Disclosures:**
Dorde Komljenovic: Nothing to disclose
Tobias Bäuerle: Nothing to disclose
Christina Engert: Nothing to disclose
Michael Uder: Nothing to disclose

### RPS 1213-5

**Impact of x-ray exposure by single- and dual-energy CT on gene expression, DNA methylation profiles and DNA integrity in peripheral blood cells**

*B. V. Becker*; Koblenz/DE

**Purpose:** Computed tomography is responsible for the majority of occupational radiation exposure in medical imaging. However, the possible impact on specific biological processes of a single low-dose CT examination remains elusive to some extent. For a better understanding of biological effects induced by medical imaging, changes of DNA integrity, DNA methylation and gene expression profiles after exposure to single- and dual-energy CT in peripheral blood cells were examined.
**Methods or Background:** Blood samples were irradiated ex vivo in a phantom with single- (80 and 150 kV) and dual-energy (80/Sn150kV) tube voltages. Differential gene expression in peripheral blood mononuclear cells was analysed six hours after irradiation using whole transcriptome sequencing. DNA methylation was analysed by means of bisulfite sequencing. DNA double strand break (DSB) frequency was studied by 53BP1+γH2AX co-immunostaining.
**Results or Findings:** Irradiated samples showed a significantly higher rate of DSBs ($p < 0.001$) and the shared upregulation of five genes, AEN, BAX, DDB2, FDXR and EDA2R. No significantly differentially methylated region specific to CT exposure was identified. Neither the analysis of gene expression, nor DSB frequency, nor methylation profiles provided any evidence for significantly increased biological effectiveness of dual-energy CT in comparison to single-energy CT spectra.
**Conclusion:** Despite steadily decreasing doses, CT diagnostics seems to remain a genotoxic stressor with impact on gene regulation and DNA integrity. However, no evidence was found that this damaging effect is aggravated by low energy photons emitted by varying X-ray spectra of CT. In addition, CT exposure did not result in detectable early changes of DNA methylation.
**Limitations:** This in vitro single-centre study focuses on an early time point after low-dose irradiation in a limited study population.
**Ethics committee approval:** Approvel by ethics commission of the Medical Association of Rhineland Palatinate, Germany (837.084.17[10918]).
**Funding for this study:** Not applicable.
**Author Disclosures:**
Benjamin Valentin Becker: Nothing to disclose

### RPS 1213-6

**Feasibility of using size-specific dose estimate (SSDE) to estimate effective dose of individual patients**

*A. Abuhaimed*[1], C. Martin[2], C. Lee[3]; [1]Riyadh/SA, [2]Glasgow/UK, [3]Rockville, MD/US
*(ahaimed@gmail.com)*

**Purpose:** The aim of this study is to investigate the relationship between size-specific dose estimate (SSDE) and a derivative form of effective dose called size-specific effective dose (SED) for individual patients.
**Methods or Background:** Values of SED resulting from CT scans of six regions over the trunk were estimated for 193 adult phantoms using NCICT software. The phantom library covers a wide range of adult sizes of various heights (150-190 cm) and weights (40-125 kg). The regions studied were chest, abdomen, pelvis, chest-abdomen, abdomen-pelvis, and the trunk. A MATLAB code developed in-house was employed to calculate the patient size in terms of water-equivalent diameter for the same phantom set used in NCICT software. This allowed SSDE values to be calculated for the CT scans investigated.
**Results or Findings:** Good correlations between SED and SSED values were observed for all scan regions studied, except the pelvis. The variations for the regions were all within ±8%, but they reached 16% for pelvis scans due to differences in colon doses. These occurred because sections of the transverse colon lay inside the scan range for some phantoms and outside for others depending on the colon position for the individual.
**Conclusion:** The strong correlations between SED and SSED values suggest that coefficients of the best-fit for the correlations could be derived, from which risk of an individual patient could be assessed based on SSED of a given scan

Friday taking into account the patient size and the gender. This may provide a simple and straightforward approach to assess SED in a manner similar to that based on k-factors.
**Limitations:** The study has been conducted using a phantom library, and may be extended to include data of patients.
**Ethics committee approval:** No ethics approval was needed.
**Funding for this study:** No funding received.
**Author Disclosures:**
Colin Martin: Nothing to disclose
Abdullah Abuhaimed: Nothing to disclose
Choonsik Lee: Nothing to disclose

### RPS 1213-7

**Evaluation of BMI's (body mass index) impact on LDRL deriving from large cohort of oncology patients undergoing three phases multiregional CT**

J. Jasieniak, *A. Kuchcinska*, P. Rybarczyk, M. Dedecjus, P. Czuchraniuk, K. Wrzesien, D. Kiprian, A. Cieszanowski; Warsaw/PL
*(akuchcinska@wp.pl)*

**Purpose:** ICRP 135 suggest that with a large cohort of patients, BMI data might be omitted, at the same time pointing out that anthropology of specific cohorts should be considered. Dose tracking systems provide statistical data usually for whole BMI adult patients distribution. The aim of study is to check the impact of BMI on mean dose and LDRL.
**Methods or Background:** Analysis of total DLP collected by DTS for whole examination: 1st phase without contrast agent (chest, abdomen, pelvis), 2nd phase after 35s (chest + abdomen), 3rd phase after 60s (abdomen+ pelvis). Cohort consists of 2110 patients divided by 6 relevant groups of the BMI: 600 patients with 18.5-24.9 BMI considered as healthy weight; 41p. BMI <18.5 underweight; 537p. 25.0-29.9 BMI overweight; 258p. 30.0-34.9 BMI obesity class 1; 83p. 35.0-39.9 BMI obesity class 2; 48p. BMI>40,0 obesity class 3 and one group of 543 for which BMI data wasn't available.
**Results or Findings:** Total DLP median and 75th percentile is: 348.3 / 398.7cGy*cm; 540.3 / 672.4cGy*cm; 1031.5 / 1272.2cGy*cm; 1576.3 / 1817.5cGy*cm; 1906.4 / 2026.2cGy*cm; 2090.4 / 2275.3cGy*cm; for underweight, normal weight, overweight and obesities class 1-3, respectively. The 75th percentile, for unknown BMI, equal to 1302.7mGy*cm is most similar to overweight group (2.4%-3.5%) difference, while it is almost double normal weight. Median in this group is equal to 825.9mGy*cm and is 20% lower than for overweight while 50% higher than in normal weight.
**Conclusion:** Calculations provided by DTS, which don't take into account BMI data, are relevant for local dose management. In order to compare LDRL between hospitals it would be beneficial to recalculate typical doses for given BMI even if large cohort of patent is available.
**Limitations:** Not applicable.
**Ethics committee approval:** Not applicable due to retrospective analysis of dose data.
**Funding for this study:** Not applicable.
**Author Disclosures:**
Agnieszka Kuchcinska: Nothing to disclose
Jakub Jasieniak: Nothing to disclose
Karolina Wrzesien: Nothing to disclose
Piotr Czuchraniuk: Nothing to disclose
Andrzej Cieszanowski: Nothing to disclose
Dorota Kiprian: Nothing to disclose
Pawel Rybarczyk: Nothing to disclose
Marek Dedecjus: Nothing to disclose

### RPS 1213-8

**Evaluation of the IVIscan detector for CT scan's wide radiation beam and radiotherapy cone-beam CT dosimetric quality control**

*C. Popotte*, C. Devic, M. Munier; Strasbourg/FR
*(christian.popotte@gmail.com)*

**Purpose:** With the advent of multi-detector scanners, z-axis collimations have increased up to 160mm. In addition, image guided radiotherapy (IGRT) based on cone-beam CT (CBCT) with collimations up to 400mm is often used for patient's positioning in highly accurate radiotherapy treatments. The literature shows that for collimations greater than 40mm, the 100mm pencil ionisation chamber does not consider a large part of the primary and scattered radiation and several methods have been proposed to overcome this problem but because of the limitation of the available tools, dosimetric quality controls are rarely performed on these wide collimations. This study introduces a new method and device in order to overcome these limitations and perform accurate dosimetric quality control on wide collimations and CBCT.
**Methods or Background:** Fibermetrix® has developed a measurement method and associated device (IVIscan®: based on a scintillating fiber placed on the CT scan's couch) allowing to integrate the dose even on wide collimations. The CTDI100, CTDIw and CTDIvol are measured and calculated on a Siemens definition EDGE and a Canon Aquilion ONE Genesis for thinnest, largest and clinical collimations. The same measurements and calculations are done on the CBCT of a Varian TrueBeam linear accelerator. We compared the results between the ionisation chamber of 100mm, 300mm and the IVIscan® device for the calculated CTDI100, CTDIW for wide CT collimations and for CBCT collimations.
**Results or Findings:** IVIscan detector showed comparable performances to currently used methods for small and large collimations with differences of less than 5% compared to the reference calculation methods for CTDIw and CTDIvol.
**Conclusion:** IVIscan is an easy-to-use tool and can be used routinely for regulatory quality control including large collimations and radiotherapy CBCT.
**Limitations:** No limitations were identified.
**Ethics committee approval:** This study was approved by an ethics committee.
**Funding for this study:** PhD scholarship funded by Fibermetrix.
**Author Disclosures:**
Christian Popotte: Research/Grant Support: scholarship funded by the FIBERMETRIX company
Clément Devic: Employee: FIBERMETRIX
Mélodie Munier: Founder: FIBERMETRIX

**08:00-09:30** **Room N**

### Research Presentation Session: Abdominal Viscera

### RPS 1201

### Advances in liver imaging

**Moderator**
C. J. Zech; Basle/CH

### RPS 1201-2

**Detecting NASH and grading fibrosis in subjects with NAFLD using non-invasive imaging biomarkers**

*S. Alsaqal*[1], P. Hockings[2], H. Ahlström[1], J. Hulthe[2], C. Schoelch[3], J. Vessby[1], A. Wanders[4], F. Rorsman[1], C. Ebeling Barbier[1]; [1]Uppsala/SE, [2]Mölndal/SE, [3]Biberach/DE, [4]Aalborg/DK
*(salem.alsaqal@radiol.uu.se)*

**Purpose:** Nonalcoholic fatty liver disease (NAFLD) is rapidly increasing world-wide. It is subdivided into nonalcoholic fatty liver (NAFL) and the more aggressive form, nonalcoholic steatohepatitis (NASH) that carries a high risk of developing fibrosis and cirrhosis. The aim of this study was to investigate the ability and to assess the repeatability of imaging biomarkers to detect NASH and significant or advanced fibrosis.
**Methods or Background:** Sixty-eight subjects with biopsy proven NAFLD (53 NASH and 15 NAFL) were examined using a wide variety of MRI techniques, including MR elastography (MRE), MR proton density fat fraction (PDFF), apparent diffusion coefficient, R1 (relaxation rate 1/T1), and R2* (relaxation rate 1/T2*). Plasma biomarkers (CK18 M30, ALT, and AST) were also measured. Thirty participants underwent a second MRI within 2-4 weeks in order to assess repeatability.
**Results or Findings:** In univariate logistic regression analysis, there was a significant difference between the NASH and NAFL groups in MRE (p=0.028), CK18 M30 (p=0.016) and ALT (p=0.047), with an area under the receiver operating characteristic curve (AUROC) of 0.74, 0.76 and 0.70, respectively. In bivariate logistic regression analysis, a model combining MRE with PDFF showed better performance in diagnosing NASH (AUROC=0.84), compared with MRE or PDFF alone (bivariate p=0.008 and 0.009, compared with univariate p=0.028 and 0.070, respectively). MRE could differentiate between F2-4 (significant and advanced fibrosis) and F0-1 (no or mild fibrosis). A bivariate model combining MRE with AST improved the detection of F2-4. The intraclass correlation coefficient for repeatability was 0.94 and 0.99 for MRE and PDFF, respectively.
**Conclusion:** MRE can potentially detect NASH and differentiate between fibrosis stages. Combining MRE with PDFF improves detection of NASH.
**Limitations:** One identified limitation of this study was participant numbers.
**Ethics committee approval:** This study received ethics committee approval.
**Funding for this study:** Antaros Medical AB, Boehringer Ingelheim, Swedish Research Council and the Swedish Heart-Lung Foundation have financially supported this study.

**Author Disclosures:**
Paul Hockings: Nothing to disclose
Alkwin Wanders: Nothing to disclose
Fredrik Rorsman: Nothing to disclose
Johan Vessby: Nothing to disclose
Salem Alsaqal: Nothing to disclose
Håkan Ahlström: Nothing to disclose
Corinna Schoelch: Employee: Boehringer Ingelheim Pharma GmbH & Co. KG
Johannes Hulthe: Nothing to disclose
Charlotte Ebeling Barbier: Nothing to disclose

### RPS 1201-3

**Changes in volumetric enhancing tumour burden on CT predicts survival outcomes in patients with neuroendocrine liver metastases after intraarterial treatments**

J. Assouline[1], *R. Cannella*[2], G. Porrello[2], L. de Mestier[1], M. Dioguardi Burgio[1], O. Hentic[1], V. Vilgrain[1], R. Duran[3], R. Maxime[1]; [1]Clichy/FR, [2]Palermo/IT, [3]Lausanne/CH

**Purpose:** To investigate whether liver enhancing tumour burden (LETB) assessed on contrast-enhanced CT could serve as an early response biomarker and help predict survival in patients with multifocal neuroendocrine liver metastases (NELM) after intraarterial treatments (IATs).
**Methods or Background:** This retrospective study included patients with NELM who underwent IATs with either transarterial embolisation or chemoembolisation. Tumour response of treated NELM was evaluated according to the RECIST 1.1 and mRECIST criteria by two readers. Using a dedicated software, LETB was measured as the viable tumour tissue having more than two standard deviations of the regions of interest placed in the nontumoral liver parenchyma. Overall survival (OS), time to unTACEable progression, hepatic and whole-body progression-free survival (PFS) were evaluated by using the multivariable Cox proportional hazards analyses, Kaplan-Meier method, and log-rank test.
**Results or Findings:** The study included 119 patients (mean age 59.8±10.7 years old, 61 [51.3%] men) who underwent 161 treatments composed of 241 intraarterial procedures. The median LETB change of -25.8% best separated OS curves (log-rank p=0.021) and whole-body PFS curves (log-rank p<0.001). A LETB change of -10% demonstrated the highest discrimination according to time to unTACEable progression (log-rank p<0.001) and hepatic PFS (log-rank P<0.001). At multivariable analyses, LETB change remained independently associated with improved OS (hazard ratio 0.56), time-to-unTACEable progression (hazard ratio 0.49), hepatic PFS (hazard ratio 0.42), and whole-body PFS (hazard ratio 0.47). Neither RECIST or mRECIST criteria were retained by multivariable analyses.
**Conclusion:** Response according to LETB change predicts survival outcomes in patients with neuroendocrine liver metastases after intraarterial treatments, with better discrimination than RECIST and mRECIST criteria.
**Limitations:** Identified limitations of this study were: the retrospective design, multiple treatments before IATs, and inclusion of both TAE and TACE.
**Ethics committee approval:** This study was IRB approved.
**Funding for this study:** No funding was received for this study.
**Author Disclosures:**
Giorgia Porrello: Nothing to disclose
Marco Dioguardi Burgio: Nothing to disclose
Olivier Hentic: Nothing to disclose
Jessica Assouline: Nothing to disclose
Rafael Duran: Nothing to disclose
Roberto Cannella: Nothing to disclose
Ronot Maxime: Nothing to disclose
Valérie Vilgrain: Nothing to disclose
Louis de Mestier: Nothing to disclose

### RPS 1201-4

**Gadoxetic acid-enhanced MR imaging predictors of mortality and hepatic decompensation in Chronic Liver Disease (CLD)**

*L. Beer*, N. Bastati-Huber, S. Pötter-Lang, M. Mandorfer, A. Ba-Ssalamah; Vienna/AT

**Purpose:** To investigate the accuracy of the functional liver imaging score (FLIS) plus spleen-craniocaudal-diameter (SCCD) for predicting hepatic decompensation and transplant free survival (TFS) in patients with chronic liver disease (CLD).
**Methods or Background:** FLIS derived from hepatobiliary phase (HBP) MRI was associated with graft survival in patients who underwent liver transplantation and linked with first hepatic decompensation and TFS in patients with advanced chronic liver disease (ACLD). 397 patients with CLD who had undergone gadoxetic acid-enhanced liver MRI were included. A FLIS was assigned based on the sum of three HBP features: hepatic enhancement, biliary excretion, and the signal intensity in the portal vein. The SCCD was measured. Patients were stratified into three groups: non-advanced CLD, compensated-advanced CLD (cACLD), and decompensated-advanced CLD (dACLD). The predictive value of SCCD and FLIS for first hepatic decompensation and TFS was investigated.
**Results or Findings:** We observed a strong positive correlation between the measured spleen volume and the SCCD (Spearman's rho=0.887; p<0.001). In patients with ACLD, the FLIS was a risk-factor for mortality (adjusted-hazard-ratio, [aHR]: 2.38, 95%CI: 1.51-43.76, p<.001). Allocating patients into three groups based on their FLIS and SCCD enabled further stratification of patients according to their risk for mortality (log-rank-test: p<.001). The SCCD was further identified as an independent risk factor for first and further hepatic decompensation in patients with ACLD (aHR: 1.10, 95%CI: 1.02-1.19, p=.01; aHR: 1.13, 95%CI: 1.05-1.22, p=.001).
**Conclusion:** The functional liver imaging score derived from gadoxetic acid-enhanced MRI in combination with the splenic craniocaudal diameter identifies patients with advanced chronic liver disease who are at increased risk of hepatic decompensation and of mortality.
**Limitations:** This retrospective single centre study needs prospective multi-centre trial validation.
**Ethics committee approval:** EK Nr:2027/2017.
**Funding for this study:** No funding was received for this study.
**Author Disclosures:**
Sarah Pötter-Lang: Nothing to disclose
Mattias Mandorfer: Nothing to disclose
Lucian Beer: Nothing to disclose
Ahmed Ba-Ssalamah: Speaker: Bayer
Nina Bastati-Huber: Nothing to disclose

### RPS 1201-5

**Gadoxetic acid MRI in cirrhotic patients: radiologic-pathologic correlation in nodules without arterial enhancement and hepatobiliary hypointense pattern**

*G. Candita*, M. Chiellini, A. Goddi, M. Marchini, D. Campani, P. De Simone, D. Cioni, E. Neri; Pisa/IT

**Purpose:** To assess HCC nodules with high signal restriction at diffusion weighted imaging (DWI), and hepatobiliary phase (HBP) hypointense appearance without arterial phase enhancement (APHE) at gadoxetic acid–enhanced MRI in patients who underwent orthotopic liver transplantation (OLT).
**Methods or Background:** Imaging data of 394 patients who had HCC diagnosed and OLT were retrospectively collected, meeting the following inclusion criteria: age >18 years, cirrhosis, MRI performed <45 days before OLT, MRI including DWI and HBP phase. Final study population included 48 patients with a mean age at OLT of 55 years. Pathological examination of the native livers was obtained. MRI studies were performed by using 1.5/3T systems. For dynamic vascular study, 0.025 mmol/kg of gadoxetic acid disodium was injected.
**Results or Findings:** At pathology 155 nodules were identified. Seventy-four were classified as HCC (mean size 24 mm; 11 as well, 27 as moderately, 14 as poorly, and 22 as undifferentiated lesions), 25 as high-grade dysplastic nodules (mean size 15 mm), and 34 as regenerative nodules (mean size 14 mm). DWI signal restriction was observed in 1/11 well differentiated HCC, in 4/27 moderately differentiated HCC, in 12/14 poorly differentiated HCC, in 22/22 undifferentiated HCC, and in 8/25 dysplastic nodules, with an overall sensitivity of 68% and specificity of 91%. HBP hypointense appearance without APHE was observed in 5/11 well differentiated HCC, in 4/27 moderately differentiated HCC, in 4/11 poorly differentiated HCC, in 5/22 undifferentiated HCC, and in 5/25 dysplastic nodules, with an overall sensitivity of 80% and specificity of 92%.
**Conclusion:** Nodules arising in cirrhotic livers with high signal restriction at DWI and HBP hypointense appearance should be considered as HCC lesions in absence of typical APHE.
**Limitations:** One identified limitation of this study was the sample size.
**Ethics committee approval:** This study received ethics committee approval.
**Funding for this study:** No funding was received for this study.
**Author Disclosures:**
Emanuele Neri: Nothing to disclose
Daniela Campani: Nothing to disclose
Gianvito Candita: Nothing to disclose
Matteo Marchini: Nothing to disclose
Paolo De Simone: Nothing to disclose
Martina Chiellini: Nothing to disclose
Antonio Goddi: Nothing to disclose
Dania Cioni: Nothing to disclose

### RPS 1201-6

**MRI extracellular volume fraction in liver fibrosis: a comparison of different blood pool measurements with MR elastography and histology**

*V. Obmann*, J. Klaus, A. Berzigotti, A. A. Peters, D. Catucci, L. Ebner, C. Gräni, A. Christe, A. Huber; Bern/CH
*(verena.obmann@insel.ch)*

**Purpose:** To analyse the performance of liver MRI extracellular volume (ECV) fraction with different blood pool measurements in comparison to the grade of liver fibrosis and MR elastography.
**Methods or Background:** A total of 41 patients planned for liver biopsy were prospectively included to undergo multiparametric MRI at 3T, including T1 mapping before, and 15 minutes after, intravenous injection of an extracellular contrast agent (Gd-DOTA) and SE-EPI based MR elastography. T1 relaxation times were measured in the liver and in different blood pool locations in the aorta, portal vein and inferior vena cava. Extracellular volume fraction was calculated using the following formula: ECV=(1-hematocrit)*(1/(T1 liver post-contrast)-1/(T1 liver pre-contrast))/(1/(T1 blood pool post-contrast)-1/(T1 blood pool pre-contrast)).
The performance of ECV to predict advanced liver fibrosis (F3-4) in comparison to early fibrosis (F0-2) was analysed with an ROC analysis. Combined predictive value of ECV and MRE for advanced liver fibrosis was analysed using a multivariate logistic regression analysis with ROC analysis.
**Results or Findings:** ECV allowed prediction of advanced fibrosis with an AUC of 0.73 (p=0.01) in the aorta, an AUC of 0.71 (p=0.02) in the portal vein and an AUC of 0.64 (p=0.12) in the inferior vena cava, while MRE had an AUC of 0.76 (p=0.005). Combination of ECV and MRE yielded a combined prediction for significant fibrosis with an AUC of 0.77 (p=0.003) in the aorta and 0.81 (p<0.001) in the portal vein.
**Conclusion:** MRI ECV has the highest predictive value for advanced liver fibrosis when measured in the aorta and in the portal vein, while measurement in the inferior vena cava is not recommended.
**Limitations:** An identified limitation is that the initial results are from a small cohort.
**Ethics committee approval:** This study received ethics committee approval.
**Funding for this study:** Funding was received from: SNF grant #320030_188591.
**Author Disclosures:**
Damiano Catucci: Nothing to disclose
Verena Obmann: Nothing to disclose
Alan Arthur Peters: Nothing to disclose
Christoph Gräni: Nothing to disclose
Jeremias Klaus: Nothing to disclose
Adrian Huber: Nothing to disclose
Lukas Ebner: Nothing to disclose
Andreas Christe: Nothing to disclose
Annalisa Berzigotti: Nothing to disclose

### RPS 1201-7

**The role of liver vessel volume ratio in chronic liver disease**

*A. J. Herold*, D. Sobotka, L. Beer, N. Bastati-Huber, S. Pötter-Lang, T. Reiberger, M. Mandorfer, A. Ba-Ssalamah, G. Langs; Vienna/AT
*(alexander.herold@meduniwien.ac.at)*

**Purpose:** In chronic liver disease (CLD), early detection is pivotal and a predictive tool for events such as decompensation or transplant-free survival could change the surveillance and evaluation of treatment in these patients. By analysing the liver vessel volume ratio (LVVR) and linking this information to clinical data we expected to not only find predictive features influencing the patient's treatment and outcome, but also identify phenotypical information that may inform our understanding of the biology, development, and early changes of CLD.
**Methods or Background:** In this retrospective study 250 patients with known CLD, who underwent gadoxetic acid enhanced MRI were included. According to the Fibrosis-4 index (FIB-4 scores) and histories of decompensation, patients were separated into 3 groups (non-advanced chronic liver disease (non-ACLD), compensated advanced chronic liver disease (c-ACLD) and decompensated advanced chronic liver disease (d-ACLD)). After successful automatic segmentation of the liver and liver vessels, Spearman and Pearson correlation, multinomial regression analysis and one-way ANOVA analysis were used to assess the association of LVVR with various established clinical and imaging parameters such as FIB-4 scores, MELD score, functional liver imaging score (FLIS) and liver functional parameters.
**Results or Findings:** The mean LVVR exhibited significant differences between all three groups. In FIB-4 scores non-ACLD could be differentiated from advanced liver disease (p<.001) and in FLIS d-ACLD from c-ACLDs (p<.001). In the multinomial regression analysis LVVR showed a verifiable impact on correct group assignation.
**Conclusion:** Liver vessel volume ratio may be valuable for distinguishing between non-advanced and advanced chronic liver disease.
**Limitations:** This was a retrospective single centre study and requires validation.
**Ethics committee approval:** The local ethics committee approved this study protocol which was performed in accordance with the Helsinki Declaration (IRB: EK Nr:2027/2017).
**Funding for this study:** Funding for this study was obtained from Novartis Pharmaceuticals Corporation (D.S., G.L.) and the Austrian Science Fund (FWF P35198).
**Author Disclosures:**
Sarah Pötter-Lang: Nothing to disclose
Alexander Johannes Herold: Nothing to disclose
Georg Langs: Nothing to disclose
Daniel Sobotka: Nothing to disclose
Mattias Mandorfer: Nothing to disclose
Lucian Beer: Nothing to disclose
Ahmed Ba-Ssalamah: Speaker: Bayer Consultant: Bayer
Nina Bastati-Huber: Nothing to disclose
Thomas Reiberger: Nothing to disclose

### RPS 1201-8

**Progression of portal hypertension in acute cellular rejection after liver transplantation**

*J. Y. Choi*; Seoul/KR

**Purpose:** This study was designed to investigate the frequency of computed tomography (CT) features indicating progression of portal hypertension and their clinical relevance in patients who experienced acute cellular rejection (ACR) after liver transplantation (LT).
**Methods or Background:** This retrospective study included 145 patients with pathologically diagnosed ACR following LT. Patients were divided into early and late ACR groups according to the time of ACR diagnosis. Two radiologists analysed the interval changes in spleen size and variceal engorgement on CT images obtained at the times of surgery and biopsy. Aggravation of splenomegaly and variceal engorgement were considered CT features associated with the progression of portal hypertension. Clinical outcomes, including responses to treatment and graft survival, were compared between patients with and without these features.
**Results or Findings:** The frequency of progression of portal hypertension was 31.7% and did not differ significantly in patients who experienced early (30.8% [29/94]) and late (33.3% [17/51]) ACR (p=0.85). In the late ACR group, CT features indicating progression of portal hypertension were significantly associated with poor response to treatment (p=0.033). Graft survival in both the early and late ACR groups did not differ significantly in patients with and without progression of portal hypertension.
**Conclusion:** CT features suggesting the progression of portal hypertension were encountered in about one-third of patients who experienced ACR after LT. Progression of portal hypertension was significantly related to poor response to treatment, particularly in the late ACR group.
**Limitations:** Due to the retrospective nature, there may be selection bias in the study populations.
**Ethics committee approval:** Institutional review board approval was obtained for this study.
**Funding for this study:** This research was supported by the Basic Science Research Program through the National Research Foundation of Korea, funded by the Ministry of Science, ICT and Future Planning.
**Author Disclosures:**
Ji Young Choi: Nothing to disclose

### RPS 1201-9

**Liver MRI: assessment of liver cirrhosis severity with extracellular volume fraction**

*N. Mesropyan*, P. A. Kupczyk, A. Isaak, C. Endler, A. M. Sprinkart, C. C. Pieper, D. Kütting, U. I. Attenberger, J. A. Luetkens; Bonn/DE

**Purpose:** To investigate the diagnostic utility of MRI-derived hepatic extracellular volume fraction (ECV) for the assessment of liver cirrhosis severity as defined by Child-Pugh classes.
**Methods or Background:** In this retrospective study, 90 participants (68 patients with liver cirrhosis and 22 control subjects) who underwent multiparametric liver MRI were identified. Hepatic T1 relaxation times and ECV were assessed. Clinical scores of liver disease severity were calculated. ANOVA followed by Turkey´s multiple comparison tests, Spearman's correlation coefficient, and ROC analysis were used for statistical analysis.
**Results or Findings:** In cirrhotic patients, hepatic native T1 increased depending on Child-Pugh class (620.5±78.9 ms [Child A] vs. 666.6±73.4 ms [Child B] vs. 828.4±91.2 ms [Child C], p<0.001). ECV was higher in cirrhotic patients compared to the controls (40.1±11.9% vs 25.9±4.5%, p<0.001) and also increased depending of Child-Pugh class (33.3±6.0% [Child A] vs. 39.6±4.9% [Child B] vs. 52.8±1.2% [Child C], p<0.001). ECV correlated with Child-Pugh score (r=0.64, p<0.001). ECV allowed differentiating between Child-Pugh classes A and B, and Child-Pugh classes B and C with an AUC of 0.785 and 0.944, p<0.001, respectively. The diagnostic performance of ECV for differentiating between Child-Pugh classes A and B, and Child-Pugh

Friday classes B and C was higher compared to hepatic native T1 (AUC: 0.651 and 0.910) and MELD score (AUC: 0.740 and 0.795), p<0.05, respectively.
**Conclusion:** MRI-derived ECV correlated well with the Child-Pugh score and showed a high diagnostic performance in differentiation between different Child-Pugh cirrhosis classes. MRI-derived ECV might be a valuable non-invasive biomarker for the assessment of liver cirrhosis severity.
**Limitations:** The small sample size with a limited number of patients with Child-Pugh class C.
**Ethics committee approval:** This study was approved by the local institutional review board that waived informed consent.
**Funding for this study:** This work has not received any funding.
**Author Disclosures:**
Patrick Arthur Kupczyk: Nothing to disclose
Julian Alexander Luetkens: Nothing to disclose
Narine Mesropyan: Nothing to disclose
Alexander Isaak: Nothing to disclose
Christoph Endler: Nothing to disclose
Alois M. Sprinkart: Nothing to disclose
Ulrike I. Attenberger: Nothing to disclose
Daniel Kütting: Nothing to disclose
Claus Christian Pieper: Nothing to disclose

**10:30-11:30** **Open Forum #1 (Radiographers)**

### Research Presentation Session: Radiographers

### RPS 1314

### Radiography service considerations: COVID-19, being green and patient-focussed

**Moderators**
M. Zanardo; Milan/IT
A. P. Parkar; Bergen/NO

### RPS 1314-3

**Mobile X-rays and COVID-19: increased utilisation of mobile X-ray during COVID-19 and the effect on radiographer dose**
*P. Yeung*; Melbourne/AU
*(Phoebe.Yeung17@gmail.com)*

**Purpose:** To assess the effect of increased utilisation of mobile X-ray units, mobile imaging of non-routine body regions and radiographer work practice changes for impact on staff radiation dose during the early stages of the COVID-19 pandemic, in Australia.
**Methods or Background:** A retrospective analysis of general radiology departments across two metropolitan hospitals was performed. Personal radiation monitor exposure reports between January 2019 and December 2020 were analysed. Statistical analysis was conducted using a Mann–Whitney U test when comparing each quarter, from 2019 to 2020. Categorical data were compared using a chi-squared test.
**Results or Findings:** Mobile X-ray use during the pandemic increased approximately 1.7-fold, with the peak usage observed in September 2020. The mobile imaging rate per month of non-routine body regions increased from approximately 6.0–7.8%. Reported doses marginally increased during Q2, Q3 and Q4 of 2020 (in comparison to 2019 data), though was not statistically significant (Q2: p=0.13; Q3: p= 0.31 and Q4 p= 0.32). In Q1, doses marginally decreased and were not statistically significant (p=0.22).
**Conclusion:** There was a substantial increase in the utilisation of mobile X-rays following the COVID-19 pandemic. Additionally, mobile X-ray work practices evolved to reduce infection transmission risk, such as imaging patients in isolation rooms. Despite the increase in the utilisation of mobile radiography, there was no statistically significant increase in radiation exposure to radiographers during the COVID-19 pandemic.
**Limitations:** These findings are the experience of one Australian health service and may not be generalisable to other health services. Staff rostering also varied leading to many radiographers being excluded.
**Ethics committee approval:** Ethics approval for this study has been provided by the Monash Health research office, our local Human Research Ethics Committee (HREC reference number: QA/70407/MonH-2020-239841(v1)).
**Funding for this study:** No funding was received for this study.
**Author Disclosures:**
Phoebe Yeung: Nothing to disclose

### RPS 1314-4

**The impact of the COVID-19 pandemic on 10 main public imaging services in Chile**
*M. Zenteno*[1], D. Zenteno[2]; [1]Santiago/CL, [2]Santiago, R./CL
*(mzentenos@gmail.com)*

**Purpose:** The aim is to know the impact of the COVID-19 pandemic on attendance at imaging services in the Chilean public health system. The change in the number of visits by imaging modality in the years 2018-2019 compared to the years 2020-2021 is studied.
**Methods or Background:** The statistics of care in 10 imaging services of the main public hospitals in Chile from 2018 to 2022 are analysed. The changes in the number of cares by the modalities of general radiology, CT, MRI, mammography and ultrasound are studied.
**Results or Findings:** It was found that in the 10 main public hospitals in Chile, care imaging services reduced between 5% and 31%, with an average reduction of 21% in care in the different modalities of imaging examinations. The most affected modality turned out to be the ultrasound, which registered a drop of between 3% and 56%, with an average of 32%.
**Conclusion:** The COVID-19 pandemic generated an average reduction of 21% in care in the 10 imaging services of the main public hospitals in Chile, with ultrasound being the most affected modality and general radiology the least affected. An effort by the state will be needed to recover the levels prior to the pandemic and resume the growth of the system in pre-pandemic years.
**Limitations:** This study shows the statistics sent by the 10 main public hospitals in Chile and does not contain the total care provided by the public sector in the years 2018-2022.
**Ethics committee approval:** This study did not require ethical approval because it involved information freely available in the public domain.
**Funding for this study:** No specific grant from any funding agency in the public, commercial or not-for-profit sectors.
**Author Disclosures:**
Marcelo Zenteno: Nothing to disclose
Diego Zenteno: Nothing to disclose

### RPS 1314-5

**Professional quality of life of Nova Scotian medical radiation and imaging professionals highly willing to work during the COVID-19 pandemic: a study of compassion satisfaction and compassion fatigue**
M. Brydon, *J. Avery*, M. Sponagle, R. Gilbert; Halifax, NS/CA
*(julieavery@nscmirtp.ca)*

**Purpose:** To profile the professional quality of life (PQL) of medical radiation and imaging professionals (MRIPs) during the COVID-19 pandemic.
**Methods or Background:** PQL is defined as the quality an individual feels in relation to their work as a helper. It is influenced by the positive (compassion satisfaction) and negative (compassion fatigue) aspects encountered in one's job. Compassion is a feeling of empathy towards another's suffering combined with the motivation to help. In healthcare, compassion is associated with increased trust in therapeutic relationships, improved outcomes for patients, increased job satisfaction, and retention of staff. Understanding the influence of ongoing crisis situations on aspects of PQL is important for identifying areas needing support and ensuring sustained contribution. This study explores aspects of compassion in MRIPs willing to work during the COVID-19 Pandemic. A self-report survey of MRIPs was conducted in Fall, 2020. PQL was measured using ProQOL-5 (compassion satisfaction and compassion fatigue). Compassion fatigue was assessed by subscales: Burnout and Secondary Trauma.
**Results or Findings:** Of 162 MRIPs, 99% reported having moderate (58%) or high (41%) compassion satisfaction. For burnout, 26% of MRIPs reported low levels and 74% reported moderate levels. For secondary trauma, 52% reported low levels and 48% reported moderate levels. Compassion satisfaction was associated with age (p≤0.021) and years in practice (p=0.019), but not gender, education or job role. Burnout was not associated with demographic factors. Secondary trauma was associated with gender (p=0.02) and age (p≤0.041).
**Conclusion:** Compassion satisfaction influences motivation to help. 99% of MRIPs reported moderate-high levels of compassion satisfaction despite moderate levels of burnout and secondary trauma. Compassion satisfaction may prove to be a dominant factor in willingness to work during the pandemic.
**Limitations:** An identified limitation of this study was the presence of social-desirability bias.
**Ethics committee approval:** Ethics committee approval was received from the IWK Health Centre REB (1025856).
**Funding for this study:** Funding was received from the Nova Scotia COVID-19 Health Research Coalition Grant.
**Author Disclosures:**
Megan Brydon: Nothing to disclose
Melissa Sponagle: Nothing to disclose
Robert Gilbert: Nothing to disclose
Julie Avery: Nothing to disclose

### RPS 1314-6

**An evaluation of the impact of the COVID-19 pandemic on the wellbeing of interventional radiographers in Ireland**

*M. L. Murphy*, M. McEntee, N. Moore, A. England; Cork/IE
*(michellelauramurphy@gmail.com)*

**Purpose:** The aim of this study was to gain insight into the wellbeing of interventional radiographers (IRs) prior to and during the COVID-19 pandemic.
**Methods or Background:** The aim of this study was to gain insight into the wellbeing of IR's. An explanatory sequential mixed methods approach, using both questionnaires and interviews, was used to explore and evaluate IR wellbeing. An electronic self-administered questionnaire was completed by forty IRs and a semi-structured interview was conducted with two IRs.
**Results or Findings:** Physical, mental and social wellbeing of IRs has deteriorated since the onset of COVID-19. All forms of wellbeing were negatively impacted with mental wellbeing being the most impacted (82.5%), closely followed by physical wellbeing (the impact of which increased to 75%) and social wellbeing (50%). Half of IRs were “highly stressed” while working during COVID-19. Physical activity levels decreased, caffeine consumption increased, and consumption of a healthy diet decreased. Most IRs (95%) had anxiety about passing the virus onto family or friends, they had a reduced level of sleep and IRs (60%) noted a deterioration in their relationships with friends. Three key themes were identified including the importance of teamwork, physical demands and the mental impact of work.
**Conclusion:** COVID-19 has subsequently had a negative effect on the wellbeing of IRs in Ireland. The implications of staff having a diminished sense of wellbeing is that productivity could reduce and subsequent burnout could lead to illness. Further work is needed to focus on and contribute to improving the wellbeing of IRs.
**Limitations:** Further research is needed to identify methods of addressing the shortcomings in support services and identifying the specific wellbeing needs of IRs.
**Ethics committee approval:** This study was approved by University College Cork Social Research Ethics Committee.
**Funding for this study:** Not applicable as this study was part of an MSc in Diagnostic Radiography.
**Author Disclosures:**
Niamh Moore: Nothing to disclose
Michelle Laura Murphy: Nothing to disclose
Andrew England: Nothing to disclose
Mark McEntee: Nothing to disclose

### RPS 1314-7

**Chest CT protocols in COVID-19 and its influence on radiation dose**

*M. C. P. Ribeiro*[1], M. Andrade[1], L. Dias[1], G. Saldanha[1], A. Gonçalves[2], J. M. Lopes[2], A. Matos[1], T. Tomas[1], L. Serôdio[1]; [1]Lisbon/PT, [2]Almada/PT
*(margarida.ribeiro@estesl.ipl.pt)*

**Purpose:** According to the American College of Radiology, for pulmonary computed tomography (CT) applied in suspected COVID-19 infection, an adjustment of the image acquisition variables (protocol) is recommended, which determines the radiation dose received by the patient. The International Atomic Energy Agency found wide variation in protocols, within and between different countries. The aim of this study was to analyse whether imaging departments have adapted their routine chest CT protocols in relation to the COVID-19 infection.
**Methods or Background:** Through records of examinations in CD-ROM, 5 protocols of chest CT in suspected COVID-19 patients in 4 imaging departments were randomly selected. The parameters which impact the CTDIvol and DLP were analysed: thickness, pitch, kV, mA, time rotation and gap. The effective and cumulative dose were calculated for each protocol. The Mann-Whitney test was applied to compare the values of DLP and CTDIvol and to compare the dose descriptors of the protocol the (Kruskal-Wallis test) was used.
**Results or Findings:** There were no significant differences between the 5 protocols of the 4 imaging departments (p=0.406) in the evaluation of the effective dose measured in (mSv). However, it was found that the lowest value was obtained in department B (2.94mSv) and the highest value in department C (6.68mSv). The differences between the protocol that presented the lowest effective dose, and the others, were not statistically significant.
**Conclusion:** The high differences found were not statistically significant regarding the dose descriptors of each protocol. Compared to the previous ones, the different imaging departments adapted their chest CT protocols for COVID-19 patients, however there is latitude for further rigorous optimisation adapted to this clinical situation.
**Limitations:** The small sample of protocols was an identified limitation.
**Ethics committee approval:** This study received ethics committee approval.
**Funding for this study:** This study received funding from the funding entity of the Instituto Politécnico de Lisboa, through the IDI&CA programme (grant agreement number 6339462).
**Author Disclosures:**
Leonor Serôdio: Nothing to disclose
Lidia Dias: Nothing to disclose
José Miguel Lopes: Nothing to disclose
Teresa Tomas: Nothing to disclose
Analia Matos: Nothing to disclose
Micael Andrade: Nothing to disclose
Margarida Carmo Pinto Ribeiro: Nothing to disclose
Afonso Gonçalves: Nothing to disclose
Gonçalo Saldanha: Nothing to disclose

### RPS 1314-8

**Importance of circular economy and green skills to radiation therapists: a European Union (EU) survey**

A. L. F. Soares[1], S. Buttigieg[2], B. Bak[3], *J. G. Couto*[2], S. L. McFadden[4], C. Hughes[4], P. McClure[4], I. Bravo[1]; [1]Porto/PT, [2]Msida/MT, [3]Poznan/PL, [4]Belfast/UK
*(jose.g.couto@um.edu.mt)*

**Purpose:** To evaluate the perceptions of radiation therapists (TR/RTTs) regarding circular economy (CE) practices relevant to the professional practice across the European Union and identify the relevant green skills.
**Methods or Background:** An electronic survey (Google forms) was distributed to TR/RTTs across the EU. Convenience sampling was used since the population is ill-defined; as such, participants were recruited by all SAFE EUROPE partners covering all of Europe and via social media to include as many participants as possible. This survey was open from 1st October 2021 to 30th November 2021. Data analysis was performed with the aid of SPSS version 22.
**Results or Findings:** Sixty-seven responses were received. 40.3% (n=27) and 47.8% (n=32) of TR/RTTs were not aware of CE and green skills, respectively. 70% (n=47) of the TR/RTTs did not receive training in CE practice. 82% (n=55) agreed that green skills should be included in RTTs education. Environment-friendly procurement, waste management and energy reduction/efficiency were the most relevant topics to be developed.
**Conclusion:** TR/RTTs have a lack of knowledge regarding CE and green skills. Further development of green skills is required to enable the implementation of CE into radiotherapy practice.
**Limitations:** The survey was in English so may have excluded non-English speakers from participating. Lack of knowledge on CE may have decreased the number of responses. The length of time to complete the survey was a further limitation.
**Ethics committee approval:** Ethical permission was sought and obtained from Ulster University, Belfast, UK. Reference number FCNUR-21-068.
**Funding for this study:** This work was co-funded by the SAFE EUROPE project under the Erasmus+ Sector Skill Alliances program (grant agreement 2018e2993/001-001).
**Author Disclosures:**
Ana Luísa Ferreira Soares: Research/Grant Support: This work was co-funded by the SAFE EUROPE project under the Erasmus+ Sector Skill Alliances program [grant agreement 2018e2993/001-001].
Isabel Bravo: Research/Grant Support: This work was co-funded by the SAFE EUROPE project under the Erasmus+ Sector Skill Alliances program [grant agreement 2018e2993/001-001].
Bartosz Bak: Research/Grant Support: This work was co-funded by the SAFE EUROPE project under the Erasmus+ Sector Skill Alliances program [grant agreement 2018e2993/001-001].
Ciara Hughes: Research/Grant Support: This work was co-funded by the SAFE EUROPE project under the Erasmus+ Sector Skill Alliances program [grant agreement 2018e2993/001-001].
Patricia McClure: Research/Grant Support: This work was co-funded by the SAFE EUROPE project under the Erasmus+ Sector Skill Alliances program [grant agreement 2018e2993/001-001].
Sandra Buttigieg: Research/Grant Support: This work was co-funded by the SAFE EUROPE project under the Erasmus+ Sector Skill Alliances program [grant agreement 2018e2993/001-001].
Sonyia Lorraine McFadden: Grant Recipient: This work was co-funded by the SAFE EUROPE project under the Erasmus+ Sector Skill Alliances program [grant agreement 2018e2993/001-001].
Jose Guilherme Couto: Research/Grant Support: This work was co-funded by the SAFE EUROPE project under the Erasmus+ Sector Skill Alliances program [grant agreement 2018e2993/001-001].

**RPS 1314-9**

**Breast cancer patient viewpoint versus staff thoughts regarding mammography and radiation therapy services**

*E. M. Metsälä*[1], T. S. Schroderus-Salo[2], L. Marmy[3], J. A. Pires Jorge[3], K. Straume[4], B. Strom[4], M. Øynes[4], L. Randle[5], S. Kivistik[5]; [1]Helsinki/FI, [2]Oulu/FI, [3]Lausanne/CH, [4]Bergen/NO, [5]Tartu/EE
*(eija.metsala@metropolia.fi)*

**Purpose:** The purpose was to compare breast cancer patients' opinions about mammography and radiation therapy services to what health care staff thought to be success factors for these services.
**Methods or Background:** Patient data was collected from breast cancer patients (n=14) by using open-ended online questionnaires via the websites and social media of national breast cancer patient organisations in four countries. In addition a web-based open-ended questionnaire was sent to breast care hospitals located in four different countries, focusing on four professional groups: diagnostic radiographers, radiation therapists, breast cancer nurses and biomedical laboratory scientists (n=23). Both data sets were analysed using deductive thematic analysis.
**Results or Findings:** In regard to both services, patients emphasise competent staff, good information and smooth-flowing, comfortable and individual services. Staff tend to put more emphasis on patient characteristics and the technical performance features of the process. Common aspects for both patients and staff are understanding the importance of aftercare and follow-up, and the fact that the patient should be given a chance to keep in close contact with care and treatment staff even after their active treatment process has finished.
**Conclusion:** Patients and health care staff view mammography and radiation therapy services somewhat differently. Patients put more emphasis on non-clinical issues and staff focus more on patient related and technical factors. Both patients and staff should be involved in planning services for breast cancer patients.
**Limitations:** Limitations associated with self-reporting instruments apply to this study.
**Ethics committee approval:** Not applicable.
**Funding for this study:** The study was supported by European Commission Erasmus+ Strategic partnership programme grant number 2020-1-EE01-KA203-077941. For the Swiss associate Partner this work was supported by the Swiss national agency MOVETIA.
**Author Disclosures:**
Eija Metsälä Metsälä: Nothing to disclose
Kjersti Straume: Nothing to disclose
José A. Pires Jorge: Nothing to disclose
Siret Kivistik: Nothing to disclose
Bergliot Strom: Nothing to disclose
Liis Randle: Nothing to disclose
Laurent Marmy: Nothing to disclose
Tanja Susanne Schroderus-Salo: Nothing to disclose
Mona Øynes: Nothing to disclose

**10:30-12:00** **Room E1**

### Research Presentation Session: GI Tract

### RPS 1301

### Inflammatory bowel disease and perianal fistula: what's new?

**Moderator**
G. Masselli; Rome/IT

**RPS 1301-2**

**Radiomics as a tool to predict surgery at 10 years in Crohn's disease**

L. Larosa, L. M. Minordi, *A. Bevere*, L. Laterza, L. Boldrini, H. E. Tran, C. Votta, A. Armuzzi, R. Manfredi; Rome/IT
*(antonio.bevere1@gmail.com)*

**Purpose:** The aim of this study is to assess the capability of radiomics to predict the need for surgery in patients with Crohn's disease (CD).
**Methods or Background:** A cohort of 30 patients with CD that had undergone one or more CT-enterographies between 2009 and 2011 was retrospectively selected. A total of 44 CT scans were examined by an expert radiologist who generated a region of interest (ROI) segmentation for each pathological intestinal tract found, obtaining 93 lesions overall for radiomic analysis. A dedicated software extracted 217 radiomic features from each ROI. Patients charts were reviewed to evaluate if patients underwent surgery in a 10-year follow-up for a binary classification. A logistic regression model was built with the selected features and evaluated by computing the area under the curve (AUC) of the receiver operating characteristic (ROC) curve. Accuracy, sensitivity, specificity, positive and negative predictive values (PPV, NPV) of the model were calculated.
**Results or Findings:** Two radiomic features were found to be statistically significant in predicting the need for surgery in the fitted logistic regression model ($p < 0.0001$): grey level histogram variance and grey level non-uniformity. This model presented an AUC of 0.83, with a confidence interval of 95% in predicting surgery. Mean values of the model performance metrics over the cross-validation iterations were: accuracy 0.78 (0.02), sensitivity 0.68 (0.14), specificity 0.86 (0.07), PPV 0.72 (0.12), NPV 0.83 (0.09).
**Conclusion:** Radiomics could be a helpful tool to identify high risk for surgery patients. Early identification of such patients may influence their treatment choice during the course of the disease, avoiding unnecessary medical therapy.
**Limitations:** Further studies are required to obtain larger and external validations of this model.
**Ethics committee approval:** Not applicable
**Funding for this study:** There is no funding to report.
**Author Disclosures:**
Luca Boldrini: Nothing to disclose
Lucrezia Laterza: Nothing to disclose
Luigi Larosa: Nothing to disclose
Huong Elena Tran: Nothing to disclose
Riccardo Manfredi: Nothing to disclose
Alessandro Armuzzi: Nothing to disclose
Antonio Bevere: Nothing to disclose
Laura Maria Minordi: Nothing to disclose
Claudio Votta: Nothing to disclose

**RPS 1301-3**

**Machine learning model incorporating computed tomography body composition features for predicting the response to mesalamine treatment in Crohn's disease**

*J. Zhang*, X. Yi; Changsha/CN
*(798844942@qq.com)*

**Purpose:** Mesalamine is a common treatment for Crohn's disease but is not effective in all patients. This study aimed to develop a machine learning model incorporating computed tomography body composition features to improve prediction of mesalamine treatment response in Crohn's disease.
**Methods or Background:** 107 patients with confirmed Crohn's disease who were treated with mesalamine were retrospectively included and separated randomly into a training and a validation group. The prediction models were developed using machine learning methods (least absolute shrinkage and selection operator, random forest, and support vector machine [SVM]) using just clinical/laboratory values (SVM-Clinic-Labtest), using computed tomography body composition features and clinical/laboratory values (SVM-Combined), or using multivariable logistic regression (LR).
**Results or Findings:** After incorporating body composition features, the SVM-Combined model showed good discrimination between the responder and non-responder groups, with an area under the curve of 0.953 (95% CI: 0.883 to 1.000) in the training group and 0.957 (95% CI: 0.957 to 1.000) in the validation group. This was significantly higher than for the SVM-Clinic-Labtest model (area: training group, 0.910 [95% CI: 0.799 to 1.000]; validation group, 0.910 [95% CI: 0.841 to 0.980]), and LR model (area: training group, 0.625 [95% CI: 0.413 to 0.837]; validation group: 0.788 [95% CI: 0.686 to 0.890]). Favourable calibration performance and clinical applicability of the machine learning model were observed using calibration and decision curve analysis.
**Conclusion:** We developed a machine learning model incorporating computed tomography body composition features along with clinical/laboratory values, which could aid in predicting mesalamine treatment response in Crohn's disease patients.
**Limitations:** Not applicable
**Ethics committee approval:** This retrospective study was approved by the ethics committee and Institutional Review Board in Xiangya Hospital of Central South University, P. R. China (IRB No.202104078).
**Funding for this study:** Not applicable
**Author Disclosures:**
Jinwei Zhang: Nothing to disclose
Xiaoping Yi: Nothing to disclose

**RPS 1301-4**

**The development and value of magnetic resonance activity evaluation index without contrast agent injury in Crohn's disease**

*H. Wu*, X-G. Peng; Nanjing/CN
*(hhseu520@163.com)*

**Purpose:** This study aimed to develop a simple magnetic resonance index of activity to evaluate Crohn's disease activity.
**Methods or Background:** Eighty-two Crohn's disease patients with terminal ileal involvement, who underwent magnetic resonance enterography, were retrospectively analysed. Magnetic resonance variables included bowel wall thickness, relative wall edema, water-fat ratio, apparent diffusion coefficient value, mucosal ulceration, enlarged lymph nodes, fistula, comb signs, abscesses, and stenosis. We used logistic regression analysis to identify magnetic resonance imaging indices independently associated with Magnetic Resonance Index of Activity (MaRIA) scores (reference standard).
**Results or Findings:** Logistic regression analysis showed that bowel wall thickness, water-fat ratio, apparent diffusion coefficient value were independently associated with MaRIA scores, the m-Clermont index was constructed as follows: 21.219 + 1.283 × thickness – 0.295 × WFR – 4.239 × ADC value (R2 = 0.783). Receiver Operating Characteristic curve (ROC) analysis of our model showed high accuracy for discriminating inactive and active Crohn's disease with 87.5% sensitivity and 100% specificity (area under the curve 0.964). For each patient, there was a good correlation between m-Clermont scores and MaRIA (R = 0.747, P < 0.001).
**Conclusion:** The accuracy and simplicity of the magnetic resonance inflammatory activity index for detecting disease activity may render it an alternative to endoscopy in the evaluation of Crohn's disease.
**Limitations:** First, the sample size is relatively small, and the data are from a single centre, so there may be selection bias. Second, the model was not externally validated to test its clinical usefulness, which is also a pity.
**Ethics committee approval:** This study has been approved by the medical ethics committee of Zhongda Hospital, Southeast University (approval number: 2021zdsyll163-p01).
**Funding for this study:** This study was funded by the National Natural Science Foundation of China (81501523, 81871412).
**Author Disclosures:**
Honghong Wu: Author: None of us have a conflict of interest.
Xin-Gui Peng: Author: None of us have a conflict of interest.

### RPS 1301-5

**Prediction of active inflammation in small intestinal Crohn's disease based on MRE imaging**

*H. Wu*; Nanjing/CN

**Purpose:** To predict Crohn's disease activity by radiomics analysis of magnetic resonance enterography contrast enhancement.
**Methods or Background:** 82 patients with terminal ileal involvement with CD who were divided into the non-mild and the moderate-severe activity group according to the magnetic resonance activity index. The model was established in the training set and verified in the test set. We used the receiver operating characteristic curve to compare the diagnostic efficacy of conventional imaging results (London index) and the radiomics model for distinguishing non-mild and moderate-severe active inflammation in CD.
**Results or Findings:** We finally got the model as: 1.248 × wavelet - HLL _firstorder_Kurtosis + 0.717 × wavelet -LLL_firstorder_90 Percentile + 1.116 × wavelet - LLL_glcm_JointAverage. The diagnostic efficacy of the radiomics model was 0.953 (sensitivity: 90.3%, specificity: 88.9%) in the training set, and 0.881 (sensitivity: 84.6%, specificity: 81.2%) in the test set. The diagnostic efficacy of visual interpretation was 0.848 (sensitivity 80.6%, specificity 77.8%) in the training set. The diagnostic performance of the radiomics model in differentiating non-mild and moderate-severe Crohn's disease activity was significantly higher than that of the radiologists' visual interpretation (AUC = 0.953 vs 0.848, P < 0.05).
**Conclusion:** The radiomics model based on contrast enhanced magnetic resonance enterography is effective in the diagnosis of active inflammation of the small intestine in Crohn's disease.
**Limitations:** The sample size is relatively small, and the data is from a single center, so there may be data bias. This study only investigated the non-mild and moderate-severe activity of the terminal ileum, but not the whole intestine.
**Ethics committee approval:** This study has been approved by the medical ethics committee of Zhongda Hospital, affiliated to Southeast University (Grant Number: 2021zdsyll163-P01).
**Funding for this study:** This project is a general project of the national natural science foundation of China (fund no.: 81501523, 81871412).
**Author Disclosures:**
Honghong Wu: Nothing to disclose

### RPS 1301-6

**The utility of diffusion weighted images in Crohn's disease acute flare**

*K. Alkhalili*, N. Holalkere; Boston, MA/US
*(kalkhalili1@tuftsmedicalcenter.org)*

**Purpose:** To evaluate the utility of DWI in evaluation of acute flare of Crohn's disease.
**Methods or Background:** A retrospective analysis of MRE exams was performed for 39 patients with established Crohn's disease with acute symptoms. MRE sequences included DWI (highest b-value of 1200 s/mm) and multiphase post-gadolinium imaging. Two radiologists qualitatively analyzed 3 groups of studies: 1) DWI/ ADC maps, 2) Post-contrast T-1 WI, and 3) combination of T1, T2, DWI, and post-contrast images. Receiver operating characteristic analysis (AUC) and descriptive statistics were performed. A p-value of <.05 was considered significant.
**Results or Findings:** A total of 156 segments/areas were evaluated for each set of sequences. Active inflammation was seen in 43 segments of small bowel, 10 of colon, and 12 of mesenteric involvement. The sensitivity, specificity, positive predictive value, negative predictive value, and accuracy in identification of acute flare were 97%, 77%, 57%, 98%, and 82% on DWI, 98%, 94%, 92%, 98% and 96% on post Gd-T1WI, and 100%, 98%, 98%, 100% and 99% when a combination of T1, T2, DWI and Post Gd-T1WI were used respectively. AUC was 0.792 for DWI, 0.956 for Post Gd-T1, and 0.992 for combination of all sequences with p<0.05. DWI alone failed to identify 11/12 cases of penetrating mesenteric disease. However, with combination of post contrast images, all 12/12 cases of penetrating disease were identified.
**Conclusion:** DWI/ADC maps offer a comparable diagnostic sensitivity to contrast enhanced images in detecting acute flare of Crohn's disease, however, they provide a limited assessment of penetrating mesenteric disease. The utility of contrast enhanced sequences can be optimized by the addition of DWI sequence, particularly when motion artifact is encountered.
**Limitations:** The qualitative approach used during interpretation of DWI/ADC imaging, the retrospective approach, and the small sample were identified as limitations in this study.
**Ethics committee approval:** This study was approved by the IRB.
**Funding for this study:** No funding was received for this study.
**Author Disclosures:**
Nagaraj Holalkere: Author: Senior Author
Kenan Alkhalili: Author: Author

### RPS 1301-7

**Submucosal fat accumulation in Crohn's disease: evaluation with sonography**

*R. Garcia Dosda*, T. Ripolles Gonzalez, M. J. Martinez Perez, L. Navarro Vilar, R. Ruiz Marco, A. A. Painel Seguel, R. A. Amat Pérez, J. M. Paredes; Valencia/ES

**Purpose:** to investigate the US features that allow us to suspect the presence of submucosal fat deposition (SFD) in the intestinal wall of patients with Crohn's disease.
**Methods or Background:** CT examinations over a period of 10 years (2011-2020) were reviewed for the presence of the SFD in the bowel wall. A measurement of less than -10 HU was regarded as indicative of fat. We included only patients who had undergone US examination 3 months before or after CT. The study cohort group comprised 67 patients, 43 men and 24 women. Wall and submucosal thickness were measured on longitudinal US sections; an average of 3 measures was used for analysis. Measurements were made on images where a continuous and similar thickness of the submucosa was seen. A ROC curve was constructed to determine the best cut-off of US submucosal wall thickness for predicting SFD in the bowel wall determined on CT.
**Results or Findings:** The SFD was present in 25 of the 67 patients (37,3%) on CT. There were significant differences between submucosal thickness of patients with SFD and patients without SFD (25,5 versus 39,4 mm). From the ROC curve, a threshold value of 31mm of submucosal thickness had the best sensitivity and specificity to suspect SFD (84% and 88,1%, respectively) (AUC, 0.902), with an odds ratio of 38,85. All patients with a submucosal thickness > 39mm had HSF (n = 16).
**Conclusion:** Based on our results, US may suspect the presence of SFD on the wall and therefore recommend its confirmation with other techniques.
**Limitations:** It is important to note that ultrasound cannot diagnose SFD in the submucosa, it can only suggest it.
**Ethics committee approval:** The institutional review board of the hospital approved this retrospective study and waived the requirement to obtain informed consent.
**Funding for this study:** There was no funding required for this study.
**Author Disclosures:**
Rosa Ana Amat Pérez: Nothing to disclose
Rubén Ruiz Marco: Nothing to disclose
Rosa Garcia Dosda: Nothing to disclose
Jose M. Paredes: Nothing to disclose
Tomas Ripolles Gonzalez: Nothing to disclose
Maria Jesus Martinez Perez: Nothing to disclose
Lidia Navarro Vilar: Nothing to disclose
Andrés Adolfo Painel Seguel: Nothing to disclose

### RPS 1301-9

**Optimizing MR enterography for inflammatory bowel disease: our initial experience in Shifa International Hospital**

B. Y. Faiz, A. Rana, *R. Kanwal*, A. Gulzar, S. Saghir, M. Shahid; Islamabad/PK
*(raanakanwal@hotmail.com)*

**Purpose:** This study aims to describe the typical radiological findings in patients with Crohn's disease to aid in the diagnosis of inflammatory bowel disease (IBD) in daily practice.

**Methods or Background:** After IRB approval, a retrospective study was performed at a single institution. 30 patients with Crohn's disease with magnetic resonance enterography were included in the study. Data was reviewed from the electronic system.
**Results or Findings:** 9 patients (30%) were female and 21 patients (70%) were male, who underwent magnetic resonance enterography showing characteristic Crohn's disease characteristics. Among these patients, post-contrast enhancement was reported in 64% of patients, whereas 60% of patients had mural wall thickening. Another common feature seen among these patients was lymphadenopathy. This was seen in 20% of the study population. Other features of lesser percentages include mural thickening (7%), 6.6% had ulceration and 5% showed DWI restricted diffusion. None of these patients had extra-intestinal manifestation or any complications at the time of presentation. 60% of the patients underwent biopsy, whereas 3 (10%) patients had ASCA positive associated Crohn's. The remaining 7 patients had neither biopsy nor a blood test to confirm the diagnosis. Among the biopsy proven cases, our study revealed 61% diagnostic accuracy for Crohn's disease. This study was limited by its small sample size, its retrospective nature, and loss to follow-up in some patients.
**Conclusion:** Magnetic resonance enterography was found to be an effective, non-invasive imaging modality for the accurate diagnosis of Crohn's disease.
**Limitations:** The small data set was a limiting factor in this study.
**Ethics committee approval:** This study was approved by an ethics commitee.
**Funding for this study:** No funding was received for this study.
**Author Disclosures:**
Amaima Gulzar: Nothing to disclose
Belqees Yawar Faiz: Nothing to disclose
Atif Rana: Nothing to disclose
Raana Kanwal: Nothing to disclose
Mina Shahid: Nothing to disclose
Sabiyal Saghir: Nothing to disclose

**10:30-12:00** **Room M 1**

### Research Presentation Session: Chest

### RPS 1304

### New insights into pulmonary abnormalities through advanced CT techniques

**Moderator**
B. Heidinger; Vienna/AT

### RPS 1304-2

**Ultra-high-resolution photon-counting CT versus conventional HRCT of the lungs: a comparison of image quality and diagnostic impact**
*V. Van Ballaer*, A. Dubbeldam, E. Muscogiuri, L. Cockmartin, W. Coudyzer, J. Coolen, W. De Wever; Leuven/BE

**Purpose:** To compare clinical image quality in chest CT between ultra-high-resolution photon-counting CT (PCCT) and conventional HRCT using visual grading analysis (VGA) scores.
**Methods or Background:** Thirty-five patients with a PCCT (Naeotom Alpha, Siemens) between Nov 2021 and Jan 2022 and with a previous conventional HRCT in the last 14 months were included. Central and peripheral airways, lung vasculature, nodules, ground-glass opacities, intra- and interlobular lines, emphysema, fissures, bullae/cysts and air trapping were evaluated on PCCT (0.4mm) versus conventional HRCT (1mm) via side-by-side reference scoring using a 5-point Likert scale (-2 to +2). Visibility and possible impact on diagnosis were assessed by three radiologists using a visual grading analysis (VGA). The median VGA scores were compared and tested using one-sample Wilcoxon signed-rank tests with hypothesised median values of 0 (same visibility) and 2 (better visibility and diagnostic impact) at a 2.5% significance level.
**Results or Findings:** Almost all lung structures had significantly better visibility on PCCT compared to HRCT (p<0.025), with the highest VGA scores for peripheral airways (1.06), intralobular lines (1.10) and centrilobular emphysema (1.20). A positive trend, though non-significant, was seen for macro-nodules and ground-glass nodules (p=0.046; N= 27 and p= 0.157; N=6 resp.). Although image quality was better, no significant impact on diagnosis (VGA-score 2) could be demonstrated.
**Conclusion:** All lung structures had superior clinical image quality on PCCT versus HRCT, being most pronounced for peripheral airways, intralobular lines and centrilobular emphysema. A significant impact on diagnosis, however, could not be demonstrated.
**Limitations:** Small population sample, of which the majority had interstitial lung disease.
**Ethics committee approval:** Approval by the Ethics Committee Research of University Hospitals Leuven.
**Funding for this study:** No funding was received for this study.
**Author Disclosures:**
Walter Coudyzer: Nothing to disclose
Johan Coolen: Nothing to disclose
Valerie Van Ballaer: Nothing to disclose
Emanuele Muscogiuri: Nothing to disclose
Lesley Cockmartin: Nothing to disclose
Walter De Wever: Nothing to disclose
Adriana Dubbeldam: Nothing to disclose

### RPS 1304-3

**Impact of photon-counting CT (PCCT) in the evaluation of interstitial lung disease (ILD): Preliminary experience in 29 patients**
Y. Gaillandre, A. Duhamel, S. Khung, A. Hutt, J. Remy, *M. Rémy-Jardin*; Lille/FR
*(martine.remy@chru-lille.fr)*

**Purpose:** To compare lung parenchyma analysis based on the ultra-high resolution (UHR) mode of a PCCT scanner with that of the high-resolution (HR) mode of a 3rd-generation dual-source CT scanner.
**Methods or Background:** 29 patients underwent a HRCT examination at T0 (collimation : 2x96x0.6mm ; pitch : 2-3 ; Sn 150 kV ; 150 mAs ) and a UHR examination at T1 (collimation : 120 x 0.2mm ; pitch : 1 ; Sn100kV / Sn140 kV ) with similar reconstructions (512 matrix; 1-mm thick sections). The rating ranged (a) from score 1 (« very sharp ») to score 4 ("marked blurring") for anatomical structures; and (b) from score 0 ("feature absent") to score 3 ("feature present and sharp") for ILD features.
**Results or Findings:** The anatomical structures were more precisely depicted at T1 with: (a) visualization of more distal bronchial divisions (median order; Q1-Q3) (T1: 9th division [9-10]; T0: 8th division [8-9]; p<0.0001); (b) greater scores of sharpness for bronchial walls (p<0.0001), right minor (p<0.0001) and major (p=0.02) fissures. The scores of visualization of micronodules (p=0.25), lines (p=0.07), bronchiectasis (p=0.5) and honeycombing (p=0.06) did not differ between T0 and T1. The scores of visualization (median ; Q1-Q3) of intralobular reticulation (T1 : score 3 [2-3]; T0: score 2 [2-2] ; p< 0.0001) and bronchiolectasis ( T1 : score 2 [0-3]; T0: score 2 [0-2] ; p<0.0001) were higher at T1. The DLP value was significantly lower at T1 (85.8 ±21.3 mGy.cm) than at T0 (132.8 ± 41.7 mGy.cm) (p<0.01).
**Conclusion:** These preliminary results demonstrate the superiority of the UHR mode.
**Limitations:** This study is based on a small number of patients.
**Ethics committee approval:** Waiver of patient informed consent
**Funding for this study:** No funding was received for this work
**Author Disclosures:**
Yann Gaillandre: Nothing to disclose
Suonita Khung: Nothing to disclose
Martine Rémy-Jardin: Research/Grant Support: Siemens Healthineers
Jacques Remy: Consultant: Siemens Healthineers
Alain Duhamel: Nothing to disclose
Antoine Hutt: Nothing to disclose

### RPS 1304-4

**Image quality assessment of chest CT scans: Initial experiences with the first photon counting CT approved for clinical use**
*M. M. Woeltjen*, J. H. Niehoff, A. E. Michael, C. Mönninghoff, J. Borggrefe, J. R. Kröger; Minden/DE

**Purpose:** This study aims to investigate the qualitative and quantitative image qualities of low dose high resolution (LD-HR) thorax CT scans with the first clinical approved photon counting CT (PCCT) scanner and its radiation dose compared to a conventional scanner with an energy-integrating detector (EID-CT).
**Methods or Background:** Patients who underwent a LD-HR CT scan with dual-source PCCT for suspicion of interstitial lung disease and had previously undergone a LD-HR CT scan with an EID-CT scanner were retrospectively identified. In 29 of these patients no change in pulmonary changes were noted in the clinical report and these patients were included in our study. For the PCCT images were reconstructed with and without quantum iterative reconstruction (QIR). Magnified images were rated by 3 senior radiologists, blinded to clinical information and the CT scanner, for qualitative image quality parameters using a 5-point Likert-scale. Moreover, the effective dose was calculated.
**Results or Findings:** Overall image quality was rated valued better in PCCT images with QIR compared to EID-CT images (4 ± 0.8 vs. 3.4 ± 1.1, p<0.001). Imaging quality was also higher in PCCT images with QIR compared to PCCT images without QIR (4 ± 0.8 vs. 3.7 ± 0.8, p<0.001). Effective radiation dose was significantly lower in PCCT examinations (1.41 ± 0.5mSv) compared to EID-CT scans (1.9 ± 0.52mGy) (p < 0.001).

**Conclusion:** PCCT LD-HR chest examinations provide better image quality with significant dose reduction compared to EID-CT scans. QIR significantly improves image quality for lung examinations with the PCCT.
**Limitations:** Low patient number. The EID-CT scanner we used was an older model (Siemens S64) that was released in 2003 and may not depict a comparison to an up-to-date EID-CT scanner.
**Ethics committee approval:** Yes.
**Funding for this study:** No.
**Author Disclosures:**
Julius Henning Niehoff: Nothing to disclose
Matthias Michael Woeltjen: Nothing to disclose
Christoph Mönninghoff: Research/Grant Support: Reports personal fees from Bayer Medical.
Jan Borggrefe: Research/Grant Support: Received honoraria for scientific lectures from Philips and Siemens.
Jan Robert Kröger: Research/Grant Support: Received research support from Philips and support for attending meetings and/or travel from Veryan.
Arwed E Michael: Nothing to disclose

### RPS 1304-5

**Pattern recognition on ultra-low-dose CT to identify the aetiology of pneumonia**

*I. A. H. Van Den Berk*[1], M. M. N. P. Kanglie[1], T. van Engelen[1], M. Hovinga-De Boer[1], W. de Monye[2], S. Bipat[1], P. M. M. Bossuyt[1], J. M. Prins[1], J. Stoker[1]; [1]Amsterdam/NL, [2]Haarlem/NL
*(inge.van.den.berk@suerte.nl)*

**Purpose:** It has been suggested that pattern recognition on CT aids in identifying the aetiology of pneumonia. We retrospectively studied the diagnostic accuracy at ultra-low-dose CT (ULDCT).
**Methods or Background:** In the OPTIMACT trial 1,208 patients with suspected non-traumatic pulmonary disease underwent ULDCT at the emergency department. All 276 patients with a definite clinical diagnosis of pneumonia were selected, of which 96 (35%) had a positive microbiology result. 60 had viral pathogens; 48 had bacterial pathogens; one had a fungus; and there were multiple pathogens in 13. Two chest radiologists independently evaluated the corresponding ULDCT for pneumonia pattern (lobar, interstitial or bronchopneumonia) and most likely pathogen.
**Results or Findings:** In the patients with bacterial pathogens a bronchopneumonia pattern was observed by both radiologists in 14/48 (29%) and by only one in 17/48 (35%); a lobar pneumonia pattern was observed by both in 15/48 (31%) and by one in 11/48 (23%); and an interstitial pneumonia pattern was observed by one in 5/45 (11%). In the patients with viral pathogens a bronchopneumonia pattern was observed by both in 23/60 (38%) and by one in 23/60 (38%); a lobar pattern was observed by both in 6/60 (10%) and by one in 6/60 (10%); and an interstitial pneumonia pattern was observed by both in 1/60 (2%) and by one in 12/60 (20%). In the patients with bacterial pathogens this was suggested by both radiologists in 34/48 (71%) and by one in 8/48 (17%). In the patients with viral pathogens this was suggested by both in 6/60 (10%) and by one in 18/60 (30%).
**Conclusion:** Although a lobar pneumonia pattern is associated with a bacterial infection and the bronchopneumonia and interstitial pneumonia patterns are seen more often with a viral infection, overall the cause of pneumonia cannot reliably be determined at CT.
**Limitations:** Retrospective.
**Ethics committee approval:** NL57923.018.16
**Funding for this study:** Innovation grant Amsterdam UMC. ZonMW: 843001806
**Author Disclosures:**
Jan M. Prins: Nothing to disclose
Shandra Bipat: Nothing to disclose
Tjitske van Engelen: Nothing to disclose
Jaap Stoker: Nothing to disclose
Maadrika Meenakshi Nirvana Phoelmatie Kanglie: Nothing to disclose
Wouter de Monye: Nothing to disclose
Inge Alma Henrica Van Den Berk: Nothing to disclose
Patrick M. M. Bossuyt: Nothing to disclose
Marieke Hovinga-De Boer: Nothing to disclose

### RPS 1304-6

**Are interstitial lung abnormalities a prognostic factor for worse outcomes in COVID-19 pneumonia?**

*D. Colombi*[1], M. Petrini[1], N. Morelli[1], M. Silva[2], G. Milanese[2], N. Sverzellati[2], E. Michieletti[1]; [1]Piacenza/IT, [2]Parma/IT
*(colombidavide@gmail.com)*

**Purpose:** To assess the association between interstitial lung abnormalities (ILA) and worse outcomes in patients affected by COVID-19 pneumonia.
**Methods or Background:** Patients with COVID-19 pneumonia who underwent chest CT at an emergency department between February 29th 2020 and March 31st 2020 were retrospectively evaluated. Patients with CT findings indeterminate or typical for COVID-19 pneumonia, with positive nasal-pharyngeal molecular swab for SARS-CoV-2, who had obtained a chest CT before the pandemic were selected. Pre-pandemic CT scans were reviewed for the presence of ILA. ILA were also assessed according to the Fleischner Society diagnostic criteria. The outcome was either death from COVID-19 or intensive care unit (ICU) admission (death/ICU). Multivariable Cox proportional hazards regression analysis was used to test the independent association between ILA and death/ICU admission.
**Results or Findings:** 121/1391 (9%) patients were included (median age 72 years-old, IQR 65-79 years-old; males 87/12, 72%). Prior CT was performed within a median time of 14 months (IQR 5-35 months). ILA were identified in 31/121 (26%) patients. 13/31 (42%) ILA displayed fibrotic CT features. A significant association between fibrotic ILA and death/ICU admission (HR 2.2, 95% CI 1.11-4.34, p=0.024) was found.
**Conclusion:** CT scans obtained before the diagnosis of COVID pneumonia are to carefully review for the presence and characterisation of ILA. Indeed, fibrotic ILA are independent risk factors for worse outcomes in patients with COVID-19 pneumonia.
**Limitations:** Retrospective analysis from a single hospital. Small number of patients included, particularly with ILA.
**Ethics committee approval:** The study is approved by the local ethics committee.
**Funding for this study:** No funding source.
**Author Disclosures:**
Emanuele Michieletti: Nothing to disclose
Marcello Petrini: Nothing to disclose
Davide Colombi: Nothing to disclose
Nicola Sverzellati: Nothing to disclose
Nicola Morelli: Nothing to disclose
Gianluca Milanese: Nothing to disclose
Mario Silva: Nothing to disclose

### RPS 1304-7

**The next generation of reference books: combining content-based image retrieval with a knowledge-based diagnostic decision support system in chest CT**

*P. Agarwal*, K. Mueller-Peltzer, E. Kotter; Freiburg/DE
*(prerana.vkag@gmail.com)*

**Purpose:** The purpose of this pilot study is to evaluate the usability and potential advantage of a combined solution of an integrated artificial intelligence-(AI) and content-based image retrieval (CBIR) web application (Contextflow) with the knowledge-based diagnostic decision support system STATdx.
**Methods or Background:** Contextflow is an AI-based CBIR application which identifies lung-specific patterns on chest CTs and identifies cases with identical or similar patterns in its repository to support an objective image assessment. The pilot enables STATdx to present differential diagnoses to the users, which are adopted to the automatically analysed patterns. Theses differential diagnoses then serve as a case-fitted reference book. This solution overcomes the traditional "black-box" problem related to AI by leaving the final decision to the radiologist. Nine radiologists (7 trainees and 2 specialists) with different levels of experience in thoracic imaging evaluated this pilot based on a pre-reading and a post-reading survey while each analysing 5 cases of lung diseases with different levels of difficulty. The surveys were recorded in a questionnaire on a Likert scale from 0-5 and analysed in a descriptive manner.
**Results or Findings:** All participants agreed that it is important to have references available while using computer assistive technology, and 66.6% (6 out of 9) of the radiologists reported increased user confidence. Overall positive feedback was given by all participants reporting a potential role of this integrative technology for training purposes (44.4% rating as high and 55.6% rating as very high).
**Conclusion:** The combination of an AI-based CBIR presenting cases with similar patterns and a linked comprehensive radiology knowledge system like STATdx can increase user confidence and help young radiologists in establishing diagnoses.
**Limitations:** Small sample size and subjective nature of the evaluation.
**Ethics committee approval:** Not applicable.
**Funding for this study:** Not applicable.
**Author Disclosures:**
Prerana Agarwal: Nothing to disclose
Katharina Mueller-Peltzer: Nothing to disclose
Elmar Kotter: Advisory Board: Contextflow Speaker: Bureau Siemens Healtineers and Abbvie

### RPS 1304-8

**Invasive pulmonary aspergillosis (IPA) in non-oncohematologic patients: differential radiological findings.**

M. T. Filigheddu, *S. Ventura Díaz*, M. Á. Gómez Bermejo, L. Gorospe Sarasua, P. Martín-Dávila, E. Gómez-García de la Pedrosa, J. Fortún-Abete; Madrid/ES
*(sofi9417vd@gmail.com)*

**Purpose:** The diagnosis of invasive pulmonary aspergillosis (IPA) can be particularly difficult in non-oncohematological patients due to the lack of well-established radiological criteria. In this study we propose a new diagnostic criteria.
**Methods or Background:** In this multicenter retrospective study we included non-oncohematologic patients admitted in four different hospitals (3 in Spain and 1 in Italy). Different IPA criteria were used according to the baseline conditions of the patients. Patients were divided into three immunosuppression groups : 1) neutropenic (non-oncohematologic), 2) severe non-neutropenic (mostly solid organ transplantion), and 3) intermediate non-neutropenic patients. Pulmonary X-Rays and CT findings were reviewed in each group, describing bronchoinvasive and angioinvasive signs.
**Results or Findings:** 146 patients were included: 9 (6.2%) neutropenic, 105 (71.9%) severe non-neutropenic, and 32 (21.9%) intermediate non-neutropenic. Bronchoinvasive signs were more frequent than angioinvasive signs (94.5% vs. 45.2%, p= 0.023). Invasive airway signs were equally distributed in all groups (88.9% vs 94.3 vs 96.9%; p=0.524). Ground-glass opacities (64.5%) and bronchial wall thickening (58%) were the most common findings. Bronchoinvasive signs were observed more frequently in patients with less immunosuppression (0% vs 41.9% vs 68.8%, p= 0.000). Nodules (100% vs 79.2% vs 40%; p=0.010), halo signs (50% vs 22.9% vs 10%, p=0.027) and other angioinvasive signs (88.9% vs 45.7% vs 31.3%; p= 0.008) were more uncommon in less immunosuppressed hosts.
**Conclusion:** Bronchoinvasive signs are more common in less immunosuppressed hosts with IPA, whereas angioinvasive forms are most frenquently seen in neutropenic and severe non-neutropenic patients. Therefore, in less neutropenic patients such as non-oncohematological patients, early recognition of bronchoinvasive signs can contribute to a prompt diagnosis in the initial stages of the disease.
**Limitations:** The retrospective nature.
**Ethics committee approval:** This study was approved by the Ethics Committee of our hospital.
**Funding for this study:** None.
**Author Disclosures:**
Miguel Ángel Gómez Bermejo: Nothing to disclose
Maria Teresa Filigheddu: Nothing to disclose
Pilar Martín-Dávila: Nothing to disclose
Jesús Fortún-Abete: Nothing to disclose
Sofía Ventura Díaz: Nothing to disclose
Luis Gorospe Sarasua: Nothing to disclose
Elia Gómez-García de la Pedrosa: Nothing to disclose

### RPS 1304-9

**Evaluation of additional silver filtration for ultra-low dose chest CT scans**

*L. J. Oostveen*, F. De Lange, I. Sechopoulos, M. Prokop; Nijmegen/NL
*(luuk.oostveen@radboudumc.nl)*

**Purpose:** To investigate the impact of additional silver filtration (Ag) in the X-ray beam for ultra-low dose (ULD) chest CT acquisitions.
**Methods or Background:** Acquisitions with and without Ag were taken at CTDIvol = 0.9 mGy (low-dose chest (LD)) and CTDIvol = 0.4 mGy (ULD) using a 320-row detector CT (Aquilion One PRISM edition, Canon Medical), all with 120 kVp, pitch 1.4, rotation 0.5 seconds, and hybrid-iterative reconstruction with lung kernel. Contrast-to-noise ratios (CNRs) for air (≈-1000 HU), Delrin (≈350 HU), and Nylon (≈100 HU) inserts, standard deviations (SDs) and noise power spectra (NPSs), and modulation transfer functions (MTFs) of a Delrin rod edge, all in a water background, were determined. The beam-hardening artefact in the shoulder region of a chest phantom was quantified with an artefact index (AI), calculated by subtracting the mean HU value in a region of interest in the artefact from the background.
**Results or Findings:** With Ag, SDs were 1.3-1.5x lower and CNR was 1.5-1.8x higher than without. Average NPS frequency with Ag was 14% lower. 20% MTF frequencies for ULD and LD were 2.5 lp/cm and 3.4 lp/cm with Ag, and 2.8 lp/cm and 3.5 lp/cm without Ag, respectively. AI for ULD and LD was 31 HU and 57 HU with Ag, and 105 HU and 86 HU without Ag, respectively.
**Conclusion:** Acquisitions with additional silver filtration have lower noise, higher CNR, less sharpness and less beam-hardening artefacts compared to normal filtration. CT acquisitions with an Ag filter can be expected to be especially suited for (ultra) low-dose chest examinations.
**Limitations:** Phantom study only.
**Ethics committee approval:** Phantom study, so not needed.
**Funding for this study:** Canon Medical Systems.
**Author Disclosures:**
Frank De Lange: Nothing to disclose
Luuk J. Oostveen: Nothing to disclose
Mathias Prokop: Speaker: Canon Medical SysteResearch/Grant Support: Canon Medical Systems
Ioannis Sechopoulos: Research/Grant Support: Canon Medical Systems

### RPS 1304-10

**Comparison of three digitally reconstructed radiograph models for ultra-low-dose CT images of the chest**

H. J. Lamb[1], *O. Paalvast*[1], M. Sevenster[2]; [1]Leiden/NL, [2]Eindhoven/NL
*(o.t.paalvast@lumc.nl)*

**Purpose:** To investigate the clinical preference for a digitally reconstructed radiograph (DRR) construction method from ultra-low-dose CT (ULDCT) scans.
**Methods or Background:** An ULDCT scan takes more time to interpret clinically than a CXR but is a more sensitive and specific modality. To aid the interpretation of a ULDCT scan, fake CXRs, or DRRs, can be generated. These offer a quick overview of an ULDCT scan in a format that is highly familiar to radiologists. Three methods of constructing DRRs ('Softmip', 'Tomogram' and 'DeepDRR') were identified using a systemic literature review. DRRs were constructed for six cases with a known absence of pathology for which both an ULDCT as well as a chest radiograph (CXR) were available. Three radiologists with 3, 8 and 12 years of experience reading CXRs reviewed the DRR and the original CXR and scored the diagnostic quality of the DRR as a whole, soft tissue, bone, mediastinum and lungs on a scale from 1 to 6. The DRRs were also presented with the original CXR to determine which method best matched the original CXR.
**Results or Findings:** 'Softmip' scored (standard deviation) 3.3 (0.9), 4.3 (1.3), 3.4 (1.3), 3.9 (0.9), 3.3 (0,9); 'Tomogram' scored 3.4 (0.9), 4.4 (1.2), 3.6 (1.1), 3.9 (0.9), 3 (1.2); and 'DeepDRR' scored 3.2 (1), 4.2 (1.2), 3.5 (1.2), 3.7 (0.9), 3.4 (0.9) for the evaluation as a whole, soft tissue, bones, mediastinum and lungs, respectively. The methods 'Softmip', 'Tomogram' and 'DeepDRR' were elected as best matching the original CXR eight, five and five times, respectively.
**Conclusion:** The 'Softmip' DRR was preferred in cases with a known absence of pathology.
**Limitations:** Small reader size and case number size.
**Ethics committee approval:** Approved by METC Leiden-Delft.
**Funding for this study:** Not applicable.
**Author Disclosures:**
Merlijn Sevenster: Nothing to disclose
Olivier Paalvast: Nothing to disclose
Hildo J. Lamb: Nothing to disclose

### RPS 1304-11

**Pulmonary tuberculosis in HIV: a study of computed tomography findings in relation to CD4 count**

*S. K N*, S. Singh, R. Dixit; New Delhi/IN
*(sowmyashree.kn4u@gmail.com)*

**Purpose:** The non-classical imaging manifestations of pulmonary tuberculosis (PTB) in HIV and its varying pattern based on the level of immune compromise leads to challenges in the early diagnosis of PTB. Our study aims to determine the spectrum of CT findings of PTB in HIV seropositive patients and to study the association of various CT findings, i.e. the radiological pattern of PTB with the level of immunosuppression.
**Methods or Background:** Relevant clinico-demographic details and CD4 counts were noted in 100 HIV seropositive patients with suspected PTB, following which chest radiograph and contrast-enhanced CT were performed. Data obtained was analysed statistically.
**Results or Findings:** A total of 71/100 cases were found to have PTB. A primary pattern of PTB with predominant extra-pulmonary involvement and miliary nodules was found in 42/71 (59.15%) cases, of which 32 (76.19%) cases had a CD4 count < 200 and only 10 (23.81%) cases had a CD4 count > 200. A typical post-primary pattern of PTB was found in the remaining 29/71 (40.85%) cases, of which 22 (75.9%) cases had CD4 > 200 and only 7 (24.1%) cases had CD4 < 200. A statistically significant association was found between the radiological pattern of PTB and the level of immunosuppression (p-value <0.0001).
**Conclusion:** CT proved to be an excellent imaging modality for the early and prompt diagnosis of PTB in HIV seropositive patients. The extent of immune compromise greatly influenced the imaging findings of PTB in HIV patients, showing a predominant primary pattern in the immunosuppressed group and a re-activation pattern in the immunocompetent group.
**Limitations:** The study also included a few patients on antiretroviral (HAART) treatment; however, the independent effect of HAART on imaging manifestation has not been studied in detail.

**Ethics committee approval:** Ethical clearance was obtained from the institutional ethics committee.
**Funding for this study:** No funding was received for this study.
**Author Disclosures:**
Sowmyashree K N: Nothing to disclose
Rashmi Dixit: Nothing to disclose
Sapna Singh: Nothing to disclose

**10:30-12:00** **Room M 2**

### Research Presentation Session: Musculoskeletal

### RPS 1310

### New methods for imaging spine and nerves

**Moderator**
C. W. A. Pfirrmann; Zurich/CH

### RPS 1310-3

**Comparison between 2D TSE and 3D SPACE in the evaluation of craniocervical ligaments**
*B-M. Chung*; Seoul/KR

**Purpose:** To evaluate the MRI findings of the craniocervical junction ligaments and compare the 2D TSE sequence and isotropic 3D SPACE sequence on cervical spine MRI.
**Methods or Background:** Eighty cervical spine MRIs were retrospectively analyzed, including 2D TSE and 3D SPACE sequences. The transverse ligament (TL) and alar ligament (AL) were evaluated by two readers using a three-point grading scale for visibility (0 = complete visualization from the odontoid process to the insertion site, well-defined margin, 1 = complete visualization, indistinct margin, 2 = incomplete visualization of ligament) and a four-point grading scale for morphology (0 = homogeneous low SI with normal thickness, 1 = high SI with normal thickness, 2 = reduced thickness, 3 = full-thickness rupture or indistinguishable from surrounding structures). The grades of the two sequences were compared. Intra-reader and inter-reader agreements were measured using Cohen's kappa and the percentage of exact agreement.
**Results or Findings:** The visibility grades of TL were significantly lower in 3D images in all reading sessions ($p < 0.001$), indicating better visualization. The morphological grades of TL were significantly lower in 3D images, and AL was significantly higher in 3D images ($p < 0.05$). The exact agreements of visibility and morphological grade of TL in 3D tended to be higher than in 2D. The agreement of the morphological grade of AL tended to be lower in 3D than in 2D.
**Conclusion:** The utilization of 3D sequences significantly improves the visibility of TL and could potentially reduce false-positive diagnoses of ligament injury.
**Limitations:** Our study population comprised symptomatic patients with neck pain or radiculopathy.
**Ethics committee approval:** This retrospective observational study was approved by our institutional review board, which waived the requirement for informed consent.
**Funding for this study:** Not applicable
**Author Disclosures:**
Bo-Mi Chung: Nothing to disclose

### RPS 1310-4

**Muscle mass index at the level of Th12 adjusted for the length of the thoracic spine as a prognostic factor for fatal outcomes among SARS-COV2 pneumonia patients**
*A. N. Bashkov*; Moskow/RU
*(abashkov@yandex.ru)*

**Purpose:** To develop a methodology for determining the muscle mass index (MMI-L) at the Th12, adjusted for the length of the thoracic spine based on the chest CT, and to estimate the impact of MMI-L on the outcome of patients with pneumonia as a prognostic factor.
**Methods or Background:** The medical data and computed tomography of the chest of 247 patients with confirmed SARS-Cov2 pneumonia were used. A correlation analysis of MMI-L and MMI adjusted for the height (MMI-H) as generally accepted was performed. A ROC analysis was made among male and female patients in different age groups in order to find cut-off values of MMI-L associated with lethality.
**Results or Findings:** A strong correlation was found between the MMI-L and MMI-H ($r = 0.861$, $p < 0.001$). The age threshold, which was associated with an increase in the likelihood of death, was 60 years in men (AUC=0.728) and 65 years in women (AUC=0.734). MMI-L = 3.37 cm2/m2 is a cut-off value, below which the probability of death in male patients under 60 years increased 26.3 times (95% CI: 4.8-143.0). In female patients, there was no statistically significant threshold value of MMI-L which would be associated with a higher risk of death.
**Conclusion:** MMI-L can be used to estimate sarcopenia-associated muscle loss, if the patient's height is unknown, as many patients are in bed condition. An MMI-L less than 3.37 cm2/m2 is a strong predictor of death in men under 60 years of age. Further work is needed to study the association between MMI-L and the outcome of pneumonia in female patients.
**Limitations:** No limitations were identified.
**Ethics committee approval:** The investigation was approved by the local committee.
**Funding for this study:** No funding was received for this study.
**Author Disclosures:**
Andrey Nikolaevich Bashkov: Nothing to disclose

### RPS 1310-5

**Compliance with lumbar spine X-ray referral guidelines: a retrospective audit of practice at one hospital in East Dorset (UK)**
P. J. Mowlem[1], B. Mayiza[2], *D. H. Ockri*[2], R. Meertens[2]; [1]Poole/UK, [2]Exeter/UK
*(Dho202@exeter.ac.uk)*

**Purpose:** X-ray referrals from general practices (GPs) can be over-requested, particularly for degenerative change in the lumbar spine (L-spine). The Pan-Dorset L-Spine Imaging Guidelines were introduced to reduce unnecessary radiation exposure. A recent audit of all GP X-ray requests at one hospital in East Dorset (UK), revealed several L-spine examinations were rejected. This prompted further investigation into the compliance with the imaging guidelines for justified and rejected L-spine requests.
**Methods or Background:** A retrospective audit of all L-spine x-ray requests between September and December 2021 inclusive were extracted from the radiology information system and identified 92 referrals. The Pan-Dorset L-Spine Imaging Guidelines were used to determine if the clinical information was justified or unjustified. The reasons for cancellation and rejection and the report findings were collated and analysed using Microsoft Excel 365.
**Results or Findings:** Of the 92 L-spine requests evaluated, 30 (32.6%) were not performed; 18 (60.0%) of these were cancelled, and the remainder were rejected. Of the remaining 62 examinations performed, 30 (48.4%) of these had both justified and unjustified clinical indications; 15 (24.2%) had justified clinical indications; and 17 (27.4%) had no justified clinical indications. The most common unjustified indication was lower back pain (67.7%); however, the location (69.0%) and duration (50.0%) were frequently not specified. Of these, degenerative change was mostly reported (61.9%).
**Conclusion:** Unjustified L-spine examinations are being performed, thus contributing to the unnecessary increase in patient dose. More diligence is required when radiographers justify or reject L-spine X-ray requests. The Pan-Dorset L-Spine Imaging Guidelines require revision to become more specific in terms of pain location and duration. In addition, the guidelines should be recirculated to GP surgeries in East Dorset to improve compliance.
**Limitations:** Only one hospital in Dorset was sampled.
**Ethics committee approval:** Not applicable.
**Funding for this study:** No funding was received for this study.
**Author Disclosures:**
Philip James Mowlem: Nothing to disclose
Dise Henrietta Ockri: Nothing to disclose
Robert Meertens: Nothing to disclose
Blessing Mayiza: Nothing to disclose

### RPS 1310-6

**Ultrasonographic and shear-wave sonoelastographic findings of Achilles tendon in patients with seronegative spondyloarthropathy and their relationship with disease activity**
*L. Aghaghazvini*, M. Parvaneh, S. T. Faezi, M. Shakiba, M. Alborzi Avanaki; Tehran/IR
*(aghaghazvini.leila@gmail.com)*

**Purpose:** Seronegative spondyloarthropathies can make enthesitis, tendinitis and tendinopathy, which could change the Achilles tendon's elastic properties. We aimed to assess the sonoelastographic findings of 82 patients with seronegative spondyloarthropathy and their relationship with clinical findings.
**Methods or Background:** A total of 82 patients [78 with ankylosing spondylitis (AS); 2 with juvenile pondyloarthritis and 2 with psoriasis + AS] [mean age: 46.1 ± 14.8 years, 56 male (68.3%)] were enrolled. Demographic data, clinical data [including presence of active enthesitis, synovitis, dactylitis and active systemic phase] and laboratory data were evaluated. Grey-scale and shear-wave elastography were performed by a Siemens [ACUSON] machine [probe 7-14 MHz] and color mapping in regions of interest were determined. A sonographic assessment of the Achilles was performed on 3 parts, the myotendinous [proximal], middle and tendinotubercle [distal]. In sonographic and sonoelastographic assessment, tendon echogenicity and peritendinous fluid, tendon calcification, tendon thickness, elastography color-mapping pattern and velocities [m/s] were measured.
**Results or Findings:** A total of 50 patients were in systemic active phase [61%]; 23 [28%] showed enthesitis active phase; and 41 [50%] had current or previous history of enthesitis. Homogeneous echogenicity, peritendinous fluid and calcification were seen in 71 [86.6%], 4 [4.9%] and 2 [2.4%] of patients, respectively. Color-mapping grade was homogeneous red in 56 [68.3%], heterogeneous red in 15 [18.3%] and heterogeneous green in 7 [8.5%] of patients. The mean tendon thickness was 3.7 ± 1 [2.3-7.4], 4.5 ± 1.3 [2.6-12.8] and 4.6 ± 1.2 [2.9-10.1] in myotendinous junction, midportion and tendinotubercle junction. The mean velocity was 15.3 ± 2.9 [7.2-19.1], 15.6 ± 2.5 [7.2-19] and 15 ± 3.2 [7.1-18.8] in the myotendinous junction, midportion and tendinotubercle junction. There was no statistically significant relationship between imaging findings and positive clinical findings and severity of disease.
**Conclusion:** Achilles sonography and sonoelastography findings don't show an association with disease activity in seronegative spondyloarthropathies.
**Limitations:** Low sample size.
**Ethics committee approval:** The study protocol was approved by the medical ethics committee of our institute.
**Funding for this study:** Not applicable.
**Author Disclosures:**
Seyedeh Tahereh Faezi: Nothing to disclose
Majid Shakiba: Nothing to disclose
Mahsa Alborzi Avanaki: Nothing to disclose
Milad Parvaneh: Nothing to disclose
Leila Aghaghazvini: Nothing to disclose

### RPS 1310-8
**Comparing the elastic moduli estimations of Siemens VTQ and VTIQ shear wave elastography technology**
*R. J. Rendle*; Exeter/UK

**Purpose:** Shear wave elastography has been validated as a reliable method differentiating the elastic moduli of normal breast tissue (23.91 ± 4.57 kPa) and abnormal breast lesions. To be applicable as a tool for evaluating the moduli of normal and abnormal articular cartilage It would need to be able to reliably measure elastic moduli of around 1MPa. This study is designed to test the upper limit of elastic moduli that can be reliably measured using the Siemens S3000 elastography software Virtual touch Quantification (VTQ) and the new Virtual touch Image Quantification (VTIQ).
**Methods or Background:** A series of aqueous gels were made with concentrations of gelatine ranging from 6% through to 18%. These gels were mechanically tested using an Instron to obtain a reference elastic modulus for each gel. The results were then compared with the mean elastic moduli measured using both the VTQ and VTIQ software on the Siemens S3000 machine. The standard error of each set of readings was calculated.
**Results or Findings:** We found that both the VTQ and VTIQ elastography settings were reliable at measuring a gel with an elastic modulus up to around 30kPa (10% gel) and VTIQ could measure the elastic moduli of gels up to 60kPa (14% gel). Higher concentration gels were not reliably estimated using either VTQ or VTIQ, however, but VTIQ performed better and had a smaller standard error.
**Conclusion:** The new VTIQ technology improved the range of elastic moduli measured by the S3000 and has less measurement error than the VTQ software.
**Limitations:** The limitations of this study include the potential for inhomogeneity in the gels, which may have added to measurement errors.
**Ethics committee approval:** N/A
**Funding for this study:** Self
**Author Disclosures:**
Richard James Rendle: Nothing to disclose

### RPS 1310-9
**Qualitative and quantitative evaluation of sacroiliac MRI: can Dixon sequences replace standard protocol in diagnosis of sacroiliitis?**
*N. B. Karatoprak (Demir)*, Z. Ozdemir, A. Sağir Kahraman, L. Karaca, S. Karatoprak, S. Yolbaş; Malatya/TR
*(nebedr@gmail.com)*

**Purpose:** To evaluate the performance of Dixon magnetic resonance imaging (MRI) sequences for the detection and assessment of active and chronic sacroiliitis compared with standard protocol.
**Methods or Background:** Active and chronic sacroiliitis findings were evaluated in 107 patients on 3-T MRI, including T1-weighted (W) and T2W Dixon sequences. Signs of active sacroiliitis were evaluated on water-only T2W Dixon images by comparing with fat-saturated (FS) T2W images and signs of chronic sacroiliitis were evaluated on in-phase, out-phase and fat-only T1W-T2W Dixon images by comparing T1W images. Signal-to-noise ratios (SNRs) and contrast-to-noise ratios (CNRs) of bone marrow oedema and fat deposition were measured for quantitative assessment.
**Results or Findings:** There were no statistically significant differences for the detection of bone marrow oedema, enthesitis and capsulitis between water-only T2W Dixon and FS T2W sequences ($p<0.05$) and for the detection of erosion, fat deposition, backfill and ankylosis between T1-T2W Dixon and T1W sequences ($p<0.05$). SNRs and CNRs were significantly higher on T2W Dixon for bone marrow oedema and fat deposition and also lower on T1W Dixon for fat deposition than the standard protocol ($p<0.05$).
**Conclusion:** The single T2W Dixon sequence can be used instead of the standard protocol for the diagnosis of active and chronic sacroiliitis.
**Limitations:** The study was planned as single centre and non-randomised. Although the number of patients was sufficient, statistical comparison couldn't be made for some rare findings.
**Ethics committee approval:** Approval for this study was granted by the Scientific Research and Publication Ethics Committee of Malatya Inonu University (decision No. 2020/97).
**Funding for this study:** No funding was received for this study.
**Author Disclosures:**
Servet Yolbaş: Nothing to disclose
Sinan Karatoprak: Nothing to disclose
Zeynep Ozdemir: Nothing to disclose
Ayşegül Sağir Kahraman: Nothing to disclose
Nur Betül Karatoprak (Demir): Nothing to disclose
Leyla Karaca: Nothing to disclose

### RPS 1310-11
**Radiofrequency echographic multi spectrometry (REMS): ultrasonographic technology for bone health status assessment of the femur and spine**
G. Dibenedetto[1], M. Urbano[1], S. Russo[1], V. Testini[2], G. Guglielmi[2], *R. Gifuni*[2]; [1]Barletta/IT, [2]Foggia/IT
*(rossella.gifuni@unifg.it)*

**Purpose:** Radiofrequency echographic multi spectrometry (REMS) technology has been introduced in order to overcome both the DXA and peripheral QUS limitations. REMS is the first radiation-free technique directly applied to the anatomical reference sites for osteoporosis diagnosis (spine and femur), the same sites investigated by the DXA. The aim of this study is to assess REMS state-of-the-art features and basic principles.
**Methods or Background:** A review of the available literature was performed, including published papers, reviews and abstracts.
**Results or Findings:** REMS has been recently included in the Italian ministerial guidelines to improve the diagnosis of osteoporosis in routine care. It is implemented in the EchoS device (Echolght SpA, Italy), and it is very easy to use. The echographic scan of L1-L4 lumbar vertebrae is performed by placing the transducer in a trans-abdominal position for 80 s. Similarly, femoral scans are performed by placing the transducer parallel to the head-neck axis of the femur, in order to visualise the typical proximal femur profile. This lasts 40 s and is guided by the software. REMS is able to automatically collect both the sequence of B-mode images and the related raw, unprocessed ultrasound signals that are automatically processed after the scan. This process allows it to retain the maximum information about the characteristics of the investigated tissues, which are normally filtered out during the conventional process of B-mode image reconstruction. The bone health status is assessed through the comparison of the analysed signal spectra with reference spectral models, and the BMD, T-Score and Z-Score values, together with the fragility score for bone quality assessment, are estimated.
**Conclusion:** REMS is a valuable approach for fast and accurate early osteoporosis diagnosis and fracture risk assessment.
**Limitations:** No limitations were identified.
**Ethics committee approval:** Not applicable.
**Funding for this study:** No funding was received for this study.
**Author Disclosures:**
Maria Urbano: Nothing to disclose
Giuseppe Guglielmi: Nothing to disclose
Valentina Testini: Nothing to disclose
Rossella Gifuni: Nothing to disclose
Graziana Dibenedetto: Nothing to disclose
Salvatore Russo: Nothing to disclose

**10:30-12:00** **Room X**

### Research Presentation Session: Imaging Informatics / Artificial Intelligence and Machine Learning

### RPS 1305

### Artificial intelligence (AI) in chest imaging: part 2

**Moderator**
G. Chassagnon; Paris/FR

### RPS 1305-2

**Overall survival prediction for stage II and stage III non-small cell lung cancer patients using a graph-based deep learning algorithm**

J. Lian[1], Y. She[2], Y. Long[1], F. Huang[1], J. Deng[2], Q. Dou[1], C. Chen[2], *V. Vardhanabhuti*[1]; [1]Hong Kong/HK, [2]Shanghai/CN

**Purpose:** Lung cancer has become the leading cause of cancer-related mortality worldwide, and predicting patient survival to provide better medical treatment can improve disease outcomes. Although current methods have produced accurate predictions, the majority of them are designed for specific datasets and cannot be easily adapted to other cases. In this study, we proposed a graph representation of CT images in non-small cell lung cancer (NSCLC) patients and used a graph convolutional neural network (GCN) model to predict overall survival in a multi-institutional dataset.
**Methods or Background:** In this retrospective study, a total of 558 patients with lung cancer (stage II and III) were included. The dataset was randomly separated into a training set with 418 patients (182 survivors and 236 deaths), a validation set with 70 patients (26 survivors and 44 deaths), and a test set with 70 patients (26 survival and 44 deaths). The airways, lung lobes, and tumours were segmented automatically from the original CT. Four critical parts of airway segments, five lung lobes, and tumours were assigned as nodes in a graph, and were connected according to their structural relationships. A pre-trained convolutional network was used to produce nodes' features, followed by a principal component analysis. A three-layer GCN model was then trained to predict survival using the lung graph as input. We also trained a radiomics model using only tumour information as a comparison. The AUC values were reported, along with the Wilcoxon rank test.
**Results or Findings:** The GCN models were significantly predictive of 5-year overall survival with an AUC of 0.7020 (p-value=0.02), compared to the radiomics feature predictor (AUC=0.5805, p-value=0.14).
**Conclusion:** The proposed GCN model achieves state-of-the art performance, outperforming conventional radiomics models in our study.
**Limitations:** Not applicable
**Ethics committee approval:** This study was approved by ethics committees at participating institutions.
**Funding for this study:** Not applicable
**Author Disclosures:**
Qi Dou: Nothing to disclose
Varut Vardhanabhuti: Nothing to disclose
Yonghao Long: Nothing to disclose
Jie Lian: Nothing to disclose
Chang Chen: Nothing to disclose
Jiajun Deng: Nothing to disclose
Yunlang She: Nothing to disclose
Fan Huang: Nothing to disclose

### RPS 1305-3

**A laboratory-medicine-like approach to the analysis of unremarkable chest radiographs using artificial intelligence**

*T. Weikert*[1], R. Sexauer[1], J. Poletti[1], J. S. Ahn[2], S. S. Lee[2], C. Breit[1], A. W. Sauter[1]; [1]Basel/CH, [2]Seoul/KR
*(thomas.weikert@usb.ch)*

**Purpose:** Single-click confirmation of unremarkable blood test results and subsequent auto-generation and -processing of reports is standard in clinical laboratory medicine. The purpose of this study is to adapt this idea to the reading of unremarkable chest radiographs in radiology.
**Methods or Background:** This retrospective study included a consecutive series of unremarkable chest radiographs from a university hospital (time period: Feb.-Apr., 2021). The PA views were processed by a deep-neural-network (ResNet34). It detects ten findings such as pneumothorax and infiltrates with excellent sensitivity (AUROC > 0.9 for all finding categories). For training, a multi-center dataset of 205k chest radiographs had been used. In this study, (1) the proportion of radiographs correctly categorized as "unremarkable" was determined. (2) Time needed to read chest-X-rays in clinical routine was determined using time stamps from the Radiology-Information-System. (3) The time taken by two radiology residents (PGY-3=R1 and PGY-5=R2) for single-click confirmation of the status "unremarkable", with AI-results available, was recorded and compared to step (2) with independent t-tests.
**Results or Findings:** Of 150 unremarkable chest radiographs, 146 (97.3%) were correctly classified by the algorithm. With AI, R1 needed 23.2s on average (SD: 10.4s); R2 needed 14.1s (SD: 13.2s) for status confirmation. Significantly more time was needed in clinical routine: 77s on average (SD: 46s, p<0.01 for both comparisons).
**Conclusion:** Reliable identification of unremarkable chest radiographs with AI is feasible. A Batch-wise interpretation of those cases seems to be time-efficient. Further research on the impact of a batch-wise reading of pre-sorted chest-X-rays on report quality are needed.
**Limitations:** Major limitations of this study are its single-centre setting and limited number of readers.
**Ethics committee approval:** This study used fully anonymized data and was conducted under the provisions of the local ethics committee.
**Funding for this study:** Not applicable
**Author Disclosures:**
Thomas Weikert: Nothing to disclose
Sanghyup Simon Lee: Employee: Lunit Inc.
Julien Poletti: Nothing to disclose
Christian Breit: Nothing to disclose
Raphael Sexauer: Nothing to disclose
Jong Seok Ahn: Employee: Lunit Inc.
Alexander Walter Sauter: Nothing to disclose

### RPS 1305-4

**Deep learning universal lesion segmentation for automated RECIST measurements on CT: comparison to manual assessment by radiologists**

*M. J. J. De Grauw*[1], B. Van Ginneken[1], B. Geisler[2], E. J. Smit[1], M. De Rooij[1], S. Schalekamp[1], M. Prokop[1]; [1]Nijmegen/NL, [2]Bremen/DE
*(max.degrauw@radboudumc.nl)*

**Purpose:** Automating aspects of RECIST evaluation can save time and potentially reduce inter-observer variability. We trained a 3D Universal Lesion Segmentation model (ULS) to estimate long and short axis diameters in CT exams based on a single click inside the lesion.
**Methods or Background:** We used the nnUnet framework to train the ULS using 3213 lesions from 1481 studies collected from eight public challenge datasets. We fine-tuned the model using masks predicted for lesions from a subset of the public DeepLesion dataset. A reader study was conducted with 128 separate DeepLesion scans. Four radiologists manually measured long- and short-axis of lesions on axial CT slices and assessed whether a lesion was eligible as target lesion.
**Results or Findings:** For 85 out of 128 scans, all readers agreed that it contained a valid RECIST target lesion. For those lesions, the relative difference between the DeepLesion measurements and the radiologists was –4.2% ± 14.2 and –0.3% ± 13.2, for the long and short axis respectively. For ULS these measures were 6% ± 17 and –5.8% ± 18.9. The mean absolute differences were 2.5 ± 3 mm and 1.9 ± 2 mm for radiologists. For ULS these measures were 4.1 ± 5.8 mm and 2.8 ± 2.7mm. For 78.8% of lesions the absolute difference between DeepLesion and ULS measurements fell within a standard deviation of the inter-radiologist variability.
**Conclusion:** Single-click measurement using ULS shows promise to simplify and speed-up RECIST evaluation in circa 80% oncological CT exams.
**Limitations:** This study used a small number of lesions in the test set, and readers measured long and short axis in all lesions, which is not required by RECIST.
**Ethics committee approval:** Not applicable
**Funding for this study:** This research was supported by the Eurostars PIANO project E113829.
**Author Disclosures:**
Mathias Prokop: Nothing to disclose
Max Jacobus Johannes De Grauw: Nothing to disclose
Benjamin Geisler: Nothing to disclose
Bram Van Ginneken: Nothing to disclose
Ewoud J. Smit: Speaker: Canon Medical Systems
Steven Schalekamp: Nothing to disclose
Maarten De Rooij: Nothing to disclose

### RPS 1305-5

**Lightweight techniques to improve generalisability of U-Net based segmentations of lung lobes**

*A. Dadras*, A. Jaziri, E. Prescher, R. Fischbach, D. El Naggar, B. Hamm, T. J. Vogl, T. Penzkofer, A. M. Bucher; Frankfurt/DE

**Purpose:** Lung lobe segmentation in chest CT is relevant to a wide range of clinical applications. Proposed pipelines tend to be vulnerable to perturbations and face performance degradation on external data sets. Therefore, we systematically analysed the combination of novel machine learning techniques (self-supervision (SSL), data augmentation (DA), attention (A)) to improve generalisability.
**Methods or Background:** We analysed three techniques (SSL, DA, A) to train a fast and fully-automated lung lobe segmentation model based on U-Net and specifically collect an in-house dataset that covers a wide range of challenging cases. As part of the RACOON project it contains 100 CT chest scans of patients with bacterial, viral or covid infection (25, 25, 50). Furthermore, we evaluated common external datasets (LUNA, IEEE). The segmentation results of our method are compared to a baseline U-Net model, trained on the same dataset and a publicly available state of the art segmentation pipeline (SOTA).
**Results or Findings:** Our model improves the baseline (Dice-Score of 92.8% vs 82.3%, p<0.001) and achieves state of the art performance (Dice-score of 92.8% vs 90.8% for SOTA, p=0.102) while using less training examples (69 vs. 231) and a leaner neural network model (4.1 + 2 s per scan). The performance varied among the different techniques (SSL: 0.841, DA: 0.878, A: 0.842). The usage of DA with expert knowledge showed the biggest impact on performance (+0.056 Dice-score).
**Conclusion:** The considered design choices manage to improve segmentation accuracy on diverse datasets without an increased computational overhead. They are not specific to lobe segmentation and can be further integrated in other medical imaging segmentation tasks.
**Limitations:** Our approach should undergo further multicentric validation.
**Ethics committee approval:** IRB approval for this multicentre study was obtained.
**Funding for this study:** RACOON is funded by the network of university medicine (BMBF grant-number: 01KX2021).
**Author Disclosures:**
Bernd Hamm: Nothing to disclose
Tobias Penzkofer: Nothing to disclose
Dina El Naggar: Nothing to disclose
Achref Jaziri: Nothing to disclose
Thomas J. Vogl: Nothing to disclose
Armin Dadras: Nothing to disclose
Ricarda Fischbach: Nothing to disclose
Erik Prescher: Nothing to disclose
Andreas Michael Bucher: Nothing to disclose

### RPS 1305-6

**Development and validation of a machine learning based CADx designed to improve patient management in lung cancer screening programmes**

P. Baudot, *C. M. Voyton*, D. Francis, A. Baili-Laya, V. Bobin, B. Renoust, Y. Liu, A. Iannessi, B. Huet; Valbonne/FR
*(charles.voyton@mediantechnologies.com)*

**Purpose:** Lung cancer screening has gained adoption worldwide following the success of the NLST and NELSON trials, although with the guidelines and tools currently available the number of indeterminate nodules and unnecessary scans endangers patient enrollment and the economic viability of screening programmes. We present the development and verification of a machine learning based computer aided diagnostic device (CADx) designed to characterise pulmonary nodules in lung cancer screening patients.
**Methods or Background:** The malignancy prediction algorithm was trained on 1,224 patients from the NLST dataset with 11,392 nodules annotated by radiologists in a multi-read approach. Reference standard data for the nodules were based on histology for cancerous findings and on stable patient follow-up (>12 months) for benign findings. The verification set contained 472 patients (330 benign, 142 cancerous) with 4,716 nodules.
**Results or Findings:** The developed AI characterisation model achieved an AUC of 0.991 (95% CI: 0.987-0.995), with 98% sensitivity at a specificity of 90%. When used for management of a screening population similar to the NLST population, assuming the same performance, this algorithm could help reduce unnecessary scans and missed cancers by 57% and 67% respectively compared to using Lung-RADS to inform patient management.
**Conclusion:** The algorithm demonstrates robust performance for the characterisation of pulmonary nodules already detected by radiologists. When deployed in a screening program, high performant characterisation tools stand to drastically improve patient management. With such AI tools, unnecessary scans and invasive procedures, and missed cancers will be decreased, which is critical if patient adherence and screening adoption is to increase to numbers like those of the successful screening efforts in prostate and breast cancer.
**Limitations:** This study includes only parenchymal solid or part solid lesions, with biased imbalance due to partial non-cancer patient sampling.
**Ethics committee approval:** This study was approved by an ethics committee.
**Funding for this study:** No funding was received for this study.
**Author Disclosures:**
Benjamin Renoust: Employee: Median technologies
Vincent Bobin: Employee: Median technologies
Charles Michael Voyton: Employee: Median technologies
Benoit Huet: Employee: Median technologies
Yan Liu: Employee: Median technologies
Pierre Baudot: Employee: Median technologies
Mme Afef Baili-Laya: Employee: Median technologies
Antoine Iannessi: Employee: Median technologies
Danny Francis: Employee: Median technologies

### RPS 1305-7

**First performance evaluation of an artificial intelligence-based computer aided detection system for pulmonary nodule evaluation in dual source photon-counting detector CT at different low dose levels**

*L. Jungblut*[1], C. Blüthgen[1], M. Polacin[1], M. A. Messerli[1], B. Schmidt[2], A. Euler[1], H. Alkadhi[1], T. Frauenfelder[1], K. Martini[1]; [1]Zurich/CH, [2]Forchheim/DE

**Purpose:** To evaluate the image quality and performance of an artificial-intelligence (AI)-based computer aided detection (CAD) system in photon-counting detector computed tomography (PCD-CT) for pulmonary nodule evaluation at different low dose levels.
**Methods or Background:** An anthropomorphic chest-phantom containing 14 pulmonary nodules of different sizes was imaged on a PCD-CT and on a conventional energy-integrating detector CT (EID-CT). Scans were performed with each of the three vendor-specific scanning-modes (QuantumPlus [Q+], Quantum [Q] and High Resolution [HR]) at decreasing matched radiation dose levels by adapting image quality (IQ) levels. Image noise was measured manually in the subcutaneous fat at eight different locations. Subjective image quality was evaluated by two readers in consensus. Nodule detection and volumetry were performed using a commercially available AI-CAD system.
**Results or Findings:** Subjective image quality was superior in PCD-CT compared to EID-CT (p<0.001) and objective image noise was similar in the Q+ and Q mode (p>0.05) and superior in the HR mode (p=0.01). Overall, the AI-CAD-system delivered comparable results for lung nodule detection and volumetry between PCD- and dose matched EID-CT (p=0.08-1.00), with a mean sensitivity of 95% for PCD-CT and of 86% for dose matched EID-CT. The HR-mode showed a sensitivity of 100% with a false positive rate of 1 even at the lowest evaluated dose level (IQ5; CDTI vol 0.41 mGy).
**Conclusion:** PCD-CT was superior to dose matched EID-CT in subjective image quality while showing lower objective image noise by comparison. Fully automatized AI-aided nodule detection and volumetry are feasible in PCD-CT, but attention has to be paid to false positive findings.
**Limitations:** No limitations were identified.
**Ethics committee approval:** This study was approved by an ethics committee.
**Funding for this study:** No funding was received for this study.
**Author Disclosures:**
Thomas Frauenfelder: Nothing to disclose
Michael Andreas Messerli: Nothing to disclose
Christian Blüthgen: Nothing to disclose
Katharina Martini: Nothing to disclose
Lisa Jungblut: Nothing to disclose
Bernhard Schmidt: Employee: Siemens
Malgorzata Polacin: Nothing to disclose
Andre Euler: Nothing to disclose
Hatem Alkadhi: Nothing to disclose

### RPS 1305-8

**Reducing clinical chest X-ray reading times by using artificial intelligence to stratify worklists into normal/abnormal categories**

*K. Hergaarden*[1], O. Hertgers[1], A. Efitorov[2], J. V. Stadelmann[2], D. Mavroeidis[3], A. Saalbach[4], S. Renisch[4], H. Schulz[4], H. J. Lamb[1]; [1]Leiden/NL, [2]Moscow/RU, [3]Eindhoven/NL, [4]Hamburg/DE
*(K.F.M.Hergaarden@LUMC.nl)*

**Purpose:** To determine the effect of automatically stratifying the reporting worklist into "normal" and "abnormal" cases on the total and per-case reading times of a typical clinical chest X-ray (CXR) worklist.
**Methods or Background:** A Deep Neural Network (DenseNet121, trained on MIMIC-CXR and NIH datasets) was used to select 100 cases with a high suspicion of (typical) chest pathology and 100 cases supposedly without pathological findings, derived from a dataset of 24,829 frontal-view chest X-ray images acquired at our hospital. A baseline (random mix) and stratified worklist were created, both consisting of 50 "normal" and 50 "abnormal" CXR images. Two radiologists independently reported on the two worklists in a simulated radiology reading environment while being blinded for the AI findings. The overall and per-case reading times were measured and compared.

**Results or Findings:** For the mixed baseline worklist the averaged total reading time was 87min 7s, with an averaged per-case reading time of 52s. For the worklist stratified by AI the averaged total reading time was 67min 48s, with a per-case reading time of 41s. An overall reading time reduction of 22,1% was found using the stratified worklist.
**Conclusion:** Artificial intelligence can be used to positively impact the reporting workflow. We demonstrated how increased reporting efficiency can be achieved through stratification of the worklist into "normal" and "abnormal" cases, leading to reduced reporting times.
**Limitations:** This is a proof of concept. To evaluate if this time reduction will hold, a larger number of imaging studies with different ratios in normal/abnormal exams is needed, while being read by radiologists of different expertise levels.
**Ethics committee approval:** A waiver of consent was obtained from the local ethics committee.
**Funding for this study:** No funding was received for this study.
**Author Disclosures:**
Heinrich Schulz: Employee: Philips N.V.
Omar Hertgers: Nothing to disclose
Steffen Renisch: Employee: Philips N.V.
Axel Saalbach: Employee: Philips N.V.
K.F.M. Hergaarden: Nothing to disclose
Aleksandr Efitorov: Employee: Philips N.V.
Joël Valentin Stadelmann: Employee: Philips N.V.
Dimitrios Mavroeidis: Employee: Philips N.V.
Hildo J. Lamb: Nothing to disclose

### RPS 1305-9

**Development of a deep learning-based model for chest X-ray quality assessment**

*R. Khansa*; Paris/FR

**Purpose:** Chest radiography is the first-line imaging modality for diagnosing thoracic pathologies. Diagnostic accuracy may be drastically reduced due to technical limitations resulting in poor image quality and thus leading to incorrect diagnosis.
**Methods or Background:** We collected 6955 frontal and lateral Chest X-rays (CXRs), performed in the supine or standing position, labelled by 7 radiologists from the radiology department of Cochin University hospital (AP-HP, Paris, France). Frontal CXR quality criteria included full anatomical coverage, lack of rotation or scapula projection, deep inspiration and optimal exposure. Lateral CXR quality criteria included full anatomical coverage, lack of rotation and lack of superimposition by the arms. A deep convolutional neural network was trained to classify CXRs as technically correct or incorrect. The model predictions were compared to a ground truth set of 1000 CXR, labelled by two chest radiology experts.
**Results or Findings:** There were no significant differences regarding age, sex, and classification for each criterion between the test, validation and training datasets (p>0,05). We first evaluated the inter-rater reliability and found good correlation for each criterion (Cohen's kappa > 0,6). The model performance was evaluated and compared to that of each observer with expert annotation as ground truth. The model performance was close to the radiologists' performance, (accuracies 80-90% compared to 83-95% for radiologists) except for the rotation criteria.
**Conclusion:** The trained model to detect quality-deficient CXRs could be used by technologists in real-time to ensure high-quality images.
**Limitations:** Not applicable
**Ethics committee approval:** Not applicable
**Funding for this study:** Not applicable
**Author Disclosures:**
Rémi Khansa: Nothing to disclose

### RPS 1305-10

**AI model uncertainty for detecting pneumothorax on chest radiographs is a strong predictor for annotator confidence**

*O. Hertgers*[1], K. Hergaarden[1], S. Renisch[2], H. Schulz[2], M. Sevenster[1], D. Mavroeidis[3], H. J. Lamb[1]; [1]Leiden/NL, [2]Hamburg/DE, [3]Eindhoven/NL
*(ohertgers@gmail.com)*

**Purpose:** To evaluate the usability of uncertainty computed from an AI model for automated diagnostic image quality verification.
**Methods or Background:** We trained a neural network model that outputs both pneumothorax status of chest radiographs (CXRs) and the uncertainty associated with this classification. The model was trained in two stages. During the first stage we used solely the outputs produced by the model predictions branch and optimized their accuracy using the cross-entropy loss. Consequently, we optimized both the model prediction output, as well as the model uncertainty output using the (Kendall and Gal, NeuIPS 2017) loss function. The model training was done using the publicly available NIH CXR dataset. We validated the model uncertainty results using a dataset of 599 representative CXRs from the PACS system of our academic hospital over the period of two years. Two resident radiologists annotated pneumothorax status (present, not present, uncertain, unable to assess) and image quality (insufficient, moderately sufficient, sufficient). In the study we considered that a CXR is of insufficient diagnostic quality when both annotators marked its pneumothorax status as uncertain or unable to assess and image quality insufficient or moderately sufficient. In total, 55 out of the 599 CXRs were of insufficient diagnostic quality.
**Results or Findings:** We used the area under the curve to validate whether the uncertainty estimated by the AI model assigns higher uncertainty scores to the 55 scans of insufficient diagnostic image quality. We obtained an AUC score of 0.964 with estimated 95% confidence interval of [0.949-0.977].
**Conclusion:** The uncertainty score of our pneumothorax model is a strong predictor for annotator confidence. AI model uncertainty can identify CXRs with insufficient diagnostic quality.
**Limitations:** Single-site validation was a limiting factor in this study.
**Ethics committee approval:** A waiver of consent was obtained from the local ethics committee.
**Funding for this study:** No funding was received for this study.
**Author Disclosures:**
Heinrich Schulz: Employee: Philips N.V.
Omar Hertgers: Nothing to disclose
Steffen Renisch: Employee: Philips N.V.
Merlijn Sevenster: Employee: Philips N.V.
K.F.M. Hergaarden: Nothing to disclose
Dimitrios Mavroeidis: Employee: Philips N.V.
Hildo J. Lamb: Nothing to disclose

### RPS 1305-11

**Impact of a content-based image retrieval system on the interpretation of chest CTs of patients with diffuse parenchymal lung disease**

*S. Röhrich*, B. H. Heidinger, G. Langs, M. Krenn, H. Prosch, R. Zhang; Vienna/AT
*(sebastian.roehrich@meduniwien.ac.at)*

**Purpose:** Content-based image retrieval systems (CBIRS) are a relatively new and potentially impactful tool for radiological reporting, but their clinical evaluation is largely missing. This study aimed at assessing the impact of CBIRS on the interpretation of chest CT scans from patients with suspected diffuse parenchymal lung disease (DPLD).
**Methods or Background:** Eight radiologists (four residents, four attending, median years reading chest CT scans 2.1±0.7 and 12±1.8, respectively) created 430 reports from 100 chest CT scans with 22 unique, clinically- and histopathologically-verified diagnoses. Reporting was done at a radiological workstation to simulate clinical routine. 216 reports were done without CBIRS and 214 with the additional support of the CBIRS.
**Results or Findings:** Reading time decreased by 31.3% (p<0.001) despite the radiologists searching for additional information more frequently when the CBIRS was available (154 [72%] vs. 95 [43%]). There was also a trend towards higher overall diagnostic accuracy (42.2% vs 34.7%, p=0.083) when the CBIRS was available.
**Conclusion:** Summarized, the CBIRS' impact on the interpretation of chest CT scans from patients with DPLD included significant improvements in reading time despite increased engagement with informational resources. Results also show a trend towards increased diagnostic accuracy.
**Limitations:** No clinical data or previous examinations were available to the participants. As we encountered delays as a result of the ongoing SARS-CoV-2 pandemic, time between the two phases ranged from 9 and 15 months for individual participants. Thus, increasing experience between phases constitutes a potential bias. However, the results for radiology residents and attending radiologists were similar, even though the latter had a markedly less pronounced relative increase in years of professional experience and finished reports.
**Ethics committee approval:** The study was approved by the institutional review board (protocol number 1288/2018).
**Funding for this study:** European Commission H2020, Project Number 780495
**Author Disclosures:**
Georg Langs: Other: Grant: Austrian Science Fund (FWF); Shareholder/Co-Founder contextflow GmbH; Speaker fees: Roche, Siemens; Research support: Siemens, Novartis, IBM, NVIDIA
Benedikt H Heidinger: Nothing to disclose
Helmut Prosch: Other: Grant: H2020 European Commission; Speakers fees: Boehringer-Ingelheim, Roche, Novartis, MSD, BMS, GSK, Chiesi, AstraZeneca; Research support: Boehringer-Ingelheim
Sebastian Röhrich: Other: Consulting activities for contextflow GmbH
Rui Zhang: Employee: Contextflow GmbH
Markus Krenn: Other: Chief Product Officer contextflow GmbH

**RPS 1305-12**

**CAD significantly increases the accuracy of pulmonary nodule detection in both concurrent and second reader paradigms**

*Y. Zhao*[1], N. Obuchowski[2], A. Johri[1], G. Hermosillo Valadez[1], L. Bogoni[1], D. R. Aberle[3]; [1]Malvern, PA/US, [2]Cleveland, OH/US, [3]Los Angeles, CA/US
*(zhaneforever@gmail.com)*

**Purpose:** To evaluate the impact of an AI-based computer-aided detection (CAD) algorithm on radiologists' accuracy for detecting pulmonary nodules in thoracic CTs when using CAD as concurrent 1st reader (Mode-1) and 2nd reader (Mode-2).
**Methods or Background:** A dual-arm MRMC study included 232 retrospectively collected multivendor chest CTs from screening and diagnostic examinations. 20 radiologists reviewed all cases twice with one-month washout, randomly doing either Mode-1 followed by "non-aided + Mode-2" or vice versa. Readers were asked to identify solid, part-solid and ground-glass nodules in the range of 3 mm to 30 mm. The consolidated marks, blinded to their source, were then reviewed independently by five expert thoracic radiologists. The reference standard was determined following final adjudication by an expert thoracic radiologist. AUCs at lobe level, sensitivities at nodule level and specificities at lobe level were computed for unaided-reader, Mode-1 and Mode-2.
**Results or Findings:** 268 nodules (182 solid) were identified in 130 cases (202 lobes). The lobe-level AUCs were 0.760, 0.809 and 0.811, for unaided-reader, Mode-1 and Mode-2 respectively, improving by 0.049 (p<0.001, 95% CI [0.024,0.074]) and 0.051 (p<0.001, 95% CI [0.032,0.070]) for Mode-1 and Mode-2 respectively. The nodule level sensitivities were 0.43, 0.519, 0.537, improving by 0.082 (p<0.0001, 95% CI [0.039,0.125]) and 0.100 (p<0.0001, 95% CI [0.063,0.137]). The lobe level specificities (958 lobes) were 0.942, 0.922 and 0.915, changing by -0.020 (95% CI [-0.031,-0.008]) and -0.027 (95% CI [-0.041,-0.013]). The mean (standard-deviation) false positive rates were 0.44 (1.04), 0.55 (1.13) and 0.65 (1.34), changing by -0.11 (95% CIs [0.038,0.197]) and -0.21 (95% CIs [0.106,0.322]).
**Conclusion:** Using AI-based CAD either as concurrent first reader or second reader significantly improved readers' accuracy in detecting solid, part-solid and ground-glass nodules with a very small increase in false positives.
**Limitations:** Not applicable
**Ethics committee approval:** Not applicable
**Funding for this study:** Not applicable
**Author Disclosures:**
Luca Bogoni: Employee: Siemens Healthineers
Yiyuan Zhao: Employee: Siemens Healthineers
Nancy Obuchowski: Consultant: Siemens Healthineers Employee: Cleveland Clinic, Cleveland/US
Abhineet Johri: Employee: Siemens Healthineers
Gerardo Hermosillo Valadez: Employee: Siemens Healthineers, Malvern/US
Denise R. Aberle: Employee: University of California at Los Angeles
Investigator: Siemens Healthineers

**10:30-12:00** **Room Z**

### Research Presentation Session: Oncologic Imaging

### RPS 1316

### Advanced imaging for oncology staging and follow-up

**Moderator**
M. E. Mayerhöfer; Vienna/AT
**Author Disclosure:**
M. E. Mayerhöfer: Speaker: Siemens, GE, BMS

**RPS 1316-2**

**A novel non-invasive approach to cancer of unknown primary: AI can detect CT morphological differences between primary tumours and metastases across tumour types**

*Z. Elkarghali*[1], N. Bogveradze[1], F. Castagnoli[2], E. K. Hong[1], F. Landolfi[3], N. Gennaro[4], A. Van Der Kolk[5], S. Trebeschi[1], R. G. H. Beets-Tan[1]; [1]Amsterdam/NL, [2]Bordighera/IT, [3]Rome/IT, [4]Lugano/CH, [5]Arnhem/NL
*(z.elkarghali@nki.nl)*

**Purpose:** Cancer of unknown primary (CUP) is a diagnosis assigned to 3-6% of cancer patients. CUP patients only receive empirical treatment, and until a definitive diagnosis could possibly be reached, the prognosis remains poor. We aimed to use AI-based methods to non-invasively distinguish between primary and metastatic lesions based on their morphology, in a pan-cancer setting.
**Methods or Background:** Lesion segmentation and radiomic feature extraction were performed on baseline CT scans from two cohorts. Cohort A consisted of 1630 routine cancer patients (n=11227 lesions) with a histopathologically-proven diagnosis. This cohort was divided into a training/finetuning set (80%, 9050 lesions) and an unseen independent validation set (20%, 2177 lesions) to evaluate the model performance on unseen data. For further validation, Cohort B was formed of real-world CUP patients (n=74 patients, 303 lesions) who later received a definitive diagnosis. We explored different machine learning classifiers and hyperparameters on the training set and validated separately on the unseen data from Cohorts A and B.
**Results or Findings:** AI algorithms were able to distinguish between primary and metastatic lesions across all tumour types both in the unseen routine cohort (AUC=0.93, 95%-CI=0.91-0.95, p<0.001) and in the real-world CUP cohort (AUC=0.82, 95%-CI=0.74-0.89, p<0.001). We could also identify lung cancer (AUC=0.67, 95%-CI=0.65-0.69, p<0.001) and colorectal lesions (AUC=0.69, 95%-CI=0.67-0.72, p<0.001) from all lesions in our cohorts.
**Conclusion:** AI-detected quantitative morphological differences between primary and metastatic lesions and between different tumour types may help non-invasively diagnose the primary tumour in patients with CUP, facilitating more efficient treatment strategies.
**Limitations:** Large-scale multicenter prospective studies are needed to evaluate the robustness and generalisability of this algorithm.
**Ethics committee approval:** The study received institutional IRB approval (IRBd20-213).
**Funding for this study:** Not applicable
**Author Disclosures:**
Eun Kyoung Hong: Nothing to disclose
Stefano Trebeschi: Nothing to disclose
Francesca Castagnoli: Nothing to disclose
Zuhir Elkarghali: Nothing to disclose
Nicolò Gennaro: Nothing to disclose
Anja Van Der Kolk: Nothing to disclose
Regina G. H. Beets-Tan: Nothing to disclose
Federica Landolfi: Nothing to disclose
Nino Bogveradze: Nothing to disclose

**RPS 1316-3**

**Magnetic resonance imaging (MRI) for staging melanoma patients in direct comparison to computed tomography (CT): results from a prospective positron-emission tomography PET/CT and PET/MRI study**

*C. Zhang*, C. P. Reinert, F. F. Seith, P. Martirosian; Tübingen/DE

**Purpose:** To directly compare the diagnostic performance of contrast-enhanced whole-body (WB) MRI to WB CT for staging melanoma patients.
**Methods or Background:** 57 patients (25f) with advanced melanoma underwent PET/CT and PET/MRI on the same day. Two blinded readers evaluated independently the CT and MRI (T1VIBE, DWI, T2HASTE) scans. A third blinded reader analysed both PET/CT and PET/MRI including pre- and follow-up examinations to define a reference standard.
**Results or Findings:** All patients had confirmed metastases. Among these, comparison of lesion-based detection rates revealed higher sensitivity of CT for lung (0.90 vs. 0.68) and abdominal lesions (0.89 vs. 0.77), whereas MRI showed higher or equal specificity (0.93 vs. 0.86 and 0.88 vs. 0.88). MRI was superior in the detection of bone metastases regarding both sensitivity (0.89 vs. 0.61) and specificity (0.93 vs. 0.88). For lymph nodes and soft tissue metastases, comparable sensitivity resulted from CT and MRI analysis (0.88), but specificity was higher for CT (0.76).
**Conclusion:** WB MRI could serve as an alternative to CT with comparable specificity for lung and abdominal metastases and equal diagnostic accuracy for bone and lymph node metastases. Advanced MR-techniques of the lung might increase the sensitivity for lung metastases.
**Limitations:** Overall inter-reader reliability was good, but disagreements in detection rate between the 2 readers analysing CT and MRI scans appeared most of all for lymph nodes and soft tissue lesions.
**Ethics committee approval:** This study was approved by the local ethics committee of the University of Tuebingen.
**Funding for this study:** This study received funding from the Wilhelm Sander Foundation.
**Author Disclosures:**
Cecilia Zhang: Nothing to disclose
Ferdinand Frederic Seith: Nothing to disclose
Christian Philipp Reinert: Nothing to disclose
Petros Martirosian: Nothing to disclose

### RPS 1316-4

**Identification of early CT imaging response predictors after CAR T-cell therapy**

*M. Winkelmann*, V. Blumenberg, V. Bücklein, M. Unterrainer, F. Vettermann, P. Bartenstein, J. Ricke, M. Subklewe, W. Kunz; Munich/DE
*(michael.winkelmann@med.uni-muenchen.de)*

**Purpose:** Chimeric antigen receptor (CAR) T-cell therapy with patient-derived T cells against tumor cells are approved for relapsed or refractory (r/r) diffuse large B-cell lymphoma (DLBCL). Anti-tumour activity differs from conventional treatment strategies or even checkpoint blockade with so far undefined response patterns. We assessed volumetric tumour burden 30 days after transfusion to identify early predictors of response at 3 months as well as progression-free (PFS) and overall survival (OS).
**Methods or Background:** Consecutive r/r DLBCL patients with CT imaging at baseline (BL), CT at 30 days (FU1) and PET/CT at 3 months (FU2) after CAR T-cell transfusion were selected. For all patients, up to 6 target lesions were 3D-segmented using Mint Lesion at all time points. The sum of target lesion volume (STLV) was used to represent tumour burden. Percent change in STLV indicated depth or response (DoR). The prognostic value of DoR-FU1 was tested for treatment response at 3 months and for stratification of PFS and OS.
**Results or Findings:** 26 patients were included (median age: 62 years, 58% male) with median BL-STLV of 84.6 ml. A positive correlation between DoR-FU1 and DoR-FU2 was observed (r=0.87; p<0.05). For patients with DoR ≥50% in FU1, no significant differences in PFS or OS were detected (p=0.60 and p=0.34). OS was numerically longer in patients with DoR ≥50% in FU1.
**Conclusion:** An early reduction in tumour burden at FU1 after CAR-T cell therapy showed a good correlation with the DoR-FU2. Conventional end points at early interim staging have limited prognostic value regarding OS. Future studies may explore the value of radiomic analysis as early response markers.
**Limitations:** Since CAR T-cells represent a new form of therapy, there is a lack of data on long-term follow-up.
**Ethics committee approval:** Ethical committee approval was obtained.
**Funding for this study:** Not applicable
**Author Disclosures:**
Marion Subklewe: Nothing to disclose
Franziska Vettermann: Nothing to disclose
Viktoria Blumenberg: Nothing to disclose
Peter Bartenstein: Nothing to disclose
Michael Winkelmann: Nothing to disclose
Marcus Unterrainer: Nothing to disclose
Wolfgang Kunz: Nothing to disclose
Veit Bücklein: Nothing to disclose
Jens Ricke: Nothing to disclose

### RPS 1316-5

**CT-based radiomics score of PET-negative residual CT Masses: a potential biomarker for the prediction of relapse-free survival in lymphoma patients showing complete metabolic response**

*S. H. Cha*, N. Y. Han, K-W. Kang, Y. Cho, D. J. Sung, B. J. Park, K. C. Sim, Y. E. Han; Seoul/KR
*(seunghacha@gmail.com)*

**Purpose:** To determine whether CT-based radiomics score of PET-negative residual CT masses (PnRCM) can predict relapse-free survival (RFS) in lymphoma patients showing complete metabolic response (CMR) after first line chemotherapy.
**Methods or Background:** A total of 247 patients who showed CMR after completion of first line chemotherapy for PET-avid lymphomas were recruited. Patients with PnRCM were selected in accordance with the Lugano criteria and 3-D segmentation was done on contrast-enhanced CT. Radiomics features for masses were extracted and radiomics scores were constructed using the least absolute shrinkage and selection operator (LASSO) analysis. Cox regression was performed with radiomics and clinical parameters (IPI score, sex, lymphoma aggressiveness, and transplantation history). The efficiency of the model was evaluated using the area under the curve (AUC).
**Results or Findings:** Among the patients included, 76 patients (30.1%) had PnRCM. Kaplan-Meier analysis showed that patients with PnRCM had significantly shorter RFS (p=0.009) than those without PnRCM. In Kaplan-Meier analysis using the cut-off value generated by maximally selected rank statistics, the high radiomics score group showed significantly shorter RFS (p=0.0016). In the Cox regression test, radiomics score (hazard ratio [HR]=3.01; p=0.002), IPI score (HR=2.00; p=0.13) and transplantation history (HR=2.46; p=0.075) were related factors for RFS. The combined model and radiomics model showed an AUC of 0.80 and 0.74 respectively, in estimating RFS.
**Conclusion:** The combined model that incorporated both clinical parameters and CT based radiomics score showed good prognostic efficacy in lymphoma patients with PnRCM.
**Limitations:** The predictive model was not validated with a separate test set due to the limitations of a small study group.
**Ethics committee approval:** This study was approved by the IRB, and the requirement for informed consent was waived.
**Funding for this study:** The authors received no financial support for the research, authorship, and/or publication of this article.
**Author Disclosures:**
Ki Choon Sim: Nothing to disclose
Na Yeon Han: Nothing to disclose
Seung Ha Cha: Nothing to disclose
Deuk Jae Sung: Nothing to disclose
Ka-Won Kang: Nothing to disclose
Yongwon Cho: Nothing to disclose
Yeo Eun Han: Nothing to disclose
Bum Jin Park: Nothing to disclose

### RPS 1316-6

**Preliminary whole-body MRI study for evaluation of minimal residual disease in a group of multiple myeloma patients**

*V. Angelini*[1], A. Villanacci[2], A. Belotti[2], M. Chiarini[2], V. Giustini[2], R. Ievoli[3], B. Frittoli[2], L. Grazioli[2]; [1]Aosta/IT, [2]Brescia/IT, [3]Ferrara/IT
*(va.angelini90@gmail.com)*

**Purpose:** This pilot study has aimed at the evaluation of minimal residual bone marrow disease (MRD) in patients (Pz) with multiple myeloma (MM) and undergoing autologous bone marrow transplantation (ASCT).
**Methods or Background:** Simultaneous (within 4 months of each other) MRD assessments by flow cytometry (MFC) and whole-body MRI (WB-MR) were retrospectively compared (the WB-MR were acquired by MY-RADS 2019 accommodations).
**Results or Findings:** The results show that the two methods of assessing residual disease are independent (Fisher exact test statistic value is 0.09284, p<0,10). There is a statistical dependence between the radiological variable and the haematological one only in Pz in either clear remission or progression of the disease (the Fisher exact test statistic value is 0.0964, p<0,1). There is statistical independence in radiologically doubtful subjects (Fisher exact test statistic value is 0.09285, p < 0.10,p<0.05, p<0,01).
**Conclusion:** Having a subgroup of Pz with an outcome, some hypotheses can be advanced: first, the possibility that for radiologically "probably in remission" Pz (RAC2) WB-MR has a greater positive predictive value (VPP) than MCF; second, that WB-MR could be more sensitive than MFC in the early diagnosis of disease recurrence. The panoramic view, the addition of functional data to the morphological data (DWI) of the WB-MRI go well with the intrinsically heterogeneous character of multiple myeloma: heterogeneous for spatially and also temporally differentiated localisations.
**Limitations:** The study was limited by the size of the patients sample and its status as a pilot study.
**Ethics committee approval:** Consent to the execution of the study was requested from the ethics committee of the hospital ASST “Spedali Civili” in Brescia, which gave a favourable opinion.
**Funding for this study:** No funds were used for this study.
**Author Disclosures:**
Valentina Angelini: Nothing to disclose
Riccardo Ievoli: Nothing to disclose
Angelo Belotti: Nothing to disclose
Luigi Grazioli: Nothing to disclose
Marco Chiarini: Nothing to disclose
Barbara Frittoli: Nothing to disclose
Alberta Villanacci: Nothing to disclose
Viviana Giustini: Nothing to disclose

### RPS 1316-7

**Ultra low dose whole-body computed tomography protocol optimisation for patients with plasma cell disorders: diagnostic accuracy and effective dose analysis from a reference centre**

*D. Tore*, A. Depaoli, O. Rampado, C. Guarnaccia, A. Santonocito, A. N. A. Serafini, G. A. Strazzarino, M. Oronzio, P. Fonio; Turin/IT
*(davide.tore91@gmail.com)*

**Purpose:** Whole-body low dose CT (WBLDCT) is the first-choice imaging technique in patients with suspected plasma cell disorder (PCD) to assess the presence of osteolytic lesions. We investigated the performances of an optimised protocol, evaluating diagnostic accuracy and patient effective dose reduction with a latest generation scanner.
**Methods or Background:** Retrospective study on 212 patients with PCD performed on a 256-rows CT scanner. First WBLDCT examinations were performed using a reference protocol with acquisition parameters obtained from the literature. A phantom study was performed for protocol optimisation for subsequent exams to minimise dose while maintaining optimal diagnostic accuracy. Images were evaluated by three readers for image quality and to detect lesions. Effective doses (E) were evaluated for each patient considering the patient dimensions and tube current modulation.

**Results or Findings:** A very good image quality was observed for both protocols by all readers with a good agreement at repeated measures ANOVA test (p>0.05). An excellent inter-rater agreement for lesion detection was achieved obtaining high values of Fleiss' kappa for all districts considered (p<0.001). The optimised protocol resulted in a 56% reduction of median DLP (151 mGycm vs 345 mGycm,), of 60% of CTDIvol, (2.2 mGy vs 0.9 mGy). The median E was 2.6 mSv for standard protocol and 1.5 mSv for the optimised one. Dose reduction was statistically significant with p<0.001.
**Conclusion:** Protocol optimisation makes ultra low dose WBLDCT feasible on latest generation CT scanners for patients with PCD with effective doses inferior to conventional skeletal survey while maintaining excellent image quality and diagnostic accuracy. Dose reduction is crucial in such patients as they are likely to undergo multiple WBLDCT during follow-up.
**Limitations:** This study was limited by its monocentric nature.
**Ethics committee approval:** The study was approved by the ethics committee.
**Funding for this study:** No funding was received for this work.
**Author Disclosures:**
Giulio Antonino Strazzarino: Nothing to disclose
Alessandro Depaoli: Nothing to disclose
Osvaldo Rampado: Nothing to disclose
Paolo Fonio: Nothing to disclose
Carla Guarnaccia: Nothing to disclose
Ambra Santonocito: Nothing to disclose
Alessandro Niccolò Annibale Serafini: Nothing to disclose
Maria Oronzio: Nothing to disclose
Davide Tore: Nothing to disclose

### RPS 1316-8

**Iodine quantification on multi-vendor DECT platforms: inter-scanner variability and impact of normalisation**

*S. Lennartz*[1], J. Cao[1], N. Pisuchpen[1], A. Parakh[1], D. V. Sahani[2], A. Kambadakone[1]; [1]Boston, MA/US, [2]Seattle, WA/US
*(simon.lennartz@uk-koeln.de)*

**Purpose:** To compare iodine quantification between different dual-energy CT (DECT) scanner types and investigate if normalisation can mitigate intra-patient, inter-scanner variability.
**Methods or Background:** 44 patients who received examinations on three different DECT scanner types during cancer follow-up between 01/2016 and 09/2020 were included. The scanner types were a dual-source (dsDECT), a rapid kVp switching (rsDECT), and a dual-layer detector DECT (dlDECT). Patients with interval increase/newly developed disease between examinations were excluded. Iodine concentrations were obtained ROI-based in the liver, pancreas, kidney, aorta, portal vein, muscle, gallbladder, and retroperitoneal fat. Absolute iodine concentration (IC) and three different normalised iodine concentrations were included: NIC(aa): normalised to aorta; NIC(pv): normalised to portal vein; NIC(all): normalised to overall iodine load. IC and NIC were compared between scanner types and median inter-scanner variability was calculated.
**Results or Findings:** IC was significantly different between scanner types in all tissues except for the kidneys and the aorta. The median inter-scanner variability of IC was highest in the liver (dsDECT vs dlDECT: 28.96 (14.28-46.87)%, dsDECT vs rsDECT: 29.08 (16.59-62.55)%, rsDECT vs dlDECT: 22.85 (7.52-33.49)%), lowest in the kidneys (dsDECT vs dlDECT: 15.76 (7.03-26.1)%, dsDECT vs rsDECT: 15.67 (8.86-25.56)%), rsDECT vs dlDECT: 10.92 (4.92-22.79)%), and intermediate for the pancreas (dsDECT vs dlDECT: 22.24 (7.06-37.93)%), dsDECT vs rsDECT: 19.86 (10.9-27.14)%), rsDECT vs dlDECT: 13.66 (7.67-30.72)%)). NIC(all) was the only normalisation approach that decreased inter-scanner variability of the pancreas and kidneys for all inter-scanner comparisons, whereas for the liver, normalisation only reduced variability between rsDECT and dlDECT (NIC(all): 11.03 (4.88-26.75)%; IC: 22.85 (7.52-33.49)%).
**Conclusion:** Inter-scanner variability of iodine quantification in DECT was partly mitigated by normalisation, yet variability in the liver remained on a high level. Large-scale studies validating iodine concentration as a longitudinal DECT imaging biomarker in a multivendor setting should be encouraged.
**Limitations:** The small, retrospective sample was identified as a limitation.
**Ethics committee approval:** Consent was waived by the IRB.
**Funding for this study:** This study received funding from the DFG (Deutsche Forschungsgemeinschaft, German Research Foundation) and Philips.
**Author Disclosures:**
Simon Lennartz: Research/Grant Support: Philips
Dushyant V Sahani: Nothing to disclose
Avinash Kambadakone: Research/Grant Support: GE Healthcare
Research/Grant Support: Philips
Jinjin Cao: Nothing to disclose
Nisanard Pisuchpen: Nothing to disclose
Anushri Parakh: Nothing to disclose

### RPS 1316-9

**Investigating the validity of computed tomography low dose simulations in a prospective intra-individual in-vivo real low dose animal study on a 3rd generation dual-source scanner**

*A. S. Brendlin*, H. Almansour, S. Afat; Tübingen/DE

**Purpose:** To investigate to which extent simulated low-dose CT data sets resemble their real-dose counterparts.
**Methods or Background:** At 120 and 80 kV, fourteen veterinarian-sedated alive pigs were scanned with the same 3rd generation dual-source scanner at 300 mAs (100%), and with mAs reduced to 50%, 25%, 10%, and 5%. Additional data sets at 50%, 25%, 10%, and 5% were simulated from the 100% images. Weighted filtered back projection (wFBP) was used for reconstructions. Objective image quality (Hounsfield units stability, noise, signal-to-noise, and contrast-to-noise ratio) was analysed by topographically matched regions of interest (ROIs) using ANOVA. A structural similarity index (SSIM) quantified the voxel-wise similarity of the volume histograms. Five blinded readers independently rated subjective overall image quality using a 5-point-Likert scale (1 = poor to 5 = excellent). An intraclass correlation coefficient (ICC) was used to quantify agreement.
**Results or Findings:** Image quality was stable down to 25% mAs in both kV groups, but image noise was overestimated at 10% and below in the simulations. Datasets ≥25% mAs had a structural similarity of ≥96 ± 2% to their real dose counterparts. Subjective image quality correlated almost perfectly (ICC≥0.89, p<0.001) in real vs. simulated datasets across all readers.
**Conclusion:** Simulated low dose CT datasets are subjectively and objectively indistinguishable from their real-dose counterparts down to 25% mAs, making them an invaluable tool to highlight the effects of radiation dose reduction on image quality in this range safely.
**Limitations:** First, we performed an animal study to avoid increased radiation exposure in humans. Second, we used wFBP to rule out confounding factors. A confirmation study using more modern reconstruction methods might be helpful. Lastly, this study was performed on a high-end 3rd generation dual-source CT scanner, so generalisability may be limited.
**Ethics committee approval:** This study was approved by the IRB.
**Funding for this study:** No funding was received for this study.
**Author Disclosures:**
Andreas Stefan Brendlin: Nothing to disclose
Saif Afat: Nothing to disclose
Haidara Almansour: Nothing to disclose

### RPS 1316-10

**Optimal keV in virtual monoenergetic imaging reconstructions for the detection of hypovascularized liver metastases on a novel photon counting dual-source CT scanner: initial experience**

S. Bette, *J. A. Decker*, P. Woźnicki, J. Becker, F. Risch, C. Wollny, C. Scheurig-Münkler, T. J. Kroencke, F. Schwarz; Augsburg/DE
*(Josua.Decker@uk-augsburg.de)*

**Purpose:** To identify the optimal keV-settings for the detection of hypovascularized liver metastases using virtual monoenergetic imaging (VMI) reconstructions on a novel dual-source photon-counting detector CT (PCD-CT).
**Methods or Background:** 20 consecutive patients with hypovascularized liver metastases who had undergone CT of the abdomen in portalvenous phase on a PCD-CT were retrospectively identified. VMI-reconstructions were performed at different keV levels (40-190 keV). Regions of interest were placed in liver metastases and liver parenchyma and in defined anatomical regions (e.g. aorta, portal vein). Mean and standard deviation of CT values (HU) were reported. Radiation dose (CTDI), image noise, tumor-to-liver ratio and contrast-to-noise ratio (CNR) were measured. Subgroup analyses for patients with higher and lower BMI values were performed.
**Results or Findings:** Image noise was significantly higher at keV levels ≤ 70 keV (P<0.001). Tumor-to-liver ratio was significantly lower at lower keV levels (≤ 70 keV) (P<0.001), resulting in better demarcation of the metastases. CNR was significantly higher at lower keV levels (≤ 70 keV) (P<0.001). The highest CNR was observed at 40 keV. Patients with lower BMI values (<24.8) showed significantly lower tumor-to-liver ratios and significantly higher CNR's (P>0.001). Noise was only slightly different between BMI values. CTDI was 8.51 (±3.93) mGy and significantly higher in patients with higher BMI values (≥24.8) than in patients with lower BMI values (P=0.007).
**Conclusion:** These first experiences with VMIs on a novel PCD-CT strongly suggest improved delineation of hypovascularized liver metastases at lower keV levels (≤ 70 keV), regardless of BMI. Whether this translates into overall improved diagnostic accuracy is currently under investigation.
**Limitations:** This study was limited by a small cohort and its status as a retrospective single-centre study.
**Ethics committee approval:** This study was approved by an ethics committee.

Friday

**Funding for this study:** This study received no funding.
**Author Disclosures:**
Judith Becker: Nothing to disclose
Franka Risch: Nothing to disclose
Stefanie Bette: Nothing to disclose
Christian Scheurig-Münkler: Nothing to disclose
Claudia Wollny: Nothing to disclose
Thomas J. Kroencke: Nothing to disclose
Florian Schwarz: Nothing to disclose
Piotr Woźnicki: Nothing to disclose
Josua A. Decker: Nothing to disclose

### RPS 1316-11

**Feasibility and dose evaluation of a dynamic liver perfusion CT protocol with latest generation scanner**

*F. Ullo*, S. Salto, D. Tore, A. Depaoli, C. Guarnaccia, A. Biondo, P. Fonio; Asti/IT
*(federica.ullo@edu.unito.it)*

**Purpose:** To evaluate the feasibility of a dynamic contrast-enhanced (DCE) whole-liver CT perfusion protocol with acceptable radiation doses in patients with metastatic cancer on a latest generation wide detector CT scanner.
**Methods or Background:** Prospective monocentric study on 25 consecutive patients with liver metastases. Exams were acquired on a 256-rows CT scanner (Revolution CT, GE Healthcare). The protocol consisted in an unenhanced upper abdomen acquisition followed by a free-breathing DCE study after injecting 50 ml of contrast with serial low-dose axial acquisitions on the upper abdomen (gantry rotation time 0.28 s, 256 x 0.625 mm collimation, 80 kV, 270 mA, total scan time 230 seconds). The study was completed with a helical thoraco-abdominal acquisition after injecting 50 ml of contrast.
36 standard quadruple-phase CT exams with thoraco-abdominal portal-phase acquisition were considered as a control group. Radiation dose was evaluated with total dose length product (DLP) and effective dose (ED), considering k (mSv/mGycm) 0.014 for thorax and 0.015 for abdomen/pelvis.
**Results or Findings:** Patients' BMIs were similar in both groups ( 26±4,24 vs 26,8±4,28 kg/m2, p=0,44). Radiation dose was not significantly different as mean DLP for DCE exams was 1381±243 mGycm and 1559±443 mGycm for quadruple-phase CTs (p=0.1); ED was 20.5±3,58 mSv in DCE group and 23.03±6.55 in control group (p=0.11). There were no significant differences in the volume of contrast infused (100 ml vs 105±17,2, p=0,052).
**Conclusion:** Latest generation CT scanners allow DCE liver CT perfusion imaging with radiation doses comparable to standard quadruple-phase CT scans.
**Limitations:** This study was limited by its status as a preliminary study on a limited patient population.
**Ethics committee approval:** The study was approved by the ethics committee.
**Funding for this study:** No funding was received for this work.
**Author Disclosures:**
Sara Salto: Nothing to disclose
Alessandro Depaoli: Nothing to disclose
Paolo Fonio: Nothing to disclose
Federica Ullo: Nothing to disclose
Carla Guarnaccia: Nothing to disclose
Davide Tore: Nothing to disclose
Andrea Biondo: Nothing to disclose

**12:30-13:30 Open Forum #1 (Radiographers)**

### Research Presentation Session: Radiographers

### RPS 1414

### How radiography work practices and personal wellbeing have been impacted by COVID-19

**Moderators**
M. F. McEntee; Cork/IE
J.-F. Meder; Paris/FR
**Author Disclosures:**
M. F. McEntee: Author: Multiple Journals, Radiography, European Journal of Radiology, British Journal of Radiography; Employee: University College Cork; Grant Recipient: Society and College of Radiographers (UK), Health Research Board (IRE), Investigator: University College Cork; Speaker: ECR
J.-F. Meder: Advisory Board: Olea; Speaker: Siemens, GE, BMS

### RPS 1414-3

**Radiographers from the frontline: the COVID-19 pandemic's effect on professionals' burnout and occupational stress level**

*D. Sipos*[1], T. Jenei[1], O. L. Kovesdi[1], Á. Kovács[2], M. Petone Csima[1]; [1]Kaposvár/HU, [2]Debrecen/HU
*(cpt.david.sipos@gmail.com)*

**Purpose:** While acquiring medical images, radiographers get in close contact with patients, therefore they are exposed to possible infection by COVID-19.
**Methods or Background:** Cross-sectional, descriptive study was carried out by purposeful, non-random sampling. We used the email addresses of nearly 3,500 radiographers registered at the Society of Hungarian Radiographers. Effort-Reward Imbalance, Maslach Burnout Inventory questionnaires with our self-designed questionnaire were available from January 2021 to March 2021. Descriptive statistics, two-sample t-test, ANOVA test, Mann-Whitney and Kruskal-Wallis test were performed at 95% probability level.
**Results or Findings:** 439 responses were included in the statistical analysis, mean age 42.01 (SD=10.90) years. Male respondent (t=2.42; p≤0.05) between 20-29 and 30-39 years (F=2.81; p≤0.05) working in the healthcare system for 1-9 and 10-19 years (F=8.67; p≤0.05), who were in quarantine during COVID-19 pandemic had significantly higher stress values. Male respondents (t=6.86; p≤0.05; t=5.23; p≤0.05), radiographers between 20-29; 30-39 years of age (F=17.19; p≤0.05; F=8.30; p≤0.05), working in the healthcare system for 1-9; 10-19 years (F=18.30; p≤0.05; F=6.58; p≤0.05), who have been examining confirmed COVID-19 infected (t=3.93; p≤0.05; t=2.08; p≤0.05) or COVID-19 suspected patient (t=2.02; p≤0.05; t=5.62 p≤0.05) and also worked at the emergency departments (t=4.48; p≤0.05; t=4.77; p≤0.05) tend to be more attached by depersonalisation and emotional exhaustion. Being in quarantine raised emotional exhaustion (t=2.11; p≤0.05) and lowered personal achievement values significantly (t=-3.79; p≤0.05).
**Conclusion:** Male radiographers below 40 years of age working in the healthcare system less than 20 years at the emergency departments are exposed to depersonalisation and emotional exhaustion. Examinations of COVID-19 confirmed and suspected patients and quarantine obligations also negatively affected emotional exhaustion level.
**Limitations:** Radiographers who did not complete our questionnaire due to their workload.
**Ethics committee approval:** Medical Research Council has approved our study (IV/672-1/2021/EKU).
**Funding for this study:** The study received no fundings.
**Author Disclosures:**
Dávid Sipos: Nothing to disclose
Árpád Kovács: Nothing to disclose
Melinda Petone Csima: Nothing to disclose
Timea Jenei: Nothing to disclose
Orsolya Liza Kovesdi: Nothing to disclose

### RPS 1414-4

**UK obstetric sonographers' experiences of the Covid-19 pandemic: burnout, role satisfaction and impact on clinical practice**

*E. Skelton*, G. Harrison, M. Rutherford, S. Ayers, C. Malamateniou; London/UK
*(emily.skelton@city.ac.uk)*

**Purpose:** To explore obstetric sonographers' experiences of performing pregnancy ultrasound scans during the pandemic, and to assess the impact on burnout, role satisfaction and clinical practice.
**Methods or Background:** An online, anonymous cross-sectional survey of a convenience sample of obstetric sonographers (n=89, 96% female, 49% in full-time NHS employment) was completed using the Oldenburg Burnout Inventory (OLBI) to evaluate burnout, CORE-10 to measure psychological distress, with additional questions to capture sonographers' experiences. Parametric statistical analyses were performed using SPSS.
**Results or Findings:** Of those who completed the OLBI, 92% and 91% met the burnout thresholds for exhaustion and disengagement, respectively. Sonographers with higher total OLBI scores perceived that Covid-19 had a greater, negative impact on their practice (p<0.05). The mean CORE-10 score of 14.49 (SD7.99) suggests mild psychological distress of respondents. A significant decrease in role satisfaction was reported from before to during the pandemic (p<0.001), associated with higher OLBI and CORE-10 scores (p<0.001). Change in role satisfaction was significantly correlated with sonographers' perception of safety whilst scanning during the pandemic (R2=0.148, p<0.001). Sixty-five sonographers (73%) responded positively to considering leaving the profession, changing their area of practice or working hours within 5 years.
**Conclusion:** Job-specific interventions are required to mitigate sonographer burnout and its consequences on workforce shortages and service provision beyond the Covid-19 pandemic.
**Limitations:** Participants self-selected and self-reported which may skew the results. Conclusions of causality cannot be drawn because of the cross-sectional design. Results cannot be widely generalised because of

Friday convenience sampling and due to the homogeneity of participant characteristics.
**Ethics committee approval:** This study was approved by the School of Health Sciences Research Ethics Committee, City University of London (ref: ETH2021-1240).
**Funding for this study:** Funding was received from the College of Radiographer's Doctoral Fellowship Award (DF017) School of Health Sciences, City University of London.
**Author Disclosures:**
Mary Rutherford: Nothing to disclose
Emily Skelton: Nothing to disclose
Gill Harrison: Nothing to disclose
Christina Malamateniou: Nothing to disclose
Susan Ayers: Nothing to disclose

### RPS 1414-5

**The gendered impact of the Covid-19 pandemic on medical imaging and radiation therapy academics**

*K. O'Donoghue*[1], N. Moore[1], L. A. Walton[2], A. England[3], C. Malamateniou[2], M. F. F. McEntee[1]; [1]Cork/IE, [2]London/UK, [3]Keele/UK
*(od.katie@hotmail.com)*

**Purpose:** Medical imaging and radiation therapy (MIRT) are female-dominated professions. During the Covid-19 pandemic, MIRT academics continued to teach academically and clinically while leading research. Whilst systematic reviews evaluated the impact of the pandemic on healthcare workers, the gendered effects on MIRT academics is unknown. This work aims to determine the gendered impact of the pandemic on the physical and mental health of MIRT academics and researchers.
**Methods or Background:** A mixed-methods electronic survey was designed on Qualtrics in English and distributed via email and online platforms to radiography academics and researchers around the world. The survey was divided into four sections: demographics, the CORE10 questionnaire for evaluating mental health, and questionnaires for physical health and general well-being assessment. The survey was open between March and May 2021. Quantitative analysis was carried out using SPSS.
**Results or Findings:** The survey reached 32 countries and 412 participants; 24.5% were male (n=97) and 76.5% were female (n=315). Females reported worse sleep quality than males and a much lower preference for remote work. A higher percentage of males (73% versus 40.5% of females) reported reduced outdoors activities. The CORE10-questionnaire found that 10.3% of males and 2.7% of females experienced severe psychological distress the week immediately before the survey was conducted.
**Conclusion:** The study has identified gender differences in the impact of Covid-19 on the mental and physical health of MIRT academics. However, males and females have experienced notable deterioration in health and well-being due to the pandemic.
**Limitations:** Due to social distancing and lockdowns, it was not possible to carry out face-to-face focus groups or interviews, which may have been beneficial for the study.
**Ethics committee approval:** Research was approved by University College Cork (CT-SREC-2020-35).
**Funding for this study:** This research was funded by the College of Radiographers Industrial Partnerships Scheme (Ref: 196).
**Author Disclosures:**
Mark F. F. McEntee: Author: University College Cork
Niamh Moore: Author: University College Cork
Katie O'Donoghue: Author: University College Cork
Lucy A Walton: Author: University College Cork
Andrew England: Author: University College Cork
Christina Malamateniou: Author: University College Cork

### RPS 1414-6

**Impact of the COVID-19 pandemic on the radiology department of a university hospital in Northern Italy**

*A. Roletto*[1], M. Zanardo[1], A. Cozzi[1], S. Schiaffino[2], S. Tritella[2], F. Susini[2], F. Gerra[2], F. Sardanelli[1]; [1]Milan/IT, [2]San Donato Milanese/IT

**Purpose:** To evaluate the impact of the first two waves of the COVID-19 pandemic on the radiology department of a university hospital in Northern Italy.
**Methods or Background:** The numbers of all radiological exams performed at the radiology department of a university hospital from March 2019 to March 2021 were collected and compared, subdividing them according to time periods, imaging modality, and setting.
**Results or Findings:** Comparing the first 12 months of the COVID-19 pandemic (March 2020 to February 2021) with the previous 12 months (March 2019 to February 2020), there was an overall 26% decrease in examinations (from 127,998 to 94,550). The most affected modality was dual energy x-ray absorptiometry (from 4,706 to 2,989, -36%), followed by ultrasonography (from 17,212 to 11,644, -32%) digital radiography (from 66,050 to 47,374, -28%), and magnetic resonance imaging (from 13,332 to 10,140, -24%). Volume surges were observed for standard chest x-ray (CXR) in outpatients (from 3,032 to 7,536, +131%), chest CT in inpatients (from 1,087 to 1,144, +15%), and bedside CXR examinations (from 11,317 to 11,823, +4%). Further subanalysis according to pandemic waves highlighted an overall 65% decrease of radiological examination during the first wave (March to May 2020), curtailed to only -3% during the June-October 2020 period, then reaching -23% during the second wave (November 2020 to February 2021).
**Conclusion:** The COVID-19 pandemic led to a marked decrease in radiological examinations during the first two pandemic waves, limited to -26% by a relatively increased activity during the inter-wave period and by the implementation of safety protocols during the second wave.
**Limitations:** Single-centre analysis.
**Ethics committee approval:** Not applicable.
**Funding for this study:** No funding was received.
**Author Disclosures:**
Andrea Cozzi: Nothing to disclose
Francesco Sardanelli: Research/Grant Support: Bracco Research/Grant Support: Bayer Healthcare Speaker: General Electric Healthcare Advisory Board: Bracco Speaker: Bayer Healthcare Speaker: Bracco Research/Grant Support: General Electric Healthcare
Andrea Roletto: Nothing to disclose
Simone Schiaffino: Other: Bracco Imaging Speaker: General Electric Healthcare
Francesco Gerra: Nothing to disclose
Federica Susini: Nothing to disclose
Moreno Zanardo: Nothing to disclose
Stefania Tritella: Nothing to disclose

### RPS 1414-7

**The experiences of diagnostic radiographers through the Covid-19 pandemic**

S. M. Naylor[1], *R. M. Strudwick*[2], J. Harvey-Lloyd[2], S. Booth[3]; [1]Derby/UK, [2]Ipswich/UK, [3]Salford/UK
*(r.strudwick@uos.ac.uk)*

**Purpose:** Diagnostic radiography plays a major role in the diagnosis and management of patients with Covid-19. This has seen an increase in the demand for imaging services. Diagnostic radiographers have been on the frontline, dealing with an unprecedented situation. The aim of this study was to explore the experiences of diagnostic radiographers working during the pandemic.
**Methods or Background:** This study used an interpretative phenomenological approach to explore the experiences of diagnostic radiographers using virtual focus groups as a method of data collection.
**Results or Findings:** Data was analysed independently by four researchers and five themes emerged from the data. Adapting to new ways of working, feelings and emotions, support mechanisms, self-protection and resilience, and professional recognition.
**Conclusion:** The adaptability of radiographers came across strongly in this study. Anxieties attributed to the provision of PPE, fear of contracting the virus and spreading it to family members were evident. The resilience of radiographers working throughout this pandemic came across strongly throughout this study. A significant factor for coping has been peer support from colleagues within the workplace. The study highlighted the lack of understanding of the role of the radiographer and how the profession is perceived by other health care professionals.
**Limitations:** The qualitative nature of this study limits the generalisability of the findings.
**Ethics committee approval:** Approval for the project was obtained from the Universities of Derby, Suffolk and Salford.
**Funding for this study:** This study was funded by the College of radiographers Industrial Partnership Scheme.
**Author Disclosures:**
Sarah Booth: Nothing to disclose
Sarah Marion Naylor: Nothing to disclose
Ruth Mary Strudwick: Nothing to disclose
Jane Harvey-Lloyd: Nothing to disclose

### RPS 1414-8

**Management and reorganisation of diagnostic imaging departments during the COVID-19 pandemic: an Italian national survey**

*A. Roletto*[1], D. Catania[1], C. Ciaralli[2], A. Cozzi[1], D. Di Feo[3], S. Durante[4], D. Pasini[2], N. Raiano[5], M. Zanardo[1]; [1]Milan/IT, [2]Rome/IT, [3]Firenze/IT, [4]Bologna/IT, [5]Naples/IT

**Purpose:** To evaluate the impact of the COVID-19 pandemic on the management of diagnostic imaging departments in Italy.
**Methods or Background:** An online survey (32 questions) was developed and promoted by the Italian Federation of Scientific Radiographers Societies (FASTeR) and sent to all 39 Italian Radiology Service Managers (RSM) identified in the RSM Committee of the Italian Federation of Radiographers and Health Professionals (FNO TSRM & PSTRP). The survey investigated RSM demographics, the number of radiographers, imaging modalities and subspecialties managed, the effects of the pandemic on the diagnostic imaging workflow (including the number of radiographers who tested positive for SARS-CoV-2), and any subsequent reorganisation of their services (including the timeframe of partial/total suspensions of activities).
**Results or Findings:** Twenty (52%) RSM from different Italian regions completed the questionnaire. Overall, 70% of respondents reported that more than 50% of radiographers of their service had at least a positive SARS-CoV-2 test during the investigated period. 70% of respondents had implemented service reorganisations in terms of space, equipment, and pathways dedicated to COVID-19-infected patients. Changes in exam scheduling and tailored extensions of the allocated time-per-exam were reported by 90% of respondents. All respondents reported a suspension of activity in at least one imaging modality or sub-specialty, dual-energy x-ray absorptiometry (55%) and breast imaging modalities (50%) were the most impacted areas.
**Conclusion:** Among the 20 diagnostic imaging services across Italy whose RSMs answered this survey, 70% had high rates of radiographers testing positive for SARS-CoV-2 and underwent a workflow reorganisation due to the COVID-19 pandemic, 90% reporting changes in exam schedules to allow room and equipment disinfection.
**Limitations:** Limited sample of RSMs; potential bias towards highly-impacted regions.
**Ethics committee approval:** Not applicable.
**Funding for this study:** No funding was received.
**Author Disclosures:**
Andrea Cozzi: Nothing to disclose
Diego Catania: Nothing to disclose
Nicola Raiano: Nothing to disclose
Stefano Durante: Nothing to disclose
Andrea Roletto: Nothing to disclose
Danilo Pasini: Nothing to disclose
Moreno Zanardo: Nothing to disclose
Carmine Ciaralli: Nothing to disclose
Daniele Di Feo: Nothing to disclose

### RPS 1414-9

**Organisational and occupational commitment among Australian radiographers journeying through COVID-19**

*C. Makanjee*, M. O'Connor, A. Alumtairi, M. Lemon, Y. Amin; Bruce/AU
*(chandra.makanjee@canberra.edu.au)*

**Purpose:** Purpose of this exploratory study was to establish organisational and occupational commitment as radiographers journeyed through the first COVID-19 wave.
**Methods or Background:** Qualitative research approach with purposefully recruiting 20 participants to acquire insights into experiences of organisational and occupational commitment through in-depth interviews.
**Results or Findings:** Radiographers were committed throughout despite the constraints faced like the associated risks. Appraisal in terms of leadership and management support. Some of the unique findings were the cross institutional resource management in order sustain finance and services. Collective decision making processes and interactions and participative management leadership. Junior staff acquiring skills and opportunities to provide leadership in terms of developing protocols. Lastly, the experiences varied from state to sate.
**Conclusion:** One of the very few studies which focused on aspects of commitment in terms of both organisation and occupation in a single study.
**Limitations:** Due to the qualitive nature cannot be generalised. However, contribute to other similar studies conducted adding to scientific knowledge contribution. Recruiting participants was challenging as there was a similar study underway at the same institution at a national level of which the researchers were not aware of.
**Ethics committee approval:** Ethics approval was obtained from the University of Canberra Human Research Ethics Committee- 4763.
**Funding for this study:** No funding was received.
**Author Disclosures:**
Yousuf Amin: Nothing to disclose
Matthew Lemon: Nothing to disclose
Chandra Makanjee: Nothing to disclose
Abdullah Alumtairi: Nothing to disclose
Mathew O'Connor: Nothing to disclose

**12:30-13:30** **Room Z**

### Research Presentation Session: Cardiac

### RPS 1403

### 4D flow in cardiac MR

**Moderator**
S. Pradella; Florence/IT

### RPS 1403-2

**Background phase correction in MR compressed sensing 4D flow**

*G. Reiter*[1], C. Reiter[1], C. Kräuter[1], N. Jin[2], D. Giese[3], M. Fuchsjäger[1], U. Reiter[1]; [1]Graz/AT, [2]Chicago, IL/US, [3]Erlangen/DE
*(gert.reiter@siemens-healthineers.com)*

**Purpose:** Background phase correction is considered to be an important preprocessing step in cardiac magnetic resonance four-dimensional (4D) flow imaging improving the precision of flow volume measurements and "mass conservation" comparisons. The purpose of the present study was to investigate if background phase correction in compressed sensing (CS) 4D flow imaging has similar impact on derived aortic and pulmonary flow volumes as in conventional 4D flow imaging.
**Methods or Background:** 27 prospectively recruited cardiac patients without known or suspected shunts underwent 3T (Magnetom Skyra, Siemens Healthcare), ECG- and navigator-gated whole-heart 4D flow imaging with and without CS employing a prototype sequence, retrospectively. Repetition/echo times as well as resolution were matched in the 4D flow protocols. For both data sets, aortic (Qa) and pulmonary (Qp) net flow volumes with and without stationary-tissue-mask-based background phase correction were evaluated using prototype software (4D Flow, Siemens Healthcare). Relationships between results were investigated by correlation analysis; means were compared employing t-test.
**Results or Findings:** Background phase correction improved the correlation between Qa and Qp from r=0.96 to r=0.98 for conventional and from r=0.95 to r=0.97 for CS 4D flow. All flow volumes decreased applying background phase correction (Qa: 77±22 ml vs. 72±20 ml, Qp: 81±23 ml vs 73±20 ml, for conventional 4D flow; Qa: 76±22 ml vs 73±18 ml, Qp: 78±22 ml vs 72±18 ml, for CS 4D flow; p<0.0001 in all cases). Aortic and pulmonary non-corrected-to-corrected flow volume differences correlated strongly between conventional and CS 4D flow imaging (r=0.94 in both cases).
**Conclusion:** Background phase correction behaves similarly in conventional and CS 4D flow imaging. Therefore, its application can be recommended also for CS 4D flow imaging.
**Limitations:** Flow volumes were only checked by mass conservation.
**Ethics committee approval:** Approval was obtained.
**Funding for this study:** Funding was received from OeNB-Anniversary-Fund No.17934, ESR Seed Grant 2020.
**Author Disclosures:**
Daniel Giese: Employee: Siemens Healthineers
Ning Jin: Employee: Siemens Healthineers
Ursula Reiter: Nothing to disclose
Gert Reiter: Employee: Siemens Healthineers
Michael Fuchsjäger: Nothing to disclose
Clemens Reiter: Nothing to disclose
Corina Kräuter: Nothing to disclose

### RPS 1403-3

**Automated estimation of mean pulmonary arterial pressure from 4D-flow MRI**

*C. Kräuter*, U. Reiter, G. Kovacs, C. Reiter, M. Masana, H. Olschewski, M. Fuchsjäger, R. Stollberger, G. Reiter; Graz/AT
*(corina.kraeuter@medunigraz.at)*

**Purpose:** Pulmonary hypertension (PH) is characterised by an elevated mean pulmonary arterial pressure (mPAP). There is a piecewise linear relationship between mPAP and the duration of vortical blood flow along the main pulmonary artery as visualised by magnetic resonance four-dimensional phase-contrast imaging (4D-flow MRI). The aim of this study was to investigate the accuracy of an automated PH-related vortex detection method to non-invasively diagnose PH and estimate elevated mPAP.
**Methods or Background:** 32 subjects with known or suspected PH (male/female 7/25; age 62±15 years) and regular heart rhythm underwent right heart catheterisation (RHC) and 4D-flow MRI of the main pulmonary artery at 3T. Pre-processing of velocity data was performed by prototype software (4DFlow, Siemens Healthcare). Automated detection and tracking of the PH-related vortex and mPAP estimation were performed by in-house software. The relationship between estimated mPAP and mPAP measured by RHC was analysed by correlation and Bland-Altman analysis. The diagnostic

Friday performance of automated mPAP estimation was investigated by receiver operating characteristic curve analysis.
**Results or Findings:** PH was diagnosed by RHC in 19 subjects. The area under the curve for PH diagnosis from automated mPAP estimation was 1.00 [0.89,1.00]. The cut-off mPAP=20 mmHg resulted in a sensitivity of 1.00 [0.82,1.00] and a specificity of 0.92 [0.64,1.00]. For all subjects with PH, automatically estimated mPAP and mPAP measured by RHC correlated strongly (r=0.94); they yielded no bias (p=0.79), and the standard deviation of differences between them was small (5 mmHg).
**Conclusion:** Automated PH-related vortex detection from 4D-flow MRI allows accurate diagnosis of PH and estimation of elevated mPAP.
**Limitations:** Small subject number; no arrhythmic patients.
**Ethics committee approval:** This study was approved by the Medical University Graz (25-044 ex 12/13).
**Funding for this study:** Funding was received from OeNB-Anniversary-Fund No.17934, DES Silicon Austria Labs.
**Author Disclosures:**
Horst Olschewski: Nothing to disclose
Ursula Reiter: Nothing to disclose
Marc Masana: Nothing to disclose
Gert Reiter: Employee: Siemens Healthcare Diagnostics GmbH, Graz, Austria
Michael Fuchsjäger: Nothing to disclose
Clemens Reiter: Nothing to disclose
Rudolf Stollberger: Nothing to disclose
Gabor Kovacs: Nothing to disclose
Corina Kräuter: Nothing to disclose

### RPS 1403-4

**Scan-rescan repeatability of MR compressed sensing 4D flow assessment of aortic and pulmonary flow volumes**

*U. Reiter*[1], C. Reiter[1], C. Kräuter[1], N. Jin[2], D. Giese[3], M. Fuchsjäger[1], G. Reiter[1]; [1]Graz/AT, [2]Chicago, IL/US, [3]Erlangen/DE
*(ursula.reiter@medunigraz.at)*

**Purpose:** Compressed sensing (CS) allows substantial acceleration of cardiac magnetic resonance four-dimensional (4D) flow imaging without significant impact on the precision of derived aortic and pulmonary flow volumes. The purpose of the present study was to investigate the scan-rescan repeatability of these measurements derived from 4D flow imaging with and without CS.
**Methods or Background:** 23 prospectively recruited cardiac patients without known or suspected shunts and regular heart rhythm underwent two consecutive 3T (Magnetom Skyra, Siemens Healthcare) retrospectively ECG- and navigator-gated whole-heart 4D flow imaging scans with a prototype sequence. For 15 subjects a CS 4D flow protocol (acceleration-factor=7.6) was employed twice, 8 subjects twice underwent a protocol without CS (parallel acquisition factor 3). Background phase corrected aortic (Qa) and pulmonary (Qp) net flow volumes were evaluated using prototype software (4DFlow, Siemens Healthcare). Relationships between the first and the second acquisition were investigated by correlation and Bland-Altman analysis. Scan-rescan repeatability was quantified by coefficients of variations (CVs).
**Results or Findings:** For all subjects net flow volumes from first and second scans correlated strongly (r=0.96 for Qa and Qp) and did not demonstrate a significant bias. Differences in cardiac intervals between scans were strongly correlated with scan-rescan differences of Qa (r=0.72) and of Qp (r=0.78). CVs in CS 4D flow acquisitions (Qa: 6%, Qp: 5%) were slightly smaller than CVs in 4D flow acquisition without CS (Qa: 7%, Qp: 7%).
**Conclusion:** Assessment of aortic and pulmonary flow volumes by 4D flow imaging with and without CS is repeatable, especially when the heart rate remains constant.
**Limitations:** Small sample size was identified as a limiting factor.
**Ethics committee approval:** This study was approved by an ethics committee.
**Funding for this study:** This study has received funding from the OeNB-Anniversary-Fund No.17934 as well as via the ESR Seed Grant 2020.
**Author Disclosures:**
Daniel Giese: Employee: Siemens Healthineers
Ning Jin: Employee: Siemens Healthineers
Ursula Reiter: Nothing to disclose
Gert Reiter: Employee: Siemens Healthineers
Michael Fuchsjäger: Nothing to disclose
Clemens Reiter: Nothing to disclose
Corina Kräuter: Nothing to disclose

### RPS 1403-5

**Compressed sensing 4D flow assessment of left ventricular flow components**

C. Reiter[1], *G. Reiter*[1], C. Kräuter[1], N. Jin[2], D. Giese[3], M. Fuchsjäger[1], U. Reiter[1]; [1]Graz/AT, [2]Chicago, IL/US, [3]Erlangen/DE
*(gert.reiter@siemens-healthineers.com)*

**Purpose:** Cardiac magnetic resonance four-dimensional (4D) flow imaging is a potential technique for derivation of new biomarkers of left ventricular (LV) function which could substantially benefit from reduction of scan times. The purpose of the present study was to investigate if acceleration of 4D flow imaging by compressed sensing (CS) can be employed to derive LV flow components.
**Methods or Background:** 20 prospectively recruited cardiac patients with regular heart rhythm underwent two consecutive 3T (Magnetom Skyra, Siemens Healthcare) retrospectively ECG- and navigator-gated whole-heart 4D flow imaging scans with prototype sequence. For 5 subjects a 4D flow protocol (parallel acquisition factor 3) was employed twice, for 15 subjects whole-heart 4D flow and a matched CS protocol (acceleration-factor=7.6) was used. LV direct, retained inflow, delayed ejection, and residual volume flow components were evaluated for all data sets using dedicated software (cvi42, Circle CVI). Relationships of the LV flow components from paired measurements with and without CS were investigated by means of correlation and Bland-Altman analysis; repeatability of LV flow component measurements was determined from paired 4D flow measurements without CS.
**Results or Findings:** Scan times were significantly shorter employing CS (301±86s vs 535±158s, p<0.01). LV flow components from CS measurements (direct=29±7%; retained=29±6%; delayed=26±6%; residual=16±4%) exhibited no significant bias compared to respective results without CS (direct=29±7%, p=0.87; retained=29±6%; p=0.97; delayed=26±5%, p=0.39; residual=17±4%, p=0.52) and correlated strongly (r=0.75-0.94). Standard deviations of differences between LV flow components from measurements with and without CS (direct=2%; retained=3%; delayed=3%; residual=3%) were similar to the repeatability of non-CS- derived LV flow component measurements (direct=3%; retained=2%; delayed=3%; residual=3%).
**Conclusion:** Compressed sensing allows acceleration of 4D flow imaging without significant impact on the evaluation of LV flow components.
**Limitations:** Small sample size.
**Ethics committee approval:** Ethics committee approval was obtained.
**Funding for this study:** Funding was received from OeNB-Anniversary-Fund No.17934, ESR Seed Grant 2020.
**Author Disclosures:**
Daniel Giese: Employee: Siemens Healthineers
Ning Jin: Employee: Siemens Healthineers
Ursula Reiter: Nothing to disclose
Gert Reiter: Employee: Siemens Healthineers
Michael Fuchsjäger: Nothing to disclose
Clemens Reiter: Nothing to disclose
Corina Kräuter: Nothing to disclose

### RPS 1403-6

**Comprehensive assessment of haemodynamics before and after aortic valve replacement in an ex-vivo swine model with 4D flow MRI**

*M. F. Balks*[1], H. Saisho[1], B. Fujita[1], T. Schaller[1], N. Sadat[1], E. Stephan[1], J. Barkhausen[1], A. Frydrychowicz[1], T. H. Oechtering[2]; [1]Lübeck/DE, [2]Madison, AL/US

**Purpose:** Secondary flow patterns deviating from main flow have been described after aortic valve replacement (AVR). The impact of the surgical procedure, i.e. aortotomy, on postoperative haemodynamics as compared to the impact of the valve itself remains unclear. Therefore, we introduce an ex-vivo model for comprehensive evaluation of haemodynamics after different types of AVR.
**Methods or Background:** 6 fresh swine aortas were anastomosed to an in-house developed piston pump pumping blood-mimicking fluid at 3l/min and 60bpm. 4D Flow MRI was acquired at 3T prior to surgery (PreSur, n=6), after sham surgery, i.e. aortotomy without valve replacement (ShSur, n=6), and after AVR: 2 mechanical valves (MecV; Standard Masters, St.Jude Medical, USA), 2 biological valves (BioV; Perimount MagnaEase, Edwards Lifesciences, USA), and 2 Ozaki aortic valve neocuspidization procedures (AVneo). Peak velocity was analysed with GTFlow (GyroTools, Switzerland). Secondary flow patterns deviating from main flow were assessed visually in comparison to PreSur.
**Results or Findings:** Normal systolic haemodynamics without secondary flow patterns could be observed before surgery. Secondary flow patterns developed after sham surgery in the ascending aorta. They were notably more pronounced after valve replacement. Most extensive secondary flow patterns were induced by BioV. They were associated with increased peak velocity in the ascending aorta compared to PreSur and ShSur (PreSur=89.6±59cm/s, ShSur=57.6±17.9cm/s, MecV=69.2±27.6cm/s, BioV=110.9±27.1cm/s, AVneo=69.8±5.3cm/s).

Friday

**Conclusion:** With this ex-vivo model, we were able to evaluate the effect of AVR and surgical pathway on aortic haemodynamics. Postoperative flow changes can be attributed not only to the implanted valve but also to the aortotomy. In future studies, we will determine the influence of setup-related valve insufficiency on systolic haemodynamics.
**Limitations:** The model did not allow analysis of diastolic flow patterns due to setup-related valve insufficiency.
**Ethics committee approval:** No ethics approval was required.
**Funding for this study:** No funding was received.
**Author Disclosures:**
Hiroyuki Saisho: Nothing to disclose
Maren Friederike Balks: Nothing to disclose
Najla Sadat: Nothing to disclose
Thekla Helene Oechtering: Nothing to disclose
Ensminger Stephan: Nothing to disclose
Tim Schaller: Nothing to disclose
Alex Frydrychowicz: Nothing to disclose
Jörg Barkhausen: Nothing to disclose
Buntaro Fujita: Nothing to disclose

### RPS 1403-7

**4D flow MRI for quantification of aortic regurgitation**
*F. Bouhajja*, O. Lozinguez, P. Garçon, Y-W. Kim, L. Kalifa, M. Zins, A. A. Azarine; Paris/FR
*(fraj.bouhajja@gmail.com)*

**Purpose:** To learn how to use four-dimensional (4D) flow MRI for the assessment of aortic valvular regurgitation (AR).
**Methods or Background:** Cardiac magnetic resonance imaging (CMR) may evaluate the morphologic and functional parameters of cardiac valves, identifying strucural abnormalities and characterising the severity of valvulopathy. The assessment of AR by echocardiography may be difficult sometimes and CMR may be a complementary technique in patients with inadequate echocardiographic image quality or discordant results. Several studies show that four-dimensional (4D) flow MRI and echocardiography presented good interobserver correlation, with good agreement in assessing AR.
**Results or Findings:** The 4D flow in the AR has the advantage to be acquired during free breathing with or without gadolinium injection. It has the ability to evaluate qualitatively any flow type (laminar, helical, vortical) in any direction and precise its eccentricity. Visualisation of the turbulent flows helps optimal positioning of the plan to perform flow measurements. It enables also comprehensive assessment of aortic flows. Quantitative evaluation is based on conventional parameters such as velocity, forward and backward flows rate, regurgitation fraction. As a quality check control the forward flow will be compared to the left ventricular stroke volume and the net flow to the pulmonary artery flow. Optimal choice of velocity encoding (Venc) and measurement pitfalls will be discussed. New markers such as wall shear stress and energy loss due to turbulent flow will be explained. Flow eccentricity and increased wall shear stress provide a better understanding of the development of aortic dilations. Indirect predictors of severe AR such as holodiastolic backward flow in the descending aorta will be discussed.
**Conclusion:** CMR using 4D flow MRI has a growing and promising role for grading AR. Few parameters and pitfalls are to consider for optimal assessment of AR in the routine clinical practice.
**Limitations:** No limitations were identified.
**Ethics committee approval:** Not applicable.
**Funding for this study:** Not applicable.
**Author Disclosures:**
Philippe Garçon: Nothing to disclose
Marc Zins: Nothing to disclose
Olivier Lozinguez: Nothing to disclose
Young-Wouk Kim: Nothing to disclose
Arshid A. Azarine: Nothing to disclose
Laurette Kalifa: Nothing to disclose
Fraj Bouhajja: Nothing to disclose

### RPS 1403-8

**Left atrial volume evaluation from MR 4D-flow data**
C. Reiter, C. Kräuter, G. Reiter, A. Schmidt, D. Scherr, M. Fuchsjäger, *U. Reiter*; Graz/AT
*(ursula.reiter@medunigraz.at)*

**Purpose:** The evaluation of left ventricular diastolic function comprises the assessment of blood and tissue velocities as well as the maximal left atrial (LA) volume. Although magnetic resonance (MR) four-dimensional phase-contrast (4D-flow) imaging allows accurate determination of these velocities, the derivation of the LA volume directly from the same 4D-flow data remains unknown. The aim of this study was to compare maximal LA volumes derived from 4D-flow data and two-dimensional cine imaging.
**Methods or Background:** 58 subjects without signs of heart failure (male/female, 34/24; age, 75±14 years) underwent MR whole-heart 4D-flow imaging and two-dimensional balanced steady-state-free-precession (bSSFP) cine imaging in 2- and 4-chamber views under breathing. End-systolic LA areas and lengths were determined from multiplanar reformatted 2- and 4-chamber 4D-flow magnitude as well as bSSFP cine images. Maximal (end-systolic) LA volume and LA volume index (LAVI) were calculated employing the area-length method. 4D-flow and cine results were compared by correlation analysis and paired t-test.
**Results or Findings:** End-systolic LA areas (4-chamber: 4D-flow 24.6±4.0 cm2 vs cine 24.4±4.9 cm2, p=0.50; 2-chamber: 4D-flow 23.3±3.7 cm2 vs cine 23.3±3.8 cm2, p=0.96) and length measurements (4-chamber: 4D-flow 58±6 mm vs cine 58±7 mm, p=0.85; 2-chamber: 4D-flow 55±7 mm vs cine 55±6 mm, p=0.83) demonstrated no significant differences. Derived maximal LA volumes (4D-flow 85±22 ml vs cine 84±21 ml, p=0.17) and LAVIs (4D-flow 45.6±11.7 ml vs cine 44.5±9.8 ml, p=0.11) exhibited no significant bias and correlated strongly (r=0.95 and 0.89, respectively).
**Conclusion:** Maximal LA volumes and LAVIs can be directly evaluated from 4D-flow data without the need of additional acquisition of bSSFP cine series.
**Limitations:** Few subjects with enlarged atria; only area-length method was investigated.
**Ethics committee approval:** Approval was obtained (Medical University of Graz, 24-126 ex 11/12).
**Funding for this study:** Funding was received from OeNB-Anniversary-Fund No.17934, Foerderstipendium MedUni Graz.
**Author Disclosures:**
Ursula Reiter: Nothing to disclose
Gert Reiter: Employee: Employee of Siemens Healthcare Diagnostics GmbH
Michael Fuchsjäger: Nothing to disclose
Daniel Scherr: Nothing to disclose
Clemens Reiter: Nothing to disclose
Albrecht Schmidt: Nothing to disclose
Corina Kräuter: Nothing to disclose

**14:00-15:00 Open Forum #1 (Radiographers)**

### Research Presentation Session: Radiographers

### RPS 1514

### The impact of software and technology advances for radiographic practice

**Moderators**
J. Potočnik; Dublin/IE
L. Donoso; Barcelona/ES

### RPS 1514-3

**Evaluation of European based radiographers' knowledge and attitude towards implementation of Artificial Intelligence in radiography**
*S. Coakley*, M. McEntee, N. Moore, R. Young; Cork/IE
*(s.coakley@hotmail.com)*

**Purpose:** The purpose of this work was to investigate the knowledge, attitudes, perceptions, and expectations of a sample of radiographers towards the integration of Artificial Intelligence (AI) into medical imaging and determine the current state of AI education within the European radiography community.
**Methods or Background:** An online survey targeting radiographers based in Europe was conducted over a ten-week period. Captured data included demographic information, data relating to participant's perceptions and understanding of AI, and AI-related educational backgrounds. Both descriptive and inferential statistical techniques were used to analyse the obtained data.
**Results or Findings:** A total of 96 valid responses were collected. Of these, 64% correctly identified the correct definition of AI, but fewer (37%) fully understood the difference between AI, machine-learning and deep-learning. The majority of participants (83%) were excited about the advancement of AI, yet, despite this, a level of apprehension remained among 29%. A severe lack of education on AI was apparent, with only 8% of participants having received AI teachings in their primary qualification.
**Conclusion:** A positive attitude towards AI implementation into the radiographic role was noted, though concern remains amongst a minority of radiographers. Hesitation may stem from the lack of technical understanding of AI technologies and the absence of AI-training within the community. Enhancements to educational programmes are required, focusing on AI principles to help boost European radiography workforce engagement and involvement in AI technologies.

**Limitations:** A risk of over-sampling of academic radiographers and an under-sampling of clinically-based radiographers may have occurred due to limited access to the ECR, which acted as a primary source for survey distribution.
**Ethics committee approval:** The Clinical Therapies Social Research and Ethical Committee of University College Cork approved the study (CT-SREC-2020-37).
**Funding for this study:** No funding was received for this study.
**Author Disclosures:**
Sarah Coakley: Nothing to disclose
Niamh Moore: Nothing to disclose
Rena Young: Nothing to disclose
Mark McEntee: Nothing to disclose

### RPS 1514-4

**Artificial Intelligence: Society of Radiographers UK professional guidance**

*T. J. T. O'Regan*[1], Y. McQuinlan[1], A. England[2], N. H. Woznitza[1], S. Goldsworthy[3], C. Currie[4], E. Skelton[1], K-Y. Chu[5], R. Tucker[6], J. Matthew[1], C. Kalinka[1], S. L. McFadden[7], C. Malamateniou[1]; [1]London/UK, [2]Keele/UK, [3]Taunton/UK, [4]Glasgow/UK, [5]Oxford/UK, [6]Derby/UK, [7]Belfast/UK

**Purpose:** To ensure safe, efficient, and effective clinical imaging services for the future, radiographers need to engage with Artificial Intelligence (AI). Work was undertaken to provide baseline guidance; to determine and summarise current recommendations and priorities for UK radiographers.
**Methods or Background:** The Society of Radiographers (SoR) AI working party undertook a six-month review of articles and resources related to diagnostic and therapeutic radiography and AI. Priorities and recommendations were formulated and presented for consultation with SoR member advisory groups, the SoR director of professional policy, and an external expert. Amendments were made and final consensus reached by the SoR working party.
**Results or Findings:** Guidance was produced and intended for use by multi-professional clinical imaging and therapeutic radiography teams. Recommendations were themed across 4 areas: 1. Educational provision is needed to build knowledge, skills, and competences for radiographers: to enable them to navigate a future where AI will be central to patient diagnosis and treatment pathways. 2. Radiography-led research in AI should address key clinical challenges and enable radiographers to co-design services. 3. The fostering of stakeholder partnerships is key to ensuring maximal contribution of radiographers, patients, and service users. 4. Clinical practitioners must be involved in the co-construction, development, implementation, validation, and audit of AI.
**Conclusion:** SoR guidance offers baseline recommendations. Priorities include a need to update educational curricula and research foci, and to forge strong clinical-academic-industry-patient partnerships.
**Limitations:** Radiographers should proactively develop AI-enabled healthcare to ensure gains of AI technologies are maximised while risks and challenges are minimised. The guidance will therefore need to be updated regularly, with repeated reviews, given the fast-changing pace of AI development and innovation.
**Ethics committee approval:** Not applicable
**Funding for this study:** No funding was received for this study.
**Author Disclosures:**
Yasmin McQuinlan: Employee: Mirada Medical Ltd Advisory Board: SoR AI Advisory Board
Richard Tucker: Nothing to disclose
Claire Currie: Nothing to disclose
Emily Skelton: Nothing to disclose
Chris Kalinka: Nothing to disclose
Sonyia Lorraine McFadden: Nothing to disclose
Nicholas Hans Woznitza: Nothing to disclose
Kwun-Ye Chu: Nothing to disclose
Andrew England: Nothing to disclose
Jacqueline Matthew: Nothing to disclose
Tracy Jane Tracy O'Regan: Nothing to disclose
Christina Malamateniou: Nothing to disclose
Simon Goldsworthy: Nothing to disclose

### RPS 1514-5

**AIRx: augmenting the teaching of radiography using artificial intelligence**

C. Chênes[1], M. Butt[2], S. Fazeli[1], B-M. Muanga[1], S. Mahamat[1], *J. Schmid*[1]; [1]Geneva/CH, [2]Lausanne/CH
*(jerome.schmid@hesge.ch)*

**Purpose:** Current teaching of radiography presents some inadequacies, explained by safety and ethical reasons. Students perform radiographs on inanimate objects, while they practice positioning on other students without acquiring X-ray images. Conversely, internships expose students to live situations, which, however, cannot be controlled from a pedagogic perspective. This study investigates the development of a simulator using artificial intelligence (AI) to detect human positioning and generate artificial yet realistic radiographs.
**Methods or Background:** Using a real but rayless radiography device, student radiographers position a subject playing the role of a patient. Our system leverages deep learning (DL) markerless approaches to detect human pose with calibrated video cameras. Using a collection of computed tomography volumes, ray casting techniques quickly produce artificial radiographs considering subject limb positioning and operator settings. Radiograph realism is enforced by a DL approach simulating polychromatic X-rays, image noise and scattering.
**Results or Findings:** Simulated radiographs for chest and pelvis were rated by experienced and student radiographers as sufficiently realistic to assess the impact of technical parameters (e.g., mAs) and human positioning (e.g. correct internal rotation of femurs). A survey on 53 students reported that the use of AI for teaching is welcomed (73%) and that the simulator could be valuable to boost skills and confidence before internships (81%).
**Conclusion:** AI opens up some new perspectives in the simulation of radiographic practice in controllable conditions (e.g. type of pathologies, implant presence), paving the way for new teaching approaches.
**Limitations:** Further development of the AIRx simulator is necessary to improve stability and user experience, as well as to support additional exams, such as shoulder radiographs, which were requested by surveyed students.
**Ethics committee approval:** Ethics committee approval was not needed.
**Funding for this study:** This research was funded by the Hasler Foundation (#19019).
**Author Disclosures:**
Christophe Chênes: Nothing to disclose
Shervin Fazeli: Nothing to disclose
Meryam Butt: Nothing to disclose
Jerome Schmid: Nothing to disclose
Bonga-Macaire Muanga: Nothing to disclose
Saleh Mahamat: Nothing to disclose

### RPS 1514-6

**Experiences of students and educators related to a postgraduate introductory module in artificial intelligence for radiographers**

*R. van de Venter*[1], E. Skelton[2], J. Matthew[2], G. Tarroni[2], S. P. Hirani[2], N. H. Woznitza[2], R. Malik[3], C. Malamateniou[2]; [1]Port Elizabeth/ZA, [2]London/UK, [3]Manchester/UK

**Purpose:** To evaluate how students and educators experienced a novel postgraduate introductory artificial intelligence module for radiographers.
**Methods or Background:** Artificial intelligence (AI) is increasingly being used in clinical imaging services. Different workforce surveys highlight AI training as vital for AI implementation, so radiographers can provide safe, effective and efficient care to patients. Despite radiography being one of the most technology-enabled professions, it has not yet proportionally invested in introducing AI training in radiography curricula. Using a participatory action research design, semi-structured interviews and a focus group, we evaluated the experiences of students (n=7) and lecturers (n=6) involved in the first running of an introductory module in AI for radiographers. Data was recorded using Microsoft Teams and analysed using a data-driven, inductive coding process to generate themes.
**Results or Findings:** Three themes were generated which revolved around the module delivery, content covered and amendments for future delivery. Participants were satisfied with the delivery and the content they had access to. But, participants have suggested amendments to the teaching and learning strategies, learning pathways and mode of delivery.
**Conclusion:** Developing educational provisions in AI for radiographers should be a priority. Module content should consider context-specific requirements for every cohort and delivery should allow flexibility to accommodate different learning styles.
**Limitations:** We recognise that these experiences are context-specific and may not necessarily be transferrable to other cultures or countries; it still remains an important evaluation of a “first-of-its-kind” programme. Testing these ideas on a bigger sample size when these provisions are available to a larger audience would be important for future research.
**Ethics committee approval:** This study was approved by the City, University of London School of Health Sciences Research Ethics Committee (REC Ref: ETH2021–0948).
**Funding for this study:** Global Challenges Research Funding (GCRF) was provided by Research England.
**Author Disclosures:**
Riaan van de Venter: Nothing to disclose
Rizwan Malik: Nothing to disclose
Emily Skelton: Nothing to disclose
Giacomo Tarroni: Nothing to disclose
Nicholas Hans Woznitza: Nothing to disclose
Jacqueline Matthew: Nothing to disclose
Christina Malamateniou: Nothing to disclose
Shashivadan P. Hirani: Nothing to disclose

### RPS 1514-7

**Radiological multimodality approach for preoperative planning in dynamic total hip arthroplasty**

*D. Polverari*, M. Zerunian, D. Caruso, G. Argento, A. Ferretti, A. Benvenga, A. Laghi; Rome/IT
*(daniele.polverari@gmail.com)*

**Purpose:** To assess the reliability of radiological multimodality preoperative study in the identification of the correct pelvic tilt necessary for a medical device for patient-specific (PSI) preoperative planning, intraoperative delivery and postoperative analysis in Total Hip Arthroplasty (THA).
**Methods or Background:** Eleven patients from January to May 2019 were prospectively included; they underwent a specific radiological preoperative assessment including x-ray and CT scan for the dynamic assessment of pelvic tilt. CT scan protocol included total hip bone to distal femur bone, knee joint and ankle joint bilaterally. All CT scans were performed with a 64--slice MDCT (Revolution-EVO, GE healthcare, WI, USA) including the same examination parameters: slice thickness 1.25mm, pitch 0.516, rotation -tube 1 sec. Reconstruction filter used was Bone Plus with W/L of 2500/500 HU. Four radiographic projections were performed including hip bone (antero-posterior e orthostatism projections) and Lumbar spine (latero-lateral projection), in different dynamic positions: standing, step -up and flexed -seated, to simulate dynamic position. All patients also performed a post-operative CT scan for the assessment of THA.
**Results or Findings:** According to the preoperative protocol, all the acquired images per patients (11/11) were suitable for personalised pelvic tilt assessment. Mean inclination preoperative planning does not differ significantly from postoperative imaging assessment (38°[44°-32°] vs 37.5°[51°-28°]) as well as from the mean absolute deviation for preoperative inclination and anteversion planning and post-operative measurement (all P>0.05). The mean absolute deviation from the planned patient--specific level of osteotomy (1.6 mm [-3/+4mm]), showed no significant differences between preoperative osteotomy planning and post-operative measurement (P>0.05) as well as mean deviation offset (3.3 mm[-6/+8mm], P>0.05).
**Conclusion:** Specific radiological assessment for PSI preoperative planning of pelvic offset resulted reproducible and accurate with surgical management.
**Limitations:** The small cohort of patients as well as the fact that there was no control group were recognized as limiting factors.
**Ethics committee approval:** The Institutional Review Board -approved this single-centre prospective study; patients signed written informed consent.
**Funding for this study:** No funding was received for this work.
**Author Disclosures:**
Damiano Caruso: Other: co-authors
Andrea Ferretti: Other: co-authors
Marta Zerunian: Other: co-authors
Daniele Polverari: Author: Author
Antonella Benvenga: Other: co-authors
Andrea Laghi: Other: co-authors
Giuseppe Argento: Other: co-authors

### RPS 1514-8

**3D-printed heart phantoms for automatic measurement of left ventricular volume**

*M. W. Kusk*; Esbjerg/DK
*(martin.weber.kusk@rsyd.dk)*

**Purpose:** To develop cheap and accurate 3D printed heart phantoms from clinical CT data, in sufficient detail to facilitate automated measurements of left ventricular volumes, based on anatomical landmarks. If succesful, such phantoms could be used to explore dose reduction potential in CT-based ejection fraction measurement.
**Methods or Background:** Two high-dose cardiac CT-series from the same patient (1 systolic and 1 diastolic) were obtained from PACS. Active contours technique was used in ITK-snap to segment myocardium, vessels and ventricles. STL-files were exported and 3D-printed using an FDM 3D-printer. Printing material was selected based on literature, to target enhancing myocardium HU values. Cavities were filled with a mix of gelatine and contrast media, targeting 400 HU at 120 kVp. The models were placed inside an anthropomorphic lung phantom and scanned at different kVP and mAs values, using synthetic ECG. Volume measurement was done using Syngo.via Cardiac Function (Siemens Healthineers).
**Results or Findings:** At all parameter combinations, the clinical software was able to automically detect the mitral and aortic valve planes and delineate the LV cavity. Hounsfield values of phantom myocardium corresponded to live enhancing myocardium. Measured phantom LV volumes in both systolic and diastolic phases differed less than 5% from the clinical source scans. Costs were 350 for the printer and under 40 Euro in materials per phantom.
**Conclusion:** Cardiac cavities and myocardium HU values can be accurately reproduced, using cheap 3D-printing technique, making phantom studies feasible.
**Limitations:** K-edge effects on enhancing myocardium could not be simulated. Right-sided hear chambers were not modelled. Movement artefact influence could not be quantified.
**Ethics committee approval:** Not applicable
**Funding for this study:** The study was supported by a research grant from the Danish Radiographers' Association ("Radiograf Rådet") and Karola Jørgensen Research fund at the hospital of Soutwest Jutland.
**Author Disclosures:**
Martin Weber Kusk: Nothing to disclose

**14:00-15:30** **Room E1**

### Research Presentation Session: Neuro

### RPS 1511

### Acute stroke

**Moderator**
S. Afat; Tübingen/DE

### RPS 1511-2

**Cost-effectiveness of endovascular thrombectomy in childhood stroke: an analysis of the Save ChildS Study**

W. G. G. Kunz, M. Wildgruber, S. Mönch, *D. M. Mehrens*; Munich/DE
*(mehrens.dirk@gmail.com)*

**Purpose:** The Save ChildS Study demonstrated that endovascular thrombectomy (EVT) is a safe treatment option for paediatric stroke patients with large vessel occlusions with high recanalisation rates. Our aim was to determine the long-term cost, health consequences and cost-effectiveness of EVT in this patient population.
**Methods or Background:** In this retrospective study, a decision-analytic Markov model estimated lifetime costs and quality-adjusted life years (QALY). Early outcome parameters were based on the entire Save ChildS Study to model the EVT group. As no randomised data exist, the Save ChildS patient subgroup with unsuccessful recanalisation was used to model the standard of care group. For modelling of lifetime estimates, paediatric and adult input parameters were obtained from the current literature. The analysis was conducted in a United States setting applying healthcare and societal perspectives. Probabilistic sensitivity analyses were performed. The willingness-to-pay (WTP) threshold was set to $100,000 per QALY.
**Results or Findings:** The model results yielded EVT as the dominant (cost-effective as well as cost-saving) strategy for paediatric stroke patients. The incremental effectiveness for the average age of 11.3 years at first stroke in the Save ChildS Study was determined as an additional 4.02 lifetime QALYs, with lifetime cost-savings that amounted to $169,982 from a healthcare perspective and $254,110 when applying a societal perspective. Acceptability rates for EVT were 96.60% and 96.66% for the healthcare and societal perspectives.
**Conclusion:** EVT for paediatric stroke patients with large vessel occlusions resulted in added QALY and reduced lifetime costs. Based on the available data in the Save ChildS Study, EVT is very likely to be a cost-effective treatment strategy for childhood stroke.
**Limitations:** The study design was retrospective with the inherent limitations of this type of design.
**Ethics committee approval:** IRB waived requirement for informed consent.
**Funding for this study:** No funding was received for this study.
**Author Disclosures:**
Dirk Mathias Mehrens: Nothing to disclose
Moritz Wildgruber: Nothing to disclose
Wolfgang Gerhard Gerhard Kunz: Nothing to disclose
Sebastian Mönch: Nothing to disclose

### RPS 1511-3

**Prevalence and clinical outcome of remnant occlusion at superior cerebellar artery after mechanical thrombectomy for basilar artery occlusion**

*B. H. Baek*, W. Yoon; Gwangju/KR
*(qorqod10@gmail.com)*

**Purpose:** The superior cerebellar artery (SCA) is one of the major arteries arising from the distal basilar artery. This study aimed to assess the prevalence of superior cerebellar artery occlusion (SCAO) on final angiography after mechanical thrombectomy for basilar artery occlusion (BAO), to determine baseline and procedural factors associated with SCAO, and to examine clinical outcomes of SCAO.

**Methods or Background:** We retrospectively analysed clinical and angiographic data from 116 patients who underwent mechanical thrombectomy for BAO. Characteristics and clinical outcomes were compared between patients with SCAO and those without SCAO. The clinical outcomes included malignant SCA infarction, in-hospital mortality, hospital length of stay, and 90-day functional outcome. Of the SCAO patients, delayed recanalisation of occluded artery was assessed with follow up CT angiography.
**Results or Findings:** Occlusion of the SCA ostium was found in 10 patients (8.6%) on final angiography after thrombectomy. Two patients had bilateral SCAOs. SCA infarction on post-treatment DWI occurred more frequently in patients with SCAO than those without it (100% vs 50.9%, P=0.002). Of 10 patients with SCAO, malignant infarction or in-hospital mortality did not occur. There were no significant differences in hospital length of stay and the rates of malignant infarction, in-hospital mortality, and 90-day functional outcome between the two groups. Of the 12 lesions of SCAO, nine showed delayed recanalisation of the occluded superior cerebellar artery.
**Conclusion:** SCAO was not uncommon after mechanical thrombectomy in patients with basilar artery occlusion. In our study, untreated SCAO after endovascular therapy did not show significant effect on clinical outcomes. Thus, leaving an occlusion of the SCA might be a reasonable treatment option after successful recanalisation of the basilar artery.
**Limitations:** This was a retrospective single centre design.
**Ethics committee approval:** The institutional review board approved this study.
**Funding for this study:** There is no funding to disclose.
**Author Disclosures:**
Byung Hyun Baek: Nothing to disclose
Woong Yoon: Nothing to disclose

### RPS 1511-6

**Assessment of MR perfusion software packages in predicting final infarct volume after mechanical thrombectomy**

*A. Bani Sadr*; Lyon/FR
*(apbanisadr@gmail.com)*

**Purpose:** Quantification of ischemic core and perfusion deficit volumes (PDV) may reveal significant variations between CT perfusion software packages. Little is known regarding MR perfusion packages (MRPP) in predicting final infarct volume (FIV) after mechanical thrombectomy (MT). We aimed to assess the accuracy of three MRPP (A: RAPID®; B: OleaSphere®; C: Philips ®) and to determine whether expert correction may improve FIV prediction.
**Methods or Background:** HIBISCUS-STROKE cohort includes acute ischemic stroke patients referred to our Stroke Center, treated with MT following admission MRI and undergoing follow-up MRI at Day 6. Admission MRI were post-processed using three MRPP to retrieve ischemic core and PDV. These outputs were corrected by an expert to provide corrected volumes. Uncorrected and corrected volumes were compared between all packages as well as to the FIV according to the recanalisation profile.
**Results or Findings:** 94 patients were included of which 67 (71.28%) had successful recanalisation. In patients with successful recanalisation, ichemic core volumes did not differ signicantly between MRPP and FIV (all P > 0.15). In patients with unsuccessful recanalisation, uncorrected PDV provided by packages A and B overestimated FIV to a lesser degree compared to package C (P=0.03 for A, P=0.12 for B) while there were no differences after correction (P≥0.99). When applying DEFUSE 3 trial criteria, corrected volumes would have led to MT in 51 patients with package A, 63 with package B and, 64 with package C.
**Conclusion:** The MRPP assessed have limited differences in ischemic core quantification but substantial differences for PDV that may impact patient selection.
**Limitations:** The small number of patients with unsuccessful recanalisation was identified as a limiting factor.
**Ethics committee approval:** The local ethics committee approved the study. All subjects signed an informed consent form.
**Funding for this study:** Funding for this study was received from RHU MARVELOUS of Université de Lyon, as part of the "Investissements d'Avenir" programme (French National Research Agency).
**Author Disclosures:**
Alexandre Bani Sadr: Nothing to disclose

### RPS 1511-7

**Intracranial carotid artery calcification subtype and collaterals in patients undergoing endovascular thrombectomy**

*S. Luijten*[1], S. van der Donk[1], K. Compagne[1], C. B. L. M. Majoie[2], W. van Zwam[3], D. Dippel[1], A. Van Der Lugt[1], B. Roozenbeek[1], D. Bos[1]; [1]Rotterdam/NL, [2]Amsterdam/NL, [3]Maastricht/NL
*(s.luijten@erasmusmc.nl)*

**Purpose:** We investigated the association of intracranial carotid artery calcification (ICAC) subtype with collateral status in patients undergoing endovascular thrombectomy (EVT) for ischemic stroke. We further investigated whether ICAC subtype modified the association between collateral status and functional outcome.
**Methods or Background:** We used data from 2701 patients with ischemic stroke undergoing EVT. Presence and subtype of ICAC was assessed on baseline non-contrast CT. Collateral status was assessed on baseline CT angiography using a visual scale from 0 (absent) to 3 (good). We investigated the association of ICAC subtype with collateral status using ordinal and binary logistic regression. Next, we assessed whether ICAC subtype modified the association between collateral status and functional outcome (modified Rankin Scale, 0-6) using a multiplicative interaction term.
**Results or Findings:** Compared to patients without ICAC, we found no association of intimal (acOR, 0.99 [95%CI: 0.80-1.22]) nor medial ICAC (acOR, 0.90 [95%CI: 0.73-1.11]) with collateral status (ordinal variable). When collateral grades were dichotomised (3 versus 0-2), we found that intimal ICAC was significantly associated with good collaterals in comparison to patients without ICAC (aOR, 1.41 [95%CI:1.06-1.89]) or with medial ICAC (aOR, 1.50 [95%CI:1.14-1.97]). The association between higher collateral grade and better functional outcome was significantly modified by ICAC subtype (acOR, 1.62 [95% CI: 1.36-1.94]) than in patients with medial ICAC (acOR, 1.26 [95% CI: 1.10-1.45]; P for interaction=0.01).
**Conclusion:** Patients with intimal ICAC are more likely to have good collaterals and benefit more from an extensive collateral circulation in terms of functional outcome after EVT.
**Limitations:** Not applicable
**Ethics committee approval:** Approval was given by the ethics committee of the Erasmus MC University Medical Center.
**Funding for this study:** The MR CLEAN Registry was partly funded by TWIN Foundation, Erasmus MC University Medical Center, Maastricht University Medical Center, and Amsterdam University Medical Center.
**Author Disclosures:**
Aad Van Der Lugt: Nothing to disclose
Kars Compagne: Nothing to disclose
Sophie van der Donk: Nothing to disclose
Sven Luijten: Nothing to disclose
Charles Bernardus Lucia Maria Majoie: Shareholder: NICO.LAB
Diederik Dippel: Nothing to disclose
Wim van Zwam: Nothing to disclose
Bob Roozenbeek: Nothing to disclose
Daniel Bos: Nothing to disclose

### RPS 1511-8

**Is the optimal Tmax threshold identifying perfusion deficit volumes variable across MR perfusion software packages? A pilot study**

*A. Bani Sadr*; Lyon/FR
*(apbanisadr@gmail.com)*

**Purpose:** Accurate quantification of ischemic core and penumbra is mandatory for late-presenting acute ischemic stroke. Previous studies reported substantial differences in quantification of penumbra depending on the MR perfusion software package used suggesting that optimal Tmax threshold may be variable. We performed a pilot study assessing perfusion deficit volumes (PDV) with different Tmax thresholds of two commonly used MR perfusion packages (A: RAPID®; B: OleaSphere®) in comparison with final infarct volume (FIV).
**Methods or Background:** HIBISCUS-STROKE cohort includes anterior circulation acute ischemic stroke patients treated by mechanical thrombectomy after baseline MRI. Mechanical thrombectomy failure was defined as mTICI score of 0. Admission MR perfusion were post-processed using two software packages with increasing Tmax thresholds (≥6s, ≥8s and ≥10s) and compared to FIV provided by follow-up MRI at Day 6.
**Results or Findings:** Eighteen patients were included. Lengthening of the threshold from ≥6s to ≥10s led to significantly smaller PDV for both packages (A: p=0.01, B: p=0.05). For package A, Tmax ≥6s and ≥8s moderately overestimated final infarct volume (median absolute difference: -9.49mL, interquartile range (IQR) [-17.54; 0.92] and 0.21mL, IQR [-8.130; 4.75]). Bland-Altmann analysis indicated that they were closer to FIV (mean absolute differences -17.44mL and, -8.39mL) and had narrower ranges of agreement while Tmax≥10s underestimated FIV (mean absolute difference 4.78mL). For package B, Tmax ≥10s was closer to FIV (median absolute difference: -10.12mL, IQR: [-17.72; -2.86]) versus -21.76mL (IQR: [-36.73; -9.53]) for Tmax ≥6s. Bland-Altmann plots confirmed these findings (mean absolute difference: 2.24mL versus 31.54mL).
**Conclusion:** Optimal Tmax threshold delineating penumbra may vary across MR perfusion software packages.
**Limitations:** This study was limited by its nature as a small population study.
**Ethics committee approval:** The local ethics committee approved the study. All subjects signed an informed consent form.
**Funding for this study:** This study received funding from RHU MARVELOUS, Université de Lyon and the "Investissements d'Avenir" programme (French National Research Agency).
**Author Disclosures:**
Alexandre Bani Sadr: Nothing to disclose

### RPS 1511-9

**Value of CT perfusion imaging in outcome prediction in patients with basilar artery occlusion**

*M. P. Fabritius*; Munich/DE

**Purpose:** Basilar artery occlusion (BAO) is associated with high morbidity and mortality. Optimal imaging and treatment strategy are still controversial and prognosis estimation challenging. We aimed to determine the predictive value of CT perfusion (CTP) parameters for functional outcome in patients with BAO in the context of endovascular treatment (EVT).
**Methods or Background:** Patients with BAO who underwent EVT were selected from a prospectively acquired cohort. Ischemic changes were assessed with the pcASPECTS on non-contrast CT, CTA source images, and CTP maps. Basilar artery on CTA score, posterior-circulation CTA score, and posterior-circulation collateral score were evaluated on CTA. Perfusion deficit volumes were quantified on CTP maps. Good functional outcome was defined as mRS ≤3 at 90 days. Statistical analysis included binary logistic regressions and receiver operating characteristics analyses.
**Results or Findings:** Among 49 patients 24 (49.0%) achieved good outcome. In multivariate analyses, Basilar artery on CTA score, pc-ASPECTS (OR range, 1.31–2.10 [95% CI, 1.00–7.24]), and perfusion deficit volumes on all CTP maps (OR range, 0.77–0.98 [95% CI, 0.63–1.00]) remained as independent outcome predictors. Cerebral blood flow deficit volume yielded the best performance for the classification of good clinical outcome with an area under the curve of 0.92 (95% CI, 0.84–0.99). Age and admission NIHSS had lower discriminatory power (AUC, <0.7).
**Conclusion:** CTP imaging parameters contain prognostic information for functional outcome in stroke patients due to BAO and may identify patients with higher risk of disability at an early stage of hospitalisation.
**Limitations:** Manual segmentations of CTP deficit volumes, the small number of patients, the retrospective nature of the study as well as patients receiving EVT and results therefore not being generalisable were identified as limitations.
**Ethics committee approval:** Approved by the Institutional Review Board of the LMU Munich according to the Declaration
of Helsinki of 2013. The requirement for written informed consent was waived.
**Funding for this study:** No funding was received for this study.
**Author Disclosures:**
Matthias Philipp Fabritius: Nothing to disclose

### RPS 1511-10

**Comparison of cerebral blood flow quantification using multi- and single-delay arterial spin labelling in ischaemic stroke**

*S. Luijten*, P. J. van Doormaal, D. Dippel, B. Roozenbeek, A. Van Der Lugt, E. Warnert; Rotterdam/NL

**Purpose:** To compare cerebral blood flow (CBF) quantification using multi- versus single-delay arterial spin labelling (ASL) in ischaemic stroke patients.
**Methods or Background:** We used data from patients with ischaemic stroke due to anterior large vessel occlusion and of whom follow-up MRI imaging (3T, GE Healthcare) at 24 hours was available. Hadamard-encoded pseudocontinuous ASL was acquired using 7 post-labelling delays (PLDs) ranging from 0.8 to 3.5s. We estimated CBF and arterial transit time (ATT) by fitting a kinetic model to all 7 PLD images and to the image with a PLD of 1.8s only, fixing the ATT to 1.3s in the latter. Mean ± SD CBF and ATT values were extracted from the infarct area delineated on diffusion-weighted imaging and a contralateral grey matter (GM) mask. We stratified patients according to recanalisation status (successful versus unsuccessful). Paired t-tests were used to compare CBF and ATT quantified from multi-PLD images, and to compare CBF quantified using multi- vs single-PLDs.
**Results or Findings:** We included 43 patients (37 successfully and 6 unsuccessfully recanalised). ATT was significantly shorter in the infarct area compared to contralateral GM (1146±157 vs. 1222±82.5 milliseconds; p<0.01) after successful recanalization, but not after unsuccessful recanalisation (1291±120 vs. 1240±74 milliseconds; p=0.19). After successful recanalisation, mean CBF in the infarct area was significantly higher when estimated using 7 PLDs compared to a single PLD of 1.8s (82.3±32.5 vs. 75.6±25.1 ml/100g/min; p=0.03), whereas mean CBF in contralateral GM was similar (51.2±19.3 vs. 52.6±15.8 ml/100g/min; p=0.65). After unsuccessful recanalisation, no significant differences were seen between CBF estimates calculated with multi- or single-PLDs in the infarct area nor the contralateral GM.
**Conclusion:** ATT varies across brain regions after successful recanalisation and may result in underestimation of CBF when using single-PLD ASL.
**Limitations:** Not applicable
**Ethics committee approval:** Not applicable
**Funding for this study:** Not applicable
**Author Disclosures:**
Esther Warnert: Nothing to disclose
Aad Van Der Lugt: Nothing to disclose
Pieter Jan van Doormaal: Nothing to disclose
Sven Luijten: Nothing to disclose
Diederik Dippel: Nothing to disclose
Bob Roozenbeek: Nothing to disclose

### RPS 1511-11

**Utility of multimodal tomography of the stroke code in the functional prognosis of vertebral-basilar ischaemic stroke**

*F. E. Dianderas Gutiérrez*, R. F. Ocete Pérez, M. Bueno Gómez, A. González García; Sevilla/ES

**Purpose:** Posterior circulation stroke is a rare but potentially "catastrophic" entity. Early diagnosis is difficult due to its non-specific symptoms. The search for prognostic predictors and treatment indications implies a challenge due to the small volume of studies. The objective of this study is to evaluate the performance of the CT scales proposed in the literature to predict the functional result in our environment.
**Methods or Background:** We selected all patients over 18 years of age who received endovascular treatment for posterior circulation stroke in our hospital within the space of three years. Two readers (an expert and a resident) independently analysed the multimodality CT studies. Scores on the different tomographic scales were statistically correlated with the mRS scale at 90 days.
**Results or Findings:** 61.8% of the patients were men, the mean age was 69 ± 13, 74.4% had hypertension. The median PC-ASPECTS was 10 and 90.4% had a favourable value (> 8). The means of BATMAN and PC-CS were 6 for both, 39.7% had a favourable BATMAN (> 7) and 60.3% had a "good" value in the PC-CS. The PC-ASPECTS scale showed statistical significance with the mRs at 90 days in its two versions (p = 0.003; p = 0.005) as well as the quantitative PC-CS (p = 0.027) and qualitative BATMAN (p = 0.009). The interobserver correlation was good for PC-CS (0.778) and very good for BATMAN (0.823).
**Conclusion:** Multimodality tomography is a useful tool in predicting the functional outcome of posterior circulation strokes (PC-ASPECTS, PC-CS quantitative and BATMAN qualitative). PC-CS and BATMAN showed good interobserver correlation. In contrast, PC-ASPECTS shows a greater dependence on the reader's experience.
**Limitations:** This work was limited by its nature as a retrospective study without a control group.
**Ethics committee approval:** An ethics committee approved this study.
**Funding for this study:** No funding was needed.
**Author Disclosures:**
Alejandro González García: Nothing to disclose
Francisco Esteban Dianderas Gutiérrez: Nothing to disclose
Rafael Felix Ocete Pérez: Nothing to disclose
Marta Bueno Gómez: Nothing to disclose

### RPS 1511-12

**Haemorrhagic transformation rates following contrast media administration in patients hospitalised with ischaemic stroke**

*F. G. Moser*[1], T. Todoran[2], M. Ryan[3], E. Baker[4], C. Gunnarsson[4], J. Kellum[5]; [1]Los Angeles, CA/US, [2]Charleston, SC/US, [3]Cincinnati, OH/US, [4]Covington, KY/US, [5]Pittsburgh, PA/US
*(Franklin.Moser@cshs.org)*

**Purpose:** Haemorrhagic transformation is a critical complication associated with ischaemic stroke and has been associated with contrast media administration. The objective of our study was to use real-world in-hospital data to evaluate the correlation between contrast media type and transformation from ischaemic to haemorrhagic stroke.
**Methods or Background:** We obtained data on inpatient admissions with a diagnosis of ischaemic stroke and a record of either iso-osmolar or low osmolar iodinated contrast media for a stroke related diagnostic test and a treatment procedure (thrombectomy, thrombolysis, or angioplasty). We performed multivariable regression analysis to assess the relationship between contrast media type and development of haemorrhagic transformation during hospitalisation, adjusting for patient characteristics, comorbid conditions, and procedure type.
**Results or Findings:** Inpatient visits with exclusive usage of either low osmolar (N=38,130) or iso-osmolar contrast media (N=4,042) were included. We observed an overall risk reduction in haemorrhagic transformation among patients who received iso-osmolar compared with low osmolar contrast media with an absolute risk reduction of 1.4% (p=0.032), relative risk reduction of 12.5%, and number needed to harm of 70. This outcome was driven primarily by patients undergoing endovascular thrombectomy, in which iso-osmolar contrast media was associated with absolute risk reduction of 4.6%, and number needed to harm of 22, compared with low osmolar contrast media.

**Conclusion:** Iso-osmolar contrast media was associated with a lower rate of haemorrhagic transformation compared to low osmolar contrast media in patients with ischaemic stroke.
**Limitations:** The limitations of this study include those which are inherent to retrospective database analyses. The data source for this study was the Premier Hospital Database, which represents 20% of all inpatient discharges in the United States.
**Ethics committee approval:** Not required
**Funding for this study:** This study received funding from General Electric Healthcare.
**Author Disclosures:**
Michael Ryan: Employee: MPR consulting
Franklin G Moser: Consultant: General electric
Canace Gunnarsson: Employee: CTI Clinical Trial & Consulting
Erin Baker: Employee: CTI Clinical Trial & Consulting
John Kellum: Consultant: General electric
Thomas Todoran: Consultant: General electric

**14:00-15:30** **Room M 2**

### Research Presentation Session: Paediatric

### RPS 1512

### Imaging the paediatric chest and abdomen: optimisation and future perspectives

**Moderator**
D. Baleva; Mistelbach/AT

### RPS 1512-2

**Ultrasound-guided percutaneous renal biopsy in paediatric population**
*A. Zafirovski*[1], M. Thaler[2], M. Zafirovska[2], M. Brovc[2], A. Kenig[2], T. Kersnik Levart[2], D. Ključevšek[2]; [1]Jesenice/SI, [2]Ljubljana/SI
*(aleksandarzafirovski5@gmail.com)*

**Purpose:** This retrospective study aims to investigate if the number of renal core samples has an impact on the number and severity of post-biopsy complications in children with percutaneous renal biopsy (PRB).
**Methods or Background:** Ultrasound-guided PRB is an invasive procedure performed on native and transplant kidneys. Clinical data were obtained from the electronic records for consecutive paediatric patients who underwent a PRB from the 1st of January 2012 to 31st of July 2021. Complications were separated into minor and major depending on further engagement. Quantitative data were presented by descriptive statistics and analysed using Pearson's chi-squared test.
**Results or Findings:** This study consisted of 223 PRB in 156 children with slight male predomination (55.77%) and average age 11.59± 5.24 years, ranging from 111 days to 19 years old. 23.71% of children had more than one biopsy. PRB of the transplanted kidney was performed in 17 children (10.89%). No complication after PRB was recorded in 75% of children. Minor complications, which required no treatment, were perinephric haematoma, haematuria, and intraparenchymal bleeding. Most common minor complication was haematuria. Only one child (0.6%) had a major complication; high-flow arterio-venous fistula was created which was treated conservatively and resulted in nephrectomy. Two core samples were taken in 62.32%, three only in 2.7%.
**Conclusion:** There was no statistical correlation between the number of core samples and complications (p>0.1931). When two core samples were taken quality sample for interpretation was reached in 100% of the cases, while in one core samples it was reached in 94.87%. There was no significant statistical difference between one or two samples regarding minor complications (p=0.875).
**Limitations:** This was a retrospective study and data was not systematically evaluated.
**Ethics committee approval:** Ethics committee approval was not required.
**Funding for this study:** The author(s) received no financial support.
**Author Disclosures:**
Anton Kenig: Nothing to disclose
Marija Zafirovska: Nothing to disclose
Tanja Kersnik Levart: Nothing to disclose
Monika Brovc: Nothing to disclose
Aleksandar Zafirovski: Nothing to disclose
Martin Thaler: Nothing to disclose
Damjana Ključevšek: Nothing to disclose

### RPS 1512-3

**Impact of improved protocol on the percutaneous renal biopsy complications in children**
*A. Zafirovski*[1], M. Thaler[2], M. Zafirovska[2], M. Brovc[2], A. Kenig[2], T. Kersnik Levart[2], D. Ključevšek[2]; [1]Jesenice/SI, [2]Ljubljana/SI
*(aleksandarzafirovski5@gmail.com)*

**Purpose:** This retrospective study aims to investigate if the number of renal core samples has an impact on the post-biopsy complications and compare their proportion of patients who develop a complication after ultrasound-guided percutaneous renal biopsy (PRB) in the same medical center in two different time periods.
**Methods or Background:** PRB is an invasive procedure performed in native and transplant kidneys. Clinical data were obtained from a published article for the first time period (1994-1999) and from electronic data for the second time period (2012-2021). Number of core samples and complications were examined. Quantitative data were presented by descriptive statistics and analysed using Pearson's chi-squared test.
**Results or Findings:** Two core biopsies were needed to yield satisfactory pathological material in 93.82% of cases in first period, one core biopsy was never performed. On the other hand, in the second period, single core biopsy was performed in 35% of total biopsies and yielded satisfactory pathological material in 94.87% of cases. Two core biopsies were performed in 62.32% of cases and yielded satisfactory material in all 139 (100%) cases. 63.2% minor complications were noted in the first period, without any major complication. 20 years later 26.9% instances had minor complications and only one child (0.6%) had a major complication that resulted in nephrectomy.
**Conclusion:** There is a reduction of minor complication rate by 36.3% between the two time periods, due to better PBR protocol, more experience, and due to reduced number of core samples. One core biopsy has become increasingly common and yields better satisfactory material than two core biopsies did in the first period.
**Limitations:** Different physicians performing the biopsy, different protocols and technological advancements in radiological equipment.
**Ethics committee approval:** Ethics committee approval was not required.
**Funding for this study:** The author(s) received no financial support.
**Author Disclosures:**
Anton Kenig: Nothing to disclose
Marija Zafirovska: Nothing to disclose
Tanja Kersnik Levart: Nothing to disclose
Monika Brovc: Nothing to disclose
Aleksandar Zafirovski: Nothing to disclose
Martin Thaler: Nothing to disclose
Damjana Ključevšek: Nothing to disclose

### RPS 1512-5

**MaRIA and Clermont MR enterography indices correlate with degree of mucosal healing in paediatric Crohn's disease**
*M. Gladkikh*[1], E. Benchimol[2], D. Mack[1], N. Mojaverian[1], K. Highmore[1], E. Miller[1], J. Davila[1]; [1]Ottawa, ON/CA, [2]Toronto, ON/CA
*(mglad050@uottawa.ca)*

**Purpose:** To evaluate if MaRIA and Clermont scores can serve as surrogates to endoscopy for assessing the degree of mucosal healing following treatment in children with CD.
**Methods or Background:** This prospective cohort study evaluated children with known or newly-diagnosed ileocolonic CD starting or changing therapy. Children underwent ileocolonoscopy, scored with simple endoscopic score for Crohn's disease (SES-CD), and MRE with DWI on the same day at two different time points (Week 0 and 12). Accuracy of MaRIA and Clermont relative to ileocolonoscopy in detecting disease change was assessed through correlational coefficients (r). Interreader agreement was calculated for both imaging scores through intraclass correlation (ICC).
**Results or Findings:** 16 children (mean age 11.5 ± 2.8) were evaluated. Global MaRIA and global Clermont strongly correlated with SES-CD in detecting the degree of mucosal healing following treatment (r=0.676 and r=0.677, p<0.005, respectively). Correlation for pooled timepoint assessments between SES-CD and global MaRIA/global Clermont was moderate (r=0.519, p<0.005 and r=0.570, p<0.001 respectively). Interrater reliability for global MaRIA and global Clermont for pooled timepoint assessments was good (ICC=0.809 and ICC=0.768, respectively, p<0.001).
**Conclusion:** MRE-based global scores correlate with endoscopic indices and may be used to monitor mucosal healing in children with CD undergoing treatment. MRE with DWI may represent a favourable alternative to colonoscopy and contrast-enhanced MRE in children due to its greater tolerability.
**Limitations:** First, the modest study sample size introduced an innate level of variability in the data. Second, all MRE examinations were performed under the colonoscopy bowel preparation protocol, which is not a common practice and may thus not be reflective of MRE and DWI assessments that occur in regular practice.

**Ethics committee approval:** The hospital Research Ethics Board approved this study.
**Funding for this study:** This study received funding from the CHAMO Innovation Fund Operating Grant.
**Author Disclosures:**
David Mack: Nothing to disclose
Elka Miller: Nothing to disclose
Maria Gladkikh: Nothing to disclose
Kerri Highmore: Nothing to disclose
Nassim Mojaverian: Nothing to disclose
Jorge Davila: Nothing to disclose
Eric Benchimol: Nothing to disclose

### RPS 1512-6

**Semi-quantitative and quantitative assessment of multiphase contrast-enhanced MRI and diffusion-weighted imaging in paediatric Crohn's disease**

*M. Gladkikh*[1], E. Benchimol[2], D. Mack[1], K. Highmore[1], E. Miller[1], J. Davila[1]; [1]Ottawa, ON/CA, [2]Toronto, ON/CA
*(mglad050@uottawa.ca)*

**Purpose:** To correlate multiphase contrast-enhanced magnetic resonance (MCE-MR) and semi-quantitative assessment of diffusion-weighted imaging (DWI) with paediatric Crohn's disease (CD) severity (assessed by endoscopy) and evaluate the optimal timing for acquisition of postcontrast sequences.
**Methods or Background:** This is an ancillary analysis of a prospective cohort study where children with CD underwent endoscopy and MCE-MR with DWI on the same day. These were repeated 12 weeks later. Semi-quantitative DWI assessment, ADC, and RCE were assessed in five intestinal segments. The correlation between Simple Endoscopic Score for CD (SES-CD) and MR parameters was evaluated through correlational coefficients (ρ). Multiple line mean graph of RCE in six acquisition phases (8-128 seconds) was constructed to assess enhancement in pathologic and normal bowel.
**Results or Findings:** 156 ileocolonic segments from 16 children (mean age 11.5 ± 2.8) were included. A moderate correlation emerged between semi-quantitative DWI assessment and SES-CD ($\rho=0.521$, $p<0.001$). A weak correlation emerged between ADC and SES-CD ($\rho=-0.232$, $p=0.005$), and RCE and SES-CD in all six phases ($\rho=0.179$ $p<0.05$; $\rho=0.335$, $\rho=0.295$, $\rho=0.308$, $\rho=0.251$, $\rho=0.324$, all $p<0.001$). Pathologic bowel segments showed a quicker pattern of enhancement than normal bowel in MCE-MR, with the greatest significance within 40 seconds of contrast administration.
**Conclusion:** Semi-quantitative DWI assessment shows a more robust correlation with degree of CD activity compared to ADC and RCE. This may indicate the futility of acquiring post-contrast images in MRE and measuring ADC values in paediatric CD.
**Limitations:** The modest sample size and extensive bowel preparation protocol may have introduced variability in the data and over-represented MR assessments.
**Ethics committee approval:** The hospital Research Ethics Board approved the parent study.
**Funding for this study:** The parent study received the CHAMO Innovation Fund Operating Grant.
**Author Disclosures:**
David Mack: Nothing to disclose
Elka Miller: Nothing to disclose
Maria Gladkikh: Nothing to disclose
Kerri Highmore: Nothing to disclose
Jorge Davila: Nothing to disclose
Eric Benchimol: Nothing to disclose

### RPS 1512-8

**Development of deep-learning AI algorithm for detecting ileocolic intussusception on ultrasonography in children**

S. Kim[1], Y. Choi[2], *J-Y. Hwang*[3], S. Lee[2], S. Lee[2], Y. Cho[2], J-E. Cheon[2]; [1]Daejeon/KR, [2]Seoul/KR, [3]Busan/KR
*(jyhwang79@gmail.com)*

**Purpose:** To develop and validate a deep learning-based artificial intelligence (AI) model for detecting ileocolic intussusception on ultrasonography in children.
**Methods or Background:** This retrospective multicentre study included grayscale US images from paediatric patients who visited ER and underwent US examination for suspicion of ileocolic intussusception. 40783 images from two tertiary hospitals (Hospital_A: N=38166, lesion (+): lesion(-)=2770:35379; Hospital_B: N=2617, lesion(+): lesion(-)=140:2477) were included. The development dataset consisted of images from Hospital_A, and was split into training, tuning, and internal test sets in a ratio of 7 : 1.5 : 1.5. External test set consisted of images from Hospital_B. The precision, recall, average precision (AP), F1-score were calculated to evaluate the performance of our model. The optimal cut-off values of confidence scores were determined using internal dataset and performance was evaluated with external dataset through per-lesion and per-patient-based analyses
**Results or Findings:** The AI model showed AP of 0.952 and 0.932 in the internal and external test set. We determined two confidence thresholds, CTopt and CTprecision as 0.557 and 0.790, from internal test set. The former yielded maximum value of F1-score, and the latter yielded maximum value of F1 score while yielding precision over 98.0%. The precision-recall with CTopt and CTprecision were 94.5% (377/399) - 90.6% (377/416) and 98.1% (264/269) - 63.5% (63.5/416) in internal test set and 95.7% (112/117) - 80.0% (112/140) and 98.4%(62/63) - 44.3% (62/140) in external test set. In external test set, the sensitivity and specificity for per-patient diagnosis was 100.0% (20/20) and 97.1% (101/104) with CTopt and 100.0% (20/20) and 99.0% (103/104) with CTprecision.
**Conclusion:** A deep learning-based AI model could detect ileocolic intussusception on grayscale US with good to excellent per-lesion and per-patient performance.
**Limitations:** Retrospective analysis of ultrasonographic images, which means only captured images were analysed in this study.
**Ethics committee approval:** This study was approved by the IRB and informed consent was waived.
**Funding for this study:** No funding was received for this study.
**Author Disclosures:**
YoungHun Choi: Nothing to disclose
Seulbi Lee: Nothing to disclose
Sewoo Kim: Nothing to disclose
Seunghyun Lee: Nothing to disclose
Jae-Yeon Hwang: Nothing to disclose
YounJin Cho: Nothing to disclose
Jung-Eun Cheon: Nothing to disclose

### RPS 1512-9

**The role of Doppler waveform indices of the foetal main pulmonary artery in the prediction of neonatal respiratory distress syndrome - a prospective cohort study**

*A. Prakash*, V. K; Bengaluru/IN
*(drarjunprakash@gmail.com)*

**Purpose:** This study investigates the role of antenatal foetal main pulmonary artery (MPA) Doppler in predicting the subsequent development of clinical Respiratory distress syndrome (RDS) in neonates.
**Methods or Background:** A prospective cohort study was conducted from November 2019 to October 2020 on 729 pregnant women between 34 to 39 weeks of gestational age, who came for routine third trimester ultrasound examination to Bangalore Medical College & Research Institute, India. The standard foetal biometric parameters, gestational age and estimated foetal weight were first obtained. The MPA Doppler indices like PI, RI, PSV, S/D ratio and Acceleration Time/Ejection Time (At/Et) were recorded. The pregnant women were followed up till delivery to look for development of RDS in the neonate. Correlation was carried out using Pearson's moment correlation equation. Determination of the optimum cut-off value for pulmonary At/Et ratio in predicting neonatal RDS was done using ROC analysis.
**Results or Findings:** Of the 729 foetuses, 342 were eligible for final analysis, of which 47 developed neonatal RDS. These fetuses had significantly higher PI and RI values, whereas At/Et ratio and PSV were significantly lower. At/Et ratio, PSV, RI and PI showed significant correlation with gestational age, of which At/Et ratio showed the most statistically significant correlation. A cut-off value of 0.2865 (sensitivity: 89.45%, specificity: 94.79%) and 0.3155 (sensitivity: 93.22%, specificity: 96.78%) for At/Et ratio predicted the development of RDS in late preterm and early term infants respectively.
**Conclusion:** Foetal MPA indices, especially At/Et ratio is a promising non-invasive tool in identifying fetuses at risk of neonatal RDS.
**Limitations:** Pregnant women with co-morbidities like hypertension and diabetes mellitus were excluded from the study. Correlation with Lecithin/Sphingomyelin ratio could have been done, thus warranting further studies.
**Ethics committee approval:** Institutional ethics committee approval was obtained.
**Funding for this study:** No funding was received for this study.
**Author Disclosures:**
Arjun Prakash: Author: Principal Investigator
Veena K: Investigator: Co-author

### RPS 1512-10

**Scoping review of the utilisation of lung ultrasound in paediatric COVID-19**

*Y. S. Nawawi*, I. Maryetty, V. Widyaningsih, I. Andharini, W. Soewondo; Surakarta/ID
*(yusufnawawi@unsyiah.ac.id)*

**Purpose:** Lung ultrasound (LUS) has been introduced as the modality of choice with high accuracy in diagnosing pneumonia. This study focuses on the potential use of LUS in cases of COVID-19 pneumonia in children by conducting a scoping review of the literature to comprehensively explore the current evidence.

**Methods or Background:** This scoping review compiled published results from various relevant studies. Studies using LUS with experimental or observational design and case series of paediatric patients suspected or confirmed COVID-19 are included in this study. Summary of the data and narrative approach were performed to describe the finding.
**Results or Findings:** This scoping review includes 33 articles from various countries. Of the 24 studies with primary data, three studies are multicentre-based, and the remaining 21 studies are based on a single centre. The number of study subjects ranged from 3 to 74 patients. The quality of the included studies was relatively low; however, LUS certainly appears to be a highly sensitive and fairly specific test for COVID-19 in paediatric. There may be LUS findings and patterns, predominantly interstitial abnormalities, that are relatively specific to COVID-19, despite the other factors may vary.
**Conclusion:** Almost all identified studies positively recognise the role of LUS as a sensitive and relatively specific modality in the diagnosis of COVID-19 pneumonia in children.
**Limitations:** Our iterative search on the role of LUS in the paediatric population with COVID-19 discovered limited number of publications than in the adult population. Even with a careful and systematic search of the literature, it is possible that not all publications can be identified due to publication bias and exclusion of publications in non-English languages.
**Ethics committee approval:** Not applicable
**Funding for this study:** Not applicable
**Author Disclosures:**
Widiastuti Soewondo: Nothing to disclose
Ida Maryetty: Nothing to disclose
Vitri Widyaningsih: Nothing to disclose
Ismiranti Andharini: Nothing to disclose
Yusuf Syaeful Nawawi: Nothing to disclose

### RPS 1512-11

**Automated airway-artery analysis in evaluating the effect of inhaled hypertonic saline in preschool children with cystic fibrosis**

*Y. Chen*[1], Q. Lv[1], E-R. Andrinopoulou[1], J-P. Charbonnier[2], L. Gallardo Estrella[2], D. Caudri[1], H. A. W. M. Tiddens[1], SHIP-CT study group[1]; [1]Rotterdam/NL, [2]Nijmegen/NL
*(y.chen.1@erasmusmc.nl)*

**Purpose:** To investigate the efficacy of inhaled hypertonic saline (HS) on airway and artery (AA) outcomes in preschool children with CF (pCwCF).
**Methods or Background:** The SHIP-CT study was an international multicentre randomised controlled trial that compared the effect of inhaled HS vs isotonic saline (IS) on lung structure as measured by chest CT in pCwCF. The manual PRAGMA-CF showed less airway-related abnormalities including bronchiectasis in the HS group over 48 weeks. An algorithm was recently developed and validated to quantify dimensions of visible AA pairs on CT. LungQ (v2.1.0.1, Thirona) automatically segments the bronchial tree and identifies segmental (G0) and distal (G1-10) airway generations. For each AA-pair, the following dimensions are quantified and analysed by a mixed effect model: diameters of airway outer wall (Aout), airway lumen wall (Alumen), airway wall thickness (Awt), artery (A), and AA-ratios were computed: AoutA, AlumenA, and AwtA.
**Results or Findings:** 113 baseline and 103 48-week CTs of 115 pCwCF (55 HS, 60 IS) were analysed (4 excluded due to inconsistent slice spacing). 13,205 AA-pairs in the HS group and 13,480 in the IS group were detected. Baseline characteristics were balanced between groups. At 48 weeks, AwtA was higher in the IS group than for the HS (mean difference 0.02; 95%CI 0.004-0.04; p=0.02). AlumenA and AoutA were not significantly different between groups. Significant changes from baseline to 48 weeks in AwtA and AlumenA between groups were observed, favoring HS (all p<0.001).
**Conclusion:** The automated AA-analysis was able to detect a large number of AA-pairs and quantified AA-dimensions on chest CT in pCwCF. A positive effect of inhaled HS at 48 weeks and the progression of airway wall thickness was observed, in line with the PRAGMA-CF results.
**Limitations:** This was a retrospective analysis.
**Ethics committee approval:** This study was approved by an ethics committee.
**Funding for this study:** Funding was received from CFFT, EFRO and Health Holland.
**Author Disclosures:**
Jean-Paul Charbonnier: Shareholder: Thirona
SHIP-CT study group: Nothing to disclose
Eleni-Rosalina Andrinopoulou: Nothing to disclose
Qianting Lv: Nothing to disclose
Daan Caudri: Nothing to disclose
Leticia Gallardo Estrella: Employee: Thirona
Harm A W M Tiddens: Consultant: Thirona Patent Holder: PRAGMA-CF
Yuxin Chen: Nothing to disclose

### RPS 1512-12

**Diagnostic accuracy of ultrasound for confirmation of peripherally inserted central catheter tips (PICC's) in infants in the neonatal intensive care unit (NICU) - a systematic review and meta-analysis**

*S. C. Doyle*, A. England, M. McEntee, N. Bergin, R. Young; Cork/IE

**Purpose:** Chest or abdominal radiography, after peripherally inserted central catheter (PICC) insertion, is the gold standard for confirming catheter tip location. The utilisation of ultrasound in catheter placement confirmation amongst the neonatal and paediatric population has been the focus of many recent studies. This work sought to establish the diagnostic accuracy of ultrasonography for the confirmation of PICC tip position amongst patients within the neonatal intensive care unit, compared to the gold standard of conventional radiography.
**Methods or Background:** A PRISMA methodology was used, searches included four (Pubmed, CINAHL, Embase, and MEDLINE) databases. Diagnostic accuracy measurements and study characteristics were extracted from all studies eligible for inclusion. The QUADAS-2 tool was employed to assess the methodological quality of the included studies. The meta-analysis used the primary outcomes of sensitivity and specificity.
**Results or Findings:** Eight studies were included, with 421 patients receiving a PICC line. The estimated pooled ultrasound sensitivity was 95.2% (95% CI: 91.94%-97.42%) and specificity was 71.4% (95% CI: 59.38%-81.60%). No significant differences between operator experience were noted.
**Conclusion:** Ultrasonography is a sensitive, specific, and timely imaging modality for confirming the position of PICCs in the neonatal intensive care unit when compared with chest or abdominal radiography. Results of this systematic review study support the use of US as the first line imaging test to confirm PICC tip position.
**Limitations:** This systematic review is limited to those studies that were published in the English language.
**Ethics committee approval:** This was entirely a literature-based study and as such ethical approval was not required.
**Funding for this study:** This study was part of a pre-registration MSc in Diagnostic Radiography.
**Author Disclosures:**
Niamh Bergin: Nothing to disclose
Rena Young: Nothing to disclose
Andrew England: Nothing to disclose
Mark McEntee: Nothing to disclose
Shauna Claire Doyle: Nothing to disclose

**14:00-15:30** **Room M 3**

### Research Presentation Session: Interventional Radiology

### RPS 1509

### Percutaneous non-vascular interventions: minimal invasive and maximal effective

**Moderator**
T. Albrecht; Berlin/DE

### RPS 1509-2

**Improved visualisation of hepatic tumours in magnetic resonance guided thermoablation using T1 inversion-recovery imaging compared to T1 volume-interpolated breath-hold sequence**

*J. Kuebler*, R. Hoffmann, P. Krumm, T. Küstner, M. T. Winkelmann, G. Gohla, K. Nikolaou; Tübingen/DE

**Purpose:** To evaluate the potential of native T1 IR imaging for delineation of liver lesions in interventional MR without the application of contrast agent.
**Methods or Background:** A total of 48 liver lesions in 41 patients with known malignancies (hepatocellular carcinoma or metastases) were treated with MR guided thermoablation between March 2020 and February 2022. T1-VIBE sequence was acquired as part of the standard imaging protocol. Additionally, T1 mapping look-locker images were acquired with eight different inversion times (TI) between 148 ms and 1743 ms. Lesion-to-liver contrast (LLC) was compared between VIBE and IR-images for each TI. Wilcoxon signed-rank test was conducted for analysis of data, statistical significance was set for p≤0.05.
**Results or Findings:** Mean LLC in T1-VIBE sequences was 0.3±0.1. In IR images mean LLC was 0.9±0.8 at TI 148 ms (p<0.001), 1.1±1.1 at TI 228 ms (p<0.001), 0.7±0.7 at TI 548 ms (p<0.001), 0.7±0.2 at TI 628 ms (p<0.001), 0.5±0.2 at TI 946 ms (p<0.001), 0.4±0.2 at TI 1025 ms (p=0.037), 0.3±0.2 at TI 1343 ms (p=0.2), and 0.2±0.2 at TI 1743 ms (p=0.08).

**Conclusion:** IR imaging is promising to provide improved visualisation compared to standard T1-VIBE sequence when using specific TI with best LLC between 148 and 628 ms. However, there is greater variance of contrast compared to T1-VIBE.
**Limitations:** Not applicable.
**Ethics committee approval:** This prospective study was approved by the local ethics committee and written informed consent was obtained.
**Funding for this study:** Not applicable.
**Author Disclosures:**
Patrick Krumm: Nothing to disclose
Konstantin Nikolaou: Nothing to disclose
Moritz T. Winkelmann: Nothing to disclose
Rüdiger Hoffmann: Nothing to disclose
Georg Gohla: Nothing to disclose
Thomas Küstner: Nothing to disclose
Jens Kuebler: Nothing to disclose

### RPS 1509-4

**Novel needle-guiding robotic system for percutaneous minimally invasive procedures: a technical feasibility study**

S. Y. Avital, J. Heidkamp, I. Spenkelink, *J. J. Futterer*; Nijmegen/NL
*(jurgenfutterer@gmail.com)*

**Purpose:** The accuracy of image guided percutaneous ablations and biopsies may be enhanced by using robotic systems for needle guidance. In this technical feasibility study, we evaluated the performance of a novel needle-guiding robotic system (ANT-C) during percutaneous CT-guided procedures in an abdominal phantom in comparison with free-hand technique. Our primary end point was the technical feasibility of the robotic system to perform accurate needle guidance. Our secondary end points were its accuracy, i.e., 3-D deviation from target centre, the number of CT scans needed to reach the target (determining radiation exposure and overall procedural duration) and the duration of needle manipulation.
**Methods or Background:** 18 gauge needles were used to target 8 fiducial markers (diameters: 8, 10 and 12 mm; median depth 63.5 mm). Our operators (an experienced interventional radiologist with over 10 years of experience, and a novice interventional radiology fellow) performed 12 robotic guided punctures and 12 free-hand punctures.
**Results or Findings:** Compared with the free-hand technique, the robotic system was more accurate with a statistically significant reduction in needle deviation from the target centre (robot: 3.63; manual: 4.93 mm; p-value<0.0096). The number of CT scans needed to reach the target was significantly reduced (robot: 1.0 ± 0.2; manual: 2.7 ± 0.9; p-value<0.001) and a significant reduction in needle manipulation time was recorded (robot: 35.5 ± 21.0; manual: 94.6 ± 35.7 Sec.; p-value<0.001).
**Conclusion:** It was technically feasible to use the ANT-C robotic system for needle guidance during percutaneous procedures, resulting in more accurate needle placement, shorter needle manipulation time and reduced radiation exposure, compared with the free-hand technique.
**Limitations:** As a technical feasibility study, the number of percutaneous punctures was limited.
**Ethics committee approval:** Not applicable.
**Funding for this study:** This study was supported by a research grant from NDR medical.
**Author Disclosures:**
Shalom Yaniv Avital: Nothing to disclose
Jan Heidkamp: Author: co-author
Ilse Spenkelink: Nothing to disclose
Jurgen J. Futterer: Author: co-author

### RPS 1509-5

**The role of volumetric assessment in predicting the success of percutaneous transhepatic biliary drainage in inoperable Klatskin tumours**

*G. K. Bahadır*, M. Özdemir; ANKARA/TR

**Purpose:** To determine the predictors of effective drainage after PTBD for inoperable type 3 and 4 Klatskin tumours.
**Methods or Background:** Thirty-four patients with type 3 and 4 Klatskin tumours who underwent PTBD were included. A ≥50% decrease in total bilirubin level within 2 weeks was considered effective drainage. The total liver volume, and 3 main hepatic sector volumes (right anterior, right posterior and left), were calculated separately using computed tomography volumetry. According to the Bismuth type and the sector in which the drainage catheter was placed, the drained liver volume was determined by virtually cutting the liver. Receiver operating characteristic (ROC) analysis was performed to determine the optimal cut-off value of liver volume for effective drainage. In addition, the relationship between liver function status before the procedure and effective drainage was investigated. Multivariate analysis was performed to determine independent predictors of effective drainage.
**Results or Findings:** Multivariate logistic regression analysis showed that drained liver volume (OR=1.10, 95% CI: 1.014-1.208; p=0.02), preprocedural INR (OR=0.001, 95% CI: < 0.001-0.389; p=0.03), and preprocedural serum albumin level (OR=1.55, 95% CI: 1.008-2.397; p=0.04) were independent factors for effective drainage. In the ROC analysis, the cut-off value of liver volume to be drained for effective drainage was 37%. The sensitivity and specificity were 81% and 73%, respectively. In the group of patients who developed cholangitis after the procedure, the drained liver volume was significantly lower than in the group without cholangitis (29% and 50.7%, respectively, p=0.003).
**Conclusion:** A drained liver volume ≥37%, high albumin and low INR values, which are indicators of better liver function, are factors that positively affect effective drainage.
**Limitations:** The retrospective design of this study, and the small number of patients, were identified limitations.
**Ethics committee approval:** This study was approved by an ethics committee.
**Funding for this study:** No funding was received for this study.
**Author Disclosures:**
Mustafa Özdemir: Nothing to disclose
Gülsüm Kübra Bahadır: Nothing to disclose

### RPS 1509-6

**Risk of persistent air leaks following percutaneous cryoablation and microwave ablation of peripheral lung tumours: a bi-institutional retrospective study**

M. Abrishami Kashani[1], M. Murphy[1], J. Saenger[1], M. M. Wrobel[1], I. Tahir[1], S. Silverman[1], P. Shyn[1], D. Pachamanova[2], *F. J. Fintelmann*[1]; [1]Boston, MA/US, [2]Wellesley, MA/US

**Purpose:** To retrospectively compare the incidence of persistent air leak (PAL) following cryoablation or microwave ablation (MWA) of lung tumours with ablation zones encompassing the pleura.
**Methods or Background:** This bi-institutional retrospective cohort study evaluated 305 lung tumours treated with cryoablation or MWA in 146 consecutive patients between 2006 and 2021. PAL was defined as air leak for more than 24 hours after chest tube placement or an enlarging postprocedural pneumothorax requiring chest tube placement. The extent of the pleural surface encompassed by the ablation zone was quantified on CT using semiautomated segmentation. A parsimonious generalised estimating equation model was developed using purposeful selection of predefined covariates to estimate PAL incidence by ablation modality. Local tumour progression (LTP) was compared between ablation modalities using Cox proportional-hazard models, with death as a competing risk.
**Results or Findings:** A total of 260 tumours (mean diameter, 13.1 mm +/- 7.4; mean distance to pleura, 3.6 mm +/- 5.2) in 116 patients (mean age, 61.1 years +/-15.3; 60 women) and 173 sessions (112 cryoablations, 61 MWA) were included. PAL occurred after 25 of 173 (15%) sessions. The incidence was significantly lower following cryoablation compared to MWA (10 [9%] vs 15 [25%]; p=.006). The odds of PAL adjusted for the number of treated tumours per session were 67% lower following cryoablation (odds ratio [OR], 0.33 [95% CI, 0.14-0.82]; p=.02) compared to MWA. There was no significant difference in LTP between ablation modalities (p=.36).
**Conclusion:** Cryoablation of peripheral lung tumours bears a lower risk of PAL compared to MWA if the ablation zone encompasses the pleura, without adversely affecting LTP.
**Limitations:** The findings of this study are limited by the retrospective design.
**Ethics committee approval:** The IRB of both institutions approved this HIPAA-compliant retrospective study.
**Funding for this study:** No funding was received for this study.
**Author Disclosures:**
Paul Shyn: Nothing to disclose
Jonathan Saenger: Nothing to disclose
Dessislava Pachamanova: Nothing to disclose
Stuart Silverman: Nothing to disclose
Florian J. Fintelmann: Nothing to disclose
Maria Marta Wrobel: Nothing to disclose
Maya Abrishami Kashani: Nothing to disclose
Ismail Tahir: Nothing to disclose
Mark Murphy: Nothing to disclose

### RPS 1509-7

**US-CT fusion-guided percutaneous radiofrequency ablation of large substernal benign thyroid nodules**

D. Orlandi[1], *U. Viglino*[1], G. Dedone[2], G. Leale[1], G. Mauri[3], G. Turtulici[1]; [1]Genova/IT, [2]Genova, GE/IT, [3]Milano/IT

**Purpose:** Our aim was to assess feasibility, safety and outcome of ultrasound (US) guided percutaneous radiofrequency (RF) ablation of large substernal benign thyroid nodules assisted by US-computed tomography (CT) fusion imaging and real-time virtual needle tracking (VT) system.
**Methods or Background:** Thirty patients (18 females, mean age 56y, range 32-76y) with 35 benign non-functioning thyroid nodules (mean volume±SD 26.8±7.6 ml; range 20-38ml) were selected for CT-US fusion guided RF

ablation. Nodules' volume was evaluated before treatment and during 12-months follow-up. Complications' rate was also evaluated. The nodules were treated using US-CT fusion imaging and real-time needle virtual tracking system with the "moving shot" technique, inserting the RF electrode in the distal part of the nodule and then moving the RF electrode backwards and upwards with steps of 5-10 s.
**Results or Findings:** US-CT fusion imaging with VT system was feasible in all cases (feasibility 100%) and it was always possible to complete the procedure as planned (technical success 100%). Minor complications occurred in 2/30 cases (6.6 %). No major complications occurred. 50% volume reduction (technique efficacy) was achieved in 93% cases, with a significant mean volume reduction at 12 months follow-up (68.7±10.8%), ($p<0.001$). VAS compression score and cosmetic score significantly improved at 12-months follow-up from 8±2 and 4±0 to 3±3 and 2±2 respectively ($p<0.05$).
**Conclusion:** The VT system could be useful in thyroid nodules ablation procedures assistance being able to track the RF electrode tip even when this is obscured by the bubbles produced by the ablative process. The combination of fusion imaging with VT assisted RF ablation represents a safe, non-surgical treatment option for patients with large substernal benign thyroid nodules.
**Limitations:** No limitations were identified.
**Ethics committee approval:** We obtained the approval of an ethic committee.
**Funding for this study:** No funding was necessary.
**Author Disclosures:**
Giacomo Leale: Nothing to disclose
Davide Orlandi: Nothing to disclose
Umberto Viglino: Nothing to disclose
Giovanni Mauri: Nothing to disclose
Giovanni Turtulici: Nothing to disclose
Giorgia Dedone: Nothing to disclose

### RPS 1509-8

### MRI-guided transurethral ultrasound ablation (TULSA) for the treatment of benign prostatic hyperplasia: early outcomes

*C. Wright*[1], M. Anttinen[2], A. Viitala[1], P. Mäkelä[1], I. Virtanen[2], T. Sainio[1], P. Taimen[2], R. Blanco Sequeiros[1], P. J Boström[2]; [1]Helsinki/FI, [2]Turku/FI
*(cwright67@gmail.com)*

**Purpose:** To investigate the early clinical safety and feasibility of MRI-guided transurethral ultrasound ablation (TULSA) for the treatment of benign prostatic hyperplasia (BPH).
**Methods or Background:** Men with lower urinary tract symptoms and in need of a surgical intervention were enrolled in this prospective, investigator-initiated, single-centre study. Patients were followed for at least 3 months. Efficacy outcomes included: uroflowmetry, PSA, and quality of life (QoL) questionnaires including EPIC-26, IPSS, and IIEF-5. MRI imaging was performed at baseline and 3-months. Adverse events (AEs) were classified according to the Clavien-Dindo scale. Any medication use pre- and post-TULSA was also monitored.
**Results or Findings:** A total of 17/20 patients have completed their 3-mo follow-up. At 3 months, the following median values were reported: prostate volume reduced by 25%, PSA reduced by 55%, post-void residual volume decreased by 24%, average flow rate increased by 100%, Qmax increased by 109%, voided volume decreased by 19%, IPSS improved from 16 to 6, IPSS QoL improved from 4 to 1, IIEF-5 improved from 17 to 21, EPIC-26 urinary incontinence domain improved from 86-97, and the EPIC-26 irritative/obstructive domain improved from 63-95. Improvements were observed in every measure despite the discontinuation of LUTS medication after TULSA. Median ablation time was 43 minutes, hospitalisation time was 24 hours and catheterisation time was 16 days. Six AEs were reported, including six grade 2 and one grade 3 event, including urinary tract infection, urinary retention, and epididymitis (grade 3), which all resolved by 3 months.
**Conclusion:** TULSA appears to be a promising surgical option for the treatment of BPH.
**Limitations:** A limitation of this study was the small sample size.
**Ethics committee approval:** Ethics committee approval was obtained and informed consent was obtained from all study participants.
**Funding for this study:** This study was funded by Profound Medical GmbH.
**Author Disclosures:**
Antti Viitala: Nothing to disclose
Peter J Boström: Speaker: Profound Medical GmbH
Pietari Mäkelä: Nothing to disclose
Ilari Virtanen: Nothing to disclose
Mikael Anttinen: Nothing to disclose
Teija Sainio: Nothing to disclose
Pekka Taimen: Nothing to disclose
Cameron Wright: Employee: Profound Medical GmbH
Roberto Blanco Sequeiros: Nothing to disclose

### RPS 1509-9

### 12-month functional and oncological outcomes of salvage TULSA for localised radio-recurrent prostate cancer

M. Anttinen, *C. Wright*, P. Mäkelä, R. Blanco Sequeiros, P. J Boström; Turku/FI
*(cwright67@gmail.com)*

**Purpose:** To evaluate the safety, functional and oncological outcomes of men undergoing MRI-guided transurethral ultrasound ablation (sTULSA) for treatment of localised radio-recurrent prostate cancer (PCa).
**Methods or Background:** In this prospective study (NCT03350529), patients underwent either whole-gland or partial sTULSA, depending on their tumour characteristics and patient preferences. Patients were followed every 3 months and adverse events (AEs, Clavien-Dindo scale), functional status questionnaires, uroflowmetry, and prostate-specific antigen (PSA) were assessed at every study visit. Disease control was assessed at 3 months (mpMRI) and 12 months by mpMRI, PSMA PET-CT, and prostate biopsy covering the treatment area plus areas suspicious on imaging.
**Results or Findings:** A total of 29 patients (median age 72 years) underwent sTULSA and 19 have completed their 12-monoth follow-up. Median baseline PSA was 3.8 ng/ml. AEs included two grade 3 events (2J stent and urethral stricture), two grade 2 events (osteitis pubis and IV antibiotics), seven grade 1 events (infection or urinary retention). Median PSA at 12 months post-TULSA for 18 available records was 0.19 ng/ml, with 5 patients having undetectable PSA. Three patients out of 30 have been diagnosed with biochemical recurrence, who also have extraprostatic disease on imaging. At 12-month biopsy follow-up, 14/16 (88%) patients were free of any PCa in the treatment region, with two out-of-field recurrences.
**Conclusion:** TULSA is a safe salvage therapy option with encouraging 12-month oncological outcomes.
**Limitations:** Limitations included the small sample size, only early-stage oncological and safety outcomes after treatment. Patients also had a diverse disease history prior to undergoing TULSA.
**Ethics committee approval:** All studies were conducted in accordance with the principles of the Declaration of Helsinki. Ethical approval was obtained for all studies and written informed consent was obtained.
**Funding for this study:** No funding was received for this study.
**Author Disclosures:**
Peter J Boström: Speaker: Profound Medical Corp.
Pietari Mäkelä: Nothing to disclose
Mikael Anttinen: Nothing to disclose
Cameron Wright: Employee: Profound Medical Corp.
Roberto Blanco Sequeiros: Nothing to disclose

### RPS 1509-10

### Percutaneous tumour ablation guided by electromagnetic navigation system: a retrospective study

*S. Ventura Díaz*, A. Olavarria Delgado, J. Cobos Alonso, R. R. Romera Sanchez, A. Palomera Rico, J. Sánchez Corral; Madrid/ES
*(sofi9417vd@gmail.com)*

**Purpose:** To assess the applicability of an electromagnetic navigation system (EMNS) as an auxiliary tool for abdominal tumour ablation.
**Methods or Background:** This study retrospectively collected patients with kidney and liver tumours treated with ablation from January 2020 to April 2021. The ablation modality was decided depending on the tumour characteristics. Subfrenic and subcapsulary lesions or tumours located less than 1 cm away from the bowel or gall bladder were categorised as high-risk. Major and minor complications were recorded. Median follow-up was 9 months after treatment.
**Results or Findings:** A total of 97 lesions were treated in 67 patients, with mean size of 18.9 mm (range 5-40). The majority (77.6%) were male, with a mean age of 66.5 years (range 20-88). 67.2% (45) were hepatocarcinomas, 20.9% (14) were liver metastases from colonic cancer, and 11.9% (8) were renal carcinomas. In total, 89 liver lesions were treated and 42% (38) of them were in high-risk locations. 91% (41) of hepatocarcinomas were treated with radiofrequency, all liver metastases with microwave and all kidney lesions with cryotheraphy. A total of 94% had a complete response within the first month, and 98% after a second ablation. Four major complications (2 active bleedings, 1 hepatic abscess and a pneumothorax) and 10 minor complications (8 autolimited bleedings and 2 autolimited neumothorax) were recorded.
**Conclusion:** EMNS (Imactis-CT®) helps radiologists reach non-visible lesions in non-contrast studies and/or with high risk locations. This reduces the number of complications regardless of radiologist experience and allows ablation as a valuable therapeutic option to many more patients.
**Limitations:** The retrospective nature of the study was an identified limitation.
**Ethics committee approval:** This study was approved by the institutional ethics committee.

**Funding for this study:** No funding was received for this study.
**Author Disclosures:**
Ana Palomera Rico: Nothing to disclose
Juan Sánchez Corral: Nothing to disclose
Sofía Ventura Díaz: Nothing to disclose
Jorge Cobos Alonso: Nothing to disclose
Rut Romera Romera Sanchez: Nothing to disclose
Andreina Olavarria Delgado: Nothing to disclose

### RPS 1509-11

**Multi-institutional study on safety and effectiveness of percutaneous RF, MW and cryo-ablation for T1 renal cancers: which is the best imaging guide?**

*L. Bertolotti*[1], F. Segato[2], F. Pagnini[1], D. Basile[3], P. Biondetti[4], A. Beltrame[3], C. Cicero[2], M. De Filippo[1]; [1]Parma/IT, [2]Bassano del Grappa (VI)/IT, [3]Orbassano (TO)/IT, [4]Milan/IT

**Purpose:** The aim of this study is to compare the outcomes and the safety of T1 renal cancers percutaneous thermal ablation techniques performed with different imaging guides. In addition, we aimed to investigate if different thermal sources influence the efficacy of the procedure.
**Methods or Background:** In this retrospective multi-institutional study, 194 renal cancers in 165 patients were treated with percutaneous image-guided thermal ablation over the period 2015-2020. All patients were staged according with TNM classification and only T1, N0, M0 were included prior to the procedure. The imaging modalities used for guiding were divided into three groups: CT (CT and CBCT), US (US and CEUS) and combined US-CT. Mean tumour size was 25 mm (range 4-56 mm). RF and MW procedures were labelled "heat", while procedures based on cryoablations were labelled "cold".
**Results or Findings:** Primary effectiveness rate, considered as the absence of residual or recurrent disease during follow-up, was: 83,5% in the CT group; 68% in the US group, and 100% in the combined US-CT group (p=0,004). Major and minor complications were, respectively: 1 and 11 in the CT group, 1 and 4 in the US group, and 0 and 3 in the combined US-TC group (p = 0,04). The mean rise in serum creatinine level was similar among the three groups. Primary effectiveness was similar among the group "heat" and the group "cold".
**Conclusion:** CT and combined US-CT guide showed a lower rate of residual or recurrent disease compared to US. US guide showed a lower rate of procedural complications.
**Limitations:** In this study, differences in general health conditions between the three groups were not investigated.
**Ethics committee approval:** Ethical approval was obtained from the ethics committees of the institutions.
**Funding for this study:** This study was not supported by any funding.
**Author Disclosures:**
Domenico Basile: Nothing to disclose
Andrea Beltrame: Nothing to disclose
Calogero Cicero: Nothing to disclose
Pierpaolo Biondetti: Nothing to disclose
Francesco Pagnini: Nothing to disclose
Massimo De Filippo: Nothing to disclose
Lorenzo Bertolotti: Nothing to disclose
Federica Segato: Nothing to disclose

**14:00-15:30** **Room O**

### Research Presentation Session: Cardiac

### RPS 1503

### Coronary artery CT for diagnosis and prognosis

**Moderator**
G. Aviram; Tel Aviv/IL

### RPS 1503-2

**Coronary CTA versus functional testing for the diagnosis of obstructive coronary artery disease: results from the collaborative meta-analysis of Cardiac CT (COME-CCT)**

P. Schlattmann[1], *V. Wieske*[2], K. K. Bressem[2], T. Götz[1], G. M. Schuetz[2], D. Andreini[3], G. Pontone[3], R. Haase[2], M. Dewey[4]; [1]Jena/DE, [2]Berlin/DE, [3]Milano/IT, [4]Berlin; On behalf of the COME-CCT Consortium/DE
*(viktoria.wieske@charite.de)*

**Purpose:** To determine the effectiveness of computed tomography angiography (CTA) and functional stress testing for diagnosis of obstructive coronary artery disease (CAD) in stable chest pain.
**Methods or Background:** 2920 patients from the international COME-CCT consortium were enrolled, in order to compare CTA with exercise electrocardiography (ECG) and single-photon emission CT (SPECT) for diagnosis of CAD (≥50% diameter stenosis) by invasive coronary angiography. Generalised linear mixed models were used, including non-diagnostic results as dependent variables, in a logistic regression model. Covariates were invasive coronary angiography, type of diagnostic method and their interactions.
**Results or Findings:** CTA showed significantly better diagnostic performance with a sensitivity of 94.6% (95% CI: 92.7–96) and specificity of 76.3% (72.2–80) compared to exercise-ECG with 54.9% (47.9–61.7) and 60.9% (53.4–66.3), SPECT with 72.9% (65–79.6) and 44.9% (36.8–53.4), respectively. The PPV of CTA was ≥50% in patients with a clinical pre-test probability of 10% or more, compared to ECG and SPECT at pre-test probabilities of ≥40% and 28%, respectively. CTA reliably excluded obstructive CAD with a post-test probability ≤15% in patients with a pre-test probability ≤74%.
**Conclusion:** In patients with stable chest pain, CTA is more effective than functional testing for the diagnosis as well as for reliable exclusion of obstructive CAD. CTA should become widely adopted in patients with an intermediate pre-test probability.
**Limitations:** Functional testing was not available in all patients (55%; n=2920). Fourteen studies (47.8%; n=1367) used CT scanners with less than 64 detector rows.
**Ethics committee approval:** The original studies were approved by the local ethics committees in the conduct of the primary studies within the COME-CCT Consortium.
**Funding for this study:** This study was funded by the joint program of the German Research Foundation (DFG) and the German Federal Ministry of Education and Research (BMBF, 01KG1110) to PS and MD; Digital Health Accelerator of the Berlin Institute of Health to MD.
**Author Disclosures:**
Robert Haase: Nothing to disclose
Theresa Götz: Nothing to disclose
Keno K. Bressem: Nothing to disclose
Georg M Schuetz: Research/Grant Support: Greports grants from the German Federal Ministry of Education and Research (BMBF), during the conduct of the study.
Peter Schlattmann: Research/Grant Support: PS has support from the German Research Foundation. Research/Grant Support: Joint programme of the German Research Foundation and the German Federal Ministry of Education and Research for the submitted work. Grant Recipient: Grants from Bayer Pharma AG. Grant Recipient: Grants from the European Union.
Viktoria Wieske: Research/Grant Support: VW reports grant support from the FP7 Program of the European Commission for the randomized multicenter DISCHARGE trial (603266-2, HEALTH-2012.2.4.-2).
Marc Dewey: Patent Holder: Professor Dewey holds a joint patent with Florian Michallek on dynamic perfusion analysis using fractal analysis (PCT/EP2016/071551). Other: Editor: Cardiac CT (Springer Nature). Other: Hands-on cardiac CT courses (www.ct-kurs.de) Institutional research agreements: Siemens, General Electric, Philips, Canon. Other: Grants: EU (603266-2) DFG (DE 1361/14-1, DE 1361/18-1, BIOQIC GRK 2260/1, Radiomics DE 1361/19-1 [428222922] and 20-1 [428223139] in SPP 2177/1), Berlin University Alliance (GC_SC_PC 27), Berlin Institute of Health (Digital Health Accelerator). Other: M.D. is European Society of Radiology (ESR) Research Chair (2019–2022) and the opinions expressed in this presentation are the author's own and do not represent the view of ESR.
Daniele Andreini: Nothing to disclose
Gianluca Pontone: Other: GP is on the speakers bureau for Medtronic and Bracco; Grant Recipient: GP reports grants from General Electric.

### RPS 1503-3

**Atherosclerotic plaque characteristics and their dynamics assessed by CT angiography in patients with acute coronary syndrome**

*A. Semenova*, I. Merkulova, M. Shariya, N. Barysheva, O. Kolesnikova, I. Staroverov, S. K. Ternovoy; Moscow/RU
*(alina.sema.147@mail.ru)*

**Purpose:** Assessment of coronary atherosclerotic plaque (ASP) features and their changes for more than 1 year in patients with acute coronary syndrome (ACS).
**Methods or Background:** This study included 40 patients aged 61.9±11 years with ACS. All patients underwent coronary CT angiography (CTA) (320-row CT scanner) 3-6 days after percutaneous coronary intervention for culprit lesions and after 17±6.2 months. We analysed the degree of stenosis, plaque type, size and signs of ASP instability (napkin-ring sign, positive remodelling of the artery, spotty calcifications, rough contour).
**Results or Findings:** There were 68 (66.7%) soft and 34 (33.3%) combined ASPs. In summary, 6 (5.9%) of plaques transformed into another type (4 from soft to combined and 2 from combined to calcified). At the end of the observation period all size characteristics of the plaques increased: stenosis degree from 58.3±16.3 to 60.4±16.1% (p=0.049), plaque burden from 74.2±11.1 to 75.9±11.2% (p=0.043), plaque length from 10.0 [7.2; 14.0] by

0.25 [0.00; 1.00] mm (p=0.008). Mean and minimal plaque density and the remodelling index were not significantly changed. The baseline incidence of various CT signs of instability in non-culprit plaques was 17-45%. Subsequent appearance or disappearance of at least one of these signs was observed in 24 plaques (24.2%). A relationship between appearance of positive remodelling and napkin-ring sign (U=0.236, p<0.001) was found, as well as between disappearance of rough contour and positive remodelling (U=0.102, p=0.008).
**Conclusion:** Changes in plaque features in the observation period reflect natural atherosclerotic lesion progression. The appearance or disappearance of at least one instability sign in non-culprit plaques was noted in a quarter of them. The relationships between appearance or disappearance of the above-mentioned instability signs reflects the processes of plaque 'destabilisation' and 'stabilisation'.
**Limitations:** An identified limitation was the small cohort of patients.
**Ethics committee approval:** This study was approved by an ethics committee.
**Funding for this study:** No funding was received for this study.
**Author Disclosures:**
Sergey K Ternovoy: Nothing to disclose
Alina Semenova: Nothing to disclose
Merab Shariya: Nothing to disclose
Nataliya Barysheva: Nothing to disclose
Olga Kolesnikova: Nothing to disclose
Irina Merkulova: Nothing to disclose
Igor Staroverov: Nothing to disclose

### RPS 1503-4

**Usefulness and clinical implications of plaque analysis and pFAI for the evaluation of cardiovascular risk**

*G. Cabrelle*, V. Pergola, S. Cattarin, A. Giorgino, C. Giraudo, G. Mattesi, R. Stramare, R. Motta; Padova/IT

**Purpose:** Coronary-computed-tomographic-angiography (CCTA) represents a non-invasive approach to analyse coronary plaques. The principal aim of this study was to compare clinical characteristics and outcomes (death, necessity of percutaneous angioplasty or by-pass procedure) of patients with different plaque compositions. The secondary aim was to analyse the correlation between the plaque density and peri-coronary-fat-attenuation-index (pFAI).
**Methods or Background:** Patients who underwent CCTA for evaluation of ischaemic heart disease in our facility were retrospectively recruited from 03/2016 to 06/2021. CCTA was done on Aquilion ONE ViSION (Toshiba Medical Systems, Otawara, Japan); pFAI was calculated with Aquarius Workstation (version 4.4.13; TeraRecon Inc., Foster City, CA). Exclusion criteria: age <18 years; history of coronary-stenting, cardiac surgery, coronary dissection, congenital-heart-disease, coronary anomalies. 372 patients were finally included (237 male, 57±15 years). Patients were divided into 3 groups: 37 (9.9%) with high attenuation plaques - HAPs (>60 HU), 137 (36.8%) with low attenuation plaques - LAPs (<29 HU and a volume ≥15 mm3 and/or 30-59 HU with a volume > 52 mm3) and 198 (53.2%) without plaques. Clinical, haematochemical, pharmacological, EKG and echocardiographic parameters were collected.
**Results or Findings:** LAPs were more numerous in elderly male patients (p<0.001). Dyslipidaemia and diabetes positively correlated with LAPs (p<0.001). Patients with LAPs had higher pFAI and more plaques (p=0.005). The overall volume of LAPs was greater than HAPs (p=0.009). A favourite localisation of LAPs in the anterior descendant artery with higher stenosis (p <0.001) was also noted. Follow-up demonstrated that LAPs independently predisposed to outcomes (p=0.04).
**Conclusion:** Plaque analysis is effective in identifying “at risk” plaques. LAPs are related to higher pFAI, supporting the hypothesis that inflammation plays a role in the plaques’ composition.
**Limitations:** Identified limitations of this study were the retrospective design and the fact that a heterogeneous population was used.
**Ethics committee approval:** This study was approved by the institutional review board.
**Funding for this study:** No funding was received for this study.
**Author Disclosures:**
Giulio Cabrelle: Nothing to disclose
Raffaella Motta: Nothing to disclose
Roberto Stramare: Nothing to disclose
Adelaide Giorgino: Nothing to disclose
Chiara Giraudo: Nothing to disclose
Simone Cattarin: Nothing to disclose
Giulia Mattesi: Nothing to disclose
Valeria Pergola: Nothing to disclose

### RPS 1503-5

**Can super resolution deep learning reconstruction upgrade the atheroma burden in CAD-RAS 0-2 patients?**

*M. Ohana*[1], F. Tatsugami[2], A. Labani[1], S. El Ghannudi[1], A. Taniguchi[3], K. Haioun[3], K. Awai[2], C. Roy[1]; [1]Strasbourg/FR, [2]Hiroshima/JP, [3]Otawara/JP

**Purpose:** In patients with non-obstructive coronary artery disease on coronary CT angiography (CCTA), the overall cardiovascular risk assessment can be based on the Agatston score and/or the number of involved coronary segments. Whether the use of a super resolution deep learning reconstruction (SR-DLR) algorithm could better discriminate minimal coronary atherosclerosis or not is unknown. Our objective is therefore to compare the number of abnormal coronary segments diagnosed on CCTA when using iterative reconstruction (IR), standard deep learning reconstruction (DLR) and SR-DLR.
**Methods or Background:** Thirty non-consecutive CAD-RADS 0-2 CCTA with absence of kinetic artifacts, coronary enhancement above 350 HU and Agatston score <500 acquired on a 4th generation 320-row scanner were retrospectively included and reconstructed using IR, DLR and SR-DLR. Three readers analysed all reconstructions in a random order and graded 11 coronary segments per reconstruction in a binary fashion (normal/abnormal), in addition to the global CAD-RADS. Variations in numbers of abnormal segments were compared at a patient level and at a population level.
**Results or Findings:** The average Agatston score was 29 ±44. The mean DLP was 68 ±24. Ten CCTA were classified as CAD-RADS 0, 10 as CAD-RADS 1 and 10 as CAD-RADS 2 using IR and DLR. With SR-DLR, 1 CCTA was upgraded from CAD-RADS 0 to 1 and 1 downgraded from CAD-RADS 2 to 1. The overall number of abnormal coronary segments was higher using SR-DLR (2.4 ±2.7) compared to DLR and IR (1.9 ±2.2 and 2.1 ±2.3, respectively, p<0.05). Per patient, the number of abnormal coronary segments was increased by a mean of 0.7 ±1.2 when using SR-DLR.
**Conclusion:** SR-DLR better identifies minimal non-obstructive coronary atherosclerosis compared to IR and DLR, which could better stratify patient's cardiovascular risk.
**Limitations:** One identified limitation was the fact that this was a retrospective preliminary study.
**Ethics committee approval:** This study was approved by an ethics committee.
**Funding for this study:** No funding was received for this study.
**Author Disclosures:**
Aissam Labani: Nothing to disclose
Soraya El Ghannudi: Nothing to disclose
Fuminari Tatsugami: Nothing to disclose
Karim Haioun: Employee: Canon Medical Systems
Catherine Roy: Nothing to disclose
Kazuo Awai: Nothing to disclose
Akira Taniguchi: Employee: Canon Medical Systems
Mickaël Ohana: Consultant: Canon Medical SysteEurope

### RPS 1503-6

**Levels of troponin T may predict the overall plaque burden of patients with chronic coronary syndrome**

*M. Vecsey-Nagy*, J. Csőre, M. Kolossvary, M. Boussoussou, B. Vattay, P. Maurovich-Horvat, B. Szilveszter; Budapest/HU
*(vnagymilan@gmail.com)*

**Purpose:** Laboratory parameters of patients with suspected coronary artery disease (CAD) may yield the potential of adding incremental value to the cardiovascular (CV) risk prediction of patients with chest pain even if acute myocardial infarction has been excluded. The level of high-sensitivity cardiac troponin T has demonstrated promising results in the setting of chronic coronary syndrome and it is plausible that it may provide valuable information regarding plaque burden in patients with chest pain and low to intermediate CV risk.
**Methods or Background:** Overall, 243 consecutive patients referred for coronary computed tomography angiography due to suspected coronary artery disease (CAD) were enrolled in a tertiary referral centre. Segment stenosis score (SSS) was calculated to characterise overall plaque burden. For each coronary segment, an estimate of worst diameter stenosis per segment was performed, scored as minimal (<25%; score: 0), mild (25-49%; score: 1), moderate (50-69%; score: 2) or severe (≥70%; score: 3). The SSS was calculated as the sum of the individual segment scores. Uni- and multivariate linear regression analyses were used to identify predictors of SSS.
**Results or Findings:** In the recruited 243 patients (mean age: 57.3±11.8, 44.0% female), 70.0% had CAD. The independent predictors of SSS were female sex (B=-0.07 [95%CI: -0.12–-0.01], p=0.02), total Agatston score (B=0.001 [95%CI: 0.001–0.001], p=<0.001) and the level of troponin T (B=-8.28 [95%CI: 3.94–12.62], p=0.048).

**Conclusion:** It is plausible that elevated troponin levels are the result of clinically silent plaque ruptures and subsequent microembolisations in patients with chronic coronary syndrome. The measurement of troponin may potentially allow for a more precise CV risk stratification of patients with stable chest pain.
**Limitations:** The fact that only low to intermediate cardiovascular risk patients were enrolled was an identified limitation.
**Ethics committee approval:** The local ethics committee approved the current research.
**Funding for this study:** No funding was received for this study.
**Author Disclosures:**
Melinda Boussoussou: Nothing to disclose
Pál Maurovich-Horvat: Nothing to disclose
Milán Vecsey-Nagy: Nothing to disclose
Marton Kolossvary: Nothing to disclose
Judit Csőre: Nothing to disclose
Bálint Szilveszter: Nothing to disclose
Borbála Vattay: Nothing to disclose

### RPS 1503-7

**Association between coronary artery disease and clinical outcome in cancer patients: a propensity score matching analysis**

*Z. Huang*; Wuhan/CN
*(304527885@qq.com)*

**Purpose:** The aim of this study was to evaluate the prognostic value of coronary computed tomography angiography (CTA) to predict the risk of all-cause mortality in cancer patients in a propensity score matching (PSM) analysis.
**Methods or Background:** A total of 331 patients who previously had cancer and underwent coronary CTA from January 2015 to December 2019 were included. Multivariate Cox proportional hazards regression analysis and propensity-score matching analysis were performed. The primary endpoint was all-cause of mortality.
**Results or Findings:** In total, 125 patients with obstructive coronary artery disease (CAD) and 206 with non-obstructive CAD during a median follow-up of 3.3 years were included in this study. After PSM, age (HR, 1.040; 95%CI, 1.001-1.081; p=0.046), smoking history (HR, 2.344; 95%CI, 1.211-4.536; p=0.011), diabetes mellitus (HR, 1.862; 95%CI, 1.053-3.292; p=0.033) and obstructive CAD (HR, 1.845; 95%CI, 1.008-3.377; p=0.047) remained significant factors for all-cause mortality.
**Conclusion:** Obstructive CAD evaluated by coronary CTA was found to be at higher risk for developing all-cause mortality in cancer patients.
**Limitations:** A number of limitations were identified. Firstly, this study was a retrospective study with potential for selection bias and missing values. To overcome these limitations we performed Cox multivariate analysis and propensity-matching analysis with adjustment for potential confounders, but we could not adjust for unmeasured potential confounders. Secondly, there was a lack of information on duration between CAD diagnosis and cancer diagnosis. Finally, the follow-up duration was relatively short to assess the long-term all-cause mortality. Nevertheless, this study suggests that coronary CTA will be instrumental in risk stratification of cancer patients. Further studies are needed to better define these observations.
**Ethics committee approval:** The present study was approved by the ethics committee of the Central Hospital of Wuhan, Tongji Medical College, Huazhong University of Science and Technology.
**Funding for this study:** No funding was received for this study.
**Author Disclosures:**
Zengfa Huang: Nothing to disclose

### RPS 1503-8

**Myocardial late contrast enhancement in patients presenting with acute chest pain syndrome: focus on obstructive coronary artery disease**

*D. Vignale*, A. Palmisano, C. Colantoni, V. Nicoletti, L. Brunetti, M. Slavich, M. Montorfano, A. Esposito; Milano, MI/IT
*(vignale.davide@hsr.it)*

**Purpose:** Acute chest pain with mild troponin rise represents a diagnostic conundrum. A recent study (doi:10.1148/radiol.211288) demonstrated the diagnostic value of a late contrast enhancement (LCE) scan in addition to coronary CT angiography (CTA) in patients with non-obstructive coronary artery disease (CAD). However, the value of LCE in patients with obstructive CAD is undetermined.
**Methods or Background:** Prospective study on 36 patients (men=20 [58%], median age 64 [IQR48-77] years) presenting to emergency department with acute chest pain or anginal equivalent with troponin rise (median 154 [IQR45-510] ng/L) undergoing CTA (to evaluate CAD status and wall motion abnormalities) with LCE for myocardial tissue characterisation.
**Results or Findings:** Fourteen (39%) patients had negative CTA: at LCE, 9 (64%) had myocarditis, 3 (21%) had Takotsubo and 2 (14%) had myocardial infarction with non-obstructed coronary arteries. Twenty-two (61%) patients had obstructive CAD (stenosis >50%) involving one vessel in 16 (73%) and two and three vessels in 3 (13.5%) each. Among these, 12 (55%) had ischaemic LCE, mostly with transmural distribution (10 [83%]) involving a median of 4 [IQR: 3-5] segments, with microvascular obstruction (MVO) in 3 (30%). Two (17%) had subendocardial LCE involving two segments. LCE was always in the territory of obstructive lesion in one-vessel CAD (9 [75%]) and in the territory of worse stenosis in multi-vessel CAD (3 [25%]). Ten (45%) patients with obstructive CAD had preserved wall thickness and no LCE, with hypokinesia in 6 (60%), and were diagnosed with non-ST elevation myocardial infarction.
**Conclusion:** This study confirms the value of LCE in patients with negative CTA. Furthermore, it shows that in patients with obstructive CAD, LCE can detect loss of viable myocardium and predictors of worse prognosis (MVO and transmurality) and helps in identifying the culprit lesion, especially in multivessel CAD, thus guiding revascularisation.
**Limitations:** The small sample size was an identified limitation.
**Ethics committee approval:** This study was approved by the IRB: CTMyoC. Written informed consent was obtained.
**Funding for this study:** Not applicable.
**Author Disclosures:**
Valeria Nicoletti: Nothing to disclose
Massimo Slavich: Nothing to disclose
Davide Vignale: Nothing to disclose
Antonio Esposito: Nothing to disclose
Caterina Colantoni: Nothing to disclose
Anna Palmisano: Nothing to disclose
Lisa Brunetti: Nothing to disclose
Matteo Montorfano: Nothing to disclose

### RPS 1503-9

**Prevalence and extent of mitral annular dysjunction in a normal population: a comprehensive morphometric analysis on CT coronary angiography**

*S. Sharma*, R. Rajagopal, P. K. Garg, P. S. Khera, T. Yadav, S. Tiwari, B. Sureka, V. S. Arunachalam; Jodhpur/IN
*(drsmilysharma@gmail.com)*

**Purpose:** The aim of the study was to retrospectively evaluate the prevalence and extent of mitral annular disjunction (MAD) in a normal population without any evidence of arrhythmias.
**Methods or Background:** Using CT coronary angiography scans of patients referred to our institute, a comprehensive 3D morphometric analysis of the presence and circumferential extent of MAD was conducted from August 2021 to January 2022. A total of 50 consecutive patients each from age groups <40 years, 40-50 years, 50-60 years, and >60 years were selected, making a total of 200 patients. Patients with evidence of arrhythmias and mitral valve disease were excluded. Systolic datasets were evaluated for the presence of fibrous separation between the posterior mitral leaflet and left ventricular myocardium by rotating the view plane around the centre of the mitral valve. The maximum degree of disjunction was recorded, if present.
**Results or Findings:** MAD was present in 122 patients (61%) with the most frequent involvement of P1 (n: 94) and P3 (n: 74) scallops of the posterior mitral valve leaflet. According to age-wise distribution, MAD was present in 53% of the patients with age <40 years, 60% of the patients with age 40-50 years, 46% of the patients with age 50-60 years, and 66 % in age >60 years. The mean distance of MAD involving P1 was 1.1+/- 1.3 mm, P2 was 0.78 +/- 1.3 mm and P3 was 0.97 +/- 1.3 mm.
**Conclusion:** MAD is a common finding in CT coronary angiography with maximal involvement of P1 and P3 scallops of the posterior mitral valve leaflet. There is no significant statistical difference in the prevalence of mitral annular disjunction in different age groups.
**Limitations:** The following limitations were identified: this was a single-centre study; patients with coronary artery disease were not excluded from the study.
**Ethics committee approval:** This study was approved by an ethics committee.
**Funding for this study:** No funding was received for this study.
**Author Disclosures:**
Sarbesh Tiwari: Nothing to disclose
Rengarajan Rajagopal: Nothing to disclose
Pawan Kumar Garg: Nothing to disclose
Pushpinder Singh Khera: Nothing to disclose
Venkata Subbaih Arunachalam: Nothing to disclose
Binit Sureka: Nothing to disclose
Smily Sharma: Nothing to disclose
Taruna Yadav: Nothing to disclose

### RPS 1503-10

**The cardiac paradigm in acute stroke evaluation: findings in dual energy CT cardiac screening**

*G. Priyadharshinee*, P. A, K. M. Athiyappan, M. Nedunchelian, S. Varadharajan; Coimbatore, Tamil Nadu/IN
*(atypicaldoc@gmail.com)*

**Purpose:** To study the spectrum of findings in additional cardiac screening during evaluation of acute multiphasic CT angiography (CTA) in ischemic stroke patients of suspected embolic aetiology, on a dual energy CT scanner.
**Methods or Background:** Cardioembolic stroke requires early detection due to differences in antithrombotic management. Currently, they are mostly detected in the subacute phase using echocardiography.
This single-centre, observational prospective open pilot study included stroke patients with suspected cardioembolic aetiology who underwent CT cardiac screening as part of the initial CTA and findings were noted. Multiphasic CT cerebral angiography was carried out with dual source DECT with bolus-tracking method, followed by venous phase cardiac screening with retrospective ECG-gated DE acquisition and half-rotation reconstruction. DE perfusion blood volume (PBV) application was used to identify potential sources of embolism. A total of 37 patients were included in the study.
**Results or Findings:** Out of the 37 patients, 14 patients had abnormal cardiac findings on CT: intra cavitary thrombus, infarcts, LV aneurysm, septal defect, valvular calcifications, tumours and aortic arch plaques. Clot detection in CT angiography was 5 out of 37 (13.5%).
**Conclusion:** Including ECG-gated cardiac screening in multiphasic cerebral CTA of acute ischemic stroke patients enhances the diagnostic yield of detecting cardioembolic causes. ECG-gated cardiac screening as a part of routine cerebral CTA during the acute phase may be a promising adjunct in cardiac thrombi detection in patients with ischemic stroke of suspected embolic aetiology.
**Limitations:** An identified limitation was the small sample size.
**Ethics committee approval:** Not applicable.
**Funding for this study:** No funding was received for this study.
**Author Disclosures:**
Gayathri Priyadharshinee: Nothing to disclose
Shriram Varadharajan: Nothing to disclose
Pudhiavan A: Nothing to disclose
Karthikeyan Muthugounder Athiyappan: Nothing to disclose
Meena Nedunchelian: Nothing to disclose

**14:00-15:30 Room X**

### Research Presentation Session: Imaging Informatics / Artificial Intelligence and Machine Learning

### RPS 1505

### Artificial intelligence (AI) in neuroimaging

**Moderator**
S. Bisdas; London/UK

### RPS 1505-2

**Real-life evaluation of the AI-based neuroradiology suite mdbrain**

J. Albert[1], M. Fernandez[1], M. Thauerer[2], F. Gärtner[3], S. Hock[4], D. Trautmann[1], S. Peters[3], K. Egger[2], *J. R. Opalka*[1]; [1]Berlin/DE, [2]Zell am See/AT, [3]Kiel/DE, [4]Erlangen/DE
*(JROpalka@t-online.de)*

**Purpose:** To assess whether the AI-based system mdbrain leads to efficiency gains when used in a real-life setting
**Methods or Background:** We asked 7 radiologists from 5 sites to assess subsequent radiological images as part of their daily routine, in total 285 (128/157 with/without the system's support, resp.). Diagnosis (Dementia/MS) and reading time were documented. Additionally, the system's subjective influence on the radiological report was surveyed.
**Results or Findings:** The median assessment time was significantly reduced by 25% when mdbrain was used ($p < 0.001$) equivalent to 1:56min. This reduction was significant for both diagnoses, and more pronounced for dementia (-57%) compared to MS cases (-13%). We further observed a strong correlation between years of experience in radiology vs reduction of reading times (R=0.76, p=0.05). Radiologists reported that mdbrain had a diagnostic impact in 118/128 AI-aided assessments. Among these cases, radiologist reported that mdbrain "reinforced their original assessment" in 76 cases, "enabled a clearer diagnosis" in 25 cases, "reported anomalies that could have been missed" in 7 cases, and "lead to confusion or less clear diagnosis" in 9 cases. Therefore, mdbrain had a positive diagnostic impact in 108 out of 128 cases (84%).
**Conclusion:** In the majority of cases (84%) the AI-based system had a positive qualitative impact on the diagnostic process. In terms of efficiency, we observed a clear drop in reading times (median -25%) whereas the effect was more pronounced in dementia (median -57%).
**Limitations:** There was limited control of the length of personal experience with the AI-system and the sample size was rather low with distribution of dementia and MS cases uneven across participants.
**Ethics committee approval:** Not applicable
**Funding for this study:** The Health Reality Lab Network is funded by the German Federal Ministry for Economic Affairs and Energy (Program: Smart Service World II).
**Author Disclosures:**
Jonas Albert: Nothing to disclose
Sönke Peters: Nothing to disclose
Jens Ruediger Opalka: Employee: mediaire GmbH
Karl Egger: Advisory Board: mediaire GmbH
Maria Fernandez: Nothing to disclose
Friederike Gärtner: Nothing to disclose
Stefan Hock: Nothing to disclose
Michael Thauerer: Nothing to disclose
Dietmar Trautmann: Nothing to disclose

### RPS 1505-3

**Comparison of 3 deep learning models to perform a multiclass CT-scan segmentation of traumatic brain lesions: trained from scratch vs pre-trained vs fine-tuned**

*C. Brossard*, J. Grèze, J-A. de Busschère, A. Attye, C. Acquitter, J-F. Payen, E. L. Barbier, P. Bouzat, B. Lemasson; Grenoble/FR
*(clement.brossard@univ-grenoble-alpes.fr)*

**Purpose:** Evaluation of 3 deep learning models to perform a multiclass CT-scan segmentation of traumatic brain lesions : trained from scratch vs trained vs fine-tuned.
**Methods or Background:** We retrieved 84 CT-scans acquisition of 29 patients acquired at the hospital CHUGA (Grenoble, France). We automatically segmented intraparenchymal (IPH), extra-axial (EAH), intraventricular hemorrhage (EAH) and oedema volumes using the CNN BLAST-CT [Monteiro, 2020], pre-trained on 184 scans. We then manually corrected these segmentations to obtain a ground truth. Then, we evaluated the performance of 3 deep learning models (DeepMedic-based architecture) to perform a multiclass CT-scan segmentation: i) a new model trained from scratch, ii) the pre-trained model from BLAST-CT and ii) the pre-trained model from BLAST-CT fine-tuned on our data set. Performances on the test data set were measured as mean Dice score for each lesion type.
**Results or Findings:** On our test data set, BLAST-CT provided Dice scores of 0.351, 0.295, 0.140, 0.108 on HIP, EAH, oedema and IVH respectively. The new model trained from scratch provided better results (0.425, 0.417, 0.510, 0.459) although outperformed by the fine-tuned model (0.538, 0.458, 0.593, 0.493).
**Conclusion:** Based on our results, it seems more efficient to fine-tune a pre-trained model in order to perform a multiclass CT-scan segmentation of traumatic brain lesions.
**Limitations:** BLAST-CT was trained on manual segmentations and CT-scans which may be different from ours because of inter-observer and inter-acquisition variabilities. Other metrics beside the Dice could be used to evaluate the segmentations.
**Ethics committee approval:** This study was reviewed and approved by the French institution Comité de protection des personnes Sud-Ouest/Outre-Mer-II.
**Funding for this study:** This work has been supported by the Fondation des Gueules Cassées (Paris, France) and the hospital CHUGA through the project Radiomic-TBI.
**Author Disclosures:**
Jules-Arnaud de Busschère: Nothing to disclose
Clément Acquitter: Nothing to disclose
Benjamin Lemasson: Nothing to disclose
Jean-François Payen: Nothing to disclose
Clément Brossard: Nothing to disclose
Pierre Bouzat: Nothing to disclose
Jules Grèze: Nothing to disclose
Emmanuel L. Barbier: Nothing to disclose
Arnaud Attye: Nothing to disclose

### RPS 1505-4

**Deep learning super-resolution MR spectroscopic imaging of brain metabolism and mutant IDH glioma**

X. Li[1], *O. Andronesi*[2]; [1]Melbourne, FL/US, [2]Charlestown, MA/US

**Purpose:** To develop deep learning super-resolution techniques for MR spectroscopic imaging (MRSI) to map tumor metabolism in patients with mutant IDH glioma.
**Methods or Background:** We developed a deep learning method based on generative adversarial network (GAN) using Unet as generator network to upsample MRSI by a factor of 4. Neural networks were trained on simulated metabolic images from 75 glioma patients. The performance of deep neuronal networks was evaluated on MRSI data measured in 20 glioma patients and 10 healthy controls at 3T with a whole-brain 3D MRSI protocol optimised for detection of D-2-hydroxyglutarate (2HG). To further enhance the structural details of metabolic maps we used prior information from high resolution anatomical MR imaging. Super-resolution MRSI was compared to ground truth by Mann-Whitney U-test of peak SNR (PSNR), structure similarity index measure (SSIM), feature based similarity index measure (FSIM), and mean-opinion-score (MOS).
**Results or Findings:** The proposed deep learning super-resolution improved PSNR by 17%, SSIM by 5%, FSIM by 7%, and MOS by 30% compared to conventional interpolation methods. In mutant IDH glioma patients the proposed method provided the highest resolution for 2HG maps that delineate clearly tumor margins and tumor heterogeneity.
**Conclusion:** Our results indicate that the proposed deep learning methods are effective in enhancing the spatial resolution of metabolite maps. The results for the MRSI data acquired in glioma patients suggest that the proposed methods have great clinical potential for image guided therapy of surgery, radiation, chemotherapy to deliver precision oncology.
**Limitations:** Not applicable
**Ethics committee approval:** Not applicable
**Funding for this study:** Not applicable
**Author Disclosures:**
Ovidiu Andronesi: Nothing to disclose
Xianqi Li: Nothing to disclose

### RPS 1505-5

**A deep learning algorithm for the automatic detection of intracranial haemorrhage on CT head imaging**

*M. Yeo*[1], B. Tahayori[1], H. K. Kok[1], J. T. Maingard[1], N. Kutaiba[1], A. Jhamb[1], M. Brooks[1], C. Barras[2], H. Asadi[3]; [1]Melbourne/AU, [2]Adelaide/AU, [3]Clayton/AU

**Purpose:** Deep learning (DL) algorithms are often used in automatic medical image analysis. This study aims to evaluate a DL implementation for the automatic detection of intracranial haemorrhage and its subtypes (extradural (EDH), intracerebral (ICH), intraventricular (IVH), subarachnoid (SAH) and subdural (SDH) haemorrhage) on non-contrast CT (NCCT) head studies.
**Methods or Background:** The DL algorithm was trained and externally validated on open-source, multi-centre retrospective data containing radiologist-annotated NCCT head studies, from research centres across Canada, the United States, Brazil and India. To improve model performance, all input NCCT images were preprocessed, using CT image windowing and adjacent slice image concatenation techniques. Additionally, a convolutional neural network-recurrent neural network (CNN-RNN) framework was used, instead of a single CNN alone. The area under the receiver operating characteristic curve (AUC-ROC) was used to evaluate model performance. To increase explainability, the developed model also output heatmap images which highlighted input image pixels which contributed most significantly to the model's final predictions.
**Results or Findings:** The training and test datasets contained 21,744 and 491 NCCT head studies, respectively, with 8,882 (40.8%) and 205 (41.8%) positive for intracranial haemorrhage. For the detection of any intracranial haemorrhage, and the subtypes of EDH, ICH, IVH, SAH and SDH, the model achieved AUC-ROCs of 0.966, 0.971, 0.983, 0.991, 0.949, and 0.953, respectively. Additionally, the generated heatmap images showed potential benefit in helping to rationalise the predictions (both correct and incorrect) produced by the model.
**Conclusion:** The DL model accurately detected intracranial haemorrhage and its subtypes, demonstrating clinical potential as a decision support tool and an automated system to improve radiologist workflow efficiency.
**Limitations:** The datasets used contained class imbalances. Additionally, the influence of haemorrhage mimics had not been evaluated in this study.
**Ethics committee approval:** Not applicable
**Funding for this study:** Not applicable
**Author Disclosures:**
Julian Tam Maingard: Nothing to disclose
Ashu Jhamb: Nothing to disclose
Mark Brooks: Nothing to disclose
Christen Barras: Nothing to disclose
Melissa Yeo: Nothing to disclose
Numan Kutaiba: Nothing to disclose
Bahman Tahayori: Nothing to disclose
Hong Kuan Kok: Nothing to disclose
Hamed Asadi: Nothing to disclose

### RPS 1505-6

**Preoperative magnetic resonance imaging radiomics for predicting early recurrence of glioblastoma**

*X. Yi*[1], Y. Fu[1], B. T. Chen[2]; [1]Changsha/CN, [2]Duarte, CA/US
*(yixiaoping@csu.edu.cn)*

**Purpose:** Early recurrence of glioblastoma after standard treatment makes patient care challenging. This study aimed to assess preoperative magnetic resonance imaging (MRI) radiomics for predicting early recurrence of glioblastoma.
**Methods or Background:** A total of 122 patients (training cohort: n = 86; validation cohort: n = 36) with pathologically confirmed glioblastoma were included in this retrospective study. Preoperative brain MRI images were analysed for both radiomics and the Visually Accessible Rembrandt Image (VASARI) features of glioblastoma. Models incorporating MRI radiomics, the VASARI parameters, and clinical variables were developed and presented in a nomogram. Performance was assessed based on calibration, discrimination, and clinical usefulness.
**Results or Findings:** The nomogram consisting of the radiomic signatures, the VASARI parameters, and blood urea nitrogen (BUN) values showed good discrimination between patients with early recurrence and those with later recurrence, with an area under the curve of 0.85 (95% CI, 0.77-0.94) in the training cohort and 0.84 (95% CI, 0.71-0.97) in a validation cohort. Decision curve analysis demonstrated favourable clinical application of the nomogram.
**Conclusion:** This study shows the potential usefulness of preoperative brain MRI radiomics in predicting the early recurrence of glioblastoma, which should help in personalised management of glioblastoma.
**Limitations:** This study had several limitations. First, this was a retrospective study at a single institution, limiting the generalisability of our study results. In addition, our sample size was still modest for a radiomic study, given the heterogeneous nature of GBM. Lastly, we used 2D texture features of the brain MRI images. A 3D approach for textural features may offer more information about the entire tumor, which may improve predictive model performance.
**Ethics committee approval:** Ethical approval was obtained from our hospital (IRB number: 201607831). Informed consent was waived.
**Funding for this study:** Not applicable
**Author Disclosures:**
Yan Fu: Nothing to disclose
Bihong T Chen: Nothing to disclose
Xiaoping Yi: Nothing to disclose

### RPS 1505-7

**Convolutional neural network-based segmentation of the posterior limb of the internal capsule correlates with motor outcome in very preterm neonates**

*M. Galijašević*, N. Gruber, M. Regodic, M. Hammerl, R. Steiger, A. E. Grams, C. Siedentopf, E. R. Gizewski, T. Janjic; Innsbruck/AT
*(malikgalijasevic@icloud.com)*

**Purpose:** To access differences in the posterior limb of the internal capsule (PLIC) myelination between very preterm neonates with favourable and those with poor motor outcome scores using convolutional neural network (CNN) based approach.
**Methods or Background:** 498 very preterm neonates received an MRI at term-equivalent age and were included in the study. Training data consisted of 100 T1 weighted images with the PLIC manually segmented by an expert. Another 398 images were segmented automatically using an in-house developed CNN. To extract morphometric parameters of PLIC on one specific level we employed a CNN performing a smoothed version of slice selection. The motor outcomes were determined using the Bayley Scale of Infant Development at 1 year of age. Infants with motor scores above 85 were regarded to have a normal motor outcome, the infants with scores below 85 were considered to have delayed outcome, whereas infants with motor scores below 70 were considered to have impaired outcome.
**Results or Findings:** On the chosen level at the boundary between the middle and upper third of the thalamus, the PLIC was significantly larger in neonates with the normal motor outcome than in the neonates with the impaired outcome ($p<0.0001$ for right side, and $p<0.01$ for left side).

**Conclusion:** Our preliminary results could indicate a significantly larger PLIC in neonates with the normal motor outcome than those with impaired motor outcome. Our research group is currently conducting further studies to evaluate prediction possibilities of CNN-based algorithms regarding outcomes in very preterm neonates.
**Limitations:** This study was limited by its status as a single centre study with only one AI method.
**Ethics committee approval:** The study was conducted in accordance with the Declaration of Helsinki, and the study protocol was approved by the local ethics committee.
**Funding for this study:** The study was funded by the University's own funds.
**Author Disclosures:**
Christian Siedentopf: Nothing to disclose
Ruth Steiger: Nothing to disclose
Marlene Hammerl: Nothing to disclose
Malik Galijašević: Nothing to disclose
Tanja Janjic: Nothing to disclose
Astrid E Grams: Nothing to disclose
Elke Ruth Gizewski: Nothing to disclose
Milovan Regodic: Nothing to disclose
Nadja Gruber: Nothing to disclose

### RPS 1505-8

**Application of automatic segmentation on super-resolution reconstruction MRI images of the abnormal fetal brain**

*T. Deprest*[1], L. Fidon[2], F. De Keyzer[3], M. Ebner[2], J. Deprest[3], P. Demaerel[3], L. De Catte[3], T. Vercauteren[2], M. Aertsen[3]; [1]Aalst/BE, [2]London/UK, [3]Leuven/BE
*(tdeprest@gmail.com)*

**Purpose:** To evaluate a novel automatic segmentation algorithm for the segmentation of different fetal brain structures by using a set of exclusively abnormal brains.
**Methods or Background:** Background: Fetal brain magnetic resonance (MR) imaging is clinically used to characterise fetal brain abnormalities. Recently algorithms have been proposed to reconstruct high resolution 3D fetal brain volumes from 2D-slices. Using these reconstructions, convolutional neural networks (CNN) have been developed for automatic image segmentation to avoid labour-intensive manual annotations, usually trained on data of normal fetal brains. Herein, we test the performance of an algorithm specifically developed for brain anomalies.
Methods: Single centre retrospective study on MR-images of sixteen randomly selected fetuses with severe central nervous system anomalies (gestation: 21-39 weeks). T2-weighted 2D-slices were converted to 3D-volumes using a super-resolution reconstruction algorithm. The acquired volumetric data was then processed by a novel CNN, trained using distributionally robust optimisation, to perform segmentations of white matter, ventricular system and cerebellum. These were compared to manual segmentation using Dice coefficient, Hausdorff distance and volume difference. Using interquartile ranges, outliers of these metrics were identified and further analysed in detail.
**Results or Findings:** The mean Dice coefficient was 96.2, 93.7 and 94.7% for white matter, ventricular system and cerebellum respectively. The Hausdorff distance was 1.1, 2.3 and 1.6 mm respectively. The volume difference was 1.6, 1.4 and 0.3 ml respectively. Of the 126 parameters there were sixteen outliers among five fetuses, discussed on a case-by-case basis.
**Conclusion:** We obtained excellent results on archived brain MRIs of fetuses with severe brain abnormalities. Analysis of the outliers shows the need to include pathologies underrepresented in the current training set. Quality control to prevent occasional errors is still needed.
**Limitations:** The fact that the number of cases for each pathology is rather small was identified as a limitation.
**Ethics committee approval:** This study was approved by an ethics committee.
**Funding for this study:** No funding was received for this study.
**Author Disclosures:**
Lucas Fidon: Nothing to disclose
Luc De Catte: Nothing to disclose
Tom Vercauteren: Nothing to disclose
Thomas Deprest: Nothing to disclose
Frederik De Keyzer: Nothing to disclose
Jan Deprest: Nothing to disclose
Michael Aertsen: Nothing to disclose
Philippe Demaerel: Nothing to disclose
Michael Ebner: Nothing to disclose

### RPS 1505-9

**Automated detection of MCI to Alzheimer's disease conversion before clinical onset by evaluation of atrophy rates**

J. Steiglechner, B. Bender, U. Ernemann, K. Scheffler, *T. Lindig*; Tübingen/DE

**Purpose:** Alzheimer's disease (AD) is the most common type of an irreversible neurodegenerative disorder, affecting millions of people. Especially early stratification of patients with mild cognitive impairment (MCI) into patients who will convert to AD remains a challenging task. We aimed to predict automatically whether MCI patients will develop the disease (MCIc) by following subjects over time and quantifying spatial atrophy rates (AR) in magnetic resonance imaging (MRI).
**Methods or Background:** 3D T1w MRIs at 3T from 276 MCI patients participating in the first period of Alzheimer's Disease National Initiative (ADNI-1) with at least two MRIs more than 60 days apart without evident artifacts were segmented by a deep-learning-based 3D-UNet into 30 anatomical regions. Z-scores of TIV-adjusted volumes were calculated compared to a normal reference population, and AR of these z-scores were calculated longitudinally per subject (AR=0 normal aging). Rolling AR were calculated as the mean AR over a half-year time window (mRAR). A 80:20 train-test-partition was used to train a logistic regression to discriminate MCIc vs MCInc.
**Results or Findings:** We found accelerated regional mRAR in MCIc. The temporal cortex and hippocampal regions showed the most striking mRAR. On the test set, of 34 MCIc, the classifier predicted 27 as true positive with a median of 1.7 Y (Q1/3=2.0/0.6Y) before conversion (sensitivity=0.79), with 5/22 false positives MCInc (stable specificity=0.77, AUC ROC=0.81).
**Conclusion:** Our method provides reliable results due to a stable specificity that can be obtained well before previous clinical diagnoses for conversions to disease. Therefore, it is suitable for use in subsequent studies.
**Limitations:** Validation in an independent sample is missing.
**Ethics committee approval:** This study has been approved by an ethics committee.
**Funding for this study:** AFI project #18052
**Author Disclosures:**
Tobias Lindig: Research/Grant Support: AFI project #18052 Founder: CEO and Co-founder of AIRAmed
Julius Steiglechner: Grant Recipient: AFI project #18052
Benjamin Bender: Founder: CTO and Co-founder of AIRAmed
Klaus Scheffler: Research/Grant Support: AFI project #18052
Ulrike Ernemann: Nothing to disclose

### RPS 1505-10

**AI-based prediction of onset of intracerebral hemorrhage: potential and limits**

*T. Rusche*, J. Wasserthal, H-C. Breit, M-N. Psychogios, E. M. Merkle, P. Sporns; Basel/CH
*(rusche.thilo@googlemail.com)*

**Purpose:** Intracerebral hemorrhage (ICH) is associated with high mortality and long-term morbidity and thus has a significant overall health-economic impact. The overall outcome is especially poor if the onset of ICH is unknown, but reliable radiological methods to determine this exactly do not exist. Therefore, the aim of our work was to investigate a machine learning (AI)-based age-prediction of ICH.
**Methods or Background:** First, we selected 7421 CT-datasets with proven ICH and trained an automated ICH-segmentation-algorithm. Afterwards, for 622 datasets (bleeding age<48h) a comparison of our AI-based age-prediction with the anamnestically known onset-times of ICH was performed. Additionally, we performed manual age-prediction by two radiologists from 117 datasets and compared the results with the AI-based approach.
**Results or Findings:** Age-prediction by both AI and radiologists was only partially accurate with a mean absolute error (in hours) of 9.77 (95%CI [8.56-11.06]) for the AI-model, 13.38 (95%CI [11.21-15.74]) for rater 1 and 11.21 (95%CI [9.61-12.90]) for rater 2. Thus, the human (rater) was significantly inferior (p=0.0067 rater1; p=0.0453 rater2) to the AI. However, the performance of the AI was equal (with no significant difference, p=0.3104) to simply taking the mean bleeding age of the dataset (15 hours) as prediction.
**Conclusion:** To our knowledge, such an AI-based approach has never been performed. Nevertheless, our results confirm the assumption that, in principle, no data on ICH-age can be extracted exactly from the image information of a CT-dataset. Possible therapy decisions (blood-pressure regulation, drug-therapy approaches) can thus only be derived to a limited extent.
**Limitations:** Limitations include the relative imprecision of the ICH-onset-data (history only) and the relatively small dataset for an AI-based study. It is possible that follow-up-studies with a significantly larger data set can provide further relevant information.

Ethics committee approval: Swissethics Project ID: 2021-01831
Funding for this study: No funding has been received for this study.
**Author Disclosures:**
Hanns-Christian Breit: Nothing to disclose
Peter Sporns: Nothing to disclose
Marios-Nikos Psychogios: Nothing to disclose
Jacob Wasserthal: Nothing to disclose
Elmar M. Merkle: Nothing to disclose
Thilo Rusche: Nothing to disclose

### RPS 1505-12
**Comparative performance of AI algorithm using MRI for the diagnosis of Alzheimer's disease: can MRI replace PET?**
D. S. Lee, H. Oh, J. Sung, K-H. Jung, *W. Jung*; Seoul/KR
*(wooseok.jung@vuno.co)*

**Purpose:** We aimed to assess and compare diagnostic performances of a structural MRI-based AI algorithm and other previously known imaging biomarkers for the diagnosis of AD.
**Methods or Background:** We included a total of 295 subjects (143 clinically diagnosed Alzheimer's disease (AD), 152 Cognitively normal (CN) subjects) who had 3D T1-weighted images. The included AD patients and CN controls were matched with gender, age, education years (p-value=1.00, 0.69 and 0.18, respectively). All included subjects (n=295) underwent structural MRI (sMRI), amyloid PET (AV45) and FDG-PET (FDG), and majority of the subjects (n=260) underwent total tau (tTAU) and phosphorylated tau (pTAU) PET. A previously developed deep learning-based AD prediction algorithm which was trained with 100 sMRIs of AD and CN subjects using Inception-v4 architecture (sMRI-based AI algorithm), was utilised for the analysis and prediction of AD using sMRI. The diagnostic performances of the sMRI-based AI algorithm and other imaging biomarkers are assessed and compared in terms of accuracy, sensitivity, specificity and AUC.
**Results or Findings:** The accuracy and sensitivity and specificity of sMRI-based AI algorithm, AV45, FDG, tTAU and pTAU was 85.8, 93.7 and 78.3 for sMRI-based AI algorithm, 76.3, 88.1 and 65.1 for AV45, 83.7, 84.6 and 82.9 for FDG, 73.5, 78.1 and 68.9 for tTAU and 74.6, 82.8 and 66.6 for pTAU, respectively. AUC was 0.95 for sMRI-based AI algorithm, 0.83 for AV45, 0.91 for FDG, 0.80 for tTAU and 0.82 for pTAU. From the comparison of ROC curves, the sMRI-based AI algorithm exhibited significantly better diagnostic performance compared to AV45, tTAU and pTAU ($p<0.001$).
**Conclusion:** The sMRI-based AI algorithm demonstrated significantly superior diagnostic performance in distinguishing AD patients from CN, compared to other imaging biomarkers.
**Limitations:** This was a retrospective study.
**Ethics committee approval:** Institutional review boards have approved this study.
**Funding for this study:** No funding was received for this study.
**Author Disclosures:**
HyunWoo Oh: Employee: VUNO
Wooseok Jung: Nothing to disclose
Jinkyeong Sung: Employee: VUNO
Kyu-Hwan Jung: Founder: VUNO
Dong Soo Lee: Employee: VUNO

### RPS 1505-11
**MRI-based prediction of AD progression from MCI using deep learning**
D. S. Lee, H. Oh, J. Sung, K-H. Jung, *W. Jung*; Seoul/KR
*(wooseok.jung@vuno.co)*

**Purpose:** The aim of this study is to investigate the prediction performance of a deep learning algorithm in predicting conversion of MCI to dementia.
**Methods or Background:** This study included 284 MCI patients (144 early MCI (EMCI) and 140 late MCI (LMCI) patients) who underwent 3D T1-weighted MRI. Among them, 52 MCI patients went through conversion from MCI to AD within 3 years from being diagnosed with MCI. A previously developed deep learning-based AD prediction model was utilised for the analysis of structural MRI. The output of the model was presented as an AD score. AD scores from the deep learning model were compared between EMCI vs. LMCI patients and patients who converted to AD vs. those who did not. Thereafter, the performance of AD score in predicting conversion of MCI to dementia was analysed and compared with that of amyloid β PET.
**Results or Findings:** The average AD scores were 0.38 for EMCI and 0.60 for LMCI ($p<0.001$) and 0.69 for patients who converted to AD and 0.41 for patients who did not ($p<0.001$). When a cut-off of 0.38 was applied for AD score, accuracies and AUC were 0.83 and 0.77 for AD score and 0.92 and 0.82 for amyloid β PET in predicting dementia conversion from MCI (p=0.140 and 0.270 for difference). Accuracies of AD score and amyloid-β PET were 0.73 and 0.91 for EMCI (p=0.280), 0.85 and 0.93 for LMCI (p=0.292). There was no statistically significant difference in prediction performance between the two modalities.
**Conclusion:** A deep learning-based AD prediction model using MRI showed comparable performance in predicting conversion of MCI to dementia in both EMCI and LMCI patients to amyloid β PET.
**Limitations:** This was a retrospective study.
**Ethics committee approval:** Institutional review boards have approved this study.
**Funding for this study:** No funding was received for this study.
**Author Disclosures:**
HyunWoo Oh: Employee: VUNO
Wooseok Jung: Nothing to disclose
Jinkyeong Sung: Employee: VUNO
Kyu-Hwan Jung: Founder: VUNO
Dong Soo Lee: Employee: VUNO

**14:00-15:30** **Room Z**

### Research Presentation Session: Breast

### RPS 1502

### Early diagnosis and breast lesions characterisation

**Moderator**
E. Giannotti; Nottingham/UK

### RPS 1502-2
**Simultaneous multiparametric [18F]-fluoroethylcholine PET-MRI for the diagnosis of breast cancer**
*P. Clauser*[1], L. Grana Lopez[2], I. Bolengo[3], S. Rasul[1], P. Kapetas[1], R-I. Milos[1], T. H. Helbich[1], P. A. Baltzer[1]; [1]Vienna/AT, [2]Lugo/ES, [3]Milan/IT
*(clauser.p@hotmail.it)*

**Purpose:** To assess the diagnostic performance of simultaneous multiparametric [18F]-fluoroethylcholine (FEC) PET-MRI of the breast.
**Methods or Background:** This is a prospective single-centre study. Lesions classified as BI-RADS 3, 4, 5 on mammography, tomosynthesis and/or ultrasound and no contraindications to [18F]-fluoroethylcholine PET-MRI were included. Histology was considered as reference standard. Hybrid [18F]-fluoroethylcholine PET-MRI of the breast was performed in a prone position with a dedicated 16-channel MRI breast coil. Two breast fellows (R1, R2) evaluated contrast-enhanced (CE) MRI using BI-RADS, measured the apparent diffusion coefficient (ADC) and qualitatively evaluated FEC-uptake using a four-point scale. A nuclear medicine specialist measured the maximum standardized FEC-uptake vale ($SUV_{max}$) of the CE-MRI findings. The area under the curve (AUC) was calculated for quantitative measurements. Sensitivity and specificity were calculated using three reading methods: CE-MRI, CE-MRI+ADC, CE-MRI+ADC+PET-FEC.
**Results or Findings:** 101 patients (mean age 52.3 years, standard deviation 12.0) with 122 lesions were included (38 benign, 84 malignant). AUC for PET-FEC was 0.859; AUC for ADC were 0.927 (R1) and 0.891 (R2, $P>0.101$). Sensitivity ranged from 92.9% to 98.1%, with no significant difference between reading methods. Specificity improved from MRI to MRI+ADC (R1: 48.6% to 76.3%, P=0.013; R2: 81.6% to 86.8%, P=0.537). Specificity improved with MRI+ADC+PET-FEC for R1 (76.3% to 86.8%, P=0.172). PET-FEC did not influence the performance of R2.
**Conclusion:** Breast MRI has a very high sensitivity. Simultaneous multiparametric [18F]-fluoroethylcholine PET-MRI of the breast can improve the specificity of CE-MRI.
**Limitations:** An identified limitation was that this was a feasibility study with a small sample size.
**Ethics committee approval:** This prospective study was approved by the local ethics committee. Patients gave written informed consent.
**Funding for this study:** This study received funding from the Austrian National Bank Jubiläumsfonds, project number 17186.
**Author Disclosures:**
Pascal A.T. Baltzer: Nothing to disclose
Thomas H. Helbich: Nothing to disclose
Lucia Grana Lopez: Nothing to disclose
Ruxandra-Iulia Milos: Nothing to disclose
Sazan Rasul: Nothing to disclose
Panagiotis Kapetas: Nothing to disclose
Isabella Bolengo: Nothing to disclose
Paola Clauser: Speaker: Siemens healthineers

### RPS 1502-3

**Dynamic contrast-enhanced MRI and intravoxel incoherent motion diffusion-weighted imaging for predicting breast cancer recurrence**

*W. Tsai*; Taipei/TW

**Purpose:** To evaluate the diagnostic performance of the combination of dynamic contrast-enhanced magnetic resonance imaging (DCE-MRI), intravoxel incoherent motion (IVIM) diffusion-weighted imaging (DWI), and pathology factors for predicting the risk of breast cancer recurrence as given by four gene assays.
**Methods or Background:** This retrospective study included 289 women (mean age, 49+10 years) with biopsy-proven breast cancer undergoing 1.5T DCE-MRI and IVIM between January 2014 and August 2017. Included patients were assigned into two groups: the training dataset consisting of 202 (70%) patients and the testing dataset consisting of 87 (30%) patients. Subgroup analysis of 158 oestrogen-positive, progesterone-positive, HER-2 negative breast cancer patients also was conducted. Thirty-six DCE-MRI and IVIM parameters and 7 pathology factors were obtained. ANOVA and Chi-square test were used for feature selection. Multiple linear regression (of the training data) was used for each risk scores prediction model. Log rank test was used for the comparison of survival risk groups. The research versions of PAM50, Mammaprint, OncotypeDX and EPClinic recurrence risk scores based on microarray data were used.
**Results or Findings:** For patients in the testing dataset, the radiomic-pathology models had high accuracy and area under the curve (AUC) values in distinguishing between high versus intermediate/low recurrence risk: PAM50 (84%, 0.93), MammaPrint (80%, 0.83), OncotypeDX (82%, 0.91), and EPclinic (78%, 0.89). For oestrogen-positive/progesterone-positive HER-2 negative patients, the radiomic-pathology models had acceptable accuracy and AUC: PAM50 (79%, 0.82), OncotypeDX (72%, 0.82), and EPClinic (74%, 0.80).
**Conclusion:** A combination of dynamic contrast-enhanced MRI, intravoxel incoherent motion imaging, and pathology features predicted breast cancer recurrence risk with high diagnostic performance.
**Limitations:** The study was retrospective.
**Ethics committee approval:** This retrospective study was approved by the institutional review board.
**Funding for this study:** This study is supported by the Ministry of Health and Welfare, Surcharge of Tobacco Products (CCGII Program).
**Author Disclosures:**
WanChen Tsai: Nothing to disclose

### RPS 1502-4

**A machine learning (ML) ensemble based on radiomics to reduce the biopsy rate of ultrasound-detected suspicious breast masses**

M. Interlenghi[1], C. Salvatore[2], *V. Magni*[1], G. Caldara[2], E. Schiavon[1], A. Cozzi[1], S. Schiaffino[3], I. Castiglioni[1], F. Sardanelli[3]; [1]Milan/IT, [2]Pavia/IT, [3]San Donato Milanese/IT

**Purpose:** To develop a model based on radiomics to support decision making for ultrasound-detected suspicious breast lesions.
**Methods or Background:** From a retrospective 2015-2019 series of ultrasound-guided core needle biopsies performed by four board-certified breast radiologists using six ultrasound systems from three vendors, we collected 821 images of 834 suspicious breast masses from 819 patients (806 women and 13 men), resulting into 404 malignant and 430 benign lesions at histopathology. A balanced image set of biopsy-proven benign (n=299) and malignant (n=299) lesions were used for training and cross-validation of ensembles of different machine learning algorithms, using histopathology as the reference standard. External testing was performed on two further datasets including a total of 236 lesions.
**Results or Findings:** Based on the majority vote of the classifiers (>80% of the votes for a valid prediction of benign lesion), the ensemble of support vector machines showed a reduction in the biopsy rate of benign lesions of 15–18%, when tested on the two external datasets: 1) 123 lesions (51 malignant and 72 benign) obtained from the same ultrasound systems used for training, resulting into a 45.4% positive predictive value (PPV) (95% confidence interval [CI] 35.8–55.2%) versus a 41.5% radiologists' PPV (p<0.005), and a 96.1% sensitivity (95% CI 86.5–99.5%); 2) 113 lesions (54 malignant and 59 benign) obtained from two ultrasound systems different from those used for training, resulting into a 51.6% PPV (95% CI 40.4–60.6%) versus a 47.8% radiologists' PPV (p<0.005), and a 94.4% sensitivity (95% CI 84.6–98.8%).
**Conclusion:** Our model can support radiologists towards decision of follow-up versus biopsy for suspicious breast lesions, providing an over 15% reduction in the biopsy rate while still guaranteeing very high sensitivity.
**Limitations:** The retrospective design of this study was an identified limitation.
**Ethics committee approval:** This study received ethics committee approval.
**Funding for this study:** No funding was received for this study.
**Author Disclosures:**
Andrea Cozzi: Nothing to disclose
Elia Schiavon: Nothing to disclose
Isabella Castiglioni: Other: DeepTrace Technologies S.R.L.
Francesco Sardanelli: Speaker: Bayer Healthcare Advisory Board: Bracco Research/Grant Support: Bracco Speaker: General Electric Healthcare Research/Grant Support: General Electric Healthcare Research/Grant Support: Bayer Healthcare Speaker: Bracco
Christian Salvatore: CEO: DeepTrace Technologies S.R.L.
Simone Schiaffino: Other: Bracco Imaging Speaker: General Electric Healthcare
Matteo Interlenghi: Other: DeepTrace Technologies S.R.L.
Gabriele Caldara: Nothing to disclose
Veronica Magni: Nothing to disclose

### RPS 1502-5

**Evaluation of a deep learning-based artificial intelligence system for breast asymmetry detection**

*L. Li*[1], R. Ouyang[1], X. Lin[1], J. Ma[1], M. Wu[1], Z. Cao[2], Y. Tang[2], P. Chang[2]; [1]Shenzhen/CN, [2]Palo Alto, CA/US
*(LL_smu@163.com)*

**Purpose:** To explore the value of an artificial intelligence system in the detection of breast asymmetrical lesions in mammograms and its comparison with junior radiologists.
**Methods or Background:** The mammograms of 100 patients with asymmetrical lesions confirmed by mammography were retrospectively analysed. According to the criteria in the fifth edition of BI-RADS, two senior radiologists specialising in breast imaging diagnosis manually segmented the area of the asymmetrical lesions. A deep learning-based AI system was trained to detect asymmetrical lesions. We evaluate the performance of the AI system on these mammograms and compare it with the interpretation of a junior radiologist, in terms of sensitivity, specificity, and the accuracy of lesion detection and suspicious malignancy classification.
**Results or Findings:** Of the 400 mammogram images, 157 were positive and 243 were negative of asymmetrical lesions. The results of the AI system, in terms of sensitivity, specificity and accuracy, were 0.65, 0.82 and 75.3% respectively. The junior radiologist achieved 0.84, 0.98 and 92.5% respectively. Of the 33 asymmetrical lesion cases, 8 needed biopsy tests to identify the malignancy of the lesions. For this suspicious malignancy classification task, the sensitivity, specificity and accuracy of the AI were 0.75, 0.92 and 88.8%, while the junior radiologist's were 0.50, 0.64 and 60.6%.
**Conclusion:** The current AI model is inferior to the junior radiologists in detecting breast asymmetrical lesions (p<0.05). However, for malignancy classification of suspicious asymmetrical lesions, the AI exceeded the level of the junior radiologist in diagnostic sensitivity, specificity and accuracy (p<0.05). In particular, the AI's superior specificity rate indicates that AI is exceptionally good at identifying those benign asymmetrical lesions.
**Limitations:** The size of the database is limited and this is a single-centre research.
**Ethics committee approval:** Not applicable.
**Funding for this study:** No funding was received for this work.
**Author Disclosures:**
Jie Ma: Author: Department of Radiology, Shenzhen People's Hospital(The Second Clinical Medical College, Jinan University; The First Affiliated Hospital), 518020, Shenzhen, Guangdong, CHINA
Peng Chang: Author: PAII Inc, Bethesda, MD
Mingxiang Wu: Author: Department of Radiology, Shenzhen People's Hospital(The Second Clinical Medical College, Jinan University; The First Affiliated Hospital), 518020, Shenzhen, Guangdong, CHINA
Lin Li: Author: Department of Radiology, Shenzhen People's Hospital(The Second Clinical Medical College, Jinan University; The First Affiliated Hospital), 518020, Shenzhen, Guangdong, CHINA
Yuxing Tang: Author: PAII Inc, Bethesda, MD
Rushan Ouyang: Author: Department of Radiology, Shenzhen People's Hospital(The Second Clinical Medical College, Jinan University; The First Affiliated Hospital), 518020, Shenzhen, Guangdong, CHINA
Zhenjie Cao: Author: PAII Inc, Bethesda, MD
Xiaohui Lin: Author: Department of Radiology, Shenzhen People's Hospital(The Second Clinical Medical College, Jinan University; The First Affiliated Hospital), 518020, Shenzhen, Guangdong, CHINA

### RPS 1502-6

**Breast composition may predict breast cancer features and cancer-specific survival**

*E. U. Ekpo*[1], I. Kanbayti[2], M. McEntee[3], W. Rae[1]; [1]Sydney/AU, [2]Jeddah/SA, [3]Cork/IE
*(ernest.ekpo@sydney.edu.au)*

**Purpose:** To assess the association between breast composition and histopathologic features of breast cancer (BC), and the prognostic utility of mammographic density (MD) in women diagnosed with BC.
**Methods or Background:** MD of 297 BC-affected women was measured using BI-RADS and LIBRA. The association between MD phenotypes and histopathologic features of BC was assessed. A set of 33 global radiomic features were extracted from the ipsilateral mammogram, and receiver-operating characteristic curve analyses were performed to assess the abilities of these features to predict BC characteristics. Kaplan-Meier analysis and Cox-proportional hazards models were used to assess BC-specific survival due to MD.
**Results or Findings:** MD showed no association with histopathologic features of BC. Tumours were commonly found in dense breast regions of patients with higher medians of percent density (PD) (p=0.001), dense area (p=0.02), and lower medians of non-dense area (p<0.001). MD (BI-RADS 4) was associated with 5-fold increased odds of tumours developing within dense areas (OR, 4.99, 95% CI: 0.93-25.9). Tumours in dense regions exhibited HER2+ and in-situ characteristics (p≤0.05). Histogram-based features predicted progesterone status and tumour size (AUC range: 0.65–0.71 and 0.65–0.67) respectively. GLRLM-based features predicted lymph-node status among younger women (AUC range: 0.710–0.863), and fractal features predicted tumour size among patients with low PD (AUC: 0.704). The 5-year cancer-specific survival for women with low MD (<20%) was 0.35 (95% CI :0.13 – 0.97) compared to those with high MD (0.87; 95% CI: 0.79 – 0.96). Low MD increased the risk of death from BC (HR, 5.17, 95% CI: 1.97–13.5).
**Conclusion:** Global radiomic features from the ipsilateral breast mammogram predict lymph-node status and tumour size, and low MD is associated with poor BC-specific survival.
**Limitations:** Our sample size was small due to the small number of cancer events.
**Ethics committee approval:** This study received ethics committee approval from the University of Sydney (IRB: 2019/459).
**Funding for this study:** Funding was received from the SOAR Prize, University of Sydney.
**Author Disclosures:**
Ibrahem Kanbayti: Nothing to disclose
Ernest Usang Ekpo: Nothing to disclose
William Rae: Nothing to disclose
Mark McEntee: Nothing to disclose

### RPS 1502-9

**Digital breast tomosynthesis combined with contrast-enhanced spectral mammography helps reveal anatomic causes of artefacts and benign enhancing findings on subtracted contrast images.**

*O. Weaver*, M. E. Scoggins, B. E. Adrada, E. Arribas, W. T. Yang; Houston, TX/US
*(ooweaver@mdanderson.org)*

**Purpose:** Contrast-enhanced spectral mammography (CESM) is a functional 2-dimensional (2D) mammographic technique that is susceptible to superimposition artefacts, which can affect both low energy (LE) and subtracted contrast images (SCI). Benign enhancing findings (BEF) on SCI can create artefacts and mimic malignancy. Digital breast tomosynthesis (DBT) may resolve some artefacts and reveal BEF in both components of CESM.
**Methods or Background:** Bilateral diagnostic CESM/DBT combination studies were performed for patients enrolled in four IRB-approved research protocols, using a full-field digital 2D/3D mammography system with CESM capabilities (Senographe Pristina, GE Healthcare). CESM and DBT exposures were done in quick succession under the same compression in standard views. The instances of DBT resolving artefacts and BEF on SCI were prospectively recorded and retrospectively analysed for the types of BEF, their imaging characteristics, and the anatomic cause.
**Results or Findings:** Among 73 consecutive patients imaged with CESM/DBT, DBT resolved 18 BEF on SCI in 12 patients. The BEF imaging features on SCI were mass enhancement (44%, 8/18), focal non-mass enhancement (39%, 7/18), and linear non-mass enhancement (17%, 3/18). 15/18 (83%) BEF were seen on one view only. The anatomic structures mimicking pathology on SCI were vascular structures (56%, 10/18) and enhancing skin lesions (44%, 8/18).
**Conclusion:** CESM/DBT enables exact correlation of 2D, DBT, and CESM findings. This can improve the specificity of CESM and avoid additional imaging and biopsies by resolving artefacts and BEF on SCI. Yet the addition of DBT increases the radiation dose by ~66%. Further combined efforts of clinical researchers and equipment manufacturers are needed to investigate the CESM/DBT combination, and to minimise the radiation dose.
**Limitations:** Single institution experience, uniform cohort of patients, research settings.
**Ethics committee approval:** This study was IRB-approved.
**Funding for this study:** GE Healthcare, NIH/NCI Cancer Centre Support Grant P30 CA016672 provided funding for this study.
**Author Disclosures:**
Beatriz E Adrada: Nothing to disclose
Marion E Scoggins: Nothing to disclose
Wei Tse Yang: Other: Elsevier
Olena Weaver: Equipment Support Recipient: GE Healthcare Research/Grant Support: Brown Foundation, Little Green Book Foundation, GE Healthcare in-kind grant.
Elsa Arribas: Shareholder: Volumetric Inc Advisory Board: Volumetric Inc.

### RPS 1502-10

**An effective approach for breast tumour segmentation based on Mask R-CNN architecture**

N. Sirjani[1], *M. Ghelichoghli*[1], M. Gity[1], M. K. Tarzamni[2], V. Ashkani Chenarlogh[1], A. Shabanzadeh[1], A. Akhavan[1]; [1]Karaj/IR, [2]Tabriz/IR
*(m.g31_mesu@yahoo.com)*

**Purpose:** The precise delineation of breast lesions' borders is significant since determining the malignancy of a lesion is critically reliant on the lesion's morphological features. Therefore, accurate detection of the lesion boundaries can assist in automated breast tumours' classification. So, this study has utilised the Mask R-CNN model as a practical approach to segment breast lesions.
**Methods or Background:** In this study, we used Mask R-CNN architecture to segment breast lesions in ultrasound images automatically. Mask R-CNN is a deep neural network for object instance segmentation that separates objects in an image and has two stages. The first stage proposes candidate object bounding boxes. The second stage predicts the object's class, refines the bounding box, and generates a binary mask on each region of interest (RoI). For training and evaluating the model, we utilised the combination of four datasets (public and private datasets). We selected 410 images per class. We used 90% of the dataset in the training stage and 10% in the testing stage.
**Results or Findings:** This model was tested with 82 images. Dice coefficient (DC), Jaccard coefficient (JC), and mean squared error (MSE) are used to evaluate the network performance. We achieved 0.88 of DC, 0.81 of JC, and 0.018 of MSE on the test dataset.
**Conclusion:** The attained results demonstrate that the Mask R-CNN can precisely delineate the borders of breast lesions. Consequently, it can help the radiologists and other automatic algorithms have a brighter view of lesions' morphology.
**Limitations:** No limitations were identified in this study.
**Ethics committee approval:** Med Fanavaran Plus Co. approved this study.
**Funding for this study:** Med Fanavaran Plus Co. funded this study.
**Author Disclosures:**
Vahid Ashkani Chenarlogh: Author: Artificial intelligence researcher at Med Fanavaran Plus Co
Masoumeh Gity: Author: Radiologist
Mohammad Kazem Tarzamni: Nothing to disclose
Mostafa Ghelichoghli: Author: Artificial intelligence researcher at Med Fanavaran Plus Co
Ardavan Akhavan: Author: Researcher at Med Fanavaran Plus Co
Nasim Sirjani: Author: Artificial intelligence researcher at Med Fanavaran Plus Co
Ali Shabanzadeh: Author: Radiologist

### RPS 1502-11

**Comparison of artificial intelligence computer-aided detection enhanced synthesised mammograms versus original digital mammograms alone and in combination with tomosynthesis images**

*T. Uematsu*; Shizuoka/JP

**Purpose:** To assess reader performance when artificial intelligence computer-aided detection enhanced synthesised mammograms (AI CAD SM) versus original full-field digital mammograms (DM) are used alone or in combination with digital breast tomosynthesis images (DBT).
**Methods or Background:** AI CAD SM has enhanced various structural patterns detected and extracted by AI CAD like technology from DBT images. A retrospective reader study compared the performance of multi-reader (n=4) reading multi-case (n=388) with 84 cancer cases, 83 benign cases, and 221 normal or benign cases with negative 1-year follow-up results. Each reading was independently interpreted with four reading modes: DM, AI CAD SM, DM with DBT, and AI CAD SM with DBT. Accuracy of probability of malignancy ratings and five-category forced breast imaging reporting and data system ratings were evaluated by using areas under the receiver operating characteristic curve (AUC) in the random-reader analysis.
**Results or Findings:** Probability of malignancy based mean AUC values for DM, AI CAD SM, DM with DBT, and AI CAD SM with DBT were 0.871, 0.902, 0.895, and 0.909 respectively. The mean AUC of AI CAD SM was significantly

Friday higher (P=.002) than that of DM. The mean AUC of AI CAD SM alone and DM with DBT did not differ significantly (P=.264). The mean AUC of AI CAD SM with DBT was higher than that of DM with DBT and it approached significance (P=.066).
**Conclusion:** AI CAD SM alone was superior to DM alone. AI CAD SM with DBT was also superior to DM with DBT although not statistically significant. AI CAD SM can replace DM in both screening and clinical setting.
**Limitations:** This was a preliminary single-site retrospective study with a single manufacturer.
**Ethics committee approval:** Ethical approval was received for this study.
**Funding for this study:** Funding for this study was provided by FUJIFILM.
**Author Disclosures:**
Takayoshi Uematsu: Nothing to disclose

**16:00-17:00** **Open Forum #1 (Radiographers)**

### Research Presentation Session: Radiographers

### RPS 1614

### Advancing radiographic practice and training needs

**Moderators**
J. Portelli; Msida/MT
F. M. H. M. Vanhoenacker; Antwerp/BE

### RPS 1614-3

**Role expansion in ultrasound: radiographers in European Federation of Radiographer Societies (EFRS) countries**
G. Harrison[1], M. R. V. Pedersen[2], *B. Kraus*[3], R. Martins Dos Santos[4]; [1]London/UK, [2]Odense/DK, [3]Vienna/AT, [4]Coimbra/PT

**Purpose:** Variability within radiographer performed ultrasound practice in EFRS countries has been previously reported. In 2019/2020 several EFRS surveys evaluated radiographers working in ultrasound. These results aimed to investigate the prevalence of radiographer extended and advanced practice ultrasound roles and explore priorities for future career development.
**Methods or Background:** Participants were recruited to an online cross-sectional survey by national societies of the EFRS and social media. Mixed questions were included, with some Likert scale options to explore opinions. Descriptive statistics, chi square test and theme analysis were used to assess the results.
**Results or Findings:** Radiographers from 58% of EFRS countries responded to the survey (n=561), predominantly the UK, Ireland and Spain (81%). Most undertook teaching (83.4%), fewer engaged in research, audit and leadership. UK sonographers were more likely to communicate findings to patients (83.4%) and/or doctors (94.6%) than other respondents. Smaller numbers refer for further examinations (UK=45.6%, non-UK=12.5%). A significant difference ($p<0.0001$) was seen between those that do and do not perform ultrasound in their country when asked if radiographers should provide the final report on scans they perform. Commonly radiographers reported working in ultrasound to challenge themselves, increase knowledge, skills, responsibility and independent working. Most enjoyed ultrasound, but many reported feeling undervalued. Future priorities varied between the UK, Ireland and other countries.
**Conclusion:** Whilst many radiographers are independently performing ultrasound examinations, few outside the UK independently report and communicate the results to patients. Advanced practice skills including audit, research and leadership were less common amongst radiographers in ultrasound than teaching others. Priorities highlighted in this study reflect previous work: legislation enabling independent regulated ultrasound practice, additional training opportunities and support from clinical colleagues to develop the role.
**Limitations:** Limitations will be discussed, including survey language.
**Ethics committee approval:** Not applicable.
**Funding for this study:** Not applicable.
**Author Disclosures:**
Malene Roland Vils Pedersen: Nothing to disclose
Rute Martins Dos Santos: Nothing to disclose
Barbara Kraus: Nothing to disclose
Gill Harrison: Nothing to disclose

### RPS 1614-4

**European radiographers' experience in ultrasound: roles, support and legal responsibility**
R. Santos[1], G. Harrison[2], *B. Kraus*[3], M. R. V. Pedersen[4]; [1]Coimbra/PT, [2]London/UK, [3]Wolkersdorf/AT, [4]Vejle/DK

**Purpose:** The practice of ultrasound by radiographers in Europe is well documented. Detailed individual radiographer involvement in ultrasound needed further exploration, therefore, as part of a suite of surveys the European Federation of Radiographer Societies (EFRS) this study investigated the examinations performed, extent of the role and legal responsibilities of radiographers performing ultrasound.
**Methods or Background:** EFRS national societies (n=38) disseminated an online survey to members, along with social media recruitment of participants. The survey, included a mix of closed questions, free text options, and scale responses.
**Results or Findings:** Responses were received from 561 radiographers: mean age 33.5 years, with 13.5 years ultrasound experience. Radiographers performed ultrasound in most respondent countries (92%). Reasons for not undertaking ultrasound included lack of available education or legislation and/or limited/no support from medical colleagues. Academic level 7 education (diploma or master's) were most frequently cited for radiographers working within ultrasound. Common ultrasound examinations performed by radiographers were general medical, gynaecological, vascular and obstetrics. Of those undertaking ultrasound, 97.4% work independently; 70.4% provide full interpretative reports, whilst 52% of those, predominantly in UK and Ireland, give advice on further investigations. Over 50% of radiographers take responsibility for the report. 72% had professional indemnity insurance.
**Conclusion:** Radiographers are involved in a wide range of ultrasound examinations, however ultrasound education, level of reporting and support for independent practice vary across countries. Recommendations to support radiographer career development in ultrasound, in conjunction with legislative and radiology support, include team working, co-development of educational programmes, clear clinical protocols, up-skilling existing practitioners and auditing practice.
**Limitations:** There was self-selection bias, inter-country differences and the survey was written in English, leading to potential for misinterpretation.
**Ethics committee approval:** Not applicable.
**Funding for this study:** Not applicable.
**Author Disclosures:**
Malene Roland Vils Pedersen: Nothing to disclose
Barbara Kraus: Nothing to disclose
Gill Harrison: Nothing to disclose
Rute Santos: Nothing to disclose

### RPS 1614-5

**Ultrasound evaluation of the patellar tendon in sports practitioners and non-sports practitioners: the radiographer's role**
*Â. P. G. Pissarra*[1], B. Vicente[1], L. P. V. Ribeiro[2], S. I. Rodrigues[1], R. P. P. Almeida[1], A. F. C. L. Abrantes[1], O. Lesyuk[3], J. Pinheiro[3]; [1]Faro/PT, [2]Parchal/PT, [3]São Brás de Alportel/PT
*(anagomes22@sapo.pt)*

**Purpose:** Patellar tendon (PT) injuries are one of the most common overuse injuries in sports. Ultrasound is a well-established technique that has proven to be an ideal choice to characterise and evaluate this tendon. The aim of this study was to evaluate PT dimensions in sports practitioners (SP), namely cyclists and volleyball players (VP), and non-sports practitioners (NSP).
**Methods or Background:** A sample of 115 individuals was recruited, of which 37 were cyclists, 38 VP, and 40 NSP. Length, thickness, and width of the PT were bilaterally measured using a longitudinal and transverse probe approach. The individuals were placed in supine with a 30º flexion of the knee.
**Results or Findings:** Results obtained demonstrate that cyclists and VP present increased values for PT length and thickness, since that statistically significant differences were found in length and thickness bilateral measurements between SP and NSP ($p<0.003$). Concerning physical activity level, significant differences were found, mostly between low and high levels, in the three dimensions measured bilaterally ($p<0.004$). PT length, thickness, and width dimensions of both limbs' present correlations with gender and height, highlighting moderated to strong intensity values in the PT length ($0.520<r<0.601$). Positive correlations of weak to moderate intensity between the length and thickness dimensions with physical activity level in both limbs were valid ($0.281<r<0.422$).
**Conclusion:** Physical activity stimulates the development and stabilisation of the patellar tendon, preventing associated pathologies. The evaluation of the PT through ultrasound is reliable and allows its measurement and characterisation in several clinical situations, highlighting the radiographer's fundamental role in the musculoskeletal field.
**Limitations:** Sample size.

Friday

**Ethics committee approval:** Ethics committee approvall and written consent was delivered to the participants.
**Funding for this study:** No funding was received.
**Author Disclosures:**
Joao Pinheiro: Nothing to disclose
António Fernando Caldeira Lagem Abrantes: Nothing to disclose
Rui Pedro Pereira Almeida: Nothing to disclose
Bianca Vicente: Nothing to disclose
Sónia Isabel Rodrigues: Nothing to disclose
Ângela Patrícia Gomes Pissarra: Nothing to disclose
Oksana Lesyuk: Nothing to disclose
Luís Pedro Vieira Ribeiro: Nothing to disclose

### RPS 1614-6

**Characteristics associated with high interpretation performance amongst radiography advanced practitioners (RAPs) involved in breast cancer screening**

*N. Lay*[1], C. Ski[1], P. Brennan[2], R. M. Strudwick[1]; [1]Ipswich/UK, [2]Sydney/AU
*(noellefgeclerkin@gmail.com)*

**Purpose:** We propose a method to optimise cancer detection through identification of key RAP characteristics that enhance mammographic interpretation. A database of performance levels will also be provided to act as a benchmark for interpretation standards. The objectives are to: i. identify reader characteristics that promote optimal image interpretation; ii. create an evidence-base from which standards for interpretation can be proposed.
**Methods or Background:** The performance of 50 UK-based RAPs reading, a cloud-based test set of 60 mammographic cases with known results, will be assessed. Participants will be asked to complete a questionnaire to record their characteristics. Sensitivity, specificity and receiver operating characteristics (ROC) values will be established for each RAP and correlation techniques will be used to explore associations between characteristics and image interpretation accuracy. Percentile values on performance levels for each of the three metrics will be provided, serving as initial benchmarks for future evaluations.
**Results or Findings:** This project is ongoing. The aim is to present preliminary data on factors that promote optimum performance of RAPs. Collaborators are invited to be a part of this study which should form the basis for optimising image interpretation. Causal agents linked to best performance will be identified as well as baseline values for diagnostic efficacy. The author will also present a framework for the ongoing assessment of standards, available to all mammographic readers through the cloud.
**Conclusion:** An exploration of casual agents that impact optimal interpretation as well as RAP-based standards will be developed to promote breast screen reading efficacy.
**Limitations:** This is a pilot study, however it will establish system to optimise breast cancer screening interpretation.
**Ethics committee approval:** In progress.
**Funding for this study:** Ongoing/self funded.
**Author Disclosures:**
Patrick Brennan: Nothing to disclose
Noelle Lay: Nothing to disclose
Ruth Mary Strudwick: Nothing to disclose
Chantal Ski: Nothing to disclose

### RPS 1614-7

**An investigation of radiographers' perception about their role in providing psychosocial support to cancer patients**

J. Azzopardi, *J. L. Portelli*, S. M. Mercieca; Msida/MT
*(jonathan.portelli@um.edu.mt)*

**Purpose:** The purpose of this study was to investigate radiographers' perception about their role in identifying and providing psychosocial support to cancer patients receiving radiotherapy.
**Methods or Background:** The study comprised of a prospective, non-experimental design that involved the distribution and collection of a paper questionnaire to all radiographers working at a radiotherapy department in Malta. The self-designed questionnaire contained 19 questions, including a few that were incorporated from other research studies following respective authors' permission.
**Results or Findings:** Twenty one from twenty six radiographers completed the questionnaire anonymously, resulting in a response rate of 80.7%. All participants perceived the provision of psychosocial care to be highly important (71.4%, n=15/21) or important (28.6%, n=6/21). The majority of radiographers (85.71%, n=18/21) saw their primary role as care coordinators, linking patients to the right support resources. Participants felt most confident in providing counselling and support for treatment-related symptoms and least confident in delivering psychosocial support. Lack of training (95.2%) was identified as the most common obstacle to providing psychosocial support, followed by a lack of use of a screening tool (85.7%), an absence of environmental space (76.2%), and a limited understanding in the field (61.9%).
**Conclusion:** Despite radiographers acknowledging that the provision of psychosocial support was an important part of their role, this study identified various barriers that may be restricting them from doing so. In this regard, adequate training, the use of a psychosocial screening instrument and the implementation of clear referral process are recommended so as to enhance radiographers' ability to provide psychosocial support to their patients.
**Limitations:** Small sample size restricts generalisation of the study findings.
**Ethics committee approval:** This study was approved by the University of Malta Research Ethics Committee. Application number-5863_300620_Jade Azzopardi.
**Funding for this study:** No funding was received for this study.
**Author Disclosures:**
Jonathan Loui Portelli: Nothing to disclose
Jade Azzopardi: Nothing to disclose
Susan Mercieca Mercieca: Nothing to disclose

### RPS 1614-8

**CT head scans for final year students: too great an expectation?**

*V. T. Major*[1], S. Ryan[2], D. O'Leary[3]; [1]Middlesex/UK, [2]Hatfield/UK, [3]Newcastle/UK
*(vickimajor@hotmail.co.uk)*

**Purpose:** Following on a previous presentation at ECR 2019, we present part one of a longitudinal study examining the need for further CT postgraduate programmes. Two further factors are examined: professional body graduate attribute requirements for non-contrast CT head imaging, and the length of time taken for graduate radiographers to undertake CT scanning post-qualification.
**Methods or Background:** This study set out to identify training requirements for UK CT radiographers by investigating whether social and/or academic factors have an influence on students' approach toward CT dose optimisation both before and after qualification since the variation in CT doses across countries are primarily attributable to local choices regarding technical parameters. In-depth interviews were analysed in a systematic and rigorous manner through thematic analysis following the Braun and Clarke qualitative data analysis framework.
**Results or Findings:** Three themes were identified: education, dose optimisation, and culture. Student comments from the three themes respectively are shown: "(…) if you get stuck doing one thing one way you think it is the only way to do this thing"; "Would like to have had protocols explanation of why they change"; "students just helping patients getting on and off table, hard to learn anything". Mind maps of themes can be seen in figures. Two years post qualification, a third of the students hadn't worked in CT.
**Conclusion:** Whilst participants may have been aware of how to optimise dose from theory, many did not have the experience to do so in practice, with many not ever being allowed to optimise parameters under direct supervision. The question arises: is it reasonable to expect final year students to scan CT heads?
**Limitations:** Part 1 of a longitudinal study.
**Ethics committee approval:** This study was approved by the University of Hertfordshire Ethics Committee with Delegated Authority HSK/UH/02331 and aHSK/UH/02331.
**Funding for this study:** No funding was received for this study.
**Author Disclosures:**
Sean Ryan: Nothing to disclose
Victoria Teresa Major: Nothing to disclose
Desiree O'Leary: Nothing to disclose

### RPS 1614-9

**Building bridges among students and young radiographers in Nigeria based on previous ECR sponsored experience**

*A. A. C. Igwegbe*, F. B. Nkubli; Maiduguri/NG
*(igwegbea@gmail.com)*

**Purpose:** To highlight the lessons learned and experiences gained from previous attendance of the European Congress of Radiology (ECR) as an "Invest in the Youth" and "Shape your Skills" programme recipient. To highlight how the experience gained has been transferred to the young generations of radiographers in Nigeria, thus building bridges across the globe.
**Methods or Background:** Realist review of series of activities and events embarked upon based on experience from previous ECR attended both onsite and online will be presented.
**Results or Findings:** Talk shows and awareness creation to student radiographers during special days like student health week world radiography day celebrations have resulted in growing interest in ECR related activities. Series of side events held with the Nigerian Association of Radiography students (NARS) during the General conference in 2019 and 2020. Interest among young generation of radiographers including students in "Invest in the youth" and "Shape your skills" programme has increased over the years. This is evident by the current number of applications from Nigeria.

**Conclusion:** In conclusion, the efforts of the sponsors of the "Invest in the youth" and "Shape your skills" programmes in building the next generation of radiographers are highly commendable. However, we would like to make a passionate appeal to the sponsors that the quota for the "Shape your skills" be increased for young professionals in resource poor settings especially those affected by armed conflict and the current COVID-19 pandemic.
**Limitations:** Extensive empirical data was not collected from participants at our events to get their opinions. This does not however negate the relevance of this presentation.
**Ethics committee approval:** Not applicable.
**Funding for this study:** Not applicable.
**Author Disclosures:**
Aiman Amin Chidera Igwegbe: Author: speaker Owner: Speaker
Flavious Bobuin Nkubli: Author: Author

**16:00-17:30** **Room E1**

### Research Presentation Session: Chest

### RPS 1604
### The post-COVID lung

**Moderator**
A. Snoeckx; Antwerp/BE
**Author Disclosure:**
A. Snoeckx: Advisory Board: Agfa; Speaker: Samsung

### RPS 1604-2
**Quantitative chest CT analysis to measure short-term sequelae of COVID-19 pneumonia: a monocentric prospective study**
E. Lanza[1], *A. Ammirabile*[2], M. Casana[1], D. Pocaterra[1], F. M. P. Tordato[1], C. Lisi[2], G. Messana[2], L. Balzarini[1], P. Morelli[1]; [1]Rozzano/IT, [2]Pieve Emanuele/IT
*(angela.ammirabile@humanitas.it)*

**Purpose:** Quantitative CT analysis (QCT) has been implemented to predict the prognosis of patients affected by COVID-19. In this prospective monocentric trial performed during the first wave of the Italian COVID-19 pandemic, we assessed the evolution of respiratory symptoms at short-term follow-up and correlated them with abnormal QCT results.
**Methods or Background:** From March to May 2020, all patients hospitalised for COVID-19 pneumonia received a non-contrast chest CT upon admission and a clinical-radiological follow-up after 30-60 days. A quantitative analysis of segmented CT structures was performed, subdividing the total lung volume into hyperinflated, normally aerated, poorly aerated (%PAL), and non-aerated (%NNL); compromised lung volume (%CL=%PAL+%NNL) was additionally computed.
**Results or Findings:** Our study included 282 patients with complete clinical and radiological data. Median lung QCT parameters were: 11% %CL and 9% %PAL at admission, 5 %CL and 4 %PAL at follow-up. The median differences were 5% delta%CL and 3% delta%PAL. At control, 6 (2%) patients lamented residual dyspnoea, 13 (4%) reported generalised chest pain and 3 (1%) had residual uncontrollable coughing. After binomial logistic regression analyses, %deltaCL (OR<-0.01) and %deltaPAL (OR<-0.01) were significant predictors of residual dyspnoea and thoracalgia was correlated with %deltaPAL (OR=1.11).
**Conclusion:** We reported a correlation between changing lung abnormalities measured by QCT, and residual symptoms at short-term follow up after COVID-19 pneumonia. QCT was able to quantify the extent of residual lung damage and may be used as an objective metric for the measurement of COVID-19 sequelae.
**Limitations:** Relatively small cohort of patients with short-term follow-up, lack of an established treatment regimen in the early pandemic phase, lack of instrumental pulmonary function tests at follow-up.
**Ethics committee approval:** The IRB of Humanitas Research Hospital approved this study.
**Funding for this study:** Not applicable
**Author Disclosures:**
Costanza Lisi: Nothing to disclose
Federica Maria Pilar Tordato: Nothing to disclose
Angela Ammirabile: Nothing to disclose
Maddalena Casana: Nothing to disclose
Ezio Lanza: Nothing to disclose
Daria Pocaterra: Nothing to disclose
Paola Morelli: Nothing to disclose
Luca Balzarini: Nothing to disclose
Gaia Messana: Nothing to disclose

### RPS 1604-4
**Radiologic-pathologic correlation in 1-year follow-up patients after COVID-19 infection**
*D. Cozzi*, E. Cavigli, S. Luvarà, A. Bindi, C. Moroni, S. Tomassetti, V. Pasini, C. E. Comin, V. Miele; Firenze/IT
*(dilettacozzi@gmail.com)*

**Purpose:** The aim is to evaluate the characteristics of post-COVID-19 interstitial lung changes, with the unique opportunity to evaluate radiologic-pathologic correlations using HRCT and transbronchial lung cryobiopsy specimens.
**Methods or Background:** These are preliminary results of HRCT features of post-COVID-19 ILD. Authors collected data of hospitalised patients at baseline, then at 6 (+/-1) and 12 (+/-1) months after discharge. HRCT changes at 6 months involving more than 5% of the total lung volume were considered significant. Patients with significant HRCT changes underwent BAL and/or cryobiopsy and a subsequent follow-up with HRCT and lung function evaluation at 18 (+/-1) months.
**Results or Findings:** At the time of the present interim analysis, 139 patients from our university hospital were enrolled (enrollment is still ongoing). Median age was 67 years (range 18-87), 85 were males (61.1%). After 1-year follow-up, HRCT significant changes (both fibrotic-like and non-fibrotic) were detected in 53 subjects (38.1%): of them, only 2/53 (3.8%) patients have parenchymal progression of the disease, stability in 33/53 (62.3%) and improvement of lung involvement in 18/53 (33.9%). Cryobiopsies were performed in 6 patients, showing some discordance with radiological appearance. In particular, biopsy find 2 cases of histological UIP/early-UIP where HRCT demonstrate an NSIP/OP pattern. Cryobiopsy confirm 1 case of HRCT UIP-probable pattern and 3 cases of NSIP/OP/HP pattern.
**Conclusion:** This preliminary analysis confirms that after COVID-19 infection a large minority of patients develops interstitial lung changes mostly with NSIP/OP or (early) UIP pattern. The hypothesis is that the infection could be a trigger for a possible underlying latent interstitial disease, in predisposed subjects.
**Limitations:** Small simple size; patients with fibrotic-like changes (without histologic confirm), may need a longer follow-up to determine whether the fibrotic-like changes are permanent, progressive, or reversible.
**Ethics committee approval:** This study was approved by an ethics committee.
**Funding for this study:** No funding was received.
**Author Disclosures:**
Alessandra Bindi: Nothing to disclose
Valeria Pasini: Nothing to disclose
Camilla Eva Comin: Nothing to disclose
Vittorio Miele: Nothing to disclose
Silvia Luvarà: Nothing to disclose
Sara Tomassetti: Nothing to disclose
Edoardo Cavigli: Nothing to disclose
Chiara Moroni: Nothing to disclose
Diletta Cozzi: Nothing to disclose

### RPS 1604-5
**Inflammatory burden and persistent CT lung abnormalities in COVID-19 patients**
*F. Monelli*[1], G. Besutti[1], M. Ottone[1], L. Spaggiari[1], D. Giovanni[2], G. Ghidoni[2], P. Giorgi Rossi[1], P. Pattacini[1]; [1]Reggio Emilia/IT, [2]Modena/IT
*(mofilippo@hotmail.it)*

**Purpose:** Inflammatory burden is associated with COVID-19 severity and outcomes. Residual computed tomography (CT) lung abnormalities have been reported in COVID-19 pneumonia survivors. The study aim was to evaluate the association between inflammatory burden during COVID-19 and short- and medium-term residual abnormalities on follow-up CT.
**Methods or Background:** Follow-up CT scans, performed 2-3 and 6-7 months after COVID-19 pneumonia by survivors who experienced respiratory failure or had baseline CT extent of disease >40%, were retrospectively reviewed to evaluate residual non-fibrotic and fibrotic CT abnormalities. C-reactive protein (CRP) curves describing inflammatory burden during the clinical course were built, and CRP peaks, velocities of increase, and integrals were calculated. Other putative determinants were age, sex, mechanical ventilation, lowest $PaO_2/FiO_2$ ratio, D-dimer peak, and length of stay.
**Results or Findings:** Out of the 259 included patients (median age 65 years; 30.5% females), 202 (78%) and 100 (38.6%) had residual, predominantly non-fibrotic, CT abnormalities at 2-3 and 6-7 months, respectively. In age- and sex-adjusted models, best CRP predictors for residual abnormalities were CRP peak (odds ratio [OR] for one standard deviation [SD] increase=1.79; 95% confidence interval [CI]=1.23-2.62) at 2-3 months and CRP integral (OR for one SD increase=2.24; CI=1.53-3.28) at 6-7 months.
**Conclusion:** Mechanical ventilation and LOS were mediators of the relationship between CRP and residual abnormalities. D-dimer, though associated with outcomes, was not a mediator of CRP.

**Limitations:** No data on symptoms and function. Selection of severe cases only.
**Ethics committee approval:** The study was approved by the AVEN Ethics Committee (855/2020/OSS/AUSLRE).
**Funding for this study:** Funding was received from the Italian Ministry of Health (COVID-2020-12371808).
**Author Disclosures:**
Lucia Spaggiari: Nothing to disclose
Pierpaolo Pattacini: Nothing to disclose
Filippo Monelli: Nothing to disclose
Dolci Giovanni: Nothing to disclose
Giulia Ghidoni: Nothing to disclose
Paolo Giorgi Rossi: Nothing to disclose
Giulia Besutti: Nothing to disclose
Marta Ottone: Nothing to disclose

### RPS 1604-6

**Structural and functional pulmonary assessment in severe COVID-19 patients at 12 months after discharge**

*E. Mercanzin*[1], P. A. Bonaffini[1], A. Corsi[1], A. Caroli[2], A. Arrigoni[2], C. Conti[2], G. Imeri[2], F. Di Marco[2], S. Sironi[1]; [1]Milan/IT, [2]Bergamo/IT
*(e.mercanzin@campus.unimib.it)*

**Purpose:** To assess clinical status, pulmonary function tests (PFT), and radiological findings evolution from 3 to 12 months post-discharge in patients surviving severe COVID-19 pneumonia. To investigate the relation between functional and radiological findings.
**Methods or Background:** Severe COVID-19 survivors, admitted between February 25th and May 2nd, 2020 and then undergone repeated pulmonary assessment and chest CT scans at 3 and 12 months post-discharge, were prospectively included. Patients with chronic pulmonary disease or COVID-19-unrelated complications were excluded. The pulmonary assessment included PFT, laboratory testing, and symptoms. Pathological patient-reported outcomes were also registered. Unenhanced CT were analysed quantitatively (% compromised lung volume) and qualitatively (main pattern: ground-glass opacity/GGO, consolidation, reticular configuration). Patients were subsequently divided into groups based on their radiological trends.
**Results or Findings:** Seventy-one patients were included. At 12-month post-discharge, all showed significantly improved laboratory tests and PFT. D-dimer values were low in patients with normal CT and higher in improved or stable abnormal CT (median values 213 vs 329 vs 1000 ng/mL, respectively). Sixty-three patients underwent CT: 14/63 findings remained negative at 12 months, 1/49 (2%) normalised, 40/49 (82%) improved, 7/49 (14%) stable, 1/49 (2%) worsened. The compromised lung volume reduced compared with 3 months post-discharge (12.3 vs 14.4 %, $p < 0.001$). The CT pattern changed, with GGO reduction and reticular configuration increase.
**Conclusion:** PFT were normal in most COVID-19 survivors 12 months post-discharge. CT structural abnormalities persisted (although mostly improved over time) and were associated with higher D-dimer values.
**Limitations:** Limited sample size; acute and follow-up CT data not available for a few patients; no patient-reported outcomes at 3 months post-discharge; no pre-COVID structural and functional findings.
**Ethics committee approval:** The study was approved by the Comitato Etico di Bergamo.
**Funding for this study:** Partial grant support was received from Brembo SpA (Curno, Bergamo, Italy; "Progetto TrexUno").
**Author Disclosures:**
Sandro Sironi: Nothing to disclose
Alberto Arrigoni: Nothing to disclose
Elisa Mercanzin: Nothing to disclose
Pietro Andrea Bonaffini: Nothing to disclose
Fabiano Di Marco: Nothing to disclose
Andrea Corsi: Nothing to disclose
Caterina Conti: Nothing to disclose
Anna Caroli: Nothing to disclose
Gianluca Imeri: Nothing to disclose

### RPS 1604-7

**Follow-up CT in patients with confirmed COVID-19 lung injury on the example of outpatient triage CT centres in St. Petersburg**

*D. I. Kuplevatskaya*, V. И. Kuplevatsky, N. Berezina, M. Cherkashin; Saint Petersburg/RU

**Purpose:** To assess the features of COVID-19 lung injury regression and to work out an algorithm of monitoring patients in different time periods after the disease.
**Methods or Background:** Retrospective analysis of 1759 low-dose control CT scans was conducted in patients with a previous confirmed SARS-CoV-2 lung injury. All control CT studies were distributed to periods (pr), according to the time of primary study: Ipr-3-9 weeks after the primary CT, IIpr-3-4 months, IIIpr-5-7 months, IVpr-8-12 months. The degree of involvement of each lung in addition to overall extent of lung involvement was also evaluated according to generally accepted CT score (CT1-CT4).
**Results or Findings:** The follow-up CT changers were distributed by severity of lung injury: grade I (Igr), abnormalities with ground-glass opacity (GGO) low intensity; grade II (IIgr), scattered low intensity GGO with liner and reticular pattern; grade III (IIIgr), IIgr with subsegmental and lobular atelectases; grade IV (IVgr), opacities with a "crazy-paving" patterns, atelectases, bronchiectases. In 84.7% of follow-up CT abnormalities remained after the COVID-19 lung injury. In patients with lung injury CT3 and CT4 in 33,25% of follow-up CT IIIgr and 25.21% IVgr of residual abnormalities were observed in the IIpr. CT1 and CT2 lung involvement showed 51,85% Igr follow-up CT and 37,03% IIdg of residual changes in the IIpr, while in 41,66% of such patients IIgr persisted in the IIIpr. Significant differences were found in the severity of changes in the lungs depending on the primary CT (p=0.00225).
**Conclusion:** The follow-up CT showed the necessity of dynamic CT in patients with COVID-19 CT3 and CT4 lung injury. Monitoring of lung abnormalities will allow timely rehabilitation and possibly prevent the manifestations of lung fibrosis.
**Limitations:** Not applicable.
**Ethics committee approval:** Not applicable.
**Funding for this study:** Not applicable.
**Author Disclosures:**
Mikhail Cherkashin: Nothing to disclose
Natalia Berezina: Nothing to disclose
Vladimyr Игоревич Kuplevatsky: Nothing to disclose
Daria Igorevna Kuplevatskaya: Nothing to disclose

### RPS 1604-11

**Follow-up CT scan at 6-7 months in a cohort of 405 severe COVID-19 pneumonia survivors: preliminary results of a multicentre study**

*F. Monelli*[1], G. Besutti[1], S. Schirò[2], E. Bonelli[1], L. Spaggiari[1], N. Sverzellati[2], P. Pattacini[1]; [1]Reggio Emilia/IT, [2]Parma/IT
*(mofilippo@hotmail.it)*

**Purpose:** Studies on COVID-19 pneumonia follow-up reported a variably high prevalence of residual chest CT abnormalities including fibrotic-like changes, heterogeneously defined. Our aim is to describe follow-up chest CT features in a prospective cohort of severe COVID-19 survivors.
**Methods or Background:** All consecutive COVID-19 survivors who received a follow-up CT scan 6-7 months after severe COVID-19 pneumonia in two Italian hospitals (Reggio Emilia and Parma) were enrolled. Individual chest CT findings were retrospectively collected by two experienced radiologists, and categorised as: resolution (no residual or trivial CT abnormalities); residual non-fibrotic abnormalities; residual fibrotic abnormalities, and post-ventilatory abnormalities. When available, 12 months follow-up CT scans were also reviewed.
**Results or Findings:** Of 405 included patients (234 from Reggio Emilia and 171 from Parma), 225 (55.6%; 57.9% and 53.8% in the two cohorts) had complete resolution at 6-7 months, 152 (37.5%) had residual non-fibrotic abnormalities, while residual fibrotic and post-ventilatory abnormalities were found in 18 (4.4%) and 10 (2.5%) patients, respectively. In the non-fibrotic group, prevalent findings were ground-glass opacities (GGO) (n=130/152), mainly barely visible (n=110/130) rather than overt (n=20/130), and bronchiectasis (n=52/152). In the fibrotic group, honeycombing was found in 2 patients only, while subpleural reticulation (15/18), bronchiectasis (16/18) and GGO (14/18) were frequent. Median CT extension was 20% (IQR=10%-30%), 30% (IQR=20-39%), and 45% (32.5%-60%), in the non-fibrotic, fibrotic, and post-ventilatory group, respectively. Of the 25 patients with available 12 month follow-up CT scans, 14 had complete resolution, 7 had persistent non-fibrotic and 4 persistent fibrotic abnormalities.
**Conclusion:** Residual abnormalities 6-7 months after severe COVID-19 pneumonia are frequent (approximately 45%), but largely predominantly non-fibrotic, suggesting a slow resolution rather than persistent fibrotic changes.
**Limitations:** An identified limitation is that there was scarce data at 12 months follow-up.
**Ethics committee approval:** Ethical approval was received from AVEN Ethics Committee (855/2020/OSS/AUSLRE).
**Funding for this study:** Funding was received from the Italian Ministry of Health (COVID-2020-12371808).
**Author Disclosures:**
Silvia Schirò: Nothing to disclose
Lucia Spaggiari: Nothing to disclose
Pierpaolo Pattacini: Nothing to disclose
Filippo Monelli: Nothing to disclose
Nicola Sverzellati: Nothing to disclose
Efrem Bonelli: Nothing to disclose
Giulia Besutti: Nothing to disclose

**RPS 1604-12**

**Ultra-low-dose CT (ULDCT) vs low-dose CT (LDCT) of the chest in post-COVID-19 patients: an intrapatient comparison**

*C. Wassipaul*, D. Kifjak, F. Prayer, R-I. Milos, M. Winter, M. Weber, H. Ringl, H. Prosch, B. H. Heidinger; Vienna/AT

**Purpose:** To prospectively assess the diagnostic accuracy of ULDCT compared to LDCT for post-COVID-19 lung changes.
**Methods or Background:** We included 155 consecutive post-COVID-19 patients referred for LDCT of the chest until January 2021 into this prospective study. All participants received both an LDCT and an unenhanced ULDCT. CT examinations were performed with a 3rd-generation 256-row MD-DSCT system with tin-filtration and tube current modulation enabled. LDCTs were performed with standard imaging parameters of 110kV/66ref.mAs and ULDCTs at 100kV/50ref.mAs. All CT images were evaluated by four radiologists (two expert radiologists, two residents) with regard to subjective image quality, the presence of residuals from COVID-19 pneumonia. Sensitivity and specificity were calculated.
**Results or Findings:** 47 of the 155 participants (30.3%) showed lung abnormalities consistent with post-COVID-19 changes. Sensitivity and specificity of ULDCT compared to LDCT for detecting post-COVID-19 changes were 88.6% and 93.3%, respectively. The mean total dose length product (DLP) for ULDCTs was 13.7 mGy*cm (range 5.6-54.5) and therefore one tenth of LDCTs with 138.2 mGy*cm (range 53.7-499.2). Mean effective dose of ULDCTs was 0.23 mSv, corresponding to approximately twice the mean effective dose of CXRs in two views (0.1 mSv).
**Conclusion:** ULDCT has a high sensitivity and specificity for detecting post-COVID-19 lung changes at about twice the mean effective dose of a CXR. Therefore, ULDCT might be a viable alternative to LDCT in the follow-up of COVID-19 patients.
**Limitations:** Objective image quality was not evaluated.
**Ethics committee approval:** This study was approved by the IRB of Medical University of Vienna (EK-Nr.2254/2018).
**Funding for this study:** Institution has research support/grants from Siemens Healthineers.
**Author Disclosures:**
Helmut Ringl: Research/Grant Support: Institution has/had research support/grants from Siemens Healthineers.
Benedikt H Heidinger: Research/Grant Support: Institution has research support/grants pending from Siemens Healthineers.
Helmut Prosch: Research/Grant Support: Institution has research support/grants from Siemens Healthineers.
Florian Prayer: Research/Grant Support: Institution has research support/grants from Siemens Healthineers.
Christian Wassipaul: Other: The Department of Biomedical Imaging and Image-guided Therapy of Medical University of Vienna received, among others, funding from Siemens Healthineers (Erlangen, Germany). The Department of Biomedical Imaging and Image-guided Therapy of Medical University of Vienna employed Christian Wassipaul as research assistant for one year.
Daria Kifjak: Research/Grant Support: Institution has research support/grants from Siemens Healthineers.
Ruxandra-Iulia Milos: Research/Grant Support: Institution has research support/grants from Siemens Healthineers.
Melanie Winter: Research/Grant Support: Institution has research support/grants from Siemens Healthineers.
Michael Weber: Nothing to disclose

**16:00-17:30** **Room E2**

### Research Presentation Session: Abdominal Viscera

### RPS 1601

### How to improve your abdominal imaging protocols?

**Moderator**
G. Ferraioli; Pavia/IT
**Author Disclosure:**
G. Ferraioli: Speaker: Canon ultrasound, Fujifilm ultrasound, Mindray Ultrasound, Philips Ultrasound, Seemens Ultrasound

**RPS 1601-2**

**Low-dose CT of the abdomen: initial experience on a novel photon-counting detector CT and comparison with energy-integrating detector CT**

*J. A. Decker*, S. Bette, N. Lubina, K. Rippel, F. Braun, F. Risch, C. Scheurig-Muenkler, T. J. Kroencke, F. Schwarz; Augsburg/DE
*(Josua.Decker@uk-augsburg.de)*

**Purpose:** To analyse the quantitative and qualitative image quality of low-dose computed tomography (CT) scans of the abdomen performed on a novel photon-counting detector (PCD) CT in comparison to a traditional CT with an energy-integrating detector (EID).
**Methods or Background:** Consecutive patients with clinically indicated low-dose CT were scanned on the PCD-CT and compared to an EID-CT-cohort matched for BMI. Radiation dose, noise (standard deviation [SD] of CT values) and signal-to-noise ratio (SNR) were measured for each patient. Additionally, image quality and conspicuity of four defined abdominal structures (adrenals, mesenteric vessels, ureter and renal pelvis) were assessed on 5-point Likert-scales (1=very poor quality/not detectable; 5=excellent quality/differentiability).
**Results or Findings:** Twenty patients (mean age 46.2 [range: 19-77]; 13 men) were included. There was no significant difference in radiation dose between PCD-CT and EID-CT (1.61 vs. 1.45 mGy; p=0.21). Noise was significantly lower (24.89±3.30 HU vs. 31.43±5.57 HU, p<0.001) and SNR significantly higher (2.08±0.28 vs. 1.48±0.35; p<0.001) on the PCD-CT. Subjective image quality was substantially higher (4.02±0.28 vs. 3.08±0.59; p<0.001) and conspicuity better for the renal pelvis, ureters and mesenteric vessels on the PCD-CT. There was no significant difference in the conspicuity of the adrenals. With increasing BMI (1st to 4th BMI quartile), noise increased and SNR decreased more strongly on the EID-CT than on the PCD-CT (deltaNoise: 39% vs. 2%, deltaSNR: -33% vs. -7% for EID-CT vs. PCD-CT, respectively) while radiation dose increased comparably (70 vs. 59%).
**Conclusion:** Low-dose CT scans of the abdomen performed on a novel PCD-CT exhibit reduced noise, higher SNR, increased subjective image quality and superior conspicuity of abdominal fine structures compared to scans in comparable patients on an EID-CT.
**Limitations:** Identified limitations were: the small cohort; retrospective single-centre study.
**Ethics committee approval:** This study was approved by an ethics committee.
**Funding for this study:** No funding was received for this study.
**Author Disclosures:**
Franka Risch: Nothing to disclose
Stefanie Bette: Nothing to disclose
Franziska Braun: Nothing to disclose
Thomas J. Kroencke: Nothing to disclose
Florian Schwarz: Nothing to disclose
Christian Scheurig-Muenkler: Nothing to disclose
Katharina Rippel: Nothing to disclose
Josua A. Decker: Nothing to disclose
Nora Lubina: Nothing to disclose

**RPS 1601-4**

**Are dilution and slow injection with fluoroscopic triggering technique the solution to mitigating arterial-phase artifacts in gadoxetic acid enhanced liver MRI?**

*R. Ambros*, S. Pötter-Lang, A. Messner, A. Kristic, J. C. Hodge, N. Bastati-Huber, A. Ba-Ssalamah; Vienna/AT

**Purpose:** To evaluate the effect of dilution and slow injection of gadoxetic acid (GA) using automated fluoroscopic triggering on the frequency of arterial-phase artifacts.
**Methods or Background:** Three independent readers evaluated 1985 Liver MRIs routinely done with a fixed bolus of 10 ml GA diluted with 10 ml saline,

Friday injected at 1 mL/s using automated fluoroscopic triggering on a 3T MR machine. All readers graded severity of artifacts and their impact on diagnostic performance on a 5-point-scale system. Two readers assessed the type of artifacts: Gibbs, TSM or both. One reader evaluated arterial-phase acquisition timing and the presence of ascites, pleural effusions and cirrhosis.
**Results or Findings:** A total of 1793 exams (male =852 (49.7%), female =863 (50.3%); with a mean age of 56.3 years), were included. In 366 (20.4%) exams there was ascites, in 319 (17.8%) pleural effusions and liver cirrhosis in 352 (19.6%). Arterial-phase images of diagnostic quality included 1163 (64.9%) images without artifacts, 415 (23,1%) with minimal, and 171 (9,5%) with moderate artifacts. The exams were only non-diagnostic in 44 patients (2,5%), 39 (2,2%) with severe arterial-phase artifacts and 5 (0,3%) with uninterpretable images. The inter-rater agreement (Kappa =0.670, p<0.001) was substantial. Acquisition timing for AP imaging was optimal in 1567 (87,4%) exams. The number of artifacts found was significantly higher in the presence of ascites (p=0.002) and pleural effusions (p=<0.001), as well as in advanced age (p=0.006). A higher BMI (p=0,022) was found in subjects with moderate artifacts, as compared to no artifacts.
**Conclusion:** The combination of a diluted and slowly injected bolus of gadoxetic acid using MRI fluoroscopic triggering provides properly-timed arterial phase imaging, and reduced severe or non-diagnostic artifacts in the arterial phase to only 2.5%.
**Limitations:** The study was limited due to its nature as a retrospective, single-centre study.
**Ethics committee approval:** This study is IRB-approved.
**Funding for this study:** No funding was received for this study.
**Author Disclosures:**
Sarah Pötter-Lang: Nothing to disclose
Raphael Ambros: Nothing to disclose
Jacqueline C. Hodge: Nothing to disclose
Ahmed Ba-Ssalamah: Consultant: Bayer AG Speaker: Bayer AG
Nina Bastati-Huber: Nothing to disclose
Alina Messner: Nothing to disclose
Antonia Kristic: Nothing to disclose

### RPS 1601-5

**A feasibility study of "dark-blood" angiography using dual-layer detector CT in pre-operative staging of pancreatic cancer**

*K. Si*[1], X. Zhang[2], P. Lei[1], J. Li[1], C. Ding[2], P. Han[1], X. Li[1]; [1]Wuhan/CN, [2]Shanghai/CN

**Purpose:** To investigate the diagnostic impact and performance of a newly developed material decomposition method, "dark-blood" on contrast-enhanced dual-layer detector CT in preoperative staging of pancreatic cancer compared with conventional images (CI).
**Methods or Background:** Eleven patients with pancreatic carcinoma (3 women, age: 63±16 years) who underwent contrast-enhanced CT scans on spectral dual-layer detector CT (IQon, Philips Healthcare) were retrospectively analysed. Dark-blood images in late arterial phase were derived from spectral based images using a two-material decomposition method, where the first material was defined as the content of a region of interest placed in the coeliac artery and superior mesenteric artery for each patient. Two abdominal radiologists with 7 and 12 years' experience independently determined the tumour-vessel anatomy: 1. the type of involved vessels (intact, irregular or deformed) according to the tumour-vessel relationship; 2. the degree of tumour-vessel interface (abutment: ＜180°; encasement: ≥180°) involved, a third senior radiologist with 18 years' experience in abdominal radiology made the final diagnosis in the case of any disagreements.
**Results or Findings:** 48 involved arteries (18 abutment, 30 encasement) of 11 pancreatic cancer patients were detected in both CI and dark-blood images. The vessel walls of all abutment arteries were determined as intact in CI, while in dark-blood images 5 abutment arteries of 18 (27.8%) were determined as contour irregularity, which would influence the diagnosis as "borderline resectable" instead of "resectable" according to NCCN criteria. In 30 encasement arteries, there were 28 deformed, 1 intact and 1 irregular contour in dark-blood images, compared with 25 deformed and 5 intact in CI.
**Conclusion:** Dark-blood material decomposition method, which enhanced the visibility of vessel walls, could potentially improve diagnostic performance for preoperative staging of pancreatic cancer.
**Limitations:** The number of patients is small.
**Ethics committee approval:** This study was approved by an ethics committee.
**Funding for this study:** No funding was received for this study.
**Author Disclosures:**
Jing Li: Nothing to disclose
Chengyu Ding: Nothing to disclose
Ping Han: Nothing to disclose
Keke Si: Nothing to disclose
Xiaohui Zhang: Nothing to disclose
Xin Li: Nothing to disclose
Ping Lei: Nothing to disclose

### RPS 1601-6

**Value of dual-layer spectral-detector CT in reducing intestinal peristalsis-related streak artifacts on the liver**

*S. Grosu*[1], Z. J. Wang[2], M. Obmann[2], M. Sugi[2], Y. Sun[2], B. M. Yeh[2]; [1]Munich/DE, [2]San Francisco, CA/US

**Purpose:** To evaluate different image reconstructions at dual-layer spectral-detector CT for reducing intestinal peristalsis-related streak artifacts on the liver.
**Methods or Background:** We retrospectively evaluated 220 contrast-enhanced abdominal dual-energy CT scans in 131 consecutive patients (mean age: 68±10 years, 120 men) who underwent routine clinical dual-layer spectral-detector CT imaging (120kVp, 40keV, 200keV, virtual non-contrast (VNC), iodine images). Two independent readers evaluated bowel peristalsis streak artifacts on the liver qualitatively on a five-point Likert scale (1=none to 5=severe) and quantitatively by depth of streak artifact extension into the liver and measurements of Hounsfield Unit and iodine concentration differences from normal liver. Artifact severity between image reconstructions were compared by Wilcoxon signed-rank and paired t-tests.
**Results or Findings:** Streak artifacts on the liver were seen in 51/208 (25%) CT scans and involved the left lobe only in 49/51 (96%), the right liver lobe only in 0/51 (0%), and both liver lobes in 2/51 (4%) scans. Artifact frequency was lower in iodine than in 120kVp images (scans 18/208 vs. 51/208, p<0.001). Artifact severity was less in iodine than in 120kVp images (median score 1 vs. 3, p<0.001). Streak artifact extension into the liver was shorter in iodine than 120kVp images (mean length 2±4 vs. 12±5 mm, p<0.001). ROI measurements differed significantly between bright streak artifacts and the neighboring unaffected liver parenchyma in 120kVp, 40keV, 200keV and VNC images (p<0.001, each), but not in iodine images (p=0.23).
**Conclusion:** Iodine image reconstructions at dual-layer spectral-detector CT substantially reduced peristalsis-related streak artifacts on the liver.
**Limitations:** One DECT scanner model, limiting the applicability of our results to other DECT scanner models. Proportion of men in our study population disproportionately high.
**Ethics committee approval:** Institutional Review Board approval was obtained.
**Funding for this study:** Philips Healthcare; National Institutes of Health
**Author Disclosures:**
Zhen Jane Wang: Nothing to disclose
Mark Sugi: Nothing to disclose
Markus Obmann: Nothing to disclose
Yuxin Sun: Nothing to disclose
Sergio Grosu: Nothing to disclose
Benjamin M Yeh: Nothing to disclose

### RPS 1601-8

**Virtual non-contrast imaging of clinical photon-counting detector CT: accuracy in the abdomen**

*V. Mergen*[1], L. Jungblut[1], T. D. J. Sartoretti[1], M. Petersilka[2], S. Bickel[1], K. Higashigaito[1], H. Alkadhi[1], A. Euler[1]; [1]Zurich/CH, [2]Forchheim/DE

**Purpose:** To assess the accuracy of virtual non-contrast images (VNC) of the abdomen acquired on clinical dual-source photon-counting detector CT (PCD-CT).
**Methods or Background:** Seventy consecutive patients (mean age 73±11, 20 female) undergoing a triphasic examination on a dual-source PCD-CT (Naeotom Alpha, Siemens) were included. VNC images from the arterial and portal venous phases were reconstructed. CT attenuation was measured in the aorta, liver, spleen, kidney, urinary bladder, paravertebral musculature, subcutaneous fat and spongious bone. Attenuation of the VNC images was compared to the true unenhanced images (reference standard). Absolute error of attenuation (HUerror) was computed. Image noise texture was assessed by measuring the noise power spectrum (NPS) in a cylindrical water phantom.
**Results or Findings:** NPS showed similar noise texture among VNC and true unenhanced images. Mean HUerror was less than 10HU for all regions except subcutaneous fat and spongious bone. Smallest HUerror was found in the liver (2.4±2.3HU) for the arterial and in the spleen (2.4±1.8HU) for the venous VNC images. For liver and spleen, HUerror was less than 5HU in 86% of the cases. Highest HUerror was 24.3±3.0HU/23.5±3.1HU in subcutaneous fat and 56.3±28.8HU/58.6±38.2HU for arterial and venous VNC images, respectively.
**Conclusion:** Virtual non-contrast imaging of PCD-CT from arterial and portal venous phase demonstrated high accuracy with small error ranges compared with true unenhanced images in parenchymal organs of the abdomen.
**Limitations:** A limitation of this study was that the impact of lesion detection and classification was not assessed.
**Ethics committee approval:** This study was IRB approved.
**Funding for this study:** VM: research grant of the SAMS and Bangerter-Rhyner-Foundation funded this study.

**Author Disclosures:**
Victor Mergen: Nothing to disclose
Kai Higashigaito: Nothing to disclose
Martin Petersilka: Employee: Siemens Healthcare GmbH, Forchheim, Germany
Thomas Daniel Jean Sartoretti: Nothing to disclose
Lisa Jungblut: Nothing to disclose
Steven Bickel: Nothing to disclose
Andre Euler: Nothing to disclose
Hatem Alkadhi: Nothing to disclose

### RPS 1601-9

**Quantum iterative reconstruction for abdominal photon-counting detector CT: assessment of image quality and lesion conspicuity in a phantom and patients**

*A. Euler*[1], T. Sartoretti[1], V. Mergen[1], A. Landsmann[1], D. N. Nakhostin[1], K. Higashigaito[1], R. Raupach[2], M. Eberhard[3], H. Alkadhi[1]; [1]Zurich/CH, [2]Erlangen/DE, [3]Unterseen/CH

**Purpose:** To investigate the image quality and the optimal strength level of a novel iterative reconstruction QIR (quantum iterative reconstruction) for virtual monoenergetic images (VMI) and polychromatic images (T3D) in a phantom and patients in portal venous abdominal PCD-CT.
**Methods or Background:** In this retrospective study, noise power spectrum (NPS) was measured in a water-filled phantom. Consecutive oncologic patients who received a portal venous abdominal CT on a clinical PCD-CT between March and April 2021 were included. VMI at 60keV and T3D were reconstructed without QIR (QIR-off) and with QIR at four levels (QIR1-4). Global noise index (GNI), CNR, and voxel-wise CT attenuation differences were measured. Noise and texture, artefacts, diagnostic confidence, and overall quality were assessed qualitatively. Conspicuity of hypodense liver lesions was rated by four readers. Parametric and non-parametric tests were used.
**Results or Findings:** In the phantom, NPS showed unchanged noise texture across reconstructions. Fifty patients (mean age, 59 years ±16, 31 women) were included. GNI was reduced from QIR-off to QIR-4 by 45% and by 44% for 60keV and T3D, respectively (P<.001). CNR of the liver improved from QIR-off to QIR-4 by 74% from 11±2.2 to 19.1±4.5 and by 69% from 11.5±2.5 to 19.4±5.3 for 60 keV and T3D,respectively (P<.001). No evidence of a difference was found in mean attenuation of fat and liver (P=.79–P=.84) and on a voxel-wise basis among reconstructions. Qualitatively, QIR-4 outperformed all other reconstructions in every category for 60keV and T3D (P<.001–P=.01). All four readers rated QIR-4 superior for lesion conspicuity (P<.001–P=.04).
**Conclusion:** In portal venous abdominal PCD-CT, high levels of QIR improved image quality by reducing noise and improving CNR and lesion conspicuity without compromising image texture or CT attenuation values.
**Limitations:** No limitation were identified.
**Ethics committee approval:** This study was approved by a local ethics committee.
**Funding for this study:** No funding was received for this study.
**Author Disclosures:**
Victor Mergen: Nothing to disclose
Matthias Eberhard: Nothing to disclose
Rainer Raupach: Employee: Siemens Healthineers
Kai Higashigaito: Nothing to disclose
Anna Landsmann: Nothing to disclose
Thomas Sartoretti: Nothing to disclose
Dominik Nader Nakhostin: Nothing to disclose
Andre Euler: Nothing to disclose
Hatem Alkadhi: Nothing to disclose

### RPS 1601-11

**Comparison of iterative reconstruction and deep learning imaging reconstruction in detection of hypoattenuating liver metastasis**

*N. Liu*, P. Lu, J. Gao; Zheng Zhou/CN
*(1193355391@qq.com)*

**Purpose:** To evaluate the impact of deep learning image reconstruction (DLIR) on liver metastasis detection compared with iterative reconstruction.
**Methods or Background:** Forty-five patients (23 men) underwent abdominal scans with data sets reconstructed using ASiR-V 60%, and DLIR (low, medium, and high strength levels) at 1.25mm slice thickness. Metastases were defined by histopathologic analysis or progression and regression. After a portal venous pass with ASiR-V 60% images, a 70% reduced radiation dose pass was added with DLIR images. One reviewer scored ASiR-V image quality and marked findings. Two additional independent reviewers noted whether marked findings were present on DLIR images and assigned scores for relative conspicuity. Quantitative metrics (image noise, contrast to noise ratio [CNR], and signal to noise ratio [SNR]) and qualitative parameters (sharpness, artefacts, and overall image quality) were compared. Formal statistical inference for size-specific dose estimate (SSDE) was made (paired t tests), with Bonferroni adjustment.
**Results or Findings:** Two independent reviewers identified all ASiR-V image lesions (n=98) on DLIR images, scoring them as equal to or better than DLIR at low, medium and high strength levels for conspicuity in 96.9% (95 of 98), 97.9% (96 of 98) and 98.9% (97 of 98) , respectively. Compared to ASiR-V 60% images, DLIR images at medium or high strength levels showed similar or lower image noise, similar or higher CNR and SNR, similar scores in artefacts, and better perceived scores in sharpness and overall image quality. For ASIR and MBIR, SSDE was 14.6 mGy ±4.3 versus 4.7 mGy ± 2.1 (P< .001) respectively.
**Conclusion:** Liver CT images reconstructed with DLIR may allow up to 70% radiation dose reduction compared with the dose with ASiR-V 60%, without compromising depiction of liver metastases or image quality.
**Limitations:** Not applicable.
**Ethics committee approval:** Not applicable.
**Funding for this study:** Not applicable.
**Author Disclosures:**
Nana Liu: Nothing to disclose
Peijie Lu: Nothing to disclose
Jianbo Gao: Nothing to disclose

**16:00-17:30** **Room F1**

### Research Presentation Session: Head and Neck

### RPS 1608

### Thyroid and parathyroid gland imaging

**Moderator**
E. Gotsiridze; Tbilisi/GE

### RPS 1608-2

**Deep convolutional neural network with transfer learning for automated classification of thyroid nodules on ultrasound**

Y-J. Kim, *P. Y. Choi*; Seoul/KR
*(phillipchoi007@gmail.com)*

**Purpose:** To train and validate deep learning (DL) classification models for automatically detecting and differentiating malignant thyroid nodules from benign nodules on ultrasound (US) images and to compare their performances to those of experienced radiologists.
**Methods or Background:** US images of thyroid nodules of patients who underwent US-guided fine-needle aspiration biopsy (FNAB) at our institution between January 2010 and March 2020 were retrospectively reviewed. A total of 15409 images from 7321 patients (mean age, 60±13) were randomly grouped into a training (n=12327) and validation (n=3082) set. Independent internal [n=432; mean age, 50±14] and external [n=168; mean age, 52±15] test datasets were also acquired. Four experienced radiologists independently classified the images of internal and external test datasets. Images of thyroid nodules were automatically segmented via DL algorithm, which were subsequently trained using three different image-classification DL models (VGG16, VGG19 and ResNet). The accuracy, sensitivity, specificity, and areas under the receiver operating characteristic curve (AUC) of DL models were calculated for the internal and external test datasets and compared with diagnoses by four radiologists.
**Results or Findings:** All three DL models demonstrated higher diagnostic performances than four radiologists in both internal (AUC, 0.83–0.86 vs 0.71–0.76) and external (AUC, 0.80–0.83 vs 0.60–0.80) test datasets. VGG16 model demonstrated the highest diagnostic performance in both internal (AUC, 0.86; sensitivity, 91.8%; specificity, 73.2%) and external (AUC, 0.83; sensitivity, 78.6%; specificity, 76.8%) test datasets.
**Conclusion:** The DL models overall demonstrated higher diagnostic performances than experienced radiologists in distinguishing benign from malignant thyroid nodules on US images. DL classification models may augment radiologists' diagnosis.
**Limitations:** The reference standard was cytologic samples from FNAB.
**Ethics committee approval:** Approved by the IRBs of Seoul St. Mary's and Yeouido St. Mary's hospitals.
**Funding for this study:** Fund was received from the National Research Foundation of Korea funded by Ministry of Education (2021R1I1A1A01040285).
**Author Disclosures:**
Philip Yangsean Choi: Nothing to disclose
Yeon-Jae Kim: Nothing to disclose

### RPS 1608-3

**Efficiency of biopsy criteria in thyroid nodules: the 2017 European Thyroid Imaging Reporting and Data System versus the 2017 American College of Radiology Thyroid Imaging management guidelines**

*G. Dolz*, V. Perez-Riverola, M. Prenafeta Moreno, R. Monmany, K. El-Hamshari, H. Peris, O. Vázquez, E. Granell; Sabadell/ES

**Purpose:** Determine the diagnostic efficiency and assess malignancy risk stratification of the 2017 European Thyroid Imaging Reporting and Data System (EU-TIRADS) in comparison to the 2017 American College of Radiology Thyroid Imaging Reporting and Data System (ACR-TIRADS) management guidelines.
**Methods or Background:** Retrospective study reviewing all fine-needle aspiration biopsies (FNAB) of thyroid nodules done following the EU-TIRADS at our center from January 2018 to December 2020. We evaluated the ultrasound characteristics of all the nodules and regrouped them according to ACR-TIRADS. The ACR-TIRADS criteria for FNAB differ slightly from those recommendations made by European guidelines. Low-risk lesions (TR3) require larger dimensions for FNAB in ACR-TIRADS compared to EU-TIRADS (25 vs 20mm). Regarding intermediate risk lesions (TR4), the interpretation of ultrasound characteristics is somewhat different in ACR-TIRADS. We analysed FNABs done under the EU-TIRADS criteria that did not meet the ACR-TIRADS criteria and calculated the rate of malignancy for all this FNABs and for each subgroup in both systems.
**Results or Findings:** Of 589 thyroid nodules biopsied, ACR-TIRADS criteria for biopsy were unmet in 188 (31.9%): 33 nodules did not meet the size condition, and 155 were not candidates for FNAB because of their ultrasound characteristics. FNABs were inconclusive in 20 patients; in the remaining 168, only 1 (0.6%) was malignant, which was an ACR-TR3 mixed cystic and solid hypoechoic nodule (papillary thyroid carcinoma). None of the nodules reclassified as ACR-TR2 were malignant.
**Conclusion:** Using ACR-TIRADS would have decreased the number of FNABs by 31.9%; only 1 malignant nodule would have been missed (0.6%). Most of the nodules that did not meet ACR-TIRADS criteria were mixed cystic-solid isoechoic lesions (ACR-TR2), and all of them were benign.
**Limitations:** Most of the pathological results are from FNAB, without having surgical confirmation.
**Ethics committee approval:** Not applicable.
**Funding for this study:** No funding was received for this study.
**Author Disclosures:**
Roser Monmany: Nothing to disclose
Esther Granell: Nothing to disclose
Helena Peris: Nothing to disclose
Victor Perez-Riverola: Nothing to disclose
Khaled El-Hamshari: Nothing to disclose
Mario Prenafeta Moreno: Nothing to disclose
Olaya Vázquez: Nothing to disclose
Guillem Dolz: Nothing to disclose

### RPS 1608-4

**TIRADS, SRE and SWE in the indeterminate thyroid nodule characterisation: which is the best?**

*G. Del Gaudio*, D. Fresilli, V. Dolcetti, P. Pacini, G. Polti, G. T. Lucarelli, O. Guiban, V. Cantisani, C. Catalano; Rome/IT
*(g.d.gaudio@gmail.com)*

**Purpose:** To determine Shear Wave Elastography (SWE) and Strain Ratio (SRE) accuracy alone and with TIRADS classification for indeterminate thyroid nodules at FNAC characterisation.
**Methods or Background:** Preoperative staging neck ultrasound was performed on 160 patients candidates for thyroidectomy with 160 indeterminate nodules and were classified using K-TIRADS score. Subsequently, elastosonographic evaluation was performed by means of quantitative (SWE expressed in kPa) and semi-quantitative (SRE) and compared with post-thyroidectomy histological results. The main aim was to asses diagnostic performance of each method, alone and in combination. 2x2 contingency tables and ROC curve analysis were made. To evaluate statistically significant differences (P-value <0.05), Bonferroni test was used. Liu test was used to calculate cut-off values to maximise SWE and SRE sensitivity and specificity.
**Results or Findings:** Sensitivity, specificity, PPV and NPV were respectively 71.5%, 82.5%, 62.5%, 87.5% for K-TIRADS baseline US, 85.8%, 94.2%, 85.8%, 94.2% for SRE and 57.2%, 79.5%, 53.4%, 81.8% for SWE (kPa expressed). SRE evaluation showed the best diagnostic accuracy compared to the SWE (kPa expressed) (p<0.05) and to the K-TIRADS (p>0.05). The association of SRE with conventional ultrasound with K-TIRADS score increased sensitivity (93% vs 71.5%) but decreased the specificity than conventional US alone (76.5% vs 82.5%).
**Conclusion:** Strain US-Elastography (SRE) used in combination with K-TIRADS improves negative predictive value and sensitivity in the thyroid nodule charaterisation with indeterminate cytology. Nevertheless, further multicentre studies on larger population are needed.
**Limitations:** Still small population study, the lack of interoperator variability computation.
**Ethics committee approval:** All procedures performed were in accordance with the ethical standards of the institutional and/or national research committee.
**Funding for this study:** This research received no external funding.
**Author Disclosures:**
Daniele Fresilli: Nothing to disclose
Giovanni Del Gaudio: Nothing to disclose
Patrizia Pacini: Nothing to disclose
Olga Guiban: Nothing to disclose
Giorgia Polti: Nothing to disclose
Giuseppe Tiziano Lucarelli: Nothing to disclose
Vincenzo Dolcetti: Nothing to disclose
Vito Cantisani: Other: Lecturer fee from Bracco, Samsung and Toshiba.
Carlo Catalano: Nothing to disclose

### RPS 1608-5

**Contrast-enhanced ultrasonography for differential diagnosis of benign and malignant thyroid lesions: single-institutional prospective study of qualitative and quantitative CEUS characteristics**

*H. Petrasova*; Brno/CZ
*(petrasovahana@gmail.com)*

**Purpose:** To extend and revise the diagnostic value of contrast enhanced ultrasonography (CEUS) for differentiation between malignant and benign thyroid nodules.
**Methods or Background:** This single-institution prospective study aims to compare CEUS qualitative and objective quantitative parameters in benign and malignant thyroid nodules. Consecutive cohort of 100 patients was examined by CEUS, 68 out of them were further analysed in detail. All included patients underwent cytological and/or histopathological verification of the diagnosis.
**Results or Findings:** Fifty-five (81%) of thyroid nodules were benign and 13 (19%) were malignant. Ring enhancement was strongly associated with a benign diagnosis (positive predictive value 100%) and heterogeneous enhancement was associated with malignancy (positive predictive value 72,7%). The shape of the TIC (time-intensity curve) curves was more often identical in the benign lesion (98.2%) than in malignant lesions (69.2%), p=0.004.
**Conclusion:** This study suggests that CEUS enhancement patterns were significantly different in benign and malignant lesions, and that ring enhancement was very helpful to identify benign lesions, whereas heterogeneous enhancement was helpful to detect malignant lesions.
**Limitations:** Generally, there is a disagreement about the clinical value of CEUS on the thyroid in the present literature. Overlapping data between CEUS qualitative and quantitative evaluation parameters and criteria of benign and malignant nodules indicate a limitation in the interpretation of tumor microvascularity. So far, there is no established standard for examination methodology, thus it is difficult to compare the results of different studies.
**Ethics committee approval:** Study design and protocol were approved by the institutional Ethics Committee (05-110516/EK).
**Funding for this study:** This research was funded by Ministry of Health, Czech Republic, conceptual development of research organisation (FNBr, 65269705) and (MMCI 00209805).
**Author Disclosures:**
Hana Petrasova: Nothing to disclose

### RPS 1608-6

**Diagnostic accuracy and interobserver agreement of ACR Thyroid Imaging Reporting and Data System (TI-RADS) among radiologists with different experience levels**

*M. T. El-Diasty*, L. H. Meriky, A. O. Aludaini, M. Sabri, L. Hefni, A. T. Alharthy, A. Badeeb; Jeddah/SA
*(meldiasty@hotmail.com)*

**Purpose:** To determine the diagnostic accuracy and interobserver variability in assessment of TI-RADS ultrasound features, final categories, and recommendations for nodule biopsy.
**Methods or Background:** This retrospective study was approved by the institutional review board with waiver of the informed consent. We searched our database for patients who underwent thyroidectomy between 2016 and 2019 with available histopathological results and preoperative ultrasound. Ultrasound images of the selected cases were collected, anonymised, and distributed among 2 third-year radiology trainees (readers 1 & 2) and 2 board certified radiologists (readers 3 & 4 with 10 and 13 years of experience, respectively). All the readers performed the analysis independently and were blinded to the clinical data. Diagnostic performance of TIRADS was calculated for each reader. The Fleiss kappa (κ) statistics were applied to assess interobserver agreement of TI-RADS scoring results for thyroid nodules as well as the recommendations for biopsy.
**Results or Findings:** After exclusion, a final cohort of 177 patients were included. 91 (51.4%) of the nodules were benign and 86 (48.6%) were malignant. Reader 4 achieved the best diagnostic performance (AUC = 0.766 compared to 0.53, 0.6 & 0.732 for readers 1,2 & 3 respectively). The interobserver agreement among all readers was poor for both the TI-RADS category and biopsy recommendation (κ=0.16 and 0.2 respectively). Individual readers comparison showed good agreement between reader 1 and 2 for the TI-RADS category and recommendations (κ=0.747 and 0.79). Fair agreement was found between the other individual readers comparisons.
**Conclusion:** Our results showed better diagnostic performance for experienced readers. However, the overall interobserver agreement regarding TIRADS assessment categories and recommendations was poor, which may be related to variability in experience level. Further larger studies are required to validate this assumption.
**Limitations:** Retrospective; single centre.
**Ethics committee approval:** This study was approved by an ethics committee.
**Funding for this study:** No funding was received for this study.
**Author Disclosures:**
Ahmed Tarek Alharthy: Nothing to disclose
Lujain Hefni: Nothing to disclose
Mohamed T. El-Diasty: Nothing to disclose
Lama Hassan Meriky: Nothing to disclose
Afnan Omer Aludaini: Nothing to disclose
Mohammad Sabri: Nothing to disclose
Arwa Badeeb: Nothing to disclose

### RPS 1608-7

**Extracellular volume fraction using dual-energy CT for diagnosing cervical lymph nodes metastasis from papillary thyroid cancer**
*Y. Zhou*[1], Z. Sheng[1], X. Chen[2], X-Q. Xu[1], F-Y. Wu[1]; [1]Nanjing/CN, [2]Shanghai/CN
*(siyan_njmu@163.com)*

**Purpose:** To evaluate the value of extracellular volume (ECV) fraction using dual-energy CT (DECT) as compared with single-energy CT (SECT) for diagnosing cervical lymph nodes (LNs) metastasis from papillary thyroid cancer (PTC).
**Methods or Background:** One hundred and fifty-seven cervical LNs (81 non-metastatic and 76 metastatic) were retrospectively analysed. Fifty-nine cervical LNs (27 non-metastatic and 32 metastatic) diagnosis were affected by cervical root artifact using contrast-enhanced CT images. ECV fraction was calculated using an equilibrium-phase iodine map derived from DECT and SECT. Pearson correlation coefficient and Bland-Altman analysis were applied to evaluate correlations between SECT and DECT derived ECV fractions. Receiver operating characteristic (ROC) curves analysis was performed to assess the diagnostic performance.
**Results or Findings:** ECV fraction using DECT was strong correlated to that using SECT (r=0.883; p<0.001) with a small bias (-0.4). Metastatic cervical LNs showed significantly higher SECT and DECT derived ECV fraction than non-metastatic cervical LNs (25.45% vs 39.18% and 22.53% vs 42.41%; p both <0.001). Optimal diagnostic performance was achieved with an AUC of 0.793 and 0.813, setting SECT and DECT derived ECV fraction of 34.99% and 36.45% as cut-off value. In LNs diagnosis affected by cervical root artifact group, SECT and DECT derived ECV fraction still obtained favourable efficiency with an AUC of 0.716 and 0.756.
**Conclusion:** The correlation between DECT and SECT derived ECV fraction was strong. ECV fraction can help to diagnose metastatic cervical LNs from PTC, especially in LNs diagnosis affected by cervical root artifact group.
**Limitations:** Use of iodinated contrast agents; increased radiation exposure.
**Ethics committee approval:** Ethical approval was obtained and the requirement of written informed consent was waived.
**Funding for this study:** This study received funding from the National Natural Science Foundation of China (82171928) and Natural Science Foundation of Jiangsu Province (BK20201494).
**Author Disclosures:**
Xingbiao Chen: Nothing to disclose
Fei-Yun Wu: Nothing to disclose
Xiao-Quan Xu: Nothing to disclose
Zhihong Sheng: Nothing to disclose
Yan Zhou: Nothing to disclose

### RPS 1608-8

**Contrast-enhanced ultrasonography with Sonazoid in small lateral neck lymph nodes metastasis of papillary thyroid carcinoma: excellent diagnostic performance**
*L. Xiao*, L. Liu; Guangzhou/CN
*(xiaoll@sysucc.org.cn)*

**Purpose:** This study aims to prospectively assess the diagnostic value of contrast enhanced ultrasonography CEUS with Sonazoid in metastatic small lateral neck lymph nodes (LLNs) of patients with papillary thyroid carcinoma (PTC), and explore the clinical significance of the enhancement degree of LLNs in post-vascular phase (PostVP-CEUS).
**Methods or Background:** This study prospectively enrolled 64 consecutive PTC patients with 71 small LLNs suspected by conventional US according to the 2015 ATA guidelines from October 2020 to September 2021, and the 52 metastatic LNs and 19 benign LNs all received CEUS a week before biopsy or surgery. The sensitivity, specificity, and accuracy of US and CEUS features were calculated and the diagnostic performance of CEUS and PostVP-CEUS was compared.
**Results or Findings:** CEUS correctly diagnosed 18 LNs more than conventional US. Among the 19 benign LLNs, 16 manifested centrifugal (6, 31.6%) or overall (10, 52.6%) enhancement in artery phase; 18 LLNs presented iso-enhancement in post-vascular phase (94.7%). Among the 52 metastatic LLNs, 46 manifested centripetal (25, 48.0%) or asynchronous (21, 40.0%) enhancement in artery phase; 51 LLNs were no- (12, 23.1%), hypo- (10,19.2%), or focal-enhanced (29,55.7%) in post-vascular phase. The sensitivity, specificity, and accuracy of CEUS and PostVP-CEUS was 96.2% and 98.1%, 94.7% and 94.7%, 95.4% and 96.4%, respectively, without significant difference (p-value＞0.05).
**Conclusion:** CEUS with Sonazoid has excellent performance in diagnosing small LLNs of PTC patients compared with US; the diagnostic value of PostVP-CEUS has no difference from CEUS.
**Limitations:** This study is a single-centre study and the sample size was small, lacking of normal LNs as control group.
**Ethics committee approval:** This prospective study was approved by institutional review board of Sun Yat-sen University cancer center.
**Funding for this study:** No funding was received for this study.
**Author Disclosures:**
Longzhong Liu: Nothing to disclose
Lingli Xiao: Nothing to disclose

### RPS 1608-9

**Comparative study of MRI features and pathological features of thyroid nodules**
*X. Y. Liu*, B. J. Wang, L. Zhang, D. T. Ma, X. Kong, Z. B. Ma, Y. Z. Xie, X. J. Li; Taian/CN
*(liuxiaoyan0522@163.com)*

**Purpose:** To analyse the MRI features and pathological basis of thyroid nodules, and to explore the relationship between MRI and pathological features of thyroid nodules, so as to improve the diagnostic level of benign and malignant thyroid nodules.
**Methods or Background:** Twenty-eight patients with thyroid nodules confirmed by surgery and pathology were analysed retrospectively. The patients were examined by conventional plain scan, enhanced scan and diffusion weighted imaging (DWI) before surgery, and were divided into benign nodule group and malignant nodule group according to pathological results. The morphology, boundary, texture, signal type, accompanying features, enhancement mode and diffusion weighted limitation degree of the lesions were observed. The results were compared with intraoperative pathology.
**Results or Findings:** There were 62 nodules on MRI images of 28 patients with thyroid nodules. 15 lesions were accompanied by cystic degeneration (24.2%),13 lesions were associated with calcification (21.0%). There were 43 cases (69.4%) with mild to moderate enhancement and 19 cases (39.6%) with obvious enhancement. DWI showed obvious diffusion limitation in 28 lesions (45.2%). There were 17 benign nodules (27.4%) and 45 malignant nodules (72.6%). Univariate analysis showed that there were differences in boundary (P=0.002), TIWI signal (P=0.018), enhancement degree (P=0.003) and DWI limitation (P=0.001) between the benign and malignant groups. Multivariate regression analysis showed that enhancement signal degree (P=0.003, OR value 0.095) and DWI limitation (P=0.008,OR value 12.109) were independent predictors of malignant thyroid nodules.
**Conclusion:** Thyroid malignant nodules showed morphologic hemodynamic characteristics on MRI, showing mild to moderate enhancement and significantly limited on DWI. Routine thyroid scan combined with enhancement and DWI can improve the detection rate of malignant nodules.
**Limitations:** The sample amount is small.
**Ethics committee approval:** Under approval.
**Funding for this study:** Funding was received from the Youth Science Foundation Project of National Natural Science Foundation of China (grant no. 81903010).
**Author Disclosures:**
Xue Kong: Nothing to disclose
Litao Zhang: Nothing to disclose
Xiu Juan Li: Nothing to disclose
De Ting Ma: Nothing to disclose
Zhen Bo Ma: Nothing to disclose
Xiao Yan Liu: Nothing to disclose
Yuan Zhong Xie: Nothing to disclose
Bao Jian Wang: Nothing to disclose

### RPS 1608-10

**US-elastography with different techniques for thyroid nodule characterisation: systematic review and meta-analysis**

*P. Pacini*, G. Polti, E. Polito, O. Guiban, D. Fresilli, V. Cantisani, C. Catalano; Rome/IT

**Purpose:** To evaluate US-elastosonography (USE) diagnostic performance for characterisation thyroid nodules.
**Methods or Background:** Pubmed and Embase databases were searched from January 2010 to July 2021. Four reviewers checked studies, evaluated the articles' evidence quality level and extracted the data. The overall diagnostic accuracy of qualitative USE, semi-quantitative USE and quantitative USE were evaluated calculating their pooled sensitivity, specificity and area under the curve (AUC). All statistical tests were performed using Metadisc and Medcal software package.
**Results or Findings:** 72 studies with 13505 patients and 14015 thyroid nodules undergoing elastography were included. The pooled sensitivity, specificity, AUC and 84% (95% CI, 0.83-0.85), 81% (95% CI, 0.80-0.83) and 89% (95% CI, 0.85-0.93) for qualitative USE; 83% (95% CI, 0.81-0.84), 81% (95% CI, 0.80-0.82) and 0.93 (95% CI, 0.89-0.97) for semi- quantitative USE and 78% (95% CI, 0.76-0.79), 0.81 (95% CI, 0.80-0.82) and 0.87 (95% CI, 0.85-0.89) for quantitative USE. The positive likelihood ratios (PLR) and negative likelihood ratios (NLR) were 4.7 (95% CI, 3.5–6.3) and 0.24 (95% CI, 0.17–0.34) for qualitative USE; 6.5 (95% CI, 4.2– 10.1) and 0.19 (95% CI, 0.13–0.27) for semi-quantitative USE; 4.4 (95% CI, 3.6-5.5) and 0.28 (95% CI, 0.24-0.33) for quantitative USE. Semi-quantitative USE AUC was statistically higher than quantitative USE one (p-value>0.05).
**Conclusion:** Strain-based USE shows the best diagnostic performance compared to SWE with a statistically significant result.
**Limitations:** Heterogeneity of the articles included; the non-univocal qualitative SE score to use (score 1-2; score 1-4 or score 1-5) and different Strain Ratio cut-off; high pooled malignancy rate (33%) deriving from the studies published by research institutes considered as a reference centre for thyroid pathology.
**Ethics committee approval:** All procedures performed were in accordance with the ethical standards of the institutional and/or national research committee.
**Funding for this study:** Not applicable.
**Author Disclosures:**
Daniele Fresilli: Nothing to disclose
Patrizia Pacini: Nothing to disclose
Olga Guiban: Nothing to disclose
Giorgia Polti: Nothing to disclose
Eleonora Polito: Nothing to disclose
Vito Cantisani: Nothing to disclose
Carlo Catalano: Nothing to disclose

### RPS 1608-11

**Macrocalcifications do not alter malignancy risk within the 2017 European Thyroid Imaging Reporting and Data System (EU-TIRADS) when present in non-high suspicion thyroid nodules**

*A. C. Sánchez*, V. Perez-Riverola, M. Prenafeta Moreno, O. Vazquez Muiños, M. Bello Cueto, M. Rigla; Sabadell/ES

**Purpose:** To determine the malignancy risk of thyroid nodules with isolated macrocalcifications (MC) and/or rim calcifications when present in non-high suspicion thyroid nodules.
**Methods or Background:** Restrospective study reviewing all fine-needle aspiration biopsies (FNAB) of thyroid nodules done in our center from January 2014 to December 2020. We rejected the nodules with previous FNAB. We selected the nodules with MC (echogenic foci >1 mm in size with posterior shadowing and/or peripheral dystrophic calcifications) and classify them in groups following the 2017 European Thyroid Imaging Reporting and Data System (EU-TIRADS). We calculated the rate of malignancy and compare them with the rest of the nodules for each subgroup, using Chi-square comparison tests.
**Results or Findings:** Of 1536 biopsied thyroid nodules from January 2014 to December 2020, 1224 met criteria for EU-TIRADS 3 (Low Risk) or EU-TIRADS 4 (Intermediate Risk). 5 nodules were unclassifiable following EU-TIRADS due to their circumferential calcifications restricting further sonographic assessment. The remaining 307 were classified in other EU-TIRADS category or had previous FNAB and therefore they were excluded. From 1224 nodules selected, we classified 943 as EU-TIRADS 3 and 281 as EU-TIRADS 4. Of the 943 EU-TR3 nodules, 59 of them had macrocalcifications and 884 did not. The malignancy rate in these two groups was 5.7% and 3.4% respectively. Of the 281 EU-TR4 nodules, 40 of them had macrocalcifications and 241 did not. The malignancy rate in these two groups was 20.7% and 21.8%. A p value <0.05 indicated statistical significance. The results were not significantly different in both groups (TR3 p=0.358, TR4 p=0.894).
**Conclusion:** Macrocalcifications in thyroid nodules do not increase the malignancy risk in a statistically significant way when present in NHSTN.
**Limitations:** Pandemics.
**Ethics committee approval:** The study was approved by an ethics committee.
**Funding for this study:** Funding was received from the Parc Tauli Hospital.
**Author Disclosures:**
Olalla Vazquez Muiños: Nothing to disclose
Mario Prenafeta Moreno: Nothing to disclose
Ana Cecilia Sánchez: Nothing to disclose
Mercedes Rigla: Nothing to disclose
Victor Perez-Riverola: Nothing to disclose
María Bello Cueto: Nothing to disclose

### RPS 1608-12

**MRI and 4D-CT diagnostic performance in preoperative localisation of parathyroid adenoma correlated with surgical and pathological findings**

*A. Yahav-Dovrat*[1], D. Fischer[2], D. Manor[1], M. Mekel[1], A. Eran[1]; [1]Haifa/IL, [2]Nahariya/IL
*(anat.yahav.dovrat@gmail.com)*

**Purpose:** To evaluate the diagnostic performance of 3T MRI in comparison with 4D CT for presurgical localisation of parathyroid adenomas.
**Methods or Background:** Accurate localisation of parathyroid adenomas is critical in planning the surgical approach in patients with primary hyperparathyroidism. Magnetic resonance imaging (MRI), 4D computed tomography (4D CT), ultrasound (US) and 99mTc-sestamibi scan have all been shown to have good diagnostic performance for this purpose. Both 4D CT and MRI are able to give a diagnosis together with a good demonstration of related anatomy. MRI has the advantage of using non-ionising radiation and causing fewer allergies to intravenous contrast. A prospective single-centre study was conducted. Patients with a clinical and laboratory diagnosis of hyperparathyroidism completed both a dynamic MRI and a 4D CT scan. The images were evaluated by a neuroradiologist and later correlated with surgical outcomes. Any other imaging data available (99mTc-sestamibi scan or US) were also recorded and analysed.
**Results or Findings:** Twenty one hyperfunctioning glands were surgically proven. Eighteen of which were detected by MRI with no false positive results (sensitivity 85.7%, PPV 100%). Sixteen adenomas were detected by 4D CT, with one suspected lesion surgically proven to be a normal parathyroid gland (Sensitivity 76.2%, PPV 94.2%). Two of the adenomas not apparent on MRI were detected on 4D CT, while 4 adenomas were detected by MRI alone. When combining the two methods, the sensitivity rose to 87.0%.
**Conclusion:** Our study suggests that both MRI and 4D CT are good diagnostic tools in the case of hyperparathyroidism. Combining the methods may be of benefit.
**Limitations:** Our sample size was suboptimal and therefore the results need further affirmation by future studies.
**Ethics committee approval:** The study was approved by the local ethics committee.
**Funding for this study:** No funding was received.
**Author Disclosures:**
Anat Yahav-Dovrat: Nothing to disclose
Doron Fischer: Nothing to disclose
David Manor: Nothing to disclose
Ayelet Eran: Nothing to disclose
Michal Mekel: Nothing to disclose

**16:00-17:30** **Room M 1**

### Research Presentation Session: Breast

### RPS 1602

### Breast ultrasound in 2022

**Moderator**
P. Kapetas; Vienna/AT

### RPS 1602-2

**Contrast enhanced ultrasound (CEUS) with microbubble for preoperative assessment of sentinel lymph nodes in breast cancer patients improved surgical planning during COVID-19 pandemic**

*P. K. G. Palanisamy*, J. Weeks, J. Rait, C-M. Marsh, C. Kam, K. Cox; Maistone/UK
*(p.palanisamy@nhs.net)*

**Purpose:** During the COVID-19 pandemic, breast cancer treatment was affected by lack of operating resources. Standard B-mode ultrasound together with CEUS core biopsy of sentinel lymph nodes (SLN) provide enhanced radiological assessment of the axilla. At a time of crisis, this study aimed to assess whether CEUS SLN biopsy improved theatre utilisation and in reducing patient exposure.

**Methods or Background:** Between March 2020 and January 2022, prospective data was collected on 174 patients. Patients had a normal B-mode axillary ultrasound, CEUS SLN core biopsy and following clinical/ tumour features: pre-menopausal, ER-,Her-2+,T3 tumours, multifocal tumours, multicentric tumours.
**Results or Findings:** SLN were visualised in 156/174 (89%) and successfully biopsied in 127/174 (72%). The median age was 53 and median tumour size 30mm. 34 (40%) of patients had ER- or Her-2+ breast cancer. Of those with successful SLN core biopsy, 22 had neo-adjuvant chemotherapy, 15 primary endocrine treatment and 4 had incomplete data. 86 patients had primary surgical treatment and the sensitivity was 50%, specificity 98% and NPV 79% with a 35% prevalence of LN metastases in this group. Of the 16 patients with malignant SLN on core biopsy, 9 had 2 or more LN macrometastases found at the end of surgical treatment, 5 patients had single LN macrometastasis, 1 had a single micrometastasis and 1 a 1.5 mm deposit in peri-nodal fat.
**Conclusion:** The addition of CEUS SLN core biopsy to standard B-mode ultrasound improved surgical treatment planning. Directing those with malignant SLN to axillary lymph node dissection rather than surgical SLN excision avoided a second axillary procedure for 14 patients. Although, 2 patients were overtreated for micrometastatic disease, enhanced axillary staging with CEUS allowed better use of theatre resources during the crisis.
**Limitations:** There was incomplete data in 4 patients.
**Ethics committee approval:** Ethics committee approval was not required.
**Funding for this study:** No funding was received for this study.
**Author Disclosures:**
Karina Cox: Nothing to disclose
Priya Karuppana Gounder Palanisamy: Nothing to disclose
Clare-Marie Marsh: Nothing to disclose
Jaideep Rait: Nothing to disclose
Cheuk Kam: Nothing to disclose
Jennifer Weeks: Nothing to disclose

### RPS 1602-3

**Value of imaging in women presenting with breast pain: a cross-sectional study**

*C. Payne*, T. Zachari; Edinburgh/UK

**Purpose:** Breast pain is one of the most common symptoms prompting referral to specialist breast services; however there is limited evidence to support best radiological practice. We aimed to determine the patient outcomes following initial investigation by mammography and/or ultrasound to establish the value of imaging in these patients.
**Methods or Background:** Retrospective analysis of 297 patients presenting to the symptomatic breast clinic with breast pain and a normal clinical examination who were referred for imaging between September 2020 and April 2021.
**Results or Findings:** In total only 1 cancer was identified, this did not correspond to the site of pain and therefore was classed as incidental. 22% (n=66) of patients required additional imaging (including compression/ magnification views, digital breast tomosynthesis or ultrasound). 11 biopsies were performed with 8 normal/benign pathological findings and 2 cases of indeterminate nature, which included a radial scar and flat epithelial atypia. No patients had a treatable cause for their pain identified on imaging.
**Conclusion:** Imaging in patients with breast pain in the absence of other symptoms results in a cancer detection rate equal to or below the screening incidence (0.3% vs 0.7%) and may result in unnecessary additional investigations and biopsies.
**Limitations:** Our study included all types of breast pain and a relatively small sample size (n=16) had unilateral focal breast pain. We propose that further research on a larger group of patients with focal breast pain should be performed to establish whether there is a higher cancer detection rate in this subgroup.
**Ethics committee approval:** Not applicable
**Funding for this study:** Not applicable
**Author Disclosures:**
Theodora Zachari: Nothing to disclose
Catherine Payne: Nothing to disclose

### RPS 1602-4

**Lymphatic mapping using ultrasound microbubbles prior to lymphaticovenous anastomosis surgery for upper extremity lymphoedema**

*S. Jang*, C. Lee, G. Hesley, J. Knudsen, N. Brinkman, V. Fahradyan, N. Tran; Rochester, MN/US
*(jang.samuel@mayo.edu)*

**Purpose:** Lymphaticovenous anastomosis (LVA) surgery is an effective surgical treatment for secondary lymphoedema in the extremities, but indocyanine green (ICG) fluorescent lymphography, the reference standard for imaging target lymphatic vessels, has several limitations. We evaluated whether contrast-enhanced ultrasound (CEUS) can identify target lymphatic vessels for LVA surgery in the upper extremities.
**Methods or Background:** In this retrospective review, CEUS with intradermal injection of microbubbles was used prior to LVA surgery in the upper extremities between October 2019 and September 2021. All patients had secondary upper extremity lymphoedema from breast cancer treatment. Technical success was defined as lymphatics identified by CEUS that led to successful LVAs.
**Results or Findings:** All 11 patients were women (mean age: 56 years ± 8). The median number of microbubble injection sites was 11 (range: 8-14). CEUS identified lymphatic vessels in all 11 women, including in 6 women where ICG fluorescent lymphography failed to identify any targets. A total of 35 explorations (median: 3 per patient, range: 2-4) were performed, and 24 LVAs (median 3 per patient, range: 0-4) were created. Of the anastomosis, 33% (8/24) was mapped by both CEUS and ICG fluorescent lymphography, 58% (14/24) was mapped by CEUS only, and 8% (2/24) was mapped by ICG fluorescent lymphography only. Of the 33 explorations on targets mapped by CEUS, anastomosis could be made in 22 sites for a technical success rate of 67% (22/33). Seven women had at least one additional LVA created from the use of CEUS.
**Conclusion:** CEUS is a promising tool for identifying lymphatic vessels in the upper extremities, especially when ICG fluorescent lymphography fails to identify targets or cannot be used.
**Limitations:** Larger prospective study may further delineate the role of CEUS.
**Ethics committee approval:** A waiver was received from the institutional review board.
**Funding for this study:** No funding was received for this study.
**Author Disclosures:**
Vahe Fahradyan: Nothing to disclose
Gina Hesley: Nothing to disclose
Nho Tran: Nothing to disclose
Samuel Jang: Nothing to disclose
Nathan Brinkman: Nothing to disclose
Christine Lee: Nothing to disclose
John Knudsen: Nothing to disclose

### RPS 1602-5

**Second-look ultrasound for MRI-detected lesions: MRI features, detection rate and sonographic findings**

*S. Güven*[1], B. Hekimoğlu[1], A. T. Arıkök[1], I. Durur Subasi[2]; [1]Ankara/TR, [2]Istanbul/TR
*(seldaaatezel@hotmail.com)*

**Purpose:** The purpose of this study was to predict the second-look US detection rate (SUDR) of initially MRI-detected breast lesions by using MRI features and to compare second-look US findings of benign and malignant lesions.
**Methods or Background:** 110 MRI-detected lesions (80 patients), subsequently underwent second-look US between February 2016 and July 2019, were retrospectively reviewed. The lesions were not detected in previous mammography and bilateral breast US. The chi-square and Fischer exact tests were used to evaluate the associations between MRI features and pathology results with SUDR and second-look US findings with malignancy rates. Significant variables in the univariate analyses were evaluated with multivariate analysis. Additionally, the performance of MRI features in SUDR prediction was evaluated with receiver operating characteristic analysis.
**Results or Findings:** While 28.8% of the lesions with a second-look US correlate were malignant, 96.1% of the lesions without a sonographic correlation were benign. In univariate analyses, lesion type (mass-non mass-focus), pathology result (benign-malignant), MRI BI-RADS category, and kinetic curve were significantly associated with SUDR. In multivariate analysis, MRI BI-RADS category (odds ratio: 11.896, $p<0.001$) and lesion type (odds ratio: 9.470, $p=0.001$) were significantly associated with SUDR. Combined compound scoring with these factors could predict SUDR [Area Under Curve: 0.712, %95 Confidence Interval: 0.617-0.807, $p<0.001$]. Furthermore, lesions with irregular shapes and margins were more often malignant in the analysis of US findings ($p<0.001$). In subgroup analysis, this association was significant only in sonographic correlations of masses.
**Conclusion:** Combined compound scoring with MRI BI-RADS category and lesion type could predict SUDR. Besides, second-look US could give additional diagnostic information to distinguish between benign and malignant masses.
**Limitations:** This was a singlecentre retrospective study. MRI-guided biopsy was not available at our institution.
**Ethics committee approval:** Approved by the institutional review board (107/17).
**Funding for this study:** Not applicable
**Author Disclosures:**
Selda Güven: Nothing to disclose
Ata Türker Arıkök: Nothing to disclose
Baki Hekimoğlu: Nothing to disclose
Irmak Durur Subasi: Nothing to disclose

### RPS 1602-6

**Elastography and diffusion-weighted imaging in breast cancer**

*I. Ş. S. Örgüç*, Ç. R. Açar, B. Mutlu; Manisa/TR
*(sebnemorguc@hotmail.com)*

**Purpose:** Diffusion weighted imaging and elastography are widely accepted methods in the evaluation of breast masses, however there is very limited data comparing the two methods. The purpose of this study is to compare MRI ADC values of the breast masses with quantitative elastography based on ultrasound shear wave measurements.
**Methods or Background:** We retrospectively evaluated 130 patients with histopathologically proven breast masses. The mean age of the patients was 51 years (range 34 – 78 years). Shear wave elastography was measured in kiloPascals (kPa9) unites which is a quantitative measure of tissue stiffness. DWI were obtained using a 1.5-T MRI system.
**Results or Findings:** ADC values were strongly inversely correlated with elasticity. In our study the cut-off value of ADC was 1.015 x 10-3 cm2 /sec (p=0.01) to achieve the sensitivity of 72% and specificity 75% and cut-off value of elasticity was 114.50 kPa to achieve to sensivity of 75% and spesificity 72% to discriminate between the malignant and benign breast lesions. Elasticity was inversely strongly correlated with ADC ( r = -0,46, p<0.01) according to Pearson Correlation.
**Conclusion:** Tissue density correlated with stiffness of breast tumours as measured by DWIC MRI and shear wave elastography. The relationship was linear.
**Limitations:** Retrospective design of the study is one of the limitations of our study. Misregistration of the two measurements is another possible limitation. Manually drown ROIs on the ADC map may show variations, and may not correspond exactly to the ROI which is also manually drown on the elastographic image. Fusion imaging technologies may ensure more accurate registration of the ROIs used in two modalities.
**Ethics committee approval:** Approval for the study was obtained from the Ethics Committee of Manisa Celal Bayar University. Written informed consent was obtained from all patients.
**Funding for this study:** No funding was received for this study.
**Author Disclosures:**
Ihsan Şebnem Sebnem Örgüç: Nothing to disclose
Batuhan Mutlu: Nothing to disclose
Çağdaş Rıza Açar: Nothing to disclose

### RPS 1602-8

**Evaluation of additional MRI-detected breast lesions using the quantitative analysis of contrast-enhanced ultrasound scans and its comparability with dynamic contrast-enhanced MRI findings of the breast**

*K. E. Lee*, O. Woo, S. E. Song, K. R. Cho, B. K. Seo; Seoul/KR

**Purpose:** To assess the diagnostic performance of contrast-enhanced ultrasound (CEUS) for additional magnetic resonance (MR)-detected enhancing lesions and to determine whether or not kinetic pattern results comparable to dynamic contrast-enhanced magnetic resonance imaging (DCE-MRI) of the breast can be obtained using the quantitative analysis of CEUS.
**Methods or Background:** In this singlecentre prospective study, a total of 71 additional MR-detected breast lesions were included. CEUS examination was performed, and lesions were categorised according to the breast imaging-reporting and data system (BI-RADS). The sensitivity, specificity, and diagnostic accuracy of CEUS were calculated by comparing the BI-RADS category to the final pathology results. The degree of agreement between CEUS and DCE-MRI kinetic patterns was evaluated using weighted kappa.
**Results or Findings:** On CEUS, 46 lesions were assigned as BI-RADS category 4B, 4C, or 5, while 25 lesions category 3 or 4A. The diagnostic performance of CEUS for enhancing lesions on DCE-MRI was excellent, with 84.9% sensitivity, 94.4% specificity, and 97.8% positive predictive value. A total of 57/71 (80%) lesions had correlating kinetic patterns and showed good agreement (weighted kappa = 0.66) between CEUS and DCE-MRI. Benign lesions showed excellent agreement (weighted kappa = 0.84), and intraductal carcinoma (IDC) showed good agreement (weighted kappa = 0.62).
**Conclusion:** The diagnostic performance of CEUS for additional MR-detected breast lesions was excellent. Accurate kinetic pattern assessment, fairly comparable to DCE-MRI, can be obtained for benign and IDC lesions using CEUS.
**Limitations:** The benign breast lesions were not confirmed by pathology, therefore, excluded. Furthermore, the small sample size may limit statistical power.
**Ethics committee approval:** The study was approved by the Institutional Review Board (IRB).
**Funding for this study:** This research received no specific grant from any funding agency.
**Author Disclosures:**
Okhee Woo: Nothing to disclose
Bo Kyoung Seo: Nothing to disclose
Kyung Eun Lee: Nothing to disclose
Sung Eun Song: Nothing to disclose
Kyu Ran Cho: Nothing to disclose

### RPS 1602-10

**Identifying non-mass-like breast lesions on automated breast volume scanner images: analysis of the features analysis and diagnostic performance**

*L. Jing*, Q. Lu, L. Yan, F. Zheng, P. Wang, B. Huang, W. Wang; Shanghai/CN
*(luxiajing2018@163.com)*

**Purpose:** To analyse the clinical and imaging findings of non-mass-like breast lesions on automated breast volume scanner (ABVS) images and to evaluate their diagnostic values in the differentiation of benign from malignant lesions.
**Methods or Background:** From Dec. 2014 to Apr. 2020, a total of 113 women with 118 histologically proven NML breast lesions were retrospectively enrolled. The clinical and ABVS findings were compared using the chi-square test, Fisher's exact test and independent samples t test. Binary logistic regression analysis was performed to select independent predictors of malignancy. A linear discriminant analysis classifier was employed to classify benign and malignant lesions.
**Results or Findings:** According to the postoperative pathology, 39/118 and 79/118 lesions were classified as benign and malignant, respectively. Age, nipple discharge, calcification, the retraction phenomenon and the distribution pattern were significantly different between the two groups (all p<0.05). In the binary logistic regression analysis, calcification (odds ratio [OR]: 6.420; 95% confidence interval [CI]: 2.189, 18.831; p=0.001), retraction phenomenon (OR: 5.882; 95% CI: 1.411, 24.513; p=0.015), and segmental distribution (OR: 19.347; 95% CI: 2.396, 156.241; p=0.005) were independent risk factors for malignancy, with high positive predictive values (82.8%, 89.3%, 90.9%, respectively). The combination of these three features yields a sensitivity of 84.8%, specificity of 66.7% and accuracy of 78.8% in linear discriminant analysis.
**Conclusion:** Calcification, the retraction phenomenon and the segmental distribution pattern may aid in the differential diagnosis of non-mass-like lesions. Combined diagnosis with these features can improve the diagnostic performance.
**Limitations:** This was a retrospective study, and the sample size was relatively small.
**Ethics committee approval:** The hospital's institutional review board approved this study and waived the need for informed consent from all patients.
**Funding for this study:** This work has not received any funding.
**Author Disclosures:**
Lixia Yan: Nothing to disclose
Fengyang Zheng: Nothing to disclose
Peilei Wang: Nothing to disclose
Beijian Huang: Nothing to disclose
Wenping Wang: Nothing to disclose
Qing Lu: Nothing to disclose
Luxia Jing: Nothing to disclose

### RPS 1602-11

**Evaluation of computer-aided diagnosis in breast ultrasonography: improvement in diagnostic performance of inexperienced radiologists**

*G. Muscogiuri*, L. Nicosia, G. Signorelli, F. Pesapane, A. C. Bozzini, E. Cassano, M. Montesano, A. Latronico, L. Meneghetti; Milan/IT
*(giulia.muscogiuri@gmail.com)*

**Purpose:** To evaluate if a computer-aided diagnosis (CAD) system on mammary ultrasound (US) can improve the diagnostic performance of inexperienced radiologists.
**Methods or Background:** We collected ultrasound images of 256 breast lesions, acquired with a linear high-frequency probe. We asked two experienced and two inexperienced radiologists to retrospectively review the US features of each breast lesion according to the Breast Imaging Reporting and Data System (BI-RADS) categories. A CAD examination with S-DetectTM software (Samsung Healthcare, Seoul, South Korea) was conducted retrospectively by another uninvolved radiologist, blinded to the BI-RADS values previously attributed to the lesions. Diagnostic performances of experienced and inexperienced radiologists and CAD were compared and the interobserver agreement among radiologists was calculated.
**Results or Findings:** The diagnostic performance of the experienced group in terms of sensitivity was significantly higher than CAD (p<0.001). Conversely, the diagnostic performance of the inexperienced group in terms of both sensitivity and specificity was significantly lower than CAD (p<0.001). We obtained an excellent agreement in the evaluation of the lesions among the two expert radiologists (Kappa coefficient: 88.7%), and among the two non-expert radiologists (Kappa coefficient: 84.9%).

**Conclusion:** Our results showed that S-detect has greater sensitivity, specificity, NPV and PPV than inexperienced radiologists. The US CAD system is a useful additional tool to improve the diagnostic performance of the inexperienced radiologists, eventually reducing the number of unnecessary biopsies. Moreover, it is a valid second opinion in case of experienced radiologists.
**Limitations:** There is some limitation of our study: it is a retrospective study, the S-Detect cannot evaluate the non-mass breast lesions and also, the small number of cases.
**Ethics committee approval:** This retrospective study was notified to the ethics committee and was approved by the Institutional Review Board.
**Funding for this study:** No funding was received for this study.
**Author Disclosures:**
Luca Nicosia: Author: Author
Giulia Muscogiuri: Author: Author
Antuono Latronico: Author: Author
Anna Carla Bozzini: Author: Author
Giulia Signorelli: Author: Author
Filippo Pesapane: Author: Author
Enrico Cassano: Author: Author
Lorenza Meneghetti: Author: Author
Marta Montesano: Author: Author

### RPS 1602-12

**Malignant lesions characterised as BI-RADS 4A in ultrasound, a subtype analysis**
*S. Sefidbakht*[1], L. Ataei Rooyani[1], A. Hajati[1], P. Pishdad[1], S. Tahmasebi[1], H. Hosseinpour[1], B. Bijan[2]; [1]Shiraz/IR, [2]Sacramento, CA/US
*(SepidehSefidbakht@yahoo.com)*

**Purpose:** To report on molecular subtypes of breast cancer initially categorised as BI-RADS 4A in ultrasound.
**Methods or Background:** A retrospective review was done on PACS and HIS database of a subspecialty breast clinic for malignant lesions categorised as BI-RADS 4A in ultrasound. Various characteristics including demographic data, ultrasound description, major histologic characteristics and molecular subtypes were recorded and compared with lesions characterised as BI-RADS 5 using chi-squared test.
**Results or Findings:** Out of 3049 ultrasound-guided breast biopsies pathology and IHC results were available in 2076 patients. There were 873 patients categorised as BI-RADS 4A; 49 of which turned out malignant. These included 6 non-mass lesions including two luminal cancers with extensive DCIS, one HER2-positive, two HER2-positive luminal b DCIS, all the HER2-positive cancers in young women who underwent ultrasound prior to mammogram. 12 were medullary cancers, 9 with triple negative molecular subtype. 11 cases of invasive papillary cancer were also categorised as BI-RADS 4A; in 4 of which a prominent cystic component was not visible, presenting as a lobular mass. 5 were triple negative cancers without medullary features presenting as non-shadowing lobular hypoechoic masses. Out of 9 luminal A cancers categorised as BI-RADS 4A only one was luminal A and 7 had Ki67 above 30%.
**Conclusion:** Rate of papillary and medullary cancer was significantly higher and luminal a cancer significantly lower in patients categorised as BI-RADS 4A comparing to those categorised as BI-RADS 5. Also the age of patients with less typical appearance for malignancy in ultrasound was significantly lower than those accurately categorised.
**Limitations:** Patients with underestimated risk of malignancy were younger and many of them didn't have mammograms; this can affect the risk assessment by the radiologist.
**Ethics committee approval:** The university ethics committee approved this retrospective study.
**Funding for this study:** No funding was received for this study.
**Author Disclosures:**
Bijan Bijan: Nothing to disclose
Parisa Pishdad: Nothing to disclose
Lobat Ataei Rooyani: Nothing to disclose
Azadeh Hajati: Nothing to disclose
Sepideh Sefidbakht: Nothing to disclose
Sedigheh Tahmasebi: Nothing to disclose
Hamidreza Hosseinpour: Nothing to disclose

**16:00-17:30** **Room M 2**

### Research Presentation Session: Vascular

### RPS 1615

### Advances in thoracoabdominal and peripheral vascular CT

**Moderator**
J. Kettenbach; Wiener Neustadt/AT

### RPS 1615-2

**Low volume contrast media protocol for CT angiography of the aorta: prospective development and evaluation with photon-counting detector CT**
K. Higashigaito, V. Mergen, M. Eberhard, A. Euler, S. Rätzer, B. Zanini, *H. Alkadhi*; Zurich/CH

**Purpose:** To develop and evaluate a low-volume contrast media (CM) protocol for thoracoabdominal CT angiography (CTA) of the aorta with first generation photon-counting detector CT (PCD-CT).
**Methods or Background:** 100 consecutive patients (mean age 74.5±8.4years, 17 women) were prospectively included who underwent thoracoabdominal CTA of the aorta with first-generation PCD-CT at 120kV and who underwent previous thoracoabdominal CTA with energy-integrating detector (EID)-CT using automated tube voltage selection. Tube current-time product in PCD-CT was modified in each patient to obtain equal volume CT-dose-index (CTDIvol) as with EID-CT. In PCD-CT, virtual monoenergetic images (VMI) were reconstructed (5keV-intervals, 40-55keV). Attenuation of the aorta, noise and contrast-to-noise ratio (CNR) was determined. Subjective image quality was rated. In the first cohort (40 patients), the same CM-protocol was used for PCD-CT and EID-CT (volume 70ml, flow rate 4ml/s, 370mg/ml). Increase of CNR in PCD-CT compared to EID-CT in this cohort was quantified and served as reference for CM-volume reduction in the second cohort (60 patients).
**Results or Findings:** Similar CTDIvol between PCD-CT and EID-CT was observed (both, 4.9±1.9mGy) (p>0.05). In the first cohort, VMI at 40-50keV showed significantly higher CNR compared to EID-CT (all, p<0.05). VMI at 50 and 55keV showed highest subjective image scores. VMI at 50keV was selected as ideal keV-level (best trade-off between subjective and objective image quality), showing 25% increase of CNR compared to EID-CT. CM-volume in the second cohort was reduced by 25% (52.5ml, 3ml/s). In the second cohort similar CNR and subjective image quality was observed between VMI at 50keV from PCD-CT using low-volume CM-protocol compared with EID-CT using normal CM-protocol (both, p-value>0.05).
**Conclusion:** PCD-CTA of the aorta with generation of VMI increases CNR, which can be translated into a low-volume CM-protocol resulting in diagnostic image quality.
**Limitations:** No limitations were identified.
**Ethics committee approval:** This study was approved by an ethics committee.
**Funding for this study:** No funding was received for this study.
**Author Disclosures:**
Victor Mergen: Nothing to disclose
Matthias Eberhard: Nothing to disclose
Bettina Zanini: Nothing to disclose
Kai Higashigaito: Nothing to disclose
Susan Rätzer: Nothing to disclose
Andre Euler: Nothing to disclose
Hatem Alkadhi: Nothing to disclose

### RPS 1615-3

**Lower extremity run-off CT angiography on a novel dual-source photon counting detector CT: image quality comparison with a second generation dual-source energy-integrating detector CT**
*K. Rippel*, J. A. Decker, D. Popp, F. Braun, T. J. Kroencke, F. Schwarz, C. Scheurig-Muenkler; Augsburg/DE

**Purpose:** To compare image quality of a novel dual-source photon-counting detector CT with a second generation dual-source CT with energy integrating detector (EID-CT) in patients who undergo lower extremity run-off CT angiography.
**Methods or Background:** Here, we present initial data from 40 consecutive patients who underwent CTA on a novel dual-source photon counting detector CT (NAEOTOM Alpha, Siemens Healthineers, Erlangen, Germany) (PCD-CT group). For comparison, 40 patients matched for sex, age, height and BMI who underwent a similar scan on a second generation dual-source CT scanner (Somatom Definition Flash, Siemens Healthineers) were retrospectively included as a control group. Basic demographic data were compared. Virtual monoenergetic imaging (VMI) reconstructions in different keV settings (range:

40-120) were performed. Enhancement and noise were measured for each of 13 vascular segments as mean and standard deviation of CT values in ROI's in the abdominal aorta and downstream vascular segments as well as the standard deviation within surrounding air, respectively. Signal- and contrast-to-noise-ratio were calculated.
**Results or Findings:** There were no significant differences between both groups in sex ratio (26 male, 14 female in both groups), age (70.5±10.0 vs 68.0±10.7 years) or BMI (27.9±6.5 vs 27.7±5.8 kg/m²). When comparing CNR and SNR with the control group, the VMI-reconstructions in the 40-55 keV range were clearly superior (e.g. in the superficial femoral artery SNR: 23.60±13.43 vs 17.96±12.94 / CNR: 24.66±12.73 vs14.17±14.75; p<0.001).
**Conclusion:** In comparison with a second generation dual-source EIDCT, lower extremity run-off studies on the novel dual-source PCD-CT demonstrate significantly higher signal- and contrast-to-noise-ratios, particularly for low keV VMI-reconstructions. These routinely available reconstructions represent a substantial improvement in objective image quality of CT angiographies.
**Limitations:** The retrospective character of this study is a limitation.
**Ethics committee approval:** This study was approved by an ethics committee.
**Funding for this study:** No funding was received for this study.
**Author Disclosures:**
Christian Scheurig-Muenkler: Nothing to disclose
Franziska Braun: Nothing to disclose
Thomas J. Kroencke: Nothing to disclose
Florian Schwarz: Nothing to disclose
Katharina Rippel: Nothing to disclose
Josua A. Decker: Nothing to disclose
Daniel Popp: Nothing to disclose

### RPS 1615-5

**The applied research of diagnose fibrosing mediastinitis by "one-stop" pulmonary angiography of dual-layer detector spectral CT**
*X. Zhou*, M. M. Zhong, G. Huang; Lanzhou/CN

**Purpose:** To explore the clinical application of double-layer detector spectral CT pulmonary angiography (CTPA) in the diagnosis of fibrosing mediastinitis.
**Methods or Background:** The CTPA data of 30 patients diagnosed with fibrosing mediastinitis were retrospectively analysed. The degree of compression and stenosis of pulmonary arteries, veins, and bronchi were measured on conventional images, the iodine density and Z effective values of lung segments were measured to evaluate the pulmonary perfusion, analysed and compared CT multi-parameter data with SPECT ventilation/perfusion imaging data.
**Results or Findings:** The pulmonary arteries, veins, and bronchi of 30 patients were narrowed in varying degrees, which was surrounded and compressed by proliferative soft tissue of mediastinum and hilar. The iodine density and Z effective values reflecting pulmonary perfusion parameters were correlated with the degree of pulmonary artery stenosis. The more severe pulmonary artery stenosis, the lower the iodine density and Z effective values of corresponding pulmonary segment (P<0.001). The right superior pulmonary vein and the primary branch of the double superior pulmonary vein were more easily involved and the degree of stenosis was more serious (P<0.001). 16 cases of SPECT pulmonary ventilation/perfusion imaging showed different degrees of pulmonary perfusion and ventilation damage. CT iodine density values showed more abnormal perfusion lung segments than SPECT (P<0.05).
**Conclusion:** Dual-layer detector spectral CTPA can accurately evaluate the degree of pulmonary vascular and bronchial stenosis in patients with fibrosing mediastinitis, and can also accurately quantify the degree of pulmonary perfusion damage. It can be used as a "one-stop" imaging evaluation method for the diagnosis of fibrosing mediastinitis.
**Limitations:** Not applicable.
**Ethics committee approval:** Not applicable.
**Funding for this study:** Not applicable.
**Author Disclosures:**
Gang Huang: Nothing to disclose
Ma Ming Zhong: Nothing to disclose
Xing Zhou: Nothing to disclose

### RPS 1615-6

**Dual-energy CT as an adjunct to evaluate the significance of type-II endoleaks after endovascular aneurysm repair**
*S. Charalambous*, K. Perisinakis, N. Kontopodis, A. E. Papadakis, G. Prekatsounis, T. G. Maris, C. Ioannou, A. H. Karantanas, D. K. Tsetis; Iraklion/GR

**Purpose:** Persistent type-II endoleaks (T2EL) after endovascular aneurysm repair (EVAR) require lifelong imaging surveillance to avoid potential life-threatening complications. The aim of the study was to examine the value of dual-energy CT imaging as an adjunct to differentiate aggressive from benign T2EL after EVAR.
**Methods or Background:** Study participants were consecutive patients referred for CT at 1-month after EVAR. CT imaging acquisition included a dual-energy CT angiography (DECTA) and a delayed single-energy CT (SECT) imaging. Patients diagnosed with T2EL were re-examined at 6-months post-EVAR to assess the aneurysm sac growth (ASG). Upon ASG recorded, patients were categorised as having benign (group A) or suspicious for aggressive (group B) T2EL. DECTA image data were employed to calculate the normalised effective atomic number (NZeff), the normalised iodine concentration (NIC), the slope (S) of HUendoleak/HUaorta against monochromatic energy, the dual-energy index (DEI) and an improvised endoleak index (EI) for each T2EL. Statistical analysis was employed to compare all above parameters regarding their ability to differentiate aggressive from benign T2EL.
**Results or Findings:** Among 40 patients examined at 1-month after EVAR, 14 patients were diagnosed with T2EL. Nine patients were assigned to group A and five patients to group B. NZeff and EI were found to be significantly lower in group A. There were no significant differences in NIC, DEI, and S values between groups A and B. NZeff was found to have the highest power to discriminate aggressive T2EL with an area-under-curve of 86.7%, showing 100% specificity and 60% sensitivity.
**Conclusion:** The use of DECT imaging at 1-month after EVAR may provide quantitative indices able to discriminate aggressive from benign T2ELs after EVAR and could therefore provide decision support tools to assist in patient management.
**Limitations:** Small sample size.
**Ethics committee approval:** This study was approved by an ethics committee.
**Funding for this study:** No funding was received.
**Author Disclosures:**
Giorgos Prekatsounis: Nothing to disclose
Thomas G. Maris: Nothing to disclose
Konstantinos Perisinakis: Nothing to disclose
Christos Ioannou: Nothing to disclose
Dimitrios K Tsetis: Nothing to disclose
Antonios E Papadakis: Nothing to disclose
Nikos Kontopodis: Nothing to disclose
Stavros Charalambous: Nothing to disclose
Apostolos H. Karantanas: Nothing to disclose

### RPS 1615-8

**Personalised high-pitch CTPA facilitates a reduction of contrast agent volume**
*R. Varga*, A. E. Strassl, H. Prosch, R. Schernthaner; Vienna/AT

**Purpose:** Despite recent developments in computed tomography pulmonary angiography (CTPA), high volumes of contrast agent are still required due to haemodynamic differences in between patients. The aim of this study was to identify the optimal scan delay for each patient using test bolus technique in order to reduce the volume of contrast agent.
**Methods or Background:** All patients included in this prospective trial (n=109) were referred to CTPA due to suspected pulmonary embolism (PE). The study group (n=78) underwent CTPA with 20ml Iomeron 400 i.v using test bolus technique. Only standard exclusion criteria for CTPA were applied; no study-related exclusion criteria were defined. The retrospectively selected control group (n=31) underwent CTPA using our standard protocol (bolus tracking with 50 ml Iomeron 400 i.v). Two independent readers measured the attenuation in the pulmonary trunk (PT) and the superior vena cava (SVC). For statistical purposes, unpaired t-tests and intraclass correlation coefficients (ICCs) were calculated.
**Results or Findings:** PE was detected in 14 patients (12.8%). According to both readers, the attenuation in the PT was significantly (p<0.001) lower in the study group (mean 352±145 HU) compared to the control group (mean 520±180 HU), with excellent interreader agreement (ICC 0.968). However, a higher percentage of patients in the study group reached diagnostic attenuation values of >200 HU in the PT, according to reader 1 (96.2% vs 93.5%) and reader 2 (92.3% vs 90.3%). In addition, the attenuation in the SVC was significantly (p<0.001) higher in the control group (mean1128±786 HU) compared to the study group (mean 382±149 HU), indicating suboptimal contrast agent timing.
**Conclusion:** Personalised high-pitch CTPA facilitates an optimised scan delay and allows a significant reduction in contrast agent volume while maintaining diagnostic attenuation of the pulmonary arteries.
**Limitations:** No limitations were identified.
**Ethics committee approval:** This study was approved by an ethics committee.
**Funding for this study:** Not applicable.
**Author Disclosures:**
Helmut Prosch: Nothing to disclose
Andreas Eduard Strassl: Nothing to disclose
Ruediger Schernthaner: Nothing to disclose
Raoul Varga: Nothing to disclose

**RPS 1615-10**

**Determination of aortic pulsatility on ECG-gated CT angiography in patients with abdominal aneurysms**

*M. Huber*, D. M. Fontanini, P. Sótonyi, C. Csobay-Novák; Budapest/HU

**Purpose:** Recently, the toolbox of aortic reconstruction has been expanded with grafts suitable for endovascular treatment of the aortic arch. Systolo-diastolic diameter changes of the aorta in the fixation zones may pose problems regarding sizing of the endograft. The aim of this study is to determine the aortic pulsatility in patients with abdominal aortic aneurysms.
**Methods or Background:** In this retrospective study, analyses of readily available CTA images were performed of 31 patients treated for abdominal aortic aneurysms. Reconstructions of the raw ECG gated dataset at 30 and 90% of the R-R cycle were used. After lumen segmentation, total aortic cross-sectional area was measured in a plane perpendicular to the centreline. 12 measurements were completed both in diastole and systole in the following six zones: Z0, Z3, Z5, Z6, Z8, Z9. Effective diameters were calculated from systolic and diastolic cross-sectional areas to determine absolute (ds-dd; mm) and relative pulsatility [(ds-dd)/dd; %].
**Results or Findings:** We performed 24 measurements per patient, 744 in total. The mean pulsatility values at each measurement point were as follows: Z0: 0.7±0.75 mm, Z3: 1.0±0.55 mm, Z5: 0.95±0.6 mm, Z6: 0.8±0.65 mm, Z8: 0.65±0.95 mm, Z9: 0.9±0.9 mm.
**Conclusion:** Based on the measured results, the mean pulsatility of patients with abdominal aortic aneurysms is in a submillimetric range, which is less than 5% of the diastolic diameter. It is probably not relevant for stent graft sizing as an average oversize of 10-20% is used in planning. According to our previous studies, aortic pulsatility in patients with aneurysms is similar to that of patients with non-dilated vessels.
**Limitations:** There were no limitations.
**Ethics committee approval:** Approved by the Semmelweis University Regional and Institutional Committee of Science and Research Ethics (95/2021).
**Funding for this study:** This study is not funded.
**Author Disclosures:**
Máté Huber: Nothing to disclose
Daniele Mariastefano Fontanini: Nothing to disclose
Csaba Csobay-Novák: Nothing to disclose
Péter Sótonyi: Nothing to disclose

**RPS 1615-11**

**Potential utility of ECG-gated CT angiography for investigation of the elastic properties of the ascending thoracic aorta**

*V. V. Saushkin*, V. V. Shipulin, D. Panfilov, B. Bazarbekova, B. Kozlov, S. Sazonova; Tomsk/RU
*(saushkin.vv@outlook.com)*

**Purpose:** The aim of the study was to compare the elastic properties (EP) of normal and dilated ascending aorta (AA) measured by electrocardiographic-gated computed tomography angiography (ECG-gated CTA).
**Methods or Background:** The study enrolled 41 patients (aged 63±10 years; 15 men), which were divided on group 1 (n=22, aged 64±7) with AA diameter (AAd) >45mm (dilatation and aneurysm) and group 2 (n=19, aged 63±9) with AAd<45mm (normal). All patients underwent ECG-gated CTA. CTA datasets were reformatted into 10-phases. We evaluated EP of AA as systolic and diastolic difference of AAd, cross-sectional areas, aortic segments length, and calculated strain, compliance (Com) and distensibility (Dis) of the aortic wall according to [Zubair V, 2020].
**Results or Findings:** In group 1 for both Sinus of Valsalva (SV) and AA all calculated EP parameters, except ST, were lower, comparing with group 2: SVcom 0,39 (-1.23; 4.68) vs 1,01 (0.79; 2.11), p< 0,05; SVdis 0.18(-2.20; 4.99) vs 4.09(1.69;5.20), p<0.05; AAcom 1.17 (-0.99; 2.94) vs 1.79 (1.02; 3.35), p<0.05; AAdis 0.48 (-2.5; 0.78) vs 4.7 (2.41; 6.58), p<0.05. We also divided group 2 into 2 subgroups: subgroup 1 (n=10) with AAdiameter<50 mm (dilatation) and subgroup 2 (n=12) with AAdiameter>50 mm (aneurysm). EP of the aorta between these subgroups did not differ significantly: SVcom 0.63 (-7.68; 3.34) vs 0.39 (-0.91; 6.61), p=0.29; SVdis 0.40 (-2.40; 5.32) vs 0.14(-2.18; 1.85), p=0.67; AAcom 3.03(-0.17; 4.01) vs 1.08 (-4.1; 4.26), p=0.49; AAdis 0.55(-2.23; 0.79) vs -0.20(-2.50; 1.03), p=0.23.
**Conclusion:** ECG-gated CTA allows evaluating of EP of AA and gives additional information, which potentially may have prognostic value in patients with AA dilatation or aneurysm. Patients with AA dilatation and aneurysm have worse AA EP comparing with normal AA, but do not differ from each other.
**Limitations:** Data analysis depending on the patient's gender was not performed.
**Ethics committee approval:** Approved by the Ethics Committee.
**Funding for this study:** The study was supported by a grant from the Russian Science Foundation (project № 21-15-00160).
**Author Disclosures:**
Botazhan Bazarbekova: Nothing to disclose
Svetlana Sazonova: Nothing to disclose
Vladimir V. Shipulin: Nothing to disclose
Dmitriy Panfilov: Nothing to disclose
Viktor V. Saushkin: Nothing to disclose
Boris Kozlov: Nothing to disclose

**RPS 1615-12**

**Can we finally skip unenhanced acquisitions prior to CT angiography of aortic stent patients? Comparison of 2- and 3-material decomposition reconstructions of photon-counting detector CT datasets**

*J. A. Decker*, S. Bette, C. Scheurig-Muenkler, B. Jehs, F. Risch, F. Braun, C. Wollny, T. J. Kroencke, F. Schwarz; Augsburg/DE
*(Josua.Decker@uk-augsburg.de)*

**Purpose:** To assess the image quality of virtual-unenhanced series derived by two-material (2MD) or three-material decomposition (3MD) reconstructions of arterial scans of the aorta on a novel photon-counting detector CT (PCD-CT) in comparison with true non-contrast scans (TNC).
**Methods or Background:** Twenty consecutive patients with clinically indicated CT of the aorta after endovascular aneurysm repair were scanned on a novel PCD-CT (Naeotom alpha, SiemensHealthineers, Erlangen, Germany). Scans were acquired as unenhanced series and in arterial phase; 2MD- and 3MD-reconstructions were performed using the arterial phase dataset; virtually unenhanced series generated from both reconstructions. Standard-deviation of CT-values (noise) was measured on 6 regions of interest in the aorta. Two experienced interventional radiologists compared all series regarding image quality, contrast-removal and potential removal of calcifications and stent parts using a 5-point Likert-scale (5=excellent/no removal of stent-parts/calcium to 1=non-diagnostic/complete removal of stent-parts/calcium). Subjective diagnostic suitability of virtual unenhanced series was assessed as: no, partial or full suitability for the replacement of TNC series.
**Results or Findings:** Image noise was higher in 2MD- (20.2±4.9) and 3MD-reconstructions (18.1±4.7) than in TNC-series (14.2±1.7; p<0.001). Subjective image quality was substantially higher in 3MD-reconstructions than in 2MD-reconstructions (4.2±0.9 vs. 2.5±0.6; p<0.001). Contrast-removal was considered complete for all patients in both 2MD- and 3MD-reconstructions. Unlike in 2MD-reconstructions, in 3MD-reconstructions only minuscule parts of stents (4.7±0.7 vs. 3.8±1.2;p=0.003) and atherosclerotic calcifications (4.6±0.5 vs. 3.0±0.6;p<0.001) were erroneously subtracted. The expert readers found 92.5% of 3MD-reconstructions as suited (27.5% partially suited) to replace TNC-series.
**Conclusion:** 3MD-reconstructions of arterial phase PCD-CT datasets show high diagnostic quality with full contrast removal and only minimal erroneous subtractions of stent parts and calcifications. Using these to replace additional unenhanced acquisitions would significantly reduce radiation doses.
**Limitations:** Small sample-size, retrospective single-centre study.
**Ethics committee approval:** This study was approved by an ethics committee. Informed consent was obtained.
**Funding for this study:** No funding was received for this study.
**Author Disclosures:**
Franka Risch: Nothing to disclose
Stefanie Bette: Nothing to disclose
Bertram Jehs: Nothing to disclose
Christian Scheurig-Muenkler: Nothing to disclose
Franziska Braun: Nothing to disclose
Claudia Wollny: Nothing to disclose
Thomas J. Kroencke: Nothing to disclose
Florian Schwarz: Nothing to disclose
Josua A. Decker: Nothing to disclose

**16:00-17:30** **Room M 3**

### Research Presentation Session: Neuro

### RPS 1611

### Neuroimaging in brain tumours

**Moderator**
D. Chourmouzi; Thessaloniki/GR

### RPS 1611-2

**Dural angioleiomyoma: a new tumour type with frequent GJA4 mutation and a distinct DNA methylation profile**

*T. Pierre*[1], A. Tauziède-Espariat[1], M. Wassef[1], D. Castel[1], P. Sievers[2], J. Grill[1], F. Riant[1], C. S. Balleyguier[1], P. Varlet[1]; [1]Paris/FR, [2]Heidelberg/DE
*(p_thibaut@hotmail.fr)*

**Purpose:** The International Society for the Study of Vascular Anomalies has defined four vascular lesions in the central nervous system (CNS): arteriovenous malformations, cavernous malformations, venous malformations, and telangiectasias.
**Methods or Background:** From a retrospective central radiological (by a senior neuroradiologist) and histopathological (by a senior neuropathologist) review of 202 CNS vascular lesions, we identified three cases of unclassified vascular anomalies. Interestingly, they shared the same radiological and histopathological features evoking the cavernous subtype of angioleiomyomas described in the soft tissue. We grouped them together with four additional similar cases from our clinicopathological network and performed combined molecular analyses (whole-exome-sequencing (WES) analysis and DNA-methylation profiling). Moreover, cases were compared with a cohort of 5 angioleiomyomas of soft tissue.
**Results or Findings:** The seven lesions had the same histopathology (cavernous angioma features with muscular component) and neuroimaging criteria (solitary extra-axial, dural-based lesion with heterogeneous enhancement mimicking meningioma). WES analysis identified a p.Gly41Cys GJA4 mutation in 3/6 cases. This mutation was reported in hepatic haemangiomas and cutaneous venous malformations. Most DNA methylation profiles were not classifiable using the CNS brain tumour (version 12.5), and sarcoma (version 12.2) Classifiers, but by unsupervised t-SNE and hierarchical clustering analyses, 5/6 lesions grouped together and formed a distinct epigenetic group separated from the clusters of soft tissue angioleiomyomas, other vascular tumours and meningiomas.
**Conclusion:** Dural angioleiomyomas represent a single radiological and histomolecular tumour type, with frequent GJA4 mutations and a distinct DNA methylation pattern corresponding to the suggested new terminology of "dural angioleiomyomas". They are a rare and benign lesion that radiologists need to know among the differential diagnoses of meningioma.
**Limitations:** The cohort was relatively small, due to low frequency and small number of lesions operated because of their often being interpretated as meningioma.
**Ethics committee approval:** Written informed consent was obtained.
**Funding for this study:** Funding was received from Etoile de Martin and Les Boucles du Cœur.
**Author Disclosures:**
Florence Riant: Nothing to disclose
Thibaut Pierre: Nothing to disclose
Michel Wassef: Nothing to disclose
Philipp Sievers: Nothing to disclose
Corinne S. Balleyguier: Nothing to disclose
Arnault Tauzièdе-Espariat: Nothing to disclose
Jacques Grill: Nothing to disclose
David Castel: Nothing to disclose
Pascale Varlet: Nothing to disclose

### RPS 1611-3

**Clinical applicability of whole brain SLOW-editing compared to MEGA-editing for the evaluation of the IDH-mutation status in glioma patients at 7T**

*G. Weng*, J. Slotboom, P. Radojewski; Bern/CH
*(wegodem@gmail.com)*

**Purpose:** Comparison of the novel spectral editing method (SLOW-editing) with two different MEGA-editing based methods for the detection of 2HG in glioma patients at 7T.
**Methods or Background:** 12 patients with suspected glioma have been prospectively examined. Then, surgery and neuropathological analysis to determine the IDH-status have been performed in all patients. All MRI, MRS(I) measurements were performed on a Siemens MAGNETOM Terra 7T MR-scanner in clinical-mode using a Nova 1Tx32Rx head-coil. The proposed SLOW-ESPI, as well as the classical MEGA-SVS/CSI methods were performed on all 12 patients during the same examination.
**Results or Findings:** Thanks to lower artifacts, SLOW-EPSI has better spectral quality compared to MEGA-SVS/CSI in many cases. When using MEGA-SVS and -CSI, there are one and two ambiguous cases, respectively, mainly due to water contamination and B0/B1 inhomogeneities. In contrast, there is no ambiguous case using SLOW-EPSI. 12 patients with suspected glioma were included in this prospective study. SLOW-editing for detection of 2HG signal showed 91.7% (11/12) accuracy whereas the other methods resulted in 58.3% (7/12, MRSI-CSI at 7T), 75% (9/12, MRSI-SVS at 7T), respectively with ambiguous cases included.
**Conclusion:** SLOW-editing shows its advantage in detecting 2HG and predicting IDH-status in vivo at 7T over MEGA-editing. The EPSI-based SLOW-editing can be performed within 10 minutes in clinical mode.
**Limitations:** SLOW-editing does not work at low fields ≤ 1.5T, and it might work at 3T but needs to be investigated.
**Ethics committee approval:** Approval was given by the KEK (Bern Switzerland, approval number 2019-00503).
**Funding for this study:** The research is supported by the Swiss National Science Foundation (SNSF-182569) and the European Union's Horizon 2020 research and innovation program under grant agreement No 813120.
**Author Disclosures:**
Johannes Slotboom: Patent Holder: SLOW-editing
Piotr Radojewski: Nothing to disclose
Guodong Weng: Patent Holder: SLOW-editing

### RPS 1611-4

**Conventional MRI features can predict the molecular subtype of adult grade 2-3 intracranial diffuse gliomas**

*A. Lasocki*[1], M. Buckland[2], K. Drummond[1], H. Wei[2], J. Xie[1], M. Christie[1], A. Neal[1], F. Gaillard[1]; [1]Melbourne/AU, [2]Sydney/AU

**Purpose:** Molecular markers have become key to classifying low grade (grade 2-3) intracranial gliomas (LGG), leading to research into correlating imaging features with genotype ("radiogenomics"). Few studies have specifically characterised LGG as one of the three key molecular subtypes. Our study investigated the accuracy of combining multiple conventional MRI features to predict LGG molecular subtype, aiming to developing a practical predictive algorithm.
**Methods or Background:** LGG diagnosed between 2007 and 2013 were identified. Two neuroradiologists independently assessed nine conventional MRI features. Features with interobserver agreement yielding $\kappa \geq 0.6$ proceeded to consensus assessment. MRI features were correlated with genotype, classified as IDH-mutant and 1p/19q-codeleted (IDHmut/1p19qcodel), IDH-mutant and 1p/19q-intact (IDHmut/1p19qint), or IDH-wildtype (IDHwt). Additional molecular markers of glioblastoma were also noted for IDHwt tumours.
**Results or Findings:** 119 patients met the study criteria. T2-FLAIR mismatch sign was the most predictive feature across subtypes ($p<0.001$). All 30 tumours with >50% mismatch were IDHmut/1p19qint, and all 7 with 25-50% mismatch, including enhancing tumours. Well-defined tumour margins correlated with IDHmut/1p19qint status on univariate analysis ($p<0.001$), but there was no longer an association once T2-FLAIR mismatch was accounted for. Enhancement ($p=0.001$), necrosis ($p=0.002$) and haemorrhage ($p=0.027$) correlated with IDHwt status, especially molecular glioblastoma. 7 of 10 tumours with calcifications were IDHmut/1p19qcodel ($p=0.008$).
**Conclusion:** T2-FLAIR mismatch strongly predicts IDHmut/1p19qint even with a lower threshold of ≥25% mismatch. When tumours exhibit <25% mismatch, secondary features include enhancement, necrosis and haemorrhage (predicting IDHwt, especially "molecular glioblastoma"), and calcifications (IDHmut/1p19qcodel). Well-defined margins are no longer predictive after accounting for mismatch.
**Limitations:** Not applicable
**Ethics committee approval:** Institutional Human Research Ethics Committee approval was obtained.
**Funding for this study:** This study was supported by a RANZCR research grant in 2018. Dr Arian Lasocki was supported by a Peter MacCallum Cancer Foundation Discovery Partner Fellowship.
**Author Disclosures:**
Arian Lasocki: Nothing to disclose
Michael Christie: Nothing to disclose
Kate Drummond: Nothing to disclose
Jing Xie: Nothing to disclose
Andrew Neal: Nothing to disclose
Heng Wei: Nothing to disclose
Frank Gaillard: Nothing to disclose
Michael Buckland: Nothing to disclose

### RPS 1611-5

**Clinical and pathologic characteristics of long-term oligodendroglioma survivors**

*B. Nan*[1], A. Rashiwala[2], A. Krayyem[3], E. B. Wang[4], A. Darbandi[5], A. Amer[5], J. M. Johnson[2]; [1]Valhalla, NY/US, [2]Houston, TX/US, [3]Austin, TX/US, [4]Dallas, TX/US, [5]Chicago, IL/US

**Purpose:** Oligodendrogliomas are reported to have a 75% survival time of 47 months and a 5-year survival rate of 70.8%. They are classified by their genetics (IDH mutation, 1p/19q co-deletion). To better understand factors influencing prognosis, we conducted a large retrospective single-institution study using a novel method to identify long-term survivor (LTS) characteristics.
**Methods or Background:** Using a combination of regular expression (RegEx) search and natural language processing (NLP), data extracted from the radiology information system (RIS) was used to identify patients with a pathology-confirmed diagnosis of oligodendroglioma or astrocytoma. Reports were extracted using RegEx, and overall survival (OS) was obtained by utilising the first CT/MRI reports and last MRI on record. To verify the accuracy of this method, we performed a chart review where OS was defined as the date of diagnosis to the current date or date of death.
**Results or Findings:** Following inclusion criteria, 269 patients were obtained. NLP was validated with a mean absolute error of 149.8 days. ANOVA analysis of tumours revealed no difference in OS of patients within the 4 genetic classifications: IDH mutated 1p/19q co-deletion, IDH non-mutated co-deleted, IDH mutated no co-deletion, IDH non-mutated no-codeletion (p=.34). When classifying by pathology, a significant difference in OS was seen between oligodendroglioma patients (1633.39 days), astrocytoma (2192.06 days), and mixed pathology (2512.76 days) (p<.001).
**Conclusion:** Our study provides insight regarding the genetic and pathological classification of oligodendrogliomas. Pathology showed that oligodendrogliomas have significant lower OS than astrocytoma and mixed-tumour counterparts. Genetics showed no significant OS based on genetic classification suggesting the need for further analysis.
**Limitations:** Limitations to this study include controlling for treatment options such as radiation.
**Ethics committee approval:** The IRB of UT MDACC approved this study.
**Funding for this study:** Not applicable
**Author Disclosures:**
Azad Darbandi: Nothing to disclose
Jason Michael Johnson: Grant Recipient: Blue Earth Diagnostics Consultant: Kura Oncology Consultant: InformAI
Ahmad Amer: Nothing to disclose
Abhi Rashiwala: Nothing to disclose
Ethan Bocheng Wang: Nothing to disclose
Brian Nan: Nothing to disclose
Apollo Krayyem: Nothing to disclose

### RPS 1611-6

**The impact of tumour location on survival in glioblastoma patients**

*A. Amer*, A. Darbandi, E. B. Wang, B. Ciavarra, K. R. Lano, A. Krayyem, H. Alhasan, J. M. Johnson; Houston, TX/US

**Purpose:** Glioblastoma (GBM) is an aggressive brain tumour with a mean survival of 14 months. We conducted a large retrospective single-institution study to identify the impact of GBM tumour location on patient prognosis.
**Methods or Background:** Using regular expression (RegEx) search and natural language processing (NLP) in Python, data extracted from the radiology information system (RIS) was used to identify 4425 patients who presented at MD Anderson Cancer Center from 2006-2021 with pathology-confirmed GBM. We defined overall survival (OS) as the time between the first CT/MRI report mentioning GBM and the last, a method verified using chart review. We compared OS of patients with GBMs involving different lobes to each other, including those with multilobe involvement.
**Results or Findings:** Frontal lobe tumours showed an average survival of 785.64±1034.64 (n=579), parietal lobes 653.90±823.20 days (n=195), temporal lobes 668.04±838.87 (n=383), and occipital lobes 498.091±456.29 days (n=47). Non-parametric t testing was done to compare OS. Multilobe analysis showed that frontal lobe involvement had better OS than non-frontal lobe involvement (656.82±887.78 vs 601.493±722.140 [p<.05]). Conversely, occipital lobe involvement was associated with significantly lower survival than non-occipital lobe involvement (516.57±622.26 vs 655.12±848.01 [p<.01]).
**Conclusion:** Our study is the largest single-institution study of the impact of GBM tumour location on OS. We demonstrate that location has an impact on patient survival. Frontal lobe involvement was associated with better prognosis and occipital with worse. This may be for a number of reasons. Frontal lobe tumours are more amenable to surgical resection. Furthermore, patients typically become symptomatic earlier, allowing for earlier intervention. Prior studies have also suggested a potential biomarker difference between lobes, which will be investigated in future analyses of our dataset.
**Limitations:** Extent of involvement was not considered.
**Ethics committee approval:** The IRB of UT MDACC approved this study.
**Funding for this study:** Not applicable
**Author Disclosures:**
Azad Darbandi: Nothing to disclose
Kinsey Read Lano: Nothing to disclose
Jason Michael Johnson: Grant Recipient: Blue Earth Diagnostics Consultant: InformAI Consultant: Kura Oncology
Ahmad Amer: Nothing to disclose
Ethan Bocheng Wang: Nothing to disclose
Hamza Alhasan: Nothing to disclose
Bronson Ciavarra: Nothing to disclose
Apollo Krayyem: Nothing to disclose

### RPS 1611-7

**Histologic validation of multi-echo perfusion MRI to evaluate brain tumour microstructure**

*F. Sanvito*[1], C. Raymond Guzman[2], N. S. Cho[2], A. Hagiwara[3], S. Bastianello[1], B. M. Ellingson[2]; [1]Pavia/IT, [2]Los Angeles, CA/US, [3]Tokyo/JP
*(francesco.sanvito.1992@gmail.com)*

**Purpose:** To extract quantitative MRI markers from multi-echo DSC perfusion and correlate them with cell density and cell size.
**Methods or Background:** In a classic DSC perfusion sequence, T1 and T2* leakage effects determine the post-bolus signal, and are influenced by tissue geometry (i.e. cell density, cell size). Multi-echo perfusion MRI enables to disentangle T2* and T1 components that contribute to the classic DSC curve. We retrospectively selected patients with: diagnosis of primary brain tumour, availability of multi-echo DSC perfusion and DWI datasets, availability of histopathological images from targeted biopsies. Post-processing allowed to distinguish pure T2* component from pure T1 component, and to perform a DCE analysis on the latter. The quantitative MRI markers obtained will be compared to histopathological data.
**Results or Findings:** The following novel MRI quantitative maps were successfully computed voxelwise: ΔR2* at steady state (reflecting T2* leakage effects), ΔR1 at steady state (reflecting T1 leakage effects), transverse relaxivity at tracer equilibrium (TRATE, reflecting the combination of T2* and T1 leakage effects). In addition, VE and kTRANS were computed from the DCE analysis, and the percentage of signal recovery (PSR) was computed from the second echo of the multi-echo DSC (comparable to a classic single-echo DSC sequence).
**Conclusion:** Histopathological validation will assess the usefulness of the novel multi-echo derived quantitative maps for the non-invasive prediction of tumour microstructure. This would be particularly relevant for: 1) differential diagnosis between brain tumours with different cell size and cell density (e.g. lymphoma vs glioblastoma); 2) treatment response assessment (as pre-existing studies proved that cell shrinkage is an early event in treatment response).
**Limitations:** This was a retrospective analysis and the cohort included only gliomas.
**Ethics committee approval:** Local ethics committee approval was received.
**Funding for this study:** Grants: RSG-15-003-01-CCE, NIH/NCI 1P50CA211015-01A1, NIH/NCI 1R21CA223757-01, NIH-NIGMS Training Grant GM008042.
**Author Disclosures:**
Nicholas S. Cho: Nothing to disclose
Stefano Bastianello: Nothing to disclose
Catalina Raymond Guzman: Nothing to disclose
Benjamin M. Ellingson: Nothing to disclose
Akifumi Hagiwara: Nothing to disclose
Francesco Sanvito: Nothing to disclose

### RPS 1611-8

**Radiological and pathological predictors of post-surgical evolution of pituitary neuroendocrine tumours. A retrospective analysis of 125 patients**

*J. M. Castro*, T. Arguello Gordillo, L. Martínez Gauffin, L. Concepción Aramendia, A. M. Picó, J. Abarca Olivas, F. I. Aranda López; Alicante/ES
*(jose_miguel2552@hotmail.com)*

**Purpose:** We retrospectively analysed laboratory, pathological, and imaging characteristics of 125 patients with large pituitary adenomas to identify post-surgical prognosis-defining features.
**Methods or Background:** This was a retrospective cohort study including all patients diagnosed with pituitary adenomas who were treated surgically and followed up at a tertiary hospital between 2012 and 2022. Radiological variables from the first MRI that were collected were tumour dimensions, volume, sphenoidal, cavernous sinus invasion, tumour signal intensity on diffusion- and T2-weighted imaging (signal intensity ratio in T2-weighted sequences between the pituitary tumour and corpus callosum) and diffusion restriction. Activity and secretory type of adenomas were also collected.

Pathological variables included for analysis were immunohistochemistry results, citokeratins and KI-67 status. Tumour remnants and time until recurrence were obtained from successive MRI scans. Statistically significant variables on univariate analysis were subjected to multivariate analysis to obtain an adjusted relative risk. Time to recurrence was analysed through Mantel-Cox and Kaplan-Meyer.
**Results or Findings:** Tumours with a higher volume than 5cm3, cavernous and sphenoidal sinus invasion were significantly associated with tumour recurrence RR (1.7, 2.1, 2.8), IC (1.1-2.7, 1.5-2.9, 1.8-4.3) respectively, with p<0.05. High Ki-67 did not reach statistical significance, with a RR 1.7 (0.4-7.3) p>0.05; however tumours with sinus invasion and high KI-67 showed earlier recurrence (RR 9.5, p>0.05) as did those with a higher tumour signal intensity on T2-weighted sequences (RR 15.2, p<0.001). Respecting secretory type, those with ACTH and GH overproduction had earlier recurrence rates on Mantel-Cox test (RR 18.42, p<0.05).
**Conclusion:** Sinus invasion, ki-67 and T2 signal ratio higher than 2, were associated with a higher rate of recurrence. Time until recurrence varied according to these variables, tumour volume and type of hormone status.
**Limitations:** Some of the patients were followed for shorter durations of time, leading to censored data.
**Ethics committee approval:** This study was approved by an ethics committee.
**Funding for this study:** No funding was received for this study.
**Author Disclosures:**
Thalia Arguello Gordillo: Nothing to disclose
Javier Abarca Olivas: Nothing to disclose
Lucía Martínez Gauffin: Nothing to disclose
Luis Concepción Aramendia: Nothing to disclose
José Miguel Castro: Nothing to disclose
Antonio Miguel Picó: Nothing to disclose
Francisco Ignacio Aranda López: Nothing to disclose

### RPS 1611-9

### Comparative diagnostic accuracy of amide proton transfer-weighted imaging and dynamic susceptibility contrast perfusion in the distinction between brain radiation necrosis and tumour progression

*M. Bensemain*[1], S. Casagranda[2], J. Jacob[3], C. Valery[3], S. Lehericy[3], L. Nichelli[3]; [1]Nancy/FR, [2]La Ciotat/FR, [3]Paris/FR

**Purpose:** Stereotactic radiosurgery (SRS) is an effective therapy for brain metastases. The distinction between tumour progression and radionecrosis is a clinical challenge and currently relies on Dynamic Susceptibility Contrast (DSC) perfusion. Amide Proton Transfer-weighted (APTw) imaging enables measurement of the chemical exchange saturation transfer (CEST) contrast between mobile protein/peptide amide protons and bulk water. The aim of this study was to compare the diagnostic accuracy of APTw imaging and DSC perfusion in the distinction between metastasis recurrence and radionecrosis.
**Methods or Background:** 22 brain pre-irradiated lesions were prospectively examined at 3 Tesla. Diagnosis of tumour progression or radionecrosis was assessed by either (i) histological examination or (ii) minimum 6 months' follow-up or (iii) CT-PET scan. APTw sequence was acquired with a 3D snapshot-GRE, a B1 value of 2.22 μT and a Duty Cycle of 55%. DSC perfusion was acquired after a single dose of gadolinium-chelated contrast agent and a low flip angle. APTw, fuild suppressed (F.S.) APTw and rCBV maps were obtained after post-processing.
**Results or Findings:** Among 22 lesions, 10 were evaluated as radionecrosis and 12 as tumoural progression. Area under the ROC Curve (AUC) were 0.641 for rCBV metrics (0.506-0.776) and 0.966 for APTw metrics (0.93-1). DSC perfusion and APTw discriminated cerebral lesions with a specificity of 90% and a sensitivity of 66.7% and 100% respectively. Concerning F.S. APTw metric, the AUC was 1 (1-1).
**Conclusion:** APTw metrics are more accurate than rCBV values in the distinction between tumour recurrence and radio-induced tissue changes in brain metastasis. Fluid suppression enhance diagnostic accuracy of APTw imaging.
**Limitations:** Despite the encouraging results of the F.S. APTw metric, these must be explored on a larger patient cohort.
**Ethics committee approval:** This study was approved by the local ethic committee.
**Funding for this study:** No funding has been provided.
**Author Disclosures:**
Lucia Nichelli: Nothing to disclose
Stefano Casagranda: Research/Grant Support: Olea Medical
Charles Valery: Nothing to disclose
Julian Jacob: Nothing to disclose
Mehdi Bensemain: Nothing to disclose
Stephane Lehericy: Nothing to disclose

### RPS 1611-10

### Introduction of intra-arterial administration of [68Ga]Ga-PSMA and [68Ga]Ga-DOTA-TATE in CNS tumors; a high potential approach for future development of novel theranostic treatment strategies

I. J. Pruis, F. A. Verburg, P. J. van Doormaal, R. Balvers, E. M. Bos, M. van den Bent, M. Smits, *S. E. Veldhuijzen van Zanten*; Rotterdam/NL
*(s.veldhuijzenvanzanten@erasmusmc.nl)*

**Purpose:** We here introduce the principle of theranostics and intra-arterial (IA) administration of radionuclides to the field of neuro-oncology in order to improve current – and explore novel – treatment strategies.
**Methods or Background:** Seven patients receiving palliative care for meningioma (MG, n=2), brain metastasis from lung carcinoma (BM, n=2) and glioblastoma (GBM, n=3), received intravenous (IV) and separate IA administration of [68Ga]Ga-DOTA-TATE (in MG patients) or [68Ga]Ga-PSMA-11 (in BM/GBM patients), followed by diagnostic PET-imaging at respectively 60 and 90 (DOTA-TATE), or 90, 165 and 240 minutes post-injection (PSMA). Standardised uptake values were calculated for tumour, contralateral healthy brain and liver. Tumour-to-liver (T/L) ratios were calculated using OLINDA software.
**Results or Findings:** All seven patients showed positive uptake in tumour with low background signal (SUV mean in healthy brain ranging from <0.01 – 0.13) indicating selective targeting. Each lesion showed a notable increase in uptake after IA (IV) administration: SUVmax 42.0 (14.0) and 77.1 (27.7) for [68Ga]Ga-DOTA-TATE in the MG patients, SUVmax values of 155.4 (17.1) and 257.3 (12.0), and 133.7(5.8), 216.0 (9.0) and 108.1 (9.0) (versus 5.8, 9.0 and 9.0) for [68Ga]Ga-PSMA-11 in BM and GBM patients, respectively. No increase in SUV values was observed for healthy brain and liver. T/L ratios increased with respectively a factor 3 in the MG patients; a factor 6 and 16 in BM patients; and 14, 16 and 26 in GBM patients, resulting in much more favourable target-to-non-target exposure ratios in case of therapy.
**Conclusion:** Selective IA administrative leads to a strong, significant and relevant increase in CNS tumour uptake thus opening up new avenues for more effective therapeutic use of theranostic tracers in patients, many of whom are without effective options for further treatment.
**Limitations:** This was an exploratory study.
**Ethics committee approval:** Ethics committee approval was obtained.
**Funding for this study:** Funding for this study was received from the Semmy Foundation.
**Author Disclosures:**
Frederik A. Verburg: Nothing to disclose
Pieter Jan van Doormaal: Nothing to disclose
Sophie E.M. Veldhuijzen van Zanten: Nothing to disclose
Rutger Balvers: Nothing to disclose
Ilanah Johanna Pruis: Nothing to disclose
Eelke M Bos: Nothing to disclose
Martin van den Bent: Nothing to disclose
Marion Smits: Nothing to disclose

### RPS 1611-11

### Do we match? An evaluation of the T2/FLAIR mismatch sign for oligodendroglioma diagnosis

*J. M. Brunelli*, I. d. S. Alves, C. S. Barbosa, C. B. F. Leite, S. S. Alves, C. Amancio, C. d. C. Leite; Sao Paulo/BR
*(juliamartinsbrunelli@gmail.com)*

**Purpose:** The 2016 revision of the World Health Organization classification of tumours of the central nervous system considers absence of codeletion of chromosomes 1p and 19q as suggestive of the diagnosis of oligodendroglioma. The T2-fluid-attenuated inversion recovery (FLAIR) mismatch sign has been considered a highly specific imaging biomarker for IDH-mutant, 1p/19q non-codeleted low-grade glioma. We aimed to test and validate if neuroradiologists' performance could predict 1p/19q status based on the mismatch sign.
**Methods or Background:** The study included patients with low grade gliomas who have histopathological confirmation (through biopsy/surgical resection), molecular study and imaging tests in our database (n=86). Preoperative imaging was retrospectively assessed by two independent neuroradiologists and one fellow radiologist, blinded to the 1p/19q status, to assess presence/absence of "T2–FLAIR mismatch" sign. κ statistics were calculated to determine interobserver agreement between the 3 reviewers, and the Fisher exact test was used to determine the association between 1p/19q status and the presence of T2-FLAIR mismatch. The analysis was performed by using SSPS statistics.
**Results or Findings:** Analysis demonstrated moderate interreader agreement for the T2–FLAIR mismatch sign [κ = 0.584 (0.478–0.691)]. The T2-FLAIR mismatch sign was present in 8 cases (9.3%) and had a positive predictive value of 75%, negative predictive value of 96%, a sensitivity of 66%, and a specificity of 97% (p<0.00001).

**Conclusion:** Among low-grade gliomas, T2-FLAIR mismatch sign represents a highly specific imaging biomarker for the IDH-mutant, 1p/19q non-codeleted molecular subtype. The main difference between the readers was observed because of the imprecise definition of homogeneity on these sequences that characterise the T2/FLAIR mismatch.
**Limitations:** The small number of the IDH-mutant, 1p/19q non-codeleted molecular subtype tumours was a limitation in this study.
**Ethics committee approval:** This study was approved by an ethics committee (CAAE 98395118.0.0000.5461).
**Funding for this study:** No funding was received for this study.
**Author Disclosures:**
Cristyano Bismark Ferreira Leite: Nothing to disclose
Camila Silva Barbosa: Nothing to disclose
Isabela dos Santos Alves: Nothing to disclose
Julia Martins Brunelli: Nothing to disclose
Camila Amancio: Nothing to disclose
Claudia da Costa Leite: Nothing to disclose
Samya Saraiva Alves: Nothing to disclose

### RPS 1611-12

**Effect of radiation therapy on meningioma enhancement on PET CT**

*D. Abeysekera*, A. Darbandi, H. Pokhylevych, K. R. Lano, L. Flynt, M. F. McAleer, S. McGovern, O. Mawlawi, J. M. Johnson; Houston, TX/US

**Purpose:** To assess the ability of 68Ga-DOTATATE PET CT imaging to measure responses to radiation therapy in meningiomas.
**Methods or Background:** It is well-recognised that postoperative radiation improves disease-free survival in patients with meningiomas. Currently, guidelines for grading treatment response in those undergoing radiation therapy are significantly limited. Adult patients with any meningioma of at least 10 mm measurable residual disease with planned radiation therapy underwent 68Ga-DOTATATE PET CT imaging 30 days prior to radiation therapy initiation and then at 12 +/- 4 weeks following radiation therapy conclusion. Quantitative analysis of lesions DOTATATE activity was performed using MIM (MIM Software Inc, Beachwood, OH) on pre- and post-treatment scans.
**Results or Findings:** Three patients received external beam radiation ranging from 50.4-60 Gy. One received proton therapy to a dose of 59 Gy. One received Gamma Knife only (14 Gy) and one received both with culmulative dose of 64.4 Gy. PET data was analysed via a non-parametric paired t-test (Wilcoxon signed-rank test). Max SUV in patients before and after radiation was 8.53±5.59 and 7.61±5.43 respectively, with no significant difference between groups (p=.162). No significant difference was found between mean SUV (p=.303), TLG SUV (p=.240), total SUV (p=.240), or volume (p=.442) before and after radiation.
**Conclusion:** This study suggests there is little to no measured metabolic response to radiation therapy in meningiomas as measured by 68Ga-DOTATATE PET CT imaging. Currently, treatment for meningioma is mostly observation and surgery when clinically indicated; adjuvant radiotherapy/radiosurgery is used for atypical and anaplastic meningiomas. Further studies on effects of radiation therapy for meningiomas can provide further insight on treatment guidelines.
**Limitations:** This was an imaging-only study without traditional two-year follow-up.
**Ethics committee approval:** The IRB of UT MDACC approved this study.
**Funding for this study:** This was an internally funded study. No external funding was received.
**Author Disclosures:**
Azad Darbandi: Nothing to disclose
Dylan Abeysekera: Nothing to disclose
Kinsey Read Lano: Nothing to disclose
Lesley Flynt: Nothing to disclose
Halyna Pokhylevych: Nothing to disclose
Mary Frances McAleer: Nothing to disclose
Jason Michael Johnson: Consultant: Kura Oncology Consultant: InformAI
Grant Recipient: Blue Earth Diagnostics
Susan McGovern: Nothing to disclose
Osama Mawlawi: Nothing to disclose

### Saturday, July 16

**08:00-09:00** **Open Forum #1 (Radiographers)**

### Research Presentation Session: Radiographers

### RPS 1714

### Professional challenges for radiography

**Moderators**
P. Cornacchione; Rome/IT
J. C. Vilanova; Girona/ES

### RPS 1714-3

**Incidents of workplace harassment: reporting mechanisms & support services available to radiographers in the Republic of Ireland**

L. Langan, R. Young, *M. F. F. McEntee*; Cork/IE
*(mark.mcentee@ucc.ie)*

**Purpose:** Health care workers experiences of workplace harassment are well documented across the world. There appears to be a lack of up-to-date data available on issues relating to workplace harassment amongst radiographers. Therefore, this study aimed to explore the incident rate, reporting mechanisms and support services associated with workplace harassment.
**Methods or Background:** A questionnaire was designed by adapting a WHO (2003) survey on violence in the health sector. Following ethical approval and a pilot phase, radiographers across the ROI were invited to participate via a snowballing methodology using Social Media and direct contact.
**Results or Findings:** A total of 60 responses were recorded. Verbal abuse was the most frequent form of harassment (65%), followed by acts of microaggression (48%) sexual & gender-based harassment (41.6%) and physical assault (15%). Over 78% were aware of the reporting procedures in place concerning workplace harassment. The majority of participants (78.3%) reported that they had not received official training on how to utilise reporting mechanisms, with 73% stating that they would be more likely to report an incident if they knew how. Almost 90% felt that support offered to them following work-related harassment was inadequate.
**Conclusion:** The vulnerability of radiographers to workplace harassment is demonstrated throughout this research. Emphasis is placed on the need for departments to implement strategies to protect employees from such incidents.
**Limitations:** The limited-time frame of this research did not allow for a thorough analysis to be conducted on a larger cohort. As a result, the sample size and findings do not represent the wider population of radiographers in ROI.
**Ethics committee approval:** Ethical approval was granted from the Social Research Ethics Committee in UCC (CT-SREC-2020-39).
**Funding for this study:** No funding was received for this study.
**Author Disclosures:**
Mark F. F. McEntee: Nothing to disclose
Rena Young: Nothing to disclose
Lisa Langan: Nothing to disclose

### RPS 1714-4

**Radiographers' perceived workload**

*S. E. de Labouchere*[1], E. M. Metsälä[2]; [1]Lausanne/CH, [2]Helsinki/FI

**Purpose:** These past years radiology has seen an increse in examination demands and in workload. Increased flow of patients and heavy workloads have been shown to have numerous negative outcomes on work well-being. The aim of this study was to assess radiographers' perceived workload.
**Methods or Background:** For this cross sectional designed study conducted as part of a MSc degree in 2019, data was gathered via an online survey sent to diagnostic radiographers in Western Switzerland. Perceived workload was determined using the validated translated version of the NASA-RTLX. Descriptive statistics were established.
**Results or Findings:** Response rate was 23.9% (n=150). Radiographers' percieved workload was above average (6.48/0-10 scale). Mental, physical, temporal demand and performance all scored above 7/0-10 scale, with temporal demand being the highest score. The increased number of examinations and the increased pressure to reduce time both have an impact on radiographers' perceived temporal workload.
**Conclusion:** These results show that radiographers have high perceived workload. Misalignment between radiographers' willingness to have more time with their patients and the induced time pressures, could lead to an increase in staff turnover. Staff shortage can contribute to higher perceived workload since understaffing will automatically induce more work and may lead to error and negative impacts on patient safety. To decrease perceived physical workload and strain, ergonomic principals must be encouraged through adequate training and work organisation. Departments must take actions that will increase staff in radiological departments to decrease perceived high workload and guarantee quality of care in the future.
**Limitations:** Limitations linked to using self-reported surveys, analysis of likert scales and the understandability of some statements.
**Ethics committee approval:** Approval was obtained through head of departments.
**Funding for this study:** No funding was received. Conducted during a MSc program of the University of Applied Sciences of Western Switzerland.
**Author Disclosures:**
Eija Metsälä Metsälä: Nothing to disclose
Stephanie Elaine de Labouchere: Nothing to disclose

### RPS 1714-5

**Impact of work interruptions on stress level of health care workers and professional quality of life: single institute experience**

*D. Sipos*, A. Miovecz, N. Szalai, A. Farkas, N. Ambrus, G. Bajzik, F. Lakosi, I. Repa; Kaposvár/HU
*(cpt.david.sipos@gmail.com)*

**Purpose:** Interruptions while working may adversely affect the level of stress and professional quality of life of healthcare workers. Our aim was to assess the impact of workplace distractions at our institution.
**Methods or Background:** 61 respondents completed our self-designed and internationally validated Effort Reward Imbalance and Professional Quality of Life Scale questionnaire. We also observed the amount and the reason of daily phone calls. Results were analysed using descriptive statistics, two-sample T-test, ANOVA, Mann Whitney and Kruskal-Wallis tests ($p \le 0.05$).
**Results or Findings:** Male respondents (p=0.026) over 40 years of age (p=0.020) working as a physician (p=0.004) had significantly higher stress values compared to other professions. Coffee and smoking habits did not, but the presence of background noise increased stress levels (p=0.004). The values of Compassion Satisfaction dimension were significantly elevated in the group of women (p=0.004), those over 40 years of age (p=0.008), and those who assist 25 or more phone calls per day (p=0.019). Male gender (p=0.05), administration and physician positions (p=0.030; p=0.001), the presence of background noise (p=0.05) affected significantly negatively on burnout dimension. Regarding compassion fatigue dimension the values of non-smoking workers were found to be elevated (p=0.003).
**Conclusion:** Men over the age of 40 who experience background noise had significantly higher stress values. Profession, gender, age and background noise had significant impact on an individual's professional quality of life.
**Limitations:** Among the limitations of the study belong co-workers who did not fill out our questionnaire.
**Ethics committee approval:** Approval obtained from the Institutional Ethics Committee.
**Funding for this study:** This study has no fundings.
**Author Disclosures:**
Adam Miovecz: Nothing to disclose
Andrea Farkas: Nothing to disclose
Nelli Ambrus: Nothing to disclose
Nora Szalai: Nothing to disclose
Dávid Sipos: Nothing to disclose
Gábor Bajzik: Nothing to disclose
Ferenc Lakosi: Nothing to disclose
Imre Repa: Nothing to disclose

### RPS 1714-6

**The lived experiences of radiographers imaging trauma patients in Gauteng, South Africa**

*S. Wahid*, S. Lewis, Y. Casmod; Johannesburg/ZA
*(shab0703@gmail.com)*

**Purpose:** To explore South African diagnostic radiographers' lived experiences when imaging trauma patients.
**Methods or Background:** Healthcare workers who work with trauma patients experience changes in their psychological functioning. Since diagnostic radiographers image trauma patients as part of their work routine, they may have similar experiences. However, limited studies were found on radiographers' experience imaging trauma patients. Therefore this qualitative, explorative and descriptive phenomenological study explored diagnostic radiographers' lived experiences when imaging trauma patients. Data was collected through one-on-one in-depth interviews from 20 diagnostic radiographers in both the private and public healthcare sectors in Gauteng, South Africa. Detailed notes were taken during the interviews and interviews were audio-recorded. The data was transcribed and underwent thematic analysis. Trustworthiness and ethical principles were adhered to throughout the study.
**Results or Findings:** Participants conceptualised trauma differently, experiencing both positive and negative effects to imaging trauma patients. They also shared their varying degrees of preparedness to image trauma patients and their coping mechanisms.

**Conclusion:** Participants viewed imaging of patients during the COVID-19 pandemic, road accidents, gender-based violence, burns and paediatric injuries to be traumatic. They experienced emotional saturation, desensitisation, detachment and vicarious traumatisation but evidenced post-traumatic growth and professional commitment. They shared the mechanisms they employ to deal with imaging trauma patients.
**Limitations:** In-depth interviews were conducted virtually, and therefore non-verbal communication could not be assessed.
**Ethics committee approval:** Approval received from the University of Johannesburg's Faculty of Health Science's research ethics committee: REC-184-2019.
**Funding for this study:** No funding was received.
**Author Disclosures:**
Shantel Lewis: Author: University of Johannesburg, South Africa
Yasmin Casmod: Author: University of Johannesburg, South Africa
Shabnam Wahid: Author: University of Johannesburg, South Africa

### RPS 1714-7

**Supporting radiographers in clinical education of students**
*R. Young*, M. McEntee, D. Bennett; Cork/IE
*(rena.young@ucc.ie)*

**Purpose:** Optimisation of clinical education in radiography is crucial to ensure competent graduates that provide safe patient care. Radiographers play a key role in student education. This role is multi-faceted, often requiring skills not closely aligned to their primary priority of healthcare service provision. There is a lack of research on clinical training in radiography in Europe. A greater understanding is needed of factors influencing radiographers' ability to effectively undertake clinical education in the context of the challenging "real-life" clinical department. This study aimed to develop our current understanding of how radiographers view student supervision in their clinical setting by undertaking a national study in Ireland to contribute to the evidence base for Europe.
**Methods or Background:** A mixed-methods, anonymous survey was developed in line with best practice and distributed electronically via networks associated with the radiographer community. Descriptive statistics were generated and thematic analysis was conducted on optional free-text comments.
**Results or Findings:** There were 217 respondents, representative of the range of radiographer grades, with 57.1% having greater than 6 years' experience of student supervision. Although the majority reported positive attitudes, a significant minority did not feel adequately trained in the tasks required. Time pressures from clinical workload and perceived lack of organisational support, along with lack of guidance on expectations were challenges highlighted. Additionally, there is a perceived academia-clinical divide to be bridged.
**Conclusion:** Effective clinical supervision is dependent on collaborative engagement and support at all levels including the clinical department, academic and healthcare institutions, and national organisations. The findings highlight implications for educational supports, practice, policy and future research.
**Limitations:** The study was undertaken in the context of one European country (Ireland).
**Ethics committee approval:** Ethical approval was obtained through University College Cork Social Research Ethics Committee.
**Funding for this study:** No funding was received for this study.
**Author Disclosures:**
Deirdre Bennett: Nothing to disclose
Rena Young: Nothing to disclose
Mark McEntee: Nothing to disclose

### RPS 1714-8

**Exploring and standardising research ethics processes for radiography research across Europe**
A. England[1], S. Bockhold[2], J. McNulty[2], L. A. Rainford[2], M. F. F. McEntee[1], C. S. d. Reis[3], N. Mekis[4], H. Precht[5], A. Santos[6], P. Bezzina[7], V. G. Syrgiamiotis[8], E. Abdurakman[9], D. Flinton[10], S. Willis[11], R. Khine[12], C. A. Beardmore[10], J. Woodley[13], N. Drey[10], T. J. T. O'Regan[10], R. Harris[10], *C. Malamateniou*[10]; [1]Cork/IE, [2]Dublin/IE, [3]Lausanne/CH, [4]Ljubljana/SI, [5]Odense/DK, [6]Coimbra/PT, [7]Msida/MT, [8]Athens/GR, [9]Leicester/UK, [10]London/UK, [11]Cambridge/UK, [12]High Wycombe/UK, [13]Bristol/UK
*(christina.malamateniou@city.ac.uk)*

**Purpose:** To evaluate and report the research ethics processes and related challenges for radiographers undertaking research.
**Methods or Background:** An online survey was distributed electronically to radiographers. Convenience sampling was used but respondents were also invited through professional networks. A variety of open- and closed-ended questions were employed, to document the research ethics processes/related challenges within their country. Numerical data was described using descriptive statistics; inferential statistics were used for comparisons/correlations. Qualitative responses were analysed thematically.
**Results or Findings:** 288 questionnaires were received from radiographers involved in research within Europe; 56 were excluded due to incomplete responses. The remaining 232 participants represented 33 (61%) of the European nations. 95.7% (n = 222) of respondents indicated that ethical approval was required prior to commencing research projects and 94.8% (n = 220) felt it was important to have research ethics approval before analysing patient data. Most respondents (69%) noted it was a requirement to report research ethics procedures/reference numbers in research outputs. Respondents indicated some uncertainty around processes for reporting incidental findings, risk assessments and incentives. Seven themes emerged regarding the challenges for research ethics: 1. Onerous and time-consuming processes for approval; 2. Complexity/lack of guidelines/procedures for applications; 3. Lack of training, experience and knowledge of the applicant; 4. Lack of standardisation and consistency of the application process; 5. Difficulties accessing data; 6. Cost of applications; 7. Difficulties adhering to rules/regulations.
**Conclusion:** There is broad alignment with research ethics principles and processes across Europe, likewise, challenges exist which highlight areas for improvement, and these are again broadly similar.
**Limitations:** This was a self-administered survey and was not representative of all European countries. Hence results may not be representative of the wider picture in Europe. Responses may have disproportionately come from clinical/academia with a stronger research culture.
**Ethics committee approval:** This study was approved by City, University of London (ETH1920-0977).
**Funding for this study:** No funding was received for this study.
**Author Disclosures:**
Helle Precht: Nothing to disclose
Ricardo Khine: Nothing to disclose
Sophie Bockhold: Nothing to disclose
Claudia Sa dos Reis: Nothing to disclose
Mark F. F. McEntee: Nothing to disclose
Nejc Mekis: Nothing to disclose
Nicholas Drey: Nothing to disclose
Rachel Harris: Employee: UK SCoR
Louise A. Rainford: Nothing to disclose
Vassilis Georgios Syrgiamiotis: Nothing to disclose
Sophie Willis: Nothing to disclose
Julie Woodley: Nothing to disclose
Jonathan McNulty: Nothing to disclose
Dave Flinton: Nothing to disclose
Edwin Abdurakman: Nothing to disclose
Paul Bezzina: Nothing to disclose
Charlotte A. Beardmore: Nothing to disclose
Andrew England: Board Member: EFRS
Tracy Jane Tracy O'Regan: Employee: UK SCoR
Christina Malamateniou: Nothing to disclose
Adelino Santos: Nothing to disclose

**08:00-09:00** **Open Forum #3 (ESR)**

### Poster Presentation Session

### PP 17

### Imaging genitourinary complications and pathologies

**Moderator**
L. E. Derchi; Genoa/IT

### PP 17-2

**Vascular complications in renal transplant: Doppler ultrasound evaluation and the potential therapeutic role of interventional radiology**
DOI: 10.26044/ecr2022/18838

*T. Cobo Ruiz*, D. Herrán de la Gala, D. Castanedo Vázquez, A. Pérez del Barrio, P. Sanz Bellón, P. Menéndez Fernández-Miranda, A. Fernandez Florez; Santander/ES

**Purpose:** To describe and illustrate the symptoms and imaging appearance using Doppler ultrasound of vascular complications in renal transplant. To review the therapeutic options in case of vascular complications analysing especially the role of interventional radiology.
**Methods or Background:** Kidney transplantation is the treatment of choice for end-stage renal disease, with better quality of life and longer life expectancy demonstrated. Doppler ultrasound is an excellent tool for the evaluation of kidney transplantation not only in the immediate postoperative but also for long-term follow-up. In this poster, we will review the appearance of vascular

Saturday renal transplant complications at imaging, typical presenting symptoms, and treatment options.
**Results or Findings:** Vascular complications occur in less than 10% of renal transplants, but we must be able to recognize them due to the potential kidney loss that they may entail. The main vascular complications in renal transplant are: segmental infarction, renal artery stenosis, external iliac artery stenosis, renal vein stenosis, renal artery thrombosis, renal vein thrombosis, pseudoaneurysm, arteriovenous fistula, compartment syndrome, torsion of the transplanted kidney. The role of endoluminal techniques in the treatment of these complications is constantly increasing. Although early vascular complications, such as renal artery or vein thrombosis usually require urgent surgical treatment, most late vascular complications, such as renal artery stenosis or post-biopsy iatrogenic complications are successfully treated using interventional radiology techniques.
**Conclusion:** Ultrasonography is a useful tool to evaluate anatomical characteristics and vascular Doppler flow in renal transplant, being the initial imaging modality used to evaluate vascular patency and potential complications arising in the renal vasculature, among others. Many vascular renal transplant complications may be potentially treatable if detected early, and the interventional radiologist has an important therapeutic role in these cases.
**Limitations:** No limitations were identified in this study.
**Ethics committee approval:** Not applicable.
**Funding for this study:** No funding was received for this study.
**Author Disclosures:**
Alejandro Fernandez Florez: Nothing to disclose
Amaia Pérez del Barrio: Nothing to disclose
Teresa  Cobo Ruiz: Nothing to disclose
Dario Herrán de la Gala: Nothing to disclose
Pablo Menéndez Fernández-Miranda: Nothing to disclose
Pablo Sanz Bellón : Nothing to disclose
David Castanedo Vázquez : Nothing to disclose

### PP 17-3

**Renal cell carninoma with vascular extension: what to look for**
*R. Alonso González*, S. Morón Hodge, C. Martín Hervás, S. Agudo Fernandez, M. E. Garcia Fernandez, M. Alvarez Maestro; Madrid/ES

**Purpose:** To review TNM and Mayo Clinic classification for intravascular extension of renal cell carcinoma (RCC). To revise radiological findings employed to differentiate bland and tumour thrombus and signs that may indicate complex vascular surgery. To illustrate renal tumours with vascular wall invasion by different cases from our institution.
**Methods or Background:** Intravascular tumour thrombus is seen at presentation in approximately 10% of patients with RCC. In many cases radical nephrectomy with thrombectomy is the only therapeutic option. Sometimes it has to be performed with inferior vena cava resection. These surgeries entail high complexity and require multidisciplinary teams. Radiological evaluation to detect vascular extension is crucial for an adequate surgical planning. Initial imaging may be done with CT. Thrombus level, type of thrombus and ancillary findings that have been associated with vascular wall invasion and complex surgery need to be specified.
**Results or Findings:** We have retrospectively reviewed patients with RCC and vascular extension treated at our institution from 2011-2021. A total number of 18 patients had vascular invasion. Most of them were clear cell carcinomas (15 cases) with Mayo level 1 (7 patients) and Mayo 4 (5 patients). Urography, US, CT and MRI images will be displayed with pathologic correlation. To determine thrombus level, we will revise TNM (8th Ed.) and Mayo Clinic classifications. We will review radiological findings that may aid in the differentiation between bland and tumour thrombus, as well as morphologic and quantitative criteria that associate complex vascular surgery.
**Conclusion:** Thorough radiological evaluation followed by prompt surgery is vital for an adequate management of patients with RCC and vascular invasion. Radiologic evaluation must include thrombus level, type of thrombus and vascular invasion likelihood criteria related with complex vascular surgery.
**Limitations:** No limitations were identified.
**Ethics committee approval:** Not applicable.
**Funding for this study:** No funding was received for this study.
**Author Disclosures:**
Sergio Agudo Fernandez: Nothing to disclose
Carmen Martín Hervás: Nothing to disclose
Rodrigo Alonso González: Nothing to disclose
Sara Morón Hodge: Nothing to disclose
Maria Eugenia Garcia Fernandez: Nothing to disclose
Mario Alvarez Maestro: Nothing to disclose

### PP 17-4

**Initial findings of prostatic artery occlusion (PAO) with Onyx® in a canine model for the management of benign prostatic hyperplasia**
*V. Lucas Cava*[1], F. M. Sánchez-Margallo[1], J. R. Lima-Rodríguez[1], L. Dávila-Gómez[1], V. García-Rodríguez[2], M. Rodríguez-Romero[1], F. Sun[1]; [1]Cáceres/ES, [2]Badajoz/ES

**Purpose:** To evaluate the technical feasibility, effectiveness, and safety of prostatic artery occlusion (PAO) with a liquid embolic agent in a canine model.
**Methods or Background:** Five adult male Beagle dogs (5.00±0.71 years) underwent PAO with Onyx-18. MRI evaluation was performed immediately before, and at 1 week, 2 weeks, 1 month, and 3 months after PAO. Onxy cast and recanalisation of the occluded arteries were documented by CT and angiography immediately after PAO, and at 1 month and 3 months follow-ups. All dogs were closely inspected for potential procedure-related complications. Prostate volume reduction and prostate infarction size were statistically compared.
**Results or Findings:** PAO procedures were performed successfully on both sides in all dogs. Onyx reflux resulting in occlusion of the internal pudendal artery occurred in one dog, and was confirmed by CT. No major PAO-related complications were observed in any dogs during 3 months. Compared with baseline data, MRI study showed a significant prostate shrinkage in all animals, which started at 2 weeks after PAO, with a maximal prostate volume reduction at 1 month, and kept stable until 3 months. Prostate infarction was detected bilaterally in all animals, and then decreased statistically at 2 weeks and 1 month. Recanalisation was observed in 7 and 8 prostate sides, respectively at 1 and 3 months after PAO.
**Conclusion:** PAO with Onyx® is a safe and effective procedure that may induce a significant prostate shrinkage due to the local ischaemia. As an alternative technique, PAO may have potential in clinical practice in the management of symptomatic benign prostatic hyperplasia (BPH).
**Limitations:** The limitations was the small sample size.
**Ethics committee approval:** Institutional Ethic Committee of Animal Experimentation from JUMISC.
**Funding for this study:** Funding was received from: Grants IB18129 and GR 18199 from Consejería de Economía, Ciencia y Agenda Digital, Junta de Extremadura, and FEDER.
**Author Disclosures:**
Fei Sun: Nothing to disclose
Virginio García-Rodríguez: Nothing to disclose
Luis Dávila-Gómez: Nothing to disclose
Vanesa Lucas Cava: Nothing to disclose
Miguel Rodríguez-Romero: Nothing to disclose
Juan Rafael Lima-Rodríguez: Nothing to disclose
Francisco Miguel Sánchez-Margallo: Nothing to disclose

### PP 17-5

**Endometriosis and pelvic innervation: what the abdominal radiologist needs to know**
DOI: 10.26044/ecr2022/12130

*A. P. Bavaresco*, A. P. C. Moura, F. Silva, F. O. Zorzenoni, M. I. Novis; São Paulo/BR

**Purpose:** This study aims to summarise the somatic and visceral neural anatomy in a practical way, to illustrate the main findings of neural involvement by deep pelvic endometriosis.
**Methods or Background:** A survey of images from magnetic resonance imaging (MRI) of patients with endometriosis in the radiology department of our institution over three years, with a literature review.
**Results or Findings:** Although rare, the involvement of the pelvic nerves by deep endometriosis (DE) has high clinical relevance and its early diagnosis and adequate treatment can avoid functional and sensory limitations. The pelvic neural anatomy is divided into somatic and visceral nerves. The main somatic nerves that have an endopelvic course include the lumbosacral plexus, the sciatic, pudendal, and obturator nerves, responsible for innervation of the lower limbs and pelvic floor. Pelvic visceral innervation is performed by the superior hypogastric plexus, hypogastric nerves and inferior hypogastric plexus, responsible for innervating the vagina, cervix, uterus, fallopian tubes, bladder and rectum. DE can affect the nerves by extension of other pelvic lesions or as an isolated finding. MRI findings include infiltrative and retractile tissue, sometimes with haematic content, blood cysts, diffuse neural root thickening, restricted diffusion, signs of denervation with muscle atrophy and, rarely, bone extension with oedema.
**Conclusion:** Diagnosis of neural injuries from DE is a challenge for abdominal radiologists. The recognition of details of pelvic neuroanatomy allows the radiologist to identify or at least suspect neural endometriosis and supply relevant information to the surgeon. Highlighting neural involvement is crucial not only for surgical planning, but also for informing and discussing possible complications with patients.

**Limitations:** Case number.
**Ethics committee approval:** Not registered.
**Funding for this study:** No funding was received for this study.
**Author Disclosures:**
Ana Paula Bavaresco: Nothing to disclose
Ana Paula Carvalhal Moura: Nothing to disclose
Fernando Ometto Zorzenoni: Nothing to disclose
Flavio Silva: Nothing to disclose
Maria Inês Novis: Nothing to disclose

### PP 17-6

**Magnetisation transfer imaging of ovarian cancer correlates with tissue cellularity and can detect microstructural changes following neoadjuvant chemotherapy**

*S. Deen*; Cambridge/UK

**Purpose:** To investigate the relationship between magnetisation transfer (MT) and tissue macromolecules in high-grade serous ovarian cancer (HGSOC) and whether there is a detectable change in magnetisation transfer ratio (MTR) following neoadjuvant chemotherapy (NACT).
**Methods or Background:** In this prospective observational study, twelve HGSOC patients were imaged at 3T before treatment and five of these patients were also imaged after three cycles of NACT. MTR was compared to tissue histology and immunohistochemistry, quantified with semi-automated histology analysis software. Tumour cellularity was used as a marker of intracellular macromolecular concentration and the extracellular proteins collagen IV and laminin were used as markers of extracellular macromolecular concentration. The Shapiro-Wilk test was first used to assess for normality of data. Spearman's rank-order and Pearson's correlation tests were then used to compare MTR with tissue quantifications.
**Results or Findings:** The mean treatment-naïve tumour MTR was 21.9 ± 3.1% (mean ± S.D.). MTR had a positive correlation with cellularity, rho = 0.56 ($P < 0.05$) and a negative correlation with tumour volume, rho = -0.72 ($P = 0.01$). MTR did not correlate with collagen IV or laminin quantification ($P = 0.40$ and $P = 0.90$ respectively). For those patients imaged before and after NAC, an increase in MTR was observed in each case with mean MTR 20.6 ± 3.1% pre-treatment and 25.6 ± 3.4% post-treatment ($P = 0.06$).
**Conclusion:** These results suggest that in treatment-naïve HGSOC, MTR is reflective of cellularity and therefore intracellular macromolecular concentration. MT may also detect the HGSOC response to NACT.
**Limitations:** Small sample of patients, however, the appropriate statistical tests were applied to account for this.
**Ethics committee approval:** This study was approved by the South Cambridge Research Ethics Committee (Reference: 15/EE/0378).
**Funding for this study:** Funding was received for this study by Cancer Research UK (CRUK: C19212/A27150, C19212/A16628), CRUK Cambridge Centre (C9685/A25177) & NIHR Cambridge Biomedical Research Centre.
**Author Disclosures:**
Surrin Deen: Nothing to disclose

### PP 17-8

**Adrenal masses: radiologic-pathologic correlation**

DOI: 10.26044/ecr2022/12789

*A. M. H. Hernández García-Calvo*, C. Sánchez Muñoz, C. Ruiz De Castañeda Zamora, M. J. Risco Fernández, X. Aragón, I. Cifuentes García, M. M. Merideño García, A. Calero Ortega, E. F. Berríos; Toledo/ES

**Purpose:** Describe the main imaging characteristics of adrenal pathology and its histological correlation.
**Methods or Background:** At present, with the growing demand for radiological tests such as computed tomography (CT), there has been a significant increase in the incidental detection of adrenal tumours, with adenomas and metastases being the most frequent pathologies.
The indications for imaging studying the adrenal glands are patients with impaired adrenal function, evaluation of incidentally found masses, and identification of metastatic disease in cancer patients. There is a vast spectrum of adrenal lesions, such as benign and malignant masses, hemorrhage, infections, pseudotumours, and adrenal hyperplasia. With the use of CT, two criteria differentiate benign adenomas from malignant lesions: the intracellular lipid content in the mass and vascular enhancement differences.
**Results or Findings:** We will use a classification based on the main imaging findings to facilitate radiological diagnosis, distinguishing the following groups: small adrenal masses, large solid masses, cystic lesions, and others. We provide CT, ultrasound, and MRI images to describe and illustrate the most common features of common and rare adrenal lesions that may suggest a specific diagnosis. Furthermore, we show the histopathological findings of these adrenal affections to highlight the capacity of histology in the differentiation of lesions indistinguishable by imaging, which is essential for the subsequent therapeutic management of the patient.
**Conclusion:** Radiologists play a fundamental role in the detection and characterisation of adrenal lesions in symptomatic and asymptomatic patients. Ultrasound, CT, and magnetic resonance imaging allow the diagnosis in most cases. Consequently, it is crucial to know the main imaging findings to differentiate benign from malignant masses and guide the differential diagnosis that allows adequate patient therapeutic management.
**Limitations:** No limitations were identified in this study.
**Ethics committee approval:** An ethics committee approval is not required.
**Funding for this study:** No funding is required for this study.
**Author Disclosures:**
Andrea Calero Ortega: Nothing to disclose
Ximena Aragón: Nothing to disclose
María Montaña Merideño García: Nothing to disclose
Ana María Hernández Hernández García-Calvo: Nothing to disclose
César Sánchez Muñoz: Nothing to disclose
Esnelly Francismaría Berríos: Nothing to disclose
Cecilia Ruiz De Castañeda Zamora: Nothing to disclose
Maria José Risco Fernández: Nothing to disclose
Irene Cifuentes García: Nothing to disclose

### PP 17-10

**How to prepare CT scanners from a radiology department for radiomic studies**

*M. Aymerich*[1], M. Riveira[1], M. D. C. Sebastia Cerqueda[2], A. Mesa-Alvarez[3], G. Tardaguila[1], R. Berenguer Serrano[4], S. Sabater Marti[4], A. Lopez-Medina[1], M. M. Otero-Garcia[1]; [1]Vigo/ES, [2]Barcelona/ES, [3]Oviedo/ES, [4]Albacete/ES

**Purpose:** To describe the preparation of five CT scanners to perform radiomic studies, selecting the most stable and robust features for these machines.
**Methods or Background:** Radiomic studies are based on the use of quantitative biomarkers or features for their use in prognosis and diagnosis of several pathologies. However, this field presents a lack of standardisation. There are several initiatives regarding this topic and quality in radiomic research starts with the use of textural phantoms to analyse the response of the features when different protocols or scanners are used. In this study, the most robust features are selected in terms of repeatability and reproducibility for five scanners using a CCR textural phantom, ARIA software for segmentation and registration, and Quibim Precision platform for the extraction of 91 features.
**Results or Findings:** From the test-retest analysis without repositioning, 42.9% of the features were repeatable, fulfilling that ICC>0.9 and wCV<1% for the 80% of the scanners. In the intra-CT study, 38.5% of the features satisfied the reproducibility criteria based on CCC>0.9 and wCV<10%. In the inter-CT comparison, 45.1% of the features were reproducible across the scanners. Finally, 25.3% (23/91) features were considered as both repeatable and reproducible biomarkers, suitable for radiomic studies.
**Conclusion:** Repeatability and reproducibility studies with textural phantoms are the first step for quality radiomics research, assessing the most robust features and helping in high-dimensionality reduction of variables for further model training.
**Limitations:** Filtering conditions were chosen to obtain a similar amount of features in each selection. Different conditions would lead to other datasets. Moreover, shape features were not analysed since volumes of interest were imposed.
**Ethics committee approval:** This study received the approval of the Galician Local Ethics Committee (2019/230).
**Funding for this study:** This work was not funded and had no commercial interests.
**Author Disclosures:**
Sebastia Sabater Marti: Nothing to disclose
Mercedes Riveira: Nothing to disclose
Gonzalo Tardaguila: Nothing to disclose
Roberto Berenguer Serrano: Nothing to disclose
Maria Aymerich: Nothing to disclose
Milagros M. Otero-Garcia: Nothing to disclose
Maria Del Carmen Sebastia Cerqueda: Nothing to disclose
Antonio Lopez-Medina: Nothing to disclose
Alicia Mesa-Alvarez: Nothing to disclose

**08:00-09:00** **Open Forum #4 (ESR)**

### Research Presentation Session: Imaging Informatics / Artificial Intelligence and Machine Learning

### RPS 1705a

### Evaluation of artificial intelligence (AI) systems

**Moderator**
A. Alberich-Bayarri; Valencia/ES
**Author Disclosure:**
A. Alberich-Bayarri: Board Member: QUIBIM SL; CEO: QUIBIM SL; Share holder: QUIBIM SL

### RPS 1705a-3

**Retrospective evaluation of an AI based software tool for chest x-ray quality assurance purposes on Elisabeth-TweeSteden Hospital study sample**

*N. Ramanauskas*[1], E. R. Ranschaert[2], N. Bielskienė[1], D. Barušauskas[1]; [1]Vilnius/LT, [2]Ghent/BE
*(naglisr@gmail.com)*

**Purpose:** To evaluate the performance of Oxipit “ChestEye Quality” tool for quality assurance purposes in chest x-ray reporting on Elisabeth-TweeSteden Hospital (Tilburg, the Netherlands) retrospective data sample.
**Methods or Background:** A retrospective sample of anonymised chest x-ray studies from the time period of 10.02.2021-15.09.2021 (n = 15655) was selected. The studies were processed by the solution which identified the cases with the most potential for a missed finding based on the output of the solution and the results of the automatic evaluation of the radiologists final report using a custom made chest x-ray report natural language processing tool. A radiologist has evaluated the cases flagged by the software to determine if there is a clinically significant radiological finding detectable in the image which was not appropriately described in the radiologist report. The final performance metrics were evaluated to quantify the number of cases with significant radiological findings missed in the radiologists report and flagged by the solution.
**Results or Findings:** A total of 14 studies (0,0894 %) were evaluated as containing clinically significant radiological findings detectable in the image on retrospective review which were not appropriately described in the final radiologist report. 8 studies contained nodular opacities and 6 studies contained an area of consolidation.
**Conclusion:** The results indicate that AI based software used for quality assurance purposes is able to detect studies where a clinically significant radiological finding is not appropriately described by the reporting radiologist. In a prospective setting this could help to prevent a significant number of radiological errors.
**Limitations:** This was a retrospective study.
**Ethics committee approval:** In progress.
**Funding for this study:** No funding was received for this study.
**Author Disclosures:**
Naglis Ramanauskas: Founder: Oxipit
Neringa Bielskienė: Employee: Oxipit
Erik R. Ranschaert: Advisory Board: Oxipit Other: Visiting professor at Ghent University Other: Radiologist at ETZ hospital (Tilburg)
Darius Barušauskas: Founder: Oxipit

### RPS 1705a-4

**Retrospective evaluation of an AI based software tool for chest x-ray quality assurance purposes on Vilnius University Santaros clinics (Vilnius, Lithuania) study sample**

*N. Ramanauskas*, J. Razanskas, J. Stankeviciene, J. Dementaviciene, N. Bielskienė, D. Barušauskas, J. Bialopetravičius; Vilnius/LT
*(naglisr@gmail.com)*

**Purpose:** To evaluate the performance of Oxipit “ChestEye Quality” tool for quality assurance purposes in chest x-ray reporting on Vilnius University Santaros clinics retrospective data sample.
**Methods or Background:** A retrospective sample of anonymised chest x-ray studies from the time period of 15.02.2020-01.08.2020 (n = 52818) was selected. The studies were processed by the solution which identified the cases with the most potential for a missed finding based on the output of the solution and the results of the automatic evaluation of the radiologists final report using a custom made chest x-ray report natural language processing tool. A radiologist has evaluated the cases flagged by the software to determine if there is a clinically significant radiological finding detectable in the image, which was not appropriately described in the radiologist report. The final performance metrics were evaluated to quantify the number of cases with significant radiological findings missed in the radiologists report and flagged by the solution.
**Results or Findings:** A total of 42 studies (0,795 %) were evaluated as containing clinically significant radiological findings detectable in the image on retrospective review which were not appropriately described in the final radiologist report. 10 studies contained nodular opacities, 13 studies contained an area of consolidation, 19 studies contained a pneumothorax, 5 studies contained malposition of central venous line.
**Conclusion:** The results indicate that AI based software used for quality assurance purposes is able to detect studies where a clinically significant radiological finding is not appropriately described by the reporting radiologist. In a prospective setting this could help to prevent a significant number of radiological errors.
**Limitations:** This was a retrospective study.
**Ethics committee approval:** In progress.
**Funding for this study:** No funding was received for this study.
**Author Disclosures:**
Jolanta Stankeviciene: Nothing to disclose
Naglis Ramanauskas: Founder: Oxipit
Neringa Bielskienė: Employee: Oxipit
Jurate Dementaviciene: Nothing to disclose
Jonas Bialopetravičius: Founder: Oxipit
Darius Barušauskas: Founder: Oxipit
Jonas Razanskas: Employee: Oxipit

### RPS 1705a-5

**Incorrect application of feature selection when using cross-validation can lead to bias**

A. Demircioglu, *D. Bos*; Essen/DE

**Purpose:** Radiomics often deal with high-dimensional datasets. Since often external cohorts are not available, cross-validation is applied to obtain an estimation on the generalisability of the models. Unfortunately, if feature selection is applied incorrectly before the cross-validation, it can result in data leakage and thus to biased results. An experiment is conducted to measure the extend of this bias.
**Methods or Background:** Ten publicly available datasets were used to apply feature selection incorrectly before a 10-fold cross-validation on the one hand, and to apply it correctly inside the cross-validation. Models were evaluated using AUC-ROC, as well as accuracy.
**Results or Findings:** Incorrect feature selection before cross-validation led to biases of up to 0.15 in AUC-ROC and 0.17 in accuracy.
**Conclusion:** Highly biased results can be trained if feature selection and cross-validation are incorrectly applied.
**Limitations:** No external cohort were available for further validating the resulting bias, although it can be expected that results of the cross-validation generalised to external cohorts.
**Ethics committee approval:** All data used in this study were previously published, therefore ethical approval was waived by the local Ethics Committee.
**Funding for this study:** No funding was received for this study.
**Author Disclosures:**
Denise Bos: Nothing to disclose
Aydin Demircioglu: Nothing to disclose

### RPS 1705a-6

**Selective on-site retraining for efficient performance drop compensation due to domain shift**

*O. Hertgers*[1], K. Hergaarden[1], A. Efitorov[2], J. V. Stadelmann[2], D. Mavroeidis[3], A. Saalbach[4], S. Renisch[4], H. Schulz[4], H. J. Lamb[1]; [1]Leiden/NL, [2]Moscow/RU, [3]Eindhoven/NL, [4]Hamburg/DE
*(ohertgers@gmail.com)*

**Purpose:** Compensating the performance drop of AI models due to deployment in clinical workflows different from the training environment by using dedicated ensembling technique and re-training parts of the model with a limited number of images.
**Methods or Background:** We trained Convolutional Neural Networks (CNN) for the classification of chest x-rays with respect to occurrence of pneumothorax on ChestXray-14, MIMIC-CXR and CheXpert datasets. Variation of architecture and hyperparameters yielded a total of nine pretrained CNNs, which were ensembled by a single fully connected layer trained on the mentioned datasets. By minimising this last layer’s number of parameters, we enabled retraining on a small number of images. For performance evaluation, 542 chest x-rays from clinical routine from our hospital were annotated by radiologists and split into a training set (118 positive/316 negative pneumothorax) and a test set (30 positive/78 negative). After establishing the baseline performance on the test set, the model was retrained on the training set, and performance was reevaluated on the test set.

**Results or Findings:** We obtained an AUC of 0.868 on the MIMIC-CXR data. The performance dropped to 0.542 when applying the model directly on the clinical data. By selective retraining the performance increased to an AUC of 0.915. The difference between the original and retrained AUC values stems presumably from different samplings of the overall case distribution.
**Conclusion:** We demonstrated a time-efficient workflow to adapt laboratory trained AI models to the clinical data distribution they will be used with. This is done by retraining a small ensembling layer using limited dataset to counteract the drop in performance that occurs, thus making the AI predictions more reliable and clinically usable.
**Limitations:** Single-site validation.
**Ethics committee approval:** A waiver of consent was obtained from the local ethics committee.
**Funding for this study:** No funding was received for this study.
**Author Disclosures:**
Heinrich Schulz: Employee: Philips N.V.
Omar Hertgers: Nothing to disclose
Steffen Renisch: Employee: Philips N.V.
Axel Saalbach: Employee: Philips N.V.
K.F.M. Hergaarden: Nothing to disclose
Aleksandr Efitorov: Employee: Philips N.V.
Joël Valentin Stadelmann: Employee: Philips N.V.
Dimitrios Mavroeidis: Employee: Philips N.V.
Hildo J. Lamb: Nothing to disclose

### RPS 1705a-7

**Stand-alone detection performance of two commercial deep learning algorithms in a UK breast screening workflow**
*S. Hickman*, Y. Huang, N. Payne, R. Black, A. N. Priest, F. J. Gilbert; Cambridge/UK
*(sh2040@cam.ac.uk)*

**Purpose:** To evaluate the performance of deep learning (DL) algorithms in a triennial breast screening programme.
**Methods or Background:** Two DL algorithms (DL-1&DL-2) were tested retrospectively on full-field digital mammograms of 50-70-year-olds from a UK National Health Service screening centre and compared with reader performance. Ground truth for normals was negative three-yearly mammogram, and histopathology for cancer cases. Thresholds were pre-specified at UK consensus reader performance of 96% specificity.
**Results or Findings:** In 2017 18,831 women were screened of which 10,052 cases were included with either a normal subsequent examination (Covid19 delayed screening round), screen-detected cancer diagnosis 138 (7.3/1000) or interval cancers diagnosis 71 (3.8/1000). At consensus reader specificity of 96.1% sensitivity was 68.4%, 59.3% (51.7-66.0), 58.9% (51.7-65.6) for consensus reading, DL-1, DL-2 respectively. Reader 1 achieved 95.5% and 63.6% specificity and sensitivity, respectively. The AUC for DL-1 and DL-2 was 0.875 and 0.874 and APRC was 0.454, 0.487 respectively. DL-1 and DL-2 picked up 13.0% and 22.2% next round cancers as well as 18.3% and 19.7% interval cancers respectively, at the assigned threshold. With the threshold at 90% specificity the respective sensitivities for DL-1 and DL-2 were 70.8% and 71.3%. The proportion of next round cancers detected were 33.3%, and 31.5%, and interval cancers were 33.8%, and 43.7%, for DL-1 and DL-2 respectively.
**Conclusion:** At 96.1% specificity threshold the systems show similar performance, but inferior to consensus and first reader performance. Stand-alone AI could be used at 90% threshold with second reader arbitrating to 95% specificity. Benchmarking performance with screening cohorts establishes appropriate thresholds before prospective testing.
**Limitations:** One screening site and majority of cases from one manufacture.
**Ethics committee approval:** Approvals obtained: HRA-REC 20/LO/0104HRA, HRA-CAG 20/CAG/0009, PHE-RAC BSPRAC_090.
**Funding for this study:** Funding was received from the CRUK grant - C543/A26884 and the NIHR Cambridge Biomedical Research Centre.
**Author Disclosures:**
Fiona J. Gilbert: Other: F.J.G receives research support from Hologic, GE Healthcare, Bayer; F.J.G has research collaborations with Vara, ScreenPoint, Lunit, Volpara; F.J.G undertakes consultancy for DeepMind/Alphabet Inc. and Kheiron
Nicholas Payne: Nothing to disclose
Sarah Hickman: Other: S.E.H has research collaborations with Vara, ScreenPoint, Volpara, Lunit
Richard Black: Nothing to disclose
Andrew N Priest: Nothing to disclose
Yuan Huang: Nothing to disclose

### RPS 1705a-8

**Comparative testing of three artificial intelligence algorithms interval cancer detection performance and the importance of application context in evaluating such algorithms for mammographic screening**
*S. Hickman*, N. Payne, R. Black, Y. Huang, A. Priest, F. J. Gilbert; Cambridge/UK

**Purpose:** To evaluate the performance of three different Deep Learning (DL) algorithms, designed for various automated mammography screen reading tasks, applied for interval cancer (IC) detection.
**Methods or Background:** DL algorithms were independently tested on two-view digital screening mammograms collected from 2011-2020 at one UK National Health Service Breast Screening Programme site on predominantly (>90%) Philips machines. The dataset consisted of ICs with a histopathological diagnosis as well as normal age and year matched controls at a ratio of 1:3. Pre-specified thresholds were used to evaluate performance of algorithms designed for different screening tasks. The thresholds were European consensus (98%), UK consensus (96%), and adapted reading specificity (90%) and expected IC detection sensitivity (30%). An application-specific threshold for each algorithm was also applied.
**Results or Findings:** 8308 images containing 528 IC cases (25.42%) were used for testing all three DL algorithms and results are listed in a consistent order. At 98%, specificity, sensitivity was 15.91%, 11.74%, 15.53%; at 96% it was 23.49%, 19.51%, 23.86%; and at 90% it was 37.50%, 31.44%, 35.80%. At 30% sensitivity, specificity was 93.87%, 90.77%, 92.45%. The area under the receiver operating characteristic curve (AUC) was 0.72, 0.71, 0.71. At the pre-specified company threshold, the specificity and sensitivity were 75.86% and 57.77%, 98.52% and 9.66%, 60.17% and 69.32%. Combined AUC performance was 0.746.
**Conclusion:** DL algorithms could play a role in the earlier detection of cancers. How algorithms are designed for specific screening tasks should be considered when determining thresholds for use and their application. In addition, this study demonstrates how a combination of algorithms could improve performance.
**Limitations:** Small study cohort without the class-imbalance of routine screening from one screening site and predominantly one vendor.
**Ethics committee approval:** Ethics committee approval was obtained: HRA-REC ref.20/LO/0104; HRA-CAG ref.20/CAG/0009; PHE-RAC ref.BSPRAC_090.
**Funding for this study:** Funding was received from: CRUK:C543/A26884; NIHR-Cambridge BRC-1215-20014.
**Author Disclosures:**
Andrew Priest: Nothing to disclose
Yuan Huang: Nothing to disclose
Fiona J. Gilbert: Research/Grant Support: Hologic, GE Healthcare, Bayer, Vara, ScreenPoint, Volpara, and Lunit Consultant: DeepMind/Alphabet Inc. and Kheiron
Sarah Hickman: Research/Grant Support: Vara, ScreenPoint, Volpara, and Lunit
Nicholas Payne: Nothing to disclose
Richard Black: Nothing to disclose

**08:00-09:00** **Room D**

### Research Presentation Session: Oncologic Imaging

### RPS 1716

### Pancreatic malignancies

**Moderator**
A. Zaheer; Baltimore, MD/US

### RPS 1716-2

**Missed and misinterpreted findings of pancreatic cancer on MRI: prevalence of early features/findings**
S. Hoogenboom[1], M. M. L. Engels[2], J. van Hooft[3], M. Wallace[2], C. Bolan[2], J. Legout[2], *A. Chuprin*[2]; [1]Amsterdam/NL, [2]Jacksonville, FL/US, [3]Leiden/NL

**Purpose:** To highlight the prevalence of findings associated with pre-diagnostic pancreatic cancer on MRI.
**Methods or Background:** As part of a single-centre, retrospective, case-control study of patients diagnosed with pancreatic ductal adenocarcinoma (PDAC) between 2010-2016, a cohort of 27 patients who had an MRI within 3 years prior to diagnosis were matched with 103 healthy controls for age, gender, modality, contrast use, and date of imaging. Two board-certified radiologists independently interpreted each imaging exam, blinded to case/control status. The prevalence of missed PDAC and related features were evaluated.
**Results or Findings:** A focal pancreatic mass was suspected in 63% of cases by the two radiologists, with an increased detection rate closer to eventual diagnosis. The interobserver agreement was substantial (kappa 0.66). MRI-findings, including disruption or dilatation (>3 mm) of the pancreatic duct, had 100% specificity for pre-diagnostic PDAC and similar sensitivities among the reviewers, between 41% to 52% (p<0.0001). Focal pancreatic atrophy was seen in 35% of cases and none of the controls. Other findings significantly associated with pre-diagnostic PDAC included diffuse pancreatic atrophy and the presence of perivascular soft tissue. A completely normal pancreas was seen in 5.6% of cases and 59.2% of controls.
**Conclusion:** In patients who underwent an MRI up to 3 years before diagnosis of PDAC, a suspected mass was found in as many as 63% on secondary review by radiologists, an astonishing statistic. In pursuit of detecting PDAC at a more curable stage, this study suggests paying extra attention to highly specific findings.
**Limitations:** Retrospective design and selection bias.
**Ethics committee approval:** This study was approved by the Mayo Clinic IRB approval #18-002403.
**Funding for this study:** Funding was received from the Champions for Hope, Funk-Zitiello Foundation.
**Author Disclosures:**
Sanne Hoogenboom: Nothing to disclose
Jeanin van Hooft: Nothing to disclose
Anthony Chuprin: Nothing to disclose
Candice Bolan: Nothing to disclose
Jordan Legout: Nothing to disclose
Megan Maria Lynn Engels: Nothing to disclose
Michael Wallace: Nothing to disclose

### RPS 1716-3

### 18F-FDG PET/MRI enables early chemotherapy response prediction in pancreatic ductal adenocarcinoma

F. N. Harder, G. Kaissis, *F. Jungmann*, F. Lohöfer, S. Ziegelmayer, M. Schwaiger, M. Makowski, M. Eiber, R. Braren; Munich/DE

**Purpose:** Pancreatic ductal adenocarcinoma (PDAC) is characterised by a low response rate to chemotherapy and an overall poor prognosis. In this prospective exploratory study, we evaluated the feasibility of [18F]fluorodeoxyglucose ([18F]FDG) PET/MRI-based early chemotherapy response prediction in PDAC at two weeks upon therapy onset.
**Methods or Background:** In a mixed cohort, seventeen patients treated with chemotherapy (FOLFIRINOX- or Gemcitabine-based) in neoadjuvant or palliative intent were enrolled. All patients were imaged by [18F]FDG PET/MRI before and two weeks after onset of chemotherapy. Response per RECIST1.1 was then assessed at 3 months. [18F]FDG PET/MRI-derived parameters (MTV50%, TLG50%, MTV2.5, TLG2.5, SUVmax, SUVpeak, ADCmax, ADCmean and ADCmin) were assessed, using multiple t-test, Man-Whitney-U test and Fisher's exact test for binary features.
**Results or Findings:** At 72±43 days, twelve patients were classified as responders and five patients as non-responders. An increase in ΔMTV50% and ΔADCmean (≥ 20% and 15%, respectively) and a decrease in ΔTLG50% (≤ 20%) at 2 weeks after chemotherapy onset enabled excellent prediction and discrimination of responders and non-responders, respectively. Parameter combinations (ΔTLG50% and ΔADCmax or ΔMTV50% and ΔADCmax) further improved discrimination (ROC-AUC = 0.96-1.00; sensitivity = 0.92-1.00; specificity = 1.00).
**Conclusion:** Multiparametric [18F]FDG PET/MRI-derived parameters, in particular indicators of a change in tumour glycolysis and cellularity, may enable very early chemotherapy response prediction in PDAC.
**Limitations:** Small sample size; single institution study; RECIST limitations.
**Ethics committee approval:** This study was approved by an ethics committee: Protocol Nr. 181 17S.
**Funding for this study:** Funding was received from: German Research Foundation (DFG) SFB824, Project C6; German Cancer Consortium (DKTK); Technical University of Munich.
**Author Disclosures:**
Rickmer Braren: Nothing to disclose
Sebastian Ziegelmayer: Nothing to disclose
Matthias Eiber: Nothing to disclose
Friederike Jungmann: Nothing to disclose
Marcus Makowski: Nothing to disclose
Felix N. Harder: Nothing to disclose
Georgios Kaissis: Nothing to disclose
Fabian Lohöfer: Nothing to disclose
Markus Schwaiger: Nothing to disclose

### RPS 1716-5

### Prediction of greater pancreatic neuroendocrine tumour aggressiveness by increased stiffness

*E. Gültekin*, C. Wetz, J. Braun, D. Geisel, C. Furth, B. Hamm, I. Sack, S. R. Marticorena Garcia; Berlin/DE

**Purpose:** To evaluate the diagnostic performance of multifrequency MR-Elastography (MRE) in distinguishing pancreatic neuroendocrine tumours (PNETs) from healthy pancreatic tissue and to assess the prediction of tumour aggressiveness by correlating PNET stiffness with PET derived asphericity.
**Methods or Background:** 13 patients with PNET were prospectively compared to 13 age-/sex-matched heathy volunteers. Multifrequency MRE with tomoelastography-postprocessing provided high-resolution maps of shear wave speed (SWS in m/s). SWS of pancreatic neuroendocrine tumour (PNET-T) were compared with nontumourous pancreatic tissue in PNET patients (PNET-NT) and heathy pancreatic tissue (CTR). Diagnostic performance of MRE was evaluated by ROC-AUC analysis. PNET-SWS correlations were calculated with Pearson's r.
**Results or Findings:** SWS was higher in PNET-T (2.02±0.61 m/s) compared to PNET-NT (1.31±0.18 m/s; p<0.01) and CTR (1.26±0.09 m/s; p<0.01). An SWS-cutoff of 1.46 m/s distinguished PNET-T from PNET-NT (AUC=0.89; sensitivity=0.85; specificity=0.92) and a cutoff of 1.49 m/s differentiated CTR from PNET-T (AUC=0.96; sensitivity=0.92; specificity=1.00). SWS of PNET-T was positively correlated with PET derived asphericity (r=0.81; p=0.01).
**Conclusion:** Multifrequency MRE provides quantitative imaging markers for the detection of PNET and the prediction of greater tumour aggressiveness by increased stiffness.
**Limitations:** A small sample size without further analysis of PNET subgroups was investigated. Furthermore, for ethical reasons, no prospective tumour biopsies were performed directly before MRE. Instead, diagnoses were confirmed by routine clinical histopathology and PET/CT and PET/MRI.
**Ethics committee approval:** The study was conducted according to the guidelines of the Declaration of Helsinki, and approved by the local Ethics Committee of Charité, Universitätsmedizin Berlin.
**Funding for this study:** The authors gratefully acknowledge support from the German Research Foundation (SFB 1340 to B.H., I.S., J.B., and S.R.M.G.; BIOQIC GRK 2260 to I.S.; project number 467843609 to S.R.M.G.).
**Author Disclosures:**
Jürgen Braun: Nothing to disclose
Christian Furth: Nothing to disclose
Bernd Hamm: Nothing to disclose
Ingolf Sack: Nothing to disclose
Christoph Wetz: Nothing to disclose
Stephan Rodrigo Marticorena Garcia: Nothing to disclose
Dominik Geisel: Nothing to disclose
Emin Gültekin: Nothing to disclose

### RPS 1716-6

### Detailed MRI subtyping of cystic morphology in serous and mucinous cystic neoplasms of the pancreas: a retrospective evaluation

*F. M. Kubicka*[1], Q. Tan[1], P. Jurmeister[2], B. Hamm[1], M. M. Wagner[1]; [1]Berlin/DE, [2]Munich/DE

**Purpose:** Current radiology guidelines for cystic pancreatic neoplasms differentiate between four main morphologic types (unilocular, microcystic, macrocystic, cyst with solid component). We evaluated a more detailed MRI subtyping of the cystic morphology in patients with serous cystic neoplasms (SCN) and mucinous cystic neoplasms (MCN).
**Methods or Background:** Our retrospective single-centre study included MR examinations of pathologically confirmed SCN (n = 45) and MCN (n = 21) within the years 2005-2020. We designed an MRI reading template with graphic illustration of seven subtypes of cystic patterns (micro, macro, micro-macro, honeycomb, micro-honeycomb, macro-honeycomb, micro-macro-honeycomb) and three additional features (septal centralisation, scar, cyst-in-cyst sign). Interobserver variability and diagnostic performance was evaluated by three independent readers.
**Results or Findings:** Based on the current guidelines, the majority of SCN were microcystic (28/45; 62%) and the majority of MCNs macrocystic (13/21; 62%). Several SCN, however, were also classified as macrocystic (16/45; 36%) and therefore potentially suspicious of MCN. Using the more detailed subtyping, 11/16 (69%) of these SCN showed at least one of the following imaging findings: honeycomb-components, centralisation, scar. These findings were not detected in MCN and therefore had a high negative predictive value for MCN (100%). In contrast, cyst-in-cyst was only found in MCN (5/21; 24%). Interobserver agreement was excellent for honeycomb components (kappa = 0.86), good for centralisation (kappa = 0.66) and cyst-in-cyst sign (kappa = 0.70) and moderate for scar (kappa = 0.57).
**Conclusion:** Detailed MRI subtyping allows for improved differentiation between SCN and MCN, especially in SCN with macrocystic components.
**Limitations:** Limitations of the study include the retrospective patient selection and the single-centre design.

**Ethics committee approval:** The institutional ethics committee approved this study.
**Funding for this study:** Funded by the Stiftung Charité.
**Author Disclosures:**
Philipp Jurmeister: Nothing to disclose
Bernd Hamm: Nothing to disclose
Moritz Moritz Wagner: Nothing to disclose
Qinxuan Tan: Nothing to disclose
Felix Maximilian Kubicka: Nothing to disclose

### RPS 1716-8

**Prognostic effect of sarcopenia and myosteatosis in patients with resectable pancreatic ductal adenocarcinoma**

*H. Ahn*, D. W. Kim, K. W. Kim; Seoul/KR
*(ypc99093@naver.com)*

**Purpose:** Pancreatic ductal adenocarcinoma (PDAC) is a dismal disease with high muscle loss rate. We aimed to investigate the prognostic effect of sarcopenia and myosteatosis in patients with resectable PDAC, by using muscle quality map on preoperative computed tomography (CT).
**Methods or Background:** This study included 347 patients with resectable PDAC, underwent successful upfront surgery. Preoperative CT-based muscle quality map was generated to measure skeletal muscle area (SMA), further classified into normal attenuating muscle area (NAMA) and low attenuating muscle area. Skeletal muscle index (SMI) was used as an sarcopenia index. As there is no widely used index of myosteatosis on muscle quality map, we evaluated optimal index of myosteatosis and diagnostic cutoffs. Survival analysis was carried out using univariate and multivariate Cox regression according to muscle type; normal muscle type (nMT), sarcopenic (sMT), myosteatotic (mMT), combined (cMT).
**Results or Findings:** NAMA/SMA had the lowest correlation with SMI (Pearson's r = 0.32), and determined as the optimal index of myosteatosis. Either having sarcopenia or myosteatosis is prognostic for poor overall survival (OS), even adjusted by clinicopathologic factors (hazard ratio [HR] compared with nMT: sMT=1.58 [95% CI, 1.05/2.38], mMT=1.50 [95% CI, 1.00/2.25], and cMT=1.00 [95% CI, 1.12/2.46]). Regarding recurrence free survival (RFS), preoperative sarcopenia and myosteatosis found to be associated in the univariable analysis, albeit only the mMT showed significantly higher tumour recurrence compared with nMT in the multivariable analysis (HR, 1.49 [95% CI, 1.01/2.20]).
**Conclusion:** Presence of sarcopenia or myosteatosis is associated with poor OS and RFS after upfront surgery in patients with resectable PDAC.
**Limitations:** We measured muscle parameters in a single timepoint on preoperative CT. Longitudinal change of muscle after surgery needs to be further investigated.
**Ethics committee approval:** Approved by the Institutional Review Board of Asan Medical Center.
**Funding for this study:** Not applicable.
**Author Disclosures:**
Hyemin Ahn: Nothing to disclose
Dong Wook Kim: Nothing to disclose
Kyung Won Kim: Nothing to disclose

**08:00-09:00** **Room E1**

### Research Presentation Session: Chest

### RPS 1704

### Airways, emphysema and lung cysts

**Moderator**
M. Simic; Zagreb/HR

### RPS 1704-2

**Detection of progressive airway disease on chest computed tomography in a phase III cystic fibrosis study cohort using a fully automated method to analyse airway and artery dimensions**

*Q. Lv*[1], L. Gallardo Estrella[2], E-R. Andrinopoulou[1], P. Ciet[1], J-P. Charbonnier[2], M. Kemner van de Corput[1], M. De Bruijne[1], H. A. W. M. Tiddens[1]; [1]Rotterdam/NL, [2]Nijmegen/NL
*(l.qianting@erasmusmc.nl)*

**Purpose:** Cystic Fibrosis (CF) lung disease is characterised by progressive airway wall thickening (Awt) and bronchiectasis on chest computed tomography (CT). A fully automated airway-artery (AA) method was validated for the detection and monitoring of Awt and bronchiectasis on chest-CT. We hypothesised the AA-method is sensitive to detect progression of Awt and bronchiectasis in CF.
**Methods or Background:** LungQ-AA software (v2.3.1rc, Thirona B.V., The Netherlands) fully automatically identifies the airway tree, matching arteries, and airway generation (G) for each AA-pair. For each AA-pair outer airway wall diameter (Aout), Awt and paired artery diameter (A) are measured and Aout/A and Awt/A are calculated and presented from segmental bronchi (G1) up to the last visible airways generation (G20). LungQ-AA %Bronchiectasis is defined as %AA-pairs with Aout/A>1.5 and correlated to PRAGMA-CF[1] %Bronchiectasis for validation. We analysed CTs of 190 CF patients who participated in the phase III Ataluren study[1]. Mixed-effects models were used to investigate differences in Aout/A and Awt/A over 48 weeks in each generation adjusted for gender, age, weight, and height.
**Results or Findings:** From G1 to G20, 104,965 AA-pairs (Mean±SD: 317±147) were identified on 332 CT scans of 190 patients: baseline 45,828 AA-pairs (156 patients) and follow-up 59,137 AA-pairs (176 patients). Over 48 weeks, Aout/A and Awt/A increased (all p<0.0001). The Aout/A progression was significant for G1-G6 (adjusted p<0.001) and for Awt/A for G3, G4, G6 (adjusted p<0.005). LungQ-AA %Bronchiectasis correlated to PRAGMA-CF %Bronchiectasis (r =0.49, p<0.001).
**Conclusion:** The automated AA-method successfully detected and measured airway dimensions of a large number of AA-pairs on CTs of CF patients and showed progression of airway wall thickening and bronchiectasis over 48 weeks in a large phase III clinical trial.
**Limitations:** Reference
[1] Tiddens, 2020, PLoS One.
**Ethics committee approval:** Mixed case.
**Funding for this study:** Funding was received from the PPP grant.
**Author Disclosures:**
Jean-Paul Charbonnier: Shareholder: JPC: is Head of product development and shareholder of Thirona
Eleni-Rosalina Andrinopoulou: Nothing to disclose
Pierluigi Ciet: Nothing to disclose
Qianting Lv: Nothing to disclose
Mariëtte Kemner van de Corput: Nothing to disclose
Leticia Gallardo Estrella: Employee: LGE is scientist working at Thirona
Harm A W M Tiddens: Consultant: HT: Director ErasmusMC Lunganalysis, Consultant for Novartis, Insmed, Thirona, Translate Bio
Marleen De Bruijne: Nothing to disclose

### RPS 1704-3

**Magnetic resonance imaging detects improvement in upper and lower airway abnormalities in adults with cystic fibrosis treated with novel CFTR-modulator therapy**

*L. Wucherpfennig*, M. O. Wielpütz, S. Triphan, S. Wege, H-U. Kauczor, C. P. Heussel, M. Eichinger; Heidelberg/DE
*(lena.wucherpfennig@med.uni-heidelberg.de)*

**Purpose:** Previous studies showed that novel CFTR modulator therapy (CFTRm) significantly improved lung function in patients with cystic fibrosis (CF). However, the effects of CFTRm on lung disease as well as on chronic rhinosinusitis (CRS) depicted by magnetic resonance imaging (MRI) are little understood.
**Methods or Background:** 52 adults with CF (31±9y, range 19-55y) underwent chest MRI twice, before (MRI1) and after (MRI2) at least one month of Kaftrio therapy (n=19), CFTRm other than Kaftrio (n=8) and without CFTRm (controls, n=25; time difference between MRI1 and MRI2: 34±18month). 25 patients also underwent MRI of the paranasal sinuses twice before and after Kaftrio (n=9), other CFTRm (n=5) and without CFTRm (n=11). MRI scans were visually assessed in consensus by two readers using the validated CF-MRI and CRS-MRI scoring systems. Forced expiratory volume in 1s percent predicted (FEV1%) was measured by spirometry in conjunction with MRI.
**Results or Findings:** The CF-MRI score decreased significantly from MRI1 to MRI2 only in patients treated with Kaftrio (-11.4±4.6, p<0.001), mainly due to reduction of mucus plugging (-5.2±1.5, p<0.001) and bronchiectasis/wall thickening (-3.3±2.2, p<0.001), whereas perfusion score did not differ (-0.4±1.7, p=0.298). The CRS-MRI score also decreased only in patients with Kaftrio from MRI1 to MRI2 (-6.9±3.0, p=0.001), mainly due to a reduction of mucopyoceles (maxillary sinus: -0.9±1.0, p=0.001). In patients treated with Kaftrio, the decrease in the CF-MRI score correlated with an increase in FEV1% (r=-0.547, p<0.001).
**Conclusion:** MRI detected beneficial effects of Kaftrio on structural lung disease, and improvements were accompanied by an increase in FEV1%. Further, we could demonstrate improvements of chronic rhinosinusitis after Kaftrio therapy.
**Limitations:** As a limitation our study was performed unicentrically and children were not included.
**Ethics committee approval:** This retrospective study was approved by the institutional ethics committee (S-646/2016).
**Funding for this study:** No funding was received for this study.

**Author Disclosures:**
Mark O. Wielpütz: Nothing to disclose
Lena Wucherpfennig: Nothing to disclose
Claus Peter Heussel: Nothing to disclose
Hans-Ulrich Kauczor: Nothing to disclose
Sabine Wege: Nothing to disclose
Simon Triphan: Nothing to disclose
Monika Eichinger: Nothing to disclose

### RPS 1704-4

**Ten years of cumulative effective dose (CED) data in Cystic Fibrosis (CF): the evolution of low dose CT techniques in CF thoracic imaging and impact on radiation exposure**

K. P. Sheahan, *A. T. O'Mahony*, C. Crowley, D. Morrissey, H. Ibrahim, A. McMahon, O. O'Connor, M. A. Maher, B. Plant; Cork/IE
*(aomahony@ucc.ie)*

**Purpose:** As the life expectancy of patients with CF extends, the effect of ionising radiation exposure is of increasing concern. This paper directly quantifies and evaluates CED in the era of low dose CT techniques and CFTR modulator therapy.
**Methods or Background:** Retrospective observational study at a nationally designated specialist CF tertiary care centre. A study period from 01/01/2010-31/01/2021, included 180 patients who were >18 years and attending this facility alone for the entire study duration. Clinical data including demographics, transplant status and modulator status were recorded. CED data for all modalities was taken from actual institutional imaging, institutional averages if not possible and estimated using up to date dose data from published literature where unavailable. Number of studies and individual CED were recorded. Segregated according to thoracic or extra-thoracic and within these groups by modality. Data split into either pre-or post-modulator where applicable.
**Results or Findings:** 139 of 180 commenced modulator treatments, 15 of 180 receiving lung with two liver transplants. 82% of patients received <25 mSv over the study period. Pre-modulator mean number of studies/year/person was 2.65 (1.52 Thoracic, 1.13 Extra-thoracic). Equating to an annual CED/person of 1.488 mSv (0.841 mSv Thoracic, 0.647 mSv Extra-thoracic) with CT contributing 64% of dose received. Post-modulator mean number of studies/year/person was 2.98 (1.78 Thoracic, 1.20 Extra-thoracic). This equates to an annual CED/person of 1.484 mSv (0.7485 mSv Thoracic, 0.736 mSv Extra-thoracic) with CT contributing 71%.
**Conclusion:** There was a five-fold increase in thoracic CT scanning. However, there was no increased radiation exposure and in fact a net CED reduction. Increased abdomen-pelvic imaging in CF is having a relative proportional effect on CED.
**Limitations:** Retrospective. Not all actual doses. Duration to commencement of modulator variable and presented as a mean.
**Ethics committee approval:** Institutional review board approval was obtained.
**Funding for this study:** No funding was received for this study.
**Author Disclosures:**
Alexander Tobias O'Mahony: Nothing to disclose
Barry Plant: Nothing to disclose
Kevin Patrick Sheahan: Nothing to disclose
Michael Anthony Maher: Nothing to disclose
Owen O'Connor: Nothing to disclose
Hisham Ibrahim: Nothing to disclose
David Morrissey: Nothing to disclose
Aisling McMahon: Nothing to disclose
Claire Crowley: Nothing to disclose

### RPS 1704-5

**Visual and quantitative CT-based emphysema and lung cancer: a systematic review and meta-analysis**

*X. Yang*, H. Wisselink, R. Vliegenthart, M. A. Heuvelmans, H. Groen, M. Vonder, M. Dorrius, G. de Bock; Groningen/NL
*(x.yang@umcg.nl)*

**Purpose:** To investigate whether visual and quantitative CT-based emphysema are comparably associated with risk of lung cancer.
**Methods or Background:** PubMed, Embase and Cochrane were searched by two independent reviewers for studies on association between CT-based emphysema (visually or quantitatively assessed based on HU) and lung cancer risk. Emphysema was measured as a dichotomous and continuous variable (%Low Attenuation Area). Associations of emphysema severity (trace, mild and moderate-severe by visual and quantitative analysis) and subtype (only by visual) with lung cancer were also identified. Adjusted odds ratios, risk ratios or hazard ratios were derived and combined to estimate overall and stratified pooled ORs (pORs) with 95% confidence intervals (95%CIs).
**Results or Findings:** 21 out of 3,343 screened studies with 25 study subsets were included. The overall pOR for lung cancer given the presence of emphysema was 2.28 (95%CI: 2.01-2.60, I2=31.1%, 19 study subsets) in dichotomous analysis and 1.02 (95%CI: 1.01-1.02, I2=0%, 6 study subsets) per 1% increase of %LAA. Studies with visual emphysema yielded comparable results with that of quantitative CT emphysema: pOR 2.26 (95%CI: 1.94-2.64, I2=48.4%; 12 study subsets) versus pOR 2.25 (95%CI: 1.82-2.77, I2=0%; 9 study subsets), respectively. Based on 6 studies including 1,716 participants, pORs of emphysema severity for lung cancer ranged from 2.48-4.50 for visual assessment and 1.90-2.45 for quantitative CT. Compared with no emphysema, centrilobular emphysema was significantly associated with lung cancer (pOR: 2.19, 95%CI: 1.50-3.22, I2=0 %), whereas paraseptal emphysema was not (pOR: 1.12, 95%CI: 0.62-2.01, I2=65.6 %).
**Conclusion:** Both visual and quantitative CT-based emphysema are associated with the risk of lung cancer and this risk increases with emphysema severity. Regarding subtype, only centrilobular emphysema shows an association with lung cancer.
**Limitations:** Few studies reported severity and subtype of emphysema.
**Ethics committee approval:** Not applicable.
**Funding for this study:** Funding was received from the Royal Netherlands Academy Arts and Sciences.
**Author Disclosures:**
Monique Dorrius: Nothing to disclose
Marjolein A. Heuvelmans: Nothing to disclose
Geertruida de Bock: Nothing to disclose
Xiaofei Yang: Nothing to disclose
Rozemarijn Vliegenthart: Nothing to disclose
Harry Groen: Nothing to disclose
Marleen Vonder: Nothing to disclose
Hendrik Wisselink: Nothing to disclose

### RPS 1704-6

**Understanding chronic respiratory disease: quantifying mechanical properties of trachea/radiology meets engineering mechanics**

*J. Gawlitza*[1], A. Jung[2], R. Kose[2], S. Diebels[2], A. Bücker[3]; [1]Munich/DE, [2]Saarland/DE, [3]Homburg/DE
*(joshua.gawlitza@tum.de)*

**Purpose:** Chronic obstructive pulmonary disease (COPD) usually leads to noxious-induced airway obstruction that is also quantifiable by computed tomography (CT). At present, there is no data on the mechanical properties of tracheae and to what extent e.g. chronic lymphocyte infiltration with concomitant remodeling affects respiratory mechanics. The aim of this basic research work was to establish a methodology to investigate tracheae for their biomechanical properties and to create a CT-based, finite element model (FEM) to simulate respiratory mechanics.
**Methods or Background:** 8 tracheae were obtained from body donors (6♀/2♂), three with known severe COPD, within 24 hours after death. Scans were performed on a dual-source CT (Somatom Force, Siemens) and FEM with separate representation of cartilage braces and ligamentous connective tissue was created (Synopsys Simpleware ScanIP). Biomechanical measurement of tracheae was performed using tensile tests. To classify different material properties, individual cartilage braces were tested in isolation in a separate compression test.
**Results or Findings:** In tensile tests on composite samples of the trachea a linear stiffening behaviour could be derived for larger deformations. Stiffness between 10-30 MPa occurred. Stiffness of COPD patients' trachea (Ø15MPa) was distinctly lower when compared to the other samples (Ø25MPa). Through combination of mechanical experiments and CT scan, a FEM was created and with parameter identification the biological parameters were determined. There, the annular ligaments were significantly stiffer than the cartilages.
**Conclusion:** In this work, the biomechanical properties of trachea were determined and the first biomechanically realistic digital trachea model for airway simulation was calculated with the acquired CT images. Lower stiffness of the examined COPD trachea might be a first biomechanical correlate for radiological findings such as tracheal collapse, merging applied pathomechanics and imaging.
**Limitations:** Low sample size; heterogeneous group.
**Ethics committee approval:** Ethics committee approval was received.
**Funding for this study:** No funding was received for this study.
**Author Disclosures:**
Rebecca Kose: Nothing to disclose
Anne Jung: Nothing to disclose
Stefan Diebels: Nothing to disclose
Arno Bücker: Nothing to disclose
Joshua Gawlitza: Speaker: Bracco Imaging

**RPS 1704-7**
**Semi-automatic assessment of cystic pulmonary changes in children with juvenile systemic sclerosis**
*G. Fichera*, R. Stramare, R. Motta, F. Zulian, C. Giraudo; Padua/IT

**Purpose:** To apply a semi-automatic quantification of cystic pulmonary changes in paediatric patients with juvenile systemic sclerosis (JSSc) and to perform a longitudinal quantitative evaluation of such changes at follow-up.
**Methods or Background:** Children with JSSc referring to our tertiary centre who performed at least one computed tomography (CT) were included. One radiologist with four years of experience in paediatric imaging performed all segmentations by 3D Slicer (www.slicer.org). In particular, the lung segmenter and CT analyser tools were used and the volume affected by cystic changes was considered in the range -1050 – -950 Hu. To evaluate the reliability of the method, a second reader performed the same measurements and the intraclass correlation coefficient (ICC) was computed. For the longitudinal assessment, aiming to reduce the bias due to the growth of children during the interval between the two CT scans, a ratio between the volume affected by cystic changes and the overall inflated lungs volume was applied. Then the ratios at the two-time intervals were compared using the paired Student's t-test. The applied level of significance was 0.05 for all analyses.
**Results or Findings:** Ten patients (4 female; mean age 7.8±4.6 years old) were examined at diagnosis and seven (70%) underwent a follow-up CT. At diagnosis, on average, the bilateral inflated volume was of 1085±475 cc3 and the volume affected by cystic changes was of 183.82 cc3 (16.8%). The method demonstrated high interrater reliability in the quantification of cystic changes (ICC=0.832). At follow-up, the proposed ratio demonstrated a statistically significant increase of the cystic changes (ratio 0.11 vs 0.19, p=0.009).
**Conclusion:** The proposed semi-automatic quantification of cystic changes in paediatric patients with JSSc demonstrated to be a reliable tool at diagnosis and follow-up.
**Limitations:** Not applicable.
**Ethics committee approval:** This study was approved by an ethics committee.
**Funding for this study:** No funding was received for this work.
**Author Disclosures:**
Giulia Fichera: Nothing to disclose
Raffaella Motta: Nothing to disclose
Francesco Zulian: Nothing to disclose
Roberto Stramare: Nothing to disclose
Chiara Giraudo: Nothing to disclose

**RPS 1704-8**
**Longitudinal thoracic CT study in Birt-Hogg-Dubé syndrome: progression of cysts and relation with prognosis**
*S. M. Cho*, J. Choe, E. J. Chae; Seoul/KR
*(sumin943@gmail.com)*

**Purpose:** This study aimed to investigate whether pulmonary cyst progression after long-term follow-up of thoracic CT in patients with Birt-Hogg-Dubé syndrome (BHD) and to investigate risk factors for pneumothorax in patients with BHD.
**Methods or Background:** Our retrospective cohort included 44 patients with BHD (18 men; mean age 54.2 ± 11.6 years). We evaluated whether cysts progress via visual assessment and quantitative measurement on the initial and serial thoracic CT images. We also evaluated whether the pulmonary function test (PFT) progresses. The visual assessment included size, location, number, shape, distribution, presence of the visible wall, fissural or subpleural cysts, and air-cuff signs. In 26 patients available 1-mm thin-section data, the quantitative assessment was performed with the volume of the low attenuation area (LAA) using in-house software. Risk factors for pneumothorax were analysed using multiple regression analysis.
**Results or Findings:** On visual assessment, the largest cyst in both lungs showed a significantly increase in size (right: 5.00 mm, p < 0.001, 95% CI; 2.697~7.303, left: 5.11 mm, p = 0.001, 95% CI; 2.372~7.855, retrospectively) on the last CT. On quantitative assessment, cysts showed a slow increase in size on the dot plot on serial thoracic CT. In 33 patients with available PFT data, the annual change of FEV1/FVC was -0.65% (p=0.054, 95% CI; -1.308~0.013), which did not show a significant difference but showed decreasing tendency. The family history of pneumothorax was the risk factor for the development of pneumothorax.
**Conclusion:** Pulmonary cysts in patients with BHD increase in size on longitudinal thoracic CT. The risk factor for pneumothorax in BHD patients is the family history of pneumothorax.
**Limitations:** This study is a retrospective cohort study conducted in a single centre.
**Ethics committee approval:** This retrospective study received institutional review board approval.
**Funding for this study:** No funding was received for this study.
**Author Disclosures:**
Su Min Cho: Author: writing, data collection, performed analysis
Eun Jin Chae: Author: Performed analysis, wrote the paper
Jooae Choe: Author: Data analysis

**08:00-09:00** **Room E2**

### Research Presentation Session: Breast

### RPS 1702

### How to improve lymph node detection and characterisation

**Moderator**
P. Clauser; Vienna/AT
**Author Disclosure:**
P. Clauser: Speaker: siemens healthineers

**RPS 1702-2**
**Utility of novel silicone-specific dual-energy CT material classification method in diagnosing silicone breast implant rapture and detecting silicone in regional lymph nodes**
*M. Yalon*, K. N. Glazebrook, L. D. Viers, S. Leng, M. Johnson, C. McCollough, J. G. Fletcher; Rochester, MN/US

**Purpose:** To compare the performance of virtual non-contrast and silicone-specific dual-energy CT (DECT) material classification for identifying silicone associated with breast implant rupture.
**Methods or Background:** This prospective study included patients with silicone breast implants undergoing MRI. DECT images were reconstructed using two methods: a) conventional virtual non-contrast (VNC) and b) silicone specific. A non-reader breast radiologist established the reference standard for extracapsular and nodal silicone on MRI. Three blinded radiologists reviewed the randomised DECT images reconstructed using the two material classification methods on two separate sessions to detect extracapsular and nodal silicone.
**Results or Findings:** Forty-seven female patients with 89 implants were included. Extracapsular silicone was identified in 12/12 patients and 35/35 lymph nodes, relatively to the MR gold standard. Pooled reader data demonstrated that the silicone-specific classification had higher AUC in detecting extracapsular (0.925 vs 0.754; p= 0.065) and nodal silicon (0.813 vs 0.729; p= 0.284), but these trends were not significant. Silicon-specific material classification resulted in significant improvement in detection of extracapsular and nodal silicone for one of three readers [0.957 vs 0.716; p= 0.024 and 0.797 vs 0.644; p= 0.024, respectively]. Interobserver agreement for detection of extracapsular silicon overlapped (ICC silicone: 0.74 95%CI [0.62-0.83]; ICC VNC: 0.81 95%CI [0.72- 0.88]).
**Conclusion:** Silicone-specific material classification tends to show improved reader confidence in detecting both extracapsular and nodal silicon, with statistical significance shown for one reader. Silicone-specific DECT material classification offers a promising alternative to MR for patients undergoing breast implant evaluation.
**Limitations:** Number of cases.
**Ethics committee approval:** IRB approved.
**Funding for this study:** No funding was received for this study.
**Author Disclosures:**
Katrina N Glazebrook: Nothing to disclose
Mariana Yalon: Nothing to disclose
Lyndsay D Viers: Nothing to disclose
Joel G. Fletcher: Research/Grant Support: Siemens Healthcare Institutional grant
Shuai Leng: Nothing to disclose
Johnson, Matthew P. Matthew Johnson: Nothing to disclose
Cynthia McCollough: Research/Grant Support: Siemens Healthcare Institutional grant

**RPS 1702-3**
**Radiomics nomogram for prediction of axillary lymph node metastasis in contrast-enhanced cone-beam breast CT**
*Y. Zhu*, Y. Zhang, Y. Ma, A. Liu, H. Li, J. Ma, Y. Wang, Z. Ye; Tianjin/CN
*(zhuyueqiang1985@126.com)*

**Purpose:** To develop a radiomics nomogram based on contrast-enhanced cone-beam breast CT (CE-CBBCT) images for preoperative prediction of axillary lymph node (ALN) metastasis in breast cancer patients.
**Methods or Background:** 169 patients with breast cancer confirmed by surgical excision pathology were divided into training (n=113) and validation

(n=56) cohorts. 851 radiomics features were extracted from tumours on CE-CBBCT, features selection were performed using two-sample t test and least absolute shrinkage and selection operator, and radiomics score was constructed with support vector machine. Multivariate logistic regression analysis was used to establish a radiomics nomogram based on the combination of radiomics score and independent clinicopathologic risk factors for identifying whether the tumour presented ALN metastasis. The performance of the radiomics nomogram was assessed by its discrimination, calibration, and clinical usefulness with independent validation.
**Results or Findings:** 67 of 169 patients were confirmed with ALN metastasis. The radiomics nomogram, comprising pathological grade, Ki-67 index and radiomics score, showed good performance in predicting which tumours presented ALN metastasis (AUC=0.837 with 95% confidence interval 0.774-0.900 in the validation cohort) and good calibration. Decision curve analysis confirmed the clinical utility of the radiomics nomogram.
**Conclusion:** CE-CBBCT based radiomics nomogram has potential to be used as a non-invasive tool in predicting ALN metastasis from preoperative images, and could therefore be useful to assist clinicians in preoperative decision-making. This could be especially important in CBBCT, since it could help address the issue of limited axilla coverage.
**Limitations:** The cohort was relatively small and only mass lesions were enrolled.
**Ethics committee approval:** The institutional review board approved this study and written informed consent was obtained from all patients.
**Funding for this study:** Funding was received from the National Key R&D Program of China (No. 2017YFC0112600, 2017YFC0112601), Tianjin Science and Technology Major Project (No. 19ZXDBSY00080).
**Author Disclosures:**
Yue Ma: Nothing to disclose
Yueqiang Zhu: Nothing to disclose
Haijie Li: Nothing to disclose
Zhaoxiang Ye: Nothing to disclose
Juanwei Ma: Nothing to disclose
Yafei Wang: Nothing to disclose
Aidi Liu: Nothing to disclose
Yuwei Zhang: Nothing to disclose

### RPS 1702-4

### Preoperative axillary LN staging by breast expert vs general radiologist: area for improvement

*S. Sefidbakht*, P. Iranpour, R. Jalli, S. Tahmasebi, P. Pishdad, F. Zarei; Shiraz/IR
*(SepidehSefidbakht@yahoo.com)*

**Purpose:** To prospectively re-evaluate axillary LNs for preoperative axillary staging in patients referred for biopsy of breast masses to a tertiary breast clinic. To compare the accuracy of US done by breast imaging expert (BI) to general radiologist (GR) in the diagnosis of malignant axillary LNs. To identify areas of improvement in GR training in preoperative staging of the axilla.
**Methods or Background:** The axillae were re-evaluated in 655 patients with B4b-B5 masses in the breast who were referred for biopsy of the breast and/or axillary LN. The number, size, and morphological characteristics of additional LNs detected on expert ultrasound were compared to initial US and surgery results in patients who underwent surgery without NAC.
**Results or Findings:** Out of 655 pateints who entered the study, initially 98 patients turned out malignant and underwent surgery (BCT or MRM) and SLNB 7/or ALND without NAC. In 75 patients additional obviously malignant (29) or suspicious (46) LN were detected in expert US. In surgery in 58 patients the additional LNs were proven to be malignant. Out of 58 surgery-proven new LNs, 23 were located in level 1, 8 in level 3, and 16 were either small but round with or without evidence of extra-LN invasion or showed focal cortical thickening level 2 lymph nodes. In 11 patients number of obviously malignant lymph nodes in level 2 were underestimated.
**Conclusion:** There is a significant added value to BI-performed axillary LN staging (58/98). This specifically includes level 1 and 3LN. The GR will benefit from training in evaluating these areas, detailed morphological evaluation of the smaller lymph nodes, and clinical significance of the exact number of involved lymph nodes in clinical decision making.
**Limitations:** Exact ultrasound-surgery correlation for LNs is challenging.
**Ethics committee approval:** The institutional ethics committee approved this retrospective blinded study.
**Funding for this study:** No funding was received.
**Author Disclosures:**
Parisa Pishdad: Nothing to disclose
Fariba Zarei: Nothing to disclose
Sepideh Sefidbakht: Nothing to disclose
Sedigheh Tahmasebi: Nothing to disclose
Reza Jalli: Nothing to disclose
Poya Iranpour: Nothing to disclose

### RPS 1702-5

### The value of dual-layer detector computed tomography for preoperative diagnosis of axillary sentinel lymph node in breast cancer

L. H. Jun[1], *X. Zhang*[1], X. Liying[1], H. Jinbai[2]; [1]Wuhan/CN, [2]Jingzhou/CN

**Purpose:** To investigate the benefits of spectral dual-layer detector computed tomography (DLCT) for preoperative diagnosis of axillary sentinel lymph node (SLNs) metastases in breast cancer.
**Methods or Background:** In this prospective study, twenty-eight patients with breast cancer (age: 53.4 ± 9.0 years) performed dual-phase contrast enhanced CT on a spectral dual-layer detector during August 2019 to June 2020 before surgery. Spectral quantitative parameters including λHU (in Hounsfield units per kiloelectron-volt), nIC (normalized iodine concentration), and Zeff (Z-effective value) in both arterial and delay phase were compared between metastatic and non-metastatic SLNs using the McNemar test. The diagnostic performance of spectral parameters were analysed by receiver operating characteristic curves.
**Results or Findings:** In total, 103 SLNs (23 metastatic, 80 nonmetastatic) were matched in operation with preoperatively labeled SLNs on DLCT images. All spectral parameters (λHu, nIC, and Zeff) during both arterial and delay phase were greater in metastatic than nonmetastatic SLNs (all $P \leq 0.001$). Logistic regression analyses showed that λHu in delay phase was the best single parameter for the detection of metastatic SLNs on a per-lymph node basis, with AUC of 0.761, accuracy of 79.6% (82/103), sensitivity of 82.6% (19/23), and specificity of 78.8% (63/80).
**Conclusion:** The spectral quantitative parameters of DLCT, such as the λHu, demonstrated high diagnostic performances in the differentiation of metastatic and non-metastatic SLNs in breast cancer.
**Limitations:** Single-centre study with limited size of samples.
**Ethics committee approval:** This study has been approved by the Medical Ethics Committee of Central South Hospital of Wuhan University.
**Funding for this study:** This study was supported by Clinical research and development project of Science and Technology Innovation Cultivation Fund of Central South Hospital of Wuhan University (grant number lcyf202109).
**Author Disclosures:**
Li Hui Jun: Nothing to disclose
Huang Jinbai: Nothing to disclose
Xu Liying: Nothing to disclose
Xiaohui Zhang: Nothing to disclose

### RPS 1702-6

### Accuracy of 14G core biopsy in the diagnosis of axillary lymph node metastasis from breast cancer

*C. Bellini*[1], G. Bicchierai[1], F. Amato[2], A. Ventimiglia[3], D. De Benedetto[1], F. Di Naro[1], V. Miele[1], J. Nori[1]; [1]Florence/IT, [2]Palermo/IT, [3]Naples/IT
*(1chiarabellini@gmail.com)*

**Purpose:** To assess the diagnostic accuracy of 14G core biopsy (CB) in the diagnosis of axillary lymph nodes (LN) metastasis from breast cancer.
**Methods or Background:** We retrospectively screened from June 2017 to August 2021 women with histological diagnosis of breast cancer who underwent 14G CB of suspicious axillary LNs (n=152); we excluded 57 patients who underwent neo-adjuvant chemotherapy. We compared histologic results of CB with the histology from axillary LN dissection, our gold standard, to evaluate the diagnostic performance of CB.
**Results or Findings:** Out of 95 LN CB, 95.8% resulted positive for breast cancer infiltration while 4.2% negative. Sensitivity of CB in our study was 95.79% (CI 95%: 89.57%-98.84%), PPV 100% and accuracy 94.79% (CI 95%: 88.26%-98.29%). The mean US diameter of true-positive (TP) LN was 16.1 mm (range 4-40 mm), while of false-negative (FN) LN was 6.5 mm (range 5-8 mm).
**Conclusion:** 14G CB of axillary LN showed a high accuracy in the diagnosis of breast cancer metastasis. FN results in our sample could be explained by sampling error due to small diameter of LN.
**Limitations:** Retrospective studies; sample bias.
**Ethics committee approval:** Not needed.
**Funding for this study:** No funding was reveiced for this study.
**Author Disclosures:**
Giulia Bicchierai: Nothing to disclose
Federica Di Naro: Nothing to disclose
Jacopo Nori: Nothing to disclose
Antonio Ventimiglia: Nothing to disclose
Vittorio Miele: Nothing to disclose
Francesco Amato: Nothing to disclose
Chiara Bellini: Nothing to disclose
Diego De Benedetto: Nothing to disclose

**RPS 1702-7**

**Simultaneous multiparametric [18F]-fluoroethylcholine PET-MRI in the local staging of breast cancer**

*P. Clauser*[1], I. Bolengo[2], L. Grana Lopez[3], S. Rasul[1], P. Kapetas[1], R-I. Milos[1], T. H. Helbich[1], P. A. Baltzer[1]; [1]Vienna/AT, [2]Milan/IT, [3]Lugo/ES
*(clauser.p@hotmail.it)*

**Purpose:** To assess the role of [18F]-fluoroethylcholine (FEC) PET-MRI in the local staging of breast cancer.
**Methods or Background:** This prospective, single-centre study was approved by the ethics committee and patients gave written informed consent. Patients with suspicious lesions on conventional imaging (mammography, tomosynthesis, ultrasound) and no contraindications to [18F]-fluoroethylcholine-PET-MRI were included. Histology was considered as reference standard for breast lesions and lymph-nodes. Breast FEC-PET-MRI was performed in a prone position with a dedicated coil. Data on staging with conventional imaging were collected. Two breast fellows (R1, R2) evaluated the PET-MRI examinations. Lesion detection and diagnostic performance for lymph-node status were evaluated.
**Results or Findings:** 79 patients (mean age 55 years, standard deviation 11.9) with 83 lesions were included (76 invasive carcinomas [IC]), seven ductal carcinomas in situ [DCIS]). Four patients had bilateral tumours, two were not detected on conventional imaging (one monofocal, one multifocal). Conventional imaging correctly staged 57/58 monofocal (98%), 9/15 (60%) multifocal and 8/10 (80%) multicentric tumours. FEC-PET-MRI correctly staged 58/58 monofocal (100%), 14/15 multifocal (93%) and 10/10 (100%) multicentric tumours. The only false negative was a grade 3 DCIS with a non-suspicious FEC-uptake. Two/58 (3%) monofocal tumours showed a non-suspicious FEC uptake (one IC Luminal-A, one IC Luminal-B Her2 negative). Sensitivity and specificity for lymph-node metastasis were 43% and 89% with conventional imaging. Sensitivity was 50% (R1) and 46% (R2) with MRI, 79% (R1) and 70% (R2) with FEC-PET. Specificity was 96% (R1 and R2) with MRI, 81% (R1) and 74% (R2) with FEC-PET.
**Conclusion:** [18F]-fluoroethylcholine PET-MRI improves local staging of breast cancer, in particular the detection of lymph-node metastasis.
**Limitations:** Single centre, sample size.
**Ethics committee approval:** Approved by the ethics committee. Patients gave written informed consent.
**Funding for this study:** Received funding by the Austrian National Bank Jubiläumsfonds project n.17186.
**Author Disclosures:**
Pascal A.T. Baltzer: Nothing to disclose
Thomas H. Helbich: Nothing to disclose
Lucia Grana Lopez: Nothing to disclose
Ruxandra-Iulia Milos: Nothing to disclose
Sazan Rasul: Nothing to disclose
Panagiotis Kapetas: Nothing to disclose
Isabella Bolengo: Nothing to disclose
Paola Clauser: Speaker: Siemens healthineers

**08:00-09:00** **Room K**

### Research Presentation Session: Neuro

### RPS 1711

### Neurodegenerative diseases: Parkinson's

**Moderator**
J. M. García Santos; Murcia/ES

**RPS 1711-2**

**Automated midbrain volumetry: potential tool to differentiate progressive supranuclear palsy from Parkinson's disease**

*F. J. Mendoza Ferradas*, D. P. Martín Antonio, M. Calvo Imirizaldu, P. Dominguez Echavarri, R. Garcia de Eulate, M. Vidorreta Díaz de Cerio, M. A. Fernández Seara, M. R. I. Luquin, G. Martí-Andrés; Pamplona/ES
*(fmendozaf@unav.es)*

**Purpose:** To evaluate the precision of automated volumetry in brain MRI to differentiate Progressive Supranuclear Palsy (PSP) from Parkinson's disease (PD) in comparison with planimetric measurements.
**Methods or Background:** A retrospective study analysed 141 patients with the clinical diagnosis of Parkinsonism paired by age and sex and with a healthy control group (HCF) (PSP n = 47, PE n = 68, HCG=26) who underwent brain MRI. Patients without T1 MPRAGE sequences (1 mm thin slice) were excluded, leaving 83 patients (PSP n = 29, PE n = 54). The different subclinical types of PSP were PSP-RS (19), PSP-P (5), PSP-PGF (4), and PSP-F (1). Two independent radiologists blind to diagnosis performed a planimetric assessment with measurement of the width of the midbrain and bridge in the sagittal plane, drawing perpendicular lines (method of Luke et al.), and calculated the midbrain-pons ratio (MP). The volumes of the midbrain and the pons were obtained in an automated and normalised way using SyngoviaBrainMorphometry software, Siemens. The intraclass correlation coefficient (ICC) was used to assess interobserver agreement and ROC curves to assess the diagnostic precision of the different biomarkers to differentiate PSP from PD.
**Results or Findings:** The brainstem planimetric analysis that best-discriminated PSP from PD was the midbrain with an area under the curve (AUC) of 0.99, followed by the MP ratio with an AUC of 0.96. The planimetric measurements presented very good reproducibility with an ICC of > 0.75. The automated midbrain volume presented an AUC of 0.86, while the volumetric MP ratio had an AUC of 0.66.
**Conclusion:** The automated segmentation of the brainstem is a robust and fast-application technique, showing the midbrain volume a remarkable diagnostic performance in the differentiation of PSP from PD.
**Limitations:** Not applicable.
**Ethics committee approval:** This study was approved by an ethics committee.
**Funding for this study:** No funding was received for this study.
**Author Disclosures:**
Gloria Martí-Andrés: Nothing to disclose
Pablo Dominguez Echavarri: Nothing to disclose
María A. Fernández Seara: Nothing to disclose
Marta Vidorreta Díaz de Cerio: Nothing to disclose
Francisco Javier Mendoza Ferradas: Nothing to disclose
Daiana Paula Martín Antonio: Nothing to disclose
Marta Calvo Imirizaldu: Nothing to disclose
Reyes Garcia de Eulate: Nothing to disclose
María Rosario Isabel Luquin: Nothing to disclose

**RPS 1711-3**

**Imaging the primary motor cortex in hereditary spastic paraplegia**

*G. Donatelli*, I. Ricca, F. Bianchi, D. Frosini, V. Montano, E. Del Prete, A. Tessa, M. Mancuso, F. M. Santorelli; Pisa/IT

**Purpose:** The differential diagnosis between Hereditary Spastic Paraplegia (HSP), Primary Lateral Sclerosis (PLS) and Amyotrophic Lateral Sclerosis (ALS) can be challenging, especially in the early steps of assessment of adult patients with a lower limb onset upper motor neuron (UMN) syndrome when family history and gene testing are mute. These diseases share the UMN impairment, but the neuropathology showed different patterns of neuronal involvement: while HSP is primarily an axonopathy, in PLS and ALS the primary motor cortex is often involved. In ALS, moreover, the UMN impairment is associated with increased iron-laden microglia in the primary motor cortex (PMC); the abnormal iron deposition can be detected in vivo using T2*-weighted MR images and appears as an hypointense rim. Here we employed 3T T2*-weighted images to investigate the radiological appearance of the PMC in HSP patients and, for comparison, in PLS and ALS.
**Methods or Background:** We retrospectively included 3T T2*-weighted MR scans of 23 HSP patients, 7 PLS patients with lower limb onset and 92 ALS patients, 8 of which had a lower limb onset UMN syndrome (UMN-ALS). The signal intensity of the PMC was visually rated as isointense, mildly or marked reduced compared to the post-central cortex.
**Results or Findings:** Most HSP patients had normal signal intensity (86%); on the contrary, all the PLS, most UMN-ALS patients (75%) and 42% of the remaining ALS patients had marked PMC hypointensity.
**Conclusion:** Iron sensitive imaging of the PMC could provide useful information in the differential diagnosis of sporadic adult-onset UMN syndromes, as the hypointense rim often seen in PLS and UMN-ALS patients is apparently rare in HSP patients.
**Limitations:** The small number of HSP, PLS and UMN-ALS patients.
**Ethics committee approval:** The local ethics committee has been informed about this study.
**Funding for this study:** No funding was received for this study.
**Author Disclosures:**
Filippo Maria Santorelli: Nothing to disclose
Michelangelo Mancuso: Nothing to disclose
Graziella Donatelli: Nothing to disclose
Ivana Ricca: Nothing to disclose
Alessandra Tessa: Nothing to disclose
Daniela Frosini: Nothing to disclose
Eleonora Del Prete: Nothing to disclose
Francesca Bianchi: Nothing to disclose
Vincenzo Montano: Nothing to disclose

**RPS 1711-6**
**Diagnostic performance of the magnetic resonance parkinsonism index in differentiating progressive supranuclear palsy from Parkinson's disease: an updated systematic review and meta-analysis**
*S. Kim*, C. Suh, W. H. Shim, S. J. Kim; Seoul/KR

**Purpose:** Progressive supranuclear palsy (PSP) and Parkinson's disease (PD) are difficult to differentiate especially in the early stages. We aimed to investigate the diagnostic performance of the magnetic resonance parkinsonism index (MRPI) in differentiating PSP from PD.
**Methods or Background:** A systematic literature search of PubMed-MEDLINE and EMBASE was performed to identify original articles evaluating the diagnostic performance of the MRPI in differentiating PSP from PD published up to 20 February 2021. The pooled sensitivity and specificity were calculated using the bivariate random-effects model. The area under the curve (AUC) was calculated using a hierarchical summary receiver operating characteristic (HSROC) model. Meta-regression was performed to explain the effects of heterogeneity.
**Results or Findings:** A total of 14 original articles involving 484 PSP patients and 1243 PD patients were included. T1-weighted images were used to calculate the MRPI in all studies. Among the 14 studies, 9 studies used 3D T1-weighted images. The pooled sensitivity and specificity for the diagnostic performance of the MRPI in differentiating PSP from PD were 96% (95% CI, 87-99%) and 98% (95% CI, 91-100%), respectively. The area under the HSROC curve was 0.99 (95% CI, 0.98-1.00). Meta-regression showed the association of the magnet field strength with heterogeneity.
**Conclusion:** The MRPI could accurately differentiate PSP from PD and support the implementation of appropriate management strategies for patients with PSP.
**Limitations:** There was heterogeneity among the selected studies. We performed meta-regression analysis and studies using 3.0 T MRI showed significantly higher sensitivity (100%) and specificity (100%) than those of studies using 1.5 T MRI (sensitivity of 98% and specificity of 97%) (P < 0.01).
**Ethics committee approval:** Ethics committe approval was not required because of the type of this study (meta-analysis).
**Funding for this study:** No funding was received for this study.
**Author Disclosures:**
Seongken Kim: Author: First Author
Sang Joon Kim: Author: Co-Author
Woo Hyun Shim: Author: Co-Author
ChongHyun Suh: Author: Corresponding Author

**RPS 1711-8**
**Microstructural but not macrostructural cortical degeneration in Parkinson's disease with mild cognitive impairment: evidence from NODDI investigation**
*X. Bai*, T. Guo, X. Guan, M. Zhang; Hangzhou/CN
*(baixueqin1@126.com)*

**Purpose:** This study aimed to investigate the cortical microstructural and morphological degenerative patterns in patients with Parkinson's disease (PD) and mild cognitive impairment (MCI) using Neurite Orientation Dispersion and Density Imaging (NODDI) and T1-weighted imaging.
**Methods or Background:** A total of 108 subjects, including 38 patients with normal cognition (PD-NC), 38 PD-MCI, and 32 healthy controls (HC), were included. PD-MCI was diagnosed according to the MDS Task Force level II criteria. Cortical microstructural alterations were evaluated by using NODDI with gray matter-based spatial statistics (GBSS). Cortical thickness analyses were performed using the FreeSurfer software.
**Results or Findings:** For cortical microstructural analyses, compared with HC, PD-NC showed lower orientation dispersion index (ODI) in bilateral cingulate, supplementary motor area, right paracentral lobule and precuneus; while PD-MCI showed widespread lower ODI throughout bilateral frontal, parietal, occipital and right temporal areas and regional lower neurite density index (NDI), predominantly in left frontal area and cingulate. However, for cortical thickness analyses, there were no group difference in any vertex in the between-group comparisons. Besides, the NDI values within the areas with lower NDI were associated with the Montreal Cognitive Assessment (MoCA) scores in the PD patients (r = 0.240, P = 0.041).
**Conclusion:** Microstructural but not macrostructural cortical degeneration in Parkinson's disease with mild cognitive impairment, suggesting that cortical microstructural alterations may be much more sensitive than morphological changes to cortical degeneration in PD-MCI.
**Limitations:** Our study is a cross-sectional study with relatively small sample size.
**Ethics committee approval:** All the subjects signed informed consent forms in accordance with the approval of the Medical Ethics Committee of the local Hospital.
**Funding for this study:** This study was supported by the National Natural Science Foundation of China (Grant Nos. 81971577, 82001767).
**Author Disclosures:**
Xueqin Bai: Nothing to disclose
Xiaojun Guan: Nothing to disclose
Minming Zhang: Nothing to disclose
Tao Guo: Nothing to disclose

**08:00-09:00** **Room M 1**

### Research Presentation Session: Abdominal Viscera

### RPS 1701

### Surveillance and workup of hepatocellular carcinoma (HCC)

**Moderator**
G. Brancatelli; Palermo/IT
**Author Disclosure:**
G. Brancatelli: Consultant: Bayer Healthcare; Speaker: Bracco, GE Healthcare, Guerbet

**RPS 1701-2**
**Outcomes of LI-RADS US-2 subthreshold observations**
*J. R. Tse*, L. Shen, L. S. Yoon, K. Bird, A. Kamaya; Stanford, CA/US

**Purpose:** To determine the imaging outcomes of US-2 observations.
**Methods or Background:** In this retrospective study, we evaluated 175 adult patients (70 women, 105 men) with US-2 subthreshold observations on HCC surveillance ultrasound. Inclusion criteria was ≥2-year imaging follow-up (n=138) or <2-year follow-up with diagnostic characterisation (n=37) with either CT/MRI (n=34) or orthotopic liver transplant (n=3). At ultrasound, the observation outcome was defined as resolved if it was no longer present, stable if it was re-identified and remained subcentimeter, or progressed if it grew to ≥10 mm. At CT/MRI, the observation outcome was defined as resolved if it was not re-identified. Otherwise, the appropriate CT/MRI LI-RADS category was assigned.
**Results or Findings:** Of 175 patients, 173 (99%) had US-2 observations that were either stable (n=68), resolved (n=85), no correlate at OLT (n=3), or classified as LR-1 or LR-2 at CT/MRI (n=17). 2 (1%) observations were LR-3 at CT/MRI. No observations progressed on ultrasound follow-up or were classified as LR-4 or higher at CT/MRI. 8 patients developed HCC after a median of 2.0 years that were metachronous to the US-2 observation; all HCCs were unequivocally in a separate location and were preceded by a surveillance ultrasound that showed resolution of the US-2 observation.
**Conclusion:** US-2 subthreshold observations are unlikely to progress or become HCC. Approximately half of them resolve at follow-up imaging.
**Limitations:** This was a singlecentre retrospective study with relatively small sample size.
**Ethics committee approval:** This study was approved by the Institutional Review Board.
**Funding for this study:** No funding was received for this study.
**Author Disclosures:**
Luke S Yoon: Nothing to disclose
Luyao Shen: Nothing to disclose
Justin Ruey Tse: Nothing to disclose
Kristen Bird: Nothing to disclose
Aya Kamaya: Nothing to disclose

**RPS 1701-3**
**What factors are associated with increased risk for peritoneal seeding after radiofrequency ablation for hepatocellular carcinoma**
*H. Ryu*, T. U. Kim; Yangsan/KR

**Purpose:** To evaluate the incidence and risk factors associated with extrahepatic seeding after percutaneous radiofrequency ablations (RFA) for hepatocellular carcinoma (HCC), especially focused on viable tumour from previous locoregional treatment including transarterial chemoembolisation (TACE) and RFA.
**Methods or Background:** Between June 2012 and December 2019, 290 patients (mean age, 67.9 years ± 9.74; 223 men) with 383 HCCs (mean size, 15.9 mm±5.49) undergoing RFA were included in this retrospective study. Among patients, 158 patients had history of previous treatment (mean number, 1.3±1.8) with 109 viable HCCs. Kaplan-Meier method was used to estimate cumulative seeding metastasis after RFA. Independent factors affecting seeding metastasis were investigated by using multivariable Cox proportional hazard regression.

**Results or Findings:** Incidence of seeding metastasis per patient was 3.1% (9/290) and per tumour was 4.4% (17/383). The mean time interval after recent RFA until first detection of seeding metastasis was 807.2 days ±121.7 (range: 81-1961). Independent risk factors for seeding metastasis include subcapsular tumour location (HR, 4.2; 95% CI: 1.4, 13.0; p=.012) and RFA for viable HCC from previous locoregional treatment (HR, 4.5; 95% CI: 1.7. 12.3; p=.003).
**Conclusion:** Extrahepatic seeding metastasis after RFA is a rare delayed complication. Subcapsular HCC, and viable HCC from previous locoregional treatment are potential risk factors for seeding metastasis.
**Limitations:** The limitations of this study were as follows. First, this was retrospective study conducted at a single institution where RFA was performed by one experienced radiologist, thus the results may not be generalisable. Second some potential risk factors were not analysed such as tumour differentiation. Finally relative small number of patients with seeding metastasis were included.
**Ethics committee approval:** Institutional Review Board approval was obtained.
**Funding for this study:** The authors state that this work has not received any funding.
**Author Disclosures:**
Hwaseong Ryu: Nothing to disclose
Tae Un Kim: Nothing to disclose

### RPS 1701-4

**Value of intravoxel incoherent motion for preoperative evaluation of liver regeneration in hepatocellular carcinoma**

*Q. Li*; Chengdu/CN

**Purpose:** To explore the role intravoxel incoherent motion (IVIM) to preoperatively assess liver regeneration.
**Methods or Background:** 54 HCC patients who had undergone IVIM before hepatectomy, preoperative and postoperative CT examination were retrospectively included. A semiautomatic CT volumetric analysis software drew the outline of the total functional liver, preoperative future liver remnant volume, and postoperative liver remnant volume. And the regeneration index (RI) and parenchymal hepatic resection rate were then manually calculated. The relation between diffusion parameters and fibrosis stage was assessed using Spearman correlation analysis. Logistic regressions analysis were used for exploring factors associated with higher RI. ROC analyses were performed to evaluate the diagnostic performance of IVIM.
**Results or Findings:** Significant negative relations showed between D value and fibrosis stage (r =-0.361, p=0.007), between D* value and fibrosis stage (r =-0.457, p=0.001). At multivariate analysis, only higher D value (OR, 8.131; 95% CI, 1.094-60.424; p=0.041) was associated with higher RI. D value and D* value for the diagnosis of higher RI showed good differentiation power with AUC of 0.843 (95% CI, 0.712–0.974) and 0.740 (95% CI, 0.599-0.880).
**Conclusion:** D value and D* value worked as reliable preoperative predictors for evaluating the capacity of liver regeneration for HCC patients undergoing hepatectomy.
**Limitations:** First, the retrospective design may cause selection bias and limit its feasibility. Second, due to the retrospective nature, the interval between postoperative CT image and surgery is not uniformed, ranging from 35–300 days. Nevertheless, it has proved that the first week after operation is quantitatively important in the process of liver regeneration, and then the speed of regeneration slow down significantly. Thus, the time interval of our study was in the tolerable range.
**Ethics committee approval:** This retrospective study was conducted was approved by the Institutional Review Board.
**Funding for this study:** No funding was received for this study.
**Author Disclosures:**
Qian Li: Nothing to disclose

### RPS 1701-5

**Radiomics-based on multisequence MRI for hepatocellular carcinoma prediction in hepatobiliary phase hypointense nodules without arterial phase hyperenhancement: a multicentre study**

*S-Y. Youn*, J-I. Choi, Y. Nam; Seoul/KR

**Purpose:** Hepatobiliary phase (HBP) hypointense nodules without arterial phase hyperenhancement (APHE) at gadoxetic acid–enhanced MRI may indicate hepatocellular carcinoma (HCC) or nonmalignant cirrhosis-associated nodules. This study aimed to develop a radiomics prediction model based on multisequence MRI to predict HCC in HBP hypointense nodules without APHE.
**Methods or Background:** This retrospective multicentre study included pathologic confirmed HBP hypointense nodules without APHE (less than 3 cm) in patients with chronic liver disease or cirrhosis screened between January 2008 and June 2016. Radiomics features were separately extracted from the T2-weighted images (T2WIs), T1-weighted images (T1WIs), HBPs, and apparent diffusion coefficient (ADC) maps. A support vector machine (SVM) model with 8-fold cross validation was built by using multisequence radiomic features.
**Results or Findings:** A total of 278 patients with 291 lesions (199 HCCs and 92 nonmalignant cirrhosis-associated nodules) in eight institutions were used. 420 radiomics features are extracted in four MRI sequences. 54, 10, 21 and 33 significant radiomics features were selected in T2WI, T1WI, ADC map and HBP, respectively. Using SVM with c value of 0.0007, mean area under the curve (AUC) to predict HCC was 0.797 (range, 0.740-0.890).
**Conclusion:** A radiomics model based on multisequence MRI serves as an effective quantitative approach to predict HCC in HBP hypointense nodules without APHE.
**Limitations:** This was a retrospective study.
**Ethics committee approval:** This study was approved by the Institutional Review Board and informed consent was waived.
**Funding for this study:** This research was supported by National Research Foundation of Korea (NRF) funded by the Ministry of Education (2019R1FI1A106056613).
**Author Disclosures:**
Seo-Yeon Youn: Nothing to disclose
Yoonho Nam: Nothing to disclose
Joon-Il Choi: Nothing to disclose

Saturday

### RPS 1701-6

**Value of precontrast and portal venous phases for evaluating atypical hepatocellular carcinoma mimicking arterioportal shunt**

*M. Song*; Seoul/KR
*(amy4090@caumc.or.kr)*

**Purpose:** To evaluate the value of precontrast phase (PP) and portal venous phase (PVP) for differentiation of small hypervascular hepatocellular carcinomas (HCCs) without delayed washout from arterioportal (AP) shunts in high-risk patients of HCC.
**Methods or Background:** A total of 122 lesions (73 AP shunts and 49 HCCs) detected on quadriphasic CT in 101 patients with chronic liver disease were analysed. All lesions showed arterial enhancement and isodensity on delayed phase (DP) with exclusion of typical features of AP shunts. Lesion morphologic features on biphasic CT (AP and DP), Alpha-fetoprotein values and coexistent HCC were evaluated. The qualitative/quantitative analyses of lesion attenuation on quadriphasic CT were performed. Diagnostic performances for prediction of AP shunts over HCC were compared among the biphasic CT, triphasic CT (adding PP or PVP) and quadriphasic CT.
**Results or Findings:** In multivariate analysis, the presence of concomitant HCC, visual hypodensity on PP and visual hyperdensity on PVP were independent predictors for HCCs rather than AP shunts. Additional review of PP and PVP revealed significantly improved diagnostic performance yielding the highest diagnostic performance.
**Conclusion:** Hypodensity on PP and hyperdensity on PVP are significant predictive features in differentiating atypical small hypervascular HCC from AP shunts in patients with high-risk of HCC. Careful evaluation of the PP and PVP may reduce underdiagnosis and lead to earlier diagnosis of atypical small HCCs.
**Limitations:** First, our study was retrospective and has small case number. Second, pathological proofs weren't available for most of the lesions. Third, the fixed time delay after intravenous contrast injection without customisation. Fourth, the visual assessment
of the lesion's attenuation is subjective.
**Ethics committee approval:** Ethical approval was granted by the Chung-Ang University Hospital ethics committee
**Funding for this study:** This study was supported by a grant from the Central Medical Service (CMS) research fund.
**Author Disclosures:**
Minkyo Song: Nothing to disclose

### RPS 1701-7

**Positive predictive value of hepatocellular carcinoma surveillance ultrasound depends on presence of cirrhosis**

*J. R. Tse*, L. Shen, T. Tiyarattanachai, K. Bird, T. Liang, L. S. Yoon, A. Kamaya; Stanford, CA/US

**Purpose:** To determine how the presence of cirrhosis and modality (CT versus MRI) for diagnostic characterisation affect positive predictive values (PPV) of US-3 observations.
**Methods or Background:** In this retrospective study, 225 adult patients (100 women, 125 men) high-risk for hepatocellular carcinoma (HCC) from 2017-2021 had an US-3 observation on surveillance ultrasound and underwent diagnostic characterisation with CT (93; 41%), MRI (130; 58%), or contrast-enhanced ultrasound (2; 1%). US-3 observations included focal observations ≥10 mm in 216 patients and venous thrombi in 9 patients. PPV were calculated using diagnostic characterisation as the reference standard.
**Results or Findings:** Of 225 patients, 116 (52%) had cirrhosis and 109 (48%) did not. Most non-cirrhotic patients had hepatitis B virus (n=100). Overall PPV was 33% (27-39%) for at least intermediate probability of cancer and 15% (10-20%) for at least probable cancer. Cirrhosis significantly affected the PPV of cancer. For at least intermediate probability of cancer, PPV increased from

15% (8-21%) in non-cirrhotic patients to 51% (42-60%) in cirrhotic patients (p<0.001). For at least probable cancer, PPV increased from 4% (0-7%) to 26% (18-34%; p<0.001). CT and MRI were similar at identifying a correlate (p=0.470). At multivariable analysis, cirrhosis was the most important predictor of at least probable cancer (p<0.001; odds ratio OR 20.4), followed observation size (p<0.001; OR 2.65) and age (p=0.004; OR 1.05). Alpha-fetoprotein, visualisation score, and modality (CT vs MRI) were not significant predictors.
**Conclusion:** PPV for at least probable cancer in US-3 observations decreases from about 1 in 4 among cirrhotic patients to 1 in 25 among non-cirrhotic patients. Modality did not affect PPV.
**Limitations:** This was a single-institution, retrospective study that evaluated positive predictive value only.
**Ethics committee approval:** This study is IRB approved.
**Funding for this study:** No funding was received for this study.
**Author Disclosures:**
Thodsawit Tiyarattanachai: Nothing to disclose
Luke S Yoon: Nothing to disclose
Luyao Shen: Nothing to disclose
Tie Liang: Nothing to disclose
Justin Ruey Tse: Nothing to disclose
Kristen Bird: Nothing to disclose
Aya Kamaya: Nothing to disclose

**08:00-09:00** **Room M 2**

### Research Presentation Session: Imaging Informatics / Artificial Intelligence and Machine Learning

### RPS 1705b

### Artificial intelligence in brain imaging

**Moderator**
S. Bisdas; London/UK

### RPS 1705b-2

**Comprehensive deep-learning model on CT brain outperforms radiologist performance in the majority of findings**
J. C. Y. Seah[1], C. Tang[1], M. G. Vasimalla[1], G. Bottrell[1], H. T. Pham[2], Q. Buchlak[1], J. Hall[1], S. Edelstein[1], *P. Brotchie*[1]; [1]Sydney/AU, [2]Ho Chi Minh/VN

**Purpose:** To assess the performance of a comprehensive CT brain (CTB) deep-learning model that has been trained on data from multiple sites across Australia.
**Methods or Background:** Whilst numerous deep-learning models for CT brain are available for use, they have been limited to detection of only a single or a small number of findings (<15). This is the first study of a comprehensive learning model for CT brain capable of assessing up to 212 findings. A total of 2,848 CTB cases from adults (≥18 years) who had at least one thin-slice (≤1.5mm thick) non-contrast CTB were included in the test dataset. This was ground-truthed by 5 radiologists and adjudicated by a neuroradiologist. 32 radiologists read each case to assess baseline radiologist performance. The difference in AUC was calculated for each of the findings.
**Results or Findings:** When adjusted for multiple hypotheses (across 212 CTB findings), the performance of the deep-learning model was superior to the average radiologist performance in 65% of CTB findings. The radiologist was superior in 3% of findings, and 22% were inconclusive. Statistical measurements showed radiologist performance had a macroaveraged AUC of 0·680 across all the clinical findings, compared with the deep-learning model's standalone performance AUC of 0·896.
**Conclusion:** The comprehensive deep-learning model performed better than the radiologist in a majority of findings on a large set of non-contrast CTB studies.
**Limitations:** This study was a comparison of radiologist performance to model performance based on AUCs. To determine its usefulness and its utility in improving the performance of radiologists in reporting findings on CT brain studies will need to be tested.
**Ethics committee approval:** University of Notre Dame Human Research Ethics Committee (2020-127S) and Bellberry Human Research Ethics Committee (2021-03-259) approval.
**Funding for this study:** Fully funded by Annalise.ai.
**Author Disclosures:**
Simon Edelstein: Employee: Annalise.ai
Hung Thai Pham: Employee: Annalise.ai
Jarrel Chen Yi Seah: Employee: Annalise.ai
Michael Gagarin Vasimalla: Employee: Annalise.ai
Cyril Tang: Employee: Annalise.ai
Georgie Bottrell: Employee: Annalise.ai
Peter Brotchie: Employee: Annalise.ai
Quin Buchlak: Employee: Annalise.ai
Jonathan Hall: Employee: Annalise.ai

### RPS 1705b-3

**A cautionary tale about lesion detection performance evaluation in multiple sclerosis**
*V. Munoz Ramirez*[1], P. Rubini[1], B. Lambert[1], H. Dehaene[1], P. Roca[1], A. Attye[2], S. Doyle[1], A. Tucholka[1]; [1]La Tronche/FR, [2]Grenoble/FR
*(veronica@pixyl.ai)*

**Purpose:** Address the discordance between classical metrics used to rank neuroimaging solutions for lesion segmentation and their applicability in clinical practice.
**Methods or Background:** The metrics used to rank neuroimaging solutions are often centred around classical scores such as Dice and sensitivity, whereas when assessing neurological illnesses like multiple sclerosis, the detection of lesions is of greater clinical importance than their delineation. Also, an algorithm could present great sensitivity in general, but systematically miss lesions in one of the McDonald regions. We trained a 3D CNN model with two different MR scans datasets. The first one contained 15289 lesions and the second one 18700 lesions from which 634 and 840 were present in the infratentorial region, respectively. We evaluated the performance of both models on a curated dataset of 29 FLAIR scans from open source databases (ISBI and MSSEG challenges). The images were annotated by expert neuroanatomists.
**Results or Findings:** The Dice score of both models was very similar (0.676 and 0.662). The overall lesion sensitivity increased by 18% (0.60 for the first model and 0.71 for the second one) but, more strikingly, the lesion sensitivity proper to the infratentorial region improved by 82.35% for the second model.
**Conclusion:** When evaluating the performance of lesion detection algorithms in multiple sclerosis it is important to evaluate detection over delineation performance and measure separately by McDonald's region.
**Limitations:** Both models are still deficient in the infratentorial region and more data is needed for efficient detection. Infratentorial lesions are the least abundant type of lesions from the McDonald regions; this poses an issue of class-imbalance that should be addressed during model-training.
**Ethics committee approval:** The open source data was used in conformity with their respective user agreements.
**Funding for this study:** This work has been fully funded by Pixyl S. A.
**Author Disclosures:**
Pauline Roca: Employee: Pixyl
Veronica Munoz Ramirez: Employee: Pixyl
Benjamin Lambert: Employee: Pixyl
Harmonie Dehaene: Employee: Pixyl
Alan Tucholka: Employee: Pixyl
Senan Doyle: CEO: Pixyl
Pascal Rubini: Employee: Pixyl
Arnaud Attye: Consultant: Pixyl

### RPS 1705b-4

**Radiomics-based machine learning methods for the prediction of acoustic neuroma response to Cyberknife treatment: a multicenter study**
I. Bossi Zanetti[1], R. Pascuzzo[1], *S. Gonella*[1], D. Aquino[1], P. Soda[2], S. Morlino[1], E. De Martin[1], S. Papa[1], L. Fariselli[1]; [1]Milan/IT, [2]Rome/IT

**Purpose:** To predict acoustic neuroma response to radiosurgery, using machine learning (ML) methods based on radiomic features extracted from pre-treatment MRI.
**Methods or Background:** Patients with acoustic neuroma treated with radiosurgery from 2004 to 2016 were retrospectively evaluated. Inclusion criteria were having contrast-enhanced T1-weighted MR images available before and at 24 and 36 months after treatment. Clinical, audiometric and treatment data were collected at the same time points. Lesions were classified as stable, reduced or increased according to the volume variation assessed on the pre- and post-radiosurgery MRI. Tumours were semi-automatically segmented on the pre-treatment MRI using 3DSlicer. Pre-treatment images were pre-processed in three steps: uniformity correction; deskulling; Z-score transformation. For each patient, 851 features were extracted using PyRadiomics. We trained ML methods to predict the lesion increase at the two time points, using a nested cross-validation (CV) scheme. On the training set of each outer loop, feature selection was performed using LASSO and the selected features were used as input to build four ML classification methods: SVM, RF, NNet and XGBoost. To overcome class imbalance during training SMOTE was used. Finally, trained models were tested on the corresponding held-out patients in each outer loop to evaluate balanced accuracy, sensitivity and specificity.
**Results or Findings:** We included 95 patients treated with Cyberknife®. The NNet was the best algorithm for predicting the response at 24 months (accuracy 71%, sensitivity 60%, specificity 82%) and 36 months (accuracy 66%, sensitivity 50%, specificity 83%).
**Conclusion:** Preliminary results indicate that the proposed methods have great potential in distinguishing, before radiosurgery, patients with tumour volume increase from patients with stable or reduced lesions.
**Limitations:** Subsequent studies are ongoing to assess the robustness and reproducibility of these results
**Ethics committee approval:** Ethical committee approval was obtained in 2019.
**Funding for this study:** 0
**Author Disclosures:**
Paolo Soda: Nothing to disclose
Sergio Papa: Nothing to disclose
Elena De Martin: Nothing to disclose
Domenico Aquino: Nothing to disclose
Sara Morlino: Nothing to disclose
Isa Bossi Zanetti: Nothing to disclose
Riccardo Pascuzzo: Nothing to disclose
Silvia Gonella: Nothing to disclose
Laura Fariselli: Nothing to disclose

### RPS 1705b-5

**Segmentation of MS lesions: accuracy of mdbrain 4.5 versus a pool of human experts**
T. Dalbis, J. Grilo, S. Hitziger, W. X. Ling, J. R. Opalka, *A. Lemke*; Berlin/DE

**Purpose:** The diagnosis of multiple sclerosis (MS) requires the assessment of lesion load from brain MRIs. Traditionally, MS lesions are manually annotated by radiologists, a process that is inefficient and error prone. The AI-software mdbrain leverages deep learning to automatically segment MS lesions. Here, we assess the accuracy of the lesion-segmentation algorithm of mdbrain version 4.5 compared to SPM-SLS and to the inter-rater performance of 4 experts.
**Methods or Background:** mdbrain uses a deep neural network to segment lesions from a FLAIR scan. The network was trained using 280 annotated FLAIRs. Performances were tested on a separate dataset of 30 FLAIRs annotated by 4 experts. To assess segmentation accuracy, we computed the lesion-wise F1 score between each algorithm (mdbrain and SPM-SLS) and rater, averaged across raters. The inter-rater F1 was computed by comparing the annotation of each rater against the remaining 3. F1 scores were also computed for different lesion classes separately.
**Results or Findings:** mdbrain achieved an F1 score of 0.72, which was larger than SPM-SLS (F1=0.55) but slightly smaller than the inter-rater (F1=0.75). F1 scores of mdbrain were larger than the inter-rater for juxtacortical (mdbrain F1=0.75; inter-rater F1=0.72) and infratentorial lesions (mdbrain F1=0.58; inter-rater F1=0.55), but smaller for periventricular (mdbrain F1=0.74; inter-rater F1=0.77) and deep-white matter lesions (mdbrain F1=0.70; inter-rater F1=0.76). An average time of 2 minutes was required by mdbrain to process a single scan (GPU-equipped machine).
**Conclusion:** The AI-software mdbrain 4.5 achieved a lesion-segmentation accuracy comparable to a pool of human experts and considerably higher than SPM-SLS.
**Limitations:** Not applicable.
**Ethics committee approval:** Not applicable.
**Funding for this study:** Not applicable.
**Author Disclosures:**
Tiziano Dalbis: Employee: mediaire GmbH
Wen Xin Ling: Employee: mediaire GmbH
Jens Ruediger Opalka: Board Member: mediaire GmbH
Sebastian Hitziger: Employee: mediaire GmbH
Joana Grilo: Employee: mediaire GmbH
Andreas Lemke: CEO: mediaire GmbH

### RPS 1705b-7

**K-space based deep learning reconstruction empowers 50% acceleration of MR spine imaging: a prospective multicentre, multireader trial**
*L. N. Tanenbaum*[1], S. Bash[2], M. Thomas[3], M. Fung[3], M. Lebel[3];
[1]Riverside, CT/US, [2]Woodland Hills, CA/US, [3]Waukesha, WI/US
*(nuromri@gmail.com)*

**Purpose:** This prospective multicentre, multireader study evaluates the impact on perceived image quality of 50% scan-time reduced spine MRI reconstructed with deep learning (DL).
**Methods or Background:** With IRB approval and patient consent, 50 consecutive patients underwent standard-of-care (SOC) and accelerated (FAST) spine MRI exams acquired from a GE 3T Architect scanner. DL processing of the FAST scan data set (FAST-DL) was performed using an FDA-cleared CNN-based DL image-enhancement product: Air Recon DLTM. The k-space based tool offers powerful denoising, sharpness enhancement and elimination of some artifacts, such as truncation ringing. Two neuroradiologists were presented with the different image series as paired, side-by-side datasets. Datasets were blinded and randomized in sequence and left-right display order. Image features were preference rated on a 5-point Likert scale for: (1) overall IQ, (2) anatomy conspicuity, (3) cord/CSF/lesion contrast, (4) sharpness, (5) apparent SNR, (6) artefacts and (7) aberrations.
**Results or Findings:** FAST-DL was qualitatively better than SOC and FAST across all 6 categories, ($\chi 2$ (4, n = 52), p = < .01). Very high inter-rater agreement was observed, with mean score difference = -0.26, 95% CI = -1.26, 0.74.
**Conclusion:** DL enables 50% spine MRI scan time reduction as well as what radiologists perceive as enhanced image quality with benefits in SNR, image sharpness and artifact reduction over SOC and FAST images without DL processing, providing gains in efficiency and portending practice utility for routine use.
**Limitations:** Small sample size.
**Ethics committee approval:** This study was approved by an ethics committee.
**Funding for this study:** No funding was received for this study.
**Author Disclosures:**
Marc Lebel: Employee: GE Healthcare
Mary Thomas: Employee: GE Healthcare
Suzie Bash: Advisory Board: Subtle Meical, Cortechs
Lawrence Neil Tanenbaum: Advisory Board: Subtle Medical Speaker: GE Healthcare
Maggie Fung: Employee: GE Healthcare

### RPS 1705b-8

**mdbrain vs. FreeSurfer & SPM: repeatability and performance of different methods for brain volumetry**
*J. R. Opalka*, P. Ferrera Bertran, A. Lemke; Berlin/DE
*(JROpalka@t-online.de)*

**Purpose:** To test and compare the repeatability and diagnostic accuracy of different academic and commercial brain volumetry solutions with different methodologies with and without Deep Learning (DL).
**Methods or Background:** Brain volumetry measurements were carried out with the open-source software packages FreeSurfer (v6.0.0) and SPM (v12) and were compared against a commercially available software solution, mdbrain (v4.4), based on DeepLearning technology. The MIRIAD cohort included 45 patients with confirmed Alzheimer's disease, and 23 age-matched healthy controls served as a data set. Furthermore, back-to-back scans (n=178) carried out on the same day were included. Images were acquired on a 1.5T MR-scanner using standard 3D-T1w images. Brain volumetry was performed for several regions, including the whole brain, grey and white matter, all lobes, the hippocampus and all ventricles. All systems were compared in terms of repeatability and performance. The performance was quantified using ROC analysis by calculating the corresponding AUCs.
**Results or Findings:** For the repeatability tests, the DL-based mdbrain showed a significantly better stability as compared to FreeSurfer and SPM for all analyzed regions (e.g. mean deviations to reference for whole brain 0.06+/-0.09% vs. 0.43+/-0.87% vs. 0.12+-0.06%, mean +/-SD). Performance analysis also yielded higher AUC values for mdbrain and SPM (mean value whole brain 0.96 & 0.95) vs. FreeSurfer (0.77).
**Conclusion:** As compared to FreeSurfer and SPM, mdbrain showed signs of better repeatability for all of the evaluated regions. This is reflected in improved mean values and a lower overall error. Taking into account the shorter evaluation time of <5min for the DL-based approach vs. ~10h for FreeSurfer and ~30min for SPM, mdbrain appears to be a valuable tool to enable the routine application of brain volumetry in clinical practice.
**Limitations:** Not applicable.
**Ethics committee approval:** Not applicable.
**Funding for this study:** Not applicable.
**Author Disclosures:**
Pere Ferrera Bertran: Employee: mediaire GmbH
Jens Ruediger Opalka: Board Member: mediaire GmbH
Andreas Lemke: CEO: mediaire GmbH

**08:00-09:00** **Room M 3**

### Research Presentation Session: Head and Neck

### RPS 1708

### Head and neck oncology

**Moderator**
D. Farina; Brescia/IT
**Author Disclosure:**
D. Farina: Consultant: Siemens; Speaker: Bayer

### RPS 1708-3

**Outcomes of head and neck fine-needle aspiration cytology results: consultant vs. sonographer**
*A. Khan*, R. Dave, P. Brennan, J. Bekker; Portsmouth/UK
*(abeera.khan1@nhs.net)*

**Purpose:** Fine-needle aspiration is the first line diagnostic test for thyroid nodules, cervical lymph nodes and salivary gland lesions. The aim of this study is to compare the success rate of FNAC done by a head and neck sonographer who was trained by in-house head and neck radiologists, compared to the success rates of FNAC performed by consultants and trainees. The standard range of non-diagnostic yield (in percentage) from several studies has been quoted as between 10-30%. Our aim is to train sonographers in doing ultrasound and FNAC to help with the acute and outpatient workload.
**Methods or Background:** Retrospective analysis of FNAC outcomes done by a sonographer over a 13-month period were included.
**Results or Findings:** 182 FNAC were done by a sonographer from 1 January 2021 to 1 February 2022. This involved sampling of pathological cervical lymph nodes, thyroid, and salivary gland lesions. Out of 182 studies, 70.8% showed conclusive results and 29.2% were inconclusive. The results were then compared with a standard cytology assessment set by RCR. As per RCR Audit Live, FNAC of thyroid nodules should have a 70% diagnostic yield. A comparison was made with the previous FNAC audit done by consultants and trainees within the department which showed non-diagnostic sample yield 27.2% of 1222 FNA done over a 4-year period.
**Conclusion:** The diagnostic yield of FNAC done by a sonographer is the same as the FNAC outcomes done by consultants and trainees in the first audit cycle and meets the standard set by RCR Audit Live and several other published studies.
**Limitations:** No limitations were identified.
**Ethics committee approval:** Not required.
**Funding for this study:** No funding was received for this study.
**Author Disclosures:**
Jasper Bekker: Nothing to disclose
Abeera Khan: Nothing to disclose
Roma Dave: Nothing to disclose
Peter Brennan: Nothing to disclose

### RPS 1708-4

**Agreement of TIRADS and ATA as ultrasonographic thyroid nodules scoring systems and their relationship with pathology findings**
*L. Aghaghazvini*, N. Shahbazifar, M. Shakiba, M. Alborzi Avanaki, A. Mohammadi, M. Tohidi; Tehran/IR
*(aghaghazvini.leila@gmail.com)*

**Purpose:** The agreement of two main ultrasound-based thyroid assessment systems, i.e.TIRADS and ATA, isn't well known. We aim to identify this agreement with pathology.
**Methods or Background:** All nodules were examined using high-resolution ultrasound, and then FNA was done. In ultrasound, all descriptors for determining TIRADS and ATA scores were evaluated. Nodules with non-diagnostic cytology in FNA were excluded, while suspicious/malignant cytology was confirmed by excisional biopsy and pathology. Benign lesions were confirmed by follow-up imaging within one year. The agreement of TIRADS and ATA and their relationship with pathology were determined.
**Results or Findings:** 200 patients (52.5% male) with a mean age of 53±9.2 years (36-68) were enrolled. Twenty-seven (13.5%) nodules were malignant [PTC:14, FTC:11, MTC:2]. The Kappa coefficient of agreement between TIRADS and ATA was 0.96 (P<0.001). 5 cases showed non-concordance(2.5%): 4 nodules with high ATA and TIRADS of 4 (2 benign and 2 PTC), and one FTC nodule with low ATA and TIRADS of 4. Considering pathology, 106 benign nodules showed ATA of low or very low and TIRADS of 2 or 3; 10 PTC nodules showed high ATA and TIRADS of 5; 2 MTC nodules showed high ATA and TIRADS of 5; 45 benign nodules showed high ATA and TIRADS of 5; 20 benign nodules showed intermediate ATA and TIRADS of 4; 10 FTC nodules showed intermediate ATA and TIRADS of 4; and 2 PTC nodules showed intermediate ATA and TIRADS of 4.
**Conclusion:** TIRADS and ATA can be used interchangeably due to high agreement.
**Limitations:** Unsatisfactory sample and pathology results.
**Ethics committee approval:** The project was approved by the ethics committee of the Tehran University of Medical Sciences.
**Funding for this study:** No funding to dislose.
**Author Disclosures:**
Majid Shakiba: Nothing to disclose
Ario Mohammadi: Nothing to disclose
Nesa Shahbazifar: Nothing to disclose
Mahsa Alborzi Avanaki: Nothing to disclose
Mohadese Tohidi: Nothing to disclose
Leila Aghaghazvini: Nothing to disclose

### RPS 1708-5

**Predictive value of 18F-FDG PET / CT in BRAFV600E mutation in patients with differentiated thyroid cancer**
*Z. Jiang*; Guilin/CN
*(jzw18377330355@163.com)*

**Purpose:** To explore the value of 18F-FDG PET / CT parameters in predicting BRAFV600E mutation in patients with differentiated thyroid cancer (DTC).
**Methods or Background:** The clinical data of 141 patients with DTC confirmed by surgery were analysed retrospectively. All patients underwent 18F-FDG PET / CT and BRAFV600E mutation detection. The differences of clinical characteristics (age, gender, pathological type, tumour diameter, lymph node metastasis, extrathyroidal extension, clinical stage, Ki-67) and PET/CT parameters (maximum standardised uptake value (SUV max) and metabolic tumour volume（MTV) between patients of BRAFV600E mutation and BRAFV600E wild type were analysed using an independent-sample t test, a $\chi^2$ test and a Fisher exact test. The receiver operating characteristic (ROC) curve was used to obtain the best boundary point for MTV to predict BRAFV600E mutation. A logistic regression model was utilised to analyse the variables predicting the mutation status of BRAFV600E.
**Results or Findings:** There were 74 (52.5%) patients with BRAFV600E mutation and 67 (47.5%) patients with wild type. There were significant differences between the two groups in gender, extrathyroidal extension, lymph node metastasis, pathological type, clinical stage and MTV (all $P < 0.05$). ROC curve analysis showed that the best cut-off values for MTV prediction of BRAFV600E mutation were 12.0cm3 and the area under ROC curve (AUC) was 0.64. Logistic multivariate analysis showed that extrathyroidal extension, lymph node metastasis and MTV were independent factors predicting BRAFV600E mutation ($P < 0.05$).
**Conclusion:** MTV has a certain reference value in predicting BRAFV600E mutation in patients with DTC.
**Limitations:** This study did not analyse other types of gene mutations, such as the TERT gene, which need to be discussed in future research.
**Ethics committee approval:** The protocol was approved by the Ethics Committee of Affiliated Hospital of Guilin Medical College.
**Funding for this study:** Affiliated Hospital of Guilin Medical College（2019GLMU1A009)
**Author Disclosures:**
Zewen Jiang: Author: Image analysis and data statistics

### RPS 1708-6

**Use of artificial intelligence in management of head-neck tumours: a multidisciplinary survey**
*S. Lusi*, C. Giannitto, G. Mercante, M. Scorsetti, A. Santoro, G. Spriano, L. Balzarini, L. S. Politi; Milan/IT

**Purpose:** The purpose of this study was to conduct a survey among the specialists involved in the H&N tumour board (radiologists, ENT surgeons, oncologists, radiotherapists) to evaluate the perception, fields of application and interest in AI.
**Methods or Background:** We have developed an online questionnaire consisting of 12 questions, which has been distributed since March 2021 to February 2022 through the European Society of Head and Neck Radiology (ESHNR), the head-neck section of the Italian Medical Radiology Society (SIRM), the Italian Society of ENT and Head and Neck Surgery (SIOeChCF) and social media. The areas investigated concerned personal information, academic roles, the use of AI, perceived relevance and applicative sectors.
**Results or Findings:** 102 participants with different levels of experience responded: professors (8.8%), assistant doctors (45.2%), residents (31.3%), doctoral students (14.7%). 32.3% of the participants were aged between 30 and 49; 28.6% between 40 and 49; 16.2% between 50 and 59; 12.5% under 30; and 10.37% over 60 years. Radiologists accounted for the largest share (53.9%), followed by ENT (36.2%), oncologists (3.9%), radiotherapists (3.9%) and bioinformaticians (1.9%). The most relevant AI applications were imaging data quantification (70.4%), diagnosis computer-assisted (46.5%) and outcome predictions (32.4%). 33.3% of the participants considered the use of AI as very important; 50.8% important; 4.5% essential; 8.3% not fundamental; and 3% irrelevant. Participants showed interest in future applications in precision diagnosis and preoperative planning (72%), together with the choice of more adequate treatment (58.1%) and time-saving in patient management (52.9%).
**Conclusion:** The results of this survey demonstrate a relevant interest in AI in the multidisciplinary management of H&N cancers, in particular for precision diagnosis, surgical planning and personalised treatment.
**Limitations:** No limitations were identified.
**Ethics committee approval:** No need for the approval.
**Funding for this study:** No funding for the study.
**Author Disclosures:**
Caterina Giannitto: Nothing to disclose
Armando Santoro: Nothing to disclose
Giuseppe Mercante: Nothing to disclose
Letterio Salvatore Politi: Nothing to disclose
Giuseppe Spriano: Nothing to disclose
Stefano Lusi: Nothing to disclose
Marta Scorsetti: Nothing to disclose
Luca Balzarini: Nothing to disclose

### RPS 1708-7

**HEad and neCK TumOR segmentation and outcome prediction: The HECKTOR challenge**

V. Andrearczyk[1], V. Oreiller[1], M. Jreige[2], H. Elhalawani[3], S. Boughdad[2], C. Chez Le Rest[4], J. O. Prior[2], M. Hatt[5], *A. Depeursinge*[1]; [1]Sierre/CH, [2]Lausanne/CH, [3]Cleveland, OH/US, [4]Poitiers/FR, [5]Brest/FR
*(adrien.depeursinge@hevs.ch)*

**Purpose:** Automatic segmentation and radiomics on 2-[18F]fluoro-2-deoxyglucose Positron Emission Tomography (FDG-PET) / Computed Tomography (CT) images and clinical data can contribute in optimizing patient management for head and neck cancer. We present the outcomes after the first two editions (2020/2021) of the HECKTOR-MICCAI challenge and discuss the future goals.
**Methods or Background:** We collected FDG-PET/CT images with clinical data from 5/6 centers in 2020/2021, totaling 254/325 cases. Primary tumors were delineated by experts according to established guidelines. The HECKTOR challenge was organized at the MICCAI 2020 and 2021 conferences to evaluate algorithms on tumor segmentation and prediction of Progression-Free Survival (PFS).
**Results or Findings:** Simple, well-designed ensembles of 3D U-Nets obtained the best segmentation results (Dice coefficient of 0.76/0.78). PET images contained the most predictive information with the tumor metabolic uptake acting as tumor detection. In association with CT providing additional tissue characteristics, multi-modal models provided the best results. Some nodal metastases were hard to differentiate from primary tumors. A high inter-algorithm agreement was obtained, with some cases incorrectly segmented by all algorithms. For the PFS prediction, approaches relying on deep learning and/or standard radiomics were proposed. Best performance (C-index of 0.72) was achieved without using the expert contours.
**Conclusion:** The growing interest in the challenge is matched by the growth of the consortium and dataset. We conducted various post-challenge analyses, leading the path for new challenge designs and tasks. The challenge will be organized in 2022 with more than 1000 cases.
**Limitations:** The gold-standard segmentation used in this study was expert manual annotation on the PET/CT fusion image without contrast. This can lead to errors and could explain the saturation in segmentation results.
**Ethics committee approval:** Obtained for all collected data.
**Funding for this study:** SNSF VISIBLE (205320 179069)
SPHN IMAGINE
HASLER MSXplain
HASLER LOCALITY
**Author Disclosures:**
Mathieu Hatt: Nothing to disclose
Valentin Oreiller: Nothing to disclose
Adrien Depeursinge: Nothing to disclose
Sarah Boughdad: Nothing to disclose
Mario Jreige: Nothing to disclose
John O Prior: Nothing to disclose
Vincent Andrearczyk: Nothing to disclose
Catherine Chez Le Rest: Nothing to disclose
Hesham Elhalawani: Nothing to disclose

### RPS 1708-8

**Can quantitative diffusion-weighted MR imaging be useful as non-invasive prognostic marker to predict histologic type in uveal melanomas?: preliminary results**

*C. Ini*, P. V. Foti, G. Broggi, R. Caltabiano, R. Farina, C. Spatola, A. Russo, S. Palmucci, A. Basile; Catania/IT
*(corrado.ini@gmail.com)*

**Purpose:** The prognosis of uveal melanomas (UMs) is related to several factors, among which are histopathologic features. Histologically, UM can be classified as spindle cell, mixed cell and epithelioid cell type, the latter being related to a more severe prognosis. The diagnosis of UM is almost exclusively clinical, whereas tumour-sampling procedures are not routinely performed due to their invasiveness and related complications. The aim of our study was to retrospectively assess the correlation between apparent diffusion coefficient (ADC) values and the histologic type of uveal melanomas, in order to verify the clinical value of diffusion-weighted magnetic resonance imaging (DWI) as non-invasive prognostic marker.
**Methods or Background:** 16 uveal melanomas that had undergone MR examination (with conventional and DWI sequences) and subsequent primary enucleation without any previous radiotherapy treatment were retrospectively selected. The ADC value of the tumor was compared with histologic type. For the final analysis, histologic type was dichotomised as epithelioid versus non-epithelioid cell type (including spindle cell and mixed cell type). The data were compared by using the independent t test.
**Results or Findings:** Histologic type was as follows: epithelioid cell type (n = 3), non-epithelioid cell type (n = 13; spindle cell n = 7, mixed cell type n = 6). The overall ADC value of UMs was 1.06 ± 0.22 × 10-3mm2/s. The mean ADC value of UMs was 1.17 ± 0.12 × 10-3mm2/s in the epithelioid cell type group and 1.03 ± 0.23 × 10-3mm2/s in the non-epithelioid cell type group. No significant difference between the two groups was found (t = 0.4, p = 0.34).
**Conclusion:** Quantitative measurement of DWI-ADC of UMs is not accurate enough to non-invasively select patients with epithelioid cell type.
**Limitations:** Retrospective.
**Ethics committee approval:** Under evaluation.
**Funding for this study:** No funding was received for this study.
**Author Disclosures:**
Renato Farina: Nothing to disclose
Andrea Russo: Nothing to disclose
Pietro Valerio Foti: Nothing to disclose
Rosario Caltabiano: Nothing to disclose
Corrado Ini: Nothing to disclose
Corrado Spatola: Nothing to disclose
Antonio Basile: Nothing to disclose
Giuseppe Broggi: Nothing to disclose
Stefano Palmucci: Nothing to disclose

**08:00-09:00** **Room X**

### Research Presentation Session: Interventional Radiology

### RPS 1709

### Arterial and venous interventions

**Moderator**
R. Schernthaner; Vienna/AT
**Author Disclosure:**
R. Schernthaner: Grant Recipient: Siemens Healthineers

### RPS 1709-2

**Initial clinical experience of intraprocedural aortic aneurysm sac embolisation using shape memory polymer devices during endovascular aneurysm repair**

A. Massmann[1], *F. Frenzel*[1], R. Shayesteh-Kheslat[1], P. Berg[2], A. Buecker[1], P. Fries[1]; [1]Homburg/DE, [2]Kevelaer/DE

**Purpose:** Feasibility of periprocedural aortic aneurysm sac embolisation using a novel shape memory polymer embolic device.
**Methods or Background:** The embolic scaffold is a self-expanding porous polymer structure, which expands during contact with blood and has been shown to support aneurysm shrinkage in animal studies. Retrospective analysis of 18 consecutive patients (88.9% male, mean age 72±9 years) treated at two centres in Germany, which were candidates for elective endovascular thoracic or abdominal aortic aneurysm repair and non-calcified iliac access vessels to enable a parallel wire approach to the aneurysm lumen using large transfemoral access sheaths were included. The aneurysm sacs were implanted with shape memory polymer devices immediately following aortic endograft placement.
**Results or Findings:** Technical success was achieved in all patients. Mean baseline aortic aneurysm sac volume was 195±117 mL, and the perfused aneurysm volume was 97±60 mL. Patients were implanted with a mean of 24±12 shape memory polymer devices (range, 5-45, corresponding to 6.25-56.25 mL expanded embolic material volume per patient). At 11±7 months (range, 3-24 months) follow-up, mean change in aneurysm sac volume of 16 patients was −30 ± 21 mL (p = .0006); two patients have not yet reached 3-month follow-up. Sac regression was even observed in the presence of type II endoleaks in 6 patients and 2 small type IA endoleaks, without necessity for further intervention to date. No morbidity or mortality related to sac treatment with shape memory polymer devices occurred.
**Conclusion:** Usage of shape memory polymer devices for active aortic aneurysm sac management appears to be safe and feasible in this initial small case series. Prospective studies are needed to further evaluate effectiveness.
**Limitations:** Not applicable.
**Ethics committee approval:** Not applicable.
**Funding for this study:** Not applicable.
**Author Disclosures:**
Peter Fries: Nothing to disclose
Arno Buecker: Nothing to disclose
Patrick Berg: Nothing to disclose
Roushanak Shayesteh-Kheslat: Nothing to disclose
Felix Frenzel: Nothing to disclose
Alexander Massmann: Advisory Board: Shape Memory Medical

### RPS 1709-3

### Diagnostic performance of digital variance angiography in reduced radiation lower limb examinations

*I. Góg*, M. Gyánó, Z. Mihály, P. Legeza, J. P. P. Kiss, S. Osváth, K. Szigeti, B. Nemes, P. Sótonyi; Budapest/HU
*(gogistvan@gmail.com)*

**Purpose:** In previous clinical studies Digital Variance Angiography (DVA) provided higher contrast-to-noise ratio and better image quality than Digital Subtraction Angiography (DSA). The aim of this study was to investigate the diagnostic performance of DVA in reduced radiation lower limb examinations.
**Methods or Background:** We enrolled 30 patients undergoing diagnostic lower limb x-ray angiography. In three anatomical regions duplicated series were made by normal and reduced dose x-ray protocol (N: normal 1,2 microGy/frame, R: reduced 0,36 microGy/frame). The diagnostic value of DSA and DVA images was evaluated in a task-based survey. Six readers were asked to identify the arteries and evaluate the degree of stenosis. The endpoints were the number of recognised arteries, and the sensitivity and specificity of DVA-R images compared to DSA-N.
**Results or Findings:** There was no significant difference in the overall number of recognised arteries (DSA-N: 5.56±0.01, DVA-R: 5.46±0.01) and in the proportion of arteries suitable for diagnosis (DSA-N: 92.3±0.1 %, DVA-R: 93.5±0.1 %). DVA-R reproduced the DSA-N diagnostic categories with 0.84 sensitivity and 0.80 specificity. When the discordant decisions were supervised and the valid diagnostic category was determined by an expert, the accuracy of DSA-N and DVA-R was identical in the abdominal and femoral regions, but DVA-R had significantly higher accuracy in the crural region (91% vs 80%).
**Conclusion:** DVA allows a very substantial (70%) reduction of DSA-related radiation exposure in lower limb angiographiy without compromising the diagnostic value of images, therefore this technology might increase the safety of these endovascular procedures.
**Limitations:** Single-centre study in dedicated anatomical regions.
**Ethics committee approval:** The study was approved by the Hungarian National Institute of Pharmacy and Nutrition.
**Funding for this study:** Funding was received from the European Union's Horizon 2020 EIC Accelerator program and the National Research, Development and Innovation Fund of the Ministry of Innovation and Technology of Hungary.
**Author Disclosures:**
Janos Pal P. Kiss: Employee: Kinepict Health Ltd.
Krisztián Szigeti: CEO: Kinepict Health Ltd.
Balázs Nemes: Nothing to disclose
István Góg: Employee: Kinepict Health Ltd.
Szabolcs Osváth: CEO: Kinepict Health Ltd.
Péter Legeza: Employee: Kinepict Health Ltd.
Marcell Gyánó: Employee: Kinepict Health Ltd.
Péter Sótonyi: Advisory Board: Kinepict Health Ltd.
Zsuzsanna Mihály: Employee: Kinepict Health Ltd.

### RPS 1709-4

### Unreported venous thromboembolism on routine CT imaging of the abdomen and pelvis and the possibility of earlier diagnosis and intervention

*N. A. O'Halloran*, C. Lehane, E. O'Malley, G. J. O'Sullivan; Galway/IE

**Purpose:** The true prevalence of venous thromboembolism (VTE) is underestimated because many cases are not apparent clinically. About half of VTEs in cancer patients are incidentally detected without clinical suspicion of VTE. Incidental VTEs are mostly diagnosed by multidetector CT (MDCT) scans requested for oncologic staging. However, VTE on routine imaging may be overlooked by non-vascular specialist radiologists. The aim of this study was to evaluate if VTE may be detected earlier in asymptomatic patients on routine abdominal CT imaging.
**Methods or Background:** All patients who underwent intervention for lower limb and pelvic VTE between 2008 and 2021 and who had a CT abdomen/pelvis one month prior to intervention were identified. These studies were reviewed by a vascular interventional radiologist to assess for unreported deep venous disease.
**Results or Findings:** We identified 514 patients who underwent venous intervention for inferior vena cava or iliofemoral venous disease who had previous CT investigation. The mean interval time from first CT to radiological intervention was 2.2 years (range 1 month-10 years). Patients underwent a mean of 4.3 studies prior to intervention. 65% of retrospectively reviewed CTs demonstrated IVC or ileofemoral venous disease. The presence of venous pathology was described in 14% of reports provided by non-vascular specialist consultant radiologists.
**Conclusion:** Deep venous pathology is often under-reported on CT abdomen/pelvis. The high rate of venous intervention demonstrated in this study in this patient cohort highlights the importance of including abdominal and pelvic veins as a review area when reporting all CTs to allow for earlier and therefore more effective intervention and reducing patient morbidity.
**Limitations:** Single centre study.
**Ethics committee approval:** This study was approved by an ethics committee.
**Funding for this study:** No funding was received for this study.
**Author Disclosures:**
Ciannait Lehane: Nothing to disclose
Niamh A O'Halloran: Nothing to disclose
Eoin O'Malley: Nothing to disclose
Gerry J O'Sullivan: Nothing to disclose

### RPS 1709-5

### Risk factors associated with difficult-retrieval inferior vena cava filters (D-RIVCF)

*E. Vila-Trias Jover*, E. Serrano Alcalá, N. Macías, F. Zarco, F. Gómez Muñoz, D. Corominas Muñoz, A. Lopez Rueda; Barcelona/ES
*(elivilatrias@gmail.com)*

**Purpose:** The objective is to analyse which variables are associated with D-RIVCF.
**Methods or Background:** Thromboembolic disease, including deep vein thrombosis and pulmonary embolism, is a frequent pathology associated with high morbidity and mortality rate. The initial treatment is anticoagulation, but is associated with an increased bleeding risk. In those patients in whom anticoagulation is not recommended, placement of temporary IVCF would be indicated. IVCF are not free of complications, so they should be removed as soon as the indication that led to their placement ceases.
**Results or Findings:** Single-centre, observational, retrospective study of patients undergoing inferior vena cava (IVC) filters removal between May 2015 and May 2021. The primary endpoint was D-RIVCF, defined as a procedure requiring more than five minutes of fluoroscopy. Demographic variables and comorbidities were collected. The type of filter, time from placement (<45 days vs >45 days) and the angiographic variables angulation with respect to IVC (>15º), hook against wall and embeded legs(>3mm) were analysed. A total of 109 patients (mean age 65 years old, 64.2% males) met the criteria for analysis. Mean fluoroscopy time for removal was 12.2 minutes (0.3-132.9 minutes), in 49.5% (n54) of cases >5mins of fluoroscopy were necessary. Univariate analysis showed significant differences between the two groups in the variables hook against IVC wall (D-RIVCF 44.4% vs 23.6%; p = 0.027), embebed legs (D-RIVCF20.4% vs 3.6%; p = 0.008) and time from placement (D-RIVCF 51.9% vs 5.5%; p = 0.006).
**Conclusion:** This retrospective study shows a significant association between D-IVCFR in patients with embedded legs, hook against IVC wall and time from placement. Understanding and identifying the variables that hinder removal helps better planning of the procedure.
**Limitations:** The limitations were the retrospective nature of the study and the lack of long-term follow-up.

**Ethics committee approval:** The study protocol was approved by the local Clinical Research Ethics Committee (registration number HCB.2021.0729).
**Funding for this study:** No funding was received for this study.
**Author Disclosures:**
Federico Zarco: Nothing to disclose
Napoleón Macías: Nothing to disclose
Elena Serrano Alcalá: Nothing to disclose
Fernando Gómez Muñoz: Nothing to disclose
Daniel Corominas Muñoz: Nothing to disclose
Antonio Lopez Rueda: Nothing to disclose
Elisabet Vila-Trias Jover: Nothing to disclose

### RPS 1709-6

**Volume-flow-guided angioplasty of dysfunctional autologous arteriovenous fistula: the VOLA Study**

*P. Filippou*[1], E. Efthymiou[1], O. Moschovaki Zeiger[1], K. Palialexis[1], K. Katsanos[2], P. M. Kitrou[2], D. Filippiadis[1], S. C. Spiliopoulos[1], E. Brountzos[1]; [1]Athens/GR, [2]Patras/GR
*(panagiof@yahoo.gr)*

**Purpose:** To investigate the use of volume-flow-guided angioplasty (VFA) using sequential intraprocedural duplex ultrasound (DUS), in dysfunctional autologous arteriovenous fistula (AVF) as a quantifiable functional endpoint of endovascular treatment.
**Methods or Background:** The study prospectively investigated 20 consecutive patients (23 lesions; 16 men; mean age 67±16 years) with dysfunctional AVF undergoing fluoroscopically-guided, balloon angioplasty between June 2019-May 2020. Primary endpoints were outcome quantification using sequential DUS VF analysis following each dilation, 6-months target lesion re-intervention (TLR)-free rate, procedural success (postprocedural VF value≥to the baseline steady-state access) and correlation between procedural success and TLR-free rate. Secondary endpoints included 6-months lesion late lumen loss (LLL), correlation between balloon diameter and intraprocedural VF values, and correlation between VF and LLL at 6 months.
**Results or Findings:** Mean VF increase was 168.5%±102.5% (range: 24.24%-493.33%). Procedural success was 80%a (16/20 cases). VFA improved procedural success by 20% (4/20 cases) compared to standard assessment (<30% residual stenosis and palpable thrill). TLR-free rate was 78.3%, 67.3% at 6 and 12 months. Significantly less TLR was noted in cases of procedural success (82.4% vs 66.7% 6-months; p=0.041). Unweighted linear regression showed significant correlation between diameter of balloon and VF (146.9±42.3ml/min VF gain per mm of balloon diameter; p=0.001, R2=0.23) and a significant correlation between LLL and VF decline at follow-up (102.0±34.6ml/min loss per mm of LLL; p=0.01, R2=0.35). Optimal VF cut off value and percentile increase to predict access failure were 720 ml/min (sensitivity 58.3%, specificity 71.4%) and 153% (sensitivity 66.7%, specificity 85.7%); respectively.
**Conclusion:** Intraprocedural VF-guidance seems to improve AVF angioplasty outcomes.
**Limitations:** Single-centre study; small sample size.
**Ethics committee approval:** The study was approved by the Hospital's Scientific Board and was registered in a publically available database (clinicaltrials. gov: NCT04430478).
**Funding for this study:** This study was not funded.
**Author Disclosures:**
Ornella Moschovaki Zeiger: Nothing to disclose
Konstantinos Palialexis: Nothing to disclose
Dimitrios Filippiadis: Nothing to disclose
Elias Brountzos: Nothing to disclose
Konstantinos Katsanos: Nothing to disclose
Stavros C. Spiliopoulos: Nothing to disclose
Panagiotis M Kitrou: Nothing to disclose
Panagiotis Filippou: Nothing to disclose
Evgenia Efthymiou: Nothing to disclose

### RPS 1709-7

**A single-centre experience of percutaneous arteriovenous fistula creation with an RF-based EndoAVF System**

M. Theofanis, *V. Taki*, L. Balta, P. M. Kitrou, D. Karnabatidis, G. Georgopoulou, M. Papasotiriou, E. Papachristou, D. Goumenos; Patras/GR

**Purpose:** To retrospectively estimate the safety and efficacy of percutaneous arteriovenous fistula (pAVF) creation with the WavelinQ™ 4Fr EndoAVF System.
**Methods or Background:** From February 2018 to June 2020, 30 pAVF were created in 30 consecutive patients (30/30 male, age: 55.3±13.6years). 21/30 patients (70%) were already on hemodialysis using a central venous catheter. Outcome evaluations were technical success, complications, and cannulation rate. Secondary outcome evaluations included the number of secondary procedures needed for cannulation, maintenance time to cannulation and pAVF survival.
**Results or Findings:** Technical success was 100%. Complication rate was 6.7% (2/30) including a pseudoaneurysm of the brachial artery that occured immediately after sheath removal and an aneurysm of the anastomosis 17 days post-procedure, which was isolated with a covered stent placed in the arterial side. Mean follow-up was 547±315.7 days (range: 14-1071). Cannulation rate was 86.7% (26/30). Mean time to cannulation was 61.3±32.5 days (range: 15-135). Mean follow-up after cannulation was 566.2±252.7 days (range: 35-1041 days). Four pAVFs were thrombosed after cannulation with two of them successfully declotted. Sixteen interventions were needed to achieve cannulation after the index procedure in 15 patients (overall 0.53 procedures/patient). Seven conservative endovascular interventions (following cannulation) were performed during the follow-up period in 6 patients (overall 0.27 procedures/patient, 0.17 procedures/patient-years). For the pAVF that were cannulated, survival was 96.1% at one year, and 82% at two and three years according to Kaplan Meier survival analysis.
**Conclusion:** This primary experience suggests that pAVF creation is secure, successfully performed with high maturation and long-term patency rates. Large-scale prospective studies are needed to affirm the results.
**Limitations:** Retrospective study.
**Ethics committee approval:** Ethics commitee approval was not needed.
**Funding for this study:** No funding was received.
**Author Disclosures:**
Dimitrios Goumenos: Nothing to disclose
Vasiliki Taki: Nothing to disclose
Michail Theofanis: Nothing to disclose
Evangelos Papachristou: Consultant: BD
Marios Papasotiriou: Nothing to disclose
Georgia Georgopoulou: Nothing to disclose
Dimitrios Karnabatidis: Consultant: BD
Panagiotis M Kitrou: Consultant: BD
Lamprini Balta: Nothing to disclose

### RPS 1709-8

**Evaluation of the effectiveness and safety of local fibrinolysis guided by catheter as part of a multidisciplinary management of acute pulmonary thromboembolism**

*A. Portillo Perdomo*, A. Lorenzo, M. Lafuente Sánchez, J. Solaz Solaz, S. Buso Gil, L. G. Chávez Marroquin, M. D. F. Ferrer Puchol, E. Esteban Hernández; Alzira/ES

**Purpose:** To assess the efficacy and safety of catheter-guided local fibrinolysis in acute pulmonary thromboembolism (APT) with hemodynamic stability and right ventricular dysfunction (RVD).
**Methods or Background:** Retrospective descriptive study in adult intensive care unit (ICU). Catheter-guided local fibrinolysis (pigtail 5F catheter) was performed with a bolus of 250,000 IU urokinase followed by continuous infusion of 100,000 IU/hour until arteriographic control at 24 and/or 48h. After treatment, and before hospital discharge, a follow-up echocardiography was performed. Clinical success was defined by no progression to shock, decreased thrombotic load and systolic pulmonary artery pressure (PAPs), improvement in RVD, and survival to ICU discharge. Safety variables were major bleeding events and procedure-related complications.
**Results or Findings:** 23 patients were included (mean age 64 ± 14 years). Clinical success was obtained in 91.3% of the patients. Angiographic control revealed a reduction in thrombotic load in 91.3% of cases. The mean PAPs (mmHg) decreased from 54 to 34 (P-value = 3.616e-07). In the ultrasound control, an improvement in DVD was observed in all cases with an increase in the mean TAPSE (mm) from 13.8 to 19.6 mm (p-value = 0.002293) and an increase in mean St from 7.9 to 12. 5 cm/s (p-value = 0.01881), 95% CI. Survival to hospital discharge was 95.7%. One patient progressed to shock. There were two minor bleeding complications.
**Conclusion:** Catheter-guided local fibrinolysis is effective for the treatment of PE in our case series by significantly reducing thrombotic load, PAPs, and RVD, thus avoiding progression to hemodynamic instability, and without associating life-threatening complications or mortality related to the procedure.
**Limitations:** Retrospective study with limited sample. Diagnostic techniques limitations.
**Ethics committee approval:** Approved by the ethics committe Hospital de la Ribera.
**Funding for this study:** No funding was neccesary.
**Author Disclosures:**
Abraham Portillo Perdomo: Nothing to disclose
Luis Gregorio Chávez Marroquin: Nothing to disclose
Matilde Lafuente Sánchez: Nothing to disclose
Silvia Buso Gil: Nothing to disclose
MARIA DOLORES FERRER Ferrer Puchol: Nothing to disclose
Alberto Lorenzo: Nothing to disclose
Jorge Solaz Solaz: Nothing to disclose
Enrique Esteban Hernández: Nothing to disclose

**10:30-11:30** **Open Forum #3 (ESR)**

### Research Presentation Session: Imaging Informatics / Artificial Intelligence and Machine Learning

### RPS 1805b

### Artificial intelligence (AI) in prostate imaging

**Moderator**
S. Morozov; Liège/BE
**Author Disclosure:**
S. Morozov: Employee: Osimis S.A.

### RPS 1805b-3

**Metadata-independent classification of MRI sequences using convolutional neural networks: successful application to prostate MRI**
*G. L. Baumgärtner*, C. Hamm, P. Asbach, N. L. Beetz, M. Rudolph, F. Dräger, K. P. Froböse, F. Biesmann, T. Penzkofer; Berlin/DE
*(georg.baumgaertner@charite.de)*

**Purpose:** To develop and test a convolutional neural network that recognises the type and anatomical orientation of MRI volumes solely from imaging without relying on DICOM metadata. Automatic sequence detection would have a wide variety of applications in AI, conventional reporting or quality assurance.
**Methods or Background:** The underlying dataset included 31,602 prostate MRI volumes of 10 different sequence types (localiser, axial T1, axial/coronal/sagittal T2, axial ADC map, axial DWI, axial DWI high b-value, axial DWI exponential, axial contrast-enhanced T1 dynamic/subtraction) from 1243 patients. The images were rescaled to a common size of 64 x 64 x 64 voxels. A 3D ResNet18 was trained to differentiate the sequence classes using a standard training/validation/hold-out test set strategy.
**Results or Findings:** The ResNet18 model was able to consistently classify the MRI sequences of the hold-out test set with an accuracy of 99.7% (i.e. less than 1 in 340 volumes is misclassified). The sensitivities of the individual sequences were between 97.4% and 100%.
**Conclusion:** The developed ResNet successfully distinguishes different sequences with very high precision. The trained model can quickly assign sequences to large data sets with unknown naming and/or metadata standards. In the context of automatic sequence identification, the trained model shows a number of potential clinical and scientific applications, such as managing data from multiple sources with inconsistent naming standards, quality assurance of imaging protocols, and identification of appropriate imaging studies for study cohorts.
**Limitations:** Considering that this study is limited to a prostate MRI dataset (which itself is a benefit), nevertheless further studies should investigate the applicability to more heterogeneous data sets, including imaging volumes from different modalities such as CT.
**Ethics committee approval:** This study was approved by the local IRB. Patient consent was waived.
**Funding for this study:** Financial support was received from the Berlin Institute of Health.
**Author Disclosures:**
Nick Lasse Beetz: Nothing to disclose
Charlie Hamm: Nothing to disclose
Madhuri Rudolph: Nothing to disclose
Franziska Dräger: Nothing to disclose
Tobias Penzkofer: Grant Recipient: Berlin Institute of Health (Clinician Scientist Grant, Platform Grant) Other: fees for a book translation (Elsevier). Research/Grant Support: AGO, Aprea AB, ARCAGY-GINECO, Astellas Pharma Global Inc. (APGD), Astra Zeneca, Clovis Oncology, Inc., Dohme Corp, Holaira, Incyte Corporation, Karyopharm, Lion Biotechnologies, Inc., MedImmune, Merck Sharp, Millennium Pharmaceuticals, Inc., Morphotec Inc., NovoCure Ltd., PharmaMar S.A. and PharmaMar USA, Inc., Roche, Siemens Healthineers, and TESARO Inc.
Konrad Philip Froböse: Nothing to disclose
Patrick Asbach: Nothing to disclose
Georg Lukas Baumgärtner: Shareholder: Siemens AG
Felix Biesmann: Nothing to disclose

### RPS 1805b-4

**Characterisation of prostate cancer at multiparametric MRI using machine learning: development and assessment of a zone-specific computer-aided diagnosis system**
*T. Jaouen*, R. Souchon, O. Rouviere; Lyon/FR
*(tristan.jaouen@inserm.fr)*

**Purpose:** Develop a zone-specific computer-aided diagnostic (CAD) system capable of performing well on a variety of magnetic resonance (MR) scanners, and retrospectively assess its performances in an external cohort of patients.
**Methods or Background:** Multiparametric MR imaging is recommended prior to prostate biopsies but lacks specificity. A CAD system based on quantitative imaging could help radiologists. The CAD extracted quantitative features from regions-of-interest (ROIs) of the peripheral and the transition zone of the prostate. Normalisation methods were investigated to handle vendor-dependent features such as T2-weighted signal and apparent diffusion coefficient. Logistic regression models combined the most efficient features to characterise International Society of Urological Pathology (ISUP) grade ≥2 cancers in these regions. Models were developed and selected in a prospectively-maintained training data set of 290 patients imaged on four MR scanners and treated by prostatectomy at our institution, and then in a refinement data set of 114 consecutive patients referred for prostate biopsy and imaged on two of the previous scanners. Finally, models combined in the CAD were compared to the Prostate Imaging-Reporting and Data System version 2 (PI-RADSv2) in an external test data set in which a radiologist delineated lesions targeted at biopsy for 119 random patients referred for prostate biopsy.
**Results or Findings:** In the test data set, the area under the receiver operating characteristic curve (AUC) for characterizing ISUP≥2 cancers was 85.8% (95%CI: 81-91) for PI-RADSv2. The CAD AUC was 86.1% (95%CI: 79-93; p-value=1). The CAD sensitivity was 93% (95% CI: 89-100), its specificity was 70% (95%CI: 64-76).
**Conclusion:** The ROI-based zone-specific CAD provided robust results similar to those of a specialised uro-radiologist.
**Limitations:** Not applicable
**Ethics committee approval:** This study has been approved by the local IRB. Informed consent was obtained from all patients.
**Funding for this study:** Grant RHU PERFUSE (ANR-17-RHUS-0006), European Patent Application EP21305545.2; there are no other conflicts of interest.
**Author Disclosures:**
Rémi Rémi Souchon: Grant Recipient: Grant RHU PERFUSE (ANR-17-RHUS-0006) Patent Holder: European Patent Application EP21305545.2
Tristan Jaouen: Patent Holder: European Patent Application EP21305545.2 Grant Recipient: Grant RHU PERFUSE (ANR-17-RHUS-0006)
Olivier Rouviere: Grant Recipient: Grant RHU PERFUSE (ANR-17-RHUS-0006) Patent Holder: European Patent Application EP21305545.2

### RPS 1805b-5

**Interactive deep-learning-based real-time three-dimensional lesion segmentation from single point seeds in prostate MRI**
*A. Schrader*, N. B. Netzer, V. Saase, M. Görtz, M. Hohenfellner, H-P. Schlemmer, D. Bonekamp; Heidelberg/DE

**Purpose:** MRI-directed targeted biopsy has become the standard for prostate cancer diagnosis and relies on lesion segmentations. Manual segmentation is time-consuming and suffers from limited inter-reader reliability. We propose a real-time deep learning (DL) inference model for interactive lesion segmentation from placement of small lesion seed points and allowing for lesion volume adjustments.
**Methods or Background:** 1848 manually segmented lesions from 914 patients were used for CNN training in 5-fold cross-validation. Testing was performed on a subsequent test set containing 427 lesions from 236 patients. Lesion positions were indicated by small oblate spheres placed randomly inside training segmentations. 3D-UNet ensemble was trained with T2w images and lesion seeds as input, predicting full segmentations by minimising boundary CE and dice loss. For testing, seeds were placed at the center-of-mass of ground truth ROIs, then probability map thresholds were set to A) a fixed value of 0.5, corresponding to default lesion proposals; B) custom thresholds, matching lesion volume to ground truth volume, comparable to interactive fine-tuning of thresholds by users. Overlap between CNN and manual segmentations was quantified with Dice coefficients.
**Results or Findings:** Median Dice coefficient was A) 0.63 (0.51-0.72) for fixed thresholds and B) 0.70 (0.60-0.78) for volume-matched thresholds. For PROSTATEx, median Dice on significant lesions was A) 0.68 (0.58-0.73) and B) 0.71 (0.66-0.76). Processing and prediction took on average 247ms per lesion.

**Conclusion:** This real-time DL annotation assistance system achieves Dice scores approaching the range of inter-radiologist agreement, which is improved further by user thresholding. Fast prediction times allow repeated real-time seed placement and interactive volume adjustment.
**Limitations:** Similar studies for automatic segmentations should be undertaken for ADC/DCE and systems should be evaluated by multiple users during manual interaction.
**Ethics committee approval:** Informed consent was waived for this retrospective study.
**Funding for this study:** Not applicable
**Author Disclosures:**
Magdalena Görtz: Nothing to disclose
David Bonekamp: Other: Honorarium/Consultancy fees, payment for lectures: Bayer Vital Speaker: Bayer Vital
Markus Hohenfellner: Nothing to disclose
Adrian Schrader: Nothing to disclose
Nils Bastian Netzer: Nothing to disclose
Victor Saase: Nothing to disclose
Heinz-Peter Schlemmer: Speaker: Siemens, Curagita, Profound, Bayer Grant Recipient: BMBF, Deutsche Krebshilfe, Dietmar-Hopp-Stiftung, Roland-Ernst-Stiftung Consultant: Curagita, Bayer Board Member: Curagita Other: Honorarium/Consultancy fees, travel support: Siemens, Curagita, Profound, Bayer

### RPS 1805b-6

**AI assisted biparametric MRI surveillance of prostate cancer: feasibility study**

*C. Roest*[1], T. Kwee[1], A. Saha[2], J. J. Futterer[2], D. Yakar[1], H. Huisman[2]; [1]Groningen/NL, [2]Nijmegen/NL

**Purpose:** To evaluate the feasibility of automatic longitudinal analysis of consecutive bi-parametric MRI (bpMRI) scans to detect clinically significant (cs) prostate cancer (PCa).
**Methods or Background:** This retrospective study included a multi-center data set of 1539 patients who underwent bpMRI (T2+DWI) between 2014 and 2020, of whom 105 patients underwent at least two consecutive bpMRI before biopsy without pathologically confirmed csPCa prior to follow-up. A deep learning prostate cancer detection model was developed and trained to produce a heatmap of all PIRADS≥2 lesions across baseline and current studies. The aligned heatmaps for each patient's baseline and current examination were used to extract differential volumetric and likelihood features reflecting explainable changes between examinations. A logistic classifier was trained to predict from these features csPCa (ISUP>1) at the time of the current examination according to biopsy. A model trained on the current study only was developed for comparison. An extended model was developed incorporating clinical parameters (PSA density and age). Cross-validation was performed to assess the detection performance of the models on unseen data. The diagnostic performance of the best model was compared to the radiologist scores. Diagnostic accuracies are compared using likelihood ratio tests and ROC analysis.
**Results or Findings:** The model including baseline and current study (AUC 0.73 CI: 0.49 0.89) performed better than the current only model (AUC 0.70 CI: 0.42 0.86), and significantly (P=0.002) improved fit. Adding clinical variables further improved diagnostic performance (AUC 0.79 CI: 0.60 0.94). The extended surveillance model's performance was comparable to that of the radiologist (AUC 0.69 CI: 0.52 0.86).
**Conclusion:** Our proposed AI-assisted surveillance of prostate MRI can pick up explainable, diagnostically relevant changes with promising diagnostic accuracy.
**Limitations:** Not applicable
**Ethics committee approval:** Not applicable
**Funding for this study:** This study was funded by a grant from Siemens Healthineers.
**Author Disclosures:**
Henkjan Huisman: Research/Grant Support: Siemens Healthineers
Thomas Kwee: Nothing to disclose
Anindo Saha: Nothing to disclose
Derya Yakar: Grant Recipient: Health Holland Consultant: Astellas Grant Recipient: Siemens Healthineers
Jurgen J. Futterer: Grant Recipient: Siemens Healthineers
Christian Roest: Grant Recipient: Siemens Healthineers

### RPS 1805b-7

**Accelerated T2-weighted TSE imaging of the prostate using deep learning image reconstruction: a prospective comparison with standard T2-weighted TSE imaging**

*S. Gassenmaier*[1], S. Afat[1], D. Nickel[2], M. Mostapha[3], J. Herrmann[1], H. Almansour[1], K. Nikolaou[1], A. Othman[1]; [1]Tuebingen/DE, [2]Erlangen/DE, [3]Princeton, NY/US

**Purpose:** To investigate the impact of deep learning image reconstruction (DLR) in accelerated T2-weighted TSE imaging of the prostate on image quality, diagnostic confidence, and PI-RADS T2 score and overall PI-RADS score as compared to standard T2 TSE imaging.
**Methods or Background:** 60 patients undergoing 3T multiparametric MRI (mpMRI) for evaluation of prostate cancer were prospectively enrolled in this institutional review board–approved study between October 2020 and March 2021. After acquisition of standard T2 TSE imaging (T2S), the novel T2 TSE sequence with DLR (T2DLR) was applied in three planes. Overall, acquisition time for T2S resulted in 10:21 min versus 3:50 min for T2DLR. Image evaluation was performed by two radiologists independently in a blinded random order using a Likert scale ranging from 1-4 (4 best) and applying the following criteria: noise levels, artifacts, overall image quality, diagnostic confidence, and lesion conspicuity. Additionally, T2 and PI-RADS scoring were performed.
**Results or Findings:** Mean patient age was 69±9 years (range, 49–85 years). Noise levels as well as extent of artifacts were evaluated to be significantly improved in T2DLR versus T2S by both readers (p<0.05). Overall image quality was also evaluated to be superior in T2DLR versus T2S in all three acquisition planes (p=0.005 - <0.001). Lesion conspicuity was rated by both readers with a median of 4 in T2DLR versus a median of 3 in T2S (p=0.001 and <0.001, respectively).
**Conclusion:** Accelerated T2-weighted TSE imaging of the prostate in three planes including DLR is feasible, with a significant improvement of image quality, and allows an acquisition time reduction of more than 60%.
**Limitations:** The fact that there was no DLR of DCE or DWI was identified as a limitation of this study.
**Ethics committee approval:** IRB approval was obtained. Written informed consent was obtained from all patients.
**Funding for this study:** No funding was received for this study.
**Author Disclosures:**
Judith Herrmann: Nothing to disclose
Konstantin Nikolaou: Nothing to disclose
Saif Afat: Nothing to disclose
Haidara Almansour: Nothing to disclose
Mahmoud Mostapha: Employee: Siemens Medical Solutions USA Inc.
Dominik Nickel: Employee: Siemens Healthcare GmbH
Ahmed Othman: Nothing to disclose
Sebastian Gassenmaier: Nothing to disclose

**10:30-11:30** **Room M 2**

### Research Presentation Session: Neuro

### RPS 1811

### Advanced neuroimaging in various diseases

### RPS 1811-1

**Moderator**

S. Haller; Geneva/CH

**Author Disclosure:**
S. Haller: Advisory Board: EPAD; Consultant: WYSS, SPINEART; Speaker: Philips Samsung Siemens Esaote Bracco

### RPS 1811-2

**Distinct brain structural-functional network topological coupling explains different outcomes in tinnitus patients treated with sound therapy**

*Q. Chen*, H. Lv, Z. Wang; Beijing/CN
*(chenqian8319@163.com)*

**Purpose:** Topological properties, which serve as the core of the neural network, and their couplings can reflect different therapeutic effects in tinnitus patients. We hypothesised that tinnitus patients with different outcomes after sound therapy (narrowband noise) would have distinct brain network topological alterations.

**Methods or Background:** Diffusion tensor imaging and resting-state functional magnetic resonance imaging were prospectively performed in 60 patients with idiopathic tinnitus and 57 healthy controls (HCs). Graph-theoretical network analyses of structural connectivity (SC), functional connectivity (FC) and SC and FC coupling were performed. Associations between clinical performance and graph-theoretical features were also analysed.
**Results or Findings:** Treatment was effective (effective group; EG) in 28 patients and ineffective (ineffective group; IG) in 32 patients. For FC, the patients in the EG showed higher local efficiency than patients in the IG. For SC, patients in both the EG and IG displayed lower normalised characteristic path length, characteristic path length and global efficiency than the HCs. More importantly, patients in the IG had higher coupling than the HCs, whereas there was no difference in coupling between patients in the EG and HCs. Additionally, there were significant associations between the SC features and clinical performance in patients in the EG.
**Conclusion:** Our findings demonstrate that tinnitus patients exhibited significant brain network topological alterations, especially in the structural brain network. More importantly, patients who demonstrated different curative effects showed distinct SC-FC topological coupling properties. SC-FC coupling could be an indicator that could be used to predict prognoses in patients with idiopathic tinnitus before sound therapy.
**Limitations:** We didn't conduct it as a longitudinal study.
**Ethics committee approval:** This study was approved by the ethics committees of Beijing Friendship Hospital, Capital Medical University.
**Funding for this study:** No. [2015] 160 from the Beijing Scholars Program.
**Author Disclosures:**
ZhenChang Wang: Nothing to disclose
Han Lv: Nothing to disclose
Qian Chen: Nothing to disclose

### RPS 1811-4

**Intraspinal stem cell infusion in amyotrophic lateral sclerosis patients: longitudinal analysis of MR spectroscopy ratios, cortical thickness, clinical parameters and survival**

*C. Trejo Gallego*[1], N. Rodríguez Albacete[1], F. Iniesta Martinez[1], J. M. Plasencia Martinez[1], C. Vázquez Olmos[1], M. Blanquer[1], S. Martínez Pérez[2], J. M. Garcia Santos[1]; [1]Murcia/ES, [2]Alicante/ES

**Purpose:** Evaluate metabolic changes in the precentral gyrus and cortical thickness (CT) after autologous bone marrow mononuclear cell (ABMMC) intraspinal infusion in amyotrophic lateral sclerosis (ALS) patients and its clinical correlation.
**Methods or Background:** In this phase II clinical trial, 63 patients were randomized in three groups with different interventions each: intraspinal (G1) or intrathecal (G2) injection of ABMMC, or intrathecal (G3) injection of saline. Patients were followed with 1.5 T MR, performed before treatment and 12 months later. The protocol included univoxel MR spectroscopy at both hand knobs and Brain Volume BRAVO, to extract region-wise CT through surface-based morphometry analysis. Clinical surveillance included scales to evaluate functional status, muscular strength and respiratory function, as well as duration and disease progression. For statistical analysis we used SPSS 20.0.0 for Windows and JASP 0.14.
**Results or Findings:** NAA/Cho and NAA/Cho Cr in the dominant hemisphere increased 12 months after intraspinal infusion. NAA/Cho Cr in the dominant hemisphere before treatment was lower in G1 than in G2 patients. Symptoms progression before treatment was greater in the G1 patients as compared with the other groups. Besides, NAA/Cr and NAA/Cho Cr in the non-dominant hemisphere and rate of progression in the G1 patients demonstrated a negative correlation. Regarding CT in the G1 patients, 3-month MRIs showed a reduction in some points of the frontal and temporo-occipital lobes in the dominant hemisphere, as well as in the parietal and frontal lobes of the non-dominant hemisphere, which, however, increased after 12 months.
**Conclusion:** Our results go along with those of previous investigations regarding the potential trophic effect of ABMMC intraspinal injection in ALS patients .
**Limitations:** Small number of patients in the 12 month follow-up and heterogeneous distribution of the disease within each group.
**Ethics committee approval:** Obtained.
**Funding for this study:** Absent.
**Author Disclosures:**
Nicolás Rodríguez Albacete: Nothing to disclose
Juana María Plasencia Martinez: Nothing to disclose
Jose Maria Garcia Santos: Nothing to disclose
Carmen Trejo Gallego: Nothing to disclose
Carlos Vázquez Olmos: Nothing to disclose
Miguel Blanquer: Nothing to disclose
Salvador Martínez Pérez: Nothing to disclose
Francisca Iniesta Martinez: Nothing to disclose

### RPS 1811-5

**Validation of automated MS lesion detection in two independent cohorts**

*P. Ngum*[1], Y. Forslin[2], C. Cananau[2], J. Koikkalainen[1], R. Ouellette[2], E. Sandberg[2], F. Piehl[2], F. Barkhof[3], T. Granberg[2]; [1]Tampere/FI, [2]Stockholm/SE, [3]London/UK
*(peter.ngum@combinostics.com)*

**Purpose:** To examine the performance of the cNeuro® cMRI software in detecting MS lesions from T2-FLAIR images using two independent cohorts.
**Methods or Background:** The Karolinska Institutet (KI) cohort contained data from 39 relapsing-remitting MS patients on anti-CD-20 therapy with manual segmentation (19 males, 44.7±8.2 years). The MICCAI-2016 cohort contained data from 38 MS patients (8 males, 46.9±10.2 years, four sites, four scanners) with manual segmentations by seven experts used for generating a consensus ground truth. Lesions were detected using the cNeuro® cMRI software (Combinostics, Finland). Several metrics were used to evaluate performance against the ground truth: F1 score = 2*(SENS*PPV)/(SENS+PPV) for lesion detection (lesions > 3 mm3), a correlation coefficient for lesion counts and lesion volumes, and an absolute value of difference in the lesion counts (median and 95th percentile) (lesions > 10 mm3).
**Results or Findings:** The F1 score was 0.61 (KI) and 0.64 (MICCAI-2016) when compared with the ground truth. When each expert was compared with all other experts, the experts had F1 scores 0.545-0.706, while the corresponding value for automatic detection was 0.552. The correlation coefficient was 0.88 (0.92 without one outlier) (KI), 0.91 (MICCAI-2016) for lesion counts and R=0.97 (KI) and R=0.96 (MICCAI-2016) for lesion volumes. The median (95th percentile) for the absolute difference in lesion counts was 8 (28.6) (KI) and 5.5 (26.6) (MICCAI-2016).
**Conclusion:** The results show comparable overall performance, F1 score 0.61 and 0.64, in two independent cohorts. As F1 score for automated detection was within the range of seven experts (0.552 vs. 0.545-0.706), the results suggest that image quantification tools are reaching the detection level comparable to human experts.
**Limitations:** A limited number of cases was used.
**Ethics committee approval:** The study was approved by the Swedish Ethical Review Authority.
**Funding for this study:** Supported by grants provided by Medical Diagnostics Karolinska.
**Author Disclosures:**
Russell Ouellette: Nothing to disclose
Carmen Cananau: Nothing to disclose
Elisabeth Sandberg: Nothing to disclose
Juha Koikkalainen: Founder: Combinostics Oy
Frederik Barkhof: Consultant: Combinostics Oy
Peter Ngum: Employee: Combinostics Oy
Yngve Forslin: Nothing to disclose
Fredrik Piehl: Nothing to disclose
Tobias Granberg: Nothing to disclose

### RPS 1811-6

**Comparison of quick-brain and routine-brain image quality measured via MRQy, an open-source quality-control tool for imaging**

*B. Ciavarra*, D. Abeysekera, J. Wood, B. J. Reed, N. Swanston, J. Yung, K-P. Hwang, J. M. Johnson; Houston, TX/US

**Purpose:** To evaluate quantitative performance measures between "quick" brain (QB) protocol versus standard-of-care (SOC) protocol in patients at high risk of motion.
**Methods or Background:** QB sequences reduce scanning time, motion and patient sedation needs. Quantitative imaging measures were provided by MRQy, an open-source quality-control tool for imaging data. The SOC imaging group consisted of ER and inpatients receiving brain MR. QB patients were those suspected not to tolerate the SOC examination. Both studies included Ax T1, T2, T2 FLAIR, T2*, DWI, T1 post and 3D T1 post. Imaging metrics between the groups were analysed using a student's t-test.
**Results or Findings:** Mean imaging time for the SOC study (420 exams) was 25 min versus 12 min for the QB study (185 scans). Both studies included Ax T1, T2, T2 FLAIR, T2*, DWI, T1 post and 3D T1 post. The QB version utilises GRE sequences and added acceleration. Comparison of mean, range, and variance for the two groups showed no statistical difference for 3D T1 post, T2 and T2 FLAIR imaging (P > .01). Variance within measures of SNR were greater for quick sequences (T2: 0.07, 3D T1: 0.05, T1: 0.08, FLAIR 0.06) versus traditional sequences (T2: 0.06, 3D T1: 0.05, T1: 0.06, FLAIR 0.06).
**Conclusion:** The QB study reduced imaging time from ~25 to ~12 minutes without significant change in mean, range and variance of foreground intensity values, though variability within measures of SNR were slightly higher in the quick scans compared to SOC studies. Thus, the gain in speed is a trade-off for potential decrease in quantitative imaging metrics.
**Limitations:** Study relies on quantitative measures, not qualitative, which may not correlate with radiologists' quality impression.

**Ethics committee approval:** IRB of UT MDACC approved this study.
**Funding for this study:** Not applicable.
**Author Disclosures:**
Brandy Jean Reed: Nothing to disclose
John Wood: Nothing to disclose
Dylan Abeysekera: Nothing to disclose
Ken-Pin Hwang: Nothing to disclose
Jason Michael Johnson: Consultant: Kura Oncology Grant Recipient: Blue Earth Diagnostics Consultant: InformAI
Nancy Swanston: Nothing to disclose
Joshua Yung: Nothing to disclose
Bronson Ciavarra: Nothing to disclose

### RPS 1811-7

**Radiomics in MRI in the study of patients with multiple sclerosis**

*M. Stubos*, L. Calderoni, I. Gandin, A. Sartori, M. Ukmar, M. A. A. Cova; Trieste/IT

**Purpose:** The aim of this study was to evaluate the contribution of radiomics through the texture analysis in magnetic resonance imaging (MRI) in patients with MS and compare the apparently normal white matter of patients with MS with the white matter of healthy controls.
**Methods or Background:** A retrospective study on 25 patients with MS (24 with a relapsing-remitting type and 1 with a primary-progressive type) and 25 healthy controls who underwent a 3T MRI was performed. Through a dedicated software, ROIs were placed in the following areas: bilateral frontal, parietal and temporal lobes, thalami and cerebellar peduncles, genu and splenium of the corpus callosum and the lateral ventricles. Finally, features on which to develop a logistic model were chosen.
**Results or Findings:** Statistically significant differences ($p<0.05$/nfeature) were found between the two groups, especially in the frontal and parietal lobes and in the splenium of the corpus callosum. In the temporal lobe no statistically significant difference was found. As far as the thalami, the middle cerebellar peduncles and the genu are concerned, the model did not show an optimal performance.
**Conclusion:** This study allowed the identification of the statistically significative differences between the apparently normal white matter of patients with MS and the white matter of healthy controls through radiomics. The chosen features can become a classification model and be further used to implement machine learning in order to achieve the possibility of an early diagnoses between several different white matter diseases.
**Limitations:** Not applicable.
**Ethics committee approval:** This study was approved by an ethics committee.
**Funding for this study:** Not applicable.
**Author Disclosures:**
Luca Calderoni: Nothing to disclose
Maja Ukmar: Nothing to disclose
Arianna Sartori: Nothing to disclose
Maria Assunta A. Cova: Nothing to disclose
Ilaria Gandin: Nothing to disclose
Melania Stubos: Nothing to disclose

### RPS 1811-8

**Left inferior frontal gyrus neuroplastisity during novel speech-sound learning: an fMRI and DTI study**

*S. S. AL Otaibi*[1], G. Meyer[2], S. Wuerger[2]; [1]Taif/SA, [2]Liverpool/UK
*(dahhasi@gmail.com)*

**Purpose:** While the functional and structural changes that occur when we learn new language skills are well documented, relatively little is known about the time course of these changes, especially in the short term. Here we present a combined functional magnetic resonance (fMRI) and diffusion tensor imaging (DTI) study that tracks functional and microstructural change over three days of learning phonetic categorisation in a new language.
**Methods or Background:** Twenty adult native English-speaking participants were scanned before and after attending a new language (Arabic) training course of one hour for three consecutive days. During the course, the participants were trained to do two tasks: phonetic discrimination and a pronunciation task.
**Results or Findings:** Behavioural results show significant performance improvements on both tasks after training. Functional imaging analysis provides evidence for (i) significant pre-training blood-oxygenation-level-dependent (BOLD) signal differences between native and non-native languages in the left inferior frontal gyrus (IFG) but not in any other language-related areas; and (ii) significant post-training BOLD signal increases in the left IFG. This increase is correlated with the participants' behavioural performance change. Microstructural (DTI) analysis in the left IFG shows a significant post-training increase in fractional anisotropy (FA) after just three days of training. In functional connectivity, positive correlation was observed between the left inferior frontal gyrus and two areas (the right cerebellum and the left middle occipital gyrus) and a negative correlation in the right lingual gyrus.
**Conclusion:** These findings indicate that short-term speech-sound learning recruits the same brain area and the same functional network that is being used for processing the L1. The microstructural changes could indicate an early stage of myelination process in the left IFG.
**Limitations:** No follow-up scan.
**Ethics committee approval:** University of Liverpool Ethics [ref# 3384].
**Funding for this study:** University of Taif.
**Author Disclosures:**
Sahal Saad AL Otaibi: Nothing to disclose
Sophie Wuerger: Nothing to disclose
Georg Meyer: Nothing to disclose

**10:30-12:00** **Room E1**

### Research Presentation Session: Oncologic Imaging

### RPS 1816

### Genito-urinary tumours

**Moderator**
D. Prezzi; London/UK

### RPS 1816-2

**Repeatability of quantitative individual lesion and total disease multiparametric whole-body MRI measurements in prostate cancer bone metastases**

*R. Donners*[1], A. Candito[2], M. Blackledge[2], M. Rata[2], C. Messiou[1], D-M. Koh[1], N. Tunariu[1]; [1]Sutton/UK, [2]London/UK
*(ricardo.donners@usb.ch)*

**Purpose:** To assess the repeatability of quantitative multiparametric whole-body MRI (mpWB-MRI) parameters in advanced prostate cancer (APC) bone metastases.
**Methods or Background:** 10 APC patients were scanned twice on the same day on Siemens Aera 1.5T MRI. MpWB-MRI included DWI (b50 and b900) and gradient-echo 2-point DIXON sequences. ADC and relative fat-fraction (rFF) maps were calculated. A radiologist measured up to 10 target bone metastases per study. Means of ADC, b900 signal intensity (SI), b900 SI normalised versus the conus medullaris, rFF and maximum diameter(MD) on rFF images for each target lesion and averages across all targets per patient were recorded. On commercially available post-processing software the total disease volume (tDV in ml) was manually delineated on b900 images and mean global (g)ADC was derived. Same-day repeatability coefficients (RCs) and coefficients of variance (CoVs) were calculated.
**Results or Findings:** 73 individual targets (median MD 26 mm, range 10-56 mm) were included. Lesion ADC RC was 12.5%, CoV 4.5%, b900 SI RC 137%, CoV 49.5%, normalised b900 SI RC 110%, CoV 39.9%, rFF RC 35.5% (3.2 units), CoV 12.8% and target MD RC 16.3% (4.5 mm), CoV 5.9%. Patient target lesion average ADC RC was 6.4%, CoV 2.3%, b900 SI RC 104%, CoV 37.5% and normalised b900 SI RC 39.6%, CoV 14.3%. Target average rFF RC was 18.5% (1.8 units), COV 6.7%, average MD RC 4.8% (1.3 mm), CoV 1.7%. tDV segmentation RC was 6.4%, CoV 2.3 % and mean gADC RC 5.3%, CoV 1.9%.
**Conclusion:** Individual APC bone metastases' ADC and MD, average target ADC, rFF and MD and tDV and gADC show good repeatability, which is good technical validation and evidence to support further development as imaging biomarkers.
**Limitations:** Not applicable
**Ethics committee approval:** Local ethics# 1406
**Funding for this study:** Funding was received from Prostate Cancer UK, the Swiss Society of Radiology as well as the University of Basel.
**Author Disclosures:**
Christina Messiou: Nothing to disclose
Matthew Blackledge: Nothing to disclose
Dow-Mu Koh: Nothing to disclose
Nina Tunariu: Nothing to disclose
Ricardo Donners: Nothing to disclose
Antonio Candito: Nothing to disclose
Mihaela Rata: Nothing to disclose

### RPS 1816-3

**Hyperpolarised 13C-pyruvate MRI as a non-invasive read-out of tumour grade in renal cell carcinoma**

*S. Ursprung*[1], R. Woitek[2], M. McLean[2], M. Crispin-Ortuzar[2], A. Priest[2], K. Brindle[2], E. Sala[2], G. Stewart[2], F. A. Gallagher[2]; [1]Tübingen/DE, [2]Cambridge/UK

**Purpose:** There is an unmet clinical need to differentiate indolent and aggressive renal cell cancers (RCC) to improve treatment stratification (surveillance vs surgery). However, conventional imaging and single tumour biopsies cannot determine tumour grade sufficiently accurately. Metabolic changes reflecting aggressiveness could be used to assess RCC. Hyperpolarised 13C-pyruvate MRI has shown promise to measure grade-dependent alterations in other cancers. This study aimed to prospectively correlate the metabolic phenotype of RCC with tumour grade and validate the findings on histology.
**Methods or Background:** Six participants with treatment-naïve clear cell RCC underwent preoperative hyperpolarised 13C-pyruvate MRI and multiparametric 1H-MRI. The conversion of pyruvate to lactate (kPL) was derived from the metabolite signals to create metabolic maps within the tumour. Patient-specific 3D-printed moulds enabled multi-regional, image-guided tissue sampling. Forty-four samples were stained for MCT1 and MCT4, membrane transporters of pyruvate and lactate, to determine their role in 13C-pyruvate metabolism. RNA expression of lactate dehydrogenase, catalysing the conversion between pyruvate and lactate, was measured in each tumour.
**Results or Findings:** The kPL correlated positively with tumour grade (P=0.009). Its intratumoural heterogeneity correlated with the tumour grade of co-localised biopsies (P=0.03, corrected for patient dependency). The kPL also correlated with the expression of MCT1 (P=0.016), the transporter taking up pyruvate, providing mechanistic evidence for signal generation. The RNA expression of lactate dehydrogenase trended towards a positive correlation with kPL. The kPL was not correlated with physiological MRI measures of diffusivity, perfusion, and oxygenation as well as histological cell density.
**Conclusion:** Hyperpolarised 13C-pyruvate MRI may serve to assess tumour grade in RCC. Furthermore, it may guide biopsies to the most aggressive sub-regions of a tumour, enabling more accurate treatment stratification.
**Limitations:** Larger studies are needed to confirm these findings.
**Ethics committee approval:** Cambridge-South REC: 15/EE/0378
**Funding for this study:** Funding was received from Cancer Research UK.
**Author Disclosures:**
Kevin Brindle: Nothing to disclose
Andrew Priest: Nothing to disclose
Ramona Woitek: Nothing to disclose
Mary McLean: Nothing to disclose
Evis Sala: Shareholder: Lucida Medical Advisory Board: Amazon Speaker: GSK and Siemens
Stephan Ursprung: Nothing to disclose
Grant Stewart: Consultant: Pfizer, Merck, EUSA Pharma and CSurgical Research/Grant Support: Pfizer, AstraZeneca and Intuitive Surgical Speaker: Pfizer
Ferdia Aidan Gallagher: Grant Recipient: GSK Research/Grant Support: GE Healthcare Consultant: AstraZeneca
Mireia Crispin-Ortuzar: Nothing to disclose

### RPS 1816-4

**Early reduction in spectral dual-layer detector CT parameters as favourable imaging biomarkers in patients with metastatic renal cell carcinoma**

*A. Drljevic-Nielsen*[1], J. Mains[1], K. Thorup[1], M. Brun Andersen[2], F. R. Rasmussen[1], F. Donskov[1]; [1]Aarhus/DK, [2]Herlev-Gentofte/DK
*(askadrlj@rm.dk)*

**Purpose:** Baseline spectral dual-layer detector CT (DL-CT) parameters have shown prognostic ability in patients with metastatic renal cell carcinoma (mRCC). Whether early change in DL-CT parameters is associated with patient outcomes is unknown.
**Methods or Background:** DL-CT scans were performed at baseline and after one month of checkpoint immunotherapy or tyrosine-kinase inhibitor therapy. Scans were reconstructed to conventional CT and DL-CT series, and used for assessment of Hounsfield Units (HU), iodine concentration (IC), and the effective atomic number (Zeffective) in the combined RECISTv.1.1 target lesions. The relative changes from baseline to one month, defined as ΔIC(combined), ΔZeffective(combined) and ΔHU(combined), were assessed categorically (cut-off: lower quartile) and associated with progression-free survival (PFS), overall survival (OS) and objective response rate.
**Results or Findings:** Overall, 115 and 104 mRCC patients were included at baseline and one month, respectively. Following one month of treatment, median IC (combined) decreased from 2.3 to 1.2 mg/ml,(p<0.001), Zeffective (combined) from 8.5 to 8.0,(p<0.001), and HU (combined) from 86.0 to 64.0 HU, (p<0.001). After adjustments for treatments, histology and baseline factors, the largest reductions in ΔIC (combined) (HR 0.47, 95% CI: 0.24-0.94, p=0.033) and ΔZeffective (combined) (HR=0.43, 95% CI: 0.21-0.87, p=0.019) were independently associated with favorable OS; the largest reduction in ΔZeffective (combined) was associated with higher response (OR=2.79, 95% CI: 1.12-6.94, p=0.027). No associations were found between ΔHU (combined) and outcomes.
**Conclusion:** Early reductions at one month in DL-CT derived ΔIC (combined) and ΔZeffective (combined) are associated with favourable outcomes in patients with mRCC. This information may reassure physicians and patients about treatment strategy.
**Limitations:** Cardiac output and patient size can affect the contrast enhancement of the tissue and thus the quantification of DL-CT parameters.
**Ethics committee approval:** The study has been approved by the regional ethics committee (no. 1-10-72-242-17).
**Funding for this study:** Funding was received from Ipsen (Finn Rasmussen and Frede Donskov have shared last authorship).
**Author Disclosures:**
Frede Donskov: Grant Recipient: FD reports research grants from Ipsen, Pfizer, MSD and The Health Research Foundation, Central Denmark Region
Master of Science Kennet Thorup: Nothing to disclose
Aska Drljevic-Nielsen: Research/Grant Support: ADN reports recieving a research grant from Ipsen
Michael Brun Andersen: Speaker: MBA reports teaching fees from Philips and Boehringer Ingelheim
Jill Mains: Nothing to disclose
Finn R Rasmussen: Nothing to disclose

### RPS 1816-5

**Evaluation of immunotherapy response with CT histogram analysis in metastatic Renal Cell Carcinoma**

*S. Cankaya*, L. Damgaci; Ankara/TR

**Purpose:** To evaluate the value of histogram analysis in immunotherapy response assessment and predicting prognosis in metastatic Renal Cell Carcinoma (RCC) patients treated with nivolumab.
**Methods or Background:** 27 metastatic RCC patients treated with nivolumab evaluated. CT scans that are analysed are non-enhanced basal CT which is before the drug administration, non-enhanced first control CT which is 3 months after the drug administration and non-enhanced second control CT which is 6 months after the drug administration. CT scans were evaluated based on iRECIST criteria, then target lesions were selected and measured for each patient. Each target lesion was segmented using software (Olea Sphere 3.0-SP 23 Olea Medicals, La Ciotat, France) with free hand technique and the entire lesion was obtained sequantially as VOI. Using this VOI, size, first order parameters and shape parameters were studied for each target lesion via software. Patients dichotomised into two groups as objective responders and non-objective responders according to iRECIST criteria, then histogram analysis parameters were compared between the groups using Wilcoxon, paired t, Mann Whitney u, Kruskal Wallis tests.
**Results or Findings:** According to first CT scans measurements at 3rd month control, objective responders have lower values of volume, 90th percentile and at 6th months measurements skewness values are lower in objective responders. According to second CT scans measurements objective responders have lower values of skewness. We also researched the correlation between histogram analysis parameters and IMDC scores, which is a prognostic model. We found a positive correlation between IMDC scores and median, volume, 90th percentile values.
**Conclusion:** Histogram analysis might be promising quantitative imaging tool in response assesment and predicting prognosis of metastatic RCC patients treated with immunotherapy.
**Limitations:** The nature of this study as singlecentred, the measurements being obtained by a single researcher, the free hand technique used, as well as the small sample size were identified as limitations.
**Ethics committee approval:** Ankara City Hospital Ethics Committee approved this study, as E2-21-491 numbered research.
**Funding for this study:** No funding was received for this study.
**Author Disclosures:**
Sıla Cankaya: Nothing to disclose
Lale Damgaci: Nothing to disclose

### RPS 1816-6

**Validation of PI-RADS v2 for detection of prostate cancer among readers with different degree of experience using US/MRI fusion guided prostate biopsy as reference standard**

*J. Yu*, H. Le, S. Winks, A. Fulcher, M. A. Turner; Richmond, VA/US
*(jinxing.yu@vcuhealth.org)*

**Purpose:** To evaluate diagnostic performance of prostate multi-parametric MRI (mp-MRI) using PI-RADS v2 in detection of prostate cancer (PCa) among radiologists with different levels of experience.
**Methods or Background:** Between 07/1/2018 and 12/31/2020, 462 patients underwent prostate mp-MRI and then US/MRI fusion target biopsies. In order to be considered suitable candidates for study, patients had to have at least one cancer-suspicious region (CSR) at mp-MRI and no prior history of PCa treatment. All CSRs at MRI were assigned PI-RADS scores based on PI-RADS

v2 when studies were interpreted by two GU radiologists, one with 15 years (reader 1) and the other 3 years (reader 2) in reading prostate MRI. Results were compared to histopathology obtained by MR/ultrasound fusion guided biopsies. Statistical analysis was applied to the data collected.
**Results or Findings:** US/MRI fusion prostate biopsies of 513 lesions in 462 patients were performed and revealed 396 PCa (77% tumour detection rate). In PI-RADS 5, 4 and 3 lesions, detection rates of PCa was 97%, 87% and 50% for reader 1, and 90%, 88% and 36% for reader 2, respectively. There was a significant difference between reader 1 and reader 2 regarding detection of PCa for PI-RADS 3 lesions, p<0.01 (50% vs. 36%, P=0.0054). When considering high cancer-suspicious lesions (PI-RADS 4,5), diagnostic performances were similar between reader 1 and 2 with no statistically significant difference (P>0.05).
**Conclusion:** Tumour detection rate of 77% amongst lesions deemed suspicious for PCa at mp-MRI using PI-RADS v2 was demonstrated supporting PI-RADS v2 as a reliable and replicable reporting system for detection of PCa. Among them, cancer detection rate in PI-RADS 3 lesions increased with greater reader experience and detection rate in PI-RADS 4 and 5 was similar between readers.
**Limitations:** The retrospective nature of the study was identified as a limitation.
**Ethics committee approval:** This study was approved by an ethics committee.
**Funding for this study:** No funding was received for this study.
**Author Disclosures:**
Hong Le: Nothing to disclose
Ann Fulcher: Nothing to disclose
Sarah Winks: Nothing to disclose
Mary Ann Turner: Nothing to disclose
Jinxing Yu: Nothing to disclose

### RPS 1816-8

### Repeatability and reproducibility of fat-fraction in prostate cancer patients with bone metastases: intra and inter-reader agreement of single-slice and volumetric first-order features

*C. Saeli*, G. M. Agazzi, M. Ravanelli, D. Farina, R. Maroldi; Brescia/IT

**Purpose:** 1) To assess repeatability and reproducibility of "fat-fraction" parameter (FF%) in whole-body magnetic resonance of patients with bone metastases from castration-resistant prostate cancer (RCPC). 2) To evaluate correlation of FF% between single-slice and volumetric segmentation. 3) To assess volumetric first-order radiomic features reproducibility.
**Methods or Background:** Thirty-four patients were randomly selected from the database of the BonEnza phase III trial. Imaging was performed on a 1.5T scanner, with MET-RADS-P compliant protocol. For each patient, a Small Active Lesion (SAL, < 10 mm) and a Large Active Lesion (LAL, >10 mm) were identified. Manual segmentation on the most representative slice (blinded choice) was performed once by R1 and twice by R2. Intra- and interrater agreement of single-slice and volumetric FF% on SAL and LAL and first-order features were evaluated with interclass correlation coefficient (ICC). Spearman's correlation coefficient was used for single-slice and volumetric measurements correlation. "3DSlicer" was used for segmentation, "Python library pyradiomics" for first-order feature extraction, "R" for statistics.
**Results or Findings:** Regarding FF%, intra-reader ICC for SAL and LAL was 0.914 and 0.971 on single-slice measurements; 0.957 and 0.897 on volumetric measurements; interreader ICC for SAL and LAL was 0.641 and 0.805 on single-slice measurements and 0.762 and 0.883 on volumetric measurements. Correlation between FF% measured on single slice and volume was 0.817 for SAL and 0.649 for LAL. Regarding first-order features, 10Percentile, Median, Minimum, RootMeanSquared had inter- and intra-rater agreement >0.75 for both LAL and SAL, 90Percentile for LAL only.
**Conclusion:** In mRCPC, FF% on bone metastases is reproducible, especially when considering lesions >10 mm with a volumetric approach. First-order features showed good reproducibility too.
**Limitations:** Small sample size and manual segmentation were identified as limitations.
**Ethics committee approval:** NCT identifier 03336983
**Funding for this study:** No funding was received for this study.
**Author Disclosures:**
Davide Farina: Nothing to disclose
Marco Ravanelli: Nothing to disclose
Giorgio Maria Agazzi: Nothing to disclose
Roberto Maroldi: Nothing to disclose
Chiara Saeli: Nothing to disclose

### RPS 1816-9

### The estimated volume index (EVI); a new practical approach for treatment response monitoring in advanced ovarian cancer patients treated with neoadjuvant chemotherapy

*E. Berardi*, C. Rijsemus, N. Pereira da Silva, M. Engbersen, W. van Driel, C. Lok, R. van Stein, M. Lahaye, R. G. H. Beets-Tan; Amsterdam/NL
*(berardi.eva@gmail.com)*

**Purpose:** Neoadjuvant chemotherapy (NACT) is used in ovarian cancer (OC) patients with peritoneal metastasis (PM). Current standards like RECIST are difficult to apply in the daily clinic because peritoneal lesions are challenging to measure, especially after NACT. This study aims to evaluate a new standardised scoring system for monitoring chemotherapy response: "The Estimated Volume Index (EVI)".
**Methods or Background:** Ovarian cancer patients with PM treated with NACT followed by surgery were included from 2012-2017. A retrospective analysis of CT-scans before and after NACT was performed by an experienced reader and a reader with no experience in staging PM. EVI categorises patients according to the subjective overall response based on the response present in the majority of 7 regions as defined by the "Dutch Region Score". The response is classified based on the percentage of lesions decreased or progressed for each region: progressive, mixed, stable, partial, good or complete. To validate EVI, results were compared to the median overall survival (OS).
**Results or Findings:** 58 ovarian cancer patients were included with a median age of 70 years. Twenty-four out of 58 patients (41%) had a complete resection and 34/58 (59%) an incomplete debulking. Median OS was 29.0 months. For reader 1 median OS correlated significantly (p<0.001) with the EVI scoring. The median OS for patients with progressive disease or mixed response was 8 months, stable disease 25 months and partial, good or complete response was 49 months (p<0.001). The interrater reliability agreement was 0.338 (p<0.001).
**Conclusion:** These preliminary results show that EVI, in experienced hands, can help quantify treatment response in advanced OC patients. EVI provides vital information concerning the prognosis and might help in selecting the optimal treatment for each patient.
**Limitations:** Sample size and the retrospective data used were identified as limitations.
**Ethics committee approval:** This study was approved by an ethics committee.
**Funding for this study:** Not applicable
**Author Disclosures:**
Eva Berardi: Nothing to disclose
Nuno Pereira da Silva: Nothing to disclose
Max Lahaye: Nothing to disclose
Willemien van Driel: Nothing to disclose
Christianne Lok: Nothing to disclose
Charlotte Rijsemus: Nothing to disclose
Regina G. H. Beets-Tan: Nothing to disclose
Maurits Engbersen: Nothing to disclose
Ruby van Stein: Nothing to disclose

### RPS 1816-10

### CT radiomic based models to predict residual disease and early recurrence in primary debulked ovarian cancer

*G. Aluffi*[1], G. Avesani[2], A. Perazzolo[2], C. Panico[2], H. E. Tran[2], L. Boldrini[2], F. Botta[2], A. Fagotti[2], R. Manfredi[2]; [1]Verona/IT, [2]Rome/IT
*(greg.aluffi@gmail.com)*

**Purpose:** To build radiomic models based on preoperative contrast-enhanced CT images for predicting residual tumor (RT) and early recurrence (ER) (<1year) after debulking surgery.
**Methods or Background:** In this retrospective multicentre study we enrolled 227 patients with ovarian cancer (OC) who underwent debulking surgery with an available preoperative CT. Manual segmentation of the primary ovarian tumour was performed. 217 radiomic features were extracted from the contoured lesion using MODDICOM software. Feature stability was assessed with ANOVA. ComBat method was used to harmonise parameters that proved unstable due to image heterogeneity (different slice thickness and different scanners). Patients were randomly grouped into a testing set and a validation test. Wilcoxon-SSMann-Whitney and Pearson analysis were performed to select features in the training set, while the synthetic minority oversampling technique (SMOTE) was used to balance our data set. Machine learning models (Decision Tree, Random Forest, XGBoost, Logistic Regression with elastic net regularisation) were developed using alternatively all the 217 features or the features selected with Wilcoxon-Mann-Whitney and Pearson analysis. Machine learning model performances were finally estimated by 3-fold cross-validation (CV).

**Results or Findings:** The models built using all the extracted features showed lower predictive performance than those built using only the selected features. Best results were obtained with Decision Tree model predicting RT (mean AUC: 0.61, CI: 0.09) and with XGBoost model predicting ER (mean AUC: 0.60, CI: 0.07).
**Conclusion:** Our multicentre study, which analysed a relatively sizable "real world" data set, showed unsatisfying results of the radiomic models both for RT and ER. Further studies are needed to build a reliable radiomic models for the decision making process.
**Limitations:** The retrospective non-consecutive study design was identified as a limiting factor.
**Ethics committee approval:** Informed consent was obtained from all patients for using the data of their studies in the clinical trial.
**Funding for this study:** No funding was received for this study.
**Author Disclosures:**
Giacomo Avesani: Nothing to disclose
Francesca Botta: Nothing to disclose
Luca Boldrini: Nothing to disclose
Alessio Perazzolo: Nothing to disclose
Huong Elena Tran: Nothing to disclose
Riccardo Manfredi: Nothing to disclose
Camilla Panico: Nothing to disclose
Anna Fagotti: Nothing to disclose
Gregorio Aluffi: Nothing to disclose

### RPS 1816-12
**Histogram analysis for characterisation of indeterminate adrenal nodules**
M. Affes[1], *A. Masmoudi*[1], M. Attia[1], I. Baccouche[1], S. Kchaou[1], H. Neji[2], S. Hantous-Zannad[2]; [1]Tunis/TN, [2]Ariana/TN
*(masmoudi.abd@gmail.com)*

**Purpose:** Evaluate the performance of CT histogram analysis method to differentiate lipid-poor adrenal adenomas from adrenal metastases in neoplastic context.
**Methods or Background:** 94 patients and 115 adrenal nodules (32 lipid-poor adenomas, 83 metastases) were included. We used a software to calculate the percentage of negative voxel in the lesions. The measure of the volume was hand-traced made slice-by-slice for the extraction of the volume of interest (VOI). On unenhanced CT-scan, standard deviation (SD) of the mean attenuation values has been noted to assess noise effect. We calculated the sensitivity and specificity of percentage of negative pixels to distinguish between adenomas and metastases.
**Results or Findings:** 68.7% of lipid-poor adenomas, and 15.7% of metastases showed more than 10% negative pixels on unenhanced CT-scan. By comparison, on enhanced CT-scan, we didn't find any metastases and only 3.1% of lipid-poor adenomas, showed the percentage of negative pixels above 10%. For the 10% negative pixel percentage threshold, our sensitivity and specificity were 68.7% and 84.3% respectively for the diagnosis of adenoma. After excluding 24 nodules in 21 patients because of a large SD (> 20 of HU values), we would detect 56.5% of lipid-poor adenomas and 7.5% of metastases on unenhanced CT with the same threshold. We aimed to find the threshold of percentage of negative pixel by establishing a ROC curve. We found that 12% corresponded to the most specific threshold with an acceptable sensitivity (respectively 95.6% and 56.5%).
**Conclusion:** No biopsy-proven diagnosis; we used adrenal washout CT to differentiate benign and malignant nodules.
**Limitations:** The CT histogram method with a threshold value of >12% negative pixels can identify many benign adrenal nodules with attenuation values >10 HU on unenhanced CT with extremely high specificity.
**Ethics committee approval:** Nothing to disclose
**Funding for this study:** Nothing to disclose
**Author Disclosures:**
Salma Kchaou: Nothing to disclose
Henda Neji: Nothing to disclose
Saoussen Hantous-Zannad: Nothing to disclose
Meriem Affes: Nothing to disclose
Monia Attia: Nothing to disclose
Abdellatif Masmoudi: Nothing to disclose
Ines Baccouche: Nothing to disclose

**10:30-12:00** **Room M 1**

### Research Presentation Session: Chest

### RPS 1804

### The evolution of COVID imaging

**Moderator**
E. Kocova; Hradec Kralove/CZ

### RPS 1804-2
**Long-term follow-up of pulmonary arterial circulation after hospitalisation for SARS-CoV-2 pneumonia: dual-energy CT angiographic study in 79 patients**
I. Mohamed, A. Duhamel, J. Giordano, A. Ego, N. Fonne, J. Remy, *M. Rémy-Jardin*; Lille/FR
*(martine.remy@chru-lille.fr)*

**Purpose:** To evaluate pulmonary vascular abnormalities more than 6 months after hospitalisation for SARS-CoV-2 pneumonia.
**Methods or Background:** In a cohort of 739 patients having been hospitalised for SARS-CoV-2 pneumonia between March 2020 and April 2021 (T0 period), 222 patients remaining symptomatic more than 6 months after the initial infection underwent a delayed specialised follow-up. The eligibility criteria for the long-term assessment of pulmonary circulation (T1 period) included: (a) a dual-energy CT angiographic (CTA) examination obtained with the same equipment; and (b) interpretable lung perfusion images. 143 patients were excluded because (a) chest CTA had been obtained <6 months after pneumonia (n=126); (b) there was a non-interpretable lung perfusion (n=17). The final study group included 79 patients with morphologic and lung perfusion imaging at T1 (mean ± SD between T0 and T1: 7.9 ±1.7 months).
**Results or Findings:** At T1, morphologic images showed (a) complete resolution of acute PE (12/79;15.2%) and newly developed features of chronic PE (3/79; 3.8%); (b) newly diagnosed acute PE (2/79; 2.5%). Lung perfusion was abnormal in 69 patients (87.4%), depicting (a) perfusion defects of 3 types: patchy defects (n=60; 76%); areas of non-systematised hypoperfusion (n=27; 34.2%) and/or PE-type defects (n=14; 17.7%) with (2/14) and without (12/14) endoluminal filling defects; and (b) areas of increased perfusion in 59 patients (74.9%), superimposed on ground-glass opacities (58/59) and/or areas of vascular tree-in-bud (5/59).
**Conclusion:** Delayed follow-up showed newly developed CT features of acute and chronic PE but also two types of perfusion abnormalities, suggestive of persistent hypercoagulability as well as unresolved/sequelae of the widespread microangiopathy described in the acute phase of the disease.
**Limitations:** This study is a single-centre evaluation, based on a single, dual-energy CT technology.
**Ethics committee approval:** Waiver of patient-informed consent.
**Funding for this study:** No funding was received for this work.
**Author Disclosures:**
Islam Mohamed: Nothing to disclose
Alice Ego: Nothing to disclose
Martine Rémy-Jardin: Research/Grant Support: Siemens Healthineers
Nicolas Fonne: Nothing to disclose
Jessica Giordano: Nothing to disclose
Jacques Remy: Consultant: Siemens Healthineers
M. Alain Duhamel: Nothing to disclose

### RPS 1804-3
**The impact of vaccination on the severity of COVID-19 pneumonia: effectiveness of mRNA and adenovirus vector vaccines and comparison between vaccinated and unvaccinated patients**
*S. Vicini*[1], A. Iannarelli[1], S. Ruggiero[1], D. M. Bellini[1], M. Rengo[1], C. Catalano[2], I. Carbone[1]; [1]Latina/IT, [2]Rome/IT
*(simone.vicini@gmail.com)*

**Purpose:** The purpose of our study was to evaluate and compare the severity of COVID-19 pneumonia on chest CT imaging in unvaccinated and vaccinated COVID-19 individuals, along with the impact of different types of vaccines.
**Methods or Background:** Retrospective observational study on COVID-19 positive patients with respiratory symptoms, and chest CT to evaluate lung involvement. Unvaccinated and vaccinated patients were included. Each CT exam was interpreted by 3 radiologists with the attribution of a score from 0 to 5 for each lobe (for a maximum value of 25) based on the percentage of parenchymal involvement according to Chang et al. Morphological patterns of lung involvement were also evaluated. Scores and characteristics were compared between vaccinated and unvaccinated patients and mRNA and adenovirus vector vaccines.

**Results or Findings:** 467 patients were analysed, including 216 unvaccinated and 251 vaccinated (167 mRNA vaccine; 84 adenovirus vaccine). Unvaccinated patients showed a median CT score of 10/25 compared to the median score of 5/25 in the vaccinated (3/25 mRNA vaccine; 6/25 adenovirus vaccine) (P <0.05). Considering a value >= 15 of the score as a cut-off, a diagnostic accuracy with AUC of 0.98, sensitivity of 100% and specificity of 93%, was obtained in predicting admission to intensive care unit (ICU). Logistic regression analysis identified complete vaccination as a protective factor with respect to a score >= 15 (OR = 15.2).
**Conclusion:** Complete vaccination was found to be a protective factor in preventing the onset of severe COVID-19 pneumonia at imaging.
**Limitations:** Retrospective nature. The study was conducted in a single region in Italy. No possibility to evaluate all the types of vaccines; limited to the types of vaccines distributed in our region.
**Ethics committee approval:** Approved by our institutional ethical review board.
**Funding for this study:** No funding was received.
**Author Disclosures:**
Iacopo Carbone: Nothing to disclose
Simone Vicini: Nothing to disclose
Marco Rengo: Nothing to disclose
Sergio Ruggiero: Nothing to disclose
Davide Maria Bellini: Nothing to disclose
Carlo Catalano: Nothing to disclose
Angelo Iannarelli: Nothing to disclose

### RPS 1804-4

**Does submillisievert chest CT imaging impair diagnostic value in patients with suspected SARS-CoV-2 infection**

*H-M. Thieß*, K. K. Bressem, L. C. Adams, J. L. Vahldiek, S. M. Niehues; Berlin/DE
*(martin@hjts.de)*

**Purpose:** The purpose of this study is to analyse image quality and confounding factors that affect image quality in patients suspected of SARS-CoV-2 infection. Also, the impact on reproducibility of quality ratings of low-dose chest CT protocols is evaluated.
**Methods or Background:** We retrospectively included 100 randomly selected low-dose chest CT scans of patients suspected of SARS-CoV-2 infection in two centres. Two radiologists rated image quality based on a Likert scale. Reasons for reduced ratings were evaluated. In order to allow for analysis of inter- and intra-reader reliability, ratings were repeated after three weeks. Additionally, radiation dose was analysed.
**Results or Findings:** The median effective radiation dose of the analysed scans was in the submillisievert range (0.53 mSv, IQR: 0.35 mSv) and the majority of scans received optimal quality ratings. Those with reduced ratings (38%) most commonly demonstrated noticeable artefacts (63%), high noise (39%) or lack of sharpness (18%). Only one scan was rated as non-diagnostic due to excessive artefacts caused by lowered arms. Inter-reader and intra-reader reliability showed almost perfect agreement (Cohen's kappa of 0.82 and 0.87).
**Conclusion:** Submillisievert low-dose chest CT demonstrates appropriate image quality with almost perfect inter-reader and intra-reader agreement in patients suspected of SARS-CoV-2 infection. Image quality was mostly impaired by unavoidable artefacts due to low dose, without limiting clinical assessment of lung parenchyma.
**Limitations:** The major limitation of our study is the retrospective image evaluation of two centers with limited sample size.
**Ethics committee approval:** This study was approved by the institutional review board (EA4/140/17).
**Funding for this study:** The author(s) received no financial support for the research, authorship and/or publication of this article.
**Author Disclosures:**
Janis Lucas Vahldiek: Nothing to disclose
Keno K. Bressem: Nothing to disclose
Lisa C. Adams: Nothing to disclose
Hans-Martin Thieß: Nothing to disclose
Stefan Markus Niehues: Other: Stefan Markus Niehues declares relationships with the following companies: Vital Images, CanonMedical Systems, Guerbet, Bracco Imaging, Teleflex/Vidacare.

### RPS 1804-5

**Possible alterations of imaging patterns in computed tomography for Delta-VOC of SARS-CoV-2**

*C. Yueksel*, M. J. Saehn, M. Kleines, J. C. Brokmann, C. K. Kuhl, D. Truhn, A. Ritter, P. Isfort, M. Schulze-Hagen; Aachen/DE
*(cyueksel@ukaachen.de)*

**Purpose:** Typical findings for COVID-19 in computed tomography (CT) have been described as bilateral, multifocal ground-glass opacities (GGOs), consolidations and reticulation. Round pulmonary masses or nodules with a halo sign are considered uncommon. The authors recently observed several patients with COVID-19 pneumonia presenting with these uncommon findings. This may indicate alterations of CT morphology as variants fluctuate and is to be retrospectively analysed.
**Methods or Background:** 161 initial CTs of patients with confirmed SARS-CoV-2 infection (RT-PCR within 2 days of CT) examined between January 2021 and 15th of September 2021 were included. Patients with invasive ventilation and patients with insufficient virus typing were excluded. CTs were assessed for signs established to be typical for COVID-19, as well as other pulmonary findings. Results were compared using Mann-Whitney U tests, students' t-tests, descriptive statistics and Fisher exact tests.
**Results or Findings:** After exclusion, 86/161 patients/CTs were included. 22 patients had a viral genome profile consistent with Delta-VOC, 39 patients with Alpha-VOC and 25 patients with non-VOC SARS-CoV-2. Three Delta-VOC-patients demonstrated multiple round consolidations with a surrounding halo sign, whereas no Alpha-VOC-patients (p=0.043) or non-VOC-patients (p=0.095) demonstrated these findings.
**Conclusion:** In this study 13.6% of Delta-VOC-patients presented with bilateral round consolidations with halo signs. This was not an established imaging pattern in COVID-19 pneumonia yet. Based on these results Delta-VOC might cause a divergence in CT-morphologic phenotype.
**Limitations:** Low recruitment count. Selection bias (e.g. vaccination prioritisation).
**Ethics committee approval:** Approved 10.12.2021: EK 488/21
**Funding for this study:** No funding was received for this study.
**Author Disclosures:**
Andreas Ritter: Nothing to disclose
Peter Isfort: Nothing to disclose
Jörg Christian Brokmann: Nothing to disclose
Can Yueksel: Nothing to disclose
Marwin -Jonathan Saehn: Nothing to disclose
Michael Kleines: Nothing to disclose
Christiane K. Kuhl: Nothing to disclose
Maximilian Schulze-Hagen: Nothing to disclose
Daniel Truhn: Nothing to disclose

### RPS 1804-6

**Impact of vaccination on COVID-related pneumonia: use of "CT severity score" in vaccinated and non-vaccinated patients during SARS-CoV-2 infection**

*G. M. Masci*, G. Bonito, A. Izzo, S. Lucchese, S. Ciaglia, L. Marchitelli, F. Iafrate, C. Catalano, P. Ricci; Rome/IT
*(giorgio.masci.93@gmail.com)*

**Purpose:** To investigate the effectiveness of COVID-19 vaccination during SARS-CoV-2 infection using the CT Severity Score (CTSS) to compare the degree of pulmonary involvement between vaccinated and non-vaccinated patients.
**Methods or Background:** 1033 SARS-CoV-2+ patients (673 vaccinated and 360 non-vaccinated), matched for clinical and demographic characteristics, who underwent a chest CT scan were enrolled. Images were evaluated by two readers with 3 and 15 years of experience using the CTSS (0-25), resulting from the sum of lobar scores based on the percentage of involvement (0:0%; 1: < 5%; 2:5–25%; 3:26–50%; 4:51–75%; 5: > 75%). The CTSS was compared between vaccinated and non-vaccinated patients, also considering the type of vaccine and the number of doses received.
**Results or Findings:** The CTSS resulted significantly lower in vaccinated compared to non-vaccinated patients (p<0.0001). The data was confirmed also when patients with no evidence of pneumonia (CTSS=0) were not considered in the analysis(p<0.0001). Moreover, when patients were stratified based on the number of doses received, non-vaccinated subjects showed higher CTSS than patients with 1, 2 and 3 doses (p<0.0001); patients with 3 doses obtained lower CTSS compared to those with 1 and 2 doses (p=0.03). No difference of CTSS was found between the different types of vaccine received.
**Conclusion:** The CTSS allowed for differentiating the degree of pulmonary involvement during COVID-19 infection between vaccinated and non-vaccinated patients, demonstrating milder cases of disease following vaccination, suggesting its effectiveness in reducing the severity of COVID-19 pneumonia.

**Limitations:** The main limitations were represented by the retrospective nature of the study, which did not allow for establishing a cause-effect relationship between vaccination and the lower pulmonary involvement, and the unavailable data on the time interval between the date of vaccination and the infection.
**Ethics committee approval:** Ethical approval was obtained for this study.
**Funding for this study:** Not applicable.
**Author Disclosures:**
Giacomo Bonito: Nothing to disclose
Livia Marchitelli: Nothing to disclose
Paolo Ricci: Nothing to disclose
Antonella Izzo: Nothing to disclose
Sonia Lucchese: Nothing to disclose
Giorgio Maria Masci: Nothing to disclose
Franco Iafrate: Nothing to disclose
Carlo Catalano: Nothing to disclose
Simone Ciaglia: Nothing to disclose

### RPS 1804-7

**Diagnostic performance in differentiating COVID-19 from other viral pneumonias on CT imaging: multi-reader analysis compared with an artificial intelligence model**

*F. Rizzetto*, L. Berta, G. Zorzi, L. A. Carbonaro, A. Cincotta, A. Torresin, P. E. Colombo, A. Vanzulli; Milan/IT
*(Francesco.rizzetto@unimi.it)*

**Purpose:** To evaluate the diagnostic performance in differentiating COVID-19 pneumonia from other viral pneumonias on CT imaging by comparing radiologists and a radiomics-based artificial intelligence (R-AI) model.
**Methods or Background:** Chest CT images of 1028 patients with positive swab for SARS-CoV-2 (n=646) and other respiratory viruses (n=382) were used to develop a R-AI classifier to discriminate between COVID-19 pneumonia (COVID) and pneumonia from other viruses (non-COVID). The model was trained with 808 CT images; the remaining 220 CT images (151 COVID-19, 69 non-COVID-19) were used as independent validation dataset, applying a threshold on the predicted values of 0.5. Four readers (three radiologists with >10 years experience and one radiology resident with 3 years experience) were enrolled to blindly evaluate the independent validation dataset using the CO-RADS score. A high-suspicion scenario (CO-RADS 3 considered as COVID) and a low-suspicion scenario (CO-RADS 3 considered as non-COVID) were simulated. Specificity (SP), sensitivity (SE) and accuracy (ACC) were calculated for human readers and R-AI model. Inter-reader agreement was also assessed with ordinal-weighted Fleiss' kappa (k).
**Results or Findings:** The R-AI model achieved SE=79%, SP=78% and ACC=79% in distinguishing COVID from non-COVID pneumonia on the validation dataset. Inter-reader agreement in assigning CO-RADS was good (k=0.68, IC95% 0.63-0.72) and diagnostic performance was averaged between readers. In particular, the readers obtained SE=83%, SP=65% and ACC=78% in the high-suspicion scenario and SE=68%, SP=88% and ACC=74% in the low-suspicion scenario. Excluding the less experienced reader did not significantly change these results.
**Conclusion:** A radiomics-based artificial intelligence model may provide comparable diagnostic performance to human readers in distinguishing COVID-19 pneumonia from other viral pneumonias on CT imaging.
**Limitations:** Generalisability of the results should be assessed in a multicentre setting.
**Ethics committee approval:** Local ethical committee approved the study.
**Funding for this study:** Not applicable.
**Author Disclosures:**
Francesco Rizzetto: Nothing to disclose
Luca Berta: Nothing to disclose
Luca Alessandro Carbonaro: Nothing to disclose
Giulia Zorzi: Nothing to disclose
Angelo Vanzulli: Nothing to disclose
Alberto Torresin: Nothing to disclose
Antonino Cincotta: Nothing to disclose
Paola Enrica Colombo: Nothing to disclose

### RPS 1804-8

**A combined risk model using imaging and clinical parameters in COVID-19 patients from a nationwide German cohort to predict disease progression**

*R. Armbruster*[1], J. Wailzer[1], A. Dadras[1], M. J. Saehn[2], B. Hamm[3], T. Vogl[1], T. Penzkofer[3], D. Pinto[1], A. M. Bucher[1]; [1]Frankfurt/DE, [2]Aachen/DE, [3]Berlin/DE
*(rebecca.armbruster@kgu.de)*

**Purpose:** COVID-19 infections are on a steep rise in early 2022 and new mutations are spreading rapidly. Machine-learning models predicting severe disease courses remain of interest to efficiently allocate limited medical resources.
**Methods or Background:** In this analysis, we included 551 PCR-confirmed COVID cases from 10 university hospitals out of a nationwide collection of 3065 COVID cases across all 36 German university hospitals (age: 60.62±14.87; female/male: 172/372; known comorbidities: 1.68±1.47[min-max: 0-7]). We combined outcome variables in a binary disease-severity score and trained predictors for severe disease progression. Our cohort included 129 severe (ICU admission and invasive ventilation) and 422 non-severe cases. We leveraged 31 structured CT-reporting items per patient. Complementary clinical parameters included 18 additional features as input variables (9 anamnestic parameters, 4 vital parameters, oxygen therapy type, IL-6, lymphocyte count, CRP and D-dimers). We generated classifiers (RandomForest, GradientBoosting) to estimate the likelihood of severe outcomes for hospitalised patients and, additionally, for all location-specific folds to test generalisability.
**Results or Findings:** Predicting the highest treatment status, we achieve an accuracy of 73.19% and increase this by 15 percentage-points when predicting the highest ventilatory needs (88.41%). By combining these two metrics, we achieve an accuracy of 94.93%. Compared to models trained on image-derived parameters only, we improve by 8 percentage-points. Location-specific train/test folds achieved an averaged accuracy of 94.64±0.03% and show that our model generalises among different hospitals.
**Conclusion:** By using baseline parameters complementary to image analysis, the model accurately predicts COVID-19-patients' disease progression at the time of the first CT scan after hospital admission. Our tool will be made available for public use.
**Limitations:** This preliminary analysis should be expanded to include datasets of further centers of the nationwide RACOON network.
**Ethics committee approval:** IRB approval was obtained.
**Funding for this study:** RACOON is funded by the Network of University Medicine (BMBF-grant-number: 01KX202).
**Author Disclosures:**
Bernd Hamm: Nothing to disclose
Thomas Vogl: Nothing to disclose
Tobias Penzkofer: Nothing to disclose
Marwin -Jonathan Saehn: Nothing to disclose
Armin Dadras: Nothing to disclose
Jasmin Wailzer: Nothing to disclose
Daniel Pinto: Nothing to disclose
Rebecca Armbruster: Nothing to disclose
Andreas Michael Bucher: Nothing to disclose

### RPS 1804-9

**Risk stratification of hospitalised patients with COVID-19 pneumonia by chest radiograph scoring in a tertiary hospital in Johannesburg, South Africa**

*H. C. Labuschagne*, H. Moodley, J. Venturas; Johannesburg/ZA
*(chrisjanlab@live.com)*

**Purpose:** To compare Brixia scores of patients admitted to hospital with COVID-19 pneumonia in a middle- to lower-income country and develop predictive models of clinical outcome using Brixia scores and clinical and laboratory data.
**Methods or Background:** Retrospective cross-sectional analysis of adults with RT-PCR confirmed COVID-19 pneumonia admitted at a tertiary hospital in Johannesburg, South Africa, from 1 May - 30 June 2020. Two radiologists, blinded to all clinical information, generated Brixia scores independently. These were compared to clinical parameters, length of stay, and clinical outcomes. Inter-rater agreement was assessed. Multivariable logistic regression identified variables predictive of mortality.
**Results or Findings:** There were 263 patients, 51% were male (median age 47 years [IQR = 20]). Hypertension (38.4%), diabetes (25.1%), obesity (19.4%) and HIV (15.6%) were the commonest co-morbidities. The median length of stay (n = 258) was 7.5 days (IQR = 7). Fifty (19%) patients died, median age 55 years (IQR = 23), versus survivors, 46 years (IQR = 20; p=0.01). One or more co-morbidities conferred a higher death rate (23% versus 9.2% without) (p = 0.01). The median Brixia score for the deceased was higher (14.5) than for discharged patients (9.0) (p = 0.00). Inter-rater agreement was good (ICC 0.77; 95% CI 0.6-0.85; p = 0.00). Models combining Brixia score, age, male gender and obesity as well as Brixia score and CRP predicted the highest risk for mortality (AUC = 0.78).
**Conclusion:** We developed the first South African models incorporating Brixia scores, clinical features and a serological marker, which is a promising risk-stratification tool in a resource-limited setting.
**Limitations:** Single-centre study with no control group. Two general radiologists allocated Brixia scores.
**Ethics committee approval:** Obtained from the Human Research Ethics committee (certificate number M2011113) of the University of the Witwatersrand.
**Funding for this study:** No funding was received for this study.

**Author Disclosures:**
Hendrik Christiaan Labuschagne: Nothing to disclose
Halvani Moodley: Nothing to disclose
Jacqueline Venturas: Nothing to disclose

### RPS 1804-10

**Chest CT characteristics are strongly predictive of mortality in patients with COVID-19 pneumonia: a multicentric cohort study**

N. Malécot[1], J. Chrusciel[2], S. Sanchez[2], H-P. Leveque[3], E. Parizel[4], J. Pradel[2], *D. Geindreau*[5], M. Schertz[6], M. Cavet[7]; [1]Nimes/FR, [2]Troyes/FR, [3]Belfort/FR, [4]Metz/FR, [5]London/UK, [6]Illkirch/FR, [7]Paris/FR

**Purpose:** The aim of this study was to determine whether chest-computed tomography (CT) characteristics had any prognostic value in patients with COVID-19.
**Methods or Background:** A retrospective analysis of COVID-19 patients who underwent a chest CT-scan was performed in four medical centres. The prognostic value of chest CT results was assessed using a multivariable survival analysis. The characteristics included in the model were the degree of lung involvement, ground-glass opacities, nodular consolidations, linear consolidations, a peripheral topography, a predominantly inferior lung involvement, pleural effusion and crazy paving. The model was also adjusted on age, sex, and the centre in which the patient was hospitalised. The primary endpoint was 30-day in-hospital mortality. A second model used a composite endpoint of admission to an intensive care unit or 30-day in-hospital mortality.
**Results or Findings:** A total of 515 patients with available follow-up information were included. Advanced age, a degree of pulmonary involvement ≥50% (Hazard Ratio 2.25 [95% CI: 1.378-3.671], p = 0.001), nodular consolidations and pleural effusions were associated with lower 30-day in-hospital survival rates. An exploratory subgroup analysis showed a 60.6% mortality rate in patients over 75 with ≥50% lung involvement on a CT-scan.
**Conclusion:** Chest CT findings such as the percentage of pulmonary involvement ≥50%, pleural effusion and nodular consolidation were strongly associated with 30-day mortality in COVID-19 patients
**Limitations:** In the context of the first outbreak peak of the epidemic, many patients did not have access to an RT-PCR at the time of admission. Hence, for some patients, inclusion was decided based on the clinical presentation combined with CT scan findings.
**Ethics committee approval:** The study was declared to the French national register of studies using healthcare data under declaration number MR0210190520.
**Funding for this study:** Academic Radiology. Available 20 January 2022.
**Author Disclosures:**
Jean Pradel: Nothing to disclose
Stéphane Sanchez: Nothing to disclose
Mathieu Schertz: Nothing to disclose
Jan Chrusciel: Nothing to disclose
Elisabeth Parizel: Nothing to disclose
Nicolas Malécot: Nothing to disclose
Henri-Paul Leveque: Nothing to disclose
Madeleine Cavet: Nothing to disclose
Damien Geindreau: Nothing to disclose

### RPS 1804-11

**Text mining of radiology reports to calculate "number needed to image" for the detection of pulmonary infection shows seasonality and real-time course of the COVID-19 pandemic**

*T. Heye*, M. Segeroth, E. M. Merkle, J. Vosshenrich; Basle/CH
*(tobias.heye@usb.ch)*

**Purpose:** To investigate the "number needed to image" in chest radiographs and CTs for detecting pulmonary infections if clinicians suspect pneumonia.
**Methods or Background:** A total of 88'864 reports (62'803 chest radiographs; 26'061 CTs performed in the emergency room; years 2012-3/2022) were included from the radiological information system. Using a text search algorithm, first, all chest radiographs/CT examinations containing the clinical question "pulmonary infection/pneumonia" in the referring physicians order were identified (28'715 chest radiographs; 7'887 CTs). Secondly, the algorithm searched for the written negation of the presence of a pulmonary infection "no pulmonary infiltrate" in the report. After excluding examinations negative for pulmonary infections, the remainder of the reports were searched for the positive statement of a pulmonary infection. If none of the conditions applied, the examination was labeled "other" regarding diagnosis.
**Results or Findings:** From 1/2014 to 2/2020 the average pulmonary infections detected by chest radiographs ranged from 55.3-62.9 per month while 241.1-303.6 chest radiographs were negative for pulmonary infection, yielding a 15.5-18.4% detection rate. There were clear seasonal changes with maximum detection counts ranging from 89-104 in the winter months compared to 31-46 in the summer. For CT, there was a sharp increase in average monthly pulmonary infection detection in 2020/21, ranging between 68.8-72.2 (vs. 13.8-21.2, 2014 to 2019). However, detection rates remained similar (2014-2019: 38.2-49.3% vs. 2020-2021: 41.6-44.8%). The course of the positive pulmonary infection CT curve 2020-2022 correlates well with local waves of the COVID-19 pandemic.
**Conclusion:** Text mining of radiology reports allows for extraction of diagnosis, thus delivering a metric to track the trend of such diagnosis in real time. As demonstrated the seasonality and pandemic course of pulmonary infections can be revealed.
**Limitations:** Text search algorithm may not have 100% accuracy.
**Ethics committee approval:** Not applicable.
**Funding for this study:** Not applicable.
**Author Disclosures:**
Tobias Heye: Nothing to disclose
Martin Segeroth: Nothing to disclose
Elmar M. Merkle: Nothing to disclose
Jan Vosshenrich: Nothing to disclose

**10:30-12:00** **Room M 3**

### Research Presentation Session: Abdominal Viscera

### RPS 1801

### New techniques in abdominal imaging

**Moderator**
M. Maas; Amsterdam/NL

### RPS 1801-2

**Value of dual-energy CT late arterial phase iodine maps for the diagnosis of acute bowel ischaemia: initial results**

*C. Booz*[1], T. D'Angelo[2], I. Yel[1], V. Koch[1], T. Vogl[1]; [1]Frankfurt/DE, [2]Messina/IT

**Purpose:** To evaluate diagnostic accuracy, diagnostic confidence and image quality of dual-energy CT (DECT) late arterial (LA) phase iodine mapping for assessment of ABI compared to portal venous (PV) iodine maps and LA conventional CT series.
**Methods or Background:** Data from 142 patients (72 men) who had undergone DECT based on a standardised three-phasic scan protocol due to clinical suspicion of ABI were included. One board-certified radiologist manually performed region-of-interest (ROI) measurements in bowel segments on LA and PV DECT iodine maps as well as LA conventional series, both in surgery-confirmed ischaemic and non-ischaemic bowel loops. Receiver operating characteristic (ROC) curve analysis was performed. Additionally, subjective visual image rating was carried out. Surgical data served as the reference standard.
**Results or Findings:** DECT-based iodine uptake values showed significant differences in LA phases between ischaemic (0.71 ± 0.28 mg/mL) and non-ischaemic bowel loop (4.94 ± 1.86 mg/mL) (p<.001), as well as in PV phases (ischaemic: 1.13 ± 1.12 mg/ml vs. non-ischaemic: 2.65 ± 0.90 mg/ml) (p<.001). In conventional LA series, CT values yielded significant difference between ischaemic (40.19 ± 12.00 HU) and non-ischaemic (55.63 ± 8.97 HU) (p<.001). Iodine quantification on LA phase revealed an area under the curve (AUC) of 0.982, significantly higher compared to the CT values evaluated on conventional series (0.828) and PV phase iodine quantification (0.852). The optimal LA phase iodine density threshold was 1.24 mg/mL, providing a sensitivity of 100% and specificity of 97%. LA iodine maps were rated significantly better than PV iodine maps (p<.001) regarding image quality and diagnostic confidence.
**Conclusion:** Application of LA phase DECT iodine maps increases diagnostic accuracy, diagnostic confidence and image quality for assessement of ABI.
**Limitations:** Single-centre study.
**Ethics committee approval:** This study was approved by an ethics committee.
**Funding for this study:** No funding was received for this study.
**Author Disclosures:**
Christian Booz: Speaker: Siemens Healthineers (past)
Ibrahim Yel: Nothing to disclose
Thomas Vogl: Nothing to disclose
Vitali Koch: Nothing to disclose
Tommaso D'Angelo: Nothing to disclose

### RPS 1801-3

**Liver and spleen MR elastography, T1/T2 mapping for detection-staging of liver fibrosis and prediction of oesophageal varices**

*G. M. Özyurt*, K. Esen, E. Ucbilek, F. D. Apaydın; Mersin/TR
*(gokhanmertozyurt@gmail.com)*

**Purpose:** To compare the stiffness measurements of liver and spleen with MR elastography (MRE) and T1/T2 mapping and to evaluate the relationship of spleen stiffness with oesophageal varices and clinical fibrosis score.
**Methods or Background:** We prospectively evaluated 75 cirrhotic patients and 25 healthy volunteers with MRE, T1/T2 mapping. Fibrosis stage, splenic/liver volume and the presence of oesophageal varices were also determined.
**Results or Findings:** Liver MRE showed a significant moderate positive correlation with T1 mapping (r=0.51 and p=0.000) and T2 mapping (r=0.30, p=0.009). Liver stiffness , T1 and T2 mapping and spleen volume were found to be statistically significant in detecting fibrosis (p<0.05). There was a significant moderate positive correlation with spleen MRE and T2 mapping (r=0.37 and p=0.001). Although there was a statistically significant difference between patient and control group in spleen T1 mapping (p=0.000), a correlation wasn't detected between spleen MRE and T1 mapping in the patient group. In the comparison of the patient and the control groups the cut-off values for liver MRE, liver T1 mapping and liver T2 mapping were 2.6 kPA (AUC=0.97), 619 ms (AUC=0.90) and 52.5 ms (AUC=0.62), respectively. The cut-off value for the prediction of oesophageal varices in the patient group was 8.65 kPA (AUC=0.834, p<0.0001) and 607 cm3 (AUC=0.790, p<0.0001) for spleen MRE and spleen volume, respectively.
**Conclusion:** Liver MRE showed a positive moderate correlation with liver T1 and T2 mapping. In the prediction of oesophageal varices, spleen volume showed a similar diagnostic performance with spleen MRE.
**Limitations:** This study was limited by the inability to use contrast agent.
**Ethics committee approval:** Approval was obtained from Mersin University Clinical Research Ethics Committee, with the board decision number 2021/99.
**Funding for this study:** This study was supported by the research fund of Mersin University in Turkey, with project number 2021-1-TP3-4168.
**Author Disclosures:**
Gökhan Mert Özyurt: Other: data collection
Feramuz Demir Apaydın: Consultant: data collection
Kaan Esen: Consultant: data collection
Enver Ucbilek: Consultant: patient's info

### RPS 1801-4

**Assessing pancreatic fibrosis and treatment response with dynamic contrast-enhanced MRI: an experimental chronic pancreatitis animal model induced by dibutyltin dichloride**

*Y. Lu*, T. Zhang, D. Wang; Shanghai/CN
*(lym1007@sjtu.edu.cn)*

**Purpose:** To evaluate whether quantitative DCE-MRI allows accessing pancreatic fibrosis and the anti-fibrotic effect of curcumin in a rat model of chronic pancreatitis (CP).
**Methods or Background:** The CP model was induced by injecting dibutyltin dichloride (DBTC). Curcumin was administrated from the next day of DBTC injection. DCE-MRI was performed in five groups on an 11.7 T MR scanner: the control group (n=10); CP for 2 weeks (n=15); CP for 4 weeks (n=15); CP + curcumin for 2 weeks (n=15); CP + curcumin for 4 weeks (n=15). DCE-MRI quantitative parameters (Ktrans, Ve, and Vp) derived from an extended Tofts model. Pancreatic fibrosis was determined by Sirius Red stain.
**Results or Findings:** Ktrans and Vp significantly correlated with the fibrotic area of the pancreas (r = - 0.619 and - 0.450). Ktrans in rats 4 weeks after DBTC injection was significantly lower than in CP 2 weeks rats and control rats (0.30 ± 0.06 min-1 vs 0.49 ± 0.09 vs. 0.62 ± 0.09, respectively). Vp in CP 4 weeks was also significantly lower in rats that underwent DBTC injection than in control rats (0.048 ± 0.010 min-1 vs 0.065 ± 0.011 min-1, respectively). Ktrans and Vp in rats with daily curcumin treatment for 4 weeks were significantly lower than CP 4 weeks rats (Ktrans, 0.51 ± 0.09 vs 0.30 ± 0.06; Vp, 0.064 ± 0.015 vs 0.048 ± 0.010).
**Conclusion:** DCE-MRI parameters (Ktrans and Vp) could be helpful for monitoring pancreatic fibrosis progression and investigating the anti-fibrotic response of curcumin.
**Limitations:** We located the ROIs in pancreatic tail portion only.
**Ethics committee approval:** This study was approved by our Institutional Animal Care and Use Committee.
**Funding for this study:** DCE-MRI parameters (Ktrans and Vp) have potential to evaluate pancreatic fibrosis and non-invasively assess the anti-fibrotic treatment response of curcumin.
**Author Disclosures:**
Tingting Zhang: Nothing to disclose
Yimei Lu: Nothing to disclose
Dengbin Wang: Nothing to disclose

### RPS 1801-5

**Non-alcoholic fatty pancreatic disease determined by unenhanced CT: evaluation of the association with hepatosteatosis, visceral adiposity and epicardial adipose tissue volume**

*G. K. Bahadır*, Y. C. Güneş, A. Sözeri, M. Vural; Ankara/TR

**Purpose:** To investigate the relationship between CT-quantified pancreatic steatosis with epicardial adipose tissue volume (EATV), hepatosteatosis and visceral adiposity.
**Methods or Background:** A total of 61 patients with CT coronary angiography (CCTA) and unenhanced abdomen CT were included. The NAFPD group was defined if the difference between the pancreas-splenic attenuation was ≤ -5 Hounsfield Unit (HU). Total fat tissue area (TFA), visceral fat tissue area (VFA), subcutaneous fat tissue area (SFA), and hepatosteatosis of patients with and without non-alcoholic fatty pancreatic disease (NAFPD) were calculated using non-contrast CT. The EAT volume was measured by a semi-automated method using CCTA.
**Results or Findings:** EATV (115.36 ± 50.62 mL and 77.02 ± 36.04 mL, p = 0.002 ), TFA (44.71 ± 17.56 cm2 and 33.19 ± 20.33 cm2, p = 0.022 ), VFA (21.81 ± 9.27 cm2 and 15.05 ± 8.61 cm2, p = 0.006), hepatosteatosis ($\chi 2(1)$ = 5.951, p = 0.015) and patient age (54.06 ± 10.92 and 45.83 ±15.01, p= 0.046) were significantly higher in patients with NAFPD than in patients without NAFPD. In multiple logistic regression analyses, NAFPD was significantly associated with hepatosteatosis (OR=4.809, 95% CI=1.281-18.050, p=0.02) and EATV (OR=1.017, 95% CI=1.002-1.033, p=0.024). A cut-off value of EATV ≥ 97.37 mL estimated NAFPD with a sensitivity and specificity of 80% and 77%, respectively. The difference between the pancreas-splenic attenuation was moderately correlated with EATV (r=-0.511, p<0.001).
**Conclusion:** High EATV values and hepatosteatosis are independent factors associated with NAFPD. EATV ≥ 97.37 mL had 80% sensitivity and 77% specificity for prediction of NAFPD.
**Limitations:** The small number of cases.
**Ethics committee approval:** Not applicable.
**Funding for this study:** No funding was received for this work.
**Author Disclosures:**
Murat Vural: Nothing to disclose
Asiye Sözeri: Nothing to disclose
Yasin Celal Güneş: Nothing to disclose
Gülsüm Kübra Bahadır: Nothing to disclose

### RPS 1801-6

**Bowel preparation in MRI for detection of endometriosis: comparison of the effect of an enema, no additional medication and intravenous butylscopolamine on image quality**

*I. Ciggaar*[1], O. D. F. Henneman[1], S. Oei[1], I. J. S. M. L. I. Vanhooymissen[1], M. Blikkendaal[1], S. Bipat[2]; [1]The Hague/NL, [2]Amsterdam/NL

**Purpose:** To compare the effect of three different patient-preparation strategies for reducing bowel motion on image quality in pelvic MRI.
**Methods or Background:** Retrospective study in which 95 consecutive patients undergoing pelvic MRI were subdivided based on preparation type for reduction of bowel motion. Group 1 (N=31) fasted 4 hours and applied an enema (Bisacodyl 10 mg); group 2 (N = 32) received no medication; and group 3 (N = 32) received intravenous butylscopolamine (Buscopan® 50 mg). Image quality was reviewed by visual assessment of delineation (3-point-scale) of pelvic structures: uterus, adnexa, bladder, rectum, sigmoid, uterosacral ligaments, round ligaments and small bowel. As a secondary endpoint, the presence of rectal-wall oedema was evaluated. Interobserver agreement was calculated, as well as relative diagnostic odds ratios (RDOR) for the protocols to provide an outcome in the best delineation category.
**Results or Findings:** Interobserver agreement proportions varied from 0.48 - 1.00. The rectum and sigmoid colon, respectively, have a RDOR of 5.4 and 2.6 when butylscopolamine is applied compared to Bisacodyl (P=0.051; P = 0.008), and a RDOR of 4.2 and 5.7 with Bisacodyl compared to no medical preparation (P=0.006; P<0.01). Small bowel delineation was significantly better with butylscopolamine compared to Bisacodyl (P=0.007). There was no significant difference in delineation of the other structures between protocols. There is a significant higher chance of observing rectal wall oedema with Bisacodyl compared to the other protocols (resp. RDOR 0.055 and 0.051 for the butylscopolamine and 'no medication' protocol; both P<0.001).
**Conclusion:** Butylscopolamine provides better delineation of the small bowel and rectosigmoid compared to Bisacodyl, which, in turn, provides better delineation of the rectosigmoid compared to no medication. Moreover, Bisacodyl causes rectal wall oedema in the majority of cases.
**Limitations:** Retrospective study design.
**Ethics committee approval:** Not applicable.
**Funding for this study:** Not applicable.

Saturday

**Author Disclosures:**
Shandra Bipat: Nothing to disclose
Isabeau Ciggaar: Nothing to disclose
Stanley Oei: Nothing to disclose
Mathijs Blikkendaal: Nothing to disclose
Onno Dirk Fransiscus Henneman: Nothing to disclose
Inge Jeanne Suzanne Marleen Lovely Inge Vanhooymissen: Nothing to disclose

### RPS 1801-7

**Growth kinetics of pancreatic neuroendocrine tumours by histopathologic grade**

*J. R. Tse*, L. Shen, L. S. Yoon, A. Kamaya; Stanford, CA/US

**Purpose:** To determine if growth kinetics of pancreatic neuroendocrine tumours (PNETs) are associated with histologic grade.
**Methods or Background:** 50 treatment-naïve PNETs from 50 adult patients (57±14 years; 29 women, 21 men) from 2010-2021 with serial CT/MRI separated by at least 3 months and histopathology were included for analysis. Orthogonal dimensions of PNETs were measured for each PNET. Growth kinetics were assessed with volume doubling time (VDT) using the modified Schwartz equation. Qualitative imaging features were evaluated by two abdominal radiologists blinded to histopathology. Histopathologic grade was assigned using the 2017 WHO classification system.
**Results or Findings:** Of 50 PNETs, 38 (76%) were grade 1, and 12 (24%) were grade 2-3. Median VDT for grade 1 PNETs was 1.8 years (interquartile range IQR 1.4-6.4 years) while median VDT for grade 2-3 PNETs was 9.4 years (IQR 3.7-70 years; p=0.003). Grade 2-3 PNETs were more likely to have local mass effect (defined as vascular involvement, main duct dilation, or distal pancreatic atrophy; 58% vs 21%; p=0.027), radiologic evidence of metastases at baseline imaging (50% vs 13%; p=0.014) and VDT <3 years (75% vs 18%), compared to grade 1 PNETs. Baseline size, presence of calcifications and tumour vascularity relative to the rest of the pancreas were not associated with grade. At multivariable analysis, VDT was the most significant predictor of PNET grade (odds ratio OR 3.9), followed by radiologic evidence of metastases (OR 2.1) and local mass effect (OR 1.6).
**Conclusion:** High-growth kinetics defined by volume doubling time are associated with higher-grade pancreatic neuroendocrine tumours.
**Limitations:** Single-centre, retrospective study with a small sample size.
**Ethics committee approval:** Approved by the institutional review board.
**Funding for this study:** No funding was received for this study.
**Author Disclosures:**
Luke S Yoon: Nothing to disclose
Luyao Shen: Nothing to disclose
Justin Ruey Tse: Nothing to disclose
Aya Kamaya: Nothing to disclose

### RPS 1801-8

**An imaging spectrum of IgG4-RD with analysis of post-treatment imaging response**

*N. S. Hegde*; Mumbai/IN

**Purpose:** Immunoglobulin G4-related disease (IgG4) is now a well recognised fibro-inflammatory systemic disease. The imaging manifestations of IgG4-RD are broad and variable depending on the organ involved. However, when there are simultaneous findings of typical of IgG4-RD in multiple organs, then the possibility of IgG4-RD should be raised. Hence, the objective behind this study is to understand the imaging spectrum of IgG4-RD in multiple organs in histopathological proven cases, analyse the post-treatment imaging response in these cases and to address imaging differentials for the same.
**Methods or Background:** Imaging features (CT and MRI based on the organ involved) in 15 histopathologically proven cases of IgG4-related disease with varied organ involvement were retrospectively analysed and characterised with respect to each organ. Post-treatment imaging response in these after a month of corticosteroid therapy was assessed.
**Results or Findings:** Images from 10 patients were retrospectively evaluated. Multiple patients had abdominal findings with involvement of the pancreas, biliary tree, genito-urinary system, intra-abdominal vasculature and the bowel in our study. Besides this, we had a couple of patients with IgG4-RD presenting with varied lung appearances. A few patients with head and neck involvement were also analysed with involvement of salivary glands and lymph nodes, and cervical vasculature and orbital pseudo-tumours were also seen. Post-treatment imaging revealed gradual regression of the inflammation.
**Conclusion:** IgG4-RD is an inflammatory sclerosing disease process. Most patients usually present with simultaneous involvement of a few organs or with isolated organ involvement. It shows good response to steroids. Awareness of the imaging spectrum of the same should prevent a wrong diagnosis.
**Limitations:** Some patients were lost to follow-up, and hence post-treatment imaging was not available for them.
**Ethics committee approval:** Not applicable.
**Funding for this study:** Not applicable.
**Author Disclosures:**
Nikrish S Hegde: Nothing to disclose

### RPS 1801-9

**Effect of an online reference system on the diagnosis of rare abdominal tumours**

*M. M. Woeltjen*, J. H. Niehoff, S. Saeed, A. Mendorf, R. Kullmann, C. Mönninghoff, J. Borggrefe, J. R. Kröger; Minden/DE

**Purpose:** This study aims to investigate the effect of the online reference system (ORS) STATdx Elsevier (Amsterdam, NL) as a diagnostic tool regarding the correct diagnosis, the subjective confidence of the radiologists and its influence on the invested time in the diagnosis of rare abdominal tumours.
**Methods or Background:** 101 patients with rare tumour entities or atypic manifestations and histological secured diagnosis were retrospectively included. A blinded rereading was performed by three radiologists with different levels of experience (8, 5 and 2 years). First, an ad-hoc diagnosis was made and immediately afterwards a second diagnosis was established with the help of an ORS. Additionally, the subjective confidence of the diagnosis was recorded with a 5-point Likert scale and the used time was noted.
**Results or Findings:** The experienced reader (correct diagnosis, CD, 46.5%), as well as the advanced reader (CD 45.5%) made the correct diagnosis more frequently compared to the less experienced reader (CD 25.7%). A relevant improvement in the correct diagnostic frequency was only achieved by the advanced reader (CD with ORS 57.4%). The experienced reader (CD with ORS 47.5%) and the less experienced reader (CD with ORS 27.7%) showed less benefit. The subjective confidence increased significantly when the ORS was used (3,9 ± 0,9 vs. 3,2 ± 0,9; p < 0,001). The invested time when using the ORS was significantly increased compared to the time needed for an ad-hoc diagnosis (3,7 ± 2,0 min vs. 2,2 ± 1,1 min; p < 0,001).
**Conclusion:** The effect of the ORS on the correct diagnosis differs with the experience of the radiologists, where especially the advanced reader showed an improvement. The used ORS system increased the diagnostic confidence significantly at a significant increased expenditure of time.
**Limitations:** No limitations were identified.
**Ethics committee approval:** This study was approved by an ethics committee.
**Funding for this study:** No funding was received for this study.
**Author Disclosures:**
Julius Henning Niehoff: Nothing to disclose
Matthias Michael Woeltjen: Nothing to disclose
Saher Saeed: Nothing to disclose
Ruth Kullmann: Nothing to disclose
Alexander Mendorf: Nothing to disclose
Christoph Mönninghoff: Research/Grant Support: Reports personal fees from Bayer Medical outside the submitted work.
Jan Borggrefe: Research/Grant Support: Received honoraria for scientific lectures from Philips and Siemens.
Jan Robert Kröger: Research/Grant Support: Received research support from Philips and support for attending meetings and/or travel from Veryan.

### RPS 1801-10

**Single vs. multi-level comparison of visceral fat estimation on abdominal CT**

T. H. Chan, C. K. Cheng, F. Huang, *V. Vardhanabhuti*; Hong Kong/HK

**Purpose:** CT contains body composition data that can be quantitatively measured and used as imaging biomarkers. Traditionally because of the labour-intensive nature of segmenting visceral abdominal fat, these have been done manually at a single-slice level. With new deep-learning techniques, automated whole-volume segmentation is now possible. The purpose of this study is to compare the accuracy of single-level estimation of visceral fat estimation to multi-level combinations between L1-L5 and whole visceral abdominal fat volume as the reference standard.
**Methods or Background:** We retrospectively collected 130 cases from our institutional database who had received whole-body non-contrast CT scans. All scans were manually segmented for visceral fat by a board-certified radiologist. We compared single levels and different combinations of multi-vertebral levels (e.g. L1+L2, L1+L2+L3, etc.), with a total of 31 combinations. Whole visceral abdominal fat volume segmented for the abdominal and pelvic region was used as the reference standard. R2 correlation analysis was performed.
**Results or Findings:** The R2 correlation ranged from 0.7769 – 0.9534. As expected the highest R2 correlation was achieved using all slice levels L1-L5. Using a single-slice level at L3 (generally regarded as the previous standard) achieved an R2 of 0.9183. Using combined L1+L3+L5 could achieve an R2 of 0.9501.

**Conclusion:** With the advent of deep-learning automated segmentation, it now becomes possible to segment visceral abdominal fat at whole volume. We systematically compared multiple levels combination of visceral fat estimation and showed that multi-level combinations achieved higher correlation, but whether these differences would amount to meaningful clinical significance needs further exploration.
**Limitations:** The clinical significance of these differences needs to be further explored. For example, if there are statistical differences in the prediction of subsequent risk i.e. metabolic syndrome, etc.
**Ethics committee approval:** Approved by the local institution.
**Funding for this study:** No funding was received for this study.
**Author Disclosures:**
Varut Vardhanabhuti: Nothing to disclose
Chak Kong Cheng: Nothing to disclose
Tak Hon Chan: Nothing to disclose
Fan Huang: Nothing to disclose

### RPS 1801-11

**The integrative role of magnetic resonance cholangiopancreatography and percutaneous transhepatic cholangiography in malignant obstructive jaundice**

*O. M. A. O. Hamada*; Cairo/EG
*(omaritotefa@hotmail.com)*

**Purpose:** MRCP and PTC are established techniques for the evaluation of intra-hepatic and extra-hepatic bile ducts in patients with malignant hepato-biliary diseases. PTC has procedure-related complications. MRCP is accurate, non-invasive and safe. The aim of this study was to assess the over-added outcome of the integration of MRCP and PTC in the diagnosis of malignant biliary obstruction.
**Methods or Background:** This study was conducted on 60 patients (36 males and 24 females) with malignant obstructive jaundice. Their ages ranged between 36 and 85 years with a mean age of 59.9 years. All the 60 examined cases in our study were subjected to MRCP and PTC to detect the diagnosis, the level of obstruction and the degree of obstruction.
**Results or Findings:** Both MRCP and PTC were accurate in the detection of the malignant biliary dilatation and the level of obstruction. MRCP provided adequate information in the diagnosis of the malignant lesions with a sensitivity of 86.6%, with low sensitivity, to detect the degree of obstruction whether complete or partial, 12.5% compared to PTC. MRCP detected other related malignant features in 40% of cases. PTC-related complications were detected in 12 out of 60 cases (20%). In 11 patients more than one puncture had to be performed during PTC to delineate the whole biliary system.
**Conclusion:** MRCP is an accurate and non-invasive procedure for the diagnosis of malignant obstructive jaundice and the level of biliary obstruction. PTC is accurate in the detection of the level and the degree of biliary obstruction, but the possibility of complications reserves PTC for therapeutic procedures.
**Limitations:** No limitations were identified.
**Ethics committee approval:** An approval of the study was obtained from the Menoufia University Academic and Ethical Committee. Every patient signed an informed written consent for acceptance of the examination.
**Funding for this study:** No funding was received for this study.
**Author Disclosures:**
Omar Mohamed Atef Omar Hamada: Nothing to disclose

**10:30-12:00** **Room N**

### Research Presentation Session: Paediatric

### RPS 1812

### Neuroimaging: from foetus to adolescent

**Moderator**
M. Blouet; Caen/FR

### RPS 1812-2

**Quantitative MRI of the human fetal ganglionic eminence: neuroradiological insights into a transient brain structure**

*M. Stümpflen*[1], C. Mitter[1], E. Schwartz[1], M. C. Diogo[2], B. Pfeiler[1], V. Schmidbauer[1], E. Krampl-Bettelheim[1], D. Prayer[1], G. Kasprian[1]; [1]Vienna/AT, [2]Almada/PT

**Purpose:** Failure of fetal interneuron migration arising from the ganglionic eminence (GE) may lead to neuropsychiatric and neurodevelopmental disorders. Early detection of alterations of this transient brain structure at prenatal stages may improve the MRI phenotyping of neurodevelopmental diseases. This atlas-based fetal MRI study aimed to quantitatively assess longitudinal development of the GE.
**Methods or Background:** In this retrospective IRB-approved singlecentre study, postprocessing was conducted based on semiautomated segmentation of super-resolution fetal brain 1.5T and 3T MR data sets. After assessment of data quality, a longitudinal, quantitative atlas-based analysis of the ganglionic eminence was conducted by several raters.
**Results or Findings:** A total of 112 patients (gestational age 19-39 weeks, mean 27.5 GW) without structural brain anomalies, cardiac defects, fetal growth restriction, and/or poor super-resolution image quality were included and analysed. In the observed time interval, the volume of the ganglionic eminence ranged from 1,100.25 mm3 to 53.25 mm3 (mean 572.31 mm3, SD 232.01) with average volumes continuously decreasing from 19 to 39 GW. For each gestational day, a volumetric reduction of 3.59 mm3 (95% CI 2.45 – 4.73) within the GE was detected.
**Conclusion:** Super-resolution based quantitative MR volumetry allowed to analyse the continuous decline in size of the GE from 19GW onwards - initially documenting a physiological degenerative process in the developing human brain. The first set of reference values of this structure was provided, enabling radiologists to objectively quantify GE development using fetal MRI.
**Limitations:** Potential risk of selection bias (exclusion of fetuses with severe comorbidities) was identified as a limitation.
**Ethics committee approval:** This study was approved by an ethics committee (EK-Nr. 1585/2021).
**Funding for this study:** No funding was received for this study.
**Author Disclosures:**
Victor Schmidbauer: Nothing to disclose
Christian Mitter: Nothing to disclose
Ernst Schwartz: Nothing to disclose
Mariana Cardoso Diogo: Nothing to disclose
Birgit Pfeiler: Nothing to disclose
Daniela Prayer: Nothing to disclose
Gregor Kasprian: Nothing to disclose
Marlene Stümpflen: Nothing to disclose
Elisabeth Krampl-Bettelheim: Nothing to disclose

### RPS 1812-3

**In utero tractography of the ganglionic eminence in the human fetal brain**

*C. Mitter*, M. Stümpflen, P. Brugger, D. Prayer, G. Kasprian; Vienna/AT

**Purpose:** In the fetal brain GABAergic basal ganglia projection neurons and cortical interneurons proliferate within the ganglionic eminence (GE), a transient developmental structure located adjacent to the lateral ventricle, which demonstrates a highly anisotropic microstructure. We used in utero DTI-based tractography to investigate the 3D morphology and diffusion parameters of the GE in the developing human brain in vivo during the second trimester.
**Methods or Background:** We included non-motion degraded in utero DTI examinations of 15 unsedated fetuses (21–27 gestational weeks, GW) with normal brain development. Orthogonal axial diffusion tensor sequences were performed using a 1.5T superconducting MR unit. FA maps were geometrically co-registered with multiplanar T2-weighted MR sequences. The GE was anatomically defined using a multiple ROI approach and visualised using a FACT algorithm.
**Results or Findings:** Tractography of the GE allowed the 3D visualisation of anisotropic diffusion within the GE as C-shaped streamlines, forming thick “pseudofiber” tracts along the wall of the lateral ventricle. Pseudofibers showed an anterior-posterior orientation along the body of the lateral ventricle and a superior-inferior orientation in front of the trigone. Co-registered T2 weighted sequences confirmed the location of pseudofibers to the cell dense GE compartment. Mean FA and ADC values were established for GE pseudofiber tracts.
**Conclusion:** DTI based tractography can be used to visualise pseudofiber tracts within the GE in living unsedated fetuses in utero as early as 21 GW. Since the anisotropic microstructure of the GE may be related to tangential migration of developing neurons, normal values for GE diffusion parameters in healthy fetuses might be of value in the prenatal diagnosis of neurodevelopmental disorders.
**Limitations:** Preselection of cases to ensure minimal fetal motion was identified as a limitation.
**Ethics committee approval:** The study was approved by the Ethics Committee of the Medical University of Vienna.
**Funding for this study:** No funding was received for this study.
**Author Disclosures:**
Christian Mitter: Nothing to disclose
Peter Brugger: Nothing to disclose
Daniela Prayer: Nothing to disclose
Gregor Kasprian: Nothing to disclose
Marlene Stümpflen: Nothing to disclose

### RPS 1812-4

**Fetal 4D flow MRI of the thoracic aorta at 3 Tesla using Doppler-ultrasound gating**

J. Knapp, M. Tavares de Sousa, F. Kording, S-T. Antoni, J. Yamamura, *A. Lenz*, G. Adam, P. Bannas, B. Schönnagel; Hamburg/DE
*(a.lenz@uke.de)*

**Purpose:** To evaluate the feasibility of Doppler-ultrasound (DUS) gated 4D flow MRI of the thoracic aorta in human fetuses at 3T.
**Methods or Background:** 4D flow MRI of the thoracic aorta was performed at 3T in four healthy fetuses and in six fetuses with vascular pathologies (gestational week 30–38). Direct cardiac gating of the fetal heart was performed using an MR-compatible DUS-sensor. Source images (1.8–2.5x1.8–2.5x2.0–2.5 $mm^3$) of the thoracic aorta were obtained in parasagittal orientation using compressed sensing and free maternal breathing. Blood flow in the great thoracic vessels was visualised by streamlines. Flow volumes and velocities were quantified in the ascending aorta, descending aorta, main pulmonary artery, and ductus arteriosus.
**Results or Findings:** DUS-gated 4D flow MRI was successfully performed in 7/10 fetuses. Three data sets of fetuses with cardiovascular pathologies could not be analysed due to image artefacts caused by fetal movement or maternal breathing. Blood flow in the great thoracic vessels was successfully visualised in the remaining seven fetuses. Streamline-visualisation revealed narrowing of the aortic isthmus and excluded double aortic arch in one case, respectively. Time-velocity curves based on 4D flow MRI measurements demonstrated typical arterial blood flow patterns with early systolic peaks and low-positive diastolic blood velocities. Average blood flow and velocity in the descending aorta was 27.7±6.3 cm/s and 8.3±4.5 ml/s, respectively.
**Conclusion:** Fetal 4D flow MRI at 3T using DUS-gating was feasible and allowed for visualisation and quantification of blood flow in the fetal thoracic aorta. Further prospective studies are warranted to determine the diagnostic value of DUS-gated fetal 4D flow MRI in congenital cardiovascular disease.
**Limitations:** The fact that there was no comparison with fetal echocardiography, as well as the small study population were identified as limitations.
**Ethics committee approval:** The study was approved by the local research ethics committee.
**Funding for this study:** No funding was received for this study.
**Author Disclosures:**
Sven-Thomas Antoni: Employee: northh medical GmbH
Manuela Tavares de Sousa: Shareholder: northh medical GmbH
Fabian Kording: Founder: northh medical GmbH
Janine Knapp: Nothing to disclose
Alexander Lenz: Nothing to disclose
Jin Yamamura: Shareholder: northh medical GmbH
Peter Bannas: Nothing to disclose
Björn Schönnagel: Shareholder: northh medical GmbH
Gerhard Adam: Nothing to disclose

### RPS 1812-5

**In utero effects of prenatal alcohol exposure on the human fetal brain: an atlas-based MRI study**

*M. Stümpflen*[1], E. Schwartz[1], M. C. Diogo[2], S. Glatter[1], B. Pfeiler[1], V. Schmidbauer[1], E. Krampl-Bettelheim[1], D. Prayer[1], G. Kasprian[1]; [1]Vienna/AT, [2]Almada/PT

**Purpose:** Numerous postnatal imaging studies have shown structural brain anomalies in patients suffering from fetal alcohol spectrum disorders, potentially resulting in long-lasting behavioural changes. This atlas-based fetal MRI study aimed to identify regional effects of prenatal alcohol exposure (PAE) on human fetal brain development.
**Methods or Background:** This IRB approved, prospective single-center study identified pregnant women referred for fetal MRI with variable amounts of alcohol intake during gestation using two standardised, anonymised questionnaires (TACE and PRAMS). Postprocessing generated super-resolution imaging and semiautomated atlas-based segmentations. After visual inspection, assessment of data quality and manual correction, an atlas-based analysis of twelve fetal brain structures was performed. Linear models were applied with an additional factor to account for varying gestational ages and corrected for multiple comparisons using the Benjamini-Hochberg procedure.
**Results or Findings:** After excluding 476 subjects with structural brain anomalies and/or poor super-resolution image quality, a total of 24 patients (26 observations) with and 52 age- and gender-matched controls without PAE (gestational age 21-37 weeks, mean 27.4 GW) were included and analysed. In fetuses with PAE the corpus callosum ($p<0.001$) showed significantly larger volumes, whereas the periventricular/germinal zone ($p=0.001$) showed smaller volumes compared to controls.
**Conclusion:** This study systematically documented the selective effects of PAE on regional brain volumes at prenatal stages: besides the reduction of the periventricular zone, an increased regional growth of the corpus callosum was found, indicating a change in the developmental dynamics of the normal trajectory of interhemispheric connectivity - even with minor prenatal alcohol exposure (1-3 standardised drinks/week).
**Limitations:** The potential risk of selection bias (exclusion of fetuses with severe comorbidities) and reporting bias (underreporting of alcohol consumption) were identified as limitations.
**Ethics committee approval:** This study was approved by an ethics committee (EK-Nr.2199/2017).
**Funding for this study:** This study has received funding through the Austrian Research Fund grant I3925-B27 in collaboration with the French National Research Agency (ANR).
**Author Disclosures:**
Victor Schmidbauer: Nothing to disclose
Sarah Glatter: Nothing to disclose
Ernst Schwartz: Nothing to disclose
Mariana Cardoso Diogo: Nothing to disclose
Birgit Pfeiler: Nothing to disclose
Daniela Prayer: Nothing to disclose
Gregor Kasprian: Nothing to disclose
Marlene Stümpflen: Nothing to disclose
Elisabeth Krampl-Bettelheim: Nothing to disclose

### RPS 1812-6

**Normogram on transfontannelle Doppler indices and cerebral blood flow velocities in healthy preterm and term neonates within 72 hours of life**

*K. M. Limbad*, B. K. Kapadia, C. Mehta; Vadodara/IN
*(Kajalsradiology@gmail.com)*

**Purpose:** To generate normal reference data for anterior and middle cerebral artery blood flow velocity and resistance index in preterm and term neonates as a baseline. Grey-scale ultrasound for brain parenchyma is non-invasive, portable, inexpensive, and gives real time assessments. However, many cerebral lesions are circulatory in origin so it is important to study Doppler, which provides the ideal tool for diagnosis, follow-up and management of brain damage caused by perinatal asphyxia, infection, developmental and cerebrovascular disorders.
**Methods or Background:** An observational study of transfontanelle and transtemporal pulsed wave Doppler of 715 neonates born at gestational age 27-42 weeks within 72 hours of life. Parameters measured were peak systolic velocity (PSV), end diastolic velocity (EDV), time average maximum and mean velocities (TAMAX & TAMEAN), pulsatility index (PI) and resistive index (RI) in anterior and middle cerebral arteries.
**Results or Findings:** The mean PSV, EDV and TAMAX were 33.47±9.8, 11.08±4.62, 16.95±5.34 for ACA and 34.15±9.25, 11.16±4.35 and 18.64±5.86 for MCA. The mean PI and RI were 1.39±0.26, 0.69±0.06 for ACA and 1.35±0.27, 0.68±0.07 for MCA.
**Conclusion:** This is the largest study to establish a normative database for above mentioned indices. CRBV are directly proportional to gestational age and birth weight. Higher RI seen in preterm as compared to term neonates. No statistically significant differences on Doppler parameters in relation to type of delivery, sex and postnatal age. No correlation was observed between Doppler indices and gestation as component velocities all increase with advancing gestation. These data are important for facilitating the correct interpretation of abnormal findings.
**Limitations:** No major limitation was identified. Machine to machine variation in values was seen to a minor degree.
**Ethics committee approval:** This study was approved by an ethics committee.
**Funding for this study:** No extra funding was required.
**Author Disclosures:**
Bhautik Kantilal Kapadia: Nothing to disclose
Chetan Mehta: Nothing to disclose
Kajal Manojkumar Limbad: Nothing to disclose

### RPS 1812-7

**A novel nomogram based on volumetric quantitative MRI and clinical features for the prediction of neonatal intracranial hypertension**

*Y. Qin*, Y. Liu, S. Yue, L. Weihua; Changsha/CN
*(yanqin1607@163.com)*

**Purpose:** To develop a nomogram based on quantitative magnetic resonance imaging data and clinical parameters for predicting neonatal intracranial hypertension.
**Methods or Background:** Neonatal intracranial hypertension is a serious threat to the health of neonates. The diagnosis of the NICH is quite difficult in clinical situation due to the unspecific clinical manifestations. And the lumbar puncture is an invasive operation. The clinical doctors and anxious parents are eager to find a noninvasive technology to detect NICH timely and accurately. A total of 117 neonates who were suspicious of intracranial lesions were

Saturday included. We utilised the quantitative MRI to obtain the volumetric data . The nomogram was constructed by incorporating the volumetric data and clinical features by multivariable logistic regression. The performance of the nomogram was evaluated by discrimination, calibration curve, and decision curve.
**Results or Findings:** The clinical and volumetric quantitative MRI features, including postmenstrual age, weight, mode of delivery and the gray matter volume, were significantly associated with neonatal intracranial hypertension risk. The nomogram showed satisfactory discrimination with an AUC of 0.76. Favourable clinical utility was observed using decision curve analysis.
**Conclusion:** The nomogram, incorporating the clinical and volumetric quantitative MRI features, provided an individualised prediction of neonatal intracranial hypertension risk and helped decision making and guidance of early diagnosis and treatment for neonatal intracranial hypertension.
**Limitations:** First, our sample size was relatively small and had a limited portion of non-ICH group. Second, our model was not externally validated yet due to the limited dataset. Further studies to collect larger cohorts of subjects are needed to validate the performance of our developed model.
**Ethics committee approval:** Our study was approved by the institutional ethics committee of our hospital.
**Funding for this study:** Our study was supported by the National Natural Science Foundation of China (NSFC,81671676).
**Author Disclosures:**
Yang Liu: Nothing to disclose
Liao Weihua: Nothing to disclose
Yan Qin: Nothing to disclose
Shaojie Yue: Nothing to disclose

### RPS 1812-9

**Dual-layer spectral CT improves image quality of paediatric cerebral unenhanced CT scan**

Z. Tan[1], X. Zhang[2], Y. Ming[1], *J. Wang*[1]; [1]Wuhan/CN, [2]Shanghai/CN
*(jjwinflower@126.com)*

**Purpose:** To evaluate the image quality and optimal energies of virtual monoenergetic images (VMIs) from dual-layer spectral detector computed tomography (DLCT) in unenhanced paediatric cerebral scan.
**Methods or Background:** Twenty-three consecutive unenhanced cerebral scans on a DLCT (IQon, Philips Healthcare) in children (age: 6.09±2.55 years) were retrospectively analysed. CI and VMIs (range: 40-100 KeV, interval 5 KeV) were reconstructed. Gray matter (GM) and white matter (WM) noise, signal-to-noise ratio (SNR), contrast-to-noise ratio (CNR), posterior fossa and subcalvarial artifact index (PFAI, SAI) were calculated. Two radiologists independently determined the image quality using 5-point Likert-type scale: the GM-WM differentiation (GWMA), the subcalvarial space (SAA), the beam hardening artifacts in posterior fossa (PFAA) and the overall diagnostic quality. Student's t-test, ANOVA and Wilcoxon test were used to determine statistical significance. Intra-class correlation coefficient (ICC) was used to assess the interrater agreement.
**Results or Findings:** Compared with CI, significant superior CNR and noise of GM and WM were observed in VMIs at all 40-100 KeV levels (all P<0.001). The SAI and PFAI were lowest in VMI at 100 KeV. Besides, statistical difference was detected for PFAI compared to CI (P<0.001). In subjective ratings, the observers reported optimal GWMA at 50-55 KeV, optimal SAA and PFAA at 100 KeV, best assessment of overall diagnostic quality at 55 KeV, all of which were significantly better compared with CI (all P<0.001). Good ICC was observed between two radiologists (overall ICC: 0.764).
**Conclusion:** VMI scan significantly improves image quality of pediatric cerebral scan compared with CI. The optimal energy-level for the supratentorial brain was 55 KeV, while for subcalvarial space and posterior fossa 100 keV.
**Limitations:** Not applicable
**Ethics committee approval:** Not applicable
**Funding for this study:** Not applicable
**Author Disclosures:**
Zhengwu Tan: Nothing to disclose
Yang Ming: Nothing to disclose
Jing Wang: Nothing to disclose
Xiaohui Zhang: Nothing to disclose

### RPS 1812-10

**Gadolinium-based contrast agents can cross non-nervous cell membranes rather than remain confined to the extracellular compartment of the central nervous system**

*F. F. Zennaro*[1], R. Sala[2], C. Covino[3], D. Zampieri[1], D. Zanon[1], M. Crosera[1], N. Maximova[1]; [1]Trieste/IT, [2]Parma/IT, [3]Milan/IT
*(fzennaro@mac.com)*

**Purpose:** The human cerebrospinal fluid (CSF) changes completely every 6 hours. Human microglia is one of the slowest dividing immune cells. The main goal is to find the cause of everlasting gadolinium (Gd) persistence in CSF of paediatric cancer survivors after GBCA.
**Methods or Background:** Study group: 68 paediatric patients with haematological malignancies with Dotarem-enhanced MRI, underwent lumbar puncture. Control group: 47 paediatric patients with haematological malignancies, never exposed to the GBCA, underwent lumbar puncture. CSF samples of both groups were analysed for gadolinium, iron and ferritin concentration. In vitro siderotic and control human oligodendrocyte (HOG) cell line models were developed and cultured with calcein-labelled Dotarem. Intracellular amount of Gd and iron in both cell lines was performed by ICP-Mass Spectrometry. Single-cell uptake of Dotarem was performed using confocal fluorescence microscopy.
**Results or Findings:** The mean number of Gd-enhanced MRI was 1.8 (1 – 8). The mean interval between the last MRI and lumbar puncture was 2.8 (0.3 – 12) years. Mean (± SD) CSF Gd concentration was 0.065 (0.199) µg/L. We detected 0.001 µg/L Gd concentration in the CSF of the patients who had undergone a single Gd-enhanced MRI 12 years before the lumbar puncture. We found differences (p<0.05) comparing CSF Gd concentration in low, moderate and severe systemic siderosis. We confirmed that Gd deposition in glia increased in iron overload. We documented transmembrane passage of gadolinium in two glial cells lines, with and without iron overload.
**Conclusion:** Neurodegenerative diseases have been increasingly diagnosed in paediatric patients in recent years. Gd brain storage along with other metals such as iron could accelerate the onset of degenerative symptoms.
**Limitations:** The fact that this was a singlecentre study, the paediatric population, as well as the small sample number were identified as limitations.
**Ethics committee approval:** This study was approved by the bioethics committee of IRCCS Burlo Garofolo Trieste (reference no. 1105/2015).
**Funding for this study:** No funding was received for this study.
**Author Disclosures:**
Roberto Sala: Nothing to disclose
Natalia Maximova: Nothing to disclose
Floriana Floriana Zennaro: Nothing to disclose
Davide Zanon: Nothing to disclose
Matteo Crosera: Nothing to disclose
Cesare Covino: Nothing to disclose
Daniele Zampieri: Nothing to disclose

### RPS 1812-12

**MRI of the optic nerve: evaluation of reference values in children and adolescents**

T. Ahrens, P-C. Krüger, K. Glutig, M. Waginger, *H-J. Mentzel*; Jena/DE

**Purpose:** Few studies exist for MRI evaulation of the dimensions of the optic nerve (OND) in children and adolescents. Published reference values are only comparable to a limited extent. The aim of this study was to use routine MRI for the estimation of OND and to establish a simple measurement protocol.
**Methods or Background:** Routine skull MRI at 1.5 T and 3.0 T of 164 children and adolescents (88m, 76f) aged 0-18 years (median 8.3 years) were evaluated retrospectively. OND in sagittal and axial orientation was determined 3 and 10 mm posterior to the lamina cribrosa sclerae (measuring points I and II) and immediately before entering the optic chiasm (measuring point III). Evaluation was performed without optic nerve sheath. For statistical analysis of the measured values, the patients were divided into five age groups.
**Results or Findings:** Evaluating the influence of various variables (hemisphere, gender, orientation level, field strength), individual statistically significant differences were found. The reference values were only given for measuring point I and amounted to 1.9-2.8 mm in the sagittal orientation plane and 1.5-3.1 mm in the axial plane. It could be shown that OND increases with age, with most of the growth being completed by the age of six.
**Conclusion:** The collected reference data were comparable with existing studies. So, the OND can be measured in children using a routine MRI. For exact measurements, thin-slice 3D T2-weighted MRI with isovoxels would have to be carried out.
**Limitations:** MRI was performed at both 1.5 and 3 T. Despite strong exclusion criteria, no typical normal collective was recorded.
**Ethics committee approval:** The local ethics committee approved this retrospective study.
**Funding for this study:** There was no funding.
**Author Disclosures:**
Theresa Ahrens: Nothing to disclose
Hans-Joachim Mentzel: Nothing to disclose
Paul-Christian Krüger: Nothing to disclose
Katja Glutig: Nothing to disclose
Matthias Waginger: Nothing to disclose

**10:30-12:00** **Room X**

### Research Presentation Session: Imaging Informatics / Artificial Intelligence and Machine Learning

### RPS 1805a

### Artificial intelligence (AI) in breast imaging

**Moderator**
C. Van Ongeval; Leuven/BE

### RPS 1805a-3

**Preparing screening mammograms for Artificial Intelligence using deep learning**

*S. Salas Aguirre*, C. E. Haro Mares, M. Iñiguez, R. Ramírez, A. Sánchez, E. U. Moya; Guadalajara/MX
*(so_sarmx@hotmail.com)*

**Purpose:** We collaborate with AI experts to design together two deep learning architectures (models) to classify the mammography projections in terms of left or right (L/R) and Craniocaudal and Mediolateral oblique considering the most important anatomical regions and compare it with the model feature maps.
**Methods or Background:** We use the Curated Breast Imaging Subset (CBIS) to train, validate and test in the mini-MIAS database. Our work is totally reproducible due to the fact that we use open data sources, and we provide all the labels and code.
**Results or Findings:** ProjectionNet, devoted to classify between CC and MLO projections, archives a test accuracy of 99.5 and AUC of 0.999 in comparison to MobileNet with 97.7 and AUC of 0.997. LRNet, capable of classifying the left and right breast in the MLO projection, obtained a test accuracy of 98.8 and AUC 0.999. whereas a MobileNet gets an accuracy of 79.8 and AUC of 0.80. The saliency-maps show that the proposed CNNs highlight the anatomical regions of the breast (pectoral muscle, nipple, edges and perimeter). In contrast, the MobileNet map focuses mainly in broad areas with empty space. For the segmentation model we present preliminary results in a test set where it is possible to see how is removed the non-relevant features of the image.
**Conclusion:** By preparing the mammography screening images for the AI, we address a fundamental step to allow the classification by breast area segmentation and the classification as CC/MLO and L/R projections.
**Limitations:** This study is limited by the lack of access to more mammography data from unlabelled projections and more diverse sources.
**Ethics committee approval:** We have followed most of the applicable recommendations of prediction model study Risk of Bias Assesment Tool (PROBAST) to make explainable and responsible AI.
**Funding for this study:** Funding was received from the Jalisco Goverment.
**Author Disclosures:**
Claudia Eunice Haro Mares: Investigator: Instituto Mexicano del Seguro Social
Radamés Ramírez: Investigator: Universidad Autónoma de Guadalajara
Eduardo Ulises Moya: Investigator: Gobierno de Jalisco
Abraham Sánchez: Investigator: Gobierno de Jalisco
Sofia Salas Aguirre: Investigator: Instituto Mexicano del Seguro Social
Marco Iñiguez: Investigator: Universidad Autónoma de Guadalajara
Investigator: Universidad Autónoma de Guadalajara

### RPS 1805a-4

**AI-based strategy to reduce the recall rate and consensus meeting workload of double reading in breast cancer screening with digital mammography: a retrospective evaluation**

K. Hamm[1], *D. Hellingman*[2], L. Kotrini[1], B. Vetter[1], T. Jordan[1], C. Entrup[3], M. Engelke[4], N. Janssen[5], B. Schubotz[1]; [1]Chemnitz/DE, [2]Forchheim/DE, [3]Koblenz/DE, [4]Hamburg/DE, [5]Nijmegen/NL
*(daan.hellingman@siemens-healthineers.com)*

**Purpose:** To evaluate an autonomous artificial intelligence (AI) based triaging strategy in breast cancer screening as compared to independent double reading with consensus.
**Methods or Background:** A consecutive cohort of 37674 digital mammography screening exams (including 210 screen-detected, 45 interval, and 110 next-round screen-detected (NRSD) cancers) were retrospectively collected from a German screening site. An AI system (Transpara, ScreenPoint Medical) computed a cancer risk score (from 1 to 10) for each exam. Double reading of all exams was compared with an autonomous AI triaging strategy; no human reading is performed for the least suspicious exams classified by AI (score 1-6), only exams with score 7-10 are double read, and the top 1% most suspicious exams as classified by AI are automatically recalled. Cancer detection rate (CDR), recall rate (RR), and consensus workload were evaluated.
**Results or Findings:** Double reading of all exams resulted in a CDR of 5.6/1000, RR of 4.4% (1655/37674), and consensus workload of 4255 exams. A total of 27562 exams (73.2%), including 5 screen-detected cancers and 636 false-positive recalls, had an AI score of 1-6. AI found 5 additional cancers (2 interval cancers and 3 NRSD cancers) and 173 additional false-positive recalls in the 1% most suspicious exams. Autonomous AI triaging resulted in a similar CDR (5.6/1000), 28.0% lower RR (down to 3.2%), and 45.1% reduction of consensus workload compared to standard double reading.
**Conclusion:** Not reading exams with AI score 1-6 can reduce radiologists' RR and workload in screening at the cost of missing some screen-detected cancers. However, recalling the top 1% might compensate this loss in CDR. AI-assisted double reading of all exams can likely lower the RR and increase the CDR, but prospective studies should confirm this.
**Limitations:** This study was limited by its status as a retrospective study.
**Ethics committee approval:** Not applicable
**Funding for this study:** Not applicable
**Author Disclosures:**
Birgit Schubotz: Nothing to disclose
Daan Hellingman: Employee: Siemens Healthcare GmbH
Natasja Janssen: Employee: Screenpoint Medical BV
Martin Engelke: Nothing to disclose
Klaus Hamm: Nothing to disclose
Torsten Jordan: Nothing to disclose
Christian Entrup: Nothing to disclose
Bert Vetter: Nothing to disclose
Larissa Kotrini: Nothing to disclose

### RPS 1805a-5

**The use of deep learning techniques in craniocaudal views in mammography**

*R-E. Meetescu*, M. S. Șerbănescu, L. M. Florescu, G. C. Camen, R. V. Teica, C. T. Streba, I. Gheonea; Craiova/RO
*(raluca.meetescu@gmail.com)*

**Purpose:** The objective of the study was, using deep learning and artificial intelligence techniques, to determine if the pathology depicted on a mammogram is either benign or malignant. A total of 559 patients underwent breast ultrasound, mammography, and ultrasound-guided breast biopsy. The patients were divided into three categories: benign, ductal carcinomas, and non-ductal carcinomas, based on the histopathological results.
**Methods or Background:** Using only the craniocaudal view of the mammograms, we performed pre-processing and segmentation algorithms. An algorithm was used to remove the areola and the adjacent skin, given the large variability of the tissue. Therefore, patients with breast lesions close to the skin were removed from the study. The remaining breast image was resized on the Y axis to a square image and then resized to 512 × 512 pixels. To identify the lesion, a variable square of 322,622 pixels was searched inside every image. No information was lost during each image rotation. For data augmentation, each image was rotated 360 times and a crop of 227 × 227 pixels was saved, resulting in a total of 201,240 images. The images were cropped at this size because the deep learning algorithm transfer learning used from AlexNet network has an input image size of 227 × 227.
**Results or Findings:** On 100 runs of the algorithm, the mean accuracy was 95.8344% ± 6.3720% and mean AUC 0.9910% ± 0.0366%.
**Conclusion:** Based on the results on mammograms in the craniocaudal view, the proposed solution can be used as a non-invasive and highly precise computer-aided system based on deep learning that can classify breast lesions based on changes identified.
**Limitations:** Patients with recent trauma and lesions next to the areola were not included in the study.
**Ethics committee approval:** This study was approved by an ethics committee.
**Funding for this study:** Our study received no funding.
**Author Disclosures:**
Rossy Vladut Teica: Nothing to disclose
Georgiana Cristiana Camen: Nothing to disclose
Ioana Gheonea: Nothing to disclose
Raluca-Elena Meetescu: Nothing to disclose
Mircea Sebastian Șerbănescu: Nothing to disclose
Lucian Mihai Florescu: Nothing to disclose
Costin Teodor Streba: Nothing to disclose

### RPS 1805a-6

**Breast lesion recognition through machine learning: MammoWave clinical trial data**

*S. P. Rana*[1], M. Dey[1], D. Álvarez Sánchez-Bayuela[2], C. R. Castellano[2], R. Giovanetti González[2], L. M. Cruz Hernandez[2], P. M. Aguilar Angulo[2], L. Papini[3], L. Sani[3], A. Vispa[3], M. Ghavami[1], S. Dudley[1], G. Tiberi[3]; [1]London/UK, [2]Toledo/ES, [3]Perugia/IT
*(soumyarana08@gmail.com)*

**Purpose:** MammoWave is a microwave imaging apparatus for breast lesion detection, able to function in air with two antennas rotating in azimuth plane and operating within band 1-9 GHz. Supervised Machine learning (SML) algorithms can be applied to understand trends from the frequency spectrum collected through MammoWave in response to stimulus, allowing automatic recognition of breasts without lesion, benign, and malignant lesions. The performance is determined by statistically measured detection rates.
**Methods or Background:** The study comprises 327 breasts (from 180 patients) collected in Toledo Hospital, Spain, in the context of a multicentric international clinical trial (enrollment just ended). All breasts have their own correspondent output of radiologist study review (i.e. gold standard), obtained from echography and/or mammography and/or MRI, plus radiological/histological follow up where required. MammoWave examinations are performed measuring complex S21 in multi-bistatic fashion where magnitudes reveal diverse patterns when reflected from tissues without lesion, benign, and malignant lesions. SML has been applied to train the model and recognise different breast patterns.
**Results or Findings:** According to radiologist study review, 247 breasts without lesions and 80 breasts with lesions underwent MammoWave examination. Radiological/histological follow up confirmed that 21 were benign lesions and 59 malignant lesions. The proposed SML model achieved a no lesion detection-rate of 69%, a benign lesion detection-rate of 95%, and a malignant lesion detection-rate of 86%.
**Conclusion:** The proposed SML from MammoWave data can discriminate breasts without lesion, benign, and malignant lesions at a high rate.
**Limitations:** We didn't consider patients' pre-menstrual information.
**Ethics committee approval:** This study was approved by the Ethical Committee of Toledo Hospital, Spain (7/10/2019). Additionally, Approval was received from the Spanish Agency for Medicines and Health Products (N. 760/19/EC of 28/02/2020). The study is registered at Clinicaltrials.gov/ct2/show/NCT04253366.
**Funding for this study:** This project has received funding from the European Union's Horizon 2020 research and innovation programme under grants agreements No: 830265, 872752.
**Author Disclosures:**
Lorenzo Sani: Shareholder: Lorenzo Sani is employee and shareholder of UBT - Umbria Bioengineering Technologies.
Sandra Dudley: Nothing to disclose
Lina Marcela Cruz Hernandez: Nothing to disclose
Daniel Álvarez Sánchez-Bayuela: Employee: Daniel Sánchez-Bayuela is employed by UBT - Umbria Bioengineering Technologies.
Cristina Romero Castellano: Nothing to disclose
Lorenzo Papini: Nothing to disclose
Maitreyee Dey: Nothing to disclose
Paul Martin Aguilar Angulo: Nothing to disclose
Mohammad Ghavami: Nothing to disclose
Alessandro Vispa: Employee: Alessandro Vispa is employed by UBT - Umbria Bioengineering Technologies.
Soumya Prakash Rana: Nothing to disclose
Gianluigi Tiberi: Shareholder: Gianluigi Tiberi is shareholder of UBT - Umbria Bioengineering Technologies.
Rubén Giovanetti González: Nothing to disclose

### RPS 1805a-7

**Can an AI-based decision referral approach improve the overall sensitivity of a breast cancer screening programme?**

*S. Bunk*[1], D. Byng[1], M. Brehmer[1], T. Töllner[2], L. Umutlu[3], K. Pinker-Domenig[4], C. Leibig[1]; [1]Berlin/DE, [2]Stade/DE, [3]Essen/DE, [4]New York, NY/US
*(stefan.bunk@vara.ai)*

**Purpose:** We developed and evaluated an AI system's decision referral approach, where very confident algorithmic assessments are performed automatically and more difficult assessments are deferred to the radiologist. This two-part system incorporates triage of normal exams, while introducing a "safety net" to maintain high sensitivity by performing predictions on cancer-positive exams.
**Methods or Background:** A representative screening program data set of a total of N=24,501 full-field mammography exams from biopsy-confirmed, screen-detected (n=2,105) plus interval breast cancers (n=2,396), and follow-up proven negatives (n=20,000) from 8 German breast cancer screening units was used to simulate the impact of the decision referral approach on screening program-level sensitivity. Images were analysed using a commercial AI system. We computed the program-level change in sensitivity based on a previously validated threshold for cancer detection derived from an operating point of 98% screen-detected cancer sensitivity for normal triaging and 99% specificity for the safety net. Upper and lower bounds of the change in sensitivity were computed using resampling methods. Absolute reduction in missed cancers and triaging rate was also calculated.
**Results or Findings:** The sensitivity of the simulated screening program was 59.7%. At the selected operating point, sensitivity of the screening programme is improved by 3.9 (2.8–5.2) percentage points. The AI system detected 20.5% of cancers missed by radiologists, while also offering automation potential with a triaging rate of 50.7% for each radiologist.
**Conclusion:** By combining triaging with algorithmic detections of highly suspicious lesions that would otherwise have been missed by radiologists, a decision referral approach demonstrates improvement of sensitivity for the screening program.
**Limitations:** Not applicable
**Ethics committee approval:** Approval by an ethics committee was not necessitated due to the retrospective and fully anonymised nature of the mammography studies.
**Funding for this study:** Funding was received from Vara (Berlin, Germany).
**Author Disclosures:**
Stefan Bunk: Employee: Vara
Thilo Töllner: Nothing to disclose
Katja Pinker-Domenig: Grant Recipient: NIH/NCI Cancer Center Support Grant Grant Recipient: Breast Cancer Research Foundation Advisory Board: Vara
Danalyn Byng: Employee: Vara
Moritz Brehmer: Employee: Vara
Lale Umutlu: Grant Recipient: Siemens Healthcare & Research Advisory Board: Bayer Healthcare Advisory Board: Vara
Christian Leibig: Employee: Vara

### RPS 1805a-8

**Detection of breast arterial calcifications on mammograms with deep learning**

*N. Mobini*[1], F. Riva[1], M. Codari[2], M. G. Ienco[1], D. Capra[1], A. Cozzi[1], R. M. Trimboli[1], G. Baselli[1], F. Sardanelli[1]; [1]Milan/IT, [2]Menlo Park, CA/US
*(nazanin.mobini@gmail.com)*

**Purpose:** Breast arterial calcifications (BAC) are found in about 12% of women and have been suggested as a sex-specific cardiovascular disease (CVD) biomarker that might improve female CVD risk stratification. In this study, we aimed to implement and evaluate the performance of a deep convolutional neural network (CNN) model for automatic detection of BACs.
**Methods or Background:** Four-view mammograms were included in this retrospective study and labelled as BAC positive (BAC+) or BAC negative (BAC-) by 3 readers. The study included 1493 women (169 BAC+), aged 59.1±9.9 years. A 16-layer pre-trained Visual Geometry Group (VGG16) model was modified in its convolutional end to learn high-level features for detecting vascular calcifications, discriminating BAC+ from BAC- images. To account for class imbalance during model training, we randomly undersampled the majority BAC- class in the training set to reach a 30% BAC+ prevalence; the validation and test sets remained intact to reflect the BAC real prevalence. The training, validation, and test sets consisted of 1552 (365 BAC+), 896 (69 BAC+), and 908 (78 BAC+) images. Accuracy, geometric-mean (G-mean), and area under the curve (AUC) were used to assess the diagnostic performance at image-level.
**Results or Findings:** The resulting accuracy, G-mean, and mean AUC were 0.96, 0.95, and 0.98 for the training, 0.91, 0.82, and 0.86 for the validation, and 0.92, 0.83, and 0.85 for the test set, respectively.
**Conclusion:** Our model showed promising performances in BAC detection, confirmed by an AUC of 0.85 in the testing. A reliable automatic tool for BAC detection could allow large-scale studies on women's CVD risk stratification leveraging the mammographic screening programme.
**Limitations:** Increased BAC+ prevalence in the training set due to majority class undersampling was identified as a limitation of this study.
**Ethics committee approval:** The study was approved by the local Ethics Committee.
**Funding for this study:** No funding was received for this study.
**Author Disclosures:**
Andrea Cozzi: Nothing to disclose
Giuseppe Baselli: Nothing to disclose
Francesco Sardanelli: Grant Recipient: Bracco Grant Recipient: General Electric Speaker: Bracco Advisory Board: Bracco Speaker: General Electric Speaker: Bayer Advisory Board: General Electric Advisory Board: Bayer Grant Recipient: Bayer
Nazanin Mobini: Nothing to disclose
Francesca Riva: Nothing to disclose
Marina Codari: Nothing to disclose
Maria Giovanna Ienco: Nothing to disclose
Rubina Manuela Trimboli: Nothing to disclose
Davide Capra: Nothing to disclose

### RPS 1805a-9

**Can a screening mammography teaching file with AI improve trainee interpretation skills?**

R. Seidel[1], S. Pacilè[2], *P. Fillard*[2], E. Krupinski[1]; [1]Atlanta, GA/US, [2]Valbonne/FR

**Purpose:** Determine if completion of a screening teaching file (TF) augmented by AI software can improve radiology trainees' ability to identify malignancy on a 2D digital mammogram. Assess the trainees' perception of AI software for screening mammography.
**Methods or Background:** A pre-test, TF and post-test were created from an AI training dataset. Trainees were randomised into control or intervention groups. The intervention group completed the pre-test, AI-augmented TF, and post-test. The control group completed a pre-test and post-test, and was offered the opportunity to complete the AI-augmented TF. For each case, participants were asked to mark the single most suspicious lesion (if any), assign a forced BI-RADS and Level of Suspicion (LOS) score on a scale of 0 to 100%. Area Under the Curve (AUC) analysis was used to compare pre- and post-test performance. Participants were asked to provide feedback via survey.
**Results or Findings:** For the control group (n=9), average pre-test AUC was .874 and average post-test AUC was .891. For the intervention group (n=9), average pre-test AUC was .848 and average post-test AUC was .910. The AUC difference for the intervention group was .062 (-0.04, 0.17, 95% CI). The AUC difference for the control group was .018 (-0.08, 0.11, 95% CI). Though the difference in delta AUC between the control and intervention groups was not significant, performance in the intervention group improved more than in the control group. 14 out of 18 participants did some or all of the teaching file cases. 93% of participants agreed or strongly agreed with “The teaching file was a valuable educational tool.”
**Conclusion:** Reviewing a TF with AI software may improve trainees' ability to detect malignancy. Trainees felt the AI system served as a valuable educational tool.
**Limitations:** This study's small sample was identified as a limitation.
**Ethics committee approval:** This work is exempt from ethics committee approval.
**Funding for this study:** No funding was received for this study.
**Author Disclosures:**
Pierre Fillard: Founder: Therapixel
Serena Pacilè: Employee: Therapixel
Rebecca Seidel: Consultant: Therapixel
Elizabeth Krupinski: Nothing to disclose

### RPS 1805a-10

**Implementation of lesion detection box-derived radiomics signature in computer-aided decision support system for breast cancer ultrasound imaging**

*Z. A. Magnuska*[1], B. Theek[1], M. Darguzyte[1], M. Palmowski[2], E. Stickeler[1], V. Schulz[1], F. Kiessling[1]; [1]Aachen/DE, [2]Baden-Baden/DE
*(zmagnuska@ukaachen.de)*

**Purpose:** The goal of our study was to develop a CAD system for breast lesion detection and classification based on radiomics signatures (RS). YOLOv3 and Viola-Jones-based lesion detection boxes were evaluated to identify the lesions. Then, RS resulting from detection boxes and manually obtained segments were compared.
**Methods or Background:** The data pool of breast US images (199 malignant and 143 benign cases) was used to prepare eight different data sets (including pre-processed and spatially augmented images). Viola-Jones and YOLOv3 algorithms were trained for lesion detection. Three separate RS were obtained using the features extracted from the automatically outlined breast lesion detection boxes and manually delineated segments. The classification models were established and evaluated concerning the accuracy, sensitivity, specificity, and area under the Receiver Operating Characteristic curve (AUROC).
**Results or Findings:** After training on a dataset including logarithmic derivatives of US images, we found that YOLOv3 obtains better results in breast lesion detection (IoU: 0.528 ± 0.113; LE: 0.123 ± 0.028) than the Viola-Jones framework (IoU: 0.421 ± 0.01; LE: 0.066 ± 0.023). Interestingly, the classification model trained with RS derived from YOLOv3 detection boxes achieved higher overall accuracy (89.58%), sensitivity (90.00%), specificity (88.89%), and AUROC (0.90) than RS extracted from manually obtained segments (80.00%; 83.33%; 75.00%; 0.87).
**Conclusion:** Deriving RS from the detection box is a promising technique for building a breast lesion classification model, which might reduce the need for lesion segmentation in the future design of CAD systems.
**Limitations:** A bigger dataset would strengthen the study findings.
**Ethics committee approval:** Study approved by the Institutional Review Board of University Clinic Aachen, RWTH Aachen University (protocol code EK 066/18, 07 March 2018).
**Funding for this study:** DFG, research group FOR2591: project 321137804; Research Training Group 2375 "Tumor-targeted Drug Delivery"
**Author Disclosures:**
Fabian Kiessling: Nothing to disclose
Elmar Stickeler: Nothing to disclose
Benjamin Theek: Nothing to disclose
Milita Darguzyte: Nothing to disclose
Volkmar Schulz: Nothing to disclose
Zuzanna Anna Magnuska: Nothing to disclose
Moritz Palmowski: Nothing to disclose

### RPS 1805a-11

**A multicentric study to evaluate the effectiveness of machine learning over thermal images for detecting suspected breast cancer**

*S. Sampangi*, A. Singh, V. Bhat, A. Sigamani; Bangalore/IN
*(dr_sudhakar79@yahoo.com)*

**Purpose:** Machine learning (AI) over mammography has shown to enhance the accuracy of interpretation and reduce diagnosic errors. In this study, we evaluate a new technique called Thermalytix that uses machine learning over thermal images. If Thermalytix is found to be non-inferior to standard screening methods, a new affordable, accessible, radiation-free screening test would be available for the developing world to address the issue of low survival rates.
**Methods or Background:** A prospective two-centre study of Thermalytix was conducted to evaluate the sensitivity of Thermalytix for detecting malignant breasts. Subjects with possible symptoms of breast cancer took a Thermalytix test before standard imaging tests. Radiologist reports of mammography and breast ultrasound were blinded and compared with auto-generated Thermalytix AI scores. An ROC curve for Thermalytix was obtained for different cut-off points on Thermalytix scores. An operating point was determined for 10% non-inferiority margin.
**Results or Findings:** 258 symptomatic women were enrolled. 63 women (24.4%) were diagnosed with malignant breast cancer on histopathology. The AUROC of Thermalytix was 0.845. At Youden's Index, Thermalytix showed a sensitivity of 82.5% (95% CI, 73.2 to 91.9) and specificity of 80.5 % (95% CI, 75.0 to 86.1). Diagnostic mammogram had a sensitivity of 92% (95% CI, 80.7 to 97.8) and a specificity of 45.9% (95% CI, 34.3 to 57.9) when BI-RADS 3 was considered test-positive. For women under 45 years, Thermalytix sensitivity was 87.0% and specificity was 80.6%.
**Conclusion:** Thermalytix™ was found to be 10% non-inferior in sensitivity to mammography. It also showed good performance on women younger than 45 years.
**Limitations:** Only symptomatic population was included in the study, as Clinical Breast Examination is the standard population screening method in LMICs.
**Ethics committee approval:** EC Approval number: NHH/MEC-CL-2017-466, obtained from Mazumdar Cancer Center and HCG Hospital.
**Funding for this study:** Funding was received from NIRAMAI Health Analytix.
**Author Disclosures:**
Akshita Singh: Nothing to disclose
Venkataramana Bhat: Nothing to disclose
Sudhakar Sampangi: Nothing to disclose
Alben Sigamani: Nothing to disclose

### RPS 1805a-12

**Breast tissue segmentation in digital mammography using deep learning: one method for multiple views and vendors**

*S. Verboom*[1], M. Caballo[1], M. Broeders[1], J. Teuwen[2], I. Sechopoulos[1]; [1]Nijmegen/NL, [2]Amsterdam/NL
*(sarah.verboom@radboudumc.nl)*

**Purpose:** To develop an automatic segmentation algorithm of digital mammograms (DMs) into background, breast, and pectoral muscle that is applicable to cranio-caudal (CC) and medio-lateral oblique (MLO) projections and can generalise across varied models of different vendors.
**Methods or Background:** Two data sets were used: 247 diagnostic DM exams and 253 screening DM exams, totalling 1000 CC and 1000 MLO processed images, of which 379 (37.9%) and 985 (98.5%) contained a pectoral muscle respectively. The images were acquired with 6 different DM system models from three vendors: Siemens (13%), GE (36%), and Hologic (51%). The multi-class segmentation was done by a U-Net trained with a subset of 600 mammograms and a multi-class weighted focal loss. Several types of data augmentation were used during training, to generalise across model types, including a random look-up table, random elastic and gamma transformations. The model was tested on a subset of 244 processed mammograms.
**Results or Findings:** Segmentation of the test set resulted in dice scores of (mean ± std. dev.) 0.997 ± 0.004, 0.986 ± 0.016, 0.851 ± 0.248 for background, breast, and pectoral muscle, respectively. The pectoral muscle segmentation resulted in a higher dice score for MLO views (0.952 ± 0.072) than for CC views (0.597 ± 0.337). Among different model types, the mean overall DICE scores ranged from 0.987 to 0.995 for the different system models.

**Conclusion:** The developed method yielded accurate overall segmentation results independent of view and was able to generalise well over mammograms acquired by different models. Segmentation of the pectoral muscle in cranio-caudal views requires further optimisation.
**Limitations:** The segmentation method is based on mammograms from a single population.
**Ethics committee approval:** Ethical approval was waived for this retrospective study.
**Funding for this study:** This study forms part of the aiREAD project funded by NWO/KWF/TKI.
**Author Disclosures:**
Mireille Broeders: Nothing to disclose
Marco Caballo: Nothing to disclose
Sarah Verboom: Nothing to disclose
Jonas Teuwen: Nothing to disclose
Ioannis Sechopoulos: Nothing to disclose

**10:30-12:00** **Room Z**

### Research Presentation Session: Interventional Radiology

### RPS 1809

### Interventional ablation therapy outside the liver

**Moderator**
B. Lucey; Galway/IE

### RPS 1809-2

**Does ramping power protocol reduce complications in patients treated with image-guided microwave ablation for renal tumours?**
*E. Lucertini*[1], I. Monfardini[2], P. Della Vigna[3], G. Bonomo[3], D. Maiettini[3], G. M. Varano[3], N. Camisassi[3], F. Orsi[3], G. Mauri[3]; [1]Rome/IT, [2]Brescia/IT, [3]Milan/IT

**Purpose:** Microwave ablation (MWA) of renal tumors can produce subcapsular haematomas as a possible complication, due to the increase of intra-organ pressure. A progressive power increase could reduce the intra-organ pressure during MWA, and thus the number of subcapsular haematomas. The purpose of this study was to compare subcapsular haematoma rate and outcome of patients with renal tumour treated with MWA using ramping power technique with patients treated with standard fixed power protocol MWA.
**Methods or Background:** 167 patients (181 procedures) were included in the study: group 1 (82 patients/ 88 procedures) underwent ramping power MWA, starting at 30/40W and gradually increasing power to the desired maximum value, while group 2 (85 patients/93 procedures) underwent standard MWA with a fixed power value. All patients underwent contrast-enhanced CT immediately after the ablation and 24h later, to identify immediate complications and results of the ablation.
**Results or Findings:** Subcapsular haematoma occurred in a significantly lower number of procedures in group 1 compared to group 2 (1/88 (1.1%) vs 8/93 (8.6%), p=0.038). No significant differences were found in the number of overall complications (9/88 (10.2%) in group 1 vs 16/93 (17.2%), p=0.287). Complete ablation at 24h was achieved in 83/88 procedures (94,3%) in group 1 and in 85/93 procedures (91,4% ) in group 2 (p=0.915).
**Conclusion:** A lower incidence of subcapsular haematomas in kidney tumours treated with MWA can be obtained using ramping protocol instead of standard MWA, maintaining a similar efficacy.
**Limitations:** Limiting factors in this study were retrospective evaluation, pathological heterogeneities in renal tumours not having been considered as well as the choice of the ramping protocol being empirical and subjective.
**Ethics committee approval:** Ethics committee approval has been obtained.
**Funding for this study:** No funding was received for this study.
**Author Disclosures:**
lorenzo Monfardini: Nothing to disclose
Daniele Maiettini: Nothing to disclose
Paolo Della Vigna: Nothing to disclose
Gianluca Maria Varano: Nothing to disclose
Guido Bonomo: Nothing to disclose
Giovanni Mauri: Nothing to disclose
Franco Orsi: Nothing to disclose
Nicola Camisassi: Nothing to disclose
Elena Lucertini: Nothing to disclose

### RPS 1809-3

**Radiofrequency ablation for treatment of abdominal wall endometriosis**
*S. A. A. Mahdavi Anari*, Z. Haghighi; Tehran/IR
*(mahdavi0ali@yahoo.com)*

**Purpose:** Abdominal wall endometriosis (AWE) is a rare condition that usually develops in a surgical scar resulting from a Caesarean section. Its treatment options consist of the excision of the lesion and/or hormonal therapies, although wide surgical excision is the treatment of choice in the literature. Here we review a case series of abdominal wall endometriosis treated with radio-frequency ablation (RFA) under ultrasound guidance.
**Methods or Background:** 8 patients were included in this study, all of them presenting with palpable mass(es) in the abdominal wall close to a cesarean scar. The patients underwent full diagnostic workup including ultrasound and MRI. Written informed consents were obtained. Before RFA, the patient's pain level was assessed using a validated visual analogue scale (VAS) for pain assessment. RFA was performed under general anaesthesia with Neuro N50 generator with 500KHZ up to 99°C temperature for a maximum of 10 minutes. The patients' pain level was assessed one week, one month and 4 months after the procedure.
**Results or Findings:** Before the procedure mean VAS was 8.2. One week after the procedure, this was reduced to 2.3, after one month to 1.5 and after 4 months to 1.2. Four patients reported no pain after one month (VAS=0). No major complications was observed except for moderate erythema at RFA site resolving after one week.
**Conclusion:** Medical management of AWE often results in temporary relief. Wide surgical excision may create a defect in the abdominal wall and may increase the risk of hernia formation. RFA is used in many solid and superficial organs such as the thyroid. However, there are no studies in the literature on the role of RFA in the treatment of pain related to endometriosis. Here we suggest that RFA could be considered as an alternative treatment for AWE.
**Limitations:** No limitations were identified.
**Ethics committee approval:** This study was approved by an ethics committee.
**Funding for this study:** No funding was received for this study.
**Author Disclosures:**
Seyed Ali Akbar Mahdavi Anari: Speaker: main investigator
Zahra Haghighi: Investigator: main investigator

### RPS 1809-4

**Early assessment of MRI signal intensity of irreversible electroporation in pancreatic cancer**
*O. W. Kozak*, S. Hac, T. Gorycki, K. Skrobisz, B. Brzeska, J. Pienkowska, M. Studniarek; Gdansk/PL
*(oliwiak@gumed.edu.pl)*

**Purpose:** Most of patients with pancreatic cancer are diagnosed in the advanced stage of disease. Only 15-20% of them are qualified for pancreatoduodenectomy. Irreversible Electroporation (IRE) of pancreas is an increasingly used method for unresectable pancreatic cancer that can be used in cytoreduction followed by surgical treatment and shows promising results in palliative care. It is claimed that IRE is not causing thermal effect comparing to radiofrequency and microwave ablation. Early assessment of signal intensity changes after IRE could provide information on actual processes occurring in the ablation zone.
**Methods or Background:** So far, we have retrospectively analysed the MRI studies of 26 patients (10 F, 16 M, aged 35-71) with unresectable pancreatic cancer, performed on 1,5T and 3T scanners one day before IRE procedure and up to 7 days after. The standard protocol for pancreas was performed including subtraction series. The qualitative and quantitive assessment of obtained images was performed.
**Results or Findings:** The signal intensity in the ablated zone on T1 FatSat images after contrast media administration with subtraction was in the range 0-36. The ADC values after IRE were significantly higher compared to pre-IRE measurements. The signs of thermal effect, e.g. areas of needles placement, were found in 76% of patients.
**Conclusion:** Results of this study could facilitate the interpretation of MRI studies after IRE. Lower SI values in the ablated zone on T1 FAT SAT CM images with subtraction could be linked with a better completeness of the procedure. The growth of ADC values after IRE is associated with water displacement to intercellular space what is a sign of procedure success.
**Limitations:** The small group of patients and the fact that examinations were performed on different scanners were identified as limiting factors.
**Ethics committee approval:** There is ethics committee approval for this project.
**Funding for this study:** There was no external funding of this project.

**Author Disclosures:**
Joanna Pienkowska: Nothing to disclose
Stanislaw Hac: Nothing to disclose
Beata Brzeska: Nothing to disclose
Tomasz Gorycki: Nothing to disclose
Oliwia Wiktoria Kozak: Nothing to disclose
Katarzyna Skrobisz: Nothing to disclose
Michal Studniarek: Nothing to disclose

### RPS 1809-5

**Imaging and response characteristics of Desmoid tumours following percutaneous cryoablation**

*E. W. Johnston*, C. Messiou, M. Smith, D. Strauss, A. Hayes, C. Benson, S. Gennatas, R. Jones, N. Fotiadis; London/UK

**Purpose:** A prospective phase 2 trial has shown that image guided cryoablation is effective and safe in pre-treated Desmoid tumours. However, imaging appearances following treatment can be complex and the optimal method to evaluate response remains to be defined.
**Methods or Background:** A retrospective analysis of 10 patients with symptomatic progressive disease undergoing cryoablation procedures. Quantitative imaging metrics were derived from T2 weighted STIR images, before and after treatment: i) normalised T2 weighted signal intensity (T2nSI), ii) long axis diameter (LAD) iii) segmented tumour volumes at baseline iv) volume estimation using the prolate ellipsoid formula (length x width x height x 0.52) at all timepoints (PEV). Response was interpreted for LAD and PEV using RECIST 1.1. Symptomatic response to treatment was evaluated at clinical follow-up, and percentage agreement with both objective metrics (LAD and PEV) calculated.
**Results or Findings:** PEV showed near perfect correlation with full segmented tumour volume (Spearman's r 0.99, p<0.0001). Best objective response based upon post-treatment scans (median 15, IQR 7 – 23 months) was as follows: LAD: 6 stable disease (SD), 1 CR (complete response), 1 partial response (PR) 2 PD (progressive disease). Objective response rate (ORR) = 2/10, 30% agreement with symptomatic response. STV: 1CR, 6PR, 1SD, 2PD. ORR = 7/10, 80% agreement with symptomatic response. Changes in signal intensity were unpredictable, with no statistically significant differences between baseline MRI, 6-month follow-up MRI, and most recent MRI (median 255, IQR 157 – 575) days.
**Conclusion:** Our study suggests volumetric assessment of Desmoid tumours following cryoablation is more concordant with clinical outcome than single long axis measurement and can be estimated without labour intensive segmentation. We also found that T2nSI is unpredictable following cryoablation.
**Limitations:** Sample size was identified as a limitation of this study.
**Ethics committee approval:** This study was approved by an institutional service evaluation committee.
**Funding for this study:** Not applicable
**Author Disclosures:**
Spyridon Gennatas: Nothing to disclose
Christina Messiou: Nothing to disclose
Dirk Strauss: Nothing to disclose
Robin Jones: Nothing to disclose
Charlotte Benson: Nothing to disclose
Edward William Johnston: Nothing to disclose
Andrew Hayes: Nothing to disclose
Nicos Fotiadis: Nothing to disclose
Myles Smith: Nothing to disclose

### RPS 1809-6

**Safety of MR-guided High Intensity Focused Ultrasound (MRgHIFU) in the treatment of bone metastases**

*V. D'Agostino*[1], M. P. Aparisi Gomez[2], C. Gasperini[1], F. Vendetti[1], A. Bazzocchi[1]; [1]Bologna/IT, [2]Valencia/ES
*(valerio.dagostino123@gmail.com)*

**Purpose:** A distance greater than 1 cm between the target lesion and sensitive structures is highly recommended in MRgHIFU treatment of bone lesions. Our aim is to evaluate the safety and effectiveness of MRgHIFU treatment in bone metastases located in close proximity (< 1 cm) of sensitive structures.
**Methods or Background:** 88 patients (45.8% female, 54.2% male; mean age 58±11.6) with 107 bone metastases were treated. Close proximity between lesion and sensitive structures was established on pre-treatment MR exams. VAS score at baseline, 3 and 6 months after treatment was assessed in all patients. MR/CT imaging was performed before and 3-6 months after treatment. Primary endpoint was to assess major and minor complications involving sensitive structures. Secondary endpoints were assessment of treatment response, tumour control and impact of the proximity of sensitive structures on treatment efficacy.
**Results or Findings:** 107 metastases were treated with MRgHIFU (Primary tumors: 37,3% breast, 19% kidney, 10,7% prostate, 33% others). Close proximity between lesion and sensitive structures was recorded for 117 sites (31 tendon/enthesis/ligament, 16 vessels, 48 joints, 3 skin, 19 nerves). No adverse events over grade 2 were documented. VAS at baseline was 5.9±2.7, with a significant reduction at 3 months (2.57±2.5, p<0.0001) and at 6 months (2.82±2.83, p<0.0001). Imaging follow-up at 3 and 6 months was available for 49 patients. Complete response was seen in 11 cases, partial response in 14, stability in 20 and progression in 4. Proximity to sensitive structures did not affect the efficacy of treatment (p<0.01).
**Conclusion:** MRgHIFU can be safely and effectively performed on lesions located at < 1 cm from sensitive structures, widening the range of applications and allowing currently ineligible patients according to recommendations to benefit from this treatment.
**Limitations:** Partial drop-out in follow-up was identified as a limitation.
**Ethics committee approval:** Not applicable
**Funding for this study:** Not applicable
**Author Disclosures:**
Chiara Gasperini: Nothing to disclose
Alberto Bazzocchi: Nothing to disclose
Maria Pilar Aparisi Gomez: Nothing to disclose
Francesco Vendetti: Nothing to disclose
Valerio D'Agostino: Nothing to disclose

### RPS 1809-7

**Electrochemotherapy (ECT) with bleomycin in the treatment of bone metastases**

*N. Papalexis*, G. Peta, M. Miceli, E. Costantino, L. Campanacci, G. Facchini; Bologna/IT
*(nicolaspapalexis@gmail.com)*

**Purpose:** The primary goal of this study is to evaluate the efficacy and safety of ECT with bleomycin on bone metastases, assessing results in radiologic response, pain reduction, and improved quality of life.
**Methods or Background:** Between 2009 and 2020, we enrolled 85 cancer patients (mean age 60 years) with bone metastases. 43 patients had lesions at the upper limb, 13 at the lower limb, 21 pelvis, and 8 thorax and vertebrae. The treatment was performed using ECT, through the application of an electric pulse to the tumour mass allowing a local increased bleomycin delivery into the cells. Intravenous bolus injection of bleomycin (15 mg/m2) was performed, followed by the application of 8 electric pulses of 1000V/cm between each couple of electrodes (8 minutes after the end of drug injection) generated by Cliniporator VITAE (Igea S.p.A., Carpi, Italy) using dedicated electrodes. The procedures were performed under CT or fluoroscopic guidance.
**Results or Findings:** 73 patients received a single course of ECT, 9 patients 2, 2 patients 3, and 1 patient 4. All the evaluable patients reported a decrease in pain from 30% to 100% (average 57% of pain relief) after the local treatment with ECT. 76% of evaluable patients reported a pain reduction of more than 50% after ECT treatment. No general complications related to the treatment were encountered. Local complications to treatment have been observed in two cases: skin necrosis.
**Conclusion:** Our data provide evidence that the ECT approach is feasible and safe in patients with bone metastatic lesions. Patients reported radiology response and an improvement in usual activities and overall health.
**Limitations:** The observational nature of the study with the lack of a control group was identified as a limiting factor.
**Ethics committee approval:** We received approval from the ethics committee of our institute.
**Funding for this study:** Not applicable
**Author Disclosures:**
Marco Miceli: Nothing to disclose
Errani Costantino: Nothing to disclose
Laura Campanacci: Nothing to disclose
Giancarlo Facchini: Nothing to disclose
Giuliano Peta: Nothing to disclose
Nicolas Papalexis: Nothing to disclose

### RPS 1809-8

**Correlation of hormone indices and collimated C-arm CT findings for adrenal venous sampling in primary hyperaldosteronism**

*L. S. Becker*, M. Hinrichs, T. Werncke, C. Dewald, S. K. Maschke, J. B. Hinrichs, F. Wacker, B. C. Meyer; Hannover/DE

**Purpose:** Evaluation of feasibility and effect of a novel approach to adrenal venous sampling (AVS) analysis by combining established selective cortisol and aldosterone indices with the acquisition of a collimated C-arm CT (CACT_Coll).
**Methods or Background:** Overall, 107 consecutive patients (62m; 54±10 years) undergoing 111 AVS procedures without hormonal stimulation from July 2013 to February 2020 in a single institution were retrospectively analysed. Hormone levels were sequentially measured in suspected adrenal veins and right iliac vein, and selectivity indices (SI) computed. Successful AVS procedures were defined by stand-alone SI_Cortisol and/or SI_Aldosterone ≥2.0 as well as SI_Cortisol and/or SI_Aldosterone ≥1.1 combined with a positive right-sided CACT_Coll of the adrenals (n=80; opacified right adrenal vein). Radiation exposure of CACT was measured via dose area product (DAP) and compared to an age-/weight-matched cohort (n=66).
**Results or Findings:** Preliminary success rates (SI_Cortisol and/or SI_Aldosterone≥ 2.0) amounted to 99.1% (left) and 72.1% (right). These could be significantly increased to a 90.1% success rate on the right, by combining an adjusted SI of 1.1 with a positive CACT_Coll, proving the correct sampling position. Sensitivity for stand-alone collimated CACT (CACT_Coll) was 0.93, with 74/80 acquired CACTColl confirming selective cannulation by adrenal vein enhancement. Mean DAP_Coll_CACT measured 2414 ± 958 µGyxm2, while mean DAP_Full-FOV_CACT in the matched cohort measured 8766 ± 1956 µGyxm2 (p<0.001).
**Conclusion:** Collimated CACT in AVS procedures is feasible and leads to a significant increase in success rates of (right-sided) selective cannulation and may in combination with adapted hormone indices, offer a successful alternative to previously published AVS analysis algorithms with lower radiation exposure compared to a full-FOV CACT.
**Limitations:** The retrospective nature of the study and the evaluation of a singular procedure in one individual centre were identified as limitations. A larger population combined with a multi-centric approach, prospective study design and correlation with post-surgical results should be considered.
**Ethics committee approval:** Not applicable
**Funding for this study:** Not applicable
**Author Disclosures:**
Marie Hinrichs: Nothing to disclose
Bernhard C Meyer: Nothing to disclose
Cornelia Dewald: Nothing to disclose
Sabine Katharina Maschke: Nothing to disclose
Lena Sophie Becker: Nothing to disclose
Thomas Werncke: Nothing to disclose
Frank Wacker: Nothing to disclose
Jan B Hinrichs: Nothing to disclose

### RPS 1809-9

**Metal artifact reduction algorithm for monitoring and assessing ablation zones during CBCT guided pulmonary microwave ablation in an ex vivo porcine model: a qualitative and quantitative study**

*M. Öztürk*[1], P. Laeseke[2], S. Schäfer[2]; [1]Samsun/TR, [2]Madison, WI/US

**Purpose:** To assess the value of a metal artifact reduction (MAR) algorithm for assessing ablation zones during cone-beam computed tomography (CBCT) guided pulmonary microwave ablation in an ex vivo porcine model.
**Methods or Background:** Six microwave ablations were performed in ex vivo porcine lungs using a single 17-gauge antenna for 5 minutes at 65W under CBCT guidance. Ablation zone growth was monitored at 1-minute-intervals using CBCT. An immediate post-procedure CBCT scan was obtained after the probe was removed. Images acquired during the ablations with the probe-in-place were reconstructed using a MAR algorithm (MAR image). The unprocessed images (non-MAR image) were compared to MAR images using a subjective image quality assessment scale from 1 (severe artifact, no ablation zone visible) to 4 (minimal artifact, ablation zone clearly visible) and by comparing image noise and signal to noise ratio (SNR) in several regions around the ablation probe.
**Results or Findings:** Mean image quality score of MAR images (mean scores from 1st to 5th minute: 1.04±0.58 – 3.08±0.72) was significantly higher than the mean image quality score of non-MAR images (mean scores from 1st to 5th minute: 3.54±0.72 – 3.75±0.61, p<0.001 for each). The quantitative analysis demonstrated significant image noise reduction at either side of the ablation probe (p<0.001 and p=0.002) and at the tip of the needle (p<0.001) at MAR images. SNR of the MAR images was significantly higher than the SNR of non-MAR images (p<0.001 – p=0.002).
**Conclusion:** A metal artifact reduction algorithm improved image noise and SNR around the ablation probe and subjectively improved image quality during CBCT guided pulmonary microwave ablation in an ex vivo porcine model.
**Limitations:** The lack of histopathological reference as well as this work's status as an ex vivo study with unpredictable results regarding in vivo settings were identified as limitations.
**Ethics committee approval:** This study was approved by an ethics committee.
**Funding for this study:** No funding was received for this study.
**Author Disclosures:**
Sebastian Schäfer: Employee: Siemens Healthineers
Paul Laeseke: Consultant: NeuWave Medical
Mesut Öztürk: Nothing to disclose

### RPS 1809-10

**Long-term outcomes in percutaneous cryoablation of tumours in solitary kidneys**

*F. Segato*[1], C. Cicero[2], A. Casarin[2], A. Celia[2], S. Cernic[3], F. Stacul[3], L. Balestreri[4], A. Pinzani[5], G. Mansueto[1]; [1]Verona/IT, [2]Bassano del Grappa/IT, [3]Trieste/IT, [4]Aviano/IT, [5]Serenissima/IT

**Purpose:** The purpose of this study was to evaluate the impact of percutaneous cryoablation of tumours in Patients with solitary kidney in terms of renal function, treatment-related complications, and oncological outcomes.
**Methods or Background:** We have performed, as Italian groups, a multicentre retrospective study on patients with solitary kidney affected by renal cancer. From December 2009 to June 2018, all Patients underwent percutaneous renal cryoablation. Their characteristics regarding age, tumour's size, PADUA score and presence of recurrence were collected. Serum creatinine concentration and estimated glomerular filtration rate (eGFR) were measured before and after treatment for the evaluation of renal function.
**Results or Findings:** Thirty-two Patients Patients, mean age 70 years (range, 62-80 years), with 32 tumours (mean maximum diameter, 2.7 cm; range, 1.7-4.5 cm) were treated with percutaneous cryoablation procedures with a mean follow-up period of 20 months (range, 12-84 months). There was no significant change in creatinine pre- and post-ablation. None of the patients needed dialysis. One grade 3 or greater complications (Clavien-Dindo score) occurred after the 32 procedures (4% complication rate). The median hospital stay was 1.8 days (range, 1-3 days). The local tumour control rate was 89%, patients with tumours smaller than 4 cm had a lower probability of recurrence (p=0.01).
**Conclusion:** Percutaneous cryoablation is effective and safe in the management of renal tumours in patients with a solitary kidney. This technique showed a low morbidity rate with preservation of renal function and good local oncological control.
**Limitations:** The retrospective nature of the study was identified as a limitation.
**Ethics committee approval:** This retrospective chart review study involving human participants was in accordance with the ethical standards of the institutional and national research committee and with the 1964 Helsinki Declaration and its later amendments or comparable ethical standards.
**Funding for this study:** Not applicable
**Author Disclosures:**
Alessandro Pinzani: Nothing to disclose
Giancarlo Mansueto: Nothing to disclose
Stefano Cernic: Nothing to disclose
Calogero Cicero: Nothing to disclose
Antonio Celia: Nothing to disclose
Andrea Casarin: Nothing to disclose
Federica Segato: Nothing to disclose
Fulvio Stacul: Nothing to disclose
Luca Balestreri: Nothing to disclose

### RPS 1809-12

**Optimising CT-guided bone biopsies in cancer patients**

*R. Donners*[1], N. Fotiadis[1], I. Figueiredo[2], M. Blackledge[2], D. Westaby[2], C. Guo[2], M. d. l. D. F. Maza[2], D-M. Koh[1], N. Tunariu[2]; [1]London/UK, [2]Surrey/UK

**Purpose:** To optimise CT-guided bone biopsies in cancer patients by identifying laboratory, imaging and procedure parameters associated with biopsy success.
**Methods or Background:** 113 CT-guided bone biopsies performed in cancer patients by an interventional radiologist in one institution were retrospectively included. Routine blood parameters and tumour marker levels were recorded. In addition to the non-contrast (NC) biopsy CTs (113), contrast-enhanced (CE)-CTs (48) and PET/CTs (47) performed within four weeks of biopsy were reviewed; lesion location, diameter, lesion-to-cortex distance and NC-CT appearance (dense sclerosis, ground-glass, normal bone, mixed or lytic) were recorded. Mean NC-CT, CE-CT HU and PET SUVmax were derived from biopsy tract and lesion segmentations. Needle diameter, tract length and number of samples were noted. Comparisons between tumour-positive/negative and next-generation sequencing (NGS)-feasible/non-feasible biopsies determined significant (p<0.05) laboratory, imaging and procedural parameter differences.
**Results or Findings:** 76% of biopsies were tumour-positive. NGS was feasible in 28/42 cases (67%). Neither laboratory blood parameters, nor size, location or lesion-to-cortex distance affected biopsy success (p>0.099). Lytic lesions showed 89% PPV for a tumour-positive biopsy. Excluding lytic lesions, ground-glass (277 ± 142 HU) had 91% PPV for tumour and 88% PPV for NGS-feasibility (each p<0.025). NC-CT lesion HU was significantly lower in positive biopsies (p=0.017). A 528 HU cut-off yielded 86% PPV. Biopsy tract HU and SUVmax tract and lesion measurements were not associated biopsy success (each p>0.068). Needle diameter, tract length and number of samples were non-significant factors (each p>0.107).

**Conclusion:** In non-lytic bone lesions, ground-glass CT areas should be selected for CT-guided bone marrow biopsies in cancer patients.
**Limitations:** Not applicable
**Ethics committee approval:** Not applicable
**Funding for this study:** This study received support via the Prostate Cancer UK Research Innovation Award (reference RIA18-ST2-023), the Foundation of the Swiss Society of Radiology for Research, as well as the Research Fund for excellent Junior Researchers of the University of Basel.
**Author Disclosures:**
Christina Guo: Nothing to disclose
Daniel Westaby: Nothing to disclose
Matthew Blackledge: Nothing to disclose
Maria de los Dolores Fenor Maza: Nothing to disclose
Ines Figueiredo: Nothing to disclose
Nina Tunariu: Nothing to disclose
Ricardo Donners: Nothing to disclose
Nicos Fotiadis: Nothing to disclose
Dow-Mu Koh: Nothing to disclose

**12:30-13:30** **Open Forum #4 (ESR)**

### Poster Presentation Session

### PP 19

### Breast diseases and paediatric imaging

**Moderator**
W. Sanderink; Nijmegen/NL

### PP 19-2

**Calcifications with suspicious morphology: CESM findings and radiologic-pathologic correlation**
DOI: 10.26044/ecr2022/12330

*D. Calvo Gijón*, C. Gómez De Las Heras, P. Escobar Casas, B. Fernandez Gordillo, R. Aznar Méndez; Sevilla/ES

**Purpose:** To describe the most common contrast-enhanced spectral mammography (CESM) features of calcifications with suspicious morphology in comparison with the final diagnosis.
**Methods or Background:** Patients with suspicious breast calcifications evaluated by image-guided biopsy who had undergone CESM imaging were retrospectively selected from the institutional breast cancer database. Morphologic features and enhancement characteristics on CESM were analysed according to the MRI BI-RADS lexicon by two radiologists in consensus. The findings were correlated with the final diagnosis in the subsequent histopathologic examination. The statistical significance was assessed with the chi-square test ($p \leq 0.05$).
**Results or Findings:** 127 women were included. Pathology showed 41 benign and 86 malignant lesions, of which 44 were ductal carcinomas in situ (DCIS), 8 invasive ductal carcinoma and 34 mixed invasive and DCIS lesions. In post-contrast recombined images, 14 of 41 benign lesions (34.1%) and 77 of 86 malignant lesions (89.5%) showed contrast enhancement ($p < 0.001$); 10 of 14 benign lesions showed mild enhancement (71.4%), and 49 of 77 malignant lesions (63.6%) showed moderate/marked enhancement ($p = 0.014$). Of the 91 lesions who showed enhancement, 73 showed nonmasslike enhancement (80.2%). The most common enhancement pattern was heterogeneous in both benign and malignant lesions (72.7% and 74.2%, respectively) ($p = 0.188$). Enhancement distribution in the malignant lesions was most commonly segmental (41.9%), while benign lesions showed a focal, regional and multiple enhancement in equal proportions (27.3%) ($p = 0.189$).
**Conclusion:** CESM combines the evaluation of breast microcalcifications in the low-energy mammographic image with contrast enhancement information in the recombined image. The presence of enhancement and the intensity have been associated with the result of malignancy.
**Limitations:** Retrospective design and limited sample size. Subjective assessment of imaging features.
**Ethics committee approval:** Ethical approval was obtained from local Institutional Review Board.
**Funding for this study:** No funding was received for this study.
**Author Disclosures:**
Cristina Gómez De Las Heras: Nothing to disclose
Borja Fernandez Gordillo: Nothing to disclose
Daniel Calvo Gijón: Nothing to disclose
Pilar Escobar Casas: Nothing to disclose
Rafael Aznar Méndez: Nothing to disclose

### PP 19-3

**Imaging features of breast disease in PHTS women**
*A. Hoxhaj*, M. Drissen, J. Vos, N. Hoogerbrugge, R. M. Mann; Nijmegen/NL

**Purpose:** Women with PTEN Hamartoma Tumour Syndrome (PHTS) have increased breast cancer (BC) lifetime risk (67-85%). Moreover, some studies state that between 67-75% of female PHTS carriers develop benign breast lesions (BBL), which might hinder cancer detection. In this study, we investigated imaging features of breast disease in PHTS women.
**Methods or Background:** This retrospective study included 65 PHTS women (aged ≥18 years) who visited our university medical centre, a national PHTS expert centre, between 2001 and 2021. Breast MRI examinations were independently re-read by two radiologists.
**Results or Findings:** In 21/65 (32%) women, 35 different BCs were diagnosed. The median age at diagnosis of the first BC was 40 years [range: 24-59]. At MRI, BCs had malignant features with irregular shapes and margins, fast initial enhancement, plateau or washout in the delayed phase, and diffusion restriction. Eleven out of 21 (52.4%) women who developed BC were also diagnosed with BBL. Overall, 23/65 (35%) women were diagnosed with 89 pathologically-confirmed BBL. At MRI, BBL showed round to oval masses, with circumscribed margins and dark internal septation, slow/medium initial enhancement and persistent/plateau enhancement in the delayed phase.
**Conclusion:** The high prevalence of BC in our cohort is in line with literature findings. BBL were instead somewhat less frequent than previously reported in literature but were commonly present in women who also developed BC. We conclude that BBL have a role in hindering BC detection. However, since imaging characteristics are typical, a close examination of the multiparametric MRI features is essential in order to detect BC early and avoid unnecessary biopsy.
**Limitations:** Possible selection bias.
**Ethics committee approval:** Study was approved by the review board of the Radboudumc.
**Funding for this study:** Funding was received for this study by VIDI grant ZonMw.
**Author Disclosures:**
Alma Hoxhaj: Nothing to disclose
Meggie Drissen: Nothing to disclose
Janet Vos: Nothing to disclose
Ritse Maarten Mann: Nothing to disclose
Nicoline Hoogerbrugge: Nothing to disclose

### PP 19-4

**Unilateral axillary lymphadenopathy after COVID-19 vaccination: a pictorial review**
DOI: 10.26044/ecr2022/16862

*A. T. Teixeira*, R. Ribeiro, A. E. A. G. Costa, M. Faustino, J. P. A. Lopes, I. Cerejo, Z. T. d. Seabra; Vila Franca de Xira/PT

**Purpose:** To provide a pictorial review about the development of homolateral axillary lymphadenopathy following covid-19 vaccination, using imaging cases from our institution. To present the main recommendations for its management, especially in the context of breast cancer surveillance. To highlight the role of the radiologist in its diagnosis and follow-up.
**Methods or Background:** With the unprecedented range of covid-19 vaccination, the development of ipsilateral axillary lymphadenopathy has become an increasingly encountered side effect, evident over different imaging methods. This represents a challenge for the radiologist with an impact on breast imaging due to its ability for mimicking metastatic disease.
**Results or Findings:** Lymphadenopathy imaging findings related to COVID-19 vaccination are mostly consistent with a reactive scenario, with oval morphology, diffuse or focal cortical thickening and preserved hilum. Seen mainly up to 5 and 6 weeks after vaccination, lymphadenopathy may remain after 10 weeks, with decreased lymph nodes' size and residual cortical thickening. Nonetheless, the relation may not be so clear. This led the European Society of Breast Imaging to present a list of 10 recommendations adjusted to different clinical statuses, including women attending screening programmes and cases with known breast cancer or history of breast cancer. The key points consist in obtaining vaccination data, and scheduling breast examinations, if possible, before the first dose or at least 12 weeks after the last dose of the vaccine.
**Conclusion:** As the vaccination campaign proceeds, COVID-19 vaccine-induced lymphadenopathy will continue to be a reality. This reinforces the radiologist's role for the proper schedule of imaging exams and its correct interpretation, providing adequate follow-up if necessary, as the goal is to reduce unnecessary anxiety, additional imaging and invasive procedures while maintaining the suitable quality of breast cancer surveillance.
**Limitations:** Not applicable.
**Ethics committee approval:** Not applicable.
**Funding for this study:** Not applicable.

**Author Disclosures:**
Zita Teresa de Seabra: Nothing to disclose
Rui Ribeiro: Nothing to disclose
António Emanuel Almeida Gonçalves Costa: Nothing to disclose
Isabel Cerejo: Nothing to disclose
Madalena Faustino: Nothing to disclose
Ana Teresa Teixeira: Nothing to disclose
João Pedro Antunes Lopes: Nothing to disclose

### PP 19-5

**Performance and dose dependency of computer-aided detection (CAD) of pulmonary nodules in tin-filtered paediatric ultra low dose chest CT**

*P. J. Kuhl*, P. Gruschwitz, J. F. Heidenreich, S. Veldhoen; Würzburg/DE

**Purpose:** To evaluate the diagnostic performance of a computer-aided detection system for pulmonary nodules and its dependence on radiation dose in paediatric ULDCT.
**Methods or Background:** 382 consecutive ULDCT scans were retrospectively reviewed. Scans with present inflammatory consolidations were excluded. Two study groups were built from remaining 254 data sets based on the scan protocol applied: with identical tube voltage of 100kV, the reference mAs (ref.mAs) was set to 30mAs in 72 patients (m=34; 11.0±4.8 years) whereas 182 patients (m=108; 11.6±5.1 years) were scanned using 96ref.mAs. Three radiologists assessed the data sets for pulmonary nodules and each nodule was categorised by size (2-3mm;>3mm). Findings were compared to the CAD results and total CAD errors were quantified. Objective image noise (OIN) was measured. Radiation dose was estimated using size-specific dose estimates (SSDE).
**Results or Findings:** Using the 30ref.mAs setup, radiation dose was significantly lower compared to the 96ref.mAs group (SSDE 0.15±0.09 vs. 0.49±0.19mGy; p<0.001). OIN was significantly higher in all measured regions (p<0.001). Detection rates differed significantly for pulmonary nodules 2-3mm (0.24 vs. 0.48;p=0.002) and >3mm (0.44 vs. 0.71;p=0.007). False positive findings per scan did not differ significantly (0.33 vs. 0.39;p=0.53). No significant correlation between radiation dose and CAD errors was found.
**Conclusion:** Lowering the parameters for automatic tube current modulation in paediatric ULDCT results in significant dose reduction but also in reduced detection rates especially of small pulmonary nodules, suggesting a dose dependency of evaluated CAD system. However, CAD can assist radiologists as second observer tool even in paediatric ULDCT, especially in detection of nodules >3mm.
**Limitations:** The evaluated CAD system was initially trained on CT scans of adults. Our findings are not to be generalised to the totality of CAD due to the different work principles.
**Ethics committee approval:** Approved by an ethics committee.
**Funding for this study:** Not applicable.
**Author Disclosures:**
Simon Veldhoen: Nothing to disclose
Philipp Gruschwitz: Nothing to disclose
Julius Frederik Heidenreich: Nothing to disclose
Philipp Josef Kuhl: Nothing to disclose

### PP 19-7

**Use of arterial spin labelling (ASL) perfusion in the evaluation of paediatric patiens with new onset focal neurological deficit in acute settings**

*F. Dellepiane*[1], M. Resaz[2], D. Tortora[2], F. Sertorio[2], A. Ramaglia[2], M. Severino[2], A. Rossi[2]; [1]Rome/IT, [2]Genoa/IT

**Purpose:** To present the additional value of ASL perfusion in the evaluation of children with new-onset acute neurological symptoms.
**Methods or Background:** Arterial spin labelling (ASL) is a perfusion MRI technique that uses magnetically labelled water molecules in blood stream as an endogenous tracer, thereby obviating the use of exogenous tracers such as gadolinium-based contrast media (DSC/DCE-MRI) as well as radiation exposure (SPECT, CTP, PET). These advantages make ASL an attractive technique in children with a host of conditions including brain neoplasms, cerebrovascular diseases, and epilepsy. ASL also has an increasing role in the initial evaluation of patients with new-onset acute neurological deficit, allowing the neuroradiologist to strongly suggest a specific diagnosis in otherwise doubtful cases.
**Results or Findings:** Increased ASL signal can be found in patients with post-ictal states, often without or with very subtle changes on diffusion-weighted imaging (DWI). During seizures, uncontrolled electrical activity increases the metabolic demand from the affected brain area, in turn resulting in increased perfusion. Conversely, post-critical paralysis (i.e. Todd's palsy) presents with diffuse hypoperfusion of the affected hemisphere, reflecting temporary vasoconstriction. Hemiplegic migraine can present with both hyper- and hypoperfusion of the affected hemisphere depending on timing; migraine aura is associated with vasoconstriction and hypoperfusion, while the subsequent headache is characterised by vasodilatation and hyperperfusion. In all these conditions, the perfusion abnormality typically resolves on follow-up studies in the next few days.
**Conclusion:** In acute neurological disorders, ASL can be the only abnormal sequence in an otherwise unremarkable MRI study, allowing to suggest a specific diagnosis as well as to monitor evolution.
**Limitations:** ASL is still not widely available.
**Ethics committee approval:** This study is in accordance with the ethical standards of the Giannina Gaslini Institute and the 1964 Helsinki declaration.
**Funding for this study:** No funding was received for this study.
**Author Disclosures:**
Martina Resaz: Nothing to disclose
Domenico Tortora: Nothing to disclose
Andrea Rossi: Nothing to disclose
Mariasavina Severino: Nothing to disclose
Fiammetta Sertorio: Nothing to disclose
Antonia Ramaglia: Nothing to disclose
Francesco Dellepiane: Nothing to disclose

### PP 19-8

**Feasibility of contrast enhanced voiding urosonography for the assessment of vesico-ureteral reflux in children with UTI**

*S. Tamasi*, R. D'Arcangelo, I. Capaldo, R. Mamone, F. Savoia, C. Pecoraro, G. di Iorio, M. Zeccolini; Naples/IT

**Purpose:** Urinary tract infection (UTI) is a common query in paediatric radiology due to possible associated vesico-ureteral reflux (VUR) and the high risk for developing long-term sequelae. Current guidelines recommend renal ultrasound as a screening test after febrile urinary tract infection, with voiding cystourethrogram (VCUG) only if the ultrasound is abnormal. Contrast enhanced voiding urosonography (ce-VUS) is an emergent technique performed using ultrasound contrast agent (SonoVue Bracco) that allows examination of the urinary tract, without radiation. The aim of this study is to validate ce-VUS in the study of vesico-ureteral reflux in paediatric patients to replace it with the traditional x-ray technique.
**Methods or Background:** The study group consisted of 210 children with history of relapsing UTIs between November 2016 and October 2020: 170 underwent VCUG and 142 contrast-enhanced voiding (ce-VUS) in order to determine the sensitivity, specificity of ce-VUS as well as the number and grading of vesico-ureteral reflux. The imaging protocol consisted of an ultrasound exam of the kidneys and bladder in the supine position before and after the bladder filling with intravescical contrast media and at the end of the urinary phase by performing scan of the ureters.
**Results or Findings:** VCUG revealed the presence of VUR in 148 out of 170 (28%), while ce-VUS allowed the visualisation of VUR in 110 out of 142 children (77%). VURs of low degree (I or II) detected by ce-VUS were in majority (33%, 12% respectively) vs VCUG (20%, 14% respectively).
**Conclusion:** Ce-VUS method has got high sensitivity and specificity in the diagnostics of vesico-ureteral reflux. It can be used in the same patients and in the same pathological conditions as the VCUG, but without the use of ionising radiation.
**Limitations:** Not applicable.
**Ethics committee approval:** Not applicable.
**Funding for this study:** Not applicable.
**Author Disclosures:**
Giovanni di Iorio: Nothing to disclose
Carmine Pecoraro: Nothing to disclose
Iolanda Capaldo: Nothing to disclose
Rosamunda D'Arcangelo: Nothing to disclose
Rosanna Mamone: Nothing to disclose
Sonia Tamasi: Nothing to disclose
Fabio Savoia: Nothing to disclose
Massimo Zeccolini: Nothing to disclose

### PP 19-9

**Diagnostic accuracy of post-mortem imaging compared to conventional autopsy in identifying the cause of death in children: a systematic review**

*S. Z. Almutairi*[1], M. Cohen[2], A. C Offiah[2]; [1]Alkharj/SA, [2]Sheffield/UK

**Purpose:** To assess the diagnostic accuracy of post-mortem imaging compared to conventional autopsy in identifying the cause of death in children.
**Methods or Background:** Systematic reviews were undertaken to identify relevant English-language articles published in the literature up until July 2021. The inclusion and exclusion criteria for the studies were developed in accordance with the Patient/Population, Intervention, Comparison, Outcome (PICO) model. Studies reporting the diagnostic accuracy of various imaging modalities, or those in which sufficient data were present to enable this to be calculated, were included. The methodological quality of the included studies was analysed using the QUADAS-2 tool.
**Results or Findings:** The initial electronic database search identified 1,915 potentially relevant publications. Out of these, 24 studies were available for qualitative synthesis. The overall mean sensitivities and specificities were 72.56 and 84.9 for post-mortem magnetic resonance imaging (PMMR), 58.54

and 94.34 for post-mortem computed tomography (PMCT), and 36.9 and 98.4 for post-mortem radiography (PM radiography), respectively. In fetuses, stillbirths, and neonates, the mean sensitivity and specificity for microfocus-CT (micro-CT) were 91 and 98.5, respectively.
**Conclusion:** PMCT and PMMR can be supplementary to autopsy or used as an alternative option in cases where parents refuse autopsy. Micro-CT provides accurate information on fetuses, stillbirths, and neonates, similar to that provided by autopsy.
**Limitations:** The overall sample sizes for the imaging modalities in the available studies differed from one another; the results would have been more accurate if the sample sizes were similar for each imaging modality.
**Ethics committee approval:** The study was registered with the PROSPERO international database of prospectively registered systematic reviews with a health-related outcome (registration number CRD42021264098).
**Funding for this study:** Funding was received for this study by Prince Sattam Bin Abdulaziz University and the University of Sheffield.
**Author Disclosures:**
Marta Cohen: Author: The authors of this manuscript declare no relationships with any companies whose products or services may be related to the subject matter of the article.
Salman Ziyad Almutairi: Author: The authors of this manuscript declare no relationships with any companies whose products or services may be related to the subject matter of the article.
Amaka C Offiah: Author: The authors of this manuscript declare no relationships with any companies whose products or services may be related to the subject matter of the article.

### PP 19-10
**Evaluation of pectus excavatum deformity through computed tomography in a paediatric population**
*D. A. Santos*, J. I. C. Malhão, M. C. P. Ribeiro, F. I. G. Batalha; Lisbon/PT

**Purpose:** Pectus excavatum (PE) is an abnormality that involves the rib cage more common in children. This study aims to analyse the contribution of CT and MPR images from CT scan comparatively to chest x-ray in the diagnosis of PE, in a paediatric population and the Haller index (HI) as a model to assess this pathology.
**Methods or Background:** 38 thoracic CT(M=32,F=6) and 33 chest X-ray was retrospectively reviewed from the same patients (M=28, F=5) reported with PE, between January 2018 to May 2021. The RadiAnt®DICOM Viewer software was used. The length and width of the thorax and width of the heart were extracted starting from the posterior surface of the sternum to the anterior border of the vertebral bodyn(axial view), and from thoracic vertebras 3-12 selecting sagital and coronal MPR images. The same anatomic references were used in the x-ray measurements.
**Results or Findings:** The correlation between the increase in heart width with the increase of the HI value was $\rho = 0.4$ ($p < 0.05$) indicating a moderate positive correlation, while the value of chest wall diameter and the increase of the HI had a weak negative correlation ($\rho = -0.01$; $p > 0.05$). The HI mean standard error based on CT images was 0.4 and the value based on chest radiographs was 1.4. Sagittal MPR were an asset reliable for the HI's characterisation and confirmation of measurements made in axial views. All the values from the clinical report were validated.
**Conclusion:** There is an advantage of using the CT scan, which provides a higher accuracy of measurements, yielding, in addiction, a greater anatomical detail in the diagnostic of PE deformity aiming correctly inform the surgeon. The HI is a good index to assess the PE.
**Limitations:** A retrospective study nulling the choice of protocol parameters.
**Ethics committee approval:** This study was approved by an ethics committee.
**Funding for this study:** Not applicable.
**Author Disclosures:**
Margarida Carmo Pinto Ribeiro: Nothing to disclose
Joana Isabel Cortiçadas Malhão: Nothing to disclose
Daniela Antunes Santos: Nothing to disclose
Filomena Isabel Gonçalves Batalha: Nothing to disclose

### PP 19-11
**Foetal MRI of cerebellar abnormalities: a pictorial review**
*A. M. Bozzato*[1], F. M. Murru[2], A. Spezzacatene[2], M. Cesarotto[1], A. Di Giusto[1], M. Quadrifoglio[2], T. Stampalija[2], C. Granata[2], M. A. A. Cova[1]; [1]Trieste, TS/IT, [2]Trieste/IT

**Purpose:** Illustrate the fetal MRI appearance of the normal prenatal cerebellum. Review the fetal MRI patterns of the main cerebellar abnormalities.
**Methods or Background:** The cerebellar development is protracted, extending from the fifth week of gestation through the second postnatal year of life. The extended period of development makes the cerebellum vulnerable to a broad range of abnormalities. Prenatal ultrasound (US) is the first prenatal imaging method for the assessment of cerebellar abnormalities, because it is a safe, non-invasive, and low-cost modality. However, fetal MRI, usually performed after the twentieth week of gestation, has emerged as a widely used secondary technique to evaluate suspected US features with a higher accuracy for the detection of posterior fossa abnormalities compared to that of US (87,7% vs 65.4%, respectively).
**Results or Findings:** We describe a state-of-the-art fetal MRI protocol, including multiplanar single-shot T2-weighted sequences, acquired at different echo times, and FFE T1 and T2-weighted sequences. We review the normal prenatal development of the cerebellum from the twentieth week of gestation. We explain the main cerebellar abnormalities amenable to prenatal diagnosis: Dandy-Walker malformation, vermian agenesis, vermian hypoplasia or dysplasia, cerebellar agenesis or hypoplasia, pontocerebellar hypoplasia, cerebellar atrophy, rhombencephalosynapsis, macrocerebellum, unilateral cerebellar damage, mega cisterna magna, posterior fossa arachnoid cyst, Blake pouch cyst, Smith-Lemli-Opitz syndrome, venous drainage anomalies (e.g. aneurysm of the Vein of Galen), and occipital meningocele.
**Conclusion:** Fetal MRI plays a key role in the evaluation of suspected US features of cerebellar abnormalities; therefore, fetal radiologists have to be familiar to the fetal MRI appearance of the normal prenatal cerebellum and the most common MRI findings of these diseases to reach the correct diagnosis.
**Limitations:** N/A
**Ethics committee approval:** N/A
**Funding for this study:** N/A
**Author Disclosures:**
Anita Spezzacatene: Nothing to disclose
Matteo Cesarotto: Nothing to disclose
Tamara Stampalija: Nothing to disclose
Alessandro Marco Bozzato: Nothing to disclose
Anna Di Giusto: Nothing to disclose
Flora Maria Murru: Nothing to disclose
Maria Assunta A. Cova: Nothing to disclose
Claudio Granata: Nothing to disclose
Mariachiara Quadrifoglio: Nothing to disclose

**14:00-15:00** **Room M 2**

### Research Presentation Session: Chest

### RPS 2004

### Nodules, airway and extra pulmonary findings

**Moderator**
A. Poellinger; Bern/CH

### RPS 2004-2
**Risk factors for presence of pulmonary nodules in a Dutch general population: a cross-sectional study**
*J. Cai*, M. Vonder, Y. Du, G. J. Pelgrim, M. Rook, G. Kramer, H. Groen, R. Vliegenthart, G. de Bock; Groningen/NL
*(caicai597@gmail.com)*

**Purpose:** The aim was to determine risk factors for the presence of pulmonary nodules in a general Dutch population.
**Methods or Background:** Participants aged ≥ 45 years from the Dutch Lifelines cohort who had undergone LDCT were included. Information on socio-demographic, medical history, smoking exposures and lifestyle factors were obtained from the Lifelines self-reported data. A participant was considered as having pulmonary nodules if at least one noncalcified nodule with a volume ≥ 30mm3 was detected. Multivariable logistic regression analysis was used to identify independent risk factors for the presence of pulmonary nodules.
**Results or Findings:** In total, 7458 participants were included. 2926 (39.2%) participants had pulmonary nodules and 42.7% of them were never-smokers. Males accounted for 44.0% of the total population and 1433 (49.0%) had nodules. The median age was 58.18 years (IQR 52.18-64.39). After adjusting for socio-demographic, medical history, smoking and lifestyle variables, it was found that former-smoker (pack-year <20: OR = 1.20, 95%CI: 1.08, 1.33), former-smoker (pack-year ≥20: OR = 1.51, 95%CI: 1.23, 1.84), current-smoker (pack-year ≥20: OR = 1.62, 95%CI: 1.34, 1.96), and those having a family history of lung cancer (OR = 1.17, 95%CI: 1.00, 1.37) had a significantly increased risk for the presence of pulmonary nodules in the general population.
**Conclusion:** In this population-based cohort, 40% had pulmonary nodules. Risk factors were former and current smoking status and having a family history of lung cancer.
**Limitations:** The causality between those factors and the presence of pulmonary nodules cannot be established due to the cross-sectional design.
**Ethics committee approval:** The ImaLife study was approved by the medical ethics committee of the University Medical Center Groningen, the Netherlands.
**Funding for this study:** No funding was received for this study.

**Author Disclosures:**
Mieneke Rook: Nothing to disclose
Jiali Cai: Nothing to disclose
Yihui Du: Nothing to disclose
Geertruida de Bock: Nothing to disclose
Rozemarijn Vliegenthart: Nothing to disclose
Harry Groen: Nothing to disclose
Marleen Vonder: Nothing to disclose
Gerdien Kramer: Nothing to disclose
Gert Jan Pelgrim: Nothing to disclose

### RPS 2004-3

**Standardising criteria and follow-up for frequent actionable incidental fundings in lung cancer screening**

M. McInnis[1], M. Ang[1], Y. Leung[1], G. Darling[1], M. Tammemagi[2], D. L. Langer[1], *H. C. Schmidt*[1]; [1]Toronto, ON/CA, [2]St. Catherines, ON/CA

**Purpose:** Identification of actionable incidental findings (AIFs) during lung cancer screening can contribute positively to patient outcomes, but can also lead to additional investigations. To help optimise investigations of AIFs, this study assessed the frequency and type of the most commonly reported AIFs from the first-year pilot of the Ontario Lung Screening Program (OLSP). Multidisciplinary input was then used to standardise criteria to guide follow-up.
**Methods or Background:** OLSP participants received low-dose computed tomography (LDCT) at participating sites. AIF descriptions and recommended management, as identified by site radiologists, were reviewed by a fellowship-trained thoracic radiologist, classified for frequency and anatomic distribution, and correlated with patient characteristics. A multidisciplinary working group defined criteria for 'actionable' versus 'non-actionable' incidental findings for the seven most frequent AIFs, and provided management recommendations.
**Results or Findings:** 1624 LDCTs were performed between June 2017 – May 2018. 534 AIFs were reported in 389 LDCTs. The most frequent AIF was coronary artery calcification (CAC) (n=96, 18.0%), followed by interstitial disease (n=39, 7.3%), emphysema (n=38, 7.1%), liver and kidney lesions (n=29, 5.3% and n=26, 4.9% respectively). AIFs were associated with higher Lung-RADs scores (p<0.0001). Management recommendations were most diverse for CAC and ascending aortic dilatation. The seven most frequent AIFs comprised 44% of all AIFs; retrospective application of working group criteria for 'actionable' findings results in at least 25% fewer of these AIFs.
**Conclusion:** This work provides pragmatic guidance for the most frequently reported AIFs in lung cancer screening. When applied, criteria may substantively decrease follow-up for findings unlikely to have clinical significance, and help standardise patient follow-up.
**Limitations:** We did not follow up the outcome of the AIFs, nor the costs to establish final diagnosis.
**Ethics committee approval:** Not applicable.
**Funding for this study:** Funding was provided through Cancer Care Ontario (Ontario Health).
**Author Disclosures:**
Yvonne Leung: Nothing to disclose
Deanna L. Langer: Nothing to disclose
Michelle Ang: Nothing to disclose
Martin Tammemagi: Nothing to disclose
Micheal McInnis: Nothing to disclose
Gail Darling: Nothing to disclose
Heidi C. Schmidt: Nothing to disclose

### RPS 2004-4

**Opportunistic osteoporosis screening using chest CT with artificial intelligence**

*J. Yang*, F. Yang; Wuhan/CN

**Purpose:** To explore the feasibility of the attenuation values of all thoracic vertebrae and the first lumbar vertebra measured by artificial intelligence on non-enhanced chest CT to do osteoporosis screening.
**Methods or Background:** The patients were divided into three groups: normal group, osteopenia group and osteoporosis group according to the results of DXA. And the attenuation values (HU) of all thoracic and the first vertebrae were measured using artificial intelligence. The correlation between attenuation values and BMD values was analysed, and the diagnostic efficacy of attenuation values on osteopenia or osteoporosis risk was further evaluated.
**Results or Findings:** CT values of each thoracic vertebrae and the first lumbar vertebrae presented high predictive ability and diagnostic efficacy for osteopenia or osteoporosis. After clinical data correction, with every 10HU increase in CT values, the risk of osteopenia or osteoporosis decreased by 32%~44% and 61%~80%, respectively. And the combined diagnostic efficacy of all thoracic vertebrae was higher than that of a single vertebra. The AUC of recognising osteopenia or osteoporosis from normal group was 0.831and 0.972, respectively.
**Conclusion:** The routine chest CT with AI is of great value in opportunistic screening for osteopenia or osteoporosis, which can quickly screen the population at high risk of osteoporosis without increasing radiation dose, thus reducing the incidence of osteoporotic fracture.
**Limitations:** This study was a retrospective study of a single-centre, and the generalisability of the results may be limited to some extent. In the future, prospective studies are necessary to consolidate our results.
**Ethics committee approval:** All procedures for this retrospective study were in accordance with the Declaration of Helsinki but formal consent was not required for this type of study.
**Funding for this study:** The study was supported by the National Natural Science Foundation of China (Grant No.81571373, No.81601217, No.82001491).
**Author Disclosures:**
Fan Yang: Nothing to disclose
Jinrong Yang: Nothing to disclose

### RPS 2004-5

**Effect of nodule location on diagnostic performance of deep learning-based nodule detection system**

*S. You*, J. Sun; Suwon/KR
*(seulgi88322@gmail.com)*

**Purpose:** To investigate the diagnostic performance of commercially available deep learning-based nodule detection (DLD) system according to the nodule location on chest radiographs (CXRs).
**Methods or Background:** We used a dataset of 300 CXRs (100 normal and 200 abnormal images with 216 nodules). We divided the intrathoracic region on CXR into two regions: non-danger zone (NDZ) and danger zone (DZ). DZ included the lung apices and parahilar, paramediastinal, and retrodiaphragmatic areas, where nodules may be missed. The abnormal images included 107 NDZ and 109 DZ nodules. Eight observers (two thoracic radiologists (TRs), two non-thoracic radiologists (NTRs), and four residents) interpreted each radiograph with and without the DLD system. The metrics of lesion localisation fraction (LLF; the number of lesion localisations divided by the total number of lesions) were used to evaluate the diagnostic performance according to nodule location.
**Results or Findings:** The DLD system showed a lower LLF for the detection of DZ nodules (64.2%) than that of NDZ nodules (83.2%, p=0.0016). The TRs showed similar LLF regardless of nodule location (DZ, 81.7%; NDZ, 84.6%; p=0.4170). The NTRs and residents showed poorer performance in detection of DZ nodules than NDZ nodules (NTR : DZ, 56.4%; NDZ, 77.1%; p<0.0001; Residents : DZ, 56.7%; NDZ, 75.5%; p<0.0001). With the DLD system, the LLF of residents significantly improved from 56.7% to 65.6% (p=0.0072) in detecting DZ nodules.
**Conclusion:** The performance of the DLD system was lower in the detection of danger zone nodules. Nonetheless, the deep learning-based nodule detection system can help less experienced observers detect pulmonary nodules in the danger zone.
**Limitations:** Since our study focused on per-nodule detection according to the location of nodule, per-image specificity was not evaluated.
**Ethics committee approval:** Our institutional review board approved this study.
**Funding for this study:** No funding was received for this study.
**Author Disclosures:**
Joosung Sun: Nothing to disclose
Seulgi You: Nothing to disclose

### RPS 2004-6

**The potential reduction in nodule follow up using volumetric analysis**

*A. G. Tolliday*, C. S. Johns, S. Matthews, M. Bull, M. Kamil, O. D. Evans, S. Saha, S. Anwar; Sheffield/UK
*(toli.day@live.com)*

**Purpose:** Introduction: pulmonary nodules are common and result in patient anxiety and sizeable healthcare costs. According to the British Thoracic Society (BTS) guidelines for the investigation and management of pulmonary nodules (2015) using volumetry can reduce follow up imaging as further scans are not required if: nodule volume <80 mm3, nodule volume is stable at 12 months. The aim is to assess potential impact of nodule volumetry vs 2D measurements in a large teaching hospital.
**Methods or Background:** Solid nodules discussed over 6 months at the pulmonary nodule MDT were retrospectively assessed using Enterprise Imaging (AGFA) pulmonary nodule volumetry tool. Volume was measured at baseline, 3, 12 and 24 months. Cases with incomplete imaging/ tool error or prior malignancy were excluded.
**Results or Findings:** 61/84 (73%) cases were excluded. Specifically, tool failure accounted for exclusion of 46/61 patients (55% of total group); 45 on initial scan and 1 on 12 month scan. 39 cases were successfully measured at baseline, 7 (18%) had volume <80 mm3 and could have been discharged. 23 cases were successfully measured at 12 months, 16 (70%) had stable nodule volume and could have been discharged. 7 (30%) cases required further workup, of which the final diagnoses were 5 benign and 2 malignant. One malignant nodule was stable at 12 months but grew at 24 months.

**Conclusion:** Volumetric analysis of pulmonary nodules could greatly reduce follow-up requirements.
The BTS guidelines failed to capture one malignancy.
**Limitations:** Tool failure was a significant limiting factor.
**Ethics committee approval:** Not applicable – retrospective service evaluation.
**Funding for this study:** Not applicable.
**Author Disclosures:**
Matthew Bull: Nothing to disclose
Anna Gabriela Tolliday: Nothing to disclose
Oscar Daniel Evans: Nothing to disclose
Chris S Johns: Nothing to disclose
Sadia Anwar: Nothing to disclose
Mohamed Kamil: Nothing to disclose
Shironjit Saha: Nothing to disclose
Sue Matthews: Nothing to disclose

### RPS 2004-7
**Automated liver attenuation measurement in COVID-19 patients: a link between lung and liver**
A. P. Gonchar, Y. Shumskaya, I. Blokhin, M. Mnatsakanyan, A. Pogromov, R. V. Reshetnikov, S. Morozov, *V. Gombolevskiy*; Moscow/RU
*(g_victor@mail.ru)*

**Purpose:** We aimed to assess the effect of COVID-19-associated pneumonia on liver attenuation with an automated liver attenuation measurement software in chest CT scans.
**Methods or Background:** Retrospective cohort study. Data from outpatients who underwent two chest CT scans (baseline and follow-up) for COVID-19-associated pneumonia from January to July 2020 were analysed. We compared three subgroups per lung damage severity on follow-up scans (0%, <25%, or >25% of the lung volume) with the baseline without pulmonary changes, analysed the correlation between liver attenuation <40HU and increased ALT, AST. Automated liver attenuation measurement with batch processing was performed by an in-house developed software «CTLiverExam».
**Results or Findings:** The software successfully analysed 499 patients. Among all subgroups, there was no statistically significant difference between liver densities in baseline and follow-up CT scans. We found an association between liver attenuation at first CT and the lung involvement on a second (95% CI 1.755-6.429; OR 3.359; p<0.001). The odds for reduced liver attenuation were 2.2 times higher for subgroup #3 vs. subgroup #1 (p=0.026). Association between decreased liver attenuation and elevated ALT, AST levels was significant among all subgroups: 0.243 (p<0.0001) and 0.205 (p<0.0001) for the baseline and 0.192 (p<0.0001) and 0.120 (p=0.0074) for the follow-up.
**Conclusion:** Decreased liver attenuation may favour the development of pulmonary lesions in COVID-19 patients. Additionally, automated liver attenuation measurement with batch processing could be used in chest CT.
**Limitations:** There was a strict threshold for reduced liver attenuation: <40 HU.
**Ethics committee approval:** Approved by an independent ethics committee (Russian Society of Radiology).
**Funding for this study:** This study is a part of research No. USIS: AAAA-A20-120071090058-7, “Scientific Support of the Capital's Healthcare”, 2020–2022.
**Author Disclosures:**
Alexandr Pogromov: Nothing to disclose
Anna P. Gonchar: Nothing to disclose
Marina Mnatsakanyan: Nothing to disclose
Sergey Morozov: Nothing to disclose
Roman V. Reshetnikov: Nothing to disclose
Yuliya Shumskaya: Nothing to disclose
Ivan Blokhin: Nothing to disclose
Victor Gombolevskiy: Nothing to disclose

### RPS 2004-8
**MDCT Vs MRI in bronchiectasis: a non-inferiority trial**
*K. S. Lokesh*, M. Jana, A. S. Bhalla, A. Kumar Gupta, P. M. Naranje, S. K. Kabra, V. Hadda; New Delhi/IN
*(drlokesh.aiims@gmail.com)*

**Purpose:** To compare and evaluate the usefulness of MRI Vs CT in bronchiectasis, to evaluate the reproducibility of CT and MRI scores, to compare MRI and CT scores with pulmonary function tests (PFT), to optimise the MRI protocol in bronchiectasis.
**Methods or Background:** In this prospective study, 25 patients between 7-21 years of age with a clinical/radiological diagnosis of bronchiectasis underwent MDCT and MRI chest. MRI and CT scoring was performed using modified Bhalla-Helbich’s score by two independent radiologists. A final consensus score was recorded both in CT and MRI. The overall image quality of different MRI sequences to identify the pathologies was also assessed. Appropriate statistical tests were used for inter-observer agreements, and correlation amongst CT and MRI; as well as CT, MRI and PFT.
**Results or Findings:** Strong agreement (ICC 0.80-0.95) between CT and MRI was seen for extent and severity of bronchiectasis, number of bullae, sacculation/abscess, emphysema, collapse/ consolidation, mucus plugging, and mosaic perfusion. Overall CT and MRI scores had perfect concordance (ICC 0.978). Statistically significant (p-value <0.01) intraobserver and interobserver agreement for all CT and MRI score parameters was seen. A strong negative correlation was seen between total CT and MRI severity scores and FEV1, FVC, FEF 25-75%. DWI MR, with an ADC cut-off of 1.62 x 10-3 mm3/sec had a sensitivity of 70% and specificity of 75% in detecting true mucus plugs.
**Conclusion:** MRI with DWI can be considered in the diagnostic algorithm for assessment of lung changes in bronchiectasis as a radiation-free non-invasive method of imaging in children, especially in follow-up.
**Limitations:** The limitation is the small sample size, and non-inclusion of children less than 7 years.
**Ethics committee approval:** This study was approved by the Aiims Ethics committee.
**Funding for this study:** Funding was received for this study by Aiims, New Delhi.
**Author Disclosures:**
Priyanka Mahadeorao Naranje: Nothing to disclose
Ashu Seith Bhalla: Nothing to disclose
Vijay Hadda: Nothing to disclose
Sushil K Kabra: Nothing to disclose
Arun Kumar Gupta: Nothing to disclose
Manisha Jana: Nothing to disclose
Kumar Sharma Lokesh: Nothing to disclose

**14:00-15:30** **Room E1**

### Research Presentation Session: Breast

### RPS 2002
### Advances in mammography

**Moderator**
A. A. Marzogi; Makkah/SA

### RPS 2002-2
**Automated detection and BI-RADS classification of breast masses in digital mammographic views, enhanced by subtracting temporally consecutive mammograms**
*G. Skouroumouni*[1], K. Loizidou[2], C. Pitris[2], C. Nikolaou[1]; [1]Limassol/CY, [2]Nicosia/CY
*(galskour@hotmail.com)*

**Purpose:** Improve the detection and BI-RADS classification of breast masses, by exploiting the subtraction of temporally consecutive mammograms and machine learning.
**Methods or Background:** For this retrospective study, 80 pairs of full-field digital mammograms were collected, with either no or BI-RADS benign masses (normal population) and BI-RADS suspicious masses (suspicious population), in the most recent mammograms. Pre-processing, image registration and post-processing were applied to effectively subtract the images. The remaining regions were identified as normal tissue or true masses and, subsequently, the true masses were classified as BI-RADS benign or suspicious. For comparison, state-of-the-art temporal analysis was also performed.
**Results or Findings:** Temporal subtraction improved the contrast ratio ~2 times, compared to the most recent mammographic view. The breast masses were detected with 99.9% accuracy (0.98 AUC) and then classified as BI-RADS benign vs suspicious with 98% accuracy (0.98 AUC), using an artificial neural network. The improvement using temporal subtraction was statistically significant compared to the state-of-the-art temporal analysis (p<0.05).
**Conclusion:** With further improvements, the proposed algorithm has the potential to substantially contribute to the development of automated computer-aided diagnosis systems, with significant impact on patient prognosis.
**Limitations:** The BI-RADS classification of masses not only varies from one radiologist to another but might also be disproved by follow-up or pathology. Also, the adoption of the BI-RADS classification as the ground truth, without any confirmation by follow-up or pathology, limits the generalisability of the tool.
**Ethics committee approval:** This study was approved by the Cyprus National Bioethics Committee.
**Funding for this study:** This study was funded by the European Union's Horizon 2020 research and innovation program under grant agreement No. 739551 (KIOS CoE) and by the Republic of Cyprus through the Directorate General for European Programs, Coordination and Development.

**Author Disclosures:**
Kosmia Loizidou: Nothing to disclose
Costas Pitris: Nothing to disclose
Christos Nikolaou: Nothing to disclose
Galateia Skouroumouni: Nothing to disclose

### RPS 2002-3

**Density and breast cancer risk score in a screening cohort using participant-completed digital questionnaires**

*N. Payne*[1], A. Antoniou[1], T. Carver[1], D. Parmar[2], P. Pharoah[1], F. J. Gilbert[1]; [1]Cambridge/UK, [2]London/UK
*(np520@cam.ac.uk)*

**Purpose:** Dense breast tissue elevates the risk of developing breast cancer and can mask suspicious areas on screening mammograms. Supplemental imaging could result in earlier detection but needs to be targetted to those who will benefit. This interim analysis of the questionnaire and mammographic density data collected in the BRAID (breast screening – risk adapted imaging for density) trial assesses the questionnaire completion-rate and the distribution of multifactorial breast cancer risk.
**Methods or Background:** Women aged 50-70 years with dense breast tissue (BIRADS C/D) attending screening at four UK sites were invited to join the trial. Following online consent, they were asked to complete an online questionnaire covering their cancer family history and questionnaire-based breast cancer risk factors. Where data were incomplete women were contacted and asked for the missing information on attendance for supplemental imaging. The questionnaire-based risk factors and mammographic density were used to obtain the risk of developing breast cancer within a five-year period using the CanRisk web-services.
**Results or Findings:** 1,553 questionnaires were completed by women aged between 50 and 70. The multifactorial 5-year breast risks followed a log-normal distribution. 187 [12.1%] participants were found to be at low risk (<1%), 684 [44.2%] at average risk (1-1.66%), 666 [42.1%] at high risk (1.67-6%), and 9 [0.6%] at very high risk (>6%). Less than 5% of participants contacted the research team with queries when entering data using the self-administered questionnaire.
**Conclusion:** While high breast density alone conveys an elevated risk, multifactorial risk-assessment using CanRisk results in high levels of risk-stratification and risk-reclassification among women with BIRADS C/D. With finite resources for a population screening programme, supplemental imaging could be targeted to those at increased risk of developing the disease.
**Limitations:** Not applicable
**Ethics committee approval:** HRA REC reference 19/LO/0350
**Funding for this study:** CRUK C543/A26884; NIHR Cambridge BRC-1215-20014
**Author Disclosures:**
Fiona J. Gilbert: Nothing to disclose
Dharmishta Parmar: Nothing to disclose
Timothy Carver: Author: The BOADICEA model which has been licensed by Cambridge Enterprise
Antonis Antoniou: Author: The BOADICEA model which has been licensed by Cambridge Enterprise
Nicholas Payne: Nothing to disclose
Paul Pharoah: Nothing to disclose

### RPS 2002-4

**Bridging the gap in automatic breast density estimation for screening personalisation**

*R. Samperna*[1], N. Moriakov[1], N. Karssemeijer[1], J. Teuwen[2], R. M. Mann[1]; [1]Nijmegen/NL, [2]Amsterdam/NL

**Purpose:** Personalised breast cancer screening allows for more accurate and cost-effective women care. Breast density is a well-known risk factor for future development of breast cancer and may be used to stratify women to alternative screening modalities (e.g. breast MRI). Automated software-based density classes are commonly used in mammography, but they correlate poorly with MRI breast density estimations for dense breasts. To obtain a more homogeneous automatic breast density assessment across modalities it is thus important to match density classes between modalities.
**Methods or Background:** 510 patients with a total of 1039 studies were selected from the breast screening database available at our institution based on the availability of paired mammogram and MRI studies (max. +/- 1 month in between). Volpara (version 1.5.4) and a deep learning-based segmentation solution were used to calculate breast densities for mammograms and MRIs respectively. Standard Volpara density grade (VDG) thresholds were applied to mammographic breast densities. From the mammographic VDG classes distribution, (A = 19.5%, B = 23.5%, C= 35.7%, D =21.3%) using a quantile transform, we calculated the optimised matching thresholds for MRI.
**Results or Findings:** Using the adjusted thresholds for MRI, 74.4% (95% CI: [71.6% - 77.1%]) of studies showed an exact match between mammographic and MRI VDG classes (Cohen's weighted kappa: 0.87). For dense cases (VDG D) in mammography, 81.1% (95% CI: [76.1% - 86%]) of corresponding MRI studies showed a perfect VDG class match.
**Conclusion:** Matching of VDG thresholds in MRI facilitates comparing density assessment across modalities. However, approximately 20% of cases are rated as less dense on MRI than on mammography.
**Limitations:** The mammographic VDG classes distribution does not reflect a realistic screening distribution.
**Ethics committee approval:** The Ethics Committee of Radboudumc approved the study.
**Funding for this study:** Funding was received from EFRO Oost-Nederland.
**Author Disclosures:**
Nikita Moriakov: Nothing to disclose
Jonas Teuwen: Nothing to disclose
Ritse Maarten Mann: Consultant: Seno Medical Grant Recipient: Siemens Advisory Board: Siemens Healthcare Speaker: Bayer HealthCare
Riccardo Samperna: Nothing to disclose
Nico Karssemeijer: Shareholder: Volpara Health Technologies Ltd. (Wellington, New Zealand) Owner: Screenpoint Shareholder: QView Medical Inc. (Los Altos, California)

### RPS 2002-5

**Missed cancer detection in mammography with artificial intelligence**

*G. Park*, B. J. Kang; Seoul/KR

**Purpose:** To evaluate whether artificial intelligence (AI) can improve the missed cancer detection in mammography screening.
**Methods or Background:** A total of 204 consecutive women diagnosed with breast cancer between 2018 and 2020 were included. Mammograms at the time of diagnosis and at the time of preceding screening were reviewed by two radiologists and were classified into true negative and false negative (missed cancer and minimal findings) by consensus. Mammograms were also analysed with a deep learning-based AI system. The AI system remarks the site of suspected malignancy with grayscale and assigns an abnormality score. Student's t-test and one-way ANOVA were used to compare the abnormality score between groups.
**Results or Findings:** Of the 204 cases, 137 were classified as true negative and 67 as false negative. Among the false negative cases, 34 were classified as missed cancer and 33 as minimal findings. The AI system accurately detected 27 cases from 34 missed cancers and 18 from 33 minimal findings. The abnormality scores between missed cancer and minimal findings detected by AI on the preceding mammograms showed significant difference (p<0.05; 56 vs 33.8, respectively). Asymmetry was the most common finding among the seven cases which could not be detected by AI in the missed cases (5/7).
**Conclusion:** This retrospective study showed that the AI system correctly identified 13% of missed cancer (27/204) on the preceding screening mammography. The AI system facilitated cancer diagnosis by helping to easily detect missed or minimal findings on screening mammography.
**Limitations:** Only a single AI system was used for analysis. The review process of mammograms was highly informed. Therefore, the number of false negatives might have been exaggerated. Also,
**Ethics committee approval:** This study was approved by the Institutional Review Board.
**Funding for this study:** No funding was received for this study.
**Author Disclosures:**
GaEun Park: Nothing to disclose
Bong Joo Kang: Nothing to disclose

### RPS 2002-7

**Artificial intelligence model for personalising educational mammography test sets for diagnosticians**

Z. Gandomkar, P. Brennan, *M. E. Suleiman*, M. T. Rickard; Camperdown NSW/AU

**Purpose:** Proposing an artificial intelligence (AI) model for personalising educational mammography test sets for diagnosticians.
**Methods or Background:** Data from 260 radiologists, 12 breast physicians, and 207 radiology trainees while interpreting mammographic screening cases were collected. Each reader interpreted one to six test sets, each containing 60 mammographic cases (20 cancer, 40 normal) and provided a rating of 1 (confident normal) to 5 (confident abnormal) to each case. Using Matrix factorisation technique, a model (M1) was built to predict the rating provided by a reader to a case based on the user's previous interactions with other cases as well as other user interaction data. The leave-one-out cross-validation was used for the assessment and an AUC of the model for predicting the difficult-to-interpret cancer cases for an observer was found. A difficult-to-interpret cancer case was defined as an abnormal case, rated as 2 or less. For comparison, a baseline model (M2), which only considered user (average rating from a user to all interpreted cases) and case (average rating provided by all users to a case) bias, was also built.

**Results or Findings:** The mean AUC of M1 and M2 were 0.87±0.21 and 0.73±0.16, respectively. The paired, two-sided Wilcoxon signed-rank test showed the difference between the two models is significant (p<0.001), demonstrating that our AI-based model can better predict the rating given by a reader to an abnormal case compared with the baseline model.
**Conclusion:** Artificial intelligence can be used to predict difficult-to-interpret cases and hence provide educational test sets, customised for a learner.
**Limitations:** The study is a retrospective study. A prospective study will be required in the future to investigate how the model should be updated as the user progresses and interacts with the customised educational materials.
**Ethics committee approval:** This study was approved by the ethics committee of the University of Sydney (2019/013).
**Funding for this study:** Not applicable
**Author Disclosures:**
Patrick Brennan: CEO: CEO and Co-founder Detected-X (A University of Sydney-affiliated start-up)
Mary Theresa Rickard: Founder: Co-Founder and Chief Medical Officer at Detected-X (A University of Sydney-affiliated start-up)
Ziba Gandomkar: Employee: Chief Scientific Officer at Detected-X (A University of Sydney-affiliated start-up)
Mo'ayyad E Suleiman: Founder: Co-Founder and Chief Technology Officer at Detected-X (A University of Sydney-affiliated start-up)

### RPS 2002-8

**The opportunity for AI to evaluate mammography positioning criteria at a population level**

*N. Sharma*, R. Heathcote-Watson, A. Nielsen Moody, M. Fletcher; Leeds/UK
*(Nisha.sharma2@nhs.net)*

**Purpose:** The purpose of this study was to evaluate the feasibility of automated image quality assessments in a UK-based symptomatic mammography clinic, by comparing the visual assessments from a consensus of radiographers with automated AI software results.
**Methods or Background:** This was a retrospective audit of 50 symptomatic mammograms, which included 200 images (100 CC and 100 MLO). Radiographers reviewed image quality criteria based on UK guidelines. The mammograms were processed by densitas® qualityAI™ algorithms to assess positioning criteria including pectoralis length and shape; IMF missing; IR placement; posterior tissues missing; and exaggeration. Manual and automated assessments were compared using Cohen's kappa for positioning errors with a frequency greater than n>5.
**Results or Findings:** The AI algorithms assessed similar positioning criteria to the UK image quality review. Agreement was the highest when assessing CC posterior tissue missing (kappa=0.954, 95%CI: 0.756, 1.00) and the lowest when assessing CC exaggeration (kappa 0.700, 95%CI: 0.502, 0.898). Agreement for MLO assessments ranged from substantial (IMF missing, kappa=0.711, 95%CI: 0.513, 0.909) to almost perfect (pectoralis length, kappa=0.889, 95%CI: 0.691, 1.00).
**Conclusion:** This study demonstrates that an automated AI software tool may be practical for clinical image quality reviews in the NHS. Further research is required to validate the algorithms using a larger sample of data.
**Limitations:** The use of a small data set of randomly selected DICOM studies presented with infrequently occurring positioning errors, which resulted in wide confidence intervals, was identified as a limitation.
**Ethics committee approval:** Ethics committee approval was waived.
**Funding for this study:** No funding was received for this study.
**Author Disclosures:**
Nisha Sharma: Nothing to disclose
Anne Nielsen Moody: Nothing to disclose
Rosie Heathcote-Watson: Nothing to disclose
Maggie Fletcher: Nothing to disclose

### RPS 2002-9

**Simultaneous digital breast tomosynthesis and mechanical imaging of a deformable physical phantom**

*R. Axelsson*, H. Tomic, A. Tingberg, S. Zackrisson, M. Dustler, P. Bakic; Malmö/SE

**Purpose:** We have combined digital breast tomosynthesis (DBT) with mechanical imaging (MI) in simultaneous acquisition, called DBTMI. DBT is known to improve accuracy of cancer detection. MI measures local stiffness on the compressed breast surface. MI can be used to distinguish malignant from benign lesions, thus reducing false positive findings. This is a preliminary study of DBTMI repeatability, using a deformable breast phantom. The phantom contains simulated lesions: solid tumours and cysts. We compared MI values of different findings in phantom images. We investigated the effect of sensor artifacts due to simultaneous acquisition.
**Methods or Background:** We performed five repeated DBTMI acquisitions of phantom images. DBT images were acquired using a clinical system and automatic exposure settings. MI data was acquired using a commercially available pressure sensor, placed on the breast support during DBT imaging. The sensor was calibrated using a vacuum device. The DBT images were preprocessed to reduce sensor artifacts. We generated composite images by overlaying MI data onto reconstructed DBT images, by matching the chest wall and breast outline.
**Results or Findings:** High pressure values (18.0±1.0 kPa for a centrally located solid tumour and 12.3±1.5 kPa for a tumour near the nipple) relative to the background (7.44±0.19 kPa) corresponded to locations of solid tumours in composite images. Simulated cysts did not produce high pressure. Variations in repeated measurements might be caused by the difference in the lesion depth. Residual artifacts have not obstructed visibility of lesions in the phantom.
**Conclusion:** We performed preliminary clinical DBTMI acquisitions of a deformable breast phantom. Composite images were produced by combining DBT images with MI data. High pressure corresponded to solid tumours in the phantom. Preliminary collection of patient DBTMI images is ongoing.
**Limitations:** Not applicable
**Ethics committee approval:** Not applicable
**Funding for this study:** Not applicable
**Author Disclosures:**
Anders Tingberg: Research/Grant Support: Siemens
Hanna Tomic: Nothing to disclose
Predrag Bakic: Nothing to disclose
Magnus Dustler: Nothing to disclose
Rebecca Axelsson: Nothing to disclose
Sophia Zackrisson: Research/Grant Support: Siemens

### RPS 2002-10

**Propagation-based phase-contrast breast imaging: an investigation of the performance of photon-counting and flat-panel x-ray detectors**

*N. Giannotti*[1], S. Tavakoli Taba[1], T. Gureyev[2], S. Lewis[1], L. Brombal[3], S. Donato[3], G. Tromba[3], D. Hausermann[2], P. Brennan[1]; [1]Sydney/AU, [2]Melbourne/AU, [3]Trieste/IT
*(nicola.giannotti@sydney.edu.au)*

**Purpose:** Breast cancer represents the leading cause of death from cancer in women worldwide. Early detection of breast tumours improves the prognosis of patients and survival rate. Propagation-based phase-contrast CT (PB-CT) is an imaging technique that uses refraction and absorption of the x-ray beam to produce images. The aim of this study was to compare the performance of photon-counting and flat-panel x-ray detectors with different pixel sizes in PB-CT breast imaging.
**Methods or Background:** Three mastectomy specimens underwent PB-CT imaging at Elettra Synchrotron (Trieste, Italy) and six at IMBL Synchrotron (Melbourne, Australia) using the PIXIRAD-8 CdTe single-photon-counting and Hamamatsu-C10900D Flat Panel Sensor respectively. Coronal PB-CT images produced at different imaging conditions were compared to absorption-based computed tomography (AB-CT) reference images acquired at similar imaging conditions to investigate the image quality performance of the two image detectors. The image quality of the different data sets was assessed by eleven readers using a visual grading characteristics (VGC) study.
**Results or Findings:** The ICC test showed a moderate/good interobserver agreement for all the mastectomy images analysed (ICC=0.626, p=<0.001). The area under the curve (AUC) showed that, when compared to AB-CT images, PIXIRAD-8 CdTe single-photon-counting detector can generate significantly higher quality PB-CT images at reduced mean glandular dose compared to flat panel PBCT detectors (AUC=0.985, p<0.001).
**Conclusion:** The main photon-counting and flat panel detectors differences are associated with the amount of dark current noise, and spatial resolution. Although the respective significance in image quality was not quantified in this study, our findings demonstrated that photon-counting detectors produce higher quality PB-CT images at pre-defined scanning and radiation dose conditions.
**Limitations:** Not applicable
**Ethics committee approval:** This study was approved by the Human Research Ethics Committee; CF15/3138-2015001340.
**Funding for this study:** NHMRC (Australia): APP1138283; International Synchrotron Access Program; SYRMA-3D project by INFN; National Scientific Committee5 for Technological and Inter-disciplinary Research; Elettra-Sincrotrone Trieste SCpA
**Author Disclosures:**
Daniel Hausermann: Nothing to disclose
Nicola Giannotti: Nothing to disclose
Giuliana Tromba: Nothing to disclose
Patrick Brennan: Nothing to disclose
Seyedamir Tavakoli Taba: Nothing to disclose
Luca Brombal: Nothing to disclose
Timur Gureyev: Nothing to disclose
Sarah Lewis: Nothing to disclose
Sandro Donato: Nothing to disclose

### RPS 2002-11

**Head-to-head comparison of contrast-enhanced mammography and contrast-enhanced MRI of the breast: a meta-analysis**

*N. Pötsch*[1], G. Vatteroni[2], P. Clauser[1], T. H. Helbich[1], P. A. Baltzer[1]; [1]Vienna/AT, [2]Milan/IT

**Purpose:** Head-to-head comparison of contrast enhanced Mammography (CEM) and breast CE-MRI regarding sensitivity, specificity and negative predictive value (NPV).
**Methods or Background:** Studies investigating the comparative diagnostic performance of CEM and CE-MRI published until April 2021 were eligible. Two readers extracted study characteristics and true positives, false positives, true negatives and false negatives of both CEM and CE-MRI. Sensitivity, specificity, NPV and positive and negative likelihood ratios (LR+, LR-) were calculated by bivariate random effects models. A Fagan nomogram was used to identify the maximum pretest probability at which posttest probabilities of a negative CEM or CE-MRI exam were in line with the 2% malignancy rate benchmark for downgrading a BI-RADS 4 to BI-RADS 3 result. I2-statistics, Deek's funnel plot testing and meta-regression were employed.
**Results or Findings:** Eight studies investigating 1161 lesions with an average cancer prevalence of 49% (range: 0.9-82.2%) were included. Sensitivity was higher for CE-MRI (97%, 95%-CI 86-100%) compared to CEM (90%, 95%-CI 75-96%), at a minor expense of specificity (CE-MRI 75%, 95%-CI 53-89%; CEM 79%, 95%-CI 58-91%). The maximum pretest probability at which both tests could rule out breast cancer was 38% for CE-MRI and 13% for CEM.
**Conclusion:** While CEM and CE-MRI show comparable diagnostic performance, CE-MRI is superior regarding sensitivity and LR and can rule out malignancy up to higher pretest probabilities than CEM.
**Limitations:** This systematic review reveals a lack of data regarding patient populations and indications, technical aspects as well as contrast media concentration and dose for CEM.
**Ethics committee approval:** Not applicable
**Funding for this study:** No funding was received for this study.
**Author Disclosures:**
Pascal A.T. Baltzer: Nothing to disclose
Nina Pötsch: Nothing to disclose
Thomas H. Helbich: Nothing to disclose
Giulia Vatteroni: Nothing to disclose
Paola Clauser: Nothing to disclose

### RPS 2002-12

**Breast tomosynthesis reduces radiation exposure in specific patient populations**

*B. Barufaldi*[1], A. Gastounioti[2], W. Mankowski[1], T. Vent[1], D. Kontos[1], E. F. Conant[1], A. Maidment[1]; [1]Philadelphia, PA/US, [2]Saint Louis, MO/US
*(bruno.barufaldi@pennmedicine.upenn.edu)*

**Purpose:** Evaluate radiation dose for a specific patient population enrolled in the breast cancer screening program from the Hospital of the University of Pennsylvania (Philadelphia, PA-USA).
**Methods or Background:** The radiation dose delivered by screening tomosynthesis (DBT) is a current concern. 660 screening exams were collected using a dual-modality imaging protocol (DM+DBT). Exams were acquired on four tomosynthesis systems (Selenia Dimensions, Hologic Inc.). Exposure and patient metadata were extracted from each DM and DBT image. Incident air kerma (IAK) and half value layer measurements were performed using a calibrated dosimeter (Accu Gold AGMS-M+, Radcal). LIBRA and DBT-derived density segmentations provided breast glandularity estimates. Average glandular dose (AGD) was calculated using the IAK scaled to the breast surface and correction factors for x-ray spectra, beam quality, thickness and glandularity following the IAEA guidelines.
**Results or Findings:** DBT shows a significant decrease in mean patient AGD for women compared to DM for [40,49] years (DM=11.78±5.74, DBT=8.69±2.04 mGy, P<0.001) and [50,64] years (DM=10.54±4.47, DBT=8.82±1.96 mGy, P<0.001). Differences in mean AGD per image are statistically significant for women [40,49] years (DM=2.45±0.96, DBT=2.17±0.53 mGy, P<0.001) but not for women [50,64] years (DM=2.26±0.88, DBT=2.20±0.52 mGy, P=0.16). Overall, mean AGD for DBT (8.61±1.97 mGy) is significantly lower than DM (10.34±4.69 mGy, P<0.001).
**Conclusion:** Screening DBT does not increase radiation dose axiomatically and dose measurements should consider patient demographics. Breast glandularity affects AGD calculations significantly, resulting in estimates potentially inconsistent with DICOM headers.
**Limitations:** Images were collected retrospectively, whereas exposure measurements were collected after the fact. The reference used to calculate AGD (Dance) considers semi-cylindrical breasts with glandularity homogeneously distributed in a central core. Non-uniform glandular distribution may affect AGD estimates.
**Ethics committee approval:** Anonymised image use approved after institutional review (protocol#825735, HIPPA–compliant with waiver of consent).
**Funding for this study:** AAPM-ResearchSeedGrant2020, NIH-U54-CA196528163313-04,CA2R01CA161749-05,R01CA177150, Komen-PDF17479714
**Author Disclosures:**
Andrew Maidment: Advisory Board: Real Time Tomography (RTT), LLC Owner: Daimroc Imaging, LLC. Equipment Support Recipient: Barco NV, Hologic Inc., and Analogic Inc.
Walter Mankowski: Nothing to disclose
Trevor Vent: Nothing to disclose
Aimilia Gastounioti: Nothing to disclose
Bruno Barufaldi: Nothing to disclose
Despina Kontos: Research/Grant Support: Hologic Inc.
Emily F. Conant: Advisory Board: Member of the iCAD advisory panel Research/Grant Support: Hologic Inc., OM1, and iCAD Speaker: AuntMinnie.com

**14:00-15:30** **Room M 1**

### Research Presentation Session: Radiographers

### RPS 2014

### Risk reduction and quality assurance in radiographic practice

**Moderators**
L. J. O. C. Lança; Lisbon/PT
A. Trianni; Trento/IT

### RPS 2014-3

**X-ray scatter correction software for improving image quality and reducing radiation dose in large anatomical regions**

*M. F. Sayed*[1], K. Knapp[2], J. Fulford[2], C. Heales[2], S. J. M. Alqahtani[1], S. Rimes[3], D. Moffatt[3]; [1]Najran/SA, [2]Exeter/UK, [3]Taunton/UK

**Purpose:** Globally, X-ray imaging is routinely used for diagnostic purposes. Scattered radiation from the patient is a major image degradation factor, as scattered X-rays appear as mislocated events. The conventional method of reducing X-ray scatter is an anti-scatter grid device but this increases the dose and requires precise positioning and alignment. Recently, new 'virtual grid' image processing software (VG) has been developed to correct scattered X-rays and overcome some of the technical issues associated with grid devices. This study aims to assess the image quality and the radiation dose of VG corrected images compared to conventional grid devices.
**Methods or Background:** A general FDR (Fujifilm) X-ray unit was used to perform anatomical phantom (KYOTO) scanning with different thicknesses of fat layers present. A standard abdomen (AP) examination was applied with images acquired sequentially with/without the physical grid (PG) and exposure factors kept constant (kVp and SID) with AEC were used. VG software was applied to non-grid images. Mean intensity values and standard deviations of selected ROIs were measured, with paired samples T-Tests undertaken to compare differences.
**Results or Findings:** The contrast-to-noise ratio (CNR) of grid-less images was increased from (3±1.56) to (5.1±1.63) after applying VG software. The CNR of images acquired with the PG was found to be higher (9.61±0.79) than VG (5.1±1.63). In terms of signal-to-noise ratio (SNR), grid-less images (42.06±4.87), VG images (32.39±5.5), and PG images (53.01±1.52). A significant difference ($p < 0.001$) was found for mean DAP between VG images (9.76±0.40 µGy.m²) and PG images (43.40±0.95 µGy.m²). The mean effective dose of VG was (0.013±0.5 mSv) and PG (0.085±0.5mSv).
**Conclusion:** VG software improves the image quality of grid-less images, with a lower radiation dose compared to PG for Phantom based imaging of the abdomen. However, PG seemed to have superior image quality to VG software.
**Limitations:** Not applicable.
**Ethics committee approval:** Not applicable.
**Funding for this study:** Funding was received for this study by Exeter University and Najran University.
**Author Disclosures:**
Karen Knapp: Author: Academic Supervisor
2nd Author Jonthan Fulford: Author: Academic Supervisor
3rd co-author Christine Heales: Author: Academic Supervisor
Saeed Jaber M Alqahtani: Author: Academic Supervisor
Drew Moffatt: Author: Academic Supervisor
Mohammad Faya Sayed: Author: student
Susan Rimes: Author: Academic Supervisor

### RPS 2014-4

**What determines a good skeletal X-ray image? Expert annotation of lateral ankle-joint positioning in 2D skeletal X-ray imaging using a standardised tool**

*O. Hertgers*[1], K. Hergaarden[1], S. Challiui[1], A. Bubberman[1], M. Brueck[2], T. Harder[2], J. von Berg[2], M. Sevenster[3], H. J. Lamb[1]; [1]Leiden/NL, [2]Hamburg/DE, [3]Eindhoven/NL
*(ohertgers@gmail.com)*

**Purpose:** To understand what determines the decision for a retake, annotating ankle-joint positioning on X-ray imaging is necessary. Additionally, this is a critical requirement for future implementations of AI-assisted pose estimation for automated quality assessment.
**Methods or Background:** We randomly sampled 500 lateral ankle-joint X-rays from the PACS system of our academic hospital. A web-based annotation tool was developed, and the images were assessed and labeled by two expert image-reading radiographers. Pediatric cases and images with obscured joint spaces were excluded. Six quality labels defined: 1) diagnostic parameters 'joint space visibility' and 'talus condyle overlap'; 2) technical positioning parameters 'flexion', 'collimation', 'medial-lateral rotation' and 'cranial-caudal rotation'. For each aspect, three values were assigned: good, poor or bad. Overall image quality was labeled 'good' or 'bad'. The overall image quality was compared with the quality labels to determine which individual labels and combinations thereof were most predictive.
**Results or Findings:** Image quality was assessed in 460 images, 40 cases meet the exclusion criteria (pediatric and joint space obscured). Regarding 'overall image quality', both readers agreed on rating 'bad' in 41% and 'good' in 42% of cases (Cohens kappa 0.66). In 87% of the cases, the overall image quality was determined by the combination of 'joint space visibility' or 'condyle overlap'. 'Joint space visibility' and 'condyle overlap' alone can explain 85% and 80% of the images respectively. 'Medial-lateral rotation' or 'cranial-caudal rotation' explain in 77% and 65% of the cases the overall image quality.
**Conclusion:** The aspects of joint-space visibility, condyle overlap, and to a lesser extent medial-lateral/cranial-caudal rotations are predictive for correct lateral ankle X-ray positioning, and thus for preventing retakes.
**Limitations:** The limitations are a single-site validation and one view position evaluated.
**Ethics committee approval:** A waiver of consent was obtained from the local ethics committee.
**Funding for this study:** No funding was received for this study.
**Author Disclosures:**
Omar Hertgers: Research/Grant Support: Koninklijke Philips N.V.
Merlijn Sevenster: Employee: Koninklijke Philips N.V.
K.F.M. Hergaarden: Research/Grant Support: Koninklijke Philips N.V.
Adriaan Bubberman: Research/Grant Support: Koninklijke Philips N.V.
Tim Harder: Employee: Koninklijke Philips N.V.
Jens von Berg: Employee: Koninklijke Philips N.V.
Matthias Brueck: Employee: Koninklijke Philips N.V.
Hildo J. Lamb: Research/Grant Support: Koninklijke Philips N.V.
Samir Challiui: Research/Grant Support: Koninklijke Philips N.V.

### RPS 2014-5

**Trabecular bone score (TBS) role in fracture risk assessment**

*L. Rodrigues*, K. Knapp, J. Meakin, C. Heales, A. G. ALQahtani, M. Gill, Z. Haji Abdullah, C. Vilela-Mansell, C. Ding; Exeter/UK
*(leerodri@gmail.com)*

**Purpose:** The clinical interest for software to predict fracture risk, like trabecular bone score (TBS), has recently increased. The additional value of TBS, when compared to traditional bone mineral density (BMD), is that it measures the bone texture and correlates it with the bone microarchitecture. This study explored the role of TBS in fracture risk assessment (FRA) compared with BMD.
**Methods or Background:** A total of 48 female participants were randomly selected from a study population. All participants had a DXA scan using a GE Lunar Prodigy (GE Healthcare, Chicago, IL), and a TBS calculated for the lumbar spine (Medimaps Group SA, Switzerland). Participants were split into three groups: control (mean age is 40.8), osteoporosis without vertebral fractures (mean age is 58.1), and osteoporosis with vertebral fractures (mean age 71.8). Pearson correlations were performed to examine the relationship between BMD-adjusted FRAX and TBS-adjusted FRAX, and logistic regression to access their fracture discrimination using SPSS (IBM, NY).
**Results or Findings:** A strong correlation between BMD-adjusted FRAX and TBS-adjusted FRAX was found for all three groups, R2 ranged from 0.895-0.947 with an R2 of 0.9429 for the whole sample. The BMD adjusted FRAX model had an odds ratio (OR) of odds were 1.189 p<.001. The TBS adjusted FRAX model had an OR of odds were 1.214 p< .001.
**Conclusion:** Our results demonstrated that there is a strong correlation between TBS-adjusted FRAX and BMD-adjusted FRAX.
The TBS-adjusted FRAX performed slightly better than the BMD-adjusted FRAX for fracture discrimination in our vertebral fracture group. Further work is required to explore the benefit of BMD and TBS-adjusted FRAX to improve the FRA in the future.
**Limitations:** Participants were all white females, results might not be generalisable.
**Ethics committee approval:** This study was approved by NRES (18/SW/0217).
**Funding for this study:** Not applicable.
**Author Disclosures:**
Karen Knapp: Nothing to disclose
Judith Meakin: Nothing to disclose
Zulfadhli Haji Abdullah: Nothing to disclose
Clement Ding: Nothing to disclose
Abdullah Ghanem ALQahtani: Nothing to disclose
Christine Heales: Nothing to disclose
Molly Gill: Nothing to disclose
Carl Vilela-Mansell: Nothing to disclose
Liliana Rodrigues: Nothing to disclose

### RPS 2014-6

**Technical ultrasonic quality assurance in Danish radiological departments**

*K. T. T. Rasmussen*[1], S. Grønnegaard Hansen[1], K. Brage[1], L. K. Sondergaard[2]; [1]Odense/DK, [2]Kolding/DK

**Purpose:** Lack of technical quality assurance in ultrasound poses a threat to patients and may lead to misdiagnosis and inappropriate treatment.
The purpose of this study was first to investigate the application of quality assurance in ultrasound in Danish radiological departments, and second to test the quality of a variety of transducers using the in-air method.
**Methods or Background:** An anonymous short questionnaire, regarding the extent of ultrasound quality assurance used in the specific department, was sent out to leaders of all Radiological Departments in Denmark. The leaders were asked to forward the questionnaire to employees responsible for ultrasound quality. Furthermore, two blinded senior year radiography students tested the transducer quality of 63 transducers (in clinical use) from different departments using the in-air method. Transducer faults were registered only when there was agreement between the two observers.
**Results or Findings:** The questionnaire was answered by 48 respondents, of which only 38% answered that their department uses a quality assurance procedure. Furthermore, 50% answered that they did not experience the lack of quality assurance as a problem but 77% had experienced transducer faults in their department. The in-air method demonstrated transducer faults in 35 out of the 63 (56%) transducers including dropout, delamination and/or lens wear.
**Conclusion:** The result of the questionnaire demonstrates a lack of technical quality assurance of ultrasound transducers in Danish radiological departments, which is further demonstrated by a high rate of faulty transducers. The in-air method, where the reverberation pattern is assessed, is an easy and inexpensive method to test the transducer for obvious faults and may be included in a quality assurance protocol.
**Limitations:** It is not possible to estimate the samplesize as the questionnaire was anonymous.
**Ethics committee approval:** Not applicable.
**Funding for this study:** No funding was received for this work.
**Author Disclosures:**
Sisse Grønnegaard Hansen: Nothing to disclose
Karen Brage: Nothing to disclose
Lis Koudal Sondergaard: Nothing to disclose
Kristina Thybo Tellquist Rasmussen: Nothing to disclose

### RPS 2014-7

**Image quality evaluation of on-centre and off-centre FOV in cardiac CT examination (spatial resolution and motion artifacts)**

*K. Tsujioka*[1], K. Yamada[2], M. Niwa[2]; [1]Toyota/JP, [2]Yokkaichi/JP
*(tsujioka@fujita-hu.ac.jp)*

**Purpose:** The heart is offset from the centre of the human body. Therefore, there is a concern that the CT image will change compared to the centre of the FOV. We conducted experiments on spatial resolution and motion artifacts in cardiac CT examinations.
**Methods or Background:** A wire phantom was used to evaluate the spatial resolution of on-centre and off-centre of the FOV. The position of moving object and X-ray tube become an important problem in the evaluation of the motion artifact in the CT. We developed a new moving phantom to solve this problem. And we compared the motion artifact in the centre and the off-centre of the FOV by using this phantom.
**Results or Findings:** Comparing the on-centre and off-centre of the FOV, the on-centre had better spatial resolution than the off-centre. The shapes of the motion artifact were changed by the position of the X-ray tube. However, according to new moving phantom, the motion artifacts of the on-centre and off-centre were possible to evaluate in same X-ray tube position. The artifact of the off-centre became bigger than on-centre of the FOV.

**Conclusion:** We evaluated the spatial resolution and motion artifacts of the off-centre of FOV. It was found that the spatial resolution is reduced and motion artifacts occur in the off-centre of FOV. From the results of this experiment, it was found that it is important to place the subject in the on-centre of FOV when performing the cardiac CT examinations.
**Limitations:** This report is based on the phantom experiment. We are planning to conduct research using human body.
**Ethics committee approval:** Our experiments have been approved by the ethics committee.
**Funding for this study:** We are not financially funded by any organisation for this report.
**Author Disclosures:**
Katsumi Tsujioka: Nothing to disclose
Masayoshi Niwa: Nothing to disclose
Kyohei Yamada: Nothing to disclose

### RPS 2014-8

**Dual-energy CT: reduction of metal artifacts in the skeletal-muscle system**

*M. Monteiro*, S. Pais; Coimbra/PT

**Purpose:** Understand whether the use of dual-energy CT is effective in reducing metallic artefacts in CT images. It is also intended to understand which level of monoenergetic reconstruction is most effective, as well as the added value of using iterative algorithms to improve the image.
**Methods or Background:** Materials and methods: Images with energies of 80 and 140Kv were acquired and the post-processing included reconstructions with 150 and 190keV. In addition to monoenergetic reconstructions, the iMAR algorithm was applied. Qualitative assessment: experienced CT radiographers performed a blind assessment about the presence of artefacts and image quality on a 5-level scale. Quantitative evaluation: in the different sets of images, several regions of interest were defined which, in average values of Hounsfield units, gave us the beam attenuation and the image noise.
**Results or Findings:** The 150keV monoenergetic images with application of the iMAR algorithm proved to be the images with the best diagnostic quality and the lowest presence of metal artifacts in both qualitative and quantitative assessment.
**Conclusion:** The reduction of artefacts and image quality were evident in the 150keV monoenergetic images and with the use of iMAR compared to images without the use of the algorithm.
**Limitations:** We did not evaluate the constitution of the different constituent materials of the metallic artefacts.
**Ethics committee approval:** Study approved by the ethics committee of the Polytechnic of Coimbra.
**Funding for this study:** This study was without financing.
**Author Disclosures:**
Mário Monteiro: Nothing to disclose
Sara Pais: Nothing to disclose

### RPS 2014-9

**Characterisation of national radiotherapy departments: organisation, occupational exposure values and local diagnostic reference levels for breast and prostate CT-planning**

*R. Guisantes*, J. Santos, A. Ferrreira; Coimbra/PT
(rafaela_guis@hotmail.com)

**Purpose:** This study aims to characterise the organisation of radiotherapy (RT) departments, analyse occupational exposure values and establish local diagnostic reference levels (DRL's) for breast and prostate CT-planning.
**Methods or Background:** Authorisation and ethical approval were obtained. A national survey was disseminated across the RT national departments. The survey presented 3 sections: equipment, staff and radiographer's roles characterisation; occupational exposure values during one month; and exposure parameters and CT does values (CTDIvol and DLP) for breast and prostate cancer CT planning. Local DRL's were based on the 75th percentile dose values.
**Results or Findings:** A response rate of 25% of the national centres (50% of the state-run centres) was obtained. All departments promote three-dimensional conformal radiation therapy (3D-CRT) and include at least intensity modulated radiation therapy (IMRT) and/or volumetric modulated arc therapy (VMAT) irradiation techniques. Half of the participating centres presented brachytherapy. Occupational exposure was collected from 54 radiographers' dosimeters, with an occupational dose value of 0 mSv. The CT-planning dose values were 13mGy and 512mGy.cm for breast and 16mGy and 689mGy.cm for prostate.
**Conclusion:** The majority of the RT national characterisation aspects were in line with the literature. The occupational values did not vary across the RT modalities. Local DRLs were established for breast and prostate CT-planning and the obtained values are similar to the recent European studies.
**Limitations:** The response rate was above desired, particularly from the private institutions.
**Ethics committee approval:** This study was approved by all the institutions' ethical committees.
**Funding for this study:** This study was performed without funding.
**Author Disclosures:**
Joana Santos: Nothing to disclose
Rafaela Guisantes: Nothing to disclose
António Ferrreira: Nothing to disclose

### RPS 2014-10

**Development of a patient face recognition system in the radiology department (demonstration study of a face mask-enabled face recognition system in a CT examination environment)**

*H. Ota*[1], T. Shibuya[1], A. Takagi[2], T. Taniguchi[2], D. Hayashi[2], T. Kubota[2], Y. Nagai[1], Y. Muramatsu[1], T. Kobayashi[1]; [1]Kashiwa/JP, [2]Kawasaki/JP
(hiroyuki.oota0220@gmail.com)

**Purpose:** Patient misidentification causes serious accidents in medical practice. Authentication systems based on facial images are used in many fields. The purpose of this study is to evaluate a newly developed face mask-enabled face recognition system in a CT room environment.
**Methods or Background:** The face recognition system is PFAS version 2.0 (Canon Medtec Supply, Kawasaki, Japan). Thirty-two volunteers participated in the demonstration. Assuming a CT examination, the patients performed the authentication task while wearing the face mask during CT room entry (walking and in a wheelchair) and positioning (supine position), and the success rate was calculated from the authentication results. Significant difference tests were conducted using the Kruskal-Wallis test on the authentication scores obtained in the three situations. In this system, an authentication score of 500 or higher is considered a successful authentication.
**Results or Findings:** Authentication scores (mean +/- standard deviation) for entering the room (walking, wheelchair) and positioning were mean 592 +/- 59, 596 +/- 60, and 567 +/- 51. No significant differences ($p>0.05$) were found between the authentication scores in each situation. Authentication success rates were similarly 100%, 97%, and 59%. The reason for the low authentication success rate during positioning was due to the angle between the camera and the examinee's face.
**Conclusion:** Volunteers demonstrated the newly developed face mask-enabled face recognition system for practical use in the radiology department. The system can be used in CT examinations by optimising the positioning of the camera for face recognition during positioning.
**Limitations:** Although an actual CT exam room was used, this was only a demonstration experiment in a single room environment.
**Ethics committee approval:** Approved by the institution's ethics review committee. Written consent obtained from the volunteer.
**Funding for this study:** There is no funding for this study.
**Author Disclosures:**
Yoshihisa Muramatsu: Nothing to disclose
Yuichi Nagai: Nothing to disclose
Hiroyuki Ota: Nothing to disclose
Tatsushi Kobayashi: Nothing to disclose
Daigo Hayashi: Research/Grant Support: Instrument Configuration
Akira Takagi: Research/Grant Support: Instrument Configuration
Canon Medtec Supply Co. Tomoyuki Kubota: Research/Grant Support: Instrument Configuration
Tomonari Taniguchi: Research/Grant Support: Instrument Configuration
Toshiyuki Shibuya: Nothing to disclose

### RPS 2014-11

**Explaining radiation risk: an investigation of approaches preferred by Irish patients**

K. Matthews, *E. B. Comiskey*; Dublin/IE
(evan.comiskey@ucdconnect.ie)

**Purpose:** Benefit-risk communication is a legal responsibility of radiographers. Several publications offer advice on how to frame benefit-risk explanations, and whether explanations are consistently offered by radiographers. Fewer papers analyse the content of risk explanations or report patient opinions on the analogies used. The current research investigates Irish patient preferences regarding detailed explanation of radiation risk.
**Methods or Background:** In an online survey, participants with a history of X-ray examination offered their opinions on risk explanations for pelvis X-rays and pelvis CT scans. Five explanation types were investigated, using comparisons with risks of background radiation, number of chest X-rays, developing cancer, sea swimming and monetary cost. Responses were summarised as percentage weighted scores. Response distributions were compared using Friedman's two-way analysis of variance to determine any significant differences in opinion regarding explanations.

**Results or Findings:** For each explanation, the 106 participants reported their opinions on ease of understanding, reassurance, alarm and level of risk. Significant differences in each of understanding, reassurance and alarm were recorded between different explanations. For all explanations, the perceived level of risk was greater for CT than for X-ray examinations.
**Conclusion:** Patients reported background radiation as the most understandable and reassuring explanation. Monetary comparison was the least alarming analogy for risk in XR but conveyed increased alarm in CT, and resulted in the greatest appreciation of risk difference between XR and CT. Mentioning a risk of cancer in explanations did not significantly increase participant alarm or reduce reassurance.
**Limitations:** Participants were recruited through patient organisations and thus distant from their imaging examination. Results from actual patients may record greater alarm with risk explanation owing to the hospital setting.
**Ethics committee approval:** The research received approval to proceed from the ethics committee of University College Dublin.
**Funding for this study:** No funding was received for this study.
**Author Disclosures:**
Evan Barry Comiskey: Nothing to disclose
Kate Matthews: Nothing to disclose

### RPS 2014-12

**NiFi: an open source application for secure and efficent handling of image data for radiographic studies**
*M. W. Kusk*; Esbjerg/DK
*(martin.weber.kusk@rsyd.dk)*

**Purpose:** In radiographic research or quality assurance studies, images are often analysed or processed outside PACS. Efficient handling, while adhering to GDPR and other data security concerns is challenging, especially in large AI data sets. Manual export from workstations, storage on USB-, or network drives are potential sources of data leaks, making mandatory logging/tracking difficult. We present implementation in a radiology department, of the opensource Apache NIFI server as solution to secure routing, processing and tracking image data.
**Methods or Background:** Following server installation, dataflows were constructed in a graphical user interface. A DICOM listener was configured to receive images, and workstations/modalities were configured to push images. Processes were constructed to handle the following: automatic anonymisation/pseudonymisation, automatic sorting and routing of images to secure drives, based on DICOM meta-information, automatic rule-based renaming of images, extraction of DICOM tags to text files, and automatically triggering postprocessing tasks.
**Results or Findings:** A 5000-image CT dataset could be pushed from a workstation followed by anonymisation and rerouting back in less than two minutes. Provided modality protocols were correctly named, pushed studies were automatically stored in project-specific folders and individual series were organised into separate folders on a secure drive, based on acquisition parameters stored in DICOM tags. It was possible to track the provenance of every single file, and no images ever left the hospital network. Once project-specific dataflows were set up and tested, no user intervention was required. The system was approved by local IT security. Learning curve for users was fast
**Conclusion:** Apache NiFi is an efficient secure data infrastructure, suited to research and quality assurance in radiographic research with large datasets, without massive expenditure.
**Limitations:** No comparison was made with commercial (paid) software solutions.
**Ethics committee approval:** Not applicable.
**Funding for this study:** No funding was received for this study.
**Author Disclosures:**
Martin Weber Kusk: Nothing to disclose

**14:00-15:30** **Room M 3**

### Research Presentation Session: Abdominal Viscera

### RPS 2001

### New techniques in ultrasound

**Moderator**
D. A. Clevert; Munich/DE
**Author Disclosure:**
D. A. Clevert: Speaker: Philips Samsung Siemens Esaote Bracco

### RPS 2001-3

**Performance of attenuation imaging and two-dimensional shear-wave elastography new technologies in the noninvasive assessment of liver disease**
*G. Ruiz-Fernández*, M. Abadía, M. Romero, E. Marín, A. García-Sánchez, J. Poza, C. Amiama, I. González-Díaz, A. Olveira; Madrid/ES
*(gloria.ruizf@gmail.com)*

**Purpose:** There's an increasing interest in accessible, reliable tools in the non-invasive assessment of liver disease. New, promising technology is under development and validation is needed.
**Methods or Background:** Consecutive adult patients programmed for percutaneous liver biopsy were prospectively included. The same day of biopsy, transient elastography and controlled-attenuation parameter (TE and CAP; Fibroscan 502 Touch, EchoSens, France), and two-dimensional shear-wave and attenuation imaging (2D-SWE and ATI; Aplio i800, Canon, Japan) were previously performed. The sonographer was blind to the Fibroscan results. Exclusion criteria were acute liver disease, focal liver lesion, and biopsy <1.5 cm in length. 16-18G Tru-cut needle was used under ultrasound guidance. Histological findings were staged according to endorsed classifications (mainly METAVIR, NASH CRN). Normally distributed quantitative variables were expressed as mean value; otherwise as median. Diagnostic performances for steatosis (fatty content >5%) and advanced fibrosis (F3-F4) were assessed with receiver operating characteristic curves (AUROC).
**Results or Findings:** 108 patients were included: 50.8 yo, female 64%, biopsy lenght 2.5 cm. Diagnoses were: metabolic-associated fatty liver 46%, autoimmune hepatitis 13%, biliary cholangitis 7%, other 34%. For steatosis, the performances were: unaided conventional sonographic diagnosis 0.85, CAP 0.85 (CI95% 0.74–0.91; Best cut-off 235 dB/m, Sens 87%, Esp 65%, PPV 81%, NPV 75%), ATI 0.93 (CI95% 0.86–0.97; Best cut-off 0.62 dB/cm/MHz, Sens 85%, Esp 75%, PPV 84%, NPV 77%). For advanced fibrosis, the performances were: TE 0.91 (CI95% 0.84–0.97; Best cut-off 10.9 kPa, Sens 65%, Esp 94%, PPV 75%, NPV 90%), 2D-SWE 0.94 (CI95% 0.86–1; Best cut-off 8.1 kPa, Sens 70%, Esp 98%, PPV 89%, NPV 92%).
**Conclusion:** Two-dimensional shear-wave and attenuation imaging technologies (Canon Aplio i800 device) are very reliable tools in the non-invasive assessment of liver disease.
**Limitations:** The limitations are heterogeneous liver diseases.
**Ethics committee approval:** This study was approved by an ethics committee. All patients signed ICF.
**Funding for this study:** No funding was received for this study.
**Author Disclosures:**
Antonio Olveira: Research/Grant Support: Canon Speaker: Canon
Irene González-Díaz: Nothing to disclose
Marta Abadía: Speaker: Canon
Clara Amiama: Nothing to disclose
Eva Marín: Nothing to disclose
Miriam Romero: Speaker: EchoSens Investigator: EchoSens
Gloria Ruiz-Fernández: Speaker: Canon
Joaquín Poza: Speaker: Canon
Araceli García-Sánchez: Nothing to disclose

### RPS 2001-5

**Contrast-enhanced ultrasound (CEUS) and contrast-enhanced computed tomography (CECT) for differentiating between mass-forming pancreatitis and pancreatic ductal adenocarcinoma: a meta-analysis**
*J. Yang*, J. Huang, Y. Zhang, Q. Lu; Chengdu/CN
*(1529350018@qq.com)*

**Purpose:** To compare the diagnostic performance of contrast-enhanced ultrasound (CEUS) and contrast-enhanced computed tomography (CECT) for differentiating MFP from PDAC and to compare the two modalities.

**Methods or Background:** A literature search was performed in the PubMed, EMBASE (Ovid), The Cochrane Library (CENTRAL), CNKI, VIP and WanFang databases to identify original studies published from inception to August 20, 2021. Studies reporting the diagnostic performances of CEUS and CECT for differentiating MFP from PDAC were included. Bayesian network meta-analysis was conducted to indirectly compare the overall diagnostic performance.
**Results or Findings:** Twenty-six studies with 1602 pancreatic masses were included. The pooled sensitivity and specificity of CEUS for MFP were 82% and 95%, respectively; and the area under the SROC curve (AUC) was 0.90 (95% CI, 0.87-92). However, the overall sensitivity and specificity of CECT were 81% and 94%; and, the SROC AUC was 0.92 (95% CI, 0.90-0.94). CEUS showed a comparable overall diagnostic accuracy in the differential diagnosis of MFP and PDAC than CECT (relative DOR 1.26, 95% CI [0.42-3.83], P>0.05).
**Conclusion:** CEUS and CECT have comparable diagnostic performance for differentiating MFP from PDAC.
**Limitations:** Most of the original studies were originally designed for examining the performance of only one of the two modalities, which could not avoid the effect of the characteristics of different patients. Although we did a Bayesian network meta-analysis to indirectly compare the overall diagnostic performance of the two modalities, further original studies should be designed to directly compare the two diagnostic tests.
**Ethics committee approval:** Institution review board approved this single-centre retrospective study and the requirement to obtain written informed consent was waived.
**Funding for this study:** Funding was received for this study from the Science and Technology Department of Sichuan Province(2018FZ0044), and the National Natural Science Foundation of China (81571697)
**Author Disclosures:**
Yonggang Zhang: Nothing to disclose
Qiang Lu: Nothing to disclose
Jiayan Huang: Nothing to disclose
Jie Yang: Nothing to disclose

### RPS 2001-6

**Integrating simulation-based learning into ultrasound training: the perspectives of radiology trainees and lessons for educators**
*J. D. Yeomans*, V. E. Whitchurch, H. Samir, D. P. A. Wardle; Bridgend/UK

**Purpose:** In recent years, simulation-based transabdominal ultrasound training has been widely adopted due to its ability to create safe and standardised training experiences and reduce patient exposure to novice radiology trainees. It has been shown to improve performance in quantitative studies, but there has been minimal qualitative investigation into perceptions of its worth. This study addresses this by investigating the perspectives of radiology trainees regarding the value of simulation-based learning in a transabdominal ultrasound training programme.
**Methods or Background:** 11 semi-structured interviews using questions modelled on the critical incident technique and the New World Kirkpatrick Model were conducted with trainees from two year groups who had experienced simulation-based learning. The interviews were thematically analysed.
**Results or Findings:** 92 codes were generated and sorted into themes. There were 3 lower-order themes: direct perceptions of content, assessment and feedback; 3 mid-order themes: learner confidence, learner motivation and relevance of content; and 2 higher-order themes: self-directed learning, and fidelity and technical factors. The findings highlighted the simulation's strengths in providing new ways to learn psychomotor skills and clinical knowledge as an adjunct to traditional training. The importance of diligent selection and curation of content, meaningful feedback and purposeful assessments was emphasised. The findings suggested empowering learners to embrace self-directed learning and adopting near-peer teaching approaches would improve trainees' experiences.
**Conclusion:** The study demonstrates there is a role for simulation in ultrasound training, but its effective implementation requires approaches that embrace self-directed learning and consider the impact of fidelity and technical factors on the learning experience.
**Limitations:** There is a possibility of recall and self-selection bias amongst interviewees. The lead researcher's reflexivity and positionality as a senior trainee within the same training scheme is acknowledged.
**Ethics committee approval:** Approved by Cwm Taf Morgannwg UHB.
**Funding for this study:** Not applicable.
**Author Disclosures:**
Hashim Samir: Nothing to disclose
Victoria Elizabeth Whitchurch: Nothing to disclose
James Declan Yeomans: Nothing to disclose
David Phillip Anthony Wardle: Nothing to disclose

### RPS 2001-8

**Valsalva manoeuvre decreases liver and spleen stiffness measured by time-harmonic ultrasound elastography**
T. Meyer[1], H. Tzschätzsch[1], B. Wellge[1], I. Sack[1], T. J. Kroencke[2], *A. Martl*[2]; [1]Berlin/DE, [2]Augsburg/DE

**Purpose:** Ultrasound elastography quantitatively measures tissue stiffness and is widely used in clinical practice to diagnose various diseases, including liver fibrosis and portal hypertension. The stiffness of soft organs has been shown to be sensitive to pressure-related diseases such as portal hypertension. Because of the coupling between tissue stiffness of abdominal organs and perfusion-related factors, simple breathing manoeuvres have altered the results of liver elastography, while other organs such as the spleen are understudied. Therefore, we investigated the effect of a standardised Valsalva manoeuvre on liver stiffness and, for the first time, on spleen stiffness, using time-harmonic elastography (THE).
**Methods or Background:** THE acquires full-field-of-view stiffness maps based on shear-wave speed (SWS), covers deep tissues and is potentially sensitive to SWS changes induced by altered pressure in the hepatosplenic system. SWS of the liver and the spleen was measured in 17 healthy volunteers at baseline and during the Valsalva manoeuvre.
**Results or Findings:** During Valsalva, SWS in the liver decreased by 2.2 % (from a median of 1.36 m/s to 1.32 m/s; p=0.021), while SWS in the spleen decreased by 5.2 % (from a median of 1.63 m/s to 1.51 m/s; p=0.00059). Furthermore, we observed that the decrease was more pronounced the higher the baseline SWS values were.
**Conclusion:** The results confirm our hypothesis that the Valsalva manoeuvre decreases liver and spleen stiffness, showing that THE is sensitive to perfusion pressure-related changes in tissue stiffness. With its extensive organ coverage and high penetration depth, THE may facilitate translation of pressure-sensitive ultrasound elastography into clinical routine.
**Limitations:** The small number of volunteers and the lack of Doppler flow parameters for correlation.
**Ethics committee approval:** Approval by the ethics committee of Charité Universitätsmedizin Berlin EA1/276/16
**Funding for this study:** German Research Foundation (SFB1340, GRK2260) and Pfizer (WP2487656).
**Author Disclosures:**
Tom Meyer: Grant Recipient: Deutsche Forschungsgemeinschaft GRK 2260 BIOQIC
Ingolf Sack: Grant Recipient: Deutsche Forschungsgemeinschaft (Grants SFB 1340 Matrix in Vision and GRK 2260 BIOQIC)
Heiko Tzschätzsch: Nothing to disclose
Thomas J. Kroencke: Nothing to disclose
Brunhilde Wellge: Grant Recipient: Pfizer (Grant number WP2487656)
Alma Martl: Nothing to disclose

### RPS 2001-10

**RLQ pain and tenderness in paediatric patients with PIMS: is it appendicitis? Ask the sonographer**
*A. Ilivitzki*; Haifa/IL
*(ailivitzki@gmail.com)*

**Purpose:** Compare the sonographic findings in paediatric patients presenting with RLQ tenderness or peritonitis due to PIMS versus appendicitis.
**Methods or Background:** Paediatric patients with PIMS may present with right lower quadrant tenderness or even peritonitis, resembling the presentation of acute complicated appendicitis. We retrospectively studied all abdominal ultrasound studies performed in our hospital for the evaluation of suspected appendicitis in two groups of paediatric patients, the first with a diagnosis of PIMS and the second with a pathologically confirmed diagnosis of complicated appendicitis. A team of two experienced paediatric radiologists reviewed and agreed on the pathologic findings.
**Results or Findings:** Altogether we had 17 paediatric patients with PIMS (mean age 8.2y ) and 22 patients with complicated appendicitis (mean age 11y ). The appendix was identified in 20 (91%) patients with appendicitis, all with signs of inflammation, and in 11 (65%) patients in the PIMS group (8 normal, 3 thickened). Mesenteric lymphadenitis was seen in 8 (36%) of the appendicitis group, all appearing reactive, and in 10 (59%) of the PIMS group, of which 9 (90%) were round, hypoechoic and in clusters (one had central necrosis). Abdominal fluid, unclear to pus, was identified in 20 (91%) of the appendicitis patients, mostly peri-appendicular and minimal. Of the PIMS group, in 7 (41%) there was a moderate to large amount of clear ascites. Gall bladder wall thickening was only noted in the PIMS group in 5 (30%) patients.
**Conclusion:** RLQ tenderness and abnormal sonographic findings may be seen both in appendicitis and PIMS. Sonographic clues to diagnosis are the appearance of the appendix and the mesenteric lymph nodes, along with the amount and consistency of the abdominal fluid.

**Limitations:** Retrospective study.
**Ethics committee approval:** We have approval of our local Helsinki committee.
**Funding for this study:** No funding was received for this study.
**Author Disclosures:**
Anat Ilivitzki: Nothing to disclose

### RPS 2001-11

**Imaging features of hepatic epithelioid hemangioendothelioma on contrast enhanced ultrasound**

*Y. Xu*, W. Tao, Z. Bin, W. Xi, J. Biao, W. Ping; Shanghai/CN

**Purpose:** To investigate the contrast-enhanced ultrasound(CEUS) features of hepatic epithelioid hemangioendothelioma (HEHE) in order to improve the ability of diagnosis.
**Methods or Background:** The images of CEUS have been retrospectively evaluated in 42 lesions in 32 patients of HEHE who were confirmed by pathology from January 2004 to June 2021.
**Results or Findings:** These 32 patients included 1 case of single type, 29 cases of multiple type and 2 cases of diffuse type. During the arterial period, 18 lesions showed overall enhancement, 6 lesions showed uneven dendritic enhancement, 16 lesions showed circular enhancement, and 2 lesions showed only slight peripheral enhancement. Three of the multiple lesions had both the overall enhancement pattern and the circular enhancement pattern in each patient. Fast in was observed in 20 lesions on CEUS, synchronous enhancement in 20 lesions and slow enhancement in 2 lesions and all lesions washed out quickly in late arterial phase or early portal phase. When the enhancement intensity reached its peak, compared to the normal hepatic parenchyma, hypoenhancement, isoenhancement and hyperenhancement were observed in 11 lesions, 11 lesions and 20 lesions respectively. All 16 circular enhancement lesions showed high enhancement, 4 of the overall enhancement lesions showed high enhancement, 5 showed low enhancement, and 9 showed equal enhancement. Among the dendritic enhancements, 2 showed equal enhancement, and 4 low enhancement. After contrast-enhanced ultrasound, the boundaries of all lesions were displayed more clearly than two-dimensional ultrasound.
**Conclusion:** CEUS is valuable in the diagnosis of HEHE.
**Limitations:** The sample size of this study is small.
**Ethics committee approval:** This study was approved by the ethics committee of Zhongshan Hospital Affiliated to Fudan University.
**Funding for this study:** Funding was received for this study by the National Natural Science Foundation of China (Grant NO.82071942); the Shanghai Shenkang Center Major Clinical Research Project (SHDC2020CR1031B); the Shanghai Key Clinical Specialty Project (shslczdzk03501); the Youth Project of Shanghai Municipal Health Commission (20194Y0473).
**Author Disclosures:**
Wang Tao: Nothing to disclose
Yadan Xu: Nothing to disclose
Wang Ping: Nothing to disclose
Ji Biao: Nothing to disclose
Zhang Bin: Nothing to disclose
Wang Xi: Nothing to disclose

**14:00-15:30** **Room Z**

### Research Presentation Session: Neuro

### RPS 2011

### Imaging in neuro-psychiatric diseases

**Moderator**
S. Van Cauter; Genk/BE

### RPS 2011-2

**Shared grey matter alterations in subtypes of addiction: a voxel-wise meta-analysis**

*M. Zhang*, X. Gao, Y. Zhang; Zhengzhou/CN
*(zmzlll4@163.com)*

**Purpose:** Numerous studies based on voxel-based morphometry (VBM) have revealed grey matter (GM) alterations in multiple brain regions for addiction. However, findings are poorly replicated and it remains elusive whether distinct diagnoses of addiction are underpinned by shared abnormalities. Our aim was to conduct a quantitative meta-analysis of structural neuroimaging studies investigating GM abnormalities in two main categories of addiction: substance use disorders (SUD) and behavioral addictions (BA).
**Methods or Background:** A systematic database search was conducted in several databases from 1st Jan 2010 to 23rd Oct 2020 to identify eligible VBM studies. Meta-analysis was performed with the Seed-based d Mapping software package to compare alternations between individuals with addiction-related disorders and healthy controls (HC).
**Results or Findings:** A total of 59 VBM studies including 2096 individuals with addiction-related disorders and 2637 HC met the inclusion criteria. Individuals with addiction-related disorders showed shared GM volume decrease in bilateral prefrontal cortex, bilateral insula, left superior temporal gyrus, and right heschl gyrus and GM increase in right lingual gyrus and right fusiform gyrus comparing with HC ($p<0.005$). Subgroup analysis found heterogeneity between SUD and BA mainly in left inferior occipital gyrus and right striatum ($p < 0.005$). Meta-regression revealed GM atrophy in left inferior frontal gyrus ($r = 0.595$, $p = 0.015$) were positively correlated with higher impulsivity.
**Conclusion:** This meta-analysis identified a concordance across subtypes of addiction in terms of the brain structural changes in prefrontal and insula areas, which may relate to higher impulsivity observed across addiction diagnoses. This concordance provides an organising model that emphasises the importance of shared neural substrates in addiction.
**Limitations:** The causality between GM decrease and development of addiction is inexplicable by means of the integration of cross-sectional studies.
**Ethics committee approval:** This study was approved by the Medical Ethics Committee of First Affiliated Hospital of Zhengzhou University.
**Funding for this study:** No funding was received.
**Author Disclosures:**
Xinyu Gao: Nothing to disclose
Mengzhe Zhang: Nothing to disclose
Yong Zhang: Nothing to disclose

### RPS 2011-3

**Functional networks stability in patients affected by autism compared to healthy controls: a possible novel biomarker from resting-state fMRI**

*L. Pasquini*[1], M. Lucignani[2], F. Bottino[2], A. I. Holodny[1], A. Napolitano[2]; [1]New York, NY/US, [2]Rome/IT

**Purpose:** Autism spectrum disorder (ASD) is associated with atypical brain networks. However, ASD cognitive dynamics are still elusive and functional connectivity (FC) biomarkers are missing. Our hypothesis was that spatial stability (SS) of functional networks, intended as FC resilience to small brain parcellation changes, would capture cognitive dynamics undetected on conventional analyses in ASD. Our aim was to investigate SS in ASD compared to healthy controls (HC).
**Methods or Background:** ASD patients and HC were selected from 3 independent datasets (ABIDE-I/II/III). Dkatlas40 was used for brain parcellation (standard parcels-SP). New modified parcels (MP) were generated by randomly moving 3% of vertices in 30% of adjacent SP (10% parcel's area variation). To evaluate FC, multiple centrality measures were calculated based on generated parcellations. SS for every measure was defined by the mean variation across generated networks. Statistical analysis (ASD vs HC) was performed with ANOVA ($p > 0.05$).
**Results or Findings:** 65 ASD patients and 80 HC were included. 100 parcellations were generated for each subject and 14500 functional networks were generated from rs-fMRI. SS of eigenvector centrality (EC) was higher in ASD compared to HC in every dataset (p 0.0021; 0.034; 0.007). On the contrary, SS of assortativity was lower (p 0.0021; 0.034; 0.007).
**Conclusion:** SS of centrality measures was significantly different in ASD compared to HC. While prior analyses demonstrated both under- and over-connectivity in ASD, SS may capture dynamics underlying both processes. SS expresses the consistency of a node's connectivity pattern across its neighborhood. In our case, it may reflect a pattern of interaction between eloquent brain areas, as a new potential biomarker of ASD.
**Limitations:** Our main limitation was the high computational costs of the analysis, partially mitigated by ad-hoc optimisation in python.
**Ethics committee approval:** Not applicable.
**Funding for this study:** Not applicable.
**Author Disclosures:**
Francesca Bottino: Nothing to disclose
Andrei Igor Holodny: Nothing to disclose
Antonio Napolitano: Nothing to disclose
Luca Pasquini: Nothing to disclose
Martina Lucignani: Nothing to disclose

### RPS 2011-5

**Functional magnetic resonance study of static and dynamic amplitude of low frequency fluctuation in smoking addicted males**

*X. Gao*, M. Zhang, Y. Zhang; Zhengzhou/CN
*(gaoxinyu0806@163.com)*

**Purpose:** Smoking is associated with altered intrinsic activity of the brain. The aim of our study was to investigate how exactly cigarette smoking affects static and temporal dynamic intrinsic brain activity in the resting state of smoking addicts and whether these changes are related to smoking behaviour.

**Methods or Background:** Based on static amplitude of low-frequency fluctuation (sALFF) and dynamic amplitude of low frequency fluctuation (dALFF), we compared the differences of static and dynamic spontaneous brain activity between smoking addicted males (n=63) and healthy controls (n=30). Pearson correlation analysis was performed between dALFF in areas showing group differences and smoking behaviour (e.g., the Fagerström test for nicotine Dependence [FTND] scores and pack-years).
**Results or Findings:** Compared with healthy controls, the value of static ALFF in the left superior/middle/inferior orbitofrontal gyrus was increased, and the variation in dynamic ALFF in the right superior temporal/middle gyrus, left orbitofrontal region, left orbital superior/middle/inferior frontal gyrus, right superior frontal gyrus and right putamen was also increased in the smoking group. It was noteworthy that the dALFF values of the right superior temporal/middle gyrus, left orbital region, right superior frontal gyrus and right putamen were positively correlated with pack-years of smoking males.
**Conclusion:** In resting state, smoking addicted males may have abnormal static and dynamic spontaneous neural activity in the prefrontal cortex (including orbital frontal lobe), putamen and superior temporal/middle gyrus, which is correlated with pack-years of smoking males. It is speculated that these changes in spontaneous brain activity may be helpful to explore the mechanism of smoking addiction and guide clinical withdrawal treatment.
**Limitations:** It's a cross-sectional study.
**Ethics committee approval:** This study was approved by First Affiliated Hospital of Zhengzhou University Research Ethics Board.
**Funding for this study:** This study was funded by the National Key Research and Development Program of China (SQ2018YFC130095).
**Author Disclosures:**
Xinyu Gao: Nothing to disclose
Mengzhe Zhang: Nothing to disclose
Yong Zhang: Nothing to disclose

### RPS 2011-6

**Young subjects at risk of psychosis show specific features of cortical thinning**

*L. Melazzini*, L. Mazzocchi, M. Paoletti, A. F. Paredes Arevalo, E. Ballante, M. Iorio, M. M. Mensi, R. Borgatti, A. Pichiecchio; Pavia/IT

**Purpose:** Psychosis is a symptom common to several mental illnesses and a defining feature of schizophrenia spectrum disorders. The onset of psychotic disorders typically occurs in adolescence or early adulthood. Neuroradiological studies have reported evidence of brain structural abnormalities in patients with overt psychosis. However, early identification of brain structural changes in young subjects at risk for developing psychosis is currently lacking.
**Methods or Background:** Brain 3D T1-weighted and 64-directions DTI HARDI images were acquired on 55 help-seeking adolescents (12-17 years old) with psychiatric disorders who referred to our Institute. Patients were classified into non-psychotic (n=20), at risk of psychosis (n=20) and psychotic (n=15) using the CAARMS tool. Cortical thickness was calculated from T1w images using FreeSurfer v.7.1.0. FSL-TBSS voxel-wise analysis was performed to study the distribution of diffusion-tensor imaging metrics in the white matter. A thorough neuropsychological test battery was adopted to investigate cognitive performance in several domains.
**Results or Findings:** In patients at risk of psychosis, left pars triangularis and left superior frontal gyrus were significantly thinner than in psychotic (p=0.020) and non-psychotic (p=0.050) patients. Also in patients at risk of psychosis, thinner right and left pars triangularis were associated with worse processing speed performance (p=0.005 and p=0.030 respectively), whereas thinner rostral anterior cingulate gyrus was significantly associated with lower processing speed/coding subtest scores (p=0.010). TBSS analysis did not show any statistically significant results.
**Conclusion:** Cortical thickness values were significantly different among the three diagnostic groups. This study also showed specific associations between structural imaging features and cognitive performance in patients at risk for psychosis. Overall, capturing the pre-psychotic phase using neuroimaging could prove useful for the adoption of preventive strategies in these individuals.
**Limitations:** The following limitations were identified: this was a cross-sectional study; preliminary results.
**Ethics committee approval:** This study was approved by an ethics committee.
**Funding for this study:** Funding was received from Ricerca Corrente 2017-2019.
**Author Disclosures:**
Laura Mazzocchi: Nothing to disclose
Renato Borgatti: Nothing to disclose
Matteo Paoletti: Nothing to disclose
Luca Melazzini: Nothing to disclose
Elena Ballante: Nothing to disclose
Martina Maria Mensi: Nothing to disclose
Anna Pichiecchio: Nothing to disclose
Melanie Iorio: Nothing to disclose
Alexandra Fabiola Paredes Arevalo: Nothing to disclose

### RPS 2011-7

**Structural and functional brain abnormalities in internet gaming disorder and attention-deficit/hyperactivity disorder: a comparative meta-analysis**

*X. Gao*, M. Zhang, Y. Zhang; Zhengzhou/CN

**Purpose:** Patients with Internet Gaming Disorder (IGD) and attention-deficit/hyperactivity disorder (ADHD) have high comorbidity, but it is still unknown whether these disorders have shared and distinctive neuroimage alterations. The aim of this meta-analysis was to identify shared and disorder-specific structural, functional, and multimodal abnormalities between IGD and ADHD.
**Methods or Background:** A systematic literature search was conducted for whole-brain voxel-based morphometry (VBM) and functional magnetic resonance imaging (fMRI) studies comparing people with IGD or ADHD with healthy controls. Regional grey matter volume (GMV) and fMRI differences were compared over the patient groups and then a quantitative comparison was performed to find abnormalities (relative to controls) between IGD and ADHD using seed-based d mapping meta-analytic methods.
**Results or Findings:** The meta-analysis contained 14 IGD VBM studies (contrasts covering 333 IGDs and 335 HCs), 26 ADHD VBM studies (1,051 patients with ADHD and 887 controls), 30 IGD fMRI studies (603 patients with IGD and 564 controls), and 29 ADHD fMRI studies (878 patients with ADHD and 803 controls). Structurally, VBM analysis showed disorder-specific GMV abnormality in the putamen among IGD subjects and orbitofrontal cortex in ADHD and shared GMV in the prefrontal cortex. Functionally, fMRI analysis discovered that IGD-differentiating increased activation in the precuneus and shared abnormal activation in anterior cingulate cortex, insular, and striatum.
**Conclusion:** IGD and ADHD have shared and special structural and functional alterations. IGD has disorder-differentiating structural alterations in the putamen and ADHD has alterations in the orbitofrontal cortex. Disorder-differentiating fMRI activations were predominantly observed in the precuneus among IGD subjects and shared impairing function connection was in the rewards circuit (including ACC, OFC, and striatum).
**Limitations:** No limitations were identified.
**Ethics committee approval:** Institutional Review Board approval was not required.
**Funding for this study:** Funding was received from the National Key Research and Development Program of China (SQ2018YFC130095).
**Author Disclosures:**
Xinyu Gao: Nothing to disclose
Mengzhe Zhang: Nothing to disclose
Yong Zhang: Nothing to disclose

### RPS 2011-8

**Functional connectivity differences between the patients with disorders of consciousness and the patients with clinical cognitive motor dissociation**

*P. Pozeg*[1], J. Jöhr[1], A. Pincherle[2], K. Diserens[1], V. Dunet[1]; [1]Lausanne/CH, [2]Luxembourg/LU
*(polona.pozeg@chuv.ch)*

**Purpose:** Recovery of consciousness depends on restoration of the anterior forebrain mesocircuit, involving thalamus, globus pallidus, striatum, and its connectivity with the brainstem and default mode network (DMN). This study investigated whether resting state fMRI (rsfMRI) functional connectivity (FC) in the brainstem-mesocircuit-DMN network is more preserved in patients with clinical cognitive motor dissociation (cCMD), i.e. behaviorally unresponsive patients with residual cognition as assessed by Motor Behavior Tool-revised (MBT-r), than in the patients with true disorders of consciousness (DOC).
**Methods or Background:** 22 cCMD and 7 DOC patients, as identified with the Coma Recovery Scale-revised and MBT-r were included in this retrospective, cross-sectional study (12 women; age: 46.8±18.7 years). RsfMRI data were acquired on 3T scanner (Siemens, Erlangen, Germany), preprocessed and analysed with the CONN toolbox-v.19. Seed-to-voxel analyses using bilateral thalami and medial prefrontal cortex (MPFC) within DMN as seeds, and independent component analysis (ICA) were performed. We correlated FC with the Level of Cognitive Functioning (LCF) and Disability Rating Scale (DRS) scores, and compared the FC between cCMD and DOC patients. The significance level was set at cluster size p-FDR-corrected < 0.05 and voxel threshold p-uncorrected < 0.001.
**Results or Findings:** LCF and DRS scores correlated with FC between bilateral thalamus, brainstem, MPFC and right frontal and temporal regions, and with FC between precuneus and MPFC within the ICA-defined DMN. cCMD patients displayed stronger FC than DOC patients between the bilateral thalamus and left frontal lobe, within MPFC, and between ICA-defined DMN and the right temporal lobe.
**Conclusion:** These findings suggest more preserved FC within brainstem-mesocircuit-DMN network in the cCMD than DOC patients, and demonstrate that increased FC within this network is associated with a better clinical outcome.
**Limitations:** No limitations were identified.

Saturday

**Ethics committee approval:** Local ethics commitee (CER-VD) approved this study (142/09).
**Funding for this study:** Funding was received from the Swiss National Science Foundation (FNS320030_189129).
**Author Disclosures:**
Jane Jöhr: Nothing to disclose
Vincent Dunet: Grant Recipient: Swiss National Science Foundation (grant nr. FNS 320030_189129)
Polona Pozeg: Nothing to disclose
Alessandro Pincherle: Nothing to disclose
Karin Diserens: Grant Recipient: Swiss National Science Foundation (grant nr. FNS 320030_189129)

### RPS 2011-9

**Disrupted functional connectivity of amygdala subregional networks in social anxiety disorder**
*X. Zhang*, S. Wang, Q. Gong; Chengdu/CN

**Purpose:** Anomalous amygdala-based networks have been reported to be centrally involved in social anxiety disorder (SAD), but findings have been heterogeneous due to small sample sizes, demographic, and methodological differences, while little is known about the amygdala networks at subregional level in SAD. Herein, we aimed to identify the alterations of amygdala subregional networks in homogenous patients with SAD to investigate whether distinct parts of the amygdala functionally interact with dissociable brain networks and contribute differently to the cerebral network substrates of SAD.
**Methods or Background:** Based on the power analysis (Cohen's d = 0.5; $\alpha = 0.05$; $1-\beta = 0.8$), 49 non-comorbid patients with SAD and 53 demography-matched healthy controls were recruited in the current study, from whom the resting-state functional MRI images were obtained. Then whole-brain functional connectivity maps of four distinct cytoarchitectonically-determined amygdala subregions (i.e. amygdalostriatal transition, basolateral, centromedial, and superficial area) and the amygdalar entirety were generated, and whole-brain voxel-wise comparisons of functional connectivity between groups were conducted using two-sample t-test with age, sex, and mean frame-wise displacement as covariates.
**Results or Findings:** SAD patients demonstrated widespread dysconnectivity in cortico-amygdalo-cerebellar circuitry, mainly including hyperconnectivity between the amygdala with default mode network/sensorimotor network and hypoconnectivity between the amygdala with visual network/cerebellum network, in which the basolateral amygdala showed most prominent effects.
**Conclusion:** For the first time, our findings suggested that distinct disruption patterns of large-scale amygdala subregional networks was implicated in SAD, and may reflect imbalanced network function of bottom-up response and top-down regulation in cognitive, emotional, and sensory domains, which could offer novel insights into the underlying pathophysiological mechanisms of SAD.
**Limitations:** No limitations were identified.
**Ethics committee approval:** This study was approved by the local Medical Research Ethics Committee.
**Funding for this study:** Funding was received from the National Natural Science Foundation of China (81621003, 81761128023, 81820108018, 82027808).
**Author Disclosures:**
Song Wang: Nothing to disclose
Qiyong Gong: Nothing to disclose
Xun Zhang: Nothing to disclose

### RPS 2011-10

**Relationship between fractional anisotropy of the external capsule and hippocampal myo-inositol levels in patients with mild cognitive impairment: evidence from DTI-1H-MRS pilot study**
*K. Valatkevičienė*[1], R. Gleiznienė[1], O. Levin[2], N. Masiulis[1]; [1]Kaunas/LT, [2]Leuven/BE

**Purpose:** Examine associations between structural and biochemical properties of brain in the aging population.
**Methods or Background:** MCI is characterised by neuro-inflammation, which is expected to elevated levels myo-inositol (mIns). In this study, we examined possible association between mIns levels in the hippocampus, prefrontal and temporal regions and microstructural integrity of the external capsule. 1H-MRS was used to quantify levels of N-acetylaspartate (NAA), choline (Cho), and myo-inositol (mIns) from left hippocampus (HPC), left medial temporal cortex (MTC), and right dorsolateral prefrontal cortex (dlPFC) from 20 MCI patients and 29 age-matched cognitively-intact older adults (non-MCI). Fractional anisotropy (FA) was estimated from whole-brain water diffusion imaging.
**Results or Findings:** Mann-Whitney test revealed lower FA values in the left external capsule of the MCI subjects as compared to non-MCI (Z = -1.942, p = 0.052) but not in other white matter (WM) tracts (all, p > 0.1). Findings revealed that increased levels of hippocampal mIns were negatively related to decreased FA in the left external capsule of MCI (r = -0.399) which was not seen in the non-MCI (r = 0.02). Contrarily, higher levels of hippocampal Cho were negatively related to lower FA in non-MCI (r = -0.455, p = 0.033) but not in MCI (r = 0.120). No associations were found between metabolites in the two other brain locations and FA.
**Conclusion:** Observations suggest that decrease microstructural organisation of left external capsule could be related to local neuro-inflammation in the hippocampus.
**Limitations:** Observations are limited by low sample size and missing data points that were excluded from the final analyses due to low quality of MRS. Therefore, findings of this pilot study should be viewed as trends.
**Ethics committee approval:** This study was approved by the Local Medical Ethics Committee for Biomedical Research (No. BE-10-7).
**Funding for this study:** Funding was received from the Research Council of Lithuania (No. S-SEN-20-5).
**Author Disclosures:**
Nerijus Masiulis: Nothing to disclose
Kristina Valatkevičienė: Nothing to disclose
Rymantė Gleiznienė: Nothing to disclose
Oron Levin: Nothing to disclose

### RPS 2011-12

**Hypothalamic paraventricular nucleus inputs to the cingulate cortex and paraventricular thalamic nucleus modulate anxiety and arousal**
*Y. Liu*; Nanchang/CN

**Purpose:** To explore the similarities and differences between the neural circuits that regulate insomnia and anxiety disorders.
**Methods or Background:** We use a series of techniques including chemo-fMRI, chemogenetics and optogenetics and prove that PVNvglut2 neurons can regulate both anxiety and wakefulness, and through two different downstream neural circuits. The PVN-PVT pathway regulates arousal but not anxiety-like behavior, while the PVN-Cg pathway is critical for regulating anxiety-like behavior but not arousal.
**Results or Findings:** 1. The whole-brain BOLD signals and functional connectivity change after activation of the glutamatergic neurons in PVN. 2. Optogenetic activation of the PVN glutamatergic neurons increases anxiety-like behaviors in mice. 3. Optogenetic activation of the PVN-Cg neural circuit increases anxiety-like behaviors in mice. 4. Optogenetic long-term synaptic depression of the PVN-Cg neural circuit reduces anxiety-like behaviors in mice. 5. Optogenetic activation of the PVN-Cg neural circuit has no effect on wakefulness in mice. 6. Optogenetic activation of the PVN-PVT neural circuit increases wakefulness. 7. Chemogenetic inhibition of the PVN-PVT neural circuit reduces wakefulness. 8. Optogenetic activation of the PVN-PVT neural circuit does not affect anxiety-like behaviors in mice.
**Conclusion:** The PVN-PVT pathway regulates arousal but not anxiety-like behavior, while the PVN-Cg pathway is critical for regulating anxiety-like behavior but not arousal. Our study may help to uncover the neuronal mechanisms for anxiety disorders and insomnia.
**Limitations:** Since the PVN is a complex nuclei which consist of different subregions containing functionally distinct neuronal subtypes, further study needs to be conducted to explore whether the PVNvglut2 neurons projecting to these targets belong to a common or distinct neuronal population.
**Ethics committee approval:** The study was approved by the Animal Ethics Committee of Wuhan University.
**Funding for this study:** Funding was received from the National Natural Science Foundation of China (under Grant Nos 81771819 and 32071140).
**Author Disclosures:**
Ying Liu: Nothing to disclose

16:00-17:00 **Open Forum #1 (Radiographers)**

### Research Presentation Session: Radiographers

### RPS 2114

### Optimising MRI practice and imaging services

**Moderators**
M. Zanardo; Milan/IT
S. Harden; Southampton/UK

### RPS 2114-3

**Comparison between the use of PET and MRI 3.0 T in meningiomas**

L. Anemoni, L. Preda, *M. S. Cadeo*, E. Orlandi, A. Mancin, I. E. Mascayano, S. Tampellini, M. E. Piazzolla; Pavia/IT
*(margherita.cadeo@cnao.it)*

**Purpose:** Positron emission tomography (PET-CT) PET-CT 68-gallium (PET-CT 68Ga-DOTATOC), in combination with traditional imaging are used in hadrontherapy both in the planning phase and follow-up. The primary endpoint of this retrospective study is the assessment of the correlation between the treatment volumes defined with PET-CT 68Ga-DOTATOC and the potential clinical impact of any discrepancies. The secondary objective is to assess the sensitivity, specificity and positive and negative predictive value of the multimethodological approach of high-field Magnetic Resonance Imaging (MRI) in the assessment of early response to particle therapy, also estimating the degree of concordance with PET-CT method and with only morphological MRI. For this study, both PET-CT and MRI images of 34 patients suffering meningioma treated at CNAO with adrotherapy were analysed. Based on histology, patients underwent PET-CT 68Ga-DOTATOC a Gross Tumor Volume (GTV) of reference PET (GTVrifPET) and another one evaluated on MRI images (GTV3T) by using a multimethodological approach of high-field MRI (3.0 T) have been defined.
**Methods or Background:** A GTVrifPET and a GTV were defined, evaluated on MRI (GTV3T) images using a multimethodological approach of high-field MRI (3.0 T). Clinical Target Volume (CTV) was also analysed in relation to GTV. In the assessment of GTV3T, the following sequence was taken into account: DWI with 7 different intensity of diffusion gradients (b-value = 0, 50, 100, 150, 200, 400, 1000 s/mm2). MRI was performed using multichannel phased-array coils.
**Results or Findings:** It was observed that, in the brain district, the GTVrifPET was volumetrically higher than the GTV3T (55.88%).
**Conclusion:** This improvement in the definition of GTV leads to an optimisation and a clear change in the definition not only of the GTV, but especially of other target volumes such as CTV and Planning Target Volume (PTV) for the radiotherapist.
**Limitations:** Not applicable
**Ethics committee approval:** Not applicable
**Funding for this study:** Not applicable
**Author Disclosures:**
Alice Mancin: Nothing to disclose
Margherita Sofia Cadeo: Nothing to disclose
Sara Tampellini: Nothing to disclose
Lorenzo Preda: Nothing to disclose
Luca Anemoni: Nothing to disclose
Ester Orlandi: Nothing to disclose
Ivonne Elenoire Mascayano: Nothing to disclose
Maria Elena Piazzolla: Nothing to disclose

### RPS 2114-4

**Comparison between the use of PET-CT and high-field MRI 3.0T in adenoid cystic carcinoma**

L. Anemoni, L. Preda, E. Orlandi, M. S. Cadeo, A. Mancin, I. E. Mascayano, S. Tampellini, *M. E. Piazzolla*; Pavia/IT
*(piazzolla@cnao.it)*

**Purpose:** Positron emission tomography (PET-CT) 11C-methionine (MET PET-CT), in combination with traditional imaging are used in hadrontherapy both in the planning phase and follow-up. The main goal of this retrospective study is the assessment of the correlation between the treatment volumes, defined with MET PET-CT, and the potential clinical impact of any discrepancies. The secondary objective is to assess the sensitivity, specificity, the positive and negative predictive value of the multimethodological approach of high-field Magnetic Resonance Imaging (MRI) in the assessment of early response to the therapy. The object of analysis were the images obtained by PET-CT and MRI of 25 patients suffering adenoid cystic carcinoma (ACC) treated at CNAO from 2012 to 2019 with hadrontherapy. Based on histology, we define Gross Tumour Volume (GTV) on MET PET-CT and also on MRI images (GTV3T) by using a multimethodological approach of high-field MRI (3.0 T)
**Methods or Background:** A GTVrifPET and a GTV were defined, evaluated on MRI (GTV3T) images using a multimethodological approach of high-field MRI (3.0 T). Clinical Target Volume (CTV) was also analysed in relation to GTV. The variation of these volumes was analysed in a descriptive way according to the definition of volume and percentage changes. In the assessment of GTV3T, the following sequence was taken into account: head-neck district, Turbo-Spin-Echo T2w sequence in the axial plane. MRI was performed using multichannel phased-array coils.
**Results or Findings:** In the head-neck district the GTVrifPET was volumetrically lower than the GTV3T(68%). GTV were used by the radiotherapist in defining the CTV.
**Conclusion:** Definition of GTV leads to an optimisation and a clear change in the definition not only of the GTV, but especially of other target volumes such as CTV and Planning Target Volume (PTV) for the radiotherapist.
**Limitations:** Not applicable
**Ethics committee approval:** Not applicable
**Funding for this study:** Not applicable
**Author Disclosures:**
Alice Mancin: Nothing to disclose
Margherita Sofia Cadeo: Nothing to disclose
Sara Tampellini: Nothing to disclose
Lorenzo Preda: Nothing to disclose
Luca Anemoni: Nothing to disclose
Ester Orlandi: Nothing to disclose
Ivonne Elenoire Mascayano: Nothing to disclose
Maria Elena Piazzolla: Nothing to disclose

### RPS 2114-5

**Challenges in prostate MR image quality: perspectives from Saudi and Irish MRI departments**

*S. Al Dahery*; Jeddah/SA
*(shrooq_talal@hotmail.com)*

**Purpose:** The aim of this study was to investigate the challenges of optimising the quality of prostate MR images acquired in Saudi and Irish MRI departments and evaluate the influencing factors.
**Methods or Background:** Axial T1W- and T2W-FSE MR images were retrospectively collected from 10 Irish (85 cases) and 7 Saudi (51 cases) hospitals. Quantitative analysis: signal-to-noise-ratio (SNR) and contrast-to-noise-ratio (CNR) were calculated based on signal intensity measurements obtained from the image background and from uniform areas of the central and transitional prostate zones, away from the prostatic urethra. The image datasets were independently evaluated by eight observers and qualitative evaluation scores were recorded. An intervention was undertaken to improve prostate MR image quality at a single Saudi centre which had recorded low quality for several criteria during phase one. Both methods were again used to evaluate the post-intervention image quality.
**Results or Findings:** Significant differences were noted in the SNR and CNR values for images acquired by using the T1W-FSE-TRA sequence between both countries. Median SNR and CNR values for T1W-FSE-TRA images acquired at Irish hospitals were higher than for those obtained from Saudi hospitals attributed to the wide range of slice thicknesses (4-9 mm) utilised across participating hospitals. Observer scores indicated a preference for images acquired using thicker slices (≥6 mm) than those obtained with (≤4.5 mm). There were no significant differences for SNR and CNR values for T2W-FSE-TRA images in both countries. Scores identified a preference for thin-slice (3.5 mm).
**Conclusion:** A multi-phase optimisation process of MR pulse sequences based on patient presentation is effective to optimise visualisation of critical anatomical structures.
**Limitations:** The small sample size was identified as a limitation.
In addition, old versions of MRI machines were used in Irish hospitals, which could be a factor that makes a difference.
**Ethics committee approval:** KSA-IRB Ministry-of-Health
**Funding for this study:** Funding was received via the Saudi Arabia Scholarship.
**Author Disclosures:**
Shrooq Al Dahery: Nothing to disclose

### RPS 2114-6

**Assessing the image quality of brain MR images taken with 1.5T and 3T scanners**

*L. Borg*, F. Zarb, K. B. Borg Grima; Msida/MT
*(leanne.borg.17@um.edu.mt)*

**Purpose:** Literature indicated that 3T magnetic resonance imaging (MRI) scanners produce images with an improved quality when compared to 1.5T scanners. However, this improvement is limited by the increased presence of artefacts. This research evaluated brain images taken with both types of scanners.
**Methods or Background:** Twenty brain scans (10 performed on a 1.5T scanner and 10 on a 3T scanner), reported as having no significant abnormalities, were included in this study. In phase one, objective measures of the signal-to-noise ratio (SNR) and contrast-to-noise ratio (CNR) were acquired and statistically analysed, for the axial 3DT1 weighted (W), T2W, fluid low attenuation inversion recovery (FLAIR), diffusion weighted imaging (DWI) and sagittal T2W sequences. Statistically significant variations in image quality were obtained for the axial T2W and FLAIR sequences ($p<0.05$), these were then included in phase two. Phase two of the study involved the latter sequences being reviewed by two radiologists using visual grading analysis (VGA).
**Results or Findings:** Phase one results indicated that for all sequences, the SNR and CNR were higher on the 3T scanner, except for the CNR of the 3DT1W sequence. Results from phase two showed that, for both sequences, images produced by the 3T scanner had a better image quality (AUC>0.5). Artefacts were potentially more common on the 1.5T scanner, however image quality variations were not statistically significant ($p>0.05$).
**Conclusion:** The results obtained from both phases indicated that, while both scanners produced images of sufficient diagnostic quality, scans obtained by the 3T scanner were found to have a superior image quality.
**Limitations:** Each participant was scanned once, randomly on either the 1.5T or 3T scanner, due to ethical reasons.
**Ethics committee approval:** This study was performed following ethical permission from the University of Malta research ethics committee.
**Funding for this study:** No funding was received for this study.
**Author Disclosures:**
Karen Borg Borg Grima: Nothing to disclose
Francis Zarb: Nothing to disclose
Leanne Borg: Nothing to disclose

### RPS 2114-7

**Results of a survey on MRI reporting radiographers in the UK**

*H. Estall*; Leicester/UK
*(helen.estall@uhl-tr.nhs.uk)*

**Purpose:** To summarise the results of a national study looking at the numbers and scope of practice of radiographers that report MRI scans in the UK.
**Methods or Background:** The demand on imaging in the UK is increasing year on year, with an increase in the demand for MRI of 48% in the last five years and an average annual increase of 10%. Issues with staffing have been found to affect reporting times. Health Education England are investing in an additional 300 reporting radiographer posts in 2021 and state that radiographer reporting is an effective, efficient and safe way of meeting rising demand. There is no central register or knowledge of the number of radiographers reporting MRI examinations nationally. A questionnaire was sent to multiple different MRI groups and trusts within the UK. This report summarises the results of this survey, including the number training, trained and in practice, their scope of practice and post qualification expectations including details of governance and audit.
**Results or Findings:** Responses were received from 46 trusts. There is huge variation in scope, sign off and post qualification support and expectations.
**Conclusion:** This survey provides an insight into the current status of MRI reporting radiographers in the UK. Although courses have been available since 2003, numbers are still low and there are significant geographical and work place practice variations. Defined standards of practice and the implementation of a central register would benefit both those in practice and those looking to implement an MRI reporting radiographer service.
(Published December 2020 - https://doi.org/10.1016/j.radi.2020.11.017)
**Limitations:** There will be some reporters that were not contacted due to the lack of a central register.
**Ethics committee approval:** Ethics committee approval was not required.
**Funding for this study:** No funding was received for this study.
**Author Disclosures:**
Helen Estall: Nothing to disclose

### RPS 2114-8

**Fractional anisotropy changes in early Parkinson's disease and Parkinson-plus syndromes: a comparative study using diffusion tensor imaging**

*R. P. Kotian*[1], P. K[2], L. H. K[3], D. R. Kotian[2], R. N[4]; [1]Ajman/AE, [2]Manipal/IN, [3]Mangalore/IN, [4]Delhi/IN
*(kotian.rahul18@gmail.com)*

**Purpose:** Conventional MRIs fail to pick up early-stage abnormalities in Parkinson's disease (PD). The dependability of diffusion tensor imaging (DTI) as a standard tool for imaging in PD, is still a debate within itself. The study aimed to compare the usefulness of DTI derived FA values between early PD and PD-plus syndromes like multiple system atrophy (MSA) and supranuclear palsy (PSP).
**Methods or Background:** We evaluated DTI derived FA in early PD and PD-plus syndromes (MSA & PSP) patients. A total 100 patients (50) early PD were compared to (50) PD-plus syndrome patients. The following regions of the brain grey and white matter were included for FA analysis: corpus callosum, centrum semiovale, pons, putamen, caudate nucleus, substantia nigra, cerebral peduncles and cerebellar peduncles. We further employed a region of interest (ROI) technique, to derive FA using a DTI protocol with b-value 1000s/mm2 and TE = 100 milliseconds at 1.5 Tesla (T).
**Results or Findings:** Early PD and PD-plus syndrome patients showed statistically significant differences in FA values in the brain at the substantia nigra, putamen and cerebellar peduncles respectively.
**Conclusion:** In conclusion, DTI derived FA values help in detecting microstructural changes in brain grey and white matter. Hence, they should be used as a standard tool for differentiating between PD and PD-plus syndromes.
**Limitations:** An identified limitation was the use of 1.5 T MRI system for obtaining DTI fractional anisotropy values.
**Ethics committee approval:** Institutional research and ethics committee approval was obtained from Kasturba Medical College; Reg No. ECR/146/Inst/KA/2020.
**Funding for this study:** Funding was received from Bombay Scientific, Mumbai, Maharashtra, India.
**Author Disclosures:**
Lakshmikanth H K: Nothing to disclose
Ravishankar N: Nothing to disclose
Prakashini K: Nothing to disclose
Disha R Kotian: Nothing to disclose
Rahul Pratap Kotian: Nothing to disclose

**16:00-17:00** **Room M 2**

### Research Presentation Session: Breast

### RPS 2102

### New developments in breast cancer diagnostics and interventions

**Moderator**
G. Ivanac; Zagreb/HR

### RPS 2102-2

**BraCoil – a wearable breast coil for 3 T MR mammography**

M. Obermann[1], *L. Nohava*[1], P. Clauser[1], S. Roat[1], R. Frass-Kriegl[1], O. Soanca[1], J. Felblinger[2], P. A. Baltzer[1], E. Laistler[1]; [1]Vienna/AT, [2]Nancy/FR
*(lena.nohava@meduniwien.ac.at)*

**Purpose:** Standard MR mammography is performed with rigid radio-frequency coils with cup-shaped molds entailing a number of conceptual drawbacks: the woman is lying uncomfortably in prone position with exposed breasts; Even adaptable RF coils are designed for large breasts, leading to poor image quality in smaller breasts; coil change and subject positioning are tedious for MR technologists; the anatomical distortion of hanging breasts leads to significant signal loss in central and posterior areas; The deformation between prone MRI and supine surgical and ultrasound examination complicates translation of imaging into treatment decisions; the axillary lymph nodes are often insufficiently covered. We present a new approach for MR mammography addressing all of these Limitations: a wearable vest-like breast coil ("BraCoil") usable in prone and supine position.

Saturday

**Methods or Background:** BraCoil is an ultra-flexible array made of 28 coil elements covering both breasts and axillae. A semi-flexible 18-channel multi-purpose coil and rigid 16-channel breast coil were used as a reference for signal-to-noise ratio (SNR) comparison in 10 healthy female volunteers. SNR maps were calculated without and with LR/HF acceleration to investigate parallel imaging performance. Two breast radiologists provided feedback on image quality and anatomical breast representation.
**Results or Findings:** A significant SNR gain up to a factor of 3 was measured with the BraCoil compared to commercial coils; acceleration factors up to 12 can be used. Excellent image quality for both small and large breasts were demonstrated.
**Conclusion:** BraCoil provided 3 times higher SNR, better parallel imaging performance and superior image quality and anatomical breast representation than standard coils, as the breast shape is well aligned with other imaging modalities and therapeutic interventions.
**Limitations:** Not applicable
**Ethics committee approval:** This study was approved by the ethics committee of the Medical University of Vienna, EK Nr. 2137/2021.
**Funding for this study:** FWF Nr. I-3618/ANR-17-CE19-0022 “BraCoil“
**Author Disclosures:**
Pascal A.T. Baltzer: Nothing to disclose
Lena Nohava: Nothing to disclose
Roberta Frass-Kriegl: Nothing to disclose
Sigrun Roat: Nothing to disclose
Elmar Laistler: Nothing to disclose
Michael Obermann: Nothing to disclose
Onisim Soanca: Nothing to disclose
Jacques Felblinger: Nothing to disclose
Paola Clauser: Nothing to disclose

### RPS 2102-3

**Percutaneous treatment of breast phyllodes tumours: a meta-analysis**
*M. L. B. V. Gil*[1], G. Oliveira Bernardes Gil[1], H. L. Couto[1], R. Sobral Monteiro-Junior[2], E. Carvalho Pessoa[3], A. Dias Salvador[1], F. Soares Cantidio[1], B. A. Coelho[2], A. Lopes da Silva Filho[1]; [1]Belo Horizonte/BR, [2]Montes Claros/BR, [3]Botucatu/BR
*(mluisabragagil@gmail.com)*

**Purpose:** To determine, through meta-analyses, the chance of recurrence of benign PT undergoing ultrasound-guided vacuum-assisted excision (US-VAE).
**Methods or Background:** Systematic review and meta-analyses, following the PRISMA standard, of studies that analysed the recurrence of benign PT, undergoing treatment by US-VAE compared with open surgical excision and follow-up for at least 12 months after the interventions. Studies found in PubMed, Scopus, Web of Science and Embase were selected. The main outcome assessed was local tumour recurrence. The pooled effect measure used was the odds radio (OR) in order to estimate the chances of recurrence of the benign PT.
**Results or Findings:** Four studies were selected, with a total of 607 participants, undergoing US-VAE (252) and surgery (355). There was local recurrence in 26 cases in the US-VAE group and 24 in the surgery group. Of the four studies, one of them was not included in the estimate because it did not presented cases of recurrence in US-VAE or surgery. Therefore, the meta-analysis comprised the grouping of three studies, resulting in a total sample of 595 patients. There was no difference in the recurrence of benign phyllodes tumours, regardless of the procedure adopted (OR=1.20; p=0.55). The heterogeneity index was inexpressive ($I^2$=0; p=0,63).
**Conclusion:** This meta-analysis suggests that US-VAE is a minimally invasive therapeutic option for benign phyllodes tumours, being a treatment as safe as standard surgical treatment. The type of treatment chosen, US-VAE or open surgery, does not impact the chance of recurrence for benign phyllodes tumours.
**Limitations:** All eligible studies were observational studies.
**Ethics committee approval:** The study was registered in PROSPERO under the number CRD 42022309782.
**Funding for this study:** No funding was received for this study.
**Author Disclosures:**
Agnaldo Lopes da Silva Filho: Nothing to disclose
Renato Sobral Monteiro-Junior: Nothing to disclose
Bertha Andrade Coelho: Nothing to disclose
Gabriel Oliveira Bernardes Gil: Nothing to disclose
Henrique Lima Couto: Nothing to disclose
Maria Luísa Braga Vieira Gil: Nothing to disclose
Eduardo Carvalho Pessoa: Nothing to disclose
Farley Soares Cantidio: Nothing to disclose
Anna Dias Salvador: Nothing to disclose

### RPS 2102-4

**Efficacy of ultrasound-guided cryoablation in the treatment of low-risk breast cancer**
M. J. Roca Navarro, *S. F. Fernández*, D. Garrido, Y. Navarro, F. García Martínez, T. Díaz de Bustamante Durbán, V. Córdoba, J. M. Oliver, C. Martí; Madrid/ES
*(susana8_94@hotmail.com)*

**Purpose:** To evaluate if in patients with HER2 luminal tumours smaller than 1.5 cm and ultrasound negative axilla, after ultrasound-guided cryoablation, there is absence of infiltrating carcinoma in the lumpectomy specimen.
**Methods or Background:** Between April and September 2021 we performed preoperative cryoablation in 20 patients (between 53 and 79 years old, mean 63) with 20 unifocal infiltrating ductal carcinomas (IDC) (between 13 mm and 4 mm, mean 8 mm). All IDC were visible on ultrasound, were low grade (10 G1, 10 G2), Luminal A or B molecular phenotype, Ki 67 between 3-30% (mean 12%) and ultrasound negative axilla. All patients were studied with mammography and tomosynthesis, staged and biopsied by ultrasound. In 7 of the 8 patients with associated intraductal carcinoma (DCIS) in the core needle biopsy (CNB), MRI was performed to rule out extensive intraductal component. All of them underwent pre-surgical marking with ferromagnetic seed and cryoablation with 17G or 14G needle at that moment, taking advantage of the same anesthesia and cutaneous access. We applied the usual protocol of triple phase: freezing-passive thawing-freezing and duration of approximately 40 minutes. Subsequently, we checked the correct placement of the seed with mammographic projection.
**Results or Findings:** Out of 20 patients with low-risk unifocal IDC, in 19 of them no cells of the invasive component were identified in the examination of the surgical specimen, only 1 patient had an infiltrating carcinoma focus smaller than 1 mm. There were no post-cryoablation complications, only a slight burn.
**Conclusion:** Cryoablation is effective in the local treatment of early low-risk IDC. The presence of isolated DIC nests close to the area of post-Cryoablation steatonecrosis does not indicate treatment failure and does not imply a change in subsequent management with respect to traditional lumpectomy.
**Limitations:** No limitations were identified.
**Ethics committee approval:** This study was not approved by an ethics committee.
**Funding for this study:** No funding was received for this study.
**Author Disclosures:**
Covadonga Martí: Nothing to disclose
Maria Jose Roca Navarro: Nothing to disclose
Srta Susana Fernández Fernández: Nothing to disclose
Jose María Oliver: Nothing to disclose
Ylenia Navarro: Nothing to disclose
Teresa Díaz de Bustamante Durbán: Nothing to disclose
Fernando García Martínez: Nothing to disclose
Diego Garrido: Nothing to disclose
Vicenta Córdoba: Nothing to disclose

### RPS 2102-5

**Role of ultrasound-guided vacuum-assisted breast biopsy (US-VABB) in the management of rad-path discordance**
G. Levrini, *G. Vatteroni*, P. Malerba, I. Bolengo, R. M. Trimboli, D. Bernardi; Rozzano/IT
*(giulia.vatteroni@gmail.com)*

**Purpose:** To assess the value of US-VABB in lesions with rad-path discordance at the first-step core-needle biopsy (CNB).
**Methods or Background:** From February 2020 to October 2021, 16 women (51 years ± 14) with 8 BI-RADS 4b and 8 BI-RADS 4c US lesions underwent US-VABB for 10 B2 and 6 B3 results at the first-step CNB. Global rate of malignancy, and according to the BI-RADS category (chi-squared with α=0.05), were evaluated and false negatives were assessed. Tru-cut 14G (Max-Core®, BARD BD) and VABB 10G (EnCor EnspireTM, BARD BD) needles were employed for CNB (≥3 samplings) and VABB (≥6 samplings). Final pathology was considered the reference standard for women with subsequent surgery, imaging follow-up in the remaining cases.
**Results or Findings:** US-VABB yielded 6 B2, 4 B3, 6 B5 (6/16, 38% rate of malignancy). Out of 6 carcinomas (5 invasive and 1 in situ), 5 were identified in BI-RADS 4c lesions (5/8, 63%) and 1 in BI-RADS 4b lesions (1/8, 13%) (p=0.039). Of the remaining 10 cases (6 B2, 60% and 4 B3, 40%), 6 underwent surgery, with a benign final pathology in 4 B3 and an upgrade to carcinoma in situ in 2 B2, for a resulting false negative rate of 13% (2/16) at US-VABB.
**Conclusion:** The use of US-VABB in the management of rad-path discordance obtained a micro-invasive malignant diagnosis in almost 40% of cases underdiagnosed at CNB, avoiding excisional surgical biopsy. The latter is still recommended when an elevated suspicion at imaging persists despite a benign result at US-VABB. Our results highlight the importance of rad-path correlation by breast radiologists.
**Limitations:** Small sample.

**Ethics committee approval:** This study was approved by an ethics committee.
**Funding for this study:** No funding was received for this study.
**Author Disclosures:**
Rubina Manuela Trimboli: Nothing to disclose
Paolo Malerba: Nothing to disclose
Daniela Bernardi: Nothing to disclose
Gabriele Levrini: Nothing to disclose
Giulia Vatteroni: Nothing to disclose
Isabella Bolengo: Nothing to disclose

### RPS 2102-6

**Marking of metastatic axillary node and BIRADS 6 lesion with an iodine-125-radiolabelled seed in breast cancer patients who received neoadjuvant chemotherapy**

*F. J. Pérez García*, A. J. Ariza Sánchez, D. Luengo Gómez, M. J. Rabaza Espigares, S. Martínez Meca, R. Sánchez Sánchez, I. Mendoza Arnau; Granada/ES
*(javipegarcia@gmail.com)*

**Purpose:** (1) To determine if the iodine-125 (I-125) marked metastatic biopsy-confirmed lymph node (MN) during axillary staging in invasive breast cancer patients eventually matches with the sentinel node (SN) and (2) to assess the potential benefits of I-125 marking of BIRADS 6 lesions.
**Methods or Background:** Between May 2018 to July 2021, 82 biopsy-confirmed invasive breast cancer with positive axillary node patients who were candidates for neoadjuvant chemotherapy were recruited. In a prospective design, we performed I-125 seed marking of both the positive node and the BI-RADS 6 lesion.
**Results or Findings:** The MN was localised during the surgical procedure in 80/82 patients (97,5%) while the SN was localised in 79/82 patients (96,34%). In 62 of 80 patients, the MN matched with the SN. 34 (54,83%) of these cases were reported as negative while 28 (45,6%) were reported as positive. Pathologic analysis of the MN correctly predicted the axillary post-neoadjuvant status in 98,75% of cases. A metallic marker was used to track the confirmed BIRADS 6 lesion in 15 patients, while in 67 of them a I-125-radiolabelled seed was placed instead. This avoided the posterior marker retrieval procedure when there was a complete radiological response.
**Conclusion:** (1) I-125 node marking in combination with a sentinel lymph node biopsy (SNLB) is a feasible technique in the intraoperative setting. Pathological assessment of the MN allows the determination of the axillary post-neoadjuvant status, thus potentially avoiding lymphadenectomy and decreasing false negatives of SLNB after neoadjuvant chemotherapy. (2) I-125 marking of BIRADS 6 lesions allows avoiding the posterior marker retrieval procedure in the case of a complete radiological response.
**Limitations:** Not applicable.
**Ethics committee approval:** The Nuclear Security Council and the ethics comittee approved this study. Written informed consent was obtained.
**Funding for this study:** No funding was received for this work.
**Author Disclosures:**
Salvador Martínez Meca: Nothing to disclose
Manuel Jesus Rabaza Espigares: Nothing to disclose
Francisco Javier Pérez García: Nothing to disclose
Rocío Sánchez Sánchez: Nothing to disclose
Adolfo Jesús Ariza Sánchez: Nothing to disclose
Inmaculada Mendoza Arnau: Nothing to disclose
David Luengo Gómez: Nothing to disclose

### RPS 2102-7

**Reduction of breast scar marker-induced metal artefacts in CT simulation: a preliminary study**

Z. ALMandhari, *H. Sulaiman*, M. ALGhafri, N. Babu, I. ALAmri; Muscat/OM
*(h.alsiyabi@cccrc.gov.om)*

**Purpose:** To develop a streaking artefact-free breast scar marker during CT simulation in radiation therapy using playdough.
**Methods or Background:** Identifying breast scar areas is crucial for an accurate delineation of the tumour lesions in CT simulation radiation therapy. Most of the CT marking wires are made of highly dense materials that cause metallic and streaking artefacts. Some CT markers are made of inflexible wires and could result in inaccuracies when marking scar borders over or under the irradiated areas. The treatment planning system may induce calculation errors because of the presence of artefacts.
Materials and methods: CT simulation images were acquired on the Siemens SOMATOM scanner using a 5 cm thick block of tissue equivalent plastic using playdough material shaped with a syringe as a cylindrical form of 2 mm x 2 mm x 30 mm (with different orientations). A multidetector spiral CT scan was performed using a collimation of 1.2 mm, 16 rows at 120 kVp and 15 mAs, as per local breast protocol. The resulted images were evaluated qualitatively (by visual assessment) and quantitatively by means of wire dimensions and Hounsfield units (HU).
**Results or Findings:** Playdough markers were clearly seen in CT images, as judged by two experienced radiation oncologists. No streaking and metallic artefacts were detected in the images. The quantitative assessment shows the same original wire dimensions (±0.2) mm and 300 (±5) HU of the playdough material.
**Conclusion:** The preliminary findings of this study are promising. They demonstrated that playdough material, as a tissue marker, is clearly visible in CT images without streaking or metallic artefacts. Further evaluation is required, performed prior to implementation in a clinical routine.
**Limitations:** Limited sample size.
**Ethics committee approval:** This study was approved by an ethics committee.
**Funding for this study:** No funding was received for this study.
**Author Disclosures:**
Mohammed ALGhafri: Nothing to disclose
Zahid ALMandhari: Nothing to disclose
Hajir Sulaiman: Nothing to disclose
Iqpal ALAmri: Nothing to disclose
Nirmal Babu: Nothing to disclose

### RPS 2102-8

**Efficacy and safety of gadopiclenol for body magnetic-resonance imaging (MRI): the PROMISE trial**

*C. K. Kuhl*; Aachen/DE
*(ckuhl@ukaachen.de)*

**Purpose:** Gadopiclenol is a new GBCA with high relaxivity, developed by Guerbet and currently under review by regulatory authorities. The PROMISE trial was designed to demonstrate the non-inferiority of gadopiclenol at 0.05 mmol/kg to gadobutrol at 0.1 mmol/kg for contrast-enhanced body MRI and its superiority to unenhanced MRI.
**Methods or Background:** This international, randomised, double-blind, controlled, cross-over study included patients with lesions in different body regions (head and neck, thorax, abdomen, pelvis and musculoskeletal). Patients (N=304) were randomised to undergo two MRIs (2 14 days interval), first with gadopiclenol, then gadobutrol, or vice versa. The primary criterion was lesion visualisation based on 3 parameters (border delineation, internal morphology and degree of contrast enhancement), assessed by 3 independent blinded readers by main body region. Overall diagnostic preference was assessed in a global matched-pairs fashion by 3 additional blinded readers. Adverse events (AEs) were collected up to one day post-second MRI.
**Results or Findings:** For all readers, and all visualisation co-criteria, the difference in mean of scores showed the non-inferiority of gadopiclenol to gadobutrol (lower limit 95%CI ≥-0.10, above the non-inferiority margin [-0.35]; p<0.0001) and its superiority over unenhanced images (p<0.0001).
For overall diagnostic preference, readers reported no preference in 74.6%-82.6% of the evaluations, preferred images with gadopiclenol in 12%-14.5% of the evaluations and gadobutrol in 5.4%-10.9% of the evaluations.
AEs were reported similarly after MRI with gadopiclenol (18.1%) and gadobutrol (20.0%). AEs were considered related to gadopiclenol for 12 patients (4.2%) and to gadobutrol for 16 patients (5.5%), mostly injection-site reactions and none serious.
**Conclusion:** MRI with gadopiclenol at 0.05 mmol/kg is non-inferior to gadobutrol at 0.1 mmol/kg for lesion visualisation in different body regions. Gadopiclenol showed a good safety profile.
**Limitations:** Diagnostic performance not evaluated.
**Ethics committee approval:** Obtained.
**Funding for this study:** Sponsored by Guerbet.
**Author Disclosures:**
Christiane K. Kuhl: Nothing to disclose

16:00-17:30 Room E1

### Research Presentation Session: Genitourinary

### RPS 2107

### Uterine masses: benign or invasive - is it always that easy?

**Moderator**
M. Basta Nikolic; Novi Sad/RS

### RPS 2107-2

**Using amide proton transfer-weighted MRI to non-invasively differentiate mismatch repair deficient and proficient tumours in endometrioid endometrial adenocarcinoma**
*X. Liu*, Y. He, Y. Li, X. Wang, H. Xue, Z. Jin; Beijing/CN

**Purpose:** To investigate the utility of three-dimensional (3D) amide proton transfer-weighted (APTw) imaging to differentiate mismatch repair deficient (dMMR) and mismatch repair proficient (pMMR) tumours in endometrioid endometrial adenocarcinoma (EEA).
**Methods or Background:** Forty-nine patients with EEA underwent T1-weighted imaging, T2-weighted imaging, 3D APTw imaging, and diffusion-weighted imaging at 3 T MRI. Image quality and measurement confidence of APTw images were evaluated on a 5-point Likert scale. APTw and apparent diffusion coefficient (ADC) values were calculated and compared between the dMMR and pMMR groups and among the three EEA histologic grades based on the Federation of Gynecology and Obstetrics (FIGO) grading system criteria. Student's t-test, analysis of variance with Scheffe post hoc test, and receiver operating characteristic analysis were performed. Statistical significance was set at $p<0.05$.
**Results or Findings:** Thirty-five EEA patients (9 with dMMR tumours and 26 with pMMR tumours) with good image quality were enrolled in quantitative analysis. APTw values were significantly higher in the dMMR group than in the pMMR group (3.2±0.3% and 2.8±0.5%, respectively; p=0.019). ADC values of the dMMR and pMMR groups were 0.874±0.104 × 10 – 3 mm2/s and 0.903±0.100 × 10 – 3 mm2/s, respectively. No significant intergroup difference was noted (p=0.476). No statistically significant differences were observed in APTw values or ADC values among the three histologic grades (p = 0.766 and p = 0.295, respectively).
**Conclusion:** APTw values may be used as potential imaging markers to differentiate dMMR from pMMR tumors in EEA.
**Limitations:** This study was a singlecentre analysis, besides, improvements in the pelvic APTw sequence are warrented.
**Ethics committee approval:** The local ethics committee has approved the project and all participants signed informed consent.
**Funding for this study:** Funding was received from the Natural Science Foundation of China (grant No. 81901829).
**Author Disclosures:**
Zhengyu Jin: Nothing to disclose
Huadan Xue: Nothing to disclose
Yonglan He: Nothing to disclose
Xinyu Liu: Nothing to disclose
Yuan Li: Nothing to disclose
Xiaoqi Wang: Employee: Philips Healthcare China

### RPS 2107-3

**Magnetic resonance imaging evaluation of adenomyosis rat model constructed by autologous endometrial implantation**
*Q. Zhang*, Y. Li, S. Wu, J. Zhang; Shanghai/CN

**Purpose:** To explore the feasibility of establishing an adenomyosis rat model by endometrial implantation and the application value of magnetic resonance imaging (MRI) in evaluating an adenomyosis rat model.
**Methods or Background:** 20 female rats were divided into control group and model group randomly. In the modelling group, one side of uterus of every rat was ligated and intercepted, and the endometrium was scraped and mixed with normal saline, which was evenly injected into the contralateral myometrium with a syringe. In the control group, one side of the rat uterus was ligated and intercepted in the same way, and normal saline was uniformly injected into the contralateral myometrium. Two months after modelling, the rats were examined by MRI, including T2-weighted axial and T1-weighted axial sequences. And the thickness of the uterus was measured. After MRI scanning, the uterus of the rats was taken for hematoxylin-eosin staining. Statistical analysis was performed via paired-samples t-test.
**Results or Findings:** In the modelling group, adenomyosis lesions were successfully formed in all rats with a success rate of 100%. MRI showed that the myometrium of rats in the modelling group was thickened with multiple hyperintense foci. The average thickness of uterine in the modelling group was higher than that in the control group, and the difference was statistically significant.
**Conclusion:** The autologous endometrial implantation method is feasible to construct a rat model of adenomyosis. Adenomyosis in rats can be clearly demonstrated by MRI, and adenomyosis in rats has similar imaging characteristics to that in humans.
**Limitations:** In this study, conventional MRI sequences were used to evaluate adenomyosis in rats without related functional sequences.
**Ethics committee approval:** This study was approved by the Animal Ethics Committee of Fudan University.
**Funding for this study:** This study was funded by the horizontal 046 project of Huashan Hospital.
**Author Disclosures:**
Junhai Zhang: Nothing to disclose
Qi Zhang: Nothing to disclose
Yajie Li: Nothing to disclose
Shiman Wu: Nothing to disclose

### RPS 2107-4

**Preoperative tumour texture analysis on MRI predicts high-risk disease in endometrial cancer**
*M. Lupinelli*, M. Miccò, B. Gui, L. Russo, L. Boldrini, M. Mangialardi, F. Fanfani, V. Pignatelli, R. Manfredi; Rome/IT
*(michela.lupinelli@gmail.com)*

**Purpose:** To evaluate the association between magnetic resonance (MR) imaging–based texture features and deep myometrial invasion (DMI), lymphovascular space invasion (LVSI) and different classes of risk (according to the risk class model proposed by the ESMO-ESGO-ESTRO Consensus Conference in 2016) in endometrial cancer (EC).
**Methods or Background:** Improved methods for preoperative risk stratification in EC are highly requested by clinicians. Imaging texture analysis is a promising diagnostic tool in various cancer types, but not largely explored in EC. This study retrospectively included 108 women with EC who underwent 1.5-T MR imaging before hysterectomy from April 2009 to May 2019. Texture features were extracted using the MODDICOM library with manual delineation of a region of interest around the whole tumour on MR images (axial T2-weighted). ROC curve was used to evaluate the diagnostic performance of training and validation sets by using a subset of the most relevant texture features tested individually in univariate analysis using Wilcoxon–Mann–Whitney.
**Results or Findings:** A total of 228 radiomics features were extracted and ultimately limited to 38 for DMI, 29 for LVSI, and 15 for risk class data sets. Accuracy, sensitivity, specificity, positive predictive value and negative predictive value were estimated at 0.78, 0.67, 0.89, 0.86 and 0.72 for DMI (AUC 0.845); 0.89, 1.00, 0.77, 0.81 and 1.00 for LVSI (AUC 0.925); and 0.86, 0.64, 0.93, 0.73 and 0.89 for prediction of low-risk vs others (intermediate/intermediate to high/high), respectively (AUC 0.837). Radiomic models showed good predictive performance in validation cohort (AUC 0.683 - 0.769).
**Conclusion:** An MRI-based radiomic model has great potential in developing advanced predictive performance in endometrial cancer.
**Limitations:** The sample study could be enlarged.
**Ethics committee approval:** The research was approved by the institutional ethics committee.
**Funding for this study:** Not applicable.
**Author Disclosures:**
Maura Miccò: Nothing to disclose
Vincenza Pignatelli: Nothing to disclose
Francesco Fanfani: Nothing to disclose
Luca Boldrini: Nothing to disclose
Benedetta Gui: Nothing to disclose
Michela Lupinelli: Nothing to disclose
Riccardo Manfredi: Nothing to disclose
Luca Russo: Nothing to disclose
Matteo Mangialardi: Nothing to disclose

### RPS 2107-5

**MRI staging of endometrial cancer: is there any room for improvement?**
*T. Neves*, M. Correia, A. P. Caetano, T. M. Cunha; Lisbon/PT

**Purpose:** To evaluate the utility of fusing T2-Weighted Images (T2WI) with Diffusion-Weighted Images (DWI) for evaluation of myometrial invasion depth with relevance in endometrial cancer staging and treatment planning.
**Methods or Background:** The authors conducted a retrospective, singlecentre study, which included 87 patients that had surgically confirmed primary endometrial cancer and had undergone preoperative pelvic MRI. Patients submitted to neoadjuvant therapy, with a history of pelvic neoplasia or treated with radiotherapy were excluded. All exams were read by an experienced radiologist dedicated to urogenital radiology and the depth of myometrial invasion was evaluated using T2WI and fused T2WI-DWI images. Both results were compared to postsurgical histopathological analysis.
**Results or Findings:** When comparing both sets of imaging (T2WI and fused T2WI-DWI images), fused images had better accuracy in diagnosing deep myometrial invasion and this difference was statistically significant (p<0.001). T2WI analysis correctly diagnosed 82.1% (70.6 - 88.7) of cases, against 92.1% correctly diagnosed with fused images (79.5 - 97.2).
**Conclusion:** Although the accuracy of detecting myometrial invasion depth with classic sequences is high, this work shows a statistically significant difference when comparing the accuracy of fused images with standard evaluation, allowing for a better diagnosis with a post-processing technique that does not add acquisition time to MRI evaluation.
**Limitations:** One limitation is related to the design of the study, since it is a retrospective, singlecentre study. Another limitation was the absence of randomisation in the analysis of T2WI images, functional sequences, and then the fused images (all morphological images were first evaluated, followed by DWI and then fused images, in that order). This could introduce some learning bias. However, within each group of studies analysed (T2WI or fused images) randomisation was ensured.
**Ethics committee approval:** This study was approved by the institutional review board.
**Funding for this study:** No funding was received for this study.
**Author Disclosures:**
Teresa Neves: Nothing to disclose
Teresa Margarida Cunha: Nothing to disclose
António Proença Caetano: Nothing to disclose
Mariana Correia: Nothing to disclose

### RPS 2107-7

**Early regression index (ERI) as response predictor to neoadjuvant chemoradiotherapy in locally advanced cervical cancer**

*S. Persiani*, L. Russo, B. Gui, M. Miccò, D. Cusumano, L. D'Erme, G. Mazzotta, R. Autorino, R. Manfredi; Rome/IT
*(salvatorepersiani@gmail.com)*

**Purpose:** Early Regression Index (ERI) is an image-based biomarker that reported interesting results in predicting pathological complete response (pCR) after neoadjuvant chemoradiotherapy (nCRT) in case of rectal cancer.
Such a parameter is generally calculated on T2-weighted MR images and consists in modelling the early tumour regression combining the tumour volume GTV volume measured at staging (Vpre) and at mid therapy (Vmid). This study aims to evaluate the feasibility of ERI in predicting pathological complete response (pCR) in Locally Advanced Cervical Cancer (LACC), starting from T2-weighted and diffusion weighted images (DWI).
**Methods or Background:** 88 patients affected by LACC (FIGO IB2-IVA) underwent 1.5 T MRI at two stages (before treatment and at mid therapy). GTV was delineated on axial T2-WI and DWI and ERI was calculated for both. Radical hysterectomy was performed for each patient within 8 weeks after nCRT: pCR was considered in case of absence of any residual tumour cells at any site (pR0). The ERI performance in identifying pCR patients was quantified calculating the area (AUC) under the Receiver Operating Characteristic (ROC) curve and measuring sensitivity and specificity at the best threshold value.
**Results or Findings:** The performance of ERI_DWI (AUC=0.81; CI 95% 0.70-0.91) are superior to those reported by ERI_T2 (AUC=0.76 with 95% CI 0.65-0.87). At the best cut-off threshold, ERI_T2 shows high specificity (97.4%) with low sensitivity (43%), while ERI_DWI high sensitivity (86.5%) and limited specificity (64.1%).
**Conclusion:** ERI is a good biomarker in case of LACC, especially if calculated considering DWI. Using this indicator, it is possible to identify non-responders early and modify the treatment accordingly.
**Limitations:** No external validation cohort was available.
**Ethics committee approval:** The workflow presented was approved by the institutional ethical committee.
**Funding for this study:** No funding was received for this study.
**Author Disclosures:**
Rosa Autorino: Nothing to disclose
Maura Miccò: Nothing to disclose
Davide Cusumano: Nothing to disclose
Giorgio Mazzotta: Nothing to disclose
Benedetta Gui: Nothing to disclose
Riccardo Manfredi: Nothing to disclose
Salvatore Persiani: Nothing to disclose
Luca D'Erme: Nothing to disclose
Luca Russo: Nothing to disclose

### RPS 2107-8

**Value of MRI in parametrial invasion assessment and association with prognosis in locally advanced cervical cancer: preliminary results**

*M. Lupinelli*[1], L. Russo[1], B. Gui[1], A. Urbano[2], M. Mangialardi[1], S. Persiani[1], T. Pasciuto[1], M. G. Ferrandina[1], R. Manfredi[1]; [1]Rome/IT, [2]Verona/IT
*(michela.lupinelli@gmail.com)*

**Purpose:** Parametrial invasion (PI) is an important prognostic factor in locally-advanced cervical cancer (LACC). To our knowledge, impact of PI on prognosis has not been investigated. The aim of this study is to evaluate the association between PI degree and prognosis, in terms of disease-free survival (DFS) and overall survival (OS).
**Methods or Background:** From 2005 to 2019, 228 patients with a pathological diagnosis of LACC undergoing neoadjuvant chemo-radiotherapy (CRT) followed by radical hysterectomy were enrolled. Staging MRI were retrospectively evaluated. Maximum PI (PImax) and parametrial length were quantitatively evaluated in millimetres, bilaterally. Then, the ratio between PImax and parametrial length was calculated (PIratio). All the analysis were performed in homogeneous subsets grouping patients according to pathological lymph nodal assessment (N- and N+) and for each patient according to the worst PI. OS and DFS curves were estimated by Kaplan-Meier product limit method. In both groups (N- and N+), correlation of PImax and PIratio with prognosis was performed using univariable Cox regression analysis provided both in terms of DFS and OS.
**Results or Findings:** Out of 228 patients, 128 (56%) patients had nonmetastatic lymph nodes (N-) and 100 (44%) had metastatic lymph nodes (N+). The median follow-up of all patients was 73 months (95% Confidence Interval (CI): 66-77). The probability of DFS and OS at five years from diagnosis were respectively 74.4% and 84.8% in N-group and 51.8% and 71.9% in N+ group. Greater PImax (hazard ratio [HR] = 1.10) and PIratio (HR=1.05) were associated with poorer overall survival in the N-group (P=0.011 and P=0.024). Greater PImax (HR=1.07) was also associated with earlier recurrence (P=0.037). These parameters did not show statistically significant correlation in the N+ group.
**Conclusion:** PImax and PIratio affect survival of N- patients with LACC.
**Limitations:** The retrospective nature of the study was identified as a limitation.
**Ethics committee approval:** The research was approved by the institutional ethics committee.
**Funding for this study:** Not applicable
**Author Disclosures:**
Tina Pasciuto: Nothing to disclose
Benedetta Gui: Nothing to disclose
Michela Lupinelli: Nothing to disclose
Riccardo Manfredi: Nothing to disclose
Salvatore Persiani: Nothing to disclose
Maria Gabriella Ferrandina: Nothing to disclose
Luca Russo: Nothing to disclose
Matteo Mangialardi: Nothing to disclose
Alessandra Urbano: Nothing to disclose

### RPS 2107-9

**Validation of a qualitative magnetic resonance imaging-based method to predict placenta accreta spectrum disorders in patients with placenta previa**

*F. Verde*, S. Maurea, P. P. Mainenti, L. Barbuto, F. Iacobellis, V. Romeo, R. Liuzzi, L. Romano, A. Brunetti; Naples/IT
*(francescoverde87@gmail.com)*

**Purpose:** To externally validate a qualitative magnetic resonance imaging (MRI) method to predict placental accreta spectrum (PAS) in patients with placenta previa (PP).
**Methods or Background:** Two qualitative MRI-based methods in interpreting PAS-related MRI findings were developed in our previous experience consisting of the presence of at least one (Method 1) or two (Method 2) of the following abnormal MRI signs: intraplacental dark bands, focal interruption of myometrial border and abnormal vascularity. These two methods were tested in an external cohort of 65 patients with PP and clinical suspicion of PAS. Placental MRI evaluation was performed by three groups of radiologists with different levels of expertise. Group 1 included radiologists with at least 5 years of experience in body diagnostic imaging who routinely evaluated MR images on the basis of the qualitative presence of common abnormal signs associated with PAS. Group 2 and 3 respectively consisted of two couples of board-certified radiologists with at least 10 and 20 years of experience in genito-urinary MRI which used both Methods 1 and 2 for imaging evaluation.
**Results or Findings:** A significant (p<0.0001) difference was found between AUC values of imaging evaluation performed by radiologists of Group 3 using Method 1 (0.71) and Method 2 (0.89). The accuracy of Method 2 by radiologists of Group 3 was also significantly (p<0.0001) higher compared to that of both Methods 1 (0.61) and 2 (0.69) by radiologists of Group 2 as well as to that of the routine evaluation by radiologists of Group 1 (0.64).

**Conclusion:** The identification of at least two abnormal MRI signs seems to be an accurate method for predicting PAS in patients with PP particularly when this method is used by more experienced radiologists.
**Limitations:** The retrospective nature of the study and the sample size were identified as limiting factors.
**Ethics committee approval:** Not applicable
**Funding for this study:** Not applicable
**Author Disclosures:**
Francesca Iacobellis: Nothing to disclose
Valeria Romeo: Nothing to disclose
Raffaele Liuzzi: Nothing to disclose
Francesco Verde: Nothing to disclose
Pier Paolo Mainenti: Nothing to disclose
Luigia Romano: Nothing to disclose
Luigi Barbuto: Nothing to disclose
Arturo Brunetti: Nothing to disclose
Simone Maurea: Nothing to disclose

### RPS 2107-10

**Abnormal cord coiling: a harbinger of adverse pregnancy outcome?**
*A. Khandelwal*, M. K. Mittal, P. Mittal; New Delhi/IN
*(ayush7khandelwal@gmail.com)*

**Purpose:** Evaluation of antenatal umbilical cord coiling index as predictor of pregnancy outcome
**Methods or Background:** After written informed consent, 200 singleton pregnant women between 18-24 weeks' gestation with amniotic fluid index between 8-24, three umbilical cord vessels and no medical comorbidities were enrolled. Longitudinal view of umbilical cord showing at least two complete segments (two arteries and one vein), excluding the two extremes was acquired. Distance between the coils was measured from inner edge of a vessel wall to outer edge of the next coil along the ipsilateral side of cord. Antenatal umbilical cord coiling index (UCI) was calculated as reciprocal of distance between a pair of coils in centimetres. 10th and 90th centile values were considered as cut offs for low and high estimates. Patients were followed up till delivery and pregnancy outcomes like gestational age at birth, mode of delivery, meconium stained liquor, birth weight, 5 min Apgar score and NICU admission were noted.
**Results or Findings:** Normal UCI was in the range of 0.43 - 0.75 with abnormal UCI seen in 34 out of 200 patients. Significant association was found between UCI and gestational age (p=0.008), mode of delivery (p=0.010), birth weight (p<0.0001) and adverse pregnancy outcomes (p<0.0001). Hypocoiling showed a significant association with preterm delivery, low birth weight and adverse pregnancy outcome while hypercoiling was significantly associated with low birth weight, Lower Segment Caesarean Section (LSCS) and adverse pregnancy outcome.
**Conclusion:** We found high specificity and negative predictive value for abnormal UCI in predicting low birth weight, LSCS, preterm gestation, indicating that a normal UCI can quite confidently rule out adverse pregnancy outcomes.
**Limitations:** This was a singlecentre study with a small sample size.
**Ethics committee approval:** Institutional ethical clearance was granted.
**Funding for this study:** No funding was received for this study.
**Author Disclosures:**
Pratima Mittal: Nothing to disclose
Ayush Khandelwal: Nothing to disclose
Mahesh Kumar Mittal: Nothing to disclose

### RPS 2107-11

**Investigation of correlation between number of echolucent zones with/without colour flow and morbidly adherent placenta**
*B. Aminzadeh*, F. Tara, N. Saghafi, M. Emadzadeh, F. Sadeghi Ardakani; Mashhad/IR
*(aminzadeh.b@gmail.com)*

**Purpose:** The accuracy of ultrasound indices in predicting the incidence of morbidly adherent placenta is still under investigation. In this study, we evaluated the sensitivity and specificity of different colour Doppler and grayscale ultrasound quantitative indices in predicting morbidly adherent placenta.
**Methods or Background:** All pregnant women over 20 weeks of gestational age having a history of at least one previous caesarean section and the presence of anterior placenta were assessed for inclusion in this research. Number of intraplacental echolucent zones (grayscale) and echolucent zones with colour flow in colour Doppler ultrasound were counted in all patients. The nonparametric receiver operating characteristic (ROC) curves, the areas under the curve (AUC), and cut-off value were also evaluated.
**Results or Findings:** A total of 120 patients were finally included in analysis, fifteen of whom had morbidly adherent placenta and the rest were normal. Based on colour Doppler ultrasonography, more than two intraplacental echolucent zones with colour flow had respective sensitivity and specificity of 93% and 98% in predicting the morbidly adherent placenta. Based on grayscale ultrasonography, more than thirteen intraplacental echolucent zones had respective sensitivity and specificity of 86% and 80% for diagnosis of morbidly adherent placenta.
**Conclusion:** Ultrasound quantitative criteria, including the number of the echolucent zones with/without colour flow showed significant sensitivity and specificity in predicting and diagnosing morbidly adherent placenta.
**Limitations:** The low number of cases with morbidly adherent placenta was the main limitation of our study, further studies with larger samples size are recommended.
**Ethics committee approval:** The ethics committee and our institutional review board approved the study (IR.MUMS.MEDICAL.REC.1398.220).
**Funding for this study:** Mashhad University of medical sciences financially supported this study.
**Author Disclosures:**
Fatemeh Sadeghi Ardakani: Nothing to disclose
Behzad Aminzadeh: Nothing to disclose
Nafiseh Saghafi: Nothing to disclose
Maryam Maryam Emadzadeh: Nothing to disclose
Fatemeh Tara: Nothing to disclose

### RPS 2107-12

**Comparing diagnostic accuracy and diagnostic confidence in the MRI interpretation of placenta accreta spectrum disorders before and after implementation of SAR/ESUR consensus framework**
*A. Khurana*[1], E. Chishti[1], L. Nelson[1], H. Kapoor[2], J. Owen[1], R. Nair[1], R. Kryscio[1], F. Chapelin[1], J. Lee[1]; [1]Lexington, KY/US, [2]Dallas, TX/US
*(draman.rad@gmail.com)*

**Purpose:** To determine if an educational intervention with the 2020 SAR/ESUR consensus article improves diagnostic efficiency and confidence of abdominal radiologists interpreting placenta accreta spectrum (PAS) MRIs.
**Methods or Background:** Three experienced radiologists blindly reviewed 20 MRIs (10 normal, 10 PAS: 1 accreta, 5 increta and 4 percreta), assigned them a diagnosis, and rated their confidence level. Afterwards, readers were provided with the SAR/ESUR consensus article detailing the 7 recommended MRI signs as well as an illustrated memory aid (intervention). A 6 – 8-week gap was employed in an effort to reduce memory bias. The readers reassessed the same set of MRIs, first objectively looking for the presence/absence of each sign, and then assigning a PAS diagnosis and re-rating confidence level. If the pre-intervention diagnosis was incorrect, change-to-correct (C2C) diagnosis or change-to-near-correct (C2NC) diagnosis was assessed. If the original diagnosis was correct, change in diagnostic confidence (CDC) was measured. Pairwise comparisons were conducted using multiple Fisher's exact tests.
**Results or Findings:** Only 1 of the 3 radiologists had significant change to correct diagnosis (33% C2C; p=0.031), significant change to near-correct diagnosis (33% C2NC, p=0.0156), but overall there was increased confidence/CDC for all radiologists (92.8% of correct cases). When comparing according to pathology, MRI interpretation of placenta increta has the largest impact after intervention (C2NC - p=0.0078; CDC - p=0.0013).
**Conclusion:** Prenatal radiological diagnosis of PAS disorders is vital for optimising antenatal care and preventing life-threatening complications. Standardised interpretation and reporting by incorporating the consensus recommended MRI signs can improve both accuracy and confidence in the PAS MRI diagnosis.
**Limitations:** To ensure uniformity in study protocol and quality, only a small single institutional sample was used. Small reader sample allowed only intrareader and precluded interreader assessment. A larger scale multicentre study is warranted.
**Ethics committee approval:** The IRB approved this study.
**Funding for this study:** No funding was received for this study.
**Author Disclosures:**
Fanny Chapelin: Nothing to disclose
Emad Chishti: Nothing to disclose
James Lee: Nothing to disclose
Richard Kryscio: Nothing to disclose
Harit Kapoor: Nothing to disclose
Rashmi Nair: Nothing to disclose
Leslie Nelson: Nothing to disclose
Aman Khurana: Nothing to disclose
Joseph Owen: Nothing to disclose

16:00-17:30 Room F1

### Research Presentation Session: Cardiac

### RPS 2103

### Coronary artery CT and myocardial perfusion

**Moderator**
M. Hrabak Paar; Zagreb/HR

### RPS 2103-2

**National trends in coronary computed tomography angiography usage: associations with invasive coronary angiography usage and healthcare costs**

*J. R. Weir-Mccall*[1], M. C. Williams[2], A. Shah[3], G. H. Roditi[4], J. Rudd[1], D. Newby[2], E. Nicol[3]; [1]Cambridge/UK, [2]Edinburgh/UK, [3]London/UK, [4]Glasgow/UK
*(jw2079@cam.ac.uk)*

**Purpose:** In 2016 the updated National Institute for Health and Care Excellence chest pain guidelines (CG95) recommended coronary computed tomography angiography (CTA) as the most cost-effective first line imaging test for the investigation of coronary artery disease. We sought to determine the impact of CG95 on the temporal trends of coronary CTA use, its association with invasive coronary angiography (ICA) usage, and healthcare costs.
**Methods or Background:** Investigations for coronary artery disease from 2012-2018 were extracted for the 42 service providers within the National Health Service of England. Annual growth rates were calculated and adjusted for population sizes. The impact of CG95 was assessed using an interrupted time-series analysis. Changes in ICA usage were compared between tertiles of CTA growth. Healthcare costs were calculated using national healthcare tariffs.
**Results or Findings:** From 2012 to 2018, 1,909,314 investigations for coronary artery disease were performed, with an annualised per capita growth rate of 4.8%. CTA use grew over the study period, while perfusion imaging fell. CG95 was associated with a rise in CTA use (exp(β) 1.10 [95% confidence interval 1.03 to 1.18], p=0.006). High ICA growth was seen in the regions with the lowest growth in CTA (12.25 [95% CI 5.09 to 15.46] angiograms/100,000 population/year) whereas ICA usage fell in those with the highest CTA growth (-1.13 [95% CI -7.07 to 5.71] angiograms/100,000 population/year). Annual healthcare spending for coronary imaging was £0.36 million/100,000 population/year, with an average annual change of -0.8%.
**Conclusion:** Imaging investigations for coronary artery disease are increasing, with the greatest growth seen in coronary CTA following CG95. Greater regional increases in coronary CTA were associated with fewer ICA. Overall healthcare costs for the chest pain pathway remained stable despite the growth in imaging.
**Limitations:** The study was observational in nature.
**Ethics committee approval:** Ethics committee approval was waived.
**Funding for this study:** No funding was received for this study.
**Author Disclosures:**
Anoop Shah: Nothing to disclose
Edward Nicol: Nothing to disclose
Giles Hannibal Roditi: Nothing to disclose
David Newby: Nothing to disclose
Jonathan R. Weir-Mccall: Nothing to disclose
James Rudd: Nothing to disclose
Michelle Claire Williams: Nothing to disclose

### RPS 2103-4

**Feasibility and diagnostic performance of a new CCTA-derived and AI-based fully automated system for detection of coronary artery disease**

*V. Brandt*[1], J. O'Doherty[2], U. J. Schoepf[1], A. Varga-Szemes[1], T. S. Emrich[1], C. Tesche[3], G. Aquino[1], B. Yacoub[1], J. A. Decker[4]; [1]Charleston, SC/US, [2]Malvern, PA/US, [3]Munich/DE, [4]Augsburg/DE
*(verena.brandt@gmx.net)*

**Purpose:** To evaluate a novel CCTA-derived fully automated artificial intelligence (AI)-based software solution for automated coronary artery segmentation and stenosis assessment using the Coronary Artery CAD-RADS.
**Methods or Background:** Image data sets of 100 patients (48% male, 48.3±10.8 years) who underwent clinically indicated CCTA were retrospectively analysed. Two readers independently evaluated CCTAs for the degree of coronary artery stenosis on a per-segment level using the 18-coronary artery segment model with subsequent CAD-RADS classification according to SCCT guidelines. A fully automated investigational AI-based software prototype by Siemens was designed and tested on the CCTA data sets and compared to human reading. Interreader agreement was assessed using Cohen's kappa. Subsequently, the diagnostic performance of the software prototype for detection of diseased coronary artery segments was assessed.
**Results or Findings:** Forty-one patients had CAD with stenosis in at least one segment. Agreement between expert readers was 0.83 for CAD-RADS and 0.89 for the identification of diseased segments. The software prototype yielded a sensitivity of 97.6% (92.8 - 100), and a negative predictive value of 96.9% (90.8 - 100) for the detection of diseased segments, respectively. The software prototype reliably detected 40 out of 41 patients with CAD. One patient who was not correctly identified had a small, calcified plaque without associated coronary artery stenosis (CAD-RADS 1). The average computational time of the software prototype was 240s per case.
**Conclusion:** The fully automated investigational AI-based software prototype demonstrated fast and reliable identification of patients with coronary artery stenosis on CCTA with high diagnostic accuracy.
**Limitations:** This was a retrospective, single-centre study with a limited number of patients. CT scans with insufficient image quality were excluded. CAD-RADS analysis by the software prototype was carried out without a vessel diameter cut-off.
**Ethics committee approval:** The protocol of this study was approved by the Institutional Review Board.
**Funding for this study:** No funding was received for this study.
**Author Disclosures:**
Verena Brandt: Nothing to disclose
Christian Tesche: Speaker: Siemens Healthineers and Heartflow Inc.
Jim O'Doherty: Employee: Siemens Healthineers
Uwe Joseph Schoepf: Speaker: Bayer, Bracco, Elucid BioImaging, General Electric, Guerbet, HeartFlow Inc., Keya Medical, and Siemens Healthineers Research/Grant Support: Bayer, Bracco, Elucid BioImaging, General Electric, Guerbet, HeartFlow Inc., Keya Medical, and Siemens Healthineers Consultant: Bayer, Bracco, Elucid BioImaging, General Electric, Guerbet, HeartFlow Inc., Keya Medical, and Siemens Healthineers
Tilman Stephan Emrich: Speaker: Siemens Healthcare
Basel Yacoub: Nothing to disclose
Josua A. Decker: Nothing to disclose
Akos Varga-Szemes: Research/Grant Support: Siemens Healthcare Consultant: Bayer and Elucid Bioimaging
Gilberto Aquino: Nothing to disclose

### RPS 2103-5

**Image quality from super resolution deep learning reconstruction on coronary CT angiography**

*J. Schuzer*[1], J. Zhou[1], L. Cai[1], M. Matsuura[2], T. Nemoto[2], H. Taguchi[2], Y. Noshi[2], M. Y. Chen[3]; [1]Vernon Hills, IL/US, [2]Otawara/JP, [3]Bethesda, MD/US

**Purpose:** To evaluate image quality from super resolution deep learning reconstruction (PIQE) trained to increase spatial resolution.
**Methods or Background:** A deep convolution neural network-based super resolution deep learning reconstruction (PIQE) was developed where ultra-high resolution CT scanner (Canon Aquilion Precision) acquired high dose data represented the training target and low dose normal resolution data were used as the training input. This super resolution neural network was optimised through different doses in the learning process. PIQE was applied on normal resolution CT data for achieving resolution restored low noise CT data from 52 coronary CT scans (Canon Aquilion ONE Prism or Genesis) at both 512x512 (PIQE-512) and 1024x1024 matrix (PIQE-1024). PIQE and conventional hybrid iterative reconstruction (AIDR) at 512x512 matrix were reviewed by an experienced cardiothoracic radiologist and evaluated on a Likert scale (5=excellent, 1=poor) for spatial resolution, noise, diagnostic confidence and overall image quality. All images were reconstructed with 0.5 mm slice thickness, 0.25 mm increment and 200 mm field of view. The data was analysed using a paired t-test.
**Results or Findings:** Overall image quality for PIQE-1024 (4.92±0.27) was higher than both AIDR (3.58±0.54) or PIQE-512 (4.63±0.48), both p<0.01. Noise was subjectively lower with PIQE-1024 (4.78±0.41) vs AIDR (3.35±0.52, p<0.01), but not significantly different than PIQE-512 (4.79±0.41, p=0.74). Spatial resolution with PIQE-1024 (4.89±0.32) was higher than either AIDR (3.40±0.50) or PIQE-512 (4.62±0.48), both p<0.01. Diagnostic confidence was improved with PIQE-1024 over AIDR (4.88±0.32 vs 3.96±0.44, p<0.01) but not over PIQE-512 (4.88±0.36, p=0.32). Signal-to-noise for PIQE-1024 (19.5±5.61) was higher than AIDR (18.4±5.07, p<0.01) and slightly lower than PIQE-512 (20.1±5.8, p<0.01).
**Conclusion:** PIQE has improved spatial resolution, noise characteristics, diagnostic confidence and image quality compared to conventional reconstruction.
**Limitations:** The was a single expert observer.
**Ethics committee approval:** The institutional review board approved this study.
**Funding for this study:** Funding was received from the National Institutes of Health.

**Author Disclosures:**
Takuya Nemoto: Employee: Canon Medical
Marcus Y Chen: Nothing to disclose
Hiroki Taguchi: Employee: Canon Medical
Yasuhiro Noshi: Employee: Canon Medical
Jian Zhou: Employee: Canon Medical
Liang Cai: Employee: Canon Medical
John Schuzer: Employee: Canon Medical
Masakazu Matsuura: Employee: Canon Medical

### RPS 2103-6

**Machine learning for time-to-event analysis in patients with suspected coronary artery disease: increased long-term prognostic value of coronary CT angiography-derived measures and clinical parameters**

*M. J. Bauer*, N. Nano, R. Adolf, A. Will, E. Hendrich, S. Martinoff, M. Hadamitzky; Munich/DE

**Purpose:** To assess the long-term prognostic value of machine learning (ML) in time-to-event analyses on coronary CT angiography (CCTA)-derived measures and clinical parameters.
**Methods or Background:** Data sets of 5457 patients (61.1±11.1 years, 66.8% male) with suspected coronary artery disease who underwent CCTA were retrospectively analysed. Major adverse cardiovascular events (MACE) were defined as composite of all-cause death, myocardial infarction, instable angina, or late revascularisation (>90 days after index scan). Demographic parameters, clinical cardiovascular risk factors, CCTA-derived plaque features, and established CCTA-derived risk scores were assessed as predictors of MACE and incorporated into two models: a) a Cox proportional hazards (CPH) model with recursive feature elimination as reference. b) a ML model based on random survival forests. Both models were trained and validated employing repeated nested cross-validation. Harrell's concordance index (C-index) was used to assess the predictive power.
**Results or Findings:** During the median follow-up of 7.3 years (IQR: 4.5 – 9.8 years), MACE was observed in 304 patients (5.6 %). The predictive power of the ML model (C-index 0.74 [95%CI 0.71-0.76]) was significantly higher compared to the CPH model (C-index 0.71 [95%CI 0.68-0.74], p=0.02). The ML model also outperformed single CCTA-derived parameters (C-indices 0.44-0.69, all p<0.001), including the segment stenosis score (C-index 0.69 [95%CI 0.66-0.72], p<0.001) and the number of segments with spotty calcifications (C-index 0.66 [95%CI 0.63-0.69], p<0.001). Similar results were observed compared to the clinical predictors (C-indices 0.48 - 0.66, all p<0.001), including patients' age (C-index 0.66 [95%CI 0.63 - 0.69], p<0.001) and the Framingham risk score (C-index 0.65 [95%CI 0.62-0.69], p<0.001).
**Conclusion:** ML-based time-to-event analysis was found to predict MACE more accurately than existing clinical or CCTA-derived metrics and a conventional Cox-based model integrating these features.
**Limitations:** This work's status as a retrospective, single-centre study was identified as a limitation.
**Ethics committee approval:** This study was approved by the local Institutional Review Board.
**Funding for this study:** Not applicable
**Author Disclosures:**
Maximilian Josef Bauer: Nothing to disclose
Nejva Nano: Nothing to disclose
Martin Hadamitzky: Nothing to disclose
Rafael Adolf: Nothing to disclose
Stefan Martinoff: Research/Grant Support: Siemens Healthineers
Albrecht Will: Nothing to disclose
Eva Hendrich: Nothing to disclose

### RPS 2103-7

**Comparison of clinical scores in predicting coronary artery disease in familial hypercholesterolaemia: a CCTA study**

*L. Dominici*[1], N. Galea[1], F. Catapano[1], L. Marchitelli[1], C. Roberti[1], M. Francone[2], C. Catalano[1]; [1]Rome/IT, [2]Milan/IT

**Purpose:** Familial hypercholesterolaemia (FH) is an autosomal dominant genetic disorder characterised by a high risk of premature ASCVD that cannot be satisfactorily predicted by common risk scores.
The aim of our study is to use Cardiac computed tomography angiography (CCTA) to evaluate the performance of DLCN, Montreal risk score and SAFEHEART-RE in predicting the severity of CAD in this population.
**Methods or Background:** From October 2013 to May 2019, we evaluated 139 consecutive subjects (±years, 82 males [%]) with clinical and genetic diagnoses of FH. Dutch Lipid Clinic Network score (DLCN), Montreal risk score and SAFEHEART-RE were calculated for each patient. Atherosclerotic burden CT scores (Agatston score, segment stenosis score [SSS]) and CAD-RADS score were computed after analysis of coronary artery calcium and degree of stenosis for all coronary segments.
**Results or Findings:** CAD-RADS <3 (non-obstructive) was found in 109 patients, while 30 patients had CCTA findings of obstructive disease (CAD-RADS ≧ 3). Agatston score and SSS were significantly higher in patients with higher Montreal score (p<001) and higher SAFEHEART-RE (p=0.047) and both scores showed a significant difference between the two CAD-RADS groups (p<0.001), whereas DLCN showed no statistically significant differences. Montreal risk score proved to be excellent in discriminating between CAD groups (AUC=0.819; 95% CI, p<0.001), followed by SAFEHEART-RE (AUC=0.725; 95% CI, p<0.001). The best cut-offs were respectively a Montreal risk score of 30 (sensitivity 52.1%, specificity 86%) and a SAFEHEART-RE value of 1.4 (sensitivity 43.3%, specificity 92%).
**Conclusion:** Montreal risk score and SAFEHEART-RE can efficaciously identify obstructive CAD and might help stratifying FH patients for prompt referral to CCTA.
**Limitations:** Not applicable
**Ethics committee approval:** The ethics committee approved this study.
**Funding for this study:** Not applicable
**Author Disclosures:**
Camilla Roberti: Nothing to disclose
Livia Marchitelli: Nothing to disclose
Marco Francone: Nothing to disclose
Federica Catapano: Nothing to disclose
Nicola Galea: Nothing to disclose
Lorenzo Dominici: Nothing to disclose
Carlo Catalano: Nothing to disclose

### RPS 2103-8

**Frequency and clinical implications of coronary anomalies and myocardial bridging on CT coronary angiography**

M. C. Williams[1], K. Leadbetter[1], *G. H. Roditi*[2], E. Van Beek[1], D. Newby[1], E. Nicol[3]; [1]Edinburgh/UK, [2]Glasgow/UK, [3]London/UK
*(giles.roditi@glasgow.ac.uk)*

**Purpose:** Coronary anomalies and myocardial bridging can be identified on coronary computed tomography angiography (CCTA). However, their frequency and clinical implications remain uncertain.
**Methods or Background:** In this post-hoc analysis of the Scottish Computed Tomography of the HEART (SCOT-HEART) trial presence of coronary artery anomalies and myocardial bridging was assessed on CCTA. Imaging and clinical information were obtained from the SCOT-HEART database.
**Results or Findings:** CCTA from 1769 patients (mean age 58±9, 56% male) were assessed. Coronary anomalies were identified in 40 (2.3%) patients with 11 morphologies. The commonest was left circumflex from right sinus with retro-aortic course (14, 0.8%), followed by absent left main stem (10, 0.6%). There were no differences in demographic characteristics, CCTA findings, myocardial infarction, or death in patients with or without anomalies. Myocardial bridging occurred in 215 (12%) patients, who were older (59±9 vs 57±9, p=0.027), more likely to be male (69% vs 55%, p<0.001) and had higher 10-year cardiovascular risk scores (18 [interquartile range 12, 26] vs 15 [10, 23], p=0.001). Patients with myocardial bridging had a higher coronary artery calcium score [34 [0, 292] vs 18 [0, 219] Agatston Units, p=0.004) and were more likely to have obstructive coronary artery disease (35% vs 24%, p<0.001). They were more likely to undergo revascularisation (21% vs 13%, p=0.002), but this was not independent of coronary artery disease. There were no differences concerning fatal or non-fatal myocardial infarction between patients with and without myocardial bridging (0.9% versus 2.5%, p=0.230).
**Conclusion:** Coronary anomalies are infrequent but myocardial bridging is common. Myocardial bridging is associated with risk factors and coronary artery disease on CCTA, but not with cardiac outcomes.
**Limitations:** The study only concerned a single country and only symptomatic cardiology clinic patients were examined.
**Ethics committee approval:** This study was approved by an ethics committee.
**Funding for this study:** Funding was received from the Chief Scientist Office of the Scottish Government and British Heart Foundation (FS/ICRF/20/26002).
**Author Disclosures:**
Edward Nicol: Nothing to disclose
Kai Leadbetter: Nothing to disclose
Giles Hannibal Roditi: Nothing to disclose
David Newby: Nothing to disclose
Edwin Van Beek: Nothing to disclose
Michelle Claire Williams: Nothing to disclose

### RPS 2103-9

**Silent myocardial infarction fatty scars detected by coronary calcium score CT scan in diabetic patients without history of coronary heart disease**

*S. Boccalini*[1], M. Teulade[1], F. Rapallo[2], E. Paquet[1], S. Charrière[1], S. A. Si-Mohamed[1], C. Bergerot[1], P. Douek[1], P. Moulin[1]; [1]Lyon/FR, [2]Genoa/IT
*(sara.boccalini@yahoo.com)*

**Purpose:** To evaluate the prevalence of intra-myocardial fatty scars (IMFS) most-likely indicating previous silent myocardial infarction (SMI) as detected on coronary artery calcium (CAC) computed tomography (CT) scans in diabetic patients without history of coronary heart disease (CHD).
**Methods or Background:** Diabetic patients screened for silent coronary insufficiency were categorised according to their CAC score in two groups comprising 242 patients with CAC=0 and 145 patients with CAC≥300. CAC-CT were retrospectively evaluated for subendorcardial and transmural IMFS of the left ventricle in all likelihood indicating history of SMI. Adipose remodelling, patients' characteristics, cardiovascular (CV) risk factors and metabolic profile were compared between different groups.
**Results or Findings:** 83 (21%) patients with IMFS were identified, 55 (37.9%) in the group CAC≥300 and 28 (11.6%) in the CAC=0 (OR=4.67; IC95%=2.78-7.84; p<0.001). Total and average surface of detected IMFS and their number per patient were similar in both groups (p=0.55; p=0.29; p=0.61). In the group CAC≥300, patients with IMFS were older (p=0.03) and had a longer-lasting diabetes (p=0.04). Patients with IMFS were older, had longer history of diabetes, reduced glomerular filtration rate, more coronary calcifications (all p<0.05), and higher prevalence of carotid plaques (OR=3.03; 95%IC=1.43-6.39, p=0.004). Higher CAC centile (OR=1.02; 95%IC=1-1.03; p=0.001) was associated with an increased risk of having IMFS with multivariate analysis.
**Conclusion:** In diabetic patients without known CHD, CT for CAC assessment provides additional information regarding IMFS, likely indicating previous unrecognised SMI.
**Limitations:** First, the absence of confirmation with another imaging modality that all the lesions are post-infarction.
Second, this is a retrospective study and, thus, it remains to be established if this marker translates in an upwards CV risk restratification especially in diabetic patients with CAC=0.
**Ethics committee approval:** This retrospective study on a prospective cohort was approved by the local ethic committee.
**Funding for this study:** No funding was received for this study.
**Author Disclosures:**
Salim Aymeric Si-Mohamed: Nothing to disclose
Fabio Rapallo: Nothing to disclose
Emilie Paquet: Nothing to disclose
Cyrille Bergerot: Nothing to disclose
Marie Teulade: Nothing to disclose
Philippe Douek: Nothing to disclose
Sara Boccalini: Nothing to disclose
Sybil Charrière: Nothing to disclose
Philippe Moulin: Nothing to disclose

### RPS 2103-11

**Dynamic myocardial perfusion imaging using quantitative iodine density maps in a 3D printed heart phantom**

*J. L. Hammel*, L. Birnbacher, M. Makowski, F. Pfeiffer, D. Pfeiffer; Munich/DE
*(johannes.hammel@tum.de)*

**Purpose:** To check whether dual-energy CT technology provides appropriate data to calculate iodine-based perfusion maps for functional assessment of coronary stenosis.
**Methods or Background:** An anatomical heart model was designed and produced using a 3D printing laser sintering technique. The dynamical phantom can be flooded with water and contrast medium. To simulate a perfusion defect, the bolus flow through the left coronary artery was blocked by approximately 75 %. Time attenuation curves (TAC) were acquired with a 64-slice single source dual-layer spectral CT scanner using a fixed tube voltage of 120 kVp and an exposure of 30 mAs for 36 timepoints. A post-processing algorithm for tracer kinetic modelling using a one compartment approach was applied.
**Results or Findings:** When averaged over a region with several voxels, dual-energy CT based dynamic perfusion imaging showed very similar results in blood flow measurements calculated from conventional TAC. The relative deviation was 0.93%. A relative deviation of 4.82% was found for flow values calculated from a single voxel. The fit accuracy of the tracer kinetic model increases for iodine-based TAC compared to conventionally derived TAC (signal to noise ratio of 10.50 and 5.45 respectively). For the differentiation of stenotic myocardium from unaffected regions the receiver operating characteristic shows an area under the curve of 0.89 and 1.00 for conventional and spectral data respectively.
**Conclusion:** Dual-energy CT and future spectral technologies like photon counting CT provide 4D iodine density data, which enables calculation of absolute quantitative perfusion maps with increased signal to noise ratio and improved ischaemia characterisation.
**Limitations:** This study presents results for a 3D-printed heart phantom with simplified anatomy. Additionally, no heart movement can be simulated.
**Ethics committee approval:** Not applicable.
**Funding for this study:** No funding was received for this study.
**Author Disclosures:**
Johannes Leopold Hammel: Nothing to disclose
Lorenz Birnbacher: Nothing to disclose
Franz Pfeiffer: Nothing to disclose
Marcus Makowski: Nothing to disclose
Daniela Pfeiffer: Nothing to disclose

**16:00-17:30** **Room M 1**

### Research Presentation Session: Imaging Informatics / Artificial Intelligence and Machine Learning

### RPS 2105

### Artificial intelligence in breast imaging

**Moderator**
J. Neubauer; Freiburg/DE

### RPS 2105-2

**Safe and effective integration of AI as supporting reader in double reading breast cancer screening**

A. Ng[1], B. Glocker[1], C. Oberije[1], G. Fox[1], F. J. Gilbert[2], *J. Nash*[1], E. Karpati[1], S. Kerruish[1], P. Kecskemethy[1]; [1]London/UK, [2]Cambridge/UK
*(jonathan.nash@kheironmed.com)*

**Purpose:** To evaluate the effectiveness and practical implications of a novel workflow of using AI as a supporting reader for the detection of breast cancer in double reading screening mammography.
**Methods or Background:** AI strategies in breast cancer screening should optimise the interaction between AI and human readers to maximise their combined benefit while ensuring patient safety and minimising clinical and operational risks. Large-scale retrospective data is used to evaluate a new paradigm of AI-supported reading. Instead of replacing a human reader, the AI serves as the second reader only if it agrees with the recall/no-recall decision of the first human reader. Otherwise, a second human reader makes an assessment, enacting standard human double reading. 280,594 participants from seven centres in two countries (UK, Hungary), and four hardware vendors (Giotto, Hologic, GE, Siemens) are included. Synthesised performance was measured via superiority/non-inferiority tests on cancer detection rate, recall rate, sensitivity, specificity, and positive predictive value. Workload was measured as arbitration rate and number of cases requiring a second human reading.
**Results or Findings:** The novel synthesised workflow was found to be superior or non-inferior on all screening metrics, almost halving arbitration and reducing the number of cases requiring second human reading by up to 87.5% compared to human double reading.
**Conclusion:** AI as a supporting reader adds a safety net in case of AI discordance while retaining screening performance of standard of care and drastically reducing workload.
**Limitations:** The second human reader would only assess cases where the AI and first human reader disagree. The impact of the change in case mix needs to be investigated further.
**Ethics committee approval:** UK HRA (REC reference: 19/HRA/0376) and ETT-TUKEB (Medical Research Council, Hungary) approval (Reg no: OGYÉI/46651-4/2020).
**Funding for this study:** Funding was received from Kheiron Medical Technologies.
**Author Disclosures:**
Cary Oberije: Employee: Kheiron Medical Technologies
Fiona J. Gilbert: Nothing to disclose
Ben Glocker: Employee: Kheiron Medical Technologies
Georgia Fox: Employee: Kheiron Medical Technologies
Peter Kecskemethy: CEO: Kheiron Medical Technologies
Annie Ng: Employee: Kheiron Medical Technologies
Edith Karpati: Employee: Kheiron Medical Technologies
Jonathan Nash: Employee: Kheiron Medical Technologies
Sarah Kerruish: Employee: Kheiron Medical Technologies

### RPS 2105-3

**Detection and classification of lesions in breast ultrasound using a deep convolutional neural network**

*C. Ruppert*[1], A. Landsmann[1], K. Borkowski[1], P. Hejduk[1], L. Jungblut[1], T. Schnitzler[2], A. Boss[1], M. Bonmarin[1], A. Ciritsis[1]; [1]Zurich/CH, [2]Aarau/CH
*(carlotta.ruppert@b-rayz.ch)*

**Purpose:** The aim of this study was to investigate the potential of a deep convolutional neural network (dCNN) to (i) detect (ii) segment and (iii) classify lesions in conventional breast ultrasound images in accordance with the Breast imaging-reporting and data system (BI-RADS).
**Methods or Background:** 3278 conventional breast ultrasound images from 1078 individual patients depicting lesions were manually segmented and classified by two radiologists according to the BI-RADS standard. A U-Net-based architecture was trained with 2510 images and validated with 768 images. The performance of the network was evaluated on a test dataset consisting of 154 excluded from the training and validation dataset. The performance of the dCNN compared to human readers was quantified using inter-rater agreement.
**Results or Findings:** For lesion detection, the network reached 70% (65%) precision and 70% (81%) recall with respect to the annotating radiologist 1 (2). The inter-rater agreement for lesion classification between the dCNN and radiologist 1 (2) was substantial (moderate) for 3 classes and almost perfect (substantial) for binary classification (benign and malignant). The inter-rater agreement between the radiologists was measured as moderate to substantial for 3 and 2 classes, respectively.
**Conclusion:** In this study, we demonstrated that the performance of the dCNN was comparable to the performance of experienced radiologists. Thus, our dCNN can serve as an observer-independent guide for subsequent clinical procedures. Furthermore, it can contribute to the standardisation of lesion classifications preventing unnecessary biopsies.
**Limitations:** The test dataset consisted of only 154 images, which were unbalanced and extracted from one single site.
**Ethics committee approval:** This retrospective study has been approved by the local ethics committee (“Kantonale Ethikkommission Zurich”; Approval Number: 2016-00064).
**Funding for this study:** Supported by the Clinical Research Priority Program (CRPP) Artificial Intelligence in oncological Imaging of the University Zurich.
**Author Disclosures:**
Karol Borkowski: Nothing to disclose
Andreas Boss: Nothing to disclose
Carlotta Ruppert: Nothing to disclose
Tician Schnitzler: Nothing to disclose
Mathias Bonmarin: Nothing to disclose
Patryk Hejduk: Nothing to disclose
Anna Landsmann: Nothing to disclose
Lisa Jungblut: Nothing to disclose
Alexander Ciritsis: Nothing to disclose

### RPS 2105-4

**Development and validation of an AI-driven mammographic breast density classification tool based on radiologist consensus**

V. Magni, M. Interlenghi, A. Cozzi, *M. Alì*, C. Salvatore, D. Fazzini, I. Castiglioni, S. Papa, F. Sardanelli; Milan/IT
*(marco.ali90@gmail.com)*

**Purpose:** To develop and clinically validate an artificial intelligence software (Trace4BDensity©) for breast density classification, in order to eliminate subjective variability in visual assessment.
**Methods or Background:** Trace4BDensity© analyses the breast density of mid-lateral oblique mammographic projections (MLO) according to the Breast Imaging-Reporting and Data System (BI-RADS) categories. The system was trained and validated internally using as reference the majority density class (mode measurement) independently assigned by 7 radiologists to a dataset of MLO mammograms obtained from centre 1. External validation was performed by 3 radiologists (whose breast density classification was closest to the statistical mode measurement of the initial 7) on a dataset of MLO mammograms obtained from centre 2. Concordance between TRACE4BDensity© and the statistical mode measurement of the 3 radiologists was calculated as a percentage, reliability by Cohen's k, both reported with their respective 95% confidence intervals (CI).
**Results or Findings:** Two datasets of 760 (of 380 women) and 384 (of 197 women) MLO mammography projections were used for internal training/validation and external validation, respectively. In the external validation dataset, Trace4BDensity© achieved an accuracy of 89.3% in distinguishing between BI-RADS categories a/b (non-dense breasts) and c/d (dense breasts), with a software/radiologist concordance of 90.4% (178/197 MLOs; CI 95% 85.3%, 94.1%) and a reliability of 0.807 (Cohen's k, CI 95% 0.667, 0.947).
**Conclusion:** Trace4BDensity© was developed and validated, proving to reliably distinguish dense from non-dense breasts according to the BI-RADS classification.
**Limitations:** Mammograms with malignant lesions were excluded. Reliability analysis was performed only by three readers. TRACE4BDensity does not provide quantitative BD measurements.
**Ethics committee approval:** Ethics committee approval was obtained for this study.
**Funding for this study:** No funding was recieved for this study.
**Author Disclosures:**
Andrea Cozzi: Nothing to disclose
Isabella Castiglioni: Consultant: DeepTrace Technologies
Sergio Papa: Nothing to disclose
Marco Alì: Consultant: Bracco Imaging
Francesco Sardanelli: Consultant: Bracco Imaging Consultant: DeepTrace Technologies
Christian Salvatore: Founder: DeepTrace Technologies CEO: DeepTrace Technologies
Deborah Fazzini: Nothing to disclose
Matteo Interlenghi: Employee: DeepTrace Technologies Founder: DeepTrace Technologies
Veronica Magni: Nothing to disclose

### RPS 2105-5

**Combining radiomics and deep learning for classification of suspicious lesions on contrast-enhanced mammography images**

*M. Beuque*[1], M. Lobbes[2], Y. van Wijk[1], Y. Widaatalla[1], S. Primakov[1], M. Majer[3], H. C. Woodruff[1], P. Lambin[1]; [1]Maastricht/NL, [2]Sittard-Geleen/NL, [3]Villejuif/FR
*(m.beuque@maastrichtuniversity.nl)*

**Purpose:** Radiomics-based and deep learning (DL) models individually achieve good classification performance (benign/malignant) on contrast-enhanced mammography (CEM). We hypothesise that combining both allows for automated detection, delineation, and improved classification of masses. Such a system can aid clinicians in their workflow and decision making.
**Methods or Background:** 921 cases acquired at our hospital (with status and subtype proven by histopathological examination or 2-year follow-up) and 212 cases provided by Institute Gustave Roussy (external validation) had masses delineated by an expert radiologist. Pre-processed recombined and low energy images were used to train a RetinaNet DL model to automatically detect, contour, and classify suspicious masses. We also trained a radiomics-based machine learning model (xgboost) to classify masses detected and contoured both by the radiologist and by the DL model. We report the detection sensitivity as well as the AUC achieved by the models separately, combined, and when the models agreed on the external validation.
**Results or Findings:** The DL model obtained a sensitivity of 90% for mass detection. Both the DL and radiomics-based models obtained an AUC of 0.86 on the manual contours which increased to 0.89 when ensembling the models. The models agreed on 79% of the lesions achieving an AUC of 0.93 on this subset. On the DL generated contours, the DL model obtained a classification AUC of 0.93, 0.94 for the radiomics-based model, and 0.95 when combining the models. The models' classification agreed on 85% of the DL contours and obtained an AUC of 0.96 on the subset.
**Conclusion:** A combination of DL and radiomics for the automated detection and classification of masses on CEM achieves clinically competitive results that can be translated into a clinical decision support system.
**Limitations:** Not applicable
**Ethics committee approval:** This study is board approved.
**Funding for this study:** Funding was received through the Marie Skłodowska-Curie grant.
**Author Disclosures:**
Marc Lobbes: Nothing to disclose
Yousif Widaatalla: Nothing to disclose
Henry Christian Woodruff: Shareholder: Radiomics
Michael Majer: Nothing to disclose
Yvonka van Wijk: Nothing to disclose
Sergey Primakov: Nothing to disclose
Manon Beuque: Nothing to disclose
Philippe Lambin: Shareholder: Radiomics

### RPS 2105-6

**On the importance of including unconfirmed cases when assessing the effect of AI on the recall rate in breast cancer screening**

A. Ng, *B. Glocker*, T. Rijken, P. Kecskemethy; London/UK

**Purpose:** To demonstrate the importance of using representative data, including unconfirmed cases (neither positive nor negative) to assess recall rate (RR) and avoid obscuring real-world performance.
**Methods or Background:** The performance of a commercially available AI system was evaluated in a large-scale retrospective study of unenriched representative real-world data (275,900 cases) from seven sites, two countries (UK, Hungary), and four device vendors (Hologic, GE, Siemens, IMS Giotto) from 2009-2019. Positives were pathology-proven malignancies. Negatives had 3-year negative follow-up results. Recall rate was assessed in two ways:

1) using the unenriched representative dataset, including positives, negatives, and unconfirmed cases, and 2) after removing unconfirmed cases and artificially scaling up negatives to reconstruct the screening cancer prevalence found in 1.
**Results or Findings:** The representative dataset included 74.6% unconfirmed cases. Cancer prevalence in method 1 versus 2 (pre-scaling) were 1.0% and 4.5%, respectively. The AI's standalone RR in method 1 versus 2 (post-scaling) was 11.5% and 9.5%, respectively, demonstrating an apparent 17.6% relative reduction when using a constructed dataset.
**Conclusion:** For the assessment of AI performance on RR, it is important to include unconfirmed cases which are likely to be more difficult for AI to assess correctly. Validating AI on non-representative, constructed datasets, excluding unconfirmed cases, may otherwise show optimistically low RR which would not translate to screening practice. Subsequent implementation would pose a significant risk for overdiagnosing patients, leading to unnecessary use of resources and unnecessary patient anxiety. Studies should use unbiased metrics with minimal truthing requirements such as RR and representative, real-world data, avoiding artificial construction, to assess AI performance in breast cancer screening.
**Limitations:** Results may not be representative for other AI systems.
**Ethics committee approval:** UK HRA (REC reference: 19/HRA/0376) and ETT-TUKEB (Hungary) approval (Reg no: OGYÉI/46651-4/2020).
**Funding for this study:** Funding was received from Kheiron Medical Technologies.
**Author Disclosures:**
Ben Glocker: Employee: Kheiron Medical Technologies
Peter Kecskemethy: CEO: Kheiron Medical Technologies
Tobias Rijken: Founder: Kheiron Medical Technologies
Annie Ng: Employee: Kheiron Medical Technologies

### RPS 2105-7

**Automatic and standardised quality control of digital mammography and tomosynthesis with deep convolutional neural networks**

P. Hejduk[1], R. Sexauer[2], *K. Borkowski*[1], N. Schmidt[2]; [1]Zurich/CH, [2]Basel/CH
*(karolbo1@02.pl)*

**Purpose:** The aim of this study was to develop and test a deep learning algorithm for the automatic determination of image quality in mammography and tomosynthesis considering a standardised set of features.
**Methods or Background:** In this retrospective study, 12,301 mammography and tomosynthesis images from 3,516 patients of three institutions (craniocaudal and mediolateral-oblique projections) have been analysed by assessing the presence of 7 key features that impact image quality. A dCNN (deep Convolutional Neural Network) was applied to train seven models on features detecting the presence of anatomical landmarks and three models for feature localisation. The validity of the models was assessed by calculation of the mean squared error in a test dataset and in comparison to reading by experienced radiologists.
**Results or Findings:** Accuracies of the dCNN models ranged between 93.0% for the classification nipple visualisation (RSME 0.84 cm) to 98.5% for the depiction the pectoralis muscle in the CC view (RSME 1.79 cm). All models showed almost perfect agreement compared to human reading with Cohen's Kappa score above 0.9.
**Conclusion:** An AI-based quality assessment system using a dCNN allows for precise, consistent and observer-independent rating of digital mammography and tomosynthesis imaging.
**Limitations:** Datasets chosen for training, validation and testing may potentially be biased due to the retrospective nature of this study. Images from not all the manufacturers of the devices were included in this study limiting number of possible variants of images used for analysis. Not all ethnicities were included in this study, however representative data was taken from the general cohort of female patients in our institutions. Only one AI architecture tested.
**Ethics committee approval:** This study was approved by the Kantonale Ethikkommission Zurich; Approval Number: 2016-00064.
**Funding for this study:** This study has been supported by the Clinical Research Priority Program Artificial Intelligence in Oncological Imaging of the University Zurich.
**Author Disclosures:**
Noemi Schmidt: Nothing to disclose
Karol Borkowski: Nothing to disclose
Patryk Hejduk: Nothing to disclose
Raphael Sexauer: Nothing to disclose

### RPS 2105-8

**Virtual abbreviated contrast enhanced MRI for breast cancer diagnostics: initial experience**

*A. Liebert*, H. Schreiter, L. A. Kapsner, S. Ohlmeyer, F. B. Laun, A. Maier, M. Uder, E. Wenkel, S. Bickelhaupt; Erlangen/DE
*(andrzej.liebert@uk-erlangen.de)*

**Purpose:** To evaluate abbreviated, non-contrast enhanced breast magnetic resonance imaging (MRI) augmented by a high-resolution AI series reproducing the image characteristics of a contrast-enhanced subtraction sequence.
**Methods or Background:** This IRB approved study included n=540 multiparametric breast MRI examinations including a multi-b-value DWI (50,750,1500 sec/mm2). Virtually subtraction series were derived for n=78 subjects applying a 2D-U-Net architecture trained using on the native acquisitions as input and the gadolinium-enhanced subtraction series as target. Two abbreviated protocols using maximum intensity projections (MIPs) of the subtraction series were diagnostically assessed. First protocol was a virtual abbreviated contrast enhanced protocol (VACE), which used the MIP of the virtual subtraction series and all non-enhanced acquisitions necessary to derive the virtual series. The second protocol was an abbreviated contrast-enhanced protocol (ABCE) using the original gadolinium-enhanced subtraction acquisition and its MIP. Diagnostic indexes, image quality and artifact strength were assessed.
**Results or Findings:** Sensitivity of 0.86 (31/36) and 0.94 (34/36), specificity of 0.81 (34/42) and 0.73 (31/42) and accuracy of 0.83 (65/78) and 0.83 (65/78) was achieved by VACE and ABCE protocols respectively. These diagnostic parameters showed statistically no significant difference. No significant difference in image quality and reading time were found between the two protocols. However a significantly higher number of artifacts could be noted for the ABCE protocol (p=0.003).
**Conclusion:** Here we demonstrate the technical feasibility of augmenting non-contrast enhanced breast MRI by a virtual contrast enhanced subtraction series in an abbreviated protocol. Further research is necessary to advance the technique.
**Limitations:** The study used acquisition only from a single scanner type (Siemens, Skyra-Fit). Due to the low number of test cases, the applied tests have a relatively small power to detect minor differences between both protocols.
**Ethics committee approval:** This IRB approved retrospective study included studies performed between 2017 and 2020.
**Funding for this study:** This study was funded via the BMBF 'SMART Select MRI' GoBioInital project.
**Author Disclosures:**
Lorenz A. Kapsner: Nothing to disclose
Andreas Maier: Nothing to disclose
Evelyn Wenkel: Nothing to disclose
Frederik Bernd Laun: Nothing to disclose
Michael Uder: Nothing to disclose
Hannes Schreiter: Nothing to disclose
Sabine Ohlmeyer: Nothing to disclose
Sebastian Bickelhaupt: Nothing to disclose
Andrzej Liebert: Nothing to disclose

### RPS 2105-9

**Post-market real-world data demonstrating use of an AI system as an extra reader to augment breast cancer detection without unnecessary recalls**

C. Oberije[1], E. Ambrozay[2], E. Karpati[1], *J. Nash*[1], A. Ng[1], G. Fox[1], B. Glocker[1], P. Kecskemethy[1]; [1]London/UK, [2]Budapest/HU
*(jonathan.nash@kheironmed.com)*

**Purpose:** To demonstrate real-world post-market benefit of artificial intelligence (AI) as an extra reader in breast cancer screening (BCS).
**Methods or Background:** A commercially available AI system was employed as an extra reader (XR) in addition to standard double reading (DR) at a BCS provider in Hungary from Apr-Sept 2021. The XR workflow involved flagging cases the AI suggested to recall which DR did not recall, i.e. positive discordant cases, for arbitration by an experienced radiologist. Detected cancers were pathology-confirmed.
**Results or Findings:** Standard DR for 3746 patients had an arbitration rate of 3.0% (114 patients), a recall rate of 6.7% (250 patients), and a cancer detection rate (CDR) of 12.5/1000 (47 cancer cases). Of the cases that were not recalled by DR, the AI flagged 396 cases to recommend recall (positive discordance rate 10.6%). Extra arbitration resulted in recalling 6 patients, all of whom were diagnosed with breast cancer. This equated to a total arbitration rate of 13.6% and 1.6/1000 increase in CDR (sum 14.1/1000). An exact simulation with a less sensitive AI operating point yielded a total arbitration rate of 5.3%, while still detecting 5 of 6 extra cancer cases.

**Conclusion:** This real-world deployment of AI increased cancer detection rates without recalling extra false positives, indicating the effectiveness of AI as extra reader. Combining the XR workflow with workflows focused on workload savings will mitigate the increased arbitration rate and optimise clinical and operational benefits. The results provide important, real-world evidence showing the benefit of using an AI reader in breast cancer screening, paving the way for innovative workflows where synergy of humans and AI achieve optimal performance for patients.
**Limitations:** This was a singlecentre analysis; only one extra arbitration reader was included.
**Ethics committee approval:** Ethics committee approval was not required.
**Funding for this study:** Funding was received from Kheiron Medical Technologies.
**Author Disclosures:**
Cary Oberije: Employee: Kheiron Medical Technologies
Annie Ng: Employee: Kheiron Medical Technologies
Ben Glocker: Employee: Kheiron Medical Technologies
Eva Ambrozay: Nothing to disclose
Georgia Fox: Employee: Kheiron Medical Technologies
Peter Kecskemethy: CEO: Kheiron Medical Technologies
Edith Karpati: Employee: Kheiron Medical Technologies
Jonathan Nash: Employee: Kheiron Medical Technologies

### RPS 2105-11
**Can a breast-screening AI solution reduce the incidence of interval cancers?**
*C. F. de Vries*[1], S. J. Colosimo[1], R. Staff[1], J. Dymiter[1], J. Yearsley[2], D. Dinneen[2], D. Harrison[3], L. Anderson[1], G. Lip[1]; [1]Aberdeen/UK, [2]London/UK, [3]St Andrews/UK

**Purpose:** Breast screening reduces breast cancer mortality. However, a small number of women present with interval cancers between screening rounds which were potentially missed by human readers. Artificial intelligence (AI) could potentially support breast cancer screening, but its impact on interval cancers is unknown. Here, the ability of a commercially available AI algorithm to detect interval cancers was evaluated.
**Methods or Background:** The performance of the AI algorithm was evaluated using UK screening data. All available interval cancers from cases between 1 April 2016 and 31 March 2019 in NHS Grampian were included (N=52). Performance was stratified by lesion visibility on the screening mammogram, grade and size.
**Results or Findings:** The AI would have recalled 14/52 (26.9%) for additional examinations. The AI indicated to recall 8/35 cases for which the lesion was not visible on screening mammograms; 3/9 cases for which the lesion was visible on review in hindsight; 2/2 cases for which the lesion was clearly visible; and 1/6 occult cases (lesion was not visible on screening or subsequent mammograms). The AI would have recalled a higher proportion of interval cancers <15 mm (7/16) than those >=15 mm (7/26), and a higher proportion of grade II (5/19) and III (7/24) tumours than DCIS (1/6).
**Conclusion:** The introduction of a breast-screening AI solution into the screening pathway may reduce the number of women presenting with interval cancers by recalling them during routine screening. This AI algorithm might detect smaller, higher-grade cancers, which could have a significant impact on morbidity and mortality.
**Limitations:** Not applicable.
**Ethics committee approval:** The study received ethical approval from the Proportionate Review Sub-committee of the London-Bloomsbury REC (20/LO/0563). PBPP approval was obtained (1920-0258).
**Funding for this study:** This work is supported by iCAIRD, which is funded by Innovate UK on behalf of UKRI.
**Author Disclosures:**
Roger Staff: Nothing to disclose
Clarisse Florence de Vries: Nothing to disclose
Deirdre Dinneen: Employee: Kheiron Medical Technologies
Joseph Yearsley: Employee: Kheiron Medical Technologies
Lesley Anderson: Nothing to disclose
David Harrison: Nothing to disclose
Gerald Lip: Nothing to disclose
Samantha Jean Colosimo: Nothing to disclose
Jaroslaw Dymiter: Nothing to disclose

### RPS 2105-12
**Machine-learning radiomics for breast-mass malignancy prediction in contrast-enhanced breast CT**
*M. Caballo*[1], A. Athanasiou[2], D. Kontos[3]; [1]Nijmegen/NL, [2]Athens/GR, [3]Philadelphia, PA/US

**Purpose:** To validate a machine-learning radiomic model in classifying benign versus malignant masses in contrast-enhanced breast CT (CE-BCT) images.
**Methods or Background:** A total of 185 masses (84 benign, 101 malignant) from 166 patient CE-BCT images were previously acquired. Each mass was manually annotated in 3D, and 174 radiomic features were extracted with a publicly available, previously validated software (CaPTk). A multi-step reduction process was applied to discard non-informative, correlated and non-robust features to variations in annotations. Using the remaining features, a logistic regression model was trained and validated with stratified nested five-fold cross-validation. Additional forward feature selection was performed in each cross-validation loop (using only the training fold examples) to further reduce the feature space and prevent overfitting. The final selected features were analysed statistically (Mann-Whitney U-test; Bonferroni correction). The model performance was evaluated using the area under the curve (AUC) of the receiver-operating characteristic, and the calibration curve.
**Results or Findings:** A total of 49/174 features were retained after the multistep reduction process. Five features, adjusted by patient age, were used to train the model for each cross-validation fold. Interquartile range (IQR), equivalent spherical perimeter (ESP) and cooccurrence contrast (CC) were selected for all folds and were statistically significant. The model resulted in an AUC=0.83 (95% Confidence Interval [0.76-0.89]) in good calibration (Hosmer-Lemeshow test: P>0.15).
**Conclusion:** The model showed promising results in classifying breast mass malignancy in CE-BCT, especially considering that it was developed only upon six features. Statistical analysis highlighted different imaging phenotypes for benign and malignant masses, with the latter showing greater contrast medium uptake (IQR), size (ESP) and heterogeneity (CC).
**Limitations:** Absence of an independent test set, which will be included in future work.
**Ethics committee approval:** Waived by our institutional review board.
**Funding for this study:** No funding was received for this study.
**Author Disclosures:**
Alexandra Athanasiou: Nothing to disclose
Marco Caballo: Nothing to disclose
Despina Kontos: Nothing to disclose

**16:00-17:30** **Room M 3**

### Research Presentation Session: Musculoskeletal

### RPS 2110

### Advances in imaging of bone lesions and muscles

**Moderator**
V. Vasilevska-Nikodinovska; Skopje/MK

### RPS 2110-2
**Radiofrequency ablation for osteoid osteoma of the hand**
F. Ruiz Santiago, A. J. Lainez Ramos-Bossini, *F. J. Pérez García*; Granada/ES
*(javipegarcia@gmail.com)*

**Purpose:** To present our technique for the treatment of osteoid osteoma of the hand using radiofrequency thermal ablation (RTA).
**Methods or Background:** Six cases of osteoid osteoma of the hand were treated at our institution. The tumors were located in the proximal (2), middle (2), distal (1) phalanx and the hamate bone (1). We used a T-wrench and inserted a Kirschner wire (1.4-1.6 mm thick) into the nidus by pressing and turning. Once the Kirschner wire was inserted, we introduced the cannula of a beveled vertebroplasty needle co-axially, using the needle as a guide. Next, we removed the wire by pulling it, grasped by the wrench. Finally, we introduced the 7 mm active tip electrode and performed dry ablation for 5 minutes at 70 degrees Celsius.
**Results or Findings:** Technical success was achieved in all 6 cases, but 1 case developed a fracture shortly after treatment, with a stiff interphalangeal joint that required arthrolysis and plaster cast of the finger. The visual analog score (VAS) improved, from a mean value of 8 (8-10) to a mean value of 1 (0-4).
**Conclusion:** Radiofrequency thermal ablation of osteoid osteoma of the hand is feasible and provides good results in terms of pain control and functional recovery. However, special care must be taken to avoid fractures due to the small size of bones in this anatomical region.
**Limitations:** The sample size is small due to the low frequency of osteoid osteoma in the hand.
**Ethics committee approval:** This study was approved by our local ethics committee (code TFG-ATME-2021).
**Funding for this study:** Not applicable.
**Author Disclosures:**
Fernando Ruiz Santiago: Nothing to disclose
Francisco Javier Pérez García: Nothing to disclose
Antonio Jesus Lainez Ramos-Bossini: Nothing to disclose

### RPS 2110-3

**Safety of MR-guided high-intensity focused ultrasound (MRgHIFU) in the treatment of osteoid osteoma**

*V. D'Agostino*[1], M. P. Aparisi Gomez[2], C. Gasperini[1], M. Perrone[1], L. Campanacci[1], A. Bazzocchi[1]; [1]Bologna/IT, [2]Auckland/NZ
*(valerio.dagostino123@gmail.com)*

**Purpose:** A distance greater than 1 cm between the target lesion and sensitive structures is highly recommended in MRgHIFU treatments of osteoid osteoma (OO). The aim of this work is to evaluate the safety of MRgHIFU treatment in OO located in close proximity (<1 cm) of sensitive structures.
**Methods or Background:** Thirty-six patients (67% males, 33% females; mean age 23±10,7yo) with OO were treated. Close proximity of the site of treatment to sensitive structures was measured in pre-treatment MR exams. VAS score at baseline and follow-up (12-24 months) after treatment was assessed in all patients. Primary endpoint was to assess major and minor complications involving sensitive structures. Secondary endpoints were assessment of treatment response and the impact of the proximity to sensitive structures on treatment efficacy.
**Results or Findings:** Close proximity of the target lesion to sensitive structures was recorded in 23 patients: 5 for tendon/enthesis/ligament, 2 vessels, 8 joints, 6 skin, 1 nerve and 1 organ (lung). No complications were experienced. VAS at baseline was 4±1,4, with a complete pain relief at clinical follow-up in 33 patients. In 3 patients, a symptom relapse was observed: one was retreated with MRgHIFU and two with CT-guided radiofrequency ablation (with pain relief at follow-up). Location in proximity to sensitive structures did not affect the efficacy of treatments (p<0.01). Only two of three lesions re-treated were in proximity of, respectively, the iliopsoas tendon and coxofemoral joint.
**Conclusion:** MRgHIFU can be safely and effectively performed even on lesions located at <1 cm from sensitive structures, suggesting a broader range of applications and allowing a higher number of patients to benefit from this mini-invasive treatment.
**Limitations:** Small sample size.
**Ethics committee approval:** Not applicable.
**Funding for this study:** Not applicable.
**Author Disclosures:**
Chiara Gasperini: Nothing to disclose
Alberto Bazzocchi: Nothing to disclose
Laura Campanacci: Nothing to disclose
Mariada Perrone: Nothing to disclose
Maria Pilar Aparisi Gomez: Nothing to disclose
Valerio D'Agostino: Nothing to disclose

### RPS 2110-4

**Analysis of results after radiofrequency ablation of osteoid osteomas**

*M. Martínez-Cachero García*, A. M. Montes García, J. Rodríguez Castro, L. Martínez Camblor, F. García Arias, A. Prieto Fernández; Oviedo/ES
*(migcachero@gmail.com)*

**Purpose:** (1) Describe the radiofrequency ablation (RFA) technique guided with computed tomography (CT) used in our centre for treatment of osteoid osteomas; (2) evaluate technical efficacy and clinical results after RFA treatment of osteoid osteomas in our hospital; and (3) assess the type and rate of complications derived from RFA treatment of osteoid osteomas in our environment.
**Methods or Background:** A retrospective observational study was made from a registry of 16 patients with osteoid osteomas treated in our centre with RFA between 2013 and 2021. The median age was 17 years, with a range between 5 and 41. Patients were evaluated 3 months after the procedure with a CT scan and were followed up for 1 year. A wide range of variables like location of the lesion, biopsy results, success rates and complications were reviewed for the subsequent statistical analysis.
**Results or Findings:** Technical success was accomplished in 15 of the 16 patients (93.75%). All patients treated with this technique showed complete remission of symptoms 48 hours after the procedure. There were no immediate or late complications. Recurrence of pain was reported by two of our patients (12.5%) 30 and 48 months after treatment respectively due to recurrence of the lesion.
**Conclusion:** Radiofrequency ablation is an effective minimally invasive technique for treatment of osteoid osteomas. The safety of this procedure is denoted due to its low rate of complications.
**Limitations:** Small sample size.
**Ethics committee approval:** This study was approved by an ethics committee.
**Funding for this study:** No funding was needed.
**Author Disclosures:**
Luis Martínez Camblor: Nothing to disclose
Miguel Martínez-Cachero García: Nothing to disclose
José Rodríguez Castro: Nothing to disclose
Ana María Montes García: Nothing to disclose
Faustino García Arias: Nothing to disclose
Amador Prieto Fernández: Nothing to disclose

### RPS 2110-6

**Bone lesion AI classifier**

*B. Rinott*, C. Zeltser Dekel, D. Militianu, E. Bercovich; Haifa/IL
*(barinott@gmail.com)*

**Purpose:** Limb radiographs present a diagnostic challenge, especially when clinical data is nonspecific. therefore, bone lesions in early stages are sometimes difficult to identify and treatment may be delayed. In recent years, advancements in the artificial intelligence field, and specifically deep neural networks (DNN), have proved to be a powerful tool that can assist in classifying images. These algorithms are based on vast databases collected and labelled by certified professionals relevant to the studied field. This study's objective was to evaluate the use of deep-learning algorithms to develop a decision support tool for primary physicians, which can classify radiographs into normal and abnormal. The main goal of the study is to create a proof of concept for future radiograph decision-support classifiers of limb radiographs.
**Methods or Background:** This retrospective study included patients aged 5-40 with upper and lower extremity radiographs. The dataset was divided into an abnormal group, which included radiographs containing bone lesions, and a normal control group. Initially 10,000 radiographs matched the original research parameters. After screening by radiologists, a total of 1469 radiographs from 351 patients were included in the study. 973 images (245 patients) were classified as normal, and 496 images (106 patients) were classified as abnormal. The radiographs were then processed and were used to train several DNNs. The best DNN was chosen based on network sensitivity and accuracy.
**Results or Findings:** The DNN which yielded the best results was found to be MobileNet, with sensitivity of 98.6% and accuracy of 91.5%.
**Conclusion:** This study is an initial proof of concept of the feasibility of a decision-support algorithm for primary care physicians in diagnosing limb radiograph abnormality.
**Limitations:** Small sample size due to low prevalence of primary bone tumors.
**Ethics committee approval:** This study was approved by an ethics committee.
**Funding for this study:** Not applicable.
**Author Disclosures:**
Eyal Bercovich: Nothing to disclose
Bar Rinott: Nothing to disclose
Carmel Zeltser Dekel: Nothing to disclose
Daniela Militianu: Nothing to disclose

### RPS 2110-7

**Feasibility of MRI in diagnosis and characterisation of intra-articular synovial masses and mass-like lesions**

*T. Hassan*, N. Mohey; Zagazig/EG
*(tamirhaq@yahoo.com)*

**Purpose:** To correlate the results of MRI with arthroscopy in characterisation of different varieties of intra-articular synovial masses and mass-like lesions.
**Methods or Background:** This observational prospective study was conducted between February 2018 and August 2019. We screened 1000 routine musculoskeletal MRI examination during this period, 32 of which showed intra-articular synovial masses/mass-like lesions. The selected 32 patients had a mean age of 49.20 ± 2.0 years; all presented with joint swelling, pain or difficulty of movement according to the joint affected. All patients underwent arthroscopy for histopathological correlation.
**Results or Findings:** The final diagnosis was synovial chondromatosis in 14 patients (43.8%); PVNS in ten patients (31.3%); and lipoma arborescens in five patients (15.6%). Synovial chondrosarcoma, synovial haematoma and synovial ganglion cyst each in one patient (3.1%). The concordance of MRI to arthroscopy was 96.6% accuracy, 91.7% sensitivity, 99% specificity, 52.3% PPV and 99.9% NPV.
**Conclusion:** Good correlation was observed between MRI and arthroscopy in the diagnosis of intra-articular synovial masses/mass-like lesions.
**Limitations:** Mixed case.
**Ethics committee approval:** Approved by the local institutional board.
**Funding for this study:** No funding was received for this study.
**Author Disclosures:**
Tamer Hassan: Nothing to disclose
Nesreen Mohey: Nothing to disclose

### RPS 2110-8

**Soft-tissue tumor reporting and data system (ST-RADS): MRI reporting guidelines with multi-institutional validation study of extremity soft-tissue tumours**

*A. Chhabra*[1], B. Amini[2]; [1]Baltimore, MD/US, [2]Houston, TX/US

**Purpose:** To our knowledge, no current guidelines exist in outlining follow-up or interventional strategies in musculoskeletal soft-tissue tumour management. To develop and validate soft-tissue tumour reporting and data system (ST-RADS).
**Methods or Background:** This is a multi-institutional cross-sectional study of soft-tissue masses. An expert consensus agreement was reached for ST-RADS categories using the terminology from the WHO classification. Adipocytic tumors, T2-hyperintense and T2-hypointense masses of extremities with a wide spectrum of histologies were assessed. MRI categories were: STRADS 0 - incomplete imaging, I – no lesion identified, II - definitely benign, III - probably benign, IV - indeterminate or suspicious for malignancy, V- highly suggestive of malignancy, and VI - known biopsy-proven malignancy or recurrence. Eight readers evaluated cases, and the ICC and AUC were calculated.
**Results or Findings:** 200 soft-tissue masses were tested. There was good inter-reader agreement with ICC= 0.72 [95% CI=0.64-0.79] and 0.69 [95% CI=0.59-0.70] for adipocytic and T2-hyperintense, and fair, 0.48 [95% CI=0.35-0.62] for T2- hypointense masses. The sensitivity and specificity for detection of malignancy were 96% and 63%, 93% and 71%, 64% and 84% for adipocytic, T2-hyperintense, and T2-hypointense masses, respectively. The AUC was 0.79-0.89.
**Conclusion:** ST-RADS guidelines using standardised terminology stratify musculoskeletal tumours into benign and malignant categories and provide a management strategy.
**Limitations:** The cases were presented by PowerPoint presentations among institutions. While this allowed for easy anonymisation and data sharing, real-time windowing and image scrolling were not available, which may have limited detailed evaluation. However, it was ensured that the representative sections, margins and extents of tumours were included in the imaging presentation. Ideally, this system should be tested and re-tested prospectively before widespread use.
**Ethics committee approval:** Retrospective IRB at local institution.
**Funding for this study:** No funding was received for this study.
**Author Disclosures:**
Behrang Amini: Nothing to disclose
Avneesh Chhabra: Grant Recipient: Image biopsy Lab Inc. Speaker: Siemens Other: Book Royalties- Jaypee, Wolters Advisory Board: Image biopsy Lab Inc. Consultant: Treace Medical Concepts Inc., Icon Medical

### RPS 2110-9

**Sarcopenia in central European patients with multiple myeloma**

*J. Neubauer*, T. D. Diallo; Freiburg/DE

**Purpose:** To evaluate the association of sarcopenia with overall survival (OS) and progression free survival (PFS) in central European patients with multiple myeloma (MM). Our hypothesis is that sarcopenia is associated with shorter overall and progression-free survival.
**Methods or Background:** This retrospective study included patients with MM who were treated in our institution over a period of 17 years and who underwent non-contrast computed tomography (CT) of the abdomen within 100 days of the date of initial diagnosis. We determined sarcopenia using axial CT reconstructions at the level of third lumbar vertebra and collected data from patients on established clinical myeloma markers. Variables were compared using the Mann-Whitney U test and Pearson Chi² test. Calculations of OS and PFS were performed using the Kaplan-Meier method and univariate Cox proportional hazard regression models. Corrected P-values <0.05 were considered to denote statistical significance.
**Results or Findings:** 226 patients were included (median age: 65 years (range 29-89); 62.8 % male gender; mean BMI: 24.69 (range 14.08-42.45)). The prevalence of sarcopenia overall at baseline was 52.7% (119/226). In univariate analyses sarcopenia was not associated with a significant reduction in overall survival (hazard ratio, 1.13; p = 0.58) or progression-free survival (HR, 1.07, p = 0.69).
**Conclusion:** The findings of this study suggest that sarcopenia has no predictive value in central European patients with multiple myeloma.
**Limitations:** This is a monocentric and retrospective study.
**Ethics committee approval:** This study is approved by the local ethics committee.
**Funding for this study:** This is an investigator initiated trial.
**Author Disclosures:**
Jakob Neubauer: Nothing to disclose
Thierno D. Diallo: Nothing to disclose

### RPS 2110-10

**COVID-19 related acute sarcopenia in pectoral and intrinsic paraspinal muscles**

*B. Akdal Dölek*, C. Aydin; Ankara/TR
*(b_akdal@yahoo.com)*

**Purpose:** Acute sarcopenia and muscle wasting are emerging as a new field of interest in patients with newly acquired inflammatory and catabolic status. It's the decrease in muscle function and mass due to a stressor event such as infection, trauma or surgery. SARS-Cov-2 infection leads to cytokine storm, the hallmark of COVID-19 infection. This proinflammatory state combined with immobility due to hospitalisation leads to decline in muscle quantity and function.
**Methods or Background:** In this research, we aimed to show this decrease in muscle mass in patients who had been hospitalised due to COVID-19 and who had lost their lives during intensive care. These patients, mostly older adults, had two chest computerised-tomography scans during their hospitalisation. We measured cross sectional area on axial chest CT of paravertebral muscles at the T12 vertebral level and of pectoralis muscles at above the aortic arch level. The pulmonary involvement was classified according to percentage of lung parenchymal extent of disease. Also, the comorbidities of each patient and laboratory results at admission were assessed.
**Results or Findings:** A total of 100 patients were first included, out of which 13 patients got excluded because of the time interval between two scans or the poor quality of images. The mean age of 87 patients (58 males, 29 females) was 71,94. The CT measurement values are the difference between the second and first scans' area of the muscle groups. Mean difference is found to be -5273 mm² of paravertebral muscles and -3629 mm² of the pectoralis muscles. The overall muscle area is decreased while overall blood levels are changed in favor of inflammatory status.
**Conclusion:** Acute sarcopenia affects hospitalised patients, and its degree is associated with the patient's pre-existing medical condition.
**Limitations:** We didn't have a control group.
**Ethics committee approval:** Pending.
**Funding for this study:** Not applicable.
**Author Disclosures:**
Ceren Aydin: Nothing to disclose
Betül Akdal Dölek: Nothing to disclose

### RPS 2110-11

**Femoral 2D-DXA and 3D-DXA analysis: PHPT effects on cortical and trabecular bone mineral density**

*N. Palladino*[1], R. Winzenrieth[2], F. Gorgoglione[1], I. Notarangelo[1], G. Orciulo[1], G. Prencipe[1], M. T. Paparella[1], E. A. Serricchio[1], G. Guglielmi[3]; [1]San Giovanni Rotondo/IT, [2]Barcelona/ES, [3]Foggia/IT
*(nicolapalladino90@gmail.com)*

**Purpose:** To evaluate the impact of parathormone (PHT) in a group of patients with hyperparathyroidism (PHPT) compared with a control group of patients with osteoporosis, femural bone mineral density (BMD) of cortical and trabecular bone were analysed.
**Methods or Background:** 144 Caucasian women and men suffering from PHPT were recruited from 2011 to 2016. BMD values detected in the femoral neck and lumbar spine and biochemical values were collected. Density, thickness, areal and cortical volumetric density were evaluated with 3D-DXA software and standardised by age, biochemical dosages, anthropometric parameters and bone parameters.
**Results or Findings:** The data showed a significant negative correlation between patients with PHPT and the control group regarding the areal bone mineral density (aBMD) and volumetric bone mineral density (vBMD), both at the level of the cortical and trabecular bone. No significant correlation was found when the thickness of the cortical bone was analysed.
**Conclusion:** In this study we found a negative effect of PHT levels on cortical, trabecular, areal and volumetric bone mineral density, whilst there were no significant effects on cortical bone thickness.
**Limitations:** The main limitations of the present study were the relative small number of patients with PHPT, which needs to be increased since the biological effects of PTH on cortical and trabecular bone is still under investigation.
**Ethics committee approval:** The study was approved by the ethics committee.
**Funding for this study:** No funding was received for this study.
**Author Disclosures:**
Nicola Palladino: Nothing to disclose
Ettore Angelo Serricchio: Nothing to disclose
Renaud Winzenrieth: Nothing to disclose
Giuseppe Guglielmi: Nothing to disclose
Francesca Gorgoglione: Nothing to disclose
Ilenia Notarangelo: Nothing to disclose
Gianluca Prencipe: Nothing to disclose
Grazia Orciulo: Nothing to disclose
Maria Teresa Paparella: Nothing to disclose

**RPS 2110-12**
**Muscle injuries and recovery: correlation MRI injury severity and return to play (RTP): a prospective study**
T. Vogl, *S. Michalik*, C. Mader, V. Koch, K. Eichler, I. Yel;
Frankfurt a. Main/DE

**Purpose:** MRI is the most sensitive tool for the diagnosis of muscle injuries. Premature return to play (RTP) leads to a high re-injury rate and prolonged period of re-convalescence. The aim of this study was to correlate MR-rated injury severity to physical conditions at the time of RTP and predict RTP by MRI.
**Methods or Background:** 26 male soccer players with non-contact hamstring injuries underwent 3T-MRI and physical examinations (passive leg raise, active knee extension) at initial injury (within 72 hours), and, after 3 weeks without physical exercise, follow-up examinations were performed. Three experienced radiologist graded the injuries (mild, moderate and severe) and aimed to perform recommendation for the re-uptake of physical activity based on MRI.
**Results or Findings:** Overall mean MRI measurements were as follows (initial examination, first follow-up): craniocaudal length of injury decreased, from 9.1 ± 4.5 cm to 5.9 ± 3.7 cm ($p < 0.01$); and the injured cross-sectional area regenerated slightly, from 16.4 ± 11.9 to 11.8 ± 11.8 cm2 ($p < 0.02$). RTP was recommended for 2/26 players based on MRI, while ranges of motion and pain in the functional examination significantly improved and aligned with the values of the contralateral healthy side ($p < 0.02$).
**Conclusion:** In the follow-up MRI the extent of muscle injury severity is often overrated due to persistent extensive signal alteration in the muscle, leading to a prolonged recovery time, while functional tests show significant improvements. The healing process of muscle injuries highlights the discrepancy between physical/functional improvements and reduced imaged-based monitoring of recovery. RTP prediction based on MRI led to prolonged absence in physical activities.
**Limitations:** The limitations of this study are e.g. the small study group and no uniform rehabilitation measure.
**Ethics committee approval:** Institutional review board approval was obtained.
**Funding for this study:** Any funding has been received.
**Author Disclosures:**
Ibrahim Yel: Nothing to disclose
Thomas Vogl: Nothing to disclose
Vitali Koch: Nothing to disclose
Christoph Mader: Nothing to disclose
Sabine Michalik: Nothing to disclose
Katrin Eichler: Nothing to disclose

**16:00-17:30 Room Z**

### Research Presentation Session: Oncologic Imaging

### RPS 2116

### Gastrointestinal tumours

**Moderator**
S. Gourtsoyianni; Athens/GR

**RPS 2116-3**
**Clinical impact of MRI compared to 68Ga-DOTATATE PET-CT in monitoring metastasised neuroendocrine tumours (NETs): can MRI replace 68Ga-DOTATATE PET-CT?**
*C. Rijsemus*, E. Berardi, M. P. Engbersen, M. Tesselaar, M. Versleijen, R. G. H. Beets-Tan, M. Lahaye; Amsterdam/NL
*(c.rijsemus@nki.nl)*

**Purpose:** To evaluate the clinical impact of DW-MRI on the management of patients with (suspicion of) metastatic neuroendocrine tumours (NETs) as compared to the standard staging tool: 68Ga-DOTATATE PET-CT.
**Methods or Background:** Patients with NETs and (suspicion of) metastasis determined on 68Ga-DOTATATE PET-CT who also underwent an abdominal DW-MRI during the disease course between August 2016 and June 2020 were included. Retrospectively, the initial original MRI and PET-CT reports were reviewed. The findings of both modalities were compared and categorised as agreed or disagreed on the course of disease (stable disease, progressive disease or response to treatment). The clinical impact was defined as a change of management. The MRI consisted of diffusion-weighted, T2-weighted, and post-contrast T1-weighted imaging of the thorax, abdomen, and pelvis. The 68Ga-DOTATATE PET-CT acquisitions were made 45 minutes after intravenous injection of the tracer.
**Results or Findings:** Twenty-three patients were included. The median time between imaging modalities was 37 days (range: 1-111 days). Twenty patients (87%) showed comparable results on both modalities: 13 patients with stable disease, 6 patients with progression and 1 patient had a good treatment response. For three patients (13%) MRI showed progressive disease compared to PET-CT. Most patients (22/23) showed no management change after MRI was performed except for one patient where DW-MRI detected a second primary colorectal tumour that needed surgical removal.
**Conclusion:** This study shows that the clinical outcome of monitoring metastasised NETs with MRI and 68Ga-DOTATATE PET-CT are comparable. Because MRI compared to PET-CT produces less radiation, is most often easier to access and is cheaper, further research is needed to determine the role of MRI in monitoring metastasised NETs.
**Limitations:** Identified limitations were the small sample size and the fact that this was retrospective research.
**Ethics committee approval:** This study has been approved by the ethics committee.
**Funding for this study:** Not applicable.
**Author Disclosures:**
Eva Berardi: Nothing to disclose
Margot Tesselaar: Nothing to disclose
Max Lahaye: Nothing to disclose
Maurits Peter Engbersen: Nothing to disclose
Charlotte Rijsemus: Nothing to disclose
Regina G. H. Beets-Tan: Nothing to disclose
Michelle Versleijen: Nothing to disclose

**RPS 2116-4**
**Contrast-enhanced computed tomography texture analysis in colon cancer: correlation with genetic markers**
C. Zanon, *G. Cabrelle*, K. D. Luong, A. Pepe, F. Crimì, E. Quaia; Padua/IT
*(giulio.cabrelle@gmail.com)*

**Purpose:** Contrast-enhanced computed tomography (CT) is the preferred imaging examination for colorectal cancer (CRC) staging. Patients with CRC should have genetic characterisation for RAS, BRAF and MMR mutations, as a predictive marker for response to therapies, and as a prognostic indicator for patient outcome. Texture analysis is a technique that extracts information that is not easily depicted by visual inspection. The purpose of the study is to determine whether contrast-enhanced CT texture features relate to, and can predict, the presence of microsatellite instability (MSI) and specific genetic mutations in CRC carcinogenesis.
**Methods or Background:** This retrospective study analysed the pre-operative CT images of patients with pathological diagnosis of CRC, who underwent testing for mutations in the KRAS, NRAS, BRAF and MMR genes. Using specific software on the CT image on venous phase of each patient, a region of interest was manually drawn along the CRC in all its volumetric extension. A total of 56 texture parameters were extracted. Texture parameters were compared between the wild-type gene group and the mutated gene group. A p-value of <0.05 was considered statistically significant.
**Results or Findings:** The study included 47 patients with stage III-IV CRC. A statistically significant difference between the microsatellite-stable group and the MSI group was found in four parameters: GLRLM RLNU with AUC of 0.725 (sensitivity 77.8%, specificity 65.8%); GLZLM SZHGE with AUC of 0.787 (sensitivity 88.9%, specificity 65.8%); GLZLM GLNU with AUC of 0.743 (sensitivity 88.9%, specificity 60.5%), GLZLM with AUC of 0.775 (sensitivity 88.9%, specificity 65.8%).
**Conclusion:** The findings support the potential role of CT texture analysis in detecting MSI CRC on pre-treatment CT scans.
**Limitations:** Identified limitations were as follows: this study was a single centre retrospective analysis; only a small group of patients were included.
**Ethics committee approval:** This study received ethics committee approval.
**Funding for this study:** No external funding was received for this study.
**Author Disclosures:**
Alessia Pepe: Nothing to disclose
Giulio Cabrelle: Nothing to disclose
Kim Duyen Luong: Nothing to disclose
Chiara Zanon: Nothing to disclose
Filippo Crimì: Nothing to disclose
Emilio Quaia: Nothing to disclose

**RPS 2116-5**
**Whether quantitative spectral parameters can predict lymph node metastasis in colorectal cancer: a preliminary study**
*W. Peng*, H. Zhang, S. Dong; Beijing/CN

**Purpose:** To explore the clinical application of quantitative spectral parameters to predict lymph node metastasis (LNM) in patients with colorectal cancer (CRC).
**Methods or Background:** This prospective study selected patients with suspicion of CRC between April 2021 and August 2021 consecutively to undergo portal venous phase dual-layer spectral computed tomography.

Patients with confirmed CRC by surgery were enrolled and divided into two groups based on LNM status. Mean attenuation on conventional 120-kVp images (HUconv), virtual noncontrast (VNC), and virtual monoenergetic images (VMIs) at 40–180 keV; iodine concentration (IC) and normalized IC (NIC) were measured for each lesion. The ratio of IC/VNC×100 (I/V) was calculated. Each parameter was compared between LNMs and non-LNMs by using the Mann-Whitney U test. Diagnostic performance was evaluated by using the area under the receiver operating characteristic curve (AUC) with sensitivity and specificity.
**Results or Findings:** There were a total of 117 patients with 60 LNMs and 57 non-LNMs. HUconv, mean attenuation on VMIs at 40–80 keV, IC and I/V were lower in the LNM group than in the non-LNM group (p<0.05 for all). Attenuation differences between the LNM and the non-LNM group on VMIs were maximal at 40 keV (P<0.001). The highest sensitivity for differentiating LNM and non-LNMs was achieved for I/V (87% [52 of 60 LNMs]), with a specificity of 89%.
**Conclusion:** Quantitative spectral parameters enabled an ideal predictive performance of LNM in patients with CRC.
**Limitations:** The lack of external validation was an identified limitation.
**Ethics committee approval:** This prospective study was approved by the local ethics committee and written informed consent was obtained from all subjects.
**Funding for this study:** General Project of National Natural Science Foundation of China funded this study.
**Author Disclosures:**
Shushan Dong: Nothing to disclose
Hongmei Zhang: Nothing to disclose
Wenjing Peng: Nothing to disclose

### RPS 2116-7

**A new standard for monitoring treatment response after neoadjuvant chemotherapy in colorectal cancer patients with peritoneal metastases: 'the estimated volume index' (EVI)**
*C. Rijsemus*, N. Pereira da Silva, E. Berardi, N. Kok, A. Aalbers, R. G. H. Beets-Tan, M. Lahaye; Amsterdam/NL

**Purpose:** Nowadays neoadjuvant chemotherapy (NACT) is given more frequently to colorectal cancer patients (CRC) with peritoneal metastases (PM). However, after NACT lesions are often difficult to score with the current RECIST-criteria because lesions are too small, have no clear borders and/or disintegrated after therapy. This study evaluates a new, imaging based, scoring system: 'the estimated volume index (EVI)'.
**Methods or Background:** Patients with PM from CRC who underwent NACT were included from January 2015 to August 2020. A CT-scan was made before and after NACT and a retrospective analysis was done by 2 readers. The EVI divides the abdomen into 7 regions (Dutch Region Score); then patients are categorised according to the overall response based on the response present in the majority of those regions. The EVI response could be applied the following way: EVI1 (progressive disease >25%), EVI2 (mixed response; more than 25% decrease and 25% increase), EVI3 (stable disease -25%<->25%), EVI4 (partial response -25%<->-50%), EVI5 (good response -50%<->-100%), EVI6 (complete response -100%). To validate EVI the results were compared to the median overall survival (OS).
**Results or Findings:** Twenty-nine patients were included (median age of 62 years). Twenty-three (79%) patients underwent a CRS-HIPEC and 6 (21%) patients had an exploratory surgery. Median OS was 35 months. For reader 1 the median OS correlated significantly (p=0.007) with the EVI scoring. For patients with progressive disease (n=5) the median OS was 6 months vs. 42 months for patients with stable disease, partial, good or complete response (p=0.01). The interrater reliability agreement was 0.672 (p<0.001).
**Conclusion:** These results show that EVI, when validated with more patients and multiple readers, could be a new practical approach for treatment response monitoring of CRC patients with PM.
**Limitations:** Identified limitations were the sample size and the use of retrospective data.
**Ethics committee approval:** This study received ethics committee approval.
**Funding for this study:** Not applicable.
**Author Disclosures:**
Eva Berardi: Nothing to disclose
Nuno Pereira da Silva: Nothing to disclose
Max Lahaye: Nothing to disclose
Charlotte Rijsemus: Nothing to disclose
Niels Kok: Nothing to disclose
Regina G. H. Beets-Tan: Nothing to disclose
Arend Aalbers: Nothing to disclose

### RPS 2116-8

**The definition of a near-complete response after neoadjuvant (chemo)radiotherapy for rectal cancer: results of an expert survey**
*P. A. Custers*, D. M. J. Lambregts, G. L. Beets, M. Maas; Amsterdam/NL

**Purpose:** To establish an internationally supported definition of a near-complete response (nCR) on endoscopy and MRI following (chemo)radiotherapy for rectal cancer.
**Methods or Background:** An online survey on the definition of a nCR was held, consisting of 21 image features derived from endoscopy, T2W-MRI and DWI, 20 statements, and 20 cases of rectal cancer patients 8-12 weeks following (chemo)radiotherapy. Ten clinical experts (7 surgeons, 3 radiologists) on organ-preservation for rectal cancer indicated agreement with the items. Consensus was defined as ≥80% agreement.
**Results or Findings:** Consensus was reached in 8/21 of features, 7/20 statements, and 6/20 cases. Respectively 86% and 80% of experts agreed that a small flat ulcer on endoscopy and small focal spots of high signal on DWI were indicative of a nCR. The experts agreed that the response on endoscopy is more decisive than the response on MRI, that biopsies are not always needed if a nCR is found, and that a suspicious residual (lateral) lymph node should be considered an incomplete response. Consensus was achieved on mandatory inclusion of nodal status in defining a nCR. The following cases were deemed a nCR: (1) a flat white scar on endoscopy, homogeneous hypo-intense fibrosis on T2W-MRI, and focal diffusion signal on DWI; (2) a flat white scar on endoscopy, heterogeneous irregular fibrosis on T2W-MRI, and a linear focal signal on DWI; and (3) a small (<1cm) flat ulcer on endoscopy, heterogeneous irregular fibrosis on T2W-MRI, and no high signal on DWI.
**Conclusion:** The definition of a nCR varies between centres. These preliminary results can be considered a first step towards defining a nCR to achieve more uniformity regarding selection of patients for organ-preservation after neoadjuvant (chemo)radiotherapy.
**Limitations:** A limitation of the study was that a small expert panel with relatively few radiologists was used.
**Ethics committee approval:** This study received Ethics committee approval: NL58095.031.16
**Funding for this study:** Not applicable.
**Author Disclosures:**
Monique Maas: Nothing to disclose
Petra A Custers: Nothing to disclose
Doenja Marina Johanna Lambregts: Nothing to disclose
Geerard L. Beets: Nothing to disclose

### RPS 2116-9

**Prognostic models for classifying rectal tumour response to therapy using radiomics and CNN features**
*G. Guido*, M. H. Soomro, G. Giunta, M. Polici, B. Bracci, G. Piccinni, I. Nacci, M. Zerunian, A. Laghi; Rome/IT
*(gisellaguido29@gmail.com)*

**Purpose:** To retrospectively evaluate the best radiomic features in predicting complete response to neoadjuvant therapy in patients affected by rectal cancer and to assess the possible correlation between them.
**Methods or Background:** A total of 109 handcrafted radiomic features and 4096 deep radiomic features were extracted from pre-treatment 3D-MRI of 43 patients, using transfer learning from a pre-trained convolutional neural network. The most widely explored 7 supervised machine learning-based classifiers and 6 different feature selection algorithms were validated and compared utilising all possible radiomic features, in order to examine their effectiveness in achieving an accurate predictive model. Cross-validation was performed in 100 rounds partitioning the data as 75% for training and 25% for testing.
**Results or Findings:** Using only handcrafted radiomic features, artificial neural network classifier and Fisher as feature selection algorithm delivered the best predictive performance on test data sets with the area under the curves (AUCs) [mean±SD] of 0.79±0.016 and 0.8±0.01, respectively. The best prognostic performance, using only deep radiomic features, was achieved by linear support vector machine (LSVM) classifier and Relief-based feature selection algorithm as 0.8±0.042 and 0.82±0.04, respectively. When using a combination of both handcrafted and deep radiomic features, almost all classifiers in combination with every feature selection algorithm generated better AUC than that obtained individually; the best AUCs were generated by the LSVM classifier and Relief-based feature selection as 0.84±0.025 and 0.87±0.013, respectively.
**Conclusion:** The best predicting models' performance was achieved by integrating both handcrafted and deep radiomic features, i.e. LSVM classifier and Relief-based feature selection algorithms in combination with all classifiers.
**Limitations:** Identified limitations were the small population sample and the retrospective nature of the study.
**Ethics committee approval:** This study was approved by our local institutional review board; written informed consent was obtained from all study participants.

**Funding for this study:** No funding was received for this study.
**Author Disclosures:**
Marta Zerunian: Nothing to disclose
Benedetta Bracci: Nothing to disclose
Ilaria Nacci: Nothing to disclose
Mumtaz Hussain Soomro: Nothing to disclose
Giulia Piccinni: Nothing to disclose
Gaetano Giunta: Nothing to disclose
Andrea Laghi: Nothing to disclose
Michela Polici: Nothing to disclose
Gisella Guido: Nothing to disclose

### RPS 2116-10

**Added value of multiparametric PET/MRI in staging of rectal cancer**

*A. J. Herold*, C. Wassipaul, M. Weber, P. Baltzer, S. Riss, A. Stift, A. Haug, A. Ba-Ssalamah, D. Tamandl; Vienna/AT
*(alexander.herold@meduniwien.ac.at)*

**Purpose:** PET/MRI has emerged as a 'one-stop-shop' examination in staging of colon and rectal cancer. The purpose of this study was to determine, whether multiparametric PET/MRI can improve locoregional staging of rectal cancer (RC) and to evaluate its prognostic value for early recurrence and death after resection.
**Methods or Background:** In this retrospective study, 49 patients with primary RC who underwent multiparametric 18-F FDG PET/MRI, followed by surgical resection, were included. T and N staging was performed on standard T2w-sequences by two readers. Various parameters were extracted from the multiparametric protocol, consisting of DWI, perfusion imaging and PET sequences, and were compared to the histopathological and radiological staging. Additionally, clinical follow-up data (tumour markers, lab results, physical examination, etc.) as well as follow-up imaging were assessed for signs of recurrence. Statistical analysis (median, mean, 90th percentile, interquartile range of parametric data), ROC curve analysis and multivariate regression analysis was performed for correlation with clinical and histological parameters, Cox regression was used for survival analysis.
**Results or Findings:** Locally advanced tumours exhibited significantly higher metabolic tumour volume (MTV) and total lesion glycolysis. Rate constant (kep) was associated with increased lymph node ratio as well as with lymphatic invasion. In the multivariate regression analysis, a combination of MTV and kep best predicted locally advanced RC. Metabolic tumour volume was associated with overall survival and recurrence. Further data will be presented at the congress.
**Conclusion:** Multiparametric PET/MRI including dynamic contrast-enhanced MR perfusion provides additional information on RC tumour biology and tumour involvement of the lymphatic system and may have prognostic value. This can improve identification of locally advanced tumours and hence help in treatment stratification.
**Limitations:** Identified limitations were that this was a single centre study with a rather small patient cohort.
**Ethics committee approval:** IRB: EK Nr: 1403/2015.
**Funding for this study:** This study was funded by the Margaretha Hehberger Foundation.
**Author Disclosures:**
Anton Stift: Nothing to disclose
Alexander Haug: Nothing to disclose
Alexander Johannes Herold: Nothing to disclose
Ahmed Ba-Ssalamah: Consultant: Bayer Speaker: Bayer
Stefan Riss: Nothing to disclose
Christian Wassipaul: Nothing to disclose
Dietmar Tamandl: Investigator: Roche
Michael Weber: Nothing to disclose
Pascal Baltzer: Nothing to disclose

### RPS 2116-11

**Diffusion weighted MRI (DWI) and carbon ion radiotherapy (CIRT): a preliminary study on DWI predictive role in CIRT re-treatment of locally recurrent rectal cancer**

*D. N. Boccuzzi*, G. Fontana, A. Barcellini, A. Vai, M. Pecorilla, G. Baroni, E. Orlandi, L. Preda, S. Imparato; Pavia/IT

**Purpose:** To assess the role of pre-treatment b=1000 s/mm2 DWI (b1000) and apparent diffusion coefficient (ADC) in outcome prediction of locally recurrent rectal cancer (LRRC) re-irradiated with CIRT.
**Methods or Background:** CIRT has proved to be safe and effective for re-irradiation of unresectable LRRC, and the availability of a pre-treatment stratification of patients could optimise treatment strategy. Clinical and radiological data from 17 consecutive LRRC patients re-irradiated with CIRT, between 2014 and 2020, were retrospectively collected. Patients were stratified as 1-year-responder (Re) and 1-year-non-responder (NRe) based on volume changes between pre-treatment and 1-year follow-up MRI. Each lesion was manually contoured on pre-treatment b1000 and ADC, and DWI features were extracted (median, inter-quartile, skewness, kurtosis). Statistically significant differences of DWI features were tested with non-paired Mann-Whitney U test ($\alpha$=0.05). Diagnostic accuracy of relevant features was explored, hence the potential biomarkers were fed to k-means clustering algorithm for automatic stratification purposes.
**Results or Findings:** The b1000 median values for Re (n=11) and NRe (n=6) were 34.0 [13.0] and 62.5 [23.9] respectively, while ADC median values were 963.0 [277.0] and 942.5 [339.0] µm2/s (median [inter-quartile]). Statistically significant differences between Re and NRe were found for all b1000 features and ADC kurtosis. Only b1000 median and inter-quartile and ADC kurtosis showed relevant diagnostic accuracy and the k-means accuracy score was 0.88.
**Conclusion:** b1000 median, inter-quartile and ADC kurtosis, showed remarkable potential for being a biomarker of CIRT response. Further investigations should be carried out on a larger cohort to confirm this data.
**Limitations:** Identified limitation were: the small sample size; non-generalisability of b1000 values, due to its sensitivity to MR scanner hardware; retrospective nature; all data from a single study centre.
**Ethics committee approval:** An independent ethics committee approved the conduction of this retrospective analysis on the 30th June 2021.
**Funding for this study:** No funding was received for this study.
**Author Disclosures:**
Guido Baroni: Nothing to disclose
Mattia Pecorilla: Nothing to disclose
Dario Nicola Boccuzzi: Nothing to disclose
Giulia Fontana: Nothing to disclose
Alessandro Vai: Nothing to disclose
Sara Imparato: Nothing to disclose
Amelia Barcellini: Nothing to disclose
Lorenzo Preda: Nothing to disclose
Ester Orlandi: Nothing to disclose

### RPS 2116-12

**Role of MRI for early tumour response evaluation in anal cancer after five weeks of chemoradiotherapy in guiding boost strategies**

*D. M. J. Lambregts*[1], E. van den Hurk[2], F. Peters[1], F. Voncken[1], B. Grotenhuis[1], C. Deijen[1], B. van Triest[1]; [1]Amsterdam/NL, [2]Maastricht/NL
*(doenja.lambregts@gmail.com)*

**Purpose:** Standard treatment for ≥cT2 and/or N+ stage anal cancer is chemoradiotherapy(CRT). At our institution, a sequential radiotherapy boost (3x1.8 Gy) is given selectively based on the interim response after 5 weeks of CRT. The aim was to assess the impact of MRI in addition to digital rectal examination (DRE) in guiding this decision.
**Methods or Background:** This was a retrospective analysis of 48 anal cancer patients treated with CRT±sequential boost. The interim response was assessed after five weeks with DRE (when feasible) and T2W-MRI+DWI. On DRE, the response was classified as insufficient when an obvious residual mass was palpable. On MRI, the response was classified as insufficient (obvious residual mass on T2W-MRI+DWI) or sufficient ([near-]complete response; fibrosis, no/minor high signal on DWI). The decision whether to boost (in case of insufficient response) was guided by the findings of DRE+MRI.
**Results or Findings:** In 16/48 patients (33%), DRE was not feasible because of pain. In 8/16 cases, MRI indicated insufficient response; 88% were boosted. In 7 cases, MRI indicated sufficient response; boost was omitted in 86%. In 1 case, MRI was inconclusive. In 21/48 patients (44%), MRI confirmed the findings of DRE; in 81% boost strategies were planned accordingly. In the remaining patients, alternative strategies were mainly guided by patient preference/comorbidities. In 11/48 patients (23%), MRI contradicted the findings of DRE. These were mostly (8/11) cases where DRE suspected residual tumour, but MRI indicated a near-complete/complete response; boost was omitted in the majority (75%).
**Conclusion:** The addition of MRI to DRE seems helpful to assess response during CRT and guide the decision whether or not to give a sequential radiotherapy boost, specifically when DRE is not feasible (±1/3) and in cases where DRE underestimates the response.
**Limitations:** Descriptive retrospective study; small cohort.
**Ethics committee approval:** Ethics committee approval was acquired for this study.
**Funding for this study:** Not applicable.
**Author Disclosures:**
Charlotte Deijen: Nothing to disclose
Baukelien van Triest: Nothing to disclose
Elke van den Hurk: Nothing to disclose
Doenja Marina Johanna Lambregts: Nothing to disclose
Francine Voncken: Nothing to disclose
Brechtje Grotenhuis: Nothing to disclose
Femke Peters: Nothing to disclose

### Sunday, July 17

**08:00-09:00** **Room D**

### Research Presentation Session: Breast

### RPS 2202

### Contrast-enhanced mammography in clinical practice

**Moderator**
C. Dromain; Lausanne/CH
**Author Disclosure:**
C. Dromain: Advisory Board: Ipsen

### RPS 2202-2

**Preliminary results of the RACER trial: diagnostic accuracy of CESM and conventional imaging as primary work-up tool after being recalled from breast cancer screening**

*L. Neeter*[1], H. Raat[2], C. Frotscher[3], K. M. Duvivier[4], T. van Nijnatten[1], M. Smidt[1], J. E. Wildberger[1], P. Nelemans[1], M. B. I. Lobbes[3]; [1]Maastricht/NL, [2]Roermond/NL, [3]Sittard-Geleen/NL, [4]Amsterdam/NL

**Purpose:** Preliminary results of a multicentre, prospective, randomised, controlled trial comparing contrast-enhanced spectral mammography (CESM) to conventional imaging in the workup of women recalled from breast cancer screening.
**Methods or Background:** Participants were randomised to either CESM or conventional imaging (i.e., FFDM and/or DBT) as primary workup tool with/without supplemental ultrasound. The primary outcome was diagnostic accuracy expressed as sensitivity and specificity. A secondary outcome was costs associated with diagnostic imaging and biopsies. Data for primary and secondary outcomes will be collected until the next screening round.
**Results or Findings:** 529 patients were enrolled in the study between 2018-2021. 264 were randomised to CESM and 265 to conventional imaging as primary work-up tool. Currently, data from 481 patients could be used for analysis. Baseline characteristics were comparable between groups. Preliminary sensitivity was 97.9% for CESM and 97.6% for conventional imaging, and specificity estimates were 74.7% for CESM and 74.4% for conventional imaging. Ultrasound was performed in 68% and 98% of the cases in the CESM and control group. Supplemental MRI was performed for inconclusive findings four times more often in the control group than in the CESM group. Histopathological examination was required in 58% and 61% of the cases in the CESM and control group.
**Conclusion:** Diagnostic accuracy of CESM is comparable with that of conventional imaging in women recalled from screening. However, in the control group more supplemental examinations were required for diagnosis. The findings support the hypothesis that CESM as initial imaging modality is more rapid and (cost)efficient.
**Limitations:** The COVID-19 pandemic increased the screening rounds interval, thereby extending the follow-up period.
**Ethics committee approval:** The ethics committee approved the study protocol and written informed consent by the patient was obtained.
**Funding for this study:** Supported by ZonMw Efficiency Studies grant and GE Healthcare.
**Author Disclosures:**
Marjolein Smidt: Research/Grant Support: Support by Servier pharmaceutical and Nutricia for microbiome studies
Lidewij Neeter: Nothing to disclose
Patty Nelemans: Nothing to disclose
Marc B I Lobbes: Other: Speaker's fee: GE Healthcare, Hologic, Tromp Medical, Guerbet and Bayer. Medical advisory boards: GE Healthcare, Hologic, Bayer, and Guerbet.
Joachim E. Wildberger: Other: Institutional grants via Clinical Trial Center Maastricht: Agfa, Bard, Bayer, Cook, GE, Philips, Optimed, Radiomics, Siemens. Speaker's fee via Maastricht UMC+: Bayer, Siemens. Disclosures all outside the submitted work.
Caroline Frotscher: Nothing to disclose
Katya M Duvivier: Nothing to disclose
Thiemo van Nijnatten: Nothing to disclose
Henricus Raat: Nothing to disclose

### RPS 2202-3

**Diagnostic accuracy of contrast-enhanced spectral mammography in the prediction of malignancy of breast lesions presenting as microcalcifications**

L. Nicosia[1], S. Palma[2], A. C. Bozzini[1], M. Montesano[1], *D. Ballerini*[3], G. Signorelli[1], V. Bagnardi[1], S. Frassoni[1], E. Cassano[1]; [1]Milan/IT, [2]Rome/IT, [3]Pavia/IT

**Purpose:** To evaluate the diagnostic accuracy of contrast-enhanced spectral mammography (CESM) in the prediction of malignancy of breast lesions presenting as microcalcifications compared to lesions presenting as masses, based on the intensity of contrastographic enhancement.
**Methods or Background:** 321 patients with 377 suspected breast lesions (BIRADS>3) undergoing CESM in the period from January 2013 to February 2022 were included. All lesions were assessed using a 4-point scale regarding the degree of intensity of constrastographic enhancement (0 = no impregnation, 1 = minimal, 2 = moderate, 3 = marked). Scores 2 and 3 were considered predictive of malignancy. Histological results were considered the gold standard in evaluating the diagnostic performance of CESM.
**Results or Findings:** The sensitivity of the intensity of the enhancement in predicting breast lesion malignancy in CESM for microcalcifications (101 lesions) was 53.3%; the specificity was 95.8%; and the positive predictive value (PPV) and negative predictive value (NPV) were 84.2% and 82.9%, respectively. The accuracy was 83.2%. For lesions that presented as masses (276 lesions), sensitivity was 82.2%; specificity was 84.2%; and PPV and negative NPV were 95.2% and 55.2%, respectively. The accuracy was 82.6%.
**Conclusion:** The sensitivity of CESM in predicting the malignancy of lesions presenting as microcalcifications is significantly worse than in predicting the malignancy of lesions presenting as masses. However, the specificity is very high. The overall diagnostic accuracy is therefore satisfactory.
**Limitations:** No limitations were identified.
**Ethics committee approval:** All patients have read and accepted an informed consent prepared in agreement with the ethics committee of our institute before undergoing the contrast-enhanced mammography examination. The study was conducted according to the guidelines of the Declaration of Helsinki.
**Funding for this study:** No funding was received for this study.
**Author Disclosures:**
Samuele Frassoni: Nothing to disclose
Luca Nicosia: Nothing to disclose
Simone Palma: Nothing to disclose
Vincenzo Bagnardi: Nothing to disclose
Anna Carla Bozzini: Nothing to disclose
Giulia Signorelli: Nothing to disclose
Daniela Ballerini: Nothing to disclose
Enrico Cassano: Nothing to disclose
Marta Montesano: Nothing to disclose

### RPS 2202-4

**Contrast-enhanced mammography and breast MRI in the assessment of response to neoadjuvant treatment**

M. Hogan, *J. V. M. Horvat*, D. Ross, S. Sevilimedu Veeravalli, M. S. Jochelson, L. Kirstein, S. Goldfarb, C. Comstock, J. Sung; New York, NY/US

**Purpose:** To investigate the utility of contrast-enhanced mammography (CEM) as an alternative to breast MRI for the evaluation of residual disease after neoadjuvant treatment in patients with breast cancer.
**Methods or Background:** This prospective study enrolled consecutive women undergoing neoadjuvant treatment for breast cancer from July 2017 - July 2019. Breast MRI and CEM exams were performed after completion of the neoadjuvant treatment and were read by two breast radiologists blinded to the results of the other modality. Residual disease and lesion size on MRI and CEM recombined and low-energy images were compared. Statistical analysis was performed using McNemar's and Leisenring's tests where appropriate. Multiple comparison adjustment was made using Bonferroni procedure. Lesion sizes were correlated using Kendall's tau correlation coefficient.
**Results or Findings:** There were 110 participants with 115 breast cancers. Residual disease (invasive cancer or ductal carcinoma in situ) was detected in 83/115 (72%) lesions on pathology, 71/115 (62%) on MRI, 55/115 (48%) on CEM recombined images and 75/115 (65%) on CEM low-energy images. When using multiple comparison adjustment ($\alpha = < 0.001$), no significant differences were detected between MRI combined with low-energy images and CEM in assessing residual disease, accuracy (MRI: 77%, CEM: 72%; p=0.125), sensitivity (MRI: 88%, CEM: 81%; p=0.031), specificity (MRI: 47%, CEM: 50%; p=>0.999), PPV (MRI: 81%, CEM: 81%; p=0.722) or NPV (MRI: 60%, CEM: 50%; p=0.041). Size correlation between pathology and imaging modalities was moderate: $\tau = 0.36$ vs 0.33 for residual disease.
**Conclusion:** Contrast-enhanced mammography is an acceptable alternative to breast MRI for the detection of residual disease after neoadjuvant treatment.
**Limitations:** Each imaging study was interpreted by only one reader. Only largest dimensions of lesions were considered in the analysis.
**Ethics committee approval:** Institutional review board-approved study.

**Funding for this study:** This study was funded by Hologic, Inc.
**Author Disclosures:**
Joao Vicente Machado Horvat: Nothing to disclose
Janice Sung: Nothing to disclose
Shari Goldfarb: Nothing to disclose
Molly Hogan: Nothing to disclose
Srinivasa Sevilimedu Veeravalli: Nothing to disclose
Maxine S Jochelson: Nothing to disclose
Laurie Kirstein: Nothing to disclose
Dara Ross: Nothing to disclose
Christopher Comstock: Nothing to disclose

### RPS 2202-5

**Comparison of the contrast enhancement of benign and malignant lesions on CESM**

*A. Ali*, L. Metaxa, T. Suaris; London/UK
*(anam.ali@nhs.net)*

**Purpose:** The aim of this study was to assess the enhancement characteristics of malignant and benign breast lesions on CESM measuring the lesional enhancement as a ratio compared with the background parenchymal enhancement.
**Methods or Background:** A retrospective analysis of patients undergoing CESM as part of cancer staging at Barts Health NHS trust between November 2020 and February 2022 was performed. All lesions seen on CESM for which there was a histopathological correlate were inluded in the analysis. Quantitative degree of enhancement was assessed using a region of interest (ROI) placed manually over of the enhancement area within the lesion. A separate ROI representative of background enhancement (excluding the enhancing lesion). ROI signal values were assessed separately for MLO and CC projections in the early phase. Enhancement calculated as ROI signal ratio: [(ROI of lesion - ROI of background) ÷ ROI of background].
**Results or Findings:** A total of 55 female patients underwent CESM, with 61 lesions detected. Amongst these, 35 (58%) were assessed to be invasive cancers, 13 (21%) were non-invasive cancers and 13 (21%) were benign. Analysis of enhancement indices showed the following mean ROI signal values: invasive cancers 1.70; non-invasive 1.35; benign 1.05.
**Conclusion:** This study has demonstrated a correlation between the degree of lesion enhancement in CESM and malignant and benign lesions. Invasive malignant lesions had a stronger degree of enhancement than benign lesions, and an intermediate signal enhancement is seen in DCIS. Quantitative analysis of enhancement levels in CESM is a feasible practice comparable to MRI in the pre-operative assessment of women with breast cancer.
**Limitations:** Premenopausal patients did not have CESM timed with cycle, possibly affecting the enhancement ratio.
**Ethics committee approval:** This has been peer reviewed locally.
**Funding for this study:** This has been obtained from Barts Charity.
**Author Disclosures:**
Tamara Suaris: Nothing to disclose
Linda Metaxa: Nothing to disclose
Anam Ali: Nothing to disclose

### RPS 2202-6

**Contrast enhanced digital mammography (CEDM) and BI-RADS 3 findings: what benefits can we get?**

*C. Charalambous*, G. Kosta, A. N. N. Chalazonitis; Athens/GR
*(christofis.ch@gmail.com)*

**Purpose:** (1) To evaluate if the cancer detection rate in our institution for BI-RADS 3 lesions was less than 2% and (2) to assess if the detection rate could be improved by using contrast-enhanced digital mammography (CEDM).
**Methods or Background:** A total of 473 BI-RADS 3 patients depicted on screening mammogram from 2018 to 2020 were collected from our files. All the patients consented to undergo CEDM and biopsy at their next hospital visit. Contrast enhancement intensity was categorised as follows: (a) type 0: no enhancement; (b) type 1: moderate or intense enhancement; and/or (c) type BE: background enhancement. Pathology results confirmed 473 lesions in total.
**Results or Findings:** 18 (3,81%) out of the 473 lesions were diagnosed as malignant. The mean age of the patients was 56.22 years. On the contrary, 455 (96.89%) lesions were benign. The mean age of these patients was 51.04. Using CEDM's technique data a total of 5 (1.95%) out of 256 lesions with Type 0 enhancement were diagnosed as malignant and 251 (98.05%) were benign. Additionally, a total of 13 (5.99%) out of 217 lesions with any type of enhancement were proved as malignant and 204 (94.01%) were proved as benign. No enhancement of a BI-RADS 3 lesion at CEDM technique shows 1.95% possibility to be malignant, which is 1.86% better than digital mammography alone.
**Conclusion:** Our study indicates CEDM' s added value in BI-RADS 3 lesions evaluation regarding the positive impact on more accurate staging in the first categorisation. The use of CEDM on a BI-RADS 3 lesion could potentially predict more accurate benign entities based on their type of enhancement and thus play a key role as first-line mammography and reduce the recall rates.
**Limitations:** Both experienced and inexperienced readers.
**Ethics committee approval:** This study was approved by an ethics committee.
**Funding for this study:** No funding was received for this study.
**Author Disclosures:**
Athanasios N. N. Chalazonitis: Nothing to disclose
Christofis Charalambous: Nothing to disclose
Georgia Kosta: Nothing to disclose

### RPS 2202-7

**Comparing the diagnostic performance of CEM and standard contrast-enhanced breast MRI: a systematic review and meta-analysis**

*L. Neeter*[1], M. M. Q. Robbe[1], T. van Nijnatten[1], H. Raat[2], J. E. Wildberger[1], M. Smidt[1], P. Nelemans[1], M. B. I. Lobbes[3]; [1]Maastricht/NL, [2]Roermond/NL, [3]Sittard-Geleen/NL

**Purpose:** To provide a systematic review and meta-analysis to evaluate the diagnostic accuracy of contrast-enhanced mammography (CEM) versus standard contrast-enhanced breast magnetic resonance imaging (CE-MRI). CE-MRI is currently considered the most accurate imaging modality for breast cancer detection, but it is not the primary imaging modality in breast imaging. Like CE-MRI, CEM enables tumour visualisation by contrast accumulation. CEM seems to be an attractive substitute for CE-MRI for various reasons.
**Methods or Background:** This systematic search focused on the diagnostic accuracy in women with suspicious breast lesions on prior imaging or physical examination and in those who have undergone both CE-MRI and CEM. CEM had to be performed on a commercially available system. The MRI sequence parameters had to be described sufficiently to ensure that the standard CE-MRI sequences were used. Pooled sensitivity, pooled specificity and diagnostic odds ratio (DOR) were estimated using bivariate mixed-effects logistic regression modeling. Hierarchical summary receiver operating characteristic curves for CEM and CE-MRI were also constructed.
**Results or Findings:** Six studies (607 patients with 775 lesions) met the predefined inclusion criteria. Pooled sensitivity was 96% for CEM and 97% for CE-MRI. Pooled specificity was 77% for both modalities. DOR was 79.5 for CEM and 122.9 for CE-MRI. Between-study heterogeneity expressed as the I2-index was substantial with values over 80%.
**Conclusion:** The diagnostic accuracy of CEM for diagnostic workup in women with suspicious breast lesions is quite similar to that of CE-MRI regarding sensitivity and specificity. However, the pooled DOR estimates indicate higher overall diagnostic performance of CE-MRI compared to CEM.
**Limitations:** Limited number of studies in meta-analysis.
**Ethics committee approval:** Not applicable.
**Funding for this study:** Supported by a ZonMw Efficiency Studies grant and GE Healthcare.
**Author Disclosures:**
Marjolein Smidt: Research/Grant Support: Support by Servier pharmaceutical and Nutricia for microbiome studies.
Lidewij Neeter: Nothing to disclose
Magretha Maria Quirien Robbe: Nothing to disclose
Patty Nelemans: Nothing to disclose
Marc B I Lobbes: Other: Speaker's fee: GE Healthcare, Hologic, Tromp Medical, Guerbet and Bayer. Medical advisory boards: GE Healthcare, Hologic, Bayer, and Guerbet.
Joachim E. Wildberger: Other: Institutional grants via Clinical Trial Center Maastricht: Agfa, Bard, Bayer, Cook, GE, Philips, Optimed, Radiomics, Siemens. Speaker's fee via Maastricht UMC+: Bayer, Siemens. Disclosures all outside the submitted work.
Thiemo van Nijnatten: Nothing to disclose
Henricus Raat: Nothing to disclose

### RPS 2202-8

**Diagnostic value of contrast-enhanced spectral mammography in the malignity prediction of breast lesions.**

*S. Palma*[1], L. Nicosia[2], M. Montesano[2], V. Bagnardi[2], S. Frassoni[2], L. Meneghetti[2], A. Latronico[2], A. C. Bozzini[2], E. Cassano[2]; [1]Rome/IT, [2]Milan/IT
*(simone.palma@outlook.it)*

**Purpose:** The aim of this study is to evaluate if the degree of contrast enhancement in contrast-enhanced spectral mammography (CESM) may predict the malignity of a breast lesion.
**Methods or Background:** Patients with suspicious breast lesions (BIRADS >3) found on digital mammography, ultrasound or magnetic resonance in a period between January 2013 and February 2022 were enrolled. All those patients underwent CESM prior to the breast biopsy. All the lesions were scored using a 4-point scale regarding the degree of contrast enhancement (0 = no contrast enhancement, 1 = minimal, 2 = moderate, 3 = marked). Scores 2 and 3 were considered predictive of malignity. The histological results were considered the gold standard in the evaluation of relationship with the degree of enhancement.

**Results or Findings:** 377 breast lesions were found in 321 patients. The sensitivity of the degree of contrast enhancement in the prediction of the malignancy of breast lesion in CESM was 78.7 %, the specificity was 90.6 %, the positive predictive value (PPV) was 94.2% and the negative predictive value (NPV) was 68.6%. The diagnostic accuracy was 82.8%. Excluding in situ malignant lesions, sensitivity was 82.5%; specificity was 90.6%; negative PPV and NPV were 93.7% and 75.3% respectively; and diagnostic accuracy was 85.5%.
**Conclusion:** The degree of contrast enhancement of the lesions in CESM may help to predict the malignancy potential of the breast lesions. CESM performance is better in predicting breast lesion with infiltrating component.
**Limitations:** No limitations were identified.
**Ethics committee approval:** All patients have read and accepted an informed consent prepared in agreement with the ethics committee of our institute before undergoing the contrast-enhanced mammography examination. The study was conducted according to the guidelines of the Declaration of Helsinki.
**Funding for this study:** No funding was received for this study.
**Author Disclosures:**
Samuele Frassoni: Nothing to disclose
Luca Nicosia: Nothing to disclose
Simone Palma: Nothing to disclose
Vincenzo Bagnardi: Nothing to disclose
Antuono Latronico: Nothing to disclose
Anna Carla Bozzini: Nothing to disclose
Enrico Cassano: Nothing to disclose
Lorenza Meneghetti: Nothing to disclose
Marta Montesano: Nothing to disclose

**08:00-09:00** **Room E1**

### Research Presentation Session: Oncologic Imaging

### RPS 2216

### Lymphnode assessment in oncological patients

**Moderator**
J. O. Barentsz; Nijmegen/NL

### RPS 2216-2

**Lymph node reporting and data system (LN-RADS) for ultrasound classification of lymph nodes**

*C. Chudobinski*[1], A. Kaczmarek[1], K. Pasicz[2], M. Pajdziński[1], M. Hanke[1], W. Kuncman[1], A. Kołacińska-Wow[1], L. Gottwald[1], A. Cieszanowski[2]; [1]Łódź/PL, [2]Warsaw/PL
*(cezary.chudobinski@wp.pl)*

**Purpose:** To assess the accuracy of a novel system of ultrasound (US) classification of lymph nodes - Lymph Node Reporting and Data System (LN-RADS) for differentiation between malignant and benign lesions.
**Methods or Background:** This retrospective multiparametric analysis comprised 512 US examinations (gray scale and colour Doppler) in oncology patients with suspected lymph node metastases. After evaluation (by two readers) of lymph node diameter, diameter ratios, cortical thickness, sinus diameter, shape, margin, echogenicity, homogeneity and vascular architecture each lesion was categorised as LN-RADS 1 (definitely benign), LN-RADS 2 (steatotic node), LN-RADS 3 (reactive node), LN-RADS 4, consisting of 4a (mild possibility of malignancy) and 4b (high possibility of malignant) and LN-RADS 5 (definitely malignant). All observations were correlated with histopathology results. The diagnostic performance of this classification system was validated using Wilcoxon rank sum test and ROC analysis. Cohen's Kappa statistic test and percent agreement were used to test interrater reliability.
**Results or Findings:** Pathology results confirmed 282 malignant and 230 benign lymph nodes. Proposed LN-RADS system yielded 84% sensitivity, 86% specificity, 85% accuracy, 88% positive predictive value, 82% negative predictive value for the diagnosis of malignant lymph node (45 false negative and 32 false positive results were noted, including 6 benign nodes classified as LN-RADS 5). Comparison of morphological features revealed that the largest area under the ROC curve was seen for cortical thickness (0.87). Cohen's Kappa test showed substantial agreement between readers (77.3%).
**Conclusion:** Proposed novel system of classification of lymph nodes (LN-RADS) showed adequate accuracy and positive predictive value, facilitating diagnosis of malignancy. However, further studies comprising large patient samples are necessary to validate our results.
**Limitations:** The proposed system incorporates one imaging modality (US) and applies only to superficial lymph nodes.
**Ethics committee approval:** This study was approved by an ethics committee.
**Funding for this study:** No funding was received for this study.
**Author Disclosures:**
Wojciech Kuncman: Nothing to disclose
Katarzyna Pasicz: Nothing to disclose
Andrzej Cieszanowski: Nothing to disclose
Adam Kaczmarek: Nothing to disclose
Agnieszka Kołacińska-Wow: Nothing to disclose
Leszek Gottwald: Nothing to disclose
Mateusz Pajdziński: Nothing to disclose
Małgorzata Hanke: Nothing to disclose
Cezary Chudobinski: Nothing to disclose

### RPS 2216-3

**Detection of metastatic lymph nodes by increased stiffness: a road to eRECIST?**

*S. R. Marticorena Garcia*, T. Elgeti, U. Grittner, B. Hamm, I. Sack, C. Neelsen; Berlin/DE
*(stephan.marticorena-garcia@charite.de)*

**Purpose:** The purpose of this study is to improve the response to treatment in solid tumours (RECIST 1.1) by adding multifrequency magnetic resonance elastography (MRE) based viscoelasticy for the detection of metastatic lymph nodes.
**Methods or Background:** In this prospective study 25 benign and 82 metastatic lymph nodes were examined by multifrequency MRE at 1.5 T using tomoelastography post-processing at frequencies of 30, 40, 50 and 60 Hz providing shear wave speed (SWS) in m/s as a surrogate of soft tissue stiffness. Short axis diameter (SAD) with a cut-off of 10 mm for determination of a benignity according to RECIST 1.1. was used as the imaging reference standard. Positron emission tomography (PET)-CT/MRI was used as the reference standard for the determination of metastatic lymph nodes of histopathologically confirmed primary tumors. Diagnostic performance of MRE was evaluated by ROC-AUC analysis, and predictive values by generalised linear mixed models and binary logistic mixed models.
**Results or Findings:** Metastatic lymph nodes (1.90±0.57 m/s) were stiffer compared to benign lymph nodes (0.98±0.20 m/s) with an excellent AUC=0.95 and best cut-off of 1.32 m/s was calculated. According to a conservative approach with a specificity of 1.0, the sensitivity (SAD / MRE / MRE+SAD, 0.56 / 0.84 / 0.88), negative predicate value (0.41 / 0.66 / 0.71) and overall accuracy (0.66 / 0.88 / 0.91) increased by using MRE and again higher combining MRE with SAD.
**Conclusion:** Non-invasive multifrequency MRE has proven to be a suitable imaging technique for quantifying the viscoelasticity of lymph nodes by generating elastograms with high resolution. Viscoelasticity provides an important contribution to the accurate determination of malignant lymph nodes, particularly in small lymph nodes with a SAD below 10 mm.
**Limitations:** No direct lymph node-histopathology was involved.
**Ethics committee approval:** This study is IRB-approved.
**Funding for this study:** Funding was received from the German Research Foundation (DFG), SFB-1340; BIOQIC-GRK-2260; 467843609.
**Author Disclosures:**
Christian Neelsen: Nothing to disclose
Bernd Hamm: Nothing to disclose
Ingolf Sack: Nothing to disclose
Ulrike Grittner: Nothing to disclose
Stephan Rodrigo Marticorena Garcia: Nothing to disclose
Thomas Elgeti: Nothing to disclose

### RPS 2216-4

**Prospective evaluation of N- and M-staging in conventional imaging, MRI and 18F-FDG PET/MRI**

*J. Morawitz*[1], N-M. Bruckmann[1], K. Jannusch[1], F. Dietzel[1], K. Herrmann[2], G. Antoch[1], L. Umutlu[1], J. Kirchner[1]; [1]Düsseldorf/DE, [2]Essen/DE

**Purpose:** To compare the diagnostic potential of whole-body MRI, whole-body 18F-FDG PET/MRI and conventional staging (CT, axillary sonography and bone scintigraphy) for N- and M- staging in newly diagnosed breast cancer.
**Methods or Background:** A total of 210 patients (age 52.6±11.9) with newly diagnosed breast cancer were prospectively included in this study. All patients underwent whole-body 18F-FDG PET/MRI, thoracoabdominal CT, whole-body scintigraphy and axillary sonography. Datasets were evaluated separately regarding lesion count, lesion localisation, and lesion characterisation. Histopathology and follow-up imaging served as reference standard. A McNemar test was used to compare diagnostic performance of 18F-FDG PET/MRI, MRI and conventional staging.

**Results or Findings:** Conventional staging determined the N-stage correctly in 191/210 (90.9%) patients with a sensitivity of 80.0%, a specificity of 99.2%, a PPV (positive predictive value) of 98.6%, a NPV (negative predictive value) of 86.9%. Corresponding results for MRI were 186/210 (88.6%), 73.3%, 100%, 100% and 83.3% and for 18F-FDG PET/MRI 192/210 (91.4%), 87.8%, 94.2%, 91.8%, and 91.1%, showing a significantly better sensitivity of 18F-FDG PET/MRI in determining malignant lymph nodes (p=0.0036 and p=0.0001). The M-stage was identified correctly in 200/210 (%) in conventional staging, with a sensitivity, specificity, PPV, NPV of 58.3%, 97.5%, 58.3% and 97.5%. Corresponding results for MRI were 204/210 (%), 75.0%, 98.5%, 75.0% and 98.5% and for 18F-FDG PET/MRI 207/210 (%), 100.0%, 98.5 %, 80.0% and 100.0%, leading to a significantly better diagnostic performance of 18F-FDG PET/MRI (p=0.02 and p=0.01).
**Conclusion:** 18F-FDG PET/MRI detects nodal and distant metastasis in significantly more patients, leading to a significatly better diagnostic performance of 18F-FDG PET/MRI than MRI alone and conventional staging in primary breast cancer patients
**Limitations:** No limitations were identified.
**Ethics committee approval:** This study was approved via an institutional research committee votum.
**Funding for this study:** Funding was received from Deutsche Forschungsgemeinschaft.
**Author Disclosures:**
Ken Herrmann: Nothing to disclose
Frederic Dietzel: Nothing to disclose
Lale Umutlu: Nothing to disclose
Julian Kirchner: Nothing to disclose
Gerald Antoch: Nothing to disclose
Janna Morawitz: Nothing to disclose
Kai Jannusch: Nothing to disclose
Nils-Martin Bruckmann: Nothing to disclose

### RPS 2216-5

**A nomogram for preoperatively predicting sentinel lymph node status in breast cancer based on DCE-MRI radiomic features and clinical factors**
*C. Wang*[1], X. Chen[1], L. Hongbing[1], Y. Liu[1], R. Meng[1], S. Liu[2], M. Wang[1], J. Ren[1], P. Zhou[1]; [1]Chengdu/CN, [2]Beijing/CN

**Purpose:** To develop a nomogram combining radiomics signature of primary tumour and fibroglandular tissue (FGT) based on pharmacokinetic dynamic contrast-enhanced magnetic resonance imaging (DCE-MRI) and clinical factors for preoperative prediction of sentinel lymph node (SLN) status in breast cancer patients.
**Methods or Background:** This study retrospectively enrolled 186 breast cancer patients undergoing pretreatment pharmacokinetic DCE-MRI with positive (n = 93) and negative (n = 93) SLN. Radiomic features were extracted from tumour and FGT, respectively. Intraclass correlation coefficients, minimal redundancy maximum relevance, least absolute shrinkage selection operator and backward stepwise multivariate logistic regression were used for feature selection. Logistic regression models of tumour and FGT were constructed. The radiomics signatures were further combined with clinical factors with independent prediction ability for combined model. Prediction performance was assessed by receiver operating characteristic (ROC), calibration and decision curve analysis. The areas under the ROC curve (AUCs) of models were corrected by 1000-times bootstrapping method and compared.
**Results or Findings:** The AUCs of tumour model and FGT model were 0.783 (95% confidence interval [CI], 0.717-0.849) and 0.680 (95% CI, 0.604-0.757), respectively. A higher AUC of 0.799 (95% CI, 0.737-0.862) was obtained by combining tumour and FGT radiomics signatures. By further combining tumour and FGT radiomics signatures with progesterone receptor (PR) status, a nomogram was developed and showed better predictive ability for SLN status (AUC 0.839 [95% CI, 0.783-0.895]). The IDI and NRI indices also showed FGT and PR improved the prediction performance of SLN status in breast cancer (all p<0.05).
**Conclusion:** A nomogram integrating DCE-MRI radiomics signature of tumour and FGT and PR expression has the potential to predict SLN status.
**Limitations:** The small sample and the singlecentre status of this study were identified as limitations.
**Ethics committee approval:** This study was approved by Sichuan cancer hospital (SCCHE2015029).
**Funding for this study:** No funding was received for this study.
**Author Disclosures:**
Yuanyuan Liu: Nothing to disclose
Jing Ren: Nothing to disclose
Peng Zhou: Nothing to disclose
Min Wang: Nothing to disclose
Chunhua Wang: Nothing to disclose
Xiaoyu Chen: Nothing to disclose
Ruirui Meng: Nothing to disclose
Siyun Liu: Nothing to disclose
Luo Hongbing: Nothing to disclose

### RPS 2216-7

**Machine learning and radiomics analysis of 18F-FDG PET/MR datasets for the prediction of therapy response of isolated limp perfusion in patients with soft-tissue sarcomas**
*J. Grueneisen*, M. Chodyla, A. Demircioglu, M. Forsting, K. Herrmann, L. Umutlu; Essen/DE

**Purpose:** To investigate the potential of PET/MR-derived parameters for the prediction of therapy response of isolated limp perfusion with melphalan and alpha-TNF (TM-ILP) in patients with soft-tissue sarcomas (STS).
**Methods or Background:** A total of 47 patients with the verification of a soft-tissue sarcoma manifestation were prospectively enrolled for an integrated 18F-FDG PET/MR examination prior to neoadjuvant TM-ILP. The study protocol comprised the acquisition of several 18F-FDG PET- and MR-derived morphological, functional and metabolic datasets. After tumour segmentation, 17748 quantitative imaging features were extracted and tested for significance by a $\chi^2$-test. Statistical modelling was performed using Random Forests and evaluated by repeated 5-fold cross-validation. Histopathological results after subsequent tumour resection served as reference standard and patients were categorised as responders/non-responders based on the grading scale by Salzer-Kuntschik.
**Results or Findings:** Histopathological analysis categorised 28 patients as therapy responders (Grade I-III) and 19 patients as non-responders (Grade IV-VI). For the differentiation between therapy response and non-response to TM-ILP, receiver operating characteristic analysis revealed an area under the curve (AUC) of 0.71. Furthermore, statistical analysis showed a positive predictive value of 76% to identify therapy responders in our patient cohort and a specificity of 74% to correctly define patients as non-responders.
**Conclusion:** Our preliminary results demonstrate the potential to predict therapy response of soft-tissue sarcomas under TM-ILP with a non-invasive imaging procedure, which has high impact on further treatment and patients' prognosis. Accordingly, besides primary tumour staging, 18F-FDG PET/MRI may have the potential to enhance the comprehensive pre-therapeutic evaluation of STS and improve patient therapy management.
**Limitations:** The limited patient cohort was a limiting factor.
**Ethics committee approval:** The study was approved by the local ethics committee.
**Funding for this study:** Not applicable
**Author Disclosures:**
Ken Herrmann: Nothing to disclose
Michael Forsting: Nothing to disclose
Johannes Grueneisen: Nothing to disclose
Lale Umutlu: Nothing to disclose
Michal Chodyla: Nothing to disclose
Aydin Demircioglu: Nothing to disclose

### RPS 2216-8

**Paediatric small round blue cell tumours from head to toe**
*L. d. A. Defendi*, R. d. O. Tostes, V. D. M. R. Nishimura, I. G. Pozzi, M. Camargo, F. Silva, R. Regacini, H. M. Lederman; São Paulo/BR
*(lariad@gmail.com)*

**Purpose:** - Clarify the histopathological concept of small round blue cell tumours; - Highlight the importance of clinical data and multidisciplinary approach in their accurate diagnosis; - Present the main imaging features of small round blue cell tumours in multiple sites and how they correlate with clinical and pathological findings.
**Methods or Background:** Small round blue cell tumours include a variety of undifferentiated neoplasms in paediatrics that share a similar morphological pattern and can occur in multiple sites. Although their definitive diagnosis relies on advanced pathological techniques, clinical and imaging findings allow the multidisciplinary team to narrow the diagnosis and establish prompt therapeutics.
**Results or Findings:** Teaching cases from our Oncology Department will be used to illustrate the following topics: - Histopathological overview: clarifying the concept of small round blue cell tumours and why they may pose a diagnostic challenge. The importance of advanced techniques. - Small round blue cell tumours from head to toe, with radiologic-pathology correlation. Epidemiological, clinical and imaging features will be presented for each of the following entities, as well as differential diagnosis and a brief review on treatment and outcomes. 1. Peripheral primitive neuroectodermal tumour 2. Central nervous system primitive neuroectodermal tumour 3. Medulloblastoma 4. Pineoblastoma 5. Retinoblastoma 6. Desmoplastic small round cell tumour 7. Neuroblastoma 8. Embryonal Rhabdomyosarcoma 9. Ewing sarcoma 10. Wilms tumour 11. Hepatoblastoma.
**Conclusion:** Small round blue cell tumours pose a challenge to pathologists and cytologists and their accurate diagnosis relies on experienced teamwork and sophisticated complementary diagnostic techniques, only available in reference centres.
**Limitations:** Not applicable (educational exhibit)
**Ethics committee approval:** Not applicable (educational exhibit)
**Funding for this study:** No funding was received.

**Author Disclosures:**
Henrique M Lederman: Nothing to disclose
Marcos Camargo: Nothing to disclose
Iona Grossman Pozzi: Nothing to disclose
Rodrigo de Oliveira Tostes: Nothing to disclose
Larissa de Andrade Defendi: Nothing to disclose
Victor Dyego Mena Romeiro Nishimura: Nothing to disclose
Frederico Silva: Nothing to disclose
Rodrigo Regacini: Nothing to disclose

**08:00-09:00 Room F1**

### Research Presentation Session: Abdominal Viscera

### RPS 2201

### New frontiers in colorectal imaging

**Moderator**
S. Nougaret; Montpellier/FR

### RPS 2201-2

**Polyp identification rate variation in symptomatic CT colonography: does professional background define performance level in a team?**
D. J. M. Tolan[1], *C. Roe*[1], V. Chillal[2], J. Taylor[1]; [1]Leeds/UK, [2]Coventry/UK

**Purpose:** PIR (polyp identification rate) defines the rate that polyps are detected at CT colonography (CTC) and is equivalent to the polyp detection rate (PDR) at colonoscopy. A higher polyp identification rate is a proxy measure of quality of interpretation particularly if combined with an adequate positive predictive value. We analysed single CTC reporting by an experienced CT Radiographer in comparison with published standards from the British Society of Gastrointestinal Radiology (BSGAR) 2021 guidelines and experienced gastrointestinal radiologists to evaluate whether this should be developed more widely in healthcare.
**Methods or Background:** Proforma reports for 1798 CT colonography examinations from 2019 were analysed using English Bowel Cancer Screening programme reporting codes. All CTC reporters with more than 100 cases were included in analysis. The PIR and PPV were calculated using the BSGAR methodology.
**Results or Findings:** 7 reporters were identified who reported 1786 examinations (mean 255, range 144-463). Mean PIR was 21.44% (range 14.58-32.10%) with one reporter exceeding the "adequate" 13% BSGAR standard and remaining 6 exceeding the "aspirational" 16% standard. All reporters were above the lower 2 standard deviation limit of the mean while one reporter exceeded the upper 3 SD limit. Mean PPV was 88.67% (range 76.47-100%) with 2 exceeding the minimum 80% BSGAR standard and 4 exceeding the aspirational 90% standard. The radiographer exceeded the aspirational PIR and minimum PPV standards.
**Conclusion:** Significant variation exists between reporters in high performing symptomatic CTC services. Individual professional background does not define performance. Robust methods should be developed to identify and support reporters with lower PIR and PPV to improve outcomes.
**Limitations:** The status of this work as a retrospective, singlecentre study was identified as a limiting factor.
**Ethics committee approval:** A service evaluation waiver was granted.
**Funding for this study:** Funding was received from the Yorkshire Cancer Research Bowel Cancer Improvement Programme.
**Author Disclosures:**
Damian John Michael Tolan: Nothing to disclose
John Taylor: Nothing to disclose
Varun Chillal: Nothing to disclose
Craig Roe: Nothing to disclose

### RPS 2201-4

**The role of diverticular disease and patient age in insufficient colon distension at CT colonography**
*A. Meshcheryakov*, O. Pugacheva, N. Gurova, I. Kieva; Moscow/RU
*(aim.radiologist@gmail.com)*

**Purpose:** The purpose of this study is to investigate the effect of different factors that can affect insufficient colon distention at CT colonography (CTC).
**Methods or Background:** We assessed the effect of clinical characteristics (body mass index, obesity, age, gender, diverticular disease, previous abdominal surgery), and procedure-related factors (type of laxative, fecal tagging agent, the technologist conducting the study) on insufficient colon distention frequency after scanning in two positions in 162 patients (53 (32.7%) men, with the median age 75 years (Q1-Q3 67-80 years)) who underwent CTC for colorectal cancer screening.
**Results or Findings:** Insufficient distention after two scans was observed in 25 cases among the 162 patients (15.4%). Multivariable analysis identified that an increase in age by 1 year increases insufficient distention chances by 1.07 times (95% CI: 1-1.14), and presence of diverticula increases insufficient distention chances by 3.01 times (95% CI: 1.16-7.81). The resulting regression model is statistically significant (p=0.001).
**Conclusion:** Chances of insufficient colon distention in CT colonography depend on patients' age and presence of diverticula. The results of our study have important implications for clinical practice, including special training of technicians that can reduce the frequency of insufficient colon distention and avoid unjustified additional scanning when distention is adequate.
**Limitations:** The retrospective design of the study was identified as a limitation. In addition, the study does not assess the influence of the insufficient distention frequency on the diagnostic effectiveness of CTC. Also, the study does not evaluate the effect of an antispasmodic on the insufficient colon distention frequency. A further limiting factor was the manual method of distention with room air.
**Ethics committee approval:** This singlecentre retrospective study was approved by the local ethics committee.
**Funding for this study:** No funding was received for this study.
**Author Disclosures:**
Irina Kieva: Nothing to disclose
Andrey Meshcheryakov: Nothing to disclose
Olga Pugacheva: Nothing to disclose
Nadezda Gurova: Nothing to disclose

### RPS 2201-5

**Neurovascular invasion and lymph node metastasis in resectable rectal cancer evaluated by intravoxel incoherent motion MR study**
C. L. Xie, P. Zhou, *X. Chen*; Chengdu/CN
*(20shenbaise@163.com)*

**Purpose:** To study whether the intravoxel incoherent motion (IVIM) parameters could be used to assess the neurovascular invasion and lymph node metastasis in resectable rectal cancer.
**Methods or Background:** Retrospective study 103 consecutive patients with rectal cancer who underwent conventional MR sequences and IVIM sequence (b=0, 50, 100, 150, 200, 300, 400, 500, 600, 800, 1000, 1200 s/mm2) and who underwent radical surgery within one week of MRI. Tumour IVIM parameters (D, D*, and f) were measured and calculated. According to the pathology, patients were divided into four groups, namely, without neurovascular invasion (group 1), only neural invasion (group 2), only vascular invasion (group 3), both neural and vascular invasion (group 4). the ROIs were determined on both diffusion-weighted and T2-weighted MR images. Mann-Whitney U tests were used to analyse the differences of parameters (D, D*, and f) between the four groups, and the difference between patients with and without lymph node metastasis.
**Results or Findings:** The F values in group 1 and group 4 were (157.04±34.47) vs (139.86±27.08) (p =0,015), and they were (161.23±29.68) vs (139.86±27.08) (p =0,009) in group 2 and group 4. Whereas, the differences of other IVIM parameters between those groups all showed no statistically significant differences (p>0.05). Besides, all of the tumour IVIM parameters (D, D*, and f) had no significant differences between patient with and without lymph node metastasis (p>0.05).
**Conclusion:** The F value of resectable rectal cancer on IVIM might be useful for the evaluation of neurovascular invasion.
**Limitations:** The sample is small.
**Ethics committee approval:** This study was approved by our ethics committee.
**Funding for this study:** There is no funding in this study.
**Author Disclosures:**
Peng Zhou: Nothing to disclose
Department of Radiology, Sichuan Cancer Hospital & Institute, Sichuan Cancer Center, School of Medicine, University of Electronic Science and Technology of China, Chengdu, China. Chao Lian Xie: Nothing to disclose
Xiaoli Chen: Nothing to disclose

### RPS 2201-6

**Predictive value of MRI texture features for KRAS mutation in colorectal cancer patients**
*R. N. Fan*, X. Chen, P. Zhou; Chengdu/CN

**Purpose:** To explore the application value of T2WI imaging-based radiomics features in predicting KRAS mutation in colorectal cancer.
**Methods or Background:** This retrospective study consisted of patients with pathologically confirmed colorectal cancer (n=99). Patients underwent KRAS mutation detection and MR-T2WI scan of magnetic resonance before treatment. The patients were divided into a training group (n=68) and a validation group (n=31). Imaging features were extracted from ROIs using AK software. The training group was dimensionally reduced by using the LASSO

algorithm, and the radiomics label was established. Univariate logistic regression, multivariate logistic regression and backward stepwise logistic regression were used to construct the rad score model, clinical variable model and clinical variable + rad score model. ROC and DCA curve were drawn to evaluate the predictability.
**Results or Findings:** In the training group, there were 36 cases of KRAS mutant and 32 cases of wild type; In the validation group, there were 16 cases of KRAS mutant and 15 cases of wild type. In the training group and the validation group, there was no significant difference in the selected clinical characteristics between the two groups with different KRAS gene mutation status (p>0.05). A total of 395 radiological features were extracted from the delineated ROIs, and three steady texture features are retained by feature selection to construct the model. The AUC of the training group was 0.789, 0.674 and 0.823; within the validation group it was 0.750, 0.633 and 0.742. The specificity of the training group was 0.812, 0.594 and 0.625; within the validation group it was 0.600, 0.467 and 0.733. The sensitivity of the training group was 0.694,0.722 and 0.889; within the validation group it was 0.875, 0.750 and 0.625.
**Conclusion:** The radiomics model based on preoperative T2WI has a certain potential in predicting KRAS status of colorectal cancer.
**Limitations:** The number of cases in our training group and validation group is not enough.
**Ethics committee approval:** This study was approved by the ethics committee.
**Funding for this study:** Funding was received from the international cooperation project of the Sichuan Science and Technology Department.
**Author Disclosures:**
Peng Zhou: Nothing to disclose
Xiaoli Chen: Nothing to disclose
Rui Na Fan: Nothing to disclose

### RPS 2201-8

**Transanal Minimally Invasive Surgery (TAMIS): a pictorial review of postoperative MRI findings**
*C. Reid*, A. M. Lee, R. Dunne, M. M. Morrin; Dublin/IE

**Purpose:** Learning Objectives
1) To provide a pictorial review of postoperative appearances of the rectum and adjacent structures following TAMIS for T1/T2 rectal tumours.
2) To highlight the potential appearances of postoperative scarring/fibrosis and the importance in distinguishing this from disease recurrence.
3) To describe the evolutional change in MR appearances of the postoperative rectum with time.
**Methods or Background:** Transanal minimally invasive surgery (TAMIS) is increasingly utilised in the management of early rectal cancers (1). Post-operative surveillance plays an important role in this patient cohort, ensuring early identification of any potential recurrence. Within our institution, a tertiary referral centre for rectal surgery, we perform an initial rectal MRI 6 months postoperatively with further MRI at 12, 18 and 24 months (2). Here we provide a pictorial review of potential postoperative appearances of the rectum.
**Results or Findings:** A wide variety of postoperative findings can be identified following TAMIS. Fibrosis and scarring along the rectal wall resection site is to be expected. Associated architectural distortion, extending to involve the mesorectal fat, mesorectal fascia and pelvic side wall can be encountered to varying degrees. These architectural changes and the degree of postoperative fibrosis is often related to preoperative tumour size and extent of resection. Fibrosis and the associated T2 signal abnormality generally demonstrate involutional change with time. Diffusion-weighted imaging can be a useful adjunct in distinguishing postoperative fibrosis from tumour recurrence.
**Conclusion:** MRI plays an important role in surveillance post-TAMIS and distinguishing tumour recurrence from benign postoperative change is challenging. A good understanding of the potential postoperative appearances of the rectum is essential in allowing the interpreting radiologist to identify areas of concern.
**Limitations:** TAMIS is a relatively new modality, long term follow-up imaging on this patient cohort is lacking.
**Ethics committee approval:** This study was approved by an ethics committee.
**Funding for this study:** No funding was received for this study.
**Author Disclosures:**
Conor Reid: Nothing to disclose
Martina M Morrin: Nothing to disclose
Aoife Michelle Lee: Nothing to disclose
Ruth Dunne: Nothing to disclose

**08:00-09:00** **Room M 1**

### Research Presentation Session: Interventional Radiology

### RPS 2209

### Interventional radiology from head to toe

**Moderator**
E. Dósa; Budapest/HU

### RPS 2209-3

**Complications and complaints in patients undergoing diagnostic cerebral digital subtraction angiography**
*M. Voormolen*, A. Mondelaers, T. Van der Zijden, T. Menovsky; Edegem/BE
*(maurits.voormolen@uza.be)*

**Purpose:** How many and which complications and complaints occur up to a month after cerebral angiography. A limitation of current studies is the lack of follow-up after hospital discharge.
**Methods or Background:** Retrospective analysis of electronic patient records between 2006-2012 of 1250 cases of patients who underwent cerebral angiography. Prospective analysis of 78 patients who had cerebral angiography from January 2020 until April 2021 with standardised questionnaires (RAND-36 and EQ-5D-5L) and VAS for pain and general health scores, respectively, pre procedure and 1 day, 1 week and 1 month post procedure.
**Results or Findings:** Complications, mainly neurological, due to cerebral angiography in retrospective analysis occurred in 0.9%. Permanent complications were seen in 0.2%. These results are slightly lower compared to literature. No significant risk factor was found. Complications occurred mainly in women and patients older than 52 years. Prospective study showed 5 patients (6,4%) with transient complaints, particularly headache and groin haematoma, up to a week post procedure. Overall, 33% of patients indicated an impact on mobility, daily life activities, self care, pain and mental health the first week after angiography. However, patients indicated significantly better health and physical scores one month post procedure compared to before procedure.
**Conclusion:** Cerebral angiography causes very limited (neurological) complications. A third of patients experience transient complaints the first week after examination. Patients feel physically and mentally better a month after cerebral angiography compared to before.
**Limitations:** Patient inclusion was limited due to COVID pandemic. Single-centre results. Not all patients returned all questionnaires in the prospective study. For retrospective analysis, patient data had to be available in the electronic patient record.
**Ethics committee approval:** This study was approved by the Medical Ethical Committee of the Antwerp University Hospital in July 2019.
**Funding for this study:** No funding was received for this study.
**Author Disclosures:**
Maurits Voormolen: Nothing to disclose
Annelies Mondelaers: Nothing to disclose
Thijs Van der Zijden: Nothing to disclose
Tomas Menovsky: Nothing to disclose

### RPS 2209-4

**Safety and efficacy of rotational thrombectomy for treatment of infrarenal arterial occlusions in a large single-centre cohort**
*C. Artzner*, I. Martin, A. Estler, K. Nikolaou, G. Grözinger; Tübingen/DE

**Purpose:** To investigate the safety and efficacy of rotational thrombectomy (RT) for the treatment of infrarenal arterial occlusion in a large single-centre cohort.
**Methods or Background:** This retrospective IRB-approved study included 397 consecutive procedures in 294 patients between April 2010 and December 2019. All patients underwent RT (Rotarex®S, Straub Medical AG) for infrarenal occlusions of native arteries, arterial stents and/or bypass grafts. Complications, clinical success, and technical success were assessed.
**Results or Findings:** Symptoms were acute, subacute, and chronic in 47.5%, 22.2% and 30.3% of patients, respectively. Rutherford categories three (23.5%) and four (32.1%) were most common. Target lesions were located in iliac arteries (7.1%), iliac/femoral arteries (5%), femoral arteries (59.4%), femoral/BTK arteries (27%) and BTK arteries (1.5%). The target lesion had the following characteristics: length >20 cm, 61.5%; after bypass surgery, 14.9%; and prior stenting, 41.4%. RT device size was 6F 88.6% and 8F 11.4%. Additional lysis after RT was required in 24.2% of cases. Subsequent balloon angioplasty or drug-eluting balloon angioplasty was performed in 89.5% of cases. Technical and clinical success rates were 90.9% and 90.4%, respectively, with an improvement in ankle-brachial index, from 0.33±0.29 to

0.81±0.25. A symptom-free walking distance of more than 200 m was achieved in 78.1% of cases after treatment. Complications occurred in 38.8% of cases, with peripheral embolism being the most common at 22.4% and requiring further treatment in 65.2% of cases. Dissections and perforations occurred less frequently in 9.6% and 2.7% of cases, respectively. RT had an overall complication rate of 6.8%.
**Conclusion:** Rotational thrombectomy is a safe and effective procedure for the treatment of acute, subacute, and chronic thrombotic occlusions of infrarenal arterial native vessels, in-stent occlusions and bypass grafts.
**Limitations:** Retrospective study design.
**Ethics committee approval:** This study was approved by an ethics committee.
**Funding for this study:** No funding was received for this study.
**Author Disclosures:**
Gerd Grözinger: Nothing to disclose
Konstantin Nikolaou: Nothing to disclose
Isabelle Martin: Nothing to disclose
Arne Estler: Nothing to disclose
Christoph Artzner: Nothing to disclose

### RPS 2209-5

**Balloon Pulmonary Angioplasty in patients with non-operable or residual chronic thromboembolic hypertension: initial 5-year experience in a national referral centre**

*A. Páez Carpio*, F. Zarco, C. Martin, E. Serrano, D. Corominas Muñoz, X. Freixa, F. Gómez Muñoz, I. Blanco, J. A. Barberà; Barcelona/ES

**Purpose:** Balloon Pulmonary Angioplasty (BPA) has become an emerging and complementary strategy for chronic thromboembolic hypertension (CTEPH) patients who are not suitable for pulmonary endarterectomy (PEA). The purpose of our study was to evaluate the efficacy and safety of BPA in patients with non-operable or residual CTEPH during the initial 5-year experience in a single national referral center.
**Methods or Background:** Fifteen consecutive, non-operable, anatomically suitable, symptomatic patients for CTEPH were identified and offered BPA between January 2017 and December 2021. Baseline assessment was performed using pulmonary haemodynamics, New York Heart Association (NYHA) functional class, 6-minute walking distance (6MWD), and N-terminal pro b-type natriuretic peptide (NT pro-BNP). Serial BPA sessions were then performed. All procedures were performed jointly by an Interventional Radiologist and an Interventional Cardiologist. The treatment effect was measured by comparing the same values before and 3-6 months after all BPA sessions. The Society of Interventional Radiology (SIR) adverse event classification was used to grade procedure-related complications.
**Results or Findings:** A total of 76 procedures were performed, with a median of 5 BPA sessions per patient (range, 4-7). Mean pulmonary arterial pressure (PAPm) (preBPA: 32+10 vs postBPA: 26+7mmHg, p=0.001), pulmonary vascular resistance (PVR) (421±193 vs 290±114 din/s/cm5, p=0.001) and NYHA functional class I-II (%) (60% vs 100%, p=0.05) were significantly improved. The 6MWD (475±101 vs 484±87, p=0.29) and NT pro-BNP (187±81 vs 159±46 pg/mL, p=0.65) tended to improve too. No deaths or major complications requiring invasive ventilation were reported. The most common complication was lung injury (mild to moderate hemoptysis or transient hypoxaemia).
**Conclusion:** In our initial experience, BPA significantly improves cardiopulmonary haemodynamics with an acceptable safety profile in patients with non-operable or residual CTEPH.
**Limitations:** The small sample was a limiting factor.
**Ethics committee approval:** This study was approved by our institution IRB.
**Funding for this study:** No funding was received for this study.
**Author Disclosures:**
Federico Zarco: Nothing to disclose
Joan A. Barberà: Nothing to disclose
Fernando Gómez Muñoz: Nothing to disclose
Daniel Corominas Muñoz: Nothing to disclose
Clara Martin: Nothing to disclose
Elena Serrano: Nothing to disclose
Xavier Freixa: Nothing to disclose
Isabel Blanco: Nothing to disclose
Alfredo Páez Carpio: Nothing to disclose

### RPS 2209-7

**Impact of collateral status on the patient's outcome after endovascular treatment of the anterior circulation acute ischaemic stroke: a singlecentre experience**

*M. Jablonska*[1], B. J. Regent[2], W. P. Dorniak[2]; [1]Poland/PL, [2]Gdańsk/PL
*(m.jablonska@aol.com)*

**Purpose:** The aim of this study is to determine the impact of baseline collateral status assessed with singlephase CT-angiography (CTA) on the outcome of endovascular treatment in patients with acute ischaemic stroke (AIS) due to large vessel occlusion presenting within 6 hours from symptom onset treated at University Clinical Centre in Gdansk, Poland.
**Methods or Background:** This study was a retrospective assessment of radiological examinations and clinical data of patients who underwent mechanical thrombectomy for AIS at our institution between November 2015 and December 2018. We included all the patients who underwent mechanical thrombectomy due to an occlusion of a large vessel in the anterior circulation, underwent baseline CTA and who were available for outcome assessment at 3 months post-procedure. Collateral status assessment was performed on singlephase CTA. Patients were divided into two groups based on the collateral status: good collaterals – score 2-3 and poor collaterals – score 0-1. The primary clinical outcome was the functional independence defined as ≤2 on the modified Rankin Scale at 90 days.
**Results or Findings:** A total of 92 patients were included in the analysis. A group with good collaterals had a significantly higher chance for favourable clinical outcome than a group with poor collaterals (64.9% vs 20.6%). The adjusted odds ratio for favourable outcome was 11.406 (95% CI: 3.452 – 37.681). Moreover, 90-day mortality was significantly lower in patients with good collaterals (3.5% vs 23.5%). Symptomatic intracranial haemorrhage occurred predominantly in patients with bad collaterals (20.5% vs 1.7%).
**Conclusion:** In this singlecentre, real-life study we proved that baseline CTA collateral status is a robust determinant of better functional outcome and lower mortality after endovascular treatment in acute ischaemic stroke.
**Limitations:** This was a singlecentre study involving singlephase CT.
**Ethics committee approval:** Not applicable
**Funding for this study:** Not applicable
**Author Disclosures:**
Bartosz Jerzy Regent: Nothing to disclose
Magda Jablonska: Nothing to disclose
Waldemar Piotr Dorniak: Nothing to disclose

### RPS 2209-8

**Arterial embolisation reduces synovial hyperaemia in haemophilia patients before total knee replacement**

*W. Foppen*, I. C. Van Der Schaaf, F. H. P. van Leeuwen, D. H. Verlind, L. F. D. van Vulpen, H. C. Vogely, M. W. Barentsz; Utrecht/NL

**Purpose:** Haemophilia is characterised by recurrent hemarthrosis due to a lack of clotting factor VIII or IX, causing end-end stage arthropathy in relatively young patients. Total knee replacement may be complicated by postoperative bleeds despite costly clotting factor therapy. Preoperative angiographic embolisation is expected to reduce synovial hyperaemia and may limit postoperative hemarthrosis. The aim of this study was to evaluate the effects of preoperative embolisation on synovial hyperaemia and compare these findings with embolisation procedures for the treatment of severe hemarthrosis.
**Methods or Background:** In this retrospective cohort study, all patients with haemophilia who underwent periarticular catheter angiography between January 2009 and December 2020 were evaluated after written informed consent was provided. Patient characteristics and complications within 3 months after surgery were extracted from patient records. Synovial hyperemia on pre- and post-embolisation angiography was scored by an experienced interventional radiologist.
**Results or Findings:** 46 angiography procedures in 27 patients were evaluated. The median age at the time of the angiography was 54.4 years (IQR 48.6-65.9). Preoperative synovial hyperaemia was observed in 22/34 joints (65%) and decreased in 14/16 (88%) joints after embolisation. Post-operative joint bleeds occurred in 6/33 joints (18%). Synovial hyperaemia was reduced by embolisation in 11/12 (92%) joints with severe joint bleeding episodes; however, residual hyperaemia after embolisation was more severe compared to preoperative embolisation procedures. No complications were observed after embolisation.
**Conclusion:** Preoperative embolisation in haemophilia safely reduces synovial hyperaemia in the majority of joints. Residual synovial hyperaemia after embolisation is more severe in joints treated for severe hemarthrosis compared to knees evaluated and treated preoperatively.
**Limitations:** Retrospective cohort study.
**Ethics committee approval:** The study was approved by the institutional review board.
**Funding for this study:** Not applicable.
**Author Disclosures:**
Irene C Van Der Schaaf: Nothing to disclose
Flora H. P. van Leeuwen: Nothing to disclose
H. Charles Vogely: Nothing to disclose
Lize F. D. van Vulpen: Advisory Board: Tremeau Pharmaceuticals Grant Recipient: CSL Behring Advisory Board: CSL Behring Grant Recipient: Griffols Advisory Board: Swedish Orphan Biovitrum BV (sobi)
David H. Verlind: Nothing to disclose
Wouter Foppen: Grant Recipient: Pfizer Grant Recipient: NovoNordisk
Maarten W Barentsz: Nothing to disclose

**08:00-09:00** **Room M 2**

### Research Presentation Session: Chest

### RPS 2204

### Pulmonary and extra-pulmonary malignancies

**Moderator**
A. R. Larici; Rome/IT

### RPS 2204-2

**Preoperative staging of advanced mediastinal tumours with cine-magnetic resonance imaging (cine-MRI): a comparison with contrast-enhanced computed tomography (CT) and histology**

*C. Lisi*, U. Cariboni, P. Rondi, F. Catapano, M. Francone, L. Monti; Milan/IT
*(costanza.lisi@hotmail.it)*

**Purpose:** Malignant neoplasms involving the mediastinum require accurate preoperative staging to evaluate the feasibility of surgical excision and the need for a combined cardio-thoracic surgical approach. CT and cine-MRI are the most common imaging modalities for T staging, without any consensus about the superiority of one over the other. The aim of our study was to compare the diagnostic accuracy of contrast-enhanced CT and cine-MRI in the preoperative assessment of mediastinal tumour invasion of nearby cardiovascular structures.
**Methods or Background:** We conducted a single-centre, prospective study on 37 patients affected by primary malignancy contacting the mediastinal structures [49% of cases were primary lungs tumours, followed by thymic neoplasms (24%) and sarcomas (11%)] who preoperatively underwent contrast-enhanced CT and cine-MRI, followed by surgery. On CT, a circumferential contact between tumour and vessels >90°, the absence of an adipose cleavage between the two and blurring of epicardial profile confirmed invasion. Cine-MRI criteria for suspected mediastinal invasion were the absence of sliding motion and of "India-ink" artefact between the mass and the adjacent structures. The histological and surgical report were used as reference standard for invasion.
**Results or Findings:** Histopathology confirmed infiltration in 17/27 cases. Cine-MRI showed a significantly higher sensitivity (94.12% vs. 17.65%) and accuracy (64.86% vs. 54.05%) and lesser specificity (40% vs. 85%) in identifying infiltration, with a greater NPV (89% vs. 55%).
**Conclusion:** Cine-MRI provides a more reliable preoperative staging of advanced mediastinal tumours by means of a higher NPV, leading to a better shaping of surgical approach and patient management.
**Limitations:** A limited number of cases were collected in a monocentric setting.
**Ethics committee approval:** This study was approved by an ethics committee.
**Funding for this study:** No funding was received for this study.
**Author Disclosures:**
Umberto Cariboni: Nothing to disclose
Lorenzo Monti: Nothing to disclose
Costanza Lisi: Nothing to disclose
Paolo Rondi: Nothing to disclose
Marco Francone: Nothing to disclose
Federica Catapano: Nothing to disclose

### RPS 2204-3

**Pulmonary MALT lymphoma: detailed CT findings, including "galaxy sign"**

*Y. Song*, K. S. Beck; Seoul/KR
*(youngsong106@gmail.com)*

**Purpose:** To describe the detailed chest CT findings, including "galaxy sign" described in sarcoidosis, of patients with pulmonary mucosa associated lymphoid tissue (MALT) lymphoma.
**Methods or Background:** From January 2011 to December 2021, 43 patients (23 men and 20 women; mean age, 57.7 years ± 13.2) with pathology-confirmed pulmonary MALT lymphoma and chest CT immediately prior to pathology confirmation were selected for this study. Chest CT scans were reviewed for various findings, including "galaxy sign" described in sarcoidosis, by two radiologists who reached a conclusion by consensus. Patients were classified as those with pulmonary involvement only (group 1) and those with pulmonary and extrapulmonary involvement (group 2), and CT findings were compared between group 1 and group 2 using the Mann-Whitney U test.
**Results or Findings:** Of a total of 43 patients, 29 patients (67.4%) presented with multiple lung lesions; 33 (76.7%) patients showed nodule or mass; 15 (34.8%) patients showed consolidation; and 4 (9.3%) patients showed both nodule/mass and consolidation on CT, with bronchocentric distribution of lesions in 32 patients (74.4%). Other CT findings were GGO component (21 patients, 48.8%), air-bronchogram (39 patients, 90.7%), perilesional spicules (34 patients, 79.7%), and “galaxy sign” (17 patients, 39.5%). GGO component was associated with intralesional interstitial thickening in 95.2%. All patients in group 2 demonstrated multiple lung lesions (p=0.031) and more patients in group 2 showed "galaxy sign" (75.0% vs. 31.4%, p=0.025).
**Conclusion:** Pulmonary MALT lymphoma should be considered in multifocal bronchocentric nodule or mass with air-bronchogram and perilesional spicules; GGO with intralesional interstitial thickening; and "galaxy sign" may also be seen.
**Limitations:** This is a retrospective, single-centre study with a small number of patients.
**Ethics committee approval:** Our institutional review board approved this retrospective study and waived the requirement for informed consent.
**Funding for this study:** No funding was received for this study.
**Author Disclosures:**
Kyongmin Sarah Beck: Nothing to disclose
Yeongran Song: Nothing to disclose

### RPS 2204-4

**Can computed tomography radiomics analysis discriminate the mediastinal lymph nodes in patients with sarcoidosis and lymphoma**

G. Durhan, S. Ardali Düzgün, *F. Atak*, J. Karakaya, İ. Irmak, M. G. Akpinar, F. Demirkazik, O. M. Ariyürek; Ankara/TR

**Purpose:** Definitive diagnosis of mediastinal lymphadenopathies in patients with sarcoidosis and lymphoma may be challenging, although some clinical and imaging findings help to discriminate. We aimed to assess the ability of computed tomography (CT) radiomics to differentiate mediastinal lymphadenopathies as sarcoidosis versus lymphoma.
**Methods or Background:** 94 lymphoma patients and 111 sarcoidosis patients with mediastinal lymph nodes >1 cm were included. Lesion segmentation was performed with dedicated software on pretreatment contrast-enhanced chest CTs (Frontier, Syngovia, Siemens Healthcare). The least absolute shrinkage and selection operator (LASSO) logistic regression algorithm was used to choose between 862 features. Of these features, seven were selected, and, using these features, the classification performance of various data-mining methods in separating groups was investigated. The success of model classification was assessed. Also, the classification performance for each of the seven features was examined by ROC analysis. The best cut-off points for each feature were determined according to Youden index. Sensitivity and specificity values were calculated.
**Results or Findings:** 7 features showed excellent discrimination between sarcoidosis and lymphoma. Wavelet HLL_glcm_ldmn, wavelet HLL_glrlm_low grey-level run emphasis, wavelet HLL_ngtdm_contrast, wavelet LHL_glrlm_low grey-level run emphasis, and wavelet LHL_ngtdm_contrast features had 100% sensitivity and specificity, with cut-off values of 0.95, 0.36, 0.06, 0.35, 0.05 and 0.18, respectively. Wavelet_LLH_glrlm_low grey level run emphasis and wavelet HLL_gldm_low grey level emphasis demonstrated 99% sensitivity and 100% specificity, with cut-off values of 0.18 and 0.09, respectively.
**Conclusion:** CT radiomics analysis can discriminate the lymph nodes of sarcoidosis and lymphoma with great performance and can be used in the diagnosis of these diseases, which can sometimes be challenging.
**Limitations:** It was a retrospective, single-centre study. Although the images were derived from various scanners, all patients had the same CT scan parameters.
**Ethics committee approval:** This study was approved by an ethics committee.
**Funding for this study:** No funding was received for this study.
**Author Disclosures:**
Orhan Macit Ariyürek: Nothing to disclose
Gamze Durhan: Nothing to disclose
Selin Ardali Düzgün: Nothing to disclose
Meltem Gulsun Akpinar: Nothing to disclose
İlim Irmak: Nothing to disclose
Jale Karakaya: Nothing to disclose
Firat Atak: Nothing to disclose
Figen Demirkazik: Nothing to disclose

### RPS 2204-5

**ARTIMES: artificial intelligence-based volumetric assessment of malignant pleural mesothelioma**

*K. Groot Lipman*, R. Wittenberg, I. Smesseim, N. Vakhidova, M. de Oliveira Taveira, R. G. H. Beets-Tan, S. Burgers, S. Trebeschi, D. de Gooijer; Amsterdam/NL
*(k.groot.lipman@nki.nl)*

**Purpose:** Malignant pleural mesothelioma (MPM) is a rare and aggressive cancer. Quantifying response to therapy is troublesome due to its crescent shape, where commonly used linear measurements do not reflect the change in tumor volume well and even the currently recommended mRECIST 1.1 does not correlate to survival adequately. Summing every tumour-containing voxel in the CT scan enables us to analyse the total tumour volume (TTV), which yields a higher correlation to survival. However, manual TTV measurement (segmentation) of MPM is time consuming.
**Methods or Background:** We developed an artificial intelligence (AI) algorithm for automatic volume quantification of MPM in CT scans of patients treated at the Netherlands Cancer Institute. Ground-truth labels consisted of corrected AI segmentations by medical experts, which were further reviewed/adjusted by an expert thoracic radiologist.
**Results or Findings:** Our AI algorithm was trained in n=97 and tested in n=24 subjects that were part of the multicenter NVALT19 trial (total n=121). We found a significant correlation between the ground-truth and the AI-predicted volumes of 0.99 (spearman r, p<0.0001), and an overlap between these volumes of 0.84 (DSC, CI: 0.79 - 0.89). Preliminary results suggest using a 25% volume increase/decrease cutoff for both progressive disease and partial response respectively. Compared to mRECIST, AI volume monitoring using these cutoffs was able to detect progression earlier in n=9/15 cases, at the same time in n=5/15 and later in n=1/15 cases.
**Conclusion:** The results suggest that our AI model reaches excellent performance in CT-based volumetry of MPM and that automatic AI volumetry is promising for the follow-up of patients with MPM.
**Limitations:** Compared to fully manually annotated CT scans, our method of correcting AI-segmentations results is an overestimation of the performance of the AI.
**Ethics committee approval:** IRB approved.
**Funding for this study:** No funding was received for this study.
**Author Disclosures:**
Sjaak Burgers: Advisory Board: Roche International (payment to institution) Investigator: Received financial support and free drugs for an investigator-initiated study by MSD
Stefano Trebeschi: Nothing to disclose
Rianne Wittenberg: Nothing to disclose
Kevin Groot Lipman: Nothing to disclose
Illaa Smesseim: Nothing to disclose
Regina G. H. Beets-Tan: Research/Grant Support: received grants outside of this work from BTG and the Dutch Organization of Science
Dianne de Gooijer: Nothing to disclose
Nargiza Vakhidova: Nothing to disclose
Mateus de Oliveira Taveira: Nothing to disclose

### RPS 2204-6

**Assessing the performance of CT Node-RADS in lung cancer with histopathological correlation**

C. Horst, A. Bille, E. Karapanagiotou, M. Grezda, V. Goh, *G. Benedetti*; London/UK
*(giulietta.benedetti@gmail.com)*

**Purpose:** Visual assessment of malignant lymph node involvement on imaging has limitations. Node-RADS was proposed to standardise and improve nodal categorisation. We aimed to assess the performance of Node-RADS scoring in early-stage non-small cell lung cancer (NSCLC) against histopathology.
**Methods or Background:** Following ethical approval, staging thoracic CTs of surgically resected NSCLC patients were visually assessed by two radiologists in consensus. The presence and location of suspicious nodes were noted; the largest visually abnormal and indeterminate node per patient was also scored as per Node-RADS and compared to histopathology.
**Results or Findings:** 65 consecutive patients (68±9 years; 34 male; all Stage I/II) who underwent surgery were included. 28% (18/65) had radiologist-detected abnormal nodes; 40% (26/65) had indeterminate nodes. 12/18 abnormal nodes (67%) were categorised ≥Node-RAD 4; 6/18 (33%) were Node-RADS ≤3. 6/18 (33%) were positive on histology, all were Node-RADS 4/5, i.e. no histopathologically positive node was scored as Node-RADS 2/3. Of the 26 radiologist-defined indeterminate nodes, 20 (77%) were Node-RADS 2 or 3, i.e., concordant; 4 were Node-RADS 4; 2 were Node-RADS 1. Histology was positive in 10/26 (39%). 2/10 (20%) positive nodes were scored Node-RADS 4; 1/10 (10%) was Node-RADS 1.
**Conclusion:** Radiologists may overestimate nodal involvement. Against a histopathology reference standard, Node-RADS appropriately decreased nodal staging in six patients. Node-RADS also upgraded indeterminate nodes correctly in two patients.
**Limitations:** Only one abnormal node per category was assessed per station per patient.
**Ethics committee approval:** Approval for this study was given by the Guy’s Cancer Cohort.
**Funding for this study:** Not applicable.
**Author Disclosures:**
Eleni Karapanagiotou: Nothing to disclose
Andrea Bille: Nothing to disclose
Mariusz Grezda Mariusz Grezda: Nothing to disclose
Vicky Goh: Nothing to disclose
Giulia Benedetti: Nothing to disclose
Carolyn Horst: Nothing to disclose

### RPS 2204-7

**Can an artificial intelligence algorithm help identify malignant nodules on chest radiographs**

S. Matthews, K. Dwivedi, A. J. Swift, J. Taylor, P. Metherall, K. Hocking, Y. Shahin, S. Holt, *C. S. Johns*; Sheffield/UK
*(chrisjohns@doctors.org.uk)*

**Purpose:** Lung cancer is common and has a poor outcome. Identification on radiographs (CXR) may help with early diagnosis and improve survival. Artificial intelligence (AI) algorithms have the potential to help. We assessed a CE marked AI algorithm that aims to identify malignant lung nodules on chest radiographs. The algorithm has not yet been tested in an unselected consecutive clinical cohort. We assessed the algorithm's performance in primary-care-referred chest radiographs, acquired in a large teaching hospital.
**Methods or Background:** A retrospective service evaluation of consecutive CXRs requested by primary care from 1/7/2020 to 26/02/21 was performed. The radiologist report was interrogated and assigned as positive or negative for a malignant nodule. Where there was disagreement between radiology report and AI algorithm, a second read was performed by specialist cardiothoracic consultant radiologists. A final endpoint of a diagnosis of cancer made in MDT over the subsequent 6 months was also used. The specificity, sensitivity, positive and negative predictive values (PPV/NPV) for the algorithm and radiologists in the identification of malignant pulmonary nodules were calculated.
**Results or Findings:** 5722 CXR were assessed. The algorithm and reporting radiologist identified 1001 and 158 potential malignant lung nodules, respectively. In total, 92 cases were diagnosed with cancer. The AI had sensitivity 0.63 (0.52-0.73); specificity 0.83 (0.82-0.84); PPV 0.06 (0.04-0.07); and NPV 0.99 (0.99-1.00). Radiologists had sensitivity 0.62 (0.51-0.72); specificity 0.98 (0.98-0.99); PPV 0.36 (0.29-0.44); and NPV 0.99 (0.99-1.00).
**Conclusion:** A CE marked AI algorithm had a similar NPV but a lower PPV and specificity than the reporting radiologist, which could potentially result in significant over investigation.
**Limitations:** Retrospective data; "real-life" challenges with ground truth.
**Ethics committee approval:** Approval for this service evaluation was granted by the local Clinical Effectiveness Unit (ref 9914) and Information Governance department.
**Funding for this study:** Not applicable.
**Author Disclosures:**
Yousef Shahin: Nothing to disclose
Jonathan Taylor: Nothing to disclose
Andrew J Swift: Nothing to disclose
Katie Hocking: Nothing to disclose
Chris S Johns: Nothing to disclose
Peter Metherall: Nothing to disclose
Sarah Holt: Nothing to disclose
Sue Matthews: Nothing to disclose
Krit Dwivedi: Nothing to disclose

**08:00-09:00** **Room M 3**

### Research Presentation Session: Neuro

### RPS 2211

### Imaging in head trauma, emergency room and of the cerebral veins

**Moderator**
U. Schwarz-Nemec; Vienna/AT

### RPS 2211-3

**Analysis of current practices in the investigation of suspected subarachnoid haemorrhage: is a negative CT head within 6 hours of onset enough to rule out SAH?**

*R. Banerjee*, M. T. Macmillan; Edinburgh/UK
*(Rohan.Banerjee@nhslothian.scot.nhs.uk)*

**Purpose:** The national institute of health and care excellence (NICE) propose that patients with a normal CT head within 6 hours of onset of symptoms require no further investigations for suspected subarachnoid haemorrhage (SAH). We aimed to assess current practice in investigating SAH and assess the accuracy of CT head for identifying SAH.
**Methods or Background:** Data was collected retrospectively on patients who underwent CT head for suspected SAH in NHS Lothian in May, June and July 2021. Patients were selected if "SAH", "subarachnoid haemorrhage" or "thunderclap" were mentioned in the CT head request. The reference standard was either lumbar puncture (LP), CT angiogram (CTA) or catheter angiogram (DSA).

**Results or Findings:** The final cohort included 157 patients. 44 (28%) patients underwent LP, 19 (12%) CTA, and 5 (3%) DSA. Median time from symptom onset to presentation was 15.52 hours, presentation to CT head request 1.78 hours, CT request to completed 0.77 hours, and completed CT to report 0.45 hours. 33 patients had a negative CT head within 6 hours of onset of symptoms, 4 of which underwent LP (12.1%), 1 CTA (3%). Patients who had CT head within 6 hours, the specificity was 85.7 and negative predictive value (NPV) 100 for detecting SAH.
**Conclusion:** Proposed NICE guidelines could save a significant number of investigations for suspected SAH. CT head is specific and has a high NPV for SAH within 6 hours.
**Limitations:** The major limitation of this study was the number of patients in the assessment of accuracy. Larger studies could further determine the accuracy of CT head in the investigation suspected SAH.
**Ethics committee approval:** Ethical approval was granted by the NHS Lothian R&D team and the local Caldicott guardian.
**Funding for this study:** No funding was received for this study.
**Author Disclosures:**
Mark Thomas Macmillan: Nothing to disclose
Rohan Banerjee: Nothing to disclose

### RPS 2211-4

**Consistency of stroke and haemorrhage diagnosis on native brain CT scans in emergency medicine by clinicians using AI compared to radiologists**

*A. N. Khoruzhaya*, E. I. Kremneva, A. K. Smorchkova, N. Kudryavtsev, A. Vladzymyrskyy; Moscow/RU
*(khoruanna69@yandex.ru)*

**Purpose:** To compare the accuracy of diagnosing stroke and intracranial hemorrhages (ICH) by clinicians using AI services and by radiologists in the emergency medical service.
**Methods or Background:** Within the framework of the Moscow Experiment on Computer Vision in Radiology, a dataset was created containing 104 anonymised native brain CT scans: 52 with the norm and 52 with the target pathology (stroke and ICH, ratio 1/1). The dataset was verified and processed by AI algorithm with known accuracy characteristics (ROC AUC 0.9). AI performed visual marking of pathological areas of ischaemia and haemorrhage in each study and calculated the probability of having a target pathology. 5 radiologists evaluated the original dataset, 5 clinicians evaluated the dataset with the result of AI work. Responses were collected using an online questionnaire.
**Results or Findings:** The first results of the study show that the sensitivity and specificity of diagnosing stroke and ICH in the radiologist group was 89% (95% CI: 87-91%) and 92% (95% CI: 90-94%), respectively. The sensitivity and specificity of diagnosing stroke and ICH in the group of clinicians using AI was 85% and 87%, respectively. When comparing sensitivity and specificity between the groups of radiologists and clinicians, a significant difference was not shown ($p>0.05$).
**Conclusion:** A study was made of the clinical scenario of using AI algorithms by clinicians to assess brain CT for the presence of stroke or ICH in order to make an accelerated decision on treatment tactics.
**Limitations:** The dataset included CT examinations from one scanner model, but had a statistically significant amount of data. Obtaining data after the washout period (1 month) will allow more reliable conclusions about the effectiveness of AI in emergency care.
**Ethics committee approval:** This study was approved by an ethics committee.
**Funding for this study:** Funding was received from the Program of the Moscow Healthcare Department.
**Author Disclosures:**
Elena Igorevna Kremneva: Nothing to disclose
Nikita Kudryavtsev: Nothing to disclose
Anastasiia Kirillovna Smorchkova: Nothing to disclose
Anton Vladzymyrskyy: Nothing to disclose
Anna Nikolaevna Khoruzhaya: Nothing to disclose

### RPS 2211-5

**Diagnostic accuracy of sagittal TSE-T2W, variable flip angle 3D TSE-T2W and high-resolution 3D heavily T2W sequences for the cerebral aqueduct and the superior medullary velum stenosis**

*A. N. Ş. Özcan*, K. Aslan; Ankara/TR
*(aysenursirinozcan@gmail.com)*

**Purpose:** This study aimed to investigate the accuracy of conventional Sagittal Turbo Spin Echo T2-weighted (Sag TSE-T2W), variable flip angle 3D TSE (VFA-3D-TSE) and high-resolution 3D heavily T2W (HR-3D-HT2W) sequences in the diagnosis of primary aqueductal stenosis (PAS) and Superior Medullary Velum Stenosis (SMV-S), and the effect of stenosis localisation on the diagnosis.
**Methods or Background:** Seventy-seven patients were included in the study. The diagnosis of the sequences was classified into three grades: grade 0 (the sequence has no diagnostic ability), grade 1 (the sequence diagnoses stenosis but does not show focal stenosis itself or membrane formation), and grade 2 (the sequence makes a definitive diagnosis). Stenosis localisations were classified as Cerebral Aquaduct (CA), Superior Medullary Velum (SMV) and SMV+CA. First, the grades were compared without making a differentiation based on localisation. Then, the effect of localisation on diagnosis was determined.
**Results or Findings:** In the sequence comparison grade 0 was found 25.4% on the Sag TSE-T2W sequence contrary to other sequence determine all cases ($p<0.05$). Grade 2 was detected by HRH-3D-T2W in 98.7% of the cases, while it was detected by VFA-3D-TSE in 69.2% of the cases, and the difference was statistically significant ($p<0.05$). When investigating localisations, the rate of grade 0 in the Sag TSE-T2W sequence was statistically higher for the SMV localisation ($p<0.05$). Localisation had no effect on diagnosis using the other sequences.
**Conclusion:** In our study, we found that the VFA-3D-TSE and HR-3D-HT2W sequences were successful in the diagnosis of stenosis. The Sag TSE-T2W sequence has limited diagnostic performance, especially at SMV localisation.
**Limitations:** The restricted number of patients is our limitation of the study.
**Ethics committee approval:** The study is part of an institutional review board-approved protocol (Ankara City Hospital Ethical Committee E1-21-1493).
**Funding for this study:** No funding was received.
**Author Disclosures:**
Ayşe Nur Şirin Özcan: Nothing to disclose
Kerim Aslan: Nothing to disclose

### RPS 2211-6

**Quantitative study of movement and image quality on ER and inpatient MRI of the brain**

*D. Abeysekera*, B. Ciavarra, J. Wood, J. Yung, K-P. Hwang, C. Walker, J. M. Johnson; Houston, TX/US

**Purpose:** To assess quantitative features of brain MRI in ER and inpatient examinations.
**Methods or Background:** The sample included 496 adults (mean age = 58.6, std 14.5) ER (40) and inpatients (456) receiving brain MR (281 male, 215 female), including Ax T1, T2, T2 FLAIR, T2*, DWI, T1 post and 3D T1 post. MRQy software provided quantitative imaging measures. Subjective measures of motion were derived by natural language processing (NLP) of radiology reports. ANOVA was assessed for demographic variables versus quantitative measures of motion and image quality. The group was separated into quartiles based on quantitative measures and metrics between selected pairs were analysed using a Student's t-test.
**Results or Findings:** Quantitative measures of motion were associated with subjective motion measures for T2 FLAIR, T1, 3D T1 post, and T1 post ($p < 0.05$) but not T2, DTI, and T2*. There was a significant association between motion and male gender ($p<0.0001$). Motion was not associated with age overall, but there was a significant difference between motion in the third age quartile (62-70, lowest motion) versus the fourth age quartile (71-92, highest motion) ($p = 0.045$) Quantitative measures of image quality (PSNR, CNR, CJV) were also significant associated with motion ($p<0.0001$).
**Conclusion:** MRQy revealed that men and older patients have increased motion and that increased motion was associated with decreases in measures of image quality, including peak signal to noise ratio of the foreground, contrast to noise ratio for shadowing and noise artifacts, and coefficient of joint variation between the foreground and background for aliasing and inhomogeneity artifacts.
**Limitations:** Subjective variables of motion and quality were extracted using NLP and were not qualitatively assessed.
**Ethics committee approval:** The IRB of UT MDACC approved this study.
**Funding for this study:** Not applicable
**Author Disclosures:**
John Wood: Nothing to disclose
Dylan Abeysekera: Nothing to disclose
Ken-Pin Hwang: Nothing to disclose
Christopher Walker: Nothing to disclose
Jason Michael Johnson: Consultant: InformAI Consultant: Kura Oncology Grant Recipient: Blue Earth Diagnostics
Joshua Yung: Nothing to disclose
Bronson Ciavarra: Nothing to disclose

### RPS 2211-8

**The clinical factors most likely to result in an abnormal CT head – a UK trauma centre experience**

*A. Haque*, K. Kow, K. S. Tsang; Stoke-on-Trent/UK
*(mushrulhaque@yahoo.co.uk)*

**Purpose:** Demand for medical imaging has significantly increased in the UK with Computed Tomography (CT) becoming the main modality used in the assessment of suspected head injuries. We looked at all such CT heads performed in our trust over the course of one month and analysed those that were abnormal to assess for specific links with the clinical factors provided in the history.
**Methods or Background:** A retrospective audit was performed looking at all inpatient CT heads performed over the course of one month between 15th October and 15th November 2021. We then analysed the reports for the presence of intracranial and/or extracranial injuries and tried to identify commonly related clinical findings.
**Results or Findings:** 535 CT heads were analysed of which 13.3% (n=71) were abnormal. Almost half of patients (45% n=242) were on some form of anticoagulation and this was found to be the most common indication for requesting a CT head. Of the abnormal scans, bruising around the ears/eyes and retrograde amnesia were the two most common clinical factors in the history provided, demonstrated in almost 30% of patients. 25% of patients found to have an abnormal CT were on some form of anticoagulation.
**Conclusion:** Within our Trust, being on anticoagulation is by far the most common indication for a CT head overall. However, facial bruising and retrograde amnesia were found to be the most common clinical factors resulting in an abnormal CT head.
**Limitations:** This audit was limited to a single centre. It would be useful to compare with other centres and their experiences of the same.
**Ethics committee approval:** Not applicable
**Funding for this study:** Not applicable
**Author Disclosures:**
Kevin Kow: Nothing to disclose
Kai Sun Tsang: Nothing to disclose
Abul Haque: Nothing to disclose

**08:00-09:00** **Room M 4**

### Research Presentation Session: Musculoskeletal

### RPS 2210

### Advances in assessing joints and ligaments of the lower extremity

**Moderator**
C. Martinoli; Genoa/IT
**Author Disclosure:**
C. Martinoli: Advisory Board: Pfizer, Philips; Consultant: Pfizer, Roche, Philips, Esaote, Canon; Speaker: Pfizer, Novonordisk, Sobi, Takeda, Philips, Canon

### RPS 2210-2

**Sonographic classification of Achilles tendon tears and its clinical significance**

*N. Singla*, S. Choudhary; Birmingham/UK
*(nehalsingla19@gmail.com)*

**Purpose:** The aim was first, to understand basic anatomy of the Achilles tendon, second, to form a standardised classification system of tears of Achilles tendon, and third, to see if the location of the tear or gap in equinus has any impact on management.
**Methods or Background:** We analysed 84 patients who presented to the emergency department following an injury and had a tear on the ultrasound. The study is a retrospective observational study from 2009-2021. The tears were further classified on the basis of location and gap of tear and its correlation with clinical outcome and management.
**Results or Findings:** Age ranges from 22-93 years were included in the study, 82% were males. The most common aetiology of tear was playing sports. 64% of the patients had a full thickness tear. 52% of patients had a tear in the proximal tendon. Chi square test results proved a significant association between age and tear location and sex and tear location. Out of the 84 patients, only 5 were managed operatively. All 5 cases had mid tendon tears. There was significant association between the location of tear and gap in equinus and patients selected for operative management. Also of the 79 patients selected for conservative management, 4 patients had re-rupture and three of those were further operated on. The fourth patient died due to other comorbidities.
**Conclusion:** We can summarise that all patients who had failed conservative management were managed operatively. The most common ultrasound findings for operative management were mid substance tear of the Achilles and a gap of at least >10mm. A further gap of >15mm were more suitable for the operation.
**Limitations:** The study was conducted during the covid pandemic and there might be other reasons of delay to surgery.
**Ethics committee approval:** This study was approved by an ethics committee.
**Funding for this study:** Not applicable
**Author Disclosures:**
Surabhi Choudhary: Nothing to disclose
Nehal Singla: Nothing to disclose

### RPS 2210-3

**Can gadolinium contrast agents be replaced with saline for direct MR arthrography of the hip? A retrospective study with arthroscopic comparison**

M. K. Meier[1], M. Wagner[2], T. D. Lerch[1], S. Steppacher[1], K. Siebenrock[1], P. Vavron[2], A. Brunner[2], E. Schmaranzer[2], *F. Schmaranzer*[1]; [1]Bern/CH, [2]St. Johann in Tirol/AT
*(florian.schmaranzer@insel.ch)*

**Purpose:** Although severe adverse events related to intraarticular injection of gadolinium-based contrast agents for direct MR arthrography (MRA) are extremely rare, use of a saline solution could bypass patient concerns and reduce costs. Thus, we compared diagnostic performance of direct MRA of the hip performed with gadolinium contrast agent and saline solution in patients undergoing hip arthroscopy.
**Methods or Background:** IRB-approved retrospective study of 140 patients (mean age 34 years, 22% females) with hip pain due to femoroacetabular impingement. 140 patients who either underwent intraarticular injection of 15-20 ml gadopentetate dimeglumine 2,0 mmol/l ("Gd-MRA"-group, n=70) or 0.9% NaCl solution ("saline-MRA"-group, n=70) for preoperative hip MRA and subsequent hip arthroscopy were age and gender matched. 1.5 T MRI was performed using multiplanar non-fs PD-w TSE sequences including standardised application of leg traction (application of 15-23 kg). Two readers assessed labrum- and femoroacetabular cartilage lesions. Arthroscopic diagnosis of chondrolabral damage served as reference. Likert scale and diagnostic performance (accuracy) was compared between groups.
**Results or Findings:** Accuracy was comparable between groups for Reader 1 / Reader 2 for labrum- (Gd-MRA 94%/96% versus saline-MRA 96%/93%; p>0.999/p =0.718), acetabular cartilage- (Gd-MRA 86%/82% versus saline-MRA 89%/ 87%; p = 0.801/p>0.999) and femoral cartilage lesions (Gd-MRA 97%/99% versus saline-MRA 97%/ 97%; both p>0.999).
**Conclusion:** Diagnostic accuracy of saline MRA in assessing chondrolabral lesions was high and comparable to gadolinium-based MRA underlining the potential role of non-gadolinium based hip MRA.
**Limitations:** One limitation is the retrospective selection of patients. Another limitation is that MR arthrography was performed with leg traction. Future studies should try to reproduce these finding using conventional direct MR arthrography.
**Ethics committee approval:** Ethics committee approval was granted.
**Funding for this study:** No funding was received for this study.
**Author Disclosures:**
Till Dominic Lerch: Nothing to disclose
Florian Schmaranzer: Nothing to disclose
Ehrenfried Schmaranzer: Nothing to disclose
Simon Steppacher: Nothing to disclose
Malin Kristin Meier: Nothing to disclose
Moritz Wagner: Nothing to disclose
Klaus Siebenrock: Nothing to disclose
Peter Vavron: Nothing to disclose
Alexander Brunner: Nothing to disclose

### RPS 2210-5

**Applications of a novel contrast agent in x-ray imaging of articular cartilage: a preliminary experience**

S. Fantoni[1], P. Cardarelli[2], A. Taibi[2], V. Cristofori[2], C. Trapella[2], A. Bazzani[1], M. Assenza[1], *D. Conti*[1], F. Baruffaldi[1]; [1]Bologna/IT, [2]Ferrara/IT
*(daniele.conti@ior.it)*

**Purpose:** The aim of this study is a preliminary assessment of cationic contrast agent CA4+ spatial distribution in cartilage of ex vivo bovine samples.
**Methods or Background:** A sub-millimeter scale investigation was conducted on osteochondral plugs (N=18), extracted from tibial and femoral distal ends of bovine stifle joints (n=2). The samples were immersed in CA4+ solution at fixed concentration for up to 26 hours, and planar images were acquired using a micro-computed tomography apparatus, at different time points. The CA4+

distribution inside the cartilage and saturation time were evaluated. Millimiter scale assessments were performed using a clinical computed tomography apparatus on distal-end tibiae of bovine stifle joints (N=3). After baseline acquisitions were obtained, samples were immersed in CA4+ baths at different concentrations (5, 10, 15mgI/ml) for up to 24 hours and imaged. No animals were sacrificed for the aims of this work.
**Results or Findings:** The distribution of CA4+, at sub-millimeter scale, differentiates into three distinct layers inside the cartilage. It should reflect the spatial distribution of proteoglycans, by which CA4+ molecules are electrostatically attracted. After 24-h diffusion, the iodine concentration reached in cartilage is approximately seven times the CA4+ bath. The saturation time is $\tau F95\%=2.61\pm0.83h$ and $\tau T95\%=3.14\pm2.39h$, for femoral and tibial samples respectively. Millimeter scale analysis with computed tomography confirms contrast enhancement of cartilage after 24-hours immersion, observed for each contrast medium concentration. Distinct contrast enhancement is reached in different cartilage regions, depending on tissue's local features. Attenuation is observed to increase at higher CA4+ concentration baths ($\Delta C5$ = 463HU±11HU, $\Delta C10$ = 517HU±13HU, $\Delta C15$ = 620HU±6HU).
**Conclusion:** CA4+ contrast agent significantly improves cartilage visualisation and its qualitative analysis. The adoption of reference assessments (i.e. histology) may enable quantitative measurements of cartilage constituents and features.
**Limitations:** There was a limited number of samples and these experimental conditions are far from physiological conditions.
**Ethics committee approval:** Not applicable
**Funding for this study:** Not applicable
**Author Disclosures:**
Marta Assenza: Nothing to disclose
Fabio Baruffaldi: Nothing to disclose
Angelo Taibi: Nothing to disclose
Daniele Conti: Nothing to disclose
Simone Fantoni: Nothing to disclose
Armando Bazzani: Nothing to disclose
Claudio Trapella: Nothing to disclose
Virginia Cristofori: Nothing to disclose
Paolo Cardarelli: Nothing to disclose

### RPS 2210-6

**(Dis)location of the anterior intermeniscal ligament: an exploratory MRI study**

*C. D. B. Visser*, M. Maas, R. Zwiers, G. Kerkhoffs; Amsterdam/NL
*(c.d.b.visser@amsterdamumc.nl)*

**Purpose:** Anterior knee pain is common, yet the aetiology is often not fully understood. We found dislocation of the anterior intermeniscal ligament (AIL) as a possible cause of anterior knee pain. Dislocation was discovered during knee arthroscopy. Literature on the anatomical characteristics of the AIL is scarce, and to our knowledge there is no data available on the clinical relevance or radiologic assessment of AIL pathology. Therefore we aim to learn more about the normal anatomy of the AIL and develop a method to identify dislocation of the AIL on MR imaging.
**Methods or Background:** Preoperative MR images of patients with a perioperatively confirmed dislocated AIL (Group 1) were compared with preoperative MR images of patients without a dislocated AIL (Group 2). Both groups consisted of 34 patients. In addition to analysing the characteristics of the AIL, spatial measurements were conducted to determine the AIL's relative position in the knee joint and identify features that could indicate a dislocated AIL.
**Results or Findings:** Dislocated ligaments were significantly thicker. The masterline was found to be a useful tool in detecting the position of the AIL. The deviation from the masterline was significantly larger in Group 1 (2.51 vs 1.41 mm). Using a cut-off point of ≥1.5mm, the masterline test yields a specificity of 74% and sensitivity of 79%. After stratifying for substantial effusion, the specificity and sensitivity were 96% and 81%, respectively.
**Conclusion:** This study contributed to the current knowledge regarding the normal anatomy of the AIL. A method was developed to identify dislocation of the AIL using MR imaging. The test is easily applicable in practice and has high sensitivity and specificity. Diagnostic accuracy is higher in the absence of substantial effusion.
**Limitations:** Not applicable
**Ethics committee approval:** Not applicable
**Funding for this study:** Not applicable
**Author Disclosures:**
Casper Dick Benjamin Visser: Nothing to disclose
Ruben Zwiers: Nothing to disclose
GinoM.M.J. Kerkhoffs: Nothing to disclose
Mario Maas: Nothing to disclose

### RPS 2210-8

**Novel experimental model to evaluate the effects of cement polymerization timing and bone density in trabecular bone penetration in total knee arthroplasty**

*M. Mattone*, S. Perotti, G. Alfieri, G. Pacchiarotti, C. M. Bruno, P. Petitti, G. Cinotti, C. Catalano; Rome/IT
*(monicamattone95@gmail.com)*

**Purpose:** Aseptic loosening of cemented prosthetic components is among the most common causes of failure in total knee arthroplasty (TKA) requiring revision surgery. The aim of this study is to analyse the results of a novel experimental model for evaluating the effects of cement polymerisation timing and bone density in trabecular bone penetration during TKA.
**Methods or Background:** Proximal tibial epiphyses were harvested during TKA. After a washing of the tibial epiphyses on the trabecular surface, they were examined with a CT scan to evaluate bone density. Each tibial epiphysis was divided into two hemiportions enclosing the medial and the lateral cancellous bone plane respectively. The two hemiplates were cemented using different polymerisation timings. The samples were subjected to a second CT scan to calculate the maximum and minimum penetration peak and the extension of cement penetration area into trabecular bone.
**Results or Findings:** The extension of the cement penetration area, calculated on 92 CT scans, was on average 69.0 mm2 in the samples processed with early polymerisation timing and 53.4 mm2 in those processed with late polymerisation timing (p<0,0001). At a depth greater than 2 mm the penetrating area was on average 7.0 mm2 and 3.2 mm2 in the samples processed with early and late polymerisation timing, respectively (p<0,02). The maximum cement penetration peak was 4.8 mm and 4.1 mm in the samples processed with early and late polymerisation timing, respectively.
**Conclusion:** The experimental model designed may be suitable to detect significant differences in terms of cement penetration area into trabecular bone using different polymerisation timings.
**Limitations:** There were few samples and intra- and inter-observer variability as well as the time-consuming method were identified as limitations.
**Ethics committee approval:** Not applicable
**Funding for this study:** No funding was received for this study.
**Author Disclosures:**
Carlo Maria Bruno: Nothing to disclose
Giulia Alfieri: Nothing to disclose
Monica Mattone: Nothing to disclose
Giacomo Pacchiarotti: Nothing to disclose
Stefano Perotti: Nothing to disclose
Gianluca Cinotti: Nothing to disclose
Paolo Petitti: Nothing to disclose
Carlo Catalano: Nothing to disclose

**08:00-09:00** **Room X**

### Research Presentation Session: Imaging Informatics / Artificial Intelligence and Machine Learning

### RPS 2205

### Integration of artificial intelligence (AI) in radiological workflow and structured reporting

**Moderator**
D. Pinto dos Santos; Cologne/DE
**Author Disclosure:**
D. Pinto dos Santos: Advisory Board: cook medical; Author: AMBOSS; Speaker: Bayer

### RPS 2205-2

**Standardising workflow nomenclature in radiology**

*K. Nairz*, N. Cihoric; Bern/CH

**Purpose:** Advocating implementation of CWL (common workflow language) principle in radiology process metrics.
**Methods or Background:** Radiology Information Systems are databases to manage and document medical, administrative, and billing information. RIS products may be structured according to the workflow, but they are usually not suited to provide current and retrospective process metrics. RIS databases also store timestamps, which either may be manually set or automatically recorded. However, the information content is usually not sufficient to allow for a depiction of the process flow. For example, even with accurate arrival time and end time of the exam, it is impossible to calculate accurate waiting times. Additionally and generally, manually set timestamps are inadequate to build a reliable model of the process flow.
**Results or Findings:** We have developed Radcount, a workflow dashboard that exhibits the progression of radiological examination and reporting in a team-specific and visually appealing manner. Radcount extracts automatically recorded and - if unavoidable - manually set time information from RIS and DICOM. Thereby we can build a comprehensive model of the patient flow and occupancy of the imaging machinery. Utilising a machine learning algorithm we are also able to predict future patient flow. Radcount is specifically tailored to our RIS environment. A main obstacle for further scaling is the lack of standardised semantic and logical infrastructure. The logical construct should be shareable and comparable between systems, institutions, within a country, and at an international level. Therefore, introduction of simple and understandable modelling language is requisite. We found CWL to be suited, because it is already used in complex computational environments. We will be presenting a clinical CWL extension for the radiology workflow.
**Conclusion:** Standardisation of workflow nomenclature is desirable and feasible in radiology.
**Limitations:** Not applicable
**Ethics committee approval:** Not applicable
**Funding for this study:** Not applicable
**Author Disclosures:**
Knud Nairz: Nothing to disclose
Nikola Cihoric: Consultant: Wemedoo AG

### RPS 2205-3

### Standardising the mpMRI prostate reporting for one-stop clinics: a novel dedicated semi-automated workflow

*F. Giganti*[1], S. Lelie[2], L. Dickinson[1], D. A. Pendse[1], N. Ramachandran[1], D. Heffernan Ho[1], A. Kirkham[1], C. M. Moore[1], C. Allen[1]; [1]London/UK, [2]Brussels/BE

**Purpose:** Our one-stop clinic provides patients with clinically suspect of prostate cancer with a multiparametric MR scan and treatment plan within one visit to the clinic. We present our results from a specifically designed workflow that allows two different radiologists to report the MR scans and contour the lesions on the same day of the MR acquisition.
**Methods or Background:** Sixty scans of men referred for clinical suspicion of prostate cancer were prospectively acquired. One amongst seven different genitourinary radiologists (all highly experienced in prostate MRI reading) reported the scan and contoured the prostate and the lesions according to their clinical rota using a semi-automated workflow (MIM®) for the first read. Only two out of seven radiologists performed the second read using a customised version of the workflow for quality assurance reasons. A dedicated structured report including a biopsy plan was created with annotated images sent to PACS for use during the multidisciplinary team meetings and targeted biopsies if needed.
**Results or Findings:** Concordance between the first and the second read occurred in 41 out of 58 scans. In the 19 discordant cases, lesion downgrade occurred in 10 patients (the majority of lesions were downgraded from Likert 3 to Likert 2). New lesions were contoured in the remaining 9 patients during the second read. 17 out of 60 patients were biopsied and cancer (Gleason ≥ 3 + 4) was found in 11 patients.
**Conclusion:** A dedicated semi-automated workflow streamlined our one-stop clinic and allowed the radiologists to perform a double reporting of prostate MR scans on the same day, contouring the lesions and producing a standardised structured report that includes the biopsy plan to assist the clinician performing the procedure.
**Limitations:** The small population and this work's status as a singlecentre study were identified as limiting factors.
**Ethics committee approval:** This study was approved by an ethics committee.
**Funding for this study:** Funding was received from the North and East London cancer network.
**Author Disclosures:**
Steven Lelie: Nothing to disclose
Daniel Heffernan Ho: Nothing to disclose
Douglas Andrew Pendse: Nothing to disclose
Caroline M Moore: Nothing to disclose
Navin Ramachandran: Nothing to disclose
Louise Dickinson: Nothing to disclose
Clare Allen: Nothing to disclose
Alex Kirkham: Nothing to disclose
Francesco Giganti: Nothing to disclose

### RPS 2205-4

### Integrating artificial Intelligence algorithms with DICOM structured reporting into clinical workflows

*K. S. Younis*; Cleveland, WI/US
*(kh.younis@gmail.com)*

**Purpose:** We present a prototype that implements a modular, cloud-based, and interoperable analysis pipeline in reporting workflows of artificial intelligence (AI) algorithms. Different parts of the pipeline can be exchanged due to the modular software architecture for prototyping/testing while ensuring the reliability of the remaining elements and applicability to different domains. We integrate image viewing, machine learning algorithms results representation, structured reporting, and evaluation by using custom user interfaces and different verified ML algorithms.
**Methods or Background:** Our implementation consists of different modules: a PACS connection, a viewer application, a machine learning (ML)-based image interpretation pipeline, a structured reporting editor, a traditional annotation/processing pipeline, and a diverse publicly available database. We use DICOM SR (TID 1500) as a standardised mechanism to store measurements reports for the ML algorithms and manual annotation to ensure consistent representation and enable repeatability of the effectiveness of evaluation. Mapping of the DICOM SR to other formats such as HL7 FHIR resources ensures the interoperability with other systems dispersed at the radiology department and hospital enterprise healthcare system. Modules can be distributed across the network or cloud-based to eliminate the need for expensive hardware and the need for moving large datasets.
**Results or Findings:** A proof of concept with sample MRI database from The Cancer Image Archive for the prostate showed that measurements data can be converted to the data store for significantly faster retrieval of relevant data, more consistent quantitative measurements comparison as compared to the traditional system, and more accurate reporting.
**Conclusion:** A standardised, modular, and network-enabled system is a viable tool for integrating artificial intelligence algorithms into clinical workflow, assessing the performance, and improving the quality of the department and the AI algorithms by monitoring and giving feedback.
**Limitations:** Not applicable
**Ethics committee approval:** Not applicable
**Funding for this study:** Not applicable
**Author Disclosures:**
Khaled Salem Younis: Employee: Philips

### RPS 2205-5

### Pilot development of a natural language processing algorithm for classification of head CT reports

*E. B. Wang*, A. Rashiwala, A. Krayyem, J. Li, J. M. Johnson; Houston, TX/US
*(Ethan.Wang@utsouthwestern.edu)*

**Purpose:** Automated classification of radiology reports is useful for medical discovery and innovation. In the oncologic community, a subset of ambulatory outpatients with cancer are screened for brain metastases using head CT with and without contrast. To assess the utility of the "without" portion of the exam, we sought to automate the classification of head CT reports as positive or negative for disease. Furthermore, we aimed to identify specific disease entities within reports.
**Methods or Background:** 739 reports were extracted on outpatients who received head CT with and without contrast at a designated cancer centre. The natural language processing model en_ner_bc5cdr_md, pretrained by SciSpacy for disease entity identification, was then applied to reports for disease extraction. Entities present in the MalaCards human disease database were extracted as well, and extracted entities were eliminated if grammatically linked to a negative modifier. Preliminary model performance was assessed on 299 reports in comparison to human observers.
**Results or Findings:** Our model obtained an accuracy of 0.803 in binary classification. Identification of specific entities yielded a sensitivity of 0.455, specificity of 0.825, accuracy of 0.686, precision of 0.611, and recall of 0.455.
**Conclusion:** Our model can classify head CT reports with good accuracy and possesses potential in extracting finer-grain data. Further development can help contribute to the detection of important radiographic findings from EHRs.
**Limitations:** Diseases referred to using specialist-specific terminology are often missed, and elimination of false positives is complicated by grammatical heterogeneity in disease negation. Nonetheless, establishing clearer links between grammar and semantics and training models on radiology-specific reports could mitigate such issues.
**Ethics committee approval:** Local IRB approval was obtained for this research. Individual patient informed consent was waived.
**Funding for this study:** This study was department-funded. No external funds were utilised.

**Author Disclosures:**
Jason Michael Johnson: Nothing to disclose
Jing Li: Nothing to disclose
Abhi Rashiwala: Nothing to disclose
Ethan Bocheng Wang: Nothing to disclose
Apollo Krayyem: Nothing to disclose

### RPS 2205-6

**Structured reporting of COVID-19 chest CT with natural language processing through a semi-supervised deep learning approach**

C. Romei, *S. C. Fanni*, E. Rocchi, C. A. A. D'Amore, F. Volpi, L. Colligiani, A. De Liperi, E. Neri; Pisa/IT
*(fannisalvatoreclaudio@gmail.com)*

**Purpose:** To evaluate the structured reporting performance of natural language processing based on a deep learning algorithm starting with free-form radiological reports of COVID-19 lung CT.
**Methods or Background:** Two hundred COVID-19 chest CT of the first wave were reviewed by two experienced radiologists. For each exam, the radiologists wrote a free-form text radiological report and coherently filled the template provided by the Italian Society of Medical and Interventional Radiology (SIRM), which was used as ground truth. Each structured report presented with 70 categorical or Boolean's variables (yes/no). Natural language processing based on a semi-supervised and not-scalable convolutional neural network (CNN) architecture was implemented. In order to analyse the free-form text reports we adopted a Question & Answering (QA) model, more specifically unified QA, to extract predefined items used to automatically fill the SIRM template. The performance of the model in filling structured reports was then compared to the template filled by the two radiologists.
**Results or Findings:** The model performance was measured using two parameters: 1. the mean accuracy for each template filled 2. F1 mean score, which is a harmonised average of sensitivity and positive predictive value (PPV) and is frequently used as an overall measure of natural language processing tools performance. As a result, our model achieved 95.8% for both parameters. For some variables the algorithm showed a suboptimal performance that was inferior to the mean value of accuracy and F1 score, mostly due to reports ambiguity in the free-form radiological reports.
**Conclusion:** Our approach achieved excellent performance for this clinical application, suitable in radiological reporting even in other fields, such as oncology or urgent care.
**Limitations:** The retrospective nature of the study and the lack of external validation were identified as limiting factors.
**Ethics committee approval:** The study was approved by the ethics committee.
**Funding for this study:** No funding was required for this study.
**Author Disclosures:**
Emanuele Neri: Nothing to disclose
Chiara Romei: Nothing to disclose
Salvatore Claudio Fanni: Nothing to disclose
Caterina Aida AIDA D'Amore: Nothing to disclose
Annalisa De Liperi: Nothing to disclose
Erika Rocchi: Nothing to disclose
Leonardo Colligiani: Nothing to disclose
Federica Volpi: Nothing to disclose

### RPS 2205-7

**Why standardisation of preinferencing image processing methods is crucial for deep learning algorithms: compelling evidence based on the variations in outputs for different inferencing workflows**

*V. K. Venugopal*, S. Gupta, R. Takhar, V. Mahajan, H. Mahajan; New Delhi/IN
*(vasanthdrv@gmail.com)*

**Purpose:** To evaluate if there are statistically significant differences in the outputs of a deep learning algorithm on two inferencing workflows, with different unit-processing methods.
**Methods or Background:** The study was performed on DeepCOVIDXR, an open-source algorithm for the detection of COVID19 on chest x-rays. It is an ensemble of convolutional neural networks developed to detect COVID-19 on frontal chest radiographs. The algorithm was evaluated using a dataset of 905 chest x-rays containing 484 COVID-positive cases (as determined via RTPCR test) and 421 COVID-negative cases. The algorithm supports both batch image processing (workflow1) and single image processing (workflow2) for running inferencing. All the x-rays were inferenced using both methods. In batch image processing, images were resized (224x224 and 331x331) and then the lung was cropped out, but in single image processing, cropping was done without resizing of images.
**Results or Findings:** We observed a significant difference in the results for the two inferencing workflows. The AUC for COVID classification was 0.632 on the bulk image processing pathway whereas it was 0.769 for the single image processing. There were discordant results in 334 studies, 164 were classified as positive in workflow1 whereas negative in workflow2 whereas 170 x-rays that were classified as negative on workflow1 were classified as positive in workflow2.
**Conclusion:** We report statistically significant differences in the results of a deep learning algorithm on using different inferencing workflows.
**Limitations:** With the rising adoption of radiology AI, it is important to understand that seemingly innocuous changes in processing pathways can lead to disastrous clinical results.
**Ethics committee approval:** Not applicable
**Funding for this study:** Not applicable
**Author Disclosures:**
Salil Gupta: Employee: Carpl.ai
Rohit Takhar: Employee: Carpl.ai
Harsh Mahajan: Other: Research Collaboration with Koninklijke Philips NV Research Other: Research collaboration with Oxipit.ai Other: Director at Mahajan Imaging Other: Research Collaboration with Subtle Medical Other: Research Collaboration with Qure.ai Other: Research Collaboration with Quibim Other: Research Collaboration with Lunit.ai Other: Research Collaboration with Predible Health Other: Research Collaboration with General Electric
Vasantha Kumar Venugopal: Other: Research Collaboration with Koninklijke Philips NV Research Other: Research Collaboration with Subtle Medical Other: Research Collaboration with General Electric Other: Research Collaboration with Qure.ai Other: Research Collaboration with Lunit.ai Consultant: Carpl.ai Other: Research Collaboration with Quibim Other: Research collaboration with Oxipit.ai Other: Research Collaboration with Predible Health
Vidur Mahajan: Other: Research Collaboration with Subtle Medical Other: Research Collaboration with Quibim Other: Research Collaboration with Koninklijke Philips NV Research Other: Research Collaboration with General Electric Other: Research collaboration with Oxipit.ai Other: Head of Research at Carpl.ai Other: Research Collaboration with Lunit.ai Other: Research Collaboration with Qure.ai Other: Research Collaboration with Predible Health

### RPS 2205-8

**Using machine learning based CAD-RADS from radiological reports and non-curated electronic medical records data for adverse cardiac events prediction**

*E. Muscogiuri*[1], A. Tariq[2], M. van Assen[1], G. Tessarin[1], I. Banerjee[2], C. N. De Cecco[1]; [1]Atlanta, GA/US, [2]Phoenix, AZ/US
*(e.muscogiuri@gmail.com)*

**Purpose:** To evaluate whether Coronary Artery Disease Reporting & Data System score (CADRADs) extracted from radiology reports and non-curated electronic medical records (EMR) data can be used to forecast 5-year risk of adverse cardiac events (ACE) using machine learning (ML).
**Methods or Background:** We included CCTA images of 2407 patients. Two groups of ACE, ischaemic (ICD-10: I20-25) and other (ICD-10: I30-39) within 5 years of CCTA were considered as outcome. A natural language processing model was used to extract CADRADs from reports. We designed a multimodal ML model that used EMR data (before CCTA exam). Two fusion models were evaluated: an early-fusion model where features from all EMR modalities were concatenated, normalised, and processed through a predictor; a late-fusion model where cardiac event probabilities, estimated using individual predictors for each EMR modality, were concatenated and passed through a meta-learner. XGBoost was used for early-fusion and as meta-learner for late-fusion. We fine-tuned hyper-parameters using grid-search. The models were evaluated using accuracy and F1 scores.
**Results or Findings:** A total of 331 (14%) and 400 (17%) patients had ischaemic or other ACE. Using CADRADs for outcome prediction resulted in an accuracy of 0.68 and 0.74 for ischaemic and other ACE. F1-scores for no events were 0.79 and 0.84 for both outcome classes and F1-scores for events were 0.32 and 0.19. The early/late-fusion models resulted in similar accuracies of 0.68/0.62 and 0.75/0.72 for ischaemic and other ACE. F1-scores for no events were 0.79/0.75 and 0.84/0.82 for both outcome classes and F1-scores for events were 0.38/0.27 and 0.43/0.3.
**Conclusion:** ML-CADRADs and EMR data could predict ACE with moderate accuracy. The EMR model shows better performance for event prediction, since more clinical data was included compared to CADRADs.
**Limitations:** The retrospective design of the study was identified as a limitation.
**Ethics committee approval:** The IRB approved the study.
**Funding for this study:** Not applicable
**Author Disclosures:**
Amara Tariq: Nothing to disclose
Marly van Assen: Nothing to disclose
Carlo Nicola De Cecco: Research/Grant Support: Siemens
Imon Banerjee: Nothing to disclose
Giovanni Tessarin: Nothing to disclose
Emanuele Muscogiuri: Nothing to disclose

**08:00-09:00** **Room Z**

### Research Presentation Session: Cardiac

### RPS 2203

### Cardiac MRI of cardiomyopathy: part 2

**Moderator**
B. Baeßler; Würzburg/DE
**Author Disclosure:**
B. Baeßler: CEO: Lernrad GmbH

### RPS 2203-2

**Cardiovascular magnetic resonance native T1 mapping in Anderson-Fabry disease: a systematic review and meta-analysis**

*A. Ponsiglione*, M. Gambardella, R. Green, V. Cantoni, C. Nappi, M. de Giorgi, M. Petretta, A. Cuocolo, M. Imbriaco; Naples/IT
*(andreaponsiglione@hotmail.it)*

**Purpose:** We aimed to determine the weighted mean native T1 values by cardiac magnetic resonance (CMR) of Anderson-Fabry disease (AFD) patients and the standardised mean differences (SMD) as compared to healthy control subjects.
**Methods or Background:** A comprehensive literature search of the PubMed, Scopus and Web of Science databases was conducted according to the PRISMA statement to retrieve original studies reporting myocardial native T1 values in AFD patients and healthy controls. A random effects model was used to calculate SMD, and meta-regression analysis was conducted to explore heterogeneity sources. Subgroup analysis was also performed according to scanner field strength and sequence type.
**Results or Findings:** From a total of 151 items, 14 articles were included in the final analysis. Overall, weighted mean native T1 values were 984±47 ms in AFD patients and 1016±26 ms in controls (P<0.0001), with a pooled SMD of -2.38. In AFD patients there was an inverse correlation between native T1 values and male gender (P=0.002) and left ventricular hypertrophy (LVH) (P<0.001). Subgroup analyses confirmed lower T1 values in AFD patients compared to controls, with a pooled SMD of -2.54, -2.28, -2.46 for studies performed on 1.5T with modified look-locker inversion recovery (MOLLI), shortened MOLLI and saturation-recovery single-shot acquisition, respectively, and of -2.41 for studies conducted on 3T.
**Conclusion:** Our findings confirm a reduction of native T1 values in AFD patients compared to healthy controls and point out that the degree of T1 shortening in AFD is influenced by gender and LVH. Although T1 mapping is useful in proving cardiac involvement in AFD patients, there is need to standardise cut-off values according to imaging equipment and protocols.
**Limitations:** AFD is a relatively rare disease, thus limiting the number of subjects included in the investigations.
**Ethics committee approval:** Not applicable.
**Funding for this study:** No funding was received for this study.
**Author Disclosures:**
Carmela Nappi: Nothing to disclose
Mario Petretta: Nothing to disclose
Roberta Green: Nothing to disclose
Alberto Cuocolo: Nothing to disclose
Valeria Cantoni: Nothing to disclose
Andrea Ponsiglione: Nothing to disclose
Michele Gambardella: Nothing to disclose
Massimo Imbriaco: Nothing to disclose
Marco de Giorgi: Nothing to disclose

### RPS 2203-3

**Comparison of classical Fabry patients and its p.D313Y and p.A143T variants by multiparametric CMR with native T1 mapping, LGE and four-chamber feature-tracking myocardial strain**

*M. Avanesov*, A. Asgari, N. Muschol, A. Köhn, E. Tahir, G. Adam, P. Kirchhof, E. Cavus, M. Patten-Hammel; Hamburg/DE
*(maxim.avanesov@gmx.de)*

**Purpose:** To assess early cardiac manifestations in classical Fabry disease patients (cFDp) and the FD-associated variants p.D313Y and p.A143T (vFDp) by multiparametric CMR using native T1 mapping, LGE and CMR feature tracking (CMR-FT) in all cardiac chambers.
**Methods or Background:** Fifty one patients with FD (cFDp=37; vFDp=14) and 14 healthy controls underwent CMR at 1.5T, including steady-state free precession (SSFP), LGE and native T1 mapping (nT1). CMR-FT was assessed using biventricular longitudinal strain (LV-GLS/RV-GLS), left ventricular circumferential and radial strain (LV-GCS/LV-GRS), and three atrial phases of longitudinal strain (LA/RATotal, LA/RAConduit and LA/RABooster).
**Results or Findings:** In cFDp, significantly reduced myocardial strain (LV-GLS: -20±4% vs. -24±3%,p=0.007; LV-GCS: -20±4% vs. -26±4%, p=0.002, LA Total -GLS: 29±10% vs. 37±6%, p=0.007; LA Conduit -GLS: 15±10% vs. 23±5%, p=0.003) and nT1 values (951±51ms vs. 1036±20ms, p<0.001) was observed compared to controls. In vFDp all parameters were comparable to controls. LV-GCS provided the closest area under the curve (AUC) to native T1 mapping (0.84 vs. 0.92, p>0.05) for discrimination of cFDp versus controls. Significantly lower LV-GLS/LV-GCS was found in male compared to female cFDp (-19±4% vs.-22±4 %,p=0.03). In six non-hypertrophied female cFDp with normal T1 times, LATotal -GLS was the only discriminating parameter with an accuracy of 86%.
**Conclusion:** Left ventricular (LV-GLS/LV-GCS) and left atrial (LATotal -GLS) CMR-FT can detect impaired cardiac mechanics of cFDp besides native T1 mapping. Non-hypertrophied female cFDp with normal T1 values might be identified by LATotal -GLS. The FD-associated variants p.D313Y/p.A143T didn't reveal any cardiac involvement by LGE, T1 mapping or CMR-FT.
**Limitations:** No T2 mapping. No regional strain. Small number of patients due to rare disease. Single post-processing vendor
**Ethics committee approval:** Institutional Review Board of Hamburg approval was obtained.
**Funding for this study:** Initiated research grant (IIR-DEU-002334) by Takeda Pharma Vertrieb GmbH & Co. KG.
**Author Disclosures:**
Maxim Avanesov: Grant Recipient: Investigator Initiated research grant (IIR-DEU-002334) by Shire International GmbH, a member of the Takeda group of companies
Anahid Asgari: Nothing to disclose
Ersin Cavus: Nothing to disclose
Paulus Kirchhof: Nothing to disclose
Monica Patten-Hammel: Grant Recipient: Investigator Initiated research grant (IIR-DEU-002334) by Shire International GmbH, a member of the Takeda group of companies
Anja Köhn: Nothing to disclose
Gerhard Adam: Nothing to disclose
Nicole Muschol: Nothing to disclose
Enver Tahir: Nothing to disclose

### RPS 2203-4

**Personalising CMR-based risk stratification for outcomes in dilated cardiomyopathy with LVEF ≥35%: cohort study with a long-term follow-up**

*S. Li*[1], W. Yang[1], D. Zhou[1], S. Piyush[2], A. Sirajuddin[3], A. E. Arai[3], S. Zhao[1], M. Lu[1]; [1]Beijing/CN, [2]Illinois, IL/US, [3]Bethesda, MD/US
*(lishuangshirley@163.com)*

**Purpose:** To identify the risk factors for adverse events in DCM patients with LVEF ≥35% and establish a scoring model to predict the adverse event risk.
**Methods or Background:** A cohort of 466 consecutive DCM patients with LVEF ≥35% who had undergone enhanced CMR imaging were enrolled in this study. The primary endpoint was a composite of SCD or aborted SCD. The secondary endpoints were all-cause mortality, heart transplantation and hospitalisation for heart failure. The risk factors for primary and secondary endpoints were identified by multivariate Cox analysis and used to create a nomogram.
**Results or Findings:** During a mean follow-up period of 79.4 ±29.5 months, a total of 40 and 61 patients reached the primary and secondary endpoints, respectively. Multivariate stepwise analyses showed that age, family history of SCD, NYHA, and LGE≥ 7.7% (HR=6.5; 95%CI, 2.8-15.4) had a significant prognostic association with the primary endpoints (all p<0.05). NYHA, LAVi >47.3ml/m2, LGE>6.9% and GLS>-8.5% (HR=2.5; 95%CI, 1.5-4.1) had significant prognostic associations with the secondary endpoints (all p<0.05). Nomograms for the adverse events were created by these factors.
**Conclusion:** LGE and GLS are new parameters that identify the risk of adverse events in DCM patients with LVEF ≥35%. The nomogram we created using these parameters provides a novel way to clinically assess and risk stratify these patients.
**Limitations:** First, this is a single-centre, retrospective study that may be subject to referral bias. A randomised, controlled trial with a larger sample size is needed to further validate the results of our study. Second, the incidence of adverse events in LGE-negative patients was not increasingly high, which limited our risk stratification of LGE-negative patients.
**Ethics committee approval:** The Ethics Committee of Fuwai Hospital approved our study (No. 2019-1236).
**Funding for this study:** Capital Clinically Characteristic Applied Research Fund (Z191100006619021).
**Author Disclosures:**
Minjie Lu: Nothing to disclose
Andrew E. Arai: Nothing to disclose
Arlene Sirajuddin: Nothing to disclose
Shuang Li: Nothing to disclose
Sharma Piyush: Nothing to disclose
Shihua Zhao: Nothing to disclose
Di Zhou: Nothing to disclose
Wenjing Yang: Nothing to disclose

### RPS 2203-6

**Disease stage-dependent changes in left ventricular and right ventricular functions and in myocardial strain in patients with pulmonary sarcoidosis**

*A. Kocharyan*, D. P. Overhoff, G. Thater, U. Ansari, J. Michels, S. O. Schönberg, T. Papavassiliu; Mannheim/DE
*(k_arpine@yahoo.com)*

**Purpose:** The goal of this study was to estimate left ventricular (LV) and right ventricular (RV) functions and to evaluate the diagnostic value of myocardial strain analysis in patients with suspected cardiac sarcoidosis, depending on the stage of pulmonary disease.
**Methods or Background:** CMR examinations of 74 patients with pulmonary sarcoidosis (stage I n=17, stage II n=44, stage III n=7, stage IV n=6) were retrospectively evaluated. LV and RV functions were estimated and strain analysis was performed by feature tracking (Circle cvi42). The results were compared to the normal values obtained from 24 healthy controls.
**Results or Findings:** RV ejection fraction (EF) was decreased within the normal range in patients with pulmonary sarcoidosis: stage I 51% (P=0.0008); stage II 54% (P=0.006); and stage III 52% (P= 0.007); stage IV 57% (P=0.5) vs. 59% in healthy controls. LV EF was significantly decreased in patients with stage IV sarcoidosis: 51% vs. 67% (P=0.0009). 3D LV global longitudinal strain (GLS) was significantly decreased in patients with stage IV sarcoidosis: -7.98 vs -12.67 (P=0.019). 2D RV global radial strain (GRS) and 2D RV global circumferential strain (GCS) were decreased in patients with stage IV sarcoidosis too: 10.37 vs 16.22 (P=0.026) and -6.13 vs -9.72 (P=0.014), respectively.
**Conclusion:** LV and RV functions and strain parameters depend on the stage of pulmonary sarcoidosis. Myocardial strain analysis may have additional diagnostic value in detection of cardiac sarcoidosis and outcome.
**Limitations:** Few patients with stage III and stage IV sarcoidosis.
**Ethics committee approval:** Not applicable.
**Funding for this study:** Not applicable.
**Author Disclosures:**
Stefan Oswald Schönberg: Nothing to disclose
Arpine Kocharyan: Nothing to disclose
Uzair Ansari: Nothing to disclose
Greta Thater: Nothing to disclose
Theano Papavassiliu: Nothing to disclose
Daniel Pasqual Overhoff: Nothing to disclose
Julia Michels: Nothing to disclose

### RPS 2203-7

**Atrial involvement in Takotsubo syndrome: a CMR feature-tracking study**

*L. Ruoli*[1], G. C. Pambianchi[1], G. Cundari[1], L. Conia[1], M. Francone[2], N. Galea[1], C. Catalano[1]; [1]Rome/IT, [2]Milan/IT

**Purpose:** Takotsubo syndrome (TTS) is still a poorly known entity, whose stress-induced reversible ventricular dysfunction is the typical hallmark. The occurrence of atrial involvement in this syndrome is still unclear. Our purpose was to evaluate atrial injury in TTS using bi-atrial strain analysis with a cardiac magnetic resonance feature tracking technique (CMR-FT).
**Methods or Background:** We retrospectively enrolled 20 patients with a clinical diagnosis of TTS who performed CMR within 20 days from the onset of symptoms. Cine sequences were analysed using CMR-FT techniques to measure global longitudinal strain (GLS%), global radial strain (GRS%) and reservoir and conduit strain of both atria. The same analysis was performed on 20 sex- and age-matched healthy controls and then compared. Statistical analysis was assessed with Mann-Witney and correlation analysis; results were considered statistically significant when p<0,05.
**Results or Findings:** All strain parameters were found to be significantly altered in TTS patients compared to controls. We found no significant difference between the right atrium and the left atrium among TTS. GLS% (-26,9±2,2 vs -16,8±3), GRS% (64,9±10,7 vs. 28,4±8,9), reservoir strain (∈s), reservoir strain rate (srs: 1,2± 0,3 vs. 0,9 0,5), conduit(∈e), conduit strain rate (sre: -1,6±0,4 vs -1,3±0,3) of the left atrium were significantly reduced in TTS patients compared to controls (p<0,03 for all parameters). Correlation analysis of GLS%, GRS% and srs also showed an intermediate correlation with FEvs% (P<0.01; r=0.62, 0.64 and 0.63).
**Conclusion:** Atrial strain analysis with CMR-FT can identify atrial involvement in TTS and could be a useful tool for the assessment of contractile dysfunction, risk stratification and monitoring of functional recovery during follow-up.
**Limitations:** Retrospective study; population represented only by females; atrial CMR-FT was performed only on longitudinal views (lack of axial view of atria in cine imaging).
**Ethics committee approval:** Local ethical committee approved our study.
**Funding for this study:** No funding was received for this study.
**Author Disclosures:**
Giacomo Carlo Pambianchi: Nothing to disclose
Giulia Cundari: Nothing to disclose
Luca Conia: Nothing to disclose
Marco Francone: Nothing to disclose
Nicola Galea: Nothing to disclose
Carlo Catalano: Nothing to disclose
Letizia Ruoli: Nothing to disclose

### RPS 2203-8

**Prognostic value of left atrial reservoir strain by cardiac magnetic resonance in left ventricular myocardial noncompaction**

*P-l. Han*, M-T. Shen, X-M. Li, Z-K. Jiang, K. Li, Z-G. Yang; Chengdu/CN
*(1136738957@qq.com)*

**Purpose:** This study aims to evaluate the potential prognostic value of left atrial (LA) strain in left ventricular myocardial noncompaction (LVNC) and the influencing factors of it.
**Methods or Background:** A total of 95 patients (57 males and 38 females; mean age 37.47±14.46 years) with a cardiac magnetic resonance (CMR) diagnosis of LVNC were included. LA volume index (LAVI), LA ejection fraction (LAEF) and LA longitudinal strains were measured and calculated. The primary outcome was incident heart failure (HF), a composite of first HF hospitalisation, hospitalisation for worsening HF and death from HF. The associations between LA performance and incident HF were evaluated by receiver operating characteristic analysis, Kaplan-Meier analysis and Cox regression analysis. The influencing factors of LA strain in LVNC were explored by linear regression analysis.
**Results or Findings:** During a median follow-up of 32.17 months, HF occurred in 13 (13.68%) patients. Patients with increased LAVI (LAVImax>38.4ml/m2, LAVIpre-a>35.0ml/m2 and LAVImin>30.1ml/m2), decreased LAEF (LA total EF<40.1%, LA passive EF<19.7%, and LA active EF<28.8%) and decreased LA longitudinal strain (εs<12.7%, εe<9.3%, and εa<4.4%) had significantly higher risks of HF (all P<0.05). In patients with LVNC, εs was independently associated with incident HF (HR=23.208 (95% CI: 2.993-179.967), P=0.003). GLS (β=-1.783 (95% CI: -2.493- -1.073), P<0.001) was significantly and independently associated with εs.
**Conclusion:** Among patients with LVNC, LA reservoir strain was an independent predictor for incident HF. GLS was an independent effector of LA reservoir strain in LVNC.
**Limitations:** This is a retrospective single-centre study with small sample size.
**Ethics committee approval:** This study was approved by the institutional review boards of our hospital.
**Funding for this study:** This work was supported by the grants from Sichuan Science and Technology Program (2020YJ0229) and 1·3·5 project for disciplines of excellence, West China Hospital, Sichuan University (ZYGD18013).
**Author Disclosures:**
Meng-Ting Shen: Nothing to disclose
Kang Li: Nothing to disclose
Pei-lun Han: Nothing to disclose
Ze-Kun Jiang: Nothing to disclose
Xue-Ming Li: Nothing to disclose
Zhi-Gang Yang: Nothing to disclose

**09:30-10:30** **Open Forum #3 (ESR)**

### Poster Presentation Session

### PP 23

### Adding up from professional issues, GI and emergency imaging

**Moderator**
J. O. Barentsz; Nijmegen/NL

### PP 23-2

**Building a no-blame culture in radiology departments and driving clinical quality to a high level: shared experience from 13 European countries**

DOI: 10.26044/ecr2022/13524

*A. Papachristodoulou*, C. Paraskevopoulou, E. Virág, A. Roncacci; Budapest/HU

**Purpose:** Team collaboration, solid communication channels based on trust and continuous optimisation of the systems is the basis to clinical excellence. The purpose of this study is to present a method to introduce no-blame culture

Sunday in radiology departments in 13 European countries and the impact on safety awareness.
**Methods or Background:** A no-blame culture has been introduced in radiology departments in a European healthcare organisation by various processes of clinical governance and quality functions: incident management; clinical audits; clinical governance calls; learning from excellence. Clinical incident analysis with active participation and open discussion between the involved personnel, identification of the root cause and agreement on the next steps, was performed monthly. During clinical audits, the centre teams had the opportunity to actively interact and propose improvement actions which were agreed and implemented. Clinical governance meetings with each country clinical management team as well as meetings with stakeholders from all countries, promoted sharing of experiences, exchange of ideas and alignment on the next steps. Excellence reports submitted by peers were used to provide positive feedback to the involved persons and the teams.
**Results or Findings:** Active team involvement and continuous open communication within and between clinical teams proved to be a positive initiative. The number of incident reports increased by 11.2% in the first 6 months and 16% in a 12 month-period. Country team’s involvement in discussion making optimised the improvement areas by 40% while the number of excellence reports was increased by 53%.
**Conclusion:** Change of culture in healthcare organisations is a timely process which requires excellent team communication, detailed planning and commitment. A no-blame approach leads to improved quality with positive outcomes on safety and experience.
**Limitations:** Pandemic restrictions limited face-to-face meetings.
**Ethics committee approval:** Not applicable.
**Funding for this study:** Not applicable.
**Author Disclosures:**
Alessandro Roncacci: Nothing to disclose
Athanasia Papachristodoulou: Nothing to disclose
Chryssa Paraskevopoulou: Nothing to disclose
Emese Virág: Nothing to disclose

### PP 23-3
**Communication dose exposure for common radiological procedures: what the patients would like know**
DOI: 10.26044/ecr2022/17900

M. Pace[1], L. Rabiolo[1], C. Nardi[2], A. Magistrelli[3], C. Granata[4], P. Toma[3], S. Colagrande[2], D. Matranga[1], *S. Salerno*[1]; [1]Palermo/IT, [2]Florence/IT, [3]Rome/IT, [4]Trieste/IT

**Purpose:** Purpose of our study is twofold. First, to investigate interest in knowing radiation dose; second, to individuate an effective communication for dose exposure.
**Methods or Background:** A cross-sectional multicentric data collection from 4 different hospitals, two general and two paediatric ones recruiting 1,084 patients (relative to paediatric). A questionnaire was administered anonymously, composed of a brief explanation of radiation use in medical procedure, a patient data section (sex, age, educational and working status), and communicating section with four followed different modalities: first, dose in number (mGy, DAP, CTDI and DLP) obtained from the x-ray apparatus. Second, dose in number with a reference range from national DRL. Third, comparison with x-ray equivalent dose and natural background. Fourth, x-ray symbol and a colorimetric scale (from green to red). The last section assesses patient's comprehension, interest in dose exposure and preferred communication way.
**Results or Findings:** 1009 patients (75 refuse to fill); 173 participants were relatives of paediatric patients. Preliminary information was declared comprehensible, and patients demonstrate interest in knowing about radiation dose exposure. Modality with symbols was the most understandable without appreciable socio-cultural differences; modality with numbers and reference levels (DRL) was preferred by patients with higher socio-cultural background. "None of those" was preferred by one-third of our sample composed of 4 different clusters: female, over 60yrs, lower socio-cultural level and not employed.
**Conclusion:** There is an interest in knowing the radiation dose exposure. Written modality is simple and effective to satisfy this interest. Iconographic modality is better understood; however, it is necessary to find a clear and effective explanatory model universally understandable.
**Limitations:** Patients are not randomised. Administration of the questionnaire before radiological examination by the staff may influence response.
**Ethics committee approval:** This study was approved by an ethics committee (Ethical Committee authorization n. 4 date 29.04.19).
**Funding for this study:** No funding was received for this study.
**Author Disclosures:**
Paolo Toma: Nothing to disclose
Sergio Salerno: Nothing to disclose
Cosimo Nardi: Nothing to disclose
Domenica Matranga: Nothing to disclose
Andrea Magistrelli: Nothing to disclose
Stefano Colagrande: Nothing to disclose
Lidia Rabiolo: Nothing to disclose
Claudio Granata: Nothing to disclose
Mario Pace: Nothing to disclose

### PP 23-4
**Revisiting the general linear model for radiology research**
DOI: 10.26044/ecr2022/13255

*J. F. Castro Pereira*, B. Gaspar, J. F. P. Sardinha, G. Saldanha; Almada/PT

**Purpose:** To elucidate radiologists doing research on the strengths and weaknesses of linear models, their assumptions and how to interpret their results using a dataset and R software as examples, but that can be extrapolated to other commonly used statistical software.
**Methods or Background:** Linear models are one of the main statistical methods used in radiological research even when the researchers are unaware they are using it: in fact t-tests and ANOVA are just linear models. Knowing the assumptions of linear models and how to diagnose when they aren’t fulfilled will in turn help radiologists become better users of the main statistical methods while doing research and ease communications between them and statisticians.
**Results or Findings:** Using a dataset to elucidate the main assumptions of the linear model: linearity, independence, equal variance and normality, we will show the main commands in R for running a linear model, how to diagnose its problems and how to interpret the coefficients and confidence intervals that result from it.
**Conclusion:** We hope that after this presentation radiologists will be better empowered to start their own data analysis projects using statistical software, understand when linear models should or should not be used and better understand the statistical jargon used when speaking with a statistical consultant during a project.
**Limitations:** No limitations were identified.
**Ethics committee approval:** Not applicable.
**Funding for this study:** No funding was received for this study.
**Author Disclosures:**
José F. Castro Pereira: Nothing to disclose
José Francisco Parreira Sardinha: Nothing to disclose
Gonçalo Saldanha: Nothing to disclose
Barbara Gaspar: Nothing to disclose

### PP 23-6
**Common and uncommon errors in emergency ultrasound: what the radiologist needs to know**
M. Di Serafino, *V. Sabatino*, F. Iacobellis, F. Verde, G. Dell'aversano Orabona, M. Caruso, D. Grimaldi, C. Rinaldo, L. Romano; Naples/IT

**Purpose:** To evaluate the common sources of diagnostic errors in emergency ultrasound (US) usually found in clinical-radiological practice and the useful tips to recognise and avoid them.
**Methods or Background:** Emergency US is a standard emergency radiological skill, often the first course of action of the emergency radiologist that plays a pivotal role in the diagnostic assessment of patients in the emergency setting. Emergency US is particularly susceptible to the errors and the emergency room is a typical scenario for malpractice claims.
**Results or Findings:** We can recognise and classify ultrasound diagnostic errors in emergency US into three groups, that are: (1) Errors dependent on: environmental factors (crowded emergency rooms, large number of examinations with or without character of appropriateness, rapid diagnosis and management); patient (morphotype, lack of cooperation); technical skill of the sonographer himself (unsuitable training emergency courses). (2) Errors of interpretation: “bad” artifacts (lateral lobe artifacts, mirror effect, doubling artifact, refraction, reverberation, image adaptation artifact; anisotropy); chest artifacts (not contextualised to the clinical data); US setting errors (B-mode and color-Doppler); anatomy and anatomical variants (pseudo-splenic haematoma, pseudo-collections of pleural, pericardial, peritoneal and retroperitoneal fluids, pseudo pneumothorax); generic and conditioning (hypertrophic diaphragmatic pillar, bladder pseudo masses, inguinal pseudo hernias, Rouleaux phenomenon). (3) Errors of underestimation: retroperitoneum (undergoing FAST exam); spleen (partial visualisation and poor attention or interest), poor quality of images produced; inconclusive and synthetic US report.

**Conclusion:** Following some rules in technique and interpretation, and always integrating emergency US findings into the broader clinical context, most misdiagnosis can be avoided, and thus patients' safety can be enhanced. Being aware of a list of common pitfalls may help to avoid misdiagnoses.
**Limitations:** No limitations were identified.
**Ethics committee approval:** None.
**Funding for this study:** No funding was received for this study.
**Author Disclosures:**
Giuseppina Dell'aversano Orabona: Nothing to disclose
Dario Grimaldi: Nothing to disclose
Francesca Iacobellis: Nothing to disclose
Francesco Verde: Nothing to disclose
Chiara Rinaldo: Nothing to disclose
Luigia Romano: Nothing to disclose
Marco Di Serafino: Nothing to disclose
Martina Caruso: Nothing to disclose
Vittorio Sabatino: Nothing to disclose

### PP 23-7

**PERC criteria: does it reliably exclude the diagnosis of a pulmonary embolism?**

*A. Sathiyakeerthy*, I. M. R. Kandil, G. Gamtkitsulashvili, N. Akter, H. Ahmad, A. Khan, R. Gauhar, M. Anwar; London/UK

**Purpose:** To clarify the reliability of the PERC scoring system in confirming the low probability in those suspected to have a PE, thereby minimising the need for CT imaging.
**Methods or Background:** Diagnosis/exclusion of PE can be challenging with overuse of CTPA imaging in diagnosis. The PE rule-out criteria (PERC) is utilised to negate the need for workup. A retrospective study was conducted at Princess Alexandra Hospital. The study period was from August–November 2020 with a total of 128 patients undergoing CTPA imaging for suspected PE, aged <50 years old (18-49 years of age). PERC scores were collated for patients.
**Results or Findings:** 38.2% of all patients had a PERC score 0 and theoretically did not need further investigation. The remaining patients had a score ≥1 (scores ranged between 0-3), thereby validating the requirement for CTPA imaging. Of these 79 patients, 83.5% did not have PE on imaging. In the patients with a confirmed PE, all had a PERC score ≥1. Reviewing correlation with D-dimer values, of those with a PERC score of 0, 93.7% had a positive d-dimer (cut off >500ng/ml) whereas 6.3% had a negative d-dimer value.
**Conclusion:** Given our study, when applying the PERC scores, it can be concluded that this scoring system reliably identified those that did not warrant further investigation. Arguably, the PERC score is more useful than D-dimer values in aiding management and could minimise the number of unnecessary irradiative imaging if used in conjunction. The average cost of CTPA imaging is £85 and potentially £3,825 could have been saved for those with a PERC value of 0 despite a positive D-dimer level.
**Limitations:** Aged >50 excluded. Clinical context is not considered in combination with values. Small sample size in a retrospective study.
**Ethics committee approval:** This study was approved by Nil.
**Funding for this study:** Funding was received for this study by Nil.
**Author Disclosures:**
Neelima Akter: Nothing to disclose
Hanif Ahmad: Nothing to disclose
Anushka Sathiyakeerthy: Nothing to disclose
Ameera Khan: Nothing to disclose
Muhammad Anwar: Nothing to disclose
Rehan Gauhar: Nothing to disclose
Gvansta Gamtkitsulashvili: Nothing to disclose
Iman Mohamed Reda Kandil: Nothing to disclose

### PP 23-8

**Role of diagnostic imaging technologists during the COVID-19 pandemic: the importance of organisation and planning in the first line**

*D. I. Ribas Mercau*; Sabadell/ES

**Purpose:** COVID-19 required changes throughout the entire health system and diagnostic imaging departments were no exception. These led to a restructuring of the working dynamics of the radiographers' staff. To ensure that these new needs were met, the staff has to be trained and distributed into different areas.
**Methods or Background:** The purpose of this poster is to describe, detail, and illustrate the different changes and decision-making carried out by the radiographers' staff due to the COVID-19, as an example of organisation and coordination in a crisis situation.
**Results or Findings:** The staff was divided into two groups. These carried out the activity alternately weekly. Each group was divided into two teams: "COVID-19" and "No COVID-19". Also, they were separated on the base of the different risk factors they had. Radiographers with potential risk factors for severe COVID-19 due to underlying pathologies were assigned to perform tests on patients without suspicion of COVID-19.
**Conclusion:** It is essential to recognise the role played by radiographers during the pandemic. They were one of the main actors on the front line, who has not only lived through grueling working hours with changing guidelines but also endured the stress and emotional pressure that this critical situation entails.
**Limitations:** The lack of experience in similar situations was the most important deficiency. This led to a constant need to adapt to different situations that presented themselves day after day. The continuous improvisation led to a stressful work scenario that included all actors at all levels.
**Ethics committee approval:** In carrying out this work, the author declares that there has been no financial financing or any conflict of interest.
**Funding for this study:** The ability to adapt for the professionals in a hard-working environment is worthy of any kind of recognition.
**Author Disclosures:**
Diego Ignacio Ribas Mercau: Nothing to disclose

### PP 23-9

**A nomogram based on multi-modal ultrasound for pre-operative prediction of microvascular invasion and recurrence of hepatocellular carcinoma**

*X. Zhong*, M. Lin, X. Xie; Guangzhou/CN

**Purpose:** To establish and validate a nomogram based on multi-modal ultrasound for preoperative prediction of microvascular invasion (MVI) and progression-free survival (PFS) of hepatocellular carcinoma (HCC).
**Methods or Background:** A total of 287 patients with HCC undergoing surgical resection were prospectively enrolled, including 210 patients in the training cohort and 77 patients in the test cohort. All patients underwent conventional ultrasound (CUS), contrast-enhanced ultrasonography (CEUS), and shear wave elastography (SWE) examination within one week before surgery. Taking the result of the histopathological examination as the reference standard, independent factors associated with MVI in HCC were determined by logistic regression and a nomogram was established and further evaluated. The Kaplan-Meier method was used to analyse the prognostic value of histologic MVI status and nomogram-predicted MVI status.
**Results or Findings:** Multivariate analysis showed that tumour maximum diameter, echogenicity, tumour shape, peritumoral enhancement in arterial phase and enhancement level in portal venous phase were independent predictors of MVI (all $P < 0.05$). The nomogram based on these variables showed good discrimination and calibration with the area under the receiver operating characteristic curve (AUC) of 0.821 (0.762-0.870) in the training cohort and 0.789 (0.681-0.874) in the test cohort. There was a significant difference in PFS between the nomogram-predicted MVI positive and the nomogram-predicted MVI negative groups in training and test cohort ($p<0.001$ and $p=0.001$ respectively).
**Conclusion:** The multimodal ultrasound features were effective imaging markers for preoperative prediction of MVI of HCC and the nomogram might be an effective tool to stratify the risk of recurrence.
**Limitations:** This study is a single-centre study, multi-centre validation is needed to verify its generalisability before clinical application.
**Ethics committee approval:** This study was approved by the ethics committee of our hospital.
**Funding for this study:** Funding was received for this study by the Natural youth science foundation of China (81901768).
**Author Disclosures:**
Xiaoyan Xie: Nothing to disclose
Xian Zhong: Nothing to disclose
Manxia Lin: Nothing to disclose

### PP 23-10

**CT texture analysis of primary and metastatic pancreatic ductal adenocarcinomas: value in assessment of histopathological grade and differences between primary and metastatic lesions**

*M. Janisch*, G. Adelsmayr, E. Janek, H. Mueller, A. Holzinger, E. Talakic, M. Fuchsjäger, H. Schöllnast; Graz/AT

**Purpose:** Despite progress in therapeutic options, prognosis of pancreatic ductal adenocarcinoma (PDAC) remains poor and is also influenced by histopathological grading. As neoadjuvant therapy may be beneficial for patients with poorly differentiated PDAC, a non-invasive method to assess histopathological grade would be valuable. Therefore we evaluated the significance of CT-texture analysis (CTTA) in assessment of histopathological grade of PDAC and compared CTTA texture features between primary and metastatic PDAC.
**Methods or Background:** This retrospective study included 120 patients with histopathologically confirmed PDAC. Sixty-five patients underwent CT-guided biopsy of primary PDAC. Fifty-five patients underwent CT-guided biopsy of hepatic PDAC metastasis. Lesions were segmented in non-contrast CT scans for CTTA. Statistical analysis was conducted for 372 textural features using Mann-Whitney-U-test, Bonferroni-Holm correction and receiver operating characteristic (ROC) analysis. P-values <0.05 were considered statistically significant.

**Results or Findings:** Three features were identified that differed significantly between histopathological G2 and G3 primary tumours. Of these, "low gray-level zone emphasis" yielded the largest AUC (0.87 +/- 0.04), reaching a sensitivity and specificity of 0.76 and 0.83, respectively, when a cut-off value of 0.482 was applied. Fifty-four features differed significantly between primary and hepatic metastatic PDAC (AUCs: 0.72-0.93).
**Conclusion:** CTTA of PDAC identified differences in texture features between primary G2 and G3 tumours that could be used for non-invasive tumour assessment. Extensive differences between the features of primary and metastatic PDAC on CTTA suggest that CTTA of a metastatic lesion may not allow conclusions to be drawn regarding the histology of the primary tumour.
**Limitations:** Due to retrospective design selection bias cannot be ruled out. Biopsy material instead of surgical specimen was used to determine histopathological grading.
**Ethics committee approval:** The ethics committee of the Medical University of Graz has approved this retrospective study.
**Funding for this study:** No funding was received.
**Author Disclosures:**
Andreas Holzinger: Nothing to disclose
Michael Fuchsjäger: Nothing to disclose
Helmut Schöllnast: Nothing to disclose
Michael Janisch: Nothing to disclose
Elmar Janek: Nothing to disclose
Emina Talakic: Nothing to disclose
Heimo Mueller: Nothing to disclose
Gabriel Adelsmayr: Nothing to disclose

### PP 23-11
**Multi-colour magnetic particle imaging for real-time detection of gastrointestinal hemorrhage**
*C. Riedel*, F. Mohn, P. Szwargulski, T. Knopp, M. Kaul, G. Adam, M. Gräser, J. Salamon; Hamburg/DE

**Purpose:** To evaluate the feasibility of detecting gastrointestinal (GI) hemorrhage in vitro by real-time 3D multi-colour magnetic particle imaging (MPI).
**Methods or Background:** Small bowel phantoms were 3D printed with a lumen measuring 13.5 mm. The surrounding wall was constructed with a hollow layer representing the vascular compartment in a perfused bowel wall. One phantom with separation of the luminal and the vascular compartment served as control. Another phantom was created with a perforation between both compartments representing the source of a bleeding. The lumen was filled with water for single-color MPI and with a tracer suspension of LS-008 (LodeSpin) for multi-colour MPI. The vascular compartment was connected to a water-filled circulatory system. Phantoms were imaged dynamically (46 Volumes/s) in the MPI scanner (Bruker/Philips) to evaluate the fluid exchange between the vascular compartment and the lumen while a bolus of 1 ml Perimag (Micromod) was injected as blood pool tracer. Similar experiments were performed using bovine small bowel specimens with GI hemorrhage simulated by an incision in the mucous membrane.
**Results or Findings:** MPI enabled real-time visualisation of the enhancing bowel wall following tracer injection for single-colour and multi-colour experiments. Leakage of the blood pool tracer into the lumen was not observed in the control experiments. In case of simulated bleeding, real-time MPI enabled the detection of the blood pool tracer in the lumen. Here, multi-colour MPI confirmed the bleeding by co-registration of both tracers in the lumen at the same location.
**Conclusion:** Both single- and multi-colour MPI are feasible to visualise GI hemorrhage in a bowel phantom as well as in bovine bowel specimens. MPI might emerge as a useful tool for the radiation-free detection of acute and chronic GI hemorrhage.
**Limitations:** Not applicable.
**Ethics committee approval:** Not applicable.
**Funding for this study:** Not applicable.
**Author Disclosures:**
Michael Kaul: Nothing to disclose
Christoph Riedel: Nothing to disclose
Tobias Knopp: Nothing to disclose
Patryk Szwargulski: Nothing to disclose
Johannes Salamon: Nothing to disclose
Gerhard Adam: Nothing to disclose
Fabian Mohn: Nothing to disclose
Matthias Gräser: Nothing to disclose

**09:30-10:30 Open Forum #4 (ESR)**

### Research Presentation Session: Imaging Informatics / Artificial Intelligence and Machine Learning

### RPS 2305a

### New trends in artificial intelligence (AI) and steps for AI standardisation

**Moderator**
M. Huisman; Utrecht/NL
**Author Disclosure:**
M. Huisman: Board Member: EuSoMII; Speaker: DeepC, Bayer, MedicalPHIT

### RPS 2305a-2
**The impact of digitalisation on COVID-19 outcomes and government responses**
*H. Heinrichs*[1], F. Müller[1], L. Rohfleisch[1], V. Schulz[1], S. Talbot[2], F. Kiessling[1]; [1]Aachen/DE, [2]Hannover/DE
*(hheinrichs@ukaachen.de)*

**Purpose:** COVID-19 has placed a significant burden on populations worldwide. Countries are suffering the consequences of the pandemic, which are affected by varying determinants, including digitalisation. While the pandemic has accelerated digital transformation in many areas of life, we know little about how digitalisation has shaped the crisis and handling thereof. In this study, we aim to investigate the role of digitalisation in the course of the pandemic.
**Methods or Background:** For this purpose, we chose the digital adoption index as a representative score to evaluate its association with COVID-19, its attributed cases, deaths, and government responses. We used an algorithm based on gradient tree boosting (GTB) to select key predicting features. A mixed-effects model was used to model the heterogeneous nature of data per point in time (March, June, September, and December). Lastly, a scoping review offered essential insights into recent literature.
**Results or Findings:** GTB found potential links between corporate and government digital developments and the stringency of measures taken by the governments. By the end of 2020, we observed that European countries had 4.8 times lower COVID-19 attributed death rates when they scored higher in digitalisation ($p<0.001$). This trend was possibly enhanced by the significant correlation between the digital adoption index and the gross national income ($r=0.749$, $p=<0.001$), as well as the development of a country ($r=0.920$, $p<0.001$).
**Conclusion:** We found that the more digitised a country, the less severe the impact of COVID-19. Our findings encourage the importance of digital transformation in the face of the current pandemic.
**Limitations:** This study is based on COVID-19 outcomes reported by countries. Differences in reporting or inaccurate reporting of COVID-19 data may have influenced the power of our model.
**Ethics committee approval:** Not applicable
**Funding for this study:** Funding was received from the Federal Ministry of Education and Research: 01GP1910A.
**Author Disclosures:**
Fabian Kiessling: Nothing to disclose
Florian Müller: Nothing to disclose
Helen Heinrichs: Nothing to disclose
Steven Talbot: Nothing to disclose
Volkmar Schulz: Nothing to disclose
Lucia Rohfleisch: Nothing to disclose

### RPS 2305a-3
**Innovation trends in radiology: is it a long way to the top?**
*L. Gorenstein*[1], S. Soffer[2], S. Apter[1], E. Konen[1], E. Klang[1]; [1]Ramat Gan/IL, [2]Ashdod/IL
*(larisagorenshtein@gmail.com)*

**Purpose:** The healthcare sector is one of the most complex industries and may benefit from the adaptation of technological innovations. Radiology has often been the portal for medical advancements. We aimed to reveal the engagement of innovation in the radiological literature compared to the general medical literature.
**Methods or Background:** We have queried the PubMed API for all available innovation-related entries published from 2000 to 2020. Papers were classified into research fields that comply with innovation: artificial intelligence (AI), virtual reality (VR) and telemedicine. Terms to classify entries into the three research fields were determined by consensus among the authors. A journal list of radiology and nuclear medicine and other medical fields was compiled using the open access Scimago Journal & Country Rank (SJR) site.
**Results or Findings:** Our search yielded 194,685 innovation-related entries published from 2000 to 2020. Most digital technology innovation-related publications were written in the last decade. AI research had a steep rise in all fields of healthcare with 20,552 publications in 2020 compared to only 2,184 publications in VR and 5,189 in telemedicine. In 2020, 2603 (38.7%) of the innovation publications were published in radiology and nuclear medicine related journals vs 1426 (21.2%) surgery, 1294 (19.2%) oncology and 566 (8.4%) internal medicine related journals.
**Conclusion:** Radiology and nuclear medicine were at the top of innovation related publications with a steep rise at the last years. While AI is at the main focus of radiology and nuclear medicine literature, VR and telemedicine had a limited number of publications. Innovation employment may help with the ever-growing workload and improve performance. Hence, understanding of innovation engagement in different medical fields is of paramount importance.
**Limitations:** The high-level look at the field taken as well as data extraction from PubMed were identified as limiting factors.
**Ethics committee approval:** As a PubMed literature review, this study required no IRB approval.
**Funding for this study:** No funding was received for this study.
**Author Disclosures:**
Eli Konen: Nothing to disclose
Larisa Gorenstein: Nothing to disclose
Sara Apter: Nothing to disclose
Shelly Soffer: Nothing to disclose
Eyal Klang: Nothing to disclose

### RPS 2305a-4

### Selecting, evaluating and monitoring artificial intelligence models for clinical use

*B. Allen*[1], K. Dreyer[2], C. Wald[3]; [1]Birmingham, AL/US, [2]Boston, MA/US, [3]Nahant, MA/US
*(bibb@mac.com)*

**Purpose:** Tools for evaluating and monitoring AI models for clinical use are currently limited. The American College of Radiology (ACR) data science institute has developed freely available resources to assist practices of all sizes select, evaluate and monitor AI in clinical practice.
**Methods or Background:** The commercial availability of radiology artificial intelligence algorithms continues to accelerate; however, regulatory approval alone may not ensure AI will function as expected in real-world clinical practice. To be savvy consumers of AI, radiologists must be able to navigate the growing market of available AI models, evaluate the models for their practices prior to purchase and monitor model performance in clinical use over time. While institutions with robust informatics infrastructure may be able to perform these functions, most practices will not be able to do so without assistance. Likewise, a recent survey of ACR members found that a majority would like to have access to tools for AI evaluation and longitudinal performance monitoring.
**Results or Findings:** To meet the growing need of the specialty the ACR has developed a catalog of USFDA cleared algorithms radiologists can use to understand the availability, use case and validation parameters of AI tools available for use in clinical practice. Additionally, the ACR is making the infrastructure and tools available to allow practices of all sizes evaluate AI models using their own patient data. Finally, the ACR has developed an AI data registry that practices can use to monitor the performance of AI models deployed in their practices.
**Conclusion:** By providing tools and infrastructure, medical specialty societies can assist radiologists select, evaluate and monitor AI for clinical practice.
**Limitations:** Similar tools may be available but require robust health system informatics infrastructure to deploy and use.
**Ethics committee approval:** Ethics committee approval was not required.
**Funding for this study:** No funding was received for this study.
**Author Disclosures:**
Christoph Wald: Nothing to disclose
Bibb Allen: Nothing to disclose
Keith Dreyer: Nothing to disclose

### RPS 2305a-5

### The Image Biomarker Standardisation Initiative (IBSI) on reproducible convolutional radiomics

P. Whybra[1], R. Schaer[2], A. Depeursinge[2], V. Andrearczyk[2], H. Müller[2], M. Vallières[3], *A. Zwanenburg*[4]; [1]Cardiff/UK, [2]Sierre/CH, [3]Sherbrooke, QC/CA, [4]Dresden/DE
*(alexander.zwanenburg@nct-dresden.de)*

**Purpose:** Convolutional filters are used to quantify characteristics in medical imaging, such as tissue heterogeneity and tissue structures. Though powerful, such filters lack reproducibility. The purpose is to standardise the computation of convolutional image filters for radiomics analyses.
**Methods or Background:** A reference manual and digital phantoms were provided to participating teams who develop radiomics software. We defined 36 tests covering common convolutional filters. Participants provided response maps (RM) for each implemented test after applying a filter to a digital phantom. We aggregated RMs into a consensus-based response map (CRM) by iteratively removing outlying contributions. Strength of consensus was classified into 4 categories (weak, moderate, strong, very strong) based on the number of RMs matching the candidate CRM (<3, 3-5, 6-9, ≥10). Moreover, candidate CRMs required an absolute majority (>50% RMs) to be accepted.
**Results or Findings:** Forty-two researchers from fourteen teams participated in this work. Over the course of 12 months, we observed an improved consensus (very strong, 0/36 to 12/36; strong, 4/36 to 8/36; moderate, 14/36 to 1/36; weak, 5/36 to 0/36). Currently (Oct. 2021), 15/36 tests do not show a majority consensus.
**Conclusion:** This work demonstrates that radiomics analyses utilising convolutional filtering can significantly differ due to implementation decisions. Through increase in participation and by resolving ambiguities, we managed to improve consensus between software packages for several filter families. However, a comprehensive standardised radiomics workflow including convolutional filters is not yet fully achieved.
**Limitations:** We did not obtain CRMs for Gabor and Riesz transform filter tests, indicating their current lack of reproducibility. Moreover, integration of convolutional filtering into a radiomics workflow is currently being assessed, with pending results.
**Ethics committee approval:** Ethics committee approval was not required.
**Funding for this study:** No extramural funding needs to be declared.
**Author Disclosures:**
Roger Schaer: Nothing to disclose
Alex Zwanenburg: Nothing to disclose
Adrien Depeursinge: Nothing to disclose
Vincent Andrearczyk: Nothing to disclose
Henning Müller: Nothing to disclose
Philip Whybra: Nothing to disclose
Martin Vallières: Nothing to disclose

### RPS 2305a-6

### Towards personalised predictions in medical imaging: Siamese networks, Gaussian processes and memory networks for colonic transit time-series data

*B. S. Kelly*, R. P. Killeen, A. Lawlor, P. Mathur; Dublin/IE
*(brendanskelly@me.com)*

**Purpose:** To use deep learning to identify radiopaque markers as part of a colonic transit study and to predict time to completion of the study on both a population- and patient-specific basis.
**Methods or Background:** This retrospective cohort study included all patients undergoing a colonic transit study in a single institution from 2010 to 2020. We trained and tested deep learning models based on a Siamese network architecture to classify images according to the presence or absence and the number of radiopaque markers and to identify interval change. We also employed time-series methods based on these data to predict the total duration of the study. Saliency maps were interrogated for explanations of false positives and false negatives.
**Results or Findings:** 568 images of 229 patients (143, 62% female, mean age 57) patients were included. For binary classification, the best performing model (Siamese DenseNET trained with contrastive loss with unfrozen weights) achieved an accuracy, precision and recall of 0.988, 0.986 and 1. On a per-patient basis, an LSTM trained on outputs of a Gaussian process regressor outperformed basic statistical curve fitting with a MAE of 1.93 compared to 5.22 ($p<0.05$).
**Conclusion:** Siamese networks perform well at identification of radiopaque markers in colonic transit studies. For time series prediction, advanced methods improved performance for personalised prediction.
**Limitations:** This work's status as a singlecentre study as well as a pilot study was identified as a limitation.
**Ethics committee approval:** Approval was received after full review from the hospital REC.
**Funding for this study:** This work was performed within the Irish Clinical Academic Training (ICAT) Programme, supported by the Wellcome Trust and the Health Research Board (Grant Number 203930/B/16/Z), the Health Service Executive National Doctors Training and Planning and the Health and Social Care, Research and Development Division, Northern Ireland and the Faculty of Radiologists, Royal College of Surgeons in Ireland.
**Author Disclosures:**
Aonghus Lawlor: Nothing to disclose
Ronan P Killeen: Nothing to disclose
Brendan S Kelly: Nothing to disclose
Prateek Mathur: Nothing to disclose

### RPS 2305a-7

**QuantImage v2: a clinician-in-the-loop cloud platform for radiomics research**

R. Schaer[1], V. Oreiller[1], D. Abler[1], H. Verma[1], J. Reichenbach[1], F. Evéquoz[1], M. Jreige[2], J. O. Prior[2], *A. Depeursinge*[1]; [1]Sierre/CH, [2]Lausanne/CH

**Purpose:** First, to allow radiologists and nuclear medicine physicians to create patient cohorts and extract radiomics features from CT/PET/MR images. Second, to allow feature exploration using visualisation tools and creating machine learning models for classification and survival tasks. This is achieved via an open-source web-based platform without the need for programming. Interactive visualisation is crucial for exploring links between radiomics features and patient outcomes but is absent from many available radiomics tools.
**Methods or Background:** We used an existing open-source web-based tool (Kheops) that enables users to create, manage and share collections of DICOM images. In addition, we developed a user-friendly companion web platform (QuantImage v2) for radiomics feature extraction and management, predictive model building and validation, as well as interactive data visualisation.
**Results or Findings:** After creating a patient cohort using Kheops, users extracted radiomics features using QuantImage v2 from CT/PET/MR images. Interactive visualisation tools assisted users in feature selection for training machine learning models, e.g. by allowing users to filter features by imaging modality, region-of-interest & feature categories. Finally, the platform enabled real-time training and comparison of predictive models for classification and survival analysis tasks using several algorithms. The iterative process of feature exploration and predictive model building allowed identifying outliers, revealed intraclass group heterogeneity, and helped novice users to build better-performing models relying on fewer predictors.
**Conclusion:** The developed platform empowers clinical researchers with no background in programming to investigate and test radiomics models via an easy-to-use web interface. The novel feature visualisation functionality helps identify salient features that produce well-performing predictive models. First user tests are encouraging, with feedback highlighting the ease of use and usefulness of the freely available tool.
**Limitations:** Currently, the platform cannot evaluate created models on independent test sets.
**Ethics committee approval:** Not applicable
**Funding for this study:** Funding was received from the SPHN.
**Author Disclosures:**
Roger Schaer: Nothing to disclose
Valentin Oreiller: Nothing to disclose
Adrien Depeursinge: Nothing to disclose
Julien Reichenbach: Nothing to disclose
Florian Evéquoz: Nothing to disclose
Himanshu Verma: Nothing to disclose
Daniel Abler: Nothing to disclose
Mario Jreige: Nothing to disclose
John O Prior: Nothing to disclose

### RPS 2305a-8

**Continual active learning for efficient image labelling while image characteristics change**

*M. Perkonigg*, J. Hofmanninger, C. J. J. Herold, H. Prosch, G. Langs; Vienna/AT

**Purpose:** To develop a machine learning method for effectively choosing examples for ground truth labelling to adapt models to new scanners and image characteristics in a continuous stream of medical imaging data.
**Methods or Background:** The proposed approach continuously trains models on a stream of imaging data by recognising shifts in image acquisition characteristics and selecting optimal examples for labelling. Those examples are stored in a rehearsal memory to keep a machine learning model up-to-date, while at the same time not forgetting previous knowledge. A style embedding is extracted from each image to assign it to a pseudo-domain. Those pseudo-domains are used to balance the rehearsal memory and training procedure. The benefits of the method were evaluated for cardiac MR image segmentation in 2D.
**Results or Findings:** A total of 7230 2D MR slices of the Multicentre, Multivendor & Multidisease Cardiac Image Segmentation Challenge (M&Ms) challenge dataset from four different scanners were used for evaluation. Results showed that the method is capable of learning on a continuous stream, while keeping the number of required manual annotations low. The mean dice scores for the four scanners were 0.81, 0.73, 0.80 and 0.68 respectively, compared to continual training on the stream without the proposed method (0.82, 0.71, 0.76, 0.56) or a static model (0.81, 0.69, 0.72, 0.34).
**Conclusion:** The proposed method was capable of training models on a continuous stream of imaging data, with a limited labelling budget and outperformed baseline methods.
**Limitations:** Variability in annotator accuracy was not evaluated in this study.
**Ethics committee approval:** Not applicable
**Funding for this study:** Funding was received from the Austrian Science Fund (FWF): P 35189, Vienna Science and Technology Fund (WWTF): LS20-065, Novartis Pharmaceuticals Corporation.
**Author Disclosures:**
Christian J. Johannes Herold: Nothing to disclose
Georg Langs: Nothing to disclose
Helmut Prosch: Nothing to disclose
Johannes Hofmanninger: Nothing to disclose
Matthias Perkonigg: Nothing to disclose

**09:30-10:30** **Room M 2**

### Research Presentation Session: GI Tract

### RPS 2301

### Novelties in intestinal imaging

**Moderator**
A. Ba-Ssalamah; Vienna/AT
**Author Disclosure:**
A. Ba-Ssalamah: Consultant: Bayer; Speaker: Bayer, Sanofi

### RPS 2301-3

**A radiomic model for identifying patients with locally advanced rectal cancer who will respond to neoadjuvant chemoradiotherapy**

*X. Yi*; Changsha/CN
*(yixiaoping@csu.edu.cn)*

**Purpose:** Early detection of non-response to neoadjuvant chemoradiotherapy (nCRT) for locally advanced colorectal cancer (LARC) remains challenging. We aimed to assess whether pretreatment radiotherapy planning computed tomography (CT) radiomics could distinguish the patients with no response or no downstaging after nCRT from those with response and downstaging after nCRT.
**Methods or Background:** Patients with LARC who were treated with nCRT were retrospectively enrolled between March 2009 and March 2019. Traditional radiological characteristics were analysed by visual inspection and radiomic features were analysed through computational methods from the pretreatment radiotherapy planning CT images. Differentiation models were constructed using radiomic methods and clinicopathological characteristics for predicting non-response to nCRT. Model performance was assessed for classification efficiency, calibration, discrimination, and clinical application.
**Results or Findings:** This study enrolled a total of 215 patients, including 151 patients in the training cohort (50 non-responders and 101 responders) and 64 patients in the validation cohort (21 non-responders and 43 responders). For predicting non-response, the model constructed with a machine learning method had higher performance with area under the curve (AUC) values of 0.92 and 0.89 as compared to the model constructed with the logistic regression method (AUC: 0.72 and 0.71 for the training and validation cohorts, respectively). Both decision curve and calibration curve analyses confirmed that the machine learning model had higher prediction performance.
**Conclusion:** Pretreatment CT radiomics achieved satisfying performance in predicting non-response to nCRT and could be helpful to assist in treatment planning for LARC patients.
**Limitations:** First, this was a retrospective study conducted at a single center. Second, our sample size was modest.
**Ethics committee approval:** This study was approved by the institutional review board of our Hospital (IRB # 201910070).
**Funding for this study:** Funding was received from Xiangya-Peking University, Wei Ming Clinical and Rehabilitation Research Fund (No. xywm2015I35).
**Author Disclosures:**
Xiaoping Yi: Nothing to disclose

### RPS 2301-4

**DW-MRI assessment of response to total neoadjuvant therapy in locally advanced rectal cancer**

*F. Marruzzo*, G. M. Masci, D. Grasso, F. Ciccarelli, L. Cosma, M. Pasculli, F. Iafrate, C. Catalano; Rome/IT

**Purpose:** To investigate the role of diffusion-weighted (DW) magnetic resonance imaging (MRI) in the assessment of response to total neoadjuvant therapy (TNT) in patients with locally advanced rectal cancer (LARC).
**Methods or Background:** In this retrospective study, patients with LARC who underwent TNT were enrolled. Pre- and post-TNT MRI examinations were evaluated and MRI-based staging, tumour volume and DWI values were analysed. Patients were classified as complete responders (pCR) and non-complete responders (non-pCR), according to post-surgical outcome. Pre-TNT DWI values were compared to pathological outcome, post-treatment downstaging and reduction of tumour volume. The diagnostic accuracy of DWI in differentiating between pCR and non-pCR groups was calculated with receiver operating characteristic (ROC) analysis.
**Results or Findings:** A total of 36 patients were analysed (pCR, n=20; non-pCR, n=16). Pre-treatment DWI values were significantly higher in in group pCR compared to non-pCR (median value of 265 [195.8, 334] and 103 [53.5, 187], respectively p=0.026), while no association was found between pre-TNT tumour volume and pathological response. DWI values correlated to loco-regional downstaging after therapy (r=0.473, p=0.048), and less significantly with the reduction of tumour volume (r=0.434, p=0.072). AUC of DWI in differentiating between pCR and non-pCR patients was 80.9%, with a sensitivity of 75% and specificity of 81.3%.
**Conclusion:** High DWI values on pre-treatment MRI were strongly associated with a better outcome in patients with LARC, both in terms of pathological response and in loco-regional downstaging after TNT, suggesting the use of DW-MRI as a potential predictive tool of response to therapy.
**Limitations:** The main limitation of this research is the relatively small size of the study population.
**Ethics committee approval:** This singlecentre analysis was approved by the local ethics committee and written informed consent was waived due to the retrospective nature of the study.
**Funding for this study:** Not applicable
**Author Disclosures:**
Francesco Marruzzo: Nothing to disclose
Damiano Grasso: Nothing to disclose
Fabio Ciccarelli: Nothing to disclose
Giorgio Maria Masci: Nothing to disclose
Laura Cosma: Nothing to disclose
Franco Iafrate: Nothing to disclose
Carlo Catalano: Nothing to disclose
Marcella Pasculli: Nothing to disclose

### RPS 2301-5

**Locoregional nodal regrowth in rectal cancer patients following a Watch-and-Wait approach: a clinical challenge?**

*B. M. Geubels*, P. A. Custers, Y. Guzmán, M. Maas, G. L. Beets, B. Grotenhuis; Amsterdam/NL
*(b.geubels@nki.nl)*

**Purpose:** To evaluate the diagnostic aspects and management of locoregional nodal regrowth in rectal cancer patients following Watch-and-Wait (W&W).
**Methods or Background:** 33 rectal cancer patients with suspicious lymph nodes (LN) on MRI during follow-up in W&W were retrospectively identified. The diagnostic trajectory and treatment outcome was analysed, with special attention to MRI details.
**Results or Findings:** The primary rectal tumour was managed with (chemo)radiation in 25 (76%) patients (of whom 17 had cN+), and with primary local excision in 8 (24%). At first restaging MRI, all 17 patients with cN+ had no remaining nodal disease. Median time to suspicious LN detection on MRI following W&W was 10 (8-23) months; 26% were new and 74% were pre-existing LN with growing or changing aspect. At time of LN detection, 26 (79%) patients had no endoscopic luminal abnormalities. After first detection, the MRI was repeated once in 13 (39%), and up to 4 times in 2 (6%) patients. In 9 (27%) patients endoscopic ultrasound guided biopsies were attempted of which 4 failed; PET-CT was performed in 5 (15%), and 12 (36%) patients underwent no additional imaging. 9 (27%) patients received (chemo)radiation and eventually 32 (97%) underwent TME-surgery. Median time between suspicious LN detection and TME-surgery was 3 (1-5) months. In 11 (34%) patients positive LN were found in the surgical TME-specimen and in 13 (39%) mesorectal tumour deposits; in 5 (15%) no tumour was found and in 3 (9%) the pathological outcome is unknown.
**Conclusion:** MRI is an essential follow-up tool to detect nodal regrowth in rectal cancer patients following W&W. Because of the difficulty to obtain histological proof and avoid an unnecessary resection, there is however still the clinical need to improve the diagnostic accuracy.
**Limitations:** The small cohort was identified as a limitation.
**Ethics committee approval:** NL58095.031.16/IRBd22-054
**Funding for this study:** No funding was received for this study.
**Author Disclosures:**
Monique Maas: Nothing to disclose
Yoelimar Guzmán: Nothing to disclose
Petra A Custers: Nothing to disclose
Brechtje Grotenhuis: Nothing to disclose
Geerard L. Beets: Nothing to disclose
Barbara Melissa Geubels: Nothing to disclose

### RPS 2301-6

**Polyp detection as a quality indicator in a CT colonography service using a reduced bowel preparation without dietary restrictons**

*S. Ruggiero*, S. Vicini, D. M. Bellini, F. Tiberia, M. Rengo, I. Carbone, A. Laghi; Latina/IT
*(s.ruggiero94@gmail.com)*

**Purpose:** To evaluate the performances of a CT colonography (CTC) service using polyp detection rate (PDR) as quality measurement.
**Methods or Background:** We retrospectively analysed consecutive patients who underwent CTC from July 2015 to September 2018. In all patients a reduce bowel preparation (100 g of Macrogol and 60 ml of hyperosmolar iodinated contrast media for fluid tagging) and no dietary restriction was administered the day before the examination. PDR was calculated, considering only polyps ≥ 6 mm, for the entire population and after the stratification in sub-groups according to age (<65 vs ≥65 y), gender and symptoms (asymptomatic vs symptomatic). All positive patients at CTC underwent endoscopy to confirm the presence of polyps. Polyps were scored according to the location, dimension and quality of the bowel preparation. Differences between sub-groups were evaluated with chi-squared test.
**Results or Findings:** In total 1.446 patients (627/819 M/F, mean age 62.45 ± 14.22 years) were analysed. Bowel preparation was optimal in 1392 patients (96.3%). PDR of total population was 9.19%. PDR was significantly higher in older (p=0.025) and males (p=0.014). PDR was not significantly different between symptomatic and asymptomatic patients (p=0.052).
**Conclusion:** CTC without diet restriction for bowel preparation is effective in detecting colorectal polyps, with PDR comparable to those previously reported in literature.
**Limitations:** It was not possible to use OC as reference standard in all participants, which could have raised ethical concerns regarding the need to refer to OC even patients with negative CTC findings. The quality of colon cleansing was assessed exclusively by subjective analysis. This reflects the current lack of a standardised objective evaluation scale for CTC.
**Ethics committee approval:** The study was approved by our institutional ethical board.
**Funding for this study:** No funding was received for this study.
**Author Disclosures:**
Iacopo Carbone: Nothing to disclose
Simone Vicini: Nothing to disclose
Marco Rengo: Nothing to disclose
Sergio Ruggiero: Nothing to disclose
Filippo Tiberia: Nothing to disclose
Andrea Laghi: Nothing to disclose
Davide Maria Bellini: Nothing to disclose

### RPS 2301-7

**DWI ratios at MR-Enterography: a new way for inflammation degree assessment in Crohn's disease patients?**

*G. Cicero*[1], A. Alibrandi[1], A. Viola[1], V. Ascenti[2], A. Blandino[1], S. Mazziotti[1]; [1]Messina/IT, [2]Milan/IT
*(gcicero@unime.it)*

**Purpose:** Magnetic resonance enterography represents the imaging modality of choice for intestinal assessment of Crohn's disease (CD) patients. Among the different sequences, particular interest has been reported on diffusion weighted imaging (DWI) and the related apparent diffusion coefficient map. However, no standardised cut-off has been established. The aim of this work is to assess inflammation degree through measurements obtained within intestinal mural thickening on DWI images at high b-values and correlation to those of mesenteric lymph nodes, spleen and psoas muscle.
**Methods or Background:** A singlecentre retrospective study was conducted on 59 patients between September 2018 and July 2021. The inclusion criteria were: adult age, proven diagnosis of CD, MRE and ileocolonoscopy performed at an interval not superior to 4 weeks. Measurements have been made by two radiologists with calculation of three ratios: Bowel/Lymph node (BL), Bowel/Spleen (BS) and Bowel/Psoas (BP) ratios. Spearman, Mann–Whitney and Jonckheere-Terpstra (J-T) tests were performed for assessing correlation among the ratios, differences between surgical and non-surgical patients, and correlation between ratios and endoscopic classes.
**Results or Findings:** In non-surgical patients a significant positive correlation has been found among the endoscopic score and the three different ratios: BS (rS= 0.464; p=0.034); BP (rS= 0.506; p=0.008); BL (rS= 0.495; p=0.010). Positive correlation was also found also for BL/BP ratio (rS= 0.387; p=0.046) and BS/BP ratio (rS= 0.550; p=0.010). The J-T test demonstrated an increasing monotonic trend for BP and BL ratios and the four -CD classes. Within the surgical patients group, a positive correlation was found between the BL-ratio and BP-ratio (rS= 0.539; p=0.014).
**Conclusion:** Bowel DWI ratios can be considered as a fast approach for assessment of active inflammation degree in non-surgical CD patients.
**Limitations:** The small sample size was a limitation.
**Ethics committee approval:** This study was approved by an ethics committee.

Sunday

**Funding for this study:** No funding was received for this study.
**Author Disclosures:**
Giuseppe Cicero: Speaker: Philips Webinar entitled "TC Spettrale: quali vantaggi?" Monday, October 4th 2021
Angela Alibrandi: Nothing to disclose
Velio Ascenti: Nothing to disclose
Silvio Mazziotti: Nothing to disclose
Alfredo Blandino: Nothing to disclose
Anna Viola: Nothing to disclose

**09:30-10:30** **Room M 3**

### Research Presentation Session: Neuro

### RPS 2311

### Contrast agents meet neuroimaging

**Moderator**
S. Pedraza; Girona/ES

### RPS 2311-2

**Gadoquatrane - a new tetrameric, high relaxivity, macrocyclic gadolinium-based contrast agent for MRI**
*H. Pietsch*, G. Jost, T. Frenzel, J. Lohrke; Berlin/DE
*(hubertus.pietsch@bayer.com)*

**Purpose:** The novel tetrameric, macrocyclic gadolinium (Gd)-based contrast agent Gadoquatrane features high stability and high relaxivity, a key property for use in contrast-enhanced MR-imaging. The present work investigates key physico-chemical characteristics and MR-Imaging features of the new Gd-chelate.
**Methods or Background:** The complex stability was tested for Gd release in human plasma over 15 days. The T1-relaxivities in water and human plasma were measured at different field strengths (up to 4.7T). A 4D MR-angiography (MRA TWIST) study was performed with a clinical 1.5T scanner in healthy Goettingen minipigs. Two different doses of BAY 1747846 (0.025 and 0.03 mmol Gd/kg) were compared to the standard dose (0.1 mmol Gd/kg) of gadoterate meglumine and gadobutrol.
**Results or Findings:** The tetrameric BAY 1747846 showed high kinetic inertness and no Gd release over 15 days in human plasma under physiological conditions. Compared to clinically available GBCAs, highly pronounced T1-relaxivities were observed in water and human plasma at field strengths used in clinical practice (r1: 47.2 mM-1·s-1 per molecule corresponding to 11.8 mM-1·s-1 per Gd at 1.41T in human plasma). In the 4D-MRA study similar signal enhancement profiles were observed for BAY 1747846 at approximately 70% reduced Gd dose compared to the standard dose of marketed macrocyclic GBCAs (gadoterate meglumine and gadobutrol).
**Conclusion:** The novel tetrameric Gd-chelate combines high stability and high relaxivity. The high relaxivity of BAY 1747846 offers the opportunity of a substantially lower Gd dose, while reaching the same MR signal enhancement compared to the marketed GBCAs gadoterate meglumine and gadobutrol at a standard dose.
**Limitations:** The animal models cannot replicate pathological conditions or non-disease related heterogenities in human patients.
**Ethics committee approval:** The study was performed with the approval of the animal welfare committee in Germany.
**Funding for this study:** The authors are employees of Bayer AG.
**Author Disclosures:**
Gregor Jost: Employee: Bayer AG Employee: Bayer AG
Thomas Frenzel: Employee: Bayer AG
Jessica Lohrke: Employee: Bayer AG
Hubertus Pietsch: Employee: Bayer AG

### RPS 2311-3

**Radiomic and volumetric analysis on the accumulation of gadolinium in the basal nuclei: a pilot study**
*G. Piga*, P. Crivelli, D. Turilli, M. Scaglione, M. Conti; Sassari/IT

**Purpose:** The purpose of this study was to evaluate whether volumetric and radiomic changes may occur in the areas of most frequent gadolinium accumulation (basal nuclei), by comparing two groups of patients, one composed of subjects suffering from multiple sclerosis and subjected to multiple administrations of gadolinium and one composed of healthy volunteers, not suffering from neurodegenerative diseases and never exposed to magnetic resonance contrast medium.
**Methods or Background:** For this retrospective study, 51 patients, aged between 30 and 40 years, were recruited to perform a follow-up of multiple sclerosis. Twenty volunteers of the same age group were then recruited as a control population, who had never performed gadolinium-based contrast agents. MRI examinations were performed with a standardised protocol, with FLAIR axial sequence acquisitions.
**Results or Findings:** The radiomic data detected didn't show statistically significant changes; skewness, uniformity, entropy and kurtosis were, respectively: -0.34 in group 1 and -0.41 in group 2 (p = 0.152), 0.32 in group 1 and 0.31 in group 2 (p = 0.576), 1.86 in group 1 and 1.88 (p = 0.562), 2.63 in group 1 and 2.71 in group 2 (p = 0.142). About the volumetric data, volume of pallidus and putamen was slightly lower in group 1 (10.51 cc) than in group 2 (13.18 cc), but this difference was not statistically significant (p = 0.124).
**Conclusion:** The data obtained show that gadolinium does not cause detectable alterations by radiomics and this supports the hypothesis that its presence does not cause clinically evident damage.
**Limitations:** The limited sample and this study's retrospective nature were identified as limitations.
**Ethics committee approval:** This study was approved by an ethics committee.
**Funding for this study:** No funding was received for this study.
**Author Disclosures:**
Davide Turilli: Nothing to disclose
Paola Crivelli: Nothing to disclose
Mariano Scaglione: Nothing to disclose
Giorgio Piga: Nothing to disclose
Maurizio Conti: Nothing to disclose

### RPS 2311-4

**Detection rate of MR myelography without intrathecal gadolinium in patients with newly diagnosed spontaneous intracranial hypotension**
S. Lee, *D. Kim*, C. Suh; Seoul/KR
*(piintoretto@gmail.com)*

**Purpose:** To evaluate the detection rate of MR myelography without intrathecal gadolinium for CSF leakage in patients with newly diagnosed spontaneous intracranial hypotension (SIH) and to validate a published scoring system for predicting CSF leakage.
**Methods or Background:** This retrospective, observational, single-institution study included patients with newly diagnosed SIH between March 2015 and April 2021. Patients were included if they (a) had newly diagnosed SIH and (b) underwent initial brain MRI and preprocedural MR myelography with two- and three-dimensional turbo spin-echo sequences. Patients who underwent spine surgery or procedures including epidural injection and acupuncture were excluded. Detection rate was defined as the proportion of patients with a true-positive MR myelography result among all patients with confirmed CSF leakage. The interobserver agreement for the MR myelography results between two radiologists was analysed using weighted kappa statistics.
**Results or Findings:** A total of 136 patients (mean age, 48 years; 70 women) with suspected SIH were included. Of these patients, 120 (88%, 120/136) were confirmed to have CSF leakage. Of the patients with confirmed CSF leakage, 90 (75%, 90/120) had epidural fluid collection. The detection rate of MR myelography for CSF leakage was 88% (105/120). The interobserver agreement between the 2 readers for detecting CSF leakage ($\kappa = 0.76$) or epidural fluid collection ($\kappa = 0.77$) on MR myelography was high. Among 24 patients with normal brain MR imaging results, 16 had CSF leakage (67%, 16/24).
**Conclusion:** Noninvasive MR myelography without intrathecal gadolinium should be considered to detect CSF leakage in patients with suspected SIH.
**Limitations:** The limitation was the retrospective and singlecentre design.
**Ethics committee approval:** Institutional Review Board approval was obtained.
**Funding for this study:** This study has received funding by the National Research Foundation of Korea.
**Author Disclosures:**
SoJeong Lee: Nothing to disclose
Dana Kim: Nothing to disclose
ChongHyun Suh: Nothing to disclose

### RPS 2311-6

**Differences in gadolinium retention of macrocyclic MR contrast agents in a rat model of subtotal renal failure**
*R. Bonafe*, A. Coppo, S. Bussi, F. Maisano, M. Kirchin, F. Tedoldi; Colleretto Giacosa/IT
*(roberta.bonafe@bracco.com)*

**Purpose:** To compare gadolinium (Gd) levels in tissues of subtotally nephrectomised (SN) rats after repeated intravenous administration of macrocyclic gadolinium-based contrast agents (GBCAs).
**Methods or Background:** Sprague-Dawley SN male rats received 16 injections of gadoteridol, gadobutrol or gadoterate meglumine at 0.6 mmol Gd/kg/adm over 4 weeks. Gadoteridol at the same dosage was also administered to healthy (non-SN) male rats. Urea and creatinine plasma levels were monitored to assess renal function. Blood, cerebrum, cerebellum, liver, femur, kidney(s), skin and peripheral nerves were harvested for Gd determination by ICP-MS at 28 and 56 days after the end of dosing.

**Results or Findings:** Urea and creatinine plasma levels were about 2-fold higher in SN rats than in healthy rats at all timepoints. At 28 days post-dosing, Gd contents in peripheral nerves of gadobutrol and gadoterate-treated SN animals were 5.4 and 7.2 times higher ($p<0.001$) than in gadoteridol-treated SN animals. Similarly, Gd levels were 5 and 6 times higher ($p<0.001$ and $p<0.01$, respectively) in kidneys of gadobutrol and gadoterate-treated SN animals than in the kidney of gadoteridol-treated SN animals. Gd levels were similar in cerebrum, cerebellum, skin and liver of SN animals receiving gadobutrol or gadoterate, being about 2-fold higher than in gadoteridol-treated SN animals. Gd levels were 1.2 and 1.5 times higher in femur of gadobutrol and gadoterate-treated SN animals than in gadoteridol-treated SN animals. Lower Gd levels were determined 56 days post-dosing both in SN and healthy rats for all GBCAs and tissues, except femur. Gd levels were higher in all tissues of gadoteridol-treated SN animals compared to gadoteridol-treated healthy animals.
**Conclusion:** Lower Gd levels were determined in SN rats in all tissues and at all timepoints for gadoteridol compared to gadobutrol and gadoterate.
**Limitations:** Not applicable
**Ethics committee approval:** Not applicable
**Funding for this study:** Not applicable
**Author Disclosures:**
Fabio Tedoldi: Employee: Bracco Imaging Spa
Miles Kirchin: Employee: Bracco Imaging Spa
Roberta Bonafe: Employee: Bracco Imaging Spa
Alessandra Coppo: Employee: Bracco Imaging Spa
Simona Bussi: Employee: Bracco Imaging Spa
Federico Maisano: Employee: Bracco Imaging Spa

### RPS 2311-7

**Clinical efficacy of reduced-dose gadobutrol vs standard-dose gadoterate in contrast-enhanced CNS imaging: an international, multicentre, prospective, cross-over trial (LEADER-75)**

B. Liu[1], M. Rosenberg[2], S. Pollice[3], Y. Cheol Weon[4], S. Peters[5], F-D. Ardellier[6], A. Boeckenhoff[7], *J. S. Endrikat*[8]; [1]Chicago, IL/US, [2]Whippany, NJ/US, [3]Andria/IT, [4]Ulsan/KR, [5]Kiel/DE, [6]Strasbourg/FR, [7]Wuppertal/DE, [8]Berlin/DE
*(jan.endrikat@bayer.com)*

**Purpose:** To explore the efficacy of a 25% reduced dose of gadobutrol (0.075 mmol/kg) for contrast-enhanced CNS MRI.
**Methods or Background:** In LEADER-75 adult patients with known/suspected CNS pathology underwent contrast-enhanced MRI with standard-dose gadoterate (0.1 mmol/kg); if an enhancing lesion was identified, a second MRI with reduced-dose gadobutrol (0.075 mmol/kg) was performed within 15 days. Three study-independent radiologists scored three primary lesion efficacy variables: contrast enhancement, border delineation, and internal morphology. The mean reader scores were used for all analyses. Noninferiority was based on reduced-dose gadobutrol minus unenhanced achieving at least 80% of the difference of standard-dose gadoterate minus unenhanced for all three efficacy variables. To substantiate the findings, a post-hoc analysis directly comparing the combined (contrast-enhanced plus unenhanced) image sets was performed.
**Results or Findings:** 141 patients received both agents and were analysed for efficacy. Noninferiority was confirmed for all three primary efficacy variables ($p\leq0.025$). The mean scores for the combined images differed by less than 1% so a post-hoc analysis for equivalence of reduced-dose gadobutrol and standard-dose gadoterate was performed using two one-sided tests with a 5% margin and supported equivalence for all three variables ($p\leq0.025$).
Number of lesions detected, diagnostic accurary and image quality were similar.
**Conclusion:** A 25% reduced dose of gadobutrol and a standard dose of gadoterate provide comparable efficacy in adults with known/suspected CNS pathology.
**Limitations:** Study treatment was not randomised, however, the studies were scored in a randomised blinded evaluation.
**Ethics committee approval:** This study was approved by local ethics committees.
**Funding for this study:** Funding was received from Bayer AG.
**Author Disclosures:**
Jan Siegfried Endrikat: Author: Bayer Employee: Bayer
Martin Rosenberg: Author: Bayer Employee: Bayer
Saverio Pollice: Author: Bayer
Benjamin Liu: Author: Bayer
Young Cheol Weon: Author: Bayer
François-Daniel Ardellier: Author: Bayer
Annette Boeckenhoff: Author: Bayer Employee: Bayer
Soenke Peters: Author: Bayer

**09:30-11:00** **Room E1**

### Research Presentation Session: Musculoskeletal

### RPS 2310

### Osteoporosis and metabolic

**Moderator**
A. Bazzocchi; Bologna/IT

### RPS 2310-3

**Diagnostic accuracy of quantitative dual-energy CT-based volumetric bone mineral density assessment for the prediction of osteoporosis-associated fractures**

L. D. Grünewald, C. Booz, *I. Yel*, V. Koch; Frankfurt am Main/DE

**Purpose:** To evaluate the predictive value of volumetric BMD assessment of the lumbar spine derived from phantomless dual-energy CT (DECT)-based volumetric material decomposition as an indicator for the 2-year occurrence risk of osteoporosis-associated fractures.
**Methods or Background:** L1 of 92 patients who had undergone third-generation dual-source DECT was retrospectively analysed with dedicated DECT postprocessing software using material decomposition. The incidence of osteoporotic fractures within 2 years after measurement was evaluated. Receiver-operating characteristic analysis was used to calculate cut-off values and logistic regression models were used to determine associations of BMD, sex and age with osteoporotic fractures.
**Results or Findings:** A DECT-derived BMD cut-off of 93.70 mg/cm³ yielded 85.45% sensitivity and 89.19% specificity for the prediction to sustain osteoporosis-associated fractures within 2 years after measurement. DECT-derived BMD was significantly associated with the occurrence of new fractures, indicating a protective effect of increased DECT-derived BMD values. Overall AUC was 0.9373 (CI 0.867-0.977, $p<0.001$) for the differentiation of patients that sustained osteoporosis-associated fractures within 2 years of BMD assessment.
**Conclusion:** Retrospective DECT-based volumetric BMD assessment can accurately predict the 2-year risk to sustain an osteoporosis-associated fracture without requiring a calibration phantom. Lower DECT-based BMD values are strongly associated with an increased risk to sustain fragility fractures.
**Limitations:** Most patients of our study cohort underwent CT of the spine following previous trauma or because of chronic pain. Therefore, due to hospital policy, many of these patients received prior x-ray imaging that could not exclude an acute vertebral fracture and caused a preselection bias towards patients that are at risk to sustain vertebral fractures.
**Ethics committee approval:** This retrospective study was approved by the institutional review board. The requirement to obtain written informed consent was waived.
**Funding for this study:** No funding was received for this study.
**Author Disclosures:**
Christian Booz: Nothing to disclose
Ibrahim Yel: Nothing to disclose
Vitali Koch: Nothing to disclose
Leon David Grünewald: Nothing to disclose

### RPS 2310-4

**Osteoporosis detection via T-score measurement and hydroxyapatite quantification in CT Imaging: a multicentric, phantom-based study**

*V. Palm*, C. P. Heussel, O. v. Stackelberg, E. W. Y. Tong, K. Eckl, W. Stiller, H-U. Kauczor, M. O. Wielpütz, S. Skornitzke; Heidelberg/DE

**Purpose:** Although a frequent comorbidity, Osteoporosis is remarkably underdiagnosed in routine computed tomography (CT) because of poor visual bone density quantification. Pre-symptomatic diagnoses could prevent irreversible complications, and reduce mortality and healthcare costs.
We aim to develop an osteoporosis and osteopaenia detection pipeline for routine CT by calculating the hydroxyapatite (HA)/cm3 and T-score derived from Hounsfield Units (HU) in vertebral bodies.
**Methods or Background:** In this prospective, multicentric trial, we performed repeated measurements on seven different CT systems of bodies with known density of HA and a commercial quantitative CT phantom to generate HU reference values for T-score calculation. We then analysed the precision of measurements and their possible correlations to the scanner type, tube voltage and reconstruction kernel.

**Results or Findings:** Our results show a scanner-independent HA measurement accuracy of 87.4±0.7%. The average difference of two different Siemens SOMATOM Definition AS scanner was 2.9% at a ground truth of 100mg HA. Kernel dependence was excluded by a mean measurement error increase of 0.08%, whereas Pearson CHI² test demonstrated a statistically significant (p-value 0.0065) association between tube voltage and measurement error with regard to the reference body. The average measurement error at a ground truth of 100mg HA increased from 1.63% at 80kV and 5.65% at 100kV to 9.1% at 120kV and 12.86% at 140kV.
**Conclusion:** Our results demonstrate a scanner-independent, high HA quantification accuracy. However, quantification is dependent on tube voltage. Subsequently, this needs to be accounted for when applying the pipeline to routine CT scans for HA acquisition.
**Limitations:** The reproducibility of our results, and how they fare against dual-energy x-ray absorptiometry (DXA) measurements, must be further evaluated.
**Ethics committee approval:** This study was approved under reference number (S-937/2020).
**Funding for this study:** This study was funded by the State Ministry of Baden-Wuerttemberg for Sciences, Research and Arts, Germany (32-5400/58/3).
**Author Disclosures:**
Mark O. Wielpütz: Nothing to disclose
Wolfram Stiller: Nothing to disclose
Claus Peter Heussel: Nothing to disclose
Hans-Ulrich Kauczor: Nothing to disclose
Oyunbileg von Stackelberg: Nothing to disclose
Elizabeth Wai Yee Tong: Nothing to disclose
Viktoria Palm: Nothing to disclose
Kira Eckl: Nothing to disclose
Stephan Skornitzke: Nothing to disclose

### RPS 2310-5

**Bone mineral density differences between femurs of scoliotic patients undergoing quantitative computed tomography analysis**
*F. Serpi*, D. Albano, R. Colombo, G. Buccimazza, C. Messina, L. M. Sconfienza; Milan/IT

**Purpose:** Unlike the arms, leg dominance does not appear to exert a significant effect on BMD between the right and left femur. Evidence of a difference between two femurs in a scoliotic patient are supposed due to the presence of a loading imbalance on the lower limbs. Our aim was to investigate if a significant difference in areal BMD (aBMD) and volumetric BMD (vBMD) exists between femurs in patients with scoliosis, and whether this difference is related to spine convexity. We also compared such differences in patients without scoliosis.
**Methods or Background:** Abdominal CT examinations were retrospectively reviewed. We used the ''asynchronous'' calibration of the CT images to obtain BMD values from QCT (Mindways Software Inc., Austin, TX). Differences between aBMD and vBMD of femurs were assessed using the Student's t-test and Wilcoxon's signed-rank, according to data distribution. Scoliosis was evaluated on the antero-posterior CT localiser to calculate the Cobb angle.
**Results or Findings:** Our final study cohort consisted of 263 subjects, 225 of them without scoliosis (85.6%) and 38 with scoliosis (14.4%). BMD comparison between left and right femur of scoliotic patients - without considering the side of scoliosis - showed no statistically significant differences at all sites. When considering the convexity or concavity of scoliotic curve, we found a statistically significant difference at total femur, for both aBMD (-0,032 g/cm2, p=0.008) and vBMD (-8,9 mg/cm3, p=0.011). Total areal and volumetric BMD values were lower on the convexity side.
**Conclusion:** The present study demonstrated that a difference in femoral BMD exists between the two femurs of scoliotic patients undergoing QCT analysis of the hip. If this data is confirmed by a larger study, bilateral femoral DXA acquisition may be proposed for these patients.
**Limitations:** The study's retrospective nature was identified as a limitation.
**Ethics committee approval:** Ethics committee approval was obtained.
**Funding for this study:** No funding was received for this study.
**Author Disclosures:**
Giorgio Buccimazza: Nothing to disclose
Carmelo Messina: Nothing to disclose
Luca Maria Sconfienza: Speaker: Samsung Medison, Esaote, Abiogen, Pfizer, Novartis, Fidia, Biolive, Janssen Cilag, Bracco, MSD
Roberta Colombo: Nothing to disclose
Francesca Serpi: Nothing to disclose
Domenico Albano: Nothing to disclose

### RPS 2310-8

**Dual-energy CT-based monitoring of treatment-induced bone marrow changes in lung cancer patients: preliminary results**
*S. Werner*[1], B. Krauss[2], M. Horger[1]; [1]Tübingen/DE, [2]Forchheim/DE

**Purpose:** To investigate lung cancer treatment-induced changes in bone marrow attenuation assessed via dual-energy CT-based virtual non-calcium (VNCa) imaging of the axial skeleton.
**Methods or Background:** We performed retrospective region-of-interest based attenuation measurements in VNCa bone marrow images of the axial skeleton derived from 93 unenhanced reduced dose dual energy CTs of the thorax and abdomen of 31 patients. Each patient had received one pretherapy baseline exam and two consecutive follow-up exams (FU1 and FU2). Concurrent haematologic laboratory data were available for every exam. Twenty-two patients receiving highly myelotoxic treatment (Group A) were compared to 9 patients receiving less toxic substances (Group B).
**Results or Findings:** Median bone marrow attenuation at baseline/FU1/FU2 was -31.8 HU (IQR 12.7)/-46.5 HU (IQR 12.5)/-46.9 HU (IQR 22.0) in Group A and -40.6 HU (IQR 12.2)/-43.8 HU (15.7)/-38.5 HU (IQR 18.5) in Group B. In both subgroups the reduction of the mean attenuation between baseline and FU1 was statistically significant; differences between FU1 and FU2 and between groups were not. Attenuation measurement results are supported by haematological laboratory data. Leukopenia rates at FU1/FU 2 were 50%/54.5% in Group A and 0%/22% in Group B. Respective anaemia rates were 90%/86.4% in Group A and 66.7%/55.6% Group B.
**Conclusion:** Both highly myelotoxic as well as - to a smaller degree - less myelotoxic systemic therapy led to a significant drop in bone marrow attenuation with no significant tendency towards subsequent elevation. The results suggest that in this clinical setting an increase in bone marrow attenuation should be regarded as suspicious for tumour infiltration.
**Limitations:** Limitations include a relatively small cohort, some heterogeneity regarding time of follow-up and treatment regimens and the lack of inter- and intrareader reliability analysis.
**Ethics committee approval:** Institutional ethics committee approval was received. Individual consent was waived.
**Funding for this study:** No funding was received for this study.
**Author Disclosures:**
Bernhard Krauss: Employee: Siemens Healthcare GmbH
Marius Horger: Nothing to disclose
Sebastian Werner: Nothing to disclose

### RPS 2310-11

**The effect of gadolinium (Gd) and gadolinium-based contrast agents (GBCAs) on osteoblasts**
*F. S. Strunz*[1], C. Stähli[2], J. T. Heverhagen[1], W. Hofstetter[1], R. Egli[1]; [1]Bern/CH, [2]Bettlach/CH
*(franziska.strunz@dbmr.unibe.ch)*

**Purpose:** Use of GBCAs in MRI is associated with Gd retention in bone tissue. Hence, to understand the long-term role of Gd on bone metabolism, effects of free and chelated Gd on osteoblasts (OBs) were studied in vitro.
**Methods or Background:** Murine OBs were cultured in αMEM supplemented with Gd (12.5-100µM), nonionic-linear (Gd-DTPA-BMA), ionic-linear (Gd-DTPA) or macrocyclic (Gd-DOTA) GBCAs (100-2000µM). Moreover, OBs were cultured with empty chelators DTPA or DOTA alone or combined with free Gd. Cell viability (XTT assay) and differentiation (alkaline-phosphatase, ALP) was analysed on day 5.
**Results or Findings:** Gd and GBCA showed significant effects on proliferation at highest concentrations tested. Free Gd inhibited OB differentiation more efficiently than chelated Gd and ALP activity was significantly reduced at 12.5µM. In comparison, GBCAs lead to significant decline of ALP activity at concentrations higher than 100µM. The nonionic-linear complex induced the strongest dose-dependent inhibition of OB differentiation followed by the ionic-linear and macrocyclic. Cultures supplemented with DTPA revealed a decrease of viability and ALP activity, this effect was neutralised by addition of Gd. In contrast, DOTA did not affect OB proliferation or differentiation. Both empty chelators reversed the inhibitory effect of Gd on OBs.
**Conclusion:** Gd and GBCA did not affect OB viability, but inhibited cell differentiation dose-dependetly. The negative effect of GBCAs on OB differentiation (Gd-DTPA-BMA>Gd-DTPA>Gd-DOTA) corresponds to their thermodynamic stability (Gd-DTPA-BMA<Gd-DTPA<Gd-DOTA), indicating that the effect is attributable to Gd release from the complex. This Gd release also explains the higher concentrations necessary to decline ALP activity in GBCA-supplemented cultures.
**Limitations:** Further studies are ongoing to understand the effect of GBCA on bone metabolism, OBs represent only one of several players.
**Ethics committee approval:** The experiments are covered by Tierversuchsbewilligung BE19/19 to WH.
**Funding for this study:** This study was supported by Bangerter-Rhyner Foundation and CTU-Grant of Bern University.
**Author Disclosures:**
Johannes T. Heverhagen: Nothing to disclose
Rainer Egli: Nothing to disclose
Franziska Silvia Strunz: Nothing to disclose
Christoph Stähli: Nothing to disclose
Willy Hofstetter: Nothing to disclose

### RPS 2310-12
**Sarcopaenia may differentiate patients with wild-type from mutant-type gastrointestinal stromal tumour**

*X. Yi*[1], Y. Fu[1], B. T. Chen[2]; [1]Changsha/CN, [2]Duarte, CA/US
*(yixiaoping@csu.edu.cn)*

**Purpose:** To evaluate whether sarcopaenia assessed on abdominal CT imaging could differentiate KIT/PDGFRA wild-type gastrointestinal stromal tumour (wt-GIST) from the mutant-type GIST (mu-GIST), and to evaluate genetic features of GIST.
**Methods or Background:** A total of 174 patients with GIST (wt-GIST=52) were retrospectively identified between January 2011 to October 2019. A sarcopaenic nomogram was constructed by multivariate logistic regression. The performance of the nomogram was evaluated by discrimination, calibration curve, and decision curve. Genomic data was obtained from our own specimens and also from the open databases cBioPortal. Data was analysed by R version 3.6.1 and clusterProfiler (http://cbioportal.org/msk-impact).
**Results or Findings:** There was a significantly higher incidence (75.0% vs 48.4%) and more severe sarcopaenia in patients with wt-GIST than in patients with mu-GIST. Multivariate logistic regression analysis showed that sarcopaenic score (fitted based on age, gender and skeletal muscle index), and muscle fat index were independent predictors for higher risk of wt-GIST ($p<0.05$ for both the training and validation cohorts). Our sarcopaenic nomogram achieved a promising efficiency with an AUC of 0.879 (95% CI: 0.816-0.943) for the training cohort, and 0.9099 (95% CI: 0.8324–0.9873) for the validation cohort with a satisfying consistency in the calibration curve. Favourable clinical usefulness was observed using decision curve analysis. The additional gene sequencing analysis based on both our data and the external data demonstrated aberrant signal pathways being closely associated with sarcopaenia in the wt-GIST.
**Conclusion:** Our study supported the use of CT-based assessment of sarcopaenia in differentiating the wt-GIST from the mu-GIST preoperatively.
**Limitations:** Our study was inherently limited by its retrospective nature and singlecentre design.
**Ethics committee approval:** This study was approved by the Institutional Review Board of our hospital (IRB#: 202009685), and the written informed consents were waived.
**Funding for this study:** Not applicable
**Author Disclosures:**
Yan Fu: Nothing to disclose
Bihong T Chen: Nothing to disclose
Xiaoping Yi: Nothing to disclose

**09:30-11:00** **Room M 1**

### Research Presentation Session: Imaging Informatics / Artificial Intelligence and Machine Learning

### RPS 2305b
### Artificial intelligence in abdominal imaging

**Moderator**
E. G. Klompenhouwer; Amsterdam/NL

### RPS 2305b-2
**Artificial intelligence support system to improve the diagnostic accuracy of radiologist in differentiating colon carcinoma and diverticulitis in computed tomography**

*S. Ziegelmayer*, H. Havrda, J. Gawlitza, M. Graf, N. Lenhart, T. Lemke, S. Reischl, R. Braren; Munich/DE

**Purpose:** Differentiating between colon carcinoma (CC) and acute diverticulitis (AD) on computed tomography (CT) images is often a difficult task, particularly when complicated changes like perforation are present. As a result, guidelines recommend colonoscopy after 4-6 weeks to exclude carcinoma. Deep learning algorithms may improve the diagnostic accuracy of radiologists distinguishing both entities. The aim of our study was to develop and evaluate a deep learning algorithm as a second reader in the differentiation of AD and CC on CT images.
**Methods or Background:** In this retrospective study portal venous CT scans of 589 patients with AD (n=269) and CC (n=320) were acquired. Patients were splitted into a training, validation and testing cohort. A bounding box was cropped for each scan including the wall thickening, mesenterial surrounding and locoregional lymph nodes. A 3D-CNN was developed and trained on the bounding boxes and validated on the testing cohort. Finally a reader study was performed with differently experienced radiologists (n=6), who classified the test dataset with and without algorithmic support.
**Results or Findings:** The CNN reached a sensitivity and specificity of 80% and 86% respectively on the test dataset in differentiating CC and AD. The mean reader sensitivity and specificity was comparable to the AI system with 78% and 86%. All readers improved with the AI support reaching a mean sensitivity and specificity of 84% and 91%.
**Conclusion:** AI support has the potential to improve the diagnostic accuracy of radiologists in differentiating AD and CC. Consequently reducing unnecessary intervall colonoscopy.
**Limitations:** This is a retrospective, monoinstitutional study. No external test dataset was available.
**Ethics committee approval:** Ethics committee approval was received.
**Funding for this study:** No funding was received for this study.
**Author Disclosures:**
Hannah Havrda: Nothing to disclose
Tristan Lemke: Nothing to disclose
Stefan Reischl: Nothing to disclose
Nicolas Lenhart: Nothing to disclose
Markus Graf: Nothing to disclose
Rickmer Braren: Nothing to disclose
Sebastian Ziegelmayer: Nothing to disclose
Joshua Gawlitza: Nothing to disclose

### RPS 2305b-3
**Predicting the progression of branch-duct intraductal papillary mucinous neoplasms using a DenseNet**

*M. Debić*, P. Mayer; Heidelberg/DE
*(manuel.debic@med.uni-heidelberg.de)*

**Purpose:** Branch-duct intraductal papillary mucinous neoplasms (BD-IPMN) represent the most common pancreatic cystic lesions and are possible precursor lesions of pancreatic cancer. Most BD-IPMN represent low-risk lesions (without worrisome features [WF] or high-risk stigmata [HRS] according to the Fukuoka criteria). Since it is difficult to predict the course of low-risk BD-IPMN, regular follow-up imaging is recommended, which can create societal costs and significant patient burden. Our objective is to identify imaging features with deep learning that may help to predict imaging progression of low-risk BD-IPMN on MRI.
**Methods or Background:** Using the patient database of our tertiary referral center, we retrospectively identified patients with low-risk BD-IPMN depicted by pancreatic MRI. A board-certified pancreatic radiologist analysed all follow-up MRI scans for progression of the lesions, defined as development of new WF/HRS or cyst growth of ≥ 20 % / ≥ 2 mm on MRI. Lesions with progression within 50 months and lesions without progression on follow-up MRI for 50 months were included. Of 70 identified lesions, 43 lesions showed no imaging progression and 27 lesions showed imaging progression. All lesions were manually segmented on the T2-HASTE-images of the baseline MRI scans. We trained a DenseNet (DN) on the segmented baseline images with the presence of imaging progression at 50 months as outcome.
**Results or Findings:** Our results indicate that the DN may predict a possible malignant progression at an early stage (Accuracy, Recall, F1 score between 60 % and 70 %).
**Conclusion:** Predicting imaging progression of low-risk BD-IPMN with deep-learning is feasible. We assume that the classification metrics might be relatively low due to our small cohort size and could be improved by larger studies.
**Limitations:** Not applicable
**Ethics committee approval:** Ethics committee approval application approved.
**Funding for this study:** Not applicable
**Author Disclosures:**
Manuel Debić: Nothing to disclose
Philipp Mayer: Nothing to disclose

### RPS 2305b-4
**External validation of A CT-based radiomics model for prediction of local tumour progression after thermal ablation In colorectal liver metastases**

*D. van der Reijd*[1], F. C. Staal[1], C. Guerendel[1], M. Busard[1], S. H. Benson[1], A. Moelker[2], C. Verhoef[2], R. G. H. Beets-Tan[1], M. Maas[1]; [1]Amsterdam/NL, [2]Rotterdam/NL
*(d.vd.reijd@nki.nl)*

**Purpose:** After thermal ablation (TA) of colorectal liver metastases (CRLM) local tumour progression (LTP) is reported in 6-46%. Early identification of patients at risk for LTP may avoid delay in additional treatment. We aimed to validate a previously published clinical-radiomics model (c-statistic: 0.78 95%CI: 0.58-0.84) to identify patients at risk for LTP in an independent external cohort.
**Methods or Background:** 53 patients (n=79 CRLM) treated with TA were retrospectively included. Clinical features were collected. Portal-venous CT images 2-8 weeks after TA were used for segmentation. Radiomics features (with different Laplacian of Gaussian (LoG) filters) as used in the previous model were extracted from the ablation zone (AZ) and a 10 mm periablational rim (PAR). The three multivariable stepwise Cox regression models (clinical-only, radiomics-only, clinical-radiomics-combined) were applied on the data and performances (concordance [c]-statistics) were compared to the previous results.
**Results or Findings:** After a median follow-up of 26 months (range 6-149), LTP occurred in 25 ablation zones (32%). Both the clinical-only and radiomics-only models demonstrated poor predictive performance with c-statistics 0.53 (95%CI: 0.41-0.65) and 0.50 (95%CI: 0.39-0.62), respectively. The combined model included T-stage, metastasis size, adjuvant chemotherapy treatment, AZ_Uniformity_LoG-1.5, AZ_Skewness, PAR_Mean_LoG-0.5, PAR_Skewness_LoG-0.5 and PAR_Uniformity_LoG-1.5, and yielded a poor predictive performance, with a c-statistic of 0.50 (95%CI: 0.39-0.62).
**Conclusion:** Previously published clinical and radiomics models could not predict LTP in CRLM after TA in an independent external cohort. This inability to reproduce the earlier findings could be explained by overtraining and scanner differences. These results underline the importance of external validation. Singlecentre radiomics models could be hospital specific and currently prohibit clinical use.
**Limitations:** The small validation cohort was identified as a limitation.
**Ethics committee approval:** This study was approved by an ethics committee.
**Funding for this study:** No funding was received for this study.
**Author Disclosures:**
Denise van der Reijd: Nothing to disclose
Femke C.R. Staal: Nothing to disclose
Monique Maas: Nothing to disclose
Corentin Guerendel: Nothing to disclose
Sean Harry Benson: Nothing to disclose
Adriaan Moelker: Nothing to disclose
Milou Busard: Nothing to disclose
Regina G. H. Beets-Tan: Nothing to disclose
C Verhoef: Nothing to disclose

### RPS 2305b-5

### Deep learning for automatic detection of the main pancreatic duct dilatation

*C. Abi Nader*[1], R. Vétil[1], L. Wood[1], F. Nicolas[1], M-M. Rohé[1], M-P. Vullierme[2]; [1]Villepinte/FR, [2]Annecy/FR

**Purpose:** Pancreatic ductal adenocarcinoma (PDAC) is an aggressive cancer with a low 5-year survival rate due to a late diagnosis. The dilatation of the main pancreatic duct caused by a stenosis due to PDAC is generally detected on portal venous phases CT scans. However, the detection of the duct dilatation, difficult to see on portal CT, can have low sensitivity. Given this context, we show how deep learning can help to automatically detect pancreatic duct dilatation.
**Methods or Background:** We used a state-of-the-art deep learning algorithm to predict the duct dilatation based on portal CT scans annotated by expert radiologists. The model was calibrated on our private cohort composed of 627 patients. These patients had a histologically confirmed PDAC diagnosis, and 41% of them were showing a dilated duct. In addition, we tested the method on a public dataset composed of 281 subjects diagnosed with either a tumour or a cyst, among which 49% had a dilated duct.
**Results or Findings:** We trained the model on 527 patients from our private cohort and validated it on 100 unseen patients, leading to a 0.95 AUC (95% CI 0.944-0.956), with 93% sensitivity and 82% specificity at the cut-off point maximizing the Youden's index. We also applied it on the 281 independent patients from the public dataset and obtained an AUC of 0.913 (95% CI 0.911-0.917), with 93% sensitivity and 75% specificity at cut-off.
**Conclusion:** Our results showed high performances on two independent cohorts, suggesting the interest of deep learning to help radiologists detecting the main pancreatic duct dilatation. Ultimately, this could lead to an earlier diagnosis of PDAC and therefore improve patients' prognosis.
**Limitations:** The approach should be further validated on multicentric data in a reader study.
**Ethics committee approval:** Not applicable
**Funding for this study:** Not applicable
**Author Disclosures:**
Marc-Michel Rohé: Employee: Guerbet
Laura Wood: Employee: Guerbet
Francois Nicolas: Employee: Guerbet
Clément Abi Nader: Employee: Guerbet
Rebeca Vétil: Employee: Guerbet
Marie-Pierre Vullierme: Nothing to disclose

### RPS 2305b-6

### Are we there yet? The value of deep learning in a multicentre-setting for response prediction of locally advanced rectal cancer to neoadjuvant chemoradiotherapy

*B. D. Wichtmann*[1], S. Albert[2], W. Zhao[2], A. Maurer[1], J. Hesser[2], F. G. Zöllner[2], U. I. Attenberger[1]; [1]Bonn/DE, [2]Mannheim/DE
*(barbara.wichtmann@ukbonn.de)*

**Purpose:** To evaluate a state-of-the-art multi-task deep learning (DL) approach in a multicentre-setting for its performance in predicting pathological complete response (pCR) of locally advanced rectal cancer (LARC) to neoadjuvant chemoradiotherapy (nCRT).
**Methods or Background:** This study included 83 LARC patients collected retrospectively at four different medical centers between 2015 and 2018. All patients underwent nCRT followed by surgery and histopathologic evaluation of tumour regression. A trained radiologist manually segmented the tumour region on pre- and post-treatment, axial, high-resolution T2-weighted (T2w) magnetic resonance images (MR-images). A Siamese network with two U-Nets joined at multiple layers was developed to predict pCR based on pre- and post-nCRT T2w-/diffusion weighted MR-images. After preprocessing, 72 patients were selected as training, 11 as validation data. To assess predictive performance, the trained network was applied to an external clinical routine dataset of 46 LARC patients acquired without study conditions.
**Results or Findings:** The training and test datasets differed significantly in terms of their composition, e.g. T-/N-staging, the time interval between initial staging, nCRT, re-staging, and surgery, as well as with respect to imaging parameters, such as resolution, echo/repetition time, flip angle and field strength. The area under the receiver operating characteristic curve was 0.60 in the external test cohort for predicting pCR.
**Conclusion:** Even though initial results are promising, the performance of the used DL model in a multicentre-setting is substantially reduced compared to a previously published single- or two-centre-setting. Before translating DL approaches into clinical routine for screening, treatment response assessment and surveillance their predictive performance remains to be verified in larger multicentre-studies.
**Limitations:** Larger training datasets along with dedicated preprocessing of low-quality datasets, including normalisation of sequence variations may improve performance.
**Ethics committee approval:** This study was approved by an ethics committee.
**Funding for this study:** This work was supported by Deutsche Forschungsgemeinschaft (grant number: DFG 410981386).
**Author Disclosures:**
Wenzhao Zhao: Nothing to disclose
Barbara Daria Wichtmann: Nothing to disclose
Jürgen Hesser: Nothing to disclose
Ulrike I. Attenberger: Nothing to disclose
Frank G Zöllner: Nothing to disclose
Angelika Maurer: Nothing to disclose
Steffen Albert: Nothing to disclose

### RPS 2305b-8

### EOB-MR based radiomics analysis to assess clinical outcomes following liver resection in colorectal liver metastases

*F. Dell'Aversana*[1], F. Grassi[1], F. De Muzio[2], C. Cutolo[3], R. Grassi[1], A. Petrillo[1]; [1]Naples/IT, [2]Campobasso/IT, [3]Fisciano/IT
*(fe.dellaversana@gmail.com)*

**Purpose:** To evaluate the efficacy of the radiomic features obtained from the EOB-MRI phase to evaluate the clinical outcomes after hepatic metastasectomy, recurrence, mutational status, histological characteristics and the surgical resection margin of the tumour.
**Methods or Background:** For each segmented volume of interest 851 radiomics features were extracted as median values using PyRadiomics. Non-parametric test, intraclass correlation, receiver operating characteristic (ROC) analysis, linear regression modelling and pattern recognition methods (support vector machine (SVM), k-nearest neighbors (KNN), artificial neural network (NNET), and decision tree (DT)) were considered.
**Results or Findings:** The best predictor to discriminate expansive versus infiltrative front of tumour growth was HLH_glcm_MaximumProbability extraxted on VIBE_FA30 (accuracy of 84%). The best predictor to discriminate tumour budding was inverse variance obtained by the original GLCM matrix extraxted on VIBE_FA30 (accuracy of 89%). The best predictor to differentiate the mucinous type of tumour was the HHL_glszm_ZoneVariance extraxted on VIBE_FA30 (accuracy of 85%). The best predictor to identify tumour recurrence was the LHL_glcm_Correlation extraxted on VIBE_FA30 (accuracy of 86%). The best linear regression model was obtained in the identification of the tumour growth front considering the height textural significant metrics by VIBE_FA10 (accuracy of 89%). Considering significant texture metrics tested with pattern recognition approaches, the best performance for each outcome was reached by a KNN in the identification of recurrence with the 3 textural

Sunday significant features extracted by VIBE_FA10 (AUC of 91%, an accuracy of 93%).
**Conclusion:** Radiomics can identify some prognostic features as biomarkers that could influence the choice of treatment in patients with colorectal cancer liver metastases.
**Limitations:** The small population size considered, the retrospective nature of the study and the manual segmentation were identified as limiting factors.
**Ethics committee approval:** The Ethical Committee board of the National Cancer Institute of Naples, IRCCS "Fondazione Pascale" approved this study.
**Funding for this study:** No external funding was received.
**Author Disclosures:**
Federica Dell'Aversana: Nothing to disclose
Antonella Petrillo: Nothing to disclose
Federica De Muzio: Nothing to disclose
Carmen Cutolo: Nothing to disclose
Roberto Grassi: Nothing to disclose
Francesca Grassi: Nothing to disclose

**09:30-11:00** **Room Z**

### Research Presentation Session: Physics in Medical Imaging

### RPS 2313

### Computed tomography (CT)

**Moderator**
S. Sawall; Heidelberg/DE

### RPS 2313-2

**Estimating the high scatter frequencies caused by coarse anti-scatter grids in x-Ray CT**

*J. F. J. Erath*[1], J. Maier[2], E. Fournié[1], K. Stierstorfer[1], M. Kachelrieß[2]; [1]Forchheim/DE, [2]Heidelberg/DE

**Purpose:** To estimate and remove high frequencies in scatter signals transmitted through coarse anti-scatter grids (ASGs).
**Methods or Background:** CT detectors with very small pixels, such as in photon-counting CT, may have anti-scatter grids with a grid spacing being an integer multiple of the detector pixel size. For example, an ASG field may comprise a 2x2 matrix of detector pixels. The shading caused by the grid affects each of the pixels within a grid field differently. Although scatter is a low frequency effect, this pixel-dependent shading results in high frequencies in the scatter signal transmitted through the ASG. These may cause ring or Moiré artifacts in the reconstructed images. We trained a modification of our deep scatter estimation (DSE, [Med. Phys. 46(1): 238-249]) network to predict the modulated scatter signal which can then be subtracted from the measured data. To train MatrixDSE, scatter signals were obtained by Monte Carlo simulation of patient CT data. To mimic the ASG modulation the simulated scatter is multiplied 2x2-pixel-wise with a random 2x2 matrix whose coefficients are within 0.5 and 1.0 that change with the detector position and view number. The MatrixDSE-corrected data are then reconstructed and evaluated in image domain.
**Results or Findings:** DSE and MatrixDSE reduce scatter artifacts from a mean (across all patients) mean absolute error of about 10 HU to about 1 HU. MatrixDSE is further able to correct the ring artifacts, that originally had a mean amplitude of about 20 HU to an amplitude of less than 1 HU.
**Conclusion:** MatrixDSE can estimate ASG-modulated scatter frequencies in CT data and correct both for the scatter artifacts and for the ring artifacts caused by the modulation.
**Limitations:** Our results are based on patient-based simulations only.
**Ethics committee approval:** This study was approved by an ethics committee.
**Funding for this study:** Funding was received from Siemens Healthineers.
**Author Disclosures:**
Eric Fournié: Employee: Siemens Healthineers
Julien Frank Josef Erath: Employee: Siemens Healthineers
Karl Stierstorfer: Employee: Siemens Healthineers
Marc Kachelrieß: Nothing to disclose
Joscha Maier: Nothing to disclose

### RPS 2313-3

**Scanning outside the localiser borders in chest CT: a multicentre study**

A. S. L. Dedulle[1], *N. Fitousi*[1], Y. De Bruecker[2], A. Houben[3], C. Vanreppelen[4], M. Willocxand[5], H. Bosmans[1]; [1]Leuven/BE, [2]Bonheiden/BE, [3]Genk/BE, [4]Sint-Truiden/BE, [5]Aalst/BE
*(niki.fitousi@qaelum.com)*

**Purpose:** Advanced dose management systems go beyond dose recording and address several quality aspects. Such an advanced functionality is the assessment of blind scan, i.e. the extent of scan length beyond the localiser borders. Without localiser information, tube current modulation (TCM) is not optimally applied, with repercussion on doses such as to the thyroid during chest CT. In this Belgian multicentre study, the blind scan in chest CT examinations was investigated.
**Methods or Background:** Anonymised data of chest CT examinations performed in two years in seven hospitals from 14 CT scanners were collected. All participating centres use the same dose management system (DOSE, Qaelum). The blind scan (upper and lower, towards neck and abdomen respectively), scan length, and other information about the examinations were extracted by DOSE and analysed. A Kruskal-Wallis test (significance level 0.05), was used to compare the data of the different centres.
**Results or Findings:** Overall, 51629 CT series were included. The centres use significantly different scan lengths ($p<0.05$), with median lengths ranging from 35 cm to 42 cm. There was lower blind scan in less than 1.5% of the series in all centres; the frequency of upper blind scan is substantial for all centers, ranging from 5% to 21%, with significantly different median values ranging from 16 mm to 20 mm. The difference in frequency of series with blind scan above 30 mm is considerable, ranging from 1% to 6%.
**Conclusion:** There are significant differences in the frequency and amount of blind scan. Results indicated that there is room for optimisation. Education on the impact of the blind scan might improve applied scan techniques.
**Limitations:** Not applicable
**Ethics committee approval:** Not applicable
**Funding for this study:** Not applicable
**Author Disclosures:**
An Saskia Luc Dedulle: Research/Grant Support: VLAIO (HBC.2016.0233) Employee: Qaelum NV
Maarten Willocxand: Nothing to disclose
Albrecht Houben: Nothing to disclose
Hilde Bosmans: Board Member: Qaelum NV
Cederic Vanreppelen: Nothing to disclose
Niki Fitousi: Employee: Qaelum NV
Yves De Bruecker: Nothing to disclose

### RPS 2313-4

**Personalised iodine contrast media injection in liver MDCT**

*F. Zanca*[1], B. Dufour[2], P. Pujadas[3], D. Racine[4], B. Rizk[5], H. Brat[2]; [1]Leuven/BE, [2]Sion/CH, [3]Madrid/ES, [4]Lausanne/CH, [5]Villars-sur-Glane/CH
*(federica.zanca@palindromo.consulting)*

**Purpose:** To validate an algorithm for personalised iodine contrast media (CM) injection in liver MDCT.
**Methods or Background:** 366 prospective patients (mean Fat Free Mass (FFM) 46.7 kg (23.0-78.3), mean age 61.3 years (18.3-95.2 years)) underwent a standardised multiphase liver CT examination (kVp range 80-120) in a multicentre imaging network. The contrast dose (gI) to be injected for an optimal liver parenchymal enhancement (40-60 Hounsfield Units (HUtarget)) was estimated prior to the exam by an in-house developed algorithm. The algorithm calculated the gI based on impedencemetric-determined FFM, exam tube voltage and contrast media concentration (350_AccupaqueTM/370_IopamirolTM mgI/ml). Lower limits were set in the algorithm to avoid off-label usage of the contrast media (AccupaqueTM : 30 gI, IopamirolTM : 0.5 ml/kg). The liver enhancement of each patient was measured by difference between portal and unenhanced parenchyma attenuation (HUliver) and compared to HUtarget through median and interquartile range (IQR). Total amount of iodine dose was estimated by median and IQR and stratified per contrast media concentration, KVp and gender.
**Results or Findings:** Median HUliver was 53.5 HU (IQR= 48.5-60.5) with 68% of patients having an enhancement between 40-60 HU. Median iodine dose was 27.8 gI (IQR = 23.7-30.2). Per contrast media type, median iodine dose was 30.5gI/26.7gI for 350/370 iodine concentration ($p<0.001$) with an associated enhancement of 57.5/53.0 HU respectively. Per 80/100/120 kVp, median iodine dose significantly increased ($p<0.01$) from 18.6/27.8/36.3gI. Per gender, women needed a significantly lower ($p<0.01$) dose than men (24.8 vs 30.1 gI).
**Conclusion:** The algorithm enabled a diagnostically appropriate and reproducible median liver enhancement of 53.5 HU, independently of patient habitus, contrast concentration or tube voltage. Lower limit iodine dose due to off-label use caused a higher than needed contrast dose for one contrast type.

**Limitations:** FFM had to be measured for each patient.
**Ethics committee approval:** Not applicable
**Funding for this study:** No funding was received for this study.
**Author Disclosures:**
Damien Racine: Nothing to disclose
Benoît Dufour: Nothing to disclose
Benoît Rizk: Nothing to disclose
Hugues Brat: Nothing to disclose
Federica Zanca: Nothing to disclose
Pilar Pujadas: Employee: GE Healthcare

### RPS 2313-5

**Optimising CT perfusion acquisition parameters based on digital perfusion phantoms: obtaining maximum precision for a specific radiation exposure setting**

*S. Skornitzke*, N. Vats, P. Mayer, H-U. Kauczor, W. Stiller; Heidelberg/DE
*(Stephan.Skornitzke@med.uni-heidelberg.de)*

**Purpose:** Determining the optimum trade-off between temporal sampling rate (Δt) and tube current, i.e. image noise, with respect to precision of CT perfusion measurements.
**Methods or Background:** Digital perfusion phantoms were simulated by forward-convolution of an arterial input function averaged over 67 patients with a deconvolution model for 10 values of blood flow (30-120ml/100ml/min), 4 values of mean transit time (4-10s) and 4 values of flow-extraction product (10-40ml/100ml/min), with 10 steps of temporal sampling rate (Δt = 0.5-5.0s) and 57 levels of added image noise (standard deviation = 1-57HU), performing 128 repetitions for each setting. Phantoms were evaluated on a commercially available workstation with deconvolution (blood flow [BFD], blood volume [BVD], flow-extraction product [FED]), maximum-slope (blood flow [BFM]) and Patlak models (blood volume [BVP], flow-extraction product [FEP]). Results were exported, mean and standard deviation (SD) over repetitions were calculated for each setting, and influence of Δt and noise was tested by analysis of covariances (ANCOVA). Optimum Δt with corresponding noise, defined by minimum SD of perfusion parameters, was determined along an iso-dose line of constant radiation exposure through reference values Δt = 2.0s and noise = 20HU.
**Results or Findings:** Average mean and SD for perfusion parameters were as follows: BFD: 102.0±52.6ml/100ml/min, BVD: 7.8±2.7ml/100ml, FED: 31.2±17.0ml/100ml/min, BFM: 50.5±8.7ml/100ml/min, BVP: 6.9±2.5/100ml/min, FEP: 23.3±9.0ml/100ml/min. ANCOVA showed a significant influence of Δt and noise on mean and SD for all parameters (p<0.0001). Optimum Δt was 1.5s (noise = 23.1HU) for BFD (95.3±43.4ml/100ml/min) and 4.5s (noise = 13.3HU) for BVD (7.6±2.1ml/100ml), FED (31.2±14.5ml/100ml/min), BFM (48.7±5.6ml/100ml/min), BVP (6.8±1.8ml/100ml) and FEP (23.4±6.8ml/100ml/min).
**Conclusion:** Optimum CT perfusion acquisition parameters differ based on perfusion model but results generally favour longer Δt, allowing for reduced image noise by increased tube current when keeping radiation dose constant.
**Limitations:** Filters reducing image noise were not investigated.
**Ethics committee approval:** This study was approved by an ethics committee.
**Funding for this study:** Funding was received via the German BMBF grant (031L0163).
**Author Disclosures:**
Neha Vats: Nothing to disclose
Wolfram Stiller: Advisory Board: Philips Medical Systems
Hans-Ulrich Kauczor: Nothing to disclose
Philipp Mayer: Nothing to disclose
Stephan Skornitzke: Shareholder: Investment funds containing stock of healthcare companies.

### RPS 2313-6

**Advanced physics-based image quality assessment of a commercial super resolution deep learning reconstruction algorithm for cardiac radiology applied to a wide volume computed tomography system**

*K. Boedeker*[1], D. Shin[1], N. Akino[1], Z. Yu[2], H. Taguchi[1]; [1]Otawara-shi, Tochigi/JP, [2]Vernon Hills, IL/US
*(kboedeker@mru.medical.canon)*

**Purpose:** Super Resolution Deep Learning Reconstruction (SR-DLR) presents a new and unique image quality performance space. The purpose of this study is to characterise a commercial SR-DLR, relative to conventional hybrid iterative reconstruction, with both standard and advanced metrics for cardiac wide volume CT acquisitions.
**Methods or Background:** A 20cm Catphan was scanned on a wide volume CT scanner (Canon Aquilion ONE Prism) with a standard cardiac 0.275 sec protocol and reconstructed with a commercial SR-DLR and hybrid iterative reconstruction. The SR-DLR uses high-dose CT data acquired on a commercial high resolution CT system (Aquilion Precision) as the training target. The Precision has a 0.25 mm detector element size, in-plane and longitudinal, at isocenter. Simulated low-dose normal resolution data was used as the training input. A task-based low contrast detectability (LCD) model observer evaluated LCD and false positive/false negatives rates associated with noise and low contrast signal. The contrast-based modulation transfer function (MTF) and Noise Power Spectra (NPS) were determined. The NEQ(f) was used to characterise dose efficiency and task performance, such as ability to determine change in object size.
**Results or Findings:** Limiting resolution for clinical field-of-field increased by 5-8 lp/cm and the magnitude of the MTF, on average, doubled while noise was simultaneously decreased by 45-50% and LCD remained equivalent between algorithms. SR-DLR resulted in a lower false positive/negative fraction. NEQ increased across all frequencies. NEQ applied to a high contrast size discrimination task demonstrated significant improvement in distinguishing two objects 1 mm different in diameter.
**Conclusion:** SR-DLR improves spatial resolution while reducing noise and maintaining LCD relative to conventional reconstruction, including false positive/false negative rates. The spatial resolution improvements increase dose efficiency and increase task performance for non-LCD tasks.
**Limitations:** The fact that a single SR-DRL algorithm was studied was identified as a limitation.
**Ethics committee approval:** Not applicable.
**Funding for this study:** Funding was received from Canon Medical Systems.
**Author Disclosures:**
Kirsten Boedeker: Employee: Canon Medical Systems
Zhou Yu: Employee: Canon Medical Systems
Hiroki Taguchi: Employee: Canon Medical Systems
Daniel Shin: Employee: Canon Medical Systems
Naruomi Akino: Employee: Canon Medical Systems

### RPS 2313-7

**Variability of water-equivalent diameter (Dw) with tube potential of CT scans**

*A. Abuhaimed*[1], C. Martin[2]; [1]Riyadh/SA, [2]Glasgow/UK
*(ahaimed@gmail.com)*

**Purpose:** The aim of this work was to study variability of the patient size, which is reported by water-equivalent diameter (Dw) for paediatric and adult patients with tube potential of CT scans.
**Methods or Background:** A library of 363 phantoms (168 paediatric and 195 adult) were used. The library was designed to represent characteristics of the whole patient population. It covers a wide range of ages from newborn baby to adult with weights of 10 to 125 kg and heights of 85 to 190 cm. Values of Dw were assessed for each phantom using a MATLAB developed in house for this purpose. The values were calculated over six regions of the trunk that represent CT protocols applied in the clinic. The regions studied were chest, abdomen, pelvis, chest-abdomen, abdomen-pelvis, and the trunk. Five equivalent monoenergetic beams, which represented tube potentials of 80–140 kVp, were used.
**Results or Findings:** The differences between Dw values assessed for the various spectra were found to be insignificant. For all phantoms, the differences were within ±2% over the scan regions, with the majority of differences being less than 1%. The differences were not affected by the size or gender of the patient.
**Conclusion:** Dw was recommended by the AAPM to replace dimension-based metrics to estimate size-specific dose estimate (SSDE) for an individual patient. Although Dw values were affected by the area attenuation, results of this study showed that impact of tube potential on Dw of the trunk regions was minimal. Since assessment of Dw requires advanced computational tools, the results suggest that energy-independent Dw values could be established and linked to effective dimeter, which can be simply measured for an individual patient.
**Limitations:** Equivalent monoenergetic beams were used to simulate various spectra.
**Ethics committee approval:** Not applicable
**Funding for this study:** No funding was received for this study.
**Author Disclosures:**
Colin Martin: Nothing to disclose
Abdullah Abuhaimed: Nothing to disclose

### RPS 2313-8

**Impact of technology on local clinical dose reference levels in MDCT**

*B. Dufour*[1], F. Zanca[1], P. Sastre[2], B. Rizk[1], D. Racine[1], H. Brat[1]; [1]Sion/CH, [2]Lyon/FR
*(benoit.dufour@groupe3r.ch)*

**Purpose:** To determine the impact of a Deep Learning (DL) image reconstruction algorithm on local clinical dose reference levels (LcDRLs) in CT.
**Methods or Background:** From September 2017 to June 2020 and after protocol optimisation according to clinical indication and body mass index (BMI) class (< 25; ≥ 25), 824 chest and 3153 abdomen CT series were collected from 6 high-end CT scanners (Group 1, G1). As of October 2020 a DL reconstruction algorithm was installed and 1222 chest and 5037 abdomen CT series collected (data till September 2021, G2). LcDRLs were calculated as

Sunday the third quartile of the median CTDIvol and DLP values for each CT scanner for G1 and G2, and compared. The evolution of the cDRLs with and without DL reconstruction was evaluated in terms of percent change. The G2 LcDRLs were also compared to Swiss NDRLs (P50 (achievable)).
**Results or Findings:** The average change in LcDRLs for G2 BMI<25 for CTDIvol/DLP was -43%/-46% for chest, -29%/-34% for abdomen. For G2 BMI≥25 average LcDRLs CTDIcol/DLP reduction was -39%/-54% for chest and -23/-26% for abdomen. Highest LcDRLs CTDIvol dose reduction for BMI < 25/≥ 25 classes was observed for emphysema (-68%/-71%) and kidney stones (-60%/-55%) clinical indications. For chest, G2 LcDRLs compared to P50 NDRLS were lower in all indications and BMI classes for CTDIvol (range 1.6–5.1 vs. 6.0 mGy) and DLP (61.7–100.8 vs. 210 mGycm). For abdomen, G2 LcDRLs were lower than P50 NDRLS in all indications and BMI classes for CTDIvol (2.2-9.0 vs 10 mGy) and DLP (96.1–425.4 vs. 470mGycm).
**Conclusion:** DL reconstruction algorithms combined with protocol stratification allows further optimisation of CT doses which remain lower than national P50 DRLs for chest and abdomen CT examinations.
**Limitations:** Not applicable
**Ethics committee approval:** Not applicable
**Funding for this study:** Not applicable
**Author Disclosures:**
Damien Racine: Other: IRA Employees
Benoît Dufour: Nothing to disclose
Benoît Rizk: Nothing to disclose
Pauline Sastre: Other: GE employees
Hugues Brat: Other: lecturer for GE Healthcare
Federica Zanca: Nothing to disclose

### RPS 2313-9

**Synthesising 4D CBCT scans from 3D CBCT phantom acquisitions**
M. Susenburger[1], P. Paysan[2], R. Savjani[2], G. Echner[1], S. Scheib[2], M. Kachelrieß[1], *J. F. J. Erath*[1]; [1]Heidelberg/DE, [2]Baden-Dättwil/CH

**Purpose:** To synthesise 4D CBCT raw data from multiple 3D CBCT scans of a thorax phantom to provide a ground truth for the evaluation of motion-compensated (MoCo) image reconstruction methods.
**Methods or Background:** A LUNGMAN thorax phantom (Kyoto Kagaku, Kyoto) is mounted on a linear motion stage to be scanned on a TrueBeam (Varian Medical Systems, Palo Alto, USA) flat detector-based CBCT system. The phantom's chest is fixated on the couch while the abdominal and the bronchial tree inserts are moved in steps of 0.8 mm into the chest using the motion stage to cover a total motion amplitude of 35 mm. At each step, we acquire CBCT scans with 900 projections over 360° each at 100 and at 125 kV tube voltage. 3D-printed tumours and metal markers are used as inserts. We sample a set of projections according to geometry and respiration of a real scan to synthesise a 4D CBCT with a known ground truth.
**Results or Findings:** The 4D CBCT synthesis results in realistic streak artifacts. It is possible to model the effect of irregular respiration of patients up to an amplitude of 35 mm. The effect of MoCo on small structures, the tumour's shape and metal artifacts can be compared to the ground truth.
**Conclusion:** The proposed method synthesises realistic 4D-CBCT motion from multiple phantom measurements. It can thus serve as a ground truth for the evaluation of image reconstruction, motion correction or motion compensation algorithms.
**Limitations:** The phantom used consists of two rigid parts that can be moved relatively to each other. The motion simulated in this study is AP motion only. Thus it does not fully represent the anatomical motion present in real patients.
**Ethics committee approval:** Not applicable
**Funding for this study:** The study was supported by Varian Medical Systems.
**Author Disclosures:**
Ricky Savjani: Employee: Varian
Julien Frank Josef Erath: Nothing to disclose
Gernot Echner: Nothing to disclose
Pascal Paysan: Employee: Varian
Stefan Scheib: Employee: Varian Medical Systems
Marc Kachelrieß: Nothing to disclose
Markus Susenburger: Nothing to disclose

### RPS 2313-10

**Comparison of 3D-printed phantom parts with a conventional anthropomorphic phantom**
*P. Kunert*, A. Giussani, S. Trinkl, G. Brix; Neuherberg/DE
*(pkunert@bfs.de)*

**Purpose:** Anthropomorphic phantoms are commercially available only for standardised sizes. 3D-printing methods can be employed for creating individualised phantoms. The properties of those phantoms in imaging and dosimetry need to be tested and verified.
**Methods or Background:** A transaxial thorax section with a thickness of 2.5 cm and the breast of a female phantom (CIRS, Norfolk, Virginia) were reproduced with a multi-material fused deposition modelling (FDM) 3D-printer (3ntr A2, Oleggio, Italy). Different tissues were segmented from CT-images of the original phantom based on their Hounsfield-units (HU). The following materials were used, based on a previous study on their tissue equivalence: polylactide (PLA) for muscle and for the lung-tissue (using 35% infill-density); polyethylene-terephthalate-glycol (PETG) for the cartilage and Granite-PLA for the bone structures. Acrylonitrile-butadiene-styrene (ABS) was used for the breast. HU values of the tissues were determined by means of CT-scans of the phantom using either the original or the printed sections and breasts; absorbed dose was measured at different locations using TLD-100 rods (Bicron-Harshaw, Cleveland, Ohio). The results were compared to one another.
**Results or Findings:** The original phantom parts were reproduced easily in their organ placement. Regarding imaging properties, CT densities were higher in muscle tissue (100 HU) and for lung tissue (140 HU) and lower in bone structures (180 HU). For the bone such differences are ascribable to the difficulty of finding an appropriate 3D-printable surrogate material. The attenuation behaviours in soft tissues can be lowered by decreasing the infill density. Absolute doses agreed within 10% difference.
**Conclusion:** Anthropomorphic phantoms can be printed with all relevant tissues. Depending on the intended use, printing materials – if available – must be carefully selected with regard to their physical properties, based on tissue reference values.
**Limitations:** Not applicable
**Ethics committee approval:** Not applicable
**Funding for this study:** Not applicable
**Author Disclosures:**
Sebastian Trinkl: Nothing to disclose
Gunnar Brix: Nothing to disclose
Patrizia Kunert: Nothing to disclose
Augusto Giussani: Nothing to disclose

### RPS 2313-11

**Proposal of SSIM suitable for 3D medical images**
*A. Hasegawa*[1], A. S. Brendlin[2]; [1]Chapel Hill, NC/US, [2]Tübingen/DE

**Purpose:** The structural similarity index (SSIM) is a valuable tool to assess the similarity between two images and can also be used to measure objective image quality if the reference image is considered perfect quality. However, it is challenging to apply it directly to images that contain noise, such as medical images. In particular, if the noise in the two images is independent, its contribution in the numerator of the contrast comparison function of SSIM will be zero because the covariance between the noises will be zero. On the other hand, in the denominator, where the variance of each image is calculated separately, the contribution of noise remains. Therefore, the SSIM is underestimated. In this study, we propose a modified SSIM for the evaluation of 3D images that contain noise, such as CT images.
**Methods or Background:** The sum of the noise variances contained in the two images appearing in the denominator of the contrast comparison function was replaced by the absolute value of the difference of the variances. To achieve this, the noise in each of the two images to be compared was calculated by subtracting the adjacent images since CT image data is composed of slice images. The ACR phantom was scanned twice at different doses between 0.6 and 32.2 mGy, and the same dose images were evaluated using the conventional and the proposed SSIMs.
**Results or Findings:** We confirmed that the lower the dose and the noisier the image, the more the SSIM was underestimated by the conventional method. In contrast, our improved method improved the underestimation.
**Conclusion:** We proposed an improved SSIM for 3D medical images. We confirmed that the SSIM could improve the underestimation.
**Limitations:** The study was performed on phantom.
**Ethics committee approval:** Not applicable
**Funding for this study:** Not applicable
**Author Disclosures:**
Andreas Stefan Brendlin: Nothing to disclose
Akira Hasegawa: Employee: AlgoMedica, Inc.

11:30-12:30 Room E1

### Research Presentation Session: Neuro

### RPS 2411

### Multiple sclerosis (MS), inflammatory and infectious diseases

**Moderator**
J. Boban; Novi Sad/RS

### RPS 2411-2

**The susceptibility of normal-appearing white matter by quantitative susceptibility mapping (QSM) as a new marker of disability progression in multiple sclerosis**

*A. P. Savoldi*, V. E. Contarino, S. Criscuolo, A. M. Petroboni, F. Lo Russo, C. M. Cinnante, F. M. Triulzi, G. Conte; Milan/IT
*(anna.savoldi@unimi.it)*

**Purpose:** To assess magnetic susceptibility of normal-appearing white matter (NAWM) and disease white matter (DWM) in Multiple Sclerosis (MS) using Quantitative Susceptibility Mapping (QSM); to correlate QSM values with clinical follow-up, cerebrospinal fluid β-amyloid1-42 (CSF Aβ) and serum neurofilament light chain (sNfL) levels.
**Methods or Background:** Fifty-nine patients with a first demyelinating episode were enrolled: 42 Relapse-Remitting Multiple Sclerosis (RRMS), 12 Progressive Multiple Sclerosis (PMS) and 6 Clinical Isolated Syndrome (CIS). They underwent neurological examination, quantification of CSF Aβ and sNfL levels, and brain MRI. They were clinically evaluated every six months and Expanded Disability Status Scale (EDSS) and Multiple Sclerosis Severity Scale (MSSS) were calculated. Volume fractions of NAWM (NAWM-VF) and DWM (DWM-VF), and QSM values of NAWM (NAWM-QSM) and DWM (DWM-QSM) were calculated on brain MR images.
**Results or Findings:** NAWM-QSM was lower than DWM-QSM at both baseline and follow-up ($p<0.001$); no difference was found between baseline and follow-up MRI in terms of NAWM-QSM ($p=0.25$) and DWM-QSM ($p=0.26$). At baseline, patients with higher NAWM-QSM showed lower CSF Aβ ($\rho=-0.34$, $p=0.009$) and higher sNfL levels ($\rho=-0.38$, $p=0.004$). NAWM-QSM was higher in PMS compared to CIS ($p=0.024$) and RRMS ($p=0.02$), NAWM-VF was lower in PMS compared to RRMS ($p=0.02$) and CIS ($p=0.001$). Disease phenotypes did not differ in terms of DWM-QSM, DWM-VF, CSF Aβ and sNfL levels. NAWM-QSM was the only independent predictor of EDSS worsening over time ($\beta=0.41$, $p=0.01$). NAWM-QSM ($\beta=0.38$, $p=0.019$) and DWM-VF ($\beta=0.41$, $p=0.006$) independently predicted MSSS.
**Conclusion:** QSM may assess early subtle microstructural changes in NAWM of MS patients and is a promising biomarker to predict disease progression.
**Limitations:** Studies with a larger cohort and longer follow-up will be needed.
**Ethics committee approval:** This study was approved by the Institutional Review Board.
**Funding for this study:** The study was supported by the Italian Ministry of Health.
**Author Disclosures:**
Claudia Maria Cinnante: Nothing to disclose
Fabio Maria Triulzi: Nothing to disclose
Stefania Criscuolo: Nothing to disclose
Francesco Lo Russo: Nothing to disclose
Anna Paola Savoldi: Nothing to disclose
Giorgio Conte: Nothing to disclose
Valeria E. Contarino: Nothing to disclose
Anna M. Petroboni: Nothing to disclose

### RPS 2411-3

**Quantitative T1-mapping of multiple sclerosis lesions using magnetic resonance fingerprinting**

*G. Donatelli*[1], P. Cecchi[1], M. Cencini[1], L. Peretti[1], G. Buonincontri[1], L. Pasquali[1], M. Tosetti[1], M. Cosottini[1], M. Costagli[2]; [1]Pisa/IT, [2]Pisa; Genoa/IT

**Purpose:** In patients with Multiple Sclerosis (MS), contrast enhancement in demyelinating lesions is considered a marker of blood-brain barrier breakdown and active inflammation. It is visible in conventional post-contrast T1-weighted MR images as the result of the contrast-related T1 relaxation time shortening (ΔT1), but it is usually not quantified. Here we used quantitative T1 mapping derived from a recent implementation of Magnetic Resonance Fingerprinting (MRF) to detect contrast enhancing lesions (EL) and measure ΔT1 in both EL and non-enhancing lesions (NEL).
**Methods or Background:** We enrolled 12 patients with relapsing-remitting MS who underwent a 3T-MRI exam of the brain for clinical purpose. Besides conventional sequences, a 3D whole brain MRF acquisition (1.1 mm isotropic voxel) was performed both before and after contrast administration. After obtaining MRF-derived quantitative T1 maps, for each exam the pre- and post-contrast T1 maps were co-registered and a ΔT1 map was obtained by voxelwise subtraction. Then, ΔT1 maps were visually inspected to detect T1-shortening related signal changes and ΔT1 was measured in EL, representative NEL and normal-appearing white matter (NAWM).
**Results or Findings:** At visual inspection of conventional images, two patients had EL, two each; three of these lesions were clearly visible on ΔT1 map. In quantitative analysis, ΔT1 was significantly higher in EL than in NEL and lower in NAWM than in both NEL and EL ($p<0.001$).
**Conclusion:** MRF-derived ΔT1 mapping reveals enhancing MS lesions with both qualitative and quantitative analyses. Moreover, ΔT1 values measured in NEL may indicate a subtle blood-brain barrier disruption in some of them.
**Limitations:** The small number of patients and the inclusion of relapsing-remitting MS only were identified as limitations.
**Ethics committee approval:** The local ethics committee approved this study.
**Funding for this study:** Funding was received from the Italian Ministry of Health and the Health Service of Tuscany (grant GR-2016-02361693).
**Author Disclosures:**
Mauro Costagli: Nothing to disclose
Michela Tosetti: Research/Grant Support: Research grant from GE Healthcare.
Matteo Cencini: Research/Grant Support: Research grant from GE Healthcare
Livia Pasquali: Nothing to disclose
Luca Peretti: Nothing to disclose
Graziella Donatelli: Nothing to disclose
Guido Buonincontri: Nothing to disclose
Paolo Cecchi: Nothing to disclose
Mirco Cosottini: Speaker: Speaker honoraria from Biogen and GE Healthcare.

### RPS 2411-4

**Imaging of cns infections in patients with acquired immuno-deficiency syndrome.**

*S. N. Naik*, H. C. Chadaga, S. Patwari, A. Kumar, V. H P, B. Singh, D. C, R. Hassan, A. A. Makai; Bangalore/IN

**Purpose:** 1. To understand the direct impact of HIV on the brain and its imaging features. 2. To understand the treatment related complications in patients with acquired immune- deficiency syndrome. 3. Understand the characteristic MR Findings to differentiate the various opportunistic infections in patients with acquired immune-deficiency syndrome.
**Methods or Background:** HIV is a highly neurotropic virus that causes both direct and indirect effects on CNS. Complications arise from the HIV infection itself, from opportunistic infections due to immune-suppressed status and treatment related complications. Also, the imaging spectrum in patients with HIV-AIDS is diverse and can seem daunting to the untrained eye, so we try to simplify it for better understanding and to aid the clinician in making the accurate diagnosis. In our study, we have selected 50 cases of patients with HIV related CNS complications, which were characterised on MRI by neuro-radiologist with 11 years of experience and co-related with CSF analysis.
**Results or Findings:** 1. HIV-encephalitis: Symmetric T2/FLAIR hyperintensities in deep white matter, spares cortical U fibers, Neuroparenchymal atrophy inappropriate for age. 2. PML: Asymmetric T2/FLAIR hyperintensities out of proportion to mass effect, affects cortical U fibers 3. CMV: Periventricular T2/FLAIR hyperintensities, thin ependymal enhancement 4. TOXOPLASMOSIS: Ring enhancing 'double target' , T2/FLAIR hyperintense with lots of oedema. 5. CRYPTOCOCCUS: Basal ganglia location T2/FLAIR hyperintense. Leptomeningeal enhancement with dilated perivascular spaces.
TREATMENT RELATED COMPLICATIONS: 1. Immune reconstitution syndrome: New enhancement and mass effect of the pre-existing white matter lesions.
**Conclusion:** Radiologist's awareness about various spectrum of imaging pattern in HIV-AIDS helps to come to an accurate diagnosis and helps the clinicians in further management.
**Limitations:** The retrospective nature of the study was identified as a limitation.
**Ethics committee approval:** This study was approved by an ethics committee.
**Funding for this study:** No funding was received for this study.
**Author Disclosures:**
Akshay Kumar: Nothing to disclose
Vikas H P: Nothing to disclose
Bhupinder Singh: Nothing to disclose
Diwakar C: Nothing to disclose
Abid Ali Makai: Nothing to disclose
Harsha C. Chadaga: Nothing to disclose
Sriram Patwari: Nothing to disclose
Soham Niren Naik: Nothing to disclose
Rosmi Hassan: Nothing to disclose

**RPS 2411-5**

**Brain structural and cerebral blood flow changes in patients recovered from COVID-19 pneumonia: a 3 months' follow up study**

*Y. Qin*, W. Zhu; Wuhan/CN
*(qinyuanyuan-1021@163.com)*

**Purpose:** To explore the impact on the brain structure and cerebral blood flow (CBF) in patients recovered from COVID-19 pneumonia 3 months after discharge.
**Methods or Background:** A cohort of 51 COVID-19 recovered patients divided into mild group (n=19, MG) and severe group (n=32, SG) according to WHO guidelines, as well as age-, sex- and education-matched healthy controls (n=31, NC) were involved. All patients had no specific neurological manifestations at acute stage and 3 months' follow up. Changes of the gray matter (GM), white mater (WM) and CBF were investigated using advanced MRI and state-of-the-art post-processing protocols.
**Results or Findings:** The severe group patients had more decreased GM thickness, decreased CBF, as well as decreased WM tract volume and length compared to NC in widespread brain regions, especially in the frontal and limbic systems. The changes in brain structure and CBF were highly correlated with the inflammatory markers.
**Conclusion:** The brain was sensitive and responsive to the impact of COVID-19, even though there were no obvious neuropsychological symptoms. The abnormalities in these brain areas need to be monitored in the process of complete recovery, which could help clinicians to understand the potential neurological sequelae of COVID-19.
**Limitations:** First, the sample size is relatively small. Second, patients had no specific neurological manifestation and so, to avoid cross-infection, the head MRI was not performed during the acute phase. Third, a follow-up study should be conducted to see whether brain anatomical and functional changes progress or regress.
**Ethics committee approval:** This prospective study was approved by the Clinical Institute Ethics Committee and written informed consent was obtained from each participant.
**Funding for this study:** This study was funded by the projects of the Natural Science Foundation of China (81873890, 81730049).
**Author Disclosures:**
Wenzhen Zhu: Nothing to disclose
Yuanyuan Qin: Nothing to disclose

**RPS 2411-6**

**Cerebral microbleeds assessment and quantification in COVID-19 patients with neurological manifestation**

*A. Napolitano*[1], A. Arrigoni[1], A. Caroli[1], A. Remuzzi[1], M. Cava[2], A. Barletta[1], M. Sessa[1], L. G. Longhi[1], S. Gerevini[1]; [1]Bergamo/IT, [2]Bosco Marengo/IT
*(angela.napolitano90@gmail.com)*

**Purpose:** Neurological manifestations of COVID-19 have been increasingly acknowledged. Concurrently, Cerebral Microbleeds (CMB) have been observed in the brain. The aim of the study was to characterise CMB patterns on susceptibility-weighted imaging (SWI) in hospitalised COVID-19 patients with neurological manifestations. CMB volume was quantified and correlated with clinical and laboratory parameters.
**Methods or Background:** Consecutive patients hospitalised with COVID-19, who showed neurological manifestations and underwent brain MRI from March to May 2020, were included. MRI was performed on a 3T scanner with a standardised protocol that included SWI. The CMB burden was assessed by a semi-automatic SWI processing procedure specifically developed for the purpose of this study. Odds ratios (OR) for cerebral microbleeds were calculated among age, sex, clinical and laboratory data by logistic regression analysis.
**Results or Findings:** Among the 1760 COVID-19 patients admitted to the ASST Papa Giovanni XXIII hospital between 1 March and 31 May 2020, 116 exhibited neurological symptoms requiring neuroimaging evaluation. 63 patients underwent brain MRI and were included. Fourteen patients had cerebral microbleeds (CMB+ group). CMB+ patients had higher prevalence of CSF inflammation (p=0.020), higher white blood cells level (p=0.020), and lower lymphocytes level (p=0.010); D-dimer (p=0.026), LDH (p=0.004), procalcitonin (p=0.002) and CRP concentration (p<0.001) were higher than the CMB- group. In multivariable logistic regression analysis, CRP (OR = 1.16, p = 0.011) showed association with CMB. Estimated CMB volume decreases with age (Rho=-0.38; p=0.18); it is positively associated with CRP (Rho=0.36; p=0.22), while negatively associated with lymphocytes (Rho=-0.52; p=0.07).
**Conclusion:** CMB is a frequent imaging finding in COVID-19 and seems to be related to pro-inflammatory status.
**Limitations:** This is a retrospective study and only hospitalised patients with neurological symptoms underwent MRI.
**Ethics committee approval:** An ethics committee approved the protocol (reg 2020-144)
**Funding for this study:** No funding was received for this study.
**Author Disclosures:**
Alberto Arrigoni: Nothing to disclose
Mariangela Cava: Nothing to disclose
Maria Sessa: Nothing to disclose
Antonino Barletta: Nothing to disclose
Anna Caroli: Nothing to disclose
Angela Napolitano: Nothing to disclose
Luca Giovanni Longhi: Nothing to disclose
Andrea Remuzzi: Nothing to disclose
Simonetta Gerevini: Nothing to disclose

**RPS 2411-7**

**Assessment of white matter alterations in patients of migraine using diffusion tensor imaging**

*C. Nanda*, N. Sachdev; New Delhi/IN
*(civunanda@gmail.com)*

**Purpose:** To assess white matter alterations in patients of migraine by using diffusion tensor imaging.
**Methods or Background:** This was an observational comparative study that was conducted in the department of Radiodiagnosis, Dr. RML hospital from 1st November 2020 to 31st March 2021. Adult migraine patients (<50 years) and 20 age- and sex-matched controls were included in the study. DTI was performed on a 3 Tesla MR scanner, along 64 isotropically distributed diffusion sensitising directions.
**Results or Findings:** Our study revealed significantly lower (p<0.05) FA values in migraine patients in the anterior limb of left internal capsule, body of corpus callosum (CC), posterior limb of right internal capsule (PLIC) and bilateral thalami and mean MD values of anterior limb of bilateral internal capsule (ALIC), posterior limb of left internal capsule, left superior longitudinal fasciculus (SLF) and bilateral cingulum, compared to healthy controls. A significant correlation (p<0.05) was found between the type of migraine, average duration of episodes, frequency of attacks and severity of migraine as per migraine disability assessment test.
**Conclusion:** Recently, there has been a paradigm shift in understanding of the pathophysiology of migraine from vascular etiology to neurological mechanisms. Changes in FA and MD values can be compared with values obtained in the healthy population, which aids in the assessment of the extent of neurological damage. Thus, DTI is an excellent technique which can elucidate the tracts involved in migraine, thereby assisting not only in diagnosis but also enabling disease prognostication and guiding treatment strategies.
**Limitations:** Our study had a few shortcomings including a small sample size due to the COVID-19 pandemic. Also, manually drawn regions of interest were used for the calculation of DTI parameters while few studies using tract-based spatial statistics analysis have been conducted.
**Ethics committee approval:** This study was approved by an ethics committee (726/19).
**Funding for this study:** No funding was received for this study.
**Author Disclosures:**
Namrita Sachdev: Nothing to disclose
Civilee Nanda: Nothing to disclose

**RPS 2411-8**

**Non-lesional sources of contrast enhancement on post-gadolinium "black-blood" 3D-T1-SPACE images in patients with multiple sclerosis**

*E. Pravatà*[1], L. Danieli[1], L. Roccatagliata[2], D. Distefano[1], E. Prodi[1], G. C. Riccitelli[1], A. Diociasi[2], A. Kaelin-Lang[1], A. Cianfoni[1]; [1]Lugano/CH, [2]Genoa/IT
*(emanuele.pravata@eoc.ch)*

**Purpose:** Contrast enhancing brain lesion (CEL) misdiagnosis in patients with MS may derive from intraparenchimal vein (iV) enhancement misinterpretation on post-gadolinium MRI. The objective of this study was to assess the risk of CEL misdiagnosis using "black-blood" 3D-T1-TSE ("SPACE") compared to 3D-T1-GRE MPRAGE and VIBE images.
**Methods or Background:** SPACE images were obtained from 232 patients with MS, clinically isolated syndrome or radiologically isolated syndrome, and compared with standard MPRAGE and VIBE images. iV contrast-to-noise ratio (CNR) was estimated at the level of the thalami. CELs were blindly detected by two expert and one beginner readers. True and false positive (TP and FP) were determined by senior readers' consensus. TP and FP frequency differences, and patient-level diagnosis probability, were tested with McNemar's and OR. CNR and morphology were compared with Mann-Whitney-U and $\chi^2$ tests, respectively.
**Results or Findings:** iV CNR was higher with SPACE than MPRAGE and VIBE (p<0.001, both). There were 66 TP and 74 FP overall. SPACE detected more TP and FP (p range <0.001-0.07), but did not increase patient's TP likelihood (OR=1-1.29, p=0.478-1). However, FP likelihood was increased (OR=3.03-3.55, p=0.008-0.027). Venous-origin FP (N=59) occurred more frequently with SPACE (p<0.001), with similar CNR and morphology features to small-sized (≤14mm3, P=0.544) TP.

**Conclusion:** Small iV may confound enhancing lesion diagnosis on post-gadolinium "black-blood" SPACE images.
**Limitations:** Not applicable
**Ethics committee approval:** This study was approved by the ethics committee of Canton Ticino.
**Funding for this study:** This study received a grant from the Advisory Board of Research of Ente Ospedaliero Cantonale, Bellinzona, Switzerland.
**Author Disclosures:**
Luca Roccatagliata: Nothing to disclose
Daniela Distefano: Nothing to disclose
Andrea Diociasi: Nothing to disclose
Emanuele Pravatà: Nothing to disclose
Alain Kaelin-Lang: Nothing to disclose
Alessandro Cianfoni: Nothing to disclose
Gianna Carla Riccitelli: Nothing to disclose
Elena Prodi: Nothing to disclose
Lucia Danieli: Grant Recipient: Grant from the Advisory Board of Research of Ente Ospedaliero Cantonale, Bellinzona, Switzerland.

**11:30-12:30** **Room E2**

### Research Presentation Session: Breast

### RPS 2402

### Breast cancer staging and response to therapy

**Moderator**
M. Nadrljanski; Belgrade/RS

### RPS 2402-2

**A model to predict upstaging to invasive carcinoma in patients preoperatively diagnosed with low-grade ductal carcinoma in situ of the breast**

*D. Ballerini*[1], L. Nicosia[2], A. C. Bozzini[2], C. Trentin[2], S. Penco[2], M. Pizzamiglio[2], G. Di Giulio[1], F. Pesapane[2], E. Cassano[2]; [1]Pavia/IT, [2]Milan/IT

**Purpose:** The purpose of our study is to create a predictive model that identifies the features, mainly based on imaging, that can predict the upgrade rate of low-grade ductal carcinoma in situ (DCIS) to invasive carcinoma or worst grade DCIS in patients undergoing vacuum-assisted breast biopsy (VABB) and subsequent surgery.
**Methods or Background:** A total of 3100 VABBs were retrospectively reviewed, among which we reported 295 low-grade DCIS who subsequently underwent surgery. We developed a nomogram for predicting the upstage at surgery, according to a multivariate logistic regression model. Patients were categorised into five classes, based on predicted probabilities (≤2%, 2–5%,5–10%, 10–25% and >25%)
**Results or Findings:** The overall upgrade rate to invasive carcinoma was 10.8%. At univariate analysis, the risk of upgrade was significantly lower in patients with greater age (p= 0.018), without post-biopsy residual lesion or with a smaller post-biopsy residual lesion size (p<0.001), and in the presence of low-grade DCIS only in specimens with microcalcifications (p= 0.002). At multivariate analysis, patients with disease in specimens without microcalcifications and with post-biopsy residual lesion were instead significantly associated with the upstage at surgery (respectively OR 0.33, 95% CI 0.13-0.83 and OR 7.14, 95% CI 1.58-32.2) According to our final nomogram, the predicted probability of upstage at surgery was lower than 2% in 58 patients.
**Conclusion:** By selecting a population with a low risk of upgrading (<2%), we may identify patients with low-grade breast cancer in which surgery may be safely spared.
**Limitations:** No limitations were identified.
**Ethics committee approval:** The study was conducted according to the guidelines of the Declaration of Helsinki and approved by the Ethics Committees of European Institute of Oncology.
**Funding for this study:** No funding was received for this study.
**Author Disclosures:**
Chiara Trentin: Nothing to disclose
Luca Nicosia: Nothing to disclose
Silvia Penco: Nothing to disclose
Anna Carla Bozzini: Nothing to disclose
Maria Pizzamiglio: Nothing to disclose
Daniela Ballerini: Nothing to disclose
Giuseppe Di Giulio: Nothing to disclose
Filippo Pesapane: Nothing to disclose
Enrico Cassano: Nothing to disclose

### RPS 2402-4

**Diagnostic performance of imaging modalities for response of ductal carcinoma in situ in breast cancer patients treated with neoadjuvant systemic therapy: a systematic review and meta-analysis**

R. Ploumen, C. M. de Mooij, S. Gommers, M. Smidt, *T. van Nijnatten*; Maastricht/NL
*(Thiemo.nijnatten@mumc.nl)*

**Purpose:** In approximately 45% of invasive breast cancer treated with neoadjuvant systemic therapy (NST), ductal carcinoma in situ (DCIS) is present. Recent studies suggest response of DCIS to NST. The purpose was to provide a systematic review and meta-analysis of the current evidence on diagnostic performance of mammography, contrast-enhanced mammography (CEM) and magnetic resonance imaging (MRI) for response evaluation of DCIS in patients treated with NST.
**Methods or Background:** PubMed and Embase were searched for studies investigating NST response of breast cancer, including information on DCIS, with the imaging modalities mammography, CEM or MRI. Screening and data extraction were performed independently by two reviewers. Response evaluation of DCIS was assessed per imaging modality. A meta-analysis was conducted on studies reporting sufficient data to calculate pooled sensitivity and specificity for detecting residual disease between two definitions of pathological complete response (pCR) (ypT0 versus ypT0/is).
**Results or Findings:** Thirty-one studies were included. Eleven mammography studies showed calcifications are frequently associated with DCIS, however, residual calcifications do not necessarily indicate residual disease. Two CEM studies and 21 MRI studies demonstrated differences among imaging findings of residual DCIS, in which approximately 50% showed residual enhancement. A meta-analysis of 16 MRI studies confirmed higher pooled sensitivity (0.86 versus 0.82) and lower pooled specificity (0.61 versus 0.68) for detection of residual disease when DCIS is considered pCR (ypT0/is), because of more false positives due to enhancement of DCIS. Combining imaging findings of calcifications and residual enhancement might improve detection of residual DCIS.
**Conclusion:** Diagnostic performance of imaging modalities for response evaluation of DCIS to NST is insufficiently accurate. Combining imaging findings of calcifications and enhancement might be promising to distinguish residual DCIS from pCR.
**Limitations:** Not applicable
**Ethics committee approval:** Not applicable
**Funding for this study:** Funding was received from the Jules Coenegracht Sr. Foundation.
**Author Disclosures:**
Marjolein Smidt: Nothing to disclose
Cornelis Maarten de Mooij: Nothing to disclose
Roxanne Ploumen: Nothing to disclose
Suzanne Gommers: Nothing to disclose
Thiemo van Nijnatten: Nothing to disclose

### RPS 2402-5

**Prediction of primary tumour and axillary lymph node response to neoadjuvant systemic therapy with dedicated breast 18F-FDG PET/MRI in breast cancer**

C. M. de Mooij[1], B. Goorts[1], M. Lobbes[2], L. F. Kooreman[1], K. Keymeulen[1], J. E. Wildberger[1], F. M. Mottaghy[3], M. Smidt[1], *T. van Nijnatten*[1]; [1]Maastricht/NL, [2]Sittard-Geleen/NL, [3]Aachen/DE
*(Thiemo.nijnatten@mumc.nl)*

**Purpose:** To investigate the diagnostic accuracy of 18F-FDG PET/MRI to determine primary tumour and axillary lymph node response to neoadjuvant systemic therapy (NST) in breast cancer patients.
**Methods or Background:** Forty-one breast cancer patients with 42 primary tumours and 26 pathologically proven clinically node-positive (cN-positive) disease were prospectively included. PET/MRI was performed before, halfway and following NST. Qualitative response evaluation following NST was based on FDG-activity on PET and residual enhancement on MRI. The quantitative variables maximum standardized uptake value (SUVmax) on PET and signal enhancement ratio (SER) on dynamic contrast enhanced (DCE)-MRI were determined on each exam and their percentage change was calculated. Receiver operating characteristic (ROC) curves were generated to determine the optimal cut-off values for quantitative variables in predicting residual disease. Diagnostic accuracy in predicting residual disease of the primary tumour in all patients, and residual axillary disease in cN-positive patients, was assessed.
**Results or Findings:** Pathological complete response (pCR) of the primary tumour occurred in 16 (38.1%) of all, and axillary pCR in 14 (53.8%) of cN-positive patients. Accuracy of the qualitative evaluation was 73% for primary tumour and 62% for axillary response. For primary tumour response, combining the percentage decrease in SUVmax and SER halfway NST achieved an accuracy of 74%. Additionally, combining the absolute as well as the percentage decrease in SUVmax halfway NST improved accuracy for axillary response prediction to 91%.

**Conclusion:** Qualitative PET/MRI shows promising results for primary tumour but not for axillary response prediction. Diagnostic accuracy for axillary response prediction can be improved by combining quantitative variables from longitudinal imaging.
**Limitations:** Small sample size and no separate evaluation per breast cancer subtype were identified as limitations.
**Ethics committee approval:** The study was approved by the local medical ethics committee.
**Funding for this study:** Academic incentive was received from the Maastricht University Medical Centre+.
**Author Disclosures:**
Marjolein Smidt: Nothing to disclose
Briete Goorts: Nothing to disclose
Loes F.S. Kooreman: Nothing to disclose
Kristien Keymeulen: Nothing to disclose
Cornelis Maarten de Mooij: Nothing to disclose
Marc Lobbes: Nothing to disclose
Joachim E. Wildberger: Nothing to disclose
Felix M. Mottaghy: Nothing to disclose
Thiemo van Nijnatten: Nothing to disclose

### RPS 2402-6

### Role of contrast-enhanced mammography (CEM) as a promising tool in preoperative staging of breast cancer

*C. Rucci*, A. R. Speranza, C. Bernardi, A. Zucchelli, M. Mattei; Rome/IT
*(carlottarucci@gmail.com)*

**Purpose:** The aim of this study is to evaluate the added value of CEM in preoperative staging of breast cancer confirmed by surgical histological sample.
**Methods or Background:** From January 2018 to June 2021, 340 patients with strongly suspected diagnosis of breast malignancy based on assessment with Digital Mammography (DM) and Ultrasound (US) (BIRADS 3, 4, 5) were retrospectively included. After performance of biopsy by core biopsy or Vacuum Assisted Breast Biopsy, patients were scheduled for primary surgery and were invited to undergo CEM as an additional preoperative procedure. Two readers with respectively 10 and 20 years of experience, blinded to pathology and clinical information, independently evaluated CEM findings. The following major endpoints were evaluated: findings of additional lesions, additional number of biopsies performed, sensitivity and specificity of CEM and changes in surgery treatments (mastectomy instead of lumpectomy or bilateral surgery instead of unilateral).
**Results or Findings:** The final population included 156 patients (26 – 83 years), 312 breasts examined for a total of 165 lesions. CEM correctly identified 21 additional lesions for a total of 186 lesions (+ 13.5%) with 21 additional biopsies and a change in surgical management in 14/156 (8.9%) patients. Regarding per breast analysis, the diagnostic accuracy, sensitivity and specificity of CEM were 94,9%, 97,6% and 91,8%, respectively.
**Conclusion:** Results imply an added value of CEM in preoperative staging of breast cancer and contribute to our knowledge on CEM as an additional imaging method to standard investigation with digital mammography and ultrasound.
**Limitations:** This was a monocentric study.
**Ethics committee approval:** The local institutional review board approved the study; all study participants provided written informed consent.
**Funding for this study:** No funding was received for this study.
**Author Disclosures:**
Mauro Mattei: Nothing to disclose
Carlotta Rucci: Nothing to disclose
Anna Rita Speranza: Nothing to disclose
Claudia Bernardi: Nothing to disclose
Alberto Zucchelli: Nothing to disclose

### RPS 2402-7

### Preoperative staging of invasive lobular carcinoma (ILC): contrast-enhanced mammography (CEM) as alternative for breast MRI?

*M. B. I. Lobbes*[1], L. Neeter[2], K. Turk[1], H. Raat[3], T. van Nijnatten[2], J. E. Wildberger[2]; [1]Sittard-Geleen/NL, [2]Maastricht/NL, [3]Roermond/NL
*(mbi.lobbes@gmail.com)*

**Purpose:** This study aims to evaluate CEM's accuracy in preoperative staging of ILC, comparing its performance to MRI.
**Methods or Background:** All ILC cases of two hospitals diagnosed between 2013-2021 were collected. For both CEM and MRI, tumour diameter was assessed. Histopathology served as gold standard. We calculated mean differences in tumour size assessment measured on CEM and MRI in Bland Altman plots. Also, we calculated the diagnostic performance of CEM and MRI to detect multifocal or contralateral breast cancers.
**Results or Findings:** In the participating centers, CEM was introduced in 2018 and 2019, identifying 379 ILC cases. 187 (49.3%) fulfilled our inclusion criteria. Mean patient age was 64.8 years (SD 10.7). Mean tumour diameter was 22.8mm (SD 17.5mm). Multifocal or contralateral breast cancer foci were confirmed in 12.8% and 10.2% of the cases, respectively. Preoperative staging was performed using MRI or CEM in 176 (94.1%) or 22 (11.8%) of the cases, respectively. Eleven cases received both examinations. Mean difference between measurements for MRI was +2.8mm (95% LOA -23.8-29.4mm), for CEM this was +2.7mm (95% LOA -22.3-27.7mm). Sensitivity and specificity of CEM (100% and 94.1%) to detect multifocal disease were superior to MRI (82.6% and 76.0%). Sensitivity and specificity of MRI to detect contralateral disease was 94.7% and 89.2%. For CEM, both sensitivity and specificity were 100%.
**Conclusion:** Accuracy of tumour size measurements is similar for CEM and MRI in ILC cases. Diagnostic accuracy of CEM to detect multifocal or contralateral breast cancer might be superior to MRI. The number of ILC cases that were evaluated using CEM was limited, which might have affected results.
**Limitations:** Limited number of ILC cases. We are currently including additional ILC cases from a third centre that had CEM since 2013.
**Ethics committee approval:** Ethics committee approval was waived.
**Funding for this study:** No funding was received for this study.
**Author Disclosures:**
Lidewij Neeter: Nothing to disclose
Thiemo van Nijnatten: Nothing to disclose
Marc B I Lobbes: Research/Grant Support: GE Healthcare Speaker: GE Healthcare, Hologic, Tromp Medical
Joachim E. Wildberger: Nothing to disclose
Henricus Raat: Nothing to disclose
Kim Turk: Nothing to disclose

### RPS 2402-8

### The kinetic and morphologic changes of conventional and ultrafast dynamic contrast-enhanced MRI in breast cancer patients after neoadjuvant chemotherapy-preliminary report

*A. Y. Park*; Seongnam-si/KR

**Purpose:** To analyse the kinetic and morphologic changes of conventional dynamic contrast-enhanced MRI (DCE-MRI) and ultrafast MRI in breast cancer patients with neoadjuvant chemotherapy (NAC) and to identify the predictive MRI factors for pathologic complete response (pCR).
**Methods or Background:** This retrospective study included 28 patients diagnosed with breast cancer who underwent NAC and operation. The kinetic and morphologic features on conventional and ultrafast DCE-MRI before and after NAC were compared with pathologic features and response.
**Results or Findings:** Twelve patients (12/28, 42.9%) showed pCR. The accuracy of MRI in predicting pCR was 0.833 in area under the receiver operating curve. Residual tumour size measured on delayed dynamic phase better predicted histologic residual tumour size, compared to that on initial phase, especially for overall invasive and in situ cancer than invasive cancer (ICC 0.703 for overall cancer and 0.421 for invasive cancer). pCR group showed smaller tumour size (p=0.02), smaller angiovolume (p=0.01), the absence of lymph node metastasis (p=0.023) on pre-NAC MRI and a more decrease on tumour size (p=0.001), angiovolume (p=0.002), maximum slope on kinetic curve of ultrafast MRI (p=0.032). Lymph node metastasis on pre-NAC MRI was an independent predictor of pathologic response, regardless or tumour size (p=0.035). Triple-negative breast cancer (TNBC) showed more angiovolume on pre-NAC MRI (p=0.015) and more delayed washout percentage on post-NAC MRI (p=0.018), compared to non-TNBC.
**Conclusion:** Small tumour size, vascularity, and the absence of lymph node metastasis were significant factors for pCR. Angiovolume and maximum slope on ultrafast MRI were significantly decreased in pCR group during NAC.
**Limitations:** The small study population and the retrospective setting were identified as limitations.
**Ethics committee approval:** The study was approved by the institutional review board.
**Funding for this study:** This work was supported by the National Research Foundation of Korea grant funded by the Korea government (No. 2020R1G1A1102372).
**Author Disclosures:**
Ah Young Park: Nothing to disclose

**13:00-14:30** **Room B**

### Research Presentation Session: Musculoskeletal

### RPS 2510a

### Ultrasound intervention and new applications of CT and MRI

**Moderator**
E. E. Drakonaki; Iraklion/GR

### RPS 2510a-2

**Elastosonography evaluation after ESWT (Extracorporeal Shock Wave Therapy) treatment in plantar fasciopathy**

*D. Fresilli*[1], G. Schillizzi[2], G. Del Gaudio[1], V. Dolcetti[1], M. Martino[1], P. Pacini[1], C. Catalano[1], V. Cantisani[1], V. D'Andrea[1]; [1]Rome/IT, [2]London/UK
*(daniele.fresilli@hotmail.it)*

**Purpose:** To evaluate elastosonography ultrasound to assess plantar fascia elasticity in patients with plantar fasciitis before and after ESWT treatment.
**Methods or Background:** 20 Patients with plantar fasciitis nonresponsive to previous noninvasive conservative treatment were enrolled. Clinical and ultrasound evaluation (including Swear Wave Elastography and Compression Elastography) were performed at baseline (T0) and at 1 month (T1) and 3 months (T2) after ESWT treatment ended. Patients were treated with 3 session, once a week of ESWT.
**Results or Findings:** At baseline, (T0) statistically significant differences were found in SWE velocity between the affected side and healthy side with higher value in healthy side with value equal to 3.8 (1.5; 5.1) ms-1 and 4.7 (4.07;7.04) ms-1 respectively (p=0,006; z=2,758), while no significant differences were found for strain ratio (p=0.656; z=0.445). One month after ESWT treatment (T1) the strain ratio of the affected side increased, with median value equal to 0.89 (0.3-1.5) at baseline to 1.16 (0.3-1.6) at 1 month and decreased at three months (T2) with median value equal to 0.82 (0.38-1.12). No statistically significant differences were found. Significant differences were found in shear wave velocity over time, with an increase of SWE velocity after shock-wave treatment (p=0.04), results showed significant differences from T0 to T2 with median value varying from 3.8 (1.5-5.1) ms-1 at baseline and 5.23 (4.55-6.74) ms-1 a three months after treatment ended respectively (p=0.003).
**Conclusion:** Shear Wave Elastography seems to be more accurate to assess soft tissue stiffness, it provides more objective results and less technical variation than compression elastography. SWE seems effective tool to assess ESWT treatments efficacy.
**Limitations:** Interoperator variability and the small study population were identified as limitations.
**Ethics committee approval:** All procedures performed were in accordance with the ethical standards of the institutional and/or national research committee.
**Funding for this study:** Not applicable
**Author Disclosures:**
Daniele Fresilli: Nothing to disclose
Giovanni Del Gaudio: Nothing to disclose
Patrizia Pacini: Nothing to disclose
Vito D'Andrea: Nothing to disclose
Milvia Martino: Nothing to disclose
Vincenzo Dolcetti: Nothing to disclose
Giuseppe Schillizzi: Nothing to disclose
Vito Cantisani: Other: Lecturer fee from Bracco, Samsung and Toshiba
Carlo Catalano: Nothing to disclose

### RPS 2510a-3

**Ultrasound-guided percutaneous needle tenotomy of the long head of biceps tendon**

*A. C. O'Brien*[1], M. Rinaldi[1], E. Lee[2], Z. Teh[3], N. Chaudhary[4], J. Papanikitas[1], R. Hughes[1], S. McElroy[5], D. McKean[1]; [1]Aylesbury/UK, [2]London/UK, [3]Cardiff/UK, [4]Glasgow/UK, [5]Great Missenden/UK

**Purpose:** Singlecentre retrospective cohort study of clinical outcomes of percutaneous ultrasound-guided needle tenotomy of the long head of biceps tendon (LHBT).
**Methods or Background:** Clinical outcomes of patients who had percutaneous ultrasound-guided needle tenotomy of the long head of biceps tendon (LHBT) were analysed. 15 patients (mean age 77, SD 6.3, range 67-87) with significant LHBT pain or instability were treated. Under local anaesthetic, LHBT tenotomy was performed using a 19 gauge white needle under continuous ultrasound monitoring until it was no longer visible. Pain was recorded before and at least 2 months post-procedure and Oxford Shoulder Scores (OSS) were recorded.
**Results or Findings:** Follow-up data obtained in 11 patients. Complete tenotomy achieved in 10 patients. One patient with incomplete percutaneous tenotomy proceeded to arthroscopic surgery. Pre-tenotomy VAS score was 8±1.1, post-tenotomy VAS at day one was 5±3.4 (p=0.0397), at one week was 4±2.8 (p=0.0053), and at 8 weeks was 2±2.0 (p=0.0001). Pre-tenotomy OSS was 19±5 and post-tenotomy at 8 weeks was 38±8 (p=0.0012). No patients had weakness in elbow flexion or limited daily activities due to LHBT tenotomy. No adverse outcomes or significant complications were reported.
**Conclusion:** Ultrasound-guided percutaneous LHBT tenotomy is a feasible, safe and effective treatment for patients with pain secondary to long head of biceps tendinosis or instability. This minimally invasive approach may be of particular relevance for elderly or infirm patients to avoid the potential risks of arthroscopic surgery.
**Limitations:** Our cohort size is relatively small but was sufficient to reach statistical significance. Larger studies will be necessary to further define the risk of complications related to this procedure.
**Ethics committee approval:** This study was a retrospective review of clinical outcomes and ethics committee approval was not required.
**Funding for this study:** No funding was received for this study.
**Author Disclosures:**
Joseph Papanikitas: Nothing to disclose
David McKean: Nothing to disclose
Neera Chaudhary: Nothing to disclose
Marta Rinaldi: Nothing to disclose
Zoe Teh: Nothing to disclose
Amy Clare O'Brien: Nothing to disclose
Elsa Lee: Nothing to disclose
Sam McElroy: Nothing to disclose
Richard Hughes: Nothing to disclose

### RPS 2510a-6

**Study the characteristic of occult rib fracture in spectral CT**

*S. P. Luo*; Tianjin/CN
*(luo4pin@163.com)*

**Purpose:** This study aimed to determine whether there was water content change in the medullary cavity of occult rib fractures by spectral CT.
**Methods or Background:** The reconstructed spectral CT data was water/HAP material images. The water content of medullary cavity in subtle and occult rib fractures and corresponding symmetrical parts of contralateral ribs were measured retrospectively, and calculated changes. By comparing with patients without trauma, the absolute value of the difference of bilateral rib water content was measured and calculated. Independent sample T test was adopted to compare the consistency of water content in normal ribs' medullary cavity. Intergroup comparison and pairwise comparison was applied on the difference of water content among subtle, occult fractures and normal ribs, followed by calculated ROC curves. p<0.05 was considered a statistically significant difference.
**Results or Findings:** 100 subtle, 47 occult fractures and 96 normal bilateral ribs were included. The water content of the medullary cavity in the subtle and occult fracture was 31.06±15.03 mg/cm3 and 27.83±11.40 mg/cm3 higher than that in the symmetrical parts, respectively. The difference of water content in normal bilateral ribs was 8.05±6.13 mg/cm3 but without difference (p=0.096). The increased water content of fractured ribs was higher than that of normal ribs (p=0.000), but there was no difference between subtle and occult fractures (p=0.497). According to the classification based on whether the ribs were fractured or not, the AUC was 0.94.
**Conclusion:** It was confirmed by spectral CT that the water content in medullary cavity increased obviously when occult rib fractures occurred.
**Limitations:** The sample size was small and the study was based on singlecentre data.
**Ethics committee approval:** This study was approved by the Tianjin hospital ethics committee: 2021YLS018.
**Funding for this study:** Not applicable
**Author Disclosures:**
Si Pin Luo: Nothing to disclose

### RPS 2510a-7

**Optimal keV-settings for virtual monoenergetic reconstructions of photon-counting detector CT datasets in patients status post posterior internal fixation of the spine**

*D. Popp*, A. X. Sinzinger, S. Bette, K. Rippel, F. Braun, J. A. Decker, C. Scheurig-Muenkler, T. J. Kroencke, F. Schwarz; Augsburg/DE
*(daniel_popp@t-online.de)*

**Purpose:** To investigate the usefulness of virtual monoenergetic imaging (VMI) reconstructions derived from scans on a novel photon-counting detector CT (PCD-CT) for artifact reduction in patients status post spinal fixation.
**Methods or Background:** Here, we present initial data from 10 patients status post posterior spinal fixation who were scanned as part of routine clinical care on a novel dual-source photon-counting detector CT (NAEOTOM Alpha, Siemens Healthineers, Erlangen, Germany). 14 sets of VMI-reconstructions were performed from 60-190 keV in 10 keV increments. The mean and the standard deviation (SD) of CT-values in 12 defined locations around a pair of pedicle screws on one vertebral level and the SD of homogenous fat were measured and used to calculate an artifact index (AI).
**Results or Findings:** The lowest AI was observed at 110 keV increasing in both lower and higher keV-VMI-reconstructions. For high-artifact regions in close proximity to the screws the difference in AI was significant at energy levels ±20 keV. For instance, between the screw heads, AI at 110 keV was 34.0±11.4 increasing to 63.1±22.7 (p=0.002) and to 54.7±18.1 (p=0.015) at 90 and 130 keV, respectively. Overall AI was highest between pedicular screw heads and dorsal to the screw heads and decreased in regions more distant to the screws.
**Conclusion:** Virtual monoenergetic reconstructions derived from PCD-CT datasets substantially decrease artifacts of spinal fixation metal and thus facilitate the evaluation of peri-implant regions. Interestingly, artifact index was lowest at energy levels ranging from 100-120 keV suggesting this as the ideal window for artifact minimisation.
**Limitations:** The small sample size, this work being a singlecentre, retrospective study as well as the lack of a control group (conventional CT) were identified as limitations.
**Ethics committee approval:** The local ethics committee approved this retrospective analysis with a waiver for informed consent.
**Funding for this study:** No funding was received for this study.
**Author Disclosures:**
Stefanie Bette: Nothing to disclose
Christian Scheurig-Muenkler: Nothing to disclose
Franziska Braun: Nothing to disclose
Andrea Xaver Sinzinger: Nothing to disclose
Thomas J. Kroencke: Nothing to disclose
Florian Schwarz: Nothing to disclose
Katharina Rippel: Nothing to disclose
Josua A. Decker: Nothing to disclose
Daniel Popp: Nothing to disclose

### RPS 2510a-8

**Dynamic MRI measurement allows reliable control of success after surgical correction of patellofemoral instability and maltracking**

*K-J. Maas*, M. L. Warncke, M. Krause, K-H. Frosch, G. Adam, G. H. Welsch, F. O. Henes, J. Frings, T. Dust; Hamburg/DE
*(k.maas@uke.de)*

**Purpose:** To evaluate the applicability of dynamic MRI to control surgical correction of patella maltracking in patients with patellofemoral instabilities.
**Methods or Background:** Patients who presented with symptoms of patellofemoral instability (PFI) and patellar maltracking (PM) between December 2019 and November 2020 were included. Inclusion criteria were reported patellar dislocation and/or persistent feeling of instability after dislocation, a positive (reverse) J-sign as well as clinically increased mediolateral translation. Exclusion criteria were PFI without PM and a limited range of motion (<0° extension, <90° flexion). All patients were examined using a 3 T MRI with repeated active flexion (40°) and full extension of the affected knee joint preoperatively and at least 3 months after the surgical treatment. Common anatomical risk factors for PM (tibial-tuberosity-to-trochlear-groove-(TT-TG)-distances, trochlea-sulcus-angle (TSA), trochlea-sulcus-depth (TSD), lateral-inclination-angle (LTI), Caton-Deschamps-ratio (CDR), Insall-Salvati-ratio (ISR)) were analysed using static MRI sequences. Dynamic measurement of dynamic mediolateral translation (dMPT) and patella tilt (dPT) were assessed.
**Results or Findings:** 18 Patients (3 males, 15 females, average 23 years) were included in the study. Primarily addressed pathologies for PM were lateralised-tibial-tubercle, trochlea dysplasia, patella alta and valgus deformity. After 3 months both, the dMPT (12.3±7.5 mm vs 5.4±4.5 mm p=0,028, r=0,585) and the patellotrochlear distance in extension were significantly reduced (15.86 ± 7.55 mm vs 10.7 ± 5.55 mm, p=0,02, r=0,350). Postoperative TT-TG, TT-PCL and LTI were significantly improved after surgery. The preoperative TT-TG correlated with dMPT (p=0,040, r=0,519).
**Conclusion:** Dynamic MRI is a reliable tool to control surgical success, following surgical correction of patella maltracking. Dynamic patellar translation correlated with TT-TG, which enhances its clinical value.
**Limitations:** Not applicable
**Ethics committee approval:** Not applicable
**Funding for this study:** Not applicable
**Author Disclosures:**
Götz Hannes Welsch: Nothing to disclose
Karl-Heinz Frosch: Nothing to disclose
Matthias Krause: Nothing to disclose
Jannik Frings: Nothing to disclose
Tobias Dust: Nothing to disclose
Frank Oliver Henes: Nothing to disclose
Kai-Jonathan Maas: Nothing to disclose
Gerhard Adam: Nothing to disclose
Malte Lennart Warncke: Nothing to disclose

### RPS 2510a-10

**T2 mapping of paediatric patellar chondromalacia**

*E. Voronkova*, P. Menshchikov, I. Melnikov, A. Manzhurtsev, M. Ublinskiy, D. Vorobyev, D. Kupriyanov, T. Akhadov; Moscow/RU
*(elena_voronkova13@mail.ru)*

**Purpose:** T2 mapping is seen to be promising method for detection and staging of chondromalacia. Various biochemical and biophysical processes might be involved in cartilage degradation in different cartilage zones. Thus, the aim of the study was the investigation of T2 relaxation times separately in the deep, intermediate and superficial layers depending on the severity of chondromalacia.
**Methods or Background:** 171 (15.1±1.8 years) patients with mild and severe patellar chondromalacia and 51 healthy volunteers (14.7±2.2 years) underwent MRI examination including axial T2 mapping (TSE, 6 TE from 13 to 78 ms, voxel size 0.4×0.4×3 mm). T2 were quantified from whole cartilage and with layer segmentation. One-vs-rest logistic regression was used to create the classification model for chondromalacia severity determination.
**Results or Findings:** In the superficial layer, no differences between groups were found. In the deep and intermediate layer, the T2 significantly increases with the degree of chondromalacia. By contrast, for the whole cartilage only severe chondromalacia shows significant increase in T2 values. Sensitivity and specificity of the created classification model increases with the growth of the feature number from 58% and 52% for the whole cartilage assessment to 69% and 61%.
**Conclusion:** Consideration of the differences in the water concentration, collagen matrix organisation and anisotropy in the different cartilage zones by the segmentation into layers can significantly increase the clinical efficiency of the T2 mapping. This approach increases sensitivity and specificity of chondromalacia stage determination by 17% compared with whole cartilage assessment.
**Limitations:** The main limitation of our study is unbalanced patient distribution by groups (the number of subjects with mild chondromalacia is 3-5 times higher than in the severe and control group).
**Ethics committee approval:** The study was approved by CRIEPST ethic committete.
**Funding for this study:** This work was supported by the RSF 21-75-00068 grant.
**Author Disclosures:**
Petr Menshchikov: Employee: joint scientific work
Maxim Ublinskiy: Nothing to disclose
Andrei Manzhurtsev: Nothing to disclose
Denis Vorobyev: Nothing to disclose
Elena Voronkova: Nothing to disclose
Dmitry Kupriyanov: Employee: joint scientific work
Tolibjohn Akhadov: Nothing to disclose
Ilya Melnikov: Nothing to disclose

### RPS 2510a-11

**Comparison of standard and post-processed motion-insensitive propeller MRI sequences using a deep learning-based convolutional neural network for assessment of the shoulder joint**

*M. Kaniewska*, J. M. Getzmann, E. Deininger-Czermak, R. Guggenberger; Zurich/CH
*(malwina.kaniewska@usz.ch)*

**Purpose:** Compare image quality and diagnostic confidence of standard and post-processed motion-insensitive propeller MRI sequences using a deep learning-based convolutional neural network (DL-CNN) for assessment of the shoulder joint.
**Methods or Background:** Image quality and diagnostic confidence of standard propeller and sequences using a DL-CNN post-processing were assessed. Analysis of shoulder structures was performed. Signal-to-noise (SNR) and contrast-to-noise (CNR) ratios were calculated. Wilcoxon signed-rank test was used for comparison.

**Results or Findings:** 25 MRI of the shoulder joint (18-80y) were analysed. Mean acquisition time for standard vs DL-CNN propeller MR sequences was 16 min vs 7 min 30 s. Images acquired with standard vs DL-CNN sequences showed a perfect image quality in 20% and 80%, and a good image quality in 66% and 17% of all cases. There was overall a higher diagnostic confidence for evaluation of shoulder structures DL-CNN sequences. Pathologies were rated slightly higher for degeneration of the cartilage (n=4) and degree of subscapularis tendon injury (n=3) in standard sequences. Delineation of the subacromial bursa was possible in 16 cases only with DL-CNN. There was excellent agreement between sequences in evaluation of the AC joint. SNR and CNR was higher for DL-CNN sequences with significant difference compared to standard sequences ($p<0.05$).
**Conclusion:** Propeller MRI sequences of the shoulder joint based on a DL-CNN show a higher diagnostic performance and superior image quality compared to standard sequences resulting in higher diagnostic confidence. Due to significantly shorter scan times and higher SNR and CNR compared to standard sequences, use in clinical routine should be considered.
**Limitations:** This was a preliminary study of 25 patients comparing qualitative and quantitative imaging findings without intraoperative or arthroscopic reference standard.
**Ethics committee approval:** This prospective study received approval from the local ethical committee and informed consent from all patients.
**Funding for this study:** No funding was received for this study.
**Author Disclosures:**
Roman Guggenberger: Nothing to disclose
Eva Deininger-Czermak: Nothing to disclose
Malwina Kaniewska: Nothing to disclose
Jonas Martin Getzmann: Nothing to disclose

### RPS 2510a-12
**Whole body photon counting detector CT showing promising results in metal artifact reduction compared to DECT**
*A-S. Björkman*, H. Gauffin, S. K. Koskinen; Linköping/SE
*(annsofi.bn@gmail.com)*

**Purpose:** To evaluate if a new type of CT with photon-counting detector (PCD-CT) could improve image quality.
**Methods or Background:** Metal artifacts impair image quality in postsurgical imaging. A new type of CT with photon-counting detector may reduce these artifacts. A bovine knee with a fracture and stainless-steel plate and screws was imaged in a whole-body research PCD-CT at 120kV and 140kV and in a dual source CT (DSCT) at single energy Sn150kV (SE) and dual energy 80/Sn150kV (DE) (SOMATOM Count Plus and SOMATOM Force, Siemens Healthcare, Germany). Different sharp kernels (Br59-Br64) were used. PCD-CT images were reconstructed as 72 and 150keV and DSCT images with and without metal artifact reduction algorithm (iMAR). iMAR and tin filter was unavailable on the PCD-CT. Four image sets for each scanner were analysed. Four radiologists rated the anonymised images on a 5-point scale regarding visualisation of the fracture line, bony structure close to and further from the screws as well as metal artifact severity (maximum 5x13=65 points). Mean scores were analysed using the Friedman test and the Wilcoxon signed-rank test with Bonferroni's correction. P-values of ≤0.0001 were considered statistically significant.
**Results or Findings:** PCD-CT 140 kV 150 keV Br60 (40 points) and Br64 (38 points) images got the highest mean grades and were significantly better than SE Sn150 Br59 (33 points) but not DE -0.3 Br59 iMAR (35 points) images. PCD-CT 72keV images got the lowest grade (19 points) and were statistically worse than all the rest.
**Conclusion:** PCD-CT images reconstructed as high keV were better than or as good as, the best DSCT images even without the presumed advantage of tin filter and metal artifact reducing algorithms. PCD-CT is a promising method to reduce metal artifacts.
**Limitations:** This was an animal specimen study.
**Ethics committee approval:** Not applicable
**Funding for this study:** Funding was received via ALF grants.
**Author Disclosures:**
Håkan Gauffin: Nothing to disclose
Seppo K. Koskinen: Nothing to disclose
Ann-Sofi Björkman: Nothing to disclose

**13:00-14:30** **Room D**

### Research Presentation Session: Genitourinary

### RPS 2507
### Male pelvis and bladder

**Moderator**
A. Tsili; Ioannina/GR

### RPS 2507-2
**Strain elastography in establishing the aetiology of chordee in young hypospadiac males: a work in progress report**
*A. Prakash*, M. Bhat, A. Bhat; Jaipur/IN

**Purpose:** Hypospadias is a common congenital anomaly and presence of chordee in hypospadias necessitates modification in technique depending on the severity of chordee. A preoperative establishment of the tissue responsible for the chordee can aid the decision making and prognostication process. The purpose of our study was to evaluate the factors responsible for chordee in each individual case by assessing the stiffness of the ventral penile tissues.
**Methods or Background:** We evaluated nine cases of hypospadias using B-mode ultrasound and strain elastography. Strain elastography was used to determine the stiffness of ventral penile tissues including urethral plate, spongiosum, the tunica albuginea and the corpora cavernosa. Clinical data regarding the severity and type of deformity, and the subsequent treatment course were recorded.
**Results or Findings:** Strain elastography was able to determine the stiffness of spongiosal tissue and urethral plate. Three patients with mild chordee showed an average strain elastography value of the spongiosal tissue as 0.5. Four patients had moderate chordee and had an average spongiosal strain value of 2.775. Two patients with severe chordee and showed an average spongiosal strain value of 2.8. In addition, the corporal bodies were stiff in 4 cases (avg strain 3.8) with mild to moderate chordee. However, the cases with severe chordee showed reduced corporal stiffness (0.75). The findings of our study correlate well with clinical findings and showed a relationship between the severity of chordee and the stiffness of spongiosal tissue. An interesting finding of reduced corporal stiffness in cases of severe chordee was also seen, suggesting a causal relationship between the two.
**Conclusion:** A preoperative diagnosis of the presence, aetiology and severity of chordee aids in better clinical decision making and patient counselling.
**Limitations:** The fact that cases were limited and age-matched controls were not evaluated was identified as a limitation.
**Ethics committee approval:** Ethics committee approval was granted.
**Funding for this study:** No funding was received for this study.
**Author Disclosures:**
Mahakshit Bhat: Nothing to disclose
Aparna Prakash: Nothing to disclose
Amilal Bhat: Nothing to disclose

### RPS 2507-3
**Network analysis integrating microRNA expression profiling with MRI biomarkers and clinical data for prostate cancer early detection: a proof-of-concept study**
*M. Pecoraro*, E. Messina, M. Bicchetti, M. Pisciotti, C. Catalano, V. Panebianco; Rome/IT
*(pecoraro.martina1@gmail.com)*

**Purpose:** MRI of the prostate is the gold-standard for the detection of clinically significant prostate cancer (csPCa). Nonetheless, MRI still misses around 11% of clinically significant disease. The aim was to comprehensively integrate tissue and circulating microRNA profiling, MRI biomarkers and clinical data to implement early detection.
**Methods or Background:** In this prospective cohort study 76 biopsy-naïve patients underwent MRI and MRI-directed biopsy. A sentinel sample of 15 patients was selected for a pilot molecular analysis. Weighted gene co-expression network analysis was applied to identify microRNAs drivers of csPCa. Mi-croRNA-target gene-interaction maps were constructed, and enrichment analysis performed. The ANOVA on ranks test was used. The diagnostic power of each miRNA was evaluated in terms of the ROC probability curve analysis.
**Results or Findings:** Disease status was associated with under-expression of the miRNA profiled; a correlation was found with ADC (r= -0.51, p=0.02) and normalised ADC values (r=-0.64, p=0.002). Overexpression of miRNAs from plasma was associated with csPCa (r=0.72; p=0.02), and with PI-RADS assessment score (r=0.73; p=0.02); a linear correlation was found with biomarkers of diffusion and perfusion. Among the 800 profiled microRNA, eleven were identified as to correlate with PCa, among which hsa-miR-548a-3p, miR-138-5p and has-miR-520d-3p were confirmed using the RT-qPCR approach on an additional validation cohort of ten subjects. ROC analysis showed an accuracy >90%.
**Conclusion:** Provided a validation of the identified microRNAs on a larger cohort, we propose a diagnostic paradigm shift that sees molecular data and MRI biomarkers as pre-biopsy triage of patients at risk for PCa. This approach will allow for accurate patient allocation to biopsy, and for stratification into risk-group categories.
**Limitations:** The small sample size of this study was identified as a limitation.
**Ethics committee approval:** Institutional ethics committee approval received.
**Funding for this study:** No funding was received for this study.
**Author Disclosures:**
Emanuele Messina: Nothing to disclose
Marco Bicchetti: Nothing to disclose
Valeria Panebianco: Nothing to disclose
Martina Pecoraro: Nothing to disclose
Martina Pisciotti: Nothing to disclose
Carlo Catalano: Nothing to disclose

### RPS 2507-4

**Diagnostic performance of MRI and US in suspicion of penile fracture**
*P. Spiesecke*, J. Mang, T. Fischer, B. Hamm, M. H. Lerchbaumer; Berlin/DE

**Purpose:** The acute rupture of the tunica albuginea of the corpora cavernosa is known as penile fracture (PF). While rapid surgical therapy leads to improved functional outcome, the role of imaging prior to surgery is still under discussion. The aim of this study was to gain further knowledge concerning the role of imaging (magnetic resonance imaging [MRI] and ultrasound [US]) in the diagnostic assessment of patients with suspicion of PF.
**Methods or Background:** MRI and US examinations performed in our institution between 2000 and 2021 were compared to imaging reports with either intraoperative finding or final clinical diagnosis. Inclusion criteria were a) patient age ≥18 years, b) available information on trauma history and clinical findings on admission, and c) confirmed final urological diagnosis. Besides diagnostic accuracy, typical imaging findings such as penile haematoma, tear of the tunica albuginea including location and involvement of corpus spongiosum were reported.
**Results or Findings:** Overall, 46 out of 88 patients included (54.5%) had a confirmed diagnosis of PF, predominantely right-sided. A total of 69 MRI and 31 US examinations were included. Sensitivity and specificity were 91.9% (95%-CI: 78.7 – 97.2%) and 90.6% (95%-CI: 75.8 – 96.8%) for MRI and 71.4% (95%-CI: 95% - 45.4 - 88.3%) and 100.0% (95%-CI: 81.6 – 100.0%) for US, respectively. Overall, both modalities detected penile haematoma disproportionately often compared with the frequency of penile fractures.
**Conclusion:** The findings suggest that MRI is more suitable to confirm a PF and determine the exact localisation of a tunica albuginea tear, while US is a adequate tool for ruling out a PF.
**Limitations:** A limitation of our study is its retrospective design, although this is a rational approach when investigating a rare pathology such as PF.
**Ethics committee approval:** This study was approved by the local ethics committee.
**Funding for this study:** No funding was received for this study.
**Author Disclosures:**
Bernd Hamm: Nothing to disclose
Paul Spiesecke: Nothing to disclose
Markus Herbert Lerchbaumer: Nothing to disclose
Josef Mang: Nothing to disclose
Thomas Fischer: Nothing to disclose

### RPS 2507-5

**A multivariate correlation analysis for determining the clinical and pathology features associated with VI-RADS assessment of bladder cancer in a multicentric prospective validation study**
M. Bicchetti, M. Pecoraro, S. Lucciola, E. Messina, C. Catalano, V. Panebianco, *A. Dehghanpour*; Rome/IT
*(ad48ad@yahoo.com)*

**Purpose:** To determine Vesical Imaging-Reporting and Data System (VI-RADS) score accuracy in predicting muscle-invasive bladder cancer (BCa), in a multicentric national setting. To correlate the clinical and pathology features of bladder cancer with VI-RADS scoring.
**Methods or Background:** Patients with BCa suspicion were offered MRI before TURBT. According to VI-RADS, a cutoff of ≥3 or ≥4 was assumed to define muscle-invasive bladder cancer (MIBC). Trans-urethral resection of the tumour (TURBT) and/or cystectomy reports were compared with preoperative VI-RADS scores to assess accuracy of MRI for discriminating between non-muscle-invasive (NMIBC) vs MIBC. Sensitivity, specificity, PPV and NPV were calculated. Performance was assessed by ROC curve analysis. The univariate analysis was performed including clinical and pathology data and the overall VI-RADS categories. A multivariate logistic regression model, adjusted for age and sex was implemented to determine the clinico-pathological features that had independent effect on MIBC.
**Results or Findings:** 148 patients were enrolled (median-age [IQR] was 71 [64-77]). MRI showed sensitivity, specificity, PPV, and NPV for discriminating NMIBC from MIBC of about 89-91%, 88-90%, 75-78%, and 95-8%, respectively (according to the different cut-off). AUC was 0.93-0.95. In the multivariable logistic regression model, being assigned with a preoperative VI-RADS score of 4-5 was associated with an increased probability of muscle-invasiveness at final pathology. Other positive predictors were represented by macro-haematuria and smoking.
**Conclusion:** VI-RADS assessment scoring proved to be an accurate preoperative tool in predicting bladder cancer invasiveness, in a multicentric setting where MRI acquisition and reporting-related biases can be overcome. In addition, it proved to be an independent predictor of muscle-invasiveness, which might implicate a shift towards a more aggressive selection approach of patients' candidate to deep TURBT, to avoid delayed time to cystectomy.
**Limitations:** Small sample size.
**Ethics committee approval:** Ethics committee approval was obtained.
**Funding for this study:** No funding was received.
**Author Disclosures:**
Emanuele Messina: Nothing to disclose
Marco Bicchetti: Nothing to disclose
Valeria Panebianco: Nothing to disclose
Ailin Dehghanpour: Nothing to disclose
Martina Pecoraro: Nothing to disclose
Sara Lucciola: Nothing to disclose
Carlo Catalano: Nothing to disclose

### RPS 2507-6

**Radiomics-based machine learning for the prediction of lymph node metastases in bladder cancer**
*E. K. Gresser*[1], P. Woznicki[2], K. Messmer[1], W. Kunz[1], D. Puhr-Westerheide[1], J. Ricke[1], D. Nörenberg[3], A. Buchner[1], G. Schulz[1]; [1]Munich/DE, [2]Augsburg/DE, [3]Mannheim/DE
*(eva.gresser@med.uni-muenchen.de)*

**Purpose:** To evaluate the diagnostic potential of quantitative radiomics features extracted from preoperative CT scans to detect lymph node (LN) metastases of patients with bladder cancer and to compare its performance to radiologists' assessment.
**Methods or Background:** This retrospective analysis included 404 patients with bladder cancer who had received a preoperative contrast-enhanced CT examination of the pelvis within 3 months of radical cystectomy. Of these, 101 patients had histologically proven LN metastases and 303 were metastasis-free. Iliac, obturator, and perivesical LN were semi-automatically segmented (in total n=1918 LN) and each LN was visually assessed for the presence of metastasis. The dataset was split into training (250 patients), validation (53 patients), and test (121 patients) cohorts. We compared machine learning models trained on extracted quantitative radiomics features with end-to-end deep learning methods and visual radiologists' assessment to assess the value of radiomics for non-invasive prediction of LN metastases.
**Results or Findings:** The radiomics model trained with histopathology labels achieved a predictive AUC=0.87 for the presence of LN metastases (CI: 0.75-0.99), compared with an AUC=0.80 (CI: 0.66-0.94) for radiologists' assessment. The radiomics model numerically outperformed the deep learning model (AUC=0.70, p<0.05). At the same sensitivity threshold of 0.67, our model had a specificity of 0.95 (CI: 0.87-1.0) compared to 0.89 for visual radiologists' assessment (CI: 0.78-0.97).
**Conclusion:** Our proposed radiomics model trained with histopathology labels performed non-inferior to radiologists on the challenging task of detecting pelvic lymph node metastases of bladder cancer.
**Limitations:** There was no external validation cohort.
**Ethics committee approval:** This study was approved by an ethics committee.
**Funding for this study:** No funding was received for this study.
**Author Disclosures:**
Alexander Buchner: Nothing to disclose
Gerald Schulz: Nothing to disclose
Katharina Messmer: Nothing to disclose
Daniel Puhr-Westerheide: Nothing to disclose
Piotr Woznicki: Nothing to disclose
Wolfgang Kunz: Nothing to disclose
Dominik Nörenberg: Nothing to disclose
Eva Kristina Gresser: Nothing to disclose
Jens Ricke: Nothing to disclose

### RPS 2507-7

**Is transperineal ultrasound a reliable tool in the evaluation of postprostatectomy urinary incontinence?**

*A. Colarieti*[1], N. Shaida[2], N. Thiruchelvam[2], T. Barrett[2]; [1]Milan/IT, [2]Cambridge/UK
*(anna.colarieti@gmail.com)*

**Purpose:** Assess the feasibility of transperineal ultrasound (TPUS) pre/post robot-assisted radical prostatectomy (RARP), during pelvic floor contraction (PFC) and Valsalva (VS) manoeuvre.
**Methods or Background:** 98 patients undergoing RARP for prostate cancer were scanned with TPUS preoperatively and at four time-points postoperatively (3, 6, 9, 12 months). Images were performed using a low frequency curvilinear array abdominal transducer, with TPUS images and real-time video acquired in the dorsal recumbent and standing positions at rest, during PFC and during VS manoeuver. The feasibility of the technique was assessed and TPUS urodynamic measurements were evaluated including the bladder neck angle at rest and following PFC/VS, and the degree of bladder neck ascent/descent, which was recorded as a resultant vector (in mm).
**Results or Findings:** Preoperative measurements for each of the proposed parameters were technically feasible in more than 85% of patients. At postoperative scans the technical feasibility of assessing parameters pre- and post-PFC and VS manoeuvre was high in both the supine and standing positions (>90%). The average membranous urethral length reduced postoperatively (12 mm at 12 months), compared to preoperatively (15.2 mm). Postoperatively there was a general trend for an increase in the average angle of change during PFC (17.1°) and VS (16.2°) and a increase in the degree of bladder ascent post PFC and increasing bladder descent post-VS with increasing postoperative time. At 12 months the average bladder neck vector movement was 7.2 mm (supine) and 8.5 mm (standing) ascent during PFC, and -5.5 mm (supine) and 6.5 mm (standing) descent during VS manoeuver.
**Conclusion:** TPUS offers a reliable, accurate, noninvasive, cost-effective modality for the evaluation of postoperative continence and may be a useful adjunct for guiding pelvic floor exercises in order to preserve continence.
**Limitations:** These findings are considered preliminary.
**Ethics committee approval:** This study was not approved by an ethics committee.
**Funding for this study:** No funding was received for this study.
**Author Disclosures:**
Anna Colarieti: Nothing to disclose
Nikesh Thiruchelvam: Nothing to disclose
Nadeem Shaida: Nothing to disclose
Tristan Barrett: Nothing to disclose

### RPS 2507-8

**MRI grading: prediction of prostate cancer aggressiveness**

L. Schimmöller, M. Boschheidgen, T. Ullrich, I. Esposito, P. Albers, G. Antoch, *F. Ziayee*; Düsseldorf/DE
*(farid.ziayee@med.uni-duesseldorf.de)*

**Purpose:** To evaluate the value of multiparametric MRI (mpMRI) for the prediction of prostate cancer (PCA) aggressiveness.
**Methods or Background:** In this single centre cohort study consecutive patients with histologically confirmed PCA were retrospectively enrolled. Four different ISUP grade groups (1, 2, 3, and 4-5) were defined and fifty patients per group were included. Several qualitative and quantitative clinical (age, PSA, PSAD, percentage of PCA infiltration) and mpMRI parameters (ADC value, signal increase on high b-value images, PCA diameter, extraprostatic extension [EPE], cross-zonal growth) were evaluated and correlated within the four groups. Based on combined descriptors, MRI grading groups (mG1-mG3) were defined to predict PCA aggressiveness.
**Results or Findings:** In total 200 patients (mean age 68 years, median PSA value 8.1ng/ml) were analysed. Between the four groups, statistically significant differences could be shown for age, PSA, PSAD, and for MRI parameters cross-zonal growth, high b-value signal increase, EPE, and ADC ($p < 0.01$). All examined parameters revealed a significant correlation with the histopathologic biopsy ISUP grade groups ($p < 0.01$), except PCA diameter ($p = 0.09$). A mixed linear model demonstrated the strongest prediction of the respective ISUP grade group for the MRI grading system ($p < 0.01$) compared to single parameters.
**Conclusion:** MpMRI yields relevant pre-biopsy information about PCA aggressiveness. A combination of quantitative and qualitative parameters (MRI grading groups) provided the best prediction of the biopsy ISUP grade group and may improve clinical pathway and treatment planning. Due to the high prevalence of higher-grade PCA in patients within mG3 a timely re-biopsy seems indicated in cases of negative or post-biopsy low-grade PCA.
**Limitations:** Before using MRI grading groups, one should verify that these definitions are suitable for the individual settings. It might be interesting to prove MRI grading in a prospective study design.
**Ethics committee approval:** This study was approved by an ethics committee.
**Funding for this study:** No funding was received.
**Author Disclosures:**
Farid Ziayee: Nothing to disclose
Tim Ullrich: Nothing to disclose
Peter Albers: Nothing to disclose
Matthias Boschheidgen: Nothing to disclose
Irene Esposito: Nothing to disclose
Lars Schimmöller: Nothing to disclose
Gerald Antoch: Nothing to disclose

### RPS 2507-9

**Comparison of distinguishing capability from malignant to benign prostatic areas among CEST Imaging, DWI and combined discriminators**

T. Ueda[1], *Y. Ohno*[1], K. Yamamoto[2], M. Yui[2], M. Ikedo[2], S. Hanamatsu[1], H. Ikeda[1], K. Murayama[1], H. Toyama[1]; [1]Toyoake/JP, [2]Otawara, Tochigi/JP
*(yohno@fujita-hu.ac.jp)*

**Purpose:** To compare the capability for distinguishing malignant from benign areas among CEST imaging, DWI at standard and super high b values and combined discriminators in suspected prostatic cancer patients.
**Methods or Background:** 60 suspected prostatic cancer patients underwent T2WI, DWI at b value as 1500 (DWI1500) and 3000 (DWI3000) s/mm2 and CEST imaging at a 3T MR system and pathological examinations. According to the pathological results, 56 areas were determined as malignant, and 56 out of 664 areas were computationally selected as benign. On each CEST imaging, magnetisation transfer ratio asymmetry (MTRasym) at 3.5 ppm map was generated from z-spectra by pixel-by-pixel analyses. Then, MTRasym and ADCs from DWI1500 (ADC1500) and DWI3000 (ADC3000) in each area were determined. Student's t-test was performed to compare all quantitative indexes between two areas. Multivariate regression analyses were performed to investigate the discriminating factors. Then, diagnostic performance was compared among all methods by ROC analysis. Finally, sensitivity, specificity and accuracy were compared among all methods by McNemar's test.
**Results or Findings:** Each index had significant difference between two areas ($p<0.05$). Multivariate regression analyses identified MTRasym (Odds ratio [OR]: 1.01, $p=0.007$) and ADC3000 (OR: 0.00003, $p<0.0001$) as significant for combined quantitative discriminators. Area under the curve (AUC) and accuracy of combined quantitative predictors were significantly better than those of others ($p<0.05$).
**Conclusion:** CEST imaging is considered as valuable as both DWIs and should best be combined with DWI3000 for distinguishing between malignant and benign areas in suspected prostatic cancer patients.
**Limitations:** Not applicable
**Ethics committee approval:** This study was approved by the Institutional Review Board of Fujita Health University School of Medicine.
**Funding for this study:** This study was financially supported by Canon Medical Systems Corporation.
**Author Disclosures:**
Masato Ikedo: Employee: Canon Medical SysteCorporation
Kazuhiro Murayama: Research/Grant Support: Canon Medical SysteCorporation
Satomu Hanamatsu: Nothing to disclose
Kaori Yamamoto: Employee: Canon Medical SysteCorporation
Hirotaka Ikeda: Nothing to disclose
Hiroshi Toyama: Research/Grant Support: Canon Medical SysteCorporation
Takahiro Ueda: Nothing to disclose
Masao Yui: Employee: Canon Medical SysteCorporation
Yoshiharu Ohno: Research/Grant Support: Canon Medical SysteCorporation

13:00-14:30 Room E1

### Research Presentation Session: Neuro

### RPS 2511

### Brain tumour: glioma

**Moderator**
S. Van Cauter; Genk/BE

### RPS 2511-2

**Predictive accuracy of T2-FLAIR mismatch sign for the IDH-mutant, 1p/19q non-codeleted low grade glioma: an updated systematic review and meta-analysis**

*Y. Do*, S. Cho, B. S. Choi, S. H. H. Baik, Y. J. Bae, L. Sunwoo, C. Jung, J. H. Kim; Seongnam/KR

**Purpose:** To evaluate the diagnostic performance of T2-fluid-attenuated inversion recovery (FLAIR) mismatch sign for prediction of a patient with 1p/19q non-codeleted low grade glioma, and identify the causes responsible for the heterogeneity.
**Methods or Background:** A systematic literature search in the Ovid-MEDLINE and EMBASE databases was performed for studies reporting the relevant topic before November 17, 2020. The pooled sensitivity and specificity values were calculated. Meta-regression analyses were also performed to determine factors influencing heterogeneity.
**Results or Findings:** For all the 10 included cohorts from 8 studies, the pooled sensitivity was 40% (95% CI 28–53%), and the pooled specificity was 100% (95% CI 95–100%). In the hierarchic summary receiver operating characteristic curve, the difference between the 95% confidence and prediction regions was relatively large, indicating heterogeneity among the studies. Higgins I2 statistics demonstrated considerable heterogeneity in sensitivity (I2 = 83.5%) and specificity (I2 = 95.83%). Among the potential covariates, it seemed that none of factors was significantly associated with study heterogeneity in the joint model. However, the specificity was increased with all the factors based on the differences of the composition of the detailed tumours.
**Conclusion:** The T2-FLAIR mismatch sign is a near-perfect specific marker of IDH mutation and 1p/19q non-codeletion.
**Limitations:** First, we assumed that MRI sequence acquisition parameters could be confounders in interpreting the presence of T2-FLAIR mismatch sign; however, most studies did not present the detailed parameters. Second, the definition of T2-FLAIR mismatch sign was not identical across the studies, despite being similar. Third, all cohorts of the included studies were retrospective, resulting in a risk of selection bias.
**Ethics committee approval:** The requirement was waived because of the retrospective nature of this study.
**Funding for this study:** This study was supported by a grant from the Seoul National University Bundang Hospital Research Fund.
**Author Disclosures:**
Sung Hyun H Baik: Nothing to disclose
Cheolkyu Jung: Nothing to disclose
Byung Se Choi: Nothing to disclose
Yun Jung Bae: Nothing to disclose
Jae Hyoung Kim: Nothing to disclose
Leonard Sunwoo: Nothing to disclose
Sejin Cho: Nothing to disclose
Yoonah Do: Nothing to disclose

### RPS 2511-3

**18 F-FLT compared to 11 C-MET positron emission tomography (PET) in the differential diagnosis between recurrent intracranial tumours and treatment-induced changes**

*I. Huq*, J. Hellström, P. Witt Nyström, E. Blomquist, S. Libard, T. Danfors, J. Sorensen, J. Wikström, R. Raininko; Uppsala/SE
*(ishita.huq@surgsci.uu.se)*

**Purpose:** Irradiation and chemotherapy may cause contrast enhancing tissue changes difficult to differentiate from a recurrent tumour on MRI. PET with tumour specific markers may be helpful. This prospective study aimed to compare the newer marker 18 F-fluorothymidine (FLT) with 11 C-methionine (MET) in the discrimination between a recurrent intracranial tumour and treatment-induced changes with PET.
**Methods or Background:** 20 patients with intracranial tumours (17 high-malignant tumours) treated with surgery, radiotherapy and in 15 cases chemotherapy, presenting with a new enhancing lesion on MRI were recruited. After standard examinations, all patients underwent MET-PET and FLT-PET. The results were compared to the definite diagnosis achieved by histopathology or follow-up at 6 months. Measurements were made by two independent observers. The means of their measurements were used in the analyses. The mean standardised uptake value (SUV) of the area of maximum uptake in the suspected tumour was normalised to the mean SUV of the contralateral cortex (T/N ratio). Diagnostic efficacy was determined through receiver-operating characteristic (ROC) curve analysis.
**Results or Findings:** 11 patients had tumour recurrence and 9 patients had treatment-induced changes. 6 patients (5 tumour recurrence, one treatment-induced change) had histopathological diagnosis. Data of one MET-PET were lost. The area under the curve (AUC) was 0.85 for FLT-PET and 0.77 for MET-PET. T/N ratios 3.60 and 1.64 were chosen as cut-off values in FLT-PET and MET-PET, respectively. 17/20 lesions (85%) were correctly diagnosed with FLT-PET and 14/19 lesions (74%) with MET-PET. Sensitivity and specificity for tumour recurrence were 70% and 100% with FLT-PET and 80% and 67% with MET-PET, respectively.
**Conclusion:** Results in this small material indicate that FLT may be a useful marker in discriminating recurrent tumours from treatment-induced changes.
**Limitations:** Not applicable
**Ethics committee approval:** This study was approved by an ethics committee.
**Funding for this study:** Not applicable
**Author Disclosures:**
Torsten Danfors: Nothing to disclose
Sylwia Libard: Nothing to disclose
Ishita Huq: Nothing to disclose
Raili Raininko: Nothing to disclose
Jussi Hellström: Nothing to disclose
Petra Witt Nyström: Nothing to disclose
Johan Wikström: Nothing to disclose
Jens Sorensen: Nothing to disclose
Erik Blomquist: Nothing to disclose

### RPS 2511-4

**Comparison of intravoxel incoherent motion imaging and dynamic susceptibility contrast perfusion in differentiating intracranial tumour recurrence from treatment-induced changes**

*J. Hellström*, I. Huq, P. Witt Nyström, E. Blomquist, S. Libard, R. Raininko, J. Wikström; Uppsala/SE
*(hellstrom@gmail.com)*

**Purpose:** Intravoxel incoherent motion (IVIM) is a MRI technique by which perfusion measures can be obtained without contrast agent administration. In this prospective study, we compared IVIM with dynamic susceptibility contrast (DSC) perfusion MRI for differentiation of intracranial tumour recurrence from treatment-induced changes.
**Methods or Background:** 60 patients, previously treated with radiotherapy or radiochemotherapy for intracranial tumours (48 high-malignant tumours), with a new contrast enhancing lesion, or with increase in size of a known contrast enhancing lesion, were included. The final diagnosis was the diagnosis at 6 months after inclusion from either neuropathological examination (n=19) or clinical diagnosis based on all available clinical and radiological follow-up (n=41). The patients were examined at 1.5 T with routine MRI sequences, DSC perfusion for calculation of blood volume (rCBV) and a multiple b-value diffusion-weighted sequence for calculation of the perfusion fraction (f) using IVIM technique. Measurements of rCBV and f were made in perfusion hot spots in the area of contrast enhancement by two independent observers. Means of their measures were used in analyses. Measures of rCBV were normalised to normal appearing contralateral white matter.
**Results or Findings:** Forty-four patients had tumour recurrence and 16 had treatment-induced changes. Mean normalised rCBV was 3.52 for tumours and 1.79 for treatment-induced changes (p=0.002). Mean f was 0.090 for tumours and 0.058 for treatment-induced changes (p=0.002). ROC analysis showed area under the curve (AUC) of 0.77 (rCBV) and 0.72 (f). Cut-off values of 2.26 for rCBV and 0.073 for f yielded equal values for sensitivity (73%), specificity (75%), and accuracy (73%).
**Conclusion:** Estimation of perfusion with IVIM and DSC yields similar differential diagnostic value for differentiation between intracranial tumour recurrence and treatment-induced changes.
**Limitations:** Not applicable
**Ethics committee approval:** Regional ethics committee approval was obtained.
**Funding for this study:** Not applicable
**Author Disclosures:**
Sylwia Libard: Nothing to disclose
Ishita Huq: Nothing to disclose
Raili Raininko: Nothing to disclose
Jussi Hellström: Nothing to disclose
Petra Witt Nyström: Nothing to disclose
Johan Wikström: Nothing to disclose
Erik Blomquist: Nothing to disclose

### RPS 2511-5

**Subventricular zone involvement as an adverse prognostic factor for glioblastoma, IDH-wild type: survival model update in a brain tumour registry**

*J. Kim*, J. E. Park; Seoul/KR
*(jjeun.kim62@gmail.com)*

**Purpose:** To define subventricular zone involvement (SVI) and evaluate the prognostic value of by using a pre-existing survival nomogram in a prospective brain tumour registry.
**Methods or Background:** This retrospective study identified patients with newly diagnosed glioblastoma from a prospective brain tumour registry (NCT NCT02619890). The subventricular zone involvement (SVI) was defined as presence of contrast-enhancing lesion within 5 mm of ventricular wall, assessed by two neuroradiologists indepedently. The associations of SVI with overall survival (OS) were evaluated and a survival model was created with clinical predictors - age, sex, Karnofsky performance status, extent of resection, and MGMT promoter methylation status by using multivariable Cox proportional hazard regression analysis. Performance of the SVI-updated survival model was compared with that from the RTOG nomogram (doi:10.1093/neuonc/now208) using c-index and area under the curve (AUC) of the time-dependent receiver operating characteristic analysis.
**Results or Findings:** A total of 203 patients (median age, 60 years; 91 women) were evaluated. SVI was adversely associated with OS (hazard ratio, 1.98 [95% CI: 1.30, 3.02; P=.001]). The overall performance was superior in the SVI-updated model compared with the RTOG model (c-index, 0.706 vs 0.684). Moreover, the SVI-updated model had an added value to the RTOG nomogram from 6-month (AUC 0.763 vs 0.725), 12-month (AUC 0.830 vs 0.706), and 24-month (AUC 0.792 vs 0.781).
**Conclusion:** Subventricular zone involvement is an independent adverse prognostic factor in patients with IDH-wild type glioblastoma. The approach has advantages in terms of survival model update and guiding clinical management.
**Limitations:** This was a retrospective study.
**Ethics committee approval:** This study was approved by the institutional review board of Asan Medical Center.
**Funding for this study:** No funding was received for this study.
**Author Disclosures:**
Ji Eun Park: Nothing to disclose
Jieun Kim: Nothing to disclose

### RPS 2511-6

**Magnetic resonance imaging-based risk stratification in diffuse glioma, not otherwise specified, achieves pathologic-level prognostication**

*E. Jang*, H. S. Kim, J. E. Park; Seoul/KR
*(kabi121342@gmail.com)*

**Purpose:** To identify whether imaging-based risk stratification enables prognostication in patients with diffuse glioma, NOS (Not Otherwise Specified).
**Methods or Background:** Data in patients classified as diffuse glioma, NOS through the revised 2021 WHO classification between January 2011 and December 2020 were included. From presurgical CT and MRI, integrative analysis was performed by two neuroradiologists to assign gliomas to the three imaging-based risks considering tumour location, margin, contrast-enhancement pattern, necrosis, hemorrhage/calcification, internal cyst, and T2/FLAIR mismatch. The three risks included imaging-based oligodendroglioma, isocitrate dehydrogenase (IDH)-mutant, and 1p/19q-codeleted (low-risk); astrocytoma, IDH-mutant (intermediate-risk); and glioblastoma, IDH-wildtype (high-risk). Progression free survival (PFS) and overall survival (OS) of each risk were shown using Kaplan-Meier estimates. Performance of the imaging-based survival model with other predictors - age at diagnosis, extent of resection, adjuvant treatment, histological type and WHO grade - was compared with that of the historical molecular-based survival model (N Engl J Med 2015 DOI:10.1056/NEJMoa1402121) using the time-dependent receiver-operating-characteristic analysis.
**Results or Findings:** A total of 220 patients (median age, 46 years; inter-quartile range, 35–55 years; 130 males) were evaluated. Distinct prognostic stratification was achieved according to the three imaging-based risks (log-rank test, p<.001). The imaging-based survival model achieved high prognostic values with 1-year area under the curve (AUC) of 0.792 for PFS and 0.676 for OS, similar to the molecular-based model (AUC 0.74 for PFS and 0.87 for OS). For long-term prognostication, the imaging-based survival model showed high performance in both PFS (3-year AUC 0.826) and OS (5-year AUC 0.841).
**Conclusion:** Imaging-based risk stratification achieves molecular pathologic-level prognostic performance in diffuse glioma, NOS, which aids to guide patient referral when molecular diagnosis is not available.
**Limitations:** It was a retrospective study.
**Ethics committee approval:** This study was approved by the institutional review board of Asan Medical Center.
**Funding for this study:** No funding was received for this study.
**Author Disclosures:**
Eunbee Jang: Nothing to disclose
Ji Eun Park: Nothing to disclose
Ho Sung Kim: Nothing to disclose

### RPS 2511-7

**Multiparametric MRI for T2 FLAIR mismatch sign in IDH mutant 1p19q non-codeleted astrocytoma**

*J. P. Jen*, M. Patel, U. Pohl, S. Nagaraju, V. Sawlani; Birmingham/UK

**Purpose:** The 2021 WHO classification amalgamates histological and molecular biomarkers for the diagnosis of diffuse glioma. T2-FLAIR mismatch is a validated radiological correlate for the diagnosis of IDH mutant 1p19q non-codeleted astrocytoma. Central FLAIR suppression may be inhomogeneous, corresponding to regions of intra-tumoural heterogeneity, the true nature of which remains uncertain.
**Methods or Background:** 128 IDH mutant diffuse gliomas were reviewed (2014-2019) for T2-FLAIR mismatch sign, including multiparametric MRI, and correlated with molecular and histological data.
**Results or Findings:** 9 showed true T2-FLAIR mismatch, all astrocytomas, grade II 5/9 (55%) and grade III 4/9 (45%), median follow-up 7.1 ± 3.3 years. Multiparametric MRI was performed in 6. 3/6 showed a normal index choline/creatine ratio (Cho/Cr), the other half showed mildly raised Cho/Cr 1.37 - 1.56 on short TE single voxel spectroscopy. 2/6 showed significantly raised peak Cho/Cr 2.14 - 2.33 on short TE 2D chemical shift imaging (CSI). These abnormal areas on CSI were highly visually correlated with intra-tumoural ADC/T2 hypointensity and FLAIR hyperintensity. None showed hyperperfusion (relative cerebral blood volume, rCBV >2.0) or enhancement.
**Conclusion:** T2-FLAIR mismatch is infrequently seen in astrocytoma, but 100% specific when present. Correlating standard structural MRI with intra-tumoural choline mapping on spectroscopy best characterised regions of intra-tumoural heterogeneity, suggesting the greatest volume of cellularity/activity in specific areas of the core. These areas were targeted for biopsy, and also corresponded to areas of recurrence if not completely debulked. Structural MRI and spectroscopy are complementary to an integrated molecular and histological grading approach in the new WHO 2021 classification.
**Limitations:** Small numbers due to tumour rarity. Full molecular profile not always available, e.g. CDKN2A/B.
**Ethics committee approval:** Approval was obtained from the institution's Research Governance Office.
**Funding for this study:** No funding was received for this study.
**Author Disclosures:**
Markand Patel: Nothing to disclose
Jian Ping Jen: Nothing to disclose
Santhosh Nagaraju: Nothing to disclose
Ute Pohl: Nothing to disclose
Vijay Sawlani: Nothing to disclose

### RPS 2511-8

**Machine learning-based radiomics can distinguish between glioblastoma and metastasis**

*M. Patel*, M. Kershaw, S. Meade, H. Benghiat, P. Sanghera, V. Wykes, I. Ughratdar, V. Sawlani; Birmingham/UK
*(markandpatel@gmail.com)*

**Purpose:** Brain metastases occur in approximately 30% of patients with systemic malignancy. When presenting as a single lesion, brain metastasis can have similar conventional imaging appearances to glioblastoma. The aim of this study was to investigate the accuracy of radiomics and machine learning for distinguishing between glioblastoma and brain metastasis, on contrast-enhanced T1-weighted imaging (CE-T1WI).
**Methods or Background:** Retrospective analysis was undertaken of 106 treatment-naïve lesions (53 IDH-wildtype glioblastoma, 53 brain metastases) with a diameter greater than 10 mm. Lesions were manually segmented on volumetric CE-T1WI and after pre-processing steps, 107 quantitative shape-based, first order and second order radiomic features were extracted for each lesion using PyRadiomics. A multi-step nested data processing and machine learning pipeline was created to perform feature selection within bootstrapped cross-validated recursive feature elimination with a random forest algorithm, followed by five-fold cross-validation with a Naive Bayes classifier to validate the final model.
**Results or Findings:** The most predictive features included two shape-based features (least axis length, sphericity), and one first order feature (energy). The glioblastoma group showed significantly higher least axis length, lower sphericity, and higher energy (p<0.001). The combined final model had an AUC of 0.97, accuracy of 88.7% (81.1-94.0), sensitivity 88.7% (77.0-95.7), specificity 88.7% (77.0-95.7), positive predictive value 88.7% (78.6-94.4) and negative predictive value 88.7% (78.6-94.4).

**Conclusion:** A machine learning-based radiomics model using shape-based and first order features from a single whole tumour segmentation mask on CE-T1WI can differentiate between IDH-wildtype glioblastoma and metastasis with a moderately high accuracy.
**Limitations:** Multi-institutional data and the use of clinical features, semantic features, multiple sequences or advanced parameters and perilesional environment mask may help improve diagnostic accuracy further.
**Ethics committee approval:** Approval was obtained from the institution's Research Governance Office.
**Funding for this study:** This research did not receive any specific funding.
**Author Disclosures:**
Markand Patel: Nothing to disclose
Marie Kershaw: Nothing to disclose
Helen Benghiat: Nothing to disclose
Sara Meade: Nothing to disclose
Ismail Ughratdar: Nothing to disclose
Victoria Wykes: Nothing to disclose
Paul Sanghera: Nothing to disclose
Vijay Sawlani: Nothing to disclose

### RPS 2511-11

**The influence of pre- and postsurgery volumetric MRI on high grade gliomas**

*M. T. Fernandez Taranilla*, E. Salvador, P. Martin Medina, A. Hilario, J. Sepulveda, V. M. Pérez-García, J. Perez-Beteta, D. Molina-Garcia, A. Ramos Gonzalez; Madrid/ES
*(maiteftr@hotmail.com)*

**Purpose:** Glioblastoma is still the most common malignant brain tumour with a very short expected survival; even with early treatment. The main goal of this study is to determine if there are differences in survival depending on residual tumour volume (RTV) and preoperative tumour volumes. The secondary goals were to determine if outcomes are different for patients who underwent partial resection vs complete resection and to identify if certain tumour locations were associated with better outcomes.
**Methods or Background:** 128 primary wild-type glioblastoma patients from different institutions were retrospectively reviewed. Pre- and post-contrast 3D T1 weighted images were evaluated to determine pre- and postoperative tumor volumes. Kaplan-Meier analysis was performed on both volumes, type of surgery and tumour's location. Spearman correlations were used to investigate the association between postoperative tumour volume and survival.
**Results or Findings:** There were no significant differences in survival in patients with higher preoperative tumour volumes, however, patients with RTV< 7cc had an increased survival of 9,30 months compared with higher RTV (p<0,001). Extent of resection >51% increased survival by 10 months compared with lower percentages of resection (p<0,001). Total and subtotal resected patients increased survival 10 months compared with just biopsied (p<0,001). Basal ganglia and corpus callosum were the locations with the worst survival compared with brain lobules locations (p=0,003). Eloquent and near eloquent regions had worse survival compared with tumour in non-eloquent areas.
**Conclusion:** Patients with RTV< 7cc exhibit significant better survival independently of presurgery tumor volume. Even subtotal resection could increase survival when total resection is not possible and location of the tumour is an important prognostic factor of final outcome.
**Limitations:** The main limitation of the study is its retrospective design, which may cause a selection bias.
**Ethics committee approval:** This study was approved by an ethics committee (CEIm code 20/406).
**Funding for this study:** No funding was received for this study.
**Author Disclosures:**
Patricia Martin Medina: Nothing to disclose
Amaya Hilario: Nothing to disclose
Julian Perez-Beteta: Nothing to disclose
Ana Ramos Gonzalez: Nothing to disclose
David Molina-Garcia: Nothing to disclose
Elena Salvador: Nothing to disclose
Maria Teresa Fernandez Taranilla: Nothing to disclose
Juan Sepulveda: Nothing to disclose
Víctor M Pérez-García: Nothing to disclose

### RPS 2511-12

**Fast high-resolution metabolic imaging in glioma patients with non-cartesian compressed-sense MR spectroscopic imaging at 7 Tesla**

A. Klauser[1], B. Strasser[2], W. Bogner[2], B. Thapa[3], D. Cahill[4], J. Dietrich[4], E. Uhlmann[4], T. Batchelor[4], *O. Andronesi*[3]; [1]Geneva/CH, [2]Vienna/AT, [3]Charlestown, MA/US, [4]Boston, MA/US

**Purpose:** Magnetic resonance spectroscopic imaging (MRSI) can measure metabolic alterations in glioma for tumour typing and grading, including isocitrate dehydrogenase mutations. High-resolution MRSI can probe tumour margins for surgical resection and intratumour heterogeneity for treatment response. Standard high-resolution MRSI is prohibitively long and strategies for accelerating data acquisition are needed. In this work we developed a new MRSI method that combines fast non-cartesian k-space trajectories with compressed-sense acceleration at 7 Tesla.
**Methods or Background:** A new sequence (ECcentric Circle ENcoding TRajectorIes for Compressed-sensing - ECCENTRIC) was implemented on a 7T MRI (Terra, Siemens, Erlangen, Germany) with an excite-acquire scheme, 0.9 ms echo-time, 27° excitation flip-angle and 250ms repetition-time. The field-of-view was 220x220x105 mm3 with a 3.4x3.4x3.4 mm3 spatial resolution. The acquisition time was 9 min 20 s. MRSI data were reconstructed with a low-rank compressed-sense model and fitted with LCModel. 9 glioma patients (2 GBM, 6 anaplastic astrocytoma, 1 oligodendroglioma) were scanned.
**Results or Findings:** High-resolution metabolic maps provide structural details for tumour boundary and heterogeneity similar to anatomical imaging. In addition to typical metabolites such a N-acetyl-aspartate and choline, good quality maps were obtained for glutamine, glutamate, glycine and myo-inositol.
**Conclusion:** ECCENTRIC MRSI is a new tool for high-resolution metabolic imaging for brain tumours and other neurological diseases. MRSI has high specificity for molecular mechanisms of disease and may enable precision medicine in patients. MRSI can be repeated without limitations for safety risks, does not require radioactive tracers and is a cheaper alternative than other molecular imaging modalities.
**Limitations:** Not applicable
**Ethics committee approval:** The research protocol was approved by the institutional ethics committee and written informed consent was given by all subjects before participation.
**Funding for this study:** This research was supported by the Swiss National Science Foundation (grant number: IZSEZ0 188859), and the National Institutes of Health through National Cancer Institute grant R01-CA255479.
**Author Disclosures:**
Tracy Batchelor: Nothing to disclose
Wolfgang Bogner: Nothing to disclose
Ovidiu Andronesi: Nothing to disclose
Daniel Cahill: Nothing to disclose
Jorg Dietrich: Nothing to disclose
Bijaya Thapa: Nothing to disclose
Antoine Klauser: Nothing to disclose
Bernhard Strasser: Nothing to disclose
Erik Uhlmann: Nothing to disclose

**13:00-14:30** **Room F1**

### Research Presentation Session: Musculoskeletal

### RPS 2510b

### Spine (musculoskeletal) and peripheral nerves

**Moderator**
C. Zini; Florence/IT

### RPS 2510b-2

**Diagnostic value of water-fat separated images and CT-like images extracted from a single ultra-short echo time sequence for the evaluation of vertebral fractures and degenerative changes of the spine**

*G. C. Feuerriegel*[1], S. Kronthaler[1], Y. N. Leonhardt[1], M. Renz[1], K. Weiss[2], M. Makowski[1], D. C. Karampinos[1], B. J. Schwaiger[1], A. S. Gersing[1]; [1]Munich/DE, [2]Hamburg/DE

**Purpose:** To evaluate the performance of single echo Dixon water-fat imaging and computed tomography (CT)-like imaging based on a single-TE UTE (sUTE) MR sequence for imaging of vertebral fractures as well as degenerative bone changes of the spine in comparison to conventional CT and MR sequences.
**Methods or Background:** 30 patients with CT images of acute vertebral fractures were examined using a 3T-MRI, including a sUTE sequence as well as short-tau-inversion-recovery (STIR) and T1-weighted sequences. Dixon water-fat separation was performed by solving the smoothness-constrained inverse water-fat problem. By removing the unwanted low-frequency phase terms, additional CT-like susceptibility-weighted images were created. Two radiologists evaluated the semi-quantitative and quantitative morphological features of fractures and degenerative changes on CT and MR-images.

**Results or Findings:** Of 58 fractures detected, 24 were identified correctly as acute fractures with an oedema detected on the water-fat separated UTE images, using STIR and T1w sequences as standard of reference (κ 1.00 (95% confidence interval (CI) 1.00-1.00). For morphological assessment the overall agreement between CT-like SW-images and CT was substantial to excellent (Genant: κ 0.90 (95%CI 0.54 - 1.00); AO/Magerl: κ 0.75 (95%CI 0.43-1.00); Osteophytes: κ 0.81 (95%CI 0.51-1.00); Sclerosis: κ 0.82 (95%CI 0.60-1.00)). Overall inter-reader agreement was substantial to almost perfect (κ 0.88 (95%CI 0.73-1.00).
**Conclusion:** Detection of vertebral fractures as well as the morphological assessment of fractures and degenerative bone changes were feasible and accurate using water-fat separated images as well as CT-like SW-images, both derived from the sUTE-Dixon technique. Simultaneously extracting water-fat images and CT-like images from one single MR sequence could be highly useful for clinical examinations due to a reduction of overall examination times and radiation exposure.
**Limitations:** sUTE Echo-time was 0.14ms, which might not be achievable on older scanners.
**Ethics committee approval:** This study was approved by an ethics committee.
**Funding for this study:** Not applicable
**Author Disclosures:**
Benedikt Jakob Schwaiger: Nothing to disclose
Martin Renz: Nothing to disclose
Alexandra Sophia Gersing: Nothing to disclose
Kilian Weiss: Nothing to disclose
Georg Constantin Feuerriegel: Nothing to disclose
Marcus Makowski: Nothing to disclose
Sophia Kronthaler: Nothing to disclose
Dimitrios C. Karampinos: Nothing to disclose
Yannik Niklas Leonhardt: Nothing to disclose

### RPS 2510b-3

**Prospective deployment of a novel deep learning MRI reconstruction for accelerated spine imaging: an analysis of interchangeability, image quality and diagnostic confidence**

*H. Almansour*[1], S. Afat[1], J. Herrmann[1], D. Nickel[2], A. Othman[1]; [1]Tübingen/DE, [2]Erlangen/DE

**Purpose:** To introduce a novel deep-learning-based T1- and T2-weighted turbo-spin-echo reconstructions (TSE-DL) in spine MRI and investigate its interchangeability regarding pathology detection and its impact on image-quality and diagnostic confidence compared to standard turbo-spin-echo (TSE) sequences.
**Methods or Background:** Fifty patients with various spinal pathologies were prospectively enrolled. Each patient underwent TSE and TSE-DL sequences using a 3-fold and 4-fold acceleration. The number of signal averages was reduced in TSE-DL images to a factor of "1". Image evaluation was performed by two radiologists in a blinded and a randomised manner. An interchangeability analysis and an image quality-based analyses were performed. Interchangeability was tested using the individual-equivalence-index regarding various spinal pathologies, which were analysed on each vertebra. A decrease in interobserver agreement of ≥5% when one reader used TSE-DL compared with when both readers used the standard protocol was considered clinically significant. Interreader and intrareader agreement were assessed using Cohen's-Kappa. Image-quality criteria were: sharpness of anatomical structures (intervertebral discs, spinal canal, facet joints and neuroforamina), artifacts, noise, overall image-quality, and diagnostic confidence.
**Results or Findings:** Mean patient age was 46±18 years. Thirty-eight exams were performed at 1.5T and twelve exams at 3T. TSE-DL enabled up to 73% reduction of acquisition time. A high-degree of interchangeability between both protocols was noted (individual-equivalence-index ranged between 1-3 %). For TSE-DL, noise was rated significantly superior by both readers (p<0.01). There was no statistically significant difference between both protocols regarding all other image quality criteria.
**Conclusion:** The data-driven TSE-DL is clinically feasible and interchangeable with standard TSE for detecting various spinal pathologies. TSE-DL provided excellent image quality and diagnostic confidence with a significant reduction of examination time by ≥ 70%. The DL technique might set the stage for ultra-fast spine MRI.
**Limitations:** The singlecentre/vendor nature of the study was identified as a limitation.
**Ethics committee approval:** Ethics committee approval for this study is available.
**Funding for this study:** Not applicable
**Author Disclosures:**
Judith Herrmann: Author: Co-author of possible publication of this project. The prototype sequence was provided by Siemens Healthineers GmbH
Saif Afat: Author: Co-author of possible publication of this project. The prototype sequence was provided by Siemens Healthineers GmbH
Haidara Almansour: Author: Co-author of possible publication of this project. The prototype sequence was provided by Siemens Healthineers GmbH
Dominik Nickel: Employee: Employee at Siemens Healthineers
Ahmed Othman: Author: Co-author of possible publication of this project. The prototype sequence was provided by Siemens Healthineers GmbH

### RPS 2510b-4

**"Ghost sign" and "mushroom sign" on preoperative cervical spine MRI: new signs of ruptured disc with intraoperative correlations**

*E. K. Khil*, I. Choi, S. A. Lee; Hwaseong-si/KR
*(nizzinim@gmail.com)*

**Purpose:** To correlate preoperative MRI and intraoperative findings of herniated disc in cervical spine (C-spine) and to compare the diagnostic performance of two new MRI signs in the diagnosis of the ruptured disc.
**Methods or Background:** This retrospective study included patients who underwent C-spine MRI and surgery for disc pathology with intraoperative confirmation of ruptured disc (September 1, 2016 to January 31, 2021). Two radiologists evaluated whether the disc was extruded or ruptured during two reading sessions: session 1 without signs (criterion 1) and session 2 with additional two new MRI signs (sign 1= ghost; sign 2= mushroom) suggesting ruptured disc (criterion 2). All ruptured discs evaluated on session 2 were included in extruded discs. Using surgical findings as the reference standard, the diagnostic performance of two criteria was analysed by the validity and McNemar's test. Interobserver agreement was measured for each MRI sign.
**Results or Findings:** A total of 91 patients with 131 discs were enrolled (mean age, 56.02±12.93; range 26-88; 62 men), out of these, 62 were surgically confirmed with ruptured disc. When the MRI diagnosis was based on Criterion 1 alone, extruded disc was diagnosed with 62.9-79.0% sensitivity, 81.2-95.7% specificity, and 80.2% accuracy; for ruptured disc, 35.5-45.2% sensitivity, 94.2-97.1% specificity, 67.9-71.0% accuracy were found. However, based on criterion 2, extruded disc was diagnosed with 96.8% sensitivity, 72.5-78.3% specificity, and 84.0-87.0% accuracy; the same values were noted for ruptured disc. For ruptured disc, sensitivity and accuracy values were significantly different between criteria 1 and 2 (p<0.001). Interobserver agreements were substantial (0.74-0.79 vs 0.68-0.79 in sign 1 and 2, p<0.001, respectively).
**Conclusion:** Detection of "ghost sign" and "mushroom sign" on preoperative MRI can lead to more accurate diagnosis of clinically ruptured disc.
**Limitations:** The retrospective nature of the study was identified as a limiting factor.
**Ethics committee approval:** This study was approved by the IRB.
**Funding for this study:** No funding was received for this study.
**Author Disclosures:**
Eun Kyung Khil: Nothing to disclose
Seun Ah Lee: Nothing to disclose
Il Choi: Nothing to disclose

### RPS 2510b-5

**A novel MRI method for assessment of fatty infiltration of the paraspinal muscles in patients with lumbar spinal stenosis, reliability and association with patient reported pain and disability**

*H. B. Banitalebi*[1], K. Storheim[2], T. Å. Myklebost[3], A. Espeland[4], A. Negård[1], C. Hellum[2], E. Hermansen[3]; [1]Lorenskog/NO, [2]Oslo/NO, [3]Ålesund/NO, [4]Bergen/NO
*(hasanb@medisin.uio.no)*

**Purpose:** To evaluate the reliability of a novel MRI method for assessment of fatty infiltration of the paraspinal muscles (FIPM) and its association with the clinical symptoms of lumbar spinal stenosis (LSS).
**Methods or Background:** Axial T2-weighted routine MR images of 243 patients with symptomatic LSS were evaluated by four independent investigators. Muscle fat index (MFI) was calculated by dividing the signal intensity of the psoas muscle with that of the multifidus and erector spinae. FIPM was assessed by both MFI and Goutallier Classification System (GCS). Observer reliability was assessed by Intraclass Correlation Coefficient (ICC) for MFI and Gwet's Agreement Coefficient (AC1) for GCS. Univariate and multivariate regression analyses (adjusted for age, sex, weight, and smoking) were performed to assess the associations between FIPM, and pain and disability reported by the Oswestry Disability Index (ODI), the Zurich Claudication Questionnaire (ZCQ) and a numeric rating scale for back and leg pain.

**Results or Findings:** Inter- and intraobserver reliability was good or excellent for MFI (ICC range 0.87 to 0.99) but varied from fair to almost perfect for GCS (AC1 range 0.35 to 0.93). The results of the regression analyses suggested associations between FIPM assessed by both methods and the clinical measures. Compared to GCS, regression models including MFI suggested slightly stronger association with ODI and ZCQ.
**Conclusion:** MFI is a highly reliable quantitative method for evaluation of FIPM by using routine spine MRI, demonstrating associations with pain and disability in patients with LSS.
**Limitations:** Including patients with symptomatic LSS may lead to an overestimation of the associations between FIPM and clinical symptoms.
**Ethics committee approval:** This study was approved by the Norwegian National Ethics Committees (Reference number: 2011/2034 Central region).
**Funding for this study:** Funding was received from the Liaison Committee for Education, Research and Innovation in Central Norway and Sophies Minde Foundation in Norway.
**Author Disclosures:**
Tor Åge Myklebost: Nothing to disclose
Erland Hermansen: Nothing to disclose
Anne Negård: Nothing to disclose
Hasan Banitalebi Banitalebi: Nothing to disclose
Kjersti Storheim: Nothing to disclose
Christian Hellum: Nothing to disclose
Ansgar Espeland: Nothing to disclose

### RPS 2510b-6

**Radiomics of spinal muscles as predictor of allograft rejection in lung transplant**

*A. Modugno*, G. Negro, A. Dell'Amore, R. Stramare, F. Calabrese, F. Rea, C. Giraudo; Padua/IT
*(modugno.a@libero.it)*

**Purpose:** To assess the role of muscle composition and radiomics in predicting allograft rejection in lung transplant (LT).
**Methods or Background:** The last available chest computed tomography before surgery of patients undergoing LT in our tertiary center was retrospectively examined. One radiologist with four years of experience in chest and musculoskeletal imaging segmented the spinal muscles of each patient at the level of the 11th dorsal vertebra covering a volume of 1 cm height by an open source software (3D Slicer, www.slicer.org). The same software was applied to extract 33 radiomic features of first and second order. Factor analysis was applied to select highly correlating features; the prognostic value of the selected features for allograft rejection was investigated by logistic regression analysis. If a significant prognostic role emerged, then the diagnostic value of the model was computed by ROC curves. Muscle composition expressed in Hounsfield Unit (Hu) was collected by the same software. The applied level of significance was 0.05 for all analyses.
**Results or Findings:** Ninety-seven patients were examined (29 women; mean age 50.4±13 yrs old). Thirty-seven patients underwent LT for idiopathic pulmonary fibrosis. Overall, 21 patients showed allograft rejection. The mean Hu value of the examined spinal muscles was 36.6±8. The following features were selected by the factor analysis: cluster prominence, lmc2, gray level non-uniformity normalised, median, kurtosis, gray level non-uniformity, and inverse variance. The radiomics-based model including also Hu demonstrated that only the feature lmc2 acts as a predictor of allograft rejection (p=0.021). The model showed 76.6% accuracy and the lmc2 value of 0.19 demonstrated 81% sensitivity and 64.5% specificity in predicting LT rejection.
**Conclusion:** The radiomic feature lmc2 was demonstrated to be a predictor of allograft rejection in LT.
**Limitations:** Not applicable
**Ethics committee approval:** Not applicable
**Funding for this study:** Not applicable
**Author Disclosures:**
Andrea Dell'Amore: Nothing to disclose
Giacomo Negro: Nothing to disclose
Roberto Stramare: Nothing to disclose
Chiara Giraudo: Nothing to disclose
Fiorella Calabrese: Nothing to disclose
Antonella Modugno: Nothing to disclose
Federico Rea: Nothing to disclose

### RPS 2510b-7

**Correlation and association between lumbar spinal stenosis and paraspinal muscle morphology changes**

F. I. Rozalli, Y. T. Lim, W. Y. Chan, *F. B. Fadzli*, T. Li Kuo, K. Rahmat; Kuala Lumpur/MY
*(farhana.fadzli@yahoo.com)*

**Purpose:** To investigate the association between the lumbar paraspinal muscle fatty infiltration and muscle size with the severity of LSS. To examine the diagnostic accuracy of qualitative visual grading in evaluating paraspinal muscle fatty infiltration.
**Methods or Background:** Lumbar spinal stenosis (LSS) is a degenerative disc disease that commonly affects older adults. The relationship between morphometric changes of lumbar paraspinal muscles with LSS is scarcely studied. Seventy symptomatic patients who underwent lumbosacral magnetic resonance imaging (MRI) were enrolled. Severity of lumbar central canal stenosis (LCCS) at L3/L4 to L5/S1 levels was graded as mild (Grade 1), moderate (Grade 2) and severe (Grade 3). Paraspinal muscle morphometric parameters including visual grading of fatty infiltration, fatty infiltration percentage (FI%) and functional muscle cross-sectional area (FCSA) of erector spinae (ES) and multifidus (MF) muscles were measured at L3/L4 to L5/S1 levels using simplified 3-tier classification. Associations between the paraspinal muscle parameters and severity of LSS were examined. Diagnostic accuracy of the visual grading of fatty infiltration was determined using quantitative FI% as reference.
**Results or Findings:** There were statistically significant low positive correlations (rs < 0.3) observed between the visually graded paraspinal muscle fatty infiltration and severity of LCCS in ES at L4/L5 and MF at L4/L5 and L5/S1. Significant association was also found between the severity of LCCS and FI% at those same levels. FCSA of MF was smaller in patients with Grade 3 LCCS compared to other grades at L4/L5 and L5/S1. Qualitative visual grading of fatty infiltration was accurate on all paraspinal muscles compared with quantitative measurement.
**Conclusion:** There were significant associations between the severity of LSS and paraspinal muscle fatty infiltration as well as MF atrophy at L4/L5 and L5/S1.
**Limitations:** The small number of patients was identified as a limitation.
**Ethics committee approval:** This study was approved by an ethics committee (MEDIC No:201782-5456).
**Funding for this study:** No funding was received for this study.
**Author Disclosures:**
Yi Ting Lim: Nothing to disclose
Tan Li Kuo: Nothing to disclose
Farhana Binti Fadzli: Nothing to disclose
Kartini Rahmat: Nothing to disclose
Faizatul Izza Rozalli: Nothing to disclose
Wai Yee Chan: Nothing to disclose

### RPS 2510b-8

**Evolution and prognostic value of intervertebral disc T2-mapping values after ozone chemonucleolysis in patients with lumbar disc herniation**

*F. Bruno*, V. Pagliei, F. Sgalambro, L. Pertici, F. Arrigoni, A. Splendiani, C. Masciocchi; L'Aquila/IT
*(federico.bruno.1988@gmail.com)*

**Purpose:** To assess the validity of the MR T2-mapping sequence in estimating the modifications of the intervertebral disc (IVD) treated by O2 - O3 chemodiscolysis and its possible role in predicting clinical outcomes.
**Methods or Background:** 40 patients with Low Back Pain (LBP) (22 males, 18 females; mean age 46.15 years) were enrolled for percutaneous CT-guided O2-O3 chemodiscolysis. Thirty-one sex- and age-matched patients, served as controls and were treated by CT-guided periradicular injections. All patients were assessed clinically trough the Visual Analogue Scale (VAS) and the Oswestry Disability Index (ODI), and radiologically, by using a 3Tscanner, for evaluating the intervertebral disc area (IDA) and the T2-mapping values of the IVD before, at 1-month, and 6-months follow-up.
**Results or Findings:** The mean pre-treatment T2 relaxation time values were 38.80±4.51 ms, 44.05±0.91 ms, and 45.45±14.11 ms for the anterior annulus fibrosus (aAF), nucleus pulposus (NP), and posterior annulus fibrosus (pAF), respectively, with a significant increase at the level of the NP (p<0,05) at the 1-month follow-up. The 6-months follow-up showed a reduction with normalisation of intradiscal T2 relaxation time values showed a significant correlation of NP values with both the reduction of IDA (0.81, p<0.001) and the improvement of clinical scores (0.86, p<0.001). In the control group, despite the clinical improvement, we did not find significant IVA reduction nor significant T2 values changes after treatment.
**Conclusion:** T2-mapping may be a useful indicator to predict disc chrinkage and the clinical response to CT-guided 02 - 03 injection.
**Limitations:** The study population was limited.
**Ethics committee approval:** This study was approved by the IRB.
**Funding for this study:** No fuding was received for this study.
**Author Disclosures:**
Ferruccio Sgalambro: Nothing to disclose
Alessandra Splendiani: Nothing to disclose
Francesco Arrigoni: Nothing to disclose
Carlo Masciocchi: Nothing to disclose
Leonardo Pertici: Nothing to disclose
Federico Bruno: Nothing to disclose
Valeria Pagliei: Nothing to disclose

### RPS 2510b-9

**Appropriateness evaluation of lumbar spine magnetic resonance imaging referrals from two Irish university hospitals**

*A. H. Alanazi*, L. Rainford, A. Cradock; Dublin/IE

**Purpose:** The study aims to determine the percentage of Lumbar Spine Magnetic Resonance Imaging (LSMRI) referrals which were not indicated for scanning and compare radiology clinical decisions to iRefer adherence.
**Methods or Background:** Clinical indications were extracted from 1021 LSMRI referrals. Two review groups were involved: three MRI radiologists and three MRI radiographers. Radiologists were asked to assign the referrals' appropriateness as indicated or not indicated for imaging based on their clinical judgement. The radiographers were asked to follow iRefer guidelines to assign the appropriateness. Majority voting for each referral was applied to both review groups and agreement between reviewers in each group was measures using Kappa analysis. Logistic regression analysis was used to identify specialties associated with high rates of referring indicated referrals.
**Results or Findings:** 21.7% and 11.9% of the referrals were found not indicated for MRI for radiologists and radiographers, respectively. Not indicated referrals from general practitioners reached 17%-18% of the total referrals received from them. Agreement between radiologists was fair: Kappa =0.23, and between radiographers was also fair: Kappa = 0.26. Neurosurgery was associated with the highest rate of referring indicated referrals across both review groups: oncology referrals raised the highest number of open comments.
**Conclusion:** Findings indicate the importance of both guidelines adherence and clinical judgement to optimise practice. Involvement of MRI radiographers in referrals auditing can offer substantial support to radiology services.
**Limitations:** Selection bias may existed as the data was retrospectively collected. Very limited written comments were provided on not indicated referrals by reviewers, therefore, the ability to follow-up on reviewer decisions was limited.
**Ethics committee approval:** Ethical approval were obtained from the relevant institutional review from University College Dublin (Reference Numbers : LS-E-19-171-Alanazi-Rainford) and (LS-E-19-69-Alanazi-Rainford).
**Funding for this study:** This research did not receive any funding.
**Author Disclosures:**
Louise Rainford: Nothing to disclose
Andrea Cradock: Nothing to disclose
Ali Hasayan Alanazi: Nothing to disclose

### RPS 2510b-10

**Ultra high-resolution US of the great auricular nerve: normal anatomy and pathological findings in parotidectomised patients**

R. Picasso, F. Zaottini, *F. Pistoia*, S. Sanguinetti, M. Pansecchi, L. Tovt, C. Martinoli; Genoa/IT

**Purpose:** The aim of this study was twofold: i) to describe the normal ultrasound (US) appearance of the Great Auricular Nerve (GAN) and its divisional branches along their course across the posterior triangle of the neck and in the face region; ii) to investigate the potential of US in disclosing pathological findings affecting the GAN in patients with sensory disturbances in its territory following partial or total parotidectomy.
**Methods or Background:** The effectiveness of US in recognising the GAN and its divisional branches was firstly tested by injecting latex under US guidance in n:2 face specimens. A series of n:30 healthy volunteers was then evaluated with a 33-9MHz US probe to establish an appropriate scanning protocol for the GAN and its divisional branches. Finally, a cohort of n:18 patients with sensory disturbances in the territory of the GAN following partial or total parotidectomy was examined with US.
**Results or Findings:** In cadavers, US-guided latex injection confirmed the correct identification of the GAN and its anterior and posterior divisional branches. In 86.7% of volunteers the GAN was seen splitting in an anterior and a posterior branch along its course superficial to the sternocleidomastoid. At this level, GAN diameter averaged 1.3±0.2 mm. In 13.3% of volunteers the nerve bifurcated before emerging from the posterior margin of the muscle. In both cases, the posterior branch resulted significantly larger than the anterior (p<.001). Pathologic abnormalities of the GAN were detected and characterised in all of the patients.
**Conclusion:** Ultra high-resolution US is an effective diagnostic tool to identify the GAN and its terminal branches and allows to detect nerve pathologic changes in patients with iatrogenic damage following parotidectomy.
**Limitations:** The small number of patients examined was identified as a limitation.
**Ethics committee approval:** The present study is in accordance with the Helsinki criteria.
**Funding for this study:** Not applicable
**Author Disclosures:**
Federico Zaottini: Nothing to disclose
Carlo Martinoli: Nothing to disclose
Luca Tovt: Nothing to disclose
Michelle Pansecchi: Nothing to disclose
Sara Sanguinetti: Nothing to disclose
Riccardo Picasso: Nothing to disclose
Federico Pistoia: Nothing to disclose

### RPS 2510b-11

**Novel demonstration of peripheral nerves with high-resolution tomographic ultrasound and high-field MR-tractography – a comparison study**

*S. A. Jengojan*[1], T. Matarić[1], G. Bodner[1], P. Sorgo[2], G. Kasprian[1]; [1]Vienna/AT, [2]Krems an der Donau/AT
*(suren.jengojan@meduniwien.ac.at)*

**Purpose:** High-resolution ultrasound (HRUS), and MR-Neurography (MR-N) are the imaging gold standard for diagnosing mononeuropathies. Recently, Tomographic Ultrasound (tUS) has made it possible to transform standard ultrasound systems into a tomographic imaging device comparable to multiplanar imaging techniques such as CT and MRI. The purpose of this prospective multimodal study is to compare the volumetric results of 3D tUS to MR-N (including MR-Tractography).
**Methods or Background:** 10 healthy volunteers and 10 patients with peripheral neuropathies underwent high-resolution tUS and high-field MRN. A PIUR tUS infinity system was used to convert a sequence of 2D ultrasound images into high-resolution tomographic 3D ultrasound volume. Peripheral nerves were reconstructed using an automatised tracking algorithm. MRI was performed using a high-field 3 Tesla MR system. Deterministic tractography was used to perform the nerve tract reconstruction (b-values of 0 and 700 s/mm2, 16 diffusion encoding directions).
**Results or Findings:** The results from the MR-N (including MR-T) and the tUS provided comparable results. In cases of heavily pathologically altered nerves tUS was able to depict the structurally altered nerve in 3D, while MR-T failed to do so, due to changes in the nerve anisotropic characteristics. 3D reconstruction of small nerves (diameter >2mm) was superior using tUS.
**Conclusion:** tUS can reliably visualise normal peripheral nerves, as well as pathologies. tUS can therefore be considered a reliable technique for diagnostic assessment of peripheral nerve pathologies, and therefore could be used as a complement to MR-N in the imaging of more superficial peripheral nerves and their pathologies, due to its high-resolution ultrasound-based approach.
**Limitations:** The limitation of our study is the small number of study participants.
**Ethics committee approval:** This study was approved by the local ethics committee.
**Funding for this study:** This study was funded by the Medical University of Vienna.
**Author Disclosures:**
Gerd Bodner: Author: Co-Author
Philipp Sorgo: Author: Co-Author
Suren Armeni Jengojan: Author: First Author
Teofil Matarić: Author: Co-Author
Gregor Kasprian: Author: Co-Author

### RPS 2510b-12

**CT-like MR-derived images for the assessment of craniosynostosis and other pathologies of the paediatric skull**

*Y. N. Leonhardt*, S. Kronthaler, G. C. Feuerriegel, D. C. Karampinos, B. J. Schwaiger, M. Makowski, I. I. I. Koerte, K. Woertler, A. S. Gersing; Munich/DE
*(yannik.leonhardt@tum.de)*

**Purpose:** To evaluate the diagnostic value of CT-like images based on a 3D T1SGRE sequence for the visualisation of the paediatric skull and for the identification of pathologies such as prematurely fused cranial sutures or fractures.
**Methods or Background:** In 20 patients with suspected craniosynostosis (mean age 1.26±1.38 years, 10 females), MR imaging was performed including CT-like images and 3D reconstructions derived from a T1SGRE. Image quality and osseous pathologies were evaluated by two radiologists. The results were compared to the diagnosis derived from the other imaging modalities (radiography, CT, ultrasound) and from the intraoperative findings. Additionally, we included two more patients that presented with skull fractures (0.5 and 6.3 years, both male).
**Results or Findings:** Of the 22 patients, 8 had a metopic, 4 a coronal and 2 a sagittal craniosynostosis. Two patients showed a complex combination of craniosynostoses. The agreement between the diagnosis made based on the T1SGRE and the final diagnosis was substantial (Cohen's κ 0.89[0.67, 1.00] for

Sunday radiologist 1 and 0.78 [0.50, 1.00] for radiologist 2). Of the patients with skull fractures, one presented with a "ping pong" skull fracture and one with a fracture of the right temporal bone. Both radiologists could identify the fractures correctly.
**Conclusion:** The visualisation of the paediatric skull and the assessment of sutures and fractures using a CT-like T1SGRE MR-sequence is feasible and comparable to other imaging modalities and may help to reduce the radiation exposure in paediatric patients.
**Limitations:** There was an average acquisition time of 4 minutes 55 seconds, termination of the image acquisition due to motion artifacts or agitation of the child being a possibility. Moreover, our patient cohort size was fairly small.
**Ethics committee approval:** This study was approved by the local institutional review board (Ethics Commission of the Medical Faculty, TU Munich).
**Funding for this study:** No funding was received for this study.
**Author Disclosures:**
Benedikt Jakob Schwaiger: Nothing to disclose
Alexandra Sophia Gersing: Nothing to disclose
Georg Constantin Feuerriegel: Nothing to disclose
Marcus Makowski: Nothing to disclose
Sophia Kronthaler: Nothing to disclose
Dimitrios C. Karampinos: Nothing to disclose
Inga Inga Inga Koerte: Nothing to disclose
Yannik Niklas Leonhardt: Nothing to disclose
Klaus Woertler: Nothing to disclose

**13:00-14:30** **Room K**

### Research Presentation Session: Breast

### RPS 2502

### What is new in screening

**Moderator**
G. Ivanac; Zagreb/HR

### RPS 2502-3

**The performance of automated breast ultrasound, hand-held ultrasound, digital breast tomosynthesis and mammography in breast cancer detection: a comparative study in 2000 women**
*A. Vourtsis*, A. Kachulis; Athens/GR

**Purpose:** This study prospectively evaluated the performance of automated breast ultrasound (ABUS), hand-held breast ultrasound (HHUS), digital breast tomosynthesis (DBT) as adjunct to digital mammography in women with dense breasts.
**Methods or Background:** Under an IRB protocol that required a written consent, a total of 2000 women with heterogeneously dense or extremely dense breasts (aged 52.2±9.4 years, range: 34-88 years) were recruited. Concurrent 2D mammography in the MLO projection, DBT in MLO and CC projection, ABUS and HHUS were performed. All examinations were independently evaluated by two experienced radiologists.
**Results or Findings:** This study cohort revealed 53 cancers (36 invasive carcinomas and 17 DCIS) of which 45/53 cancers were seen on mammography (29 invasive and 16 DCIS). ABUS detected 43/53 cancers (36 invasive and 7 DCIS), of which 7 invasive carcinomas were not seen on mammography. DBT identified 47/53 cancers (32 invasive and 15 DCIS). ABUS detected 4 invasive carcinomas not seen on DBT, but 9 cases of DCIS not seen on ABUS were found on DBT; ABUS identified 1 case of DCIS that was not evident on DBT. Both ABUS and HHUS showed an excellent agreement. Overall, ABUS detected an additional 3.5 invasive cancers per 1000 women, while 35.6% (16/45) of cancers seen on mammography and 31.9% (15/47) seen on DBT were DCIS.
**Conclusion:** The outcome of this prospective study shows that ABUS detected additional invasive cancers not seen on mammography or on DBT in women with dense breasts. ABUS showed a comparable performance with HHUS in breast cancer detectability.
**Limitations:** Prospective evaluation for the assessment of interval cancer rates is needed.
**Ethics committee approval:** IRB approval number 219/08-Sep-2019
**Funding for this study:** Invenia ABUS Research Grant Proposals (“Invenia CFP”), GE, Investigator Sponsored Research Data Form Grant number 16745041186
**Author Disclosures:**
Athina Vourtsis: Grant Recipient: General Electric (GE) Consultant: General Electric (GE) Advisory Board: Volpara Solutions Other: Research collaborator with Screen Point B.V. Company and with Q.V.C.A. company
Aspasia Kachulis: Nothing to disclose

### RPS 2502-4

**Contrast-enhanced mammography in the work-up of screening recalls: first results of a prospective two-centre study**
*A. Cozzi*[1], S. Schiaffino[2], C. G. Monaco[2], L. Menicagli[2], V. Magni[1], D. Spinelli[1], M. Fanizza[3], G. Di Giulio[3], F. Sardanelli[2]; [1]Milan/IT, [2]San Donato Milanese/IT, [3]Pavia/IT
*(andrea.cozzi@gmail.com)*

**Purpose:** To evaluate if a work-up strategy based on contrast-enhanced mammography (CEM) can reduce the biopsy rate of screening recalls.
**Methods or Background:** Recalled women aged 40–80 were enrolled to undergo CEM alongside standard assessment (SA: tomosynthesis, additional views, and/or ultrasound). Exclusion criteria were breast symptoms, breast implants, allergy to iodinated contrast agents, renal failure, and pregnancy. One of five radiologists (6–30 years of experience) independently evaluated SA or CEM, recommending biopsy or 2-year follow-up. The McNemar's test was used to compare the biopsy rate of the CEM-based work-up with the SA-based biopsy rate. Sensitivity, specificity, positive and negative predictive values (PPV, NPV) with their 95% confidence intervals (CIs) were calculated for recombined CEM (rCEM, i.e., assessing only the contrast enhancement) for lesions with available biopsy report.
**Results or Findings:** Between January 2019 and July 2021, 206 women (median age 57 years, interquartile range 50–65) were enrolled at two centres, with 223 suspicious findings. Overall, 134/223 findings were referred for biopsy, 85/223 by both SA and rCEM, 44/223 by SA alone, 5/223 by rCEM alone. The 90/223 biopsy rate of rCEM (40.4%, 95% CI 34.1–46.9%) was lower (p<0.001) than the 129/223 biopsy rate of SA (57.8%, 51.3–64.1%). Considering the 127/134 biopsies with available reports (48 benign and 79 malignant lesions) rCEM showed 87.3% sensitivity (78.0–93.8%), 64.6% specificity (49.5–77.8%), 80.2% PPV (73.3–85.7%), and 75.6% NPV (62.6–85.2%), with 8/10 false negatives being ductal carcinoma in situ, all of them detectable on low-energy CEM images.
**Conclusion:** Compared to SA, the rCEM-based work-up would have avoided biopsy for 39/223 (17.5%) suspicious findings. The inclusion of low-energy images in exam interpretation may provide optimal overall CEM sensitivity.
**Limitations:** This work's nature as a non-randomised study was identified as a limitation.
**Ethics committee approval:** This study was approved by an ethics committee.
**Funding for this study:** This study was funded by GE Healthcare.
**Author Disclosures:**
Andrea Cozzi: Nothing to disclose
Francesco Sardanelli: Grant Recipient: Bayer Healthcare Speaker: Bracco Advisory Board: Bracco Grant Recipient: General Electric Healthcare Grant Recipient: Bracco Speaker: General Electric Healthcare Speaker: Bayer Healthcare
Cristian Giuseppe Monaco: Nothing to disclose
Diana Spinelli: Nothing to disclose
Simone Schiaffino: Speaker: General Electric Healthcare Other: Bracco Imaging
Marianna Fanizza: Nothing to disclose
Giuseppe Di Giulio: Nothing to disclose
Veronica Magni: Nothing to disclose
Laura Menicagli: Nothing to disclose

### RPS 2502-5

**The effects of time of reading, hours of sleep, and high reporting volumes without breaks on radiological diagnostic efficacy when detecting breast cancer**
A. S. Alshabibi, *M. E. Suleiman*, R. Heard, K. Tapia, P. Brennan; Sydney/AU

**Purpose:** The effects of time of day, previous night's sleep, and excessive workloads on vigilance and general performance are well understood across many domains, but few studies have evaluated their effects on radiologist performance when diagnosing breast cancer.
**Methods or Background:** Radiologists read test sets at conference workshops. In study 1, we analysed 197 reading assessments using five test sets with timestamps to determine whether time of day influenced specificity, lesion sensitivity, and JAFROC. Study 2 examined 133 reading assessments to determine whether sensitivity, specificity, lesion sensitivity, ROC AUC, and JAFROC were influenced by the previous night's hours of sleep. Study 3 assessed the performance of 10 radiologists on three series of 20 test cases presented without breaks to determine whether the order in which a series was read (i.e. fixed-series sequence) affected sensitivity, specificity, lesion sensitivity, or ROC AUC. We also examined interactions between the fixed-series sequence and radiologist experience (annual number of readings).
**Results or Findings:** 1) Adjusting for experience and fellowship, we found lower specificity in the late morning and late afternoon than the early morning and mid-afternoon. 2) Less experienced radiologists with ≤6 hours of sleep had lower ROC values than those with >6 hours, but experienced radiologists were unaffected. 3) Linear interactions between experience and fixed-series sequences were found for sensitivity and lesion sensitivity - experienced readers improved during the last test case series while less experienced readers deteriorated. Specificity and ROC AUC were unaffected.
**Conclusion:** Time of day, previous night's sleep, and high reporting volume without breaks can affect radiologist performance, with important implications for planning radiology schedules and fostering accurate reporting.
**Limitations:** The test sets interactions may not fully reflect clinical practice.
**Ethics committee approval:** Approval was obtained from the Human Research Ethics Committee, Project No. 2019/169.
**Funding for this study:** Not applicable
**Author Disclosures:**
Kriscia Tapia: Nothing to disclose
Patrick Brennan: Nothing to disclose
Abdulaziz Saad Alshabibi: Nothing to disclose
Robert Heard: Nothing to disclose
Mo'ayyad E Suleiman: Nothing to disclose

### RPS 2502-6

**"Earlier than Early" breast cancer detection in BRCA carriers using computerised quantitative analysis of consecutive DCE-MRI scans: an AI-based feasibility study**

*D. Anaby*, G. Zimmerman Moreno, N. Nissan, E. Friedman, M. Sklair Levy; Ramat Gan/IL

**Purpose:** Facilitate early diagnosis of breast cancer (BC) in BRCA-carriers based on computational analysis of breast MRI scans.
**Methods or Background:** BRCA1/2 mutation carriers are at a high-risk for developing BC, likely to be high-grade with an aggressive progression and an early age onset. A surveillance scheme including annual MRI (from 25 years of age) reduces advanced-stage diagnosis. In a retrospective visualisation of breast MRI scans from ~1 year prior to the diagnosis of BC in 42 BRCA-carriers, enhancing lesions were visualised in ~60% of the cases. However, cancer was revealed only ~1 year later at the next annual MRI scan. We hypothesised that quantitative computational analysis of MRI with AI techniques may enable focusing and flagging radiologically suspicious regions ~1 year before diagnosis. Breast MRI scans of the following BRCA-carrier groups at two time-points were considered: (1) 42 patients at BC diagnosis and ~1 year before (2) 40 cancer-free patients at consecutive time-points, ~1 year apart. Tumours were delineated and ~250 "curveology" and "deep radiomics" features were extracted from corresponding regions in the scans from ~1 year before. A selection of 10 features was performed using feature's AUCs (Area Under ROC Curve), followed by Jansen-Shannon divergence analysis. The predictive power was tested by an SVM model on the full- and reduced-sets of features.
**Results or Findings:** Using the 10-feature set, in 34/42 (~80%) cases, BC was successfully identified in the scans ~1 year prior to diagnosis. In the comparison group 13/40 (33%) cancer-free carriers were misclassified as positive. This limitation is subject to future work.
**Conclusion:** These preliminary results support the feasibility of "Earlier than Early" BC diagnosis in BRCA-carriers, potentially improving the detection abilities of an expert's visual inspection significantly.
**Limitations:** The study's small cohort was identified as a limiting factor.
**Ethics committee approval:** Smc 141492
**Funding for this study:** No funding was received for this study.
**Author Disclosures:**
Debbie Anaby: Nothing to disclose
Gali Zimmerman Moreno: Nothing to disclose
Eitan Friedman: Nothing to disclose
Noam Nissan: Nothing to disclose
Miri Sklair Levy: Nothing to disclose

### RPS 2502-7

**Comparison of the performance of radiologists and radiographers in the double reading of screening mammograms in a national breast screening programme**

*J. James*, E. Michalopoulou, I. Darker, Y. Chen; Nottingham/UK
*(jonathan.james@nuh.nhs.uk)*

**Purpose:** To compare the performance of radiologists and radiographer readers in a national breast screening programme.
**Methods or Background:** Workforce issues have meant that appropriately trained radiographers (technologists) are used as readers in the UK breast screening programme (NHSBSP) where double reading of mammograms is standard of care. It has been suggested that radiographers may exhibit a decrease in specificity with an increase in recall rates and reduced PPV compared to radiologists. However, previous studies took no account of the experience of readers within each professional group.
**Results or Findings:** Individual performance and experience data for 224 radiologists and 177 radiographers, participating as readers in the NHSBSP in England, were obtained for the years 2015 and 2016. Mean cancer detection rates (CDR) for radiologist and radiographer readers were 7.8 and 7.5 per 1000 women screened respectively. CDR did not vary significantly by professional group or years of experience (p=.075 and p=.871 respectively). Mean recall rates for radiologists and radiographer readers were 5.0% and 5.2% respectively. Recall rates showed no significant variation based on the readers' professional group (p=.627), but did decrease with increasing reader experience for all readers regardless of professional group (p=.001). There was no significant variation in PPV between radiologists and radiographers (p=.417) with a PPV of 17.1% and 16.1% for radiologists and radiographers respectively. There was a significant increase in PPV with increasing years of experience regardless of a reader's professional group (p=.02).
**Conclusion:** There were no significant differences in the performance of radiographers and radiologists as readers in the UK breast screening programme, supporting the approach to routinely use radiographer readers in the double reading workflow.
**Limitations:** Findings are likely only generalisable to screening programmes where double reading is the standard of care.
**Ethics committee approval:** Ethics committee approval was waived (audit of current practice).
**Funding for this study:** No funding was received for this study.
**Author Disclosures:**
Iain Darker: Nothing to disclose
Eleni Michalopoulou: Nothing to disclose
Jonathan James: Speaker: GE Healthcare
Yan Chen: Nothing to disclose

### RPS 2502-8

**The yield of breast cancer surveillance in women with PTEN Hamartoma Tumour Syndrome**

*A. Hoxhaj*[1], M. Drissen[2], J. Vos[2], N. Hoogerbrugge[2], R. M. Mann[1]; [1]Amsterdam/NL, [2]Nijmegen/NL
*(a.hoxhaj@nki.nl)*

**Purpose:** Women with PTEN Hamartoma Tumour Syndrome (PHTS) have an increased breast cancer (BC) lifetime risk (67-85%). Therefore, expert opinion-based surveillance guidelines suggest annual magnetic resonance imaging (MRI) and mammography starting from age 25 and 30, respectively. We investigated the yield of BC surveillance in PHTS women.
**Methods or Background:** This retrospective study included PHTS women who visited our expert centre. Performance measures of combined modalities, MRI alone and mammography alone were analysed, along with measures of first vs follow-up rounds. Characteristics of BCs detected during vs outside surveillance were compared.
**Results or Findings:** During a total follow-up time of 134 women-years, 156 surveillance rounds were performed among 39 women (median age at first examination: 38 years [range, 24-70]). During surveillance, 7/39 women were diagnosed with BC (cancer detection rate, 44.9 per 1000, 95% CI [19.8- 93.8]). The median age at first BC detection was 43 years (range, 31-55). The specificity of the overall programme was significantly higher in the follow-up rounds as compared to the first-round (86.5%, 95% CI [78.4, 92.0] vs 68.4%, 95% CI [51.2,82.0]). Sensitivity of mammography alone was 50.0%, while sensitivity of MRI alone was 100%. BCs detected during surveillance were more often early-stage as compared to BCs detected outside surveillance ($p<0.005$).
**Conclusion:** Our findings support offering annual BC surveillance to PHTS women from age 25 onwards, as surveillance contributes to the detection of early-stage BC. The specificity of the overall program was moderate in the first round but increased in follow-up rounds. The additional value of mammography was limited.
**Limitations:** The limited number of patients precluded the evaluation of potential variations in the surveillance yield over time. There was also possible selection bias.
**Ethics committee approval:** The study was approved by the review board of the Radboud university medical centre.
**Funding for this study:** VIDI grant ZonMw
**Author Disclosures:**
Alma Hoxhaj: Nothing to disclose
Meggie Drissen: Nothing to disclose
Janet Vos: Nothing to disclose
Ritse Maarten Mann: Nothing to disclose
Nicoline Hoogerbrugge: Nothing to disclose

### RPS 2502-9

**Mammographic breast compression practice: vendor, screening and diagnostic mammography differences**

*A. Brameier*[1], M. G. van Lier[2], L. Kerschke[1], B. Hurtienne[1], J. de Groot[2], W. Heindel[1]; [1]Münster/DE, [2]Enschede/NL

**Purpose:** There is currently no consensus on how much mammographic breast compression should be applied for optimal results. Detailed studies on compression practice in Germany are not available. We aimed to evaluate mammographic compression practice differences between screening and diagnostic examinations and between mammographic systems of different vendors within the screening programme in Germany.

**Methods or Background:** Mammographic compression parameters were retrospectively (October 2018 – September 2019) obtained for craniocaudal and mediolateral oblique views from three sites: site-1 (screening, Hologic 3Dimensions), site-2 (screening, Siemens Healthineers Mammomat Revelation) and site-3 (clinic, Hologic Selenia Dimensions). Data were analysed using (generalised) linear mixed models.
**Results or Findings:** In total 58,098 mammographic views were included for analysis (site-1: n=29,634 views, site-2: n=24,534 views, site-3: 3,930 views). The compression force (marginal mean ± standard error) (site-1: 109.52±0.23 N, site-3: 84.92±0.64N, p<0.001), pressure (site-1: 11.99±0.05 kPa, site-3: 10.99±0.14 kPa, p<0.001) and dose (site-1: 1.75±0.01 mGy, site-3: 1.69±0.02 mGy, p=0.005) were higher in screening site-1 as compared to diagnostic site-3. The breast thickness (site-1: 62.40±0.20 mm, site-3: 63.34±0.46 mm, p=0.058) was lower for screening examinations. Vendor comparison revealed a large average dose difference (site-1: 1.75±0.01 mGy, site-2: 0.87±0.01 mGy, p<0.001). Against expectations, in site-1, with the highest average dose, the average force (site-1: 109.54±0.20 N, site-2: 99.13±0.21 N, p<0.001) and pressure (site-1: 11.97±0.05 kPa, site-2: 10.49±0.06 kPa, p<0.001) were also notably higher than in site-2. The breast thickness was lower in site-2 (site-1: 62.28±0.19 mm, site-2: 59.47±0.22 mm, p<0.001).
**Conclusion:** Mammographic compression parameters differ between diagnostic and screening examinations as well as between vendors within the screening program, which may impact image comparability and patient experience.
**Limitations:** No correlation with image quality was performed. Despite similar technician training between sites, mammographic procedure execution may differ.
**Ethics committee approval:** This study was approved by the local ethics committee.
**Funding for this study:** This study was funded by the INTERREG V-A-project “InMediValue” (122207).
**Author Disclosures:**
Monique G.J.T.B. van Lier: Employee: Holland Innovative Employee: Sigmascreening
Brigitte Hurtienne: Nothing to disclose
J.E. de Groot: Employee: Holland Innovative Employee: Sigmascreening
Walter Heindel: Nothing to disclose
Anika Brameier: Nothing to disclose
Laura Kerschke: Nothing to disclose

### RPS 2502-10

### A pilot analysis to determine impact of actual time between screens on interval cancer rates in the English Breast Screening Programme

*M. G. Wallis*[1], A. Murphy[2], K. Mellor[2], M. Press[2], D. Vulkan[3], S. W. Duffy[3], J. Walton[2], O. Kearins[2]; [1]Cambridge/UK, [2]Birmingham/UK, [3]London/UK

**Purpose:** Automated links between cancer registry and screening services allocate a screening history to all English breast cancers. Data for women screened between 2010 and 2016 show that the 24 to <36month interval cancer rate is paradoxically only slightly higher than that of 12 to <24months. When plotted by month, the rate plateaus between 24 and 28 months and falls after 33 months. It has been presumed that this is a function of actual time between screen and re-screen/invitation. In preparation for more detailed analysis, we piloted a correction to address this hypothesis.
**Methods or Background:** Interval cancer data for women screened from 2008 to 2009 were obtained from the National Cancer Registration Analysis Service. Months since last invitation for the women invited for screening in January through March of 2011, 2012 and 2013 were obtained from breast screening repository data and used to correct the population denominator to recalculate interval cancer rates.
**Results or Findings:** 5.5% of women were invited within 24 months of their previous invitation, 13.0% by 30 months and 97.4% by 36 months. Crude interval cancer rates were 0.56 per 1000 women screened at 0-<12 months, 1.20 for 12-<24 months and 1.37 for 24-<36 months. After correcting the denominator, the rates were 0.56, 1.24 and 1.73 per 1000 respectively and no plateau was seen.
**Conclusion:** Correcting for actual time between screens has a measurable impact on interval cancer rates. This needs to be accounted for in comparisons of interval cancer rates. It could also be an important confounder in all screening trials.
**Limitations:** In this pilot, the screening year was not the same as the years used to derive the correction.
**Ethics committee approval:** Not applicable
**Funding for this study:** Not applicable
**Author Disclosures:**
Matthew G. Wallis: Nothing to disclose
Stephen W Duffy: Nothing to disclose
Daniel Vulkan: Nothing to disclose
Alison Murphy: Nothing to disclose
Jackie Walton: Nothing to disclose
Mike Press: Nothing to disclose
Olive Kearins: Nothing to disclose
Kim Mellor: Nothing to disclose

### RPS 2502-11

### Breast cancer screening: tomosynthesis plus synthesised mammograms versus digital mammography: first results of the TOSYMA RCT

W. Heindel, S. Weigel, L. Kerschke, S. Baier, A. Sommer, J. Czwoydzinski, H. Lenzen, *H-W. Hense*, J. Gerß; Münster/DE

**Purpose:** To assess whether digital breast tomosynthesis plus synthesised 2D mammograms (DBT+s2D) increases screening efficacy as compared to standard 2D full-field digital mammography (2D-FFDM).
**Methods or Background:** The prospective randomised controlled TOSYMA study (ClinicalTrials.gov identifier: NCT03377036) was designed as a diagnostic superiority trial comparing DBT+s2D and 2D-FFDM in the quality-controlled, population-based, biennual German mammography screening program. Seventeen screening units recruited women of the eligible age group, 50-69 years, attending the routine mammography screening program. Women were assigned by 1:1 randomisation to either the intervention (DBT+s2D) or the control arm (2D-FFDM). Based on a multivendor approach, DBT systems of 5 medical device manufacturers were used. An adaptive design with one interim analysis was applied to compare two hierarchically ordered primary endpoints between the study arms: 1) the detection rate of invasive breast cancers, and 2) the 24-months cumulative incidence of invasive interval cancers in screen-negative women.
**Results or Findings:** Between July 2018 and December 2020, a total of 99,689 women were recruited. Study results regarding the difference in screen-detected invasive cancer detection rates between DBT+s2D and 2D-FFDM and further secondary outcomes will be presented.
**Conclusion:** First study results will provide evidence regarding the expected increase in invasive cancer detection by DBT. This increase, however, needs to be balanced against the potential for overdiagnosis.
**Limitations:** For this purpose, follow-up data on interval cancers will be evaluated in an additional funding period of the study until 2025.
**Ethics committee approval:** The study protocol has been reviewed and approved by the medical ethical committees (2016-132-f-S, Ärztekammer Westfalen-Lippe und der Westfälischen Wilhelms-Universität).
**Funding for this study:** This work is supported by the Deutsche Forschungsgemeinschaft (DFG - German Research Council - Project No. HE 1646/5-1).
**Author Disclosures:**
Hans-Werner Hense: Nothing to disclose
Horst Lenzen: Nothing to disclose
Stefanie Weigel: Nothing to disclose
Joachim Gerß: Nothing to disclose
Jörg Czwoydzinski: Nothing to disclose
Sonja Baier: Nothing to disclose
Walter Heindel: Nothing to disclose
Alexander Sommer: Nothing to disclose
Laura Kerschke: Nothing to disclose

### RPS 2502-12

### MammoWave - a novel microwave screening system for early breast cancer detection: implications in future breast screening

*D. Álvarez Sánchez-Bayuela*[1], C. Romero Castellano[1], L. M. Cruz Hernandez[1], P. M. Aguilar Angulo[1], R. Giovanetti González[1], M. D. P. Sánchez Camacho González Carrato[1], G. Tiberi[2]; [1]Toledo/ES, [2]London/UK

**Purpose:** The present work aims to analyse the current breast screening protocol of a reference hospital and identify its potential improvements by the introduction of a novel microwave-based imaging system (MammoWave) for early breast cancer detection.
**Methods or Background:** A biennial mammography-based (tomosynthesis) breast screening programme in asymptomatic women aged 45 to 69 years is carried out in our centre, based on a double-reading by two independent radiologists, who agree a final BI-RADS assessment if initial consensus is not achieved. MammoWave was proposed as complementary test to a set of volunteers, whose output about the microwave exam has been collected by an on-site questionnaire. The Net Promoter Score (NPS) was asked to evaluate overall participants' satisfaction with the microwave exam.

**Results or Findings:** Currently, frequent mammographic false positives (mostly on dense breasts) require a re-call for the patient to ask for unnecessary additional exams. MammoWave could be a promising tool for overcoming this key limitation, due to the overall good system's performance and the results of the on-site questionnaires, which show an excellent acceptance by 155 trial participants, with an average NPS of 9.50 (out of 10) and with all women (100%) reassured about this novel technology and willing to recommend the exam.
**Conclusion:** This microwave system shows an acceptance with an excellent average NPS. Its introduction could avoid key issues that tomosynthesis cannot solve currently: re-calls and patient psychological stress, additional unnecessary mammography exams that lead to x-ray overdose and, consequently, higher costs for health systems.
**Limitations:** Not applicable
**Ethics committee approval:** This study was approved by the ethics committee of the Toledo Hospital Complex, Spain on 7/10/2019. Approval was also received from the Spanish Agency for Medicines and Health Products N. 760/19/EC on 28/02/2020. The study is registered at Clinicaltrials.gov/ct2/show/NCT04253366.
**Funding for this study:** Funding was received through the EU Horizon 2020 programme (Grants No: 830265, 872752).
**Author Disclosures:**
Lina Marcela Cruz Hernandez: Nothing to disclose
Daniel Álvarez Sánchez-Bayuela: Investigator: Virgen De La Salud Hospital (Toledo) Employee: Umbria Bioengineering Technologies
Paul Martin Aguilar Angulo: Nothing to disclose
Cristina Romero Castellano: Nothing to disclose
Gianluigi Tiberi: Founder: Umbria Bioengineering Technologies
María Del Pilar Sánchez Camacho González Carrato: Nothing to disclose
Rubén Giovanetti González: Nothing to disclose

**13:00-14:30** **Room N**

### Research Presentation Session: Chest

### RPS 2504

### Fibrotic lung diseases

**Moderator**
N. Sverzellati; Parma/IT
**Author Disclosure:**
N. Sverzellati: Advisory Board: Boehringer Ingelheim; Consultant: Coreline; Research Grant/Support: Chiesi; Speaker: Boehringer Ingelheim

### RPS 2504-2

**Artificial Intelligence-based detection of asbestosis in CT-scans**
*K. Groot Lipman*[1], D. de Gooijer[1], T. N. Boellaard[1], F. van der Heijden[2], R. G. H. Beets-Tan[1], Z. Bodalal[1], S. Trebeschi[1], S. Burgers[1]; [1]Amsterdam/NL, [2]Enschede/NL
*(k.groot.lipman@nki.nl)*

**Purpose:** The diagnosis of asbestosis lacks diagnostic criteria and generally depends on the multidisciplinary team conference, a discussion that often illustrates high interobserver variability. In the Netherlands, patients are eligible for financial compensation when a panel of three independent pulmonologists diagnoses asbestosis, which increases the time to diagnosis. We aimed to develop a rapid, automatic, non-invasive method supporting the diagnosis of asbestosis using primarily CT imaging with addition of pulmonary function tests (PFT).
**Methods or Background:** We designed an Artificial Intelligence (AI) system to diagnose asbestosis based on CT scans of Dutch patients with high occupational exposure to asbestos. The ground-truth for the algorithm consisted of the verdict of three independent expert pulmonologists. If there was no unanimity, the uncertainty was reflected in the ground-truth for the AI model. The regions of interest to the AI model were visualised through saliency maps. We compared the AI to three PFT metrics: vital capacity (VC), forced vital capacity (FVC), and diffusing capacity for carbon monoxide (DLCO).
**Results or Findings:** We trained the AI on CT scans in the training set (n=415). On the independent test set (n=88), the AI system yielded an AUC of 0.87 (p<0.001). Combining the AI and the highest predictive PFT (DLCO, AUC=0.85, p<0.001), the prediction yielded 0.95 AUC (p<0.001), 84% accuracy, 77% sensitivity, and 91% specificity, 91% PPV, and 78% NPV.
**Conclusion:** Our AI system to classify asbestosis solely based on CT scans reached excellent performance. Adding the DLCO to the AI classification score yielded the highest diagnostic performance. Prospective validation of this score is ongoing.
**Limitations:** Ground truth consisted of non-invasive law-driven classification of asbestosis instead of biopsy.
**Ethics committee approval:** This study was approved by the institutional board.
**Funding for this study:** No funding was received for this study.
**Author Disclosures:**
Zuhir Bodalal: Nothing to disclose
Sjaak Burgers: Nothing to disclose
Stefano Trebeschi: Nothing to disclose
Thierry N. Boellaard: Nothing to disclose
Kevin Groot Lipman: Nothing to disclose
Regina G. H. Beets-Tan: Nothing to disclose
Dianne de Gooijer: Nothing to disclose
Ferdi van der Heijden: Nothing to disclose

### RPS 2504-3

**MRI ultra short echo-time sequence in systemic sclerosis-interstitial lung disease assessment and correlations with pulmonary function tests: comparison with CT**
*N. Landini*[1], P. Ciet[2], M. Orlandi[1], T. Benkert[3], C. Nardi[4], S. Colagrande[4], M. Matucci-Cerinic[4], G. Morana[1]; [1]Treviso/IT, [2]Rotterdam/NL, [3]Erlangen/DE, [4]Florence/IT

**Purpose:** Interstitial Lung Disease (ILD) is a major complication of Systemic Sclerosis (SSc). Ultra Short Echo-Time Magnetic Resonance Imaging (UTE-MRI) sequences were developed for lung parenchyma. We compared a UTE-MRI sequence with CT in ILD assessment and Pulmonary Function Tests (PFT) correlations.
**Methods or Background:** 27 SSc patients with ascertained or suspected ILD underwent UTE-MRI and CT. Two thoracic radiologists evaluated in consensus the extents of ILD, Ground Glass Opacities (GGO), Reticulations, Honeycombing and Consolidation. The extents were computed as the mean percentage of lung involvement evaluated at five levels, to the nearest 5%. ILDs were divided in limited (<20%) or extensive (>20%), according to the literature. The ANOVA test was adopted to compare CT and MRI extents. The Cohen's k was exploited for the agreement in distinguishing between limited and extensive disease. The Pearson correlation coefficient r was adopted for alterations extents correlations with PFT.
**Results or Findings:** Mean ILD, GGO, reticulations extents on UTE-MRI were 14.6%, 10.4%, 2.3%, while on CT they were 17.0%, 8.5%, 8.2%. Honeycombing and Consolidation extents were <1% and considered no further. ILD and GGO extents on UTE-MRI and CT were similar (p>0.5), contrarily to reticulations (p<0.04). UTE-MRI and CT were concordant in identifying limited and extensive disease in 24 patients, k=0.70. CT ILD, GGO and reticulation extents correlations with Forced Vital Capacity (FVC) were r=-0.65 (p<0.0003), -0.59 (p<0.002) and -0.49 (p<0.01), with Diffusion Lung Carbon Monoxide (Dlco) were r=-0.70 (p<0.0001), -0.58 (p<0.002) and -0.66 (p<0.0002). UTE-MRI ILD, GGO and reticulation extents correlations with FVC were r=-0.65 (p<0.0003), -0.61 (p<0.002), -0.23 (p>0.5), while with DLco they were r=-0.68 (p<0.0001), -0.59 (p<0.002), -0.48 (p<0.02).
**Conclusion:** UTE MRI identified limited and extensive SSc-ILD similarly to CT. ILD and GGO extents assessment, as well their correlations with PFT, are also comparable.
**Limitations:** No limitations were identified.
**Ethics committee approval:** This study was approved by an ethics committee.
**Funding for this study:** No funding was received for this study.
**Author Disclosures:**
Nicholas Landini: Nothing to disclose
Giovanni Morana: Nothing to disclose
Pierluigi Ciet: Nothing to disclose
Martina Orlandi: Nothing to disclose
Cosimo Nardi: Nothing to disclose
Stefano Colagrande: Nothing to disclose
Thomas Benkert: Nothing to disclose
Marco Matucci-Cerinic: Nothing to disclose

### RPS 2504-4

**Lung CT-based elastic registration technique in longitudinal evaluation of idiopathic pulmonary fibrosis**
*H. Sun*; Changchun/CN
*(dr_hssun@163.com)*

**Purpose:** To assess the value of the elastic registration method in evaluating the development of lung fibrosis in patients with idiopathic pulmonary fibrosis (IPF), which is based on the detection of lung contraction by elastic registration of chest high-resolution CT images.
**Methods or Background:** Patients with IPF who had undergone at least two supine high-resolution CT plain scans with pulmonary function tests (PFTs) within 3 months between January 2015 and May 2021 were retrospectively studied. Elastic registration was performed on baseline and follow-up CT images to obtain deformation maps of the whole lung. Jacobian determinants were calculated from the deformation fields and represented on colour maps to describe morphological and functional deterioration. Correlations of Jacobian values with PFT and pulmonary vascular changes were assessed by Spearman correlation method.
**Results or Findings:** A total of 69 patients were included in the research. Jacobian maps demonstrated constriction of the lung parenchyma marked at the posterior lung base in patients with IPF who deteriorated on visual and functional assessment. The Jacobian logarithm was significantly reduced in patients with deterioration compared to patients with stable disease. Jacobian values were positively correlated with changes in forced vital capacity ($p<0.001$), diffusing capacity for carbon monoxide ($p<0.001$), pulmonary vascular volume ($p<0.001$) and the number of pulmonary vascular branches ($p<0.001$).
**Conclusion:** Elastic registration based on CT scans is helpful to assess the morphological and functional deterioration of lung lesions in patients with IPF and correlates with changes in pulmonary vascular volume and the number of vascular branches.
**Limitations:** This was a retrospective study with an uneven range of time intervals between baseline and follow-up CT scans.
**Ethics committee approval:** This study was approved by the China-Japan Friendship Hospital ethics committee.
**Funding for this study:** Funding was received from the Chinese Academy of Medical Sciences as well as from the Science and Technology Innovation in Medicine and Health Project (Major Collaborative Innovation Project 2018-I2M-1-0001).
**Author Disclosures:**
Haishuang Sun: Nothing to disclose

### RPS 2504-5

### HRCT chest reporting in patients with suspected idiopathic pulmonary fibrosis (IPF) as per ATS / ERS guidelines

*U. S. Umer*, A. Hamid, S. Alam, S. Yasmin, N. Gul, A. Sharif, M. Asif, M. M. Abdullah, Z. Sohail; Peshawar/PK
*(ummara_81@hotmail.com)*

**Purpose:** To evaluate reporting of HRCT chest in patients with suspected idiopathic pulmonary fibrosis (IPF) in accordance with ATS/ERS/JRS/ALAT guidelines.
**Methods or Background:** Diagnosis of IPF requires exclusion of known causes of interstitial lung disease (ILD) and usual interstitial pneumonia (UIP) pattern on HRCT. Guidelines for the diagnosis of IPF published by the American Thoracic Society/European Respiratory Society/Japanese Respiratory Society/Latin American Thoracic Association (ATS/ERS/JRS/ALAT) in September 2018 provided new criteria for UIP patterns on HRCT; four patterns, "UIP pattern", "probable UIP", "indeterminate for UIP" and "alternative diagnosis". In the appropriate clinical context, presence of typical or probable UIP on HRCT is sufficient for a diagnosis of IPF. 100% of thoracic HRCT reports in patients with suspected IPF/ ILD should be categorised into one of four categories as per ATS/ERS guidelines. This audit was done by retrospectively assessing HRCT reports in the Radiology Department of Rehman Medical Institute Peshawar over a period of one year from January 2019 to December 2019 in suspected IPF/ ILD. A total of 60 patients' reports from the hospital's database were assessed.
**Results or Findings:** Out of 60 patient reports 42 (72%) of the HRCT reports had been reported according to ATS/ESR guidelines. Among these 42 reports (76%) were labelled as definite UIP/probable UIP pattern and 10 (24%) were of alternative diagnoses, with definite diagnosis or differentials. 32 (53.3%) of our patients were males and 28 (46.6%) were females, at a mean age of 60 years.
**Conclusion:** We concluded from our results that only 72% reports of HRCT chest were in accordance with ATS/ERS/JRS/ALAT guidelines, which did not meet the required target of 100%. Radiologists' training is planned and a re-audit would be done after 1 year.
**Limitations:** A larger group study should be done.
**Ethics committee approval:** This study was approved by an ethics committee.
**Funding for this study:** No funding was received for this study.
**Author Disclosures:**
Muhammad Muhammad Abdullah: Nothing to disclose
Muhammad Asif: Nothing to disclose
Aliya Sharif: Nothing to disclose
Zamara Sohail: Nothing to disclose
Ayesha Hamid: Nothing to disclose
Saira Yasmin: Nothing to disclose
Nida Gul: Nothing to disclose
Ummara Siddique Umer: Nothing to disclose
Shahjehan Alam: Nothing to disclose

### RPS 2504-6

### Pulmonary ultrashort TE MR imaging: comparison with high-resolution CT for the assessment of idiopathic pulmonary fibrosis

*X. Yang*, M. Liu, H. Dai, C. Wang; Beijing/CN
*(yxy18161659153@126.com)*

**Purpose:** To determine the feasibility of pulmonary MR imaging with ultrashort echo time (UTE-MRI) for lung assessment using high-resolution computed tomography (HRCT) as the reference standard, for idiopathic pulmonary fibrosis (IPF).
**Methods or Background:** Lung MR imaging was performed with a 1.5T system as UTE-MRI acquisitions. Two radiologists independently evaluated HRCT and MR images for various morphologic abnormalities of IPF. The image quality difference between HRCT and UTE-MRI was compared with a Wilcoxon-rank sum test. Kappa and weighted kappa analysis were used to measure intra- and interobserver and intermethod agreements. Sensitivity and specificity were used to assess the performance of UTE-MRI for detecting pulmonary fibrosis.
**Results or Findings:** A total of 35 patients with IPF who underwent UTE-MRI and HRCT were included in this study. The image quality of HRCT was higher than UTE-MRI(HRCT vs UTE-MRI: $4.9\pm0.3$ vs $4.1\pm 0.7$, $p<0.0001$). Moreover, interobserver agreements of HRCT and UTE-MRI for evaluating the image signs of pulmonary fibrosis were determined as substantial and excellent (HRCT: $0.727\leq k\leq 1$, $P<0.0001$; UTE-MRI: $0.719\leq k\leq 0.824$, $p<0.0001$). In addition, reticular (97.1%, k=0.654), honeycombing (83.3%, k=0.625), traction bronchiectasis (94.1%, k=0.640) were also well visualised on UTE-MRI. The sensitivity of UTE-MRI in the identification of pulmonary fibrosis (n=35) was 97.2%, when compared with HRCT.
**Conclusion:** UTE-MRI is inferior to HRCT in imaging the details of lung parenchymal. However, our study does show that there is a potential role for the UTE sequence as an alternative radiation-free imaging modality in patients with IPF.
**Limitations:** The sample size was small.
**Ethics committee approval:** Institutional review board approval was obtained for prospective imaging analysis. All study participants provided written informed consent.
**Funding for this study:** No funding was received for this study.
**Author Disclosures:**
Min Liu: Nothing to disclose
Chen Wang: Nothing to disclose
Huaping Dai: Nothing to disclose
Xiaoyan Yang: Nothing to disclose

### RPS 2504-7

### Prognostic implication of baseline HRCT findings on pulmonary function impairment in the course of fibrotic interstitial lung disease

*K. Akbari*, D. Lang, B. Lamprecht, F. Fellner; Linz/AT
*(kaveh.akbari@kepleruniklinikum.at)*

**Purpose:** To evaluate the association of high-resolution computed tomography (HRCT) features with the change of pulmonary function test (PFT) biomarkers after 6, 12 and 24 months in fibrotic interstitial lung disease patients.
**Methods or Background:** HRCT findings of 106 consecutive ILD-board patients were semi-quantitatively evaluated in a standardised approach: six distinct lung regions were scored for nodules, reticulation, honeycombing, traction bronchiectasis, consolidations, ground glass opacities and emphysema. Forced vital capacity (FVC), and diffusion capacity for carbon monoxide (DLCO) were assessed at the time of the imaging and after 6, 12 and 24 months. Association of the baseline HRCT scores and the change of the PFT biomarkers were evaluated using Spearman correlation coefficients and graphical presentation.
**Results or Findings:** The extent of nodules, ground glass opacities and consolidations on the initial imaging correlate with PFT improvement; reticulation, traction bronchiectasis and honeycombing with worsening of PFT biomarkers.
**Conclusion:** The extent of typical features of fibrosis like reticulation, traction bronchiectasis and honeycombing in initial HRCT imaging predict a progressive fibrosing phenotype, while inflammatory findings like ground glass opacities and consolidations correlate with PFT improvement and may suggest a response to anti-inflammatory therapies.
**Limitations:** The main limitations that need to be addressed are the retrospective and explorative approach and the limited sample size.
**Ethics committee approval:** This evaluation was based on data from the ILD-registry of Kepler University Hospital Linz, as approved by the ethics comittee of the Federal State of Upper-Austria (EK Number. 1-26-17).
**Funding for this study:** No external funding was used.
**Author Disclosures:**
Kaveh Akbari: Nothing to disclose
David Lang: Nothing to disclose
Franz Fellner: Nothing to disclose
Bernd Lamprecht: Nothing to disclose

### RPS 2504-8

**Potential of photon-counting detector CT for radiation dose reduction in the assessment of interstitial lung disease in patients with scleroderma compared to energy-integrated detector CT**

*L. Jungblut*, A. Landsmann, V. Englmaier, V. Mergen, A. Euler, H. Alkadhi, T. Frauenfelder, K. Martini; Zurich/CH
*(lisa.jungblut@usz.ch)*

**Purpose:** To evaluate the potential of photon-counting detector computed tomography (PCD-CT) in radiation dose reduction compared to conventional energy-integrated detector CT (EID-CT) based on image quality and assessment of interstitial lung disease in scleroderma patients (Scc-ILD).
**Methods or Background:** Forty-five patients with scleroderma who underwent a non-contrast chest CT on a first-generation, clinical dual-source PCD-CT and a non-contrast split-dose EID-CT scan within one year were retrospectively included. The split-dose images on EID-CT were generated with two tubes at a fixed tube voltage at 100 kV and adjusted amperage to generate 66% and 33% dose scans. The 100% dose scan was generated out of the two split-dose images. PCD-CT scans were performed in the high-resolution (HR) mode at 120 kV. Images were assessed qualitatively by independent readers (overall image quality, noise, fibrosis assessment) on a five-point Likert scale. Quantitative assessment was done by noise measurements in subcutaneous fat, defined as the standard deviation of attenuation.
**Results or Findings:** The PCD-CT and the 33% dose EID-CT scans resulted in similar dose values (0.73 vs. 0.77 mGy*cm, p=0.177) while there was significantly less dose in PCD-CT than in 66% dose EID-CT (0.73 vs. 1.54 mGy*cm, p<0.001). Quantitatively, PCD-CT was comparable to 100% dose EID-CT scan (130.64 vs. 128.61 HU, p=0.734) and performed significantly superior (p<0.001) to the 60% dose scan (p<0.001). Diagnostic performance for the detection of Scc-ILD of PCD-CT was comparable to the 100% dose scan and superior to the 66% and 33% dose scan (p<0.001).
**Conclusion:** PCD-CT allows for 66% dose reduction in the assessment of Scc-ILD while maintaining image quality and diagnostic performance compared to EID-CT.
**Limitations:** The singlecentredness of the study was identified as a limitation.
**Ethics committee approval:** This study was approved by an ethics committee.
**Funding for this study:** No funding was received for this study.
**Author Disclosures:**
Thomas Frauenfelder: Nothing to disclose
Victor Mergen: Nothing to disclose
Anna Landsmann: Nothing to disclose
Katharina Martini: Nothing to disclose
Lisa Jungblut: Nothing to disclose
Vanessa Englmaier: Nothing to disclose
Andre Euler: Nothing to disclose
Hatem Alkadhi: Nothing to disclose

### RPS 2504-11

**Assessment of interstitial lung disease in systemic sclerosis using semiquantitative and quantitative HRCT analysis**

*F. Tiralongo*, S. Palmucci, F. Galioto, L. Reali, L. A. Mauro, A. Vancheri, G. Sambataro, C. Vancheri, A. Basile; Catania/IT
*(tiralongofrancesco91@hotmail.it)*

**Purpose:** To investigate relationship between HRCT indexes and Pulmonary Function Tests (PFTs), and between HRCT indexes and semi-quantitative analysis in Systemic Sclerosis (SSc) ILD patients.
**Methods or Background:** 22 SSc patients having at least one HRCT and PFTs (acquired nearest to the CT) were retrospectively investigated. Patients were classified as having a diffused (dcSSc) or limited (lcSSc) form of the disease according to their skin involvement, and divided in early stage or intermediate/late stage. A semi-quantitative analysis was obtained applying Warrick and Wells scores to the various HRCT alterations. Histogram-based analysis was performed providing HRCT indexes – kurtosis, skewness, High Attenuation Areas (HAA%) and mean lung density (MLD). Strength of association between HRCT indexes and PFTs values, semiquantitative scores and HRCT indexes were investigated using Pearson correlation. Stages of diseases were analysed using a U-test. ANOVA variance was performed to investigate the fluctuation of mean values of HRCT indexes, semiquantitative scores and PFTs.
**Results or Findings:** Moderate correlations were found between kurtosis and PFTs, and between skewness and PFTs. Also HAA% and PFTs showed moderate correlation. We found inverse strong correlation between MLD and DLCO, and a moderate correlation between MLD and FVC. Comparing early stage versus longstanding group – the U-test do not show statistical differences for HRCT indexes. Moderate correlation was found between Warrick scale and HRCT indexes. A good correlation was observed between MLD and HAA% and global extent of disease assessed by Wells score; weak correlation was found for kurtosis and skewness. ANOVA analysis, including mean values for FVC, HRCT indexes and Wells score, reported an F value of 1712.71.
**Conclusion:** In line with the literature, our results provide evidence that quantitative indexes could be a useful tool in disease severity evaluation.
**Limitations:** Not applicable
**Ethics committee approval:** Not applicable
**Funding for this study:** Not applicable
**Author Disclosures:**
Federica Galioto: Nothing to disclose
Letizia Antonella Mauro: Nothing to disclose
Antonio Basile: Nothing to disclose
Gianluca Sambataro: Nothing to disclose
Linda Reali: Nothing to disclose
Ada Vancheri: Nothing to disclose
Stefano Palmucci: Nothing to disclose
Francesco Tiralongo: Nothing to disclose
Carlo Vancheri: Nothing to disclose

### RPS 2504-12

**HRCT correlation with clinical and cryobiopsy findings in cases of interstitial lung diseases (ILD)**

*B. R. Chaudhari*[1], S. Nandikoor[1], A. Mathur[1], J. Shah[2]; [1]Bangalore/IN, [2]Mumbai/IN
*(bhush1991@gmail.com)*

**Purpose:** This study aimed to analyse and categorise interstitial lung diseases on radiological patterns on HRCT, and to correlate radiological patterns of ILDs with clinical symptoms and with histopathological patterns on cryobiopsy.
**Methods or Background:** In a prospective observational study from November 2018 to May 2020, 50 patients who had signs and symptoms of ILD who later underwent HRCT and transbronchial cryobiopsy for further diagnosis were enrolled. The clinical symptoms and HRCT findings were noted. The HRCT findings were then classified into different patterns. Lobar involvement and mediastinal lymphadenopathy were also noted and a radiological diagnosis was given. The patient later underwent transbronchial cryobiopsy. Histopathological diagnosis was considered as gold standard and the radiological diagnosis was correlated to it and the clinical symptoms and the diagnostic accuracy of HRCT was calculated.
**Results or Findings:** On HRCT pattern and histopathological correlation, it was found that 34 patients were correctly diagnosed on HRCT giving a positive predictive value of 68% (p<0.001). When analysing each subtype of ILD, it was found that the diagnostic accuracy of HRCT in diagnosing UIP was 78%, HSP was 90%, NSIP was 80% and HSP-NSIP was 90%.
**Conclusion:** A multidisciplinary approach is the key to diagnosing interstitial lung diseases. In spite of a small study at a single centre, HRCT did show adequate accuracy in diagnosing certain types of ILD. The trend seen in this study can be a stepping stone for initiating larger studies where a larger sample size is given and more types of ILD are studied and that can help us understand the radiological characteristics further and can guide the radiologist to narrow down a particular diagnosis.
**Limitations:** The small sample size, this work's nature as a single center study and the fact that the present study is limited to transbronchial cryobiopsies and lacks consideration of its failure rate were identified as limitations.
**Ethics committee approval:** This study was approved by an ethics committee.
**Funding for this study:** No funding was received for this study.
**Author Disclosures:**
Abhinav Mathur: Nothing to disclose
Jessicka Shah: Nothing to disclose
Shrivalli Nandikoor: Nothing to disclose
Bhushan Rajendra Chaudhari: Nothing to disclose

**13:00-14:30** **Room O**

### Research Presentation Session: Cardiac

### RPS 2503

### Procedural planning and prognosis of CT prior to TAVI/TAVR

**Moderator**
R. Salgado; Antwerp/BE

### RPS 2503-2

**More than annulus sizing: risk prediction by comprehensive quantitative analysis of preprocedural CTA provides superior prediction of mortality compared to conventional risk scores in TAVR**

*T. S. Emrich*, G. Aquino, J. A. Decker, V. Brandt, U. J. Schoepf, A. Varga-Szemes; Charleston, SC/US
*(emrich@musc.edu)*

**Purpose:** To assess the predictive value of a comprehensive, preprocedural cardiac CT-based risk score for mortality in transcatheter aortic valve replacement (TAVR) patients.
**Methods or Background:** TAVR patients (n=168; 78.5±8.8 years, 52.4% male) were retrospectively evaluated in this 24-months follow up study. Mortality was the primary endpoint. Pre-TAVR CTAs were analysed to calculate left and right ventricular long axis strain, left atrial ejection fraction, tricuspid annular plane systolic excursion, pulmonary artery- and tricuspid annular diameter. A binary score was created for each parameter and summed up into a comprehensive CT score ranging between 0 and 6. Groups were defined as follows: score 0 (Gr 0), 1-3 (Gr 1) and >3 (Gr 2). Survival analysis was done by Kaplan-Meier analysis and Cox-regression. Harrell's c-index was used to evaluate risk models.
**Results or Findings:** There were 38 deaths (22%) over the 24-month follow-up. Mortality was significantly different among the CT risk score groups: survival was 100% in Gr 0, 81% in Gr 1 and 44% in Gr 2 (Logrank p<0.001 for both). CT risk score (HR 4.6; 95% CI 2.2-9.4; p<0.001) was independently associated with mortality after multivariate adjustments for clinical parameters. When adjusted for the Society of Thoracic Surgeons (STS) risk score, CT Score (HR 4.3;95% CI 2.2-8.4; p<0.001) remained significantly predictive. Adding CT score to the STS score significantly improved its c-index from 0.637 to 0.765.
**Conclusion:** The presented pre-TAVR CT score independently predicts post-TAVR mortality and is superior to clinical parameters only. CTA-based functional assessment improves risk prediction and has the potential to influence post-treatment surveillance in TAVR patients.
**Limitations:** The nature of this work as a singlecentre, retrospective study was identified as a limitation.
**Ethics committee approval:** The study was approved by the local IRB committee (Pro00092078).
**Funding for this study:** Not applicable
**Author Disclosures:**
Verena Brandt: Nothing to disclose
Uwe Joseph Schoepf: Research/Grant Support: Bayer, Guerbet, Elucid, Siemens Healthcare, Bracco, HeartFlow
Tilman Stephan Emrich: Speaker: Siemens Healthineers
Josua A. Decker: Nothing to disclose
Akos Varga-Szemes: Research/Grant Support: Elucid, Siemens Healthcare
Gilberto Aquino: Nothing to disclose

### RPS 2503-4

**Combined Coronary CT-Angiography (cCTA) and TAVI planning: value of CT-FFR in cCTAs without morphological signs of obstructive coronary artery disease**

*R. F. Gohmann*, P. Seitz, K. Pawelka, L. Heiser, C. D. Krieghoff, C. Lücke, S. Gottschling, M. Abdel-Wahab, M. Gutberlet; Leipzig/DE

**Purpose:** To analyse the ability of machine-learning (ML)-based CT-derived fractional flow reserve (CT-FFR) to correctly classify coronary CT-angiography (cCTA) studies without morphological signs of obstructive coronary artery disease (CAD) acquired during pre-TAVI evaluation.
**Methods or Background:** Background: CAD is a frequent comorbidity in patients undergoing TAVI. Current guidelines recommend its assessment before TAVI. If significant CAD can be excluded on cCTA, invasive coronary angiography (ICA) may be avoided. Patients prior to TAVI frequently have a high load of plaque and a high calcium score, making the exclusion of significant stenoses difficult, particularly for non-expert readers. It has been proposed that CT-FFR may guide reader confidence in such cCTAs.
Methods: 116 patients without significant stenosis (≥50% diameter) on cCTA as part of pre-TAVI CT were included. These patients were examined with an electrocardiogram-gated CT scan of the heart and high-pitch scan of the vascular access route. All patients were re-evaluated with ML-based CT-FFR (threshold=0.80) blinded to cCTA. ICA served as the standard of reference.
**Results or Findings:** ML-based CT-FFR was successfully performed in 94.0% (109/116) of all patients, including 436 vessels. With CT-FFR 76 patients and 126 vessels were falsely reclassified as having significant CAD, respectively. With CT-FFR 2 patients and no vessels were correctly reclassified as having significant CAD, thus the reclassification on patient level was only coincidental.
**Conclusion:** ML-based CT-FFR could potentially be used as a guide to the less experienced cCTA reader. However, unselectively applied, CT-FFR may vastly increase the number of false positive ratings compared to morphological scoring in patients before TAVI.
**Limitations:** The retrospective nature of the study was identified as a limitation.
**Ethics committee approval:** Ethics committee approval was passed.
**Funding for this study:** No funding was received for this study.
**Author Disclosures:**
Sebastian Gottschling: Nothing to disclose
Christian Lücke: Nothing to disclose
Mohamed Abdel-Wahab: Nothing to disclose
Matthias Gutberlet: Nothing to disclose
Patrick Seitz: Nothing to disclose
Konrad Pawelka: Nothing to disclose
Linda Heiser: Nothing to disclose
Christian Dominik Krieghoff: Nothing to disclose
Robin F. Gohmann: Nothing to disclose

### RPS 2503-5

**Preliminary results on the comparison of resting full-cycle ratio and CT fractional flow reserve in patients with severe aortic valve stenosis**

*M. C. Langenbach*, K. Klein, I. L. Langenbach, D. Maintz, A. Bunck, H. Wienemann; Cologne/DE
*(marcel.langenbach@me.com)*

**Purpose:** The value of CT-derived fractional flow reserve (CT-FFR) or resting full-circle ration (RFR) in patients with severe aortic valve stenosis (AS) undergoing transcatheter aortic valve replacement (TAVR) is unknown. Therefore, the purpose of the study was to evaluate the diagnostic performance of CT-FFR derived from TAVR-planning CT compared to invasive RFR in patients with relevant AS and intermediate coronary stenosis.
**Methods or Background:** Preliminary results on patients with relevant AS undergoing ICA with pressure wire assessment and routine contrast-enhanced pre-TAVR CTA without an additional coronary CTA. CT-FFR analysis was performed using on-site CT-FFR software (Siemens Healthineers, Inc) by two radiologists experienced in cardiac imaging.
**Results or Findings:** 23 patients were investigated using RFR and CT-FFR. The mean age of the subjects was 81.0 ±5.6 years. Of these patients, 9 (39.1%) were female. No complications were observed during CTA or pressure wire assessment. The RFR showed a significant correlation with CT-FFR (Pearson correlation coefficient, R =0.72, p<0.001). The optimal CT-FFR cut-off value for RFR ≤0.89 prediction was 0.765. Per-patient receiver operator curve using RFR ≤ 0.89 as the reference standard analysis showed a larger area under the curve (AUC) for CT-FFR 0.81 (95% CI 0.62–0.99, p=0.013) compared with that for CTA >50% 0.56 (95% CI 0.32–0.80) and CTA >70% 0.72 (95% CI 0.38–0.85).
**Conclusion:** Preliminary results indicate non-invasive CT-FFR assessed by routine pre-TAVR CTA as a feasible method superior to qualitative analysis of the coronary arteries in CT and ICA in assessing the hemodynamic relevance of coronary lesions in patients with relevant AS. This might help to reduce the number of unnecessary pre-TAVR ICA in the presence of intermediate coronary stenosis.
**Limitations:** The preliminary nature of the results, the pilot character of the study, as well as its small cohort and retrospective nature were identified as limitations.
**Ethics committee approval:** This study was approved by an ethics committee.
**Funding for this study:** No funding was received for this study.
**Author Disclosures:**
Isabel Luisa Langenbach: Nothing to disclose
David Maintz: Nothing to disclose
Konstantin Klein: Nothing to disclose
Marcel Christian Langenbach: Nothing to disclose
Alexander Bunck: Nothing to disclose
Hendrik Wienemann: Nothing to disclose

### RPS 2503-6

**Impact of ML-based coronary computed tomography angiography-derived fractional flow reserve on decision-making in patients with severe aortic stenosis undergoing transcatheter aortic valve replacement**

*V. Brandt*[1], G. Aquino[1], U. J. Schoepf[1], R. Bekeredjian[2], A. Varga-Szemes[1], T. S. Emrich[1], R. R. Bayer[1], C. Tesche[3], J. A. Decker[4]; [1]Charleston, SC/US, [2]Stuttgart/DE, [3]Munich/DE, [4]Augsburg/DE

**Purpose:** To evaluate feasibility and diagnostic performance of CCTA-derived fractional flow reserve (CT-FFR) for detection of haemodynamically significant coronary artery disease (CAD) in patients with severe aortic stenosis (AS) undergoing transcatheter aortic valve replacement (TAVR) to potentially avoid additional pre-TAVR invasive coronary angiography (ICA).
**Methods or Background:** Patients with severe AS (n=95, 78.6±8.8 years, 53% female) undergoing preprocedural TAVR-CT followed by ICA were included and retrospectively analysed. CCTA datasets were evaluated using CAD-RADS classification. CT-FFR measurements were computed using an on-site machine-learning algorithm. A combined algorithm was developed for decision-making to determine if ICA is needed based on pre-TAVR CCTA: (1) all patients with CAD-RADS ≥4 are referred for ICA; (2) patients with CAD-RADS 2 and 3 are evaluated utilising CT-FFR and sent to ICA if CT-FFR ≤0.80; (3) patients with CAD-RADS <2 or CAD-RADS <4 and normal CT-FFR are not referred for ICA.
**Results or Findings:** In 12 patients (13%), significant CAD was diagnosed in ICA and treated with PCI. Twenty-eight patients (30%) showed CT-FFR ≤0.80 and 24 (86%) of those were reported to have a maximum stenosis ≥50% during ICA. Using the proposed algorithm, haemodynamically significant CAD could be identified with a sensitivity, specificity, and positive and negative predictive value of 100%, 78%, 40% and 100%, respectively, potentially decreasing the number of necessary ICAs by 65 (68%).
**Conclusion:** Combination of CT-FFR and CAD-RADS is able to identify haemodynamically significant CAD pre-TAVR and bears potential to significantly reduce the number of needed ICAs.
**Limitations:** The retrospective, singlecentre nature of the study was identified as a limitation; in addition, invasive FFR was not performed in all coronary lesions as this decision is at the discretion of the cardiologist. Thus, angiographic stenosis assessment on ICA served as the reference standard.
**Ethics committee approval:** This study was approved by an ethics committee (IRB number #Pro00092078).
**Funding for this study:** No funding was received for this study.
**Author Disclosures:**
Verena Brandt: Nothing to disclose
Christian Tesche: Speaker: Siemens Healthineers and Heartflow Inc.
Uwe Joseph Schoepf: Consultant: Bayer, Bracco, Elucid BioImaging, Guerbet, HeartFlow Inc., Keya Medical, and Siemens Healthineers Speaker: Bayer, Bracco, Elucid BioImaging, Guerbet, HeartFlow Inc., Keya Medical, and Siemens Healthineers
Raffi Bekeredjian: Nothing to disclose
Tilman Stephan Emrich: Speaker: Siemens Healthineers
Josua A. Decker: Nothing to disclose
Richard Robert Bayer: Research/Grant Support: Bayer, Siemens, and HeartFlow Inc.
Akos Varga-Szemes: Research/Grant Support: Siemens Healthineers Consultant: Bayer and Elucid Bioimaging
Gilberto Aquino: Nothing to disclose

### RPS 2503-7

**Diagnostic accuracy of CCTA for the evaluation of obstructive CAD in patients referred for TAVI: a systematic review and meta-analysis**

A. N. A. Serafini, *G. A. Strazzarino*, D. Tore, M. Gatti, R. Faletti; Turin/IT

**Purpose:** To evaluate the diagnostic accuracy of coronary computed tomography angiography (CCTA) for the evaluation of obstructive coronary artery disease (CAD) in patients referred for transcatheter aortic valve implantation (TAVI).
**Methods or Background:** EMBASE, PubMed/MEDLINE and CENTRAL were searched for studies reporting accuracy of CCTA for the evaluation of obstructive CAD compared to invasive coronary angiography (ICA). The QUADAS-2 tool was used to assess the risk of bias. A bivariate random-effects model was used to analyse, pool and plot the diagnostic performance measurements across studies. Pooled sensitivity, specificity, positive (+LR) and negative (-LR) likelihood ratio and diagnostic odds ratio (DOR) and hierarchical summary ROC curve (HSROC) were evaluated. Prospero id: CRD42021252527.
**Results or Findings:** Fourteen studies (2533 patients) were included. In the intention-to-diagnose patient-level analysis sensitivity and specificity for CCTA were 97% (94–98%) and 68% (56–68%) respectively, and +LR and -LR were 3.0 (2.1–4.3) and 0.05 (0.03–0.09), with DOR 60 (30–121). The HSROC had AUC = 0.96 (0.94–0.98). No difference in sensitivity was found when comparing single-heartbeat scanner CTs to others [96% (91–98%) vs 97% (94–99%); p=0.37], whereas the specificity was higher [81% (67–90%) vs 58% (43–71%); p<0.0001].
**Conclusion:** CCTA proved to have an excellent diagnostic accuracy for assessing obstructive CAD in patients referred for TAVI. Routine CCTA in the pre-TAVI work-up could save more than 40% of ICAs. The use of single-heartbeat scanners can further improve such data.
**Limitations:** A relatively low number of studies met the selection criteria. Only a fraction of the studies reported the analyses at patient level, either by considering non-analysable segments as positive or by excluding them. Almost all studies included are retrospective cohorts, only one is prospective and none a randomised controlled study. In addition, many of the included studies are small in size.
**Ethics committee approval:** Not applicable
**Funding for this study:** Not applicable
**Author Disclosures:**
Giulio Antonino Strazzarino: Nothing to disclose
Marco Gatti: Nothing to disclose
Riccardo Faletti: Nothing to disclose
Alessandro Niccolò Annibale Serafini: Author: Not to dissclosure
Davide Tore: Nothing to disclose

### RPS 2503-8

**Combined coronary CT-angiography and TAVI planning for ruling out significant coronary artery disease: added value of machine-learning-based CT-FFR**

*R. F. Gohmann*, K. Pawelka, P. Seitz, L. Heiser, C. D. Krieghoff, C. Lücke, S. Gottschling, M. Abdel-Wahab, M. Gutberlet; Leipzig/DE

**Purpose:** To analyse the ability of machine-learning (ML)-based CT-derived fractional flow reserve (CT-FFR) to further improve the diagnostic performance of coronary CT-angiography (cCTA) for ruling out significant CAD during pre-TAVI evaluation.
**Methods or Background:** Background: CAD is a frequent comorbidity in patients undergoing TAVI. If significant CAD can be excluded on cCTA, invasive coronary angiography (ICA) may be avoided. Although cCTA is a very sensitive test, it is limited by relatively low specificity and positive predictive value, particularly in high-risk patients.
Methods: 460 patients (79.6±7.4 years) undergoing pre-TAVI CT were included and examined with an electrocardiogram-gated CT scan of the heart and high-pitch scan of the vascular access route. Images were evaluated for significant CAD. Patients routinely underwent ICA (388/460), which was omitted at the discretion of the local Heart Team if CAD could be effectively ruled out on cCTA (72/460). CT examinations in which CAD could not be ruled out (CAD+) (n=272) underwent additional ML-based CT-FFR.
**Results or Findings:** ML-based CT-FFR was successfully performed in 79.4% (216/272) of all CAD+ patients and correctly reclassified 17 patients as CAD negative. CT-FFR was not feasible in 20.6% because of reduced image quality (37/56) or anatomic variants (19/56). Sensitivity, specificity, positive predictive value, and negative predictive value were 94.9%, 52.0%, 52.2%, and 94.9%, respectively. The additional evaluation with ML-based CT-FFR increased accuracy by Δ+3.4% (CAD+: Δ+6.0%) and raised the total number of examinations negative for CAD to 43.9% (202/460).
**Conclusion:** ML-based CT-FFR may further improve the diagnostic performance of cCTA by correctly reclassifying a considerable proportion of TAVI-patients with morphological signs of obstructive CAD on cCTA. Thereby, CT-FFR has the potential to further reduce the need for ICA in this challenging elderly group of patients before TAVI.
**Limitations:** The retrospective nature of the study was identified as a limitation.
**Ethics committee approval:** Ethics committee approval was passed.
**Funding for this study:** No funding was received for this study.
**Author Disclosures:**
Sebastian Gottschling: Nothing to disclose
Christian Lücke: Nothing to disclose
Mohamed Abdel-Wahab: Nothing to disclose
Matthias Gutberlet: Nothing to disclose
Patrick Seitz: Nothing to disclose
Konrad Pawelka: Nothing to disclose
Linda Heiser: Nothing to disclose
Christian Dominik Krieghoff: Nothing to disclose
Robin F. Gohmann: Nothing to disclose

### RPS 2503-9

**Multiparametric CT-characterisation of myocardial tissue remodelling in patients with severe aortic stenosis candidate to transcatheter aortic valve implantation**

*C. Gnasso*[1], A. Sorrentino[1], A. Palmisano[1], D. Vignale[1], F. Spiritigliozzi[1], D. Margonato[2], E. Agricola[1], F. De Cobelli[1], A. Esposito[1]; [1]Milan/IT, [2]Monza/IT
*(gnasso.chiara@hsr.it)*

**Purpose:** Case-control study aimed to investigate structural adaptations of the myocardium on CT examination in Low Flow Low Gradient (LFLG) and High Gradient (HG) aortic stenosis (AS) patients candidate for transcatheter aortic valve implantation (TAVI).

Sunday

**Methods or Background:** Eighty-eight consecutive patients candidate to TAVI, 19 (22%) LFLG e 69 (78%) HG according to echocardiographic data, were enrolled. A multiparametric TAVI-planning CT protocol was implemented including a time-resolved volumetric reconstruction of aortic valve (AV) and cardiac chambers and 5-minutes late contrast enhancement scan with left ventricle (LV) extracellular volume (ECV) map reconstruction. Quantitative CT-derived variables were: AV area, AV calcium score (AVCS), AV regurgitant volume (AVRV), LV myocardial mass and ECV, ventricles volumes (end systole, ESV; and end diastole, EDV) and ejection fraction (EF). Measurements were generated by semi-automatic post-processing on dedicated softwares.
**Results or Findings:** Mean gradient in LFLG group was 30 mmHg [IQR, 22.5 – 35.5] vs 46 mmHg [IQR, 41 – 53] in HG group (p<0.0001). At CT-scan, mean ESV on overall population was 70.8 ml (±54,9), EDV 161 ml (±55), mean AVRV was 16.5 ml (±20,9), mean LV mass 162.5 g (±48,5) and AVCS was 2738 (±1994). There was a significant difference in ECV (35% vs 28%, p=0.001), LV-EF (45% vs 63%, p=0.037), AVA (1 cm2 vs 0.90 cm2; p=0.036) and global longitudinal strain (-12% vs -16%, p<0.05) between LFLG and HG groups. ECV had a significant negative correlation with LV-EF (R=-0,253 p=0.017), mean transvalvular gradient (R=-0,415; p<0.001) and AV calcium score (R=-0.226; p=0.035).
**Conclusion:** Improved CT-scan protocol may provide additional information about myocardial remodelling and tissue characterisation in patients with different AS functional stages.
**Limitations:** Further studies are needed to assess the prognostic value of this approach.
**Ethics committee approval:** This study was approved by an ethics committee and consent was signed.
**Funding for this study:** No funding was received for this study.
**Author Disclosures:**
Davide Margonato: Nothing to disclose
Davide Vignale: Nothing to disclose
Alberto Sorrentino: Nothing to disclose
Antonio Esposito: Nothing to disclose
Anna Palmisano: Nothing to disclose
Francesco De Cobelli: Nothing to disclose
Francesco Spiritigliozzi: Nothing to disclose
Eustachio Agricola: Nothing to disclose
Chiara Gnasso: Nothing to disclose

### RPS 2503-10

### Preoperative computed tomography evaluation of SuPRarEnal Aortic burDen predicts postprocedural acute kidney injury after transcatheter aortic valve replacement: the SPREAD-AKI study

*U. Viglino*, V. De Marzo, J. Matos, M. Pigati, M. Vercellino, G. Crimi, M. Balbi, I. Porto, S. Seitun; Genoa/IT

**Purpose:** Determine the impact of suprarenal aortic atheroma burden (AB) on AKI, and the potential role of preoperative multislice computed tomography (PO-MSCT) in evaluating the suprarenal aortic atherosclerosis and preoperative risk of AKI.
**Methods or Background:** Acute kidney injury (AKI) is a potential complication of transcatheter aortic valve replacement (TAVR). Athero-embolisation linked to catheter manipulation in the supra-renal aorta is a possible pathogenetic mechanism of AKI after TAVR. We collected PO-MSCT, baseline, procedural, and post-procedural characteristics of 222 consecutive patients who underwent TAVR at a single, high-volume, Italian centre. PO-MSCT was performed using a dedicated TAVR protocol with an ECG-triggered high-pitch spiral acquisition using a second-generation dual-source CT system. We recorded aortic valve calcium score (AV-CS), aortic plaque bidimensional measurements, total renal volume (TRV), presence of significant (≥50%) renal artery stenosis (RAS), suprarenal AB (quantified using a "plaque analysis" software). Calcific plaque was subcategorised on a voxel-level basis into 3 strata: low-(351-700 HU), mid-(701-1000 HU), and high-calcium (>1000 HU, termed 1K plaque). Multivariate logistic regression analysis adjusted for other univariate predictors (male sex, baseline eGFR, baseline ejection-fraction, baseline mean aortic gradient, and RAS) was done.
**Results or Findings:** Patients who developed AKI had higher suprarenal AB (17.6±5.1% vs. 13.9±4.3%, p<0.001), TRV indexed for BSA, mid-calcium plaque, 1K plaque and more postprocedural major/life-threatening bleedings. 3-knots spline curve analysis identified percent of suprarenal AB > 15.0% as the optimal threshold to predict an increased risk of AKI.
**Conclusion:** Suprarenal AB is associated with AKI, and this association is strengthened as the percentage of calcified plaque increases. Quantitative and qualitative preoperative MSCT assessment of aortic atherosclerosis may help in early identification of patients at high risk for AKI who could benefit from higher perioperative surveillance.
**Limitations:** No limitations were identified.
**Ethics committee approval:** This study was approved by an ethics committee approval.
**Funding for this study:** No funding was received for this study.
**Author Disclosures:**
Sara Seitun: Nothing to disclose
Joao Matos: Nothing to disclose
Manrico Balbi: Nothing to disclose
Umberto Viglino: Nothing to disclose
Maria Pigati: Nothing to disclose
Vincenzo De Marzo: Nothing to disclose
Gabriele Crimi: Nothing to disclose
Italo Porto: Nothing to disclose
Matteo Vercellino: Nothing to disclose

### RPS 2503-11

### Cerebral embolisation after percutaneous transfemoral aortic valve implantation (TAVI) and impact on neurocognitive function in the RETORIC study

*F. I. Suhai*[1], A. Varga[1], B. Szilveszter[1], A. I. Nagy[1], M. Vecsey-Nagy[1], J. Karady[2], A. Jermendy[1], P. Maurovich-Horvat[1]; [1]Budapest/HU, [2]Boston, MA/US
*(suhaiimi987@gmail.com)*

**Purpose:** To evaluate the predictors, occurrence and distribution of TAVI-related ischaemic brain lesions using diffusion MRI, and to assess the impact of these lesions on patients' neurocognitive function.
**Methods or Background:** We investigated 113 consecutive patients from the prospective arm of the RETORIC study who underwent brain MRI one week and 6 months after TAVI. To determine the occurrence and distribution of periprocedural cerebral ischaemic lesions averaged diffusion-weighted images (trace) and mean diffusivity (MD) maps from the DTI dataset were used. We evaluated the aortic CT angiography scans performed before TAVI implantation and assessed the periprocedural factors. Multivariate linear regression analysis was performed to identify the independent predictors of TAVI-related ischaemic lesions. A neurocognitive evaluation was performed before TAVR, at discharge, and at 6- and 12-month follow-up.
**Results or Findings:** After TAVI, a total of 944 new cerebral ischaemic lesions were detected in 104 patients (92%). The median ischaemic lesion volume was 257 μl (interquartile range 97.1-718.8 μl) with a median lesion number of 6 (2-10) per patient. Most lesions (796/944, 84%) were subcortical. The vast majority of ischaemic lesions were clinically silent (95%); 5% of patients had stroke, proven by MRI. The number of valve positioning and ballon predilatation during TAVI implantation showed a significant correlation with total ischaemic brain lesion volume (p<0.05), and balloon predilatation showed a correlation with stroke incidence (p<0.05) on multivariate analysis.
**Conclusion:** Although periprocedural ischaemic lesions are frequent, most of them are clinically silent and did not have a significant impact on neurocognitive functions. The number of valve positioning and predilatation showed correlation with total ischaemic brain lesion volume and balloon predilatation during TAVI was associated with stroke.
**Limitations:** The grafts were mainly self-expanding grafts.
**Ethics committee approval:** This study was approved by the local ethics committee.
**Funding for this study:** No funding was received for this study.
**Author Disclosures:**
Pál Maurovich-Horvat: Nothing to disclose
Milán Vecsey-Nagy: Nothing to disclose
Ferenc Imre Suhai: Nothing to disclose
Andrea Varga: Nothing to disclose
Anikó Ilona Nagy: Nothing to disclose
Adam Jermendy: Nothing to disclose
Bálint Szilveszter: Nothing to disclose
Julia Karady: Nothing to disclose

### RPS 2503-12

### Impact of computed tomography defined sarcopaenia on long-term outcomes of older adults undergoing transcatheter aortic valve implantation

*D. Tore*, A. Depaoli, L. Allois, A. Biondo, S. Salto, F. Ullo, G. Gallone, F. D'Ascenzo, P. Fonio; Turin/IT
*(davide.tore91@gmail.com)*

**Purpose:** To evaluate the role of Computed tomography (CT)-defined sarcopaenia for risk stratification in candidates to transcatheter aortic valve implantation (TAVI) comparing two definitions of CT-defined sarcopaenia complying with European Working Group on Sarcopaenia in Older People (EWGSOP-2) guidelines.
**Methods or Background:** 391 consecutive TAVI patients with preprocedural CT scan were included (81±6 years, 57.5% male, STS-PROM score 4.4±3.6%) and abdominal muscle retrospectively quantified. Two definitions of CT-defined sarcopaenia previously adopted in TAVI studies were compared (psoas muscle area [PMA] at the L4 vertebra level: "PMA-sarcopenia"; indexed skeletal muscle area at the L3 vertebra level: "SMI- sarcopaenia"). The primary

Sunday endpoint was long-term all-cause mortality. Secondary endpoints were Valve Academic Research Consortium-2-defined in-hospital and 30-day outcomes.
**Results or Findings:** SMI- and PMA-sarcopaenia were present in 192 (49.1%) and 117 (29.9%) patients, respectively. After a median of 24 (12-30) months follow-up, 83 (21.2%) patients died. PMA- (adj-HR 1.81, 95%CI 1.12-2.93, p=0.015), but not SMI-sarcopaenia (adj-HR 1.23, 95%CI 0.76-2.00, p=0.391), was associated with all-cause mortality independently of age, sex and in-study outcome predictors. PMA-defined sarcopaenia provided additive prognostic value over current post-TAVI mortality risk estimators including STS-PROM (p=0.001), Euroscore II (p=0.025), Charlson index (p=0.025) and TAVI2-score (p=0.020). Device success, early safety, clinical efficacy and 30-day all-cause death were unaffected by sarcopaenia status regardless of definition.
**Conclusion:** PMA-sarcopaenia (but not SMI-sarcopaenia) is highly predictive of worse clinical outcomes among TAVI patients, suggesting its use as a reference standard. The prognostic information provided by PMA-sarcopaenia is independent of the tools currently adopted to predict post-TAVI mortality in clinical practice.
**Limitations:** This work's status as a monocentric retrospective study was identified as a limitation.
**Ethics committee approval:** Not applicable
**Funding for this study:** No funding was received for this work.
**Author Disclosures:**
Sara Salto: Nothing to disclose
Luca Allois: Nothing to disclose
Alessandro Depaoli: Nothing to disclose
Fabrizio D'Ascenzo: Nothing to disclose
Paolo Fonio: Nothing to disclose
Guglielmo Gallone: Nothing to disclose
Federica Ullo: Nothing to disclose
Davide Tore: Nothing to disclose
Andrea Biondo: Nothing to disclose

**13:00-14:30** **Room X**

### Research Presentation Session: Head and Neck

### RPS 2508

### Imaging: squamous cell carcinoma

**Moderator**
M. Ravanelli; Brescia/IT

### RPS 2508-2

**Comparison of image quality and diagnostic performance among DWI obtained by FASE and EPI sequences and reconstructed with and without deep learning reconstruction in suspected head and neck tumour**

H. Ikeda[1], *Y. Ohno*[1], K. Yamamoto[2], K. Murayama[1], M. Yui[2], S. Hanamatsu[1], A. Iwase[3], T. Ueda[1], H. Toyama[1]; [1]Toyoake/JP, [2]Otawara, Tochigi/JP, [3]Toyoake, Aichi/JP
*(yohno@fujita-hu.ac.jp)*

**Purpose:** To compare the capability of DWI obtained by FASE and EPI sequences with and without deep learning reconstruction (DLR) for image quality and diagnostic performance improvements in patients suspected head and neck tumours.
**Methods or Background:** As part of an in vitro study, a QIBA phantom was scanned at a 3T scanner by DWIs with both methods and reconstructed with and without DLR. Then, ADC of each phantom was evaluated on all data sets. As part of an in vivo study, 41 patients (malignant vs benign: 17 vs 24) were obtained T2WI and both DWIs. Then, each DWI was reconstructed with and without DLR. SNR and ADC of each suspected lesion was measured. Moreover, deformation ratio (DR) of each suspected lesion was calculated as follows: (ROI area difference between each DWI and T2WI)/ (ROI area on T2WI). As part of the in vitro study, correlation of ADC between each DWI and standard reference was statistically assessed. As part of the in vivo study, each index was compared among all datasets by Student t-test. Following a ROC-based positive test, diagnostic performance was compared among all datasets via McNemar's test.
**Results or Findings:** Each correlation coefficient was excellent on in vitro study ($0.95<r<0.99$, $p<0.0001$). Within the in vivo study, DRs and SNRs of FASE with and without DLR were significantly improved as compared with EPI with or without DLR ($p<0.05$). When applying each threshold value, FASE with and without DLR were significantly more specific and accurate than EPI with and without DLR ($p<0.05$).
**Conclusion:** FASE is superior to EPI for image quality and diagnostic performance improvements on DWI in this setting.
**Limitations:** Not applicable
**Ethics committee approval:** This retrospective study was approved by the Institutional Review Board of Fujita Health University School of Medicine, Japan.
**Funding for this study:** This study was financially supported by Canon Medical Systems Corporation.
**Author Disclosures:**
Kazuhiro Murayama: Grant Recipient: Murayama got research grants from Canon Medical SysteCorporation.
Satomu Hanamatsu: Nothing to disclose
Kaori Yamamoto: Employee: Yamamoto is an employee of Canon Medical SysteCorporation.
Hirotaka Ikeda: Nothing to disclose
Hiroshi Toyama: Grant Recipient: Hiroshi Toyama got research grants from Canon Medical SysteCorporation.
Takahiro Ueda: Nothing to disclose
Masao Yui: Employee: Yui is an employee of Canon Medical SysteCorporation.
Yoshiharu Ohno: Grant Recipient: Yoshiharu Ohno got research grants from Canon Medical SysteCorporation.
Akiyoshi Iwase: Nothing to disclose

### RPS 2508-3

**Early response prediction of multiparametric functional MRI and 18F-FDG-PET in patients with head and neck squamous cell carcinoma treated with (chemo)radiation**

*R. Martens*[1], T. Koopman[1], C. Lavini[1], T. Van de Brug[1], C. René Leemans[1], R. de Bree[2], P. De Graaf[1], R. Boellaard[1], J. A. Castelijns[1]; [1]Amsterdam/NL, [2]Utrecht/NL

**Purpose:** To assess early (chemo)radiotherapy-induced tumoral changes with functional (DWI-, intravoxel-incoherent-motion-, DCE-) MRI and 18F-FDG-PET/CT-imaging and the prognostic value of extracted parameters on locoregional recurrence-free survival (LRFFS), distant-metastasis-free survival (DMFS) and overall-survival (OS) in patients with advanced-staged head and neck cancer.
**Methods or Background:** Fifty-seven patients with histopathologically proven HNSCC and curative (chemo)radiotherapy were prospectively included. Functional-imaging-parameters were extracted from primary tumours prior to and 10-days after treatment initiation (intratreatment). Univariate and multivariate analysis (LASSO-logistic-regression) were performed to construct prognostic models and risk stratification for 2-year LRFFS, DMFS and OS (log-rank test). Model performance was measured by cross-validated area-under-the-receiver-operating-characteristic-curve (AUC).
**Results or Findings:** Median follow-up time was 31 months. The best prognostic model for LRFFS contained pretreatment imaging parameters ADC_kurtosis, Kep, SUV_peak and the intratreatment change (Δ) of imaging-parameters Δ-ADC_skewness, Δ-f, Δ-SUV_peak and Δ-total lesion glycolysis (TLG) (AUC=0.81). Clinical parameters did not enhance LRFFS-prediction. Best DMFS-model contained pretreatment ADC_kurtosis and SUV_peak (AUC=0.88). Best OS-model contained gender, HPV-status, N-stage, pretreatment ADC_skewness, D and f, and metabolic active tumour volume (MATV), SUV_mean and SUV_peak (AUC=0.82). Risk stratification in high/medium/low-risk using these models was significantly prognostic for LRFFS, DMFS and OS ($p \leq 0.003$).
**Conclusion:** Intratreatment functional imaging parameters capture early tumoral changes that provide only prognostic information regarding LRFFS. The best prognostic LRFFS-model are the combination of pretreatment, intratreatment and Δ-functional imaging parameters; for DMFS only pretreatment functional imaging-parameters, and for OS the combination of HPV-status, gender and only pretreatment functional-imaging parameters. Accurate clinically applicable risk stratification calculators may enhance personalised treatment management early during-treatment.
**Limitations:** Population-based AIF caused a systematic error, which did not affect change of specific parameters. Variable interval of pretreatment imaging after presentation may have caused intratumoral changes.
**Ethics committee approval:** Local ethics committee approval.
**Funding for this study:** Funding was received from the Netherlands Organisation for Health Research and Development, grant 10-10400-98-14002.
**Author Disclosures:**
Jonas A. Castelijns: Nothing to disclose
C. René Leemans: Nothing to disclose
Pim De Graaf: Nothing to disclose
Cristina Lavini: Nothing to disclose
Remco de Bree: Nothing to disclose
Roland Martens: Nothing to disclose
Thomas Koopman: Nothing to disclose
Tim Van de Brug: Nothing to disclose
Ronald Boellaard: Nothing to disclose

### RPS 2508-4

**MRI-based assessment of the mylohyoid muscle in squamous cell cancer of the floor of mouth, a 7-point score method: T2wi vs STIR and T1+Gdwi**

J. De Groodt, *E. Radin*, F. Degrassi, L. Calderan, A. V. Marcuzzo, G. Tirelli, M. A. A. Cova; Trieste/IT

**Purpose:** We aimed to identify preoperative MR imaging characteristics of mylohyoid muscle that could predict insecure (positive or close) margin in surgically treated floor of mouth squamous cell carcinomas.
**Methods or Background:** Between March 2010 and March 2021, 81 consecutive patients with diagnosis of squamous cell carcinoma of the floor of mouth underwent surgical intervention at our institution. Among these, 52 with preoperative MR imaging and postoperative pathologic reports after surgery were included. Two neuroradiologists evaluated retrospectively the preoperative MR imaging scans to predict the tumour spread through the mylohyoid muscle using a 7-point scale respectively in T2w, STIR and T1w+Gd images: (1) distance between tumour and mylohyoid ≥ 5 mm; (2) distance < 5 mm but no contact; (3) tumour in contact with mylohyoid; (4) bulging of mylohyoid; (5) thinned and/or irregular mylohyoid; (6) full-thickness signal alteration; (7) signal alteration trough mylohyoid in submandibular space. Among the subgroup of score 4-5-6-7 (31) a single sequence blind assessment was repeated to evaluate which one has the highest accuracy.
**Results or Findings:** A score of >4 could predict an insecure margin with sensibility of 90.91% (C.I. 0.59±0.99) and specificity of 60.98% (C.I. 0.45±0.75), resulting in a limited positive predictive value (38.46%, C.I. 0.21±0.59), but a remarkable negative predictive value (96.15%, C.I. 0.78±0.99). The interobserver agreement of MR imaging scores was excellent, as proved by a Cohen's Kappa Coefficient of 0.83 (C.I. 0.72±0.95). In the subsequent monoparametric assessment, T2wi showed the highest specificity (78%, C.I. 0.88±0.62), while T1wi+Gd the lowest (53%, C.I. 0.69±0.37).
**Conclusion:** The presurgery T2wi MR imaging scoring-system for the mylohyoid muscle is a promising predictor of the surgical margin in floor of mouth squamous cell carcinoma.
**Limitations:** Small population.
**Ethics committee approval:** Not applicable.
**Funding for this study:** Not applicable.
**Author Disclosures:**
Erik Radin: Nothing to disclose
Alberto Vito Marcuzzo: Nothing to disclose
Giancarlo Tirelli: Nothing to disclose
Jasmina De Groodt: Nothing to disclose
Loretta Calderan: Nothing to disclose
Maria Assunta A. Cova: Nothing to disclose
Ferruccio Degrassi: Nothing to disclose

### RPS 2508-5

**Radiomics model to predict radiation-induced temporal lobe injury of nasopharyngeal carcinoma before treatment**

*D. Bao*, Y-f. Zhao, D. Luo; Beijing/CN
*(baodan1020@sina.com)*

**Purpose:** To develop and validate a radiomics-based model for predicting radiation-induced temporal lobe injury (RTLI) in nasopharyngeal carcinoma (NPC) by using MR imaging before treatment.
**Methods or Background:** A total of 216 patients with NPC between January 2017 and May 2021 were retrospectively reviewed. Patients were randomly allocated to a training (n=156) and validation cohort (n=60). A total of 1316 radiomics features were extracted from pretreatment contrast-enhanced T1- or T2/FS-weighted MRI. A radiomics signature was generated by using the least absolute shrinkage and selection operator (LASSO) regression algorithm, Pearson correlation analysis, and univariate logistic analysis. Clinical features were selected with logistic regression analysis. Further validation of the radiomics signature as an independent biomarker was performed by using multivariate logistic regression. A radiomics nomogram was constructed and assessed with respect to calibration, discrimination, reclassification, and clinical usefulness.
**Results or Findings:** The radiomics signature, composed of three radiomics features, was significantly associated with RTLI. The proposed radiomics model demonstrated favourable discrimination in both the training (AUC, 0.88) and validation cohort (AUC, 0.93), outperforming clinical prediction model (p<0.05). When combining radiomics and clinical features, higher AUC were achieved (AUC 0.93, 0.97), as well as a better calibration and improved accuracy of the prediction of RTLI. The clinical-radiomics model showed excellent predictive performance of RTLI in patients within different clinical-pathologic subgroups.
**Conclusion:** A radiomics model derived from pretreatment MR of the temporal lobe has good performance for predicting RTLI in NPC and may help to improve clinical decision making.
**Limitations:** This is a singlecentre retrospective study.
**Ethics committee approval:** The institutional review board approved this retrospective study, and the requirement to obtain informed consent was waived (institutional ethics approval number 21/278-2949).
**Funding for this study:** Funding was received from the non-profit Central Research Institute Fund of the Chinese Academy of Medical Sciences (2019XK320073).
**Author Disclosures:**
Dan Bao: Nothing to disclose
Yan-feng Zhao: Nothing to disclose
Dehong Luo: Nothing to disclose

### RPS 2508-6

**Iterative metal artifact reduction improves image quality and lesion detection in CT imaging of head and neck cancer**

*I. Burck*, E. J. Fillmann, J-E. Scholtz, B. Bodelle, D. Pinto, R. Sader, T. Stöver, T. J. Vogl, S. Martin; Frankfurt a. Main/DE

**Purpose:** To evaluate an iterative metal artifact reduction (iMAR) algorithm in patients with head and neck cancer.
**Methods or Background:** We included 49 patients (33 men and 16 women; mean age, 62.3 years) with dental hardware who underwent CT for staging of head and neck cancer. CT images were post-processed with standard iterative reconstruction (ADMIRE) and dedicated iMAR technique for improved metal artifact reduction. Evaluation of quantitative image quality parameters included signal-to-noise (SNR) and contrast-to-noise ratios (CNR) of tumour lesions as well as densities of the streaking artifacts (dark bands). Qualitative image quality was assessed by two radiologists using 5-point Likert scores. Interobserver agreement was calculated using Cohen's Kappa.
**Results or Findings:** SNR and CNR values of tumour lesions significantly improved using the iMAR technique compared to standard image reconstructions (SNR 3.5 vs 4.1; CNR 5.9 vs 4.6; p<0.02). The density of the streaking artifacts increased from 83.7 HU to 79.1 HU (p<0.01). Moreover, iMAR images were rated superior regarding image quality, artefact reduction, and lesion delineation (all p<0.01). Interrater agreement was excellent for overall image quality (κ=0.86), artifact reduction (κ=0.82), and lesion delineation (κ=0.76).
**Conclusion:** Our data suggest that image reconstructions with iMAR technique substantially improves image quality and lesion detection of head and neck cancer in patients with dental hardware.
**Limitations:** Our study followed a retrospective study design. We evaluated a limited sample size and did not compare our data to iMAR algorithms from other vendors, non-iterative MAR algorithms or dual-energy CT with monoenergetic reconstructions.
**Ethics committee approval:** Ethics approval was obtained by the institutional review board (Nr. 20-911).
**Funding for this study:** No funding was received for this study.
**Author Disclosures:**
Simon Martin: Nothing to disclose
Jan-Erik Scholtz: Nothing to disclose
Timo Stöver: Nothing to disclose
Boris Bodelle: Nothing to disclose
Thomas J. Vogl: Nothing to disclose
Elena Jasmin Fillmann: Nothing to disclose
Daniel Pinto: Nothing to disclose
Robert Sader: Nothing to disclose
Iris Burck: Nothing to disclose

### RPS 2508-7

**Comparison between path tracing and volume rendering 3D reconstructions in postsurgical assessment of head and neck complex surgery, followed by bone flap reconstruction in oncologic patients**

*N. Cardobi*, R. Nocini, G. Molteni, V. Favero, A. Fior, D. Marchioni, S. A. Montemezzi, M. D'Onofrio; Verona/IT
*(nicolo.cardobi@gmail.com)*

**Purpose:** The purpose of this study is to compare Path Tracing (PT) to traditional Volume Rendering technique (VR) in postoperative assessment of head and neck complex surgery followed by free flap bone reconstructions.
**Methods or Background:** In this retrospective study, 39 patients (mean age 58.18) who underwent head and neck complex surgery with free flaps bony reconstructions were included. All exams were acquired using a 64 Multi-Detector CT (MDCT). The images were independently reviewed by four observers, respectively a radiologist, 1 head and neck surgeon and 2 otorhinolaryngologists with different degrees of experience in bone flap reconstructive surgery. Every observer evaluated the images according a 5-point Likert scale. The parameters evaluated were image quality, anatomical accuracy, bone flap evaluation and metal artifact. Mean and medial values for all the parameters across the observers were calculated. Scores of both reconstruction methods were compared using a Wilcoxon matched-pairs signed rank test. Interreader agreement was calculated using Spearman's rank correlation coefficient.
**Results or Findings:** PT was considered significantly superior to VR 3D reconstructions by all readers (p<0.0001). Interreader agreement was moderate to strong across three readers with a maximum agreement for image quality of ρ = 0.546 (VR) and 0.537 (PT), for anatomical accuracy of ρ = 0.518

(VR) and 0.320 (PT), for bone flap evaluation of ρ = 0.657 (VR) and 0.670 (PT), for metal artifacts of ρ = 0.596 (VR) and ρ = 0.628 (PT). The fourth reader showed no significant agreement between the other observers.
The agreement was stronger with PT images compared to VR images.
**Conclusion:** PT reconstructions significantly improve the postsurgical evaluation of bone free flap reconstructions following major head and neck surgery.
**Limitations:** The study's retrospective nature was identified as a limitation.
**Ethics committee approval:** Ethics committee approval was obtained.
**Funding for this study:** No funding was received for this study.
**Author Disclosures:**
Nicolò Cardobi: Nothing to disclose
Andrea Fior: Nothing to disclose
Mirko D'Onofrio: Nothing to disclose
Daniele Marchioni: Nothing to disclose
Stefania Anna Montemezzi: Nothing to disclose
Vittorio Favero: Nothing to disclose
Riccardo Nocini: Nothing to disclose
Gabriele Molteni: Nothing to disclose

### RPS 2508-8

**Determining Human Papilloma Virus-status of head and neck squamous cell carcinomas using magnetic resonance imaging: a systematic review of the current literature**

*H. J. van der Hulst*, R. W. Jansen, W. Schats, R. G. H. Beets-Tan, M. van den Brekel, J. A. Castelijns; Amsterdam/NL
*(h.vd.hulst@nki.nl)*

**Purpose:** This systematic review was conducted with the objective of exploring the value of magnetic resonance imaging (MRI) on discerning HPV-status.
**Methods or Background:** Most-studied distinction within in the Head and Neck Squamous-Cell Carcinoma (HNSCC) is derived from an infection with high-risk human papillomaviruses (HPVs). HPV+ tumours are characterised by a significantly more favorable outcome. Non-invasive methods with the ability to reliably detect HPV-subtype would significantly advance prognostication and treatment planning. A wide-scoped systematic search was conducted in the PubMed (MEDLINE), Embase and Web of Science databases for original articles published until 20.08.2020 on MRI prediction of HPV-status in primary HNSCC lesions. Standardised mean differences were calculated as well as Tau-squared estimates of total heterogeneity and Higgins inconsistency index (I2 test).
**Results or Findings:** A total of 15 MRI studies were included, encompassing 10 studies describing diffusion-weighted imaging (DWI), 2 on Dynamic Contrast Enhanced (DCE) features and 5 studies reporting MRI-derived radiomics features and/or models. Lower mean ADC value was overall correlated with HPV+ HNSCC in 6 of the 10 articles. Overall volume and ADCminimum were inconsistent or non-significant. The amount of research on DCE and IVIM parameters is still too limited to draw conclusions, though some value of Ktrans could be inferred. Radiomic features have been moderately successful in discerning HPV status to some extent stand-alone and/or combined in models in 3 of 5 studies, a variety of different radiomic approached was utilised.
**Conclusion:** MRI is able to depict imaging differences between HPV-subtypes. Mean ADC is currently the most established stand-alone parameter. Radiomic models are not yet structured, but have the potential of providing options for the future to depict more complex tumour biology.
**Limitations:** Study design: review.
**Ethics committee approval:** Not applicable.
**Funding for this study:** Not applicable.
**Author Disclosures:**
Jonas A. Castelijns: Nothing to disclose
Winnie Schats: Nothing to disclose
Robin W. Jansen: Nothing to disclose
Regina G. H. Beets-Tan: Nothing to disclose
Hedda Joanne van der Hulst: Nothing to disclose
Michiel van den Brekel: Nothing to disclose

### RPS 2508-9

**Dynamic contrast-enhanced MRI in post-treatment head and neck squamous cell carcinoma: are MRI time signal intensity curves useful for differentiating recurrence from benign changes?**

*J. De Tobel*, V. Lenoir, B. Delattre, M. Becker; Geneva/CH
*(jannick.detobel@gmail.com)*

**Purpose:** To evaluate the diagnostic performance of different time signal intensity curve (TIC) types to distinguish head and neck squamous cell carcinoma (HNSCC) recurrence from benign post-treatment changes.
**Methods or Background:** 156 consecutive HNSCC patients treated with radiotherapy ± surgery (n=132) versus surgery only (n=24) underwent DCE-MRI examinations during follow-up. Histopathology and/or follow-up formed the standard of reference. TICs measured in regions of interest (ROIs) were classified into type 1-5. TIC type distribution and diagnostic performance were evaluated.
**Results or Findings:** Among the 261 ROIs analysed, 54 corresponded to recurrences and 207 to benign changes. There were 201/261 (77%) type 2, 52/261 (20%) type 3 and 8/261 (3%) type 4 TICs. Type 1 and 5 were not observed. TIC type distribution differed in recurrences versus benign changes (p=0.006). Treatment type (radiotherapy ± surgery versus surgery only) did not affect TIC type distribution in recurrences (p=1.000), but did in benign changes (p=0.007). Overall sensitivity, specificity, positive predictive value and negative predictive value to detect recurrence were 61%, 19%, 16%, 65% for type 2, 35%, 84%, 37%, 83% for type 3 and 4%, 97%, 25%, 79% for type 4, respectively.
**Conclusion:** Treatment type affects TIC type distribution in benign post-treatment changes but not in recurrences. Due to their low positive and negative predictive values, the utility of TICs to detect recurrent HNSCC is limited.
**Limitations:** The studied ROIs only represented selected suspicious parts of the lesions. Furthermore, we only focused on TIC type and we did not analyse quantitative DCE parameters.
**Ethics committee approval:** This study was approved by the institutional ethics committee. Written informed consent was waived due to its retrospective nature.
**Funding for this study:** This study was part of an ongoing clinical research project supported by the Swiss National Science Foundation (SNSF) under grant SNSF No 320030_173091/1.
**Author Disclosures:**
Jannick De Tobel: Nothing to disclose
Minerva Becker: Nothing to disclose
Vincent Lenoir: Nothing to disclose
Bénédicte Delattre: Nothing to disclose

### RPS 2508-10

**MRI detection of suspected nasopharyngeal carcinoma: a systematic review and meta-analysis**

*V. V. Gorolay*[1], N. N. Niles[1], R. Huo[1], N. Ahmadi[1], K. Hanneman[2], E. Thompson[1], M. V. Chan[1]; [1]Sydney/AU, [2]Toronto, ON/CA

**Purpose:** Although endoscopic biopsy is recommended for primary diagnosis of nasopharyngeal carcinoma (NPC), a proportion of lesions are hidden from endoscopic view. Magnetic resonance imaging (MRI) has an established role in locoregional staging and has been shown to detect endoscopically occult tumours. This meta-analysis investigates the diagnostic performance of MRI for detection of NPC.
**Methods or Background:** A systematic review was performed until May 2021 of studies which examined the diagnostic performance of MRI for detection of NPC in patients at-risk or suspected of having NPC. Studies comparing the diagnostic accuracy of the index test (MRI) with a reference standard (histopathology) were included. The primary outcome was accuracy for detection of NPC. Random effects models were used to pool outcomes for sensitivity, specificity, positive likelihood ratio (LR) and negative LR. Bias and applicability were assessed using the modified QUADAS-2 tool.
**Results or Findings:** Eight studies were included involving 1092 patients of whom 333 were diagnosed with NPC. MRI demonstrated a pooled sensitivity of 97.8% (95% CI; 93.5%-99.3%), specificity of 94.2% (95% CI; 89.8%-96.8%), negative LR of 0.02 (95% CI; 0.01-0.06), positive LR of 17.00 (95% CI; 9.56-30.25) for detection of NPC.
**Conclusion:** This study demonstrates that MRI has a high pooled sensitivity, specificity and negative predictive value for detection of NPC. MRI may be an important diagnostic tool for high-risk patients prior to endoscopic biopsy, and avoid biopsy in patients with a low post-test probability of disease.
**Limitations:** The main limitation of this review is risk of selection bias due to inclusion of retrospective studies and one case-control study, with limited reporting of study randomisation strategy. Most studies were performed in regions where NPC is endemic.
**Ethics committee approval:** Not required.
**Funding for this study:** None received.
**Author Disclosures:**
Vineet Vijay Gorolay: Nothing to disclose
Kate Hanneman: Nothing to disclose
Ruth Huo: Nothing to disclose
Michael Vinchill Chan: Nothing to disclose
Naomi Natasha Niles: Nothing to disclose
Navid Ahmadi: Nothing to disclose
Elizabeth Thompson: Nothing to disclose

### RPS 2508-11

**Evaluation of the causal relationship between imaging-defined nodal features and event free survival in patients with oropharyngeal carcinoma**

*S. A. Qureshi*, S. Tahir, R. Frood, Z. Iyizoba, S. Vaidyanathan, R. Prestwich, A. Scarsbrook; Leeds/UK
*(saadqureshi12@icloud.com)*

**Purpose:** To assess the causal relationship between radiologically-defined nodal features assessed on pretreatment computed tomography (CT) or magnetic resonance imaging (MRI) and event free survival in patients with oropharyngeal carcinoma.
**Methods or Background:** Patients with oropharyngeal carcinoma referred to a specialist tertiary centre between 2011 and 2017 were included. The number of abnormal lymph nodes, the presence of necrotic nodes, matted lymph nodes and radiologically determined extracapsular spread (ECS) were assessed by a radiologist with 1 year's experience and reviewed by a radiologist with 7 years' experience. A directed acyclic graph was constructed to identify the confounders, competing exposures and mediators for each of the imaging features. Cox regression models were created for each imaging feature and direct adjustment for confounders and competing exposures performed.
**Results or Findings:** 352 patients (273 male, 79 female, median age 57 years [range 24-84 years]) were included with a median follow-up period of 31 months (range 2-104 months). Eighty-six patients (24.4%) had an event (progression/relapse/death) during follow-up. 280 patients had baseline MRI and 72 patients had baseline CT. 49 patients had matted nodes, 227 had necrotic nodes, 130 had radiologically determined ECS and the median number of lymph nodes was 2 (range 1 to 10). The presence of matted lymph nodes had the highest hazard ratio, 1.73 (p = 0.048).
**Conclusion:** Out of the nodal imaging features explored the presence of matted nodes had the highest HR for event free survival with a 73% higher likelihood of an event.
**Limitations:** The lack of data surrounding socioeconomic status, alcohol intake and the limited numbers of non-white ethnic groups are likely to introduce bias into the models.
**Ethics committee approval:** Formal ethics committee approval was waived as this study represents evaluation of routine clinical service.
**Funding for this study:** No funding was received.
**Author Disclosures:**
Sriram Vaidyanathan: Nothing to disclose
Robin Prestwich: Nothing to disclose
Sanna Tahir: Nothing to disclose
Saad Ahmad Qureshi: Nothing to disclose
Andrew Scarsbrook: Nothing to disclose
Zsuzsanna Iyizoba: Nothing to disclose
Russell Frood: Nothing to disclose

### RPS 2508-12

**Magnetic resonance imaging for assessing cartilage invasion in recurrent laryngeal carcinoma after transoral laser microsurgery**

*P. Rondi*, M. Ravanelli, D. Lancini, N. Di Meo, C. Pessina, C. Piazza, D. Farina; Brescia/IT
*(paolo.rondi92@gmail.com)*

**Purpose:** To assess the diagnostic performance of MRI, acquired with surface coils, in detecting cartilage invasion in recurrences occurring after transoral laser microsurgery (TLM).
**Methods or Background:** 30 histologically proven laryngeal cancer recurrences after TLM were studied on a 1.5 T MR scanner using surface coils. All patients underwent salvage total or supracricoid laryngectomy. MRI protocol consisted of TSE T2, TSE T1, DWI and 3D post-Gadolinium VIBE sequences (radial k-space sequences were used in uncooperative patients). Two expert head and neck radiologists performed the analysis to assess inter-operator agreement.
**Results or Findings:** Cartilage invasion was detected in 18/30 cases (13 thyroid cartilages, 3 cricoid, 2 arytenoid). Interobserver agreement for all cartilage invasion was good (Choen's kappa 0.76). Overall diagnostic performance reported a sensitivity of 76%, specificity of 93%, PPV of 72% and NPV of 94%. For thyroid, cricoid and arytenoid, sensitivity was 82%, 100% and 33%, respectively; specificity was 79%, 100% and 96%, respectively; PPV was 69%,100% and 56%, respectively and NPV was 88% 100% and 93%, respectively.
**Conclusion:** MR with surface coils is sufficiently accurate in detecting cartilage invasion by recurrent laryngeal cancer after TLM. The high NPV may be exploited to increase the possibility to personalise treatment patients allowing voice-sparing surgical procedures in selected cases.
**Limitations:** Low number of cases, which influence the statistical power of our results. The entity of cartilage infiltration was not graded and minimal lysis was considered in the same way as full-thickness invasion.
**Ethics committee approval:** This retrospective study has been approved by the institutional review board of our hospital, in agreement with ethical standards of Helsinki declaration.
**Funding for this study:** No founding was provided for this study.
**Author Disclosures:**
Nunzia Di Meo: Nothing to disclose
Carlotta Pessina: Nothing to disclose
Davide Farina: Nothing to disclose
Paolo Rondi: Nothing to disclose
Cesare Piazza: Nothing to disclose
Marco Ravanelli: Nothing to disclose
Davide Lancini: Nothing to disclose

**13:00-14:30** **Room Z**

### Research Presentation Session: Interventional Radiology

### RPS 2509

### Interventional management of malignant liver lesions

**Moderator**
T. K. Helmberger; Munich/DE

### RPS 2509-2

**Can microwave ablation of HCC modulate systemic inflammation and immunity? An analysis of factors affecting neutrophil-to-lymphocyte ratio and correlation with local tumour progression**

*A. Della Corte*[1], C. Sallemi[2], I. Monfardini[2], D. Santangelo[1], R. Pennella[1], F. Ratti[1], S. Gusmini[1], L. Aldrighetti[1], F. De Cobelli[1]; [1]Milan/IT, [2]Brescia/IT
*(dellacorte.angelo@hsr.it)*

**Purpose:** Microwave ablation (MWA) is known to induce changes in the immune landscape after treatment of Hepatocellular Carcinoma (HCC). This study aims at identifying which technique- and disease-related factors may predict post-treatment changes in neutrophil-to-lymphocyte-ratio (NLR) as well as their impact on local tumour progression (LTP).
**Methods or Background:** From October 2018 to August 2020, 144 consecutive patients underwent MWA of 164 HCCs with a 2450Hz/100W generator. Fourty-four nodules (40 patients) met inclusion criteria for analysis (percutaneous approach, availability of pre- and post-treatment inflammatory markers, follow-up > 6 months, technique efficacy, absence of complications). Data were collected regarding technique, disease and tumour-related features, as well as NLR prior to therapy and at 1-month follow-up.
**Results or Findings:** After a median follow-up of 22 months, LTP occurred in 17 nodules (38.6%). At univariate analysis, risk factors for LTP included 1-month NLR increase (HR=3.3, p=0.038) and non-viral cirrhosis (HR=3.4, p=0.025). A multivariate model confirmed that 1-month NLR increase and non-viral cirrhosis were the only independent predictors (p=0.04 and p=0.03, respectively). NLR increase occurred in 23 patients (57.5%). In this subgroup, higher rates of female gender (p=0.026), higher mean baseline NLR (p<0.0001) and lower mean delivered energy (p=0.008) were observed. Upon ROC curve analysis, an energy cut-off > 1296 J/mm allowed prediction of NLR decrease at first follow-up with 74% sensitivity and 64% specificity (AUC=0.73).
**Conclusion:** Systemic inflammation and immunity contribute to development of LTP after MWA, as NLR increase in response to ablation was an independent predictor. The amount of delivered energy seems to influence change in NLR, reinforcing the concept of immune ablation.
**Limitations:** There was a limited population size and this was a retrospective study.
**Ethics committee approval:** The study was approved by the IRBs in full respect of the declaration of Helsinki and later amendments.
**Funding for this study:** No funding was received for this study.
**Author Disclosures:**
Angelo Della Corte: Nothing to disclose
lorenzo Monfardini: Nothing to disclose
Luca Aldrighetti: Nothing to disclose
Domenico Santangelo: Nothing to disclose
Simone Gusmini: Nothing to disclose
Renato Pennella: Nothing to disclose
Claudio Sallemi: Nothing to disclose
Francesco De Cobelli: Nothing to disclose
Francesca Ratti: Nothing to disclose

### RPS 2509-3

**Prevalence and clinical significance of clinically evident portal hypertension in patients with hepatocellular carcinoma undergoing transarterial chemoembolisation**

*L. Müller*[1], A. Mähringer-Kunz[1], F. Stoehr[1], D. Pinto dos Santos[2], M. B. B. Pitton[1], C. Düber[1], R. Kloeckner[1], F. Hahn[1]; [1]Mainz/DE, [2]Cologne/DE

**Purpose:** Clinically evident portal hypertension (CEPH) is a estimate of the hepatic vein pressure gradient. CEPH was previously identified as a prognostic factor for patients with hepatocellular carcinoma (HCC). However, little is known about the prognostic influence of CEPH on the long-term outcome of patients with HCC that undergo transarterial chemoembolisation (TACE). This study investigated the prevalence and prognostic influence of CEPH in a Western population of patients with HCC undergoing TACE.
**Methods or Background:** This study included 349 treatment-naïve patients that received initial TACE treatment at our tertiary care center between January 2010 and November 2020. We assessed the influence of CEPH on median overall survival (OS). We compared the effects of CEPH to those of well-known prognostic factors.
**Results or Findings:** CEPH was present in 227 (65.0%) patients. The median OS times were 10.3 months for patients with CEPH and 16.7 months for patients without CEPH (log rank p=0.079). Median OS without a present surrogate was 16.7 months, while patients with one respectively more than two present CEPH surrogates had a median OS of 10.7 months and 9.4 months (log rank p=0.083). Of the CEPH defining factors, only ascites reached significance in a univariate analysis.
**Conclusion:** CEPH was present in almost two thirds of the patients with HCC undergoing TACE. In our study, patients with CEPH had an impaired survival. However, no significance was reached in univariate analysis and multivariate analysis yielded other factors that seem to be more important for OS stratification. Thus, when TACE treatment is oncologically reasonable, patients should not be excluded from TACE treatment due to the presence of CEPH alone.
**Limitations:** The singlecentre nature of the study and its retrospective design were identified as limitations.
**Ethics committee approval:** This study was approved by the ethics committee of the Medical Association of Rhineland Palatinate, Mainz, Germany (permit number 2021-15984).
**Funding for this study:** Not applicable
**Author Disclosures:**
Daniel Pinto dos Santos: Nothing to disclose
Roman Kloeckner: Nothing to disclose
Michael Bernhard B Pitton: Nothing to disclose
Fabian Stoehr: Nothing to disclose
Christoph Düber: Nothing to disclose
Aline Mähringer-Kunz: Nothing to disclose
Felix Hahn: Nothing to disclose
Lukas Müller: Nothing to disclose

### RPS 2509-4

**Laser-induced thermotherapy (LITT) versus microwave ablation (MWA) in hepatocellular carcinoma (HCC): therapy response and survival rates**

*T. J. Vogl*, H. Adwan, J. Trojan, T. Gruber-Rouh; Frankfurt a. Main/DE

**Purpose:** To retrospectively compare CT-guided microwave ablation (MWA) with MR-guided laser-induced thermal ablation (LITT) of hepatocellular carcinoma (HCC) regarding therapy response and overall survival (OS).
**Methods or Background:** In total, 303 patients (64f, 239m) were treated with 521 sessions of thermal ablation: 250 patients (52f, 198m; mean: 66±10 years) with 445 CT-guided MWAs and 53 patients (12f, 41m; mean: 67.5±8 years) with 76 MR-guided LITTs. Diameter of HCC lesions, technical success, complete ablation, local tumour progression and OS rates were evaluated in all cases.
**Results or Findings:** Mean tumour diameter was 2.16 cm in the MWA group vs 2.4 cm in the LITT group. Technical success was achieved in all ablations in both groups. Complete ablation was achieved in 97.7% of the tumours in the MWA group and in 98.7% in the LITT group. In the MWA group 6% of the patients and in the LITT group 3.8% developed local tumour progression. The 1-, 3-, and 5-year OS rates starting at the date of ablation were 86.6%, 53.4% and 40.4% in the MWA group and 85%, 37.7% and 17% in the LITT group, respectively. (p-value: 0.001). No peri-procedural deaths were reported in both groups.
**Conclusion:** Image-guided thermal ablation like LITT and MWA are both effective and safe for the local treatment of HCC. Patients in the MWA group had an overall longer survival time, but with higher rate of local tumour progression than the patients in the LITT-group.
**Limitations:** The retrospective study design was identified as a limitation.
**Ethics committee approval:** The approval of the institutional review board was obtained.
**Funding for this study:** No funding was received for this study.
**Author Disclosures:**
Jörg Trojan: Nothing to disclose
Thomas J. Vogl: Nothing to disclose
Tatjana Gruber-Rouh: Nothing to disclose
Hamzah Adwan: Nothing to disclose

### RPS 2509-6

**Treatment of primary and secondary liver tumours using microwave ablation (MWA): MR elastography as response parameter**

*T. J. Vogl*, M. Dosch, B. Panahi, L. Basten; Frankfurt a. Main/DE

**Purpose:** To determine hepatic tissue response to microwave ablation (MWA) of liver tumours using MR elastography (MRE).
**Methods or Background:** After 3.5 (0-15) transarterial chemoembolisations on average, 51 patients (28m/23f; median: 61 years; range: 32-87) underwent MWA of hepatocellular carcinoma (HCC) (n=20) and liver metastases of different origin (n=31) receiving MRI+MRE prior to and 24h-post MWA. Retrospective lesion analysis included pre-/post-ablative tumour size measurement, MAP T1/T2 and stiffness via MRI+MRE. For assessing the effectiveness, the following parameters were established: ablation excess, ablation intensity, cumulative energy during MWA. Four regions-of-interest (ROI) were established to determine tissue ablation response: entire liver, tumour, ablation zone, healthy paraenchyma.
**Results or Findings:** HCC vs metastases showed significantly increased duration of MWA, cumulative energy applied, tumour stiffness pre-MWA. Total liver stiffness increased from 3.3±1.5 kPa to 3.4±1.6 kPa, total liver MAPT1 from 607.9±90.3 ms to 645.7±75.2 ms, total liver MAPT2 from 66.2±10.5 ms to 68.9±10.9 ms. Healthy paraenchyma stiffness increased from 3.5±2.3 kPa to 3.6±2.2 kPa, healthy paraenchyma MAPT1 from 568.8±94.1ms to 606.8±107.2 ms, MAPT2 from 64.5±16.7 ms to 69.4±23.1 ms. Average ablation zone width (10.1 mm ±11.1 mm) correlated with distance to closest blood vessel (r=0.476). Ablation zone stiffness strongly depended on its area (r=0.511). MWA intensity correlated with post-MWA tumour stiffness (r=0.322) ablation zone thickness (r=0.421) and healthy paraenchyma stiffness (r=0.322). Cumulative energy applied moderately correlated to tumour stiffness pre-MWA (r=0.391), ablation halo stiffness (r=0.382), ablated tumour area (r=0.277) and healthy paraenchyma stiffness post-MWA (r=0.436).
**Conclusion:** MRE+MRI offers benefits for evaluating MWA response and detects early changes.
**Limitations:** The fact that there was no follow-up, the small cohort, the lack of a control-group, as well as different cancer and treatment stages (some had chemotherapy/some hepatectomy) and different stages in MWA protocol (but no big influence on investigated tumour) being involved were identified as limiting factors.
**Ethics committee approval:** The approval of an ethics committee was obtained.
**Funding for this study:** This research did not receive any specific grant from public, commercial, or non-profit sectors.
**Author Disclosures:**
Lajos Basten: Nothing to disclose
Max Dosch: Nothing to disclose
Bita Panahi: Nothing to disclose
Thomas J. Vogl: Nothing to disclose

### RPS 2509-7

**Radiofrequency ablation (RFA) of hepatocellular carcinoma (HCC): CT texture analysis of the ablated area to predict local recurrence**

*D. Autelitano*, L. Geraci, L. Tomaiuolo, M. Todesco, V. Rossi, C. Longo, F. Cicalò, G. Aluffi, M. D'Onofrio; Verona/IT
*(danieleaute91@live.it)*

**Purpose:** To investigate the role of Computed Tomography (CT) texture analysis in the risk assessment of local recurrence after HCC ablation.
**Methods or Background:** Patients treated with percutaneous liver ablation were retrospectively enrolled between January 2015 and December 2018. CT texture analysis was performed both on the core and borders of the ablation area 1-2 months after procedure. Tumours were grouped according to the onset of local recurrence at follow-up (persistence, recurrence-free, short- or long-term recurrence). ANOVA/Kruskal-Wallis tests and a multivariable Cox regression model were used to assess differences in texture parameters and which parameters were predictive of recurrence risk.
**Results or Findings:** 263 tumours were treated in 200 patients; 98 patients (72±9 years, 83 men) met the inclusion criteria for a total of 151 HCCs considered independently. 68 HCCs reported no disease recurrence, 32 persistent disease, 19 short-term and 32 long-term recurrence. Median follow-up was 121 [range: 29-1680] days. VenSkewness (p=0.02) and VenKurtosis (p=0.01) of the ablation core were predictive of short-term recurrence. VenHUmean (p=0.02) and VenGLRLM_HGRE (p=0.02) of the core were independent predictors of tumour recurrence. ArtEntropy of ablation border predicted the recurrence risk (p=0.04) and values higher than 3.71 reported an increased recurrence incidence (p=0.05). ArtHUstd (p=0.01), LateHUmean (p=0.04), LateGLRLM_HGRE (p=.003), LateGLZLM_HGZE (p<0.01) and LateGLZLM_SZHGE (p=.02) of ablation border were independent predictors of local recurrence risk.
**Conclusion:** CT texture analysis of the ablation area performed at 1-2 months follow-up allows to estimate the risk of local recurrence of hepatocellular carcinoma treated by radiofrequency ablation; one may be able to modify clinical-therapeutic decisions accordingly.
**Limitations:** Not applicable
**Ethics committee approval:** Not applicable
**Funding for this study:** Not applicable
**Author Disclosures:**
Luisa Tomaiuolo: Nothing to disclose
Marco Todesco: Nothing to disclose
Chiara Longo: Nothing to disclose
Luca Geraci: Nothing to disclose
Mirko D'Onofrio: Nothing to disclose
Francesco Cicalò: Nothing to disclose
Daniele Autelitano: Nothing to disclose
Vittoria Rossi: Nothing to disclose
Gregorio Aluffi: Nothing to disclose

### RPS 2509-8

**Immunonutritive scoring for patients with hepatocellular carcinoma undergoing transarterial chemoembolisation: evaluation of the CALLY index**

*L. Müller*[1], F. Hahn[1], A. Mähringer-Kunz[1], D. Pinto dos Santos[2], M. B. B. Pitton[1], C. Düber[1], R. Kloeckner[1], F. Stoehr[1]; [1]Mainz/DE, [2]Cologne/DE

**Purpose:** The novel CRP-albumin-lymphocyte index (CALLY) is an improved immunonutritive scoring system, based on serum C-reactive protein (CRP), serum albumin, and the lymphocyte count. It showed promise as a prognostic index for patients with hepatocellular carcinoma (HCC) undergoing resections. This study evaluated the prognostic ability of the CALLY index for patients with HCC undergoing transarterial chemoembolisation (TACE).
**Methods or Background:** We retrospectively identified 280 treatment-naïve patients with HCC that underwent an initial TACE at our institution, between 2010 and 2020. We compared the CALLY to established risk factors in univariate and multivariate regression analyses for associations with median overall survival (OS).
**Results or Findings:** A low CALLY was associated with a low median OS (low vs high CALLY: 9.0 vs 24.0 months, p<0.001). In the multivariate analysis, the CALLY remained an independent prognostic predictor (p=0.008). Furthermore, all factors of the CALLY reached significance in univariate and in-depth multivariate analyses. However, the concordance index (C-Index) of the CALLY (0.60) was similar to the C-indices of established immunonutritive and inflammation scoring systems (range: 0.54 to 0.63).
**Conclusion:** In conclusion, the CALLY showed promise as a stratification tool for patients with HCC undergoing TACE. Notably, the CALLY index was not superior to other immunonutritive and inflammation scoring systems in predicting the median OS. Thus, future studies should reevaluate the mathematical calculation of the index, particularly the contributions of individual parameters.
**Limitations:** This was a singlecentre study with a retrospective design.
**Ethics committee approval:** This analysis of clinical data was approved by the Ethics Committee of the Medical Association of Rhineland Palatinate, Mainz, Germany (permit number 2021-15666).
**Funding for this study:** Not applicable
**Author Disclosures:**
Daniel Pinto dos Santos: Nothing to disclose
Roman Kloeckner: Nothing to disclose
Michael Bernhard B Pitton: Nothing to disclose
Fabian Stoehr: Nothing to disclose
Christoph Düber: Nothing to disclose
Aline Mähringer-Kunz: Nothing to disclose
Felix Hahn: Nothing to disclose
Lukas Müller: Nothing to disclose

### RPS 2509-9

**Augmented reality-guided percutaneous thermal ablation of small hepatic tumours: first clinical experience in human patients worldwide**

*L. A. Solbiati*[1], T. Ierace[1], R. Iezzi[2], V. Pedicini[1]; [1]Rozzano/IT, [2]Rome/IT
*(lusolbia@gmail.com)*

**Purpose:** To assess feasibility and precision of augmented reality as only guidance modality for percutaneous interventional procedures.
**Methods or Background:** Fifteen hepatic malignancies (9 HCCs, and 6 metastases from breast (3) and pancreas (2) carcinomas) in 8 patients, with a size ranging from 0.7 to 3.0 cm underwent percutaneous ablation using microwaves for 14/15 lesions and radiofrequency (1/15), under the only guidance of augmented reality (R.A.W. Endosight, Milan, Italy) during patient's free breathing. After applying radiopaque orientation reference sensors on abdominal skin before acquiring preliminary CT scans, segmentation and 3D reconstruction of CT scans were performed with dedicated software. Through commercially available smartglasses (Oculus Rift-S, Facebook Technologies, USA) paired with Zed Mini cameras (Stereolabs, San Francisco, USA), 3D visualisation of target tumour, ablation device (through markers with no repetitive pattern applied to introducer coaxial needle), and device tip-to-target geometrical center trajectory line was achieved and superimposed over the patient's body.
**Results or Findings:** Time needed for segmentation and 3D reconstruction ranged from 12 to 17.2 minutes, and for tumour targeting from 3.2 to 5.7 minutes. Targeting accuracy ranged from 2.1 to 4.5 mm (mean 3.2 + 0.7). One ablation device insertion was performed for each target tumour. Technical success was achieved for all tumours. No intra- or periprocedural, major or minor adverse events occurred.
**Conclusion:** Augmented reality alone can guide interventional procedures in real clinical conditions with great precision, quickly and with significant reduction of radiation dose to patients and operators compared to CT-guided procedures.
**Limitations:** This was a singlecentre study with a small number of patients.
**Ethics committee approval:** This study was performed at two tertiary referral centres for liver diseases with the approval of the local Institutional Ethics Committees. Written informed consent was obtained from all patients prior to treatment.
**Funding for this study:** No funding was received for this study.
**Author Disclosures:**
Vittorio Pedicini: Nothing to disclose
Tiziana Ierace: Nothing to disclose
Roberto Iezzi: Nothing to disclose
Luigi Alessandro Solbiati: Nothing to disclose

### RPS 2509-10

**Comparison of combined chemoembolisation and microwave ablation with chemoembolisation alone in patients with hepatocellular carcinoma beyond the Milan criteria: a propensity scoring matching study**

*Y-d. Xiao*; Changsha/CN

**Purpose:** To compare the treatment efficacy and downstaging rate of combined transarterial chemoembolisation (TACE) and microwave ablation (MWA) with TACE alone in patients with unresectable hepatocellular carcinoma (HCC) beyond the Milan Criteria (MC).
**Methods or Background:** A total of 231 patients with unresectable HCC beyond the MC who underwent either TACE-MWA (n=91) or TACE alone (n=140) at four medical institutions were included. Factors potentially influencing overall survival (OS) and progression free survival (PFS) were included in the Cox regression analysis. Propensity score matching (PSM) was performed between patients treated with TACE-MWA and TACE alone. Differences in OS and PFS were compared with the log-rank test. Patients who met the University of California, San Francisco (UCSF) criteria were considered to assess the probability of downstaging to the MC. Downstaging rate was compared between the two groups.
**Results or Findings:** In multivariate analysis, patients treated with TACE alone was an independent predictor of poor PFS (p=0.011) and OS (p<0.001). After one-to-one PSM, a total of 146 patients were matched. Both PFS (p=0.043) and OS (p=0.002) were significantly higher in patients treated with TACE-MWA than those of TACE alone. The downstaging rate was also higher in patients with TACE-MWA than those of TACE alone (p=0.039).
**Conclusion:** TACE-MWA may offer a survival benefit in terms of OS and PFS in HCC patients beyond the MC compared with TACE alone. Additionally, TACE-MWA may provide a higher probability of downstaging to the MC than TACE alone, which may increase the chance for HCC patients to receive liver transplant.
**Limitations:** First, this is a retrospective study with a small sample size. Second, the survival benefit of liver transplant after successful downstaging was not evaluated.

**Ethics committee approval:** This study was approved by the institutional review board of our hospitals.
**Funding for this study:** Not applicable
**Author Disclosures:**
Yu-dong Xiao: Nothing to disclose

### RPS 2509-12
**Does lipiodol retention pattern influence the treatment efficacy of computed tomography guided thermal ablation for hepatocellular carcinoma: a propensity scoring matching study**
*Y-d. Xiao*; Changsha/CN

**Purpose:** To investigate whether the lipiodol retention pattern can influence treatment efficacy of computed tomography (CT)-guided thermal ablation for hepatocellular carcinoma (HCC).
**Methods or Background:** Between June 2014 and September 2020, a total of 198 patients with 280 HCC lesions who underwent transarterial lipiodol injection (TLI) plus thermal ablation at three medical institutions were included. The lipiodol retention pattern was classified into complete retention and incomplete retention based on the unenhanced CT at the time of ablation. The primary outcome was local recurrence-free survival (LRFS) for lesions, and the secondary outcome was overall survival (OS) for patients. Propensity score matching (PSM) was performed using a caliper width of 0.1 between two groups. The differences in LRFS and OS between the two groups were compared with the log-rank test.
**Results or Findings:** A total of 133 lesions presented with a complete lipiodol retention pattern, while 147 lesions presented with incomplete retention pattern. After a PSM analysis of the baseline characteristics of the lesions, 121 pairs of lesions were matched in each group. The LRFS was significantly higher for lesions with complete retention than for lesions with incomplete retention (p=0.030). After a PSM analysis of the baseline characteristics of patients, 74 pairs of patients were matched in each group. There was no significant difference in OS between the two groups (p=0.456).
**Conclusion:** The lipiodol retention pattern may influence thermal ablation for HCC lesions. However, a survival benefit of the lipiodol retention pattern for HCC patients was not observed and needs further confirmation.
**Limitations:** The retrospective study design, the limited follow-up period and the small sample size were identified as limiting factors.
**Ethics committee approval:** This retrospective study was approved by the institutional review board of our hospitals.
**Funding for this study:** Not applicable
**Author Disclosures:**
Yu-dong Xiao: Nothing to disclose

**15:00-16:00** **Room B**

### Research Presentation Session: Breast

### RPS 2602

### Improving breast cancer staging and pre-operative imaging

**Moderator**
D. Baditescu; Bucharest/RO

### RPS 2602-2
**The CEDM performance in the presurgical evaluation of DCIS**
*E. Savi*, G. Bicchierai, C. Bellini, D. De Benedetto, F. Di Naro, J. Nori, V. Miele; Florence/IT
*(elena.savi121@gmail.com)*

**Purpose:** Ductal Carcinoma in Situ (DCIS) is a malignat proliferation of ductal epithelial cells without the invasion of the basement membrane. It can be considered as a precursor of invasive carcinoma and a diagnosis of DCIS lead to an increased risk of developing new breast cancer. The aim of our study was to estimate the role of Contrast-Enhanced Digital Mammography (CEDM) in presurgical evaluation of DCIS, without association with microinvasive or invasive carcinoma, evaluating its sensitivity and specificity in identifying index lesions and any additional lesions.
**Methods or Background:** All patients diagnosed with DCIS, who performed CEDM as a preoperative staging method, afferent over a three-year period, from 2017 to 2020, were included. Patients with a high background parenchymal enhancement (BPE) or a post-biotic haematoma, as potential confounding elements for the enhancing evaluation, were excluded.
**Results or Findings:** 109 patients for 111 total index lesions were evaluated, of which 70 were microcalcifications, 33 a nodule, 8 a distortion. CEDM led 16 (14,8%) patients to undergo a new biopsy: 8 lesions were B2, 4 were a B3 and 2 were a B5 (DCIS). CEDM changed the surgical approach in 6 (5,50%) patients. In the histological post-operative examination 4.5% (5/111) of the lesions were G1, 63% (70/111) G2 and 32.5% (36/111) G3. It has been shown that for a nodule-like index lesion or opacity the risk of nonenhancing is statistically (p 0.004) lower than for microcalcifications. In the case of a G3 lesion, the risk of nonenhancing was significantly (p 0.0002) lower than the other types of grading, with a sensitivity of 91.67% CI95% 77.53-98.25%.
**Conclusion:** In our experience, CEDM has shown to have an important role in the presurgical staging of pure DCIS with good sensitivity and specificity, in particular showing a high significance in the individualisation of higher grade G3 DCIS.
**Limitations:** A larger study group should be required.
**Ethics committee approval:** This study was approved by an ethics committee.
**Funding for this study:** No funding was received for this study.
**Author Disclosures:**
Elena Savi: Nothing to disclose
Giulia Bicchierai: Nothing to disclose
Federica Di Naro: Nothing to disclose
Diego De Benedetto: Nothing to disclose
Jacopo Nori: Nothing to disclose
Vittorio Miele: Nothing to disclose
Chiara Bellini: Nothing to disclose

### RPS 2602-3
**Breast lesions magnetic localisation: a European large scale evaluation in a national cancer institute**
*C. Depretto*, C. De Berardinis, G. Della Pepa, L. Suman, C. Ferranti, M. Marchesini, G. Pruneri, S. Folli, G. P. Scaperrotta; Milan/IT
*(cathie.dp@gmail.com)*

**Purpose:** The purpose of our study is to evaluate on a large scale the performance of pre-operative magnetic seed localisation of non-palpable breast lesions.
**Methods or Background:** We prospectively collected data on all patients undergoing image-guided magnetic seed localisation from September 2019 to October 2021. We analysed imaging findings, histological results, and type of surgery. The primary outcome was the successful localisation rate. Secondary outcomes were: the successful placement rate, the ease of percutaneous positioning, the procedural complications, and the reintervention rate.
**Results or Findings:** A total of 550 magnetic seeds were placed in 539 patients by four radiologists under ultrasound (506) or stereotactic (44) guidance. All seeds were detectable transcutaneously in all breasts sizes and at all depths by seven surgeons with a success rate of 100%. 96% of the seeds were correctly placed into the target lesions (only 4% were dislocated). All radiologists have shown good compliance during the procedure, and there were no complications or safety issues. The re-operation rate was 6.3 %.
**Conclusion:** Image-guided localisation with magnetic seeds is an easy, safe, reliable, and effective method for localising non-palpable breast lesions. Both radiologists and surgeons reflected that the technology was intuitive to use and that it can be widely applied as the first option in pre-operative localisation in breast units.
**Limitations:** The trial is a single-centre study. Additionally, it was conducted by dedicated breast cancer radiologists and surgeons, and it may not be generalisable to all institutions.
**Ethics committee approval:** Institutional review board approved this prospective study, and informed consent was obtained for all patients.
**Funding for this study:** The authors received no financial support for the research.
**Author Disclosures:**
Gianmarco Della Pepa: Nothing to disclose
Giancarlo Pruneri: Nothing to disclose
Claudia De Berardinis: Nothing to disclose
Gianfranco Paride Scaperrotta: Nothing to disclose
Claudio Ferranti: Nothing to disclose
Laura Suman: Nothing to disclose
Catherine Depretto: Nothing to disclose
Monica Marchesini: Nothing to disclose
Secondo Folli: Nothing to disclose

### RPS 2602-4

**Predictive value of automated breast volume scanner in the preoperative evaluation of breast tissues planned for conservative mastectomy and prepectoral reconstruction**

*A. A. D'Angelo*, G. Macrì, L. Barone Adesi, M. Salgarello, G. Ciriello, S. Palma, F. Ferrara, P. Belli, R. Manfredi; Rome/IT
*(anna.dangelo05@gmail.com)*

**Purpose:** The prepectoral direct-to-implant (DTI) breast reconstruction following conservative mastectomy is associated with good oncological outcomes. The mastectomy skin flap thickness is the most important factor in predicting outcome and complications. First aim of the study was to evaluate the predictive value of the Automated Breast Volume Scanner (ABVS) compared to mammography, handheld ultrasound (HHUS) and MRI in the preoperative evaluation of breast tissue thickness. Secondary aim was to compare the breast thickness obtained 6 months after surgery to the previous preoperative as well as intraoperative findings to investigate a change in thickness.
**Methods or Background:** Between June 2020 and June 2021 patients eligible for conservative mastectomies and DTI reconstruction were consecutively enrolled in this prospective single-institutional study. The inclusion criteria were: age 18 years old or older, preoperative imaging evaluations performed in our center. Measurements of breast tissue thickness were obtained by ABVS, HHUS, mammography and MRI. The intraoperative breast tissue coverage measurements were the gold standard. The Bland-Altman analysis and plot were used.
**Results or Findings:** 75 patients were enrolled. 93 breast mastectomies were performed. The difference between ABVS and intraoperative measurements was -0,084 mm (-2,5%). Mammography and MRI tended to overestimate mastectomy skin flap thickness of about respectively 11.41% and 1,22 mm (+ 10,4%). HHUS showed a good level of agreement with an overall overestimation of + 9,42%. Follow-up was conducted on 28 patients corresponding to 36 measurements obtained through ABVS and HHUS. The breast tissue thickness tended to decrease after surgery (-2,6 mm (- 32,4%) with ABVS and - 1,75 mm (- 23,6%) with HHUS).
**Conclusion:** ABVS could be the modality of choice for the preoperatory evaluation of breast tissue thickness in DTI reconstruction.
**Limitations:** Only a small sample size was followed up.
**Ethics committee approval:** This study was approved by an ethics committee.
**Funding for this study:** No funding was received for this study.
**Author Disclosures:**
Marzia Salgarello: Nothing to disclose
Simone Palma: Nothing to disclose
Giulia Macrì: Nothing to disclose
Francesca Ferrara: Nothing to disclose
Liliana Barone Adesi: Nothing to disclose
Riccardo Manfredi: Nothing to disclose
Giovanna Ciriello: Nothing to disclose
Paolo Belli: Nothing to disclose
Anna Anna D'Angelo: Nothing to disclose

### RPS 2602-5

**Multi-detector CT chest: can it replace contrast enhanced spectral mammography in breast cancer patients candidate for CT staging?!**

*A-D. Salwa Yahya Ahmed*, A. F. Moustafa, E. Faker, A. Abd ElLatif, A. H. Radwan, R. W. Abdel Hamid; Cairo/EG

**Purpose:** To compare contrast-enhanced MDCT chest and CESM in detection of multifocal, multicentric or bilateral breast disease in patients candidate for CT chest as a part metastatic workup with feasibility of MDCT chest to be a replacing diagnostic tool allowing locoregional and systemic staging with a single examination, reducing costs and excess patients radiation exposure.
**Methods or Background:** This study included 113 female patients candidate for contrast-enhanced multidetector computed tomography (MDCT) of the chest as part of their metastatic workup (stage II or higher). Contrast-enhanced spectral mammography (CESM) and multi-detector CT chest were performed for all patients.
**Results or Findings:** The study is a retrospective study that included 113 female breast cancer patients candidate for CT staging. 106 of the patients had unilateral carcinoma and 7 of them had bilateral cancer. A total of 120 breasts were evaluated. The CT findings were correlated with CESM findings regarding the multiplicity and bilaterality of the disease. The sensitivity, specificity, PPV, NPV, and accuracy of the CT in the detection of multiplicity were 97.44%, 100%, 100%, 95.45%, and 98.33% respectively and the sensitivity, specificity, PPV, NPV, and accuracy of the CT in the detection of bilateral disease were 68.18%, 97.96%, 88.24%, 93.20%, and 92.50% respectively.
**Conclusion:** Breast cancer patients candidate for MDCT chest as a part of their metastatic workup can omit further need for CESM.
**Limitations:** No limitations were identified.
**Ethics committee approval:** This study was approved by IRB of Baheya centre for early diagnosis and treatment of breast cancer.
**Funding for this study:** No funding was received for this study.
**Author Disclosures:**
Ahmed Abd ElLatif: Nothing to disclose
Al-Dhurani Salwa Yahya Ahmed: Nothing to disclose
Rasha Wessam Abdel Hamid: Nothing to disclose
Eman Faker: Nothing to disclose
Amira Hamed Radwan: Nothing to disclose
AFarouk Moustafa: Nothing to disclose

### RPS 2602-7

**The relationship between combined 18F-FDG PET/MRI parameters of primary tumour and clinical-pathological factors in breast cancer patients with stage T1 tumours**

U. Aydos, O. Kurukahvecioğlu, P. Uyar Göçün, *L. Ö. Atay*; Ankara/TR
*(ozlematay@gazi.edu.tr)*

**Purpose:** To evaluate the association of combined quantitative PET/MRI parameters of primary tumour with the clinical and histopathological findings in newly diagnosed breast invasive ductal carcinoma (IDC) patients with clinical stage T1 tumours.
**Methods or Background:** The data of 102 patients with IDC who underwent 18F-FDG PET/MRI for primary staging were evaluated retrospectively. Maximum standardised uptake value (SUVmax), metabolic tumour volume (MTV), total lesion glycolysis (TLG) and minimum apparent diffusion coefficient (ADCmin) values of primary tumours were obtained from PET/MR images. The SUVmax/ADCmin, MTV/ADCmin and TLG/ADCmin ratios were calculated to obtain combined quantitative PET/MRI parameters. The status of nodal and distant metastasis and histopathological data were also recorded.
**Results or Findings:** Metabolic PET parameters and combined imaging parameters were significantly higher in patients with axillary lymph node metastasis compared to those without metastasis ($p<0.001$, for all). For the comparison between patients with and without extraaxillary nodal metastasis, it was found that only MTV/ADCmin and TLG/ADCmin demonstrated significant differences between two groups ($p < 0.05$ for both), with higher values in metastatic group. Although metabolic PET parameters and combined imaging parameters had higher values in patients with distant metastasis compared to those without distant metastasis, only combined PET/MRI parameters demonstrated significant differences between two groups ($p<0.001$ for all). SUVmax/ADCmin had significantly higher values in grade 3 tumours, in hormone receptor negative tumours, in tumours with HER2 overexpression and with higher Ki-67 indices (> 20%) ($p < 0.05$ for all).
**Conclusion:** The combination of quantitative parameters that related to tumour metabolism and cellularity correlated with clinical and histopathological findings in IDC patients with early stage breast tumors. Combined parameters simultaneously obtained by both PET and MRI seem to be a promising tool in predicting poor prognostic factors.
**Limitations:** Limitations were identified retrospective.
**Ethics committee approval:** This study was not approved by an ethics committee.
**Funding for this study:** No funding was received for this study.
**Author Disclosures:**
Pınar Uyar Göçün: Nothing to disclose
Uguray Aydos: Nothing to disclose
Osman Kurukahvecioğlu: Nothing to disclose
Lütfiye Özlem Atay: Nothing to disclose

### RPS 2602-8

**Influence of receptor expression and molecular subtypes on baseline 18F-FDG uptake in breast cancer: systematic review and meta-analysis**

C. M. de Mooij, R. Ploumen, P. Nelemans, C. Mitea, F. M. Mottaghy, M. Smidt, *T. van Nijnatten*; Maastricht/NL
*(Thiemo.nijnatten@mumc.nl)*

**Purpose:** The purpose of this study was to investigate the relationship between immunohistochemical expression of estrogen receptor (ER), human epidermal growth factor 2 (HER2) receptor, and subtypes based on the expression of these receptors, with 18F-FDG uptake measured by positron emission tomography (PET) in breast cancer patients by performing a systematic review and meta-analysis.
**Methods or Background:** PubMed and Embase were searched for studies that compared maximum standardised uptake values (SUVmax) between breast cancer patients positive and negative for ER or HER2, or that compared SUVmax between subtypes based on ER and HER2 status. Two reviewers independently screened the studies and extracted the data. Summary standardised mean differences (SMD) and 95% confidence intervals (CI) were calculated.

**Results or Findings:** Thirty-nine studies describing 5564 patients were included for final analysis. Mean SUVmax was significantly higher in 1462 ER-negative patients than in 3337 ER-positive patients (SMD 0.66, 95% CI 0.55 - 0.77, P < 0.0001) and significantly lower in 3278 HER2-negative patients than in 1526 HER2-positive patients (SMD -0.27, 95% CI -0.45 ~ -0.08, P = 0.0046). Additionally, mean SUVmax was significantly lower in 275 ER-positive/HER2-negative patients than in 194 HER2-positive patients (SMD -0.26, 95% CI -0.45 ~ -0.07, P = 0.0062), significantly lower in 334 ER-positive/HER2-negative patients than in 189 triple negative patients (SMD -0.95, 95% CI -0.18 ~ -0.72, P < 0.0001), and significantly lower in 494 HER2-positive than in 614 triple negative patients (SMD -0.39, -0.62 ~ -0.17, P = 0.0005).
**Conclusion:** The uptake of 18F-FDG measured by SUVmax is significantly influenced by ER and HER2 status in breast cancer patients. Moreover, mean SUVmax significantly differs between subtypes with uptake in ascending order from ER-positive/HER-negative, HER2-positive and triple negative breast cancer.
**Limitations:** No limitations were identified.
**Ethics committee approval:** This study was not approved by an ethics committee
**Funding for this study:** No funding was received for this study.
**Author Disclosures:**
Marjolein Smidt: Nothing to disclose
Cornelis Maarten de Mooij: Nothing to disclose
Roxanne Ploumen: Nothing to disclose
Patty Nelemans: Nothing to disclose
C. Mitea Cristina Mitea: Nothing to disclose
Felix M. Mottaghy: Nothing to disclose
Thiemo van Nijnatten: Nothing to disclose

**15:00-16:00** **Room C**

### Research Presentation Session: Genitourinary

### RPS 2607

### Diffuse renal pathologies and stone disease: are we better in the meantime?

**Moderator**
A. Ljimani; Düsseldorf/DE

### RPS 2607-2

**Diffusion kurtosis imaging and arterial spin labelling for the noninvasive evaluation of persistent post-contrast acute kidney injury**
*B. Wang*[1], Y. Wang[1], L. Li[1], J. Guo[2], P-Y. Wu[2], H. Zhang[1], H. Zhang[3]; [1]Taiyuan/CN, [2]Beijing/CN, [3]Hangzhou/CN
*(wangbin_915@163.com)*

**Purpose:** We investigated whether diffusion kurtosis imaging (DKI) and arterial spin labelling (ASL) facilitated the assessment of serial alterations in persistent post-contrast acute kidney injury (PC-AKI)
**Methods or Background:** We randomly divided 24 rats into four PC-AKI groups (days 1, 3, 7, and 13, n = 6/group), with an additional six control animals. We conducted functional magnetic resonance imaging, DKI, and ASL analyses. Mean kurtosis (MK), axial kurtosis (Ka), mean diffusivity (MD), fractional anisotropy (FA), radial kurtosis (Kr), and renal blood flow (RBF) maps were normalised to baseline to calculate adjusted △RBF, △MK, △Ka, △MD, △FA, and △Kr values. We also investigated urinary neutrophil gelatinase associated lipocalin (NGAL), serum cystatin C (CysC), aquaporin-2 (AQP2), hypoxia-inducible factor-1 (HIF-1α), and histological indices.
**Results or Findings:** In the inner stripe of the outer medulla, when compared with controls, decreased △FA and △MD levels were observed on days 1, 3, and 7, and a distinct elevation in △MK and △Kr on days 1-13, and a persistent decrease in △RBF on days 1-13, and a prominent increase in △Ka on days 7 and 13 in PC-AKI animals (all p<0.05). △Ka and △MK were positively correlated with AQP-2 (r=0.8086, p<0.0001 and r=0.7314, p<0.0001, respectively), and △RBF was highly correlated with HIF-1α (r=-0.7592, p<0.0001). Moreover, both CysC and NGAL were significantly elevated in PC-AKI animals when compared with controls from days 1-13 (all p<0.05). Renal histological data indicated severe tubular and glomerular injury at days 1-13 in all PC-AKI groups.
**Conclusion:** ASL and DKI may be noninvasively and longitudinally used to detect PC-AKI and predict further outcomes.
**Limitations:** Our study was limited by baseline variations in renal functional parameters among rats.
**Ethics committee approval:** This study was approved by the Animal Care and Use Committee at our institute.
**Funding for this study:** Funding was received for this study by the National Scientific Foundation of China.
**Author Disclosures:**
Yongfang Wang: Nothing to disclose
Pu-Yeh Wu: Nothing to disclose
Hui Zhang: Nothing to disclose
Lina Li: Nothing to disclose
Jinxia Guo: Nothing to disclose
Bin Wang: Nothing to disclose
Hong Zhang: Nothing to disclose

### RPS 2607-3

**Diagnostic value of contrast-enhanced ultrasound in renal grafts: a 12 year tertial referral centre experience**
M. H. Lerchbaumer, T. Fischer, B. Hamm, *P. Spiesecke*; Berlin/DE

**Purpose:** Sonography (including Doppler) is an established modality both for short-term follow-up. To clarify unclear B-Mode Sonography findings, contrast-enhanced ultrasound (CEUS) allows more detailed evaluation of the microcirculation. Therefore, the aim of the present study is to investigate the diagnostic performance of CEUS in the diagnostic of pathologies of renal transplants.
**Methods or Background:** A systematic query of CEUS examinations of renal transplants between 2008 and 2020 in our institution was performed. Following inclusion criteria were definded: I) patient age ≥ 18 years and II) confirmation of diagnosis by biopsy and histopathology or imaging follow-up by either CEUS, contrast-enhanced computed tomography (ceCT), contrast-enhanced magnetic resonance imaging (ceMRI), angiography or intraoperative finding. Exclusion criteria were: I) study examination of CEUS and II) another indication than dedicated examination of renal transplant.
**Results or Findings:** Overall, N = 78 patients were included in the statistical analysis. Analysis revealed high sensitivity (92.2%, 95%-confidence interval [CI] 81.5 – 96.9%) and high specificity (88.9%, 95%-CI 71.9 – 96.1%). Sensitivity and specificity resulted in the sub-cohort of tumors (n = 23 participants) in 100% (95%-CI 80.6 – 100.0%) and 74.1% (95%-CI 35.9 – 91.8%) and in a sub-cohort with non-focal findings (n = 55 participants) in 72.7% (95%-CI 43.4 – 90.3%) and 95.5% (95%-CI 84.9 – 98.7%), respectively.
**Conclusion:** Ultrasound contrast agents do not impair renal function and are therefore attractive to use in patients after renal transplant. Due to the superficial location of the grafts, they are much more accessible to US than autologous kidneys, which again advocates the use of CEUS. Our study results suggest a relevant role for CEUS in the clarification of findings requiring contrast agent application in renal transplants.
**Limitations:** The study is retrospective.
**Ethics committee approval:** This study was approved by the local ethics committee.
**Funding for this study:** No funding was received for this study.
**Author Disclosures:**
Bernd Hamm: Nothing to disclose
Paul Spiesecke: Nothing to disclose
Markus Herbert Lerchbaumer: Nothing to disclose
Thomas Fischer: Nothing to disclose

### RPS 2607-4

**Association of adipose tissue volume with kidney function parameters**
*K. Mueller-Peltzer*[1], B. Mujaj[1], R. Lorbeer[2], A. Peters[3], C. L. Schlett[1], F. Bamberg[1]; [1]Freiburg/DE, [2]Munich/DE, [3]Neuherberg/DE
*(katharina.mueller-peltzer@uniklinik-freiburg.de)*

**Purpose:** To determine the relationship of adipose tissue (AT) compartments assessed by magnetic resonance imaging (MRI) and renal function parameters in a western general population without history of prior renal function impairment.
**Methods or Background:** From the general population, 377 subjects underwent T1-DIXON whole-body 3Tesla-MRI. Visceral (VAT) and subcutaneous (SAT) adipose tissue were quantified by a semi-automatic algorithm. Serum creatinine (Cr), cystatin-C (CysC) and urine microalbumin (uMA) were quantified using standardised tests. Estimated glomerular filtration rates (e-GFR) were calculated based on creatinine (e-GFRcr) and cystatin-C (e-GFRcys).
**Results or Findings:** After multivariate adjustment for known risk factors including measures of obesity, VAT volume was inversely associated with eGFRcys (ß:–2.77, 95%CI:[-5.15; -0.39], p=<0.02), and positively associated with CysC (ß:0.03, 95%CI:[0; 0.05], p=0.02). In contrast, SAT was not associated with CysC, uMA and eGFRcys (all p>0.096). Stratified analyses according to body mass index (BMI) yielded confirmation of these findings also in obese subjects (for BMI >30, e-GFRcys: ß:-7.49, 95%CI:[-13.43; -1.56], CysC: ß:0.08, 95%CI:[0.01; 0.15], p<0.05 respectively). Stratified analyses according to sex did not provide any gender differences.

**Conclusion:** VAT is positively associated with CysC and inversely associated with eGFRcys, which might suggest a direct involvement of VAT in increased metabolism of CysC and therefore in impaired kidney function. Volumetric fat quantification by MRI may provide additional information for better risk stratification regarding renal function impairment in obesity.
**Limitations:** The single-centre study population is limited to 377 subjects, the results need to be confirmed in a larger study population.
**Ethics committee approval:** The local institutional ethics committee approved this study.
**Funding for this study:** Funding was received for this study by Helmholtz-Zentrum-München, German Research Center for Environmental Health, German Federal Ministry of Education and Research, State of Bavaria. German Research Foundation (Project ID 245222810), German Centre for Diabetes Research, German Centre for Cardiovascular Disease Research.
**Author Disclosures:**
Annette Peters: Nothing to disclose
Roberto Lorbeer: Nothing to disclose
Blerim Mujaj: Nothing to disclose
Christopher L. Schlett: Nothing to disclose
Fabian Bamberg: Nothing to disclose
Katharina Mueller-Peltzer: Nothing to disclose

### RPS 2607-6

**Diagnostics of radiographic contrast-induced nephropathy by functional renal MR perfusion imaging**
*A. Ljimani*, T. Thiel, J. Schweitzer, T. Xia, E. Bechler, B. Valentin, F. Boege, H-J. Wittsack, R. Westenfeld; Düsseldorf/DE

**Purpose:** The purpose of our study was to evaluate changes of perfusion and oxygenation properties in renal tissue due to radiographic contrast media (XCM) by arterial spin labelling (ASL) and blood oxygen level dependent (BOLD) MRI.
**Methods or Background:** 15 patients with history of severe chronic kidney disease (CKD) (5 females and 10 males, mean age 76.3 ± 8.1 years) undergoing coronarography with administration of XCM were scanned pre and post-intervention on a clinical 3T MR-Scanner. Renal clinical parameters were collected for all included patients before and 48 h post-intervention. For renal ASL, paracoronal FAIR-TrueFisp sequence (TR/TE 4.0/2.0 ms, TI 1200 ms, 30 averages) were acquired. For renal BOLD, paracoronal multi-GRE (TR 100,0ms; 6 TE: 2.46ms, 4.92ms, 7.38ms, 9.84ms, 12.30ms, 14.75ms). Parameter maps were quantified for both methods and region-of-interest-based analysis was performed. The results were analysed by R and correlated to renal clinical parameters.
**Results or Findings:** Considering the renal damage after the intervention, the patients were divided into two groups: patient without renal involvement (non-CIN, n = 10) and with contrast-induced nephropathy (CIN) group (n = 5). Mean renal blood flows measured by ASL in cortex and medulla show a significant decrease before and after administration of XCM in patients with CIN ($p<0.05$). Resembling results occur for BOLD. Interestingly, perfusion values correlated significantly with acquired renal clinical parameters of the CKD patients ($p<0.05$).
**Conclusion:** Perfusion and oxygenation properties in renal tissue assessed by functional MR perfusion imaging might reflect renal changes due to administration of nephrotoxic XCM and might be a useful tool for the improvement of renal surveillance in high-risk patients.
**Limitations:** No limitations were identified.
**Ethics committee approval:** The study was approved by the Ethics Committee of HHU Düsseldorf.
**Funding for this study:** No funding was received for this study.
**Author Disclosures:**
Julian Schweitzer: Nothing to disclose
Birte Valentin: Nothing to disclose
Eric Bechler: Nothing to disclose
Thomas Thiel: Nothing to disclose
Ralf Westenfeld: Nothing to disclose
Alexandra Ljimani: Nothing to disclose
Toagetu Xia: Nothing to disclose
Hans-Jörg Wittsack: Nothing to disclose
Friedrich Boege: Nothing to disclose

### RPS 2607-7

**Can virtual unenhanced images in dual-energy CT replace true unenhanced examination in measuring urinary stone volume?**
*Y. Cheng*, J. Li, J. Guo; Xi'an/CN
*(1291068604@qq.com)*

**Purpose:** To evaluate the accuracy of using virtual unenhanced (VUE) images in dual-energy CT to measure the volume of urinary stones in comparison with true unenhanced (TUE) examination.
**Methods or Background:** 83 patients with urinary stones who underwent unenhanced and contrast-enhanced CTU were included (Table 1). TUE and VUE images at nephrographic (VUE(NP)) and excretory phase (VUE(EP)) were analysed. Stone volume was calculated using the tool provided by AW.4.7. VUE(EP) images were divided into three groups according to the degree of residual iodine (Fig 1). Wilcoxon test was used to compare the stone volume between TUE and VUE groups.
**Results or Findings:** 104 urinary stones were detected on both the TUE images and VUE images. Stone volumes determined on the VUE(NP) images and VUE(EP) images with none or mild iodine residuals were systematically smaller than those of the TUE images with volume measurement differences of 17%, 16%, and 22% for the above three types of images, respectively. However, stone volume on the VUE(EP) images with severe iodine residuals were not different from the TUE images (Table 2 and Figure 2) due to the contribution of the iodine residuals.
**Conclusion:** Compared with the TUE images, VUE images in dual-energy CT underestimate the volume of urinary stones by about 20%, and the incomplete removal of iodine in VUE(EP) images may artificially reduce the volume measurement difference.
**Limitations:** The study has a small sample.
**Ethics committee approval:** This study was approved by the Institutional Review Board of the first author’s affiliation and written informed consent was obtained from all patients.
**Funding for this study:** This work was supported by the Key R&D Program of Shaanxi Province Universities and Colleges [NO. 2020GXLH-Y-026]. 3D Printing Medical Research Funding Project of the First Affiliated Hospital of Xi'an Jiaotong University [NO. XJTU1AF-3D-2018-003].
**Author Disclosures:**
Yannan Cheng: Nothing to disclose
Jianying Li: Nothing to disclose
Jianxin Guo: Nothing to disclose

### RPS 2607-8

**Improvement of diagnostics of myeloma specific renal changes by functional MRI-biomarkers**
*B. Valentin*, R. Zukovs, L. Prehm, T. Thiel, H. Wittsack, A. Boquoi, R. Fenk, A. Ljimani; Düsseldorf/DE
*(birte.valentin@med.uni-duesseldorf.de)*

**Purpose:** To evaluate multiparametric functional renal MRI (mpMRI) for the assessment of myeloma specific (MM) renal changes.
**Methods or Background:** 37 MM-patients and 10 age-matched healthy volunteers without any history of renal damage were examined on a 3T whole-body MR-scanner (MAGNETOM Prisma, Siemens Healthineers, Germany) by mpMRI-protocol including the following sequences: diffusion weighted and tensor imaging (DWI/DTI), arterial spin labelling (ASL) and blood oxygenation level dependent (BOLD). The frequently used clinical parameters, including glomerular filtration rate (eGFR), total 24-hour proteinuria (TPU), serum albumin, affected immunoglobulin (IG) and free light chains (FLCs) levels were correlated with assessed mpMRI parameter. Patients were divided in groups due to their renal impairment due to MM.
**Results or Findings:** All acquired MR-parameters correlated significantly with eGFR, respectively ($p<0.05$). Medullary BOLD correlated significantly with IG and FLCs, and medullary FA with TPU and IG levels, respectively ($p<0.05$). Significant difference between mean medullary BOLD, ASL and DTI values could be shown in patients with severe renal impairment compared to less impaired patients. BOLD, medullary DWI and DTI values of healthy controls were significantly different from MM patients.
**Conclusion:** mpMRI exhibit a good correlation with frequently used clinical parameters for evaluation of MM specific renal changes. Especially the possibility to differentiate between mild and severe renal MM damage indicate mpMRI as potential important diagnostic tool for assessment of MM specific renal changes.
**Limitations:** No limitations were identified.
**Ethics committee approval:** The study was conducted according to the guidelines of the Declaration of Helsinki and approved by the Ethics Committee of Heinrich Heine University Düsseldorf.
**Funding for this study:** A.L. is supported by an internal research grant of the local Research Committee of the Medical Faculty of Heinrich-Heine-University Düsseldorf.
**Author Disclosures:**
Roland Fenk: Nothing to disclose
Amelie Boquoi: Nothing to disclose
Birte Valentin: Nothing to disclose
Thomas Thiel: Nothing to disclose
Lukas Prehm: Nothing to disclose
Alexandra Ljimani: Nothing to disclose
Romans Zukovs: Nothing to disclose
Hansjoerg Wittsack: Nothing to disclose

**15:00-16:00** **Room D**

### Research Presentation Session: Neuro

### RPS 2611

### Imaging and interventions in neurovascular diseases

**Moderator**
D. P. Auer; Nottingham/UK

### RPS 2611-2

**Diffusion tensor imaging in transcatheter aortic valve implantation; association of metric changes and cerebral ischaemic injury**

*A. Varga*, G. Gyebnár, F. I. Suhai, C. Póka, S. Borzsák, P. Maurovich-Horvat, B. Merkely; Budapest/HU

**Purpose:** To assess diffusion tensor imaging (DTI) metric changes in the corpus callosum (CC) and cingulum, and correlate these changes with ischaemic lesion load (ILL) following transcatheter aortic valve implantation (TAVI).
**Methods or Background:** 78TAVI subjects had DTI post-TAVI (≤10days) and at 6 months (males 56%, age 79 years±6). The number, volume of post-TAVI ischaemic brain lesions (IBL) were recorded. Average DTI scalar metrics (fractional anisotropy=FA; mean, axial, radial diffusivities=MD, AD, RD, respectively) were calculated in 7 regions (genu, body, splenium of CC, letf/right cingulate gyri (CG) and parahippocampal gyri (PHG)) and were compared with the Student's paired t-test ($p<0.007$) and ANOVA covarying for sex, age ($p<0.05$) and ILL ($p<0.0167$).
**Results or Findings:** IBLs were frequent (95%). In 4 regions significant AD reduction was detected (genu, body, splenium, right PHG; $p \leq 0.0023$), with a significant decrease of MD (body of CC: $p=0.0006$) and FA (splenium: $p<0.0001$). Significant RD and MD reduction was found in both CG ($p \leq 0.0053$). ANOVA confirmed significant effect of female sex on AD and MD reduction (body, right CG) and AD reduction (left CG); $p \leq 0.0254$. Significant effect of ILL on the following DTI metric changes was also found (AD, MD-body; MD-left CG; $p \leq 0.0050$ for all).
**Conclusion:** The trend of DTI metric changes is opposite to the general trends reported in various neurodegenerative conditions, suggesting that TAVI might improve cerebral microstructural integrity, especially in women and lower ILL.
**Limitations:** 1) DTI is a mathematical representation of the underlying structure, not always reflecting true anatomy. 2) Only 7 tracts with reliable manual correction of the automated segmentation were studied. 3) Possible selection bias as patients with an improved general/cognitive state are more likely to participate at follow-ups.
**Ethics committee approval:** Medical Research Council registration number: 034489-004/2016/OTIG
**Funding for this study:** Funding was received from the National Research, Development and Innovation Office (NVKP-16-1-2016-0017).
**Author Disclosures:**
Csenge Póka: Nothing to disclose
Pál Maurovich-Horvat: Nothing to disclose
Bela Merkely: Nothing to disclose
Ferenc Imre Suhai: Nothing to disclose
Andrea Varga: Nothing to disclose
Sarolta Borzsák: Nothing to disclose
Gyula Gyebnár: Nothing to disclose

### RPS 2611-3

**Brush sign and collateral supply as potential markers of infarct growth after successful thrombectomy**

*A. Bani Sadr*, D. Pavie, L. Mechtouff, M. Cappucci, M. Hermier, L. Derex, T-H. Cho, N. Nighoghossian, Y. Berthezene; Lyon/FR
*(apbanisadr@gmail.com)*

**Purpose:** To investigate the impact of brush sign and cerebral collateral status on infarct growth after successful thrombectomy.
**Methods or Background:** The HIBISCUS-STROKE cohort includes patients with acute ischaemic stroke treated with thrombectomy after MRI triage and, undergoing a follow-up MRI at Day-6 including FLAIR images to quantify final infarct volume (FIV). Successful revascularisation was defined as a mTICI score ≥ 2B. Infarct growth was calculated by subtracting the final FLAIR images from the admission DWI after co-registration and, considered significant (SIG) when expansion > 11.6mL. Brush sign was assessed on T2* images and was defined as abnormal visualization of medullary veins. Cerebral collateral status was assessed using the hypoperfusion intensity ratio, which is the volume of Tmax ≥ 10s divided by the volume of Tmax ≥ 6s. Good collaterals were defined by a hypoperfusion intensity ratio < 0.4.
**Results or Findings:** Eighty-eight patients were included, of whom 30 (34.1%) had a brush sign and, 42 (47.7%) good collaterals. The brush sign was associated with higher relative cerebral blood volume ($p=0.05$), larger penumbra ($p<0.001$), infarct growth ($p=0.01$) and, larger FIV ($p=0.02$). Patients with good collaterals had a smaller ischaemic core ($p<0.001$), larger penumbra ($p=0.05$) and, lower FIV ($p<0.001$). Collateral status was not associated with brush sign ($p=0.18$) or infarct growth ($p=0.72$). Nineteen (21.6%) patients experienced SIG. Univariate regressions indicated that brush sign (OR: 4.86, 95% CI: [1.69; 14.93], $p=0.004$) and haemorrhagic transformation (OR: 1.61; 95% CI: [1.05; 2.65], $p=0.04$) were predictive of SIG. In multivariate regression, only the brush sign remained predictive of SIG (OR: 5.21, 95% CI: [1.61-18.78], $p=0.007$).
**Conclusion:** Brush sign is a predictor of infarct growth after successful thrombectomy.
**Limitations:** The design was retrospective and monocentric.
**Ethics committee approval:** This study was approved by an ethics committee.
**Funding for this study:** Funding was received from the French National Research Agency (ANR-16-RHUS-0009).
**Author Disclosures:**
Yves Berthezene: Nothing to disclose
Dylan Pavie: Nothing to disclose
Marc Hermier: Nothing to disclose
Laura Mechtouff: Nothing to disclose
Norbert Nighoghossian: Nothing to disclose
Matteo Cappucci: Nothing to disclose
Tae-Hee Cho: Nothing to disclose
Laurent Derex: Nothing to disclose
Alexandre Bani Sadr: Nothing to disclose

### RPS 2611-5

**Sinovenous outflow patency predicts radiosurgical outcomes of dural arteriovenous fistulas in the transverse-sigmoid sinus**

*Y-S. Hu*[1], C-A. Wu[2], C-J. Lin[2], H. Wu[2], C-B. Luo[2], C-C. Lee[2], H-C. Yang[2], W-Y. Chung[2], W-Y. Guo[2]; [1]New Taipei/TW, [2]Taipei/TW

**Purpose:** Sinovenous outflow plays a role in clinical presentation, classification, and therapeutic strategy of transverse-sigmoid sinus dural arteriovenous fistulas (TSDAVFs). Therefore, we conducted the study to explore the impact of sinovenous outflow patency on radiosurgical outcomes of TSDAVFs.
**Methods or Background:** We retrospectively (1995–2020) included 83 TSDAVFs treated with radiosurgery. Two neuroradiologists blinded to the therapeutic outcomes served as imaging evaluators on pretreatment angiography and MRI. The sinovenous outflow of TSDAVF was scored using combined conduit score (CCS), ranging from 0 (total occlusion) to 8 (full patency). The patients' follow-up MRI and angiography images were evaluated for the presence of TSDAVF obliteration. Cox regression and Kaplan–Meier analyses were performed to determine the correlations between the variables and outcomes.
**Results or Findings:** Among the 83 cases, 60 (72%) achieved obliteration after radiosurgery at a median latency period of 24.5 months. After adjustment for aggressive presentation, cortical venous reflux, straight sinus reflux, and optic nerve sheath enlargement, a CCS of >6 remained predictive of TSDAVF obliteration (hazard ratio: 2.335, $p = 0.007$). The estimated 36-month probabilities of obliteration were 80% and 53.6% for TSDAVFs with a CCS of >6 and ≤6, respectively.
**Conclusion:** TSDAVFs with a CCS of >6, indicating a nearly patent sinovenous outflow, were more likely to be obliterated after radiosurgery. The evaluation of sinovenous outflow patency may help in predicting treatment outcomes and making therapeutic decisions.
**Limitations:** The small sample size and retrospective design may have led to selection bias.
**Ethics committee approval:** This study was approved by an ethics committee.
**Funding for this study:** Funding was received from Taipei Veterans General Hospital (grant number: V110C-056) and Taiwan's Ministry of Science and Technology (grant number: MOST-109-2628-B-010-014-MY2).
**Author Disclosures:**
Cheng-Chia Lee: Nothing to disclose
Chao-Bao Luo: Nothing to disclose
Chung-Jung Lin: Nothing to disclose
Yong-Sin Hu: Nothing to disclose
Huai-Che Yang: Nothing to disclose
Chia-An Wu: Nothing to disclose
Wan-Yuo Guo: Nothing to disclose
Hsiumei Wu: Nothing to disclose
Wen-Yuh Chung: Nothing to disclose

### RPS 2611-6

**Impact of the laboratory results and initial CT findings on cerebral vasospasm and intrahospital mortality in aneurysmal subarachnoid haemorrhage**

*V. Opancina*; Kragujevac/RS

**Purpose:** The main objective of this study was to examine the laboratory values and initial head CT findings, associated with an increased or decreased risk of cerebral vasospasm and intrahospital mortality, which develop after aneurysmal subarachnoid haemorrhage.
**Methods or Background:** Aneurysmal subarachnoid haemorrhage is a type of spontaneous haemorrhagic stroke which is caused by a ruptured cerebral aneurysm. Cerebral vasospasm is its most severe complications. The study was designed as a cross-sectional study. It included all patients ≥18 years, with 1st time diagnosed aneurysmal subarachnoid haemorrhage, treated with endovascular embolization, at our institution, during 2017-2012. The following variables were examined: socio-demographic data, CT findings, laboratory analysis (maximum recorded and nadir values): blood count, coagulation tests and biochemistry analysis. The impact of these variables on the two main outcomes was investigated by univariate and multivariate logistic regression.
**Results or Findings:** In total, 66 patients were enrolled. The univariate analysis showed that intraventricular haemorrhage in CT, maximum recorded INR and leucocytes were strongly associated with cerebral vasospasm increasing its chances by 3.5 (95%CI=1.28-9.81), 4.1 (95%CI=1.45-11.56), 6.7 (95%CI=2.10-21.71). The multivariate analysis showed that delayed ischaemic neurological deficit, haemorrhage in the 4th ventricle, and the maximum recorded urea were strongly associated with intrahospital mortality, increasing its chances by 16.3, 12, and 12.6 times, respectively.
**Conclusion:** The effect of elevated INR on cerebral vasospasm in our study may reflect a similar effect of systemic inflammation on the coagulation cascade, and may have a therapeutic effect. Monitoring of renal function and urea levels in patients with aneurysmal subarachnoid haemorrhage is important as maximum recorded urea is significant risk factor for intrahospital mortality, as well as intraventricular haemorrhage.
**Limitations:** This was a unicentric study.
**Ethics committee approval:** This study was approved by the Institutional Ethics Committe and formed part of my PhD thesis.
**Funding for this study:** Not applicable
**Author Disclosures:**
Valentina Opancina: Nothing to disclose

### RPS 2611-7

**Mobile commercial consumer-grade device as an option for telemedicine - evaluation of diagnostic performance and efficiency in intracranial arterial aneurysm detection**

*E. Can*, E. Sodemann, J. Kolck, J. Stöckel, C. Güttler, T. Walter-Rittel, A. Maaßen, G. Bohner, G. Böning; Berlin/DE
*(elif.can@charite.de)*

**Purpose:** To evaluate a commercially available mobile device for use in highly specialised telemedicine for detection of intracranial arterial aneurysm.
**Methods or Background:** Six radiologists with 3 different levels of experience retrospectively evaluated 60 CT angiographies for the presence of intracranial arterial aneurysm, with confirmed positive findings in 30 cases. Each radiologists reviewed the angiography datasets twice: once on a dedicated medical-grade workstation and again, after an interval of 3 months, on a commercially available mobile consumer-grade device.
Diagnostic performance, reading efficiency, and subjective scorings including diagnostic confidence were analysed and compared.
**Results or Findings:** Diagnostic performance was comparable on both devices regardless of the readers' experience levels, and no significant differences in sensitivity (66-87.5%) and specificity (79.4-87%) (p = 0.157-1) were found. The results obtained with both devices were also comparable in terms of subjective assessment across all reader groups. However, reading efficiency was significantly poorer when the mobile device was used.
**Conclusion:** Diagnostic performance was comparable on both devices, while efficiency was lower on the mobile device.
**Limitations:** Since the same datasets were read twice by each radiologist for comparability, a memory effect may theoretically occur. To minimise such an effect, a 3-month waiting interval was chosen and the datasets were presented in a different order during the second reading.
**Ethics committee approval:** Ethics committee approval is given.
**Funding for this study:** There is no funding for this study.
**Author Disclosures:**
Elisa Sodemann: Nothing to disclose
Juliane Stöckel: Nothing to disclose
Elif Can: Nothing to disclose
Georg Bohner: Nothing to disclose
Christopher Güttler: Nothing to disclose
Johannes Kolck: Nothing to disclose
Thula Walter-Rittel: Nothing to disclose
Georg Böning: Nothing to disclose
Anna Maaßen: Nothing to disclose

### RPS 2611-8

**Novel imaging for acute stroke - bioelectromagnetic tomography is safe and sensitive to detect stroke lesions**

M. Vosko[1], S. Sahebdivan[2], C. Brunner[3], W. Struhal[3], T. Henriksson[2], *F. Romero-Hinrichsen*[2], C. El-Salloum[2], S. Semenov[2], S. Pearce[4]; [1]Linz/AT, [2]Vienna/AT, [3]Tulln/AT, [4]Fremont, CA/US

**Purpose:** Acute stroke is a neurological emergency regarding rapid therapy based on brain imaging differentiation between haemorrhagic and ischaemic lesions. Computerised tomography and magnetic resonance imaging are method of the choice, but mostly located in hospital facilities. Bioelectromagnetic tomography (BET) seems to be able to detect brain lesion due to dielectric properties of brain tissue without radiation.
**Methods or Background:** We performed a singlecentre, limited scale exploratory feasibility clinical study using a BET in 10 volunteers and 30 patients with acute stroke after clinical examination and reference imaging: computerised tomography (CT) or magnetic resonance (MRI). Safety, sensitivity and specificity of BET was analysed.
**Results or Findings:** 40 participant were enrolled in the study, of whom 10 healthy volunteers (HV) and 30 patients with acute stroke (6 haemorrhagic and 24 ischaemic). 5/10 HV measurements had to be excluded from the further analysis due to measurement mistake. In 5 HV, 5 valid BET scans were obtained and all five were true normal without signs of ischaemic, nor haemorrhage. Among all patients, 9 scans in ICH and 38 scans in AIS patients underwent image processing. BET was true positive in 9/9 ICH scans and true positive in 37/38 AIS scans, 1 scan was non conclusive. The haemorrhagic exclusion analysis showed a 100% of sensitivity, 97.7% of specificity, 98.1% of accuracy, and 0.0% false omission rate.
**Conclusion:** Electromagnetic tomography is a safe and feasible method used in acute stroke patients. Characteristic BET-patterns were calculated for cerebral ischaemia and haemorrhagic stroke.
**Limitations:** Further studies are needed to examine/confirm the sensitivity and specificity in acute stroke patients with different locations and size of cerebral lesions.
**Ethics committee approval:** Approved by Ethikkommission des Landes Oberösterreich on 25.04.2017
**Funding for this study:** The study was privately funded by EMTensor GmbH.
**Author Disclosures:**
Milan Vosko: Investigator: Principal Investigator of conducted clinical study for EMTensor GmbH
Christian El-Salloum: Employee: EMTensor GmbH
Walter Struhal: Investigator: Investigator of conducted clinical study for EMTensor GmbH
Francisco Romero-Hinrichsen: Employee: EMTensor GmbH
Stephen Pearce: CEO: EMTensor Inc. is the parent company to EMTensor GmbH
Serguei Semenov: Board Member: EMTensor GmbH
Cornelia Brunner: Investigator: Investigator of conducted clinical study for EMTensor GmbH
Sahar Sahebdivan: Employee: EMTensor GmbH
Tommy Henriksson: Employee: EMTensor GmbH

15:00-16:00 Room E1

### Research Presentation Session: Abdominal Viscera

### RPS 2601

### Advances in hepatobiliary imaging

**Moderator**
B. J. Op de Beeck; Antwerp/BE

### RPS 2601-2

**Hepatocellular Adenomas: Gd-EOB-DTPA-enhanced MR imaging features based on 2017 classification system**

*J. R. Tse*[1], E. Felker[2], B. Naini[2], D. Lu[2], S. Raman[2]; [1]Stanford/US, [2]Los Angeles, CA/US

**Purpose:** To determine Gd-EOB-DTPA-enhanced MR features of hepatic adenomas (HCAs) based on the 2017 genotypic classification system.
**Methods or Background:** Histologically proven HCAs evaluated with Gd-EOB-DTPA-enhanced MRI from 2010-2021 were reviewed. HCAs were re-subtyped per 2017 criteria into hepatocyte nuclear factor-1alpha mutated (H-HCA), inflammatory (I-HCA), beta-catenin exon 3 (B-HCA), mixed inflammatory and beta-catenin exon 3 (BI-HCA), sonic hedgehog (SH-HCA), and unclassified (U-HCA). Two fellowship-trained abdominal radiologists independently reviewed images with discrepancies resolved by a third. Signal intensity (SI) ratios of HCAs relative to liver were measured on dynamic phases. Qualitative and quantitative data were correlated with subtype using Fisher's exact test, one-way ANOVA, and logistic regression.
**Results or Findings:** 65 HCAs from 56 adult patients (37±13 years; 49 women, 7 men) were evaluated: 16 H-HCAs, 31 I-HCAs, 6 B-HCA, 4 BI-HCA, 5 SH-HCA, and 3 U-HCA. H-HCAs had intralesional steatosis (15/16; 94%; $p<0.0001$) and portal venous "washout" (15/16; 95%; $p<0.0001$). I-HCAs had moderate T2 hyperintensity (16/31; 52%; $p=0.001$) and atoll sign (18/31; 58%; $p<0.001$). B-HCA/BI-HCAs were large (10.1+/-6.8 cm; $p<0.0001$), necrotic (6/10; 60%; $p<0.001$), hemorrhagic (4/10; 40%; $p=0.039$), and predominantly affected males (5/8; 63%; $p<0.0001$). On hepatobiliary phase, 8/10 (80%) B-HCA/BI-HCAs were isointense or hyperintense, compared to 3/55 (5%; $p<0.0001$) of all other HCAs. SI ratios of B-HCA/BI-HCAs were the highest at 1.04+/-0.21 and different from all other subtypes, including H-HCA (0.46+/-0.12; $p<0.0001$), I-HCA (0.70+/-0.09; $p<0.0001$), SH-HCA (0.75+/-0.10; $p<0.001$), and U-HCA (0.76+/-0.13; $p=0.009$).
**Conclusion:** B-HCA/BI-HCAs were iso- to hyperintense relative to liver on the hepatobiliary phase while almost all other HCAs, including SH-HCAs, were hypointense.
**Limitations:** Singlecentre, retrospective study with relatively small sample size and selection bias with only pathologically-proven HCAs.
**Ethics committee approval:** This study was approved by the Institutional Review Board.
**Funding for this study:** No funding was received for this study.
**Author Disclosures:**
Bita Naini: Nothing to disclose
David Lu: Nothing to disclose
Steven Raman: Nothing to disclose
Ely Felker: Nothing to disclose
Justin Ruey Tse: Nothing to disclose

### RPS 2601-3

**Comparative study of MR elastography and dynamic contrast-enhanced imaging in the staging of NAFLD fibrosis**

*H. Ren*, D. Yang, H. Xu, Z. Yang; Beijing/CN
*(hao_ren@ccmu.edu.cn)*

**Purpose:** The purpose of this study was to evaluate the preliminary study of the DCE-MRI of exchange model on the staging of liver fibrosis and to compare the differential ability of DCE imaging and MRE in the staging of liver fibrosis.
**Methods or Background:** The subjects were NAFLD patients who underwent MRE and DCE in our hospital. All patients had liver biopsy results. Spearman rank correlation coefficient was used to compare the correlation between MRE and DCE and liver fibrosis. ROC was used to evaluate the efficacy of DCE and MRE in the diagnosis of liver fibrosis.
**Results or Findings:** The correlation coefficient between PS parameters of DCE and liver fibrosis was -0.76, the correlation coefficient between FP parameters of DCE and liver fibrosis was -0.75, and the correlation coefficient between liver stiffness and liver fibrosis of MRE was 0.83. Some parameters of DCE (PS, FP) were slightly higher in AUC than in MRE stiffness values in the diagnosis of the presence or absence of liver fibrosis, but there was no statistical difference.
**Conclusion:** MRE has good diagnostic efficacy for all stages of liver fibrosis in NAFLD. DCE has certain advantages over MRE in the diagnosis of early fibrosis, and DCE is expected to be a new tool for the noninvasive assessment of liver fibrosis.
**Limitations:** This study is a retrospective study with less data.
**Ethics committee approval:** All procedures in this study were in accordance with the Ethical Standards of the Institutional Research Committee and with the 1964 Helsinki Declaration and its later amendments or comparable ethical standards.
**Funding for this study:** Funding was received from the National Natural Science Foundation of China (No. 61871276) and the National Science and Technology Major Project (2017ZX0203202-003).
**Author Disclosures:**
Hao Ren: Nothing to disclose
Dawei Yang: Nothing to disclose
Hui Xu: Nothing to disclose
Zhenghan Yang: Nothing to disclose

### RPS 2601-5

**Colorectal liver metastases: MRI appearances before and after chemotherapy**

*D. van der Reijd*, E. Soykan, B. C. Heeres, D. M. J. Lambregts, K. Kuhlmann, T. Buffart, R. G. H. Beets-Tan, M. Maas, E. G. Klompenhouwer; Amsterdam/NL
*(d.vd.reijd@nki.nl)*

**Purpose:** To investigate to what extent colorectal liver metastases (CRLM) show typical imaging characteristics, such as arterial rim enhancement, moderate hyperintensity to liver on T2W-MRI, diffusion restriction and non-enhancement in hepatobiliary phase, on gadoxetic-acid-enhanced MRI before and after chemotherapy.
**Methods or Background:** From November 2015 until February 2021, we retrospectively identified 247 patients with gadoxetic-acid-enhanced MRI before and/or after chemotherapy for pathologically proven CRLM. 664 CRLM were included, with a maximum of 5 CRLM per patient. MRIs with low image quality and mucinous CRLM were excluded. MRI pre-chemotherapy was available in 654 CRLM(n=157 patients), MRI post-chemotherapy in 160 CRLM(n=49 patients) and both pre- and post-therapy MRI in 150 CRLM(n=41 patients). Two expert radiologists assessed size, location, enhancement patterns, signal intensity on multiple sequences, hyperintensity on DWI.
**Results or Findings:** On pre-chemotherapy MRI, only 58% of CRLM showed peripheral rim enhancement, the others appeared homogenous (30%) or heterogeneous (12%) on arterial and portal-venous-phase. On T2W-MRI CRLM presented moderately hyperintense (94%), isointense (5%) and severely hyperintense (1%). Diffusion restriction was found in 87% of CRLM. On post-chemotherapy MRI, CRLM showed similar patterns on contrast series with peripheral enhancement (47%), homogeneous (32%) or heterogeneous (8%) appearance, or disappeared (13%). On T2W-MRI, CRLM presented less often moderately hyperintense (76%) and more often isointense (24%). Post-chemotherapy, diffusion restriction was found in 60% of CRLM. All CRLM (100%) were unenhancing on hepatobiliary phase, both before and after chemotherapy.
**Conclusion:** CRLM do not always show typical imaging characteristics on gadoxetic-acid-enhanced MRI like arterial rim enhancement and diffusion restriction, especially after chemotherapy.
**Limitations:** Not all metastases were pathologically proven; at least 1 per patient.
**Ethics committee approval:** IRB approval was granted.
**Funding for this study:** No funding was received for this study.
**Author Disclosures:**
Denise van der Reijd: Nothing to disclose
Monique Maas: Nothing to disclose
Elisabeth Genevieve Klompenhouwer: Nothing to disclose
Ezgi Soykan: Nothing to disclose
Tineke Buffart: Nothing to disclose
Koert Kuhlmann: Nothing to disclose
Birthe Christina Heeres: Nothing to disclose
Doenja Marina Johanna Lambregts: Nothing to disclose
Regina G. H. Beets-Tan: Nothing to disclose

### RPS 2601-6

**New release of Esaote QElaXto 2D-Shear Wave Elastography (SWE) to evaluate liver stiffness in diffuse chronic liver diseases**

*L. Maiocchi*, M. Colaneri, A. Raimondi, C. Filice; Pavia/IT
*(L.Maiocchi@smatteo.pv.it)*

**Purpose:** To evaluate the agreement between the reference standard method Transient Elastography (TE; Fibroscan, Echosense, Paris, France) and the new release of QElaXto 2D-SWE (Esaote, Genoa, Italy) in liver stiffness (LS) measurement and to determine the cut off value to rule-out compensated advanced chronic liver disease (cACLD).

**Methods or Background:** This is a singlecentre study. We evaluated 166 patients with different etiologies of chronic liver disease (CLD) with QElaXto 2D-SWE and TE. The fibrosis stage was assessed with the "rule of 5" for TE and the "rule of 4" for QElaXto 2D-SWE. Cohen's kappa test was used for the agreement between the 2 techniques. The Receiver Operating Characteristic (ROC) curve analysis was used to identify the cut-off value to rule-out cACLD, using TE as the reference method.
**Results or Findings:** We found a good concordance between the 2 techniques (weighted k = 0.81). The best cut-off value to rule-out cACLD using QElaXto 2D-SWE was 6.9kPa (AUROC 0.93, 95%CI 0.88-0.99).
**Conclusion:** There is a good concordance between QElaxto 2D-SWE and TE in assessing LS. Since the cut-off of 6.9kpa is lower than the one proposed by the "rule of 4", we suggest the implementation of in-house cut-off, which should be evaluated by further analyses.
**Limitations:** Firstly, the patients had different CLD etiologies, mostly HCV and NAFLD. Secondly, the majority of patients (74%) had a fibrosis stage of F0-F1 and F2, with only few patients with F3, which might have limited our analysis.
**Ethics committee approval:** All the subjects agreed to undergo elastographic measurements and signed the informed consent; study was approved by the local Ethics Committee and performed in accordance with the Helsinki Declaration of 1975.
**Funding for this study:** No funding was received for the study execution.
**Author Disclosures:**
Ambra Raimondi: Nothing to disclose
Laura Maiocchi: Nothing to disclose
Carlo Filice: Nothing to disclose
Marta Colaneri: Nothing to disclose

### RPS 2601-7

**High resolution 4D flow MRI as a novel approach to multi-dimensional evaluation of hepatic arterial haemodynamics: in vitro optimisation and volunteer feasibility study**
*I. P. Dimov*[1], C. Tous[1], N. Li[1], M. Barat[1], T. Bomberna[2], N. Jin[3], G. Moran[4], A. Tang[1], G. Soulez[1]; [1]Montreal, QC/CA, [2]Ghent/BE, [3]Cleveland, OH/US, [4]Oakville, ON/CA

**Purpose:** Evaluate in-vitro and in-vivo the ability of 4D flow MRI to non-invasively measure flow in the branches of the common hepatic artery.
**Methods or Background:** A dynamic, 3D printed hepatic artery phantom perfused with pulsatile flow was scanned with 2D phase-contrast (PC) and 4D flow at 0.6-, 0.8- and 1-mm isotropic resolution with navigator acceptance windows of ±2 to ±8 mm. Twenty volunteers were scanned at 0.8- and 1-mm 4D flow with a ±4 mm acceptance window and 2D PC. Haemodynamic parameters at the common hepatic, proper hepatic, gastroduodenal, left and right hepatic branches were extracted. Mean percent error (MPE) compared to ground truth was calculated in-vitro; percent visibility of vascular branches and interobserver agreement with Cohen's kappa and coefficients of variation (CV) were evaluated in volunteers.
**Results or Findings:** MPE of average area, flow, velocity and peak velocity at the highest spatial resolution and narrowest acceptance window were -8.0 %, -4.7%, -2.6%, and -5.7%, respectively. These changed to -10.4%, -6.6%, -1.9%, and -8.8% at the widest navigator acceptance window and to 49.9%, 30.6%, -14.6%, and -17.6% at the lowest spatial resolution. 4D flow at 0.8- and 1-mm resolution were successful in 14 and 18 volunteers, with overall vessel visibilities of 90% and 71%, interobserver agreement of kappa = 0.66 and kappa = 0.70 and CVs of 20.0% and 30.7% for area, 34.9% and 51.9% for flow, 26.5% and 44.7% for average velocity and 36.9% and 39.4% for peak velocity, respectively.
**Conclusion:** 4D flow with high spatial resolution and moderate motion suppression can accurately and reproducibly measure hepatic arterial flow.
**Limitations:** Not applicable
**Ethics committee approval:** The CHUM Research Ethics Board approved this study.
**Funding for this study:** Funding was provided by the Canadian Institutes of Health Research, the TransMedTech Institute, and Siemens Healthineers, Canada.
**Author Disclosures:**
Ning Jin: Employee: Cardiovascular R&D, Siemens Medical Solutions USA, Inc., Cleveland, Ohio, USA
Tim Bomberna: Nothing to disclose
Cyril Tous: Nothing to disclose
Ning Li: Nothing to disclose
Gilles Soulez: Nothing to disclose
An Tang: Nothing to disclose
Ivan P Dimov: Nothing to disclose
Gerald Moran: Employee: Siemens Healthineers, Canada
Maxime Barat: Nothing to disclose

**15:00-16:00** **Room F1**

### Research Presentation Session: Paediatric

### RPS 2612

### Imaging in children: augmenting accuracy and reducing risk

**Moderator**
L. E. Derchi; Genoa/IT

### RPS 2612-2

**Pharmacokinetics, safety and efficacy of a new gadolinium-based contrast agent, gadopiclenol, in paediatric patients (2-17 years) undergoing MRI**
*S. B. Tsvetkova*; Plovdiv/BG

**Purpose:** To evaluate the pharmacokinetic (PK) profile, safety and efficacy of gadopiclenol, a new high-relaxivity gadolinium-based contrast agent, in children aged 2-17 years.
**Methods or Background:** Children scheduled to undergo contrast-enhanced MRI of the Central Nervous System (CNS cohort) or other organs (Body cohort) were included in 3 age groups (12-17, 7-11 and 2-6 years) and four blood samples were collected after gadopiclenol administration (0.05 mmol/kg). A population PK modeling was used with the CNS cohort and adult subjects from a previous study. Adverse events (AEs) were recorded, and efficacy was assessed for all children.
**Results or Findings:** Sixty children in the CNS cohort and 20 in the Body cohort were included. The two-compartment model with linear elimination from the central compartment developed in adults was suitable for children. PK parameters were similar between adults and children. Terminal half-life was 1.82 h for adults and 1.29 to 1.77 h for children. Median clearance ranged from 0.08 L/h/kg in adults and 12-17 years to 0.12 L/h/kg in 2-6 years. Median central and peripheral volume of distribution were 0.11-0.12 L/kg and 0.06 L/kg, respectively, for adults and children. Simulations of plasma concentrations showed minor differences and median area under the curve was 590 mg.h/L for adults and 403 to 582 mg.h/L for children. Two patients (2.5%) experienced AEs considered related to gadopiclenol (non-serious): a mild QT interval prolongation and a moderate maculopapular rash. As for diagnostic efficacy, there was no difference among the paediatric age groups.
**Conclusion:** Gadopiclenol PK in children aged 2-17 years seems similar to that observed in adults. Thus, there is no indication for age-based dose adaptation. Gadopiclenol had a good safety profile in these patients.
**Limitations:** Not applicable.
**Ethics committee approval:** Not applicable.
**Funding for this study:** Funding was received for this study by Guerbet.
**Author Disclosures:**
Silvia Bogdanova Tsvetkova: Investigator: Guerbet

### RPS 2612-3

**Combined metabolic and functional tumour volumes on PET/MRI in neuroblastoma: complementary or redundant information by 18F-FDG uptake and diffusion restriction?**
*M. Chaika*, S. Maennlin, S. Gassenmaier, S. Warmann, C. Urla, M. Esser, I. Tsiflikas, S. Gatidis, J. F. Schäfer; Tübingen/DE

**Purpose:** In neuroblastoma, high metabolic 18F-FDG-tumour-volume is associated with highly viable tissue and poor prognosis. MR-studies including diffusion-weighted imaging have shown that extent of diffusion restriction is related to tumour cell differentiation and response to chemotherapy. The purpose was to evaluate the correlation between standard-uptake-value and apparent-diffusion-coefficient in neuroblastic tumours by voxel-wise-analysis.
**Methods or Background:** Prospective, observational PET/MRI study, all patients diagnosed with NB at baseline and after chemotherapy were included. After image-registration/and tumour-segmentation, metabolic and functional tumour volumes were calculated from ADC and SUV values using a dedicated software allowing voxel-wise analysis. Under the means of thresholds each voxel was assigned to one of 3 virtual-tissue-groups: highly viable (lowADC and highSUV), possibly benign (highADC and lowSUV), and intermediate with highADC and highSUV or lowADC and lowSUV. Additionally, values of ADC and SUV were correlated after clustering of multiple Gaussian distributions.

**Results or Findings:** From 43 PET/MR, 17 examinations of 14 patients met the inclusion criteria. Eight examinations at baseline, 9 for response assessment. The proportion of tumour volumes were 16, 35, 48% (v ,b, i) at baseline and 6, 68, 26% after treatment, respectively. ADC and SUV showed a negative correlation prior and after to treatment (R=-0.332, -0.401; P<0.0001) in the cluster, which contains more than 70% highly viable voxels. Patients with progression under therapy had a relevant part in this cluster. In contrast, ADC and SUV correlated positively in the cluster containing possibly benign/intermediate voxels (R=0.326, 0.295; P<0.0001).
**Conclusion:** Voxel-wise-analysis of ADC and SUV can quantify different quality of tumour-tissue before and after therapy. Only in volumes, which correspond to highly viable tissue, expected negative correlation of lowADC and highSUV was found representing redundant information.
**Limitations:** Positive correlation in "equivocal" voxels could be due to cell differentiation or inflammatory/necrotic processes. Further investigations in larger cohorts have to validate this hypothesis.
**Ethics committee approval:** No required.
**Funding for this study:** No funding required.
**Author Disclosures:**
Maryanna Chaika: Author: PET/MRI, multiparametric analysis, Syngovia by Siemens (Erlangen)
Michael Esser: Nothing to disclose
Simon Maennlin: Nothing to disclose
Cristian Urla: Nothing to disclose
Sergios Gatidis: Nothing to disclose
Jürgen F Schäfer: Nothing to disclose
Steven Warmann: Nothing to disclose
Ilias Tsiflikas: Nothing to disclose
Sebastian Gassenmaier: Nothing to disclose

### RPS 2612-4

**Prognostic impact of CT severity score in childhood cancer with SARS-CoV-2**

M. Romeih, *A. Refaat*, I. Zaky, M. R. M. K. Ibrahim; Cairo/EG
*(amal.mohamed@57357.org)*

**Purpose:** CT chest severity score (CTSS) is a semi-quantitative measure done to correlate the severity of the pulmonary involvement on the CT with the severity of the disease. Aim to describe CTSS of the COVID-19 infection in paediatric oncology patients (POP), to find a cut-off value of CTSS that can differentiate mild cases that can be managed at home and moderate to severe cases that need hospital care.
**Methods or Background:** A retrospective cohort study included 64 (POP) with confirmed COVID-19 infection. They were classified clinically into mild, moderate, and severe groups. CT findings were evaluated for lung involvement and CTSS was calculated and range from 0 (clear lung) to 20 (all lung lobes were affected).
**Results or Findings:** Overall, 89% of patients had haematological malignancies and 92% were under active oncology treatment. The main CT findings were ground-glass opacity (70%) and consolidation patches (62.5%). In total, 85% of patients had bilateral lung involvement, ROC curve showed that the area under the curve of CTSS for diagnosing severe type was 0.842 (95% CI 0.737-0.948). The CTSS cut-off of 6.5 had 90.9% sensitivity and 69% specificity, with 41.7% (PPV) and 96.9% (NPV). According to the Kaplan-Meier analysis, mortality risk was higher in patients with CT score > 7 than in those with CTSS < 7.
**Conclusion:** Paediatric oncology patients, especially those with haematological malignancies, are more vulnerable to COVID-19 infection. Chest CT severity score > 6.5 (about 35% lung involvement) can be used as a predictor of the need for hospitalisation.
**Limitations:** No comparative group including normal immunocompetent paediatric patients, so the generalisability in the paediatric group was not being allowed.
**Ethics committee approval:** Approved by the Ethics Committee of Children Cancer Hospital 57357 Cairo Egypt.
**Funding for this study:** This study was funded by CCH 57357 and (AFNCI).
**Author Disclosures:**
Marwa Romeih: Nothing to disclose
Iman Zaky: Nothing to disclose
Mary Rabea Mahrous Khalil Ibrahim: Nothing to disclose
Amal Refaat: Nothing to disclose

### RPS 2612-5

**Accelerating whole-body MRI for follow-up and prevention of paediatric patients with cancer and cancer predisposition syndromes: preliminary results**

*J. Herrmann*, M. Esser, I. Tsiflikas, J. F. Schäfer; Tübingen/DE
*(judith.herrmann@med.uni-tuebingen.de)*

**Purpose:** Over the last decades, whole-body MRI (WB-MRI) has gained significant importance for staging of paediatric patients with cancer or cancer predisposition syndromes (CPS). One major disadvantage of WB-MRI is its long acquisition time. Revolutionary technological developments enabled an acceleration of MR-sequences. The purpose of our study was to evaluate the feasibility and diagnostic confidence of novel MR sequences to accelerate WB-MRI in paediatric patients.
**Methods or Background:** A total of 90 patients (inclusion criteria: ≥7 years-old, diagnosis of cancer or CPS) are to be included in this institutional review board-approved, prospective study. To date, 28 patients were included, who underwent WB-MRI staging at a 3T MR scanner with our standard WB-MRI protocol including a standard T2-weighted (T2w) Turbo-Inversion Recovery-Magnitude (TIRM) in coronal orientation and a post-contrast T1-weighted (T1w) VIBE DIXON. Additionally a T2w HASTE in coronal orientation with either compressed sensing (CS) or deep learning (DL) and a T1w VIBE DIXON with CS are acquired and compared to the standard sequences. Three readers independently evaluated the images of the sequences concerning image quality and diagnostic confidence (image quality, sharpness, noise, artifacts) on a Likert-Scale ranging from 1 to 5.
**Results or Findings:** In all examined patients, HASTE and VIBE with DL and CS were successfully acquired. The first results showed comparable image quality, extent of artifacts and sharpness, as well as reduced noise compared to standard sequences. For a normal sized adolescent, scan time could be reduced from 9:39 min (TIRM) to at least 3:30 min (HASTE).
**Conclusion:** Using new MRI sequences with CS and DL for WB-MRI in paediatric patients is feasible and may allow for a remarkable time saving in paediatric WB imaging.
**Limitations:** Preliminary results.
**Ethics committee approval:** Approved by the local ethics committee.
**Funding for this study:** No funding was received.
**Author Disclosures:**
Judith Herrmann: Nothing to disclose
Michael Esser: Nothing to disclose
Jürgen F Schäfer: Nothing to disclose
Ilias Tsiflikas: Nothing to disclose

### RPS 2612-6

**Dose levels for paediatric fluoroscopic imaging in the Netherlands**

*G. O. Croes*[1], M. F. Boomsma[1], I. M. Nijholt[1], M. Greuter[2], C. Jeukens[3], A. Vegter[4], G. Streekstra[5], A. J. Dam[1], J. Siegersma[2]; [1]Zwolle/NL, [2]Groningen/NL, [3]Maastricht/NL, [4]Emmen/NL, [5]Amsterdam/NL
*(g.o.croes@isala.nl)*

**Purpose:** The objective of this study was to assess paediatric fluoroscopic dose levels based on exposures in Dutch clinical practice, as the current paediatric fluoroscopic DRLs need an update according to European Guidelines on DRLs for Paediatric Imaging (PiDRL) and are only available for the age categories 0, 1 and 5 years.
**Methods or Background:** Dose area product (DAP)-values were retrospectively collected from paediatric patients (0-18y) who had undergone a fluoroscopic examination between 1-1-2017 and 1-6-2021 in nine Dutch hospitals (total 1047 examinations). The protocols involved were those recommended in the PiDRL guidelines: micturating cystourethrography (MCU), upper gastro-intestinal (UpperGI) and lower gastro-intestinal with contrast enema (LowerGI). In case the period of the provided data was shorter than the time interval of the study, dose information data was partly duplicated to meet the chosen study time interval. In accordance with the PiDRL recommendations for sparse data the 75th percentile of the median DAP values of each hospital were fitted to an exponential dose-age curve which values were compared to European DRLs.
**Results or Findings:** For MCU the proposed DRL curve was a factor 3.7 to 12 lower compared to the current Dutch DRLs (0y: 30 µGy•m2, 1y: 70 µGy•m2 and 5y: 80 µGy•m2). The proposed DRL curve was for UpperGI a factor 2.5 to 4 and for LowerGI a factor 1.4 to 7 lower compared to the current European DRLs.
**Conclusion:** Our study shows that the current dose levels for paediatric fluoroscopic imaging are substantially lower than the current DRLs for MCU, UpperGI and LowerGI. Therefore, our data indicates that determination of new national DRLs for MCU, UpperGI and LowerGI seems absolutely necessary.
**Limitations:** Not applicable.
**Ethics committee approval:** Not applicable.
**Funding for this study:** Not applicable.
**Author Disclosures:**
Jenny Siegersma: Nothing to disclose
Goswin Otmar Croes: Nothing to disclose
Alie Vegter: Nothing to disclose
Cécile Jeukens: Nothing to disclose
Marcel Greuter: Nothing to disclose
Alida Johanna Dam: Nothing to disclose
Martijn Franklin Boomsma: Nothing to disclose
Geert Streekstra: Nothing to disclose
Ingrid M. Nijholt: Nothing to disclose

### RPS 2612-7

**Diagnostic value of abdominal follow-up sonography in paediatric polytrauma patients with inconspicuous initial computed tomography**

*S-C. V. Alt*, K. Wintges, P. Bannas, J. Herrmann, J. M. Weinrich; Hamburg/DE

**Purpose:** Ultrasound (US) is recommended for follow-up (FU) examinations in paediatric polytrauma patients. However, the impact of FU-US in patients with inconspicuous initial computed tomography (CT) remains unclear. We therefore aimed to evaluate the impact and therapeutic relevance of FU-US in paediatric polytrauma patients with inconspicuous initial abdominal CT.
**Methods or Background:** We retrospectively identified all paediatric polytrauma patients who underwent initial CT between February 2009 and May 2019. We included only patients with both inconspicuous CT without abdominal injuries or free intraabdominal fluid and standardised FU-US. For the establishment of the final clinical diagnosis, the discharge report, other imaging examinations and surgical records were reviewed and served as standard of reference.
**Results or Findings:** We identified 217 paediatric polytrauma patients (98 girls; age 9.7±4.8 years) who underwent abdominal CT. One hundred and thirty-three patients (61%) had inconspicuous CT findings and underwent FU-US (59 girls; age 9.2±4.9 years) within 1.4±0.9 days. FU-US revealed no specific posttraumatic pathologies, only minimal intraabdominal fluid in 34/128 patients (26%), which was solely present as pelvic fluid in 29/34 of patients (85%) and within the Koller and/or Morison pouch in the remaining patients. The presence of intraabdominal fluid had no impact on further clinical management and no subsequent parenchymal injury was detected.
**Conclusion:** FU-US in paediatric polytrauma patients with inconspicuous initial abdominal CT did not reveal additional relevant findings and thus had no impact on clinical management. Hence, FU-US in paediatric polytrauma patients with inconspicuous initial abdominal CT is not routinely required. However, in case of equivocal CT findings or abnormal clinical/laboratory findings it may still be considered as a non-invasive and ionising radiation-free FU-imaging method.
**Limitations:** Single centre study.
**Ethics committee approval:** Yes.
**Funding for this study:** None.
**Author Disclosures:**
Peter Bannas: Nothing to disclose
Sophie-Charlotte Victoria Alt: Nothing to disclose
Kristofer Wintges: Nothing to disclose
Julius Matthias Weinrich: Nothing to disclose
Jochen Herrmann: Nothing to disclose

### RPS 2612-8

**Assessment of cancer risk associated with increased radiation field sizes for neonates undergoing chest radiography**

*W. Elshami*[1], M. M. Abuzaid[1], T. Akudjedu[2], H. O. Tekin[1]; [1]Sharjah/AE, [2]Bournemouth/UK
*(welshami@sharjah.ac.ae)*

**Purpose:** Cancer risk is associated with ionising radiation and is related directly to the increasing absorbed radiation dose. Studies indicate that with transition to digital radiography, there has been poor collimation practice consequently resulting in electronic image cropping by some radiographers during paediatric radiography. The advantages of collimation should be investigated to disseminate convenient use among radiographers and create awareness.
**Methods or Background:** The study examined the extent of the effect of collimation on cancer risk to neonates undergoing anteroposterior (AP) chest examination using Monte Carlo Simulation. Using Monte Carlo Simulations, series of cancer risk were measured for neonate underwent AP chest radiography. The minimum radiation field size in the series was set in line with the European guidelines. The field size was increased by 1cm for 5 times. Monte Carlo Simulations were employed for the computation of cancer risk serially (increasing by 1cm) on all sides and in the head-to-feet direction from the minimum field size recommended by the European guidelines.
**Results or Findings:** Increasing the field size increases cancer risk to the breast, colon, liver, lung cancer. Similarly, the risk of leukemia and other cancers increased.
**Conclusion:** Using optimum field size is recommended to reduce unnecessary radiation doses. Therefore, radiographers should be aware of the consequences of excessive field size in cancer risk.
**Limitations:** This is an experimental study.
**Ethics committee approval:** This is an experimental study and exempted from ethical approval.
**Funding for this study:** No funding was received for this study.
**Author Disclosures:**
Theophilus Akudjedu: Nothing to disclose
Wiam Elshami: Nothing to disclose
Huseyin Ozan Tekin: Nothing to disclose
Mohamed M. Abuzaid: Nothing to disclose

**15:00-16:00** **Room G**

### Research Presentation Session: Imaging Informatics / Artificial Intelligence and Machine Learning

### RPS 2605

### Artificial intelligence (AI) in neuroradiology and head and neck imaging

**Moderator**
S. Islam; London/UK

### RPS 2605-2

**Investigating data fusion and training strategies of artificial intelligence for the diagnosis of Parkinson's disease with Dopamine SPECT imaging**

*J. Schmid*[1], A. Arrigo[2], G. Favre-Gillioz[2], N. Nicastro[1], V. Garibotto[1]; [1]Geneva/CH, [2]Lausanne/CH
*(jerome.schmid@hesge.ch)*

**Purpose:** Visual inspection of dopamine transporter SPECT imaging improves diagnostic confidence for Parkinson's disease (PD) but depends on readers' level of expertise. While artificial Intelligence (AI) can provide valuable support, its performance heavily depends on availability and quality of training data. We aim to investigate the performance of AI to detect PD in heterogenous imaging datasets, collected with different equipment and at various locations.
**Methods or Background:** We selected two local SPECT datasets (avg. n=115 images each) that differed in equipment and imaging characteristics, and a larger one (n=598) from the Parkinson Progression Markers Initiative (PPMI). Then we built a deep learning approach and assessed its performance when trained and tested on each dataset individually with data preprocessing (spatial normalization and image augmentation). Finally, we studied the fusion of the different datasets using balanced merging and transfer learning (TL).
**Results or Findings:** For local datasets, data preprocessing improved the average accuracy (acc) of 84% by +5% – especially for the most heterogenous dataset. The training combining all datasets resulted in accuracies ranging from 88% to 96% when tested on each dataset. The best performance was measured with the augmented PPMI dataset (acc. 96%), which was successfully used to initialise the training of the local datasets based on TL.
**Conclusion:** The developed AI showed very promising results, although intra-dataset variability in small datasets hindered AI performance, which could be mitigated with data preprocessing.
**Limitations:** Issues related to size imbalance and image heterogeneities between datasets could not be totally addressed, demanding further investigation, especially in advanced transfer learning and fusion of imaging and patient data.
**Ethics committee approval:** This study was approved by the canton of Geneva Ethics Committee (CER 12-006R).
**Funding for this study:** This research did not receive any funding from public, commercial, or not-for-profit sectors.
**Author Disclosures:**
Guillaume Favre-Gillioz: Nothing to disclose
Adrien Arrigo: Nothing to disclose
Jerome Schmid: Nothing to disclose
Valentina Garibotto: Nothing to disclose
Nicolas Nicastro: Nothing to disclose

### RPS 2605-3

**Whole 3D tumour delineations are required for MR-based radiomic models predictive of locoregional control in oropharyngeal cancer**

*P. Bos*[1], M. van den Brekel[1], M. Taghavi[1], A. Al-Mamgani[1], S. Waktola[1], H. J. W. L. Aerts[2], R. G. H. Beets-Tan[1], J. A. Castelijns[1], B. Jasperse[1]; [1]Amsterdam/NL, [2]Boston, MA/US

**Purpose:** Clinical implementation of radiomics is hampering due to several factors. One of these factors is the need for time-consuming whole tumour delineations. Substitution of faster, easier or readily available delineations might increase clinical adoption of radiomics. Therefore, this study investigates if alternative delineations can substitute 3D whole-tumour contours in the prediction of locoregional control (LRC) in oropharyngeal squamous cell carcinoma (OPSCC) tumours.
**Methods or Background:** Radiomic features were extracted from postcontrast T1-weighted MRIs of 177 OPSCC primary tumours using six different manual delineation strategies. Logistic regression and recursive feature elimination were used to build models predictive of LRC. Models were trained and tested based on data from each separate delineation (method 1), trained and tested based on features extracted by an experienced observer and tested on alternative delineations (method 2). Additionally, method 2 was repeated with the exclusion of shape features (method 3). Prediction performance was assessed using area under the receiver operation characteristic (AUC) curve.
**Results or Findings:** Models trained on delineations performed by an experienced observer perform better (AUC: 0.74) compared to alternative delineations in method 1 (AUC: 0.40-0.56) as well in method 2 (AUC: 0.56-0.67). Removal of shape features decreased model performance of the expert radiologist (AUC: 0.54). Testing this model on alternative delineation shows higher performances on alternative tumour contours, such as the largest axial diameter (AUC: 0.63) or a sphere of a diameter of 4mm placed in the most solid tumour area (AUC: 0.72). Since these models were only able to predict one class, prediction using these models is worthless.
**Conclusion:** Fast, easier or readily available tumour contours cannot substitute full 3D tumour delineations in radiomic models predictive of LRC in OPSCC.
**Limitations:** Not applicable.
**Ethics committee approval:** This study was approved by an ethics committee (IRBd18047).
**Funding for this study:** Funding was received for this study by Verwelius Foundation, and by Willem Meindert De Hoop Stichting.
**Author Disclosures:**
Jonas A. Castelijns: Nothing to disclose
Abrahim Al-Mamgani: Nothing to disclose
Marjaneh Taghavi: Nothing to disclose
Regina G. H. Beets-Tan: Nothing to disclose
Hugo J W L Aerts: Other: Stockholder Sphera Genospace, outside submitted work
Bas Jasperse: Nothing to disclose
Selam Waktola: Nothing to disclose
Michiel van den Brekel: Nothing to disclose
Paula Bos: Nothing to disclose

### RPS 2605-4

### Myelin age: automated estimation of myelin maturation on brain MRIs in infancy and early childhood

*T. Akıncı D'Antonoli*, A. Todea, A. Datta, B. Stieltjes, F. H. Prüfer, N. B. Slavova, J. Wasserthal; Basel/CH

**Purpose:** Determination of myelin development is an essential part of pediatric neuroradiology practice. Our goal is to automate the estimation of myelin maturation with a deep learning algorithm.
**Methods or Background:** T1w and T2w MRI images of the brain from 1002 children aged 0-36 months were retrospectively evaluated (01.01.2011-01.01.2021). A Convolutional Neural Network was trained to predict "myelin age". A 2D input was generated from side-by-side MRI slices of the whole brain. Images were randomly resized by 20% to prevent the network from learning the age-dependent brain size. 778 images with normal myelin maturation, where chronological age was identical to "myelin age," were selected for the training set. 224 images containing both healthy and sick subjects were chosen for the test set. 5-fold cross-validation was applied for hyperparameter optimisation. The test set was also evaluated by two radiologists to compare the model's performance with that of the human rater.
**Results or Findings:** Myelin age within 1-month of chronological age is considered normal, and lower myelin age is considered delayed. The model had a mean absolute error of 1.5 months. The model achieved 93% accuracy in predicting myelin age in children with normal myelin maturation (212 cases). In children with delayed myelin maturation, the algorithm only showed 28% accuracy (12 cases). However, this task was inherently ambiguous, as human raters achieved only 52% agreement in detecting delayed cases.
**Conclusion:** Our model can predict the corresponding myelin maturation age in infancy and early childhood with high accuracy. When myelin maturation was delayed, both the raters and the algorithm showed lower performance.
**Limitations:** The study included a scarcity of cases with delayed myelination.
**Ethics committee approval:** This study was approved by EKNZ.
**Funding for this study:** No funding was received for this study.
**Author Disclosures:**
Bram Stieltjes: Nothing to disclose
Alexandre Datta: Nothing to disclose
Tuğba Akıncı D'Antonoli: Nothing to disclose
Jacob Wasserthal: Nothing to disclose
Alexandra Todea: Nothing to disclose
Friederike Helene Prüfer: Nothing to disclose
Nedelina Bozhidarova Slavova: Nothing to disclose

### RPS 2605-5

### A deep learning system for the automated quantification and screening of suspected ventriculomegaly from 2D ultrasound images of the fetal brain

*H. Shankar*[1], S. Jain[1], D. Singh[2], N. Hegde[3], R. PS[3], R. Kumar[1], P. Radhakrishnan[1], A. Vasudeva[3], S. K. K. Devalla[4]; [1]Bangalore/IN, [2]Chandigarh/IN, [3]Manipal/IN, [4]Singapore/SG
*(hari.shankar@originhealth.ai)*

**Purpose:** Ventriculomegaly (dilated cerebral ventricles) is a relatively common finding on prenatal ultrasound and a soft marker requiring a specialist referral for a detailed search of associated anomalies. We propose a deep learning system (DLS) for the automated quantification and screening of suspected ventriculomegaly to assist novice operators to provide timely referrals.
**Methods or Background:** On 514 2D US images (298 mid-trimester pregnancies; normal [N]/unilateral ventriculomegaly [VM]: 259/39), we trained (ground-truth: caliper points provided by fetal medicine specialists [FMS]) a DLS to automatically predict the caliper points for measuring the atrial width (AW) of the lateral ventricles. The predicted AW measurements were then classified into normal or suspected VM based on clinical guidelines (ISUOG). The suspected VM cases were further classified into prominent, mild, and severe categories. We assessed the DLS performance (vs. FMS) in the automated measurement (mean error [ME] and screening (sensitivity [Sn], specificity [Sp], accuracy [Ac]; with 95% CI).
**Results or Findings:** On an independent test set of 226 images (186 cases), the MEs (in mm) in DLS AW measurements were 0.47+-0.56 (normal, 143 cases), 0.41+-0.37 (prominent, 18 cases), 0.71+-0.77 (mild, 20 cases), and 0.77+-0.97 (severe, 5 cases). Further, the normal and suspected VM cases were discriminated with a Sn, Sp, and Ac of 95.18% (92.82 - 97.53%), 95.74% (94.03 - 97.44%), and 95.53% (94.14 - 96.91%), respectively.
**Conclusion:** We successfully developed and validated a DLS for the automated quantification and screening of suspected VM cases. Its clinical translation can help expecting mothers in low-resource/remote settings to receive timely referrals for detailed examination.
**Limitations:** Bilateral VM cases were excluded from the study. The study had a limited dataset size (only mid-trimester cases).
**Ethics committee approval:** This study received the IRB approval from both the tertiary centres and data were anonymised (tenets of the declaration of Helsinki).
**Funding for this study:** Not applicable.
**Author Disclosures:**
Roopa PS: Nothing to disclose
Divya Singh: Nothing to disclose
Nivedita Hegde: Nothing to disclose
Sripad Krishna Krishna Devalla: Founder: Origin Health
Hari Shankar: Nothing to disclose
Akhila Vasudeva: Nothing to disclose
Radhika Kumar: Nothing to disclose
Prathima Radhakrishnan: Nothing to disclose
Shefali Jain: Nothing to disclose

### RPS 2605-7

### External validation of a single centre MR-based radiomic model predictive of locoregional control in oropharyngeal cancer

*P. Bos*, R. Martens, P. De Graaf, M. van de Wiel, J. J. van Griethuysen, B. Jasperse, R. G. H. Beets-Tan, R. Boellaard, J. A. Castelijns; Amsterdam/NL
*(pa.bos@nki.nl)*

**Purpose:** Radiomic signatures as imaging biomarker to assist the clinician and radiologist in treatment decisions is full of development. However, multi-centric validation of these radiomic signatures is needed to prove generalisability for clinical implementation. This study aims to validate a previously published single-centre radiomic signature to predict locoregional control (LRC) in oropharyngeal squamous cell carcinomas (OPSCC) patients using an independent external dataset.
**Methods or Background:** The validation cohort consists of 149 OPSCC patients. Radiomic features were extracted from primary tumour volumes on postcontrast T1-MRI. ComBat harmonisation was performed to transform these features towards the feature space of the published dataset. Performances were assessed using area under the curve (AUC). Statistical differences between patient demographics were calculated using the Independent Student t-test.
**Results or Findings:** Clinical characteristics were comparable between the published and validation cohort for age, gender, smoking status, nodal status. Differences were shown for positive HPV tumor status (44% vs 69%, p=0.001), low T-stage (53% vs 33%, p<0.001) and cancer subsite (p<0.053). Both cohorts had a LRC rate of 80% of the patients. Performance of the published radiomic model (AUC: 0.74) decreased when the model was validated in an external cohort (AUC: 0.5).

**Conclusion:** The previously published single-centre radiomic model is not generalisable when validating on an independent external dataset. Differences between cohorts regarding patient population, scan acquisition or pre-processing might induce a too large source of noise limiting reproducibility. Therefore standardisation in scan protocols is highly recommended.
**Limitations:** Not applicable.
**Ethics committee approval:** This study was approved by an ethics committee (IRBd19-240).
**Funding for this study:** Funding was received for this study by Verwelius Foundation, and by Willem Meindert De Hoop Stichting.
**Author Disclosures:**
Jonas A. Castelijns: Nothing to disclose
Joost J.M. van Griethuysen: Nothing to disclose
Pim De Graaf: Nothing to disclose
Roland Martens: Nothing to disclose
Regina G. H. Beets-Tan: Nothing to disclose
Ronald Boellaard: Nothing to disclose
Bas Jasperse: Nothing to disclose
Paula Bos: Nothing to disclose
Mark van de Wiel: Nothing to disclose

**15:00-16:00** **Room K**

### Research Presentation Session: Head and Neck

### RPS 2608

### Imaging: technical, vascular and infectious issues

**Moderator**
M. Schernthaner; Vienna/AT

### RPS 2608-2

**Accelerated 3D MR imaging for the semiautomatic airway segmentation in patients with obstructive sleep apnea syndrome**

*U. Grzyska*[1], A. Ibbeken[1], F. Zell[1], C. Hagen[1], A. Steffen[1], H. Kooijman[2], T. M. Buzug[1], J. Barkhausen[1], A. Frydrychowicz[1]; [1]Lübeck/DE, [2]Hamburg/DE

**Purpose:** There is an increasing interest for simultaneous anatomic analysis and 3D airway segmentation in patients with obstructive sleep apnea syndrome (OSAS). Hence, we sought to analyse image and semiautomated segmentation quality of a 3D MRI protocol in patients with OSAS.
**Methods or Background:** 25 patients with OSAS (45±13 years, 11 female, BMI 27.6±4.5) and an apnea-hypopnea index (AHI) of 15.4±10 were examined after IRB approval and consent using an isotropic 3D T1 TSE sequence on a 3T MR scanner (Ingenia, v5.4, Philips) with a 16-channel surface coil. The effective isotropic resolution was 0.58mm. The field of view was adapted to each individual's anatomy and was typically 200x250x180mm. Parallel imaging with an effective factor b=4.5 was applied to achieve a clinically acceptable acquisition time below 3 min. Each sequence was repeated 6 times in different jaw positions partially with a protrusion spint. 3D airway segmentation was performed using ITK-SNAP (www.itksnap.org, v3.8.0, open-source). Diagnostic image quality of reference structures was visually graded from 1 (very good) to 5 (unusable) in the nasopharynx, oropharynx, hypopharynx, and larynx; apparent signal-to-noise and contrast-to-noise ratios were quantified.
**Results or Findings:** Good or very good image quality was confirmed in 74/100 stations (74%), only 22/100 and 4/100 stations showed moderate or significant artifacts. No study was unusable. Semi-automatic 3D segmentation of the pharynx and proximal larynx was successfully performed in 24/25 datasets with only few post-processings; in one case, nearly complete manual segmentation was required due to artifacts.
**Conclusion:** Accelerated 3D T1 TSE MRI is well suited for simultaneous anatomical imaging and semi-automatic airway segmentation in OSAS patients. Swallowing artifacts may in some cases compromise quality such that manual segmentation or post-processing is required instead of semi-automatic segmentation.
**Limitations:** Not applicable.
**Ethics committee approval:** Not applicable.
**Funding for this study:** Not applicable.
**Author Disclosures:**
Alina Ibbeken: Nothing to disclose
Fenja Zell: Nothing to disclose
Ulrike Grzyska: Nothing to disclose
Thorsten M Buzug: Nothing to disclose
Hendrik Kooijman: Nothing to disclose
Christina Hagen: Nothing to disclose
Alex Frydrychowicz: Nothing to disclose
Armin Steffen: Nothing to disclose
Jörg Barkhausen: Nothing to disclose

### RPS 2608-4

**Evaluation of image quality and lesion detection using iterative metal artifact reduction in CT imaging of head and neck abscess**

*S. S. Martin*, F. Kouakoua, J-E. Scholtz, T. Stöver, T. J. Vogl, I. Burck; Frankfurt a. Main/DE
*(simartin@outlook.com)*

**Purpose:** The purpose of this study was to evaluate the impact of a CT-based iterative metal artifact (iMAR) algorithm on image quality in patients with head/neck abscess and dental hardware.
**Methods or Background:** Dual-energy CT images of 80 patients with dental hardware and head/neck abscess were reconstructed with an iMAR algorithm. Two senior radiologists specialised with 6 and 10 years of experience evaluated standard and iMAR reconstructed CT images for subjective and objective image quality, metal artifacts, and delineation of the abscess based on a 5-point-rating-scale. Interobserver agreement was calculated using the Cohens Kappa.
**Results or Findings:** Compared with standard CT images, iMAR showed a significant artefact reduction from -480 HU to 53 HU in all patients ($P < 0.001$). Subgroup analysis of 38 patients with abscess and metal artifacts on equivalent anatomic levels showed a significantly higher objective and subjective noise reduction for iMAR images ($P = 0.005$ and $P = 0.028$). Furthermore, iMAR images were rated superior regarding image quality, artifact reduction, and abscess delineation. Interrater agreement was excellent for overall image quality ($\kappa = .88$), artefact reduction ($\kappa = .91$), and lesion delineation ($\kappa = .95$).
**Conclusion:** CT image reconstructions using iMAR significantly improve image quality and lesion delineation of head/neck abscess in patients with dental hardware.
**Limitations:** This is a retrospective study with a limited sample size. iMAR algorithms from different vendors were not included. Furthermore, non-iterative MAR algorithms or dual-energy CT with monoenergetic reconstructions were not assessed in the current study.
**Ethics committee approval:** The institutional review board of our university hospital approved this study with a waiver for informed consent (Nr. 20-911).
**Funding for this study:** No funding was received for this study.
**Author Disclosures:**
Jan-Erik Scholtz: Nothing to disclose
Timo Stöver: Nothing to disclose
Thomas J. Vogl: Nothing to disclose
Fouad Kouakoua: Nothing to disclose
Simon S. Martin: Speaker: Siemens Healthineers
Iris Burck: Nothing to disclose

### RPS 2608-5

**Angiographic carotid computed tomography image quality assessment for left arm versus right arm injections**

*R. Kanwal*, M. Saeed Wahla, Z. G. Khan; Islamabad/PK
*(raanakanwal@hotmail.com)*

**Purpose:** To assess the differences in image quality of carotid computed tomographic angiography (CTA) of patients injected with contrast material into their left arms versus patients injected with contrast material into their right arms.
**Methods or Background:** After IRB approval, a total of 500 consecutive carotid angiography of patients were retrospectively examined in the period extending from Jan 2019 to Jun 2021. We randomly selected 250 patients for each right and left arm contrast injection. Patients with perivenous streak artifacts and contrast material reflux into the veins of the neck and upper thorax were included. Images were reviewed on PACS. Relationships between the injection site and presence of perivenous streak artifacts and venous reflux were determined.
**Results or Findings:** Out of 500 patients, 121 (24.2%) were female, whereas, 379 (75.8%) were male with a mean age of 55 years. Perivenous streak artefact was seen in 17.7% (89 patients) in right arm group, whereas left arm patients showed this artefact in 24.1% (121 patients). Similarly, reflux was reported in both arms with right being 11.5% (58) and left 19.5% (98). Venous reflux into the neck and upper thoracic veins was also more frequent with left arm injections. We found that perivenous streak artefact and reflux were more common in patients who were injected with contrast into left arm as compared to that into right arm.

**Conclusion:** Perivenous beam hardening streak artefacts and venous reflux could not be prevented with injection. However, patients who were injected with contrast material in their right arms showed fewer artefacts, thus allowing for better quality images on CTA.
**Limitations:** This study had a small sampling size.
**Ethics committee approval:** This study was approved by an ethics committee.
**Funding for this study:** No funding was received for this study.
**Author Disclosures:**
Raana Kanwal: Nothing to disclose
Zeeshan Ghias Khan: Nothing to disclose
Madiha Saeed Wahla: Nothing to disclose

### RPS 2608-6

**Mapping of facial artery and vein with high-resolution ultrasound**

*F. Pistoia*, R. Picasso, F. Zaottini, A. Ascoli, A. Carobbio, A. Ioppi, S. Sanguinetti, F. Marchi, C. Martinoli; Genoa/IT
*(federicopistoia1@gmail.com)*

**Purpose:** Surgical interventions involving the face carry a not high but significant risk of facial vessels iatrogenic lesions. Computed tomography angiography is the noninvasive imaging tool generally employed in preoperative vascular mapping. The study aimed to evaluate the feasibility of high-resolution Ultrasound (US) in mapping facial artery and facial veins and to identify US-based anatomical landmarks useful to avoid facial vessel iatrogenic lesions.
**Methods or Background:** Facial artery and vein of n=45 healthy volunteers (mean age 33) were scanned using an ultra-high frequency, 8mm footprint, 22-8MHz hockey stick. The ability of US in studying facial artery and the origin of their facial branches and facial veins has been checked. Furthermore, 4 fixed points have been identified along facial artery and facial vein course which could be potentially used as landmarks in the operative setting to avoid facial vessel iatrogenic lesions.
**Results or Findings:** High-resolution US was able to map facial artery and the origin of its facial branches, and facial vein in all 45 volunteers. The 4 points identified as landmarks demonstrated a statistically significant reproducibility ($P<0.01$) among the healthy volunteers.
**Conclusion:** High-resolution US is suitable to assess and map facial artery and veins. The use of US-based landmarks could potentially reduce the risk of facial vessel iatrogenic lesions. Finally, US enables a real-time evaluation in the operative room.
**Limitations:** The study was limited by a small number and low mean age of the studied population.
**Ethics committee approval:** The study was conducted according to the declaration of Helsinki.
**Funding for this study:** No funding was received for this study.
**Author Disclosures:**
Filippo Marchi: Nothing to disclose
Federico Zaottini: Nothing to disclose
Carlo Martinoli: Nothing to disclose
Andrea Carobbio: Nothing to disclose
Alessandro Ascoli: Nothing to disclose
Sara Sanguinetti: Nothing to disclose
Riccardo Picasso: Nothing to disclose
Federico Pistoia: Nothing to disclose
Alessandro Ioppi: Nothing to disclose

### RPS 2608-7

**Increasing sensitivity and specificity for diagnosing giant cell arteritis with a multimodal algorithm: moving beyond temporal artery biopsy by mobilising multiple visual diagnostic tools**

*A. Lecler*, F. Charbonneau, J-C. Sadik, J. Savatovsky, G. Clavel; Paris/FR
*(alecler@for.paris)*

**Purpose:** Accurate diagnosis of giant cell arteritis (GCA) is crucial to prevent serious ischaemic complications. We assessed whether a combination of multiple diagnostic imaging tests could improve diagnostic performance.
**Methods or Background:** This IRB-approved prospective single-center study enrolled participants presenting with a suspected diagnosis of GCA from December 2014 to October 2017. Participants underwent high-resolution 3T Magnetic Resonance Imaging (MRI), temporal and extra-cranial arteries ultrasonography (US) and retinal angiography (RA), prior to a temporal artery biopsy (TAB). Diagnostic accuracy of each imaging modality alone, then a combination of several imaging modalities, was evaluated. Several algorithms were constructed to test optimal combinations using the McNemar test.
**Results or Findings:** Forty five patients (24 women and 21 men, mean age 75.4+/-16 years) were enrolled. 43/45 (96%) patients had ophthalmological symptoms. Diagnosis of GCA was confirmed in 25/45 (56%) patients. Sensitivity and specificity of MRI, US and RA alone were of 100% [CI95% 86-100], 86% [CI95% 65-97], 88% [CI95% 69-97], and 84% [CI95% 60-97], 94% [CI95% 70-100] and 74% [CI95% 49-91], respectively. Sensitivity, specificity, positive predictive and negative predictive values ranged from 0.95-1 [CI95% 0.77-1.0], 0.67-1[CI95% 0.38-1.0], 0.81-1[CI95% 0.61-1.0] and 0.91-1[CI95% 0.59-1.0] when combining several imaging tests, respectively. The diagnostic algorithm with the overall best diagnostic performance was the one starting with MRI, followed either by US or RA, with a sensitivity and specificity of 100 %[CI95% 78-100].
**Conclusion:** We showed that a combination of several imaging examinations was highly predictive for diagnosing GCA and could be used in daily practice.
**Limitations:** First, the overall number of patients remains low. Second, this study was performed in a tertiary centre specialising in ophthalmological and neurological diseases.
**Ethics committee approval:** This study was approved by an IRB. Signed informed consent was obtained from all subjects.
**Funding for this study:** No funding was received for this study.
**Author Disclosures:**
Frédérique Charbonneau: Nothing to disclose
Jean-Claude Sadik: Nothing to disclose
Gaëlle Clavel: Nothing to disclose
Julien Savatovsky: Nothing to disclose
Augustin Lecler: Nothing to disclose

### RPS 2608-8

**MRI in odontogenic neck infections: diagnostic accuracy and reliability**

*J. Heikkinen*, V. Jokihaka, J. Nurminen, J. Velhonoja, H. Irjala, T. Soukka, M. J. Nyman, K. Mattila, J. Hirvonen; Turku/FI
*(jaheik4@gmail.com)*

**Purpose:** Magnetic resonance imaging (MRI) has high diagnostic accuracy in evaluating the extent of disease in odontogenic infections. We sought to characterise the prevalence and reliability of MRI findings in these patients and whether MRI can identify the tooth responsible for the infection.
**Methods or Background:** A retrospective cohort study reviewed 106 emergency neck MRI scans of patients with odontogenic infections. We studied whether MRI can accurately identify the abscess and the underlying infected tooth/teeth and associated bony changes in a blinded multi-reader setting. Also, we correlated various MRI edema patterns and abscess diameter to clinical results and outcomes, such as the surgical approach (intraoral vs. extraoral).
**Results or Findings:** The study included 106 patients with odontogenic infections. Of these, 77 patients (73%) had one or more abscesses. Imaging showed sensitivity, specificity, and accuracy of 0.95, 0.84, and 0.92, respectively, for MRI diagnosis of an odontogenic abscess. MRI showed bone marrow edema in the majority of patients, and multi-reader assessment showed good reliability. MRI was also able to accurately predict the causative tooth. Patients without recent dental procedures had more severe illnesses. Among imaging findings, mediastinal edema was the strongest predictor for extraoral surgery.
**Conclusion:** Emergency neck MRI can accurately describe the source and extent of abscess formation in odontogenic infections. These results should increase the utility and reliance of MRI in clinical decision-making.
**Limitations:** This study was retrospective in nature.
**Ethics committee approval:** The hospital district board's permission was obtained. Institutional review board (IRB) review (approval or waiver) was not sought because the national legislature does not require it for retrospective studies of existing data.
**Funding for this study:** This work was supported by Sigrid Jusélius Foundation, Turku University Foundation, and Turku University Hospital.
**Author Disclosures:**
Tero Soukka: Nothing to disclose
Jaakko Heikkinen: Nothing to disclose
Jarno Velhonoja: Nothing to disclose
Jussi Hirvonen: Nothing to disclose
Mikko Juhani Nyman: Nothing to disclose
Kimmo Mattila: Nothing to disclose
Heikki Irjala: Nothing to disclose
Viljami Jokihaka: Nothing to disclose
Janne Nurminen: Nothing to disclose

15:00-16:00 Room N

### Research Presentation Session: Chest

### RPS 2604

### Lung nodules

**Moderator**
M. F. Boomsma; Nigtevecht/NL

### RPS 2604-3

**An integrated nomogram combined clinical-semantic-radiomic features to predict invasive pulmonary adenocarcinomas in subjects with subsolid nodules**
*F-Z. Wu*; Kaohsiung/TW
*(cmvwu1029@gmail.com)*

**Purpose:** Patients with persistent subsolid nodules (SSNs) have a high incidence of lung adenocarcinoma spectrum lesions. Pre-operative early diagnosis of invasive pulmonary adenocarcinomas could lead to avoiding extensive advanced cancer treatment and overdiagnosis in lung cancer screening programme.
**Methods or Background:** A total of 260 patients with 260 SSNs confirmed with surgically pathologic proof were retrospectively reviewed from February 2016 to March 2020. The study subjects (N=260) were divided into the training cohort (N=195) and the validation cohort (N=65) with a ratio of 3:1. In this study, our purpose was to develop and to validate LASSO-derived nomogram integrating clinical-semantic-radiomic features, and compare the predictive ability with clinical-semantic, semantic, and radiologists' performance.
**Results or Findings:** In the training cohort of 195 SSNs, there were 106 invasive lesions and 89 pre-invasive lesions. We developed a LASSO-derived combined nomogram prediction model based on six predictors to predict invasive pulmonary adenocarcinoma lesions in SSNs. The Hosmer-Lemeshow test yielded P values of 0.394 and 0.787 in the training and validation cohorts respectively, which indicated good calibration power in this model.
**Conclusion:** In conclusion, this nomogram could help clinicians to identify invasive pulmonary adenocarcinoma lesions for guidance of the personalised medicine and making more informed decisions in SSNs management
**Limitations:** This study had limitations. First, this study was a retrospective study, and only surgically resected SSNs were enrolled. Therefore, the selection bias was inherent to our study design.
**Ethics committee approval:** The study protocol was approved by the Institutional Review Board Committee of Kaohsiung Veterans General Hospital, Kaohsiung, Taiwan as No. VGHKS 19-CT6-02.
**Funding for this study:** This work was supported by the grants of Taiwan's Ministry of Science and Technology (MOST 108-2314-B-075B-008-, MOST 109-2314-B-075B-006-, MOST 110-2314-B-075B-008-).
**Author Disclosures:**
Fu-Zong Wu: Nothing to disclose

### RPS 2604-4

**How can we help improve the identification of lung cancers mimicking unresolved community acquired pneumonias?**
*F. Dadnam*; Cardiff/UK

**Purpose:** The aim of this study was to evaluate whether high-risk patients who were radiologically diagnosed with community-acquired pneumonia (CAP) were having follow-up chest x-ray imaging as per The British Thoracic Society (BTS).
**Methods or Background:** The BTS has published guidelines on high-risk patients having follow-up imaging, particularly those over the age of 50 and smokers are considered high risk for lung cancers mimicking an unresolved pneumonia. The standard practice is that 100% of these patients should have follow-up thoracic imaging to identify infective resolution or not. Data was collected retrospectively on high-risk adult patients radiologically diagnosed with a CAP. The reports of these patients were then reviewed to identify if a radiologist had: made a clear diagnosis of infective changes and/or clearly stated whether follow-up would be advised. These patients' records were then reviewed to see if follow-up imaging had been arranged.
**Results or Findings:** 55 patients were identified with a CAP on CXR. Only 23 (42%) of these had follow-up imaging as per BTS guidelines. Of the 55 identified, 13 had a written report which identified both a diagnosis of pneumonia and appropriate advice for follow-up and 11 of those had this arranged (85%).
**Conclusion:** We identified that national standards are not being met, but the results do demonstrate that by having a trained radiologist providing a diagnosis and recommendation leads to a higher incidence of appropriate follow-up, if not arranged by the hospital then picked up by their community physician. This simple modification can lead to greater adherence to national standards and earlier diagnosis of lung cancers and therefore improved patient outcomes.
**Limitations:** The study included a small data set.
**Ethics committee approval:** The study was submitted to the Audit team.
**Funding for this study:** No funding was received for this study.
**Author Disclosures:**
Farah Dadnam: Nothing to disclose

### RPS 2604-5

**Lung nodule morphology: effect of deep learning versus iterative reconstruction at different dose levels**
*C. Franck*[1], F. Zanca[2], K. Carpentier[1], H. El Addouli[1], M. C. Niekel[1], M. Spinhoven[1], A. Van Hoyweghen[1], A. Snoeckx[1]; [1]Edegem/BE, [2]Heverlee/BE

**Purpose:** The research objective was to assess the value of deep learning image reconstruction (DLIR) compared to iterative reconstruction (ASIR-V) in terms of lung nodule morphology perception in chest CT.
**Methods or Background:** CT images were acquired using an anthropomorphic chest phantom (Lungman, Kyoto Kagaku) containing 6 spherical, 6 lobulated and 6 spiculated 3D printed solid nodules (volume range 28-392 $mm^3$) at six dose levels (0.2, 0.4, 0.8, 1.5, 3, 6 mGy). The images were reconstructed with ASIR-V 60% and DLIR (TrueFidelity high level) at 1.25 mm slice thickness. Images were randomly read by 5 experienced chest radiologists who were asked to score the nodule's image quality (IQ) on a five-point scale (1=poor, 5=excellent). Readers were blinded for dose and reconstruction algorithm. Percent score frequency was calculated per reconstruction algorithm and dose level. An IQ of 3 was considered diagnostic.
**Results or Findings:** Overall, IQ was higher for TrueFidelity compared to ASIR-V, with 94% of the DLIR cases having an IQ ≥ 3, versus 84% for ASIR-V. Spherical nodules had a significant better IQ score compared to lobulated nodules, for both reconstructions (p=0.003). When stratifying per dose level, the percent frequency of IQ ≥ 3 for ASIR-V/DLIR algorithms was: 0.2 mGy 26%/70%, 0.4 mGy 80%/94%, 0.8 mGy 96%/100%, 1.5 mGy 100%/100%, 3.6 mGy 100%/99%, 6.4 mGy 100%/100%.
**Conclusion:** Lung nodule morphology perception in chest CT performs equally or better with TrueFidelity compared to ASIR-V, for all nodule types. At very low dose levels (0.2-0.8 mGy) DLIR outperforms ASIR-V, while at higher dose levels (≥1.5mGy), DLIR and ASIR-V are comparable in terms of nodule perception.
**Limitations:** Only phantom images were used in the study.
**Ethics committee approval:** Not applicable.
**Funding for this study:** Not applicable.
**Author Disclosures:**
Astrid Van Hoyweghen: Nothing to disclose
Caro Franck: Nothing to disclose
Annemie Snoeckx: Nothing to disclose
Maarten Christian Niekel: Nothing to disclose
Maarten Spinhoven: Nothing to disclose
Haroun El Addouli: Nothing to disclose
Ken Carpentier: Nothing to disclose
Federica Zanca: Nothing to disclose

### RPS 2604-6

**Ultrashort echo time pulmonary MRI: nodule detection and lung-RADS classification versus standard- or low-dose CTs**
*Y. Ohno*[1], D. Takenaka[2], T. Yoshikawa[2], M. Yui[3], K. Yamamoto[3], T. Ueda[1], H. Ikeda[1], K. Murayama[1], H. Toyama[1]; [1]Toyoake/JP, [2]Akashi/JP, [3]Otawara/JP
*(yohno@fujita-hu.ac.jp)*

**Purpose:** To compare capabilities of pulmonary MRI with ultrashort echo time (UTE) with those of standard or low-dose thin-section CTs for lung-RADS classification.
**Methods or Background:** In this prospective study, standard and low-dose chest CTs (270 mA and 60 mA) and MRI with UTE were used to examine consecutive participants who met ACR appropriateness criteria for lung cancer screening with low-dose CT. Probability of nodule presence was assessed for all methods with a 5-point visual scoring system by two board-certified radiologists. All nodules were then evaluated in terms of their lung-RADS classification using each method. To compare nodule detection capability of the three methods, consensus for performances was rated by using jackknife free-response receiver operating characteristic analysis, and sensitivity was compared by means of McNemar's test. Moreover, weighted kappa statistics were used to determine the agreement of lung-RADS classification obtained with each method with standard reference generated from standard-dose CT evaluated by two radiologists who were not included in image analysis session.
**Results or Findings:** 205 participants with 1073 nodules were enrolled. Sensitivity of pulmonary MRI with UTE (sensitivity=87.9%) was higher than standard-dose CT (sensitivity=87.1%, p=0.008) and low-dose CT (sensitivity=87.0%, p=0.004), with no evidence of a difference in figure of merit among them (p=0.25). Agreements for lung-RADS between all modalities and standard reference were almost perfect (standard-dose CT: κ=0.82, p<0.001; low-dose CT: κ=0.82, p<0.001; pulmonary MRI with UTE: κ=0.81, p<0.001).

**Conclusion:** In a lung cancer screening population, pulmonary MRI with UTE was comparable to standard or low-dose CTs for Lung-RADS classification.
**Limitations:** Not applicable.
**Ethics committee approval:** This retrospective study was approved by Institutional Review Boards of Kobe University Graduate School of Medicine and Fujita Health University School of Medicine in Japan.
**Funding for this study:** This study was financially supported by Canon Medical Systems Corporation.
**Author Disclosures:**
Kazuhiro Murayama: Research/Grant Support: Canon Medical SysteCorporation
Kaori Yamamoto: Employee: Canon Medical SysteCorporation
Hirotaka Ikeda: Nothing to disclose
Hiroshi Toyama: Research/Grant Support: Canon Medical SysteCorporation
Takahiro Ueda: Nothing to disclose
Daisuke Takenaka: Nothing to disclose
Masao Yui: Employee: Canon Medical SysteCorporation
Takeshi Yoshikawa: Nothing to disclose
Yoshiharu Ohno: Research/Grant Support: Canon Medical SysteCorporation

### RPS 2604-7

**Contextual lung nodule detection with adjacent slices: raising hope for early detection and reducing false positives per scan**
*S. Lopez*, K. Mannevy, Y. Diascorn, B. Padovani; Nice/FR

**Purpose:** We aim to detect lung nodules a year before actual diagnosis while reducing the number of false positives per scan (FP/scan).
**Methods or Background:** We apply 2D RetinaNet to detect lung nodules integrating adjacent slices on axial axis. Then, we apply a 3D false positive reduction algorithm and merge results. The algorithms had been trained and validated on 888 CT scans from LIDC dataset and tested on CT scans of 1198 patients from NLST subset, taken the year of diagnosis and also a year before. We only consider nodules with a diameter below 60mm.
**Results or Findings:** On NLST subset, we detect 96% of malignant nodules and 79% of benign nodules the year of diagnosis. A year before NLST diagnosis, we detect 93% of already visible malignant nodules and 78% of benign nodules. The missed nodules were close to the mediastinum.
In both cases, the number of false positives per scan is only 6, which is half of the false positive rate in the state of the art article (https://www.ersnet.org/news-and-features/news/artificial-intelligence-diagnose-lung-cancer-a-year-earlier/). The total process takes less than 2 minutes per scan, which could be done in parallel with a radiologist's analysis.
**Conclusion:** Our contextual approach helped to detect all reported malignant nodules from NLST subset the year of diagnosis and a year before, when they were not near to the mediastinum. The false positive rate is half compared with state of the art for a similar sensitivity.
**Limitations:** The missed malignant nodules should be addressed in a separate algorithm to ensure a detection of 100% of malignant nodules.
In addition, the algorithm should be tested on a different population and more recent images.
**Ethics committee approval:** Not applicable.
**Funding for this study:** This study has been achieved with grants by Idex scholarship UCA-Jedi and a subsidy by AstraZeneca.
**Author Disclosures:**
Bernard Padovani: Nothing to disclose
Yann Diascorn: Nothing to disclose
M. Kostia Mannevy: Nothing to disclose
Stéphanie Lopez: Nothing to disclose

### RPS 2604-8

**Diagnostic validation of a deep-learning nodule detection algorithm in low-dose chest CT: determination of optimised dose thresholds in a virtual screening scenario**
*A. A. Peters*, A. Christe, A. Huber, V. Obmann, J. T. Heverhagen, L. Ebner; Bern/CH

**Purpose:** This study was performed to evaluate the effect of dose reduction on the performance of a deep-learning (DL)-based computer-aided diagnosis (CAD) system regarding pulmonary nodule detection in a virtual screening scenario.
**Methods or Background:** 68 anthropomorphic chest phantoms were equipped with 329 nodules (150 ground-glass, 179 solid) of four sizes (5 mm, 8 mm, 10 mm, 12 mm) and scanned with nine tube voltage/current combinations. The examinations were analyzed by a commercially available DL-based CAD system. The results were compared by Cochran's Q test or chi-square test. Logistic regression was performed to evaluate the impact of tube voltage, tube current, nodule size, nodule density and nodule localisation.
**Results or Findings:** The combination with the lowest effective dose (E) and unimpaired detection rate was 80kV/50mAs (sensitivity: 97.9%, mean false-positive rate (FPR): 1.9, mean E: 0.66 mSv). Logistic regression revealed that tube voltage and current had the greatest impact on the detection rate, while nodule size, density and localisation had no significant influence on the software's performance.
**Conclusion:** The optimal tube voltage/current combination proposed in this study (80kV/50mAs) is comparable to the proposed combinations in similar studies. Tube voltage and tube current have a significant impact on the performance of the DL-based CAD software in pulmonary nodule detection regardless of their size, composition or localisation.
**Limitations:** The study used chest phantoms instead of actual patients (e.g. no influences of background pathologies such as fibrosis, emphysema, pneumonia or pleural effusion). Relatively small group sizes of the different dose combinations. Only perfectly spherical lesions with a homogenous density were used in this study, which do not resemble the average lung cancer, which may also present as an irregular or part-solid lesion.
**Ethics committee approval:** Not applicable (phantom study).
**Funding for this study:** No funding was received for this study.
**Author Disclosures:**
Johannes T. Heverhagen: Grant Recipient: Bayer Healthcare AG, Guerbet AG, Siemens Healthineers, Bracco Imaging Spa (all outside the submitted work)
Verena Obmann: Nothing to disclose
Alan Arthur Peters: Nothing to disclose
Adrian Huber: Nothing to disclose
Lukas Ebner: Nothing to disclose
Andreas Christe: Nothing to disclose

**15:00-16:00 Room O**

### Research Presentation Session: Cardiac

### RPS 2603

### The importance of epicardial and perivascular fat

**Moderator**
R. Vliegenthart; Groningen/NL
**Author Disclosure:**
R. Vliegenthart: Research Grant/Support: Siemens Healthineers; Speaker: Bayer, Siemens Healthineers

### RPS 2603-2

**Epicardial adipose tissue attenuation and fat attenuation index: ex-vivo and in-vivo measurements with photon-counting CT**
*V. Mergen*[1], E. Ried[1], T. Allmendinger[2], T. Sartoretti[1], K. Higashigaito[1], R. Manka[1], A. Euler[1], H. Alkadhi[1], M. Eberhard[1]; [1]Zurich/CH, [2]Forchheim/DE
*(victor.mergen@usz.ch)*

**Purpose:** To define the attenuation of epicardial adipose tissue (EAT) including calculation of the fat attenuation index (FAI) on virtual monoenergetic images (VMI) from first-generation dual-source PCD-CT ex-vivo and in-vivo patients.
**Methods or Background:** A multi-energy CT phantom at two sizes with an insert mimicking adipose tissue was imaged on a first-generation PCD-CT and on an energy-integrating detector CT (EID-CT) at 120kV, as reference. In addition, 30 patients who underwent ECG-gated unenhanced and contrast-enhanced coronary CT-angiography with the same PCD-CT without or minimal coronary calcifications were included. Virtual monoenergetic images (VMI) from PCD-CT data were reconstructed at 55-80keV in 5keV-intervals. We measured fat attenuation in the phantom and EAT attenuation of patients on both unenhanced and contrast-enhanced scans. The FAI of the RCA, LAD, and CX were calculated in patients.
**Results or Findings:** In the phantom, attenuation values of fat increased with keV-level (34% increase for the small size, r=0.98, p<.001; and 22% for the large size, r=0.99, p<.001). Fat attenuation values on 70keV VMI were similar to those measured on EID-CT. In patients, EAT attenuation was significantly lower in unenhanced compared to contrast-enhanced scans at all keV-levels (p<.05). Significant correlations were found between EAT attenuation and keV-levels in unenhanced (r=0.74, p<.001) and in contrast-enhanced CT scans (r=0.67, p<.001). Significant correlations were also found between the FAI and keV-levels for the RCA (r=0.38, p<.001), LAD (r=0.34, p<.001), and CX (r=0.24, p=.001).
**Conclusion:** Spectral behaviour of EAT determined on a PCD-CT indicates a dependency of attenuation on keV-levels and contrast enhancement. 70keV VMI are most appropriate for measuring pericoronary inflammation and yield comparability with polychromatic images.
**Limitations:** Sample size with 30 patients is rather small. We did not assess the effect of different tube voltages on PCAT attenuation.

**Ethics committee approval:** This study was approved by an ethics committee.
**Funding for this study:** Funding was received for this study by V.Mergen: research grant of the SAMS and Bangerter-Rhyner-Foundation.
**Author Disclosures:**
Victor Mergen: Nothing to disclose
Matthias Eberhard: Nothing to disclose
Emanuel Ried: Nothing to disclose
Thomas Allmendinger: Employee: Siemens Healthineers GmbH, Forchheim, Germany
Kai Higashigaito: Nothing to disclose
Robert Manka: Nothing to disclose
Thomas Sartoretti: Nothing to disclose
Andre Euler: Nothing to disclose
Hatem Alkadhi: Nothing to disclose

### RPS 2603-3

**Additive value of epicardial adipose tissue quantification to coronary CT angiography derived plaque characterisation and CT fractional flow reserve for the prediction of lesion-specific ischaemia**

*V. Brandt*[1], J. A. Decker[2], U. J. Schoepf[1], A. Varga-Szemes[1], T. S. Emrich[1], G. Aquino[1], U. Ebersberger[3], R. Bekeredjian[4], C. Tesche[3]; [1]Charleston, SC/US, [2]Augsburg/DE, [3]Munich/DE, [4]Stuttgart/DE
*(verena.brandt@gmx.net)*

**Purpose:** Epicardial adipose tissue (EAT) from coronary CT angiography (CCTA) is strongly associated with coronary artery disease (CAD). We investigated the additive value of EAT volume to coronary plaque quantification and CT-derived fractional flow reserve (CT-FFR) to predict lesion-specific ischaemia.
**Methods or Background:** Patients (n=128, 60.6±10.5 years, 61% male) with suspected CAD who had undergone invasive coronary angiography (ICA) and CCTA were retrospectively analysed. EAT volume and plaque measures were derived from CCTA using a semi-automatic software approach, while CT-FFR was calculated using a machine-learning algorithm. The predictive value and discriminatory power of EAT volume, plaque measures, and CT-FFR to identify ischaemic CAD were assessed using invasive FFR as the reference standard.
**Results or Findings:** Fifty-five of 152 lesions showed ischaemic CAD by invasive FFR. EAT volume, CCTA ≥50% stenosis, and CT-FFR were significantly different in lesions with and without hemodynamic significance (all p<0.05). Multivariate analysis revealed predictive value for lesion-specific ischaemia of these parameters: EAT volume (OR 2.93, p=0.021), CCTA ≥50% (OR 4.56, p=0.002), and CT-FFR (OR 6.74, p<0.001). ROC analysis demonstrated incremental discriminatory value with the addition of EAT volume to plaque measures alone (AUC 0.84 vs. 0.62, p<0.05). CT-FFR (AUC 0.89) showed slightly superior performance over EAT volume with plaque measures (AUC 0.84), however, without significant difference (p>0.05).
**Conclusion:** EAT volume is significantly associated with ischaemic CAD. The combination of EAT volume with plaque quantification demonstrates a predictive value for lesion-specific ischaemia similar to that of CT-FFR. Thus, EAT assessment has the potential to improve the therapeutic management of CAD.
**Limitations:** Retrospective study with a limited number of patients. No follow-up.
**Ethics committee approval:** This study was approved by an ethics committee. IRB number #Pro-81880.
**Funding for this study:** No funding was received for this study.
**Author Disclosures:**
Verena Brandt: Nothing to disclose
Christian Tesche: Speaker: Siemens Healthineers and Heartflow Inc.
Ullrich Ebersberger: Nothing to disclose
Uwe Joseph Schoepf: Research/Grant Support: Bayer, Bracco, Elucid BioImaging, General Electric, Guerbet, HeartFlow Inc., Keya Medical, and Siemens Healthineers Consultant: Bayer, Bracco, Elucid BioImaging, General Electric, Guerbet, HeartFlow Inc., Keya Medical, and Siemens Healthineers Speaker: Bayer, Bracco, Elucid BioImaging, General Electric, Guerbet, HeartFlow Inc., Keya Medical, and Siemens Healthineers
Raffi Bekeredjian: Nothing to disclose
Tilman Stephan Emrich: Speaker: Siemens Healthineers and Heartflow Inc.
Josua A. Decker: Nothing to disclose
Akos Varga-Szemes: Research/Grant Support: Siemens Healthineers Consultant: Bayer and Elucid Bioimaging
Gilberto Aquino: Nothing to disclose

### RPS 2603-4

**Potential role of epicardial adipose tissue as a biomarker of anthracycline cardiotoxicity**

*C. B. Monti*[1], S. Schiaffino[2], D. Capra[1], M. Zanardo[1], M. D. M. Galimberti Ortiz[1], P. Spagnolo[2], F. Secchi[1], F. Sardanelli[1]; [1]Milan/IT, [2]San Donato Milanese/IT
*(caterinab.monti@gmail.com)*

**Purpose:** We investigated the radiodensity of epicardial (EAT), subcutaneous (SAT) and visceral adipose tissue (VAT) before and after treatment with anthracyclines in a population of breast cancer (BC) patients, and in controls not treated with anthracyclines, to detect a potential role of EAT density as a biomarker of changes related to chemotherapy cardiotoxicity.
**Methods or Background:** We reviewed BC patients treated with anthracyclines who underwent CT before (CT-t0) and after (CT-t1) chemotherapy, and age- and sex-matched controls who underwent two CT examinations at comparable intervals. On non-contrast scans, EAT was segmented contouring the pericardium and thresholding between -190 and -30 Hounsfield Units (HU), and SAT and VAT with two regions of interest with 15-mm diameter thresholded between -195 and -45 HU.
**Results or Findings:** 32 female patients and 32 controls were included. There were no differences in age (p=0.439) and follow-up duration (p=0.162) between patients and controls. Between CT-t0 and CT-t1, EAT density decreased in BC patients (-66 HU, IQR -71 to -63 HU, versus -71 HU, IQR -75 to -66 HU, p=0.003), while it did not vary in controls (p=0.955). SAT density increased from CT-t0 to CT-t1 in BC patients (-107 HU, IQR -111 to -105 HU, versus -105 HU, IQR -110 to -100 HU, p=0.014), whereas it did not change in controls (p=0.477). VAT density did not vary in either BC patients (p=0.911) or controls (p=0.627).
**Conclusion:** EAT density appears to be influenced by anthracycline treatment for BC, well-known for its cardiotoxicity, shifting towards lower values indicative of a less active metabolism.
**Limitations:** The study is retrospective and single-centre.
**Ethics committee approval:** This retrospective study was approved by the local Ethics Committee.
**Funding for this study:** No funding was received for this study.
**Author Disclosures:**
Francesco Secchi: Nothing to disclose
Francesco Sardanelli: Grant Recipient: General Electric Healthcare, Bayer, Bracco Speaker: General Electric Healthcare, Bayer, Bracco Advisory Board: General Electric Healthcare, Bayer, Bracco
Caterina Beatrice Monti: Nothing to disclose
Maria Del Mar Galimberti Ortiz: Nothing to disclose
Simone Schiaffino: Speaker: General Electric Healthcare Other: Bracco Imaging
Moreno Zanardo: Nothing to disclose
Pietro Spagnolo: Nothing to disclose
Davide Capra: Nothing to disclose

### RPS 2603-5

**Left atrial enhancing epicardial adipose tissue volume fraction on cardiac CT: an independent predictor of atrial fibrillation 1-Year recurrence following pulmonary vein isolation**

*A. T. Huber*, S. Fankhauser, C. Gräni, A. Lam, T. Reichlin; Bern/CH
*(adrian.huber@insel.ch)*

**Purpose:** To investigate the predictive value of an increased left atrial enhancing adipose tissue volume fraction (E-EATvf) on cardiac CT to predict 1-year atrial fibrillation recurrence following pulmonary vein isolation.
**Methods or Background:** 208 consecutive with pulmonary vein isolation were included. The left atrial epicardial adipose tissue (EAT) volume was segmented between -15 HU and -195 HU on pre-procedural cardiac CT scans, including a pre-contrast and an arterial phase scan. The left atrial E-EATvf was defined as the ratio of the segmented enhancing left atrial EAT volume in the arterial phase and the total EAT volume in the native phase. Association of a E-EATvf above the mean with 1-year atrial fibrillation recurrence was analysed based on a univariate and multivariate model.
**Results or Findings:** Atrial fibrillation recurrence occurred in 76 patients after 1 year (37%). Patients with a left atrial E-EATvf above the mean (E-EATvf above 0.33) had a significantly higher atrial fibrillation recurrence after 1-year (Log-Rank p=0.003). In multivariate analysis, E-EATvf remained a significant predictor of atrial fibrillation recurrence (p=0.018) after correction for age, sex, body mass index, left atrial volume, EAT volume and type of atrial fibrillation.
**Conclusion:** An increased left atrial E-EATvf on cardiac CT is a strong predictor of 1-year atrial fibrillation recurrence, independent of age, sex, body mass index, left atrial volume, EAT volume and type of atrial fibrillation.
**Limitations:** Retrospective study design.

**Ethics committee approval:** This study was approved by the institutional review board.
**Funding for this study:** No funding was received for this study.
**Author Disclosures:**
Tobias Reichlin: Nothing to disclose
Christoph Gräni: Nothing to disclose
Severin Fankhauser: Nothing to disclose
Anna Lam: Nothing to disclose
Adrian Thomas Huber: Nothing to disclose

**15:00-16:00** **Room X**

### Research Presentation Session: Hybrid, Molecular and Translational Imaging

### RPS 2606

### Translational, hybrid and molecular imaging

**Moderator**
J. Ferda; Plzen/CZ
**Author Disclosure:**
J. Ferda: Advisory Board: Siemens Photon counting project; Board Member: EJR

### RPS 2606-2

**Effects of anti-TNF-therapy on inflammatory, structural and osteoblastic activity lesions in radiographic axial spondylarthritis: a prospective proof-of-concept study using PET/MRI of SJI and spine**
*N-M. Bruckmann*[1], C. Rischpler[2], J. Kirchner[1], L. Umutlu[2], K. Herrmann[2], G. Antoch[1], X. Baraliakos[3]; [1]Düsseldorf/DE, [2]Essen/DE, [3]Herne/DE

**Purpose:** Assess the effect of TNF-inhibitors on inflammatory and post-inflammatory lesions in relation to the effects on osteoblastic activity assessed by Na[18F]F PET/MRI in the axial skeleton of r-axSpA patients prior (baseline, BL) and up to 6 months after (follow-up, FU) treatment.
**Methods or Background:** Clinically active r-axSpA patients (11 male, 5 female, mean age 38.6±12.0 years) prospectively underwent Na[18F]F PET/MR images of the SIJ (n=16 patients) and the whole spine (n=10 patients) at BL and FU. Three independent blinded readers (1 for PET/MRI, 2 for conventional MRI) evaluated all images. Inflammation (bone marrow edema, BME), structural lesions (fat lesions (FL), sclerosis, erosions and ankylosis) and Na[18F]F uptake were recorded on the level of sacroiliac joint quadrants (SIJ-Q) and vertebral quadrants (VQ). Chi-Square test was applied for comparisons between time points.
**Results or Findings:** Overall, 11 male and 5 female patients (mean age 38.6±12.0 years) were followed up over a mean of 4.6 (3-6) months. A total of 128 SIJ-Q and 920 VQs were analysed at each timepoint. At BL, Na[18F]F uptake was found in 96.0% SIJ-Qs with BME, 94.2% with sclerosis and 88.3% with FL at BL. At FU, 65.3% of SIJ-Q with BME ($p<0.01$), 33.8% with sclerosis ($p=0.23$) and 24.5% with FL ($p=0.01$) showed decrease of 1 Na[18F]F uptake. In VQ, Na[18F]F uptake was found in 81.5% with sclerosis, 41.9% with FL and 33.7% with BME at BL. At FU, 73.5% VQ with BME, 53.3% with FL and 55.6% with sclerosis showed decrease of Na[18F]F uptake.
**Conclusion:** Anti-TNF treatment led to a significant decrease of osteoblastic activity within 4 months, which was most prominent at sites with inflammation. These data support the early anti-inflammatory treatment initiation for prevention of radiographic progression in axSpA.
**Limitations:** No limitations were identified.
**Ethics committee approval:** This study was not yet approved by an ethics committee (Votum).
**Funding for this study:** No funding was received for this study.
**Author Disclosures:**
Ken Herrmann: Nothing to disclose
Christoph Rischpler: Nothing to disclose
Lale Umutlu: Nothing to disclose
Julian Kirchner: Nothing to disclose
Xenofon Baraliakos: Nothing to disclose
Gerald Antoch: Nothing to disclose
Nils-Martin Bruckmann: Nothing to disclose

### RPS 2606-5

**Interrelation between cardiac and brain small vessel disease: a quantitative PET-MRI study**
*B. Mazini*, M. Dietz, M. Bénédicte, R. Corredor-Jerez, J. O. Prior, V. Dunet; Lausanne/CH
*(bianca.mazini@chuv.ch)*

**Purpose:** Beyond large vessel occlusions, small vessel disease (SVD) plays a crucial role in cardiac and brain ischaemia. However, little is known about potential interrelation between both. We aimed at assessing the interrelation between cardiac and brain SVD by using quantitative Rb-82 cardiac PET/CT and brain MRI.
**Methods or Background:** We retrospectively evaluated 186 patients without cardiac/brain large vessel disease, of whom 29 had pure cardiac SVD and 157 had no cardiac SVD as defined by cardiac perfusion PET/CT and coronarography. All underwent both a cardiac Rb-82 PET/CT and a brain 1.5T or 3T MRI (Siemens Healthcare, Erlangen, Germany). Left-ventricle myocardial blood flow (LV-MBF) and flow reserve (LV-MFR) were recorded from Rb-82 PET/CT, while Fazekas score, white matter lesion (WMab) volume, deep grey matter lesion (GMab) volume, and brain morphometry using the MorphoBox prototype software were derived from T1-/T2-weighted images. Groups were compared with Kruskal-Wallis test, and the potential interrelation between heart and brain SVD markers was assessed using Spearman's correlation coefficient.
**Results or Findings:** Compared with healthy controls, patients with cardiac SVD had lower stress LV-MBF and MFR ($p<0.0019$) but similar Fazekas scores and WMab volumes ($p>0.45$). In patients with cardiac SVD, but not in controls, increased rest LV-MBF was associated with left-putamen Z-score reduction (rho=–0.62, $p=0.033$), right-thalamus (rho=0.64, $p=0.026$) and right-pallidum (rho=0.60, $p=0.039$) GMab volume increase. Decreased stress LV-MBF was associated with left-caudate Z-score reduction (rho=0.69, $p=0.014$) while decreased LV-MFR was associated with left- (rho=0.75, $p=0.005$) and right- (rho=0.59, $p=0.045$) putamen Z-score reduction and increased right-thalamus GMab volume (rho=–0.72, $p=0.009$).
**Conclusion:** This retrospective study data supports the hypothesis of an association between cardiac and brain SVD, especially regarding deep grey matter alterations.
**Limitations:** The study is a retrospective study. Patients' subgroup analysis.
**Ethics committee approval:** This study was approved by an ethics committee.
**Funding for this study:** No funding was received for this study.
**Author Disclosures:**
Vincent Dunet: Nothing to disclose
Marechal Bénédicte: Nothing to disclose
Ricardo Corredor-Jerez: Nothing to disclose
John O Prior: Nothing to disclose
Matthieu Dietz: Nothing to disclose
Bianca Mazini: Nothing to disclose

### RPS 2606-6

**Performance and value of 18F-FDG PET/CT in patients with fever of unknown origin: a saudi tertiary care centre retrospective study**
*A. Fathala*; Riyadh/SA

**Purpose:** Fever of unknown origin (FUO) is a diagnostic challenge. The aim of this study was to determine the diagnostic value of 18F-fluorodeoxyglucose (18FDG) positron emission tomography/computed tomography (PET/CT) in patients who present with FUO.
**Methods or Background:** Overall, 105 consecutive patients (61 men and 44 women) with a mean (± standard deviation) age of 51 (± 35) years and a diagnosis of FUO underwent 18FDG PET/CT scans. The value of 18FDG PET/CT in determining the etiology of FUO was assessed.
**Results or Findings:** PET/CT results were classified into four groups: group 1 comprised patients with true-positive results (n = 51; 49%), in whom abnormal 18FDG uptake identified the final diagnosis; group 2, patients with false-positive results (n = 24; 23%), in whom 18FDG uptake was not consistent with final diagnosis; group 3, patients with true-negative results (n = 10; 9.5%), in whom the 18FDG uptake was normal and no final disease was established; and group 4, patients with false-negative results (n = 20; 19%), in whom 18FDG uptake was normal and final disease was established. Of the 51 patients with true-positive PET/CT results, 51% had an infection, 35% had malignancies, and 14% had inflammatory processes. The sensitivity, specificity, positive and negative predictive values, and accuracy were 72%, 29%, 68% and 33%, and 58%, respectively.
**Conclusion:** Our findings show that 18FDG PET/CT contributes to the establishment of the final diagnosis of FUO in many patients (72%). Our results support the use of 18FDG PET/CT in the initial assessment of FUO.

**Limitations:** The study was retrospective and was set in a single tertiary care center with a relatively small number of patients, and referral bias cannot be excluded.
**Ethics committee approval:** This study was not approved by an ethics committee.
**Funding for this study:** No funding was received for this study.
**Author Disclosures:**
Ahmed Fathala: Nothing to disclose

### RPS 2606-7

**Estimation of early therapy effects within the tumour microenvironment using S100A9-specific in-vivo imaging**

*A. Helfen*[1], A. Schnepel[1], M. Masthoff[1], M. Gerwing[1], W. Heindel[1], M. Wildgruber[2], M. Eisenblaetter[3]; [1]Münster/DE, [2]Munich/DE, [3]Freiburg/DE

**Purpose:** The prognosis of cancer is strongly dependent on the immune cell infiltration within the emerging tumour microenvironment (TME). The protein complex S100A8/S100A9 has been recognised as an essential regulator of the TME and is associated with poor overall prognosis. In this study, we evaluated early therapy effects on the TME in syngeneic murine breast cancer via S100A9-specific in-vivo imaging.
**Methods or Background:** Murine 4T1 cells were implanted orthotopically in female BALB/c mice (n = 43). Tumour size-adapted fluorescence reflectance imaging was performed 0 and 24 h ahead of conventional chemo- (Doxorubicin, n=20), anti-angiogenic therapy (Bevacizumab, n=20) or placebo (NaCl, n=19). A second in-vivo imaging was performed 5 days after therapy began. Furthermore, early changes of S100A9-activity after one week under immune checkpoint inhibition (anti-PD-L1, n=7 vs. rat IgG2b as isotype control, n=3) were evaluated. Results were correlated via immunohistochemistry and FACS.
**Results or Findings:** While there were no differences in tumour growth over the entire study period (p=0.48), tumours treated with Bevacizumab had significantly lower fluorescence intensities (FI) than tumours under Doxorubicin therapy (2.60 vs. 15.65 AU, p < 0.0001). FI for Doxorubicin were significantly increased as compared to placebo (8.95 AU, p=0.01). Under anti-PD-L1 treatment S100A9 activity significantly decreased compared with the control group (224.29 AU and 124.57 AU, p=0.0264).
**Conclusion:** S100A9-specific imaging enables the early detection of therapeutic effects via a visualisation of immune cell activity in the TME, even before clinically detectable changes in tumour size occur. Therefore, it may serve as a non-invasive imaging biomarker for early therapeutic effects.
**Limitations:** Further experiments are needed for a translational approach.
**Ethics committee approval:** All animal experiments were approved by the responsible authorities of North Rhine-Westfalia (Protocol 84-02.04.2017.A011).
**Funding for this study:** This study was funded by CiM Cluster of Excellence/DFG, PP-2017-01.
**Author Disclosures:**
Anne Helfen: Nothing to disclose
Annika Schnepel: Nothing to disclose
Moritz Wildgruber: Nothing to disclose
Michel Eisenblaetter: Nothing to disclose
Mirjam Gerwing: Nothing to disclose
Walter Heindel: Nothing to disclose
Max Masthoff: Nothing to disclose

### RPS 2606-8

**Characterisation of morphologic and metabolic response in CAR-T cell treatment of lymphoma**

*M. Winkelmann*, V. Blumenberg, V. Bücklein, M. Unterrainer, F. Vettermann, P. Bartenstein, J. Ricke, M. Subklewe, W. Kunz; Munich/DE
*(michael.winkelmann@med.uni-muenchen.de)*

**Purpose:** Chimeric antigen receptor (CAR) T-cell therapy uses patient-derived tumour antigen-directed T cells for targeted elimination of cancer cells. We assessed different imaging response criteria and parameters of morphologic and metabolic response to identify predictors of overall survival (OS).
**Methods or Background:** Consecutive lymphoma patients with CT imaging at baseline and PET/CT at 3 months after CAR T-cell infusion were selected. Overall response was determined based on Lugano criteria with up to 6 target lesions. The sum of the product of diameters (SPD) was used to represent initial tumour burden and its per cent change over time (depth of response, DoR). Deauville Scores at 3 months were applied as metabolic response parameters. Additional response criteria (RECIL, LYRIC) were applied to compare the overall response status.
**Results or Findings:** 29 patients were included (median age: 64 years, 59% male) with mean baseline SPD of 6,363 mm^2. According to Lugano criteria, 11 patients (38%) had overall progressive disease, 2 patients (7%) a partial response and 16 patients showed a complete response (55%). Discordance in overall response classification was observed when applying other response criteria. DoR, Deauville scores, overall response according to RECIL and Lugano criteria at 3 months were each able to stratify OS (all with p<0.05).
**Conclusion:** Imaging response criteria for lymphoma show considerable discordance in the context of CAR T-cell therapy. Our data indicate a potential prognostic value of quantitative imaging parameters for OS stratification, which should be further investigated as novel imaging endpoints.
**Limitations:** Since CAR T-cells represent a new form of therapy, there is a lack of data on long-term follow-up.
**Ethics committee approval:** Ethical committee approval was obtained.
**Funding for this study:** No funding was received for this study.
**Author Disclosures:**
Marion Subklewe: Nothing to disclose
Franziska Vettermann: Nothing to disclose
Viktoria Blumenberg: Nothing to disclose
Peter Bartenstein: Nothing to disclose
Michael Winkelmann: Nothing to disclose
Marcus Unterrainer: Nothing to disclose
Wolfgang Kunz: Nothing to disclose
Veit Bücklein: Nothing to disclose
Jens Ricke: Nothing to disclose

**15:00-16:00** **Room Z**

### Research Presentation Session: Vascular

### RPS 2615

### Cervical and cranial vascular imaging

**Moderator**
E. Johansson; Umea/SE

### RPS 2615-3

**Relaxation-enhanced angiography without contrast and triggering (REACT) for the assessment of carotid artery stenosis in acute ischaemic stroke**

U. C. I. Hoyer[1], S. Lennartz[1], N. Abdullayev[1], F. Fichter[1], T. Persigehl[1], C. Kabbasch[1], J. Borggrefe[2], K. Weiss[3], *L. Pennig*[1]; [1]Cologne/DE, [2]Minden/DE, [3]Hamburg/DE

**Purpose:** To compare the detection of internal carotid artery (ICA) stenosis and plaques as well as the image quality of extracranial carotid arteries between a novel relaxation-enhanced angiography without contrast and triggering (REACT) sequence and contrast-enhanced magnetic resonance angiography (CE-MRA) in acute ischaemic stroke (AIS).
**Methods or Background:** This was a retrospective, single-centre study of 105 consecutive AIS patients (65.3±18.7 years, 42 females) who received a stroke protocol at 3T in clinical routine including Compressed SENSE (CS) accelerated (factor 4) flow-independent 3D isotropic REACT (fixed scan time: 02:46 min) and CS accelerated (factor 6) 3D CE-MRA. Three radiologists assessed scans for the presence of ICA stenosis and plaques (including hyper-/hypointense signal) with concomitant diagnostic confidence (DC) using 3-point scales (3=excellent). Vessel quality, artifacts, and image noise of extracranial carotid arteries were rated on 5-point scales (5=excellent/none).
**Results or Findings:** REACT achieved a sensitivity of 89.8% and specificity of 95.2% for any and of 93.5%/95.8% for clinically relevant (≥50%) ICA stenosis while yielding a to CE-MRA comparable DC (mean 2.76±0.45 vs. 2.72±0.49, P=.0305). REACT showed an almost perfect intersequence agreement with CE-MRA regarding the assessment of the disease grade (Cohen's Kappa of 0.90). Using REACT, readers detected more plaques overall (n=57.3 vs. 47.7, P=.0001) and plaques of hyperintense signal (n=12.3 vs. 5.7, P=.024) with higher DC (2.77±0.47 vs. 2.57±0.66; P <.0001) compared to CE-MRA. Vessel quality of all segments combined (4.61±0.66 vs. 4.58±0.68, P=.0299) and artifacts (4.51±0.70 vs. 4.44±0.73, P>.05) were comparable between the sequences with REACT showing a lower image noise (4.43±0.67 vs. 4.25±0.71, P<.0001).
**Conclusion:** In AIS, REACT provides a high sensitivity and specificity for detection of ICA stenosis and an improved depiction of plaques while yielding an equal vessel quality compared to CE-MRA.
**Limitations:** Retrospective study design.

**Ethics committee approval:** This study was approved by an ethics committee.
**Funding for this study:** No funding was received.
**Author Disclosures:**
Simon Lennartz: Research/Grant Support: Philips Healthcare
Christoph Kabbasch: Nothing to disclose
Florian Fichter: Nothing to disclose
Lenhard Pennig: Research/Grant Support: Philips Healthcare
Nuran Abdullayev: Research/Grant Support: Philips Healthcare
Kilian Weiss: Employee: Philips Healthcare
Ulrike Cornelia Isabel Hoyer: Nothing to disclose
Jan Borggrefe: Speaker: Philips Healthcare
Thorsten Persigehl: Nothing to disclose

### RPS 2615-5

**T1 mapping derived by STAGE may help assess the severity of oedema in patients with ischaemic stroke**
*L. Shen*, S. Xia; Tianjin/CN

**Purpose:** The decrease of cerebral blood flow is inevitably accompanied by tissue oedema. The increase in water content of brain tissue is manifested as a prolonged T1 relaxation time. Our purpose was to evaluate the relative T1 (rT1) value in different ischaemic regions and the relationship between rT1 value and ischaemia in patients with ischaemic stroke use a noninvasive quantitative T1 mapping derived by STAGE .
**Methods or Background:** We restrospectively included eighty-three ischaemic stroke patients undergoing STAGE and DSC-PWI examination from January 2017 to December 2020. The ischaemic core and the hypoperfusion regions were segmented based on the the ADC threshold value less than 620x10-6 mm2/s and the time to max (Tmax) respectively. T1 value were measured in ischaemic and contralateral regions, the rT1 value was caculated from the ratio of T1 value on the ischaemic side to the contralateral side. One sample t-test and GEE were used to account for the change of rT1 value in the different hypoperfusion regions. The relationship between the rT1value and time to onset in ischaemic core were assesed.
**Results or Findings:** In all ischaemic regions, the mean rT1 value were different from 1 ($P<0.001$). GEE results revealed that, with hypo-Tmax>4s as a control, the rT1 value in other hypoperfusion areas increased (Wald X2=24.7, $P<0.001$). The rT1 value in the ischaemic core was correlated with the onset time ($r_s=0.45$ $P=0.004$).
**Conclusion:** T1 mapping based on STAGE may provide valuable data for understanding the pathophysiological mechanism of oedema progression in patients with ischaemic stroke.
**Limitations:** The measurement of the severity of hypoperfusion only relies on Tmax, the final tissue state cannot be determined because of lack of follow-up.
**Ethics committee approval:** Ethics approval was obtained from the local institutional review board.
**Funding for this study:** Funding was received from the National Natural Science Foundation of China (81871342).
**Author Disclosures:**
Lianfang Shen: Nothing to disclose
Shuang Xia: Nothing to disclose
